# Proteome-wide evidence for enhanced positive Darwinian selection within intrinsically disordered regions in proteins

**DOI:** 10.1186/gb-2011-12-7-r65

**Published:** 2011-07-19

**Authors:** Johan Nilsson, Mats Grahn, Anthony PH Wright

**Affiliations:** 1 School of Life Sciences, Södertörn University, SE-141 89 Huddinge, Sweden; 2 Clinical Research Center, Novum Level 5, Department of Laboratory Medicine and Center for Biosciences, Karolinska Institutet, SE-141 86 Huddinge, Sweden

## Abstract

**Background:**

Understanding the adaptive changes that alter the function of proteins during evolution is an important question for biology and medicine. The increasing number of completely sequenced genomes from closely related organisms, as well as individuals within species, facilitates systematic detection of recent selection events by means of comparative genomics.

**Results:**

We have used genome-wide strain-specific single nucleotide polymorphism data from 64 strains of budding yeast (*Saccharomyces cerevisiae *or *Saccharomyces paradoxus*) to determine whether adaptive positive selection is correlated with protein regions showing propensity for different classes of structure conformation. Data from phylogenetic and population genetic analysis of 3,746 gene alignments consistently shows a significantly higher degree of positive Darwinian selection in intrinsically disordered regions of proteins compared to regions of alpha helix, beta sheet or tertiary structure. Evidence of positive selection is significantly enriched in classes of proteins whose functions and molecular mechanisms can be coupled to adaptive processes and these classes tend to have a higher average content of intrinsically unstructured protein regions.

**Conclusions:**

We suggest that intrinsically disordered protein regions may be important for the production and maintenance of genetic variation with adaptive potential and that they may thus be of central significance for the evolvability of the organism or cell in which they occur.

## Background

Understanding the process of adaptation is of central importance for many biological questions, such as how species respond to climate changes, pathogens or other environmental perturbations, as well for the mechanisms underlying genetic diseases, such as cancer. Evolutionary adaptation occurs when an inheritable change in the phenotype of an organism makes it more suited to its present environment. In diseases like cancer, adaptive mutations allow individual cells within multi-cellular organisms to thrive at the expense of neighbouring cells by over-riding the normal cellular controls that restrict cell growth and division. At the molecular level such phenotypic changes are the result of mutational processes acting on either protein-coding or non-coding DNA sequences. Although the neutral theory of evolution [1] predicts the vast majority of mutations to be either deleterious or neutral, recent years have seen a sharp increase in publications indentifying the action of positive Darwinian selection on genes in various species [2]. The rapidly increasing number of completely sequenced genomes, along with improved bioinformatic methodologies for detecting evidence of selection [3-5], has enabled large-scale scanning of genes or genetic elements for evidence of positive selection. In particular, comparative approaches using sets of genomes from closely related species, or strains within a species, have proven powerful in detecting genes or genetic regions under recent positive selection [6-8].

SNPs are the most abundant source of genetic variation affecting populations. SNPs found within a protein-coding region may be classified as synonymous SNPs or non-synonymous SNPs, depending on whether the encoded amino acid is altered in the alternative DNA sequence variants. Non-synonymous SNPs in coding sequences, together with SNPs in gene regulatory regions, are believed to have the highest impact on phenotype [9] and hence they are suitable targets for studies on adaptation. However, a major task is still to understand which of the 10 million or so SNPs in the human genome are of functional significance. There is therefore a need for approaches that help to predict the subclass of SNPs that are more likely to be of adaptive significance. The relevance of this task is underscored by the International HapMap Project, which uses genetic variation as a tool to better understand the molecular basis of human disease as well as the mechanisms underlying pharmaceutical therapy [10].

Evolvability is often described as an organism's capacity to generate heritable phenotypic variation [11-13]. This capacity may either entail a reduction in the potential lethality of mutations or a reduction in the number of mutations required to generate phenotypically novel traits [14-17]. At the molecular level, non-synonymous SNPs in a protein-coding gene may result in structural changes in the encoded protein, which may cause phenotypic changes and an increased potential for evolutionary innovation, either directly or in future environments [15]. Proteins consist of conformationally structured regions, containing α-helices and β-sheets, as well as intrinsically disordered regions that are conformationally flexible. Intrinsically disordered protein regions (IDRs) have been a recent focus of attention [18-21]. IDRs are abundant in the eukaryotic proteome, with an estimated 50 to 60% of all *Saccharomyces cerevisiae *proteins containing at least one disordered segment comprising more than 30 amino acid residues [22]. Interestingly, IDRs occur more frequently in eukaryotes than in bacteria or archea, perhaps suggesting a role in the evolution of eukaryotes [23]. To our knowledge, the relationship between recent adaptation and the different types of structural domains within proteins has not been systematically studied.

The budding yeast *S. cerevisiae *is one of the best-studied model organisms at the molecular level. It was the first eukaryotic genome to be fully sequenced [24], and it has a well-annotated proteome [25]. The relatively small sizes of fungal genomes, along with recent advances in whole genome sequencing, have facilitated the establishment of multiple yeast genome sequences [26-29]. From an evolutionary perspective, the short generation time of yeasts combined with the strong environmental selective pressures to which they are exposed facilitate the detection of recent selection events in these organisms. Indeed, different budding yeast species display a surprisingly high level of genome diversity that is comparable to that observed within the family of chordates [27]. The *Saccharomyces *Genome Resequencing Project has resulted in genomic sequences of multiple strains of *S. cerevisiae *and its close relative, *Saccharomyces paradoxus *[30]. Studying polymorphism and divergence between the genomes of *S. cerevisiae *and *S. paradoxus *strains thus provides an excellent opportunity to identify genes or genetic regions likely to be under positive Darwinian selection.

In this study, we performed genome-wide analyses of SNPs identified in the *Saccharomyces *Genome Resequencing Project that lie within protein coding genes and used phylogenetic and population genetic methods to detect evidence of selection acting either on entire protein-coding genes or on individual codon sites within genes. Interestingly, we found a stronger association of both genes and codons under positive selection with intrinsically disordered protein regions compared to regions of regular secondary or tertiary structure. Furthermore, a higher degree of positive selection was found to act on proteins belonging to different functional and structural protein categories that are characterized by a high average IDR content. The biological significance of these findings is discussed in the context of the structure, function and evolvability of proteins.

## Results

### The frequency of codon sites under positive selection is enhanced in protein regions with intrinsically disordered structure

**Figure 1:**
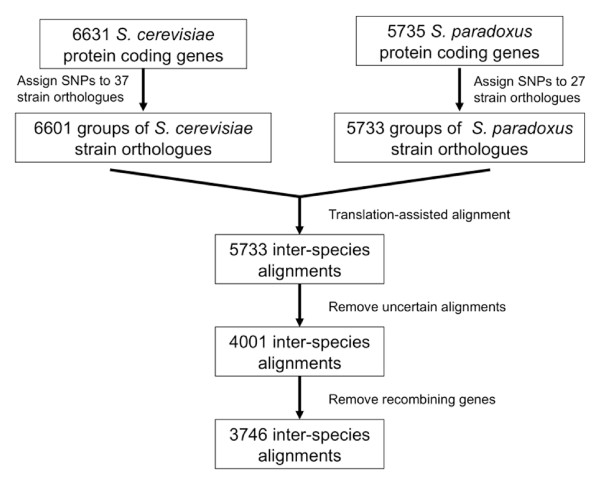
**Flow chart illustrating the initial processing of the source data**. The diagram show the steps involved in creating multiple alignments including *S. cerevisiae *and *S. paradoxus *strains as well as the number of genes involved at each step. Filtering steps for removal of uncertain alignments are also shown. See Materials and methods for details.

The Fixed Effects Likelihood (FEL) method was used to predict codon sites under selection in the coding regions of 3,746 *S. cerevisiae *protein coding genes, for which inter-species alignments could be reliably constructed and for which no recombination events were predicted in the 37 *S. cerevisiae *and 27 *S. paradoxus *genome sequences used (Figure 1). One or more codon sites were predicted to be under selection in 3,421 of these genes. As expected, the total number of sites predicted to be under positive selection (7,561 sites) was considerably lower than the number of sites predicted to be under negative selection (178,408 sites).

**Figure 2:**
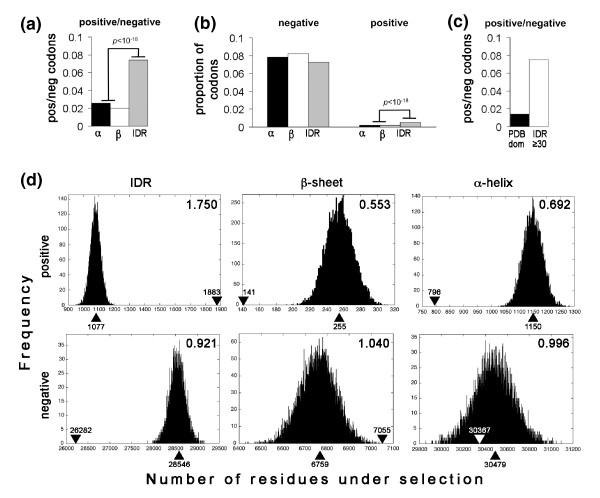
**Codon sites under positive selection are over-represented in gene regions encoding intrinsically disordered regions of proteins**. **(a) **The ratio of positive to negative sites is higher in IDRs than in regions of regular protein structure. The ratio of positive to negative sites is shown for protein regions predicted to have α-helical (α), β-sheet (β) or intrinsically disordered (IDR) protein conformation. The *P*-value shows the significance of the difference between the ratio associated with IDRs in relation to regions of regular structure (a χ^2 ^test was used to test the null hypothesis that there is no difference between the ratios associated with different protein conformation classes). **(b) **The proportion of codons under selection is enhanced in IDRs for positively selected sites but not negatively selected sites. Annotations are as for (a). Differences between the frequencies of negative sites in regions of different protein conformation were not significant. **(c) **The ratio of positive to negative sites is higher in long IDRs than in structured protein domains. The ratio of positive to negative sites is shown for protein regions within known protein domains (PDB dom) or predicted intrinsically disordered protein regions of at least 30 residues in length (IDR ≥30). The frequency of positively selected codons in IDR ≥30 and PDB dom is 0.0055 and 0.0011, respectively, while the equivalent frequencies for negatively selected codons are 0.0728 and 0.0750, respectively. **(d) **Codons under positive selection are significantly more frequent in IDRs than expected in relation to an empirically generated random distribution of selected sites. The panels show empirical frequency distributions (histograms) predicted for a random distribution of positively and negatively selected sites within protein regions with intrinsically disordered structure (IDR), β-sheet and α-helix conformation, generated by 10,000 randomization trials. The median of each distribution is shown associated with upward-pointing arrowheads and the observed number of selected sites together with downward-pointing arrowheads. The ratio of the observed number of sites in relation to the median of the random distribution is shown in the upper right corner of each panel. The ratio is significantly different from unity in all cases (*P *≤ 10^-3^) except for negative sites in α-helical regions.

To investigate whether the pattern of selection on individual codon sites is correlated with the structural context of the encoded amino acids, the frequency of positively and negatively selected sites in IDRs as well as structured regions (α-helices and β-strands) was compared. Regions of regular secondary structure and IDRs were predicted using PSIPRED and VSL2, respectively. Frequency differences were assessed by a χ^2 ^test. The ratio of positive to negative sites was approximately three-fold higher in IDRs compared to regions of regular secondary structure, for which the ratio was similar in α-helical and β-strand regions (Figure 2a). To investigate whether the higher ratio of positive to negative sites in IDRs was mainly due to an excess of positive sites or a depletion of negative sites, the mean proportion of positively and negatively selected codon sites in the three structural conformation states was investigated. Interestingly, the proportion of negatively selected sites was not significantly lower in IDRs compared to regions of regular secondary structure, whereas the proportion of positively selected sites was almost threefold higher in IDRs (Figure 2b). We thus conclude that there was a strong enrichment of positively selected sites in IDRs compared to regions of regular secondary structure, whereas the distribution of negatively selected sites was similar in regions of structured and disordered conformation. Simulation experiments have suggested that selective forces might act more strongly on longer IDRs (≥30 amino acid residues) compared to shorter disordered sequences or secondary structure elements [31]. Further, it has been suggested that selective forces affecting long IDRs might be similar to those affecting the tertiary structure domains of proteins [32]. We therefore calculated the ratio of predicted positive to negative codon sites in tertiary structure domains and IDRs that were 30 or more residues in length. Figure 2c shows that the relative frequency of positive selection in long IDRs is greater that in regions of tertiary structure. This is due to an elevated frequency of positively selected codons in the long IDRs.

To independently test whether the observed frequency differences were greater than would be expected by chance, a randomization test was performed. Briefly, the test entailed sampling a number of selected sites, equivalent to the number of sites found for each of the three conformational states individually, from the combined set of selected sites. The number of sites under either positive or negative selection in each such sample was then calculated. The procedure was repeated 10,000 times to obtain an empirical distribution of the number of selected sites expected by chance. The null hypothesis that the actual number of sites under selection for each conformational state belonged to the derived distributions of selected sites was assessed by a *t*-test. The results showed a significant (*P *≤ 0.001) difference between the observed frequencies of selected sites in different conformational states and the empirically generated random distributions in all cases except in the case of negatively selected sites in α-helical regions. Figure 2d (left panel) shows the derived distributions from each randomization test along with the observed number of positively and negatively selected sites (downward-pointing arrowheads) for IDRs. The figure provides independent support for a strong enrichment of positively selected sites in IDRs and a small but significant depletion of negatively selected sites in these regions. The relative difference between the number of observed (downward-pointing arrowheads) and expected (upward-pointing arrowheads) sites under selection was much greater for positively than for negatively selected sites, as shown by the ratio of the two values (top right corner in each panel). The enrichment level for positively selected sites in IDRs is almost ten-fold higher than the under-representation level of negatively selected sites in the same regions. Hence, the distribution was considerably less skewed for negatively selected sites. The trend was exactly the opposite for regions with α-helical (right panels) and β-sheet (middle panels) conformation. Positively selected sites are under-represented in these regions. Again the extent of positive site under-representation is much greater than the deviation level for negative sites, which differ little, if at all, from the empirically generated value expected for a random distribution within the α-helical and β-sheet conformational classes. Based on the proteome-wide analysis of codons under selection, we thus concluded that there is a strong bias in the distribution of positively selected sites between gene regions encoding regular and disordered protein structure.

We next investigated whether a similar bias in the distribution of codons under selection could be observed at the level of intact genes. To this end, a non-overlapping sliding window of 25 codons was moved across each aligned gene in the analyzed data set, and the number of positively selected codon sites within each window was counted. The predicted IDR content within each window was also calculated. Each window containing at least one positive site thus generated a data point and for genes resulting in at least five such data points the correlation between IDR content and the number of codons under positive selection was assessed by calculation of Spearman's rank correlation coefficient (*P *≤ 0.05). Again, the correlation between degree of disorder and incidence of positive selection was obvious. For the genes analyzed, a significant positive correlation between IDR content and positively selected codon sites was observed in 528 genes, whereas a significant negative correlation was found in only 28 genes. These results thus suggest that the correlation between positively selected sites and gene regions encoding IDRs can be extended to the level of intact genes and proteins.

### Intrinsically disordered protein regions have a higher proportion of fixed non-synonymous polymorphisms

**Figure 3:**
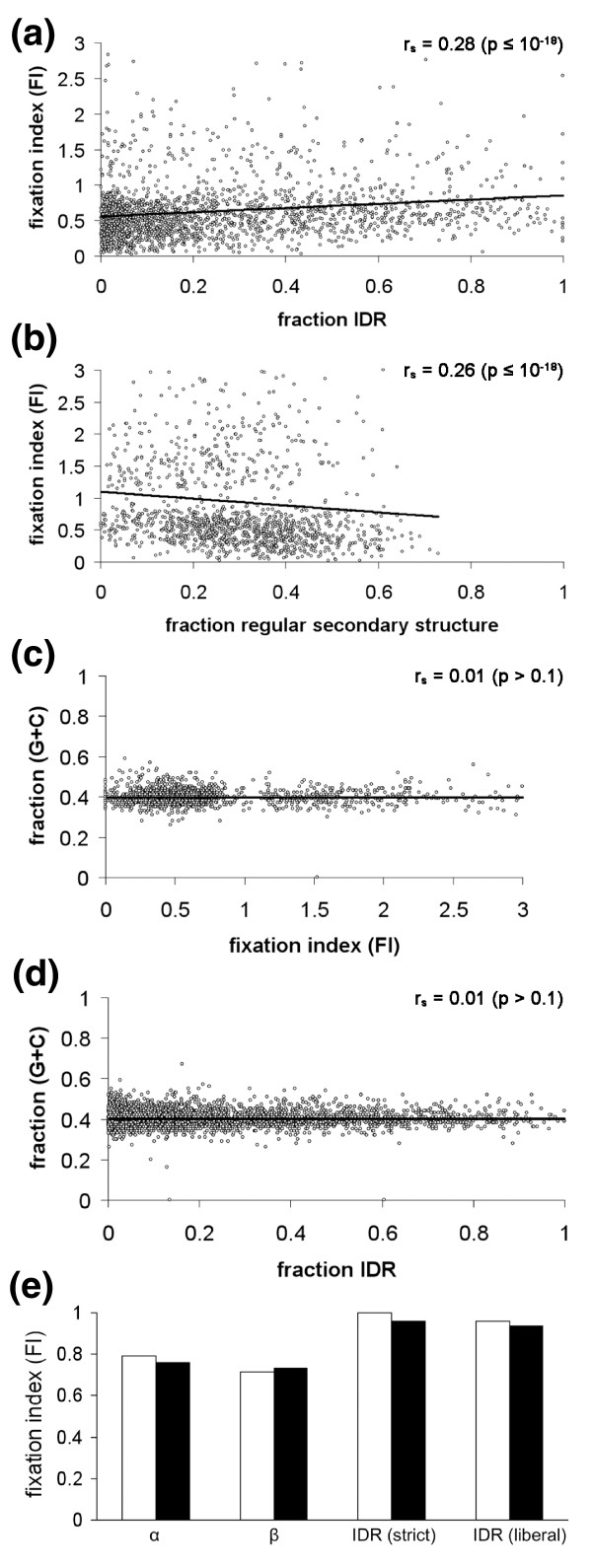
**Relative levels of species-specific fixation of variant SNP alleles in each gene are correlated with the level of intrinsically disordered region content in the corresponding proteins**. **(a, b) **Scatter plot showing the fixation index (FI) for genes, calculated by the McDonald-Kreitman test (see Materials and methods), is positively correlated with the fraction of IDR (a) and negatively correlated with the fraction of regular secondary structure (b) in the corresponding proteins. Spearman's rank correlation coefficients (r_S_) and associated *P*-values are shown. **(c, d) **The (G+C) content of genes is not correlated with their FI (c) or with the fraction of IDR in the corresponding proteins (d). Spearman's rank correlation coefficients (r_S_) and associated *P*-values are shown. **(e) **The mean FI corresponding to all IDRs studied is higher than that for all α-helical regions or β-sheet regions studied. The FI for concatenated tracts of predicted α-helical (α), β-sheet (β) and IDRs are plotted. Values are shown for IDR predictions using confidence thresholds of 0.8 (strict) or 0.5 (liberal) (see Materials and methods for details). Open bars designate results obtained for the non-filtered data set while the filled bars designate the data set after removal of outliers (see Materials and methods for details).

Having observed that intrinsically disordered protein regions were enriched in codon sites under positive selection, we next used an alternative approach to investigate whether enhanced positive selection in genes with high IDR content could be observed at the level of intact genes. The McDonald-Kreitman test was used to estimate the degree of selection acting on the 3,746 aligned *S. cerevisiae *and *S. paradoxus *protein coding genes by means of the fixation index (FI; see Materials and methods for details). Similar to the codon level, a minority of genes were predicted to be under positive selection (FI > 1; 128 genes under a *P*-value threshold of 0.05), while a larger number were predicted to be under negative selection (FI < 1; 519 genes under a *P*-value threshold of 0.05). Figure 3a shows the FI as a function of IDR content for each of the analyzed genes and the equivalent plot for regular secondary structure regions is shown in Figure 3b. Spearman's rank correlation coefficient was calculated to assess the correlation between secondary structure content and FI values, and a *t*-test was used to determine its statistical significance. Consistent with our results at the individual codon level, there was a significant (*P *≤ 10^-18^) tendency for FI and IDR content to be correlated (*r_s _*= 0.28). A negative correlation of similar magnitude was seen between FI and regular secondary structure content (*r_s _*= -0.26, *P *≤ 10^-18^). As a negative control, we similarly assessed the level of correlation between (G+C) content and FI (Figure 3c), and between (G+C) content and IDR content (Figure 3d). No significant correlation was found with *r_s _*values of 0.01 for correlation of (G+C) content with both FI and IDR content. Removal of 63 outliers (genes with a fixation index deviating more than three standard deviations from the mean of the entire data set) did not significantly affect any of the obtained results (data not shown).

A Mann-Whitney *U *test was also performed in order to independently test the significance of the correlation between FI values and IDR content. Genes were sorted into two equally sized groups according to the level of their FI value (the median FI value was 0.42 after removal of outliers). The null hypothesis of equal secondary structure content in the resulting data sets was then tested. There was a significantly higher IDR content in the dataset containing higher FI values (*P *≤ 10^-15^). No significant difference in FI or IDR content (*P *> 0.5) was found between subsets when the dataset was divided in the same way into subsets of high and low (G+C) content (the median G+C value was 0.42). Thus, we conclude that there is a higher proportion of fixed non-synonymous polymorphism in IDRs than in other protein regions, again suggesting an enhanced level of positive selection in these regions.

A potential problem with the analyses presented above is the fact that most genes did not obtain a statistically significant FI value at the chosen level of significance, and hence were discarded from the analysis. To assure that this did not prejudice the overall conclusion, we performed an alternative, proteome-wide analysis. Three composite alignments were created by concatenating protein regions from all 3,746 aligned genes that are predicted to be α-helix, β-strand or IDR. The overall FI was then calculated for each of the three concatenated alignments. Figure 3e shows the resulting overall FI for each composite alignment. In accordance with our previous observations, the overall FI value was close to 1.0 in the IDRs, indicating an overall balance between positive and negative selection acting within these regions. These results were very similar whether a strict or a liberal confidence value was used in the IDR predictions (see Materials and methods). In protein regions with regular secondary structure, the overall FI value was lower than 1.0, indicating an overall bias towards purifying selection acting on these regions. Thus, the data support enhanced positive selection in IDRs even when data from all the gene alignments are studied.

Finally, as an independent assessment of the distribution bias of positively selected polymorphic sites within genes, a non-overlapping window of 25 codons was moved over all the gene alignments, and a regional FI was calculated within each such window. The correlation between the resulting FI and IDR content was estimated by Spearman's rank correlation coefficient. The number of genes with a positive correlation between intrinsic disorder and FI (329 genes) was about an order of magnitude higher than the number of genes where a negative correlation was observed (39 genes), again suggesting a positive correlation between intrinsic disorder and degree of positive selection within proteins.

### Intrinsically disordered regions are not depleted in functional sites

**Figure 4:**
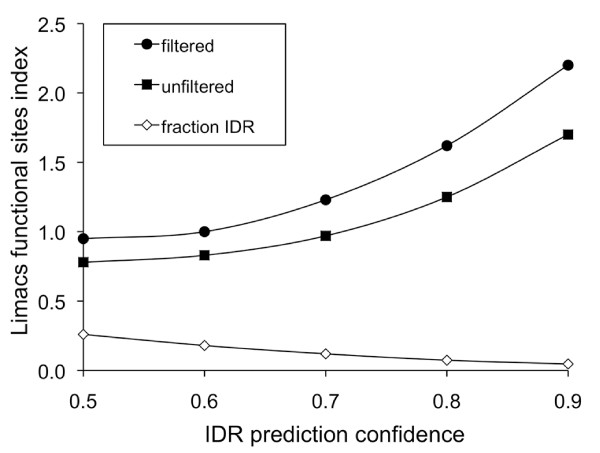
**Functional amino acid residues are not under-represented in intrinsically disordered regions within proteins**. The Limacs functional sites index calculated for mapped Pfam domains within IDRs is plotted against different confidence value thresholds used for prediction of IDRs. The mean fraction of residues predicted to be in IDRs relative to structured regions, at different prediction threshold values, is indicated by open diamonds (default threshold used in the study was 0.8). The corresponding Limacs functional sites index is shown without filtering (filled squares) or after filtering to remove multiple examples of the same Pfam domain (filled circles; see Materials and methods for details).

Given the higher frequency of positively selected amino acid-altering substitutions observed in IDRs, we wanted to further exclude the possibility that this was merely a consequence of a lower level of functional sites in these regions. To this end, we compared the distribution of predicted functional sites between IDRs and non-IDRs using the Limacs functional sites index, for which values show the ratio of functional sites in IDRs in relation to their level in non-IDRs (see Materials and methods). Although we might have expected most annotated functional domains studied by this method to consist mainly of regular secondary structure elements, previous studies have shown that conserved disordered regions occur frequently in annotated protein domains [33]. The mean IDR content in mapped Pfam domains was shown to be about 26%, using a confidence value threshold of 0.5 for IDR prediction (compared to a content of about 44% for the entire proteome). Using a more stringent confidence value threshold (0.8) the equivalent values for IDR content were 7.4% and 26%, respectively. As shown in Figure 4, the Limacs functional sites index was close to or in excess of 1.0 for most IDR prediction parameter settings, suggesting that functional sites are at least as frequent in IDRs as they are in non-IDRs. Somewhat higher relative levels of functional sites were detected in IDRs after filtering the IDR and non-IDR data sets by removing duplicate examples of Pfam domains that occur in two or more proteins in order to prevent possible bias from Pfam domains that are found in many proteins. The Limacs functional sites index increases for both the filtered and non-filtered data sets as the stringency for IDR prediction is increased. Thus, the high relative identification of Limacs sites in IDRs cannot be accounted for by their preferential occurrence in falsely identified IDRs at low stringency levels. Taken together with the relatively high level of negatively selected codons in IDRs and the relatively high FI for polymorphisms in IDRs, these data provide independent evidence that the high levels of apparent adaptive genetic variation predicted for IDRs is not a consequence of reduced negative selection acting on amino acid residues located in IDRs.

### Positively selected sites are over-represented in a subset of functional protein categories

**Figure 5:**
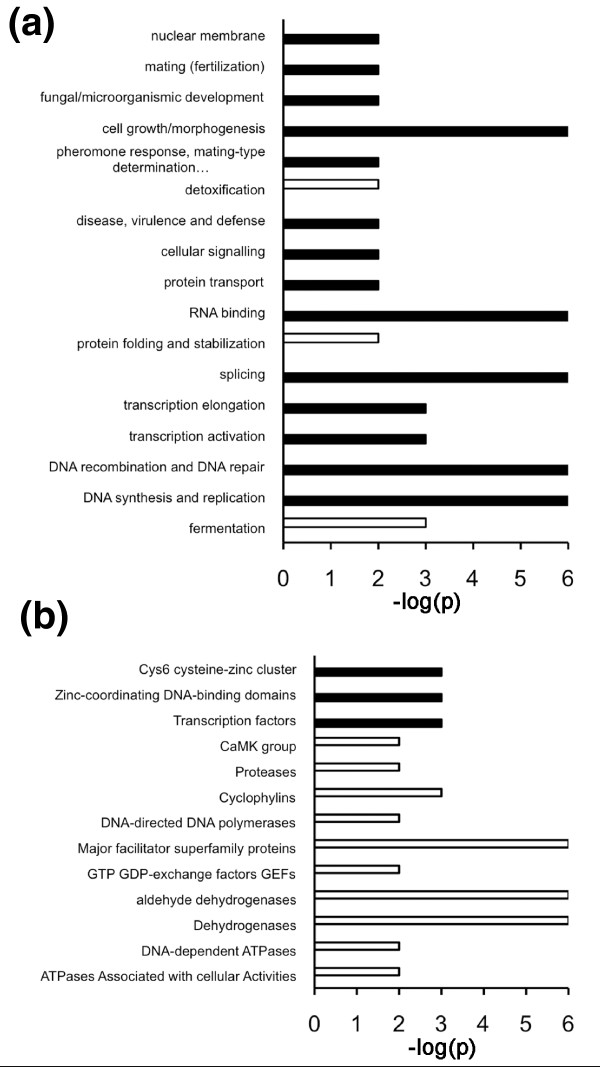
**Specific protein categories are significantly over-represented in their content of codon sites under positive or negative selection**. **(a) **Functional categories of the MIPS FunCat proteins that show significant (*P *≤ 0.01) enrichment of codon sites under positive (filled bars) or negative (open bars) selection. **(b) **Functional categories of the MIPS ProteinCat proteins that show significant (*P *≤ 0.01) enrichment of codon sites under positive (filled bars) or negative (open bars) selection.

To determine the generality of enhanced positive selection in IDRs, we next wanted to investigate how codon sites under positive and negative selection are distributed between different functional classes of proteins. To this end, we used two alternative protein annotation schemes from the Munich Information Center for Protein Sequences (MIPS), FunCat and ProteinCat [34]. A randomization test was employed to detect whether a statistically significant excess of selected sites occurred in any of the subcategories in either catalogue. Figure 5 shows categories significantly enriched in positively (filled bars) or negatively (open bars) selected residues, using a *P*-value threshold of 0.01. In FunCat (Figure 5a), statistical support for positively selected residues is found in proteins involved in both cell growth and morphogenesis, including mating, cell signaling, virulence and defense, as well as various aspects of nucleic acid biology, including the replication, repair, recombination and transcription of DNA. Enrichment of negatively selected residues was observed for a smaller number of categories, including conserved metabolic processes, such as fermentation and detoxification, as well as for protein folding and stabilization. In ProteinCat (Figure 5b), fewer categories were enriched in positively selected sites but all are associated with transcription factors. Most categories are enriched in negatively selected residues and mainly represent different categories of enzymes. The clearest common conclusion from analysis of both catalogues is that transcription factors tend to be enriched in positively selected amino acid residues.

### Protein categories with a high propensity for positive selection have a high average IDR content

**Figure 6:**
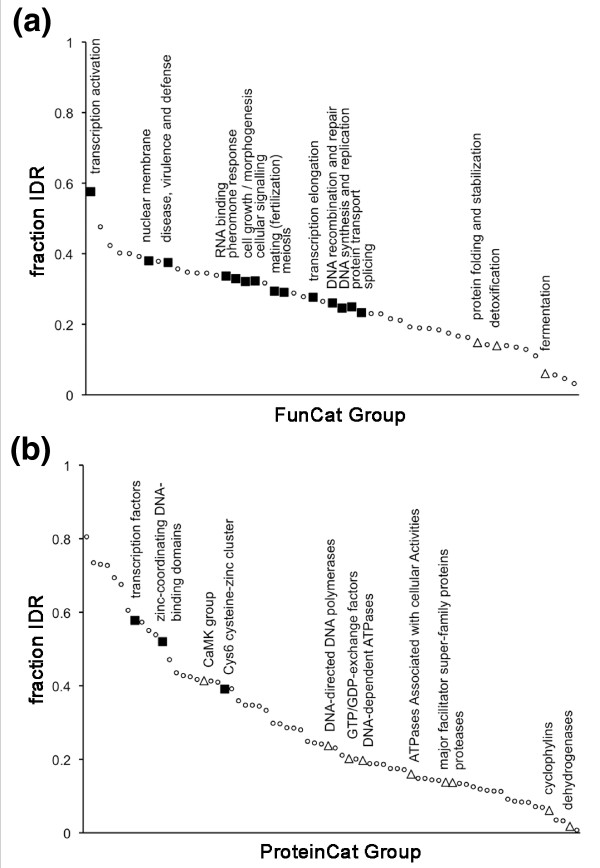
**Protein categories enriched in codon sites under positive selection tend to have higher average levels of intrinsically disordered regions compared to categories enriched in sites under negative selection**. **(a) **MIPS FunCat categories are plotted in a rank according to their IDR content (small open circles). Categories from Figure 5a that are enriched in codon sites under positive (filled squares) or negative (open triangles) selection are plotted with a larger symbol. **(b) **MIPS ProteinCat classes, including those enriched in codon sites under selection (Figure 5b), are plotted as in (a).

Given the correlation between positive selection and both the IDR content of proteins and their functional categorization, we were interested to test directly whether the average IDR content of protein categories is generally correlated with their content of positively or negatively selected sites. To investigate this, the major categories in FunCat and ProteinCat were sorted into ranks according to their average IDR content (Figure 6). The ranks of values for FunCat (Figure 6a) and ProteinCat (Figure 6b) categories show clearly that categories enriched in positively selected sites (filled squares) tend to have higher average IDR contents while the reverse is true for categories enriched in negatively selected sites (open triangles). Transcription factor categories that are significantly enriched in positively selected sites lie closest to the top of both category ranks. We conclude that transcription factors may provide good examples of proteins in which IDRs play an important role in functional adaptation.

## Discussion

Here we show evidence for association between positive adaptive selection and regions of proteins with a low intrinsic propensity for secondary structure formation. This conclusion is based on the study of how genetic variation within 64 strains of *S. cerevisiae *and *S. paradoxus *affects the amino acid sequence of about two-thirds of the proteins within the yeast proteome. Since we cannot reconstruct the evolutionary history of these strains, it is relevant to discuss issues that influence the robustness of our conclusions.

Firstly, we have addressed whether the conclusions we draw could be influenced by the selection of gene alignments for study since we have not studied all genes. Genes were mainly excluded from the study based on uncertainty of the alignments. For the analysis shown, we required a level of 70% amino acid identity in proteins translated from the aligned genes. Reducing this threshold to 60% did not increase the number of proteins appreciably, probably because many of the low quality alignments result from incomplete genome sequences for one or more of the strains. An increase of the threshold to 80% identity, however, led to the exclusion of a further 800 gene alignments. Importantly, the use of these different thresholds for selection of gene alignments for study did not significantly influence the conclusions drawn.

Secondly, we have used different approaches to identify evidence of natural selection since each individual method may be subject to potential drawbacks. While the accuracy of maximum likelihood methods for identifying codons under selection has been questioned recently [35,36], the McDonald-Kreitman approach is an insensitive method for detecting positive selection because evidence of positive selection is often cancelled out by negative selection, which is much more common. Indeed, the recent study by Liti *et al*. [30] did not find any statistical support for the existence of individual genes under positive selection when McDonald-Kreitman data were corrected for random effects associated with multiple testing. We have not corrected the data in our analysis since the aim was to study the overall association of protein structure with propensity for positive or negative selection rather than to identify individual genes under selection. The fact that we identify evidence for similar patterns of positive and negative selection at the level of codons using the FEL method and at the level of intact genes or gene regions using the McDonald-Kreitman test strongly supports the conclusion that the propensity for positive selection is enhanced in the IDRs of proteins. Nowaza *et al*. [36] have pointed to the utility of correlating bioinformatic predictions of codon sites under positive selection with biochemical data. Our observation that predicted evidence of positive selection tends to correlate with IDRs in proteins will be a useful parameter to test in other systems.

Thirdly, we have used several alternative strategies and statistical tests, including permutation tests of empirical significance levels, to assess the significance of the associations we have observed in the different tests for positive and negative selection. In all cases these tests provide statistical support for the association between positive selection and IDRs in proteins.

Fourthly, we have used alternative approaches to study the possibility that the increased frequency of positively selected residues in IDRs is the result of reduced negative selection in these regions due to the fact that they might be less important for protein function. This hypothesis would fit well with preconceptions about protein structure that have stated that structured conformation correlates with functional significance. Most importantly, however, this explanation of our results is contradicted by our data, since the frequency of negatively selected codons is not significantly reduced in IDRs relative to protein regions with a structured conformation. Consistently, other recent reports also suggest that IDRs are under negative selection at a level that may even exceed the level for secondary structure elements [31,32]. To further address the issue, we used the Limacs method to independently predict the relative frequency of functionally import amino acid residues in IDRs in relation to regions of structured conformation. The results are consistent with our codon selection data and show that the predicted frequency of functionally important residues is similar in IDRs and regions with structured conformation.

Other approaches to assess the robustness of our conclusion that the IDRs in proteins are particularly susceptible to positive selection are to test whether protein classes predicted to have high adaptive potential make biological sense, whether they are generally characterized by proteins with high IDR content, as well as whether such proteins are associated with molecular mechanisms that could explain their higher adaptive potential. To test whether particular protein categories are enriched in proteins predicted to be under positive or negative selection, we used the FunCat and ProteinCat catalogues of yeast proteins. Several FunCat categories showed significant over-representation of proteins predicted to be under positive selection. These included functions associated with development, mating, and morphogenesis that contain known targets for adaptive selection. Other categories have to do with virulence, defense and cell signaling as well as many categories related to DNA functions, including transcription. Most of these categories contain many proteins that are potentially relevant targets for adaptive mutation. Fewer FunCat categories showed significant evidence of negative selection but these include classes containing highly conserved proteins involved in detoxification, fermentation and protein folding. The ProteinCat categories that are significantly enriched in proteins predicted to be under negative or positive selection also make sense. Most of the categories under negative selection contain enzymes, which are known to be high in structured regions under negative selection. The three categories enriched in proteins predicted to be under positive selection are all categories containing transcription factors. Individual transcription factors have been suggested to be under positive selection previously [37-39]. Furthermore, transcription factors have been shown to evolve faster than other protein classes in yeast [40]. As predicted by our model, FunCat and ProteinCat categories that are over-represented in proteins predicted to be under positive selection also have a high average IDR content while the reverse is true for categories associated with negative selection.

Our data suggest that the conformational flexibility of IDRs, which might potentially translate to a functional flexibility, could represent a generally evolvable characteristic. IDRs might represent a conformational ground state that provides proteins with an intrinsic ability to adapt new functionality. According to this view, protein regions with regular structure would tend to favor structural and functional specialization but at a cost in terms of evolvability. Consistent with this, experimental studies suggest that naturally occurring proteins are not maximally stable, but rather that they seem to exhibit the minimal level of stability necessary for the environment in which they function [41]. Furthermore, *in silico *studies have shown that strong selection for structural stability would be expected to lead to reduced evolution of novel protein functions [42,43].

A key question is thus whether there is evidence to support a link between the conformational flexibility of IDRs and functional flexibility? The widespread involvement of IDRs in interactions between protein partners involved in a diverse range of biological functions provides such evidence [44]. Further, IDRs have, as predicted, been shown to adopt different conformations upon interaction with different binding partners [21]. It has long been recognized that alterations affecting gene regulation provide a powerful opportunity for evolution of phenotypic differences between organisms and hence for the adaptation of organisms to new environments [45]. Much attention has focused on studies of adaptive changes that affect the sequence of *cis*-acting regulatory elements in gene promoters, enhancers or silencers [46,47]. However, recent studies have suggested that mutations in *trans*-acting components, including transcription factors and co-regulators, are also important for the evolutionary adaptation of transcription networks [48-50]. Protein interactions involving transcriptional components have been suggested to play a role in such evolutionary processes [46,51]. Several studies have shown evidence of adaptive changes in the protein interaction domains of transcription factor proteins [37-39] and previous computational studies have independently shown that transcription factors have a high IDR content [20,52]. Interestingly, transcription factor activation domains have been shown to be IDRs [53,54] that interact with other proteins by two-step target-assisted folding mechanisms in which their intrinsically unstructured nature plays an important role [55,56].

## Conclusions

Taken together with previous knowledge, our results thus provide strong evidence for the involvement of IDRs in evolutionary adaptation. Such IDRs are sometimes associated with transcription factors, where they have been relatively well studied, but it is likely that IDRs involved in adaptation may be found in a much broader range of protein classes. The genome-wide nature of the study suggests that the conclusions are significant to most if not all of the proteome. The adaptive nature of IDRs gives a new perspective for understanding the potential adaptive significance of gene variants that arise in nature and medicine.

## Materials and methods

### Retrieval of genomic sequences and polymorphism information

Plain text files containing the *S. cerevisiae *and *S. paradoxus *reference genome sequences, genomic coordinates of identified SNPs in each of the sequenced isolates (37 *S. cerevisiae *strains and 27 *S. paradoxus *strains), and genomic coordinates of the protein coding genes in *S. cerevisiae *were downloaded on 1 September 2007 from the Sanger ftp site [57]. The strains studied are listed in Additional file 1. Only confirmed polymorphisms were used in the subsequent analyses. Details of synonymous and non-synonymous SNPs in *S. cerevisiae *and *S. paradoxus *strains are described per protein-coding sequence and per chromosome in Additional files 2, 3, 4, 5, 6 and 7.

### Retrieval of protein coding genes in S. cerevisiae and S. paradoxus

The coding sequences of all annotated *S. cerevisiae *protein coding genes in the *Saccharomyces *Genome Database [58] were extracted from the reference genome sequence, and reverse complemented for genes where transcription occurs from the lower strand. For *S. paradoxus*, we retrieved the genomic coordinates of genes inferred previously based on synteny and sequence similarity of predicted ORFs in the *S. paradoxus *genome to annotated genes in the *S. cerevisiae *genome [28]. The corresponding coding sequences were extracted from the *S. paradoxus *reference genome sequence and tested for their coding potential in six possible ORFs using the *sixpack *method of the EMBOSS sequence analysis package [59]. When necessary, the coding sequence was reverse complemented and/or shifted to yield a translatable ORF. No mitochondrial genes were included in the analysis.

### Alignment of orthologous genes

For each *S. cerevisiae *gene where a *S. paradoxus *orthologue could be inferred, a multiple sequence alignment was created consisting of all strain orthologues for which at least one sequence contained at least one SNP relative to the respective reference genome sequence. To assure that the alignment did not result in any frameshifts, translation-assisted alignments were created using *DIALIGN-T *[60]. To ensure that subsequent analyses were not affected by the occurrence of uncertain alignments, orthologous protein coding gene alignments were filtered at different stringency thresholds based on the level of sequence identity in the alignments of the translated sequences and alignments below the filtering threshold were removed (60%, 4,029 alignments; 70%, 4,001 alignments; 80%, 3,198 alignments). Subsequent analyses were performed on each of the three datasets to determine whether the choice of filtering threshold altered the conclusions drawn from subsequent analyses. The choice of filtering criteria did not significantly influence subsequent analysis and results using the alignments with ≥70% are shown in the paper. Details of the DNA sequence alignments used in the study are available on request. The methods used to detect selection assume that all sites in each gene share the same phylogeny [61] and therefore alignments where recombination events were predicted by the *GENECONV *method [62] using the '/r' option (only silent sites analyzed), and calculating global *P*-values based on Bonferroni-corrected Karlin-Altschul *P*-values were removed from the subsequent analysis.

### Prediction of structured and intrinsically disordered protein regions

The *PSIPRED *[63] and *VSL2 *[64] methods were used to predict the occurrence of structured and disordered protein regions, respectively, in all *S. cerevisiae *proteins. The protein-coding DNA sequences used as well as the protein sequences translated from them are provided in Additional files 8 and 9, respectively. The *VSL2 *method was among the best performing in the CASP7 assessment of IDR prediction algorithms [65], and performs particularly well in predicting short disordered regions [64]. Both methods rely on evolutionary information derived from PSI-BLAST generated profiles. For the PSI-BLAST searches we used a filtered version of the Uniref90 database (release 12.8) where transmembrane regions, coiled-coil regions and low-complexity regions had been removed using the *pfilt *program [66]. Each PSI-BLAST search was performed for three iterations, with an E-value threshold of 0.001 for inclusion in a multi-pass model, against the reduced Uniref90 database. A position-specific scoring matrix was produced (option -Q) and this was used as input to the *PSIPRED *and *VSL2 *algorithms. Amino acid residues predicted by *PSIPRED *to belong to state 'helix' or 'extended beta' with a confidence value equal to or larger than 8 were chosen for subsequent analysis of regular secondary structures (Additional files 10 and 11). For prediction of disordered residues, we used both a strict confidence value threshold of 0.8 and a liberal threshold value of 0.5 (Additional files 12 and 13). Any residue sites for which predictions of disordered and regular structure overlapped were removed. Except where stated, the strict confidence value (0.8) was used for IDR prediction in the data shown in the paper. The mean fraction of residues reliably predicted to be in α-helical, β-strand, and intrinsically disordered conformation was 26%, 6% and 23%, respectively. Since the sequences studied using these selection criteria represent only 55% of amino acid residues, all analyses were also performed using the liberal confidence threshold (0.5) for IDR detection (44% of residues identified as IDR, 76% of residues included in analyses). None of the overall conclusions were affected by use of reduced-stringency IDR prediction criteria (0.5). We obtained 1,191 protein regions mapping to known structured domains in the protein database (PDB) and corresponding to 643 yeast proteins from the PFAM database (version 25.0) [67].

### Phylogenetic test for selection

Amino acid residues under selection in inter-strain/species alignments were identified using a codon-based maximum likelihood method implemented in the *HyPhy *software package [68]. Each alignment was analyzed separately for codon sites under selection using the FEL method. A neighbor-joining tree was built for each alignment under the Tajima-Nei model of nucleotide substitution [69], and the tree topology along with the alignment were used as input. The HKY85 model of nucleotide substitution was used [70]. In the FEL method, a likelihood ratio test is performed to estimate the rates of synonymous (α) and non-synonymous (β) substitution at each codon site. If the synonymous substitution rate is higher than the non-synonymous rate (α > β), this is indicative of negative selection, whereas a higher non-synonymous substitution rate (β > α) indicates the action of positive selection. The default threshold value of *P *≤ 0.1 was used to reject the null-hypothesis of α = β at a codon site.

### Population genetic test for selection

To detect genes under selection, the multiple sequence alignments of all orthologous protein coding genes were subjected to the McDonald-Kreitman test [71] as implemented in the *MKtest *program of the *libsequence *package [72]. This test investigates the correlation of polymorphisms within species and their divergence between species, and also distinguishes between synonymous and non-synonymous sites. In a sequence having no evolutionary constraints, the ratio of non-synonymous and synonymous sites that are fixed between species (dN/dS) should be roughly equal to the ratio of non-synonymous and synonymous sites that are polymorphic within a species (pN/pS), according to the neutral theory of evolution [73]. We refer to the ratio (dN/dS)/(pN/pS) as the fixation index (FI). When negative selection is acting on a locus, non-synonymous mutations are unlikely to become fixed, although they might still contribute to polymorphism within a species. Thus, the ratio (dN/dS) is expected to be lower than the ratio (pN/pS), yielding an FI <1. However, if positive selection is acting on a locus, non-synonymous mutations are expected to spread rapidly through the population, thus having a greater effect on divergence than on polymorphism. In this case, the ratio (dN/dS) is expected to be higher than the ratio (pN/pS), yielding an FI >1. We calculated the FI for each gene and performed a Fisher's exact test of the null hypothesis of independence between the two ratios (dN/dS) and (pN/pS). Rejection of the null hypothesis at the 5% significance level was taken as an indication of either negative or positive selection, depending on the FI value (Additional file 14). The occurrence of slightly deleterious mutations is known to cause an underestimation of the level of adaptive evolution [74], and a frequently used approach to control for some of the effects of these mutations is to remove polymorphisms segregating at low frequencies. Thus, all SNPs occurring in less than 15% of the strains within a species were removed, as this has been demonstrated to be an appropriate threshold [75]. Additionally, average proteome McDonald-Kreitman tests were performed by merging all aligned codons encoding amino acid residues reliably predicted to be in regions of α-helical, β-sheet or intrinsically disordered conformation, and performing the calculations described above on each of the three resulting composite alignments (Additional file 15). The (G+C) content of each *S. cerevisiae *gene was calculated using the *geecee *program of the EMBOSS package.

### Prediction and analysis of functionally important amino acid residues

We applied the Limacs method [76,77] to predict the occurrence of functionally important sites in the amino acid sequence of translated *S. cerevisiae *genes. Given a multiple sequence alignment, Limacs uses a template library for prediction of functionally important sites in the alignment. Since the method is based on known functional sites in conserved functional domains [77], we constrained the analysis to mapped Pfam domains [78]. All annotated Pfam domains were mapped to the translated yeast genes using reverse position-specific BLAST (RPS-BLAST). Domains that mapped to at least one of the yeast proteins were subjected to analysis by Limacs to predict the location of functional sites. To score positive as a functional site, sites were required to have a query column versus template pattern score (QT score) of at least 0.95, a QT Z-score of at least 1, and randomization scores QRn and TRn lower than 0.01. The distribution of predicted functional sites in mapped Pfam domains was analyzed for residues in regions of intrinsically disordered and regular conformation, and differences in the distribution were assessed by a χ^2 ^test, where a 2 × 2 contingency table of functional sites in IDRs (LI) and non-IDRs (LnI), and of non-functional sites in IDRs (nLI) and in non-IDRs (nLnI) was built. The Limacs functional sites index (LI/nLI)/(LnI/nLnI) was used to indicate whether there was a relative abundance (index above one) or depletion (index below one) of predicted functional sites in IDRs compared to non-IDRs. Predicted functional sites in each gene are listed in Additional file 16. To ensure the robustness of the obtained results, the comparison was repeated using various IDR prediction reliability cutoff values. Furthermore, to avoid bias from over-represented domains, a conservative filtering procedure was also applied, in which only one of the mapped protein regions was analyzed in cases where a domain mapped to more than one protein.

### Assessment of differences in degree of selection between protein regions of regular and intrinsically disordered structure

Spearman's rank correlation coefficient (*r_S_*) was calculated to assess the correlation between secondary structure content and FI in genes where the McDonald-Kreitman test rejected the null hypothesis of neutral evolution. In the same manner, the correlation was assessed between (G+C) content and secondary structure content, and between (G+C) content and FI. Because of the large sample size (*n*), a normal distribution was assumed and the statistical significance was determined by calculating z = r_S_√n - 1.

The intra-genic correlation between secondary structure content and FI was assessed for each gene by a sliding-window analysis, where structure content and FI were calculated within non-overlapping windows of size 25 codons for all aligned orthologous genes. Spearman's rank correlation coefficient was calculated for the resulting data points, indicating the correlation of structure content and FI for each individual gene. Only informative windows were analyzed, that is, regions for which a FI value could be calculated. If the number of informative windows from a gene alignment was more than 30, a normal distribution was assumed and an approximate z-value was calculated as above. In cases where the number of informative windows was fewer than 30 but more than 5, significance was assessed by consulting a table of pre-calculated critical *r_S _*values. If the analysis produced five or fewer informative windows, no correlation analysis was performed on the gene.

The distribution of codon sites predicted to be under positive or negative selection was likewise assessed between the IDR, α-helix and β-sheet conformational states. A series of 2 × 2 contingency tables was generated describing the frequencies of two types of codon sites in two different structure states, and for each table the difference in distribution was assessed by a χ^2 ^test. To independently investigate whether an observed difference in the distribution of a given type of codon in a certain conformational state could be expected by chance, a randomization procedure was applied. The number of codon sites, *n*, of a given type *C *in the investigated structure state *S *was counted and summed over all genes, as was the total number of codon sites, *N*, in conformational state *S*. The randomization test entailed performing 10,000 re-samples, where *N *codon sites were randomly chosen from any gene, and counting the resulting number of sites, *n'*, of type *C*. The observed number of type *C *sites, *n*, in intrinsically disordered regions was then compared to the simulated distribution of *n' *values, and the null hypothesis of equal distribution in disordered regions and regions with secondary structure was rejected by a two-tailed *t*-test (*P *≤ 0.001).

The intra-genic correlation between occurrence of type *C *sites and secondary structure content was assessed for each gene by a sliding-window analysis, where non-overlapping windows of size 25 codons were moved over the gene, counting the number of type *C *sites and calculating the secondary structure content in each window. Spearman's rank correlation coefficient was calculated to assess the correlation between secondary structure content and type *C *codon site distribution, and the statistical significance was estimated as described above, discarding windows with no type *C *codons.

### Assessment of differences in degree of selection between functional categories of proteins

We adopted the MIPS FunCat and ProteinCat annotation schemes [34] to assign each gene into one or more functional categories. Excess or depletion of codon sites under positive selection in a given functional category was assessed by a randomization test. In a category with a sum of *N *codon sites over all constituent genes, the observed number of sites under selection, *n*, was compared to the empirical distribution of the number of selected sites, which was derived by choosing *N *codon sites from random genes in any functional category and counting the number of sites under selection, *n*', then repeating this process 10,000 times. A two-tailed *t*-test was performed to estimate if the observed number of sites under positive selection in a certain category deviated significantly from the random expectation.

## Abbreviations

FEL: fixed effects likelihood; FI: fixation index (calculated in the McDonald-Kreitman test); IDR: intrinsically disordered region; MIPS: Munich Information Center for Protein Sequences; ORF: open reading frame; SNP: single nucleotide polymorphism.

## Competing interests

The authors declare that they have no competing interests.

## Authors' contributions

JN was involved in the conception and planning of the study, carried out the bioinformatic studies and was involved in interpretation of results and drafting the manuscript. MG was involved in the conception and planning of the study as well as interpretation of results and preparation of the manuscript. AW was involved in the conception and planning of the study as well as interpretation of results and writing the manuscript. All authors read and approved the final manuscript.

## Supplementary Material

Additional file 1 **Yeast strain isolates used in the study**. Click here for file

Additional file 2 **Non-synonymous SNPs in *S. cerevisiae *genes studied**. The nature of each amino acid change for each changed amino acid in each strain is shown for each of 3,639 genes. Click here for file

Additional file 3 **Non-synonymous SNPs in *S. paradoxus *genes studied**. The nature of each amino acid change for each changed amino acid in each strain is shown for each of 3,691 genes. Click here for file

Additional file 4 **Synonymous SNPs in *S. cerevisiae *genes studied**. The identity of each affected amino acid in each affected strain is shown for each of 3,737 genes. Click here for file

Additional file 5 **Synonymous SNPs in *S. paradoxus *genes studied**. The identity of each affected amino acid in each affected strain is shown for each of 3,756 genes. Click here for file

Additional file 6 **Chromosomal location of protein-coding region SNPs in the different strains of *S***. ***cerevisiae***. The direction of each gene as well as the base change in each SNP and its affect on codons and encoded amino acids is shown. Click here for file

Additional file 7 **Chromosomal location of protein-coding region SNPs in the different strains of *S. paradoxus***. The direction of each gene as well as the base change in each SNP and its affect on codons and encoded amino acids is shown. Click here for file

Additional file 8 **FastA file containing the ORF DNA sequence of all analyzed *S. cerevisiae *genes**. Strand, phase, chromosone and genomic start/finish coordinates are specified in the header line. Click here for file

Additional file 9 **FastA file containing the protein sequence translated from the ORFs of all analyzed *S. cerevisiae *genes**. Click here for file

Additional file 10 **Fraction of amino acid residues for each protein that are predicted by the PSIPRED method to adopt α-helical conformation, using a confidence value threshold of 8**. Click here for file

Additional file 11 **Fraction of amino acid residues for each protein that are predicted by the PSIPRED method to adopt β-sheet conformation, using a confidence value threshold of 8**. Click here for file

Additional file 12 **Fraction of amino acid residues for each protein that are predicted by the VSL2 method to adopt intrinsically disordered conformation, using a confidence value threshold of 0.8**. Click here for file

Additional file 13 **Fraction of amino acid residues for each protein that are predicted by the VSL2 method to adopt intrinsically disordered conformation, using a confidence value threshold of 0.5**. Click here for file

Additional file 14 **Fixation index of each investigated protein coding gene along with the associated *P*-value**. The FI was calculated using the MKtest program and is defined as the ratio (dN/dS)/(pN/pS). Click here for file

Additional file 15 **Fixation index calculated for the merged aligned regions in α-helical, β-strand, or intrinsically disordered conformation from all analyzed proteins, with or without removal of genes with a fixation index deviating more than three standard deviations from the mean of the entire data set**. Click here for file

Additional file 16 **Amino acid positions of functional sites in each investigated protein as predicted by the Limacs method for known pfam domains mapped to the proteins**. Click here for file
