# Supplementary material for: Proteome-wide evidence for enhanced positive Darwinian selection within intrinsically disordered regions in proteins
Source: Genome Biol. 2011 Jul 19;12(7):R65. doi: 10.1186/gb-2011-12-7-r65 (PMC3218827; doi:10.1186/gb-2011-12-7-r65)
Supplement: Additional file 1 — Yeast strain isolates used in the study. [file gb-2011-12-7-r65-S1.PDF]

Table S1. Yeast strain isolates used in the study

S. cerevisiae strains#	*S. paradoxus* strains#
REF*	REF*
273614X	A12
322134S	A4
378604X	CBS432
BC187	CBS5829
DBVPG1106	DBVPG4650
DBVPG1373	DBVPG6304
DBVPG1788	IFO1804
DBVPG1853	KPN3828
DBVPG6040	KPN3829
DBVPG6044	N_17
DBVPG6765	N_43
K11	N_44
L_1374	N_45
L_1528	Q32_3
NCYC110	Q59_1
NCYC361	Q62_5
S288c	Q89_8
SK1	Q95_3
UWOPS03_461_4	S36_7
UWOPS05_217_3	T21_4
UWOPS05_227_2	UFRJ50791
UWOPS83_787_3	UFRJ50816
UWOPS87_2421	UWOPS91_917_1
W303	Y6_5
Y12	Y7
Y55	YPS138
Y9	Z1_1
YGPM	
YIIc17_E5	
YJM975	
YJM978	
YJM981	
YPS128	
YPS606	
YS2	
YS4	
YS9	

The strain isolates used are described in Liti G et al Nature 458, 337-41, 2009
* Refers to the reference strain to whose sequence the different strain isolates were compared
