# Supplementary material for: Proteome-wide evidence for enhanced positive Darwinian selection within intrinsically disordered regions in proteins
Source: Genome Biol. 2011 Jul 19;12(7):R65. doi: 10.1186/gb-2011-12-7-r65 (PMC3218827; doi:10.1186/gb-2011-12-7-r65)
Supplement: Additional file 2 — Non-synonymous SNPs in S. cerevisiae genes studied. The nature of each amino acid change for each changed amino acid in each strain is shown for each of 3,639 genes. [file gb-2011-12-7-r65-S2.RTF]

ID:YAL001C	AA:145		322134S:I>L	AA:240		UWOPS83_787_3:K>T	AA:380		322134S:D>G	AA:425		273614X:P>L		YS9:P>L	AA:435		273614X:R>H		322134S:R>H		DBVPG1106:R>H		DBVPG1373:R>H		DBVPG1788:R>H		DBVPG6765:R>H		K11:R>H		L_1374:R>H		L_1528:R>H		SK1:R>H		UWOPS03_461_4:R>H		UWOPS05_217_3:R>H		UWOPS83_787_3:R>H		UWOPS87_2421:R>H		Y12:R>H		Y55:R>H		YIIc17_E5:R>H		YJM975:R>H		YJM978:R>H		YS4:R>H		YS9:R>H	AA:495		273614X:N>D		K11:N>D		NCYC110:N>D		SK1:N>D		UWOPS03_461_4:N>D		UWOPS05_217_3:N>D		UWOPS83_787_3:N>D		Y12:N>D		Y55:N>D		YPS606:N>D		YS9:N>D	AA:537		322134S:G>D		DBVPG1106:G>D		DBVPG1373:G>D		DBVPG1788:G>D		DBVPG6765:G>D		L_1374:G>D		L_1528:G>D		YJM975:G>D		YJM978:G>D		YS4:G>D	AA:651		322134S:H>R	AA:710		NCYC110:K>Q	AA:731		273614X:D>N		DBVPG1853:D>N		DBVPG6044:D>N		SK1:D>N		UWOPS05_217_3:D>N		UWOPS05_227_2:D>N		Y55:D>N		YPS128:D>N	AA:823		UWOPS05_217_3:L>V		UWOPS05_227_2:L>V	AA:1098		DBVPG1106:V>L		DBVPG6765:V>L		L_1528:V>L		YIIc17_E5:V>L		YJM975:V>L		YJM981:V>L	AA:1127		UWOPS05_217_3:S>L		UWOPS05_227_2:S>L	AA:1141		273614X:T>S		378604X:T>S		SK1:T>S		UWOPS05_217_3:T>S		UWOPS05_227_2:T>S		Y12:T>S		Y55:T>S		YPS128:T>S		YPS606:T>S	AA:1157		273614X:Y>F		378604X:Y>F		DBVPG1853:Y>F		SK1:Y>F		Y12:Y>F		Y55:Y>F		YPS128:Y>F		YPS606:Y>F	AA:1160		273614X:T>I		378604X:T>I		DBVPG1853:T>I		K11:T>I		SK1:T>I		Y12:T>IID:YAL005C	AA:83		DBVPG6044:A>G		SK1:A>G		UWOPS05_227_2:A>G		Y55:A>G	AA:208		322134S:F>S		BC187:F>S		DBVPG1106:F>S		DBVPG1373:F>S		DBVPG6044:F>S		DBVPG6765:F>S		K11:F>S		L_1528:F>S		NCYC110:F>S		S288c:F>S		SK1:F>S		UWOPS05_217_3:F>S		UWOPS05_227_2:F>S		UWOPS83_787_3:F>S		YGPM:F>S		YJM975:F>S		YPS128:F>S		YPS606:F>S	AA:265		DBVPG1106:E>D	AA:353		DBVPG1106:G>V	AA:418		322134S:S>P		378604X:S>P		DBVPG6040:S>P		DBVPG6044:S>P		DBVPG6765:S>P		NCYC110:S>P		NCYC361:S>P		SK1:S>P		UWOPS05_217_3:S>P		UWOPS83_787_3:S>P		UWOPS87_2421:S>P		Y55:S>P		Y9:S>P		YGPM:S>P		YIIc17_E5:S>P		YJM978:S>P		YPS606:S>P	AA:422		322134S:F>S		378604X:F>S		DBVPG6040:F>S		DBVPG6044:F>S		DBVPG6765:F>S		NCYC110:F>S		NCYC361:F>S		SK1:F>S		UWOPS05_217_3:F>S		UWOPS83_787_3:F>S		UWOPS87_2421:F>S		Y55:F>S		Y9:F>S		YGPM:F>S		YIIc17_E5:F>S		YJM978:F>S		YPS606:F>S	AA:616		322134S:A>SID:YAL007C	AA:4		K11:S>C	AA:28		322134S:S>I	AA:30		322134S:A>V		DBVPG1788:A>V		YJM975:A>V	AA:91		NCYC110:D>N		Y55:D>N	AA:95		DBVPG1853:K>R		NCYC361:K>R		YS2:K>RID:YAL008W	AA:19		K11:K>M		SK1:K>M	AA:45		L_1374:G>E	AA:79		BC187:Q>E		DBVPG1106:Q>E		DBVPG1788:Q>E		DBVPG6765:Q>E		L_1528:Q>E		UWOPS05_217_3:Q>E		YPS606:Q>E	AA:85		BC187:T>N		DBVPG1106:T>N		DBVPG1788:T>N		DBVPG6765:T>N		L_1528:T>N		UWOPS05_217_3:T>N		UWOPS87_2421:T>N		Y55:T>N		YPS606:T>N	AA:115		DBVPG6040:V>IID:YAL009W	AA:63		322134S:R>H		378604X:R>H		DBVPG1106:R>H		DBVPG1373:R>H		DBVPG1788:R>H		DBVPG1853:R>H		DBVPG6765:R>H		L_1374:R>H		UWOPS87_2421:R>H		YJM975:R>H		YS2:R>H		YS4:R>H	AA:65		273614X:A>V	AA:103		322134S:L>F		DBVPG1106:L>F		DBVPG1373:L>F		DBVPG1788:L>F		DBVPG1853:L>F		DBVPG6040:L>F		DBVPG6044:L>F		DBVPG6765:L>F		L_1374:L>F		NCYC110:L>F		NCYC361:L>F		UWOPS03_461_4:L>F		UWOPS87_2421:L>F		Y55:L>F		YIIc17_E5:L>F		YJM975:L>F		YPS128:L>F		YPS606:L>F		YS2:L>F		YS4:L>F		YS9:L>F	AA:114		322134S:F>L	AA:140		W303:T>	AA:232		UWOPS87_2421:W>-ID:YAL010C	AA:53		DBVPG6040:P>S	AA:83		322134S:K>N		378604X:K>N		DBVPG6040:K>N		DBVPG6765:K>N		L_1374:K>N		L_1528:K>N		NCYC110:K>N		UWOPS03_461_4:K>N		UWOPS05_217_3:K>N		Y55:K>N		YJM975:K>N		YJM981:K>N		YPS606:K>N		YS2:K>N	AA:105		L_1528:P>S	AA:109		273614X:F>Y	AA:114		273614X:A>G		322134S:A>G		DBVPG6040:A>G		DBVPG6765:A>G		L_1374:A>G		L_1528:A>G		NCYC110:A>G		SK1:A>G		UWOPS03_461_4:A>G		Y55:A>G		YJM975:A>G		YJM981:A>G		YPS606:A>G		YS2:A>G	AA:116		SK1:T>M	AA:162		322134S:S>N		378604X:S>N		DBVPG1373:S>N		DBVPG1788:S>N		DBVPG6765:S>N		L_1374:S>N		L_1528:S>N		YIIc17_E5:S>N		YJM978:S>N		YJM981:S>N		YS2:S>N	AA:166		YJM978:Q>P	AA:343		322134S:P>S		378604X:P>S		DBVPG1106:P>S		DBVPG1373:P>S		DBVPG1788:P>S		DBVPG1853:P>S		DBVPG6040:P>S		DBVPG6765:P>S		YIIc17_E5:P>S		YJM978:P>S	AA:360		322134S:S>L		378604X:S>L		DBVPG1106:S>L		DBVPG1373:S>L		DBVPG1788:S>L		DBVPG6040:S>L		DBVPG6044:S>L		DBVPG6765:S>L		L_1528:S>L		Y55:S>L		YIIc17_E5:S>L		YJM978:S>L	AA:395		UWOPS05_227_2:E>D	AA:483		378604X:A>T		DBVPG1106:A>T		DBVPG1373:A>T		DBVPG1853:A>T		DBVPG6765:A>T		L_1374:A>T		L_1528:A>T		YJM981:A>TID:YAL011W	AA:35		UWOPS05_217_3:D>G		UWOPS05_227_2:D>G	AA:86		UWOPS87_2421:N>D	AA:266		DBVPG6040:P>S	AA:326		BC187:V>I		DBVPG1373:V>I		DBVPG1788:V>I		DBVPG6765:V>I		L_1374:V>I		L_1528:V>I		YJM975:V>I		YJM981:V>I		YS9:V>I	AA:334		YPS128:T>A		YPS606:T>A	AA:338		UWOPS05_217_3:A>E	AA:345		YJM975:D>Y		YJM981:D>Y		YS9:D>Y	AA:388		DBVPG6040:T>A		NCYC361:T>A		YPS606:T>I		YS9:T>A	AA:408		UWOPS05_217_3:G>S	AA:411		DBVPG6040:S>A		UWOPS05_217_3:S>A		YS9:S>A	AA:473		322134S:V>I		DBVPG1106:V>I		DBVPG1788:V>I		DBVPG6765:V>I		L_1374:V>I		L_1528:V>I		UWOPS87_2421:V>I		YJM975:V>I		YJM981:V>I	AA:481		322134S:D>G		DBVPG1106:D>G		DBVPG1788:D>G		DBVPG6040:D>G		DBVPG6765:D>G		K11:D>G		L_1528:D>G		UWOPS05_217_3:D>G		UWOPS87_2421:D>G		Y55:D>G		YJM975:D>G		YJM981:D>G		YS9:D>GID:YAL013W	AA:83		273614X:L>P		DBVPG1373:L>P		DBVPG1788:L>P		DBVPG6040:L>P		DBVPG6765:L>P		L_1374:L>P		YJM975:L>P		YJM981:L>P	AA:329		322134S:C>R		DBVPG1373:C>R		DBVPG1788:C>R		DBVPG6765:C>R		L_1374:C>R		L_1528:C>R		SK1:C>R		UWOPS87_2421:C>R		Y55:C>R		YIIc17_E5:C>R		YJM978:C>R		YPS128:C>R		YPS606:C>R	AA:333		YJM978:E>G	AA:365		DBVPG1373:L>F		DBVPG1788:L>F		DBVPG6765:L>F		L_1374:L>F		L_1528:L>F		SK1:L>F		UWOPS87_2421:L>F		YIIc17_E5:L>F		YS9:L>F	AA:401		273614X:H>ID:YAL016W	AA:32		NCYC110:S>P		Y55:S>P	AA:94		DBVPG6044:A>S		NCYC110:A>S		Y55:A>S	AA:181		DBVPG6044:V>I		Y55:V>I	AA:241		YS9:N>I	AA:312		L_1528:S>G	AA:494		273614X:R>-	AA:546		YS9:V>E	AA:593		YPS128:A>V	AA:633		YIIc17_E5:L>-ID:YAL018C	AA:46		273614X:H>Q		BC187:H>Q		DBVPG1788:H>Q		DBVPG6765:H>Q		YJM981:H>Q	AA:50		273614X:V>I	AA:52		YS9:S>N	AA:54		NCYC110:T>I		Y55:T>I	AA:175		YPS606:V>I	AA:182		K11:A>V		SK1:A>V		Y12:A>V		Y55:A>V		YPS606:A>V		YS4:A>V		YS9:A>V	AA:297		DBVPG6044:D>N		Y55:D>N	AA:323		UWOPS05_217_3:P>AID:YAL019W	AA:4		DBVPG6044:S>P		K11:S>P		Y12:S>P		Y55:S>P		YPS128:S>P		YPS606:S>P	AA:64		DBVPG6040:K>T	AA:224		DBVPG6044:E>D		NCYC110:E>D		Y55:E>D	AA:330		DBVPG6044:D>N		Y55:D>N	AA:335		DBVPG6044:A>T		Y55:A>T	AA:419		DBVPG1853:N>K	AA:420		DBVPG6044:N>K		Y55:N>K	AA:430		UWOPS03_461_4:A>T		UWOPS05_227_2:A>T		YS2:A>T	AA:470		378604X:I>T		BC187:I>T		DBVPG1373:I>T		DBVPG1788:I>T		DBVPG6040:I>T		DBVPG6044:I>T		DBVPG6765:I>T		L_1374:I>T		NCYC361:I>T		UWOPS03_461_4:I>T		Y12:I>T		Y55:I>T		YPS128:I>T		YPS606:I>T		YS4:I>T		YS9:I>T	AA:513		DBVPG6044:N>K		Y55:N>K	AA:534		UWOPS03_461_4:F>L		UWOPS05_217_3:F>L	AA:544		DBVPG6044:I>V		UWOPS87_2421:I>V		Y12:I>V		Y55:I>V		YPS128:I>V		YPS606:I>V		YS4:I>V		YS9:I>V	AA:599		YS4:M>V	AA:620		UWOPS87_2421:P>L	AA:651		Y9:Y>-	AA:663		SK1:D>H	AA:861		DBVPG1853:S>PID:YAL020C	AA:21		UWOPS05_217_3:E>-	AA:120		NCYC110:Q>E		Y55:Q>E		YPS606:Q>E		YS4:Q>E	AA:130		K11:E>G		Y12:E>G		Y55:E>G		YPS606:E>G		YS4:E>G	AA:172		K11:V>I		UWOPS05_217_3:V>I		UWOPS87_2421:V>I		Y12:V>I		Y55:V>I		YPS606:V>I		YS4:V>I	AA:181		UWOPS83_787_3:A>P	AA:192		UWOPS05_227_2:M>T	AA:208		UWOPS05_227_2:L>P	AA:224		UWOPS05_227_2:V>A	AA:226		UWOPS05_227_2:L>P	AA:237		K11:A>T		UWOPS05_227_2:A>T		YPS606:A>T		YS4:A>T		YS9:A>T	AA:248		UWOPS05_217_3:G>C		Y12:A>S		YPS128:A>S		YPS606:A>S	AA:276		K11:T>I		UWOPS03_461_4:T>I		UWOPS05_227_2:T>I		UWOPS87_2421:T>I		YPS606:T>I		YS4:T>I		YS9:T>I	AA:308		UWOPS83_787_3:P>RID:YAL022C	AA:46		UWOPS05_217_3:D>E		UWOPS05_227_2:D>E	AA:182		DBVPG6044:M>V		UWOPS05_227_2:M>V		UWOPS87_2421:M>V		Y55:M>V		YPS606:M>V	AA:190		UWOPS87_2421:G>V	AA:255		DBVPG1853:V>M	AA:427		273614X:M>I	AA:517		273614X:R>TID:YAL023C	AA:32		SK1:S>N	AA:54		Y55:A>V	AA:76		L_1374:L>F	AA:222		378604X:N>Y	AA:436		SK1:V>G	AA:489		YS4:G>S	AA:498		322134S:S>C	AA:630		L_1528:I>V	AA:753		322134S:I>LID:YAL025C	AA:150		YS9:R>I	AA:250		273614X:Q>E		322134S:Q>E		378604X:Q>E		BC187:Q>E		DBVPG1373:Q>E		DBVPG1788:Q>E		DBVPG6040:Q>E		DBVPG6765:Q>E		L_1374:Q>E		L_1528:Q>E		UWOPS87_2421:Q>E		Y55:Q>E		Y9:Q>E		YJM975:Q>E		YPS128:Q>E		YPS606:Q>E		YS4:Q>E		YS9:Q>EID:YAL027W	AA:21		W303:F>S	AA:27		SK1:I>M		UWOPS87_2421:I>M		YPS606:I>M		YS4:I>M	AA:34		DBVPG6044:T>N		UWOPS83_787_3:T>N		Y55:T>N	AA:77		DBVPG6040:I>V		DBVPG6044:I>V		K11:I>V		SK1:I>V		UWOPS83_787_3:I>V		UWOPS87_2421:I>V		Y55:I>V		YPS606:I>V		YS4:I>V	AA:111		DBVPG6040:I>T		DBVPG6044:I>T		DBVPG6765:I>M		K11:I>T		SK1:I>T		UWOPS83_787_3:I>T		UWOPS87_2421:I>T		Y55:I>T		YPS606:I>T		YS4:I>T	AA:146		YIIc17_E5:Y>S	AA:157		DBVPG6044:N>D		Y55:N>D	AA:199		UWOPS03_461_4:V>I		UWOPS87_2421:V>I	AA:207		Y55:G>W	AA:214		DBVPG1853:T>I		DBVPG6040:T>I		DBVPG6044:T>I		K11:T>I		SK1:T>I		Y55:T>I		Y9:T>I		YPS128:T>I		YPS606:T>I		YS4:T>I	AA:223		UWOPS03_461_4:V>LID:YAL028W	AA:3		DBVPG1853:N>Y	AA:12		DBVPG6044:G>D		K11:G>D		UWOPS05_217_3:G>D		UWOPS05_227_2:G>D		Y12:G>D		Y55:G>D		YPS606:G>D	AA:19		DBVPG6044:D>G		K11:D>G		NCYC110:D>G		Y12:D>G		Y55:D>G		YPS606:D>G	AA:47		UWOPS03_461_4:H>Q		UWOPS05_217_3:H>Q		UWOPS05_227_2:H>Q	AA:70		Y9:H>	AA:128		YS9:P>S	AA:148		YS9:E>K	AA:160		NCYC361:D>V	AA:163		NCYC361:P>T	AA:170		DBVPG1373:A>S	AA:182		UWOPS03_461_4:N>D	AA:184		UWOPS03_461_4:K>E	AA:189		L_1528:S>T	AA:222		DBVPG1373:A>T		DBVPG1853:A>T		DBVPG6040:A>T		NCYC110:A>T		SK1:A>T		UWOPS03_461_4:A>T		UWOPS87_2421:A>T		Y55:A>T		Y9:A>T		YPS606:A>T		YS9:A>T	AA:225		Y9:P>L	AA:235		Y9:I>V	AA:259		DBVPG1853:I>V	AA:286		DBVPG6040:A>V		Y9:A>V	AA:297		DBVPG1788:I>N		DBVPG1853:I>N		DBVPG6040:I>N		DBVPG6765:I>N		L_1528:I>N		UWOPS83_787_3:I>N		Y9:I>N		YIIc17_E5:I>N		YPS606:I>N	AA:340		DBVPG1373:P>S	AA:345		YS9:T>	AA:446		Y55:T>A	AA:496		DBVPG1373:T>I	AA:525		BC187:V>I		DBVPG1373:V>I		DBVPG1788:V>I		DBVPG6765:V>I		SK1:V>I		UWOPS83_787_3:V>I		UWOPS87_2421:V>I		Y55:V>I		YJM975:V>I		YPS128:V>I		YS4:V>I		YS9:V>IID:YAL032C	AA:13		DBVPG1853:Q>E		NCYC110:Q>E		UWOPS05_227_2:Q>E		UWOPS87_2421:Q>E		Y12:Q>E		Y55:Q>E		YPS128:Q>E		YPS606:Q>E	AA:22		UWOPS05_227_2:S>P		UWOPS87_2421:S>P	AA:103		DBVPG6044:N>S		K11:N>S		Y12:N>S		Y55:N>S		YPS128:N>S	AA:191		DBVPG6044:D>G		K11:D>G		Y55:D>G		Y9:D>G	AA:229		DBVPG6040:A>S	AA:252		Y9:N>S	AA:288		273614X:I>V		322134S:I>V		DBVPG6044:I>V		SK1:I>V		UWOPS03_461_4:I>V		UWOPS05_217_3:I>V		UWOPS87_2421:I>V		Y12:I>V		Y55:I>V		Y9:I>V		YPS128:I>V		YS2:I>V	AA:291		DBVPG6044:G>S		Y55:G>S	AA:356		DBVPG1853:A>P	AA:380		DBVPG1853:->Q		DBVPG6044:->Q		NCYC110:->Q		UWOPS03_461_4:->Q		UWOPS05_217_3:->Q		UWOPS83_787_3:->Q		Y55:->Q		Y9:->Q		YPS128:->Q		YPS606:->Q		YS4:->QID:YAL033W	AA:155		DBVPG1853:I>TID:YAL034C	AA:29		UWOPS05_227_2:R>H	AA:74		K11:N>S		SK1:N>S		UWOPS83_787_3:N>S		Y55:N>S		YPS606:N>S	AA:90		322134S:H>N	AA:114		322134S:A>T	AA:196		DBVPG6765:P>L	AA:204		UWOPS05_227_2:R>I	AA:216		SK1:R>G	AA:314		322134S:K>N	AA:402		SK1:G>R	AA:413		SK1:L>IID:YAL034W-A	AA:48		UWOPS03_461_4:S>F		UWOPS05_217_3:S>F		UWOPS05_227_2:S>F	AA:118		378604X:N>D	AA:123		DBVPG6040:S>T	AA:131		DBVPG6765:V>L	AA:182		NCYC361:E>G	AA:196		378604X:D>EID:YAL035W	AA:216		322134S:K>R		DBVPG6044:K>R		K11:K>R		NCYC110:K>R		Y55:K>R		YPS606:K>R		YS4:K>R		YS9:K>R	AA:230		DBVPG1106:R>L		DBVPG1373:R>L		DBVPG6044:R>L		DBVPG6765:R>L		K11:R>L		L_1374:R>L		NCYC110:R>L		SK1:R>L		Y55:R>L		YIIc17_E5:R>L		YPS606:R>L		YS4:R>L		YS9:R>L	AA:267		YS9:A>V	AA:336		DBVPG6044:G>E		NCYC110:G>E		UWOPS03_461_4:G>E		Y55:G>E		Y9:G>E		YPS606:G>E	AA:376		DBVPG6044:H>Q		K11:H>Q		NCYC110:H>Q		UWOPS03_461_4:H>Q		UWOPS05_227_2:H>Q		Y55:H>Q		Y9:H>Q		YPS606:H>Q	AA:419		YS9:T>N	AA:480		YS2:H>	AA:487		YS2:R>	AA:496		322134S:I>L	AA:792		DBVPG1853:V>A	AA:835		K11:V>I		Y9:V>I		YPS128:V>IID:YAL036C	AA:222		DBVPG6765:F>L	AA:354		W303:L>FID:YAL037W	AA:26		273614X:G>R	AA:29		273614X:E>D	AA:32		273614X:T>S	AA:46		BC187:I>S		DBVPG1373:I>S		DBVPG1788:I>S	AA:70		DBVPG6040:V>I		DBVPG6044:V>I		NCYC361:V>I		UWOPS05_227_2:V>I		UWOPS83_787_3:V>I		Y12:V>I		Y55:V>I		Y9:V>I		YIIc17_E5:V>I		YPS128:V>I	AA:74		YJM981:C>S	AA:75		DBVPG6044:S>P		Y12:S>P		Y55:S>P		Y9:S>P		YPS128:S>P	AA:103		DBVPG1106:V>A		L_1374:V>A	AA:168		YPS128:S>-	AA:185		DBVPG1106:M>V	AA:198		K11:Q>P	AA:209		SK1:S>N	AA:219		YS2:D>N		YS4:D>N	AA:256		DBVPG1853:R>K		DBVPG6040:R>K		K11:R>K		SK1:R>K		UWOPS83_787_3:R>K		Y9:R>K		YPS606:R>K		YS2:R>K		YS4:R>K	AA:257		DBVPG6044:M>I		Y55:M>IID:YAL038W	AA:19		YS9:R>G	AA:341		YJM978:P>ID:YAL039C	AA:37		YJM978:P>T	AA:256		YPS606:D>N	AA:263		DBVPG6044:S>P		SK1:S>P		UWOPS03_461_4:S>P		Y12:S>P		Y55:S>P		YPS606:S>P		YS4:S>PID:YAL040C	AA:15		DBVPG1373:R>S		DBVPG1788:R>S		DBVPG6040:R>S		DBVPG6765:R>S		L_1528:R>S		SK1:R>S		UWOPS05_227_2:R>S		UWOPS83_787_3:R>S		YJM975:R>S		YPS606:R>S		YS2:R>S		YS4:R>S	AA:27		DBVPG1106:A>V		DBVPG1373:A>V		DBVPG1788:A>V		DBVPG6040:A>V		DBVPG6765:A>V		L_1528:A>V		YJM975:A>V		YJM981:A>V	AA:34		DBVPG1106:S>A		DBVPG1373:S>A		DBVPG1788:S>A		DBVPG6040:S>A		L_1528:S>A		YJM975:S>A		YJM981:S>A	AA:50		DBVPG6044:S>G		Y55:S>G	AA:51		YS2:A>E		YS4:A>E	AA:87		DBVPG6044:T>A		Y55:T>A	AA:227		YJM981:S>L	AA:277		YS4:M>I		YS9:M>I	AA:316		DBVPG6044:L>V		Y55:L>V	AA:345		YS4:I>V		YS9:I>V	AA:359		NCYC361:K>-	AA:370		YS4:K>Q		YS9:K>Q	AA:391		UWOPS05_217_3:S>L	AA:493		Y9:S>C	AA:574		UWOPS05_217_3:S>N	AA:579		322134S:T>IID:YAL041W	AA:41		W303:D>	AA:51		273614X:R>W		SK1:R>W	AA:58		YS4:P>R	AA:92		DBVPG1373:K>N	AA:93		YJM981:S>F	AA:97		YJM981:N>S	AA:141		273614X:L>-	AA:290		UWOPS03_461_4:E>D		UWOPS05_217_3:E>D	AA:314		Y9:T>M	AA:366		322134S:Y>N	AA:385		SK1:H>Y	AA:554		SK1:P>S	AA:685		YS4:T>R	AA:690		YIIc17_E5:A>T		YS2:A>T		YS4:A>T	AA:731		DBVPG1373:V>A	AA:733		YIIc17_E5:P>S		YS2:P>S	AA:778		DBVPG6044:S>N		Y55:S>N	AA:835		UWOPS05_227_2:N>KID:YAL042W	AA:46		DBVPG6044:G>R		SK1:G>R		UWOPS03_461_4:G>R		UWOPS05_217_3:G>R		UWOPS05_227_2:G>R		Y55:G>R		YPS128:G>R		YPS606:G>R		YS4:G>R	AA:48		YS4:F>Y	AA:110		YS9:S>	AA:122		322134S:H>R	AA:296		Y9:N>	AA:297		Y9:T>	AA:349		322134S:V>A		378604X:V>A		DBVPG1373:V>A		DBVPG1788:V>A		DBVPG1853:V>A		DBVPG6044:V>A		DBVPG6765:V>A		K11:V>A		SK1:V>A		UWOPS03_461_4:V>A		UWOPS05_217_3:V>A		Y55:V>A		Y9:V>A		YIIc17_E5:V>A		YPS606:V>A		YS4:V>AID:YAL043C	AA:3		YS9:S>F	AA:23		NCYC361:H>Y		YS4:E>V	AA:176		BC187:V>A		DBVPG1106:V>A		DBVPG1373:V>A		DBVPG1788:V>A		DBVPG6765:V>A		L_1374:V>A		L_1528:V>A		NCYC361:V>A		YJM981:V>A	AA:182		L_1374:K>N	AA:203		SK1:K>R	AA:212		Y9:R>K	AA:344		UWOPS05_217_3:N>Y	AA:397		DBVPG1853:Q>L	AA:465		322134S:S>T		DBVPG1373:S>T		DBVPG1853:S>T		DBVPG6044:S>T		DBVPG6765:S>T		L_1528:S>T		NCYC110:S>T		NCYC361:S>T		SK1:S>T		UWOPS05_217_3:S>T		Y55:S>T		YIIc17_E5:S>T		YJM981:S>T		YPS128:S>T		YS9:S>T	AA:526		322134S:M>I		DBVPG1373:M>I		DBVPG1853:M>I		DBVPG6765:M>I		L_1528:M>I		YJM981:M>I	AA:551		UWOPS05_227_2:E>D	AA:568		322134S:I>M		DBVPG1373:I>M		DBVPG1853:I>M		DBVPG6044:I>M		DBVPG6765:I>M		L_1374:I>M		L_1528:I>M		NCYC110:I>M		NCYC361:I>M		SK1:I>M		UWOPS05_217_3:I>M		UWOPS05_227_2:I>M		Y55:I>M		YJM981:I>M		YPS606:I>M	AA:652		UWOPS05_217_3:L>V		UWOPS05_227_2:L>V	AA:686		Y9:N>HID:YAL044C	AA:14		273614X:A>T		322134S:A>T		378604X:A>T		DBVPG1106:A>T		DBVPG1373:A>T		DBVPG1853:A>T		DBVPG6044:A>T		DBVPG6765:A>T		K11:A>T		NCYC361:A>T		S288c:A>T		UWOPS03_461_4:A>T		UWOPS05_217_3:A>T		UWOPS87_2421:A>T		Y12:A>T		Y9:A>T		YGPM:A>T		YPS606:A>T		YS4:A>T	AA:73		273614X:S>A		322134S:S>A		378604X:S>A		DBVPG1373:S>A		DBVPG1853:S>A		DBVPG6044:S>A		DBVPG6765:S>A		K11:S>A		L_1374:S>A		NCYC361:S>A		UWOPS05_217_3:S>A		UWOPS87_2421:S>A		Y12:S>A		Y9:S>A		YGPM:S>A		YIIc17_E5:S>A		YPS606:S>A		YS4:S>A	AA:90		273614X:S>A		322134S:S>A		378604X:S>A		DBVPG1853:S>A		DBVPG6044:S>A		DBVPG6765:S>A		K11:S>A		L_1374:S>A		NCYC361:S>A		UWOPS87_2421:S>A		Y12:S>A		YGPM:S>A		YIIc17_E5:S>A		YPS606:S>A		YS4:S>A		YS9:S>AID:YAL044W-A	AA:51		322134S:V>A		378604X:V>A		DBVPG1373:V>A		DBVPG1788:V>A		DBVPG1853:V>A		DBVPG6765:V>A		L_1374:V>A		L_1528:V>A		NCYC361:V>A		YS4:V>A		YS9:V>A	AA:96		YIIc17_E5:Q>ID:YAL046C	AA:12		DBVPG6044:S>Y		Y55:S>Y	AA:23		Y9:T>R		YIIc17_E5:T>RID:YAL047C	AA:5		NCYC361:W>C	AA:128		Y9:V>A	AA:164		YPS128:E>K	AA:216		YPS128:A>V	AA:238		BC187:L>F		DBVPG1373:L>F		DBVPG1788:L>F		DBVPG1853:L>F		DBVPG6765:L>F		L_1374:L>F		NCYC361:L>F		SK1:L>F		UWOPS03_461_4:L>F		Y9:L>F		YPS128:L>F		YS4:L>F	AA:251		BC187:T>A		DBVPG1373:T>A		DBVPG1788:T>A		DBVPG1853:T>A		DBVPG6765:T>A		L_1374:T>A		NCYC361:T>A		SK1:T>A		UWOPS03_461_4:T>A		Y55:T>A		Y9:T>A		YPS128:T>A		YS4:T>A	AA:259		DBVPG1853:E>G	AA:267		DBVPG6765:I>S		L_1374:I>S	AA:284		BC187:V>A		DBVPG1373:V>A		DBVPG1788:V>A		DBVPG1853:V>A		DBVPG6765:V>A		L_1528:V>A		NCYC361:V>A		SK1:V>A		Y9:V>A	AA:302		BC187:I>N		DBVPG1373:I>N		DBVPG1788:I>N		DBVPG1853:I>N		DBVPG6765:I>N		L_1374:I>N		L_1528:I>N		NCYC361:I>N		S288c:I>N		SK1:I>N		UWOPS03_461_4:I>N		W303:I>N		Y55:I>N		Y9:I>N		YPS128:I>N		YS4:I>N	AA:315		SK1:R>K		Y9:R>T	AA:320		DBVPG6044:K>R		Y55:K>R	AA:435		YIIc17_E5:V>L	AA:469		YIIc17_E5:S>N	AA:478		DBVPG6044:L>F		Y55:L>F	AA:495		322134S:E>-	AA:511		378604X:V>L		DBVPG1106:V>L		DBVPG1373:V>L		DBVPG1788:V>L		DBVPG1853:V>L		DBVPG6040:V>L		DBVPG6765:V>L		L_1374:V>L		L_1528:V>L		SK1:V>L		YS4:V>L	AA:549		322134S:S>A		378604X:S>A		DBVPG1106:S>A		DBVPG1788:S>A		DBVPG1853:S>A		DBVPG6040:S>A		DBVPG6765:S>A		L_1374:S>A		L_1528:S>A		SK1:S>A		YJM978:S>A		YS4:S>A	AA:560		Y12:G>S		YIIc17_E5:G>SID:YAL048C	AA:39		UWOPS05_227_2:P>S	AA:173		YS9:N>S	AA:316		273614X:R>K		378604X:R>K		DBVPG1788:R>K		DBVPG1853:R>K		DBVPG6765:R>K		K11:R>K		L_1528:R>K		NCYC361:R>K		SK1:R>K		YS2:R>K	AA:424		DBVPG6040:Q>R	AA:426		YS2:T>IID:YAL049C	AA:46		DBVPG6765:V>I	AA:96		DBVPG1373:F>L		DBVPG1788:F>L		DBVPG6765:F>L		YJM975:F>L		YJM981:F>L	AA:104		UWOPS05_217_3:T>A	AA:127		K11:V>L	AA:145		NCYC361:G>CID:YAL054C	AA:30		322134S:A>S		378604X:A>S		DBVPG1373:A>S		DBVPG1853:A>S		DBVPG6765:A>S		L_1374:A>S		NCYC361:A>S		SK1:A>S		UWOPS05_217_3:A>S		Y9:A>S		YIIc17_E5:A>S		YJM975:A>S	AA:75		NCYC361:D>E	AA:113		SK1:P>S	AA:114		UWOPS03_461_4:K>E	AA:185		UWOPS83_787_3:G>V	AA:249		273614X:G>S	AA:272		273614X:V>I	AA:277		DBVPG6765:K>N	AA:404		UWOPS03_461_4:E>K		UWOPS05_217_3:E>K		UWOPS05_227_2:E>K	AA:453		YS4:W>R	AA:629		322134S:S>N		DBVPG1373:S>N		DBVPG6040:S>N		DBVPG6765:S>N		L_1374:S>N		NCYC361:S>N		SK1:S>N		YJM975:S>N		YS9:S>N	AA:668		YPS606:L>-ID:YAL055W	AA:2		UWOPS05_217_3:P>S	AA:4		DBVPG6044:P>S		Y55:P>S	AA:12		UWOPS83_787_3:T>S	AA:23		UWOPS05_217_3:A>T		UWOPS05_227_2:A>T	AA:32		UWOPS87_2421:Y>F	AA:44		DBVPG6044:A>T		Y55:A>T	AA:47		DBVPG1853:P>S		DBVPG6044:P>S		K11:P>S		Y55:P>S		YIIc17_E5:P>S		YPS128:P>S		YPS606:P>S		YS4:P>S	AA:49		UWOPS05_227_2:G>D	AA:82		UWOPS05_227_2:A>V	AA:98		UWOPS87_2421:M>I	AA:103		378604X:G>E		DBVPG1106:G>E		DBVPG1788:G>E		DBVPG6040:G>E		DBVPG6765:G>E		L_1374:G>E		L_1528:G>E		SK1:G>E		Y55:G>E		YS9:G>E	AA:129		378604X:R>K		DBVPG1106:R>K		DBVPG1788:R>K		DBVPG6040:R>K		DBVPG6765:R>K		L_1374:R>K		L_1528:R>K		SK1:R>K		YS9:R>K	AA:170		322134S:V>I		378604X:V>I		DBVPG1106:V>I		DBVPG6040:V>I		DBVPG6765:V>I		L_1374:V>I		NCYC361:V>I		SK1:V>I		UWOPS05_227_2:V>I		YS9:V>I	AA:172		UWOPS87_2421:D>NID:YAL059W	AA:6		DBVPG1373:R>-		DBVPG1853:R>-		DBVPG6040:R>-		DBVPG6765:R>-		L_1528:R>-		NCYC110:R>-		SK1:R>-		UWOPS03_461_4:R>-		Y55:R>-		YPS128:R>-		YPS606:R>-		YS4:R>-		YS9:R>-	AA:15		UWOPS03_461_4:I>T	AA:16		DBVPG6765:L>P	AA:29		UWOPS03_461_4:I>M	AA:83		UWOPS03_461_4:A>V	AA:87		YS4:K>N	AA:98		BC187:E>K	AA:102		BC187:D>A		DBVPG1106:D>A		DBVPG1373:D>A		DBVPG1853:D>A		DBVPG6040:D>A		DBVPG6044:D>A		DBVPG6765:D>A		K11:D>A		S288c:D>A		SK1:D>A		UWOPS03_461_4:D>A		W303:D>A		Y55:D>A		YJM978:D>A		YS4:D>A		YS9:D>A	AA:167		273614X:A>E		BC187:A>E		DBVPG1106:A>E		DBVPG1373:A>E		DBVPG6040:A>E		DBVPG6765:A>E		L_1528:A>E		YJM978:A>E	AA:171		273614X:S>G		BC187:S>G		DBVPG1106:S>G		DBVPG1373:S>G		DBVPG1853:S>G		DBVPG6040:S>G		DBVPG6765:S>G		L_1528:S>G		SK1:S>G		YJM978:S>G	AA:174		UWOPS03_461_4:N>D		Y9:N>D	AA:177		DBVPG1853:K>N	AA:195		YPS128:V>AID:YAL060W	AA:61		K11:K>N	AA:163		YS9:E>Q	AA:172		273614X:V>I		322134S:V>I		DBVPG1106:V>I		DBVPG6040:V>I		DBVPG6765:V>I		L_1374:V>I		L_1528:V>I		SK1:V>I		YJM978:V>I	AA:204		DBVPG1106:C>S	AA:222		322134S:I>V		378604X:I>V		DBVPG1106:I>V		DBVPG1853:I>V		DBVPG6040:I>V		DBVPG6765:I>V		K11:I>V		L_1374:I>V		L_1528:I>V		SK1:I>V		UWOPS05_227_2:I>V		UWOPS87_2421:I>V		YJM978:I>V		YS9:I>V	AA:245		378604X:H>Q		UWOPS05_227_2:H>Q	AA:262		YJM978:D>E	AA:284		378604X:K>R		DBVPG1106:K>R		DBVPG1373:K>R		DBVPG1853:K>R		DBVPG6040:K>R		DBVPG6765:K>R		L_1374:K>R		L_1528:K>R		SK1:K>R		UWOPS83_787_3:K>R		YPS606:K>R		YS9:K>R	AA:322		DBVPG6044:A>D		K11:A>D		NCYC110:A>D		W303:A>D		Y55:A>D		YGPM:A>D		YS4:A>D	AA:336		378604X:A>T		DBVPG1373:A>T		DBVPG1853:A>T		DBVPG6044:A>T		DBVPG6765:A>T		L_1528:A>T		NCYC110:A>T		SK1:A>T		UWOPS83_787_3:A>T		Y55:A>T		YPS128:A>T		YPS606:A>T		YS9:A>T	AA:360		DBVPG1853:E>QID:YAL061W	AA:5		YS4:A>E	AA:19		273614X:K>E		DBVPG1373:K>E		DBVPG1788:K>E		DBVPG6765:K>E		L_1374:K>E		YJM975:K>E		YJM978:K>E		YJM981:K>E	AA:22		DBVPG1853:H>Y		DBVPG6044:H>R		NCYC110:H>R		UWOPS03_461_4:H>R		UWOPS05_217_3:H>R		Y55:H>R		YIIc17_E5:H>R		YPS606:H>R		YS4:H>R	AA:34		273614X:E>A		DBVPG1373:E>A		DBVPG1788:E>A		DBVPG1853:E>A		DBVPG6044:E>A		DBVPG6765:E>A		L_1528:E>A		NCYC110:E>A		SK1:E>A		UWOPS03_461_4:E>A		UWOPS05_217_3:E>A		Y55:E>A		Y9:E>A		YIIc17_E5:E>A		YJM975:E>A		YJM978:E>A		YJM981:E>A		YPS606:E>A		YS4:E>A		YS9:E>A	AA:85		SK1:G>S	AA:114		SK1:N>K	AA:149		273614X:R>G		BC187:R>G		DBVPG1373:R>G		DBVPG1788:R>G		DBVPG6765:R>G		L_1374:R>G		L_1528:R>G		SK1:R>G		YJM975:R>G		YJM978:R>G		YJM981:R>G	AA:152		UWOPS83_787_3:M>I	AA:160		YS9:V>A	AA:165		YS4:P>L	AA:296		UWOPS83_787_3:H>R	AA:326		SK1:A>T	AA:353		UWOPS83_787_3:D>N	AA:376		DBVPG6044:P>S	AA:384		UWOPS83_787_3:R>GID:YAL062W	AA:42		UWOPS05_227_2:R>K	AA:235		UWOPS03_461_4:L>VID:YAR002C-A	AA:3		BC187:L>S	AA:105		Y12:E>-	AA:111		Y12:Q>HID:YAR002W	AA:10		DBVPG1373:S>N	AA:12		322134S:T>I		BC187:T>I		DBVPG1373:T>I		DBVPG6765:T>I		L_1374:T>I		L_1528:T>I		UWOPS87_2421:T>I		YJM975:T>I		YJM978:T>I		YS4:T>I		YS9:T>I	AA:27		322134S:H>R		BC187:H>R		DBVPG1373:H>R		DBVPG1853:H>R		DBVPG6044:H>R		DBVPG6765:H>R		L_1374:H>R		L_1528:H>R		NCYC361:H>R		SK1:H>R		UWOPS05_217_3:H>R		UWOPS05_227_2:H>R		Y55:H>R		YJM975:H>R		YJM978:H>R		YPS128:H>R		YPS606:H>R		YS4:H>R		YS9:H>R	AA:158		322134S:G>R		BC187:G>R		DBVPG1373:G>R		DBVPG1788:G>R		DBVPG6765:G>R		L_1528:G>R		SK1:G>R		UWOPS05_217_3:G>R		UWOPS05_227_2:G>R		UWOPS87_2421:G>R		Y55:G>R		YJM975:G>R		YJM978:G>R		YJM981:G>R		YPS128:G>R		YPS606:G>R		YS4:G>R	AA:164		378604X:E>K	AA:165		Y55:G>R	AA:226		322134S:T>A		BC187:T>A		DBVPG1106:T>A		DBVPG1373:T>A		DBVPG1788:T>A		DBVPG1853:T>A		DBVPG6044:T>A		DBVPG6765:T>A		L_1528:T>A		UWOPS05_217_3:T>A		UWOPS05_227_2:T>A		Y55:T>A		YJM975:T>A		YJM978:T>A		YJM981:T>A		YPS128:T>A		YS4:T>A	AA:253		YJM978:A>P		YPS128:A>T		YPS606:A>T	AA:285		DBVPG6044:S>N		Y55:S>N	AA:309		K11:K>R		SK1:K>R	AA:327		DBVPG6044:T>A		Y55:T>A	AA:347		DBVPG1106:N>K		DBVPG1373:N>K		DBVPG1788:N>K		DBVPG6765:N>K	AA:396		DBVPG1373:A>T	AA:406		YPS128:N>K		YPS606:N>K	AA:444		DBVPG1853:E>G	AA:496		DBVPG1106:S>P		DBVPG1373:S>P		DBVPG1788:S>P		DBVPG6765:S>P		YJM975:S>P		YJM978:S>P		YS4:S>PID:YAR003W	AA:22		DBVPG6765:T>M		L_1374:T>M	AA:98		K11:L>	AA:112		UWOPS03_461_4:R>P		UWOPS05_227_2:R>P	AA:142		273614X:V>I		DBVPG6044:V>I		Y55:V>I	AA:163		YPS128:S>G	AA:165		Y9:T>	AA:211		DBVPG1853:K>NID:YAR007C	AA:8		BC187:R>K		DBVPG1373:R>K		DBVPG1788:R>K		DBVPG6044:R>K		K11:R>K		L_1374:R>K		L_1528:R>K		UWOPS05_227_2:R>K		UWOPS87_2421:R>K		Y55:R>K		YIIc17_E5:R>K		YJM978:R>K	AA:160		YS9:S>L	AA:342		NCYC110:R>L	AA:475		322134S:F>LID:YAR008W	AA:29		S288c:T>A	AA:45		Y55:L>F	AA:54		DBVPG6044:N>	AA:67		DBVPG1106:D>H		DBVPG1788:D>H		DBVPG6765:D>H		L_1374:D>H		YIIc17_E5:D>H	AA:86		S288c:N>Y	AA:87		DBVPG6044:D>A		Y55:D>A	AA:128		K11:K>N	AA:135		DBVPG6040:L>P	AA:168		322134S:D>N		BC187:D>N		DBVPG1106:D>N		DBVPG1788:D>N		DBVPG6765:D>N		L_1528:D>N		YIIc17_E5:D>N		YJM975:D>N	AA:188		Y12:A>P		Y9:A>P	AA:236		K11:I>KID:YAR014C	AA:29		322134S:G>C		DBVPG1788:G>C		DBVPG6765:G>C		L_1374:G>C		L_1528:G>C		YIIc17_E5:G>C		YS2:G>C		YS9:G>C	AA:89		273614X:V>M	AA:141		Y12:R>G	AA:153		DBVPG6044:L>F		NCYC110:L>F		Y55:L>F	AA:201		YS9:D>A	AA:216		UWOPS87_2421:D>E	AA:223		DBVPG6044:D>E		NCYC110:D>E		Y55:D>E	AA:349		273614X:S>I	AA:372		Y12:K>R		Y9:K>R	AA:439		273614X:A>G		DBVPG1373:A>G		DBVPG1788:A>G		DBVPG6040:A>G		DBVPG6044:A>G		DBVPG6765:A>G		K11:A>G		L_1374:A>G		L_1528:A>G		UWOPS03_461_4:A>G		UWOPS87_2421:A>G		W303:A>G		Y9:A>G		YIIc17_E5:A>G		YPS606:A>G		YS4:A>G	AA:524		YS2:F>L	AA:527		DBVPG1788:G>A		DBVPG6765:G>A	AA:532		YS2:D>N	AA:565		273614X:S>G		378604X:S>G		DBVPG1853:S>G		DBVPG6040:S>G		DBVPG6044:S>G		SK1:S>G		UWOPS05_227_2:S>G		UWOPS83_787_3:S>G		Y12:S>G		Y55:S>G		Y9:S>G		YPS606:S>G		YS4:S>G		YS9:S>G	AA:596		SK1:I>V	AA:599		273614X:S>T		DBVPG6044:S>T		Y55:S>T	AA:601		273614X:G>E		378604X:G>E		DBVPG1853:G>E		DBVPG6040:G>E		DBVPG6044:G>E		SK1:G>E		UWOPS05_227_2:G>E		Y12:G>E		Y55:G>E		Y9:G>E		YPS606:G>E		YS4:G>E		YS9:G>E	AA:607		273614X:P>A		378604X:P>A		DBVPG1373:P>A		DBVPG1788:P>A		DBVPG1853:P>A		DBVPG6040:P>A		DBVPG6044:P>A		DBVPG6765:P>A		L_1528:P>A		S288c:P>A		SK1:P>A		UWOPS05_227_2:P>A		UWOPS83_787_3:P>A		W303:P>A		Y12:P>A		Y55:P>A		Y9:P>A		YGPM:P>A		YIIc17_E5:P>A		YJM975:P>A		YPS606:P>A		YS2:P>A		YS4:P>A		YS9:P>A	AA:655		DBVPG6040:A>V		UWOPS05_227_2:A>V	AA:673		DBVPG6044:S>C		NCYC110:S>C		Y55:S>C	AA:700		DBVPG6044:Q>R		NCYC110:Q>R		Y55:Q>RID:YAR015W	AA:15		UWOPS03_461_4:V>M		UWOPS05_217_3:V>M		UWOPS05_227_2:V>M	AA:80		DBVPG6765:A>V	AA:127		UWOPS05_217_3:G>R	AA:202		YS9:C>	AA:226		UWOPS05_217_3:T>I		UWOPS05_227_2:T>IID:YAR018C	AA:7		DBVPG6765:F>L		L_1528:F>L		NCYC361:F>L		YS4:F>L	AA:43		378604X:I>M	AA:191		DBVPG6765:I>V		L_1528:I>V	AA:217		UWOPS83_787_3:G>WID:YAR019C	AA:54		DBVPG1853:K>Q	AA:166		W303:G>V	AA:198		YS4:L>F	AA:218		DBVPG6044:Y>H		NCYC110:Y>H		Y55:Y>H	AA:259		378604X:K>N	AA:321		322134S:P>A		378604X:P>A		DBVPG1788:P>A		DBVPG1853:P>A		DBVPG6040:P>A		DBVPG6044:P>A		DBVPG6765:P>A		K11:P>A		S288c:P>A		UWOPS05_217_3:P>A		UWOPS87_2421:P>A		Y55:P>A		YGPM:P>A		YJM975:P>A		YPS606:P>A		YS4:P>A		YS9:P>A	AA:346		322134S:S>P	AA:375		YIIc17_E5:R>H	AA:397		UWOPS05_227_2:N>Y	AA:487		NCYC361:E>-	AA:507		NCYC361:K>R	AA:631		DBVPG6044:A>T		NCYC110:A>T		Y55:A>T	AA:668		UWOPS05_217_3:A>G		UWOPS05_227_2:A>G	AA:700		K11:M>I	AA:819		YIIc17_E5:P>R	AA:851		DBVPG1106:S>T		DBVPG1373:S>T		DBVPG1788:S>T		DBVPG6040:S>T		DBVPG6044:S>T		DBVPG6765:S>T		K11:S>T		L_1374:S>T		L_1528:S>T		SK1:S>T		UWOPS83_787_3:S>T		Y55:S>T		Y9:S>T		YIIc17_E5:S>T		YPS606:S>T	AA:955		378604X:L>FID:YAR035W	AA:170		YJM978:P>S	AA:230		SK1:S>T	AA:292		UWOPS05_217_3:E>K		UWOPS05_227_2:E>K	AA:294		YJM981:T>	AA:427		SK1:R>H		Y55:R>H	AA:502		YIIc17_E5:G>A		YPS606:G>A	AA:605		UWOPS03_461_4:G>S	AA:660		DBVPG1853:T>A		DBVPG6044:T>A		NCYC110:T>A		SK1:T>A		UWOPS05_217_3:T>A		UWOPS05_227_2:T>A		Y55:T>A		YPS606:T>A	AA:664		BC187:A>TID:YAR062W	AA:31		UWOPS05_217_3:N>D		UWOPS05_227_2:N>D		W303:N>D	AA:35		DBVPG1106:E>D	AA:43		UWOPS05_217_3:P>Q		W303:P>Q	AA:60		W303:S>R	AA:107		DBVPG6040:D>N	AA:131		DBVPG6040:K>I	AA:160		DBVPG6040:G>DID:YBL005W	AA:56		322134S:Q>R		BC187:Q>R		DBVPG6765:Q>R		L_1374:Q>R		L_1528:Q>R		NCYC361:Q>R		UWOPS03_461_4:Q>R		UWOPS05_217_3:Q>R		UWOPS87_2421:Q>R		YJM978:Q>R		YS9:Q>R	AA:97		UWOPS83_787_3:N>S	AA:119		UWOPS83_787_3:T>A	AA:138		UWOPS83_787_3:T>K	AA:289		YS9:L>V	AA:293		UWOPS05_217_3:C>R		UWOPS05_227_2:C>R	AA:358		UWOPS03_461_4:L>F		UWOPS05_217_3:L>F		UWOPS05_227_2:L>F	AA:363		DBVPG6044:S>L		SK1:S>L	AA:375		Y12:H>R	AA:466		YJM978:G>R	AA:550		UWOPS87_2421:N>D	AA:570		UWOPS03_461_4:D>E		UWOPS05_217_3:D>E	AA:600		322134S:K>E	AA:688		DBVPG1853:W>C	AA:788		UWOPS03_461_4:Y>C		UWOPS05_217_3:Y>C	AA:799		UWOPS03_461_4:E>G		UWOPS05_217_3:E>G	AA:879		YIIc17_E5:S>	AA:885		273614X:A>T		322134S:A>T		BC187:A>T		DBVPG1373:A>T		DBVPG1788:A>T		DBVPG1853:A>T		DBVPG6765:A>T		K11:A>T		L_1374:A>T		SK1:A>T		UWOPS03_461_4:A>T		UWOPS05_217_3:A>T		UWOPS83_787_3:A>T		Y55:A>T		YJM975:A>T		YJM978:A>T		YPS606:A>T		YS2:A>T		YS4:A>T	AA:916		273614X:N>S		322134S:N>S		BC187:N>S		DBVPG1373:N>S		DBVPG1853:N>S		DBVPG6765:N>S		L_1374:N>S		UWOPS87_2421:N>S		YJM975:N>S		YJM981:N>S		YS2:N>S		YS4:N>S	AA:970		DBVPG1853:N>S	AA:971		273614X:T>IID:YBL006C	AA:39		322134S:H>D		DBVPG1373:H>D		DBVPG1788:H>D		DBVPG6044:H>D		DBVPG6765:H>D		L_1528:H>D		NCYC361:H>D		SK1:H>D		UWOPS83_787_3:H>D		UWOPS87_2421:H>D		Y55:H>D		YJM975:H>D		YJM978:H>D		YPS128:H>D		YPS606:H>D		YS9:H>D	AA:96		UWOPS05_217_3:Q>H	AA:116		DBVPG6765:K>R	AA:167		DBVPG1373:S>P		DBVPG1788:S>P		DBVPG1853:S>P		DBVPG6040:S>P		DBVPG6044:S>P		DBVPG6765:S>P		L_1528:S>P		NCYC110:S>P		SK1:S>P		UWOPS03_461_4:S>P		Y55:S>P		YIIc17_E5:S>P		YJM975:S>P		YPS606:S>P		YS4:S>PID:YBL007C	AA:106		DBVPG6044:A>T		NCYC110:A>T		SK1:A>T		Y55:A>T	AA:141		273614X:P>A		BC187:P>A		DBVPG1373:P>A		DBVPG6765:P>A		L_1374:P>A		YIIc17_E5:P>A		YJM981:P>A	AA:151		UWOPS05_227_2:P>S	AA:157		UWOPS03_461_4:S>P		UWOPS05_217_3:S>P		UWOPS05_227_2:S>P	AA:195		UWOPS05_227_2:P>S	AA:387		YS2:K>Q	AA:407		UWOPS03_461_4:A>G		UWOPS05_217_3:A>G	AA:544		YS9:A>V	AA:559		Y12:A>T	AA:576		378604X:E>-	AA:634		NCYC110:V>I		SK1:V>I		Y55:V>I	AA:640		UWOPS03_461_4:V>I		UWOPS05_217_3:V>I	AA:643		322134S:A>V		DBVPG1373:A>V		DBVPG1853:A>V		DBVPG6040:A>V		DBVPG6765:A>V		YJM975:A>V		YS4:A>V		YS9:A>V	AA:666		K11:V>I	AA:726		322134S:P>R		DBVPG1853:P>R		L_1528:P>R		UWOPS87_2421:P>R		YJM975:P>R		YS4:P>R		YS9:P>R	AA:869		YS2:G>A	AA:880		273614X:D>A		DBVPG1373:D>A		DBVPG6040:D>A		DBVPG6044:D>A		DBVPG6765:D>A		K11:D>A		L_1374:D>A		L_1528:D>A		NCYC361:D>A		SK1:D>A		UWOPS03_461_4:D>A		UWOPS05_217_3:D>A		UWOPS83_787_3:D>A		Y12:D>A		Y55:D>A		YJM975:D>A		YJM978:D>A		YPS128:D>A		YPS606:D>A		YS2:D>A	AA:885		UWOPS83_787_3:Q>-	AA:886		DBVPG6044:R>K		SK1:R>K	AA:941		273614X:P>R		DBVPG1373:P>R		DBVPG6765:P>R		L_1374:P>R		L_1528:P>R		NCYC361:P>R		YJM975:P>R		YJM978:P>R	AA:994		UWOPS03_461_4:G>A		UWOPS05_217_3:G>A	AA:997		DBVPG6044:M>I		SK1:M>I		Y55:M>I	AA:1036		UWOPS03_461_4:M>T		UWOPS05_217_3:M>T	AA:1186		DBVPG1373:D>G		DBVPG1788:D>G		DBVPG6044:D>G		DBVPG6765:D>G		L_1374:D>G		SK1:D>G		UWOPS03_461_4:D>G		UWOPS05_217_3:D>G		UWOPS83_787_3:D>G		Y12:D>G		Y55:D>G		Y9:D>G		YJM978:D>G		YJM981:D>G		YS9:D>GID:YBL009W	AA:123		DBVPG1373:V>A		DBVPG6040:V>A		UWOPS87_2421:V>A		YJM978:V>A	AA:149		UWOPS87_2421:F>Y	AA:173		UWOPS83_787_3:S>P	AA:180		UWOPS03_461_4:S>G	AA:183		Y9:K>R	AA:233		Y9:N>S	AA:244		DBVPG1853:P>S		DBVPG6765:P>S		YJM978:P>S		YJM981:P>S	AA:461		273614X:S>G		322134S:S>G		DBVPG1373:S>G		DBVPG6765:S>G		L_1374:S>G		L_1528:S>G		UWOPS87_2421:S>G		YJM978:S>G		YJM981:S>G	AA:463		K11:V>I	AA:472		DBVPG6044:V>L		SK1:V>L		Y55:V>L	AA:489		UWOPS03_461_4:F>Y	AA:569		YPS606:D>		YS9:D>A	AA:572		YS9:Y>S	AA:590		322134S:S>R		DBVPG1373:S>R		DBVPG1853:S>R		DBVPG6765:S>R		L_1374:S>R		L_1528:S>R		UWOPS83_787_3:S>R		UWOPS87_2421:S>R		YJM978:S>RID:YBL010C	AA:10		UWOPS03_461_4:V>A		UWOPS05_217_3:V>A	AA:54		UWOPS05_227_2:C>R	AA:186		YS9:R>C	AA:194		DBVPG6765:Q>R		L_1528:Q>R		YJM975:Q>R		YJM981:Q>R		YS9:Q>R	AA:198		UWOPS03_461_4:A>V		UWOPS05_217_3:A>V		UWOPS05_227_2:A>V	AA:224		DBVPG6765:S>G		UWOPS05_217_3:S>G		YJM975:S>G		YJM981:S>G		YS9:S>GID:YBL011W	AA:10		273614X:F>S		378604X:F>S		DBVPG1373:F>S		DBVPG6044:F>S		DBVPG6765:F>S		K11:F>S		L_1528:F>S		SK1:F>S		UWOPS05_217_3:F>S		UWOPS05_227_2:F>S		UWOPS83_787_3:F>S		Y12:F>S		Y55:F>S		YJM975:F>S		YJM981:F>S		YPS128:F>S		YPS606:F>S		YS2:F>S	AA:125		273614X:P>A		378604X:P>A		DBVPG1106:P>A		DBVPG1373:P>A		DBVPG1853:P>A		DBVPG6044:P>A		DBVPG6765:P>A		NCYC110:P>A		SK1:P>A		UWOPS05_217_3:P>A		UWOPS05_227_2:P>A		UWOPS83_787_3:P>A		UWOPS87_2421:P>A		W303:P>A		Y55:P>A		YJM981:P>A		YPS606:P>A		YS2:P>A	AA:157		DBVPG1853:T>I	AA:217		DBVPG6044:L>I		NCYC110:L>I		SK1:L>I		Y55:L>I	AA:324		DBVPG6044:P>S		NCYC110:P>S		SK1:P>S		Y55:P>S	AA:362		378604X:R>G		UWOPS87_2421:R>G	AA:365		YS4:Y>H	AA:528		UWOPS83_787_3:S>P	AA:710		DBVPG6040:L>S	AA:730		DBVPG1853:G>SID:YBL013W	AA:18		378604X:Y>F		DBVPG1373:Y>F		DBVPG1788:Y>F		DBVPG6040:Y>F		NCYC110:Y>F		SK1:Y>F		UWOPS87_2421:Y>F		Y55:Y>F		YJM975:Y>F		YJM978:Y>F		YPS606:Y>F		YS2:Y>F		YS9:Y>F	AA:58		378604X:C>S		DBVPG1788:C>S		DBVPG6040:C>S		DBVPG6044:C>S		DBVPG6765:C>S		SK1:C>S		UWOPS03_461_4:C>S		UWOPS05_217_3:C>S		UWOPS05_227_2:C>S		UWOPS87_2421:C>S		Y55:C>S		YJM975:C>S		YJM978:C>S		YPS606:C>S		YS2:C>S		YS9:C>S	AA:119		378604X:G>E		DBVPG1788:G>E		DBVPG6040:G>E		DBVPG6765:G>E		UWOPS87_2421:G>E		YJM978:G>E		YJM981:G>E		YS2:G>E		YS9:G>E	AA:127		YJM978:G>S	AA:132		NCYC361:A>T		UWOPS83_787_3:A>T	AA:249		DBVPG1853:Q>R	AA:284		DBVPG1853:H>Y	AA:285		UWOPS83_787_3:W>R	AA:306		UWOPS05_217_3:F>	AA:313		BC187:R>GID:YBL014C	AA:33		UWOPS05_217_3:T>I		UWOPS05_227_2:T>I	AA:39		378604X:N>K		DBVPG1373:N>K		DBVPG1788:N>K		DBVPG6765:N>K		L_1528:N>K		NCYC110:N>K		S288c:N>K		SK1:N>K		UWOPS05_217_3:N>K		UWOPS05_227_2:N>K		UWOPS83_787_3:N>K		W303:N>K		Y55:N>K		YIIc17_E5:N>K		YJM975:N>K		YJM978:N>K		YJM981:N>K		YPS128:N>K		YPS606:N>K		YS9:N>K	AA:98		DBVPG6044:D>G		NCYC110:D>G		SK1:D>G		Y55:D>G	AA:171		YPS128:A>V		YPS606:A>V	AA:229		273614X:R>Q		378604X:R>Q		BC187:R>Q		DBVPG1373:R>Q		DBVPG1788:R>Q		DBVPG6765:R>Q		NCYC361:R>Q		YJM975:R>Q		YJM981:R>Q		YS9:R>Q	AA:234		BC187:T>A	AA:249		SK1:G>E		UWOPS05_217_3:G>E		UWOPS83_787_3:G>E		Y55:G>E	AA:261		273614X:V>M		378604X:V>M		BC187:V>M		DBVPG1373:V>M		DBVPG1788:V>M		DBVPG6765:V>M		L_1374:V>M		NCYC361:V>M		YJM975:V>M		YJM981:V>M		YS9:V>M	AA:299		YS9:D>N	AA:318		YS9:I>V	AA:508		DBVPG6044:I>V		NCYC110:I>V		SK1:I>V		Y55:I>V	AA:531		UWOPS87_2421:F>C	AA:560		K11:P>L		Y12:P>L		Y9:P>L	AA:581		378604X:A>T		DBVPG1373:A>T		DBVPG1788:A>T		DBVPG6040:A>T		L_1374:A>T		NCYC110:A>T		SK1:A>T		UWOPS05_217_3:A>T		UWOPS05_227_2:A>T		UWOPS83_787_3:A>T		Y55:A>T		YJM975:A>T		YJM981:A>T	AA:619		Y12:E>A		Y9:E>A	AA:663		273614X:L>V		322134S:L>V		378604X:L>V		DBVPG1373:L>V		DBVPG1788:L>V		DBVPG6040:L>V		DBVPG6765:L>V		NCYC110:L>V		UWOPS05_217_3:L>V		UWOPS05_227_2:L>V		UWOPS83_787_3:L>V		Y12:L>V		Y55:L>V		Y9:L>V		YJM975:L>V		YJM981:L>V		YPS128:L>V		YS9:L>V	AA:666		NCYC110:S>A		Y55:S>A	AA:782		378604X:E>K		YS9:E>K	AA:821		UWOPS87_2421:S>N	AA:834		DBVPG1373:Q>R		DBVPG6765:Q>R		L_1528:Q>RID:YBL015W	AA:102		SK1:I>F	AA:211		NCYC361:D>	AA:379		W303:H>	AA:392		W303:N>	AA:526		YPS128:D>G		YPS606:D>GID:YBL016W	AA:75		YJM978:F>	AA:334		322134S:Y>H		DBVPG1106:Y>H		DBVPG1373:Y>H		DBVPG1788:Y>H		DBVPG6765:Y>H		L_1374:Y>H		L_1528:Y>H		NCYC361:Y>H		UWOPS87_2421:Y>H		YJM975:Y>H		YJM981:Y>H		YS4:Y>H		YS9:Y>HID:YBL019W	AA:7		BC187:T>M		DBVPG1373:T>M		DBVPG1788:T>M		DBVPG6765:T>M		L_1374:T>M		YJM975:T>M		YJM981:T>M	AA:42		UWOPS05_217_3:S>C	AA:85		BC187:T>N		DBVPG1106:T>N		DBVPG1373:T>N		DBVPG1788:T>N		DBVPG6765:T>N		L_1374:T>N		L_1528:T>N		UWOPS87_2421:T>N		YJM975:T>N		YJM981:T>N	AA:99		DBVPG1788:P>A		UWOPS87_2421:P>A	AA:123		UWOPS05_217_3:T>P	AA:132		UWOPS83_787_3:I>S		YPS128:I>S		YPS606:I>S	AA:146		UWOPS05_217_3:D>E	AA:154		UWOPS05_217_3:E>A	AA:169		UWOPS87_2421:V>L	AA:194		DBVPG1106:M>L		DBVPG1788:M>L		DBVPG6044:M>L		DBVPG6765:M>L		L_1374:M>L		L_1528:M>L		SK1:M>L		UWOPS83_787_3:M>L		UWOPS87_2421:M>L		Y12:M>L		Y55:M>L		YJM981:M>L		YPS128:M>L		YPS606:M>L		YS4:M>L		YS9:M>L	AA:197		DBVPG1106:L>I		DBVPG1788:L>I		DBVPG6044:L>I		DBVPG6765:L>I		L_1374:L>I		L_1528:L>I		SK1:L>I		UWOPS83_787_3:L>I		UWOPS87_2421:L>I		Y12:L>I		Y55:L>I		YJM981:L>I		YPS128:L>I		YPS606:L>I		YS4:L>I		YS9:L>I	AA:286		UWOPS03_461_4:D>G	AA:342		273614X:A>T		BC187:A>T		DBVPG1106:A>T		DBVPG1788:A>T		DBVPG6765:A>T		L_1374:A>T		L_1528:A>T		YJM975:A>T		YJM978:A>T	AA:357		YPS128:V>L		YPS606:V>L	AA:364		273614X:L>V		BC187:L>V		DBVPG1106:L>V		DBVPG1788:L>V		DBVPG1853:L>V		DBVPG6765:L>V		L_1374:L>V		L_1528:L>V		YJM975:L>V		YJM978:L>V		YPS128:L>P		YPS606:L>P	AA:369		L_1528:E>A	AA:376		273614X:P>A		322134S:P>A		378604X:P>A		BC187:P>A		DBVPG1106:P>A		DBVPG1788:P>A		DBVPG1853:P>A		DBVPG6044:P>A		DBVPG6765:P>A		L_1374:P>A		L_1528:P>A		SK1:P>A		UWOPS03_461_4:P>A		UWOPS05_217_3:P>A		UWOPS83_787_3:P>A		Y55:P>A		YJM975:P>A		YJM978:P>A		YPS128:P>A		YPS606:P>A		YS4:P>A	AA:387		YS9:N>D	AA:391		UWOPS83_787_3:H>D	AA:392		273614X:N>D		322134S:N>D		BC187:N>D		DBVPG1106:N>D		DBVPG1788:N>D		DBVPG1853:N>D		DBVPG6765:N>D		L_1374:N>D		L_1528:N>D		YJM975:N>D		YJM978:N>D	AA:406		YIIc17_E5:S>F	AA:419		YIIc17_E5:T>P	AA:459		YJM975:I>M	AA:468		YPS606:D>NID:YBL020W	AA:28		DBVPG1853:M>I	AA:57		UWOPS05_217_3:A>T	AA:165		273614X:P>	AA:279		NCYC361:Y>F	AA:288		UWOPS03_461_4:H>Y		UWOPS05_227_2:H>Y	AA:335		YJM978:L>-	AA:394		YIIc17_E5:Q>	AA:450		NCYC110:S>	AA:505		YJM975:F>L		YJM981:F>L	AA:525		UWOPS03_461_4:W>RID:YBL021C	AA:12		UWOPS05_217_3:S>IID:YBL023C	AA:32		Y9:G>E	AA:80		SK1:E>D		W303:E>D		Y55:E>D	AA:253		YJM978:K>N	AA:267		YJM975:M>T	AA:274		YJM975:V>A	AA:286		YJM975:Y>S	AA:399		UWOPS03_461_4:P>A		UWOPS05_217_3:P>A	AA:433		YJM978:N>I	AA:780		YGPM:Q>HID:YBL024W	AA:42		YPS128:K>E	AA:43		DBVPG1106:T>A	AA:50		DBVPG6044:D>N		SK1:D>N		W303:D>N	AA:319		DBVPG1788:A>S		DBVPG1853:A>S		DBVPG6765:A>S		L_1374:A>S		YS4:A>S	AA:376		273614X:S>P		DBVPG1373:S>P		DBVPG1853:S>P		DBVPG6040:S>P		DBVPG6044:S>P		DBVPG6765:S>P		NCYC110:S>P		SK1:S>P		UWOPS05_217_3:S>P		UWOPS83_787_3:S>P		UWOPS87_2421:S>P		Y55:S>P		YJM981:S>P		YPS128:S>P		YS4:S>P	AA:485		UWOPS87_2421:N>K	AA:574		Y9:F>L	AA:668		BC187:P>S		DBVPG1106:P>S		DBVPG6040:P>S		L_1374:P>S		L_1528:P>S		NCYC110:P>S		SK1:P>S		UWOPS83_787_3:P>S		W303:P>S		YIIc17_E5:P>S		YJM975:P>S		YJM978:P>S		YS9:P>SID:YBL025W	AA:44		322134S:E>D		BC187:E>D		DBVPG1106:E>D		DBVPG1373:E>D		DBVPG6044:E>D		DBVPG6765:E>D		L_1374:E>D		L_1528:E>D		NCYC110:E>D		SK1:E>D		UWOPS03_461_4:E>D		UWOPS83_787_3:E>D		Y55:E>D		YJM975:E>D		YJM978:E>D		YPS128:E>D		YPS606:E>DID:YBL028C	AA:84		273614X:R>TID:YBL029W	AA:29		W303:C>W	AA:31		YS9:V>A	AA:51		DBVPG1853:E>A	AA:69		322134S:T>I	AA:77		K11:N>K	AA:88		DBVPG6044:N>I		NCYC110:N>I		SK1:N>I		UWOPS05_217_3:N>I		UWOPS05_227_2:N>I		UWOPS83_787_3:N>I		UWOPS87_2421:N>I		W303:N>I		Y55:N>I		YPS128:N>I		YPS606:N>I	AA:101		YJM975:L>	AA:124		378604X:A>V		DBVPG6040:A>V	AA:147		322134S:P>S		378604X:P>S		BC187:P>S		DBVPG1106:P>S		DBVPG1373:P>S		DBVPG1788:P>S		DBVPG6044:P>S		DBVPG6765:P>S		K11:P>S		L_1374:P>S		L_1528:P>S		NCYC110:P>S		NCYC361:P>S		SK1:P>S		UWOPS05_217_3:P>S		UWOPS05_227_2:P>S		UWOPS83_787_3:P>S		UWOPS87_2421:P>S		W303:P>S		Y55:P>S		YJM978:P>S		YPS128:P>S		YPS606:P>S		YS4:P>S	AA:170		UWOPS87_2421:H>N	AA:173		YS4:A>P	AA:174		BC187:I>T	AA:185		378604X:F>L	AA:222		DBVPG1373:E>K	AA:228		DBVPG6765:S>L		L_1374:S>L	AA:254		NCYC361:Y>N	AA:259		322134S:P>R		BC187:P>R		DBVPG1106:P>R		DBVPG1373:P>R		DBVPG1788:P>R		DBVPG6765:P>R		L_1374:P>R		L_1528:P>R		NCYC361:P>R		YJM975:P>R	AA:260		UWOPS83_787_3:S>A	AA:324		L_1528:K>NID:YBL030C	AA:36		DBVPG1373:V>I		DBVPG1788:V>I		DBVPG6765:V>I		L_1528:V>I		NCYC361:V>I		YJM981:V>I	AA:71		DBVPG1373:L>V		DBVPG1788:L>V		DBVPG6765:L>V		L_1528:L>V		NCYC361:L>V		YJM975:L>V		YJM981:L>V	AA:217		273614X:L>I		DBVPG1373:L>I		DBVPG6765:L>I		L_1528:L>I		YJM975:L>I		YJM981:L>I	AA:273		273614X:R>K		DBVPG1373:R>K		DBVPG1788:R>K		DBVPG6765:R>K		L_1528:R>K		YJM975:R>K		YJM981:R>KID:YBL031W	AA:20		BC187:G>D		DBVPG6765:G>D		L_1374:G>D		L_1528:G>D		NCYC110:G>D		NCYC361:G>D		SK1:G>D		UWOPS03_461_4:G>D		UWOPS05_217_3:G>D		UWOPS05_227_2:G>D		UWOPS83_787_3:G>D		W303:G>D		Y55:G>D		YJM975:G>D		YJM978:G>D		YJM981:G>D		YS2:G>D		YS9:G>D	AA:70		NCYC110:Q>	AA:81		UWOPS03_461_4:N>K		UWOPS05_217_3:N>K		UWOPS05_227_2:N>K	AA:98		378604X:H>Y	AA:111		NCYC361:H>Y	AA:147		YS2:T>A		YS9:T>A	AA:179		322134S:M>T		K11:M>T	AA:185		322134S:I>V		378604X:I>V		BC187:I>V		DBVPG1373:I>V		DBVPG1853:I>V		DBVPG6044:I>V		K11:I>V		L_1374:I>V		NCYC361:I>V		SK1:I>V		UWOPS05_217_3:I>V		UWOPS05_227_2:I>V		UWOPS83_787_3:I>V		W303:I>V		Y55:I>V		YJM978:I>V		YJM981:I>V		YPS128:I>V		YS2:I>V		YS9:I>V	AA:208		378604X:P>H	AA:220		NCYC110:H>L	AA:229		NCYC110:A>V	AA:241		322134S:I>T		378604X:I>T		BC187:I>T		DBVPG1373:I>T		DBVPG1853:I>T		DBVPG6044:I>T		L_1374:I>T		SK1:I>T		UWOPS03_461_4:I>T		UWOPS05_217_3:I>T		UWOPS05_227_2:I>T		UWOPS83_787_3:I>T		W303:I>T		Y55:I>T		YJM978:I>T		YJM981:I>T		YPS128:I>T	AA:280		YS9:T>	AA:328		378604X:T>A		SK1:T>A		Y55:T>AID:YBL032W	AA:74		273614X:A>S		BC187:A>S		DBVPG1853:A>S		DBVPG6040:A>S		DBVPG6044:A>S		DBVPG6765:A>S		L_1528:A>S		NCYC110:A>S		NCYC361:A>S		UWOPS83_787_3:A>S		W303:A>S		Y55:A>S		YJM981:A>S		YPS128:A>S		YPS606:A>S	AA:109		DBVPG6044:D>G		NCYC110:D>G		SK1:D>G		W303:D>G		Y55:D>G	AA:177		378604X:A>D	AA:253		322134S:P>S	AA:263		L_1528:L>M	AA:372		BC187:E>K		DBVPG1788:E>K		L_1374:E>K		L_1528:E>K		YJM975:E>K		YS2:E>KID:YBL033C	AA:22		NCYC361:G>V		UWOPS03_461_4:S>N		UWOPS05_217_3:S>N	AA:81		Y9:R>T	AA:112		DBVPG6044:R>T	AA:131		YIIc17_E5:A>S	AA:178		L_1528:P>H	AA:231		378604X:D>E	AA:330		UWOPS03_461_4:Q>-		UWOPS05_217_3:Q>-	AA:338		DBVPG6040:S>PID:YBL036C	AA:35		DBVPG6044:V>A		SK1:V>A		Y55:V>A	AA:36		273614X:N>T	AA:112		273614X:H>R		DBVPG1373:H>R		L_1374:H>R		L_1528:H>R		UWOPS83_787_3:E>-		YJM978:H>R	AA:222		Y12:G>R	AA:243		DBVPG6044:D>E		Y55:D>EID:YBL038W	AA:11		322134S:S>A		DBVPG1373:S>A		DBVPG1788:S>A		DBVPG1853:S>A		DBVPG6044:S>A		DBVPG6765:S>A		L_1374:S>A		SK1:S>A		UWOPS05_217_3:S>A		UWOPS05_227_2:S>A		Y55:S>A		YJM978:S>A		YS9:S>A	AA:21		273614X:E>G		322134S:E>G		DBVPG1373:E>G		DBVPG1788:E>G		DBVPG1853:E>G		DBVPG6044:E>G		DBVPG6765:E>G		L_1374:E>G		SK1:E>G		Y55:E>G		YJM978:E>G		YS9:E>G	AA:218		273614X:I>F		UWOPS05_217_3:I>FID:YBL041W	AA:100		YJM975:D>G	AA:181		YIIc17_E5:P>A	AA:215		YJM981:I>LID:YBL045C	AA:10		UWOPS03_461_4:V>I	AA:25		UWOPS03_461_4:P>L	AA:31		YIIc17_E5:Q>K	AA:39		DBVPG6765:A>T		L_1374:A>T		YS9:A>T	AA:112		DBVPG1373:S>A		DBVPG6765:S>A		L_1374:S>A		YS9:S>A	AA:287		YS4:S>LID:YBL049W	AA:3		Y9:L>V	AA:32		DBVPG6044:H>Y		SK1:H>Y		Y55:H>YID:YBL051C	AA:9		L_1528:A>S	AA:22		DBVPG1373:I>L		L_1374:I>L		UWOPS05_227_2:I>T	AA:48		SK1:V>A		UWOPS05_227_2:V>A		UWOPS87_2421:V>A		Y55:V>A		YPS606:V>A		YS2:V>A		YS4:V>A	AA:70		UWOPS87_2421:N>D	AA:152		K11:S>N	AA:196		378604X:S>N		DBVPG1106:S>N		DBVPG1373:D>H		DBVPG1788:D>H		DBVPG6040:D>H		DBVPG6765:S>N		K11:D>H		L_1374:S>N		L_1528:S>N		NCYC110:D>H		SK1:D>H		UWOPS05_227_2:D>H		UWOPS83_787_3:D>H		UWOPS87_2421:D>H		Y12:D>H		Y55:D>H		YJM975:D>H		YJM981:D>H		YPS128:D>H		YPS606:D>H		YS2:D>H		YS4:D>H		YS9:S>N	AA:200		UWOPS05_227_2:K>Q	AA:210		L_1374:M>I		UWOPS05_227_2:S>C	AA:242		DBVPG6040:N>T	AA:270		Y12:V>I	AA:274		378604X:A>T		DBVPG1106:A>T		DBVPG1373:A>T		DBVPG1788:A>T		DBVPG6040:A>T		DBVPG6765:A>T		K11:A>T		NCYC110:A>T		SK1:A>T		UWOPS05_227_2:A>T		UWOPS83_787_3:A>T		Y12:A>T		Y55:A>T		Y9:A>T		YJM975:A>T		YJM981:A>T		YPS128:A>T		YPS606:A>T		YS4:A>T		YS9:A>T	AA:420		K11:V>F	AA:487		YS9:Q>E	AA:522		YS9:S>F	AA:553		273614X:T>S		322134S:T>S		378604X:T>S		DBVPG1106:T>S		DBVPG1788:T>S		DBVPG6765:T>S		K11:T>S		L_1374:T>S		L_1528:T>S		NCYC361:T>S		SK1:T>S		UWOPS03_461_4:T>S		UWOPS83_787_3:T>S		UWOPS87_2421:T>S		Y12:T>S		Y55:T>S		Y9:T>S		YJM975:T>S		YPS606:T>S		YS9:T>S	AA:564		NCYC361:Q>H	AA:569		UWOPS05_227_2:V>L	AA:597		K11:Q>R	AA:646		K11:L>-ID:YBL052C	AA:6		L_1528:N>D		UWOPS87_2421:N>K	AA:53		YJM978:S>T	AA:65		K11:G>D	AA:69		L_1528:I>T	AA:78		NCYC110:S>P		SK1:S>P		Y55:S>P	AA:95		BC187:K>R	AA:96		DBVPG1373:M>V		DBVPG1788:M>V		DBVPG6765:M>V		L_1528:M>V		NCYC361:M>V		YJM978:M>V	AA:100		DBVPG6040:S>T		DBVPG6044:S>T		K11:S>T		NCYC110:S>T		SK1:S>T		Y55:S>T		YPS606:S>T	AA:112		YPS606:E>K	AA:124		L_1528:K>N	AA:142		K11:V>I	AA:167		YPS606:D>N	AA:172		DBVPG1853:N>S		YS4:N>S	AA:181		DBVPG6044:N>S		NCYC110:N>S		SK1:N>S		Y55:N>S	AA:291		YS4:P>A	AA:336		DBVPG6040:D>E		Y12:D>E		Y9:D>E		YPS606:D>E	AA:391		K11:P>L		UWOPS03_461_4:P>S		Y9:P>L	AA:392		378604X:Y>H		BC187:Y>H		DBVPG1106:Y>H		DBVPG6765:Y>H		YJM975:Y>H		YJM978:Y>H	AA:480		378604X:D>Y		BC187:D>Y		DBVPG1106:D>Y		DBVPG1373:D>Y		DBVPG1788:D>Y		DBVPG6765:D>Y		YJM975:D>Y		YJM978:D>Y	AA:483		DBVPG6040:R>K		K11:R>K		NCYC110:R>K		SK1:R>K		UWOPS03_461_4:R>K		UWOPS87_2421:R>K		Y12:R>K		Y9:R>K		YPS606:R>K		YS9:R>K	AA:492		UWOPS87_2421:D>N	AA:502		378604X:I>M		BC187:I>M		DBVPG1106:I>M		DBVPG1373:I>M		DBVPG1788:I>M		DBVPG6040:I>M		DBVPG6765:I>M		K11:I>M		NCYC110:I>M		SK1:I>M		UWOPS87_2421:I>M		Y12:I>M		Y9:I>M		YJM975:I>M		YJM978:I>M		YPS606:I>M		YS9:I>M	AA:585		NCYC110:M>V		SK1:M>V	AA:595		DBVPG6765:D>E	AA:615		K11:N>K	AA:653		DBVPG6765:T>N		NCYC110:T>P		SK1:T>P	AA:746		YS9:T>I	AA:753		Y12:N>D	AA:754		DBVPG6040:A>G		UWOPS87_2421:A>G		YPS606:A>G		YS2:A>G	AA:777		DBVPG1106:E>G		DBVPG1788:E>G	AA:784		Y12:D>E	AA:790		Y12:S>N	AA:809		SK1:E>K	AA:810		Y12:S>NID:YBL054W	AA:185		UWOPS03_461_4:P>S	AA:206		K11:S>L	AA:239		DBVPG6044:A>V		SK1:A>V		Y55:A>V	AA:261		273614X:N>S		322134S:N>S		BC187:N>S		DBVPG1106:N>S		DBVPG1853:N>S		DBVPG6040:N>S		DBVPG6044:N>S		K11:N>S		L_1528:N>S		NCYC361:N>S		SK1:N>S		UWOPS83_787_3:N>S		UWOPS87_2421:N>S		Y55:N>S		YJM975:N>S		YPS128:N>S		YPS606:N>S	AA:290		378604X:S>L		BC187:S>L		DBVPG1106:S>L		DBVPG1373:S>L		DBVPG6765:S>L		YJM975:S>L	AA:324		322134S:P>R	AA:410		273614X:D>E		322134S:D>E		378604X:D>E		DBVPG1106:D>E		DBVPG1373:D>E		DBVPG1788:D>E		DBVPG6040:D>E		DBVPG6765:D>E		L_1374:D>E		L_1528:D>E	AA:445		273614X:R>C		378604X:R>C		DBVPG1106:R>C		DBVPG1373:R>C		DBVPG1788:R>C		DBVPG6044:R>C		DBVPG6765:R>C		K11:R>C		L_1374:R>C		L_1528:R>C		NCYC110:R>C		NCYC361:R>C		SK1:R>C		UWOPS03_461_4:R>C		UWOPS05_227_2:R>C		UWOPS87_2421:R>C		Y55:R>C		YPS128:R>C		YPS606:R>C		YS4:R>C	AA:462		NCYC361:D>N	AA:490		NCYC110:H>R	AA:505		378604X:N>K		DBVPG1106:N>K		DBVPG1373:N>K		DBVPG1788:N>K		DBVPG6765:N>K		L_1374:N>K		L_1528:N>K		YJM975:N>KID:YBL056W	AA:157		Y9:F>S	AA:206		273614X:H>Y	AA:256		YJM975:G>D	AA:299		378604X:S>P	AA:302		BC187:Q>K	AA:324		L_1528:S>N	AA:328		378604X:F>L	AA:358		SK1:D>G	AA:369		L_1528:D>G	AA:378		L_1528:S>T	AA:381		L_1528:D>G	AA:429		DBVPG1853:S>N	AA:430		K11:N>D	AA:439		L_1528:N>D	AA:442		378604X:D>Y	AA:445		L_1528:D>N	AA:462		L_1528:S>F	AA:467		L_1528:I>VID:YBL057C	AA:9		DBVPG1373:K>E	AA:37		DBVPG6044:T>M		SK1:T>M		Y55:T>MID:YBL058W	AA:9		UWOPS05_227_2:I>T	AA:48		YS2:Q>R	AA:64		UWOPS05_227_2:A>T		UWOPS83_787_3:A>T	AA:68		YS2:E>G	AA:88		UWOPS05_227_2:C>Y		UWOPS83_787_3:C>Y		YPS128:C>Y		YPS606:C>Y		YS2:C>Y	AA:92		273614X:G>R	AA:188		273614X:F>S		378604X:F>S		DBVPG6044:F>S		DBVPG6765:F>S		NCYC110:F>S		UWOPS83_787_3:F>S		Y55:F>S		YJM975:F>S		YJM981:F>S		YPS128:F>S		YS2:F>S	AA:194		NCYC110:H>Q		UWOPS83_787_3:H>Q		Y12:H>Q		Y55:H>Q		YPS128:H>Q		YS2:H>Q	AA:214		378604X:A>V	AA:300		378604X:T>K		DBVPG1373:T>K		DBVPG1788:T>K		DBVPG6765:T>K		L_1528:T>K		UWOPS05_217_3:T>K		UWOPS83_787_3:T>K		Y12:T>K		Y55:T>K		YJM975:T>K	AA:341		Y55:D>N	AA:343		UWOPS05_217_3:E>V		UWOPS83_787_3:E>V	AA:364		UWOPS05_217_3:H>R		UWOPS83_787_3:H>R		Y55:H>R		YPS606:H>R	AA:370		UWOPS03_461_4:T>S		UWOPS05_217_3:T>S		YPS606:T>SID:YBL060W	AA:3		YS4:A>P	AA:53		273614X:N>	AA:73		DBVPG6044:T>I		NCYC110:T>I		SK1:T>I		Y55:T>I	AA:82		Y12:G>S		Y9:G>S	AA:103		DBVPG6044:S>L		NCYC110:S>L		SK1:S>L		Y55:S>L	AA:108		L_1528:G>D	AA:146		UWOPS83_787_3:L>S	AA:174		DBVPG6044:I>K		DBVPG6765:I>K		K11:I>K		L_1528:I>K		SK1:I>K		UWOPS03_461_4:I>K		UWOPS05_217_3:I>K		Y12:I>K		Y55:I>K		Y9:I>K		YIIc17_E5:I>K		YJM981:I>K		YS4:I>K	AA:209		UWOPS03_461_4:N>S		UWOPS05_217_3:N>S	AA:213		DBVPG1106:D>N	AA:246		273614X:R>C	AA:269		YIIc17_E5:C>Y	AA:278		DBVPG1106:I>T		DBVPG1788:I>T		DBVPG6765:I>T		K11:I>T		L_1528:I>T		SK1:I>T		UWOPS03_461_4:I>T		UWOPS05_217_3:I>T		UWOPS83_787_3:I>T		Y55:I>T		Y9:I>T		YJM975:I>T		YPS606:I>T	AA:388		BC187:M>T		DBVPG1788:M>T		DBVPG6765:M>T		L_1528:M>T		YJM975:M>T	AA:402		SK1:P>S		Y55:P>S	AA:412		BC187:T>A		DBVPG1788:T>A		DBVPG6765:T>A		L_1528:T>A		YJM975:T>A	AA:419		UWOPS03_461_4:S>T		UWOPS05_227_2:S>T	AA:519		DBVPG6044:N>K		NCYC110:N>K		SK1:N>K		Y55:N>K	AA:538		DBVPG1853:H>R	AA:591		378604X:K>N	AA:657		W303:W>R	AA:665		UWOPS05_227_2:N>YID:YBL061C	AA:49		NCYC361:N>S		UWOPS83_787_3:N>S		UWOPS87_2421:N>S	AA:91		SK1:E>K	AA:95		273614X:V>D		378604X:V>D		DBVPG1853:V>D		DBVPG6040:V>D		NCYC361:V>D		SK1:V>D		UWOPS87_2421:V>D		YIIc17_E5:V>D		YPS128:V>D		YPS606:V>D		YS4:V>D		YS9:V>D	AA:103		378604X:H>R	AA:113		L_1528:S>I	AA:226		YS2:A>T		YS9:A>T	AA:238		378604X:D>G	AA:245		L_1374:V>A	AA:351		322134S:L>F	AA:442		DBVPG1788:G>R	AA:514		YS9:G>V	AA:577		UWOPS83_787_3:N>Y	AA:596		K11:P>S		UWOPS03_461_4:P>S		UWOPS05_227_2:P>S		UWOPS83_787_3:P>S		UWOPS87_2421:P>S	AA:653		322134S:L>S		378604X:L>S		DBVPG1788:L>S		DBVPG6040:L>S		DBVPG6044:L>S		K11:L>S		L_1528:L>S		NCYC110:L>S		SK1:L>S		UWOPS03_461_4:L>S		UWOPS05_227_2:L>S		UWOPS83_787_3:L>S		Y12:L>S		Y55:L>S		YIIc17_E5:L>S		YS2:L>S		YS4:L>S	AA:662		DBVPG6044:I>T		K11:I>T		NCYC110:I>T		SK1:I>T		UWOPS03_461_4:I>T		UWOPS05_227_2:I>T		UWOPS83_787_3:I>S		Y12:I>T		Y55:I>T		YIIc17_E5:I>T		YS2:I>S		YS4:I>S	AA:673		UWOPS83_787_3:S>P		YS2:S>P		YS4:S>P	AA:678		DBVPG1106:S>NID:YBL066C	AA:310		DBVPG6044:D>N		K11:D>N		SK1:D>N		UWOPS05_227_2:D>N		UWOPS83_787_3:D>N		Y12:D>N		Y55:D>N		Y9:D>N		YPS128:D>N		YPS606:D>N		YS9:D>N	AA:529		DBVPG1853:D>N		W303:I>V	AA:549		DBVPG6044:H>L		NCYC110:H>L		SK1:H>L		Y55:H>L	AA:555		W303:K>E	AA:571		UWOPS87_2421:A>S	AA:655		L_1528:A>T	AA:706		BC187:A>V		DBVPG1788:A>V		DBVPG6765:A>V	AA:730		DBVPG6040:N>S	AA:789		YPS128:P>L	AA:814		DBVPG6044:T>A		SK1:T>A		Y55:T>A	AA:823		YJM978:R>-	AA:861		W303:A>S	AA:874		W303:G>V	AA:925		273614X:I>M		DBVPG1853:I>M		UWOPS05_217_3:I>M		UWOPS83_787_3:I>M		YPS128:I>M		YPS606:I>M	AA:949		DBVPG1853:K>R	AA:1000		DBVPG6040:D>G		K11:D>G		Y12:D>G		YIIc17_E5:D>G		YS2:D>G		YS4:D>GID:YBL068W	AA:71		YIIc17_E5:G>V	AA:90		378604X:Q>K		DBVPG1373:Q>K		DBVPG1788:Q>K		DBVPG6040:Q>K		DBVPG6044:Q>K		DBVPG6765:Q>K		L_1374:Q>K		L_1528:Q>K		S288c:Q>K		SK1:Q>K		UWOPS03_461_4:Q>K		UWOPS05_217_3:Q>K		UWOPS83_787_3:Q>K		Y55:Q>K		Y9:Q>K		YIIc17_E5:Q>K		YJM975:Q>K		YJM981:Q>K		YPS128:Q>K		YPS606:Q>K		YS4:Q>K		YS9:Q>K	AA:216		UWOPS03_461_4:K>R		UWOPS05_217_3:K>R		UWOPS05_227_2:K>R	AA:250		Y9:E>V	AA:254		Y9:K>NID:YBL069W	AA:32		UWOPS05_217_3:A>S		UWOPS05_227_2:A>S	AA:124		UWOPS03_461_4:L>P		UWOPS05_217_3:L>P		UWOPS05_227_2:L>P	AA:133		273614X:E>K	AA:365		L_1528:Y>-	AA:398		YS2:D>N		YS9:D>N	AA:402		Y9:D>VID:YBL072C	AA:45		UWOPS03_461_4:S>T		UWOPS05_217_3:S>T		UWOPS05_227_2:S>T		UWOPS83_787_3:S>TID:YBL074C	AA:3		UWOPS05_217_3:T>A	AA:24		YPS606:V>I	AA:27		DBVPG6040:N>D	AA:105		322134S:Y>H	AA:131		UWOPS03_461_4:R>W	AA:166		DBVPG1853:A>T		DBVPG6040:A>T		DBVPG6044:A>T		SK1:A>T		UWOPS05_217_3:A>T		Y55:A>T		YIIc17_E5:A>T	AA:309		UWOPS05_217_3:M>I	AA:336		DBVPG6044:D>H		SK1:D>H		Y55:D>HID:YBL075C	AA:20		DBVPG6040:S>F		K11:S>F		YIIc17_E5:S>F		YS2:S>F	AA:33		DBVPG1853:N>I	AA:50		Y12:G>V	AA:107		Y12:G>V	AA:348		YS4:V>I	AA:444		K11:E>D	AA:449		YIIc17_E5:K>M	AA:471		UWOPS83_787_3:Q>H	AA:517		273614X:V>F	AA:567		UWOPS05_217_3:R>I	AA:639		L_1528:E>DID:YBL078C	AA:75		YIIc17_E5:A>GID:YBL080C	AA:3		322134S:R>Q		DBVPG1373:R>Q		DBVPG6765:R>Q		L_1374:R>Q		YIIc17_E5:R>Q		YJM975:R>Q		YJM978:R>Q	AA:44		DBVPG1853:K>-	AA:60		DBVPG1373:P>S	AA:131		YPS128:K>N		YPS606:K>N	AA:145		378604X:A>T		UWOPS83_787_3:A>T		UWOPS87_2421:A>T		Y9:A>T		YS9:A>T	AA:245		YPS606:P>S	AA:313		322134S:P>Q		BC187:P>Q		DBVPG1788:P>Q		DBVPG6765:P>Q		L_1374:P>Q		YJM975:P>Q		YS2:P>Q	AA:333		322134S:I>M		BC187:I>M		DBVPG1373:I>M		DBVPG1788:I>M		DBVPG6044:I>M		L_1374:I>M		SK1:I>M		UWOPS03_461_4:I>M		UWOPS83_787_3:I>M		Y55:I>M		YJM975:I>M		YS2:I>M		YS4:I>M	AA:352		DBVPG6044:N>S		SK1:N>S	AA:409		322134S:K>R		BC187:K>R		DBVPG1373:K>R		DBVPG1788:K>R		DBVPG1853:K>R		DBVPG6765:K>R		L_1374:K>R		YS2:K>R	AA:415		273614X:A>P		DBVPG6040:A>P		NCYC361:A>P		S288c:A>P		SK1:A>P		UWOPS03_461_4:A>P		UWOPS83_787_3:A>P		W303:A>P		YGPM:A>P	AA:448		273614X:E>K		BC187:E>K		DBVPG1373:E>K		DBVPG1788:E>K		DBVPG1853:E>K		DBVPG6765:E>K		L_1374:E>K		NCYC361:E>K		SK1:E>K		UWOPS03_461_4:E>K		UWOPS83_787_3:E>K	AA:475		NCYC361:V>A		UWOPS83_787_3:V>IID:YBL081W	AA:22		YS9:L>S	AA:77		YS9:P>L	AA:81		YS4:A>G	AA:115		322134S:H>R		YS4:H>R	AA:121		YS4:N>K	AA:123		DBVPG6044:N>S		NCYC110:N>S		SK1:N>S		UWOPS87_2421:N>S		Y55:N>S	AA:167		378604X:S>N		UWOPS87_2421:S>N	AA:169		UWOPS87_2421:N>S	AA:170		UWOPS05_217_3:N>S	AA:171		YPS128:N>S		YPS606:N>S	AA:173		UWOPS05_217_3:N>S		UWOPS05_227_2:N>S	AA:174		YPS128:N>S		YPS606:N>S	AA:177		322134S:S>N		378604X:S>N		DBVPG1373:S>N		DBVPG6044:S>N		NCYC110:S>N		UWOPS87_2421:S>N		Y55:S>N		Y9:S>N		YS4:S>N	AA:245		YS4:P>L	AA:277		YS9:T>N	AA:304		YS9:P>R	AA:346		DBVPG1853:S>YID:YBL082C	AA:236		378604X:I>M		SK1:I>M		UWOPS03_461_4:I>M		UWOPS05_227_2:I>M		Y55:I>M		Y9:I>M		YS4:I>M	AA:245		SK1:T>I		Y55:T>I	AA:320		378604X:V>A		YS4:V>AID:YBL084C	AA:28		NCYC361:I>V	AA:31		DBVPG6044:Q>E		NCYC110:Q>E		Y55:Q>E	AA:210		DBVPG6044:I>T		SK1:I>T		Y55:I>T	AA:274		YS9:S>P	AA:421		K11:S>N	AA:451		DBVPG1853:N>D	AA:480		YS9:K>N	AA:490		DBVPG1373:M>I		DBVPG1788:M>I		YJM978:M>I		YJM981:M>I	AA:535		DBVPG1853:T>A		YPS128:T>A		YPS606:T>A		YS2:T>A		YS9:T>A	AA:537		YS9:P>RID:YBL086C	AA:51		273614X:A>V	AA:53		DBVPG6044:D>G		NCYC110:D>G		SK1:D>G		Y55:D>G	AA:72		YS9:V>L	AA:137		UWOPS05_217_3:A>S		UWOPS05_227_2:A>S	AA:149		K11:S>F	AA:221		DBVPG1853:Y>S	AA:265		K11:G>S		NCYC110:G>S		SK1:G>S		UWOPS03_461_4:G>S		UWOPS83_787_3:G>S		UWOPS87_2421:G>S		Y12:G>S		Y55:G>S		YPS128:G>S		YS2:G>S		YS4:G>S		YS9:G>S	AA:268		UWOPS83_787_3:A>T	AA:290		UWOPS87_2421:T>A	AA:310		DBVPG6765:Y>H		YJM978:Y>H	AA:317		YS9:P>S	AA:386		Y12:S>G		Y9:S>G		YS9:S>G	AA:439		DBVPG6044:S>F		NCYC110:S>F		SK1:S>F		Y12:S>F		Y55:S>F		Y9:S>F		YPS606:S>F		YS2:S>F		YS9:S>FID:YBL087C	AA:119		378604X:G>VID:YBL089W	AA:32		378604X:G>A	AA:92		UWOPS83_787_3:C>W	AA:142		DBVPG6044:I>M		DBVPG6765:I>M		L_1374:I>M		NCYC110:I>M		SK1:I>M		UWOPS87_2421:I>M		Y55:I>M		Y9:I>M		YPS128:I>M		YPS606:I>M		YS4:I>M	AA:196		UWOPS87_2421:P>L	AA:337		Y9:S>N	AA:343		273614X:A>E		DBVPG6044:A>E		NCYC110:A>E		NCYC361:A>E		SK1:A>E		UWOPS03_461_4:A>E		UWOPS05_227_2:A>E		UWOPS83_787_3:A>E		Y55:A>E		Y9:A>E		YPS128:A>E		YS4:A>E	AA:385		YS9:K>E	AA:416		SK1:T>M		Y55:T>M		YS4:T>MID:YBL090W	AA:16		UWOPS03_461_4:G>D		UWOPS05_227_2:G>D	AA:50		UWOPS83_787_3:Q>HID:YBL091C	AA:86		322134S:V>D		378604X:V>D		DBVPG1853:V>D		DBVPG6044:V>D		DBVPG6765:V>D		L_1374:V>D		NCYC110:V>D		NCYC361:V>D		SK1:V>D		UWOPS03_461_4:V>D		UWOPS05_217_3:V>D		UWOPS87_2421:V>D		W303:V>D		Y55:V>D		YIIc17_E5:V>D		YJM975:V>D		YJM978:V>D		YPS128:V>D		YPS606:V>D		YS4:V>D	AA:163		Y55:P>Q	AA:231		DBVPG6040:I>M	AA:258		DBVPG6040:I>SID:YBL093C	AA:52		378604X:L>S	AA:199		NCYC110:G>-ID:YBL095W	AA:99		Y12:S>N	AA:181		322134S:S>-	AA:199		W303:N>S	AA:244		378604X:M>I		DBVPG6044:M>I		NCYC110:M>I		YS4:M>IID:YBL098W	AA:8		273614X:I>L	AA:141		DBVPG1853:D>G	AA:154		K11:K>R	AA:155		378604X:T>A		DBVPG1853:T>A		DBVPG6044:T>A		SK1:T>A		UWOPS87_2421:T>A		YPS128:T>A		YPS606:T>A		YS4:T>A		YS9:T>A	AA:250		378604X:D>G		YS4:D>GID:YBL099W	AA:340		273614X:P>S		DBVPG1106:P>S		DBVPG6040:P>S		DBVPG6765:P>S		K11:P>S		L_1374:P>S		L_1528:P>S		NCYC361:P>S		S288c:P>S		SK1:P>S		UWOPS83_787_3:P>S		UWOPS87_2421:P>S		W303:P>S		Y12:P>S		Y55:P>S		Y9:P>S		YGPM:P>S		YJM975:P>S		YJM978:P>S		YJM981:P>S		YPS128:P>S		YPS606:P>S		YS4:P>S		YS9:P>S	AA:463		UWOPS05_227_2:Q>	AA:489		UWOPS03_461_4:H>P	AA:509		DBVPG1106:L>IID:YBL102W	AA:56		K11:T>ID:YBL103C	AA:20		YIIc17_E5:N>T	AA:103		DBVPG1853:S>P		K11:S>P		SK1:S>P	AA:104		YIIc17_E5:S>P	AA:276		378604X:G>V		Y12:G>V		Y9:G>V		YS4:G>V	AA:372		Y12:V>F		Y9:V>F	AA:388		DBVPG6044:Q>K		SK1:Q>K	AA:414		273614X:S>T		322134S:S>T		378604X:S>T		DBVPG1853:S>T		DBVPG6040:S>T		DBVPG6044:S>T		NCYC361:S>T		SK1:S>T		UWOPS03_461_4:S>T		UWOPS05_227_2:S>T		Y12:S>T		Y9:S>T		YPS128:S>T		YPS606:S>T		YS4:S>T	AA:422		Y12:E>Q		Y9:E>Q	AA:429		YS4:V>I	AA:435		Y12:A>T		Y9:A>T		YS4:A>TID:YBL104C	AA:38		UWOPS83_787_3:E>V	AA:43		378604X:I>V	AA:169		DBVPG1853:R>G	AA:363		DBVPG6044:N>S		SK1:N>S	AA:428		DBVPG6040:V>I	AA:443		378604X:I>V		BC187:I>V		DBVPG1106:I>V		DBVPG1373:I>V		DBVPG1788:I>V		DBVPG1853:I>V		DBVPG6040:I>V		DBVPG6044:I>V		DBVPG6765:I>V		K11:I>V		L_1528:I>V		NCYC110:I>V		NCYC361:I>V		SK1:I>V		UWOPS03_461_4:I>V		UWOPS83_787_3:I>V		Y12:I>V		Y55:I>V		Y9:I>V		YPS606:I>V		YS4:I>V	AA:481		BC187:E>D		DBVPG1788:E>D		DBVPG6765:E>D		Y55:E>D	AA:549		DBVPG6040:I>M	AA:593		L_1528:T>K	AA:777		DBVPG1853:T>S	AA:867		322134S:V>L	AA:961		322134S:G>-	AA:1026		DBVPG6040:V>IID:YBL107C	AA:30		378604X:T>R		YS4:T>R	AA:56		UWOPS03_461_4:D>Y	AA:76		DBVPG6044:K>R		SK1:K>R	AA:115		DBVPG1373:G>D		L_1528:G>D		YJM978:G>D	AA:139		273614X:A>E		378604X:A>E		DBVPG6044:A>E		NCYC361:A>E		SK1:A>E		UWOPS03_461_4:A>E		YPS128:A>E		YPS606:A>E		YS4:A>E	AA:143		NCYC361:E>D	AA:172		DBVPG6044:G>S		SK1:G>SID:YBR002C	AA:113		K11:R>H	AA:147		K11:T>I	AA:170		UWOPS03_461_4:H>Y	AA:172		DBVPG6765:M>I		YS2:M>I	AA:186		322134S:D>G		DBVPG1853:D>G		DBVPG6765:D>G		L_1528:D>G		YJM978:D>G		YJM981:D>G		YS2:D>GID:YBR003W	AA:28		Y12:A>S	AA:38		UWOPS03_461_4:P>H	AA:140		DBVPG1106:P>	AA:207		DBVPG1106:S>	AA:210		322134S:A>T		DBVPG1373:A>T		DBVPG6765:A>T		L_1528:A>T		YJM981:A>T	AA:221		YS4:G>D		YS9:G>D	AA:419		DBVPG6044:K>R		NCYC110:K>R		SK1:K>R		Y55:K>R	AA:428		DBVPG6044:N>H		NCYC110:N>H		SK1:N>H		Y55:N>H	AA:433		UWOPS03_461_4:T>A	AA:461		UWOPS87_2421:L>IID:YBR004C	AA:17		YS2:Y>N	AA:60		Y9:S>L	AA:159		UWOPS05_227_2:S>N	AA:187		322134S:T>S		378604X:T>S		BC187:T>S		DBVPG1788:T>S		DBVPG1853:T>S		DBVPG6044:T>S		DBVPG6765:T>S		K11:T>S		SK1:T>S		UWOPS05_227_2:T>S		UWOPS83_787_3:T>S		W303:T>S		Y55:T>S		Y9:T>S		YGPM:T>S		YJM975:T>S		YJM978:T>S		YPS128:T>S		YPS606:T>S		YS4:T>S	AA:199		322134S:S>P		BC187:S>P		DBVPG1788:S>P		DBVPG1853:S>P		DBVPG6044:S>P		DBVPG6765:S>P		SK1:S>P		Y55:S>P		YJM975:S>P		YJM978:S>P		YPS128:S>P		YPS606:S>P		YS4:S>P	AA:212		322134S:A>T		BC187:A>T		DBVPG1788:A>T		DBVPG1853:A>T		DBVPG6044:A>T		DBVPG6765:A>T		SK1:A>T		Y55:A>T		YJM975:A>T		YJM978:A>T		YPS128:A>T		YPS606:A>T		YS4:A>T	AA:254		YPS128:S>A		YPS606:S>A	AA:307		DBVPG1788:G>R	AA:323		YPS128:L>S	AA:395		YPS128:P>S		YPS606:P>SID:YBR005W	AA:10		UWOPS05_227_2:A>T	AA:14		YPS606:V>A	AA:17		DBVPG1853:A>T	AA:41		DBVPG1373:I>R		DBVPG1788:I>R		DBVPG1853:I>R		DBVPG6765:I>R		L_1374:I>R		NCYC110:I>R		UWOPS05_217_3:I>R		Y12:I>L		Y55:I>R		YJM978:I>R		YS4:I>R	AA:52		DBVPG1373:V>I		DBVPG1853:V>I		DBVPG6765:V>I		L_1374:V>I		YJM978:V>I		YS4:V>IID:YBR006W	AA:301		K11:A>	AA:374		UWOPS03_461_4:G>A	AA:384		DBVPG6044:S>T		SK1:S>T		Y55:S>T	AA:395		DBVPG1788:E>AID:YBR007C	AA:5		BC187:L>I		DBVPG1106:L>I		DBVPG1788:L>I		DBVPG6765:L>I		YJM975:L>I		YJM978:L>I	AA:18		Y12:D>N		Y9:D>N	AA:47		BC187:S>N	AA:79		UWOPS03_461_4:L>Q		UWOPS05_227_2:L>Q	AA:154		Y12:V>L	AA:179		378604X:G>C		DBVPG1788:G>C		DBVPG6765:G>C		L_1528:G>C		YJM975:G>C		YS9:G>C	AA:184		YJM975:M>T		YS9:M>T	AA:205		273614X:R>S		322134S:R>S		378604X:R>S		DBVPG1373:R>S		DBVPG1788:R>S		DBVPG6044:R>S		DBVPG6765:C>Y		L_1374:R>S		L_1528:R>S		S288c:R>S		UWOPS05_227_2:R>S		UWOPS87_2421:R>S		W303:R>S		Y55:R>S		Y9:R>S		YJM975:R>S		YPS606:R>S		YS9:R>S	AA:208		UWOPS05_227_2:P>L	AA:234		273614X:T>I		322134S:T>I		378604X:T>I		DBVPG1373:T>I		DBVPG1788:T>I		DBVPG1853:T>I		DBVPG6044:T>I		DBVPG6765:T>I		L_1374:T>I		L_1528:T>I		SK1:T>I		UWOPS05_227_2:T>I		Y55:T>I		YJM975:T>I		YPS606:T>I		YS9:T>I	AA:256		273614X:T>I		DBVPG1106:T>I		DBVPG1373:T>I		DBVPG1788:T>I		DBVPG6765:T>I		L_1374:T>I		L_1528:T>I		YJM975:T>I		YS9:T>I	AA:327		322134S:T>P		378604X:T>P		BC187:T>P		DBVPG1106:T>P		DBVPG1373:T>P		DBVPG1788:T>P		DBVPG1853:T>P		DBVPG6044:T>P		DBVPG6765:T>P		L_1374:T>P		L_1528:T>P		SK1:T>P		UWOPS05_227_2:T>P		UWOPS87_2421:T>P		Y55:T>P		Y9:T>P		YGPM:T>P		YJM975:T>P		YPS606:T>P		YS9:T>P	AA:452		K11:K>E		Y12:K>E	AA:481		Y9:G>V	AA:495		378604X:H>R		DBVPG1788:H>R		DBVPG1853:H>R		DBVPG6765:H>R		L_1374:H>R		YJM975:H>R		YS4:H>R		YS9:H>R	AA:521		322134S:S>I	AA:539		DBVPG1788:H>Q		DBVPG6765:H>Q		L_1374:H>Q		YJM975:H>Q		YJM978:H>Q		YJM981:H>Q		YS9:H>Q	AA:597		K11:K>R	AA:700		273614X:R>K		322134S:R>K		378604X:R>K		DBVPG1106:R>K		DBVPG1788:R>K		DBVPG6044:R>K		DBVPG6765:R>K		K11:R>K		SK1:R>K		Y12:R>K		Y55:R>K		Y9:R>K		YJM975:R>K		YJM978:R>K		YJM981:R>K		YPS606:R>K		YS9:R>KID:YBR008C	AA:23		YPS606:S>F	AA:29		378604X:Q>R		DBVPG1788:Q>R		DBVPG6765:Q>R		W303:Q>R		YJM975:Q>R	AA:49		YPS606:P>H	AA:137		UWOPS05_227_2:G>C	AA:199		UWOPS83_787_3:V>I	AA:268		YS2:W>-	AA:320		UWOPS87_2421:R>K	AA:331		273614X:L>F		DBVPG1373:L>F		DBVPG1788:L>F		DBVPG1853:L>F		DBVPG6044:L>F		DBVPG6765:L>F		NCYC110:L>F		SK1:L>F		UWOPS05_227_2:L>F		UWOPS83_787_3:L>F		UWOPS87_2421:L>F		Y55:L>F		YJM975:L>F		YJM978:L>F		YJM981:L>F		YPS128:L>F		YPS606:L>F		YS2:L>F		YS4:L>F		YS9:L>F	AA:372		273614X:S>N		378604X:S>N		DBVPG6765:S>N	AA:450		YJM978:V>F	AA:473		273614X:K>T		378604X:K>T		DBVPG1106:K>T		DBVPG1373:K>T		DBVPG6765:K>T		L_1374:K>T		L_1528:K>T		YJM975:K>T		YJM978:K>T		YJM981:K>T		YS4:K>T		YS9:K>T	AA:502		378604X:D>E		DBVPG1106:D>E		DBVPG1373:D>E		DBVPG1788:D>E		DBVPG6765:D>E		L_1374:D>E		L_1528:D>E		YJM975:D>E		YJM978:D>E		YS4:D>E		YS9:D>E	AA:507		DBVPG1853:K>E	AA:541		378604X:T>A		DBVPG1106:T>A		DBVPG1788:T>A		DBVPG1853:T>A		DBVPG6044:T>A		DBVPG6765:T>A		K11:T>A		L_1374:T>A		L_1528:T>A		NCYC110:T>A		SK1:T>A		UWOPS03_461_4:T>A		UWOPS83_787_3:T>A		UWOPS87_2421:T>A		Y55:T>A		YJM975:T>A		YJM981:T>A		YPS128:T>A		YPS606:T>A		YS4:T>A		YS9:T>AID:YBR009C	AA:47		UWOPS03_461_4:I>F	AA:68		YJM978:R>IID:YBR011C	AA:89		UWOPS05_217_3:G>V	AA:112		YS9:K>N	AA:171		YS9:D>N	AA:173		YS9:E>DID:YBR014C	AA:23		378604X:V>A	AA:31		UWOPS87_2421:N>S	AA:42		DBVPG6044:H>Y		NCYC110:H>Y		SK1:H>Y		Y55:H>Y	AA:78		W303:K>E	AA:96		YPS128:E>Q		YPS606:E>Q	AA:129		322134S:Y>H		378604X:Y>H		BC187:Y>H		DBVPG1373:Y>H		DBVPG1788:Y>H		DBVPG1853:Y>H		DBVPG6765:Y>H		L_1374:Y>H		YJM981:Y>H		YS2:Y>H	AA:185		YS2:K>EID:YBR016W	AA:61		DBVPG1853:G>AID:YBR017C	AA:100		YPS128:D>E		YPS606:D>E	AA:107		UWOPS05_227_2:N>Y	AA:319		L_1374:V>A		YS4:V>A	AA:403		DBVPG1788:S>F	AA:559		YS9:K>E	AA:564		YS9:I>L	AA:566		YS9:L>V	AA:574		K11:A>S	AA:576		YS9:K>-	AA:742		DBVPG6765:T>A	AA:753		BC187:T>I	AA:856		322134S:N>Y	AA:891		273614X:T>I	AA:900		YPS128:I>V		YPS606:I>VID:YBR018C	AA:187		UWOPS05_227_2:C>G	AA:195		UWOPS05_227_2:V>G	AA:203		YPS606:D>G	AA:207		K11:R>C	AA:211		K11:T>I		UWOPS05_227_2:T>A		Y12:T>I	AA:267		BC187:V>M	AA:305		BC187:G>D	AA:340		YS9:P>HID:YBR019C	AA:62		UWOPS87_2421:H>L		YS9:S>I	AA:69		DBVPG6040:D>Y	AA:70		DBVPG6040:L>F	AA:160		DBVPG6044:T>N		SK1:T>N		Y55:T>N	AA:179		YJM975:N>D	AA:184		DBVPG6044:S>G		SK1:S>G		Y55:S>G	AA:263		DBVPG1853:Q>K		DBVPG6040:Q>K		DBVPG6044:Q>K		SK1:Q>K		UWOPS03_461_4:Q>K		UWOPS05_217_3:Q>K		UWOPS05_227_2:Q>K		UWOPS83_787_3:Q>K		UWOPS87_2421:Q>K		Y12:Q>K		Y55:Q>K		YIIc17_E5:Q>K		YPS128:Q>K		YPS606:Q>K	AA:370		DBVPG6040:R>H	AA:442		YJM975:C>R		YJM981:C>R	AA:518		378604X:M>I		DBVPG1853:M>I		K11:M>I		SK1:M>I		UWOPS05_217_3:M>I		UWOPS83_787_3:M>I		Y55:M>I		Y9:M>I		YPS128:M>I		YPS606:M>I	AA:600		SK1:C>G		Y55:C>G	AA:677		DBVPG6765:N>KID:YBR020W	AA:138		Y9:L>Q	AA:139		BC187:K>Q	AA:141		273614X:L>F	AA:282		378604X:T>I		NCYC361:T>I	AA:297		378604X:L>P		DBVPG6044:L>P		DBVPG6765:L>P		K11:L>P		L_1528:L>P		NCYC110:L>P		NCYC361:L>P		UWOPS05_217_3:L>P		UWOPS05_227_2:L>P		UWOPS83_787_3:L>P		Y12:L>P		Y55:L>P		YIIc17_E5:L>P		YPS128:L>P		YPS606:L>P		YS9:L>P	AA:313		YJM981:F>I	AA:359		YJM981:V>D	AA:414		NCYC110:A>E		SK1:A>E		Y55:A>E	AA:502		NCYC110:Y>ID:YBR021W	AA:33		NCYC361:N>H	AA:75		K11:I>V	AA:83		273614X:D>	AA:88		NCYC361:G>A	AA:229		DBVPG6044:K>R		SK1:K>R		UWOPS05_217_3:K>R		UWOPS87_2421:K>R		Y12:K>R		Y55:K>R		YPS606:K>R	AA:304		K11:Q>	AA:450		YS9:A>G	AA:484		YS9:C>G	AA:508		Y12:F>S		Y9:F>S	AA:617		DBVPG1106:V>E	AA:625		Y12:E>Q	AA:628		DBVPG1106:H>QID:YBR022W	AA:48		YPS128:K>I	AA:66		SK1:I>M		UWOPS05_217_3:I>M		UWOPS05_227_2:I>M		Y55:I>M		YS2:I>M		YS9:I>M	AA:73		L_1374:N>K	AA:76		378604X:L>V	AA:114		K11:A>T	AA:124		K11:S>L		YS9:S>L	AA:157		YS9:K>RID:YBR024W	AA:29		SK1:G>A		Y55:G>A	AA:53		K11:Q>K		SK1:Q>K		UWOPS05_227_2:Q>K		Y12:Q>K		Y55:Q>K		YIIc17_E5:Q>K		YPS128:Q>K		YPS606:Q>K	AA:89		322134S:L>P	AA:269		UWOPS87_2421:Q>K	AA:302		YIIc17_E5:->WID:YBR025C	AA:165		YS9:E>D	AA:259		378604X:G>VID:YBR026C	AA:19		Y12:H>Q		Y9:H>Q	AA:87		DBVPG6044:E>Q		Y55:E>Q	AA:213		NCYC361:F>L	AA:222		DBVPG1853:D>E		UWOPS83_787_3:D>E		Y55:D>E	AA:248		L_1528:I>V	AA:317		DBVPG1373:N>T	AA:334		DBVPG1853:Y>D		SK1:Y>D		UWOPS03_461_4:Y>D		UWOPS83_787_3:Y>D		Y55:Y>D		YPS606:Y>D		YS9:Y>DID:YBR028C	AA:31		273614X:N>D		322134S:N>D		BC187:N>D		DBVPG6044:N>D		DBVPG6765:N>D		SK1:N>D		UWOPS03_461_4:N>D		UWOPS05_217_3:N>D		UWOPS87_2421:N>D		Y55:N>D		YIIc17_E5:N>D		YJM978:N>D		YPS606:N>D	AA:63		273614X:S>T	AA:98		273614X:R>P		BC187:R>P		DBVPG1853:R>P		DBVPG6044:R>P		DBVPG6765:R>P		SK1:R>P		UWOPS03_461_4:R>P		UWOPS05_217_3:R>P		Y55:R>P		YIIc17_E5:R>P		YJM975:R>P		YJM978:R>P		YPS128:R>P		YPS606:R>P	AA:180		Y9:K>N	AA:262		W303:S>I	AA:309		UWOPS87_2421:D>Y	AA:315		SK1:N>K		Y55:N>K	AA:340		DBVPG6040:S>I	AA:359		YIIc17_E5:K>R	AA:500		UWOPS05_217_3:N>DID:YBR030W	AA:144		BC187:A>T		DBVPG1373:A>T		DBVPG6044:A>T		DBVPG6765:A>T		K11:A>T		L_1374:A>T		L_1528:A>T		NCYC361:A>T		SK1:A>T		UWOPS05_217_3:A>T		UWOPS83_787_3:A>T		UWOPS87_2421:A>T		Y12:A>T		Y55:A>T		YJM978:A>T		YPS606:A>T	AA:195		YS4:I>T	AA:248		273614X:D>G		BC187:D>G		DBVPG1788:D>G		DBVPG1853:D>G		DBVPG6765:D>G		K11:D>G		L_1374:D>G		L_1528:D>G		SK1:D>G		UWOPS83_787_3:D>G		UWOPS87_2421:D>G		W303:D>G		Y12:D>G		Y55:D>G		YJM978:D>G		YPS606:D>G		YS4:D>G	AA:255		L_1374:A>V		YJM978:A>V	AA:293		DBVPG1853:K>N	AA:295		273614X:Y>H		BC187:Y>H		DBVPG1788:Y>H		DBVPG6765:Y>H		L_1374:Y>H		L_1528:Y>H		SK1:Y>H		UWOPS83_787_3:Y>H		UWOPS87_2421:Y>H		W303:Y>H		Y12:Y>H		Y55:Y>H		YIIc17_E5:Y>H		YJM978:Y>H		YPS128:Y>H		YPS606:Y>H		YS4:Y>H	AA:302		273614X:I>T		BC187:I>T		DBVPG1788:I>T		DBVPG6765:I>T		K11:I>T		L_1374:I>T		L_1528:I>T		SK1:I>T		UWOPS83_787_3:I>T		UWOPS87_2421:I>T		W303:I>T		Y12:I>T		Y55:I>T		YIIc17_E5:I>T		YJM978:I>T		YPS128:I>T		YPS606:I>T		YS4:I>T	AA:405		YPS128:D>G	AA:473		BC187:A>T		YS4:A>T	AA:504		BC187:N>K		DBVPG1373:N>K		DBVPG6040:N>K		DBVPG6044:N>K		DBVPG6765:N>K		NCYC361:N>K		SK1:N>K		UWOPS03_461_4:N>K		UWOPS83_787_3:N>K		UWOPS87_2421:N>K		Y12:N>K		YIIc17_E5:N>K		YJM975:N>K		YJM978:N>K		YS9:N>K	AA:518		UWOPS87_2421:N>K	AA:529		DBVPG6040:M>V		NCYC361:M>V		Y12:M>V		YS9:M>V	AA:537		DBVPG6044:V>A		SK1:V>AID:YBR031W	AA:29		Y12:P>T	AA:168		YS4:A>S	AA:333		YJM978:V>IID:YBR033W	AA:3		DBVPG6044:H>L		NCYC110:H>L		SK1:H>L		Y55:H>L	AA:19		YPS128:R>H		YPS606:R>H	AA:40		DBVPG6044:R>K		NCYC110:R>K		SK1:R>K		Y55:R>K	AA:46		YS2:T>M	AA:54		DBVPG1106:H>R		DBVPG1788:H>R		DBVPG6040:H>R		DBVPG6044:H>R		DBVPG6765:H>R		NCYC110:H>R		NCYC361:H>R		SK1:H>R		UWOPS03_461_4:H>R		UWOPS05_217_3:H>R		UWOPS83_787_3:H>R		Y55:H>R		Y9:H>R		YIIc17_E5:H>R		YJM978:H>R		YPS128:H>R		YPS606:H>R		YS2:H>R		YS4:H>R	AA:101		DBVPG1106:G>A		DBVPG1788:G>A		DBVPG6044:G>A		DBVPG6765:G>A		NCYC110:G>A		NCYC361:G>A		SK1:G>A		UWOPS03_461_4:G>V		UWOPS05_217_3:G>V		UWOPS83_787_3:G>A		Y55:G>A		Y9:G>A		YIIc17_E5:G>A		YJM978:G>A		YPS128:G>A		YPS606:G>A		YS2:G>A		YS4:G>A	AA:106		DBVPG6044:I>N		NCYC361:I>N		SK1:I>N		UWOPS03_461_4:I>N		UWOPS05_217_3:I>N		UWOPS83_787_3:I>N		Y55:I>N		Y9:I>N		YPS128:I>N		YPS606:I>N	AA:121		DBVPG6044:R>K		SK1:R>K		Y55:R>K	AA:151		NCYC361:V>G		UWOPS83_787_3:V>G	AA:177		DBVPG1106:I>T		DBVPG1788:I>T		DBVPG1853:I>T		DBVPG6044:I>T		DBVPG6765:I>T		K11:I>T		NCYC361:I>T		SK1:I>T		UWOPS05_217_3:I>T		UWOPS83_787_3:I>T		UWOPS87_2421:I>T		Y55:I>T		YIIc17_E5:I>T		YJM978:I>T		YPS128:I>T		YPS606:I>T		YS4:I>T		YS9:I>T	AA:181		UWOPS05_217_3:D>N	AA:188		YS9:F>S	AA:204		UWOPS03_461_4:G>V		UWOPS05_217_3:G>V	AA:206		YPS128:D>N	AA:220		DBVPG1106:G>A	AA:232		DBVPG1853:Q>K	AA:235		UWOPS05_217_3:P>T	AA:238		DBVPG6044:E>D		SK1:E>D	AA:262		DBVPG1853:S>N	AA:265		YS9:T>S	AA:274		DBVPG6044:K>Q		SK1:K>Q		Y55:K>Q	AA:282		322134S:N>H	AA:299		DBVPG1106:F>C		DBVPG1788:F>C		DBVPG1853:F>C		DBVPG6040:F>C		DBVPG6765:F>C		K11:F>C		L_1374:F>C		UWOPS03_461_4:F>C		UWOPS05_227_2:F>C		UWOPS83_787_3:F>C		UWOPS87_2421:F>C		Y9:F>C		YIIc17_E5:F>C		YJM975:F>C		YJM978:F>C		YPS128:F>C		YPS606:F>C		YS4:F>C		YS9:F>C	AA:302		UWOPS83_787_3:K>N	AA:329		UWOPS87_2421:S>Y	AA:338		DBVPG6044:S>P		SK1:S>P		Y55:S>P	AA:384		UWOPS83_787_3:S>R	AA:393		DBVPG6040:E>G		K11:E>G		Y9:E>G	AA:398		UWOPS03_461_4:I>V	AA:399		K11:D>N		Y9:D>N	AA:402		DBVPG6044:R>T		SK1:R>T		Y55:R>T	AA:432		DBVPG6040:S>P		K11:S>P		Y9:S>P		YPS128:S>P		YPS606:S>P	AA:433		DBVPG6044:T>K		SK1:T>K		Y55:T>K	AA:438		DBVPG6044:R>H		SK1:R>H		UWOPS03_461_4:R>H		UWOPS87_2421:R>H		Y55:R>H	AA:440		DBVPG6044:S>N		SK1:S>N		UWOPS03_461_4:S>N		UWOPS87_2421:S>N		Y55:S>N	AA:451		K11:I>S		YPS128:I>S		YPS606:I>S	AA:467		DBVPG6044:T>S		SK1:T>S		Y55:T>S	AA:526		DBVPG6040:L>R		K11:L>R	AA:529		BC187:Q>E		DBVPG1106:Q>E		DBVPG1373:Q>E		DBVPG6040:Q>E		DBVPG6044:Q>E		DBVPG6765:Q>E		L_1374:Q>E		SK1:Q>E		UWOPS03_461_4:Q>E		UWOPS05_227_2:Q>E		UWOPS87_2421:Q>E		Y55:Q>E		YIIc17_E5:Q>E		YJM975:Q>E		YPS128:Q>E		YPS606:Q>E		YS2:Q>E		YS4:Q>E		YS9:Q>E	AA:532		DBVPG1106:S>F	AA:533		DBVPG6040:P>S		K11:P>S		YPS128:P>S		YPS606:P>S		YS2:P>S		YS4:P>S	AA:549		DBVPG6040:D>N	AA:579		YJM975:N>D	AA:584		YJM975:S>R	AA:591		YJM975:S>R	AA:596		UWOPS03_461_4:H>N		UWOPS05_227_2:H>N		UWOPS87_2421:H>N		YS4:H>N	AA:601		DBVPG6044:E>K		SK1:E>K		Y55:E>K	AA:634		YS4:V>I	AA:654		UWOPS05_217_3:G>E		UWOPS05_227_2:G>E		UWOPS87_2421:G>E		YS4:G>E	AA:671		DBVPG6044:E>K		SK1:E>K		Y55:E>K	AA:672		YS4:I>N	AA:675		UWOPS05_217_3:R>K		UWOPS05_227_2:R>K		UWOPS87_2421:R>K	AA:694		YS4:S>L	AA:713		YPS606:S>L	AA:752		UWOPS03_461_4:T>A		UWOPS05_217_3:T>A		UWOPS05_227_2:T>A		UWOPS87_2421:T>A	AA:760		DBVPG6040:A>P		YPS128:A>P		YPS606:A>P	AA:791		DBVPG6040:I>V		YPS128:I>V		YPS606:I>V	AA:814		DBVPG6044:G>V		SK1:G>V		Y55:G>V	AA:840		YS4:E>-	AA:875		DBVPG1853:K>N	AA:883		DBVPG6040:N>ID:YBR034C	AA:3		NCYC110:K>E	AA:15		NCYC110:K>-	AA:65		NCYC110:V>G	AA:96		Y9:A>V	AA:291		DBVPG6044:D>G		NCYC110:D>G		SK1:D>G		Y55:D>G		YS4:F>IID:YBR035C	AA:67		YS9:S>R	AA:170		378604X:N>D	AA:192		W303:V>FID:YBR037C	AA:43		YS9:N>K	AA:124		DBVPG6040:M>I	AA:161		322134S:L>V		DBVPG1853:L>V		DBVPG6040:L>V		DBVPG6044:L>V		K11:L>V		NCYC110:L>V		SK1:L>V		UWOPS83_787_3:L>V		Y12:L>V		Y55:L>V		YPS606:L>V	AA:169		DBVPG1853:K>N	AA:211		Y9:E>-ID:YBR039W	AA:247		YS9:L>V	AA:291		YJM975:T>ID:YBR040W	AA:89		DBVPG6044:I>V		NCYC110:I>V		SK1:I>V		Y55:I>V	AA:170		UWOPS05_227_2:L>Q	AA:192		Y12:V>D	AA:216		UWOPS83_787_3:V>LID:YBR041W	AA:39		DBVPG6040:N>D		DBVPG6044:N>D		K11:N>D		SK1:N>D		UWOPS05_227_2:N>D		Y55:N>D		YPS606:N>D	AA:221		DBVPG6040:D>Y	AA:384		YPS128:K>R		YPS606:K>R	AA:552		K11:Y>-	AA:560		K11:I>T	AA:587		K11:T>P	AA:590		K11:L>-	AA:594		K11:L>SID:YBR043C	AA:17		DBVPG6044:C>Y		NCYC110:C>Y		SK1:C>Y		Y55:C>Y	AA:20		273614X:N>D	AA:43		UWOPS87_2421:N>H	AA:75		YS2:Y>-	AA:88		YIIc17_E5:Q>P	AA:258		322134S:R>H	AA:262		YS9:R>K	AA:269		YS9:V>G	AA:302		UWOPS03_461_4:E>D	AA:334		YJM978:A>V	AA:375		SK1:R>W		Y55:R>W	AA:446		322134S:Q>E		378604X:Q>E		DBVPG1853:Q>E		DBVPG6044:Q>E		DBVPG6765:Q>E		L_1528:Q>E		NCYC110:Q>E		SK1:Q>E		UWOPS03_461_4:Q>E		UWOPS05_227_2:Q>E		Y12:Q>E		Y55:Q>E		Y9:Q>E		YJM975:Q>E		YPS128:Q>E		YS2:Q>E	AA:662		S288c:S>IID:YBR045C	AA:57		DBVPG6765:D>G	AA:71		UWOPS83_787_3:E>D		YPS128:E>D		YPS606:E>D	AA:131		YPS128:S>F		YPS606:S>F	AA:147		322134S:D>N		DBVPG1106:D>N		DBVPG1788:D>N		DBVPG1853:D>N		DBVPG6044:D>N		DBVPG6765:D>N		L_1374:D>N		NCYC110:D>N		SK1:D>N		UWOPS87_2421:D>N		Y55:D>N		YIIc17_E5:D>N		YJM975:D>N		YJM978:D>N		YJM981:D>N		YS2:D>N		YS9:D>N	AA:170		322134S:E>D		DBVPG1106:E>D		DBVPG1788:E>D		DBVPG1853:E>D		DBVPG6765:E>D		L_1374:E>D		UWOPS87_2421:E>D		YJM975:E>D		YJM978:E>D		YJM981:E>D		YS2:E>D		YS9:E>D	AA:172		YPS128:A>V		YPS606:A>V	AA:176		YJM975:N>D	AA:194		273614X:Y>H	AA:254		Y9:D>H	AA:287		DBVPG1373:F>L	AA:303		UWOPS05_217_3:A>G		UWOPS87_2421:A>G		YPS128:A>G		YPS606:A>G	AA:319		DBVPG6044:S>L		NCYC110:S>L		SK1:S>L		Y55:S>L	AA:325		273614X:T>I	AA:391		DBVPG6044:D>N		NCYC110:D>N		SK1:D>N		Y55:D>N	AA:430		UWOPS05_227_2:D>N		YPS128:D>N		YPS606:D>N	AA:521		322134S:T>I		378604X:T>I		DBVPG1106:T>I		DBVPG1373:T>I		DBVPG1788:T>I		DBVPG6044:T>I		DBVPG6765:T>I		NCYC110:T>I		SK1:T>I		UWOPS05_227_2:T>I		UWOPS87_2421:T>I		Y55:T>I		YIIc17_E5:T>I		YJM975:T>I		YJM978:T>I	AA:523		322134S:M>T		378604X:M>T		DBVPG1106:M>T		DBVPG1373:M>T		DBVPG1788:M>T		DBVPG6044:M>T		DBVPG6765:M>T		NCYC110:M>T		SK1:M>T		UWOPS05_227_2:M>T		UWOPS87_2421:M>T		Y55:M>T		YIIc17_E5:M>T		YJM975:M>T		YJM978:M>T		YPS128:M>T		YPS606:M>TID:YBR046C	AA:45		L_1528:T>M	AA:76		YS2:V>L	AA:83		DBVPG6765:T>I	AA:87		273614X:V>I		DBVPG1106:V>I		DBVPG1373:V>I		DBVPG1788:V>I		DBVPG1853:V>I		DBVPG6765:V>I		K11:V>I		L_1374:V>I		UWOPS03_461_4:V>I		UWOPS05_217_3:V>I		UWOPS05_227_2:V>I		UWOPS83_787_3:V>I		Y9:V>I		YIIc17_E5:V>I		YJM975:V>I		YPS128:V>I		YPS606:V>I	AA:107		UWOPS87_2421:S>T	AA:206		273614X:L>S		Y12:L>S		Y9:L>S	AA:271		DBVPG6040:P>R	AA:289		DBVPG1106:E>K		DBVPG1373:E>K		DBVPG1788:E>K		DBVPG6765:E>K		L_1374:E>K		L_1528:E>K		YJM978:E>K		YJM981:E>K		YS9:E>K	AA:333		UWOPS05_227_2:P>TID:YBR047W	AA:174		DBVPG1853:K>QID:YBR050C	AA:10		378604X:D>V	AA:24		UWOPS05_217_3:K>I	AA:28		DBVPG6765:A>V	AA:54		BC187:I>M	AA:165		UWOPS05_217_3:R>Q		UWOPS05_227_2:R>Q	AA:179		UWOPS83_787_3:I>T	AA:190		YS2:P>L	AA:197		322134S:T>A		378604X:T>A		BC187:T>A		DBVPG1106:T>A		DBVPG1373:T>A		DBVPG1788:T>A		DBVPG1853:T>A		DBVPG6044:T>A		DBVPG6765:T>A		L_1374:T>A		NCYC110:T>A		SK1:T>A		UWOPS05_227_2:T>A		UWOPS83_787_3:T>A		Y55:T>A		YIIc17_E5:T>A		YS2:T>A	AA:225		322134S:N>S		378604X:N>S		BC187:N>S		DBVPG1106:N>S		DBVPG1373:N>S		DBVPG1788:N>S		DBVPG1853:N>S		DBVPG6044:N>S		DBVPG6765:N>S		L_1374:N>S		NCYC110:N>S		SK1:N>S		UWOPS05_227_2:N>S		UWOPS83_787_3:N>S		Y55:N>S		YIIc17_E5:N>S		YJM978:N>S		YS2:N>S		YS4:N>S	AA:255		UWOPS05_227_2:H>Y	AA:276		UWOPS05_227_2:V>M		YS4:V>M	AA:294		322134S:V>L		378604X:V>L		DBVPG1106:V>L		DBVPG1373:V>L		DBVPG1788:V>L		DBVPG1853:V>L		DBVPG6765:V>L		L_1374:V>L		YIIc17_E5:V>L		YJM978:V>LID:YBR052C	AA:14		L_1528:I>T	AA:44		L_1528:K>RID:YBR053C	AA:3		378604X:S>R		DBVPG1373:S>R		DBVPG1788:S>R		DBVPG1853:S>R		DBVPG6765:S>R		L_1374:S>R	AA:65		YPS128:F>I		YPS606:F>I	AA:73		DBVPG6044:G>A		Y55:G>A	AA:96		DBVPG1373:P>S		DBVPG1788:P>S		DBVPG6765:P>S		L_1528:P>S	AA:139		YS9:L>F	AA:158		DBVPG6044:G>E		NCYC110:G>E		Y55:G>E	AA:183		UWOPS05_227_2:D>N	AA:219		UWOPS03_461_4:N>D	AA:332		L_1374:N>D	AA:338		DBVPG1106:K>R		DBVPG1373:K>R		DBVPG6044:K>R		DBVPG6765:K>R		K11:K>R		L_1374:K>R		L_1528:K>R		NCYC110:K>R		SK1:K>R		UWOPS03_461_4:K>R		UWOPS05_227_2:K>R		Y55:K>R		YIIc17_E5:K>R		YJM978:K>R		YPS606:K>R		YS4:K>R		YS9:K>RID:YBR054W	AA:49		DBVPG6044:I>V		SK1:I>V		Y55:I>V	AA:205		322134S:I>V		378604X:I>V		DBVPG1106:I>V		DBVPG1373:I>V		DBVPG1788:I>V		DBVPG1853:I>V		DBVPG6044:I>V		DBVPG6765:I>V		L_1528:I>V		SK1:I>V		UWOPS03_461_4:I>V		UWOPS05_217_3:I>V		UWOPS83_787_3:I>V		Y55:I>V		YIIc17_E5:I>V		YJM975:I>V		YJM978:I>V		YS4:I>VID:YBR055C	AA:20		YPS128:G>S		YPS606:G>S	AA:37		DBVPG6044:D>G		SK1:D>G		Y55:D>G	AA:61		UWOPS87_2421:D>N	AA:90		UWOPS87_2421:D>N	AA:99		322134S:N>Y	AA:135		UWOPS87_2421:N>S	AA:151		UWOPS03_461_4:S>A		UWOPS05_217_3:S>A	AA:248		DBVPG6044:A>V		SK1:A>V		Y55:A>V	AA:284		378604X:L>I		DBVPG1853:L>I	AA:289		322134S:V>I		378604X:V>I		DBVPG1373:V>I		DBVPG1788:V>I		DBVPG1853:V>I		DBVPG6040:V>I		DBVPG6044:V>I		DBVPG6765:V>I		K11:V>I		L_1528:V>I		SK1:V>I		UWOPS03_461_4:V>I		UWOPS05_217_3:V>I		UWOPS05_227_2:V>I		Y12:V>I		Y55:V>I		Y9:V>I		YJM975:V>I		YJM978:V>I		YJM981:V>I		YPS128:V>I		YS9:V>I	AA:306		DBVPG6044:S>A		SK1:S>A		Y55:S>A	AA:342		UWOPS03_461_4:K>M		UWOPS05_217_3:K>M		UWOPS05_227_2:K>M		Y12:G>C	AA:393		L_1528:K>E	AA:407		322134S:L>R		378604X:L>R		BC187:L>R		DBVPG1788:L>R		DBVPG1853:L>R		DBVPG6765:L>R		YJM978:L>R	AA:449		Y9:E>D	AA:565		322134S:V>L		BC187:V>L		DBVPG1373:V>L		DBVPG6765:V>L		YIIc17_E5:V>L		YJM975:V>L	AA:611		YS4:D>H	AA:715		UWOPS05_227_2:D>N	AA:743		DBVPG6044:K>N		NCYC110:K>N		SK1:K>N		Y55:K>N	AA:751		K11:G>D		Y12:G>D		YS2:G>D		YS9:G>D	AA:786		YS2:K>EID:YBR056W	AA:6		322134S:R>G	AA:27		DBVPG1853:P>	AA:34		322134S:D>E	AA:48		YJM978:G>S	AA:172		UWOPS83_787_3:G>D	AA:192		DBVPG6765:S>L	AA:194		DBVPG1373:S>N	AA:201		YJM975:H>L	AA:224		UWOPS87_2421:G>C	AA:294		DBVPG1853:S>C	AA:309		UWOPS83_787_3:A>S	AA:373		W303:W>CID:YBR057C	AA:22		UWOPS87_2421:A>T	AA:117		YJM975:A>V		YJM978:A>V	AA:134		UWOPS87_2421:T>A	AA:254		DBVPG1106:K>R	AA:261		Y12:A>S		Y9:A>S	AA:262		DBVPG1106:K>M	AA:305		DBVPG1106:V>G	AA:330		DBVPG1106:Q>R	AA:331		DBVPG1853:I>T	AA:366		UWOPS03_461_4:N>DID:YBR060C	AA:38		DBVPG6044:R>G		NCYC110:R>G		SK1:R>G		Y55:R>G	AA:54		322134S:G>D		378604X:G>D		DBVPG1788:G>D		DBVPG6044:G>D		DBVPG6765:G>D		K11:G>D		NCYC110:G>D		SK1:G>D		UWOPS83_787_3:G>D		Y12:G>D		Y55:G>D		Y9:G>D		YJM975:G>D		YPS128:G>D		YPS606:G>D	AA:111		DBVPG6044:D>G		SK1:D>G		Y55:D>G	AA:145		DBVPG1373:V>I		DBVPG1788:V>I		DBVPG6040:V>I		DBVPG6765:V>I		L_1374:V>I		YJM975:V>I		YS2:V>A		YS9:V>I	AA:154		YS2:V>M	AA:155		DBVPG6044:T>I		SK1:T>I		Y55:T>I	AA:618		UWOPS03_461_4:N>KID:YBR061C	AA:71		UWOPS87_2421:S>N	AA:76		SK1:D>E	AA:92		NCYC361:H>Y	AA:144		YS9:I>LID:YBR065C	AA:8		DBVPG1106:P>S		DBVPG1373:P>S		DBVPG1788:P>S		DBVPG6044:P>S		DBVPG6765:P>S		L_1374:P>S		L_1528:P>S		SK1:P>S		UWOPS03_461_4:P>S		UWOPS87_2421:P>S		Y55:P>S		YIIc17_E5:P>S	AA:9		322134S:P>S	AA:50		DBVPG1106:K>R	AA:99		DBVPG6044:V>I		SK1:V>I		Y55:V>I	AA:153		K11:T>N	AA:232		DBVPG1788:W>G	AA:328		DBVPG6044:F>L		SK1:F>L		Y55:F>L	AA:339		YS9:D>A	AA:350		322134S:D>A		DBVPG1373:D>A		DBVPG1788:D>A		DBVPG6044:D>A		DBVPG6765:D>A		L_1528:D>A		NCYC110:D>A		SK1:D>A		UWOPS03_461_4:D>A		UWOPS05_227_2:D>A		Y55:D>A		YIIc17_E5:D>A		YJM975:D>A		YJM978:D>A		YPS128:D>AID:YBR066C	AA:100		YS4:S>P	AA:107		UWOPS03_461_4:N>S		UWOPS05_227_2:N>S	AA:176		YS4:T>A	AA:219		K11:S>NID:YBR067C	AA:129		SK1:K>E		Y55:K>E	AA:162		DBVPG6040:S>FID:YBR068C	AA:14		SK1:E>A		Y55:E>A	AA:30		273614X:N>H	AA:86		DBVPG6040:T>M	AA:125		YS2:G>V	AA:130		DBVPG1373:I>V		DBVPG6765:I>V		L_1374:I>V		L_1528:I>V		SK1:I>V		UWOPS03_461_4:I>V		UWOPS05_217_3:I>V		UWOPS05_227_2:I>V		UWOPS83_787_3:I>V		UWOPS87_2421:I>V		Y55:I>V		YIIc17_E5:I>V		YJM975:I>V		YJM978:I>V		YPS128:I>V		YPS606:I>V		YS4:I>V	AA:139		DBVPG1106:E>V		DBVPG1373:E>V		DBVPG6040:E>V		DBVPG6765:E>V		K11:E>V		L_1374:E>V		SK1:E>V		UWOPS03_461_4:E>V		UWOPS05_217_3:E>V		UWOPS05_227_2:E>V		UWOPS83_787_3:E>V		UWOPS87_2421:E>V		W303:E>V		Y55:E>V		Y9:E>V		YIIc17_E5:E>V		YJM975:E>V		YJM978:E>V		YPS128:E>V		YPS606:E>V		YS4:E>V	AA:146		Y9:A>D	AA:178		YS4:V>L	AA:181		SK1:Y>F		Y55:Y>F	AA:203		DBVPG1106:G>W		DBVPG1373:G>W		DBVPG1788:G>W		K11:G>W		L_1528:G>W		SK1:G>W		UWOPS03_461_4:G>W		UWOPS05_217_3:G>W		UWOPS83_787_3:G>W		UWOPS87_2421:G>W		W303:G>W		Y55:G>W		Y9:G>W		YGPM:G>W		YIIc17_E5:G>W		YPS128:G>W		YPS606:G>W		YS2:G>W		YS4:G>W	AA:222		SK1:F>S		Y55:F>S	AA:341		UWOPS05_217_3:I>L	AA:342		YS4:L>F	AA:385		322134S:A>S	AA:540		DBVPG6044:N>S		NCYC110:N>S		SK1:N>S		UWOPS03_461_4:N>S		UWOPS83_787_3:N>S		UWOPS87_2421:N>S		Y55:N>SID:YBR069C	AA:16		L_1528:Q>P	AA:60		UWOPS83_787_3:Q>P		UWOPS87_2421:Q>P	AA:65		L_1528:E>K	AA:72		DBVPG1373:R>G		DBVPG6765:R>G	AA:87		L_1528:K>R	AA:205		DBVPG1106:V>A		DBVPG1373:V>A		DBVPG1853:V>A		DBVPG6765:V>A		L_1374:V>A		YS9:V>A	AA:263		YPS128:R>K		YPS606:R>K	AA:271		378604X:H>R		DBVPG1106:H>R		DBVPG6765:H>R		L_1374:H>R		YIIc17_E5:H>R		YJM978:H>R		YPS128:H>R		YPS606:H>R		YS9:H>R	AA:472		378604X:R>K	AA:501		UWOPS83_787_3:A>S	AA:578		SK1:L>V		Y55:L>V	AA:606		NCYC110:R>L	AA:617		378604X:F>LID:YBR070C	AA:49		UWOPS83_787_3:K>E		UWOPS87_2421:K>E	AA:102		DBVPG6044:H>Q		NCYC110:H>Q		SK1:H>Q		UWOPS03_461_4:H>Q		UWOPS05_227_2:H>Q		Y55:H>Q	AA:113		378604X:K>E		DBVPG1373:K>E		DBVPG6040:K>E		DBVPG6044:K>E		DBVPG6765:K>E		K11:K>E		L_1374:K>E		SK1:K>E		UWOPS03_461_4:K>E		UWOPS05_227_2:K>E		UWOPS83_787_3:K>E		UWOPS87_2421:K>E		Y55:K>E		YIIc17_E5:K>E		YJM978:K>E		YPS128:K>E		YS4:K>E	AA:116		DBVPG6044:E>Q		SK1:E>Q		Y55:E>Q	AA:172		DBVPG1373:F>L		DBVPG1788:F>L		DBVPG1853:F>L		DBVPG6765:F>L		L_1374:F>L		UWOPS87_2421:F>L		YIIc17_E5:F>L		YJM978:F>L		YPS128:F>L		YPS606:F>L		YS4:F>L	AA:173		UWOPS87_2421:W>R	AA:235		SK1:I>V		Y55:I>VID:YBR071W	AA:8		UWOPS05_227_2:S>P	AA:56		L_1374:Q>P	AA:146		UWOPS87_2421:Y>F	AA:158		DBVPG6040:F>L	AA:161		Y9:N>	AA:178		NCYC110:N>	AA:181		YPS128:S>YID:YBR072W	AA:4		DBVPG6040:N>Y	AA:39		L_1374:L>I	AA:93		DBVPG6040:V>A	AA:148		378604X:V>F		DBVPG1106:V>F		DBVPG1373:V>F		DBVPG1853:V>F		DBVPG6765:V>F		L_1528:V>F		YJM975:V>F	AA:180		Y12:A>E	AA:181		273614X:N>S		378604X:N>S		DBVPG1106:N>S		DBVPG1373:N>S		DBVPG1788:N>S		DBVPG1853:N>S		DBVPG6040:N>S		DBVPG6044:N>S		DBVPG6765:N>S		L_1528:N>S		SK1:N>S		UWOPS05_217_3:N>S		UWOPS87_2421:N>S		Y55:N>S		YJM975:N>S		YPS606:N>S		YS9:N>SID:YBR073W	AA:44		378604X:V>A		DBVPG6044:V>A		SK1:V>A		UWOPS05_217_3:V>A		UWOPS05_227_2:V>A		UWOPS83_787_3:V>A		UWOPS87_2421:V>A		YS4:V>A	AA:66		378604X:S>T		DBVPG6044:S>T		SK1:S>T		UWOPS05_217_3:S>T		UWOPS05_227_2:S>T		UWOPS83_787_3:S>T		UWOPS87_2421:S>T		YS4:S>T	AA:100		378604X:S>N		YS4:S>N	AA:114		DBVPG1788:L>F	AA:165		DBVPG1106:S>N		DBVPG1373:S>N		DBVPG1788:S>N		DBVPG1853:S>N		DBVPG6765:S>N		L_1528:S>N		YIIc17_E5:S>N		YJM975:S>N		YJM978:S>N	AA:264		Y55:P>H	AA:314		UWOPS83_787_3:M>I	AA:325		UWOPS83_787_3:L>S	AA:335		UWOPS83_787_3:F>L	AA:418		DBVPG1106:L>	AA:422		DBVPG6765:S>Y	AA:633		DBVPG1106:K>R		DBVPG1853:K>R		DBVPG6765:K>R		L_1528:K>R	AA:701		UWOPS03_461_4:G>A		UWOPS05_227_2:G>A		UWOPS83_787_3:G>A	AA:748		UWOPS83_787_3:P>A	AA:807		W303:H>D	AA:836		UWOPS03_461_4:Q>H		UWOPS05_227_2:Q>H		UWOPS83_787_3:Q>H	AA:846		SK1:S>R		Y55:S>R	AA:904		BC187:E>D		DBVPG1106:E>D		DBVPG1373:E>D		DBVPG1788:E>D		DBVPG1853:E>D		DBVPG6765:E>D		L_1374:E>D		L_1528:E>D		UWOPS05_217_3:E>D		UWOPS05_227_2:E>D		UWOPS83_787_3:E>D		Y55:E>D		YJM978:E>D		YPS128:E>D		YPS606:E>DID:YBR077C	AA:20		UWOPS05_217_3:Y>H	AA:38		DBVPG1373:I>L		DBVPG1788:I>L		DBVPG6765:I>L		YJM975:I>L	AA:86		378604X:D>Y	AA:91		DBVPG6044:H>L		NCYC361:H>L		SK1:H>L		UWOPS05_227_2:H>L		UWOPS87_2421:H>LID:YBR082C	AA:130		YS9:T>IID:YBR084W	AA:165		322134S:A>T		YS4:A>T	AA:219		YIIc17_E5:A>T	AA:233		SK1:E>K	AA:311		L_1374:M>I	AA:332		YS4:V>A	AA:335		YS4:P>A	AA:392		UWOPS03_461_4:E>G		UWOPS05_217_3:E>G	AA:431		K11:K>N	AA:516		UWOPS03_461_4:R>G		UWOPS05_217_3:R>G	AA:520		UWOPS03_461_4:P>S		UWOPS05_217_3:P>S	AA:530		273614X:D>G		322134S:D>G		BC187:D>G		DBVPG1853:D>G		DBVPG6044:D>G		DBVPG6765:D>G		L_1374:D>G		NCYC110:D>G		UWOPS03_461_4:D>G		UWOPS05_217_3:D>G		Y12:D>G		YIIc17_E5:D>G		YJM975:D>G		YJM978:D>G		YJM981:D>G		YPS128:D>G		YPS606:D>G		YS9:D>G	AA:574		Y9:T>	AA:603		DBVPG6040:A>T	AA:605		YIIc17_E5:S>C	AA:613		322134S:E>K	AA:627		NCYC361:K>E	AA:645		UWOPS03_461_4:R>H		UWOPS05_217_3:R>H	AA:699		273614X:N>Y		DBVPG1853:N>Y		DBVPG6040:N>Y		DBVPG6765:N>Y		L_1374:N>Y		L_1528:N>Y		NCYC361:N>Y		UWOPS83_787_3:N>Y		Y55:N>Y		YJM975:N>Y		YJM978:N>Y		YJM981:N>Y		YS9:N>Y	AA:882		UWOPS03_461_4:V>L	AA:889		UWOPS83_787_3:K>TID:YBR085C-A	AA:9		378604X:S>N		DBVPG1373:S>N		DBVPG1788:S>N		DBVPG6040:S>N		DBVPG6044:S>N		DBVPG6765:S>N		L_1374:S>N		SK1:S>N		UWOPS05_227_2:S>N		UWOPS83_787_3:S>N		UWOPS87_2421:S>N		Y12:S>N		Y55:S>N		YIIc17_E5:S>N		YJM975:S>N		YJM978:S>N		YPS128:S>N		YPS606:S>N		YS4:S>NID:YBR085W	AA:116		273614X:F>L		BC187:F>L		DBVPG1106:F>L		DBVPG1373:F>L		DBVPG1788:F>L		DBVPG1853:F>L		DBVPG6765:F>L		L_1528:F>L		Y55:F>L		YJM978:F>L		YJM981:F>L		YS4:F>L	AA:232		UWOPS03_461_4:T>S		UWOPS05_217_3:T>S		UWOPS05_227_2:T>SID:YBR087W	AA:30		YS4:S>A	AA:55		S288c:A>E	AA:268		DBVPG1106:R>M		DBVPG1788:R>MID:YBR089C-A	AA:10		YIIc17_E5:P>S	AA:64		322134S:R>K		378604X:R>K		DBVPG6044:R>K		SK1:R>K		Y12:R>K		Y55:R>K		Y9:R>K		YPS606:R>K	AA:98		UWOPS05_227_2:R>LID:YBR092C	AA:3		DBVPG6765:K>E	AA:212		UWOPS05_217_3:G>V	AA:281		YS2:E>K	AA:325		378604X:E>V	AA:333		YS4:L>F	AA:343		322134S:N>Y	AA:423		322134S:D>YID:YBR093C	AA:36		DBVPG1373:D>Y		DBVPG1788:D>Y		DBVPG6765:D>Y		L_1528:D>Y		YJM975:D>Y		YJM978:D>Y		YS4:D>Y	AA:43		YJM978:G>C	AA:48		DBVPG1788:Y>F		UWOPS83_787_3:Y>F	AA:104		YJM978:G>S	AA:133		YS4:D>V	AA:160		UWOPS83_787_3:V>F		YS2:V>F	AA:187		378604X:L>F	AA:194		YS2:T>S	AA:225		UWOPS05_227_2:D>G	AA:311		322134S:S>A		NCYC361:S>A	AA:466		DBVPG6044:R>I		NCYC110:R>I		SK1:R>I		Y55:R>IID:YBR094W	AA:59		Y9:T>	AA:127		273614X:P>Q		BC187:P>Q		DBVPG1106:P>Q		DBVPG1373:P>Q		DBVPG1788:P>Q		DBVPG1853:P>Q		DBVPG6040:P>Q		DBVPG6765:P>Q		L_1374:P>Q		L_1528:P>Q		YJM981:P>Q		YS4:P>Q	AA:137		YS9:V>I	AA:255		W303:E>K	AA:297		DBVPG6040:E>A	AA:393		YS4:D>G	AA:648		YS9:I>F	AA:652		YS9:T>A	AA:682		YS9:L>S	AA:703		YS9:T>I	AA:732		YS9:N>IID:YBR095C	AA:17		DBVPG6044:S>G		NCYC361:S>G		SK1:S>G		UWOPS83_787_3:S>G		Y55:S>G		YPS128:S>G		YPS606:S>G	AA:21		BC187:N>S		DBVPG1788:N>S		DBVPG6765:N>S		L_1528:N>S		YS4:N>S	AA:47		UWOPS87_2421:S>F	AA:97		322134S:E>G	AA:103		DBVPG1788:T>R		DBVPG6765:T>R		L_1374:T>R		L_1528:T>R		UWOPS87_2421:T>R		YIIc17_E5:T>R		YS9:T>R	AA:111		K11:R>G	AA:140		YJM975:D>E		YS2:D>E	AA:155		DBVPG1373:D>N		DBVPG6044:D>N		DBVPG6765:D>N		L_1374:D>N		L_1528:D>N		NCYC361:D>N		SK1:D>N		UWOPS03_461_4:D>N		UWOPS83_787_3:D>N		UWOPS87_2421:D>N		Y55:D>N		YIIc17_E5:D>N		YJM975:D>N		YJM978:D>N		YPS128:D>N		YPS606:D>N		YS2:D>N		YS4:D>N		YS9:D>N	AA:248		UWOPS05_227_2:A>SID:YBR096W	AA:155		DBVPG6765:F>LID:YBR097W	AA:32		273614X:Q>	AA:47		UWOPS87_2421:P>H	AA:134		273614X:T>A		DBVPG1373:T>A		DBVPG1788:T>A		DBVPG1853:T>A		DBVPG6044:T>A		DBVPG6765:T>A		L_1374:T>A		L_1528:T>A		NCYC110:T>A		SK1:T>A		UWOPS03_461_4:T>A		UWOPS05_227_2:T>A		W303:T>A		Y12:T>A		Y55:T>A		YJM975:T>A		YJM978:T>A		YPS128:T>A		YS2:T>A		YS4:T>A		YS9:T>A	AA:164		273614X:T>S		DBVPG1373:T>S		DBVPG1788:T>S		DBVPG1853:T>S		DBVPG6765:T>S		L_1374:T>S		L_1528:T>S		UWOPS87_2421:T>S		YJM975:T>S		YS2:T>S		YS4:T>S		YS9:T>S	AA:308		UWOPS05_227_2:I>V	AA:349		DBVPG6044:Y>H		NCYC110:Y>H		SK1:Y>H		Y55:Y>H	AA:357		UWOPS05_227_2:H>R	AA:371		273614X:G>S		DBVPG1373:G>S		DBVPG1788:G>S		DBVPG1853:G>S		DBVPG6765:G>S		L_1374:G>S		L_1528:G>S		YJM978:G>S	AA:384		YPS128:I>L	AA:414		273614X:I>V		BC187:I>V		DBVPG1373:I>V		DBVPG1788:I>V		DBVPG1853:I>V		DBVPG6044:I>V		DBVPG6765:I>V		L_1374:I>V		L_1528:I>V		SK1:I>V		UWOPS83_787_3:I>V		YIIc17_E5:I>V		YJM975:I>V		YJM978:I>V		YPS128:I>V	AA:479		DBVPG1373:D>G		YJM978:D>G		YS4:D>G	AA:499		UWOPS05_227_2:N>S	AA:525		DBVPG1373:T>A		DBVPG6765:T>A		L_1374:T>A		L_1528:T>A		YIIc17_E5:T>A		YJM975:T>A		YJM978:T>A		YS4:T>A	AA:569		DBVPG6040:S>N		Y12:S>N		Y9:S>N	AA:657		322134S:Q>E		DBVPG1373:Q>E		DBVPG1788:Q>E		DBVPG6765:Q>E		UWOPS87_2421:Q>E		YIIc17_E5:Q>E		YJM975:Q>E		YS4:Q>E	AA:754		UWOPS83_787_3:F>L	AA:764		UWOPS03_461_4:P>S		UWOPS05_227_2:P>S	AA:775		322134S:S>N	AA:811		DBVPG6044:S>N		NCYC110:S>N		SK1:S>N		Y55:S>N	AA:851		378604X:I>R		BC187:I>R		DBVPG1106:I>R		DBVPG1373:I>R		DBVPG6044:I>R		DBVPG6765:I>R		NCYC110:I>R		S288c:I>R		SK1:I>R		UWOPS03_461_4:I>R		UWOPS05_227_2:I>R		UWOPS83_787_3:I>R		W303:I>R		Y12:I>R		Y55:I>R		YGPM:I>R		YIIc17_E5:I>R		YJM975:I>R		YPS606:I>R		YS4:I>R	AA:889		YIIc17_E5:D>N	AA:1032		L_1528:S>R	AA:1131		K11:V>I	AA:1229		DBVPG6044:P>R		SK1:P>R		Y55:P>R	AA:1275		DBVPG6044:A>T		SK1:A>T		Y55:A>T	AA:1348		YPS128:A>S		YPS606:A>S	AA:1377		UWOPS87_2421:L>I	AA:1385		UWOPS03_461_4:S>N		UWOPS05_227_2:S>N	AA:1415		YJM978:H>YID:YBR098W	AA:18		YJM978:S>N	AA:25		Y9:P>A	AA:36		Y9:S>-	AA:41		UWOPS05_217_3:E>K	AA:47		YS4:V>A	AA:70		273614X:D>N		322134S:D>N		BC187:D>N		DBVPG1373:D>N		DBVPG1788:D>N		DBVPG6765:D>N		L_1528:D>N		YIIc17_E5:D>N		YS4:D>N	AA:138		273614X:H>R		DBVPG1373:H>R		DBVPG1788:H>R		DBVPG1853:H>R		DBVPG6765:H>R		L_1528:H>R		YJM975:H>R		YS4:H>R	AA:157		DBVPG1373:L>F		DBVPG6765:L>F	AA:173		273614X:G>R		322134S:G>R		BC187:G>R		DBVPG1373:G>R		DBVPG1788:G>R		DBVPG1853:G>R		DBVPG6765:G>R		L_1528:G>R		SK1:G>R		UWOPS05_217_3:G>R		UWOPS05_227_2:G>R		Y55:G>R		Y9:G>R		YJM975:G>R		YPS128:G>R		YS4:G>R	AA:176		273614X:K>E		BC187:K>E		DBVPG1373:K>E		DBVPG1788:K>E		DBVPG1853:K>E		DBVPG6765:K>E		L_1528:K>E		SK1:K>E		UWOPS05_217_3:K>E		UWOPS05_227_2:K>E		Y55:K>E		YJM975:K>E		YJM978:K>E		YS4:K>E	AA:182		DBVPG6044:S>T		SK1:S>T		UWOPS05_217_3:S>T		Y55:S>T	AA:205		DBVPG1853:H>N		YS4:H>N	AA:208		DBVPG6044:P>S		NCYC110:P>S		Y55:P>S	AA:223		DBVPG6044:P>A		NCYC110:P>A		SK1:P>A		Y55:P>A	AA:240		BC187:T>K		DBVPG1373:T>K		DBVPG1788:T>K		DBVPG1853:T>K		DBVPG6044:T>K		DBVPG6765:T>K		L_1528:T>K		NCYC110:T>K		UWOPS03_461_4:T>K		UWOPS05_217_3:T>K		UWOPS05_227_2:T>K		Y55:T>K		YIIc17_E5:T>K		YJM975:T>K		YJM978:T>K		YS4:T>K	AA:265		BC187:I>M		DBVPG1373:I>M		DBVPG1788:I>M		DBVPG1853:I>M		DBVPG6765:I>M		L_1528:I>M		YIIc17_E5:I>M		YJM975:I>M		YJM978:I>M		YS4:I>M	AA:375		UWOPS03_461_4:K>R		UWOPS05_227_2:K>R	AA:420		DBVPG6044:Q>L		NCYC110:Q>L		SK1:Q>L	AA:556		Y9:F>SID:YBR099C	AA:43		Y9:N>D	AA:48		DBVPG6040:N>SID:YBR101C	AA:6		UWOPS05_217_3:Q>K	AA:119		DBVPG1373:L>F		SK1:L>F		Y55:L>F	AA:201		K11:T>I	AA:250		UWOPS03_461_4:I>V		UWOPS05_217_3:I>V		UWOPS05_227_2:I>V	AA:261		DBVPG1373:L>SID:YBR104W	AA:134		273614X:L>I	AA:187		NCYC110:D>ID:YBR105C	AA:36		UWOPS05_217_3:V>I		UWOPS05_227_2:V>I	AA:53		SK1:F>L		Y55:F>L	AA:86		YS9:P>L	AA:124		322134S:R>K		DBVPG1788:R>K		DBVPG6765:R>K		UWOPS05_217_3:R>K		UWOPS05_227_2:R>K	AA:144		DBVPG6765:V>I	AA:164		DBVPG6040:S>AID:YBR106W	AA:91		DBVPG6040:E>-	AA:125		NCYC361:S>YID:YBR107C	AA:15		273614X:S>P		BC187:S>P		DBVPG6765:S>P		L_1374:S>P		NCYC110:S>P		SK1:S>P		Y55:S>P		YJM981:S>P		YS4:S>P	AA:22		273614X:K>Q		BC187:K>Q		DBVPG6765:K>Q		L_1374:K>Q		L_1528:K>Q		NCYC110:K>Q		SK1:K>Q		UWOPS83_787_3:K>Q		Y55:K>Q		YJM981:K>Q		YS4:K>Q	AA:44		DBVPG6044:P>A		NCYC110:P>A		SK1:P>A		UWOPS83_787_3:P>A		Y55:P>A	AA:54		273614X:I>F		BC187:I>F		DBVPG6765:I>F		L_1374:I>F		L_1528:I>F		YJM981:I>F		YS4:I>F	AA:55		UWOPS83_787_3:R>L	AA:137		273614X:I>V		BC187:I>V		DBVPG1373:I>V		DBVPG1853:I>V		DBVPG6044:I>V		DBVPG6765:I>V		L_1374:I>V		L_1528:I>V		SK1:I>V		UWOPS03_461_4:I>V		UWOPS05_217_3:I>V		UWOPS05_227_2:I>V		UWOPS83_787_3:I>V		UWOPS87_2421:I>V		Y55:I>V		YJM975:I>V		YJM981:I>V		YPS128:I>V		YS4:I>V	AA:230		YS9:N>KID:YBR109C	AA:68		UWOPS05_217_3:E>DID:YBR110W	AA:3		YS9:L>-	AA:34		K11:N>	AA:117		UWOPS83_787_3:V>F	AA:159		YS9:I>T	AA:222		DBVPG6044:A>V		SK1:A>V		Y55:A>V	AA:253		BC187:N>D		DBVPG1106:N>D		DBVPG1373:N>D		DBVPG1853:N>D		DBVPG6765:N>D		UWOPS87_2421:N>D		YJM981:N>D	AA:264		DBVPG1373:G>A	AA:288		DBVPG6765:K>E	AA:349		UWOPS87_2421:C>	AA:448		322134S:I>VID:YBR111C	AA:8		273614X:V>L		322134S:V>L		BC187:V>L		DBVPG1853:V>L		DBVPG6044:V>L		DBVPG6765:V>L		L_1374:V>L		L_1528:V>L		SK1:V>L		UWOPS03_461_4:V>L		UWOPS05_217_3:V>L		UWOPS05_227_2:V>L		Y55:V>L		YJM981:V>L		YPS128:V>L		YPS606:V>L		YS4:V>L		YS9:V>L	AA:35		322134S:A>T		BC187:A>T		DBVPG1853:A>T		DBVPG6765:A>T		L_1374:A>T		L_1528:A>T		YJM981:A>T		YS4:A>T		YS9:A>T	AA:47		DBVPG6040:I>F	AA:121		YJM978:G>A	AA:123		SK1:D>N		Y55:D>NID:YBR115C	AA:6		K11:V>A	AA:87		DBVPG1106:F>V		DBVPG1373:F>V		L_1528:F>V		YJM975:F>V		YJM981:F>V	AA:108		322134S:K>E		DBVPG1106:K>Q		DBVPG1373:K>Q		DBVPG6044:K>Q		DBVPG6765:K>Q		K11:K>Q		L_1528:K>Q		SK1:K>Q		UWOPS03_461_4:K>E		UWOPS05_217_3:K>E		UWOPS83_787_3:K>E		UWOPS87_2421:K>E		Y55:K>Q		YJM975:K>Q		YJM978:K>Q		YJM981:K>Q		YPS606:K>E		YS4:K>Q	AA:305		YJM978:V>M	AA:397		SK1:P>S		Y55:P>S	AA:399		378604X:D>V		DBVPG1106:D>V		DBVPG1853:D>V		DBVPG6765:D>V		UWOPS05_217_3:D>V		UWOPS05_227_2:D>V		Y9:D>V		YIIc17_E5:D>V		YS2:D>V	AA:504		DBVPG1106:G>C	AA:594		Y12:G>C	AA:604		DBVPG6044:V>I		SK1:V>I		Y55:V>I	AA:850		NCYC110:R>L		SK1:R>L		Y55:R>L	AA:1184		DBVPG1373:A>T		DBVPG1788:A>T		DBVPG6765:A>T		YJM975:A>T		YS4:A>T		YS9:A>T	AA:1254		UWOPS83_787_3:D>N	AA:1271		YIIc17_E5:Y>F	AA:1280		YIIc17_E5:A>PID:YBR119W	AA:2		YJM978:S>P	AA:37		273614X:P>L		BC187:P>L		DBVPG1788:P>L		DBVPG6765:P>L		L_1374:P>L		L_1528:P>L		YJM978:P>L	AA:43		W303:H>	AA:49		UWOPS83_787_3:S>L	AA:61		K11:G>R	AA:67		273614X:I>V		BC187:I>V		DBVPG1788:I>V		DBVPG6765:I>V		L_1374:I>V		L_1528:I>V		YJM978:I>V	AA:109		273614X:G>E		378604X:G>E		BC187:G>E		DBVPG1788:G>E		DBVPG1853:G>E		DBVPG6044:G>E		DBVPG6765:G>E		K11:G>E		L_1374:G>E		L_1528:G>E		SK1:G>E		UWOPS03_461_4:G>E		Y12:G>E		Y55:G>E		Y9:G>E		YJM981:G>E		YPS128:G>E		YS4:G>E	AA:113		Y12:T>I		Y9:T>I	AA:131		DBVPG1853:E>K	AA:156		YIIc17_E5:C>Y	AA:271		273614X:V>I		BC187:V>I		DBVPG1853:V>I		DBVPG6765:V>I		YIIc17_E5:V>IID:YBR120C	AA:35		Y9:V>L	AA:43		Y9:V>L	AA:143		DBVPG1788:V>IID:YBR121C	AA:151		378604X:K>I	AA:161		378604X:D>G	AA:329		YS9:R>T	AA:419		UWOPS05_217_3:P>S		UWOPS05_227_2:P>S	AA:442		UWOPS05_217_3:A>G		UWOPS05_227_2:A>G	AA:476		Y55:G>D	AA:496		W303:V>F	AA:631		273614X:N>D		378604X:N>D		DBVPG1106:N>D		DBVPG1373:N>D		DBVPG6765:N>D		K11:N>D		L_1374:N>D		UWOPS03_461_4:N>D		UWOPS05_217_3:N>D		UWOPS87_2421:N>D		Y12:N>D		Y9:N>D		YPS128:N>D		YPS606:N>D		YS2:N>D	AA:652		UWOPS03_461_4:D>N		UWOPS05_217_3:D>NID:YBR122C	AA:94		YS9:S>F	AA:132		SK1:K>E		Y55:K>E		Y9:K>E		YPS606:K>E		YS4:K>E	AA:161		YS9:E>DID:YBR123C	AA:22		DBVPG6044:P>S		NCYC110:P>S		Y55:P>S	AA:96		BC187:S>R		DBVPG1106:S>R		DBVPG1788:S>R		DBVPG6765:S>R		YJM975:S>R		YS9:S>R	AA:113		K11:E>D	AA:220		DBVPG1106:I>T		DBVPG1788:I>T		YIIc17_E5:I>T		YJM975:I>T	AA:277		SK1:D>N		Y55:D>N	AA:310		DBVPG1373:K>E		DBVPG1853:K>E		DBVPG6765:K>E		L_1374:K>E		YIIc17_E5:K>E		YJM975:K>E	AA:327		UWOPS03_461_4:A>T		UWOPS05_217_3:A>T		UWOPS05_227_2:A>T	AA:373		K11:G>C		UWOPS03_461_4:G>S		UWOPS05_217_3:G>S	AA:385		DBVPG1373:Y>F	AA:413		UWOPS03_461_4:V>A		UWOPS05_217_3:V>A		UWOPS05_227_2:V>A	AA:421		UWOPS87_2421:G>S	AA:469		W303:W>C	AA:478		DBVPG6044:I>M		SK1:I>M		Y55:I>M	AA:529		273614X:T>A		DBVPG1373:T>A		DBVPG1788:T>A		DBVPG1853:T>A		DBVPG6765:T>A		L_1528:T>A		YJM981:T>A		YS9:T>A	AA:545		DBVPG6040:M>I	AA:551		378604X:E>-	AA:563		DBVPG6044:D>E		SK1:D>E		Y55:D>E	AA:566		322134S:G>D	AA:647		YPS128:V>FID:YBR125C	AA:98		DBVPG1788:R>L		DBVPG6765:R>L		L_1374:R>L	AA:103		UWOPS87_2421:G>V		YPS606:V>I	AA:206		Y9:K>I	AA:321		K11:L>IID:YBR126C	AA:54		UWOPS03_461_4:L>S	AA:93		UWOPS03_461_4:L>M	AA:487		UWOPS05_217_3:S>PID:YBR128C	AA:32		UWOPS03_461_4:D>A	AA:65		DBVPG6765:M>I	AA:87		UWOPS03_461_4:N>I		UWOPS05_217_3:N>I		UWOPS05_227_2:N>I	AA:92		UWOPS03_461_4:R>K		UWOPS05_217_3:R>K		UWOPS05_227_2:R>K		Y9:R>K		YIIc17_E5:R>K		YS4:R>K	AA:121		DBVPG6040:C>R		DBVPG6044:C>R		SK1:C>R		UWOPS03_461_4:C>R		UWOPS05_217_3:C>R		UWOPS05_227_2:C>R		Y12:C>R		Y55:C>R		Y9:C>R		YIIc17_E5:C>R		YPS128:C>R	AA:165		378604X:W>C	AA:194		DBVPG6040:I>V		DBVPG6044:I>V		SK1:I>V		UWOPS03_461_4:I>V		UWOPS05_217_3:I>V		UWOPS05_227_2:I>V		Y55:I>V		YIIc17_E5:I>V		YPS128:I>V	AA:246		W303:R>K	AA:336		YS2:P>SID:YBR129C	AA:41		DBVPG6044:N>S		SK1:N>S		Y55:N>S	AA:47		UWOPS03_461_4:A>T		UWOPS05_217_3:A>T	AA:122		DBVPG6040:I>T	AA:124		DBVPG6040:K>R	AA:135		UWOPS05_227_2:M>I	AA:151		UWOPS05_227_2:S>I	AA:221		Y9:I>T	AA:230		Y9:K>T	AA:235		Y9:A>T	AA:238		Y9:Q>H	AA:264		K11:K>T		Y9:K>T		YIIc17_E5:K>T	AA:268		SK1:I>F		Y55:I>F	AA:275		UWOPS05_217_3:D>N		UWOPS05_227_2:D>N		UWOPS83_787_3:D>NID:YBR130C	AA:69		YS2:N>S	AA:140		S288c:S>F	AA:157		273614X:N>K	AA:178		UWOPS05_217_3:K>E	AA:206		UWOPS05_217_3:D>N		UWOPS05_227_2:D>N	AA:304		DBVPG6044:A>T		SK1:A>T		Y55:A>T	AA:388		K11:N>D	AA:403		322134S:V>G	AA:415		322134S:G>R		378604X:G>R		DBVPG1106:G>R		DBVPG6040:G>R		DBVPG6765:G>R		K11:G>R		L_1374:G>R		L_1528:G>R		SK1:G>R		UWOPS05_217_3:G>R		UWOPS83_787_3:G>R		Y55:G>R		YJM981:G>R		YPS606:G>R		YS9:G>R	AA:419		YPS606:V>IID:YBR132C	AA:1		322134S:M>L	AA:53		YS4:P>S	AA:81		378604X:F>S		BC187:F>S		DBVPG1788:F>S		DBVPG1853:F>S		DBVPG6044:F>S		DBVPG6765:F>S		L_1374:F>S		NCYC361:F>S		SK1:F>S		UWOPS03_461_4:F>S		Y12:F>S		Y55:F>S		Y9:F>S		YJM975:F>S		YJM978:F>S		YPS606:F>S		YS4:F>S	AA:147		L_1374:F>L	AA:203		DBVPG1853:I>V	AA:256		DBVPG1788:H>Y		YJM975:H>Y	AA:375		DBVPG1788:N>H		DBVPG6765:N>H		YIIc17_E5:N>H		YS9:N>H	AA:547		Y9:G>D	AA:555		322134S:I>V		DBVPG1106:I>V		DBVPG1373:I>V		DBVPG6765:I>V		L_1374:I>V		L_1528:I>V		NCYC361:I>V		YIIc17_E5:I>V		YJM975:I>V		YS9:I>VID:YBR133C	AA:23		322134S:R>C	AA:55		YS9:V>D	AA:296		DBVPG6044:S>P		SK1:S>P		Y55:S>P	AA:313		273614X:L>V	AA:326		DBVPG1788:D>E		DBVPG6044:D>E		DBVPG6765:D>E		L_1528:D>E		SK1:D>E		UWOPS03_461_4:D>E		UWOPS05_217_3:D>E		UWOPS05_227_2:D>E		Y55:D>E		YIIc17_E5:D>E		YJM975:D>E		YPS606:D>E	AA:375		378604X:A>V		Y9:A>V	AA:554		BC187:V>A		DBVPG1106:V>A		DBVPG6765:V>A		L_1374:V>A		SK1:V>A		Y55:V>A		YJM975:V>A		YJM981:V>A		YPS128:V>A		YPS606:V>A		YS4:V>A		YS9:V>A	AA:609		DBVPG6044:K>Q	AA:622		YS4:E>G	AA:666		BC187:D>N		DBVPG1106:D>N		DBVPG1373:D>N		DBVPG6765:D>N		YJM975:D>N		YJM981:D>N	AA:772		UWOPS87_2421:I>V	AA:828		378604X:->CID:YBR136W	AA:83		UWOPS83_787_3:Y>H	AA:85		UWOPS83_787_3:P>R	AA:132		UWOPS03_461_4:K>N		UWOPS05_217_3:K>N		UWOPS05_227_2:K>N	AA:141		UWOPS03_461_4:P>S		UWOPS05_217_3:P>S		UWOPS05_227_2:P>S	AA:166		YPS606:L>S	AA:209		273614X:T>A		322134S:T>A		BC187:T>A		DBVPG1373:T>A		DBVPG1788:T>A		DBVPG6765:T>A		L_1374:T>A		YJM975:T>A		YJM978:T>A		YS4:T>A	AA:228		UWOPS05_217_3:S>T	AA:233		273614X:A>P		322134S:A>P		BC187:A>P		DBVPG6765:A>P		L_1374:A>P		L_1528:A>P		YJM975:A>P		YJM978:A>P		YS4:A>P	AA:318		Y9:H>Q	AA:428		UWOPS05_227_2:A>S	AA:518		322134S:I>V		BC187:I>V		DBVPG1106:I>V		DBVPG1373:I>V		DBVPG1788:I>V		DBVPG1853:I>V		DBVPG6044:I>V		DBVPG6765:I>V		L_1374:I>V		L_1528:I>V		NCYC110:I>V		UWOPS05_217_3:I>V		UWOPS05_227_2:I>V		Y55:I>V		YJM975:I>V		YJM981:I>V	AA:536		DBVPG1106:S>N		DBVPG6765:S>N		L_1374:S>N		YJM975:S>N	AA:667		322134S:L>S		NCYC361:L>S	AA:710		DBVPG6044:I>T		SK1:I>T		Y55:I>T	AA:714		Y9:G>E	AA:741		Y9:L>R	AA:743		Y9:E>A	AA:863		273614X:N>S		BC187:N>S		DBVPG1106:N>S		DBVPG1373:N>S		DBVPG1788:N>S		DBVPG6765:N>S		L_1528:N>S		NCYC361:N>S		YIIc17_E5:N>S		YJM975:N>S	AA:1248		DBVPG1106:N>H	AA:1480		W303:Q>L	AA:1491		W303:D>V		YJM978:D>E	AA:1499		W303:M>K	AA:1503		W303:M>K	AA:1505		W303:D>G	AA:1518		UWOPS03_461_4:F>Y	AA:1524		322134S:K>E		DBVPG1373:K>E		DBVPG1788:K>E		L_1374:K>E		L_1528:K>E		NCYC361:K>E		YJM975:K>E		YJM978:K>E	AA:1542		322134S:A>T	AA:1589		DBVPG6044:R>K		Y55:R>K	AA:1603		YJM978:T>	AA:1678		Y9:M>T	AA:1681		DBVPG6044:Q>H		Y55:Q>H	AA:1821		YJM978:T>N	AA:1861		UWOPS05_227_2:A>	AA:1905		YJM975:L>-	AA:1913		YS4:Q>P	AA:1930		L_1528:Y>-	AA:2080		NCYC110:K>M	AA:2167		378604X:V>A		BC187:V>A		DBVPG1788:V>A		DBVPG1853:V>A		DBVPG6765:V>A		K11:V>A		L_1374:V>A		L_1528:V>A		NCYC361:V>A		UWOPS05_227_2:V>A		UWOPS87_2421:V>A		Y9:V>A		YPS128:V>A		YS4:V>AID:YBR137W	AA:101		W303:S>L	AA:110		322134S:S>G		378604X:S>G		BC187:S>G		DBVPG6044:S>G		DBVPG6765:S>G		NCYC361:S>G		S288c:S>G		SK1:S>G		UWOPS05_217_3:S>G		W303:S>G		Y12:S>G		Y55:S>G		YGPM:S>G		YJM975:S>G		YJM978:S>G		YJM981:S>G		YPS128:S>G		YPS606:S>G	AA:156		YS4:D>ID:YBR138C	AA:7		BC187:Q>H		DBVPG1373:Q>H		DBVPG1853:Q>H		DBVPG6044:Q>H		DBVPG6765:Q>H		L_1374:Q>H		L_1528:Q>H		NCYC361:Q>H		SK1:Q>H		UWOPS83_787_3:Q>H		UWOPS87_2421:Q>H		Y55:Q>H		YJM975:Q>H		YPS128:Q>H		YPS606:Q>H	AA:96		BC187:N>T		DBVPG1373:N>T		DBVPG1788:N>T		DBVPG6765:N>T		L_1374:N>T		L_1528:N>T		NCYC361:N>T	AA:165		DBVPG6044:D>E		NCYC110:D>E		SK1:D>E		Y55:D>E	AA:240		W303:V>L	AA:290		BC187:R>K		DBVPG1373:R>K		DBVPG1788:R>K		DBVPG6044:R>K		DBVPG6765:R>K		L_1374:R>K		L_1528:R>K		NCYC110:R>K		SK1:R>K		Y55:R>K		YJM975:R>K		YS4:R>K	AA:448		BC187:I>T		DBVPG1373:I>T		DBVPG1788:I>T		DBVPG6044:I>T		DBVPG6765:I>T		L_1374:I>T		L_1528:I>T		UWOPS05_217_3:I>T		Y55:I>T		YJM975:I>T		YJM978:I>T		YPS128:I>T		YPS606:I>T		YS9:I>T	AA:502		Y12:W>-		Y9:W>-ID:YBR139W	AA:14		378604X:I>T		Y12:I>T	AA:16		DBVPG1853:I>V	AA:20		NCYC361:S>T	AA:95		K11:S>P	AA:302		DBVPG6044:K>E		NCYC110:K>E		SK1:K>E		Y55:K>E	AA:362		W303:Q>R	AA:364		YPS128:T>A		YPS606:T>A	AA:394		UWOPS83_787_3:Q>	AA:407		273614X:V>E	AA:460		YS4:N>S	AA:497		DBVPG1106:A>G		DBVPG1373:A>G		DBVPG1788:A>G		DBVPG6765:A>G		L_1374:A>G		L_1528:A>G		YS4:A>GID:YBR141C	AA:105		DBVPG1853:I>M	AA:117		Y9:L>F	AA:122		DBVPG6044:T>I		SK1:T>I		Y55:T>I	AA:171		322134S:C>R		DBVPG1106:C>R		DBVPG1373:C>R		DBVPG6765:C>R		YJM975:C>R		YJM978:C>R	AA:175		YPS128:V>M	AA:196		322134S:N>K		DBVPG1106:N>K		DBVPG1373:N>K		DBVPG6765:N>K		YJM975:N>K		YJM978:N>K		YPS128:N>K	AA:220		YPS128:R>K	AA:223		SK1:N>S		Y55:N>S	AA:286		322134S:G>C		DBVPG1106:G>C	AA:312		273614X:S>P		322134S:S>P		378604X:S>P		DBVPG1106:S>P		DBVPG6040:S>P		DBVPG6765:S>P		L_1374:S>P		L_1528:S>P		NCYC361:S>P		S288c:S>P		UWOPS05_227_2:S>P		UWOPS83_787_3:S>P		W303:S>P		Y12:S>P		Y9:S>P		YGPM:S>P		YIIc17_E5:S>P		YJM975:S>P		YJM978:S>P		YPS128:S>P		YPS606:S>PID:YBR145W	AA:7		UWOPS03_461_4:P>L	AA:26		UWOPS03_461_4:D>Y	AA:172		NCYC110:I>M		SK1:I>M		Y55:I>M	AA:233		NCYC110:G>D		SK1:G>D		Y55:G>DID:YBR146W	AA:97		273614X:K>R		378604X:K>R		BC187:K>R		DBVPG1106:K>R		DBVPG1373:K>R		DBVPG1788:K>R		DBVPG1853:K>R		DBVPG6040:K>R		DBVPG6044:K>R		DBVPG6765:K>R		K11:K>R		L_1528:K>R		SK1:K>R		UWOPS03_461_4:K>R		UWOPS05_227_2:K>R		UWOPS83_787_3:K>R		Y55:K>R		YJM975:K>R		YPS128:K>R		YPS606:K>R		YS4:K>R	AA:115		BC187:N>K	AA:248		UWOPS87_2421:K>IID:YBR147W	AA:14		K11:S>N	AA:51		DBVPG1106:V>A		DBVPG6765:V>A		L_1528:V>A		YS4:V>A	AA:85		UWOPS83_787_3:L>S	AA:115		DBVPG1788:P>S		DBVPG1853:P>S		DBVPG6044:P>S		DBVPG6765:P>S		L_1374:P>S		L_1528:P>S		SK1:P>S		UWOPS03_461_4:P>S		UWOPS05_227_2:P>S		UWOPS83_787_3:P>S		Y55:P>S		YPS128:P>S		YS4:P>S	AA:155		DBVPG6765:T>R		YS4:T>RID:YBR148W	AA:5		BC187:A>T		DBVPG1788:A>T		DBVPG6040:A>T		DBVPG6765:A>T		L_1374:A>T		YJM975:A>T		YJM978:A>T		YS4:A>T		YS9:A>T	AA:50		BC187:S>G		DBVPG1788:S>G		DBVPG6040:S>G		DBVPG6765:S>G		L_1374:S>G		UWOPS03_461_4:S>G		YJM975:S>G		YJM978:S>G		YS2:S>G		YS4:S>G	AA:58		273614X:D>N		DBVPG6040:D>N		DBVPG6044:D>N		K11:D>N		SK1:D>N		Y55:D>N		Y9:D>N		YPS128:D>N		YPS606:D>N	AA:68		UWOPS03_461_4:N>K	AA:106		DBVPG6044:V>G		SK1:V>G		UWOPS83_787_3:V>G		Y55:V>G	AA:137		Y9:F>L	AA:151		DBVPG1373:N>K		DBVPG1788:N>K		DBVPG6765:N>K		L_1374:N>K		YJM975:N>K		YJM978:N>K		YS2:N>K		YS4:N>K		YS9:N>K	AA:172		DBVPG6044:N>Y		NCYC110:N>Y		SK1:N>Y		Y55:N>Y	AA:180		DBVPG1788:S>I	AA:189		UWOPS03_461_4:Q>E	AA:203		322134S:E>G		BC187:E>G		DBVPG1373:E>G		DBVPG1788:E>G		DBVPG6765:E>G		L_1528:E>G		YJM975:E>G		YJM978:E>G		YS2:E>G		YS9:E>G	AA:211		322134S:Y>C		BC187:Y>C		DBVPG1373:Y>C		DBVPG1788:Y>C		DBVPG6765:Y>C		L_1528:Y>C		YJM975:Y>C		YJM978:Y>C		YS2:Y>C		YS4:Y>C		YS9:Y>C	AA:244		DBVPG6044:T>I		NCYC110:T>I		SK1:T>I		Y55:T>I	AA:286		YPS606:N>K	AA:335		UWOPS05_227_2:L>F	AA:341		322134S:Q>L		DBVPG1373:Q>L		DBVPG6040:Q>L		DBVPG6765:Q>L		L_1528:Q>L		YJM975:Q>L		YJM978:Q>L		YS9:Q>L	AA:351		322134S:D>N		DBVPG1373:D>N		DBVPG6040:D>N		DBVPG6765:D>N		L_1528:D>N		YJM978:D>N		YS9:D>N	AA:355		DBVPG6040:S>C	AA:356		UWOPS05_227_2:I>V		YPS128:I>V		YPS606:I>V	AA:365		273614X:L>M		322134S:L>M		378604X:L>M		DBVPG1373:L>M		DBVPG6040:L>M		DBVPG6044:L>M		DBVPG6765:L>M		K11:L>M		L_1528:L>M		SK1:L>M		UWOPS05_227_2:L>M		Y55:L>M		YJM978:L>M		YPS128:L>M		YPS606:L>M		YS9:L>M	AA:372		DBVPG6765:K>R		YJM978:K>R		YS9:K>R	AA:381		273614X:E>D		378604X:E>D	AA:394		UWOPS05_227_2:T>I		YPS128:T>I		YPS606:T>I	AA:433		DBVPG6040:E>K	AA:436		DBVPG6765:L>V	AA:449		UWOPS05_227_2:S>T	AA:460		378604X:D>N		BC187:D>N		DBVPG1373:D>N		DBVPG6044:D>N		DBVPG6765:D>N		K11:D>N		SK1:D>N		UWOPS05_227_2:D>N		UWOPS83_787_3:D>N		Y55:D>N		YIIc17_E5:D>N		YJM978:D>N		YPS128:D>N		YPS606:D>N	AA:471		YPS128:I>V		YPS606:I>V	AA:515		DBVPG6765:K>E	AA:549		YJM978:L>	AA:551		DBVPG1373:M>I	AA:592		UWOPS03_461_4:E>K		UWOPS05_227_2:E>KID:YBR149W	AA:104		BC187:V>I		DBVPG1853:V>I		DBVPG6765:V>I		L_1374:V>I		YJM978:V>I		YS4:V>I		YS9:V>I	AA:140		378604X:K>R		DBVPG6040:K>R	AA:157		DBVPG1853:G>R	AA:187		SK1:R>K		Y55:R>K	AA:290		322134S:V>IID:YBR151W	AA:87		273614X:Q>RID:YBR154C	AA:2		273614X:D>H	AA:148		322134S:E>KID:YBR155W	AA:8		L_1528:G>A	AA:114		K11:C>Y		Y12:C>Y		YS4:C>R	AA:187		322134S:P>Q		BC187:P>Q		DBVPG1106:P>Q		DBVPG1373:P>Q		DBVPG6765:P>Q		L_1374:P>Q		L_1528:P>Q	AA:220		UWOPS03_461_4:R>H		UWOPS05_227_2:R>H	AA:258		YS4:P>	AA:316		SK1:P>L		Y55:P>L	AA:331		DBVPG6040:I>S	AA:341		SK1:D>N		Y55:D>N	AA:355		YS4:N>I	AA:382		DBVPG1106:R>S		DBVPG1373:R>S		L_1374:R>SID:YBR156C	AA:60		UWOPS03_461_4:L>F	AA:67		UWOPS87_2421:M>I	AA:80		DBVPG6044:K>R		SK1:K>R		Y55:K>R	AA:100		DBVPG1788:D>A	AA:139		Y9:H>Y	AA:158		BC187:E>D		DBVPG1106:E>D		DBVPG1373:E>D		DBVPG1788:E>D		DBVPG1853:E>D		DBVPG6044:E>D		DBVPG6765:E>D		L_1374:E>D		L_1528:E>D		SK1:E>D		Y55:E>D		YJM978:E>D	AA:159		Y9:S>G	AA:173		BC187:V>I		DBVPG1788:V>I		DBVPG1853:V>I		DBVPG6044:V>I		L_1528:V>I		SK1:V>I		YJM978:V>I	AA:187		DBVPG1788:T>I	AA:216		BC187:V>I	AA:265		BC187:V>A		DBVPG1106:V>A		DBVPG1373:V>A		DBVPG1788:V>A		DBVPG1853:V>A		DBVPG6044:V>A		DBVPG6765:V>A		L_1374:V>A		L_1528:V>A		SK1:V>A		UWOPS87_2421:V>A		YJM978:V>A	AA:276		UWOPS87_2421:S>Y	AA:279		UWOPS03_461_4:N>K	AA:289		DBVPG1788:S>N	AA:322		DBVPG6044:G>S		SK1:G>S	AA:343		L_1528:K>N	AA:349		UWOPS87_2421:S>P	AA:438		DBVPG1853:P>S	AA:441		DBVPG1853:E>Q	AA:621		UWOPS87_2421:I>M	AA:698		BC187:S>P		DBVPG1373:S>P		DBVPG1853:S>P		DBVPG6765:S>P		L_1374:S>P		L_1528:S>P		SK1:S>P		Y55:S>P		YJM978:S>PID:YBR157C	AA:70		Y9:E>K	AA:72		K11:L>R		Y12:L>R	AA:90		K11:T>R	AA:121		YJM975:R>H	AA:130		DBVPG1788:P>T	AA:153		273614X:N>D		BC187:N>D		DBVPG1373:N>D		DBVPG1788:N>D		DBVPG1853:N>D		DBVPG6044:N>D		DBVPG6765:N>D		L_1374:N>D		L_1528:N>D		NCYC110:N>D		SK1:N>D		UWOPS05_227_2:N>D		UWOPS83_787_3:N>D		UWOPS87_2421:N>D		Y55:N>D		Y9:N>D		YJM975:N>D		YPS606:N>D	AA:212		DBVPG1788:S>RID:YBR159W	AA:7		YJM975:L>F	AA:61		273614X:T>S		322134S:T>S		DBVPG1788:T>S		DBVPG6765:T>S		L_1528:T>S		YJM975:T>S		YS9:T>S	AA:66		273614X:A>V		322134S:A>V		DBVPG1788:A>V		DBVPG6765:A>V		L_1528:A>V		UWOPS83_787_3:A>V		UWOPS87_2421:A>V		YJM975:A>V		YS9:A>V	AA:168		322134S:N>D		DBVPG1853:N>D		DBVPG6765:N>D		NCYC361:N>D		UWOPS83_787_3:N>D		UWOPS87_2421:N>D	AA:232		322134S:G>S		DBVPG1853:G>S		DBVPG6044:G>S		NCYC361:G>S		SK1:G>S		UWOPS83_787_3:G>S		Y55:G>S		YS2:G>S	AA:309		DBVPG6044:V>I		SK1:V>I		Y55:V>I	AA:337		YPS128:L>-ID:YBR160W	AA:79		NCYC110:S>F	AA:84		UWOPS03_461_4:L>I	AA:231		YS9:P>LID:YBR162C	AA:7		DBVPG1106:M>I		DBVPG1373:M>I		DBVPG1788:M>I		DBVPG6765:M>I		L_1528:M>I		UWOPS83_787_3:M>I	AA:80		YJM978:E>D	AA:155		Y12:Q>R	AA:175		L_1528:T>I	AA:177		273614X:D>A		DBVPG1373:D>A		DBVPG1788:D>A		DBVPG1853:D>V		DBVPG6044:D>V		DBVPG6765:D>A		L_1374:D>A		L_1528:D>V		UWOPS83_787_3:D>V		UWOPS87_2421:D>V		Y55:D>V		YJM975:D>A		YJM978:D>A		YPS128:D>V		YPS606:D>VID:YBR162W-A	AA:7		273614X:R>K		322134S:R>K		DBVPG6040:R>K		DBVPG6765:R>K		L_1528:R>K		YJM975:R>K		YJM981:R>K	AA:63		322134S:Y>NID:YBR163W	AA:3		SK1:G>C		Y55:G>C	AA:5		273614X:A>T		DBVPG1373:A>T		DBVPG1853:A>T		DBVPG6765:A>T		L_1374:A>T		L_1528:A>T		SK1:A>T		Y55:A>T		YJM975:A>T		YS2:A>T		YS4:A>T	AA:10		273614X:Y>H		DBVPG1373:Y>H		DBVPG1853:Y>H		DBVPG6765:Y>H		L_1374:Y>H		L_1528:Y>H		SK1:Y>H		Y55:Y>H		YJM975:Y>H		YS2:Y>H		YS4:Y>H	AA:45		YS4:T>I	AA:54		L_1374:A>G	AA:55		YPS128:S>L		YPS606:S>L	AA:76		SK1:N>S		Y55:N>S		YPS128:N>S		YPS606:N>S	AA:98		L_1374:P>L	AA:152		UWOPS87_2421:Q>R	AA:170		UWOPS03_461_4:K>T		UWOPS05_227_2:K>T	AA:190		L_1528:T>N	AA:277		273614X:R>K		322134S:R>K		BC187:R>K		DBVPG1788:R>K		DBVPG6765:R>K		L_1528:R>K		UWOPS03_461_4:R>K		UWOPS05_227_2:R>K		UWOPS83_787_3:R>K		UWOPS87_2421:R>K		YJM978:R>K	AA:348		YPS128:F>L		YPS606:F>L	AA:369		322134S:Q>K		BC187:Q>K		DBVPG1373:Q>K		DBVPG1788:Q>K		DBVPG1853:Q>K		DBVPG6765:Q>K		L_1528:Q>K		YJM975:Q>K		YJM978:Q>K		YS2:Q>K	AA:370		DBVPG6044:R>K		SK1:R>K		Y55:R>K	AA:410		YS2:S>T	AA:418		322134S:G>D	AA:425		322134S:T>A		BC187:T>A		DBVPG1373:T>A		DBVPG1788:T>A		DBVPG6765:T>A		L_1374:T>A		L_1528:T>A		YJM975:T>A		YJM978:T>A		YS2:T>A	AA:452		DBVPG6044:T>K		SK1:T>K		Y55:T>K	AA:515		273614X:P>T		BC187:P>T		DBVPG1373:P>T		DBVPG1788:P>T		DBVPG1853:P>T		DBVPG6765:P>T		L_1528:P>T		YS2:P>T		YS9:P>T	AA:539		BC187:E>D		L_1374:E>D		YJM975:E>D		YJM978:E>D	AA:545		YS2:F>VID:YBR165W	AA:26		YJM978:L>I	AA:41		UWOPS83_787_3:V>M		UWOPS87_2421:V>M	AA:57		273614X:Y>C	AA:109		UWOPS03_461_4:P>	AA:168		UWOPS83_787_3:I>V	AA:175		DBVPG1853:S>I	AA:210		UWOPS03_461_4:M>V		UWOPS05_217_3:M>V	AA:244		YS2:I>T	AA:266		UWOPS03_461_4:R>L		UWOPS05_217_3:R>LID:YBR166C	AA:38		322134S:G>S		378604X:G>S		DBVPG1373:G>S		DBVPG1788:G>S		DBVPG6040:G>S		DBVPG6044:G>S		DBVPG6765:G>S		L_1374:G>S		L_1528:G>S		SK1:G>S		UWOPS03_461_4:G>S		UWOPS05_227_2:G>S		UWOPS83_787_3:G>S		Y12:G>S		Y55:G>S		Y9:G>S		YPS128:G>S		YPS606:G>S		YS4:G>S	AA:56		DBVPG1373:A>V		DBVPG1788:A>V		DBVPG6040:A>V		DBVPG6765:A>V		L_1374:A>V		L_1528:A>V	AA:91		DBVPG1373:T>M		DBVPG1788:T>M		DBVPG6040:T>M		DBVPG6765:T>M		L_1374:T>M		L_1528:T>M		UWOPS03_461_4:T>M		UWOPS83_787_3:T>M		YJM975:T>M		YJM978:T>M		YPS128:T>M		YPS606:T>M	AA:207		YS4:Y>H		YS9:Y>H	AA:313		DBVPG6044:N>D		SK1:N>D		UWOPS03_461_4:N>D		UWOPS83_787_3:N>D		Y55:N>D		YPS128:N>D		YPS606:N>D	AA:315		DBVPG6044:N>D		SK1:N>D		Y55:N>D	AA:383		DBVPG6044:I>M		SK1:I>M		Y55:I>M	AA:443		273614X:D>E		322134S:D>E		BC187:D>E		DBVPG1373:D>E		DBVPG1853:D>E		DBVPG6044:D>E		DBVPG6765:D>E		L_1374:D>E		L_1528:D>E		NCYC361:D>E		SK1:D>E		UWOPS03_461_4:D>E		UWOPS83_787_3:D>E		UWOPS87_2421:D>E		Y55:D>E		YJM975:D>E		YPS128:D>H		YPS606:D>H		YS4:D>E		YS9:D>E	AA:449		273614X:K>Q		322134S:K>Q		BC187:K>Q		DBVPG1373:K>Q		DBVPG1853:K>Q		DBVPG6765:K>Q		L_1374:K>Q		L_1528:K>Q		NCYC361:K>Q		UWOPS03_461_4:K>Q		UWOPS83_787_3:K>Q		UWOPS87_2421:K>Q		YJM975:K>Q		YS4:K>QID:YBR167C	AA:15		UWOPS05_227_2:V>I		UWOPS83_787_3:V>I	AA:55		322134S:V>I	AA:58		322134S:Q>H		DBVPG1373:Q>H		DBVPG1788:Q>H		DBVPG6044:Q>H		DBVPG6765:Q>H		L_1528:Q>H		SK1:Q>H		UWOPS05_227_2:Q>H		UWOPS83_787_3:Q>H		UWOPS87_2421:Q>H		Y55:Q>H		YJM975:Q>H		YJM978:Q>H		YPS606:Q>HID:YBR168W	AA:172		UWOPS05_227_2:E>G	AA:192		273614X:I>V		322134S:I>V		BC187:I>V		DBVPG1106:I>V		DBVPG1373:I>V		DBVPG1788:I>V		DBVPG6040:I>V		DBVPG6765:I>V		L_1528:I>V		YJM978:I>V		YS2:I>V	AA:209		273614X:T>I		322134S:T>I		BC187:T>I		DBVPG1106:T>I		DBVPG1373:T>I		DBVPG1788:T>I		DBVPG6040:T>I		DBVPG6765:T>I		L_1528:T>I		YJM978:T>I		YS2:T>I	AA:239		DBVPG6044:I>M		NCYC110:I>M		SK1:I>M		Y55:I>M	AA:302		UWOPS87_2421:G>S	AA:359		273614X:G>E		322134S:G>E		BC187:G>E		DBVPG1788:G>E		DBVPG1853:G>E		DBVPG6044:G>E		DBVPG6765:G>E		NCYC110:G>E		SK1:G>E		UWOPS03_461_4:G>E		UWOPS87_2421:G>E		Y55:G>E		YJM975:G>E		YJM978:G>E		YPS128:G>E		YPS606:G>E		YS2:G>E		YS4:G>E		YS9:G>E	AA:366		UWOPS03_461_4:P>L	AA:371		UWOPS03_461_4:W>S	AA:389		273614X:K>R		322134S:K>R		BC187:K>R		DBVPG1788:K>R		DBVPG1853:K>R		DBVPG6044:K>R		DBVPG6765:K>R		NCYC110:K>R		SK1:K>R		UWOPS03_461_4:K>R		UWOPS87_2421:K>R		Y55:K>R		YJM975:K>R		YJM978:K>R		YS2:K>R	AA:395		273614X:S>N		322134S:S>N		BC187:S>N		DBVPG1788:S>N		DBVPG6765:S>N		YJM975:S>N		YJM978:S>N		YS2:S>NID:YBR169C	AA:31		L_1374:V>L	AA:113		UWOPS87_2421:F>L	AA:234		W303:G>V	AA:248		DBVPG1788:Q>H		DBVPG6765:Q>H	AA:466		YJM975:G>W	AA:468		DBVPG1373:K>N		DBVPG1788:K>N		DBVPG1853:K>N		DBVPG6044:K>N		DBVPG6765:K>N		L_1374:K>N		L_1528:K>N		UWOPS03_461_4:K>N		Y55:K>N	AA:481		YS9:K>Q	AA:565		UWOPS83_787_3:A>S	AA:684		DBVPG1853:D>N	AA:688		UWOPS83_787_3:E>Q		UWOPS87_2421:E>Q	AA:690		YS9:M>IID:YBR170C	AA:80		UWOPS83_787_3:D>N	AA:117		YS4:A>T		YS9:A>T	AA:126		DBVPG1373:D>Y	AA:182		DBVPG1853:K>N	AA:185		Y12:N>I	AA:186		Y55:G>R	AA:364		DBVPG1106:R>G		DBVPG1373:R>G		DBVPG1788:R>G		DBVPG6765:R>G		L_1528:R>G		YJM975:R>G	AA:437		322134S:I>R	AA:482		DBVPG1853:V>I		UWOPS87_2421:V>D	AA:510		DBVPG6044:N>S		SK1:N>S		Y55:N>S	AA:518		DBVPG6044:N>H		SK1:N>H	AA:544		UWOPS87_2421:W>RID:YBR172C	AA:53		BC187:A>T		DBVPG1106:A>T		DBVPG1853:A>T		DBVPG6765:A>T		UWOPS87_2421:A>T		YJM978:A>T		YS2:A>T	AA:169		DBVPG1106:S>G		DBVPG1853:S>G		DBVPG6765:S>G		L_1374:S>G		L_1528:S>G		SK1:S>G		UWOPS87_2421:S>G		Y55:S>G		YJM975:S>G		YS9:S>G	AA:271		BC187:D>E		DBVPG1373:D>E		DBVPG1853:D>E		DBVPG6765:D>E		L_1374:D>E		L_1528:D>E		UWOPS03_461_4:D>E		UWOPS87_2421:D>E		YJM975:D>E		YS9:D>E	AA:276		W303:T>S	AA:289		BC187:D>G		DBVPG1373:D>G		DBVPG1853:D>V		DBVPG6765:D>G		L_1374:D>G		L_1528:D>G		UWOPS87_2421:D>G		YJM975:D>G		YS9:D>G	AA:356		L_1528:E>K	AA:364		NCYC361:E>A		Y9:E>V	AA:418		DBVPG1853:Q>K	AA:439		273614X:T>I		322134S:T>I		BC187:T>I		DBVPG1373:T>I		DBVPG6765:T>I		YJM975:T>I	AA:482		DBVPG6044:G>S		NCYC110:G>S		SK1:G>S		Y55:G>S	AA:485		322134S:S>G		BC187:S>G		DBVPG1788:S>G		DBVPG1853:S>G		DBVPG6765:S>G		YJM975:S>G	AA:576		DBVPG6044:T>A		SK1:T>A		UWOPS83_787_3:T>A		Y55:T>A	AA:626		SK1:T>A		Y55:T>A	AA:704		322134S:E>DID:YBR175W	AA:9		378604X:G>E		DBVPG1373:G>E		DBVPG1788:G>E		DBVPG6765:G>E		UWOPS87_2421:G>E		YS9:G>E	AA:58		NCYC361:F>S		YIIc17_E5:F>S	AA:315		UWOPS87_2421:V>IID:YBR176W	AA:36		273614X:F>V		322134S:F>V		DBVPG1106:F>V		DBVPG1373:F>V		DBVPG1788:F>V		DBVPG1853:F>V		DBVPG6044:F>V		DBVPG6765:F>V		L_1374:F>V		SK1:F>V		UWOPS03_461_4:F>V		UWOPS87_2421:F>V		Y55:F>V		Y9:F>V		YJM975:F>V		YPS128:F>V		YPS606:F>V		YS2:F>V		YS9:F>V	AA:40		DBVPG6044:P>L		SK1:P>L		Y55:P>L	AA:65		322134S:V>I		DBVPG1373:V>I		DBVPG1788:V>I		DBVPG6765:V>I		L_1374:V>I		YJM975:V>I		YJM978:V>I		YS2:V>I		YS9:V>I	AA:89		322134S:Y>H		DBVPG1373:Y>H		DBVPG1788:Y>H		L_1374:Y>H		YJM975:Y>H		YJM978:Y>H		YS2:Y>H		YS9:Y>H	AA:125		YS4:D>G	AA:196		UWOPS83_787_3:E>Q		UWOPS87_2421:E>QID:YBR177C	AA:52		UWOPS83_787_3:V>I		UWOPS87_2421:V>I	AA:85		DBVPG6044:K>R		SK1:K>R	AA:117		DBVPG1106:E>K		DBVPG1373:E>K		DBVPG1788:E>K		DBVPG6044:E>K		L_1528:E>K		SK1:E>K		UWOPS03_461_4:E>K		UWOPS05_227_2:E>K		UWOPS87_2421:E>K		YJM978:E>K		YPS606:E>K		YS2:E>K	AA:119		YPS606:D>N	AA:132		DBVPG1106:K>E		DBVPG1373:K>E		DBVPG1788:K>E		DBVPG6044:K>E		L_1528:K>E		SK1:K>E		UWOPS03_461_4:K>E		UWOPS05_227_2:K>E		UWOPS87_2421:K>E		YIIc17_E5:K>E		YJM978:K>E		YPS606:K>E		YS2:K>E	AA:170		L_1528:I>V	AA:194		UWOPS03_461_4:G>W	AA:384		BC187:R>Q		DBVPG1373:R>Q		DBVPG1788:R>Q		DBVPG1853:R>Q		DBVPG6765:R>Q		L_1528:R>Q		NCYC361:R>Q		YS2:R>QID:YBR179C	AA:14		Y12:P>S	AA:118		DBVPG1373:N>D	AA:166		L_1528:H>Q	AA:256		L_1528:M>L	AA:272		DBVPG1788:E>Q	AA:278		L_1528:P>S	AA:281		L_1528:G>E	AA:397		YGPM:Y>-	AA:409		YGPM:K>N	AA:417		DBVPG6044:Y>F		SK1:Y>F		Y12:Y>F		Y55:Y>F		YPS128:Y>F	AA:418		YGPM:H>R	AA:429		322134S:R>G		378604X:R>G		DBVPG1788:R>G		DBVPG6044:R>G		DBVPG6765:R>G		K11:R>G		L_1374:R>G		L_1528:R>G		SK1:R>G		UWOPS05_227_2:R>G		UWOPS83_787_3:R>G		Y55:R>G		YIIc17_E5:R>G		YPS128:R>G	AA:438		UWOPS83_787_3:S>N	AA:454		W303:N>D	AA:573		UWOPS03_461_4:A>T	AA:662		273614X:A>E		BC187:A>E		DBVPG1373:A>E		DBVPG6040:A>E		DBVPG6765:A>E		UWOPS83_787_3:A>E		UWOPS87_2421:A>E		YJM975:A>E		YS9:A>E	AA:677		W303:G>D	AA:774		273614X:Q>E	AA:788		L_1528:N>K	AA:818		DBVPG1853:E>K	AA:825		L_1528:S>N	AA:835		378604X:S>CID:YBR180W	AA:7		UWOPS83_787_3:Q>E	AA:52		322134S:I>T		DBVPG1373:I>T		DBVPG6044:I>T		DBVPG6765:I>T		L_1374:I>T		SK1:I>T		UWOPS83_787_3:I>T		Y12:I>T		Y55:I>T		Y9:I>T		YIIc17_E5:I>T		YJM975:I>T		YJM978:I>T		YS2:I>T	AA:128		YIIc17_E5:N>I	AA:142		L_1528:D>N	AA:150		UWOPS03_461_4:A>S		UWOPS05_227_2:A>S	AA:168		273614X:A>S		BC187:A>S		DBVPG6765:A>S		L_1374:A>S		YJM975:A>S		YS2:A>S	AA:264		273614X:N>K	AA:305		273614X:D>G		BC187:D>G		DBVPG6765:D>G		L_1374:D>G		L_1528:D>E		NCYC110:D>G		SK1:D>G		Y55:D>G		Y9:D>G		YJM975:D>G		YPS606:D>G	AA:308		NCYC110:D>G		SK1:D>G		Y55:D>G	AA:322		L_1528:K>E	AA:323		UWOPS03_461_4:I>T		UWOPS05_227_2:I>T	AA:332		L_1528:I>M	AA:347		YPS606:N>H	AA:356		NCYC110:L>W		SK1:L>W		Y55:L>W	AA:398		322134S:R>C	AA:468		322134S:A>V	AA:504		322134S:F>L		BC187:F>L		DBVPG1106:F>L		DBVPG1788:F>L		DBVPG6040:F>L		DBVPG6765:F>L		L_1374:F>L		L_1528:F>L		YJM975:F>L		YJM978:F>L		YS2:F>L	AA:528		322134S:A>V		BC187:A>V		DBVPG1106:A>V		DBVPG1788:A>V		DBVPG1853:A>V		DBVPG6040:A>V		DBVPG6765:A>V		L_1374:A>V		L_1528:A>V		YJM975:A>V		YJM978:A>V		YS2:A>VID:YBR181C	AA:16		K11:F>I	AA:46		322134S:K>MID:YBR182C	AA:102		YIIc17_E5:R>M	AA:125		DBVPG6765:S>P		L_1374:S>P		L_1528:S>P		UWOPS03_461_4:S>P		UWOPS05_227_2:S>P		Y12:S>P		Y9:S>P		YJM975:S>P		YJM978:S>P		YPS606:S>P		YS2:S>P	AA:146		DBVPG6044:A>T		Y55:A>T	AA:187		UWOPS83_787_3:G>E	AA:189		DBVPG1853:F>L	AA:202		UWOPS83_787_3:H>Y	AA:274		YJM975:P>L		YJM978:P>L	AA:284		UWOPS83_787_3:D>N	AA:366		UWOPS03_461_4:P>S		UWOPS05_227_2:P>S	AA:369		Y9:M>I	AA:408		UWOPS83_787_3:K>N		UWOPS87_2421:K>N	AA:418		378604X:R>-ID:YBR183W	AA:81		DBVPG1373:V>IID:YBR185C	AA:13		DBVPG1853:P>L	AA:15		UWOPS83_787_3:S>F	AA:63		UWOPS83_787_3:F>Y	AA:71		YPS128:L>F	AA:119		UWOPS83_787_3:A>S	AA:168		UWOPS87_2421:I>M	AA:188		NCYC361:P>AID:YBR186W	AA:2		NCYC110:S>N		SK1:S>N		Y55:S>N	AA:18		NCYC361:K>E	AA:25		NCYC110:E>V		SK1:E>V		Y55:E>V	AA:132		322134S:F>I	AA:147		273614X:S>P		DBVPG1373:S>P		DBVPG1788:S>P		DBVPG6040:S>P		DBVPG6765:S>P		L_1528:S>P		NCYC361:S>P		YJM978:S>P		YS9:S>P	AA:179		273614X:M>V		322134S:M>I		DBVPG1373:M>V		DBVPG1788:M>V		DBVPG6040:M>V		DBVPG6765:M>V		L_1528:M>V		NCYC361:M>V		UWOPS03_461_4:M>V		UWOPS05_217_3:M>V		YJM978:M>V		YPS128:M>V		YPS606:M>V		YS9:M>V	AA:182		273614X:V>A		DBVPG1373:V>A		DBVPG1788:V>A		DBVPG6040:V>A		DBVPG6765:V>A		L_1528:V>A		NCYC361:V>A		UWOPS03_461_4:V>A		UWOPS05_217_3:V>A		UWOPS87_2421:V>A		YJM978:V>A		YPS128:V>A		YPS606:V>A		YS9:V>A	AA:197		273614X:D>G		322134S:D>G		DBVPG1373:D>G		DBVPG1788:D>G		DBVPG1853:D>G		DBVPG6040:D>G		DBVPG6044:D>G		DBVPG6765:D>G		L_1528:D>G		NCYC361:D>G		SK1:D>G		UWOPS03_461_4:D>G		UWOPS05_217_3:D>G		Y55:D>G		Y9:D>G		YJM978:D>G		YPS128:D>G		YPS606:D>G		YS9:D>G	AA:229		UWOPS87_2421:N>S	AA:241		NCYC110:L>	AA:259		K11:F>V	AA:288		YPS128:R>K		YPS606:R>K	AA:292		DBVPG1853:T>I	AA:300		YPS128:I>N		YPS606:I>N	AA:340		BC187:G>C		DBVPG1373:G>C		DBVPG6765:G>C		YJM975:G>C		YJM978:G>C		YS2:G>C	AA:343		Y9:T>A	AA:365		YJM978:F>L	AA:367		Y9:E>D	AA:387		Y9:E>D	AA:499		BC187:V>I		DBVPG1106:V>I		DBVPG6765:V>I		L_1374:V>I		UWOPS83_787_3:V>I		YJM978:V>I	AA:525		UWOPS03_461_4:T>P	AA:544		UWOPS83_787_3:D>HID:YBR191W	AA:17		NCYC110:R>	AA:84		NCYC361:Y>S	AA:93		DBVPG6044:V>F	AA:119		DBVPG6044:A>DID:YBR193C	AA:27		W303:G>V	AA:161		YJM981:T>I	AA:167		BC187:Y>N		DBVPG1373:Y>N		DBVPG6765:Y>N		L_1528:Y>N		YJM975:Y>N		YJM978:Y>N		YJM981:Y>N	AA:186		SK1:L>V		UWOPS05_217_3:L>V		UWOPS05_227_2:L>V		Y55:L>V	AA:190		YS9:S>I	AA:212		SK1:I>F		Y55:I>F	AA:218		322134S:S>P		BC187:S>P		DBVPG1106:S>P		DBVPG1373:S>P		DBVPG1853:S>P		DBVPG6765:S>P		L_1528:S>P		SK1:S>P		UWOPS05_217_3:S>P		UWOPS05_227_2:S>P		UWOPS87_2421:S>P		Y55:S>P		YJM978:S>P		YJM981:S>P		YS2:S>PID:YBR195C	AA:58		BC187:T>A		DBVPG1106:T>A		DBVPG1373:T>A		DBVPG1788:T>A		DBVPG6040:T>A		DBVPG6765:T>A		L_1528:T>A		YJM975:T>A		YJM978:T>A	AA:71		NCYC361:S>F	AA:75		YPS606:P>S	AA:126		Y9:F>S	AA:182		DBVPG6044:G>D		NCYC110:G>D		SK1:G>D		Y55:G>D	AA:223		K11:G>S	AA:236		YIIc17_E5:E>D	AA:309		DBVPG1853:S>F	AA:352		SK1:F>LID:YBR197C	AA:28		BC187:E>K		DBVPG1106:E>K		DBVPG1788:E>K		DBVPG6765:E>K		YJM978:E>K		YJM981:E>K		YS9:E>K	AA:43		UWOPS05_227_2:D>N	AA:68		BC187:T>S		DBVPG1106:T>S		DBVPG1373:T>S		DBVPG1853:T>S		DBVPG6044:T>S		DBVPG6765:T>S		L_1528:T>S		SK1:T>S		UWOPS05_227_2:T>S		Y55:T>S		YJM978:T>S		YJM981:T>S		YPS606:T>S		YS9:T>S	AA:103		L_1528:A>V	AA:160		DBVPG1853:N>K	AA:171		BC187:P>S		DBVPG1106:P>S		DBVPG1373:P>S		DBVPG1853:P>S		DBVPG6040:P>S		DBVPG6765:P>S		L_1528:P>S		YJM975:P>S		YJM978:P>S		YJM981:P>S		YS9:P>S	AA:218		273614X:->L		BC187:->L		DBVPG1373:->L		DBVPG1853:->L		DBVPG6040:->L		DBVPG6765:->L		L_1528:->L		YJM975:->L		YJM978:->L		YS9:->LID:YBR199W	AA:26		DBVPG1853:V>A		YS2:V>A	AA:27		DBVPG1106:L>F		YJM978:L>F	AA:73		YS4:A>T		YS9:A>T	AA:79		DBVPG1853:I>V	AA:83		YJM978:L>I	AA:200		DBVPG6044:G>D		NCYC110:G>D		SK1:G>D		UWOPS03_461_4:G>D		UWOPS05_217_3:G>D		UWOPS05_227_2:G>D		Y55:G>D	AA:223		YS9:S>G	AA:229		DBVPG6040:Y>D	AA:280		YPS128:N>S		YPS606:N>S	AA:344		Y9:T>S	AA:348		Y9:W>R	AA:414		DBVPG1788:Y>C		DBVPG6765:Y>C		YJM975:Y>C	AA:450		UWOPS03_461_4:T>I		UWOPS05_217_3:T>I		UWOPS05_227_2:T>IID:YBR201W	AA:28		Y9:G>S	AA:41		322134S:V>M	AA:95		Y9:C>W	AA:109		K11:T>I	AA:136		UWOPS87_2421:N>	AA:145		322134S:A>V		BC187:A>V		DBVPG1106:A>V		DBVPG1853:A>V		DBVPG6044:A>V		DBVPG6765:A>V		NCYC110:A>V		SK1:A>V		UWOPS03_461_4:A>V		UWOPS05_217_3:A>V		UWOPS05_227_2:A>V		UWOPS83_787_3:A>V		W303:A>V		Y55:A>V		YJM975:A>V		YJM978:A>V		YJM981:A>V		YPS606:A>V		YS2:A>V	AA:169		UWOPS03_461_4:W>C		UWOPS05_217_3:W>C		UWOPS05_227_2:W>C		UWOPS83_787_3:W>C	AA:210		322134S:T>AID:YBR202W	AA:90		YJM978:N>K	AA:372		YS4:T>I	AA:552		322134S:V>G		378604X:V>G		DBVPG1106:V>G		DBVPG1373:V>G		DBVPG1788:V>G		DBVPG1853:V>G		DBVPG6765:V>G		K11:V>G		NCYC110:V>G		S288c:V>G		SK1:V>G		UWOPS03_461_4:V>G		UWOPS83_787_3:V>G		UWOPS87_2421:V>G		W303:V>G		Y12:V>G		Y55:V>G		Y9:V>G		YGPM:V>G		YIIc17_E5:V>G		YJM975:V>G		YJM978:V>G		YJM981:V>G		YPS128:V>G		YPS606:V>G		YS2:V>G		YS4:V>G	AA:629		UWOPS83_787_3:D>E	AA:655		UWOPS83_787_3:A>T	AA:706		NCYC110:D>N		SK1:D>N		Y55:D>N	AA:805		UWOPS87_2421:D>N	AA:828		K11:T>I	AA:833		UWOPS05_227_2:V>A		YPS128:V>AID:YBR203W	AA:31		322134S:S>N		DBVPG1853:S>N		DBVPG6040:S>N		DBVPG6044:S>N		DBVPG6765:S>N		K11:S>N		L_1374:S>N		NCYC361:S>N		SK1:S>N		UWOPS05_217_3:S>N		UWOPS05_227_2:S>N		Y12:S>N		Y55:S>N		Y9:S>N		YJM975:S>N		YJM978:S>N		YJM981:S>N		YPS128:S>N	AA:70		K11:M>T	AA:248		DBVPG6040:L>	AA:290		DBVPG6044:H>Y		NCYC110:H>Y		SK1:H>Y		Y55:H>Y	AA:301		DBVPG6765:S>I	AA:332		NCYC361:S>N	AA:433		Y12:L>M	AA:446		L_1374:S>F	AA:502		DBVPG1373:L>I		DBVPG6765:L>I		L_1374:L>I		W303:L>I		YJM981:L>I		YS2:L>I		YS4:L>I	AA:508		UWOPS05_217_3:H>	AA:624		UWOPS83_787_3:G>E	AA:708		322134S:K>T	AA:744		YPS128:R>T		YPS606:R>T	AA:745		DBVPG1853:H>N	AA:802		BC187:Y>F		DBVPG1106:Y>F		DBVPG1373:Y>F		DBVPG1788:Y>F		DBVPG6765:Y>F		L_1528:Y>F		YJM978:Y>F		YJM981:Y>F		YS9:Y>F	AA:819		DBVPG1853:R>K	AA:834		DBVPG1106:L>P		DBVPG1373:L>P		DBVPG1788:L>P		DBVPG1853:L>P		DBVPG6044:L>P		DBVPG6765:L>P		L_1528:L>P		SK1:L>P		UWOPS05_217_3:L>P		UWOPS05_227_2:L>P		UWOPS83_787_3:L>P		Y12:L>P		Y55:L>P		Y9:L>P		YJM981:L>P		YPS128:L>P		YPS606:L>P		YS4:L>P		YS9:L>P	AA:879		DBVPG1853:D>N	AA:921		DBVPG1106:G>E		DBVPG1373:G>E		DBVPG1788:G>E		DBVPG1853:G>E		DBVPG6040:G>E		DBVPG6044:G>E		DBVPG6765:G>E		L_1528:G>E		S288c:G>E		SK1:G>E		UWOPS05_217_3:G>E		UWOPS05_227_2:G>E		UWOPS83_787_3:G>E		W303:G>E		Y12:G>E		Y55:G>E		YGPM:G>E		YJM975:G>E		YPS606:G>E		YS4:G>EID:YBR204C	AA:22		DBVPG1106:T>A		DBVPG1373:T>A		DBVPG1788:T>A		L_1528:T>A		UWOPS05_217_3:T>A		UWOPS05_227_2:T>A		YJM975:T>A		YJM978:T>A	AA:25		DBVPG1106:E>V		DBVPG1373:E>V		DBVPG1788:E>V		L_1528:E>V		YJM978:E>V	AA:54		DBVPG6765:I>L	AA:63		322134S:E>V		DBVPG1106:E>V		DBVPG1373:E>V		DBVPG1788:E>V		DBVPG6044:E>V		L_1528:E>V		SK1:E>V		UWOPS05_217_3:E>V		UWOPS05_227_2:E>V		W303:E>V		Y55:E>V		YJM975:E>V		YJM978:E>V		YPS128:E>V		YPS606:E>V	AA:69		322134S:N>T		DBVPG1106:N>T		DBVPG1373:N>T		DBVPG1788:N>T		L_1528:N>T		YJM975:N>T		YJM978:N>T	AA:85		322134S:G>S		DBVPG1106:G>S		DBVPG1373:G>S		DBVPG1788:G>S		DBVPG6044:G>S		L_1528:G>S		SK1:G>S		UWOPS05_217_3:G>S		Y55:G>S		YJM978:G>S	AA:110		322134S:D>E		DBVPG1106:D>E		DBVPG1373:D>E		DBVPG1788:D>E		L_1528:D>E		YJM978:D>E	AA:144		322134S:V>I	AA:212		DBVPG1106:Q>R		DBVPG1788:Q>R		DBVPG1853:Q>R		DBVPG6040:Q>R		YJM975:Q>R		YJM978:Q>R		YS4:Q>R		YS9:Q>R	AA:227		DBVPG1853:V>L	AA:238		YS2:R>I	AA:259		YS4:Q>K	AA:335		UWOPS87_2421:I>M		YS2:I>T	AA:370		DBVPG1853:I>V	AA:373		UWOPS05_217_3:A>T		UWOPS05_227_2:A>T		UWOPS87_2421:S>YID:YBR205W	AA:44		YS2:S>-	AA:166		322134S:F>L	AA:267		UWOPS05_227_2:F>I	AA:281		UWOPS03_461_4:D>N		UWOPS05_217_3:D>N		UWOPS05_227_2:D>NID:YBR207W	AA:18		322134S:K>E		378604X:K>E		BC187:K>E		DBVPG1106:K>E		DBVPG1373:K>E		DBVPG1788:K>E		DBVPG6040:K>E		DBVPG6044:K>E		DBVPG6765:K>E		L_1374:K>E		S288c:K>E		SK1:K>E		UWOPS05_227_2:K>E		UWOPS83_787_3:K>E		UWOPS87_2421:K>E		Y55:K>E		YJM975:K>E		YJM978:K>E		YJM981:K>E		YPS128:K>E		YPS606:K>E		YS4:K>E		YS9:K>E	AA:36		322134S:D>G		378604X:D>G		BC187:D>G		DBVPG1106:D>G		DBVPG1373:D>G		DBVPG1788:D>G		DBVPG6044:D>G		L_1374:D>G		S288c:D>G		SK1:D>G		UWOPS05_227_2:D>G		UWOPS83_787_3:D>G		UWOPS87_2421:D>G		Y55:D>G		YJM975:D>G		YJM978:D>G		YJM981:D>G		YPS128:D>G		YPS606:D>G		YS4:D>G		YS9:D>G	AA:103		DBVPG1373:Y>H	AA:228		322134S:Q>E		DBVPG1106:Q>E		DBVPG1373:Q>E		DBVPG6044:Q>E		L_1374:Q>E		S288c:Q>E		SK1:Q>E		UWOPS05_217_3:Q>E		UWOPS83_787_3:Q>E		UWOPS87_2421:Q>E		W303:Q>E		Y12:Q>E		Y55:Q>E		YJM975:Q>E		YJM981:Q>E		YPS128:Q>E		YPS606:Q>E		YS4:Q>E	AA:272		DBVPG1106:A>S		DBVPG1373:A>S		DBVPG6765:A>S		W303:A>S		Y55:A>S		YJM975:A>S	AA:334		322134S:E>G		378604X:E>G		DBVPG1373:E>G		DBVPG6044:E>G		DBVPG6765:E>G		L_1374:E>G		SK1:E>G		UWOPS05_227_2:E>G		UWOPS87_2421:E>G		W303:E>G		Y55:E>G		YPS128:E>G		YPS606:E>G		YS4:E>G		YS9:E>G	AA:371		YS4:N>K	AA:396		UWOPS05_217_3:M>I		UWOPS05_227_2:M>I	AA:399		378604X:G>E		DBVPG1373:G>E		DBVPG6044:G>E		DBVPG6765:G>E		SK1:G>E		UWOPS05_217_3:G>E		UWOPS05_227_2:G>E		UWOPS83_787_3:G>E		UWOPS87_2421:G>E		W303:G>E		Y12:G>E		Y55:G>E		YPS128:G>E		YPS606:G>E		YS4:G>E		YS9:G>E	AA:401		378604X:C>Y		DBVPG1373:C>Y		DBVPG6044:C>Y		DBVPG6765:C>Y		L_1374:C>Y		SK1:C>Y		UWOPS05_217_3:C>Y		UWOPS05_227_2:C>Y		UWOPS83_787_3:C>Y		UWOPS87_2421:C>Y		W303:C>Y		Y12:C>Y		Y55:C>Y		YPS128:C>Y		YPS606:C>Y		YS4:C>Y		YS9:C>Y	AA:431		UWOPS05_217_3:H>Q		UWOPS05_227_2:H>Q	AA:443		YS4:P>SID:YBR210W	AA:28		YS2:I>T	AA:98		W303:T>MID:YBR212W	AA:87		Y9:S>I	AA:108		DBVPG6044:Q>R		NCYC110:Q>R		SK1:Q>R	AA:154		UWOPS05_217_3:N>S	AA:159		273614X:T>K	AA:185		YS2:S>A	AA:300		DBVPG1788:D>N	AA:322		Y12:P>S		Y9:P>S	AA:325		322134S:A>T	AA:329		UWOPS05_217_3:A>V	AA:351		UWOPS05_217_3:N>K	AA:377		DBVPG1853:R>S	AA:390		W303:R>T	AA:433		DBVPG6044:S>C		NCYC110:S>C		SK1:S>C	AA:518		UWOPS05_217_3:H>D		UWOPS05_227_2:H>D	AA:568		378604X:T>N		DBVPG1373:T>N		DBVPG1788:T>N		DBVPG6040:T>N		DBVPG6044:T>N		DBVPG6765:T>N		L_1374:T>N		L_1528:T>N		NCYC110:T>N		SK1:T>N		UWOPS03_461_4:T>N		UWOPS05_217_3:T>N		UWOPS05_227_2:T>N		UWOPS87_2421:T>N		W303:T>N		Y55:T>N		YJM978:T>N		YS2:T>N	AA:584		UWOPS03_461_4:L>P		UWOPS05_217_3:L>P		UWOPS05_227_2:L>P		UWOPS87_2421:L>PID:YBR213W	AA:15		BC187:K>R		DBVPG1373:K>R		DBVPG1853:K>R		DBVPG6040:K>R		DBVPG6044:K>R		L_1374:K>R		L_1528:K>R		NCYC361:K>R		SK1:K>R		UWOPS83_787_3:K>R		UWOPS87_2421:K>R		Y55:K>R		YJM975:K>R		YJM978:K>R		YPS606:K>R		YS4:K>R	AA:33		YS4:I>M	AA:61		DBVPG1373:E>K		DBVPG1788:E>K		DBVPG1853:E>K		L_1374:E>K		L_1528:E>K		NCYC361:E>K		Y55:E>K		YJM975:E>K		YJM978:E>K		YS2:E>K		YS4:E>K		YS9:E>K	AA:68		UWOPS83_787_3:E>K		UWOPS87_2421:E>K	AA:80		DBVPG1373:P>S		DBVPG1788:P>S		DBVPG6040:P>S		L_1374:P>S		L_1528:P>S		NCYC361:P>S		Y55:P>S		YJM975:P>S		YJM978:P>S		YS2:P>S		YS9:P>S	AA:102		UWOPS87_2421:D>E		YS4:D>NID:YBR214W	AA:30		SK1:M>I		Y9:M>I	AA:68		273614X:C>S		BC187:C>S		DBVPG1106:C>S		DBVPG1373:C>S		DBVPG1788:C>S		DBVPG6040:C>S		DBVPG6044:C>S		DBVPG6765:C>S		L_1528:C>S		NCYC110:C>S		NCYC361:C>S		SK1:C>S		UWOPS83_787_3:C>S		W303:C>S		Y55:C>S		YPS128:C>S		YS2:C>S		YS9:C>S	AA:74		DBVPG1788:P>A		DBVPG6765:P>A	AA:184		YS2:C>G	AA:203		W303:P>S	AA:320		378604X:G>D		BC187:G>D		DBVPG1106:G>D		DBVPG1373:G>D		DBVPG1788:G>D		DBVPG6040:G>D		DBVPG6765:G>D		L_1374:G>D		L_1528:G>D		NCYC361:G>D		Y55:G>D		YJM978:G>D		YJM981:G>D		YS2:G>D	AA:523		K11:G>DID:YBR217W	AA:56		UWOPS05_217_3:Q>L	AA:76		K11:T>A		UWOPS87_2421:T>S	AA:77		YJM978:Y>N	AA:80		DBVPG6044:E>G		NCYC110:E>G	AA:83		DBVPG6044:I>T		NCYC110:I>T		UWOPS05_217_3:I>T		UWOPS05_227_2:I>T		UWOPS83_787_3:I>T	AA:138		DBVPG6044:K>E		L_1374:K>E		NCYC110:K>E	AA:162		DBVPG6044:I>V		NCYC110:I>V	AA:174		K11:E>K		UWOPS83_787_3:E>VID:YBR220C	AA:116		Y9:G>V	AA:240		DBVPG6765:K>Q		UWOPS05_227_2:K>N	AA:254		UWOPS05_227_2:T>P	AA:285		YS4:R>C	AA:288		UWOPS05_227_2:I>M	AA:336		UWOPS83_787_3:L>F	AA:545		322134S:K>N	AA:551		W303:S>IID:YBR221C	AA:14		DBVPG1373:A>T	AA:40		L_1528:T>P	AA:170		NCYC110:P>S	AA:193		W303:L>F	AA:211		Y9:L>F	AA:226		273614X:E>D		378604X:E>D		DBVPG1788:E>D		DBVPG6044:E>D		DBVPG6765:E>D		NCYC110:E>D		NCYC361:E>D		UWOPS05_227_2:E>D		UWOPS87_2421:E>D		Y55:E>D		YJM975:E>D		YPS128:E>D		YS4:E>D	AA:262		YS9:Q>LID:YBR223C	AA:7		273614X:F>L		378604X:F>L		BC187:F>L		DBVPG1373:F>L		DBVPG1788:F>L		DBVPG6044:F>L		DBVPG6765:F>L		L_1374:F>L		L_1528:F>L		NCYC361:F>L		SK1:F>L		UWOPS83_787_3:F>L		UWOPS87_2421:F>L		Y55:F>L		YJM981:F>L		YS9:F>L	AA:14		UWOPS83_787_3:R>K	AA:50		273614X:V>I		378604X:V>I		BC187:V>I		DBVPG1373:V>I		DBVPG1788:V>I		DBVPG6044:V>I		DBVPG6765:V>I		L_1374:V>I		L_1528:V>I		Y55:V>I		YJM981:V>I	AA:69		YPS128:E>D	AA:72		378604X:E>G		DBVPG1373:E>G		DBVPG6044:E>G		DBVPG6765:E>G		L_1374:E>G		L_1528:E>G		UWOPS83_787_3:E>G		W303:E>G		Y55:E>G		YJM981:E>G	AA:76		DBVPG6765:T>M	AA:77		YIIc17_E5:A>T	AA:81		DBVPG6044:V>A	AA:99		DBVPG6765:V>I	AA:238		SK1:I>L	AA:261		DBVPG6044:N>K	AA:298		UWOPS05_217_3:S>N		UWOPS05_227_2:S>N	AA:313		DBVPG6044:I>M	AA:329		YS9:L>V	AA:444		322134S:A>T		378604X:A>T		DBVPG1106:A>T		DBVPG1788:A>T		DBVPG6040:A>T		DBVPG6044:A>T		DBVPG6765:A>T		L_1374:A>T		UWOPS87_2421:A>T		W303:A>T		Y55:A>T		YJM975:A>T		YJM978:A>T		YJM981:A>T		YPS606:A>T		YS9:A>TID:YBR227C	AA:10		DBVPG6044:F>L		NCYC110:F>L		SK1:F>C		UWOPS03_461_4:F>L		UWOPS05_217_3:F>L	AA:77		NCYC361:K>-	AA:82		K11:R>K		Y12:R>K		YIIc17_E5:R>K	AA:96		273614X:R>K		DBVPG1373:R>K		DBVPG6765:R>K		L_1374:R>K		NCYC361:R>K		Y55:R>K		YS9:R>K	AA:202		NCYC361:I>V	AA:227		UWOPS87_2421:V>D	AA:300		UWOPS87_2421:Q>L	AA:309		DBVPG6044:S>L	AA:439		UWOPS87_2421:N>I	AA:497		BC187:V>A		DBVPG1106:V>A		DBVPG1373:V>A		DBVPG1788:V>A		DBVPG6044:V>A		DBVPG6765:V>A		L_1374:V>A		L_1528:V>A		UWOPS87_2421:V>A		Y55:V>A		YJM981:V>A		YPS128:V>A		YPS606:V>A		YS4:V>A		YS9:V>A	AA:509		BC187:I>VID:YBR228W	AA:11		DBVPG1788:P>L	AA:134		273614X:E>D		DBVPG1373:E>D		DBVPG6765:E>D		L_1528:E>D		UWOPS03_461_4:E>D		UWOPS05_227_2:E>D		Y55:E>D		YJM978:E>D		YS2:E>D		YS4:E>D		YS9:E>D	AA:170		UWOPS03_461_4:P>S		UWOPS05_227_2:P>S	AA:194		273614X:T>I	AA:198		Y9:E>V	AA:201		Y9:R>-	AA:240		YS4:L>F	AA:241		Y12:C>	AA:257		UWOPS83_787_3:R>S	AA:266		273614X:M>I		322134S:M>I		DBVPG1373:M>I		DBVPG6040:M>I		DBVPG6765:M>I		K11:M>I		L_1374:M>I		L_1528:M>I		SK1:M>I		UWOPS05_227_2:M>I		UWOPS83_787_3:M>I		UWOPS87_2421:M>I		W303:M>I		Y12:M>I		Y55:M>I		Y9:M>I		YJM975:M>I		YJM978:M>I		YPS606:M>I		YS2:M>I	AA:284		273614X:M>T		322134S:M>T		DBVPG6040:M>T		DBVPG6765:M>T		L_1374:M>T		L_1528:M>T		UWOPS05_227_2:M>T		UWOPS83_787_3:M>T		Y55:M>T		YJM975:M>T		YJM978:M>T		YS2:M>T	AA:289		Y12:T>PID:YBR230C	AA:3		SK1:A>S		Y9:A>S	AA:42		DBVPG6044:S>P		YJM975:N>H	AA:47		UWOPS03_461_4:G>A	AA:62		273614X:V>L	AA:72		DBVPG6044:V>I	AA:102		DBVPG6765:P>S		YJM978:P>S	AA:121		YPS128:G>S		YPS606:G>S	AA:130		DBVPG1106:R>K		DBVPG1373:R>K		L_1374:R>K		L_1528:R>K		YJM975:R>KID:YBR231C	AA:3		DBVPG1106:E>K		L_1374:E>K	AA:20		UWOPS03_461_4:G>D		UWOPS87_2421:K>I	AA:38		Y55:G>D	AA:50		DBVPG6044:D>E	AA:200		DBVPG1788:K>N		Y55:K>N		YJM975:K>N	AA:210		BC187:R>Q		DBVPG1106:R>Q		DBVPG1788:R>Q		DBVPG6765:R>Q		L_1374:R>Q		Y55:R>Q		YJM975:R>Q		YJM978:R>Q		YJM981:R>Q	AA:259		DBVPG6040:V>M	AA:300		BC187:S>N		DBVPG1788:S>N		DBVPG1853:S>N		DBVPG6765:S>N		L_1374:S>N		W303:S>N		Y55:S>N		YJM975:S>N		YJM978:S>N		YJM981:S>NID:YBR233W	AA:17		DBVPG6044:V>A	AA:49		UWOPS05_217_3:A>T	AA:255		DBVPG6040:H>R	AA:263		DBVPG1373:P>S		DBVPG6765:P>S	AA:286		YJM978:A>S		YJM981:A>S	AA:303		YS9:P>	AA:304		YS9:N>ID:YBR233W-A	AA:78		DBVPG6044:L>FID:YBR236C	AA:51		322134S:P>A		SK1:P>A		YPS128:P>A		YPS606:P>A	AA:58		378604X:I>T		DBVPG1106:I>T		DBVPG1788:I>T		DBVPG1853:I>T		DBVPG6765:I>T		L_1528:I>T		Y55:I>T		YS2:I>T		YS4:I>T	AA:70		DBVPG1106:A>E		DBVPG1788:A>E		DBVPG1853:A>E		DBVPG6765:A>E		L_1528:A>E		Y55:A>E		YS2:A>E	AA:265		DBVPG6044:A>T		NCYC110:A>T	AA:347		W303:E>V	AA:386		SK1:R>K		Y12:R>K		Y9:R>KID:YBR237W	AA:39		273614X:H>N		378604X:H>N		BC187:H>N		DBVPG1373:H>N		DBVPG6765:H>N		K11:H>N		L_1528:H>N		NCYC110:H>N		SK1:H>N		UWOPS05_217_3:H>N		UWOPS05_227_2:H>N		UWOPS83_787_3:H>N		Y12:H>N		Y55:H>N		YPS128:H>N		YPS606:H>N		YS2:H>N	AA:53		273614X:G>A		378604X:G>A		BC187:G>A		DBVPG1373:G>A		DBVPG6765:G>A		L_1528:G>A		Y55:G>A		YS2:G>A	AA:76		273614X:N>S		378604X:N>S		BC187:N>S		DBVPG1373:N>S		DBVPG6765:N>S		L_1528:N>S		W303:N>S		Y55:N>S		YS2:N>S	AA:133		273614X:N>I		378604X:N>I		BC187:N>I		DBVPG1373:N>I		DBVPG6765:N>I		L_1528:N>I		W303:N>I		Y55:N>I		YS2:N>I	AA:165		K11:V>I	AA:217		YIIc17_E5:N>K	AA:231		DBVPG6044:M>V	AA:238		DBVPG1373:L>F		DBVPG6765:L>F		W303:L>F		Y55:L>F	AA:333		DBVPG6040:G>S	AA:359		YS9:S>F	AA:434		UWOPS03_461_4:K>R		UWOPS05_217_3:K>R	AA:453		YS9:L>V	AA:475		378604X:V>I		DBVPG1373:V>I		DBVPG1788:V>I		DBVPG6765:V>I		L_1374:V>I		L_1528:V>I		W303:V>I		Y55:V>I		YS9:V>I	AA:496		YS9:L>V	AA:501		378604X:H>Y		DBVPG1373:H>Y		DBVPG1788:H>Y		DBVPG6765:H>Y		K11:H>D		L_1374:H>Y		L_1528:H>Y		W303:H>Y		Y55:H>Y		YS4:H>Y		YS9:H>Y	AA:524		YS9:V>A	AA:647		DBVPG6044:E>K		NCYC110:E>K	AA:719		BC187:S>N		DBVPG1373:S>N		DBVPG6765:S>N		UWOPS05_227_2:S>N		UWOPS87_2421:S>N		W303:S>N		YJM978:S>N		YS9:S>N	AA:722		BC187:R>H		DBVPG1373:R>H		DBVPG6765:R>H		UWOPS05_227_2:R>H		UWOPS87_2421:R>H		W303:R>H		YJM978:R>H		YS9:R>H	AA:728		DBVPG6044:V>E		NCYC110:V>E		SK1:V>E		Y12:V>E		YIIc17_E5:V>E		YPS128:V>E		YPS606:V>E	AA:731		SK1:S>F		Y12:S>FID:YBR239C	AA:5		YJM975:D>E	AA:19		YJM975:A>T	AA:90		DBVPG1788:N>S		DBVPG1853:N>S		DBVPG6765:N>S		L_1374:N>S		L_1528:N>S		W303:N>S		Y55:N>S		YJM975:N>S	AA:101		DBVPG6044:M>I	AA:119		Y55:G>R	AA:212		273614X:I>M		DBVPG1373:I>M		DBVPG1788:I>M		DBVPG1853:I>M		DBVPG6044:I>M		DBVPG6765:I>M		L_1374:I>M		L_1528:I>M		UWOPS05_217_3:I>M		UWOPS05_227_2:I>M		Y55:I>M		YJM975:I>M	AA:259		L_1528:S>P	AA:261		NCYC361:N>T		UWOPS87_2421:N>S	AA:329		273614X:E>G		DBVPG1106:E>G		DBVPG1373:E>G		DBVPG6765:E>G		L_1528:E>G		W303:E>G		Y55:E>G		YJM975:E>G		YJM981:E>G		YS2:E>G		YS9:E>G	AA:358		YJM978:H>Y	AA:488		UWOPS05_227_2:K>EID:YBR240C	AA:2		DBVPG6765:V>I	AA:4		K11:S>N		SK1:S>N	AA:15		DBVPG6044:S>P	AA:83		UWOPS87_2421:Q>-	AA:88		DBVPG6044:K>N	AA:127		UWOPS05_227_2:E>D	AA:129		DBVPG6044:T>A	AA:267		NCYC110:E>G	AA:298		SK1:N>SID:YBR242W	AA:3		Y55:A>T		YJM981:A>T	AA:85		DBVPG6040:S>G		DBVPG6044:S>G		K11:S>G		SK1:S>G		UWOPS03_461_4:S>G		UWOPS05_217_3:S>G		UWOPS05_227_2:S>G		UWOPS83_787_3:S>G		UWOPS87_2421:S>G		Y12:S>G		YIIc17_E5:S>G		YPS128:S>G		YPS606:S>G		YS4:S>G	AA:98		L_1374:R>C	AA:173		UWOPS87_2421:Y>-	AA:187		YIIc17_E5:F>YID:YBR243C	AA:104		273614X:G>A	AA:139		273614X:T>S	AA:383		UWOPS03_461_4:D>G		UWOPS05_217_3:D>G		UWOPS05_227_2:D>GID:YBR244W	AA:52		NCYC110:K>NID:YBR246W	AA:2		DBVPG1373:D>N		DBVPG1853:D>N	AA:4		DBVPG1853:I>S	AA:28		UWOPS03_461_4:K>R		UWOPS05_227_2:K>R	AA:83		UWOPS03_461_4:A>V		UWOPS05_227_2:A>V	AA:95		DBVPG1373:R>G		DBVPG1853:R>G		DBVPG6044:R>G		K11:R>G		NCYC110:R>G		SK1:R>G		UWOPS03_461_4:R>G		UWOPS05_217_3:R>G		UWOPS05_227_2:R>G		UWOPS87_2421:R>G		Y12:R>G		YIIc17_E5:R>G		YPS606:R>G	AA:179		322134S:G>D	AA:190		322134S:G>E		DBVPG1373:G>E		DBVPG1853:G>E		DBVPG6044:G>E		K11:G>E		NCYC110:G>E		SK1:G>E		UWOPS03_461_4:G>E		UWOPS05_217_3:G>E		UWOPS05_227_2:G>E		UWOPS83_787_3:G>E		UWOPS87_2421:G>E		Y12:G>E		Y9:G>E		YIIc17_E5:G>E		YPS606:G>E	AA:280		K11:M>L	AA:289		DBVPG1373:A>E		K11:A>E		NCYC110:A>E		NCYC361:A>E		SK1:A>E		UWOPS05_227_2:A>E		UWOPS83_787_3:A>E		Y12:A>E		Y9:A>E	AA:316		YS2:E>K		YS9:E>KID:YBR247C	AA:60		NCYC110:R>K	AA:67		L_1528:K>R	AA:74		273614X:E>D	AA:109		273614X:A>G		SK1:A>G		UWOPS03_461_4:A>G		YPS128:A>G		YPS606:A>G		YS4:A>G	AA:154		YS9:S>R	AA:466		UWOPS83_787_3:R>S	AA:473		DBVPG6040:E>G		DBVPG6044:E>G		NCYC110:E>G		NCYC361:E>G		SK1:E>G		UWOPS83_787_3:E>G		Y12:E>G		Y9:E>G		YPS128:E>GID:YBR248C	AA:59		DBVPG6044:N>S	AA:98		DBVPG6765:K>R		Y55:K>R	AA:156		DBVPG1373:E>G	AA:157		273614X:K>N	AA:160		DBVPG6765:N>H	AA:270		YS2:G>C	AA:291		YS2:A>SID:YBR249C	AA:14		378604X:K>N		K11:K>N		SK1:K>N		Y12:K>N	AA:17		SK1:Q>R	AA:225		Y9:M>VID:YBR251W	AA:88		UWOPS03_461_4:S>C		UWOPS05_227_2:S>C	AA:277		378604X:E>KID:YBR252W	AA:3		DBVPG6040:A>T		K11:A>T		SK1:A>T		Y12:A>T		YS4:A>TID:YBR253W	AA:26		DBVPG6044:N>S		NCYC110:N>S	AA:31		YPS606:A>T	AA:39		DBVPG6044:G>D		K11:G>D		NCYC110:G>D		NCYC361:G>D		SK1:G>D		UWOPS05_217_3:G>D		UWOPS05_227_2:G>D		UWOPS83_787_3:G>D		UWOPS87_2421:G>D		YIIc17_E5:G>D		YPS606:G>D		YS4:G>D	AA:42		DBVPG6044:A>T		K11:A>T		NCYC110:A>T		SK1:A>T		UWOPS05_217_3:A>T		UWOPS05_227_2:A>T		UWOPS83_787_3:A>T		UWOPS87_2421:A>T		YIIc17_E5:A>T		YPS606:A>T	AA:62		K11:T>I		SK1:T>I	AA:111		YS4:C>RID:YBR254C	AA:142		SK1:V>I	AA:150		SK1:Y>H	AA:170		SK1:R>L	AA:176		UWOPS05_227_2:->EID:YBR256C	AA:9		378604X:M>I		DBVPG6044:M>I		NCYC110:M>I		SK1:M>I		UWOPS05_227_2:M>I		UWOPS83_787_3:M>I		UWOPS87_2421:M>I		YPS128:M>I		YPS606:M>I	AA:87		DBVPG6044:Q>K		NCYC110:Q>K		UWOPS05_227_2:Q>K		UWOPS83_787_3:Q>K		UWOPS87_2421:Q>K		YPS128:Q>K		YPS606:Q>K		YS4:Q>K	AA:188		YPS128:D>N		YPS606:D>N	AA:214		L_1374:S>TID:YBR257W	AA:10		DBVPG6040:D>E		DBVPG6044:D>E		SK1:D>E		UWOPS05_217_3:D>E		UWOPS87_2421:D>E		Y12:D>E		YIIc17_E5:D>E		YPS128:D>E	AA:63		DBVPG6044:V>A		UWOPS05_217_3:V>A	AA:105		DBVPG6040:K>R		SK1:K>R		Y12:K>R	AA:117		DBVPG6044:D>G	AA:129		DBVPG6040:I>RID:YBR258C	AA:30		DBVPG6044:S>F	AA:35		DBVPG6044:N>S		SK1:N>S		UWOPS05_227_2:N>S		Y12:N>S		YIIc17_E5:N>S	AA:98		DBVPG1853:G>D	AA:128		UWOPS83_787_3:Q>H		UWOPS87_2421:Q>H	AA:136		YIIc17_E5:E>-ID:YBR259W	AA:15		YPS128:G>C	AA:42		UWOPS05_227_2:T>A	AA:126		DBVPG6044:V>A		K11:V>A		NCYC110:V>A		SK1:V>A		UWOPS05_227_2:V>A		UWOPS83_787_3:V>A		YIIc17_E5:V>A		YPS128:V>A		YS4:V>A	AA:139		DBVPG6044:A>T		NCYC110:A>T		UWOPS05_227_2:A>T		YPS128:A>T		YPS606:A>T	AA:159		K11:I>V		SK1:I>V		UWOPS83_787_3:I>L		YIIc17_E5:I>V		YS4:I>V	AA:162		UWOPS83_787_3:K>R	AA:188		DBVPG6044:M>T		K11:M>T		NCYC110:M>T		SK1:M>T		UWOPS05_227_2:M>T		UWOPS83_787_3:M>T		Y9:M>T		YIIc17_E5:M>T		YPS128:M>T		YPS606:M>T		YS4:M>T	AA:199		DBVPG6044:E>K		NCYC110:E>K		UWOPS83_787_3:E>K		YPS128:E>K		YPS606:E>K	AA:257		K11:A>T		SK1:A>T		Y9:A>T		YS4:A>T	AA:268		UWOPS87_2421:F>L	AA:276		K11:E>K		SK1:E>K		UWOPS83_787_3:E>K		Y9:E>K		YPS128:E>K		YPS606:E>K		YS4:E>K	AA:301		NCYC361:I>V	AA:336		SK1:K>N		Y9:K>N	AA:366		DBVPG6044:T>M		NCYC110:T>M	AA:380		YPS128:P>S		YPS606:P>S	AA:393		NCYC110:H>R		SK1:H>R		UWOPS05_227_2:H>R		Y9:H>R		YIIc17_E5:H>R		YPS128:H>R		YS4:H>R	AA:400		YS4:Y>H	AA:404		NCYC110:I>V		NCYC361:I>V		SK1:I>V		UWOPS05_227_2:I>V		Y9:I>V		YIIc17_E5:I>V		YPS128:I>V		YS4:I>V	AA:438		NCYC361:K>R	AA:466		Y9:F>C	AA:491		YPS128:I>M		YPS606:I>M	AA:548		Y12:D>Y	AA:575		YS9:Q>R	AA:633		UWOPS05_217_3:A>T		UWOPS05_227_2:A>T	AA:674		DBVPG6040:K>N		DBVPG6044:K>N		SK1:K>N		UWOPS05_217_3:K>N		UWOPS05_227_2:K>N		Y12:K>N		YPS128:K>N		YPS606:K>N		YS9:K>NID:YBR260C	AA:114		DBVPG1106:Q>L	AA:255		DBVPG1106:Q>L	AA:366		273614X:S>N		SK1:S>N		UWOPS05_227_2:S>N		UWOPS87_2421:S>N		Y9:S>N		YIIc17_E5:S>N		YS9:S>N	AA:405		273614X:A>T	AA:420		UWOPS05_227_2:T>I	AA:423		YPS128:I>T		YPS606:I>T	AA:623		YS9:L>SID:YBR261C	AA:100		378604X:E>V		DBVPG6765:E>V		YJM978:E>V	AA:120		UWOPS05_217_3:E>-	AA:177		UWOPS87_2421:D>N	AA:202		UWOPS83_787_3:A>VID:YBR262C	AA:65		YS2:N>D		YS9:N>DID:YBR263W	AA:4		DBVPG6044:R>K	AA:20		DBVPG1106:L>I	AA:55		DBVPG6044:I>V	AA:205		SK1:V>L	AA:228		378604X:G>E		SK1:G>E		UWOPS05_217_3:G>E		YPS128:G>E		YPS606:G>E	AA:276		YS9:F>L	AA:283		YS9:S>F	AA:411		NCYC110:G>RID:YBR264C	AA:18		378604X:T>S	AA:53		378604X:S>A		DBVPG6044:S>A		NCYC110:S>A		SK1:S>A		UWOPS05_217_3:S>A		UWOPS87_2421:S>A		Y12:S>A		YIIc17_E5:S>A		YPS128:S>A		YPS606:S>A	AA:119		378604X:I>L		DBVPG6044:I>L		NCYC110:I>L		SK1:I>L		UWOPS87_2421:I>L		Y12:I>L		YIIc17_E5:I>L		YPS606:I>L		YS2:I>L	AA:131		378604X:H>D		DBVPG6044:H>D		NCYC110:H>D		SK1:H>D		UWOPS87_2421:H>D		Y12:H>D		Y9:H>D		YIIc17_E5:H>D		YPS606:H>D	AA:136		YS2:T>I	AA:140		L_1528:E>A		Y55:E>A	AA:156		DBVPG6044:N>D	AA:157		SK1:F>SID:YBR265W	AA:102		378604X:A>V		DBVPG6040:A>V		K11:A>V		SK1:A>V		UWOPS87_2421:A>V		Y9:A>V		YIIc17_E5:A>V		YPS128:A>V		YS4:A>V	AA:148		NCYC110:A>	AA:255		273614X:E>D		322134S:E>D		DBVPG1373:E>D		DBVPG6040:E>D		DBVPG6044:E>D		DBVPG6765:E>D		L_1528:E>D		S288c:E>D		SK1:E>D		UWOPS03_461_4:E>D		UWOPS05_227_2:E>D		UWOPS83_787_3:E>D		UWOPS87_2421:E>D		W303:E>D		Y55:E>D		YIIc17_E5:E>D		YJM975:E>D		YJM978:E>D		YPS128:E>D		YS4:E>D	AA:307		L_1528:I>VID:YBR267W	AA:6		YS4:V>I	AA:12		DBVPG1106:C>R	AA:18		322134S:S>A		378604X:S>A		BC187:S>A		DBVPG1373:S>A		DBVPG6040:S>A		DBVPG6044:S>A		DBVPG6765:S>A		L_1374:S>A		UWOPS03_461_4:S>A		UWOPS05_217_3:S>A		UWOPS05_227_2:S>A		UWOPS83_787_3:S>A		Y12:S>A		Y9:S>A		YJM975:S>A		YJM981:S>A		YS2:S>A		YS4:S>A		YS9:S>A	AA:23		UWOPS83_787_3:R>W	AA:56		DBVPG6040:A>V	AA:115		SK1:S>R	AA:185		YS9:R>S	AA:222		UWOPS05_217_3:Y>-	AA:267		378604X:N>D		UWOPS05_217_3:N>D		UWOPS05_227_2:N>D	AA:268		DBVPG6044:S>N		NCYC110:S>N	AA:269		UWOPS87_2421:N>S	AA:378		SK1:A>TID:YBR268W	AA:11		378604X:I>V	AA:16		378604X:I>V		DBVPG6044:I>V		DBVPG6765:I>V		NCYC110:I>V		SK1:I>V		UWOPS05_217_3:I>V		UWOPS05_227_2:I>V		UWOPS87_2421:I>V		YPS128:I>V	AA:38		DBVPG6040:L>F	AA:62		UWOPS87_2421:V>AID:YBR269C	AA:30		UWOPS05_227_2:T>N	AA:97		K11:E>KID:YBR270C	AA:11		322134S:A>T	AA:15		322134S:S>A		378604X:S>A		DBVPG1373:S>A		DBVPG1853:S>A		DBVPG6044:S>A		DBVPG6765:S>A		L_1374:S>A		L_1528:S>A		SK1:S>A		UWOPS05_227_2:S>A		Y55:S>A		Y9:S>A		YJM978:S>A		YJM981:S>A		YPS128:S>A		YS2:S>A	AA:94		YJM978:S>N		YJM981:S>N	AA:96		DBVPG6044:G>R	AA:107		YJM975:G>V	AA:167		DBVPG6044:N>S		K11:N>S		SK1:N>S		UWOPS03_461_4:N>S	AA:215		K11:P>S	AA:229		322134S:P>S		378604X:P>S		NCYC361:P>S		YS4:P>S	AA:273		UWOPS87_2421:S>C	AA:346		UWOPS03_461_4:W>R		UWOPS05_227_2:W>R	AA:373		DBVPG6765:V>A	AA:386		322134S:I>V		YS2:I>V	AA:481		DBVPG1788:S>R		Y55:S>R		YJM975:S>RID:YBR271W	AA:24		DBVPG6044:E>G	AA:46		YS4:E>Q	AA:166		273614X:E>D		378604X:E>D		DBVPG1106:E>D		DBVPG1373:E>D		DBVPG1853:E>D		DBVPG6044:E>D		DBVPG6765:E>D		L_1374:E>D		L_1528:E>D		SK1:E>D		UWOPS87_2421:E>D		Y12:E>D		Y55:E>D		Y9:E>D		YJM978:E>D		YJM981:E>D		YPS128:E>D		YS2:E>D	AA:206		Y12:P>S		Y9:P>S	AA:248		BC187:A>V		UWOPS05_227_2:A>V	AA:352		378604X:V>I	AA:417		YJM978:V>AID:YBR272C	AA:79		DBVPG6044:V>I	AA:161		378604X:E>V	AA:179		UWOPS03_461_4:P>S		UWOPS05_227_2:P>S	AA:248		DBVPG1373:I>V	AA:419		UWOPS03_461_4:G>E		UWOPS05_227_2:G>EID:YBR273C	AA:20		378604X:E>Q		BC187:E>Q		DBVPG1373:E>Q		DBVPG1788:E>Q		DBVPG6765:E>Q		L_1528:E>Q		UWOPS87_2421:E>Q		Y55:E>Q		YJM981:E>Q		YPS606:E>Q		YS2:E>Q	AA:90		SK1:E>G	AA:106		378604X:A>T		DBVPG1373:A>T		DBVPG1788:A>T		DBVPG6765:A>T		L_1528:A>T		UWOPS05_227_2:A>T		UWOPS83_787_3:A>T		UWOPS87_2421:A>T		Y55:A>T		YJM981:A>T		YPS128:A>T		YPS606:A>T		YS2:A>T		YS4:A>T	AA:116		Y9:G>R	AA:248		322134S:V>L		378604X:V>L		DBVPG1373:V>L		DBVPG1788:V>L		DBVPG6044:V>L		DBVPG6765:V>L		K11:V>L		L_1528:V>L		UWOPS05_227_2:V>L		UWOPS83_787_3:V>L		UWOPS87_2421:V>L		Y55:V>L		YJM975:V>L		YJM978:V>L		YPS128:V>L		YPS606:V>L	AA:323		322134S:H>R		378604X:H>R		DBVPG1373:H>R		DBVPG6044:H>R		DBVPG6765:H>R		K11:H>R		L_1528:H>R		UWOPS83_787_3:H>R		UWOPS87_2421:H>R		YJM975:H>R		YJM978:H>R	AA:352		DBVPG6044:S>T	AA:364		UWOPS83_787_3:N>Y	AA:426		L_1374:H>L	AA:436		322134S:K>E		378604X:K>E		DBVPG6044:K>E		DBVPG6765:K>E		K11:K>E		L_1374:K>E		L_1528:K>E		UWOPS05_227_2:K>E		UWOPS83_787_3:K>E		YJM978:K>EID:YBR274W	AA:9		YIIc17_E5:L>F	AA:23		YJM975:Q>R	AA:35		YJM975:Q>R	AA:58		YJM975:S>G	AA:69		YJM975:Q>R	AA:85		DBVPG1853:N>D	AA:127		UWOPS83_787_3:S>R	AA:147		UWOPS83_787_3:N>D	AA:299		UWOPS83_787_3:L>I	AA:357		YJM978:Q>L	AA:430		K11:P>ID:YBR276C	AA:56		322134S:Q>K	AA:91		DBVPG6040:C>R	AA:111		UWOPS05_227_2:N>K		YPS606:N>K	AA:122		L_1374:L>M	AA:128		SK1:A>D		UWOPS05_227_2:A>D		W303:A>D		YIIc17_E5:A>D		YPS606:A>D	AA:139		UWOPS05_227_2:H>Y	AA:207		322134S:R>Q		378604X:R>Q		NCYC110:R>Q		Y55:R>Q		YJM975:R>Q		YJM981:R>Q		YS2:R>Q	AA:214		322134S:M>I		378604X:M>I		NCYC110:M>I		Y55:M>I		YJM975:M>I		YJM981:M>I		YS2:M>I	AA:253		UWOPS05_227_2:G>E	AA:257		UWOPS05_227_2:Y>H	AA:275		322134S:Q>P		K11:Q>K		SK1:Q>K		W303:Q>K		YIIc17_E5:Q>K		YPS128:Q>K	AA:285		322134S:N>H	AA:299		322134S:S>A	AA:304		DBVPG1853:V>M		K11:V>M		SK1:V>M	AA:331		322134S:E>Q	AA:345		322134S:N>S	AA:357		378604X:T>P		DBVPG1106:T>P		DBVPG1373:T>P		K11:T>P		SK1:T>P		UWOPS87_2421:T>P		W303:T>P		Y55:T>P		Y9:T>P		YPS128:T>P		YS2:T>P	AA:362		DBVPG1373:E>K	AA:373		378604X:N>I		K11:N>I		SK1:N>I		UWOPS87_2421:N>I		W303:N>I		YPS128:N>I	AA:424		378604X:D>G	AA:479		378604X:E>G		SK1:E>G		UWOPS05_227_2:E>G		UWOPS87_2421:E>G		W303:E>G		Y12:E>G		Y9:E>G		YIIc17_E5:E>G		YPS128:E>G		YPS606:E>G	AA:496		Y12:H>Q		Y9:H>Q	AA:515		378604X:L>F	AA:528		378604X:P>T	AA:548		YPS128:L>S		YPS606:L>S	AA:605		YIIc17_E5:A>D	AA:645		Y55:N>H		YJM975:N>H		YJM978:N>H	AA:705		DBVPG6765:D>N		L_1528:D>N		Y55:D>N		YJM975:D>N		YS9:D>N	AA:706		378604X:K>N	AA:783		DBVPG6044:N>H		NCYC110:N>HID:YBR278W	AA:21		Y9:A>S	AA:38		S288c:T>P	AA:61		Y9:S>L	AA:193		UWOPS03_461_4:V>A		UWOPS05_217_3:V>A		UWOPS87_2421:V>AID:YBR279W	AA:59		DBVPG6044:D>N	AA:211		DBVPG6044:T>A		NCYC110:T>A	AA:344		Y9:N>I	AA:420		DBVPG6044:A>D		NCYC110:A>D		UWOPS05_227_2:A>D		UWOPS83_787_3:A>D		UWOPS87_2421:A>D	AA:423		DBVPG6044:P>T		NCYC110:P>T		UWOPS05_227_2:P>T		UWOPS83_787_3:P>T		UWOPS87_2421:P>T	AA:429		UWOPS05_227_2:V>FID:YBR280C	AA:11		322134S:P>S		378604X:P>S		DBVPG6040:P>S		DBVPG6044:P>S		UWOPS05_227_2:P>S		UWOPS87_2421:P>S		Y9:P>S		YIIc17_E5:P>S		YPS128:P>S	AA:16		322134S:S>P		378604X:S>P		DBVPG6040:S>P		DBVPG6044:S>P		UWOPS05_227_2:S>P		UWOPS87_2421:S>P		Y9:S>P		YIIc17_E5:S>P		YPS128:S>P	AA:25		DBVPG6044:P>S	AA:75		273614X:G>C		322134S:G>C		DBVPG1373:G>C		DBVPG6044:G>C		DBVPG6765:G>C		UWOPS05_227_2:G>C		UWOPS87_2421:G>C		Y55:G>C		Y9:G>C		YIIc17_E5:G>C		YJM975:G>C		YJM978:G>C		YPS128:G>C	AA:101		273614X:V>L		322134S:V>L		DBVPG1373:V>L		DBVPG6040:V>L		DBVPG6044:V>L		DBVPG6765:V>L		K11:V>L		UWOPS05_227_2:V>L		UWOPS87_2421:V>L		Y55:V>L		Y9:V>L		YPS128:V>L	AA:110		DBVPG6044:K>R	AA:127		YS2:S>C	AA:136		322134S:S>T		YS2:S>L	AA:141		UWOPS87_2421:S>G	AA:157		UWOPS87_2421:F>I	AA:173		YIIc17_E5:D>E	AA:179		UWOPS87_2421:K>M	AA:237		UWOPS87_2421:N>Y	AA:302		378604X:S>N		DBVPG1373:S>G	AA:312		SK1:M>I		W303:M>I		Y12:M>I		YPS128:M>I		YPS606:M>I	AA:344		L_1528:A>V	AA:361		UWOPS05_227_2:F>S	AA:381		YIIc17_E5:T>A	AA:406		YS2:S>I	AA:518		DBVPG1788:L>I	AA:532		DBVPG1853:G>A	AA:547		UWOPS05_227_2:N>I	AA:601		YS9:R>T	AA:630		YS4:G>VID:YBR281C	AA:3		378604X:D>H		DBVPG1373:D>H		DBVPG1853:D>H	AA:78		DBVPG6044:G>R		K11:G>R		SK1:G>R		UWOPS05_217_3:G>R		UWOPS05_227_2:G>R		UWOPS83_787_3:G>R		UWOPS87_2421:G>R		Y12:G>R		YIIc17_E5:G>R		YPS128:G>R		YPS606:G>R	AA:110		378604X:P>S		DBVPG1373:P>S		DBVPG1853:P>S	AA:138		DBVPG6044:I>V	AA:140		322134S:F>S	AA:163		DBVPG6765:D>N	AA:235		K11:R>S	AA:245		DBVPG1373:R>Q	AA:407		DBVPG1373:S>T	AA:423		DBVPG6044:L>S		SK1:L>S		UWOPS03_461_4:L>S		UWOPS05_217_3:L>S		Y12:L>S		Y9:L>S		YPS128:L>S		YPS606:L>S	AA:441		DBVPG1373:L>V	AA:455		Y55:D>V	AA:541		UWOPS87_2421:C>F	AA:598		UWOPS83_787_3:E>K	AA:637		DBVPG6044:Q>R	AA:641		322134S:W>C		YS2:W>C	AA:675		378604X:N>K	AA:684		UWOPS03_461_4:S>A		UWOPS05_217_3:S>A		UWOPS05_227_2:S>A	AA:815		UWOPS83_787_3:V>IID:YBR282W	AA:73		K11:N>SID:YBR283C	AA:7		DBVPG1373:I>T	AA:181		DBVPG6765:A>T	AA:387		K11:A>V	AA:408		DBVPG6044:M>I		NCYC110:M>I	AA:479		YS9:A>TID:YBR284W	AA:66		DBVPG6765:S>N	AA:100		YPS128:A>T		YPS606:A>T	AA:147		DBVPG6044:R>Q		NCYC110:R>Q		SK1:R>Q	AA:180		DBVPG1853:F>S	AA:195		YS9:N>T	AA:207		Y55:F>V	AA:283		UWOPS03_461_4:L>I		UWOPS05_227_2:L>I	AA:307		DBVPG6044:D>G		NCYC110:D>G		SK1:D>G	AA:355		322134S:L>P		DBVPG1853:L>P		DBVPG6765:L>P		UWOPS83_787_3:L>P		YJM975:L>P		YJM978:L>P		YJM981:L>P		YS2:L>P		YS9:L>P	AA:519		UWOPS05_217_3:D>G	AA:526		UWOPS05_217_3:K>R	AA:567		YPS128:A>S		YPS606:A>S	AA:602		322134S:A>T		BC187:A>T		DBVPG1106:A>T		DBVPG6765:A>T		K11:A>T		L_1528:A>T		UWOPS05_217_3:A>T		Y55:A>T		Y9:A>T		YJM975:A>T		YJM981:A>T		YS2:A>T	AA:650		322134S:K>N		BC187:K>N		DBVPG1106:K>N		DBVPG6765:K>N		K11:K>N		L_1528:K>N		UWOPS05_217_3:K>N		Y12:K>N		Y55:K>N		Y9:K>N		YJM975:K>N		YJM981:K>N		YS2:K>N	AA:728		YS2:S>R	AA:771		322134S:S>T		BC187:S>T		DBVPG1106:S>T		DBVPG6040:S>T		DBVPG6765:S>T		L_1374:S>T		UWOPS83_787_3:S>T		YS2:S>T	AA:786		UWOPS87_2421:S>R	AA:788		378604X:L>IID:YBR285W	AA:43		YIIc17_E5:S>I	AA:44		NCYC110:P>S	AA:55		322134S:H>D		DBVPG1373:H>D		DBVPG1788:H>D		DBVPG6040:H>D		DBVPG6044:H>D		K11:H>D		NCYC110:H>D		S288c:H>D		SK1:H>D		UWOPS05_217_3:H>D		UWOPS05_227_2:H>D		UWOPS83_787_3:H>D		UWOPS87_2421:H>D		W303:H>D		Y12:H>D		Y55:H>D		YIIc17_E5:H>D		YJM975:H>D		YJM978:H>D		YPS128:H>D		YS4:H>D		YS9:H>D	AA:129		NCYC110:R>-ID:YBR286W	AA:46		YJM975:N>K	AA:124		322134S:V>A		BC187:V>A		DBVPG1788:V>A		DBVPG1853:V>A		DBVPG6765:V>A		SK1:V>A		UWOPS05_217_3:V>A		UWOPS83_787_3:V>A		Y55:V>A		YJM975:V>A		YJM981:V>A		YPS128:V>A		YPS606:V>A		YS2:V>A	AA:217		SK1:D>H	AA:238		DBVPG1788:K>E		YS2:K>E		YS4:K>E	AA:294		DBVPG6044:Q>K		SK1:Q>K		UWOPS05_217_3:Q>K		YPS128:Q>K		YPS606:Q>KID:YBR287W	AA:224		UWOPS87_2421:R>G	AA:228		YJM975:S>Y	AA:229		K11:F>L	AA:245		378604X:T>A		DBVPG1853:T>A		DBVPG6044:T>A		DBVPG6765:T>A		K11:T>A		NCYC110:T>A		SK1:T>A		UWOPS87_2421:T>A		Y12:T>A		Y55:T>A		Y9:T>A		YIIc17_E5:T>A		YJM975:T>A		YS2:T>A	AA:266		UWOPS83_787_3:V>GID:YBR288C	AA:68		YPS606:V>L	AA:75		YPS606:W>C	AA:85		UWOPS03_461_4:G>C		UWOPS05_217_3:G>C		UWOPS05_227_2:G>C	AA:100		YS2:L>P	AA:190		BC187:N>S		DBVPG6765:N>S		UWOPS03_461_4:N>S		UWOPS05_217_3:N>S		UWOPS05_227_2:N>S		UWOPS83_787_3:N>S		UWOPS87_2421:N>S		Y55:N>S		YIIc17_E5:N>S		YPS128:N>S		YPS606:N>S		YS4:N>S	AA:205		YIIc17_E5:R>K	AA:307		DBVPG6044:S>N		NCYC110:S>N		SK1:S>N		UWOPS03_461_4:S>N		UWOPS05_227_2:S>N		UWOPS83_787_3:S>N		Y12:S>N		YIIc17_E5:S>N		YPS128:S>N		YPS606:S>N		YS4:S>N	AA:337		UWOPS05_227_2:E>D	AA:405		273614X:T>I		322134S:T>S	AA:427		DBVPG6044:K>N		SK1:K>N		UWOPS03_461_4:K>N		UWOPS05_227_2:K>N		UWOPS83_787_3:K>N		Y12:K>N		YIIc17_E5:K>N		YPS128:K>N		YPS606:K>N		YS4:K>N	AA:446		DBVPG6040:G>RID:YBR290W	AA:14		UWOPS03_461_4:T>I		UWOPS05_227_2:T>I	AA:62		UWOPS03_461_4:G>V		UWOPS05_227_2:G>V	AA:76		DBVPG6040:E>K	AA:116		YPS128:F>Y	AA:119		YIIc17_E5:L>I	AA:313		UWOPS83_787_3:Y>CID:YBR291C	AA:36		Y55:A>S	AA:97		322134S:M>L		L_1528:M>L		SK1:M>L		UWOPS03_461_4:M>L		UWOPS05_217_3:M>L		Y9:M>L		YIIc17_E5:M>L		YPS606:M>L	AA:101		SK1:S>R		UWOPS03_461_4:S>R		UWOPS05_217_3:S>R		UWOPS83_787_3:S>R		Y9:S>R		YIIc17_E5:S>R		YPS606:S>R	AA:110		UWOPS83_787_3:R>K	AA:248		UWOPS83_787_3:T>P	AA:250		UWOPS83_787_3:Y>CID:YBR293W	AA:91		BC187:I>T	AA:99		378604X:L>I	AA:139		UWOPS05_217_3:N>K	AA:153		DBVPG6044:S>F		NCYC110:S>F		SK1:S>F		YPS606:S>F	AA:197		UWOPS83_787_3:T>A	AA:203		YJM978:L>Q	AA:210		UWOPS03_461_4:I>V		UWOPS05_217_3:I>V		UWOPS83_787_3:I>V		YIIc17_E5:I>V		YPS606:I>V	AA:215		YJM978:F>L	AA:217		UWOPS83_787_3:L>S	AA:257		UWOPS83_787_3:F>S	AA:271		NCYC110:T>I		SK1:T>I	AA:379		BC187:V>I		DBVPG1106:V>I		DBVPG1373:V>I		DBVPG1853:V>I	AA:384		UWOPS03_461_4:I>T		UWOPS05_217_3:I>T	AA:385		YIIc17_E5:S>-	AA:455		322134S:S>L		BC187:S>L		DBVPG1853:S>L		DBVPG6765:S>L		K11:S>L		L_1374:S>L		L_1528:S>L		NCYC110:S>L		NCYC361:S>L		SK1:S>L		UWOPS03_461_4:S>L		UWOPS05_217_3:S>L		UWOPS83_787_3:S>L		UWOPS87_2421:S>L		Y12:S>L		Y55:S>L		YIIc17_E5:S>L		YPS128:S>L		YPS606:S>L		YS4:S>LID:YBR296C	AA:24		378604X:I>L	AA:30		378604X:A>E	AA:40		378604X:R>T	AA:45		DBVPG6040:W>-	AA:279		YS4:E>D	AA:293		UWOPS87_2421:N>D		Y12:N>D	AA:326		DBVPG6040:L>V	AA:386		Y55:K>R	AA:413		NCYC361:T>S	AA:417		378604X:A>T		YIIc17_E5:A>T	AA:494		378604X:V>A		DBVPG1788:V>A		DBVPG6040:V>A		DBVPG6765:V>A		NCYC361:V>A		SK1:V>A		UWOPS05_217_3:V>A		UWOPS05_227_2:V>A		UWOPS87_2421:V>A		YIIc17_E5:V>A		YPS606:V>A		YS4:V>A	AA:498		378604X:T>I	AA:554		DBVPG1853:A>SID:YBR298C	AA:18		UWOPS03_461_4:H>N		UWOPS05_227_2:E>D	AA:38		DBVPG6044:E>-		NCYC110:E>-	AA:45		UWOPS03_461_4:F>V		UWOPS05_227_2:F>V		UWOPS83_787_3:F>V	AA:46		SK1:D>G	AA:50		SK1:L>H		YS4:L>H	AA:58		273614X:I>T		322134S:I>T		DBVPG6044:I>T		NCYC110:I>T		UWOPS03_461_4:I>T		UWOPS05_227_2:I>T		UWOPS83_787_3:I>T		UWOPS87_2421:I>T		Y55:I>T		YPS606:I>T		YS2:I>T		YS4:I>T	AA:66		UWOPS03_461_4:V>F		UWOPS05_227_2:V>F		UWOPS83_787_3:V>F	AA:122		DBVPG6044:A>S		NCYC110:A>S		UWOPS03_461_4:A>S		UWOPS05_217_3:A>S		UWOPS05_227_2:A>S		UWOPS87_2421:A>S		Y55:A>S		YPS606:A>S		YS4:A>S	AA:130		DBVPG1788:Q>H	AA:147		322134S:V>A		YS2:V>A	AA:157		YS2:Y>C	AA:163		378604X:V>F	AA:171		322134S:S>F		DBVPG6044:S>F		NCYC110:S>F		SK1:S>F		UWOPS03_461_4:S>F		UWOPS05_217_3:S>F		UWOPS05_227_2:S>F		UWOPS83_787_3:S>F		UWOPS87_2421:S>F		YPS128:S>F		YPS606:S>F		YS2:S>F	AA:222		YS4:T>A	AA:232		YS4:L>M	AA:247		273614X:A>T		BC187:A>T		DBVPG1788:A>T		DBVPG1853:A>T		DBVPG6765:A>T		K11:A>T		L_1374:A>T		Y9:A>T		YJM975:A>T		YJM981:A>T	AA:261		YJM978:Q>H	AA:268		UWOPS03_461_4:E>D		UWOPS05_217_3:E>D		UWOPS05_227_2:E>D		UWOPS83_787_3:E>D		UWOPS87_2421:E>D		YPS128:E>D		YPS606:E>D	AA:300		273614X:V>A	AA:315		Y9:T>I	AA:328		273614X:S>T		DBVPG1788:S>T		DBVPG1853:S>T		L_1374:S>T		SK1:S>T	AA:367		DBVPG6040:C>F	AA:378		322134S:A>T	AA:383		322134S:Y>N	AA:415		322134S:I>V		SK1:I>V		Y12:I>V		Y9:I>V		YS4:I>V	AA:419		UWOPS03_461_4:A>G		UWOPS05_217_3:A>G		UWOPS05_227_2:A>G		UWOPS87_2421:A>G		YPS128:A>G	AA:422		DBVPG1853:Y>H		YS4:Y>H	AA:430		UWOPS03_461_4:A>T		UWOPS05_217_3:A>T		UWOPS05_227_2:A>T	AA:434		UWOPS03_461_4:A>V		UWOPS05_217_3:A>V		UWOPS05_227_2:A>V	AA:438		UWOPS03_461_4:I>V		UWOPS05_217_3:I>V		UWOPS05_227_2:I>V	AA:455		K11:K>E	AA:460		K11:A>S		SK1:A>S		UWOPS83_787_3:A>S		YPS606:A>S	AA:466		Y55:A>V		YPS606:A>T	AA:475		YPS606:P>L	AA:484		273614X:I>M		322134S:I>M		378604X:I>M		BC187:I>M		DBVPG1788:I>M		Y12:I>M		Y9:I>M		YS4:I>M	AA:489		YPS606:L>P	AA:498		YPS606:R>H	AA:506		UWOPS05_217_3:V>I		UWOPS05_227_2:V>I	AA:508		UWOPS05_217_3:V>I		UWOPS05_227_2:V>I	AA:584		273614X:A>V	AA:588		Y12:I>N		Y9:I>N	AA:593		YS4:P>S	AA:594		378604X:K>-	AA:595		YS4:E>D	AA:607		DBVPG6040:N>S		Y55:N>S		YIIc17_E5:N>S		YJM978:N>S		YS4:N>S		YS9:N>S	AA:609		273614X:S>P		DBVPG1373:S>LID:YBR301W	AA:7		NCYC361:I>V	AA:55		322134S:V>A		DBVPG1853:V>A		DBVPG6765:V>A		K11:V>A		L_1374:V>A		SK1:V>A		UWOPS05_217_3:V>A		UWOPS83_787_3:V>A		Y9:V>A		YJM975:V>A		YJM981:V>A		YPS128:V>A		YPS606:V>A		YS2:V>A	AA:92		DBVPG1853:I>F	AA:100		DBVPG1853:S>T		L_1374:S>T		Y55:S>T		YS4:S>TID:YCL004W	AA:62		322134S:K>N		378604X:K>N		DBVPG1853:K>N		DBVPG6044:K>N		K11:K>N		SK1:K>N		UWOPS05_217_3:K>N		UWOPS05_227_2:K>N		UWOPS83_787_3:K>N		Y12:K>N		YPS128:K>N		YS4:K>N		YS9:K>N	AA:81		DBVPG1788:I>T	AA:237		273614X:I>T	AA:270		DBVPG1853:A>T	AA:394		UWOPS87_2421:Q>K	AA:440		DBVPG1853:N>S	AA:478		273614X:K>N	AA:481		YJM975:A>T	AA:500		322134S:S>-ID:YCL005W	AA:66		YJM978:F>I	AA:120		Y9:R>P	AA:247		DBVPG1788:N>SID:YCL010C	AA:72		DBVPG6040:S>P	AA:74		322134S:M>V	AA:78		L_1528:S>P	AA:99		DBVPG6044:A>P		NCYC110:A>P		YPS128:A>P	AA:123		DBVPG6044:A>V		NCYC110:A>V	AA:180		378604X:R>W		DBVPG6040:R>W		DBVPG6044:R>W		NCYC110:R>W		UWOPS05_217_3:R>W		Y9:R>W		YPS128:R>W	AA:192		UWOPS87_2421:T>A	AA:253		322134S:L>S		378604X:L>S		DBVPG6044:L>S		DBVPG6765:L>S		L_1528:L>S		UWOPS05_217_3:L>S		Y55:L>S		Y9:V>L		YJM975:L>S		YPS128:L>SID:YCL011C	AA:36		L_1374:S>T	AA:60		Y9:D>H	AA:64		NCYC110:G>D	AA:69		YS4:H>Y	AA:230		322134S:M>I		DBVPG1106:M>I		DBVPG1373:M>I		DBVPG1788:M>I		DBVPG1853:M>I		DBVPG6044:M>I		DBVPG6765:M>I		L_1528:M>I		SK1:M>I		UWOPS05_227_2:M>I		Y55:M>I		YIIc17_E5:M>I		YPS128:M>I		YS4:M>I	AA:311		DBVPG1853:D>N	AA:321		DBVPG6044:G>S		YIIc17_E5:G>S		YPS128:G>S	AA:323		DBVPG1853:A>T	AA:338		YGPM:T>I	AA:416		UWOPS87_2421:G>AID:YCL016C	AA:23		273614X:E>K		BC187:E>K		DBVPG6765:E>K		L_1374:E>K		L_1528:E>K		SK1:E>K		Y55:E>K		YJM978:E>K		YS4:E>K	AA:35		DBVPG6044:H>R	AA:36		K11:Q>K		YPS606:Q>K	AA:104		YS9:T>I	AA:133		DBVPG6040:D>G	AA:139		UWOPS83_787_3:S>P		YIIc17_E5:S>P	AA:148		YIIc17_E5:E>-	AA:183		DBVPG6044:D>N	AA:221		NCYC361:D>V	AA:277		YIIc17_E5:I>L	AA:360		UWOPS83_787_3:S>CID:YCL017C	AA:8		DBVPG1106:R>K		DBVPG1373:R>K		DBVPG1788:R>K		DBVPG6765:R>K		L_1374:R>K		Y55:R>K		YS2:R>K		YS9:R>K	AA:14		NCYC110:S>F	AA:22		UWOPS05_217_3:A>V		UWOPS05_227_2:A>V	AA:50		UWOPS03_461_4:E>-	AA:58		UWOPS03_461_4:A>S	AA:368		YPS128:D>N		YPS606:D>NID:YCL021W-A	AA:66		L_1528:S>N	AA:69		322134S:Y>H		DBVPG6040:Y>H		YPS128:Y>H		YPS606:Y>H	AA:85		Y12:V>A		YIIc17_E5:V>AID:YCL025C	AA:7		DBVPG1853:L>P		DBVPG6044:L>P		K11:L>P		NCYC361:L>P		SK1:L>P		UWOPS03_461_4:L>P		UWOPS05_217_3:L>P		UWOPS05_227_2:L>P		UWOPS83_787_3:L>P		UWOPS87_2421:L>P		Y12:L>P		YPS606:L>P		YS4:L>P	AA:16		UWOPS03_461_4:S>G		UWOPS05_227_2:S>G	AA:24		DBVPG1853:G>E		DBVPG6044:G>E		K11:G>E		NCYC361:G>E		SK1:G>E		UWOPS03_461_4:G>E		UWOPS05_217_3:G>E		UWOPS05_227_2:G>E		UWOPS83_787_3:G>E		YPS606:G>E		YS4:G>E	AA:32		SK1:F>I	AA:74		DBVPG1853:V>I	AA:92		DBVPG1853:Q>R		K11:Q>R		UWOPS83_787_3:Q>R		Y9:Q>R		YPS606:Q>R	AA:215		UWOPS05_217_3:P>L	AA:316		273614X:A>V		378604X:A>V		BC187:A>V		DBVPG1373:A>V		DBVPG6044:A>V		DBVPG6765:A>V		K11:A>V		NCYC110:A>V		SK1:A>V		UWOPS03_461_4:A>V		UWOPS05_217_3:A>V		UWOPS05_227_2:A>V		UWOPS83_787_3:A>V		Y55:A>V		YIIc17_E5:A>V		YJM978:A>V		YPS128:A>V		YPS606:A>V		YS9:A>V	AA:324		273614X:F>Y	AA:355		L_1374:Y>C	AA:418		L_1374:S>C	AA:530		Y12:A>SID:YCL026C-A	AA:12		YS4:T>A	AA:78		BC187:E>G		DBVPG1106:E>G		DBVPG1373:E>G		DBVPG1788:E>G		DBVPG6044:E>G		DBVPG6765:E>G		L_1528:E>G		NCYC110:E>G		UWOPS03_461_4:E>G		UWOPS05_227_2:E>G		Y55:E>G		YJM975:E>G		YJM981:E>G	AA:141		DBVPG6044:L>F		NCYC110:L>F		Y55:L>F	AA:168		YPS128:S>C		YPS606:S>C	AA:186		UWOPS03_461_4:N>S		UWOPS05_227_2:N>SID:YCL028W	AA:55		K11:D>E	AA:199		NCYC110:S>F	AA:230		YPS606:Y>C	AA:238		DBVPG6044:N>S		NCYC110:N>S	AA:360		378604X:Q>H		DBVPG6044:Q>H		NCYC110:Q>H		Y9:Q>H		YPS606:Q>H		YS4:Q>H	AA:383		DBVPG6044:Q>H		NCYC110:Q>H		YPS606:Q>HID:YCL029C	AA:59		S288c:T>S	AA:131		W303:S>I	AA:138		DBVPG6044:D>G	AA:224		YIIc17_E5:M>I	AA:353		UWOPS03_461_4:K>M		UWOPS05_227_2:K>M	AA:357		DBVPG6044:L>S		NCYC361:L>S		UWOPS03_461_4:L>S		UWOPS05_227_2:L>S		UWOPS83_787_3:L>S		Y12:L>S		Y9:L>S		YIIc17_E5:L>S		YPS606:L>S	AA:360		DBVPG6044:P>S		NCYC110:P>S	AA:392		DBVPG6044:T>A		NCYC110:T>AID:YCL030C	AA:33		DBVPG6044:S>L		NCYC110:S>L	AA:49		UWOPS83_787_3:V>F	AA:63		UWOPS03_461_4:D>N	AA:80		DBVPG6044:A>G		NCYC361:A>G		UWOPS83_787_3:A>G		YPS128:A>G		YPS606:A>G	AA:86		UWOPS03_461_4:T>I	AA:88		273614X:E>D		322134S:E>D		BC187:E>D		DBVPG1373:E>D		DBVPG1788:E>D		DBVPG6044:E>D		DBVPG6765:E>D		L_1374:E>D		L_1528:E>D		NCYC361:E>D		SK1:E>D		UWOPS03_461_4:E>D		UWOPS83_787_3:E>D		Y12:E>D		Y55:E>D		Y9:E>D		YJM978:E>D		YPS128:E>D		YPS606:E>D	AA:122		BC187:D>H	AA:172		BC187:K>E		DBVPG6044:K>E		NCYC361:K>E		SK1:K>E		UWOPS83_787_3:K>E		YIIc17_E5:K>E		YJM975:K>E		YJM978:K>E		YPS128:K>E		YPS606:K>E	AA:202		UWOPS05_217_3:N>I	AA:211		UWOPS05_217_3:T>A	AA:356		UWOPS03_461_4:P>S		UWOPS05_217_3:P>S	AA:469		YS2:L>M	AA:553		YS9:Y>S	AA:583		NCYC361:T>SID:YCL031C	AA:5		DBVPG6044:D>E	AA:13		Y55:F>I	AA:71		DBVPG6044:V>I	AA:139		UWOPS05_217_3:N>I		UWOPS05_227_2:N>I		UWOPS83_787_3:N>I	AA:153		L_1528:N>K	AA:161		Y55:T>R	AA:202		DBVPG6044:E>G		L_1528:V>I	AA:289		UWOPS05_227_2:A>VID:YCL032W	AA:4		UWOPS05_217_3:G>S	AA:62		UWOPS05_217_3:E>D		UWOPS05_227_2:E>D		UWOPS83_787_3:E>D	AA:100		YIIc17_E5:R>G	AA:146		273614X:D>E		322134S:D>E		BC187:D>E		DBVPG6040:D>E		DBVPG6044:D>E		K11:D>E		NCYC361:D>E		SK1:D>E		UWOPS03_461_4:D>E		UWOPS05_227_2:D>E		UWOPS83_787_3:D>E		Y55:D>E		YIIc17_E5:D>E		YJM975:D>E		YJM981:D>E		YPS606:D>E		YS2:D>E		YS4:D>E	AA:153		DBVPG6044:S>N	AA:180		DBVPG6040:V>A	AA:247		UWOPS03_461_4:P>S		UWOPS05_227_2:P>S		UWOPS83_787_3:P>S	AA:286		YS9:V>L	AA:339		UWOPS03_461_4:N>Y		UWOPS83_787_3:N>YID:YCL033C	AA:9		DBVPG6044:V>A		SK1:V>A		UWOPS03_461_4:V>A		UWOPS05_217_3:V>A		UWOPS83_787_3:V>A		Y12:V>A		YPS606:V>A	AA:19		DBVPG6044:R>-	AA:26		322134S:Q>HID:YCL034W	AA:48		NCYC110:T>S	AA:114		NCYC110:E>A	AA:119		273614X:R>S	AA:141		UWOPS03_461_4:E>K		UWOPS05_217_3:E>K		UWOPS05_227_2:E>K	AA:170		SK1:G>S	AA:196		DBVPG6044:S>N		NCYC110:S>N		SK1:S>N		YPS606:S>N	AA:202		DBVPG1373:R>G	AA:253		DBVPG6044:I>T		NCYC110:I>T	AA:324		YJM978:R>KID:YCL036W	AA:1		SK1:M>I		Y9:M>I	AA:4		378604X:Q>E	AA:31		NCYC110:P>H		SK1:P>H		Y9:P>H		YPS606:P>H	AA:49		SK1:N>S		Y9:N>S		YPS606:N>S		YS9:N>S	AA:94		378604X:D>E	AA:116		DBVPG1853:K>R	AA:131		DBVPG6040:D>Y	AA:145		YS9:L>R	AA:147		DBVPG1853:Q>L		SK1:Q>L		Y9:Q>L		YS9:Q>L	AA:165		YJM978:K>E		YJM981:K>E	AA:179		L_1374:T>M	AA:198		378604X:D>	AA:219		YJM978:I>N	AA:245		YJM981:N>H	AA:461		UWOPS05_217_3:H>	AA:465		YS2:N>T	AA:473		YS2:L>-	AA:480		322134S:V>I		DBVPG1106:V>I		DBVPG6765:V>I		L_1374:V>I		NCYC110:V>I		UWOPS03_461_4:V>I		UWOPS05_227_2:V>I		Y55:V>I		YIIc17_E5:V>I		YPS128:V>I		YPS606:V>I		YS2:V>I		YS4:V>I		YS9:V>I	AA:512		YS2:V>E	AA:524		YS2:V>F	AA:532		YS2:G>V	AA:535		YS2:S>C	AA:560		SK1:T>I		UWOPS03_461_4:T>I		UWOPS05_227_2:T>I	AA:561		K11:S>N	AA:565		DBVPG6044:T>AID:YCL038C	AA:79		Y9:K>N	AA:100		322134S:V>A		YS9:V>A	AA:150		DBVPG1373:N>D	AA:271		378604X:V>A		BC187:V>A		DBVPG1106:V>A		DBVPG1373:V>A		DBVPG1853:V>A		DBVPG6765:V>A		L_1374:V>A		L_1528:V>A		SK1:V>A		UWOPS03_461_4:V>A		UWOPS05_217_3:V>A		UWOPS87_2421:V>A		Y55:V>A		Y9:V>A		YIIc17_E5:V>A		YJM978:V>A		YPS606:V>A	AA:286		SK1:E>QID:YCL039W	AA:19		NCYC361:A>T		UWOPS05_217_3:A>T		UWOPS83_787_3:A>T		UWOPS87_2421:A>T		YIIc17_E5:A>T		YPS128:A>T		YPS606:A>T	AA:50		322134S:N>D		DBVPG1853:N>D		NCYC361:N>D		SK1:N>D		UWOPS05_217_3:N>D		UWOPS05_227_2:N>D		UWOPS83_787_3:N>D		UWOPS87_2421:N>D		Y9:N>D		YIIc17_E5:N>D		YJM975:N>D		YJM978:N>D		YJM981:N>D		YPS128:N>D		YPS606:N>D		YS2:N>D		YS4:N>D	AA:92		K11:A>V		NCYC361:A>V		SK1:A>V		YS2:A>V	AA:101		NCYC110:W>R	AA:103		DBVPG1106:Q>	AA:110		NCYC110:P>S	AA:221		YS4:T>	AA:224		DBVPG1853:E>G	AA:260		DBVPG6040:P>A	AA:310		UWOPS87_2421:L>M		Y9:L>M	AA:323		378604X:T>S	AA:341		YS9:S>Y	AA:370		YS9:L>-	AA:395		DBVPG1853:V>A	AA:400		YS9:M>K	AA:409		Y9:S>P	AA:410		322134S:A>T		YS4:A>T	AA:413		YS9:S>-	AA:416		322134S:L>P		BC187:L>P		DBVPG1106:L>P		DBVPG1853:L>P		DBVPG6040:L>P		DBVPG6044:L>P		DBVPG6765:L>P		L_1528:L>P		SK1:L>P		UWOPS03_461_4:L>P		UWOPS05_217_3:L>P		Y55:L>P		YIIc17_E5:L>P		YJM975:L>P		YJM978:L>P		YJM981:L>P		YPS128:L>P		YPS606:L>P		YS4:L>P	AA:530		UWOPS05_217_3:N>K	AA:538		YIIc17_E5:D>	AA:556		SK1:Y>C	AA:563		UWOPS03_461_4:Q>R	AA:574		DBVPG6044:F>L	AA:580		UWOPS03_461_4:L>R	AA:591		DBVPG1853:L>M	AA:613		322134S:T>N		BC187:T>N		DBVPG1853:T>N		DBVPG6040:T>N		DBVPG6765:T>N		L_1528:T>N		SK1:T>N		Y55:T>N		YIIc17_E5:T>N		YJM975:T>N		YJM981:T>N		YS4:T>N	AA:614		Y12:T>A		YS9:T>A	AA:641		322134S:M>K	AA:698		DBVPG6040:S>F	AA:727		NCYC361:M>IID:YCL043C	AA:5		YS2:A>T	AA:16		Y55:L>F	AA:146		DBVPG1106:V>A		DBVPG6765:V>A		L_1374:V>A		Y55:V>A	AA:151		YS9:A>T	AA:224		NCYC361:A>S	AA:227		NCYC361:D>A	AA:334		DBVPG6765:A>V		L_1528:A>V		Y55:A>V	AA:415		UWOPS03_461_4:T>A		UWOPS05_217_3:T>A	AA:454		NCYC361:V>A	AA:481		UWOPS03_461_4:K>R		UWOPS05_217_3:K>R		UWOPS05_227_2:K>R	AA:506		UWOPS03_461_4:D>E		UWOPS05_217_3:D>E		UWOPS05_227_2:D>EID:YCL044C	AA:10		SK1:N>K	AA:30		Y9:I>V	AA:69		DBVPG1373:F>I	AA:139		378604X:I>V	AA:142		NCYC110:R>H		Y55:R>H	AA:246		Y9:K>R	AA:263		Y9:N>I	AA:270		Y9:D>A	AA:286		Y9:I>V	AA:341		DBVPG1373:T>A		YJM978:T>A		YJM981:T>A	AA:400		Y12:D>Y	AA:402		DBVPG6044:P>S		SK1:P>S		Y55:P>S	AA:404		UWOPS83_787_3:P>SID:YCL045C	AA:61		DBVPG1373:N>K		K11:N>S	AA:64		NCYC110:E>D		SK1:E>D	AA:115		DBVPG1373:Y>F		DBVPG1788:Y>F		DBVPG1853:Y>F		DBVPG6765:Y>F		K11:Y>F		L_1374:Y>F		NCYC110:Y>F		SK1:Y>F		UWOPS03_461_4:Y>F		UWOPS05_227_2:Y>F		Y12:Y>F		Y55:Y>F		YIIc17_E5:Y>F		YJM975:Y>F		YJM978:Y>F		YJM981:Y>F		YPS128:Y>F		YPS606:Y>F		YS4:Y>F	AA:150		K11:N>D		NCYC110:N>D		SK1:N>D		UWOPS03_461_4:N>D		UWOPS05_217_3:N>D		UWOPS05_227_2:N>D		Y12:N>D		YPS128:N>D		YPS606:N>D		YS4:N>D	AA:247		YS4:S>L	AA:263		DBVPG6044:L>F		K11:L>F		SK1:L>F		UWOPS03_461_4:L>F		UWOPS05_217_3:L>F		UWOPS05_227_2:L>F		UWOPS83_787_3:L>F		Y12:L>F		YIIc17_E5:L>F		YPS606:L>F	AA:342		DBVPG6765:N>S		L_1528:N>S		YJM975:N>S		YJM978:N>S	AA:362		DBVPG1853:K>N		DBVPG6040:D>N	AA:384		DBVPG6044:R>K		NCYC110:R>K		SK1:R>K		UWOPS03_461_4:R>K		UWOPS05_217_3:R>K		UWOPS83_787_3:R>K		YIIc17_E5:R>K	AA:423		DBVPG6044:S>N		NCYC110:S>N		SK1:S>N	AA:497		NCYC110:I>V		SK1:I>V	AA:549		DBVPG1853:S>A	AA:560		322134S:V>M	AA:631		K11:T>A	AA:662		YJM975:T>P	AA:666		YJM975:P>SID:YCL049C	AA:21		BC187:S>Y	AA:30		DBVPG6044:L>F		SK1:L>F	AA:38		UWOPS05_227_2:D>N	AA:39		K11:G>S	AA:48		K11:D>N		UWOPS03_461_4:D>N		UWOPS05_227_2:D>N		UWOPS83_787_3:D>N		Y9:D>N		YPS128:D>N		YPS606:D>N	AA:70		K11:H>Q		UWOPS03_461_4:H>Q		UWOPS05_227_2:H>Q		UWOPS83_787_3:H>Q		Y9:H>Q		YPS128:H>Q		YPS606:H>Q	AA:117		DBVPG6044:D>E		K11:D>E		L_1528:D>N		SK1:D>E		UWOPS03_461_4:D>E		UWOPS05_227_2:D>E		UWOPS83_787_3:D>E		Y9:D>E		YPS128:D>E		YPS606:D>E	AA:137		DBVPG6765:S>-		L_1528:S>-		YS9:S>-	AA:140		273614X:Y>N	AA:144		DBVPG6044:S>L		SK1:S>L	AA:181		YIIc17_E5:T>S	AA:194		DBVPG6044:R>K		K11:R>K		UWOPS03_461_4:R>K		UWOPS05_217_3:R>K		UWOPS05_227_2:R>K		Y9:R>K		YIIc17_E5:R>K		YPS128:R>K		YPS606:R>K		YS9:R>K	AA:199		DBVPG6044:V>I	AA:206		DBVPG6044:D>Y		K11:D>Y		UWOPS03_461_4:D>Y		UWOPS05_217_3:D>Y		UWOPS05_227_2:D>Y		Y9:D>Y		YIIc17_E5:D>Y		YPS128:D>Y		YPS606:D>Y		YS9:D>Y	AA:253		K11:L>S		UWOPS03_461_4:L>S		UWOPS05_217_3:L>S		Y9:L>S		YIIc17_E5:L>S		YPS128:L>S		YPS606:L>S		YS9:L>S	AA:305		YS9:I>FID:YCL050C	AA:100		YIIc17_E5:E>GID:YCL051W	AA:2		YIIc17_E5:P>S	AA:9		UWOPS83_787_3:V>M	AA:26		L_1528:R>I	AA:95		UWOPS87_2421:P>R	AA:108		322134S:L>V		DBVPG6040:L>V		YIIc17_E5:L>V		YS4:L>V	AA:121		378604X:P>	AA:175		322134S:N>S		DBVPG6040:N>S		K11:N>S		YIIc17_E5:N>S		YS4:N>S	AA:221		Y12:D>E	AA:239		322134S:A>P		DBVPG1853:A>P		DBVPG6040:A>P		DBVPG6044:A>P		UWOPS03_461_4:A>P		UWOPS05_227_2:A>P		UWOPS83_787_3:A>P		UWOPS87_2421:A>P		YPS606:A>P		YS4:A>P	AA:274		K11:S>T	AA:314		UWOPS87_2421:F>I	AA:317		322134S:S>G		DBVPG1853:S>G		DBVPG6040:S>G		DBVPG6044:S>G		UWOPS83_787_3:S>G		UWOPS87_2421:S>G		YIIc17_E5:S>G		YPS606:S>G	AA:360		DBVPG6040:Q>K	AA:422		DBVPG6044:A>V		SK1:A>V	AA:432		DBVPG1853:V>A		DBVPG6040:V>A		K11:V>A		UWOPS03_461_4:V>A		UWOPS05_217_3:V>A		Y12:V>A		YIIc17_E5:V>A	AA:436		K11:N>D		Y12:N>D	AA:439		DBVPG6044:G>A		SK1:G>A	AA:536		UWOPS03_461_4:F>I		UWOPS05_217_3:F>I	AA:552		Y12:S>N	AA:554		322134S:D>G		DBVPG6040:D>G		DBVPG6044:D>G		K11:D>G		SK1:D>G		UWOPS03_461_4:D>G		UWOPS05_217_3:D>G		UWOPS83_787_3:D>G		UWOPS87_2421:D>G		Y12:D>G		YIIc17_E5:D>G		YPS128:D>G		YPS606:D>G	AA:558		UWOPS03_461_4:K>R		UWOPS05_217_3:K>R	AA:568		UWOPS05_217_3:K>EID:YCL052C	AA:48		Y12:G>E		YIIc17_E5:G>E	AA:66		DBVPG6044:A>T		NCYC110:A>T		SK1:A>T	AA:104		YIIc17_E5:S>G	AA:191		YJM978:D>N	AA:192		UWOPS87_2421:G>S	AA:243		322134S:P>S		DBVPG1853:P>S		K11:P>S		Y12:P>S		Y9:P>S		YIIc17_E5:P>S		YS4:P>S	AA:281		L_1528:S>F	AA:331		DBVPG1788:A>T	AA:362		DBVPG1373:R>H		DBVPG1788:R>H		Y55:R>H		YJM975:R>H		YJM978:R>H	AA:404		378604X:F>L		DBVPG6765:F>LID:YCL055W	AA:4		UWOPS87_2421:Q>	AA:29		DBVPG6044:I>M	AA:48		322134S:D>E	AA:145		322134S:R>K		DBVPG6044:R>K		Y12:R>K		YPS128:R>K		YPS606:R>KID:YCL056C	AA:59		YS4:I>VID:YCL057W	AA:151		YPS128:V>M	AA:189		322134S:S>T		DBVPG6040:S>T	AA:234		UWOPS83_787_3:N>D		YPS128:N>D	AA:266		YJM975:A>T	AA:308		273614X:E>K		322134S:E>K		BC187:E>K		DBVPG1373:E>K		DBVPG1853:E>K		DBVPG6040:E>K		DBVPG6044:E>K		DBVPG6765:E>K		K11:E>K		L_1374:E>K		NCYC110:E>K		UWOPS05_227_2:E>K		UWOPS83_787_3:E>K		Y12:E>K		YIIc17_E5:E>K		YPS128:E>K		YPS606:E>K		YS4:E>K	AA:411		NCYC110:N>S	AA:466		NCYC110:D>G		UWOPS83_787_3:D>G	AA:469		322134S:R>G	AA:488		K11:P>S	AA:614		322134S:D>E		DBVPG1853:D>E		DBVPG6040:D>E		YIIc17_E5:D>E		YS9:D>E	AA:668		322134S:A>T		DBVPG1853:A>T		DBVPG6040:A>T		DBVPG6044:A>T		NCYC110:A>T		YIIc17_E5:A>T		YPS128:A>T		YS9:A>TID:YCL059C	AA:221		Y12:S>T	AA:304		322134S:I>V		K11:I>V		YIIc17_E5:I>VID:YCL063W	AA:36		DBVPG6040:I>V	AA:74		378604X:S>N		Y12:S>N	AA:111		SK1:V>L	AA:116		378604X:P>S		DBVPG1373:P>S		DBVPG6765:P>S		K11:P>S		L_1528:P>S		SK1:P>S		UWOPS03_461_4:P>S		UWOPS05_227_2:P>S		UWOPS83_787_3:P>S		Y12:P>S		Y55:P>S		YPS128:P>S		YS2:P>S		YS4:P>S		YS9:P>S	AA:118		UWOPS03_461_4:D>N	AA:180		UWOPS03_461_4:K>Q	AA:185		DBVPG6040:R>K	AA:210		378604X:T>A		DBVPG6040:T>A		K11:T>A		UWOPS05_227_2:T>A		UWOPS83_787_3:T>A		UWOPS87_2421:T>A		Y12:T>A		YPS128:T>A		YPS606:T>A	AA:255		K11:T>P		UWOPS05_227_2:T>P		Y12:T>P		YPS128:T>P		YPS606:T>P	AA:288		K11:S>N		SK1:S>C	AA:295		273614X:R>K		378604X:R>K		DBVPG1106:R>K		DBVPG1373:R>K		DBVPG1788:R>K		DBVPG1853:R>K		DBVPG6044:R>K		DBVPG6765:R>K		K11:R>K		SK1:R>K		UWOPS05_227_2:R>K		UWOPS87_2421:R>K		Y12:R>K		Y55:R>K		YPS128:R>K		YPS606:R>K		YS4:R>K	AA:330		378604X:S>P		DBVPG1853:S>P		K11:S>P		Y12:S>P		YPS128:S>P		YPS606:S>P	AA:378		YPS128:K>R		YPS606:K>RID:YCL064C	AA:37		YPS128:K>T	AA:58		DBVPG1853:R>K		DBVPG6044:R>K		NCYC110:R>K		UWOPS03_461_4:R>K		UWOPS05_217_3:R>K		UWOPS05_227_2:R>K		Y12:R>K		YIIc17_E5:R>K		YPS128:R>K		YPS606:R>K	AA:59		SK1:S>F	AA:196		NCYC361:Y>H	AA:264		Y55:D>H	AA:267		322134S:E>-ID:YCL066W	AA:28		SK1:K>E	AA:61		YJM981:L>F	AA:73		378604X:S>T		DBVPG1853:S>T		K11:S>T		UWOPS83_787_3:S>T		UWOPS87_2421:S>T		YIIc17_E5:S>T		YPS128:S>T		YPS606:S>T	AA:103		Y12:Y>ID:YCL068C	AA:171		DBVPG1373:L>I		DBVPG6040:L>I		DBVPG6044:L>I		DBVPG6765:L>I		L_1528:L>I		SK1:L>I		UWOPS05_217_3:L>I		UWOPS05_227_2:L>I		UWOPS83_787_3:L>I		Y12:L>I		Y55:L>I		Y9:L>I		YIIc17_E5:L>I		YJM978:L>I		YPS128:L>I		YPS606:L>I	AA:207		DBVPG6040:H>D	AA:252		Y12:H>L		Y9:H>LID:YCR002C	AA:3		273614X:P>S		K11:P>S		SK1:P>S		YPS606:P>S	AA:26		SK1:L>M	AA:212		UWOPS03_461_4:S>L	AA:230		UWOPS03_461_4:I>VID:YCR004C	AA:49		322134S:K>E		SK1:K>E	AA:69		DBVPG6044:E>K	AA:172		YIIc17_E5:P>S	AA:232		UWOPS05_227_2:A>S	AA:242		UWOPS05_227_2:S>FID:YCR005C	AA:14		UWOPS03_461_4:S>L	AA:75		YJM978:G>V	AA:160		UWOPS05_217_3:Q>H	AA:206		YS2:A>SID:YCR008W	AA:7		DBVPG6044:N>H	AA:63		SK1:K>R	AA:93		SK1:M>I	AA:124		DBVPG6040:S>T	AA:243		322134S:T>S		378604X:T>S		DBVPG1853:T>S		SK1:T>S		UWOPS05_217_3:T>S		UWOPS83_787_3:T>S		Y12:T>S		Y9:T>S		YIIc17_E5:T>S		YS2:T>S		YS4:T>S	AA:261		Y12:Q>H		Y9:Q>H	AA:281		322134S:C>G		378604X:C>G		DBVPG1853:C>G		DBVPG6044:C>G		SK1:C>G		UWOPS05_217_3:C>G		UWOPS83_787_3:C>G		UWOPS87_2421:C>G		Y12:C>G		Y9:C>G		YIIc17_E5:C>G		YPS128:C>G		YS4:C>G	AA:306		L_1528:K>R	AA:308		L_1528:R>TID:YCR009C	AA:88		DBVPG6765:N>S		L_1528:N>S		Y55:N>S	AA:109		YJM978:D>Y	AA:221		DBVPG1853:T>AID:YCR010C	AA:31		L_1528:V>A	AA:280		DBVPG6044:R>KID:YCR011C	AA:8		NCYC110:L>F	AA:29		322134S:K>E		378604X:K>E		DBVPG1853:K>E		DBVPG6044:K>E		K11:K>E		L_1528:K>E		NCYC110:K>E		SK1:K>E		UWOPS03_461_4:K>E		UWOPS05_217_3:K>E		UWOPS05_227_2:K>E		Y12:K>E		YIIc17_E5:K>E		YPS128:K>E		YPS606:K>E	AA:36		BC187:T>A	AA:112		Y12:A>T	AA:239		322134S:V>M		DBVPG1788:V>M		DBVPG6044:V>M		DBVPG6765:V>M		NCYC361:V>M		SK1:V>M		UWOPS03_461_4:V>M		UWOPS05_227_2:V>M		UWOPS83_787_3:V>M		Y12:V>M		Y9:V>M		YIIc17_E5:V>M		YPS606:V>M		YS4:V>M	AA:374		DBVPG1373:L>S	AA:494		NCYC361:K>I	AA:501		378604X:K>-	AA:529		Y9:G>A	AA:534		Y9:E>D	AA:545		Y9:V>L	AA:616		YS4:K>E	AA:666		378604X:D>H	AA:680		UWOPS87_2421:T>I	AA:747		378604X:E>V	AA:751		UWOPS03_461_4:S>R	AA:870		322134S:Y>S	AA:873		322134S:K>EID:YCR015C	AA:66		UWOPS83_787_3:V>I	AA:104		YIIc17_E5:Q>R		YPS606:Q>R	AA:107		378604X:K>N	AA:124		K11:E>D		NCYC110:E>D		UWOPS05_227_2:E>D		UWOPS83_787_3:E>D		Y9:E>D		YIIc17_E5:E>D		YPS128:E>D		YPS606:E>D	AA:164		UWOPS87_2421:G>C	AA:185		UWOPS03_461_4:C>Y		UWOPS05_227_2:C>Y		YIIc17_E5:C>Y		YPS128:C>Y		YPS606:C>Y	AA:206		378604X:I>V	AA:265		378604X:E>GID:YCR016W	AA:28		NCYC110:K>E	AA:76		DBVPG6044:P>L	AA:118		322134S:T>I		SK1:T>I	AA:210		322134S:E>K		SK1:E>K	AA:212		322134S:K>R		K11:K>R		SK1:K>R		Y12:K>R	AA:242		DBVPG1788:Q>H		L_1374:Q>HID:YCR017C	AA:85		YS2:Y>H	AA:229		322134S:S>N		NCYC110:S>N		SK1:S>N		UWOPS83_787_3:S>N		YIIc17_E5:S>N	AA:252		378604X:E>G	AA:253		NCYC110:K>N		UWOPS83_787_3:K>N	AA:259		322134S:A>T		SK1:A>T	AA:275		UWOPS83_787_3:N>S	AA:329		322134S:T>M		SK1:T>M	AA:449		DBVPG6044:A>E	AA:468		UWOPS05_217_3:L>-	AA:508		UWOPS05_217_3:M>L		UWOPS05_227_2:M>L	AA:571		YJM975:Q>P	AA:634		273614X:I>V		322134S:I>V		378604X:I>V		DBVPG1788:I>V		DBVPG6765:I>V		K11:I>V		L_1374:I>V		L_1528:I>V		SK1:I>V		UWOPS05_217_3:I>V		UWOPS05_227_2:I>V		Y55:I>V		Y9:I>V		YIIc17_E5:I>V		YJM978:I>V		YPS128:I>V	AA:708		YPS128:K>E	AA:710		W303:M>I	AA:791		YS9:Y>H	AA:831		YS2:I>L	AA:873		378604X:R>K		DBVPG6044:R>K		K11:R>K		NCYC110:R>K		NCYC361:R>K		SK1:R>K		UWOPS05_227_2:R>K		UWOPS87_2421:R>K		Y55:R>K		Y9:R>K		YJM978:R>K		YPS128:R>K		YPS606:R>K		YS2:R>K		YS4:R>K		YS9:R>K	AA:905		Y55:A>V		YJM978:A>V		YS2:A>V	AA:933		UWOPS05_227_2:F>LID:YCR018C	AA:23		BC187:M>I		DBVPG1788:M>I		L_1374:M>I		YJM975:M>I		YS9:M>I	AA:33		UWOPS03_461_4:I>T		UWOPS05_217_3:I>T	AA:67		Y9:A>S	AA:96		BC187:N>H		DBVPG1373:N>H		DBVPG1788:N>H		DBVPG6765:N>H		L_1374:N>H		L_1528:N>H		UWOPS03_461_4:N>H		UWOPS05_217_3:N>H		Y55:N>H		YJM975:N>H		YJM978:N>H	AA:130		UWOPS03_461_4:A>V		UWOPS05_217_3:A>V	AA:141		SK1:K>NID:YCR020C	AA:99		NCYC361:T>I	AA:128		SK1:A>S		YIIc17_E5:A>S	AA:198		SK1:F>YID:YCR020C-A	AA:2		YIIc17_E5:D>N	AA:7		YPS128:S>P	AA:17		UWOPS05_227_2:S>F	AA:27		SK1:S>G	AA:75		UWOPS05_227_2:E>A	AA:84		YPS128:M>IID:YCR021C	AA:50		378604X:V>I		DBVPG1853:V>I		DBVPG6040:V>I		DBVPG6044:V>I		DBVPG6765:V>I		K11:V>I		SK1:V>I		UWOPS05_217_3:V>I		UWOPS05_227_2:V>I		UWOPS83_787_3:V>I		UWOPS87_2421:V>I		Y12:V>I		Y55:V>I		Y9:V>I		YIIc17_E5:V>I		YPS128:V>I		YPS606:V>I		YS4:V>I	AA:141		NCYC361:S>N		Y12:A>V		Y9:A>V	AA:148		DBVPG6044:D>N	AA:192		YS4:V>I	AA:197		YS4:T>A	AA:278		YPS128:R>K		YPS606:R>K	AA:286		SK1:S>P		YS2:S>P	AA:296		DBVPG6040:D>G	AA:307		K11:E>D	AA:324		DBVPG1853:A>TID:YCR023C	AA:107		NCYC361:G>R	AA:110		SK1:R>H	AA:200		DBVPG1106:L>F		DBVPG6765:L>F		L_1374:L>F		L_1528:L>F		NCYC361:L>F		Y55:L>F		YJM975:L>F	AA:208		UWOPS03_461_4:F>S	AA:212		UWOPS03_461_4:G>S	AA:229		273614X:D>N		378604X:D>N		DBVPG1106:D>N		DBVPG1853:D>N		DBVPG6040:D>N		DBVPG6044:D>N		DBVPG6765:D>N		L_1374:D>N		L_1528:D>N		NCYC361:D>N		SK1:D>N		UWOPS03_461_4:D>N		UWOPS83_787_3:D>N		Y12:D>N		Y55:D>N		Y9:D>N		YJM978:D>N		YPS128:D>N		YPS606:D>N	AA:258		UWOPS83_787_3:R>H	AA:276		273614X:I>N		DBVPG1853:I>N		DBVPG6044:I>N		SK1:I>N		UWOPS83_787_3:I>N		Y12:I>N		Y9:I>N		YPS128:I>N		YPS606:I>N		YS2:I>N	AA:280		273614X:D>N	AA:282		DBVPG6044:E>G	AA:406		UWOPS83_787_3:G>D	AA:458		DBVPG6040:V>F	AA:473		322134S:Y>C		BC187:Y>C		DBVPG1373:Y>C		DBVPG1853:Y>C		DBVPG6040:Y>C		DBVPG6044:Y>C		DBVPG6765:Y>C		K11:Y>C		L_1374:Y>C		L_1528:Y>C		SK1:Y>C		UWOPS05_217_3:Y>C		UWOPS83_787_3:Y>C		Y55:Y>C		Y9:Y>C		YJM975:Y>C		YJM978:Y>C		YPS128:Y>C		YPS606:Y>C		YS2:Y>C		YS4:Y>C		YS9:Y>C	AA:580		UWOPS83_787_3:S>N	AA:583		DBVPG6044:A>T		K11:A>T		SK1:A>T		UWOPS05_217_3:A>T		UWOPS83_787_3:A>T		Y12:A>T		YPS606:A>TID:YCR024C	AA:9		UWOPS87_2421:K>R	AA:18		378604X:I>V		UWOPS87_2421:T>A	AA:106		YS9:L>W	AA:139		DBVPG1853:S>T	AA:148		DBVPG6040:A>G		UWOPS05_227_2:A>S	AA:166		DBVPG6044:L>Q	AA:197		DBVPG6044:T>N	AA:199		DBVPG1853:T>M	AA:266		DBVPG6040:N>T	AA:285		UWOPS05_227_2:D>Y	AA:296		DBVPG6044:I>V		NCYC110:I>V		UWOPS05_227_2:I>V	AA:315		W303:Q>R	AA:324		273614X:I>L		378604X:I>L		Y12:I>L		Y9:I>L		YIIc17_E5:I>LID:YCR026C	AA:29		DBVPG1788:D>H	AA:107		SK1:H>R		Y12:H>R		YS4:H>R	AA:120		SK1:F>L		Y12:F>L		YS4:F>L	AA:177		DBVPG1788:G>S	AA:212		K11:V>I		SK1:V>I	AA:311		322134S:L>S		DBVPG6044:N>K		K11:N>K		SK1:N>K		UWOPS05_217_3:N>K		Y12:N>K		Y55:N>K		Y9:N>K		YPS606:N>K		YS2:N>K	AA:326		DBVPG6044:N>D		Y55:N>D	AA:345		DBVPG1853:S>G	AA:427		UWOPS83_787_3:V>I	AA:436		BC187:E>K		DBVPG1106:E>K		DBVPG1373:E>K		DBVPG1788:E>K		DBVPG6765:E>K		L_1374:E>K		YJM978:E>K	AA:439		BC187:R>G		DBVPG1106:R>G		DBVPG1373:R>G		DBVPG1788:R>G		DBVPG6765:R>G		L_1374:R>G		YJM978:R>G	AA:444		DBVPG6044:S>A		Y55:S>A	AA:496		Y12:G>E	AA:519		L_1528:Q>-	AA:627		Y12:E>G		Y9:E>G	AA:688		273614X:L>P		DBVPG1853:L>P		DBVPG6765:L>P		L_1374:L>P		L_1528:L>P		SK1:L>P		UWOPS05_217_3:L>P		Y12:L>P		Y55:L>P		Y9:L>P		YJM975:L>P		YJM978:L>P		YPS128:L>P		YPS606:L>P		YS9:L>P	AA:697		DBVPG6765:S>R		L_1374:S>R		L_1528:S>R		SK1:S>R		Y55:S>R		YJM975:S>R		YJM978:S>R		YS9:S>R	AA:712		UWOPS05_217_3:V>M	AA:713		Y12:G>EID:YCR027C	AA:6		L_1528:M>I	AA:42		UWOPS05_217_3:V>L	AA:63		DBVPG6044:D>E		Y55:D>E	AA:87		K11:G>S		SK1:G>S	AA:92		322134S:I>M		BC187:I>M		DBVPG1788:I>M		DBVPG6765:I>M		L_1528:I>M		YJM975:I>M		YJM978:I>M	AA:136		DBVPG1853:S>G	AA:137		273614X:T>IID:YCR028C	AA:2		UWOPS87_2421:M>L	AA:23		UWOPS03_461_4:A>T		UWOPS05_217_3:A>T	AA:90		YJM978:V>F	AA:109		DBVPG1788:T>M	AA:180		YS2:M>L	AA:183		DBVPG6765:S>G		L_1528:S>G	AA:224		273614X:D>N	AA:325		YS4:T>M	AA:333		S288c:S>T	AA:361		S288c:D>N	AA:392		S288c:I>V	AA:426		UWOPS83_787_3:D>H	AA:463		UWOPS87_2421:K>M	AA:476		322134S:G>RID:YCR033W	AA:58		YS4:V>A	AA:92		YJM978:S>G	AA:141		UWOPS05_217_3:N>K		UWOPS05_227_2:N>K		UWOPS83_787_3:N>K		Y12:N>K		Y55:N>K		YPS128:N>K		YPS606:N>K		YS9:N>K	AA:149		YJM975:M>I	AA:166		Y12:P>S		YS9:P>S	AA:243		UWOPS05_217_3:T>I		UWOPS05_227_2:T>I	AA:262		K11:T>N		UWOPS05_217_3:T>N		UWOPS05_227_2:T>N		Y12:T>N		YS2:T>N		YS9:T>N	AA:275		DBVPG6044:L>F		Y55:L>F	AA:286		DBVPG1373:L>F	AA:289		DBVPG6044:P>R		Y55:P>R	AA:324		K11:A>V		UWOPS05_217_3:A>V		Y12:A>V		YS9:A>V	AA:331		UWOPS05_217_3:I>T	AA:365		YS9:D>N	AA:375		DBVPG6044:S>N		K11:S>N		Y12:S>N		Y55:S>N		YS9:S>N	AA:381		YS2:G>R	AA:395		YS4:S>N	AA:439		UWOPS03_461_4:T>S		YS9:T>S	AA:442		Y55:N>D	AA:446		YS9:S>I	AA:482		273614X:E>K		BC187:E>K		DBVPG1373:E>K		DBVPG1853:E>K		DBVPG6765:E>K		L_1528:E>K		NCYC361:E>K		SK1:E>K		UWOPS03_461_4:E>K		UWOPS87_2421:E>K		Y12:E>K		Y55:E>K		YJM975:E>K		YJM981:E>K		YPS128:E>K		YPS606:E>K		YS2:E>K		YS4:E>K	AA:489		273614X:K>Q		YS4:K>Q	AA:490		BC187:A>T	AA:635		YJM978:P>S	AA:644		273614X:H>Y		378604X:H>Y		BC187:H>Y		DBVPG1106:H>Y		DBVPG1373:H>Y		DBVPG1788:H>Y		DBVPG1853:H>Y		L_1528:H>Y		SK1:H>Y		UWOPS83_787_3:H>Y		UWOPS87_2421:H>Y		Y12:H>Y		Y55:H>Y		Y9:H>Y		YPS128:H>Y		YPS606:H>Y		YS2:H>Y		YS4:H>Y	AA:724		YS4:L>H	AA:757		DBVPG1788:S>N		DBVPG6765:S>N		SK1:S>N		YS4:S>N	AA:766		378604X:E>D		DBVPG1788:E>D		DBVPG6765:E>D		NCYC110:E>D		SK1:E>D		UWOPS05_227_2:E>D		UWOPS87_2421:E>D		Y12:E>D		Y55:E>D		YPS606:E>D		YS4:E>D	AA:806		322134S:Q>K		378604X:Q>K		DBVPG1788:Q>K		DBVPG6765:Q>K		NCYC110:Q>K		SK1:Q>K		UWOPS05_227_2:Q>K		UWOPS87_2421:Q>K		Y12:Q>K		Y55:Q>K		Y9:Q>K		YPS128:Q>K		YPS606:Q>K		YS4:Q>K	AA:831		322134S:V>I		378604X:V>I		DBVPG1788:V>I		DBVPG6765:V>I		NCYC110:V>I		SK1:V>I		UWOPS05_227_2:V>I		UWOPS87_2421:V>I		Y12:V>I		Y55:V>I		Y9:V>I		YPS128:V>I		YPS606:V>I		YS4:V>I	AA:836		YPS128:N>S		YPS606:N>S	AA:846		322134S:G>E		DBVPG1788:G>E		DBVPG6040:G>E		DBVPG6765:G>E		NCYC110:G>E		SK1:G>E		UWOPS05_227_2:G>E		UWOPS87_2421:G>E		Y12:G>E		Y55:G>E		Y9:G>E		YPS128:G>E		YPS606:G>E		YS4:G>E	AA:851		322134S:D>A		DBVPG6040:D>A		NCYC110:D>A		UWOPS05_227_2:D>A		UWOPS87_2421:D>A		Y12:D>V		Y55:D>A		Y9:D>V		YPS128:D>A		YPS606:D>A	AA:855		273614X:A>G		322134S:A>G		DBVPG1788:A>G		DBVPG6040:A>G		DBVPG6765:A>G		NCYC110:A>G		SK1:A>G		UWOPS05_227_2:A>G		UWOPS87_2421:A>G		Y12:A>G		Y55:A>G		Y9:A>G		YPS128:A>G		YPS606:A>G		YS4:A>G	AA:862		UWOPS05_227_2:P>S	AA:919		322134S:G>R	AA:948		378604X:L>S		DBVPG1853:L>S		DBVPG6044:L>S		NCYC110:L>S		UWOPS03_461_4:L>S		UWOPS87_2421:L>S		Y55:L>S		Y9:L>S		YPS128:L>S		YPS606:L>S	AA:998		DBVPG6044:N>S		Y55:N>S	AA:1023		273614X:I>V		378604X:I>V		BC187:I>V		DBVPG1853:I>V		DBVPG6044:I>V		UWOPS03_461_4:I>V		UWOPS87_2421:I>V		Y55:I>V		Y9:I>V		YPS128:I>V		YPS606:I>V		YS4:I>V	AA:1035		YS9:L>	AA:1045		DBVPG1853:M>I	AA:1046		YS4:A>V	AA:1048		BC187:P>S	AA:1114		378604X:H>Q		Y9:H>Q	AA:1174		Y9:G>SID:YCR035C	AA:51		Y12:D>N		Y9:D>N		YS2:D>N	AA:102		322134S:A>S		BC187:A>S		DBVPG1106:A>S		DBVPG1373:A>S		DBVPG6765:A>S		L_1528:A>S		SK1:A>S		YIIc17_E5:A>S		YS4:A>S	AA:161		DBVPG6040:H>Q	AA:182		YS9:D>E	AA:194		YIIc17_E5:E>Q	AA:363		L_1528:V>M		SK1:V>M		YS2:V>MID:YCR037C	AA:9		K11:Y>F	AA:21		DBVPG6040:D>E	AA:55		DBVPG6765:P>L	AA:72		378604X:S>P	AA:167		Y9:K>-	AA:169		Y9:K>T		YIIc17_E5:K>R		YJM978:K>N	AA:314		K11:S>N	AA:352		K11:S>T		UWOPS87_2421:S>T		Y12:S>T		Y9:S>T		YPS128:S>T		YPS606:S>T	AA:881		378604X:K>R		DBVPG6044:K>R		UWOPS03_461_4:K>R		UWOPS05_227_2:K>R		UWOPS83_787_3:K>R		Y55:K>R		Y9:K>R		YPS606:K>R		YS9:K>RID:YCR038C	AA:140		UWOPS03_461_4:L>F	AA:205		W303:I>L	AA:275		DBVPG1373:Y>H	AA:281		DBVPG1788:N>K	AA:333		UWOPS03_461_4:Q>L		UWOPS05_227_2:Q>L	AA:340		273614X:R>G	AA:360		Y12:K>N	AA:375		UWOPS05_227_2:D>N	AA:401		273614X:L>P		DBVPG6040:L>P		DBVPG6044:L>P		DBVPG6765:L>P		K11:L>P		L_1528:L>P		NCYC110:L>P		UWOPS05_227_2:L>P		Y12:L>P		Y55:L>P		Y9:L>P		YIIc17_E5:L>P		YJM975:L>P		YJM981:L>P		YPS606:L>P	AA:588		SK1:V>I	AA:598		UWOPS03_461_4:Q>H		UWOPS05_227_2:Q>HID:YCR039C	AA:66		378604X:K>NID:YCR042C	AA:43		DBVPG6765:T>S	AA:157		Y9:Y>H	AA:182		YJM975:I>V	AA:183		322134S:E>Q		NCYC361:E>Q	AA:206		YS9:W>R	AA:240		DBVPG6765:S>N		YIIc17_E5:S>N	AA:243		DBVPG6044:R>K		NCYC110:R>K		UWOPS03_461_4:R>K		UWOPS05_227_2:R>K		Y55:R>K		Y9:R>K		YPS128:R>K		YPS606:R>K	AA:285		K11:D>E		NCYC110:D>E		UWOPS03_461_4:D>E		UWOPS05_227_2:D>E		Y12:D>E		Y55:D>E		Y9:D>E		YPS128:D>E		YPS606:D>E	AA:297		NCYC110:E>D		Y12:E>D		Y55:E>D		Y9:E>D	AA:339		YS9:S>N	AA:401		YIIc17_E5:D>Y	AA:403		UWOPS83_787_3:E>D	AA:444		Y12:T>I		Y9:T>I	AA:455		YIIc17_E5:V>F	AA:488		YS2:M>L	AA:554		YIIc17_E5:K>R	AA:611		DBVPG6044:I>V		K11:I>V		UWOPS05_217_3:I>V		UWOPS05_227_2:I>V		UWOPS83_787_3:I>V		Y12:I>V		Y55:I>V		YPS128:I>V		YPS606:I>V		YS2:I>V	AA:712		Y12:V>M	AA:713		DBVPG1106:V>L		YJM978:V>L		YJM981:V>L	AA:726		UWOPS83_787_3:L>V	AA:820		DBVPG6044:S>N		K11:S>N		UWOPS05_227_2:S>N		UWOPS83_787_3:S>N		Y12:S>N		Y55:S>N		Y9:S>N		YPS128:S>N		YPS606:S>N	AA:935		Y12:G>V	AA:940		DBVPG6044:I>V		Y55:I>V	AA:958		YIIc17_E5:N>D	AA:969		YIIc17_E5:Q>P	AA:981		YIIc17_E5:N>Y	AA:1014		YIIc17_E5:V>I	AA:1015		DBVPG6044:R>C	AA:1032		DBVPG6044:K>E		K11:K>E		UWOPS03_461_4:K>E		UWOPS05_217_3:K>E		UWOPS05_227_2:K>E		UWOPS83_787_3:K>E		Y12:K>E		Y9:K>E		YPS128:K>E		YPS606:K>E	AA:1050		NCYC361:N>Y	AA:1057		L_1374:S>A		NCYC361:S>A		SK1:S>A	AA:1075		UWOPS03_461_4:A>S		UWOPS05_217_3:A>S		UWOPS05_227_2:A>S	AA:1087		DBVPG1106:H>N	AA:1098		K11:I>M		Y12:I>M		Y9:I>M	AA:1114		DBVPG6044:M>I	AA:1121		K11:K>Q	AA:1122		DBVPG6044:N>Y	AA:1145		K11:S>C		Y12:S>C		Y9:S>C	AA:1155		K11:L>P	AA:1271		UWOPS03_461_4:T>A		UWOPS05_227_2:T>A		YPS128:T>A	AA:1281		K11:M>I	AA:1289		DBVPG1106:L>R		YIIc17_E5:L>R		YJM975:L>R	AA:1292		K11:V>F	AA:1372		Y9:V>I	AA:1403		DBVPG1373:N>K		DBVPG6040:N>K		L_1528:N>K		UWOPS05_217_3:N>K		UWOPS05_227_2:N>K		Y55:N>K		Y9:N>K		YS4:N>KID:YCR043C	AA:1		DBVPG6044:M>R	AA:117		UWOPS05_217_3:G>E		UWOPS05_227_2:G>EID:YCR045C	AA:63		322134S:M>L	AA:97		DBVPG6044:N>S		NCYC110:N>S		YPS128:N>S	AA:357		BC187:D>N		DBVPG1373:D>NID:YCR046C	AA:60		K11:K>R		SK1:K>R		YS9:K>R	AA:78		DBVPG6044:I>V		K11:I>V		SK1:I>V		UWOPS83_787_3:I>V		Y55:I>V		Y9:I>V		YPS128:I>V		YS9:I>V	AA:84		Y9:K>-	AA:130		378604X:I>V		K11:I>V		SK1:I>V		Y12:I>V		Y9:I>V		YPS128:I>V		YS9:I>V	AA:143		Y9:R>-ID:YCR047C	AA:6		UWOPS05_217_3:E>V	AA:17		K11:S>I	AA:41		322134S:E>G	AA:85		DBVPG6765:T>S	AA:119		YS4:A>V	AA:144		378604X:L>F	AA:222		L_1528:N>T	AA:242		UWOPS05_217_3:A>VID:YCR048W	AA:18		SK1:R>S	AA:24		BC187:A>P		DBVPG1373:A>P		DBVPG1788:A>P		DBVPG1853:A>P		L_1528:A>P		YJM981:A>P	AA:60		DBVPG6044:T>S		Y55:T>S	AA:66		DBVPG6044:T>S		UWOPS83_787_3:T>S		Y55:T>S		YPS606:T>S	AA:78		L_1528:E>K	AA:102		DBVPG1853:F>L	AA:123		UWOPS05_217_3:P>S	AA:127		UWOPS05_217_3:D>V	AA:157		K11:T>A	AA:163		273614X:T>A		322134S:T>A		DBVPG6044:T>A		K11:T>A		SK1:T>A		UWOPS05_227_2:T>A		Y55:T>A		YPS606:T>A		YS2:T>A	AA:181		UWOPS05_217_3:K>T	AA:234		322134S:M>I	AA:257		DBVPG1853:I>T	AA:266		322134S:V>I		YPS606:V>I	AA:321		L_1528:I>V	AA:406		YS9:V>G	AA:431		K11:C>S	AA:433		L_1528:Q>E	AA:466		NCYC110:M>V		Y55:M>V	AA:524		YS4:S>T	AA:564		DBVPG6044:M>I		NCYC110:M>I		Y55:M>I	AA:581		378604X:L>V	AA:596		DBVPG6044:V>I		NCYC110:V>I		Y55:V>IID:YCR050C	AA:2		378604X:V>A		DBVPG6044:V>A		NCYC110:V>A		SK1:V>A		UWOPS83_787_3:V>A		UWOPS87_2421:V>A		Y55:V>A		YPS606:V>A	AA:14		L_1528:I>M	AA:20		DBVPG6044:T>I		NCYC110:T>I		Y55:T>I	AA:35		378604X:R>T	AA:91		L_1528:M>I	AA:92		YS4:E>V	AA:94		L_1528:W>-ID:YCR051W	AA:19		378604X:H>R		SK1:H>R		Y12:H>R		Y9:H>R	AA:22		BC187:R>H		DBVPG1106:R>H		DBVPG1373:R>H		DBVPG1788:R>H		DBVPG6765:R>H		YJM978:R>H		YJM981:R>H		YS9:R>H	AA:25		DBVPG6044:K>N		SK1:K>N		UWOPS03_461_4:K>N		UWOPS05_217_3:K>N		UWOPS83_787_3:K>N		UWOPS87_2421:K>N		Y12:K>N		Y55:K>N		Y9:K>N	AA:29		DBVPG1106:T>S		DBVPG1373:T>S	AA:30		UWOPS05_217_3:P>Q	AA:100		YS4:V>A	AA:129		DBVPG6044:A>T		Y55:A>T	AA:130		UWOPS87_2421:D>E		YS2:D>E		YS4:D>E	AA:153		YJM975:D>	AA:205		YS9:G>V	AA:215		YJM978:E>DID:YCR052W	AA:65		DBVPG1373:K>R	AA:87		NCYC361:R>C		UWOPS83_787_3:R>C	AA:165		378604X:S>I		BC187:S>I		DBVPG1373:S>I		DBVPG6040:S>I		DBVPG6044:S>I		DBVPG6765:S>I		K11:S>I		L_1528:S>I		NCYC361:S>I		SK1:S>I		UWOPS05_217_3:S>I		UWOPS05_227_2:S>I		UWOPS83_787_3:S>I		UWOPS87_2421:S>I		Y12:S>I		Y55:S>I		YIIc17_E5:S>I		YJM975:S>I		YJM978:S>I		YPS606:S>I		YS2:S>I	AA:178		378604X:S>N	AA:293		Y12:A>T		YPS606:A>T		YS2:A>T	AA:350		378604X:I>T		DBVPG1853:I>T		DBVPG6040:I>T		DBVPG6044:I>T		K11:I>T		UWOPS05_217_3:I>T		UWOPS05_227_2:I>T		UWOPS87_2421:I>T		Y55:I>T		YPS128:I>T		YPS606:I>T		YS2:I>T	AA:365		378604X:A>T	AA:426		UWOPS05_217_3:P>S		UWOPS05_227_2:P>S	AA:447		DBVPG1788:D>GID:YCR053W	AA:23		L_1528:E>D	AA:102		YS4:N>	AA:150		YS9:E>G	AA:416		UWOPS03_461_4:H>YID:YCR057C	AA:4		378604X:D>Y	AA:37		YPS128:V>I	AA:44		DBVPG1853:N>K	AA:128		Y9:N>I	AA:229		SK1:D>E		YS4:D>E	AA:235		DBVPG1788:D>E		DBVPG6044:D>E		DBVPG6765:D>E		K11:D>E		L_1528:D>E		NCYC110:D>E		SK1:D>E		UWOPS05_217_3:D>E		UWOPS05_227_2:D>E		UWOPS87_2421:D>E		Y55:D>E		Y9:D>E		YJM975:D>E		YJM978:D>E		YJM981:D>E		YS4:D>E	AA:284		DBVPG6765:F>S	AA:398		UWOPS83_787_3:R>K	AA:451		SK1:D>V	AA:463		Y9:G>D	AA:487		K11:V>I	AA:549		YS9:Q>R	AA:583		UWOPS05_227_2:I>V	AA:618		YS9:S>T	AA:628		YS9:E>K	AA:732		DBVPG1106:E>G		DBVPG1373:E>G	AA:741		YJM975:M>I		YJM978:M>I		YJM981:M>I	AA:765		YJM978:L>S	AA:788		DBVPG1373:E>G		L_1374:E>G	AA:869		DBVPG6044:K>E		NCYC110:K>E		Y55:K>E	AA:878		UWOPS03_461_4:E>D		UWOPS05_217_3:E>D		UWOPS05_227_2:E>D	AA:885		K11:V>A		UWOPS03_461_4:V>A		UWOPS05_217_3:V>A		UWOPS05_227_2:V>A		UWOPS87_2421:V>A		Y12:V>A		Y9:V>A	AA:912		K11:S>F		Y9:S>F	AA:918		DBVPG1853:N>D		DBVPG6040:N>D		DBVPG6044:N>D		K11:N>D		NCYC110:N>D		UWOPS05_227_2:N>D		UWOPS83_787_3:N>D		UWOPS87_2421:N>D		Y12:N>D		Y55:N>D		Y9:N>D		YPS606:N>DID:YCR059C	AA:52		NCYC110:H>Q	AA:65		273614X:G>S		322134S:G>S		DBVPG6044:G>S		K11:G>S		NCYC110:G>S		SK1:G>S		UWOPS05_217_3:G>S		UWOPS83_787_3:G>S		YPS606:G>S		YS4:G>S	AA:66		Y9:V>F	AA:69		273614X:S>F	AA:70		DBVPG1106:L>F		Y9:L>F	AA:74		UWOPS83_787_3:D>E	AA:87		DBVPG1106:E>-	AA:89		DBVPG1106:M>I	AA:106		DBVPG1853:E>GID:YCR060W	AA:19		UWOPS83_787_3:L>M	AA:32		273614X:T>A		DBVPG6044:T>A		NCYC110:T>A		Y55:T>A	AA:34		UWOPS83_787_3:Q>H	AA:41		378604X:Y>F		UWOPS03_461_4:Y>F		UWOPS05_217_3:Y>F		UWOPS05_227_2:Y>F	AA:66		273614X:R>W		DBVPG1853:R>W	AA:71		DBVPG6044:A>P		NCYC110:A>P		Y55:A>P	AA:88		K11:Q>R		Y9:Q>R		YPS606:Q>R	AA:99		YPS606:V>LID:YCR061W	AA:5		UWOPS05_217_3:V>I		UWOPS05_227_2:V>I	AA:50		322134S:S>P		K11:S>P		L_1528:S>P		NCYC110:S>P		NCYC361:S>P		SK1:S>P		UWOPS05_217_3:S>P		UWOPS05_227_2:S>P		UWOPS87_2421:S>P		Y12:S>P		Y55:S>P		YS4:S>P	AA:55		UWOPS05_217_3:V>I		UWOPS05_227_2:V>I	AA:103		NCYC110:R>H		Y55:R>H	AA:122		UWOPS05_217_3:A>V		UWOPS05_227_2:A>V	AA:139		Y9:L>I	AA:144		DBVPG1853:S>T	AA:154		UWOPS05_217_3:N>S		UWOPS05_227_2:N>S	AA:198		UWOPS83_787_3:E>D	AA:200		Y12:R>L	AA:223		DBVPG6040:S>F		K11:S>F		Y12:S>F	AA:239		DBVPG1853:D>G		L_1528:D>E	AA:258		L_1528:D>E	AA:276		L_1528:N>D	AA:302		L_1528:T>A	AA:308		L_1528:M>K	AA:312		322134S:H>Q		SK1:H>Q	AA:325		L_1528:T>S	AA:329		UWOPS05_217_3:F>I	AA:380		K11:A>G		L_1528:A>G		UWOPS83_787_3:A>G		Y12:A>G		Y55:A>G		Y9:A>G	AA:488		L_1528:V>I	AA:489		NCYC110:V>L		Y55:V>L	AA:557		L_1528:A>TID:YCR065W	AA:3		378604X:N>K	AA:12		UWOPS83_787_3:H>Q		UWOPS87_2421:H>Q		Y55:H>Q		YPS128:H>Q	AA:67		NCYC110:P>	AA:305		NCYC110:V>I		Y55:V>I	AA:316		UWOPS03_461_4:P>Q	AA:320		273614X:N>D		378604X:N>D		DBVPG1788:N>D		DBVPG1853:N>D		DBVPG6765:N>D		K11:N>D		L_1374:N>D		NCYC110:N>D		SK1:N>D		UWOPS03_461_4:N>D		UWOPS83_787_3:N>D		Y55:N>D		YIIc17_E5:N>D	AA:394		Y55:Q>PID:YCR066W	AA:120		273614X:W>L		DBVPG1373:W>L		L_1374:W>L		SK1:W>L		Y55:W>L		Y9:W>L		YJM978:W>L		YS2:W>L	AA:170		Y55:A>T	AA:239		K11:R>K		YS4:R>K	AA:281		322134S:M>K	AA:345		322134S:T>P	AA:400		NCYC110:R>K		Y55:R>K	AA:441		YS2:E>K		YS4:E>K	AA:445		SK1:D>V	AA:464		K11:S>L		NCYC110:S>L		Y55:S>L		YPS128:S>L		YS4:S>L	AA:469		YS2:L>SID:YCR068W	AA:34		UWOPS03_461_4:A>G		UWOPS05_217_3:A>G	AA:70		378604X:A>G	AA:133		378604X:L>M		BC187:L>M		DBVPG1106:L>M		DBVPG1788:L>M		DBVPG6765:L>M		L_1374:L>M		L_1528:L>M		UWOPS05_217_3:L>M		Y55:L>M		YIIc17_E5:L>M		YJM975:L>M	AA:253		UWOPS05_217_3:D>Y	AA:290		273614X:I>T		322134S:I>T		DBVPG1106:I>T		DBVPG1788:I>T		DBVPG6044:I>T		DBVPG6765:I>T		L_1374:I>T		L_1528:I>T		NCYC110:I>T		NCYC361:I>T		SK1:I>T		UWOPS05_217_3:I>T		Y55:I>T		Y9:I>T		YJM975:I>T		YPS128:I>T		YPS606:I>T		YS2:I>T	AA:338		YIIc17_E5:A>P	AA:422		YIIc17_E5:V>M	AA:425		DBVPG1106:D>	AA:429		Y55:S>N	AA:450		BC187:Q>R		DBVPG1788:Q>R		DBVPG1853:Q>R		DBVPG6765:Q>R		Y55:Q>R		YJM975:Q>R		YS2:Q>R	AA:473		UWOPS05_217_3:D>N		UWOPS05_227_2:D>N	AA:493		UWOPS05_217_3:T>K		UWOPS05_227_2:T>K	AA:501		BC187:T>S		YJM975:T>S		YJM981:T>SID:YCR069W	AA:45		378604X:A>V	AA:50		273614X:I>T		DBVPG1373:I>T		L_1374:I>T	AA:86		NCYC110:M>I	AA:103		DBVPG6044:I>V		NCYC110:I>V	AA:114		NCYC361:N>D		SK1:N>D	AA:128		NCYC361:A>V		SK1:A>V	AA:154		273614X:E>G		BC187:E>G		DBVPG6044:E>G		DBVPG6765:E>G		K11:E>G		L_1374:E>G		NCYC110:E>G		NCYC361:E>G		SK1:E>G		UWOPS05_217_3:E>G		UWOPS05_227_2:E>G		Y55:E>G		Y9:E>G		YIIc17_E5:E>G		YJM978:E>G		YPS606:E>G	AA:277		UWOPS05_227_2:T>SID:YCR073W-A	AA:47		DBVPG6044:S>G	AA:48		DBVPG1853:M>I	AA:63		DBVPG6044:A>T	AA:65		DBVPG1853:A>D		DBVPG6765:A>D		NCYC361:A>D		Y55:A>D	AA:74		YS2:G>D	AA:98		DBVPG6044:I>V	AA:177		K11:V>MID:YCR076C	AA:18		322134S:G>C		DBVPG1106:G>C		DBVPG1373:G>C		DBVPG1788:G>C		DBVPG6765:G>C		L_1374:G>C		L_1528:G>C		SK1:G>C		Y55:G>C		YJM981:G>C	AA:22		378604X:V>A	AA:33		UWOPS05_217_3:C>F	AA:40		YJM981:K>E	AA:50		322134S:S>P		DBVPG1106:S>P		DBVPG1373:S>P		DBVPG1788:S>P		DBVPG1853:S>P		DBVPG6765:S>P		L_1528:S>P		SK1:S>P		Y55:S>P		YJM981:S>P	AA:105		UWOPS03_461_4:V>L		UWOPS05_217_3:V>L	AA:110		UWOPS03_461_4:K>R		UWOPS05_217_3:K>R	AA:116		UWOPS03_461_4:L>F	AA:169		322134S:G>R		DBVPG1106:G>R		DBVPG1373:G>R		DBVPG1788:G>R		DBVPG1853:G>R		DBVPG6040:G>R		DBVPG6765:G>R		L_1374:G>R		L_1528:G>R		NCYC361:G>R		SK1:G>R		UWOPS03_461_4:G>R		UWOPS05_217_3:G>R		Y12:G>R		Y55:G>R		YIIc17_E5:G>R		YJM975:G>R		YJM981:G>R		YPS128:G>R		YPS606:G>R		YS2:G>R		YS4:G>R	AA:183		322134S:G>R		DBVPG1106:G>R		DBVPG1373:G>R		DBVPG1788:G>R		DBVPG1853:G>R		DBVPG6765:G>R		L_1374:G>R		L_1528:G>R		NCYC361:G>R		SK1:G>R		UWOPS03_461_4:G>R		UWOPS05_217_3:G>R		Y55:G>R		YJM975:G>R		YJM981:G>R		YPS128:G>R		YPS606:G>R	AA:246		DBVPG6040:G>RID:YCR077C	AA:39		NCYC110:D>E	AA:63		DBVPG6044:S>N		NCYC110:S>N	AA:95		BC187:T>M		YJM978:T>M		YJM981:T>M		YS9:T>M	AA:136		UWOPS83_787_3:A>V	AA:160		UWOPS05_227_2:N>S	AA:180		DBVPG6044:P>S		NCYC110:P>S	AA:184		DBVPG1788:A>T		DBVPG6765:A>T		L_1374:A>T		NCYC361:A>T		SK1:A>T		Y55:A>T		YJM978:A>T		YJM981:A>T	AA:246		UWOPS87_2421:S>G	AA:436		DBVPG1106:S>P		DBVPG1373:S>P		DBVPG1788:S>P		DBVPG1853:S>P		DBVPG6040:S>P		DBVPG6765:S>P		L_1374:S>P		L_1528:S>P		NCYC110:S>P		SK1:S>P		UWOPS87_2421:S>P		Y55:S>P		YIIc17_E5:S>P		YJM975:S>P		YPS128:S>P		YPS606:S>P	AA:452		YJM975:Q>R	AA:513		DBVPG1106:I>V		DBVPG1788:I>V		L_1374:I>V	AA:688		273614X:V>D		378604X:V>D		DBVPG1106:V>D		DBVPG1788:V>D		DBVPG1853:V>D		DBVPG6040:V>D		DBVPG6765:V>D		K11:V>D		L_1374:V>D		NCYC361:V>D		S288c:V>D		SK1:V>D		UWOPS05_217_3:V>D		UWOPS05_227_2:V>D		UWOPS83_787_3:V>D		UWOPS87_2421:V>D		W303:V>D		Y55:V>D		YGPM:V>D		YIIc17_E5:V>D		YJM981:V>D		YPS128:V>D		YPS606:V>D	AA:736		DBVPG1853:N>S	AA:750		DBVPG6044:V>LID:YCR082W	AA:82		NCYC361:V>A	AA:117		UWOPS05_217_3:D>N		UWOPS05_227_2:D>NID:YCR083W	AA:3		L_1528:F>Y	AA:5		L_1528:K>R	AA:10		L_1528:M>T	AA:74		L_1528:D>E	AA:92		YIIc17_E5:C>S	AA:118		L_1528:T>AID:YCR086W	AA:114		YIIc17_E5:S>P	AA:169		NCYC110:N>ID:YCR087C-A	AA:47		UWOPS05_217_3:N>S		UWOPS05_227_2:N>S	AA:70		Y9:K>Q	AA:84		DBVPG1373:H>Q		DBVPG1853:H>Q		UWOPS03_461_4:H>Q		YPS606:H>QID:YCR088W	AA:17		NCYC361:A>E	AA:27		W303:D>N	AA:58		DBVPG1106:L>S		DBVPG1853:L>S		DBVPG6044:L>S		DBVPG6765:L>S		K11:L>S		L_1374:L>S		SK1:L>S		UWOPS05_227_2:L>S		UWOPS87_2421:L>S		W303:L>S		Y55:L>S		Y9:L>S		YJM975:L>S		YJM981:L>S		YPS128:L>S		YPS606:L>S	AA:289		UWOPS05_227_2:P>	AA:291		322134S:S>F	AA:312		SK1:K>I	AA:329		322134S:N>K	AA:342		UWOPS05_217_3:P>S		UWOPS05_227_2:P>S	AA:348		YPS128:G>D	AA:392		DBVPG1373:P>S		DBVPG1788:P>S		DBVPG6765:P>S		L_1528:P>S		W303:P>S		Y55:P>S		YIIc17_E5:P>S	AA:402		DBVPG1373:A>T		DBVPG6765:A>T		W303:A>T		Y55:A>T		YIIc17_E5:A>T	AA:432		DBVPG1788:P>	AA:444		378604X:D>E		DBVPG1373:D>E		DBVPG6044:D>E		DBVPG6765:D>E		W303:D>E		Y55:D>E		YIIc17_E5:D>E		YS4:D>E	AA:467		DBVPG6765:P>L		L_1528:P>L	AA:473		378604X:A>T	AA:503		DBVPG6040:A>V	AA:525		378604X:A>E	AA:532		DBVPG6044:K>N	AA:562		UWOPS03_461_4:I>V		UWOPS05_217_3:I>V		UWOPS05_227_2:I>VID:YCR090C	AA:90		UWOPS05_217_3:T>A	AA:151		378604X:C>FID:YCR091W	AA:36		UWOPS05_227_2:A>G	AA:43		BC187:N>D		DBVPG6040:N>D		DBVPG6044:N>D		L_1528:N>D		SK1:N>D		UWOPS05_227_2:N>D		UWOPS83_787_3:N>D		UWOPS87_2421:N>D		W303:N>D		Y9:N>D		YPS128:N>D		YPS606:N>D		YS4:N>D	AA:76		L_1528:P>L	AA:81		DBVPG6044:V>A		SK1:V>A		UWOPS83_787_3:V>A		UWOPS87_2421:V>A		YPS128:V>A		YPS606:V>A		YS4:V>A	AA:107		W303:S>N	AA:122		Y12:R>H	AA:148		YJM975:S>G		YJM978:S>G	AA:170		Y55:P>L	AA:179		YS9:M>K	AA:256		Y12:I>K	AA:341		DBVPG1373:M>V		DBVPG1788:M>V		DBVPG1853:M>V		DBVPG6044:M>V		DBVPG6765:M>V		K11:M>V		L_1374:M>V		NCYC110:M>V		S288c:M>V		UWOPS03_461_4:M>V		UWOPS05_227_2:M>V		W303:M>V		Y12:M>V		Y55:M>V		Y9:M>V		YGPM:M>V		YJM975:M>V		YPS606:M>V		YS4:M>V	AA:405		DBVPG6044:M>I		NCYC110:M>I	AA:472		SK1:I>V	AA:492		UWOPS87_2421:I>V	AA:498		YS2:R>G	AA:618		Y12:I>MID:YCR095C	AA:20		W303:K>T	AA:103		378604X:T>S		Y12:T>S	AA:338		DBVPG1853:R>K		SK1:R>K		UWOPS83_787_3:R>K		YPS128:R>K		YPS606:R>K		YS4:R>K	AA:345		UWOPS05_227_2:E>DID:YDL001W	AA:93		DBVPG1853:T>I	AA:154		UWOPS87_2421:L>V	AA:351		UWOPS03_461_4:W>R	AA:353		322134S:E>D	AA:360		UWOPS03_461_4:Y>S	AA:430		273614X:H>RID:YDL002C	AA:81		SK1:T>I		UWOPS87_2421:T>I	AA:202		UWOPS03_461_4:S>P		Y12:S>FID:YDL003W	AA:36		273614X:G>R	AA:112		YS2:S>P	AA:176		BC187:L>I		DBVPG1788:L>I		DBVPG6765:L>I		L_1528:L>I		YJM975:L>I	AA:254		K11:D>G		YS4:D>G	AA:264		273614X:V>I		DBVPG6040:V>I		DBVPG6044:V>I		K11:V>I		NCYC110:V>I		SK1:V>I		Y55:V>I		YIIc17_E5:V>I		YPS606:V>I	AA:300		273614X:I>T		DBVPG1373:I>T		DBVPG1788:I>T		DBVPG1853:I>T		DBVPG6040:I>T		DBVPG6044:I>T		DBVPG6765:I>T		K11:I>T		L_1528:I>T		NCYC110:I>T		SK1:I>T		UWOPS05_217_3:I>T		UWOPS05_227_2:I>T		Y55:I>T		Y9:I>T		YIIc17_E5:I>T		YJM975:I>T		YPS606:I>T		YS2:I>T		YS4:I>T		YS9:I>T	AA:318		YS2:S>A	AA:358		L_1374:T>S	AA:379		378604X:P>S		DBVPG1373:P>S		DBVPG1788:P>S		DBVPG1853:P>S		DBVPG6040:P>S		DBVPG6044:P>S		DBVPG6765:P>S		K11:P>S		L_1528:P>S		NCYC110:P>S		SK1:P>S		UWOPS05_217_3:P>S		UWOPS05_227_2:P>S		Y55:P>S		Y9:P>S		YIIc17_E5:P>S		YJM975:P>S		YPS606:P>S		YS2:P>S		YS4:P>S	AA:415		L_1374:T>A	AA:439		L_1374:F>L	AA:445		273614X:G>D		378604X:G>D		DBVPG1373:G>D		DBVPG1788:G>D		DBVPG1853:G>D		DBVPG6040:G>D		DBVPG6044:G>D		DBVPG6765:G>D		K11:G>D		L_1528:G>D		NCYC110:G>D		SK1:G>D		UWOPS05_217_3:G>D		UWOPS83_787_3:G>D		UWOPS87_2421:G>D		Y55:G>D		Y9:G>D		YIIc17_E5:G>D		YJM978:G>D		YS4:G>D	AA:452		UWOPS05_217_3:P>S	AA:462		273614X:R>G		378604X:R>G		BC187:R>G		DBVPG1373:R>G		DBVPG1788:R>G		DBVPG1853:R>G		DBVPG6040:R>G		DBVPG6044:R>G		DBVPG6765:R>G		K11:R>G		L_1528:R>G		NCYC110:R>G		SK1:R>G		UWOPS05_217_3:R>G		UWOPS83_787_3:R>G		UWOPS87_2421:R>G		Y55:R>G		Y9:R>G		YIIc17_E5:R>G		YS4:R>G	AA:482		378604X:S>P		BC187:S>P		DBVPG1373:S>P		DBVPG1788:S>P		DBVPG1853:S>P		DBVPG6040:S>P		DBVPG6765:S>P		L_1528:S>P		YJM975:S>P		YJM978:S>P		YS4:S>P	AA:549		DBVPG1373:G>E	AA:562		L_1374:R>SID:YDL004W	AA:85		273614X:S>T		K11:S>T		UWOPS87_2421:S>T		Y12:S>T		Y9:S>T		YPS606:S>T	AA:145		273614X:A>DID:YDL005C	AA:13		DBVPG1853:T>A		DBVPG6765:T>A		L_1374:T>A		YIIc17_E5:T>A		YJM975:T>A		YJM978:T>A		YJM981:T>A		YS4:T>A	AA:116		YIIc17_E5:E>D	AA:158		Y9:G>D	AA:191		273614X:S>N		BC187:S>N		DBVPG1788:S>N		DBVPG1853:S>N		DBVPG6040:S>N		DBVPG6765:S>N		L_1374:S>N		SK1:S>N		UWOPS05_227_2:S>N		UWOPS83_787_3:S>N		Y55:S>N		Y9:S>N		YJM975:S>N		YJM981:S>N		YPS606:S>N		YS4:S>N	AA:197		SK1:N>D		Y55:N>D	AA:211		DBVPG6040:T>I	AA:225		273614X:V>A		BC187:V>A		DBVPG1788:V>A		DBVPG1853:V>A		DBVPG6040:V>A		DBVPG6044:V>A		DBVPG6765:V>A		L_1374:V>A		SK1:V>A		UWOPS05_227_2:V>A		UWOPS83_787_3:V>A		Y55:V>A		YJM975:V>A		YPS606:V>A		YS4:V>A		YS9:V>A	AA:282		273614X:I>T		BC187:I>T		DBVPG1788:I>T		DBVPG1853:I>T		DBVPG6040:I>T		DBVPG6044:I>T		DBVPG6765:I>T		NCYC361:I>T		SK1:I>T		UWOPS05_227_2:I>T		Y55:I>T		YPS128:I>T		YS4:I>T		YS9:I>T	AA:288		UWOPS05_227_2:S>G	AA:307		K11:E>Q	AA:315		273614X:D>N		322134S:D>N		BC187:D>N		DBVPG1788:D>N		DBVPG6044:D>N		DBVPG6765:D>N		K11:D>N		NCYC361:D>N		SK1:D>N		Y55:D>N		YJM975:D>N		YPS128:D>N		YS4:D>N		YS9:D>N	AA:321		DBVPG6044:N>S		K11:N>S		SK1:N>S		Y55:N>S		YPS128:N>S	AA:358		BC187:N>H		DBVPG1788:N>H		DBVPG6765:N>H		L_1374:N>H		NCYC361:N>H		YJM975:N>H		YS2:N>H		YS9:N>H	AA:361		DBVPG6044:N>S		K11:N>S		SK1:N>S		Y55:N>S		YPS128:N>SID:YDL006W	AA:73		UWOPS05_217_3:L>I	AA:148		378604X:V>DID:YDL007W	AA:268		NCYC110:F>V	AA:372		DBVPG1106:S>N		UWOPS03_461_4:S>G		UWOPS05_217_3:S>GID:YDL008W	AA:20		YJM978:S>G	AA:28		UWOPS83_787_3:N>S	AA:32		322134S:I>N		BC187:I>N		DBVPG1373:I>N		DBVPG1788:I>N		DBVPG6044:I>N		DBVPG6765:I>N		L_1374:I>N		NCYC110:I>N		NCYC361:I>N		SK1:I>N		UWOPS05_217_3:I>N		UWOPS05_227_2:I>N		UWOPS83_787_3:I>N		YIIc17_E5:I>N		YJM978:I>N		YPS606:I>N	AA:115		DBVPG1853:V>F	AA:129		DBVPG1853:G>DID:YDL010W	AA:45		DBVPG1106:S>N		DBVPG1788:S>N		DBVPG6765:S>N		L_1374:S>N		L_1528:S>N		YIIc17_E5:S>N		YJM975:S>N	AA:53		273614X:G>A		DBVPG1106:G>A		DBVPG1788:G>A		DBVPG6044:G>A		DBVPG6765:G>A		L_1374:G>A		L_1528:G>A		NCYC110:G>A		NCYC361:G>A		SK1:G>A		UWOPS03_461_4:G>A		UWOPS05_217_3:G>A		UWOPS05_227_2:G>A		UWOPS83_787_3:G>A		UWOPS87_2421:G>A		Y55:G>A		YIIc17_E5:G>A		YJM975:G>A		YPS128:G>A		YPS606:G>A	AA:85		YJM975:V>L	AA:93		YIIc17_E5:S>T	AA:160		NCYC361:E>D	AA:178		YS2:T>AID:YDL013W	AA:167		UWOPS05_217_3:V>E	AA:210		BC187:A>V		DBVPG1788:A>V		DBVPG6765:A>V	AA:326		UWOPS03_461_4:A>E		UWOPS05_227_2:A>E	AA:354		W303:L>	AA:383		273614X:L>V	AA:398		273614X:G>V	AA:421		273614X:E>D	AA:434		273614X:N>I	AA:480		273614X:T>A	AA:496		273614X:L>-	AA:501		NCYC361:L>MID:YDL014W	AA:47		YS2:A>S	AA:78		322134S:G>S		NCYC361:G>S		YIIc17_E5:G>SID:YDL015C	AA:6		DBVPG1788:K>-	AA:30		K11:K>R	AA:52		273614X:S>T		DBVPG1106:S>T		DBVPG1373:S>T		DBVPG1788:S>T		DBVPG1853:S>T		DBVPG6040:S>T		DBVPG6044:S>T		DBVPG6765:S>T		K11:S>T		L_1528:S>T		NCYC361:S>T		SK1:S>T		UWOPS05_217_3:S>T		UWOPS83_787_3:S>T		UWOPS87_2421:S>T		Y55:S>T		YIIc17_E5:S>T		YJM978:S>T		YPS128:S>T	AA:69		UWOPS03_461_4:D>N		UWOPS05_217_3:D>N	AA:102		273614X:F>I	AA:105		UWOPS03_461_4:L>V		UWOPS05_217_3:L>V		Y9:I>L	AA:116		DBVPG1853:H>R	AA:235		UWOPS05_217_3:G>V	AA:261		YS4:W>C	AA:281		YPS128:T>A		YPS606:T>AID:YDL017W	AA:25		UWOPS03_461_4:D>N		UWOPS05_217_3:D>N	AA:54		L_1528:T>S	AA:135		UWOPS05_217_3:L>I	AA:233		322134S:P>T		378604X:P>T		BC187:P>T		DBVPG1373:P>T		DBVPG6765:P>T		K11:P>T		L_1528:P>T		NCYC110:P>T		NCYC361:P>T		SK1:P>T		UWOPS03_461_4:P>T		UWOPS05_217_3:P>T		UWOPS05_227_2:P>T		UWOPS83_787_3:P>T		UWOPS87_2421:P>T		Y12:P>T		Y55:P>T		Y9:P>T		YJM975:P>T		YJM978:P>T		YJM981:P>T		YPS128:P>T		YPS606:P>T		YS2:P>T		YS4:P>T		YS9:P>T	AA:297		378604X:S>T		DBVPG6040:S>T		DBVPG6044:S>T		K11:S>T		L_1374:S>T		NCYC110:S>T		NCYC361:S>T		SK1:S>T		UWOPS03_461_4:S>T		UWOPS05_217_3:S>T		UWOPS05_227_2:S>T		UWOPS83_787_3:S>T		UWOPS87_2421:S>T		Y55:S>T		Y9:S>T		YPS128:S>T		YPS606:S>T		YS2:S>T		YS4:S>T		YS9:S>T	AA:335		DBVPG6040:I>M		DBVPG6044:I>M		NCYC110:I>M		SK1:I>M		Y55:I>M	AA:366		DBVPG6040:S>T		DBVPG6044:S>T		NCYC110:S>T		SK1:S>T		Y55:S>TID:YDL018C	AA:4		W303:L>-	AA:16		W303:Q>H	AA:42		322134S:E>D		DBVPG6040:E>D		DBVPG6044:E>D		NCYC110:E>D		SK1:E>D		Y55:E>D		YPS128:E>D		YPS606:E>D		YS4:E>D	AA:82		YPS128:G>D		YPS606:G>D	AA:119		DBVPG6765:N>D		L_1528:N>DID:YDL020C	AA:74		378604X:S>L		BC187:S>L		DBVPG1788:S>L		DBVPG6765:S>L		L_1374:S>L		YJM975:S>L		YJM978:S>L		YS4:S>L	AA:225		BC187:D>H	AA:247		NCYC361:V>A		UWOPS03_461_4:V>A		UWOPS05_217_3:V>A		UWOPS87_2421:V>A		Y12:V>A		Y9:V>A		YPS606:V>A		YS4:V>A	AA:261		NCYC361:R>H		YJM978:R>C	AA:333		DBVPG6040:T>I	AA:427		378604X:T>A		BC187:T>A		DBVPG1106:T>A		DBVPG1373:T>A		DBVPG1788:T>A		DBVPG6040:T>A		DBVPG6765:T>A		NCYC361:T>A		SK1:T>A		UWOPS03_461_4:T>A		UWOPS05_217_3:T>A		UWOPS87_2421:T>A		Y12:T>A		Y55:T>A		Y9:T>A		YIIc17_E5:T>A		YJM975:T>A		YPS128:T>A		YPS606:T>A		YS2:T>A		YS4:T>A		YS9:T>A	AA:429		DBVPG1373:S>P	AA:434		SK1:N>S		Y55:N>S	AA:444		UWOPS03_461_4:L>H		UWOPS05_217_3:L>HID:YDL021W	AA:2		YJM978:T>I	AA:114		UWOPS03_461_4:M>I		UWOPS05_227_2:M>IID:YDL022W	AA:336		UWOPS03_461_4:Q>RID:YDL027C	AA:3		YJM978:K>R	AA:5		YPS128:P>T		YPS606:P>T	AA:6		SK1:V>A		W303:V>A		Y55:V>A	AA:47		K11:S>L		SK1:S>L		UWOPS03_461_4:S>L		UWOPS05_217_3:S>L		UWOPS87_2421:S>L		W303:S>L		Y55:S>L		YPS606:S>L		YS9:S>L	AA:71		YJM981:S>P	AA:90		K11:S>P		SK1:S>P		UWOPS03_461_4:S>P		UWOPS05_217_3:S>P		UWOPS05_227_2:S>P		UWOPS83_787_3:S>P		UWOPS87_2421:S>P		W303:S>P		Y55:S>P		YPS606:S>P		YS9:S>P	AA:134		L_1374:I>L	AA:140		DBVPG6040:I>M		YS4:I>M	AA:167		DBVPG1373:F>I		DBVPG1788:F>I		DBVPG6040:F>I		DBVPG6765:F>I		L_1374:F>I		L_1528:F>I		NCYC361:F>I		SK1:F>I		UWOPS03_461_4:F>I		UWOPS05_217_3:F>I		UWOPS05_227_2:F>I		UWOPS83_787_3:F>I		W303:F>I		Y55:F>I		YJM978:F>I		YPS128:F>I		YPS606:F>I		YS4:F>I		YS9:F>I	AA:248		DBVPG1853:Q>P	AA:260		L_1528:L>M	AA:315		UWOPS03_461_4:L>V		UWOPS05_217_3:L>V		UWOPS05_227_2:L>V	AA:336		273614X:I>V		DBVPG6040:I>V		DBVPG6044:I>V		NCYC110:I>V		SK1:K>E		UWOPS03_461_4:K>E		UWOPS05_217_3:K>E		W303:K>E		Y12:I>V		Y55:K>E		YPS128:K>E		YPS606:K>E		YS4:I>V		YS9:I>V	AA:346		DBVPG6040:F>V	AA:383		UWOPS87_2421:T>KID:YDL028C	AA:12		UWOPS83_787_3:L>-	AA:16		UWOPS03_461_4:T>I		UWOPS05_227_2:T>I	AA:97		UWOPS83_787_3:L>F	AA:110		322134S:M>V	AA:146		273614X:A>S		K11:A>S		SK1:A>S		UWOPS05_217_3:A>S		UWOPS05_227_2:A>S		W303:A>S		Y55:A>S		YPS606:A>S		YS2:A>S	AA:297		SK1:N>T		Y55:N>T	AA:338		YS9:I>V	AA:364		YS9:I>V	AA:367		YPS606:N>Y	AA:624		378604X:H>L	AA:684		UWOPS03_461_4:K>N		UWOPS05_217_3:K>N	AA:709		UWOPS87_2421:K>R	AA:763		YJM975:K>EID:YDL029W	AA:117		DBVPG1853:K>R	AA:181		UWOPS87_2421:L>-	AA:199		322134S:R>HID:YDL030W	AA:46		Y12:T>I		YS9:T>I	AA:60		UWOPS87_2421:Y>H	AA:110		K11:P>S		UWOPS83_787_3:P>S		UWOPS87_2421:P>S		Y12:P>S		YPS128:P>S		YPS606:P>S		YS9:P>S	AA:131		378604X:E>K		L_1528:E>K	AA:152		UWOPS83_787_3:R>K		UWOPS87_2421:R>K	AA:234		K11:L>V		UWOPS03_461_4:L>V		UWOPS83_787_3:L>V		Y9:L>V		YPS606:L>V		YS9:L>V	AA:254		322134S:A>E		K11:A>E		UWOPS03_461_4:A>E		UWOPS83_787_3:A>E		Y9:A>E		YPS606:A>E	AA:291		DBVPG6044:T>R		SK1:T>R		W303:T>R		Y55:T>R	AA:318		UWOPS03_461_4:E>A		UWOPS05_227_2:E>A	AA:329		BC187:N>K		DBVPG1106:N>K		DBVPG1853:N>K		DBVPG6044:N>K		DBVPG6765:N>K		L_1528:N>K		NCYC110:N>K		SK1:N>K		W303:N>K		Y55:N>K		YJM978:N>K		YS9:N>K	AA:378		273614X:D>G		322134S:D>G		UWOPS87_2421:D>G		Y12:D>G	AA:406		UWOPS83_787_3:P>H	AA:447		273614X:I>L		322134S:I>L		UWOPS03_461_4:I>L		UWOPS05_227_2:I>L		UWOPS83_787_3:I>L		UWOPS87_2421:I>L		Y12:I>L		YPS606:I>L	AA:477		Y12:M>I	AA:484		273614X:T>A	AA:491		273614X:P>S		UWOPS03_461_4:P>S		UWOPS05_227_2:P>S		UWOPS87_2421:P>S		Y12:P>S		YPS606:P>SID:YDL033C	AA:88		K11:K>E		NCYC110:K>E		SK1:K>E		UWOPS05_227_2:K>E		UWOPS87_2421:K>E		Y12:K>E		Y55:K>E		YPS128:K>E		YS9:K>E	AA:97		DBVPG6040:V>F	AA:116		K11:S>N		Y12:S>N	AA:177		UWOPS87_2421:R>K	AA:190		DBVPG6044:S>F		NCYC110:S>F		SK1:S>F	AA:325		273614X:M>I		DBVPG1788:M>I		DBVPG6044:M>I		DBVPG6765:M>I		L_1528:M>I		NCYC110:M>I		SK1:M>I		UWOPS03_461_4:M>I		UWOPS05_227_2:M>I		UWOPS83_787_3:M>I		UWOPS87_2421:M>I		W303:M>I		Y55:M>I		Y9:M>I		YIIc17_E5:M>I		YPS606:M>I		YS4:M>I		YS9:M>I	AA:372		DBVPG6044:D>N		NCYC110:D>N		SK1:D>N		W303:D>N		Y55:D>N	AA:381		Y9:A>GID:YDL036C	AA:7		UWOPS05_227_2:L>S	AA:11		273614X:F>L		DBVPG6044:F>L		NCYC110:F>L		SK1:F>L		UWOPS03_461_4:F>L		UWOPS05_227_2:F>L		Y55:F>L	AA:92		YJM978:Q>L	AA:116		Y9:R>K	AA:159		NCYC110:T>S		SK1:T>S		W303:T>S		Y55:T>S	AA:184		UWOPS05_217_3:T>A	AA:252		BC187:K>R		L_1528:K>R		YJM978:K>R	AA:285		SK1:V>L		W303:V>L		Y55:V>L	AA:295		UWOPS05_217_3:R>S	AA:377		DBVPG6044:I>T		SK1:I>T		W303:I>T		Y55:I>T	AA:397		DBVPG1853:S>G		DBVPG6044:S>G		K11:S>G		SK1:S>G		W303:S>G		Y12:S>G		Y55:S>G		Y9:S>G		YPS128:S>G		YPS606:S>G		YS4:S>G	AA:407		W303:Q>L	AA:461		273614X:R>IID:YDL042C	AA:39		DBVPG1853:I>K	AA:42		YS4:D>G	AA:58		UWOPS05_217_3:R>K	AA:65		DBVPG6044:P>T		SK1:P>T		UWOPS83_787_3:P>T		W303:P>T		Y55:P>T		YPS128:P>T	AA:136		DBVPG6040:L>F	AA:245		YJM981:D>E	AA:299		K11:N>D	AA:320		273614X:K>N		322134S:K>N		378604X:K>N		DBVPG6044:K>N		K11:K>N		SK1:K>N		UWOPS03_461_4:K>N		W303:K>N		Y55:K>N		Y9:K>N		YPS128:K>N		YPS606:K>N		YS9:K>N	AA:416		YS4:G>S	AA:422		YPS128:G>C		YPS606:G>C	AA:424		273614X:M>V		322134S:M>V		378604X:M>V		DBVPG6044:M>V		K11:M>V		UWOPS83_787_3:M>V		UWOPS87_2421:M>V		W303:M>V		Y55:M>V		Y9:M>V		YPS128:M>V		YPS606:M>V		YS9:M>V	AA:527		DBVPG6044:T>A		W303:T>A		Y55:T>AID:YDL044C	AA:3		322134S:R>K		DBVPG6044:R>K		NCYC110:R>K		SK1:R>K		UWOPS03_461_4:R>K		UWOPS05_227_2:R>K		UWOPS83_787_3:R>K		W303:R>K		Y55:R>K		Y9:R>K		YPS128:R>K		YPS606:R>K	AA:22		UWOPS03_461_4:C>Y		UWOPS05_227_2:C>Y	AA:31		DBVPG6044:D>N		NCYC110:D>N		SK1:D>N		Y55:D>N		Y9:D>N		YPS128:D>N		YPS606:D>N	AA:40		273614X:V>A		322134S:V>A		DBVPG1373:S>A		DBVPG6044:V>A		NCYC110:V>A		SK1:V>A		UWOPS03_461_4:V>A		UWOPS05_227_2:V>A		UWOPS83_787_3:V>A		Y55:V>A		Y9:V>A		YPS128:V>A		YPS606:V>A	AA:63		DBVPG6044:A>T		NCYC110:A>T		SK1:A>T		Y55:A>T		Y9:A>T		YPS128:A>T		YPS606:A>T	AA:94		Y9:S>N	AA:258		DBVPG1853:S>A	AA:295		UWOPS03_461_4:G>V	AA:353		UWOPS83_787_3:V>L	AA:372		UWOPS83_787_3:L>F	AA:383		YJM978:N>Y	AA:390		378604X:A>S	AA:437		NCYC361:T>SID:YDL045C	AA:112		DBVPG1853:T>A	AA:119		YS9:F>Y	AA:124		UWOPS05_217_3:S>L	AA:174		273614X:A>T		DBVPG1853:A>T		DBVPG6040:A>T		DBVPG6044:A>T		K11:A>T		SK1:A>T		UWOPS03_461_4:A>T		UWOPS05_217_3:A>T		Y55:A>T		Y9:A>T		YPS606:A>T	AA:177		YS9:P>S	AA:201		Y12:N>T	AA:252		273614X:I>V		322134S:I>V		DBVPG1853:I>V		DBVPG6040:I>V		DBVPG6044:I>V		SK1:I>V		UWOPS03_461_4:I>V		UWOPS05_217_3:I>V		UWOPS05_227_2:I>V		UWOPS83_787_3:I>V		Y55:I>V		Y9:I>V		YPS128:I>V		YPS606:I>V		YS4:I>V	AA:263		273614X:R>H		DBVPG6040:R>H		DBVPG6044:R>H		SK1:R>H		UWOPS03_461_4:R>H		UWOPS05_217_3:R>H		UWOPS05_227_2:R>H		UWOPS83_787_3:R>H		Y12:R>H		Y55:R>H		Y9:R>H		YPS128:R>H		YPS606:R>H		YS4:R>H	AA:294		322134S:V>I		UWOPS05_227_2:V>I		UWOPS83_787_3:V>I		Y9:V>I		YPS128:V>I		YPS606:V>I		YS4:V>I	AA:295		L_1528:D>NID:YDL045W-A	AA:48		273614X:S>N		322134S:S>N		DBVPG6040:S>N		Y9:S>N		YS4:S>NID:YDL046W	AA:4		DBVPG1106:S>G		DBVPG1788:S>G		DBVPG1853:S>G		DBVPG6044:S>G		DBVPG6765:S>G		L_1374:S>G		L_1528:S>G		NCYC110:S>G		NCYC361:S>G		SK1:S>G		UWOPS03_461_4:S>G		UWOPS05_217_3:S>G		UWOPS05_227_2:S>G		UWOPS83_787_3:S>G		UWOPS87_2421:S>G		Y55:S>G		YJM975:S>G		YJM981:S>G		YPS128:S>G		YPS606:S>G	AA:11		DBVPG1853:L>F	AA:96		K11:R>C	AA:103		K11:L>F	AA:109		K11:L>V	AA:114		NCYC361:E>K	AA:116		K11:N>K	AA:147		UWOPS05_217_3:Y>-	AA:155		K11:T>S	AA:169		BC187:I>V		DBVPG1788:I>V		YJM978:I>V		YJM981:I>VID:YDL047W	AA:47		UWOPS03_461_4:P>R	AA:282		UWOPS05_217_3:P>HID:YDL048C	AA:2		378604X:L>P		BC187:L>P		DBVPG1373:L>P		DBVPG1853:L>P		DBVPG6765:L>P		L_1374:L>P		L_1528:L>P		NCYC361:L>P		SK1:L>P		UWOPS03_461_4:L>P		UWOPS05_217_3:L>P		UWOPS05_227_2:L>P		W303:L>P		YJM975:L>P		YJM978:L>P		YJM981:L>P		YPS128:L>P		YPS606:L>P	AA:7		W303:F>V	AA:38		UWOPS05_217_3:P>S		UWOPS05_227_2:P>S	AA:45		BC187:T>S	AA:67		UWOPS87_2421:T>I	AA:204		UWOPS03_461_4:T>S		UWOPS05_217_3:T>S	AA:219		Y12:P>L	AA:225		DBVPG6044:P>S		NCYC110:P>S		SK1:P>S		Y55:P>S	AA:243		Y9:S>F	AA:247		DBVPG1853:S>P	AA:343		NCYC361:S>I	AA:348		DBVPG6040:Q>P		DBVPG6044:Q>P		NCYC110:Q>P		SK1:Q>P		UWOPS03_461_4:Q>P		UWOPS05_217_3:Q>P		Y55:Q>P	AA:400		YS4:S>A	AA:412		L_1374:H>Y	AA:415		DBVPG1106:R>I		DBVPG1788:R>I		DBVPG1853:R>I		DBVPG6765:R>I		L_1374:R>I		L_1528:R>I		NCYC361:R>I		UWOPS83_787_3:R>I		YJM978:R>I		YS2:R>IID:YDL049C	AA:267		UWOPS03_461_4:H>N		UWOPS05_217_3:H>NID:YDL051W	AA:13		YJM981:P>	AA:243		UWOPS87_2421:N>K	AA:257		DBVPG6765:E>D	AA:262		W303:D>NID:YDL052C	AA:25		BC187:F>L		DBVPG1106:F>L		DBVPG1373:F>L		DBVPG1788:F>L		DBVPG6765:F>L		L_1528:F>L		NCYC361:F>L		SK1:F>L		UWOPS03_461_4:F>L		UWOPS05_227_2:F>L		UWOPS87_2421:F>L		Y55:F>L		YJM975:F>L		YJM978:F>L		YPS128:F>L	AA:130		DBVPG1106:R>S		NCYC361:R>S	AA:240		DBVPG1853:D>EID:YDL053C	AA:20		YIIc17_E5:T>A		YJM975:T>A	AA:22		YS4:S>T	AA:68		UWOPS05_217_3:S>A		UWOPS05_227_2:S>A	AA:73		UWOPS05_227_2:P>S	AA:131		DBVPG1853:A>S	AA:138		273614X:M>T		322134S:M>T		DBVPG1106:M>T		DBVPG1373:M>T		DBVPG1853:M>T		DBVPG6044:M>T		DBVPG6765:M>T		L_1374:M>T		L_1528:M>T		SK1:M>T		UWOPS03_461_4:M>T		UWOPS05_227_2:M>T		UWOPS87_2421:M>T		Y55:M>T		YIIc17_E5:M>T		YJM975:M>T		YPS606:M>T		YS4:M>T	AA:161		DBVPG6044:T>I		SK1:T>I		Y55:T>IID:YDL056W	AA:9		DBVPG6044:R>K		K11:R>K		NCYC110:R>K		SK1:R>K		UWOPS03_461_4:R>K		Y12:R>K		Y55:R>K		YPS128:R>K		YPS606:R>K	AA:123		378604X:V>M	AA:265		DBVPG6765:Q>	AA:267		DBVPG6765:Q>	AA:269		DBVPG1106:V>G		DBVPG1373:V>G		DBVPG1853:V>G		DBVPG6044:V>G		L_1374:V>G		L_1528:V>G		NCYC110:V>G		SK1:V>G		Y55:V>G		YIIc17_E5:V>G		YJM975:V>G		YJM978:V>G		YPS606:V>G	AA:569		DBVPG1373:H>Y	AA:577		DBVPG1373:L>M	AA:612		DBVPG1373:Q>H	AA:700		DBVPG1373:K>E		DBVPG6040:K>E		K11:K>E		NCYC110:K>E		SK1:K>E		Y55:K>E		YPS128:K>E		YPS606:K>E		YS4:K>EID:YDL057W	AA:6		DBVPG1373:V>A	AA:41		DBVPG6765:A>T		YIIc17_E5:A>T	AA:64		DBVPG6040:C>Y	AA:150		DBVPG6040:T>I	AA:177		273614X:E>G		DBVPG1373:E>G		DBVPG6040:E>G		DBVPG6765:E>G		SK1:E>G		UWOPS05_227_2:E>G		UWOPS83_787_3:E>G		Y55:E>G		YPS606:E>G		YS4:E>G	AA:213		UWOPS05_227_2:F>L	AA:215		273614X:A>T	AA:235		YS4:S>N	AA:303		UWOPS03_461_4:G>D		UWOPS05_227_2:G>D	AA:310		YS4:R>GID:YDL058W	AA:167		DBVPG6040:A>T	AA:256		DBVPG6765:N>D	AA:274		YS4:N>	AA:361		273614X:V>I		322134S:V>I		DBVPG1373:V>I		DBVPG1853:V>I		DBVPG6040:V>I		DBVPG6044:V>I		DBVPG6765:V>I		L_1528:V>I		UWOPS83_787_3:V>I		Y12:V>I		YIIc17_E5:V>I		YJM975:V>I		YJM981:V>I		YPS128:V>I		YS2:V>I	AA:370		273614X:E>V	AA:457		DBVPG6044:D>N		SK1:D>N		Y55:D>N	AA:472		UWOPS05_227_2:S>G		UWOPS87_2421:S>G		YPS128:S>G		YS4:S>G	AA:615		K11:E>K	AA:646		UWOPS05_227_2:F>L	AA:756		DBVPG6040:T>A	AA:765		DBVPG6040:I>T		K11:I>T		NCYC110:I>T		SK1:I>T		UWOPS83_787_3:I>T		Y55:I>T		YPS128:I>T		YS4:I>T	AA:847		DBVPG6040:G>E		SK1:G>E		Y12:G>E		Y55:G>E		YPS128:G>E		YPS606:G>E		YS4:G>E	AA:924		DBVPG6044:E>K		SK1:E>K		UWOPS03_461_4:E>K		UWOPS05_217_3:E>K		UWOPS05_227_2:E>K		UWOPS83_787_3:E>K		Y12:E>K		Y55:E>K		YPS128:E>K		YPS606:E>K		YS4:E>K	AA:948		UWOPS03_461_4:S>A		UWOPS05_217_3:S>A	AA:1076		DBVPG6040:K>I	AA:1103		322134S:T>K	AA:1156		YPS606:D>N	AA:1207		DBVPG1853:V>D	AA:1253		DBVPG6040:V>I		DBVPG6044:V>I		K11:V>I		NCYC110:V>I		SK1:V>I		UWOPS05_227_2:V>I		Y12:V>I		Y55:V>I		Y9:V>I		YPS606:V>I		YS4:V>I		YS9:V>I	AA:1264		W303:Q>	AA:1304		YPS606:E>D	AA:1319		DBVPG6040:I>V		K11:I>V		Y12:I>V		Y9:I>V		YS9:I>V	AA:1418		UWOPS03_461_4:V>I		UWOPS05_227_2:V>I	AA:1455		UWOPS03_461_4:L>I		UWOPS05_227_2:L>I	AA:1461		DBVPG6040:N>S		DBVPG6044:N>S		SK1:N>S		UWOPS03_461_4:N>S		UWOPS05_227_2:N>S		UWOPS87_2421:N>S		Y12:N>S		Y55:N>S		Y9:N>S		YPS128:N>S		YPS606:N>S		YS4:N>S	AA:1561		DBVPG6044:N>S		SK1:N>S	AA:1575		YPS128:R>M	AA:1581		DBVPG6040:G>S		DBVPG6044:G>S		SK1:G>S		Y12:G>S		YPS128:G>S		YS4:G>S	AA:1600		DBVPG6044:I>V		SK1:I>V		UWOPS05_217_3:I>V		UWOPS05_227_2:I>V		YPS128:I>V		YS4:I>V	AA:1606		YPS128:T>A	AA:1614		YPS128:D>G	AA:1661		DBVPG6044:R>S		SK1:R>S		UWOPS05_217_3:R>S		UWOPS05_227_2:R>S		UWOPS83_787_3:R>S		Y55:R>S		YS2:R>S	AA:1677		DBVPG6044:E>K		SK1:E>K		Y55:E>K	AA:1701		UWOPS05_217_3:D>H		UWOPS05_227_2:D>H	AA:1753		UWOPS05_217_3:E>K		UWOPS05_227_2:E>KID:YDL059C	AA:2		Y9:T>S	AA:19		DBVPG6044:G>S		SK1:G>S		Y12:G>S		Y55:G>S		YPS606:G>S	AA:35		YIIc17_E5:W>L	AA:36		L_1528:N>S	AA:40		YIIc17_E5:A>S	AA:96		322134S:R>Q	AA:99		YPS606:V>I	AA:216		YPS128:L>I	AA:222		L_1528:S>N	AA:229		YPS128:L>FID:YDL060W	AA:55		UWOPS05_227_2:K>R		UWOPS83_787_3:K>R		YPS128:K>R		YS4:K>R	AA:63		DBVPG1853:A>T	AA:65		DBVPG6044:R>K		SK1:R>K		UWOPS03_461_4:R>K		UWOPS05_227_2:R>K		UWOPS83_787_3:R>K		Y55:R>K		YPS128:R>K		YS4:R>K	AA:112		DBVPG1373:G>E		DBVPG1788:G>E		DBVPG6044:G>E		DBVPG6765:G>E		SK1:G>E		UWOPS03_461_4:G>E		UWOPS05_227_2:G>E		UWOPS83_787_3:G>E		Y12:G>E		Y55:G>E		YPS128:G>E		YS4:G>E	AA:117		UWOPS03_461_4:E>K		UWOPS05_227_2:E>K	AA:266		YS4:F>	AA:312		Y9:S>Y	AA:328		273614X:S>G		378604X:S>G		NCYC110:S>G		SK1:S>G		UWOPS83_787_3:S>G		UWOPS87_2421:S>G		Y55:S>G		Y9:S>G		YPS128:S>G		YPS606:S>G		YS4:S>G	AA:381		NCYC110:P>S		SK1:P>S		Y55:P>S	AA:422		273614X:G>A		UWOPS87_2421:G>A		YPS128:G>A		YPS606:G>A	AA:439		UWOPS03_461_4:D>N	AA:548		DBVPG1853:E>GID:YDL063C	AA:66		YJM978:A>V	AA:131		DBVPG1853:R>K		DBVPG6044:R>K		SK1:R>K		UWOPS05_227_2:R>K		UWOPS83_787_3:R>K		UWOPS87_2421:R>K		Y55:R>K		Y9:R>K		YS4:R>K		YS9:R>K	AA:193		DBVPG6044:I>M		NCYC110:I>M		SK1:I>M		UWOPS03_461_4:S>G		UWOPS83_787_3:I>M		Y12:I>M		Y55:I>M		Y9:I>M	AA:209		DBVPG1853:E>D	AA:340		DBVPG1853:I>M		K11:I>M		NCYC110:I>M		SK1:I>M		UWOPS03_461_4:I>M		UWOPS83_787_3:I>M		UWOPS87_2421:I>M		Y12:I>M		Y55:I>M		YPS606:I>M		YS4:I>M	AA:473		DBVPG1853:V>I		K11:V>I		SK1:V>I		UWOPS87_2421:V>I		Y12:V>I		Y55:V>I		YPS128:V>I		YPS606:V>I	AA:531		YPS128:Q>R		YPS606:Q>R	AA:534		UWOPS83_787_3:V>I	AA:544		YPS128:N>H		YPS606:N>H	AA:619		DBVPG6044:N>S		NCYC110:N>S		SK1:N>S		Y55:N>SID:YDL065C	AA:3		Y9:N>K	AA:73		UWOPS87_2421:D>Y	AA:120		273614X:H>R		322134S:H>R		DBVPG6044:H>R		DBVPG6765:H>R		L_1528:H>R		SK1:H>R		UWOPS03_461_4:H>R		UWOPS83_787_3:H>R		UWOPS87_2421:H>R		Y55:H>R		Y9:H>R		YPS606:H>R		YS4:H>R		YS9:H>R	AA:124		273614X:E>D		UWOPS03_461_4:E>D		UWOPS83_787_3:E>D	AA:127		273614X:C>S		322134S:C>S		DBVPG6044:C>S		DBVPG6765:C>S		SK1:C>S		UWOPS03_461_4:C>F		UWOPS05_227_2:C>F		UWOPS83_787_3:C>S		UWOPS87_2421:C>S		Y12:C>S		Y55:C>S		Y9:C>S		YPS606:C>S		YS4:C>S		YS9:C>S	AA:140		322134S:V>L		DBVPG6044:V>L		DBVPG6765:V>L		L_1528:V>L		SK1:V>L		UWOPS03_461_4:V>L		UWOPS05_227_2:V>L		UWOPS83_787_3:V>L		UWOPS87_2421:V>L		Y12:V>L		Y55:V>L		Y9:V>L		YPS606:V>L		YS9:V>L	AA:245		UWOPS05_217_3:G>S	AA:311		UWOPS05_217_3:N>S		UWOPS05_227_2:N>SID:YDL066W	AA:40		DBVPG6044:D>E		SK1:D>E		Y55:D>E	AA:137		K11:V>L	AA:138		DBVPG1106:P>SID:YDL067C	AA:2		322134S:T>A		DBVPG1788:T>A		DBVPG1853:T>A		DBVPG6040:T>A		DBVPG6044:T>A		K11:T>A		SK1:T>A		UWOPS03_461_4:T>A		UWOPS83_787_3:T>A		UWOPS87_2421:T>A		Y55:T>A		Y9:T>A		YIIc17_E5:T>A		YPS606:T>A		YS9:T>AID:YDL069C	AA:6		UWOPS05_227_2:V>A	AA:20		378604X:Y>N		UWOPS05_227_2:Y>N		UWOPS83_787_3:Y>N		Y12:Y>N		Y9:Y>N		YPS128:Y>N		YPS606:Y>N	AA:22		UWOPS83_787_3:P>S		Y9:P>S		YPS128:P>S		YPS606:P>S	AA:25		UWOPS05_227_2:T>S		Y12:T>S	AA:38		YS4:E>G	AA:57		DBVPG6044:I>L		SK1:I>L		UWOPS05_227_2:I>L		UWOPS83_787_3:I>L		Y12:I>L		Y55:I>L		Y9:I>L		YPS128:I>L		YPS606:I>L	AA:71		UWOPS83_787_3:R>K		UWOPS87_2421:R>K	AA:90		378604X:E>A	AA:183		378604X:N>H	AA:212		378604X:S>PID:YDL070W	AA:23		YPS128:T>S	AA:239		UWOPS05_217_3:M>I	AA:240		NCYC110:P>S	AA:253		UWOPS03_461_4:S>T		UWOPS05_217_3:S>T		UWOPS05_227_2:S>T	AA:265		UWOPS83_787_3:P>A	AA:279		Y9:I>T	AA:281		K11:E>G	AA:288		DBVPG6044:V>I		K11:V>I		NCYC110:V>I		SK1:V>I		UWOPS03_461_4:V>I		UWOPS05_217_3:V>I		UWOPS05_227_2:V>I		UWOPS83_787_3:V>I		UWOPS87_2421:V>I		Y12:V>I		Y55:V>I		Y9:V>I		YPS606:V>I	AA:338		DBVPG6044:N>I		NCYC110:N>I		SK1:N>I		Y55:N>I	AA:375		UWOPS03_461_4:N>K	AA:390		DBVPG1853:D>H	AA:400		YS4:Q>H	AA:502		UWOPS05_227_2:H>R	AA:512		DBVPG1853:A>S		DBVPG6044:A>S		SK1:A>S		Y55:A>S	AA:574		DBVPG1853:R>I		DBVPG6044:R>I		NCYC110:R>I		SK1:R>I		UWOPS03_461_4:R>I		UWOPS05_217_3:R>I		UWOPS87_2421:R>I		Y55:R>I		Y9:R>I		YPS128:R>IID:YDL072C	AA:10		UWOPS87_2421:A>S	AA:12		YS4:L>Q	AA:54		273614X:V>I		378604X:V>I		DBVPG1106:V>I		DBVPG1788:V>I		DBVPG6044:V>I		DBVPG6765:V>I		K11:V>I		L_1374:V>I		NCYC110:V>I		SK1:V>I		UWOPS03_461_4:V>I		UWOPS05_217_3:V>I		Y12:V>I		Y55:V>I		YIIc17_E5:V>I		YJM975:V>I		YJM978:V>I		YPS128:V>I		YPS606:V>I		YS4:V>I	AA:68		YS4:R>K	AA:132		L_1374:T>A	AA:194		DBVPG6765:K>N	AA:202		DBVPG6044:I>M		K11:I>M		NCYC110:I>M		SK1:I>M		UWOPS03_461_4:I>M		UWOPS05_227_2:I>M		UWOPS83_787_3:I>M		UWOPS87_2421:I>M		Y12:I>M		Y55:I>M		YPS128:I>M		YPS606:I>MID:YDL073W	AA:24		DBVPG6040:M>I		DBVPG6044:M>I		NCYC110:M>I		SK1:M>I		UWOPS05_217_3:M>I		UWOPS83_787_3:M>I		Y12:M>I		Y55:M>I	AA:68		YS9:L>S	AA:140		YS4:I>L	AA:223		DBVPG1853:F>L		DBVPG6044:F>L		NCYC110:F>L		SK1:F>L		UWOPS03_461_4:F>L		UWOPS05_217_3:F>L		UWOPS05_227_2:F>L		UWOPS83_787_3:F>L		UWOPS87_2421:F>L		YPS128:F>L		YPS606:F>L	AA:310		273614X:D>N		322134S:D>N		DBVPG1373:D>N		DBVPG6765:D>N		YIIc17_E5:D>N		YJM975:D>N		YJM978:D>N		YS2:D>N	AA:313		YS4:I>V	AA:329		YPS128:T>A		YPS606:T>A	AA:358		YPS128:E>V	AA:374		UWOPS87_2421:N>S	AA:381		NCYC110:R>G		SK1:R>G		Y55:R>G	AA:510		YPS128:K>N		YPS606:K>N	AA:514		DBVPG1853:S>N		K11:S>N		NCYC110:S>N		SK1:S>N		UWOPS03_461_4:S>N		UWOPS05_227_2:S>N		UWOPS83_787_3:S>N		Y12:S>N		Y55:S>N		YPS128:S>N		YPS606:S>N	AA:544		K11:S>N		Y12:S>N	AA:557		UWOPS03_461_4:D>N		UWOPS05_227_2:D>N	AA:559		Y9:I>V	AA:673		DBVPG1853:P>H	AA:691		BC187:D>E		DBVPG6765:D>E		YJM975:D>E	AA:695		UWOPS83_787_3:D>E	AA:710		DBVPG1853:N>T	AA:735		YS9:K>R	AA:769		DBVPG1853:I>N	AA:775		Y12:N>S		Y9:N>S	AA:782		DBVPG1373:Y>S		DBVPG1853:Y>S		DBVPG6040:Y>S		DBVPG6044:Y>S		DBVPG6765:Y>S		L_1528:Y>S		NCYC110:Y>S		SK1:Y>S		UWOPS03_461_4:Y>S		UWOPS05_227_2:Y>S		Y55:Y>S		Y9:Y>S		YIIc17_E5:Y>S		YPS128:Y>S		YPS606:Y>S		YS9:Y>S	AA:792		UWOPS03_461_4:E>K		UWOPS05_227_2:E>K	AA:794		DBVPG6765:I>L	AA:814		DBVPG6044:A>T		SK1:A>T		UWOPS03_461_4:A>T		UWOPS05_227_2:A>T		Y55:A>T		Y9:A>T		YPS128:A>T		YPS606:A>T	AA:841		DBVPG6044:T>A		NCYC110:T>A		SK1:T>A		Y55:T>A	AA:884		UWOPS03_461_4:I>V		UWOPS05_227_2:I>V	AA:903		DBVPG6044:A>P		NCYC110:A>P		SK1:A>P		UWOPS03_461_4:A>P		UWOPS05_227_2:A>P		UWOPS83_787_3:A>P		UWOPS87_2421:A>P		Y55:A>P		YPS128:A>P		YPS606:A>PID:YDL076C	AA:50		NCYC110:Q>P		SK1:Q>P		Y55:Q>P	AA:160		273614X:K>T		DBVPG1853:K>T		NCYC110:K>T		SK1:K>T		UWOPS03_461_4:K>T		UWOPS05_227_2:K>T		UWOPS83_787_3:K>T		Y55:K>T		Y9:K>T		YIIc17_E5:K>T		YPS128:K>T		YPS606:K>T	AA:186		DBVPG1106:F>L	AA:193		YPS128:G>D		YPS606:G>D	AA:249		NCYC110:S>P		SK1:S>P		Y55:S>P	AA:269		Y9:S>NID:YDL077C	AA:16		YS9:T>A	AA:125		YJM978:N>Y	AA:138		UWOPS03_461_4:M>L		UWOPS05_227_2:M>L	AA:305		DBVPG1788:D>V	AA:420		L_1374:P>L	AA:470		Y9:S>L	AA:506		BC187:C>Y		K11:S>P		YJM975:C>Y		YJM978:C>Y	AA:539		YS4:H>D	AA:556		NCYC110:L>S	AA:626		UWOPS03_461_4:E>A		UWOPS05_217_3:E>A		UWOPS05_227_2:E>A		UWOPS83_787_3:E>A	AA:632		DBVPG6044:H>R		K11:H>R		NCYC110:H>R		SK1:H>R		UWOPS03_461_4:H>R		UWOPS05_217_3:H>R		UWOPS05_227_2:H>R		UWOPS83_787_3:H>R		UWOPS87_2421:H>R		Y55:H>R		Y9:H>R		YPS128:H>R		YPS606:H>R	AA:643		YS9:M>T	AA:700		DBVPG6044:S>P		K11:S>P		NCYC110:S>P		SK1:S>P		UWOPS03_461_4:S>P		UWOPS05_217_3:S>P		UWOPS05_227_2:S>P		UWOPS83_787_3:S>P		Y55:S>P		YPS128:S>P		YPS606:S>P		YS2:S>P		YS9:S>P	AA:703		322134S:Q>H	AA:739		YS4:S>R	AA:747		YS4:G>R	AA:765		322134S:I>S	AA:767		YS2:T>S		YS9:T>S	AA:848		YS4:H>Y	AA:877		YS4:S>P		YS9:S>P	AA:945		DBVPG6044:T>S		SK1:T>S		UWOPS03_461_4:T>S		UWOPS05_227_2:T>S		Y55:T>S		Y9:T>S		YPS128:T>S		YPS606:T>S	AA:962		Y9:L>V	AA:978		YIIc17_E5:V>F	AA:1006		YS4:E>DID:YDL078C	AA:282		UWOPS05_217_3:G>SID:YDL080C	AA:2		Y9:N>D	AA:14		273614X:C>Y		378604X:C>Y		BC187:C>Y		DBVPG1373:C>Y		DBVPG6765:C>Y		NCYC110:C>Y		NCYC361:C>Y		SK1:C>Y		UWOPS03_461_4:C>Y		UWOPS05_217_3:C>Y		UWOPS05_227_2:C>Y		Y55:C>Y		Y9:C>Y		YIIc17_E5:C>Y		YJM978:C>Y		YJM981:C>Y		YPS128:C>Y		YPS606:C>Y	AA:37		DBVPG1853:S>P	AA:102		YJM975:E>G	AA:144		DBVPG6044:A>T		NCYC110:A>T		SK1:A>T		Y55:A>T	AA:211		DBVPG1853:V>A	AA:259		UWOPS87_2421:G>V	AA:344		DBVPG1853:I>L		DBVPG6765:I>L		K11:I>L		L_1374:I>L		SK1:I>L		Y55:I>L		YIIc17_E5:I>L		YJM981:I>L		YPS128:I>L		YPS606:I>L	AA:356		DBVPG1853:Q>R	AA:414		DBVPG6765:N>S		YJM981:N>S	AA:448		YIIc17_E5:F>S	AA:520		YS9:F>L	AA:521		UWOPS87_2421:G>V	AA:569		YIIc17_E5:D>E	AA:580		Y12:Q>L	AA:592		273614X:N>D		322134S:N>D		378604X:N>D		BC187:N>D		DBVPG1373:N>D		DBVPG1788:N>D		DBVPG1853:N>D		DBVPG6044:N>D		DBVPG6765:N>D		L_1374:N>D		L_1528:N>D		SK1:N>D		UWOPS05_217_3:N>D		UWOPS05_227_2:N>D		UWOPS83_787_3:N>D		UWOPS87_2421:N>D		Y55:N>D		Y9:N>D		YPS128:N>D		YS9:N>DID:YDL081C	AA:50		DBVPG1373:D>EID:YDL084W	AA:43		273614X:N>S		DBVPG6044:N>S		NCYC361:N>S		SK1:N>S	AA:47		378604X:T>A		BC187:T>A		DBVPG1106:T>A		DBVPG1373:T>A		DBVPG1853:T>A		DBVPG6765:T>A		L_1528:T>A		W303:T>A		YJM978:T>A		YJM981:T>A	AA:59		YS4:I>V		YS9:I>V	AA:438		UWOPS03_461_4:I>V	AA:444		NCYC361:L>VID:YDL085C-A	AA:12		273614X:Q>H	AA:66		YS9:T>MID:YDL085W	AA:17		DBVPG6044:F>C		NCYC110:F>C		SK1:F>C	AA:51		DBVPG6765:K>E		L_1374:K>E	AA:90		322134S:A>T	AA:199		273614X:S>N		DBVPG6044:S>N		NCYC110:S>N		SK1:S>N		Y12:S>N		Y55:S>N	AA:381		L_1528:I>V	AA:403		L_1528:N>D	AA:447		YJM975:D>Y		YJM978:D>Y	AA:448		Y9:M>IID:YDL086W	AA:31		378604X:P>S	AA:133		YS9:G>A	AA:145		378604X:V>I		BC187:V>I		DBVPG1373:V>I		DBVPG1853:V>I		DBVPG6765:V>I		L_1528:V>I		YJM975:V>I		YJM981:V>I	AA:157		YS9:H>L	AA:159		SK1:R>K		UWOPS87_2421:R>K		Y55:R>K		YS9:R>K	AA:163		UWOPS03_461_4:L>H		UWOPS05_217_3:L>H	AA:166		YS9:N>TID:YDL087C	AA:27		DBVPG6044:H>Y		SK1:H>Y		Y55:H>Y	AA:33		UWOPS05_217_3:Q>K	AA:148		UWOPS83_787_3:Q>-	AA:160		378604X:S>T		DBVPG1106:S>T		DBVPG1373:S>T		DBVPG1788:S>T		DBVPG1853:S>T		DBVPG6040:S>T		DBVPG6044:S>T		DBVPG6765:S>T		K11:S>T		L_1374:S>T		L_1528:S>T		SK1:S>T		UWOPS05_217_3:S>T		UWOPS83_787_3:S>T		UWOPS87_2421:S>T		Y55:S>T		Y9:S>T		YIIc17_E5:S>T		YPS128:S>T		YPS606:S>T		YS9:S>T	AA:174		UWOPS05_217_3:S>N	AA:249		DBVPG1106:S>N		DBVPG1373:S>N		DBVPG1788:S>N		DBVPG6765:S>N		L_1374:S>N		L_1528:S>NID:YDL088C	AA:47		Y55:H>R	AA:71		378604X:A>V		DBVPG1106:A>V		DBVPG1373:A>V		DBVPG6040:A>V		DBVPG6765:A>V		L_1374:A>V		NCYC361:A>V		UWOPS83_787_3:A>V		UWOPS87_2421:A>V		Y55:A>V		YIIc17_E5:A>V		YJM975:A>V		YJM978:A>V		YPS128:A>V		YPS606:A>V	AA:207		DBVPG1106:T>S		DBVPG1373:T>S		DBVPG1788:T>S		DBVPG1853:T>S		DBVPG6765:T>S		SK1:T>S		UWOPS03_461_4:T>S		UWOPS83_787_3:T>S		Y55:T>S		Y9:T>S		YIIc17_E5:T>S		YJM975:T>S		YJM978:T>S		YJM981:T>S		YPS128:T>S		YS2:T>S	AA:217		UWOPS83_787_3:R>K	AA:312		YS2:F>L	AA:378		273614X:R>H		DBVPG1106:R>H		DBVPG1373:R>H		DBVPG1788:R>H		DBVPG1853:R>H		DBVPG6765:R>H		UWOPS03_461_4:R>H		UWOPS05_227_2:R>H		UWOPS83_787_3:R>H		YJM978:R>H		YPS128:R>H		YPS606:R>H	AA:380		YS2:S>P	AA:478		YIIc17_E5:F>LID:YDL089W	AA:122		273614X:N>S		378604X:N>S		BC187:N>S		DBVPG1106:N>S		DBVPG1853:N>S		DBVPG6044:N>S		DBVPG6765:N>S		L_1374:N>S		L_1528:N>S		SK1:N>S		Y55:N>S		YJM975:N>S		YJM978:N>S		YPS128:N>S		YPS606:N>S	AA:227		DBVPG6765:D>V		YJM975:D>V	AA:357		DBVPG6044:E>V		NCYC110:E>V		SK1:E>V		Y55:E>V	AA:427		322134S:Y>F		BC187:Y>F		DBVPG1373:Y>F		DBVPG6765:Y>F		L_1528:Y>F		YIIc17_E5:Y>F		YJM975:Y>F		YJM978:Y>F		YS4:Y>F	AA:465		YPS128:K>R		YPS606:K>RID:YDL090C	AA:16		SK1:N>D		Y55:N>D	AA:29		SK1:R>K		Y55:R>K	AA:83		W303:H>Y	AA:122		YS4:R>G		YS9:R>G	AA:181		UWOPS87_2421:D>N		YS2:D>N		YS9:D>N	AA:220		UWOPS05_217_3:A>S	AA:263		DBVPG1853:F>L	AA:280		UWOPS03_461_4:V>F	AA:288		UWOPS03_461_4:S>I	AA:291		YJM978:Q>R	AA:293		UWOPS05_217_3:Q>R	AA:297		Y12:G>V	AA:323		Y12:A>T	AA:331		273614X:N>S		DBVPG1373:N>S		DBVPG1853:N>S		DBVPG6765:N>S		L_1374:N>S		L_1528:N>S		YJM975:N>S		YJM978:N>S		YS9:N>S	AA:351		UWOPS87_2421:L>F	AA:386		L_1374:P>A	AA:431		UWOPS03_461_4:S>R		UWOPS87_2421:S>R		Y12:S>R		Y9:S>RID:YDL091C	AA:120		UWOPS05_217_3:T>M	AA:121		273614X:C>F	AA:123		DBVPG6044:E>D		SK1:E>D		Y55:E>D		YPS128:E>D		YPS606:E>D	AA:158		DBVPG1106:G>R		L_1374:G>R	AA:223		322134S:D>GID:YDL098C	AA:12		UWOPS05_227_2:E>K	AA:53		Y12:S>L	AA:164		DBVPG1106:K>I	AA:176		L_1528:S>NID:YDL100C	AA:14		SK1:T>S		Y55:T>S	AA:49		YS2:K>Q	AA:116		DBVPG6765:D>N	AA:155		378604X:G>D		DBVPG1106:G>D		YJM975:G>D	AA:195		YJM975:N>DID:YDL101C	AA:4		L_1528:S>A	AA:148		BC187:D>E		DBVPG1788:D>E		DBVPG1853:D>E		L_1374:D>E		YJM975:D>E		YJM978:D>E		YS4:D>E	AA:153		BC187:S>G		DBVPG1788:S>G		DBVPG6765:S>G		L_1374:S>G		L_1528:S>G		SK1:S>G		UWOPS05_227_2:S>G		UWOPS83_787_3:S>G		Y12:S>G		Y55:S>G		YJM975:S>G		YJM978:S>G		YPS128:S>G		YPS606:S>G		YS4:S>G	AA:179		UWOPS05_227_2:A>S	AA:296		YS2:R>K	AA:466		UWOPS87_2421:D>EID:YDL102W	AA:33		K11:H>R	AA:86		378604X:M>I		NCYC361:M>I		UWOPS83_787_3:M>I		YS2:M>I	AA:89		BC187:Q>R		DBVPG6765:Q>R		L_1528:Q>R		YJM975:Q>R	AA:162		NCYC361:V>I	AA:190		UWOPS05_227_2:A>V	AA:222		DBVPG1853:H>Y	AA:351		L_1528:A>T	AA:367		378604X:P>H		DBVPG1853:P>H	AA:690		DBVPG6044:R>S	AA:789		UWOPS05_217_3:V>I	AA:1065		DBVPG1853:S>N		Y9:S>N		YS4:S>NID:YDL104C	AA:3		378604X:S>A		K11:S>A		UWOPS03_461_4:S>A		UWOPS05_227_2:S>A		UWOPS83_787_3:S>A		YS9:S>A	AA:8		DBVPG6044:G>V		SK1:G>V		Y55:G>V	AA:13		378604X:D>Y		UWOPS83_787_3:D>Y	AA:23		DBVPG1106:C>Y		DBVPG6044:F>S		DBVPG6765:F>S		K11:F>S		SK1:F>S		UWOPS03_461_4:F>S		UWOPS05_217_3:F>S		UWOPS83_787_3:F>S		Y55:F>S		YPS128:F>S		YS9:F>S	AA:30		UWOPS03_461_4:R>K		UWOPS05_227_2:R>K	AA:105		UWOPS83_787_3:E>K	AA:107		UWOPS83_787_3:I>V	AA:222		YS4:K>E	AA:273		DBVPG1788:K>R		DBVPG6765:K>R	AA:323		Y55:S>F	AA:348		DBVPG6765:F>L	AA:381		DBVPG1373:S>NID:YDL105W	AA:6		W303:I>M	AA:56		L_1374:D>E	AA:154		322134S:A>S		378604X:A>S		BC187:A>S		DBVPG1106:A>S		DBVPG1373:A>S		DBVPG1853:A>S		DBVPG6765:A>S		L_1374:A>S		YJM975:A>S	AA:247		UWOPS05_217_3:E>Q	AA:267		DBVPG1853:I>VID:YDL106C	AA:39		DBVPG6765:N>H		Y12:N>I		YJM975:N>H		YJM981:N>H	AA:88		UWOPS03_461_4:A>T		UWOPS05_227_2:A>T	AA:127		YS9:N>I	AA:157		322134S:D>E		DBVPG6040:D>E		DBVPG6044:D>E		NCYC361:D>E		SK1:D>E		UWOPS83_787_3:D>E		Y55:D>E	AA:161		NCYC361:T>K		UWOPS83_787_3:T>K	AA:164		K11:N>K		Y12:N>K	AA:232		Y12:I>T	AA:246		Y12:V>A	AA:285		YJM978:T>S	AA:291		K11:D>N		Y12:D>N	AA:331		DBVPG1373:K>N		DBVPG1788:K>N	AA:472		YIIc17_E5:N>S	AA:497		YIIc17_E5:N>D	AA:518		L_1374:N>S	AA:521		UWOPS03_461_4:D>N		UWOPS05_217_3:D>N		UWOPS05_227_2:D>NID:YDL107W	AA:11		YGPM:T>A	AA:57		322134S:E>Q		378604X:E>Q		DBVPG1106:E>Q		DBVPG1788:E>Q		DBVPG6765:E>Q		L_1374:E>Q		YIIc17_E5:E>Q		YS4:E>Q		YS9:E>Q	AA:60		DBVPG1853:I>V		UWOPS05_227_2:I>V		YPS128:I>V		YPS606:I>V	AA:135		273614X:Y>N		378604X:Y>N	AA:144		273614X:E>K		322134S:E>K		378604X:E>K		DBVPG1106:E>K		DBVPG1788:E>K		DBVPG6044:E>K		DBVPG6765:E>K		L_1374:E>K		SK1:E>K		UWOPS05_227_2:E>K		Y55:E>K		YJM975:E>K		YJM978:E>K		YPS128:E>K		YS4:E>K		YS9:E>K	AA:170		322134S:T>I		DBVPG1788:T>I		YIIc17_E5:T>I	AA:186		273614X:N>K		K11:N>S		Y12:N>S		Y9:N>S	AA:225		DBVPG1853:I>V	AA:250		UWOPS83_787_3:L>FID:YDL108W	AA:65		YS9:N>H	AA:181		YIIc17_E5:H>	AA:200		UWOPS03_461_4:L>V	AA:254		DBVPG6040:K>N		UWOPS87_2421:K>N	AA:285		UWOPS05_227_2:L>IID:YDL110C	AA:8		K11:R>K		YS9:R>I	AA:65		L_1528:A>D	AA:67		K11:D>N	AA:70		L_1528:D>H	AA:128		L_1528:N>KID:YDL111C	AA:2		DBVPG6044:S>F		Y55:S>F	AA:113		DBVPG1373:K>M		DBVPG1788:K>M		DBVPG1853:K>M		DBVPG6765:K>M		L_1374:K>M		UWOPS83_787_3:K>M		YJM975:K>M	AA:152		Y9:T>A	AA:170		UWOPS87_2421:P>H	AA:207		Y12:E>D		Y9:E>D	AA:250		S288c:M>IID:YDL114W	AA:4		YS2:K>E	AA:23		378604X:V>A		DBVPG6040:V>A		K11:V>A		Y9:V>A	AA:30		BC187:Y>C	AA:40		273614X:T>I		BC187:T>I		DBVPG1373:T>I		DBVPG1788:T>I		DBVPG6040:T>I		DBVPG6044:T>I		DBVPG6765:T>I		L_1528:T>I		SK1:T>I		UWOPS83_787_3:T>I		Y55:T>I		YS9:T>I	AA:75		UWOPS03_461_4:T>A	AA:83		UWOPS03_461_4:N>K	AA:173		YPS128:E>D		YPS606:E>D	AA:204		BC187:S>P		DBVPG1788:S>P		DBVPG6040:S>P		DBVPG6044:S>P		L_1374:S>P		L_1528:S>P		SK1:S>P		UWOPS03_461_4:S>P		UWOPS83_787_3:S>P		Y55:S>P		Y9:S>P		YIIc17_E5:S>P		YJM975:S>P		YJM978:S>P		YPS128:S>P		YPS606:S>P		YS4:S>P		YS9:S>P	AA:264		Y9:A>VID:YDL115C	AA:204		273614X:D>H	AA:205		UWOPS03_461_4:D>N		UWOPS05_227_2:D>N	AA:211		DBVPG6765:N>S	AA:215		322134S:P>S	AA:232		322134S:Q>H	AA:234		YS9:Q>RID:YDL116W	AA:22		BC187:F>Y		DBVPG1373:F>Y		DBVPG1788:F>Y		DBVPG6765:F>Y		K11:F>Y		NCYC361:F>Y		SK1:F>Y		UWOPS03_461_4:F>Y		UWOPS83_787_3:F>Y		Y55:F>Y		Y9:F>Y		YIIc17_E5:F>Y		YJM978:F>Y		YPS128:F>Y		YPS606:F>Y		YS2:F>Y	AA:52		YJM978:D>N	AA:213		DBVPG1106:C>S		DBVPG1373:C>S		DBVPG1788:C>S		DBVPG6044:C>S		DBVPG6765:C>S		SK1:C>S		UWOPS83_787_3:C>S		Y55:C>S		YJM978:C>S	AA:271		273614X:I>V		DBVPG1106:I>V		DBVPG1373:I>V		DBVPG6040:I>V		DBVPG6044:I>V		DBVPG6765:I>V		SK1:I>V		UWOPS03_461_4:I>V		UWOPS05_217_3:I>V		UWOPS83_787_3:I>V		Y12:I>V		Y55:I>V		YIIc17_E5:I>V		YJM978:I>V		YPS128:I>V		YPS606:I>V		YS2:I>V		YS4:I>V	AA:395		273614X:C>F		322134S:C>F		BC187:C>F		DBVPG1106:C>F		DBVPG1373:C>F		DBVPG1788:C>F		DBVPG6040:C>F		DBVPG6044:C>F		NCYC110:C>F		SK1:C>F		YIIc17_E5:C>F		YPS128:C>F		YPS606:C>F		YS4:C>F	AA:422		UWOPS03_461_4:L>W	AA:454		273614X:D>N		322134S:D>N		BC187:D>N		DBVPG1373:D>N		DBVPG1788:D>N		DBVPG6040:D>N	AA:479		273614X:T>S	AA:553		273614X:N>Y	AA:605		UWOPS87_2421:S>N	AA:608		DBVPG6044:S>N		SK1:S>N		UWOPS03_461_4:S>N		UWOPS87_2421:S>N		Y55:S>N		Y9:S>N		YPS128:S>N		YS4:S>NID:YDL117W	AA:7		BC187:S>C		L_1374:S>C		L_1528:S>C		YJM975:S>C		YJM978:S>C		YS2:S>C		YS4:S>C	AA:44		Y9:S>F		YJM975:S>F	AA:49		YJM975:K>Q	AA:63		L_1374:N>S	AA:89		L_1374:F>C	AA:107		BC187:E>K		DBVPG1373:E>K		DBVPG6765:E>K		L_1528:E>K		YJM978:E>K		YS2:E>K	AA:114		L_1374:K>M	AA:127		YPS606:V>E	AA:148		322134S:Y>H		L_1528:Y>H		YJM975:Y>H		YJM978:Y>H	AA:310		273614X:I>V		322134S:I>V		378604X:I>V		DBVPG1106:I>V		DBVPG1373:I>V		DBVPG1788:I>V		DBVPG6040:I>V		DBVPG6044:I>V		DBVPG6765:I>V		L_1528:I>V		NCYC361:I>V		SK1:I>V		UWOPS03_461_4:I>V		UWOPS05_217_3:I>V		Y12:I>V		YJM975:I>V		YPS606:I>V		YS2:I>V	AA:321		322134S:A>T		DBVPG1106:A>T		DBVPG1373:A>T		DBVPG1788:A>T		DBVPG6040:A>T		DBVPG6765:A>T		L_1528:A>T		NCYC361:A>T		YJM975:A>T		YJM978:A>T		YS2:A>T	AA:332		YS9:L>I	AA:369		DBVPG6040:L>F	AA:421		NCYC361:N>S	AA:554		YJM978:A>T	AA:557		378604X:W>S		DBVPG1853:W>S	AA:587		NCYC361:P>S	AA:608		UWOPS05_227_2:A>S	AA:662		DBVPG1106:L>S	AA:757		DBVPG1853:V>I	AA:813		273614X:V>I		322134S:V>I		BC187:V>I		DBVPG1373:V>I		DBVPG1788:V>I		DBVPG1853:V>I		DBVPG6044:V>I		DBVPG6765:V>I		K11:V>I		L_1528:V>I		NCYC110:V>I		SK1:V>I		UWOPS03_461_4:V>I		UWOPS83_787_3:V>I		UWOPS87_2421:V>I		Y55:V>I		YIIc17_E5:V>I		YJM978:V>I		YPS606:V>I		YS4:V>I	AA:854		SK1:G>E		Y55:G>EID:YDL119C	AA:91		DBVPG1853:R>G	AA:236		Y12:L>S	AA:290		322134S:F>L		BC187:F>L		DBVPG1373:F>L		DBVPG6040:F>L		DBVPG6765:F>L		K11:F>L		L_1374:F>L		L_1528:F>L		SK1:F>L		UWOPS03_461_4:F>L		UWOPS83_787_3:F>L		Y12:F>L		Y55:F>L		Y9:F>L		YJM975:F>L		YPS128:F>L		YPS606:F>LID:YDL120W	AA:54		DBVPG1853:S>C	AA:76		DBVPG6040:E>K		UWOPS87_2421:E>KID:YDL121C	AA:17		UWOPS03_461_4:I>V		UWOPS05_227_2:I>V	AA:43		YS9:A>S	AA:73		DBVPG6044:S>G		Y55:S>G	AA:75		DBVPG6765:G>D	AA:77		DBVPG6044:D>G		Y55:D>G	AA:123		YS9:E>DID:YDL123W	AA:47		UWOPS83_787_3:L>Q	AA:85		NCYC110:N>S		SK1:N>S		Y55:N>S	AA:125		DBVPG6040:H>PID:YDL124W	AA:23		DBVPG6044:G>V		NCYC110:G>V		SK1:G>V		Y55:G>V	AA:83		DBVPG1853:A>E		DBVPG6044:A>E		K11:A>E		NCYC110:A>E		SK1:A>E		Y12:A>E		Y55:A>E		YPS128:A>E		YPS606:A>E	AA:92		273614X:P>T		BC187:P>T		DBVPG1373:P>T		DBVPG1853:P>T		DBVPG6044:P>T		DBVPG6765:P>T		K11:P>T		L_1374:P>T		NCYC110:P>T		SK1:P>T		Y12:P>T		Y55:P>T		Y9:P>T		YJM975:P>T		YJM978:P>T		YPS128:P>T		YPS606:P>T	AA:94		L_1528:I>M	AA:96		273614X:M>V		BC187:M>V		DBVPG1373:M>V		DBVPG1853:M>V		DBVPG6044:M>V		DBVPG6765:M>V		K11:M>V		L_1374:M>V		NCYC110:M>V		SK1:M>V		Y12:M>V		Y55:M>V		Y9:M>V		YJM975:M>V		YJM978:M>V		YJM981:M>V		YPS128:M>V		YPS606:M>V	AA:102		273614X:D>E		322134S:D>E		BC187:D>E		DBVPG1373:D>E		DBVPG1853:D>E		DBVPG6044:D>E		DBVPG6765:D>E		K11:D>E		L_1374:D>E		L_1528:D>E		NCYC110:D>E		NCYC361:D>E		SK1:D>E		UWOPS87_2421:D>E		Y12:D>E		Y55:D>E		Y9:D>E		YIIc17_E5:D>E		YJM975:D>E		YJM978:D>E		YJM981:D>E		YPS128:D>E		YPS606:D>E		YS2:D>E	AA:130		273614X:V>A		322134S:V>A		BC187:V>A		DBVPG1373:V>A		DBVPG1853:V>A		DBVPG6044:V>A		DBVPG6765:V>A		K11:V>A		L_1374:V>A		L_1528:V>A		NCYC110:V>A		NCYC361:V>A		SK1:V>A		UWOPS05_227_2:V>A		Y55:V>A		Y9:V>A		YJM975:V>A		YJM978:V>A		YJM981:V>A		YPS128:V>A		YPS606:V>A	AA:133		273614X:L>F		BC187:L>F		DBVPG1373:L>F		DBVPG1853:L>F		DBVPG6044:L>F		DBVPG6765:L>F		K11:L>F		L_1374:L>F		NCYC110:L>F		NCYC361:L>F		SK1:L>F		Y12:L>F		Y55:L>F		Y9:L>F		YJM975:L>F		YJM978:L>F		YJM981:L>F		YPS128:L>F		YPS606:L>F	AA:188		NCYC361:N>D	AA:204		273614X:V>L		322134S:V>L		BC187:V>L		DBVPG1373:V>L		DBVPG1853:V>L		DBVPG6044:V>L		DBVPG6765:V>L		K11:V>L		L_1374:V>L		L_1528:V>L		NCYC110:V>L		NCYC361:V>L		SK1:V>L		UWOPS05_227_2:V>L		UWOPS87_2421:V>L		Y12:V>L		Y55:V>L		Y9:V>L		YIIc17_E5:V>L		YJM975:V>L		YJM978:V>L		YJM981:V>L		YPS128:V>L		YPS606:V>L		YS9:V>L	AA:246		DBVPG6765:R>C	AA:303		322134S:G>D		BC187:G>D		DBVPG1373:G>D		DBVPG1853:G>D		DBVPG6765:G>D		L_1374:G>D		L_1528:G>D		NCYC361:G>D		SK1:G>D		UWOPS05_227_2:G>D		UWOPS87_2421:G>D		Y12:G>D		Y55:G>D		Y9:G>D		YJM978:G>D		YJM981:G>D		YPS128:G>D		YPS606:G>D		YS9:G>D	AA:310		Y9:Q>RID:YDL126C	AA:196		K11:G>V	AA:214		273614X:D>H	AA:227		273614X:R>T	AA:271		273614X:E>D	AA:317		273614X:D>N	AA:322		YIIc17_E5:K>N	AA:405		378604X:D>V	AA:429		YS9:A>S	AA:493		BC187:D>E		DBVPG1788:D>E		DBVPG6765:D>E		L_1374:D>E		L_1528:D>E		YS2:D>E	AA:808		UWOPS87_2421:N>K	AA:835		YS4:S>RID:YDL127W	AA:23		DBVPG6040:L>	AA:108		UWOPS05_217_3:N>K	AA:154		Y12:T>R		Y9:T>R	AA:180		273614X:N>K		DBVPG1106:N>K		DBVPG1788:N>K		DBVPG1853:N>K		DBVPG6765:N>K		L_1374:N>K		L_1528:N>K		YIIc17_E5:N>K		YJM975:N>K		YJM978:N>K		YJM981:N>K		YS9:N>K	AA:231		DBVPG1853:I>T	AA:247		YS4:F>L	AA:264		DBVPG1853:R>W	AA:288		UWOPS87_2421:K>NID:YDL128W	AA:13		DBVPG1373:S>N		NCYC361:S>N		YIIc17_E5:S>N	AA:22		DBVPG1373:T>I	AA:115		DBVPG1853:K>E		DBVPG6765:K>E		L_1374:K>E		NCYC361:K>E		YIIc17_E5:K>E		YJM975:K>E		YJM978:K>E		YS4:K>E		YS9:K>E	AA:140		322134S:L>F		DBVPG1106:L>F		DBVPG1853:L>F		DBVPG6765:L>F		K11:L>F		L_1374:L>F		NCYC110:L>F		NCYC361:L>F		SK1:L>F		UWOPS05_217_3:L>F		UWOPS05_227_2:L>F		Y55:L>F		YIIc17_E5:L>F		YJM975:L>F		YJM978:L>F		YS4:L>F		YS9:L>F	AA:186		K11:G>D	AA:196		NCYC110:I>V		Y55:I>V	AA:204		W303:S>A	AA:293		NCYC110:V>I		SK1:V>I		Y55:V>I	AA:315		YJM978:M>I	AA:329		273614X:V>I		322134S:V>I		DBVPG1106:V>I		DBVPG1373:V>I		DBVPG1788:V>I		DBVPG6765:V>I		L_1374:V>I		NCYC361:V>I		YIIc17_E5:V>I		YJM975:V>I		YS9:V>I	AA:359		DBVPG1788:T>I	AA:389		273614X:I>VID:YDL130W-A	AA:19		YIIc17_E5:A>V		YS9:A>V	AA:72		Y12:K>R		Y9:K>RID:YDL131W	AA:412		YIIc17_E5:L>F		YS4:L>FID:YDL132W	AA:8		DBVPG6044:S>A		SK1:S>A	AA:169		DBVPG1106:S>N		DBVPG1373:S>N		DBVPG6765:S>N		SK1:S>N		UWOPS03_461_4:S>N		UWOPS05_217_3:S>N		UWOPS83_787_3:S>N		Y55:S>N		YJM978:S>N		YPS128:S>N		YPS606:S>N		YS4:S>N		YS9:S>N	AA:184		DBVPG1106:G>E		DBVPG1373:G>E		DBVPG6765:G>E		SK1:G>E		UWOPS03_461_4:G>E		UWOPS05_217_3:G>E		UWOPS83_787_3:G>E		Y12:G>E		Y55:G>E		YJM975:G>E		YJM978:G>E		YPS128:G>E		YPS606:G>E		YS4:G>E		YS9:G>E	AA:221		YJM975:I>M		YJM978:I>M	AA:247		DBVPG1853:H>R	AA:342		YS4:N>S	AA:381		YS4:K>R	AA:403		YS9:P>TID:YDL133W	AA:19		BC187:A>T		DBVPG6765:A>T		L_1374:A>T		NCYC361:A>T		UWOPS83_787_3:A>T		Y55:A>T		YIIc17_E5:A>T		YJM975:A>T		YJM978:A>T		YJM981:A>T		YS2:A>T	AA:137		YIIc17_E5:P>S	AA:158		DBVPG1853:L>V	AA:162		UWOPS05_217_3:L>I	AA:216		L_1374:N>S	AA:301		322134S:S>N		DBVPG6765:S>N		Y55:S>NID:YDL134C	AA:27		YJM975:L>R		YJM978:L>R	AA:289		L_1528:N>D	AA:347		YS2:L>F	AA:356		Y12:G>VID:YDL135C	AA:58		K11:E>D		Y12:E>D		Y9:E>D	AA:139		DBVPG1853:K>RID:YDL137W	AA:32		Y9:T>	AA:69		UWOPS03_461_4:G>SID:YDL139C	AA:4		322134S:N>S		378604X:N>S		BC187:N>S		DBVPG6765:N>S		L_1374:N>S		L_1528:N>S		SK1:N>S		UWOPS03_461_4:N>S		UWOPS87_2421:N>S		Y55:N>S		YIIc17_E5:N>S		YS9:N>S	AA:145		322134S:N>K		DBVPG1373:N>K		L_1374:N>K		UWOPS87_2421:N>K		Y55:N>K	AA:171		322134S:D>N		DBVPG1373:D>N		DBVPG6765:D>N		L_1374:D>N		UWOPS87_2421:D>N		Y55:D>N		YS9:D>N	AA:196		DBVPG6044:T>I		SK1:T>I		UWOPS05_217_3:T>I	AA:205		DBVPG6044:I>M		SK1:I>M		UWOPS03_461_4:I>M		UWOPS05_217_3:I>MID:YDL143W	AA:140		378604X:H>Y	AA:155		273614X:S>Y	AA:234		322134S:I>V	AA:429		DBVPG1373:A>T	AA:445		SK1:S>F	AA:525		UWOPS83_787_3:A>EID:YDL144C	AA:31		273614X:V>I		DBVPG6765:V>I		Y55:V>I	AA:93		Y12:V>A		YIIc17_E5:V>A		YPS128:V>A	AA:114		273614X:P>S		DBVPG1788:P>S		DBVPG6040:P>S		DBVPG6044:P>S		DBVPG6765:P>S		L_1374:P>S		SK1:P>S		UWOPS03_461_4:P>S		W303:P>S		Y12:P>S		Y55:P>S		YIIc17_E5:P>S		YJM975:P>S		YJM978:P>S		YJM981:P>S		YPS128:P>S	AA:216		YS4:L>S	AA:319		DBVPG6044:A>T		SK1:A>T		W303:A>T	AA:350		DBVPG6044:T>A		SK1:T>AID:YDL146W	AA:15		YJM978:Q>R	AA:34		DBVPG1853:E>G	AA:153		K11:T>I	AA:161		SK1:N>S	AA:165		273614X:S>P		322134S:S>P		DBVPG1373:S>P		DBVPG1788:S>P		DBVPG1853:S>P		DBVPG6040:S>P		DBVPG6765:S>P		K11:S>P		UWOPS03_461_4:S>P		W303:S>P		Y55:S>P		YIIc17_E5:S>P		YJM978:S>P		YS4:S>P		YS9:S>P	AA:169		273614X:T>A		322134S:T>A		DBVPG1373:T>A		DBVPG1788:T>A		DBVPG1853:T>A		DBVPG6040:T>A		DBVPG6765:T>A		K11:T>A		UWOPS03_461_4:T>A		W303:T>A		Y55:T>A		YIIc17_E5:T>A		YJM978:T>A		YS4:T>A		YS9:T>A	AA:299		YS9:M>T	AA:325		DBVPG1373:R>H		DBVPG1788:R>H		DBVPG1853:R>H		DBVPG6040:R>H		DBVPG6765:R>H		K11:R>H		SK1:R>H		UWOPS87_2421:R>H		Y12:R>H		Y55:R>H		Y9:R>H		YJM978:R>H		YS9:R>H	AA:349		YJM978:L>F	AA:447		NCYC361:I>VID:YDL148C	AA:76		UWOPS03_461_4:A>T	AA:111		W303:G>V	AA:157		K11:D>E		Y12:D>E	AA:164		W303:G>S	AA:214		378604X:V>F	AA:232		378604X:M>I	AA:360		DBVPG1373:D>Y	AA:362		378604X:G>D		DBVPG1853:G>D		DBVPG6040:G>D		DBVPG6765:G>D		K11:G>D		L_1374:G>D		UWOPS05_217_3:G>D		UWOPS87_2421:G>D		W303:G>D		Y55:G>D		YJM978:G>D	AA:481		K11:M>I	AA:496		DBVPG6765:G>D	AA:605		UWOPS87_2421:D>N		YS4:D>N		YS9:D>N	AA:618		YPS128:T>I		YPS606:T>I	AA:695		NCYC361:T>SID:YDL149W	AA:54		DBVPG6765:H>Y	AA:61		DBVPG6044:E>K		NCYC110:E>K		SK1:E>K	AA:62		Y55:D>H	AA:107		DBVPG6044:L>P		NCYC110:L>P		SK1:L>P	AA:111		DBVPG1788:T>M		DBVPG6040:T>M		DBVPG6765:T>M		L_1374:T>M		W303:T>M		Y55:T>M		YIIc17_E5:T>M		YJM978:T>M		YJM981:T>M	AA:178		DBVPG1373:S>R		DBVPG1788:S>R		DBVPG6040:S>R		L_1374:S>R		Y55:S>R		YIIc17_E5:S>R		YJM978:S>R		YJM981:S>R	AA:198		378604X:R>H		UWOPS87_2421:R>H	AA:202		DBVPG1373:D>N		DBVPG1788:D>N		DBVPG6040:D>N		L_1374:D>N		Y55:D>N		YIIc17_E5:D>N	AA:215		322134S:H>Y		378604X:H>Y		DBVPG1373:H>Y		DBVPG1788:H>Y		DBVPG1853:H>Y		DBVPG6040:H>Y		K11:H>Y		L_1374:H>Y		UWOPS87_2421:H>Y		Y55:H>Y		Y9:H>Y		YIIc17_E5:H>Y		YJM975:H>Y		YPS128:H>Y		YS2:H>Y		YS9:H>Y	AA:225		DBVPG1373:P>S		DBVPG1788:P>S		DBVPG6040:P>S		L_1374:P>S		Y55:P>S		YIIc17_E5:P>S		YJM975:P>S		YS2:P>S	AA:240		DBVPG6044:G>V		NCYC110:G>V		SK1:G>V	AA:278		L_1528:N>Y	AA:280		DBVPG6044:P>S		SK1:P>S	AA:390		DBVPG1373:I>T		YJM975:I>T	AA:482		273614X:P>L	AA:521		DBVPG6044:P>S		NCYC110:P>S		SK1:P>S	AA:527		273614X:T>P	AA:738		YJM975:P>L	AA:758		BC187:E>D		DBVPG1106:E>D		DBVPG1373:E>D		DBVPG1788:E>D		DBVPG6765:E>D		L_1374:E>D		L_1528:E>D		W303:E>D		Y55:E>D		YJM975:E>D		YS9:E>D	AA:790		273614X:E>G		BC187:E>G		DBVPG1106:E>G		DBVPG1373:E>G		DBVPG1788:E>G		DBVPG6765:E>G		L_1374:E>G		L_1528:E>G		W303:E>G		Y55:E>G		YJM975:E>G		YS9:E>G	AA:792		K11:S>N		Y12:S>N	AA:878		YS4:P>AID:YDL150W	AA:139		DBVPG6044:E>D		K11:E>D		Y9:E>D		YPS128:E>D		YPS606:E>D		YS4:E>D	AA:274		UWOPS87_2421:K>N	AA:276		273614X:A>V		BC187:A>V		DBVPG1373:A>V		DBVPG1788:A>V		DBVPG6040:A>V		DBVPG6765:A>V		Y55:A>V		YS2:A>V	AA:374		YS9:K>-	AA:379		YS2:V>M	AA:391		YS9:L>V	AA:409		YS9:L>V	AA:411		YS9:R>CID:YDL151C	AA:47		DBVPG6044:F>L		K11:F>L		Y9:F>L		YPS128:F>L		YPS606:F>L		YS4:F>LID:YDL153C	AA:3		DBVPG1373:R>H		DBVPG1788:R>H		DBVPG6765:R>H		L_1374:R>H		L_1528:R>H		W303:R>H		Y55:R>H		YJM975:R>H	AA:32		378604X:K>I	AA:53		DBVPG6044:S>P		SK1:S>P	AA:140		DBVPG1373:A>G		DBVPG1788:A>G		DBVPG6765:A>G		L_1374:A>G		L_1528:A>G		Y55:A>G		YIIc17_E5:A>G	AA:174		DBVPG1373:V>A		DBVPG1788:V>A		DBVPG6765:V>A		L_1374:V>A		L_1528:V>A		Y55:V>A		YIIc17_E5:V>A	AA:221		UWOPS05_217_3:A>T		UWOPS87_2421:A>T	AA:237		K11:L>I	AA:313		UWOPS05_227_2:G>D	AA:334		273614X:Q>E		DBVPG1373:Q>E		DBVPG1788:Q>E		DBVPG1853:Q>E		DBVPG6044:Q>E		DBVPG6765:Q>E		L_1374:Q>E		L_1528:Q>E		NCYC110:Q>E		NCYC361:Q>E		SK1:Q>E		UWOPS05_217_3:Q>E		UWOPS05_227_2:Q>E		YPS128:Q>E	AA:344		273614X:G>E		DBVPG1373:G>E		DBVPG1788:G>E		DBVPG6044:G>E		DBVPG6765:G>E		L_1374:G>E		L_1528:G>E		NCYC110:G>E		SK1:G>E		UWOPS05_217_3:G>E		UWOPS05_227_2:G>E		YIIc17_E5:G>E		YPS128:G>E	AA:347		273614X:Q>H		DBVPG1373:Q>H		DBVPG1788:Q>H		DBVPG1853:Q>H		DBVPG6040:Q>H		DBVPG6765:Q>H		L_1374:Q>H		L_1528:Q>H		UWOPS05_217_3:Q>H	AA:387		DBVPG6044:T>R		NCYC110:T>R		SK1:T>R		UWOPS05_227_2:T>R	AA:425		L_1528:Q>H	AA:491		UWOPS05_227_2:E>QID:YDL154W	AA:19		273614X:G>E		378604X:G>E		BC187:G>E		DBVPG1373:G>E		DBVPG1788:G>E		DBVPG6765:G>E		L_1374:G>E		UWOPS03_461_4:G>E		Y12:G>E		Y9:G>E		YIIc17_E5:G>E		YJM978:G>E		YJM981:G>E		YPS128:G>E		YPS606:G>E	AA:31		W303:N>	AA:40		273614X:D>E		BC187:D>E		DBVPG1373:D>E		DBVPG1788:D>E		DBVPG6765:D>E		L_1374:D>E		UWOPS03_461_4:D>E		UWOPS05_227_2:D>E		YIIc17_E5:D>E		YJM978:D>E		YJM981:D>E	AA:79		273614X:T>S	AA:83		273614X:K>Q		DBVPG1373:K>Q		DBVPG1788:K>Q		DBVPG6765:K>Q		L_1374:K>Q		UWOPS03_461_4:K>Q		UWOPS05_227_2:K>Q		YIIc17_E5:K>Q		YJM978:K>Q		YJM981:K>Q		YPS128:K>Q		YPS606:K>Q	AA:122		DBVPG1373:E>G		DBVPG6765:E>G		YJM978:E>G		YJM981:E>G	AA:214		DBVPG1788:D>G		DBVPG6040:D>G		L_1374:D>G		L_1528:D>G		W303:D>G		Y55:D>G	AA:258		273614X:H>N		BC187:H>N		DBVPG1373:H>N		DBVPG1788:H>N		DBVPG6040:H>N		DBVPG6765:H>N		K11:H>N		L_1374:H>N		L_1528:H>N		UWOPS03_461_4:H>N		UWOPS05_227_2:H>N		UWOPS83_787_3:H>N		W303:H>N		Y55:H>N		YS9:H>N	AA:261		273614X:P>S	AA:291		K11:A>P	AA:297		273614X:S>F		L_1374:S>F	AA:332		273614X:S>T		BC187:S>T		DBVPG1373:S>T		DBVPG6765:S>T		L_1374:S>T		UWOPS03_461_4:S>T		UWOPS05_227_2:S>T		W303:S>T		Y55:S>T		YIIc17_E5:S>T		YJM981:S>T	AA:383		273614X:D>N		BC187:D>N		L_1374:D>N		W303:D>N		Y55:D>N		YIIc17_E5:D>N	AA:385		273614X:A>G		378604X:A>G		BC187:A>G		DBVPG1373:A>G		DBVPG6765:A>G		UWOPS05_227_2:A>G		W303:A>G		Y12:A>G		Y55:A>G		YIIc17_E5:A>G		YJM981:A>G		YS2:A>G	AA:440		273614X:N>D		BC187:N>D		DBVPG1788:N>D		UWOPS05_227_2:N>D		Y55:N>D		YIIc17_E5:N>D		YS2:N>D	AA:454		DBVPG6044:Q>R	AA:455		273614X:I>L		BC187:I>L		DBVPG1788:I>L		Y55:I>L		YIIc17_E5:I>L	AA:461		273614X:M>L		378604X:M>L		BC187:M>L		DBVPG1373:M>L		DBVPG1788:M>L		UWOPS05_227_2:M>L		Y12:M>L		Y55:M>L		Y9:M>L		YIIc17_E5:M>L		YJM981:M>L	AA:465		DBVPG1373:D>N		DBVPG6765:D>N		YJM981:D>N	AA:468		W303:T>	AA:493		YIIc17_E5:L>I	AA:498		DBVPG6044:L>F	AA:524		DBVPG6044:L>F		SK1:L>F	AA:575		273614X:F>L	AA:585		273614X:A>T		DBVPG1788:A>T		DBVPG6040:A>T		L_1528:A>T		W303:A>T		Y55:A>T		YIIc17_E5:A>T		YS9:A>T	AA:590		DBVPG6040:V>A	AA:617		YS9:S>N	AA:632		DBVPG6040:E>A		YS9:E>A	AA:634		YS9:N>S	AA:659		W303:I>T	AA:771		322134S:N>H		DBVPG1788:N>H		DBVPG6040:N>H		DBVPG6765:N>H		L_1374:N>H		L_1528:N>H		UWOPS03_461_4:N>H		UWOPS05_217_3:N>H		UWOPS83_787_3:N>H		W303:N>H		Y55:N>H		YJM978:N>H		YPS128:N>H		YPS606:N>H		YS9:N>H	AA:786		YPS128:Q>K	AA:823		NCYC110:Y>H		SK1:Y>H	AA:847		DBVPG6040:R>K	AA:851		DBVPG6040:V>I	AA:854		322134S:Q>KID:YDL155W	AA:50		DBVPG6040:T>S	AA:73		Y9:R>K	AA:110		L_1528:P>L	AA:115		L_1528:G>E	AA:122		L_1528:E>G	AA:141		L_1528:E>D	AA:174		L_1528:E>D	AA:326		UWOPS03_461_4:L>M		UWOPS05_227_2:L>M	AA:406		YS2:L>-ID:YDL156W	AA:66		UWOPS83_787_3:T>A	AA:130		DBVPG1373:D>E		DBVPG1788:D>E		DBVPG6765:D>E		L_1374:D>E		L_1528:D>E		Y55:D>E		YIIc17_E5:D>E		YJM978:D>E	AA:136		Y55:E>G	AA:165		322134S:M>I		BC187:M>I		DBVPG1373:M>I		DBVPG1788:M>I		DBVPG6765:M>I		L_1374:M>I		L_1528:M>I		Y55:M>I		YIIc17_E5:M>I		YJM978:M>I	AA:245		YJM978:G>S	AA:253		UWOPS03_461_4:D>N	AA:272		UWOPS87_2421:N>H		YS4:N>H	AA:279		YS4:V>L	AA:365		DBVPG1853:N>K		DBVPG6044:N>K		NCYC110:N>K	AA:368		BC187:K>E		DBVPG1373:K>E		DBVPG1788:K>E		DBVPG1853:K>E		DBVPG6044:K>E		DBVPG6765:K>E		L_1528:K>E		NCYC110:K>E		UWOPS03_461_4:K>E		UWOPS87_2421:K>E		W303:K>E		YJM975:K>E		YS2:K>E		YS4:K>E	AA:383		BC187:I>V		DBVPG1788:I>V		DBVPG6765:I>V		L_1528:I>V		W303:I>V		YJM975:I>V		YJM978:I>V		YS2:I>V	AA:430		DBVPG6044:E>G		NCYC110:E>G		SK1:E>GID:YDL157C	AA:15		BC187:E>K		Y55:E>K	AA:106		273614X:Y>S		378604X:Y>S		DBVPG6765:Y>S		YIIc17_E5:Y>S		YJM978:Y>SID:YDL159W	AA:25		YJM978:N>K	AA:95		UWOPS03_461_4:P>S	AA:109		DBVPG1373:S>T		DBVPG1788:S>T		DBVPG6765:S>T		L_1374:S>T		Y55:S>T		YJM978:S>T		YS2:S>T	AA:117		DBVPG6044:P>L		NCYC110:P>L		SK1:P>L	AA:214		UWOPS03_461_4:S>F	AA:437		UWOPS03_461_4:K>E	AA:480		UWOPS03_461_4:H>RID:YDL160C	AA:218		DBVPG1788:P>S		L_1528:P>S	AA:286		W303:A>D	AA:301		Y12:K>N	AA:444		UWOPS83_787_3:Q>L	AA:467		DBVPG6044:P>S		NCYC110:P>S		SK1:P>S	AA:507		DBVPG1373:->QID:YDL161W	AA:38		DBVPG6044:S>L		NCYC110:S>L		SK1:S>L	AA:47		YJM978:Y>N	AA:49		W303:S>N	AA:333		YS2:Q>P	AA:335		YS2:Q>RID:YDL163W	AA:24		UWOPS83_787_3:S>P		YPS606:S>P	AA:62		YS2:A>T	AA:76		DBVPG1106:D>G		Y55:D>GID:YDL165W	AA:31		273614X:G>R	AA:82		UWOPS03_461_4:P>L		UWOPS05_227_2:P>L	AA:91		322134S:P>A	AA:153		UWOPS87_2421:M>KID:YDL166C	AA:26		DBVPG1853:F>L		DBVPG6044:F>L		NCYC110:F>L		NCYC361:F>L		UWOPS05_217_3:F>L		UWOPS05_227_2:F>L		UWOPS83_787_3:F>L		UWOPS87_2421:F>L		Y12:F>L		YPS128:F>L		YPS606:F>L		YS4:F>L	AA:72		UWOPS05_217_3:P>S		UWOPS05_227_2:P>S	AA:88		Y55:V>L	AA:126		378604X:D>V	AA:186		Y12:P>S		Y9:P>S	AA:196		DBVPG6044:S>R		NCYC110:S>R		SK1:S>RID:YDL167C	AA:11		L_1528:A>V	AA:24		K11:A>T	AA:28		L_1528:F>Y	AA:38		UWOPS05_227_2:C>F	AA:42		DBVPG6044:T>A		K11:T>A		SK1:T>A		UWOPS03_461_4:T>A		UWOPS05_217_3:T>A		UWOPS87_2421:T>A		Y12:T>A		Y9:T>A		YPS606:T>A		YS9:T>A	AA:59		DBVPG1853:S>C	AA:137		SK1:S>F	AA:143		322134S:M>I		DBVPG1373:M>I		DBVPG1788:M>I		DBVPG6040:M>I		DBVPG6044:M>I		DBVPG6765:M>I		L_1528:M>I		NCYC361:M>I		SK1:M>I		UWOPS05_217_3:M>I		UWOPS05_227_2:M>I		UWOPS83_787_3:M>I		Y12:M>I		Y55:M>I		YIIc17_E5:M>I		YPS128:M>I		YPS606:T>A	AA:146		SK1:I>N		UWOPS03_461_4:I>N		UWOPS05_217_3:I>N		UWOPS05_227_2:I>N		UWOPS87_2421:I>N		Y9:I>N		YPS128:I>N		YPS606:I>N		YS9:I>N	AA:170		UWOPS83_787_3:T>I	AA:428		322134S:P>H		DBVPG1788:P>H	AA:444		YS9:N>D	AA:465		YS9:S>T	AA:514		YS9:I>V	AA:524		Y12:N>S		YS9:N>S	AA:535		DBVPG6040:H>N	AA:544		YPS128:I>N	AA:654		DBVPG1106:N>S		DBVPG1373:N>S		DBVPG1788:N>S		DBVPG6765:N>S		L_1528:N>S		NCYC361:N>S		Y55:N>S		YJM975:N>S		YJM981:N>S	AA:673		DBVPG6044:M>I		SK1:M>I	AA:704		YJM981:G>DID:YDL168W	AA:4		UWOPS83_787_3:A>S	AA:191		UWOPS03_461_4:T>	AA:198		UWOPS05_227_2:D>E	AA:220		UWOPS05_217_3:G>S		UWOPS05_227_2:G>S	AA:238		L_1528:S>F	AA:253		SK1:A>T		UWOPS05_217_3:A>T	AA:281		YJM981:K>R	AA:285		Y55:D>G	AA:304		UWOPS87_2421:A>P	AA:306		322134S:G>D		DBVPG1106:G>D		DBVPG1788:G>D		DBVPG6040:G>D		L_1374:G>D		Y55:G>D		YIIc17_E5:G>D		YJM975:G>D		YJM981:G>D	AA:307		UWOPS87_2421:E>G		YS9:E>G	AA:317		YS9:V>I	AA:338		322134S:G>S		DBVPG1106:G>S		DBVPG1788:G>S		DBVPG6040:G>S		L_1374:G>S		Y55:G>S		YIIc17_E5:G>S		YJM975:G>S	AA:347		YS9:A>VID:YDL170W	AA:50		L_1528:S>P	AA:53		DBVPG1853:K>T	AA:54		YPS606:Q>E	AA:92		322134S:G>S		BC187:G>S		DBVPG1373:G>S		DBVPG6040:G>S		DBVPG6044:G>S		DBVPG6765:G>S		K11:G>S		L_1374:G>S		L_1528:G>S		UWOPS03_461_4:G>S		UWOPS05_227_2:G>S		Y55:G>S		Y9:G>S		YIIc17_E5:G>S		YJM975:G>S		YJM978:G>S		YJM981:G>S		YPS128:G>S		YPS606:G>S		YS4:G>S		YS9:G>S	AA:209		273614X:K>R		322134S:K>R		DBVPG1373:K>R		DBVPG1788:K>R		DBVPG6040:K>R		DBVPG6765:K>R		L_1374:K>R		Y55:K>R		YIIc17_E5:K>R		YJM975:K>R		YJM978:K>R		YJM981:K>R	AA:241		273614X:I>V		322134S:I>V		DBVPG1373:I>V		DBVPG1788:I>V		DBVPG6040:I>V		DBVPG6765:I>V		Y55:I>V		YIIc17_E5:I>V		YJM975:I>V		YJM978:I>V	AA:248		YS9:N>S	AA:249		UWOPS05_227_2:H>R	AA:293		273614X:I>N	AA:315		YS9:N>I	AA:388		Y12:M>K	AA:428		UWOPS87_2421:L>Q		Y12:L>Q	AA:512		L_1528:H>QID:YDL173W	AA:122		DBVPG6044:G>E		SK1:G>E	AA:150		378604X:N>D		DBVPG1106:N>D		DBVPG6765:N>D		Y55:N>D		YJM978:N>D		YJM981:N>D	AA:266		322134S:F>L		DBVPG1106:F>L		DBVPG1373:F>L		DBVPG6765:F>L		K11:F>L		SK1:F>L		UWOPS03_461_4:F>L		UWOPS05_227_2:F>L		UWOPS87_2421:F>L		Y55:F>L		YPS606:F>L		YS9:F>LID:YDL174C	AA:109		UWOPS83_787_3:K>M	AA:128		UWOPS05_227_2:A>T	AA:215		UWOPS03_461_4:K>N	AA:398		UWOPS03_461_4:P>A		UWOPS05_227_2:P>A		YPS606:P>AID:YDL175C	AA:37		YS2:R>T	AA:155		UWOPS03_461_4:K>E		UWOPS05_227_2:K>E	AA:236		DBVPG6044:S>F		SK1:S>F	AA:342		DBVPG1373:K>EID:YDL176W	AA:54		YJM978:F>Y	AA:59		DBVPG1788:P>L	AA:123		Y12:F>S	AA:140		UWOPS87_2421:C>W	AA:155		Y12:P>L	AA:209		UWOPS05_217_3:Y>	AA:211		UWOPS87_2421:L>R	AA:223		UWOPS05_217_3:P>	AA:386		DBVPG6044:S>F		NCYC110:S>F		SK1:S>F	AA:463		DBVPG1373:D>G		YJM975:D>G	AA:502		UWOPS05_217_3:Y>-	AA:638		Y12:A>T		Y9:A>T	AA:677		UWOPS83_787_3:L>IID:YDL177C	AA:73		BC187:H>N		DBVPG1373:H>N		DBVPG1788:H>N		DBVPG1853:H>N		L_1374:H>N		NCYC361:H>N		Y55:H>N		YIIc17_E5:H>N	AA:85		BC187:A>T		DBVPG1373:A>T		DBVPG1788:A>T		DBVPG1853:A>T		L_1374:A>T		NCYC361:A>T		Y55:A>T		YIIc17_E5:A>T	AA:166		378604X:G>CID:YDL178W	AA:81		UWOPS83_787_3:A>T	AA:169		YS9:K>N	AA:178		UWOPS05_217_3:N>K	AA:200		UWOPS05_217_3:C>Y	AA:360		DBVPG6044:T>K		SK1:T>KID:YDL179W	AA:123		DBVPG1788:G>D		L_1528:G>D	AA:178		YIIc17_E5:F>L	AA:273		DBVPG1853:V>I	AA:275		322134S:M>VID:YDL180W	AA:47		SK1:Q>R	AA:103		273614X:V>I		322134S:V>I		BC187:V>I		DBVPG1106:V>I		DBVPG1788:V>I		DBVPG6765:V>I		L_1374:V>I		L_1528:V>I		NCYC361:V>I		Y55:V>I		YIIc17_E5:V>I	AA:182		DBVPG1106:T>S	AA:207		322134S:D>E		BC187:D>E		DBVPG1106:D>E		DBVPG1373:D>E		DBVPG1788:D>E		DBVPG6765:D>E		L_1374:D>E		UWOPS83_787_3:D>E		Y55:D>E		YJM975:D>E		YPS128:D>E		YPS606:D>E	AA:209		DBVPG6044:S>N		SK1:S>N	AA:342		273614X:V>A		BC187:V>A		DBVPG1106:V>A		DBVPG1373:V>A		DBVPG1788:V>A		DBVPG1853:V>A		DBVPG6765:V>A		L_1374:V>A		L_1528:V>A		Y55:V>A	AA:349		273614X:I>V		BC187:I>V		DBVPG1106:I>V		DBVPG1373:I>V		DBVPG1788:I>V		DBVPG1853:I>V		DBVPG6044:I>V		DBVPG6765:I>V		L_1374:I>V		L_1528:I>V		NCYC110:I>V		SK1:I>V		UWOPS03_461_4:I>V		UWOPS05_217_3:I>V		UWOPS05_227_2:I>V		UWOPS87_2421:I>V		YPS128:I>V		YS4:I>V		YS9:I>V	AA:440		UWOPS05_217_3:S>G		UWOPS05_227_2:S>G	AA:448		YPS128:E>G	AA:488		UWOPS87_2421:Y>H		YS9:Y>H	AA:497		Y12:D>G	AA:520		Y12:R>K	AA:535		UWOPS87_2421:L>F		YS9:L>F	AA:539		273614X:T>R		BC187:T>R		DBVPG1106:T>R		DBVPG1788:T>R		DBVPG6765:T>R		YJM975:T>R	AA:545		UWOPS05_217_3:V>I		UWOPS05_227_2:V>IID:YDL182W	AA:2		378604X:T>I	AA:264		DBVPG6044:D>E		SK1:D>E	AA:361		UWOPS05_217_3:Q>	AA:384		UWOPS05_217_3:D>ID:YDL183C	AA:23		YS9:R>M	AA:53		YS9:E>K	AA:70		DBVPG1788:R>I		DBVPG6765:R>I		L_1528:R>I		Y55:R>I		YJM975:R>I		YJM978:R>I		YJM981:R>I		YS4:R>I		YS9:R>G	AA:102		UWOPS05_217_3:S>T		UWOPS05_227_2:S>T	AA:236		Y12:H>NID:YDL186W	AA:17		YPS606:T>S	AA:75		UWOPS87_2421:S>Y	AA:197		273614X:P>S		322134S:P>Q	AA:215		YS9:R>G	AA:223		SK1:P>L	AA:259		273614X:A>V		DBVPG1106:A>V		DBVPG1853:A>V		L_1374:A>V		L_1528:A>V		YS4:A>V	AA:265		DBVPG6044:D>Y		SK1:D>YID:YDL188C	AA:13		L_1528:D>N	AA:113		378604X:V>IID:YDL189W	AA:111		BC187:A>T		DBVPG1788:A>T		DBVPG1853:A>T		DBVPG6040:A>T		DBVPG6765:A>T		L_1528:A>T		UWOPS83_787_3:A>T		UWOPS87_2421:A>T		Y12:A>T		Y55:A>T		YJM975:A>T		YPS128:A>T		YPS606:A>T		YS4:A>T		YS9:A>T	AA:115		BC187:E>D	AA:170		DBVPG1373:S>N		DBVPG1788:S>N		DBVPG1853:S>N		DBVPG6765:S>N		L_1528:S>N		UWOPS83_787_3:S>N		UWOPS87_2421:S>N		Y55:S>N		YPS128:S>N		YS4:S>N		YS9:S>N	AA:175		W303:Q>L	AA:206		Y12:N>S	AA:258		YS4:Y>H	AA:300		322134S:L>I		BC187:L>I		DBVPG1106:L>I		DBVPG1373:L>I		DBVPG6765:L>I		L_1528:L>I		Y12:L>I		YS4:L>I		YS9:L>I	AA:377		DBVPG6044:T>S		NCYC110:T>S		SK1:T>S	AA:386		DBVPG6044:N>I		NCYC110:N>I		SK1:N>I	AA:407		DBVPG6044:Q>H		NCYC110:Q>H		SK1:Q>H	AA:448		DBVPG1853:V>IID:YDL193W	AA:64		UWOPS87_2421:D>E	AA:74		Y12:N>S		Y9:N>S	AA:107		378604X:L>F	AA:113		273614X:L>S		378604X:L>S		DBVPG1373:L>S		DBVPG1788:L>S		DBVPG6765:L>S		L_1374:L>S		L_1528:L>S		Y55:L>S	AA:122		273614X:N>H		378604X:N>H		DBVPG1373:N>H		DBVPG1788:N>H		DBVPG6765:N>H		L_1374:N>H		L_1528:N>H		UWOPS87_2421:N>H		Y12:N>H		Y55:N>H		YS4:N>H	AA:243		UWOPS05_227_2:N>D	AA:251		K11:T>I		Y9:T>I	AA:255		UWOPS05_227_2:N>D	AA:276		UWOPS05_227_2:R>GID:YDL198C	AA:3		YS4:H>Q	AA:117		UWOPS03_461_4:G>V	AA:251		YS4:R>K	AA:262		UWOPS03_461_4:V>I		UWOPS05_227_2:V>IID:YDL199C	AA:8		273614X:S>T	AA:161		DBVPG1853:P>L	AA:187		DBVPG1373:P>A	AA:246		DBVPG1373:C>Y		DBVPG1788:C>Y		DBVPG1853:C>Y		DBVPG6765:C>Y		Y55:C>Y		YPS128:C>Y	AA:247		DBVPG6044:I>V		SK1:I>V	AA:250		DBVPG1853:R>L	AA:258		DBVPG1373:V>I		DBVPG1788:V>I		DBVPG1853:V>I		DBVPG6765:V>I		Y55:V>I	AA:282		DBVPG6765:R>K	AA:335		YS2:N>K	AA:383		UWOPS05_217_3:R>-	AA:449		273614X:A>V		378604X:A>V		BC187:A>V		DBVPG1373:A>V		DBVPG1788:A>V		DBVPG1853:A>V		DBVPG6040:A>V		DBVPG6765:A>V		L_1528:A>V		Y55:A>V		YJM978:A>V	AA:465		SK1:T>R	AA:518		DBVPG1853:V>A	AA:544		378604X:S>Y	AA:559		K11:E>K	AA:568		UWOPS03_461_4:T>I		UWOPS05_217_3:T>I	AA:641		W303:T>PID:YDL200C	AA:7		Y12:E>K	AA:9		UWOPS87_2421:G>C		Y12:G>C	AA:86		YPS128:T>I		YPS606:T>I	AA:103		YS2:P>S	AA:136		273614X:V>A		322134S:V>A		378604X:V>A		DBVPG1373:V>A		DBVPG1788:V>A		DBVPG1853:V>A		DBVPG6765:V>A		K11:V>A		L_1528:V>A		NCYC110:V>A		SK1:V>A		UWOPS05_217_3:V>A		UWOPS05_227_2:V>A		Y55:V>A		YIIc17_E5:V>A		YJM978:V>A		YJM981:V>AID:YDL201W	AA:24		Y12:N>D		Y9:N>D	AA:74		UWOPS03_461_4:Q>R	AA:116		UWOPS87_2421:A>V	AA:214		DBVPG6040:V>I	AA:244		L_1528:E>D	AA:250		DBVPG6044:E>G		NCYC110:E>G		SK1:E>GID:YDL202W	AA:30		UWOPS03_461_4:F>L		UWOPS05_217_3:F>L	AA:121		YJM975:K>NID:YDL204W	AA:15		DBVPG1373:N>S	AA:120		DBVPG1373:A>V		DBVPG1788:A>V		L_1374:A>V	AA:136		322134S:N>D		Y55:N>D		YS4:N>D	AA:169		YJM978:T>S	AA:182		YJM978:D>G	AA:195		Y12:R>M		Y9:R>M	AA:292		YJM975:E>K	AA:313		DBVPG6040:T>I		Y12:T>I		Y9:T>IID:YDL207W	AA:33		DBVPG6044:P>H		SK1:P>H	AA:52		378604X:K>N	AA:59		273614X:V>I		378604X:V>I		BC187:V>I		DBVPG1106:V>I		DBVPG1373:V>I		DBVPG1788:V>I		DBVPG6765:V>I		L_1374:V>I		Y55:V>I		YJM975:V>I		YJM978:V>I		YJM981:V>I	AA:65		273614X:A>V		378604X:A>V		BC187:A>V		DBVPG1106:A>V		DBVPG1373:A>V		DBVPG1788:A>V		DBVPG6765:A>V		L_1374:A>V		UWOPS05_227_2:A>V		UWOPS83_787_3:A>V		Y12:A>V		Y55:A>V		YJM975:A>V		YJM978:A>V		YJM981:A>V		YPS606:A>V	AA:114		UWOPS83_787_3:A>T		YPS606:A>T	AA:146		Y12:I>V	AA:149		Y12:I>N	AA:168		UWOPS87_2421:R>G	AA:201		DBVPG6040:K>R	AA:210		378604X:E>Q		DBVPG1106:E>Q		DBVPG1373:E>Q		DBVPG1788:E>Q		DBVPG1853:E>Q		DBVPG6040:E>Q		L_1374:E>Q		L_1528:E>Q		UWOPS87_2421:E>Q		Y55:E>Q		YJM975:E>Q		YJM978:E>Q		YS2:E>Q		YS4:E>Q	AA:273		322134S:K>N		BC187:K>N		DBVPG1106:K>N		DBVPG1373:K>N		DBVPG1853:K>N		L_1374:K>N		L_1528:K>N		Y55:K>N		YJM975:K>N		YJM978:K>N		YS2:K>N	AA:531		YPS128:E>DID:YDL208W	AA:8		273614X:S>N		DBVPG1106:S>N		DBVPG1853:S>N		DBVPG6765:S>N		K11:S>N		SK1:S>N		UWOPS03_461_4:S>N		UWOPS83_787_3:S>N		Y55:S>N		Y9:S>N		YJM975:S>N		YPS128:S>N		YS2:S>N		YS4:S>N		YS9:S>N	AA:84		YS9:L>-	AA:94		DBVPG6040:I>M	AA:153		UWOPS03_461_4:K>N		UWOPS05_227_2:K>NID:YDL209C	AA:108		DBVPG1788:V>A		YIIc17_E5:V>A		YJM975:V>A		YJM978:V>A	AA:238		322134S:L>P	AA:268		L_1374:K>M		YJM975:K>M		YJM978:K>M	AA:271		YPS128:E>K		YPS606:E>K	AA:276		YPS128:T>R		YPS606:T>R	AA:280		UWOPS87_2421:A>S		YPS128:A>S		YPS606:A>S	AA:297		DBVPG6044:E>G		SK1:E>G		UWOPS03_461_4:E>G		UWOPS83_787_3:E>G	AA:312		BC187:I>S		YS4:I>SID:YDL210W	AA:10		DBVPG1853:K>T	AA:30		DBVPG1853:L>F	AA:119		SK1:I>T	AA:122		YS9:V>E	AA:198		DBVPG1853:S>R	AA:210		378604X:V>M		DBVPG1106:V>M		L_1374:V>M		Y55:V>M	AA:248		K11:G>S	AA:323		UWOPS83_787_3:I>V		UWOPS87_2421:I>V	AA:331		DBVPG1853:C>S	AA:448		DBVPG6044:A>T		SK1:A>T	AA:454		378604X:L>M	AA:514		273614X:T>S		BC187:T>S		DBVPG6765:T>S		L_1528:T>S		YS4:T>S	AA:525		Y9:I>V	AA:559		273614X:A>D	AA:567		378604X:S>FID:YDL211C	AA:29		SK1:S>G	AA:34		273614X:T>K		378604X:T>K		BC187:T>K		DBVPG1106:T>K		DBVPG1853:T>K		DBVPG6765:T>K		K11:T>K		L_1528:T>K		SK1:T>K		Y55:T>K		YS4:T>K	AA:43		DBVPG1853:E>V		YS4:E>V	AA:67		YPS128:A>V		YPS606:A>V	AA:87		378604X:C>Y		DBVPG1106:C>Y		DBVPG1373:C>Y		DBVPG1853:C>Y		DBVPG6765:C>Y		L_1528:C>Y		Y55:C>Y		YIIc17_E5:C>Y		YJM978:C>Y		YS4:C>Y	AA:95		UWOPS05_217_3:I>V		UWOPS05_227_2:I>V	AA:134		DBVPG6044:T>A		SK1:T>A	AA:161		DBVPG6044:E>K		SK1:E>K	AA:174		378604X:T>S		DBVPG1106:T>S		DBVPG1373:T>S		DBVPG1853:T>S		DBVPG6044:T>S		DBVPG6765:T>S		K11:T>S		L_1528:T>S		SK1:T>S		UWOPS03_461_4:T>S		UWOPS05_227_2:T>S		Y55:T>S		YIIc17_E5:T>S		YJM978:T>S		YS4:T>S	AA:211		378604X:T>A		DBVPG1106:T>A		DBVPG1373:T>A		DBVPG1853:T>A		DBVPG6044:T>A		DBVPG6765:T>A		K11:T>A		L_1528:T>A		SK1:T>A		UWOPS03_461_4:T>A		UWOPS05_227_2:T>A		YIIc17_E5:T>A		YJM978:T>A		YS4:T>A	AA:217		K11:R>K	AA:251		UWOPS03_461_4:K>N		UWOPS05_227_2:K>N	AA:291		UWOPS03_461_4:T>I		UWOPS05_227_2:T>I	AA:320		YJM978:P>SID:YDL212W	AA:66		DBVPG6765:V>L	AA:170		DBVPG1853:E>D	AA:174		273614X:Q>E		378604X:Q>E		DBVPG1788:Q>E		DBVPG1853:Q>E		DBVPG6040:Q>E		DBVPG6044:Q>E		K11:Q>E		L_1374:Q>E		L_1528:Q>E		NCYC110:Q>E		SK1:Q>E		UWOPS87_2421:Q>E		Y12:Q>E		Y55:Q>E		YIIc17_E5:Q>E		YJM978:Q>E		YPS128:Q>EID:YDL213C	AA:5		UWOPS03_461_4:E>K	AA:25		Y55:E>K	AA:28		DBVPG6044:Q>H		SK1:Q>H	AA:132		DBVPG6044:M>I		SK1:M>I	AA:206		UWOPS05_227_2:G>-ID:YDL214C	AA:2		UWOPS05_227_2:S>L	AA:16		YJM978:T>S	AA:20		W303:L>S	AA:43		DBVPG6044:G>D		SK1:G>D	AA:44		K11:V>A		YPS128:V>A	AA:45		UWOPS87_2421:N>I	AA:65		K11:L>F		YPS128:L>F	AA:98		DBVPG1106:K>N		DBVPG1373:K>N		DBVPG1788:K>N		DBVPG1853:K>N		DBVPG6044:K>N		DBVPG6765:K>N		K11:K>N		L_1528:K>N		NCYC361:K>N		SK1:K>N		Y55:K>N		YJM978:K>N		YPS128:K>N	AA:137		UWOPS05_227_2:N>Y	AA:170		UWOPS87_2421:T>A	AA:174		273614X:L>P		DBVPG1106:L>P		DBVPG1373:L>P		DBVPG1788:L>P		DBVPG6765:L>P		L_1374:L>P		L_1528:L>P		Y55:L>P		YJM978:L>P		YS9:D>E	AA:184		UWOPS87_2421:N>S	AA:193		UWOPS87_2421:H>P	AA:212		K11:T>I		UWOPS87_2421:T>A	AA:214		UWOPS83_787_3:H>Q		YPS128:H>Q	AA:222		UWOPS05_227_2:E>D	AA:249		UWOPS83_787_3:S>L	AA:257		Y9:R>I	AA:273		273614X:Y>F		DBVPG1373:Y>F		DBVPG1788:Y>F		DBVPG6765:Y>F		L_1374:Y>F		Y55:Y>F		YJM975:Y>F		YJM978:Y>F	AA:280		273614X:I>M		DBVPG1106:I>M		DBVPG1373:I>M		DBVPG6765:I>M		L_1374:I>M		Y55:I>M		YIIc17_E5:I>M		YJM975:I>M		YJM978:I>M	AA:295		UWOPS05_227_2:N>T		UWOPS83_787_3:N>T	AA:310		UWOPS03_461_4:I>V		UWOPS05_227_2:I>V	AA:405		UWOPS87_2421:I>F	AA:536		Y12:Y>C	AA:612		DBVPG1106:D>N		DBVPG1788:D>N		DBVPG6765:D>N		L_1374:D>N		L_1528:D>N		Y55:D>N		YJM978:D>N		YS4:D>N	AA:621		YS9:R>K	AA:628		DBVPG1106:K>T		DBVPG1788:K>T		DBVPG6040:K>T		DBVPG6765:K>T		L_1374:K>T		L_1528:K>T		Y55:K>T		YJM978:K>T		YS4:K>T	AA:637		YS9:S>T	AA:638		DBVPG1106:P>S		DBVPG1788:P>S		DBVPG6040:P>S		DBVPG6765:P>S		L_1374:P>S		L_1528:P>S		Y55:P>S		YJM978:P>S	AA:649		Y55:Q>K	AA:659		UWOPS03_461_4:C>S		UWOPS05_227_2:C>S		UWOPS83_787_3:C>SID:YDL216C	AA:48		UWOPS87_2421:S>N	AA:79		YJM975:D>A		YJM978:D>A	AA:92		UWOPS83_787_3:S>L	AA:97		UWOPS83_787_3:T>I	AA:135		W303:E>A	AA:149		YS9:V>F	AA:184		YS9:Y>H	AA:185		UWOPS03_461_4:D>N		UWOPS05_227_2:D>N		Y9:D>N	AA:196		Y9:Q>H	AA:221		DBVPG6044:I>N		SK1:I>N		UWOPS03_461_4:I>S		UWOPS05_227_2:I>S		UWOPS83_787_3:I>N		Y12:I>S		Y9:I>S	AA:231		Y9:E>Q	AA:240		UWOPS87_2421:T>A	AA:289		UWOPS83_787_3:Y>F	AA:293		DBVPG1853:M>I		K11:M>I		YPS606:M>I	AA:307		Y12:T>A	AA:366		322134S:E>Q		BC187:E>Q		DBVPG1106:E>Q		YIIc17_E5:E>Q		YS2:E>Q	AA:393		YS2:S>P	AA:397		UWOPS03_461_4:F>I		UWOPS05_227_2:F>I	AA:402		UWOPS03_461_4:K>N		UWOPS05_227_2:K>N	AA:410		BC187:R>K		DBVPG1106:R>K		DBVPG1373:R>K		DBVPG1788:R>K		DBVPG6765:R>K		L_1528:R>K		Y55:R>K		YIIc17_E5:R>K		YJM978:R>K		YS2:R>KID:YDL217C	AA:83		DBVPG1853:A>TID:YDL218W	AA:91		YJM978:V>I		YJM981:V>I	AA:122		Y55:D>N	AA:217		DBVPG6044:A>T		SK1:A>T	AA:235		YPS606:G>E	AA:259		SK1:T>	AA:285		NCYC361:G>D	AA:312		DBVPG6044:N>D		SK1:N>DID:YDL219W	AA:16		DBVPG1788:D>HID:YDL222C	AA:176		378604X:R>H		K11:R>H		SK1:R>H		UWOPS83_787_3:R>H		UWOPS87_2421:R>H		Y12:R>H	AA:255		378604X:I>V		BC187:I>V		DBVPG1788:I>V		DBVPG6044:I>V		K11:I>V		L_1374:I>V		L_1528:I>V		SK1:I>V		UWOPS03_461_4:I>V		UWOPS05_217_3:I>V		UWOPS05_227_2:I>V		UWOPS83_787_3:I>V		UWOPS87_2421:I>V		Y55:I>V		Y9:I>V		YPS606:I>V	AA:261		NCYC361:V>A	AA:306		378604X:T>SID:YDL224C	AA:34		DBVPG1853:A>V	AA:62		DBVPG1853:G>C	AA:67		UWOPS05_217_3:P>L		UWOPS05_227_2:P>L	AA:122		YS4:S>P	AA:337		Y9:G>D	AA:402		378604X:V>L	AA:411		Y9:H>L	AA:426		UWOPS83_787_3:S>N		UWOPS87_2421:S>N		YPS606:S>N	AA:470		Y9:L>SID:YDL226C	AA:27		378604X:M>I		DBVPG1853:M>I		DBVPG6044:M>I		DBVPG6765:M>I		K11:M>I		L_1528:M>I		SK1:M>I		UWOPS05_217_3:M>I		UWOPS05_227_2:M>I		UWOPS87_2421:M>I		Y12:M>I		Y55:M>I		YPS128:M>I		YPS606:M>I		YS4:M>I		YS9:M>I	AA:147		273614X:A>P		DBVPG1853:A>P	AA:178		DBVPG1106:A>S	AA:329		DBVPG6040:H>Y	AA:341		YS2:K>R	AA:350		378604X:D>A	AA:351		Y12:D>N		Y9:D>NID:YDL227C	AA:8		UWOPS03_461_4:I>V		UWOPS05_227_2:I>V	AA:128		W303:P>S	AA:189		273614X:A>T		BC187:A>T		DBVPG1373:A>T		DBVPG1788:A>T		L_1374:A>T		Y12:A>T	AA:222		UWOPS05_227_2:D>Y	AA:223		273614X:S>G		BC187:S>G		DBVPG1373:S>G		DBVPG6765:S>G		K11:S>G		L_1374:S>G		SK1:S>G		UWOPS87_2421:S>G		Y12:S>G	AA:277		Y12:D>H	AA:392		DBVPG6040:Q>R	AA:395		DBVPG1853:Y>C		YS4:Y>C		YS9:Y>C	AA:405		322134S:S>L		DBVPG6040:S>L		DBVPG6044:S>L		DBVPG6765:S>L		K11:S>L		L_1374:S>L		L_1528:S>L		NCYC110:S>L		NCYC361:S>L		SK1:S>L		UWOPS05_217_3:S>L		Y12:S>L		Y55:S>L		YJM975:S>L		YJM978:S>L		YPS606:S>L		YS2:S>L		YS4:S>L		YS9:S>L	AA:469		DBVPG6040:C>S	AA:475		DBVPG6040:L>H		DBVPG6765:L>H		L_1528:L>H		Y55:L>H		YS2:L>H	AA:501		DBVPG1853:W>R	AA:517		YJM975:F>L		YJM978:F>LID:YDL229W	AA:18		W303:Y>	AA:31		SK1:I>V	AA:32		YS9:A>S	AA:45		YS9:A>V	AA:596		378604X:R>KID:YDL230W	AA:50		UWOPS05_227_2:P>L		YPS606:P>L	AA:72		UWOPS05_217_3:K>R		UWOPS05_227_2:K>R	AA:91		273614X:G>R		BC187:G>R		DBVPG1373:G>R		DBVPG6765:G>R		L_1374:G>R		L_1528:G>R		Y55:G>R		YJM975:G>R		YJM981:G>R	AA:107		273614X:R>H		DBVPG6765:R>H		L_1528:R>H	AA:126		DBVPG6044:I>V		SK1:I>V	AA:153		UWOPS05_217_3:D>N		UWOPS05_227_2:D>NID:YDL231C	AA:14		378604X:P>L	AA:33		DBVPG6765:N>K		L_1528:N>K	AA:68		DBVPG6044:K>N		NCYC110:K>N		SK1:K>N	AA:75		DBVPG6765:D>G		L_1528:D>G	AA:144		UWOPS87_2421:N>K	AA:154		UWOPS87_2421:A>S	AA:158		Y55:A>V		YJM975:A>V	AA:170		Y9:W>R	AA:183		UWOPS87_2421:V>I	AA:212		UWOPS05_217_3:W>C	AA:219		378604X:I>V		DBVPG1853:I>V		NCYC110:I>V		NCYC361:I>V		SK1:I>V		UWOPS05_227_2:I>V		UWOPS87_2421:I>V		Y12:I>V		Y9:I>V		YPS128:I>V		YPS606:I>V		YS4:I>V		YS9:I>V	AA:335		YPS128:V>I		YPS606:V>I	AA:427		273614X:T>S		378604X:T>S		DBVPG1373:T>S		DBVPG1853:T>S		DBVPG6044:T>S		K11:T>S		L_1528:T>S		NCYC110:T>S		SK1:T>S		UWOPS05_217_3:T>S		UWOPS05_227_2:T>S		Y12:T>S		Y55:T>S		YJM975:T>S		YJM978:T>S		YPS606:T>S	AA:508		322134S:E>K	AA:578		UWOPS05_227_2:G>D	AA:691		DBVPG6040:K>N	AA:729		UWOPS83_787_3:E>A	AA:731		UWOPS83_787_3:H>R	AA:763		K11:V>I	AA:889		UWOPS03_461_4:A>T		UWOPS05_227_2:A>T	AA:899		322134S:D>Y	AA:1043		DBVPG1853:N>T	AA:1066		YPS128:S>N		YPS606:S>N	AA:1075		273614X:P>H		BC187:P>H		DBVPG1373:P>H		DBVPG6040:P>H		L_1528:P>H		W303:P>H		Y55:P>H		YJM978:P>H		YJM981:P>H	AA:1122		UWOPS03_461_4:E>-		UWOPS05_217_3:E>-		UWOPS05_227_2:E>-ID:YDL233W	AA:2		YS4:Y>	AA:34		YS9:P>S	AA:100		YJM981:P>	AA:118		YJM981:A>	AA:178		UWOPS05_217_3:D>N		UWOPS05_227_2:D>N	AA:212		K11:T>I	AA:267		DBVPG6044:F>L		NCYC110:F>L		SK1:F>L	AA:310		322134S:R>S	AA:351		DBVPG6044:N>D		NCYC110:N>D		SK1:N>D	AA:357		K11:T>A		Y9:T>A	AA:450		UWOPS05_217_3:D>G		UWOPS05_227_2:D>G	AA:455		BC187:K>M		DBVPG1788:K>M		L_1374:K>M		W303:K>M		Y55:K>M		YJM975:K>MID:YDL234C	AA:10		UWOPS83_787_3:K>-	AA:21		YS9:D>N	AA:81		DBVPG1106:N>Y	AA:125		UWOPS05_217_3:T>A		UWOPS05_227_2:T>A	AA:224		DBVPG6044:L>P		SK1:L>P		UWOPS03_461_4:L>P		UWOPS05_217_3:L>P		UWOPS05_227_2:L>P		UWOPS83_787_3:L>P		YPS606:L>P	AA:251		YJM978:T>P	AA:327		SK1:N>T	AA:360		DBVPG1853:D>N		UWOPS03_461_4:S>P		UWOPS05_217_3:S>P		UWOPS05_227_2:S>P		Y9:D>N	AA:367		DBVPG6044:G>E		SK1:G>E	AA:439		DBVPG1853:E>K	AA:446		UWOPS83_787_3:N>S	AA:481		K11:L>F		Y9:L>F	AA:489		UWOPS03_461_4:T>I		UWOPS05_217_3:T>I		UWOPS05_227_2:T>I	AA:511		DBVPG1853:I>V		UWOPS03_461_4:I>V		UWOPS05_217_3:I>V		UWOPS05_227_2:I>V	AA:687		YS2:L>S	AA:698		YS2:M>T	AA:714		YS4:T>A	AA:725		UWOPS03_461_4:T>A		UWOPS05_217_3:T>A	AA:726		DBVPG1853:L>MID:YDL237W	AA:7		Y9:C>Y	AA:44		322134S:D>N		378604X:D>N		BC187:D>N		DBVPG1106:D>N		DBVPG1373:D>N		DBVPG1788:D>N		DBVPG1853:D>N		DBVPG6044:D>N		DBVPG6765:D>N		K11:D>N		L_1374:D>N		NCYC110:D>N		S288c:D>N		SK1:D>N		UWOPS03_461_4:D>N		UWOPS05_217_3:D>N		UWOPS05_227_2:D>N		UWOPS83_787_3:D>N		W303:D>N		Y55:D>N		Y9:D>N		YGPM:D>N		YIIc17_E5:D>N		YJM975:D>N		YPS128:D>N		YS4:D>N	AA:57		378604X:E>K		BC187:E>A		DBVPG1106:E>A		DBVPG1373:E>A		DBVPG1788:E>A		DBVPG6765:E>A		L_1374:E>A		W303:E>A		Y55:E>A		Y9:E>K		YIIc17_E5:E>A		YJM975:E>A		YJM981:E>A	AA:100		UWOPS03_461_4:P>L		UWOPS05_217_3:P>L		UWOPS05_227_2:P>L	AA:190		BC187:A>S		DBVPG1373:A>S		DBVPG1853:A>S		DBVPG6044:A>S		DBVPG6765:A>S		K11:A>S		L_1374:A>S		L_1528:A>S		NCYC110:A>S		SK1:A>S		UWOPS05_217_3:A>S		UWOPS05_227_2:A>S		UWOPS87_2421:A>S		Y55:A>S		Y9:A>S		YIIc17_E5:A>S		YPS128:A>S		YS4:A>S	AA:262		Y9:T>M	AA:279		DBVPG1853:S>N		DBVPG6044:S>N		K11:S>N		SK1:S>N		UWOPS05_217_3:S>N		UWOPS87_2421:S>N		Y9:S>N		YPS128:S>N	AA:374		DBVPG6044:G>S		SK1:G>S	AA:379		UWOPS87_2421:N>KID:YDL246C	AA:108		W303:S>RID:YDR002W	AA:135		UWOPS83_787_3:C>Y	AA:156		UWOPS05_227_2:W>-ID:YDR003W	AA:15		L_1528:Q>E	AA:18		273614X:N>D		Y9:N>D	AA:24		BC187:A>T		DBVPG1788:A>T		DBVPG6765:A>T	AA:140		L_1528:E>D	AA:161		L_1374:S>N	AA:198		NCYC361:D>E		YPS606:D>EID:YDR004W	AA:39		DBVPG1373:R>K		DBVPG1788:R>K		DBVPG6040:R>K		DBVPG6044:R>K		DBVPG6765:R>K		L_1374:R>K		NCYC361:R>K		SK1:R>K		UWOPS03_461_4:R>K		UWOPS05_227_2:R>K		Y55:R>K		Y9:R>K		YJM978:R>K		YPS128:R>K		YS4:R>K	AA:110		273614X:L>V		K11:L>V		Y9:L>V		YS4:L>V	AA:151		K11:L>I	AA:177		DBVPG6044:P>S		SK1:P>S		Y55:P>S	AA:278		DBVPG6044:P>L		SK1:P>L		UWOPS87_2421:P>L		Y55:P>L	AA:286		UWOPS05_217_3:H>Q	AA:344		273614X:N>H		DBVPG6040:N>H		UWOPS03_461_4:N>H		UWOPS05_227_2:N>H		UWOPS83_787_3:N>H		Y12:N>H		YIIc17_E5:N>H		YS4:N>H	AA:378		Y9:K>N	AA:411		Y9:I>L	AA:447		Y9:Q>P	AA:453		Y9:T>P	AA:455		UWOPS83_787_3:R>QID:YDR006C	AA:19		BC187:S>N		DBVPG1373:S>N		DBVPG6765:S>N		L_1528:S>N		UWOPS87_2421:S>N	AA:27		YS2:P>L		YS9:P>L	AA:31		DBVPG6044:N>S		SK1:N>S		Y55:N>S	AA:54		273614X:P>S		DBVPG6044:P>S		SK1:P>S		Y55:P>S		Y9:P>S	AA:74		273614X:G>D		DBVPG6044:G>D		SK1:G>D		UWOPS03_461_4:G>D		Y55:G>D		Y9:G>D	AA:75		DBVPG1853:D>G	AA:132		DBVPG6765:S>L	AA:146		378604X:N>D	AA:166		378604X:E>D	AA:265		YS9:R>C	AA:272		YJM978:N>Y	AA:301		UWOPS05_217_3:S>L	AA:340		UWOPS05_217_3:Q>R	AA:354		DBVPG6044:D>N		NCYC110:D>N		SK1:D>N		Y55:D>N	AA:442		DBVPG6044:N>D		NCYC110:N>D		SK1:N>D		Y55:N>D	AA:447		BC187:V>I		DBVPG1106:V>I		DBVPG1373:V>I		DBVPG1788:V>I		DBVPG6765:V>I		L_1528:V>I		NCYC361:V>I		UWOPS87_2421:V>I	AA:526		UWOPS03_461_4:S>F		UWOPS05_217_3:S>F		UWOPS05_227_2:S>F	AA:586		YS4:T>A	AA:653		UWOPS05_217_3:D>H	AA:673		NCYC110:N>K	AA:682		NCYC110:K>R	AA:720		DBVPG1106:V>F	AA:725		DBVPG1106:V>F	AA:770		UWOPS05_217_3:S>I	AA:846		K11:T>AID:YDR012W	AA:19		322134S:A>V	AA:67		YS9:T>S	AA:133		UWOPS05_227_2:S>	AA:154		YS9:T>I	AA:158		YS9:S>F	AA:356		BC187:A>T		DBVPG1106:A>T		DBVPG1373:A>T		DBVPG1788:A>T		DBVPG6040:A>T		DBVPG6044:A>T		DBVPG6765:A>T		K11:A>T		L_1528:A>T		SK1:A>T		UWOPS03_461_4:A>T		UWOPS05_227_2:A>T		YPS606:A>TID:YDR013W	AA:73		378604X:F>LID:YDR014W	AA:10		378604X:V>I		SK1:V>I		Y55:V>I	AA:63		378604X:K>R		DBVPG6044:K>R		NCYC110:K>R		SK1:K>R		Y55:K>R	AA:66		378604X:D>N		DBVPG6044:D>N		NCYC110:D>N		SK1:D>N		Y55:D>N	AA:74		378604X:A>T		DBVPG6044:A>T		K11:A>T		NCYC110:A>T		SK1:A>T		UWOPS03_461_4:A>T		UWOPS83_787_3:A>T		UWOPS87_2421:A>T		Y12:A>T		Y55:A>T		YIIc17_E5:A>T		YS9:A>T	AA:117		Y12:G>D	AA:137		YS9:T>K	AA:151		UWOPS03_461_4:T>K	AA:194		322134S:S>A		DBVPG6040:S>A	AA:203		UWOPS03_461_4:S>F	AA:214		273614X:I>K	AA:245		DBVPG1853:N>S		Y9:N>S	AA:250		322134S:G>V		DBVPG6040:G>V	AA:286		UWOPS03_461_4:T>A		UWOPS83_787_3:T>A	AA:382		273614X:K>N		378604X:K>N		DBVPG1853:K>N		K11:K>N		SK1:K>N		UWOPS03_461_4:K>N		UWOPS83_787_3:K>N		Y12:K>N		Y55:K>N		YIIc17_E5:K>N		YPS128:K>N		YPS606:K>N		YS4:K>N	AA:441		DBVPG1853:I>V		K11:I>V	AA:447		UWOPS83_787_3:L>I		YPS128:L>I		YPS606:L>I	AA:450		273614X:A>D		SK1:A>D		UWOPS83_787_3:A>D		Y55:A>D		YIIc17_E5:A>D		YPS128:A>D		YPS606:A>D		YS4:A>D	AA:454		273614X:C>R		378604X:C>S		SK1:C>R		Y55:C>R		YIIc17_E5:C>R		YS4:C>R	AA:477		SK1:D>G		Y55:D>G	AA:530		273614X:N>S	AA:560		Y55:K>R		YS2:K>R		YS4:K>RID:YDR016C	AA:4		UWOPS03_461_4:S>T	AA:33		K11:E>D	AA:81		NCYC110:P>L		SK1:P>L		Y55:P>L	AA:88		DBVPG1853:L>SID:YDR018C	AA:90		YS2:E>D		YS9:E>D	AA:132		DBVPG6765:V>I	AA:174		322134S:K>E		DBVPG1106:K>E		DBVPG1373:K>E		DBVPG6040:K>E		DBVPG6044:K>E		DBVPG6765:K>E		L_1374:K>E		L_1528:K>E		SK1:K>E		UWOPS03_461_4:K>E		UWOPS05_217_3:K>E		UWOPS05_227_2:K>E		UWOPS83_787_3:K>E		Y9:K>E		YIIc17_E5:K>E		YJM978:K>E		YJM981:K>E		YPS606:K>E		YS4:K>E		YS9:K>E	AA:189		Y9:C>Y	AA:208		322134S:A>V	AA:242		DBVPG6040:Q>R		DBVPG6044:Q>R		SK1:Q>R		UWOPS83_787_3:Q>R		Y9:Q>R		YIIc17_E5:Q>R		YPS128:Q>R		YPS606:Q>R		YS9:Q>R	AA:298		UWOPS05_217_3:V>I		UWOPS05_227_2:V>I	AA:311		273614X:R>K		YIIc17_E5:R>K	AA:349		322134S:S>N	AA:351		UWOPS05_217_3:A>G		UWOPS05_227_2:A>G	AA:397		273614X:->R		YPS128:->RID:YDR020C	AA:17		YS4:G>-	AA:77		322134S:H>NID:YDR022C	AA:26		UWOPS03_461_4:P>S		UWOPS05_227_2:P>S		YPS128:P>H		YPS606:P>H	AA:27		322134S:S>G		YS9:S>G	AA:38		UWOPS03_461_4:S>P		UWOPS05_227_2:S>P	AA:41		DBVPG6040:T>M	AA:43		273614X:G>R		Y9:G>R	AA:44		DBVPG1106:S>I	AA:53		UWOPS05_227_2:I>M	AA:61		UWOPS05_227_2:N>S	AA:96		YPS606:S>A	AA:153		DBVPG6044:S>L		NCYC110:S>L		SK1:S>L		UWOPS05_227_2:S>L		Y12:S>L		Y55:S>L		YPS128:S>L		YPS606:S>L	AA:190		YS9:Q>RID:YDR030C	AA:9		DBVPG6044:V>L		K11:V>L		SK1:V>L		UWOPS05_217_3:V>L		UWOPS05_227_2:V>L		UWOPS83_787_3:V>L		Y12:V>L		Y55:V>L		YIIc17_E5:V>L		YPS128:V>L		YPS606:V>L	AA:21		DBVPG6044:T>S		K11:T>S		SK1:T>S		Y12:T>S		Y55:T>S		YPS128:T>S		YPS606:T>S	AA:54		UWOPS05_217_3:T>A		UWOPS05_227_2:T>A	AA:116		UWOPS05_217_3:D>N		UWOPS05_227_2:D>N	AA:125		UWOPS05_217_3:Y>S		UWOPS05_227_2:Y>S	AA:133		K11:N>D		NCYC110:N>D		SK1:N>D		UWOPS83_787_3:N>D		Y55:N>D		YPS128:N>D		YPS606:N>D	AA:287		YIIc17_E5:Q>K	AA:333		DBVPG6044:R>G		K11:R>G		NCYC110:R>G		NCYC361:R>G		SK1:R>G		UWOPS03_461_4:R>G		UWOPS05_227_2:R>G		UWOPS83_787_3:R>G		Y12:R>G		Y55:R>G		YIIc17_E5:R>G		YPS128:R>G		YPS606:R>G	AA:365		YS9:I>V	AA:393		K11:L>M		Y12:L>M		YS9:L>M	AA:438		UWOPS83_787_3:V>I	AA:453		273614X:E>K		K11:E>K		Y12:E>KID:YDR031W	AA:44		DBVPG6040:I>T		K11:I>T		UWOPS05_227_2:I>T		Y12:I>T		Y9:I>T		YIIc17_E5:I>T		YPS128:I>T		YPS606:I>T	AA:64		DBVPG6044:S>N		SK1:S>N		Y55:S>N	AA:88		SK1:L>V		Y55:L>VID:YDR032C	AA:93		DBVPG6765:R>C		YJM978:R>C	AA:153		DBVPG1106:M>I	AA:185		273614X:E>Q	AA:192		S288c:Y>CID:YDR033W	AA:199		YS4:D>N		YS9:D>N	AA:287		UWOPS05_217_3:V>I	AA:316		DBVPG1853:K>MID:YDR034C	AA:2		UWOPS05_227_2:F>L		UWOPS83_787_3:F>L	AA:21		Y12:L>F	AA:99		DBVPG1853:G>S		DBVPG6044:G>S		NCYC110:G>S		NCYC361:G>S		SK1:G>S		UWOPS05_227_2:G>S		UWOPS83_787_3:G>S		UWOPS87_2421:G>S		Y55:G>S		YPS606:G>S	AA:114		DBVPG1853:Q>E		DBVPG6044:Q>E		NCYC110:Q>E		NCYC361:Q>E		SK1:Q>E		UWOPS05_227_2:Q>E		UWOPS83_787_3:Q>E		UWOPS87_2421:Q>E		Y55:Q>E		YPS606:Q>E	AA:124		UWOPS05_227_2:A>T	AA:135		UWOPS87_2421:N>I	AA:239		UWOPS87_2421:R>Q	AA:242		DBVPG1853:L>S		DBVPG6044:L>S		NCYC361:L>S		SK1:L>S		UWOPS03_461_4:L>S		UWOPS83_787_3:L>S		UWOPS87_2421:L>S		Y12:L>S		Y55:L>S		YIIc17_E5:L>S		YPS606:L>S	AA:256		NCYC361:G>A		UWOPS83_787_3:G>A		YPS606:G>A	AA:330		Y12:S>T	AA:361		YJM978:K>-	AA:519		273614X:N>S	AA:534		Y12:D>E	AA:760		BC187:K>R		DBVPG1788:K>R		DBVPG1853:K>R		DBVPG6044:K>R		DBVPG6765:K>R		K11:K>R		L_1374:K>R		L_1528:K>R		NCYC110:K>R		SK1:K>R		UWOPS03_461_4:K>R		UWOPS05_217_3:K>R		UWOPS87_2421:K>R		Y55:K>R		YJM975:K>R		YJM978:K>R		YJM981:K>R		YS4:K>R		YS9:K>RID:YDR041W	AA:145		L_1374:R>M	AA:154		UWOPS87_2421:S>L	AA:160		Y9:E>K		YIIc17_E5:E>KID:YDR043C	AA:31		378604X:E>G		DBVPG6044:E>G		NCYC110:E>G		SK1:E>G		Y55:E>G	AA:148		273614X:S>FID:YDR044W	AA:94		YS4:R>C	AA:200		DBVPG6044:T>ID:YDR045C	AA:11		273614X:M>T	AA:19		DBVPG6044:S>N		Y55:S>N	AA:21		DBVPG1853:V>I	AA:106		322134S:R>SID:YDR046C	AA:1		UWOPS05_227_2:M>I		UWOPS87_2421:M>I	AA:2		BC187:S>P		DBVPG6765:S>P	AA:76		UWOPS05_227_2:G>S		UWOPS87_2421:G>S	AA:241		273614X:V>I		DBVPG1373:V>I		DBVPG1853:V>I		DBVPG6044:V>I		L_1374:V>I		NCYC110:V>I		SK1:V>I		UWOPS05_227_2:V>I		Y12:V>I		Y55:V>I		YIIc17_E5:V>I		YPS128:V>I	AA:278		DBVPG6044:G>D		NCYC110:G>D		SK1:G>D		Y55:G>D	AA:401		378604X:M>L		DBVPG6044:M>L		NCYC110:M>L		SK1:M>L		Y55:M>L	AA:464		UWOPS83_787_3:W>L	AA:465		378604X:S>T		DBVPG6044:S>T		NCYC110:S>T		SK1:S>T		Y55:S>T	AA:526		W303:K>Q	AA:543		Y9:I>L	AA:561		L_1374:P>SID:YDR047W	AA:44		DBVPG6044:N>K		NCYC110:N>K		SK1:N>K		Y55:N>K	AA:55		UWOPS87_2421:A>T	AA:111		UWOPS03_461_4:E>K	AA:233		DBVPG6044:L>S		SK1:L>S		Y55:L>S	AA:271		DBVPG1853:C>S	AA:294		YS9:N>D	AA:362		Y12:K>Q		YIIc17_E5:K>Q		YPS606:K>Q		YS2:K>QID:YDR050C	AA:179		YS9:E>QID:YDR051C	AA:107		YJM978:S>I	AA:164		YJM978:W>C	AA:241		K11:D>EID:YDR052C	AA:28		DBVPG1853:A>T	AA:65		DBVPG6044:L>F		SK1:L>F		Y55:L>F	AA:84		273614X:S>P	AA:220		DBVPG1853:H>Q	AA:231		273614X:A>T		DBVPG6044:A>T		K11:A>T		NCYC110:A>T		SK1:A>T		UWOPS03_461_4:A>T		UWOPS05_227_2:A>T		Y12:A>T		Y55:A>T		Y9:A>T		YIIc17_E5:A>T		YPS606:A>T	AA:388		DBVPG1853:N>I	AA:389		DBVPG1373:F>I		DBVPG6765:F>I		L_1374:F>I		YJM975:F>I	AA:453		DBVPG1853:E>D	AA:464		DBVPG6044:V>I		K11:V>I		SK1:V>I		UWOPS03_461_4:V>I		UWOPS05_217_3:V>I		UWOPS87_2421:V>I		Y12:V>I		Y55:V>I		Y9:V>I		YPS128:V>I		YPS606:V>I	AA:535		DBVPG6040:K>T	AA:555		378604X:T>A		BC187:T>A		DBVPG6044:T>A		K11:T>A		SK1:T>A		UWOPS05_217_3:T>A		UWOPS87_2421:T>A		Y55:T>A		Y9:T>A		YIIc17_E5:T>A		YPS128:T>A		YPS606:T>A	AA:693		YS9:I>NID:YDR055W	AA:33		378604X:I>V	AA:63		Y12:G>D	AA:301		SK1:E>V	AA:407		273614X:G>S		BC187:G>S		DBVPG6044:G>S		L_1374:G>S		L_1528:G>S		NCYC110:G>S		SK1:G>S		UWOPS05_227_2:G>S		Y12:G>S		Y55:G>S		YJM978:G>S		YPS128:G>S		YPS606:G>S		YS4:G>S		YS9:G>S	AA:433		YPS128:V>I		YPS606:V>IID:YDR056C	AA:12		378604X:A>S	AA:58		273614X:V>A		BC187:V>A		DBVPG6044:V>A		SK1:V>A		Y55:V>A		Y9:V>A		YIIc17_E5:V>A		YPS128:V>A		YPS606:V>A		YS2:V>A		YS4:V>A	AA:110		378604X:D>N		DBVPG1373:D>N		DBVPG1788:D>N		DBVPG6765:D>N		YJM975:D>N		YS9:D>N	AA:193		DBVPG6765:Q>LID:YDR057W	AA:43		UWOPS03_461_4:D>Y		UWOPS05_217_3:D>Y		UWOPS05_227_2:D>Y	AA:141		L_1528:R>K	AA:179		UWOPS03_461_4:V>A	AA:186		DBVPG6765:I>T		L_1374:I>T		YJM975:I>T		YJM981:I>T	AA:204		YJM981:I>T	AA:261		UWOPS03_461_4:P>S	AA:267		273614X:G>D		DBVPG1373:G>D		DBVPG1788:G>D		DBVPG6044:G>D		SK1:G>D		UWOPS03_461_4:G>D		UWOPS83_787_3:G>D		UWOPS87_2421:G>D		Y12:G>D		Y55:G>D		YIIc17_E5:G>D	AA:270		DBVPG1373:S>P		DBVPG1788:S>P	AA:371		DBVPG6044:N>D		SK1:N>D		Y55:N>D	AA:400		BC187:H>R		UWOPS83_787_3:H>R	AA:445		SK1:E>K		Y55:E>K	AA:480		273614X:N>D		BC187:N>D		K11:N>D		NCYC110:N>D		SK1:N>D		UWOPS05_217_3:N>D		Y12:N>D		Y55:N>D		YPS606:N>D		YS4:N>DID:YDR059C	AA:95		DBVPG6040:S>T	AA:127		378604X:I>T		DBVPG1106:I>T		DBVPG1373:I>T		DBVPG1788:I>T		DBVPG6765:I>T		L_1374:I>T		L_1528:I>TID:YDR061W	AA:24		UWOPS05_227_2:P>S	AA:54		UWOPS05_227_2:D>N	AA:114		YS4:Q>-	AA:159		BC187:V>A	AA:197		273614X:I>T		378604X:I>T		BC187:I>T		DBVPG1788:I>T		DBVPG1853:I>T		DBVPG6040:I>T		DBVPG6044:I>T		DBVPG6765:I>T		L_1374:I>T		SK1:I>T		UWOPS05_227_2:I>T		UWOPS87_2421:I>T		Y12:I>T		Y55:I>T		YIIc17_E5:I>T		YJM975:I>T		YJM978:I>T		YPS128:I>T		YS4:I>T	AA:213		378604X:S>N		YJM975:S>N	AA:214		273614X:G>A		DBVPG1853:G>A		DBVPG6044:G>A		UWOPS87_2421:G>A		Y55:G>A		YIIc17_E5:G>A		YS4:G>A	AA:257		273614X:M>I		DBVPG1853:M>I		UWOPS05_227_2:M>I		UWOPS83_787_3:M>I		UWOPS87_2421:M>I		Y55:M>I		YIIc17_E5:M>I		YS4:M>I	AA:269		273614X:E>Q		DBVPG1853:E>Q		DBVPG6044:E>Q		K11:E>Q		UWOPS83_787_3:E>Q		UWOPS87_2421:E>Q		Y12:E>Q		Y55:E>Q		YIIc17_E5:E>Q		YS4:E>Q	AA:276		273614X:N>D		DBVPG1853:N>D		UWOPS87_2421:N>D		Y12:N>D		Y55:N>D		YIIc17_E5:N>D		YS4:N>D	AA:315		UWOPS87_2421:S>-	AA:380		DBVPG6040:D>N	AA:435		322134S:E>D		378604X:E>D		DBVPG1373:E>D		DBVPG1788:E>D		DBVPG1853:E>D		K11:E>D		L_1374:E>D		L_1528:E>D		NCYC361:E>D		SK1:E>D		UWOPS05_227_2:E>D		UWOPS83_787_3:E>D		Y12:E>D		YJM978:E>D		YS4:E>D		YS9:E>D	AA:442		UWOPS05_227_2:K>E	AA:445		UWOPS83_787_3:G>D	AA:452		UWOPS05_227_2:S>I	AA:457		NCYC361:Q>H	AA:512		UWOPS87_2421:H>	AA:525		BC187:R>K		UWOPS83_787_3:R>K	AA:537		378604X:M>I		BC187:M>I		K11:M>I		UWOPS05_227_2:M>I		UWOPS83_787_3:M>I		Y12:M>I		Y55:M>I		Y9:M>I		YPS128:M>I		YPS606:M>I		YS4:M>IID:YDR063W	AA:48		UWOPS83_787_3:D>V		YPS128:D>V		YPS606:D>V	AA:60		273614X:S>N		DBVPG1106:S>N		DBVPG1788:S>N		DBVPG6044:S>N		DBVPG6765:S>N		K11:S>N		NCYC110:S>N		NCYC361:S>N		SK1:S>N		UWOPS03_461_4:S>N		UWOPS83_787_3:S>N		Y55:S>N		YJM975:S>N		YJM978:S>N		YPS128:S>N		YPS606:S>N		YS2:S>N		YS4:S>N	AA:136		NCYC361:D>E		YJM975:D>E	AA:145		273614X:Q>RID:YDR065W	AA:45		322134S:I>K		DBVPG1788:I>K		DBVPG1853:I>K		DBVPG6765:I>K		L_1374:I>K		SK1:I>K		Y55:I>K		YJM975:I>K		YPS128:I>K		YPS606:I>K		YS4:I>K		YS9:I>K	AA:68		Y55:L>S	AA:92		YPS128:K>R		YPS606:K>R	AA:96		322134S:K>E		DBVPG1373:K>E		DBVPG1788:K>E		DBVPG1853:K>E		DBVPG6040:K>E		DBVPG6765:K>E		SK1:K>E		Y55:K>E		YJM975:K>E		YPS128:K>E		YPS606:K>E		YS9:K>E	AA:178		YS2:G>R		YS4:G>R	AA:229		YPS128:I>V		YPS606:I>V	AA:261		YPS128:A>T		YPS606:A>T	AA:279		273614X:V>I		322134S:V>I		DBVPG1373:V>I		DBVPG1788:V>I		DBVPG1853:V>I		DBVPG6044:V>I		DBVPG6765:V>I		L_1374:V>I		SK1:V>I		UWOPS03_461_4:V>I		UWOPS05_227_2:V>I		Y55:V>I		YPS128:V>I		YPS606:V>I		YS2:V>I		YS4:V>I		YS9:V>I	AA:299		YPS128:Q>K		YPS606:Q>K	AA:337		DBVPG6044:R>K		Y55:R>KID:YDR068W	AA:45		DBVPG1373:E>G		DBVPG1788:E>G		DBVPG6040:E>G		L_1528:E>G		SK1:E>G		YJM975:E>G		YJM978:E>G	AA:126		UWOPS05_217_3:S>R	AA:129		UWOPS05_217_3:N>D	AA:141		273614X:N>S		BC187:N>S		DBVPG1853:N>S		NCYC110:N>S		UWOPS03_461_4:N>S		UWOPS87_2421:N>S		Y12:N>S		Y9:N>S		YPS128:N>S		YPS606:N>S	AA:145		DBVPG1853:N>H	AA:161		273614X:L>V	AA:259		273614X:E>D		DBVPG1373:E>D		UWOPS87_2421:E>D		Y12:E>D		YJM975:E>D	AA:268		322134S:A>V		DBVPG1106:A>V		DBVPG1373:A>V		DBVPG6765:A>V		L_1374:A>V		YJM975:A>V		YJM978:A>V	AA:280		YPS606:V>A	AA:286		DBVPG1788:K>-ID:YDR070C	AA:46		BC187:G>D		NCYC361:G>D	AA:50		273614X:S>T		DBVPG6765:S>TID:YDR071C	AA:26		YS2:L>F	AA:32		322134S:G>V	AA:61		DBVPG1373:R>IID:YDR073W	AA:14		NCYC361:T>I	AA:38		DBVPG6044:T>N		Y55:T>N	AA:42		DBVPG6044:T>N		Y55:T>N	AA:46		DBVPG6044:T>A		Y55:T>A	AA:66		K11:V>I	AA:98		DBVPG6044:S>N		Y55:S>NID:YDR074W	AA:4		Y55:T>I	AA:8		L_1374:N>T	AA:25		NCYC361:Y>S	AA:27		NCYC361:I>M	AA:41		YS2:T>S		YS9:T>S	AA:48		378604X:F>Y		BC187:F>Y		DBVPG1373:F>Y		DBVPG1853:F>Y		DBVPG6044:F>Y		DBVPG6765:F>Y		L_1374:F>Y		SK1:F>Y		UWOPS03_461_4:F>Y		UWOPS83_787_3:F>Y		Y55:F>Y		Y9:F>Y		YIIc17_E5:F>Y		YJM975:F>Y		YJM978:F>Y		YPS128:F>Y		YPS606:F>Y		YS2:F>Y		YS9:F>Y	AA:67		YS2:G>D	AA:82		378604X:R>K	AA:119		378604X:E>D	AA:126		SK1:Q>R	AA:145		378604X:S>N	AA:174		YS4:N>	AA:339		DBVPG1106:K>R	AA:358		378604X:L>-	AA:467		YS2:L>S	AA:534		UWOPS03_461_4:K>M	AA:542		SK1:Q>K		Y9:Q>K		YS4:Q>K	AA:546		273614X:N>D		BC187:N>D		DBVPG1788:N>D		DBVPG6040:N>D		DBVPG6044:N>D		DBVPG6765:N>D		L_1374:N>D		NCYC110:N>D		SK1:N>D		UWOPS03_461_4:N>D		UWOPS83_787_3:N>D		Y55:N>D		YJM975:N>D		YJM978:N>D		YPS128:N>D		YS2:N>D		YS4:N>D		YS9:N>D	AA:549		BC187:M>V		DBVPG6040:M>V		DBVPG6044:M>V		NCYC110:M>V		SK1:M>V		UWOPS03_461_4:M>V		UWOPS83_787_3:M>V		Y55:M>V		Y9:M>V		YPS128:M>V		YPS606:M>V	AA:569		BC187:A>S		DBVPG6040:A>S		DBVPG6044:A>S		NCYC110:A>S		Y55:A>S		YPS128:A>S	AA:764		DBVPG1788:S>R		DBVPG6765:S>R		L_1374:S>R		YJM978:S>R	AA:794		YJM978:T>NID:YDR075W	AA:60		YS9:L>V	AA:137		YS4:W>-	AA:155		YS9:N>S	AA:163		Y9:G>E	AA:227		UWOPS05_217_3:N>I	AA:270		UWOPS05_217_3:L>-	AA:295		UWOPS05_217_3:A>DID:YDR078C	AA:37		DBVPG6044:P>Q		NCYC110:P>Q		Y55:P>Q	AA:128		K11:E>K		SK1:E>K		Y9:E>K		YIIc17_E5:E>K		YPS128:E>K		YPS606:E>K	AA:174		DBVPG1106:V>I		DBVPG1373:V>I		DBVPG6044:V>I		NCYC110:V>I		UWOPS87_2421:V>I		Y55:V>I		YJM975:V>I		YS9:V>I	AA:208		Y9:I>VID:YDR079W	AA:18		SK1:I>L	AA:103		NCYC110:K>R		Y55:K>RID:YDR083W	AA:5		BC187:N>D		UWOPS83_787_3:N>D		YPS606:N>D	AA:33		SK1:N>K		Y12:N>K		YIIc17_E5:N>K		YS4:N>K		YS9:N>K	AA:61		SK1:P>A		Y12:P>A		YIIc17_E5:P>A		YS4:P>A		YS9:P>A	AA:78		DBVPG6044:S>N		NCYC110:S>N		Y55:S>N	AA:84		BC187:S>N		SK1:S>N		UWOPS83_787_3:S>N		Y12:S>N		Y9:S>N		YIIc17_E5:S>N		YPS606:S>N		YS4:S>N		YS9:S>N	AA:96		YS4:M>L	AA:109		L_1528:Q>K	AA:358		YS9:Q>ID:YDR084C	AA:2		DBVPG6040:D>E	AA:50		DBVPG6040:V>I	AA:138		UWOPS05_227_2:L>S	AA:162		DBVPG6044:N>S		SK1:N>S		Y12:N>S		Y55:N>S		YPS128:N>S		YS9:N>SID:YDR085C	AA:4		NCYC110:S>T		Y55:S>T	AA:28		UWOPS87_2421:L>V	AA:37		YS9:K>E	AA:39		YS9:N>D	AA:46		UWOPS87_2421:Y>-	AA:59		NCYC361:S>N		UWOPS03_461_4:S>N		UWOPS05_227_2:S>N		UWOPS83_787_3:S>N	AA:112		378604X:S>N		DBVPG1788:S>N		DBVPG6040:S>N		DBVPG6044:S>N		DBVPG6765:S>N		NCYC110:S>N		NCYC361:S>N		SK1:S>N		UWOPS03_461_4:S>N		UWOPS05_227_2:S>N		UWOPS83_787_3:S>N		UWOPS87_2421:S>N		Y12:S>N		Y55:S>N		YPS128:S>N		YS2:S>N		YS9:S>N	AA:118		DBVPG6044:Q>R		NCYC110:Q>R		Y55:Q>R	AA:119		UWOPS87_2421:Y>-	AA:139		DBVPG6040:R>T		DBVPG6044:R>T		NCYC361:R>T		SK1:R>T		UWOPS03_461_4:R>T		UWOPS05_227_2:R>T		UWOPS83_787_3:R>T		Y12:R>T		Y55:R>T		YPS128:R>T		YS2:R>T		YS9:R>T	AA:142		YS9:Q>L	AA:153		DBVPG6040:L>I		NCYC361:L>I		SK1:L>I		UWOPS83_787_3:L>I		Y12:L>I		YS2:L>I		YS9:L>I	AA:168		DBVPG6044:S>P		Y55:S>P	AA:174		DBVPG6044:F>C		UWOPS83_787_3:F>C		Y55:F>C		YPS128:F>C	AA:235		DBVPG1788:V>I	AA:253		DBVPG1853:M>I		NCYC110:E>-	AA:278		DBVPG1853:L>M		DBVPG6044:L>V		UWOPS87_2421:L>V		Y55:L>V	AA:298		DBVPG1853:K>R		DBVPG6044:K>R		K11:K>R		SK1:K>R		UWOPS05_227_2:K>R		UWOPS83_787_3:K>R		UWOPS87_2421:K>R		Y55:K>R		YIIc17_E5:K>R		YPS128:K>R		YPS606:K>R		YS9:K>R	AA:303		DBVPG1853:L>P		DBVPG6044:L>P		K11:L>P		UWOPS05_227_2:L>P		UWOPS87_2421:L>P		Y55:L>P		YIIc17_E5:L>P		YS9:L>P	AA:309		UWOPS87_2421:R>K	AA:346		322134S:R>K		DBVPG1373:R>K		DBVPG1853:R>K		DBVPG6765:R>K		K11:R>K		L_1374:R>K		L_1528:R>K		SK1:R>K		UWOPS05_227_2:R>K		UWOPS87_2421:R>K		Y55:R>K		Y9:R>K		YIIc17_E5:R>K		YPS128:R>K		YPS606:R>K	AA:352		DBVPG1853:M>I		UWOPS05_227_2:M>I		UWOPS87_2421:M>I		YPS128:M>I		YPS606:M>I	AA:364		K11:N>S		Y55:N>S		Y9:N>S	AA:404		322134S:L>S		DBVPG1853:L>S		K11:L>S		UWOPS03_461_4:L>S		UWOPS05_227_2:L>S		UWOPS87_2421:L>S		Y55:L>S		Y9:L>S		YIIc17_E5:L>S		YPS128:L>S		YPS606:L>S	AA:496		273614X:P>Q		DBVPG1853:P>S	AA:566		DBVPG1853:A>T	AA:573		SK1:N>S	AA:574		DBVPG1373:R>CID:YDR087C	AA:26		UWOPS05_217_3:K>N	AA:29		UWOPS05_217_3:L>V	AA:38		UWOPS05_217_3:K>T	AA:90		Y9:T>I		YIIc17_E5:T>I		YPS128:T>I	AA:106		UWOPS03_461_4:M>I		UWOPS05_217_3:M>I	AA:131		BC187:S>N		DBVPG1853:S>N		DBVPG6040:S>N		DBVPG6044:S>N		SK1:S>N		UWOPS03_461_4:S>N		UWOPS05_217_3:S>N		Y55:S>N		Y9:S>N		YIIc17_E5:S>N	AA:198		DBVPG1853:K>R		DBVPG6044:K>R		Y55:K>R	AA:209		YS4:A>V	AA:257		DBVPG1373:E>K	AA:264		UWOPS03_461_4:S>NID:YDR088C	AA:5		UWOPS05_217_3:S>N		UWOPS05_227_2:S>N	AA:9		322134S:E>K		DBVPG1106:E>K		DBVPG1373:E>K		DBVPG1788:E>K		DBVPG1853:E>K		DBVPG6765:E>K		K11:E>K		L_1528:E>K		SK1:E>K		UWOPS83_787_3:E>K		Y12:E>K		Y55:E>K		YJM975:E>K		YPS606:E>K	AA:59		SK1:D>G		UWOPS83_787_3:D>G	AA:64		SK1:A>T		UWOPS83_787_3:A>T		Y55:A>S		YPS606:A>S	AA:111		SK1:M>V		UWOPS05_217_3:M>V		UWOPS05_227_2:M>V		UWOPS83_787_3:M>V		Y55:M>V		YPS606:M>V	AA:147		DBVPG1788:P>L		L_1528:P>L		UWOPS05_227_2:D>G	AA:154		DBVPG1106:N>T		DBVPG1373:N>T		DBVPG1788:N>T		DBVPG6044:N>T		DBVPG6765:N>T		L_1528:N>T		SK1:N>T		UWOPS05_217_3:N>T		UWOPS05_227_2:N>T		UWOPS83_787_3:N>T		Y55:N>T		Y9:N>T		YPS606:N>T		YS9:N>T	AA:195		DBVPG6044:N>D		Y55:N>D	AA:350		UWOPS87_2421:Q>LID:YDR090C	AA:56		K11:Y>F		Y9:Y>F	AA:124		YJM975:E>-	AA:157		YS4:K>N	AA:203		DBVPG6040:L>I	AA:213		YS9:W>R	AA:250		273614X:R>Q		DBVPG1106:R>Q		DBVPG1373:R>Q		DBVPG1788:R>Q		DBVPG6040:R>Q		DBVPG6044:R>Q		DBVPG6765:R>Q		L_1374:R>Q		L_1528:R>Q		NCYC110:R>Q		SK1:R>Q		Y55:R>Q		Y9:R>Q		YJM978:R>Q		YS4:R>Q	AA:254		YS9:N>S	AA:274		YS9:N>D	AA:276		273614X:Y>H		DBVPG1106:Y>H		DBVPG1373:Y>H		DBVPG1788:Y>H		DBVPG6040:Y>H		DBVPG6044:Y>H		DBVPG6765:Y>H		L_1374:Y>H		L_1528:Y>H		NCYC110:Y>H		SK1:Y>H		Y55:Y>H		Y9:Y>H		YJM978:Y>H		YS4:Y>H	AA:289		YS9:T>AID:YDR098C	AA:24		DBVPG6044:A>T		NCYC110:A>T		SK1:A>T		Y55:A>T	AA:136		273614X:Y>F		322134S:Y>F		378604X:Y>F		DBVPG1373:Y>F		DBVPG1788:Y>F		DBVPG6044:Y>F		DBVPG6765:Y>F		K11:Y>F		L_1528:Y>F		NCYC110:Y>F		SK1:Y>F		UWOPS03_461_4:Y>F		UWOPS05_227_2:Y>F		UWOPS87_2421:Y>F		W303:Y>F		Y12:Y>F		Y55:Y>F		YJM975:Y>F		YPS128:Y>F		YPS606:Y>F		YS2:Y>F		YS4:Y>F	AA:252		YIIc17_E5:F>LID:YDR099W	AA:151		L_1528:L>-	AA:161		L_1528:A>S	AA:245		L_1528:Q>R	AA:263		378604X:P>QID:YDR100W	AA:124		NCYC361:A>TID:YDR101C	AA:29		YS9:R>G	AA:42		YS9:V>F	AA:135		K11:P>S	AA:170		273614X:L>P		DBVPG1853:L>P		DBVPG6044:L>P		K11:L>P		SK1:L>P		Y12:L>P		Y55:L>P		YPS128:L>P	AA:268		SK1:V>I		Y55:V>I	AA:487		UWOPS03_461_4:S>Y		UWOPS05_217_3:S>Y		UWOPS05_227_2:S>Y	AA:590		UWOPS83_787_3:T>SID:YDR105C	AA:76		322134S:G>V	AA:152		UWOPS83_787_3:V>F	AA:258		UWOPS03_461_4:A>S	AA:375		Y9:L>I	AA:393		YS2:T>I		YS4:T>I	AA:460		SK1:P>SID:YDR106W	AA:4		K11:T>N		Y12:T>N		YPS606:T>N		YS2:T>N		YS4:T>N	AA:11		UWOPS03_461_4:G>E		UWOPS05_217_3:G>E		UWOPS05_227_2:G>E	AA:24		DBVPG1788:C>Y	AA:50		K11:H>Y		Y12:H>Y		Y9:H>Y		YS2:H>Y	AA:63		UWOPS03_461_4:V>I		UWOPS05_227_2:V>I	AA:114		273614X:V>I	AA:118		DBVPG6044:G>S		SK1:G>S		Y55:G>S	AA:146		UWOPS87_2421:Q>E		Y9:Q>E		YS2:Q>E		YS4:Q>E	AA:156		DBVPG1373:S>C		NCYC361:S>C		UWOPS03_461_4:S>C		UWOPS05_227_2:S>C		W303:S>C	AA:208		UWOPS87_2421:E>K	AA:214		DBVPG1853:V>A		SK1:V>A		UWOPS03_461_4:V>A		UWOPS05_227_2:V>A		UWOPS83_787_3:V>A		UWOPS87_2421:V>A		Y55:V>AID:YDR107C	AA:4		DBVPG6044:G>S		K11:G>S		SK1:G>S		UWOPS05_217_3:G>S		UWOPS05_227_2:G>S		UWOPS83_787_3:G>S		Y55:G>S		YPS128:G>S		YPS606:G>S	AA:38		DBVPG6765:L>F		L_1528:L>F	AA:60		322134S:G>S	AA:144		YPS128:L>H	AA:195		DBVPG1373:A>V	AA:206		322134S:L>Q		DBVPG1853:L>Q		SK1:L>Q		UWOPS03_461_4:L>Q		UWOPS05_217_3:L>Q		UWOPS05_227_2:L>Q		UWOPS83_787_3:L>Q		Y55:L>Q		YPS128:L>Q		YPS606:L>Q	AA:398		UWOPS87_2421:G>S	AA:439		378604X:W>L	AA:457		Y9:L>QID:YDR109C	AA:2		378604X:K>E		UWOPS83_787_3:R>K		YPS128:R>K	AA:12		DBVPG6044:N>D		K11:N>D		NCYC361:N>D		SK1:N>D		UWOPS03_461_4:N>D		UWOPS87_2421:N>D		Y12:N>D		Y9:N>D		YPS606:N>D		YS2:N>D	AA:24		NCYC361:E>K		YPS606:E>K	AA:34		Y12:D>Y	AA:35		NCYC361:P>S		YPS606:P>S	AA:110		UWOPS83_787_3:G>V	AA:128		Y9:E>G	AA:140		UWOPS05_227_2:D>E	AA:231		UWOPS03_461_4:G>V	AA:283		SK1:K>R		Y55:K>R	AA:318		UWOPS83_787_3:A>P		UWOPS87_2421:A>P	AA:323		UWOPS83_787_3:A>T		UWOPS87_2421:A>T	AA:326		Y12:E>K	AA:334		YPS128:G>S		YPS606:G>S	AA:404		UWOPS83_787_3:M>L		UWOPS87_2421:M>L		Y12:M>L		YPS606:M>L	AA:408		UWOPS87_2421:A>T	AA:410		DBVPG6044:V>L		SK1:V>L		UWOPS03_461_4:V>L		UWOPS05_227_2:V>L		UWOPS83_787_3:V>L		UWOPS87_2421:V>L		Y12:V>L		Y55:V>L		YPS606:V>L		YS2:V>L		YS4:V>L		YS9:V>L	AA:423		Y12:L>S	AA:568		DBVPG1853:S>F		DBVPG6044:S>F		NCYC110:S>F		SK1:S>F		UWOPS03_461_4:S>F		UWOPS05_227_2:S>F		Y55:S>F		YS2:S>F		YS4:S>F	AA:571		DBVPG1853:T>A		DBVPG6044:T>A		NCYC110:T>A		SK1:T>A		UWOPS03_461_4:T>A		UWOPS05_227_2:T>A		UWOPS83_787_3:T>A		Y55:T>A		YPS128:T>A		YPS606:T>A		YS4:T>A	AA:573		DBVPG1853:T>A		DBVPG6044:T>A		NCYC110:T>A		SK1:T>A		UWOPS03_461_4:T>A		UWOPS05_227_2:T>A		Y55:T>A		YS4:T>A	AA:598		DBVPG6044:V>A		NCYC110:V>A		SK1:V>A		UWOPS03_461_4:V>A		UWOPS05_227_2:V>A		UWOPS87_2421:V>A		Y55:V>A	AA:605		UWOPS87_2421:L>I	AA:644		L_1374:Q>K	AA:651		DBVPG1853:R>T		DBVPG6044:R>T		NCYC110:R>T		SK1:R>T		UWOPS03_461_4:R>T		UWOPS05_227_2:R>T		Y55:R>T	AA:674		NCYC110:P>Q		SK1:P>Q		Y55:P>QID:YDR110W	AA:22		YPS128:V>G		YPS606:V>G	AA:51		DBVPG6044:M>V		NCYC110:M>V		SK1:M>V	AA:264		YGPM:F>I	AA:273		322134S:S>-	AA:320		378604X:F>Y	AA:481		DBVPG1853:E>G		DBVPG6044:E>G		K11:E>G		NCYC110:E>G		SK1:E>G		UWOPS83_787_3:E>G		Y55:E>G		Y9:E>G		YPS128:E>G		YS9:E>G	AA:508		DBVPG6044:E>G		SK1:E>G		Y55:E>G	AA:543		K11:T>I		UWOPS83_787_3:T>I		YPS128:T>I	AA:558		UWOPS05_227_2:R>KID:YDR113C	AA:1		UWOPS87_2421:M>I	AA:44		YJM975:V>I		YJM978:V>I	AA:105		322134S:S>F		DBVPG1853:S>F		DBVPG6044:S>F		K11:S>F		L_1528:S>F		NCYC110:S>F		SK1:S>F		UWOPS05_227_2:S>F		UWOPS87_2421:S>F		Y55:S>F		YJM975:S>F		YJM978:S>F		YPS606:S>F		YS2:S>F	AA:123		L_1528:R>C		YIIc17_E5:R>C		YJM975:R>C		YJM978:R>C	AA:141		DBVPG1106:A>T	AA:179		YS9:S>L	AA:200		UWOPS03_461_4:G>S		UWOPS05_227_2:G>S	AA:215		K11:G>S	AA:282		DBVPG6044:D>E		SK1:D>E		UWOPS03_461_4:D>E		UWOPS05_227_2:D>E		Y55:D>E		Y9:D>E		YPS128:D>E		YPS606:D>E		YS2:D>E	AA:327		273614X:C>R		K11:C>R		SK1:C>R		UWOPS03_461_4:C>R		UWOPS05_227_2:C>R		Y55:C>R		YPS128:C>R		YPS606:C>R		YS2:C>R		YS9:C>R	AA:335		K11:G>D		UWOPS03_461_4:G>D		UWOPS05_227_2:G>D		Y12:G>D		YPS128:G>D		YPS606:G>D		YS2:G>D	AA:364		378604X:D>V	AA:366		BC187:E>GID:YDR115W	AA:2		273614X:P>S		DBVPG1106:P>S		DBVPG6044:P>S		K11:P>S		SK1:P>S		UWOPS03_461_4:P>S		UWOPS05_227_2:P>S		UWOPS87_2421:P>S		Y12:P>S		Y55:P>S		YJM975:P>S		YJM981:P>S		YPS128:P>S		YS4:P>S	AA:5		DBVPG6044:A>V		L_1528:A>T		SK1:A>V		Y55:A>V	AA:25		L_1528:L>I	AA:40		L_1528:P>SID:YDR116C	AA:21		273614X:F>S		DBVPG6044:F>S		K11:F>S		SK1:F>S		UWOPS03_461_4:F>S		UWOPS83_787_3:F>S		Y12:F>S		Y55:F>S		Y9:F>S		YPS128:F>S	AA:37		Y12:M>V	AA:42		YJM978:P>S	AA:45		322134S:T>S	AA:140		378604X:E>D	AA:208		K11:S>G		UWOPS83_787_3:S>G		Y9:S>G		YPS128:S>G		YPS606:S>G	AA:257		322134S:D>N	AA:279		UWOPS83_787_3:G>VID:YDR117C	AA:38		UWOPS03_461_4:R>P		UWOPS05_227_2:R>P		UWOPS83_787_3:R>P		UWOPS87_2421:R>P		Y12:R>P		YPS128:R>P		YPS606:R>P	AA:152		DBVPG1853:F>Y		DBVPG6044:F>Y		SK1:F>Y		UWOPS83_787_3:F>Y		UWOPS87_2421:F>Y		Y12:F>Y		Y55:F>Y		Y9:F>Y		YPS128:F>Y	AA:168		322134S:H>Y		DBVPG1853:H>Y		DBVPG6044:H>Y		SK1:H>Y		UWOPS83_787_3:H>Y		UWOPS87_2421:H>Y		Y12:H>Y		Y55:H>Y		Y9:H>Y		YPS128:H>Y	AA:185		DBVPG1853:F>I		DBVPG6044:F>I		SK1:F>I		UWOPS83_787_3:F>I		UWOPS87_2421:F>I		Y12:F>I		Y55:F>I		Y9:F>I		YPS128:F>I	AA:187		DBVPG6044:L>F		SK1:L>F		Y55:L>F	AA:211		SK1:E>K		UWOPS03_461_4:E>K		UWOPS05_227_2:E>K		UWOPS83_787_3:E>K		UWOPS87_2421:E>K		Y12:E>K		Y55:E>K		YPS128:E>K	AA:217		DBVPG1853:V>A		Y9:V>A		YS4:V>A	AA:225		322134S:E>K	AA:227		Y12:I>T	AA:257		YJM978:L>F	AA:341		DBVPG1853:A>T		YS4:A>T	AA:411		UWOPS05_227_2:K>N	AA:487		UWOPS87_2421:V>F	AA:493		NCYC361:N>D	AA:518		YPS606:I>V	AA:521		YPS128:S>P		YPS606:S>P	AA:544		YS9:N>TID:YDR118W	AA:12		SK1:Y>N		Y55:Y>N	AA:53		K11:D>N		SK1:D>N		UWOPS03_461_4:D>N		UWOPS05_227_2:D>N		UWOPS87_2421:D>N		Y12:D>N		Y55:D>N		YPS128:D>N		YPS606:D>N	AA:98		DBVPG6040:W>L	AA:155		DBVPG1373:S>N	AA:180		K11:Y>H		Y12:Y>H	AA:193		Y12:N>S	AA:227		DBVPG6044:K>E		SK1:K>E		Y55:K>E	AA:266		YJM978:K>E	AA:305		322134S:E>K	AA:310		UWOPS05_217_3:R>M		YPS128:R>K		YPS606:R>K	AA:374		378604X:Q>R	AA:375		DBVPG6044:K>N		SK1:K>N		Y55:K>N	AA:380		S288c:T>A	AA:430		DBVPG1106:L>V		YJM975:L>V	AA:452		DBVPG6044:L>P		SK1:L>P		Y55:L>P	AA:466		DBVPG6044:D>E		SK1:D>E		UWOPS83_787_3:D>E		Y55:D>E		Y9:D>E		YPS606:D>E	AA:516		DBVPG6044:I>V		SK1:I>V		UWOPS83_787_3:I>V		Y55:I>V		YPS606:I>V	AA:584		322134S:G>E		BC187:G>E		DBVPG1106:G>E		DBVPG1373:G>E		DBVPG1853:G>E		DBVPG6044:G>E		DBVPG6765:G>E		NCYC361:G>E		SK1:G>E		UWOPS83_787_3:G>E		Y55:G>E		YJM975:G>E		YPS128:G>E		YPS606:G>E	AA:588		DBVPG1853:S>G		DBVPG6044:S>G		SK1:S>G		Y55:S>G		YPS128:S>G		YPS606:S>G	AA:601		YS9:E>D	AA:604		DBVPG1853:E>K	AA:650		DBVPG1853:Q>R		SK1:Q>R		UWOPS03_461_4:Q>R		UWOPS05_217_3:Q>R		UWOPS05_227_2:Q>R		YPS128:Q>R		YPS606:Q>R		YS9:Q>R	AA:651		L_1528:N>KID:YDR120C	AA:3		DBVPG1853:G>S		NCYC110:G>S		SK1:G>S		UWOPS87_2421:G>S		Y55:G>S	AA:82		UWOPS05_217_3:N>I	AA:127		273614X:S>Y	AA:159		NCYC110:E>K		SK1:E>K		Y55:E>K	AA:203		UWOPS05_217_3:T>S		UWOPS05_227_2:T>S		UWOPS87_2421:T>S		YPS606:T>S	AA:410		DBVPG1373:D>G		YJM978:D>G	AA:415		UWOPS03_461_4:R>H		UWOPS05_217_3:R>H		UWOPS05_227_2:R>H	AA:444		UWOPS87_2421:V>L	AA:446		UWOPS03_461_4:K>Q	AA:514		DBVPG1853:A>P	AA:517		378604X:G>R		DBVPG1106:G>R		DBVPG1373:G>R		DBVPG1788:G>R		DBVPG1853:G>R		DBVPG6044:G>R		K11:G>R		L_1374:G>R		SK1:G>R		UWOPS05_217_3:G>R		UWOPS05_227_2:G>R		UWOPS83_787_3:G>R		UWOPS87_2421:G>R		Y55:G>R		Y9:G>R		YJM975:G>R		YPS128:G>R		YPS606:G>R		YS4:G>R	AA:522		DBVPG1853:T>I	AA:526		DBVPG1853:E>QID:YDR121W	AA:102		273614X:I>T	AA:118		YS9:Y>C	AA:133		DBVPG1853:G>D		SK1:G>D		UWOPS03_461_4:G>D		UWOPS05_217_3:G>D		UWOPS05_227_2:G>D		Y55:G>D		Y9:G>D	AA:150		273614X:V>A		DBVPG1373:V>A		DBVPG1853:V>A		DBVPG6765:V>A		L_1528:V>A		SK1:V>A		UWOPS03_461_4:V>A		UWOPS05_217_3:V>A		Y9:V>A		YIIc17_E5:V>A		YJM978:V>A		YPS128:V>A		YPS606:V>A	AA:155		YIIc17_E5:V>IID:YDR122W	AA:74		YIIc17_E5:A>G		YJM975:A>G		YJM978:A>G	AA:154		DBVPG1853:A>T	AA:212		YS4:M>I	AA:266		Y9:V>I	AA:293		322134S:I>L		378604X:I>L		DBVPG1373:I>L		DBVPG1853:I>L		DBVPG6044:I>L		K11:I>L		UWOPS05_217_3:I>L		UWOPS05_227_2:I>L		UWOPS83_787_3:I>L		Y55:I>L		Y9:I>L		YJM978:I>L		YPS128:I>L		YPS606:I>L		YS4:I>L	AA:469		DBVPG6044:N>K		UWOPS05_217_3:N>K		UWOPS05_227_2:N>K		UWOPS87_2421:N>K		Y55:N>K		Y9:N>K		YPS606:N>K	AA:473		DBVPG6044:S>G		UWOPS05_217_3:S>G		Y55:S>G	AA:596		DBVPG6044:S>N		NCYC110:S>N		SK1:S>N		UWOPS05_227_2:S>N		Y55:S>N	AA:617		DBVPG1106:G>E		DBVPG6765:G>E		YJM975:G>E		YJM978:G>E	AA:626		273614X:P>T	AA:638		UWOPS05_227_2:F>L	AA:666		273614X:K>T		322134S:K>T		DBVPG1373:K>T	AA:671		YJM978:T>K	AA:756		YPS606:Y>H	AA:807		322134S:M>V		BC187:M>V		DBVPG1106:M>V		DBVPG1373:M>V		DBVPG1853:M>V		DBVPG6765:M>V		L_1374:M>V		NCYC110:M>V		SK1:M>V		Y55:M>V		YJM975:M>V		YPS606:M>V	AA:873		UWOPS05_227_2:S>F	AA:949		322134S:P>	AA:959		DBVPG6044:E>G		NCYC110:E>G		SK1:E>G		UWOPS05_227_2:E>G		Y55:E>G		YPS128:E>G		YPS606:E>G		YS4:E>GID:YDR123C	AA:87		UWOPS03_461_4:T>S		UWOPS05_217_3:T>S		UWOPS05_227_2:T>S	AA:89		YPS128:A>T		YPS606:A>T	AA:106		DBVPG6044:A>T		K11:A>T		SK1:A>T		UWOPS03_461_4:A>T		UWOPS05_227_2:A>T		UWOPS83_787_3:A>T		Y55:A>T		YPS128:A>T		YPS606:A>T		YS4:A>T	AA:149		DBVPG6044:E>D		K11:E>D		SK1:E>D		UWOPS03_461_4:E>D		UWOPS05_227_2:E>D		UWOPS83_787_3:E>D		YPS128:E>D		YS4:E>D	AA:190		UWOPS05_217_3:P>L	AA:258		UWOPS03_461_4:R>K		UWOPS05_217_3:R>KID:YDR125C	AA:1		YPS128:M>K		YPS606:M>K		YS9:M>K	AA:18		YS9:I>T	AA:161		DBVPG6044:M>L		SK1:M>L		Y55:M>L		YS9:M>L	AA:171		UWOPS05_217_3:S>F	AA:193		DBVPG1853:K>E	AA:350		DBVPG6044:G>E		NCYC110:G>E		SK1:G>E		Y55:G>E	AA:362		DBVPG6044:A>G		NCYC110:A>G		SK1:A>G		Y55:A>G		YPS606:A>GID:YDR126W	AA:26		Y12:T>A		YS4:T>A	AA:38		DBVPG6040:Q>	AA:51		273614X:L>F		BC187:L>F		DBVPG1106:L>F		DBVPG1373:L>F		DBVPG1788:L>F		DBVPG6044:L>F		DBVPG6765:L>F		NCYC110:L>F		SK1:L>F		UWOPS03_461_4:L>F		UWOPS83_787_3:L>F		Y12:L>F		Y55:L>F		YJM975:L>F		YJM978:L>F		YPS128:L>F		YS4:L>F	AA:56		YPS128:L>I	AA:185		YS4:S>P	AA:206		DBVPG6044:S>N		NCYC110:S>N		SK1:S>N		Y55:S>N	AA:284		UWOPS87_2421:A>SID:YDR132C	AA:7		YS2:V>A		YS9:V>A	AA:9		UWOPS03_461_4:T>I		UWOPS05_227_2:T>I	AA:73		K11:T>S		NCYC361:T>S		YPS606:T>S		YS9:T>S	AA:92		K11:E>A		NCYC361:E>A		YS9:E>A	AA:187		BC187:S>P		DBVPG1373:S>P		DBVPG1788:S>P		DBVPG6044:S>P		DBVPG6765:S>P		K11:S>P		L_1528:S>P		NCYC361:S>P		SK1:S>P		Y55:S>P		YJM978:S>P		YPS128:S>P		YPS606:S>P		YS2:S>P	AA:264		K11:K>R	AA:291		DBVPG1788:D>Y	AA:305		322134S:I>V		BC187:I>V		DBVPG1373:I>V		DBVPG1788:I>V		DBVPG1853:I>V		DBVPG6044:I>V		DBVPG6765:I>V		K11:I>V		L_1528:I>V		SK1:I>V		UWOPS03_461_4:I>V		UWOPS05_217_3:I>V		UWOPS87_2421:I>V		Y55:I>V		YIIc17_E5:I>V		YJM975:I>V		YJM978:I>V	AA:446		K11:L>F	AA:449		322134S:R>K		DBVPG6044:R>K		K11:R>K		NCYC110:R>K		SK1:R>K		UWOPS03_461_4:R>K		UWOPS05_217_3:R>K		UWOPS05_227_2:R>K		UWOPS87_2421:R>K		Y12:R>K		Y55:R>K		YPS128:R>K		YPS606:R>K		YS9:R>KID:YDR134C	AA:14		W303:A>S	AA:67		378604X:->Q		DBVPG6040:->Q		DBVPG6765:->Q		L_1528:->Q		NCYC110:->Q		SK1:->Q		Y55:->Q		Y9:->Q		YJM975:->Q		YPS128:->Q		YPS606:->Q		YS4:->QID:YDR137W	AA:13		322134S:E>G	AA:45		DBVPG1373:H>N		DBVPG1788:H>N		DBVPG6765:H>N		L_1374:H>N		L_1528:H>N		YJM978:H>N	AA:71		DBVPG1373:V>A		L_1528:V>A		YJM978:V>A	AA:100		DBVPG1853:E>G	AA:258		378604X:V>I		DBVPG1106:V>I		DBVPG1373:V>I		DBVPG1788:V>I		DBVPG1853:V>I		DBVPG6765:V>I		YJM975:V>I	AA:281		UWOPS83_787_3:V>E	AA:498		Y12:T>A	AA:569		DBVPG1788:F>V	AA:601		DBVPG6044:F>L		SK1:F>L		Y55:F>L	AA:607		273614X:I>V		378604X:I>V		DBVPG1373:I>V		DBVPG1788:I>V		DBVPG1853:I>V		DBVPG6765:I>V		L_1374:I>V		YJM975:I>V		YJM981:I>V	AA:618		DBVPG6044:E>K		SK1:E>K		Y55:E>K		YPS128:E>K		YPS606:E>KID:YDR138W	AA:12		273614X:I>L		378604X:I>L		DBVPG1373:I>L		DBVPG1788:I>L		DBVPG1853:I>L		DBVPG6765:I>L		YJM978:I>L		YJM981:I>L	AA:18		UWOPS05_227_2:T>A	AA:43		273614X:V>I		322134S:V>I		DBVPG1373:V>I		DBVPG1788:V>I		DBVPG1853:V>I		DBVPG6044:V>I		DBVPG6765:V>I		SK1:V>I		UWOPS05_227_2:V>I		Y12:V>I		Y55:V>I		YJM978:V>I		YPS128:V>I		YPS606:V>I		YS9:V>I	AA:53		273614X:K>E		DBVPG1373:K>E		DBVPG1788:K>E		DBVPG1853:K>E		DBVPG6044:K>E		DBVPG6765:K>E		L_1374:K>E		SK1:K>E		UWOPS87_2421:K>E		Y55:K>E		YJM978:K>E	AA:295		UWOPS05_217_3:I>F		UWOPS05_227_2:I>F	AA:318		UWOPS05_217_3:E>G		UWOPS05_227_2:E>G	AA:332		UWOPS83_787_3:T>I	AA:367		UWOPS83_787_3:L>F	AA:444		UWOPS05_217_3:N>	AA:502		Y9:S>Y	AA:555		DBVPG1373:A>T		YJM978:A>T		YJM981:A>T	AA:593		K11:D>N	AA:614		UWOPS05_217_3:E>K		UWOPS05_227_2:E>K	AA:733		DBVPG6044:E>G		SK1:E>GID:YDR140W	AA:9		Y12:C>G	AA:36		378604X:K>E		DBVPG1788:K>E	AA:99		UWOPS83_787_3:F>I	AA:103		378604X:I>V		DBVPG1788:I>V	AA:130		UWOPS05_217_3:C>S	AA:138		NCYC110:R>G		Y55:R>G	AA:208		DBVPG6040:K>IID:YDR142C	AA:2		378604X:L>F	AA:53		273614X:E>Q		378604X:E>Q		DBVPG1788:E>Q		YJM975:E>Q		YJM981:E>Q		YS9:E>Q	AA:94		L_1528:E>D	AA:269		273614X:L>P		378604X:L>P		DBVPG1373:L>P		DBVPG6765:L>P		YJM978:L>PID:YDR143C	AA:9		273614X:N>D		322134S:N>D		DBVPG1373:N>D		DBVPG1788:N>D		DBVPG6040:N>D		DBVPG6044:N>D		DBVPG6765:N>D		K11:N>D		L_1374:N>D		L_1528:N>D		SK1:N>D		UWOPS87_2421:N>D		Y55:N>D		Y9:N>D		YIIc17_E5:N>D		YJM975:N>D		YJM978:N>D		YPS128:N>D		YPS606:N>D		YS4:N>D	AA:75		DBVPG6044:S>F		SK1:S>F		Y55:S>F	AA:77		322134S:S>N		DBVPG1373:S>N		DBVPG1788:S>N		DBVPG6765:S>N		L_1374:S>N		L_1528:S>N		NCYC361:S>N		YIIc17_E5:S>N		YJM975:S>N		YJM978:S>N	AA:85		NCYC361:P>L	AA:128		K11:M>I	AA:154		L_1528:V>D	AA:312		DBVPG1853:R>Q	AA:351		SK1:M>V		Y55:M>V	AA:364		DBVPG1853:G>S	AA:383		BC187:V>I		DBVPG1106:V>I		DBVPG1788:V>I		DBVPG1853:V>I		DBVPG6765:V>I		L_1374:V>I		L_1528:V>I		YIIc17_E5:V>I		YJM975:V>I		YJM981:V>I		YS9:V>I	AA:410		UWOPS03_461_4:P>T	AA:457		YS9:L>V	AA:479		YS9:T>S	AA:482		DBVPG6044:G>R		SK1:G>R		Y55:G>R	AA:546		Y12:N>S	AA:583		DBVPG1853:S>TID:YDR147W	AA:8		YIIc17_E5:T>	AA:42		DBVPG1853:R>W	AA:51		322134S:E>K	AA:138		378604X:T>I		BC187:T>I		DBVPG6765:T>I		L_1374:T>I		YIIc17_E5:T>I		YS9:T>I	AA:147		Y12:V>I	AA:268		378604X:V>A		DBVPG1373:V>A		DBVPG6765:V>A		L_1374:V>A		YIIc17_E5:V>A		YS9:V>A	AA:287		L_1528:L>I	AA:319		L_1528:S>T	AA:337		UWOPS05_227_2:F>L	AA:341		DBVPG6044:G>S		NCYC110:G>S		Y55:G>S	AA:455		DBVPG6044:I>M		NCYC110:I>M		SK1:I>M		Y55:I>M	AA:471		UWOPS05_227_2:T>M	AA:481		L_1528:N>D	AA:503		273614X:S>T		BC187:S>T		DBVPG1373:S>T		DBVPG1788:S>T		DBVPG1853:S>T		DBVPG6765:S>T		L_1528:S>T		YJM975:S>T		YJM978:S>T		YS9:S>T	AA:534		L_1528:F>LID:YDR148C	AA:135		UWOPS83_787_3:E>V	AA:152		L_1374:A>S	AA:451		DBVPG6040:K>RID:YDR151C	AA:47		NCYC110:I>L		SK1:I>L		Y55:I>L	AA:76		378604X:E>K	AA:84		YJM975:W>S		YJM978:W>S	AA:150		UWOPS03_461_4:S>N		UWOPS05_227_2:S>N	AA:297		SK1:A>V		Y55:A>V	AA:306		SK1:N>Y		Y55:N>YID:YDR153C	AA:77		K11:V>A	AA:102		K11:W>-	AA:177		322134S:S>P		BC187:S>P		DBVPG1106:S>P		DBVPG1373:S>P		DBVPG1788:S>P		DBVPG1853:S>P		DBVPG6044:S>P		DBVPG6765:S>P		K11:S>P		NCYC361:S>P		SK1:S>P		UWOPS05_227_2:S>P		UWOPS87_2421:S>P		Y12:S>P		Y55:S>P		Y9:S>P		YIIc17_E5:S>P		YJM975:S>P		YJM981:S>P		YPS128:S>P		YS9:S>P	AA:213		YPS128:S>N	AA:257		DBVPG1853:S>F	AA:299		YS9:S>N	AA:307		DBVPG1853:T>A	AA:322		273614X:S>N		378604X:S>N		BC187:S>N		DBVPG1853:S>N		DBVPG6040:S>N		DBVPG6044:S>N		DBVPG6765:S>N		K11:S>N		UWOPS05_227_2:S>N		UWOPS83_787_3:S>N		Y12:S>N		Y55:S>N		Y9:S>N		YJM975:S>N		YJM978:S>N		YJM981:S>N		YPS606:S>N		YS9:S>N	AA:326		DBVPG1853:D>A	AA:345		Y9:P>S	AA:352		DBVPG6044:I>M		SK1:I>M		Y55:I>MID:YDR156W	AA:120		DBVPG6044:T>I		NCYC110:T>I		SK1:T>I		Y55:T>IID:YDR159W	AA:26		YIIc17_E5:T>I	AA:120		UWOPS05_227_2:R>K	AA:155		DBVPG1853:D>H	AA:277		UWOPS05_217_3:P>H	AA:549		K11:E>G		NCYC110:E>G		UWOPS03_461_4:E>G		UWOPS05_217_3:E>G		Y12:E>G		Y55:E>G		Y9:E>G		YPS606:E>G	AA:552		NCYC110:L>F		Y55:L>F	AA:563		NCYC110:K>E		Y55:K>E	AA:583		YS4:Y>H	AA:627		378604X:H>Q		K11:H>Q		NCYC110:H>Q		SK1:H>Q		UWOPS03_461_4:H>Q		UWOPS05_217_3:H>Q		UWOPS05_227_2:H>Q		Y12:H>Q		Y55:H>Q		Y9:H>Q		YPS606:H>Q	AA:641		L_1528:G>R	AA:643		378604X:L>P		YPS606:L>P	AA:673		378604X:E>Q		K11:E>Q		NCYC110:E>Q		SK1:E>Q		UWOPS03_461_4:E>Q		UWOPS05_217_3:E>Q		UWOPS05_227_2:E>Q		UWOPS87_2421:E>Q		Y12:E>Q		Y55:E>Q		Y9:E>Q		YPS606:E>Q	AA:728		UWOPS05_217_3:E>K	AA:735		DBVPG1853:V>I		DBVPG6040:V>I		DBVPG6044:V>I		NCYC110:V>I		SK1:V>I		UWOPS03_461_4:V>I		UWOPS05_217_3:V>I		UWOPS05_227_2:V>I		UWOPS87_2421:V>I		Y12:V>I		Y55:V>I		YPS606:V>I		YS4:V>I	AA:744		DBVPG6040:I>V		UWOPS03_461_4:I>V		UWOPS05_227_2:I>V		UWOPS87_2421:I>V		Y12:I>V	AA:745		YPS606:S>G	AA:792		DBVPG1106:S>F	AA:887		378604X:R>Q		DBVPG6040:R>Q		DBVPG6044:R>Q		SK1:R>Q		UWOPS03_461_4:R>Q		UWOPS05_217_3:R>Q		UWOPS87_2421:R>Q		Y12:R>Q		YPS128:R>Q		YPS606:R>Q	AA:902		UWOPS03_461_4:K>N		UWOPS05_217_3:K>N		YPS128:K>N		YPS606:K>N	AA:906		DBVPG1853:N>D	AA:952		DBVPG6040:I>V		DBVPG6044:I>V		SK1:I>V		UWOPS05_217_3:I>V		UWOPS87_2421:I>V		Y55:I>V		YPS128:I>V		YPS606:I>V	AA:1143		DBVPG1788:D>Y		DBVPG6765:D>Y	AA:1148		L_1528:S>I	AA:1260		L_1528:Q>KID:YDR161W	AA:40		DBVPG6040:N>S	AA:47		UWOPS87_2421:V>M	AA:60		YS2:K>E	AA:104		L_1528:M>V	AA:129		DBVPG6040:V>M		DBVPG6044:V>M		NCYC361:V>M		SK1:V>M		UWOPS05_217_3:V>M		UWOPS87_2421:V>M		Y55:V>M		YPS606:V>M	AA:203		UWOPS87_2421:G>S	AA:276		YS9:I>M	AA:329		DBVPG6044:V>I		SK1:V>I		UWOPS03_461_4:V>I		UWOPS05_217_3:V>I		UWOPS83_787_3:V>I		UWOPS87_2421:V>I		Y55:V>I		YPS128:V>I		YPS606:V>IID:YDR162C	AA:25		YIIc17_E5:V>A	AA:27		YIIc17_E5:N>K	AA:41		K11:Q>R		Y9:Q>R	AA:142		DBVPG1853:K>R	AA:146		UWOPS83_787_3:G>S	AA:172		K11:E>Q		Y9:E>Q	AA:201		UWOPS03_461_4:I>TID:YDR165W	AA:58		273614X:T>I		DBVPG1373:T>I		L_1528:T>I		YIIc17_E5:T>I	AA:96		DBVPG6044:L>I		NCYC110:L>I		UWOPS03_461_4:L>I		UWOPS05_227_2:L>I		UWOPS83_787_3:L>I		UWOPS87_2421:L>I		Y55:L>I		YPS128:L>I		YPS606:L>I		YS2:L>I	AA:119		YS2:A>V	AA:203		DBVPG6044:L>W		NCYC110:L>W		SK1:L>W		Y55:L>W	AA:232		W303:I>V	AA:281		273614X:N>S		322134S:N>S		BC187:N>S		DBVPG1373:N>S		DBVPG6040:N>S		DBVPG6765:N>S		L_1528:N>S		SK1:N>S		UWOPS03_461_4:N>S		UWOPS83_787_3:N>S		UWOPS87_2421:N>S		Y55:N>S		Y9:N>S		YJM975:N>S		YJM978:N>S		YPS128:N>S		YPS606:N>S		YS9:N>S	AA:337		DBVPG1853:M>I		DBVPG6044:M>I		SK1:M>I		UWOPS03_461_4:M>I		UWOPS05_227_2:M>I		UWOPS83_787_3:M>I		Y55:M>I		Y9:M>I		YPS128:M>I		YPS606:M>I	AA:352		Y9:T>S		YPS128:T>S		YPS606:T>S	AA:357		DBVPG6044:V>I		SK1:V>I		Y55:V>I	AA:377		UWOPS03_461_4:S>F		UWOPS05_227_2:S>F	AA:385		YS9:K>EID:YDR167W	AA:36		322134S:N>K	AA:45		YS9:K>-	AA:67		YS9:F>S	AA:98		Y9:T>I	AA:107		DBVPG1853:V>L	AA:182		UWOPS83_787_3:V>D	AA:201		UWOPS03_461_4:R>HID:YDR169C	AA:5		UWOPS87_2421:Q>L	AA:23		378604X:S>N		DBVPG1853:S>N		DBVPG6044:S>N		SK1:S>N		UWOPS05_227_2:S>N		UWOPS83_787_3:S>N		UWOPS87_2421:S>N		Y12:S>N		Y55:S>N		Y9:S>N		YPS128:S>N		YPS606:S>N	AA:55		378604X:M>K		DBVPG1853:M>K		DBVPG6044:M>K		SK1:M>K		UWOPS05_227_2:M>K		UWOPS83_787_3:M>K		UWOPS87_2421:M>K		Y12:M>K		Y55:M>K		Y9:M>K		YPS606:M>K	AA:76		378604X:D>N	AA:109		378604X:W>C	AA:163		378604X:V>I		YS4:E>K	AA:261		DBVPG1853:S>L	AA:334		YIIc17_E5:T>N	AA:371		YS4:N>H	AA:450		DBVPG6044:A>V		NCYC110:A>V		SK1:A>V		UWOPS05_217_3:A>V		UWOPS05_227_2:A>V		UWOPS87_2421:A>V		Y12:A>V		Y55:A>V		Y9:A>V		YS2:A>V		YS4:A>V	AA:507		YS2:L>V		YS4:L>VID:YDR171W	AA:153		NCYC361:P>Q	AA:164		DBVPG1106:A>T	AA:184		UWOPS05_227_2:A>T	AA:201		DBVPG6044:L>P		K11:L>P		SK1:L>P		UWOPS05_227_2:L>P		UWOPS83_787_3:L>P		UWOPS87_2421:L>P		Y55:L>P		YPS128:L>P		YPS606:L>P	AA:210		UWOPS83_787_3:A>V	AA:215		DBVPG1788:S>A	AA:226		K11:P>R	AA:236		UWOPS05_217_3:T>K		UWOPS05_227_2:T>K	AA:267		YPS128:R>K		YPS606:R>K	AA:290		DBVPG1853:V>M	AA:315		DBVPG1853:V>M	AA:333		UWOPS87_2421:L>IID:YDR172W	AA:109		DBVPG6044:N>S		SK1:N>S		UWOPS05_217_3:N>S		UWOPS05_227_2:N>S		UWOPS83_787_3:N>S		Y55:N>S		YPS128:N>S		YS9:N>S	AA:138		YJM978:P>L	AA:162		273614X:G>D		322134S:G>D		378604X:G>D		BC187:G>D		DBVPG1106:G>D		DBVPG6765:G>D		L_1374:G>D		L_1528:G>D		UWOPS05_217_3:G>D		UWOPS83_787_3:G>D		YIIc17_E5:G>D		YJM975:G>D		YJM978:G>D		YPS128:G>D		YS9:G>D	AA:168		UWOPS83_787_3:S>F	AA:169		DBVPG6044:D>E		SK1:D>E		UWOPS05_217_3:D>E		Y55:D>E		YS9:D>E	AA:184		L_1374:K>R	AA:202		S288c:T>I	AA:206		DBVPG6044:T>K		SK1:T>K		UWOPS05_217_3:T>K		UWOPS83_787_3:T>K		Y55:T>K		YPS128:T>K		YS9:T>K	AA:225		DBVPG6044:H>D		SK1:H>D		UWOPS05_217_3:H>D		UWOPS05_227_2:H>D		UWOPS83_787_3:H>D		UWOPS87_2421:H>D		Y55:H>D		YPS606:H>D		YS2:H>D		YS9:H>D	AA:297		DBVPG1853:R>-	AA:404		L_1528:V>I	AA:565		UWOPS03_461_4:P>S		UWOPS05_227_2:P>S	AA:681		DBVPG1853:V>IID:YDR173C	AA:85		DBVPG1853:A>T	AA:199		L_1528:N>T	AA:247		K11:M>T	AA:288		YS4:D>N		YS9:D>N	AA:294		YIIc17_E5:N>D	AA:311		DBVPG1853:D>N		DBVPG6040:D>N		K11:D>N		NCYC110:D>N		SK1:D>N		UWOPS03_461_4:D>N		UWOPS05_217_3:D>N		UWOPS83_787_3:D>N		Y55:D>N		YPS128:D>N		YS4:D>N		YS9:D>NID:YDR175C	AA:20		DBVPG6044:K>R		NCYC110:K>R		SK1:K>R		UWOPS03_461_4:K>R		UWOPS05_227_2:K>R		Y55:K>R		YPS128:K>R	AA:45		Y12:A>V		Y9:A>V	AA:198		UWOPS05_227_2:R>T	AA:296		UWOPS05_227_2:E>KID:YDR176W	AA:4		YS2:H>D	AA:122		SK1:T>A		Y55:T>A	AA:124		DBVPG6765:E>G	AA:248		YS4:G>E		YS9:G>E	AA:264		L_1528:A>T	AA:394		K11:G>D		SK1:G>D		UWOPS05_227_2:G>D		Y55:G>D		YPS128:G>D	AA:412		UWOPS05_227_2:D>E	AA:419		L_1528:L>F	AA:449		DBVPG6044:T>S		K11:T>S		SK1:T>S		UWOPS05_227_2:T>S		UWOPS83_787_3:T>S		Y55:T>S		YPS128:T>S	AA:455		L_1528:S>P	AA:467		378604X:N>S		DBVPG6044:N>S		K11:N>S		L_1528:N>S		SK1:N>S		UWOPS05_227_2:N>S		UWOPS83_787_3:N>S		Y55:N>S		YPS128:N>S		YPS606:N>S	AA:477		DBVPG1853:A>T		DBVPG6044:A>T		K11:A>T		SK1:A>T		UWOPS05_227_2:A>T		UWOPS83_787_3:A>T		Y55:A>T		YPS128:A>T		YPS606:A>T	AA:543		L_1528:V>I	AA:601		W303:D>G	AA:623		L_1528:N>TID:YDR177W	AA:49		YS9:G>D	AA:146		YJM978:R>K		YJM981:R>K	AA:150		UWOPS83_787_3:S>G	AA:195		UWOPS83_787_3:V>IID:YDR178W	AA:74		UWOPS83_787_3:I>V	AA:123		YS2:E>G		YS4:E>G	AA:145		UWOPS83_787_3:L>F	AA:166		DBVPG1788:W>G	AA:173		DBVPG1853:N>KID:YDR179C	AA:50		YJM975:I>R	AA:80		YJM978:I>F	AA:126		YS2:Y>H		YS4:Y>H	AA:136		273614X:K>E		378604X:K>E		DBVPG1788:K>E		DBVPG6040:K>E		DBVPG6765:K>E		L_1374:K>E		L_1528:K>E		SK1:K>E		UWOPS03_461_4:K>E		Y55:K>E		YJM975:K>E		YJM978:K>EID:YDR182W	AA:155		DBVPG6044:K>T		SK1:K>T		UWOPS03_461_4:K>T		UWOPS05_227_2:K>T	AA:166		UWOPS83_787_3:Q>E	AA:212		UWOPS83_787_3:D>E	AA:230		UWOPS83_787_3:T>R	AA:232		K11:P>L	AA:237		DBVPG6044:V>I		K11:V>I		SK1:V>I		UWOPS03_461_4:V>I		UWOPS05_227_2:V>I		Y55:V>I	AA:265		UWOPS83_787_3:D>N	AA:279		UWOPS83_787_3:E>K		YPS128:E>K		YPS606:E>K	AA:305		UWOPS83_787_3:I>T	AA:326		273614X:P>S	AA:350		SK1:R>K		Y55:R>K	AA:375		SK1:S>L		Y55:S>L	AA:426		YPS128:R>K		YPS606:R>KID:YDR183W	AA:88		DBVPG6044:D>E		SK1:D>E		Y55:D>E	AA:161		DBVPG6044:K>R		SK1:K>R		Y55:K>R	AA:217		DBVPG6044:T>R		SK1:T>R		Y55:T>RID:YDR184C	AA:45		Y12:D>Y		Y9:D>Y	AA:74		L_1374:L>I	AA:145		W303:T>S	AA:156		W303:T>S	AA:217		UWOPS05_227_2:I>L	AA:254		322134S:S>T		378604X:S>T		BC187:S>T		DBVPG1853:S>T		DBVPG6044:S>T		DBVPG6765:S>T		L_1374:S>T		L_1528:S>T		SK1:S>T		UWOPS05_227_2:S>T		Y55:S>T		Y9:S>T		YS2:S>TID:YDR185C	AA:92		K11:T>IID:YDR188W	AA:142		Y12:N>D	AA:393		NCYC361:D>N	AA:412		DBVPG6044:I>V		NCYC110:I>V		SK1:I>V	AA:426		YS9:L>I	AA:517		UWOPS05_217_3:I>N	AA:518		YS9:T>AID:YDR191W	AA:3		Y12:Q>E		Y9:Q>E		YPS606:Q>E	AA:15		378604X:A>V		K11:A>V		NCYC110:A>V		NCYC361:A>V		SK1:A>V		UWOPS05_217_3:A>V		UWOPS05_227_2:A>V		Y12:A>V		Y55:A>V		Y9:A>V		YPS606:A>V	AA:62		S288c:D>G	AA:68		DBVPG6044:T>A		NCYC110:T>A		SK1:T>A		Y55:T>A	AA:77		YJM975:H>R		YJM978:H>R	AA:105		YS4:V>I	AA:194		NCYC361:P>S	AA:207		DBVPG6040:P>	AA:216		DBVPG6044:I>V		SK1:I>V		Y55:I>V	AA:237		YJM975:C>S		YJM978:C>S	AA:263		273614X:M>I		BC187:M>I		DBVPG1106:M>I		DBVPG1788:M>I		DBVPG6765:M>I		L_1374:M>I		NCYC361:M>I		SK1:M>I		UWOPS05_217_3:M>I		Y55:M>I		Y9:M>I		YIIc17_E5:M>I		YJM975:M>I		YJM978:M>I		YPS606:M>I		YS9:M>IID:YDR194C	AA:32		DBVPG1853:D>V		YIIc17_E5:A>T	AA:42		BC187:N>S		DBVPG1788:N>S		L_1528:N>S		YIIc17_E5:N>S	AA:51		DBVPG1373:N>Y		DBVPG6044:N>Y		NCYC110:N>Y		SK1:N>Y		UWOPS03_461_4:N>Y		UWOPS05_227_2:N>Y		UWOPS87_2421:N>Y		Y12:N>Y		Y55:N>Y		YPS128:N>Y		YS9:N>Y	AA:71		DBVPG6044:T>R		NCYC110:T>R		SK1:T>R		Y55:T>R	AA:146		L_1528:H>Y	AA:204		L_1528:H>N		YS9:H>P	AA:370		K11:R>K	AA:387		UWOPS05_227_2:S>Y	AA:419		378604X:V>F	AA:461		UWOPS05_227_2:I>V	AA:498		UWOPS83_787_3:V>I	AA:519		YS9:V>L	AA:554		YPS606:E>D	AA:604		378604X:N>D		BC187:N>D		DBVPG1788:N>D		DBVPG1853:N>D		K11:N>D		NCYC110:N>D		SK1:N>D		UWOPS05_227_2:N>D		UWOPS87_2421:N>D		Y55:N>D		YPS128:N>D		YPS606:N>D	AA:649		Y9:R>L	AA:663		378604X:I>R		DBVPG6044:I>R		K11:I>R		NCYC110:I>R		SK1:I>R		UWOPS05_227_2:I>R		UWOPS87_2421:I>R		Y55:I>R		YPS128:I>R		YPS606:I>R		YS4:I>RID:YDR195W	AA:19		UWOPS05_227_2:T>P	AA:127		DBVPG6044:P>A		K11:P>A		SK1:P>A		UWOPS87_2421:P>A		Y55:P>A		YPS128:P>A		YPS606:P>A	AA:156		UWOPS87_2421:A>T	AA:202		UWOPS83_787_3:S>G	AA:266		DBVPG1373:A>V		DBVPG1788:A>V		K11:A>V		L_1528:A>V		SK1:A>V		UWOPS03_461_4:A>V		Y12:A>V		YPS128:A>V		YPS606:A>V	AA:294		SK1:T>A		UWOPS03_461_4:T>A		Y12:T>A		Y55:T>A		YPS128:T>A		YPS606:T>A	AA:327		YJM975:S>PID:YDR196C	AA:26		DBVPG6040:K>E	AA:45		NCYC361:N>S	AA:55		UWOPS03_461_4:K>R		UWOPS83_787_3:K>R	AA:164		322134S:N>K		DBVPG6044:N>K		NCYC110:N>K		SK1:N>K		UWOPS03_461_4:N>K		UWOPS05_227_2:N>K		UWOPS83_787_3:N>K		Y55:N>K		Y9:N>K		YPS128:N>K		YPS606:N>K		YS4:N>K	AA:169		322134S:T>A		DBVPG6040:T>A		DBVPG6044:T>A		NCYC110:T>A		SK1:T>A		UWOPS03_461_4:T>A		UWOPS05_227_2:T>A		Y55:T>A		Y9:T>A		YPS128:T>A		YPS606:T>A		YS4:T>A	AA:184		DBVPG1788:S>G		YJM975:S>GID:YDR197W	AA:67		322134S:T>I		DBVPG6044:T>I		SK1:T>I		UWOPS05_217_3:T>I		Y12:T>I		Y55:T>I		Y9:T>I		YPS128:T>I	AA:151		YIIc17_E5:T>	AA:159		UWOPS05_217_3:I>V		UWOPS05_227_2:I>V		UWOPS83_787_3:I>V	AA:175		DBVPG1373:F>L		YJM978:F>L	AA:187		L_1528:S>C	AA:189		322134S:T>A		378604X:T>A		DBVPG6044:T>A		K11:T>A		SK1:T>A		UWOPS05_217_3:T>A		UWOPS05_227_2:T>A		UWOPS83_787_3:T>A		Y12:T>A		Y9:T>A		YPS128:T>A		YPS606:T>A	AA:204		UWOPS03_461_4:N>S		UWOPS05_217_3:N>S		UWOPS05_227_2:N>S		UWOPS83_787_3:N>S	AA:210		K11:A>T		SK1:A>T		UWOPS05_217_3:A>T		UWOPS05_227_2:A>T		UWOPS83_787_3:A>T	AA:301		YPS606:T>AID:YDR201W	AA:17		DBVPG1788:S>L		YJM975:S>L		YJM978:S>L	AA:32		DBVPG6765:P>S	AA:36		Y12:L>	AA:89		DBVPG6040:D>A	AA:127		DBVPG1788:D>G		YJM975:D>G		YJM978:D>GID:YDR202C	AA:33		NCYC110:E>D	AA:98		YS4:V>F	AA:179		UWOPS03_461_4:E>K		UWOPS05_227_2:E>K	AA:219		DBVPG1373:R>-ID:YDR204W	AA:9		UWOPS83_787_3:S>P	AA:14		DBVPG6044:P>A		NCYC110:P>A		SK1:P>A		Y55:P>A	AA:34		322134S:S>T	AA:79		DBVPG1853:P>S		L_1528:P>S		YIIc17_E5:P>S		YJM975:P>S		YJM978:P>S	AA:170		BC187:F>LID:YDR205W	AA:87		Y12:R>Q		Y9:R>Q		YS4:R>Q	AA:176		UWOPS03_461_4:A>V		UWOPS05_217_3:A>V		UWOPS05_227_2:A>V	AA:210		UWOPS03_461_4:A>T		UWOPS05_217_3:A>T		UWOPS05_227_2:A>T	AA:212		378604X:H>R		DBVPG6044:H>R		K11:H>R		NCYC110:H>R		SK1:H>R		UWOPS03_461_4:H>R		UWOPS05_217_3:H>R		UWOPS05_227_2:H>R		Y12:H>R		Y55:H>R		YPS128:H>R		YPS606:H>R		YS4:H>R	AA:218		378604X:K>N	AA:220		UWOPS03_461_4:F>L		UWOPS05_217_3:F>L		UWOPS05_227_2:F>L	AA:287		L_1528:M>V	AA:456		273614X:L>V		DBVPG1106:L>V		DBVPG1788:L>V		DBVPG1853:L>V		DBVPG6040:L>V		DBVPG6765:L>V		L_1374:L>V		L_1528:L>V		YIIc17_E5:L>V		YJM975:L>V	AA:528		DBVPG6044:I>V		SK1:I>V	AA:557		UWOPS03_461_4:F>C	AA:638		273614X:N>D		378604X:N>D		DBVPG1788:N>D		DBVPG1853:N>D		DBVPG6044:N>D		DBVPG6765:N>D		K11:N>D		L_1374:N>D		SK1:N>D		UWOPS03_461_4:N>D		UWOPS05_227_2:N>D		UWOPS83_787_3:N>D		UWOPS87_2421:N>D		Y12:N>D		Y55:N>D		Y9:N>D		YJM975:N>D		YPS606:N>D		YS2:N>D		YS9:N>D	AA:709		YJM975:T>A		YJM978:T>AID:YDR206W	AA:52		322134S:A>V	AA:56		273614X:S>F	AA:176		273614X:I>S		322134S:I>S		BC187:I>S		DBVPG1373:I>S		DBVPG1788:I>S		DBVPG1853:I>S		DBVPG6040:I>S		DBVPG6044:I>S		DBVPG6765:I>S		SK1:I>S		UWOPS05_217_3:I>S		UWOPS05_227_2:I>S		UWOPS87_2421:I>S		Y55:I>S		Y9:I>S		YIIc17_E5:I>S		YJM978:I>S		YPS128:I>S		YPS606:I>S		YS4:I>S	AA:184		322134S:S>R	AA:190		YPS128:H>R		YPS606:H>R	AA:194		322134S:T>M	AA:468		273614X:V>I		DBVPG6044:V>I		SK1:V>I		Y12:V>I		Y55:V>I		YPS606:V>I	AA:501		273614X:E>G		Y12:E>G		Y9:E>G		YS4:E>G	AA:570		NCYC361:N>D	AA:590		L_1374:F>L	AA:597		378604X:S>N		DBVPG6044:S>N		K11:S>N		NCYC110:S>N		SK1:S>N		UWOPS03_461_4:S>N		UWOPS05_217_3:S>N		UWOPS05_227_2:S>N		Y12:S>N		Y55:S>N		Y9:S>N		YS4:S>N	AA:598		DBVPG1373:M>I		L_1374:M>I		NCYC361:M>I		YIIc17_E5:M>I		YJM975:M>I	AA:606		378604X:E>A	AA:637		YIIc17_E5:L>V	AA:642		UWOPS83_787_3:P>S	AA:705		DBVPG1373:N>D	AA:757		UWOPS83_787_3:G>D		UWOPS87_2421:G>D	AA:833		378604X:Y>H		BC187:Y>H		DBVPG1106:Y>H		DBVPG1853:Y>H		DBVPG6044:Y>H		DBVPG6765:Y>H		L_1528:Y>H		NCYC110:Y>H		SK1:Y>H		UWOPS03_461_4:Y>H		UWOPS05_217_3:Y>H		UWOPS05_227_2:Y>H		UWOPS83_787_3:Y>H		Y55:Y>H		Y9:Y>H		YJM975:Y>H		YJM978:Y>H		YJM981:Y>H		YPS128:Y>H		YPS606:Y>H		YS2:Y>H		YS9:Y>H	AA:853		378604X:P>H		YS9:P>H	AA:861		UWOPS83_787_3:Y>F	AA:865		UWOPS87_2421:P>S	AA:878		378604X:N>S		DBVPG6044:N>S		NCYC110:N>S		SK1:N>S		Y55:N>S		Y9:N>S		YPS128:N>S		YPS606:N>S		YS9:N>SID:YDR208W	AA:4		NCYC361:L>M	AA:14		322134S:L>I	AA:42		DBVPG1788:R>L		DBVPG6040:R>L		DBVPG6044:R>L		DBVPG6765:R>L		L_1528:R>L		NCYC110:R>L		NCYC361:R>L		SK1:R>L		UWOPS03_461_4:R>L		UWOPS05_217_3:R>L		UWOPS87_2421:R>L		Y9:R>L		YIIc17_E5:R>L		YJM975:R>L		YJM981:R>L		YPS606:R>L		YS2:R>L		YS4:R>L	AA:96		NCYC361:L>S	AA:97		YS9:N>T	AA:119		378604X:D>N		DBVPG6044:D>N		NCYC110:D>N		SK1:D>N		UWOPS03_461_4:D>N		UWOPS05_217_3:D>N		UWOPS87_2421:D>N		Y55:D>N		Y9:D>N		YPS606:D>N		YS2:D>N	AA:161		DBVPG6044:T>A		NCYC110:T>A		SK1:T>A		UWOPS87_2421:T>A		Y55:T>A	AA:176		L_1528:S>P	AA:249		UWOPS03_461_4:I>M		UWOPS05_217_3:I>M		UWOPS05_227_2:I>M		UWOPS87_2421:I>M	AA:361		DBVPG1106:R>K	AA:590		DBVPG6044:R>K		SK1:R>K		Y55:R>K	AA:742		UWOPS05_217_3:R>K		UWOPS05_227_2:R>K	AA:759		YS9:L>M	AA:768		YS9:Y>H	AA:772		378604X:P>ID:YDR210W	AA:16		YJM975:G>A		YJM978:G>A	AA:23		DBVPG1853:P>SID:YDR211W	AA:12		378604X:L>Q		DBVPG1373:L>Q		DBVPG6044:L>Q		DBVPG6765:L>Q		K11:L>Q		L_1374:L>Q		L_1528:L>Q		SK1:L>Q		UWOPS03_461_4:L>Q		UWOPS87_2421:L>Q		Y55:L>Q		YJM978:L>Q		YJM981:L>Q		YPS606:L>Q		YS9:L>Q	AA:83		YS9:A>G	AA:85		DBVPG6044:Q>L		SK1:Q>L		Y55:Q>L	AA:299		YGPM:L>S	AA:426		YJM978:L>I	AA:441		YPS606:G>SID:YDR212W	AA:171		DBVPG1373:A>S		L_1528:A>S	AA:176		K11:F>	AA:191		BC187:N>H	AA:211		BC187:K>I	AA:238		YS4:A>T	AA:269		YIIc17_E5:Q>R	AA:474		K11:V>I	AA:556		378604X:P>TID:YDR213W	AA:107		322134S:S>P		DBVPG6040:S>P		UWOPS05_217_3:S>P		UWOPS05_227_2:S>P		UWOPS83_787_3:S>P		YJM975:S>P	AA:116		273614X:T>S		DBVPG6044:T>S		NCYC110:T>S		Y55:T>S		YPS606:T>S		YS2:T>S		YS4:T>S	AA:127		273614X:I>V		DBVPG6044:I>V		NCYC110:I>V		UWOPS05_217_3:I>V		UWOPS05_227_2:I>V		UWOPS83_787_3:I>V		Y55:I>V		YPS606:I>V		YS2:I>V		YS4:I>V	AA:149		DBVPG6044:K>E		NCYC110:K>E		Y55:K>E	AA:223		DBVPG6044:L>I		NCYC110:L>I		SK1:L>I		Y55:L>I	AA:263		YS9:S>C	AA:278		273614X:C>R	AA:280		273614X:R>G	AA:475		322134S:K>M		DBVPG6040:K>M		DBVPG6044:K>R		NCYC110:K>R		SK1:K>R		Y55:K>R	AA:486		DBVPG6765:G>E		L_1374:G>E	AA:489		UWOPS05_217_3:S>G	AA:498		DBVPG6044:A>T		NCYC110:A>T		SK1:A>T		Y55:A>T	AA:522		K11:T>M	AA:528		378604X:N>I	AA:532		YJM981:E>K	AA:565		DBVPG1373:E>K		DBVPG6765:E>K		L_1374:E>K	AA:566		YS9:D>A	AA:572		YJM975:D>E		YJM981:D>E	AA:787		DBVPG1106:I>V	AA:793		YS9:V>D	AA:807		YS9:D>N	AA:811		YS9:R>H	AA:815		Y12:Q>R	AA:835		YS9:A>E	AA:846		YS9:R>K	AA:909		K11:S>F	AA:913		K11:L>F		Y12:L>FID:YDR214W	AA:73		NCYC110:V>G		W303:V>G	AA:83		NCYC110:D>E		SK1:D>E		Y55:D>E	AA:88		Y55:P>L	AA:94		378604X:N>K	AA:185		K11:K>E	AA:204		YIIc17_E5:K>R		YJM975:K>R		YJM981:K>R	AA:293		273614X:P>A		322134S:P>A		DBVPG1373:P>A		DBVPG1788:P>A		DBVPG6044:P>A		DBVPG6765:P>A		K11:P>A		L_1528:P>A		SK1:P>A		UWOPS03_461_4:P>A		UWOPS05_217_3:P>A		UWOPS83_787_3:P>A		UWOPS87_2421:P>A		Y9:P>A		YIIc17_E5:P>A		YJM975:P>A		YJM981:P>A		YPS128:P>AID:YDR217C	AA:31		YS4:G>D	AA:98		UWOPS03_461_4:D>G	AA:169		UWOPS03_461_4:K>R		UWOPS05_217_3:K>R	AA:201		K11:M>V		YS4:M>V	AA:265		DBVPG6044:R>W		K11:R>W		SK1:R>W		UWOPS05_217_3:R>W		UWOPS05_227_2:R>W		UWOPS87_2421:R>W		Y55:R>W		YPS606:R>W		YS4:R>W	AA:267		DBVPG6044:T>A		SK1:T>A		Y55:T>A	AA:270		SK1:V>I		Y55:V>I	AA:296		YJM978:N>T	AA:298		YJM978:S>-	AA:374		322134S:I>V		BC187:I>V		DBVPG1373:I>V		DBVPG1853:I>V		DBVPG6044:I>V		K11:I>V		SK1:I>V		UWOPS05_217_3:I>V		UWOPS05_227_2:I>V		Y55:I>V		YJM975:I>V		YJM978:I>V		YJM981:I>V		YPS606:I>V		YS4:I>V		YS9:I>V	AA:387		YGPM:N>S	AA:442		DBVPG6044:K>R		K11:K>R		SK1:K>R		UWOPS05_217_3:K>R		UWOPS05_227_2:K>R		Y55:K>R		YPS606:K>R		YS4:K>R	AA:472		DBVPG6044:F>L		K11:F>L		L_1528:F>L		SK1:F>L		UWOPS05_217_3:F>L		Y55:F>L		YPS606:F>L		YS4:F>L	AA:477		DBVPG6044:T>N		K11:T>N		SK1:T>N		UWOPS05_217_3:T>N		Y55:T>N		YPS606:T>N		YS4:T>N	AA:519		DBVPG1373:T>S		DBVPG1853:T>S		DBVPG6044:T>S		DBVPG6765:T>S		K11:T>S		SK1:T>S		UWOPS03_461_4:T>S		UWOPS05_227_2:T>S		Y55:T>S		Y9:T>S		YPS128:T>S		YPS606:T>S		YS4:T>S		YS9:H>P	AA:522		K11:E>K	AA:523		L_1528:V>E	AA:536		378604X:S>P	AA:551		L_1528:S>F	AA:585		L_1528:P>Q	AA:587		DBVPG6044:E>G		SK1:E>G		Y55:E>G	AA:600		DBVPG6765:V>I		L_1374:V>I	AA:622		L_1528:L>V	AA:628		322134S:T>P		DBVPG6044:T>P		K11:T>P		SK1:T>P		Y55:T>P		Y9:T>P		YPS128:T>P		YPS606:T>P	AA:649		L_1528:C>Y	AA:655		322134S:D>G		K11:D>G		Y9:D>G	AA:693		L_1528:Q>R	AA:713		Y9:E>K	AA:731		322134S:L>F		DBVPG6044:L>F		K11:L>F		SK1:L>F		Y55:L>F		Y9:L>F		YPS128:L>F		YS4:L>F	AA:740		L_1528:P>A	AA:752		K11:G>D	AA:814		K11:I>V		Y9:I>V		YS4:I>V	AA:958		UWOPS05_227_2:E>-	AA:964		UWOPS05_217_3:E>V	AA:973		YPS606:S>F	AA:995		Y12:T>I		Y9:T>I	AA:996		378604X:G>R	AA:1048		YPS606:N>D	AA:1063		DBVPG1373:A>T		DBVPG1788:A>T		DBVPG1853:A>T		DBVPG6044:A>T		K11:A>T		L_1528:A>T		SK1:A>T		UWOPS05_217_3:A>T		UWOPS05_227_2:A>T		Y12:A>T		Y55:A>T		Y9:A>T		YS9:A>T	AA:1107		UWOPS05_217_3:A>V		UWOPS05_227_2:A>V	AA:1197		UWOPS87_2421:S>N	AA:1284		322134S:T>IID:YDR219C	AA:15		DBVPG1106:R>S	AA:44		W303:C>F	AA:123		NCYC361:S>N	AA:136		UWOPS03_461_4:S>F		UWOPS05_217_3:S>F		UWOPS05_227_2:S>F		YIIc17_E5:S>F	AA:198		DBVPG6044:H>N		SK1:H>N		Y55:H>N		Y9:H>N		YS4:H>N	AA:251		DBVPG6044:M>T		K11:M>T		SK1:M>T		Y55:M>T		Y9:M>T		YPS606:M>T	AA:264		DBVPG6044:M>V		K11:M>V		SK1:M>V		Y55:M>V		Y9:M>V		YPS606:M>V	AA:376		L_1528:K>E	AA:412		YPS606:V>I	AA:415		K11:L>S		YPS606:L>S	AA:432		L_1528:N>H	AA:455		L_1528:K>NID:YDR222W	AA:5		UWOPS03_461_4:S>N		UWOPS05_227_2:S>N	AA:125		DBVPG6044:Y>F		K11:Y>F		SK1:Y>F		Y55:Y>F	AA:212		273614X:M>T		BC187:M>T		DBVPG6765:M>T		K11:M>T		L_1528:M>T		SK1:M>T		UWOPS05_217_3:M>T		Y55:M>T		Y9:M>T		YPS128:M>T		YPS606:M>T		YS4:M>T		YS9:M>T	AA:290		YPS128:V>I		YPS606:V>I	AA:328		DBVPG6040:K>N	AA:369		DBVPG6040:V>A	AA:395		DBVPG1788:C>SID:YDR225W	AA:23		YS9:A>P	AA:75		YS2:N>K		YS4:N>KID:YDR226W	AA:76		L_1528:D>G	AA:195		L_1528:K>N	AA:222		BC187:D>N		DBVPG1106:D>N		DBVPG1373:D>N		DBVPG1788:D>N		DBVPG6765:D>N		L_1374:D>N		UWOPS03_461_4:D>N		UWOPS05_217_3:D>N		UWOPS05_227_2:D>N		UWOPS83_787_3:D>N		UWOPS87_2421:D>N		YPS128:D>N		YPS606:D>NID:YDR228C	AA:8		DBVPG6044:I>T		SK1:I>T		Y55:I>T	AA:229		UWOPS03_461_4:R>T	AA:268		UWOPS05_227_2:S>F	AA:280		UWOPS03_461_4:D>H	AA:358		YS4:M>I	AA:481		YS9:N>K	AA:488		YS4:Q>R	AA:507		UWOPS05_217_3:T>A		UWOPS05_227_2:T>A	AA:545		BC187:E>K		DBVPG1106:E>K		DBVPG1373:E>K		DBVPG1788:E>K		DBVPG1853:E>K		DBVPG6040:E>K		DBVPG6044:E>K		DBVPG6765:E>K		K11:E>K		L_1374:E>K		L_1528:E>K		SK1:E>K		UWOPS05_217_3:E>K		UWOPS05_227_2:E>K		Y12:E>K		Y55:E>K		Y9:E>K		YIIc17_E5:E>K		YPS128:E>K		YPS606:E>K		YS9:E>KID:YDR229W	AA:1		YJM975:M>I	AA:12		YJM975:S>Y	AA:23		Y55:W>-	AA:116		378604X:L>F		DBVPG6040:L>F		DBVPG6044:L>F		K11:L>F		SK1:L>F		UWOPS03_461_4:L>F		UWOPS05_217_3:L>F		UWOPS05_227_2:L>F		UWOPS87_2421:L>F		Y12:L>F		Y55:L>F		Y9:L>F		YPS128:L>F		YPS606:L>F		YS2:L>F	AA:264		UWOPS87_2421:L>	AA:341		YPS128:G>R	AA:350		322134S:L>I	AA:375		Y12:P>R	AA:391		DBVPG6040:G>D		DBVPG6044:G>D		SK1:G>D		UWOPS87_2421:G>D		Y55:G>D		Y9:G>D		YPS128:G>D		YS2:G>DID:YDR232W	AA:20		YJM975:N>D	AA:53		DBVPG1106:H>N		DBVPG1373:H>N		DBVPG6040:H>N		DBVPG6765:H>N		L_1374:H>N		NCYC110:H>N		SK1:H>N		UWOPS05_217_3:H>N		UWOPS05_227_2:H>N		UWOPS83_787_3:H>N		Y55:H>N		YJM975:H>N		YPS606:H>N	AA:74		L_1374:E>K	AA:141		Y12:D>V	AA:205		DBVPG1853:D>Y	AA:397		UWOPS83_787_3:H>N		YPS128:H>N		YPS606:H>N	AA:513		378604X:S>P		DBVPG1788:S>P		DBVPG1853:S>P		DBVPG6044:S>P		NCYC361:S>P		SK1:S>P		UWOPS03_461_4:S>P		UWOPS05_217_3:S>P		UWOPS05_227_2:S>P		UWOPS87_2421:S>P		Y55:S>P		Y9:S>P		YJM981:S>P		YPS128:S>P		YPS606:S>PID:YDR233C	AA:42		L_1374:A>T	AA:87		UWOPS03_461_4:P>T		UWOPS05_217_3:P>T		UWOPS05_227_2:P>T	AA:94		NCYC110:A>V		SK1:A>V		Y55:A>V	AA:100		DBVPG1853:H>R		DBVPG6765:H>R		L_1374:H>R		L_1528:H>R		NCYC110:H>R		SK1:H>R		UWOPS03_461_4:H>R		UWOPS05_217_3:H>R		UWOPS05_227_2:H>R		Y12:H>R		Y55:H>R		YJM975:H>R		YJM978:H>R		YJM981:H>R		YPS128:H>R		YS9:H>R	AA:127		DBVPG6040:T>S	AA:153		322134S:A>G		DBVPG1853:A>G		DBVPG6765:A>G		L_1528:A>G		UWOPS03_461_4:A>G		UWOPS05_217_3:A>G		UWOPS05_227_2:A>G		YJM981:A>G		YS2:A>G		YS9:A>G	AA:194		DBVPG1106:C>G		DBVPG6765:C>G		YJM978:C>G		YJM981:C>G		YS2:C>G		YS9:C>G	AA:222		YJM978:V>M		YJM981:V>M	AA:234		DBVPG6040:S>T		NCYC110:S>T		SK1:S>T		UWOPS03_461_4:S>T		UWOPS05_217_3:S>T		UWOPS05_227_2:S>T		Y12:S>T		Y55:S>T		YPS128:S>T		YPS606:S>T	AA:236		YJM975:L>FID:YDR234W	AA:7		DBVPG6040:F>L		K11:F>L		NCYC361:F>L		UWOPS05_217_3:F>L		UWOPS05_227_2:F>L		UWOPS87_2421:F>L		YPS128:F>L		YPS606:F>L	AA:83		DBVPG1373:T>N		DBVPG6040:T>N		DBVPG6044:T>N		DBVPG6765:T>N		L_1374:T>N		NCYC110:T>N		SK1:T>N		UWOPS03_461_4:T>N		UWOPS05_217_3:T>N		UWOPS05_227_2:T>N		UWOPS87_2421:T>N		Y55:T>N		YIIc17_E5:T>N		YJM981:T>N		YPS128:T>N		YPS606:T>N		YS9:T>N	AA:287		DBVPG6040:P>L		DBVPG6044:P>L		K11:P>L		NCYC110:P>L		UWOPS03_461_4:P>L		UWOPS87_2421:P>L		Y12:P>L		Y9:P>L		YPS606:P>L	AA:415		DBVPG1853:A>T	AA:461		DBVPG6040:S>G		DBVPG6044:S>G		NCYC110:S>G		SK1:S>G		UWOPS03_461_4:S>G		UWOPS05_217_3:S>G		UWOPS83_787_3:S>G		Y55:S>G		Y9:S>G	AA:477		UWOPS03_461_4:V>I		UWOPS05_217_3:V>I	AA:626		DBVPG6044:E>V		NCYC110:E>V		SK1:E>V		Y55:E>V	AA:631		DBVPG6044:A>T		NCYC110:A>T		SK1:A>T		Y55:A>TID:YDR236C	AA:105		322134S:E>D	AA:117		Y12:V>M		Y9:V>M	AA:130		DBVPG6040:D>G		Y12:D>G		Y9:D>G		YPS128:D>G		YPS606:D>G	AA:174		UWOPS83_787_3:N>I	AA:212		YS9:Q>LID:YDR237W	AA:17		DBVPG1373:V>M		DBVPG1853:V>M		DBVPG6765:V>M		L_1374:V>M		L_1528:V>M		YIIc17_E5:V>M		YS9:V>M	AA:42		DBVPG6044:P>R		SK1:P>R	AA:139		DBVPG1373:S>G		DBVPG1788:S>G		DBVPG1853:S>G		L_1528:S>G		YIIc17_E5:S>G		YJM975:S>G		YJM981:S>G		YS9:S>GID:YDR238C	AA:261		Y12:D>A	AA:268		UWOPS05_227_2:A>S	AA:270		DBVPG1106:A>G	AA:304		YJM975:V>F	AA:470		UWOPS87_2421:E>G	AA:588		322134S:I>F	AA:597		DBVPG1106:L>V	AA:734		BC187:V>I	AA:776		W303:Q>R	AA:810		322134S:S>F	AA:832		BC187:Y>H	AA:915		YPS128:K>N	AA:936		322134S:D>E		BC187:D>E		DBVPG1106:D>E		DBVPG1373:D>E		DBVPG1788:D>E		DBVPG1853:D>E		DBVPG6044:D>E		DBVPG6765:D>E		K11:D>E		L_1374:D>E		NCYC110:D>E		SK1:D>E		UWOPS03_461_4:D>E		UWOPS05_217_3:D>E		Y55:D>E		YJM975:D>E		YJM978:D>E		YPS128:D>E		YPS606:D>E		YS4:D>E		YS9:D>E	AA:955		UWOPS83_787_3:G>VID:YDR240C	AA:38		K11:N>I		Y12:N>I	AA:68		DBVPG1106:S>I	AA:111		YPS128:M>I		YPS606:M>I	AA:197		273614X:D>N		DBVPG6040:D>N		SK1:D>N		UWOPS03_461_4:D>N		UWOPS05_227_2:D>N		UWOPS83_787_3:D>N		UWOPS87_2421:D>N		Y12:D>N		Y55:D>N		YPS606:D>N		YS4:D>N	AA:205		SK1:A>D		Y55:A>D	AA:219		DBVPG1788:F>L	AA:230		UWOPS87_2421:N>K	AA:240		DBVPG6040:N>K		DBVPG6044:N>K		K11:N>K		SK1:N>K		UWOPS03_461_4:N>K		UWOPS05_227_2:N>K		UWOPS83_787_3:N>K		Y12:N>K		Y55:N>K		YPS128:N>K		YPS606:N>K		YS4:N>K	AA:270		YPS128:T>S		YPS606:T>S	AA:309		K11:L>S	AA:313		DBVPG6040:P>A		DBVPG6044:P>A		K11:P>A		SK1:P>A		UWOPS03_461_4:P>A		UWOPS05_227_2:P>A		Y12:P>A		Y55:P>A	AA:370		DBVPG6044:P>L		SK1:P>L		Y55:P>L	AA:400		DBVPG1106:Q>P	AA:457		DBVPG6044:I>S		UWOPS83_787_3:I>S		Y55:I>S	AA:484		DBVPG6044:E>K		Y55:E>KID:YDR242W	AA:31		DBVPG6040:T>A		DBVPG6044:T>A		K11:T>A		SK1:T>A		UWOPS83_787_3:T>A		Y55:T>A		Y9:T>A	AA:69		Y9:A>T	AA:73		K11:K>R		SK1:K>R		UWOPS83_787_3:K>R		Y55:K>R		Y9:K>R	AA:108		YS9:R>K	AA:155		SK1:T>I		Y55:T>I	AA:171		YJM978:A>T	AA:210		YS9:S>F	AA:244		YS9:F>C	AA:258		YS9:V>G	AA:300		UWOPS83_787_3:V>I	AA:333		DBVPG1853:P>L	AA:385		UWOPS83_787_3:N>K	AA:388		DBVPG6040:E>K		K11:E>K		SK1:E>K		UWOPS83_787_3:E>K		Y12:E>K		Y55:E>K		YPS128:E>K		YPS606:E>K	AA:411		SK1:T>S		Y55:T>S	AA:515		K11:Q>K		UWOPS83_787_3:Q>K		Y12:Q>K		Y55:Q>KID:YDR243C	AA:56		UWOPS87_2421:Q>K		YPS606:Q>K	AA:59		K11:S>I	AA:120		BC187:W>R		DBVPG1373:W>R		DBVPG1788:W>R		DBVPG6044:W>R		DBVPG6765:W>R		K11:W>R		L_1374:W>R		L_1528:W>R		SK1:W>R		UWOPS05_227_2:W>R		UWOPS87_2421:W>R		Y55:W>R		YJM981:W>R		YPS606:W>R		YS2:W>R		YS4:W>R		YS9:W>R	AA:166		BC187:T>A		DBVPG1106:T>A		DBVPG1373:T>A		DBVPG1788:T>A		DBVPG1853:T>A		DBVPG6044:T>A		DBVPG6765:T>A		K11:T>A		L_1374:T>A		L_1528:T>A		SK1:T>A		UWOPS05_227_2:T>A		UWOPS83_787_3:T>A		UWOPS87_2421:T>A		Y55:T>A		YIIc17_E5:T>A		YPS606:T>A		YS2:T>A		YS4:T>A		YS9:T>A	AA:272		NCYC110:K>-	AA:357		NCYC361:N>I	AA:436		YPS128:D>Y		YPS606:D>Y	AA:589		UWOPS03_461_4:->YID:YDR244W	AA:5		UWOPS03_461_4:S>I		UWOPS05_217_3:S>I		UWOPS05_227_2:S>I	AA:9		DBVPG1373:G>R		DBVPG1788:G>R		DBVPG1853:G>R		DBVPG6765:G>R		YIIc17_E5:G>R		YJM975:G>R		YJM978:G>R		YJM981:G>R		YS9:G>R	AA:15		YJM975:Q>R	AA:97		YPS128:S>A	AA:129		UWOPS87_2421:S>G		YPS128:S>G	AA:135		K11:A>T		UWOPS03_461_4:A>T		UWOPS05_217_3:A>T		UWOPS05_227_2:A>T		UWOPS83_787_3:A>T	AA:234		DBVPG6044:V>I		SK1:V>I		Y55:V>I	AA:241		UWOPS83_787_3:T>A	AA:486		SK1:V>M		Y55:V>MID:YDR246W	AA:63		DBVPG1853:I>T		DBVPG6040:I>T		DBVPG6044:I>T		K11:I>T		SK1:I>T		UWOPS03_461_4:I>T		UWOPS05_227_2:I>T		UWOPS87_2421:I>T		Y55:I>T		YPS606:I>T	AA:66		YS9:T>PID:YDR247W	AA:48		YS4:G>D	AA:78		YGPM:S>W	AA:227		NCYC361:P>T	AA:241		322134S:S>N		BC187:S>N		L_1374:S>N		L_1528:S>N		NCYC361:S>N		YIIc17_E5:S>N	AA:243		YPS128:G>A		YPS606:G>A	AA:310		378604X:S>A	AA:421		DBVPG1788:H>N	AA:439		UWOPS05_217_3:S>P	AA:453		K11:R>H		Y12:R>H	AA:458		322134S:S>FID:YDR248C	AA:16		W303:L>F	AA:92		Y12:L>F	AA:116		DBVPG1106:E>A	AA:127		UWOPS87_2421:I>M	AA:175		322134S:Y>SID:YDR251W	AA:69		UWOPS03_461_4:L>H	AA:142		DBVPG6040:I>M	AA:155		BC187:T>N		DBVPG1106:T>N		DBVPG1373:T>N		DBVPG6765:T>N		L_1374:T>N		L_1528:T>N		NCYC361:T>N		YJM978:T>N	AA:246		322134S:K>R	AA:277		DBVPG1788:T>I		DBVPG6765:T>I		YIIc17_E5:T>I	AA:298		DBVPG6044:N>D		SK1:N>D		Y55:N>D	AA:412		322134S:G>E		378604X:G>E		BC187:G>E		DBVPG1373:G>E		DBVPG1788:G>E		DBVPG1853:G>E		DBVPG6765:G>E		L_1374:G>E		SK1:G>E		UWOPS83_787_3:G>E		UWOPS87_2421:G>E		Y55:G>E		YIIc17_E5:G>E		YJM978:G>E		YJM981:G>E		YPS128:G>E		YPS606:G>E		YS2:G>E		YS9:G>E	AA:434		UWOPS05_227_2:N>T	AA:442		UWOPS83_787_3:P>S	AA:513		YS2:A>T	AA:556		322134S:Q>R		BC187:Q>R		DBVPG1373:Q>R		DBVPG1853:Q>R		DBVPG6765:Q>R		L_1374:Q>R		L_1528:Q>R		YJM975:Q>R		YJM978:Q>R		YJM981:Q>R		YS9:Q>R	AA:596		Y12:S>N	AA:623		Y12:R>K	AA:640		DBVPG6044:E>D		SK1:E>D		Y55:E>D	AA:656		Y12:R>H	AA:696		322134S:N>S		BC187:N>S		DBVPG1373:N>S		DBVPG1788:N>S		DBVPG6765:N>S		L_1374:N>S		L_1528:N>S		YJM975:N>S		YJM978:N>S		YS9:N>S	AA:768		322134S:P>A	AA:770		BC187:Q>R		DBVPG1788:Q>R		DBVPG6044:Q>H		DBVPG6765:Q>R		L_1374:Q>R		L_1528:Q>R		SK1:Q>H		Y55:Q>H		YJM975:Q>R		YJM978:Q>R	AA:787		SK1:T>I		Y55:T>IID:YDR252W	AA:49		DBVPG6044:H>N		SK1:H>N		Y55:H>N	AA:65		UWOPS05_227_2:F>	AA:136		UWOPS03_461_4:G>S		UWOPS05_227_2:G>SID:YDR253C	AA:48		UWOPS03_461_4:K>E		UWOPS05_227_2:K>E	AA:50		DBVPG6044:A>T		SK1:A>T		Y55:A>T	AA:51		YS2:L>P	AA:54		UWOPS83_787_3:A>TID:YDR254W	AA:98		DBVPG6044:I>V		SK1:I>V		UWOPS05_217_3:I>V		UWOPS05_227_2:I>V		Y55:I>V	AA:121		DBVPG1853:V>I	AA:172		322134S:P>S		DBVPG1373:P>S		DBVPG1853:P>S		DBVPG6765:P>S		L_1374:P>S		YJM975:P>S		YJM978:P>S		YS9:P>S	AA:215		DBVPG6044:V>I		NCYC110:V>I		SK1:V>I		UWOPS05_217_3:V>I		UWOPS87_2421:V>I		Y55:V>I		Y9:V>I		YPS128:V>I		YPS606:V>I		YS2:V>I		YS4:V>I	AA:250		DBVPG1106:S>-	AA:280		DBVPG6044:R>Q		NCYC110:R>Q		SK1:R>Q		UWOPS05_217_3:R>Q		UWOPS87_2421:R>Q		Y55:R>Q		YPS128:R>Q		YS2:R>Q		YS4:R>QID:YDR256C	AA:13		K11:Y>S		UWOPS05_217_3:Y>S		UWOPS83_787_3:Y>S		Y55:Y>S		Y9:Y>S		YPS128:Y>S		YPS606:Y>S		YS2:Y>S	AA:287		UWOPS05_217_3:K>I	AA:303		DBVPG1853:G>E		L_1528:G>E	AA:485		UWOPS05_227_2:M>V	AA:515		UWOPS87_2421:F>LID:YDR259C	AA:4		W303:P>L	AA:54		YPS128:T>A		YPS606:T>A	AA:69		YS4:Q>L	AA:171		378604X:S>Y	AA:265		378604X:N>Y		K11:N>Y		NCYC110:N>Y		SK1:N>Y		UWOPS05_217_3:N>Y		UWOPS05_227_2:N>Y		UWOPS83_787_3:N>Y		UWOPS87_2421:N>Y		YPS128:N>Y		YPS606:N>Y	AA:295		K11:R>K	AA:303		378604X:N>S		K11:N>S		Y9:N>S	AA:328		YS9:N>T	AA:351		378604X:K>R		K11:K>R		NCYC110:K>R		SK1:K>R		UWOPS05_227_2:K>R		UWOPS87_2421:K>R		Y9:K>R		YPS128:K>R		YPS606:K>R	AA:365		378604X:K>I		K11:K>I		L_1374:K>I		L_1528:K>I		NCYC110:K>I		SK1:K>I		UWOPS05_217_3:K>I		UWOPS05_227_2:K>I		UWOPS87_2421:K>I		Y9:K>I		YPS128:K>I		YPS606:K>I		YS9:K>IID:YDR260C	AA:62		YIIc17_E5:G>E	AA:118		DBVPG6044:F>L		SK1:F>L		Y55:F>L	AA:155		DBVPG6044:P>S		SK1:P>S		UWOPS05_227_2:P>S		UWOPS83_787_3:P>S		Y12:P>S		Y55:P>S		YPS128:P>S		YPS606:Y>H		YS2:P>S		YS4:P>SID:YDR262W	AA:19		UWOPS05_227_2:M>I		UWOPS83_787_3:M>I		UWOPS87_2421:M>I		YPS128:M>I		YPS606:M>I	AA:55		UWOPS05_227_2:L>P		UWOPS83_787_3:L>P		UWOPS87_2421:L>P		YPS128:L>P		YPS606:L>P	AA:57		DBVPG6044:N>S		K11:N>S		SK1:N>S		UWOPS05_227_2:N>S		UWOPS83_787_3:N>S		UWOPS87_2421:N>S		Y55:N>S		Y9:N>S		YPS128:N>S		YPS606:N>S	AA:68		DBVPG6044:K>M		K11:K>M		SK1:K>M		UWOPS05_227_2:K>M		UWOPS83_787_3:K>M		UWOPS87_2421:K>M		Y55:K>M		Y9:K>M		YPS128:K>M		YPS606:K>M	AA:70		UWOPS05_227_2:M>K	AA:77		UWOPS83_787_3:A>T		UWOPS87_2421:A>T		YPS128:A>T		YPS606:A>T	AA:82		UWOPS05_227_2:D>G	AA:86		YS9:T>	AA:95		DBVPG6044:L>M		K11:L>M		SK1:L>M		Y55:L>M		Y9:L>M	AA:214		YPS606:D>E	AA:234		UWOPS87_2421:E>KID:YDR263C	AA:155		UWOPS03_461_4:M>T	AA:158		YS4:L>F	AA:266		UWOPS87_2421:S>T	AA:341		YS4:V>I	AA:355		DBVPG6040:M>V	AA:398		DBVPG6040:V>M	AA:421		UWOPS05_227_2:A>E		UWOPS87_2421:A>EID:YDR265W	AA:14		378604X:R>Q		UWOPS87_2421:R>Q		Y12:R>Q		Y9:R>Q	AA:23		UWOPS87_2421:A>T	AA:174		378604X:S>F		DBVPG6040:S>F		SK1:S>F		UWOPS83_787_3:S>F		UWOPS87_2421:S>F		Y12:S>F		Y55:S>F		YPS128:S>F	AA:190		378604X:K>R		DBVPG6040:K>R		NCYC110:K>E		SK1:K>E		UWOPS83_787_3:K>R		UWOPS87_2421:K>R		Y12:K>R		Y55:K>E		YS4:K>R	AA:205		UWOPS83_787_3:V>I	AA:329		YS4:P>AID:YDR268W	AA:26		273614X:L>R		322134S:L>R		DBVPG1106:L>R		DBVPG1788:L>R		DBVPG6765:L>R		L_1374:L>R		YJM978:L>R		YJM981:L>R	AA:159		SK1:D>E		UWOPS83_787_3:D>E		UWOPS87_2421:D>E		Y55:D>E		YPS128:D>E		YS4:D>E	AA:308		UWOPS83_787_3:D>GID:YDR270W	AA:134		DBVPG6040:A>V		DBVPG6044:A>V		K11:A>V		SK1:A>V		UWOPS05_217_3:A>V		UWOPS05_227_2:A>V		Y55:A>V		Y9:A>V	AA:137		273614X:M>I		DBVPG1853:M>I		DBVPG6040:M>I		DBVPG6765:M>I		K11:M>I		SK1:M>I		Y55:M>I		Y9:M>I		YS2:M>I		YS9:M>I	AA:180		DBVPG6044:S>L		SK1:S>L		Y55:S>L	AA:189		DBVPG6044:L>S		SK1:L>S		Y55:L>S	AA:279		322134S:W>S		DBVPG6040:W>S		DBVPG6044:W>S		SK1:W>S		UWOPS05_217_3:W>S		UWOPS05_227_2:W>S		UWOPS87_2421:W>S		Y9:W>S		YPS128:W>S		YPS606:W>S		YS2:W>S	AA:329		322134S:L>F		DBVPG6044:L>F		K11:L>F		NCYC110:L>F		SK1:L>F		UWOPS03_461_4:L>F		UWOPS05_217_3:L>F		UWOPS05_227_2:L>F		UWOPS87_2421:L>F		YPS128:L>F		YPS606:L>F	AA:442		K11:G>R	AA:471		NCYC110:P>L		SK1:P>L		Y55:P>L	AA:560		YS4:T>I	AA:726		YS2:L>M	AA:822		YGPM:K>I	AA:941		BC187:V>A		DBVPG1106:V>A		DBVPG1853:V>A		DBVPG6765:V>A		L_1374:V>A		YS9:V>A	AA:952		L_1528:K>R	AA:978		L_1528:R>K	AA:984		YS2:A>V	AA:998		L_1528:M>I	AA:1004		NCYC110:L>-		SK1:L>-		Y55:L>-ID:YDR272W	AA:45		K11:T>N		NCYC361:T>N		Y12:T>N		YPS128:T>N		YPS606:T>N	AA:52		K11:V>I		NCYC361:V>I		Y12:V>I		YPS128:V>I		YPS606:V>I	AA:107		UWOPS87_2421:H>Y	AA:133		UWOPS05_217_3:P>S		UWOPS05_227_2:P>S	AA:178		UWOPS05_217_3:N>K	AA:251		UWOPS83_787_3:A>V		Y12:A>V		YPS128:A>V		YPS606:A>VID:YDR275W	AA:13		322134S:S>L		UWOPS03_461_4:S>L		UWOPS05_227_2:S>L	AA:46		NCYC110:K>Q		SK1:K>Q		Y55:K>Q	AA:125		NCYC110:T>A		SK1:T>A		Y55:T>A	AA:136		273614X:V>I		BC187:V>I		DBVPG1106:V>I		DBVPG6765:V>I		L_1374:V>I		L_1528:V>I		YS9:V>I	AA:153		DBVPG1853:I>V	AA:169		NCYC110:V>A		SK1:V>A		UWOPS03_461_4:V>A		UWOPS05_217_3:V>A		UWOPS05_227_2:V>A		Y55:V>A	AA:175		322134S:L>F	AA:186		YS4:Q>-	AA:190		NCYC110:V>I		SK1:V>I		UWOPS03_461_4:V>I		UWOPS05_217_3:V>I		UWOPS05_227_2:V>I		Y55:V>I	AA:210		UWOPS05_217_3:P>L		UWOPS05_227_2:P>LID:YDR277C	AA:25		K11:S>C	AA:63		DBVPG6044:T>I		NCYC110:T>I		SK1:T>I		Y55:T>I	AA:111		378604X:S>-	AA:199		YJM981:L>V	AA:208		YJM978:R>-	AA:258		273614X:S>L	AA:301		Y12:K>NID:YDR279W	AA:47		UWOPS05_217_3:N>D		UWOPS87_2421:N>D		Y12:N>D		Y9:N>D		YPS128:N>D		YPS606:N>D	AA:108		UWOPS05_217_3:I>M	AA:143		DBVPG6044:G>S		SK1:G>S		Y55:G>S	AA:183		YS2:K>R		YS9:K>R	AA:187		DBVPG6765:A>G	AA:236		K11:E>D	AA:238		BC187:K>N		DBVPG6044:K>N		K11:K>N		SK1:K>N		UWOPS03_461_4:K>N		Y12:K>N		Y55:K>N		YPS606:K>N	AA:276		BC187:P>L		K11:P>L		UWOPS03_461_4:P>L		UWOPS05_217_3:P>L		UWOPS87_2421:P>L		Y12:P>L		YPS606:P>L		YS4:P>L	AA:282		DBVPG6044:F>L		SK1:F>L		Y55:F>L	AA:290		378604X:T>I	AA:316		BC187:R>S		DBVPG6044:R>S		DBVPG6765:R>S		K11:R>S		L_1374:R>S		SK1:R>S		UWOPS87_2421:R>S		Y12:R>S		Y55:R>S		YPS606:R>S		YS4:R>S		YS9:R>S	AA:325		BC187:T>I	AA:330		DBVPG6044:Q>P		SK1:Q>P		Y55:Q>PID:YDR280W	AA:43		DBVPG1373:E>A	AA:65		DBVPG6044:A>T		SK1:A>T		Y55:A>TID:YDR281C	AA:6		UWOPS87_2421:R>G	AA:13		DBVPG1853:K>E	AA:28		BC187:D>E		SK1:D>E		UWOPS03_461_4:D>E		UWOPS05_217_3:D>E		UWOPS83_787_3:D>E		Y55:D>E		YPS128:D>E		YPS606:D>E	AA:48		YS9:G>CID:YDR284C	AA:246		322134S:I>T		378604X:I>T		DBVPG6044:I>T		SK1:I>T		UWOPS05_227_2:I>T		UWOPS83_787_3:I>T		YPS606:I>T	AA:272		YS9:T>A	AA:282		YS9:H>RID:YDR285W	AA:110		UWOPS83_787_3:I>N	AA:126		UWOPS05_217_3:V>E	AA:219		DBVPG6044:S>C		SK1:S>C		Y55:S>C	AA:288		BC187:N>H		DBVPG6044:N>H		K11:N>H		SK1:N>H		Y55:N>H		Y9:N>H		YPS128:N>H		YPS606:N>H	AA:370		DBVPG6040:K>N		DBVPG6044:K>N		K11:K>N		NCYC110:K>N		SK1:K>N		YPS128:K>N		YPS606:K>N	AA:426		DBVPG6040:K>T		DBVPG6044:K>T		K11:K>T		NCYC110:K>T		NCYC361:K>T		SK1:K>T		YPS128:K>T		YPS606:K>T	AA:457		DBVPG6044:L>V		NCYC110:L>V		SK1:L>V	AA:477		322134S:K>Q		DBVPG6040:K>Q		DBVPG6044:K>Q		K11:K>Q		NCYC110:K>Q		NCYC361:K>Q		SK1:K>Q		UWOPS05_217_3:K>Q		YPS128:K>Q		YPS606:K>Q	AA:481		DBVPG6040:S>N		NCYC361:S>N	AA:486		322134S:V>L		DBVPG6040:V>L		DBVPG6044:V>L		K11:V>L		NCYC110:V>L		NCYC361:V>L		SK1:V>L		UWOPS05_217_3:V>L		YPS128:V>L		YPS606:V>L	AA:560		UWOPS05_217_3:E>D	AA:637		DBVPG6044:N>T		SK1:N>T		Y55:N>T	AA:638		UWOPS05_217_3:E>V	AA:706		BC187:L>F	AA:724		UWOPS05_217_3:V>M		UWOPS05_227_2:V>M	AA:733		SK1:Q>K		Y55:Q>K	AA:754		YPS128:T>K		YPS606:T>K	AA:795		SK1:S>N		Y55:S>N	AA:806		K11:S>CID:YDR286C	AA:9		YPS128:I>V		YPS606:I>VID:YDR287W	AA:36		DBVPG1853:C>Y	AA:62		322134S:N>T		DBVPG1106:N>T		DBVPG1373:N>T		DBVPG1788:N>T		DBVPG1853:N>T		DBVPG6044:N>T		DBVPG6765:N>T		L_1374:N>T		L_1528:N>T		SK1:N>T		UWOPS03_461_4:N>T		UWOPS05_217_3:N>T		Y55:N>T		YJM975:N>T		YJM978:N>T		YPS128:N>T		YPS606:N>T		YS4:N>T	AA:69		DBVPG6044:S>G		SK1:S>G		Y55:S>G	AA:71		DBVPG1853:K>T	AA:82		DBVPG1373:V>I	AA:88		322134S:G>D		DBVPG6044:G>D		SK1:G>D		UWOPS03_461_4:G>D		UWOPS05_217_3:G>D		Y12:G>D		Y55:G>D		YPS128:G>D		YPS606:G>D		YS4:G>D	AA:243		DBVPG6044:G>R		SK1:G>R		Y55:G>R	AA:259		YS4:D>	AA:288		L_1374:G>AID:YDR288W	AA:4		273614X:I>T		BC187:I>T		DBVPG1373:I>T		DBVPG1788:I>T		DBVPG1853:I>T		K11:I>T		L_1528:I>T		NCYC361:I>T		YIIc17_E5:I>T		YJM975:I>T		YJM978:I>T		YPS128:I>T		YPS606:I>T		YS9:I>T	AA:23		BC187:K>R		DBVPG6044:K>R		K11:K>R		NCYC361:K>R		SK1:K>R		Y55:K>R		YPS128:K>R		YPS606:K>R	AA:106		322134S:S>N		BC187:S>N		K11:S>N		NCYC361:S>N		SK1:S>N		UWOPS05_227_2:S>N		YPS128:S>N		YPS606:S>N	AA:153		322134S:T>N		BC187:T>N		K11:T>N		SK1:T>N		UWOPS05_227_2:T>N		Y55:T>N		YPS128:T>N		YPS606:T>N	AA:156		YS9:Y>H	AA:203		NCYC361:E>K	AA:266		322134S:L>F		BC187:L>F		K11:L>F		UWOPS05_227_2:L>F	AA:298		322134S:G>DID:YDR289C	AA:42		273614X:M>T		322134S:M>T		DBVPG1106:M>T		DBVPG1373:M>T		DBVPG1788:M>T		DBVPG6040:M>T		DBVPG6765:M>T		K11:M>T		SK1:M>T		UWOPS05_217_3:M>T		UWOPS05_227_2:M>T		Y55:M>T		Y9:M>T		YIIc17_E5:M>T		YJM978:M>T		YPS128:M>T		YPS606:M>T		YS4:M>T	AA:129		SK1:R>G		Y55:R>G	AA:179		SK1:D>N		Y55:D>N	AA:215		322134S:H>L	AA:222		UWOPS05_227_2:D>E	AA:297		DBVPG6040:S>N		DBVPG6044:S>N		K11:S>N		SK1:S>N		UWOPS05_227_2:S>N		UWOPS83_787_3:S>N		Y12:S>N		Y55:S>N		YPS128:S>N		YPS606:S>NID:YDR293C	AA:185		Y12:G>D		Y9:G>D	AA:246		378604X:D>Y	AA:339		UWOPS03_461_4:S>F	AA:377		DBVPG6044:S>C		SK1:S>C		Y55:S>C	AA:441		UWOPS03_461_4:S>G		UWOPS05_227_2:S>G	AA:522		Y9:S>P	AA:680		NCYC361:P>S	AA:684		UWOPS03_461_4:E>K		UWOPS05_227_2:E>K	AA:693		SK1:T>M		Y55:T>M	AA:698		W303:Y>-	AA:704		NCYC361:S>A	AA:800		UWOPS03_461_4:W>R	AA:812		UWOPS03_461_4:L>S	AA:827		273614X:T>S		DBVPG1853:T>S		K11:T>S		SK1:T>S		UWOPS03_461_4:T>S		UWOPS05_227_2:T>S		Y55:T>S		YJM975:T>S		YJM978:T>S		YPS128:T>S	AA:936		UWOPS03_461_4:N>S		UWOPS05_217_3:N>S	AA:1017		UWOPS03_461_4:T>S	AA:1022		UWOPS03_461_4:D>E	AA:1032		UWOPS03_461_4:Y>N	AA:1034		UWOPS03_461_4:N>S	AA:1190		DBVPG1853:S>G		DBVPG6044:S>G		K11:S>G		NCYC361:S>G		SK1:S>G		UWOPS03_461_4:S>G		UWOPS05_217_3:S>G		Y55:S>G		YPS128:S>G		YPS606:S>G	AA:1192		K11:N>K	AA:1250		DBVPG1853:V>A		K11:V>A		NCYC361:V>A		Y12:V>A		YPS128:V>A		YPS606:V>AID:YDR294C	AA:25		YPS128:E>K	AA:99		UWOPS05_217_3:W>R		UWOPS05_227_2:W>R	AA:308		DBVPG1853:I>V		DBVPG6044:I>V		SK1:I>V		UWOPS05_217_3:I>V		UWOPS05_227_2:I>V		UWOPS87_2421:I>V		Y55:I>V		YJM975:I>V		YPS128:I>V		YPS606:I>V	AA:364		UWOPS05_217_3:L>F	AA:469		DBVPG1853:N>D		SK1:N>D		UWOPS03_461_4:N>D		UWOPS05_217_3:N>D		UWOPS05_227_2:N>D		UWOPS83_787_3:N>D		Y55:N>D		Y9:N>D		YPS606:N>D		YS4:N>D	AA:473		273614X:N>S	AA:487		SK1:N>D		Y55:N>D	AA:576		K11:A>T		Y9:A>T	AA:587		SK1:A>P		Y55:A>PID:YDR296W	AA:7		W303:I>T	AA:146		DBVPG6765:E>G		YS4:E>G	AA:150		378604X:P>Q		BC187:P>Q		DBVPG1788:P>Q		DBVPG6040:P>Q		DBVPG6044:P>Q		DBVPG6765:P>Q		L_1374:P>Q		SK1:P>Q		UWOPS05_217_3:P>Q		UWOPS05_227_2:P>Q		Y55:P>Q		YJM975:P>Q		YJM981:P>Q		YS4:P>Q	AA:156		BC187:V>A		DBVPG1788:V>A		DBVPG6040:V>A		DBVPG6044:V>A		DBVPG6765:V>A		L_1374:V>A		SK1:V>A		Y55:V>A		YJM975:V>A		YJM981:V>A		YS4:V>A	AA:217		UWOPS05_217_3:P>TID:YDR297W	AA:4		UWOPS83_787_3:T>	AA:12		UWOPS03_461_4:S>P		UWOPS05_227_2:S>P	AA:28		DBVPG6044:Y>F		SK1:Y>F	AA:54		Y55:V>F	AA:63		DBVPG6044:L>F		SK1:L>F	AA:138		DBVPG1853:D>G	AA:323		273614X:E>K		378604X:E>K		DBVPG1373:E>K		DBVPG1788:E>K		DBVPG1853:E>K		DBVPG6040:E>K		DBVPG6765:E>K		K11:E>K		NCYC110:E>K		NCYC361:E>K		SK1:E>K		UWOPS03_461_4:E>K		UWOPS05_217_3:E>K		UWOPS05_227_2:E>K		UWOPS83_787_3:E>K		Y12:E>K		Y55:E>K		YJM975:E>K		YJM978:E>K		YPS128:E>K		YPS606:E>K		YS2:E>K		YS4:E>KID:YDR298C	AA:15		378604X:R>-	AA:53		DBVPG1853:A>V	AA:63		322134S:T>A	AA:128		K11:L>F	AA:137		K11:K>N	AA:162		K11:K>Q	AA:173		DBVPG1853:E>D	AA:182		UWOPS83_787_3:G>V	AA:186		K11:V>EID:YDR299W	AA:3		DBVPG1106:K>I		YIIc17_E5:K>I	AA:94		UWOPS83_787_3:V>A	AA:99		Y12:D>E	AA:105		Y12:E>K	AA:138		L_1528:G>D	AA:140		L_1528:T>M	AA:148		L_1528:H>Q	AA:154		L_1528:I>V	AA:215		L_1528:E>D	AA:222		L_1528:E>Q	AA:241		L_1528:R>Q	AA:264		DBVPG6044:H>Y		SK1:H>Y		Y55:H>Y	AA:277		L_1528:Q>K	AA:351		YPS128:Y>C	AA:415		UWOPS87_2421:R>P	AA:473		UWOPS05_217_3:A>P	AA:476		UWOPS05_217_3:E>Q	AA:504		UWOPS05_217_3:F>S	AA:510		UWOPS05_217_3:E>A	AA:513		DBVPG6040:H>Q		DBVPG6044:H>Q		SK1:H>Q		UWOPS87_2421:H>Q		Y55:H>QID:YDR300C	AA:27		YS4:K>E	AA:35		Y9:S>T	AA:101		DBVPG6040:D>G	AA:124		YS4:Q>H	AA:210		L_1528:A>T	AA:252		DBVPG1853:R>K		DBVPG6044:R>K		K11:R>K		SK1:R>K		UWOPS05_227_2:R>K		UWOPS83_787_3:R>K		Y55:R>K		YPS128:R>K	AA:261		L_1528:E>G	AA:372		Y12:T>A	AA:413		UWOPS05_227_2:D>HID:YDR304C	AA:56		UWOPS87_2421:G>VID:YDR306C	AA:38		Y9:T>A	AA:58		NCYC361:T>A		YPS606:T>A	AA:82		DBVPG6044:P>L		K11:P>L		NCYC110:P>L		NCYC361:P>L		SK1:P>L		UWOPS03_461_4:P>L		UWOPS05_217_3:P>L		UWOPS05_227_2:P>L		Y12:P>L		Y55:P>L		YPS606:P>L	AA:92		DBVPG6044:G>E		K11:G>E		NCYC110:G>E		NCYC361:G>E		SK1:G>E		UWOPS03_461_4:G>E		UWOPS05_217_3:G>E		UWOPS05_227_2:G>E		Y12:G>E		Y55:G>E		YPS606:G>E		YS4:G>E	AA:104		Y9:A>V	AA:132		DBVPG6044:K>N		K11:K>N		NCYC110:K>N		NCYC361:K>N		SK1:K>N		UWOPS03_461_4:K>N		UWOPS05_217_3:K>N		UWOPS05_227_2:K>N		Y12:K>N		Y55:K>N		YPS606:K>N	AA:135		273614X:K>T	AA:140		DBVPG6044:A>T		K11:I>V		NCYC110:A>T		NCYC361:A>T		SK1:A>T		UWOPS03_461_4:A>T		UWOPS05_217_3:A>T		UWOPS05_227_2:A>T		Y12:A>T		Y55:A>T		YPS606:A>T	AA:181		K11:G>EID:YDR308C	AA:50		YS4:V>I	AA:57		K11:N>SID:YDR309C	AA:6		YIIc17_E5:I>V	AA:71		Y12:P>L	AA:75		DBVPG6044:P>L		NCYC110:P>L		SK1:P>L		Y55:P>L	AA:92		K11:K>E	AA:116		YJM978:N>S	AA:205		NCYC110:H>Q		SK1:H>Q		Y55:H>Q	AA:253		UWOPS05_217_3:Y>C		UWOPS05_227_2:Y>C	AA:270		UWOPS05_227_2:G>S	AA:325		L_1528:K>NID:YDR311W	AA:259		NCYC361:I>F	AA:262		NCYC361:N>I	AA:276		UWOPS03_461_4:K>E	AA:277		NCYC361:N>Y	AA:279		UWOPS03_461_4:K>E	AA:288		YS4:F>L	AA:352		L_1374:N>I	AA:424		UWOPS05_227_2:L>S	AA:501		YPS606:K>R	AA:628		UWOPS05_217_3:H>Q		UWOPS05_227_2:H>Q	AA:639		Y12:N>YID:YDR312W	AA:10		UWOPS87_2421:T>M	AA:78		DBVPG6044:G>C		SK1:G>C		Y55:G>C		YS4:G>V	AA:84		Y9:M>I	AA:149		Y9:S>L	AA:152		DBVPG6044:S>P		SK1:S>P		Y55:S>P		YPS128:S>P		YPS606:S>P	AA:156		UWOPS05_217_3:D>N	AA:222		YS2:L>P	AA:247		YS2:L>-	AA:292		UWOPS03_461_4:T>I		UWOPS05_217_3:T>I	AA:340		UWOPS03_461_4:V>I		UWOPS05_217_3:V>I	AA:357		Y9:V>I	AA:369		K11:K>R	AA:440		DBVPG6044:V>I		SK1:V>I		UWOPS83_787_3:V>I		Y55:V>I		Y9:V>I		YS4:V>IID:YDR314C	AA:15		YPS128:N>S		YPS606:N>S	AA:52		YPS128:I>V		YPS606:I>V	AA:74		L_1528:R>G	AA:79		K11:K>N	AA:187		YJM978:K>M	AA:229		DBVPG6044:M>R		SK1:M>R		Y55:M>R	AA:241		Y12:T>M	AA:334		DBVPG6044:E>K		SK1:E>K		Y12:E>K		Y55:E>K		YPS128:E>K	AA:438		378604X:V>M		DBVPG1373:V>M		YIIc17_E5:V>M	AA:491		YS9:K>N	AA:505		YPS128:A>V		YPS606:A>V	AA:545		DBVPG1853:P>S		DBVPG6040:P>S	AA:567		UWOPS03_461_4:K>R	AA:582		DBVPG1373:G>R	AA:596		UWOPS03_461_4:C>Y	AA:607		DBVPG6044:K>R		K11:K>R		SK1:K>R		Y12:K>R		Y55:K>R		Y9:K>R	AA:628		Y12:H>R		Y9:H>R	AA:645		DBVPG1853:A>V		DBVPG6044:A>V		K11:A>V		SK1:A>V		Y12:A>V		Y55:A>V		Y9:A>V		YPS128:A>V		YPS606:A>V	AA:685		K11:N>KID:YDR317W	AA:25		322134S:I>T		BC187:I>T		DBVPG1106:I>T		DBVPG1373:I>T		DBVPG6044:I>T		DBVPG6765:I>T		K11:I>T		L_1528:I>T		SK1:I>T		UWOPS05_227_2:I>T		UWOPS83_787_3:I>T		Y55:I>T		YPS128:I>T		YPS606:I>T		YS2:I>T		YS4:I>T	AA:34		DBVPG6044:L>F		K11:L>F		SK1:L>F		UWOPS05_227_2:L>F		UWOPS83_787_3:L>F		UWOPS87_2421:L>F		Y55:L>F		YPS128:L>F		YPS606:L>F	AA:95		YS4:Y>C	AA:110		UWOPS03_461_4:S>F		UWOPS05_227_2:S>F	AA:149		DBVPG6044:T>A		SK1:T>A		UWOPS03_461_4:T>A		UWOPS05_227_2:T>A		Y55:T>A	AA:164		378604X:C>Y		DBVPG1373:C>Y		DBVPG1788:C>Y		DBVPG6040:C>Y		DBVPG6044:C>Y		DBVPG6765:C>Y		K11:C>Y		L_1528:C>Y		SK1:C>Y		UWOPS03_461_4:C>Y		UWOPS05_227_2:C>Y		UWOPS83_787_3:C>Y		Y55:C>Y		YJM978:C>Y		YPS128:C>Y		YPS606:C>Y		YS2:C>Y		YS9:C>Y	AA:198		DBVPG6044:I>T		SK1:I>T		Y55:I>T	AA:255		378604X:T>I		DBVPG1373:T>I	AA:261		UWOPS03_461_4:V>A	AA:268		UWOPS05_227_2:D>N	AA:274		UWOPS03_461_4:F>S	AA:319		K11:G>S		Y12:G>S	AA:329		UWOPS83_787_3:I>F		UWOPS87_2421:I>F	AA:345		K11:S>I		Y12:S>I	AA:386		DBVPG6044:A>S		NCYC110:A>S		SK1:A>S		Y55:A>S	AA:406		DBVPG6765:S>N		L_1528:S>N		YIIc17_E5:S>N		YJM975:S>NID:YDR319C	AA:45		Y9:L>F	AA:161		322134S:K>M	AA:180		322134S:C>R	AA:212		YS4:W>L	AA:222		273614X:A>T		322134S:A>T		378604X:A>T		BC187:A>T		DBVPG1106:A>T		DBVPG1788:A>T		YIIc17_E5:A>TID:YDR321W	AA:10		YJM978:T>N	AA:29		322134S:T>A	AA:287		UWOPS03_461_4:D>G		UWOPS05_217_3:D>G	AA:363		Y12:A>ID:YDR322C-A	AA:69		UWOPS03_461_4:A>T		UWOPS05_227_2:A>TID:YDR322W	AA:27		YS2:T>I	AA:68		378604X:E>Q	AA:70		378604X:I>S	AA:74		UWOPS05_227_2:F>	AA:133		DBVPG1853:D>G	AA:173		UWOPS05_227_2:V>I	AA:208		DBVPG1853:I>V		DBVPG6044:I>V		K11:I>V		NCYC110:I>V		SK1:I>V		UWOPS05_227_2:I>V		UWOPS83_787_3:I>V		YPS128:I>V		YPS606:I>V		YS9:I>V	AA:220		DBVPG1788:E>K		DBVPG6765:E>K		L_1374:E>K		YJM975:E>K		YJM978:E>K		YS2:E>K	AA:276		DBVPG1853:L>I		DBVPG6040:L>I		DBVPG6044:L>I		K11:L>I		NCYC110:L>I		SK1:L>I		UWOPS87_2421:L>I		Y55:L>I		YPS128:L>I		YPS606:L>I		YS9:L>I	AA:290		UWOPS05_227_2:V>I	AA:332		DBVPG6040:T>P	AA:343		DBVPG6040:W>R	AA:346		DBVPG1853:K>N		DBVPG6040:K>N		DBVPG6044:K>N		K11:K>N		NCYC361:K>N		SK1:K>N		UWOPS05_227_2:K>N		UWOPS87_2421:K>N		Y55:K>N		YPS128:K>N		YPS606:K>N		YS9:K>NID:YDR325W	AA:5		K11:D>H		Y12:D>H		Y9:D>H	AA:30		273614X:G>A	AA:170		UWOPS05_227_2:R>K	AA:232		DBVPG6765:N>D	AA:301		YS4:H>R	AA:387		DBVPG6765:K>R	AA:397		UWOPS05_217_3:L>F	AA:415		DBVPG1853:Y>D	AA:426		322134S:S>L		DBVPG1853:S>L		K11:S>L		SK1:S>L		UWOPS05_217_3:S>L		UWOPS83_787_3:S>L		UWOPS87_2421:S>L		Y55:S>L		YPS606:S>L	AA:439		NCYC361:G>R	AA:445		DBVPG1853:T>I	AA:510		UWOPS05_217_3:R>C	AA:517		DBVPG6044:D>N		SK1:D>N		Y55:D>N		YPS128:D>N	AA:532		DBVPG1373:D>N		YJM975:D>N	AA:536		W303:D>	AA:553		L_1528:G>D	AA:662		YS4:V>I	AA:789		UWOPS03_461_4:P>S		UWOPS05_227_2:P>S	AA:810		L_1374:T>S	AA:913		322134S:T>A		DBVPG6044:T>A		NCYC110:T>A		SK1:T>A		UWOPS83_787_3:T>A		UWOPS87_2421:T>A		Y55:T>A		Y9:T>A		YPS606:T>A	AA:953		322134S:N>S		YPS128:N>S		YPS606:N>S	AA:964		322134S:S>L		YPS128:S>L		YPS606:S>L	AA:996		YJM975:S>N		YS9:S>N	AA:998		UWOPS05_217_3:Q>H	AA:1002		322134S:T>A		DBVPG6044:T>A		NCYC110:T>A		SK1:T>A		Y55:T>A		YPS128:T>A		YPS606:T>A	AA:1035		DBVPG6040:C>R		Y9:C>RID:YDR326C	AA:59		Y9:N>D	AA:67		Y9:K>N	AA:75		Y9:G>C	AA:154		378604X:P>L		DBVPG1373:P>L		DBVPG6765:P>L		NCYC110:P>L		NCYC361:P>L		SK1:P>L		UWOPS05_227_2:P>L		Y55:P>L		Y9:P>L		YPS128:P>L		YPS606:P>L		YS4:P>L	AA:276		Y12:H>Y		Y9:H>Y	AA:298		DBVPG1373:P>R	AA:323		273614X:R>C		DBVPG6765:R>C		YIIc17_E5:R>C	AA:340		378604X:G>A		Y12:G>A		Y9:G>A		YPS128:G>A		YPS606:G>A	AA:342		UWOPS03_461_4:A>T		UWOPS05_227_2:A>T	AA:349		DBVPG6040:V>A	AA:352		UWOPS03_461_4:G>E		UWOPS05_227_2:G>E	AA:368		DBVPG6044:P>S		NCYC110:P>S		SK1:P>S		Y55:P>S	AA:369		322134S:S>L	AA:419		DBVPG6040:Q>R	AA:429		DBVPG6044:S>L		NCYC110:S>L		SK1:S>L		Y55:S>L	AA:439		DBVPG1106:E>A		YJM975:E>A	AA:469		DBVPG1106:N>K		YJM975:N>K	AA:495		273614X:T>S		322134S:T>S		BC187:T>S		DBVPG1106:T>S		DBVPG6040:T>S		DBVPG6044:T>S		DBVPG6765:T>S		NCYC110:T>S		SK1:T>S		UWOPS03_461_4:T>S		UWOPS05_227_2:T>S		Y55:T>S		YIIc17_E5:T>S		YJM975:T>S		YPS606:T>S	AA:512		UWOPS03_461_4:H>Q		UWOPS05_227_2:H>Q	AA:560		322134S:S>G	AA:777		YPS128:S>L		YPS606:S>L	AA:795		Y55:D>G	AA:797		K11:D>N	AA:974		NCYC110:T>A		SK1:T>A		Y55:T>A	AA:1121		L_1374:N>K	AA:1248		322134S:I>V	AA:1259		UWOPS03_461_4:D>E		UWOPS05_227_2:D>E	AA:1264		322134S:N>T		SK1:N>T		UWOPS03_461_4:N>T		UWOPS05_227_2:N>T		Y55:N>T		YPS128:N>T		YPS606:N>T	AA:1265		Y12:S>L		Y9:S>L	AA:1312		Y12:I>L		Y9:I>L	AA:1405		378604X:Q>L	AA:1413		322134S:I>V		DBVPG6040:I>V		K11:I>V		NCYC110:I>V		SK1:I>V		UWOPS03_461_4:I>V		UWOPS05_217_3:I>V		UWOPS05_227_2:I>V		Y12:I>V		Y55:I>V		Y9:I>V		YPS606:I>V	AA:1422		378604X:K>I	AA:1437		322134S:L>FID:YDR328C	AA:26		UWOPS87_2421:S>F	AA:49		Y9:S>L	AA:156		YS9:E>DID:YDR329C	AA:18		273614X:V>L		322134S:V>L		378604X:V>L		DBVPG6765:V>L		NCYC110:V>L		SK1:V>L		UWOPS05_227_2:V>L		Y55:V>L		YIIc17_E5:V>L		YPS606:V>L	AA:67		378604X:T>A	AA:85		YPS128:V>A	AA:112		DBVPG6765:S>P	AA:167		YPS128:D>N		YPS606:D>N	AA:177		Y9:E>D	AA:208		K11:A>T		UWOPS83_787_3:A>V		Y9:A>T	AA:236		YS2:K>R	AA:289		YPS128:N>S		YPS606:N>S	AA:335		UWOPS05_217_3:C>S	AA:378		UWOPS05_217_3:Y>HID:YDR331W	AA:4		L_1528:A>T	AA:39		UWOPS05_227_2:W>C	AA:80		UWOPS05_227_2:S>N	AA:310		YPS128:P>T		YPS606:P>T	AA:311		UWOPS03_461_4:L>F	AA:365		322134S:D>N		378604X:D>N		DBVPG6040:D>N		DBVPG6765:D>N		SK1:D>N		UWOPS03_461_4:D>N		UWOPS05_217_3:D>N		Y55:D>N		Y9:D>N		YIIc17_E5:D>N		YJM978:D>N		YPS128:D>N		YPS606:D>N		YS4:D>N		YS9:D>NID:YDR332W	AA:9		YIIc17_E5:L>	AA:87		UWOPS83_787_3:N>S	AA:117		378604X:H>Q	AA:229		UWOPS05_217_3:T>S	AA:242		DBVPG6040:S>T	AA:286		DBVPG6040:V>A	AA:319		DBVPG1853:N>S		DBVPG6044:N>S		NCYC110:N>S		SK1:N>S		Y55:N>S	AA:322		DBVPG1853:Q>R	AA:399		UWOPS05_227_2:L>Q	AA:406		SK1:I>M		Y55:I>M	AA:421		Y12:G>R	AA:445		DBVPG1853:M>V		SK1:M>V		UWOPS05_227_2:M>V		Y12:M>V		Y55:M>V	AA:446		YIIc17_E5:E>G	AA:453		Y12:S>G		YS9:S>G	AA:455		DBVPG1853:A>V	AA:509		UWOPS05_227_2:P>S	AA:541		UWOPS05_227_2:M>K	AA:610		DBVPG1853:Q>K		DBVPG6044:Q>K		K11:Q>K		SK1:Q>K		Y55:Q>K		Y9:Q>K	AA:686		YPS606:S>FID:YDR336W	AA:18		DBVPG1853:N>S		DBVPG6044:N>S		K11:N>S		SK1:N>S		UWOPS83_787_3:N>S		Y12:N>S		Y55:N>S	AA:43		UWOPS05_227_2:K>R	AA:76		UWOPS05_227_2:S>T	AA:116		YPS128:K>E		YPS606:K>E	AA:292		UWOPS03_461_4:T>IID:YDR339C	AA:36		322134S:E>D		DBVPG6044:E>D		K11:E>D		NCYC110:E>D		SK1:E>D		Y55:E>D		Y9:E>D		YPS128:E>D		YPS606:E>D	AA:57		322134S:Q>E		DBVPG6044:Q>E		K11:Q>E		NCYC110:Q>E		SK1:Q>E		UWOPS05_227_2:Q>E		Y55:Q>E		Y9:Q>E		YPS128:Q>E		YPS606:Q>EID:YDR345C	AA:50		UWOPS83_787_3:T>A		UWOPS87_2421:T>A		Y12:T>A	AA:104		NCYC110:S>T		SK1:S>T		UWOPS03_461_4:S>T		Y55:S>T	AA:107		Y9:L>V	AA:111		Y9:R>T	AA:280		Y12:A>S	AA:368		DBVPG6044:F>L		SK1:F>L		Y55:F>L	AA:392		322134S:I>V		DBVPG1853:I>V		DBVPG6044:I>V		SK1:I>V		UWOPS03_461_4:I>V		UWOPS05_227_2:I>V		UWOPS87_2421:I>V		Y55:I>V		YPS606:I>V	AA:410		273614X:W>L	AA:413		273614X:G>C	AA:459		Y9:S>A	AA:464		DBVPG1853:I>V		UWOPS05_227_2:I>V		YPS128:I>V		YPS606:I>V	AA:537		DBVPG6040:W>RID:YDR346C	AA:115		YS4:Y>C	AA:150		UWOPS83_787_3:D>Y	AA:158		DBVPG6044:P>S		NCYC110:P>S		SK1:P>S	AA:225		UWOPS03_461_4:G>C	AA:252		DBVPG1853:T>A	AA:371		DBVPG6040:L>V		UWOPS87_2421:L>V		YPS128:L>V		YPS606:L>V	AA:378		322134S:E>D	AA:380		UWOPS87_2421:E>V	AA:383		YS4:D>E	AA:454		273614X:E>-ID:YDR348C	AA:11		BC187:R>M		DBVPG6765:R>M		YJM975:R>M	AA:32		YJM978:K>I	AA:105		UWOPS87_2421:K>N	AA:180		UWOPS03_461_4:Y>C		UWOPS05_227_2:Y>C	AA:195		UWOPS03_461_4:S>G		UWOPS05_227_2:S>G	AA:202		DBVPG6040:S>P		DBVPG6044:S>P		K11:S>P		UWOPS03_461_4:S>P		UWOPS05_227_2:S>P		UWOPS83_787_3:S>P		Y55:S>P		YPS128:S>P		YPS606:S>P	AA:270		Y55:A>S	AA:331		322134S:V>L	AA:440		DBVPG1853:P>S		K11:P>S		UWOPS83_787_3:P>S		UWOPS87_2421:P>S		Y12:P>S		Y55:P>S		Y9:P>S		YPS606:P>SID:YDR350C	AA:20		DBVPG1853:P>R		UWOPS83_787_3:P>R		Y9:P>R		YIIc17_E5:P>R		YPS128:P>R		YPS606:P>R	AA:27		L_1528:S>C	AA:29		DBVPG1853:G>E		K11:G>E		UWOPS05_217_3:G>E		UWOPS05_227_2:G>E		UWOPS83_787_3:G>E		Y12:G>E		Y9:G>E		YIIc17_E5:G>E		YPS128:G>E		YPS606:G>E	AA:32		DBVPG1853:T>K		K11:T>K		UWOPS05_217_3:T>K		UWOPS05_227_2:T>K		UWOPS83_787_3:T>K		Y12:T>K		Y9:T>K		YIIc17_E5:T>K		YPS128:T>K		YPS606:T>K	AA:35		UWOPS05_217_3:P>H		UWOPS05_227_2:P>H	AA:51		DBVPG1853:I>S		K11:I>S		UWOPS05_217_3:I>S		UWOPS05_227_2:I>S		UWOPS83_787_3:I>S		Y12:I>S		Y9:I>S		YPS128:I>S		YPS606:I>S	AA:152		DBVPG1853:G>D		K11:G>D		UWOPS03_461_4:G>D		UWOPS05_227_2:G>D		Y12:G>D		Y9:G>D		YPS128:G>D		YPS606:G>D	AA:161		DBVPG6765:H>R	AA:189		DBVPG1853:R>K		UWOPS05_217_3:A>S		UWOPS05_227_2:A>S	AA:313		UWOPS03_461_4:G>R		UWOPS05_217_3:G>R		UWOPS05_227_2:G>R	AA:419		DBVPG6044:T>A		SK1:T>A		Y55:T>A	AA:448		DBVPG1853:I>V	AA:522		YPS606:L>P	AA:534		DBVPG6040:V>A	AA:551		UWOPS87_2421:H>R	AA:570		UWOPS05_227_2:V>LID:YDR352W	AA:21		UWOPS83_787_3:F>L	AA:34		YS4:T>S	AA:173		378604X:V>M		DBVPG1853:V>M		DBVPG6040:V>M		UWOPS03_461_4:V>M		UWOPS83_787_3:V>M		YIIc17_E5:V>M		YPS128:V>M		YPS606:V>M	AA:187		378604X:I>V		DBVPG1853:I>V		DBVPG6040:I>V		K11:I>V		UWOPS03_461_4:I>V		UWOPS83_787_3:I>V		Y12:I>V		Y9:I>V		YIIc17_E5:I>V		YPS128:I>V		YPS606:I>V		YS4:I>V	AA:255		YJM975:L>W	AA:256		K11:P>S		Y9:P>S	AA:280		DBVPG1853:A>VID:YDR353W	AA:150		UWOPS05_217_3:P>HID:YDR354W	AA:39		UWOPS05_227_2:C>Y	AA:294		378604X:E>K		DBVPG6040:E>K		YIIc17_E5:E>KID:YDR357C	AA:35		378604X:M>I		BC187:M>I		DBVPG1373:M>I		DBVPG1788:M>I		DBVPG1853:M>I		DBVPG6044:M>I		DBVPG6765:M>I		K11:M>I		L_1374:M>I		L_1528:M>I		SK1:M>I		UWOPS83_787_3:M>I		Y12:M>I		Y55:M>I		YPS128:M>I		YPS606:M>I	AA:64		YJM978:N>I	AA:95		378604X:I>M		DBVPG1853:I>M		DBVPG6044:I>M		K11:I>M		NCYC110:I>M		SK1:I>M		UWOPS83_787_3:I>M		Y12:I>M		Y55:I>M		YPS128:I>M		YPS606:I>M	AA:120		Y12:K>EID:YDR358W	AA:7		378604X:L>I	AA:55		DBVPG1106:A>T	AA:129		SK1:A>V		Y55:A>V	AA:266		378604X:A>T		DBVPG1106:A>T		DBVPG1373:A>T		DBVPG1853:A>T		DBVPG6044:A>T		DBVPG6765:A>T		L_1374:A>T		SK1:A>T		UWOPS83_787_3:A>T		Y55:A>T		Y9:A>T		YJM978:A>T		YPS128:A>T		YPS606:A>T		YS9:A>T	AA:293		YJM978:A>T	AA:357		DBVPG6040:S>P		DBVPG6044:S>P		K11:S>P		SK1:S>P		UWOPS05_227_2:S>P		Y55:S>P		YPS128:S>P		YPS606:S>P	AA:392		DBVPG6044:A>G		K11:A>G		SK1:A>G		UWOPS05_227_2:A>G		Y55:A>G		YPS128:A>G		YPS606:A>G	AA:426		DBVPG1373:N>S	AA:473		YJM975:Y>F		YJM978:Y>F	AA:520		K11:V>I		SK1:V>I		UWOPS03_461_4:V>I		UWOPS05_227_2:V>I		Y12:V>I		Y55:V>I		YPS128:V>I	AA:553		DBVPG1853:S>T		K11:S>T		SK1:S>T		UWOPS03_461_4:S>T		UWOPS05_227_2:S>T		Y12:S>T		Y55:S>T		YPS128:S>TID:YDR361C	AA:25		L_1528:D>E	AA:82		273614X:R>K	AA:147		Y12:S>N	AA:193		SK1:I>VID:YDR362C	AA:4		UWOPS05_227_2:I>L	AA:44		UWOPS05_227_2:K>N	AA:50		BC187:F>L		DBVPG1106:F>L		DBVPG1853:F>L		DBVPG6040:F>L		DBVPG6044:F>L		DBVPG6765:F>L		K11:F>L		SK1:F>L		UWOPS03_461_4:F>L		UWOPS05_227_2:F>L		UWOPS83_787_3:F>L		Y55:F>L		Y9:F>L		YJM978:F>L		YPS128:F>L		YPS606:F>L		YS2:F>L	AA:55		Y9:H>N	AA:79		DBVPG6040:V>I		DBVPG6044:V>I		K11:V>I		NCYC110:V>I		SK1:V>I		UWOPS03_461_4:V>I		UWOPS05_227_2:V>I		UWOPS83_787_3:V>I		Y55:V>I		Y9:V>I		YPS128:V>I		YPS606:V>I	AA:230		UWOPS87_2421:K>N		Y12:K>N		Y9:K>N	AA:308		YJM978:K>M	AA:317		UWOPS05_227_2:V>L	AA:417		DBVPG1853:T>A	AA:523		YJM975:I>V	AA:540		273614X:S>P		BC187:S>P		DBVPG1373:S>P		DBVPG6040:S>P		DBVPG6044:S>P		DBVPG6765:S>P		K11:S>P		L_1374:S>P		SK1:S>P		UWOPS05_227_2:S>P		UWOPS87_2421:S>P		Y12:S>P		Y55:S>P		YIIc17_E5:S>P		YJM975:S>P		YJM978:S>P		YPS128:S>P		YPS606:S>P		YS2:S>PID:YDR363W	AA:36		378604X:Y>F		DBVPG6040:Y>F		DBVPG6044:Y>F		K11:Y>F		SK1:Y>F		UWOPS87_2421:Y>F		Y55:Y>F		Y9:Y>F		YIIc17_E5:Y>F		YPS128:Y>F		YPS606:Y>F	AA:63		YPS128:S>P		YPS606:S>P	AA:136		378604X:F>L		NCYC110:F>L		SK1:F>L		UWOPS87_2421:F>L		Y55:F>L		Y9:F>L		YIIc17_E5:F>L		YPS128:F>L		YPS606:F>L		YS9:F>L	AA:254		YS9:E>G	AA:287		Y12:I>V		Y9:I>V	AA:321		YPS128:Q>R		YPS606:Q>R	AA:384		Y12:M>I		Y9:M>IID:YDR363W-A	AA:41		DBVPG6044:D>N		SK1:D>N		Y55:D>NID:YDR364C	AA:21		BC187:K>E	AA:49		BC187:S>P	AA:50		YS4:H>R	AA:77		BC187:D>N	AA:82		DBVPG6044:A>T		NCYC110:A>T		SK1:A>T	AA:189		K11:I>M		Y9:I>M	AA:203		YS4:Q>R	AA:206		322134S:N>S		378604X:N>S		K11:N>S		Y9:N>S		YIIc17_E5:N>S		YPS128:N>S		YS4:N>S	AA:320		YIIc17_E5:W>LID:YDR368W	AA:10		L_1374:A>G	AA:34		378604X:N>S		YIIc17_E5:N>S		YPS128:N>S		YPS606:N>S	AA:275		Y12:F>LID:YDR372C	AA:88		UWOPS87_2421:W>CID:YDR373W	AA:60		273614X:S>Y	AA:123		UWOPS05_217_3:T>AID:YDR374C	AA:76		DBVPG6044:H>Q		SK1:H>Q		Y55:H>Q	AA:83		UWOPS03_461_4:I>N		UWOPS05_217_3:I>N		UWOPS05_227_2:I>N		UWOPS83_787_3:I>N	AA:96		DBVPG6040:H>N		K11:H>N	AA:102		273614X:D>G		DBVPG1788:D>G		DBVPG6040:D>G		DBVPG6044:D>G		SK1:D>G		UWOPS03_461_4:D>G		UWOPS05_217_3:D>G		Y12:D>G		Y55:D>G		Y9:D>G		YJM975:D>G		YJM978:D>G		YPS128:D>G		YPS606:D>G	AA:142		273614X:A>S		DBVPG1373:A>S		DBVPG1788:A>S		DBVPG1853:A>S		DBVPG6040:A>S		L_1374:A>S		SK1:A>S		UWOPS03_461_4:A>S		UWOPS05_217_3:A>S		Y12:A>S		Y55:A>S		Y9:A>S		YJM975:A>S		YJM978:A>S		YPS128:A>S		YPS606:A>S	AA:292		Y9:T>SID:YDR375C	AA:251		UWOPS03_461_4:W>L	AA:258		YS9:P>S	AA:315		UWOPS05_217_3:I>M		UWOPS05_227_2:I>MID:YDR377W	AA:9		DBVPG1788:T>AID:YDR378C	AA:74		UWOPS03_461_4:R>I		UWOPS05_217_3:R>I	AA:96		273614X:N>D		322134S:N>D	AA:106		273614X:S>N		322134S:S>N		378604X:S>N		DBVPG6044:S>N		NCYC110:S>N		SK1:S>N		UWOPS03_461_4:S>N		UWOPS05_217_3:S>N		UWOPS83_787_3:S>N		Y55:S>N		YPS128:S>N		YPS606:S>N		YS4:S>NID:YDR379C-A	AA:17		UWOPS05_217_3:R>M	AA:18		DBVPG6044:A>V		NCYC110:A>V		SK1:A>V		Y55:A>V	AA:54		L_1374:T>I	AA:56		378604X:I>MID:YDR379W	AA:117		Y12:L>V	AA:138		DBVPG6044:T>K		SK1:T>K		Y55:T>K	AA:165		YJM978:N>S	AA:318		378604X:V>I		DBVPG6044:V>I		NCYC110:V>I		SK1:V>I		UWOPS05_227_2:V>I		Y55:V>I		YPS606:V>I		YS4:V>I	AA:364		DBVPG6044:S>P		NCYC110:S>P		SK1:S>P		Y55:S>P		YPS606:S>P		YS4:S>P	AA:426		DBVPG6044:P>T		NCYC110:P>T		SK1:P>T	AA:461		Y12:S>G	AA:470		Y12:H>Y	AA:501		DBVPG6044:I>M		NCYC110:I>M		SK1:I>M	AA:509		378604X:G>E		DBVPG6044:G>E		NCYC110:G>E		SK1:G>E		Y12:G>E		YPS606:G>E		YS4:G>E	AA:533		YPS606:N>D	AA:537		378604X:T>I		DBVPG6044:T>I		NCYC110:T>I		SK1:T>I		Y12:T>I		YIIc17_E5:T>I		YPS606:T>I	AA:588		SK1:Q>L		Y12:Q>R	AA:757		378604X:V>A		DBVPG6044:V>A		K11:V>A		SK1:V>A		UWOPS03_461_4:V>A		UWOPS05_217_3:V>A		UWOPS87_2421:V>A		Y55:V>A		YPS606:V>A		YS2:V>A	AA:803		UWOPS03_461_4:I>T		UWOPS05_217_3:I>T	AA:807		DBVPG6044:C>Y		NCYC110:C>Y		SK1:C>Y		Y55:C>Y	AA:934		DBVPG1373:I>NID:YDR380W	AA:158		YJM975:P>S	AA:245		UWOPS05_217_3:N>S	AA:256		UWOPS05_217_3:T>I	AA:322		378604X:C>-	AA:343		DBVPG1788:T>I	AA:374		L_1528:I>T	AA:430		YPS128:M>V		YPS606:M>VID:YDR382W	AA:93		378604X:E>QID:YDR383C	AA:125		Y12:V>I	AA:130		UWOPS05_217_3:E>K		UWOPS05_227_2:E>K		UWOPS87_2421:E>K	AA:185		273614X:S>F		DBVPG6044:S>F		SK1:S>F		Y55:S>F	AA:195		322134S:A>SID:YDR385W	AA:227		DBVPG6040:T>A	AA:252		DBVPG6040:P>S	AA:490		UWOPS87_2421:Q>R	AA:528		UWOPS83_787_3:H>NID:YDR387C	AA:25		DBVPG6040:A>T	AA:49		YS2:G>D	AA:62		SK1:G>S	AA:64		SK1:L>Q	AA:70		Y12:E>A	AA:91		YIIc17_E5:V>I	AA:141		UWOPS03_461_4:L>F		UWOPS05_217_3:L>F		UWOPS05_227_2:L>F	AA:196		YS9:D>E	AA:198		DBVPG1373:S>P	AA:225		YS9:R>G	AA:236		273614X:T>A		322134S:T>A		378604X:T>A		DBVPG1373:T>A		DBVPG1788:T>A		DBVPG6765:T>A		NCYC110:T>A		SK1:T>A		UWOPS05_217_3:T>A		UWOPS05_227_2:T>A		UWOPS83_787_3:T>A		Y12:T>A		Y55:T>A		YIIc17_E5:T>A		YPS606:T>A	AA:314		NCYC110:M>V		SK1:M>V		UWOPS05_217_3:M>V		UWOPS05_227_2:M>V		UWOPS83_787_3:M>V		Y12:M>V		Y55:M>V		YIIc17_E5:M>L		YPS606:M>V	AA:335		YPS606:S>F	AA:376		YJM978:I>T	AA:388		DBVPG1106:P>S	AA:414		DBVPG6044:A>S		Y55:A>S	AA:418		DBVPG1788:R>K	AA:437		378604X:D>N		BC187:D>N		DBVPG1373:D>N		DBVPG1788:D>N		DBVPG1853:D>N		DBVPG6044:D>N		DBVPG6765:D>N		UWOPS05_217_3:D>N		UWOPS05_227_2:D>N		UWOPS83_787_3:D>N		Y55:D>N		YIIc17_E5:D>N		YPS606:D>N	AA:444		YPS606:I>F	AA:539		378604X:R>K		DBVPG1373:R>K		DBVPG1788:R>K		DBVPG1853:R>K		DBVPG6044:R>K		DBVPG6765:R>K		SK1:R>K		UWOPS05_217_3:R>K		UWOPS05_227_2:R>K		Y55:R>K		YIIc17_E5:R>K		YPS606:R>KID:YDR388W	AA:126		273614X:E>G	AA:176		YS4:D>Y	AA:254		YS9:E>K	AA:296		YIIc17_E5:A>T	AA:317		YS4:A>V	AA:333		DBVPG6044:A>V		SK1:A>V		Y55:A>V	AA:354		YIIc17_E5:A>G	AA:406		Y12:T>I	AA:480		YS9:N>ID:YDR389W	AA:15		UWOPS05_217_3:V>D	AA:56		UWOPS03_461_4:P>	AA:92		UWOPS05_217_3:N>I	AA:164		Y12:N>S		Y9:N>S	AA:260		UWOPS05_217_3:T>A		UWOPS05_227_2:T>A		UWOPS83_787_3:T>A		Y9:T>A		YPS128:T>A		YPS606:T>A	AA:313		NCYC361:K>N	AA:418		UWOPS05_217_3:D>Y		UWOPS05_227_2:D>Y	AA:426		DBVPG6044:E>D		NCYC110:E>D		SK1:E>D		Y55:E>D	AA:447		UWOPS05_217_3:A>V		UWOPS05_227_2:A>V		UWOPS87_2421:A>V		YPS128:A>V		YPS606:A>V	AA:465		UWOPS05_227_2:S>A	AA:472		W303:D>	AA:473		YPS606:L>P	AA:497		UWOPS03_461_4:V>A		UWOPS05_227_2:V>A	AA:550		NCYC110:Q>L		SK1:Q>L		Y55:Q>L	AA:586		NCYC110:T>A		SK1:T>A		Y55:T>A	AA:601		K11:A>TID:YDR391C	AA:4		DBVPG6044:E>K		NCYC110:E>K		SK1:E>K		Y55:E>K	AA:38		UWOPS03_461_4:S>A		UWOPS05_217_3:S>A		Y9:S>A		YPS128:S>A		YPS606:S>A	AA:51		322134S:C>S		DBVPG1853:C>S		DBVPG6044:C>S		NCYC110:C>S		NCYC361:C>S		SK1:C>S		Y55:C>S	AA:62		DBVPG6040:K>E		K11:K>E		YIIc17_E5:K>E	AA:178		UWOPS03_461_4:E>K	AA:193		UWOPS83_787_3:M>L	AA:200		DBVPG6040:C>G		DBVPG6044:C>G		K11:C>G		NCYC110:C>G		SK1:C>G		UWOPS03_461_4:C>G		UWOPS83_787_3:C>G		UWOPS87_2421:C>G		Y55:C>G		Y9:C>G		YIIc17_E5:C>G		YPS128:C>G		YPS606:C>G	AA:208		DBVPG1106:C>F	AA:212		YS2:G>VID:YDR392W	AA:3		UWOPS05_227_2:D>N	AA:96		YS4:A>V	AA:151		DBVPG1106:E>A	AA:158		273614X:D>E	AA:326		YPS128:K>-ID:YDR393W	AA:20		DBVPG6040:L>F		DBVPG6044:L>F		SK1:L>F		UWOPS83_787_3:L>F		Y55:L>F	AA:24		DBVPG1853:A>P		DBVPG6040:A>P		DBVPG6044:A>P		L_1528:A>P		NCYC361:A>P		SK1:A>P		UWOPS83_787_3:A>P		Y55:A>P		YJM978:A>P		YPS128:A>P	AA:28		DBVPG1853:T>A		DBVPG6040:T>A		DBVPG6044:T>A		L_1528:T>A		NCYC361:T>A		SK1:T>A		UWOPS83_787_3:T>A		Y55:T>A		Y9:T>A		YJM978:T>A	AA:37		DBVPG1853:S>P	AA:39		Y9:Q>K	AA:41		DBVPG6040:G>D		UWOPS83_787_3:G>D		YPS128:G>D	AA:52		DBVPG1853:N>S		DBVPG6040:N>S		DBVPG6044:N>S		SK1:N>S		UWOPS83_787_3:N>S		Y55:N>S		Y9:N>S		YJM978:N>S		YPS128:N>S		YS4:N>S	AA:60		DBVPG6040:A>T		DBVPG6044:A>T		SK1:A>T		UWOPS83_787_3:A>T		Y55:A>T		Y9:A>T		YPS128:A>T	AA:77		322134S:L>I	AA:243		DBVPG1106:K>E	AA:253		DBVPG1106:Q>R	AA:258		DBVPG1106:K>E	AA:296		322134S:T>I	AA:366		DBVPG6040:E>K		K11:E>K		Y9:E>K		YIIc17_E5:E>K		YPS606:E>K		YS4:E>K	AA:370		YPS606:V>A	AA:372		NCYC110:E>Q		SK1:E>Q		Y55:E>Q	AA:380		UWOPS87_2421:C>Y	AA:387		DBVPG6040:I>T		K11:I>T		UWOPS05_217_3:I>T		UWOPS83_787_3:I>T		UWOPS87_2421:I>T		YIIc17_E5:I>T		YPS128:I>T		YPS606:I>T		YS4:I>T	AA:397		DBVPG6040:T>A		YIIc17_E5:T>A		YPS128:T>A		YPS606:T>A	AA:398		DBVPG1853:T>I	AA:401		273614X:E>G		322134S:E>G		378604X:E>G		DBVPG1106:E>G		DBVPG1373:E>G		DBVPG1788:E>G		DBVPG1853:E>G		DBVPG6040:E>A		DBVPG6765:E>G		K11:E>A		L_1528:E>G		UWOPS05_217_3:E>G		UWOPS83_787_3:E>A		UWOPS87_2421:E>A		YIIc17_E5:E>A		YPS128:E>A		YPS606:E>A		YS4:E>GID:YDR394W	AA:152		YGPM:P>LID:YDR397C	AA:14		UWOPS83_787_3:T>P	AA:101		UWOPS83_787_3:T>SID:YDR398W	AA:125		UWOPS87_2421:A>T	AA:149		UWOPS87_2421:N>S	AA:268		YJM978:R>K	AA:282		DBVPG1373:V>A		DBVPG1788:V>A		DBVPG1853:V>A		DBVPG6765:V>A		L_1374:V>A		L_1528:V>A		SK1:V>A		UWOPS83_787_3:V>A		UWOPS87_2421:V>A		Y12:V>A		Y55:V>A		YIIc17_E5:V>A		YJM978:V>A		YS4:V>A	AA:314		UWOPS87_2421:F>I	AA:340		DBVPG6765:S>P	AA:388		Y12:Q>H	AA:393		322134S:P>T		DBVPG1853:P>T		DBVPG6765:P>T		L_1528:P>T		SK1:P>T		Y12:P>T		Y55:P>T		YIIc17_E5:P>T		YJM978:P>T		YS2:P>T	AA:426		Y12:S>F	AA:437		273614X:V>E	AA:453		UWOPS83_787_3:L>M	AA:635		YS2:A>VID:YDR399W	AA:169		UWOPS87_2421:A>V		YJM978:A>V	AA:219		UWOPS05_217_3:H>RID:YDR400W	AA:231		322134S:L>F		378604X:L>F		DBVPG6044:L>F		SK1:L>F		UWOPS05_217_3:L>F		UWOPS83_787_3:L>F		Y12:L>F		Y55:L>F		Y9:L>F	AA:270		DBVPG6044:P>L		SK1:P>L		Y55:P>L	AA:285		322134S:C>-	AA:306		273614X:D>G		DBVPG1853:D>G		DBVPG6765:D>G		L_1528:D>G		YJM975:D>G		YJM978:D>G		YJM981:D>G	AA:309		UWOPS03_461_4:V>I		UWOPS05_217_3:V>I	AA:313		273614X:I>V		378604X:I>V		DBVPG1853:I>V		DBVPG6040:I>V		DBVPG6044:I>V		L_1528:I>V		SK1:I>V		UWOPS03_461_4:I>V		UWOPS05_217_3:I>V		Y12:I>V		Y55:I>V		Y9:I>V		YJM975:I>V		YJM978:I>V		YJM981:I>VID:YDR403W	AA:9		BC187:S>P		DBVPG1106:S>P		DBVPG1373:S>P		DBVPG1788:S>P		DBVPG6765:S>P		YJM978:S>P		YS2:S>P		YS4:S>P		YS9:S>P	AA:18		DBVPG6040:P>	AA:26		BC187:T>A		DBVPG1106:T>A		DBVPG1373:T>A		DBVPG1788:T>A		DBVPG6765:T>A		L_1374:T>A		YJM978:T>A		YS2:T>A		YS4:T>A		YS9:T>A	AA:84		DBVPG1853:N>H		DBVPG6040:N>H	AA:89		322134S:E>G		DBVPG1106:E>G		DBVPG1788:E>G		DBVPG1853:E>G		DBVPG6040:E>G		DBVPG6044:E>G		DBVPG6765:E>G		K11:E>G		L_1374:E>G		NCYC110:E>G		SK1:E>G		UWOPS03_461_4:E>G		UWOPS83_787_3:E>G		Y12:E>G		Y9:E>G		YJM978:E>G		YPS128:E>G		YS2:E>G		YS9:E>G	AA:125		BC187:D>G		DBVPG1106:D>G		DBVPG1373:D>G		DBVPG6765:D>G		L_1374:D>G		L_1528:D>G		YJM978:D>G		YS2:D>G		YS9:D>G	AA:146		W303:W>R	AA:154		W303:Q>L	AA:169		UWOPS05_217_3:V>F		UWOPS05_227_2:V>F	AA:182		DBVPG1853:N>I		UWOPS05_217_3:N>S		UWOPS05_227_2:N>S	AA:183		NCYC110:L>M		SK1:L>M		Y55:L>M	AA:212		YS9:L>M	AA:257		UWOPS03_461_4:I>M		UWOPS05_217_3:I>M		UWOPS05_227_2:I>M	AA:321		DBVPG1853:S>N	AA:512		DBVPG6765:M>RID:YDR404C	AA:80		DBVPG1373:K>TID:YDR405W	AA:11		K11:M>V		Y9:M>V	AA:113		K11:F>L	AA:131		DBVPG6040:M>I	AA:138		K11:L>R	AA:181		YIIc17_E5:F>L	AA:185		YIIc17_E5:T>S	AA:248		UWOPS05_217_3:R>CID:YDR408C	AA:29		DBVPG6044:E>K		NCYC110:E>K		SK1:E>K		Y55:E>K	AA:151		UWOPS03_461_4:K>M		UWOPS05_217_3:K>M	AA:199		378604X:I>V		DBVPG1853:I>V		K11:I>V		Y12:I>V		Y9:I>VID:YDR410C	AA:130		DBVPG6044:I>S		SK1:I>S		Y55:I>S	AA:193		273614X:L>V		378604X:L>V		DBVPG1106:L>V		DBVPG1373:L>V		DBVPG1788:L>V		DBVPG1853:L>V		DBVPG6044:L>V		DBVPG6765:L>V		L_1374:L>V		SK1:L>V		UWOPS03_461_4:L>V		UWOPS05_227_2:L>V		Y12:L>V		Y55:L>V		Y9:L>V		YIIc17_E5:L>V		YS4:L>V	AA:200		DBVPG1853:F>SID:YDR411C	AA:10		YIIc17_E5:L>F	AA:63		322134S:N>S		378604X:N>S		DBVPG6040:N>S		DBVPG6044:N>S		NCYC110:N>S		NCYC361:N>S		SK1:N>S		UWOPS03_461_4:N>S		UWOPS05_227_2:N>S		Y55:N>S		YIIc17_E5:N>S		YPS128:N>S		YPS606:N>S	AA:133		378604X:A>T		DBVPG6040:A>T		DBVPG6044:A>T		NCYC110:A>T		NCYC361:A>T		SK1:A>T		UWOPS03_461_4:A>T		UWOPS05_217_3:A>T		UWOPS05_227_2:A>T		Y55:A>T		YPS128:A>T		YPS606:A>T	AA:215		DBVPG6040:C>S	AA:237		DBVPG6040:G>E	AA:265		378604X:A>T		DBVPG6040:A>T		DBVPG6044:A>T		NCYC110:A>T		UWOPS03_461_4:A>T		UWOPS05_217_3:A>T		YPS128:A>T	AA:272		YS9:A>T	AA:281		UWOPS03_461_4:T>M		UWOPS05_217_3:T>M	AA:301		DBVPG1106:T>S	AA:303		Y12:G>DID:YDR412W	AA:40		YJM981:L>M	AA:152		NCYC110:S>AID:YDR414C	AA:54		DBVPG6044:I>M		NCYC110:I>M		SK1:I>M		Y55:I>M	AA:100		322134S:M>K		378604X:M>K		DBVPG6044:M>K		K11:M>K		NCYC110:M>K		SK1:M>K		UWOPS03_461_4:M>K		UWOPS05_227_2:M>K		Y55:M>K		Y9:M>K		YIIc17_E5:M>K		YPS128:M>K	AA:110		K11:I>T	AA:117		322134S:L>P	AA:131		322134S:K>N	AA:134		L_1528:Q>H	AA:148		K11:S>F	AA:168		322134S:G>A		378604X:G>A		DBVPG1106:G>A		DBVPG1853:G>A		DBVPG6044:G>A		DBVPG6765:G>A		K11:G>A		L_1374:G>A		NCYC110:G>A		SK1:G>A		UWOPS03_461_4:G>A		UWOPS05_227_2:G>A		Y55:G>A		Y9:G>A		YJM975:G>A		YJM981:G>A		YPS128:G>A	AA:214		378604X:E>D		DBVPG6044:E>D		K11:E>D		NCYC110:E>D		SK1:E>D		UWOPS03_461_4:E>D		UWOPS05_227_2:E>D		UWOPS87_2421:E>D		Y55:E>D		Y9:E>D		YPS128:E>D	AA:232		YS4:F>L	AA:236		378604X:R>K		DBVPG6044:R>K		K11:R>K		NCYC110:R>K		SK1:R>K		UWOPS03_461_4:R>K		UWOPS05_227_2:R>K		UWOPS87_2421:R>K		Y55:R>K		Y9:R>K		YPS128:R>K	AA:329		UWOPS03_461_4:I>LID:YDR415C	AA:64		378604X:F>L	AA:91		378604X:S>L		DBVPG6044:S>L		NCYC110:S>L		SK1:S>L		UWOPS05_217_3:S>L		UWOPS05_227_2:S>L		UWOPS87_2421:S>L		Y55:S>L		YPS606:S>L	AA:162		UWOPS05_217_3:V>F		UWOPS05_227_2:V>F	AA:166		DBVPG6044:T>N		SK1:T>N		Y55:T>N	AA:173		378604X:I>V		DBVPG6044:I>V		SK1:I>V		UWOPS05_217_3:I>V		UWOPS05_227_2:I>V		UWOPS87_2421:I>V		Y12:I>V		Y55:I>V		YIIc17_E5:I>V		YPS606:I>V	AA:205		YPS606:M>I	AA:260		Y9:Q>HID:YDR416W	AA:1		UWOPS83_787_3:M>T		YPS606:M>T	AA:55		DBVPG6044:K>Q		K11:K>Q		NCYC110:K>Q		SK1:K>Q		UWOPS83_787_3:K>Q		UWOPS87_2421:K>Q		YIIc17_E5:K>Q		YPS606:K>Q	AA:86		DBVPG6044:E>G		K11:E>G		NCYC110:E>G		SK1:E>G		UWOPS05_217_3:E>G		UWOPS05_227_2:E>G		UWOPS83_787_3:E>G		UWOPS87_2421:E>G		YIIc17_E5:E>G		YPS606:E>G	AA:133		DBVPG1853:A>D		DBVPG6044:A>D		K11:A>D		NCYC110:A>D		SK1:A>D		UWOPS05_217_3:A>D		UWOPS05_227_2:A>D		UWOPS83_787_3:A>D		UWOPS87_2421:A>D		Y55:A>D		YIIc17_E5:A>D		YPS606:A>D	AA:176		DBVPG6044:T>I		K11:T>I		NCYC110:T>I		SK1:T>I		UWOPS05_217_3:T>I		UWOPS05_227_2:T>I		UWOPS83_787_3:T>I		UWOPS87_2421:T>I		Y55:T>I		YIIc17_E5:T>I		YPS606:T>I	AA:201		DBVPG6044:S>G		K11:S>G		NCYC110:S>G		SK1:S>G		UWOPS05_217_3:S>G		UWOPS05_227_2:S>G		UWOPS83_787_3:S>G		UWOPS87_2421:S>G		Y55:S>G		YIIc17_E5:S>G		YPS606:S>G	AA:242		YIIc17_E5:T>P	AA:248		YIIc17_E5:E>K	AA:263		YIIc17_E5:S>R	AA:270		YIIc17_E5:L>S	AA:276		UWOPS05_217_3:A>V		UWOPS05_227_2:A>V	AA:320		YS4:D>	AA:359		YPS606:G>R	AA:363		Y12:E>G	AA:368		YPS606:F>L	AA:463		UWOPS05_227_2:I>T		UWOPS83_787_3:I>T	AA:572		DBVPG6044:M>T		K11:M>T		NCYC110:M>T		SK1:M>T		Y9:M>T		YPS128:M>T		YPS606:M>T	AA:599		DBVPG6044:Y>H		K11:Y>H		NCYC110:Y>H		SK1:Y>H		UWOPS05_217_3:Y>H		UWOPS05_227_2:Y>H		UWOPS83_787_3:Y>H		Y9:Y>H		YIIc17_E5:Y>H		YPS128:Y>H		YPS606:Y>H	AA:653		NCYC110:K>E		SK1:K>E		UWOPS05_217_3:K>E		UWOPS05_227_2:K>E		UWOPS83_787_3:K>E		Y9:K>E		YIIc17_E5:K>E		YPS128:K>E		YPS606:K>E	AA:681		UWOPS05_217_3:S>N		UWOPS05_227_2:S>N	AA:696		YJM981:V>A	AA:697		DBVPG1853:S>N	AA:737		W303:P>S	AA:774		YS2:P>S		YS4:P>S	AA:787		YJM981:F>S	AA:819		DBVPG1853:S>N	AA:842		UWOPS03_461_4:T>N		UWOPS05_227_2:T>N		Y12:T>N		Y9:T>N		YIIc17_E5:T>N		YPS128:T>N		YPS606:T>N		YS4:T>NID:YDR419W	AA:121		UWOPS05_227_2:V>M	AA:168		DBVPG6044:M>I		NCYC110:M>I		SK1:M>I		Y55:M>I	AA:185		378604X:D>G	AA:344		DBVPG6044:E>G		SK1:E>G		UWOPS05_217_3:E>G		UWOPS05_227_2:E>G		Y55:E>G	AA:385		DBVPG6040:P>S	AA:394		YJM975:S>N	AA:425		DBVPG6040:S>C	AA:456		DBVPG6040:R>-	AA:545		DBVPG6765:E>K	AA:555		378604X:Y>C		DBVPG1853:Y>C		DBVPG6044:Y>C		K11:Y>C		SK1:Y>C		UWOPS05_217_3:Y>C		UWOPS05_227_2:Y>C		Y12:Y>C		Y55:Y>C		YPS606:Y>C		YS4:Y>C	AA:561		UWOPS05_217_3:D>N	AA:564		378604X:A>T	AA:569		DBVPG1853:A>T	AA:616		DBVPG6044:K>R		SK1:K>R		Y55:K>RID:YDR421W	AA:7		378604X:P>S		DBVPG6040:P>S		NCYC361:P>S		UWOPS05_217_3:P>S		UWOPS05_227_2:P>S		UWOPS83_787_3:P>S		UWOPS87_2421:P>S		YPS128:P>S		YPS606:P>S	AA:40		UWOPS05_217_3:V>I		UWOPS05_227_2:V>I	AA:77		DBVPG6040:A>T		NCYC361:A>T		UWOPS05_217_3:A>T		UWOPS05_227_2:A>T		UWOPS87_2421:A>T		YPS128:A>T		YPS606:A>T	AA:81		DBVPG6040:P>S		NCYC361:P>S		Y12:P>S	AA:109		DBVPG1106:I>T		DBVPG1373:I>T		DBVPG1788:I>T		DBVPG6040:I>T		DBVPG6765:I>T		L_1374:I>T		L_1528:I>T		NCYC110:I>T		NCYC361:I>T		UWOPS83_787_3:I>T		Y12:I>T		Y55:I>T		YPS128:I>T		YPS606:I>T	AA:111		DBVPG6040:S>N		NCYC361:S>N		UWOPS05_217_3:S>N		UWOPS05_227_2:S>N		UWOPS83_787_3:S>N		Y12:S>N		YPS128:S>N		YPS606:S>N	AA:154		378604X:E>D		NCYC361:E>D		UWOPS05_217_3:E>D		UWOPS05_227_2:E>D		UWOPS83_787_3:E>D		Y12:E>D		Y9:E>D		YPS606:E>D	AA:163		378604X:K>E		DBVPG6040:K>E		NCYC361:K>E		UWOPS05_217_3:K>E		UWOPS05_227_2:K>E		UWOPS83_787_3:K>E		Y12:K>E		Y9:K>E		YPS606:K>E	AA:192		378604X:E>K		DBVPG6040:E>K		UWOPS05_217_3:E>K		UWOPS05_227_2:E>K		UWOPS83_787_3:E>K		Y12:E>K		Y9:E>K		YPS128:E>K		YPS606:E>K	AA:221		378604X:P>T		DBVPG6040:P>T		UWOPS05_217_3:P>T		UWOPS83_787_3:P>T		Y12:P>T		Y9:P>T		YPS128:P>T		YPS606:P>T	AA:230		378604X:D>E		DBVPG6040:D>E		UWOPS05_227_2:D>E		UWOPS83_787_3:D>E		Y12:D>E		Y9:D>E		YIIc17_E5:D>E		YPS128:D>E		YPS606:D>E	AA:290		UWOPS05_217_3:P>H		UWOPS05_227_2:P>H	AA:368		YIIc17_E5:A>T	AA:459		DBVPG6044:N>S		SK1:N>S		Y55:N>S	AA:474		UWOPS05_217_3:N>D		UWOPS05_227_2:N>D		UWOPS83_787_3:N>D		UWOPS87_2421:N>D		Y12:N>D		YIIc17_E5:N>D		YPS606:N>D	AA:502		DBVPG1853:N>K		L_1528:N>I		SK1:N>K		Y55:N>K	AA:587		UWOPS03_461_4:S>F	AA:597		YJM978:Y>N	AA:828		YS4:E>D	AA:936		DBVPG6040:N>D		UWOPS05_227_2:N>D		YIIc17_E5:N>D		YPS128:N>DID:YDR425W	AA:56		DBVPG6040:P>S	AA:96		Y12:P>	AA:109		273614X:S>N	AA:216		YS2:I>S	AA:300		Y12:P>A	AA:308		NCYC361:S>P	AA:324		UWOPS83_787_3:I>V	AA:387		378604X:K>R		UWOPS87_2421:K>R	AA:500		YS4:K>E	AA:524		DBVPG1373:K>E		DBVPG1788:K>E		YJM975:K>E		YJM978:K>E		YS4:K>E		YS9:K>E	AA:579		YJM975:N>K		YJM978:N>K		YS9:N>KID:YDR427W	AA:7		UWOPS03_461_4:I>V		UWOPS05_217_3:I>V	AA:12		DBVPG1853:S>I	AA:117		378604X:N>D		DBVPG6044:N>D		NCYC361:N>D		SK1:N>D		UWOPS03_461_4:N>D		UWOPS05_217_3:N>D		UWOPS87_2421:N>D		Y12:N>D		Y55:N>D		YPS606:N>D	AA:123		DBVPG1373:G>D	AA:170		322134S:S>NID:YDR429C	AA:139		DBVPG6040:N>S		DBVPG6044:N>S		NCYC110:N>S		SK1:N>S		UWOPS03_461_4:N>S		UWOPS05_227_2:N>S		UWOPS83_787_3:N>S		Y12:N>S		Y55:N>S		YPS128:N>S		YPS606:N>S	AA:155		DBVPG1373:G>D	AA:157		DBVPG6044:A>V		NCYC110:A>V		SK1:A>V		Y55:A>V	AA:165		DBVPG1373:V>I		L_1528:V>IID:YDR434W	AA:21		UWOPS03_461_4:L>F		UWOPS05_217_3:L>F	AA:89		DBVPG1106:R>Q		DBVPG1373:R>Q		DBVPG1788:R>Q		DBVPG1853:R>Q		DBVPG6765:R>Q		L_1374:R>Q		L_1528:R>Q		NCYC361:R>Q		UWOPS05_217_3:R>Q		UWOPS87_2421:R>Q		YJM975:R>Q		YJM978:R>Q		YJM981:R>Q	AA:201		SK1:G>V		Y55:G>V	AA:256		YS9:F>C	AA:271		SK1:A>T		UWOPS05_217_3:A>T		UWOPS05_227_2:A>T		Y55:A>T		Y9:A>T		YPS128:A>T		YPS606:A>T	AA:312		YS9:W>G	AA:315		YS9:Y>S	AA:330		DBVPG1788:L>W	AA:346		DBVPG1788:Y>S	AA:368		322134S:P>	AA:443		SK1:L>Q		Y55:L>QID:YDR435C	AA:7		378604X:Q>H	AA:11		UWOPS03_461_4:D>N		UWOPS05_217_3:D>N	AA:88		Y12:A>T	AA:98		DBVPG6044:K>E		UWOPS03_461_4:K>E		UWOPS05_217_3:K>E		UWOPS83_787_3:K>E		Y12:K>E		YIIc17_E5:K>E		YPS128:K>E		YPS606:K>E	AA:118		YIIc17_E5:M>I	AA:121		YIIc17_E5:H>Q	AA:138		K11:S>N	AA:148		BC187:I>T		DBVPG6044:I>T		K11:I>T		SK1:I>T		UWOPS83_787_3:I>T		Y12:I>T		YIIc17_E5:I>T		YPS128:I>T		YPS606:I>T		YS2:I>T	AA:158		YIIc17_E5:A>V	AA:167		YS2:G>E	AA:188		DBVPG6044:V>I		K11:V>I		SK1:V>I		UWOPS03_461_4:V>I		UWOPS05_217_3:V>I		UWOPS83_787_3:V>I		UWOPS87_2421:V>I		Y12:V>I		YIIc17_E5:V>I		YPS128:V>I		YPS606:V>I	AA:230		378604X:S>F	AA:236		K11:G>D	AA:284		UWOPS87_2421:N>S	AA:291		322134S:N>Y		NCYC361:N>Y	AA:324		YJM975:M>KID:YDR436W	AA:54		SK1:R>H		Y55:R>H	AA:67		YJM975:T>M		YJM978:T>M		YJM981:T>M	AA:76		YS4:T>A	AA:129		SK1:P>R		Y55:P>R	AA:144		NCYC361:S>N	AA:193		DBVPG1373:S>Y		DBVPG1788:S>Y		DBVPG1853:S>Y		DBVPG6765:S>Y		K11:S>Y		L_1374:S>Y		L_1528:S>Y		SK1:S>Y		UWOPS05_217_3:S>Y		UWOPS87_2421:S>Y		Y55:S>Y		Y9:S>Y		YJM975:S>Y		YJM978:S>Y		YJM981:S>Y		YPS128:S>Y		YPS606:S>Y		YS2:S>Y		YS4:S>Y	AA:241		K11:N>S	AA:273		L_1528:L>Q		YJM975:L>Q		YJM978:L>Q	AA:475		UWOPS05_217_3:A>V	AA:494		DBVPG1106:I>VID:YDR437W	AA:112		W303:F>S	AA:122		DBVPG1373:S>P		DBVPG1788:S>P		DBVPG6044:S>P		DBVPG6765:S>P		K11:S>P		L_1374:S>P		L_1528:S>P		SK1:S>P		UWOPS03_461_4:S>P		Y12:S>P		Y55:S>P		YIIc17_E5:S>P		YJM981:S>P		YPS128:S>P		YS2:S>PID:YDR438W	AA:59		SK1:T>A		Y55:T>A	AA:68		K11:S>T		SK1:S>T	AA:277		378604X:S>F		NCYC110:S>F		SK1:S>F		UWOPS87_2421:S>F		Y55:S>F		YIIc17_E5:S>F	AA:307		YS9:T>ID:YDR439W	AA:98		BC187:I>V	AA:162		DBVPG6044:G>V		SK1:G>V		Y55:G>V	AA:295		UWOPS03_461_4:G>R		UWOPS05_227_2:G>RID:YDR441C	AA:128		273614X:V>I		BC187:V>I		DBVPG1788:V>I		K11:V>I		L_1528:V>I		NCYC361:V>I		SK1:V>I		UWOPS03_461_4:V>I		UWOPS05_217_3:V>I		UWOPS05_227_2:V>I		UWOPS83_787_3:V>I		Y55:V>I		Y9:V>I		YIIc17_E5:V>I		YJM975:V>I		YPS128:V>I		YPS606:V>IID:YDR446W	AA:27		UWOPS03_461_4:D>E		UWOPS05_227_2:D>E		UWOPS87_2421:D>E	AA:37		DBVPG6040:P>Q		DBVPG6044:P>Q		NCYC110:P>Q		SK1:P>Q		UWOPS03_461_4:P>Q		UWOPS05_227_2:P>Q		UWOPS87_2421:P>Q		Y55:P>Q		YPS128:P>Q	AA:78		DBVPG6044:L>H		SK1:L>H		Y55:L>H	AA:82		K11:P>S		Y9:P>S	AA:93		DBVPG6044:A>V		SK1:A>V		Y55:A>V	AA:107		DBVPG1853:F>Y	AA:135		UWOPS87_2421:R>K	AA:165		UWOPS05_217_3:G>S		UWOPS87_2421:G>S	AA:185		BC187:S>T		DBVPG1106:S>T		DBVPG1373:S>T		DBVPG6765:S>T		L_1374:S>T		L_1528:S>T		YJM978:S>T	AA:200		BC187:L>I		DBVPG1106:L>I		DBVPG1373:L>I		DBVPG6765:L>I		L_1374:L>I		L_1528:L>I		YJM978:L>IID:YDR447C	AA:104		Y9:N>S	AA:110		L_1528:V>LID:YDR448W	AA:96		Y9:G>D	AA:205		UWOPS87_2421:L>S	AA:254		UWOPS83_787_3:F>I	AA:278		K11:A>E		Y9:A>E		YS4:A>E	AA:317		L_1528:S>F	AA:346		UWOPS05_227_2:S>N	AA:383		YGPM:L>F	AA:388		DBVPG1853:L>F	AA:401		DBVPG1853:T>I	AA:408		YGPM:S>R	AA:421		YGPM:A>GID:YDR449C	AA:274		UWOPS05_217_3:F>L		UWOPS05_227_2:F>L	AA:367		DBVPG6040:I>V		YPS128:I>V		YPS606:I>V	AA:386		DBVPG6044:L>F		SK1:L>F		Y55:L>F	AA:401		DBVPG6040:A>T		DBVPG6044:A>T		SK1:A>T		UWOPS03_461_4:A>T		UWOPS05_217_3:A>T		UWOPS05_227_2:A>T		Y55:A>T		YPS128:A>T		YPS606:A>TID:YDR451C	AA:7		322134S:V>M	AA:9		YJM978:P>S	AA:40		322134S:D>V		DBVPG1106:D>V		DBVPG1788:D>V		DBVPG1853:D>V		DBVPG6040:D>V		DBVPG6765:D>V		UWOPS03_461_4:D>V		UWOPS83_787_3:D>V		Y9:D>V		YIIc17_E5:D>V		YJM975:D>V		YJM978:D>V		YPS128:D>V		YS4:D>V		YS9:D>V	AA:70		UWOPS05_217_3:K>N	AA:136		UWOPS03_461_4:A>G	AA:157		K11:Q>R		Y12:Q>R		YIIc17_E5:Q>R		YS4:Q>R	AA:163		YS9:K>N	AA:258		YIIc17_E5:L>F	AA:353		UWOPS05_217_3:N>S		UWOPS05_227_2:N>SID:YDR452W	AA:19		K11:K>R		YIIc17_E5:K>R	AA:30		DBVPG6044:V>I		SK1:V>I		Y55:V>I	AA:71		378604X:R>K		DBVPG6044:R>K		K11:R>K		SK1:R>K		Y55:R>K	AA:127		DBVPG6044:K>E		NCYC110:K>E		SK1:K>E		Y55:K>E	AA:186		YS2:A>T	AA:422		DBVPG6044:G>R		NCYC110:G>R		Y55:G>R	AA:451		UWOPS03_461_4:D>N		YIIc17_E5:D>N		YPS128:D>N	AA:546		NCYC110:S>T		SK1:S>T		UWOPS83_787_3:S>T		Y55:S>T	AA:567		YS9:K>E	AA:574		L_1374:I>V	AA:609		DBVPG1373:E>K		DBVPG6765:E>K		YJM978:E>K		YJM981:E>K		YS9:E>K	AA:625		DBVPG1373:I>V		DBVPG6040:I>V		DBVPG6765:I>V		L_1374:I>V		NCYC110:I>V		SK1:I>V		UWOPS03_461_4:I>V		UWOPS83_787_3:I>V		UWOPS87_2421:I>V		Y55:I>V		Y9:I>V		YIIc17_E5:I>V		YJM978:I>V		YJM981:I>V		YPS606:I>V		YS9:I>V	AA:656		YJM978:P>HID:YDR453C	AA:1		UWOPS03_461_4:M>I	AA:48		L_1528:C>Y	AA:87		UWOPS03_461_4:P>S	AA:90		UWOPS03_461_4:D>N	AA:97		DBVPG6044:K>N		SK1:K>N		Y12:K>N		Y55:K>N		YIIc17_E5:K>N	AA:113		YS9:G>C	AA:117		YJM981:E>G	AA:130		YJM981:D>H	AA:146		YJM981:G>V	AA:161		YJM981:W>CID:YDR456W	AA:22		YS2:V>I	AA:31		BC187:L>P		DBVPG1106:L>P		DBVPG1373:L>P		DBVPG6765:L>P		L_1374:L>P		L_1528:L>P	AA:108		DBVPG1373:M>I	AA:371		NCYC361:E>-	AA:508		YS9:P>L	AA:520		K11:Q>P		UWOPS83_787_3:Q>P	AA:548		UWOPS05_227_2:P>S	AA:550		K11:N>S	AA:553		YIIc17_E5:T>A	AA:623		NCYC361:Q>-ID:YDR457W	AA:82		Y55:C>S		YPS606:C>S	AA:92		273614X:H>Q		UWOPS83_787_3:H>Q		Y55:H>Q		YPS606:H>Q	AA:99		UWOPS05_217_3:K>R	AA:126		273614X:L>I	AA:209		UWOPS05_217_3:L>F	AA:229		UWOPS87_2421:I>N	AA:261		UWOPS05_217_3:L>H	AA:346		L_1374:T>K	AA:482		UWOPS03_461_4:I>M	AA:686		YPS606:T>A	AA:728		DBVPG1373:D>G	AA:789		UWOPS05_227_2:Q>R	AA:806		K11:V>A	AA:810		UWOPS05_227_2:Q>R	AA:869		YS9:M>T	AA:878		DBVPG1106:R>K		DBVPG1373:R>K		DBVPG1788:R>K		DBVPG1853:R>K		DBVPG6044:R>K		DBVPG6765:R>K		K11:R>K		L_1374:R>K		NCYC361:R>K		SK1:R>K		Y55:R>K		YIIc17_E5:R>K		YS4:R>K		YS9:R>K	AA:1008		DBVPG1106:A>V		DBVPG1373:A>V		DBVPG1853:A>V		DBVPG6040:A>V		DBVPG6765:A>V		L_1374:A>V		L_1528:A>V		SK1:A>V		UWOPS05_227_2:A>V		Y55:A>V		YJM981:A>V		YS4:A>V	AA:1142		DBVPG6040:V>I	AA:1143		UWOPS03_461_4:M>V		UWOPS05_227_2:M>V	AA:1264		UWOPS03_461_4:Q>K		UWOPS05_227_2:Q>K	AA:1271		UWOPS05_227_2:C>S	AA:1327		DBVPG1373:R>K		DBVPG1788:R>K		L_1374:R>K		L_1528:R>K		NCYC110:R>K		UWOPS05_227_2:R>K		Y55:R>K		YJM978:R>K		YPS606:R>K	AA:1370		UWOPS05_227_2:F>L	AA:1572		BC187:H>Q		DBVPG6040:H>Q		DBVPG6765:H>Q		L_1528:H>Q		NCYC110:H>Q		SK1:H>Q		YIIc17_E5:H>Q		YS9:H>Q	AA:1601		BC187:T>A		DBVPG6765:T>A		L_1528:T>A		YS9:T>A	AA:1633		DBVPG6040:A>V		YIIc17_E5:A>V	AA:2022		DBVPG6765:D>N	AA:2068		378604X:E>G	AA:2193		W303:S>	AA:2306		YJM975:E>V	AA:2529		YS4:A>T	AA:2716		L_1528:G>R	AA:2879		UWOPS05_227_2:A>T	AA:3053		322134S:V>AID:YDR459C	AA:8		UWOPS87_2421:R>Q	AA:19		DBVPG6040:L>F	AA:26		Y9:L>-	AA:85		DBVPG6040:H>R		YIIc17_E5:H>R	AA:280		L_1374:R>S	AA:282		SK1:E>G		Y55:E>GID:YDR460W	AA:60		378604X:D>GID:YDR462W	AA:32		Y12:K>R	AA:89		DBVPG1788:L>I		DBVPG1853:L>I		L_1528:L>I	AA:105		YS4:L>V	AA:109		YS4:S>R	AA:111		UWOPS87_2421:R>S		YPS606:R>S	AA:112		YS4:L>M	AA:124		YS4:R>P	AA:139		YS4:T>PID:YDR464W	AA:40		UWOPS87_2421:G>A	AA:44		UWOPS03_461_4:E>D	AA:47		273614X:Y>N		378604X:Y>N		BC187:Y>N		DBVPG1373:Y>N		DBVPG1853:Y>N		DBVPG6044:Y>N		DBVPG6765:Y>N		K11:Y>N		NCYC110:Y>N		NCYC361:Y>N		SK1:Y>N		UWOPS03_461_4:Y>N		UWOPS83_787_3:Y>N		UWOPS87_2421:Y>N		Y12:Y>N		Y55:Y>N		Y9:Y>N		YJM975:Y>N		YJM978:Y>N		YPS606:Y>N		YS4:Y>N		YS9:Y>N	AA:61		UWOPS83_787_3:S>N		YPS606:S>N	AA:64		UWOPS03_461_4:M>I		UWOPS83_787_3:M>I		UWOPS87_2421:M>I		YPS606:M>I	AA:78		UWOPS03_461_4:V>I		UWOPS05_227_2:V>I		UWOPS87_2421:V>I	AA:85		K11:V>I		Y12:V>I		Y9:V>I	AA:114		K11:T>R	AA:189		DBVPG6044:T>R		SK1:T>R		Y55:T>R	AA:232		DBVPG1788:K>E	AA:249		YIIc17_E5:D>G	AA:264		YIIc17_E5:T>I	AA:269		YIIc17_E5:N>S	AA:298		YJM975:T>A		YJM978:T>A	AA:327		UWOPS03_461_4:A>T	AA:344		DBVPG6040:R>T		K11:R>T		UWOPS03_461_4:R>T		Y55:R>T		YPS128:R>T		YPS606:R>T	AA:473		DBVPG6040:A>S		UWOPS87_2421:A>S		Y55:A>S		YIIc17_E5:A>S	AA:501		L_1528:D>G	AA:547		DBVPG6044:K>E	AA:564		378604X:S>N	AA:578		UWOPS03_461_4:S>F	AA:655		DBVPG6040:P>	AA:692		DBVPG6040:R>K	AA:697		UWOPS03_461_4:E>K		UWOPS05_227_2:E>K	AA:922		273614X:K>R		BC187:K>R		DBVPG1106:K>R		DBVPG1853:K>R		DBVPG6765:K>R		K11:K>R		L_1528:K>R		NCYC110:K>R		UWOPS03_461_4:K>R		UWOPS05_227_2:K>R		UWOPS83_787_3:K>R		UWOPS87_2421:K>R		Y55:K>R		Y9:K>R		YJM975:K>R		YPS606:K>R		YS2:K>R	AA:947		UWOPS03_461_4:T>I		UWOPS05_227_2:T>I		UWOPS87_2421:T>I	AA:949		DBVPG6044:I>M		K11:I>M		NCYC110:I>M		UWOPS03_461_4:I>M		UWOPS05_227_2:I>M		UWOPS83_787_3:I>M		UWOPS87_2421:I>M		Y55:I>M		Y9:I>M		YPS606:I>M	AA:970		DBVPG6044:G>E		K11:G>E		NCYC110:G>E		Y55:G>E		Y9:G>E	AA:982		YJM975:I>K		YJM981:I>K	AA:1024		DBVPG6044:T>A		Y55:T>A	AA:1039		DBVPG6044:G>S		K11:G>S		UWOPS03_461_4:G>S		UWOPS05_227_2:G>S		UWOPS87_2421:G>S		Y12:G>S		Y55:G>S		YPS128:G>S		YPS606:G>S	AA:1074		DBVPG6044:K>E		K11:K>E		UWOPS03_461_4:K>E		UWOPS87_2421:K>E		Y12:K>E		Y55:K>E		YPS128:K>E		YPS606:K>E	AA:1091		DBVPG6044:L>V		UWOPS03_461_4:L>V		Y12:L>V		Y55:L>V		YPS128:L>V		YPS606:L>V	AA:1107		378604X:A>T		DBVPG1106:A>T		DBVPG1373:A>T		DBVPG1788:A>T		DBVPG1853:A>T		DBVPG6765:A>T		L_1374:A>T		L_1528:A>T		YJM975:A>T		YJM981:A>T		YS4:A>T	AA:1151		DBVPG6044:P>S		NCYC110:P>S		SK1:P>S		Y55:P>S	AA:1176		Y12:N>K	AA:1328		DBVPG1788:Y>H	AA:1434		UWOPS03_461_4:L>S		UWOPS05_227_2:L>SID:YDR466W	AA:3		DBVPG6040:S>P	AA:37		K11:I>F		Y12:I>F		Y9:I>F		YS2:I>F	AA:97		Y12:G>D	AA:130		UWOPS87_2421:H>N	AA:363		378604X:T>N	AA:395		UWOPS87_2421:P>L	AA:397		DBVPG6044:D>G		SK1:D>G		Y55:D>G	AA:413		DBVPG6044:A>T		K11:A>T		SK1:A>T		Y55:A>T		YPS606:A>T	AA:443		322134S:D>	AA:563		DBVPG6044:S>N		SK1:S>N		UWOPS87_2421:S>N		Y55:S>N		YPS606:S>N	AA:633		UWOPS03_461_4:I>M		UWOPS05_227_2:I>M	AA:678		322134S:A>T	AA:719		UWOPS87_2421:N>S	AA:791		DBVPG6044:S>F		SK1:S>F		Y55:S>F	AA:819		DBVPG6040:T>A		DBVPG6044:T>A		SK1:T>A		Y12:T>A		Y55:T>A		Y9:T>A		YPS606:T>A	AA:832		UWOPS03_461_4:T>ID:YDR468C	AA:74		UWOPS03_461_4:E>-	AA:79		DBVPG6044:N>D		SK1:N>D		UWOPS03_461_4:N>D		UWOPS05_227_2:N>D		UWOPS87_2421:N>D		Y12:N>D		Y55:N>D		Y9:N>D		YPS128:N>D		YS2:N>D		YS4:N>D		YS9:N>D	AA:119		Y12:A>T		Y9:A>T		YS2:A>T		YS4:A>T		YS9:A>TID:YDR469W	AA:16		DBVPG6765:P>A	AA:58		YPS128:S>P		YPS606:S>P	AA:66		DBVPG6044:V>A		K11:V>A		NCYC110:V>A		SK1:V>A		UWOPS87_2421:V>A		Y55:V>A		YIIc17_E5:V>A		YS2:V>A	AA:127		Y9:N>S	AA:133		YS2:H>P	AA:138		378604X:M>K	AA:142		UWOPS83_787_3:A>TID:YDR472W	AA:57		DBVPG1106:A>V		DBVPG1373:A>V		DBVPG6765:A>V		L_1528:A>V		YJM978:A>V	AA:65		UWOPS03_461_4:L>V		UWOPS05_227_2:L>V	AA:86		UWOPS03_461_4:K>N	AA:91		UWOPS03_461_4:G>A	AA:98		UWOPS03_461_4:L>P	AA:101		UWOPS03_461_4:L>M	AA:123		378604X:N>S		DBVPG1373:N>S		DBVPG6044:N>S		DBVPG6765:N>S		L_1528:N>S		UWOPS05_227_2:N>S		Y55:N>S		Y9:N>S		YJM978:N>S		YJM981:N>S		YPS606:N>S		YS9:N>S	AA:258		YPS128:G>S		YPS606:G>SID:YDR473C	AA:8		NCYC110:E>G		SK1:E>G		Y55:E>G		YPS606:E>G	AA:73		DBVPG6044:P>S		NCYC110:P>S		SK1:P>S		Y55:P>S	AA:104		DBVPG1373:Q>R	AA:148		Y12:K>T	AA:161		YJM978:D>G	AA:188		322134S:G>D	AA:215		UWOPS05_217_3:R>-	AA:342		YPS606:F>S	AA:427		DBVPG1788:T>S	AA:444		UWOPS03_461_4:V>I		UWOPS87_2421:V>I	AA:464		UWOPS03_461_4:Y>FID:YDR476C	AA:88		UWOPS03_461_4:K>E		UWOPS05_217_3:K>E		UWOPS05_227_2:K>E	AA:153		NCYC110:W>C		SK1:W>C		Y55:W>C	AA:155		DBVPG6040:D>H		K11:D>H		YIIc17_E5:D>N	AA:173		NCYC110:L>F		SK1:L>F		UWOPS83_787_3:L>V		Y55:L>F	AA:179		DBVPG6040:V>IID:YDR477W	AA:494		322134S:S>C	AA:545		UWOPS05_227_2:K>R	AA:549		UWOPS05_227_2:K>R	AA:556		YJM981:T>R	AA:586		NCYC361:F>I	AA:633		322134S:N>KID:YDR479C	AA:41		BC187:E>A	AA:67		273614X:G>D	AA:156		378604X:V>G	AA:164		DBVPG6044:L>H		SK1:L>H		Y55:L>H	AA:280		UWOPS03_461_4:L>S	AA:310		UWOPS03_461_4:H>L		UWOPS05_217_3:H>L		UWOPS83_787_3:H>L	AA:315		UWOPS05_217_3:E>D	AA:396		BC187:E>G	AA:406		K11:E>Q		Y12:E>Q	AA:425		UWOPS03_461_4:P>S		UWOPS05_217_3:P>S	AA:466		UWOPS03_461_4:S>G	AA:476		UWOPS03_461_4:I>L		UWOPS05_227_2:I>LID:YDR480W	AA:48		YPS128:S>T	AA:68		NCYC110:S>L		SK1:S>L		Y55:S>L	AA:83		BC187:T>I		DBVPG1106:T>I		DBVPG1373:T>I		DBVPG1853:T>I		DBVPG6040:T>I		DBVPG6765:T>I		K11:T>I		L_1374:T>I		NCYC110:T>I		SK1:T>I		UWOPS05_227_2:T>I		Y55:T>I		YJM975:T>I		YJM981:T>I		YPS128:T>I	AA:94		UWOPS05_227_2:S>P	AA:103		DBVPG6044:R>K		NCYC110:R>K		SK1:R>K		Y55:R>K	AA:152		DBVPG1373:Q>E	AA:185		UWOPS03_461_4:T>S		UWOPS05_227_2:T>S	AA:202		DBVPG6044:G>A		NCYC110:G>A		SK1:G>A		UWOPS03_461_4:G>S		Y55:G>A	AA:215		UWOPS03_461_4:H>R	AA:229		DBVPG6044:E>D		NCYC110:E>D		SK1:E>D		UWOPS03_461_4:E>D		Y55:E>D	AA:232		DBVPG6044:T>A		NCYC110:T>A		SK1:T>A		UWOPS03_461_4:T>A		Y55:T>A	AA:271		K11:L>F	AA:279		W303:N>IID:YDR482C	AA:42		SK1:R>Q		UWOPS03_461_4:R>Q		UWOPS05_227_2:R>Q		Y55:R>Q	AA:57		UWOPS05_227_2:P>L	AA:104		SK1:A>V		Y55:A>VID:YDR483W	AA:40		DBVPG1853:I>V	AA:44		DBVPG6040:F>L		UWOPS03_461_4:F>L		UWOPS05_227_2:F>L		UWOPS83_787_3:F>L		YIIc17_E5:F>L		YPS128:F>L	AA:51		DBVPG6040:I>V		DBVPG6044:I>V		SK1:I>V		UWOPS83_787_3:I>V		Y55:I>V		YIIc17_E5:I>V		YPS128:I>V	AA:57		DBVPG6040:V>F		UWOPS83_787_3:V>F		YIIc17_E5:V>F		YPS128:V>F	AA:74		DBVPG6040:S>A		UWOPS03_461_4:S>A		UWOPS05_217_3:S>A		UWOPS83_787_3:S>A		YIIc17_E5:S>A		YPS128:S>A	AA:77		DBVPG6040:S>N		DBVPG6044:S>N		NCYC110:S>N		SK1:S>N		UWOPS03_461_4:S>N		UWOPS05_217_3:S>N		UWOPS83_787_3:S>N		Y55:S>N		YPS128:S>N	AA:135		DBVPG6040:K>Q		Y12:K>Q	AA:139		YPS128:S>N	AA:147		DBVPG6044:K>R		NCYC110:K>R		SK1:K>R		UWOPS05_217_3:K>R		Y55:K>R		YPS128:K>R	AA:251		DBVPG6044:K>R		NCYC110:K>R		SK1:K>R		Y12:K>R		Y55:K>R		YPS128:K>R		YPS606:K>R	AA:266		DBVPG6044:E>D		NCYC110:E>D		SK1:E>D		UWOPS05_217_3:E>D		Y12:E>D		Y55:E>D		YPS128:E>D		YPS606:E>D	AA:282		DBVPG1853:V>A		DBVPG6044:V>A		NCYC110:V>A		SK1:V>A		Y12:V>A		Y55:V>A		YIIc17_E5:V>A	AA:309		DBVPG6044:S>N		NCYC110:S>N		SK1:S>N		Y55:S>N		YIIc17_E5:S>N		YPS128:S>N		YPS606:S>N	AA:313		YPS128:N>D		YPS606:N>D	AA:432		DBVPG6044:V>I		NCYC110:V>I		SK1:V>I		Y55:V>I	AA:440		UWOPS05_217_3:F>L		UWOPS05_227_2:F>L	AA:442		YPS128:E>Q		YPS606:E>QID:YDR484W	AA:5		DBVPG6044:K>R		NCYC110:K>R		SK1:K>R		UWOPS05_227_2:K>R		Y55:K>R		YPS128:K>R		YPS606:K>R	AA:9		DBVPG6044:S>L		NCYC110:S>L		SK1:S>L		Y55:S>L	AA:22		UWOPS03_461_4:S>T		UWOPS05_227_2:S>T	AA:29		273614X:T>R	AA:32		DBVPG6044:P>S		NCYC110:P>S		SK1:P>S		Y55:P>S	AA:39		YJM978:D>A	AA:40		273614X:C>G	AA:53		UWOPS03_461_4:F>V		UWOPS05_217_3:F>V		UWOPS05_227_2:F>V	AA:66		273614X:N>K	AA:168		322134S:D>N	AA:297		DBVPG6044:S>G		SK1:S>G		UWOPS05_217_3:S>G		Y55:S>G		YPS606:S>G	AA:322		DBVPG6044:K>R		SK1:K>R		Y55:K>R	AA:333		UWOPS05_217_3:Y>F	AA:335		UWOPS05_227_2:Q>	AA:345		UWOPS83_787_3:Q>	AA:441		DBVPG1788:N>K	AA:490		DBVPG6044:N>S		SK1:N>S		Y55:N>S	AA:522		DBVPG1788:S>I	AA:544		DBVPG1788:S>C	AA:611		DBVPG6044:A>T		SK1:A>T		Y55:A>T	AA:615		YPS128:D>NID:YDR486C	AA:14		YS2:D>N	AA:126		DBVPG1788:G>D	AA:155		DBVPG6044:A>T		NCYC110:A>T		SK1:A>T		Y55:A>TID:YDR487C	AA:114		NCYC361:R>K	AA:175		DBVPG6044:E>K		NCYC110:E>K		SK1:E>K		Y55:E>K	AA:193		UWOPS03_461_4:G>S		UWOPS05_217_3:G>S		UWOPS05_227_2:G>SID:YDR488C	AA:28		DBVPG1853:P>L	AA:45		UWOPS87_2421:T>S	AA:70		S288c:R>L	AA:71		DBVPG6044:R>P		NCYC110:R>P		SK1:R>P		Y55:R>P	AA:102		NCYC110:A>T		SK1:A>T		UWOPS03_461_4:A>T		UWOPS05_217_3:A>T		UWOPS83_787_3:A>T		Y55:A>T	AA:111		DBVPG1788:D>N	AA:115		NCYC110:K>N		SK1:K>N		UWOPS03_461_4:K>N		UWOPS05_217_3:K>N		UWOPS87_2421:K>N		Y55:K>N		Y9:K>N		YPS606:K>N	AA:150		NCYC110:T>A		NCYC361:T>A		SK1:T>A		UWOPS03_461_4:T>A		UWOPS83_787_3:T>A		UWOPS87_2421:T>A		Y55:T>A		Y9:T>A		YPS606:T>A	AA:156		DBVPG1853:G>A	AA:158		NCYC110:Q>L		SK1:Q>L		Y55:Q>L	AA:162		YJM981:N>D	AA:181		NCYC361:Q>K		UWOPS03_461_4:Q>K		UWOPS83_787_3:Q>K		YPS606:Q>K	AA:271		YS2:A>V	AA:273		DBVPG1373:L>F		DBVPG1853:L>F		DBVPG6765:L>F		L_1528:L>F		NCYC110:L>F		NCYC361:L>F		SK1:L>F		UWOPS03_461_4:L>F		UWOPS05_217_3:L>F		UWOPS83_787_3:L>F		Y55:L>F		YIIc17_E5:L>F		YPS128:L>F		YPS606:L>F		YS2:L>F		YS9:L>F	AA:312		SK1:V>A		Y55:V>A	AA:371		YS2:T>K	AA:391		322134S:T>M	AA:409		DBVPG1853:K>Q	AA:452		UWOPS03_461_4:L>V		UWOPS05_217_3:L>V	AA:497		DBVPG1853:E>K		DBVPG6044:E>G		SK1:E>G		Y55:E>G	AA:517		UWOPS03_461_4:N>K		UWOPS05_217_3:N>K	AA:522		UWOPS03_461_4:A>T		UWOPS05_217_3:A>TID:YDR489W	AA:6		DBVPG6765:D>E	AA:23		273614X:K>N		DBVPG1106:K>N		DBVPG1853:K>N		DBVPG6040:K>N		DBVPG6765:K>N		UWOPS03_461_4:K>N		UWOPS05_217_3:K>N		UWOPS83_787_3:K>N		Y12:K>N		YIIc17_E5:K>N		YJM975:K>N		YJM978:K>N		YPS128:K>N		YPS606:K>N	AA:31		DBVPG6765:T>I	AA:34		DBVPG6040:R>K	AA:93		DBVPG6044:M>I		SK1:M>I		Y55:M>I	AA:107		UWOPS03_461_4:D>N		UWOPS05_217_3:D>N	AA:112		YS9:S>F	AA:134		YS9:E>D	AA:182		YS9:Y>S	AA:185		DBVPG6044:T>A		SK1:T>A		Y55:T>A		YS9:T>A	AA:220		DBVPG6044:D>G		NCYC110:D>G		SK1:D>G		Y55:D>G	AA:255		UWOPS03_461_4:C>Y		UWOPS05_217_3:C>Y		UWOPS05_227_2:C>YID:YDR490C	AA:16		Y9:K>-	AA:44		YPS606:G>R	AA:53		YIIc17_E5:G>V	AA:84		L_1374:P>L	AA:88		YPS606:R>H	AA:187		322134S:F>I		BC187:F>I		DBVPG1106:F>I		DBVPG1373:F>I		DBVPG1788:F>I		DBVPG6040:F>I		DBVPG6044:F>I		DBVPG6765:F>I		K11:F>I		L_1374:F>I		L_1528:F>I		SK1:F>I		UWOPS03_461_4:F>I		UWOPS05_217_3:F>I		UWOPS05_227_2:F>I		W303:F>I		Y55:F>I		Y9:F>I		YGPM:F>I		YIIc17_E5:F>I		YJM981:F>I		YPS606:F>I	AA:271		K11:A>S	AA:298		YJM978:V>F	AA:326		NCYC361:I>T	AA:375		322134S:D>E		NCYC110:D>E		NCYC361:D>E		SK1:D>E		UWOPS03_461_4:D>E		UWOPS83_787_3:D>E		Y55:D>E		Y9:D>E		YIIc17_E5:D>E	AA:389		NCYC110:L>V		NCYC361:L>V		SK1:L>V		UWOPS03_461_4:L>V		UWOPS83_787_3:L>V		Y55:L>V		Y9:L>V		YIIc17_E5:L>V	AA:446		NCYC110:D>V		SK1:D>V		Y55:D>V	AA:478		UWOPS03_461_4:Q>P	AA:490		UWOPS03_461_4:R>K		UWOPS05_227_2:R>K	AA:498		UWOPS03_461_4:R>C		UWOPS05_227_2:R>C	AA:529		NCYC110:R>C		SK1:R>C		Y55:R>C	AA:558		NCYC361:N>D	AA:563		NCYC361:P>S	AA:564		UWOPS03_461_4:V>A		UWOPS05_217_3:V>A		UWOPS05_227_2:V>A	AA:565		DBVPG6044:G>S		NCYC110:G>S		SK1:G>S		Y55:G>S	AA:576		DBVPG1853:P>A		DBVPG6044:P>A		NCYC110:P>A		SK1:P>A		Y55:P>A	AA:580		DBVPG6044:E>D		DBVPG6765:E>D		K11:E>D		NCYC110:E>D		NCYC361:E>D		SK1:E>D		UWOPS03_461_4:E>D		UWOPS05_217_3:E>D		UWOPS05_227_2:E>D		UWOPS83_787_3:E>D		Y55:E>D		YPS128:E>D		YPS606:E>D		YS9:E>D	AA:583		DBVPG6044:T>I		NCYC110:T>I		SK1:T>I		Y55:T>I	AA:594		DBVPG1853:F>L		DBVPG6044:F>L		K11:F>L		NCYC110:F>L		NCYC361:F>L		SK1:F>L		UWOPS03_461_4:F>L		UWOPS05_217_3:F>L		UWOPS05_227_2:F>L		UWOPS83_787_3:F>L		Y55:F>L		YPS128:F>L		YPS606:F>L	AA:614		322134S:W>R		DBVPG1853:W>R		DBVPG6044:W>R		K11:W>R		NCYC110:W>R		NCYC361:W>R		UWOPS03_461_4:W>R		UWOPS05_217_3:W>R		UWOPS05_227_2:W>R		Y55:W>R		YPS128:W>R		YPS606:W>R	AA:615		378604X:E>V	AA:642		UWOPS83_787_3:E>V	AA:647		DBVPG6044:R>K		NCYC110:R>K		Y55:R>K	AA:652		Y12:G>V	AA:662		DBVPG1853:L>V		DBVPG6044:L>I		K11:L>V		NCYC110:L>I		NCYC361:L>V		SK1:L>I		UWOPS03_461_4:L>V		UWOPS05_217_3:L>V		UWOPS05_227_2:L>V		UWOPS83_787_3:L>V		Y12:L>V		Y55:L>I		Y9:L>V		YIIc17_E5:L>V		YPS128:L>V	AA:687		K11:D>G	AA:694		YJM975:R>S	AA:710		UWOPS05_227_2:N>Y	AA:713		DBVPG1853:I>V		UWOPS83_787_3:I>V		YPS128:I>V		YPS606:I>V	AA:735		Y12:P>R		Y9:P>R		YIIc17_E5:P>R	AA:764		DBVPG6044:D>N		NCYC110:D>N		SK1:D>N		Y55:D>NID:YDR492W	AA:25		DBVPG6044:V>M		SK1:V>M		Y55:V>M	AA:96		UWOPS87_2421:V>E	AA:125		UWOPS87_2421:L>S	AA:141		DBVPG1853:M>I	AA:201		K11:V>I		Y12:V>I		Y9:V>I	AA:242		DBVPG6044:V>F		NCYC110:V>F		SK1:V>F		Y55:V>F	AA:274		378604X:A>S		DBVPG1106:A>S		DBVPG6040:A>S		YS9:A>S	AA:298		YS2:C>S	AA:315		YS9:H>YID:YDR493W	AA:26		DBVPG1853:A>T		DBVPG6044:A>T		K11:A>T		NCYC110:A>T		SK1:A>T		UWOPS03_461_4:A>T		UWOPS05_217_3:A>T		UWOPS83_787_3:A>T		Y12:A>T		Y55:A>T		Y9:A>T		YIIc17_E5:A>T		YPS128:A>T	AA:28		DBVPG6044:V>I		NCYC110:V>I		SK1:V>I		Y55:V>I	AA:33		DBVPG6044:R>K		NCYC110:R>K		SK1:R>K		Y55:R>K	AA:71		UWOPS05_217_3:G>D	AA:76		UWOPS03_461_4:G>S		UWOPS05_217_3:G>S		UWOPS83_787_3:G>S	AA:96		YS9:D>	AA:115		YS9:P>ID:YDR494W	AA:4		SK1:S>T		Y55:S>T	AA:8		UWOPS05_217_3:C>Y		UWOPS05_227_2:C>Y	AA:65		YIIc17_E5:S>N		YPS128:S>N		YPS606:S>N	AA:66		SK1:M>K		Y55:M>K	AA:99		UWOPS83_787_3:Q>P	AA:104		DBVPG6044:A>S		SK1:A>S		Y55:A>S	AA:109		DBVPG6044:V>L		SK1:V>L		UWOPS05_217_3:V>L		UWOPS05_227_2:V>L		UWOPS83_787_3:V>L		UWOPS87_2421:V>L		Y12:V>L		Y55:V>L		Y9:V>L		YIIc17_E5:V>L		YPS128:V>L		YPS606:V>L	AA:113		UWOPS05_227_2:T>S	AA:136		YS4:D>N		YS9:D>N	AA:150		DBVPG6044:S>P		SK1:S>P		Y55:S>P	AA:172		DBVPG6044:S>N		SK1:S>N		Y55:S>N	AA:300		NCYC361:L>I		UWOPS83_787_3:L>I		UWOPS87_2421:L>I	AA:317		DBVPG1106:D>GID:YDR496C	AA:33		DBVPG1373:D>N		L_1374:D>N		YJM978:D>N		YS4:D>N	AA:37		DBVPG6765:E>D		L_1528:E>D	AA:66		DBVPG6044:E>D		SK1:E>D		Y55:E>D	AA:70		UWOPS03_461_4:E>D		UWOPS05_227_2:E>D	AA:73		DBVPG6044:E>K		SK1:E>K		Y55:E>K	AA:84		DBVPG6044:E>K		DBVPG6765:E>K		L_1528:E>K		SK1:E>K		Y55:E>K	AA:128		K11:N>S		UWOPS05_217_3:K>I	AA:155		YS9:E>D	AA:211		DBVPG6040:V>L	AA:227		DBVPG1373:N>D		DBVPG1788:N>D		DBVPG6044:N>D		DBVPG6765:N>D		L_1374:N>D		L_1528:N>D		NCYC110:N>D		SK1:N>D		UWOPS03_461_4:N>D		UWOPS05_217_3:N>D		UWOPS05_227_2:N>D		Y12:N>D		Y55:N>D		Y9:N>D		YIIc17_E5:N>D		YPS128:N>D		YPS606:N>D		YS4:N>D	AA:261		UWOPS87_2421:Q>-	AA:299		DBVPG6040:R>G	AA:338		YPS606:M>L	AA:339		YS9:I>M	AA:342		YS9:L>F	AA:485		273614X:S>N		DBVPG1788:S>N		L_1528:S>N	AA:596		DBVPG1853:S>F	AA:618		K11:T>M	AA:634		UWOPS87_2421:G>-	AA:652		SK1:A>T		Y55:A>T	AA:655		SK1:L>F		Y55:L>FID:YDR499W	AA:3		322134S:R>Q		DBVPG1788:R>Q		DBVPG1853:R>Q		DBVPG6040:R>Q		DBVPG6765:R>Q		K11:R>Q		L_1528:R>Q		SK1:R>Q		UWOPS03_461_4:R>Q		UWOPS05_227_2:R>Q		Y55:R>Q		YIIc17_E5:R>Q		YJM975:R>Q		YPS606:R>Q	AA:16		SK1:D>E		Y55:D>E	AA:38		K11:L>F	AA:45		322134S:T>A		DBVPG1788:T>A		DBVPG1853:T>A		DBVPG6040:T>A		DBVPG6765:T>A		K11:T>A		UWOPS03_461_4:T>A		UWOPS05_227_2:T>A		YJM978:T>A		YPS606:T>A	AA:65		YJM978:V>G	AA:91		SK1:K>E		Y55:K>E	AA:140		Y9:V>A	AA:171		NCYC110:P>L		SK1:P>L	AA:231		NCYC110:L>F		SK1:L>F		Y55:L>F	AA:253		NCYC110:S>P		SK1:S>P		Y55:S>P	AA:306		DBVPG1373:I>V		DBVPG1788:I>V		DBVPG6765:I>V		L_1528:I>V	AA:339		DBVPG1853:V>A	AA:432		DBVPG1373:I>V		DBVPG1788:I>V		DBVPG6765:I>V		L_1528:I>V		YJM978:I>V		YS9:I>V	AA:448		UWOPS87_2421:S>N	AA:526		DBVPG1373:G>E		DBVPG1788:G>E		DBVPG6765:G>E		L_1528:G>E		YJM978:G>E		YPS128:G>E		YPS606:G>E		YS9:G>E	AA:531		322134S:K>Q	AA:534		SK1:A>T		Y55:A>T	AA:569		L_1528:I>M	AA:631		YPS128:I>N	AA:699		YPS128:M>T		YPS606:M>T	AA:738		UWOPS03_461_4:A>S		UWOPS05_217_3:A>S		UWOPS05_227_2:A>SID:YDR501W	AA:19		DBVPG6040:P>L	AA:79		DBVPG1853:P>L	AA:156		273614X:Q>	AA:177		K11:L>S	AA:213		YJM981:Q>	AA:300		YPS128:K>R		YPS606:K>R	AA:356		378604X:R>S		DBVPG1788:R>S		DBVPG6765:R>S		YS4:R>S	AA:361		273614X:N>I		378604X:N>I		DBVPG1788:N>I		DBVPG6765:N>I		YS4:N>I	AA:392		YS9:T>	AA:396		273614X:I>S		378604X:I>S		DBVPG1788:I>S		DBVPG6765:I>S	AA:405		273614X:S>P	AA:413		378604X:D>N		DBVPG1788:D>N		DBVPG6765:D>N	AA:416		378604X:H>N	AA:457		DBVPG6044:I>M		SK1:I>M		Y55:I>M	AA:485		378604X:N>K		DBVPG1788:N>K		DBVPG6765:N>KID:YDR502C	AA:322		DBVPG6044:D>E		SK1:D>E		Y55:D>E	AA:323		YS4:T>SID:YDR505C	AA:42		UWOPS05_217_3:L>Q	AA:96		UWOPS05_217_3:P>Q	AA:97		378604X:N>S		DBVPG1373:N>S		DBVPG1788:N>S		L_1528:N>S		YS4:N>S	AA:111		273614X:E>K		378604X:E>K		DBVPG1788:E>K		DBVPG6040:E>K		DBVPG6044:E>K		DBVPG6765:E>K		L_1374:E>K		L_1528:E>K		NCYC110:E>K		S288c:E>K		SK1:E>K		UWOPS05_217_3:Q>R		W303:E>K		Y55:E>K		YPS128:E>K		YPS606:E>K		YS4:E>K		YS9:E>K	AA:141		378604X:N>H	AA:199		DBVPG1373:S>N		DBVPG1788:S>N		DBVPG6765:S>N		L_1528:S>N		UWOPS83_787_3:S>N		UWOPS87_2421:S>N		YJM978:S>N	AA:203		Y12:A>V		Y9:A>V	AA:292		322134S:E>K		DBVPG1373:E>K		DBVPG1853:E>K		DBVPG6765:E>K		L_1528:E>K		YJM975:E>K		YJM978:E>K	AA:297		322134S:V>I		DBVPG1373:V>I		DBVPG1853:V>I		DBVPG6765:V>I		L_1528:V>I		YJM975:V>I		YJM978:V>I	AA:304		DBVPG1853:D>G	AA:345		322134S:P>S		DBVPG1373:P>S		DBVPG6765:P>S		L_1528:P>S		YJM978:P>S	AA:374		273614X:V>I		322134S:V>I		378604X:V>I		DBVPG1373:V>I		DBVPG1788:V>I		DBVPG6765:V>I		YJM978:V>I		YPS128:V>I		YPS606:V>I	AA:414		273614X:V>I		322134S:V>I		378604X:V>I		BC187:V>I		DBVPG1373:V>I		DBVPG1788:V>I		DBVPG6765:V>I	AA:530		BC187:A>S		DBVPG1373:A>S		DBVPG1788:A>S		DBVPG6765:A>S		L_1374:A>S	AA:562		UWOPS03_461_4:L>S	AA:568		UWOPS03_461_4:L>S	AA:629		273614X:N>S		378604X:N>S		DBVPG1373:N>S		DBVPG1788:N>S		DBVPG6765:N>S		L_1374:N>S		L_1528:N>S		YS4:N>S		YS9:N>S	AA:729		DBVPG6040:D>E	AA:803		273614X:E>D		378604X:E>D		DBVPG1788:E>D		DBVPG6765:E>D		L_1374:E>D		L_1528:E>D		YJM978:E>D		YS4:E>DID:YDR507C	AA:377		DBVPG6044:V>I		SK1:V>I		UWOPS03_461_4:V>I		UWOPS05_227_2:V>I		UWOPS83_787_3:V>I		Y55:V>I		YIIc17_E5:V>I		YPS128:V>I		YPS606:V>I	AA:411		DBVPG1853:T>P	AA:453		DBVPG6044:S>F		SK1:S>F		Y55:S>F	AA:455		322134S:I>V		DBVPG1373:I>V		DBVPG1788:I>V		DBVPG1853:I>V		DBVPG6765:I>V		L_1528:I>V		YJM975:I>V		YJM978:I>V		YS4:I>V		YS9:I>V	AA:630		BC187:K>N		DBVPG1788:K>N		DBVPG6040:K>N		DBVPG6765:K>N		L_1528:K>N		YJM978:K>N		YS2:K>N		YS4:K>N	AA:768		YPS128:V>I		YPS606:V>I	AA:776		273614X:A>T		322134S:A>T		BC187:A>T		DBVPG1373:A>T		DBVPG1788:A>T		DBVPG6765:A>T		UWOPS87_2421:A>T		YJM978:A>T		YS2:A>T	AA:784		UWOPS87_2421:G>C	AA:848		UWOPS03_461_4:P>L		UWOPS05_217_3:P>L	AA:866		DBVPG6044:E>G		NCYC110:E>G	AA:885		273614X:I>V	AA:935		DBVPG6040:Q>E		YIIc17_E5:Q>E	AA:968		DBVPG6044:E>A		SK1:E>A		Y55:E>A		YIIc17_E5:E>A	AA:984		DBVPG1373:D>E		DBVPG6044:D>E		L_1374:D>E		SK1:D>E		Y55:D>E		YIIc17_E5:D>E		YJM978:D>E		YJM981:D>E		YS4:D>E	AA:1014		DBVPG1373:L>SID:YDR508C	AA:22		273614X:R>G		322134S:R>G		378604X:R>G		DBVPG1373:R>G		DBVPG1853:R>G		DBVPG6040:R>G		L_1374:R>G		L_1528:R>G		NCYC110:R>G		SK1:R>G		UWOPS83_787_3:R>G		UWOPS87_2421:R>G		Y12:R>G		Y55:R>G		YIIc17_E5:R>G		YJM978:R>G		YPS128:R>G		YPS606:R>G		YS4:R>G	AA:24		UWOPS87_2421:D>E	AA:25		273614X:L>V		378604X:L>V		DBVPG1373:L>V		L_1374:L>V		L_1528:L>V		YJM978:L>V		YS4:L>V	AA:42		273614X:Q>E		322134S:Q>E		DBVPG1373:Q>E		DBVPG1853:Q>E		DBVPG6040:Q>E		L_1374:Q>E		L_1528:Q>E		NCYC110:Q>E		SK1:Q>E		UWOPS05_217_3:Q>E		UWOPS87_2421:Q>E		Y12:Q>E		Y55:Q>E		YIIc17_E5:Q>E		YPS606:Q>E		YS4:Q>E	AA:77		UWOPS87_2421:L>-	AA:85		273614X:R>K		DBVPG1373:R>K		DBVPG1853:R>K		L_1374:R>K		L_1528:R>K		UWOPS87_2421:R>K		YS4:R>K	AA:143		DBVPG1853:E>G	AA:234		DBVPG6044:L>V		SK1:L>V		UWOPS05_217_3:L>I		UWOPS05_227_2:L>I		Y55:L>V	AA:262		YS9:N>S	AA:301		UWOPS87_2421:V>I	AA:322		DBVPG6040:G>V	AA:439		378604X:A>S		DBVPG1373:A>S		L_1528:A>S		YJM975:A>S	AA:451		DBVPG6044:G>S		NCYC110:G>S		SK1:G>S		UWOPS05_227_2:G>S		UWOPS87_2421:G>S		Y12:G>V		Y55:G>S		YPS128:G>S		YPS606:G>S	AA:455		UWOPS87_2421:T>A	AA:459		DBVPG6044:I>V		NCYC110:I>V		SK1:I>V		Y55:I>V	AA:484		322134S:A>V		378604X:A>V		BC187:A>V		DBVPG1373:A>V		DBVPG1853:A>V		DBVPG6040:A>V		DBVPG6765:A>V		L_1374:A>V		L_1528:A>V		UWOPS87_2421:A>V		YJM975:A>V		YS4:A>V	AA:547		YS4:V>D	AA:601		322134S:W>L		BC187:W>L		DBVPG1788:W>L		DBVPG1853:W>L		DBVPG6044:W>L		DBVPG6765:W>L		L_1528:W>L		SK1:W>L		UWOPS05_227_2:W>L		UWOPS83_787_3:W>L		UWOPS87_2421:W>L		Y55:W>L		YJM978:W>L		YJM981:W>L		YPS128:W>L		YPS606:W>L		YS4:W>L		YS9:W>L	AA:604		DBVPG1788:L>F		L_1528:L>FID:YDR511W	AA:2		UWOPS05_227_2:N>K	AA:39		DBVPG6044:G>D		SK1:G>D		Y55:G>D	AA:122		DBVPG6040:E>G		L_1374:E>G		YJM975:E>G		YJM978:E>G	AA:130		UWOPS87_2421:N>SID:YDR515W	AA:53		YS9:E>-	AA:73		SK1:L>S		Y55:L>S	AA:103		SK1:K>I		Y55:K>I	AA:105		UWOPS05_227_2:S>P	AA:127		273614X:N>I	AA:137		273614X:S>N		DBVPG6765:S>N	AA:142		DBVPG1106:L>P	AA:156		W303:H>	AA:164		DBVPG6044:N>S		K11:N>S		SK1:N>S		UWOPS05_227_2:N>S		UWOPS83_787_3:N>S		Y55:N>S		YPS128:N>S		YPS606:N>S	AA:204		273614X:A>G	AA:211		YJM975:P>R		YJM978:P>R	AA:216		273614X:A>V	AA:229		UWOPS83_787_3:N>	AA:237		DBVPG1106:K>N		DBVPG6044:K>N		K11:K>N		L_1374:K>N		SK1:K>N		UWOPS83_787_3:K>N		Y55:K>N		YJM975:K>N		YJM978:K>N		YPS128:K>N		YPS606:K>N		YS2:K>N		YS9:K>N	AA:260		UWOPS83_787_3:P>	AA:263		273614X:Y>-	AA:284		273614X:S>R	AA:302		UWOPS05_227_2:N>D	AA:303		DBVPG6044:D>E		SK1:D>E		Y55:D>E	AA:306		YS2:I>V	AA:338		YS9:Q>R	AA:344		UWOPS05_227_2:H>R	AA:349		DBVPG1373:L>F		DBVPG6040:L>F		DBVPG6044:L>F		DBVPG6765:L>F		K11:L>F		L_1374:L>F		L_1528:L>F		SK1:L>F		UWOPS03_461_4:L>F		UWOPS05_227_2:L>F		Y55:L>F		YJM975:L>F		YJM978:L>F		YPS128:L>F		YPS606:L>F		YS2:L>F		YS4:L>F	AA:360		DBVPG6044:A>V		SK1:A>V		Y55:A>V	AA:380		DBVPG6040:A>V		DBVPG6044:A>V		K11:A>V		SK1:A>V		UWOPS03_461_4:A>V		Y55:A>V		YPS128:A>V		YPS606:A>V	AA:384		DBVPG6044:N>D		SK1:N>D		Y55:N>D	AA:388		YPS128:N>S	AA:392		DBVPG1373:T>S		L_1374:T>S		YJM975:T>S		YJM978:T>S	AA:420		273614X:S>G		DBVPG1373:S>G		L_1374:S>G		L_1528:S>G		YJM975:S>G		YJM978:S>G		YS4:S>G		YS9:S>G	AA:430		YS9:Y>HID:YDR516C	AA:26		273614X:Q>E		322134S:Q>E		378604X:Q>E		BC187:Q>E		DBVPG1853:Q>E		DBVPG6040:Q>E		DBVPG6044:Q>E		DBVPG6765:Q>E		NCYC361:Q>E		SK1:Q>E		UWOPS03_461_4:Q>E		UWOPS05_217_3:Q>E		UWOPS83_787_3:Q>E		Y55:Q>E		YJM975:Q>E		YJM981:Q>E		YPS128:Q>E		YPS606:Q>E		YS9:Q>E	AA:242		YIIc17_E5:S>TID:YDR517W	AA:14		378604X:Q>K	AA:25		DBVPG6044:D>N		SK1:D>N	AA:103		YJM978:G>R	AA:165		DBVPG6044:S>N		SK1:S>N		Y55:S>N	AA:264		DBVPG6044:D>V	AA:300		YS4:P>L	AA:317		DBVPG1106:T>	AA:361		DBVPG6044:L>V		SK1:L>V		Y55:L>V	AA:362		YJM978:A>ID:YDR518W	AA:4		YS4:T>I	AA:29		DBVPG6040:A>V	AA:58		YS2:F>L	AA:90		YS9:D>V	AA:95		DBVPG6040:S>N		DBVPG6044:S>N		K11:S>N		SK1:S>N		UWOPS87_2421:S>N		Y12:S>N		Y55:S>N		Y9:S>N		YIIc17_E5:S>N		YPS128:S>N	AA:172		UWOPS05_227_2:G>D	AA:185		YS9:A>D	AA:266		273614X:E>K		322134S:E>K		378604X:E>K		DBVPG1373:E>K		DBVPG1788:E>K		DBVPG6044:E>K		DBVPG6765:E>K		K11:E>K		L_1374:E>K		SK1:E>K		UWOPS05_227_2:E>K		UWOPS87_2421:E>K		Y12:E>K		Y55:E>K		YIIc17_E5:E>K		YJM975:E>K		YPS606:E>K	AA:272		UWOPS05_227_2:Y>C	AA:319		UWOPS83_787_3:I>V	AA:393		DBVPG6044:D>N		SK1:D>N		Y55:D>N	AA:456		NCYC361:A>G	AA:511		DBVPG6044:D>N		SK1:D>N		Y55:D>NID:YDR519W	AA:2		DBVPG1853:M>L		DBVPG6044:M>L		K11:M>L		SK1:M>L		UWOPS03_461_4:M>L		Y55:M>L		Y9:M>L		YIIc17_E5:M>L		YPS128:M>L	AA:31		DBVPG6044:I>M		SK1:I>M		Y55:I>MID:YDR520C	AA:20		UWOPS87_2421:A>T	AA:23		K11:N>S	AA:120		UWOPS03_461_4:L>Q		UWOPS05_217_3:L>Q	AA:186		Y12:T>I	AA:208		L_1374:S>I	AA:351		UWOPS87_2421:I>L	AA:373		UWOPS05_227_2:F>C	AA:538		K11:N>D	AA:597		322134S:N>K		378604X:N>K		DBVPG1853:N>K		NCYC110:N>K		SK1:N>K		UWOPS83_787_3:N>K		UWOPS87_2421:N>K		Y55:N>K		YPS128:N>K		YPS606:N>K	AA:634		YJM978:S>P		YJM981:S>P	AA:679		322134S:R>C	AA:684		UWOPS05_217_3:S>GID:YDR522C	AA:32		322134S:D>N		DBVPG1788:D>N		DBVPG6765:D>N		L_1528:D>N		YJM981:D>N		YS4:D>N	AA:56		322134S:A>T		NCYC361:A>T	AA:98		322134S:Q>K		378604X:Q>K		DBVPG1106:Q>K		DBVPG1788:Q>K		DBVPG1853:Q>K		DBVPG6044:Q>K		DBVPG6765:Q>K		K11:Q>K		L_1528:Q>K		NCYC361:Q>K		SK1:Q>K		UWOPS03_461_4:Q>K		UWOPS05_217_3:Q>K		UWOPS87_2421:Q>K		Y55:Q>K		Y9:Q>K		YJM978:Q>K		YJM981:Q>K		YPS128:Q>K		YPS606:Q>K		YS4:Q>K	AA:192		DBVPG6040:K>R		DBVPG6044:K>R		NCYC361:K>R		SK1:K>R		Y55:K>R		YIIc17_E5:K>R		YJM978:K>R		YJM981:K>R		YPS128:K>R		YPS606:K>R	AA:228		Y12:D>N		YJM975:T>I		YJM978:T>I	AA:260		322134S:V>F	AA:324		322134S:V>F	AA:344		378604X:A>V		BC187:A>V		DBVPG1106:A>V		DBVPG1373:A>V		DBVPG6044:A>V		K11:A>V		L_1528:A>V		SK1:A>V		UWOPS03_461_4:A>V		UWOPS05_217_3:A>V		Y12:A>V		Y55:A>V		YIIc17_E5:A>V		YJM975:A>V		YJM978:A>V		YPS128:A>V	AA:441		273614X:E>K	AA:453		UWOPS87_2421:D>N	AA:466		378604X:S>NID:YDR523C	AA:39		NCYC361:V>G		YS2:V>G	AA:49		YS9:V>F	AA:59		322134S:E>A	AA:148		322134S:L>V	AA:258		DBVPG1853:A>T	AA:310		273614X:K>R		DBVPG1106:K>R		DBVPG1373:K>R		DBVPG6765:K>R		L_1374:K>R		L_1528:K>R		YJM975:K>R		YJM978:K>R		YS4:K>R	AA:334		Y9:S>P	AA:408		K11:N>T	AA:455		Y9:E>DID:YDR527W	AA:66		YS4:K>M	AA:118		273614X:K>I		DBVPG1788:K>I		DBVPG6765:K>I		L_1528:K>I		YS4:K>I	AA:161		273614X:D>N		DBVPG1106:D>N		DBVPG1373:D>N		DBVPG1788:D>N		DBVPG1853:D>N		DBVPG6765:D>N		L_1528:D>N		NCYC110:D>N		SK1:D>N		Y55:D>N		YJM981:D>N		YPS128:D>N		YPS606:D>N		YS4:D>N	AA:171		273614X:M>K		DBVPG1106:M>K		DBVPG1373:M>K		DBVPG1788:M>K		DBVPG1853:M>K		DBVPG6040:M>K		DBVPG6765:M>K		L_1528:M>K		NCYC110:M>K		SK1:M>K		UWOPS05_217_3:M>K		Y55:M>K		YJM981:M>K		YPS128:M>K		YPS606:M>K		YS4:M>K	AA:176		273614X:A>T		322134S:A>T		DBVPG1106:A>T		DBVPG1373:A>T		DBVPG1788:A>T		DBVPG6765:A>T		L_1528:A>T		NCYC110:A>T		SK1:A>T		Y55:A>T		YJM981:A>T		YS4:A>T	AA:192		378604X:G>E		DBVPG1106:G>E		DBVPG1373:G>E		DBVPG1853:G>E		DBVPG6040:G>E		NCYC110:G>E		UWOPS05_217_3:G>E		W303:G>E		Y55:G>E		YPS128:G>E		YPS606:G>E	AA:194		273614X:G>A		322134S:G>A		378604X:G>A		DBVPG1106:G>A		DBVPG1373:G>A		DBVPG1788:G>A		DBVPG1853:G>A		DBVPG6040:G>A		DBVPG6765:G>A		L_1528:G>A		NCYC110:G>A		S288c:G>A		UWOPS05_217_3:G>A		W303:G>A		Y55:G>A		YJM981:G>A		YPS128:G>A		YPS606:G>A		YS4:G>A	AA:237		378604X:I>S	AA:266		322134S:D>N		DBVPG1373:D>N		DBVPG6040:D>N		DBVPG6765:D>N		L_1374:D>N		L_1528:D>N		SK1:D>N		UWOPS05_217_3:D>N		Y55:D>N		YJM981:D>N		YPS128:D>N		YPS606:D>N		YS4:D>N	AA:282		K11:Y>F	AA:301		NCYC110:P>	AA:404		273614X:E>K	AA:406		273614X:S>A		322134S:S>A		BC187:S>A		DBVPG1106:S>A		DBVPG1853:S>A		DBVPG6040:S>A		DBVPG6044:S>A		K11:S>A		L_1374:S>A		NCYC361:S>A		SK1:S>A		UWOPS05_217_3:S>A		UWOPS83_787_3:S>A		Y55:S>A		YJM975:S>A		YJM978:S>A		YPS128:S>A		YS9:S>AID:YDR528W	AA:52		UWOPS87_2421:D>	AA:59		K11:T>I	AA:65		DBVPG1788:G>D		DBVPG6765:G>D	AA:120		DBVPG1106:M>K		L_1528:M>K		YJM975:M>K	AA:130		DBVPG1106:V>A		YJM975:V>A	AA:280		DBVPG1853:D>G	AA:286		UWOPS87_2421:T>A	AA:322		BC187:C>Y		DBVPG1106:C>Y		DBVPG1373:C>Y		DBVPG1788:C>Y		DBVPG1853:C>Y		DBVPG6044:C>Y		DBVPG6765:C>Y		K11:C>Y		L_1374:C>Y		SK1:C>Y		UWOPS87_2421:C>Y		Y55:C>Y		YIIc17_E5:C>Y		YJM978:C>Y		YJM981:C>Y		YS4:C>Y	AA:332		273614X:A>D		BC187:A>D		DBVPG1106:A>D		DBVPG1373:A>D		DBVPG1788:A>D		DBVPG1853:A>D		DBVPG6044:A>D		DBVPG6765:A>D		L_1374:A>D		SK1:A>D		UWOPS05_217_3:A>D		UWOPS87_2421:A>D		Y55:A>D		YIIc17_E5:A>D		YJM978:A>D		YJM981:A>D		YS4:A>D		YS9:A>D	AA:367		K11:H>Q	AA:381		273614X:L>S	AA:410		273614X:R>K		322134S:R>K		378604X:R>K		BC187:R>K		DBVPG1373:R>K		DBVPG1788:R>K		DBVPG1853:R>K		DBVPG6765:R>K		L_1374:R>K		YJM978:R>K		YJM981:R>K		YS4:R>KID:YDR530C	AA:18		DBVPG6765:K>Q	AA:69		DBVPG6044:A>T		Y55:A>T	AA:73		DBVPG6040:E>K		DBVPG6044:E>K		UWOPS05_217_3:E>K		UWOPS05_227_2:E>K		Y55:E>K		YPS128:E>K		YPS606:E>K	AA:122		DBVPG6040:T>S	AA:133		UWOPS05_217_3:D>G		UWOPS05_227_2:D>G	AA:152		UWOPS87_2421:H>Y	AA:236		YIIc17_E5:Q>P	AA:274		UWOPS87_2421:L>I	AA:276		UWOPS87_2421:I>TID:YDR531W	AA:2		322134S:P>S		DBVPG1853:P>S		K11:P>S		Y9:P>S		YS2:P>S	AA:24		UWOPS87_2421:D>E	AA:99		DBVPG1373:G>D		YJM975:G>D		YJM978:G>D	AA:111		322134S:H>Q		DBVPG1373:H>Q		DBVPG6765:H>Q		L_1528:H>Q		YJM975:H>Q		YJM978:H>Q		YS4:H>Q	AA:121		SK1:P>S		Y55:P>S	AA:246		273614X:M>I		DBVPG1788:M>I		DBVPG1853:M>I		DBVPG6044:M>I		L_1528:M>I		NCYC110:M>I		SK1:M>I		UWOPS05_217_3:M>I		UWOPS87_2421:M>I		Y55:M>I		Y9:M>I		YJM975:M>I		YJM978:M>I		YPS128:M>I		YPS606:M>I		YS4:M>I	AA:355		YS9:S>T	AA:366		273614X:S>N		BC187:S>N		DBVPG1106:S>N		DBVPG1788:S>N		DBVPG6044:S>N		L_1528:S>N		NCYC110:S>N		SK1:S>N		UWOPS05_217_3:S>N		UWOPS83_787_3:S>N		UWOPS87_2421:S>N		Y55:S>N		Y9:S>N		YJM975:S>N		YJM978:S>N		YPS128:S>N		YPS606:S>N		YS4:S>NID:YDR532C	AA:50		YPS128:A>S	AA:155		Y12:K>N	AA:161		YPS128:T>A		YPS606:T>A	AA:168		DBVPG1106:R>K		DBVPG1373:R>K		DBVPG1788:R>K		DBVPG1853:R>K
[truncated: 1,200,000 more chars]
