# Supplementary material for: Proteome-wide evidence for enhanced positive Darwinian selection within intrinsically disordered regions in proteins
Source: Genome Biol. 2011 Jul 19;12(7):R65. doi: 10.1186/gb-2011-12-7-r65 (PMC3218827; doi:10.1186/gb-2011-12-7-r65)
Supplement: Additional file 3 — Non-synonymous SNPs in S. paradoxus genes studied. The nature of each amino acid change for each changed amino acid in each strain is shown for each of 3,691 genes. [file gb-2011-12-7-r65-S3.RTF]

ID:YAL001C	AA:13		UWOPS91_917_1:V>A	AA:17		A12:N>D		DBVPG6304:N>D		UWOPS91_917_1:N>D		YPS138:N>D	AA:44		A12:H>Y		DBVPG6304:H>Y		N_43:H>Y		N_44:H>Y		N_45:H>Y		UWOPS91_917_1:H>Y		Y7:H>Y		YPS138:H>Y	AA:47		A12:F>S		DBVPG6304:F>S		N_43:F>L		N_44:F>L		N_45:F>L		YPS138:F>S	AA:48		UWOPS91_917_1:Q>H	AA:74		A12:S>P		DBVPG6304:S>P		N_43:S>P		N_44:S>P		N_45:S>P		UFRJ50816:S>P		YPS138:S>P	AA:229		UWOPS91_917_1:G>E	AA:232		UWOPS91_917_1:K>R	AA:272		IFO1804:R>M		N_44:R>M		N_45:R>M	AA:298		A12:P>S		A4:P>S		UFRJ50816:P>S		UWOPS91_917_1:P>S		YPS138:P>S	AA:338		A12:I>V		A4:I>V		YPS138:I>V	AA:401		A12:L>M		A4:L>M		DBVPG6304:L>M		UWOPS91_917_1:L>S		YPS138:L>M	AA:443		UWOPS91_917_1:S>N	AA:450		N_44:R>K		N_45:R>K	AA:466		UWOPS91_917_1:S>P	AA:506		A4:F>Y		DBVPG6304:F>Y		UFRJ50791:F>Y		UWOPS91_917_1:F>Y	AA:529		UWOPS91_917_1:S>R	AA:533		DBVPG6304:D>N		UFRJ50791:D>N	AA:555		T21_4:K>I	AA:562		DBVPG6304:N>D		UFRJ50791:N>D		UWOPS91_917_1:N>D		YPS138:N>D	AA:635		CBS432:P>S	AA:638		N_43:E>D		N_44:E>D		N_45:E>D	AA:642		UWOPS91_917_1:S>L	AA:646		CBS432:A>S	AA:647		DBVPG4650:G>S	AA:658		UWOPS91_917_1:T>N	AA:660		UWOPS91_917_1:V>I	AA:663		A4:K>T		N_43:K>T		N_44:K>T		N_45:K>T		UFRJ50791:K>T		YPS138:K>T	AA:703		A12:R>S		A4:R>S		UFRJ50816:R>S		UWOPS91_917_1:R>S		YPS138:R>S	AA:728		UWOPS91_917_1:S>	AA:781		A12:R>K		A4:R>K		UFRJ50816:R>K		YPS138:R>K	AA:869		A12:D>E		A4:D>E		N_43:D>E		N_45:D>E		UFRJ50816:D>E		YPS138:D>E	AA:876		A12:S>N		A4:S>N		UFRJ50816:S>N		YPS138:S>N	AA:913		N_45:P>S	AA:932		UWOPS91_917_1:A>T	AA:986		A12:G>E		A4:G>E		DBVPG6304:G>E		UFRJ50816:G>E		UWOPS91_917_1:G>E		YPS138:G>E	AA:987		Q62_5:D>A		T21_4:D>A	AA:1004		N_43:R>G		UWOPS91_917_1:I>L	AA:1013		N_43:C>Y		N_45:C>Y		UWOPS91_917_1:C>Y	AA:1029		A12:I>V		A4:I>V		DBVPG6304:I>V		UFRJ50816:I>V		UWOPS91_917_1:I>V	AA:1039		UWOPS91_917_1:K>E	AA:1082		A12:S>N		A4:S>N		DBVPG6304:S>N		UFRJ50816:S>N		UWOPS91_917_1:S>N	AA:1085		UWOPS91_917_1:D>N	AA:1089		N_43:T>K		N_45:T>K	AA:1097		A12:S>G		A4:S>G		UFRJ50816:S>G	AA:1098		N_43:D>N		N_45:D>N	AA:1099		A12:C>R		A4:C>R		UFRJ50816:C>R		UWOPS91_917_1:C>S	AA:1105		A12:K>N		A4:K>N		DBVPG6304:K>N		UFRJ50816:K>NID:YAL005C	AA:31		A12:D>G		N_43:D>G		N_44:D>G		N_45:D>G		UFRJ50791:D>G	AA:78		N_43:T>S		N_44:T>S		N_45:T>S	AA:392		N_43:S>T		N_44:S>T		UWOPS91_917_1:S>T		YPS138:S>T	AA:637		DBVPG6304:I>MID:YAL007C	AA:197		A12:V>I		DBVPG6304:V>I		UFRJ50791:V>I		YPS138:V>I	AA:206		N_44:F>V		N_45:F>VID:YAL008W	AA:12		A12:K>N		DBVPG6304:K>N		N_44:K>N		N_45:K>N		UFRJ50791:K>N	AA:22		N_44:E>D		N_45:E>D	AA:30		N_44:R>S		N_45:R>S	AA:62		A12:L>F		DBVPG6304:L>F		UFRJ50791:L>F		UFRJ50816:L>F	AA:119		N_44:->E		N_45:->E	AA:127		N_44:G>V		N_45:G>V	AA:170		A12:F>Y		A4:F>Y		DBVPG6304:F>Y		UWOPS91_917_1:F>Y	AA:173		A12:N>Y		DBVPG6304:N>Y		N_43:N>Y		N_44:N>Y		N_45:N>Y		UWOPS91_917_1:N>Y	AA:183		UWOPS91_917_1:A>PID:YAL009W	AA:50		N_43:N>K		N_45:N>K	AA:176		A12:S>R		A4:S>R		DBVPG6304:S>R		UWOPS91_917_1:S>R	AA:184		UFRJ50816:F>L	AA:186		A12:E>D		A4:E>D		DBVPG6304:E>D	AA:204		UFRJ50816:Y>-	AA:235		A12:I>R		A4:I>R		DBVPG6304:I>R		UFRJ50816:I>R	AA:241		UWOPS91_917_1:G>A	AA:243		UFRJ50816:F>SID:YAL010C	AA:6		DBVPG6304:L>F		IFO1804:L>F		N_44:L>F		N_45:L>F		UFRJ50791:L>F		UFRJ50816:L>F		UWOPS91_917_1:L>F		YPS138:L>F	AA:12		DBVPG6304:A>T		UFRJ50791:A>T		UFRJ50816:A>T		UWOPS91_917_1:A>T		YPS138:A>T	AA:41		A12:E>D		DBVPG6304:E>D		UFRJ50791:E>D		UFRJ50816:E>D		UWOPS91_917_1:E>D		YPS138:E>D	AA:42		KPN3829:Q>K		N_17:Q>K	AA:127		A12:P>S		DBVPG6304:P>S		IFO1804:P>S		N_43:P>S		N_44:P>S		N_45:P>S		UFRJ50791:P>S		UFRJ50816:P>S		UWOPS91_917_1:P>S		YPS138:P>S	AA:130		A12:I>V		DBVPG6304:I>V		UFRJ50791:I>V		UFRJ50816:I>V		UWOPS91_917_1:I>V		YPS138:I>V	AA:152		IFO1804:S>P		N_43:S>P		N_44:S>P		N_45:S>P	AA:163		A12:S>P		DBVPG6304:S>P		N_43:E>G		N_44:S>P		N_45:E>G		UFRJ50816:S>P		UWOPS91_917_1:S>P	AA:168		A12:N>K		DBVPG6304:N>K		UFRJ50791:N>K		UFRJ50816:N>K		YPS138:N>K	AA:285		A12:I>V	AA:319		A12:R>K		DBVPG6304:R>K		UFRJ50816:R>K	AA:323		A12:I>V		DBVPG6304:I>V		N_44:I>V		UFRJ50816:I>V		UWOPS91_917_1:I>V	AA:369		A12:D>N		DBVPG6304:D>N		UFRJ50816:D>N		UWOPS91_917_1:D>N	AA:372		UWOPS91_917_1:T>I	AA:374		A12:Q>E		A4:Q>E		DBVPG6304:Q>E		N_44:Q>E		UFRJ50816:Q>E		UWOPS91_917_1:Q>E	AA:378		N_44:L>P	AA:383		A12:S>N	AA:385		A12:S>G		DBVPG6304:S>G		UFRJ50816:S>G		UWOPS91_917_1:S>G	AA:420		UWOPS91_917_1:Y>C	AA:456		A12:I>V		A4:I>V		DBVPG6304:I>V		UFRJ50816:I>V	AA:481		N_44:Q>K		N_45:Q>KID:YAL011W	AA:83		UWOPS91_917_1:Q>L	AA:97		N_43:F>C		N_45:F>C		UWOPS91_917_1:F>C	AA:102		UWOPS91_917_1:K>T	AA:104		A12:V>A		A4:V>A		UFRJ50791:V>A		UWOPS91_917_1:V>A		YPS138:V>A	AA:114		A12:D>N		UFRJ50791:D>N		UWOPS91_917_1:D>N		YPS138:D>N	AA:117		A12:L>P		N_43:L>P		N_45:L>P		UFRJ50791:L>P		UWOPS91_917_1:L>P		YPS138:L>P	AA:120		A12:F>L		A4:F>L		UFRJ50791:F>L		UWOPS91_917_1:F>L		YPS138:F>L	AA:153		A12:H>Q		A4:H>Q		N_43:H>Q		N_45:H>Q		UFRJ50791:H>Q		UFRJ50816:H>Q		UWOPS91_917_1:H>Q		YPS138:H>Q	AA:165		N_43:R>S		N_45:R>S	AA:204		UWOPS91_917_1:L>I	AA:229		N_43:L>-		N_45:L>-	AA:247		N_43:Q>P		N_45:Q>P	AA:252		A12:L>V		UFRJ50791:L>V		UFRJ50816:L>V	AA:278		N_43:H>N		N_45:H>N	AA:280		UFRJ50791:Q>H		UFRJ50816:Q>H	AA:288		UFRJ50791:W>C		UFRJ50816:W>C	AA:340		A12:R>T	AA:385		UFRJ50816:F>V		UWOPS91_917_1:F>V		YPS138:F>V	AA:387		IFO1804:F>L		N_44:F>L	AA:414		DBVPG4650:Y>-	AA:462		DBVPG6304:R>C		UFRJ50816:R>C		UWOPS91_917_1:R>C		YPS138:R>C	AA:473		A12:M>I		DBVPG6304:M>I		UFRJ50816:M>I		YPS138:M>I	AA:494		A12:Q>H		DBVPG6304:Q>H		UFRJ50816:Q>H		UWOPS91_917_1:Q>H		YPS138:Q>H	AA:499		IFO1804:D>N		N_43:D>N		N_44:D>N		UWOPS91_917_1:D>N	AA:502		A12:I>F		DBVPG6304:I>F		UFRJ50816:I>F		UWOPS91_917_1:I>F	AA:507		A12:L>P		DBVPG6304:L>P		UFRJ50816:L>P		YPS138:L>P	AA:513		UWOPS91_917_1:F>L	AA:545		A12:K>N		DBVPG6304:K>N		UFRJ50816:K>N		YPS138:K>N	AA:556		A12:G>R		DBVPG6304:G>R		UFRJ50816:G>R	AA:605		DBVPG6304:Q>E		IFO1804:Q>E		N_43:Q>E		UFRJ50816:Q>E		UWOPS91_917_1:Q>E	AA:608		A12:I>R		DBVPG6304:I>R		IFO1804:I>R		N_43:I>R		UFRJ50816:I>R		UWOPS91_917_1:I>R	AA:610		IFO1804:V>D		N_43:V>D	AA:615		A12:F>L		DBVPG6304:F>L		UFRJ50816:F>L		UWOPS91_917_1:F>L	AA:629		T21_4:S>FID:YAL013W	AA:60		UWOPS91_917_1:L>F	AA:68		UWOPS91_917_1:Q>H	AA:172		UFRJ50816:W>C	AA:200		UFRJ50791:->Y		UFRJ50816:->Y	AA:202		UFRJ50791:I>L		UFRJ50816:I>L	AA:211		N_43:L>F		N_44:L>F		UFRJ50816:L>F		UWOPS91_917_1:L>F	AA:217		UWOPS91_917_1:F>L	AA:219		UFRJ50791:->C		UFRJ50816:->C	AA:279		DBVPG6304:Q>L		UFRJ50791:Q>L		UFRJ50816:Q>L	AA:298		N_43:D>N	AA:302		DBVPG6304:I>M		UFRJ50791:I>M		UFRJ50816:I>M		UWOPS91_917_1:I>M	AA:336		DBVPG6304:C>-		N_43:C>-		N_45:C>-		UFRJ50791:C>-		UFRJ50816:C>-		UWOPS91_917_1:C>-	AA:342		N_43:F>L		N_45:F>L	AA:373		N_43:F>L		N_45:F>L	AA:395		DBVPG6304:F>S		UFRJ50791:F>S		UFRJ50816:F>S		UWOPS91_917_1:F>SID:YAL016W	AA:6		DBVPG6304:->R		UFRJ50791:->R		UWOPS91_917_1:->G	AA:9		DBVPG6304:C>R		UFRJ50791:C>R	AA:32		UFRJ50791:R>S	AA:90		DBVPG6304:K>N		N_44:K>N		N_45:K>N		UFRJ50791:K>N		UWOPS91_917_1:K>N	AA:149		DBVPG6304:K>N		UFRJ50791:K>N		UFRJ50816:K>N		UWOPS91_917_1:K>N		YPS138:K>N	AA:156		A4:N>K		UFRJ50791:N>K		UFRJ50816:N>K		UWOPS91_917_1:N>K		YPS138:N>K	AA:175		A4:E>D		UFRJ50791:E>D		UFRJ50816:E>D		YPS138:E>D	AA:200		A4:L>F		UFRJ50791:L>F		UFRJ50816:L>F		YPS138:L>F	AA:229		A4:C>R		DBVPG6304:C>R		UFRJ50791:C>R		UFRJ50816:C>R		YPS138:C>R	AA:232		A4:I>F		UFRJ50791:I>F		UFRJ50816:I>F		YPS138:I>F	AA:240		A4:E>D		UFRJ50791:E>D		UFRJ50816:E>D		YPS138:E>D	AA:273		A4:R>S		UFRJ50816:R>S		YPS138:R>S	AA:359		UFRJ50816:->Y	AA:407		N_43:I>M		N_45:I>M	AA:520		DBVPG6304:F>L		UFRJ50816:F>L	AA:537		DBVPG6304:R>-		UFRJ50816:R>-		UWOPS91_917_1:R>-	AA:558		UWOPS91_917_1:E>D	AA:568		UWOPS91_917_1:W>-	AA:591		DBVPG6304:F>L		N_45:F>L		UFRJ50816:F>L		UWOPS91_917_1:F>L	AA:604		DBVPG6304:Q>H		UFRJ50816:Q>H	AA:621		DBVPG6304:F>I	AA:627		A12:L>V		DBVPG6304:L>VID:YAL018C	AA:8		UWOPS91_917_1:T>A	AA:11		A4:P>S		UFRJ50816:P>S	AA:13		N_43:A>T		N_45:A>T		UFRJ50816:A>T		UWOPS91_917_1:A>T	AA:15		UFRJ50816:I>L	AA:20		UWOPS91_917_1:E>D	AA:128		A4:I>V		UFRJ50816:I>V		YPS138:I>V	AA:160		A4:G>S		UFRJ50816:G>S		YPS138:G>S	AA:174		DBVPG4650:N>H		N_17:N>H		N_43:N>H		N_45:N>H		Q59_1:N>H		Q62_5:N>H		Q89_8:N>H		S36_7:N>H		T21_4:N>H		UWOPS91_917_1:N>H		Y6_5:N>H	AA:187		T21_4:Q>	AA:229		UWOPS91_917_1:G>S	AA:232		A4:I>V		UFRJ50816:I>V		YPS138:I>V	AA:249		KPN3828:F>L	AA:265		KPN3828:F>I	AA:280		N_45:L>IID:YAL019W	AA:6		N_45:->W		UWOPS91_917_1:->W	AA:10		UWOPS91_917_1:I>M	AA:40		UWOPS91_917_1:C>-	AA:61		Y7:V>L	AA:62		A12:S>R	AA:67		UWOPS91_917_1:W>-	AA:86		UWOPS91_917_1:R>S	AA:137		A12:F>L	AA:138		N_44:C>W		N_45:C>W	AA:139		A12:->Q	AA:144		UWOPS91_917_1:C>-	AA:150		UWOPS91_917_1:D>E	AA:158		A12:R>S		DBVPG6304:R>S	AA:165		UWOPS91_917_1:F>L	AA:167		A12:F>L	AA:171		A12:K>N		DBVPG6304:K>N	AA:244		DBVPG6304:T>I		UWOPS91_917_1:T>I	AA:259		DBVPG6304:H>Q		UWOPS91_917_1:H>Q	AA:266		UWOPS91_917_1:T>P	AA:281		N_44:F>C		N_45:F>C	AA:283		N_44:N>I		N_45:N>I	AA:316		DBVPG6304:R>S		UWOPS91_917_1:R>S	AA:322		UWOPS91_917_1:Y>-	AA:354		N_44:E>D		N_45:E>D		UWOPS91_917_1:E>D	AA:388		A4:P>S		DBVPG6304:P>S		UWOPS91_917_1:P>S	AA:391		UWOPS91_917_1:L>F	AA:414		A4:I>F		DBVPG6304:I>F	AA:420		A4:I>M		DBVPG6304:I>M		UWOPS91_917_1:I>M	AA:422		A4:M>I		DBVPG6304:M>I		UWOPS91_917_1:M>I	AA:425		A4:S>R		DBVPG6304:S>R	AA:435		A4:L>F		DBVPG6304:L>F	AA:477		UWOPS91_917_1:F>L	AA:503		A4:F>L	AA:570		A4:L>F	AA:609		A4:L>R		DBVPG4650:L>R		N_44:L>R		N_45:L>R		T21_4:L>R		YPS138:L>R		Z1_1:L>R	AA:615		A4:R>S		UWOPS91_917_1:R>S		YPS138:R>S	AA:657		N_45:D>G	AA:688		UWOPS91_917_1:S>C	AA:698		A4:T>I		YPS138:T>I	AA:719		A4:E>D		UWOPS91_917_1:E>D		YPS138:E>D	AA:721		A4:K>N		UWOPS91_917_1:K>N		YPS138:K>N	AA:781		DBVPG4650:C>W		Q89_8:C>W		Z1_1:C>W	AA:783		A4:C>R		UWOPS91_917_1:C>R		YPS138:C>R	AA:807		UWOPS91_917_1:V>E		YPS138:V>E	AA:852		DBVPG6304:Y>-		UWOPS91_917_1:Y>-		YPS138:Y>-	AA:864		DBVPG6304:Q>H		YPS138:Q>H	AA:884		IFO1804:G>D	AA:888		UWOPS91_917_1:V>F	AA:908		DBVPG6304:I>T		IFO1804:I>T		UWOPS91_917_1:I>T		YPS138:I>T	AA:915		UWOPS91_917_1:K>-	AA:924		DBVPG6304:E>D		UWOPS91_917_1:E>D	AA:944		DBVPG6304:N>K		UWOPS91_917_1:N>K	AA:970		DBVPG6304:L>F	AA:985		IFO1804:F>L		N_43:F>L	AA:1072		UWOPS91_917_1:Y>-	AA:1074		DBVPG6304:F>L		N_43:F>L		N_44:F>L		N_45:F>L		UWOPS91_917_1:F>L	AA:1085		DBVPG6304:S>G		KPN3829:S>N		N_17:S>N		UWOPS91_917_1:S>G		Y6_5:S>N	AA:1090		N_43:D>E		N_44:D>E		N_45:D>E	AA:1097		CBS5829:L>FID:YAL020C	AA:2		A12:F>L	AA:21		N_44:G>D	AA:57		N_44:T>A		UFRJ50791:T>A	AA:98		A12:R>K		UFRJ50791:R>K	AA:136		UWOPS91_917_1:G>S	AA:142		A12:I>V		N_43:I>V		N_44:I>V		UFRJ50791:I>V		UFRJ50816:I>V	AA:147		UWOPS91_917_1:G>A	AA:149		UWOPS91_917_1:A>P	AA:152		UWOPS91_917_1:D>E	AA:167		A12:V>I		UFRJ50791:V>I		UFRJ50816:V>I	AA:173		CBS432:R>G	AA:217		A12:F>L		UFRJ50791:F>L		UFRJ50816:F>L		UWOPS91_917_1:F>L	AA:230		A12:A>V		UFRJ50791:A>V		UFRJ50816:A>V		UWOPS91_917_1:A>V	AA:247		UFRJ50791:P>S		UFRJ50816:P>S	AA:254		A12:R>M		UFRJ50791:R>M		UFRJ50816:R>M	AA:256		A12:G>R		UFRJ50791:G>R		UFRJ50816:G>R		UWOPS91_917_1:G>R	AA:257		N_17:R>C		Q89_8:R>C		Q95_3:R>C	AA:264		A12:A>E		UFRJ50791:A>E	AA:298		A12:V>E		UWOPS91_917_1:V>E	AA:305		A12:A>V		UWOPS91_917_1:A>V	AA:315		A12:D>NID:YAL022C	AA:88		A4:I>M		DBVPG6304:I>M		YPS138:I>M	AA:106		N_44:I>V		N_45:I>V	AA:202		A4:D>G		DBVPG6304:D>G		UFRJ50791:D>G		UFRJ50816:D>G	AA:204		A4:A>T		DBVPG6304:A>T		UFRJ50791:A>T		UFRJ50816:A>T	AA:220		CBS432:R>H	AA:453		DBVPG6304:T>R		UWOPS91_917_1:T>R	AA:459		N_45:S>P	AA:468		Q32_3:N>D	AA:474		DBVPG6304:D>E	AA:479		DBVPG6304:N>D		UFRJ50816:N>D	AA:485		DBVPG6304:D>E		UWOPS91_917_1:D>E	AA:510		DBVPG6304:T>I		UFRJ50816:T>IID:YAL023C	AA:57		Q89_8:K>R	AA:216		YPS138:K>R	AA:275		A12:S>L		DBVPG6304:S>L		UFRJ50791:S>L		UWOPS91_917_1:S>L		YPS138:S>L	AA:311		N_43:I>V		N_44:I>V		N_45:I>V	AA:400		Q32_3:L>Q	AA:466		N_43:I>F		N_44:I>F		N_45:I>F	AA:540		UWOPS91_917_1:H>Y	AA:616		UWOPS91_917_1:Y>H	AA:642		DBVPG6304:M>R	AA:662		DBVPG6304:F>L	AA:710		A12:E>G		DBVPG6304:E>G		UFRJ50816:E>G	AA:741		DBVPG6304:E>VID:YAL025C	AA:4		N_43:V>M		N_45:V>M	AA:94		UWOPS91_917_1:D>E	AA:123		N_45:K>R	AA:164		UFRJ50791:R>K	AA:246		N_44:L>-ID:YAL027W	AA:45		A12:C>-		UWOPS91_917_1:C>-		YPS138:C>-	AA:85		A12:H>Q		A4:H>Q		UFRJ50816:H>Q		YPS138:H>Q	AA:87		A12:F>L		A4:F>L		UFRJ50791:F>L		UFRJ50816:F>L	AA:128		A12:S>R		A4:S>R		UFRJ50791:S>R		UFRJ50816:S>R		YPS138:S>R	AA:165		A12:->Y		YPS138:->Y	AA:201		A4:I>M		UFRJ50791:I>M		UFRJ50816:I>M		YPS138:I>M	AA:228		A4:V>I		N_43:V>I		N_45:V>I		UFRJ50791:V>I		UFRJ50816:V>I		YPS138:V>I	AA:251		N_43:Y>D		N_45:Y>DID:YAL028W	AA:32		A12:C>W		UFRJ50816:C>W	AA:46		A12:C>R		N_43:C>R		N_45:C>R		UFRJ50816:C>R	AA:53		A12:N>K		UFRJ50816:N>K	AA:75		A12:L>V		DBVPG6304:L>V		UFRJ50816:L>V	AA:81		A12:F>L		DBVPG6304:F>L		UFRJ50816:F>L		UWOPS91_917_1:F>L	AA:88		KPN3828:F>Y	AA:136		DBVPG6304:K>N		UFRJ50816:K>N	AA:140		A12:E>D		DBVPG6304:E>D		N_43:E>D		N_45:E>D		UFRJ50816:E>D		UWOPS91_917_1:E>D	AA:154		UFRJ50816:V>L	AA:193		A4:H>Q		DBVPG6304:H>Q		UFRJ50816:H>Q		UWOPS91_917_1:H>Q	AA:197		DBVPG6304:F>I		UFRJ50816:F>I		UWOPS91_917_1:F>I	AA:243		A4:V>L		DBVPG6304:V>L		UFRJ50791:V>L		UFRJ50816:V>L	AA:245		N_43:R>-		N_45:R>-	AA:260		A4:->W		DBVPG6304:->W		UFRJ50791:->W		UFRJ50816:->W	AA:264		A4:K>N		DBVPG6304:K>N		UFRJ50791:K>N		UFRJ50816:K>N	AA:276		DBVPG6304:N>K	AA:299		N_43:T>S		N_45:T>S	AA:317		A4:R>L		DBVPG6304:R>L		UFRJ50791:R>L		UFRJ50816:R>L	AA:325		N_43:R>S		N_45:R>S	AA:341		A4:V>L		DBVPG6304:V>L		UFRJ50791:V>L		UFRJ50816:V>L	AA:348		N_43:I>M		N_45:I>M	AA:364		A4:V>F	AA:376		A4:L>Q		DBVPG6304:L>Q		N_43:L>Q		N_45:L>Q		UFRJ50816:L>Q	AA:379		A4:S>I		DBVPG6304:S>I		UFRJ50816:S>I		UWOPS91_917_1:S>I	AA:383		A4:V>E		DBVPG6304:V>E		UFRJ50791:V>E		UFRJ50816:V>E	AA:389		N_43:N>D		N_45:N>D	AA:401		A4:C>-		DBVPG6304:C>-		UFRJ50791:C>-		UFRJ50816:C>-		UWOPS91_917_1:C>-	AA:419		N_43:S>A		N_45:S>A	AA:422		DBVPG6304:F>L	AA:456		UWOPS91_917_1:H>Q	AA:489		N_43:Q>K		N_45:Q>K	AA:515		A12:K>N		A4:K>N		DBVPG6304:K>N		UFRJ50791:K>N		UFRJ50816:K>N		UWOPS91_917_1:K>N	AA:517		A12:D>A		A4:D>A		DBVPG6304:D>A		UFRJ50791:D>A		UFRJ50816:D>A		UWOPS91_917_1:D>A	AA:529		N_43:->L		N_45:->LID:YAL032C	AA:2		UFRJ50816:Q>H	AA:18		N_43:E>V		N_44:E>V		N_45:E>V	AA:36		A12:V>A		A4:V>A		DBVPG6304:V>A		UFRJ50791:V>A		YPS138:V>A	AA:131		N_44:Q>L		N_45:Q>L	AA:142		UFRJ50791:S>T		UFRJ50816:S>T	AA:153		A4:T>M		DBVPG6304:T>M		UFRJ50791:T>M		UFRJ50816:T>M	AA:248		DBVPG6304:T>S		N_44:T>M	AA:274		N_44:I>V	AA:284		DBVPG6304:T>I	AA:302		DBVPG6304:N>S	AA:315		KPN3829:K>R	AA:324		UWOPS91_917_1:D>E	AA:326		UWOPS91_917_1:Q>K	AA:366		N_44:S>L		N_45:S>L	AA:376		DBVPG4650:K>RID:YAL033W	AA:1		A12:H>Q	AA:18		A12:C>S		A4:C>S		DBVPG6304:C>S		UFRJ50791:C>S		UFRJ50816:C>S		YPS138:C>S	AA:25		A12:R>-	AA:26		UFRJ50816:H>Q	AA:52		N_44:Y>-	AA:60		Y7:Q>H	AA:71		UFRJ50816:E>-	AA:101		UFRJ50816:H>Y	AA:117		A12:D>E		A4:D>E		DBVPG6304:D>E		UFRJ50791:D>E	AA:130		N_43:S>R		N_44:S>R		N_45:S>RID:YAL034C	AA:3		A4:F>Y		N_44:F>Y		N_45:F>Y		UFRJ50791:F>Y		YPS138:F>Y	AA:113		UFRJ50791:T>S		UFRJ50816:T>S		YPS138:T>S	AA:132		UWOPS91_917_1:I>V	AA:135		Q95_3:I>L	AA:169		UFRJ50791:R>H		UFRJ50816:R>H		UWOPS91_917_1:R>H		YPS138:R>H	AA:189		UWOPS91_917_1:A>V	AA:221		UWOPS91_917_1:D>G	AA:274		UWOPS91_917_1:P>A	AA:295		DBVPG6304:A>E		UFRJ50816:A>E		UWOPS91_917_1:A>E		YPS138:A>E	AA:344		UWOPS91_917_1:N>S	AA:369		UWOPS91_917_1:N>S	AA:380		A4:R>Q		DBVPG6304:R>Q		UFRJ50816:R>Q		UWOPS91_917_1:R>Q		YPS138:R>Q	AA:392		UWOPS91_917_1:K>T	AA:396		A4:D>NID:YAL034W-A	AA:3		A4:S>R		UFRJ50791:S>R	AA:65		N_44:N>K		N_45:N>K	AA:93		DBVPG4650:S>T		N_17:S>T	AA:126		YPS138:F>L	AA:137		IFO1804:C>-		N_43:C>-		N_44:C>-		N_45:C>-	AA:151		DBVPG6304:H>Q	AA:154		A12:Q>H		A4:Q>H		DBVPG6304:Q>H		YPS138:Q>H	AA:157		A12:H>D		A4:H>D		CBS432:H>D		CBS5829:H>D		DBVPG4650:H>D		DBVPG6304:H>D		IFO1804:H>D		N_43:H>D		N_44:H>D		N_45:H>D		Q32_3:H>D		Q59_1:H>D		Q95_3:H>D		Y6_5:H>D		YPS138:H>D	AA:170		A12:F>L		A4:F>L		DBVPG6304:F>L		IFO1804:F>L		N_43:F>L		N_44:F>L		N_45:F>L		UWOPS91_917_1:F>L		YPS138:F>L	AA:247		IFO1804:F>L		N_43:F>L		N_44:F>L		N_45:F>LID:YAL035W	AA:14		A12:I>L		CBS432:I>L		DBVPG6304:I>L		KPN3828:I>L		N_44:I>L		N_45:I>L		Q62_5:I>L		Q95_3:I>L		UFRJ50791:I>L		UFRJ50816:I>L		UWOPS91_917_1:I>L		Y7:I>L		YPS138:I>L	AA:19		UWOPS91_917_1:F>L	AA:42		T21_4:I>M	AA:102		DBVPG6304:F>L		UFRJ50816:F>L		YPS138:F>L	AA:135		N_44:S>R		N_45:S>R	AA:194		DBVPG6304:->Y		UFRJ50816:->Y		YPS138:->Y	AA:203		DBVPG6304:S>R		UFRJ50816:S>R		YPS138:S>R	AA:239		IFO1804:F>L		N_43:F>L		N_44:F>L		N_45:F>L	AA:301		A4:I>M		DBVPG6304:I>M		UFRJ50791:I>M		UFRJ50816:I>M		YPS138:I>M	AA:304		A4:H>Q		DBVPG6304:H>Q		UFRJ50791:H>Q		YPS138:H>Q	AA:328		IFO1804:I>M		N_43:I>M		N_45:I>M	AA:398		UWOPS91_917_1:N>K	AA:409		A4:->Y		DBVPG6304:->Y		UFRJ50791:->Y		UFRJ50816:->Y		UWOPS91_917_1:->Y		YPS138:->Y	AA:433		UWOPS91_917_1:C>W	AA:450		A12:I>M		A4:I>M		DBVPG6304:I>M		UFRJ50791:I>M		UFRJ50816:I>M		UWOPS91_917_1:I>M		YPS138:I>M	AA:472		A12:R>S		A4:R>S		DBVPG6304:R>S		UFRJ50791:R>S		UFRJ50816:R>S		YPS138:R>S	AA:486		A12:N>K		A4:N>K		DBVPG6304:N>K		IFO1804:N>K		N_43:N>K		N_44:N>K		N_45:N>K		UFRJ50816:N>K		UWOPS91_917_1:N>K		YPS138:N>K	AA:493		A12:M>I		A4:M>I		DBVPG6304:M>I		UFRJ50816:M>I		YPS138:M>I	AA:511		A12:S>R		DBVPG6304:S>R		UFRJ50816:S>R		UWOPS91_917_1:S>R		YPS138:S>R	AA:546		A12:L>F		A4:L>F		DBVPG6304:L>F		UFRJ50816:L>F		YPS138:L>F	AA:554		A12:R>S		A4:R>S		DBVPG6304:R>S		IFO1804:R>S		N_43:R>S		N_44:R>S		N_45:R>S		UFRJ50816:R>S		YPS138:R>S	AA:565		A12:Q>H		A4:Q>H		DBVPG6304:Q>H		UFRJ50816:Q>H		YPS138:Q>H	AA:599		N_43:Q>H		N_44:Q>H		N_45:Q>H	AA:612		A4:Q>L		DBVPG6304:Q>L		YPS138:Q>L	AA:620		A4:G>S		CBS432:G>S		CBS5829:G>S		DBVPG6304:G>S		N_17:G>S		N_43:G>S		N_45:G>S		Q62_5:G>S		Y6_5:G>S		Y7:G>S		YPS138:G>S		Z1_1:G>S	AA:629		Y6_5:F>I		Z1_1:F>I	AA:638		A4:D>E		DBVPG6304:D>E		YPS138:D>E	AA:682		A4:N>K		DBVPG6304:N>K		YPS138:N>K	AA:686		A4:F>L		DBVPG6304:F>L		YPS138:F>L	AA:721		A4:Q>P		DBVPG6304:Q>P		YPS138:Q>P	AA:739		A4:S>R		DBVPG6304:S>R		UFRJ50791:S>R		YPS138:S>R	AA:761		A4:F>L		DBVPG6304:F>L		UFRJ50791:F>L		UFRJ50816:F>L		YPS138:F>L	AA:802		DBVPG6304:F>L		UFRJ50791:F>L		UFRJ50816:F>L		YPS138:F>L	AA:807		IFO1804:C>-	AA:953		A12:G>A		A4:G>A		DBVPG6304:G>A		UFRJ50791:G>A		UFRJ50816:G>A	AA:959		A12:R>T		A4:R>T		CBS432:R>T		CBS5829:R>T		DBVPG6304:R>T		IFO1804:R>T		KPN3829:R>T		N_44:R>T		N_45:R>T		Q32_3:R>T		Q62_5:R>T		Q95_3:R>T		S36_7:R>T		UFRJ50791:R>T		UFRJ50816:R>T		Y7:R>T	AA:963		A12:Q>H		A4:Q>H		DBVPG6304:Q>HID:YAL037W	AA:3		A12:T>S		DBVPG6304:T>S		UFRJ50816:T>S		UWOPS91_917_1:T>S	AA:14		A12:N>S		DBVPG6304:N>S		UFRJ50816:N>S		UWOPS91_917_1:N>S	AA:21		A12:L>Q		DBVPG6304:L>Q		UFRJ50816:L>Q		UWOPS91_917_1:L>Q	AA:36		UWOPS91_917_1:C>-	AA:38		A12:R>T		DBVPG6304:R>T		UFRJ50816:R>T		YPS138:R>T	AA:45		N_43:I>M		N_45:I>M	AA:55		UWOPS91_917_1:T>A	AA:59		A12:Y>-		DBVPG6304:Y>-		YPS138:Y>-	AA:75		A12:H>P		DBVPG6304:H>P		UFRJ50791:H>P		UWOPS91_917_1:H>P		YPS138:H>P	AA:157		N_17:K>R		N_44:K>R		N_45:K>R	AA:165		UWOPS91_917_1:I>M	AA:169		A4:Y>-		DBVPG6304:Y>-		UWOPS91_917_1:Y>-		YPS138:Y>-	AA:176		A4:A>G		UWOPS91_917_1:A>G		YPS138:A>G	AA:184		A4:R>T		DBVPG6304:R>T		YPS138:R>T	AA:197		A4:I>M	AA:201		A4:I>M		DBVPG6304:I>M		YPS138:I>M	AA:203		A4:F>L		DBVPG6304:F>L		YPS138:F>L	AA:216		DBVPG4650:K>E		N_17:K>N		N_44:K>N	AA:221		T21_4:I>V	AA:224		T21_4:Q>E	AA:240		N_17:A>S		N_44:A>S	AA:253		N_17:R>-		N_44:R>-	AA:257		N_17:V>A		N_44:V>AID:YAL038W	AA:27		UFRJ50816:D>E	AA:54		A12:V>L		A4:V>L		DBVPG6304:V>L		UFRJ50816:V>L		YPS138:V>L	AA:105		UFRJ50816:W>C	AA:155		UFRJ50816:Q>H	AA:298		UFRJ50791:G>D		UWOPS91_917_1:G>D	AA:392		N_43:F>VID:YAL039C	AA:4		UWOPS91_917_1:S>P	AA:27		UWOPS91_917_1:V>L	AA:157		UWOPS91_917_1:G>S	AA:203		N_44:G>D	AA:222		A4:E>K		DBVPG6304:E>K		UFRJ50791:E>K		UWOPS91_917_1:E>K		YPS138:E>K	AA:250		N_44:S>A		N_45:S>A	AA:252		A4:A>T		DBVPG6304:A>T		UWOPS91_917_1:A>T		YPS138:A>T	AA:255		UWOPS91_917_1:G>E	AA:260		N_44:G>DID:YAL040C	AA:28		A4:A>V		UFRJ50816:A>V		YPS138:A>V	AA:42		UFRJ50816:L>V	AA:50		IFO1804:N>S		N_43:N>S		N_45:N>S	AA:91		UWOPS91_917_1:F>L	AA:183		A12:T>S		DBVPG6304:T>S		IFO1804:T>S		N_43:T>S		N_45:T>S		UFRJ50816:T>S		YPS138:T>S	AA:204		UWOPS91_917_1:N>H	AA:216		UWOPS91_917_1:T>I	AA:253		UWOPS91_917_1:E>K	AA:255		UWOPS91_917_1:D>N	AA:260		CBS5829:L>P	AA:348		CBS432:S>A		KPN3828:S>A		N_43:S>A		N_45:S>A		Q59_1:S>A		Q62_5:S>A		UWOPS91_917_1:S>A		Y6_5:S>A		Z1_1:S>A	AA:350		UWOPS91_917_1:L>F	AA:352		UWOPS91_917_1:V>I	AA:357		N_17:P>L	AA:387		UWOPS91_917_1:M>L	AA:448		IFO1804:T>AID:YAL041W	AA:36		A4:R>S		UFRJ50791:R>S		UFRJ50816:R>S	AA:42		A4:->C		CBS432:->C		DBVPG4650:->C		IFO1804:->C		KPN3829:->C		N_44:->C		N_45:->C		Q32_3:->C		UFRJ50791:->C		UFRJ50816:->C		YPS138:->C	AA:46		UFRJ50791:Y>N		UFRJ50816:Y>N		UWOPS91_917_1:Y>N		YPS138:Y>N	AA:60		UFRJ50791:E>K		UFRJ50816:E>K		YPS138:E>K	AA:78		IFO1804:L>F		N_43:L>F		N_44:L>F		N_45:L>F	AA:143		A4:S>R		UFRJ50816:S>R		YPS138:S>R	AA:169		A4:C>W		UFRJ50816:C>W		YPS138:C>W	AA:178		A4:D>E		UFRJ50816:D>E		YPS138:D>E	AA:183		A4:F>L		UFRJ50816:F>L		YPS138:F>L	AA:185		IFO1804:W>-		N_43:W>-		N_44:W>-		N_45:W>-	AA:191		A4:I>M		IFO1804:I>M		N_43:I>M		N_44:I>M		N_45:I>M		UFRJ50816:I>M		YPS138:I>M	AA:231		A4:->Y		IFO1804:->Y		N_43:->Y		N_44:->Y		UFRJ50816:->Y		YPS138:->Y	AA:270		A4:L>P		UFRJ50816:L>P		YPS138:L>P	AA:320		A4:H>Q		YPS138:H>Q	AA:408		DBVPG6304:I>M		UFRJ50816:I>M		YPS138:I>M	AA:496		Q62_5:R>L	AA:502		DBVPG6304:R>S	AA:611		DBVPG6304:Q>K		N_43:Q>H		N_45:Q>H	AA:619		DBVPG6304:Y>-	AA:621		CBS432:I>L		CBS5829:I>L		N_17:I>L		N_43:I>L		N_45:I>L		Q32_3:I>L		Q95_3:I>L		T21_4:I>L		Y7:I>L	AA:633		N_43:->K		N_45:->K	AA:694		DBVPG6304:->W		N_43:->W		N_45:->W	AA:723		DBVPG6304:R>T		IFO1804:R>T		N_43:R>T		N_45:R>T	AA:792		A4:V>L	AA:812		A4:W>CID:YAL042W	AA:13		IFO1804:S>R		N_43:S>R		N_44:S>R		N_45:S>R	AA:39		A4:E>D		YPS138:E>D	AA:77		N_43:->C		N_44:->C		N_45:->C	AA:121		UWOPS91_917_1:->Y	AA:150		DBVPG6304:R>-		YPS138:R>-	AA:171		UWOPS91_917_1:R>-	AA:180		DBVPG6304:K>N		YPS138:K>N	AA:189		DBVPG6304:T>S	AA:227		DBVPG6304:F>L		YPS138:F>L	AA:256		DBVPG6304:M>I		UFRJ50816:M>I		YPS138:M>I	AA:290		A4:L>V		DBVPG6304:L>V		UFRJ50816:L>V		UWOPS91_917_1:L>V		YPS138:L>V	AA:299		A4:E>A		DBVPG4650:E>A		DBVPG6304:E>A		KPN3829:E>A		N_43:E>A		Q32_3:E>A		S36_7:E>A		UFRJ50816:E>A		UWOPS91_917_1:E>A		YPS138:E>A	AA:307		UFRJ50816:L>H	AA:344		DBVPG6304:W>R		UFRJ50791:W>R		UFRJ50816:W>R		YPS138:W>R	AA:358		CBS432:H>Q		CBS5829:H>Q		DBVPG4650:H>Q		KPN3829:H>Q		N_17:H>Q		N_43:H>Q		N_45:H>Q		Q32_3:H>Q		Q59_1:H>Q		Q89_8:H>Q		Q95_3:H>Q		S36_7:H>Q		Y7:H>Q	AA:366		A12:F>L		A4:F>L		DBVPG6304:F>L		UFRJ50791:F>L		UFRJ50816:F>L		YPS138:F>L	AA:380		YPS138:E>D	AA:397		A12:D>N		A4:D>N		DBVPG6304:D>N		UFRJ50791:D>N		UFRJ50816:D>N		YPS138:D>N	AA:402		YPS138:E>D	AA:410		IFO1804:P>S		N_43:P>SID:YAL043C	AA:19		A12:Q>K		DBVPG6304:Q>K	AA:134		A12:S>N		DBVPG6304:S>N		UFRJ50816:S>N		UWOPS91_917_1:S>G	AA:140		A12:I>V		DBVPG6304:I>V		UFRJ50816:I>V	AA:148		UWOPS91_917_1:A>E	AA:159		UWOPS91_917_1:H>R	AA:165		A12:D>N		DBVPG6304:D>N		UFRJ50816:D>N	AA:167		UWOPS91_917_1:D>Y	AA:185		A12:H>R		DBVPG6304:H>R		UFRJ50816:H>R	AA:196		A12:R>K		DBVPG6304:R>K		UFRJ50816:R>K		UWOPS91_917_1:R>K	AA:224		A12:G>E		DBVPG6304:G>E		IFO1804:G>E		N_45:G>E		UFRJ50816:G>E		UWOPS91_917_1:G>E	AA:261		A4:R>M		UFRJ50816:R>M		UWOPS91_917_1:R>M	AA:273		UWOPS91_917_1:V>I	AA:539		UWOPS91_917_1:Q>R	AA:575		DBVPG6304:R>K		UFRJ50791:R>K		YPS138:R>K	AA:582		UWOPS91_917_1:A>T	AA:611		DBVPG6304:V>I		IFO1804:V>I		N_43:V>I		N_44:V>I		N_45:V>I		UFRJ50791:V>I		UWOPS91_917_1:V>I		YPS138:V>I	AA:634		DBVPG6304:E>G		UFRJ50791:E>G		UWOPS91_917_1:E>G		YPS138:E>G	AA:657		DBVPG6304:K>N		UFRJ50791:K>N		YPS138:K>N	AA:740		UWOPS91_917_1:P>SID:YAL044C	AA:67		N_44:S>C	AA:84		Q32_3:E>G		T21_4:E>G	AA:112		N_45:Q>K	AA:133		S36_7:S>P	AA:147		UWOPS91_917_1:S>G	AA:158		N_43:T>AID:YAL044W-A	AA:15		DBVPG6304:G>R	AA:25		DBVPG6304:W>-	AA:44		DBVPG6304:M>I	AA:61		DBVPG6304:H>P		N_44:H>R		N_45:H>R	AA:69		IFO1804:->YID:YAL046C	AA:66		IFO1804:V>I		N_44:V>I		N_45:V>I	AA:78		UFRJ50791:I>V	AA:80		UFRJ50791:E>K	AA:88		IFO1804:Q>R	AA:116		N_44:S>L		N_45:S>LID:YAL047C	AA:6		A4:S>T		UWOPS91_917_1:S>T		YPS138:S>T	AA:23		IFO1804:K>N		N_45:K>N	AA:63		A4:D>N		UFRJ50791:D>N		UWOPS91_917_1:D>N		YPS138:D>N	AA:67		A4:H>R		UFRJ50791:H>R		UWOPS91_917_1:H>R		YPS138:H>R	AA:70		A4:E>G		UFRJ50791:E>G		UWOPS91_917_1:E>G		YPS138:E>G	AA:75		UWOPS91_917_1:A>V	AA:104		A4:T>A		UFRJ50791:T>A		UFRJ50816:T>A		UWOPS91_917_1:T>A		YPS138:T>A	AA:182		UFRJ50816:K>R		UWOPS91_917_1:K>R	AA:188		UFRJ50816:I>V		UWOPS91_917_1:I>V	AA:240		UWOPS91_917_1:S>N	AA:253		UWOPS91_917_1:S>G	AA:267		A12:I>F		DBVPG6304:I>F		N_43:H>Y		N_45:H>Y	AA:297		A12:M>I		DBVPG6304:M>I		N_45:M>I	AA:341		A12:T>A		DBVPG6304:T>A	AA:361		UWOPS91_917_1:N>S	AA:369		A12:F>Y		DBVPG6304:F>Y		UWOPS91_917_1:F>Y	AA:374		A12:D>N		DBVPG6304:D>N	AA:380		A12:L>P		A4:L>P		DBVPG6304:L>P	AA:387		UWOPS91_917_1:H>Q	AA:402		UWOPS91_917_1:E>K	AA:421		UWOPS91_917_1:M>I	AA:445		CBS5829:G>R		Q62_5:G>R	AA:447		A12:R>K		A4:R>K		DBVPG6304:R>K	AA:453		A4:L>F		DBVPG6304:L>F	AA:458		A4:D>N		DBVPG6304:D>N		YPS138:D>N	AA:499		A4:T>M		DBVPG6304:T>M		YPS138:T>M	AA:502		A4:S>T		DBVPG6304:S>T		YPS138:S>T	AA:522		A4:A>T		DBVPG6304:A>T		N_43:A>T		YPS138:A>T	AA:566		A4:G>V		DBVPG6304:G>V		YPS138:G>V	AA:568		A4:S>G		DBVPG6304:S>G		N_43:S>G		YPS138:S>G	AA:571		UWOPS91_917_1:M>I	AA:574		A4:N>I		DBVPG6304:N>I		YPS138:N>I	AA:588		A4:I>M		DBVPG6304:I>M		YPS138:I>M	AA:598		A4:F>Y		DBVPG6304:F>Y		YPS138:F>Y	AA:606		A4:I>V		DBVPG6304:I>V		YPS138:I>V	AA:615		UWOPS91_917_1:S>GID:YAL048C	AA:12		A12:M>I		A4:M>I	AA:259		A12:I>T		DBVPG6304:I>T	AA:292		N_43:T>I		N_44:T>I	AA:398		UWOPS91_917_1:Y>H	AA:436		A12:N>S		UWOPS91_917_1:N>S	AA:500		UWOPS91_917_1:T>A	AA:540		A12:A>S	AA:559		CBS432:Q>L		CBS5829:Q>L		Q62_5:Q>L	AA:607		A12:N>T	AA:642		A12:V>I		A4:V>I		UWOPS91_917_1:V>I	AA:649		UWOPS91_917_1:G>RID:YAL049C	AA:7		A12:D>N		A4:D>N		UWOPS91_917_1:D>N		YPS138:D>N	AA:18		A12:E>G		A4:E>G		DBVPG6304:E>G		UWOPS91_917_1:E>G		YPS138:E>G	AA:28		A12:V>I		A4:V>I		DBVPG6304:V>I		N_43:V>I		N_45:V>I		UWOPS91_917_1:V>I		YPS138:V>I	AA:77		CBS5829:P>S	AA:152		DBVPG6304:L>F		N_45:L>F		UFRJ50791:L>F		UWOPS91_917_1:L>F	AA:180		A4:D>E		DBVPG6304:D>E		UFRJ50791:D>E	AA:181		UWOPS91_917_1:A>T	AA:193		UFRJ50791:K>R	AA:194		IFO1804:N>T	AA:207		A4:N>K		N_45:N>K		UFRJ50791:N>K	AA:225		CBS432:R>C	AA:228		A12:A>P		A4:A>P		IFO1804:A>P		N_43:A>P		N_45:A>P		UFRJ50791:A>PID:YAL054C	AA:87		UWOPS91_917_1:S>Y	AA:116		DBVPG6304:S>P		UWOPS91_917_1:G>D		YPS138:S>P	AA:588		UWOPS91_917_1:L>V	AA:603		N_43:D>N	AA:635		A4:H>Y		UWOPS91_917_1:H>Y	AA:660		A4:S>A	AA:683		A4:A>G	AA:696		UWOPS91_917_1:D>E	AA:701		UWOPS91_917_1:Q>K	AA:706		UWOPS91_917_1:S>LID:YAL055W	AA:31		A4:Y>N		DBVPG6304:Y>N		YPS138:Y>N	AA:33		IFO1804:L>-		N_45:L>-	AA:38		IFO1804:C>G		N_45:C>G	AA:85		IFO1804:H>L		N_45:H>L	AA:94		A4:S>C		DBVPG6304:S>C	AA:110		CBS432:F>L		N_17:F>L		Y7:F>L	AA:114		A4:F>Y		DBVPG6304:F>Y		IFO1804:F>Y		N_45:F>Y	AA:137		A4:Y>-		DBVPG6304:Y>-	AA:139		CBS432:I>S		IFO1804:I>S		N_17:I>S		N_44:I>S		N_45:I>S		Y7:I>S	AA:145		A4:T>N		DBVPG6304:T>N	AA:158		A4:Y>D		DBVPG6304:Y>D	AA:162		A4:D>E		DBVPG6304:D>E	AA:166		A4:N>K		DBVPG6304:N>KID:YAL059W	AA:10		IFO1804:S>P		N_44:S>P		N_45:S>P	AA:15		IFO1804:R>-		N_44:R>-		N_45:R>-	AA:20		DBVPG6304:W>-		UFRJ50791:W>-		UFRJ50816:W>-		YPS138:W>-	AA:27		IFO1804:F>V		N_43:F>V		N_44:F>V		N_45:F>V		UFRJ50791:F>V		UFRJ50816:F>V		YPS138:F>V	AA:40		DBVPG6304:Q>H		IFO1804:Q>H		N_43:Q>H		N_44:Q>H		N_45:Q>H		UFRJ50791:Q>H		UFRJ50816:Q>H		YPS138:Q>H	AA:49		N_43:C>Y	AA:61		IFO1804:F>C		N_43:F>C		N_44:F>C		N_45:F>C	AA:86		A12:F>L		DBVPG6304:F>L		IFO1804:F>L		N_43:F>L		N_44:F>L		N_45:F>L		UFRJ50791:F>L		UFRJ50816:F>L		YPS138:F>L	AA:118		A12:L>V		UFRJ50791:L>V		UFRJ50816:L>V		YPS138:L>V	AA:133		A12:S>R		UFRJ50791:S>R		UFRJ50816:S>R		YPS138:S>R	AA:150		IFO1804:V>F		N_43:V>F		N_44:V>F		N_45:V>F	AA:167		A12:L>I		UFRJ50791:L>I		UFRJ50816:L>I		YPS138:L>I	AA:168		IFO1804:I>M		N_43:I>M		N_44:I>M		N_45:I>MID:YAL060W	AA:59		A12:A>E		UWOPS91_917_1:A>E		YPS138:A>E	AA:97		UWOPS91_917_1:N>K	AA:108		A12:A>T		UWOPS91_917_1:A>T		YPS138:A>T	AA:168		DBVPG4650:H>N		UWOPS91_917_1:H>L	AA:200		UWOPS91_917_1:G>A	AA:203		UWOPS91_917_1:Q>H	AA:207		UWOPS91_917_1:Q>H	AA:220		UWOPS91_917_1:R>S	AA:301		A4:L>F		CBS432:L>F		DBVPG4650:L>F		DBVPG6304:L>F		KPN3828:L>F		N_17:L>F		N_43:L>F		Q59_1:L>F		Q89_8:L>F		Q95_3:L>F		UWOPS91_917_1:L>F		Y6_5:L>F		YPS138:L>F	AA:343		A4:Q>H		YPS138:Q>H	AA:361		UWOPS91_917_1:Q>H		YPS138:Q>H	AA:366		IFO1804:F>L		N_43:F>LID:YAL061W	AA:10		UWOPS91_917_1:T>A	AA:22		DBVPG6304:S>-		UWOPS91_917_1:S>-		YPS138:S>-	AA:29		DBVPG6304:K>E	AA:33		UWOPS91_917_1:D>V	AA:38		UWOPS91_917_1:N>K	AA:59		DBVPG6304:R>S		UWOPS91_917_1:R>S		YPS138:R>S	AA:63		DBVPG6304:T>N		UWOPS91_917_1:T>N	AA:66		DBVPG6304:W>C	AA:80		UWOPS91_917_1:Q>-	AA:188		UWOPS91_917_1:F>L	AA:190		UWOPS91_917_1:A>P	AA:204		UWOPS91_917_1:A>D	AA:212		UWOPS91_917_1:S>R	AA:222		UWOPS91_917_1:R>S	AA:262		YPS138:K>N	AA:308		DBVPG6304:Q>-		YPS138:Q>-	AA:327		IFO1804:H>Q		N_44:H>Q		N_45:H>Q	AA:333		DBVPG6304:F>L		YPS138:F>L	AA:345		DBVPG6304:Y>-		UWOPS91_917_1:Y>-		YPS138:Y>-	AA:389		DBVPG6304:F>V		UFRJ50791:F>V		YPS138:F>VID:YAL062W	AA:35		IFO1804:R>W		N_44:R>W		N_45:R>W	AA:132		CBS432:R>P		CBS5829:R>P		DBVPG4650:R>P		IFO1804:R>P		KPN3828:R>P		KPN3829:R>P		N_17:R>P		N_45:R>P		Q62_5:R>P		Q89_8:R>P		T21_4:R>P		Y6_5:R>P		Y7:R>P	AA:199		YPS138:N>D	AA:203		UWOPS91_917_1:L>F	AA:208		UWOPS91_917_1:I>M	AA:216		UWOPS91_917_1:E>D	AA:219		YPS138:L>F	AA:237		UWOPS91_917_1:D>E	AA:244		A4:H>Q		UWOPS91_917_1:H>Q		YPS138:H>Q	AA:247		IFO1804:Q>H	AA:261		A4:S>R		IFO1804:S>R		N_44:S>R		UWOPS91_917_1:S>R		YPS138:S>R	AA:268		A4:D>E		IFO1804:D>E		UWOPS91_917_1:D>E		YPS138:D>E	AA:301		UWOPS91_917_1:W>-	AA:310		UWOPS91_917_1:D>E	AA:320		A4:N>K		IFO1804:N>K		N_44:N>K		UWOPS91_917_1:N>K		YPS138:N>K	AA:324		UWOPS91_917_1:F>Y	AA:327		A4:D>Y		IFO1804:D>E		N_44:D>E		UWOPS91_917_1:D>Y		YPS138:D>Y	AA:355		N_44:R>H	AA:387		UWOPS91_917_1:S>R	AA:400		N_44:D>E	AA:443		A12:E>D		A4:E>DID:YAR002C-A	AA:67		A12:I>V		A4:I>V		UFRJ50816:I>V	AA:87		UWOPS91_917_1:D>G	AA:122		A12:L>I		A4:L>I		UFRJ50816:L>I		UWOPS91_917_1:L>I	AA:131		UWOPS91_917_1:V>D	AA:162		UWOPS91_917_1:Q>K	AA:208		UWOPS91_917_1:C>YID:YAR002W	AA:9		A12:S>R		A4:S>R		DBVPG6304:S>R		UFRJ50791:S>R		YPS138:S>R	AA:23		UWOPS91_917_1:N>K	AA:36		UWOPS91_917_1:Y>N	AA:43		A12:F>V		A4:F>V		DBVPG6304:F>V		UFRJ50791:F>V		UWOPS91_917_1:F>V		YPS138:F>V	AA:45		A12:W>R		A4:W>R		DBVPG6304:W>R		UFRJ50791:W>R		UFRJ50816:W>R		YPS138:W>R	AA:48		N_43:C>G	AA:49		UWOPS91_917_1:T>S	AA:56		N_43:I>S	AA:58		UFRJ50791:F>L		YPS138:F>L	AA:71		A12:H>N		A4:H>N		DBVPG6304:H>N		UFRJ50791:H>N		UFRJ50816:H>N		YPS138:H>N	AA:90		A12:Y>-		A4:Y>-		DBVPG6304:Y>-		KPN3828:Y>F		UFRJ50791:Y>-		UFRJ50816:Y>-		UWOPS91_917_1:Y>-	AA:98		A12:V>I		A4:V>I		DBVPG6304:V>I		UFRJ50791:V>I		UFRJ50816:V>I		UWOPS91_917_1:V>I	AA:103		A12:L>H		A4:L>H		DBVPG6304:L>H		UFRJ50791:L>H		UFRJ50816:L>H		UWOPS91_917_1:L>H	AA:106		N_17:F>L	AA:108		N_17:P>A	AA:110		DBVPG6304:I>T		UFRJ50791:I>T		UFRJ50816:I>T	AA:154		A12:Y>C		A4:Y>C		DBVPG6304:Y>C		UFRJ50791:Y>C		UFRJ50816:Y>C	AA:207		A12:A>V		A4:A>V		DBVPG6304:A>V		UFRJ50791:A>V		UFRJ50816:A>V		UWOPS91_917_1:A>V	AA:225		A12:F>L		A4:F>L		DBVPG6304:F>L		UFRJ50791:F>L		UFRJ50816:F>L		YPS138:F>L	AA:256		A12:Y>-		A4:Y>-		DBVPG6304:Y>-		UFRJ50791:Y>-		UFRJ50816:Y>-		YPS138:Y>-	AA:301		Q89_8:I>V	AA:357		Y7:L>F	AA:366		A12:C>W		IFO1804:C>W		N_44:C>W		N_45:C>W		UFRJ50791:C>W		UFRJ50816:C>W		YPS138:C>W	AA:368		A12:R>S		IFO1804:R>S		N_44:R>S		N_45:R>S		UFRJ50791:R>S		UFRJ50816:R>S		YPS138:R>S	AA:387		A12:I>V		UFRJ50791:I>V		UFRJ50816:I>V		YPS138:I>V	AA:391		A12:R>K		UFRJ50791:R>K		UFRJ50816:R>K		YPS138:R>K	AA:403		UFRJ50791:W>C	AA:406		A12:N>K		UFRJ50816:N>K		YPS138:N>K	AA:421		Y6_5:C>F	AA:428		N_45:K>E	AA:442		A12:S>G		A4:S>G		UFRJ50816:S>G		YPS138:S>G	AA:447		IFO1804:A>T		N_44:A>T		N_45:A>T	AA:462		A12:L>F		A4:L>F		IFO1804:L>F		N_45:L>F		UFRJ50816:L>F		YPS138:L>F	AA:479		IFO1804:L>M		N_44:L>M		N_45:L>M	AA:486		N_17:S>A	AA:489		A12:F>C		A4:F>C		UFRJ50816:F>C		YPS138:F>C	AA:522		A12:I>L		A4:I>L		N_17:I>L		YPS138:I>LID:YAR003W	AA:3		IFO1804:D>E		N_45:D>E	AA:51		UWOPS91_917_1:D>N	AA:75		A12:N>K		A4:N>K		DBVPG6304:N>K		UFRJ50791:N>K		UFRJ50816:N>K		UWOPS91_917_1:N>K	AA:84		UFRJ50791:S>T		UFRJ50816:S>T		UWOPS91_917_1:S>T	AA:107		A12:R>S		A4:R>S		DBVPG6304:R>S		UFRJ50791:R>S		UFRJ50816:R>S	AA:110		A12:F>L		IFO1804:F>L		N_44:F>L		N_45:F>L	AA:177		A12:M>I		A4:M>I		DBVPG6304:M>I		UFRJ50816:M>I		YPS138:M>I	AA:179		A4:F>L		DBVPG6304:F>L		UFRJ50816:F>L	AA:183		A12:D>N		A4:D>N		DBVPG6304:D>N		UFRJ50816:D>N		YPS138:D>N	AA:189		A12:->W		A4:->W		DBVPG6304:->W		UFRJ50816:->W		YPS138:->W	AA:203		A12:L>W		A4:L>W		DBVPG6304:L>W		YPS138:L>W	AA:231		A12:N>K		A4:N>K		DBVPG6304:N>K	AA:249		A12:V>A		A4:V>A		DBVPG6304:V>A		UFRJ50816:V>A		YPS138:V>A	AA:265		A4:V>D		DBVPG6304:V>D		UFRJ50816:V>D		YPS138:V>D	AA:268		A12:L>P		A4:L>P		DBVPG6304:L>P		UFRJ50816:L>P		YPS138:L>P	AA:269		N_44:P>T		N_45:P>T	AA:282		A4:->E		DBVPG6304:->E		UFRJ50816:->E		YPS138:->E	AA:301		A12:T>S		A4:T>S		DBVPG6304:T>S		UFRJ50816:T>S		YPS138:T>S	AA:304		A12:->Y		A4:->Y		DBVPG6304:->Y		UFRJ50816:->Y		YPS138:->Y	AA:333		A12:F>I		A4:F>I		DBVPG6304:F>I		YPS138:F>I	AA:346		A12:W>C		A4:W>C		DBVPG6304:W>C		YPS138:W>C	AA:400		A12:I>M		DBVPG6304:I>M		UWOPS91_917_1:I>M		YPS138:I>MID:YAR007C	AA:241		Q59_1:V>I	AA:306		UWOPS91_917_1:V>I	AA:355		N_45:Q>R	AA:370		N_45:K>E	AA:451		UWOPS91_917_1:A>T	AA:513		UWOPS91_917_1:A>T	AA:619		IFO1804:V>L		N_43:V>LID:YAR008W	AA:13		IFO1804:Y>-		N_44:Y>-		N_45:Y>-		UFRJ50816:Y>-	AA:19		UFRJ50816:R>H	AA:64		DBVPG6304:Y>-		IFO1804:Y>-		N_43:Y>-		N_44:Y>-		N_45:Y>-		UFRJ50816:Y>-	AA:89		DBVPG6304:L>-		UFRJ50816:L>-		YPS138:L>-	AA:113		DBVPG6304:T>A		IFO1804:T>A		N_43:T>A		N_44:T>A		N_45:T>A		UFRJ50816:T>A		UWOPS91_917_1:T>A		YPS138:T>A	AA:120		DBVPG6304:I>K		IFO1804:I>K		N_43:I>K		N_44:I>K		N_45:I>K		UFRJ50816:I>K		YPS138:I>K	AA:122		YPS138:S>C	AA:148		DBVPG6304:Q>H		UFRJ50816:Q>H		UWOPS91_917_1:Q>H		YPS138:Q>H	AA:179		IFO1804:N>K	AA:191		Q32_3:I>V		S36_7:I>V	AA:207		DBVPG6304:E>A		UFRJ50816:E>A		UWOPS91_917_1:E>A		YPS138:E>A	AA:261		DBVPG6304:H>Q		N_43:H>Q		N_44:H>Q		UFRJ50816:H>Q		UWOPS91_917_1:H>Q		YPS138:H>Q	AA:281		DBVPG6304:H>Q		N_43:H>Q		N_44:H>Q		UFRJ50816:H>Q		UWOPS91_917_1:H>Q		YPS138:H>Q	AA:288		DBVPG6304:M>R		UFRJ50816:M>R		UWOPS91_917_1:M>R		YPS138:M>R	AA:290		DBVPG6304:E>K		UFRJ50816:E>K		UWOPS91_917_1:E>K		YPS138:E>K	AA:294		DBVPG6304:P>T		UFRJ50816:P>T		UWOPS91_917_1:P>T		YPS138:P>TID:YAR014C	AA:28		A12:S>C		A4:S>C		DBVPG6304:S>C		UFRJ50816:S>C		YPS138:S>C	AA:33		A12:A>T		A4:A>T		DBVPG6304:A>T		UFRJ50816:A>T		UWOPS91_917_1:A>T		YPS138:A>T	AA:54		A12:A>T		A4:A>T		DBVPG6304:A>T		UFRJ50816:A>T		UWOPS91_917_1:A>T		YPS138:A>T	AA:99		N_43:T>I		N_45:T>I	AA:105		UWOPS91_917_1:E>K	AA:122		A12:T>K		A4:T>K		DBVPG6304:T>K		UFRJ50816:T>K		YPS138:T>K	AA:123		UWOPS91_917_1:S>T	AA:146		UWOPS91_917_1:S>G	AA:197		UWOPS91_917_1:R>K	AA:200		UWOPS91_917_1:A>T	AA:204		UFRJ50816:S>N	AA:215		UWOPS91_917_1:D>Y	AA:225		Q32_3:G>R	AA:230		N_43:P>T		N_44:P>T		N_45:P>T		UFRJ50816:P>T		UWOPS91_917_1:P>T		YPS138:P>T	AA:238		DBVPG6304:I>N		UFRJ50816:I>N		UWOPS91_917_1:I>N		YPS138:I>N	AA:243		A4:I>V		DBVPG6304:I>V		N_43:I>V		N_44:I>V		N_45:I>V		UFRJ50816:I>V		UWOPS91_917_1:I>V		YPS138:I>V	AA:279		UWOPS91_917_1:S>T	AA:296		UWOPS91_917_1:N>D	AA:351		A4:C>S		CBS432:C>S		CBS5829:C>S		DBVPG4650:C>S		DBVPG6304:C>S		IFO1804:C>S		N_17:C>S		N_44:C>S		N_45:C>S		Q32_3:C>S		Q62_5:C>S		Q95_3:C>S		S36_7:C>S		UFRJ50816:C>S		UWOPS91_917_1:C>S		Y7:C>S		YPS138:C>S		Z1_1:C>S	AA:359		A4:S>R		DBVPG6304:S>R		UFRJ50816:S>R		YPS138:S>R	AA:360		UWOPS91_917_1:D>Y	AA:366		IFO1804:D>N		N_44:D>N	AA:369		A4:G>D		CBS432:G>D		CBS5829:G>D		DBVPG6304:G>D		IFO1804:G>D		N_17:G>D		N_44:G>D		Q32_3:G>D		Q62_5:G>D		Q95_3:G>D		S36_7:G>D		T21_4:G>D		UFRJ50816:G>D		UWOPS91_917_1:G>D		Y6_5:G>D		Y7:G>D		YPS138:G>D		Z1_1:G>D	AA:486		A12:N>D		A4:N>D		DBVPG6304:N>D		IFO1804:N>D		N_45:N>D		UFRJ50816:N>D	AA:515		A4:E>D		DBVPG6304:E>D	AA:571		Q32_3:Q>P	AA:572		KPN3828:D>G	AA:575		YPS138:S>R	AA:589		A12:D>G		A4:D>G		DBVPG6304:D>G		UFRJ50791:D>G	AA:598		Q59_1:A>S	AA:600		N_45:H>R	AA:625		DBVPG6304:V>M	AA:639		A12:S>L		A4:S>L		DBVPG6304:S>L		UFRJ50791:S>L		YPS138:S>L	AA:695		N_44:V>F		Y7:V>F	AA:707		A12:Y>F		A4:Y>F		DBVPG6304:Y>F		UFRJ50791:Y>F		UFRJ50816:Y>F		YPS138:Y>F	AA:711		A12:N>D		A4:N>D		DBVPG6304:N>D		UFRJ50791:N>D		UFRJ50816:N>D		YPS138:N>DID:YAR015W	AA:32		DBVPG6304:Q>H	AA:57		DBVPG6304:Q>H		UFRJ50816:Q>H		YPS138:Q>H	AA:100		A4:R>G		CBS432:R>G		CBS5829:R>G		DBVPG4650:R>G		DBVPG6304:R>G		KPN3828:R>G		KPN3829:R>G		N_17:R>G		N_45:R>G		Q62_5:R>G		Q89_8:R>G		T21_4:R>G		UFRJ50816:R>G		YPS138:R>G		Z1_1:R>G	AA:151		A12:R>S		A4:R>S		DBVPG6304:R>S		UFRJ50816:R>S		YPS138:R>S	AA:154		A12:K>N		A4:K>N		DBVPG6304:K>N		N_43:K>N		N_45:K>N		UFRJ50816:K>N		YPS138:K>N	AA:170		A12:M>I		A4:M>I		DBVPG6304:M>I		N_45:M>I		UFRJ50816:M>I	AA:220		N_43:K>N		N_45:K>N	AA:228		N_45:F>I	AA:231		Q62_5:Q>P		Z1_1:Q>P	AA:236		DBVPG6304:->KID:YAR018C	AA:48		A4:D>E		DBVPG6304:D>E		UFRJ50816:D>E		UWOPS91_917_1:D>E		YPS138:D>E	AA:132		CBS432:C>W	AA:139		IFO1804:N>T		N_43:N>T		N_44:N>T		N_45:N>T		UWOPS91_917_1:N>T	AA:218		UWOPS91_917_1:P>R	AA:225		A4:N>K		DBVPG6304:N>K		UWOPS91_917_1:N>K		YPS138:N>K	AA:240		UWOPS91_917_1:G>S	AA:418		KPN3829:H>ID:YAR019C	AA:5		UWOPS91_917_1:S>T	AA:10		N_43:S>F	AA:21		A12:L>S		DBVPG6304:L>S		N_43:L>S		N_44:L>S		N_45:L>S		UFRJ50791:L>S		UFRJ50816:L>S		YPS138:L>S	AA:23		A12:T>I		DBVPG6304:T>I		UFRJ50791:T>I		UFRJ50816:T>I		YPS138:T>I	AA:25		N_43:E>V		N_44:E>V		N_45:E>V	AA:69		A12:F>S		DBVPG6304:F>S		UFRJ50791:F>S		UFRJ50816:F>S		YPS138:F>S		Z1_1:F>S	AA:71		Z1_1:R>M	AA:73		N_43:R>P		N_44:R>P		N_45:R>P		UWOPS91_917_1:R>P	AA:112		N_43:P>Q		N_44:P>Q		N_45:P>Q	AA:119		UWOPS91_917_1:V>L	AA:143		CBS432:F>C	AA:154		N_43:Y>H		N_44:Y>H		N_45:Y>H	AA:162		UWOPS91_917_1:D>N	AA:164		UWOPS91_917_1:A>T	AA:171		UWOPS91_917_1:F>L	AA:199		IFO1804:R>Q		N_43:R>Q		N_44:R>Q		N_45:R>Q	AA:207		A12:V>A		DBVPG6304:V>D		UFRJ50791:V>D		UFRJ50816:V>D		UWOPS91_917_1:V>A		YPS138:V>D	AA:219		DBVPG6304:S>F		UFRJ50791:S>F		UFRJ50816:S>F		YPS138:S>F	AA:221		UWOPS91_917_1:T>S	AA:223		IFO1804:G>C		N_43:G>C		N_44:G>C		N_45:G>C	AA:224		UWOPS91_917_1:M>V	AA:259		IFO1804:N>S		N_43:N>S		N_44:N>S		N_45:N>S	AA:262		IFO1804:I>T		N_43:I>T		N_44:I>T		N_45:I>T		UWOPS91_917_1:I>T	AA:277		UWOPS91_917_1:D>E	AA:278		A12:V>I		DBVPG6304:V>I		UFRJ50791:V>I		UFRJ50816:V>I		YPS138:V>I	AA:281		UWOPS91_917_1:T>A	AA:301		IFO1804:T>S		N_43:T>S		N_44:T>S		N_45:T>S	AA:329		A12:C>Y		A4:C>Y		DBVPG6304:C>Y		IFO1804:C>Y		N_43:C>Y		N_44:C>Y		N_45:C>Y		T21_4:C>Y		UFRJ50791:C>Y		UFRJ50816:C>Y		YPS138:C>Y	AA:342		UWOPS91_917_1:Q>E		YPS138:K>E	AA:362		UWOPS91_917_1:D>E	AA:420		IFO1804:S>P		N_43:S>P		N_44:S>P		N_45:S>P	AA:436		CBS432:T>A	AA:446		UWOPS91_917_1:I>V	AA:506		A12:R>H		A4:R>H		UFRJ50816:R>H		YPS138:R>H	AA:516		UWOPS91_917_1:Y>C	AA:535		IFO1804:S>P		N_44:S>P		N_45:S>P	AA:545		A12:A>T		A4:A>T		IFO1804:A>T		N_44:A>T		N_45:A>T		UFRJ50816:A>T		UWOPS91_917_1:A>T		YPS138:A>T	AA:555		A12:V>A		A4:V>A		IFO1804:V>A		N_43:V>A		N_44:V>A		N_45:V>A		UFRJ50816:V>A		UWOPS91_917_1:V>A		YPS138:V>A	AA:569		UWOPS91_917_1:A>V	AA:599		DBVPG4650:T>I		UWOPS91_917_1:T>N	AA:601		UWOPS91_917_1:R>C	AA:602		A12:S>L		A4:S>L		YPS138:S>L	AA:609		A4:D>N		UWOPS91_917_1:D>N		YPS138:D>N	AA:669		A4:D>E		UWOPS91_917_1:D>E		YPS138:D>E	AA:696		A4:V>I		IFO1804:V>I		N_44:V>I		N_45:V>I		UWOPS91_917_1:V>I		YPS138:V>I	AA:711		N_44:Q>L	AA:729		UFRJ50816:D>N	AA:733		A4:E>V		UFRJ50816:E>V		UWOPS91_917_1:E>V		YPS138:E>V	AA:851		DBVPG6304:I>M		UFRJ50816:I>M		UWOPS91_917_1:I>T		YPS138:I>MID:YAR035W	AA:8		UWOPS91_917_1:R>S	AA:22		UWOPS91_917_1:A>P	AA:24		A4:F>V		UFRJ50816:F>V		UWOPS91_917_1:F>V		YPS138:F>V	AA:42		A4:C>-		UFRJ50816:C>-		UWOPS91_917_1:C>-		YPS138:C>-	AA:55		A4:Q>H		UFRJ50816:Q>H		UWOPS91_917_1:Q>H		YPS138:Q>H	AA:67		UFRJ50816:W>C		YPS138:W>C	AA:125		N_45:S>I	AA:135		N_45:E>D		UFRJ50816:E>D		YPS138:E>D	AA:221		UFRJ50791:K>N		UFRJ50816:K>N		YPS138:K>N	AA:224		UFRJ50791:D>E		UFRJ50816:D>E		YPS138:D>E	AA:241		UWOPS91_917_1:E>D	AA:271		DBVPG6304:D>N		UFRJ50791:D>N		UFRJ50816:D>N		UWOPS91_917_1:D>N		YPS138:D>N	AA:285		N_45:K>N	AA:294		UWOPS91_917_1:R>L	AA:360		Q32_3:V>F	AA:411		UWOPS91_917_1:D>N	AA:412		N_44:R>W		N_45:R>W	AA:420		A4:V>L		DBVPG6304:V>L		UWOPS91_917_1:V>L		YPS138:V>L	AA:459		DBVPG4650:Q>H	AA:473		CBS432:L>F		CBS5829:L>F		DBVPG4650:L>F		Q95_3:L>F	AA:474		A4:D>E		DBVPG6304:D>E		IFO1804:D>E		N_43:D>E		N_44:D>E		N_45:D>E		UWOPS91_917_1:D>E		YPS138:D>E	AA:561		A12:D>E		A4:D>E	AA:589		A12:->Y		A4:->Y		IFO1804:->Y		N_17:->Y		N_43:->Y		N_45:->Y		S36_7:->Y		Y6_5:->Y		Y7:->Y	AA:627		N_43:H>Q	AA:642		A12:F>L		A4:F>L		UWOPS91_917_1:F>LID:YAR062W	AA:14		IFO1804:->C		N_43:->C		N_44:->C		N_45:->C	AA:16		Q89_8:S>G		Q95_3:S>G	AA:85		Q89_8:H>N		Q95_3:H>N		S36_7:H>N	AA:148		UWOPS91_917_1:W>C	AA:181		UWOPS91_917_1:A>SID:YAR066W	AA:170		N_45:->CID:YBL003C	AA:23		UWOPS91_917_1:W>C	AA:43		A12:I>M		A4:I>M		DBVPG6304:I>M		YPS138:I>M	AA:81		UWOPS91_917_1:Q>H		YPS138:Q>H	AA:85		A12:S>R		A4:S>R		DBVPG6304:S>R	AA:114		A12:R>S		A4:R>S		DBVPG6304:R>S		UWOPS91_917_1:R>S		YPS138:R>S	AA:126		UWOPS91_917_1:F>VID:YBL005W	AA:53		DBVPG6304:N>D		UFRJ50816:N>D		YPS138:N>D	AA:57		DBVPG6304:L>P		UFRJ50816:L>P		UWOPS91_917_1:L>P		YPS138:L>P	AA:60		UWOPS91_917_1:S>T	AA:65		N_44:P>L		N_45:P>L	AA:109		Q62_5:T>A	AA:119		UWOPS91_917_1:N>D	AA:121		A12:D>G		A4:D>G		DBVPG6304:D>G		UFRJ50791:D>G		UFRJ50816:D>G		UWOPS91_917_1:D>G		YPS138:D>G	AA:137		IFO1804:L>V		N_44:L>V		N_45:L>V	AA:153		A12:D>N		A4:D>N		DBVPG6304:D>N		UFRJ50791:D>N		UFRJ50816:D>N		YPS138:D>N	AA:180		IFO1804:P>S		N_43:P>S		N_44:P>S		N_45:P>S	AA:193		A12:I>L		A4:I>L		DBVPG6304:I>L		UFRJ50791:I>L		UFRJ50816:I>L		YPS138:I>L	AA:222		A12:Q>H		A4:Q>H		DBVPG6304:Q>H		UFRJ50791:Q>H		UWOPS91_917_1:Q>H		YPS138:Q>H	AA:260		UWOPS91_917_1:L>I	AA:312		A12:S>T		A4:S>T		DBVPG6304:S>T		UFRJ50791:S>T		UFRJ50816:S>T		UWOPS91_917_1:S>T	AA:315		IFO1804:A>S		N_43:A>S		N_44:A>S		N_45:A>S	AA:316		A12:P>S		A4:P>S		DBVPG6304:P>S		UFRJ50791:P>S		UFRJ50816:P>S		UWOPS91_917_1:P>S	AA:324		A12:A>S		A4:A>S		DBVPG6304:A>S		UFRJ50791:A>S		UFRJ50816:A>S		UWOPS91_917_1:A>S	AA:328		UWOPS91_917_1:N>S	AA:356		CBS432:A>E	AA:359		A12:Y>H		A4:Y>H		DBVPG6304:Y>H		IFO1804:Y>H		N_43:Y>H		N_44:Y>H		N_45:Y>H		UFRJ50816:Y>H	AA:409		A4:S>C		UFRJ50816:S>C		UWOPS91_917_1:S>C	AA:433		IFO1804:E>G	AA:460		A12:T>A		A4:T>A		DBVPG6304:T>A		UFRJ50816:T>A		UWOPS91_917_1:T>S	AA:498		CBS432:F>V	AA:513		A12:I>M		A4:I>M		DBVPG6304:I>M		UFRJ50816:I>M		UWOPS91_917_1:I>M	AA:528		A12:N>S		A4:N>S		DBVPG6304:N>S		UFRJ50791:N>S		UFRJ50816:N>S		UWOPS91_917_1:N>S	AA:555		A12:K>R		A4:K>R		DBVPG6304:K>R		UFRJ50791:K>R		UFRJ50816:K>R	AA:570		IFO1804:D>N		N_44:D>N		N_45:D>N		UWOPS91_917_1:D>N	AA:602		T21_4:F>C	AA:611		A12:R>K		A4:R>K		DBVPG6304:R>K		UFRJ50791:R>K		UFRJ50816:R>K		UWOPS91_917_1:R>K		YPS138:R>K	AA:648		A12:H>L		DBVPG6304:H>L		UFRJ50791:H>L		UWOPS91_917_1:H>L		YPS138:H>L	AA:651		UWOPS91_917_1:T>A	AA:697		A12:Q>R		DBVPG6304:Q>R		UFRJ50791:Q>R		UWOPS91_917_1:Q>R		YPS138:Q>R	AA:725		UWOPS91_917_1:G>R	AA:727		UWOPS91_917_1:P>S	AA:732		Y7:E>K	AA:799		N_43:Q>H		N_44:Q>H	AA:810		A12:D>E		A4:D>E		UFRJ50791:D>E		UFRJ50816:D>E		UWOPS91_917_1:D>E		YPS138:D>E	AA:877		UFRJ50791:V>A		UFRJ50816:V>A		UWOPS91_917_1:V>A		YPS138:V>A	AA:908		UFRJ50791:D>E		UFRJ50816:D>E		UWOPS91_917_1:D>E	AA:913		N_45:V>A	AA:920		A12:S>L		UFRJ50791:S>L		UFRJ50816:S>L		UWOPS91_917_1:S>LID:YBL006C	AA:17		A12:H>Q		A4:H>Q		DBVPG6304:H>Q		UFRJ50791:H>Q		UWOPS91_917_1:H>Q	AA:29		A12:F>L		A4:F>L		DBVPG6304:F>L		UFRJ50791:F>L		UWOPS91_917_1:F>L	AA:47		A12:G>E		A4:G>E		DBVPG6304:G>E		UFRJ50791:G>E		UWOPS91_917_1:G>E	AA:53		UWOPS91_917_1:C>W	AA:77		A12:K>E		A4:K>E		DBVPG6304:K>E		UWOPS91_917_1:K>E	AA:79		A12:D>E		A4:D>E		DBVPG6304:D>E	AA:145		A4:L>-		DBVPG6304:L>-		N_44:L>-		N_45:L>-		UFRJ50791:L>-		UWOPS91_917_1:L>-	AA:166		A4:R>S		DBVPG6304:R>S		UFRJ50791:R>S	AA:167		UWOPS91_917_1:K>-	AA:174		A4:V>E		DBVPG6304:V>E		UFRJ50791:V>E		UWOPS91_917_1:V>EID:YBL007C	AA:40		Q95_3:L>F		T21_4:L>F	AA:139		A4:C>S		UFRJ50816:C>S		UWOPS91_917_1:C>S	AA:154		UWOPS91_917_1:W>S	AA:161		UWOPS91_917_1:L>S	AA:167		UWOPS91_917_1:R>Q	AA:205		A12:L>F	AA:215		A12:R>G		A4:R>G		UFRJ50816:R>G		UWOPS91_917_1:R>G	AA:226		A12:L>F		A4:L>F		UFRJ50791:L>F		UFRJ50816:L>F		UWOPS91_917_1:L>V	AA:259		A12:->Y		A4:->Y		UFRJ50791:->Y		UWOPS91_917_1:->Y	AA:296		A12:W>C		A4:W>C		UFRJ50791:W>C		UFRJ50816:W>C		UWOPS91_917_1:W>C	AA:392		A12:W>C		A4:W>C		DBVPG6304:W>C		UFRJ50791:W>C		UFRJ50816:W>C		UWOPS91_917_1:W>C	AA:398		DBVPG6304:S>R		UFRJ50791:S>R		UFRJ50816:S>R	AA:402		UWOPS91_917_1:W>C	AA:405		A12:R>S		A4:R>S		DBVPG6304:R>S		UFRJ50791:R>S		UFRJ50816:R>S	AA:448		A12:->C		A4:->C		DBVPG6304:->C		UFRJ50791:->C		UWOPS91_917_1:->C	AA:451		Q59_1:R>-	AA:462		A12:F>I		A4:F>I		DBVPG6304:F>I		UFRJ50791:F>I	AA:467		A12:I>F		A4:I>F		DBVPG6304:I>F		IFO1804:I>F		N_44:I>F		N_45:I>F		UFRJ50791:I>F	AA:485		IFO1804:W>C		N_44:W>C		N_45:W>C	AA:488		A12:L>F		A4:L>F		DBVPG6304:L>F		IFO1804:L>F		N_44:L>F		N_45:L>F		UFRJ50791:L>F		UWOPS91_917_1:L>F	AA:495		A12:W>-		A4:W>-	AA:514		N_17:I>L	AA:521		A12:I>M		A4:I>M		DBVPG6304:I>M		UWOPS91_917_1:I>M	AA:557		A12:F>I		A4:F>I		DBVPG6304:F>I	AA:561		A12:L>F		A4:L>F		DBVPG6304:L>F		IFO1804:L>F		N_44:L>F		N_45:L>F		UFRJ50816:L>F		UWOPS91_917_1:S>R	AA:600		UWOPS91_917_1:W>-	AA:633		UWOPS91_917_1:->Q	AA:641		N_43:F>L		N_44:F>L	AA:657		A4:L>F		DBVPG6304:L>F		UFRJ50816:L>F		UWOPS91_917_1:L>F		YPS138:L>F	AA:665		N_43:F>L		N_44:F>L		N_45:F>L	AA:671		DBVPG6304:->C		UFRJ50816:->C		UWOPS91_917_1:->C		YPS138:->C	AA:698		UWOPS91_917_1:Q>-	AA:733		CBS432:F>Y		DBVPG4650:F>Y		DBVPG6304:F>Y		N_17:F>Y		N_43:F>Y		N_44:F>Y		Q32_3:F>Y		Q59_1:F>Y		Q89_8:F>Y		T21_4:F>Y		UFRJ50816:F>Y		UWOPS91_917_1:F>Y		YPS138:F>Y	AA:748		N_43:I>M		N_44:I>M		N_45:I>M	AA:752		DBVPG6304:F>L		UFRJ50816:F>L		YPS138:F>L	AA:768		DBVPG6304:->R		UFRJ50816:->R		YPS138:->R	AA:780		DBVPG6304:L>F	AA:813		N_45:L>R	AA:838		UFRJ50816:T>I	AA:842		UFRJ50816:->C	AA:855		UFRJ50816:F>L	AA:894		A4:F>L		UFRJ50816:F>L		UWOPS91_917_1:F>L		YPS138:F>L	AA:904		UFRJ50816:F>L		YPS138:F>L	AA:909		N_43:N>K		N_44:N>K		N_45:N>K	AA:924		UWOPS91_917_1:F>L	AA:938		N_43:F>L		N_44:F>L		N_45:F>L	AA:939		UWOPS91_917_1:L>I	AA:941		A4:V>L		N_43:V>L		N_44:V>L		N_45:V>L		UFRJ50816:V>L		UWOPS91_917_1:V>L		YPS138:V>L	AA:947		UFRJ50816:F>L		YPS138:F>L	AA:955		UWOPS91_917_1:S>N	AA:960		UWOPS91_917_1:Y>-	AA:971		A4:L>F		UFRJ50816:L>F		YPS138:L>F	AA:998		A4:A>T		DBVPG6304:A>T		UFRJ50791:A>T		UFRJ50816:A>T		UWOPS91_917_1:A>T	AA:1017		A4:->C		DBVPG6304:->C		UFRJ50791:->C		UFRJ50816:->C		UWOPS91_917_1:->C	AA:1039		CBS432:S>Y		CBS5829:S>Y		DBVPG4650:S>Y		KPN3829:S>Y		N_17:S>Y		Q32_3:S>Y		Q59_1:S>Y		Q95_3:S>Y	AA:1043		Q95_3:S>C		UWOPS91_917_1:N>K	AA:1045		A4:R>S		DBVPG6304:R>S		UFRJ50791:R>S		UFRJ50816:R>S	AA:1051		UWOPS91_917_1:F>L	AA:1053		UWOPS91_917_1:W>C	AA:1058		UWOPS91_917_1:F>L	AA:1068		A4:M>V		UFRJ50791:M>V		UFRJ50816:M>V	AA:1071		A4:R>W		UFRJ50791:R>W		UFRJ50816:R>W	AA:1116		A4:W>C		UFRJ50791:W>C		UWOPS91_917_1:W>C	AA:1121		UFRJ50791:F>L	AA:1127		N_43:F>L		N_44:F>L		UFRJ50791:F>L		UFRJ50816:F>L		UWOPS91_917_1:F>L	AA:1136		UWOPS91_917_1:V>I	AA:1151		IFO1804:F>V		N_17:F>V		N_44:F>V		UFRJ50791:F>V	AA:1158		UFRJ50791:F>V	AA:1159		UWOPS91_917_1:I>T	AA:1179		UWOPS91_917_1:H>N	AA:1180		UFRJ50791:->Y	AA:1185		IFO1804:F>L		N_44:F>L		N_45:F>L		Q95_3:F>L		UFRJ50791:F>L		UFRJ50816:F>L		UWOPS91_917_1:F>L	AA:1207		Y6_5:->L	AA:1217		S36_7:Q>H	AA:1221		A4:C>-		UFRJ50791:C>-		UFRJ50816:C>-		UWOPS91_917_1:C>-ID:YBL009W	AA:7		UWOPS91_917_1:A>T	AA:12		A4:A>T		DBVPG6304:A>T		IFO1804:A>T		N_43:A>T		N_44:A>T		N_45:A>T		UFRJ50816:A>T		UWOPS91_917_1:A>T	AA:109		IFO1804:D>A		N_43:D>A		N_44:D>A	AA:114		UFRJ50816:S>P	AA:119		A4:V>I		DBVPG6304:V>I		UFRJ50816:V>I	AA:130		A4:L>I		UFRJ50816:L>I		UWOPS91_917_1:L>I	AA:151		UWOPS91_917_1:T>I	AA:177		UWOPS91_917_1:R>Q	AA:184		N_43:R>C		N_45:R>C	AA:236		UFRJ50816:P>S	AA:242		UWOPS91_917_1:K>R	AA:292		KPN3828:S>A	AA:325		UWOPS91_917_1:R>H		YPS138:R>H	AA:386		IFO1804:E>K		N_43:E>K		N_44:E>K		N_45:E>K		UWOPS91_917_1:E>K	AA:393		A12:C>R		UFRJ50816:C>R		UWOPS91_917_1:C>R		YPS138:C>R	AA:402		A12:A>V		DBVPG6304:A>V		UFRJ50816:A>V		UWOPS91_917_1:A>V		YPS138:A>V	AA:426		UWOPS91_917_1:V>L	AA:464		A12:N>S		DBVPG6304:N>S		UFRJ50791:N>S		UFRJ50816:N>S		YPS138:N>S	AA:466		YPS138:L>Q	AA:475		A12:K>Q		DBVPG6304:K>Q		IFO1804:K>Q		N_43:K>Q		N_45:K>Q		UFRJ50791:K>Q		UFRJ50816:K>Q		UWOPS91_917_1:K>Q		YPS138:K>Q	AA:490		A12:D>H		DBVPG6304:D>H		N_43:D>H		N_44:D>H		N_45:D>H		UFRJ50791:D>H		UFRJ50816:D>H		UWOPS91_917_1:D>H		YPS138:D>H	AA:495		UWOPS91_917_1:L>M	AA:557		UWOPS91_917_1:M>I	AA:586		Q59_1:L>V		Y7:L>V		Z1_1:L>V	AA:618		A12:T>K		DBVPG6304:T>K		UFRJ50791:T>K		UFRJ50816:T>K		UWOPS91_917_1:T>K		YPS138:T>K	AA:623		N_43:T>I		N_44:T>I		N_45:T>I	AA:655		UFRJ50791:K>Q		UFRJ50816:K>QID:YBL010C	AA:13		DBVPG6304:S>-		UFRJ50816:S>-		YPS138:S>-	AA:18		IFO1804:K>R		N_43:K>R		N_44:K>R		N_45:K>R	AA:44		A12:L>I		DBVPG6304:L>I		IFO1804:L>I		N_44:L>I		N_45:L>I		UFRJ50816:L>I		YPS138:L>I	AA:49		A12:L>Q		A4:L>Q		DBVPG6304:L>Q		UFRJ50816:L>Q		YPS138:L>Q	AA:61		A12:K>N		A4:K>N		DBVPG6304:K>N		UFRJ50816:K>N		YPS138:K>N	AA:64		IFO1804:G>A		N_44:G>A		N_45:G>A	AA:72		A12:L>M		A4:L>M		DBVPG6304:L>M		UFRJ50816:L>M		YPS138:L>M	AA:137		N_43:F>L		N_44:F>L		N_45:F>L	AA:139		A12:Q>-		A4:Q>-		YPS138:Q>-	AA:157		A12:I>M		A4:I>M		YPS138:I>M	AA:160		A12:->C		A4:->C		YPS138:->C	AA:198		N_17:I>S	AA:215		A12:L>F		A4:L>F		YPS138:L>FID:YBL011W	AA:7		A12:T>R		A4:T>R		DBVPG6304:T>R		UFRJ50791:T>R		UWOPS91_917_1:T>R	AA:22		UWOPS91_917_1:S>F	AA:41		Q89_8:R>I	AA:46		KPN3828:T>S	AA:47		UWOPS91_917_1:K>N	AA:57		KPN3828:L>R	AA:111		UWOPS91_917_1:R>C	AA:119		A4:N>S		DBVPG6304:N>S		UWOPS91_917_1:N>S	AA:125		UWOPS91_917_1:A>V	AA:135		KPN3828:A>T	AA:158		A4:D>N		DBVPG6304:D>N	AA:173		UWOPS91_917_1:C>S	AA:178		UWOPS91_917_1:L>I	AA:191		A4:Q>L	AA:329		IFO1804:E>D		N_43:E>D		N_44:E>D		N_45:E>D		UWOPS91_917_1:E>D	AA:421		UWOPS91_917_1:L>M	AA:523		UWOPS91_917_1:N>K	AA:573		A12:K>T		A4:K>T		DBVPG6304:K>T		UFRJ50791:K>T		YPS138:K>T	AA:635		KPN3829:Q>R	AA:652		UFRJ50791:T>N		UFRJ50816:T>N		YPS138:T>N	AA:691		N_45:A>P	AA:740		IFO1804:N>D		N_43:N>D		S36_7:N>DID:YBL013W	AA:2		N_43:V>D		N_44:V>D	AA:27		A12:V>A		A4:V>A		DBVPG6304:V>A	AA:28		N_43:Q>R		N_44:Q>R	AA:60		A12:I>V		A4:I>V		DBVPG6304:I>V	AA:88		N_44:D>H	AA:112		A4:E>K		DBVPG6304:E>K	AA:126		DBVPG6304:L>P		N_44:L>P	AA:176		A12:D>E		DBVPG6304:D>E	AA:216		DBVPG6304:V>I	AA:221		A12:A>T		DBVPG6304:A>T		UWOPS91_917_1:A>T	AA:225		A12:S>P		DBVPG6304:S>P	AA:228		A12:V>L		DBVPG6304:V>L		UWOPS91_917_1:V>L	AA:241		UWOPS91_917_1:A>T	AA:253		UWOPS91_917_1:E>Q	AA:310		DBVPG6304:A>V	AA:315		A12:N>D		DBVPG6304:N>D		UWOPS91_917_1:N>D	AA:324		UWOPS91_917_1:I>V	AA:327		A12:H>Y		DBVPG6304:H>Y		UWOPS91_917_1:H>Y	AA:332		IFO1804:M>V	AA:336		A12:L>R		DBVPG6304:L>R		UFRJ50791:L>R		UWOPS91_917_1:L>R	AA:343		A12:P>Q		A4:P>Q		DBVPG6304:P>Q		UFRJ50791:P>Q		UWOPS91_917_1:P>L	AA:360		UWOPS91_917_1:R>H	AA:366		IFO1804:C>Y	AA:381		IFO1804:G>D	AA:399		A12:I>V		A4:I>V		DBVPG6304:I>V		UFRJ50791:I>VID:YBL014C	AA:21		A12:->R		A4:->R		DBVPG6304:->R	AA:27		A12:Y>-		A4:Y>-		DBVPG6304:Y>-	AA:31		A12:R>G		A4:R>G		DBVPG6304:R>G	AA:34		A12:H>Q		A4:H>Q		DBVPG6304:H>Q		N_43:H>Q		N_45:H>Q	AA:37		CBS5829:K>N	AA:42		Z1_1:->R	AA:73		DBVPG6304:S>F		UWOPS91_917_1:S>F	AA:82		A12:W>-		A4:W>-		DBVPG6304:W>-		UWOPS91_917_1:W>-	AA:110		DBVPG6304:A>T		UWOPS91_917_1:A>T	AA:132		UWOPS91_917_1:->Y	AA:151		N_44:C>W		N_45:C>W	AA:159		A12:D>E	AA:188		A12:N>K	AA:210		A4:I>M		DBVPG6304:I>M		UWOPS91_917_1:I>M	AA:223		A12:G>R		A4:G>R		DBVPG6304:G>R		UWOPS91_917_1:G>R	AA:238		UWOPS91_917_1:F>L	AA:245		A4:S>A		DBVPG6304:S>A		UFRJ50791:S>A		UWOPS91_917_1:S>A	AA:252		A12:S>R	AA:263		A4:Y>-		UWOPS91_917_1:S>C	AA:282		A4:F>Y		DBVPG6304:F>Y		UFRJ50791:F>Y	AA:285		A12:S>R	AA:287		CBS432:I>T		DBVPG4650:I>T		IFO1804:I>T		KPN3829:I>T		N_17:I>T		N_43:I>T		N_44:I>T		N_45:I>T		Q32_3:I>T		Q89_8:I>T		Q95_3:I>T		T21_4:I>T		Y6_5:I>T		Y7:I>T	AA:289		A12:D>E	AA:296		A12:F>L	AA:303		IFO1804:N>K		N_43:N>K		N_44:N>K		N_45:N>K	AA:306		A12:T>A	AA:317		KPN3828:I>V	AA:318		A12:E>K	AA:325		A12:I>L		A4:I>L	AA:327		A12:S>R		N_43:S>R		N_44:S>R	AA:349		UWOPS91_917_1:N>K	AA:380		A12:F>V		DBVPG6304:F>V		UFRJ50791:F>V	AA:390		UWOPS91_917_1:S>R	AA:415		A12:E>D		DBVPG6304:E>D		UFRJ50791:E>D	AA:418		UWOPS91_917_1:Y>-	AA:425		A12:H>L		DBVPG6304:H>L	AA:431		UWOPS91_917_1:C>W	AA:432		A12:Q>K		A4:Q>K		DBVPG6304:Q>K		UFRJ50791:Q>K	AA:474		UWOPS91_917_1:D>G	AA:475		A12:Y>-		A4:Y>-		DBVPG6304:Y>-	AA:483		A12:A>T		A4:A>T		DBVPG6304:A>T	AA:485		UWOPS91_917_1:F>V	AA:496		UWOPS91_917_1:->C	AA:503		A12:Y>S		A4:Y>S		IFO1804:Y>S		N_43:Y>S	AA:535		KPN3828:F>C		KPN3829:F>C	AA:541		UWOPS91_917_1:F>C	AA:551		A12:I>F		A4:I>F	AA:553		UWOPS91_917_1:Q>H	AA:559		IFO1804:I>M		N_44:I>M		N_45:I>M	AA:591		UWOPS91_917_1:I>M	AA:598		UWOPS91_917_1:Y>-	AA:600		A4:N>T	AA:635		IFO1804:Q>K		N_44:Q>K		N_45:Q>K		UWOPS91_917_1:Q>K	AA:637		A4:->C	AA:638		IFO1804:S>F		N_44:S>F		N_45:S>F	AA:665		A4:M>L		UWOPS91_917_1:M>L	AA:667		UWOPS91_917_1:V>D	AA:678		IFO1804:C>R		N_44:C>R		N_45:C>R	AA:696		A12:S>C	AA:708		A12:N>H		A4:N>H		IFO1804:N>H		UWOPS91_917_1:N>H	AA:713		A12:Q>H		A4:Q>H		UWOPS91_917_1:Q>H	AA:722		A12:K>Q		A4:K>Q	AA:792		IFO1804:C>W		N_45:C>W	AA:797		A12:V>L		A4:V>L		UFRJ50816:V>L	AA:814		IFO1804:F>L		N_45:F>L	AA:826		A12:S>N		A4:S>N		UFRJ50816:S>N		UWOPS91_917_1:S>N	AA:875		A12:Y>-		DBVPG6304:Y>-		UFRJ50816:Y>-		UWOPS91_917_1:Y>-		YPS138:Y>-	AA:879		DBVPG4650:->Q	AA:890		UWOPS91_917_1:L>MID:YBL015W	AA:27		A4:D>Y		DBVPG6304:D>Y		UWOPS91_917_1:D>Y	AA:29		KPN3828:Q>H	AA:58		UWOPS91_917_1:H>Q	AA:126		A4:C>W		DBVPG6304:C>W		UFRJ50816:C>W	AA:135		A4:F>L		DBVPG4650:F>L		DBVPG6304:F>L		IFO1804:F>L		KPN3828:F>L		N_17:F>L		N_43:F>L		N_44:F>L		N_45:F>L		Q59_1:F>L		Q62_5:F>L		Q89_8:F>L		Q95_3:F>L		S36_7:F>L		UFRJ50816:F>L		Y6_5:F>L		Z1_1:F>L	AA:137		A4:V>L		DBVPG6304:V>L		UFRJ50816:V>L	AA:225		A4:N>K		DBVPG6304:N>K		IFO1804:N>K		KPN3828:N>K		KPN3829:N>K		N_43:N>K		N_44:N>K		N_45:N>K		Q62_5:N>K		S36_7:N>K		UFRJ50816:N>K		UWOPS91_917_1:N>K		Y7:N>K		YPS138:N>K		Z1_1:N>K	AA:390		YPS138:H>Q	AA:458		A12:R>S		DBVPG6304:R>S		UWOPS91_917_1:R>S	AA:518		A12:F>L		A4:F>L		DBVPG6304:F>L		UWOPS91_917_1:F>L		YPS138:F>LID:YBL016W	AA:2		CBS5829:W>G	AA:67		N_44:L>R		N_45:L>R	AA:135		DBVPG6304:M>I	AA:138		DBVPG6304:Q>H		UFRJ50816:Q>H		YPS138:Q>H	AA:182		A4:I>M		DBVPG6304:I>M		UFRJ50816:I>M		YPS138:I>M	AA:185		A4:C>S		DBVPG6304:C>S		UFRJ50816:C>S		UWOPS91_917_1:C>S		YPS138:C>S	AA:213		A4:E>D		DBVPG6304:E>D		UFRJ50816:E>D		UWOPS91_917_1:E>D		YPS138:E>D	AA:231		UWOPS91_917_1:L>F	AA:234		A4:Q>H		DBVPG6304:Q>H		UFRJ50816:Q>H		UWOPS91_917_1:Q>H		YPS138:Q>H	AA:263		A4:K>N		DBVPG6304:K>N		UFRJ50816:K>N		YPS138:K>N	AA:290		A4:K>N		UFRJ50816:K>N		YPS138:K>NID:YBL019W	AA:2		A4:R>P		DBVPG6304:R>P		UFRJ50791:R>P		UFRJ50816:R>P		YPS138:R>P	AA:10		UFRJ50791:I>V		UFRJ50816:I>V	AA:32		A4:M>I		DBVPG6304:M>I		UFRJ50791:M>I		UFRJ50816:M>I		YPS138:M>I	AA:61		A4:F>L		DBVPG6304:F>L		UFRJ50791:F>L		UFRJ50816:F>L		UWOPS91_917_1:F>L		YPS138:F>L	AA:79		IFO1804:K>I		N_43:K>I	AA:82		UFRJ50791:D>E		UFRJ50816:D>E		UWOPS91_917_1:D>E	AA:85		IFO1804:C>W		N_43:C>W		N_44:C>W		N_45:C>W		UFRJ50791:C>W		UFRJ50816:C>W		UWOPS91_917_1:C>W	AA:96		DBVPG6304:N>T		UFRJ50791:N>T		UFRJ50816:N>T		YPS138:N>T	AA:109		UWOPS91_917_1:Q>P	AA:119		UWOPS91_917_1:S>G	AA:129		DBVPG6304:L>M		UFRJ50791:L>M		UWOPS91_917_1:L>M		YPS138:L>M	AA:139		CBS5829:I>M	AA:150		A12:G>V		DBVPG6304:G>V		UFRJ50791:G>V		YPS138:G>V	AA:162		A12:->C		DBVPG6304:->C		UFRJ50791:->C		UWOPS91_917_1:->C		YPS138:->C	AA:212		A12:F>L		A4:F>L		DBVPG6304:F>L		UFRJ50816:F>L		UWOPS91_917_1:F>L		YPS138:F>L	AA:277		N_44:L>F		N_45:L>F	AA:278		A12:Y>-		A4:Y>-		DBVPG6304:Y>-		YPS138:Y>-	AA:287		A12:C>S		A4:C>S		DBVPG6304:C>S		N_44:C>S		N_45:C>S		UFRJ50816:C>S		YPS138:C>S	AA:330		A12:Y>-		A4:Y>-		UFRJ50816:Y>-		YPS138:Y>-	AA:332		UFRJ50816:Y>N	AA:351		UFRJ50816:A>S	AA:363		A12:Y>-		A4:Y>-		YPS138:Y>-	AA:389		A12:S>P		A4:S>P		UFRJ50816:S>P		YPS138:S>P	AA:399		A12:F>Y		A4:F>Y		YPS138:F>Y	AA:410		A12:L>M		A4:L>M		UFRJ50816:L>M		YPS138:L>M	AA:414		UWOPS91_917_1:R>H	AA:417		A12:N>H		A4:N>H		UFRJ50816:N>H		UWOPS91_917_1:N>H		YPS138:N>H	AA:432		A12:->C		A4:->C		UFRJ50816:->C		UWOPS91_917_1:->C		YPS138:->C	AA:480		A12:F>L		A4:F>L		UFRJ50816:F>L		YPS138:F>L	AA:482		A12:R>S		A4:R>S		UWOPS91_917_1:R>S		YPS138:R>S	AA:511		A12:F>C		A4:F>C		N_43:F>C		N_44:F>C		N_45:F>C		UWOPS91_917_1:F>C		YPS138:F>CID:YBL020W	AA:10		A12:R>S		DBVPG6304:R>S		N_45:R>S		YPS138:R>S	AA:11		A12:G>C		CBS432:G>C		CBS5829:G>C		DBVPG6304:G>C		KPN3829:G>C		Q95_3:G>C		Y7:G>C		YPS138:G>C		Z1_1:G>C	AA:31		A12:K>N		DBVPG6304:K>N		UWOPS91_917_1:K>N		YPS138:K>N	AA:39		A12:Y>-		DBVPG6304:Y>-		UWOPS91_917_1:Y>-		YPS138:Y>-	AA:47		IFO1804:K>N		N_45:K>N	AA:81		IFO1804:D>E		N_45:D>E	AA:89		A12:E>D		A4:E>D		DBVPG6304:E>D		UFRJ50816:E>D		UWOPS91_917_1:E>D		YPS138:E>D	AA:140		A12:E>D		A4:E>D		DBVPG6304:E>D		UFRJ50816:E>D		UWOPS91_917_1:E>D		YPS138:E>D	AA:165		UWOPS91_917_1:W>-	AA:195		IFO1804:K>N		N_45:K>N	AA:196		A12:Y>-		A4:Y>-		DBVPG6304:Y>-		UFRJ50816:Y>-		YPS138:Y>-	AA:231		A4:S>R		DBVPG6304:S>R		UFRJ50816:S>R		UWOPS91_917_1:S>R		YPS138:S>R	AA:282		A4:L>F		DBVPG6304:L>F		YPS138:L>F	AA:289		A4:E>K		DBVPG6304:E>K		UFRJ50816:E>K		UWOPS91_917_1:E>K		YPS138:E>K	AA:301		A4:Q>R		DBVPG6304:Q>R		UFRJ50816:Q>R		YPS138:Q>R	AA:308		A4:Q>-	AA:309		N_43:N>S		N_45:N>S	AA:315		UWOPS91_917_1:G>D	AA:352		DBVPG6304:Q>K		UFRJ50816:Q>K		YPS138:Q>K	AA:362		N_43:->Y		N_45:->Y	AA:370		DBVPG6304:->Y		UFRJ50816:->Y		YPS138:->Y	AA:373		YPS138:K>N	AA:406		UWOPS91_917_1:N>K	AA:431		Y7:L>W	AA:441		N_43:L>V		N_45:L>V	AA:462		UWOPS91_917_1:F>L	AA:472		A4:Y>-		DBVPG6304:Y>-		UFRJ50791:Y>-		UFRJ50816:Y>-		UWOPS91_917_1:Y>-		YPS138:Y>-	AA:485		UWOPS91_917_1:I>L	AA:496		UWOPS91_917_1:F>L	AA:522		N_43:->E		N_45:->E	AA:551		A4:D>E		DBVPG6304:D>E		UFRJ50791:D>E		UFRJ50816:D>E		UWOPS91_917_1:D>EID:YBL021C	AA:128		DBVPG6304:E>D		UFRJ50816:E>D		YPS138:E>DID:YBL023C	AA:147		A12:D>G		DBVPG6304:D>G		IFO1804:D>E		N_44:D>E		N_45:D>E		UFRJ50791:D>G	AA:154		CBS5829:G>D		UWOPS91_917_1:G>D	AA:709		CBS432:L>F		Q95_3:L>F	AA:776		IFO1804:N>D		N_44:N>D		N_45:N>D	AA:783		DBVPG6304:N>S	AA:842		DBVPG6304:Q>H		UFRJ50816:Q>H		UWOPS91_917_1:Q>HID:YBL024W	AA:12		A12:F>L		DBVPG6304:F>L		UFRJ50816:F>L		YPS138:F>L	AA:14		UWOPS91_917_1:S>R	AA:19		A12:I>T		CBS432:I>T		DBVPG6304:I>T		N_17:I>T		N_44:I>T		N_45:I>T		Q95_3:I>T		UFRJ50816:I>T		YPS138:I>T	AA:22		N_17:S>F	AA:24		A12:L>-		DBVPG6304:L>-		UFRJ50816:L>-		YPS138:L>-	AA:35		UFRJ50816:F>I	AA:74		A12:L>F		DBVPG6304:L>F		UFRJ50816:L>F		YPS138:L>F	AA:113		A12:M>I		DBVPG6304:M>I		UFRJ50816:M>I		YPS138:M>I	AA:128		N_44:I>V	AA:139		UFRJ50816:N>K	AA:167		N_43:V>L		N_44:V>L		N_45:V>L	AA:170		A12:Q>H		DBVPG6304:Q>H		N_43:Q>H		N_44:Q>H		N_45:Q>H		UFRJ50816:Q>H		YPS138:Q>H	AA:181		A12:S>R		DBVPG6304:S>R		N_43:S>R		N_44:S>R		N_45:S>R		UFRJ50816:S>R		YPS138:S>R	AA:197		N_43:E>D		N_44:E>D		N_45:E>D	AA:231		A12:L>F		DBVPG6304:L>F		UFRJ50816:L>F		YPS138:L>F	AA:268		A12:F>L		DBVPG6304:F>L		N_43:F>L		N_44:F>L		N_45:F>L		UFRJ50791:F>L		UFRJ50816:F>L		YPS138:F>L	AA:284		A12:Q>H		DBVPG6304:Q>H		UFRJ50791:Q>H		UFRJ50816:Q>H		YPS138:Q>H	AA:286		A12:S>R		DBVPG6304:S>R		UFRJ50791:S>R		UFRJ50816:S>R		YPS138:S>R	AA:299		YPS138:->Y	AA:323		A12:L>I		DBVPG6304:L>I		UFRJ50791:L>I		UFRJ50816:L>I		YPS138:L>I	AA:350		A12:Y>D		DBVPG6304:Y>D		UFRJ50791:Y>D		UFRJ50816:Y>D		YPS138:Y>D	AA:361		A12:S>R		DBVPG6304:S>R		N_43:S>R		N_44:S>R		N_45:S>R		UFRJ50791:S>R		UFRJ50816:S>R	AA:372		A12:F>L		DBVPG6304:F>L		UFRJ50791:F>L	AA:406		DBVPG6304:S>R		UFRJ50791:S>R	AA:410		DBVPG6304:F>L		UFRJ50791:F>L		UWOPS91_917_1:F>L	AA:437		UWOPS91_917_1:L>R	AA:499		A12:H>Q		DBVPG6304:H>Q	AA:502		A12:S>R		DBVPG6304:S>R		UWOPS91_917_1:S>R	AA:518		A12:Q>H		DBVPG6304:Q>H		UFRJ50816:Q>H	AA:573		A12:->W		DBVPG6304:->W		UWOPS91_917_1:->W	AA:645		A12:D>E		A4:D>E		DBVPG6304:D>E		UWOPS91_917_1:D>E	AA:670		A12:R>S		A4:R>S		DBVPG6304:R>S		N_43:R>SID:YBL025W	AA:3		IFO1804:S>A		N_44:S>A		N_45:S>A	AA:18		KPN3828:C>R		KPN3829:C>R	AA:19		A12:M>I		A4:M>I		DBVPG6304:M>I		UFRJ50791:M>I		YPS138:M>I	AA:20		CBS5829:Y>-	AA:94		Q59_1:A>V	AA:121		IFO1804:I>MID:YBL028C	AA:11		UWOPS91_917_1:S>T	AA:32		CBS432:S>N	AA:33		UWOPS91_917_1:I>T	AA:38		A12:A>V		A4:A>V		DBVPG6304:A>V		UWOPS91_917_1:A>V		YPS138:A>V	AA:42		UWOPS91_917_1:S>F	AA:50		UWOPS91_917_1:D>V	AA:52		N_43:G>E		N_44:G>E		N_45:G>EID:YBL029W	AA:37		DBVPG6304:R>G	AA:44		IFO1804:V>D		N_43:V>D		N_44:V>D		N_45:V>D	AA:88		IFO1804:F>L		N_43:F>L		N_44:F>L		N_45:F>L		UWOPS91_917_1:F>L	AA:120		A12:K>N		DBVPG6304:K>N		UFRJ50816:K>N		UWOPS91_917_1:K>N		YPS138:K>N	AA:126		UWOPS91_917_1:V>G	AA:129		A12:F>L		DBVPG6304:F>L		UFRJ50816:F>L		UWOPS91_917_1:F>L		YPS138:F>L	AA:132		UWOPS91_917_1:Y>-	AA:151		DBVPG6304:->Y		UFRJ50816:->Y	AA:160		A12:W>G		DBVPG6304:W>G		UFRJ50816:W>G		UWOPS91_917_1:W>G		YPS138:W>G	AA:169		A12:F>L		DBVPG6304:F>L		UFRJ50816:F>L		YPS138:F>L	AA:184		A12:I>L		DBVPG6304:I>L		UFRJ50816:I>L		YPS138:I>L	AA:207		UWOPS91_917_1:L>M	AA:219		A12:F>L		DBVPG6304:F>L		UFRJ50816:F>L		YPS138:F>L	AA:222		A12:W>C		DBVPG6304:W>C		UFRJ50816:W>C		YPS138:W>C	AA:224		A12:R>S		DBVPG6304:R>S		UFRJ50816:R>S		UWOPS91_917_1:R>S		YPS138:R>S	AA:227		IFO1804:K>N		N_43:K>N		N_44:K>N		N_45:K>N	AA:262		A12:L>I		A4:L>I		DBVPG6304:L>I		UFRJ50816:L>I		YPS138:L>I	AA:289		A12:->Y		A4:->Y		UFRJ50816:->Y	AA:296		A12:Q>R		A4:Q>R		DBVPG6304:Q>R		YPS138:Q>R	AA:302		A12:F>Y		YPS138:F>Y	AA:353		UWOPS91_917_1:V>L	AA:360		UWOPS91_917_1:->KID:YBL030C	AA:284		IFO1804:I>V		N_43:I>V		N_44:I>V		N_45:I>VID:YBL031W	AA:12		UWOPS91_917_1:V>F	AA:104		A12:H>Q		DBVPG6304:H>Q		UFRJ50816:H>Q		YPS138:H>Q	AA:118		N_43:L>R		N_44:L>R	AA:123		A12:M>I		DBVPG6304:M>I		UFRJ50816:M>I		YPS138:M>I	AA:134		YPS138:F>L	AA:149		UWOPS91_917_1:D>E	AA:153		A12:C>-		DBVPG6304:C>-		UFRJ50816:C>-		YPS138:C>-	AA:157		A12:S>A		DBVPG6304:S>A		UFRJ50816:S>A		YPS138:S>A	AA:196		UWOPS91_917_1:L>F	AA:214		UWOPS91_917_1:L>V	AA:254		A12:A>G		DBVPG6304:A>G		UFRJ50816:A>G		YPS138:A>G	AA:258		N_43:C>W		N_44:C>W		N_45:C>W	AA:259		KPN3828:L>F		KPN3829:L>F	AA:299		KPN3828:L>F	AA:324		A12:I>T		DBVPG6304:I>T		N_44:I>T		N_45:I>T		YPS138:I>TID:YBL032W	AA:26		UWOPS91_917_1:A>T	AA:28		A12:G>A		DBVPG6304:G>A		UFRJ50791:G>A		YPS138:G>A	AA:49		A12:Q>H		A4:Q>H		DBVPG6304:Q>H		UFRJ50791:Q>H		UWOPS91_917_1:Q>H		YPS138:Q>H	AA:53		A12:F>L		A4:F>L		DBVPG6304:F>L		UFRJ50791:F>L		YPS138:F>L	AA:82		CBS5829:L>F	AA:90		UWOPS91_917_1:D>E	AA:137		UWOPS91_917_1:M>I	AA:153		UWOPS91_917_1:Q>H	AA:172		A4:D>E		UWOPS91_917_1:D>E		YPS138:D>E	AA:230		A4:I>F		YPS138:I>F	AA:235		IFO1804:C>W		N_43:C>W		N_45:C>W	AA:242		IFO1804:M>I		N_43:M>I		N_45:M>I	AA:244		A4:F>L		YPS138:F>L	AA:252		A4:R>S		YPS138:R>S	AA:343		A12:F>I		IFO1804:F>I		N_43:F>I		N_45:F>I		UWOPS91_917_1:F>I		YPS138:F>IID:YBL033C	AA:18		N_43:I>K		N_44:I>K	AA:212		KPN3828:D>E		KPN3829:D>E	AA:219		DBVPG6304:A>T		UFRJ50816:A>T		UWOPS91_917_1:A>T	AA:320		A12:D>E		DBVPG6304:D>E	AA:321		UWOPS91_917_1:D>N	AA:331		CBS432:H>R		CBS5829:H>R	AA:332		IFO1804:E>K		N_43:E>K		N_45:E>K		UWOPS91_917_1:E>K	AA:337		UWOPS91_917_1:N>K	AA:344		A12:I>VID:YBL036C	AA:12		A12:D>V		UFRJ50816:D>V		UWOPS91_917_1:D>V		YPS138:D>V	AA:16		A12:D>E		UFRJ50816:D>E		YPS138:D>E	AA:56		UWOPS91_917_1:V>I	AA:89		A12:N>D		DBVPG6304:N>D		UFRJ50816:N>D		YPS138:N>D	AA:94		A12:E>D		DBVPG6304:E>D		UFRJ50816:E>D		YPS138:E>D	AA:135		UWOPS91_917_1:N>D	AA:197		UFRJ50816:H>Y	AA:222		UWOPS91_917_1:G>R	AA:225		A12:Y>H		UWOPS91_917_1:Y>H	AA:231		A12:I>T		DBVPG6304:I>T		N_43:I>T		N_44:I>T		N_45:I>T		UFRJ50816:I>T		UWOPS91_917_1:I>T	AA:236		N_43:K>E	AA:249		UWOPS91_917_1:D>V	AA:255		DBVPG6304:S>N		UFRJ50816:S>N		UWOPS91_917_1:S>NID:YBL038W	AA:10		UWOPS91_917_1:->R	AA:15		A12:S>P		A4:S>P		YPS138:S>P	AA:44		UFRJ50816:P>R	AA:45		UWOPS91_917_1:K>Q	AA:48		UFRJ50816:Y>-	AA:49		A12:L>F		YPS138:L>F	AA:63		Q62_5:->S	AA:113		A4:Q>H		UFRJ50816:Q>H		UWOPS91_917_1:Q>H		YPS138:Q>H	AA:159		A4:F>L		UWOPS91_917_1:F>L		YPS138:F>L	AA:200		UWOPS91_917_1:H>N	AA:216		UWOPS91_917_1:Q>-	AA:224		UWOPS91_917_1:W>L		YPS138:W>L	AA:230		UWOPS91_917_1:P>H		YPS138:P>HID:YBL041W	AA:112		DBVPG6304:N>K		UFRJ50791:N>K		UFRJ50816:N>K	AA:144		UFRJ50816:E>Q	AA:165		DBVPG6304:D>E		UFRJ50791:D>E	AA:169		UWOPS91_917_1:Q>H	AA:208		DBVPG6304:S>R		UFRJ50791:S>R		UWOPS91_917_1:S>R	AA:221		UWOPS91_917_1:Q>H	AA:231		DBVPG6304:Y>N		UFRJ50791:Y>N	AA:241		UWOPS91_917_1:I>VID:YBL045C	AA:47		UWOPS91_917_1:A>S	AA:63		DBVPG6304:T>I		UWOPS91_917_1:T>I		YPS138:T>I	AA:70		UWOPS91_917_1:L>V	AA:76		DBVPG6304:P>H		UWOPS91_917_1:P>H		YPS138:P>H	AA:122		DBVPG6304:I>V		N_45:I>V		UWOPS91_917_1:I>V		YPS138:I>V	AA:153		UWOPS91_917_1:I>L	AA:179		UFRJ50791:L>S	AA:185		N_45:N>K		UWOPS91_917_1:N>K	AA:222		UFRJ50791:I>V		UWOPS91_917_1:I>V		YPS138:I>V	AA:224		Q89_8:K>Q	AA:277		DBVPG6304:S>L		UFRJ50791:S>L		UFRJ50816:S>L		YPS138:S>L	AA:300		A12:R>K		DBVPG6304:R>K		YPS138:R>K	AA:303		UWOPS91_917_1:H>Y	AA:348		UFRJ50791:D>N		UFRJ50816:D>N	AA:374		A12:A>V		A4:A>V		DBVPG6304:A>V		UFRJ50816:A>V		UWOPS91_917_1:A>V		YPS138:A>V	AA:400		UWOPS91_917_1:A>T	AA:410		UWOPS91_917_1:A>T	AA:452		A12:L>S		A4:L>S		DBVPG6304:L>S		N_43:L>S		UWOPS91_917_1:L>SID:YBL049W	AA:1		A12:H>R		A4:H>R		DBVPG6304:H>R		UFRJ50791:H>R		UWOPS91_917_1:H>R		YPS138:H>R	AA:110		A4:P>L		DBVPG6304:P>L		UFRJ50816:P>L		YPS138:P>L	AA:120		A4:L>F		DBVPG6304:L>F		UFRJ50816:L>F		YPS138:L>FID:YBL050W	AA:28		DBVPG6304:->C		UFRJ50791:->C		UWOPS91_917_1:->C		YPS138:->C	AA:32		UWOPS91_917_1:F>L	AA:61		CBS5829:S>T		DBVPG6304:S>T		IFO1804:S>T		KPN3828:S>T		N_44:S>T		Q32_3:S>T		Q62_5:S>T		UFRJ50791:S>T		YPS138:S>T	AA:89		DBVPG6304:S>Y		IFO1804:S>Y		N_44:S>Y		N_45:S>Y	AA:154		CBS5829:->Y		DBVPG6304:->Y		IFO1804:->Y		KPN3828:->Y		N_17:->Y		N_44:->Y		N_45:->Y		Q62_5:->Y		UFRJ50791:->Y		UFRJ50816:->Y		UWOPS91_917_1:->Y	AA:174		DBVPG6304:F>L		UFRJ50791:F>L		UFRJ50816:F>L	AA:189		DBVPG6304:I>M		UFRJ50791:I>M		UFRJ50816:I>M	AA:196		UWOPS91_917_1:Q>K	AA:226		IFO1804:F>L		N_44:F>L		N_45:F>L	AA:228		UWOPS91_917_1:W>L	AA:286		UWOPS91_917_1:F>CID:YBL051C	AA:76		UWOPS91_917_1:S>A	AA:81		A4:S>P		DBVPG6304:S>P	AA:93		UWOPS91_917_1:L>F	AA:128		IFO1804:P>S		N_44:P>S		N_45:P>S	AA:143		A4:I>M		DBVPG6304:I>M		UFRJ50791:I>M		UFRJ50816:I>M		UWOPS91_917_1:I>M		YPS138:I>M	AA:211		A4:M>I		DBVPG6304:M>I		UFRJ50791:M>I		UFRJ50816:M>I		UWOPS91_917_1:M>I		YPS138:M>I	AA:236		Q89_8:G>S	AA:353		A12:E>D		A4:E>D		UFRJ50791:E>D		UFRJ50816:E>D		YPS138:E>D	AA:441		N_44:M>V		UWOPS91_917_1:M>V		YPS138:M>V	AA:486		A4:H>D	AA:609		A12:V>I		A4:V>I		DBVPG6304:V>I		UFRJ50816:V>I		UWOPS91_917_1:V>I		YPS138:V>I	AA:634		UWOPS91_917_1:R>K	AA:646		A12:S>N		A4:S>N		DBVPG6304:S>N		UFRJ50816:S>N		UWOPS91_917_1:S>N		YPS138:S>N	AA:669		UWOPS91_917_1:P>TID:YBL052C	AA:23		Z1_1:N>T	AA:27		Z1_1:N>T	AA:31		DBVPG6304:E>G		UFRJ50791:E>G		UFRJ50816:E>G		YPS138:E>G	AA:33		Z1_1:E>D	AA:37		CBS432:E>G		DBVPG6304:E>G		UFRJ50791:E>G		UFRJ50816:E>G		YPS138:E>G	AA:69		A4:I>T		DBVPG6304:I>T		IFO1804:D>E		N_44:D>E		N_45:D>E		UFRJ50816:I>T		YPS138:I>T	AA:73		IFO1804:D>E		N_44:D>E		N_45:D>E	AA:76		DBVPG6304:L>P		UFRJ50791:L>P		UFRJ50816:L>P		YPS138:L>P	AA:85		IFO1804:E>Q		N_44:E>Q		N_45:E>Q	AA:115		DBVPG6304:S>T		UFRJ50791:S>T		UFRJ50816:S>T		YPS138:S>T	AA:122		UWOPS91_917_1:V>E	AA:127		IFO1804:I>V		N_43:I>V		N_44:I>V		N_45:I>V	AA:135		DBVPG6304:N>S	AA:149		DBVPG6304:Y>H		UFRJ50791:Y>H		UFRJ50816:Y>H		UWOPS91_917_1:Y>H		YPS138:Y>H	AA:168		CBS432:T>N		DBVPG6304:T>N		IFO1804:T>N		N_43:T>N		N_44:T>N		UFRJ50791:T>N		UFRJ50816:T>N		UWOPS91_917_1:T>N		YPS138:T>N	AA:182		A12:S>N		CBS432:S>N		DBVPG6304:S>N		IFO1804:S>N		N_43:S>N		N_44:S>N		UFRJ50816:S>N		UWOPS91_917_1:S>N		YPS138:S>N	AA:187		A12:T>S		DBVPG6304:T>S		UFRJ50816:T>S		UWOPS91_917_1:T>S		YPS138:T>S	AA:198		N_44:V>E	AA:200		A12:L>P		DBVPG6304:L>P		UFRJ50816:L>P		UWOPS91_917_1:L>P		YPS138:L>P	AA:206		DBVPG6304:E>D	AA:210		A12:K>R		A4:K>R		DBVPG6304:K>R		UFRJ50816:K>R		UWOPS91_917_1:K>R		YPS138:K>R	AA:218		UWOPS91_917_1:A>V	AA:233		IFO1804:V>L		N_43:V>L		N_44:V>L	AA:237		IFO1804:T>S		N_43:T>S		N_44:T>S	AA:281		UWOPS91_917_1:S>L	AA:285		A12:K>Q		A4:K>Q		DBVPG6304:K>Q		UFRJ50816:K>Q		UWOPS91_917_1:K>Q		YPS138:K>Q	AA:303		UWOPS91_917_1:D>N	AA:306		A12:S>C		A4:S>C		DBVPG6304:S>C		IFO1804:S>C		N_43:S>C		UFRJ50816:S>C		UWOPS91_917_1:S>C		YPS138:S>C	AA:345		UWOPS91_917_1:I>V	AA:349		A12:D>V	AA:355		IFO1804:R>C		N_43:R>C		N_45:R>C	AA:452		UFRJ50816:E>G	AA:514		A4:A>T		DBVPG6304:A>T		UFRJ50816:A>T		YPS138:A>T	AA:556		A4:R>K		DBVPG6304:R>K		UFRJ50816:R>K		UWOPS91_917_1:R>K		YPS138:R>K	AA:563		YPS138:V>A	AA:566		UWOPS91_917_1:E>K	AA:578		N_43:L>P		N_44:L>P		N_45:L>P	AA:579		KPN3828:V>A	AA:581		A4:R>G		DBVPG6304:R>G		N_43:R>G		N_44:R>G		N_45:R>G		UFRJ50791:R>G		UFRJ50816:R>G		UWOPS91_917_1:R>G		YPS138:R>G	AA:588		KPN3828:E>D	AA:595		A4:N>K		DBVPG6304:N>K		UFRJ50791:N>K		UFRJ50816:N>K		YPS138:N>K	AA:615		A4:L>F		DBVPG6304:L>F		N_43:L>F		N_44:L>F		N_45:L>F		UFRJ50791:L>F		UFRJ50816:L>F		UWOPS91_917_1:L>F		YPS138:L>F	AA:657		A4:T>S		DBVPG6304:T>S		UFRJ50791:T>S		UFRJ50816:T>S		YPS138:T>S	AA:662		N_43:Y>H	AA:663		A4:P>Q		DBVPG6304:P>Q		UFRJ50791:P>Q		UFRJ50816:P>Q		YPS138:P>Q	AA:674		A4:A>T		DBVPG6304:A>T		UFRJ50791:A>T		UFRJ50816:A>T		YPS138:A>T	AA:682		N_43:G>E		N_44:G>E		N_45:G>E		UWOPS91_917_1:G>R	AA:690		A4:P>S		DBVPG6304:P>S		UFRJ50791:P>S		UFRJ50816:P>S		YPS138:P>S	AA:700		A4:A>T		DBVPG6304:A>T		UFRJ50791:A>T		UFRJ50816:A>T		YPS138:A>T	AA:703		A4:S>L		DBVPG6304:S>L		UFRJ50791:S>L		UFRJ50816:S>L		YPS138:S>L	AA:715		UWOPS91_917_1:N>K	AA:724		UWOPS91_917_1:E>K	AA:728		N_43:S>N		N_44:S>N	AA:749		A4:V>A		DBVPG6304:V>A		UFRJ50791:V>A		UFRJ50816:V>A		YPS138:V>A	AA:751		A4:N>S		DBVPG6304:N>S		UFRJ50791:G>D		UFRJ50816:G>D		YPS138:G>D	AA:759		DBVPG6304:V>L	AA:761		YPS138:T>A	AA:770		N_43:K>R		N_44:K>R		N_45:K>R	AA:787		A4:A>E		DBVPG6304:A>E		UFRJ50816:A>E		YPS138:A>E	AA:789		A4:D>N		DBVPG6304:D>N		N_44:D>N		N_45:D>N		UFRJ50816:D>N		YPS138:D>N	AA:793		DBVPG6304:N>K		UFRJ50816:N>K		YPS138:N>K	AA:796		A4:N>H		DBVPG6304:N>H		N_44:N>H		N_45:N>H		UFRJ50816:N>H		YPS138:N>HID:YBL054W	AA:5		A12:F>L		DBVPG6304:F>L		UFRJ50791:F>L		UFRJ50816:F>L		YPS138:F>L	AA:20		A12:->S		DBVPG6304:->S		UFRJ50791:->S		UFRJ50816:->S		YPS138:->S	AA:52		A12:D>A		A4:D>A		DBVPG6304:D>A		UFRJ50791:D>A		UFRJ50816:D>A		UWOPS91_917_1:D>A		YPS138:D>A	AA:58		A12:F>L		A4:F>L		DBVPG6304:F>L		IFO1804:F>L		N_43:F>L		N_44:F>L		N_45:F>L		UFRJ50791:F>L		UFRJ50816:F>L		UWOPS91_917_1:F>L		YPS138:F>L	AA:70		CBS432:E>D	AA:77		A12:R>G		DBVPG6304:R>G		UFRJ50791:R>G		UFRJ50816:R>G		YPS138:R>G	AA:86		UWOPS91_917_1:E>G	AA:87		A12:A>P		A4:A>P		DBVPG6304:A>P		UFRJ50791:A>P		UFRJ50816:A>P		YPS138:A>P	AA:136		A12:F>L		DBVPG6304:F>L		UFRJ50791:F>L		UFRJ50816:F>L		UWOPS91_917_1:F>L	AA:156		A12:F>V		A4:F>V		DBVPG6304:F>V		UFRJ50791:F>V		UFRJ50816:F>V		UWOPS91_917_1:F>V		YPS138:F>V	AA:160		DBVPG6304:F>L		UFRJ50791:F>L		UFRJ50816:F>L	AA:193		A12:I>F		A4:I>F		DBVPG6304:I>F		UFRJ50791:I>F		UFRJ50816:I>F		UWOPS91_917_1:I>F		YPS138:I>F	AA:245		A4:A>T		DBVPG6304:A>T		IFO1804:A>T		N_43:A>T		N_44:A>T		N_45:A>T		UFRJ50791:A>T		UWOPS91_917_1:A>T		YPS138:A>T	AA:288		IFO1804:R>S		N_44:R>S		N_45:R>S	AA:315		DBVPG6304:S>F		UFRJ50791:S>F		YPS138:S>F	AA:371		IFO1804:Y>-		N_43:Y>-		N_44:Y>-	AA:373		IFO1804:V>L		N_43:V>L		N_44:V>L	AA:387		A12:H>D		A4:H>D		UFRJ50791:H>D		YPS138:H>D	AA:391		A12:S>R		IFO1804:S>R		N_43:S>R		N_44:S>R		UFRJ50791:S>R		YPS138:S>R	AA:420		A12:N>K		UFRJ50791:N>K		YPS138:N>K	AA:440		IFO1804:->Y		N_43:->Y		N_44:->Y	AA:476		UFRJ50791:W>C	AA:501		A12:H>Q		UFRJ50791:H>Q		YPS138:H>Q	AA:524		YPS138:N>KID:YBL056W	AA:3		DBVPG6304:L>V		YPS138:L>V	AA:8		UFRJ50816:W>L	AA:17		UWOPS91_917_1:T>S	AA:25		UFRJ50816:S>A	AA:34		UFRJ50816:H>Q	AA:39		N_44:G>V	AA:55		A12:Q>H		A4:Q>H		DBVPG6304:Q>H		UFRJ50791:Q>H		UFRJ50816:Q>H		YPS138:Q>H	AA:107		A12:Q>H		A4:Q>H		DBVPG6304:Q>H		IFO1804:Q>H		N_43:Q>H		N_44:Q>H		UFRJ50791:Q>H		UFRJ50816:Q>H		UWOPS91_917_1:Q>H		YPS138:Q>H	AA:117		A12:M>I		A4:M>I		DBVPG6304:M>I		UFRJ50791:M>I		UFRJ50816:M>I		UWOPS91_917_1:M>I		YPS138:M>I	AA:120		IFO1804:T>P	AA:139		IFO1804:R>S		N_43:R>S		N_44:R>S	AA:154		A4:C>W		DBVPG6304:C>W		UFRJ50791:C>W		UFRJ50816:C>W		UWOPS91_917_1:C>W		YPS138:C>W	AA:162		A4:C>W		DBVPG6304:C>W		UFRJ50791:C>W		UFRJ50816:C>W		YPS138:C>W	AA:163		UWOPS91_917_1:Q>E	AA:248		A12:->Y		A4:->Y		DBVPG6304:->Y		UFRJ50791:->Y		UFRJ50816:->Y		UWOPS91_917_1:->Y	AA:273		A12:->C		A4:->C		CBS432:->C		CBS5829:->C		DBVPG6304:->C		IFO1804:->C		N_43:->C		N_44:->C		N_45:->C		UWOPS91_917_1:->C	AA:279		A12:->W		A4:->W		DBVPG6304:->W		UWOPS91_917_1:->W	AA:316		UWOPS91_917_1:S>N	AA:376		IFO1804:L>R		N_43:L>R		N_44:L>R		N_45:L>R	AA:378		A12:C>R		A4:C>R		YPS138:C>R	AA:386		IFO1804:N>K		N_43:N>K		N_44:N>K		N_45:N>K	AA:391		A12:Q>H		A4:Q>H		DBVPG6304:Q>H		YPS138:Q>H	AA:423		A12:I>M		A4:I>M		DBVPG6304:I>M		UFRJ50816:I>M		UWOPS91_917_1:I>M		YPS138:I>M	AA:445		A12:I>M		A4:I>M		DBVPG6304:I>M		UFRJ50816:I>M		YPS138:I>M	AA:464		A12:N>K		A4:N>K		DBVPG6304:N>K		UFRJ50791:N>K		UFRJ50816:N>K		YPS138:N>KID:YBL057C	AA:78		IFO1804:I>V		N_43:I>V		N_44:I>V		N_45:I>V	AA:111		A4:A>S		DBVPG6304:A>S		UFRJ50791:A>S		UFRJ50816:A>S	AA:158		IFO1804:G>E		N_44:G>E		N_45:G>E	AA:162		A4:K>M		DBVPG6304:K>M		UFRJ50791:K>M		UFRJ50816:K>M	AA:203		IFO1804:S>A		N_44:S>A		N_45:S>A	AA:205		CBS432:T>M		N_17:T>MID:YBL058W	AA:43		A4:R>G		UFRJ50816:R>G	AA:77		IFO1804:L>R		N_43:L>R		N_44:L>R		N_45:L>R	AA:85		IFO1804:N>K		N_43:N>K		N_44:N>K		N_45:N>K	AA:97		A4:T>N		UFRJ50816:T>N	AA:127		A4:K>N	AA:130		A4:K>I		UFRJ50816:K>I	AA:154		A4:F>L		UFRJ50816:F>L	AA:169		A4:->Y		UFRJ50816:->Y	AA:173		IFO1804:Q>H	AA:194		A4:F>L		DBVPG6304:F>L		UFRJ50816:F>L	AA:203		A4:S>R		UFRJ50816:S>R	AA:219		A4:N>K		DBVPG6304:N>K		UFRJ50816:N>K	AA:231		A4:F>Y		DBVPG6304:F>Y		UFRJ50816:F>Y	AA:238		A4:F>L		DBVPG6304:F>L		UFRJ50816:F>L	AA:247		A4:L>F		DBVPG6304:L>F		UFRJ50816:L>F	AA:250		A4:I>M		DBVPG6304:I>M		UFRJ50816:I>M	AA:290		N_43:Y>-		N_44:Y>-	AA:334		A4:C>S		DBVPG6304:C>S	AA:341		A4:G>R		DBVPG6304:G>R	AA:343		A4:W>R		DBVPG6304:W>R		UWOPS91_917_1:W>R	AA:363		A4:S>T		DBVPG6304:S>T	AA:375		A4:F>L		DBVPG6304:F>L		UWOPS91_917_1:F>L	AA:380		A4:V>M		DBVPG6304:V>M		UWOPS91_917_1:V>M	AA:384		A4:I>L		CBS432:I>L		DBVPG6304:I>L		N_17:I>L		N_43:I>L		N_44:I>L		N_45:I>L		UWOPS91_917_1:I>L	AA:406		N_43:V>A		N_44:V>A		N_45:V>AID:YBL060W	AA:5		A12:->Y		A4:->Y		DBVPG6304:->Y		IFO1804:->Y		N_43:->Y		N_44:->Y		N_45:->Y		UFRJ50791:->Y		UFRJ50816:->Y		UWOPS91_917_1:->Y	AA:33		N_43:M>I		N_45:M>I	AA:49		UWOPS91_917_1:R>S	AA:55		A12:T>P		A4:T>P		UFRJ50791:T>P		UFRJ50816:T>P		YPS138:T>P	AA:63		A12:N>K		A4:N>K		UFRJ50791:N>K		UFRJ50816:N>K		YPS138:N>K	AA:86		UWOPS91_917_1:D>E	AA:103		A12:->Q		A4:->Q		UFRJ50791:->Q		UFRJ50816:->Q		YPS138:->Q	AA:112		N_43:I>S		N_44:I>S		N_45:I>S	AA:116		UWOPS91_917_1:L>I	AA:128		N_43:M>I		N_45:M>I	AA:134		UWOPS91_917_1:K>N	AA:145		UWOPS91_917_1:->Y	AA:152		N_43:->Y		N_45:->Y	AA:156		UWOPS91_917_1:Y>C	AA:161		A12:F>L		A4:F>L		UFRJ50791:F>L		UFRJ50816:F>L		YPS138:F>L	AA:186		A12:F>I		A4:F>I		UFRJ50791:F>I		UFRJ50816:F>I		YPS138:F>I	AA:188		N_43:A>S		N_44:A>S		N_45:A>S	AA:194		UWOPS91_917_1:N>K	AA:243		A12:Y>-		A4:Y>-		DBVPG6304:Y>-		UFRJ50791:Y>-		YPS138:Y>-	AA:259		UWOPS91_917_1:F>L	AA:276		UWOPS91_917_1:D>E	AA:285		CBS432:Q>H		KPN3829:Q>H	AA:295		N_43:F>L		N_45:F>L	AA:315		A12:I>M		A4:I>M		DBVPG6304:I>M		UFRJ50791:I>M		YPS138:I>M	AA:349		A12:I>T		CBS432:I>T		KPN3828:I>T		KPN3829:I>S		N_17:I>T		N_43:I>T		N_44:I>T		N_45:I>T		UFRJ50791:I>T	AA:374		N_43:F>L		N_44:F>L		N_45:F>L	AA:384		UFRJ50791:C>-	AA:391		KPN3829:L>H	AA:424		DBVPG6304:I>M		UFRJ50791:I>M		YPS138:I>M	AA:472		CBS432:I>M		KPN3828:I>M		KPN3829:I>M		N_17:I>M		N_43:I>M		N_44:I>M		N_45:I>M		UWOPS91_917_1:I>M	AA:525		A12:N>K		UFRJ50791:N>K		UFRJ50816:N>K		YPS138:N>K	AA:533		A12:N>Y		UFRJ50791:N>Y		UFRJ50816:N>Y		YPS138:N>Y	AA:550		N_43:N>K		N_44:N>K		N_45:N>K		UWOPS91_917_1:N>K	AA:558		N_43:E>D		N_44:E>D		N_45:E>D	AA:573		A12:Q>H		UFRJ50791:Q>H		UFRJ50816:Q>H	AA:577		KPN3828:S>G	AA:591		UWOPS91_917_1:W>R	AA:594		A12:A>V		N_43:A>V		N_44:A>V		UFRJ50791:A>V		UFRJ50816:A>V		UWOPS91_917_1:A>V	AA:596		A12:F>V		UFRJ50791:F>V		UFRJ50816:F>V		UWOPS91_917_1:F>V	AA:599		CBS432:V>G		KPN3828:V>G		KPN3829:V>G	AA:605		N_43:Y>-		N_44:Y>-	AA:624		CBS432:->S		KPN3828:->S		KPN3829:->S		N_17:->S		Q32_3:->S		Q59_1:->S	AA:644		CBS5829:K>N	AA:649		CBS432:K>N		KPN3828:K>N		KPN3829:K>N		N_17:K>N		Q59_1:K>N	AA:652		A12:Y>-	AA:661		UWOPS91_917_1:Q>H	AA:663		A12:H>Y		CBS432:H>Y		DBVPG6304:H>Y		IFO1804:H>Y		KPN3828:H>Y		KPN3829:H>Y		N_17:H>Y		N_43:H>Y		N_45:H>Y		Q59_1:H>Y		UFRJ50791:H>Y		UFRJ50816:H>Y		UWOPS91_917_1:H>Y	AA:668		N_43:F>Y	AA:673		DBVPG6304:I>K		UFRJ50816:I>K	AA:677		UWOPS91_917_1:->YID:YBL061C	AA:22		A12:P>S		DBVPG6304:P>S		IFO1804:P>S		N_43:P>S		N_45:P>S		UFRJ50816:P>S	AA:34		A12:K>R		DBVPG6304:K>R		UFRJ50816:K>R	AA:45		A12:T>P		DBVPG6304:T>P		UFRJ50816:T>P	AA:52		IFO1804:A>V		N_43:A>V		N_44:A>V	AA:54		N_43:S>W		N_44:S>W	AA:59		A12:A>		DBVPG6304:E>K		UFRJ50791:E>K		UFRJ50816:E>K	AA:67		Y6_5:S>F	AA:74		A12:N>D		DBVPG6304:N>D		UFRJ50791:N>D		UFRJ50816:N>D	AA:76		A12:G>S		DBVPG6304:G>S		UFRJ50791:G>S		UFRJ50816:G>S	AA:89		IFO1804:T>S		N_43:T>S		N_44:T>S		N_45:T>S	AA:143		A12:M>I		DBVPG6304:M>I		IFO1804:M>I		N_43:M>I		N_44:M>I		N_45:M>I		UFRJ50791:M>I		UFRJ50816:M>I		UWOPS91_917_1:M>I	AA:147		UWOPS91_917_1:S>G	AA:155		A12:S>R		DBVPG6304:S>R		IFO1804:S>N		N_43:S>N		N_44:S>N		N_45:S>N		UFRJ50791:S>R		UFRJ50816:S>R		UWOPS91_917_1:S>R	AA:272		UWOPS91_917_1:P>S	AA:335		A12:T>S		DBVPG6304:T>S		UFRJ50791:T>S		YPS138:T>S	AA:336		N_44:P>S		N_45:P>S	AA:457		A12:D>E		DBVPG6304:D>E		YPS138:D>E	AA:458		IFO1804:S>G		N_44:S>G		N_45:S>G	AA:604		DBVPG6304:D>G	AA:612		A12:K>Q		A4:K>Q		DBVPG6304:K>Q		YPS138:K>Q	AA:615		A12:P>S		A4:P>S		DBVPG6304:P>S		YPS138:P>S	AA:616		IFO1804:L>M		N_43:L>M		N_44:L>M		N_45:L>M	AA:691		A12:I>V		A4:I>V		DBVPG6304:I>V		N_43:I>V		N_45:I>V		UFRJ50816:I>V		YPS138:I>VID:YBL066C	AA:66		Q89_8:N>D	AA:70		A12:N>S		IFO1804:N>S		N_43:N>S		N_45:N>S		UFRJ50791:N>S		YPS138:N>S	AA:75		IFO1804:N>S		N_43:N>S		N_45:N>S	AA:171		A12:V>L		UFRJ50791:V>L		UFRJ50816:V>L	AA:188		UWOPS91_917_1:P>S	AA:190		A12:V>L		UFRJ50791:V>L		UFRJ50816:V>L		UWOPS91_917_1:V>L	AA:199		UWOPS91_917_1:G>A	AA:209		A12:T>A		UFRJ50791:T>A		UFRJ50816:T>A		UWOPS91_917_1:T>A	AA:214		A12:V>I		IFO1804:V>I		N_43:V>I		N_45:V>I		UFRJ50791:V>I		UFRJ50816:V>I		UWOPS91_917_1:V>I	AA:217		A12:S>N		UFRJ50791:S>N		UFRJ50816:S>N	AA:232		A12:T>A		UFRJ50791:T>A		UFRJ50816:T>A	AA:240		UWOPS91_917_1:S>N	AA:245		A12:V>A		IFO1804:V>A		N_45:V>A		UFRJ50791:V>A		UFRJ50816:V>A		UWOPS91_917_1:V>A	AA:282		CBS432:K>Q	AA:293		UWOPS91_917_1:V>I	AA:337		IFO1804:S>G		N_43:S>G		N_44:S>G	AA:340		A12:D>G		A4:D>G		UWOPS91_917_1:D>G	AA:348		A12:R>K		A4:R>K		IFO1804:R>K		N_43:R>K		N_44:R>K	AA:508		UWOPS91_917_1:P>S	AA:539		Q62_5:T>I		Q95_3:T>I		S36_7:T>I		T21_4:T>I		Z1_1:T>I	AA:612		A4:D>N		DBVPG6304:D>N		UFRJ50816:D>N		YPS138:D>N	AA:672		KPN3828:T>	AA:795		DBVPG4650:H>R	AA:798		A12:K>E		A4:K>E		DBVPG6304:K>E		UFRJ50816:K>E		UWOPS91_917_1:K>E		YPS138:K>E	AA:802		A12:E>D		A4:E>D		DBVPG6304:E>D		UFRJ50816:E>D		UWOPS91_917_1:E>D		YPS138:E>D	AA:836		A12:A>T		DBVPG4650:A>T		DBVPG6304:A>T		N_43:A>T		N_44:A>T		N_45:A>T		Q32_3:A>T		UFRJ50816:A>T		UWOPS91_917_1:A>T		YPS138:A>T	AA:841		Q32_3:N>Y	AA:843		UWOPS91_917_1:T>I	AA:872		A12:H>N		DBVPG6304:H>N		UFRJ50816:H>N		YPS138:H>N	AA:873		UWOPS91_917_1:I>T	AA:879		A12:N>K		DBVPG6304:N>K		UFRJ50816:N>K		UWOPS91_917_1:N>K		YPS138:N>K	AA:884		CBS432:A>T		UWOPS91_917_1:A>T	AA:921		A12:A>V		DBVPG6304:A>V		YPS138:A>V	AA:953		N_43:G>D		N_44:G>D		N_45:G>D	AA:971		A12:F>V		A4:F>V		DBVPG6304:F>V		N_43:F>V		N_44:F>V		N_45:F>V		UFRJ50791:F>V		UFRJ50816:F>V		UWOPS91_917_1:F>V	AA:1001		A12:T>A		A4:T>A		DBVPG6304:T>A		UFRJ50791:T>A		UFRJ50816:T>A		YPS138:T>A	AA:1114		A12:P>S		A4:P>S		DBVPG6304:P>S		UFRJ50816:P>S		YPS138:P>S	AA:1120		UWOPS91_917_1:L>F	AA:1133		A12:S>N		A4:S>N		DBVPG6304:S>N		UFRJ50816:S>N		UWOPS91_917_1:S>N		YPS138:S>NID:YBL068W	AA:33		N_43:->Y		N_45:->Y	AA:37		UWOPS91_917_1:K>R	AA:40		A12:N>K		A4:N>K		UFRJ50816:N>K		UWOPS91_917_1:N>K		YPS138:N>K	AA:130		IFO1804:S>R		N_43:S>R		N_45:S>R	AA:139		A12:H>Q		A4:H>Q		DBVPG6304:H>Q		UFRJ50791:H>Q		UFRJ50816:H>Q		YPS138:H>Q	AA:160		A12:F>Y		A4:F>Y		DBVPG6304:F>Y		UFRJ50791:F>Y		UFRJ50816:F>Y		YPS138:F>Y	AA:218		UWOPS91_917_1:K>N	AA:219		A12:N>K		A4:N>K		DBVPG6304:N>K		UFRJ50791:N>K		UFRJ50816:N>K		YPS138:N>K	AA:319		S36_7:I>TID:YBL069W	AA:12		N_43:F>L		N_44:F>L		N_45:F>L	AA:121		A12:T>P		DBVPG6304:T>P		UFRJ50791:T>P	AA:153		A12:->Y	AA:161		A12:D>E	AA:209		A12:->C		DBVPG6304:->C		UFRJ50791:->C	AA:232		DBVPG6304:Y>-		UFRJ50791:Y>-		UFRJ50816:Y>-		YPS138:Y>-	AA:239		DBVPG6304:L>-		IFO1804:L>-		N_43:L>-		N_44:L>-		UFRJ50791:L>-		UFRJ50816:L>-		YPS138:L>-	AA:258		DBVPG6304:Q>H		IFO1804:Q>H		N_43:Q>H		N_44:Q>H		UFRJ50816:Q>H		YPS138:Q>H	AA:351		UFRJ50816:F>L	AA:359		N_43:->C		N_44:->C		N_45:->C	AA:378		DBVPG6304:I>L		UFRJ50816:I>L		UWOPS91_917_1:I>L		YPS138:I>L	AA:396		DBVPG6304:N>Y		UFRJ50816:N>Y		UWOPS91_917_1:N>Y		YPS138:N>Y	AA:411		N_43:R>S		N_44:R>S		N_45:R>SID:YBL074C	AA:3		IFO1804:Q>R		N_43:Q>R	AA:6		A4:I>V		DBVPG6304:I>V		UFRJ50816:I>V		UWOPS91_917_1:I>V		YPS138:I>V	AA:10		UWOPS91_917_1:I>T	AA:14		DBVPG4650:Y>C		N_17:Y>C		Q59_1:Y>C		Q62_5:Y>C		Q89_8:Y>C		S36_7:Y>C		Y7:Y>C		Z1_1:Y>C	AA:16		UWOPS91_917_1:D>H	AA:21		S36_7:E>K		Y7:E>K	AA:24		CBS432:E>D		CBS5829:E>D		DBVPG4650:E>D		IFO1804:E>D		N_17:E>D		N_43:E>D		Q59_1:E>D		Q62_5:E>D		Q89_8:E>D		S36_7:E>D		UWOPS91_917_1:E>D		Y7:E>D		Z1_1:E>D	AA:33		A4:D>V		DBVPG6304:D>V		UFRJ50816:D>V		YPS138:D>V	AA:45		UWOPS91_917_1:D>N	AA:49		A4:R>K		CBS432:R>T		CBS5829:R>T		DBVPG4650:R>T		DBVPG6304:R>K		UFRJ50816:R>K		UWOPS91_917_1:R>K		YPS138:R>K	AA:82		A4:I>T		DBVPG6304:I>T		UWOPS91_917_1:I>T		YPS138:I>T	AA:104		IFO1804:S>A		N_43:S>A		N_44:S>A		N_45:S>A	AA:141		A4:H>N		DBVPG6304:H>N		N_43:H>N		N_44:H>N		N_45:H>N		UWOPS91_917_1:H>N		YPS138:H>N	AA:145		N_43:R>K		N_44:R>K		N_45:R>K	AA:177		N_43:I>L		N_44:I>L		N_45:I>L	AA:187		A4:D>N		DBVPG6304:D>N		UFRJ50816:D>N		UWOPS91_917_1:D>N		YPS138:D>N	AA:190		A4:G>E		DBVPG6304:G>E		UFRJ50816:G>E		UWOPS91_917_1:G>E		YPS138:G>E	AA:221		CBS5829:R>G		KPN3828:R>G		N_43:R>L		N_44:R>L		N_45:R>L		Q62_5:R>G	AA:238		A4:E>K	AA:262		N_43:D>N		N_44:D>N		N_45:D>N	AA:266		A4:N>D		UFRJ50816:N>D		YPS138:N>D	AA:278		IFO1804:E>D		N_43:E>D		N_44:E>D		N_45:E>D		UWOPS91_917_1:E>K	AA:290		UWOPS91_917_1:Y>F	AA:313		A4:V>I		UFRJ50816:V>I		UWOPS91_917_1:V>I		YPS138:V>I	AA:348		UWOPS91_917_1:V>AID:YBL075C	AA:31		A12:A>G		A4:A>G		UFRJ50791:A>G		UFRJ50816:A>G		YPS138:A>G	AA:46		N_17:M>T	AA:58		A12:R>K		UFRJ50791:R>K		UFRJ50816:R>K		YPS138:R>K	AA:81		A12:T>K		UWOPS91_917_1:T>A	AA:101		CBS432:T>A	AA:241		N_17:K>E	AA:326		UWOPS91_917_1:D>G	AA:338		A12:D>E		A4:D>E		DBVPG6304:D>E		UFRJ50791:D>E		UFRJ50816:D>E		UWOPS91_917_1:D>E		YPS138:D>E	AA:372		UFRJ50791:S>F		UFRJ50816:S>F	AA:398		A12:N>S	AA:547		Q32_3:E>DID:YBL078C	AA:97		UWOPS91_917_1:A>GID:YBL080C	AA:24		A4:L>R		CBS432:L>R		CBS5829:L>R		DBVPG6304:L>R		IFO1804:L>R		N_17:L>R		N_43:L>R		N_44:L>R		N_45:L>R		UFRJ50816:L>R		UWOPS91_917_1:L>R	AA:45		A4:R>Q		DBVPG6304:R>Q		UFRJ50791:R>Q		UFRJ50816:R>Q	AA:48		N_43:T>A		N_44:T>A	AA:58		UFRJ50791:N>H		UFRJ50816:N>H	AA:67		UFRJ50791:D>N		UFRJ50816:D>N	AA:76		A12:Y>F		A4:Y>F		DBVPG6304:Y>F		IFO1804:Y>F		N_43:Y>F		N_44:Y>F		N_45:Y>F		UFRJ50791:Y>F		UFRJ50816:Y>F		UWOPS91_917_1:Y>F	AA:100		A12:I>V		A4:I>V		DBVPG6304:I>V		UFRJ50816:I>V		UWOPS91_917_1:I>V	AA:116		IFO1804:H>Y		N_43:H>Y		N_44:H>Y		N_45:H>Y	AA:210		UWOPS91_917_1:M>I	AA:212		A12:M>I		A4:M>I		DBVPG6304:M>I		UFRJ50791:M>I		UFRJ50816:M>I	AA:218		UWOPS91_917_1:Q>R	AA:227		A12:T>I		A4:T>I		UFRJ50791:T>I		UFRJ50816:T>I		UWOPS91_917_1:T>I	AA:233		IFO1804:S>N		N_43:S>N		N_44:S>N		N_45:S>N	AA:271		A12:S>T		A4:S>T		Q59_1:S>L		UFRJ50791:S>T		UFRJ50816:S>T		UWOPS91_917_1:S>T	AA:280		IFO1804:G>E		N_43:G>E		N_44:G>E		N_45:G>E	AA:361		CBS5829:V>I	AA:392		A12:L>S	AA:449		Q95_3:D>E		Y7:D>E	AA:479		UWOPS91_917_1:T>A	AA:500		A12:T>N		N_44:T>A		UWOPS91_917_1:T>N	AA:518		CBS432:P>Q		N_17:P>Q		Q59_1:P>Q		Q95_3:P>Q	AA:521		A12:P>L		UFRJ50791:P>L		UWOPS91_917_1:P>LID:YBL081W	AA:1		A4:H>R		DBVPG6304:H>R		UFRJ50816:H>R		UWOPS91_917_1:H>R	AA:17		UWOPS91_917_1:H>Q	AA:53		UWOPS91_917_1:V>L	AA:56		UWOPS91_917_1:D>E	AA:64		A4:V>L		DBVPG6304:V>L		UFRJ50816:V>L	AA:66		CBS432:V>I	AA:75		UWOPS91_917_1:A>T	AA:87		A4:R>S		DBVPG6304:R>S		UFRJ50816:R>S	AA:94		A4:->C		DBVPG6304:->C		UFRJ50816:->C	AA:146		N_44:M>I		N_45:M>I	AA:149		A4:L>-		DBVPG6304:L>-		UFRJ50816:L>-	AA:152		A4:R>S		DBVPG6304:R>S		UFRJ50816:R>S	AA:191		A4:->C		DBVPG6304:->C		UFRJ50816:->C		YPS138:->C	AA:201		A4:G>S		DBVPG6304:G>S		UFRJ50816:G>S		YPS138:G>S	AA:223		A4:->Y		DBVPG6304:->Y		UFRJ50816:->Y		YPS138:->Y	AA:226		N_44:->C		N_45:->C	AA:248		A4:I>S		YPS138:I>S	AA:267		UWOPS91_917_1:R>S	AA:273		N_43:W>G		N_44:W>G		N_45:W>G	AA:280		CBS432:H>L		CBS5829:H>L		KPN3828:H>L		N_17:H>L	AA:287		N_43:G>E		N_44:G>E		N_45:G>E	AA:294		Y7:L>M	AA:305		A4:F>L		N_43:F>L		N_44:F>L		N_45:F>L		UFRJ50791:F>L		UWOPS91_917_1:F>L		YPS138:F>L	AA:307		A4:K>R		UFRJ50791:K>R		YPS138:K>R	AA:321		T21_4:Q>R	AA:331		A4:R>T		UWOPS91_917_1:R>T		YPS138:R>T	AA:337		N_43:R>S		N_44:R>S		N_45:R>S		Q62_5:R>GID:YBL082C	AA:2		A4:N>K		N_45:N>K	AA:19		A4:T>N		UFRJ50816:T>N		YPS138:T>N	AA:59		A4:A>V		CBS5829:A>V		N_43:A>V		N_45:A>V		Q32_3:A>V		Q59_1:A>V		Q62_5:A>V		Q95_3:A>V		T21_4:A>V		UFRJ50816:A>V		YPS138:A>V		Z1_1:A>V	AA:70		Q32_3:G>R		T21_4:G>R	AA:73		A4:F>Y		UFRJ50816:F>Y		UWOPS91_917_1:F>Y		YPS138:F>Y	AA:102		A4:S>A		DBVPG4650:S>T		N_43:S>T		N_44:S>T		N_45:S>T		Q59_1:S>T		Q95_3:S>T		T21_4:S>T		UFRJ50816:S>A		UWOPS91_917_1:S>A		YPS138:S>A		Z1_1:S>T	AA:111		UWOPS91_917_1:A>P		YPS138:A>G	AA:114		UWOPS91_917_1:T>I		YPS138:T>I	AA:120		N_43:L>I		N_45:L>I		UWOPS91_917_1:L>I	AA:148		UFRJ50791:L>V	AA:179		IFO1804:K>R		N_45:K>R	AA:195		UWOPS91_917_1:R>C	AA:202		A4:T>A		DBVPG6304:T>A		UWOPS91_917_1:T>A	AA:212		A4:N>D		DBVPG6304:N>D		IFO1804:N>D		N_43:N>D		N_45:N>D		UWOPS91_917_1:N>D	AA:217		A4:V>A		DBVPG6304:V>A		IFO1804:V>A		N_43:V>A		N_44:V>A		N_45:V>A		UWOPS91_917_1:V>A	AA:245		CBS432:I>M		CBS5829:I>M		DBVPG4650:I>M		IFO1804:I>M		KPN3828:I>M		N_17:I>M		N_43:I>M		N_45:I>M		Q32_3:I>M		Q59_1:I>M		Q62_5:I>M		Y7:I>M	AA:252		A12:V>A		DBVPG6304:V>A		UWOPS91_917_1:V>A	AA:258		A12:K>R		A4:K>R		DBVPG6304:K>R	AA:280		UWOPS91_917_1:L>F	AA:311		A12:H>Y		A4:H>Y		DBVPG6304:H>Y	AA:321		CBS5829:A>G		N_17:A>G	AA:345		UWOPS91_917_1:W>R	AA:383		IFO1804:E>Q		N_45:E>Q	AA:401		A4:K>Q	AA:441		A12:I>V		A4:I>V		DBVPG6304:I>V		IFO1804:I>V		N_44:I>V		N_45:I>V		UWOPS91_917_1:I>V		YPS138:I>V	AA:452		A12:Q>K		A4:Q>K		DBVPG6304:Q>K		YPS138:Q>K	AA:469		DBVPG6304:W>S		UWOPS91_917_1:W>S	AA:477		IFO1804:G>E		N_44:G>E		N_45:G>E	AA:479		UWOPS91_917_1:G>E	AA:480		Q59_1:I>V		Q62_5:I>VID:YBL084C	AA:328		CBS5829:A>T		Q32_3:A>T		Q89_8:A>T		Q95_3:A>T		T21_4:A>T		UWOPS91_917_1:T>K		Y6_5:A>T	AA:343		UWOPS91_917_1:S>P	AA:357		UFRJ50791:S>P	AA:438		UWOPS91_917_1:T>I	AA:440		CBS5829:S>P		IFO1804:S>P		N_43:S>P		N_44:S>P		N_45:S>P		Q32_3:S>P		Q62_5:S>P		Q89_8:S>P		Q95_3:S>P		T21_4:S>P		UWOPS91_917_1:S>P		Y6_5:S>P	AA:464		A4:S>T		DBVPG6304:S>T		UFRJ50791:S>T		UWOPS91_917_1:S>T	AA:484		A4:S>C		UFRJ50791:S>C		UWOPS91_917_1:S>C	AA:498		A4:G>S		UFRJ50791:G>S		UWOPS91_917_1:G>S	AA:522		A4:Q>P		UWOPS91_917_1:Q>P	AA:539		A4:I>M		UFRJ50791:I>M		UWOPS91_917_1:I>M	AA:615		A4:G>S		CBS5829:G>S		IFO1804:G>S		N_43:G>S		N_44:G>S		N_45:G>S		Q32_3:G>S		Q89_8:G>S		Q95_3:G>S		T21_4:G>S		UFRJ50791:G>S		UWOPS91_917_1:G>S	AA:655		A4:F>Y		UFRJ50791:F>Y	AA:674		A4:V>A		UFRJ50791:V>A	AA:684		A4:V>A		UFRJ50791:V>A	AA:712		UWOPS91_917_1:A>S	AA:730		UWOPS91_917_1:L>FID:YBL086C	AA:33		A12:G>R		A4:G>R		DBVPG6304:G>R		UFRJ50791:G>R		UFRJ50816:G>R	AA:95		A12:Y>F		A4:Y>F		DBVPG6304:Y>F		UFRJ50816:Y>F	AA:200		A12:S>N		A4:S>N		DBVPG6304:S>N		UFRJ50816:S>N		UWOPS91_917_1:S>N		YPS138:S>N	AA:206		A4:T>A		DBVPG6304:T>A		UFRJ50816:T>A		YPS138:T>A	AA:220		UWOPS91_917_1:I>V	AA:340		A12:A>P		UFRJ50816:A>P		UWOPS91_917_1:A>P		YPS138:A>P	AA:352		UWOPS91_917_1:A>V	AA:378		A12:T>I		A4:T>I		CBS432:T>I		CBS5829:T>I		DBVPG4650:T>I		IFO1804:T>I		N_17:T>I		N_43:T>I		N_44:T>I		N_45:T>I		Q59_1:T>I		Q62_5:T>I		Q95_3:T>I		T21_4:T>I		UFRJ50816:T>I		UWOPS91_917_1:T>I		Y7:T>I		YPS138:T>I	AA:404		UWOPS91_917_1:T>A	AA:448		IFO1804:D>A		N_43:D>A		N_44:D>A	AA:457		UFRJ50816:K>T	AA:459		N_17:N>TID:YBL089W	AA:11		A12:S>R		A4:S>R		DBVPG6304:S>R		UFRJ50791:S>R	AA:39		A12:R>C		A4:R>C		DBVPG6304:R>C		UFRJ50791:R>C	AA:53		CBS5829:->Y		IFO1804:->Y		KPN3829:->Y		N_17:->Y		N_43:->Y		N_44:->Y		N_45:->Y		Q32_3:->Y		Q59_1:->Y		Q62_5:->Y		Q89_8:->Y		Q95_3:->Y		S36_7:->Y		Y6_5:->Y	AA:85		A12:V>L		A4:V>L		DBVPG6304:V>L	AA:103		N_45:N>K	AA:124		IFO1804:R>G		N_43:R>G		N_44:R>G		N_45:R>G	AA:128		A12:M>I		A4:M>I		DBVPG6304:M>I		UFRJ50816:M>I	AA:208		A12:Q>H		A4:Q>H		DBVPG6304:Q>H		UFRJ50791:Q>H		UFRJ50816:Q>H	AA:215		A12:V>I		A4:V>I		UFRJ50791:V>I		UFRJ50816:V>I	AA:225		A12:N>K		DBVPG6304:N>K		UFRJ50791:N>K		UFRJ50816:N>K	AA:259		A12:I>L		A4:I>L		DBVPG6304:I>L		IFO1804:I>L		N_43:I>L		N_44:I>L		N_45:I>L		UFRJ50791:I>L		UFRJ50816:I>L	AA:338		IFO1804:I>T		N_44:I>T		N_45:I>T	AA:394		A12:R>S		A4:R>S		UFRJ50791:R>S		UFRJ50816:R>S	AA:407		A12:V>L		A4:V>L		UFRJ50791:V>L		UFRJ50816:V>L	AA:451		A4:Y>-ID:YBL090W	AA:59		A12:C>-		A4:C>-		UFRJ50791:C>-		UFRJ50816:C>-		YPS138:C>-	AA:64		A12:H>Q		A4:H>Q		UFRJ50791:H>Q		UFRJ50816:H>Q		YPS138:H>Q	AA:67		N_43:W>C		N_44:W>C		N_45:W>C	AA:123		A12:Y>-		A4:Y>-		UFRJ50791:Y>-		YPS138:Y>-	AA:139		UWOPS91_917_1:V>L	AA:171		N_43:I>F		N_44:I>F		N_45:I>F		UFRJ50791:I>MID:YBL091C	AA:54		A12:D>E		A4:D>E		UFRJ50791:D>E	AA:82		Q59_1:S>R	AA:237		A4:K>R		DBVPG6304:K>R		YPS138:K>R	AA:362		A4:I>L		UWOPS91_917_1:I>L		YPS138:I>L	AA:392		A4:A>V		UFRJ50791:A>V		UWOPS91_917_1:A>V		YPS138:A>V	AA:406		A12:V>L		A4:V>L		N_44:V>L		N_45:V>L		UFRJ50791:V>L		UWOPS91_917_1:V>L		YPS138:V>L	AA:413		A4:A>V		UFRJ50791:A>V		YPS138:A>V	AA:417		A4:L>I		UWOPS91_917_1:L>I		YPS138:L>I	AA:420		A12:G>D		A4:G>D		UFRJ50791:G>D		YPS138:G>DID:YBL092W	AA:96		Q32_3:N>KID:YBL093C	AA:15		UWOPS91_917_1:H>Q	AA:54		CBS432:D>E	AA:85		A12:F>L		A4:F>L		DBVPG6304:F>L		UWOPS91_917_1:F>L		YPS138:F>L	AA:131		UWOPS91_917_1:L>I	AA:220		A4:S>R		DBVPG6304:S>R		UFRJ50791:S>R		UFRJ50816:S>R		UWOPS91_917_1:S>RID:YBL095W	AA:45		A12:C>W		A4:C>W		DBVPG6304:C>W		UFRJ50791:C>W		UFRJ50816:C>W	AA:59		N_43:L>F		N_44:L>F		N_45:L>F	AA:79		A12:N>K		A4:N>K		DBVPG6304:N>K		UFRJ50791:N>K		UFRJ50816:N>K	AA:89		A12:F>L		A4:F>L		DBVPG6304:F>L		UFRJ50791:F>L		UFRJ50816:F>L	AA:93		A12:F>L		A4:F>L		DBVPG6304:F>L		UFRJ50791:F>L		UFRJ50816:F>L	AA:98		DBVPG6304:I>M	AA:126		A12:F>L		A4:F>L		DBVPG6304:F>L		UFRJ50791:F>L		UFRJ50816:F>L	AA:133		A12:R>S		A4:R>S		DBVPG6304:R>S		UFRJ50791:R>S		UFRJ50816:R>S	AA:168		UWOPS91_917_1:Q>H	AA:172		UWOPS91_917_1:F>L	AA:179		UWOPS91_917_1:K>N	AA:181		A12:Q>H		A4:Q>H		DBVPG6304:Q>H		UFRJ50791:Q>H		UFRJ50816:Q>H	AA:185		A12:F>L		A4:F>L		DBVPG6304:F>L	AA:194		UFRJ50791:->S		UFRJ50816:->S	AA:222		A12:N>K		DBVPG6304:N>K		UFRJ50791:N>K		UFRJ50816:N>K	AA:239		UWOPS91_917_1:D>E	AA:247		A12:N>Y		DBVPG6304:N>Y		UFRJ50791:N>Y		UFRJ50816:N>Y		YPS138:N>Y	AA:256		DBVPG6304:H>Q	AA:260		UWOPS91_917_1:K>N	AA:264		DBVPG6304:V>F		UFRJ50791:V>F		UFRJ50816:V>F		YPS138:V>F	AA:270		UWOPS91_917_1:R>S	AA:275		KPN3828:I>TID:YBL098W	AA:26		UWOPS91_917_1:C>W	AA:34		IFO1804:C>-		N_43:C>-		N_44:C>-		N_45:C>-	AA:137		A12:F>L		A4:F>L		DBVPG6304:F>L		UFRJ50791:F>L		UFRJ50816:F>L		UWOPS91_917_1:F>L		YPS138:F>L	AA:139		A12:R>S		A4:R>S		DBVPG6304:R>S		UFRJ50791:R>S		UFRJ50816:R>S		YPS138:R>S	AA:149		A12:->Y		A4:->Y		DBVPG6304:->Y		UFRJ50791:->Y		UFRJ50816:->Y		YPS138:->Y	AA:214		A12:K>Q		A4:K>Q		DBVPG6304:K>Q		UFRJ50816:K>Q		YPS138:K>Q	AA:278		KPN3828:H>D		KPN3829:H>D	AA:286		A12:K>N		A4:K>N		DBVPG6304:K>N		N_43:K>N		N_45:K>N		YPS138:K>N	AA:305		N_17:I>R		N_43:I>R		N_45:I>R	AA:363		A12:I>M		A4:I>M		DBVPG6304:I>M		UFRJ50816:I>M	AA:395		A12:M>T		A4:M>T		DBVPG6304:M>T		UFRJ50816:M>T		UWOPS91_917_1:M>T		YPS138:M>T	AA:433		UFRJ50816:D>V		UWOPS91_917_1:D>E	AA:445		UWOPS91_917_1:L>M	AA:464		Q62_5:T>AID:YBL099W	AA:4		UFRJ50816:T>M	AA:79		A12:K>N		DBVPG6304:K>N		UFRJ50816:K>N		YPS138:K>N	AA:155		A12:F>L		DBVPG6304:F>L		UFRJ50816:F>L		YPS138:F>L	AA:162		KPN3828:T>S	AA:175		UWOPS91_917_1:W>C	AA:246		UWOPS91_917_1:F>L	AA:263		DBVPG6304:H>Q		IFO1804:H>Q		N_43:H>Q		N_44:H>Q		N_45:H>Q		UFRJ50816:H>Q		YPS138:H>Q	AA:283		UWOPS91_917_1:S>R	AA:373		A12:S>R		DBVPG6304:S>R		UFRJ50791:S>R	AA:383		A12:S>R		DBVPG6304:S>R		IFO1804:S>R		N_44:S>R		N_45:S>R		UFRJ50791:S>R	AA:386		IFO1804:L>F		N_43:L>F		N_44:L>F		N_45:L>F	AA:388		A12:E>D		DBVPG6304:E>D		UFRJ50791:E>D	AA:393		A12:->Y		DBVPG6304:->Y		UFRJ50791:->Y	AA:413		A12:R>S	AA:429		A12:Q>-		DBVPG6304:Q>-		UFRJ50791:Q>-	AA:526		IFO1804:Q>L		N_43:Q>L		N_44:Q>L		N_45:Q>L	AA:543		UFRJ50791:G>D		UFRJ50816:G>DID:YBL102W	AA:36		A4:L>V		DBVPG6304:L>V		UFRJ50791:L>V	AA:95		UWOPS91_917_1:K>T	AA:110		UWOPS91_917_1:S>T	AA:112		UWOPS91_917_1:K>-	AA:178		N_43:K>N		N_44:K>N		N_45:K>N	AA:213		N_43:->Y		N_44:->Y		N_45:->YID:YBL103C	AA:65		A12:P>S		A4:P>S		DBVPG6304:P>S		UFRJ50791:P>S		UWOPS91_917_1:P>T	AA:72		N_43:A>S		N_44:A>S		N_45:A>S	AA:109		UFRJ50791:T>S		UFRJ50816:T>S	AA:114		DBVPG6304:A>T	AA:132		A12:N>K		A4:N>K		DBVPG6304:N>K	AA:163		A12:Q>K		A4:Q>K		DBVPG6304:Q>K		UFRJ50816:Q>K		YPS138:Q>K	AA:223		A12:L>I	AA:324		A12:Q>H	AA:358		A12:A>G	AA:387		A4:S>L		DBVPG6304:S>L		YPS138:S>L	AA:413		A4:H>N		DBVPG6304:H>N		UFRJ50791:H>N		YPS138:H>N	AA:441		A12:H>Q		A4:H>Q		UFRJ50791:H>Q		UWOPS91_917_1:H>Q		YPS138:H>Q	AA:448		UWOPS91_917_1:H>Y	AA:450		UWOPS91_917_1:P>A	AA:453		Q89_8:A>TID:YBL104C	AA:3		UWOPS91_917_1:N>K	AA:63		A12:P>L		A4:P>L		DBVPG6304:P>L		UFRJ50791:P>L		UFRJ50816:P>L		YPS138:P>L	AA:65		N_43:R>H		N_44:R>H		N_45:R>H	AA:68		A12:I>T		A4:I>T		DBVPG6304:I>T		UFRJ50791:I>T		UFRJ50816:I>T		YPS138:I>T	AA:102		A12:R>K		A4:R>K		DBVPG6304:R>K		UFRJ50791:R>K		UFRJ50816:R>K		YPS138:R>K	AA:113		UFRJ50791:N>K		UFRJ50816:N>K	AA:136		A12:G>V		A4:G>V		DBVPG6304:G>V		UFRJ50791:G>V		UFRJ50816:G>V		YPS138:G>V	AA:236		UWOPS91_917_1:V>I	AA:412		UWOPS91_917_1:P>L	AA:421		UWOPS91_917_1:T>S	AA:554		A12:A>V		CBS432:A>V		DBVPG4650:A>V		N_17:A>V		N_43:A>V		N_45:A>V		Q59_1:A>V		Q62_5:A>V		S36_7:A>V		UFRJ50791:A>V		UFRJ50816:A>V		UWOPS91_917_1:A>V		Y6_5:A>V		YPS138:A>V		Z1_1:A>V	AA:565		A12:L>F		UFRJ50791:L>F		UFRJ50816:L>F		YPS138:L>F	AA:617		A12:V>I		UFRJ50791:V>I		UFRJ50816:V>I		UWOPS91_917_1:V>I		YPS138:V>I	AA:630		UFRJ50791:R>K		UFRJ50816:R>K		YPS138:R>K	AA:660		UWOPS91_917_1:H>Q	AA:690		CBS432:D>N		DBVPG4650:D>N		N_17:D>N		N_45:D>N		Q32_3:D>N		Q59_1:D>N		Q62_5:D>N		S36_7:D>N		T21_4:D>N		Y6_5:D>N		Z1_1:D>N	AA:695		DBVPG6304:D>N		UFRJ50791:D>N		UFRJ50816:D>N		YPS138:D>N	AA:701		N_45:Y>H	AA:762		IFO1804:G>S		N_44:G>S		N_45:G>S		UFRJ50791:G>S		UFRJ50816:G>S	AA:807		IFO1804:I>T		N_44:I>T		N_45:I>T	AA:868		UWOPS91_917_1:H>Y	AA:902		A12:T>P		DBVPG6304:T>P		UFRJ50791:T>P		UFRJ50816:T>P		YPS138:T>P	AA:906		A12:L>P		DBVPG6304:L>P		UFRJ50791:L>P		UFRJ50816:L>P		UWOPS91_917_1:L>P		YPS138:L>P	AA:911		IFO1804:D>N		N_44:D>N		N_45:D>N	AA:912		A12:K>T		DBVPG6304:K>T		UFRJ50791:K>T		UFRJ50816:K>T		YPS138:K>T	AA:925		A12:A>G		DBVPG6304:A>G		UFRJ50816:A>G		YPS138:A>G	AA:931		A12:S>A		DBVPG6304:S>A		UFRJ50816:S>A		UWOPS91_917_1:S>A		YPS138:S>A	AA:933		IFO1804:A>V		N_44:A>V		N_45:A>V	AA:934		UFRJ50816:M>I		UWOPS91_917_1:M>I	AA:946		A12:A>V		YPS138:A>V	AA:950		A12:P>Q		DBVPG6304:P>Q		UFRJ50816:P>Q		YPS138:P>Q	AA:1027		UWOPS91_917_1:D>EID:YBL107C	AA:9		UWOPS91_917_1:A>T	AA:37		CBS432:R>K		CBS5829:R>K		N_17:R>K		N_43:R>K		N_44:R>K		N_45:R>K		Q32_3:R>K		Q59_1:R>K		S36_7:R>K		T21_4:R>K		UWOPS91_917_1:R>K		Y6_5:R>K		Z1_1:R>K	AA:86		A12:Q>K		A4:Q>K		DBVPG6304:Q>K		UFRJ50791:Q>K		UFRJ50816:Q>K		UWOPS91_917_1:Q>K	AA:94		UWOPS91_917_1:A>T	AA:107		UWOPS91_917_1:I>L	AA:123		A12:E>D		A4:E>D		DBVPG6304:E>D		N_43:E>D		N_44:E>D		N_45:E>D		UFRJ50791:E>D		UFRJ50816:E>D	AA:127		A12:E>A		A4:E>A		DBVPG6304:E>A		UFRJ50791:E>A		UFRJ50816:E>A		YPS138:E>A	AA:131		A12:R>K		A4:R>K		DBVPG6304:R>K		UWOPS91_917_1:R>K	AA:149		N_43:R>G		N_44:R>G		N_45:R>G	AA:156		A12:S>G		A4:S>G		DBVPG6304:S>G		UFRJ50791:S>G		UFRJ50816:S>G		YPS138:S>G	AA:186		A12:Q>R		A4:Q>R		DBVPG6304:Q>R		N_43:Q>R		N_44:Q>R		N_45:Q>R		UFRJ50791:Q>R		UFRJ50816:Q>R		UWOPS91_917_1:Q>R		YPS138:Q>R	AA:192		A12:R>C		A4:R>C		DBVPG6304:R>C		UFRJ50791:R>C		UFRJ50816:R>C		YPS138:R>C	AA:195		N_43:N>D		N_44:N>D		N_45:N>DID:YBR002C	AA:40		N_43:->Y		N_44:->Y		N_45:->Y	AA:44		A4:H>Q		DBVPG6304:H>Q		Q62_5:H>Q		UFRJ50791:H>Q		YPS138:H>Q	AA:110		A4:E>Q		DBVPG6304:E>Q		UFRJ50791:E>Q		UFRJ50816:E>Q		UWOPS91_917_1:E>Q	AA:122		A4:F>L		DBVPG6304:F>L		UFRJ50791:F>L		UFRJ50816:F>L	AA:136		A4:F>L		DBVPG6304:F>L	AA:140		N_44:R>C	AA:163		A4:F>L		DBVPG6304:F>L		UFRJ50791:F>L		UFRJ50816:F>L		UWOPS91_917_1:F>L	AA:172		UWOPS91_917_1:Q>H	AA:183		A4:F>L		DBVPG6304:F>L	AA:195		IFO1804:->C		N_43:->C		N_44:->C		N_45:->C	AA:275		Q62_5:Y>HID:YBR003W	AA:5		UWOPS91_917_1:S>Y	AA:8		A12:A>V		A4:A>V		DBVPG6304:A>V		UFRJ50791:A>V		YPS138:A>V	AA:131		YPS138:F>L	AA:371		A12:I>V		A4:I>V		IFO1804:I>V		N_43:I>V		N_44:I>V		UFRJ50816:I>V		UWOPS91_917_1:I>V	AA:374		A12:K>R		A4:K>R		UFRJ50816:K>R		UWOPS91_917_1:K>R	AA:399		UFRJ50816:E>K	AA:420		IFO1804:T>I		N_43:T>I		N_44:T>I		N_45:T>I	AA:426		UWOPS91_917_1:L>Q	AA:435		UWOPS91_917_1:V>I	AA:446		IFO1804:Q>R		N_43:Q>R		N_44:Q>R		N_45:Q>R	AA:461		IFO1804:L>I		N_43:L>I		N_44:L>I		N_45:L>IID:YBR004C	AA:13		A4:L>F		DBVPG6304:L>F		UFRJ50791:L>F		UFRJ50816:L>F		UWOPS91_917_1:L>F		YPS138:L>F	AA:16		A4:Y>-		DBVPG6304:Y>-		UFRJ50791:Y>-		UFRJ50816:Y>-		YPS138:Y>-	AA:25		A4:K>N		DBVPG6304:K>N		UFRJ50791:K>N		UFRJ50816:K>N		UWOPS91_917_1:K>N		YPS138:K>N	AA:74		DBVPG6304:Y>-		UFRJ50816:Y>-		YPS138:Y>-	AA:94		DBVPG6304:F>L		N_43:F>L		N_45:F>L		UFRJ50816:F>L		UWOPS91_917_1:F>L		YPS138:F>L	AA:109		DBVPG6304:Y>-		UFRJ50816:Y>-		UWOPS91_917_1:Y>-		YPS138:Y>-	AA:117		DBVPG6304:I>V		N_43:I>V		N_45:I>V		UFRJ50816:I>V		UWOPS91_917_1:I>V		YPS138:I>V	AA:146		UWOPS91_917_1:A>T	AA:178		A12:->Y		A4:->Y		DBVPG6304:->Y		UFRJ50816:->Y		YPS138:->Y	AA:208		UWOPS91_917_1:K>N	AA:212		UWOPS91_917_1:V>A	AA:213		A12:->C		A4:->C		DBVPG6304:->C		UFRJ50816:->C		YPS138:->C	AA:226		N_17:D>E	AA:244		IFO1804:R>W		N_43:R>W		N_44:R>W		N_45:R>W	AA:252		A12:T>S		A4:T>S		DBVPG6304:T>S		IFO1804:T>S		N_43:T>S		N_44:T>S		N_45:T>S		YPS138:T>S	AA:256		A12:N>K		A4:N>K		DBVPG6304:N>K		IFO1804:N>K		N_43:N>K		N_44:N>K		N_45:N>K		UWOPS91_917_1:N>K		YPS138:N>K	AA:260		N_17:E>V		Q62_5:E>V		Y7:E>V		Z1_1:E>V	AA:263		A12:->K		A4:->K		DBVPG6304:->K		UWOPS91_917_1:->Q		YPS138:->K	AA:265		UWOPS91_917_1:F>L	AA:288		IFO1804:D>Y		N_43:D>Y		N_44:D>Y		N_45:D>Y	AA:302		A12:N>K		A4:N>K		DBVPG6304:N>K		YPS138:N>K	AA:343		A12:E>D		A4:E>D		DBVPG6304:E>D		UFRJ50816:E>D		UWOPS91_917_1:E>D		YPS138:E>D	AA:347		UWOPS91_917_1:Q>H	AA:370		A12:E>D		A4:E>D		DBVPG6304:E>D		UFRJ50816:E>D		UWOPS91_917_1:E>D		YPS138:E>D	AA:375		UWOPS91_917_1:->C	AA:395		A12:T>R		A4:T>R		DBVPG6304:T>R		UFRJ50816:T>R		YPS138:T>R	AA:413		UWOPS91_917_1:Q>HID:YBR005W	AA:5		A4:S>L		DBVPG6304:S>L		UFRJ50791:S>L		UFRJ50816:S>L		UWOPS91_917_1:S>L		YPS138:S>L	AA:19		UWOPS91_917_1:G>D	AA:21		Y6_5:H>Y	AA:161		A4:R>Q		DBVPG6304:R>Q		IFO1804:R>Q		N_43:R>Q		N_44:R>Q		N_45:R>Q		UFRJ50791:R>Q		UFRJ50816:R>Q		UWOPS91_917_1:R>Q		YPS138:R>Q	AA:163		A4:N>T		DBVPG6304:N>T		UFRJ50791:N>T		UFRJ50816:N>T		YPS138:N>T	AA:167		UWOPS91_917_1:M>I	AA:185		A4:T>R		DBVPG6304:T>R		N_43:T>M		N_44:T>M		UFRJ50791:T>R		UFRJ50816:T>R		YPS138:T>R	AA:189		A4:L>Q		DBVPG6304:L>Q		UFRJ50791:L>Q		YPS138:L>Q	AA:195		IFO1804:A>V		N_43:A>V		N_44:A>V		N_45:A>V	AA:204		UFRJ50791:P>LID:YBR006W	AA:6		N_43:F>Y		N_44:F>Y		N_45:F>Y		UWOPS91_917_1:F>Y	AA:90		UWOPS91_917_1:M>I	AA:271		CBS432:F>Y	AA:284		UWOPS91_917_1:E>D	AA:375		N_43:P>S		N_45:P>S	AA:391		A4:M>I		DBVPG6304:M>I		UFRJ50791:M>I		UFRJ50816:M>I		UWOPS91_917_1:M>I	AA:411		A4:E>K		DBVPG6304:E>K		UFRJ50791:E>K		UFRJ50816:E>K		UWOPS91_917_1:E>GID:YBR007C	AA:20		UWOPS91_917_1:I>S	AA:31		Q59_1:C>F	AA:43		UWOPS91_917_1:R>T	AA:47		A4:P>Q		UFRJ50791:P>Q		YPS138:P>Q	AA:52		A4:->K		UFRJ50791:->K		YPS138:->K	AA:89		UFRJ50791:F>L		YPS138:F>L	AA:138		UFRJ50791:F>L		UWOPS91_917_1:F>L		YPS138:F>L	AA:166		UWOPS91_917_1:F>V	AA:176		UWOPS91_917_1:F>L	AA:189		UWOPS91_917_1:->W	AA:202		UFRJ50791:K>E		YPS138:K>E	AA:210		UWOPS91_917_1:V>L	AA:216		UFRJ50791:M>I		UWOPS91_917_1:A>G		YPS138:M>I	AA:267		UWOPS91_917_1:E>D	AA:272		UFRJ50791:S>Y		UFRJ50816:S>Y		YPS138:S>Y	AA:301		UWOPS91_917_1:N>K	AA:305		UWOPS91_917_1:R>S	AA:318		UWOPS91_917_1:N>K	AA:320		UWOPS91_917_1:I>M	AA:355		UWOPS91_917_1:C>W	AA:360		UFRJ50791:R>-		UFRJ50816:R>-		YPS138:R>-	AA:362		UFRJ50791:E>D		UFRJ50816:E>D		YPS138:E>D	AA:366		UWOPS91_917_1:->Y	AA:372		UWOPS91_917_1:F>L	AA:404		UWOPS91_917_1:P>S	AA:411		A4:R>S		UFRJ50791:R>S		UFRJ50816:R>S		UWOPS91_917_1:R>S		YPS138:R>S	AA:415		A4:T>S		UFRJ50791:T>S		UFRJ50816:T>S		YPS138:T>S	AA:427		A4:F>L		N_44:F>L		N_45:F>L		UFRJ50791:F>L		UFRJ50816:F>L		UWOPS91_917_1:F>L		YPS138:F>L	AA:430		UWOPS91_917_1:K>-	AA:477		UWOPS91_917_1:Y>-	AA:498		KPN3829:I>V	AA:500		A4:T>A		N_44:T>A		N_45:T>A		UFRJ50791:T>A		UWOPS91_917_1:T>A	AA:520		A4:S>R		UFRJ50791:S>R		UWOPS91_917_1:S>R	AA:530		KPN3829:E>G	AA:542		UFRJ50791:F>L	AA:545		UFRJ50791:W>R		UWOPS91_917_1:W>R	AA:585		A4:H>Y		UFRJ50791:H>Y		YPS138:H>Y	AA:647		N_44:R>P	AA:675		UWOPS91_917_1:C>W	AA:687		UFRJ50816:V>L		UWOPS91_917_1:V>L		YPS138:V>L	AA:713		UWOPS91_917_1:C>W	AA:715		CBS5829:F>L		Q59_1:F>L		Q62_5:F>L		Q95_3:F>L		Y7:F>L		Z1_1:F>L	AA:720		UFRJ50816:L>F		YPS138:L>F	AA:725		UWOPS91_917_1:Y>-	AA:745		DBVPG6304:K>N		N_43:K>N		N_44:K>N		N_45:K>N		UFRJ50816:K>N		UWOPS91_917_1:K>N		YPS138:K>N	AA:748		DBVPG6304:F>L		N_43:F>L		N_44:F>L		N_45:F>L		UFRJ50816:F>L		UWOPS91_917_1:F>L		YPS138:F>L	AA:750		DBVPG6304:F>S		N_43:F>S		N_44:F>S		N_45:F>S		UFRJ50816:F>S		UWOPS91_917_1:F>S		YPS138:F>SID:YBR008C	AA:17		A4:I>M		DBVPG6304:I>M		UFRJ50791:I>M		UFRJ50816:I>M		YPS138:I>M	AA:31		A4:S>A		DBVPG6304:S>A		N_43:S>A		UFRJ50791:S>A		UFRJ50816:S>A		YPS138:S>A	AA:90		UWOPS91_917_1:E>D	AA:123		UWOPS91_917_1:Q>R	AA:132		DBVPG6304:D>E		UFRJ50791:D>E		UFRJ50816:D>E	AA:176		DBVPG6304:N>K		N_43:N>K		N_45:N>K		UFRJ50816:N>K	AA:207		A4:K>Q		CBS432:K>Q		DBVPG6304:K>Q		N_43:K>Q		N_45:K>Q		UFRJ50816:K>Q		UWOPS91_917_1:K>Q	AA:226		A4:D>E		DBVPG6304:D>E		UFRJ50816:D>E		UWOPS91_917_1:D>E	AA:241		N_45:F>L	AA:257		UWOPS91_917_1:T>A	AA:318		N_43:Q>H	AA:333		UWOPS91_917_1:T>A	AA:349		A4:T>A		UFRJ50816:T>A		UWOPS91_917_1:T>A		YPS138:T>A	AA:366		N_43:K>N	AA:372		A4:Q>H		UFRJ50816:Q>H		UWOPS91_917_1:Q>L		YPS138:Q>H	AA:393		A12:D>Y		A4:D>Y		N_43:D>Y		YPS138:D>Y	AA:400		A12:K>R		A4:K>R		YPS138:K>R	AA:440		A12:H>Q		A4:H>Q		N_43:H>Q		N_45:H>Q		YPS138:H>Q	AA:455		A4:S>R		DBVPG6304:S>R		N_43:L>F		N_45:L>F		UFRJ50791:S>R		UFRJ50816:S>R		UWOPS91_917_1:S>R	AA:488		N_43:R>S		N_45:R>S	AA:510		A12:F>L		A4:F>L		DBVPG6304:F>L		N_43:F>L		N_45:F>L		YPS138:F>L	AA:515		A12:F>L		A4:F>L		DBVPG6304:F>L		YPS138:F>L	AA:531		N_43:K>T		N_45:K>TID:YBR010W	AA:62		N_17:L>VID:YBR011C	AA:54		UWOPS91_917_1:R>S	AA:170		A12:W>C		IFO1804:W>C		N_43:W>C		N_45:W>C		UWOPS91_917_1:W>C	AA:268		A12:F>L		UWOPS91_917_1:F>L	AA:287		UWOPS91_917_1:G>RID:YBR014C	AA:6		UFRJ50816:L>F	AA:25		UFRJ50816:K>N	AA:39		A4:T>I		DBVPG6304:T>I		N_43:T>I		N_44:T>I		N_45:T>I		UFRJ50791:T>I		YPS138:T>I	AA:45		A4:D>E		DBVPG6304:D>E		N_43:D>E		N_44:D>E		N_45:D>E		UFRJ50791:D>E		YPS138:D>E	AA:61		UFRJ50816:V>F	AA:68		IFO1804:F>Y		N_43:F>Y		N_44:F>Y		N_45:F>Y	AA:72		A12:Q>R		A4:Q>R		DBVPG6304:Q>R		UFRJ50791:Q>R		YPS138:Q>R	AA:81		IFO1804:K>Q		N_43:K>Q		N_44:K>Q		N_45:K>Q		UFRJ50816:K>R	AA:87		IFO1804:Q>H		N_43:Q>H		N_44:Q>H		N_45:Q>H	AA:105		A12:H>Q		A4:H>Q		DBVPG6304:H>Q		IFO1804:H>Q		N_43:H>Q		N_44:H>Q		N_45:H>Q		UFRJ50791:H>Q		YPS138:H>Q	AA:116		A12:C>S		A4:C>S		DBVPG6304:C>S		IFO1804:C>S		N_43:C>S		N_44:C>S		N_45:C>S		UFRJ50791:C>S		YPS138:C>S	AA:127		A12:F>L		A4:F>L		DBVPG6304:F>L		UFRJ50791:F>L		YPS138:F>L	AA:129		A12:V>A		A4:V>A		DBVPG6304:V>A		IFO1804:V>A		N_43:V>A		N_44:V>A		N_45:V>A		UFRJ50791:V>A		YPS138:V>A	AA:169		A12:F>C		A4:F>C		DBVPG6304:F>C		IFO1804:F>C		N_43:S>R		N_44:S>R		N_45:S>R		UFRJ50791:F>C		YPS138:F>C	AA:178		CBS432:->E		CBS5829:->E		Q59_1:->E		Q95_3:->E		T21_4:->E		UFRJ50816:->E		Y6_5:->E		Z1_1:->EID:YBR017C	AA:8		IFO1804:K>-		N_44:K>-		N_45:K>-	AA:11		UWOPS91_917_1:F>Y	AA:87		UWOPS91_917_1:->K	AA:90		A12:F>L		A4:F>L		UWOPS91_917_1:F>L	AA:98		A12:K>N		A4:K>N		UFRJ50791:K>N		UWOPS91_917_1:K>N	AA:102		DBVPG4650:T>S	AA:103		UWOPS91_917_1:E>D	AA:104		A12:N>K		A4:N>K		UFRJ50791:N>K		UFRJ50816:N>K	AA:131		UWOPS91_917_1:G>C	AA:137		UWOPS91_917_1:N>K	AA:197		A4:K>N		DBVPG6304:K>N		UFRJ50816:K>N		UWOPS91_917_1:E>Q	AA:202		A12:S>T		A4:S>T		DBVPG6304:S>T		UFRJ50791:S>T		UFRJ50816:S>T		UWOPS91_917_1:S>T	AA:219		UWOPS91_917_1:T>A	AA:222		A12:H>Q	AA:223		DBVPG6304:D>E	AA:225		IFO1804:Q>H		N_43:Q>H		N_44:Q>H		N_45:Q>H	AA:250		A12:I>M		A4:I>M		DBVPG6304:I>M		UFRJ50791:I>M		UFRJ50816:I>M	AA:271		IFO1804:M>I		N_43:M>I		N_44:M>I		N_45:M>I	AA:276		A12:Q>H		A4:Q>H		DBVPG6304:Q>H		UWOPS91_917_1:Q>H	AA:286		A12:G>S		A4:G>S		DBVPG6304:G>S		UFRJ50791:G>S		UFRJ50816:G>S		UWOPS91_917_1:G>S	AA:293		A12:C>-		A4:C>-		DBVPG6304:C>-		UFRJ50791:C>-		UFRJ50816:C>-		UWOPS91_917_1:C>-	AA:350		A12:N>K		A4:N>K		DBVPG6304:N>K		UFRJ50791:N>K		UFRJ50816:N>K		UWOPS91_917_1:N>K	AA:370		A12:I>M		A4:Y>C		DBVPG6304:Y>C		N_43:Y>C		N_44:Y>C		N_45:Y>C		UFRJ50791:Y>C		UFRJ50816:Y>C		UWOPS91_917_1:Y>C		YPS138:Y>C	AA:376		N_44:I>T		N_45:I>T	AA:413		A12:R>S		A4:R>S		UFRJ50816:R>S	AA:477		S36_7:->L	AA:489		N_17:T>S	AA:508		A12:V>D		A4:V>D		DBVPG6304:V>D		N_43:V>D		N_44:V>D		N_45:V>D		UFRJ50791:V>D		UFRJ50816:V>D		UWOPS91_917_1:V>D		YPS138:V>D	AA:546		A12:I>L		A4:I>L		DBVPG6304:I>L		UFRJ50791:I>L		UFRJ50816:I>L		YPS138:I>L	AA:553		UWOPS91_917_1:H>Q	AA:580		A4:N>D		DBVPG6304:N>D		UFRJ50791:N>D		UFRJ50816:N>D		UWOPS91_917_1:N>D		YPS138:N>D	AA:597		UWOPS91_917_1:I>V	AA:654		N_43:F>L		N_44:F>L		N_45:F>L	AA:655		A4:Q>H		DBVPG6304:Q>H		UFRJ50791:Q>H		UWOPS91_917_1:Q>H		YPS138:Q>H	AA:676		A4:Q>K		DBVPG6304:Q>K		UFRJ50791:Q>K		UWOPS91_917_1:Q>K		YPS138:Q>K	AA:686		UWOPS91_917_1:->Q	AA:697		A4:N>H		DBVPG6304:N>H		N_43:N>H		N_44:N>H		N_45:N>H		UFRJ50791:N>H		UFRJ50816:N>H		UWOPS91_917_1:N>H		YPS138:N>H	AA:701		A4:Y>-		DBVPG6304:Y>-		UFRJ50791:Y>-		UFRJ50816:Y>-		UWOPS91_917_1:Y>-		YPS138:Y>-	AA:714		A4:I>L		DBVPG6304:I>L		UFRJ50791:I>L		UFRJ50816:I>L		UWOPS91_917_1:I>L		YPS138:I>L	AA:751		A4:->K		DBVPG6304:->K		UFRJ50791:->K		UFRJ50816:->K		UWOPS91_917_1:->K		YPS138:->K	AA:772		DBVPG6304:K>N	AA:790		A4:N>K		DBVPG6304:N>K		UFRJ50791:N>K		UFRJ50816:N>K		YPS138:N>K	AA:799		A4:->Y		DBVPG6304:->Y		UFRJ50791:->Y		UFRJ50816:->Y		UWOPS91_917_1:->Y		YPS138:->Y	AA:808		N_43:M>I		N_44:M>I		N_45:M>I	AA:857		A4:V>D		DBVPG6304:V>D		UFRJ50791:V>D		UFRJ50816:V>D		YPS138:V>D	AA:858		UWOPS91_917_1:I>M	AA:864		UWOPS91_917_1:->Y	AA:885		A4:C>W		DBVPG6304:C>W		UFRJ50791:C>W		UFRJ50816:C>W		YPS138:C>W	AA:904		UWOPS91_917_1:L>MID:YBR018C	AA:2		A12:Q>H		A4:Q>H		UFRJ50816:Q>H		UWOPS91_917_1:Q>H		YPS138:Q>H	AA:5		UWOPS91_917_1:->K	AA:12		A4:Q>H		UFRJ50816:Q>H		UWOPS91_917_1:Q>H		YPS138:Q>H	AA:22		A4:W>-		UFRJ50816:W>-		UWOPS91_917_1:W>-		YPS138:W>-	AA:28		UWOPS91_917_1:G>W	AA:30		UWOPS91_917_1:T>N	AA:34		A4:K>N		UFRJ50816:K>N		YPS138:K>N	AA:53		A4:H>Q		DBVPG6304:H>Q		UFRJ50816:H>Q		UWOPS91_917_1:H>Q		YPS138:H>Q	AA:58		N_43:V>F	AA:69		N_17:S>C	AA:90		UWOPS91_917_1:->K	AA:95		A4:->G		DBVPG6304:->G		UFRJ50791:->G		UFRJ50816:->G		YPS138:->G	AA:115		DBVPG6304:->C		IFO1804:->C		N_43:->C		N_44:->C		N_45:->C		UFRJ50791:->C		UFRJ50816:->C		UWOPS91_917_1:->C		YPS138:->C	AA:130		DBVPG4650:N>S	AA:133		N_17:L>F		UWOPS91_917_1:K>T	AA:141		DBVPG6304:S>A		UFRJ50791:S>A		UFRJ50816:S>A		YPS138:S>A	AA:145		IFO1804:F>Y		N_45:F>Y	AA:196		IFO1804:C>-		N_45:C>-	AA:231		A12:N>K		DBVPG6304:N>K		UFRJ50791:N>K		UFRJ50816:N>K		YPS138:N>K	AA:244		A12:M>I		DBVPG6304:M>I		UFRJ50791:M>I		UFRJ50816:M>I		YPS138:M>I	AA:252		IFO1804:E>-		N_43:E>-		N_45:E>-		T21_4:E>K		Z1_1:E>K	AA:359		A12:R>K		DBVPG6304:R>K		N_43:R>K		N_45:R>K		UFRJ50816:R>K		YPS138:R>KID:YBR019C	AA:5		IFO1804:V>L		N_43:V>L		N_44:V>L		N_45:V>L	AA:6		A12:D>E		A4:D>E	AA:10		A12:T>I		A4:T>I	AA:20		UWOPS91_917_1:T>S	AA:50		N_43:E>K	AA:74		N_44:E>D	AA:124		CBS432:I>M	AA:179		IFO1804:T>N		N_43:T>N		N_44:T>N		N_45:T>N	AA:184		A12:F>I		A4:F>I		DBVPG6304:F>I		IFO1804:F>I		N_43:F>I		N_44:F>I		N_45:F>I		UFRJ50816:F>I		UWOPS91_917_1:F>I	AA:200		UWOPS91_917_1:->L	AA:232		A12:K>N		A4:K>N		DBVPG6304:K>N		UFRJ50816:K>N		YPS138:K>N	AA:237		A12:K>N		A4:K>N		DBVPG6304:K>N		UFRJ50816:K>N		UWOPS91_917_1:K>N		YPS138:K>N	AA:268		A12:T>R		A4:T>R		DBVPG6304:T>R		UFRJ50816:T>R		YPS138:T>R	AA:276		UWOPS91_917_1:G>C	AA:302		A12:F>V		A4:F>V		DBVPG6304:F>V		YPS138:F>V	AA:336		A4:G>R		UFRJ50816:G>R		YPS138:G>R	AA:339		UWOPS91_917_1:V>I	AA:360		A4:A>T		UFRJ50816:A>T		YPS138:A>T	AA:379		A4:W>C		UFRJ50816:W>C		UWOPS91_917_1:W>C		YPS138:W>C	AA:406		T21_4:E>Q		Z1_1:E>Q	AA:442		UFRJ50791:D>Y		UFRJ50816:D>Y		UWOPS91_917_1:D>Y		YPS138:D>Y	AA:457		UWOPS91_917_1:W>C	AA:470		IFO1804:I>M		N_43:I>M		N_44:I>M		N_45:I>M	AA:493		A4:W>-		UFRJ50791:W>-		UFRJ50816:W>-		UWOPS91_917_1:W>-		YPS138:W>-	AA:502		IFO1804:M>L		N_43:M>L		N_44:M>L		N_45:M>L	AA:526		A4:I>T		DBVPG6304:I>T		UFRJ50791:I>T		UFRJ50816:I>T		YPS138:I>T	AA:529		A4:I>T		DBVPG6304:I>T		UFRJ50791:I>T		UFRJ50816:I>T		UWOPS91_917_1:I>T		YPS138:I>T	AA:538		A4:I>R		DBVPG6304:I>R		IFO1804:I>R		N_44:I>R		N_45:I>R		UFRJ50791:I>R		UWOPS91_917_1:I>R		YPS138:I>R	AA:563		UWOPS91_917_1:N>T	AA:571		A4:E>D		DBVPG6304:E>D		UFRJ50791:E>D		UWOPS91_917_1:E>D		YPS138:E>D	AA:596		UWOPS91_917_1:Q>-	AA:608		UWOPS91_917_1:T>A	AA:621		A4:E>A		DBVPG6304:E>A		IFO1804:E>A		N_43:E>A		N_44:E>A		N_45:E>A		UFRJ50791:E>A		UFRJ50816:E>A		UWOPS91_917_1:E>A		YPS138:E>A	AA:631		A4:Q>K		DBVPG6304:Q>K		UFRJ50791:Q>K		UFRJ50816:Q>K		YPS138:Q>KID:YBR020W	AA:9		A4:V>I		DBVPG6304:V>I		UFRJ50791:V>I		UFRJ50816:V>I		YPS138:V>I	AA:10		N_45:A>T	AA:41		A4:G>D		DBVPG6304:G>D		UFRJ50791:G>D		UFRJ50816:G>D		YPS138:G>D	AA:83		UWOPS91_917_1:V>I	AA:93		UWOPS91_917_1:L>S	AA:116		UWOPS91_917_1:I>T	AA:125		UWOPS91_917_1:N>S	AA:140		A4:G>R		DBVPG6304:G>R		UFRJ50791:G>R		YPS138:G>R	AA:148		N_45:S>N	AA:193		UWOPS91_917_1:M>I	AA:195		UWOPS91_917_1:K>N	AA:223		UWOPS91_917_1:C>R	AA:302		A4:G>E		UFRJ50816:G>E		UWOPS91_917_1:G>E		YPS138:G>E	AA:327		UWOPS91_917_1:P>S	AA:349		N_43:S>F		N_44:S>F		N_45:S>F	AA:424		N_43:K>R		N_44:K>R		N_45:K>R	AA:438		N_45:D>N	AA:442		A12:E>Q		A4:E>Q		DBVPG6304:E>Q		UFRJ50816:E>Q	AA:456		UWOPS91_917_1:L>F	AA:465		UWOPS91_917_1:L>F	AA:479		N_44:P>S	AA:500		N_43:A>V		N_44:A>V		N_45:A>V	AA:526		DBVPG6304:F>Y		UFRJ50816:F>Y		UWOPS91_917_1:F>YID:YBR021W	AA:3		IFO1804:D>N		N_43:D>N		N_44:D>N		N_45:D>N	AA:30		YPS138:A>E	AA:229		A12:R>K	AA:280		YPS138:S>A	AA:302		KPN3829:D>Y	AA:420		DBVPG4650:A>T	AA:514		A12:R>K		DBVPG6304:R>K	AA:620		DBVPG6304:S>N	AA:624		N_43:Y>H		N_44:Y>H		N_45:Y>H	AA:626		N_44:H>QID:YBR022W	AA:5		A12:A>T		DBVPG6304:A>T		IFO1804:A>T		N_45:A>T		UFRJ50791:A>T		UFRJ50816:A>T		UWOPS91_917_1:A>T	AA:45		N_17:K>E	AA:53		A12:I>V		DBVPG6304:I>V		UFRJ50791:I>V		UFRJ50816:I>V		UWOPS91_917_1:I>V	AA:59		A12:S>P		DBVPG6304:S>P		UFRJ50791:S>P		UFRJ50816:S>P		UWOPS91_917_1:S>P	AA:91		UFRJ50791:H>Q		UFRJ50816:H>Q	AA:93		UWOPS91_917_1:G>E	AA:99		UWOPS91_917_1:N>D	AA:119		IFO1804:G>E		N_45:G>E	AA:121		UWOPS91_917_1:N>SID:YBR024W	AA:8		A4:Y>F		DBVPG6304:Y>F		YPS138:Y>F	AA:20		A4:T>S		DBVPG6304:T>S		YPS138:T>S	AA:30		A4:A>T	AA:47		UWOPS91_917_1:E>K	AA:50		DBVPG6304:S>N		UFRJ50816:S>N		UWOPS91_917_1:S>N		YPS138:S>N	AA:65		A4:N>S		DBVPG6304:N>S		UFRJ50791:N>S		UFRJ50816:N>S		YPS138:N>S	AA:67		CBS5829:P>L	AA:90		UWOPS91_917_1:S>N	AA:105		UFRJ50791:E>G		UFRJ50816:E>G	AA:126		A4:N>K		DBVPG6304:N>K		UFRJ50791:N>K		UFRJ50816:N>K		UWOPS91_917_1:N>K		YPS138:N>K	AA:195		A4:D>N		DBVPG6304:D>N		UFRJ50791:D>N		UFRJ50816:D>N		UWOPS91_917_1:D>N		YPS138:D>N	AA:209		A4:I>V		DBVPG6304:I>V		UFRJ50791:I>V		UFRJ50816:I>V		YPS138:I>V	AA:233		N_17:R>HID:YBR025C	AA:28		DBVPG6304:T>S	AA:64		DBVPG6304:S>I		UFRJ50791:S>I	AA:69		IFO1804:S>R		N_43:S>R		N_44:S>R		N_45:S>R	AA:127		A12:->Y		A4:->Y		DBVPG6304:->Y		UFRJ50791:->Y		YPS138:->Y	AA:163		A12:Q>H		A4:Q>H		DBVPG6304:Q>H		UFRJ50791:Q>H		YPS138:Q>H	AA:184		A4:F>L	AA:329		A12:R>S		A4:R>S		DBVPG6304:R>S		UWOPS91_917_1:R>S		YPS138:R>S	AA:340		Q59_1:N>K		S36_7:N>KID:YBR026C	AA:71		Q95_3:S>R	AA:93		DBVPG6304:L>F		UFRJ50816:L>F		YPS138:L>F	AA:114		DBVPG6304:S>R		UFRJ50816:S>R		YPS138:S>R	AA:144		UWOPS91_917_1:L>F	AA:148		UWOPS91_917_1:L>F	AA:149		N_43:->S		N_45:->S	AA:151		UWOPS91_917_1:R>S	AA:187		A12:Y>H	AA:197		A12:C>W		A4:C>W		DBVPG6304:C>W		UWOPS91_917_1:C>W		YPS138:C>W	AA:201		UWOPS91_917_1:L>V	AA:211		A12:P>A		A4:P>A		DBVPG6304:P>A		N_43:P>A		N_45:P>A		UFRJ50816:P>A		UWOPS91_917_1:P>A	AA:219		UWOPS91_917_1:L>M	AA:236		UWOPS91_917_1:->Y	AA:239		A12:H>Q		A4:H>Q		DBVPG6304:H>Q	AA:246		A12:V>I	AA:276		UWOPS91_917_1:A>G	AA:278		A12:R>S		A4:R>S		DBVPG6304:R>S		UFRJ50816:R>S	AA:297		A12:C>W		A4:C>W		UFRJ50816:C>W	AA:323		N_43:K>N	AA:325		A12:Q>H		A4:Q>H		DBVPG6304:Q>H		UFRJ50816:Q>H		UWOPS91_917_1:Q>H	AA:344		A12:->R		A4:->R		DBVPG6304:->R		KPN3828:->R		KPN3829:->R		N_17:->R		N_43:->R		UWOPS91_917_1:->R	AA:348		N_43:G>A	AA:356		A12:->C		A4:->C		DBVPG6304:->C		YPS138:->C	AA:363		A12:L>M		A4:L>M		DBVPG6304:L>M		UWOPS91_917_1:L>MID:YBR028C	AA:5		A12:L>F		A4:L>F		DBVPG6304:L>F		UFRJ50791:L>F		UFRJ50816:L>F		YPS138:L>F	AA:10		A12:M>I		A4:M>I		DBVPG6304:M>I		UFRJ50816:M>I		UWOPS91_917_1:M>I	AA:31		A12:W>R		A4:W>R		DBVPG6304:W>R		UFRJ50791:W>R		UFRJ50816:W>R		YPS138:W>R	AA:38		A12:F>I		A4:F>I		DBVPG6304:F>I		UFRJ50791:F>I		UFRJ50816:F>I		YPS138:F>I	AA:56		A12:I>F		A4:I>F		DBVPG6304:I>F		UFRJ50791:I>F		YPS138:I>F	AA:97		UWOPS91_917_1:->G	AA:111		A12:C>W		A4:C>W		DBVPG6304:C>W		N_17:C>W		N_44:C>W		N_45:C>W		UFRJ50791:C>W		UWOPS91_917_1:C>W		YPS138:C>W	AA:150		A12:I>F		A4:I>F		DBVPG6304:I>F		UFRJ50791:I>F		UWOPS91_917_1:I>F		YPS138:I>F	AA:153		UWOPS91_917_1:D>E	AA:165		A12:I>M		A4:I>M		DBVPG6304:I>M		IFO1804:I>M		N_43:I>M		N_44:I>M		N_45:I>M		UFRJ50791:I>M		YPS138:I>M	AA:170		IFO1804:Y>-		N_43:Y>-		N_44:Y>-		N_45:Y>-	AA:185		N_43:I>V	AA:190		A12:F>V		A4:F>V		DBVPG6304:F>V		UFRJ50791:F>V		UWOPS91_917_1:F>V		YPS138:F>V	AA:195		UFRJ50791:F>L	AA:231		A4:K>-		DBVPG6304:K>-		UFRJ50791:K>-		UWOPS91_917_1:K>-		YPS138:K>-	AA:285		IFO1804:M>I		N_44:M>I		N_45:M>I	AA:302		A4:->C		DBVPG6304:->C		UFRJ50791:->C		UWOPS91_917_1:->C		YPS138:->C	AA:305		IFO1804:M>I		N_44:M>I		N_45:M>I	AA:326		A4:S>R		DBVPG6304:S>R		UFRJ50791:S>R		YPS138:S>R	AA:330		A4:F>L		DBVPG6304:F>L		UFRJ50791:F>L		UWOPS91_917_1:F>L		YPS138:F>L	AA:334		IFO1804:->R		N_44:->R		N_45:->R	AA:351		A4:F>L		DBVPG6304:F>L		IFO1804:F>L		N_44:F>L		N_45:F>L		UFRJ50791:F>L		UWOPS91_917_1:F>L		YPS138:F>L	AA:365		UWOPS91_917_1:C>F	AA:379		A4:N>K		DBVPG6304:N>K		UFRJ50791:N>K		YPS138:N>K	AA:398		A12:L>F		A4:L>F		DBVPG6304:L>F		UFRJ50791:L>F		UWOPS91_917_1:L>F		YPS138:L>F	AA:418		UWOPS91_917_1:L>M	AA:420		UWOPS91_917_1:C>S	AA:429		A12:->W		A4:->W		DBVPG6304:->W		UFRJ50791:->W		UWOPS91_917_1:->W		YPS138:->W	AA:431		UWOPS91_917_1:S>R	AA:450		UWOPS91_917_1:F>L	AA:459		A12:H>Q		A4:H>Q		DBVPG6304:H>Q		UFRJ50816:H>Q		UWOPS91_917_1:H>Q		YPS138:H>Q	AA:461		A12:M>I		A4:M>I		DBVPG6304:M>I		UFRJ50816:M>I		UWOPS91_917_1:M>I		YPS138:M>I	AA:469		A12:W>-		A4:W>-		DBVPG6304:W>-		UFRJ50816:W>-		YPS138:W>-	AA:504		A12:Y>-		A4:Y>-		DBVPG6304:Y>-		IFO1804:Y>-		N_44:Y>-		N_45:Y>-		UFRJ50816:Y>-		UWOPS91_917_1:Y>-ID:YBR030W	AA:3		IFO1804:V>A		N_44:V>A		N_45:V>A	AA:13		S36_7:L>I	AA:102		A12:Q>P		A4:Q>P		UFRJ50816:Q>P		UWOPS91_917_1:Q>P		YPS138:Q>P	AA:107		Q59_1:I>V	AA:113		UWOPS91_917_1:Q>E	AA:124		UWOPS91_917_1:N>H	AA:126		UWOPS91_917_1:N>D	AA:150		UWOPS91_917_1:I>M	AA:158		CBS432:N>D		KPN3829:N>D		Q32_3:N>D		Q62_5:N>D		Q89_8:N>D		Q95_3:N>D		T21_4:N>D		UWOPS91_917_1:N>D		Y7:N>D		Z1_1:N>D	AA:185		UWOPS91_917_1:K>E	AA:210		IFO1804:A>T		N_44:A>T		N_45:A>T	AA:229		A4:N>S		DBVPG6304:N>S		UFRJ50791:N>S	AA:263		UWOPS91_917_1:D>N	AA:265		UWOPS91_917_1:D>N	AA:267		IFO1804:N>S		N_44:N>S		N_45:N>S	AA:268		UWOPS91_917_1:I>M	AA:285		UWOPS91_917_1:N>K	AA:295		IFO1804:C>R		N_44:C>R		N_45:C>R	AA:299		IFO1804:T>S	AA:312		IFO1804:P>L		N_44:P>L		N_45:P>L	AA:319		DBVPG6304:I>V		IFO1804:I>V		N_43:I>V		N_44:I>V		N_45:I>V		UFRJ50791:I>V	AA:322		IFO1804:N>S		N_43:N>S		N_44:N>S		N_45:N>S	AA:368		DBVPG6304:V>L		IFO1804:V>L		N_43:V>L		N_44:V>L		N_45:V>L		UFRJ50791:V>L	AA:411		IFO1804:D>Y		N_43:D>Y		N_44:D>Y		N_45:D>Y	AA:413		UWOPS91_917_1:P>L	AA:434		DBVPG6304:G>D		UFRJ50791:G>D	AA:446		IFO1804:S>F		N_43:S>F		N_44:S>F		N_45:S>F	AA:470		CBS432:S>L		N_17:S>L		Q62_5:S>L		S36_7:S>L		T21_4:S>L	AA:478		UWOPS91_917_1:P>T	AA:483		UWOPS91_917_1:S>F	AA:488		UWOPS91_917_1:Q>K	AA:495		IFO1804:K>R		N_43:K>R		N_44:K>R		N_45:K>R	AA:500		DBVPG6304:H>Y		UFRJ50791:H>Y	AA:516		DBVPG6304:R>C		IFO1804:R>C		N_43:R>C		N_44:R>C		N_45:R>C		UFRJ50791:R>C		UWOPS91_917_1:R>C	AA:524		DBVPG6304:H>Q		UFRJ50791:H>Q		UWOPS91_917_1:H>Q		YPS138:H>Q	AA:544		DBVPG6304:H>L		UFRJ50791:H>L		YPS138:H>L	AA:548		DBVPG6304:E>D		UFRJ50791:E>D		UWOPS91_917_1:E>D		YPS138:E>DID:YBR031W	AA:159		A4:I>V		DBVPG6304:I>V		UFRJ50791:I>V		UFRJ50816:I>V		UWOPS91_917_1:I>V	AA:301		A12:P>SID:YBR033W	AA:17		A12:Y>H		A4:Y>H		DBVPG6304:Y>H		UFRJ50816:Y>H		UWOPS91_917_1:Y>H		YPS138:Y>H	AA:26		A12:T>M		A4:T>M		DBVPG6304:T>M		UFRJ50816:T>M		UWOPS91_917_1:T>M		YPS138:T>M	AA:45		IFO1804:S>N		N_43:S>N		N_44:S>N		N_45:S>N	AA:61		A12:K>E		A4:K>E		DBVPG6304:K>E		UWOPS91_917_1:K>E		YPS138:K>E	AA:67		UWOPS91_917_1:R>T	AA:74		IFO1804:V>I		N_43:V>I		N_44:V>I		N_45:V>I	AA:81		A12:V>I	AA:88		A12:V>L		DBVPG6304:V>L		UWOPS91_917_1:V>L		YPS138:V>L	AA:98		A12:K>R		DBVPG6304:K>R		IFO1804:K>R		N_43:K>R		N_44:K>R		N_45:K>R		UWOPS91_917_1:K>R		YPS138:K>R	AA:107		IFO1804:E>K		N_43:E>K		N_44:E>K		N_45:E>K	AA:111		A12:L>S		DBVPG6304:L>S		IFO1804:L>S		N_43:L>S		N_44:L>S		N_45:L>S		UWOPS91_917_1:L>S		YPS138:L>S	AA:119		IFO1804:R>K		N_43:R>K		N_44:R>K		N_45:R>K	AA:136		A12:S>P		DBVPG6304:S>P		UFRJ50791:S>P		UWOPS91_917_1:S>P		YPS138:S>P	AA:147		IFO1804:S>N		N_43:S>N		N_44:S>N		N_45:S>N	AA:153		A12:P>S		DBVPG6304:P>S		UFRJ50791:P>S		YPS138:P>S	AA:156		A12:T>A		DBVPG6304:T>A		UFRJ50791:T>A		YPS138:T>A	AA:173		A12:R>G		DBVPG6304:R>G		UFRJ50791:R>G		UWOPS91_917_1:R>G		YPS138:R>G	AA:190		IFO1804:G>R		N_43:G>R		N_44:G>R		N_45:G>R	AA:217		UWOPS91_917_1:E>G	AA:249		A4:I>T		DBVPG6304:I>T		UFRJ50791:I>T		YPS138:I>T	AA:250		CBS432:S>F	AA:258		UWOPS91_917_1:I>V	AA:290		A4:E>K		DBVPG6304:E>K		UFRJ50791:E>K		YPS138:E>K	AA:299		A4:C>F		DBVPG6304:C>F		UFRJ50791:C>F		YPS138:C>F	AA:300		UWOPS91_917_1:S>P	AA:303		UWOPS91_917_1:K>E	AA:310		N_43:S>G		N_44:S>G		N_45:S>G	AA:320		UFRJ50791:K>R		YPS138:K>R	AA:324		A4:R>Q		DBVPG6304:R>Q		UFRJ50791:R>Q		YPS138:R>Q	AA:330		N_43:R>C	AA:333		UWOPS91_917_1:S>F	AA:336		A4:T>S		DBVPG6304:T>S		UFRJ50791:T>S		YPS138:T>S	AA:346		A4:K>T		UFRJ50791:K>T		YPS138:K>T	AA:349		A4:T>I		CBS432:T>I		KPN3829:T>I		N_17:T>I		N_43:T>I		Q59_1:T>I		UFRJ50791:T>I		YPS138:T>I	AA:356		UWOPS91_917_1:Y>F	AA:366		A4:D>N		UWOPS91_917_1:D>G	AA:370		A4:D>N		UFRJ50791:D>N		YPS138:D>N	AA:384		UWOPS91_917_1:N>S	AA:388		UWOPS91_917_1:G>D	AA:397		UWOPS91_917_1:V>M	AA:401		CBS432:I>T		DBVPG4650:I>T		KPN3829:I>T		N_17:I>T		Q59_1:I>T	AA:405		UWOPS91_917_1:S>N	AA:410		IFO1804:E>Q		N_43:E>Q		N_45:E>Q	AA:454		DBVPG6304:F>I		UFRJ50816:F>I	AA:457		A4:C>S		DBVPG6304:C>S		UFRJ50816:C>S	AA:464		DBVPG6304:S>N		UFRJ50816:S>N	AA:473		UWOPS91_917_1:R>K	AA:474		IFO1804:Q>H		N_43:Q>H		N_44:Q>H		N_45:Q>H	AA:497		UFRJ50816:I>V		YPS138:I>V	AA:530		T21_4:N>D		UFRJ50816:N>S		Y6_5:N>D		Y7:N>D		YPS138:N>S	AA:538		A4:N>D		DBVPG6304:N>D		UFRJ50816:N>D		YPS138:N>D	AA:557		IFO1804:S>L		N_43:S>L		N_44:S>L		N_45:S>L	AA:591		A12:S>R		A4:S>R		DBVPG6304:S>R		UFRJ50816:S>R		YPS138:S>R	AA:630		A12:L>S		A4:L>S		DBVPG6304:L>S		UFRJ50816:L>S		YPS138:L>S	AA:633		A12:D>N		A4:D>N		DBVPG6304:D>N		UFRJ50816:D>N		YPS138:D>N	AA:650		A12:A>V		A4:A>V		DBVPG6304:A>V		UFRJ50816:A>V		YPS138:A>V	AA:657		A12:N>D		A4:N>D		DBVPG6304:N>D		UFRJ50816:N>D		YPS138:N>D	AA:661		A12:D>N		A4:D>N		DBVPG6304:D>N		UFRJ50816:D>N		YPS138:D>N	AA:672		A12:S>I		A4:S>I		DBVPG6304:S>I		UFRJ50816:S>I		YPS138:S>I	AA:686		A12:H>Y		A4:H>Y		DBVPG6304:H>Y		UFRJ50816:H>Y		YPS138:H>Y	AA:690		N_43:T>S		N_44:T>S		N_45:T>S	AA:696		A12:A>P		A4:A>P		DBVPG6304:A>P		UFRJ50816:A>P		YPS138:A>P	AA:732		A4:S>L	AA:741		A12:E>A		A4:E>A		DBVPG6304:E>A		IFO1804:E>A		N_43:E>A		N_44:E>A		N_45:E>A		UFRJ50791:E>A		UFRJ50816:E>A		UWOPS91_917_1:E>G		YPS138:E>A	AA:744		A12:S>G		A4:S>G		DBVPG6304:S>G		UFRJ50791:S>G		UFRJ50816:S>G		YPS138:S>G	AA:757		UWOPS91_917_1:P>S	AA:760		Q59_1:A>V	AA:767		UWOPS91_917_1:Y>N	AA:780		A12:I>M		DBVPG6304:I>M		IFO1804:I>M		N_43:I>M		N_44:I>M		N_45:I>M		UFRJ50791:I>M		UFRJ50816:I>M		UWOPS91_917_1:I>M		YPS138:I>M	AA:806		IFO1804:I>V		N_43:I>V		N_44:I>V		N_45:I>V	AA:815		A4:M>I		DBVPG6304:M>I		IFO1804:M>I		N_43:M>I		N_45:M>I		UFRJ50791:M>I		UFRJ50816:M>I		UWOPS91_917_1:M>I		YPS138:M>I	AA:836		IFO1804:F>S		N_43:F>S		N_45:F>S	AA:848		A4:D>E		DBVPG6304:D>E		UFRJ50791:D>E		UFRJ50816:D>E		UWOPS91_917_1:D>E		YPS138:D>E	AA:854		A4:N>D		DBVPG6304:N>D		UFRJ50791:N>D		UFRJ50816:N>D		UWOPS91_917_1:N>D		YPS138:N>D	AA:856		UWOPS91_917_1:D>N	AA:858		UWOPS91_917_1:M>T	AA:860		UWOPS91_917_1:G>D	AA:863		A4:C>Y		DBVPG6304:C>Y		UFRJ50791:C>Y		UFRJ50816:C>Y		UWOPS91_917_1:C>Y		YPS138:C>Y	AA:881		A4:A>T		DBVPG6304:A>T		UFRJ50791:A>T		UFRJ50816:A>T		YPS138:A>T	AA:885		A4:A>T		DBVPG6304:A>T		IFO1804:A>T		N_43:A>T		N_45:A>T		UFRJ50791:A>T		UFRJ50816:A>T		UWOPS91_917_1:A>T		YPS138:A>T	AA:887		A4:L>S		DBVPG6304:L>S		UFRJ50791:L>S		UFRJ50816:L>S		UWOPS91_917_1:L>S	AA:895		A4:L>W		DBVPG6304:L>W		UFRJ50791:L>W		UFRJ50816:L>W		UWOPS91_917_1:L>WID:YBR034C	AA:6		N_43:R>T		N_45:R>T	AA:79		KPN3829:N>I	AA:98		IFO1804:I>M		N_43:I>M		N_45:I>M	AA:139		A12:F>L		A4:F>L		DBVPG6304:F>L		UFRJ50816:F>L		YPS138:F>L	AA:154		A12:W>C		A4:W>C		DBVPG6304:W>C		IFO1804:W>C		N_43:W>C		N_45:W>C	AA:181		A12:M>I		A4:M>I	AA:245		A12:E>D		A4:E>D		DBVPG6304:E>D		UFRJ50791:E>D		UFRJ50816:E>D		YPS138:E>D	AA:285		A12:N>K		A4:N>K		DBVPG6304:N>K		UFRJ50791:N>K		UFRJ50816:N>K		UWOPS91_917_1:N>K		YPS138:N>K	AA:324		UWOPS91_917_1:->L	AA:326		UWOPS91_917_1:E>D	AA:346		UWOPS91_917_1:C>WID:YBR035C	AA:50		T21_4:N>Y	AA:96		CBS432:F>L		CBS5829:F>L		KPN3829:F>L		N_17:F>L	AA:103		A4:M>I		DBVPG6304:M>I		UFRJ50816:M>I		UWOPS91_917_1:M>I		YPS138:M>I	AA:106		A4:D>E		DBVPG6304:D>E		UFRJ50816:D>E		UWOPS91_917_1:D>E		YPS138:D>E	AA:150		UWOPS91_917_1:E>D	AA:158		IFO1804:G>R		N_43:G>R		N_45:G>R	AA:176		A4:Q>H		DBVPG6304:Q>H		UFRJ50816:Q>H		UWOPS91_917_1:Q>HID:YBR037C	AA:12		A12:L>V		A4:L>V		DBVPG6304:L>V		YPS138:L>V	AA:24		A4:D>E		DBVPG6304:D>E		YPS138:D>E	AA:34		A4:L>P		YPS138:L>P	AA:38		UWOPS91_917_1:I>N	AA:54		KPN3829:E>-	AA:61		A12:Q>-	AA:115		A12:V>G		A4:V>G		DBVPG6304:V>G		UFRJ50816:V>G		YPS138:V>G	AA:168		A12:K>N		A4:K>N		DBVPG6304:K>N		UFRJ50816:K>N		YPS138:K>N	AA:178		A12:K>N		A4:K>N		DBVPG6304:K>N		UFRJ50816:K>N		UWOPS91_917_1:K>N		YPS138:K>N	AA:182		DBVPG6304:->K	AA:190		A12:V>D		DBVPG6304:V>D		UFRJ50816:V>D		UWOPS91_917_1:V>D	AA:193		A12:S>C		A4:S>C		DBVPG6304:S>C		UFRJ50816:S>C		UWOPS91_917_1:S>C		YPS138:S>C	AA:198		A12:L>V		A4:L>V		DBVPG6304:L>V		UFRJ50816:L>V	AA:251		A12:F>L		A4:F>L		DBVPG6304:F>L		UFRJ50816:F>L		YPS138:F>L	AA:293		UWOPS91_917_1:Q>E	AA:305		N_17:H>RID:YBR039W	AA:8		UWOPS91_917_1:S>N	AA:14		UFRJ50816:V>M	AA:30		UWOPS91_917_1:V>A	AA:174		UFRJ50816:L>Q		YPS138:L>QID:YBR040W	AA:17		UWOPS91_917_1:I>M	AA:74		UWOPS91_917_1:G>S	AA:76		Q32_3:E>Q		Y6_5:E>Q		Y7:E>Q	AA:95		UWOPS91_917_1:Q>R	AA:273		A4:E>K		DBVPG6304:E>K		YPS138:E>KID:YBR041W	AA:12		DBVPG6304:S>L		UWOPS91_917_1:S>L		YPS138:S>L	AA:18		DBVPG6304:F>L		UWOPS91_917_1:F>L		YPS138:F>L	AA:42		DBVPG6304:K>E		IFO1804:K>E		N_43:K>E		N_45:K>E		UWOPS91_917_1:K>E		YPS138:K>E	AA:103		UWOPS91_917_1:E>G	AA:119		A12:D>N		UFRJ50791:D>N		UWOPS91_917_1:D>N		YPS138:D>N	AA:123		IFO1804:R>K		N_43:R>K		N_44:R>K		N_45:R>K	AA:131		IFO1804:D>N		N_43:D>N		N_44:D>N		N_45:D>N	AA:212		UWOPS91_917_1:V>I	AA:233		IFO1804:E>K		N_43:E>K		N_44:E>K		N_45:E>K	AA:293		UWOPS91_917_1:V>A	AA:422		IFO1804:S>T		N_45:S>T	AA:436		A12:R>K		A4:R>K		DBVPG6304:R>K		UFRJ50816:R>K	AA:579		UWOPS91_917_1:N>K	AA:592		A12:D>N		UFRJ50816:D>N		UWOPS91_917_1:D>N	AA:595		A12:A>G		N_44:A>V		N_45:A>VID:YBR043C	AA:7		UWOPS91_917_1:Q>K	AA:21		A12:L>P		A4:L>P		KPN3828:L>P		N_17:L>P		Q32_3:L>P		Q89_8:L>P		Q95_3:L>P		T21_4:L>P		UFRJ50816:L>P		Y6_5:L>P		Y7:L>P	AA:47		A12:A>G		UWOPS91_917_1:A>G	AA:127		UWOPS91_917_1:Y>-	AA:151		IFO1804:F>L		N_43:F>L		N_44:F>L		N_45:F>L	AA:169		UWOPS91_917_1:Y>-	AA:183		A12:K>-		A4:K>-		UWOPS91_917_1:K>-	AA:271		A4:Q>-		UWOPS91_917_1:Q>-	AA:292		A12:C>-	AA:305		UWOPS91_917_1:F>V	AA:307		A12:Q>P		A4:Q>P		UWOPS91_917_1:Q>P	AA:309		A12:S>C		UWOPS91_917_1:S>C	AA:322		A12:P>T	AA:325		A12:T>N	AA:406		N_17:F>L	AA:410		A12:H>Q		UWOPS91_917_1:H>Q	AA:421		A12:N>K	AA:450		A12:W>-		A4:W>-	AA:490		UWOPS91_917_1:D>E	AA:501		A12:R>S		A4:R>S		YPS138:R>S	AA:505		A12:K>N		A4:K>N		UWOPS91_917_1:K>N		YPS138:K>N	AA:533		IFO1804:N>K		N_44:N>K	AA:547		A12:H>Q		A4:H>Q		DBVPG6304:H>Q		IFO1804:H>Q		N_43:H>Q		N_44:H>Q		N_45:H>Q		YPS138:H>Q	AA:551		A12:S>R		A4:S>R		DBVPG6304:S>R		UFRJ50816:S>R		UWOPS91_917_1:S>R		YPS138:S>R	AA:569		A12:H>Q		YPS138:H>Q	AA:576		A12:K>N		A4:K>N		DBVPG6304:K>N		UFRJ50816:K>N		YPS138:K>N	AA:577		IFO1804:C>W		N_43:C>W		N_44:C>W	AA:600		A12:N>K		A4:N>K		DBVPG6304:N>K		YPS138:N>K	AA:615		UWOPS91_917_1:N>K	AA:640		UWOPS91_917_1:F>L	AA:647		UWOPS91_917_1:I>M	AA:653		KPN3829:Q>-	AA:681		A4:Y>-		DBVPG6304:Y>-		IFO1804:Y>-		N_43:Y>-		N_44:Y>-		N_45:Y>-		UFRJ50816:Y>-		UWOPS91_917_1:Y>-		YPS138:Y>-	AA:688		IFO1804:S>G		N_43:S>G		N_44:S>G		N_45:S>GID:YBR045C	AA:17		A12:Y>-		A4:Y>-		DBVPG6304:Y>-		YPS138:Y>-	AA:29		A12:S>R		A4:S>R		DBVPG6304:S>R		N_43:S>R		N_45:S>R		YPS138:S>R	AA:33		N_43:->C	AA:50		A12:N>D		A4:N>D		DBVPG6304:N>D		YPS138:N>D	AA:58		A12:I>M		A4:I>M		DBVPG6304:I>M		YPS138:I>M	AA:121		A12:S>R		A4:S>R		DBVPG6304:S>R		UFRJ50816:S>R		YPS138:S>R	AA:139		A12:N>H		A4:N>H		DBVPG6304:N>H		UFRJ50816:N>H		YPS138:N>H	AA:176		CBS432:F>C	AA:202		N_43:M>I		N_45:M>I	AA:206		N_43:Q>H		N_45:Q>H	AA:222		UWOPS91_917_1:K>N	AA:227		A4:I>T		DBVPG6304:I>T	AA:277		A4:I>S		DBVPG6304:I>S		N_43:I>S		N_45:I>S	AA:280		UWOPS91_917_1:L>P	AA:282		UWOPS91_917_1:Q>H	AA:284		UWOPS91_917_1:I>M	AA:299		DBVPG6304:T>N		N_43:T>N		N_45:T>N	AA:307		UWOPS91_917_1:S>C	AA:308		Q62_5:Y>F	AA:328		N_43:Y>C		N_45:Y>C	AA:354		UWOPS91_917_1:->Y	AA:366		UWOPS91_917_1:L>F	AA:372		UWOPS91_917_1:N>K	AA:382		UWOPS91_917_1:M>V	AA:399		UWOPS91_917_1:R>S	AA:421		A4:K>N		DBVPG6304:K>N		YPS138:K>N	AA:424		DBVPG6304:I>S		N_43:I>S		N_44:I>S		N_45:I>S	AA:444		UWOPS91_917_1:F>L	AA:454		UWOPS91_917_1:R>S	AA:484		A4:R>C		DBVPG6304:R>C		YPS138:R>C	AA:494		UWOPS91_917_1:S>T	AA:514		A4:C>Y		DBVPG6304:C>Y		N_43:C>Y		N_44:C>Y		N_45:C>Y		UFRJ50816:C>Y		YPS138:C>Y	AA:519		A4:W>R		DBVPG6304:W>R		UFRJ50816:W>R		YPS138:W>R	AA:526		A4:M>I		DBVPG6304:M>I		UFRJ50816:M>I		YPS138:M>I	AA:531		A4:M>I		DBVPG6304:M>I		UFRJ50816:M>I		YPS138:M>I	AA:563		A4:E>D		DBVPG6304:E>D		N_43:E>D		N_44:E>D		N_45:E>D		UFRJ50816:E>D		YPS138:E>D	AA:593		N_43:R>K		N_44:R>K		N_45:R>K	AA:597		UFRJ50816:C>Y	AA:634		A4:F>L		DBVPG6304:F>L		UFRJ50816:F>L		UWOPS91_917_1:F>L		YPS138:F>LID:YBR046C	AA:38		A12:F>L	AA:81		IFO1804:W>-		N_45:W>-	AA:83		A12:D>V		A4:D>V		DBVPG6304:D>V	AA:96		A12:F>Y		A4:F>Y		DBVPG6304:F>Y		IFO1804:F>Y		N_44:F>Y		N_45:F>Y	AA:104		CBS432:N>D		CBS5829:N>D		KPN3829:N>D	AA:105		A12:F>L		A4:F>L		DBVPG6304:F>L	AA:121		IFO1804:T>S		N_43:T>S		N_44:T>S		N_45:T>S	AA:158		A12:C>W		UWOPS91_917_1:C>W	AA:189		DBVPG6304:F>L	AA:203		UWOPS91_917_1:N>K	AA:213		A12:Q>H		DBVPG6304:Q>H	AA:216		UWOPS91_917_1:I>M	AA:237		DBVPG6304:K>-		YPS138:K>-	AA:259		YPS138:C>F	AA:267		IFO1804:P>L		N_43:P>L	AA:278		DBVPG6304:K>N		YPS138:K>N	AA:291		IFO1804:C>R		N_43:C>R		N_45:C>R	AA:296		DBVPG6304:Y>-		N_43:Y>-		UWOPS91_917_1:Y>-		YPS138:Y>-	AA:314		DBVPG6304:N>Y		YPS138:N>Y	AA:330		DBVPG6304:W>C		IFO1804:W>C		N_43:W>C		UWOPS91_917_1:W>C		YPS138:W>C	AA:335		DBVPG6304:H>Q		YPS138:H>QID:YBR047W	AA:5		DBVPG6304:R>C	AA:7		UFRJ50816:S>N	AA:40		UWOPS91_917_1:T>S	AA:45		A4:A>V		UFRJ50816:A>V		YPS138:A>V	AA:69		A4:K>Q		UFRJ50816:K>Q		YPS138:K>Q	AA:98		IFO1804:V>L		N_43:V>L		N_45:V>L	AA:115		DBVPG6304:D>G		UFRJ50816:D>G	AA:137		UWOPS91_917_1:R>CID:YBR050C	AA:9		YPS138:S>R	AA:15		A4:N>K		DBVPG6304:N>K		UFRJ50816:N>K		UWOPS91_917_1:N>K		YPS138:N>K	AA:19		CBS432:R>L	AA:32		UWOPS91_917_1:I>L	AA:41		DBVPG6304:S>N		UFRJ50816:S>N		UWOPS91_917_1:S>N	AA:76		N_45:P>A	AA:107		IFO1804:I>V		N_45:I>V	AA:114		A4:C>G		UFRJ50816:C>G	AA:124		UWOPS91_917_1:S>R	AA:128		A4:S>N		IFO1804:S>N		N_45:S>N		UFRJ50816:S>N		UWOPS91_917_1:S>N	AA:201		UWOPS91_917_1:Y>N	AA:209		UWOPS91_917_1:E>K	AA:214		Q59_1:K>N		S36_7:K>N		T21_4:K>N		Y7:K>N	AA:215		A12:K>N		A4:K>N		UWOPS91_917_1:K>N	AA:231		UWOPS91_917_1:Q>K	AA:238		N_43:Y>N		N_45:Y>N		UWOPS91_917_1:Y>N	AA:244		UWOPS91_917_1:S>A	AA:245		DBVPG4650:L>I		Q59_1:L>I		S36_7:L>I		T21_4:L>I	AA:247		UWOPS91_917_1:L>F	AA:264		UWOPS91_917_1:E>D	AA:274		IFO1804:A>T		N_43:A>T		N_44:A>T		N_45:A>T	AA:284		A12:L>P	AA:294		UWOPS91_917_1:F>L	AA:302		UWOPS91_917_1:G>S	AA:318		A12:S>R		IFO1804:S>R		N_43:S>R		N_45:S>R		UWOPS91_917_1:S>R	AA:329		CBS432:->Y		KPN3828:->Y	AA:337		UWOPS91_917_1:F>C	AA:341		UWOPS91_917_1:L>FID:YBR052C	AA:3		T21_4:I>V		Y6_5:I>V	AA:14		UWOPS91_917_1:K>M	AA:18		A12:F>L		DBVPG6304:F>L		UFRJ50791:F>L		UFRJ50816:Q>R		UWOPS91_917_1:Q>R		YPS138:F>L	AA:47		A12:S>T		DBVPG6304:S>T		UFRJ50791:S>T		UFRJ50816:S>T		UWOPS91_917_1:S>T		YPS138:S>T	AA:54		A12:C>F		DBVPG6304:C>F		UFRJ50791:C>F		UFRJ50816:C>F		YPS138:C>F	AA:69		YPS138:->Y	AA:70		IFO1804:S>C		N_44:S>C		N_45:S>C	AA:75		A12:H>Q		DBVPG6304:H>Q		UFRJ50791:H>Q		UFRJ50816:H>Q		UWOPS91_917_1:H>Q		YPS138:H>Q	AA:92		UWOPS91_917_1:Q>H	AA:115		IFO1804:F>L		N_45:F>L	AA:147		UFRJ50816:K>N	AA:175		A12:L>F		DBVPG6304:L>F		UFRJ50816:L>F		YPS138:L>F	AA:179		A12:S>G		DBVPG6304:S>G		N_43:S>G		N_44:S>G		N_45:S>G		UFRJ50816:S>G		UWOPS91_917_1:S>G		YPS138:S>G	AA:182		A12:T>N		DBVPG6304:T>N		UWOPS91_917_1:T>N		YPS138:T>N	AA:208		CBS432:N>K		CBS5829:N>K		DBVPG4650:N>K		N_43:N>K		N_44:N>K		N_45:N>K		Q32_3:N>K		Q59_1:N>K		UWOPS91_917_1:N>K		Y6_5:N>K		Y7:N>KID:YBR053C	AA:11		UFRJ50791:Q>P		UFRJ50816:Q>P	AA:37		N_43:V>G	AA:59		UWOPS91_917_1:S>T	AA:60		KPN3828:W>R		Q89_8:W>-		T21_4:W>-	AA:66		A12:K>T		A4:K>T		UFRJ50791:K>T		UFRJ50816:K>T		UWOPS91_917_1:K>T	AA:70		N_43:Q>K	AA:101		IFO1804:K>I		N_43:K>I	AA:106		A12:S>T		A4:S>T		IFO1804:S>T		N_43:S>T		UFRJ50791:S>T		UWOPS91_917_1:S>T	AA:129		A12:E>D		A4:E>D		UFRJ50791:E>D		UWOPS91_917_1:E>D	AA:132		IFO1804:A>P		N_43:A>P	AA:139		A12:->Y		A4:->Y		DBVPG6304:->Y		UFRJ50791:->Y		UWOPS91_917_1:S>R		YPS138:->Y	AA:185		A12:R>W		A4:R>W		UFRJ50791:R>W		UWOPS91_917_1:R>W		YPS138:R>W	AA:188		IFO1804:F>I		N_43:F>I		N_45:F>I	AA:190		IFO1804:W>G		N_43:W>G		N_45:W>G	AA:201		DBVPG4650:F>L	AA:207		A12:V>D		A4:V>D		UFRJ50791:V>D		YPS138:V>D	AA:238		CBS432:K>N		CBS5829:K>N		N_17:K>N		Q89_8:K>N		T21_4:K>N	AA:246		A4:F>L		IFO1804:F>L		N_43:F>L		N_45:F>L		UWOPS91_917_1:F>L		YPS138:F>L	AA:262		IFO1804:E>G		N_43:E>G		N_44:E>G		N_45:E>G	AA:269		A12:H>Y		A4:H>Y		DBVPG6304:H>Y		UFRJ50816:H>Y		UWOPS91_917_1:Q>K		YPS138:H>Y	AA:273		UWOPS91_917_1:->Y	AA:283		A12:V>D		A4:V>D		DBVPG6304:W>C		UFRJ50791:V>D		UFRJ50816:V>D		UWOPS91_917_1:V>D	AA:296		A12:K>N		A4:K>N		DBVPG6304:K>N		UFRJ50816:K>N		YPS138:K>N	AA:312		A4:L>F		DBVPG6304:L>F		IFO1804:L>F		N_43:L>F		N_44:L>F		N_45:L>F		UWOPS91_917_1:L>F		YPS138:L>F	AA:321		A12:Q>H		A4:Q>H		DBVPG6304:Q>H		UFRJ50816:Q>H		YPS138:Q>H	AA:329		A12:G>S		UFRJ50791:G>S		UFRJ50816:G>S	AA:331		N_43:S>A		N_44:S>A		N_45:S>A	AA:339		N_43:K>N		N_44:K>N		N_45:K>N	AA:352		A12:F>L		A4:F>L		DBVPG6304:F>L		UFRJ50791:F>L		UFRJ50816:F>L		UWOPS91_917_1:F>L		YPS138:F>LID:YBR054W	AA:104		UWOPS91_917_1:K>R	AA:255		UWOPS91_917_1:L>F	AA:327		A12:K>EID:YBR055C	AA:7		A12:Q>R		A4:Q>R		DBVPG6304:Q>R		N_44:M>L		N_45:M>L		UFRJ50816:Q>R		UWOPS91_917_1:Q>R		YPS138:Q>R	AA:13		A12:R>K		A4:R>K		DBVPG6304:R>K		UFRJ50816:R>K	AA:28		A12:F>L		CBS432:F>L		CBS5829:F>L		DBVPG6304:F>L		IFO1804:F>L		KPN3828:F>L		KPN3829:F>L		N_44:F>L		N_45:F>L		Q32_3:F>L		Q95_3:F>L		UFRJ50816:F>L		Y7:F>L	AA:55		A12:K>Q		A4:K>Q		DBVPG6304:K>Q		IFO1804:K>Q		N_44:K>Q		N_45:K>Q		UFRJ50816:K>Q	AA:62		UWOPS91_917_1:P>R	AA:65		UWOPS91_917_1:->C	AA:79		UWOPS91_917_1:L>M	AA:88		IFO1804:Y>-		N_44:Y>-		N_45:Y>-	AA:105		A4:I>M		DBVPG6304:I>M		UFRJ50791:I>M		UFRJ50816:I>M	AA:117		IFO1804:P>H		N_44:P>H		N_45:P>H	AA:124		A4:L>F		DBVPG6304:L>F		UFRJ50791:L>F		UFRJ50816:L>F		UWOPS91_917_1:L>F		YPS138:L>F	AA:128		CBS432:K>N		CBS5829:K>N		KPN3828:K>N		KPN3829:K>N		Q32_3:K>N		Q95_3:K>N	AA:129		UWOPS91_917_1:->Y	AA:135		UWOPS91_917_1:L>H	AA:153		N_43:Y>D		UWOPS91_917_1:Y>-	AA:182		A4:W>-		DBVPG6304:W>-		UFRJ50791:W>-		UFRJ50816:W>-		YPS138:W>-	AA:183		KPN3828:I>S		KPN3829:I>S	AA:185		A4:->L		DBVPG6304:->L		UFRJ50791:->L		UFRJ50816:->L		YPS138:->L	AA:197		A4:Y>D		DBVPG6304:Y>D		IFO1804:Y>D		N_43:Y>D		N_44:Y>D		N_45:Y>D		UFRJ50791:Y>D		UFRJ50816:Y>D		UWOPS91_917_1:Y>D		YPS138:Y>D	AA:209		A4:->Y		DBVPG6304:->Y		UFRJ50791:->Y		UFRJ50816:->Y		YPS138:->Y	AA:212		A4:L>M		DBVPG6304:L>M		IFO1804:L>M		UFRJ50791:L>M		UFRJ50816:L>M		UWOPS91_917_1:L>M		YPS138:L>M	AA:216		IFO1804:F>L		N_43:F>L		N_44:F>L		N_45:F>L	AA:220		A4:N>K		DBVPG6304:N>K		UFRJ50791:N>K		UFRJ50816:N>K		UWOPS91_917_1:N>K		YPS138:N>K	AA:260		UWOPS91_917_1:I>M	AA:265		N_17:I>V	AA:295		UWOPS91_917_1:M>T	AA:309		UWOPS91_917_1:M>I	AA:319		N_43:C>-		N_44:C>-		N_45:Q>-	AA:352		A12:Y>D		A4:Y>D		DBVPG6304:Y>D		UFRJ50816:Y>D		UWOPS91_917_1:Y>D		YPS138:Y>D	AA:354		A12:Y>D		A4:Y>D		DBVPG6304:Y>D		IFO1804:V>F		N_43:V>F		N_44:V>F		N_45:V>F		UFRJ50791:Y>D		UFRJ50816:Y>D	AA:365		A12:E>D		A4:E>D		DBVPG6304:E>D		UFRJ50816:E>D		YPS138:E>D	AA:387		A12:S>C		A4:S>C		DBVPG6304:S>C		UFRJ50791:S>C		UFRJ50816:S>C	AA:391		N_43:R>W		N_44:R>W		N_45:R>W		UWOPS91_917_1:R>W	AA:394		A12:N>K		A4:N>K		DBVPG6304:N>K		UFRJ50816:N>K		UWOPS91_917_1:L>P	AA:400		A12:Y>D		A4:Y>D		DBVPG6304:Y>D		N_43:Y>D		N_44:Y>D		N_45:Y>D		UFRJ50816:Y>D		UWOPS91_917_1:Y>D	AA:406		A12:->W		A4:->W		DBVPG6304:->W		UFRJ50816:->W		UWOPS91_917_1:->W	AA:494		N_44:E>G	AA:512		A12:I>V		A4:I>V		DBVPG6304:I>V		UFRJ50791:I>V		UFRJ50816:I>V	AA:519		A12:M>I		A4:M>I		DBVPG6304:M>I		UFRJ50791:M>I		UFRJ50816:M>I	AA:520		Q32_3:I>V	AA:527		A12:Y>C		A4:Y>C		DBVPG6304:Y>C		UFRJ50791:Y>C		UFRJ50816:Y>C	AA:539		N_17:C>-	AA:544		A12:H>Q		A4:H>Q		DBVPG6304:H>Q		UFRJ50791:H>Q		UFRJ50816:H>Q		UWOPS91_917_1:H>Q	AA:551		A12:V>D	AA:577		A12:I>L		A4:I>L		DBVPG6304:I>L		UFRJ50791:I>L		UFRJ50816:I>L		YPS138:I>L	AA:580		A12:N>Y		DBVPG6304:N>Y		UFRJ50791:N>Y		UFRJ50816:N>Y		UWOPS91_917_1:N>Y		YPS138:N>Y	AA:585		UFRJ50791:Q>H		UFRJ50816:Q>H	AA:598		N_43:I>T		N_44:I>T	AA:601		A12:Y>-		A4:Y>-		DBVPG6304:Y>-		UFRJ50791:Y>-		UWOPS91_917_1:Y>-		YPS138:Y>-	AA:603		UWOPS91_917_1:C>W	AA:613		A12:I>L	AA:615		A12:L>H		A4:L>H		DBVPG6304:L>H		UFRJ50791:L>H		YPS138:L>H	AA:625		A12:V>G		A4:V>G		DBVPG6304:V>G		UFRJ50791:V>G		YPS138:V>G	AA:656		N_43:Y>S		N_45:Y>S	AA:659		A12:L>Q		A4:L>Q		DBVPG6304:L>Q		UFRJ50791:L>Q		UFRJ50816:L>Q		YPS138:L>Q	AA:671		Q59_1:->S	AA:672		A12:N>K		A4:N>K		DBVPG6304:N>K		UFRJ50791:N>K		UFRJ50816:N>K		YPS138:N>K	AA:674		UWOPS91_917_1:A>T	AA:687		A12:R>S		A4:R>S		DBVPG6304:R>S		UFRJ50791:R>S		UFRJ50816:R>S		UWOPS91_917_1:R>S		YPS138:R>S	AA:698		Y7:V>E	AA:706		A12:I>N		A4:I>N		DBVPG6304:I>N		UFRJ50791:I>N		UFRJ50816:I>N		UWOPS91_917_1:I>N		YPS138:I>N	AA:721		A12:L>-		A4:L>-		DBVPG6304:L>-		UFRJ50791:L>-		UFRJ50816:L>-		YPS138:L>-	AA:726		A12:I>L		A4:I>L		DBVPG6304:I>L		UFRJ50791:I>L		UFRJ50816:I>L		YPS138:I>L	AA:728		UWOPS91_917_1:L>-	AA:735		N_17:S>T	AA:750		A12:->W		A4:->W		DBVPG6304:->W		UFRJ50791:->W		UFRJ50816:->W		UWOPS91_917_1:->C		YPS138:->W	AA:753		A12:C>G		A4:C>G		DBVPG6304:C>G		UFRJ50791:C>G		UFRJ50816:C>G		UWOPS91_917_1:C>G		YPS138:C>G	AA:755		N_43:I>M		N_45:I>M	AA:774		A12:I>S		A4:I>S		DBVPG6304:I>S		N_43:I>S		N_45:I>S		UFRJ50791:I>S		UFRJ50816:I>S		YPS138:I>S	AA:799		UFRJ50791:L>P		UFRJ50816:L>P	AA:826		A12:F>L		A4:F>L		DBVPG6304:F>L		N_43:F>L		N_45:F>L		UFRJ50791:F>L		UFRJ50816:F>L		UWOPS91_917_1:F>L	AA:877		UWOPS91_917_1:S>R	AA:878		A12:S>T		A4:S>T		DBVPG6304:S>T		UFRJ50816:S>T		YPS138:S>TID:YBR056W	AA:24		A12:A>T		A4:A>T		DBVPG6304:A>T		UFRJ50791:A>T		UWOPS91_917_1:A>T	AA:39		IFO1804:T>A		N_43:T>A		N_44:T>A		N_45:T>A	AA:71		A12:G>D		DBVPG6304:G>D		UFRJ50791:G>D	AA:77		A12:S>N		DBVPG6304:S>N		UFRJ50791:S>N	AA:81		IFO1804:A>T	AA:93		UFRJ50816:E>D	AA:107		A12:N>D		DBVPG6304:N>D		UFRJ50791:N>D	AA:160		UFRJ50816:A>G		UWOPS91_917_1:A>G	AA:198		IFO1804:K>E		N_43:K>E		N_44:K>E		N_45:K>E	AA:207		UFRJ50791:T>	AA:211		A12:K>R		DBVPG6304:K>R	AA:221		A12:M>V		DBVPG6304:M>V		UWOPS91_917_1:M>V	AA:222		UFRJ50791:T>	AA:259		A12:E>K		DBVPG6304:E>K		UWOPS91_917_1:E>K	AA:285		A12:D>N		DBVPG6304:D>N	AA:287		UFRJ50816:G>S	AA:326		A12:D>N	AA:414		CBS432:R>K		CBS5829:R>K		DBVPG6304:R>K		N_17:R>K		N_43:R>K		N_44:R>K		Q32_3:R>K		Q62_5:R>K		Q95_3:R>K		S36_7:R>K		T21_4:R>K		UFRJ50791:R>K		UFRJ50816:R>K		UWOPS91_917_1:R>K		Y6_5:R>K		Y7:R>K	AA:430		UWOPS91_917_1:G>RID:YBR057C	AA:26		UWOPS91_917_1:F>L	AA:33		UWOPS91_917_1:Q>K	AA:86		A4:->Y		DBVPG6304:->Y		YPS138:->Y	AA:111		A4:R>S		DBVPG6304:R>S		UWOPS91_917_1:R>S	AA:116		A4:K>N		DBVPG6304:K>N		UFRJ50791:K>N	AA:127		N_17:S>I	AA:151		N_45:N>I	AA:152		UFRJ50791:F>V		UFRJ50816:F>V	AA:182		A4:F>V		DBVPG6304:F>V		UFRJ50791:F>V		UFRJ50816:F>V	AA:233		A12:I>M		DBVPG6304:I>M		IFO1804:I>M		N_43:I>M		N_44:I>M		N_45:I>M		UFRJ50816:I>M		UWOPS91_917_1:I>M	AA:239		UWOPS91_917_1:C>Y	AA:250		A12:I>T		DBVPG6304:I>T		YPS138:I>T	AA:357		A12:L>M		DBVPG6304:L>M		N_43:L>M		N_44:L>M		N_45:L>M		UFRJ50791:L>M		UFRJ50816:L>M		YPS138:L>M	AA:363		A12:S>G		DBVPG6304:S>G		UFRJ50791:S>G		UFRJ50816:S>G		UWOPS91_917_1:S>G		YPS138:S>GID:YBR058C-A	AA:21		DBVPG6304:N>S		UFRJ50791:N>S		UFRJ50816:N>S		UWOPS91_917_1:N>S	AA:25		DBVPG6304:K>N	AA:47		UWOPS91_917_1:D>E	AA:69		DBVPG6304:G>R		UFRJ50791:G>R		UFRJ50816:G>R	AA:73		IFO1804:H>Q		N_43:H>Q		N_44:H>Q		N_45:H>QID:YBR060C	AA:4		A12:I>M		A4:I>M		UFRJ50791:I>M		UFRJ50816:I>M		UWOPS91_917_1:I>M		YPS138:I>M	AA:35		A12:H>N		A4:H>N		UFRJ50791:H>N		UFRJ50816:H>N		UWOPS91_917_1:H>N		YPS138:H>N	AA:39		A12:D>V		A4:D>V		CBS432:D>V		CBS5829:D>V		KPN3829:D>V		N_44:D>V		N_45:D>V		UFRJ50791:D>V		UFRJ50816:D>V		UWOPS91_917_1:D>V		YPS138:D>V	AA:84		A12:I>M		A4:I>M		UFRJ50791:I>M		UFRJ50816:I>M		UWOPS91_917_1:I>M		YPS138:I>M	AA:89		N_44:H>R		N_45:H>R	AA:91		A12:C>-		YPS138:C>-	AA:103		A4:D>G	AA:123		A12:T>S		A4:T>S		UFRJ50791:T>S		UFRJ50816:T>S		UWOPS91_917_1:R>S		YPS138:T>S	AA:127		A12:Y>F		A4:Y>F		UFRJ50791:Y>F		UFRJ50816:Y>F		YPS138:Y>F	AA:136		CBS432:->C		CBS5829:->C		N_17:->C		N_44:->C		N_45:->C	AA:143		UWOPS91_917_1:I>F	AA:180		N_43:A>S		N_44:A>S		N_45:A>S	AA:182		A12:H>Q		A4:H>Q		UFRJ50791:H>Q		UFRJ50816:H>Q		YPS138:H>Q	AA:206		A12:->Y		A4:->Y		UFRJ50791:->Y		UWOPS91_917_1:->Y		YPS138:->Y	AA:217		A12:L>M		A4:L>M		DBVPG6304:L>M		UFRJ50791:L>M		YPS138:L>M	AA:245		A12:F>L		A4:F>L		DBVPG6304:F>L		N_43:F>L		N_44:F>L		N_45:F>L		UFRJ50791:F>L		UWOPS91_917_1:F>L		YPS138:F>L	AA:260		UWOPS91_917_1:Y>-	AA:348		A4:K>N		DBVPG6304:K>N		UFRJ50791:K>N		UWOPS91_917_1:K>N	AA:355		A4:R>T		DBVPG6304:R>T		UFRJ50791:R>T		YPS138:R>T	AA:384		UWOPS91_917_1:->C	AA:402		A4:R>W		DBVPG6304:R>W		UFRJ50791:R>W		UWOPS91_917_1:R>W		YPS138:R>W	AA:419		A4:I>N		DBVPG6304:I>N		IFO1804:I>N		N_43:I>N		N_45:I>N		UFRJ50791:I>N		YPS138:I>N	AA:443		A4:R>S		DBVPG6304:R>S		UFRJ50791:R>S		YPS138:R>S	AA:446		UWOPS91_917_1:->R	AA:534		A12:->W		A4:->W		DBVPG6304:->W		IFO1804:->W		N_44:->W		N_45:->W		UFRJ50816:->W		UWOPS91_917_1:->W		YPS138:->W	AA:541		A4:W>C		DBVPG6304:W>C		IFO1804:W>C		N_44:W>C		N_45:W>C		UFRJ50816:W>C		UWOPS91_917_1:W>C		YPS138:W>C	AA:547		UWOPS91_917_1:R>S	AA:552		A4:C>-		DBVPG6304:C>-		UFRJ50816:C>-	AA:556		A12:E>D		A4:E>D		DBVPG6304:E>D		UFRJ50816:E>D		UWOPS91_917_1:E>D		YPS138:E>D	AA:598		DBVPG4650:S>I	AA:607		A4:->G		DBVPG6304:->G		IFO1804:->G		N_43:->G		N_44:->G		N_45:->G	AA:615		A4:K>N		DBVPG6304:K>N		UWOPS91_917_1:K>NID:YBR061C	AA:11		IFO1804:F>L		N_43:F>L		N_44:F>L		N_45:F>L	AA:20		CBS5829:R>S		KPN3829:R>S	AA:27		DBVPG6304:R>S		UWOPS91_917_1:R>S	AA:35		IFO1804:R>S		N_44:R>S		N_45:R>S	AA:62		IFO1804:V>G	AA:70		N_45:R>I		UFRJ50791:R>S	AA:92		UWOPS91_917_1:I>F	AA:110		UWOPS91_917_1:N>Y	AA:112		A12:S>C		DBVPG6304:S>C		UFRJ50791:S>C	AA:132		A12:I>L		DBVPG6304:I>L		UFRJ50791:I>L		UFRJ50816:I>L	AA:143		A12:T>S		DBVPG6304:T>S		UFRJ50791:T>S		UFRJ50816:T>S		UWOPS91_917_1:T>S	AA:155		UWOPS91_917_1:F>L	AA:167		IFO1804:H>Q		N_44:H>Q		N_45:H>Q	AA:238		IFO1804:F>L		N_43:F>L		N_44:F>L		N_45:F>L	AA:243		A12:T>N		DBVPG6304:T>N		IFO1804:T>N		N_43:T>N		N_44:T>N		N_45:T>N		UFRJ50791:T>N		UFRJ50816:T>N		UWOPS91_917_1:T>N		YPS138:T>N	AA:258		IFO1804:W>C		N_43:W>C		N_44:W>C		N_45:W>C	AA:276		UWOPS91_917_1:K>N	AA:284		UWOPS91_917_1:F>IID:YBR065C	AA:8		N_43:P>L		N_45:P>L	AA:48		UWOPS91_917_1:L>H	AA:129		A12:->W		DBVPG6304:->W		N_43:->W		N_44:->W		N_45:->W		UFRJ50816:->W		UWOPS91_917_1:->W		YPS138:->W	AA:147		A12:Y>-		DBVPG6304:Y>-		UFRJ50816:Y>-		UWOPS91_917_1:Y>H		YPS138:Y>-	AA:151		N_43:R>S		N_44:R>S		N_45:R>S	AA:157		N_43:I>M		N_44:I>M		N_45:I>M	AA:172		A12:F>L		DBVPG6304:F>L		IFO1804:F>L		N_43:F>L		N_44:F>L		N_45:F>L		UFRJ50816:F>L		YPS138:F>L	AA:177		A12:F>I		DBVPG6304:F>I		UFRJ50816:F>I		YPS138:F>I	AA:180		A12:N>T		DBVPG6304:N>T		IFO1804:N>T		N_43:N>T		N_44:N>T		N_45:N>T		UFRJ50816:N>T		YPS138:N>T	AA:201		IFO1804:V>G		N_43:V>G		N_44:V>G		N_45:V>G	AA:205		A12:C>-		UFRJ50816:C>-		YPS138:C>-	AA:213		A12:W>-		YPS138:W>-	AA:221		A12:C>S		A4:C>S		IFO1804:C>S		N_43:C>S		N_44:C>S		N_45:C>S		UFRJ50791:C>S		UFRJ50816:C>S		YPS138:C>S	AA:243		A12:L>F		A4:L>F		UFRJ50791:L>F		UFRJ50816:L>F		YPS138:L>F	AA:252		A12:->Q		A4:->Q		IFO1804:->Q		N_43:->Q		N_44:->Q		N_45:->Q		UFRJ50791:->Q		UFRJ50816:->Q		YPS138:->Q	AA:257		CBS432:F>I		CBS5829:F>I		KPN3829:F>I		N_17:F>I	AA:274		A12:D>E		A4:D>E		UFRJ50791:D>E		UFRJ50816:D>E		YPS138:D>E	AA:278		UFRJ50816:M>I	AA:291		A12:K>N		A4:K>N		DBVPG6304:K>N		UFRJ50791:K>N		UFRJ50816:K>N		YPS138:K>N	AA:318		N_45:->C	AA:320		IFO1804:F>L		N_44:F>L		N_45:F>L	AA:345		A12:I>L		A4:I>L		DBVPG6304:I>L		UFRJ50791:I>L		UFRJ50816:I>L		YPS138:I>L	AA:363		A12:F>L		A4:F>L		DBVPG6304:F>L		UFRJ50791:F>L		UFRJ50816:F>L		YPS138:F>LID:YBR066C	AA:2		Q32_3:T>I		Q89_8:T>I	AA:25		A12:V>D		A4:V>D		DBVPG6304:V>D		UFRJ50791:V>D		UFRJ50816:V>D		UWOPS91_917_1:V>D	AA:43		A12:F>Y		A4:F>Y		DBVPG6304:F>Y		UFRJ50791:F>Y		UFRJ50816:F>Y		UWOPS91_917_1:F>Y	AA:63		UWOPS91_917_1:R>S	AA:84		A12:F>C		A4:F>C		DBVPG6304:F>C		UFRJ50791:F>C		UFRJ50816:F>C		UWOPS91_917_1:F>C	AA:86		A12:H>P		A4:H>P		DBVPG6304:H>P		UFRJ50791:H>P		UFRJ50816:H>P	AA:88		DBVPG6304:L>H	AA:90		UWOPS91_917_1:R>P	AA:97		IFO1804:A>D		N_43:A>D		N_44:A>D		N_45:A>D	AA:100		A12:I>M		A4:I>M		DBVPG6304:I>M		UFRJ50791:I>M		UFRJ50816:I>M		UWOPS91_917_1:I>M	AA:115		UWOPS91_917_1:I>S	AA:117		A12:Q>K		A4:Q>K		DBVPG6304:Q>K		UFRJ50816:Q>K	AA:118		IFO1804:W>-		N_43:W>-		N_44:W>-		N_45:W>-	AA:123		UWOPS91_917_1:C>W	AA:130		A12:A>V		A4:A>V		DBVPG6304:A>V		UFRJ50816:A>V		UWOPS91_917_1:A>V	AA:133		A12:S>R		A4:S>R		DBVPG6304:S>R		IFO1804:C>R		N_43:C>R		N_44:C>R		N_45:C>R		UFRJ50816:S>R		UWOPS91_917_1:S>R	AA:185		UWOPS91_917_1:W>C	AA:206		A12:F>L		A4:F>L		DBVPG6304:F>L		UFRJ50816:F>L		UWOPS91_917_1:F>L	AA:218		A12:T>A		A4:T>A		DBVPG6304:T>A		UFRJ50816:T>A		UWOPS91_917_1:T>AID:YBR067C	AA:5		N_17:S>R	AA:29		A12:S>R		A4:S>R		DBVPG6304:S>R		UFRJ50791:S>R		UFRJ50816:S>R		UWOPS91_917_1:S>R	AA:83		UFRJ50816:W>C		UWOPS91_917_1:W>C	AA:86		A12:R>S		A4:R>S		DBVPG6304:R>S		UFRJ50791:R>S		UFRJ50816:R>S	AA:89		UFRJ50816:W>-		UWOPS91_917_1:W>-	AA:102		UFRJ50816:R>S		UWOPS91_917_1:R>S	AA:106		A12:S>G		A4:S>G		DBVPG6304:S>G		UFRJ50791:S>G		UWOPS91_917_1:K>N	AA:124		DBVPG4650:N>D		Q59_1:N>D		Q95_3:N>D		T21_4:N>D		Y6_5:N>D		Z1_1:N>D	AA:134		A12:F>V		UFRJ50791:F>V	AA:136		DBVPG4650:E>K		Q59_1:E>K		Q95_3:E>K		T21_4:E>K		Z1_1:E>K	AA:150		UFRJ50816:L>F		UWOPS91_917_1:L>F	AA:154		UFRJ50816:Q>H		UWOPS91_917_1:Q>H	AA:161		IFO1804:K>-		UFRJ50816:W>C	AA:175		A12:M>I		A4:M>I		DBVPG6304:M>I		IFO1804:M>I		N_44:M>I		N_45:M>I		UFRJ50791:M>I	AA:189		UFRJ50816:L>F		UWOPS91_917_1:L>F	AA:205		Q62_5:N>I		Z1_1:N>IID:YBR068C	AA:10		A12:P>S		DBVPG6304:P>S		N_43:P>S		N_45:P>S		UFRJ50791:P>S		UWOPS91_917_1:P>S	AA:15		A12:P>A		DBVPG6304:P>A		UFRJ50791:P>A		UWOPS91_917_1:P>A	AA:30		DBVPG6304:W>C		UWOPS91_917_1:W>C	AA:36		A12:V>E	AA:45		A12:G>R		DBVPG6304:G>R		UFRJ50791:G>R	AA:46		Q62_5:I>V	AA:49		Q62_5:G>R		UWOPS91_917_1:F>L	AA:63		N_43:Q>E		N_45:Q>E	AA:78		DBVPG6304:I>L		UFRJ50791:I>L		UWOPS91_917_1:I>L	AA:128		UWOPS91_917_1:H>Q	AA:177		A12:Y>-		DBVPG6304:Y>-		N_45:Y>-		UFRJ50791:Y>-		UWOPS91_917_1:Y>-	AA:189		A12:N>K		DBVPG6304:N>K		UFRJ50791:N>K		UWOPS91_917_1:N>K	AA:210		UWOPS91_917_1:G>S	AA:213		A12:R>S		DBVPG6304:R>S		UFRJ50791:R>S	AA:234		A12:F>L		DBVPG6304:F>L		UFRJ50791:F>L		UFRJ50816:F>L	AA:244		A12:V>L		DBVPG6304:V>L		UFRJ50791:V>L		UFRJ50816:V>L		UWOPS91_917_1:V>L		YPS138:V>L	AA:254		A12:C>S		DBVPG6304:C>S		IFO1804:C>S		N_43:C>S		N_45:C>S		UFRJ50791:C>S		UFRJ50816:C>S		UWOPS91_917_1:C>S		YPS138:C>S	AA:258		A12:L>F		DBVPG6304:L>F		UFRJ50791:L>F		UFRJ50816:L>F		YPS138:L>F	AA:303		A12:C>W		DBVPG6304:C>W		UFRJ50791:C>W		UFRJ50816:C>W		YPS138:C>W	AA:336		Q32_3:W>C		Q89_8:W>C		Q95_3:W>C		T21_4:W>C		Y6_5:W>C	AA:358		DBVPG6304:R>S		IFO1804:R>S		N_43:R>S		N_45:R>S		UFRJ50791:R>S		UFRJ50816:R>S		UWOPS91_917_1:R>S		YPS138:R>S	AA:362		IFO1804:E>D		N_43:E>D		N_45:E>D	AA:366		A12:H>Q		DBVPG6304:H>Q		UFRJ50816:H>Q		YPS138:H>Q	AA:375		A12:E>D		DBVPG6304:E>D		UFRJ50791:E>D		UFRJ50816:E>D		UWOPS91_917_1:E>D		YPS138:E>D	AA:410		A12:L>V		A4:L>V		DBVPG6304:L>V		IFO1804:L>V		N_43:L>V		N_45:L>V		UFRJ50791:L>V		UFRJ50816:L>V		UWOPS91_917_1:L>V		YPS138:L>V	AA:412		UFRJ50791:S>C		UFRJ50816:S>C	AA:436		A12:E>D		A4:E>D		DBVPG6304:E>D		UFRJ50791:E>D		UFRJ50816:E>D		YPS138:E>D	AA:484		UWOPS91_917_1:S>R	AA:487		A12:T>N		A4:T>N		DBVPG6304:T>N		UFRJ50791:T>N		UFRJ50816:T>N		UWOPS91_917_1:T>N		YPS138:T>N	AA:489		A12:M>R		A4:M>R		DBVPG6304:M>R		UFRJ50791:M>R		UFRJ50816:M>R		YPS138:M>R	AA:493		A12:C>-		A4:C>-		DBVPG6304:C>-		UFRJ50791:C>-		UFRJ50816:C>-		YPS138:C>-	AA:496		UWOPS91_917_1:H>Q	AA:505		UWOPS91_917_1:P>T	AA:508		UWOPS91_917_1:H>D	AA:526		UWOPS91_917_1:D>E	AA:602		IFO1804:R>K		N_44:R>K		N_45:R>KID:YBR069C	AA:7		IFO1804:H>N		N_43:H>N		N_44:H>N		N_45:H>N	AA:10		A12:E>D		DBVPG6304:E>D		IFO1804:E>D		N_17:E>D		N_43:E>D		N_44:E>D		N_45:E>D		UFRJ50791:E>D		UFRJ50816:E>D		YPS138:E>D	AA:17		DBVPG6304:A>T		UFRJ50791:A>T		UFRJ50816:A>T		YPS138:A>T	AA:20		A12:I>N		UFRJ50791:I>N		UFRJ50816:I>N		YPS138:I>N	AA:90		A12:K>R		UFRJ50816:K>R		YPS138:K>R	AA:92		A12:F>C		A4:F>C		UFRJ50816:F>C		YPS138:F>C	AA:118		A12:->Y		A4:->Y		UFRJ50816:->Y		YPS138:->Y	AA:164		A12:K>N		A4:K>N		UFRJ50816:K>N		YPS138:K>N	AA:211		A12:R>S		A4:R>S		YPS138:R>S	AA:236		A12:D>E		A4:D>E		YPS138:D>E	AA:243		UWOPS91_917_1:I>M	AA:275		UWOPS91_917_1:F>L	AA:329		IFO1804:E>-		N_45:E>-	AA:345		IFO1804:K>N		N_45:K>N	AA:400		UWOPS91_917_1:D>E	AA:412		UFRJ50816:D>E	AA:444		UWOPS91_917_1:R>S	AA:454		A12:Q>-		A4:Q>-		UFRJ50816:Q>-		YPS138:Q>-	AA:457		A12:R>S		A4:R>S		UFRJ50816:R>S		UWOPS91_917_1:R>S		YPS138:R>S	AA:522		A12:Q>H		A4:Q>H		DBVPG6304:Q>H		IFO1804:Q>H		N_43:Q>H		N_44:Q>H		UFRJ50791:Q>H		UWOPS91_917_1:Q>H		YPS138:Q>H	AA:540		UFRJ50816:F>L	AA:554		A12:K>N		A4:K>N		DBVPG6304:K>N		UWOPS91_917_1:K>N		YPS138:K>N	AA:555		A12:G>-		UFRJ50816:G>-		YPS138:G>-	AA:557		UFRJ50816:F>L	AA:565		IFO1804:F>L		N_43:F>L		N_44:F>L		N_45:F>L	AA:580		CBS432:I>S		CBS5829:I>S	AA:582		UWOPS91_917_1:L>R	AA:585		Q59_1:Y>D	AA:589		CBS432:S>A		CBS5829:S>A		DBVPG4650:S>A		N_17:S>A		N_45:S>A		Q32_3:S>A		Q59_1:S>A		Q89_8:S>A		Q95_3:S>A		T21_4:S>A		UWOPS91_917_1:S>A		Y6_5:S>A		Y7:S>AID:YBR070C	AA:2		A12:N>K		A4:N>K		DBVPG6304:N>K		UFRJ50816:N>K		UWOPS91_917_1:N>K		YPS138:N>K	AA:7		A12:P>L		A4:P>L		DBVPG6304:P>L		UFRJ50816:P>L		YPS138:P>L	AA:14		A12:I>F		A4:I>F		DBVPG6304:I>F		N_43:I>F		N_45:I>F		UFRJ50816:I>F		UWOPS91_917_1:I>F		YPS138:I>F	AA:23		UWOPS91_917_1:N>D	AA:25		UWOPS91_917_1:F>L	AA:26		A12:I>M		A4:I>M		DBVPG6304:I>M		UFRJ50816:I>M		YPS138:I>M	AA:44		N_43:->E		N_45:->E	AA:115		CBS5829:F>Y		N_17:F>Y		N_43:F>Y		N_45:F>Y		Q59_1:F>Y		Q89_8:F>Y		Q95_3:F>Y		T21_4:F>Y		Y6_5:F>Y		Y7:F>Y		Z1_1:F>Y	AA:172		UWOPS91_917_1:S>R	AA:180		UWOPS91_917_1:Y>H	AA:197		A12:S>R		A4:S>R		DBVPG6304:S>R	AA:203		A12:L>F		A4:L>F		DBVPG6304:L>F	AA:227		A4:N>K		DBVPG6304:N>K		UWOPS91_917_1:N>K		YPS138:N>K	AA:230		A12:G>A		A4:G>A		DBVPG6304:G>A		UFRJ50816:G>A		UWOPS91_917_1:G>A		YPS138:G>A	AA:233		DBVPG6304:Q>HID:YBR071W	AA:26		A12:S>G		A4:S>G		DBVPG6304:S>G		UFRJ50816:S>G	AA:36		IFO1804:S>G		N_43:S>G		N_44:S>G		N_45:S>G		UWOPS91_917_1:S>G	AA:46		A12:K>E		A4:K>E		DBVPG6304:K>E		UWOPS91_917_1:K>E	AA:50		A12:I>T		A4:I>T		DBVPG6304:I>T		UWOPS91_917_1:I>T	AA:66		IFO1804:T>S		N_43:T>S		N_44:T>S		N_45:T>S		UWOPS91_917_1:T>S	AA:79		UWOPS91_917_1:E>K	AA:138		A12:S>T		DBVPG6304:S>T		UFRJ50816:S>T	AA:157		UWOPS91_917_1:F>L	AA:180		IFO1804:P>S		N_43:P>S		N_45:P>SID:YBR072W	AA:16		IFO1804:S>N		N_45:S>N		UFRJ50816:S>N		UWOPS91_917_1:S>N		YPS138:S>N	AA:48		A4:T>A		UWOPS91_917_1:T>A		YPS138:T>A	AA:90		UWOPS91_917_1:N>S	AA:102		UWOPS91_917_1:K>NID:YBR073W	AA:20		UFRJ50816:S>N	AA:23		A4:A>T		DBVPG6304:A>T		UFRJ50816:A>T		UWOPS91_917_1:A>T		YPS138:A>T	AA:25		UWOPS91_917_1:L>F	AA:30		A4:S>T		DBVPG6304:S>T		UFRJ50816:S>T		UWOPS91_917_1:S>T		YPS138:S>T	AA:37		N_43:I>V		N_44:I>V		N_45:I>V	AA:40		UWOPS91_917_1:A>T	AA:42		A4:V>A		DBVPG6304:V>A		UFRJ50816:V>A		UWOPS91_917_1:V>A		YPS138:V>A	AA:50		UWOPS91_917_1:I>T	AA:51		A4:S>Y		DBVPG6304:S>Y		UFRJ50816:S>Y		YPS138:S>Y	AA:54		A4:T>A		DBVPG6304:T>A		N_43:T>I		N_44:T>I		N_45:T>I		UFRJ50816:T>A		YPS138:T>A	AA:56		A4:L>F		DBVPG6304:L>F		UFRJ50816:L>F		YPS138:L>F	AA:62		DBVPG6304:P>R	AA:70		N_43:A>G		N_44:A>G		N_45:A>G	AA:101		UWOPS91_917_1:S>G	AA:102		UFRJ50816:S>N	AA:105		A4:G>S		DBVPG6304:G>S		UFRJ50816:G>S		UWOPS91_917_1:G>S		YPS138:G>S	AA:113		A4:L>F		DBVPG6304:L>F		N_43:L>F		N_44:L>F		N_45:L>F		UFRJ50816:L>F		YPS138:L>F	AA:122		UWOPS91_917_1:D>N	AA:126		UWOPS91_917_1:L>F	AA:129		Q32_3:T>I	AA:133		A4:A>V		UFRJ50816:A>V		UWOPS91_917_1:A>V		YPS138:A>V	AA:147		N_43:S>T		N_44:S>T		N_45:S>T	AA:155		A4:K>N		DBVPG6304:K>N		UFRJ50816:K>N		YPS138:K>N	AA:164		UWOPS91_917_1:T>I	AA:166		N_43:S>P		N_44:S>P		N_45:S>P	AA:167		A4:I>V		DBVPG6304:I>V		UFRJ50816:I>V		UWOPS91_917_1:I>V		YPS138:I>V	AA:169		A4:T>A		DBVPG6304:T>A		UFRJ50816:T>A		UWOPS91_917_1:T>A	AA:171		N_43:A>P		N_44:A>P		N_45:A>P	AA:187		Q59_1:M>I	AA:209		A4:S>N		DBVPG6304:S>N		UFRJ50816:S>N		UWOPS91_917_1:S>N	AA:213		Q59_1:T>S		Q95_3:T>S		UWOPS91_917_1:T>S		Y6_5:T>S		Z1_1:T>S	AA:217		UWOPS91_917_1:K>I	AA:260		DBVPG6304:K>R		UFRJ50816:K>R	AA:261		UWOPS91_917_1:F>L	AA:284		DBVPG6304:R>G		IFO1804:R>G		N_43:R>G		N_44:R>G		N_45:R>G		UFRJ50816:R>G		UWOPS91_917_1:R>G	AA:287		DBVPG6304:N>K		N_44:N>K		UFRJ50816:N>K	AA:387		DBVPG6304:S>P		UFRJ50816:S>P		UWOPS91_917_1:S>P	AA:413		UWOPS91_917_1:I>V	AA:447		IFO1804:T>A		N_43:T>A		N_44:T>A		N_45:T>A	AA:520		IFO1804:K>E		N_44:K>E		N_45:K>E	AA:562		DBVPG6304:S>G		UFRJ50816:S>G	AA:716		IFO1804:V>I		N_44:V>I		N_45:V>I	AA:747		DBVPG6304:K>R		YPS138:K>R	AA:822		A12:E>D		DBVPG6304:E>D		YPS138:E>D	AA:833		A12:T>A		DBVPG6304:T>A		YPS138:T>A	AA:837		N_44:Q>K		N_45:Q>K	AA:841		A12:E>K		DBVPG6304:E>K		YPS138:E>K	AA:859		T21_4:E>V	AA:869		A12:A>V		DBVPG6304:A>V		YPS138:A>VID:YBR077C	AA:83		A12:->C		DBVPG6304:->C		UFRJ50816:->C		UWOPS91_917_1:->C	AA:88		N_43:N>K		N_44:N>K		N_45:N>K	AA:93		N_43:H>N		N_44:H>N		N_45:H>N	AA:104		A12:Y>-		DBVPG6304:Y>-		UWOPS91_917_1:Y>-	AA:107		N_17:F>V	AA:130		A12:->W		DBVPG6304:->W		UFRJ50816:->W		UWOPS91_917_1:->W		YPS138:->W	AA:150		A12:F>L		YPS138:F>L	AA:159		A12:M>I		DBVPG6304:M>I		UFRJ50816:M>I		UWOPS91_917_1:M>I		YPS138:M>IID:YBR082C	AA:30		A12:->Y		A4:->Y		DBVPG6304:->Y		UFRJ50816:->Y		UWOPS91_917_1:->Y		YPS138:->Y	AA:34		A12:R>S		A4:R>S		DBVPG6304:R>S		UFRJ50816:R>S		UWOPS91_917_1:R>S		YPS138:R>S	AA:66		UFRJ50816:I>F	AA:67		A12:T>P		A4:T>P		DBVPG6304:T>P		YPS138:T>P	AA:74		A12:M>I		A4:M>I		DBVPG6304:M>I		UFRJ50816:M>I		YPS138:M>I	AA:76		A12:Y>-		A4:Y>-		DBVPG6304:Y>-		UFRJ50816:Y>-		UWOPS91_917_1:Y>-		YPS138:Y>-	AA:93		A12:E>D		A4:E>D		DBVPG6304:E>D		UFRJ50816:E>D		YPS138:E>DID:YBR084W	AA:31		N_43:Y>H		N_44:Y>H		N_45:Y>H	AA:93		N_44:D>N	AA:100		KPN3828:I>T		KPN3829:I>T	AA:115		A12:K>R		UFRJ50816:K>R		UWOPS91_917_1:K>R	AA:127		A12:A>G		CBS432:A>G		DBVPG4650:A>G		KPN3828:A>G		KPN3829:A>G		N_17:A>G		N_44:A>G		N_45:A>G		Q32_3:A>G		Q62_5:A>G		T21_4:A>G		UFRJ50816:A>G		Y6_5:A>G		Y7:A>G		Z1_1:A>G	AA:139		UWOPS91_917_1:D>N	AA:146		KPN3829:A>S	AA:179		A12:Y>F		UFRJ50816:Y>F	AA:254		A12:D>N		UFRJ50816:D>N		UWOPS91_917_1:D>N		YPS138:D>N	AA:277		UWOPS91_917_1:S>T	AA:293		UWOPS91_917_1:K>M	AA:325		A12:M>V		A4:M>V		DBVPG6304:M>V		UFRJ50816:M>V		UWOPS91_917_1:M>V		YPS138:M>V	AA:332		A12:V>I		A4:V>I		DBVPG6304:V>I		UFRJ50816:V>I		YPS138:V>I	AA:341		A4:K>E	AA:622		A12:S>A		IFO1804:S>A		N_43:S>A		N_45:S>A		UFRJ50816:S>A		UWOPS91_917_1:S>A		YPS138:S>A	AA:697		A12:G>D		UFRJ50816:G>D		UWOPS91_917_1:G>D		YPS138:G>D	AA:700		N_43:V>I		N_45:V>I	AA:730		N_43:G>E		N_45:G>E	AA:732		A12:I>V		UWOPS91_917_1:I>V		YPS138:I>V	AA:762		T21_4:K>E	AA:773		A12:A>T		YPS138:A>T	AA:777		A12:N>S		UWOPS91_917_1:N>S		YPS138:N>S	AA:817		CBS432:T>N		CBS5829:T>N		DBVPG4650:T>N		KPN3829:T>N		N_17:T>N		N_43:T>N		N_45:T>N		Q95_3:T>N		S36_7:T>N		Y7:T>NID:YBR085C-A	AA:82		UWOPS91_917_1:->YID:YBR085W	AA:10		UWOPS91_917_1:T>A	AA:258		A12:T>S		A4:T>S		YPS138:T>S	AA:262		KPN3829:K>R	AA:269		Q62_5:G>VID:YBR087W	AA:194		A12:C>S		A4:C>S		DBVPG6304:C>S		N_43:C>S		N_44:C>S		N_45:C>S		UFRJ50791:C>S		YPS138:C>S	AA:198		N_45:I>V	AA:260		A12:I>F		A4:I>F		UFRJ50791:I>F		UFRJ50816:I>F		UWOPS91_917_1:I>F		YPS138:I>F	AA:316		A12:M>V		A4:M>V		N_43:M>V		N_44:M>V		N_45:M>V		UFRJ50816:M>V		UWOPS91_917_1:M>V		YPS138:M>VID:YBR088C	AA:95		A12:F>L		DBVPG6304:F>L		UFRJ50791:F>L		UFRJ50816:F>L		YPS138:F>L	AA:101		A12:I>M		DBVPG6304:I>M		UFRJ50791:I>M		UFRJ50816:I>M		UWOPS91_917_1:I>M	AA:115		N_17:R>S	AA:116		A12:E>Q		A4:E>Q		DBVPG6304:E>Q		UFRJ50791:E>Q		UFRJ50816:E>Q		UWOPS91_917_1:E>Q		YPS138:E>Q	AA:137		UWOPS91_917_1:S>R	AA:168		A4:S>R		DBVPG6304:S>R		UFRJ50791:S>R		UFRJ50816:S>R		UWOPS91_917_1:S>R		YPS138:S>R	AA:212		A12:->Y		A4:D>E		DBVPG6304:->Y		UFRJ50791:->Y		UFRJ50816:D>E		YPS138:D>E	AA:257		A12:F>L		A4:F>L		N_43:F>L		N_44:F>L		N_45:F>L		UFRJ50816:F>L		UWOPS91_917_1:F>L		YPS138:F>LID:YBR089C-A	AA:7		UWOPS91_917_1:I>L	AA:10		A12:L>P		A4:L>P		DBVPG6304:L>P		UWOPS91_917_1:L>P	AA:16		UWOPS91_917_1:L>H	AA:32		A12:S>R		A4:S>R		DBVPG6304:S>R		UWOPS91_917_1:S>R	AA:51		A12:R>S		A4:R>S		DBVPG6304:R>SID:YBR091C	AA:12		A4:H>Q		UFRJ50816:H>Q	AA:17		A4:M>I		UFRJ50816:M>I		UWOPS91_917_1:M>I	AA:100		IFO1804:V>I		N_43:V>I		N_45:V>I	AA:107		IFO1804:K>I		N_43:K>I		N_45:K>I		UWOPS91_917_1:K>NID:YBR092C	AA:12		UWOPS91_917_1:G>V	AA:27		A4:->Y		DBVPG6304:->Y		IFO1804:->Y		N_43:->Y		N_44:->Y		N_45:->Y		UWOPS91_917_1:I>F	AA:69		UWOPS91_917_1:I>L	AA:85		A4:L>F		DBVPG6304:L>F	AA:163		N_43:I>S		N_44:I>S	AA:168		A4:N>K		DBVPG6304:N>K		UFRJ50816:N>K	AA:178		DBVPG6304:Q>H		IFO1804:Q>H		N_43:Q>H		N_44:Q>H		N_45:Q>H		UWOPS91_917_1:Q>H	AA:187		DBVPG6304:D>E		UFRJ50816:D>E		UWOPS91_917_1:D>E	AA:211		DBVPG6304:->Y		UFRJ50816:->Y		UWOPS91_917_1:->Y		YPS138:->Y	AA:225		DBVPG6304:I>N		N_43:I>N		N_44:I>N		N_45:I>N		UFRJ50816:I>N		YPS138:I>N	AA:242		UWOPS91_917_1:F>L	AA:261		UWOPS91_917_1:S>C	AA:271		A4:E>D		DBVPG6304:E>D		UWOPS91_917_1:E>D		YPS138:E>D	AA:285		UWOPS91_917_1:M>I	AA:290		Y7:T>P	AA:314		A4:M>I		DBVPG6304:M>I		UWOPS91_917_1:M>I		YPS138:M>I	AA:326		A4:Q>H		UWOPS91_917_1:Q>H		YPS138:Q>H	AA:387		UWOPS91_917_1:M>I	AA:401		A4:R>S		CBS432:R>S		CBS5829:R>S		DBVPG4650:R>S		KPN3829:R>S		N_17:R>S		S36_7:R>S		T21_4:R>S		Y6_5:R>S		Y7:R>S		YPS138:R>S	AA:424		CBS432:E>D		CBS5829:E>D		DBVPG4650:E>D		KPN3829:E>D		N_17:E>D		S36_7:E>D		T21_4:E>D		Y6_5:E>D		Y7:E>D	AA:440		CBS432:E>D		CBS5829:E>D		DBVPG4650:E>D		KPN3829:E>D		N_17:E>D		T21_4:E>D		Y6_5:E>D		Y7:E>DID:YBR093C	AA:22		A12:S>R		DBVPG6304:S>R	AA:54		UWOPS91_917_1:R>S	AA:62		UWOPS91_917_1:G>R	AA:69		DBVPG6304:I>L		UWOPS91_917_1:I>F	AA:114		UWOPS91_917_1:V>I	AA:119		DBVPG6304:Y>D		IFO1804:Y>D		N_44:Y>D		N_45:Y>D		UWOPS91_917_1:Y>D	AA:128		KPN3828:N>K		KPN3829:N>K	AA:134		UWOPS91_917_1:E>K	AA:185		UWOPS91_917_1:->Y	AA:222		IFO1804:L>P		N_45:L>P	AA:228		UFRJ50816:S>T	AA:234		UWOPS91_917_1:Q>H	AA:242		UWOPS91_917_1:H>Q	AA:253		Q32_3:W>L		Q59_1:W>L		Q62_5:W>L	AA:260		IFO1804:->Y		N_45:->Y		UWOPS91_917_1:->Y	AA:267		UWOPS91_917_1:L>V	AA:294		UFRJ50816:S>P		UWOPS91_917_1:S>P		YPS138:S>P	AA:314		UFRJ50816:L>F		UWOPS91_917_1:L>F		YPS138:L>F	AA:364		UFRJ50816:->C		UWOPS91_917_1:->C		YPS138:->C	AA:387		UWOPS91_917_1:N>K	AA:432		S36_7:N>H	AA:457		UWOPS91_917_1:S>IID:YBR094W	AA:49		DBVPG6304:G>S		UFRJ50816:G>S		YPS138:G>S	AA:53		UWOPS91_917_1:L>F	AA:70		UWOPS91_917_1:D>E	AA:102		DBVPG6304:T>A		N_43:T>A		N_44:T>A		N_45:T>A		UFRJ50816:T>A		YPS138:T>A	AA:166		DBVPG6304:A>S		UFRJ50816:A>S		YPS138:A>S	AA:191		DBVPG6304:K>R		N_43:K>R		N_45:K>R		UFRJ50816:K>R		UWOPS91_917_1:K>R		YPS138:K>R	AA:223		A4:N>D		CBS432:N>D		CBS5829:N>D		DBVPG4650:N>D		DBVPG6304:N>D		IFO1804:N>D		N_43:N>D		N_45:N>D		Q59_1:N>D		Q89_8:N>D		UFRJ50816:N>D		UWOPS91_917_1:N>D		Y6_5:N>D		YPS138:N>D	AA:250		UWOPS91_917_1:N>K	AA:252		A4:A>S		UFRJ50816:A>S		YPS138:A>S	AA:273		IFO1804:A>V		N_44:A>V		N_45:A>V	AA:289		A4:I>V		UFRJ50816:I>V		YPS138:I>V	AA:299		UWOPS91_917_1:E>K	AA:306		A4:L>M		IFO1804:L>M		N_44:L>M		N_45:L>M		UFRJ50816:L>M		UWOPS91_917_1:L>M		YPS138:L>M	AA:328		A4:N>S		UFRJ50816:N>S		UWOPS91_917_1:N>S		YPS138:N>S	AA:366		A4:N>S		UFRJ50816:N>S		YPS138:N>S	AA:377		A4:K>E		DBVPG6304:K>E		UFRJ50816:K>E		UWOPS91_917_1:K>E		YPS138:K>E	AA:512		A4:A>G		DBVPG6304:A>G		UFRJ50816:A>G		UWOPS91_917_1:A>G		YPS138:A>G	AA:523		A4:D>N		DBVPG6304:D>N		UFRJ50816:D>N		YPS138:D>N	AA:524		UWOPS91_917_1:V>L	AA:529		UWOPS91_917_1:M>I	AA:555		A4:P>Q		DBVPG6304:P>Q		UFRJ50816:P>Q		UWOPS91_917_1:P>Q		YPS138:P>Q	AA:587		Q32_3:A>S		Q59_1:A>S	AA:593		A4:P>S		DBVPG6304:P>S		YPS138:P>S	AA:597		A4:H>Y		DBVPG6304:H>Y		N_43:H>Y		N_44:H>Y		N_45:H>Y		UFRJ50816:H>Y		UWOPS91_917_1:H>Y		YPS138:H>Y	AA:683		IFO1804:I>V		N_43:I>V	AA:720		UWOPS91_917_1:K>-ID:YBR095C	AA:8		UWOPS91_917_1:I>V	AA:10		UWOPS91_917_1:F>Y	AA:42		UWOPS91_917_1:H>R	AA:50		DBVPG6304:S>R		UFRJ50791:S>R		UWOPS91_917_1:S>R	AA:87		IFO1804:E>D	AA:98		A12:Q>H		DBVPG6304:Q>H		UFRJ50791:Q>H		UWOPS91_917_1:Q>H	AA:109		A12:L>F		DBVPG6304:L>F		UFRJ50791:L>F		UWOPS91_917_1:L>F		YPS138:L>F	AA:128		UWOPS91_917_1:->Y	AA:131		UWOPS91_917_1:->Q	AA:148		UWOPS91_917_1:F>C	AA:158		A12:V>I		DBVPG6304:V>I		UFRJ50791:V>I		UWOPS91_917_1:V>I		YPS138:V>I	AA:205		A12:F>L		A4:F>L		DBVPG6304:F>L		UFRJ50791:F>L		YPS138:F>L	AA:235		N_44:N>I		N_45:N>I	AA:277		A12:->Y		A4:->Y		DBVPG6304:->Y		YPS138:->Y	AA:280		A12:F>S		A4:F>S		DBVPG6304:F>S		YPS138:F>S	AA:309		A4:R>S		DBVPG6304:R>S		YPS138:R>S	AA:321		N_43:F>L		N_44:F>L		N_45:F>L	AA:327		N_43:H>Q	AA:362		A4:F>L		DBVPG6304:F>L		YPS138:F>L	AA:375		A4:G>C		DBVPG6304:G>C		IFO1804:G>C		N_43:G>C		N_44:G>C		N_45:G>C		YPS138:G>C	AA:388		A4:F>L		DBVPG6304:F>L		YPS138:F>L	AA:400		IFO1804:Y>-		N_43:Y>-		N_44:Y>-		N_45:Y>-	AA:411		A4:F>L		DBVPG6304:F>L		YPS138:F>LID:YBR096W	AA:5		N_44:T>A		N_45:T>A	AA:7		DBVPG6304:V>F		IFO1804:V>F		N_44:V>F		N_45:V>F		UFRJ50816:V>F	AA:55		A4:Q>E		DBVPG6304:Q>E		IFO1804:Q>E		N_43:Q>E		N_44:Q>E		N_45:Q>E		UFRJ50816:Q>E	AA:159		A4:K>R		DBVPG6304:K>R		UFRJ50816:K>R		UWOPS91_917_1:K>R		YPS138:K>R	AA:195		A4:N>K		DBVPG6304:N>K		IFO1804:N>K		N_43:N>K		N_44:N>K		N_45:N>K		UFRJ50816:N>K		UWOPS91_917_1:N>K		YPS138:N>KID:YBR097W	AA:131		N_44:L>I	AA:194		A4:S>T		DBVPG6304:S>T		IFO1804:S>T		N_43:S>T		N_44:S>T		N_45:S>T		UFRJ50791:S>T		YPS138:S>T	AA:216		UWOPS91_917_1:K>R	AA:240		A4:T>I		DBVPG6304:T>I		UWOPS91_917_1:T>I		YPS138:T>I	AA:258		A4:R>K		DBVPG6304:R>K		UFRJ50791:R>K		UWOPS91_917_1:R>K		YPS138:R>K	AA:263		A4:E>D		DBVPG6304:E>D		UFRJ50791:E>D		UWOPS91_917_1:E>D		YPS138:E>D	AA:268		DBVPG6304:T>S	AA:327		N_43:N>S	AA:348		A12:I>T	AA:352		A12:F>L		DBVPG6304:F>L		YPS138:F>L	AA:368		A12:G>D		DBVPG6304:G>D		YPS138:G>D	AA:371		A12:D>G		DBVPG6304:D>G		YPS138:D>G	AA:373		A12:A>D		DBVPG6304:A>D		YPS138:A>D	AA:398		N_43:Q>K	AA:409		UWOPS91_917_1:Q>K	AA:433		UWOPS91_917_1:L>I	AA:500		N_44:P>Q		N_45:P>Q	AA:525		UWOPS91_917_1:S>T	AA:565		A12:E>D		A4:E>D		DBVPG6304:E>D		UFRJ50791:E>D		UFRJ50816:E>D		YPS138:E>D	AA:575		A12:T>K		UWOPS91_917_1:T>A	AA:583		UWOPS91_917_1:D>E	AA:592		A12:S>N		A4:S>N		DBVPG6304:S>N		UFRJ50791:S>N		UFRJ50816:S>N		YPS138:S>N	AA:802		N_43:I>V		N_44:I>V		N_45:I>V	AA:808		A4:N>D		DBVPG6304:N>D		UFRJ50791:N>D		YPS138:N>D	AA:819		A4:K>R		DBVPG6304:K>R		N_43:K>R		N_44:K>R		N_45:K>R		UFRJ50791:K>R		UWOPS91_917_1:K>R		YPS138:K>R	AA:844		N_45:I>T	AA:863		A4:T>I		DBVPG6304:T>I		UFRJ50791:T>I		UWOPS91_917_1:T>I		YPS138:T>I	AA:875		UWOPS91_917_1:S>G	AA:905		A4:L>P		DBVPG6304:L>P		N_45:L>P		UWOPS91_917_1:L>P		YPS138:L>P	AA:930		N_45:S>T	AA:935		A4:Y>S		CBS432:Y>S		CBS5829:Y>S		DBVPG4650:Y>S		DBVPG6304:Y>S		KPN3828:Y>S		N_17:Y>S		N_45:Y>S		Q59_1:Y>S		Q62_5:Y>S		Q89_8:Y>S		Q95_3:Y>S		UWOPS91_917_1:Y>S		Y6_5:Y>S		YPS138:Y>S		Z1_1:Y>S	AA:1005		N_43:N>S		N_45:N>S	AA:1015		A4:R>K		DBVPG6304:R>K		UWOPS91_917_1:R>K		YPS138:R>K	AA:1030		N_43:V>I		N_45:V>I	AA:1064		UWOPS91_917_1:V>M	AA:1066		A4:S>N		DBVPG6304:S>N		UWOPS91_917_1:S>N		YPS138:S>N	AA:1068		UWOPS91_917_1:K>R	AA:1072		A4:V>I		DBVPG6304:V>I		N_43:V>I		N_45:V>I		UFRJ50791:V>I		UWOPS91_917_1:V>I		YPS138:V>I	AA:1109		A4:S>N		DBVPG6304:S>N		IFO1804:S>N		N_43:S>N		N_45:S>N		UFRJ50791:S>N		UWOPS91_917_1:S>N		YPS138:S>N	AA:1115		UWOPS91_917_1:M>V	AA:1128		A4:S>F		DBVPG6304:S>F		YPS138:S>F	AA:1138		UFRJ50791:P>L	AA:1141		UWOPS91_917_1:D>E	AA:1143		UFRJ50791:F>L	AA:1148		UFRJ50791:K>I	AA:1160		A4:C>Y		DBVPG6304:C>Y		UFRJ50791:C>S		UWOPS91_917_1:C>Y		YPS138:C>Y	AA:1161		KPN3828:Q>H	AA:1164		UWOPS91_917_1:N>S	AA:1188		N_44:A>T	AA:1211		A4:I>V		DBVPG6304:I>V		UWOPS91_917_1:I>V	AA:1230		UWOPS91_917_1:K>R	AA:1237		IFO1804:L>I		N_43:L>I		N_44:L>I		N_45:L>I	AA:1288		A4:Q>K		DBVPG6304:Q>K		UFRJ50791:Q>K		YPS138:Q>K	AA:1313		DBVPG6304:Q>L		UFRJ50791:Q>L		YPS138:Q>L	AA:1315		UFRJ50791:A>V	AA:1343		DBVPG6304:I>T		UFRJ50791:I>T		YPS138:I>T	AA:1356		UFRJ50791:N>S		YPS138:N>S	AA:1362		N_43:T>A	AA:1395		IFO1804:R>P		N_43:R>P		N_44:R>P		UFRJ50791:R>P		YPS138:R>P	AA:1422		N_45:Y>N	AA:1443		DBVPG6304:A>V		UFRJ50791:A>V		YPS138:A>VID:YBR098W	AA:39		A12:Q>L		A4:Q>L		DBVPG6304:Q>L		UFRJ50816:Q>L		YPS138:Q>L	AA:51		A12:D>N		DBVPG6304:D>N		UFRJ50816:D>N		YPS138:D>N	AA:59		A12:G>E		A4:G>E		DBVPG6304:G>E		UFRJ50791:G>E		UFRJ50816:G>E		YPS138:G>E	AA:66		A12:N>D		A4:N>D		DBVPG6304:N>D		UFRJ50816:N>D		YPS138:N>D	AA:70		A12:I>V		A4:I>V		DBVPG6304:I>V		UFRJ50816:I>V		YPS138:I>V	AA:84		Q59_1:Q>R	AA:103		A12:A>G		A4:A>G		DBVPG6304:A>G		UFRJ50791:A>G		UFRJ50816:A>G		YPS138:A>G	AA:111		A12:D>G		A4:D>G		DBVPG6304:D>G		UFRJ50791:D>G		UFRJ50816:D>G		YPS138:D>G	AA:114		N_44:G>R		N_45:G>R	AA:133		KPN3829:E>D	AA:142		A12:H>N		A4:H>N		DBVPG6304:H>N		UFRJ50791:H>N		UFRJ50816:H>N		YPS138:H>N	AA:158		N_44:F>S		N_45:F>S	AA:163		A12:P>H		A4:P>H		UFRJ50791:P>H		UFRJ50816:P>H		YPS138:P>H	AA:212		A12:K>E		A4:K>E		UFRJ50791:K>E		UFRJ50816:K>E		YPS138:K>E	AA:224		A12:L>V		A4:L>V		UFRJ50791:L>V		UFRJ50816:L>V		YPS138:L>V	AA:235		IFO1804:S>N		N_44:S>N		N_45:S>N	AA:246		A12:G>R		A4:G>R		IFO1804:G>R		N_44:G>R		N_45:G>R		UFRJ50791:G>R		UFRJ50816:G>R		YPS138:G>R	AA:249		A12:E>K		A4:E>K		UFRJ50791:E>K		UFRJ50816:E>K		YPS138:E>K	AA:260		A12:N>K		A4:N>K		UFRJ50791:N>K		UFRJ50816:N>K		YPS138:N>K	AA:288		A12:R>G		A4:R>G		YPS138:R>G	AA:307		A12:D>E		A4:D>E		DBVPG6304:D>E		UFRJ50791:D>E		UFRJ50816:D>E		YPS138:D>E	AA:339		IFO1804:S>T		N_43:S>T		N_45:S>T	AA:415		A4:M>V		UFRJ50816:M>V		YPS138:M>V	AA:423		A4:A>E		DBVPG6304:A>E		UFRJ50816:A>E		YPS138:A>E	AA:433		A4:D>E		UFRJ50816:D>E		YPS138:D>E	AA:447		DBVPG6304:I>V	AA:467		A4:I>V		DBVPG6304:I>V		UFRJ50816:I>V	AA:517		A12:F>S		A4:F>S		DBVPG6304:F>S		UFRJ50816:F>S	AA:556		N_43:K>T		N_44:K>T	AA:671		UWOPS91_917_1:T>N	AA:673		N_43:T>A		N_44:T>A		N_45:T>AID:YBR099C	AA:24		A12:F>L		A4:F>L		DBVPG6304:F>L		UFRJ50816:F>L	AA:44		N_43:S>G		N_44:S>G	AA:63		A12:R>S		A4:R>S		DBVPG6304:R>S		UFRJ50816:R>SID:YBR101C	AA:3		DBVPG6304:Y>-		N_44:Y>-		N_45:Y>-		UWOPS91_917_1:Y>-	AA:22		UWOPS91_917_1:M>I	AA:29		DBVPG6304:F>L		UWOPS91_917_1:F>L	AA:41		DBVPG6304:R>S	AA:76		DBVPG6304:H>Q	AA:87		DBVPG6304:->R		UWOPS91_917_1:->R	AA:89		UWOPS91_917_1:I>V	AA:98		UWOPS91_917_1:H>Q	AA:100		UWOPS91_917_1:Q>H	AA:136		UWOPS91_917_1:->Y	AA:139		DBVPG6304:K>N		N_43:K>N		N_44:K>N		N_45:K>N		UFRJ50816:K>N	AA:155		UWOPS91_917_1:F>L	AA:157		UWOPS91_917_1:K>N	AA:173		N_43:H>Q		N_44:H>Q		N_45:H>Q	AA:176		UWOPS91_917_1:L>F	AA:199		DBVPG6304:I>F		N_43:I>F		N_44:I>F		N_45:I>F		UFRJ50791:I>F		UFRJ50816:I>F		YPS138:I>F	AA:201		CBS432:->S		DBVPG4650:->S		DBVPG6304:N>K		KPN3828:->S		N_17:->S		N_43:->S		N_44:->S		N_45:->S		Q32_3:->S		Q59_1:->S		UWOPS91_917_1:N>K	AA:223		DBVPG6304:E>D		UFRJ50791:E>D		UFRJ50816:E>D		UWOPS91_917_1:E>K		YPS138:E>D	AA:243		DBVPG6304:H>Y		UFRJ50791:H>Y		UFRJ50816:H>Y		YPS138:H>Y	AA:250		UWOPS91_917_1:W>G	AA:268		DBVPG6304:L>F		UFRJ50791:L>F		UFRJ50816:L>F		YPS138:L>F	AA:275		UWOPS91_917_1:F>L	AA:277		N_43:V>D		N_45:V>DID:YBR104W	AA:4		UWOPS91_917_1:E>D	AA:15		UWOPS91_917_1:E>Q	AA:22		IFO1804:T>A		N_44:T>A		N_45:T>A	AA:23		A12:S>T		DBVPG6304:S>T		UWOPS91_917_1:S>P		YPS138:S>T	AA:84		DBVPG4650:V>I	AA:127		N_43:M>L		N_44:M>L		N_45:M>L	AA:128		UWOPS91_917_1:S>G	AA:130		A4:Q>R		DBVPG6304:Q>R		UFRJ50791:Q>R		UWOPS91_917_1:Q>R		YPS138:Q>R	AA:314		Q59_1:A>GID:YBR105C	AA:11		A12:L>R		A4:L>R		DBVPG6304:L>R		YPS138:L>R	AA:16		A12:Q>E		A4:Q>E		DBVPG6304:Q>E		YPS138:Q>E	AA:18		A12:W>L		A4:W>L		DBVPG6304:W>L		UWOPS91_917_1:W>L		YPS138:W>L	AA:106		A12:K>R		A4:K>R		DBVPG6304:K>R		UFRJ50816:K>R		YPS138:K>R	AA:153		A4:C>G		DBVPG6304:C>G		UFRJ50816:C>G		YPS138:C>G	AA:167		A4:G>R		DBVPG6304:G>R		UFRJ50816:G>R		UWOPS91_917_1:G>R		YPS138:G>R	AA:202		A4:Q>H		DBVPG6304:Q>H		UFRJ50816:Q>H		UWOPS91_917_1:Q>H		YPS138:Q>H	AA:211		Q32_3:F>I		Q59_1:F>I	AA:235		IFO1804:C>W		N_43:C>W		N_44:C>W		N_45:C>W	AA:250		IFO1804:V>L		N_43:V>L		N_44:V>L		N_45:V>L	AA:261		A4:R>P		IFO1804:R>P		N_43:R>P		N_44:R>P		N_45:R>P		UFRJ50816:R>P		UWOPS91_917_1:R>P		YPS138:R>P	AA:279		A4:R>K		IFO1804:R>K		N_43:R>K		N_44:R>K		N_45:R>K		UWOPS91_917_1:R>K		YPS138:R>K	AA:302		IFO1804:L>F		N_43:L>F		N_44:L>F		N_45:L>F	AA:328		A12:K>N		A4:K>N		YPS138:K>NID:YBR106W	AA:94		A12:G>S		A4:G>S		DBVPG6304:G>S		UFRJ50816:G>S		UWOPS91_917_1:G>S		YPS138:G>S	AA:129		KPN3828:S>G	AA:182		A12:N>K		DBVPG6304:N>K		UFRJ50791:N>K		UFRJ50816:N>K		UWOPS91_917_1:N>K		YPS138:N>KID:YBR107C	AA:31		UWOPS91_917_1:Y>-	AA:35		A12:A>T		A4:A>T		DBVPG6304:A>T		UFRJ50816:A>T		UWOPS91_917_1:A>T		YPS138:A>T	AA:40		UFRJ50816:G>R	AA:42		N_43:Q>H		N_44:Q>H		N_45:Q>H	AA:46		UWOPS91_917_1:S>Y	AA:59		KPN3828:N>K	AA:61		Q32_3:I>S	AA:67		UWOPS91_917_1:S>R	AA:95		UWOPS91_917_1:C>W	AA:109		A12:F>V		A4:F>V		DBVPG6304:F>V		UFRJ50816:F>V		YPS138:F>V	AA:119		A12:A>T		A4:A>T		DBVPG6304:A>T		UFRJ50816:A>T		UWOPS91_917_1:A>T		YPS138:A>T	AA:137		A12:Y>N		A4:Y>N		DBVPG6304:Y>N		N_43:Y>N		N_45:Y>N		UWOPS91_917_1:Y>N		YPS138:Y>N	AA:167		A12:F>L		A4:F>L		UFRJ50816:F>L		UWOPS91_917_1:F>L		YPS138:F>L	AA:189		A12:H>Q		A4:H>Q		IFO1804:H>Q		N_43:H>Q		N_45:H>Q		UFRJ50816:H>Q		UWOPS91_917_1:H>Q		YPS138:H>Q	AA:203		A12:R>W		A4:R>W		IFO1804:R>W		N_43:R>W		N_45:R>W		UFRJ50816:R>W		UWOPS91_917_1:R>W		YPS138:R>W	AA:215		A12:I>M		A4:I>M		UFRJ50816:I>M		UWOPS91_917_1:I>M		YPS138:I>M	AA:233		UWOPS91_917_1:K>N	AA:235		A12:L>P		A4:L>P		UFRJ50791:L>P		UFRJ50816:L>P		YPS138:L>P	AA:236		UWOPS91_917_1:A>S	AA:246		A12:H>R		A4:H>R		DBVPG6304:H>R		UFRJ50791:H>R		UFRJ50816:H>R		YPS138:H>RID:YBR109C	AA:3		A12:R>S		A4:R>S		DBVPG6304:R>S		UFRJ50791:R>S		UWOPS91_917_1:R>S		YPS138:R>S	AA:33		UWOPS91_917_1:F>L	AA:40		UWOPS91_917_1:N>K	AA:49		N_43:->Y	AA:85		A12:D>E		A4:D>E		UFRJ50791:D>E		UWOPS91_917_1:D>EID:YBR110W	AA:30		A4:L>F		DBVPG6304:L>F	AA:78		N_43:T>A	AA:87		A12:L>P		A4:L>P		UWOPS91_917_1:L>P		YPS138:L>P	AA:102		UWOPS91_917_1:G>D	AA:119		A12:C>S		A4:C>S		IFO1804:C>S		N_44:C>S		N_45:C>S		UWOPS91_917_1:C>S		YPS138:C>S	AA:153		A12:T>I		A4:T>I		UWOPS91_917_1:T>I		YPS138:T>I	AA:182		N_44:V>A	AA:184		A12:I>V		A4:I>V		UWOPS91_917_1:I>V		YPS138:I>V	AA:218		UWOPS91_917_1:S>P	AA:220		A12:K>R		A4:K>R		UWOPS91_917_1:K>R		YPS138:K>R	AA:236		A12:G>A		A4:G>A		IFO1804:G>A		N_43:G>A		N_44:G>A		N_45:G>A		UWOPS91_917_1:G>A		YPS138:G>A	AA:244		UWOPS91_917_1:A>S	AA:253		CBS432:N>Y	AA:319		A4:M>V		YPS138:M>V	AA:323		A4:K>E		YPS138:K>E	AA:326		UWOPS91_917_1:D>E	AA:335		N_43:V>L	AA:417		N_43:H>D		N_45:H>D		Q62_5:H>D		Q89_8:H>D		T21_4:H>D		Z1_1:H>DID:YBR111C	AA:2		UWOPS91_917_1:L>F	AA:15		A12:H>Q		A4:H>Q		IFO1804:H>Q		N_44:H>Q		N_45:H>Q		UFRJ50791:H>Q	AA:22		UWOPS91_917_1:Q>E	AA:41		A12:D>E		A4:D>E		UFRJ50791:D>E	AA:53		A12:L>F		A4:L>F		UFRJ50791:L>F	AA:69		A12:S>R		UFRJ50791:S>R		UWOPS91_917_1:S>R	AA:120		A12:H>Q		UFRJ50791:H>Q		UWOPS91_917_1:H>Q	AA:128		UWOPS91_917_1:G>R	AA:136		A12:->Y		UFRJ50791:->Y		UWOPS91_917_1:->Y		YPS138:->Y	AA:204		A12:F>L		IFO1804:F>L		N_43:F>L		N_45:F>L		UFRJ50791:F>L		YPS138:F>L	AA:207		A12:F>V		YPS138:F>V	AA:225		A12:K>N		YPS138:K>NID:YBR115C	AA:22		IFO1804:S>R		N_17:S>R		N_43:S>R		N_44:S>R		N_45:S>R		Q95_3:S>R		UWOPS91_917_1:S>R		Y7:S>R		Z1_1:S>R	AA:25		A4:Y>-		UFRJ50816:Y>-		UWOPS91_917_1:Y>-	AA:27		A4:W>C		UFRJ50816:W>C		UWOPS91_917_1:W>C	AA:40		A4:K>-		UFRJ50816:K>-		UWOPS91_917_1:K>-	AA:42		A4:N>K		IFO1804:N>K		N_43:N>K		N_44:N>K		N_45:N>K		UFRJ50816:N>K		UWOPS91_917_1:N>K	AA:59		A4:H>Q		UFRJ50816:H>Q		YPS138:H>Q	AA:66		A4:T>S		UFRJ50816:T>S		UWOPS91_917_1:T>S		YPS138:T>S	AA:80		N_43:P>A		N_45:P>A	AA:122		CBS432:R>T		CBS5829:R>T		IFO1804:K>N		N_17:R>T		N_43:R>T		N_44:K>N		N_45:R>T		Q59_1:R>T		Q62_5:R>T		UFRJ50791:R>T		UFRJ50816:R>T		UWOPS91_917_1:R>T		Y7:R>T		YPS138:R>T	AA:151		A12:L>W		A4:L>W		DBVPG6304:L>W		UFRJ50816:L>W		UWOPS91_917_1:L>W		YPS138:L>W	AA:167		A12:N>K		A4:N>K		DBVPG6304:N>K		UFRJ50816:N>K		UWOPS91_917_1:N>K		YPS138:N>K	AA:193		UFRJ50816:->C	AA:200		A12:->Y		A4:->Y		DBVPG6304:->Y		UFRJ50816:->Y		UWOPS91_917_1:->Y		YPS138:->Y	AA:205		A12:V>A		A4:V>A		DBVPG6304:V>A		UFRJ50816:V>A		YPS138:V>A	AA:271		A12:I>M		A4:I>M		DBVPG6304:I>M		UFRJ50816:I>M		UWOPS91_917_1:I>M		YPS138:I>M	AA:327		A12:M>I		A4:M>I		CBS432:M>I		CBS5829:M>I		DBVPG4650:M>I		DBVPG6304:M>I		IFO1804:M>I		N_17:M>I		N_43:M>I		N_44:M>I		N_45:M>I		Q59_1:M>I		Q62_5:M>I		Q89_8:M>I		UFRJ50816:M>I		UWOPS91_917_1:M>I		YPS138:M>I		Z1_1:M>I	AA:360		IFO1804:->C		N_43:->C		N_44:->C		N_45:->C		UWOPS91_917_1:->C	AA:363		UWOPS91_917_1:L>H	AA:428		A12:F>L		A4:F>L		DBVPG4650:F>S		DBVPG6304:F>L		UFRJ50816:F>L		YPS138:F>L	AA:433		YPS138:F>L	AA:435		A4:K>N		DBVPG6304:K>N		UFRJ50816:K>N		UWOPS91_917_1:K>N		YPS138:K>N	AA:549		A4:K>N		DBVPG6304:K>N		UFRJ50791:K>N		UFRJ50816:K>N		UWOPS91_917_1:K>N		YPS138:K>N	AA:558		A4:C>-		DBVPG6304:C>-		UFRJ50791:C>-		UFRJ50816:C>-		UWOPS91_917_1:C>-		YPS138:C>-	AA:586		A12:->Q	AA:596		A4:H>Q		DBVPG6304:H>Q		UFRJ50791:H>Q		UFRJ50816:H>Q		YPS138:H>Q	AA:597		UWOPS91_917_1:S>C	AA:605		N_45:Q>-	AA:614		A4:K>N		DBVPG6304:K>N		UWOPS91_917_1:K>N		YPS138:K>N	AA:632		N_44:N>K		N_45:N>K	AA:638		A4:R>S		DBVPG6304:R>S		N_44:R>S		N_45:R>S		UFRJ50791:R>S		UFRJ50816:R>S		UWOPS91_917_1:R>S		YPS138:R>S	AA:660		UWOPS91_917_1:I>L	AA:666		N_44:N>T		N_45:N>T	AA:681		UWOPS91_917_1:F>L	AA:685		A4:D>H		DBVPG6304:D>H		UFRJ50791:D>H		UFRJ50816:D>H		YPS138:D>H	AA:697		A4:S>F		DBVPG6304:S>F		UFRJ50791:S>F		UFRJ50816:S>F		UWOPS91_917_1:S>F		YPS138:S>F	AA:736		A4:Q>P		DBVPG6304:Q>P		UFRJ50816:Q>P		YPS138:Q>P	AA:756		A4:F>L		UFRJ50816:F>L	AA:789		UFRJ50791:N>Y	AA:829		YPS138:C>S	AA:832		UFRJ50816:G>R		YPS138:G>R	AA:911		UWOPS91_917_1:I>M	AA:913		DBVPG4650:L>F	AA:920		UFRJ50791:C>-		UFRJ50816:C>-		UWOPS91_917_1:C>-		YPS138:C>-	AA:963		UFRJ50791:N>S		UFRJ50816:N>S		UWOPS91_917_1:N>S		YPS138:N>S	AA:979		N_43:V>D		N_45:V>D		UFRJ50791:V>D		UFRJ50816:V>D		YPS138:V>D	AA:985		UFRJ50791:H>Q		UFRJ50816:H>Q	AA:1011		UWOPS91_917_1:N>K	AA:1027		UFRJ50791:L>V		UFRJ50816:L>V		UWOPS91_917_1:L>V	AA:1039		Q32_3:I>M	AA:1058		A4:V>L		N_43:V>L		N_45:V>L		UFRJ50791:V>L		UFRJ50816:V>L		UWOPS91_917_1:V>LID:YBR119W	AA:17		DBVPG6304:V>A		YPS138:V>A	AA:29		DBVPG6304:Y>H		YPS138:Y>H	AA:40		DBVPG6304:S>P		UFRJ50816:S>P		YPS138:S>P	AA:66		DBVPG6304:I>T		N_44:I>V		N_45:I>V		UFRJ50816:I>T		YPS138:I>T	AA:91		DBVPG6304:D>E		UFRJ50816:D>E		YPS138:D>E	AA:112		DBVPG6304:I>V		UFRJ50791:I>V		UFRJ50816:I>V		YPS138:I>V	AA:131		N_43:K>N		N_44:K>N		N_45:K>N	AA:134		DBVPG6304:K>E		N_43:K>E		N_44:K>E		N_45:K>E		UFRJ50791:K>E		UFRJ50816:K>E		YPS138:K>E	AA:138		DBVPG6304:D>N		N_43:D>N		N_44:D>N		UFRJ50791:D>N		UFRJ50816:D>N		YPS138:D>N	AA:148		DBVPG6304:K>E		UFRJ50791:K>E		UFRJ50816:K>E		YPS138:K>E	AA:152		N_43:L>V		N_44:L>V		N_45:L>V	AA:154		DBVPG6304:R>H		UFRJ50791:R>H		UFRJ50816:R>H		YPS138:R>H	AA:205		DBVPG6304:Q>K		IFO1804:Q>K		N_43:Q>K		N_44:Q>K		N_45:Q>K		UFRJ50791:Q>K		UFRJ50816:Q>K		YPS138:Q>K	AA:214		DBVPG6304:S>T		UFRJ50791:S>T		UFRJ50816:S>T		YPS138:S>T	AA:217		Q62_5:P>T		Q95_3:P>T		Y6_5:P>T	AA:268		IFO1804:N>D		N_43:N>D		N_44:N>D		N_45:N>D	AA:276		UWOPS91_917_1:T>SID:YBR120C	AA:8		UWOPS91_917_1:S>C	AA:17		A12:F>L		A4:F>L		UFRJ50816:F>L		YPS138:F>L	AA:27		UWOPS91_917_1:L>-	AA:33		UWOPS91_917_1:S>C	AA:74		A12:F>L		DBVPG6304:F>L		UFRJ50816:F>L		YPS138:F>L	AA:113		A12:V>F		DBVPG6304:V>F		UWOPS91_917_1:T>P		YPS138:V>F	AA:134		UWOPS91_917_1:D>E	AA:141		UWOPS91_917_1:L>IID:YBR121C	AA:3		UWOPS91_917_1:S>R	AA:20		UWOPS91_917_1:F>L	AA:55		UWOPS91_917_1:M>I	AA:69		A12:S>R		A4:S>R		DBVPG6304:S>R		IFO1804:S>R		N_43:S>R		N_44:S>R		N_45:S>R		UFRJ50816:S>R		UWOPS91_917_1:S>R		YPS138:S>R	AA:81		A12:N>K		A4:N>K		DBVPG6304:N>K		UFRJ50816:N>K		YPS138:N>K	AA:83		A12:N>K		A4:N>K		DBVPG6304:N>K		IFO1804:N>K		N_43:N>K		N_44:N>K		N_45:N>K		UFRJ50816:N>K		UWOPS91_917_1:N>K		YPS138:N>K	AA:91		UWOPS91_917_1:W>C	AA:108		UWOPS91_917_1:->Y	AA:123		UWOPS91_917_1:S>R	AA:125		UWOPS91_917_1:Q>H	AA:158		UWOPS91_917_1:W>C	AA:163		DBVPG6304:R>S		UFRJ50791:R>S		UFRJ50816:R>S		UWOPS91_917_1:R>S		YPS138:R>S	AA:197		A12:K>N		A4:K>N		DBVPG6304:K>N		UFRJ50791:K>N		UFRJ50816:K>N		YPS138:K>N	AA:205		IFO1804:L>R		N_43:L>R		N_44:L>R		N_45:L>R	AA:221		UWOPS91_917_1:F>L	AA:226		A4:I>M		DBVPG6304:I>M		UFRJ50791:I>M		YPS138:I>M	AA:238		T21_4:Q>P	AA:258		A4:F>L		DBVPG6304:F>L		UFRJ50791:F>L		YPS138:F>L	AA:272		UWOPS91_917_1:R>S	AA:281		A4:N>K		DBVPG6304:N>K		N_43:N>K		N_44:N>K		N_45:N>K		UFRJ50791:N>K		UWOPS91_917_1:N>K		YPS138:N>K	AA:308		A12:E>D		A4:E>D		DBVPG6304:E>D		UFRJ50791:E>D		UWOPS91_917_1:E>D		YPS138:E>D	AA:319		A12:H>Q		A4:H>Q		DBVPG6304:H>Q		UFRJ50791:H>Q		UWOPS91_917_1:H>Q		YPS138:H>Q	AA:347		N_43:L>F		N_44:L>F		N_45:L>F		UWOPS91_917_1:L>F	AA:353		A12:R>S		A4:R>S		DBVPG6304:R>S		UFRJ50791:R>S		UWOPS91_917_1:R>S		YPS138:R>S	AA:380		UWOPS91_917_1:->Y	AA:390		A12:H>Q		A4:H>Q		DBVPG6304:H>Q		UWOPS91_917_1:H>Q		YPS138:H>Q	AA:422		UWOPS91_917_1:V>I	AA:433		DBVPG6304:E>Q		YPS138:E>Q	AA:553		A12:D>E		DBVPG6304:D>E		UFRJ50791:D>E		UWOPS91_917_1:D>E		YPS138:D>E	AA:557		UWOPS91_917_1:S>R	AA:589		A12:H>Q		A4:H>Q		DBVPG6304:H>Q		UFRJ50791:H>Q		UWOPS91_917_1:H>Q		YPS138:H>Q	AA:596		A12:H>Y		DBVPG6304:H>Y		UFRJ50791:H>Y		YPS138:H>Y	AA:639		A12:K>N		A4:K>N		UFRJ50791:K>N		UWOPS91_917_1:K>N		YPS138:K>N	AA:654		IFO1804:N>D		N_43:N>D		N_45:N>DID:YBR122C	AA:4		UWOPS91_917_1:V>A	AA:22		UWOPS91_917_1:->Y	AA:46		UWOPS91_917_1:C>S	AA:54		UWOPS91_917_1:Y>-	AA:57		A12:F>V		A4:F>V		YPS138:F>V	AA:98		A12:I>M		A4:I>M		DBVPG6304:I>M		N_17:I>M		N_43:I>M		N_44:I>M		N_45:I>M		UFRJ50816:I>M		YPS138:I>M	AA:108		A12:N>H		DBVPG6304:N>H		YPS138:N>H	AA:120		A12:P>S		DBVPG6304:P>S		YPS138:P>S	AA:136		N_44:V>M	AA:164		UWOPS91_917_1:->CID:YBR123C	AA:6		A12:I>F		A4:I>F		DBVPG6304:I>F	AA:8		A12:K>N		A4:K>N		DBVPG6304:K>N		UFRJ50791:K>N		UFRJ50816:K>N		UWOPS91_917_1:K>N	AA:150		A12:Q>H		A4:Q>H		DBVPG6304:Q>H		UFRJ50791:Q>H		UFRJ50816:Q>H		UWOPS91_917_1:Q>H		YPS138:Q>H	AA:246		A12:I>F		A4:I>F		DBVPG6304:I>F		UFRJ50791:I>F		UFRJ50816:I>F		YPS138:I>F	AA:310		N_45:H>Q	AA:326		A4:Q>H		DBVPG6304:Q>H		UFRJ50791:Q>H		UFRJ50816:Q>H		YPS138:Q>H	AA:339		A12:I>M		A4:I>M		DBVPG6304:I>M		UFRJ50791:I>M		YPS138:I>M	AA:367		A12:L>F		A4:L>F		DBVPG6304:L>F		UFRJ50791:L>F	AA:399		A12:F>L		A4:F>L		DBVPG6304:F>L	AA:412		N_45:R>M	AA:421		A12:R>S		A4:R>S		DBVPG6304:R>S		YPS138:R>S	AA:498		UWOPS91_917_1:Y>-	AA:508		UWOPS91_917_1:R>-	AA:522		A12:I>V		DBVPG6304:I>V		IFO1804:I>V		N_43:I>V		N_45:I>V		YPS138:I>V	AA:525		A12:->S		A4:->S		DBVPG6304:->S		YPS138:->S	AA:550		A12:V>M		A4:V>M		DBVPG6304:V>M		YPS138:V>M	AA:574		A12:R>S		A4:R>S		DBVPG6304:R>S		YPS138:R>S	AA:610		A12:->K		A4:->K		DBVPG6304:->K		YPS138:->KID:YBR125C	AA:17		UWOPS91_917_1:P>A	AA:19		DBVPG4650:L>F	AA:115		YPS138:W>C	AA:139		YPS138:F>L	AA:141		A4:L>F		UWOPS91_917_1:L>F		YPS138:L>F	AA:151		A4:Q>H		DBVPG6304:E>D		UFRJ50816:E>D		UWOPS91_917_1:E>D		YPS138:E>D	AA:241		DBVPG6304:L>F		UFRJ50816:L>F		UWOPS91_917_1:L>F		YPS138:L>F	AA:250		UWOPS91_917_1:E>D	AA:328		A12:E>D		A4:E>D		DBVPG6304:E>D		UFRJ50816:E>D		UWOPS91_917_1:E>D		YPS138:E>D	AA:355		A12:L>F		A4:L>F		DBVPG6304:L>F		UFRJ50816:L>F		YPS138:L>F	AA:390		A12:K>Q		A4:K>Q		YPS138:K>QID:YBR126C	AA:26		DBVPG6304:E>D		UFRJ50791:E>D	AA:33		A4:N>D		DBVPG6304:N>D		UFRJ50791:N>D	AA:132		UWOPS91_917_1:E>D	AA:150		N_45:->Y	AA:169		A4:H>Q	AA:203		A4:Q>H		UFRJ50791:Q>H		UFRJ50816:Q>H	AA:235		UFRJ50791:E>D		UFRJ50816:E>D	AA:389		A4:R>S		DBVPG6304:R>S		UFRJ50816:R>S		YPS138:R>SID:YBR128C	AA:41		UWOPS91_917_1:P>R	AA:42		A12:K>-		A4:K>-		DBVPG6304:K>-		UFRJ50816:K>-		YPS138:K>-	AA:58		A4:H>N		DBVPG6304:H>N		UFRJ50816:H>N		UWOPS91_917_1:H>N		YPS138:H>N	AA:59		N_44:H>R		N_45:H>R	AA:106		UFRJ50791:Y>-		UFRJ50816:Y>-		UWOPS91_917_1:Y>-	AA:114		UWOPS91_917_1:G>E	AA:122		A4:I>L		DBVPG6304:I>L		UFRJ50791:I>L		UFRJ50816:I>L		YPS138:I>L	AA:128		A4:K>R		DBVPG6304:K>R		UFRJ50791:K>R		UFRJ50816:K>R		YPS138:K>R	AA:145		A4:I>V		DBVPG6304:I>V		UFRJ50791:I>V		UFRJ50816:I>V		UWOPS91_917_1:I>V		YPS138:I>V	AA:157		A12:Q>R		A4:Q>R		DBVPG6304:Q>R		UFRJ50791:Q>R		UFRJ50816:Q>R		UWOPS91_917_1:Q>R		YPS138:Q>R	AA:222		UWOPS91_917_1:H>Q	AA:233		A12:L>V		A4:L>V		DBVPG6304:L>V		UFRJ50791:L>V		UFRJ50816:L>V		UWOPS91_917_1:L>V	AA:264		A12:->K		A4:->K		DBVPG6304:->K		UFRJ50791:->K		UFRJ50816:->K		UWOPS91_917_1:->K	AA:270		A12:E>D		A4:E>D		DBVPG6304:E>D		UFRJ50791:E>D		UFRJ50816:E>D	AA:296		A12:Y>N		A4:Y>N		DBVPG6304:Y>N		UFRJ50816:Y>N		UWOPS91_917_1:Y>N	AA:300		IFO1804:L>F		N_43:L>F		N_44:L>F		N_45:L>F	AA:314		UWOPS91_917_1:I>V	AA:317		A12:Q>H		DBVPG6304:Q>H		IFO1804:Q>H		N_43:Q>H		N_44:Q>H		N_45:Q>H		UFRJ50816:Q>H		UWOPS91_917_1:Q>HID:YBR129C	AA:21		A12:H>N		A4:H>N		DBVPG6304:H>N		UFRJ50791:H>N		UWOPS91_917_1:H>N		YPS138:H>N	AA:38		A12:->Q		A4:->Q		DBVPG6304:->Q		UFRJ50791:->Q		YPS138:->Q	AA:40		A12:K>R		A4:K>R		DBVPG6304:K>R		UFRJ50791:K>R		UWOPS91_917_1:K>R		YPS138:K>R	AA:47		Q59_1:H>Q		Q62_5:H>Q		T21_4:H>Q	AA:51		A12:I>L		DBVPG6304:I>L		Q95_3:I>S		YPS138:I>L	AA:53		A12:K>N		A4:K>N		DBVPG6304:K>N		IFO1804:K>N		N_43:K>N		N_44:K>N		N_45:K>N		UFRJ50791:K>N		YPS138:K>N	AA:127		A12:F>L		DBVPG6304:F>L		IFO1804:F>L		N_44:F>L		N_45:F>L		YPS138:F>L	AA:131		A12:L>W		DBVPG6304:L>W		YPS138:L>W	AA:136		A12:Q>E		DBVPG6304:Q>E		YPS138:Q>E	AA:202		UWOPS91_917_1:K>N	AA:227		UWOPS91_917_1:Y>-	AA:267		UWOPS91_917_1:M>L	AA:272		A12:T>S		A4:T>S		DBVPG6304:T>S		Q59_1:M>I		Q62_5:M>I		Q95_3:M>I		YPS138:T>S		Z1_1:M>I	AA:282		A12:T>N		DBVPG6304:T>N		N_44:T>N		N_45:T>N		UWOPS91_917_1:T>N		YPS138:T>N	AA:321		A12:C>-		CBS432:C>-		CBS5829:C>-		DBVPG6304:C>-		KPN3828:C>-		KPN3829:C>-		N_17:C>-		N_44:C>-		N_45:C>-		Q59_1:C>-		Q62_5:C>-		Q95_3:C>-		S36_7:C>-		T21_4:C>-		UWOPS91_917_1:C>-		Y7:C>-		YPS138:C>-		Z1_1:C>-ID:YBR130C	AA:2		A12:C>W		A4:C>W		DBVPG6304:C>W		UFRJ50816:C>W		YPS138:C>W	AA:11		N_43:I>F		N_44:I>F		N_45:I>F	AA:36		UWOPS91_917_1:S>R	AA:49		A12:->W		A4:->W		DBVPG6304:->W		UFRJ50816:->W		UWOPS91_917_1:->W		YPS138:->W	AA:52		UWOPS91_917_1:F>C	AA:112		UWOPS91_917_1:R>G	AA:129		A12:F>L		A4:F>L		N_43:F>L		N_44:F>L		N_45:F>L		UFRJ50816:F>L		UWOPS91_917_1:F>L		YPS138:F>L	AA:134		A4:H>Q		UFRJ50816:H>Q		YPS138:H>Q	AA:140		UWOPS91_917_1:L>F	AA:162		A12:L>F		A4:L>F		UFRJ50816:L>F		UWOPS91_917_1:L>F		YPS138:L>F	AA:178		A4:->W		UFRJ50816:->W		UWOPS91_917_1:->W		YPS138:->W	AA:184		A12:->E		A4:->E		N_43:->E		N_44:->E		N_45:->E		UFRJ50816:->E		UWOPS91_917_1:->E		YPS138:->E	AA:220		A4:Y>N	AA:267		T21_4:->R		Y6_5:->R	AA:280		A4:T>A		DBVPG6304:T>A		YPS138:T>A	AA:324		UWOPS91_917_1:F>L	AA:337		N_43:K>E		N_45:K>E	AA:342		N_44:->Y	AA:401		UWOPS91_917_1:F>LID:YBR132C	AA:2		A12:C>F		A4:C>F		DBVPG6304:C>F		UFRJ50816:C>F		UWOPS91_917_1:C>F		YPS138:C>F	AA:25		A12:F>L		A4:F>L		UFRJ50791:F>L		UFRJ50816:F>L		YPS138:F>L	AA:27		A12:K>N		A4:K>N		UFRJ50791:K>N		UFRJ50816:K>N		YPS138:K>N	AA:70		A4:F>V		DBVPG6304:F>V		UFRJ50791:F>V		UFRJ50816:F>V		YPS138:F>V	AA:88		A4:Q>-		DBVPG6304:Q>-		UFRJ50791:Q>-		UFRJ50816:Q>-		YPS138:Q>-	AA:156		A4:Q>-		DBVPG6304:Q>-		IFO1804:Q>-		N_43:Q>-		N_45:Q>-		UWOPS91_917_1:Q>-		YPS138:Q>-	AA:206		A4:S>R		DBVPG6304:S>R		UFRJ50791:S>R		UFRJ50816:S>R		YPS138:S>R	AA:266		A12:C>-		A4:C>-		DBVPG6304:C>-		UFRJ50816:C>-		UWOPS91_917_1:C>-		YPS138:C>-	AA:329		A12:E>D		A4:E>D		DBVPG6304:E>D		UFRJ50816:E>D		YPS138:E>D	AA:340		UWOPS91_917_1:A>T	AA:354		A12:E>D		A4:E>D		DBVPG6304:E>D		UFRJ50816:E>D		YPS138:E>D	AA:475		UFRJ50816:->Y	AA:477		UWOPS91_917_1:R>G	AA:488		UWOPS91_917_1:D>E	AA:535		A12:M>I		A4:M>I		DBVPG6304:M>I		N_43:M>I		N_45:M>I		UFRJ50816:M>I		UWOPS91_917_1:M>I		YPS138:M>I	AA:544		DBVPG6304:F>V	AA:576		UWOPS91_917_1:F>LID:YBR133C	AA:17		DBVPG6304:S>R		UWOPS91_917_1:S>R		YPS138:S>R	AA:32		YPS138:E>D	AA:33		IFO1804:V>F		N_43:V>F		N_44:V>F		N_45:V>F	AA:43		UWOPS91_917_1:I>L	AA:50		IFO1804:E>Q		N_43:E>Q		N_44:E>Q		N_45:E>Q	AA:116		CBS432:M>I		CBS5829:M>I	AA:127		A12:R>-		A4:R>-		DBVPG6304:R>-		YPS138:R>-	AA:137		A12:N>K		A4:N>K		DBVPG6304:N>K		UFRJ50816:N>K		YPS138:N>K	AA:170		A12:Q>E		A4:Q>E		DBVPG6304:Q>E		UFRJ50816:Q>E		UWOPS91_917_1:Q>E		YPS138:Q>E	AA:197		UFRJ50816:W>L	AA:206		UWOPS91_917_1:I>M	AA:237		A12:I>M		A4:I>M		DBVPG6304:I>M		UFRJ50816:I>M		UWOPS91_917_1:I>M	AA:252		Q59_1:T>P		Q62_5:T>P		Z1_1:T>P	AA:268		A12:C>W		A4:C>W		DBVPG6304:C>W		UFRJ50816:C>W		UWOPS91_917_1:C>W	AA:277		UWOPS91_917_1:I>L	AA:299		A12:W>R		A4:W>R		DBVPG6304:W>R		UFRJ50816:W>R	AA:309		A12:Q>H		A4:Q>H		DBVPG6304:Q>H		UFRJ50816:Q>H		UWOPS91_917_1:Q>H	AA:314		A12:L>F		A4:L>F		DBVPG6304:L>F		UFRJ50816:L>F		UWOPS91_917_1:L>F	AA:356		Q59_1:->K	AA:363		UWOPS91_917_1:T>P	AA:375		A12:T>R		A4:T>R		DBVPG6304:T>R		UFRJ50816:T>R	AA:377		A12:F>L		A4:F>L		DBVPG6304:F>L		UFRJ50816:F>L		UWOPS91_917_1:F>L	AA:420		A12:R>S		A4:R>S		DBVPG6304:R>S		UFRJ50816:R>S		UWOPS91_917_1:R>S	AA:451		A12:W>R		A4:W>R		DBVPG6304:W>R		UFRJ50816:W>R		UWOPS91_917_1:W>R	AA:480		UFRJ50816:F>C	AA:492		N_43:->C		N_44:->C		N_45:->C	AA:509		A12:L>F		DBVPG6304:L>F		IFO1804:L>F		N_43:L>F		N_44:L>F		N_45:L>F		UFRJ50816:L>F		UWOPS91_917_1:L>F	AA:549		UWOPS91_917_1:Y>F	AA:557		IFO1804:N>K		N_43:N>K		N_44:N>K		N_45:N>K	AA:595		A12:T>A		A4:T>A		UFRJ50791:T>A		YPS138:T>A	AA:597		A12:D>E		A4:D>E		UFRJ50791:D>E		UWOPS91_917_1:D>E		YPS138:D>E	AA:605		A12:Q>K		A4:Q>K		CBS432:Q>K		CBS5829:Q>K		KPN3829:Q>K		N_17:Q>K		N_43:Q>K		N_44:Q>K		N_45:Q>K		Q32_3:Q>K		Q59_1:Q>K		Q95_3:Q>K		S36_7:Q>K		UFRJ50791:Q>K		UWOPS91_917_1:Q>K		YPS138:Q>K	AA:613		UFRJ50791:S>P	AA:635		A12:Q>L		A4:Q>L		UFRJ50791:Q>L		UWOPS91_917_1:Q>L		YPS138:Q>L	AA:645		A12:F>L		A4:F>L		UFRJ50791:F>L		YPS138:F>L	AA:649		A12:Q>-		A4:Q>-		N_43:Q>-		N_44:Q>-		N_45:Q>-		UFRJ50791:Q>-		UFRJ50816:Q>-		UWOPS91_917_1:Q>-		YPS138:Q>-	AA:666		A12:Q>H		A4:Q>H		UFRJ50791:Q>H		UFRJ50816:Q>H		UWOPS91_917_1:Q>H		YPS138:Q>H	AA:726		UWOPS91_917_1:P>T	AA:790		A12:V>G		A4:V>G		DBVPG6304:V>G		UWOPS91_917_1:V>G	AA:799		UWOPS91_917_1:T>P	AA:807		A12:->W		A4:->W		DBVPG6304:->W		UFRJ50791:->W		UFRJ50816:->W		UWOPS91_917_1:->W	AA:818		UFRJ50791:W>C		UFRJ50816:W>CID:YBR136W	AA:23		YPS138:G>S	AA:89		UWOPS91_917_1:V>I	AA:117		A12:H>Y		DBVPG6304:H>Y		UFRJ50816:H>Y		YPS138:H>Y	AA:120		IFO1804:I>T		N_43:I>T		N_44:I>T		N_45:I>T	AA:135		N_43:V>A		N_44:V>A		N_45:V>A	AA:142		N_43:T>A		N_44:T>A		N_45:T>A	AA:154		A12:M>I		DBVPG6304:M>I		UFRJ50816:M>I		UWOPS91_917_1:M>I		YPS138:M>I	AA:168		A12:D>N		DBVPG6304:D>N		N_43:D>N		N_44:D>N		N_45:D>N		UWOPS91_917_1:D>N		YPS138:D>N	AA:177		UWOPS91_917_1:T>I	AA:219		UWOPS91_917_1:V>A	AA:235		UWOPS91_917_1:E>K	AA:257		A12:A>S		A4:A>S		DBVPG6304:A>S		YPS138:A>S	AA:288		UWOPS91_917_1:L>I	AA:316		A12:V>I		A4:V>I		DBVPG6304:V>I		UFRJ50816:V>I		UWOPS91_917_1:V>I		YPS138:V>I	AA:337		A12:Q>H		A4:Q>H		DBVPG6304:Q>H		UFRJ50816:Q>H		YPS138:Q>H	AA:367		UWOPS91_917_1:N>K	AA:375		UWOPS91_917_1:P>S	AA:381		N_45:N>S	AA:421		A4:S>N		DBVPG6304:S>N		UFRJ50816:S>N		UWOPS91_917_1:S>N	AA:432		UWOPS91_917_1:L>F	AA:452		A4:I>V		DBVPG6304:I>V		UFRJ50816:I>V	AA:459		A4:T>A		DBVPG6304:T>A		UFRJ50816:T>A		UWOPS91_917_1:T>A	AA:472		N_44:K>R		N_45:K>R	AA:483		UWOPS91_917_1:N>S	AA:507		A4:S>P		DBVPG6304:S>P		UFRJ50816:S>P		UWOPS91_917_1:S>P	AA:522		UWOPS91_917_1:L>F	AA:531		A4:K>N		IFO1804:K>N		N_44:K>N		N_45:K>N		UFRJ50816:K>N		UWOPS91_917_1:K>N	AA:568		A12:N>K		A4:N>K		UFRJ50816:N>K		UWOPS91_917_1:N>K	AA:571		T21_4:G>R	AA:581		A4:K>N		UFRJ50816:K>N	AA:667		A12:L>F		A4:L>F		UWOPS91_917_1:L>F	AA:827		A12:D>E		A4:D>E		DBVPG6304:D>E		UWOPS91_917_1:D>E		YPS138:D>E	AA:831		A12:S>G		A4:S>G		DBVPG6304:S>G		YPS138:S>G	AA:837		A12:I>V		A4:I>V		DBVPG6304:I>V		UWOPS91_917_1:I>V		YPS138:I>V	AA:943		UFRJ50816:R>Q	AA:1085		A4:V>I		YPS138:V>I	AA:1130		IFO1804:K>R	AA:1133		IFO1804:F>L	AA:1150		A12:I>V		A4:I>V		DBVPG6304:I>V		IFO1804:I>V		N_43:I>V		N_44:I>V		N_45:I>V		UFRJ50816:I>V		UWOPS91_917_1:I>V		YPS138:I>V	AA:1165		Q59_1:V>A	AA:1169		IFO1804:W>G	AA:1261		UWOPS91_917_1:F>L	AA:1317		A12:N>K		A4:N>K		UWOPS91_917_1:N>K		YPS138:N>K	AA:1393		UWOPS91_917_1:Q>R	AA:1414		A12:A>S		A4:A>S	AA:1515		UWOPS91_917_1:S>N	AA:1548		UFRJ50816:V>I	AA:1553		A4:D>N		DBVPG6304:D>N		IFO1804:D>N		N_43:D>N		N_45:D>N		UFRJ50816:D>N	AA:1557		A4:K>R		DBVPG6304:K>R	AA:1580		A4:L>I		DBVPG6304:L>I		IFO1804:L>I		N_43:L>I		N_45:L>I		UFRJ50816:L>I	AA:1585		DBVPG6304:G>D		UFRJ50816:G>D	AA:1601		UWOPS91_917_1:V>I	AA:1647		N_17:T>S	AA:1655		N_17:R>G	AA:1684		DBVPG6304:V>I	AA:1756		IFO1804:H>N		N_44:H>NID:YBR137W	AA:10		CBS5829:E>K	AA:16		A12:E>K		A4:E>K		DBVPG6304:E>K		IFO1804:E>K		N_44:E>K		N_45:E>K		YPS138:E>K	AA:52		KPN3828:E>V	AA:57		DBVPG6304:K>N	AA:125		KPN3828:Y>SID:YBR138C	AA:26		T21_4:P>R		Y6_5:P>R	AA:92		UWOPS91_917_1:T>I	AA:98		A4:I>M		UWOPS91_917_1:I>M		YPS138:I>M	AA:104		UWOPS91_917_1:->G	AA:109		A4:L>R		UFRJ50816:L>R		YPS138:L>R	AA:138		YPS138:F>L	AA:158		A12:P>R		A4:P>R		UFRJ50816:P>R		YPS138:P>R	AA:174		A12:V>F		A4:V>F		DBVPG6304:V>F		N_17:Q>H		N_43:Q>H		N_44:Q>H		N_45:Q>H		UFRJ50816:V>F		YPS138:V>F	AA:183		A12:Q>E		A4:Q>E		UFRJ50816:Q>E		YPS138:Q>E	AA:190		A12:F>Y		A4:F>Y		UFRJ50816:F>Y		YPS138:F>Y	AA:211		A12:N>K		A4:N>K		DBVPG6304:N>K		N_17:N>K		N_44:N>K		N_45:N>K		UFRJ50816:N>K		YPS138:N>K	AA:231		A12:V>F		A4:V>F		DBVPG6304:V>F		UFRJ50816:V>F		YPS138:V>F	AA:235		N_17:I>T		N_44:I>T		N_45:I>T	AA:239		A12:M>K		A4:M>K		DBVPG6304:M>K		UFRJ50816:M>K		YPS138:M>K	AA:259		A12:F>I		A4:F>I		DBVPG6304:F>I	AA:270		A12:M>R		A4:M>R		DBVPG6304:M>R		UFRJ50816:M>R		YPS138:M>R	AA:312		A12:N>I		A4:N>I		DBVPG6304:N>I		N_17:N>I		N_43:N>I		N_45:N>I		YPS138:N>I	AA:354		A12:I>M		A4:I>M		DBVPG6304:I>M		UFRJ50816:I>M		YPS138:I>M	AA:359		A12:F>L		A4:F>L		DBVPG6304:F>L		UFRJ50816:F>L		YPS138:F>L	AA:375		A12:Q>H		A4:Q>H		DBVPG6304:Q>H		YPS138:Q>H	AA:389		A12:N>K		A4:N>K		DBVPG6304:N>K		YPS138:N>K	AA:408		A12:T>P		A4:T>P		DBVPG6304:T>P		N_43:T>P		YPS138:T>P	AA:427		KPN3828:I>M	AA:449		A4:F>L		DBVPG6304:F>L		N_43:F>L	AA:453		A12:K>N		A4:K>N		DBVPG6304:K>N	AA:467		A4:H>Q		DBVPG6304:H>Q	AA:505		DBVPG6304:Q>H	AA:513		DBVPG6304:->C	AA:520		A12:Q>H		A4:Q>H		DBVPG6304:Q>H		UFRJ50816:Q>H	AA:523		DBVPG6304:F>LID:YBR139W	AA:18		IFO1804:S>F		N_43:S>F		N_44:S>F		N_45:S>F	AA:27		A12:L>I		A4:L>I		DBVPG6304:L>I		UWOPS91_917_1:L>I		YPS138:L>I	AA:31		UWOPS91_917_1:G>A	AA:59		UWOPS91_917_1:D>H	AA:162		UFRJ50791:N>T		UFRJ50816:N>T	AA:192		Y6_5:K>R	AA:194		UWOPS91_917_1:A>T	AA:236		UFRJ50791:N>K		UFRJ50816:N>K		UWOPS91_917_1:N>K		YPS138:N>K	AA:257		UFRJ50791:V>I		UFRJ50816:V>I		UWOPS91_917_1:V>I		YPS138:V>I	AA:323		N_43:A>S		N_44:A>S		N_45:A>S	AA:339		DBVPG6304:D>N		UFRJ50791:D>N		UFRJ50816:D>N		UWOPS91_917_1:D>N	AA:490		A12:S>N		A4:S>N		DBVPG6304:S>N		UFRJ50816:S>N	AA:497		UFRJ50816:G>R	AA:501		A12:L>F		A4:L>F		DBVPG6304:L>F		UFRJ50816:L>F	AA:506		DBVPG6304:N>D		N_43:N>D		N_44:N>D		N_45:N>D		UFRJ50816:N>DID:YBR141C	AA:2		T21_4:E>D	AA:9		UWOPS91_917_1:Q>H	AA:20		UWOPS91_917_1:G>-	AA:57		A12:L>F		A4:L>F		DBVPG6304:L>F		UFRJ50791:L>F		UFRJ50816:L>F		YPS138:L>F	AA:98		IFO1804:V>L		N_43:V>L		N_44:V>L		N_45:V>L	AA:109		N_43:->W		N_44:->W		N_45:->W	AA:117		A12:F>L		A4:F>L		YPS138:F>L	AA:120		A12:W>C		A4:W>C		DBVPG6304:W>C		UFRJ50791:W>C		UFRJ50816:W>C		YPS138:W>C	AA:197		A12:N>K		A4:N>K		UFRJ50791:N>K		UFRJ50816:N>K		YPS138:N>K	AA:215		A12:K>R		A4:K>R	AA:218		A12:V>L		A4:V>L		UFRJ50791:V>L		UFRJ50816:V>L	AA:224		Q95_3:T>R	AA:273		A12:F>L		A4:F>L		UFRJ50791:F>L		UFRJ50816:F>L		YPS138:F>L	AA:289		A12:->Y		A4:->Y	AA:317		A12:S>A		A4:S>A		UFRJ50791:S>A		UFRJ50816:S>A		YPS138:S>AID:YBR145W	AA:16		N_44:Y>F		N_45:Y>F	AA:172		A12:I>M		A4:I>M		YPS138:I>M	AA:211		UWOPS91_917_1:R>G	AA:216		A12:E>Q		A4:E>Q		IFO1804:E>Q		N_43:E>Q		N_44:E>Q		UWOPS91_917_1:E>Q	AA:228		UWOPS91_917_1:E>K	AA:233		A12:D>N		A4:D>N		DBVPG6304:D>NID:YBR146W	AA:4		IFO1804:R>K		N_43:R>K		N_44:R>K		N_45:R>K	AA:10		A12:R>K		A4:R>K		DBVPG6304:R>K		UFRJ50816:R>K		YPS138:R>K	AA:29		A12:T>I		A4:T>I		DBVPG6304:T>I		UFRJ50816:T>I		YPS138:T>I	AA:60		A12:R>K		A4:R>K	AA:92		A12:I>V		A4:I>V		DBVPG6304:I>V		UFRJ50816:I>V		YPS138:I>V	AA:109		A12:M>I		A4:M>I		DBVPG6304:M>I		UFRJ50816:M>I		YPS138:M>I	AA:145		A12:V>I		A4:V>I		DBVPG6304:V>I		IFO1804:V>I		N_44:V>I		N_45:V>I		UFRJ50816:V>I		UWOPS91_917_1:V>I		YPS138:V>I	AA:147		A12:T>A		A4:T>A		DBVPG6304:T>A		UFRJ50816:T>A		YPS138:T>A	AA:165		UWOPS91_917_1:S>A	AA:172		KPN3829:G>V	AA:183		UWOPS91_917_1:F>L	AA:197		UFRJ50791:M>I		UFRJ50816:M>I	AA:209		YPS138:Y>F	AA:210		KPN3829:N>I	AA:214		KPN3829:T>S	AA:226		A12:S>A		A4:S>A		DBVPG6304:S>A		UFRJ50791:S>A		UFRJ50816:S>A		YPS138:S>A	AA:227		KPN3829:I>LID:YBR147W	AA:17		A4:S>A		DBVPG6304:S>A		UFRJ50791:S>A		YPS138:S>A	AA:92		DBVPG6304:M>I	AA:107		Q89_8:K>E	AA:108		DBVPG6304:N>D	AA:139		N_43:T>I		N_44:T>I		N_45:T>I		UWOPS91_917_1:T>I	AA:148		N_43:S>N		N_44:S>N		N_45:S>N	AA:169		A12:L>F		UFRJ50791:L>F		YPS138:L>F	AA:171		Q89_8:I>V	AA:189		N_43:S>T		N_44:S>T		N_45:S>T	AA:194		A12:D>N		UFRJ50791:D>N	AA:202		A12:F>Y		A4:F>Y		UFRJ50791:F>Y	AA:203		N_43:E>G		N_44:E>G		N_45:E>G	AA:229		N_43:I>V		N_44:I>V		N_45:I>V	AA:277		A12:I>V		A4:I>V		UFRJ50791:I>V		YPS138:I>V	AA:299		UFRJ50791:P>S	AA:306		N_43:I>T		N_44:I>T		N_45:I>TID:YBR148W	AA:27		N_43:S>T		N_44:S>T		N_45:S>T	AA:50		A12:G>S		A4:G>S		DBVPG6304:G>S		UFRJ50816:G>S		YPS138:G>S	AA:54		N_43:R>T		N_44:R>T		N_45:R>T	AA:63		A12:Y>D		A4:Y>D		DBVPG6304:Y>D		UFRJ50816:Y>D		YPS138:Y>D	AA:81		N_43:R>K		N_44:R>K		N_45:R>K	AA:97		N_44:M>V	AA:100		N_43:D>E		N_44:D>E		N_45:D>E	AA:113		A12:I>K		A4:I>K		DBVPG6304:I>K		N_43:I>K		N_44:I>K		N_45:I>K		UFRJ50816:I>K	AA:117		A12:R>K		A4:R>K		DBVPG6304:R>K		UFRJ50816:R>K	AA:123		A12:T>A		A4:T>A		DBVPG6304:T>A		UFRJ50816:T>A	AA:152		A12:E>D		A4:E>D		DBVPG6304:E>D		UFRJ50816:E>D	AA:154		DBVPG4650:S>R	AA:156		N_45:L>V	AA:177		A4:H>Q		UFRJ50816:H>Q	AA:187		YPS138:I>F	AA:201		UFRJ50816:K>R		YPS138:K>R	AA:208		A4:E>V		UFRJ50816:E>V		YPS138:E>V	AA:221		N_44:E>G		N_45:E>G	AA:225		UFRJ50816:A>T		YPS138:A>T	AA:249		N_44:Q>R		N_45:Q>R	AA:254		N_44:E>V		N_45:E>V	AA:282		A4:F>S		UFRJ50816:F>S		YPS138:F>S	AA:284		YPS138:S>L	AA:290		A4:V>I		UFRJ50816:V>I		YPS138:V>I	AA:308		A4:A>E		UFRJ50816:A>E		YPS138:A>E	AA:314		A4:Y>N		N_44:Y>N		N_45:Y>N		UFRJ50816:Y>N		YPS138:Y>N	AA:328		A4:L>V		UFRJ50816:L>V		YPS138:L>V	AA:340		N_44:H>L		N_45:H>L	AA:351		N_44:L>F		N_45:L>F		YPS138:L>F	AA:368		A4:I>M		UFRJ50816:I>M	AA:372		DBVPG4650:K>N	AA:383		CBS432:K>R	AA:410		A12:V>A		A4:V>A		UFRJ50816:V>A		YPS138:V>A	AA:417		A12:S>L		A4:S>L		DBVPG4650:S>L		IFO1804:S>L		N_17:S>L		N_44:S>L		N_45:S>L		Q32_3:S>L		Q95_3:S>L		UFRJ50816:S>L		YPS138:S>L	AA:432		A12:T>A		A4:T>A		UFRJ50816:T>A		YPS138:T>A	AA:437		A12:T>A		A4:T>A		UFRJ50816:T>A		YPS138:T>A	AA:441		A12:H>Q		A4:H>Q		UFRJ50816:H>Q		YPS138:H>Q	AA:445		IFO1804:K>R		N_45:K>R	AA:451		A12:R>K		A4:R>K		UFRJ50816:R>K		YPS138:R>K	AA:465		IFO1804:L>V		N_45:L>V		UWOPS91_917_1:L>V	AA:468		UWOPS91_917_1:D>N	AA:471		A12:R>K		A4:R>K		UFRJ50816:R>K		YPS138:R>K	AA:514		UWOPS91_917_1:A>V	AA:605		UWOPS91_917_1:N>SID:YBR149W	AA:2		A12:S>T		A4:S>T		UFRJ50791:S>T		UFRJ50816:S>T	AA:96		UFRJ50791:F>V	AA:164		A4:K>N		DBVPG6304:K>N		UFRJ50816:K>N		UWOPS91_917_1:K>N		YPS138:K>N	AA:170		IFO1804:T>I	AA:208		N_17:V>I		Y6_5:V>I	AA:224		Q62_5:M>I	AA:225		A4:E>D		DBVPG6304:E>D		UFRJ50816:E>D		YPS138:E>D	AA:306		IFO1804:L>F	AA:311		A12:V>D		A4:V>D		UWOPS91_917_1:V>D		YPS138:V>DID:YBR151W	AA:35		A4:R>G		DBVPG6304:R>G		UWOPS91_917_1:R>G		YPS138:R>G	AA:45		A4:P>S		CBS5829:P>S		DBVPG4650:P>S		DBVPG6304:P>S		KPN3828:P>S		N_17:P>S		N_43:P>S		N_44:P>S		N_45:P>S		Q62_5:P>S		Q95_3:P>S		UWOPS91_917_1:P>S		YPS138:P>S	AA:58		A4:T>A		N_43:T>A		N_44:T>A		N_45:T>A		UWOPS91_917_1:T>A		YPS138:T>A	AA:76		UWOPS91_917_1:T>A	AA:105		UWOPS91_917_1:R>K	AA:119		A12:E>D		A4:E>D		UWOPS91_917_1:E>D		YPS138:E>D	AA:124		CBS5829:K>T		N_17:K>T	AA:149		UWOPS91_917_1:N>S	AA:157		A12:V>I		A4:V>I		DBVPG6304:V>I		UWOPS91_917_1:V>I		YPS138:V>I	AA:181		N_43:K>E		N_44:K>E		N_45:K>E	AA:190		A12:E>G		A4:E>G		DBVPG6304:E>G		UFRJ50816:E>G		YPS138:E>G	AA:193		A12:P>Q		DBVPG6304:P>Q		UFRJ50816:P>Q		YPS138:P>Q	AA:206		A12:L>I		A4:L>I		DBVPG6304:L>I		UFRJ50816:L>I		UWOPS91_917_1:L>I		YPS138:L>I	AA:234		A12:H>Y		A4:H>Y		DBVPG6304:H>Y		UFRJ50816:H>Y		YPS138:H>Y	AA:265		N_43:V>AID:YBR154C	AA:18		UWOPS91_917_1:Y>-	AA:42		A12:->Y		A4:->Y		DBVPG6304:->Y		UFRJ50791:->Y		UFRJ50816:->Y		YPS138:->Y	AA:103		A12:I>M		A4:I>M		DBVPG6304:I>M		UFRJ50791:I>M		UFRJ50816:I>M		UWOPS91_917_1:I>M	AA:124		UWOPS91_917_1:H>Q	AA:132		DBVPG4650:F>SID:YBR155W	AA:3		A12:P>S		A4:P>S		DBVPG6304:P>S		N_43:P>S		N_44:P>S		N_45:P>S		UWOPS91_917_1:P>S		YPS138:P>S	AA:5		A12:N>S		A4:N>S		DBVPG6304:N>S		YPS138:N>S	AA:34		N_43:D>N		N_44:D>N		N_45:D>N	AA:60		N_43:V>A		N_44:V>A		N_45:V>A		UWOPS91_917_1:V>A	AA:80		N_43:H>N		N_44:H>N		N_45:H>N	AA:96		UWOPS91_917_1:S>A	AA:110		UWOPS91_917_1:I>V	AA:118		A12:A>S		DBVPG6304:A>S		UWOPS91_917_1:A>S		YPS138:A>S	AA:193		IFO1804:L>F		N_43:L>F		N_44:L>F	AA:208		UWOPS91_917_1:V>A	AA:214		A12:Q>K		A4:Q>K		UFRJ50791:Q>K		YPS138:Q>K	AA:217		UWOPS91_917_1:V>A	AA:219		A12:D>E		A4:D>E		IFO1804:D>E		N_43:D>E		N_44:D>E		UFRJ50791:D>E		YPS138:D>E	AA:242		UWOPS91_917_1:R>H	AA:252		Q59_1:K>T	AA:255		Q59_1:L>V	AA:335		UWOPS91_917_1:K>R	AA:382		A12:R>K		A4:R>K		DBVPG6304:R>K		UFRJ50791:R>K		UWOPS91_917_1:R>K		YPS138:R>KID:YBR156C	AA:2		UFRJ50816:R>S	AA:32		UFRJ50816:K>Q	AA:38		A4:Y>N		DBVPG6304:Y>N		UWOPS91_917_1:Y>N	AA:48		IFO1804:->E		N_43:->E		N_45:->E	AA:51		A4:G>-		DBVPG6304:G>-		UWOPS91_917_1:G>-	AA:53		A4:S>G		DBVPG6304:S>G		UWOPS91_917_1:S>G	AA:55		UFRJ50816:C>G	AA:62		A12:M>I		A4:M>I		DBVPG6304:M>I		UFRJ50791:M>I		UFRJ50816:L>F		YPS138:M>I	AA:69		A4:Y>-		DBVPG6304:Y>-		IFO1804:Y>-		N_43:Y>-		N_45:Y>-		UFRJ50816:Y>-	AA:90		A4:M>I		DBVPG6304:M>I	AA:115		UFRJ50816:M>I		UWOPS91_917_1:M>I	AA:123		A4:F>L		DBVPG6304:F>L		UFRJ50816:F>L		UWOPS91_917_1:F>L	AA:135		A4:A>G		DBVPG6304:A>G	AA:142		UFRJ50816:L>M		UWOPS91_917_1:L>M	AA:157		UFRJ50816:M>I	AA:158		N_45:M>R	AA:160		A12:F>L		A4:F>L		DBVPG6304:F>L		N_45:L>R		YPS138:F>L	AA:168		UWOPS91_917_1:->Y	AA:173		UWOPS91_917_1:L>V	AA:175		A4:F>C		CBS5829:F>L		DBVPG6304:F>C		UFRJ50816:F>C	AA:187		A4:Q>H		DBVPG6304:Q>H		UFRJ50816:Q>H	AA:190		UFRJ50816:V>F		UWOPS91_917_1:V>F	AA:198		N_43:F>L		N_45:F>L	AA:232		A12:I>L		A4:I>L		DBVPG6304:I>L		UFRJ50816:I>L		YPS138:I>L	AA:247		UWOPS91_917_1:V>I	AA:272		UWOPS91_917_1:W>C	AA:300		A12:->Y		A4:->Y		DBVPG6304:->Y		IFO1804:->Y		N_43:->Y		N_44:->Y		N_45:->Y		UFRJ50816:->Y		YPS138:->Y	AA:317		UWOPS91_917_1:R>S	AA:321		A12:R>T		A4:R>T		DBVPG6304:R>T		UFRJ50816:R>T		YPS138:R>T	AA:343		A12:R>H		A4:R>H		DBVPG6304:R>H		UFRJ50816:R>H		YPS138:R>H	AA:363		A12:H>Q		A4:H>Q		DBVPG6304:H>Q		YPS138:H>Q	AA:367		A12:C>W		A4:C>W		DBVPG6304:C>W		UFRJ50816:C>W		YPS138:C>W	AA:376		A12:L>H		A4:L>H		DBVPG6304:L>H		UFRJ50816:L>H		YPS138:L>H	AA:390		IFO1804:F>L		N_43:F>L		N_44:F>L		N_45:F>L	AA:396		IFO1804:->E		N_43:->E		N_44:->E		N_45:->E	AA:434		A12:L>I		A4:L>I		DBVPG6304:L>I		YPS138:L>I	AA:454		A12:N>K		A4:N>K		DBVPG6304:N>K		YPS138:N>K	AA:465		A12:H>Q		A4:H>Q		DBVPG6304:H>Q		IFO1804:H>Q		N_43:H>Q		N_44:H>Q		N_45:H>Q		Q32_3:H>Q		UWOPS91_917_1:H>Q		YPS138:H>Q	AA:486		UWOPS91_917_1:F>L	AA:509		A12:I>M		A4:I>M		DBVPG6304:I>M		YPS138:I>M	AA:531		A12:F>L		A4:F>L		DBVPG6304:F>L		YPS138:F>L	AA:582		A12:A>P		A4:A>P		UWOPS91_917_1:A>P		YPS138:A>P	AA:594		UWOPS91_917_1:S>R	AA:595		A12:N>D		A4:N>D		UFRJ50791:N>D		YPS138:N>D	AA:622		N_44:M>I		N_45:M>I	AA:623		UWOPS91_917_1:R>L	AA:629		A4:->Y		DBVPG6304:->Y		UFRJ50791:->Y		UWOPS91_917_1:->Y		YPS138:->Y	AA:633		UWOPS91_917_1:Y>-	AA:697		A4:P>Q		DBVPG6304:P>Q		UFRJ50791:P>Q		YPS138:P>QID:YBR157C	AA:9		IFO1804:N>S		N_43:N>S		N_44:N>S		N_45:N>S	AA:73		A12:R>S		DBVPG6304:R>S		UFRJ50791:R>S		UWOPS91_917_1:R>S		YPS138:R>S	AA:93		UWOPS91_917_1:C>G	AA:109		A12:P>T		DBVPG6304:P>T		IFO1804:P>T		N_43:P>T		N_44:P>T		N_45:P>T		UFRJ50791:P>T		UFRJ50816:P>T		UWOPS91_917_1:P>T		YPS138:P>T	AA:118		A12:S>F		DBVPG6304:S>F		UFRJ50791:S>F		YPS138:S>F	AA:122		Q95_3:F>I	AA:126		A12:->W		DBVPG6304:->W		UFRJ50791:->W		YPS138:->W	AA:129		A12:C>-		DBVPG6304:C>-		UFRJ50791:C>-		UWOPS91_917_1:C>-		YPS138:C>-	AA:175		IFO1804:R>Q		N_43:R>Q		N_45:R>Q	AA:181		IFO1804:->K		N_43:->K		N_45:->K		UWOPS91_917_1:I>F	AA:199		Z1_1:I>L	AA:211		Z1_1:F>L	AA:231		DBVPG6304:C>-		UWOPS91_917_1:C>-		YPS138:C>-	AA:253		A4:K>R		DBVPG6304:K>R		UWOPS91_917_1:K>R		YPS138:K>RID:YBR159W	AA:40		UWOPS91_917_1:L>F	AA:79		UWOPS91_917_1:A>S	AA:84		UWOPS91_917_1:K>E	AA:101		A4:A>T		DBVPG6304:A>T		UFRJ50816:A>T		YPS138:A>T	AA:118		A4:A>S		DBVPG6304:A>S		YPS138:A>S	AA:125		UFRJ50816:T>A	AA:127		A4:T>S		DBVPG6304:T>S		UFRJ50816:T>S		UWOPS91_917_1:T>S		YPS138:T>S	AA:162		N_45:E>D	AA:168		DBVPG6304:D>N		UFRJ50816:D>N		YPS138:D>N	AA:196		UWOPS91_917_1:K>N	AA:235		DBVPG6304:N>S		UFRJ50816:N>S		YPS138:N>S	AA:278		N_43:K>E		N_45:K>E	AA:292		UWOPS91_917_1:D>E	AA:305		UFRJ50816:V>M		YPS138:V>M	AA:327		UWOPS91_917_1:T>SID:YBR160W	AA:240		UFRJ50816:V>AID:YBR162C	AA:3		A4:Q>K		DBVPG6304:Q>K		UFRJ50816:Q>K	AA:80		UWOPS91_917_1:F>L	AA:84		A4:H>D		DBVPG6304:H>D		UFRJ50791:H>D		UWOPS91_917_1:H>D		YPS138:H>D	AA:93		UWOPS91_917_1:R>-	AA:101		N_43:Q>H		N_45:Q>H	AA:112		UWOPS91_917_1:N>K	AA:115		A12:H>Q		A4:H>Q		UFRJ50791:H>Q		UWOPS91_917_1:H>Q		YPS138:H>Q	AA:169		A12:E>D		A4:E>D		UFRJ50791:E>D		UWOPS91_917_1:E>D		YPS138:E>D	AA:181		N_43:S>R		N_45:S>R	AA:198		A12:R>I		A4:R>I		UFRJ50791:R>I		UWOPS91_917_1:R>I		YPS138:R>I	AA:202		A12:T>K		A4:T>K		N_43:T>K		N_45:T>K		UFRJ50791:T>K		UWOPS91_917_1:T>K		YPS138:T>K	AA:208		UWOPS91_917_1:R>-	AA:317		UWOPS91_917_1:N>S	AA:350		UWOPS91_917_1:S>PID:YBR162W-A	AA:7		UWOPS91_917_1:K>R	AA:18		A4:N>K		DBVPG6304:N>K		UFRJ50791:N>K	AA:31		A12:S>G	AA:34		A12:D>E	AA:37		A12:P>A	AA:39		A12:M>VID:YBR163W	AA:15		UWOPS91_917_1:H>Q	AA:29		A12:G>E		A4:G>E		YPS138:G>E	AA:34		A12:S>C		A4:S>C		YPS138:S>C	AA:38		A12:N>S		A4:N>S		YPS138:N>S	AA:65		A12:L>F		A4:L>F		CBS432:L>F		CBS5829:L>F		N_17:L>F		N_43:L>F		N_44:L>F		N_45:L>F		Q59_1:L>F		Q62_5:L>F		Q95_3:L>F		S36_7:L>F		T21_4:L>F		UWOPS91_917_1:L>F		Y6_5:L>F		Y7:L>F		YPS138:L>F		Z1_1:L>F	AA:68		Q59_1:N>K		S36_7:N>K	AA:89		A12:N>S		A4:N>S		UWOPS91_917_1:N>S		YPS138:N>S	AA:114		T21_4:N>T	AA:145		UWOPS91_917_1:N>K	AA:178		A12:E>G		A4:E>G		YPS138:E>G	AA:192		UWOPS91_917_1:D>N	AA:207		Y7:M>R	AA:245		UWOPS91_917_1:D>N	AA:259		A12:T>K		A4:T>K		DBVPG6304:T>K		UFRJ50791:T>K		YPS138:T>K	AA:269		A12:I>V		A4:I>V		DBVPG6304:I>V		UFRJ50791:I>V		UWOPS91_917_1:I>V	AA:286		A12:M>T		A4:M>T		DBVPG6304:M>T		IFO1804:M>T		N_45:M>T		UFRJ50791:M>T		UWOPS91_917_1:M>T	AA:292		A12:E>K		A4:E>K		DBVPG6304:E>K		UFRJ50791:E>K	AA:295		UWOPS91_917_1:S>G	AA:299		A12:V>I		A4:V>I		DBVPG6304:V>I		UFRJ50791:V>I		UWOPS91_917_1:V>I	AA:312		IFO1804:S>L		N_43:S>L		N_45:S>L	AA:333		UWOPS91_917_1:E>G	AA:356		UWOPS91_917_1:D>E	AA:398		A12:V>L		A4:V>L		DBVPG6304:V>L		UFRJ50791:V>L		YPS138:V>L	AA:416		A12:T>I		A4:T>I		DBVPG6304:T>I		UFRJ50791:T>I		YPS138:T>I	AA:445		UWOPS91_917_1:E>D	AA:451		A12:P>R		A4:P>R		DBVPG6304:P>R		UFRJ50791:P>R		YPS138:P>R	AA:454		A12:C>F		A4:C>F		DBVPG6304:C>F		UFRJ50791:C>F		YPS138:C>F	AA:462		A12:Y>H		A4:Y>H		DBVPG6304:Y>H		UFRJ50791:Y>H		UWOPS91_917_1:Y>H		YPS138:Y>H	AA:519		UWOPS91_917_1:E>G	AA:523		A12:R>H		A4:R>H		N_43:R>H		N_44:R>H		N_45:R>H		UFRJ50791:R>H		UFRJ50816:R>H		UWOPS91_917_1:R>H		YPS138:R>H	AA:528		A4:V>I		UFRJ50791:V>I		UFRJ50816:V>I		YPS138:V>I	AA:565		CBS432:E>K		N_17:E>KID:YBR164C	AA:122		UWOPS91_917_1:F>L	AA:123		IFO1804:Q>H		N_45:Q>H	AA:143		UWOPS91_917_1:F>I	AA:167		A12:F>L		A4:F>L		UFRJ50816:F>L		YPS138:F>LID:YBR165W	AA:3		A12:H>Y		DBVPG6304:H>Y		N_44:H>Y		N_45:H>Y		UFRJ50816:H>Y		UWOPS91_917_1:H>Y	AA:49		A12:S>F		DBVPG6304:S>F		UFRJ50816:S>F		UWOPS91_917_1:S>F	AA:62		N_17:D>N	AA:77		A12:C>G		DBVPG6304:C>G		UFRJ50816:C>G		UWOPS91_917_1:C>G	AA:92		A12:L>F		DBVPG6304:L>F		UFRJ50816:L>F		UWOPS91_917_1:L>F	AA:94		N_44:S>L	AA:161		A4:S>G		DBVPG6304:S>G		Q59_1:S>N		UFRJ50816:S>G	AA:163		A4:S>A		DBVPG6304:S>A		UFRJ50816:S>A	AA:166		A4:L>H		DBVPG6304:L>H		N_44:L>H		N_45:L>H		UFRJ50816:L>H	AA:192		A4:V>A		DBVPG6304:V>A		N_43:V>A		N_44:V>A		N_45:V>A		UFRJ50816:V>A	AA:201		A4:I>M		DBVPG6304:I>M		UFRJ50816:I>M	AA:209		A12:G>S		A4:G>S		DBVPG6304:G>S		N_43:G>S		N_44:G>S		N_45:G>S		UFRJ50816:G>S	AA:214		CBS5829:L>F	AA:227		A4:N>S		DBVPG6304:N>S		UFRJ50816:N>S	AA:262		KPN3828:M>I		KPN3829:M>I		N_17:M>I	AA:264		A12:E>D		A4:E>D		DBVPG6304:E>D	AA:267		CBS5829:S>GID:YBR166C	AA:11		A12:S>R		DBVPG6304:S>R		UFRJ50816:S>R		UWOPS91_917_1:S>R	AA:12		N_43:R>L		N_44:R>L		N_45:R>L	AA:21		N_43:G>C		N_44:G>C		N_45:G>C	AA:38		A12:K>T		DBVPG6304:K>T		UFRJ50816:K>T		UWOPS91_917_1:K>T	AA:47		A12:L>R		DBVPG6304:L>R		UWOPS91_917_1:L>R	AA:68		UWOPS91_917_1:K>Q	AA:79		CBS432:M>I	AA:118		A12:N>K		A4:N>K		DBVPG6304:N>K		UFRJ50791:N>K		UFRJ50816:N>K	AA:141		A12:I>M		A4:I>M		DBVPG6304:I>M		UFRJ50791:I>M		UFRJ50816:I>M	AA:213		A12:Y>S		A4:Y>S		DBVPG6304:Y>S		UFRJ50791:Y>S		UFRJ50816:Y>S	AA:246		A12:Q>H		A4:Q>H		UFRJ50791:Q>H		UFRJ50816:Q>H	AA:274		A12:S>R		A4:S>R		UFRJ50791:S>R		UFRJ50816:S>R	AA:356		A12:N>K		A4:N>K	AA:442		Q32_3:D>EID:YBR167C	AA:11		A12:->L		A4:->L	AA:14		A12:F>L		A4:F>L	AA:20		A12:F>L		A4:F>L		UFRJ50816:F>L	AA:28		A12:F>V		A4:F>V		UFRJ50816:F>V	AA:33		N_43:C>G		N_44:C>G		N_45:C>G	AA:44		A12:F>L		A4:F>L		N_43:F>L		N_44:F>L		N_45:F>L		UFRJ50816:F>L	AA:72		A12:F>L		A4:F>L		UFRJ50816:F>L	AA:82		UWOPS91_917_1:->C	AA:97		N_43:Q>E		N_44:Q>E		N_45:Q>E	AA:100		UWOPS91_917_1:N>H	AA:113		UFRJ50816:Y>S	AA:117		N_43:C>S		N_44:C>S		N_45:C>S	AA:125		A4:L>H		UFRJ50816:L>H		UWOPS91_917_1:L>H	AA:141		N_43:H>Q		N_44:H>Q		N_45:H>QID:YBR168W	AA:109		UWOPS91_917_1:G>V	AA:162		N_44:L>M	AA:163		N_17:K>E		N_45:K>E	AA:176		N_44:K>R	AA:216		N_43:T>A		N_44:T>A	AA:263		N_43:E>G		N_44:E>G		N_45:E>G	AA:299		N_43:D>N		N_44:D>N		N_45:D>N	AA:323		A12:S>G		A4:S>G		DBVPG6304:S>G		UFRJ50816:S>G	AA:329		IFO1804:N>K		N_43:N>K		N_44:N>K		N_45:N>K	AA:332		A12:R>P		A4:R>P		CBS5829:R>P		DBVPG6304:R>P		IFO1804:R>P		N_17:R>P		N_43:R>P		N_44:R>P		N_45:R>P		Q32_3:R>P		Q62_5:R>P		Q95_3:R>P		T21_4:R>P		UFRJ50816:R>P		Y7:R>P		YPS138:R>P	AA:339		A12:S>T		A4:S>T		UFRJ50816:S>T		YPS138:S>T	AA:352		IFO1804:K>R		N_43:K>R		N_44:K>R		N_45:K>R	AA:359		A12:K>E		A4:K>E		UFRJ50816:K>E		YPS138:K>E	AA:375		A12:V>D		A4:V>D		IFO1804:V>D		N_43:V>D		N_44:V>D		N_45:V>D		UFRJ50816:V>D		YPS138:V>DID:YBR169C	AA:39		A12:I>F		A4:I>F		UFRJ50791:I>F		UFRJ50816:I>F		UWOPS91_917_1:I>F		YPS138:I>F	AA:46		A12:F>C		A4:F>C		UFRJ50791:F>C		YPS138:F>C	AA:61		UFRJ50791:->Y		YPS138:->Y	AA:64		UWOPS91_917_1:Q>H	AA:86		UWOPS91_917_1:F>L	AA:97		N_43:F>C		N_44:F>C		N_45:F>C	AA:98		UWOPS91_917_1:F>L	AA:150		N_44:N>I	AA:154		UWOPS91_917_1:R>C	AA:209		IFO1804:R>S		N_44:R>S	AA:217		UWOPS91_917_1:Y>-	AA:226		A12:N>S	AA:236		A12:N>D		IFO1804:N>D		N_44:N>D		UFRJ50791:N>D		UWOPS91_917_1:N>D		YPS138:N>D	AA:247		IFO1804:R>S		N_44:R>S	AA:265		IFO1804:->Y		N_44:->Y	AA:298		A12:N>K		DBVPG6304:N>K	AA:342		IFO1804:->C		N_44:->C		N_45:->C	AA:348		A12:D>E		DBVPG6304:D>E	AA:350		IFO1804:C>W		N_43:C>W		N_44:C>W	AA:354		CBS432:N>D		KPN3828:N>D		KPN3829:N>D	AA:356		IFO1804:F>C		N_43:F>C		N_44:F>C		N_45:F>C	AA:384		A12:F>L		DBVPG6304:F>L	AA:388		A12:F>L		DBVPG6304:F>L		UWOPS91_917_1:F>L	AA:421		A12:Q>H		DBVPG6304:Q>H		UWOPS91_917_1:Q>H	AA:451		A12:M>T	AA:482		N_43:N>Y		N_44:N>Y		N_45:N>Y		UWOPS91_917_1:N>Y	AA:489		UWOPS91_917_1:M>I	AA:502		A12:F>L		UFRJ50816:F>L	AA:518		A12:R>S		UFRJ50816:R>S	AA:523		N_43:D>E		N_44:D>E		N_45:D>E	AA:587		A4:T>A		UFRJ50816:T>A	AA:636		S36_7:G>V	AA:644		N_43:F>L		N_44:F>L		N_45:F>L	AA:647		A12:I>M		A4:I>M		UFRJ50816:I>M		YPS138:I>M	AA:653		YPS138:K>NID:YBR170C	AA:54		A12:V>M		A4:V>M		DBVPG6304:V>M		UFRJ50816:V>M		YPS138:V>M	AA:104		A12:T>S		A4:T>S		DBVPG6304:T>S		UFRJ50816:T>S		YPS138:I>N	AA:114		A4:M>K		DBVPG6304:M>K		N_43:M>K		N_45:M>K		UWOPS91_917_1:M>K		YPS138:M>K	AA:118		UWOPS91_917_1:H>N	AA:122		A12:->Y		A4:->Y		DBVPG6304:->Y		UFRJ50816:->Y		YPS138:V>F	AA:175		A4:C>W		DBVPG6304:C>W		YPS138:C>W	AA:203		UWOPS91_917_1:G>E	AA:219		A4:->C		DBVPG6304:->C		UWOPS91_917_1:->C		YPS138:->C	AA:228		A4:F>L		DBVPG6304:F>L		YPS138:F>L	AA:251		A12:I>M		A4:I>M		DBVPG6304:I>M		UWOPS91_917_1:I>M		YPS138:I>M	AA:255		UWOPS91_917_1:C>W	AA:260		A12:N>I		A4:N>I		DBVPG6304:N>I		N_43:N>I		N_44:N>I		UWOPS91_917_1:N>I		YPS138:N>I	AA:266		N_43:F>Y		N_44:F>Y	AA:275		A4:H>Y		DBVPG6304:H>Y		UWOPS91_917_1:H>Y		YPS138:H>Y	AA:296		UWOPS91_917_1:F>L	AA:308		A12:R>S		A4:R>S		DBVPG6304:R>S	AA:309		UWOPS91_917_1:C>W	AA:312		UWOPS91_917_1:I>R	AA:335		UWOPS91_917_1:F>L	AA:337		A12:D>E		A4:D>E		DBVPG6304:D>E	AA:345		N_43:D>E		N_44:D>E	AA:347		UWOPS91_917_1:I>M	AA:356		Q59_1:->Y		UWOPS91_917_1:->Y	AA:358		A12:N>K		A4:N>K		DBVPG6304:N>K	AA:365		N_43:R>S		N_44:R>S	AA:382		UWOPS91_917_1:D>E	AA:387		UWOPS91_917_1:F>L	AA:409		UWOPS91_917_1:Q>H	AA:415		A12:Y>N		A4:Y>N		DBVPG6304:Y>N		UFRJ50816:Y>N	AA:418		N_44:D>E	AA:449		Q59_1:F>L	AA:458		A12:F>L		A4:F>L		DBVPG6304:F>L		UFRJ50816:F>L	AA:469		A12:D>E		A4:D>E		DBVPG6304:D>E		UFRJ50816:D>E		UWOPS91_917_1:D>E	AA:473		UWOPS91_917_1:T>A	AA:484		UWOPS91_917_1:N>I	AA:496		A4:F>L		DBVPG6304:F>L		UFRJ50816:F>L	AA:504		UWOPS91_917_1:I>M	AA:532		DBVPG6304:L>F		UFRJ50816:L>F		UWOPS91_917_1:L>F		YPS138:L>F	AA:541		UWOPS91_917_1:C>F	AA:571		A4:C>W		DBVPG6304:C>W		UFRJ50816:C>W		UWOPS91_917_1:C>W		YPS138:C>WID:YBR171W	AA:15		N_17:F>V	AA:28		N_43:V>I		N_44:V>I		N_45:V>I		UWOPS91_917_1:V>I	AA:40		Q95_3:L>R	AA:54		A12:N>K		A4:N>K		DBVPG6304:N>K		N_43:N>K		N_44:N>K		N_45:N>K		UFRJ50816:N>K		UWOPS91_917_1:N>K		YPS138:N>K	AA:156		DBVPG4650:Q>H	AA:164		DBVPG4650:V>A	AA:196		DBVPG4650:L>P	AA:197		IFO1804:K>E		N_43:K>E		N_44:K>E		N_45:K>E	AA:205		IFO1804:T>I		N_43:T>I		N_44:T>I		N_45:T>IID:YBR172C	AA:23		N_43:K>M		N_45:K>M	AA:25		A4:->E		UWOPS91_917_1:->E		YPS138:->E	AA:32		A12:S>R		A4:S>R		IFO1804:S>R		N_43:S>R		N_44:S>R		N_45:S>R		UFRJ50791:S>R		UFRJ50816:S>R		UWOPS91_917_1:S>R		YPS138:S>R	AA:38		IFO1804:F>I		N_43:F>I		N_45:F>I	AA:45		UWOPS91_917_1:D>N	AA:58		N_43:T>S		N_44:T>S		N_45:T>S	AA:71		A12:F>L		YPS138:F>L	AA:83		UWOPS91_917_1:D>E	AA:114		A12:K>N		CBS432:K>N		CBS5829:K>N		DBVPG4650:K>N		KPN3828:K>N		N_17:K>N		S36_7:K>N		UWOPS91_917_1:K>N		YPS138:K>N	AA:128		N_17:->C		N_43:->C		N_44:->C		N_45:->C	AA:131		A4:N>I		N_43:->R		N_44:->R		UFRJ50791:N>I		YPS138:N>I	AA:133		A12:S>R		UWOPS91_917_1:S>R		YPS138:S>R	AA:151		N_43:R>S		N_44:R>S	AA:168		DBVPG4650:N>K		S36_7:N>K	AA:189		N_17:R>S		N_43:R>S		N_44:R>S	AA:190		A12:->L		A4:->L		YPS138:->L	AA:209		N_43:I>M		N_44:I>M	AA:244		N_17:L>V	AA:246		A12:L>H		A4:L>H		CBS432:L>H		CBS5829:L>H		DBVPG4650:L>H		KPN3828:L>H		N_17:L>H		Q32_3:L>H		S36_7:L>H	AA:248		N_43:S>R		N_44:S>R		N_45:S>R		UWOPS91_917_1:S>R	AA:259		A12:D>E		A4:D>E	AA:270		UWOPS91_917_1:W>R	AA:302		A4:R>G		DBVPG6304:R>G		UWOPS91_917_1:R>G	AA:305		A12:L>S		A4:L>S		DBVPG6304:L>S		N_43:L>S		N_44:L>S		N_45:L>S		YPS138:L>S	AA:329		A12:Q>H		A4:Q>H		DBVPG6304:Q>H		YPS138:Q>H	AA:340		A12:N>K		A4:N>K		DBVPG6304:N>K		UWOPS91_917_1:N>K		YPS138:N>K	AA:351		A12:F>S		A4:F>S		DBVPG6304:F>S		IFO1804:F>S		N_43:F>S		N_44:F>S		N_45:F>S		UWOPS91_917_1:F>S		YPS138:F>S	AA:364		DBVPG4650:R>L		IFO1804:G>V		N_43:G>V		N_44:G>V		N_45:G>V	AA:368		A12:F>C		A4:F>C		DBVPG6304:F>C		UWOPS91_917_1:F>C		YPS138:F>C	AA:384		CBS432:R>H		CBS5829:R>H		KPN3829:R>H		N_17:R>H	AA:385		A12:F>L		A4:F>L		DBVPG6304:F>L		UWOPS91_917_1:F>L		YPS138:F>L	AA:410		YPS138:I>T	AA:411		IFO1804:P>Q		N_43:P>Q		N_44:P>Q		N_45:P>Q	AA:417		UWOPS91_917_1:Q>R	AA:440		Q32_3:V>L	AA:448		A12:R>S		A4:R>S		DBVPG6304:R>S		YPS138:R>S	AA:452		CBS432:A>T		CBS5829:A>T		IFO1804:A>T		KPN3828:A>T		KPN3829:A>T		N_17:A>T		N_43:A>T		N_44:A>T		N_45:A>T		Q32_3:A>T	AA:457		CBS432:H>Y		CBS5829:H>Y		KPN3828:H>Y		KPN3829:H>Y		N_17:H>Y	AA:459		UWOPS91_917_1:V>L	AA:461		A12:S>N		A4:S>N		DBVPG6304:S>N		UWOPS91_917_1:S>N		YPS138:S>N	AA:470		IFO1804:L>V		N_43:L>V		N_44:L>V		N_45:L>V		UWOPS91_917_1:L>V	AA:482		UWOPS91_917_1:N>I	AA:487		IFO1804:->S		N_43:->S		N_44:->S		N_45:->S	AA:497		IFO1804:N>T		N_43:N>T		N_44:N>T		N_45:N>T	AA:521		N_43:Y>F		N_44:Y>F		N_45:Y>F	AA:550		UWOPS91_917_1:W>L	AA:555		IFO1804:->G		N_43:->G		N_45:->G		Q32_3:->G	AA:571		Q32_3:W>-		T21_4:W>-	AA:591		UWOPS91_917_1:T>I	AA:613		A4:R>P		UFRJ50791:R>P		UFRJ50816:R>P		YPS138:R>P	AA:617		A4:V>A		N_43:V>A		N_44:V>A		N_45:V>A		UFRJ50791:V>A		UFRJ50816:V>A		UWOPS91_917_1:V>A		YPS138:V>A	AA:630		UWOPS91_917_1:L>F	AA:633		A4:E>D		UFRJ50791:E>D		UFRJ50816:E>D		YPS138:E>D	AA:645		N_44:F>L		UWOPS91_917_1:T>R	AA:651		UWOPS91_917_1:I>M	AA:653		A12:Q>H		A4:Q>H		UFRJ50791:Q>H		UFRJ50816:Q>H		YPS138:Q>H	AA:697		A12:G>R		A4:G>R		UFRJ50791:G>R		UFRJ50816:G>R		YPS138:G>R	AA:717		A12:F>L		A4:F>L		UFRJ50791:F>L		UFRJ50816:F>L		UWOPS91_917_1:F>L		YPS138:F>LID:YBR173C	AA:4		A12:Q>H		A4:Q>H		DBVPG6304:Q>H		UFRJ50816:Q>H		UWOPS91_917_1:Q>H		YPS138:Q>H	AA:55		A4:V>A		DBVPG6304:V>A		UFRJ50816:V>A		UWOPS91_917_1:V>A		YPS138:V>A	AA:70		UWOPS91_917_1:F>L	AA:87		A4:C>W		DBVPG6304:C>W		UFRJ50816:C>W		YPS138:C>W	AA:113		N_43:F>L		N_45:F>L	AA:126		A4:R>S		DBVPG6304:R>S		UFRJ50816:R>S		YPS138:R>S	AA:138		A4:L>F		DBVPG6304:L>F		UFRJ50816:L>FID:YBR175W	AA:27		A12:F>L		A4:F>L		DBVPG6304:F>L	AA:41		A12:A>T		A4:A>T		DBVPG6304:A>T	AA:187		UWOPS91_917_1:N>	AA:249		N_43:E>G		N_45:E>G	AA:284		N_43:Y>H		N_45:Y>H	AA:287		N_43:S>G		N_45:S>G	AA:310		UWOPS91_917_1:C>RID:YBR176W	AA:4		YPS138:M>T	AA:10		A12:S>A		A4:S>A		DBVPG6304:S>A		IFO1804:S>A		N_43:S>A		N_45:S>A		UFRJ50816:S>A		UWOPS91_917_1:S>A	AA:189		A12:S>L		DBVPG6304:S>L		UFRJ50791:S>L		YPS138:S>L	AA:240		UWOPS91_917_1:S>N	AA:267		CBS5829:M>I	AA:270		UWOPS91_917_1:I>V	AA:272		DBVPG6304:T>I		UFRJ50791:T>I		UWOPS91_917_1:T>I		YPS138:T>I	AA:276		UWOPS91_917_1:K>N	AA:290		IFO1804:R>K		N_43:R>K		N_45:R>K	AA:306		A12:L>V		DBVPG6304:L>V		UFRJ50791:L>V		UWOPS91_917_1:L>I		YPS138:L>VID:YBR177C	AA:12		A12:F>L		DBVPG6304:F>L		UFRJ50816:F>L	AA:54		N_17:A>D		N_43:A>D		N_44:A>D		N_45:A>D	AA:100		A12:K>E	AA:131		UWOPS91_917_1:R>S	AA:136		A12:W>S		DBVPG6304:W>S		UFRJ50816:W>S		UWOPS91_917_1:W>S		YPS138:W>S	AA:162		A12:->R		DBVPG6304:->R		UFRJ50816:->R		YPS138:->R	AA:176		A12:I>M		DBVPG6304:I>M		UFRJ50816:I>M		YPS138:I>M	AA:197		A12:I>M		DBVPG6304:I>M		UFRJ50816:I>M		UWOPS91_917_1:I>M		YPS138:I>M	AA:212		A12:->Y		DBVPG6304:->Y		UFRJ50816:->Y		YPS138:->Y	AA:214		IFO1804:S>Y		N_44:S>Y		N_45:S>Y	AA:274		A4:M>I		DBVPG6304:M>I		UWOPS91_917_1:M>I		YPS138:M>I	AA:314		N_43:W>R		N_44:W>R		N_45:W>R	AA:348		N_43:S>R		N_44:S>R		N_45:S>R	AA:364		UWOPS91_917_1:E>D	AA:417		A12:F>L		A4:F>L		DBVPG6304:F>L		UFRJ50791:F>L		UFRJ50816:F>L		UWOPS91_917_1:F>L		YPS138:F>L	AA:433		A12:S>R		A4:S>R		DBVPG6304:S>R		UFRJ50791:S>R		UFRJ50816:S>R		UWOPS91_917_1:S>R		YPS138:S>R	AA:451		A12:->S		A4:->S		DBVPG6304:->S		UFRJ50791:->S		UFRJ50816:->S		UWOPS91_917_1:->S		YPS138:->SID:YBR179C	AA:5		UWOPS91_917_1:->W	AA:12		A12:I>T		N_45:I>T		UFRJ50816:I>T		UWOPS91_917_1:I>T	AA:34		A4:F>L		DBVPG6304:F>L		UFRJ50791:F>L		YPS138:F>L	AA:40		UFRJ50791:F>I	AA:57		A4:F>L		DBVPG6304:F>L		UFRJ50791:F>L		YPS138:F>L	AA:95		UWOPS91_917_1:W>C	AA:130		A4:F>L		DBVPG6304:F>L		UFRJ50791:F>L	AA:183		DBVPG6304:I>M		YPS138:I>M	AA:190		DBVPG6304:Y>-		YPS138:Y>-	AA:198		N_44:R>S		N_45:R>S	AA:228		A4:Y>H		DBVPG6304:Y>H		YPS138:Y>H	AA:231		A4:L>I		DBVPG6304:L>I		YPS138:L>I	AA:238		A4:F>L		DBVPG6304:F>L		YPS138:F>L	AA:247		YPS138:E>D	AA:268		N_45:L>F	AA:275		N_45:->E	AA:285		N_45:Y>H	AA:289		IFO1804:C>F		N_45:C>F	AA:309		IFO1804:W>C		N_45:W>C	AA:334		KPN3829:I>F	AA:344		IFO1804:C>W		N_45:C>W	AA:350		IFO1804:F>L		N_45:F>L	AA:355		A12:F>L		A4:F>L		DBVPG6304:F>L		YPS138:F>L	AA:362		IFO1804:Y>-		N_45:Y>-	AA:370		IFO1804:H>Q		N_45:H>Q	AA:376		DBVPG6304:H>Q	AA:378		A12:Y>-		A4:Y>-		DBVPG6304:Y>-		YPS138:Y>-	AA:405		A12:->Y		A4:->Y		DBVPG6304:->Y		YPS138:->Y	AA:418		IFO1804:A>P		N_45:A>P	AA:423		A12:I>S		A4:I>S		DBVPG6304:I>S	AA:457		A12:C>-		A4:C>-	AA:477		IFO1804:T>A		N_44:T>A		N_45:T>A	AA:593		A4:S>R		DBVPG6304:S>R		N_17:S>R		N_43:S>R		N_44:S>R		N_45:S>R		UWOPS91_917_1:S>R		YPS138:S>R	AA:612		A4:I>N		YPS138:I>N	AA:623		UWOPS91_917_1:V>D	AA:625		A4:F>Y		DBVPG6304:F>Y		UWOPS91_917_1:F>Y		YPS138:F>Y	AA:707		A4:F>L		DBVPG6304:F>L		UFRJ50816:F>L		YPS138:F>L	AA:719		N_17:Q>H		N_43:Q>H		N_45:Q>H	AA:755		A12:->C		A4:->C		DBVPG6304:->C		UFRJ50816:->C		UWOPS91_917_1:->C		YPS138:->C	AA:776		A12:M>I		A4:M>I		DBVPG6304:M>I		UFRJ50816:M>I		YPS138:M>I	AA:790		A12:I>M		A4:I>M		DBVPG6304:I>M		UFRJ50816:I>M		YPS138:I>M	AA:827		A12:W>L		A4:W>L		N_43:W>L		N_45:W>L		UFRJ50816:W>L		UWOPS91_917_1:W>L		YPS138:W>L	AA:829		N_43:H>Q		N_45:H>Q	AA:848		UWOPS91_917_1:F>L	AA:851		N_45:L>F		YPS138:L>FID:YBR180W	AA:5		A12:S>P		A4:S>P		DBVPG6304:S>P		IFO1804:S>P		N_43:S>P		N_44:S>P		N_45:S>P		UFRJ50791:S>P		UFRJ50816:S>P		UWOPS91_917_1:S>P	AA:35		UWOPS91_917_1:D>Y	AA:39		Y6_5:N>S	AA:43		DBVPG6304:D>E		UFRJ50791:D>E		UFRJ50816:D>E	AA:49		N_43:K>E		N_44:K>E	AA:52		A12:I>T		A4:I>T		DBVPG6304:I>T		IFO1804:I>T		N_43:I>T		N_44:I>T		UFRJ50791:I>T		UFRJ50816:I>T	AA:72		IFO1804:A>V		N_43:A>V		N_44:A>V		N_45:A>V	AA:82		UWOPS91_917_1:K>R	AA:90		IFO1804:D>G		N_43:D>G		N_44:D>G		N_45:D>G		UWOPS91_917_1:D>G	AA:97		A12:A>E		A4:A>E		DBVPG6304:A>E		UFRJ50791:A>E	AA:105		UFRJ50791:T>N	AA:108		A12:R>Q		A4:R>Q		DBVPG6304:R>Q		IFO1804:R>Q		N_43:R>Q		N_44:R>Q		N_45:R>Q		UFRJ50791:R>Q	AA:111		A12:V>I		A4:V>I		DBVPG6304:V>I		UFRJ50791:V>I	AA:279		CBS432:T>I		CBS5829:T>I		Q32_3:T>I		Q59_1:T>I		Q62_5:T>I		Q89_8:T>I		Q95_3:T>I		Z1_1:T>I	AA:308		IFO1804:D>E		N_43:D>E		N_44:D>E		N_45:D>E		UWOPS91_917_1:D>N	AA:321		N_43:N>S		N_44:N>S		N_45:N>S	AA:323		A4:T>I		DBVPG6304:T>I		UWOPS91_917_1:T>I		YPS138:T>I	AA:337		N_43:P>S		N_44:P>S		N_45:P>S	AA:338		UWOPS91_917_1:V>L	AA:373		N_43:V>I		N_44:V>I		N_45:V>I		UWOPS91_917_1:V>I	AA:428		UWOPS91_917_1:Y>H	AA:429		N_44:L>F		N_45:L>F	AA:431		UWOPS91_917_1:L>S	AA:436		A4:N>S		UWOPS91_917_1:N>S		YPS138:N>S	AA:443		UWOPS91_917_1:P>L	AA:534		N_44:C>S	AA:552		N_44:V>IID:YBR181C	AA:17		Z1_1:S>R	AA:27		A12:Q>E		A4:Q>E		DBVPG6304:Q>E		YPS138:Q>E	AA:48		Z1_1:L>M	AA:65		UWOPS91_917_1:W>C	AA:94		UWOPS91_917_1:E>D	AA:97		A12:D>E		A4:D>E		DBVPG6304:D>E		N_43:D>E		N_45:D>E		UWOPS91_917_1:D>E	AA:117		N_43:Q>H		N_45:Q>H	AA:147		A12:S>R		A4:S>R		DBVPG6304:F>L	AA:162		UWOPS91_917_1:Q>HID:YBR182C	AA:152		A4:I>T		DBVPG6304:I>T		YPS138:I>T	AA:162		N_43:L>F		N_45:L>F	AA:164		N_45:G>V		UWOPS91_917_1:C>W	AA:168		A4:L>F		DBVPG6304:L>F		YPS138:L>F	AA:178		N_43:P>T		N_45:P>T	AA:182		N_43:S>N		N_45:S>N	AA:187		A4:D>E		DBVPG6304:D>E		UWOPS91_917_1:D>E		YPS138:D>E	AA:198		A12:I>T		A4:I>T		DBVPG6304:I>T		UWOPS91_917_1:I>T		YPS138:I>T	AA:205		UWOPS91_917_1:->Y	AA:237		UWOPS91_917_1:N>S	AA:252		A12:K>N		DBVPG6304:K>N		UWOPS91_917_1:K>N		YPS138:K>N	AA:258		A12:L>F		A4:L>F		DBVPG6304:L>F		YPS138:L>F	AA:271		DBVPG6304:->Y	AA:287		A12:R>I		DBVPG6304:R>I		KPN3828:R>S		N_17:R>S		UWOPS91_917_1:R>I		YPS138:R>I	AA:303		A12:W>L		DBVPG6304:W>L		UWOPS91_917_1:W>L		YPS138:W>L	AA:310		IFO1804:S>T		N_43:S>T		N_45:S>T	AA:335		UWOPS91_917_1:S>R	AA:342		A12:S>N		DBVPG6304:S>N		IFO1804:S>N		N_45:S>N		UWOPS91_917_1:S>N		YPS138:S>N	AA:344		UWOPS91_917_1:L>W	AA:359		A12:N>K		DBVPG6304:N>K		IFO1804:N>K		N_45:N>K		YPS138:N>K	AA:377		Q95_3:M>I		T21_4:M>I		Y6_5:M>I	AA:391		IFO1804:A>T		N_45:A>T	AA:413		A12:Y>C		UFRJ50791:Y>C	AA:425		IFO1804:K>N		N_45:K>N	AA:433		A12:K>N		DBVPG6304:K>N		UFRJ50791:K>N	AA:450		IFO1804:S>P		N_43:S>P		N_45:S>PID:YBR183W	AA:5		A12:R>H		DBVPG6304:R>H		UFRJ50791:R>H		UFRJ50816:R>H		YPS138:R>H	AA:11		A12:N>S		DBVPG6304:N>S		UFRJ50791:N>S		UFRJ50816:N>S		UWOPS91_917_1:N>S		YPS138:N>S	AA:140		IFO1804:I>V		N_45:I>V	AA:225		N_45:I>V	AA:317		N_45:->KID:YBR185C	AA:11		UFRJ50791:I>N	AA:22		DBVPG4650:I>S	AA:26		IFO1804:A>T	AA:30		A12:F>L		A4:F>L		DBVPG6304:F>L	AA:54		IFO1804:G>D		N_43:G>D		N_44:G>D		N_45:G>D	AA:59		DBVPG6304:N>K		UFRJ50791:N>K		UWOPS91_917_1:N>K	AA:79		DBVPG6304:F>V		UFRJ50791:F>V	AA:135		N_43:P>A		N_45:P>A	AA:137		YPS138:->C	AA:180		UWOPS91_917_1:C>W	AA:204		DBVPG4650:G>C		DBVPG6304:G>C		IFO1804:G>C		N_43:G>C		N_45:G>C		UFRJ50791:G>C		UFRJ50816:G>C		UWOPS91_917_1:G>C		YPS138:G>C	AA:217		DBVPG6304:K>E		UFRJ50816:K>E		YPS138:K>E	AA:225		DBVPG6304:M>I		UFRJ50816:M>I		UWOPS91_917_1:M>I		YPS138:M>I	AA:229		DBVPG6304:K>N		UFRJ50816:K>N		UWOPS91_917_1:K>N		YPS138:K>N	AA:242		DBVPG6304:F>L		UFRJ50816:F>L		UWOPS91_917_1:F>L		YPS138:F>LID:YBR186W	AA:167		A12:E>D		A4:E>D		DBVPG6304:E>D		UFRJ50816:E>D	AA:259		IFO1804:F>L		N_43:F>L		N_44:F>L		N_45:F>L	AA:288		DBVPG4650:R>G	AA:292		A4:T>S		DBVPG6304:T>S		UFRJ50816:T>S	AA:295		DBVPG4650:F>V	AA:297		CBS5829:Q>R		Q32_3:Q>R		Q62_5:Q>R		Q89_8:Q>R		T21_4:Q>R		Y7:Q>R	AA:358		UWOPS91_917_1:S>A	AA:482		CBS432:E>K	AA:489		DBVPG4650:I>V	AA:495		DBVPG4650:R>K	AA:507		DBVPG4650:D>N	AA:508		A12:V>A		UFRJ50816:V>A	AA:510		DBVPG4650:R>W	AA:521		DBVPG4650:F>Y	AA:524		A12:T>I		DBVPG6304:T>I		UFRJ50816:T>I	AA:537		Y6_5:F>I	AA:551		A12:V>A		DBVPG6304:V>A		UFRJ50816:V>A	AA:553		DBVPG4650:S>T		UWOPS91_917_1:S>F	AA:554		A12:R>-		DBVPG6304:R>-		UFRJ50816:R>-ID:YBR193C	AA:14		UFRJ50816:W>C	AA:18		DBVPG6304:C>W		YPS138:C>W	AA:30		DBVPG6304:R>T		UFRJ50816:R>T		YPS138:R>T	AA:100		YPS138:P>R	AA:119		DBVPG6304:M>I	AA:130		DBVPG6304:N>S		IFO1804:N>S		N_43:N>S		N_44:N>S		N_45:N>S		YPS138:N>S	AA:183		A12:L>S		A4:L>S		DBVPG6304:L>S		IFO1804:L>S		N_43:L>S		N_44:L>S		N_45:L>S		UFRJ50816:L>S	AA:209		A12:G>R		A4:G>R		DBVPG6304:G>R		UFRJ50816:G>R	AA:218		N_44:->L		N_45:->LID:YBR195C	AA:3		A12:I>L		A4:L>R		DBVPG6304:L>R		UFRJ50791:L>R		UFRJ50816:L>R		UWOPS91_917_1:I>L		YPS138:I>L	AA:10		A12:G>V		A4:G>V		DBVPG6304:G>V		YPS138:G>V	AA:14		A12:V>G		A4:V>G		DBVPG6304:V>G		YPS138:V>G	AA:45		A12:C>-		A4:C>-		DBVPG6304:C>-		YPS138:C>-	AA:84		A12:T>N		A4:T>N		DBVPG6304:T>N		UWOPS91_917_1:T>N		YPS138:T>N	AA:87		A12:H>Q		A4:H>Q		DBVPG6304:H>Q		UWOPS91_917_1:H>Q		YPS138:H>Q	AA:101		A12:->L		A4:->L		DBVPG6304:->L		N_43:->L		N_44:->L		N_45:->L		UWOPS91_917_1:->L		YPS138:->L	AA:111		N_43:C>W		N_44:C>W		N_45:C>W	AA:115		UWOPS91_917_1:R>-	AA:178		UWOPS91_917_1:->W	AA:185		N_43:Y>C		N_44:Y>C		N_45:Y>C		UWOPS91_917_1:Y>C	AA:199		A12:Y>N	AA:219		A12:->C		A4:->C		DBVPG6304:->C		YPS138:->C	AA:242		A12:T>P		A4:T>P		DBVPG6304:T>P		UWOPS91_917_1:T>P		YPS138:T>P	AA:257		UWOPS91_917_1:Y>-	AA:299		A12:N>K		A4:N>K		DBVPG6304:N>K		UFRJ50791:N>K		YPS138:N>K	AA:318		A12:E>D		A4:E>D		DBVPG6304:E>D		UFRJ50791:E>D		UWOPS91_917_1:E>D	AA:333		A12:Y>-		A4:Y>-		DBVPG6304:Y>-		UFRJ50791:Y>-	AA:344		A12:Y>-		A4:Y>-		DBVPG6304:Y>-		UFRJ50791:Y>-		UWOPS91_917_1:Y>-	AA:373		UWOPS91_917_1:E>D	AA:405		DBVPG4650:Q>H	AA:416		DBVPG6304:C>W		UFRJ50791:C>W		UWOPS91_917_1:C>WID:YBR197C	AA:2		UWOPS91_917_1:A>T	AA:16		IFO1804:V>G		N_44:V>G		N_45:V>G	AA:17		UWOPS91_917_1:L>R	AA:23		A12:D>E		UFRJ50816:D>E		YPS138:D>E	AA:24		UWOPS91_917_1:F>I	AA:26		UWOPS91_917_1:V>D	AA:27		IFO1804:I>F		N_44:I>F		N_45:I>F	AA:32		A12:W>-		UFRJ50816:W>-		YPS138:W>-	AA:36		UWOPS91_917_1:I>M	AA:49		A12:A>V		UFRJ50816:A>V		YPS138:A>V	AA:51		A12:Q>H		UFRJ50816:Q>H		YPS138:Q>H	AA:56		A12:->Y		UFRJ50816:->Y		YPS138:->Y	AA:58		IFO1804:L>M		N_44:L>M	AA:60		A12:S>C		UFRJ50816:S>C		YPS138:S>C	AA:77		UWOPS91_917_1:E>D	AA:84		UWOPS91_917_1:W>-	AA:131		DBVPG4650:F>C	AA:166		UWOPS91_917_1:S>G	AA:204		UFRJ50816:I>M	AA:206		UWOPS91_917_1:H>QID:YBR199W	AA:20		DBVPG6304:S>L		UFRJ50791:S>L		UFRJ50816:S>L	AA:54		A4:T>S		DBVPG6304:T>S		UFRJ50791:T>S		UFRJ50816:T>S		YPS138:T>S	AA:59		YPS138:A>S	AA:65		DBVPG6304:T>S		UFRJ50791:T>S		UFRJ50816:T>S		UWOPS91_917_1:T>S		YPS138:T>S	AA:80		A4:F>I		DBVPG6304:F>I		N_44:F>L		N_45:F>L		UFRJ50791:F>I		UFRJ50816:F>I		UWOPS91_917_1:F>I		YPS138:F>I	AA:89		UWOPS91_917_1:D>E	AA:95		N_44:P>A		N_45:P>A	AA:107		UWOPS91_917_1:D>E	AA:108		UWOPS91_917_1:E>D	AA:120		DBVPG6304:D>N		UFRJ50791:D>N		UFRJ50816:D>N		UWOPS91_917_1:D>N		YPS138:D>N	AA:148		DBVPG6304:G>D		UFRJ50791:G>D		UFRJ50816:G>D		UWOPS91_917_1:G>D		YPS138:G>D	AA:182		DBVPG6304:D>N		YPS138:D>N	AA:205		UWOPS91_917_1:N>D	AA:223		A4:G>R		UFRJ50791:G>R		UFRJ50816:G>R		UWOPS91_917_1:G>R		YPS138:G>R	AA:226		N_44:K>N		N_45:K>N		UWOPS91_917_1:K>N	AA:228		N_44:Q>H		N_45:Q>H	AA:272		N_43:K>N		N_44:K>N		N_45:K>N	AA:294		N_43:D>E		N_44:D>E		N_45:D>E	AA:319		A12:A>V		A4:A>V		UFRJ50791:A>V		UWOPS91_917_1:A>V		YPS138:A>V	AA:334		N_43:D>E		N_44:D>E		N_45:D>E	AA:335		A12:N>K		A4:N>K		UFRJ50791:N>K		UFRJ50816:N>K		UWOPS91_917_1:N>K		YPS138:N>K	AA:357		UWOPS91_917_1:E>D	AA:362		A12:E>G		A4:E>G		UFRJ50791:E>G		YPS138:E>G	AA:363		UWOPS91_917_1:P>S	AA:412		A12:I>N		A4:I>N		CBS432:I>T		CBS5829:I>T		KPN3828:I>T		KPN3829:I>T		N_17:I>T		Q59_1:I>T		Q62_5:I>T		Q95_3:I>T		T21_4:I>T		UWOPS91_917_1:I>T		Y7:I>T		YPS138:I>N	AA:425		UWOPS91_917_1:D>G	AA:437		A12:T>S		YPS138:T>S	AA:441		N_43:N>D	AA:442		UWOPS91_917_1:L>I	AA:444		N_43:D>N	AA:462		A12:G>E		UFRJ50816:G>E		YPS138:G>EID:YBR201W	AA:15		N_43:I>V		N_45:I>V	AA:38		A12:A>T		A4:A>T		DBVPG6304:A>T		UFRJ50816:A>T	AA:43		UWOPS91_917_1:K>R	AA:65		UWOPS91_917_1:G>R	AA:106		Q59_1:V>I	AA:115		IFO1804:A>T		N_43:A>T		N_44:A>T		N_45:A>T	AA:126		CBS5829:L>F		UWOPS91_917_1:L>I	AA:145		A12:G>A		DBVPG6304:G>A		UFRJ50791:G>A		UFRJ50816:G>A		UWOPS91_917_1:G>A	AA:148		UWOPS91_917_1:I>V	AA:168		A12:G>S		A4:G>S		DBVPG6304:G>S		UFRJ50791:G>S		UFRJ50816:G>S		UWOPS91_917_1:G>S	AA:191		UWOPS91_917_1:M>K	AA:208		A12:I>T		A4:I>T		DBVPG6304:I>T		IFO1804:I>T		N_43:I>T		N_44:I>T		N_45:I>T		UFRJ50791:I>T		UFRJ50816:I>T		UWOPS91_917_1:I>TID:YBR202W	AA:30		N_44:Q>L		N_45:Q>L	AA:32		A4:S>T		DBVPG6304:S>T		UFRJ50791:S>T		UWOPS91_917_1:S>T		YPS138:S>T	AA:39		A12:R>Q		A4:R>Q		DBVPG6304:R>Q		UFRJ50791:R>Q		UWOPS91_917_1:R>Q		YPS138:R>Q	AA:49		A12:A>V		A4:A>V		DBVPG6304:A>V		UFRJ50791:A>V		YPS138:A>V	AA:100		N_44:D>E	AA:171		CBS432:V>A		CBS5829:V>A		DBVPG4650:V>A		N_17:V>A		Q32_3:V>A		Q59_1:V>A		Q89_8:V>A		T21_4:V>A	AA:210		UWOPS91_917_1:N>K	AA:456		N_43:V>A		N_44:V>A		N_45:V>A	AA:631		A4:N>T		CBS432:N>T		CBS5829:N>T		DBVPG4650:N>T		DBVPG6304:N>T		KPN3828:N>T		KPN3829:N>T		N_17:N>T		N_45:N>T		Q32_3:N>T		Q59_1:N>T		Q62_5:N>T		UWOPS91_917_1:N>T		YPS138:N>T	AA:645		A4:S>A		DBVPG6304:S>A		IFO1804:S>A		N_45:S>A		YPS138:S>A	AA:691		UWOPS91_917_1:G>C	AA:721		IFO1804:R>K		N_45:R>K	AA:817		A4:R>Q		DBVPG6304:R>Q	AA:824		UFRJ50816:S>A	AA:831		UWOPS91_917_1:P>LID:YBR203W	AA:33		A4:S>N		IFO1804:S>N		N_43:S>N		N_44:S>N		N_45:S>N		UFRJ50816:S>N		UWOPS91_917_1:S>N		YPS138:S>N	AA:36		A4:S>G		UFRJ50816:S>G		UWOPS91_917_1:S>G		YPS138:S>G	AA:57		UWOPS91_917_1:S>F	AA:59		A4:T>N		UFRJ50816:T>N		UWOPS91_917_1:T>N		YPS138:T>N	AA:62		UWOPS91_917_1:R>H	AA:90		A4:S>G		DBVPG6304:S>G		UFRJ50816:S>G		UWOPS91_917_1:S>G		YPS138:S>G	AA:103		IFO1804:S>N		N_43:S>N		N_44:S>N		N_45:S>N	AA:105		UFRJ50816:D>E	AA:198		IFO1804:K>E		N_43:K>E		N_44:K>E		N_45:K>E	AA:261		IFO1804:V>I		N_43:V>I		N_44:V>I		N_45:V>I	AA:286		A4:S>G		DBVPG6304:S>G		IFO1804:S>G		N_43:S>G		N_44:S>G		N_45:S>G		UFRJ50791:S>G		UWOPS91_917_1:S>G		YPS138:S>G	AA:288		A4:S>A		DBVPG6304:S>A		UFRJ50791:S>A		UWOPS91_917_1:S>A		YPS138:S>A	AA:302		IFO1804:T>S		N_43:T>S		N_44:T>S		N_45:T>S	AA:306		IFO1804:P>L		N_43:P>L		N_44:P>L		N_45:P>L	AA:330		A4:P>S		DBVPG6304:P>S		IFO1804:P>S		N_43:P>S		N_44:P>S		N_45:P>S		UFRJ50791:P>S		UWOPS91_917_1:P>S		YPS138:P>S	AA:411		A12:N>T		A4:N>T		CBS432:N>T		CBS5829:N>T		DBVPG4650:N>T		DBVPG6304:N>T		IFO1804:N>T		N_17:N>T		N_43:N>T		N_44:N>T		N_45:N>T		Q59_1:N>T		Q89_8:N>T		Q95_3:N>T		T21_4:N>T		UFRJ50791:N>T		UFRJ50816:N>T		UWOPS91_917_1:N>T		Y6_5:N>T		Y7:N>T		YPS138:N>T	AA:447		A12:P>S		A4:P>S		DBVPG6304:P>S		UFRJ50791:P>S		UFRJ50816:P>S		YPS138:P>S	AA:478		IFO1804:N>Y	AA:493		UWOPS91_917_1:R>K	AA:495		A12:K>E		A4:K>E		DBVPG6304:K>E		UFRJ50816:K>E		YPS138:K>E	AA:557		A12:M>L		A4:M>L		DBVPG6304:M>L		UFRJ50791:M>L		UFRJ50816:M>L		YPS138:M>L	AA:577		A12:L>I		A4:L>I		DBVPG6304:L>I		UFRJ50791:L>I		UFRJ50816:L>I	AA:582		UWOPS91_917_1:T>S	AA:667		A12:H>Y		A4:H>Y		DBVPG6304:H>Y		UFRJ50791:H>Y		UFRJ50816:H>Y		YPS138:H>Y	AA:674		CBS432:N>S		DBVPG4650:N>S		KPN3829:N>S		N_17:N>S		Q32_3:N>S		Q62_5:N>S		Q95_3:N>S		Y7:N>S	AA:680		CBS432:V>I		CBS5829:V>I		DBVPG4650:V>I		KPN3829:V>I		N_17:V>I		Q32_3:V>I		Q62_5:V>I		Q95_3:V>I		Y7:V>I	AA:682		A12:K>E		A4:K>E		DBVPG6304:K>E		UFRJ50816:K>E		YPS138:K>E	AA:686		KPN3829:P>S	AA:747		UWOPS91_917_1:H>R	AA:765		CBS432:N>S	AA:771		N_44:F>L		N_45:F>L	AA:803		A12:S>N		A4:S>N		UFRJ50816:S>N		UWOPS91_917_1:S>N		YPS138:S>N	AA:854		UFRJ50816:T>S	AA:883		UFRJ50816:I>M	AA:894		N_44:S>N		N_45:S>N	AA:922		CBS432:A>D		CBS5829:A>D		DBVPG4650:A>D		N_17:A>D		Q59_1:A>D		Q62_5:A>D		Q95_3:A>D		T21_4:A>D		Y6_5:A>D		Y7:A>D		Z1_1:A>DID:YBR204C	AA:23		N_45:A>S	AA:45		A12:F>V		DBVPG6304:F>V		YPS138:F>V	AA:55		S36_7:Y>N	AA:66		A12:F>L		DBVPG6304:F>L		UFRJ50816:F>L		YPS138:F>L	AA:75		A12:->Y		DBVPG6304:->Y		IFO1804:->Y		N_43:->Y		UFRJ50816:->Y		YPS138:->Y	AA:80		A12:N>H		DBVPG6304:N>H		UFRJ50816:N>H		YPS138:N>H	AA:104		A12:K>N		A4:K>N		DBVPG6304:K>N		UFRJ50816:K>N		YPS138:K>N	AA:110		A12:V>I		A4:V>I		DBVPG6304:V>I		UFRJ50816:V>I		YPS138:V>I	AA:113		A12:L>H		A4:L>H		DBVPG6304:L>H		UFRJ50816:L>H		YPS138:L>H	AA:140		S36_7:I>M	AA:150		A4:N>K		DBVPG6304:N>K		UFRJ50816:N>K		YPS138:N>K	AA:153		A4:K>Q		DBVPG6304:K>Q		UFRJ50816:K>Q		YPS138:K>Q	AA:157		A4:E>K		DBVPG6304:E>K		UFRJ50816:E>K		YPS138:E>K	AA:161		IFO1804:I>F		N_43:I>F		N_45:I>F	AA:163		A12:R>K		A4:R>K		DBVPG6304:R>K		UFRJ50816:R>K		YPS138:R>K	AA:171		A12:W>R		A4:W>R		DBVPG6304:W>R		UFRJ50816:W>R		YPS138:W>R	AA:183		A12:C>F		A4:C>F		DBVPG6304:C>F		UFRJ50816:C>F		YPS138:C>F	AA:200		IFO1804:R>-		N_43:R>-		N_44:R>-		N_45:R>-	AA:226		UFRJ50791:P>T		UFRJ50816:P>T	AA:240		A12:F>L		A4:F>L		DBVPG6304:F>L		UFRJ50791:F>L		UFRJ50816:F>L		YPS138:F>L	AA:251		UWOPS91_917_1:M>I	AA:265		A12:I>F		A4:I>F		DBVPG6304:I>F		UFRJ50791:I>F		UFRJ50816:I>F		UWOPS91_917_1:I>F		YPS138:I>F	AA:276		A12:L>F		A4:L>F		DBVPG6304:L>F		UFRJ50791:L>F		UFRJ50816:L>F		UWOPS91_917_1:L>F		YPS138:L>F	AA:319		A12:L>H		A4:L>H		DBVPG6304:L>H		UFRJ50791:L>H		UFRJ50816:L>H		UWOPS91_917_1:L>H		YPS138:L>H	AA:337		A12:N>S		A4:N>S		DBVPG6304:N>S		UFRJ50791:N>S		UFRJ50816:N>S		UWOPS91_917_1:N>S		YPS138:N>S	AA:373		A12:L>F		DBVPG6304:L>F		N_44:L>F		N_45:L>F		UFRJ50791:L>F		YPS138:L>FID:YBR205W	AA:52		A12:A>G		A4:A>G		DBVPG6304:A>G		UWOPS91_917_1:A>G	AA:53		S36_7:Q>R	AA:54		UWOPS91_917_1:S>N	AA:70		A4:T>S	AA:88		A12:A>G		A4:A>G		DBVPG6304:A>G		UFRJ50816:A>G		UWOPS91_917_1:A>G		YPS138:A>G	AA:119		CBS432:Y>C	AA:127		A12:E>D		UFRJ50816:E>D	AA:145		A12:T>A		A4:T>A		DBVPG6304:T>A		UFRJ50816:T>A		UWOPS91_917_1:T>A		YPS138:T>A	AA:162		A12:N>D		A4:N>D		DBVPG6304:N>D		UFRJ50816:N>D		YPS138:N>D	AA:373		A12:T>I		A4:T>I		YPS138:T>I	AA:377		A12:G>D		A4:G>D		YPS138:G>DID:YBR207W	AA:5		A12:N>D		YPS138:N>D	AA:40		A4:D>E		DBVPG6304:D>E		UFRJ50791:D>E		UFRJ50816:D>E		YPS138:D>E	AA:45		A4:V>A		UWOPS91_917_1:V>A	AA:52		N_44:A>T		N_45:A>T	AA:77		A4:G>D		DBVPG6304:G>D		UFRJ50791:G>D		UFRJ50816:G>D		YPS138:G>D	AA:85		A4:A>T		DBVPG6304:A>T		N_44:A>T		N_45:A>T		UFRJ50791:A>T		UFRJ50816:A>T		UWOPS91_917_1:A>T		YPS138:A>T	AA:101		Q59_1:E>G	AA:120		UWOPS91_917_1:T>A	AA:268		UWOPS91_917_1:V>I	AA:313		A12:I>V		DBVPG6304:I>V		UWOPS91_917_1:I>V	AA:432		CBS5829:H>	AA:460		A12:S>G		DBVPG6304:S>G		YPS138:S>G	AA:465		A12:N>S		DBVPG6304:N>S		UWOPS91_917_1:N>S		YPS138:N>SID:YBR210W	AA:2		N_43:S>T		N_44:S>T		N_45:S>T	AA:46		UWOPS91_917_1:K>R	AA:55		A12:A>G		DBVPG6304:A>G		UFRJ50791:A>G		YPS138:A>G	AA:85		CBS432:A>T		CBS5829:A>T		N_17:A>T		N_43:A>T		N_44:A>T		N_45:A>T		Q59_1:A>T	AA:92		YPS138:T>K	AA:93		UWOPS91_917_1:Y>H	AA:132		A12:A>V		DBVPG6304:A>V		YPS138:A>VID:YBR212W	AA:5		A12:N>T		A4:N>T		CBS5829:N>T		IFO1804:N>T		KPN3828:N>T		N_17:N>T		N_45:N>T		Q32_3:N>T		Q59_1:N>T		Q89_8:N>T		S36_7:N>T		UFRJ50816:N>T		UWOPS91_917_1:N>T		Y6_5:N>T		YPS138:N>T	AA:9		A12:S>P		A4:S>P		UFRJ50816:S>P		UWOPS91_917_1:S>P		YPS138:S>P	AA:11		A12:R>K		A4:R>K		UFRJ50816:R>K		UWOPS91_917_1:R>K		YPS138:R>K	AA:98		Y6_5:S>W	AA:102		UFRJ50816:D>G	AA:122		Y6_5:T>A	AA:151		IFO1804:P>S		N_45:P>S	AA:243		UWOPS91_917_1:N>D	AA:301		UWOPS91_917_1:H>Q	AA:308		UWOPS91_917_1:E>D	AA:360		IFO1804:P>	AA:379		A4:M>V	AA:448		A4:A>P	AA:464		A4:N>S	AA:472		A4:T>S		DBVPG6304:T>S	AA:500		IFO1804:Q>L		N_43:Q>L		N_44:Q>L		N_45:Q>L	AA:508		A4:V>M		DBVPG6304:V>M		UWOPS91_917_1:V>M	AA:513		A4:H>Q		DBVPG6304:H>Q		UWOPS91_917_1:H>Q	AA:520		UWOPS91_917_1:N>D	AA:526		A4:S>R		DBVPG6304:S>R		UWOPS91_917_1:S>R	AA:533		UWOPS91_917_1:G>S	AA:534		A4:D>E		DBVPG6304:D>E		UWOPS91_917_1:D>E	AA:544		UWOPS91_917_1:S>P	AA:550		CBS432:S>G		UWOPS91_917_1:S>N	AA:559		A4:S>N		DBVPG6304:S>N	AA:565		A4:P>H		DBVPG6304:P>H		UWOPS91_917_1:P>H	AA:573		UWOPS91_917_1:S>N	AA:578		A4:M>L		DBVPG6304:M>L		UWOPS91_917_1:M>L		YPS138:M>L	AA:586		CBS432:Q>P		CBS5829:Q>P		DBVPG6304:Q>P		IFO1804:Q>P		KPN3828:Q>P		N_17:Q>P		N_43:Q>P		N_44:Q>P		N_45:Q>P		Q62_5:Q>P		Q95_3:Q>P		UWOPS91_917_1:Q>P		YPS138:Q>P	AA:591		A4:L>P		DBVPG6304:L>P		YPS138:L>P	AA:603		A4:M>I		DBVPG6304:M>I		UFRJ50816:M>I		YPS138:M>I	AA:608		DBVPG6304:T>S		UFRJ50816:T>S		YPS138:T>S	AA:612		UWOPS91_917_1:N>I	AA:613		KPN3828:M>T	AA:617		DBVPG6304:L>M		IFO1804:L>V		N_43:L>V		N_44:L>V		N_45:L>V		UFRJ50816:L>M		UWOPS91_917_1:L>M	AA:623		IFO1804:V>I		N_43:V>I		N_44:V>I		N_45:V>I	AA:625		KPN3828:P>L	AA:627		DBVPG6304:P>T		UFRJ50816:P>T		UWOPS91_917_1:P>T		YPS138:P>T	AA:632		KPN3828:N>Y	AA:637		KPN3828:Y>F	AA:647		UWOPS91_917_1:L>V	AA:650		A4:A>V		DBVPG6304:A>V		UFRJ50816:A>V		YPS138:A>V	AA:654		A4:S>N		DBVPG6304:S>N		UFRJ50816:S>N		YPS138:S>N	AA:662		IFO1804:N>Y	AA:667		YPS138:R>KID:YBR213W	AA:62		A12:D>N		A4:D>N		DBVPG6304:D>N		UFRJ50791:D>N		UFRJ50816:D>N		YPS138:D>N	AA:63		IFO1804:H>Q		N_43:H>Q		N_44:H>Q		N_45:H>Q	AA:79		A12:N>D		DBVPG6304:N>D		IFO1804:N>D		N_43:N>D		N_44:N>D		N_45:N>D		UFRJ50791:N>D		UFRJ50816:N>D		UWOPS91_917_1:N>D		YPS138:N>D	AA:82		DBVPG4650:K>E	AA:101		A12:D>E		DBVPG6304:D>E		UFRJ50791:D>E		UFRJ50816:D>E		UWOPS91_917_1:D>E		YPS138:D>E	AA:183		N_43:N>S	AA:225		UWOPS91_917_1:K>R	AA:235		UWOPS91_917_1:Y>HID:YBR214W	AA:2		A4:T>A		DBVPG6304:T>A		N_43:T>A		N_45:T>A		UWOPS91_917_1:T>A	AA:6		T21_4:N>S	AA:13		T21_4:N>I	AA:23		T21_4:R>S	AA:70		A12:A>T		A4:A>T		DBVPG6304:A>T	AA:194		UWOPS91_917_1:G>D	AA:232		UWOPS91_917_1:N>D	AA:279		UWOPS91_917_1:I>T	AA:321		UWOPS91_917_1:G>D	AA:423		YPS138:S>A	AA:471		A4:G>S		DBVPG6304:G>S		UFRJ50816:G>S		UWOPS91_917_1:G>SID:YBR217W	AA:26		A12:A>T		DBVPG6304:A>T		UWOPS91_917_1:A>T		YPS138:A>T	AA:76		A12:A>T		CBS432:A>T		DBVPG6304:A>T		IFO1804:A>T		N_43:A>T		N_44:A>T		N_45:A>T		Q32_3:A>T		UWOPS91_917_1:A>T	AA:78		IFO1804:E>G		N_43:E>G		N_44:E>G		N_45:E>G	AA:82		IFO1804:V>L		N_43:V>L		N_44:V>L		N_45:V>L	AA:85		A12:I>V		DBVPG6304:I>V		UFRJ50791:I>V	AA:92		A12:H>Q		DBVPG6304:H>Q		IFO1804:H>Q		N_43:H>Q		N_44:H>Q		N_45:H>Q		UWOPS91_917_1:H>Q	AA:126		IFO1804:M>I		N_43:M>I		N_44:M>I		N_45:M>I	AA:135		Q89_8:L>F	AA:142		KPN3828:K>RID:YBR220C	AA:13		Z1_1:->Y	AA:109		A4:H>Q		N_44:H>Q		N_45:H>Q		YPS138:H>Q	AA:117		A4:E>D		YPS138:E>D	AA:226		UWOPS91_917_1:K>N		YPS138:K>N	AA:240		DBVPG6304:F>L		N_43:Q>K		N_44:Q>K		N_45:Q>K		UWOPS91_917_1:F>L		YPS138:F>L	AA:267		UWOPS91_917_1:->C		YPS138:->C	AA:317		UWOPS91_917_1:I>M	AA:331		DBVPG6304:Y>-		UWOPS91_917_1:Y>-		YPS138:Y>-	AA:335		DBVPG6304:N>K		UWOPS91_917_1:N>K		YPS138:N>K	AA:369		DBVPG6304:Q>H		YPS138:Q>H	AA:377		UWOPS91_917_1:H>R	AA:383		IFO1804:Q>H		N_43:Q>H		N_44:Q>H		N_45:Q>H	AA:406		A4:I>M		DBVPG6304:I>M		IFO1804:I>M		KPN3828:I>M		N_17:I>M		N_43:I>M		N_44:I>M		N_45:I>M		UWOPS91_917_1:I>M		YPS138:I>M	AA:419		A4:Q>E		DBVPG6304:Q>E		UWOPS91_917_1:Q>E		YPS138:Q>E	AA:493		UWOPS91_917_1:N>K	AA:495		UWOPS91_917_1:Q>H	AA:520		UWOPS91_917_1:->L	AA:536		A12:Q>H		A4:Q>H		DBVPG6304:Q>H		YPS138:Q>H	AA:553		A12:->C		A4:->C		DBVPG6304:->C		UFRJ50816:->C		YPS138:->C	AA:561		A12:H>Q		A4:H>Q		DBVPG6304:H>Q		YPS138:H>QID:YBR221C	AA:6		A12:D>H		DBVPG6304:D>H		N_43:D>H		N_45:D>H		UFRJ50816:D>H		UWOPS91_917_1:D>H	AA:53		IFO1804:D>A	AA:98		A12:F>L		DBVPG6304:F>L		UFRJ50816:F>L		UWOPS91_917_1:F>L		YPS138:F>L	AA:99		Q59_1:R>H	AA:194		YPS138:R>S	AA:223		UWOPS91_917_1:F>L	AA:245		A12:H>Q		A4:H>Q		DBVPG6304:H>Q		IFO1804:H>Q		N_44:H>Q		N_45:H>Q		UFRJ50816:H>Q		UWOPS91_917_1:H>Q		YPS138:H>Q	AA:254		UWOPS91_917_1:Y>-	AA:277		A4:C>W		YPS138:C>W	AA:299		A4:I>M		YPS138:I>M	AA:303		UWOPS91_917_1:F>L	AA:313		CBS5829:T>A	AA:330		YPS138:C>W	AA:335		A4:E>D		UWOPS91_917_1:E>D		YPS138:E>D	AA:353		A12:C>-		A4:C>-		YPS138:C>-ID:YBR223C	AA:16		A4:Q>H		DBVPG6304:Q>H		IFO1804:Q>H		N_44:Q>H		N_45:Q>H		UFRJ50816:Q>H	AA:32		IFO1804:S>N		N_44:S>N		N_45:S>N		Q32_3:T>A	AA:37		A4:V>G	AA:38		CBS5829:F>L		Q62_5:F>L	AA:46		IFO1804:A>G		N_44:A>G		N_45:A>G	AA:50		A4:L>I		DBVPG6304:L>I		UFRJ50816:L>I	AA:81		IFO1804:V>G	AA:152		A4:M>I	AA:168		A4:L>F		DBVPG6304:L>F		IFO1804:L>F		N_44:L>F		N_45:L>F		UWOPS91_917_1:N>K		YPS138:N>K	AA:193		A4:Y>D		DBVPG6304:Y>D		UWOPS91_917_1:Y>D	AA:208		A4:H>Q		CBS5829:H>Q		DBVPG6304:H>Q		IFO1804:H>Q		N_17:H>Q		N_44:H>Q		N_45:H>Q		Q32_3:H>Q		Q62_5:H>Q		Q89_8:H>Q		T21_4:H>Q		UWOPS91_917_1:H>Q		Z1_1:H>Q	AA:221		DBVPG6304:N>K		UWOPS91_917_1:->Y		YPS138:N>K	AA:223		UWOPS91_917_1:F>L	AA:230		UWOPS91_917_1:R>-	AA:290		UWOPS91_917_1:A>S	AA:292		DBVPG6304:C>Y		UWOPS91_917_1:C>Y		YPS138:C>Y	AA:307		CBS432:F>L		DBVPG4650:F>L		DBVPG6304:F>L		IFO1804:F>L		N_45:F>L		Q32_3:F>L		Q89_8:F>L		S36_7:F>L		UWOPS91_917_1:F>L		YPS138:F>L		Z1_1:F>L	AA:335		UWOPS91_917_1:Q>H	AA:342		DBVPG6304:K>N		YPS138:K>N	AA:345		DBVPG6304:R>G	AA:351		Q32_3:A>T		Q89_8:A>T		Z1_1:A>T	AA:354		UWOPS91_917_1:V>L	AA:367		UWOPS91_917_1:S>R		YPS138:S>R	AA:380		UWOPS91_917_1:L>F		YPS138:L>F	AA:396		CBS5829:H>Q		UWOPS91_917_1:H>R		YPS138:H>R	AA:401		UWOPS91_917_1:L>-		YPS138:L>-	AA:413		UWOPS91_917_1:E>V		YPS138:E>V	AA:417		UWOPS91_917_1:Q>E	AA:420		N_43:K>T		N_44:K>TID:YBR227C	AA:14		A4:T>S		DBVPG6304:T>S		UFRJ50791:T>S	AA:18		UWOPS91_917_1:M>R	AA:28		A4:V>A		DBVPG6304:V>A		UFRJ50791:V>A	AA:31		A4:S>R		CBS432:S>R		DBVPG6304:S>R		N_17:S>R		N_43:S>R		N_44:S>R		N_45:S>R		Q32_3:S>R		Q89_8:S>R		S36_7:S>R		UFRJ50791:S>R		Y7:S>R		Z1_1:S>R	AA:39		DBVPG6304:W>S		UFRJ50816:W>S		UWOPS91_917_1:W>S		YPS138:W>S	AA:56		UWOPS91_917_1:I>M	AA:71		A4:D>E		DBVPG6304:D>E		UFRJ50791:D>E		UFRJ50816:D>E		UWOPS91_917_1:D>E	AA:81		N_43:N>K		N_44:N>K		N_45:N>K	AA:86		A4:A>G		DBVPG6304:A>G		UFRJ50791:A>G		UFRJ50816:A>G	AA:114		Q89_8:R>H		Y6_5:R>H		Y7:R>H	AA:128		A4:P>H		DBVPG6304:P>H		UFRJ50791:P>H		UFRJ50816:P>H		YPS138:P>H	AA:130		UWOPS91_917_1:L>F	AA:139		UWOPS91_917_1:Q>H	AA:150		A4:K>Q		DBVPG6304:K>Q		UFRJ50816:K>Q		YPS138:K>Q	AA:154		N_43:F>L		N_44:F>L		N_45:F>L	AA:162		UWOPS91_917_1:Y>-	AA:223		UWOPS91_917_1:R>S	AA:247		A4:S>R		DBVPG6304:S>R		UFRJ50816:S>R		UWOPS91_917_1:S>R		YPS138:S>R	AA:252		A4:E>D		DBVPG6304:E>D		UFRJ50816:E>D		YPS138:E>D	AA:253		UWOPS91_917_1:K>N	AA:257		A4:S>R		DBVPG6304:S>R		UFRJ50816:S>R		UWOPS91_917_1:S>R		YPS138:S>R	AA:263		A4:F>V		DBVPG6304:F>V		UFRJ50816:F>V		UWOPS91_917_1:F>L		YPS138:F>V	AA:270		UWOPS91_917_1:L>F	AA:293		N_43:N>K		N_44:N>K		N_45:N>K		UWOPS91_917_1:N>K	AA:305		UWOPS91_917_1:G>R	AA:316		UWOPS91_917_1:L>P	AA:321		N_43:N>D		N_44:N>D		N_45:N>D	AA:325		DBVPG6304:N>K		N_43:N>K		N_44:N>K		N_45:N>K		UFRJ50816:N>K		UWOPS91_917_1:N>K		YPS138:N>K	AA:329		DBVPG6304:F>L		N_43:F>L		N_44:F>L		N_45:F>L		UFRJ50816:F>L		YPS138:F>L	AA:383		DBVPG6304:T>P		UFRJ50816:T>P		YPS138:T>P	AA:436		DBVPG6304:E>-		UFRJ50816:E>-		YPS138:E>-ID:YBR228W	AA:7		UWOPS91_917_1:E>Q	AA:10		N_43:F>I		N_45:F>I	AA:117		A4:V>M		DBVPG6304:V>M		UFRJ50791:V>M		UFRJ50816:V>M		YPS138:V>M	AA:127		A4:R>Q		DBVPG6304:R>Q		UFRJ50791:R>Q		UFRJ50816:R>Q		YPS138:R>Q	AA:132		DBVPG6304:I>L	AA:138		A4:M>I		DBVPG6304:M>I		UFRJ50791:M>I		UFRJ50816:M>I		YPS138:M>I	AA:146		N_17:Q>K		Q62_5:Q>K	AA:239		A4:A>T		DBVPG6304:A>T		UFRJ50791:A>T		UFRJ50816:A>T		YPS138:A>T	AA:244		UWOPS91_917_1:N>K	AA:267		UWOPS91_917_1:E>V	AA:281		UWOPS91_917_1:N>K	AA:287		UWOPS91_917_1:E>D	AA:304		A4:K>N		DBVPG6304:K>N		UFRJ50791:K>N		UFRJ50816:K>N		YPS138:K>NID:YBR230C	AA:7		DBVPG6304:G>R		N_43:G>R		N_44:G>R		N_45:G>R		UFRJ50791:G>R		YPS138:G>R	AA:13		UWOPS91_917_1:N>S	AA:29		UWOPS91_917_1:->Y	AA:41		UWOPS91_917_1:H>R	AA:58		UWOPS91_917_1:Q>R	AA:78		A12:V>I		A4:V>I		DBVPG6304:V>I		YPS138:V>I	AA:104		UWOPS91_917_1:F>L	AA:118		UWOPS91_917_1:V>F	AA:130		UWOPS91_917_1:F>LID:YBR231C	AA:29		N_43:N>K		N_45:N>K	AA:37		A12:G>S		UFRJ50816:G>S		YPS138:G>S	AA:44		A12:V>M		UFRJ50816:V>M		YPS138:V>M	AA:47		A12:->Y		UFRJ50816:->Y		YPS138:->Y	AA:50		A12:V>D		N_43:V>D		N_45:V>D		UFRJ50816:V>D		YPS138:V>D	AA:136		A12:R>S		UFRJ50816:R>S		YPS138:R>S	AA:154		A12:D>E		UFRJ50816:D>E		YPS138:D>E	AA:200		IFO1804:Y>-		N_43:Y>-		N_45:Y>-	AA:202		A12:C>-	AA:219		A12:->Y		UFRJ50816:->Y		UWOPS91_917_1:F>L	AA:231		UWOPS91_917_1:Q>H	AA:303		UWOPS91_917_1:Y>NID:YBR233W	AA:34		UWOPS91_917_1:E>G	AA:47		UWOPS91_917_1:E>D	AA:59		UFRJ50791:S>P		UFRJ50816:S>P	AA:71		UWOPS91_917_1:M>L	AA:87		A12:A>T		DBVPG6304:A>T		UFRJ50791:A>T		UFRJ50816:A>T		YPS138:A>T	AA:96		UWOPS91_917_1:T>S	AA:144		DBVPG6304:G>R		UFRJ50816:G>R		UWOPS91_917_1:G>E	AA:222		UFRJ50816:M>T	AA:254		N_43:L>I		N_45:L>I	AA:317		DBVPG6304:L>V		N_43:L>V		N_44:L>V		N_45:L>V		UFRJ50816:L>V		UWOPS91_917_1:L>V		YPS138:L>VID:YBR233W-A	AA:14		A4:N>D		DBVPG6304:N>D		YPS138:N>D	AA:38		A4:H>R		DBVPG6304:H>R		YPS138:H>R	AA:91		UWOPS91_917_1:S>NID:YBR236C	AA:49		UWOPS91_917_1:R>S	AA:54		UWOPS91_917_1:H>Q	AA:55		N_17:P>A	AA:57		UWOPS91_917_1:G>W	AA:74		UWOPS91_917_1:Q>H	AA:98		CBS432:I>F		CBS5829:I>F		N_43:I>F		Q95_3:I>F		T21_4:I>F		Y6_5:I>F		Y7:I>F	AA:104		UWOPS91_917_1:I>L	AA:120		UWOPS91_917_1:C>R	AA:143		N_43:F>L		UFRJ50791:F>L		YPS138:F>L	AA:206		DBVPG6304:C>-		UFRJ50791:C>-		UWOPS91_917_1:C>-		YPS138:C>-	AA:226		DBVPG6304:I>M		UFRJ50791:I>M		YPS138:I>M	AA:283		DBVPG6304:I>L		UFRJ50791:I>L		YPS138:I>L	AA:317		UWOPS91_917_1:N>KID:YBR237W	AA:28		A12:Q>L		DBVPG6304:Q>L	AA:48		UWOPS91_917_1:T>S	AA:50		UWOPS91_917_1:S>N	AA:52		N_43:G>E		N_44:G>E		N_45:G>E	AA:57		N_43:A>T		N_45:A>T	AA:78		N_43:F>Y		N_44:F>Y		N_45:F>Y	AA:83		A12:E>G		DBVPG6304:E>G		UFRJ50791:E>G	AA:100		N_43:R>K		N_44:R>K		N_45:R>K	AA:110		DBVPG6304:D>E		UFRJ50791:D>E	AA:117		N_43:H>L		N_44:H>L		N_45:H>L	AA:124		N_43:H>N		N_44:H>N		N_45:H>N	AA:129		DBVPG6304:A>T		UFRJ50791:A>T		UWOPS91_917_1:A>T	AA:134		DBVPG6304:E>K		UFRJ50791:E>K	AA:153		DBVPG6304:I>T		UFRJ50791:I>T	AA:156		DBVPG6304:T>S		UFRJ50791:T>S		UWOPS91_917_1:T>S	AA:171		DBVPG6304:A>E		UFRJ50791:A>E		UWOPS91_917_1:A>E	AA:186		DBVPG6304:S>R		UFRJ50791:S>R	AA:196		IFO1804:K>R		N_43:K>R		N_44:K>R		N_45:K>R	AA:263		DBVPG6304:S>P		YPS138:S>P	AA:278		IFO1804:S>F		N_43:S>F		N_44:S>F		N_45:S>F	AA:294		UWOPS91_917_1:R>H	AA:348		IFO1804:E>G		N_43:E>G		N_44:E>G		N_45:E>G	AA:355		UWOPS91_917_1:G>E	AA:360		A12:I>V		A4:I>V		DBVPG6304:I>V		UFRJ50791:I>V		UWOPS91_917_1:I>V		YPS138:I>V	AA:361		N_44:R>K		N_45:R>K	AA:362		UWOPS91_917_1:S>P	AA:382		A12:A>T		A4:A>T		DBVPG6304:A>T		UFRJ50791:A>T		UWOPS91_917_1:A>T		YPS138:A>T	AA:482		A12:K>M		A4:K>M		DBVPG6304:K>M		UFRJ50791:K>M		UFRJ50816:K>M		YPS138:K>M	AA:497		A12:L>V		A4:L>V		DBVPG6304:L>V		UFRJ50816:L>V		UWOPS91_917_1:L>V		YPS138:L>V	AA:502		N_43:G>E	AA:505		UFRJ50816:E>K	AA:539		UWOPS91_917_1:D>N	AA:554		N_43:T>A		N_45:T>A		UWOPS91_917_1:T>A	AA:596		A12:V>L		UFRJ50816:V>L		UWOPS91_917_1:V>L	AA:641		IFO1804:S>T		N_43:S>T		N_44:S>T		N_45:S>T	AA:651		DBVPG6304:A>V		UFRJ50816:A>V	AA:657		DBVPG6304:V>A		IFO1804:V>A		N_43:V>A		N_44:V>A		N_45:V>A		UFRJ50816:V>A	AA:661		UFRJ50816:H>D	AA:668		Q89_8:E>Q	AA:669		IFO1804:F>S		N_43:F>S		N_44:F>S		N_45:F>S	AA:688		YPS138:E>V	AA:762		UWOPS91_917_1:D>N	AA:784		KPN3828:T>		YPS138:T>S	AA:789		YPS138:T>S	AA:818		UWOPS91_917_1:D>E	AA:844		T21_4:S>AID:YBR239C	AA:28		UFRJ50816:T>K		UWOPS91_917_1:T>K		YPS138:T>K	AA:64		UWOPS91_917_1:P>S	AA:73		YPS138:H>Q	AA:91		UFRJ50816:F>V	AA:136		CBS5829:F>L	AA:159		UWOPS91_917_1:Q>H	AA:178		UWOPS91_917_1:C>-	AA:182		UWOPS91_917_1:R>S	AA:190		UWOPS91_917_1:F>S	AA:197		A12:->S		N_43:->S		UFRJ50816:->S		UWOPS91_917_1:->S		YPS138:->S	AA:202		A12:F>L		UFRJ50791:F>L		UFRJ50816:F>L		YPS138:F>L	AA:205		UWOPS91_917_1:A>S	AA:209		N_43:S>R	AA:222		N_43:Q>H	AA:229		UWOPS91_917_1:->Q	AA:232		UWOPS91_917_1:S>R	AA:250		UWOPS91_917_1:->C	AA:262		N_43:V>I		N_45:V>I		UWOPS91_917_1:I>S	AA:283		N_44:E>-	AA:284		UWOPS91_917_1:M>I	AA:288		N_43:Q>H	AA:291		A12:L>-		A4:L>-		DBVPG6304:L>-		UFRJ50791:L>-		UFRJ50816:L>-	AA:312		Q32_3:R>S	AA:338		UWOPS91_917_1:R>S	AA:361		A12:G>R		A4:G>R		DBVPG6304:G>R		UFRJ50791:G>R		UFRJ50816:G>R	AA:366		UFRJ50791:D>E		UFRJ50816:D>E	AA:379		S36_7:N>K	AA:431		A4:H>Q		DBVPG6304:H>Q		UFRJ50791:H>Q		UFRJ50816:H>Q	AA:480		A12:G>D		A4:G>D		DBVPG6304:G>D		UFRJ50816:G>DID:YBR240C	AA:2		UWOPS91_917_1:V>A	AA:24		A12:->Y		DBVPG6304:->Y	AA:57		A12:->Y		DBVPG6304:->Y		UWOPS91_917_1:->Y	AA:62		UWOPS91_917_1:F>L	AA:80		Q59_1:M>K	AA:117		A12:Q>H		A4:Q>H		DBVPG6304:Q>H		UWOPS91_917_1:Q>H	AA:120		UWOPS91_917_1:A>S	AA:128		A12:Q>H	AA:132		UWOPS91_917_1:S>R	AA:206		A4:L>M		UFRJ50816:L>M		UWOPS91_917_1:L>M	AA:227		A4:F>Y		UFRJ50816:F>Y		UWOPS91_917_1:F>Y	AA:229		UWOPS91_917_1:R>L	AA:232		IFO1804:H>Q		N_44:H>Q		N_45:H>Q		UWOPS91_917_1:H>Q	AA:234		A4:M>I		UFRJ50816:M>I		UWOPS91_917_1:M>I	AA:286		UWOPS91_917_1:A>S	AA:374		UWOPS91_917_1:Q>R	AA:392		IFO1804:I>M		N_43:I>M		N_44:I>M	AA:419		IFO1804:T>A		N_43:T>A	AA:425		UWOPS91_917_1:K>N	AA:433		A4:R>C		UFRJ50791:R>C		UFRJ50816:R>C		UWOPS91_917_1:R>CID:YBR242W	AA:5		A12:V>I		DBVPG6304:V>I		UFRJ50791:V>I		UWOPS91_917_1:V>I		YPS138:V>I	AA:10		CBS432:L>S		CBS5829:L>S		DBVPG4650:L>S		IFO1804:L>S		N_17:L>S		N_43:L>S		N_45:L>S		Q62_5:L>S		Q89_8:L>S		Y7:L>S		Z1_1:L>S	AA:21		A12:C>R		DBVPG6304:C>R		IFO1804:C>R		N_43:C>R		UFRJ50791:C>R		UWOPS91_917_1:C>R		YPS138:C>R	AA:43		A12:P>S		DBVPG6304:P>S		UFRJ50791:P>S		UFRJ50816:P>S		YPS138:P>S	AA:46		A12:S>N		DBVPG6304:S>N		UFRJ50791:S>N		UFRJ50816:S>N		UWOPS91_917_1:S>N		YPS138:S>N	AA:97		N_45:N>I	AA:146		UWOPS91_917_1:N>K	AA:219		A12:S>G		A4:S>G		DBVPG6304:S>G		UFRJ50816:S>G		YPS138:S>G	AA:223		A12:M>I		A4:M>I		DBVPG6304:M>I		UFRJ50816:M>I		UWOPS91_917_1:M>I		YPS138:M>I	AA:226		UWOPS91_917_1:R>Q	AA:231		N_44:D>N		N_45:D>N	AA:236		A12:I>V		A4:I>V		UFRJ50816:I>V		UWOPS91_917_1:I>V		YPS138:I>VID:YBR243C	AA:5		A12:P>T		A4:P>T		DBVPG6304:P>T		UFRJ50816:P>T		UWOPS91_917_1:P>T	AA:25		N_45:N>D		UWOPS91_917_1:N>Y	AA:74		UWOPS91_917_1:M>I	AA:75		DBVPG6304:K>N		UFRJ50791:K>N		UFRJ50816:K>N	AA:114		A4:S>T		DBVPG6304:S>T		UFRJ50791:S>T		UFRJ50816:S>T	AA:119		UWOPS91_917_1:Q>E	AA:167		A4:N>Y		DBVPG6304:N>Y		UFRJ50791:N>Y		UFRJ50816:N>Y		UWOPS91_917_1:N>Y	AA:179		A12:->Y		A4:->Y		DBVPG6304:->Y		UFRJ50791:->Y		UFRJ50816:->Y	AA:249		A12:I>K		A4:I>K		DBVPG6304:I>K		UFRJ50791:I>K		UFRJ50816:I>K	AA:262		UWOPS91_917_1:M>L	AA:267		IFO1804:W>-		N_45:W>-	AA:292		A12:E>D		A4:E>D		DBVPG6304:E>D		UFRJ50791:E>D		UFRJ50816:E>D		UWOPS91_917_1:E>D	AA:313		A12:F>L		A4:F>L		DBVPG6304:F>L		UFRJ50816:F>L		UWOPS91_917_1:F>L	AA:358		IFO1804:E>D		N_44:E>D		N_45:E>D	AA:365		A4:E>D		DBVPG6304:E>D		UFRJ50791:E>D		UFRJ50816:E>D		UWOPS91_917_1:E>D	AA:424		CBS5829:N>K		Y7:N>KID:YBR244W	AA:6		N_45:Y>H	AA:9		UWOPS91_917_1:E>K	AA:25		UWOPS91_917_1:G>R	AA:54		UWOPS91_917_1:Q>E	AA:58		A4:L>F		DBVPG6304:L>F		UWOPS91_917_1:L>F		YPS138:L>F	AA:60		CBS432:I>V		KPN3828:I>V		UWOPS91_917_1:I>V	AA:109		A4:N>S		UWOPS91_917_1:N>S		YPS138:N>S	AA:135		A4:F>S		YPS138:F>S	AA:143		A12:F>Y		A4:F>Y		YPS138:F>Y	AA:150		T21_4:S>PID:YBR246W	AA:29		A12:I>V		A4:I>V		YPS138:I>V	AA:34		A12:M>T		A4:M>T		CBS432:M>T		CBS5829:M>T		KPN3828:M>T		N_43:M>T		Q89_8:M>T		S36_7:M>T		T21_4:M>T		UWOPS91_917_1:M>T		Y6_5:M>T		YPS138:M>T		Z1_1:M>T	AA:56		A12:K>R		A4:K>R		YPS138:K>R	AA:101		A12:D>N		A4:D>N		UFRJ50791:D>N		UWOPS91_917_1:D>N		YPS138:D>N	AA:104		N_45:T>R	AA:190		A12:E>K		UFRJ50791:E>K		UFRJ50816:E>K		YPS138:E>K	AA:195		KPN3828:P>L		Q95_3:P>L		Z1_1:P>L	AA:229		UFRJ50791:L>V	AA:294		UFRJ50816:V>I	AA:309		A12:V>I		A4:V>I		UFRJ50816:V>I		YPS138:V>I	AA:317		A12:H>N		A4:H>N		UFRJ50816:H>N		UWOPS91_917_1:H>N		YPS138:H>NID:YBR247C	AA:81		N_43:T>I		N_44:T>I		N_45:T>I	AA:91		A12:K>N		A4:K>N		UFRJ50816:K>N	AA:102		UWOPS91_917_1:W>C	AA:107		A12:L>H		A4:L>H		IFO1804:L>V		N_43:L>V		N_44:L>V		N_45:L>V		UFRJ50816:L>H		UWOPS91_917_1:L>V	AA:127		A12:I>M		A4:I>M		UFRJ50816:I>M		YPS138:I>M	AA:133		UWOPS91_917_1:R>S	AA:135		A12:->Y		A4:->Y		CBS432:->Y		CBS5829:->Y		IFO1804:->Y		KPN3828:->Y		N_17:->Y		N_43:->Y		N_45:->Y		Q62_5:->Y		Q95_3:->Y		UFRJ50816:->Y		UWOPS91_917_1:->Y		YPS138:->Y	AA:193		A12:->W		A4:->W		IFO1804:->W		N_43:->W		N_44:->W		N_45:->W		UFRJ50791:->W		UWOPS91_917_1:->W		YPS138:->W	AA:230		IFO1804:->W		N_43:->W		N_44:->W		N_45:->W	AA:248		A12:W>C		A4:W>C		YPS138:W>C	AA:255		IFO1804:W>C		N_43:W>C		N_44:W>C		N_45:W>C	AA:322		A12:D>E		A4:D>E		UFRJ50791:D>E		YPS138:D>E	AA:451		A4:V>IID:YBR248C	AA:16		A4:F>L		DBVPG6304:F>L		IFO1804:F>L		N_43:F>L		N_44:F>L		N_45:F>L		UFRJ50816:F>L	AA:41		A4:->Y		DBVPG6304:->Y		UFRJ50816:->Y		UWOPS91_917_1:->Y	AA:72		A4:Q>H		DBVPG6304:Q>H		IFO1804:Q>H		N_43:Q>H		N_45:Q>H		UFRJ50816:Q>H		UWOPS91_917_1:Q>H	AA:152		A12:R>S		A4:R>S		DBVPG6304:R>S		UFRJ50791:R>S		UFRJ50816:R>S		UWOPS91_917_1:R>S		YPS138:R>S	AA:163		A12:->C		A4:->C		DBVPG6304:->C		UFRJ50791:->C		UFRJ50816:->C		UWOPS91_917_1:->C		YPS138:->C	AA:225		UWOPS91_917_1:D>E	AA:230		UWOPS91_917_1:K>N	AA:255		A12:Y>-		DBVPG6304:Y>-		UFRJ50791:Y>-		UFRJ50816:Y>-		YPS138:Y>-	AA:340		N_43:R>S		N_44:R>S	AA:376		Q89_8:E>G	AA:384		UWOPS91_917_1:F>Y	AA:385		A12:C>W		DBVPG6304:C>W		UFRJ50791:C>W		UFRJ50816:C>W		YPS138:C>W	AA:386		N_43:N>K		N_44:N>K	AA:392		A12:L>W		DBVPG6304:L>W		UFRJ50791:L>W		UFRJ50816:L>W		YPS138:L>W	AA:398		UFRJ50791:F>L		UFRJ50816:F>L	AA:429		CBS432:I>L		CBS5829:I>L		N_17:I>L		T21_4:I>L		Y6_5:I>L	AA:455		UWOPS91_917_1:T>I	AA:498		A12:K>N		DBVPG6304:K>N		UWOPS91_917_1:K>N		YPS138:K>N	AA:504		A12:D>E		CBS432:D>E		CBS5829:D>E		DBVPG6304:D>E		KPN3828:D>E		N_17:D>E		N_44:D>E		N_45:D>E		Q62_5:D>E		Q89_8:D>E		Q95_3:D>E		T21_4:D>E		Y6_5:D>E		Y7:D>E		YPS138:D>E	AA:551		UWOPS91_917_1:D>EID:YBR249C	AA:4		UWOPS91_917_1:V>L	AA:14		A12:S>R		A4:S>R		DBVPG6304:S>R		UFRJ50816:S>R		UWOPS91_917_1:S>R		YPS138:S>R	AA:17		A12:S>P		A4:S>P		DBVPG6304:S>P		UFRJ50816:S>P		UWOPS91_917_1:S>P		YPS138:S>P	AA:33		A12:R>S		A4:R>S		DBVPG6304:R>S		UFRJ50816:R>S		UWOPS91_917_1:R>S		YPS138:R>S	AA:81		UWOPS91_917_1:V>F	AA:91		A12:G>R		A4:G>R		DBVPG6304:G>R		UFRJ50816:G>R		YPS138:G>R	AA:137		Q95_3:D>E	AA:149		A12:M>I		A4:M>I		DBVPG6304:M>I		UFRJ50816:M>I		UWOPS91_917_1:M>I	AA:224		A4:Q>H		DBVPG6304:Q>H	AA:278		CBS432:Q>E		CBS5829:Q>E		N_17:Q>E		Q62_5:Q>E	AA:303		CBS432:N>K		CBS5829:N>K		N_17:N>KID:YBR251W	AA:5		UWOPS91_917_1:Q>KID:YBR252W	AA:16		A4:D>G		DBVPG6304:D>G		N_17:D>G		N_43:D>G		N_45:D>G		UFRJ50816:D>G	AA:128		UFRJ50791:V>F		UFRJ50816:V>FID:YBR253W	AA:30		N_43:I>M		N_44:I>M		N_45:I>M	AA:91		A4:I>M		DBVPG6304:I>M		N_43:I>M		N_44:I>M		N_45:I>M		UFRJ50816:I>M	AA:105		A4:K>E		DBVPG6304:K>E		UFRJ50816:K>E		UWOPS91_917_1:K>E	AA:119		A12:Q>K		A4:Q>K		DBVPG6304:Q>K		UFRJ50816:Q>K		UWOPS91_917_1:Q>KID:YBR254C	AA:16		CBS432:E>D		CBS5829:E>D		N_17:E>D		N_43:E>D		N_44:E>D		N_45:E>D	AA:82		UWOPS91_917_1:K>N	AA:84		A12:I>N		A4:I>N		YPS138:I>N	AA:99		UWOPS91_917_1:R>S	AA:132		N_44:->Y		N_45:->Y	AA:142		A12:F>L		A4:F>L		YPS138:F>L	AA:145		A12:I>F		A4:I>F		YPS138:I>FID:YBR256C	AA:31		A4:Q>H		DBVPG6304:Q>H		N_43:Q>H		N_44:Q>H		N_45:Q>H		YPS138:Q>H	AA:33		A4:->Y		DBVPG6304:->Y		YPS138:->Y	AA:40		UWOPS91_917_1:N>K	AA:63		CBS432:V>D		Q89_8:V>D		Q95_3:V>D		Y6_5:V>D	AA:68		UWOPS91_917_1:D>E		YPS138:D>E	AA:74		Z1_1:G>A	AA:99		N_43:Y>-		N_44:Y>-		N_45:Y>-		YPS138:Y>-	AA:129		UWOPS91_917_1:S>R	AA:157		T21_4:D>E	AA:169		A12:W>C		A4:W>C		YPS138:W>C	AA:196		A12:N>K		A4:N>K		N_43:N>K		N_44:N>K		N_45:N>K		UWOPS91_917_1:N>K		YPS138:N>K	AA:224		T21_4:I>FID:YBR257W	AA:27		UFRJ50816:K>N	AA:45		IFO1804:K>R		N_43:K>R		UFRJ50816:K>R	AA:52		UFRJ50816:K>E	AA:67		UFRJ50816:E>G	AA:79		UFRJ50816:R>Q	AA:82		UFRJ50816:N>S	AA:101		UFRJ50816:E>K	AA:138		UFRJ50816:S>C	AA:219		A12:Q>E	AA:235		A12:S>P	AA:236		N_43:I>MID:YBR258C	AA:3		A12:M>I		A4:M>I		DBVPG6304:M>I		UWOPS91_917_1:M>I	AA:13		A12:Q>H		A4:Q>H		DBVPG6304:Q>H		N_43:Q>H		N_45:Q>H		UWOPS91_917_1:Q>H	AA:96		N_43:P>R		N_45:P>R	AA:111		CBS432:P>Q		Q62_5:P>Q		Q89_8:P>Q		Z1_1:P>Q	AA:112		A12:W>R		A4:W>R	AA:117		A12:F>L		A4:F>L		DBVPG6304:F>L	AA:125		A12:S>T		A4:S>T		DBVPG6304:S>T		UWOPS91_917_1:S>TID:YBR259W	AA:11		UWOPS91_917_1:R>K	AA:60		A4:I>V		N_43:I>V		N_45:I>V		UFRJ50791:I>V		UWOPS91_917_1:I>V	AA:69		A4:T>A		UFRJ50791:T>A		UWOPS91_917_1:T>A	AA:81		A4:V>I		UFRJ50791:V>I	AA:94		A4:R>H		UFRJ50791:R>H		UWOPS91_917_1:R>H	AA:110		UWOPS91_917_1:I>V	AA:118		UWOPS91_917_1:A>V	AA:159		A4:I>V		DBVPG6304:I>V		UFRJ50791:I>V		YPS138:I>V	AA:177		A4:E>G		DBVPG6304:E>G		UFRJ50791:E>G		YPS138:E>G	AA:181		A4:Y>H		DBVPG6304:Y>H		UFRJ50791:Y>H		YPS138:Y>H	AA:220		A4:L>I		DBVPG6304:L>I		IFO1804:L>I		N_43:L>I		N_44:L>I		N_45:L>I		UFRJ50791:L>I		YPS138:L>I	AA:243		IFO1804:F>I		N_43:F>I		N_44:F>I		N_45:F>I	AA:257		YPS138:I>M	AA:262		A12:D>N		A4:D>N		DBVPG6304:D>N		Q95_3:D>N		UFRJ50791:D>N		UFRJ50816:D>N		YPS138:D>N	AA:273		A12:H>P		A4:H>P		DBVPG6304:H>P		UFRJ50791:H>P		UFRJ50816:H>P		YPS138:H>P	AA:274		KPN3829:Y>S	AA:277		A12:D>E		A4:D>E		DBVPG6304:D>E		UFRJ50791:D>E		UFRJ50816:D>E		YPS138:D>E	AA:296		A12:K>R		A4:K>R		DBVPG6304:K>R		UFRJ50791:K>R		UFRJ50816:K>R		YPS138:K>R	AA:315		A12:R>Q		A4:R>Q		DBVPG6304:R>Q		UFRJ50791:R>Q		UFRJ50816:R>Q		YPS138:R>Q	AA:337		A4:R>G		DBVPG6304:R>G		UFRJ50791:R>G		UFRJ50816:R>G		YPS138:R>G	AA:344		UWOPS91_917_1:E>D	AA:350		UWOPS91_917_1:M>I	AA:366		A4:T>A		DBVPG6304:T>A		UFRJ50791:T>A		UFRJ50816:T>A		YPS138:T>A	AA:368		A4:L>S		DBVPG6304:L>S		UFRJ50791:L>S		UFRJ50816:L>S		YPS138:L>S	AA:379		CBS5829:M>I	AA:383		KPN3828:T>A		Q95_3:T>A		Y6_5:T>A		Y7:T>A		Z1_1:T>A	AA:388		A4:S>N		DBVPG6304:S>N		N_43:S>N		N_44:S>N		N_45:S>N		UFRJ50791:S>N		UFRJ50816:S>N		YPS138:S>N	AA:390		A4:I>N		DBVPG6304:I>N		KPN3828:I>T		Q95_3:I>T		UFRJ50791:I>N		UFRJ50816:I>N		Y6_5:I>T		Y7:I>T		YPS138:I>N		Z1_1:I>T	AA:412		UWOPS91_917_1:Q>R	AA:415		CBS432:V>L	AA:426		N_43:L>P		N_44:L>P		N_45:L>P	AA:439		A4:V>I		CBS432:V>I		DBVPG6304:V>I		KPN3828:V>I		KPN3829:V>I		N_43:V>I		N_44:V>I		N_45:V>I		Q95_3:V>I		UFRJ50791:V>I		UFRJ50816:V>I		UWOPS91_917_1:V>I		Y6_5:V>I		Y7:V>I		YPS138:V>I		Z1_1:V>I	AA:443		A4:L>I		DBVPG6304:L>I		UFRJ50791:L>I		UFRJ50816:L>I		YPS138:L>I	AA:459		A4:I>V		DBVPG6304:I>V		YPS138:I>V	AA:475		A4:K>T		DBVPG6304:K>T		UFRJ50791:K>T		UFRJ50816:K>T		YPS138:K>T	AA:481		A4:F>S		DBVPG6304:F>S		UFRJ50791:F>S		UFRJ50816:F>S		YPS138:F>S	AA:482		UWOPS91_917_1:K>M	AA:507		CBS432:N>K		Q95_3:N>K		T21_4:N>K		Y6_5:N>K		Y7:N>K		Z1_1:N>K	AA:509		A4:I>V		DBVPG6304:I>V		UFRJ50791:I>V		UFRJ50816:I>V		YPS138:I>V	AA:526		CBS432:E>K		N_43:E>K		N_44:E>K		N_45:E>K		Q95_3:E>K		T21_4:E>K		Y6_5:E>K		Y7:E>K		Z1_1:E>K	AA:540		A4:S>N		DBVPG6304:S>N		UFRJ50791:S>N		UFRJ50816:S>N		UWOPS91_917_1:S>N	AA:576		UFRJ50791:V>I		UFRJ50816:V>I		UWOPS91_917_1:V>I	AA:595		UFRJ50791:N>D		UFRJ50816:N>D		UWOPS91_917_1:N>D	AA:597		UWOPS91_917_1:T>A	AA:624		UFRJ50791:S>P		UFRJ50816:S>P		YPS138:S>P	AA:627		UFRJ50791:T>S		UFRJ50816:T>S		UWOPS91_917_1:T>S		YPS138:T>S	AA:630		A4:E>K		UFRJ50791:E>K		UFRJ50816:E>K		UWOPS91_917_1:E>K		YPS138:E>K	AA:633		A4:A>V		UFRJ50791:A>V		UFRJ50816:A>V		YPS138:A>V	AA:638		UWOPS91_917_1:G>S	AA:649		A4:T>N		CBS432:T>N		CBS5829:T>N		IFO1804:T>N		N_45:T>N		Q95_3:T>N		T21_4:T>N		UFRJ50791:T>N		UFRJ50816:T>N		UWOPS91_917_1:T>N		Y6_5:T>N		YPS138:T>N	AA:652		IFO1804:T>A		N_45:T>A	AA:663		A4:E>K		UFRJ50791:E>K		UFRJ50816:E>K		UWOPS91_917_1:E>K		YPS138:E>K	AA:674		A4:K>N		IFO1804:K>N		N_45:K>N		UFRJ50791:K>N		UFRJ50816:K>N		UWOPS91_917_1:K>N		YPS138:K>N	AA:682		A4:S>T		UFRJ50816:S>T		UWOPS91_917_1:S>T		YPS138:S>TID:YBR260C	AA:8		A4:V>F		DBVPG6304:V>F	AA:12		A4:Q>K		DBVPG6304:Q>K		UWOPS91_917_1:Q>K	AA:135		UWOPS91_917_1:N>K	AA:146		Q32_3:F>C	AA:172		A4:N>H		DBVPG6304:N>H		UFRJ50816:N>H		UWOPS91_917_1:N>H		YPS138:N>H	AA:180		A4:Y>H		DBVPG6304:Y>H		UFRJ50816:Y>H		UWOPS91_917_1:Y>H		YPS138:Y>H	AA:182		A4:R>S		DBVPG6304:R>S		UFRJ50816:R>S		UWOPS91_917_1:R>S		YPS138:R>S	AA:218		UWOPS91_917_1:N>K	AA:263		A4:S>R		DBVPG6304:S>R		UFRJ50816:S>R		YPS138:S>R	AA:271		UWOPS91_917_1:R>S	AA:341		UWOPS91_917_1:->C	AA:366		UWOPS91_917_1:F>Y	AA:446		A4:R>S		DBVPG6304:R>S		UWOPS91_917_1:R>I	AA:458		N_43:I>S	AA:473		A12:F>L		A4:F>L		DBVPG6304:F>L		YPS138:F>L	AA:474		N_43:R>-	AA:505		A12:F>L		A4:F>L		DBVPG6304:F>L		UWOPS91_917_1:F>L		YPS138:F>L	AA:574		N_17:C>R	AA:591		A12:D>E		A4:D>E		DBVPG6304:D>E		UFRJ50816:D>E		UWOPS91_917_1:D>E		YPS138:D>E	AA:633		UWOPS91_917_1:D>Y	AA:657		A12:G>S		A4:G>S		DBVPG6304:G>S		UFRJ50816:G>S		YPS138:G>SID:YBR261C	AA:39		A12:K>N		A4:K>N		DBVPG6304:K>N		IFO1804:K>N		N_44:K>N		N_45:K>N		UFRJ50791:K>N		YPS138:K>N	AA:57		UFRJ50791:M>I	AA:64		UWOPS91_917_1:L>I	AA:135		A12:D>E		A4:D>E		DBVPG6304:D>E		IFO1804:D>E		N_43:D>E		N_44:D>E		N_45:D>E		UFRJ50791:D>E		UWOPS91_917_1:D>E		YPS138:D>E	AA:157		IFO1804:Y>S		N_43:Y>S		N_44:Y>S		N_45:Y>S	AA:221		A12:I>M		A4:I>M		N_43:I>M		N_44:I>M		N_45:I>M		UWOPS91_917_1:I>M		YPS138:I>MID:YBR262C	AA:2		DBVPG6304:G>A		UFRJ50791:G>A		UFRJ50816:G>A		YPS138:G>A	AA:26		DBVPG6304:P>T		UFRJ50791:P>T		UFRJ50816:P>T		YPS138:P>T	AA:83		DBVPG6304:E>D		UFRJ50791:E>D		UFRJ50816:E>D		YPS138:E>D	AA:103		UWOPS91_917_1:Q>HID:YBR263W	AA:17		DBVPG6304:R>S		UFRJ50816:R>S		YPS138:R>S	AA:200		UWOPS91_917_1:A>E	AA:227		A12:K>Q		A4:K>Q		UFRJ50816:K>Q		UWOPS91_917_1:K>Q		YPS138:K>Q	AA:255		N_43:H>Y	AA:274		UWOPS91_917_1:M>I	AA:351		A12:R>K		UFRJ50816:R>K		UWOPS91_917_1:R>K		YPS138:R>KID:YBR264C	AA:10		A12:->K		A4:->K		DBVPG6304:->K		UFRJ50816:->K		UWOPS91_917_1:->K		YPS138:->K	AA:50		Q89_8:C>F	AA:88		A4:L>F		DBVPG6304:L>F		UFRJ50816:L>F		UWOPS91_917_1:L>F	AA:107		N_45:R>S	AA:116		A12:N>K		A4:N>K		DBVPG6304:N>K		UFRJ50816:N>K		UWOPS91_917_1:N>K	AA:123		N_45:H>Q	AA:135		N_45:C>G		UWOPS91_917_1:C>G	AA:148		A12:G>V		A4:G>V		DBVPG6304:G>V		UFRJ50816:G>V	AA:154		A12:F>L		A4:F>L		DBVPG6304:F>L		UFRJ50816:F>L		UWOPS91_917_1:F>L	AA:158		N_45:G>S	AA:161		N_45:R>P	AA:163		A12:T>A		A4:T>A		DBVPG6304:T>A		UFRJ50816:T>A	AA:175		CBS5829:R>P		N_17:R>P		N_45:R>P	AA:187		DBVPG6304:->WID:YBR265W	AA:79		N_43:L>I		N_44:L>I		N_45:L>I	AA:129		A12:S>G		DBVPG6304:S>G		YPS138:S>G	AA:132		A12:D>E		DBVPG6304:D>E		UWOPS91_917_1:D>E		YPS138:D>E	AA:183		A12:A>S		DBVPG6304:A>S		UWOPS91_917_1:A>S		YPS138:A>S	AA:266		A12:M>I		A4:M>I		DBVPG6304:M>I		UWOPS91_917_1:M>I		YPS138:M>I	AA:279		UWOPS91_917_1:R>C	AA:281		UWOPS91_917_1:V>AID:YBR267W	AA:6		UWOPS91_917_1:V>I	AA:17		A4:N>S		UFRJ50791:N>S		UFRJ50816:N>S		YPS138:N>S	AA:46		A4:S>P		UFRJ50816:S>P		UWOPS91_917_1:S>P		YPS138:S>P	AA:51		UWOPS91_917_1:D>V	AA:62		DBVPG6304:T>S		IFO1804:T>S		UFRJ50791:T>S		UFRJ50816:T>S		UWOPS91_917_1:T>S		YPS138:T>S	AA:66		UWOPS91_917_1:D>E	AA:69		IFO1804:K>R	AA:71		DBVPG6304:V>I		UFRJ50816:V>I		UWOPS91_917_1:V>I		YPS138:V>I	AA:106		IFO1804:S>T		N_45:S>T	AA:127		DBVPG6304:K>E		IFO1804:K>E		N_45:K>E		YPS138:K>E	AA:130		DBVPG6304:E>K		UFRJ50816:E>K		YPS138:E>K	AA:132		DBVPG6304:E>K		N_45:E>K		UFRJ50816:E>K		YPS138:E>K	AA:173		N_45:F>L	AA:211		DBVPG6304:I>V		YPS138:I>V	AA:283		Q59_1:S>N		Q95_3:S>N		T21_4:S>N		Y7:S>N	AA:284		IFO1804:D>N	AA:291		UWOPS91_917_1:E>D	AA:356		UWOPS91_917_1:V>I	AA:358		A12:A>T		DBVPG6304:A>T		UFRJ50816:A>T		UWOPS91_917_1:A>T	AA:369		UWOPS91_917_1:D>NID:YBR268W	AA:11		A4:V>I		DBVPG6304:V>I		IFO1804:V>I		N_43:V>I		N_44:V>I		N_45:V>I		UFRJ50816:V>I		UWOPS91_917_1:V>I		YPS138:V>I	AA:21		A4:Q>P		UWOPS91_917_1:Q>P		YPS138:Q>P	AA:64		N_43:D>N		N_45:D>N	AA:79		A4:E>D		DBVPG6304:E>D		UFRJ50816:E>D		UWOPS91_917_1:E>D		YPS138:E>DID:YBR269C	AA:7		A12:S>C		A4:S>C		N_44:S>C		UFRJ50816:S>C		YPS138:S>C	AA:15		A12:P>L		A4:P>L		UFRJ50816:P>L		YPS138:P>L	AA:20		N_44:L>P		N_45:L>P	AA:35		A12:K>N		A4:K>N		DBVPG6304:K>N		UFRJ50816:K>N		UWOPS91_917_1:K>N		YPS138:K>N	AA:42		N_45:K>N	AA:61		UWOPS91_917_1:->W	AA:63		UWOPS91_917_1:C>W	AA:86		A12:S>P		DBVPG6304:S>P		UFRJ50816:S>P		YPS138:S>P	AA:101		UWOPS91_917_1:R>S	AA:127		A12:A>P		A4:A>P		DBVPG6304:R>S		UFRJ50816:A>P		UWOPS91_917_1:R>S		YPS138:A>PID:YBR270C	AA:17		UWOPS91_917_1:V>I	AA:25		A4:I>V		IFO1804:I>V		N_44:I>V		N_45:I>V		UFRJ50791:I>V		UFRJ50816:I>V		UWOPS91_917_1:I>V		YPS138:I>V	AA:35		UWOPS91_917_1:Q>H	AA:49		UWOPS91_917_1:E>D	AA:65		T21_4:E>D		Y6_5:E>D	AA:73		A12:D>E		A4:D>E		IFO1804:D>E		N_43:D>E		N_44:D>E		N_45:D>E		UFRJ50791:D>E		YPS138:D>E	AA:80		UWOPS91_917_1:->Y	AA:84		A12:I>N		A4:I>N		UFRJ50791:I>N		YPS138:I>N	AA:88		A12:N>K		A4:N>K		UFRJ50791:N>K		YPS138:N>K	AA:116		A12:C>Y		UFRJ50791:C>Y		UWOPS91_917_1:C>Y		YPS138:C>Y	AA:135		A12:F>L		UFRJ50791:F>L		YPS138:F>L	AA:137		N_43:S>R		N_44:S>R		N_45:S>R	AA:157		A12:Q>H		UFRJ50791:Q>H		UWOPS91_917_1:M>L		YPS138:Q>H	AA:166		N_43:F>Y		N_44:F>Y		N_45:F>Y	AA:195		A12:N>K		UFRJ50791:N>K		UWOPS91_917_1:N>K		YPS138:N>K	AA:212		A12:Y>-		UFRJ50791:Y>-		YPS138:Y>-	AA:238		A12:Q>H		UFRJ50791:Q>H		UWOPS91_917_1:Q>H	AA:248		A12:->Y		UFRJ50791:->Y		UWOPS91_917_1:->Y		YPS138:->Y	AA:291		A12:R>S	AA:295		UWOPS91_917_1:K>N	AA:333		N_43:->Y		N_45:->Y	AA:355		A12:Q>H		UWOPS91_917_1:Q>H		YPS138:Q>H	AA:433		UWOPS91_917_1:H>Q	AA:439		KPN3828:->L	AA:448		A12:I>F		A4:I>F		YPS138:I>F	AA:466		YPS138:I>M	AA:473		UWOPS91_917_1:R>I	AA:475		UWOPS91_917_1:K>N	AA:488		A12:R>S		A4:R>S		UFRJ50816:R>S		YPS138:R>S	AA:517		UWOPS91_917_1:W>C	AA:535		A12:G>A		UFRJ50816:G>A		YPS138:G>AID:YBR271W	AA:15		A12:V>A		DBVPG6304:V>A		UFRJ50816:V>A		YPS138:V>A	AA:19		N_44:Q>K		N_45:Q>K	AA:21		A12:D>N		DBVPG6304:D>N		UFRJ50816:D>N		YPS138:D>N	AA:34		A12:Q>L		DBVPG6304:Q>L		UWOPS91_917_1:Q>L		YPS138:Q>L	AA:53		A12:T>N		DBVPG6304:T>N		UFRJ50816:T>N		YPS138:T>N	AA:74		A12:I>V		DBVPG6304:I>V		UFRJ50816:I>V		UWOPS91_917_1:I>V		YPS138:I>V	AA:93		A12:D>N		A4:D>N		DBVPG6304:D>N		UFRJ50816:D>N		UWOPS91_917_1:D>N		YPS138:D>N	AA:97		A12:L>P		A4:L>P		DBVPG6304:L>P		UFRJ50816:L>P		UWOPS91_917_1:L>P		YPS138:L>P	AA:129		N_44:L>W		UFRJ50816:L>W	AA:130		A12:K>E		A4:K>E		DBVPG6304:K>E		N_45:K>E		Q62_5:K>E		UWOPS91_917_1:K>E		Y6_5:K>E		YPS138:K>E		Z1_1:K>E	AA:155		A12:V>A		A4:V>A		DBVPG6304:V>A		UWOPS91_917_1:V>A		YPS138:V>A	AA:162		N_45:C>S	AA:165		UWOPS91_917_1:F>L	AA:166		N_45:N>S	AA:193		A12:I>V		A4:I>V		DBVPG6304:I>V		UWOPS91_917_1:I>V	AA:209		A12:Y>H		A4:Y>H		DBVPG6304:Y>H	AA:222		Z1_1:T>	AA:242		A4:L>V		DBVPG6304:L>V		UWOPS91_917_1:L>V	AA:248		A4:S>C		DBVPG6304:S>C		IFO1804:S>C		N_45:S>C		UWOPS91_917_1:S>C	AA:284		A4:I>M		DBVPG6304:I>M		UWOPS91_917_1:I>M	AA:313		A12:T>I		A4:T>I		DBVPG6304:T>I		UWOPS91_917_1:T>I	AA:321		A4:S>G		DBVPG6304:S>G		UWOPS91_917_1:S>G	AA:376		DBVPG6304:R>K		IFO1804:R>K		KPN3828:R>K		N_45:R>K		UWOPS91_917_1:R>K	AA:389		KPN3828:N>D	AA:406		UWOPS91_917_1:V>A	AA:408		A12:K>EID:YBR272C	AA:8		UWOPS91_917_1:N>H	AA:17		A12:I>S		DBVPG6304:I>S		UWOPS91_917_1:I>S		YPS138:I>S	AA:23		A12:H>Q		UWOPS91_917_1:H>Q		YPS138:H>Q	AA:24		T21_4:R>I	AA:38		N_44:F>V	AA:70		A12:T>P		YPS138:T>P	AA:75		CBS432:W>C	AA:80		N_44:Q>P	AA:86		IFO1804:Y>N		N_43:Y>N		N_44:Y>N		N_45:Y>N	AA:94		N_44:Y>H	AA:98		A12:F>L		YPS138:F>L	AA:105		A12:N>K		YPS138:N>K	AA:111		A12:F>L		YPS138:F>L	AA:137		A12:K>I		IFO1804:K>I		N_43:K>I		N_45:K>I		YPS138:K>I	AA:246		A12:N>K		A4:N>K		YPS138:N>K	AA:285		CBS432:E>V	AA:307		A12:I>S		A4:I>S		YPS138:I>S	AA:323		A12:F>I		A4:F>I	AA:358		UFRJ50791:L>H	AA:369		A12:C>S		A4:C>S		DBVPG6304:C>S		UFRJ50791:C>S		YPS138:C>S	AA:416		IFO1804:I>V		N_43:I>V		N_44:I>V		N_45:I>V	AA:418		IFO1804:C>S		N_43:C>S		N_44:C>S		N_45:C>S	AA:422		A12:T>S		CBS5829:->S		Q89_8:->S		YPS138:T>S	AA:425		A12:L>I		A4:L>I		DBVPG6304:L>I		IFO1804:L>I		N_43:L>I		N_44:L>I		N_45:L>I		UFRJ50791:L>I		YPS138:L>I	AA:430		IFO1804:->K		N_43:->K		N_44:->K		N_45:->K	AA:432		A12:F>L		A4:F>L		DBVPG6304:F>L		YPS138:F>L	AA:452		A12:->Y		A4:->Y		DBVPG6304:->Y		UFRJ50791:->Y		UWOPS91_917_1:->Y		YPS138:->Y	AA:475		UWOPS91_917_1:H>QID:YBR273C	AA:21		A12:W>C		DBVPG6304:W>C		UFRJ50791:W>C		UFRJ50816:W>C	AA:36		A12:N>Y		DBVPG6304:N>Y		UFRJ50791:N>Y		UFRJ50816:N>Y	AA:49		A12:F>S		UFRJ50791:F>S		UFRJ50816:F>S		UWOPS91_917_1:F>S	AA:98		Z1_1:G>V	AA:128		IFO1804:K>M		N_44:K>M		N_45:K>M		UWOPS91_917_1:K>M	AA:135		A12:G>D		DBVPG6304:G>D		UFRJ50816:G>D	AA:138		IFO1804:Q>H		N_44:Q>H		N_45:Q>H	AA:150		A12:->Y		DBVPG6304:->Y		UFRJ50816:->Y		UWOPS91_917_1:->Y	AA:157		A12:F>L		A4:F>L		DBVPG6304:F>L		UFRJ50816:F>L	AA:192		A12:H>R		A4:H>R		DBVPG6304:H>R		UFRJ50816:H>R		YPS138:H>R	AA:202		A12:->C		A4:->C		DBVPG6304:->C		UFRJ50816:->C		YPS138:->C	AA:205		A12:E>D		A4:E>D		DBVPG6304:E>D		IFO1804:E>D		N_43:E>D		N_44:E>D		N_45:E>D		UFRJ50816:E>D		UWOPS91_917_1:E>D		YPS138:E>D	AA:209		A4:Q>K		DBVPG6304:Q>K		UFRJ50816:Q>K		UWOPS91_917_1:Q>K		YPS138:Q>K	AA:226		A4:->Y		DBVPG6304:->Y		UFRJ50816:->Y		UWOPS91_917_1:->Y		YPS138:->Y	AA:252		A4:->Y		DBVPG6304:->Y		UFRJ50816:->Y		YPS138:->Y	AA:302		A12:I>M		A4:I>M		DBVPG6304:I>M		IFO1804:I>M		N_43:I>M		N_44:I>M		N_45:I>M		UFRJ50791:I>M		UFRJ50816:I>M		YPS138:I>M	AA:309		A12:F>L		A4:F>L		DBVPG6304:F>L		UFRJ50791:F>L		UFRJ50816:F>L		YPS138:F>L	AA:332		A12:R>C		A4:R>C		DBVPG6304:R>C		UFRJ50791:R>C		UFRJ50816:R>C		YPS138:R>C	AA:345		A12:N>K		A4:N>K		DBVPG6304:N>K		UFRJ50791:N>K		UFRJ50816:N>K		YPS138:N>K	AA:357		UWOPS91_917_1:Y>-	AA:378		UWOPS91_917_1:D>N	AA:379		IFO1804:Q>-		N_43:Q>-		N_44:Q>-	AA:412		A12:N>K	AA:414		UWOPS91_917_1:H>YID:YBR274W	AA:6		A12:I>M		A4:I>M		DBVPG6304:I>M		UFRJ50791:I>M		UFRJ50816:I>M	AA:58		IFO1804:S>T		N_44:S>T		N_45:S>T	AA:67		A12:V>I		A4:V>I		DBVPG6304:V>I		UFRJ50791:V>I		UFRJ50816:V>I	AA:114		Q62_5:D>N	AA:166		A4:K>Q		DBVPG6304:K>Q		YPS138:K>Q	AA:224		A12:I>V		DBVPG6304:I>V		YPS138:I>V	AA:236		IFO1804:I>M		N_43:I>M		N_44:I>M		N_45:I>M	AA:273		A12:N>K		DBVPG6304:N>K		IFO1804:N>K		N_43:N>K		N_44:N>K		YPS138:N>K	AA:326		A12:S>F	AA:353		A12:V>I		DBVPG6304:V>I		UFRJ50816:V>I		UWOPS91_917_1:V>I	AA:367		A12:M>I		DBVPG6304:M>I		UFRJ50816:M>I		UWOPS91_917_1:M>I	AA:395		A12:H>Y		UFRJ50816:H>Y	AA:400		A12:N>D		IFO1804:N>D		N_43:N>D		N_44:N>D		N_45:N>D		UFRJ50816:N>D		UWOPS91_917_1:N>D	AA:427		N_45:I>M	AA:434		A12:K>E		UFRJ50816:K>E	AA:459		N_43:S>G		N_44:S>G		N_45:S>G	AA:477		N_44:G>D		N_45:G>D	AA:492		UWOPS91_917_1:E>DID:YBR276C	AA:40		IFO1804:E>Q		N_43:E>Q		N_44:E>Q		N_45:E>Q	AA:60		A12:C>Y		A4:C>Y		DBVPG6304:C>Y		IFO1804:C>Y		N_43:C>Y		N_44:C>Y		N_45:C>Y		YPS138:C>Y	AA:72		UWOPS91_917_1:N>S	AA:76		A12:C>F		A4:C>F		UWOPS91_917_1:C>F	AA:82		UWOPS91_917_1:D>Y	AA:90		IFO1804:P>T		N_43:P>T		N_44:P>T		N_45:P>T	AA:107		CBS432:M>R		N_17:M>R	AA:138		UWOPS91_917_1:F>I	AA:169		IFO1804:C>S		N_44:C>S		N_45:C>S		UWOPS91_917_1:C>S	AA:174		IFO1804:Y>D	AA:206		A4:I>F		UWOPS91_917_1:I>F		YPS138:I>F	AA:222		UWOPS91_917_1:G>E	AA:224		A4:Q>H		DBVPG6304:Q>H		IFO1804:Q>H		N_43:Q>H		N_45:Q>H		UWOPS91_917_1:Q>H		YPS138:Q>H	AA:227		DBVPG6304:L>I		YPS138:L>I	AA:268		A4:->Y		DBVPG6304:->Y		UWOPS91_917_1:->Y		YPS138:->Y	AA:279		A4:Q>H	AA:283		UWOPS91_917_1:Q>K	AA:295		CBS432:E>K		Y6_5:E>K	AA:296		DBVPG6304:K>N		UFRJ50791:K>N		YPS138:K>N	AA:319		A4:C>-		DBVPG6304:C>-		UFRJ50791:C>-		YPS138:C>-	AA:346		A4:Y>D		DBVPG6304:Y>D		UFRJ50791:Y>D		YPS138:Y>D	AA:356		IFO1804:H>R		N_43:H>R		N_44:H>R		N_45:H>R	AA:357		A4:R>K		DBVPG6304:R>K		UFRJ50791:R>K		YPS138:R>K	AA:368		A4:T>N		DBVPG6304:T>N		UFRJ50791:T>N		YPS138:T>N	AA:380		A4:->C		DBVPG6304:->C		UFRJ50791:->C		YPS138:->C	AA:387		A4:->K		DBVPG6304:->K		UFRJ50791:->K		YPS138:->K	AA:425		A4:R>S		DBVPG6304:R>S		UFRJ50791:R>S		YPS138:R>S	AA:496		A4:H>N		DBVPG6304:H>N		UFRJ50791:H>N		UFRJ50816:H>N		UWOPS91_917_1:H>N	AA:546		A12:->K		A4:->K		DBVPG6304:->K		UFRJ50816:->K		UWOPS91_917_1:->K	AA:552		A12:->C		A4:->C		DBVPG6304:->C		UFRJ50816:->C		UWOPS91_917_1:->C	AA:572		N_17:C>F	AA:603		A12:I>T		A4:I>T		DBVPG6304:I>T		UFRJ50816:I>T		UWOPS91_917_1:I>T		YPS138:I>T	AA:608		N_17:I>L	AA:617		A12:L>F		A4:L>F		DBVPG6304:L>F		UFRJ50816:L>F		UWOPS91_917_1:L>F		YPS138:L>F	AA:620		DBVPG4650:E>A		Q62_5:E>A		S36_7:E>A		Y6_5:E>A	AA:626		A12:S>Y		A4:S>Y		DBVPG6304:S>Y		UFRJ50816:S>Y		UWOPS91_917_1:S>Y		YPS138:S>Y	AA:630		Q62_5:N>H		Z1_1:N>H	AA:637		A12:->Y		A4:->Y		DBVPG6304:->Y		IFO1804:->Y		N_43:->Y		N_45:->Y		UFRJ50816:->Y		YPS138:->Y	AA:646		IFO1804:S>T		N_43:S>T		N_45:S>T	AA:650		A12:L>H		A4:L>H		YPS138:L>H	AA:654		A12:->C		A4:->C		DBVPG6304:->C		IFO1804:->C		UFRJ50816:->C		UWOPS91_917_1:->C		YPS138:->C	AA:665		CBS5829:->E	AA:667		UWOPS91_917_1:A>D	AA:680		UWOPS91_917_1:Y>N	AA:707		UWOPS91_917_1:V>I	AA:715		IFO1804:C>Y		N_43:C>Y		N_45:C>Y	AA:748		A12:S>N	AA:753		A12:F>L		UWOPS91_917_1:F>L		YPS138:F>L	AA:755		UWOPS91_917_1:Y>-	AA:793		UWOPS91_917_1:M>L	AA:805		N_43:N>D		N_45:N>DID:YBR278W	AA:11		A12:A>T		DBVPG6304:A>T		IFO1804:A>T		N_43:A>T		N_44:A>T		N_45:A>T		YPS138:A>T	AA:99		A12:A>S		A4:A>S		DBVPG6304:A>S		UWOPS91_917_1:A>S		YPS138:A>S	AA:116		A12:E>G		A4:E>G		DBVPG6304:E>G		YPS138:E>G	AA:130		A12:I>V		A4:I>V		DBVPG6304:I>V		UWOPS91_917_1:I>V		YPS138:I>V	AA:133		DBVPG6304:E>K	AA:137		A12:E>G		A4:E>G		YPS138:E>G	AA:146		IFO1804:N>H		N_43:N>H		N_44:N>H		N_45:N>H		UWOPS91_917_1:N>H	AA:154		A12:S>N		A4:S>N		DBVPG6304:S>N		UWOPS91_917_1:S>N		YPS138:S>N	AA:157		A12:Y>H		A4:Y>H		DBVPG6304:Y>H		IFO1804:Y>H		N_44:Y>H		N_45:Y>H		UWOPS91_917_1:Y>H		YPS138:Y>H	AA:162		UWOPS91_917_1:S>F	AA:164		A12:P>S		A4:P>S		DBVPG6304:P>S		YPS138:P>S	AA:184		CBS5829:H>D		KPN3828:H>D		N_17:H>D	AA:185		IFO1804:S>T		N_44:S>T		N_45:S>T	AA:198		N_17:T>AID:YBR279W	AA:14		IFO1804:Q>R		N_43:Q>R		N_44:Q>R		N_45:Q>R	AA:28		IFO1804:V>I		N_43:V>I		N_44:V>I		N_45:V>I	AA:37		A12:S>P		DBVPG6304:S>P		UWOPS91_917_1:S>P		YPS138:S>P	AA:163		A12:G>R		DBVPG6304:G>R		YPS138:G>R	AA:230		Q95_3:D>A	AA:248		UWOPS91_917_1:N>K	AA:258		UWOPS91_917_1:D>Y	AA:307		A12:R>K		DBVPG6304:R>K		UFRJ50791:R>K		UWOPS91_917_1:R>K		YPS138:R>K	AA:368		A12:E>D	AA:379		A12:V>G		DBVPG6304:V>G		UFRJ50791:V>G		YPS138:V>G	AA:399		Q89_8:T>R		Y6_5:T>R	AA:413		UWOPS91_917_1:A>G	AA:421		A12:P>S		DBVPG6304:P>S		UFRJ50791:P>S		YPS138:P>S	AA:429		N_43:D>G	AA:437		A4:S>L		DBVPG6304:S>L		UFRJ50791:S>L		UWOPS91_917_1:S>L		YPS138:S>L	AA:447		UWOPS91_917_1:E>DID:YBR280C	AA:20		UWOPS91_917_1:A>T	AA:37		UWOPS91_917_1:I>M	AA:59		A4:->Y		UWOPS91_917_1:->Y	AA:67		A4:N>S		YPS138:N>S	AA:73		A4:M>I	AA:79		A4:C>S		YPS138:C>S	AA:89		A4:R>S		UWOPS91_917_1:R>S	AA:112		A4:Q>-		UWOPS91_917_1:Q>-		YPS138:Q>-	AA:133		A4:->E		IFO1804:->E		N_43:->E		N_45:->E		UWOPS91_917_1:->E		YPS138:->E	AA:137		A4:N>K		IFO1804:N>K		N_43:N>K		N_45:N>K		UWOPS91_917_1:N>K		YPS138:N>K	AA:142		A4:H>D		YPS138:H>D	AA:224		A12:D>H		A4:D>H		YPS138:D>H	AA:235		A12:N>K		A4:N>K		DBVPG6304:N>K		IFO1804:N>K		N_43:N>K		N_44:N>K		N_45:N>K		UFRJ50791:N>K		YPS138:N>K	AA:252		IFO1804:R>S		N_43:R>S		N_44:R>S		N_45:R>S	AA:257		A12:I>M		A4:I>M		DBVPG6304:I>M		N_43:I>M		N_44:I>M		N_45:I>M		UFRJ50791:I>M		YPS138:I>M	AA:262		A12:->Y		A4:->Y		YPS138:->Y	AA:268		N_43:R>S		N_44:R>S		N_45:R>S	AA:283		A12:F>L		A4:F>L		DBVPG6304:F>L		UFRJ50791:F>L		YPS138:F>L	AA:286		A12:T>P		A4:T>P		DBVPG6304:T>P		UFRJ50791:T>P		YPS138:T>P	AA:303		N_43:V>G		N_44:V>G		N_45:V>G	AA:310		A4:S>I		UFRJ50791:S>I	AA:313		A12:F>S		A4:F>S		DBVPG6304:F>S		UFRJ50791:F>S		YPS138:F>S	AA:323		A12:L>P		A4:L>P		DBVPG6304:L>P		UFRJ50791:L>P	AA:356		A12:L>F		A4:L>F		DBVPG6304:L>F		UFRJ50791:L>F		YPS138:L>F	AA:369		YPS138:S>R	AA:410		YPS138:K>N	AA:422		CBS5829:W>C		N_17:W>C		Q95_3:W>C		Y6_5:W>C		Y7:W>C	AA:506		A12:C>W		DBVPG6304:C>W		UFRJ50791:C>W		YPS138:C>W	AA:530		IFO1804:K>E		N_45:K>E	AA:560		A12:K>T		UWOPS91_917_1:K>T	AA:586		N_43:P>S		N_45:P>S	AA:589		UWOPS91_917_1:F>V	AA:603		UWOPS91_917_1:C>W	AA:606		DBVPG6304:E>VID:YBR281C	AA:59		A12:S>P		A4:S>P		DBVPG6304:S>P		UFRJ50816:S>P		UWOPS91_917_1:S>P		YPS138:S>P	AA:63		A12:W>C		A4:W>C		DBVPG6304:W>C		UFRJ50816:W>C		YPS138:W>C	AA:115		UWOPS91_917_1:E>D	AA:170		A12:S>N		IFO1804:S>N		KPN3829:S>N		N_17:S>N		N_43:S>N		N_45:S>N		Y6_5:S>N		YPS138:S>N	AA:198		A12:K>Q		YPS138:K>Q	AA:200		A12:W>S		YPS138:W>S	AA:254		A12:Q>H	AA:272		N_43:N>K	AA:294		A12:F>L		YPS138:F>L	AA:299		A12:->Y		YPS138:->Y	AA:323		DBVPG6304:W>-		YPS138:W>-	AA:359		IFO1804:I>L		N_43:I>L		N_45:I>L	AA:393		DBVPG6304:R>S		UFRJ50816:R>S		YPS138:R>S	AA:443		A12:H>N		A4:H>N		DBVPG6304:H>N		UFRJ50816:H>N		YPS138:H>N	AA:503		YPS138:N>K	AA:505		A12:F>L		A4:F>L		DBVPG6304:F>L		UFRJ50791:F>L		UFRJ50816:F>L		YPS138:F>L	AA:509		A12:L>H		A4:L>H		DBVPG6304:L>H		UFRJ50791:L>H		UFRJ50816:L>H		YPS138:L>H	AA:529		A12:M>I		A4:M>I		DBVPG6304:M>I		UFRJ50791:M>I		UFRJ50816:M>I		YPS138:M>I	AA:535		A12:I>V		A4:I>V		DBVPG6304:I>V		N_43:I>V		N_44:I>V		N_45:I>V		UFRJ50791:I>V		UFRJ50816:I>V		UWOPS91_917_1:I>V		YPS138:I>V	AA:539		A4:Y>-		YPS138:Y>-	AA:571		A12:Y>-		A4:Y>-		DBVPG6304:Y>-		UFRJ50791:Y>-		UWOPS91_917_1:Y>-		YPS138:Y>-	AA:673		DBVPG6304:S>R		N_43:S>R		N_44:S>R		YPS138:S>R	AA:701		DBVPG6304:K>N		N_43:K>N		N_44:K>N		YPS138:K>N	AA:709		DBVPG6304:->E		YPS138:->E	AA:726		N_44:N>D	AA:732		CBS432:E>V	AA:767		N_43:F>L		N_44:F>L	AA:772		A12:E>D		A4:E>D		DBVPG6304:E>D		N_43:E>D		YPS138:E>D	AA:776		CBS432:N>K	AA:778		A12:L>F		A4:L>F		DBVPG6304:L>F		YPS138:L>F	AA:784		N_43:N>K	AA:787		UWOPS91_917_1:->YID:YBR282W	AA:131		A12:V>I		DBVPG6304:V>I		UWOPS91_917_1:V>I		YPS138:V>I	AA:134		UWOPS91_917_1:Q>R	AA:137		A12:T>A		DBVPG6304:T>A		YPS138:T>AID:YBR283C	AA:27		A12:F>L		A4:F>L		DBVPG6304:F>L		YPS138:F>L	AA:56		N_43:E>D		N_44:E>D		N_45:E>D	AA:78		N_43:I>M		N_44:I>M		N_45:I>M	AA:120		A4:S>R		DBVPG6304:S>R	AA:135		A4:E>D		DBVPG6304:E>D	AA:144		CBS5829:R>S		Q59_1:R>S		Q89_8:R>S		Q95_3:R>S		Y7:R>S	AA:167		CBS5829:N>K		N_43:N>K		N_44:N>K		N_45:N>K		Q59_1:N>K		Q89_8:N>K		Q95_3:N>K		Y7:N>K	AA:261		DBVPG6304:K>N		YPS138:K>N	AA:268		IFO1804:->K	AA:291		UWOPS91_917_1:R>I	AA:303		A4:N>K		DBVPG6304:N>K		IFO1804:N>K		N_44:N>K		N_45:N>K		UWOPS91_917_1:N>K		YPS138:N>K	AA:322		T21_4:D>E	AA:330		A12:Q>H		A4:Q>H		DBVPG6304:Q>H		YPS138:Q>H	AA:335		UWOPS91_917_1:Q>R	AA:337		A12:K>N		A4:K>N		DBVPG6304:K>N		YPS138:K>N	AA:355		A12:K>N		A4:K>N		DBVPG6304:K>N		YPS138:K>N	AA:366		CBS432:N>K		CBS5829:N>K		Q89_8:N>K		S36_7:N>K		Y7:N>K	AA:374		IFO1804:S>G		N_43:S>G		N_44:S>G		N_45:S>G	AA:398		A12:->K		A4:->K		DBVPG6304:->K		UWOPS91_917_1:->K		YPS138:->K	AA:439		UWOPS91_917_1:Q>E	AA:443		DBVPG4650:R>L	AA:444		UWOPS91_917_1:E>QID:YBR284W	AA:14		IFO1804:R>T		N_43:R>T		N_44:R>T		N_45:R>T	AA:55		CBS432:D>E	AA:61		CBS432:R>Q		CBS5829:R>Q		DBVPG4650:R>Q		IFO1804:R>Q		KPN3828:R>Q		N_17:R>Q		N_43:R>Q		N_44:R>Q		N_45:R>Q		Q59_1:R>Q		Q62_5:R>Q		Q95_3:R>Q		T21_4:R>Q		UWOPS91_917_1:R>Q		Y6_5:R>Q		Y7:R>Q		Z1_1:R>Q	AA:77		UWOPS91_917_1:N>T	AA:114		IFO1804:P>L		N_43:P>L		N_44:P>L		N_45:P>L	AA:119		N_43:K>Q		N_44:K>Q		N_45:K>Q	AA:153		N_45:K>N	AA:161		UWOPS91_917_1:I>V	AA:180		A4:R>C		DBVPG6304:R>C	AA:187		A4:N>K		N_45:N>K		UWOPS91_917_1:N>K	AA:209		A4:D>G		DBVPG6304:D>G	AA:212		CBS432:M>T	AA:216		UWOPS91_917_1:F>L	AA:218		UWOPS91_917_1:D>G	AA:221		UWOPS91_917_1:N>I	AA:226		N_45:K>R	AA:243		UWOPS91_917_1:S>R	AA:273		A4:Q>E		DBVPG6304:Q>E	AA:302		A4:R>Q		DBVPG6304:R>Q	AA:309		A4:M>T		CBS432:M>T		CBS5829:M>T		DBVPG6304:M>T		N_17:M>T		N_44:M>T		N_45:M>T		Q59_1:M>T		Q62_5:M>T		Q95_3:M>T		S36_7:M>T		T21_4:M>T		Y6_5:M>T		Y7:M>T	AA:312		A4:R>K		DBVPG6304:R>K	AA:343		CBS432:K>Q		Q62_5:K>Q		T21_4:K>Q		Y6_5:K>Q		Z1_1:K>Q	AA:362		A4:I>V		DBVPG6304:I>V		UFRJ50816:I>V	AA:390		A4:I>V		DBVPG6304:I>V		UFRJ50791:I>V		UFRJ50816:I>V	AA:413		N_44:F>L		N_45:F>L	AA:475		UFRJ50791:P>S		UFRJ50816:P>S		YPS138:P>S	AA:481		UWOPS91_917_1:G>S	AA:492		N_43:L>F		N_44:L>F		N_45:L>F	AA:500		N_43:A>S		N_44:A>S		N_45:A>S	AA:518		UFRJ50791:Q>E		UFRJ50816:Q>E		UWOPS91_917_1:Q>E		YPS138:Q>E	AA:546		UFRJ50791:N>D		UFRJ50816:N>D		UWOPS91_917_1:N>D		YPS138:N>D	AA:563		UWOPS91_917_1:V>A	AA:572		UFRJ50791:N>H		UFRJ50816:N>H		UWOPS91_917_1:N>H		YPS138:N>H	AA:591		A12:T>I		UFRJ50791:T>I		UFRJ50816:T>I		YPS138:T>I	AA:766		Q62_5:T>A	AA:769		IFO1804:V>I		N_44:V>I		N_45:V>I	AA:773		Q62_5:Y>D	AA:775		A12:C>S		A4:C>S		DBVPG6304:C>S		UFRJ50791:C>S		UFRJ50816:C>S		UWOPS91_917_1:C>S		YPS138:C>S	AA:795		A12:D>G		A4:D>G		DBVPG6304:D>G		IFO1804:D>G		N_44:D>G		N_45:D>G		UFRJ50791:D>G		UFRJ50816:D>G		UWOPS91_917_1:D>G		YPS138:D>GID:YBR285W	AA:12		DBVPG6304:S>N		UFRJ50816:S>N		YPS138:S>N	AA:21		UWOPS91_917_1:P>A	AA:73		A4:A>S		DBVPG6304:A>S		UFRJ50816:A>S		YPS138:A>S	AA:80		A4:K>N		DBVPG6304:K>N		UFRJ50791:K>N		UFRJ50816:K>N		UWOPS91_917_1:K>N		YPS138:K>N	AA:98		A4:H>R		DBVPG6304:H>R		UFRJ50791:H>R		UFRJ50816:H>R		UWOPS91_917_1:H>R		YPS138:H>R	AA:104		A4:D>A		DBVPG6304:D>A		UFRJ50791:D>A		UFRJ50816:D>A		YPS138:D>A	AA:114		A4:L>P		DBVPG6304:L>P		UFRJ50791:L>P		UFRJ50816:L>P		UWOPS91_917_1:L>P		YPS138:L>P	AA:127		UWOPS91_917_1:Q>RID:YBR286W	AA:11		A12:A>V		A4:A>V		UFRJ50816:A>V		UWOPS91_917_1:A>V		YPS138:A>V	AA:42		A12:F>L		A4:F>L		CBS432:F>L		CBS5829:F>L		DBVPG4650:F>L		IFO1804:F>L		KPN3828:F>L		KPN3829:F>L		N_43:F>L		N_44:F>L		N_45:F>L		Q62_5:F>L		S36_7:F>L		T21_4:F>L		UFRJ50816:F>L		Y6_5:F>L		YPS138:F>L		Z1_1:F>L	AA:77		A12:D>E		A4:D>E		UFRJ50816:D>E		YPS138:D>E	AA:127		N_43:D>G	AA:199		A12:G>S		A4:G>S		DBVPG6304:G>S		UFRJ50816:G>S	AA:202		A12:D>E		A4:D>E		DBVPG6304:D>E		UFRJ50816:D>E	AA:275		UWOPS91_917_1:N>D	AA:285		UWOPS91_917_1:D>E	AA:375		UFRJ50816:K>E		UWOPS91_917_1:K>E		YPS138:K>E	AA:446		UWOPS91_917_1:G>E	AA:487		N_43:I>V		N_45:I>V	AA:516		A4:E>K		UFRJ50816:E>K		YPS138:E>K	AA:527		KPN3828:Q>K		Q95_3:Q>K		T21_4:Q>K		Y7:Q>KID:YBR287W	AA:103		A4:R>K		DBVPG6304:R>K		N_43:R>K		N_44:R>K		N_45:R>K		UFRJ50816:R>K		YPS138:R>K	AA:145		UFRJ50791:S>	AA:217		UFRJ50791:L>H		UFRJ50816:L>H	AA:233		UWOPS91_917_1:S>N	AA:250		A12:V>I		DBVPG6304:V>I		UFRJ50791:V>I		UFRJ50816:V>I		UWOPS91_917_1:V>I		YPS138:V>IID:YBR288C	AA:2		UFRJ50816:Q>H		UWOPS91_917_1:Q>H		YPS138:Q>H	AA:29		UWOPS91_917_1:R>S	AA:30		A12:Q>E		UFRJ50816:Q>E		UWOPS91_917_1:Q>E		YPS138:Q>E	AA:41		A12:C>W		IFO1804:C>W		N_44:C>W		N_45:C>W		UFRJ50816:C>W		UWOPS91_917_1:C>W		YPS138:C>W	AA:127		UFRJ50816:Q>E	AA:137		DBVPG6304:F>V		UFRJ50816:F>V		YPS138:F>V	AA:186		DBVPG6304:->Y		UWOPS91_917_1:->Y		YPS138:->Y	AA:199		Q62_5:G>A		T21_4:G>A	AA:219		IFO1804:I>M		N_44:I>M		N_45:I>M	AA:237		IFO1804:R>G		N_44:R>G		N_45:R>G	AA:244		DBVPG6304:M>I		YPS138:M>I	AA:246		DBVPG6304:I>M		UWOPS91_917_1:I>M		YPS138:I>M	AA:259		DBVPG6304:K>R		UWOPS91_917_1:K>R		YPS138:K>R	AA:287		KPN3828:N>D	AA:289		DBVPG6304:F>I		UWOPS91_917_1:F>I		YPS138:F>I	AA:303		A12:L>F		DBVPG6304:L>F		YPS138:L>F	AA:314		UWOPS91_917_1:L>M	AA:342		A12:I>M		UFRJ50816:I>M		UWOPS91_917_1:I>M		YPS138:I>M	AA:351		N_43:R>W		N_44:R>W		N_45:R>W	AA:402		N_43:->G		N_44:->G		N_45:->G	AA:408		N_43:->K		N_44:->K		N_45:->K		UWOPS91_917_1:Y>-	AA:426		KPN3828:I>N		KPN3829:I>N		N_17:I>N		Q59_1:I>N		Q62_5:I>N	AA:435		UWOPS91_917_1:I>L	AA:440		A12:A>T		CBS5829:Q>-		DBVPG4650:Q>-		N_43:A>T		N_44:A>T		N_45:A>T		UFRJ50816:A>T		UWOPS91_917_1:A>T		YPS138:A>TID:YBR290W	AA:12		A12:M>L		A4:M>L		UFRJ50791:M>L		UFRJ50816:M>L		YPS138:M>L	AA:18		UWOPS91_917_1:G>D	AA:37		N_43:T>I		N_45:T>I	AA:49		UFRJ50791:E>Q		UFRJ50816:E>Q	AA:85		UWOPS91_917_1:K>Q	AA:101		A4:T>S		DBVPG6304:T>S		UFRJ50791:T>S		UFRJ50816:T>S		UWOPS91_917_1:T>S	AA:175		Y6_5:G>R	AA:194		Y6_5:G>EID:YBR291C	AA:32		UWOPS91_917_1:K>N	AA:39		A4:F>L		DBVPG6304:F>L		UWOPS91_917_1:F>L		YPS138:F>L	AA:61		IFO1804:C>F	AA:67		A4:R>S		DBVPG6304:R>S		UWOPS91_917_1:R>S		YPS138:R>S	AA:78		A4:K>N	AA:113		UWOPS91_917_1:N>K	AA:115		UWOPS91_917_1:L>F	AA:125		DBVPG6304:Q>H		IFO1804:Q>H		N_43:Q>H		N_44:Q>H		N_45:Q>H		YPS138:Q>H	AA:130		UWOPS91_917_1:K>T	AA:157		IFO1804:S>R		N_43:S>R		N_44:S>R		N_45:S>R	AA:159		A4:L>F		UFRJ50816:L>F		YPS138:L>F	AA:195		Y7:->S	AA:208		A4:S>R		UFRJ50816:S>R		YPS138:S>R	AA:282		A4:P>S		UFRJ50816:P>S		UWOPS91_917_1:P>S		YPS138:P>SID:YBR293W	AA:23		A4:G>R	AA:42		A4:L>S	AA:143		A4:R>T		YPS138:R>T	AA:219		UFRJ50816:P>Q	AA:223		A4:V>I		UFRJ50816:V>I		UWOPS91_917_1:V>I		YPS138:V>I	AA:243		UWOPS91_917_1:S>F	AA:324		A4:S>N		UFRJ50816:S>N		UWOPS91_917_1:S>N		YPS138:S>N	AA:329		UWOPS91_917_1:F>I	AA:333		A4:L>S		UFRJ50816:L>S		UWOPS91_917_1:L>S		YPS138:L>S	AA:398		A4:I>V		UFRJ50816:I>V		UWOPS91_917_1:I>V		YPS138:I>V	AA:420		UWOPS91_917_1:W>G	AA:485		UWOPS91_917_1:S>G	AA:488		A4:R>H		UWOPS91_917_1:R>H		YPS138:R>H	AA:499		A4:K>N		DBVPG6304:K>N		YPS138:K>N	AA:506		IFO1804:K>R		N_43:K>R	AA:520		IFO1804:E>Q		N_43:E>Q	AA:551		IFO1804:G>C		N_43:G>C	AA:555		A4:D>E		DBVPG6304:D>E		YPS138:D>EID:YBR296C	AA:67		A12:->C		DBVPG6304:->C		UFRJ50791:->C		UFRJ50816:->C	AA:71		UWOPS91_917_1:T>S	AA:87		Q62_5:->W		T21_4:->W	AA:92		UWOPS91_917_1:W>C	AA:99		UWOPS91_917_1:I>M	AA:115		A12:N>K		DBVPG6304:N>K		UFRJ50791:N>K		UFRJ50816:N>K	AA:144		UWOPS91_917_1:S>R	AA:155		A12:I>M		DBVPG6304:I>M		UFRJ50791:I>M		UFRJ50816:I>M		UWOPS91_917_1:I>M	AA:162		UWOPS91_917_1:H>P	AA:206		UWOPS91_917_1:N>D	AA:215		UWOPS91_917_1:D>N	AA:225		A12:R>S		DBVPG6304:R>S		N_43:R>S		N_45:R>S		UFRJ50816:R>S		UWOPS91_917_1:R>S	AA:229		A12:->Y		UFRJ50816:->Y	AA:231		A12:->C		UFRJ50816:->C	AA:238		A12:->C		UFRJ50816:->C		UWOPS91_917_1:->C	AA:243		UFRJ50816:W>C		UWOPS91_917_1:W>C	AA:247		UFRJ50816:C>-		UWOPS91_917_1:C>-	AA:250		UFRJ50816:Q>H		UWOPS91_917_1:Q>H	AA:280		UFRJ50816:C>W		UWOPS91_917_1:C>W	AA:295		UWOPS91_917_1:M>K	AA:299		IFO1804:H>Q		N_43:H>Q		N_44:H>Q		N_45:H>Q		UFRJ50816:H>Q		UWOPS91_917_1:H>Q		YPS138:H>Q	AA:315		UWOPS91_917_1:E>D	AA:318		UWOPS91_917_1:N>K	AA:335		UWOPS91_917_1:V>E	AA:337		UWOPS91_917_1:K>-	AA:444		A12:S>R		UFRJ50816:S>R		YPS138:S>R	AA:459		Q62_5:R>S		Z1_1:R>S	AA:520		A12:E>D		DBVPG6304:E>D		IFO1804:E>D		N_43:E>D		N_44:E>D		N_45:E>D		UFRJ50816:E>DID:YBR298C	AA:7		Y6_5:W>-	AA:16		DBVPG6304:G>R	AA:17		Y6_5:S>R	AA:26		Q62_5:R>T	AA:37		Y6_5:C>G	AA:39		A4:W>-		DBVPG4650:W>-		DBVPG6304:W>-		IFO1804:W>-		N_17:W>-		N_43:W>-		N_44:W>-		N_45:W>-		Q32_3:W>-		Q62_5:W>-		T21_4:W>-		UFRJ50791:W>-		UFRJ50816:W>-		Y6_5:W>-	AA:51		Y6_5:N>K	AA:69		A4:W>C		DBVPG6304:W>C		UFRJ50816:W>C	AA:78		A4:S>R		DBVPG6304:S>R		UFRJ50816:S>R	AA:104		A4:Y>-		DBVPG6304:Y>-		UFRJ50816:Y>-	AA:108		CBS432:S>R		IFO1804:S>R		N_43:S>R		N_44:S>R		N_45:S>R	AA:112		A4:C>W		CBS432:C>W		DBVPG6304:C>W		N_43:C>-		N_44:C>-		N_45:C>-		Q32_3:C>W		T21_4:C>W		UFRJ50816:C>W	AA:135		DBVPG6304:H>Q	AA:138		A12:K>N		A4:K>N		DBVPG6304:K>N		UFRJ50816:K>N	AA:171		A12:T>S		A4:T>S		DBVPG6304:T>S		UFRJ50816:T>S	AA:177		CBS432:->Y	AA:190		A12:I>L		A4:I>L		DBVPG6304:I>L		UFRJ50816:I>L	AA:196		UWOPS91_917_1:->C	AA:205		A12:S>R		A4:S>R		DBVPG6304:S>R		UFRJ50791:S>R		UFRJ50816:S>R		UWOPS91_917_1:S>R	AA:210		UWOPS91_917_1:I>M	AA:223		CBS432:N>K	AA:237		CBS432:L>F		UWOPS91_917_1:L>F	AA:251		UWOPS91_917_1:Y>-	AA:292		UWOPS91_917_1:Q>H	AA:313		A12:S>Y		DBVPG6304:S>Y		UFRJ50791:S>Y		UFRJ50816:S>Y	AA:315		UWOPS91_917_1:F>L	AA:324		CBS432:W>-		UWOPS91_917_1:W>-	AA:330		CBS432:Y>-	AA:331		IFO1804:C>W		N_43:C>W		N_45:C>W	AA:332		CBS432:K>N		UWOPS91_917_1:K>N	AA:341		IFO1804:S>R		N_43:S>R		N_45:S>R	AA:348		UWOPS91_917_1:->Q	AA:403		CBS432:W>C	AA:409		CBS432:L>F	AA:424		CBS432:K>R	AA:445		A12:Y>-		DBVPG6304:Y>-		UFRJ50791:Y>-		UFRJ50816:Y>-		UWOPS91_917_1:Y>-		YPS138:Y>-	AA:447		A12:R>S		DBVPG6304:R>S		UFRJ50791:R>S		UFRJ50816:R>S		UWOPS91_917_1:R>S		YPS138:R>S	AA:458		A12:S>R		DBVPG6304:S>R	AA:481		A12:Q>H		DBVPG6304:Q>H		UFRJ50791:Q>H		UFRJ50816:Q>H		UWOPS91_917_1:Q>H		YPS138:Q>H	AA:505		CBS432:L>F	AA:518		A12:C>W		DBVPG6304:C>W		UFRJ50816:C>W		UWOPS91_917_1:C>W		YPS138:C>W	AA:525		IFO1804:S>R	AA:544		IFO1804:S>C		N_43:S>C		N_44:S>C		N_45:S>C	AA:572		CBS432:M>I		IFO1804:M>I		N_43:M>I		N_44:M>I		N_45:M>IID:YCL001W	AA:9		A12:M>V		A4:M>V		DBVPG6304:M>V		UFRJ50791:M>V		YPS138:M>V	AA:11		UWOPS91_917_1:D>G	AA:16		A12:T>A		A4:T>A		DBVPG6304:T>A		UFRJ50791:T>A		YPS138:T>A	AA:22		A12:N>H		A4:N>H	AA:39		UWOPS91_917_1:T>AID:YCL004W	AA:39		N_45:N>S	AA:51		UFRJ50791:Q>R	AA:112		A12:K>N		A4:K>N		DBVPG6304:K>N		UFRJ50791:K>N		UWOPS91_917_1:K>N		YPS138:K>N	AA:239		Q59_1:K>E		T21_4:K>E	AA:242		A12:M>I		A4:M>I		DBVPG6304:M>I		UFRJ50791:M>I		UFRJ50816:M>I		UWOPS91_917_1:M>I		YPS138:M>I	AA:260		A12:A>S		A4:A>S		UFRJ50791:A>S		UFRJ50816:A>S		YPS138:A>S	AA:265		A12:L>M		A4:L>M		UFRJ50791:L>M		UWOPS91_917_1:L>M		YPS138:L>M	AA:279		IFO1804:L>Q		N_44:L>Q		N_45:L>Q	AA:349		YPS138:A>V	AA:436		A12:S>N		UFRJ50791:S>N	AA:439		T21_4:D>E	AA:496		A12:E>K		A4:E>K		DBVPG6304:E>K		UFRJ50791:E>K		UWOPS91_917_1:E>K	AA:503		A12:K>E		A4:K>E		DBVPG6304:K>E		N_43:K>E		N_44:K>E		N_45:K>E		UFRJ50791:K>E		UWOPS91_917_1:K>E	AA:506		A12:I>V		A4:I>V		DBVPG6304:I>V		N_43:I>V		N_44:I>V		N_45:I>V		UWOPS91_917_1:I>VID:YCL005W	AA:18		A12:L>V		A4:L>V	AA:77		T21_4:A>P	AA:109		A12:S>G		A4:S>G		DBVPG6304:S>G		YPS138:S>G	AA:124		N_45:E>G	AA:150		A12:M>L		A4:M>L		DBVPG6304:M>L		YPS138:M>L	AA:164		CBS432:A>V		CBS5829:A>V		DBVPG4650:A>V		KPN3828:A>V	AA:175		A4:P>A		CBS432:P>A		CBS5829:P>A		DBVPG4650:P>A		DBVPG6304:P>A		IFO1804:P>A		KPN3828:P>A		N_45:P>A		UWOPS91_917_1:P>A		YPS138:P>A	AA:222		A12:A>T		A4:A>T		YPS138:A>TID:YCL010C	AA:34		UWOPS91_917_1:K>E	AA:40		A12:S>A		A4:S>A		DBVPG6304:S>A		UFRJ50816:S>A		YPS138:S>A	AA:87		A12:L>I		A4:L>I		DBVPG6304:L>I		UFRJ50816:L>I	AA:96		A12:F>L		A4:F>L		DBVPG6304:F>L		UFRJ50816:F>L	AA:101		A12:F>L		A4:F>L		DBVPG6304:F>L		N_43:F>L		N_45:F>L		UFRJ50816:F>L		UWOPS91_917_1:F>L	AA:169		IFO1804:F>L		N_43:F>L		N_45:F>L	AA:186		A12:Q>H		A4:Q>H		DBVPG6304:Q>H		N_43:D>E		N_45:D>E		UFRJ50816:Q>H	AA:188		UWOPS91_917_1:->Y	AA:222		UWOPS91_917_1:->C	AA:246		UWOPS91_917_1:Y>-ID:YCL011C	AA:5		UFRJ50816:G>A		YPS138:G>A	AA:17		UFRJ50816:I>M		UWOPS91_917_1:I>M		YPS138:I>M	AA:25		UFRJ50816:K>R		UWOPS91_917_1:K>R		YPS138:K>R	AA:44		DBVPG6304:I>L		UFRJ50816:I>L		YPS138:I>L	AA:52		N_43:V>I		N_44:V>I		N_45:V>I	AA:70		UWOPS91_917_1:->C	AA:111		DBVPG6304:S>R		YPS138:S>R	AA:158		N_44:F>L		N_45:F>L	AA:165		DBVPG6304:D>E	AA:170		N_44:S>C		N_45:S>C	AA:175		DBVPG6304:E>D		UWOPS91_917_1:E>D		YPS138:E>D	AA:183		N_44:->Y		N_45:->Y	AA:192		DBVPG6304:I>L		N_45:->Y		UWOPS91_917_1:I>L		YPS138:I>L	AA:200		DBVPG6304:R>S		YPS138:R>S	AA:231		DBVPG6304:R>S	AA:251		DBVPG6304:Y>-		N_45:Y>-		UFRJ50816:Y>-		UWOPS91_917_1:Y>-	AA:329		N_45:S>T	AA:332		UWOPS91_917_1:P>RID:YCL016C	AA:13		A12:F>I		DBVPG6304:F>I		IFO1804:F>L		N_17:F>L		N_43:F>L		UFRJ50791:F>I		UWOPS91_917_1:F>V		YPS138:F>I	AA:20		A12:D>E		DBVPG6304:D>E		UFRJ50791:D>E		UWOPS91_917_1:D>E		YPS138:D>E	AA:38		IFO1804:D>A		N_17:D>A		N_43:D>A	AA:40		A12:F>L		DBVPG6304:F>L		IFO1804:F>L		N_17:F>L		N_43:F>L		UFRJ50791:F>L		UWOPS91_917_1:F>L		YPS138:F>L	AA:51		A12:D>E		DBVPG6304:D>E		UFRJ50791:D>E	AA:70		DBVPG4650:G>C		T21_4:G>C	AA:87		Q59_1:V>F	AA:106		A12:W>C		DBVPG6304:W>C		UFRJ50791:W>C		UWOPS91_917_1:W>C	AA:112		UWOPS91_917_1:L>P	AA:121		UWOPS91_917_1:V>G	AA:136		CBS432:F>L		CBS5829:F>L		DBVPG4650:F>L		N_17:F>L		Q62_5:F>L		Q95_3:F>L		T21_4:F>L		Y7:F>L	AA:166		A12:P>H		A4:P>H		DBVPG6304:P>H		UWOPS91_917_1:P>H	AA:182		A12:->W		DBVPG6304:->W		UWOPS91_917_1:->W	AA:196		A12:E>A		A4:E>A		DBVPG6304:E>A		N_45:E>A		UWOPS91_917_1:E>A	AA:207		UWOPS91_917_1:A>T	AA:232		A12:->K		A4:->K		UWOPS91_917_1:->K	AA:245		A12:L>I		A4:L>I	AA:253		UWOPS91_917_1:->E	AA:294		IFO1804:Q>H		N_45:Q>H	AA:299		UWOPS91_917_1:K>N	AA:309		A12:F>L		A4:F>L	AA:343		A4:E>D		UWOPS91_917_1:E>D		YPS138:E>D	AA:381		IFO1804:H>Q		N_45:H>QID:YCL017C	AA:24		DBVPG6304:Q>H		UFRJ50816:Q>H		YPS138:Q>H	AA:36		DBVPG6304:R>S		UFRJ50816:R>S		YPS138:R>S	AA:59		CBS432:->S		CBS5829:->S		DBVPG4650:->S		KPN3828:->S		KPN3829:->S		Q59_1:->S		Q62_5:->S		Q89_8:->S		Q95_3:->S		UWOPS91_917_1:->S		Y7:->S		Z1_1:->S	AA:71		IFO1804:D>E		N_43:D>E		N_45:D>E	AA:117		DBVPG6304:S>R		UFRJ50816:S>R		UWOPS91_917_1:S>R		YPS138:S>R	AA:131		DBVPG6304:->C		IFO1804:->C		KPN3829:->C		N_43:->C		N_45:->C		Q89_8:->C		UFRJ50816:->C		UWOPS91_917_1:->C		YPS138:->C	AA:206		IFO1804:N>K		N_43:N>K		N_45:N>K	AA:209		YPS138:N>K	AA:211		DBVPG6304:L>F		UFRJ50816:L>F		UWOPS91_917_1:L>F		YPS138:L>F	AA:238		N_43:Q>H		N_44:Q>H		N_45:Q>H	AA:292		A12:I>M		YPS138:I>M	AA:363		N_44:K>N		N_45:K>N	AA:380		A12:H>Q		UWOPS91_917_1:H>Q		YPS138:H>QID:YCL021W-A	AA:52		UFRJ50816:T>A		YPS138:T>A	AA:56		UFRJ50816:T>A		YPS138:T>A	AA:64		UWOPS91_917_1:K>R	AA:66		UWOPS91_917_1:N>S	AA:78		UFRJ50816:F>L		YPS138:F>L	AA:86		IFO1804:R>G		N_43:R>G		N_44:R>G		N_45:R>G		UWOPS91_917_1:R>K	AA:101		DBVPG6304:T>I		UFRJ50816:T>I		YPS138:T>I	AA:106		N_44:A>T		N_45:A>TID:YCL025C	AA:43		A12:I>M		A4:I>M		DBVPG6304:I>M		N_43:I>M		UFRJ50816:I>M	AA:46		A12:D>E		A4:D>E		DBVPG4650:D>E		DBVPG6304:D>E		UFRJ50816:D>E	AA:65		UFRJ50816:D>E	AA:92		A12:L>M		A4:L>M		DBVPG6304:L>M		UFRJ50816:L>M		UWOPS91_917_1:L>M	AA:134		A12:M>I		A4:M>I		DBVPG6304:M>I		YPS138:M>I	AA:143		N_43:E>D	AA:158		A12:L>F		A4:L>F		DBVPG6304:L>F		YPS138:L>F	AA:188		A12:N>K		A4:N>K		DBVPG6304:N>K		UWOPS91_917_1:N>K		YPS138:N>K	AA:272		CBS5829:S>R	AA:277		UWOPS91_917_1:Q>H	AA:306		YPS138:->G	AA:313		A12:S>R		A4:S>R		N_43:S>R		N_45:S>R		UWOPS91_917_1:S>R		YPS138:S>R	AA:323		A12:K>N		A4:K>N		UWOPS91_917_1:K>N		YPS138:K>N	AA:390		UWOPS91_917_1:N>K	AA:402		A4:I>M		DBVPG6304:I>M		YPS138:I>M	AA:433		UWOPS91_917_1:D>Y	AA:454		N_45:I>L	AA:472		A4:R>S		DBVPG6304:R>S		UFRJ50816:R>S		UWOPS91_917_1:R>S		YPS138:R>S	AA:474		A4:H>Q		DBVPG6304:H>Q		UFRJ50816:H>Q		UWOPS91_917_1:H>Q		YPS138:H>Q	AA:491		CBS432:S>R		DBVPG4650:S>R		N_17:S>R		Q95_3:S>R		Y7:S>R	AA:521		A4:V>G		DBVPG6304:V>G		UFRJ50816:V>G		UWOPS91_917_1:V>G		YPS138:V>G	AA:537		UWOPS91_917_1:F>V	AA:547		A4:R>S		DBVPG6304:R>S		UFRJ50816:R>S		UWOPS91_917_1:R>S		YPS138:R>S	AA:575		A4:E>D		DBVPG6304:E>D		UFRJ50816:E>D		YPS138:E>D	AA:617		A4:S>R		DBVPG6304:S>R		UFRJ50816:S>R		UWOPS91_917_1:S>R		YPS138:S>R	AA:626		A4:F>Y		N_43:F>Y		N_45:F>Y		UFRJ50816:F>YID:YCL026C-A	AA:11		N_43:K>N	AA:19		IFO1804:S>G	AA:29		IFO1804:H>N		N_43:H>N		N_45:H>N	AA:40		A12:S>G		A4:S>G		DBVPG6304:S>G		UFRJ50816:S>G		UWOPS91_917_1:S>G		YPS138:S>G	AA:42		UWOPS91_917_1:F>L	AA:51		N_17:I>M	AA:63		UWOPS91_917_1:R>-	AA:75		UFRJ50816:T>S	AA:107		IFO1804:I>S		N_43:I>S		N_45:I>S	AA:110		CBS432:S>R	AA:150		IFO1804:W>C		N_43:W>C		N_45:W>C	AA:178		A12:D>H		A4:D>H		DBVPG6304:D>H		UFRJ50816:D>H		YPS138:D>HID:YCL028W	AA:39		UWOPS91_917_1:M>K	AA:70		A12:S>G		A4:S>G		DBVPG6304:S>G	AA:98		A12:A>T	AA:160		DBVPG6304:S>G		N_44:S>G		YPS138:S>G	AA:192		A12:G>S		A4:G>S		DBVPG6304:G>S		YPS138:G>S	AA:221		N_44:G>D	AA:333		A12:Y>N		CBS5829:Y>N		DBVPG4650:Y>N		DBVPG6304:Y>N		IFO1804:Y>N		N_17:Y>N		Q59_1:Y>N		T21_4:Y>N		UFRJ50816:Y>N		UWOPS91_917_1:Y>N		YPS138:Y>N	AA:346		A12:V>A		DBVPG6304:V>A		IFO1804:V>A		UFRJ50816:V>A		UWOPS91_917_1:V>A		YPS138:V>A	AA:352		A12:T>P		DBVPG6304:T>P		UFRJ50816:T>P		UWOPS91_917_1:T>P		YPS138:T>P	AA:371		UWOPS91_917_1:N>K	AA:372		IFO1804:Q>K	AA:374		T21_4:S>Y	AA:375		A12:S>N		DBVPG6304:S>N		UFRJ50816:S>N		UWOPS91_917_1:S>N		YPS138:S>NID:YCL029C	AA:76		N_43:L>F		N_44:L>F		N_45:L>F	AA:155		A4:F>Y		UFRJ50816:F>Y		UWOPS91_917_1:F>Y	AA:164		A4:D>A		UFRJ50816:D>A		UWOPS91_917_1:D>A	AA:186		A4:Q>E		UFRJ50816:Q>E		UWOPS91_917_1:Q>E	AA:250		N_43:A>T		N_45:A>T	AA:291		DBVPG6304:G>R		UWOPS91_917_1:G>R	AA:302		N_45:L>H	AA:347		IFO1804:->W		N_44:->W		N_45:->W	AA:348		UWOPS91_917_1:->Y	AA:358		UWOPS91_917_1:L>V	AA:431		A12:E>D		DBVPG6304:E>D		UFRJ50816:E>DID:YCL030C	AA:2		A4:N>K		UFRJ50791:L>M		UFRJ50816:L>M		UWOPS91_917_1:N>K	AA:4		UWOPS91_917_1:I>F	AA:38		N_45:V>I	AA:40		UWOPS91_917_1:->Y	AA:88		UWOPS91_917_1:S>R	AA:92		DBVPG6304:H>Q		UFRJ50791:H>Q		UFRJ50816:H>Q		UWOPS91_917_1:H>Q	AA:101		DBVPG6304:N>I		UFRJ50791:N>I		UFRJ50816:N>I	AA:106		UWOPS91_917_1:V>G	AA:129		DBVPG6304:V>I		UFRJ50791:V>I		UFRJ50816:V>I		UWOPS91_917_1:V>I	AA:134		UWOPS91_917_1:C>S	AA:145		UWOPS91_917_1:H>Q	AA:171		UWOPS91_917_1:N>K	AA:207		N_45:A>S		UWOPS91_917_1:A>S	AA:306		A12:C>-		YPS138:C>-	AA:316		A12:R>S		A4:R>S		YPS138:R>S	AA:324		Q59_1:N>H	AA:367		A12:L>I		A4:L>I	AA:369		A12:->E		A4:->E	AA:422		N_45:L>F	AA:464		A4:I>M		DBVPG6304:I>M		UWOPS91_917_1:I>M	AA:505		A4:R>S		DBVPG6304:R>S		UWOPS91_917_1:R>S	AA:516		N_17:F>L		N_44:F>L		N_45:F>L	AA:555		A4:H>Q		DBVPG6304:H>Q	AA:558		A4:M>I		UWOPS91_917_1:M>I	AA:612		A4:R>S		CBS432:R>S		CBS5829:R>S		DBVPG4650:R>S		DBVPG6304:R>S		N_17:R>S		N_43:R>S		N_44:R>S		N_45:R>S		Q89_8:R>S		Q95_3:R>S		UWOPS91_917_1:R>S	AA:679		DBVPG6304:I>M	AA:684		A4:L>F		DBVPG6304:L>F		IFO1804:L>F		N_17:L>F		N_43:L>F		N_44:L>F		N_45:L>F		UWOPS91_917_1:L>F	AA:689		A4:F>L		DBVPG6304:F>L		UWOPS91_917_1:F>L	AA:706		A4:I>M		DBVPG6304:I>M	AA:787		A4:->WID:YCL031C	AA:65		UFRJ50791:I>F	AA:111		A12:H>Q		A4:H>Q		DBVPG6304:H>Q		UFRJ50791:H>Q		UFRJ50816:H>Q		UWOPS91_917_1:H>Y	AA:120		A12:I>M		A4:I>M		DBVPG6304:I>M		UFRJ50791:I>M	AA:125		A4:W>R		DBVPG6304:W>R		UFRJ50791:W>R		UFRJ50816:W>R	AA:175		N_45:S>R	AA:186		A12:D>E		A4:D>E		DBVPG6304:D>E		UFRJ50791:D>E		UFRJ50816:D>E		UWOPS91_917_1:D>E	AA:196		UWOPS91_917_1:Q>K	AA:197		N_44:C>W		N_45:C>W	AA:207		A12:L>I		A4:L>I		DBVPG6304:L>I		UFRJ50791:L>I		UFRJ50816:L>I	AA:218		A4:Y>-		DBVPG6304:Y>-		UFRJ50791:Y>-		UFRJ50816:Y>-	AA:222		A4:F>L		DBVPG6304:F>L		UFRJ50791:F>L		UFRJ50816:F>L		UWOPS91_917_1:F>L	AA:236		A4:V>F		DBVPG6304:V>F		UFRJ50791:V>F		UFRJ50816:V>F		UWOPS91_917_1:V>FID:YCL032W	AA:3		UWOPS91_917_1:D>N	AA:7		UWOPS91_917_1:T>N		YPS138:T>N	AA:11		UWOPS91_917_1:G>S	AA:41		YPS138:I>V	AA:101		UWOPS91_917_1:D>N	AA:127		UWOPS91_917_1:S>Y	AA:153		IFO1804:T>S		N_45:T>S		UFRJ50791:T>S		UWOPS91_917_1:T>S		YPS138:T>S	AA:193		UFRJ50816:H>N	AA:238		CBS432:A>T		CBS5829:A>T		DBVPG4650:A>T		IFO1804:A>T		KPN3828:A>T		KPN3829:A>T		N_44:A>T		N_45:A>T		Q32_3:A>T		Q59_1:A>T		Q62_5:A>T		S36_7:A>T		T21_4:A>T		Y6_5:A>T		Y7:A>T		Z1_1:A>TID:YCL033C	AA:17		IFO1804:V>A		N_43:V>A		N_44:V>A		N_45:V>A		UWOPS91_917_1:V>A	AA:23		KPN3828:Q>E		N_17:Q>E		Q62_5:Q>E	AA:30		IFO1804:F>L		N_43:F>L		N_45:F>L	AA:48		DBVPG6304:C>-	AA:84		DBVPG6304:Q>R		UWOPS91_917_1:Q>R	AA:109		UWOPS91_917_1:F>L	AA:130		UWOPS91_917_1:V>G	AA:154		UWOPS91_917_1:K>NID:YCL034W	AA:26		A12:T>A		A4:T>A		DBVPG6304:T>A		UFRJ50791:T>A	AA:46		UFRJ50791:N>K	AA:118		A12:P>A		A4:P>A		DBVPG6304:P>A		UFRJ50791:P>A		UWOPS91_917_1:P>A	AA:133		A12:N>S		A4:N>S		DBVPG6304:N>S		UFRJ50791:N>S	AA:139		IFO1804:S>R		N_44:S>R	AA:167		A4:G>R		DBVPG6304:G>R		UFRJ50791:G>R		UWOPS91_917_1:G>R	AA:169		A4:S>N		UFRJ50791:S>N	AA:181		IFO1804:N>D		N_44:N>D		N_45:N>D	AA:308		DBVPG6304:G>S	AA:313		UWOPS91_917_1:D>G	AA:314		DBVPG6304:I>T	AA:344		UWOPS91_917_1:M>IID:YCL035C	AA:65		A12:F>L		A4:F>L		DBVPG6304:F>L		UFRJ50791:F>L		UFRJ50816:F>L		YPS138:F>L	AA:107		A12:F>I		A4:F>I		DBVPG6304:F>I		UFRJ50791:F>I		UFRJ50816:F>I		YPS138:F>IID:YCL036W	AA:2		A4:H>Q		DBVPG6304:H>Q		YPS138:H>Q	AA:42		A4:A>T		DBVPG6304:A>T		YPS138:A>T	AA:53		KPN3828:P>T	AA:83		Y6_5:T>M	AA:88		DBVPG6304:A>S		YPS138:A>S	AA:105		UWOPS91_917_1:T>S	AA:128		N_43:Q>E		N_44:Q>E		N_45:Q>E	AA:154		N_44:A>T	AA:161		DBVPG6304:S>A		N_43:S>A		N_44:S>A		N_45:S>A		UWOPS91_917_1:S>A		YPS138:S>A	AA:169		DBVPG6304:E>K		YPS138:E>K	AA:200		UWOPS91_917_1:A>T	AA:202		UWOPS91_917_1:G>R	AA:210		N_45:M>I	AA:218		UWOPS91_917_1:A>V	AA:227		UFRJ50791:G>S		YPS138:G>S	AA:245		UWOPS91_917_1:N>S	AA:254		UWOPS91_917_1:M>I	AA:413		N_43:H>Y		N_45:H>Y	AA:444		A4:G>S		UFRJ50791:G>S		UFRJ50816:G>S	AA:526		A12:H>N		A4:H>NID:YCL038C	AA:29		A12:->Y		A4:V>L		DBVPG6304:V>L		UFRJ50791:->Y		UFRJ50816:->Y		UWOPS91_917_1:V>L		YPS138:->Y	AA:57		UWOPS91_917_1:N>D	AA:79		UWOPS91_917_1:F>L	AA:145		A12:G>A		A4:G>A		CBS5829:G>A		N_17:G>A		N_43:G>A		N_44:G>A		N_45:G>A		Q62_5:G>A		T21_4:G>A		UFRJ50791:G>A		UFRJ50816:G>A		UWOPS91_917_1:G>A		Y6_5:G>A		YPS138:G>A	AA:163		A12:H>Q		A4:H>Q		CBS432:H>Q		CBS5829:H>Q		DBVPG4650:H>Q		N_17:H>Q		N_43:H>Q		N_44:H>Q		N_45:H>Q		Q59_1:H>Q		Q62_5:H>Q		T21_4:H>Q		UFRJ50791:H>Q		UWOPS91_917_1:H>Q		Y6_5:H>Q		YPS138:H>Q		Z1_1:H>Q	AA:188		A12:F>L		A4:F>L		IFO1804:F>L		N_43:F>L		N_44:F>L		N_45:F>L		UFRJ50791:Q>H		YPS138:F>L	AA:218		A12:M>I		A4:M>I		IFO1804:M>I		N_43:M>I		N_44:M>I		N_45:M>I		UFRJ50791:M>I		UWOPS91_917_1:M>I		YPS138:M>I	AA:253		UWOPS91_917_1:Y>N	AA:271		A12:D>Y		A4:D>Y		UWOPS91_917_1:D>Y		YPS138:D>Y	AA:279		UWOPS91_917_1:P>L	AA:291		A12:I>S		A4:I>S		YPS138:I>S	AA:300		IFO1804:D>E		N_43:D>E		N_44:D>E		N_45:D>E	AA:305		A12:Y>-		A4:Y>-		UWOPS91_917_1:Y>-		YPS138:Y>-	AA:313		A12:->C		A4:->C		YPS138:->C	AA:357		N_45:H>P	AA:373		A12:->Y		A4:->Y		IFO1804:->Y		N_43:->Y		N_44:->Y		N_45:->Y		UWOPS91_917_1:->Y		YPS138:->Y	AA:510		IFO1804:F>L		N_17:F>L		N_43:F>L		N_44:F>L		N_45:F>LID:YCL039W	AA:4		Y6_5:T>I	AA:18		N_43:T>A		N_44:T>A	AA:25		A12:G>C		DBVPG6304:G>C		UFRJ50816:G>C	AA:47		N_43:S>T	AA:75		DBVPG6304:S>T		N_43:S>G		UFRJ50816:S>T		UWOPS91_917_1:S>T		YPS138:S>T	AA:79		DBVPG6304:Q>H		UFRJ50816:Q>H		UWOPS91_917_1:Q>H		YPS138:Q>H	AA:91		UWOPS91_917_1:L>F	AA:100		KPN3828:E>G		KPN3829:E>G	AA:110		KPN3828:P>S		KPN3829:P>S	AA:113		CBS432:T>A		CBS5829:T>A		DBVPG4650:T>A		DBVPG6304:T>A		KPN3828:T>A		KPN3829:T>A		N_17:T>A		N_43:T>A		Q32_3:T>A		Q59_1:T>A		Q89_8:T>A		T21_4:T>A		UFRJ50816:T>A		UWOPS91_917_1:T>A	AA:115		CBS432:T>A	AA:138		N_43:S>P		N_44:S>P	AA:174		DBVPG6304:A>G		UFRJ50816:A>G		UWOPS91_917_1:A>G		YPS138:A>G	AA:179		N_44:P>S	AA:357		A12:V>A		A4:V>A		DBVPG6304:V>A		YPS138:V>A	AA:404		A4:C>S		DBVPG6304:C>S		UWOPS91_917_1:C>S		YPS138:C>S	AA:410		A12:T>A		A4:T>A		IFO1804:T>A		N_43:T>A		N_45:T>A		UWOPS91_917_1:T>A		YPS138:T>A	AA:435		DBVPG6304:S>Y		UWOPS91_917_1:S>Y		YPS138:S>Y	AA:456		DBVPG6304:Q>L		UWOPS91_917_1:Q>L		YPS138:Q>L	AA:475		UWOPS91_917_1:V>I	AA:511		DBVPG6304:V>L		UWOPS91_917_1:V>L		YPS138:V>L	AA:555		DBVPG6304:N>K		IFO1804:N>K		N_43:N>K		N_45:N>K		UWOPS91_917_1:N>K		YPS138:N>K	AA:589		DBVPG6304:N>T	AA:592		UWOPS91_917_1:I>V	AA:611		DBVPG6304:L>I	AA:719		KPN3828:A>TID:YCL043C	AA:5		A12:M>I	AA:7		Y7:G>V	AA:18		A12:G>C	AA:41		A12:E>D	AA:51		CBS5829:->Q	AA:94		UWOPS91_917_1:S>R	AA:110		A4:L>F		IFO1804:L>F		N_43:L>F		N_44:L>F		N_45:L>F		UFRJ50816:L>F		UWOPS91_917_1:L>F		YPS138:L>F	AA:145		KPN3828:D>N		Q32_3:D>N		Q59_1:D>N	AA:152		A12:I>N	AA:164		A12:W>C		A4:W>C		UFRJ50816:W>C		UWOPS91_917_1:W>C		YPS138:W>C	AA:181		A12:R>S		A4:R>S		UFRJ50816:R>S		UWOPS91_917_1:R>S		YPS138:R>S	AA:217		IFO1804:L>F		N_43:L>F		N_44:L>F		N_45:L>F	AA:233		A12:I>M		IFO1804:I>M		N_43:I>M		N_44:I>M		N_45:I>M		UFRJ50816:I>M		YPS138:I>M	AA:305		A12:V>F		DBVPG6304:V>F		IFO1804:V>F		N_43:V>F		N_44:V>F		UWOPS91_917_1:V>F		YPS138:V>F	AA:339		UWOPS91_917_1:I>L	AA:355		N_43:V>I		N_44:V>I	AA:377		DBVPG6304:W>C	AA:387		DBVPG6304:L>F		UWOPS91_917_1:L>F	AA:443		IFO1804:S>R		N_43:S>R		N_44:S>R		UWOPS91_917_1:P>R	AA:460		IFO1804:H>Q		N_43:H>Q		N_44:H>Q	AA:464		A12:M>I		DBVPG6304:M>I		IFO1804:M>I		N_43:M>I		N_44:M>I		UFRJ50816:M>I	AA:501		IFO1804:S>I		N_44:S>I	AA:512		UWOPS91_917_1:Q>-	AA:519		A12:I>V		UWOPS91_917_1:D>NID:YCL044C	AA:2		A4:M>I		UFRJ50816:M>I		UWOPS91_917_1:M>I		YPS138:M>I	AA:5		S36_7:F>V	AA:12		S36_7:S>R	AA:14		UFRJ50816:P>A		UWOPS91_917_1:P>A		YPS138:P>A	AA:140		CBS432:F>L		KPN3829:F>L	AA:219		UFRJ50791:I>M		UWOPS91_917_1:I>M	AA:293		UFRJ50791:K>N		UWOPS91_917_1:K>N	AA:300		N_43:V>L		N_44:V>L		N_45:V>L	AA:301		UFRJ50791:S>N		UWOPS91_917_1:S>N	AA:307		UFRJ50791:F>L		UWOPS91_917_1:F>L	AA:339		A4:V>E		UWOPS91_917_1:V>E	AA:340		Q89_8:E>V		Y6_5:E>V	AA:364		UWOPS91_917_1:->SID:YCL045C	AA:3		UWOPS91_917_1:N>S	AA:5		A4:P>Q		DBVPG6304:P>Q		UFRJ50816:P>Q		UWOPS91_917_1:P>Q	AA:15		A4:R>-		DBVPG6304:R>-		UFRJ50816:R>-		UWOPS91_917_1:R>-	AA:65		A4:C>G		DBVPG6304:C>G		UFRJ50816:C>G		UWOPS91_917_1:C>G	AA:109		A4:F>Y		DBVPG6304:F>Y		UFRJ50816:F>Y	AA:124		A4:Q>H		DBVPG6304:Q>H		IFO1804:Q>H		N_45:Q>H		UFRJ50816:Q>H	AA:154		CBS5829:G>R		IFO1804:G>R		N_17:G>R		Q89_8:G>R	AA:183		IFO1804:K>N		N_43:K>N		N_44:K>N	AA:197		DBVPG4650:->C		Q62_5:->C		T21_4:->C		Z1_1:->C	AA:207		A4:L>M		DBVPG4650:L>M		IFO1804:L>M		N_43:L>M		N_44:L>M		Q62_5:L>M		T21_4:L>M		UWOPS91_917_1:L>M		Z1_1:L>M	AA:213		A4:R>S	AA:230		A4:W>-	AA:246		IFO1804:F>L		N_43:F>L		N_44:F>L	AA:259		Q32_3:M>V	AA:270		IFO1804:->Y		N_43:->Y		N_44:->Y		Q32_3:->Y		Q62_5:->Y		T21_4:->Y		Z1_1:->Y	AA:278		A4:K>N	AA:302		UWOPS91_917_1:Y>-	AA:323		A12:V>L		A4:V>L		UWOPS91_917_1:V>L	AA:331		Q62_5:M>I		Z1_1:M>I	AA:344		UWOPS91_917_1:->Y	AA:364		UWOPS91_917_1:N>Y	AA:380		A12:M>I		A4:M>I		N_44:M>I		UWOPS91_917_1:M>I	AA:389		IFO1804:I>V		N_43:I>V		N_44:I>V		N_45:I>V	AA:394		UWOPS91_917_1:F>V	AA:397		A12:L>I		A4:L>I		YPS138:L>I	AA:400		Q59_1:G>R	AA:406		CBS432:F>L	AA:408		A12:->S		A4:->S		UWOPS91_917_1:->S	AA:413		CBS432:F>V		CBS5829:F>V		N_17:F>V		Q32_3:F>V		Q62_5:F>V		T21_4:F>V		Z1_1:F>V	AA:425		A12:Q>K		A4:Q>K		YPS138:Q>K	AA:427		UWOPS91_917_1:M>R	AA:441		IFO1804:I>F		N_44:I>F		N_45:I>F	AA:443		IFO1804:P>T		N_44:P>T		N_45:P>T	AA:459		A4:K>M	AA:465		UWOPS91_917_1:H>Q	AA:467		A12:K>Q		YPS138:K>Q	AA:470		UWOPS91_917_1:N>K	AA:474		A12:M>I		A4:M>I		YPS138:M>I	AA:477		UWOPS91_917_1:N>K	AA:486		A4:F>L	AA:487		A12:Q>E		YPS138:Q>E	AA:490		A4:Y>F	AA:497		IFO1804:Q>H		N_44:Q>H		N_45:Q>H	AA:508		CBS432:->C	AA:509		A4:K>N	AA:518		A4:E>D	AA:525		A12:Q>H		UFRJ50816:Q>H		UWOPS91_917_1:Q>H		YPS138:Q>H	AA:538		A12:K>N		UFRJ50816:K>N		UWOPS91_917_1:K>N		YPS138:K>N	AA:582		UFRJ50816:K>N		UWOPS91_917_1:K>N		YPS138:K>N	AA:612		UFRJ50816:->Y		YPS138:->Y	AA:641		N_44:->G		UFRJ50816:->G		YPS138:->G	AA:663		A12:V>L		DBVPG6304:V>L		UFRJ50816:V>L		UWOPS91_917_1:V>L		YPS138:V>L	AA:726		N_44:P>A		N_45:P>A	AA:757		CBS5829:C>S	AA:770		A12:D>E		DBVPG6304:D>E		UFRJ50816:D>EID:YCL049C	AA:51		CBS432:T>K		DBVPG4650:T>K		N_17:T>K	AA:57		N_44:Q>H		N_45:Q>H	AA:92		N_43:A>G		N_44:A>G		N_45:A>G	AA:107		A12:I>L		A4:I>L		DBVPG6304:I>L	AA:109		N_43:K>R		N_44:K>R		N_45:K>R	AA:141		IFO1804:->W		N_43:->W		N_44:->W		N_45:->W	AA:144		A12:F>L		A4:F>L		DBVPG6304:F>L		IFO1804:F>L		N_43:F>L		N_44:F>L		N_45:F>L	AA:147		A12:P>T		A4:P>T		DBVPG6304:P>T	AA:193		A12:F>L		A4:F>L		DBVPG6304:F>L		IFO1804:F>L		N_43:F>L		N_44:F>L		N_45:F>L		UWOPS91_917_1:F>L	AA:216		A12:I>L		DBVPG6304:I>L	AA:238		A12:H>Q		DBVPG6304:H>Q		UFRJ50791:H>Q		UFRJ50816:H>Q		UWOPS91_917_1:H>Q	AA:271		A12:A>S		DBVPG6304:A>S		N_44:K>N		N_45:K>N		UFRJ50791:A>S		UFRJ50816:A>S	AA:272		UWOPS91_917_1:V>D	AA:278		A12:W>-		DBVPG6304:W>-		UFRJ50791:W>-		UFRJ50816:W>-		UWOPS91_917_1:W>-	AA:291		UWOPS91_917_1:R>P	AA:303		IFO1804:Y>H		N_17:Y>H		N_43:Y>H		N_45:Y>HID:YCL050C	AA:8		UWOPS91_917_1:F>L	AA:11		A4:R>S		DBVPG6304:R>S		UFRJ50816:R>S		UWOPS91_917_1:R>S	AA:20		A12:->Y		A4:->Y		DBVPG6304:->Y		IFO1804:->Y		N_44:->Y		N_45:->Y		UFRJ50816:->Y		UWOPS91_917_1:->Y	AA:51		UWOPS91_917_1:E>D	AA:83		UWOPS91_917_1:W>C	AA:87		UWOPS91_917_1:Q>H	AA:111		N_43:N>S	AA:116		A12:F>L		A4:F>L	AA:119		A12:W>C		A4:W>C		CBS432:W>C		N_17:W>C		N_43:W>C		Q89_8:W>C		Q95_3:W>C		T21_4:W>C		UWOPS91_917_1:W>C	AA:127		UWOPS91_917_1:D>E	AA:138		A12:S>R		A4:S>R		UWOPS91_917_1:S>R	AA:162		UWOPS91_917_1:N>Y	AA:170		N_43:->C	AA:211		A12:L>M		A4:L>M		UWOPS91_917_1:L>M	AA:226		UWOPS91_917_1:Y>-	AA:229		IFO1804:F>L		N_43:F>L	AA:305		A4:S>R		CBS432:S>R		IFO1804:S>R		N_43:S>R		N_45:S>R		Q89_8:S>R		Q95_3:S>R		T21_4:S>R		UWOPS91_917_1:S>R		YPS138:S>RID:YCL051W	AA:68		CBS432:V>F		Q62_5:V>F		Y6_5:V>F		Z1_1:V>F	AA:169		A4:D>N		DBVPG6304:D>N		UFRJ50816:D>N	AA:186		A4:I>F		DBVPG6304:I>F		UFRJ50816:I>F	AA:190		A4:T>S		DBVPG6304:T>S		UFRJ50816:T>S	AA:287		N_44:T>A		N_45:T>A	AA:320		A12:G>D		A4:G>D		DBVPG6304:G>D		N_44:G>D		N_45:G>D		UFRJ50816:G>D		YPS138:G>D	AA:326		DBVPG6304:D>E	AA:333		A12:T>A		A4:T>A		UFRJ50816:T>A		YPS138:T>A	AA:342		A12:P>S		A4:P>S		DBVPG6304:P>S		UFRJ50816:P>S		YPS138:P>S	AA:425		A12:M>I		A4:M>I		DBVPG6304:M>I		UFRJ50791:M>I		UFRJ50816:M>I		YPS138:M>I	AA:430		DBVPG4650:N>S	AA:455		N_43:V>I		N_44:V>I		N_45:V>I	AA:467		A12:N>D		A4:N>D	AA:469		A12:L>F		A4:L>F		UFRJ50791:L>F		YPS138:L>F	AA:483		N_43:Q>R		N_44:Q>R		N_45:Q>R	AA:556		CBS432:T>I		Q95_3:T>I		Z1_1:T>I	AA:576		IFO1804:S>F		N_43:S>F		N_44:S>F		N_45:S>FID:YCL052C	AA:26		UWOPS91_917_1:->Y	AA:63		CBS5829:E>-		KPN3828:E>-		Q59_1:E>-	AA:90		UWOPS91_917_1:N>K	AA:95		UWOPS91_917_1:C>S	AA:124		CBS432:F>I		CBS5829:F>I		DBVPG4650:F>I		IFO1804:F>I		KPN3828:F>I		N_45:F>I		Q59_1:F>I		Q89_8:F>I		Q95_3:F>I	AA:133		UWOPS91_917_1:L>F	AA:149		UWOPS91_917_1:K>N	AA:167		A4:Q>H		DBVPG6304:Q>H		UFRJ50791:Q>H		YPS138:Q>H	AA:192		A12:N>D		A4:N>D		DBVPG6304:N>D		UFRJ50791:N>D		UFRJ50816:N>D		YPS138:N>D	AA:203		A12:W>-		A4:W>-		DBVPG6304:W>-		UFRJ50791:W>-		UFRJ50816:W>-		YPS138:W>-	AA:210		A12:M>I		A4:M>I		DBVPG6304:M>I		UFRJ50791:M>I		UFRJ50816:M>I		YPS138:M>I	AA:243		A12:F>L		DBVPG6304:F>L		UFRJ50791:F>L		UFRJ50816:F>L		YPS138:F>L	AA:261		A12:F>L		DBVPG6304:F>L		N_43:F>L		N_44:F>L		N_45:F>L		UFRJ50816:F>L		UWOPS91_917_1:F>L		YPS138:F>L	AA:292		UWOPS91_917_1:F>L	AA:304		DBVPG6304:R>S		UFRJ50816:R>S		UWOPS91_917_1:R>S	AA:327		UWOPS91_917_1:F>L	AA:353		A12:Q>H		DBVPG6304:Q>H		UFRJ50816:Q>H		YPS138:Q>H	AA:389		YPS138:S>R	AA:395		YPS138:L>F	AA:407		N_44:I>T		N_45:I>T	AA:408		UWOPS91_917_1:E>G	AA:411		A12:Y>D		A4:Y>D		DBVPG6304:Y>D		UFRJ50816:Y>D		UWOPS91_917_1:Y>DID:YCL055W	AA:3		UFRJ50791:R>L		UWOPS91_917_1:R>L	AA:6		DBVPG6304:Q>K		UWOPS91_917_1:Q>K	AA:17		N_44:I>T		N_45:I>T	AA:24		UWOPS91_917_1:G>S	AA:27		DBVPG6304:N>T		N_43:N>I		N_44:N>I		N_45:N>I		UFRJ50791:N>T	AA:41		N_43:S>G		N_44:S>G		N_45:S>G	AA:48		CBS432:L>S		UWOPS91_917_1:L>S	AA:51		CBS5829:L>P		DBVPG6304:L>P		N_43:L>P		N_44:L>P		N_45:L>P		Q62_5:L>P		Q89_8:L>P		Q95_3:L>P		UFRJ50791:L>P		UWOPS91_917_1:L>P		Y6_5:L>P		YPS138:L>P		Z1_1:L>P	AA:81		N_45:I>V	AA:346		DBVPG6304:K>RID:YCL056C	AA:8		IFO1804:C>S		N_43:C>S		N_44:C>S		N_45:C>S	AA:18		A12:P>R		A4:P>R		DBVPG6304:P>R	AA:23		N_43:N>T		N_44:N>T		N_45:N>T	AA:24		UWOPS91_917_1:Q>H	AA:27		UWOPS91_917_1:Q>H	AA:47		A12:F>L		A4:F>L		CBS432:F>L		CBS5829:F>L		DBVPG6304:F>L		KPN3828:F>L		N_43:F>L		N_44:F>L		N_45:F>L		Q62_5:F>L		Q89_8:F>L		Q95_3:F>L		UWOPS91_917_1:F>L		Y6_5:F>L	AA:51		A12:F>L		A4:Y>C		DBVPG6304:F>L		UWOPS91_917_1:Y>C	AA:52		UWOPS91_917_1:Q>E	AA:64		A12:->Y		A4:->Y		DBVPG6304:->Y	AA:68		A12:N>Y		A4:N>Y		CBS432:N>Y		CBS5829:N>Y		DBVPG6304:N>Y		KPN3828:N>Y		N_43:N>Y		N_44:N>Y		N_45:N>Y		Q62_5:N>Y		Q89_8:N>Y		Q95_3:N>Y		UWOPS91_917_1:N>Y		Y6_5:N>Y	AA:88		A12:A>E		A4:A>E		DBVPG6304:A>EID:YCL057C-A	AA:7		A12:E>K		YPS138:E>K	AA:45		CBS5829:E>D		N_17:E>D		Q62_5:E>D		T21_4:E>D		Y6_5:E>D	AA:50		UWOPS91_917_1:K>N	AA:52		A12:H>Q		A4:H>Q		DBVPG6304:H>Q		UWOPS91_917_1:H>Q		YPS138:H>QID:YCL057W	AA:4		A12:F>L		A4:F>L		DBVPG6304:F>L		UFRJ50791:F>L		UFRJ50816:F>L		UWOPS91_917_1:F>L		YPS138:F>L	AA:12		IFO1804:S>P		N_43:S>P		N_44:S>P		N_45:S>P	AA:30		A12:A>V		A4:A>V		DBVPG6304:A>V		UFRJ50791:A>V		UFRJ50816:A>V		UWOPS91_917_1:A>V		YPS138:A>V	AA:100		A12:M>L		A4:M>L		DBVPG6304:M>L		UFRJ50791:M>L		UFRJ50816:M>L		UWOPS91_917_1:M>L		YPS138:M>L	AA:122		UWOPS91_917_1:E>G	AA:131		A12:Y>H		A4:Y>H		DBVPG6304:Y>H		UFRJ50816:Y>H		UWOPS91_917_1:Y>H		YPS138:Y>H	AA:151		A12:M>I		A4:M>I		DBVPG6304:M>I		UFRJ50816:M>I		YPS138:M>I	AA:159		A4:I>V	AA:181		N_44:I>V	AA:232		KPN3828:D>G	AA:334		N_43:F>V		N_45:F>V	AA:341		DBVPG6304:K>R		UFRJ50816:K>R		YPS138:K>R	AA:351		UWOPS91_917_1:D>N	AA:386		A4:A>T		DBVPG6304:A>T		UFRJ50816:A>T		YPS138:A>T	AA:403		UFRJ50816:F>I	AA:406		A4:A>T		CBS432:A>T		DBVPG6304:A>T		N_17:A>T		N_43:A>T		N_45:A>T		Q62_5:A>T		Q95_3:A>T		UFRJ50816:A>T		UWOPS91_917_1:A>T		Y7:A>T		YPS138:A>T	AA:410		A4:E>Q		DBVPG6304:E>Q		UFRJ50816:E>Q		UWOPS91_917_1:E>Q		YPS138:E>Q	AA:412		DBVPG6304:K>R		UFRJ50816:K>R		UWOPS91_917_1:K>R		YPS138:K>R	AA:429		N_45:D>N	AA:566		A12:D>N		A4:D>N		YPS138:D>N	AA:667		N_45:N>D	AA:689		DBVPG6304:D>AID:YCL059C	AA:16		UWOPS91_917_1:I>F	AA:26		A4:F>L		UWOPS91_917_1:F>L		YPS138:F>L	AA:30		A4:L>S		UWOPS91_917_1:L>S		YPS138:L>S	AA:53		N_17:F>L		N_45:F>L	AA:69		UWOPS91_917_1:S>G	AA:91		UWOPS91_917_1:E>D	AA:112		A4:F>L		DBVPG6304:F>L		UFRJ50791:F>L		YPS138:F>L	AA:136		A4:F>L		DBVPG6304:F>L		UFRJ50791:F>L		YPS138:F>L	AA:223		DBVPG6304:Q>H		UFRJ50791:Q>H		UFRJ50816:Q>H		UWOPS91_917_1:Q>H		YPS138:Q>H	AA:238		UWOPS91_917_1:H>Q	AA:293		N_43:F>I		N_45:F>IID:YCL063W	AA:3		A12:T>N		A4:T>N		UFRJ50816:T>N		YPS138:T>N	AA:5		UWOPS91_917_1:A>S	AA:27		A4:W>R	AA:38		Q62_5:Q>H		T21_4:Q>H	AA:88		A4:H>N		UFRJ50816:H>N		UWOPS91_917_1:H>N	AA:107		A4:E>Q		UFRJ50816:E>Q	AA:183		A4:P>T		UFRJ50816:P>T		YPS138:P>T	AA:202		A4:S>R		UFRJ50816:S>R		YPS138:S>R	AA:252		A12:V>M		YPS138:V>M	AA:352		Q89_8:T>S	AA:394		UWOPS91_917_1:P>S	AA:409		UWOPS91_917_1:M>VID:YCL064C	AA:15		A12:E>D		A4:E>D		YPS138:E>D	AA:19		A12:R>I		A4:R>I		CBS5829:S>R		UFRJ50791:R>I		YPS138:R>I	AA:20		A4:F>V	AA:21		UWOPS91_917_1:N>K	AA:79		UWOPS91_917_1:C>-	AA:80		A12:W>R		A4:W>R		DBVPG6304:W>R		UFRJ50791:W>R		YPS138:W>R	AA:83		IFO1804:H>N		N_44:H>N		N_45:H>N	AA:112		A12:S>R		DBVPG6304:S>R		UFRJ50791:S>R		YPS138:S>R	AA:137		A12:N>K		DBVPG6304:N>K		UFRJ50791:N>K		YPS138:N>K	AA:156		A12:P>A		DBVPG6304:P>A		UFRJ50791:P>A		YPS138:P>A	AA:169		IFO1804:Q>L		N_43:Q>L		N_44:Q>L		N_45:Q>L	AA:192		A12:H>Q		DBVPG6304:H>Q		UFRJ50791:H>Q		YPS138:H>Q	AA:256		A12:G>D		DBVPG6304:G>D		YPS138:G>D	AA:273		A12:N>K		A4:N>K		DBVPG6304:N>K		UFRJ50791:N>K		YPS138:N>K	AA:275		A4:D>E	AA:302		IFO1804:R>S		N_43:R>S		N_45:R>S	AA:303		A12:S>T		A4:S>T		DBVPG6304:S>T		N_44:H>Y		N_45:H>Y		UFRJ50791:S>T		YPS138:S>T	AA:309		A12:L>F		DBVPG6304:L>F		UFRJ50791:L>F		YPS138:L>F	AA:332		A4:K>R	AA:336		CBS5829:F>C		N_17:F>C		Q95_3:F>C		Z1_1:F>C	AA:337		CBS432:Q>H		Y6_5:Q>H	AA:339		A12:K>N		A4:K>N		N_43:K>N		N_45:K>N		UFRJ50791:K>N		YPS138:K>N	AA:355		N_43:F>S		N_45:F>SID:YCL066W	AA:78		A12:R>S		A4:R>S		DBVPG6304:R>S		UFRJ50791:R>S		UFRJ50816:R>S	AA:104		A12:R>S		A4:R>S		UFRJ50791:R>S		UFRJ50816:R>S	AA:109		A12:T>S		A4:T>S		DBVPG6304:T>S		UFRJ50791:T>S		UFRJ50816:T>SID:YCL068C	AA:39		A12:->Y		UFRJ50791:->Y		UFRJ50816:->Y		UWOPS91_917_1:->Y		YPS138:->Y	AA:113		UWOPS91_917_1:F>I	AA:142		A12:I>M		UFRJ50791:I>M		UFRJ50816:I>M		UWOPS91_917_1:I>M	AA:160		UWOPS91_917_1:C>-	AA:188		UWOPS91_917_1:G>CID:YCR002C	AA:56		A4:K>N		DBVPG6304:K>N		UFRJ50791:K>N		UFRJ50816:K>N		UWOPS91_917_1:K>N	AA:88		UWOPS91_917_1:T>N	AA:95		N_43:D>N		N_44:D>N		N_45:D>N	AA:165		UWOPS91_917_1:A>P	AA:178		A4:N>K		DBVPG6304:N>K		UFRJ50791:N>K		UFRJ50816:N>K	AA:233		DBVPG6304:Q>H	AA:283		A12:G>-		A4:G>-		DBVPG6304:G>-		UFRJ50791:G>-		UWOPS91_917_1:G>-	AA:300		A12:M>I		A4:M>I		DBVPG6304:M>I		IFO1804:M>I		N_43:M>I		N_44:M>I		N_45:M>I		UWOPS91_917_1:M>I	AA:304		A12:I>M		A4:I>M		DBVPG6304:I>M		UFRJ50791:I>M		UWOPS91_917_1:I>M	AA:313		A12:R>S		A4:R>S		DBVPG6304:R>S		UFRJ50816:R>S	AA:318		A12:->C		A4:->C		DBVPG6304:->C		UFRJ50816:->C		UWOPS91_917_1:->CID:YCR004C	AA:25		A12:R>S		DBVPG6304:R>S		UFRJ50816:R>S		YPS138:R>S	AA:82		A12:K>N		DBVPG6304:K>N		N_44:K>N		N_45:K>N		YPS138:K>N	AA:102		A12:E>D		DBVPG6304:E>D		YPS138:E>D	AA:158		A12:I>M		DBVPG6304:I>M		YPS138:I>M	AA:178		A12:K>N		DBVPG6304:K>N		YPS138:K>N	AA:218		A12:S>N		DBVPG6304:S>N		IFO1804:S>N		N_43:S>N		N_44:S>N		N_45:S>N		UFRJ50816:S>N		YPS138:S>N	AA:231		N_43:L>F		N_45:L>FID:YCR005C	AA:47		N_43:->K		N_44:->K		N_45:->K	AA:91		A12:->C		DBVPG6304:->C		UFRJ50816:->C		YPS138:->C	AA:153		A12:F>L		DBVPG6304:F>L		UFRJ50816:F>L		YPS138:F>L	AA:205		A12:M>I		DBVPG6304:M>I		UFRJ50816:M>I		UWOPS91_917_1:M>I		YPS138:M>I	AA:216		A12:I>F		A4:I>F		DBVPG6304:I>F		N_43:Q>H		N_44:Q>H		UFRJ50816:I>F		UWOPS91_917_1:I>F		YPS138:I>F	AA:221		A12:F>L		DBVPG6304:F>L		UFRJ50816:F>L		YPS138:F>L	AA:225		N_43:T>A		N_44:T>A	AA:238		A12:W>C		DBVPG6304:W>C		UFRJ50816:W>C		YPS138:W>C	AA:290		UWOPS91_917_1:->C	AA:304		A12:H>Y		A4:H>Y		DBVPG6304:H>Y		IFO1804:H>Y		N_43:H>Y		N_44:H>Y		N_45:H>Y		UFRJ50816:H>Y		UWOPS91_917_1:H>Y		YPS138:H>Y	AA:318		UWOPS91_917_1:D>E	AA:342		IFO1804:L>F		N_43:L>F		N_44:L>F		N_45:L>F	AA:352		UWOPS91_917_1:F>L	AA:397		IFO1804:H>Q		N_43:H>Q		N_44:H>Q		N_45:H>Q	AA:405		A12:->Y		A4:->Y		DBVPG6304:T>P		N_44:T>P		N_45:T>P		UFRJ50816:T>P		UWOPS91_917_1:->Y		YPS138:->Y	AA:407		UFRJ50816:I>MID:YCR008W	AA:3		A12:G>D		DBVPG6304:G>D		UFRJ50816:G>D		YPS138:G>D	AA:11		A12:I>L		UFRJ50816:I>L		YPS138:I>L	AA:62		A12:A>V		A4:A>V		DBVPG6304:A>V		UFRJ50816:A>V		YPS138:A>V	AA:77		A12:V>I		A4:V>I		DBVPG6304:V>I		UFRJ50816:V>I		YPS138:V>I	AA:79		N_43:T>N		N_44:T>N		N_45:T>N	AA:84		A12:A>T		A4:A>T		DBVPG6304:A>T		UFRJ50816:A>T		YPS138:A>T	AA:91		A12:M>I	AA:160		N_43:C>Y		N_44:C>Y		N_45:C>Y	AA:247		UWOPS91_917_1:S>P	AA:248		N_43:N>K		N_44:N>K	AA:273		A12:T>A		A4:T>A		UFRJ50816:T>A		UWOPS91_917_1:T>A		YPS138:T>A	AA:276		A12:N>S		A4:N>S		UFRJ50816:N>S		UWOPS91_917_1:N>S		YPS138:N>S	AA:298		A12:A>V		A4:A>V		UWOPS91_917_1:A>V		YPS138:A>V	AA:350		Q62_5:Y>F	AA:518		DBVPG6304:I>V	AA:548		CBS5829:E>G		Q32_3:E>G	AA:550		A12:G>D		A4:G>D		DBVPG6304:G>D		UFRJ50791:G>D	AA:580		N_43:N>DID:YCR009C	AA:13		A4:H>Q		DBVPG6304:H>Q		UFRJ50816:H>Q	AA:39		N_43:L>F		N_44:L>F	AA:43		A12:C>W		A4:C>W		DBVPG6304:C>W		UFRJ50816:C>W	AA:46		A12:S>R		A4:S>R		DBVPG6304:S>R		UFRJ50816:S>R	AA:76		A12:W>C		A4:W>C		DBVPG6304:W>C		UFRJ50816:W>C	AA:79		A12:S>R		A4:S>R		DBVPG6304:S>R		UFRJ50816:S>R	AA:112		A12:H>Q		A4:H>Q		DBVPG6304:H>Q		UFRJ50816:H>Q		UWOPS91_917_1:H>Q	AA:117		A12:F>L		A4:F>L		DBVPG6304:F>L		UFRJ50816:F>L		UWOPS91_917_1:F>L		YPS138:F>L	AA:186		A12:I>M		A4:I>M		DBVPG6304:I>M		UFRJ50816:I>M		UWOPS91_917_1:I>M		YPS138:I>M	AA:250		A12:H>Y		A4:H>YID:YCR010C	AA:31		A12:V>A		DBVPG6304:V>A		UFRJ50791:V>A		UFRJ50816:V>A	AA:68		A12:E>D		DBVPG6304:E>D		UFRJ50791:E>D		UFRJ50816:E>D		UWOPS91_917_1:E>D	AA:79		A12:H>Q		DBVPG6304:H>Q		UFRJ50791:H>Q		UFRJ50816:H>Q		UWOPS91_917_1:H>Q	AA:88		A12:E>D		DBVPG6304:E>D		N_44:E>D		N_45:E>D		UFRJ50791:E>D		UFRJ50816:E>D		UWOPS91_917_1:E>D	AA:172		DBVPG6304:L>F		N_44:L>F		N_45:L>F		UFRJ50791:L>F		UWOPS91_917_1:L>F	AA:177		UWOPS91_917_1:E>Q	AA:185		DBVPG6304:Q>H		UFRJ50791:Q>H	AA:232		DBVPG6304:F>L		UFRJ50791:F>L		YPS138:F>L	AA:243		DBVPG6304:Q>H		UFRJ50791:Q>H		YPS138:Q>H	AA:284		YPS138:H>RID:YCR011C	AA:3		IFO1804:L>F		N_43:L>F		N_44:L>F		N_45:L>F	AA:10		UFRJ50791:K>M		UFRJ50816:K>M		UWOPS91_917_1:K>M		YPS138:K>M	AA:45		UWOPS91_917_1:S>I	AA:65		UFRJ50791:F>L		UFRJ50816:F>L	AA:145		N_44:K>N		N_45:K>N	AA:236		A12:I>M		UWOPS91_917_1:I>M		YPS138:I>M	AA:258		N_44:F>C		N_45:F>C	AA:283		A12:L>F		UFRJ50791:L>F		UWOPS91_917_1:L>F		YPS138:L>F	AA:287		A12:Q>H		UFRJ50791:Q>H		UWOPS91_917_1:Q>H		YPS138:Q>H	AA:308		CBS432:C>W	AA:317		A12:F>L		DBVPG6304:F>L		UFRJ50791:F>L		YPS138:F>L	AA:350		A12:S>P		DBVPG6304:S>P		UFRJ50791:S>P		YPS138:S>P	AA:361		N_45:P>L	AA:409		UWOPS91_917_1:N>K	AA:427		DBVPG6304:I>M		UWOPS91_917_1:I>M	AA:430		UWOPS91_917_1:K>N	AA:432		T21_4:->C	AA:440		UWOPS91_917_1:I>L	AA:442		A12:H>Q		DBVPG6304:H>Q		UFRJ50791:H>Q	AA:489		A12:S>R		DBVPG6304:S>R		UFRJ50791:S>R		UFRJ50816:S>R	AA:498		UWOPS91_917_1:Q>H	AA:539		UWOPS91_917_1:->Y	AA:542		A12:Q>H		A4:Q>H		DBVPG6304:Q>H		UFRJ50791:Q>H		UFRJ50816:Q>H		UWOPS91_917_1:Q>H	AA:559		N_44:Q>H		N_45:Q>H	AA:583		A12:D>E		A4:D>E		DBVPG6304:D>E		UFRJ50816:D>E	AA:628		N_44:T>A		N_45:T>A	AA:667		UWOPS91_917_1:->L	AA:693		CBS5829:->C	AA:712		N_44:C>S		N_45:C>S	AA:732		A12:C>-		UFRJ50791:C>-		YPS138:C>-	AA:744		A12:N>K		UFRJ50791:N>K		UWOPS91_917_1:N>K		YPS138:N>K	AA:753		UFRJ50791:R>S		UWOPS91_917_1:R>S		YPS138:R>S	AA:761		UFRJ50791:G>E		UWOPS91_917_1:G>E		YPS138:G>E	AA:767		UFRJ50791:->Y		YPS138:->Y	AA:804		A4:N>Y		UFRJ50791:N>Y		YPS138:N>Y	AA:852		UWOPS91_917_1:Y>-	AA:867		UWOPS91_917_1:K>Q	AA:871		UWOPS91_917_1:W>C	AA:876		UWOPS91_917_1:->Y	AA:881		N_44:F>L		N_45:F>L	AA:907		UWOPS91_917_1:R>S	AA:915		UWOPS91_917_1:N>H	AA:925		UWOPS91_917_1:N>K	AA:957		A4:Q>H		DBVPG6304:Q>H		IFO1804:Q>H		N_44:Q>H		N_45:Q>H		UFRJ50791:Q>H		UFRJ50816:Q>H		UWOPS91_917_1:Q>H	AA:965		IFO1804:C>W	AA:996		A4:W>-		DBVPG6304:W>-		UFRJ50791:W>-		UFRJ50816:W>-		YPS138:W>-	AA:1020		UWOPS91_917_1:W>-	AA:1026		IFO1804:C>R		N_44:C>R		N_45:C>R		UWOPS91_917_1:C>RID:YCR015C	AA:15		A12:P>Q		A4:P>Q		DBVPG6304:P>Q		UFRJ50791:P>Q		UFRJ50816:P>Q		YPS138:P>Q	AA:20		UWOPS91_917_1:V>L	AA:26		DBVPG6304:F>L		UFRJ50816:F>L		YPS138:F>L	AA:55		CBS432:S>N		KPN3828:S>N		Q59_1:S>N	AA:63		DBVPG6304:I>F	AA:66		UWOPS91_917_1:R>Q	AA:111		UWOPS91_917_1:I>L	AA:118		UWOPS91_917_1:E>K	AA:125		UWOPS91_917_1:V>D	AA:151		UWOPS91_917_1:E>D	AA:206		A4:L>V		DBVPG6304:L>V		UWOPS91_917_1:L>V		YPS138:L>V	AA:213		A4:K>N		DBVPG6304:K>N		UWOPS91_917_1:K>N		YPS138:K>N	AA:218		UWOPS91_917_1:R>S	AA:220		UWOPS91_917_1:F>L	AA:223		A4:T>A		DBVPG6304:T>A		YPS138:T>A	AA:244		A4:E>D		DBVPG6304:E>D		YPS138:E>D	AA:261		A4:W>R		DBVPG6304:W>R		UWOPS91_917_1:W>R		YPS138:W>R	AA:273		CBS432:I>N		CBS5829:I>N		T21_4:I>N		Y6_5:I>N		Y7:I>N	AA:285		A4:F>L		DBVPG6304:F>L		UWOPS91_917_1:F>L		YPS138:F>L	AA:288		A4:R>G		DBVPG6304:R>G		YPS138:R>G	AA:294		A4:->Y		DBVPG6304:->Y		UWOPS91_917_1:->Y		YPS138:->Y	AA:306		N_43:D>E		N_44:D>E		N_45:D>E	AA:310		A4:K>Q	AA:312		A4:->R		DBVPG6304:->R		N_43:->R		N_44:->R		N_45:->R		YPS138:->R	AA:314		UWOPS91_917_1:N>I	AA:318		A4:H>N		DBVPG6304:H>N		UWOPS91_917_1:H>N		YPS138:H>NID:YCR016W	AA:31		A12:S>T		A4:S>T		UWOPS91_917_1:S>T	AA:94		UWOPS91_917_1:N>D	AA:119		A12:P>S		A4:P>S		UWOPS91_917_1:P>S	AA:136		CBS432:K>N		Q59_1:K>N	AA:142		UWOPS91_917_1:S>F	AA:177		UWOPS91_917_1:L>I	AA:217		A12:I>V		A4:I>V		DBVPG6304:I>V		UFRJ50816:I>V		YPS138:I>V	AA:225		A12:P>S		A4:P>S		DBVPG6304:P>S		UFRJ50816:P>S		YPS138:P>S	AA:237		A12:A>G		A4:A>G		DBVPG6304:A>G		UFRJ50816:A>G		YPS138:A>G	AA:276		A12:E>G		DBVPG6304:E>G		UFRJ50816:E>G		YPS138:E>GID:YCR017C	AA:11		IFO1804:M>I		N_44:M>I		N_45:M>I	AA:21		A12:->Y		DBVPG6304:->Y		YPS138:->Y	AA:31		UWOPS91_917_1:L>F	AA:82		A12:L>I		DBVPG6304:L>I		YPS138:L>I	AA:154		A12:E>K		DBVPG6304:E>K		YPS138:E>K	AA:239		N_45:T>I	AA:240		A12:Y>D		DBVPG6304:Y>D		UWOPS91_917_1:Y>D	AA:249		CBS5829:->L		N_17:->L		Y7:->L	AA:254		A12:V>G		DBVPG6304:V>G		UWOPS91_917_1:V>G		YPS138:V>G	AA:257		A12:C>S		DBVPG6304:C>S		N_45:C>S		UWOPS91_917_1:C>S		YPS138:C>S	AA:260		UWOPS91_917_1:I>F	AA:274		A12:D>V		DBVPG6304:D>V		UWOPS91_917_1:D>V		YPS138:D>V	AA:277		N_45:G>R		UWOPS91_917_1:K>N	AA:299		UWOPS91_917_1:R>C	AA:303		Y7:Y>-	AA:338		A12:K>N		A4:K>N		DBVPG6304:K>N		UWOPS91_917_1:K>N		YPS138:K>N	AA:349		A12:Y>F		A4:Y>F		DBVPG6304:Y>F		UFRJ50816:Y>F		UWOPS91_917_1:Y>F		YPS138:Y>F	AA:363		A12:M>I		A4:M>I		DBVPG6304:M>I		UFRJ50816:M>I		UWOPS91_917_1:M>I		YPS138:M>I	AA:366		A12:D>E		A4:D>E		DBVPG6304:D>E		IFO1804:D>E		N_45:D>E		UFRJ50816:D>E		UWOPS91_917_1:D>E		YPS138:D>E	AA:370		UWOPS91_917_1:I>M	AA:378		UWOPS91_917_1:N>T	AA:389		UWOPS91_917_1:K>Q	AA:397		IFO1804:H>Q		N_45:H>Q	AA:401		A12:V>I		A4:V>I		DBVPG6304:V>I		IFO1804:V>I		N_45:V>I		UFRJ50816:V>I		YPS138:V>I	AA:406		IFO1804:K>N		N_45:K>N	AA:423		A4:G>A		DBVPG6304:G>A		UFRJ50816:G>A		UWOPS91_917_1:G>A		YPS138:G>A	AA:426		UWOPS91_917_1:->Q	AA:442		IFO1804:Q>H		N_44:Q>H		N_45:Q>H	AA:454		IFO1804:T>S		N_43:T>S		N_44:T>S		N_45:T>S	AA:462		A4:M>I		DBVPG6304:M>I		UFRJ50816:M>I		YPS138:M>I	AA:468		IFO1804:F>L		N_43:F>L		N_44:F>L		N_45:F>L	AA:474		A4:S>R		DBVPG6304:S>R		UFRJ50816:S>R		YPS138:S>R	AA:475		UWOPS91_917_1:G>C	AA:500		A4:S>R		DBVPG6304:S>R		UFRJ50816:S>R		YPS138:S>R	AA:505		DBVPG6304:F>L		UFRJ50816:F>L		YPS138:F>L	AA:518		IFO1804:I>V		N_43:I>V		N_44:I>V		N_45:I>V	AA:525		IFO1804:T>S		N_43:T>S		N_44:T>S		N_45:T>S	AA:537		CBS432:I>M	AA:572		Q32_3:K>N	AA:573		A4:R>S		DBVPG6304:R>S		UFRJ50816:R>S		YPS138:R>S	AA:617		UWOPS91_917_1:N>D	AA:621		A4:K>M		DBVPG6304:K>M		UFRJ50816:K>M		YPS138:K>M	AA:627		N_43:E>V		N_44:E>V	AA:629		A4:L>R		DBVPG6304:L>R		UFRJ50816:L>R		YPS138:L>R	AA:632		A4:E>D		DBVPG6304:E>D		UFRJ50816:E>D		YPS138:E>D	AA:637		A4:E>K		DBVPG6304:E>K		UFRJ50816:E>K		UWOPS91_917_1:E>Q		YPS138:E>K	AA:639		A4:W>R		DBVPG6304:W>R		UFRJ50816:W>R		YPS138:W>R	AA:642		N_43:I>M		N_44:I>M		N_45:I>M	AA:666		A4:->L		DBVPG6304:->L		N_43:->L		N_44:->L		N_45:->L		YPS138:->L	AA:683		A4:->Y		DBVPG6304:->Y		YPS138:->Y	AA:713		UWOPS91_917_1:F>L	AA:733		UWOPS91_917_1:E>Q	AA:770		UWOPS91_917_1:F>L	AA:771		A12:K>N		A4:K>N	AA:800		N_44:K>N	AA:801		UWOPS91_917_1:Y>-	AA:811		A12:S>R		A4:S>R	AA:814		N_43:T>N		N_44:T>N		N_45:T>N	AA:882		N_44:S>R		N_45:S>R	AA:906		A12:G>E		A4:G>E		YPS138:G>E	AA:908		Q59_1:P>S	AA:915		A12:M>I		A4:M>I		YPS138:M>IID:YCR018C	AA:21		A12:S>T		DBVPG6304:S>T		UFRJ50816:S>T		UWOPS91_917_1:S>T		YPS138:S>T	AA:32		UWOPS91_917_1:G>R	AA:41		UWOPS91_917_1:S>R	AA:43		A4:F>C		DBVPG6304:F>C		UFRJ50816:F>C		UWOPS91_917_1:F>C		YPS138:F>C	AA:95		DBVPG6304:Y>N		UFRJ50816:Y>N		YPS138:Y>N	AA:101		DBVPG6304:I>L		UFRJ50816:I>L		YPS138:I>L	AA:108		DBVPG6304:I>V		N_43:I>V		N_44:I>V		N_45:I>V		UFRJ50816:I>V		UWOPS91_917_1:I>V		YPS138:I>V	AA:113		YPS138:F>Y	AA:126		UWOPS91_917_1:F>L	AA:157		N_43:N>K	AA:159		A4:->C		DBVPG6304:->C		UFRJ50816:->C		UWOPS91_917_1:->C		YPS138:->C	AA:182		UWOPS91_917_1:N>KID:YCR020C	AA:8		UWOPS91_917_1:K>-	AA:19		A12:K>N		DBVPG6304:K>N		UFRJ50816:K>N		YPS138:K>N	AA:26		UWOPS91_917_1:N>K	AA:40		A12:S>R		DBVPG6304:S>R		UFRJ50816:S>R		YPS138:S>R	AA:52		N_43:F>V		N_44:F>V		N_45:F>V	AA:78		A12:->Y		DBVPG6304:->Y		UFRJ50816:->Y		YPS138:->Y	AA:133		A12:V>L		DBVPG6304:V>L		UFRJ50816:V>L		YPS138:V>L	AA:137		A12:R>S		YPS138:R>S	AA:179		A12:Q>H		DBVPG6304:Q>H		UFRJ50816:Q>H		YPS138:Q>H	AA:188		A12:T>K		CBS432:T>K		CBS5829:T>K		DBVPG6304:T>K		N_17:T>K		N_44:T>K		N_45:T>K		Q59_1:T>K		UFRJ50816:T>K		YPS138:T>K	AA:196		DBVPG6304:F>V		UFRJ50816:F>V		YPS138:F>VID:YCR020C-A	AA:7		CBS432:Q>H		CBS5829:Q>H	AA:14		A12:K>N		A4:K>N		DBVPG6304:K>N		UFRJ50816:K>N		UWOPS91_917_1:K>N		YPS138:K>N	AA:20		N_43:L>R		N_44:L>R		N_45:L>R	AA:28		A12:->W		A4:->W		DBVPG6304:->W		UFRJ50816:->W		YPS138:->W	AA:61		CBS432:N>K		CBS5829:N>K	AA:69		UWOPS91_917_1:I>V	AA:72		A12:K>N		A4:K>N		DBVPG6304:K>N		UFRJ50816:K>N		YPS138:K>N	AA:75		N_17:Y>-	AA:78		A12:I>N		A4:I>N		DBVPG6304:I>N		UFRJ50816:I>N		UWOPS91_917_1:I>V		YPS138:I>NID:YCR021C	AA:8		A12:L>F		YPS138:L>F	AA:48		A12:R>S		YPS138:R>S	AA:99		A12:->Y		DBVPG6304:->Y		YPS138:->Y	AA:108		A12:N>S		DBVPG6304:N>S		YPS138:N>S	AA:116		A12:H>Q		DBVPG6304:H>Q		YPS138:H>Q	AA:170		DBVPG6304:T>A		IFO1804:T>A		N_43:T>A		N_44:T>A		N_45:T>A		UFRJ50816:T>A		YPS138:T>A	AA:201		IFO1804:->Y	AA:213		CBS432:F>L		UFRJ50816:F>S	AA:245		DBVPG6304:Q>H		UFRJ50791:Q>H		UFRJ50816:Q>H		YPS138:Q>H	AA:272		DBVPG6304:P>S	AA:280		DBVPG6304:K>N		UFRJ50791:K>N		UFRJ50816:K>N		YPS138:K>N	AA:288		N_44:Q>R		N_45:Q>R	AA:294		CBS432:->C	AA:333		N_44:H>Q		N_45:H>QID:YCR023C	AA:18		A12:D>E		A4:D>E		DBVPG6304:D>E		IFO1804:D>E		N_43:D>E		N_45:D>E		UWOPS91_917_1:H>D		YPS138:H>D	AA:37		UWOPS91_917_1:M>L	AA:54		UWOPS91_917_1:K>N	AA:81		A4:T>N		DBVPG6304:T>N	AA:135		UWOPS91_917_1:D>E	AA:141		UWOPS91_917_1:P>L	AA:149		UWOPS91_917_1:->Y	AA:150		A4:K>N		DBVPG6304:K>N		YPS138:K>N	AA:195		A12:C>-		DBVPG6304:C>-		N_44:C>-		UWOPS91_917_1:C>-		YPS138:C>-	AA:243		A12:H>Q		DBVPG6304:H>Q		UWOPS91_917_1:H>Q		YPS138:H>Q	AA:244		IFO1804:N>T		N_45:N>T	AA:252		UWOPS91_917_1:I>V	AA:273		A12:T>S		DBVPG6304:T>S		YPS138:T>S	AA:278		A12:P>L		A4:P>L		DBVPG6304:P>L		UWOPS91_917_1:C>G		YPS138:C>G	AA:288		UWOPS91_917_1:W>L	AA:292		A12:R>I		DBVPG6304:R>I		YPS138:R>I	AA:322		A12:I>F		A4:I>F		DBVPG6304:I>F		YPS138:I>F	AA:341		A12:I>N		A4:I>N		CBS432:I>N		CBS5829:I>N		DBVPG4650:I>N		DBVPG6304:I>N		IFO1804:I>N		KPN3829:I>N		N_17:I>N		N_44:I>N		N_45:I>N		Q62_5:I>N		S36_7:I>N		YPS138:I>N	AA:392		UWOPS91_917_1:F>Y	AA:404		UWOPS91_917_1:->Y	AA:408		A12:H>Q		UWOPS91_917_1:H>Q	AA:428		UWOPS91_917_1:K>N	AA:433		UWOPS91_917_1:F>L	AA:441		UWOPS91_917_1:Y>-	AA:447		IFO1804:H>Y		N_43:H>Y		N_44:H>Y		UWOPS91_917_1:H>Y	AA:482		A12:N>K		A4:N>K		UWOPS91_917_1:N>K		YPS138:N>K	AA:489		A12:Q>H		A4:Q>H		N_43:Q>H		N_44:Q>H		YPS138:Q>H	AA:504		UWOPS91_917_1:->C	AA:505		A12:K>N		A4:K>N		YPS138:K>N	AA:538		A12:R>S		A4:R>S		UWOPS91_917_1:R>S	AA:540		A12:D>E		A4:D>E		IFO1804:D>E		N_43:D>E		N_45:D>E		UWOPS91_917_1:D>E	AA:543		UWOPS91_917_1:->Y	AA:570		A12:E>D		A4:E>D	AA:575		A12:K>N		A4:K>N		DBVPG6304:K>N	AA:577		A12:->C		A4:->C		DBVPG6304:->CID:YCR024C	AA:11		UFRJ50816:I>K		UWOPS91_917_1:I>K		YPS138:I>K	AA:30		DBVPG6304:L>V		UFRJ50816:L>V		YPS138:L>V	AA:44		N_43:F>L		N_45:F>L		UWOPS91_917_1:F>L	AA:48		DBVPG6304:Y>-		UFRJ50816:Y>-		YPS138:Y>-	AA:58		DBVPG6304:H>Q		UFRJ50816:H>Q		UWOPS91_917_1:H>Q		YPS138:H>Q	AA:61		DBVPG6304:C>R		UFRJ50816:C>R		YPS138:C>R	AA:81		DBVPG6304:F>L		UFRJ50816:F>L		UWOPS91_917_1:F>L		YPS138:F>L	AA:103		UWOPS91_917_1:H>Q	AA:111		A12:W>R		A4:W>R		DBVPG6304:W>R		UFRJ50816:W>R		UWOPS91_917_1:W>R		YPS138:W>R	AA:114		A12:S>R		DBVPG6304:S>R		N_43:S>R		N_45:S>R		UFRJ50816:S>R		UWOPS91_917_1:S>R		YPS138:S>R	AA:131		UWOPS91_917_1:F>S	AA:137		A12:S>P		DBVPG6304:S>P		UFRJ50816:S>P		UWOPS91_917_1:S>P		YPS138:S>P	AA:209		CBS432:I>L		KPN3828:I>L		KPN3829:I>L		S36_7:I>L		T21_4:I>L	AA:210		N_43:Y>D		N_44:Y>D	AA:223		A12:K>N		A4:K>N		DBVPG6304:K>N		UWOPS91_917_1:K>N	AA:245		A12:K>N		A4:K>N		DBVPG6304:K>N		UWOPS91_917_1:K>N	AA:248		A12:W>C		A4:W>C		DBVPG6304:W>C		N_43:W>C		N_44:W>C	AA:255		UWOPS91_917_1:S>F	AA:261		A12:S>C		A4:S>C		DBVPG6304:S>C	AA:266		UWOPS91_917_1:->Y	AA:269		A12:S>R		A4:S>R		DBVPG6304:S>R	AA:323		A12:V>A		A4:V>A	AA:325		A12:L>F		A4:L>F		UWOPS91_917_1:L>F	AA:329		A12:H>L		A4:H>L	AA:337		UWOPS91_917_1:S>T	AA:338		KPN3828:->E		KPN3829:->E		S36_7:->E		T21_4:->E	AA:339		A12:P>T		A4:P>T	AA:346		Q62_5:S>T	AA:355		UWOPS91_917_1:G>R	AA:360		A12:->K		A4:->K		UWOPS91_917_1:->K	AA:369		A4:I>K		CBS5829:I>K		KPN3828:I>K		KPN3829:I>K		N_43:I>K		N_44:I>K		N_45:I>K		T21_4:I>K		UWOPS91_917_1:I>K	AA:382		N_43:N>Y		N_44:N>Y		N_45:N>Y	AA:405		A4:N>D		N_43:N>D		N_44:N>D		N_45:N>D		UWOPS91_917_1:N>D		YPS138:N>D	AA:407		A4:->Q		UWOPS91_917_1:->Q		YPS138:->Q	AA:427		A4:N>H		YPS138:N>H	AA:429		UWOPS91_917_1:Y>H	AA:451		A4:Y>-		YPS138:Y>-	AA:468		UWOPS91_917_1:Y>-	AA:498		YPS138:F>L	AA:520		IFO1804:V>L		N_43:V>L		N_45:V>L		YPS138:V>LID:YCR026C	AA:7		N_45:Q>K	AA:11		UWOPS91_917_1:A>T	AA:18		A12:E>D		A4:E>D		DBVPG6304:E>D		UFRJ50816:E>D		YPS138:E>D	AA:29		UWOPS91_917_1:P>T	AA:31		DBVPG6304:P>A		N_43:P>A		N_45:P>A		UFRJ50816:P>A		UWOPS91_917_1:P>A		YPS138:P>A	AA:56		A12:P>H		DBVPG6304:P>H		UFRJ50816:P>H		YPS138:P>H	AA:65		A12:Y>D		DBVPG6304:Y>D		UFRJ50816:Y>D		YPS138:Y>D	AA:69		UWOPS91_917_1:C>G	AA:71		A12:->S		A4:->S		DBVPG6304:->S		UFRJ50816:->S		UWOPS91_917_1:->S		YPS138:->S	AA:120		CBS5829:->Y	AA:146		CBS5829:A>V		T21_4:A>V		Z1_1:A>V	AA:152		UWOPS91_917_1:I>S	AA:169		A12:I>F		UFRJ50816:I>F		UWOPS91_917_1:I>F		YPS138:I>F	AA:171		N_44:E>Q		N_45:E>Q	AA:214		N_43:V>F	AA:280		A12:I>M		A4:I>M		DBVPG6304:I>M		UWOPS91_917_1:S>T	AA:315		UWOPS91_917_1:I>M	AA:328		N_45:N>D	AA:333		A4:S>R	AA:340		A4:F>I	AA:353		UWOPS91_917_1:N>H	AA:369		UWOPS91_917_1:C>W	AA:386		A4:E>D		DBVPG6304:E>D	AA:422		S36_7:R>S	AA:433		N_45:F>L	AA:436		UWOPS91_917_1:->Y	AA:443		CBS432:G>S		DBVPG4650:G>S		KPN3829:G>S		Q62_5:G>S	AA:468		A12:D>E		A4:D>E		DBVPG6304:D>E	AA:483		A12:R>S		A4:R>S		DBVPG6304:R>S		UWOPS91_917_1:R>S	AA:485		A12:H>Q		A4:H>Q		DBVPG6304:H>Q	AA:490		A12:E>G		A4:E>G		DBVPG6304:E>G	AA:503		A12:N>Y		A4:N>Y		CBS432:N>Y		CBS5829:N>Y		DBVPG4650:N>Y		DBVPG6304:N>Y		IFO1804:N>Y		KPN3829:N>Y		N_44:N>Y		N_45:N>Y		Q59_1:N>Y		Q62_5:N>Y		Q89_8:N>Y		S36_7:N>Y		T21_4:N>Y		UWOPS91_917_1:N>Y	AA:522		A4:G>D		DBVPG6304:G>D		UWOPS91_917_1:G>D	AA:531		A4:K>E		DBVPG6304:K>E	AA:554		A4:I>L		DBVPG6304:I>L	AA:585		UWOPS91_917_1:I>M	AA:596		DBVPG6304:->K		UFRJ50816:->K		UWOPS91_917_1:->K	AA:613		UWOPS91_917_1:K>E	AA:618		UWOPS91_917_1:Q>H	AA:639		UWOPS91_917_1:->Y	AA:645		IFO1804:I>V		N_44:I>V		N_45:I>V		UFRJ50816:I>V	AA:648		UFRJ50816:F>I	AA:652		IFO1804:R>K		N_44:R>K		N_45:R>K	AA:700		DBVPG6304:P>L		IFO1804:P>L		N_43:P>L		N_44:P>L		N_45:P>L		UFRJ50816:P>L		UWOPS91_917_1:P>L	AA:706		N_43:->G		N_44:->G		N_45:->G	AA:709		N_43:S>I		N_44:S>I		N_45:S>I	AA:713		DBVPG6304:R>C		UFRJ50816:R>C		UWOPS91_917_1:R>C		YPS138:R>C	AA:716		DBVPG6304:I>V		UFRJ50816:I>V		YPS138:I>V	AA:719		DBVPG6304:K>R		UFRJ50816:K>R		UWOPS91_917_1:K>R		YPS138:K>R	AA:722		DBVPG6304:F>L		UFRJ50816:F>L		YPS138:F>L	AA:731		UWOPS91_917_1:E>D	AA:738		UWOPS91_917_1:K>NID:YCR027C	AA:28		DBVPG6304:H>Q		UWOPS91_917_1:H>Q		YPS138:H>Q	AA:32		DBVPG6304:F>L		UFRJ50816:F>L		YPS138:F>L	AA:33		UFRJ50816:N>K	AA:43		UFRJ50816:Y>-	AA:65		N_43:F>L		N_45:F>L	AA:67		UFRJ50816:D>E	AA:78		UFRJ50816:Q>H	AA:83		Z1_1:T>I	AA:85		UWOPS91_917_1:E>D	AA:98		UFRJ50816:Q>K	AA:114		DBVPG6304:Y>N		YPS138:Y>N	AA:149		N_43:M>I		N_44:M>I		N_45:M>I	AA:207		N_43:G>A		UFRJ50816:K>EID:YCR028C	AA:4		A12:R>S		DBVPG6304:R>S		UFRJ50816:R>S		UWOPS91_917_1:R>S		YPS138:R>S	AA:35		UFRJ50816:V>F	AA:37		UWOPS91_917_1:F>L	AA:43		A12:S>R		DBVPG6304:S>R		UFRJ50816:S>R		UWOPS91_917_1:S>R	AA:60		UWOPS91_917_1:->G	AA:90		UWOPS91_917_1:->R	AA:109		UWOPS91_917_1:C>-	AA:167		A4:Y>-		UFRJ50816:Y>-	AA:168		Q62_5:E>D		Q89_8:E>D		Q95_3:E>D		Y7:E>D	AA:249		A4:D>H		DBVPG6304:D>H		UFRJ50816:D>H		YPS138:D>H	AA:269		N_43:M>L		N_44:M>L		N_45:M>L	AA:282		A4:K>N		DBVPG6304:K>N		UFRJ50816:K>N		YPS138:K>N	AA:298		A4:K>-		DBVPG6304:K>-		UFRJ50816:K>-		UWOPS91_917_1:K>-		YPS138:K>-	AA:303		Q59_1:N>T	AA:349		A4:Y>-		DBVPG6304:Y>-		UWOPS91_917_1:Y>-		YPS138:Y>-	AA:360		A4:I>L		DBVPG6304:I>L		YPS138:I>L	AA:475		UWOPS91_917_1:->L	AA:489		DBVPG6304:F>C		UFRJ50816:F>C		YPS138:F>C	AA:495		DBVPG6304:R>T		UFRJ50816:R>T		UWOPS91_917_1:R>T		YPS138:R>T	AA:505		UWOPS91_917_1:S>R	AA:508		S36_7:F>Y		Y6_5:F>YID:YCR033W	AA:52		IFO1804:T>N		N_45:T>N	AA:73		UWOPS91_917_1:G>E	AA:100		UWOPS91_917_1:S>P	AA:117		A12:S>P		DBVPG6304:S>P		UFRJ50816:S>P		UWOPS91_917_1:S>P		YPS138:S>P	AA:127		Z1_1:M>I	AA:130		A12:N>T		DBVPG6304:N>T		UFRJ50816:N>T		UWOPS91_917_1:N>T		YPS138:N>T	AA:132		CBS5829:I>T		KPN3829:I>T		N_17:I>T		N_43:I>T		N_45:I>T		T21_4:I>T		UFRJ50816:I>T		UWOPS91_917_1:I>T		Y6_5:I>T		Z1_1:I>T	AA:135		A12:N>S		UFRJ50816:N>S		UWOPS91_917_1:N>S	AA:244		A12:S>N		A4:S>N		DBVPG6304:S>N		UFRJ50791:S>N		UFRJ50816:S>N		YPS138:S>N	AA:254		IFO1804:P>S		N_43:P>S		N_45:P>S	AA:286		CBS432:L>W	AA:315		IFO1804:P>S		N_43:P>S		N_45:P>S	AA:322		A4:P>L	AA:329		IFO1804:D>A	AA:341		A12:K>T		A4:K>T		DBVPG6304:K>T		UFRJ50791:K>T		UFRJ50816:K>T	AA:343		UWOPS91_917_1:D>G	AA:352		Q59_1:V>L	AA:357		IFO1804:I>L		N_43:I>L		N_45:I>L	AA:358		A12:P>A		A4:P>A		DBVPG6304:P>A		UFRJ50791:P>A		UFRJ50816:P>A	AA:361		UWOPS91_917_1:R>K	AA:362		A12:K>R		A4:K>R		DBVPG6304:K>R		UFRJ50791:K>R		UFRJ50816:K>R	AA:369		UWOPS91_917_1:V>A	AA:399		UWOPS91_917_1:V>I	AA:401		A12:K>E		DBVPG6304:K>E		UFRJ50816:K>E		UWOPS91_917_1:K>E	AA:421		A12:D>G		DBVPG6304:D>G		UFRJ50816:D>G		UWOPS91_917_1:D>G	AA:426		UWOPS91_917_1:Y>H	AA:432		Z1_1:K>E	AA:439		A12:T>M		DBVPG6304:T>M		UFRJ50816:T>M	AA:454		A12:A>V		DBVPG6304:A>V		UFRJ50816:A>V	AA:501		A12:V>L		DBVPG6304:V>L		UFRJ50816:V>L	AA:543		A12:R>K		DBVPG6304:R>K		N_45:R>K		UFRJ50816:R>K		UWOPS91_917_1:R>K	AA:584		A12:T>S		A4:T>S		DBVPG6304:T>S		YPS138:T>S	AA:662		KPN3828:A>E	AA:670		A12:F>S		A4:F>S		DBVPG6304:F>S		UFRJ50791:F>S		UWOPS91_917_1:F>S		YPS138:F>S	AA:697		A12:H>N		A4:H>N		DBVPG6304:H>N		UFRJ50791:H>N		YPS138:H>N	AA:752		A12:T>I		A4:T>I		DBVPG6304:T>I		IFO1804:T>I		N_45:T>I		UFRJ50791:T>I		UWOPS91_917_1:T>I		YPS138:T>I	AA:766		UWOPS91_917_1:D>G	AA:779		UWOPS91_917_1:V>A	AA:784		A12:V>G		DBVPG6304:V>G		UFRJ50791:V>G		UWOPS91_917_1:V>G		YPS138:V>G	AA:792		UWOPS91_917_1:N>K	AA:794		A12:L>P		A4:L>P		DBVPG6304:L>P		UFRJ50791:L>P		YPS138:L>P	AA:812		A12:T>A		DBVPG6304:T>A		UFRJ50791:T>A		UWOPS91_917_1:T>A		YPS138:T>A	AA:817		A12:C>G		DBVPG6304:C>G		UFRJ50791:C>G		YPS138:C>G	AA:818		CBS5829:A>S	AA:821		A12:V>A		DBVPG6304:V>A		YPS138:V>A	AA:822		IFO1804:N>D		N_45:N>D	AA:831		CBS5829:I>T	AA:832		A12:E>G		DBVPG6304:E>G		UFRJ50791:E>G		UWOPS91_917_1:E>G		YPS138:E>G	AA:834		A12:K>R		DBVPG6304:K>R		UFRJ50791:K>R		UWOPS91_917_1:K>R		YPS138:K>R	AA:852		DBVPG6304:P>S		UFRJ50791:P>S		YPS138:P>S	AA:855		IFO1804:S>G		N_43:S>G		N_45:S>G	AA:864		CBS5829:D>E		IFO1804:D>E		N_43:D>E		N_45:D>E		Q89_8:D>E		Q95_3:D>E	AA:974		UWOPS91_917_1:A>V	AA:1004		A12:I>V		DBVPG6304:I>V		UFRJ50816:I>V	AA:1014		Q59_1:D>G	AA:1046		A12:S>P		A4:S>P		DBVPG6304:S>P		IFO1804:S>P		N_43:S>P		N_45:S>P		UFRJ50816:S>P		UWOPS91_917_1:S>P	AA:1051		UWOPS91_917_1:T>A	AA:1100		N_44:S>T	AA:1111		A12:V>A		A4:V>A		DBVPG6304:V>A		UWOPS91_917_1:V>A		YPS138:V>A	AA:1175		N_43:A>G		N_44:A>G		N_45:A>G	AA:1184		A4:H>R		DBVPG6304:H>R		UFRJ50816:H>R		UWOPS91_917_1:H>R		YPS138:H>R	AA:1226		KPN3829:N>T		Y6_5:N>TID:YCR035C	AA:16		A12:->Y		A4:->Y		DBVPG6304:->Y		UFRJ50791:->Y		UFRJ50816:->Y		YPS138:->Y	AA:19		N_45:->C	AA:35		IFO1804:C>W		N_43:C>W		N_45:C>W	AA:54		N_43:T>A	AA:66		A12:->Y		A4:->Y		CBS5829:->Y		DBVPG6304:->Y		IFO1804:->Y		N_43:->Y		N_45:->Y		S36_7:->Y		T21_4:->Y		UWOPS91_917_1:->Y		Y6_5:->Y		YPS138:->Y	AA:96		A12:Y>H		A4:Y>H		UFRJ50816:Y>H		UWOPS91_917_1:Y>H		YPS138:Y>H	AA:119		A12:T>A		A4:T>A		UFRJ50816:T>A		UWOPS91_917_1:T>A		YPS138:T>A	AA:157		A12:->Y		A4:->Y		UFRJ50816:->Y		UWOPS91_917_1:->Y		YPS138:->Y	AA:200		UWOPS91_917_1:I>S	AA:219		A12:N>K		A4:N>K		YPS138:N>K	AA:267		A12:S>R		A4:S>R		YPS138:S>R	AA:288		A12:I>L		A4:I>L		UWOPS91_917_1:I>L		YPS138:I>L	AA:308		UWOPS91_917_1:N>K	AA:311		UWOPS91_917_1:F>L	AA:350		UWOPS91_917_1:F>I	AA:352		A12:->Y		DBVPG6304:->Y		N_43:->Y		UWOPS91_917_1:->Y		YPS138:->Y	AA:366		A12:Q>H		DBVPG6304:Q>H		YPS138:Q>H	AA:391		A12:T>S		DBVPG6304:T>S		UFRJ50816:T>S		YPS138:T>SID:YCR037C	AA:12		A12:T>A		DBVPG6304:T>A		YPS138:T>A	AA:17		A12:N>H		DBVPG6304:N>H		YPS138:N>H	AA:28		A12:M>I		A4:M>I		DBVPG6304:M>I		N_17:N>K		N_43:N>K		N_44:N>K		N_45:N>K		UFRJ50816:M>I		YPS138:M>I	AA:44		A12:F>L		DBVPG6304:F>L		UFRJ50816:F>L		YPS138:F>L	AA:83		N_43:->Q		N_45:->Q	AA:84		DBVPG6304:F>L	AA:91		N_43:->Y		N_45:->Y	AA:131		A12:S>R		A4:S>R		DBVPG6304:S>R		UFRJ50791:S>R		UFRJ50816:S>R		YPS138:S>R	AA:141		A12:->C		A4:->C		DBVPG6304:->C		N_17:->C		N_43:->C		N_45:->C		UFRJ50791:->C		UFRJ50816:->C		YPS138:->C	AA:164		A12:E>D		A4:E>D		DBVPG6304:E>D		N_43:E>D		N_45:E>D		UFRJ50791:E>D		UFRJ50816:E>D		YPS138:E>D	AA:183		A12:N>K		A4:N>K		DBVPG6304:N>K		UFRJ50791:N>K		UFRJ50816:N>K		YPS138:N>K	AA:184		UWOPS91_917_1:F>L	AA:187		UWOPS91_917_1:->W	AA:210		N_44:N>K		N_45:N>K	AA:268		UWOPS91_917_1:V>I	AA:272		A4:D>A		DBVPG6304:D>A		UFRJ50791:D>A		UFRJ50816:D>A		UWOPS91_917_1:D>G		YPS138:D>A	AA:276		UWOPS91_917_1:W>C	AA:281		A4:->C		DBVPG6304:->C		UFRJ50791:->C		UFRJ50816:->C		UWOPS91_917_1:->C		YPS138:->C	AA:303		A4:S>R		YPS138:S>R	AA:306		DBVPG6304:->Y		UWOPS91_917_1:->Y	AA:309		A4:->Y		DBVPG6304:->Y		UFRJ50791:->Y		UFRJ50816:->Y		UWOPS91_917_1:->Y		YPS138:->Y	AA:318		A4:->Y		CBS5829:->Y		DBVPG6304:->Y		N_17:->Y		N_43:->Y		N_44:->Y		N_45:->Y		Q32_3:->Y		Q59_1:->Y		UFRJ50791:->Y		UFRJ50816:->Y		UWOPS91_917_1:->Y		Y7:->Y		YPS138:->Y	AA:351		A4:->Y		DBVPG6304:->Y		UFRJ50791:->Y		UFRJ50816:->Y		UWOPS91_917_1:->Y		YPS138:->Y	AA:355		UWOPS91_917_1:C>-	AA:376		UWOPS91_917_1:N>K	AA:399		UFRJ50791:T>A		UFRJ50816:T>A		YPS138:T>A	AA:414		DBVPG6304:N>H		UFRJ50791:N>H		UFRJ50816:N>H	AA:427		IFO1804:S>R		N_43:S>R		N_44:S>R		N_45:S>R	AA:436		DBVPG6304:Q>H		IFO1804:Q>H		N_43:Q>H		N_44:Q>H		N_45:Q>H		UFRJ50816:Q>H	AA:478		A12:I>N		DBVPG6304:I>N		UFRJ50816:I>N	AA:508		A12:S>R		DBVPG6304:S>R		UFRJ50816:S>R	AA:543		A12:F>L		DBVPG6304:F>L		IFO1804:F>L		N_43:F>L		N_45:F>L		UFRJ50816:F>L	AA:568		IFO1804:N>Y		N_43:N>Y		N_45:N>Y	AA:623		A12:Q>H		A4:Q>H		DBVPG6304:Q>H		YPS138:Q>H	AA:628		A12:Y>-		A4:Y>-		DBVPG6304:Y>-		UWOPS91_917_1:Y>-		YPS138:Y>-	AA:658		DBVPG6304:S>R		N_43:S>R		N_45:S>R		UFRJ50791:S>R	AA:665		A12:D>E		A4:D>E		DBVPG6304:D>E		UFRJ50791:D>E		UFRJ50816:D>E		UWOPS91_917_1:D>E		YPS138:D>E	AA:711		A12:R>S		A4:R>S		DBVPG6304:R>S		N_43:R>S		N_45:R>S		UFRJ50791:R>S		UFRJ50816:R>S		UWOPS91_917_1:R>S		YPS138:R>S	AA:726		A12:C>-		A4:C>-		DBVPG6304:C>-		UFRJ50791:C>-		UFRJ50816:C>-		YPS138:C>-	AA:816		A12:D>E		A4:D>E		DBVPG6304:D>E		UFRJ50791:D>E		UFRJ50816:D>E		YPS138:D>E	AA:868		A12:W>C		A4:W>C		DBVPG6304:W>C		UFRJ50791:W>C		UFRJ50816:W>C		UWOPS91_917_1:W>C		YPS138:W>C	AA:880		A12:I>M		A4:I>M		DBVPG6304:I>M		UFRJ50791:I>M		UFRJ50816:I>M		UWOPS91_917_1:I>M		YPS138:I>M	AA:914		A12:S>R		A4:S>R		DBVPG6304:S>R		UFRJ50816:S>R		UWOPS91_917_1:S>R		YPS138:S>R	AA:919		A12:K>N		A4:K>N		DBVPG6304:K>N		N_43:K>N		N_45:K>N		UFRJ50791:K>N		UFRJ50816:K>N		UWOPS91_917_1:K>N		YPS138:K>N	AA:926		A12:E>D		A4:E>D		DBVPG6304:E>D		UFRJ50816:E>D		UWOPS91_917_1:E>D		YPS138:E>D	AA:957		A12:->Y		A4:->Y		DBVPG6304:->Y		UFRJ50816:->Y		UWOPS91_917_1:->Y		YPS138:->YID:YCR038C	AA:54		DBVPG6304:K>N		N_43:K>N		N_45:K>N		YPS138:K>N	AA:201		DBVPG6304:I>T		UFRJ50816:I>T		YPS138:I>T	AA:229		UWOPS91_917_1:->W	AA:249		A12:L>F		DBVPG6304:L>F		UFRJ50816:L>F		YPS138:L>F	AA:252		Z1_1:Q>H	AA:266		N_43:I>F	AA:289		A12:Y>H		DBVPG6304:Y>H		UFRJ50816:Y>H		YPS138:Y>H	AA:299		CBS432:L>V		CBS5829:L>V		N_17:L>V		N_43:L>V		N_45:L>V		Q32_3:L>V		Q59_1:L>V		Q62_5:L>V		Q89_8:L>V		Q95_3:L>V		T21_4:L>V		Z1_1:L>V	AA:317		A12:K>N		DBVPG6304:K>N		YPS138:K>N	AA:326		A12:Q>E		DBVPG6304:Q>E		N_43:Q>E		YPS138:Q>E	AA:331		A12:E>Q		DBVPG6304:E>Q		YPS138:E>Q	AA:333		A12:K>N		A4:K>N		UFRJ50816:K>N		YPS138:K>N	AA:376		A12:E>Q		A4:E>Q		DBVPG4650:E>Q		N_17:E>Q		N_43:E>Q		N_45:E>Q		UFRJ50816:E>Q		YPS138:E>Q		Z1_1:E>Q	AA:381		N_43:C>-		N_45:C>-	AA:402		UWOPS91_917_1:F>L	AA:419		N_43:D>E		N_45:D>E	AA:429		A12:K>N		A4:K>N		UFRJ50816:K>N		YPS138:K>N	AA:466		UWOPS91_917_1:V>M	AA:489		A12:Y>H		A4:Y>H		DBVPG6304:Y>H		UFRJ50816:Y>H		YPS138:Y>H	AA:496		A12:S>R		A4:S>R		DBVPG6304:S>R		UFRJ50816:S>R		YPS138:S>R	AA:504		A12:E>A		A4:E>A		DBVPG6304:E>A		UFRJ50816:E>A		UWOPS91_917_1:E>A		YPS138:E>A	AA:512		A12:Y>-		A4:Y>-		DBVPG6304:Y>-		UFRJ50816:Y>-		YPS138:Y>-ID:YCR039C	AA:21		DBVPG6304:C>W		UFRJ50816:C>W		YPS138:C>W	AA:200		Q32_3:K>IID:YCR042C	AA:6		DBVPG6304:Q>H	AA:13		KPN3828:S>T		N_17:S>T		N_44:S>T		N_45:N>S		Q32_3:S>T		Q89_8:S>T		Q95_3:S>T	AA:52		A12:N>K		DBVPG6304:N>K		UFRJ50816:N>K		YPS138:N>K	AA:76		A12:Y>-		DBVPG6304:Y>-		UFRJ50816:Y>-		YPS138:Y>-	AA:117		A12:H>Q		DBVPG6304:H>Q		UFRJ50816:H>Q	AA:161		YPS138:H>Q	AA:264		A12:I>M		A4:I>M		DBVPG6304:I>M		YPS138:I>M	AA:282		CBS432:M>I	AA:290		A12:Q>H		A4:Q>H		DBVPG6304:Q>H		N_43:Q>H		N_44:Q>H		N_45:Q>H		YPS138:Q>H	AA:298		A12:->Y		A4:->Y		DBVPG6304:->Y		YPS138:->Y	AA:309		CBS432:H>Q		KPN3828:H>Q		KPN3829:H>Q		N_43:H>Q		N_44:H>Q		N_45:H>Q		Q95_3:H>Q		T21_4:H>Q		Y6_5:H>Q		Y7:H>Q	AA:311		UWOPS91_917_1:I>M	AA:315		A12:K>N		DBVPG6304:K>N		UWOPS91_917_1:K>N		YPS138:K>N	AA:371		A12:I>M		A4:I>M		DBVPG6304:I>M	AA:388		A12:F>L		A4:F>L		CBS432:F>L		DBVPG6304:F>L		KPN3828:F>L		KPN3829:F>L		N_17:F>L		Q95_3:F>L		T21_4:F>L		UFRJ50791:F>L		Y6_5:F>L		YPS138:F>L	AA:391		A12:I>M		A4:I>M		DBVPG6304:I>M		YPS138:I>M	AA:399		A12:F>L		A4:F>L		DBVPG6304:F>L		UFRJ50791:F>L		UFRJ50816:F>L		YPS138:F>L	AA:410		A12:V>I		A4:V>I		DBVPG6304:V>I		UFRJ50791:V>I		UFRJ50816:V>I		UWOPS91_917_1:V>I		YPS138:V>I	AA:418		A12:I>T		A4:I>T		DBVPG6304:I>T		YPS138:I>T	AA:430		A12:F>L		A4:F>L		N_43:F>L		N_44:F>L		N_45:F>L		UFRJ50791:F>L		UFRJ50816:F>L		UWOPS91_917_1:F>L		YPS138:F>L	AA:451		UWOPS91_917_1:K>E	AA:463		A12:W>-		A4:W>-		UFRJ50791:W>-		UFRJ50816:W>-		UWOPS91_917_1:W>-		YPS138:W>-	AA:468		UWOPS91_917_1:F>L	AA:477		N_43:L>F		N_45:L>F	AA:480		A12:M>I		A4:M>I		DBVPG6304:M>I		UFRJ50791:M>I		UFRJ50816:M>I		UWOPS91_917_1:M>I		YPS138:M>I	AA:523		N_43:->C		N_45:->C		UWOPS91_917_1:->W	AA:540		N_43:->W	AA:554		N_43:K>T		N_45:K>T	AA:565		A12:T>K		A4:T>K		DBVPG6304:T>K		YPS138:T>K	AA:585		A12:Q>R		A4:Q>R		DBVPG6304:Q>R		KPN3829:I>T		UFRJ50791:Q>R		UFRJ50816:Q>R		UWOPS91_917_1:Q>R		YPS138:Q>R	AA:600		UWOPS91_917_1:L>-	AA:606		A12:Y>-		A4:Y>-		DBVPG6304:Y>-		UFRJ50791:Y>-		UFRJ50816:->G		UWOPS91_917_1:Y>-		YPS138:Y>-	AA:641		A12:->Y		A4:->Y		DBVPG6304:->Y		UFRJ50816:->Y		YPS138:->Y	AA:663		A4:M>V		DBVPG6304:M>V		UWOPS91_917_1:M>V		YPS138:M>V	AA:696		A4:C>W		DBVPG6304:C>W		UWOPS91_917_1:C>W	AA:709		A12:M>I		A4:M>I		DBVPG6304:M>I		UWOPS91_917_1:M>I		YPS138:M>I	AA:779		A12:S>R		A4:S>R		UWOPS91_917_1:S>R		YPS138:S>R	AA:785		IFO1804:R>S		N_43:R>S		N_44:R>S		N_45:R>S	AA:790		A12:H>Q		A4:H>Q		DBVPG6304:H>Q		IFO1804:H>Q		N_43:H>Q		N_44:H>Q		N_45:H>Q		YPS138:H>Q	AA:883		IFO1804:H>Y		N_43:H>Y		N_44:H>Y		N_45:H>Y	AA:888		IFO1804:N>D		N_43:N>D		N_44:N>D		N_45:N>D	AA:918		A12:Q>H		A4:Q>H		DBVPG6304:Q>H		UFRJ50791:Q>H		UFRJ50816:Q>H		YPS138:Q>H	AA:921		A12:I>S		A4:I>S		DBVPG6304:I>S		UFRJ50791:I>S		UFRJ50816:I>S		YPS138:I>S	AA:947		A12:R>S		A4:R>S		DBVPG6304:R>S		UFRJ50791:R>S		UFRJ50816:R>S		YPS138:R>S	AA:954		CBS432:R>L	AA:974		A12:N>K		A4:N>K		DBVPG6304:N>K		UFRJ50791:N>K		UFRJ50816:N>K		YPS138:N>K	AA:975		CBS5829:F>Y		DBVPG4650:F>Y		KPN3828:F>Y		KPN3829:F>Y		Q95_3:F>Y		Y6_5:F>Y	AA:994		A12:I>V		A4:I>V		DBVPG6304:I>V		UFRJ50791:I>V		UFRJ50816:I>V		YPS138:I>V	AA:1009		N_45:I>M	AA:1011		A12:L>I		A4:L>I		DBVPG6304:L>I		UFRJ50791:L>F		UFRJ50816:L>F		YPS138:L>I	AA:1064		Q32_3:F>C	AA:1073		A12:K>N		A4:K>N		DBVPG6304:K>N		UWOPS91_917_1:K>N		YPS138:K>N	AA:1107		N_45:Y>C	AA:1127		A12:L>V		UFRJ50816:L>V		YPS138:L>V	AA:1135		N_45:R>S	AA:1148		DBVPG6304:L>I		UFRJ50791:L>I		UFRJ50816:L>I	AA:1166		DBVPG6304:Y>D		UFRJ50791:Y>D		UFRJ50816:Y>D		YPS138:Y>D	AA:1211		N_44:W>R		N_45:W>R	AA:1214		DBVPG4650:S>R		N_17:S>R		N_44:S>R		N_45:S>R		Q32_3:S>R		Q59_1:S>R		Q89_8:S>R		Y6_5:S>R		Z1_1:S>R	AA:1275		UWOPS91_917_1:F>L	AA:1280		UWOPS91_917_1:G>E	AA:1331		Q32_3:T>S	AA:1355		UWOPS91_917_1:L>F	AA:1363		N_44:S>R		N_45:S>R	AA:1364		UWOPS91_917_1:G>R	AA:1372		UWOPS91_917_1:S>R	AA:1401		A4:N>K		DBVPG6304:N>K		UFRJ50791:N>K		YPS138:N>K	AA:1405		N_44:S>R		N_45:S>R	AA:1412		A12:H>N		A4:H>N		DBVPG6304:H>N		UFRJ50791:H>N		YPS138:H>NID:YCR043C	AA:39		N_43:->Y		N_44:->Y		N_45:->Y	AA:76		A4:N>S		DBVPG6304:N>S		N_43:N>S		N_44:N>S		N_45:N>S		UFRJ50791:N>S		UFRJ50816:N>S		UWOPS91_917_1:N>S		YPS138:N>S	AA:102		UWOPS91_917_1:T>N	AA:128		A4:H>Q		DBVPG6304:H>Q		UFRJ50791:H>Q		UFRJ50816:H>QID:YCR045C	AA:16		N_45:K>Q	AA:77		A12:N>K		A4:N>K		DBVPG6304:N>K		N_43:N>K		N_44:N>K		N_45:N>K		UWOPS91_917_1:N>K		YPS138:N>K	AA:124		A12:G>E		A4:G>E		DBVPG6304:G>E		N_44:G>E		N_45:G>E		YPS138:G>E	AA:169		A12:M>I		A4:M>I		DBVPG6304:M>I		UWOPS91_917_1:M>I		YPS138:M>I	AA:211		A12:S>R		DBVPG6304:S>R		YPS138:S>R	AA:227		CBS432:H>Q		N_17:H>Q		Q32_3:H>Q		Q59_1:H>Q	AA:255		A12:P>T	AA:267		A12:E>D		DBVPG6304:E>D		UWOPS91_917_1:E>D	AA:352		UFRJ50791:E>D		UFRJ50816:E>D	AA:397		N_44:C>W		N_45:C>W		UFRJ50791:C>W		UFRJ50816:C>W		UWOPS91_917_1:C>W	AA:415		DBVPG6304:->K		UFRJ50791:->K		UFRJ50816:->K		YPS138:->K	AA:427		UWOPS91_917_1:G>-	AA:430		UWOPS91_917_1:H>N	AA:447		UFRJ50791:V>G		UFRJ50816:V>GID:YCR046C	AA:42		A12:Q>R	AA:61		A12:Q>H	AA:62		UWOPS91_917_1:Q>H	AA:76		A12:K>E		CBS432:K>E		CBS5829:K>E		DBVPG4650:K>E		KPN3828:K>E		N_17:K>E		N_43:K>E		N_45:K>E		Q32_3:K>E		Q89_8:K>E		UWOPS91_917_1:K>E		Y6_5:K>E	AA:78		A12:V>I		N_43:V>I		N_45:V>I		UWOPS91_917_1:V>I	AA:123		Q59_1:F>I		Y6_5:F>I	AA:138		A4:L>F		UWOPS91_917_1:L>F	AA:140		A4:R>S		UWOPS91_917_1:R>S	AA:161		UWOPS91_917_1:P>SID:YCR047C	AA:48		N_17:Q>L	AA:59		A12:H>Q		DBVPG6304:H>Q		UWOPS91_917_1:H>Q		YPS138:H>Q	AA:80		A12:K>N		DBVPG6304:K>N		IFO1804:K>N		N_44:K>N		N_45:K>N		UWOPS91_917_1:K>N		YPS138:K>N	AA:90		A12:W>-		DBVPG6304:W>-		YPS138:W>-	AA:117		A12:W>C		DBVPG6304:W>C		UWOPS91_917_1:W>C		YPS138:W>C	AA:157		A12:D>E		DBVPG6304:D>E		UFRJ50791:D>E		UWOPS91_917_1:D>E		YPS138:D>E	AA:166		N_17:K>-	AA:186		A12:Q>E		DBVPG6304:Q>E		IFO1804:Q>E		N_43:Q>E		N_44:Q>E		N_45:Q>E		UFRJ50791:Q>E		UWOPS91_917_1:Q>E		YPS138:Q>E	AA:190		A12:K>R		DBVPG6304:K>R		UFRJ50791:K>R		UWOPS91_917_1:K>R		YPS138:K>R	AA:193		IFO1804:C>-		N_43:C>-		N_44:C>-		N_45:C>-	AA:199		IFO1804:Y>-		N_43:Y>-		N_44:Y>-		N_45:Y>-	AA:205		A12:H>Q		DBVPG6304:H>Q		UFRJ50791:H>Q		UWOPS91_917_1:H>Q		YPS138:H>Q	AA:232		UWOPS91_917_1:Q>HID:YCR048W	AA:5		N_45:K>E	AA:9		UFRJ50791:Q>H		UWOPS91_917_1:Q>H	AA:48		UWOPS91_917_1:S>L	AA:57		A4:A>T		UFRJ50791:A>T		UWOPS91_917_1:A>T	AA:73		N_17:H>Q	AA:76		UWOPS91_917_1:G>V	AA:94		A4:R>Q		UWOPS91_917_1:R>Q		YPS138:R>Q	AA:158		A4:L>V		YPS138:L>V	AA:172		A4:E>D		UWOPS91_917_1:E>D		YPS138:E>D	AA:235		N_45:L>W	AA:241		CBS432:L>V	AA:242		UWOPS91_917_1:C>S	AA:254		A4:K>R		YPS138:K>R	AA:288		YPS138:N>D	AA:290		CBS432:N>S		N_17:N>S		N_44:N>S		N_45:N>S		Q32_3:N>S		Q59_1:N>S	AA:341		CBS432:S>P		CBS5829:S>P		DBVPG4650:S>P		N_17:S>P		N_44:S>P		N_45:S>P		Q32_3:S>P		Q59_1:S>P		Q62_5:S>P		T21_4:S>P		YPS138:S>P	AA:433		DBVPG6304:Q>E		UFRJ50816:Q>E		UWOPS91_917_1:Q>E	AA:513		UFRJ50791:H>Y		UFRJ50816:H>Y	AA:564		N_45:M>IID:YCR050C	AA:77		UWOPS91_917_1:F>LID:YCR051W	AA:73		N_43:T>S		N_45:T>S	AA:100		N_43:V>A		N_44:V>A		N_45:V>A		UWOPS91_917_1:V>E	AA:134		A4:V>L		DBVPG6304:V>L		UFRJ50816:V>L		YPS138:V>L	AA:135		N_43:D>N		N_44:D>N	AA:146		A12:R>K		A4:R>K		DBVPG6304:R>K		UFRJ50816:R>K		UWOPS91_917_1:R>K		YPS138:R>K	AA:148		T21_4:L>P	AA:158		Q59_1:T>P	AA:165		IFO1804:E>Q		N_43:E>Q		N_44:E>Q		N_45:E>Q	AA:217		DBVPG6304:G>D		N_43:G>D		N_44:G>D		N_45:G>D		YPS138:G>D	AA:218		KPN3829:S>CID:YCR052W	AA:3		A12:V>A		A4:V>A		DBVPG6304:V>A		N_44:V>A		N_45:V>A		UFRJ50791:V>A		YPS138:V>A	AA:24		A12:D>G		A4:D>G		UFRJ50791:D>G		YPS138:D>G	AA:44		UFRJ50791:D>N	AA:86		A4:P>S		YPS138:P>S	AA:119		A4:T>P		N_44:T>P		Q62_5:T>P		UFRJ50791:T>P		UWOPS91_917_1:T>P		YPS138:T>P	AA:165		A4:I>V		UFRJ50791:I>V		UWOPS91_917_1:I>V		YPS138:I>V	AA:180		UWOPS91_917_1:E>G	AA:195		IFO1804:Y>C	AA:200		Q32_3:V>L	AA:201		IFO1804:V>M		N_44:V>M		N_45:V>M	AA:297		A4:S>N		DBVPG6304:S>N		UFRJ50816:S>N	AA:303		A4:E>K		DBVPG6304:E>K		UFRJ50816:E>K		UWOPS91_917_1:E>K	AA:306		IFO1804:T>I		N_43:T>I		N_44:T>I		N_45:T>I	AA:329		A4:N>K		DBVPG6304:N>K		UFRJ50816:N>K		UWOPS91_917_1:N>K	AA:344		A4:K>N		DBVPG6304:K>N		UFRJ50816:K>N	AA:390		A4:G>E		DBVPG6304:G>E		UFRJ50816:G>E		UWOPS91_917_1:G>E	AA:426		A4:I>L		DBVPG6304:I>L		UFRJ50816:I>L		UWOPS91_917_1:I>L	AA:433		IFO1804:T>A		N_43:T>A		N_44:T>A		N_45:T>A	AA:453		N_43:N>IID:YCR053W	AA:72		Q95_3:Q>K	AA:82		A4:D>N		DBVPG6304:D>N		UFRJ50816:D>N	AA:149		A12:D>G		A4:D>G		DBVPG6304:D>G		UFRJ50816:D>G	AA:236		DBVPG4650:V>I	AA:349		UWOPS91_917_1:E>G	AA:355		A12:N>D		A4:N>D		DBVPG6304:N>D		UFRJ50816:N>D		UWOPS91_917_1:N>D	AA:379		Q32_3:D>G		Q62_5:D>G		Q89_8:D>G		T21_4:D>G		Y6_5:D>G	AA:445		A4:V>I		DBVPG6304:V>I		UFRJ50816:V>I		YPS138:V>I	AA:493		UWOPS91_917_1:I>M	AA:494		UFRJ50816:E>GID:YCR057C	AA:23		UWOPS91_917_1:V>I	AA:106		N_43:E>D		N_44:E>D		N_45:E>D		UWOPS91_917_1:E>D	AA:155		N_43:C>-		N_44:C>-		N_45:C>-	AA:207		UFRJ50816:K>N		YPS138:K>N	AA:209		UWOPS91_917_1:I>M	AA:221		DBVPG6304:->Q	AA:263		UWOPS91_917_1:I>M	AA:271		A4:->W		DBVPG6304:->W		UFRJ50816:->W		UWOPS91_917_1:->W		YPS138:->W	AA:293		A4:K>N		DBVPG6304:K>N		N_43:K>N		N_44:K>N		N_45:K>N		UFRJ50816:K>N		YPS138:K>N	AA:301		A4:H>Q		DBVPG6304:H>Q		UFRJ50816:H>Q		UWOPS91_917_1:H>Q		YPS138:H>Q	AA:340		A4:C>-		DBVPG6304:C>-		N_43:C>-		N_44:C>-		N_45:C>-		UFRJ50816:C>-		UWOPS91_917_1:C>-		YPS138:C>-	AA:404		A4:N>K		DBVPG6304:N>K		UWOPS91_917_1:N>K		YPS138:N>K	AA:416		N_44:A>P		N_45:A>P	AA:440		UWOPS91_917_1:T>S	AA:465		UWOPS91_917_1:N>K	AA:490		A12:L>F		DBVPG6304:L>F		UFRJ50791:L>F		UWOPS91_917_1:L>F		YPS138:L>F	AA:525		A12:F>L		DBVPG6304:F>L		UFRJ50791:F>L		YPS138:F>L	AA:594		A12:->Y		A4:->Y		DBVPG6304:->Y		UFRJ50791:->Y		UFRJ50816:->Y		YPS138:->Y	AA:612		A12:D>H		A4:D>H		DBVPG6304:D>H		UFRJ50791:D>H		UFRJ50816:D>H	AA:725		A12:C>G		A4:C>G		UFRJ50816:C>G		UWOPS91_917_1:C>G		YPS138:C>G	AA:747		A12:Y>-		A4:Y>-		DBVPG6304:Y>-		UFRJ50816:Y>-		UWOPS91_917_1:Y>-		YPS138:Y>-	AA:769		A12:R>L	AA:786		UWOPS91_917_1:K>-	AA:790		UWOPS91_917_1:L>V	AA:809		IFO1804:->K		N_43:->K		N_45:->K	AA:811		A12:K>N		A4:K>N		DBVPG6304:K>N		UFRJ50791:K>N		UWOPS91_917_1:K>N		YPS138:K>N	AA:849		A12:N>K		A4:N>K		DBVPG6304:N>K		UFRJ50791:N>K		UWOPS91_917_1:N>K		YPS138:N>K	AA:852		N_17:Q>E		N_45:Q>E	AA:867		A12:A>S		A4:A>S		DBVPG6304:A>S		IFO1804:A>S		N_43:A>S		N_45:A>S		UFRJ50791:A>S		UWOPS91_917_1:A>S		YPS138:A>S	AA:896		A12:A>D		A4:A>D		DBVPG6304:A>D		UFRJ50791:A>D		YPS138:A>D	AA:914		A12:->R		A4:->R		DBVPG6304:->R		IFO1804:->R		N_43:->R		N_45:->R		UFRJ50791:->R		YPS138:->R	AA:920		IFO1804:F>V		N_43:F>V		N_45:F>VID:YCR059C	AA:8		A4:D>H		UFRJ50791:D>H		UFRJ50816:D>H	AA:35		IFO1804:H>Q		N_44:H>Q		UFRJ50816:H>Q	AA:52		IFO1804:G>V		N_44:G>V		N_45:G>V	AA:111		DBVPG6304:H>Y		UFRJ50816:H>Y	AA:124		IFO1804:S>R	AA:187		N_43:A>G		N_44:A>G		N_45:A>G	AA:192		A4:S>R		DBVPG6304:S>R		UFRJ50816:S>R	AA:227		CBS432:D>E		KPN3828:D>E		KPN3829:D>E		N_17:D>E		S36_7:D>E	AA:255		A4:V>M		DBVPG6304:V>M	AA:257		A4:V>D		DBVPG6304:V>D		UWOPS91_917_1:V>DID:YCR060W	AA:2		DBVPG6304:S>N	AA:7		A4:V>E		DBVPG6304:V>E		UFRJ50791:V>E		UWOPS91_917_1:V>E	AA:19		UWOPS91_917_1:L>Q	AA:59		A4:R>Q		DBVPG6304:R>Q		IFO1804:R>Q		N_43:R>Q		N_44:R>Q		N_45:R>Q		UFRJ50791:R>Q		UWOPS91_917_1:R>Q		YPS138:R>Q	AA:89		UFRJ50791:A>T	AA:93		IFO1804:P>S		N_43:P>S		N_44:P>S		N_45:P>SID:YCR061W	AA:19		UWOPS91_917_1:M>K	AA:43		A12:S>G	AA:55		A12:T>A		A4:T>A		UWOPS91_917_1:T>A	AA:77		A12:P>A		A4:P>A		UWOPS91_917_1:P>A	AA:92		CBS5829:T>A	AA:104		A4:A>P		IFO1804:A>P		N_43:A>P		N_45:A>P		UWOPS91_917_1:A>P	AA:113		CBS5829:L>V	AA:117		CBS432:G>A		CBS5829:G>A		IFO1804:G>A		N_43:G>A		N_45:G>A		Q89_8:G>A		T21_4:G>A	AA:151		A4:M>I		UWOPS91_917_1:M>I	AA:155		UWOPS91_917_1:V>M	AA:163		A4:D>E		UWOPS91_917_1:D>E	AA:186		CBS432:F>L	AA:190		CBS432:V>A	AA:199		CBS432:S>L	AA:208		A4:S>T		IFO1804:S>T		N_43:S>T		N_45:S>T		UFRJ50791:S>T		UWOPS91_917_1:S>T	AA:258		A4:V>A		IFO1804:V>A		N_43:V>A		N_45:V>A		UFRJ50791:V>A		UWOPS91_917_1:V>A	AA:278		UWOPS91_917_1:Y>C	AA:328		Y7:Y>F	AA:398		UWOPS91_917_1:L>F	AA:597		UWOPS91_917_1:P>SID:YCR063W	AA:7		A4:R>K		DBVPG6304:R>K		UFRJ50791:R>K		UFRJ50816:R>K	AA:58		IFO1804:Q>K		N_43:Q>K		N_44:Q>K		N_45:Q>KID:YCR065W	AA:12		Y7:Q>K	AA:168		A4:V>I	AA:204		A4:D>G		YPS138:D>G	AA:233		A4:G>D		YPS138:G>D	AA:249		A4:I>V		YPS138:I>V	AA:262		Q59_1:S>Y	AA:290		IFO1804:M>I		N_43:M>I		N_44:M>I		N_45:M>I		YPS138:M>I	AA:296		UWOPS91_917_1:N>S	AA:323		UWOPS91_917_1:D>N	AA:338		YPS138:A>T	AA:349		CBS5829:S>A		N_43:S>A		Q95_3:S>A		UWOPS91_917_1:S>A		YPS138:S>A	AA:420		UFRJ50791:S>T		UWOPS91_917_1:S>T		YPS138:S>T	AA:439		UWOPS91_917_1:D>E	AA:457		UFRJ50791:R>K		UFRJ50816:R>K		YPS138:R>K	AA:483		CBS432:R>GID:YCR066W	AA:54		A12:T>A		A4:T>A		DBVPG6304:T>A		IFO1804:T>A		N_43:T>A		N_44:T>A		Q95_3:T>A		UFRJ50816:T>A		UWOPS91_917_1:T>A		YPS138:T>A	AA:83		A4:I>V	AA:115		A12:P>S		A4:P>S		DBVPG6304:P>S		UFRJ50791:P>S		UFRJ50816:P>S	AA:120		UWOPS91_917_1:L>V	AA:123		A4:V>L		CBS5829:V>L		DBVPG6304:V>L		IFO1804:V>L		N_43:V>L		N_44:V>L		N_45:V>L		Q95_3:V>L		UFRJ50791:V>L		UFRJ50816:V>L		UWOPS91_917_1:V>L		YPS138:V>L	AA:125		UFRJ50791:S>P		UFRJ50816:S>P	AA:181		A12:P>S		DBVPG6304:P>S		UFRJ50791:P>S		UFRJ50816:P>S	AA:218		CBS5829:E>K		N_43:E>K		N_45:E>K		Q95_3:E>K	AA:227		A12:P>L		DBVPG6304:P>L		UFRJ50791:P>L		UFRJ50816:P>L	AA:234		A12:D>E	AA:345		A12:M>L		DBVPG6304:M>L		UFRJ50791:M>L		YPS138:M>L	AA:349		A12:N>S		DBVPG6304:N>S		N_45:N>S		UFRJ50791:N>S		YPS138:N>S	AA:366		A12:R>K		DBVPG6304:R>K		UFRJ50791:R>K		UFRJ50816:R>K		YPS138:R>K	AA:390		A12:M>L		DBVPG6304:M>L		UFRJ50791:M>L		UFRJ50816:M>L		YPS138:M>L	AA:392		A12:Q>R		DBVPG6304:Q>R		N_45:Q>R		UFRJ50791:Q>R		UFRJ50816:Q>R		YPS138:Q>R	AA:406		N_45:I>M	AA:417		A12:P>A		DBVPG6304:P>A		UFRJ50791:P>A		UFRJ50816:P>A		YPS138:P>A	AA:425		N_45:G>D	AA:428		Q95_3:Q>E	AA:429		N_45:V>IID:YCR068W	AA:5		A12:R>H		A4:R>H		DBVPG6304:R>H		UFRJ50816:R>H	AA:7		IFO1804:S>L		N_43:S>L		N_44:S>L		N_45:S>L	AA:15		IFO1804:L>S		N_43:L>S		N_44:L>S		N_45:L>S	AA:17		A12:P>L		A4:P>L		DBVPG6304:P>L		UFRJ50816:P>L	AA:22		IFO1804:L>I		N_43:L>I		N_44:L>I		N_45:L>I	AA:37		A12:S>N		A4:S>N		DBVPG6304:S>N		UFRJ50816:S>N	AA:40		A12:L>S		A4:L>S		DBVPG6304:L>S		IFO1804:L>S		N_43:L>S		N_44:L>S		N_45:L>S		UFRJ50816:L>S	AA:68		A12:V>L		A4:V>L		DBVPG6304:V>L		UFRJ50816:V>L	AA:95		A12:I>T		A4:I>T		DBVPG6304:I>T		N_43:I>T		N_44:I>T		N_45:I>T		UFRJ50816:I>T	AA:161		N_43:H>Y		N_44:H>Y		N_45:H>Y	AA:326		DBVPG6304:M>I		N_44:M>I		N_45:M>I		UFRJ50791:M>I		UWOPS91_917_1:M>IID:YCR069W	AA:4		DBVPG6304:K>R		IFO1804:K>R		N_44:K>R		N_45:K>R	AA:25		DBVPG6304:E>G	AA:36		DBVPG6304:Q>R	AA:42		DBVPG6304:S>N		IFO1804:S>T		N_43:S>T		N_44:S>T		N_45:S>T	AA:56		Q32_3:F>I	AA:61		DBVPG6304:Q>K		IFO1804:Q>E		N_43:Q>E		N_44:Q>E		N_45:Q>E		YPS138:Q>K	AA:114		DBVPG6304:N>D		YPS138:N>D	AA:120		N_45:K>E	AA:130		YPS138:D>E	AA:163		KPN3829:P>L	AA:177		CBS5829:D>N	AA:184		N_43:G>D		N_44:G>D		N_45:G>D	AA:215		DBVPG6304:Q>K		YPS138:Q>K	AA:239		N_43:T>S		N_44:T>S		N_45:T>S	AA:259		DBVPG6304:K>E		YPS138:K>E	AA:265		N_43:D>G		N_44:D>G		N_45:D>G	AA:274		DBVPG6304:A>T		N_43:A>T		N_44:A>T		N_45:A>T		YPS138:A>T	AA:277		DBVPG6304:K>N		YPS138:K>N	AA:301		N_44:F>S		N_45:F>S	AA:302		A12:I>T		DBVPG6304:I>T		YPS138:I>TID:YCR071C	AA:11		A12:C>F		DBVPG6304:C>F		IFO1804:C>F		N_44:C>F		N_45:C>F		UFRJ50816:C>F	AA:30		A12:->C		DBVPG6304:->C		UFRJ50816:->C		UWOPS91_917_1:->C	AA:39		A12:V>F	AA:47		A12:N>K		DBVPG6304:N>K		UFRJ50816:N>K		UWOPS91_917_1:N>K	AA:100		A12:F>V		DBVPG6304:F>V		UFRJ50816:F>V		UWOPS91_917_1:F>V		YPS138:F>V	AA:113		A12:->C		DBVPG6304:->C		UFRJ50816:->C		UWOPS91_917_1:->C		YPS138:->C	AA:114		N_44:I>V		N_45:I>V	AA:132		A12:K>N		DBVPG6304:K>N		N_44:K>N		N_45:K>N		UFRJ50816:K>N		UWOPS91_917_1:K>N		YPS138:K>NID:YCR073W-A	AA:63		N_43:A>T		N_45:A>T	AA:66		UFRJ50791:A>T		UFRJ50816:A>T	AA:80		UFRJ50791:C>S		UFRJ50816:C>S	AA:99		N_45:Q>E		UFRJ50791:Q>E		UFRJ50816:Q>E	AA:165		A12:G>D		UFRJ50791:G>D		UFRJ50816:G>D	AA:292		N_45:V>AID:YCR076C	AA:17		UWOPS91_917_1:K>N	AA:18		UWOPS91_917_1:W>R	AA:21		UWOPS91_917_1:V>I	AA:40		A4:S>T		DBVPG6304:S>T		N_44:S>T		UWOPS91_917_1:S>T	AA:44		A4:K>E		UWOPS91_917_1:K>E	AA:72		UWOPS91_917_1:I>L	AA:74		A4:I>F	AA:84		A4:S>N		UWOPS91_917_1:S>N	AA:94		N_44:R>L		N_45:R>L	AA:121		UWOPS91_917_1:I>M	AA:129		N_45:L>F		UFRJ50791:L>F		UWOPS91_917_1:L>F	AA:133		N_45:R>S	AA:144		UWOPS91_917_1:S>C	AA:214		UWOPS91_917_1:G>C	AA:220		N_44:->W		N_45:->W	AA:232		UFRJ50791:->E		UWOPS91_917_1:->E		YPS138:->EID:YCR077C	AA:37		N_44:F>V	AA:44		YPS138:P>S	AA:50		YPS138:Q>H	AA:97		CBS432:F>I	AA:110		UWOPS91_917_1:I>M	AA:115		IFO1804:W>R		N_44:W>R		N_45:W>R	AA:127		IFO1804:S>R		N_44:S>R		N_45:S>R	AA:143		UWOPS91_917_1:->R	AA:180		UWOPS91_917_1:I>M	AA:187		UWOPS91_917_1:K>-	AA:198		UWOPS91_917_1:R>S	AA:200		N_44:E>G		N_45:E>G	AA:210		UWOPS91_917_1:L>M	AA:241		DBVPG6304:W>C		N_44:V>F		N_45:V>F		UWOPS91_917_1:W>C	AA:284		UWOPS91_917_1:I>N	AA:374		DBVPG6304:D>E	AA:400		DBVPG6304:S>C	AA:463		N_43:R>S		N_44:R>S		N_45:R>S	AA:466		N_43:Q>H		N_44:Q>H		N_45:Q>H	AA:584		DBVPG6304:Y>-		UWOPS91_917_1:Y>-	AA:617		DBVPG6304:H>Q		UWOPS91_917_1:H>Q	AA:655		N_43:G>-		N_44:G>-		UWOPS91_917_1:G>-	AA:775		A12:F>S		A4:F>S		UWOPS91_917_1:F>SID:YCR082W	AA:12		A12:A>V		A4:A>V		IFO1804:A>V		N_45:A>V		UFRJ50816:A>V	AA:54		A12:H>Y		A4:H>Y		UFRJ50816:H>Y	AA:70		UWOPS91_917_1:A>V	AA:106		UWOPS91_917_1:A>V	AA:114		Q62_5:A>EID:YCR083W	AA:10		A12:M>T		A4:M>T		DBVPG6304:M>T		UFRJ50791:M>T		UFRJ50816:M>T		UWOPS91_917_1:M>T	AA:14		IFO1804:P>S		N_43:P>S		N_44:P>S		N_45:P>S	AA:18		A12:V>I		A4:V>I		DBVPG6304:V>I		UFRJ50791:V>I	AA:118		A12:G>A		A4:G>A		DBVPG6304:G>A		IFO1804:G>A		N_43:G>A		N_44:G>A		N_45:G>A		UFRJ50791:G>A		UFRJ50816:G>A		UWOPS91_917_1:G>A		YPS138:G>AID:YCR086W	AA:66		UWOPS91_917_1:K>N	AA:72		CBS432:E>G	AA:74		UWOPS91_917_1:I>V	AA:89		UWOPS91_917_1:S>NID:YCR087C-A	AA:13		A4:H>Q		DBVPG6304:H>Q		UFRJ50791:H>Q		UWOPS91_917_1:H>Q		YPS138:H>Q	AA:24		A4:I>M		DBVPG6304:I>M		UFRJ50791:I>M		UWOPS91_917_1:I>M		YPS138:I>M	AA:57		UWOPS91_917_1:Y>-	AA:74		A4:L>M	AA:85		DBVPG6304:F>V		N_43:F>V		N_45:F>V		UFRJ50791:F>V		UWOPS91_917_1:F>V		YPS138:F>V	AA:125		DBVPG6304:L>P		YPS138:L>P	AA:133		UWOPS91_917_1:C>SID:YCR088W	AA:62		UWOPS91_917_1:T>A	AA:93		A12:M>L		A4:M>L		N_44:M>L		UFRJ50816:M>L		UWOPS91_917_1:M>L		YPS138:M>L	AA:155		A12:A>T		A4:A>T		UFRJ50816:A>T		UWOPS91_917_1:A>T		YPS138:A>T	AA:174		A4:S>F	AA:192		UWOPS91_917_1:S>P	AA:253		A12:T>A		A4:T>A		DBVPG6304:T>A		UFRJ50791:T>A		UFRJ50816:T>A		UWOPS91_917_1:T>A	AA:293		A12:M>K		A4:M>K		DBVPG6304:M>K		UFRJ50791:M>K		UFRJ50816:M>K		UWOPS91_917_1:M>K	AA:327		A12:N>S		A4:N>S		UWOPS91_917_1:N>S	AA:338		A12:K>E		A4:K>E		DBVPG6304:K>E		IFO1804:K>E		N_44:K>E		N_45:K>E		UFRJ50791:K>E		UFRJ50816:K>E		UWOPS91_917_1:K>E	AA:344		IFO1804:N>K		N_44:N>K		N_45:N>K	AA:346		UWOPS91_917_1:S>F	AA:356		A12:K>R		A4:K>R		DBVPG6304:K>R		UFRJ50791:K>R		UWOPS91_917_1:K>R	AA:400		IFO1804:V>I	AA:405		A4:I>T		CBS432:I>T		DBVPG6304:I>T		IFO1804:I>T		N_44:I>T		Q59_1:I>T		Q89_8:I>T		T21_4:I>T		UWOPS91_917_1:I>T	AA:426		IFO1804:A>E	AA:447		CBS432:P>L		KPN3829:P>L	AA:450		DBVPG6304:P>S		UFRJ50816:P>S	AA:457		N_43:A>D		N_45:A>D	AA:475		N_43:S>R		N_45:S>R	AA:487		N_43:Q>K		N_45:Q>K	AA:490		UFRJ50791:A>E		UFRJ50816:A>E	AA:492		DBVPG6304:E>G		UFRJ50791:E>G		UFRJ50816:E>G		UWOPS91_917_1:E>G	AA:505		DBVPG6304:P>A		N_43:P>A		N_44:P>S		N_45:P>A		UFRJ50791:P>A		UFRJ50816:P>A		UWOPS91_917_1:P>A	AA:553		CBS432:V>I		KPN3828:V>IID:YCR090C	AA:31		A12:->Y		UWOPS91_917_1:->Y		YPS138:->Y	AA:93		A12:V>F		YPS138:V>F	AA:103		A12:Q>-		YPS138:Q>-	AA:137		UWOPS91_917_1:N>Y	AA:139		A12:R>S		N_43:R>S		UWOPS91_917_1:R>S		YPS138:R>S	AA:144		UWOPS91_917_1:P>H	AA:165		UWOPS91_917_1:->CID:YCR091W	AA:36		CBS432:G>A		KPN3828:G>A		N_17:G>A		Q32_3:G>A		Q59_1:G>A		Q62_5:G>A		Q95_3:G>A		Y6_5:G>A		Y7:G>A	AA:55		N_17:S>A	AA:73		A12:H>Q		A4:H>Q		UFRJ50816:H>Q		UWOPS91_917_1:H>Q		YPS138:H>Q	AA:89		UFRJ50816:W>L	AA:97		A4:L>F		UFRJ50816:L>F		UWOPS91_917_1:L>F		YPS138:L>F	AA:112		A4:I>T		CBS432:I>T		DBVPG4650:I>T		IFO1804:I>T		N_17:I>T		N_43:I>T		N_44:I>T		N_45:I>T		Q32_3:I>T		Q59_1:I>T		Q95_3:I>T		UFRJ50816:I>T		YPS138:I>T	AA:115		IFO1804:S>P		N_43:S>P		N_44:S>P		N_45:S>P	AA:143		UWOPS91_917_1:R>T	AA:146		IFO1804:L>P		N_43:L>P		N_44:L>P		N_45:L>P		UFRJ50816:L>P		UWOPS91_917_1:L>P		YPS138:L>P	AA:153		UWOPS91_917_1:L>F	AA:161		UWOPS91_917_1:S>C	AA:173		A4:N>D		UFRJ50791:N>D		UFRJ50816:N>D		YPS138:N>D	AA:226		IFO1804:E>D		N_43:E>D		N_44:E>D		N_45:E>D	AA:231		A12:V>I		A4:V>I		DBVPG6304:V>I		UFRJ50791:V>I		UFRJ50816:V>I		YPS138:V>I	AA:249		IFO1804:G>E		N_43:G>E		N_44:G>E		N_45:G>E	AA:254		A12:K>R		DBVPG6304:K>R		UFRJ50791:K>R		UFRJ50816:K>R		YPS138:K>R	AA:261		A12:K>N		DBVPG6304:K>N		IFO1804:K>N		N_43:K>N		N_44:K>N		N_45:K>N		UFRJ50791:K>N		UFRJ50816:K>N		YPS138:K>N	AA:286		A12:F>L		DBVPG6304:F>L		UFRJ50791:F>L		UFRJ50816:F>L		YPS138:F>L	AA:296		A12:G>E		DBVPG6304:G>E		UFRJ50791:G>E		UFRJ50816:G>E		UWOPS91_917_1:G>E		YPS138:G>E	AA:324		A12:Q>R		DBVPG6304:Q>R		UFRJ50791:Q>R		UFRJ50816:Q>R		UWOPS91_917_1:Q>R		YPS138:Q>R	AA:426		DBVPG6304:D>E		UFRJ50791:D>E		UWOPS91_917_1:D>E		YPS138:D>E	AA:465		DBVPG6304:I>V		IFO1804:I>V		N_44:I>V		UFRJ50791:I>V		UWOPS91_917_1:I>V	AA:529		CBS5829:T>A		Z1_1:T>A	AA:626		A12:N>K		UFRJ50791:N>K	AA:643		A12:N>T	AA:677		CBS432:L>I	AA:683		N_44:S>N		N_45:S>N	AA:700		A12:S>A		UWOPS91_917_1:S>A	AA:707		UWOPS91_917_1:H>YID:YCR095C	AA:36		A12:W>-		A4:W>-		DBVPG6304:W>-		UFRJ50791:W>-		YPS138:W>-	AA:81		DBVPG6304:S>T	AA:92		A12:I>V		A4:I>V		DBVPG6304:I>V		N_43:I>V		N_44:I>V		N_45:I>V		UFRJ50816:I>V		YPS138:I>V	AA:106		A12:P>T		A4:P>T		DBVPG6304:P>T		UFRJ50816:P>T		YPS138:P>T	AA:142		A12:S>T		A4:S>T		DBVPG6304:I>M		UFRJ50816:S>T		YPS138:I>M	AA:157		N_43:L>V		N_44:L>V		N_45:L>V	AA:159		A12:Y>-		A4:Y>-		DBVPG6304:Y>-		UFRJ50816:Y>-		YPS138:Y>-	AA:169		N_43:F>L		N_44:F>L		N_45:F>L	AA:219		A12:C>F		A4:C>F		UFRJ50816:C>F	AA:270		UFRJ50816:->W	AA:319		A12:W>-		A4:W>-		UFRJ50816:W>-		YPS138:W>-	AA:331		A12:I>F		A4:I>F		DBVPG4650:I>F		N_44:I>F		N_45:I>F		Q32_3:I>F		Q59_1:I>F		Q95_3:I>F		T21_4:I>F		UFRJ50816:I>F		Y7:I>F		YPS138:I>F	AA:334		Q32_3:F>L		Q59_1:F>L		Q95_3:F>L		Y7:F>LID:YDL001W	AA:6		A12:N>S		DBVPG6304:N>S		UFRJ50791:N>S		UFRJ50816:N>S		YPS138:N>S	AA:19		N_43:L>P		N_44:L>P		N_45:L>PID:YDL002C	AA:21		N_43:->W	AA:159		DBVPG6304:F>L	AA:163		DBVPG6304:Q>H	AA:180		A4:L>W		DBVPG6304:L>W	AA:204		CBS432:H>R		CBS5829:H>RID:YDL003W	AA:6		UWOPS91_917_1:P>T	AA:43		N_17:I>M	AA:54		A12:S>T		DBVPG6304:S>T		YPS138:S>T	AA:62		A12:D>E		DBVPG6304:D>E		UFRJ50816:D>E		UWOPS91_917_1:D>E	AA:105		A12:R>K		DBVPG6304:R>K		N_43:R>K		N_44:R>K		N_45:R>K		UFRJ50816:R>K		UWOPS91_917_1:R>K		YPS138:R>K	AA:144		UFRJ50791:E>Q		UFRJ50816:E>Q	AA:147		N_44:D>N		N_45:D>N	AA:207		UWOPS91_917_1:T>S	AA:254		A12:H>Q		DBVPG6304:H>Q		UFRJ50791:H>Q		UFRJ50816:H>Q		UWOPS91_917_1:H>Q		YPS138:H>Q	AA:257		A12:D>N		DBVPG6304:D>N		UFRJ50791:D>N		UFRJ50816:D>N		YPS138:D>N	AA:261		A12:N>D		DBVPG6304:N>D		UFRJ50791:N>D		UFRJ50816:N>D		YPS138:N>D	AA:299		A12:A>T		DBVPG6304:A>T		UFRJ50791:A>T		UFRJ50816:A>T		UWOPS91_917_1:A>T		YPS138:A>T	AA:311		A12:K>N		DBVPG6304:K>N		IFO1804:K>N		N_43:K>N		N_44:K>N		N_45:K>N		UFRJ50791:K>N		UFRJ50816:K>N		UWOPS91_917_1:K>N		YPS138:K>N	AA:322		DBVPG6304:T>A	AA:333		A12:K>N		DBVPG6304:K>N		UFRJ50791:K>N		UFRJ50816:K>N		YPS138:K>N	AA:349		YPS138:N>S	AA:369		A12:A>T		DBVPG6304:A>T		IFO1804:A>T		N_43:A>T		N_44:A>T		N_45:A>T		UFRJ50791:A>T		UFRJ50816:A>T		UWOPS91_917_1:A>T		YPS138:A>T	AA:371		UWOPS91_917_1:S>N	AA:379		IFO1804:I>V		N_43:I>V		N_44:I>V		N_45:I>V	AA:411		A12:L>F		DBVPG6304:L>F		IFO1804:L>F		N_43:L>F		N_44:L>F		N_45:L>F		UFRJ50791:L>F		UWOPS91_917_1:L>F		YPS138:L>F	AA:416		IFO1804:D>E		N_43:D>E		N_44:D>E		N_45:D>E	AA:421		A12:A>V		DBVPG6304:A>V		YPS138:A>V	AA:432		T21_4:T>I	AA:447		A12:E>D		A4:E>D		DBVPG6304:E>D		UFRJ50816:E>D		YPS138:E>D	AA:453		A12:D>G		A4:D>G		DBVPG6304:D>G		UFRJ50816:D>G		UWOPS91_917_1:D>G		YPS138:D>G	AA:481		N_17:K>N	AA:498		A4:N>K		DBVPG6304:N>K		UFRJ50816:N>K		YPS138:N>K	AA:505		UWOPS91_917_1:V>I	AA:512		A4:T>R		UFRJ50816:T>R		UWOPS91_917_1:T>R		YPS138:T>R	AA:550		A4:K>E		DBVPG6304:K>E		UFRJ50816:K>E		UWOPS91_917_1:K>E		YPS138:K>E	AA:552		A4:D>N		DBVPG6304:D>N		UFRJ50816:D>N		UWOPS91_917_1:D>N		YPS138:D>NID:YDL004W	AA:85		T21_4:N>K	AA:122		DBVPG6304:L>V		IFO1804:L>V		N_43:L>V		N_45:L>V		UFRJ50816:L>V	AA:148		IFO1804:V>L		N_43:V>L		N_45:V>LID:YDL005C	AA:3		A4:I>V		DBVPG6304:I>V		N_43:I>V		N_44:I>V		N_45:I>V		UFRJ50791:I>V		UFRJ50816:I>V		UWOPS91_917_1:I>V		YPS138:I>V	AA:9		CBS432:->Y		CBS5829:->Y		DBVPG4650:->Y		KPN3829:->Y		N_17:->Y	AA:19		DBVPG6304:V>D	AA:27		A4:I>V		DBVPG6304:I>V		UFRJ50791:I>V		UFRJ50816:I>V		UWOPS91_917_1:I>V		YPS138:I>V	AA:42		A4:I>T		DBVPG6304:I>T		UFRJ50791:I>T		UFRJ50816:I>T		YPS138:I>T	AA:48		A4:N>D		DBVPG6304:N>D		UFRJ50791:N>D		UFRJ50816:N>D		YPS138:N>D	AA:57		N_44:Y>-		N_45:Y>-	AA:68		A4:I>L		DBVPG6304:I>L		UFRJ50791:I>L		UFRJ50816:I>L		YPS138:I>L	AA:79		A4:I>M		DBVPG6304:I>M		N_44:I>M		N_45:I>M		UFRJ50791:I>M		UFRJ50816:I>M	AA:99		UFRJ50791:V>I		UFRJ50816:V>I	AA:104		N_44:A>P		N_45:A>P		UWOPS91_917_1:A>P	AA:129		N_44:I>M		N_45:I>M	AA:134		UWOPS91_917_1:G>A	AA:161		UFRJ50791:F>L		UFRJ50816:F>L	AA:168		A4:T>P		DBVPG6304:T>P		UFRJ50791:T>P		UFRJ50816:T>P		UWOPS91_917_1:T>P		YPS138:T>P	AA:174		N_44:K>N		N_45:K>N	AA:181		UWOPS91_917_1:->C	AA:189		UWOPS91_917_1:I>L	AA:202		A12:L>R		A4:L>R		DBVPG6304:L>R		UFRJ50816:L>R		YPS138:L>R	AA:207		A12:H>Q		A4:H>Q		UFRJ50816:H>Q		YPS138:H>Q		Z1_1:H>L	AA:221		N_44:I>T	AA:235		A12:R>S		A4:R>S		UFRJ50816:R>S		UWOPS91_917_1:R>S		YPS138:R>S	AA:239		A12:L>F		A4:L>F		UFRJ50816:L>F		UWOPS91_917_1:L>F		YPS138:L>F	AA:250		UWOPS91_917_1:C>-	AA:259		A12:V>L		YPS138:V>L	AA:270		UWOPS91_917_1:L>F	AA:273		A12:L>Q		N_44:L>Q		N_45:L>Q		YPS138:L>Q	AA:288		A12:F>I		UWOPS91_917_1:F>I		YPS138:F>I	AA:291		A12:F>S		UWOPS91_917_1:F>S		YPS138:F>S	AA:300		A12:F>V		UWOPS91_917_1:F>V		YPS138:F>V	AA:320		A12:K>N		YPS138:K>N	AA:330		A12:Q>K		YPS138:Q>K	AA:354		A12:L>-		DBVPG6304:L>-		N_44:L>-		N_45:L>-		YPS138:L>-	AA:406		N_44:I>V		N_45:I>VID:YDL006W	AA:4		UWOPS91_917_1:H>Y	AA:11		UWOPS91_917_1:P>S	AA:13		Q32_3:T>K	AA:99		UWOPS91_917_1:T>I	AA:131		A12:H>D		DBVPG6304:H>D		N_43:H>D		N_44:H>D		N_45:H>D		UFRJ50791:H>D		UFRJ50816:H>D		UWOPS91_917_1:H>D		YPS138:H>D	AA:194		Q59_1:M>TID:YDL007W	AA:34		YPS138:K>N	AA:329		IFO1804:N>ID:YDL008W	AA:26		DBVPG6304:A>P		UFRJ50791:A>P		UFRJ50816:A>P		YPS138:A>P	AA:31		N_43:L>P		N_45:L>PID:YDL010W	AA:3		A4:P>S		DBVPG6304:P>S		UFRJ50791:P>S		YPS138:P>S	AA:7		A4:R>K		DBVPG6304:R>K		UFRJ50791:R>K		YPS138:R>K	AA:43		A4:I>V		DBVPG6304:I>V		UFRJ50791:I>V		YPS138:I>V	AA:46		A4:S>T		DBVPG6304:S>T		N_45:S>T		UFRJ50791:S>T		YPS138:S>T	AA:114		A4:I>N		DBVPG6304:I>N		UFRJ50791:I>N		YPS138:I>N	AA:153		N_43:I>V		N_44:I>V		N_45:I>V	AA:218		DBVPG6304:G>S		UFRJ50816:G>SID:YDL013W	AA:23		A12:T>A		A4:T>A		DBVPG6304:T>A	AA:78		A12:T>M		A4:T>M		DBVPG6304:T>M		UFRJ50791:T>M		UWOPS91_917_1:T>M	AA:82		A12:E>G		A4:E>G	AA:126		A12:L>M		A4:L>M		DBVPG6304:L>M		N_43:L>M		N_44:L>M		N_45:L>M		UFRJ50791:L>M		UWOPS91_917_1:L>M		YPS138:L>M	AA:142		UWOPS91_917_1:L>I	AA:163		S36_7:V>I		T21_4:V>I	AA:217		DBVPG4650:S>P	AA:234		UWOPS91_917_1:A>S	AA:262		CBS432:M>T		IFO1804:M>I		N_17:M>I		N_43:M>I		N_44:M>I		N_45:M>I		Q62_5:M>T	AA:290		UWOPS91_917_1:S>G	AA:322		UWOPS91_917_1:A>V	AA:330		UWOPS91_917_1:Q>R	AA:356		IFO1804:E>V		N_43:E>V		N_44:E>V		N_45:E>V	AA:385		UWOPS91_917_1:D>N	AA:396		DBVPG6304:V>AID:YDL014W	AA:43		UWOPS91_917_1:A>S	AA:65		UFRJ50791:A>G		UFRJ50816:A>G	AA:79		UFRJ50791:A>T		UFRJ50816:A>T	AA:196		UWOPS91_917_1:V>IID:YDL015C	AA:2		A12:K>-		A4:K>-		DBVPG6304:K>-		UFRJ50816:K>-		YPS138:K>-	AA:8		A12:E>D		A4:E>D		DBVPG6304:E>D		UFRJ50816:E>D		UWOPS91_917_1:E>D		YPS138:E>D	AA:11		UWOPS91_917_1:S>T	AA:13		UWOPS91_917_1:M>K	AA:34		A12:->E		DBVPG6304:->E		IFO1804:->E		N_43:->E		N_45:->E		UFRJ50816:->E		UWOPS91_917_1:->E	AA:42		A12:K>N		DBVPG6304:K>N		UFRJ50816:K>N		UWOPS91_917_1:K>N	AA:62		A12:K>N		DBVPG6304:K>N		UFRJ50816:K>N	AA:71		UWOPS91_917_1:D>A	AA:103		A12:K>N		DBVPG6304:K>N		UFRJ50816:K>N	AA:128		A12:E>G		IFO1804:E>G		N_43:E>G		N_44:E>G		N_45:E>G		UFRJ50816:E>G		UWOPS91_917_1:E>G	AA:136		A12:->W		IFO1804:->W		N_43:->W		N_44:->W		N_45:->W		UFRJ50816:->W		UWOPS91_917_1:->W	AA:138		A12:E>D		IFO1804:E>D		N_43:E>D		N_44:E>D		N_45:E>D		UFRJ50816:E>D		UWOPS91_917_1:E>D	AA:155		A12:W>-		UFRJ50816:W>-	AA:161		IFO1804:E>D		N_43:E>D		N_44:E>D		N_45:E>D	AA:257		N_43:N>D	AA:265		UWOPS91_917_1:G>A	AA:291		A12:F>L		A4:F>L		DBVPG6304:F>L		UFRJ50816:F>L		UWOPS91_917_1:F>L		YPS138:F>LID:YDL017W	AA:15		Y6_5:I>V	AA:54		A12:T>S		A4:T>S		UFRJ50816:T>S		UWOPS91_917_1:T>S	AA:56		UWOPS91_917_1:K>R	AA:120		DBVPG4650:L>I	AA:194		UWOPS91_917_1:S>I	AA:363		A12:N>D		DBVPG6304:N>D		UFRJ50816:N>D	AA:407		A12:L>M		DBVPG6304:L>M		UFRJ50791:L>M		UFRJ50816:L>M		UWOPS91_917_1:L>M	AA:478		A12:L>I		DBVPG6304:L>I		UFRJ50791:L>I		UFRJ50816:L>I		UWOPS91_917_1:L>I		YPS138:L>I	AA:489		DBVPG6304:V>I		YPS138:V>IID:YDL018C	AA:2		A12:H>P		A4:H>P		UFRJ50791:H>P		UFRJ50816:H>P		UWOPS91_917_1:H>P		YPS138:H>P	AA:3		N_44:I>V		N_45:I>V	AA:12		Q59_1:K>E	AA:15		A12:Y>-		A4:Y>-		UFRJ50791:Y>-		UFRJ50816:Y>-		YPS138:Y>-	AA:29		A12:T>A		A4:T>A		DBVPG6304:T>A		UFRJ50791:T>A		UFRJ50816:T>A		YPS138:T>A	AA:54		N_43:I>M		N_44:I>M		N_45:I>M	AA:67		A12:F>L		A4:F>L		DBVPG6304:F>L		N_43:F>L		N_44:F>L		N_45:F>L		UFRJ50791:F>L		UFRJ50816:F>L		YPS138:F>L	AA:114		N_43:I>F		N_44:I>F		N_45:I>F	AA:126		N_43:A>T		N_44:A>T		N_45:A>T	AA:140		N_43:F>L		N_44:F>L		N_45:F>L	AA:174		UFRJ50791:H>DID:YDL020C	AA:8		A12:I>M		A4:I>M		DBVPG6304:I>M		UFRJ50816:I>M	AA:21		UWOPS91_917_1:->Y	AA:32		UWOPS91_917_1:P>T	AA:38		IFO1804:S>C		N_45:S>C	AA:73		DBVPG6304:->L	AA:106		A12:R>S		A4:R>S		DBVPG6304:R>S		UFRJ50816:R>S		YPS138:R>S	AA:119		N_45:R>L	AA:160		A12:F>L		A4:F>L		UFRJ50816:F>L		YPS138:F>L	AA:191		A12:->C		A4:->C		UFRJ50791:->C		UFRJ50816:->C		YPS138:->C	AA:227		A12:K>N		A4:K>N		UFRJ50791:K>N		UFRJ50816:K>N		YPS138:K>N	AA:236		A12:I>S		A4:I>S		UFRJ50791:I>S		UFRJ50816:I>S		UWOPS91_917_1:I>S		YPS138:I>S	AA:240		A4:L>R		UFRJ50791:L>R		UFRJ50816:L>R		UWOPS91_917_1:L>R		YPS138:L>R	AA:310		A12:F>L		A4:F>L		UFRJ50791:F>L		UFRJ50816:F>L		UWOPS91_917_1:F>L		YPS138:F>L	AA:316		A4:I>V		CBS432:I>V		IFO1804:I>V		N_17:I>V		N_45:I>V		UFRJ50791:I>V		UFRJ50816:I>V		UWOPS91_917_1:I>V		YPS138:I>V	AA:352		UWOPS91_917_1:R>L	AA:362		A4:F>L		UFRJ50791:F>L		UFRJ50816:F>L		YPS138:F>L	AA:363		CBS432:L>F	AA:385		A4:E>D		DBVPG6304:E>D		UFRJ50816:E>D		YPS138:E>D	AA:388		YPS138:P>L	AA:392		A4:I>M		DBVPG6304:I>M		UFRJ50816:I>M		YPS138:I>M	AA:424		A4:I>M		DBVPG6304:I>M		UFRJ50816:I>M		UWOPS91_917_1:I>M		YPS138:I>M	AA:430		N_43:V>G		N_45:V>G	AA:455		A4:K>N		DBVPG6304:K>N		UFRJ50791:K>N		UFRJ50816:K>N		UWOPS91_917_1:K>N		YPS138:K>N	AA:490		T21_4:Q>-	AA:499		DBVPG4650:W>C	AA:508		IFO1804:E>D		N_43:E>D		N_45:E>D	AA:516		A4:M>I		DBVPG6304:M>I		IFO1804:M>I		N_43:M>I		N_45:M>I		UFRJ50791:M>I		UFRJ50816:M>I		UWOPS91_917_1:M>IID:YDL021W	AA:5		A12:R>K	AA:56		A12:K>N		DBVPG6304:K>N		UFRJ50816:K>N		YPS138:K>N	AA:61		A12:K>R		UFRJ50816:K>R		UWOPS91_917_1:K>R	AA:88		UFRJ50816:K>E		UWOPS91_917_1:K>E		YPS138:K>E	AA:108		UWOPS91_917_1:S>C	AA:142		DBVPG6304:E>K		N_43:E>K		N_44:E>K		N_45:E>K		UFRJ50816:E>K		UWOPS91_917_1:E>K		YPS138:E>K	AA:225		DBVPG6304:I>V		UWOPS91_917_1:I>V		YPS138:I>V	AA:227		UWOPS91_917_1:K>EID:YDL027C	AA:18		A4:R>-		DBVPG6304:R>-		UFRJ50791:R>-		YPS138:R>-	AA:26		IFO1804:V>I		N_44:V>I		N_45:V>I	AA:59		A4:R>S		DBVPG6304:R>S		UFRJ50791:R>S		YPS138:R>S	AA:62		A4:T>S		DBVPG6304:T>S		UFRJ50791:T>S		YPS138:T>S	AA:64		A4:L>S		DBVPG6304:L>S		UFRJ50791:L>S		YPS138:L>S	AA:68		Q32_3:E>Q		Q95_3:E>Q	AA:87		A4:V>A		DBVPG6304:V>A		N_44:V>A		N_45:V>A		UFRJ50791:V>A		YPS138:V>A	AA:90		DBVPG6304:N>T	AA:122		A4:D>E		DBVPG6304:D>E		N_44:D>E		N_45:D>E		UFRJ50791:D>E		YPS138:D>E	AA:165		A4:H>L		N_43:H>L		N_44:H>L		N_45:H>L		YPS138:H>L	AA:185		A4:S>T		DBVPG6304:S>T		UFRJ50791:S>T		UWOPS91_917_1:S>T	AA:187		A4:F>Y		DBVPG6304:F>Y		UFRJ50791:F>Y		UWOPS91_917_1:F>Y	AA:191		DBVPG4650:H>D	AA:194		N_43:N>K		N_44:N>K		N_45:N>K	AA:212		A4:L>I		CBS432:L>I		CBS5829:L>I		DBVPG4650:L>I		DBVPG6304:L>I		UFRJ50816:L>I		UWOPS91_917_1:L>I		Y6_5:L>I	AA:220		UWOPS91_917_1:->Y	AA:229		A4:C>G		DBVPG6304:C>G		UFRJ50816:C>G	AA:248		A4:F>L		DBVPG6304:F>L		UFRJ50816:F>L	AA:295		A4:W>C	AA:301		N_43:F>Y		N_44:F>Y		N_45:F>Y	AA:311		A4:->W		DBVPG6304:->W		UFRJ50816:->W		UWOPS91_917_1:->W	AA:336		UWOPS91_917_1:C>S	AA:343		Y6_5:->Y	AA:345		A4:L>V		DBVPG6304:L>V		IFO1804:L>F		N_43:L>F		N_44:L>F		N_45:L>F		UFRJ50791:L>V		UFRJ50816:L>V		UWOPS91_917_1:L>V	AA:369		UWOPS91_917_1:F>L	AA:381		A4:W>-		DBVPG6304:W>-		UFRJ50791:W>-		UFRJ50816:W>-ID:YDL028C	AA:15		N_45:H>P	AA:24		UFRJ50816:L>F		YPS138:L>F	AA:32		A4:N>K		UFRJ50816:N>K		YPS138:N>K	AA:52		Z1_1:S>T	AA:55		A4:Q>H		UFRJ50816:Q>H		YPS138:Q>H	AA:153		A4:S>N		UFRJ50816:S>N		YPS138:S>N	AA:168		A4:S>R		UFRJ50816:S>R		YPS138:S>R	AA:178		A12:R>S		A4:R>S		UFRJ50816:R>S		YPS138:R>S	AA:193		A12:F>L		A4:F>L		UFRJ50816:F>L	AA:199		A12:C>R		A4:C>R		UFRJ50816:C>R	AA:223		Z1_1:I>M	AA:234		A12:A>T		A4:A>T		UFRJ50816:A>T	AA:264		A12:Q>H		A4:Q>H		UFRJ50816:Q>H	AA:286		IFO1804:N>I		N_43:N>I		N_45:N>I	AA:344		A12:F>C		A4:F>C		UFRJ50791:F>C	AA:389		A12:I>M		A4:I>M		DBVPG6304:I>M		UFRJ50791:I>M	AA:398		A12:M>L		A4:M>L		CBS432:M>L		DBVPG6304:M>L		IFO1804:M>L		N_43:M>L		N_45:M>L		UFRJ50791:M>L		UWOPS91_917_1:M>L		Y6_5:M>L		Z1_1:M>L	AA:427		A12:M>I		A4:M>I		DBVPG6304:M>I		UFRJ50791:M>I		UWOPS91_917_1:M>I		YPS138:M>I	AA:470		UFRJ50791:I>F	AA:490		A12:R>-		A4:R>-		YPS138:R>-	AA:617		UWOPS91_917_1:S>R	AA:623		UFRJ50816:I>M	AA:633		A4:Y>-		UFRJ50816:Y>-		YPS138:Y>-	AA:639		A4:I>M		UFRJ50816:I>M		YPS138:I>M	AA:648		A4:Y>N		N_43:Y>N		N_45:Y>N		UFRJ50816:Y>N	AA:688		A4:W>C		UFRJ50791:W>C		UFRJ50816:W>C	AA:706		A4:L>I		UFRJ50791:L>I		UFRJ50816:L>I		UWOPS91_917_1:L>I	AA:749		UWOPS91_917_1:I>SID:YDL030W	AA:7		A12:R>K		A4:R>K		DBVPG6304:R>K		UFRJ50791:R>K		UFRJ50816:R>K	AA:54		A12:I>M		A4:I>M		DBVPG6304:I>M		KPN3828:I>L		UFRJ50791:I>M		UFRJ50816:I>M		UWOPS91_917_1:I>M	AA:95		DBVPG4650:R>K		Y6_5:R>K	AA:117		A12:Q>K		DBVPG6304:Q>K		UFRJ50816:Q>K	AA:119		DBVPG6304:F>L	AA:123		N_43:L>F		N_45:L>F	AA:129		N_43:N>D		N_45:N>D	AA:142		Q32_3:A>V		Q95_3:A>V	AA:205		A4:D>N		DBVPG6304:D>N		UFRJ50816:D>N		YPS138:D>N	AA:213		A4:D>E		DBVPG6304:D>E		UFRJ50816:D>E		YPS138:D>E	AA:214		A12:K>E	AA:221		A4:P>S		DBVPG6304:P>S		UFRJ50816:P>S		YPS138:P>S	AA:227		UFRJ50816:D>N	AA:251		A12:F>L	AA:253		A4:R>G		DBVPG6304:R>G		UFRJ50816:R>G		YPS138:R>G	AA:260		IFO1804:T>I		N_45:T>I	AA:264		CBS432:F>L	AA:277		A12:T>A	AA:334		A4:R>H		DBVPG6304:R>H		UFRJ50816:R>H		YPS138:R>H	AA:368		A4:L>P		DBVPG6304:L>P		UFRJ50816:L>P		YPS138:L>P	AA:377		A12:G>V	AA:384		A12:D>G	AA:386		A12:P>L		IFO1804:P>S		N_45:P>S	AA:420		A4:E>K		UFRJ50816:E>K		YPS138:E>K	AA:422		A4:R>C		UFRJ50816:R>C		YPS138:R>C	AA:430		UFRJ50816:I>V		YPS138:I>V	AA:472		A12:K>E	AA:479		DBVPG6304:G>E		UFRJ50816:G>EID:YDL033C	AA:17		UWOPS91_917_1:M>I	AA:39		UWOPS91_917_1:N>K	AA:49		A12:H>Q		A4:H>Q		DBVPG6304:H>Q		UFRJ50816:H>Q		UWOPS91_917_1:H>Q	AA:112		A12:S>R		A4:S>R		DBVPG6304:S>R		UFRJ50816:S>R		UWOPS91_917_1:S>R	AA:137		UWOPS91_917_1:Y>-	AA:161		N_43:R>S		N_45:R>S	AA:164		A12:F>L		A4:F>L		DBVPG6304:F>L		UFRJ50816:P>A		UWOPS91_917_1:F>L		YPS138:F>L	AA:166		A12:M>I		A4:M>I		DBVPG6304:M>I		UFRJ50816:M>I		UWOPS91_917_1:M>I	AA:181		A4:N>K		UWOPS91_917_1:N>K	AA:205		N_45:D>E	AA:255		Y7:L>W	AA:262		UWOPS91_917_1:A>G	AA:267		N_45:E>V	AA:330		Y6_5:I>M	AA:348		A12:S>F		A4:S>F		DBVPG6304:S>F		UFRJ50791:S>F		UFRJ50816:S>F		UWOPS91_917_1:S>F		YPS138:S>F	AA:371		A12:L>-		A4:L>-		DBVPG6304:L>-		UFRJ50791:L>-		UFRJ50816:L>-		YPS138:L>-	AA:384		A12:I>T		A4:I>T		DBVPG6304:I>T		UFRJ50791:I>T		UFRJ50816:I>T		YPS138:I>T	AA:392		A12:C>W		A4:C>W		DBVPG6304:C>W		UFRJ50791:C>W		UFRJ50816:C>W		UWOPS91_917_1:C>W		YPS138:C>W	AA:410		UWOPS91_917_1:K>N	AA:411		A12:L>F		A4:L>F		DBVPG6304:L>F		UFRJ50791:L>F		UFRJ50816:L>F		YPS138:L>F	AA:417		CBS432:W>G	AA:422		N_43:S>I		N_44:S>I		N_45:S>IID:YDL036C	AA:2		UWOPS91_917_1:S>F	AA:4		A12:->W		A4:->W		UFRJ50791:->W		UFRJ50816:->W		UWOPS91_917_1:->W	AA:7		A12:F>L		A4:F>L		UFRJ50791:F>L		UFRJ50816:F>L		UWOPS91_917_1:F>L	AA:15		UWOPS91_917_1:P>R	AA:32		CBS432:C>S		DBVPG4650:C>S		N_17:C>S		S36_7:C>S	AA:47		A12:Q>R		A4:Q>R		UFRJ50791:Q>R		UFRJ50816:Q>R		UWOPS91_917_1:Q>R	AA:49		UWOPS91_917_1:R>S	AA:54		A4:T>P		DBVPG6304:T>P		UFRJ50791:T>P		UFRJ50816:T>P		UWOPS91_917_1:T>P	AA:61		A12:L>P		UWOPS91_917_1:L>P	AA:66		N_44:T>S		N_45:T>S	AA:75		A4:L>H		DBVPG6304:L>H		UFRJ50791:L>H		UFRJ50816:L>H		UWOPS91_917_1:L>H	AA:83		A4:F>Y		DBVPG6304:F>Y		UFRJ50791:F>Y		UFRJ50816:F>Y		UWOPS91_917_1:F>Y	AA:93		UWOPS91_917_1:S>T	AA:109		N_44:R>G		N_45:R>G	AA:112		A4:S>C		DBVPG6304:S>C		UFRJ50791:S>C		UFRJ50816:S>C	AA:114		A4:R>P		DBVPG6304:R>P		UFRJ50791:R>P		UFRJ50816:R>P		UWOPS91_917_1:R>P	AA:149		N_17:N>K	AA:162		A4:A>S		DBVPG6304:A>S		UFRJ50791:A>S		UFRJ50816:A>S	AA:183		UWOPS91_917_1:H>Y	AA:257		CBS5829:R>S		DBVPG4650:R>S		IFO1804:R>S		KPN3828:R>S		N_17:R>S		N_44:R>S		N_45:R>S		T21_4:R>S		UWOPS91_917_1:R>S	AA:267		A4:Y>S		CBS5829:Y>S		DBVPG4650:Y>S		DBVPG6304:Y>S		IFO1804:Y>S		KPN3828:Y>S		N_17:Y>S		N_44:Y>S		N_45:Y>S		T21_4:Y>S		UFRJ50791:Y>S	AA:272		UWOPS91_917_1:K>N	AA:284		IFO1804:L>P		N_44:L>P		N_45:L>P	AA:285		A4:M>I		DBVPG6304:M>I		UFRJ50791:M>I		UWOPS91_917_1:M>I	AA:287		DBVPG6304:M>I		IFO1804:M>I		N_44:M>I		N_45:M>I		UFRJ50791:M>I		UWOPS91_917_1:M>I	AA:292		A4:Y>-		DBVPG6304:Y>-		UFRJ50791:Y>-	AA:359		UWOPS91_917_1:->Y	AA:370		UFRJ50791:I>N	AA:393		UWOPS91_917_1:T>A	AA:397		DBVPG6304:F>S		UFRJ50791:F>S		UWOPS91_917_1:F>S	AA:406		IFO1804:S>C		N_44:S>C		N_45:S>C		UWOPS91_917_1:S>C	AA:420		A12:I>S		DBVPG6304:M>I		UFRJ50791:M>IID:YDL042C	AA:12		A4:N>K		UFRJ50791:N>K	AA:42		A4:F>V		UFRJ50791:F>V	AA:70		UWOPS91_917_1:N>K	AA:73		UWOPS91_917_1:L>F	AA:97		UFRJ50791:D>N	AA:112		DBVPG4650:K>I	AA:117		UFRJ50791:S>N	AA:124		N_43:I>M		N_45:I>M		UFRJ50791:I>M	AA:153		UWOPS91_917_1:R>S	AA:181		N_43:P>A		N_45:P>A	AA:211		UWOPS91_917_1:F>L	AA:224		UWOPS91_917_1:->Y	AA:252		A12:W>-		A4:W>-		UFRJ50791:W>-	AA:255		A12:N>S		A4:N>S		UFRJ50791:F>L	AA:270		A12:Y>-		A4:Y>-	AA:328		A12:E>D		A4:E>D		UFRJ50791:E>D		YPS138:E>D	AA:420		A12:K>N	AA:430		UWOPS91_917_1:H>L	AA:491		A4:H>D		N_43:H>D		N_45:H>D		UFRJ50791:H>D		UFRJ50816:H>D		UWOPS91_917_1:H>D		YPS138:H>D	AA:513		A4:Q>H		UFRJ50791:Q>H		UFRJ50816:Q>H		UWOPS91_917_1:Q>H		YPS138:Q>H	AA:515		CBS432:S>R	AA:525		UWOPS91_917_1:Q>H	AA:539		A12:D>G		A4:D>G		UFRJ50791:D>G		UFRJ50816:D>G		YPS138:D>G	AA:557		A12:S>G		A4:S>G		IFO1804:S>G		N_43:S>G		N_44:S>G		N_45:S>G		UFRJ50791:S>G		UFRJ50816:S>G		YPS138:S>GID:YDL044C	AA:30		A12:N>S		A4:N>S		DBVPG6304:N>S		UFRJ50816:N>S		UWOPS91_917_1:N>S		YPS138:N>S	AA:36		A12:F>V		A4:F>V		DBVPG6304:F>V		UFRJ50791:F>V		UFRJ50816:F>V		YPS138:F>V	AA:41		A12:Y>S		A4:Y>S		DBVPG6304:Y>S		IFO1804:Y>S		KPN3828:Y>S		KPN3829:Y>S		N_43:Y>S		N_44:Y>S		N_45:Y>S		Q89_8:Y>S		UFRJ50791:Y>S		UFRJ50816:Y>S		Y6_5:Y>S		Y7:Y>S		YPS138:Y>S	AA:87		A12:G>R		A4:G>R		DBVPG6304:G>R		UFRJ50791:G>R		YPS138:G>R	AA:111		A12:I>M		DBVPG6304:I>M		UFRJ50791:I>M		UWOPS91_917_1:I>M		YPS138:I>M	AA:123		Y7:H>D	AA:126		UWOPS91_917_1:F>V	AA:142		UWOPS91_917_1:W>G	AA:158		A12:->E		DBVPG6304:->E		UFRJ50791:->E		UWOPS91_917_1:->E	AA:167		A12:F>I		DBVPG6304:F>I		UFRJ50791:F>I	AA:176		IFO1804:S>A		N_43:S>A		N_44:S>A		N_45:S>A	AA:224		A12:V>E		UFRJ50791:V>E		YPS138:V>E	AA:233		A12:G>C		UFRJ50791:G>C		YPS138:G>C	AA:237		UFRJ50791:D>E	AA:251		A12:D>E		UFRJ50791:D>E		YPS138:D>E	AA:285		IFO1804:F>L		N_43:F>L		N_44:F>L		N_45:F>L	AA:295		KPN3828:C>S		KPN3829:C>S		Y6_5:C>S		Y7:C>S	AA:398		UFRJ50791:L>P	AA:438		DBVPG4650:C>W		N_44:C>W		N_45:C>W		Q89_8:C>W		UFRJ50791:C>W		Y7:C>W		YPS138:C>WID:YDL045C	AA:10		A12:->Y		A4:->Y		DBVPG6304:->Y		YPS138:->Y	AA:31		UWOPS91_917_1:F>L	AA:57		A12:D>E		A4:D>E		DBVPG6304:D>E		N_44:D>E		N_45:D>E		UFRJ50791:D>E		Y7:D>E		YPS138:D>E	AA:138		A12:W>C		A4:W>C		DBVPG6304:W>C		UFRJ50791:W>C		UFRJ50816:W>C		UWOPS91_917_1:W>C	AA:140		DBVPG6304:C>-		UFRJ50791:C>-		UFRJ50816:C>-	AA:142		UWOPS91_917_1:P>T	AA:160		UWOPS91_917_1:K>N	AA:227		A12:E>D		A4:E>D		DBVPG6304:E>D		UFRJ50791:E>D		UFRJ50816:E>D	AA:246		A12:P>T		A4:P>T		DBVPG6304:P>T		UFRJ50791:P>T		UFRJ50816:P>T	AA:284		A12:G>E		A4:G>E		DBVPG6304:G>E		UFRJ50791:G>E		UFRJ50816:G>E	AA:295		A12:Y>N		A4:Y>N		DBVPG6304:Y>N		UFRJ50791:Y>N		UFRJ50816:Y>N		YPS138:Y>NID:YDL045W-A	AA:5		A12:P>SID:YDL046W	AA:4		A12:S>G		A4:S>G		DBVPG6304:S>G		UFRJ50791:S>G		UWOPS91_917_1:S>G		YPS138:S>G	AA:6		A12:K>R		A4:K>R		DBVPG6304:K>R		UFRJ50791:K>R		UWOPS91_917_1:K>R		YPS138:K>R	AA:9		A12:L>F		A4:L>F		CBS5829:L>F		DBVPG4650:L>F		DBVPG6304:L>F		KPN3828:L>F		N_43:L>F		N_44:L>F		N_45:L>F		UFRJ50791:L>F		UWOPS91_917_1:L>F		Y6_5:L>F		Y7:L>F		YPS138:L>F	AA:55		UWOPS91_917_1:L>V	AA:63		UWOPS91_917_1:N>D		YPS138:N>D	AA:68		Y7:V>FID:YDL047W	AA:4		UWOPS91_917_1:R>K	AA:13		UWOPS91_917_1:I>VID:YDL048C	AA:3		N_43:V>I		N_44:V>I		N_45:V>I	AA:33		UWOPS91_917_1:H>Q	AA:34		UWOPS91_917_1:N>K	AA:41		A12:S>R	AA:54		A12:I>F	AA:62		A12:K>N	AA:87		DBVPG6304:Q>H		YPS138:Q>H	AA:107		A4:F>Y		DBVPG6304:F>Y		UWOPS91_917_1:F>Y		YPS138:F>Y	AA:155		UWOPS91_917_1:S>F	AA:161		UWOPS91_917_1:K>I	AA:177		A4:S>F		DBVPG6304:S>F		YPS138:S>F	AA:184		DBVPG6304:D>E	AA:225		A4:R>-		DBVPG6304:R>-		YPS138:R>-	AA:236		A4:M>I		DBVPG6304:M>I		YPS138:M>I	AA:247		A4:->Y		DBVPG6304:->Y		YPS138:->Y	AA:254		A4:I>M		DBVPG6304:I>M		YPS138:I>M	AA:263		DBVPG6304:D>E	AA:306		A4:C>W		DBVPG6304:C>W		YPS138:C>W	AA:346		IFO1804:C>S		N_45:C>S	AA:355		A12:G>-	AA:357		A12:G>E		A4:G>E		DBVPG6304:G>E		IFO1804:G>E		N_45:G>E		YPS138:G>E	AA:391		IFO1804:C>W		N_45:C>W	AA:405		IFO1804:R>S	AA:434		A4:Q>H		DBVPG6304:Q>H		UFRJ50816:Q>H		YPS138:Q>H	AA:459		A12:W>C		A4:W>C		DBVPG6304:W>C		UFRJ50816:W>C		YPS138:W>CID:YDL049C	AA:7		UWOPS91_917_1:I>L	AA:28		A12:R>P		DBVPG6304:R>P		IFO1804:R>P		N_43:R>P		N_44:R>P		N_45:R>P		UFRJ50791:R>P		UWOPS91_917_1:R>P	AA:42		UWOPS91_917_1:Y>D	AA:53		A12:L>F		DBVPG6304:L>F		UFRJ50791:L>F	AA:55		A12:N>K		DBVPG6304:N>K		UFRJ50791:N>K	AA:60		N_43:S>T		N_44:S>T		N_45:S>T	AA:61		UWOPS91_917_1:V>E	AA:89		A12:S>R		DBVPG6304:S>R		UFRJ50791:S>R	AA:106		UWOPS91_917_1:R>Q	AA:144		DBVPG6304:P>R	AA:151		A12:I>M		DBVPG6304:I>M	AA:155		A12:C>-		DBVPG6304:C>-		IFO1804:C>-		N_43:C>-		N_44:C>-		N_45:C>-	AA:160		A12:L>F		DBVPG6304:L>F	AA:177		A12:E>A		DBVPG6304:E>A	AA:222		A12:H>Q		DBVPG6304:H>Q	AA:225		KPN3829:H>L	AA:253		A12:C>W		CBS432:C>W		CBS5829:C>W		DBVPG4650:C>W		DBVPG6304:C>W		IFO1804:C>W		N_17:C>W		N_43:C>W		N_45:C>W		Q32_3:C>W		Q95_3:C>W		UFRJ50816:C>W		Y7:C>WID:YDL051W	AA:4		UWOPS91_917_1:T>I	AA:146		UWOPS91_917_1:S>N	AA:153		N_44:K>R	AA:187		A12:A>T		A4:A>T		DBVPG6304:A>T		UFRJ50816:A>T		UWOPS91_917_1:A>T	AA:205		A12:E>K	AA:238		UWOPS91_917_1:S>NID:YDL052C	AA:108		A12:H>Q		A4:H>Q		DBVPG6304:H>Q		UFRJ50816:H>Q	AA:143		A12:R>H		A4:R>H		DBVPG6304:R>H		IFO1804:R>H		N_43:R>H		N_45:R>H		UFRJ50816:R>H		UWOPS91_917_1:R>H	AA:165		DBVPG6304:T>S	AA:215		A12:H>Q		A4:H>Q		YPS138:H>Q	AA:260		IFO1804:P>L		N_45:P>L	AA:302		CBS432:H>QID:YDL053C	AA:24		A12:->R		A4:->R		UFRJ50816:->R		UWOPS91_917_1:->R		YPS138:->R	AA:37		UWOPS91_917_1:A>S	AA:69		A12:N>T		A4:N>T		DBVPG6304:N>T		UFRJ50816:N>T	AA:73		UFRJ50816:I>F	AA:75		A12:R>S		A4:R>S		DBVPG6304:R>S		UFRJ50816:R>S		UWOPS91_917_1:R>S	AA:116		A12:Y>-		A4:Y>-		DBVPG6304:Y>-		IFO1804:Y>-		N_43:Y>-		N_44:Y>-		N_45:Y>-		UFRJ50816:Y>-		UWOPS91_917_1:Y>-		YPS138:Y>-	AA:130		UWOPS91_917_1:L>F	AA:146		A12:F>L		A4:F>L		DBVPG6304:F>L		UFRJ50816:F>L		UWOPS91_917_1:F>L		YPS138:F>L	AA:165		UFRJ50816:R>K	AA:175		A12:S>P		A4:S>P		DBVPG6304:S>P		UFRJ50816:S>P		YPS138:S>PID:YDL056W	AA:163		A4:V>A		DBVPG6304:V>A		IFO1804:V>A		N_43:V>A		N_44:V>A		N_45:V>A		UFRJ50791:V>A		UFRJ50816:V>A		YPS138:V>A	AA:234		DBVPG6304:S>P		UFRJ50791:S>P		UFRJ50816:S>P	AA:247		Y6_5:N>K	AA:335		DBVPG6304:A>S		UWOPS91_917_1:A>S		YPS138:A>S	AA:481		A4:R>K		DBVPG6304:R>K		YPS138:R>K	AA:565		UWOPS91_917_1:M>I	AA:624		A12:R>K	AA:642		UWOPS91_917_1:D>N	AA:670		UWOPS91_917_1:L>I	AA:679		N_44:S>G		N_45:S>G	AA:685		A12:D>G		A4:D>G		DBVPG6304:D>G		YPS138:D>G	AA:746		UWOPS91_917_1:D>N	AA:752		A12:V>E		A4:V>E		YPS138:V>E	AA:785		UWOPS91_917_1:K>R	AA:825		CBS5829:A>T	AA:834		UWOPS91_917_1:A>P	AA:839		A12:->Y		A4:->YID:YDL057W	AA:5		A12:Y>H		A4:Y>H		DBVPG6304:Y>H		UFRJ50816:Y>H		UWOPS91_917_1:Y>H	AA:18		N_45:I>T	AA:25		A4:K>N		DBVPG6304:K>N		UFRJ50816:K>N		YPS138:K>N	AA:32		IFO1804:T>A		N_45:T>A	AA:33		A12:M>T		A4:M>T		DBVPG6304:M>T		UFRJ50816:M>T		UWOPS91_917_1:M>T		YPS138:M>T	AA:36		A12:L>S		A4:L>S		DBVPG6304:L>S		UFRJ50816:L>S		UWOPS91_917_1:L>S		YPS138:L>S	AA:48		A12:E>D		A4:E>D		DBVPG6304:E>D		UFRJ50816:E>D		YPS138:E>D	AA:86		A4:T>A		DBVPG6304:T>A		UFRJ50816:T>A		UWOPS91_917_1:T>A		YPS138:T>A	AA:121		N_44:T>A	AA:137		A4:S>T		DBVPG6304:S>T		UFRJ50816:S>T		UWOPS91_917_1:S>T		YPS138:S>T	AA:139		A4:R>K		DBVPG6304:R>K		UFRJ50816:R>K		YPS138:R>K	AA:149		N_43:G>S		N_44:G>S		N_45:G>S	AA:177		A4:A>E		DBVPG6304:A>E		UFRJ50816:A>E		UWOPS91_917_1:A>E		YPS138:A>E	AA:179		A4:P>Q		DBVPG6304:P>Q		UFRJ50816:P>Q		UWOPS91_917_1:P>Q		YPS138:P>Q	AA:220		DBVPG6304:N>K		UFRJ50816:N>K		UWOPS91_917_1:N>K		YPS138:N>K	AA:242		DBVPG6304:P>S		UFRJ50816:P>S	AA:271		DBVPG6304:R>G		UFRJ50816:R>G	AA:277		UWOPS91_917_1:E>K	AA:283		N_43:E>K		N_44:E>K		N_45:E>K	AA:301		N_43:V>A		N_44:V>A		N_45:V>AID:YDL058W	AA:11		CBS5829:Q>P		N_17:Q>P		Q32_3:Q>P		Q62_5:Q>P		Q95_3:Q>P		T21_4:Q>P		UWOPS91_917_1:Q>P	AA:168		DBVPG6304:M>V		N_43:M>V		N_45:M>V		UWOPS91_917_1:M>V		YPS138:M>V	AA:393		UWOPS91_917_1:P>S	AA:467		A12:S>N		A4:S>N		DBVPG6304:S>N		IFO1804:S>N		N_43:S>N		N_44:S>N		N_45:S>N		UFRJ50791:S>N		UFRJ50816:S>N		UWOPS91_917_1:S>N	AA:475		IFO1804:N>Y		N_43:N>Y		N_44:N>Y		N_45:N>Y	AA:485		A12:G>V		A4:G>V		DBVPG6304:G>V		UFRJ50791:G>V		UFRJ50816:G>V	AA:545		UWOPS91_917_1:D>N	AA:636		UWOPS91_917_1:K>R	AA:675		N_43:D>N		N_44:D>N	AA:706		UWOPS91_917_1:E>Q	AA:718		IFO1804:G>D		N_43:G>D		N_45:G>D		UWOPS91_917_1:G>D	AA:754		A4:K>E		DBVPG6304:K>E		IFO1804:K>E		N_43:K>E		N_45:K>E		UFRJ50816:K>E		YPS138:K>E	AA:758		A4:E>G		DBVPG6304:E>G		IFO1804:E>G		N_43:E>G		UFRJ50816:E>G		UWOPS91_917_1:E>G		YPS138:E>G	AA:769		UWOPS91_917_1:K>I	AA:772		A4:Q>E		DBVPG6304:Q>E		IFO1804:Q>E		N_43:Q>E		UFRJ50816:Q>E		UWOPS91_917_1:Q>E		YPS138:Q>E	AA:803		UWOPS91_917_1:R>K	AA:815		IFO1804:A>T		N_43:A>T		N_44:A>T		N_45:A>T	AA:820		UWOPS91_917_1:D>N	AA:832		UWOPS91_917_1:S>N	AA:850		A4:T>S		UFRJ50816:T>S		YPS138:T>S	AA:878		A4:A>T		UFRJ50816:A>T		YPS138:A>T	AA:885		A4:S>N		IFO1804:S>N		KPN3828:S>N		N_43:S>N		N_44:S>N		N_45:S>N		Q32_3:S>N		Q95_3:S>N		UFRJ50816:S>N		UWOPS91_917_1:S>N		YPS138:S>N	AA:913		UFRJ50816:A>T		UWOPS91_917_1:A>T		YPS138:A>T	AA:929		N_43:H>Y	AA:941		CBS432:S>R		CBS5829:S>R		DBVPG4650:S>R		KPN3828:S>R		N_17:S>R		N_43:S>R		N_44:S>R		N_45:S>R		Q32_3:S>R		Q95_3:S>R		UFRJ50816:S>R		YPS138:S>R	AA:976		YPS138:S>A	AA:990		YPS138:N>K	AA:991		N_43:E>G		N_44:E>G		N_45:E>G	AA:994		YPS138:I>T	AA:998		UFRJ50816:D>N		UWOPS91_917_1:D>N		YPS138:D>N	AA:1012		N_43:N>S		N_44:N>S		N_45:N>S		UFRJ50816:N>S	AA:1015		UWOPS91_917_1:T>I	AA:1029		UWOPS91_917_1:E>K	AA:1033		UWOPS91_917_1:A>D	AA:1037		UWOPS91_917_1:K>T	AA:1051		UWOPS91_917_1:D>E	AA:1061		UWOPS91_917_1:K>E	AA:1073		UWOPS91_917_1:S>N	AA:1086		N_43:S>G		N_45:S>G		UWOPS91_917_1:S>G	AA:1093		A4:T>A		UFRJ50816:T>A		UWOPS91_917_1:T>A	AA:1098		UFRJ50816:K>Q	AA:1103		UWOPS91_917_1:A>T	AA:1115		A4:V>I		N_43:V>I		N_45:V>I		UFRJ50816:V>I		UWOPS91_917_1:V>I	AA:1124		A4:E>D		UFRJ50816:E>D		UWOPS91_917_1:E>D		YPS138:E>D	AA:1127		Q32_3:I>N		Q95_3:I>N	AA:1142		KPN3828:S>N	AA:1145		A4:G>S	AA:1155		UWOPS91_917_1:E>G	AA:1169		A4:D>N		DBVPG6304:D>N		UFRJ50816:D>N		UWOPS91_917_1:D>N		YPS138:D>N	AA:1177		KPN3828:E>K		Q32_3:E>K		Q95_3:E>K	AA:1181		A4:Q>E		DBVPG6304:Q>E		UFRJ50816:Q>E		UWOPS91_917_1:Q>E		YPS138:Q>E	AA:1187		IFO1804:S>N		N_43:S>N		N_44:S>N		N_45:S>N	AA:1188		A4:S>T		DBVPG6304:S>T		UFRJ50816:S>T		YPS138:S>T	AA:1189		UWOPS91_917_1:T>N	AA:1201		A4:E>K		DBVPG6304:E>K		UFRJ50816:E>K		YPS138:E>K	AA:1208		A4:K>R		DBVPG6304:K>R		UFRJ50816:K>R		YPS138:K>R	AA:1212		IFO1804:G>S		N_43:G>S		N_44:G>S		N_45:G>S	AA:1221		N_45:K>E	AA:1237		UWOPS91_917_1:E>K	AA:1253		UWOPS91_917_1:I>M	AA:1259		N_43:K>R	AA:1261		A4:R>K		DBVPG6304:R>K		UFRJ50816:R>K		YPS138:R>K	AA:1271		KPN3828:E>K		Q32_3:E>K		Q95_3:E>K	AA:1279		DBVPG6304:N>D		UFRJ50816:N>D		UWOPS91_917_1:N>D		YPS138:N>D	AA:1283		DBVPG6304:T>A		IFO1804:T>A		N_43:T>A		N_44:T>A		N_45:T>A		UFRJ50816:T>A		UWOPS91_917_1:T>A		YPS138:T>A	AA:1288		N_43:K>M	AA:1297		IFO1804:Q>E		N_43:Q>E		N_44:Q>E		N_45:Q>E	AA:1303		UWOPS91_917_1:R>K	AA:1310		UWOPS91_917_1:I>T	AA:1314		UWOPS91_917_1:N>I	AA:1331		DBVPG6304:S>A		UFRJ50816:S>A		YPS138:S>A	AA:1344		CBS432:G>R		CBS5829:G>R		DBVPG4650:G>R		KPN3828:G>R		N_17:G>R		Q32_3:G>R		Q89_8:G>R		Q95_3:G>R	AA:1347		DBVPG6304:V>A		UFRJ50816:V>A	AA:1356		N_45:N>I	AA:1360		N_45:S>G	AA:1386		YPS138:K>Q	AA:1389		N_44:T>P	AA:1395		DBVPG6304:N>I		UFRJ50816:N>I		YPS138:N>I	AA:1410		DBVPG6304:T>A		UFRJ50816:T>A		YPS138:T>A	AA:1458		A12:K>E		DBVPG6304:K>E		UFRJ50816:K>E		UWOPS91_917_1:K>E		YPS138:K>E	AA:1467		A12:S>C		DBVPG6304:S>C		UFRJ50816:S>C		YPS138:S>C	AA:1501		UWOPS91_917_1:Q>K	AA:1508		UWOPS91_917_1:K>E	AA:1527		DBVPG6304:I>T		IFO1804:I>T		N_43:I>T		N_44:I>T		N_45:I>T		UFRJ50816:I>T	AA:1537		IFO1804:Q>R		N_43:Q>R		N_44:Q>R		N_45:Q>R	AA:1550		KPN3829:Q>H	AA:1559		A12:H>Q		DBVPG6304:H>Q		UFRJ50816:H>Q		UWOPS91_917_1:H>Q		YPS138:H>Q	AA:1568		IFO1804:E>K		N_43:E>K		N_44:E>K		N_45:E>K	AA:1609		UWOPS91_917_1:K>T	AA:1617		A12:C>R		DBVPG6304:C>R		IFO1804:C>R		N_43:C>R		N_44:C>R		N_45:C>R		UWOPS91_917_1:C>R		YPS138:C>R	AA:1648		UWOPS91_917_1:D>H	AA:1649		N_43:S>N		N_45:S>N	AA:1655		UWOPS91_917_1:Q>K	AA:1668		A12:V>L		DBVPG6304:V>L		N_43:V>L		N_45:V>L		UFRJ50791:V>L		UWOPS91_917_1:V>L		YPS138:V>L	AA:1670		A12:L>V		DBVPG6304:L>V		YPS138:L>V	AA:1686		A12:Y>C		DBVPG6304:Y>C		UFRJ50791:Y>C		UWOPS91_917_1:Y>C		YPS138:Y>C	AA:1691		A12:T>A		A4:T>A		DBVPG6304:T>A		UFRJ50791:T>A		UWOPS91_917_1:T>A		YPS138:T>A	AA:1698		N_43:R>G		N_45:R>G	AA:1699		UWOPS91_917_1:E>G	AA:1709		A12:S>F		DBVPG6304:S>F		UFRJ50791:S>F		YPS138:S>F	AA:1719		A12:E>K		A4:E>K		DBVPG6304:E>K		UFRJ50791:E>K		UWOPS91_917_1:E>K		YPS138:E>K	AA:1726		A12:A>T		A4:A>T		DBVPG6304:A>T		UFRJ50791:A>T		UWOPS91_917_1:A>T		YPS138:A>T	AA:1754		UWOPS91_917_1:K>R	AA:1784		IFO1804:D>EID:YDL059C	AA:51		UWOPS91_917_1:S>R	AA:105		A12:L>I		CBS432:L>I		CBS5829:L>I		DBVPG6304:L>I		N_43:L>I		N_44:L>I		N_45:L>I		S36_7:L>I		UFRJ50816:L>I		UWOPS91_917_1:L>I		YPS138:L>I	AA:121		A12:G>S		DBVPG6304:G>S		UFRJ50816:G>S		YPS138:G>S	AA:127		N_43:C>W		N_44:C>W		N_45:C>W	AA:149		A12:S>P		DBVPG6304:S>P		N_43:S>P		N_44:S>P		N_45:S>P		UFRJ50816:S>P		UWOPS91_917_1:S>P		YPS138:S>P	AA:163		UWOPS91_917_1:Y>-	AA:218		A12:R>P		DBVPG6304:R>P		IFO1804:R>P		N_43:R>P		N_44:R>P		N_45:R>P		UFRJ50816:R>P		UWOPS91_917_1:R>PID:YDL060W	AA:52		DBVPG4650:L>M	AA:107		A12:S>T		A4:S>T		DBVPG6304:S>T		UWOPS91_917_1:S>T	AA:114		IFO1804:M>L		N_44:M>L		N_45:M>L	AA:158		UWOPS91_917_1:V>L	AA:227		A4:Y>F		DBVPG6304:Y>F		IFO1804:Y>S	AA:259		UWOPS91_917_1:V>I	AA:271		Q32_3:P>S	AA:289		N_17:T>I		N_45:T>I	AA:310		A4:R>S		DBVPG6304:R>S		IFO1804:R>K		N_43:R>K		N_44:R>K		N_45:R>K		UWOPS91_917_1:R>S	AA:319		A4:M>T		DBVPG6304:M>T	AA:325		A4:D>G		DBVPG6304:D>G	AA:354		UWOPS91_917_1:T>M	AA:385		UWOPS91_917_1:Q>K	AA:387		UWOPS91_917_1:S>P	AA:388		IFO1804:K>E	AA:417		A4:A>T		DBVPG6304:A>T	AA:419		A4:Q>E		DBVPG6304:Q>E	AA:444		CBS5829:A>T		KPN3829:A>T		N_17:A>T		Q32_3:A>T		Q62_5:A>T		Z1_1:A>T	AA:452		N_44:E>D		N_45:E>D	AA:552		DBVPG6304:V>I		IFO1804:V>I		N_45:V>I	AA:562		IFO1804:N>K		N_45:N>K	AA:572		Q95_3:Q>E	AA:615		IFO1804:V>I		N_43:V>I		N_45:V>I	AA:675		A4:I>T		DBVPG6304:I>T		UWOPS91_917_1:I>T		YPS138:I>TID:YDL063C	AA:3		A12:I>N		A4:I>N		DBVPG6304:I>N		UFRJ50791:I>N		UWOPS91_917_1:S>A	AA:44		A12:I>L		A4:I>L		DBVPG6304:I>L		UFRJ50791:I>L	AA:85		A12:F>L		A4:F>L		DBVPG6304:F>L		UFRJ50791:F>L	AA:101		A12:N>H		DBVPG6304:N>H	AA:147		IFO1804:H>R	AA:168		DBVPG4650:Q>H		N_43:V>F		N_44:V>F		N_45:V>F	AA:179		A12:F>L		A4:F>L		DBVPG6304:F>L		UWOPS91_917_1:P>R	AA:187		UWOPS91_917_1:I>T	AA:189		A12:L>P		DBVPG4650:L>P		N_44:L>P		N_45:L>P		UWOPS91_917_1:L>P	AA:205		A12:C>-		A4:C>-		DBVPG6304:C>-		UWOPS91_917_1:C>-	AA:236		A4:R>S		DBVPG6304:R>S		N_43:R>S		N_44:R>S		N_45:R>S		UWOPS91_917_1:R>S	AA:252		A4:F>L		UWOPS91_917_1:F>L	AA:258		A4:F>V		DBVPG4650:F>V		N_43:F>V		N_44:F>V		N_45:F>V		UWOPS91_917_1:F>V	AA:265		UWOPS91_917_1:L>F	AA:270		N_45:D>G	AA:285		UWOPS91_917_1:F>V	AA:299		A4:F>L		UWOPS91_917_1:F>L	AA:322		A4:Y>N		UWOPS91_917_1:Y>N	AA:350		UWOPS91_917_1:L>F	AA:371		A4:R>I		N_43:R>I		N_45:R>I		UWOPS91_917_1:R>I	AA:379		UWOPS91_917_1:->Y	AA:389		N_43:K>N		N_45:K>N	AA:402		A4:E>D		N_43:E>D		N_44:E>D		N_45:E>D	AA:432		UWOPS91_917_1:F>L	AA:440		UWOPS91_917_1:I>L	AA:471		N_43:C>G		N_44:C>G		N_45:C>G	AA:473		A12:F>L	AA:478		A12:L>F		DBVPG6304:L>F		UWOPS91_917_1:L>F		YPS138:L>F	AA:486		UWOPS91_917_1:->Y	AA:487		Q32_3:R>Q	AA:493		A12:K>R		DBVPG6304:K>R		UWOPS91_917_1:K>R		YPS138:K>R	AA:509		A12:->W		DBVPG6304:->W		UWOPS91_917_1:->W		YPS138:->W	AA:514		Q32_3:F>S	AA:559		UWOPS91_917_1:T>I	AA:570		A12:S>T		DBVPG6304:S>T		N_43:S>T		N_44:S>T		N_45:S>T		UWOPS91_917_1:S>T		YPS138:S>T	AA:574		UWOPS91_917_1:H>Q	AA:611		A12:R>S		A4:R>S		DBVPG6304:R>S		UWOPS91_917_1:R>S		YPS138:R>SID:YDL064W	AA:44		UWOPS91_917_1:G>S	AA:46		UWOPS91_917_1:N>D	AA:48		UWOPS91_917_1:A>VID:YDL065C	AA:2		A12:L>W		DBVPG6304:L>W		UFRJ50791:L>W		YPS138:L>W	AA:26		A12:H>N		DBVPG6304:H>N		UFRJ50791:H>N		UWOPS91_917_1:H>N		YPS138:H>N	AA:30		A12:I>V		DBVPG6304:I>V		IFO1804:I>V		N_43:I>V		N_44:I>V		N_45:I>V		UFRJ50791:I>V		UWOPS91_917_1:I>V		YPS138:I>V	AA:52		UWOPS91_917_1:->Y	AA:105		A12:S>R		DBVPG6304:S>R		YPS138:S>R	AA:129		N_17:L>R	AA:162		DBVPG6304:->Y	AA:173		UWOPS91_917_1:A>T	AA:197		A12:C>-		A4:C>-		CBS432:C>-		DBVPG4650:C>-		DBVPG6304:C>-		IFO1804:C>-		N_43:C>-		N_44:C>-		N_45:C>-		Q32_3:C>-		Q89_8:C>-		UWOPS91_917_1:C>-		YPS138:C>-	AA:205		UWOPS91_917_1:S>T	AA:235		UWOPS91_917_1:S>R	AA:244		UWOPS91_917_1:F>V	AA:257		A12:T>S		A4:T>S		DBVPG6304:T>S		UFRJ50791:T>S	AA:268		IFO1804:Q>R		N_43:Q>R	AA:272		A12:H>D		A4:H>D		DBVPG6304:H>D		UFRJ50791:H>D	AA:275		A12:->Y		A4:->Y		DBVPG6304:->Y		UFRJ50791:->Y	AA:277		A12:W>-		A4:W>-		DBVPG6304:W>-		IFO1804:W>-		N_43:W>-		UFRJ50791:W>-	AA:278		CBS5829:V>A		N_17:V>A		Q62_5:V>A	AA:282		A12:F>V		A4:F>V		DBVPG6304:F>V		UFRJ50791:F>V		UWOPS91_917_1:F>V	AA:283		IFO1804:I>F		N_43:I>F	AA:287		IFO1804:Y>-		N_43:Y>-	AA:299		A12:L>V		A4:L>V		DBVPG6304:L>V		UFRJ50791:L>V	AA:303		UWOPS91_917_1:I>S	AA:325		UWOPS91_917_1:F>LID:YDL066W	AA:13		DBVPG6304:R>H		UFRJ50816:R>H	AA:178		A4:A>T		DBVPG6304:A>T		UWOPS91_917_1:A>T	AA:216		UWOPS91_917_1:K>R	AA:251		UWOPS91_917_1:A>T	AA:396		CBS432:A>T	AA:402		CBS432:N>I	AA:427		DBVPG4650:S>TID:YDL067C	AA:9		A12:S>R		DBVPG6304:S>R		UFRJ50816:S>R		UWOPS91_917_1:S>R	AA:20		UWOPS91_917_1:N>K	AA:39		A12:K>N		DBVPG6304:K>N		UFRJ50816:K>N		UWOPS91_917_1:K>N		YPS138:K>N	AA:42		UWOPS91_917_1:D>EID:YDL069C	AA:12		N_43:->L		N_45:->L		UFRJ50791:S>C		UFRJ50816:S>C		YPS138:S>C	AA:36		N_43:A>D		N_45:A>D	AA:47		Y7:->Y	AA:51		UFRJ50791:V>L		UWOPS91_917_1:V>L		YPS138:V>L	AA:52		N_43:L>I		N_45:L>I	AA:54		N_43:T>A		N_45:T>A		UFRJ50791:T>A		UWOPS91_917_1:T>A		YPS138:T>A	AA:56		UWOPS91_917_1:V>F	AA:72		UWOPS91_917_1:->K	AA:74		UWOPS91_917_1:L>F	AA:88		UFRJ50791:R>P		UWOPS91_917_1:R>P		YPS138:R>P	AA:91		UWOPS91_917_1:H>Q	AA:113		N_43:I>M		N_44:I>M		N_45:I>M	AA:121		UFRJ50791:L>R		YPS138:L>R	AA:127		UWOPS91_917_1:D>E	AA:130		UWOPS91_917_1:F>C	AA:134		UWOPS91_917_1:L>F	AA:158		N_43:H>Q		N_44:H>Q		UFRJ50791:H>Q		UFRJ50816:H>Q		UWOPS91_917_1:H>Q		YPS138:H>Q	AA:178		UFRJ50791:P>T		UFRJ50816:P>T		UWOPS91_917_1:P>T		YPS138:P>T	AA:207		UWOPS91_917_1:F>I	AA:209		UWOPS91_917_1:E>D	AA:214		UWOPS91_917_1:N>I	AA:216		A4:R>K		UFRJ50791:R>K		UFRJ50816:R>K		YPS138:R>KID:YDL070W	AA:15		KPN3828:L>	AA:16		IFO1804:L>M		N_43:L>M		N_44:L>M		N_45:L>M	AA:19		A4:R>P		DBVPG6304:R>P		UWOPS91_917_1:R>P		YPS138:R>P	AA:23		IFO1804:S>T		N_43:S>T		N_44:S>T		UWOPS91_917_1:S>T	AA:36		A4:S>G		DBVPG6304:S>G		UFRJ50791:S>G		UFRJ50816:S>G		YPS138:S>G	AA:38		DBVPG6304:K>R	AA:41		A4:I>V		DBVPG6304:I>V		UFRJ50791:I>V		UFRJ50816:I>V		UWOPS91_917_1:I>V		YPS138:I>V	AA:122		UWOPS91_917_1:P>L	AA:123		IFO1804:A>T		N_43:A>T		N_44:A>T	AA:128		UWOPS91_917_1:I>M	AA:143		UWOPS91_917_1:N>S	AA:179		DBVPG6304:M>V	AA:183		IFO1804:L>F		N_43:L>F		N_44:L>F	AA:216		A4:S>P		DBVPG6304:S>P		IFO1804:S>P		N_43:S>P		N_44:S>P		YPS138:S>P	AA:271		A12:I>V		A4:I>V		DBVPG6304:I>V		YPS138:I>V	AA:307		YPS138:I>V	AA:317		N_44:T>A		N_45:T>A	AA:329		A12:I>L		A4:I>L		DBVPG6304:I>L		N_44:I>L		N_45:I>L		YPS138:I>L	AA:342		A12:Y>F		A4:Y>F		DBVPG6304:Y>F		YPS138:Y>F	AA:371		UWOPS91_917_1:I>V	AA:376		N_44:M>I		N_45:M>I	AA:388		A12:N>D		A4:N>D		DBVPG6304:N>D		YPS138:N>D	AA:390		A12:L>F		A4:L>F		DBVPG6304:L>F		YPS138:L>F	AA:503		N_45:G>D	AA:505		N_45:K>T	AA:522		UWOPS91_917_1:S>Y	AA:529		A4:E>K		DBVPG6304:E>K		YPS138:E>K	AA:597		N_44:N>T		N_45:N>T		UWOPS91_917_1:N>TID:YDL072C	AA:29		A12:F>L		A4:F>L		CBS5829:F>L		DBVPG6304:F>L		IFO1804:F>L		KPN3829:F>L		N_17:F>L		N_43:F>L		N_45:F>L		UFRJ50791:F>L		UFRJ50816:F>L		UWOPS91_917_1:F>L	AA:44		UWOPS91_917_1:L>F	AA:85		KPN3828:H>Q	AA:89		IFO1804:Q>H		N_43:Q>H		N_45:Q>H	AA:95		IFO1804:C>-		N_43:C>-		N_44:C>-		N_45:C>-	AA:104		KPN3828:E>G	AA:109		A4:H>Y		DBVPG6304:H>Y		UFRJ50791:H>Y		UFRJ50816:H>Y	AA:123		KPN3828:R>K	AA:127		IFO1804:->Q	AA:129		KPN3828:Q>-	AA:136		A4:H>N		DBVPG6304:H>N		UFRJ50791:H>N		UFRJ50816:H>N	AA:138		KPN3828:V>D	AA:144		A4:K>-		DBVPG6304:K>-		UFRJ50791:K>-		UFRJ50816:K>-	AA:188		A4:K>N		DBVPG6304:K>N		N_43:K>N		N_44:K>N		N_45:K>N		UFRJ50791:K>N		UFRJ50816:K>NID:YDL073W	AA:7		A12:N>T		A4:N>T		DBVPG6304:N>T		N_43:N>T		N_44:N>T		UFRJ50791:N>T		UFRJ50816:N>T		UWOPS91_917_1:N>T		YPS138:N>T	AA:55		A12:N>I		A4:N>I		DBVPG6304:N>I		UFRJ50816:N>I		YPS138:N>I	AA:73		UWOPS91_917_1:V>I	AA:79		A12:G>D		A4:G>D		CBS432:G>D		DBVPG4650:G>D		DBVPG6304:G>D		IFO1804:G>D		KPN3828:G>D		KPN3829:G>D		N_17:G>D		N_43:G>D		N_44:G>D		N_45:G>D		Q59_1:G>D		Q95_3:G>D		UFRJ50816:G>D		UWOPS91_917_1:G>D		YPS138:G>D		Z1_1:G>D	AA:115		A12:A>P		A4:A>P		DBVPG6304:A>P		UFRJ50816:A>P		UWOPS91_917_1:A>P		YPS138:A>P	AA:125		IFO1804:T>I		N_43:T>I		N_44:T>I		N_45:T>I	AA:144		IFO1804:F>Y		N_43:F>Y		N_44:F>Y		N_45:F>Y	AA:161		A12:V>I		A4:V>I		DBVPG6304:V>I		UFRJ50816:V>I		YPS138:V>I	AA:178		IFO1804:F>L		N_43:F>L		N_44:F>L		N_45:F>L		UWOPS91_917_1:F>L	AA:189		A12:K>T		A4:K>T		DBVPG6304:K>T		IFO1804:K>T		N_43:K>T		N_44:K>T		N_45:K>T		UFRJ50816:K>T		UWOPS91_917_1:K>T		YPS138:K>T	AA:195		A12:E>Q		A4:E>Q		DBVPG6304:E>Q		N_43:E>Q		N_44:E>Q		N_45:E>Q		UFRJ50816:E>Q		UWOPS91_917_1:E>Q		YPS138:E>Q	AA:240		A12:N>S		A4:N>S		DBVPG6304:N>S		UFRJ50816:N>S		UWOPS91_917_1:N>S		YPS138:N>S	AA:243		UWOPS91_917_1:A>T	AA:261		UWOPS91_917_1:Q>H	AA:264		CBS432:H>	AA:265		A12:A>T		A4:A>T		DBVPG6304:A>T		YPS138:A>T	AA:269		UWOPS91_917_1:E>D	AA:273		A12:T>S		A4:T>S		DBVPG6304:T>S		YPS138:T>S	AA:275		UWOPS91_917_1:F>L	AA:277		N_43:K>R		N_44:K>R		N_45:K>R	AA:291		Q62_5:S>T	AA:302		A12:Q>R		A4:Q>R		DBVPG6304:Q>R		YPS138:Q>R	AA:319		A12:S>A		A4:S>A		DBVPG6304:S>A		YPS138:S>A	AA:333		A12:T>A		A4:T>A		DBVPG6304:T>A		YPS138:T>A	AA:355		A12:I>T		A4:I>T		DBVPG6304:I>T		YPS138:I>T	AA:356		IFO1804:T>I		N_43:T>I		N_44:T>I		N_45:T>I	AA:363		KPN3829:S>L	AA:369		A12:S>G		A4:S>G		DBVPG6304:S>G		YPS138:S>G	AA:371		A12:N>S	AA:373		A12:N>S		A4:N>S		DBVPG6304:N>S		YPS138:N>S	AA:375		A12:A>V		A4:A>V		DBVPG6304:A>V		YPS138:A>V	AA:412		A12:L>S	AA:418		A12:M>V		A4:M>V		DBVPG6304:M>V		UFRJ50816:M>V		YPS138:M>V	AA:460		A4:S>P		DBVPG6304:S>P		IFO1804:S>P		N_43:S>P		N_44:S>P		UFRJ50791:S>P		UFRJ50816:S>P		YPS138:S>P	AA:466		A4:H>Q		DBVPG6304:H>Q		UFRJ50791:H>Q		UFRJ50816:H>Q		YPS138:H>Q	AA:500		CBS5829:V>I		DBVPG4650:V>I		KPN3828:V>I		KPN3829:V>I		N_17:V>I		N_45:V>I		Q59_1:V>I		Q95_3:V>I	AA:529		DBVPG6304:I>V	AA:544		A12:S>I	AA:553		N_45:F>V	AA:559		A4:I>V		DBVPG6304:I>V		UFRJ50791:I>V		UFRJ50816:I>V		YPS138:I>V	AA:567		N_45:I>T	AA:586		A4:A>T	AA:619		CBS5829:H>R		KPN3828:H>R		KPN3829:H>R		Q59_1:H>R	AA:658		A12:S>G		A4:S>G		DBVPG6304:S>G		UFRJ50791:S>G		UFRJ50816:S>G		YPS138:S>G	AA:660		A12:V>A		A4:V>A		DBVPG6304:V>A		UFRJ50791:V>A		UFRJ50816:V>A		YPS138:V>A	AA:680		N_45:S>L	AA:709		A12:S>G	AA:719		N_45:S>T	AA:726		A12:S>G		A4:S>G		DBVPG6304:S>G	AA:788		CBS432:L>F	AA:790		A4:K>N		DBVPG6304:K>N	AA:817		N_17:V>I		N_45:V>I	AA:832		A4:R>K		DBVPG6304:R>K	AA:833		N_17:I>M		N_45:I>M	AA:863		A4:S>N		DBVPG6304:S>N		UFRJ50791:S>N	AA:864		UWOPS91_917_1:A>V	AA:871		UWOPS91_917_1:M>T	AA:874		N_45:F>S	AA:876		A4:F>S		DBVPG6304:F>S		N_45:F>S		UFRJ50791:F>S		UFRJ50816:F>S	AA:879		A4:N>D		UFRJ50791:N>D		UFRJ50816:N>D	AA:883		UWOPS91_917_1:G>D	AA:896		A4:K>N		UFRJ50791:K>N		UFRJ50816:K>N	AA:900		UFRJ50791:D>Y		UFRJ50816:D>Y	AA:902		UWOPS91_917_1:H>Q	AA:905		UFRJ50791:P>R		UFRJ50816:P>R	AA:911		UWOPS91_917_1:S>N	AA:912		UFRJ50791:V>L		UFRJ50816:V>L	AA:914		N_45:N>D		UWOPS91_917_1:N>D	AA:938		UWOPS91_917_1:M>I	AA:966		UWOPS91_917_1:S>GID:YDL076C	AA:35		A4:I>N		DBVPG6304:I>N		UWOPS91_917_1:I>N		YPS138:I>N	AA:45		A4:F>Y		DBVPG6304:F>Y		YPS138:F>Y	AA:50		IFO1804:I>N		N_43:I>N		N_44:I>N		N_45:I>N		UWOPS91_917_1:I>N	AA:53		IFO1804:F>L		N_44:F>L		N_45:F>L		UWOPS91_917_1:F>L	AA:65		N_17:->Y		N_45:->Y	AA:66		A4:->Y		DBVPG6304:->Y		IFO1804:->Y		N_43:->Y		N_44:->Y		N_45:->Y		UWOPS91_917_1:->Y		YPS138:->Y	AA:78		A4:->E		DBVPG6304:->E		YPS138:->E	AA:120		IFO1804:V>G		N_43:V>G		N_44:V>G	AA:135		A4:S>F		DBVPG6304:S>F		IFO1804:S>F		N_43:S>F		N_44:S>F		N_45:S>F		UWOPS91_917_1:S>F		YPS138:S>F	AA:172		IFO1804:W>C		N_43:W>C		N_44:W>C	AA:175		A12:S>R		A4:S>R		DBVPG6304:S>R		UWOPS91_917_1:S>R		YPS138:S>R	AA:207		A12:C>F		A4:V>D		DBVPG6304:V>D		IFO1804:C>F		N_43:C>F		N_44:C>F		N_45:C>F		UWOPS91_917_1:C>S		YPS138:V>D	AA:260		UWOPS91_917_1:L>F	AA:281		UWOPS91_917_1:S>IID:YDL077C	AA:25		A4:S>R		DBVPG6304:S>R		UFRJ50816:S>R	AA:38		A4:M>I		DBVPG6304:M>I		UFRJ50816:M>I		UWOPS91_917_1:M>I	AA:67		UWOPS91_917_1:F>C	AA:85		A4:S>R		DBVPG6304:S>R		UFRJ50816:S>R		UWOPS91_917_1:S>R		YPS138:S>R	AA:88		A4:H>Q		DBVPG6304:H>Q		UFRJ50816:H>Q		UWOPS91_917_1:H>Q		YPS138:H>Q	AA:113		A4:R>-		DBVPG6304:R>-		T21_4:->W		UFRJ50816:R>-		YPS138:R>-	AA:117		UWOPS91_917_1:A>T	AA:128		A4:V>F		DBVPG6304:V>F		UFRJ50816:V>F		YPS138:V>F	AA:145		DBVPG4650:I>M	AA:155		DBVPG6304:->C		UFRJ50791:->C		UFRJ50816:->C		UWOPS91_917_1:I>M		YPS138:->C	AA:159		UWOPS91_917_1:D>E	AA:167		A4:Q>H		DBVPG6304:Q>H		UFRJ50816:Q>H		YPS138:Q>H	AA:217		UWOPS91_917_1:Y>-	AA:220		A4:->K		DBVPG6304:->K		UFRJ50816:->K		YPS138:->K	AA:236		UWOPS91_917_1:Q>-	AA:245		UWOPS91_917_1:I>M	AA:254		A12:L>H		A4:L>H		DBVPG6304:L>H		UFRJ50791:L>H		UFRJ50816:L>H		YPS138:L>H	AA:295		A12:E>G		A4:E>G		UFRJ50816:E>G		YPS138:E>G	AA:311		A12:P>R		A4:P>R		UFRJ50816:P>R		YPS138:P>R	AA:314		N_43:A>V		N_44:A>V		N_45:A>V	AA:325		A12:K>N		A4:K>N		UFRJ50816:K>N		YPS138:K>N	AA:335		A12:F>I		A4:F>I		UFRJ50816:F>I		YPS138:F>I	AA:388		A12:E>V		A4:E>V		UFRJ50816:E>V		UWOPS91_917_1:E>V		YPS138:E>V	AA:405		UWOPS91_917_1:I>S	AA:430		N_45:W>G	AA:431		UWOPS91_917_1:S>Y	AA:459		UWOPS91_917_1:I>T	AA:474		UWOPS91_917_1:T>S	AA:538		A12:Q>H		A4:Q>H		DBVPG6304:Q>H	AA:542		A12:->C		A4:->C		DBVPG6304:->C		UFRJ50816:->C		UWOPS91_917_1:->C	AA:547		UWOPS91_917_1:K>E	AA:606		A12:Q>H		A4:Q>H		DBVPG6304:Q>H		UFRJ50791:Q>H		UWOPS91_917_1:Q>H	AA:639		UWOPS91_917_1:K>N	AA:668		A4:V>A		DBVPG6304:V>A		UFRJ50791:V>A	AA:669		UWOPS91_917_1:Y>-	AA:673		A4:K>N		DBVPG6304:K>N		IFO1804:K>N		N_43:K>N		N_44:K>N		N_45:K>N		UFRJ50791:K>N		UWOPS91_917_1:K>N	AA:698		UWOPS91_917_1:F>L	AA:735		N_44:E>G	AA:743		UWOPS91_917_1:C>W	AA:751		UWOPS91_917_1:K>R	AA:785		A12:I>M		DBVPG6304:I>M		UFRJ50791:I>M		YPS138:I>M	AA:787		UWOPS91_917_1:Y>-	AA:827		DBVPG4650:I>M		Q89_8:I>M	AA:830		UWOPS91_917_1:E>Q	AA:855		UWOPS91_917_1:F>L	AA:865		DBVPG4650:I>M		KPN3828:I>M		Q89_8:I>M	AA:880		UWOPS91_917_1:C>-	AA:891		A12:F>I		DBVPG6304:F>I		UFRJ50791:F>I		UFRJ50816:F>I		YPS138:F>I	AA:918		A12:G>A		DBVPG6304:G>A		UFRJ50791:G>A		UFRJ50816:G>A		YPS138:G>A	AA:951		IFO1804:I>V		N_43:I>V		N_44:I>V		N_45:I>V	AA:964		UWOPS91_917_1:G>S	AA:965		DBVPG4650:F>Y		Y7:F>Y	AA:972		IFO1804:S>C		N_43:S>C		N_44:S>C		N_45:S>C	AA:977		A4:C>S		DBVPG6304:C>S		UFRJ50791:C>S		UFRJ50816:C>S		YPS138:C>S	AA:980		A4:K>Q		DBVPG6304:K>Q		UFRJ50791:K>Q		UFRJ50816:K>Q		YPS138:K>Q	AA:1001		IFO1804:L>P		N_43:L>P		N_44:L>P		N_45:L>P	AA:1006		UWOPS91_917_1:R>S	AA:1021		UFRJ50791:C>S		UFRJ50816:C>S	AA:1024		UWOPS91_917_1:->Y	AA:1032		UWOPS91_917_1:Q>H	AA:1048		UWOPS91_917_1:S>CID:YDL078C	AA:15		A12:L>F		DBVPG6304:L>F		UFRJ50791:D>E		UFRJ50816:L>F		YPS138:D>E	AA:20		A12:F>L		N_43:F>L		N_44:F>L		N_45:F>L		UFRJ50791:F>L		UWOPS91_917_1:F>L		YPS138:F>L	AA:29		A12:F>L		UFRJ50791:F>L		UWOPS91_917_1:F>L		YPS138:F>L	AA:54		A12:Q>H		UFRJ50791:Q>H		UWOPS91_917_1:Q>H		YPS138:Q>H	AA:60		Q62_5:R>S		Z1_1:R>S	AA:77		N_43:->Y		N_44:->Y		N_45:->Y	AA:81		N_43:C>W		N_44:C>W		N_45:C>W		UFRJ50791:C>W		UWOPS91_917_1:C>W		YPS138:C>W	AA:100		YPS138:S>R	AA:108		UFRJ50791:H>Q		UFRJ50816:H>Q	AA:123		UWOPS91_917_1:F>L	AA:129		Q59_1:D>N	AA:148		N_43:S>R		N_44:S>R		N_45:S>R	AA:150		UFRJ50791:Y>H		UFRJ50816:Y>H		UWOPS91_917_1:Y>H		YPS138:Y>H	AA:163		UFRJ50791:S>R		UFRJ50816:S>R		UWOPS91_917_1:S>R		YPS138:S>R	AA:181		N_43:F>L		N_44:F>L		N_45:F>L	AA:185		UWOPS91_917_1:I>M	AA:191		UFRJ50791:F>L		UFRJ50816:F>L		UWOPS91_917_1:F>L		YPS138:F>L	AA:220		UFRJ50791:R>S		UFRJ50816:R>S		YPS138:R>S	AA:225		UFRJ50791:N>K		UFRJ50816:N>K		YPS138:N>K	AA:234		UFRJ50816:C>-		UWOPS91_917_1:C>-		YPS138:C>-	AA:238		DBVPG6304:K>N		UFRJ50791:K>N		UFRJ50816:K>N		UWOPS91_917_1:K>N		YPS138:K>N	AA:258		N_44:I>M	AA:269		N_44:S>C		N_45:S>C	AA:301		UWOPS91_917_1:I>M	AA:306		UWOPS91_917_1:L>H	AA:319		Q59_1:V>A	AA:327		UWOPS91_917_1:->CID:YDL080C	AA:50		A12:D>G		A4:D>G		DBVPG6304:D>G		UFRJ50791:D>G		YPS138:D>G	AA:57		A12:S>R		A4:S>R		DBVPG6304:S>R		UFRJ50791:S>R		UWOPS91_917_1:S>R		YPS138:S>R	AA:59		A12:W>C		A4:W>C		DBVPG6304:W>C		UFRJ50791:W>C		YPS138:W>C	AA:76		IFO1804:N>K		N_43:N>K		N_45:N>K	AA:80		DBVPG6304:R>S	AA:131		A12:R>S		DBVPG6304:R>S		UFRJ50791:R>S		UWOPS91_917_1:R>S		YPS138:R>S	AA:157		A12:Q>H		DBVPG6304:Q>H		UFRJ50791:Q>H	AA:172		IFO1804:S>R		N_43:S>R		N_44:S>R		N_45:S>R	AA:194		UWOPS91_917_1:R>L	AA:202		DBVPG6304:S>R		IFO1804:S>R		N_43:S>R		N_44:S>R		N_45:S>R		UWOPS91_917_1:S>R	AA:209		IFO1804:I>V	AA:210		A12:H>Q		DBVPG6304:H>Q	AA:247		A12:Q>H		DBVPG6304:Q>H		UWOPS91_917_1:Q>H	AA:279		A12:->Y		UWOPS91_917_1:->Y	AA:312		A12:L>F	AA:340		A12:N>H		UWOPS91_917_1:N>H	AA:345		N_43:Y>-		N_44:Y>-		N_45:Y>-	AA:349		A12:E>D		UWOPS91_917_1:E>D	AA:406		A12:S>R		DBVPG6304:S>R		YPS138:S>R	AA:425		A12:->Y		DBVPG6304:->Y		YPS138:->Y	AA:427		A12:Q>H		DBVPG6304:Q>H		UWOPS91_917_1:Q>H		YPS138:Q>H	AA:577		KPN3829:I>V	AA:604		UFRJ50816:I>F	AA:621		UWOPS91_917_1:I>FID:YDL081C	AA:51		UWOPS91_917_1:->Y	AA:84		DBVPG6304:F>L		N_44:F>L		N_45:F>L		UFRJ50791:F>L		UFRJ50816:F>L		UWOPS91_917_1:F>LID:YDL084W	AA:181		A4:R>K		DBVPG6304:R>K		UFRJ50791:R>K		UWOPS91_917_1:R>K		YPS138:R>K	AA:195		CBS432:L>S	AA:314		UWOPS91_917_1:K>RID:YDL085C-A	AA:24		UWOPS91_917_1:L>Q	AA:39		UWOPS91_917_1:R>S	AA:57		IFO1804:F>L		N_45:F>L	AA:67		A12:S>C		A4:S>C		UFRJ50816:S>CID:YDL085W	AA:5		IFO1804:L>F	AA:17		UWOPS91_917_1:F>L	AA:20		A4:S>I		DBVPG6304:S>I		UFRJ50791:S>I		UWOPS91_917_1:S>I		YPS138:S>I	AA:382		CBS5829:P>A		Q95_3:P>A	AA:403		UWOPS91_917_1:D>N	AA:450		A4:N>K		DBVPG6304:N>K		UFRJ50791:N>K		UWOPS91_917_1:N>K		YPS138:N>K	AA:468		A4:R>K		DBVPG6304:R>K		N_43:R>K		N_44:R>K		N_45:R>K		UFRJ50791:R>K		UWOPS91_917_1:R>K		YPS138:R>KID:YDL086W	AA:39		Q32_3:I>L		Q59_1:I>L		Q62_5:I>L	AA:117		KPN3829:P>	AA:183		N_44:M>IID:YDL087C	AA:18		UWOPS91_917_1:R>S	AA:21		A12:V>F		A4:Q>H		DBVPG6304:Q>H		UFRJ50816:V>F		UWOPS91_917_1:Q>H		YPS138:Q>H	AA:54		A12:->C		DBVPG6304:->C		UFRJ50816:->C		UWOPS91_917_1:->C	AA:85		A12:L>I		A4:L>I		DBVPG4650:L>I		DBVPG6304:L>I		IFO1804:L>I		N_17:L>I		N_43:L>I		N_44:L>I		N_45:L>I		S36_7:L>I		UFRJ50816:L>I		UWOPS91_917_1:L>I		YPS138:L>I	AA:104		A12:D>E		DBVPG6304:D>E		UFRJ50816:D>E		UWOPS91_917_1:D>E		YPS138:D>E	AA:119		A12:A>S		A4:A>S		DBVPG6304:A>S		UFRJ50816:A>S		YPS138:A>S	AA:124		A12:M>L		A4:M>L		DBVPG6304:M>L		UFRJ50816:M>L		YPS138:M>L	AA:159		N_43:K>N		N_44:K>N		N_45:K>N	AA:177		A4:L>V		DBVPG6304:L>V		UFRJ50816:L>V		YPS138:L>V	AA:184		N_45:L>P	AA:188		A4:L>H		DBVPG6304:L>H		UFRJ50816:L>H		UWOPS91_917_1:L>H		YPS138:L>H	AA:227		A4:I>M		DBVPG6304:I>M		UWOPS91_917_1:I>M		YPS138:I>M	AA:251		N_43:A>E		N_45:A>E	AA:252		N_17:L>W	AA:262		A4:H>R		DBVPG6304:H>R		UWOPS91_917_1:H>R		YPS138:H>RID:YDL088C	AA:8		N_45:->K		UWOPS91_917_1:->K	AA:11		UWOPS91_917_1:I>V	AA:20		A4:F>I		DBVPG6304:F>I		YPS138:F>I	AA:47		CBS5829:P>L		Z1_1:P>L	AA:52		N_44:E>D		N_45:E>D	AA:56		N_
[truncated: 1,200,000 more chars]
