# Supplementary material for: Proteome-wide evidence for enhanced positive Darwinian selection within intrinsically disordered regions in proteins
Source: Genome Biol. 2011 Jul 19;12(7):R65. doi: 10.1186/gb-2011-12-7-r65 (PMC3218827; doi:10.1186/gb-2011-12-7-r65)
Supplement: Additional file 4 — Synonymous SNPs in S. cerevisiae genes studied. The identity of each affected amino acid in each affected strain is shown for each of 3,737 genes. [file gb-2011-12-7-r65-S4.RTF]

ID:YAL001C	AA:15		DBVPG6044:S>S		Y55:S>S	AA:52		DBVPG1373:C>C		DBVPG1788:C>C		DBVPG6044:C>C		DBVPG6765:C>C		L_1374:C>C		UWOPS03_461_4:C>C		UWOPS05_217_3:C>C		UWOPS05_227_2:C>C		Y55:C>C		YIIc17_E5:C>C		YJM975:C>C		YS9:C>C	AA:91		UWOPS03_461_4:S>S		UWOPS05_217_3:S>S		UWOPS05_227_2:S>S	AA:92		YPS606:L>L	AA:108		NCYC110:N>N		Y55:N>N	AA:151		UWOPS05_217_3:L>L		UWOPS05_227_2:L>L	AA:205		K11:Q>Q		SK1:Q>Q		Y12:Q>Q	AA:258		322134S:Y>Y		DBVPG1853:Y>Y		DBVPG6765:Y>Y		K11:Y>Y		L_1374:Y>Y		L_1528:Y>Y		NCYC110:Y>Y		SK1:Y>Y		UWOPS05_217_3:Y>Y		UWOPS05_227_2:Y>Y		UWOPS83_787_3:Y>Y		Y12:Y>Y		Y55:Y>Y		YIIc17_E5:Y>Y		YJM978:Y>Y		YPS606:Y>Y		YS9:Y>Y	AA:344		DBVPG1788:K>K	AA:368		DBVPG1853:F>F		K11:F>F		SK1:F>F		UWOPS05_217_3:F>F		UWOPS83_787_3:F>F		Y55:F>F		YS9:F>F	AA:386		322134S:T>T		UWOPS05_217_3:N>N		UWOPS05_227_2:N>N	AA:388		273614X:G>G		K11:G>G		SK1:G>G		UWOPS05_217_3:G>G		UWOPS83_787_3:G>G		Y12:G>G		Y55:G>G		YS9:G>G	AA:427		273614X:G>G		UWOPS83_787_3:G>G		YS9:G>G	AA:541		SK1:N>N		Y12:N>N	AA:590		273614X:L>L		322134S:L>L		DBVPG1373:L>L		DBVPG1853:L>L		DBVPG6044:L>L		DBVPG6765:L>L		NCYC110:L>L		SK1:L>L		UWOPS83_787_3:L>L		Y55:L>L		YJM978:L>L		YPS606:L>L		YS4:L>L		YS9:L>L	AA:603		273614X:V>V		DBVPG1853:V>V		SK1:V>V	AA:617		322134S:G>G	AA:630		DBVPG6044:A>A		NCYC110:A>A		Y55:A>A	AA:701		273614X:S>S	AA:728		YS4:T>T	AA:753		DBVPG6044:T>T		Y55:T>T	AA:827		Y55:L>L	AA:939		SK1:N>N	AA:977		273614X:G>G		DBVPG1853:G>G		DBVPG6040:G>G		SK1:G>G		UWOPS05_217_3:G>G		UWOPS05_227_2:G>G		UWOPS83_787_3:G>G		Y12:G>G		Y55:G>G		YPS128:G>G	AA:1064		DBVPG6044:K>K		Y55:K>K	AA:1094		DBVPG6044:V>V		Y55:V>V	AA:1106		273614X:P>P		378604X:P>P		DBVPG6044:P>P		SK1:P>P		UWOPS05_217_3:P>P		UWOPS05_227_2:P>P		UWOPS83_787_3:P>P		Y12:P>P		Y55:P>P		YPS128:P>P	AA:1113		L_1528:L>L	AA:1117		273614X:C>C		378604X:C>C		DBVPG1106:C>C		DBVPG6765:C>C		L_1528:C>C		SK1:C>C		UWOPS83_787_3:C>C		UWOPS87_2421:C>C		Y12:C>C		Y55:C>C		YIIc17_E5:C>C		YJM975:C>C		YJM981:C>C		YPS128:C>C	AA:1120		273614X:V>V		378604X:V>V		SK1:V>V		Y12:V>VID:YAL005C	AA:23		DBVPG1373:R>R	AA:30		BC187:D>D		DBVPG1106:D>D		DBVPG1373:D>D		UWOPS87_2421:D>D		YIIc17_E5:D>D		YJM978:D>D		YPS606:D>D	AA:71		UWOPS87_2421:L>L	AA:99		322134S:P>P		BC187:P>P		DBVPG1106:P>P		DBVPG1373:P>P		DBVPG6044:P>P		SK1:P>P		UWOPS05_227_2:P>P		UWOPS87_2421:P>P		Y55:P>P		YGPM:P>P		YJM978:P>P		YPS128:P>P		YPS606:P>P	AA:134		322134S:G>G		BC187:G>G		DBVPG1106:G>G		DBVPG1373:G>G		DBVPG6044:G>G		K11:G>G		L_1528:G>G		SK1:G>G		UWOPS05_217_3:G>G		UWOPS05_227_2:G>G		Y55:G>G		YGPM:G>G		YJM978:G>G		YPS128:G>G		YPS606:G>G	AA:262		322134S:T>T		DBVPG1106:T>T		DBVPG1373:S>S		DBVPG6040:T>T		DBVPG6044:T>T		DBVPG6765:T>T		K11:T>T		L_1528:T>T		NCYC110:T>T		UWOPS05_217_3:T>T		UWOPS05_227_2:T>T		UWOPS83_787_3:T>T		YJM975:T>T		YPS128:T>T		YPS606:T>T	AA:302		K11:L>L	AA:319		YPS128:R>R		YPS606:R>R	AA:324		UWOPS87_2421:D>D	AA:327		322134S:Q>Q		DBVPG6040:Q>Q		DBVPG6765:Q>Q		L_1528:Q>Q	AA:338		DBVPG6044:T>T		K11:T>T		NCYC110:T>T		Y55:T>T		Y9:T>T	AA:344		UWOPS83_787_3:Q>Q	AA:345		DBVPG1373:K>K		K11:K>K	AA:349		UWOPS05_217_3:D>D		UWOPS05_227_2:D>D		YPS128:D>D		YPS606:D>D	AA:362		DBVPG1373:P>P		DBVPG6044:P>P		K11:P>P		NCYC110:P>P		Y55:P>P	AA:383		UWOPS83_787_3:S>S	AA:430		DBVPG1373:D>D	AA:445		DBVPG1373:A>A	AA:482		DBVPG1373:I>I	AA:484		DBVPG1373:N>N	AA:492		DBVPG1373:T>T	AA:535		UWOPS03_461_4:S>S		UWOPS05_217_3:S>S	AA:580		DBVPG1373:T>T		DBVPG1853:T>T		DBVPG6765:T>T		NCYC361:T>T		UWOPS03_461_4:T>T		UWOPS05_217_3:T>T		Y55:T>T		YIIc17_E5:T>T		YJM978:T>T		YJM981:T>T		YPS128:T>T		YPS606:T>T		YS9:T>T	AA:582		YJM978:A>A		YJM981:A>AID:YAL007C	AA:2		NCYC110:I>I		Y55:I>I	AA:42		UWOPS83_787_3:E>E	AA:184		NCYC110:L>L		Y55:L>L	AA:198		DBVPG1853:A>A		DBVPG6040:A>AID:YAL008W	AA:15		K11:L>L		SK1:L>L	AA:16		BC187:N>N		DBVPG1106:N>N		DBVPG1788:N>N		DBVPG6765:N>N		L_1374:N>N		L_1528:N>N		UWOPS05_227_2:N>N		Y55:N>N		YPS606:N>N	AA:50		K11:G>G		SK1:G>G		Y12:G>G		YS9:G>G	AA:58		DBVPG6040:L>L	AA:73		UWOPS05_217_3:G>G		YPS606:G>G	AA:108		BC187:L>L		DBVPG1106:L>L		DBVPG1788:L>L		DBVPG6040:L>L		DBVPG6765:L>L		L_1528:L>L		UWOPS05_217_3:L>L		UWOPS87_2421:L>L		Y55:L>L		YPS606:L>L	AA:117		322134S:G>G		BC187:G>G		DBVPG1106:G>G		DBVPG1788:G>G		DBVPG6765:G>G		L_1528:G>G		UWOPS05_217_3:G>G		UWOPS87_2421:G>G		Y55:G>G		YPS606:G>G	AA:141		322134S:L>L		BC187:L>L		DBVPG1106:L>L		DBVPG1788:L>L		DBVPG6040:L>L		DBVPG6765:L>L		L_1374:L>L		L_1528:L>L		SK1:L>L		UWOPS05_217_3:L>L		UWOPS87_2421:L>L		Y12:L>L		Y55:L>L		YPS606:L>L		YS9:L>LID:YAL009W	AA:33		273614X:S>S		322134S:S>S		378604X:S>S		DBVPG1106:S>S		DBVPG1373:S>S		DBVPG1788:S>S		DBVPG1853:S>S		DBVPG6040:S>S		DBVPG6044:S>S		DBVPG6765:S>S		L_1374:S>S		NCYC361:S>S		UWOPS03_461_4:S>S		UWOPS05_217_3:S>S		Y55:S>S		YJM975:S>S		YPS128:S>S		YPS606:S>S		YS2:S>S		YS4:S>S		YS9:S>S	AA:39		DBVPG1106:R>R	AA:68		322134S:Q>Q		378604X:Q>Q		DBVPG1106:Q>Q		DBVPG1373:Q>Q		DBVPG1788:Q>Q		DBVPG1853:Q>Q		DBVPG6765:Q>Q		L_1374:Q>Q		UWOPS87_2421:Q>Q		YJM975:Q>Q		YS4:Q>Q	AA:100		322134S:V>V		DBVPG1106:V>V		DBVPG1373:V>V		DBVPG1788:V>V		DBVPG1853:V>V		DBVPG6040:V>V		DBVPG6765:V>V		L_1374:V>V		NCYC361:V>V		UWOPS87_2421:V>V		YIIc17_E5:V>V		YS2:V>V		YS4:V>V	AA:114		DBVPG1106:F>F		DBVPG1373:F>F		DBVPG1788:F>F		DBVPG1853:F>F		DBVPG6765:F>F		L_1374:F>F		UWOPS87_2421:F>F		YIIc17_E5:F>F		YJM975:F>F		YS4:F>F	AA:157		YS9:Q>Q	AA:241		DBVPG6040:A>A		DBVPG6044:A>A		NCYC110:A>A		NCYC361:A>A		Y55:A>AID:YAL010C	AA:148		322134S:Y>Y		378604X:Y>Y		DBVPG1373:Y>Y		DBVPG1788:Y>Y		DBVPG6044:Y>Y		DBVPG6765:Y>Y		L_1374:Y>Y		L_1528:Y>Y		NCYC110:Y>Y		Y55:Y>Y		YJM975:Y>Y		YJM981:Y>Y		YPS606:Y>Y		YS2:Y>Y	AA:170		DBVPG6044:K>K		NCYC110:K>K		Y55:K>K	AA:283		DBVPG6040:S>S	AA:327		UWOPS05_217_3:N>N	AA:333		UWOPS05_227_2:P>P	AA:335		322134S:S>S		378604X:S>S		DBVPG1106:S>S		DBVPG1373:S>S		DBVPG1788:S>S		DBVPG6765:S>S		YIIc17_E5:S>S		YJM978:S>S		YS2:S>S	AA:354		322134S:N>N		378604X:N>N		DBVPG1106:N>N		DBVPG1373:N>N		DBVPG1788:N>N		DBVPG6040:N>N		DBVPG6044:N>N		DBVPG6765:N>N		Y55:N>N		YIIc17_E5:N>N		YJM978:N>N		YPS128:N>N		YPS606:N>N	AA:379		DBVPG6044:L>L		NCYC110:L>L		Y55:L>L	AA:382		DBVPG6044:L>L		Y55:L>L	AA:397		322134S:R>R		378604X:R>R		DBVPG1106:R>R		DBVPG1373:R>R		DBVPG6044:R>R		DBVPG6765:R>R		L_1374:R>R		L_1528:R>R		Y55:R>R		YPS128:R>R		YPS606:R>R	AA:418		DBVPG6044:L>L		NCYC110:L>L		Y55:L>L		Y9:L>L	AA:446		Y9:L>L	AA:470		Y9:A>A	AA:492		K11:S>S		Y9:S>S		YPS128:S>S		YPS606:S>S	AA:494		DBVPG6044:->-		NCYC110:->-		Y55:->-ID:YAL011W	AA:15		L_1528:E>E	AA:75		322134S:G>G		BC187:G>G		DBVPG1373:G>G		DBVPG1788:G>G		DBVPG1853:G>G		DBVPG6765:G>G		L_1374:G>G		L_1528:G>G		UWOPS87_2421:G>G		YIIc17_E5:G>G		YJM981:G>G		YS2:G>G		YS9:G>G	AA:76		DBVPG6044:L>L		Y55:L>L	AA:90		322134S:T>T		BC187:T>T		DBVPG1373:T>T		DBVPG1788:T>T		DBVPG1853:T>T		DBVPG6044:T>T		DBVPG6765:T>T		L_1374:T>T		L_1528:T>T		UWOPS05_217_3:T>T		UWOPS05_227_2:T>T		UWOPS87_2421:T>T		Y55:T>T		YIIc17_E5:T>T		YJM981:T>T		YPS128:T>T		YS9:T>T	AA:108		YPS128:S>S	AA:118		DBVPG6044:F>F		K11:F>F		UWOPS05_217_3:F>F		UWOPS05_227_2:F>F		Y55:F>F	AA:140		322134S:P>P		DBVPG1373:P>P		DBVPG1788:P>P		DBVPG1853:P>P		DBVPG6765:P>P		L_1374:P>P		L_1528:P>P		YJM981:P>P		YS9:P>P	AA:172		Y55:F>F	AA:175		322134S:K>K		DBVPG1373:K>K		DBVPG1788:K>K		DBVPG1853:K>K		DBVPG6765:K>K		L_1374:K>K		L_1528:K>K		Y55:K>K		YJM981:K>K		YS9:K>K	AA:179		UWOPS05_217_3:I>I		UWOPS05_227_2:I>I	AA:211		322134S:E>E		BC187:E>E		DBVPG1373:E>E		DBVPG1788:E>E		DBVPG1853:E>E		DBVPG6765:E>E		L_1374:E>E		L_1528:E>E		Y55:E>E		YJM981:E>E		YS9:E>E	AA:244		273614X:L>L		K11:L>L	AA:260		273614X:K>K	AA:294		322134S:E>E		BC187:E>E		DBVPG1373:E>E		DBVPG1788:E>E		DBVPG6765:E>E		L_1374:E>E		L_1528:E>E		UWOPS05_217_3:E>E		YJM975:E>E		YS9:E>E	AA:312		273614X:F>F		BC187:F>F		DBVPG1373:F>F		DBVPG1788:F>F		DBVPG6765:F>F		L_1374:F>F		L_1528:F>F		UWOPS05_217_3:F>F		Y55:F>F		YJM975:F>F		YJM981:F>F		YS9:F>F	AA:390		YPS606:A>A	AA:392		DBVPG1373:S>S		DBVPG1788:S>S		DBVPG6765:S>S		UWOPS87_2421:S>S		YJM975:S>S		YJM981:S>S	AA:415		UWOPS05_217_3:Q>Q	AA:424		BC187:P>P		L_1374:P>P		L_1528:P>P	AA:517		322134S:E>E		DBVPG1106:E>E		DBVPG1788:E>E		DBVPG1853:E>E		DBVPG6765:E>E		L_1374:E>E		L_1528:E>E		UWOPS87_2421:E>E		Y55:E>E		YJM975:E>E		YJM981:E>E		YS9:E>E	AA:553		322134S:G>G		DBVPG1106:G>G		DBVPG1788:G>G		DBVPG1853:G>G		DBVPG6765:G>G		L_1374:G>G		L_1528:G>G		NCYC110:G>G		UWOPS05_217_3:G>G		UWOPS87_2421:G>G		Y55:G>G		YJM975:G>G		YJM981:G>G		YS9:G>G	AA:602		322134S:A>AID:YAL013W	AA:46		DBVPG6765:S>S	AA:69		273614X:K>K		DBVPG1373:K>K		DBVPG1788:K>K		DBVPG6040:K>K		DBVPG6765:K>K		L_1374:K>K		YJM975:K>K		YJM981:K>K	AA:172		DBVPG6765:P>P	AA:183		DBVPG6765:L>L	AA:334		L_1374:P>P		L_1528:P>P		YIIc17_E5:P>P	AA:341		DBVPG1373:C>C		DBVPG1788:C>C		DBVPG6765:C>C		L_1374:C>C		L_1528:C>C		SK1:C>C		YIIc17_E5:C>C		YJM978:C>C		YS9:C>C	AA:395		Y55:E>EID:YAL016W	AA:85		NCYC110:A>A		Y55:A>A		Y9:A>A	AA:91		Y9:E>E	AA:133		DBVPG6044:E>E		Y55:E>E	AA:136		DBVPG6044:V>V		Y55:V>V	AA:224		YS9:L>L	AA:244		DBVPG6044:Q>Q		Y55:Q>Q		Y9:Q>Q		YPS128:Q>Q		YPS606:Q>Q	AA:266		YS9:L>L	AA:305		Y55:S>S		Y9:S>S		YPS128:S>S		YPS606:S>S		YS9:S>S	AA:339		Y55:A>A	AA:353		UWOPS87_2421:D>D	AA:355		L_1528:S>S	AA:460		UWOPS87_2421:E>E		Y55:E>E		YPS128:E>E		YPS606:E>E	AA:463		Y55:P>P	AA:490		NCYC361:V>V	AA:514		YS9:R>R	AA:528		UWOPS87_2421:L>L		Y12:L>L		Y55:L>L		YPS128:L>L	AA:530		UWOPS87_2421:E>E		Y12:E>E		Y55:E>E		YPS128:E>E	AA:535		UWOPS87_2421:R>R		Y12:R>R		Y55:R>R		YPS128:R>R	AA:541		Y12:A>A		Y55:A>A		YPS128:A>A	AA:559		DBVPG6044:L>L		UWOPS05_217_3:L>L		UWOPS05_227_2:L>L		Y55:L>L	AA:574		DBVPG6044:R>R		L_1374:R>R		NCYC361:R>R		UWOPS05_217_3:R>R		UWOPS05_227_2:R>R		UWOPS87_2421:R>R		Y12:R>R		Y55:R>R		YPS128:R>RID:YAL018C	AA:28		273614X:L>L		378604X:L>L		BC187:L>L		DBVPG1788:L>L		DBVPG6765:L>L		L_1374:L>L		YIIc17_E5:L>L		YJM981:L>L	AA:64		273614X:D>D		378604X:D>D		BC187:D>D		DBVPG1788:D>D		DBVPG6765:D>D		L_1374:D>D		NCYC110:D>D		Y55:D>D		YJM981:D>D		YPS606:D>D	AA:66		273614X:F>F		378604X:F>F		BC187:F>F		DBVPG1788:F>F		DBVPG6765:F>F		L_1374:F>F		YJM981:F>F	AA:100		YS9:L>L	AA:125		K11:L>L		YS9:L>L	AA:131		Y55:F>F	AA:164		K11:E>E		Y12:E>E		YS9:E>E	AA:240		DBVPG6044:K>K		K11:K>K		Y12:K>K		Y55:K>K		YPS606:K>K		YS4:K>K		YS9:K>K	AA:259		K11:H>H		Y12:H>H		YS9:H>H	AA:273		YPS606:I>I		YS4:I>I	AA:312		DBVPG6044:I>I		NCYC110:I>I		Y55:I>I		YPS606:I>IID:YAL019W	AA:48		K11:R>R		Y12:R>R	AA:72		YPS606:E>E	AA:83		DBVPG6044:P>P		K11:P>P		NCYC110:P>P		Y12:P>P		Y55:P>P	AA:88		K11:T>T	AA:89		YPS606:L>L	AA:139		DBVPG6044:L>L		NCYC110:L>L		Y55:L>L	AA:180		YPS606:Q>Q	AA:192		DBVPG6044:N>N		K11:N>N		NCYC110:N>N		Y12:N>N		Y55:N>N		YPS606:N>N	AA:198		DBVPG6044:L>L		K11:L>L		NCYC110:L>L		Y12:L>L		Y55:L>L		YPS606:L>L	AA:230		DBVPG6044:L>L		K11:L>L		NCYC110:L>L		Y55:L>L		YS2:L>L	AA:237		SK1:L>L		YS9:L>L	AA:247		DBVPG6044:A>A		Y55:A>A	AA:249		SK1:D>D		YS9:D>D	AA:289		DBVPG6044:D>D		UWOPS05_227_2:D>D		Y55:D>D		YPS606:D>D		YS2:D>D	AA:292		YPS606:I>I	AA:294		YS2:D>D	AA:352		DBVPG6765:R>R	AA:353		UWOPS05_227_2:L>L		Y12:L>L		YS2:L>L		YS4:L>L		YS9:L>L	AA:360		Y12:S>S		YS2:S>S		YS4:S>S	AA:373		Y12:K>K		YS2:K>K		YS4:K>K		YS9:K>K	AA:405		YS2:G>G	AA:440		UWOPS03_461_4:T>T		UWOPS05_227_2:T>T	AA:472		DBVPG6044:N>N		UWOPS03_461_4:N>N		Y12:N>N		Y55:N>N		YPS128:N>N		YPS606:N>N		YS4:N>N		YS9:N>N	AA:474		UWOPS87_2421:T>T		Y12:T>T		YPS128:T>T		YPS606:T>T		YS4:T>T		YS9:T>T	AA:531		UWOPS03_461_4:D>D		UWOPS05_217_3:D>D		UWOPS87_2421:D>D		Y12:D>D		YPS128:D>D		YPS606:D>D		YS4:D>D		YS9:D>D	AA:536		DBVPG6044:A>A		Y12:A>A		Y55:A>A		YPS128:A>A		YPS606:A>A		YS9:A>A	AA:564		UWOPS87_2421:R>R	AA:644		Y12:P>P		Y9:P>P	AA:677		UWOPS03_461_4:T>T		UWOPS05_217_3:T>T	AA:729		K11:L>L		UWOPS03_461_4:L>L		UWOPS05_217_3:L>L		Y12:L>L	AA:825		Y55:I>I	AA:827		322134S:K>K		SK1:K>K	AA:862		K11:S>S		UWOPS03_461_4:S>S		UWOPS05_217_3:S>S		UWOPS83_787_3:S>S		UWOPS87_2421:S>S		Y12:S>S		Y55:S>S		YS2:S>S		YS9:S>S	AA:864		K11:S>S		UWOPS83_787_3:S>S		UWOPS87_2421:S>S		Y12:S>S		YS2:S>S		YS9:S>S	AA:870		Y55:A>A	AA:917		K11:S>S		UWOPS83_787_3:S>S		Y12:S>S		YS2:S>S		YS9:S>S	AA:920		K11:T>T		UWOPS83_787_3:T>T		Y12:T>T		YS2:T>T		YS9:T>T	AA:927		UWOPS87_2421:L>L	AA:930		Y12:N>N	AA:969		UWOPS83_787_3:V>V	AA:1008		322134S:L>L		DBVPG6044:L>L		K11:L>L		NCYC110:L>L		UWOPS05_217_3:L>L		UWOPS83_787_3:L>L		YPS606:L>L		YS4:L>L	AA:1058		DBVPG6044:R>R		NCYC110:R>R		Y55:R>R	AA:1066		UWOPS05_217_3:K>K		YPS606:K>K	AA:1068		DBVPG6044:V>V		NCYC110:V>V		Y55:V>V	AA:1071		UWOPS05_217_3:T>T		YPS606:T>T	AA:1075		DBVPG6044:T>T		K11:T>T		NCYC110:T>T		Y55:T>T		YS4:T>TID:YAL020C	AA:9		K11:S>S		Y12:S>S		YPS128:S>S	AA:51		UWOPS05_217_3:L>L	AA:111		SK1:R>R	AA:156		K11:T>T		NCYC110:T>T		Y12:T>T		Y55:T>T		YPS606:T>T		YS4:T>T	AA:161		K11:Q>Q		UWOPS05_217_3:Q>Q		Y12:Q>Q		Y55:Q>Q		YPS606:Q>Q		YS4:Q>Q	AA:168		Y12:L>L		YS4:L>L	AA:180		YJM981:V>V	AA:182		UWOPS05_217_3:V>V		UWOPS05_227_2:V>V		Y55:V>V	AA:198		UWOPS83_787_3:G>G	AA:202		UWOPS83_787_3:V>V	AA:205		UWOPS83_787_3:S>S	AA:207		UWOPS83_787_3:R>R	AA:210		UWOPS05_227_2:T>T	AA:212		YS9:F>F	AA:241		UWOPS83_787_3:T>T	AA:242		K11:V>V		YPS606:V>V		YS4:V>V	AA:244		UWOPS83_787_3:S>S	AA:253		K11:L>L		UWOPS03_461_4:L>L		UWOPS05_227_2:L>L		UWOPS83_787_3:L>L		UWOPS87_2421:L>L		Y55:L>L		YPS606:L>L		YS4:L>L		YS9:L>L	AA:268		Y55:T>T	AA:270		UWOPS83_787_3:S>S	AA:295		UWOPS83_787_3:G>G	AA:310		K11:L>L	AA:317		K11:S>S		YS4:S>SID:YAL022C	AA:17		DBVPG1853:V>V	AA:77		DBVPG6044:S>S		NCYC110:S>S		Y55:S>S	AA:95		DBVPG1853:I>I		DBVPG6044:I>I		DBVPG6765:I>I		NCYC110:I>I		UWOPS05_227_2:I>I		Y55:I>I	AA:100		DBVPG1853:Q>Q		DBVPG6044:Q>Q		DBVPG6765:Q>Q		NCYC110:Q>Q		UWOPS05_227_2:Q>Q		Y55:Q>Q		YPS606:Q>Q	AA:110		DBVPG1788:T>T		DBVPG1853:T>T		DBVPG6044:T>T		DBVPG6765:T>T		NCYC110:T>T		UWOPS05_227_2:T>T		Y55:T>T		YJM978:T>T		YPS606:T>T		YS4:T>T	AA:139		DBVPG6044:R>R		NCYC110:R>R		UWOPS05_227_2:R>R		Y55:R>R		YPS606:R>R	AA:149		DBVPG6044:N>N		Y55:N>N	AA:194		273614X:T>T	AA:212		DBVPG1853:G>G		DBVPG6044:G>G		UWOPS05_227_2:G>G		UWOPS87_2421:G>G		Y55:G>G		YPS606:G>G	AA:216		YPS606:G>G	AA:232		273614X:A>A		322134S:A>A		DBVPG1373:A>A		DBVPG1788:A>A		DBVPG1853:A>A		DBVPG6040:A>A		DBVPG6044:A>A		DBVPG6765:A>A		L_1374:A>A		L_1528:A>A		SK1:A>A		UWOPS05_227_2:A>A		UWOPS87_2421:A>A		Y55:A>A		YIIc17_E5:A>A		YJM981:A>A		YS9:A>A	AA:237		DBVPG1853:S>S		DBVPG6044:S>S		UWOPS05_227_2:S>S		UWOPS87_2421:S>S		Y55:S>S	AA:243		322134S:G>G	AA:264		UWOPS05_217_3:S>S		UWOPS05_227_2:S>S	AA:269		UWOPS05_217_3:S>S		UWOPS05_227_2:S>S	AA:328		DBVPG6040:L>L	AA:330		UWOPS03_461_4:L>L		UWOPS05_217_3:L>L	AA:477		BC187:L>L		YJM975:L>L		YJM981:L>L	AA:488		Y55:G>G	AA:496		Y55:T>T	AA:501		SK1:G>G	AA:508		UWOPS03_461_4:F>F		UWOPS05_217_3:F>F	AA:517		Y9:R>RID:YAL023C	AA:17		Y55:I>I	AA:42		UWOPS05_217_3:E>E		YPS128:E>E	AA:148		Y55:Y>Y		YJM978:Y>Y		YPS128:Y>Y		YPS606:Y>Y		YS4:Y>Y	AA:156		YPS128:A>A		YPS606:A>A	AA:194		322134S:S>S		DBVPG1788:S>S		SK1:S>S		Y55:S>S		YIIc17_E5:S>S		YJM975:S>S		YJM978:S>S		YPS128:S>S		YPS606:S>S		YS9:S>S	AA:338		273614X:G>G		322134S:G>G		BC187:G>G		DBVPG1373:G>G		DBVPG1788:G>G		DBVPG6040:G>G		DBVPG6765:G>G		L_1374:G>G		SK1:G>G		UWOPS05_217_3:G>G		YJM975:G>G		YPS128:G>G		YPS606:G>G		YS2:G>G		YS9:G>G	AA:349		DBVPG6040:V>V		UWOPS05_217_3:V>V		YS9:V>V	AA:356		UWOPS05_217_3:L>L	AA:363		UWOPS05_217_3:S>S	AA:380		UWOPS05_217_3:Y>Y	AA:454		Y9:N>N	AA:492		UWOPS05_217_3:L>L		Y9:L>L	AA:499		UWOPS03_461_4:L>L		UWOPS05_217_3:L>L	AA:508		273614X:E>E		DBVPG6040:E>E		UWOPS03_461_4:E>E		UWOPS05_217_3:E>E		Y55:E>E		Y9:E>E		YPS128:E>E		YPS606:E>E	AA:517		DBVPG6040:K>K		UWOPS03_461_4:K>K		UWOPS05_217_3:K>K		Y55:K>K		Y9:K>K		YPS128:K>K		YPS606:K>K	AA:533		DBVPG6040:R>R		NCYC110:R>R		Y55:R>R		YPS606:R>R	AA:536		273614X:P>P	AA:555		UWOPS03_461_4:L>L		UWOPS05_217_3:L>L	AA:584		L_1374:P>P		YJM975:P>P		YJM981:P>P	AA:624		UWOPS87_2421:T>T	AA:657		DBVPG6044:L>L		NCYC110:L>L		UWOPS03_461_4:L>L		UWOPS05_217_3:L>L		Y55:L>L		YPS128:L>L	AA:674		UWOPS03_461_4:Y>Y		UWOPS05_217_3:Y>Y	AA:733		YS2:F>FID:YAL025C	AA:40		DBVPG1853:R>R	AA:102		DBVPG6044:K>K		UWOPS83_787_3:K>K		Y55:K>K	AA:117		DBVPG1853:Q>Q	AA:153		DBVPG6044:L>L		UWOPS83_787_3:L>L		Y55:L>L	AA:210		UWOPS87_2421:E>E	AA:291		DBVPG6040:Y>Y		SK1:Y>Y		Y9:Y>Y		YS4:Y>YID:YAL027W	AA:59		DBVPG6040:P>P		DBVPG6044:P>P		K11:P>P		SK1:P>P		UWOPS83_787_3:P>P		Y55:P>P		YPS606:P>P		YS4:P>P	AA:96		YIIc17_E5:S>S	AA:119		DBVPG6040:A>A		DBVPG6044:A>A		K11:A>A		SK1:A>A		UWOPS83_787_3:A>A		Y55:A>A		YPS606:A>A		YS4:A>A	AA:123		DBVPG6040:T>T		DBVPG6044:T>T		K11:T>T		SK1:T>T		UWOPS03_461_4:T>T		UWOPS83_787_3:T>T		UWOPS87_2421:T>T		Y55:T>T		Y9:T>T		YPS606:T>T	AA:129		DBVPG6040:S>S		DBVPG6044:S>S		K11:S>S		SK1:S>S		UWOPS83_787_3:S>S		Y55:S>S		YPS606:S>S		YS4:S>S		YS9:S>S	AA:144		DBVPG1853:L>L		DBVPG6040:L>L		DBVPG6044:L>L		K11:L>L		SK1:L>L		UWOPS03_461_4:L>L		UWOPS83_787_3:L>L		Y55:L>L		Y9:L>L		YPS128:L>L		YPS606:L>L		YS4:L>L		YS9:L>L	AA:151		DBVPG1853:E>E		DBVPG6040:E>E		DBVPG6044:E>E		K11:E>E		SK1:E>E		UWOPS03_461_4:E>E		UWOPS83_787_3:E>E		UWOPS87_2421:E>E		Y55:E>E		Y9:E>E		YPS606:E>E		YS4:E>E		YS9:E>E	AA:161		DBVPG1853:K>K		DBVPG6040:K>K		DBVPG6044:K>K		K11:K>K		SK1:K>K		UWOPS03_461_4:K>K		UWOPS83_787_3:K>K		UWOPS87_2421:K>K		Y55:K>K		Y9:K>K		YPS128:K>K		YPS606:K>K		YS9:K>K	AA:168		DBVPG1853:Y>Y		DBVPG6040:Y>Y		DBVPG6044:Y>Y		K11:Y>Y		SK1:Y>Y		UWOPS03_461_4:Y>Y		UWOPS83_787_3:Y>Y		UWOPS87_2421:Y>Y		Y55:Y>Y		YPS128:Y>Y		YPS606:Y>Y		YS9:Y>Y	AA:184		DBVPG1853:G>G		DBVPG6040:G>G		DBVPG6044:G>G		K11:G>G		SK1:G>G		UWOPS83_787_3:G>G		UWOPS87_2421:G>G		Y55:G>G		Y9:G>G		YPS128:G>G		YPS606:G>G		YS9:G>G	AA:215		UWOPS83_787_3:P>P	AA:242		DBVPG1853:V>V		DBVPG6040:V>V		DBVPG6044:V>V		K11:V>V		NCYC110:V>V		SK1:V>V		UWOPS03_461_4:V>V		UWOPS87_2421:V>V		Y55:V>V		Y9:V>V		YPS128:V>V		YPS606:V>V		YS4:V>V	AA:254		DBVPG1853:Q>Q		DBVPG6040:Q>Q		K11:Q>Q		NCYC110:Q>Q		SK1:Q>Q		UWOPS03_461_4:Q>Q		UWOPS87_2421:Q>Q		Y55:Q>Q		Y9:Q>Q		YPS128:Q>Q		YS4:Q>QID:YAL028W	AA:14		DBVPG6044:G>G		NCYC110:G>G		Y12:G>G		Y55:G>G		YPS606:G>G	AA:28		DBVPG6044:K>K		NCYC110:K>K		UWOPS05_217_3:K>K		UWOPS05_227_2:K>K		Y12:K>K		Y55:K>K	AA:71		Y12:E>E	AA:79		Y12:E>E	AA:95		DBVPG6044:Y>Y		NCYC110:Y>Y		UWOPS03_461_4:Y>Y		UWOPS87_2421:Y>Y		Y12:Y>Y		Y55:Y>Y		YPS606:Y>Y	AA:137		Y12:T>T		YPS606:T>T	AA:140		NCYC361:G>G		Y12:G>G		YPS606:G>G	AA:141		DBVPG6044:P>P		NCYC110:P>P		Y55:P>P	AA:142		Y12:F>F		YPS606:F>F	AA:168		DBVPG6044:L>L		NCYC110:L>L		Y55:L>L	AA:175		UWOPS03_461_4:A>A		UWOPS87_2421:A>A		YPS606:A>A	AA:187		UWOPS87_2421:L>L	AA:189		NCYC110:S>S		UWOPS03_461_4:S>S		UWOPS87_2421:S>S		Y12:S>S		Y55:S>S		YPS606:S>S	AA:218		UWOPS87_2421:K>K	AA:237		DBVPG6040:K>K	AA:278		NCYC110:S>S	AA:282		NCYC110:P>P	AA:284		UWOPS87_2421:S>S	AA:322		UWOPS83_787_3:S>S		Y55:S>S	AA:327		DBVPG6040:T>T	AA:462		YS4:A>A	AA:505		YPS128:A>AID:YAL032C	AA:42		DBVPG6044:D>D		NCYC110:D>D		Y12:D>D		Y55:D>D		YPS128:D>D		YPS606:D>D	AA:52		Y12:E>E	AA:123		DBVPG6044:L>L		K11:L>L		UWOPS05_217_3:L>L		UWOPS05_227_2:L>L		UWOPS87_2421:L>L		Y55:L>L		YPS128:L>L		YS2:L>L	AA:133		YS2:K>K	AA:143		K11:V>V		SK1:V>V	AA:226		Y55:K>K	AA:282		UWOPS03_461_4:R>R		UWOPS05_217_3:R>R	AA:300		DBVPG6044:D>D		UWOPS03_461_4:D>D		UWOPS05_217_3:D>D		Y55:D>D		Y9:D>D	AA:341		DBVPG6044:N>N		NCYC110:N>N		Y55:N>N	AA:373		UWOPS03_461_4:K>KID:YAL033W	AA:2		UWOPS83_787_3:V>V		Y55:V>V		YPS606:V>V	AA:8		UWOPS03_461_4:Y>Y		UWOPS05_227_2:Y>Y	AA:88		Y9:C>C	AA:108		DBVPG6044:D>D		NCYC110:D>D		Y55:D>D		YPS606:D>DID:YAL034C	AA:37		K11:S>S		SK1:S>S		UWOPS05_227_2:S>S		UWOPS83_787_3:S>S	AA:87		K11:V>V		SK1:V>V	AA:116		K11:S>S		SK1:S>S	AA:170		BC187:N>N		DBVPG1373:N>N		DBVPG1788:N>N		DBVPG6040:N>N		DBVPG6765:N>N		L_1374:N>N		YIIc17_E5:N>N		YJM981:N>N		YS9:N>N	AA:209		DBVPG6044:L>L		NCYC110:L>L		Y55:L>L	AA:233		SK1:G>G	AA:336		DBVPG6044:L>L		K11:L>L		NCYC110:L>L		SK1:L>L		UWOPS03_461_4:L>L		Y55:L>L	AA:341		DBVPG6044:L>L		NCYC110:L>L		Y55:L>L	AA:369		322134S:L>LID:YAL034W-A	AA:33		L_1374:E>E	AA:69		DBVPG6040:E>E		DBVPG6044:E>E		SK1:E>E		UWOPS03_461_4:E>E		UWOPS05_217_3:E>E		UWOPS05_227_2:E>E		Y55:E>E		Y9:E>E		YPS606:E>E	AA:111		SK1:D>D	AA:120		SK1:L>L	AA:153		UWOPS05_227_2:R>R	AA:203		DBVPG6044:V>V		UWOPS03_461_4:V>V		UWOPS05_227_2:V>V		Y55:V>V	AA:217		DBVPG1373:L>L	AA:279		K11:E>E		SK1:E>E		UWOPS03_461_4:E>E		Y55:E>EID:YAL035W	AA:10		273614X:Q>Q		DBVPG6765:Q>Q		L_1374:Q>Q		L_1528:Q>Q		YJM981:Q>Q	AA:34		DBVPG6044:N>N		Y55:N>N	AA:112		YS4:R>R		YS9:R>R	AA:138		K11:E>E		YPS606:E>E	AA:145		K11:S>S	AA:169		322134S:G>G	AA:191		322134S:R>R	AA:242		K11:K>K		YPS606:K>K	AA:385		DBVPG6044:A>A		NCYC110:A>A		Y55:A>A		Y9:A>A		YPS606:A>A	AA:394		UWOPS03_461_4:S>S		UWOPS05_227_2:S>S	AA:416		DBVPG6044:T>T		NCYC110:T>T		Y55:T>T	AA:432		DBVPG1853:G>G	AA:437		DBVPG1853:G>G		DBVPG6044:G>G		K11:G>G		NCYC110:G>G		SK1:G>G		UWOPS03_461_4:G>G		UWOPS05_227_2:G>G		Y55:G>G		Y9:G>G		YPS606:G>G	AA:486		Y9:L>L	AA:587		K11:N>N		SK1:N>N		UWOPS03_461_4:N>N		UWOPS05_227_2:N>N		YPS128:N>N		YPS606:N>N		YS4:N>N	AA:615		YPS128:L>L		YPS606:L>L	AA:621		YPS128:S>S		YPS606:S>S	AA:640		SK1:V>V		YPS128:V>V		YPS606:V>V		YS4:V>V	AA:656		SK1:L>L		YS4:L>L	AA:679		DBVPG6044:L>L		K11:L>L		NCYC110:L>L		Y55:L>L		Y9:L>L		YPS128:L>L		YPS606:L>L		YS4:L>L	AA:712		DBVPG6044:L>L		K11:L>L		NCYC110:L>L		Y55:L>L		Y9:L>L		YPS128:L>L		YPS606:L>L		YS4:L>L	AA:715		UWOPS03_461_4:A>A		UWOPS05_217_3:A>A	AA:719		UWOPS03_461_4:S>S		UWOPS05_217_3:S>S	AA:758		K11:Q>Q		UWOPS03_461_4:Q>Q		UWOPS05_217_3:Q>Q		Y9:Q>Q		YPS128:Q>Q		YPS606:Q>Q	AA:787		DBVPG6044:V>V		NCYC110:V>V		Y55:V>V	AA:795		UWOPS05_217_3:A>A	AA:819		DBVPG1373:A>A	AA:828		DBVPG6044:I>I		K11:I>I		Y55:I>I		Y9:I>I		YPS128:I>I		YS2:I>I	AA:856		YS2:K>K	AA:862		378604X:A>A		K11:A>A		Y9:A>A		YPS128:A>A		YS2:A>A	AA:887		378604X:L>L		K11:L>L		Y9:L>L		YPS128:L>L		YS2:L>L	AA:917		DBVPG6044:V>VID:YAL036C	AA:12		DBVPG6044:E>E		Y55:E>E		YPS606:E>E	AA:19		YPS606:Q>Q	AA:34		DBVPG6044:K>K		Y55:K>K		YPS606:K>K	AA:41		DBVPG6044:E>E		Y55:E>E	AA:81		UWOPS05_227_2:L>L	AA:99		UWOPS05_227_2:T>T	AA:102		DBVPG6044:T>T	AA:185		DBVPG6044:K>K		Y55:K>K	AA:201		Y9:L>L	AA:202		UWOPS03_461_4:G>G		UWOPS05_227_2:G>G	AA:206		DBVPG6044:I>I		Y55:I>I	AA:211		SK1:S>S	AA:255		DBVPG6044:L>L		Y55:L>L	AA:282		YS4:E>E	AA:289		YS4:D>D	AA:292		UWOPS03_461_4:N>N		UWOPS05_227_2:N>N		Y9:N>N		YPS606:N>N	AA:307		YS4:F>F	AA:314		378604X:R>R		BC187:R>R		DBVPG1853:R>R		DBVPG6040:R>R		DBVPG6044:R>R		DBVPG6765:R>R		L_1528:R>R		UWOPS03_461_4:R>R		UWOPS05_227_2:R>R		Y55:R>R		Y9:R>R		YPS606:R>R		YS9:R>R	AA:339		YS4:L>L	AA:360		K11:D>D		SK1:D>D		UWOPS03_461_4:D>D		Y9:D>D		YPS606:D>DID:YAL037W	AA:39		DBVPG6040:Y>Y	AA:40		DBVPG1373:E>E		DBVPG1788:E>E	AA:57		SK1:S>S	AA:74		DBVPG6040:C>C		NCYC361:C>C		UWOPS05_227_2:C>C		YIIc17_E5:C>C	AA:95		NCYC361:N>N	AA:102		UWOPS05_227_2:F>F	AA:105		DBVPG6040:Q>Q	AA:128		NCYC361:L>L	AA:144		BC187:Q>Q	AA:145		UWOPS05_227_2:Y>Y	AA:149		UWOPS05_227_2:N>N	AA:157		UWOPS05_227_2:S>S	AA:173		K11:D>D		NCYC361:D>D		SK1:D>D	AA:174		UWOPS05_227_2:C>C	AA:187		YS9:Y>Y	AA:265		UWOPS83_787_3:G>GID:YAL038W	AA:359		YPS128:I>I		YPS606:I>I	AA:389		SK1:A>A		YPS128:A>A		YPS606:A>AID:YAL039C	AA:41		SK1:E>E	AA:79		Y55:I>I	AA:134		UWOPS03_461_4:G>G		UWOPS05_217_3:G>G	AA:138		273614X:E>E		SK1:E>E		Y12:E>E		YPS128:E>E		YS4:E>E	AA:154		273614X:P>P	AA:189		SK1:I>I		Y12:I>I		YPS128:I>I	AA:217		DBVPG6044:V>V		Y55:V>V	AA:219		DBVPG6044:D>D		SK1:D>D		Y12:D>D		Y55:D>D		YPS128:D>D		YPS606:D>D		YS4:D>D	AA:247		YJM978:A>A	AA:267		DBVPG6044:S>S		SK1:S>S		UWOPS03_461_4:S>S		Y12:S>S		Y55:S>S		YPS606:S>S		YS4:S>SID:YAL040C	AA:5		DBVPG6044:K>K		UWOPS05_227_2:K>K		UWOPS83_787_3:K>K		Y55:K>K		YPS606:K>K	AA:28		DBVPG6044:A>A		Y55:A>A	AA:60		UWOPS05_227_2:K>K	AA:75		YPS606:N>N	AA:82		DBVPG1106:F>F		DBVPG1373:F>F		DBVPG1788:F>F		DBVPG6040:F>F		DBVPG6044:F>F		DBVPG6765:F>F		L_1528:F>F		NCYC110:F>F		UWOPS05_217_3:F>F		UWOPS05_227_2:F>F		Y55:F>F		YJM975:F>F		YJM981:F>F		YPS606:F>F		YS2:F>F		YS4:F>F	AA:84		DBVPG1106:L>L		DBVPG1373:L>L		DBVPG1788:L>L		DBVPG6040:L>L		DBVPG6044:L>L		DBVPG6765:L>L		L_1528:L>L		NCYC110:L>L		UWOPS05_227_2:L>L		Y55:L>L		YJM981:L>L		YPS606:L>L		YS2:L>L		YS4:L>L	AA:131		DBVPG6044:P>P		UWOPS05_217_3:P>P		Y55:P>P		YJM975:L>L		YPS128:P>P		YPS606:P>P	AA:163		DBVPG6044:K>K		NCYC110:K>K		Y55:K>K	AA:179		YS2:N>N		YS4:N>N		YS9:N>N	AA:191		DBVPG1373:T>T	AA:325		YPS128:E>E	AA:464		DBVPG6044:S>S		UWOPS05_217_3:S>S		Y55:S>S		Y9:S>S		YPS128:S>S		YPS606:S>S	AA:496		322134S:N>N		DBVPG1373:N>N		DBVPG6765:N>N		NCYC361:N>N		UWOPS87_2421:N>N		YJM978:N>N		YJM981:N>N		YS4:N>N	AA:506		322134S:Q>Q		DBVPG1373:Q>Q		DBVPG6044:Q>Q		DBVPG6765:Q>Q		NCYC361:Q>Q		SK1:Q>Q		UWOPS05_217_3:Q>Q		UWOPS87_2421:Q>Q		Y55:Q>Q		Y9:Q>Q		YJM978:Q>Q		YJM981:Q>Q		YPS128:Q>Q		YPS606:Q>Q	AA:510		322134S:I>I		DBVPG1373:I>I		DBVPG6765:I>I		NCYC361:I>I		SK1:I>I		UWOPS87_2421:I>I		YJM978:I>I		YJM981:I>I		YS4:I>I	AA:537		322134S:S>S		DBVPG1373:S>S		NCYC361:S>S		SK1:S>S		YJM981:S>S	AA:543		322134S:V>V		DBVPG1373:V>V		NCYC361:V>V		SK1:V>V		YJM981:V>V	AA:550		322134S:H>H		DBVPG1373:H>H		NCYC361:H>H		SK1:H>H		YJM981:H>HID:YAL041W	AA:10		UWOPS03_461_4:G>G	AA:11		273614X:T>T	AA:15		273614X:D>D	AA:18		Y55:P>P		YPS606:P>P	AA:37		Y12:V>V		Y9:V>V		YS4:V>V	AA:62		Y12:P>P		Y55:P>P		Y9:P>P		YPS606:P>P		YS4:P>P	AA:82		UWOPS03_461_4:L>L	AA:156		UWOPS03_461_4:D>D		UWOPS05_217_3:D>D	AA:182		378604X:F>F		DBVPG1853:F>F		DBVPG6044:F>F		UWOPS05_217_3:F>F		Y55:F>F		YPS128:F>F		YPS606:F>F		YS2:F>F		YS9:F>F	AA:211		DBVPG1853:A>A	AA:215		UWOPS05_217_3:E>E	AA:227		322134S:S>S		378604X:S>S		DBVPG1373:S>S		DBVPG1853:S>S		DBVPG6040:S>S		DBVPG6044:S>S		UWOPS05_217_3:S>S		UWOPS83_787_3:S>S		Y55:S>S		YJM975:S>S		YJM978:S>S		YPS128:S>S		YPS606:S>S		YS2:S>S		YS9:S>S	AA:230		L_1528:Q>Q	AA:245		UWOPS03_461_4:S>S		UWOPS05_217_3:S>S	AA:269		DBVPG6044:P>P		Y55:P>P	AA:273		378604X:S>S		DBVPG1853:S>S		DBVPG6044:S>S		UWOPS03_461_4:S>S		UWOPS05_217_3:S>S		Y55:S>S		Y9:S>S		YPS128:S>S		YPS606:S>S		YS9:S>S	AA:284		UWOPS03_461_4:K>K		UWOPS05_217_3:K>K	AA:297		DBVPG1373:L>L	AA:307		UWOPS03_461_4:L>L		UWOPS05_217_3:L>L	AA:327		YPS606:D>D	AA:344		DBVPG6044:L>L		UWOPS03_461_4:L>L		UWOPS05_217_3:L>L		Y55:L>L	AA:409		UWOPS03_461_4:K>K		UWOPS05_217_3:K>K	AA:419		Y9:L>L	AA:473		DBVPG6044:N>N		Y55:N>N	AA:492		YS2:F>F	AA:546		K11:N>N		YS2:N>N	AA:548		BC187:T>T		DBVPG1373:T>T		YJM981:T>T	AA:552		YS9:G>	AA:608		DBVPG6044:E>E		K11:E>E		Y55:E>E		YS2:E>E	AA:613		DBVPG6044:L>L		K11:L>L		UWOPS03_461_4:L>L		UWOPS05_217_3:L>L		UWOPS05_227_2:L>L		Y55:L>L		YPS606:L>L		YS2:L>L		YS4:L>L	AA:626		UWOPS03_461_4:I>I		UWOPS05_217_3:I>I		UWOPS05_227_2:I>I	AA:648		DBVPG6044:L>L		K11:L>L		UWOPS05_227_2:L>L		Y55:L>L		YPS606:L>L		YS2:L>L		YS4:L>L	AA:652		BC187:N>N	AA:689		UWOPS05_217_3:T>T		UWOPS05_227_2:T>T	AA:692		DBVPG6044:S>S		Y55:S>S	AA:698		UWOPS05_217_3:P>P		UWOPS05_227_2:P>P	AA:699		BC187:T>T	AA:765		DBVPG6044:R>R		Y55:R>R	AA:773		UWOPS05_227_2:N>N	AA:842		273614X:A>A		322134S:A>A		SK1:A>A		UWOPS05_227_2:A>A		Y55:A>A		YJM975:A>A		YPS128:A>A		YS9:A>AID:YAL042W	AA:33		SK1:C>C	AA:38		322134S:L>L	AA:90		DBVPG6044:D>D		UWOPS03_461_4:D>D		UWOPS05_217_3:D>D		UWOPS05_227_2:D>D		UWOPS83_787_3:D>D		Y55:D>D		YPS128:D>D		YPS606:D>D	AA:131		YS4:A>A	AA:146		UWOPS83_787_3:A>A	AA:151		UWOPS03_461_4:Q>Q		UWOPS05_217_3:Q>Q		UWOPS05_227_2:Q>Q	AA:165		UWOPS83_787_3:D>D	AA:195		DBVPG6765:V>V	AA:225		K11:P>P		UWOPS05_217_3:P>P		UWOPS05_227_2:P>P		UWOPS83_787_3:P>P	AA:228		SK1:P>P	AA:246		K11:S>S	AA:265		DBVPG6044:S>S		Y55:S>S	AA:331		DBVPG6044:H>H		K11:H>H		SK1:H>H		UWOPS03_461_4:H>H		UWOPS05_217_3:H>H		Y55:H>H		YIIc17_E5:H>H		YPS606:H>H		YS4:H>H	AA:346		322134S:T>T	AA:357		YS4:F>F	AA:411		UWOPS03_461_4:G>G		UWOPS05_217_3:G>GID:YAL043C	AA:7		DBVPG1373:E>E	AA:14		K11:T>T	AA:43		K11:K>K	AA:46		SK1:L>L	AA:47		BC187:P>P		DBVPG1373:P>P		DBVPG1788:P>P		DBVPG6765:P>P		L_1374:P>P		UWOPS03_461_4:P>P		UWOPS05_217_3:P>P		YJM975:P>P		YPS606:P>P	AA:59		DBVPG6044:V>V		Y55:V>V	AA:93		BC187:L>L		DBVPG1373:L>L		DBVPG1788:L>L		DBVPG6765:L>L		L_1374:L>L		L_1528:L>L		NCYC361:L>L		SK1:L>L		Y9:L>L		YJM975:L>L		YJM981:L>L	AA:163		UWOPS03_461_4:I>I		UWOPS05_217_3:I>I	AA:165		DBVPG1106:V>V	AA:205		UWOPS03_461_4:A>A	AA:212		BC187:R>R		DBVPG1106:R>R		DBVPG1373:R>R		DBVPG1788:R>R		DBVPG6765:R>R		L_1374:R>R		L_1528:R>R		NCYC361:R>R		SK1:R>R		UWOPS03_461_4:R>R		Y55:R>R		YJM981:R>R		YPS128:R>R		YPS606:R>R		YS2:R>R		YS9:R>R	AA:223		BC187:E>E		DBVPG1106:E>E		DBVPG1373:E>E		DBVPG1788:E>E		DBVPG6765:E>E		L_1374:E>E		L_1528:E>E		NCYC361:E>E		UWOPS03_461_4:E>E		YJM981:E>E		YS2:E>E		YS9:E>E	AA:254		BC187:L>L	AA:255		DBVPG1106:S>S	AA:262		SK1:V>V		YPS128:V>V	AA:269		DBVPG1106:E>E		DBVPG1788:E>E		DBVPG6765:E>E		L_1374:E>E		L_1528:E>E		NCYC361:E>E		YJM981:E>E		YS9:E>E	AA:285		322134S:R>R		DBVPG1106:R>R		DBVPG1788:R>R		DBVPG6044:R>R		DBVPG6765:R>R		L_1374:R>R		NCYC361:R>R		SK1:R>R		UWOPS03_461_4:R>R		Y55:R>R		YJM981:R>R		YPS128:R>R		YS9:R>R	AA:296		322134S:K>K		DBVPG1106:K>K		DBVPG1788:K>K		DBVPG6765:K>K		NCYC361:K>K	AA:315		DBVPG6044:K>K		Y55:K>K	AA:356		322134S:K>K		DBVPG1106:K>K		DBVPG1373:K>K		DBVPG1788:K>K		DBVPG1853:K>K		DBVPG6044:K>K		DBVPG6765:K>K		L_1374:K>K		NCYC361:K>K		UWOPS05_217_3:K>K		Y55:K>K		YJM981:K>K		YPS128:K>K		YS9:K>K	AA:359		322134S:S>S		DBVPG1106:S>S		DBVPG1373:S>S		DBVPG1788:S>S		DBVPG1853:S>S		DBVPG6044:S>S		DBVPG6765:S>S		L_1374:S>S		NCYC361:S>S		UWOPS05_217_3:S>S		Y55:S>S		YJM981:S>S		YS9:S>S	AA:398		DBVPG6044:L>L		Y55:L>L	AA:432		YS9:Y>Y	AA:471		UWOPS05_217_3:G>G	AA:519		322134S:A>A		DBVPG1373:A>A		DBVPG1853:A>A		DBVPG6765:A>A		L_1528:A>A		YJM981:A>A	AA:553		322134S:S>S		DBVPG1106:S>S		DBVPG1373:S>S		DBVPG1853:S>S		DBVPG6044:S>S		L_1528:S>S		NCYC110:S>S		NCYC361:S>S		Y55:S>S		YJM981:S>S		YPS606:S>S	AA:561		NCYC361:A>A	AA:596		DBVPG1373:L>L	AA:608		322134S:A>A		DBVPG1106:A>A		DBVPG1373:A>A		DBVPG6044:A>A		DBVPG6765:A>A		L_1374:A>A		L_1528:A>A		NCYC110:A>A		NCYC361:A>A		UWOPS05_217_3:A>A		UWOPS05_227_2:A>A		Y55:A>A		YJM981:A>A		YPS606:A>A		YS4:A>A	AA:630		YIIc17_E5:L>L	AA:636		YPS606:R>R	AA:660		322134S:E>E		DBVPG1106:E>E		DBVPG6044:E>E		DBVPG6765:E>E		L_1374:E>E		L_1528:E>E		NCYC110:E>E		UWOPS05_217_3:E>E		UWOPS05_227_2:E>E		Y55:E>E		YJM978:E>E		YJM981:E>E		YPS606:E>E		YS4:E>E	AA:742		322134S:F>F		DBVPG1106:F>F		DBVPG1373:F>F		DBVPG1853:F>F		DBVPG6765:F>F		L_1374:F>F		L_1528:F>F		YJM978:F>F		YJM981:F>F		YS4:F>F	AA:753		322134S:P>P		DBVPG1106:P>P		DBVPG1373:P>P		DBVPG1853:P>P		DBVPG6044:P>P		DBVPG6765:P>P		L_1374:P>P		L_1528:P>P		NCYC361:P>P		YJM978:P>P		YJM981:P>P		YPS606:P>P		YS4:P>PID:YAL044C	AA:99		378604X:E>E		UWOPS87_2421:E>E	AA:128		DBVPG6044:V>V		NCYC110:V>V		Y55:V>V	AA:130		322134S:N>N		378604X:N>N		DBVPG1373:N>N		DBVPG1788:N>N		DBVPG1853:N>N		DBVPG6765:N>N		L_1374:N>N		NCYC361:N>N		UWOPS87_2421:N>N		YS4:N>NID:YAL044W-A	AA:31		K11:A>A	AA:35		K11:Y>Y	AA:36		DBVPG6044:N>N		NCYC110:N>N		Y55:N>N	AA:37		K11:D>D	AA:39		Y9:H>H		YIIc17_E5:H>H	AA:43		K11:G>G	AA:50		UWOPS03_461_4:N>N		UWOPS05_217_3:N>N	AA:80		K11:S>S	AA:97		K11:L>LID:YAL046C	AA:22		DBVPG1373:L>L		DBVPG1788:L>L		DBVPG1853:L>L		DBVPG6765:L>L		L_1528:L>L		NCYC361:L>L		YS9:L>L	AA:77		DBVPG1853:F>F		DBVPG6765:F>F		L_1528:F>F	AA:115		DBVPG1788:P>P		DBVPG1853:P>P		DBVPG6765:P>P		YJM975:P>PID:YAL047C	AA:24		DBVPG1788:S>S		DBVPG1853:S>S		DBVPG6765:S>S		L_1374:S>S		NCYC361:S>S		SK1:S>S		UWOPS05_217_3:S>S		YJM975:S>S	AA:80		UWOPS03_461_4:V>V		UWOPS05_217_3:V>V	AA:87		UWOPS03_461_4:R>R		UWOPS05_217_3:R>R	AA:126		YPS128:K>K	AA:153		SK1:H>H	AA:169		BC187:A>A		DBVPG1373:A>A		DBVPG6765:A>A		L_1374:A>A		NCYC361:A>A		YJM975:A>A		YJM981:A>A	AA:191		YPS128:V>V	AA:203		YS4:I>I	AA:221		BC187:D>D		DBVPG1373:D>D		DBVPG1788:D>D		DBVPG6765:D>D		L_1374:D>D		NCYC361:D>D		SK1:D>D		YJM981:D>D	AA:233		UWOPS03_461_4:P>P		Y9:P>P	AA:243		BC187:S>S		DBVPG1373:S>S		DBVPG1788:S>S		DBVPG1853:S>S		DBVPG6765:S>S		L_1374:S>S		NCYC361:S>S		SK1:S>S	AA:316		YPS128:L>L	AA:319		YPS128:E>E	AA:355		DBVPG1373:Q>Q		DBVPG1788:Q>Q		DBVPG1853:Q>Q		DBVPG6040:Q>Q		DBVPG6765:Q>Q		L_1374:Q>Q		L_1528:Q>Q		SK1:Q>Q		Y9:Q>Q		YPS128:Q>Q		YS4:Q>Q	AA:371		DBVPG1373:V>V		DBVPG1788:V>V		DBVPG1853:V>V		DBVPG6040:V>V		DBVPG6044:V>V		DBVPG6765:V>V		L_1374:V>V		L_1528:V>V		S288c:V>V		SK1:V>V		W303:V>V		Y55:V>V		Y9:V>V		YPS128:V>V		YS4:V>V	AA:379		YS4:L>L	AA:390		DBVPG1373:E>E		DBVPG1788:E>E		DBVPG1853:E>E		DBVPG6040:E>E		DBVPG6765:E>E		L_1374:E>E		L_1528:E>E		SK1:E>E		YPS128:E>E		YS4:E>E	AA:425		YS4:L>L	AA:438		UWOPS05_217_3:N>N		YIIc17_E5:N>N	AA:445		SK1:L>L		YS4:L>L	AA:446		UWOPS05_217_3:E>E		YIIc17_E5:E>E	AA:450		DBVPG1106:L>L		DBVPG1373:L>L		DBVPG1788:L>L		DBVPG1853:L>L		DBVPG6040:L>L		DBVPG6765:L>L		L_1374:L>L		L_1528:L>L		SK1:L>L		UWOPS05_217_3:L>L		YIIc17_E5:L>L		YPS606:L>L		YS4:L>L	AA:534		322134S:T>T		378604X:T>T		DBVPG1106:T>T		DBVPG1373:T>T		DBVPG1788:T>T		DBVPG1853:T>T		DBVPG6040:T>T		DBVPG6765:T>T		L_1374:T>T		L_1528:T>T		SK1:T>T		YS4:T>T	AA:553		YJM978:P>P	AA:563		322134S:L>L		378604X:L>L		DBVPG1106:L>L		DBVPG1373:L>L		DBVPG1788:L>L		DBVPG1853:L>L		DBVPG6040:L>L		DBVPG6765:L>L		L_1374:L>L		L_1528:L>L		SK1:L>L		UWOPS05_217_3:L>L		UWOPS05_227_2:L>L		Y12:L>L		YIIc17_E5:L>L		YJM978:L>L		YS4:L>L	AA:566		L_1374:T>T	AA:571		322134S:L>L		378604X:L>L		DBVPG1106:L>L		DBVPG1373:L>L		DBVPG1788:L>L		DBVPG1853:L>L		DBVPG6040:L>L		DBVPG6765:L>L		L_1374:L>L		L_1528:L>L		SK1:L>L		YJM978:L>L		YS4:L>L	AA:616		YS4:N>N	AA:617		UWOPS05_217_3:L>L		UWOPS05_227_2:L>LID:YAL048C	AA:2		322134S:T>T		378604X:T>T		DBVPG1106:T>T		DBVPG1373:T>T		DBVPG1853:T>T		DBVPG6765:T>T		L_1528:T>T		SK1:T>T		YJM975:T>T		YJM978:T>T		YJM981:T>T	AA:16		322134S:V>V		378604X:V>V		DBVPG1106:V>V		DBVPG1373:V>V		DBVPG1853:V>V		DBVPG6040:V>V		DBVPG6765:V>V		L_1528:V>V		SK1:V>V		YJM975:V>V		YJM978:V>V		YJM981:V>V	AA:37		378604X:V>V		DBVPG1106:V>V		DBVPG1853:V>V		DBVPG6040:V>V		DBVPG6765:V>V		L_1528:V>V		SK1:V>V		YJM975:V>V		YJM978:V>V		YJM981:V>V	AA:43		DBVPG1106:I>I		DBVPG1853:I>I		DBVPG6040:I>I		DBVPG6765:I>I		L_1528:I>I		SK1:I>I		YJM975:I>I		YJM978:I>I		YJM981:I>I		YS9:I>I	AA:55		UWOPS05_227_2:P>P	AA:94		DBVPG1106:V>V		DBVPG1373:V>V		DBVPG1853:V>V		DBVPG6040:V>V		DBVPG6765:V>V		L_1528:V>V		SK1:V>V		UWOPS05_227_2:V>V		Y12:V>V		Y9:V>V		YJM975:V>V		YJM978:V>V		YPS128:V>V		YPS606:V>V		YS4:V>V	AA:111		UWOPS05_227_2:V>V	AA:133		UWOPS05_227_2:E>E	AA:135		UWOPS05_227_2:S>S	AA:157		DBVPG1373:E>E		DBVPG1853:E>E		DBVPG6040:E>E		DBVPG6765:E>E		L_1528:E>E		SK1:E>E		YJM975:E>E		YJM978:E>E	AA:182		273614X:A>A		378604X:A>A		DBVPG1373:A>A		DBVPG1853:A>A		DBVPG6040:A>A		DBVPG6765:A>A		K11:A>A		L_1528:A>A		SK1:A>A		Y9:A>A		YPS128:A>A		YPS606:A>A	AA:198		378604X:L>L		DBVPG1373:L>L		DBVPG1853:L>L		DBVPG6040:L>L		DBVPG6765:L>L		L_1528:L>L		SK1:L>L		YJM975:L>L		YPS606:L>L	AA:205		273614X:A>A		378604X:A>A		DBVPG1373:A>A		DBVPG1853:A>A		DBVPG6040:A>A		DBVPG6765:A>A		K11:A>A		L_1528:A>A		SK1:A>A		Y9:A>A		YPS606:A>A		YS9:A>A	AA:218		273614X:D>D		K11:D>D		YS9:D>D	AA:285		378604X:A>A		DBVPG1788:A>A		DBVPG1853:A>A		DBVPG6040:A>A		DBVPG6765:A>A		L_1528:A>A		SK1:A>A	AA:313		378604X:L>L		DBVPG1788:L>L		DBVPG1853:L>L		DBVPG6765:L>L		L_1528:L>L		SK1:L>L		YS2:L>L	AA:318		378604X:V>V		DBVPG1788:V>V		DBVPG6765:V>V		L_1528:V>V		NCYC361:V>V		SK1:V>V	AA:335		YS2:L>L	AA:416		378604X:Q>Q		DBVPG1788:Q>Q		DBVPG1853:Q>Q		DBVPG6040:Q>Q		DBVPG6765:Q>Q		L_1528:Q>Q		NCYC361:Q>Q		SK1:Q>Q		YS2:Q>Q	AA:508		378604X:Q>Q		DBVPG1788:Q>Q		DBVPG1853:Q>Q		DBVPG6040:Q>Q		DBVPG6765:Q>Q		L_1374:Q>Q		L_1528:Q>Q		NCYC361:Q>Q		SK1:Q>Q		YJM978:Q>Q	AA:541		DBVPG1788:V>V		DBVPG1853:V>V		DBVPG6040:V>V		DBVPG6765:V>V		L_1374:V>V		L_1528:V>V		NCYC361:V>V		SK1:V>V		YJM978:V>V	AA:548		DBVPG1788:T>T		DBVPG1853:T>T		DBVPG6040:T>T		DBVPG6765:T>T		L_1374:T>T		L_1528:T>T		NCYC361:T>T		SK1:T>T		UWOPS05_227_2:T>T		YJM978:T>T		YPS128:T>T	AA:598		DBVPG1788:L>L		DBVPG6765:L>L		L_1374:L>L		L_1528:L>L		SK1:L>L		YJM978:L>L	AA:602		DBVPG1788:F>F		DBVPG6765:F>F		L_1374:F>F		L_1528:F>F		YJM978:F>F	AA:613		DBVPG1788:G>G		DBVPG6765:G>G		L_1374:G>G		L_1528:G>G		SK1:G>G		YJM978:G>G	AA:617		DBVPG1788:P>P		DBVPG6765:P>P		L_1374:P>P		L_1528:P>P		NCYC361:P>P		SK1:P>P		YJM978:P>P	AA:638		YIIc17_E5:G>G	AA:644		DBVPG1788:V>V		DBVPG6765:V>V		L_1374:V>V		L_1528:V>V		SK1:V>V		UWOPS05_217_3:V>V		YJM978:V>V	AA:647		NCYC361:C>C		YIIc17_E5:C>C		YPS128:C>C	AA:658		DBVPG1373:S>SID:YAL049C	AA:144		DBVPG1373:G>G		DBVPG1788:G>G		DBVPG1853:G>G		DBVPG6765:G>G		SK1:G>G		YJM975:G>G		YJM981:G>G	AA:165		273614X:A>A	AA:215		273614X:A>A		322134S:A>A		BC187:A>A		DBVPG1373:A>A		DBVPG1788:A>A		DBVPG1853:A>A		DBVPG6765:A>A		SK1:A>A		UWOPS87_2421:A>A		Y12:A>A		Y9:A>A		YIIc17_E5:A>A		YJM975:A>A		YS4:A>A	AA:246		UWOPS87_2421:V>VID:YAL054C	AA:11		DBVPG6044:L>L		Y55:L>L	AA:44		322134S:T>T		378604X:T>T		DBVPG1373:T>T		DBVPG1853:T>T		DBVPG6765:T>T		L_1374:T>T		NCYC361:T>T		SK1:T>T		UWOPS05_217_3:T>T		Y9:T>T		YJM975:T>T		YPS606:T>T	AA:116		322134S:G>G		DBVPG6765:G>G		L_1374:G>G		NCYC361:G>G		SK1:G>G		YJM975:G>G	AA:119		YS9:S>S	AA:145		273614X:T>T		322134S:T>T		378604X:T>T		DBVPG1853:T>T		DBVPG6765:T>T		L_1374:T>T		NCYC361:T>T		SK1:T>T		UWOPS05_217_3:T>T		Y9:T>T		YJM975:T>T		YPS606:T>T		YS4:T>T	AA:159		Y9:G>G	AA:165		UWOPS03_461_4:T>T	AA:201		UWOPS03_461_4:E>E		UWOPS83_787_3:E>E	AA:213		378604X:G>G		DBVPG6765:G>G		NCYC361:G>G		SK1:G>G		UWOPS05_217_3:G>G		YJM975:G>G		YJM978:G>G		YJM981:G>G		YS9:G>G	AA:249		YS4:G>G	AA:291		SK1:L>L	AA:301		UWOPS03_461_4:K>K		UWOPS83_787_3:K>K	AA:389		273614X:T>T		K11:T>T		Y9:T>T		YIIc17_E5:T>T		YPS128:T>T	AA:391		273614X:A>A		DBVPG1853:A>A		K11:A>A		Y9:A>A		YIIc17_E5:A>A		YPS128:A>A		YS2:A>A	AA:413		378604X:A>A		DBVPG1788:A>A		DBVPG1853:A>A		DBVPG6765:A>A		SK1:A>A		UWOPS03_461_4:A>A		UWOPS05_217_3:A>A		YJM978:A>A		YJM981:A>A		YPS128:A>A		YPS606:A>A		YS2:A>A		YS4:A>A		YS9:A>A	AA:444		DBVPG1788:E>E		DBVPG6765:E>E		SK1:E>E		YJM975:E>E		YJM978:E>E		YJM981:E>E		YS9:E>E	AA:486		273614X:V>V		DBVPG1373:V>V		DBVPG1788:V>V		DBVPG1853:V>V		DBVPG6765:V>V		SK1:V>V		Y9:V>V		YJM975:V>V		YJM978:V>V		YJM981:V>V		YPS606:V>V		YS2:V>V		YS4:V>V		YS9:V>V	AA:496		UWOPS83_787_3:F>F	AA:515		DBVPG1373:N>N		DBVPG1788:N>N		DBVPG6765:N>N		SK1:N>N		UWOPS03_461_4:N>N		UWOPS05_217_3:N>N		UWOPS83_787_3:N>N		YJM975:N>N		YJM978:N>N		YJM981:N>N	AA:525		YPS606:V>V	AA:547		DBVPG1853:Y>Y		YPS606:Y>Y		YS4:Y>Y	AA:561		NCYC110:A>A	AA:569		DBVPG1853:I>I		YPS606:I>I		YS4:I>I	AA:595		UWOPS83_787_3:I>I	AA:636		Y9:D>D	AA:673		322134S:S>S		378604X:S>S		DBVPG1373:S>S		DBVPG1853:S>S		DBVPG6040:S>S		DBVPG6765:S>S		K11:S>S		L_1374:S>S		NCYC361:S>S		SK1:S>S		Y9:S>S		YJM975:S>S		YPS128:S>S		YS4:S>S		YS9:S>SID:YAL055W	AA:19		DBVPG6040:G>G	AA:27		378604X:T>T		DBVPG1106:T>T		DBVPG1788:T>T		DBVPG1853:T>T		DBVPG6040:T>T		DBVPG6044:T>T		DBVPG6765:T>T		K11:T>T		L_1374:T>T		L_1528:T>T		SK1:T>T		UWOPS05_217_3:T>T		UWOPS05_227_2:T>T		UWOPS83_787_3:T>T		UWOPS87_2421:T>T		Y55:T>T		YIIc17_E5:T>T		YPS128:T>T		YPS606:T>T		YS4:T>T		YS9:T>T	AA:36		UWOPS05_217_3:T>T		UWOPS05_227_2:T>T	AA:38		DBVPG1853:A>A		DBVPG6044:A>A		K11:A>A		Y55:A>A		YIIc17_E5:A>A		YPS128:A>A		YPS606:A>A		YS4:A>A	AA:67		UWOPS87_2421:S>S	AA:76		UWOPS05_227_2:K>K	AA:117		378604X:Y>Y		DBVPG1106:Y>Y		DBVPG1788:Y>Y		DBVPG6040:Y>Y		DBVPG6765:Y>Y		L_1374:Y>Y		L_1528:Y>Y		SK1:Y>Y		UWOPS05_227_2:Y>Y		UWOPS83_787_3:Y>Y		YS9:Y>Y	AA:126		322134S:S>S		378604X:S>S		DBVPG1106:S>S		DBVPG1788:S>S		DBVPG6040:S>S		DBVPG6765:S>S		L_1374:S>S		L_1528:S>S		SK1:S>S		YS9:S>S	AA:138		322134S:L>L		378604X:L>L		DBVPG1106:L>L		DBVPG1788:L>L		DBVPG6040:L>L		DBVPG6765:L>L		L_1374:L>L		NCYC361:L>L		SK1:L>L		UWOPS05_227_2:L>L		YS9:L>L	AA:151		322134S:A>A		378604X:A>A		DBVPG1106:A>A		DBVPG1788:A>A		DBVPG6040:A>A		DBVPG6765:A>A		L_1374:A>A		NCYC361:A>A		SK1:A>A		YS9:A>A	AA:153		322134S:V>V		378604X:V>V		DBVPG1106:V>V		DBVPG1788:V>V		DBVPG1853:V>V		DBVPG6040:V>V		DBVPG6765:V>V		K11:V>V		L_1374:V>V		NCYC361:V>V		SK1:V>V		Y12:V>V		Y9:V>V		YIIc17_E5:V>V		YPS128:V>V		YPS606:V>V		YS4:V>V		YS9:V>V	AA:162		378604X:V>VID:YAL059W	AA:14		YPS128:T>T		YPS606:T>T	AA:22		DBVPG1106:K>K		DBVPG1373:K>K		DBVPG1853:K>K		DBVPG6040:K>K		DBVPG6765:K>K		L_1528:K>K		NCYC110:K>K		Y55:K>K		YPS128:K>K		YPS606:K>K		YS9:K>K	AA:43		DBVPG1106:E>E		DBVPG1373:E>E		DBVPG1853:E>E		DBVPG6040:E>E		DBVPG6765:E>E		L_1528:E>E		NCYC110:E>E		Y55:E>E		YPS128:E>E		YPS606:E>E		YS9:E>E	AA:130		273614X:S>S		BC187:S>S		DBVPG1106:S>S		DBVPG1373:S>S		DBVPG1853:S>S		DBVPG6040:S>S		DBVPG6765:S>S		K11:S>S		SK1:S>S		UWOPS03_461_4:S>S		YJM978:S>S		YPS128:S>S		YS4:S>S		YS9:S>S	AA:139		UWOPS03_461_4:N>N	AA:144		DBVPG1853:G>G		UWOPS03_461_4:G>G	AA:187		273614X:D>D		BC187:D>D		DBVPG1106:D>D		DBVPG1373:D>D		DBVPG6040:D>D		DBVPG6765:D>D		L_1528:D>D		YJM978:D>DID:YAL060W	AA:23		Y55:I>I	AA:63		Y55:S>S	AA:89		DBVPG1853:V>V		K11:V>V		UWOPS03_461_4:V>V		UWOPS05_227_2:V>V		UWOPS87_2421:V>V		YS9:V>V	AA:104		273614X:A>A		322134S:A>A		DBVPG1106:A>A		DBVPG6040:A>A		DBVPG6044:A>A		DBVPG6765:A>A		L_1374:A>A		L_1528:A>A		NCYC361:A>A		SK1:A>A		Y55:A>A		YJM975:A>A		YJM978:A>A	AA:118		273614X:K>K		322134S:K>K		DBVPG1106:K>K		DBVPG6040:K>K		DBVPG6044:K>K		DBVPG6765:K>K		L_1374:K>K		L_1528:K>K		NCYC361:K>K		SK1:K>K		Y55:K>K		YJM975:K>K		YJM978:K>K	AA:120		273614X:C>C		322134S:C>C		DBVPG1106:C>C		DBVPG6040:C>C		DBVPG6765:C>C		L_1374:C>C		L_1528:C>C		NCYC361:C>C		SK1:C>C		YJM975:C>C		YJM978:C>C	AA:151		DBVPG6044:V>V		Y55:V>V	AA:159		273614X:P>P		322134S:P>P		378604X:P>P		DBVPG1106:P>P		DBVPG1853:P>P		DBVPG6040:P>P		DBVPG6765:P>P		K11:P>P		L_1374:P>P		L_1528:P>P		SK1:P>P		YJM978:P>P		YS9:P>P	AA:194		322134S:L>L		DBVPG1106:L>L		DBVPG1853:L>L		DBVPG6040:L>L		DBVPG6765:L>L		L_1374:L>L		L_1528:L>L		SK1:L>L		YJM978:L>L	AA:199		322134S:G>G		378604X:G>G		DBVPG1106:G>G		DBVPG1853:G>G		DBVPG6040:G>G		DBVPG6044:G>G		DBVPG6765:G>G		K11:G>G		L_1374:G>G		L_1528:G>G		SK1:G>G		UWOPS05_227_2:G>G		UWOPS87_2421:G>G		Y55:G>G		YJM978:G>G		YS9:G>G	AA:214		322134S:A>A		DBVPG1106:A>A		DBVPG1853:A>A		DBVPG6040:A>A		DBVPG6765:A>A		L_1374:A>A		L_1528:A>A		SK1:A>A		YJM978:A>A		YS9:A>A	AA:251		UWOPS83_787_3:L>L	AA:272		322134S:V>V		DBVPG1106:V>V		DBVPG1853:V>V		DBVPG6040:V>V		DBVPG6765:V>V		L_1374:V>V		L_1528:V>V		SK1:V>V		UWOPS83_787_3:V>V		YPS128:V>V		YS9:V>V	AA:274		378604X:F>F	AA:298		322134S:V>V		378604X:V>V		DBVPG1106:V>V		DBVPG1853:V>V		DBVPG6040:V>V		DBVPG6765:V>V		L_1528:V>V		SK1:V>V		UWOPS83_787_3:V>V		YPS128:V>V		YPS606:V>V		YS9:V>VID:YAL061W	AA:55		378604X:E>E		DBVPG1373:E>E		DBVPG1788:E>E		DBVPG1853:E>E		DBVPG6765:E>E		L_1528:E>E		NCYC110:E>E		SK1:E>E		UWOPS03_461_4:E>E		UWOPS05_217_3:E>E		Y55:E>E		YIIc17_E5:E>E		YJM975:E>E		YJM978:E>E		YPS606:E>E		YS4:E>E		YS9:E>E	AA:81		DBVPG1853:E>E		UWOPS03_461_4:E>E		UWOPS05_217_3:E>E		UWOPS83_787_3:E>E		YPS606:E>E		YS4:E>E	AA:98		YPS606:E>E	AA:100		UWOPS03_461_4:T>T	AA:103		378604X:C>C		NCYC110:C>C		Y55:C>C		YIIc17_E5:C>C	AA:105		DBVPG1788:D>D		YS9:D>D	AA:108		DBVPG1853:R>R		YS4:R>R	AA:139		YIIc17_E5:A>A		YPS606:A>A	AA:151		UWOPS03_461_4:V>V	AA:183		273614X:R>R		BC187:R>R		DBVPG1373:R>R		DBVPG1788:R>R		DBVPG6765:R>R		L_1374:R>R		L_1528:R>R		SK1:R>R		YJM975:R>R		YJM978:R>R		YJM981:R>R	AA:190		UWOPS83_787_3:G>G	AA:241		273614X:T>T		BC187:T>T		DBVPG1373:T>T		DBVPG1788:T>T		DBVPG6765:T>T		L_1374:T>T		L_1528:T>T		SK1:T>T		YJM981:T>T		YS4:T>T	AA:245		YIIc17_E5:A>A	AA:266		DBVPG6044:F>F		NCYC110:F>F		Y55:F>F	AA:270		UWOPS87_2421:G>G	AA:283		UWOPS87_2421:T>T	AA:287		273614X:T>T		BC187:T>T		DBVPG1373:T>T		DBVPG6040:T>T		DBVPG6044:T>T		DBVPG6765:T>T		L_1374:T>T		SK1:T>T		UWOPS05_217_3:T>T		UWOPS83_787_3:T>T		UWOPS87_2421:T>T		Y55:T>T		YJM981:T>T		YS4:T>T		YS9:T>T	AA:289		DBVPG6040:V>V		DBVPG6044:V>V		Y55:V>V	AA:295		UWOPS87_2421:G>G	AA:308		UWOPS05_217_3:L>L		UWOPS87_2421:L>L	AA:350		SK1:N>N	AA:356		DBVPG1373:D>D		L_1528:D>D		SK1:D>D		UWOPS83_787_3:D>D		UWOPS87_2421:D>D		YJM978:D>D		YJM981:D>D	AA:369		DBVPG1373:T>T		L_1528:T>T		SK1:T>T		UWOPS87_2421:T>T		YJM978:T>T		YJM981:T>TID:YAL062W	AA:14		K11:I>I		Y12:I>I		YS2:I>I		YS9:I>I	AA:18		UWOPS03_461_4:V>V		UWOPS05_227_2:V>V		UWOPS87_2421:V>V	AA:23		322134S:I>I		DBVPG1373:I>I		DBVPG6765:I>I		L_1374:I>I		NCYC361:I>I		SK1:I>I		YJM975:I>I	AA:36		UWOPS03_461_4:I>I		UWOPS05_227_2:I>I	AA:39		UWOPS87_2421:V>V	AA:42		UWOPS87_2421:R>R	AA:63		322134S:Y>Y		DBVPG6765:Y>Y		L_1374:Y>Y		NCYC361:Y>Y		SK1:Y>Y		UWOPS03_461_4:Y>Y		UWOPS87_2421:Y>Y		Y12:Y>Y		YJM975:Y>Y		YS2:Y>Y	AA:76		YS2:G>G	AA:85		322134S:N>N		DBVPG6765:N>N		L_1374:N>N		NCYC361:N>N		SK1:N>N		UWOPS03_461_4:N>N		UWOPS87_2421:N>N		Y12:N>N		YJM975:N>N		YS2:N>N	AA:94		273614X:F>F		322134S:F>F		L_1374:F>F		NCYC361:F>F		SK1:F>F		YJM975:F>F		YJM978:F>F	AA:117		Y12:D>D		YS2:D>D	AA:137		273614X:L>L		322134S:L>L		DBVPG1788:L>L		DBVPG6765:L>L		L_1374:L>L		NCYC361:L>L		SK1:L>L		YJM975:L>L		YJM978:L>L	AA:141		273614X:I>I		322134S:I>I		DBVPG1788:I>I		DBVPG6765:I>I		L_1374:I>I		NCYC361:I>I		SK1:I>I		YJM975:I>I		YJM978:I>I	AA:191		YPS128:P>P	AA:193		273614X:A>A		322134S:A>A		DBVPG1788:A>A		DBVPG6765:A>A		SK1:A>A		YJM975:A>A		YJM978:A>A	AA:198		273614X:L>L		322134S:L>L		DBVPG1788:L>L		DBVPG6765:L>L		NCYC361:L>L		SK1:L>L		UWOPS03_461_4:L>L		YJM975:L>L		YJM978:L>L	AA:204		YS9:A>A	AA:235		273614X:L>L		DBVPG1788:L>L		DBVPG6765:L>L		K11:L>L		SK1:L>L		UWOPS83_787_3:L>L		Y12:L>L		YJM978:L>L		YPS128:L>L		YS9:L>L	AA:238		K11:I>I		Y12:I>I		YS9:I>I	AA:245		UWOPS03_461_4:V>V		UWOPS83_787_3:V>V	AA:246		YPS128:S>S	AA:309		NCYC110:D>D		Y55:D>D	AA:315		273614X:A>A		DBVPG1788:A>A		DBVPG1853:A>A		DBVPG6765:A>A		K11:A>A		SK1:A>A		UWOPS05_217_3:A>A		UWOPS83_787_3:A>A		YJM978:A>A		YS2:A>A		YS9:A>A	AA:318		DBVPG1853:N>N	AA:330		NCYC110:A>A		Y55:A>A	AA:336		K11:V>V		NCYC110:V>V		UWOPS05_217_3:V>V		UWOPS83_787_3:V>V		Y55:V>V		YPS128:V>V		YPS606:V>V		YS2:V>V		YS9:V>V	AA:343		K11:G>G		NCYC110:G>G		UWOPS05_217_3:G>G		UWOPS83_787_3:G>G		Y55:G>G		YPS128:G>G		YPS606:G>G		YS2:G>G		YS9:G>G	AA:362		273614X:A>A		DBVPG1788:A>A		DBVPG6765:A>A		K11:A>A		SK1:A>A		UWOPS05_217_3:A>A		UWOPS83_787_3:A>A		YJM978:A>A		YPS606:A>A		YS2:A>A		YS9:A>A	AA:372		NCYC110:K>K		Y55:K>K	AA:400		DBVPG1853:V>V		NCYC110:V>V		Y55:V>V		YPS606:V>V	AA:404		K11:L>L		UWOPS05_217_3:L>L		YS9:L>L	AA:438		DBVPG6044:A>A		NCYC110:A>A		Y55:A>A		YPS606:A>A	AA:439		273614X:N>N		DBVPG1853:N>N		DBVPG6765:N>N		SK1:N>N	AA:455		UWOPS05_217_3:D>DID:YAR002C-A	AA:56		DBVPG1853:D>D	AA:74		YPS128:D>D		YPS606:D>D	AA:131		BC187:V>V		DBVPG1373:V>V		DBVPG1788:V>V		DBVPG6765:V>V		L_1528:V>V		YJM978:V>V	AA:213		273614X:F>F		BC187:F>F		DBVPG1106:F>F		DBVPG1373:F>F		DBVPG1788:F>F		DBVPG1853:F>F		DBVPG6765:F>F		UWOPS05_217_3:F>F		UWOPS05_227_2:F>F		Y55:F>F		YJM978:F>FID:YAR002W	AA:3		NCYC361:R>R	AA:6		322134S:L>L		BC187:L>L		DBVPG1373:L>L		DBVPG6044:L>L		DBVPG6765:L>L		L_1374:L>L		L_1528:L>L		UWOPS05_217_3:L>L		UWOPS87_2421:L>L		Y55:L>L		YIIc17_E5:L>L		YJM975:L>L		YJM978:L>L		YS4:L>L		YS9:L>L	AA:33		378604X:F>F	AA:151		322134S:K>K		BC187:K>K		DBVPG1373:K>K		DBVPG1788:K>K		DBVPG6765:K>K		L_1528:K>K		UWOPS87_2421:K>K		YJM975:K>K		YJM978:K>K		YS4:K>K	AA:238		322134S:P>P		BC187:P>P		DBVPG1106:P>P		DBVPG1373:P>P		DBVPG1788:P>P		DBVPG6765:P>P		L_1528:P>P		YJM975:P>P		YJM978:P>P		YJM981:P>P		YS4:P>P		YS9:P>P	AA:274		UWOPS05_217_3:A>A	AA:300		322134S:S>S		DBVPG1373:S>S		DBVPG1788:S>S		DBVPG6765:S>S		L_1528:S>S		YJM975:S>S		YJM978:S>S		YS4:S>S		YS9:S>S	AA:381		DBVPG6044:T>T		Y55:T>T	AA:436		DBVPG1853:P>P		DBVPG6044:P>P		Y55:P>P	AA:502		DBVPG1788:S>S		DBVPG6044:S>S		DBVPG6765:S>S		Y55:S>S		YJM975:S>S		YJM978:S>S		YS4:S>S	AA:509		273614X:F>F		DBVPG6044:F>F		Y55:F>F	AA:510		UWOPS05_217_3:D>DID:YAR003W	AA:139		UWOPS03_461_4:D>D		UWOPS05_227_2:D>D	AA:167		SK1:D>D	AA:179		YPS128:K>K	AA:186		322134S:V>V		DBVPG1373:V>V		DBVPG1788:V>V		DBVPG6040:V>V		DBVPG6765:V>V		L_1528:V>V		UWOPS87_2421:V>V		YJM978:V>V		YS4:V>V		YS9:V>V	AA:252		DBVPG6765:V>V	AA:277		DBVPG1373:N>N	AA:306		UWOPS05_227_2:R>R	AA:314		UWOPS83_787_3:E>E	AA:374		UWOPS05_227_2:V>V	AA:390		DBVPG1373:A>A		DBVPG6765:A>A		L_1528:A>A		UWOPS87_2421:A>A		YIIc17_E5:A>A		YJM975:A>A		YJM978:A>A		YS9:A>AID:YAR007C	AA:39		BC187:G>G		DBVPG1373:G>G		DBVPG1788:G>G		DBVPG6765:G>G		L_1528:G>G		UWOPS87_2421:G>G		YJM978:G>G	AA:42		DBVPG6044:S>S		Y55:S>S	AA:80		YPS606:I>I	AA:93		BC187:R>R		DBVPG1373:R>R		DBVPG6765:R>R		L_1528:R>R		UWOPS87_2421:R>R		YJM978:R>R	AA:120		UWOPS03_461_4:L>L	AA:140		322134S:S>S		BC187:S>S		DBVPG1373:S>S		DBVPG6765:S>S		UWOPS87_2421:S>S		YJM978:S>S		YS9:S>S	AA:177		322134S:N>N		DBVPG1373:N>N		DBVPG1853:N>N		DBVPG6765:N>N		UWOPS03_461_4:N>N		UWOPS87_2421:N>N		Y55:N>N		YJM978:N>N		YPS606:N>N		YS9:N>N	AA:209		Y55:I>I	AA:231		DBVPG1373:G>G		YJM978:G>G		YS9:G>G	AA:236		273614X:T>T		DBVPG1373:T>T		DBVPG1788:T>T		DBVPG6765:T>T		L_1528:T>T		UWOPS03_461_4:T>T		Y55:T>T		YJM978:T>T		YPS606:T>T		YS9:T>T	AA:253		273614X:K>K	AA:287		YS9:E>E	AA:315		273614X:S>S	AA:352		Y12:G>G	AA:372		273614X:G>G		UWOPS05_217_3:G>G		UWOPS05_227_2:G>G		Y55:G>G	AA:386		DBVPG1853:G>G		YPS128:G>G		YPS606:G>G	AA:398		273614X:L>L		DBVPG1106:L>L		DBVPG1373:L>L		DBVPG1788:L>L		DBVPG1853:L>L		DBVPG6765:L>L		K11:L>L		L_1528:L>L		UWOPS05_217_3:L>L		UWOPS05_227_2:L>L		Y55:L>L		YIIc17_E5:L>L		YJM978:L>L		YPS128:L>L		YPS606:L>L		YS4:L>L		YS9:L>L	AA:427		273614X:K>K		DBVPG1106:K>K		DBVPG1788:K>K		DBVPG6765:K>K		K11:K>K		UWOPS05_217_3:K>K		UWOPS05_227_2:K>K		Y55:K>K		YS4:K>K		YS9:K>K	AA:482		NCYC110:A>A		Y55:A>A	AA:496		322134S:L>L		DBVPG1106:L>L		DBVPG1373:L>L		DBVPG1788:L>L		DBVPG1853:L>L		DBVPG6765:L>L		K11:L>L		L_1528:L>L		NCYC110:L>L		UWOPS03_461_4:L>L		UWOPS05_217_3:L>L		Y55:L>L		YIIc17_E5:L>L		YJM978:L>L		YPS128:L>L		YS4:L>L		YS9:L>L	AA:502		322134S:T>T		DBVPG1106:T>T		DBVPG1373:T>T		DBVPG1788:T>T		DBVPG1853:T>T		DBVPG6765:T>T		K11:T>T		L_1528:T>T		NCYC110:T>T		UWOPS03_461_4:T>T		UWOPS05_217_3:T>T		Y55:T>T		YIIc17_E5:T>T		YJM978:T>T		YPS128:T>T		YS4:T>T		YS9:T>T	AA:507		YPS128:K>K	AA:557		322134S:E>E		DBVPG1373:E>E		DBVPG1853:E>E		DBVPG6044:E>E		DBVPG6765:E>E		K11:E>E		L_1528:E>E		NCYC110:E>E		UWOPS03_461_4:E>E		UWOPS05_217_3:E>E		UWOPS87_2421:E>E		Y55:E>E		YIIc17_E5:E>E		YPS128:E>E		YS4:E>E	AA:595		322134S:T>T		DBVPG1373:T>T		DBVPG6765:T>T		L_1528:T>T		UWOPS87_2421:T>TID:YAR008W	AA:2		BC187:P>P		DBVPG1788:P>P		DBVPG6044:P>P		L_1374:P>P		L_1528:P>P		Y55:P>P		YIIc17_E5:P>P	AA:41		Y55:L>L	AA:220		DBVPG1853:L>L		DBVPG6040:L>L		SK1:L>L		UWOPS05_217_3:L>L		Y12:L>L		Y9:L>L		YPS128:L>L		YPS606:L>L		YS9:L>L	AA:246		UWOPS05_217_3:T>T	AA:253		DBVPG1853:V>V		DBVPG6040:V>V		SK1:V>V		UWOPS05_217_3:V>V		Y12:V>V		Y9:V>V		YPS128:V>V		YPS606:V>V		YS9:V>V	AA:264		DBVPG1853:T>T		DBVPG6040:T>T		SK1:T>T		UWOPS05_217_3:T>T		Y12:T>T		Y9:T>T		YPS128:T>T		YPS606:T>T		YS9:T>TID:YAR014C	AA:12		DBVPG6765:S>S		L_1528:S>S		YS9:S>S	AA:34		K11:L>L	AA:62		322134S:I>I		DBVPG1788:I>I		DBVPG6765:I>I		L_1374:I>I		L_1528:I>I		YIIc17_E5:I>I		YS9:I>I	AA:139		273614X:S>S		378604X:S>S	AA:179		378604X:S>S	AA:233		YPS606:D>D	AA:259		YS2:L>L	AA:308		UWOPS03_461_4:G>G	AA:355		378604X:Q>Q		DBVPG1373:Q>Q		DBVPG1788:Q>Q		DBVPG6044:Q>Q		DBVPG6765:Q>Q		L_1374:Q>Q		UWOPS03_461_4:Q>Q		UWOPS87_2421:Q>Q		Y12:Q>Q		Y55:Q>Q		YIIc17_E5:Q>Q		YJM978:Q>Q		YPS606:Q>Q		YS4:Q>Q	AA:412		L_1374:S>S		L_1528:S>S	AA:420		Y9:D>D	AA:452		UWOPS03_461_4:D>D	AA:623		DBVPG1788:H>H		DBVPG6765:H>H		YIIc17_E5:H>H	AA:645		273614X:Y>Y		378604X:Y>Y		DBVPG1853:Y>Y		DBVPG6040:Y>Y		DBVPG6044:Y>Y		NCYC110:Y>Y		SK1:Y>Y		UWOPS05_227_2:Y>Y		UWOPS83_787_3:Y>Y		Y12:Y>Y		Y55:Y>Y		Y9:Y>Y		YPS606:Y>Y		YS4:Y>Y		YS9:Y>Y	AA:667		273614X:L>L		DBVPG6044:L>L		NCYC110:L>L		Y55:L>L	AA:682		273614X:L>L		378604X:L>L		DBVPG1853:L>L		DBVPG6040:L>L		DBVPG6044:L>L		NCYC110:L>L		SK1:L>L		UWOPS05_227_2:L>L		UWOPS83_787_3:L>L		Y12:L>L		Y55:L>L		Y9:L>L		YPS606:L>L		YS4:L>L		YS9:L>LID:YAR015W	AA:12		NCYC361:L>L	AA:16		322134S:A>A		DBVPG1106:A>A		DBVPG1788:A>A		DBVPG6765:A>A		L_1528:A>A		NCYC361:A>A		YJM978:A>A		YS2:A>A	AA:34		322134S:V>V		DBVPG1106:V>V		DBVPG1788:V>V		DBVPG1853:V>V		DBVPG6765:V>V		L_1528:V>V		NCYC361:V>V		UWOPS03_461_4:V>V		UWOPS05_217_3:V>V		UWOPS05_227_2:V>V		UWOPS83_787_3:V>V		Y55:V>V		YJM978:V>V		YS2:V>V	AA:38		YPS606:R>R	AA:85		Y12:I>I	AA:88		322134S:Y>Y		DBVPG1106:Y>Y		DBVPG1788:Y>Y		DBVPG1853:Y>Y		DBVPG6765:Y>Y		L_1528:Y>Y		NCYC361:Y>Y		UWOPS03_461_4:Y>Y		UWOPS05_217_3:Y>Y		UWOPS05_227_2:Y>Y		UWOPS83_787_3:Y>Y		Y55:Y>Y		YJM978:Y>Y		YPS128:Y>Y		YPS606:Y>Y		YS2:Y>Y	AA:105		322134S:R>R		DBVPG1788:R>R		DBVPG6044:R>R		DBVPG6765:R>R		L_1528:R>R		NCYC110:R>R		NCYC361:R>R		UWOPS03_461_4:R>R		UWOPS05_217_3:R>R		UWOPS05_227_2:R>R		Y55:R>R		YS2:R>R	AA:152		322134S:Q>Q		DBVPG1788:Q>Q		L_1528:Q>Q		UWOPS03_461_4:Q>Q		UWOPS05_217_3:Q>Q		UWOPS05_227_2:Q>Q		UWOPS83_787_3:Q>Q		YJM975:Q>Q		YS2:Q>Q	AA:171		DBVPG1373:D>D		DBVPG1788:D>D		L_1528:D>D		NCYC361:D>D		YIIc17_E5:D>D		YJM975:D>D	AA:177		UWOPS83_787_3:A>A	AA:179		DBVPG1788:A>A	AA:206		DBVPG1373:A>A		L_1528:A>A		NCYC361:A>A		YIIc17_E5:A>A		YJM975:A>A	AA:216		DBVPG1373:T>T		L_1528:T>T		NCYC361:T>T		YIIc17_E5:T>T		YJM975:T>TID:YAR018C	AA:91		DBVPG1373:D>D		DBVPG1788:D>D	AA:105		273614X:Y>Y		DBVPG1373:Y>Y		DBVPG1788:Y>Y		DBVPG6044:Y>Y		DBVPG6765:Y>Y		L_1374:Y>Y		L_1528:Y>Y		NCYC361:Y>Y		Y55:Y>Y		YIIc17_E5:Y>Y		YJM975:Y>Y	AA:109		Y9:G>G	AA:150		273614X:L>L		DBVPG6044:L>L		Y55:L>L	AA:168		DBVPG6765:N>N		L_1528:N>N		YJM975:N>N	AA:198		YS9:H>H	AA:253		273614X:Y>Y		DBVPG6044:Y>Y		SK1:Y>Y		UWOPS83_787_3:Y>Y		Y12:Y>Y		Y55:Y>Y		Y9:Y>Y	AA:326		UWOPS03_461_4:V>V		UWOPS05_217_3:V>V		Y55:V>V	AA:380		322134S:Y>Y		DBVPG1373:Y>Y		DBVPG6765:Y>Y		NCYC361:Y>Y		UWOPS05_217_3:Y>Y		UWOPS83_787_3:Y>Y		YIIc17_E5:Y>Y		YJM975:Y>Y		YPS606:Y>Y		YS4:Y>Y		YS9:Y>Y	AA:382		UWOPS05_217_3:R>RID:YAR019C	AA:5		DBVPG1788:A>A		YJM975:A>A		YJM978:A>A	AA:66		DBVPG1788:D>D		DBVPG6765:D>D		L_1528:D>D		NCYC361:D>D		UWOPS87_2421:D>D		YIIc17_E5:D>D		YJM975:D>D		YJM978:D>D		YJM981:D>D		YS4:D>D		YS9:D>D	AA:73		DBVPG1788:L>L		DBVPG6765:L>L		L_1528:L>L		NCYC361:L>L		UWOPS87_2421:L>L		YIIc17_E5:L>L		YJM975:L>L		YJM978:L>L		YJM981:L>L	AA:103		DBVPG6044:A>A		NCYC110:A>A		Y55:A>A	AA:130		322134S:T>T		DBVPG1788:T>T		DBVPG6040:T>T		DBVPG6044:T>T		DBVPG6765:T>T		L_1528:T>T		NCYC110:T>T		NCYC361:T>T		Y55:T>T		YJM975:T>T		YJM978:T>T		YJM981:T>T		YS4:T>T	AA:137		DBVPG6040:L>L		DBVPG6044:L>L		NCYC110:L>L		Y55:L>L	AA:145		UWOPS83_787_3:R>R	AA:154		SK1:L>L	AA:255		322134S:K>K		DBVPG1788:K>K		DBVPG6040:K>K		DBVPG6044:K>K		DBVPG6765:K>K		K11:K>K		L_1528:K>K		NCYC361:K>K		UWOPS87_2421:K>K		Y55:K>K		YIIc17_E5:K>K		YJM975:K>K		YJM981:K>K		YPS606:K>K		YS4:K>K	AA:340		322134S:L>L		DBVPG1788:L>L		DBVPG6040:L>L		DBVPG6765:L>L		K11:L>L		UWOPS87_2421:L>L		YJM975:L>L		YS4:L>L		YS9:L>L	AA:392		UWOPS05_217_3:Y>Y		UWOPS05_227_2:Y>Y		YPS606:Y>Y	AA:398		DBVPG6044:H>H		Y55:H>H	AA:435		DBVPG6765:C>C		YS9:C>C	AA:498		DBVPG6044:S>S		K11:S>S		NCYC110:S>S		Y55:S>S	AA:536		DBVPG6044:N>N		K11:N>N		NCYC110:N>N		Y55:N>N	AA:546		DBVPG6044:I>I		K11:I>I		NCYC110:I>I		Y55:I>I	AA:558		DBVPG6044:P>P		K11:P>P		NCYC110:P>P		Y55:P>P	AA:563		DBVPG6044:P>P		K11:P>P		NCYC110:P>P		Y55:P>P	AA:565		K11:R>R	AA:577		DBVPG6044:R>R		NCYC110:R>R		Y55:R>R	AA:587		DBVPG6044:R>R		NCYC110:R>R		Y55:R>R	AA:598		UWOPS05_217_3:S>S		UWOPS05_227_2:S>S		YPS606:S>S	AA:599		322134S:N>N		DBVPG1788:N>N		DBVPG6040:N>N		DBVPG6765:N>N	AA:638		322134S:V>V		DBVPG1106:V>V		DBVPG1373:V>V		DBVPG1788:V>V		DBVPG1853:V>V		DBVPG6040:V>V		DBVPG6044:V>V		DBVPG6765:V>V		L_1374:V>V		NCYC110:V>V		NCYC361:V>V		SK1:V>V		UWOPS05_217_3:V>V		UWOPS05_227_2:V>V		UWOPS87_2421:V>V		Y12:V>V		Y55:V>V		YJM978:V>V		YJM981:V>V		YPS606:V>V		YS2:V>V		YS9:V>V	AA:703		YPS606:D>D	AA:722		SK1:K>K		UWOPS83_787_3:K>K		Y12:K>K	AA:724		K11:N>N	AA:761		SK1:F>F		UWOPS83_787_3:F>F	AA:764		SK1:K>K		UWOPS83_787_3:K>K	AA:894		DBVPG1106:L>L		K11:L>L	AA:952		SK1:R>R		Y12:R>R		Y9:R>RID:YAR035W	AA:70		SK1:S>S		UWOPS03_461_4:S>S		UWOPS05_217_3:S>S		Y55:S>S	AA:79		DBVPG1853:A>A		DBVPG6765:A>A		NCYC361:A>A		SK1:A>A		UWOPS03_461_4:A>A		UWOPS05_217_3:A>A		Y55:A>A		Y9:A>A		YIIc17_E5:A>A		YS9:A>A	AA:104		DBVPG1853:K>K		NCYC361:K>K		Y9:K>K	AA:122		BC187:V>V		DBVPG1853:V>V		K11:V>V		NCYC361:V>V		SK1:V>V		Y55:V>V		Y9:V>V		YS9:V>V	AA:129		YIIc17_E5:T>	AA:132		SK1:I>I		Y55:I>I	AA:143		DBVPG1853:T>T		K11:T>T		NCYC361:T>T		SK1:T>T		UWOPS05_217_3:T>T		UWOPS05_227_2:T>T		Y55:T>T		YS9:T>T	AA:148		378604X:T>T		DBVPG1853:T>T		K11:T>T		NCYC361:T>T		SK1:T>T		UWOPS05_217_3:T>T		UWOPS05_227_2:T>T		Y55:T>T		YS9:T>T	AA:163		DBVPG1853:L>L	AA:253		378604X:R>R		K11:R>R		UWOPS05_217_3:R>R		UWOPS05_227_2:R>R	AA:275		K11:L>L	AA:278		SK1:V>V		Y55:V>V	AA:390		UWOPS87_2421:S>S	AA:409		UWOPS83_787_3:L>L	AA:475		273614X:G>G		NCYC110:G>G		SK1:G>G		UWOPS03_461_4:G>G		UWOPS05_217_3:G>G		Y55:G>G		YIIc17_E5:G>G		YJM975:G>G		YJM981:G>G		YPS606:G>G		YS9:G>G	AA:504		273614X:S>S		DBVPG6040:S>S		L_1374:S>S		NCYC110:S>S		SK1:S>S		UWOPS03_461_4:S>S		UWOPS05_217_3:S>S		YIIc17_E5:S>S		YJM975:S>S		YJM978:S>S		YJM981:S>S		YPS606:S>S		YS9:S>S	AA:521		NCYC110:K>K		SK1:K>K		UWOPS03_461_4:K>K		UWOPS05_217_3:K>K		UWOPS05_227_2:K>K		Y55:K>K		YIIc17_E5:K>K		YPS606:K>K	AA:532		273614X:H>H	AA:542		DBVPG1853:G>G		DBVPG6044:G>G		NCYC110:G>G		SK1:G>G		UWOPS03_461_4:G>G		UWOPS05_217_3:G>G		UWOPS05_227_2:G>G		UWOPS87_2421:G>G		Y55:G>G		YIIc17_E5:G>G		YPS606:G>G	AA:548		BC187:H>H	AA:558		YPS606:Q>Q	AA:583		UWOPS87_2421:L>L	AA:607		YS2:G>G	AA:627		273614X:Q>Q		BC187:Q>Q		DBVPG1788:Q>Q		DBVPG6040:Q>Q		DBVPG6765:Q>Q		L_1374:Q>Q		YJM975:Q>Q		YJM981:Q>Q		YS9:Q>Q	AA:649		273614X:Q>Q		378604X:Q>Q		BC187:Q>Q		DBVPG1788:Q>Q		DBVPG6040:Q>Q		DBVPG6765:Q>Q		L_1374:Q>Q		YJM975:Q>Q		YJM981:Q>Q		YS9:Q>Q	AA:668		273614X:S>S		378604X:S>S		BC187:S>S		DBVPG1788:S>S		DBVPG1853:S>S		DBVPG6040:S>S		DBVPG6044:S>S		DBVPG6765:S>S		L_1374:S>S		NCYC110:S>S		SK1:S>S		UWOPS05_217_3:S>S		UWOPS05_227_2:S>S		Y55:S>S		YJM975:S>S		YJM981:S>S		YPS606:S>S		YS9:S>SID:YAR062W	AA:14		322134S:T>T		DBVPG6040:T>T	AA:16		322134S:R>R		DBVPG6040:R>R	AA:28		DBVPG1106:V>V	AA:40		UWOPS05_217_3:E>E		UWOPS05_227_2:E>E		W303:E>E	AA:47		322134S:T>T		DBVPG6040:T>T	AA:54		UWOPS05_217_3:I>I		UWOPS05_227_2:I>I		W303:I>I	AA:62		W303:P>P	AA:84		DBVPG1106:S>S	AA:86		DBVPG1106:A>A	AA:88		DBVPG1106:S>S	AA:105		W303:S>S	AA:116		DBVPG1106:D>D	AA:140		DBVPG6040:T>T	AA:145		DBVPG6040:T>T		W303:T>T	AA:148		322134S:F>F		DBVPG1788:F>F		DBVPG6040:F>F		DBVPG6765:F>F		W303:F>F		YIIc17_E5:F>F	AA:152		322134S:S>S		DBVPG1788:S>S		DBVPG6765:S>S		YIIc17_E5:S>S	AA:154		322134S:E>E		DBVPG1106:E>E		DBVPG1788:E>E		DBVPG6765:E>E		W303:E>E		YIIc17_E5:E>EID:YAR066W	AA:7		Y55:K>K	AA:12		Y55:V>V		YS2:V>V	AA:62		SK1:Y>Y		Y55:Y>Y	AA:125		SK1:A>A		Y55:A>AID:YBL003C	AA:53		322134S:L>L		BC187:L>L		DBVPG1853:L>L		DBVPG6765:L>L		YJM975:L>L		YJM978:L>L		YS9:L>L	AA:103		322134S:T>TID:YBL005W	AA:69		322134S:H>H		BC187:H>H		DBVPG6765:H>H		L_1374:H>H		L_1528:H>H		NCYC361:H>H		SK1:H>H		UWOPS03_461_4:H>H		UWOPS05_217_3:H>H		UWOPS87_2421:H>H		YJM978:H>H		YPS606:H>H		YS9:H>H	AA:75		SK1:S>S	AA:101		UWOPS83_787_3:Q>Q	AA:190		322134S:Q>Q		BC187:Q>Q		DBVPG6765:Q>Q		L_1374:Q>Q		L_1528:Q>Q		NCYC361:Q>Q		SK1:Q>Q		Y55:Q>Q		YJM978:Q>Q		YJM981:Q>Q		YPS606:Q>Q	AA:229		UWOPS05_217_3:A>A	AA:282		SK1:F>F		Y55:F>F	AA:315		DBVPG1853:A>A	AA:346		DBVPG1788:I>I		DBVPG1853:I>I		DBVPG6765:I>I		L_1374:I>I		UWOPS05_217_3:I>I		UWOPS05_227_2:I>I		YJM975:I>I		YJM978:I>I		YJM981:I>I		YS2:I>I	AA:348		UWOPS05_217_3:G>G		UWOPS05_227_2:G>G	AA:385		DBVPG6044:Q>Q		SK1:Q>Q	AA:387		DBVPG1788:F>F		DBVPG1853:F>F		DBVPG6765:F>F		L_1374:F>F		YJM975:F>F		YJM978:F>F		YS2:F>F		YS4:F>F		YS9:F>F	AA:399		DBVPG1788:S>S		DBVPG1853:S>S		DBVPG6044:S>S		DBVPG6765:S>S		L_1374:S>S		SK1:S>S		UWOPS03_461_4:S>S		UWOPS05_217_3:S>S		UWOPS05_227_2:S>S		Y55:S>S		YJM975:S>S		YJM978:S>S		YS2:S>S		YS4:S>S	AA:424		DBVPG6044:L>L		SK1:L>L		UWOPS03_461_4:L>L		UWOPS05_217_3:L>L		UWOPS05_227_2:L>L		Y55:L>L	AA:496		DBVPG1853:K>K		YS4:K>K	AA:550		DBVPG6044:N>N		SK1:N>N		Y55:N>N	AA:554		YS4:S>S	AA:576		DBVPG6044:L>L		SK1:L>L		UWOPS03_461_4:L>L		UWOPS05_217_3:L>L		Y55:L>L		YPS128:L>L		YPS606:L>L	AA:582		DBVPG6044:T>T		SK1:T>T		Y55:T>T	AA:611		DBVPG1853:K>K	AA:638		DBVPG6044:L>L		SK1:L>L		UWOPS05_217_3:L>L		Y55:L>L		YPS606:L>L	AA:647		273614X:Y>Y		DBVPG1373:Y>Y		DBVPG1788:Y>Y		DBVPG1853:Y>Y		DBVPG6765:Y>Y		L_1528:Y>Y		YJM975:Y>Y		YJM978:Y>Y		YS9:Y>Y	AA:651		UWOPS87_2421:T>T	AA:660		UWOPS03_461_4:N>N		UWOPS05_217_3:N>N	AA:679		273614X:K>K		BC187:K>K		DBVPG1373:K>K		DBVPG1788:K>K		DBVPG1853:K>K		DBVPG6765:K>K		L_1528:K>K		YJM978:K>K		YS4:K>K		YS9:K>K	AA:762		UWOPS03_461_4:C>C		UWOPS05_217_3:C>C	AA:784		L_1374:L>L	AA:846		UWOPS83_787_3:D>D		UWOPS87_2421:D>D	AA:862		322134S:D>D		BC187:D>D		DBVPG1373:D>D		DBVPG1853:D>D		DBVPG6765:D>D		L_1374:D>D		SK1:D>D		UWOPS03_461_4:D>D		UWOPS05_217_3:D>D		UWOPS83_787_3:D>D		UWOPS87_2421:D>D		Y55:D>D		YJM975:D>D		YJM978:D>D		YPS606:D>D		YS2:D>D		YS4:D>D	AA:889		273614X:N>N		322134S:N>N		BC187:N>N		DBVPG1373:N>N		DBVPG1788:N>N		DBVPG1853:N>N		DBVPG6765:N>N		K11:N>N		L_1374:N>N		SK1:N>N		UWOPS03_461_4:N>N		UWOPS05_217_3:N>N		UWOPS83_787_3:N>N		Y55:N>N		YJM975:N>N		YJM978:N>N		YPS606:N>N		YS2:N>N		YS4:N>N	AA:954		273614X:V>V		322134S:V>V		DBVPG1373:V>V		DBVPG1853:V>V		DBVPG6765:V>V		K11:V>V		SK1:V>V		UWOPS03_461_4:V>V		UWOPS05_217_3:V>V		UWOPS83_787_3:V>V		UWOPS87_2421:V>V		Y55:V>V		YJM975:V>V		YJM981:V>V		YPS606:V>V		YS2:V>VID:YBL006C	AA:7		322134S:G>G		DBVPG1373:G>G		DBVPG1788:G>G		DBVPG6765:G>G		L_1528:G>G		SK1:G>G		UWOPS03_461_4:G>G		UWOPS87_2421:G>G		Y55:G>G		YJM975:G>G		YJM978:G>G		YPS128:G>G		YPS606:G>G		YS9:G>G	AA:13		DBVPG1373:A>A	AA:16		YPS128:S>S		YPS606:S>S	AA:20		322134S:K>K		DBVPG1373:K>K		DBVPG1788:K>K		DBVPG6040:K>K		DBVPG6765:K>K		L_1528:K>K		NCYC361:K>K		SK1:K>K		UWOPS83_787_3:K>K		UWOPS87_2421:K>K		Y55:K>K		Y9:K>K		YIIc17_E5:K>K		YJM975:K>K		YJM978:K>K		YPS128:K>K		YPS606:K>K		YS9:K>K	AA:35		UWOPS83_787_3:T>T	AA:56		DBVPG6044:S>S		SK1:S>S		Y55:S>S	AA:76		DBVPG6044:A>A		SK1:A>A		Y55:A>A	AA:97		DBVPG1788:L>L	AA:112		DBVPG1373:T>T		DBVPG1788:T>T		DBVPG1853:T>T		DBVPG6044:T>T		DBVPG6765:T>T		L_1528:T>T		SK1:T>T		Y55:T>T		YJM975:T>T		YPS606:T>T	AA:127		DBVPG1853:A>AID:YBL007C	AA:7		Y9:I>I	AA:39		DBVPG6044:D>D		NCYC110:D>D		SK1:D>D		Y55:D>D	AA:126		DBVPG6044:Y>Y		NCYC110:Y>Y		SK1:Y>Y		Y55:Y>Y	AA:135		273614X:S>S		BC187:S>S		DBVPG1373:S>S		DBVPG6765:S>S		L_1374:S>S		NCYC110:S>S		SK1:S>S		UWOPS03_461_4:S>S		UWOPS05_217_3:S>S		UWOPS05_227_2:S>S		Y55:S>S		YIIc17_E5:S>S		YJM981:S>S		YPS606:S>S	AA:258		YPS606:V>V	AA:295		YS2:L>L	AA:300		YPS606:N>N	AA:351		YS2:A>A	AA:396		273614X:L>L		322134S:L>L		DBVPG1373:L>L		DBVPG1788:L>L		DBVPG1853:L>L		DBVPG6765:L>L		L_1374:L>L		NCYC110:L>L		SK1:L>L		YJM975:L>L		YJM978:L>L		YS2:L>L		YS4:L>L	AA:433		SK1:N>N	AA:458		UWOPS05_217_3:D>D	AA:492		322134S:P>P		DBVPG1373:P>P		DBVPG1853:P>P		DBVPG6040:P>P		DBVPG6765:P>P		L_1374:P>P		YJM978:P>P		YS4:P>P	AA:519		322134S:G>G		DBVPG1373:G>G		DBVPG1853:G>G		DBVPG6040:G>G		DBVPG6765:G>G		L_1374:G>G		NCYC110:G>G		SK1:G>G		UWOPS83_787_3:G>G		Y55:G>G		YJM978:G>G		YPS606:G>G		YS4:G>G		YS9:G>G	AA:522		DBVPG1373:H>H		DBVPG6040:H>H		DBVPG6765:H>H		L_1374:H>H		YJM978:H>H	AA:534		DBVPG1853:A>A	AA:541		NCYC110:E>E		SK1:E>E		Y55:E>E	AA:552		322134S:F>F		DBVPG1373:F>F		DBVPG1853:F>F		DBVPG6040:F>F		DBVPG6765:F>F		L_1374:F>F		NCYC110:F>F		SK1:F>F		UWOPS05_217_3:F>F		Y55:F>F		YJM978:F>F		YS4:F>F		YS9:F>F	AA:633		DBVPG1853:S>S		YS4:S>S	AA:663		NCYC110:N>N		SK1:N>N		Y55:N>N	AA:701		UWOPS03_461_4:L>L		UWOPS05_217_3:L>L	AA:722		UWOPS03_461_4:I>I		UWOPS05_217_3:I>I	AA:743		UWOPS03_461_4:V>V		UWOPS05_217_3:V>V	AA:828		DBVPG6044:P>P		SK1:P>P		Y55:P>P	AA:913		273614X:T>T		DBVPG1373:T>T		DBVPG6040:T>T		DBVPG6765:T>T		L_1374:T>T		L_1528:T>T		NCYC361:T>T		YJM975:T>T		YJM978:T>T	AA:967		273614X:V>V		DBVPG1373:V>V		DBVPG6044:V>V		DBVPG6765:V>V		K11:V>V		L_1374:V>V		L_1528:V>V		NCYC361:V>V		SK1:V>V		UWOPS83_787_3:V>V		Y12:V>V		YJM975:V>V		YJM978:V>V		YPS128:V>V	AA:990		DBVPG6044:S>S		SK1:S>S		Y55:S>S	AA:1067		Y12:G>G		Y9:G>G	AA:1076		UWOPS03_461_4:G>G		UWOPS05_217_3:G>G	AA:1103		DBVPG1373:G>G		DBVPG1788:G>G		DBVPG6044:G>G		DBVPG6765:G>G		NCYC361:G>G		SK1:G>G		UWOPS03_461_4:G>G		UWOPS05_217_3:G>G		UWOPS83_787_3:G>G		Y12:G>G		Y55:G>G		Y9:G>G		YJM978:G>G		YJM981:G>G	AA:1117		UWOPS03_461_4:T>T		UWOPS05_217_3:T>T	AA:1158		UWOPS03_461_4:V>V		UWOPS05_217_3:V>V	AA:1188		DBVPG6044:I>I		SK1:I>I		Y55:I>I	AA:1201		DBVPG6044:G>G		NCYC110:G>G		SK1:G>G		Y55:G>G	AA:1243		322134S:G>G		DBVPG1373:G>G		DBVPG6765:G>G		L_1374:G>G		YJM978:G>G		YJM981:G>GID:YBL009W	AA:5		DBVPG1788:A>A	AA:9		DBVPG1373:Q>Q		DBVPG1788:Q>Q		DBVPG1853:Q>Q		DBVPG6040:Q>Q		DBVPG6044:Q>Q		DBVPG6765:Q>Q		L_1374:Q>Q		SK1:Q>Q		UWOPS05_217_3:Q>Q		UWOPS05_227_2:Q>Q		UWOPS83_787_3:Q>Q		UWOPS87_2421:Q>Q		Y55:Q>Q		YJM975:Q>Q		YPS606:Q>Q	AA:125		YPS606:S>S	AA:138		UWOPS03_461_4:K>K		UWOPS05_227_2:K>K	AA:145		UWOPS03_461_4:T>T		UWOPS05_227_2:T>T	AA:222		DBVPG6044:L>L		NCYC110:L>L		SK1:L>L		Y55:L>L	AA:260		UWOPS03_461_4:S>S	AA:277		DBVPG6044:I>I		K11:I>I		NCYC110:I>I		SK1:I>I		Y55:I>I	AA:332		273614X:C>C		322134S:C>C		DBVPG1373:C>C		DBVPG6765:C>C		UWOPS87_2421:C>C		YJM978:C>C		YJM981:C>C	AA:340		273614X:D>D		322134S:D>D		DBVPG1373:D>D		DBVPG6765:D>D		UWOPS87_2421:D>D		YJM978:D>D		YJM981:D>D	AA:342		UWOPS03_461_4:V>V	AA:386		273614X:V>V		322134S:V>V		DBVPG1373:V>V		DBVPG6765:V>V		L_1528:V>V		UWOPS83_787_3:V>V		UWOPS87_2421:V>V		YJM978:V>V		YJM981:V>V	AA:408		273614X:S>S		322134S:S>S		DBVPG1373:S>S		DBVPG6765:S>S		L_1528:S>S		UWOPS03_461_4:S>S		UWOPS83_787_3:S>S		UWOPS87_2421:S>S		YJM978:S>S		YJM981:S>S	AA:426		273614X:S>S		322134S:S>S		DBVPG1373:S>S		DBVPG6765:S>S		L_1528:S>S		UWOPS87_2421:S>S		YJM978:S>S		YJM981:S>S	AA:450		273614X:E>E		322134S:E>E		DBVPG1373:E>E		DBVPG6044:E>E		DBVPG6765:E>E		L_1528:E>E		SK1:E>E		UWOPS83_787_3:E>E		UWOPS87_2421:E>E		Y55:E>E		YJM978:E>E		YJM981:E>E	AA:485		DBVPG6044:L>L		SK1:L>L		Y55:L>L	AA:508		UWOPS03_461_4:L>L	AA:514		273614X:C>C		322134S:C>C		DBVPG1373:C>C		DBVPG6765:C>C		L_1374:C>C		L_1528:C>C		UWOPS87_2421:C>C		YJM978:C>C		YJM981:C>C	AA:555		YPS606:F>F	AA:559		322134S:D>D		DBVPG1373:D>D		DBVPG6765:D>D		L_1374:D>D		L_1528:D>D		SK1:D>D		UWOPS03_461_4:D>D		UWOPS83_787_3:D>D		UWOPS87_2421:D>D		Y55:D>D		YJM978:D>D		YJM981:D>D	AA:562		322134S:K>K		DBVPG1373:K>K		DBVPG6765:K>K		L_1374:K>K		L_1528:K>K		SK1:K>K		UWOPS03_461_4:K>K		UWOPS83_787_3:K>K		UWOPS87_2421:K>K		Y55:K>K		YJM978:K>K		YJM981:K>K	AA:574		SK1:F>F		UWOPS03_461_4:F>F		UWOPS05_217_3:F>F		Y55:F>F	AA:621		SK1:A>A		Y55:A>A	AA:666		YPS128:D>DID:YBL010C	AA:47		UWOPS03_461_4:S>S		UWOPS05_217_3:S>S		UWOPS05_227_2:S>S	AA:79		DBVPG1853:S>S		DBVPG6765:S>S		L_1528:S>S		YJM975:S>S		YJM981:S>S		YS2:S>SID:YBL011W	AA:82		273614X:P>P		378604X:P>P		DBVPG1106:P>P		DBVPG1373:P>P		DBVPG6765:P>P		L_1528:P>P		W303:P>P		YS2:P>P	AA:85		UWOPS03_461_4:F>F	AA:158		DBVPG1106:D>D	AA:215		DBVPG1788:A>A		DBVPG1853:A>A		YJM981:A>A	AA:226		378604X:A>A		DBVPG1106:A>A		DBVPG1373:A>A		DBVPG1788:A>A		DBVPG1853:A>A		DBVPG6765:A>A		L_1374:A>A		UWOPS87_2421:A>A		YJM981:A>A	AA:289		378604X:C>C		DBVPG1788:C>C		DBVPG1853:C>C		DBVPG6044:C>C		DBVPG6765:C>C		L_1374:C>C		NCYC110:C>C		SK1:C>C		UWOPS87_2421:C>C		Y55:C>C		YJM981:C>C	AA:307		378604X:F>F		DBVPG1788:F>F		DBVPG1853:F>F		DBVPG6044:F>F		DBVPG6765:F>F		L_1374:F>F		NCYC110:F>F		SK1:F>F		UWOPS05_217_3:F>F		UWOPS05_227_2:F>F		UWOPS87_2421:F>F		Y55:F>F		YJM981:F>F	AA:326		YPS128:T>T		YPS606:T>T	AA:333		378604X:E>E		DBVPG1106:E>E		DBVPG1788:E>E		DBVPG1853:E>E		DBVPG6044:E>E		DBVPG6765:E>E		L_1374:E>E		NCYC110:E>E		SK1:E>E		UWOPS05_217_3:E>E		UWOPS05_227_2:E>E		UWOPS87_2421:E>E		Y55:E>E		YJM981:E>E		YPS128:E>E		YPS606:E>E	AA:397		378604X:K>K		UWOPS87_2421:K>K	AA:465		BC187:K>K	AA:486		273614X:N>N		322134S:N>N		378604X:N>N		BC187:N>N		DBVPG1788:N>N		DBVPG6765:N>N		YJM975:N>N		YJM978:N>N		YPS128:N>N	AA:514		YPS128:Y>Y	AA:538		273614X:T>T		322134S:T>T		BC187:T>T		DBVPG1788:T>T		DBVPG6044:T>T		DBVPG6765:T>T		NCYC110:T>T		SK1:T>T		UWOPS83_787_3:T>T		Y55:T>T		YJM975:T>T		YJM978:T>T		YPS128:T>T	AA:540		273614X:S>S		322134S:S>S		BC187:S>S		DBVPG1788:S>S		DBVPG6044:S>S		DBVPG6765:S>S		NCYC110:S>S		NCYC361:S>S		SK1:S>S		UWOPS83_787_3:S>S		Y55:S>S		YJM975:S>S		YJM978:S>S		YPS128:S>S	AA:602		322134S:L>L		378604X:L>L		BC187:L>L		DBVPG1788:L>L		DBVPG6765:L>L		NCYC361:L>L		YJM975:L>L		YJM978:L>L	AA:615		DBVPG6044:D>D		NCYC110:D>D		SK1:D>D		UWOPS83_787_3:D>D		Y55:D>D		YPS128:D>D	AA:623		YJM975:R>R	AA:647		YJM978:R>R	AA:660		DBVPG6044:S>S		NCYC110:S>S		SK1:S>S		Y55:S>S	AA:674		DBVPG6044:N>N		NCYC110:N>N		SK1:N>N		Y55:N>NID:YBL013W	AA:43		DBVPG6044:F>F		SK1:F>F		Y55:F>F	AA:61		YPS606:V>V	AA:81		UWOPS83_787_3:L>L		YPS606:L>L	AA:152		UWOPS87_2421:L>L		YS9:L>L	AA:175		378604X:L>L		DBVPG1788:L>L		DBVPG6040:L>L		DBVPG6765:L>L		NCYC361:L>L		SK1:L>L		UWOPS83_787_3:L>L		Y55:L>L		YJM975:L>L		YJM978:L>L		YJM981:L>L		YPS128:L>L		YPS606:L>L		YS9:L>L	AA:223		DBVPG1788:F>F		DBVPG6040:F>F		DBVPG6765:F>F		NCYC361:F>F		SK1:F>F		UWOPS83_787_3:F>F		Y55:F>F		YJM975:F>F		YJM978:F>F		YJM981:F>F		YPS128:F>F		YPS606:F>F	AA:228		DBVPG1373:L>L		DBVPG1788:L>L		DBVPG6765:L>L		YJM975:L>L		YJM978:L>L		YJM981:L>L	AA:281		BC187:K>K	AA:296		YPS128:K>K		YPS606:K>K	AA:325		SK1:L>L		Y55:L>L	AA:343		UWOPS03_461_4:P>P		UWOPS05_227_2:P>P		UWOPS83_787_3:P>P		Y12:P>P	AA:353		322134S:D>D		BC187:D>D		DBVPG1373:D>D		DBVPG1853:D>D		DBVPG6040:D>D		DBVPG6765:D>D		L_1528:D>D		YJM975:D>D		YJM981:D>DID:YBL014C	AA:34		378604X:T>T		DBVPG1373:T>T		DBVPG1788:T>T		DBVPG6765:T>T		L_1528:T>T		YJM975:T>T		YJM978:T>T		YJM981:T>T		YS2:T>T	AA:46		378604X:V>V		DBVPG1373:V>V		DBVPG1788:V>V		DBVPG6765:V>V		L_1528:V>V		NCYC110:V>V		SK1:V>V		UWOPS83_787_3:V>V		Y55:V>V		YJM975:V>V		YJM978:V>V		YJM981:V>V		YPS128:V>V		YPS606:V>V		YS9:V>V	AA:91		378604X:S>S		DBVPG1373:S>S		DBVPG1788:S>S		DBVPG6765:S>S		L_1528:S>S		NCYC361:S>S		YJM975:S>S		YJM978:S>S		YJM981:S>S		YS9:S>S	AA:133		UWOPS83_787_3:G>G		YPS128:G>G	AA:407		DBVPG1373:R>R		DBVPG6044:R>R		DBVPG6765:R>R		L_1374:R>R		SK1:R>R		UWOPS05_217_3:R>R		UWOPS83_787_3:R>R		Y55:R>R		YJM975:R>R		YJM981:R>R		YPS128:R>R		YPS606:R>R	AA:409		378604X:D>D		DBVPG1373:D>D		DBVPG6044:D>D		DBVPG6765:D>D		L_1374:D>D		SK1:D>D		UWOPS05_217_3:D>D		UWOPS83_787_3:D>D		Y55:D>D		YJM975:D>D		YJM981:D>D		YPS128:D>D		YPS606:D>D	AA:422		378604X:I>I		DBVPG1373:I>I		DBVPG6044:I>I		DBVPG6765:I>I		L_1374:I>I		NCYC110:I>I		SK1:I>I		UWOPS05_217_3:I>I		UWOPS83_787_3:I>I		Y55:I>I		YJM975:I>I		YJM981:I>I		YPS128:I>I		YPS606:I>I	AA:491		DBVPG6044:S>S		NCYC110:S>S		SK1:S>S		Y55:S>S	AA:572		UWOPS05_217_3:P>P		UWOPS05_227_2:P>P		UWOPS83_787_3:P>P	AA:596		378604X:I>I		DBVPG1373:I>I		DBVPG1788:I>I		DBVPG6040:I>I		YJM975:I>I		YJM981:I>I	AA:605		UWOPS05_217_3:S>S		UWOPS05_227_2:S>S		UWOPS83_787_3:S>S	AA:631		NCYC110:S>S		Y55:S>S	AA:745		273614X:A>A		322134S:A>A		378604X:A>A		BC187:A>A		DBVPG1373:A>A		DBVPG1788:A>A		DBVPG6040:A>A		DBVPG6765:A>A		L_1374:A>A		L_1528:A>A		NCYC110:A>A		SK1:A>A		UWOPS03_461_4:A>A		UWOPS05_217_3:A>A		UWOPS83_787_3:A>A		Y55:A>A		YJM978:A>A		YJM981:A>A		YPS128:A>A		YS9:A>A	AA:786		K11:S>S		NCYC110:S>S		SK1:S>S		UWOPS03_461_4:S>S		UWOPS83_787_3:S>S		UWOPS87_2421:S>S		Y55:S>S		YPS128:S>S	AA:833		UWOPS03_461_4:T>T	AA:838		UWOPS03_461_4:A>A	AA:870		273614X:S>S		322134S:S>S		378604X:S>S		BC187:S>S		DBVPG1373:S>S		DBVPG1788:S>S		DBVPG6040:S>S		DBVPG6765:S>S		L_1374:S>S		L_1528:S>S		NCYC361:S>S		YJM978:S>S		YJM981:S>S	AA:892		UWOPS03_461_4:G>G		UWOPS05_217_3:G>GID:YBL015W	AA:35		DBVPG1373:Y>Y		DBVPG1788:Y>Y		DBVPG6044:Y>Y		DBVPG6765:Y>Y		L_1528:Y>Y		NCYC361:Y>Y		SK1:Y>Y		UWOPS05_217_3:Y>Y		UWOPS05_227_2:Y>Y		UWOPS87_2421:Y>Y		W303:Y>Y		Y55:Y>Y		YJM978:Y>Y		YJM981:Y>Y		YPS128:Y>Y		YS9:Y>Y	AA:37		L_1528:G>G	AA:79		UWOPS05_217_3:P>P	AA:81		Y9:E>E	AA:296		DBVPG1788:H>H		DBVPG6040:H>H		DBVPG6765:H>H		L_1374:H>H		UWOPS05_217_3:H>H		UWOPS05_227_2:H>H		UWOPS87_2421:H>H		YJM978:H>H		YS4:H>H	AA:349		DBVPG1373:R>R		DBVPG1788:R>R		DBVPG6040:R>R		DBVPG6765:R>R		L_1374:R>R		YS4:R>R	AA:355		UWOPS05_217_3:N>N		UWOPS05_227_2:N>N	AA:380		W303:A>	AA:491		YS9:K>K	AA:494		YS9:C>CID:YBL016W	AA:42		DBVPG6044:K>K		SK1:K>K		UWOPS83_787_3:K>K		W303:K>K		Y55:K>K		YPS606:K>K	AA:44		YPS606:I>I	AA:47		378604X:F>F		DBVPG1788:F>F		DBVPG6765:F>F		L_1528:F>F		NCYC361:F>F		UWOPS87_2421:F>F		YJM978:F>F		YJM981:F>F	AA:123		YPS606:R>R	AA:224		SK1:P>P		UWOPS83_787_3:P>P		W303:P>P		Y55:P>P	AA:263		Y9:L>L	AA:303		DBVPG6765:A>A		L_1528:A>A	AA:310		322134S:Q>Q		DBVPG1106:Q>Q		DBVPG1373:Q>Q		DBVPG1788:Q>Q		DBVPG6765:Q>Q		K11:Q>Q		L_1374:Q>Q		L_1528:Q>Q		NCYC361:Q>Q		UWOPS87_2421:Q>Q		YJM975:Q>Q		YJM981:Q>Q		YS4:Q>Q		YS9:Q>Q	AA:316		322134S:N>N		DBVPG1106:N>N		DBVPG1373:N>N		DBVPG1788:N>N		DBVPG6765:N>N		L_1374:N>N		L_1528:N>N		NCYC361:N>N		UWOPS87_2421:N>N		YJM975:N>N		YJM981:N>N		YS4:N>N		YS9:N>NID:YBL019W	AA:12		BC187:K>K		DBVPG1373:K>K		DBVPG1788:K>K		DBVPG1853:K>K		DBVPG6765:K>K		L_1374:K>K		YJM975:K>K		YJM981:K>K	AA:73		DBVPG1788:V>V	AA:276		S288c:N>N	AA:297		YS9:T>T	AA:380		273614X:F>F		322134S:F>F		BC187:F>F		DBVPG1106:F>F		DBVPG1788:F>F		DBVPG1853:F>F		DBVPG6040:F>F		DBVPG6765:F>F		L_1374:F>F		L_1528:F>F		YJM975:F>F		YJM978:F>F	AA:389		UWOPS83_787_3:R>R	AA:409		273614X:Q>Q		322134S:Q>Q		BC187:Q>Q		DBVPG1106:Q>Q		DBVPG1788:Q>Q		DBVPG1853:Q>Q		DBVPG6040:Q>Q		DBVPG6765:Q>Q		L_1374:Q>Q		L_1528:Q>Q		YJM975:Q>Q		YJM978:Q>Q	AA:470		273614X:F>F		322134S:F>F		BC187:F>F		DBVPG1788:F>F		DBVPG1853:F>F		DBVPG6040:F>F		DBVPG6765:F>F		L_1374:F>F		L_1528:F>F		UWOPS03_461_4:F>F		UWOPS05_217_3:F>F		UWOPS83_787_3:F>F		UWOPS87_2421:F>F		YJM978:F>F		YPS606:F>F	AA:494		YJM978:G>G	AA:513		273614X:S>S		322134S:S>S		BC187:S>S		DBVPG1106:S>S		DBVPG1788:S>S		DBVPG1853:S>S		DBVPG6040:S>S		DBVPG6765:S>S		L_1374:S>S		UWOPS87_2421:S>SID:YBL020W	AA:12		322134S:S>S		BC187:S>S		DBVPG1373:S>S		DBVPG6040:S>S		DBVPG6044:S>S		DBVPG6765:S>S		L_1374:S>S		L_1528:S>S		SK1:S>S		UWOPS05_217_3:S>S		UWOPS83_787_3:S>S		UWOPS87_2421:S>S		W303:S>S		Y12:S>S		Y55:S>S		Y9:S>S		YJM975:S>S		YJM978:S>S		YJM981:S>S		YPS128:S>S		YS4:S>S	AA:16		UWOPS83_787_3:L>L	AA:25		Y12:F>F	AA:33		322134S:T>T	AA:81		DBVPG6044:I>I		NCYC110:I>I		SK1:I>I		W303:I>I		Y55:I>I	AA:100		DBVPG6044:T>T		NCYC110:T>T		SK1:T>T		W303:T>T		Y55:T>T	AA:132		UWOPS05_217_3:Q>Q	AA:164		DBVPG1373:E>E		DBVPG6044:E>E		DBVPG6765:E>E		L_1374:E>E		L_1528:E>E		NCYC110:E>E		NCYC361:E>E		SK1:E>E		UWOPS05_217_3:E>E		Y55:E>E		YJM978:E>E	AA:167		DBVPG1373:F>F		DBVPG1788:F>F		DBVPG6044:F>F		DBVPG6765:F>F		L_1374:F>F		L_1528:F>F		NCYC110:F>F		NCYC361:F>F		SK1:F>F		UWOPS05_217_3:F>F		W303:F>F		Y55:F>F		YJM978:F>F	AA:228		DBVPG1106:G>G		DBVPG1373:G>G		DBVPG6044:G>G		DBVPG6765:G>G		L_1528:G>G		NCYC110:G>G		NCYC361:G>G		SK1:G>G		UWOPS83_787_3:G>G		W303:G>G		Y55:G>G		YJM978:G>G	AA:239		273614X:C>C		DBVPG1373:C>C		DBVPG6765:C>C		L_1528:C>C		NCYC361:C>C		YJM978:C>C	AA:256		UWOPS03_461_4:T>T		UWOPS05_227_2:T>T	AA:266		273614X:N>N		DBVPG1106:N>N		DBVPG1373:N>N		L_1528:N>N		NCYC361:N>N		UWOPS03_461_4:N>N		YJM978:N>N	AA:268		NCYC361:E>E	AA:281		NCYC361:F>F	AA:323		378604X:A>A		K11:A>A		YS2:A>A	AA:331		UWOPS83_787_3:L>L	AA:407		273614X:L>L		DBVPG1106:L>L		DBVPG1373:L>L		DBVPG1788:L>L		DBVPG6765:L>L		YS2:L>L	AA:442		DBVPG1853:H>H		K11:H>H		YPS606:H>H	AA:470		273614X:G>G		322134S:G>G		DBVPG1106:G>G		DBVPG1373:G>G		DBVPG1788:G>G		DBVPG1853:G>G		DBVPG6765:G>G		L_1374:G>G		YJM975:G>G		YPS606:G>G		YS2:G>G	AA:497		322134S:L>L		378604X:L>L		DBVPG1106:L>L		DBVPG1373:L>L		DBVPG1788:L>L		DBVPG1853:L>L		DBVPG6765:L>L		L_1374:L>L		YJM975:L>L		YJM981:L>L		YPS606:L>L		YS2:L>L	AA:540		UWOPS83_787_3:N>N		YPS128:N>N		YPS606:N>NID:YBL021C	AA:22		273614X:N>N	AA:74		K11:S>S		UWOPS87_2421:S>S		Y9:S>S		YS4:S>S	AA:130		K11:L>L		SK1:L>L		W303:L>L		Y55:L>L		YS2:L>L	AA:133		273614X:Q>QID:YBL023C	AA:62		YJM975:D>D		YJM981:D>D	AA:281		YPS128:L>L		YPS606:L>L	AA:297		SK1:I>I		W303:I>I		Y55:I>I	AA:322		NCYC361:G>G		UWOPS83_787_3:G>G	AA:327		SK1:R>R		W303:R>R		Y55:R>R	AA:407		378604X:E>E	AA:422		DBVPG1788:E>E		DBVPG6765:E>E		L_1374:E>E		L_1528:E>E		UWOPS03_461_4:E>E		UWOPS05_217_3:E>E		W303:E>E		Y55:E>E		YJM975:E>E		YJM978:E>E	AA:481		DBVPG1373:E>E		DBVPG1788:E>E		DBVPG6765:E>E		L_1374:E>E		L_1528:E>E		NCYC361:E>E		UWOPS03_461_4:E>E		UWOPS05_217_3:E>E		UWOPS05_227_2:E>E		UWOPS83_787_3:E>E		W303:E>E		Y55:E>E		YJM975:E>E		YJM978:E>E		YPS128:E>E	AA:506		BC187:Y>Y		DBVPG1373:Y>Y		DBVPG1788:Y>Y		DBVPG6765:Y>Y		L_1374:Y>Y		L_1528:Y>Y		UWOPS03_461_4:Y>Y		UWOPS05_217_3:Y>Y		UWOPS05_227_2:Y>Y		YJM975:Y>Y		YJM978:Y>Y	AA:521		BC187:G>G		DBVPG1373:G>G		DBVPG1788:G>G		DBVPG6044:G>G		DBVPG6765:G>G		L_1528:G>G		NCYC361:G>G		UWOPS03_461_4:G>G		UWOPS05_217_3:G>G		UWOPS05_227_2:G>G		W303:G>G		Y55:G>G		YIIc17_E5:G>G		YJM975:G>G		YJM978:G>G		YPS128:G>G	AA:581		BC187:R>R		DBVPG1106:R>R		DBVPG1373:R>R		DBVPG1788:R>R		DBVPG6765:R>R		L_1528:R>R		YIIc17_E5:R>R		YJM975:R>R		YJM978:R>R	AA:605		YS4:L>L	AA:667		BC187:V>V		DBVPG1106:V>V		DBVPG1373:V>V		DBVPG1788:V>V		DBVPG6044:V>V		DBVPG6765:V>V		L_1528:V>V		SK1:V>V		UWOPS05_227_2:V>V		UWOPS83_787_3:V>V		W303:V>V		Y55:V>V		YJM975:V>V		YJM978:V>V		YPS128:V>V		YS2:V>V	AA:674		BC187:L>L		DBVPG1106:L>L		DBVPG1373:L>L		DBVPG1788:L>L		DBVPG6044:L>L		DBVPG6765:L>L		L_1528:L>L		SK1:L>L		UWOPS05_227_2:L>L		UWOPS83_787_3:L>L		W303:L>L		Y55:L>L		YJM975:L>L		YJM978:L>L		YPS128:L>L		YS2:L>L	AA:696		BC187:A>A		DBVPG1106:A>A		DBVPG1788:A>A		DBVPG6765:A>A		L_1528:A>A		UWOPS05_227_2:A>A		UWOPS83_787_3:A>A		YJM975:A>A		YJM978:A>A		YPS128:A>A		YS2:A>A	AA:706		BC187:S>S		DBVPG1106:S>S		DBVPG1788:S>S		DBVPG6765:S>S		L_1528:S>S		UWOPS83_787_3:S>S		YJM975:S>S		YJM978:S>S		YPS128:S>S		YS2:S>S	AA:710		YS4:N>N	AA:737		UWOPS83_787_3:Q>Q		YPS128:Q>Q	AA:789		DBVPG6044:V>V		SK1:V>V		W303:V>V		Y55:V>V	AA:806		BC187:T>T		DBVPG1106:T>T		DBVPG6765:T>T		L_1374:T>T		L_1528:T>T		YJM975:T>T		YJM978:T>T		YJM981:T>T		YPS128:T>T		YS2:T>T		YS9:T>T	AA:825		BC187:L>L		DBVPG1106:L>L		DBVPG6765:L>L		L_1374:L>L		L_1528:L>L		UWOPS83_787_3:L>L		YIIc17_E5:L>L		YJM975:L>L		YJM978:L>L		YJM981:L>L		YS2:L>L		YS9:L>LID:YBL024W	AA:44		NCYC361:L>L		UWOPS05_217_3:L>L		UWOPS83_787_3:L>L	AA:59		UWOPS05_217_3:C>C		UWOPS83_787_3:C>C	AA:143		YPS128:A>A	AA:144		L_1374:V>V	AA:165		DBVPG1788:P>P		DBVPG6044:P>P		DBVPG6765:P>P		L_1374:P>P		L_1528:P>P		NCYC110:P>P		SK1:P>P		W303:P>P		Y12:P>P		Y55:P>P		YIIc17_E5:P>P		YJM975:P>P		YS4:P>P		YS9:P>P	AA:170		DBVPG1788:L>L		DBVPG6044:L>L		DBVPG6765:L>L		L_1374:L>L		L_1528:L>L		NCYC110:L>L		SK1:L>L		UWOPS05_217_3:L>L		UWOPS05_227_2:L>L		W303:L>L		Y12:L>L		Y55:L>L		YIIc17_E5:L>L		YJM975:L>L		YS4:L>L		YS9:L>L	AA:221		YPS128:A>A	AA:269		YJM981:N>N		YS4:N>N	AA:284		DBVPG6040:L>L		L_1528:L>L	AA:324		L_1528:A>A	AA:367		UWOPS05_217_3:D>D		UWOPS05_227_2:D>D		UWOPS83_787_3:D>D	AA:369		UWOPS05_217_3:G>G		UWOPS05_227_2:G>G		UWOPS83_787_3:G>G	AA:426		DBVPG1853:T>T	AA:427		DBVPG1373:P>P		DBVPG6765:P>P	AA:499		273614X:V>V		DBVPG1373:V>V		DBVPG1853:V>V		DBVPG6040:V>V		DBVPG6765:V>V		L_1528:V>V		YJM975:V>V		YS9:V>V	AA:509		273614X:D>D		DBVPG1373:D>D		DBVPG1853:D>D		DBVPG6040:D>D		L_1528:D>D		UWOPS87_2421:D>D		YIIc17_E5:D>D		YJM975:D>D		YS9:D>D	AA:647		UWOPS05_217_3:A>A		UWOPS87_2421:A>A	AA:674		BC187:G>G		DBVPG1106:G>G		L_1374:G>G		L_1528:G>G		YJM975:G>G		YJM978:G>G		YS9:G>GID:YBL025W	AA:5		DBVPG6044:V>V		NCYC110:V>V		SK1:V>V		Y55:V>V	AA:30		322134S:K>K		BC187:K>K		DBVPG1106:K>K		DBVPG1373:K>K		DBVPG6044:K>K		DBVPG6765:K>K		L_1374:K>K		L_1528:K>K		NCYC110:K>K		SK1:K>K		UWOPS03_461_4:K>K		UWOPS83_787_3:K>K		Y55:K>K		YJM978:K>K		YPS128:K>K		YPS606:K>K		YS4:K>K	AA:66		UWOPS03_461_4:I>I		UWOPS83_787_3:I>I		YPS128:I>I		YPS606:I>I	AA:83		322134S:Y>Y		378604X:Y>Y	AA:113		UWOPS03_461_4:I>IID:YBL028C	AA:6		DBVPG6044:R>R		SK1:R>R		Y55:R>R	AA:15		DBVPG6044:S>S		SK1:S>S		Y55:S>S	AA:20		YPS128:G>G		YPS606:G>G	AA:43		273614X:K>K	AA:53		YPS128:E>E		YPS606:E>E	AA:63		DBVPG6044:E>E		SK1:E>E		Y55:E>E	AA:104		BC187:T>T		DBVPG1373:T>T		DBVPG6044:T>T		DBVPG6765:T>T		SK1:T>T		UWOPS05_217_3:T>T		UWOPS83_787_3:T>T		Y55:T>T		YPS606:T>TID:YBL029W	AA:60		322134S:L>L		DBVPG1106:L>L		DBVPG1788:L>L		DBVPG1853:L>L		DBVPG6765:L>L		L_1374:L>L		L_1528:L>L		NCYC110:L>L		SK1:L>L		UWOPS05_227_2:L>L		UWOPS83_787_3:L>L		W303:L>L		Y55:L>L		YJM978:L>L		YPS128:L>L		YPS606:L>L	AA:69		BC187:T>T	AA:74		Y12:N>N		Y9:N>N	AA:109		NCYC110:A>A		SK1:A>A		UWOPS05_217_3:A>A		UWOPS05_227_2:A>A		UWOPS83_787_3:A>A		UWOPS87_2421:A>A		W303:A>A		Y55:A>A		YPS128:A>A		YPS606:A>A	AA:137		322134S:T>T		BC187:T>T		DBVPG1106:T>T		DBVPG1373:T>T		DBVPG1788:T>T		DBVPG6765:T>T		L_1374:T>T		L_1528:T>T		YJM978:T>T	AA:139		DBVPG6044:S>S		NCYC110:S>S		SK1:S>S		W303:S>S		Y55:S>S	AA:158		DBVPG6044:P>P		NCYC110:P>P		SK1:P>P		UWOPS05_217_3:P>P		UWOPS05_227_2:P>P		UWOPS83_787_3:P>P		UWOPS87_2421:P>P		W303:P>P		Y55:P>P		YPS128:P>P		YPS606:P>P	AA:190		322134S:S>S		BC187:S>S		DBVPG1106:S>S		DBVPG1373:S>S		DBVPG1788:S>S		DBVPG6044:S>S		DBVPG6765:S>S		L_1374:S>S		L_1528:S>S		NCYC361:S>S		SK1:S>S		UWOPS05_217_3:S>S		UWOPS05_227_2:S>S		UWOPS87_2421:S>S		W303:S>S		Y55:S>S		YJM978:S>S	AA:212		Y9:N>N	AA:219		UWOPS87_2421:N>N	AA:238		L_1528:E>E	AA:264		L_1528:S>S	AA:277		L_1528:L>L	AA:280		UWOPS05_217_3:V>V		UWOPS05_227_2:V>V	AA:281		L_1528:L>L	AA:293		L_1528:E>E	AA:301		L_1528:Q>Q	AA:304		L_1528:L>L	AA:307		L_1528:P>P	AA:320		L_1528:E>E	AA:329		L_1528:L>L	AA:335		L_1528:V>V	AA:347		273614X:T>T		322134S:T>T		378604X:T>T		DBVPG1373:T>T		DBVPG1853:T>T		DBVPG6765:T>T		L_1528:T>T		SK1:T>T		UWOPS05_217_3:T>T		UWOPS05_227_2:T>T		UWOPS83_787_3:T>T		Y55:T>T		YJM975:T>T		YPS128:T>T		YPS606:T>T	AA:352		YIIc17_E5:G>G	AA:361		L_1528:L>L	AA:365		L_1528:R>RID:YBL030C	AA:3		DBVPG1373:S>S		DBVPG1788:S>S		DBVPG6765:S>S		L_1528:S>S		NCYC110:S>S		NCYC361:S>S		SK1:S>S		UWOPS83_787_3:S>S		UWOPS87_2421:S>S		W303:S>S		Y55:S>S		Y9:S>S		YJM981:S>S		YPS128:S>S		YS2:S>S	AA:11		DBVPG1373:L>L		DBVPG1788:L>L		DBVPG6765:L>L		L_1528:L>L		NCYC110:L>L		NCYC361:L>L		SK1:L>L		UWOPS83_787_3:L>L		UWOPS87_2421:L>L		W303:L>L		Y55:L>L		Y9:L>L		YJM981:L>L		YPS128:L>L		YS2:L>L	AA:48		DBVPG1373:K>K		DBVPG1788:K>K		DBVPG6044:K>K		DBVPG6765:K>K		K11:K>K		L_1528:K>K		NCYC361:K>K		SK1:K>K		UWOPS83_787_3:K>K		W303:K>K		Y12:K>K		Y55:K>K		Y9:K>K		YJM975:K>K		YJM981:K>K		YPS128:K>K		YS2:K>K	AA:167		273614X:A>A		DBVPG1373:A>A		DBVPG1788:A>A		DBVPG6765:A>A		L_1528:A>A		YJM975:A>A		YJM981:A>A	AA:197		UWOPS83_787_3:V>V	AA:238		DBVPG6765:T>T	AA:293		SK1:L>L		W303:L>L		Y55:L>L	AA:306		273614X:D>D		DBVPG1373:D>D		DBVPG1788:D>D		DBVPG6765:D>D		L_1528:D>D		YJM975:D>D		YJM981:D>DID:YBL031W	AA:48		DBVPG6044:L>L		DBVPG6765:L>L		L_1374:L>L		L_1528:L>L		NCYC361:L>L		SK1:L>L		W303:L>L		Y55:L>L		YJM975:L>L		YJM978:L>L		YJM981:L>L		YS2:L>L		YS9:L>L	AA:60		UWOPS03_461_4:A>A		UWOPS05_217_3:A>A		UWOPS05_227_2:A>A	AA:61		DBVPG1853:S>S	AA:75		UWOPS03_461_4:Y>Y		UWOPS05_217_3:Y>Y		UWOPS05_227_2:Y>Y	AA:191		378604X:T>T		DBVPG6044:T>T		SK1:T>T		W303:T>T		Y55:T>T	AA:255		378604X:R>RID:YBL032W	AA:55		UWOPS05_217_3:A>A	AA:145		322134S:D>D		DBVPG6040:D>D		NCYC110:D>D		SK1:D>D		UWOPS03_461_4:D>D		UWOPS05_217_3:D>D		UWOPS83_787_3:D>D		Y55:D>D		YPS128:D>D		YPS606:D>D	AA:163		NCYC361:V>V	AA:209		273614X:G>G		322134S:G>G		378604X:G>G		BC187:G>G		DBVPG1373:G>G		DBVPG1853:G>G		DBVPG6044:G>G		DBVPG6765:G>G		L_1374:G>G		NCYC110:G>G		NCYC361:G>G		SK1:G>G		UWOPS03_461_4:G>G		UWOPS05_217_3:G>G		UWOPS83_787_3:G>G		W303:G>G		Y55:G>G		YJM975:G>G		YJM978:G>G		YPS606:G>G		YS2:G>G	AA:294		DBVPG1373:V>V		DBVPG1788:V>V		DBVPG6765:V>V		L_1374:V>V		L_1528:V>V		YJM975:V>V		YJM978:V>V		YS2:V>V	AA:326		378604X:L>L	AA:358		DBVPG1788:S>S	AA:363		322134S:F>FID:YBL033C	AA:33		378604X:L>L		K11:L>L		Y9:L>L		YIIc17_E5:L>L	AA:58		Y9:N>N	AA:89		378604X:D>D		DBVPG6044:D>D		K11:D>D		NCYC110:D>D		SK1:D>D		W303:D>D		Y55:D>D		Y9:D>D		YIIc17_E5:D>D		YPS128:D>D	AA:94		DBVPG6044:G>G		NCYC110:G>G		SK1:G>G		W303:G>G		Y55:G>G	AA:99		378604X:K>K		DBVPG6044:K>K		K11:K>K		NCYC110:K>K		SK1:K>K		W303:K>K		Y55:K>K		YIIc17_E5:K>K	AA:149		DBVPG1373:Y>Y	AA:156		DBVPG6044:S>S		NCYC110:S>S		SK1:S>S		W303:S>S		Y55:S>S	AA:185		273614X:G>G		378604X:G>G		DBVPG1373:G>G		DBVPG1788:G>G		DBVPG6044:G>G		DBVPG6765:G>G		K11:G>G		L_1528:G>G		NCYC110:G>G		NCYC361:G>G		SK1:G>G		UWOPS03_461_4:G>G		UWOPS05_217_3:G>G		UWOPS05_227_2:G>G		W303:G>G		Y55:G>G		YIIc17_E5:G>G		YJM975:G>G		YJM978:G>G		YJM981:G>G		YPS128:G>G		YS9:G>G	AA:200		UWOPS03_461_4:G>G		UWOPS05_217_3:G>G	AA:205		273614X:E>E		DBVPG1373:E>E		DBVPG1788:E>E		DBVPG6044:E>E		DBVPG6765:E>E		NCYC110:E>E		NCYC361:E>E		SK1:E>E		UWOPS03_461_4:E>E		UWOPS05_217_3:E>E		W303:E>E		Y55:E>E		YJM978:E>E		YJM981:E>E		YS9:E>E	AA:209		DBVPG6044:A>A		NCYC110:A>A		SK1:A>A		W303:A>A		Y55:A>A	AA:236		DBVPG6044:S>S		NCYC110:S>S		SK1:S>S		W303:S>S		Y55:S>S	AA:247		273614X:I>I		378604X:I>I		DBVPG1373:I>I		DBVPG1788:I>I		DBVPG6040:I>I		DBVPG6765:I>I		L_1528:I>I		NCYC361:I>I		YIIc17_E5:I>I		YJM978:I>I		YJM981:I>I		YS9:I>I	AA:322		L_1528:T>T	AA:329		DBVPG6044:N>N		SK1:N>N		W303:N>N		Y55:N>NID:YBL036C	AA:3		DBVPG6765:T>T		L_1374:T>T		L_1528:T>T		YJM975:T>T		YJM978:T>T	AA:8		YPS128:D>D		YPS606:D>D	AA:138		YPS128:L>L		YPS606:L>L	AA:236		273614X:A>A		BC187:A>A		DBVPG1373:A>A		L_1374:A>A		YJM978:A>A	AA:238		NCYC361:V>V		YS9:V>V	AA:252		DBVPG6044:N>N		Y55:N>NID:YBL038W	AA:34		DBVPG1853:A>A		YS9:A>A	AA:42		273614X:A>A		322134S:A>A		DBVPG1373:A>A		DBVPG1788:A>A		DBVPG1853:A>A		DBVPG6044:A>A		DBVPG6765:A>A		L_1374:A>A		SK1:A>A		Y55:A>A		YJM978:A>A		YS9:A>A	AA:48		UWOPS05_227_2:V>V	AA:49		273614X:Q>Q		DBVPG1373:Q>Q		DBVPG1788:Q>Q		DBVPG1853:Q>Q		DBVPG6044:Q>Q		DBVPG6765:Q>Q		SK1:Q>Q		Y55:Q>Q		YJM978:Q>Q		YS9:Q>Q	AA:60		DBVPG1853:T>T	AA:86		273614X:S>S		DBVPG1853:S>S		DBVPG6044:S>S		DBVPG6765:S>S		UWOPS05_217_3:S>S		UWOPS83_787_3:S>S		Y55:S>S		YJM975:S>S		YJM978:S>S		YS9:S>S	AA:141		273614X:R>R		DBVPG1853:R>R		DBVPG6044:R>R		DBVPG6765:R>R		L_1374:R>R		L_1528:R>R		SK1:R>R		UWOPS05_217_3:R>R		UWOPS83_787_3:R>R		Y55:R>R		YJM975:R>R		YJM978:R>R	AA:162		273614X:R>R		DBVPG1853:R>R		DBVPG6044:R>R		DBVPG6765:R>R		L_1374:R>R		L_1528:R>R		SK1:R>R		UWOPS05_217_3:R>R		Y12:R>R		Y55:R>R		YJM975:R>R		YJM978:R>R		YPS606:R>R	AA:171		273614X:K>K		DBVPG1853:K>K		DBVPG6044:K>K		DBVPG6765:K>K		L_1374:K>K		L_1528:K>K		SK1:K>K		UWOPS05_217_3:K>K		Y55:K>K		YJM975:K>K		YJM978:K>K		YPS606:K>K	AA:179		YJM975:V>VID:YBL041W	AA:43		DBVPG6040:A>A	AA:103		273614X:L>L		322134S:L>L		378604X:L>L		DBVPG1788:L>L		DBVPG6040:L>L		DBVPG6044:L>L		DBVPG6765:L>L		L_1374:L>L		L_1528:L>L		SK1:L>L		UWOPS83_787_3:L>L		UWOPS87_2421:L>L		Y55:L>L		YJM975:L>L	AA:111		273614X:N>N		322134S:N>N		378604X:N>N		DBVPG1788:N>N		DBVPG6044:N>N		DBVPG6765:N>N		L_1374:N>N		L_1528:N>N		SK1:N>N		Y55:N>N		YJM975:N>N	AA:153		DBVPG1373:E>E	AA:196		UWOPS83_787_3:V>VID:YBL045C	AA:45		UWOPS03_461_4:S>S	AA:101		322134S:F>F		DBVPG6765:F>F		L_1374:F>F		YJM978:F>F		YS9:F>F	AA:194		DBVPG1853:E>E		YS4:E>E	AA:215		DBVPG6044:K>K		SK1:K>K		Y55:K>K	AA:275		YPS128:Y>Y	AA:293		UWOPS05_217_3:P>P		UWOPS05_227_2:P>P	AA:344		YPS128:I>I	AA:386		UWOPS05_217_3:D>D	AA:408		UWOPS05_217_3:K>KID:YBL049W	AA:3		378604X:L>L		DBVPG1373:L>L		DBVPG1788:L>L		DBVPG6765:L>L		L_1374:L>L		L_1528:L>L		NCYC361:L>L		YJM975:L>L		YS9:L>L	AA:66		YPS606:R>R	AA:113		378604X:L>L		YS9:L>LID:YBL050W	AA:49		UWOPS83_787_3:L>L	AA:89		UWOPS03_461_4:S>S		UWOPS05_227_2:S>S	AA:104		273614X:I>I		322134S:I>I		BC187:I>I		DBVPG1373:I>I		DBVPG1788:I>I		DBVPG1853:I>I		DBVPG6765:I>I		K11:I>I		L_1374:I>I		SK1:I>I		UWOPS03_461_4:I>I		UWOPS05_227_2:I>I		UWOPS83_787_3:I>I		UWOPS87_2421:I>I		Y55:I>I		YS9:I>I	AA:174		SK1:Y>Y		Y55:Y>Y	AA:182		SK1:S>S		UWOPS83_787_3:S>S		Y55:S>S	AA:201		SK1:Y>Y		UWOPS83_787_3:Y>Y		Y55:Y>Y		YPS606:Y>Y	AA:280		UWOPS83_787_3:I>I		YPS606:I>IID:YBL051C	AA:54		L_1528:E>E	AA:55		YPS606:N>N		YS2:N>N		YS4:N>N	AA:62		DBVPG1106:Q>Q		DBVPG1373:Q>Q		DBVPG1788:Q>Q		DBVPG6040:Q>Q		DBVPG6765:Q>Q		L_1374:Q>Q		L_1528:Q>Q		SK1:Q>Q		UWOPS05_227_2:Q>Q		UWOPS87_2421:Q>Q		Y55:Q>Q		YJM978:Q>Q		YPS606:Q>Q		YS2:Q>Q		YS4:Q>Q	AA:84		UWOPS87_2421:P>P	AA:85		DBVPG1106:N>N		DBVPG1373:N>N		DBVPG1788:N>N		DBVPG6040:N>N		DBVPG6765:N>N		L_1374:N>N		L_1528:N>N		SK1:N>N		Y55:N>N		YJM978:N>N		YPS606:N>N		YS2:N>N		YS9:N>N	AA:102		YPS606:L>L	AA:154		NCYC110:R>R		SK1:R>R		Y55:R>R	AA:158		378604X:V>V		DBVPG1106:V>V		DBVPG1373:V>V		DBVPG1788:V>V		DBVPG6040:V>V		DBVPG6765:V>V		K11:V>V		L_1528:V>V		NCYC110:V>V		SK1:V>V		UWOPS05_227_2:V>V		UWOPS83_787_3:V>V		UWOPS87_2421:V>V		Y12:V>V		Y55:V>V		YJM975:V>V		YJM978:V>V		YPS128:V>V		YPS606:V>V		YS2:V>V		YS4:V>V		YS9:V>V	AA:189		DBVPG1106:S>S		DBVPG1373:S>S		DBVPG1788:S>S		DBVPG6040:S>S		DBVPG6765:S>S		YJM975:S>S		YJM981:S>S	AA:212		YS4:N>N	AA:213		UWOPS05_227_2:Q>Q		UWOPS83_787_3:Q>Q	AA:215		UWOPS05_227_2:F>F		UWOPS83_787_3:F>F		YPS128:F>F		YPS606:F>F	AA:251		UWOPS05_227_2:L>L		UWOPS83_787_3:L>L		UWOPS87_2421:L>L	AA:255		YPS128:S>S		YPS606:S>S	AA:291		378604X:L>L		YS9:L>L	AA:320		378604X:K>K		DBVPG1106:K>K		DBVPG1373:K>K		DBVPG6040:K>K		DBVPG6765:K>K		L_1374:K>K		L_1528:K>K		SK1:K>K		UWOPS05_227_2:K>K		UWOPS83_787_3:K>K		UWOPS87_2421:K>K		Y55:K>K		YJM975:K>K		YJM981:K>K		YPS128:K>K		YPS606:K>K	AA:332		378604X:S>S		DBVPG1106:S>S		DBVPG1373:S>S		DBVPG6040:S>S		DBVPG6765:S>S		L_1374:S>S		L_1528:S>S		YJM975:S>S		YJM981:S>S		YS9:S>S	AA:375		DBVPG6044:Q>Q		SK1:Q>Q		Y55:Q>Q	AA:480		378604X:A>A		DBVPG1106:A>A		DBVPG6044:A>A		DBVPG6765:A>A		L_1528:A>A		SK1:A>A		UWOPS03_461_4:A>A		UWOPS83_787_3:A>A		UWOPS87_2421:A>A		Y12:A>A		Y55:A>A		Y9:A>A		YPS606:A>A		YS9:A>A	AA:483		322134S:P>P		378604X:P>P		DBVPG1106:P>P		DBVPG6765:P>P		L_1528:P>P		YS9:P>P	AA:499		UWOPS87_2421:Q>Q	AA:579		K11:S>S		Y12:S>S		Y9:S>S	AA:584		273614X:Y>Y		322134S:Y>Y		378604X:Y>Y		DBVPG1106:Y>Y		DBVPG1373:Y>Y		DBVPG1788:Y>Y		DBVPG6765:Y>Y		L_1374:Y>Y		L_1528:Y>Y		NCYC361:Y>Y		SK1:Y>Y		UWOPS83_787_3:Y>Y		UWOPS87_2421:Y>Y		Y12:Y>Y		Y55:Y>Y		Y9:Y>Y		YJM975:Y>Y		YS9:Y>Y	AA:588		322134S:G>G		K11:G>G		SK1:G>G		UWOPS83_787_3:G>G		Y12:G>G		Y55:G>G		Y9:G>G	AA:592		YS9:A>A	AA:593		322134S:N>N	AA:595		YS9:Q>QID:YBL052C	AA:9		273614X:S>S		378604X:S>S		BC187:S>S		DBVPG1373:S>S		DBVPG1788:S>S		DBVPG6765:S>S		L_1528:S>S		NCYC361:S>S		YJM975:S>S		YJM981:S>S	AA:22		273614X:E>E		378604X:E>E		BC187:E>E		DBVPG1373:E>E		DBVPG1788:E>E		DBVPG6765:E>E		L_1528:E>E		NCYC361:E>E		YJM975:E>E		YJM981:E>E	AA:25		K11:D>D	AA:53		BC187:S>S		YJM975:S>S		YJM981:S>S	AA:109		L_1528:E>E	AA:133		L_1528:S>S	AA:152		273614X:L>L		378604X:L>L		BC187:L>L		DBVPG1373:L>L		DBVPG1788:L>L		DBVPG1853:L>L		DBVPG6044:L>L		DBVPG6765:L>L		K11:L>L		L_1374:L>L		L_1528:L>L		NCYC110:L>L		NCYC361:L>L		SK1:L>L		UWOPS03_461_4:L>L		UWOPS05_227_2:L>L		Y12:L>L		Y55:L>L		YJM978:L>L		YPS606:L>L		YS4:L>L		YS9:L>L	AA:154		Y12:G>G	AA:195		K11:P>P		YS4:P>P	AA:220		L_1528:K>K	AA:227		273614X:N>N		378604X:N>N		BC187:N>N		DBVPG1788:N>N		DBVPG6044:N>N		DBVPG6765:N>N		K11:N>N		L_1374:N>N		L_1528:N>N		NCYC110:N>N		SK1:N>N		UWOPS03_461_4:N>N		UWOPS05_227_2:N>N		Y12:N>N		Y55:N>N		YJM978:N>N		YPS606:N>N		YS4:N>N	AA:250		YS4:N>N	AA:255		DBVPG6044:K>K		SK1:K>K		Y12:K>K		Y55:K>K		Y9:K>K	AA:258		273614X:G>G		378604X:G>G		BC187:G>G		DBVPG1788:G>G		DBVPG6040:G>G		DBVPG6765:G>G		L_1374:G>G		L_1528:G>G		YJM975:G>G		YJM978:G>G		YS9:G>G	AA:280		YS9:Y>Y	AA:329		273614X:P>P		BC187:P>P		DBVPG1106:P>P		DBVPG6765:P>P		L_1374:P>P		YJM975:P>P		YJM978:P>P	AA:383		BC187:E>E		YJM975:E>E	AA:415		378604X:N>N		DBVPG1106:N>N		DBVPG6765:N>N		UWOPS03_461_4:N>N		UWOPS87_2421:N>N		YJM975:N>N		YJM978:N>N		YS4:N>N		YS9:N>N	AA:430		378604X:Y>Y		DBVPG1106:Y>Y		DBVPG1788:Y>Y		DBVPG6040:Y>Y		DBVPG6765:Y>Y		K11:Y>Y		NCYC110:Y>Y		SK1:Y>Y		UWOPS03_461_4:Y>Y		UWOPS87_2421:Y>Y		Y55:Y>Y		Y9:Y>Y		YJM975:Y>Y		YPS606:Y>Y		YS9:Y>Y	AA:437		UWOPS87_2421:F>F	AA:528		BC187:T>T		YJM975:T>T	AA:569		378604X:L>L		BC187:L>L		DBVPG1106:L>L		DBVPG1373:L>L		DBVPG1788:L>L		DBVPG6040:L>L		DBVPG6765:L>L		K11:L>L		L_1374:L>L		Y12:L>L		Y9:L>L		YJM978:L>L		YS2:L>L		YS9:L>L	AA:647		NCYC110:E>E		SK1:E>E		Y9:E>E	AA:661		Y9:S>S	AA:710		DBVPG1106:C>C		DBVPG1788:C>C	AA:712		378604X:T>T		BC187:T>T		DBVPG1106:T>T		DBVPG1373:T>T		DBVPG1788:T>T		L_1374:T>T		L_1528:T>T		YJM975:T>T		YJM978:T>T	AA:757		378604X:D>D		BC187:D>D		DBVPG1106:D>D		DBVPG1373:D>D		DBVPG1788:D>D		DBVPG6765:D>D		L_1374:D>D		L_1528:D>D		YJM975:D>D		YJM978:D>D		YS9:D>D	AA:763		Y12:D>D	AA:765		Y12:T>T	AA:792		DBVPG1106:D>D		DBVPG1373:D>D		DBVPG1788:D>D		DBVPG6765:D>D		L_1374:D>D		L_1528:D>D		SK1:D>D		Y12:D>D		YJM975:D>D		YJM978:D>D		YPS606:D>D		YS2:D>D		YS4:D>D		YS9:D>D	AA:795		SK1:D>D	AA:823		Y12:I>IID:YBL054W	AA:2		L_1528:T>T	AA:3		378604X:L>L		DBVPG1373:L>L		DBVPG1788:L>L		DBVPG6765:L>L		L_1374:L>L	AA:38		L_1528:S>S	AA:46		L_1528:A>A		NCYC361:A>A		SK1:A>A		UWOPS05_227_2:A>A		UWOPS83_787_3:A>A		Y9:A>A	AA:48		L_1528:S>S	AA:66		YPS128:E>E		YPS606:E>E	AA:74		NCYC361:N>N		UWOPS83_787_3:N>N	AA:79		L_1528:D>D	AA:82		L_1528:D>D	AA:84		L_1528:I>I	AA:88		L_1528:H>H	AA:98		L_1528:K>K	AA:104		L_1528:F>F	AA:122		L_1528:G>G	AA:128		273614X:K>K		378604X:K>K		BC187:K>K		DBVPG1106:K>K		DBVPG1373:K>K		DBVPG6044:K>K		DBVPG6765:K>K		K11:K>K		L_1374:K>K		L_1528:K>K		NCYC361:K>K		SK1:K>K		UWOPS03_461_4:K>K		UWOPS83_787_3:K>K		Y55:K>K		YJM975:K>K		YPS606:K>K		YS2:K>K	AA:133		L_1528:D>D	AA:204		DBVPG1106:T>T	AA:219		UWOPS03_461_4:F>F	AA:226		L_1528:S>S	AA:230		L_1528:T>T	AA:245		K11:S>S		UWOPS87_2421:S>S	AA:278		K11:R>R		UWOPS87_2421:R>R	AA:390		UWOPS03_461_4:F>F		UWOPS05_227_2:F>F	AA:409		K11:K>K		NCYC361:K>K		UWOPS83_787_3:K>K		UWOPS87_2421:K>K		YPS128:K>K		YPS606:K>K		YS4:K>K	AA:439		K11:Y>Y		NCYC361:Y>Y		YPS128:Y>Y		YPS606:Y>Y		YS4:Y>Y	AA:447		YS4:S>S	AA:452		K11:N>N		NCYC361:N>N		YPS128:N>N		YPS606:N>N	AA:455		273614X:C>C		378604X:C>C		DBVPG1106:C>C		DBVPG1373:C>C		DBVPG1788:C>C		DBVPG6044:C>C		DBVPG6765:C>C		K11:C>C		L_1374:C>C		L_1528:C>C		NCYC110:C>C		NCYC361:C>C		SK1:C>C		UWOPS03_461_4:C>C		UWOPS05_227_2:C>C		UWOPS87_2421:C>C		Y55:C>C		YIIc17_E5:C>C		YPS128:C>C		YPS606:C>C		YS4:C>C	AA:457		273614X:D>D		378604X:D>D		DBVPG1106:D>D		DBVPG1373:D>D		DBVPG1788:D>D		DBVPG6765:D>D		L_1374:D>D		L_1528:D>D		UWOPS87_2421:D>D	AA:503		YIIc17_E5:S>SID:YBL056W	AA:70		DBVPG6044:A>A		NCYC110:A>A		SK1:A>A		UWOPS03_461_4:A>A		Y55:A>A	AA:85		DBVPG6044:E>E		NCYC110:E>E		SK1:E>E		UWOPS03_461_4:E>E		Y55:E>E	AA:122		YIIc17_E5:A>A	AA:142		YIIc17_E5:S>S	AA:182		273614X:V>V	AA:222		L_1528:N>N	AA:225		L_1528:E>E	AA:228		L_1528:F>F	AA:239		L_1528:C>C	AA:282		L_1528:G>G	AA:298		L_1528:E>E	AA:300		L_1528:E>E	AA:302		L_1528:Q>Q	AA:304		L_1528:F>F	AA:305		DBVPG1853:E>E		DBVPG6044:E>E		K11:E>E		NCYC110:E>E		SK1:E>E		UWOPS03_461_4:E>E		UWOPS05_227_2:E>E		UWOPS83_787_3:E>E		UWOPS87_2421:E>E		Y12:E>E		Y55:E>E		YIIc17_E5:E>E		YPS128:E>E		YPS606:E>E		YS4:E>E	AA:314		L_1528:I>I	AA:319		L_1528:V>V	AA:333		273614X:D>D	AA:335		273614X:D>D		322134S:D>D		378604X:D>D		BC187:D>D		DBVPG1106:D>D		DBVPG1373:D>D		DBVPG1853:D>D		DBVPG6040:D>D		K11:D>D		L_1528:D>D		SK1:D>D		UWOPS03_461_4:D>D		UWOPS05_227_2:D>D		UWOPS83_787_3:D>D		UWOPS87_2421:D>D		Y12:D>D		Y55:D>D		YIIc17_E5:D>D		YJM975:D>D		YPS128:D>D		YPS606:D>D		YS4:D>D	AA:339		L_1528:F>F	AA:344		K11:K>K	AA:355		SK1:D>D	AA:384		378604X:G>G	AA:388		L_1528:L>L	AA:398		L_1528:G>G	AA:412		L_1528:N>N	AA:415		L_1528:Y>Y	AA:429		L_1528:S>S	AA:433		L_1528:A>A	AA:436		L_1528:T>T	AA:442		L_1528:D>DID:YBL057C	AA:61		YS9:L>L	AA:70		DBVPG6044:S>S		NCYC110:S>S		SK1:S>S		UWOPS03_461_4:S>S		Y55:S>S		Y9:S>S		YIIc17_E5:S>S		YPS606:S>S	AA:76		UWOPS03_461_4:S>S	AA:120		DBVPG1373:L>LID:YBL058W	AA:5		L_1528:P>P	AA:11		UWOPS05_227_2:Q>Q	AA:83		378604X:H>H	AA:101		DBVPG6044:L>L		NCYC110:L>L		UWOPS05_227_2:L>L		UWOPS83_787_3:L>L		Y55:L>L		YPS128:L>L		YPS606:L>L		YS2:L>L	AA:105		DBVPG6044:G>G		NCYC110:G>G		UWOPS05_227_2:G>G		UWOPS83_787_3:G>G		Y55:G>G		YPS128:G>G		YPS606:G>G		YS2:G>G	AA:109		273614X:P>P	AA:120		378604X:S>S	AA:160		378604X:T>T		DBVPG6765:T>T		YJM975:T>T		YJM981:T>T	AA:165		273614X:P>P		378604X:P>P		DBVPG6044:P>P		DBVPG6765:P>P		NCYC110:P>P		UWOPS83_787_3:P>P		Y12:P>P		Y55:P>P		YJM975:P>P		YJM981:P>P		YPS128:P>P		YPS606:P>P		YS2:P>P	AA:178		NCYC110:R>R		UWOPS83_787_3:R>R		Y12:R>R		Y55:R>R		YPS128:R>R		YS2:R>R	AA:228		273614X:R>R	AA:265		UWOPS05_217_3:E>E		UWOPS83_787_3:E>E	AA:312		UWOPS05_217_3:R>R		UWOPS83_787_3:R>R		Y12:R>R	AA:314		UWOPS05_217_3:G>G		UWOPS83_787_3:G>G		Y12:G>G	AA:321		UWOPS05_217_3:S>S		UWOPS83_787_3:S>S		Y12:S>S		Y55:S>S	AA:358		UWOPS05_217_3:G>G		UWOPS83_787_3:G>G	AA:394		UWOPS03_461_4:Y>Y		UWOPS05_217_3:Y>Y		Y55:Y>Y		YPS606:Y>Y	AA:416		DBVPG6044:S>S		SK1:S>S		UWOPS03_461_4:S>S		UWOPS05_217_3:S>S		Y55:S>S		YPS606:S>S	AA:421		322134S:R>R		BC187:R>R		DBVPG6765:R>RID:YBL060W	AA:26		BC187:S>S	AA:28		Y12:S>S		Y9:S>S		YS4:S>S	AA:61		Y12:K>K		Y9:K>K		YPS128:K>K		YS4:K>K	AA:90		Y12:Q>Q		Y9:Q>Q	AA:124		DBVPG6044:N>N		SK1:N>N		UWOPS03_461_4:N>N		UWOPS05_217_3:N>N		UWOPS83_787_3:N>N		Y12:N>N		Y55:N>N		YIIc17_E5:N>N		YS4:N>N	AA:142		DBVPG6044:P>P		SK1:P>P		Y55:P>P	AA:145		DBVPG6044:L>L		SK1:L>L		Y55:L>L	AA:148		DBVPG6044:T>T		SK1:T>T		UWOPS03_461_4:T>T		UWOPS05_217_3:T>T		Y12:T>T		Y55:T>T		Y9:T>T	AA:159		DBVPG6044:I>I		SK1:I>I		Y55:I>I	AA:193		K11:H>H		SK1:H>H		UWOPS03_461_4:H>H		UWOPS05_217_3:H>H		Y12:H>H		Y55:H>H		Y9:H>H		YIIc17_E5:H>H		YS4:H>H	AA:201		UWOPS03_461_4:I>I		UWOPS05_217_3:I>I		Y9:I>I		YIIc17_E5:I>I	AA:204		K11:S>S		SK1:S>S		UWOPS03_461_4:S>S		UWOPS05_217_3:S>S		Y55:S>S		Y9:S>S		YIIc17_E5:S>S		YS4:S>S	AA:220		UWOPS03_461_4:S>S		UWOPS05_217_3:S>S	AA:238		SK1:Y>Y		YIIc17_E5:Y>Y	AA:255		YIIc17_E5:S>S	AA:273		UWOPS03_461_4:R>R		UWOPS05_217_3:R>R	AA:297		SK1:S>S		Y55:S>S	AA:340		SK1:L>L		Y55:L>L	AA:373		YPS606:R>R	AA:390		UWOPS03_461_4:S>S	AA:422		NCYC110:N>N		SK1:N>N		Y55:N>N	AA:517		NCYC361:P>P		Y12:P>P	AA:523		Y12:P>P		Y9:P>P	AA:525		Y12:L>L		Y9:L>L	AA:559		L_1528:F>F	AA:564		K11:N>N		NCYC361:N>N	AA:614		K11:R>R	AA:615		UWOPS05_227_2:L>L	AA:622		378604X:T>T	AA:638		K11:K>K		YS4:K>K	AA:648		NCYC361:N>N	AA:672		UWOPS05_227_2:L>L	AA:674		378604X:Y>Y		K11:Y>Y		UWOPS03_461_4:Y>Y		UWOPS05_227_2:Y>Y		YS4:Y>Y	AA:683		378604X:G>G		K11:G>G		UWOPS03_461_4:G>G		YS4:G>GID:YBL061C	AA:27		UWOPS05_227_2:L>L		YPS606:L>L		YS4:L>L	AA:57		Y12:T>T		YIIc17_E5:T>T	AA:100		YS2:P>P	AA:214		Y12:V>V	AA:219		YIIc17_E5:A>A	AA:252		K11:L>L		YPS128:L>L		YPS606:L>L		YS2:L>L		YS9:L>L	AA:287		K11:G>G		NCYC361:G>G		YIIc17_E5:G>G		YPS128:G>G		YPS606:G>G		YS2:G>G	AA:337		DBVPG6044:A>A		SK1:A>A	AA:353		DBVPG6044:S>S		K11:S>S		NCYC361:S>S		SK1:S>S		UWOPS03_461_4:S>S		UWOPS83_787_3:S>S		YPS128:S>S		YPS606:S>S		YS2:S>S	AA:368		DBVPG6044:L>L		K11:L>L		NCYC361:L>L		SK1:L>L		UWOPS03_461_4:L>L		UWOPS83_787_3:L>L		YPS128:L>L		YPS606:L>L	AA:404		Y9:V>V	AA:422		YS9:G>G	AA:478		DBVPG1853:A>A		K11:A>A		NCYC110:A>A		SK1:A>A		UWOPS03_461_4:A>A		UWOPS83_787_3:A>A		Y9:A>A	AA:503		DBVPG1853:F>F		UWOPS87_2421:F>F		Y9:F>F	AA:513		DBVPG1853:K>K		K11:K>K		UWOPS87_2421:K>K		Y9:K>K	AA:514		NCYC110:G>G		SK1:G>G	AA:532		DBVPG1853:N>N		K11:N>N		UWOPS87_2421:N>N		Y9:N>N		YS9:N>N	AA:536		273614X:R>R		322134S:R>R		DBVPG1853:R>R		K11:R>R		NCYC110:R>R		SK1:R>R		UWOPS05_227_2:R>R		UWOPS83_787_3:R>R		UWOPS87_2421:R>R		Y55:R>R		Y9:R>R	AA:579		UWOPS05_227_2:G>G		UWOPS83_787_3:G>G		UWOPS87_2421:G>G		Y9:G>G	AA:599		UWOPS03_461_4:D>D		UWOPS05_227_2:D>D	AA:606		UWOPS87_2421:Q>Q	AA:608		K11:N>N		UWOPS03_461_4:N>N		UWOPS05_227_2:N>N		UWOPS83_787_3:N>N		UWOPS87_2421:N>N	AA:677		DBVPG6044:T>T		K11:T>T		NCYC110:T>T		SK1:T>T		UWOPS03_461_4:T>T		UWOPS05_227_2:T>T		UWOPS83_787_3:T>T		Y55:T>T		YIIc17_E5:T>T		YS2:T>T	AA:687		UWOPS83_787_3:G>G		YS2:G>G		YS4:G>G	AA:697		K11:->-		NCYC110:->-		SK1:->-		UWOPS03_461_4:->-		UWOPS05_227_2:->-		UWOPS83_787_3:->-		Y12:->-		Y55:->-		YIIc17_E5:->-		YPS606:->-		YS2:->-		YS4:->-ID:YBL066C	AA:89		Y9:I>I		YS4:I>I		YS9:I>I	AA:162		DBVPG6044:S>S		NCYC110:S>S		SK1:S>S		Y55:S>S	AA:218		K11:L>L		UWOPS05_227_2:L>L		UWOPS83_787_3:L>L		Y9:L>L		YS2:L>L		YS4:L>L		YS9:L>L	AA:233		UWOPS05_227_2:L>L	AA:296		DBVPG6044:P>P		K11:P>P		SK1:P>P		UWOPS05_227_2:P>P		UWOPS83_787_3:P>P		Y55:P>P		Y9:P>P		YPS606:P>P		YS9:P>P	AA:320		K11:T>T	AA:392		DBVPG6044:S>S		NCYC110:S>S		SK1:S>S		Y55:S>S		YPS128:S>S		YPS606:S>S	AA:403		DBVPG6044:S>S		NCYC110:S>S		SK1:S>S		Y55:S>S	AA:418		YPS128:T>T		YPS606:T>T	AA:421		DBVPG6044:S>S		NCYC110:S>S		SK1:S>S		Y55:S>S	AA:471		DBVPG6044:F>F		NCYC110:F>F		SK1:F>F		Y55:F>F	AA:516		DBVPG6044:E>E		NCYC110:E>E		SK1:E>E		UWOPS05_227_2:E>E		Y55:E>E	AA:523		UWOPS05_227_2:D>D	AA:605		DBVPG6044:D>D		NCYC110:D>D		SK1:D>D		Y55:D>D	AA:720		YIIc17_E5:Q>Q	AA:744		DBVPG6044:F>F		SK1:F>F		UWOPS87_2421:F>F		Y55:F>F		YPS128:F>F	AA:765		DBVPG6040:S>S	AA:769		378604X:G>G	AA:771		Y9:L>L		YIIc17_E5:L>L		YS4:L>L	AA:803		Y9:R>R		YIIc17_E5:R>R		YS4:R>R	AA:815		UWOPS05_227_2:S>S	AA:836		DBVPG6040:R>R		Y9:R>R		YIIc17_E5:R>R		YS2:R>R		YS4:R>R	AA:842		DBVPG6044:V>V		SK1:V>V		UWOPS83_787_3:V>V		Y55:V>V		Y9:V>V		YIIc17_E5:V>V		YPS128:V>V		YS2:V>V		YS4:V>V	AA:853		DBVPG6044:T>T		SK1:T>T		Y55:T>T		YPS128:T>T	AA:862		DBVPG6044:L>L		SK1:L>L		UWOPS05_217_3:L>L		Y12:L>L		Y55:L>L		Y9:L>L		YIIc17_E5:L>L		YPS128:L>L		YS2:L>L		YS4:L>L	AA:970		DBVPG1373:S>S	AA:977		DBVPG1853:P>P	AA:978		DBVPG6040:A>A		YS4:A>A	AA:996		273614X:T>T	AA:1016		DBVPG1853:N>N		DBVPG6040:N>N		DBVPG6044:N>N		K11:N>N		SK1:N>N		UWOPS05_217_3:N>N		UWOPS05_227_2:N>N		UWOPS83_787_3:N>N		Y12:N>N		Y55:N>N		YIIc17_E5:N>N		YPS128:N>N		YPS606:N>N		YS2:N>N		YS4:N>N	AA:1037		DBVPG1853:S>S	AA:1083		UWOPS05_217_3:T>T		UWOPS05_227_2:T>T		UWOPS87_2421:T>T	AA:1109		DBVPG6040:K>K		DBVPG6044:K>K		K11:K>K		SK1:K>K		UWOPS05_217_3:K>K		UWOPS05_227_2:K>K		UWOPS83_787_3:K>K		Y55:K>K		YIIc17_E5:K>K		YPS128:K>K		YPS606:K>K		YS4:K>K	AA:1131		DBVPG6040:S>S		K11:S>S		YS4:S>S	AA:1138		UWOPS87_2421:G>GID:YBL068W	AA:102		UWOPS03_461_4:P>P		UWOPS05_217_3:P>P		UWOPS05_227_2:P>P	AA:123		YPS128:L>L		YPS606:L>L	AA:135		DBVPG6044:H>H		SK1:H>H		Y55:H>H	AA:159		K11:A>A		Y9:A>A		YIIc17_E5:A>A		YS4:A>A	AA:194		K11:A>A		Y9:A>A		YIIc17_E5:A>A		YS2:A>A		YS4:A>A	AA:230		DBVPG6044:L>L		K11:L>L		UWOPS03_461_4:L>L		UWOPS05_217_3:L>L		UWOPS05_227_2:L>L		UWOPS87_2421:L>L		Y55:L>L		Y9:L>L		YPS128:L>L		YPS606:L>L		YS2:L>L		YS4:L>L	AA:256		Y9:V>V	AA:307		UWOPS83_787_3:E>E	AA:326		YPS606:P>PID:YBL069W	AA:101		DBVPG6040:S>S		K11:S>S		UWOPS87_2421:S>S		YIIc17_E5:S>S	AA:118		273614X:T>T	AA:136		Y9:G>G	AA:180		K11:L>L		YIIc17_E5:L>L	AA:261		UWOPS87_2421:R>R	AA:280		DBVPG1853:Y>Y	AA:353		UWOPS83_787_3:F>F	AA:354		DBVPG1853:G>G		YPS606:G>G	AA:357		DBVPG6040:I>I		K11:I>I		YIIc17_E5:I>I		YS2:I>I	AA:385		K11:G>G		YIIc17_E5:G>G		YS2:G>G	AA:429		L_1528:F>FID:YBL074C	AA:6		378604X:F>F		DBVPG6040:F>F		SK1:F>F		Y55:F>F		YIIc17_E5:F>F		YPS606:F>F	AA:39		YPS606:G>G	AA:43		DBVPG6765:V>V	AA:57		322134S:G>G	AA:62		DBVPG6765:C>C	AA:76		YJM978:G>G	AA:88		YPS606:K>K	AA:113		378604X:H>H		DBVPG1853:T>T		DBVPG6040:H>H		DBVPG6044:H>H		SK1:H>H		UWOPS05_217_3:H>H		Y55:H>H		YIIc17_E5:H>H		YPS606:H>H	AA:207		DBVPG1853:T>T		UWOPS05_217_3:T>T	AA:258		DBVPG6044:K>K		SK1:K>K		Y55:K>K		YPS606:K>K	AA:264		YS2:L>L	AA:339		YPS128:D>DID:YBL075C	AA:11		378604X:T>T		DBVPG6040:T>T		K11:T>T		SK1:T>T		UWOPS03_461_4:T>T		Y55:T>T		YIIc17_E5:T>T		YPS128:T>T		YS2:T>T	AA:35		YPS128:T>T	AA:38		378604X:S>S		DBVPG6040:S>S		YIIc17_E5:S>S	AA:42		378604X:F>F		DBVPG6040:F>F		YIIc17_E5:F>F	AA:49		YJM975:I>I	AA:52		DBVPG1853:A>A		SK1:A>A		Y55:A>A	AA:57		YJM975:A>A	AA:66		YJM975:F>F	AA:72		YJM975:I>I	AA:114		378604X:P>P		DBVPG6040:P>P		YIIc17_E5:P>P	AA:182		378604X:G>G		DBVPG1853:G>G		DBVPG6040:G>G		DBVPG6044:G>G		NCYC110:G>G		SK1:G>G		UWOPS05_217_3:G>G		UWOPS05_227_2:G>G		Y12:G>G		YIIc17_E5:G>G	AA:211		378604X:D>D		DBVPG6040:D>D		DBVPG6044:D>D		NCYC110:D>D		SK1:D>D		UWOPS05_217_3:D>D		UWOPS05_227_2:D>D		Y12:D>D		YIIc17_E5:D>D	AA:249		Y12:K>K	AA:281		273614X:G>G		378604X:G>G		DBVPG1853:G>G		DBVPG6040:G>G		NCYC110:G>G		NCYC361:G>G		SK1:G>G		UWOPS05_217_3:G>G		UWOPS05_227_2:G>G		Y12:G>G		YPS606:G>G		YS4:G>G	AA:307		273614X:L>L		378604X:L>L		DBVPG1853:L>L		DBVPG6040:L>L		DBVPG6044:L>L		NCYC110:L>L		NCYC361:L>L		SK1:L>L		UWOPS05_217_3:L>L		UWOPS05_227_2:L>L		Y12:L>L		YPS606:L>L	AA:315		378604X:V>V		DBVPG1853:V>V		DBVPG6040:V>V		DBVPG6044:V>V		NCYC110:V>V		SK1:V>V		UWOPS05_217_3:V>V		UWOPS05_227_2:V>V		Y12:V>V		YPS606:V>V		YS4:V>V	AA:363		378604X:P>P	AA:396		BC187:P>P	AA:417		NCYC361:T>T	AA:463		K11:P>P		YPS128:P>P		YPS606:P>P		YS4:P>P	AA:488		K11:A>A		YPS128:A>A		YPS606:A>A		YS4:A>A	AA:491		DBVPG6040:K>K		DBVPG6044:K>K		SK1:K>K		UWOPS03_461_4:K>K		UWOPS05_227_2:K>K		UWOPS83_787_3:K>K		Y55:K>K		YIIc17_E5:K>K	AA:529		DBVPG1853:R>R		DBVPG6040:R>R		DBVPG6044:R>R		K11:R>R		SK1:R>R		UWOPS03_461_4:R>R		UWOPS05_227_2:R>R		UWOPS83_787_3:R>R		Y55:R>R		YIIc17_E5:R>R		YPS128:R>R		YPS606:R>R		YS4:R>R	AA:542		DBVPG1853:S>S		DBVPG6040:S>S		DBVPG6044:S>S		K11:S>S		SK1:S>S		UWOPS03_461_4:S>S		UWOPS05_227_2:S>S		UWOPS83_787_3:S>S		Y55:S>S		YIIc17_E5:S>S		YPS128:S>S		YPS606:S>S		YS4:S>S	AA:571		K11:A>A		YPS128:A>A		YPS606:A>A		YS4:A>A	AA:577		K11:D>D		YPS128:D>D		YPS606:D>D		YS4:D>D	AA:606		DBVPG1853:T>T		DBVPG6040:T>T		DBVPG6044:T>T		K11:T>T		SK1:T>T		UWOPS03_461_4:T>T		UWOPS05_217_3:T>T		UWOPS83_787_3:T>T		Y55:T>T		YIIc17_E5:T>T		YPS128:T>T		YPS606:T>T		YS4:T>T	AA:632		273614X:S>S		DBVPG1853:S>S		DBVPG6040:S>S		DBVPG6044:S>S		SK1:S>S		UWOPS03_461_4:S>S		UWOPS05_217_3:S>S		UWOPS83_787_3:S>S		Y55:S>S		YIIc17_E5:S>S		YPS128:S>S		YPS606:S>S		YS4:S>S	AA:639		DBVPG1853:E>E		DBVPG6044:E>E		SK1:E>E		UWOPS03_461_4:E>E		UWOPS05_217_3:E>E		Y55:E>EID:YBL078C	AA:10		DBVPG1853:P>P	AA:37		DBVPG1853:E>E	AA:53		SK1:A>A		UWOPS03_461_4:A>A		UWOPS05_227_2:A>A		Y55:A>A	AA:103		UWOPS05_217_3:G>G	AA:113		DBVPG1853:K>K		UWOPS05_217_3:N>NID:YBL080C	AA:37		378604X:T>T		K11:T>T		UWOPS87_2421:T>T		Y12:T>T		Y9:T>T		YS9:T>T	AA:44		YJM975:K>K		YJM978:K>K	AA:56		378604X:L>L		K11:L>L		UWOPS87_2421:L>L		Y12:L>L		Y9:L>L		YS9:L>L	AA:85		378604X:F>F		K11:F>F		UWOPS87_2421:F>F		Y12:F>F		Y9:F>F		YS9:F>F	AA:132		UWOPS03_461_4:I>I		UWOPS05_227_2:I>I	AA:135		273614X:A>A		378604X:S>S		DBVPG6040:A>A		SK1:A>A		UWOPS83_787_3:A>A		UWOPS87_2421:S>S		YS9:S>S	AA:150		UWOPS03_461_4:I>I		UWOPS05_227_2:I>I	AA:169		BC187:K>K		DBVPG6765:K>K		L_1374:K>K		YS2:K>K	AA:198		BC187:Q>Q		DBVPG6765:Q>Q		L_1374:Q>Q		UWOPS83_787_3:Q>Q		YJM975:Q>Q		YS2:Q>Q	AA:251		322134S:I>I		BC187:I>I		DBVPG1788:I>I		DBVPG6044:I>I		DBVPG6765:I>I		L_1374:I>I		SK1:I>I		Y55:I>I		YJM975:I>I		YS2:I>I	AA:275		322134S:E>E		BC187:E>E		DBVPG1788:E>E		DBVPG6765:E>E		L_1374:E>E		YJM975:E>E		YS2:E>E	AA:357		YS2:D>D	AA:367		YS2:Y>Y	AA:430		DBVPG6040:V>V	AA:453		BC187:Q>Q		DBVPG1373:Q>Q		DBVPG1788:Q>Q		DBVPG1853:Q>Q		DBVPG6040:Q>Q		DBVPG6765:Q>Q		L_1374:Q>Q		YPS606:Q>Q	AA:471		DBVPG6040:A>A	AA:490		NCYC110:Q>Q		SK1:Q>QID:YBL081W	AA:51		378604X:P>P		NCYC110:P>P		SK1:P>P		UWOPS87_2421:P>P		Y12:P>P		Y55:P>P		YPS128:P>P		YPS606:P>P		YS4:P>P	AA:77		378604X:P>P		NCYC110:P>P		SK1:P>P		UWOPS05_217_3:P>P		UWOPS87_2421:P>P		Y12:P>P		Y55:P>P	AA:84		378604X:Q>Q		DBVPG6044:Q>Q		NCYC110:Q>Q		SK1:Q>Q		UWOPS05_217_3:Q>Q		UWOPS87_2421:Q>Q		Y12:Q>Q		Y55:Q>Q		YPS606:Q>Q		YS4:Q>Q		YS9:Q>Q	AA:92		NCYC110:S>S		SK1:S>S		Y55:S>S	AA:111		UWOPS05_217_3:T>T	AA:148		322134S:T>T	AA:185		DBVPG6044:N>N		NCYC110:N>N		Y55:N>N	AA:192		UWOPS05_217_3:R>R		UWOPS05_227_2:R>R	AA:199		378604X:S>S		Y9:S>S	AA:219		UWOPS87_2421:E>E		YPS128:E>E		YPS606:E>E	AA:243		YPS128:T>T		YPS606:T>T	AA:244		UWOPS05_217_3:N>N		UWOPS05_227_2:N>N		UWOPS87_2421:N>N	AA:247		DBVPG6040:T>T	AA:266		322134S:S>S		DBVPG6765:S>S		L_1374:S>S		L_1528:S>S		YJM975:S>S	AA:339		378604X:G>G		K11:G>G		Y9:G>G		YS4:G>GID:YBL082C	AA:13		YS9:L>L	AA:38		NCYC361:F>F	AA:46		UWOPS05_217_3:V>V		UWOPS05_227_2:V>V	AA:118		SK1:E>E		Y55:E>E	AA:164		378604X:L>L		SK1:L>L		UWOPS03_461_4:L>L		UWOPS05_217_3:L>L		Y55:L>L		Y9:L>L		YPS128:L>L		YPS606:L>L		YS4:L>L	AA:201		378604X:L>L		UWOPS03_461_4:L>L		Y9:L>L		YPS128:L>L		YPS606:L>L		YS4:L>L	AA:305		YS4:L>L	AA:374		DBVPG6044:F>F		NCYC361:F>F		SK1:F>F		UWOPS05_227_2:F>F		YS9:F>F	AA:397		YS9:P>P	AA:409		378604X:S>S		DBVPG6044:S>S		NCYC361:S>S		SK1:S>S		UWOPS05_227_2:S>S		YPS128:S>S		YPS606:S>S	AA:430		DBVPG6044:L>L		NCYC361:L>L		SK1:L>L		UWOPS05_227_2:L>L		YPS128:L>L		YPS606:L>L	AA:435		DBVPG6044:Q>Q		NCYC110:Q>Q		NCYC361:Q>Q		SK1:Q>Q		UWOPS05_227_2:Q>Q		YPS128:Q>Q		YPS606:Q>Q	AA:437		DBVPG6044:S>S		NCYC110:S>S		NCYC361:S>S		SK1:S>S		UWOPS05_227_2:S>S		YPS128:S>S		YPS606:S>S	AA:452		DBVPG1373:S>S		DBVPG6044:S>S		L_1374:S>S		NCYC361:S>S		SK1:S>S		UWOPS05_227_2:S>S		YJM975:S>S		YPS128:S>S		YPS606:S>S		YS4:S>STable S4. Synonymous SNPs in S. cerevisiae genes studied. The identity of each affected amino acid in each affected strain is shown for each of 3737 genes.ID:YBL084C	AA:73		UWOPS05_217_3:L>L		Y9:L>L	AA:106		UWOPS03_461_4:G>G		UWOPS05_217_3:G>G		Y9:G>G		YPS128:G>G		YPS606:G>G	AA:193		Y9:A>A		YS4:A>A	AA:204		273614X:K>K		DBVPG1106:K>K		DBVPG1788:K>K		DBVPG6044:K>K		DBVPG6765:K>K		SK1:K>K		UWOPS03_461_4:K>K		UWOPS05_217_3:K>K		Y55:K>K		Y9:K>K		YPS128:K>K		YPS606:K>K		YS4:K>K	AA:268		DBVPG1853:P>P	AA:282		UWOPS05_217_3:F>F	AA:350		YJM978:T>T	AA:378		378604X:N>N		DBVPG1853:N>N		Y12:N>N	AA:379		DBVPG6765:N>N	AA:388		K11:N>N	AA:401		K11:L>L	AA:503		DBVPG1853:P>P		YS2:P>P	AA:628		273614X:L>L		L_1374:L>L	AA:695		DBVPG6044:A>A		Y55:A>A	AA:705		DBVPG1853:L>L	AA:730		YPS128:I>I		YPS606:I>I	AA:742		K11:K>KID:YBL086C	AA:30		378604X:S>S	AA:43		UWOPS87_2421:T>T	AA:74		K11:H>H		Y12:H>H	AA:129		YS2:V>V	AA:180		DBVPG1853:F>F	AA:201		DBVPG6044:R>R		K11:R>R		NCYC110:R>R		SK1:R>R		UWOPS03_461_4:R>R		UWOPS05_227_2:R>R		UWOPS83_787_3:R>R		UWOPS87_2421:R>R		Y55:R>R		YPS128:R>R		YPS606:R>R		YS4:R>R		YS9:R>R	AA:232		UWOPS83_787_3:T>T	AA:256		YS9:G>G	AA:311		L_1374:Q>Q	AA:409		DBVPG6044:S>S		NCYC110:S>S		SK1:S>S		UWOPS03_461_4:S>S		UWOPS87_2421:S>S		Y55:S>S		Y9:S>S		YPS128:S>S		YS2:S>S		YS9:S>S	AA:433		Y12:S>S		Y9:S>S		YPS606:S>S		YS2:S>S		YS9:S>S	AA:465		DBVPG6044:S>S		NCYC110:S>S		SK1:S>S		Y12:S>S		Y9:S>S		YPS606:S>S		YS2:S>S		YS9:S>SID:YBL087C	AA:9		DBVPG1853:T>T	AA:67		UWOPS03_461_4:P>P		UWOPS05_217_3:P>P		UWOPS05_227_2:P>P	AA:89		DBVPG6044:D>D		SK1:D>D		Y55:D>DID:YBL089W	AA:7		UWOPS83_787_3:S>S	AA:10		322134S:L>L		378604X:L>L		BC187:L>L		DBVPG6044:L>L		DBVPG6765:L>L		K11:L>L		L_1374:L>L		L_1528:L>L		SK1:L>L		UWOPS83_787_3:L>L		UWOPS87_2421:L>L		Y55:L>L		Y9:L>L		YJM975:L>L		YPS606:L>L		YS4:L>L	AA:22		322134S:L>L	AA:53		UWOPS83_787_3:L>L		UWOPS87_2421:L>L		YS4:L>L	AA:72		DBVPG6044:L>L		SK1:L>L		Y55:L>L		Y9:L>L	AA:86		UWOPS83_787_3:F>F	AA:328		UWOPS03_461_4:S>S		UWOPS05_227_2:S>S	AA:332		DBVPG6044:L>L		NCYC110:L>L		NCYC361:L>L		SK1:L>L		UWOPS03_461_4:L>L		UWOPS05_227_2:L>L		UWOPS87_2421:L>L		Y55:L>L		Y9:L>L		YPS128:L>L	AA:346		DBVPG6044:Q>Q		NCYC110:Q>Q		SK1:Q>Q		Y55:Q>Q	AA:366		YS4:T>T	AA:370		273614X:L>L	AA:440		DBVPG1853:G>G		SK1:G>G		Y55:G>G		YS4:G>G	AA:458		DBVPG1853:S>S		NCYC361:S>S		SK1:S>S		UWOPS05_217_3:S>S		UWOPS05_227_2:S>S		UWOPS83_787_3:S>S		Y55:S>S		YS4:S>SID:YBL090W	AA:19		DBVPG6040:T>T		K11:T>T		UWOPS87_2421:T>T		YPS606:T>T	AA:21		DBVPG6040:D>D		K11:D>D		UWOPS03_461_4:D>D		UWOPS05_227_2:D>D		UWOPS87_2421:D>D		YPS606:D>D		YS4:D>DID:YBL091C	AA:41		YPS606:K>K	AA:50		SK1:K>K	AA:56		DBVPG6040:K>K		NCYC361:K>K		UWOPS87_2421:K>K		YS4:K>K	AA:164		UWOPS03_461_4:T>T		UWOPS05_217_3:T>T	AA:185		273614X:V>V		DBVPG1853:L>L		UWOPS03_461_4:L>L		UWOPS05_217_3:L>L	AA:220		378604X:L>L		DBVPG6040:L>L		DBVPG6044:L>L		K11:L>L		NCYC110:L>L		SK1:L>L		UWOPS03_461_4:L>L		UWOPS05_217_3:L>L		UWOPS83_787_3:L>L		Y9:L>L		YPS128:L>L		YPS606:L>L	AA:226		273614X:A>A	AA:227		DBVPG6044:T>T		NCYC110:T>T		SK1:T>T	AA:243		273614X:I>I		DBVPG6044:I>I		NCYC110:I>I		SK1:I>I		UWOPS03_461_4:I>I		UWOPS05_217_3:I>I		UWOPS83_787_3:I>I		YPS128:I>I	AA:284		UWOPS05_217_3:G>G	AA:339		273614X:L>L		378604X:L>L		DBVPG6044:L>L		K11:L>L		NCYC361:L>L		SK1:L>L		UWOPS05_217_3:L>L		UWOPS83_787_3:L>L		Y9:L>LID:YBL092W	AA:63		UWOPS83_787_3:T>ID:YBL093C	AA:5		322134S:V>V		378604X:V>V		DBVPG6044:V>V		UWOPS87_2421:V>V		Y9:V>V		YPS128:V>V		YPS606:V>V		YS4:V>VID:YBL095W	AA:18		322134S:L>L		K11:L>L		NCYC110:L>L		NCYC361:L>L		SK1:L>L		UWOPS05_217_3:L>L		UWOPS87_2421:L>L		Y9:L>L		YPS606:L>L		YS4:L>L	AA:24		322134S:T>T		K11:T>T		NCYC110:T>T		NCYC361:T>T		SK1:T>T		UWOPS05_217_3:T>T		UWOPS87_2421:T>T		Y9:T>T		YPS128:T>T		YPS606:T>T		YS4:T>T	AA:104		322134S:Y>Y	AA:126		273614X:D>D		322134S:D>D		NCYC110:D>D		NCYC361:D>D		SK1:D>D		UWOPS03_461_4:D>D		UWOPS05_217_3:D>D		UWOPS87_2421:D>D		Y12:D>D		Y9:D>D		YPS128:D>D		YPS606:D>D	AA:138		273614X:L>L		322134S:L>L		NCYC361:L>L		UWOPS03_461_4:L>L		UWOPS05_217_3:L>L		UWOPS87_2421:L>L		Y12:L>L		Y9:L>L		YPS128:L>L		YPS606:L>L	AA:144		273614X:N>N		322134S:N>N		NCYC361:N>N		UWOPS03_461_4:N>N		UWOPS05_217_3:N>N		UWOPS87_2421:N>N		Y12:N>N		Y9:N>N		YPS128:N>N	AA:166		NCYC110:G>G		SK1:G>G	AA:252		378604X:N>N		DBVPG6044:N>N		NCYC110:N>N		YS4:N>N	AA:257		378604X:E>E		DBVPG6044:E>E		NCYC110:E>E		UWOPS87_2421:E>E		YPS128:E>E		YPS606:E>E		YS4:E>EID:YBL098W	AA:30		DBVPG1106:L>L		DBVPG1373:L>L		DBVPG1788:L>L		DBVPG6040:L>L		DBVPG6044:L>L		DBVPG6765:L>L		L_1374:L>L		L_1528:L>L		NCYC361:L>L		SK1:L>L		UWOPS03_461_4:L>L		UWOPS05_217_3:L>L		UWOPS05_227_2:L>L		UWOPS87_2421:L>L		Y12:L>L		YIIc17_E5:L>L		YJM978:L>L		YJM981:L>L		YPS128:L>L		YPS606:L>L		YS9:L>L	AA:87		378604X:D>D		DBVPG1853:D>D		DBVPG6040:D>D		DBVPG6044:D>D		K11:D>D		SK1:D>D		UWOPS03_461_4:D>D		UWOPS05_217_3:D>D		UWOPS87_2421:D>D		YPS128:D>D		YPS606:D>D		YS4:D>D		YS9:D>D	AA:97		378604X:Y>Y		DBVPG1853:Y>Y		DBVPG6040:Y>Y		DBVPG6044:Y>Y		K11:Y>Y		SK1:Y>Y		UWOPS05_217_3:Y>Y		UWOPS87_2421:Y>Y		YPS128:Y>Y		YPS606:Y>Y		YS4:Y>Y		YS9:Y>Y	AA:124		378604X:T>T		YS4:T>T	AA:278		UWOPS83_787_3:D>D	AA:295		SK1:V>V	AA:345		UWOPS87_2421:S>S		Y12:S>S		YPS128:S>S		YPS606:S>S		YS4:S>S	AA:367		DBVPG6044:T>T		NCYC110:T>T	AA:372		273614X:R>R		DBVPG1373:R>R		DBVPG1788:R>R		DBVPG6044:R>R		DBVPG6765:R>R		L_1374:R>R		NCYC110:R>R		UWOPS05_217_3:R>R		UWOPS83_787_3:R>R		UWOPS87_2421:R>R		Y12:R>R		Y55:R>R		YIIc17_E5:R>R		YJM975:R>R		YJM978:R>R		YPS128:R>R		YPS606:R>R		YS4:R>R		YS9:R>R	AA:395		322134S:L>L		378604X:L>L		UWOPS05_217_3:L>L		UWOPS87_2421:L>L		Y12:L>L		YPS606:L>L		YS4:L>L	AA:445		322134S:S>S		UWOPS03_461_4:S>S		UWOPS05_217_3:S>S		UWOPS83_787_3:S>S		UWOPS87_2421:S>S		YPS606:S>S		YS4:S>SID:YBL099W	AA:124		273614X:L>L		378604X:L>L		DBVPG1373:L>L		DBVPG6765:L>L		L_1374:L>L		NCYC361:L>L		UWOPS87_2421:L>L		Y55:L>L		YIIc17_E5:L>L	AA:134		273614X:V>V		378604X:V>V		DBVPG1373:V>V		DBVPG6044:V>V		DBVPG6765:V>V		L_1374:V>V		SK1:V>V		UWOPS87_2421:V>V		Y55:V>V		YIIc17_E5:V>V		YPS128:V>V		YPS606:V>V	AA:192		273614X:A>A		378604X:A>A		DBVPG1373:A>A		DBVPG6040:A>A		DBVPG6044:A>A		DBVPG6765:A>A		K11:A>A		L_1374:A>A		NCYC361:A>A		SK1:A>A		UWOPS03_461_4:A>A		UWOPS05_227_2:A>A		UWOPS87_2421:A>A		Y55:A>A		YIIc17_E5:A>A		YJM981:A>A		YPS128:A>A		YPS606:A>A	AA:302		UWOPS03_461_4:L>L	AA:343		YPS128:L>L		YPS606:L>L	AA:387		273614X:I>I		DBVPG1106:I>I		DBVPG6040:I>I		DBVPG6765:I>I		K11:I>I		L_1528:I>I		S288c:I>I		SK1:I>I		UWOPS03_461_4:I>I		UWOPS83_787_3:I>I		UWOPS87_2421:I>I		W303:I>I		Y12:I>I		Y55:I>I		Y9:I>I		YGPM:I>I		YJM975:I>I		YJM981:I>I		YPS128:I>I		YS4:I>I		YS9:I>I	AA:407		273614X:S>S		DBVPG1106:S>S		DBVPG6765:S>S		L_1528:S>S		Y55:S>S		YJM975:S>S		YS9:S>SID:YBL102W	AA:38		273614X:L>L		DBVPG1106:L>L		DBVPG1853:L>L		DBVPG6040:L>L		UWOPS03_461_4:L>L		UWOPS05_217_3:L>L		Y12:L>L	AA:63		DBVPG6044:D>D		SK1:D>D	AA:86		DBVPG6044:C>C		SK1:C>C		UWOPS83_787_3:C>C	AA:89		UWOPS03_461_4:L>L		UWOPS05_217_3:L>L	AA:94		DBVPG6044:C>C		SK1:C>C	AA:117		UWOPS03_461_4:T>T		UWOPS05_217_3:T>T		YPS606:T>T	AA:135		DBVPG1853:A>A	AA:154		UWOPS05_217_3:A>AID:YBL103C	AA:54		DBVPG1373:G>G	AA:150		UWOPS87_2421:T>T	AA:374		273614X:A>A		322134S:A>A		378604X:A>A		DBVPG1853:A>A		DBVPG6040:A>A		DBVPG6044:A>A		NCYC361:A>A		SK1:A>A		UWOPS03_461_4:A>A		UWOPS05_227_2:A>A		Y12:A>A		Y9:A>A		YPS128:A>A		YPS606:A>A		YS4:A>A	AA:381		322134S:N>N		378604X:N>N	AA:419		273614X:H>H		322134S:H>H		378604X:H>H		DBVPG1853:H>H		DBVPG6040:H>H		UWOPS03_461_4:H>H		UWOPS05_217_3:T>T		Y12:H>H		Y9:H>H		YPS128:H>H		YPS606:H>H		YS4:H>HID:YBL104C	AA:177		378604X:Q>Q	AA:190		273614X:T>T	AA:233		UWOPS83_787_3:I>I	AA:234		YPS606:Y>Y	AA:242		DBVPG6044:Y>Y		SK1:Y>Y	AA:258		378604X:G>G		K11:G>G		UWOPS03_461_4:G>G		YPS606:G>G		YS4:G>G		YS9:G>G	AA:283		DBVPG6044:A>A		NCYC110:V>V		SK1:V>V	AA:342		378604X:A>A		DBVPG6040:A>A		K11:A>A		UWOPS03_461_4:A>A		Y12:A>A		YPS606:A>A		YS4:A>A		YS9:A>A	AA:358		DBVPG6044:S>S		SK1:S>S	AA:405		Y12:A>A		Y9:A>A	AA:486		BC187:V>V		DBVPG1788:V>V		DBVPG6765:V>V		Y55:V>V	AA:579		DBVPG1853:G>G	AA:605		DBVPG1853:E>E		DBVPG6040:E>E	AA:608		YPS128:I>I	AA:610		DBVPG1853:L>L		DBVPG6040:L>L		Y9:L>L	AA:660		DBVPG1853:G>G	AA:759		YPS128:L>L		YPS606:L>L	AA:776		NCYC110:S>S		SK1:S>S	AA:886		273614X:Q>Q		DBVPG1373:Q>Q		L_1528:Q>Q		YIIc17_E5:Q>Q	AA:927		378604X:S>S		NCYC361:S>S		SK1:S>S		UWOPS83_787_3:S>S		Y12:S>S		Y9:S>S		YPS606:S>SID:YBL107C	AA:51		273614X:L>L		378604X:L>L		DBVPG6044:L>L		SK1:L>L		YPS606:L>L	AA:88		NCYC361:N>N	AA:114		273614X:C>C		378604X:C>C		DBVPG6044:C>C		NCYC361:C>C		SK1:C>C		UWOPS03_461_4:C>C		YPS128:C>C		YPS606:C>C		YS4:C>C	AA:139		L_1528:A>A		YS9:A>A	AA:175		DBVPG6044:R>R		SK1:R>RID:YBR002C	AA:19		DBVPG1788:N>N	AA:45		322134S:F>F		DBVPG1788:F>F		DBVPG6765:F>F		L_1374:F>F		SK1:F>F		UWOPS83_787_3:F>F		Y55:F>F		YJM978:F>F	AA:117		322134S:A>A	AA:177		322134S:V>V		DBVPG6765:V>V		L_1528:V>V		YJM978:V>V		YJM981:V>V		YS2:V>V	AA:182		DBVPG6044:G>G		SK1:G>G		Y55:G>G	AA:198		YJM978:G>GID:YBR003W	AA:2		DBVPG1373:F>F	AA:24		SK1:S>S	AA:65		YPS128:G>G	AA:69		UWOPS87_2421:P>P	AA:94		UWOPS03_461_4:L>L		UWOPS05_227_2:L>L	AA:174		NCYC361:K>K	AA:226		322134S:G>G		DBVPG1373:G>G		DBVPG1788:G>G		DBVPG1853:G>G		DBVPG6044:G>G		DBVPG6765:G>G		L_1528:G>G		NCYC110:G>G		NCYC361:G>G		SK1:G>G		UWOPS03_461_4:G>G		Y55:G>G		YJM981:G>G		YPS606:G>G		YS4:G>G	AA:229		DBVPG6044:T>T		NCYC110:T>T		SK1:T>T		Y55:T>T		YPS606:T>T	AA:233		YS4:S>S		YS9:S>S	AA:256		DBVPG1788:F>F		DBVPG1853:F>F		YS4:F>F		YS9:F>F	AA:259		NCYC361:L>L	AA:267		322134S:D>D		DBVPG1106:D>D		DBVPG1373:D>D		DBVPG1788:D>D		DBVPG1853:D>D		DBVPG6765:D>D		L_1528:D>D		NCYC361:D>D		UWOPS03_461_4:D>D		YJM981:D>D		YPS606:D>D		YS4:D>D		YS9:D>D	AA:320		322134S:T>T		DBVPG1106:T>T		DBVPG1373:T>T		DBVPG1853:T>T		DBVPG6765:T>T		L_1528:T>T		NCYC361:T>T		UWOPS03_461_4:T>T		YJM981:T>T		YPS606:T>T		YS2:T>T		YS4:T>T		YS9:T>T	AA:324		NCYC361:T>T		UWOPS03_461_4:T>T		YPS606:T>T	AA:335		DBVPG1373:A>A	AA:346		273614X:I>I		DBVPG1106:I>I		DBVPG1373:I>I		DBVPG1788:I>I		DBVPG1853:I>I		DBVPG6765:I>I		L_1528:I>I		NCYC361:I>I		UWOPS03_461_4:I>I		UWOPS05_217_3:I>I		YJM975:I>I		YJM981:I>I		YPS128:I>I		YPS606:I>I		YS4:I>I		YS9:I>I	AA:401		273614X:P>P		DBVPG1106:P>P		DBVPG1373:P>P		DBVPG1788:P>P		DBVPG1853:P>P		YJM975:P>P		YJM981:P>P		YS2:P>P		YS4:P>P		YS9:P>P	AA:430		UWOPS03_461_4:I>I	AA:461		DBVPG1853:L>L	AA:469		273614X:L>L		322134S:L>L		DBVPG1373:L>L		DBVPG6765:L>L		YJM975:L>LID:YBR004C	AA:121		322134S:L>L		BC187:L>L		DBVPG1788:L>L		DBVPG6765:L>L		L_1374:L>L		SK1:L>L		UWOPS87_2421:L>L		Y55:L>L		YJM975:L>L		YPS128:L>L		YPS606:L>L		YS4:L>L	AA:128		YPS128:F>F		YPS606:F>F	AA:156		DBVPG6044:F>F		SK1:F>F		Y55:F>F		YPS128:G>G		YPS606:G>G	AA:232		DBVPG1853:I>I	AA:297		322134S:L>L		DBVPG1373:L>L		DBVPG1788:L>L		DBVPG1853:L>L		DBVPG6044:L>L		DBVPG6765:L>L		SK1:L>L		Y55:L>L		YJM975:L>L		YJM978:L>L		YPS128:L>L		YPS606:L>L		YS4:L>L	AA:302		DBVPG1853:Q>Q	AA:312		YPS128:K>K		YPS606:K>K	AA:388		YS2:R>RID:YBR005W	AA:89		DBVPG1373:Q>Q		DBVPG1853:Q>Q		DBVPG6765:Q>Q		YS4:Q>Q	AA:136		NCYC110:P>P		SK1:P>P		UWOPS87_2421:P>P		Y55:P>P		YPS128:P>P	AA:142		NCYC110:E>E		SK1:E>E		UWOPS87_2421:E>E		Y55:E>E		YPS128:E>EID:YBR006W	AA:11		273614X:L>L		322134S:L>L		378604X:L>L		DBVPG1106:L>L		DBVPG1853:L>L		DBVPG6044:L>L		DBVPG6765:L>L		L_1374:L>L		L_1528:L>L		UWOPS03_461_4:L>L		UWOPS83_787_3:L>L		YJM981:L>L		YPS606:L>L	AA:20		DBVPG6044:S>S		UWOPS83_787_3:S>S		YPS606:S>S	AA:75		322134S:T>T		DBVPG1106:T>T		DBVPG1853:T>T		DBVPG6044:T>T		DBVPG6765:T>T		L_1528:T>T		SK1:T>T		UWOPS83_787_3:T>T		Y55:T>T		YJM981:T>T	AA:127		UWOPS83_787_3:A>A	AA:130		DBVPG6044:A>A		SK1:A>A		UWOPS83_787_3:A>A		Y55:A>A	AA:171		DBVPG1106:T>T		DBVPG1853:T>T		DBVPG6044:T>T		DBVPG6765:T>T		L_1374:T>T		L_1528:T>T		NCYC110:T>T		SK1:T>T		UWOPS87_2421:T>T		Y55:T>T		YPS606:T>T	AA:174		DBVPG1106:A>A		DBVPG1853:A>A		DBVPG6044:A>A		DBVPG6765:A>A		L_1374:A>A		L_1528:A>A		NCYC110:A>A		SK1:A>A		UWOPS87_2421:A>A		Y55:A>A		YPS606:A>A	AA:198		DBVPG1106:A>A		DBVPG1853:A>A		DBVPG6765:A>A		L_1374:A>A		L_1528:A>A	AA:250		DBVPG1788:L>L		DBVPG1853:L>L		DBVPG6765:L>L		L_1374:L>L		L_1528:L>L		YS9:L>L	AA:260		BC187:K>K		DBVPG1788:K>K		DBVPG1853:K>K		DBVPG6044:K>K		DBVPG6765:K>K		L_1374:K>K		L_1528:K>K		NCYC110:K>K		SK1:K>K		UWOPS87_2421:K>K		Y55:K>K		YJM981:K>K		YS9:K>K	AA:268		BC187:N>N		DBVPG1853:N>N		DBVPG6765:N>N		L_1374:N>N		L_1528:N>N		YJM981:N>N		YS9:N>N	AA:295		DBVPG6044:G>G		NCYC110:G>G		SK1:G>G		Y55:G>G	AA:318		UWOPS87_2421:L>L	AA:328		BC187:G>G		DBVPG1373:G>G		DBVPG1788:G>G		DBVPG1853:G>G		DBVPG6765:G>G		L_1374:G>G		L_1528:G>G		YJM981:G>G		YS9:G>G	AA:381		BC187:V>V		DBVPG1373:V>V		DBVPG1788:V>V		DBVPG1853:V>V		DBVPG6765:V>V		L_1374:V>V		L_1528:V>V		YJM981:V>V		YS4:V>V		YS9:V>V	AA:401		BC187:L>L		DBVPG1373:L>L		DBVPG1788:L>L		DBVPG1853:L>L		DBVPG6765:L>L		L_1374:L>L		L_1528:L>L		YJM981:L>L		YS4:L>L		YS9:L>L	AA:406		UWOPS87_2421:S>SID:YBR007C	AA:16		UWOPS87_2421:S>S	AA:76		DBVPG6044:E>E		NCYC110:E>E		Y55:E>E	AA:160		DBVPG6044:V>V		Y55:V>V		YPS606:V>V	AA:207		DBVPG6765:N>N	AA:340		YPS606:S>S	AA:350		BC187:N>N		DBVPG1106:N>N		DBVPG1373:N>N		DBVPG1788:N>N		DBVPG6765:N>N		L_1374:N>N		L_1528:N>N		YJM975:N>N		YS9:N>N	AA:355		DBVPG1853:P>P	AA:371		UWOPS05_227_2:S>S	AA:483		378604X:L>L		DBVPG1788:L>L		DBVPG1853:L>L		DBVPG6765:L>L		L_1374:L>L		YJM975:L>L		YS4:L>L		YS9:L>L	AA:516		UWOPS05_227_2:C>C	AA:570		YS9:L>L	AA:648		273614X:Y>Y		322134S:Y>Y		378604X:Y>Y		DBVPG1106:Y>Y		DBVPG1788:Y>Y		DBVPG6765:Y>Y		YJM975:Y>Y		YJM978:Y>Y		YJM981:Y>Y		YS4:Y>Y	AA:679		DBVPG6044:G>G		SK1:G>G		Y55:G>GID:YBR008C	AA:16		UWOPS05_227_2:L>L	AA:60		378604X:S>S		DBVPG1788:S>S		DBVPG6765:S>S		W303:S>S		YJM975:S>S	AA:68		SK1:S>S	AA:157		DBVPG1853:I>I	AA:167		378604X:R>R		DBVPG1788:R>R		DBVPG6765:R>R		W303:R>R		YS2:R>R	AA:198		UWOPS05_227_2:I>I	AA:254		Y12:V>V	AA:392		DBVPG1853:G>G	AA:409		UWOPS05_227_2:G>G	AA:510		378604X:P>P		DBVPG1106:P>P		DBVPG1373:P>P		DBVPG1788:P>P		DBVPG6765:P>P		L_1374:P>P		L_1528:P>P		YJM975:P>P		YJM978:P>P		YS4:P>P		YS9:P>P	AA:537		DBVPG1106:P>P		DBVPG1788:P>P		DBVPG6765:P>P		L_1374:P>P		L_1528:P>P		YJM975:P>P		YJM978:P>P		YJM981:P>P		YS4:P>P		YS9:P>P	AA:544		DBVPG1106:S>S		DBVPG1788:S>S		DBVPG6765:S>S		L_1374:S>S		L_1528:S>S		YJM975:S>S		YS4:S>S		YS9:S>S	AA:546		DBVPG1106:T>T		DBVPG1788:T>T		DBVPG6765:T>T		L_1374:T>T		L_1528:T>T		YJM975:T>T		YS4:T>T		YS9:T>TID:YBR009C	AA:33		273614X:T>T		378604X:T>T		BC187:T>T		DBVPG1106:T>T		DBVPG1788:T>T		DBVPG1853:T>T		DBVPG6044:T>T		DBVPG6765:T>T		L_1528:T>T		NCYC110:T>T		SK1:T>T		UWOPS03_461_4:T>T		UWOPS05_217_3:T>T		UWOPS05_227_2:T>T		UWOPS87_2421:T>T		Y55:T>T		YJM978:P>P		YPS128:T>T		YPS606:T>T		YS4:T>T		YS9:T>T	AA:49		YJM978:G>GID:YBR010W	AA:107		DBVPG6044:D>D		NCYC110:D>D		SK1:D>D		Y55:D>DID:YBR011C	AA:21		378604X:E>E		DBVPG1373:E>E		DBVPG1788:E>E		DBVPG6765:E>E		W303:E>E		YJM975:E>E		YJM981:E>E	AA:137		DBVPG6044:Q>Q		NCYC110:Q>Q		SK1:Q>Q		Y55:Q>Q	AA:182		378604X:L>L		BC187:L>L		DBVPG1373:L>L		DBVPG6765:L>L		YJM975:L>L		YJM981:L>L		YS2:L>L	AA:224		UWOPS87_2421:H>H	AA:236		DBVPG1788:S>S	AA:243		UWOPS87_2421:L>L	AA:250		DBVPG1853:D>DID:YBR014C	AA:19		UWOPS87_2421:L>L	AA:38		UWOPS03_461_4:I>I		UWOPS05_217_3:I>I		UWOPS05_227_2:I>I	AA:118		UWOPS03_461_4:L>L		UWOPS05_217_3:L>L		UWOPS05_227_2:L>L	AA:176		YS2:K>K	AA:182		322134S:D>D		378604X:D>D		BC187:D>D		DBVPG1106:D>D		DBVPG1373:D>D		DBVPG1788:D>D		DBVPG1853:D>D		DBVPG6765:D>D		L_1528:D>D		UWOPS03_461_4:D>D		UWOPS05_217_3:D>D		UWOPS87_2421:D>D		YJM981:D>D		YS2:D>D		YS4:D>DID:YBR016W	AA:23		W303:P>P	AA:109		DBVPG6040:G>G		Y12:G>G		Y9:G>GID:YBR017C	AA:42		UWOPS03_461_4:L>L		UWOPS05_217_3:L>L		UWOPS87_2421:L>L	AA:127		378604X:V>V		DBVPG1788:V>V		L_1528:V>V	AA:161		378604X:E>E		DBVPG1788:E>E		L_1528:E>E		W303:A>A	AA:282		UWOPS83_787_3:L>L		YPS128:L>L		YPS606:L>L	AA:335		DBVPG6044:E>E		SK1:E>E		Y55:E>E	AA:411		UWOPS83_787_3:T>T		YPS128:T>T		YPS606:T>T	AA:428		UWOPS83_787_3:E>E		YPS128:E>E		YPS606:E>E	AA:479		DBVPG6044:R>R		NCYC110:R>R		SK1:R>R	AA:488		YS9:R>R	AA:598		DBVPG6044:L>L		K11:L>L		L_1374:L>L		SK1:L>L		UWOPS03_461_4:L>L		UWOPS05_217_3:L>L		UWOPS87_2421:L>L		Y12:L>L		YIIc17_E5:L>L	AA:619		K11:L>L	AA:702		DBVPG6040:L>L		SK1:L>L		UWOPS03_461_4:L>L		UWOPS05_217_3:L>L		UWOPS05_227_2:L>L		Y12:L>L		Y55:L>L		Y9:L>L		YPS128:L>L		YPS606:L>L	AA:712		UWOPS03_461_4:S>S		UWOPS05_217_3:S>S		UWOPS05_227_2:S>S	AA:726		W303:S>S	AA:813		YPS128:H>H		YPS606:H>H	AA:861		UWOPS83_787_3:E>E	AA:863		YPS128:T>T		YPS606:T>T	AA:880		UWOPS83_787_3:V>V	AA:895		378604X:N>N	AA:900		L_1528:I>IID:YBR018C	AA:48		378604X:P>P	AA:52		DBVPG6044:P>P		SK1:P>P		Y55:P>P	AA:168		K11:E>E		SK1:E>E		Y12:E>E		Y55:E>E	AA:185		SK1:A>A		Y55:A>A	AA:197		UWOPS05_227_2:Q>Q		UWOPS87_2421:Q>Q	AA:295		YS9:G>G	AA:301		DBVPG6040:L>L		K11:L>L		UWOPS03_461_4:L>L		UWOPS05_227_2:L>L		UWOPS83_787_3:L>L		UWOPS87_2421:L>L		Y12:L>L		Y55:L>L		YIIc17_E5:L>L		YPS606:L>L	AA:325		DBVPG6040:A>A		DBVPG6044:A>A		SK1:A>A		UWOPS03_461_4:A>A		UWOPS05_227_2:A>A		UWOPS83_787_3:A>A		UWOPS87_2421:A>A		Y55:A>A		YIIc17_E5:A>A		YPS606:A>A	AA:356		DBVPG6040:L>L		DBVPG6044:L>L		SK1:L>L		UWOPS03_461_4:L>L		UWOPS05_227_2:L>L		UWOPS83_787_3:L>L		UWOPS87_2421:L>L		Y12:L>L		Y55:L>L		YIIc17_E5:L>L		YPS606:L>L		YS9:L>L	AA:363		DBVPG6040:L>L		DBVPG6044:L>L		SK1:L>L		UWOPS03_461_4:L>L		UWOPS05_227_2:L>L		UWOPS83_787_3:L>L		Y12:L>L		Y55:L>L		YIIc17_E5:L>L		YPS606:L>L		YS9:L>LID:YBR019C	AA:59		DBVPG1853:T>T		Y12:T>T		YPS128:T>T		YPS606:T>T	AA:147		322134S:N>N		DBVPG1373:N>N		DBVPG6765:N>N		L_1528:N>N		NCYC361:N>N		YS9:N>N	AA:204		YJM975:I>I	AA:211		YS4:I>I	AA:240		YPS128:R>R		YPS606:R>R	AA:269		NCYC361:N>N	AA:353		K11:F>F	AA:382		DBVPG1853:T>T	AA:530		SK1:T>T		Y55:T>T	AA:559		SK1:S>S		UWOPS05_217_3:S>S		Y55:S>S		Y9:S>S		YPS128:S>S		YPS606:S>S	AA:575		UWOPS05_217_3:I>I	AA:609		DBVPG6040:K>K		K11:K>K		SK1:K>K		UWOPS05_217_3:K>K		Y55:K>K		Y9:K>K		YIIc17_E5:K>K		YPS128:K>K		YPS606:K>K	AA:647		DBVPG6044:Y>Y		SK1:Y>Y		Y55:Y>Y	AA:650		UWOPS05_217_3:D>D	AA:664		378604X:I>I		DBVPG6040:I>I		DBVPG6044:I>I		K11:I>I		SK1:I>I		UWOPS05_217_3:I>I		Y55:I>I		Y9:I>I		YIIc17_E5:I>I		YPS128:I>I		YPS606:I>IID:YBR020W	AA:121		DBVPG6044:S>S		SK1:S>S		Y55:S>S	AA:180		DBVPG6765:L>L	AA:201		DBVPG6044:I>I		NCYC110:I>I		SK1:I>I		UWOPS05_217_3:I>I		UWOPS83_787_3:I>I		Y55:I>I		YIIc17_E5:I>I		YPS128:I>I		YPS606:I>I	AA:334		UWOPS03_461_4:S>S		UWOPS05_217_3:S>S		UWOPS05_227_2:S>S	AA:345		K11:L>L		Y12:L>L		YIIc17_E5:L>L		YS9:L>L	AA:363		NCYC110:A>A		SK1:A>A		Y55:A>A		YPS606:A>A	AA:413		K11:T>T		UWOPS03_461_4:T>T		UWOPS05_217_3:T>T		UWOPS05_227_2:T>T		UWOPS83_787_3:T>T		Y12:T>T		YIIc17_E5:T>T		YS9:T>T	AA:419		NCYC110:D>D		SK1:D>D		Y55:D>D		YPS128:D>D		YPS606:D>D	AA:460		UWOPS03_461_4:S>S		UWOPS05_217_3:S>S		UWOPS05_227_2:S>S	AA:466		K11:T>T		UWOPS05_217_3:T>T		UWOPS05_227_2:T>T		UWOPS83_787_3:T>T		Y12:T>T		YIIc17_E5:T>T		YS9:T>T	AA:510		DBVPG6044:L>L		SK1:L>L		Y55:L>L	AA:514		SK1:I>I		Y55:I>IID:YBR021W	AA:128		K11:N>N		UWOPS05_217_3:N>N		UWOPS83_787_3:N>N		UWOPS87_2421:N>N		Y12:N>N		YIIc17_E5:N>N	AA:191		DBVPG6044:P>P		SK1:P>P		Y55:P>P	AA:194		DBVPG6044:N>N		SK1:N>N		UWOPS05_217_3:N>N		UWOPS87_2421:N>N		Y12:N>N		Y55:N>N	AA:219		DBVPG6044:K>K		NCYC110:K>K		SK1:K>K		Y55:K>K	AA:351		DBVPG6044:V>V		NCYC110:V>V		SK1:V>V		Y55:V>V	AA:363		YS9:I>I	AA:405		DBVPG6044:I>I		NCYC110:I>I		SK1:I>I		UWOPS05_217_3:I>I		UWOPS83_787_3:I>I		Y55:I>I		Y9:I>I	AA:432		YIIc17_E5:I>I		YPS606:I>I	AA:476		K11:L>L	AA:493		UWOPS03_461_4:G>G		UWOPS05_217_3:G>G		UWOPS05_227_2:G>G	AA:526		K11:L>L		YPS606:L>L	AA:538		K11:A>A		YPS606:A>A	AA:550		DBVPG6044:G>G		SK1:G>G		UWOPS03_461_4:G>G		UWOPS05_217_3:G>G		UWOPS05_227_2:G>G		UWOPS83_787_3:G>G		UWOPS87_2421:G>G		Y12:G>G		Y55:G>G		Y9:G>G		YIIc17_E5:G>G	AA:556		K11:Y>Y	AA:559		K11:Y>YID:YBR022W	AA:2		DBVPG1373:S>S	AA:21		SK1:L>L		UWOPS05_217_3:L>L		UWOPS83_787_3:L>L		Y55:L>L		YPS128:L>L		YPS606:L>L		YS2:L>L		YS9:L>L	AA:41		SK1:L>L		UWOPS05_217_3:L>L		Y55:L>L		YPS128:L>L		YS2:L>L		YS9:L>L	AA:168		DBVPG6765:G>GID:YBR024W	AA:66		K11:E>E		SK1:E>E		UWOPS05_217_3:E>E		Y12:E>E		Y55:E>E		YIIc17_E5:E>E		YPS128:E>E		YPS606:E>E	AA:93		DBVPG6044:T>T		K11:T>T		SK1:T>T		UWOPS05_217_3:T>T		UWOPS05_227_2:T>T		Y12:T>T		Y55:T>T		YIIc17_E5:T>T		YPS128:T>T		YPS606:T>T	AA:154		DBVPG6044:C>C		SK1:C>C		UWOPS05_217_3:C>C		UWOPS05_227_2:C>C		Y55:C>C		YIIc17_E5:C>C		YPS128:C>C		YPS606:C>C	AA:174		DBVPG6044:D>D		SK1:D>D		UWOPS05_217_3:D>D		UWOPS05_227_2:D>D		Y55:D>D		YIIc17_E5:D>D		YPS128:D>D		YPS606:D>D	AA:201		UWOPS05_217_3:L>L		UWOPS05_227_2:L>L		YIIc17_E5:L>L	AA:251		YPS128:L>L		YPS606:L>L	AA:263		UWOPS87_2421:G>G		YPS128:G>G		YPS606:G>GID:YBR025C	AA:44		UWOPS03_461_4:P>P	AA:71		DBVPG6040:D>D		DBVPG6044:D>D		K11:D>D		SK1:D>D		UWOPS03_461_4:D>D		UWOPS83_787_3:D>D		UWOPS87_2421:D>D		Y12:D>D		Y55:D>D		YIIc17_E5:D>D		YS4:D>D	AA:134		BC187:H>H		YJM981:H>H	AA:147		YPS128:I>I		YPS606:I>I	AA:185		UWOPS83_787_3:E>E		UWOPS87_2421:E>E	AA:220		DBVPG6040:F>F		K11:F>F		Y12:F>F		Y9:F>F	AA:260		W303:D>D	AA:354		DBVPG6040:D>D		K11:D>D		NCYC361:D>D		UWOPS05_227_2:D>D		UWOPS83_787_3:D>D		UWOPS87_2421:D>D		Y9:D>D		YPS128:D>D		YPS606:D>D		YS4:D>D	AA:390		YS4:G>GID:YBR026C	AA:22		DBVPG6044:S>S		SK1:S>S		Y55:S>S	AA:71		YS4:G>G	AA:88		Y12:P>P	AA:121		L_1528:L>L	AA:130		DBVPG6044:N>N		Y55:N>N		YS4:N>N	AA:131		L_1528:Y>Y	AA:146		L_1528:N>N	AA:157		DBVPG6044:N>N		Y55:N>N	AA:159		L_1528:C>C	AA:183		L_1528:A>A		NCYC361:K>K	AA:205		DBVPG1853:S>S		UWOPS83_787_3:S>S		Y55:S>S	AA:207		DBVPG1853:I>I		UWOPS83_787_3:I>I		Y55:I>I	AA:217		L_1528:A>A	AA:221		L_1528:E>E	AA:224		L_1528:Y>Y	AA:231		L_1528:S>S	AA:233		L_1528:S>S	AA:239		L_1528:T>T	AA:241		Y55:A>A	AA:264		L_1528:G>G		NCYC361:G>G	AA:268		L_1528:A>A	AA:271		L_1528:A>A	AA:275		L_1528:E>E	AA:287		DBVPG1853:S>S		Y12:S>S	AA:307		DBVPG1373:G>G		DBVPG1788:G>G		DBVPG1853:G>G		SK1:G>G		UWOPS03_461_4:G>G		UWOPS83_787_3:G>G		Y55:G>G		YJM978:G>G		YPS606:G>G		YS4:G>G		YS9:G>G	AA:321		DBVPG1853:K>K		SK1:K>K		Y55:K>K	AA:343		UWOPS03_461_4:E>E		UWOPS05_217_3:E>E		UWOPS83_787_3:E>E		YPS606:E>E		YS9:E>E	AA:352		UWOPS03_461_4:N>N		UWOPS05_217_3:N>N		UWOPS83_787_3:N>N		YPS606:N>N	AA:366		SK1:G>G		Y55:G>GID:YBR028C	AA:5		273614X:L>L		DBVPG6040:L>L		UWOPS87_2421:L>L	AA:124		DBVPG6044:N>N		SK1:N>N		UWOPS87_2421:N>N		Y55:N>N		YPS128:N>N		YPS606:N>N	AA:137		DBVPG1853:G>G	AA:157		273614X:K>K		DBVPG6040:K>K	AA:174		DBVPG1853:K>K	AA:221		NCYC361:F>F		UWOPS83_787_3:F>F	AA:270		W303:T>T	AA:286		UWOPS87_2421:N>N	AA:322		NCYC361:I>I		UWOPS83_787_3:I>I	AA:325		SK1:T>T		Y55:T>T	AA:354		YIIc17_E5:D>D	AA:356		Y12:L>L		YS4:L>L		YS9:L>L	AA:397		L_1528:A>A	AA:416		UWOPS03_461_4:T>T		UWOPS05_217_3:T>T	AA:460		UWOPS87_2421:T>TID:YBR030W	AA:49		NCYC361:G>G		UWOPS05_217_3:G>G		UWOPS83_787_3:G>G		UWOPS87_2421:G>G		YS9:G>G	AA:129		273614X:R>R		DBVPG6044:R>R		K11:R>R		SK1:R>R		Y12:R>R		Y55:R>R		YS4:R>R	AA:147		273614X:E>E		DBVPG6044:E>E		K11:E>E		NCYC361:E>E		SK1:E>E		UWOPS83_787_3:E>E		Y12:E>E		Y55:E>E		YPS606:E>E		YS4:E>E	AA:156		K11:A>A		NCYC361:A>A		UWOPS83_787_3:A>A		UWOPS87_2421:A>A		Y12:A>A		YPS606:A>A	AA:160		DBVPG6044:A>A		SK1:A>A		Y55:A>A	AA:205		K11:G>G	AA:217		DBVPG1373:V>V		DBVPG1788:V>V	AA:225		273614X:H>H		BC187:H>H		DBVPG1853:H>H		DBVPG6765:H>H		K11:H>H		L_1374:H>H		L_1528:H>H		NCYC361:H>H		SK1:H>H		UWOPS83_787_3:H>H		UWOPS87_2421:H>H		W303:H>H		Y12:H>H		Y55:H>H		YJM978:H>H		YPS606:H>H		YS4:H>H	AA:230		DBVPG1788:P>P	AA:233		273614X:K>K		BC187:K>K		DBVPG1788:K>K		DBVPG6765:K>K		K11:K>K		L_1374:K>K		L_1528:K>K		SK1:K>K		UWOPS83_787_3:K>K		UWOPS87_2421:K>K		W303:K>K		Y12:K>K		Y55:K>K		YJM978:K>K		YPS606:K>K		YS4:K>K	AA:253		YPS128:L>L		YPS606:L>L	AA:255		273614X:A>A		Y12:A>A	AA:264		SK1:L>L		Y55:L>L	AA:267		273614X:N>N		Y12:N>N	AA:297		273614X:N>N		Y12:N>N		YPS128:N>N		YPS606:N>N	AA:313		273614X:D>D		Y12:D>D	AA:366		BC187:K>K		DBVPG1788:K>K		DBVPG6765:K>K		L_1374:K>K		L_1528:K>K		SK1:K>K		UWOPS87_2421:K>K		W303:K>K		Y55:K>K		YIIc17_E5:K>K		YJM975:K>K		YJM978:K>K		YS4:K>K	AA:387		YPS128:G>G	AA:388		UWOPS03_461_4:L>L	AA:411		BC187:D>D		DBVPG1788:D>D		DBVPG6765:D>D		L_1528:D>D		UWOPS03_461_4:D>D		UWOPS87_2421:D>D		W303:D>D		YIIc17_E5:D>D		YJM975:D>D		YJM978:D>D		YPS128:D>D		YS4:D>D	AA:430		DBVPG6040:D>D		DBVPG6044:D>D		NCYC361:D>D		SK1:D>D		Y12:D>D		Y55:D>D		YS9:D>D	AA:463		BC187:V>V		YIIc17_E5:V>V		YS4:V>V	AA:469		DBVPG6044:F>F		SK1:F>F		Y55:F>F	AA:490		BC187:L>L		UWOPS83_787_3:L>L		YIIc17_E5:L>L	AA:501		DBVPG6044:L>L		SK1:L>L	AA:548		DBVPG6040:L>L		NCYC361:L>L		Y12:L>L		YS9:L>LID:YBR031W	AA:32		322134S:P>P		UWOPS03_461_4:P>P		UWOPS05_217_3:P>P	AA:161		UWOPS87_2421:K>K	AA:175		YIIc17_E5:H>H	AA:216		UWOPS03_461_4:V>V		UWOPS05_227_2:V>V	AA:228		DBVPG1373:A>A		DBVPG6040:A>A		W303:A>A		Y9:A>A		YJM978:A>A		YPS606:A>A		YS4:A>A	AA:279		DBVPG1853:H>H		DBVPG6040:H>H		DBVPG6044:H>H		NCYC110:H>H		NCYC361:H>H		SK1:H>H		UWOPS03_461_4:H>H		UWOPS05_217_3:H>H		UWOPS05_227_2:H>H		UWOPS83_787_3:H>H		W303:H>H		Y55:H>H		Y9:H>H		YPS606:H>H		YS4:H>H	AA:283		DBVPG6044:T>T		NCYC110:T>T		NCYC361:T>T		SK1:T>T		UWOPS83_787_3:T>T		Y55:T>T	AA:293		DBVPG1853:S>S		DBVPG6044:S>S		DBVPG6765:S>S		NCYC110:S>S		SK1:S>S		UWOPS05_217_3:S>S		UWOPS05_227_2:S>S		UWOPS83_787_3:S>S		W303:S>S		Y55:S>S		Y9:S>S		YJM978:S>S		YPS606:S>S		YS2:S>S		YS4:S>S		YS9:S>S	AA:309		DBVPG6040:R>R	AA:325		DBVPG6040:L>L		Y9:L>L		YS4:L>L		YS9:L>L	AA:341		UWOPS05_217_3:S>SID:YBR033W	AA:7		DBVPG6044:N>N		NCYC110:N>N		SK1:N>N		UWOPS03_461_4:N>N		UWOPS05_217_3:N>N		Y55:N>N	AA:14		UWOPS03_461_4:R>R	AA:83		DBVPG6044:E>E		NCYC110:E>E		SK1:E>E		UWOPS03_461_4:E>E		UWOPS05_217_3:E>E		Y55:E>E		YPS128:E>E		YPS606:E>E	AA:105		NCYC110:S>	AA:114		DBVPG6044:D>D		NCYC361:D>D		SK1:D>D		UWOPS83_787_3:D>D		UWOPS87_2421:D>D		Y55:D>D		YPS128:D>D		YPS606:D>D		YS4:D>D	AA:139		DBVPG6044:R>R		NCYC361:R>R		SK1:R>R		UWOPS83_787_3:R>R		UWOPS87_2421:R>R		Y55:R>R		YS4:R>R	AA:171		K11:R>R	AA:211		UWOPS03_461_4:G>G		UWOPS05_217_3:G>G	AA:235		DBVPG6044:P>P		SK1:P>P	AA:252		DBVPG6044:R>R		SK1:R>R		Y55:R>R	AA:254		YJM978:R>R	AA:257		DBVPG6044:S>S		SK1:S>S		Y55:S>S	AA:258		NCYC361:L>L		UWOPS83_787_3:L>L	AA:259		DBVPG1106:S>S	AA:262		YS9:S>S	AA:287		YS9:I>I	AA:293		DBVPG6040:N>N		DBVPG6044:N>N		K11:N>N		SK1:N>N		UWOPS03_461_4:N>N		UWOPS05_227_2:N>N		UWOPS83_787_3:N>N		UWOPS87_2421:N>N		Y55:N>N		Y9:N>N		YPS128:N>N		YPS606:N>N		YS4:N>N		YS9:N>N	AA:340		322134S:N>N	AA:343		DBVPG6044:A>A		SK1:A>A		Y55:A>A	AA:394		BC187:K>K		DBVPG1106:K>K		DBVPG1373:K>K		L_1374:K>K		YIIc17_E5:K>K		YJM975:K>K		YJM978:K>K	AA:448		DBVPG6044:L>L		SK1:L>L		Y55:L>L	AA:476		UWOPS03_461_4:S>S		UWOPS87_2421:S>S		YS4:S>S	AA:484		DBVPG6044:K>K		SK1:K>K		Y55:K>K	AA:490		DBVPG6044:T>T		SK1:T>T		Y55:T>T	AA:498		DBVPG6040:L>L		DBVPG6044:L>L		K11:L>L		SK1:L>L		Y55:L>L		YPS128:L>L		YS2:L>L	AA:501		BC187:A>A		DBVPG1106:A>A		DBVPG1373:A>A		DBVPG6765:A>A		L_1374:A>A		UWOPS03_461_4:A>A		UWOPS05_227_2:A>A		UWOPS87_2421:A>A		YIIc17_E5:A>A		YJM975:A>A		YS9:A>A	AA:503		DBVPG6040:E>E		DBVPG6044:E>E		SK1:E>E		Y55:E>E		YPS128:E>E		YS2:E>E	AA:505		UWOPS03_461_4:S>S		UWOPS05_227_2:S>S	AA:508		DBVPG6040:L>L		K11:L>L		YPS128:L>L		YPS606:L>L		YS2:L>L		YS4:L>L	AA:566		378604X:T>T	AA:599		YJM975:V>V	AA:656		UWOPS05_217_3:H>H		UWOPS05_227_2:H>H		UWOPS87_2421:H>H	AA:686		DBVPG6040:H>H		DBVPG6044:H>H		SK1:H>H		Y55:H>H		YPS606:H>H	AA:704		UWOPS05_217_3:L>L		UWOPS05_227_2:L>L		UWOPS87_2421:L>L	AA:793		378604X:L>L	AA:829		UWOPS03_461_4:L>L		UWOPS05_217_3:L>L		UWOPS05_227_2:L>L		UWOPS87_2421:L>L	AA:843		L_1528:R>R		YS2:R>R	AA:873		UWOPS87_2421:S>S	AA:897		UWOPS83_787_3:K>KID:YBR034C	AA:11		NCYC110:T>T	AA:32		NCYC110:I>I		SK1:I>I		Y55:I>I	AA:41		DBVPG6044:V>V		SK1:V>V		Y55:V>V	AA:86		NCYC110:G>G	AA:100		DBVPG6040:V>V		DBVPG6044:V>V		NCYC110:V>V		SK1:V>V		UWOPS03_461_4:V>V		UWOPS05_227_2:V>V		Y55:V>V	AA:123		DBVPG1853:F>F	AA:215		K11:V>V	AA:280		DBVPG1853:P>P	AA:287		NCYC361:T>T		UWOPS83_787_3:T>T	AA:332		UWOPS03_461_4:I>IID:YBR035C	AA:8		NCYC110:T>T		SK1:T>T		Y55:T>T	AA:60		UWOPS83_787_3:F>F	AA:73		UWOPS83_787_3:R>R		Y9:R>R		YS2:R>R	AA:154		K11:S>S	AA:166		Y9:L>L	AA:194		DBVPG6044:L>L		K11:L>L		NCYC110:L>L		UWOPS83_787_3:L>L		Y55:L>L		Y9:L>L		YPS128:L>L		YPS606:L>LID:YBR037C	AA:29		DBVPG1853:T>T		DBVPG6040:T>T		DBVPG6044:T>T		K11:T>T		NCYC110:T>T		SK1:T>T		Y55:T>T		Y9:T>T		YPS606:T>T		YS4:T>T	AA:62		DBVPG6044:G>G		SK1:G>G		Y55:G>G		YPS606:G>G	AA:91		DBVPG6040:F>F		Y9:F>F		YS4:F>F	AA:118		NCYC110:P>P		SK1:P>P		UWOPS83_787_3:P>P		Y55:P>P	AA:133		DBVPG6044:N>N		NCYC110:N>N		SK1:N>N		UWOPS83_787_3:N>N		Y55:N>N	AA:139		DBVPG6044:S>S		NCYC110:S>S		SK1:S>S		UWOPS83_787_3:S>S		Y55:S>S	AA:156		DBVPG6040:L>L		Y12:L>L		Y9:L>L	AA:180		322134S:T>T	AA:225		DBVPG1853:T>T	AA:228		YPS606:N>N	AA:249		DBVPG6040:E>E		DBVPG6044:E>E		K11:E>E		NCYC110:E>E		SK1:E>E		UWOPS83_787_3:E>E		Y12:E>E		Y55:E>E		YPS606:E>E	AA:255		DBVPG1853:A>A	AA:266		UWOPS87_2421:V>V	AA:294		273614X:F>F		DBVPG1853:F>F		DBVPG6040:F>F		DBVPG6044:F>F		K11:F>F		NCYC110:F>F		SK1:F>F		UWOPS83_787_3:F>F		UWOPS87_2421:F>F		Y12:F>F		Y55:F>F		YPS606:F>FID:YBR039W	AA:27		273614X:T>T		DBVPG6044:T>T		K11:T>T		NCYC110:T>T		SK1:T>T		UWOPS05_227_2:T>T		UWOPS83_787_3:T>T		Y55:T>T		YPS128:T>T		YPS606:T>T	AA:87		273614X:N>N	AA:93		DBVPG6044:L>L		NCYC110:L>L		SK1:L>L		Y55:L>L	AA:138		273614X:A>A		DBVPG6044:A>A		NCYC110:A>A		SK1:A>A		UWOPS03_461_4:A>A		UWOPS05_227_2:A>A		UWOPS83_787_3:A>A		YPS606:A>A	AA:146		273614X:K>K		DBVPG6044:K>K		NCYC110:K>K		SK1:K>K		UWOPS03_461_4:K>K		UWOPS05_227_2:K>K		UWOPS83_787_3:K>K		YPS606:K>K	AA:192		DBVPG6040:Y>Y		UWOPS05_227_2:Y>Y		YPS128:Y>Y		YPS606:Y>Y	AA:205		DBVPG6044:S>S		NCYC110:S>S		SK1:S>S		Y55:S>S	AA:238		UWOPS05_227_2:V>V	AA:257		UWOPS05_227_2:Q>QID:YBR040W	AA:3		K11:A>A		YS2:A>A	AA:20		UWOPS05_217_3:L>L		UWOPS05_227_2:L>L	AA:95		UWOPS05_227_2:Q>Q	AA:124		273614X:D>D		DBVPG1853:D>D		DBVPG6044:D>D		NCYC110:D>D		SK1:D>D		UWOPS05_227_2:D>D		UWOPS83_787_3:D>D		Y12:D>D		Y55:D>D	AA:148		273614X:L>L	AA:167		NCYC110:T>T	AA:205		UWOPS05_227_2:L>L	AA:227		273614X:S>S		DBVPG1853:S>S		UWOPS83_787_3:S>S	AA:233		DBVPG1853:I>I		DBVPG6044:I>I		NCYC110:I>I		SK1:I>I		UWOPS05_227_2:I>I		UWOPS83_787_3:I>I		Y12:I>I		Y55:I>I	AA:240		YPS606:K>K	AA:274		DBVPG1853:I>I		K11:I>I	AA:298		UWOPS05_227_2:V>VID:YBR041W	AA:32		YJM978:S>S	AA:38		DBVPG1106:G>G		DBVPG1373:G>G		DBVPG6765:G>G		YJM978:G>G	AA:45		DBVPG6044:D>D		SK1:D>D		Y55:D>D	AA:73		DBVPG6040:R>R	AA:109		UWOPS03_461_4:L>L		UWOPS05_217_3:L>L	AA:133		UWOPS03_461_4:N>N		UWOPS05_217_3:N>N	AA:139		UWOPS87_2421:Y>Y	AA:180		UWOPS87_2421:L>L	AA:184		DBVPG6040:N>N		UWOPS03_461_4:N>N		UWOPS05_217_3:N>N		UWOPS83_787_3:N>N		YPS128:N>N	AA:239		DBVPG1853:N>N		DBVPG6044:N>N		SK1:N>N		UWOPS87_2421:N>N		Y55:N>N	AA:245		DBVPG1853:G>G		DBVPG6044:G>G		NCYC110:G>G		SK1:G>G		UWOPS87_2421:G>G		Y55:G>G		YS9:G>G	AA:251		DBVPG1853:P>P		DBVPG6044:P>P		NCYC110:P>P		SK1:P>P		UWOPS87_2421:P>P		Y55:P>P		YS9:P>P	AA:260		UWOPS03_461_4:T>T		UWOPS05_217_3:T>T	AA:264		DBVPG1106:P>P		DBVPG1373:P>P		YJM978:P>P	AA:274		DBVPG1853:S>S		DBVPG6044:S>S		NCYC110:S>S		SK1:S>S		UWOPS87_2421:S>S		Y55:S>S		YS9:S>S	AA:340		YS9:T>T	AA:347		DBVPG6044:E>E		NCYC110:E>E		SK1:E>E		Y55:E>E	AA:405		273614X:T>T	AA:420		273614X:Y>Y		DBVPG1853:Y>Y		K11:Y>Y		UWOPS05_217_3:Y>Y		UWOPS05_227_2:Y>Y	AA:428		273614X:L>L		DBVPG1853:L>L		K11:L>L	AA:477		UWOPS05_227_2:S>S	AA:484		273614X:N>N		DBVPG1853:N>N		K11:N>N		Y12:N>N		YS2:N>N	AA:487		DBVPG1853:E>E	AA:545		DBVPG1106:T>T		DBVPG1788:T>T		DBVPG6044:T>T		DBVPG6765:T>T		L_1374:T>T		SK1:T>T		YIIc17_E5:T>T		YJM975:T>T		YPS128:T>T	AA:617		273614X:T>T	AA:639		UWOPS03_461_4:N>N		UWOPS05_217_3:N>N		UWOPS05_227_2:N>N	AA:641		322134S:T>T		DBVPG1788:T>T		DBVPG6765:T>T		UWOPS03_461_4:T>T		UWOPS05_227_2:T>T		YIIc17_E5:T>T		YJM975:T>T		YPS128:T>T		YS2:T>T	AA:653		UWOPS03_461_4:V>V		UWOPS05_217_3:V>V		UWOPS05_227_2:V>VID:YBR043C	AA:123		273614X:G>G		378604X:G>G		DBVPG1788:G>G		DBVPG1853:G>G		DBVPG6765:G>G		L_1374:G>G		L_1528:G>G		UWOPS05_227_2:G>G		YIIc17_E5:G>G		YJM975:G>G		YJM978:G>G		YPS128:G>G		YPS606:G>G		YS4:G>G	AA:138		YS4:F>F	AA:169		UWOPS05_227_2:L>L	AA:244		322134S:A>A		378604X:A>A		DBVPG1788:A>A		DBVPG1853:A>A		DBVPG6044:A>A		DBVPG6765:A>A		L_1374:A>A		SK1:A>A		UWOPS05_217_3:A>A		UWOPS05_227_2:A>A		UWOPS87_2421:A>A		Y55:A>A		YIIc17_E5:A>A		YJM975:A>A		YJM978:A>A		YPS128:A>A		YPS606:A>A		YS2:A>A		YS4:A>A	AA:254		378604X:L>L		DBVPG1788:L>L		DBVPG1853:L>L		DBVPG6765:L>L		L_1374:L>L		YIIc17_E5:L>L		YJM975:L>L		YJM978:L>L		YS2:L>L		YS4:L>L	AA:280		322134S:T>T		378604X:T>T		DBVPG1788:T>T		DBVPG1853:T>T		DBVPG6765:T>T		L_1374:T>T		SK1:T>T		UWOPS05_217_3:T>T		UWOPS05_227_2:T>T		UWOPS83_787_3:T>T		UWOPS87_2421:T>T		Y55:T>T		YIIc17_E5:T>T		YJM978:T>T		YPS128:T>T		YPS606:T>T		YS2:T>T		YS4:T>T	AA:326		378604X:T>T		DBVPG1788:T>T		DBVPG1853:T>T		DBVPG6765:T>T		SK1:T>T		Y55:T>T		YJM978:T>T		YPS128:T>T		YPS606:T>T		YS2:T>T	AA:331		UWOPS03_461_4:N>N		UWOPS05_227_2:N>N	AA:359		378604X:S>S		DBVPG1788:S>S		DBVPG1853:S>S		DBVPG6765:S>S		L_1374:S>S		YJM975:S>S		YPS128:S>S		YPS606:S>S		YS2:S>S	AA:383		378604X:S>S		DBVPG1788:S>S		DBVPG1853:S>S		DBVPG6765:S>S		YJM975:S>S		YS2:S>S	AA:414		YPS128:S>S		YPS606:S>S	AA:420		UWOPS03_461_4:L>L		UWOPS05_227_2:L>L	AA:442		378604X:I>I		DBVPG1853:I>I		DBVPG6765:I>I		L_1528:I>I		Y12:I>I		Y9:I>I		YJM975:I>I		YS2:I>I	AA:489		378604X:T>T		DBVPG1853:T>T		DBVPG6765:T>T		L_1528:T>T		YJM975:T>T		YS2:T>T	AA:496		UWOPS03_461_4:T>T		UWOPS05_227_2:T>T	AA:560		UWOPS05_227_2:V>V	AA:588		378604X:I>I		DBVPG1373:I>I		DBVPG1853:I>I		DBVPG6040:I>I		DBVPG6765:I>I		K11:I>I		L_1528:I>I		YJM978:I>I		YPS128:I>I		YPS606:I>I		YS2:I>I	AA:608		378604X:L>L		DBVPG1373:L>L		DBVPG1853:L>L		DBVPG6044:L>L		DBVPG6765:L>L		L_1528:L>L		NCYC110:L>L		SK1:L>L		UWOPS05_227_2:L>L		Y55:L>L		YJM978:L>L		YS2:L>L	AA:613		UWOPS05_227_2:P>P	AA:644		UWOPS83_787_3:G>G	AA:648		DBVPG6044:G>G		NCYC110:G>G		SK1:G>G		Y55:G>G	AA:650		UWOPS05_227_2:A>A	AA:680		DBVPG1106:D>D		DBVPG1373:D>D		DBVPG1788:D>D		DBVPG1853:D>D		DBVPG6040:D>D		DBVPG6044:D>D		DBVPG6765:D>D		K11:D>D		NCYC110:D>D		SK1:D>D		UWOPS83_787_3:D>D		Y12:D>D		Y55:D>D		YJM978:D>D		YPS128:D>D		YPS606:D>D		YS2:D>D	AA:684		UWOPS83_787_3:L>LID:YBR045C	AA:39		322134S:L>L		BC187:L>L		DBVPG1106:L>L		DBVPG1788:L>L		DBVPG1853:L>L		DBVPG6765:L>L		L_1374:L>L		YIIc17_E5:L>L		YJM975:L>L		YJM978:L>L		YJM981:L>L		YS9:L>L	AA:112		322134S:G>G		BC187:G>G		DBVPG1106:G>G		DBVPG1788:G>G		DBVPG1853:G>G		DBVPG6765:G>G		L_1374:G>G		YIIc17_E5:G>G		YJM975:G>G		YJM978:G>G		YJM981:G>G		YS9:G>G	AA:121		322134S:R>R		BC187:R>R		DBVPG1106:R>R		DBVPG1788:R>R		DBVPG1853:R>R		DBVPG6765:R>R		L_1374:R>R		YIIc17_E5:R>R		YJM978:R>R		YJM981:R>R		YS9:R>R	AA:188		YS2:P>P	AA:199		YS2:H>H	AA:207		DBVPG1106:L>L		DBVPG1373:L>L		DBVPG1788:L>L		DBVPG1853:L>L		DBVPG6765:L>L		L_1374:L>L		YJM978:L>L		YS9:L>L	AA:215		UWOPS05_217_3:I>I	AA:265		DBVPG6044:T>T		NCYC110:T>T		SK1:T>T		Y55:T>T	AA:305		L_1374:D>D	AA:324		YPS128:T>T		YPS606:T>T	AA:395		378604X:S>S		BC187:S>S		DBVPG1106:S>S		DBVPG1373:S>S		DBVPG1788:S>S		DBVPG1853:S>S		DBVPG6765:S>S		UWOPS05_227_2:S>S		YJM975:S>S		YJM978:S>S		YPS128:S>S		YPS606:S>S	AA:482		322134S:L>L		378604X:L>L		BC187:L>L		DBVPG1106:L>L		DBVPG1373:L>L		DBVPG1788:L>L		DBVPG6765:L>L		YJM975:L>L		YJM978:L>L	AA:484		DBVPG6044:I>I		NCYC110:I>I		SK1:I>I		Y55:I>I	AA:507		DBVPG1788:K>K	AA:512		322134S:D>D		378604X:D>D		DBVPG1106:D>D		DBVPG1373:D>D		DBVPG1788:D>D		DBVPG6044:D>D		DBVPG6765:D>D		NCYC110:D>D		SK1:D>D		UWOPS05_227_2:D>D		UWOPS87_2421:D>D		Y55:D>D		YIIc17_E5:D>D		YJM975:D>D		YJM978:D>D	AA:559		YPS128:Y>Y		YPS606:Y>YID:YBR046C	AA:22		273614X:V>V		322134S:V>V		378604X:V>V		BC187:V>V		DBVPG1106:V>V		DBVPG1373:V>V		DBVPG1788:V>V		DBVPG6765:V>V		K11:V>V		L_1374:V>V		L_1528:V>V		NCYC110:V>V		SK1:V>V		Y55:V>V		Y9:V>V		YIIc17_E5:V>V		YJM975:V>V		YPS606:V>V	AA:38		NCYC110:L>L		SK1:L>L		Y55:L>L	AA:54		273614X:F>F		322134S:F>F		DBVPG1106:F>F		DBVPG1373:F>F		DBVPG1788:F>F		DBVPG1853:F>F		DBVPG6765:F>F		K11:F>F		L_1374:F>F		L_1528:F>F		NCYC110:F>F		SK1:F>F		UWOPS03_461_4:F>F		UWOPS05_217_3:F>F		UWOPS05_227_2:F>F		UWOPS83_787_3:F>F		Y55:F>F		Y9:F>F		YIIc17_E5:F>F		YJM975:F>F		YPS128:F>F		YPS606:F>F	AA:64		YPS606:P>P	AA:216		YPS128:K>K		YPS606:K>K	AA:277		YPS128:I>I		YPS606:I>IID:YBR047W	AA:21		YPS128:V>V		YPS606:V>V	AA:22		UWOPS03_461_4:L>L		UWOPS05_227_2:L>L	AA:35		322134S:A>A		378604X:A>A		BC187:A>A		DBVPG1106:A>A		DBVPG1373:A>A		DBVPG1853:A>A		DBVPG6765:A>A		YJM975:A>A		YJM978:A>A		YPS128:A>A		YPS606:A>A		YS2:A>A	AA:47		322134S:S>S		378604X:S>S		BC187:S>S		DBVPG1106:S>S		DBVPG1373:S>S		DBVPG1853:S>S		DBVPG6765:S>S		YJM975:S>S		YJM978:S>S		YS2:S>S	AA:94		Y12:P>P	AA:95		UWOPS87_2421:D>DID:YBR050C	AA:75		DBVPG6044:V>V		SK1:V>V		Y55:V>V	AA:101		322134S:I>I		378604X:I>I		BC187:I>I		DBVPG1373:I>I		DBVPG1788:I>I		DBVPG1853:I>I		DBVPG6765:I>I		L_1528:I>I		YIIc17_E5:I>I		YJM975:I>I		YJM981:I>I		YS4:I>I	AA:122		UWOPS05_217_3:T>T		UWOPS05_227_2:T>T		UWOPS83_787_3:T>T	AA:138		DBVPG6044:D>D		SK1:D>D		Y55:D>D	AA:191		YPS606:S>S	AA:194		UWOPS05_227_2:L>L	AA:211		322134S:C>C		378604X:C>C		BC187:C>C		DBVPG1106:C>C		DBVPG1373:C>C		DBVPG1788:C>C		DBVPG1853:C>C		DBVPG6765:C>C		L_1374:C>C		YIIc17_E5:C>C		YJM978:C>C		YS2:C>C		YS4:C>C	AA:249		DBVPG6044:G>G		NCYC110:G>G		SK1:G>G		UWOPS83_787_3:G>G		Y55:G>G	AA:251		UWOPS05_227_2:Y>Y	AA:278		UWOPS83_787_3:R>R	AA:310		YS4:E>E	AA:336		L_1374:L>LID:YBR052C	AA:4		L_1528:V>V	AA:7		L_1528:L>L	AA:9		378604X:Y>Y		DBVPG1106:Y>Y		DBVPG1373:Y>Y		DBVPG1853:Y>Y		DBVPG6040:Y>Y		DBVPG6765:Y>Y		L_1528:Y>Y		YIIc17_E5:Y>Y		YJM975:Y>Y	AA:11		DBVPG1788:V>V	AA:20		L_1528:E>E	AA:22		L_1528:E>E	AA:26		L_1528:I>I	AA:34		L_1528:E>E	AA:36		L_1528:F>F	AA:38		L_1528:V>V	AA:41		L_1528:V>V	AA:51		378604X:E>E		DBVPG1106:E>E		DBVPG1373:E>E		DBVPG1788:E>E		DBVPG1853:E>E		DBVPG6040:E>E		DBVPG6765:E>E		K11:E>E		L_1528:E>E		SK1:E>E		Y12:E>E		Y55:E>E		YIIc17_E5:E>E		YJM975:E>E		YPS128:E>E		YPS606:E>E		YS4:E>E	AA:59		L_1528:A>A	AA:63		L_1528:A>A	AA:69		L_1528:I>I	AA:73		L_1528:E>E	AA:90		378604X:G>G		DBVPG1373:G>G		DBVPG1788:G>G		DBVPG1853:G>G		DBVPG6040:G>G		DBVPG6765:G>G		K11:G>G		L_1528:G>G		SK1:G>G		Y12:G>G		Y55:G>G		YIIc17_E5:G>G		YJM975:G>G		YPS128:G>G		YPS606:G>G		YS4:G>G	AA:93		UWOPS05_227_2:P>P	AA:113		L_1528:F>F	AA:115		SK1:G>G		Y55:G>G	AA:175		L_1528:G>GID:YBR053C	AA:36		378604X:T>T		DBVPG1373:T>T		DBVPG1788:T>T		DBVPG1853:T>T		DBVPG6765:T>T		L_1374:T>T		L_1528:T>T	AA:48		UWOPS87_2421:E>E	AA:84		YPS128:P>P		YPS606:P>P	AA:87		UWOPS87_2421:L>L	AA:149		378604X:S>S		DBVPG1106:S>S		DBVPG1373:S>S		DBVPG1853:S>S		DBVPG6765:S>S		L_1374:S>S		L_1528:S>S		YIIc17_E5:S>S		YJM978:S>S		YS4:S>S	AA:170		YS2:P>P	AA:244		YPS606:N>N	AA:262		DBVPG1106:D>D		DBVPG1373:D>D		DBVPG6044:D>D		DBVPG6765:D>D		L_1374:D>D		L_1528:D>D		NCYC110:D>D		Y55:D>D		YIIc17_E5:D>D		YJM978:D>D		YS4:D>D	AA:313		DBVPG6044:F>F		NCYC110:F>F		Y55:F>FID:YBR054W	AA:14		Y12:E>E	AA:49		YS9:I>I	AA:87		UWOPS03_461_4:G>G		UWOPS05_217_3:G>G		UWOPS05_227_2:G>G	AA:102		378604X:T>T		DBVPG1106:T>T		DBVPG1373:T>T		DBVPG1788:T>T		DBVPG1853:T>T		DBVPG6040:T>T		DBVPG6044:T>T		K11:T>T		L_1528:T>T		SK1:T>T		UWOPS03_461_4:T>T		UWOPS05_217_3:T>T		UWOPS05_227_2:T>T		UWOPS83_787_3:T>T		Y55:T>T		YJM975:T>T	AA:105		K11:E>E	AA:106		DBVPG6044:H>H		SK1:H>H		UWOPS03_461_4:H>H		UWOPS05_217_3:H>H		UWOPS05_227_2:H>H		Y55:H>H	AA:210		273614X:V>V		Y12:V>V		YPS128:V>V		YPS606:V>V	AA:223		K11:F>F	AA:246		322134S:L>L		DBVPG1373:L>L		DBVPG1788:L>L		DBVPG1853:L>L		DBVPG6765:L>L		L_1528:L>L		YJM975:L>L		YJM978:L>L		YS4:L>L	AA:249		273614X:L>L		322134S:L>L		378604X:L>L		DBVPG1106:L>L		DBVPG1373:L>L		DBVPG1788:L>L		DBVPG1853:L>L		DBVPG6044:L>L		DBVPG6765:L>L		K11:L>L		L_1528:L>L		SK1:L>L		UWOPS03_461_4:L>L		UWOPS05_217_3:L>L		UWOPS05_227_2:L>L		UWOPS83_787_3:L>L		UWOPS87_2421:L>L		Y12:L>L		Y55:L>L		Y9:L>L		YJM975:L>L		YJM978:L>L		YPS128:L>L		YPS606:L>L		YS4:L>L	AA:290		322134S:S>S		DBVPG1106:S>S		DBVPG1373:S>S		DBVPG1853:S>S		DBVPG6044:S>S		DBVPG6765:S>S		L_1528:S>S		SK1:S>S		UWOPS03_461_4:S>S		UWOPS05_227_2:S>S		UWOPS83_787_3:S>S		Y55:S>S		YJM975:S>S		YJM978:S>S		YS4:S>S	AA:309		UWOPS87_2421:L>L	AA:323		DBVPG6044:A>A		SK1:A>A		UWOPS05_227_2:A>A		Y55:A>A	AA:331		BC187:K>K		DBVPG1106:K>K		DBVPG1373:K>K		DBVPG1853:K>K		DBVPG6765:K>K		L_1528:K>K		UWOPS83_787_3:K>K		YJM975:K>K		YJM978:K>K		YS4:K>K		YS9:K>KID:YBR055C	AA:77		DBVPG1788:K>K	AA:78		UWOPS03_461_4:L>L		UWOPS05_217_3:L>L	AA:94		DBVPG1788:S>S		DBVPG6040:S>S		DBVPG6765:S>S		L_1374:S>S		YJM975:S>S	AA:98		YPS128:S>S	AA:125		UWOPS03_461_4:A>A		UWOPS05_217_3:A>A	AA:149		DBVPG6044:N>N		NCYC110:N>N		SK1:N>N		UWOPS87_2421:P>P		Y55:N>N	AA:187		DBVPG1373:N>N		DBVPG1788:N>N		DBVPG1853:N>N		DBVPG6040:N>N		DBVPG6044:N>N		DBVPG6765:N>N		L_1374:N>N		L_1528:N>N		SK1:N>N		UWOPS03_461_4:N>N		Y55:N>N		YJM981:N>N		YS9:N>N	AA:204		UWOPS03_461_4:L>L	AA:213		378604X:N>N		DBVPG1373:N>N		DBVPG1788:N>N		DBVPG1853:N>N		DBVPG6040:N>N		DBVPG6765:N>N		L_1528:N>N		YJM975:N>N		YJM981:N>N		YS9:N>N	AA:222		378604X:D>D		DBVPG1373:D>D		DBVPG1788:D>D		DBVPG1853:D>D		DBVPG6040:D>D		DBVPG6765:D>D		L_1528:D>D		SK1:D>D		UWOPS03_461_4:D>D		Y55:D>D		YJM975:D>D		YJM981:D>D		YS9:D>D	AA:313		322134S:A>A		378604X:A>A		DBVPG1373:A>A		DBVPG1788:A>A		DBVPG1853:A>A		DBVPG6040:A>A		DBVPG6044:A>A		DBVPG6765:A>A		L_1528:A>A		SK1:A>A		UWOPS03_461_4:A>A		UWOPS05_217_3:A>A		UWOPS05_227_2:A>A		Y55:A>A		YJM975:A>A		YJM978:A>A		YJM981:A>A		YS9:A>A	AA:344		322134S:A>A		378604X:A>A		BC187:A>A		DBVPG1373:A>A		DBVPG1788:A>A		DBVPG1853:A>A		DBVPG6040:A>A		DBVPG6765:A>A		YJM975:A>A		YJM978:A>A		YJM981:A>A		YS9:A>A	AA:507		273614X:Q>Q	AA:618		YS4:Q>Q	AA:672		322134S:L>L		378604X:L>L		BC187:L>L		DBVPG1373:L>L		DBVPG1788:L>L		DBVPG1853:L>L		DBVPG6765:L>L		K11:L>L		L_1374:L>L		L_1528:L>L		SK1:L>L		UWOPS05_227_2:L>L		Y12:L>L		Y55:L>L		YIIc17_E5:L>L		YJM975:L>L		YJM978:L>L		YS4:L>L	AA:690		NCYC110:T>T		SK1:T>T		Y55:T>T	AA:710		322134S:K>K		378604X:K>K		DBVPG1373:K>K		DBVPG1788:K>K		DBVPG1853:K>K		DBVPG6044:K>K		DBVPG6765:K>K		K11:K>K		L_1374:K>K		L_1528:K>K		NCYC110:K>K		SK1:K>K		UWOPS05_227_2:K>K		Y12:K>K		Y55:K>K		YJM975:K>K		YJM978:K>K		YS2:K>K		YS4:K>K	AA:781		DBVPG6044:F>F		NCYC110:F>F		SK1:F>F		Y55:F>F	AA:788		322134S:S>S		378604X:S>S		DBVPG1373:S>S		DBVPG1788:S>S		DBVPG1853:S>S		DBVPG6044:S>S		DBVPG6765:S>S		L_1374:S>S		L_1528:S>S		NCYC110:S>S		SK1:S>S		UWOPS05_227_2:S>S		Y12:S>S		Y55:S>S		YJM978:S>S		YS2:S>S		YS4:S>S		YS9:S>S	AA:845		273614X:L>L		YPS606:L>L	AA:864		273614X:D>D		YPS606:D>D	AA:878		273614X:S>S		322134S:S>S		378604X:S>S		BC187:S>S		DBVPG1373:S>S		DBVPG1788:S>S		DBVPG1853:S>S		DBVPG6044:S>S		DBVPG6765:S>S		L_1374:S>S		NCYC110:S>S		SK1:S>S		UWOPS03_461_4:S>S		UWOPS05_227_2:S>S		Y55:S>S		YPS606:S>SID:YBR056W	AA:19		322134S:G>G		DBVPG6040:G>G		DBVPG6765:G>G		L_1528:G>G		NCYC110:G>G		SK1:G>G		UWOPS05_227_2:G>G		Y55:G>G		YJM975:G>G		YJM978:G>G		YPS128:G>G		YPS606:G>G	AA:81		NCYC110:A>A		SK1:A>A		Y55:A>A	AA:189		NCYC110:H>H		SK1:H>H		Y55:H>H	AA:200		SK1:F>F		Y55:F>F	AA:249		DBVPG1373:Y>Y		DBVPG1853:Y>Y		DBVPG6040:Y>Y		DBVPG6765:Y>Y		L_1528:Y>Y		NCYC110:Y>Y		SK1:Y>Y		UWOPS87_2421:Y>Y		Y55:Y>Y		YJM975:Y>Y	AA:262		NCYC110:P>P		SK1:P>P		UWOPS83_787_3:P>P		UWOPS87_2421:P>P		Y55:P>P	AA:274		YPS606:P>P	AA:292		UWOPS83_787_3:I>I	AA:305		NCYC110:K>K		SK1:K>K		Y55:K>K	AA:314		BC187:K>K		DBVPG1106:K>K		DBVPG1373:K>K		DBVPG1853:K>K		DBVPG6765:K>K		L_1528:K>K		UWOPS05_227_2:K>K		UWOPS87_2421:K>K		YJM975:K>K		YPS606:K>K	AA:327		UWOPS05_227_2:A>A	AA:423		NCYC110:E>E		SK1:E>E		Y55:E>EID:YBR057C	AA:129		DBVPG6044:T>T		NCYC110:T>T		SK1:T>T		UWOPS03_461_4:T>T		UWOPS05_217_3:T>T		UWOPS05_227_2:T>T		Y55:T>T	AA:171		YPS128:A>A		YPS606:A>A	AA:181		DBVPG6044:E>E		SK1:E>E		UWOPS03_461_4:E>E		UWOPS05_217_3:E>E		Y55:E>E	AA:213		Y12:R>R		Y9:R>R		YS4:R>R	AA:244		DBVPG1853:L>L	AA:275		DBVPG1853:S>S	AA:297		YPS128:E>E	AA:311		DBVPG1106:T>T	AA:312		DBVPG6044:N>N		NCYC110:N>N		SK1:N>N		UWOPS03_461_4:N>N		UWOPS05_217_3:N>N		Y55:N>N		YPS128:N>NID:YBR058C-A	AA:7		DBVPG6040:S>S		UWOPS83_787_3:S>S		YPS128:S>S		YPS606:S>S		YS4:S>S	AA:30		L_1528:T>T	AA:35		DBVPG6040:V>V		DBVPG6044:V>V		SK1:V>V		UWOPS03_461_4:V>V		UWOPS87_2421:V>V		Y55:V>V		YPS128:V>V		YPS606:V>V		YS4:V>VID:YBR060C	AA:46		322134S:L>L		378604X:L>L		DBVPG1788:L>L		DBVPG6044:L>L		DBVPG6765:L>L		NCYC110:L>L		SK1:L>L		UWOPS03_461_4:L>L		UWOPS83_787_3:L>L		Y55:L>L		YJM975:L>L		YPS128:L>L		YPS606:L>L	AA:227		DBVPG1788:L>L	AA:282		BC187:N>N		DBVPG1373:N>N		DBVPG1788:N>N		DBVPG6040:N>N		DBVPG6765:N>N		L_1374:N>N		L_1528:N>N		SK1:N>N		UWOPS05_227_2:N>N		UWOPS83_787_3:N>N		Y55:N>N		YIIc17_E5:N>N		YJM975:N>N		YJM978:N>N		YPS128:N>N		YS9:N>N	AA:289		BC187:P>P		DBVPG1373:P>P		DBVPG1788:P>P		DBVPG6040:P>P		DBVPG6765:P>P		L_1374:P>P		L_1528:P>P		YIIc17_E5:P>P		YJM975:P>P		YJM978:P>P		YS9:P>P	AA:296		DBVPG6044:I>I		SK1:I>I		Y55:I>I	AA:307		DBVPG6044:E>E		SK1:E>E		Y55:E>E	AA:345		YS2:Y>Y	AA:349		BC187:L>L		DBVPG1788:L>L		DBVPG6044:L>L		DBVPG6765:L>L		L_1374:L>L		L_1528:L>L		SK1:L>L		Y55:L>L		YIIc17_E5:L>L		YJM975:L>L		YJM978:L>L		YPS128:L>L		YS9:L>L	AA:387		DBVPG6044:E>E		NCYC361:E>E		SK1:E>E		UWOPS05_227_2:E>E		Y55:E>E		YPS128:E>E		YPS606:E>E	AA:400		DBVPG6044:V>V		SK1:V>V		UWOPS05_227_2:V>V		UWOPS83_787_3:V>V		Y55:V>V		YPS128:V>V		YPS606:V>V	AA:503		DBVPG6044:T>T		SK1:T>T		Y55:T>T	AA:551		BC187:R>R		DBVPG1106:R>R		DBVPG1373:R>R		DBVPG1788:R>R		DBVPG1853:R>R		DBVPG6765:R>R		L_1374:R>R		L_1528:R>R		YJM975:R>R		YJM978:R>R		YJM981:R>R	AA:582		DBVPG6044:F>F		SK1:F>F		Y55:F>F	AA:602		UWOPS03_461_4:V>V	AA:613		UWOPS03_461_4:L>LID:YBR061C	AA:22		DBVPG6044:R>R		SK1:R>R	AA:48		K11:V>V	AA:65		322134S:L>L		378604X:L>L		DBVPG1788:L>L		DBVPG6765:L>L		K11:L>L		L_1374:L>L		L_1528:L>L		UWOPS03_461_4:L>L		UWOPS83_787_3:L>L		UWOPS87_2421:L>L		YIIc17_E5:L>L		YJM975:L>L		YJM981:L>L		YPS128:L>L		YPS606:L>L	AA:86		UWOPS87_2421:P>P	AA:118		UWOPS03_461_4:A>A	AA:132		322134S:L>L		378604X:L>L		DBVPG1373:L>L		DBVPG1788:L>L		DBVPG6765:L>L		K11:L>L		L_1374:L>L		L_1528:L>L		NCYC361:L>L		UWOPS03_461_4:L>L		UWOPS83_787_3:L>L		UWOPS87_2421:L>L		YJM981:L>L		YPS128:L>L		YPS606:L>L	AA:138		322134S:Y>Y		378604X:L>L		DBVPG1373:Y>Y		DBVPG1788:Y>Y		DBVPG6765:Y>Y		L_1374:L>L		L_1528:Y>Y		NCYC361:Y>Y		YJM981:Y>Y	AA:198		378604X:L>L		DBVPG1373:L>L		L_1374:L>L		L_1528:L>L		NCYC361:L>L		YJM975:L>L		YJM981:L>L		YS9:L>L	AA:200		378604X:A>A		DBVPG1373:A>A		L_1528:A>A		NCYC361:A>A		YJM975:A>A		YJM981:A>A		YS9:A>A	AA:275		322134S:S>S		378604X:S>S		DBVPG1373:S>S		DBVPG6044:S>S		K11:S>S		NCYC110:S>S		NCYC361:S>S		SK1:S>S		UWOPS03_461_4:S>S		UWOPS83_787_3:S>S		UWOPS87_2421:S>S		Y55:S>S		YJM975:S>S		YJM978:S>S		YPS128:S>S		YPS606:S>S		YS9:S>S	AA:308		K11:R>RID:YBR065C	AA:48		DBVPG6044:T>T		SK1:T>T		Y55:T>T	AA:67		DBVPG6044:Q>Q		SK1:Q>Q		Y55:Q>Q	AA:68		DBVPG1373:R>R	AA:73		322134S:C>C	AA:106		YIIc17_E5:N>N	AA:140		DBVPG6044:K>K		NCYC110:K>K		SK1:K>K		Y55:K>K	AA:150		YPS606:G>G	AA:198		DBVPG1373:K>K		DBVPG6044:K>K		DBVPG6765:K>K		L_1528:K>K		NCYC110:K>K		SK1:K>K		UWOPS03_461_4:K>K		UWOPS05_227_2:K>K		UWOPS87_2421:K>K		Y55:K>K		Y9:K>K		YJM975:K>K		YPS128:K>K		YPS606:K>K		YS2:K>K		YS9:K>K	AA:213		DBVPG1373:S>S		DBVPG6765:S>S		L_1528:S>S		Y9:S>S		YJM975:S>S		YPS606:S>S	AA:218		Y9:K>K		YPS128:K>K		YPS606:K>K	AA:316		YS9:L>L	AA:319		322134S:L>L	AA:321		322134S:Q>Q		DBVPG6044:Q>Q		NCYC110:Q>Q		SK1:Q>Q		UWOPS03_461_4:Q>Q		UWOPS05_227_2:Q>Q		Y55:Q>QID:YBR066C	AA:143		322134S:K>K		Y12:K>K		Y9:K>K	AA:174		UWOPS03_461_4:I>I		UWOPS05_227_2:I>I	AA:187		Y9:G>G	AA:190		UWOPS05_227_2:Q>Q		UWOPS87_2421:Q>QID:YBR067C	AA:2		DBVPG1373:S>S		DBVPG6765:S>S		L_1528:S>S		SK1:S>S		UWOPS83_787_3:S>S		UWOPS87_2421:S>S		Y55:S>S		YIIc17_E5:S>S		YJM978:S>S		YJM981:S>S		YPS128:S>S		YPS606:S>S	AA:16		SK1:L>L		Y55:L>L	AA:63		DBVPG1373:Y>Y		DBVPG1788:Y>Y		DBVPG1853:Y>Y		DBVPG6765:Y>Y		L_1374:Y>Y		SK1:Y>Y		UWOPS03_461_4:Y>Y		UWOPS83_787_3:Y>Y		Y55:Y>Y		Y9:Y>Y		YIIc17_E5:Y>Y		YJM978:Y>Y		YJM981:Y>Y		YPS128:Y>Y		YPS606:Y>Y		YS9:Y>Y	AA:85		DBVPG1373:A>A		DBVPG1788:A>A		DBVPG1853:A>A		DBVPG6765:A>A		L_1374:A>A		YIIc17_E5:A>A		YJM981:A>A		YS9:A>A	AA:197		DBVPG6765:S>S	AA:203		UWOPS03_461_4:A>A		UWOPS83_787_3:A>AID:YBR068C	AA:33		YS4:S>S	AA:58		UWOPS03_461_4:F>F		UWOPS05_217_3:F>F		UWOPS05_227_2:F>F	AA:62		273614X:E>E		DBVPG1373:E>E		DBVPG6765:E>E		L_1374:E>E		UWOPS03_461_4:E>E		UWOPS05_217_3:E>E		UWOPS05_227_2:E>E		UWOPS83_787_3:E>E		UWOPS87_2421:E>E		YIIc17_E5:E>E		YJM975:E>E		YJM978:E>E		YPS128:E>E		YPS606:E>E	AA:83		YS4:E>E	AA:107		DBVPG1373:T>T		DBVPG6765:T>T		SK1:T>T		UWOPS03_461_4:T>T		UWOPS05_217_3:T>T		UWOPS05_227_2:T>T		UWOPS83_787_3:T>T		UWOPS87_2421:T>T		Y55:T>T		YIIc17_E5:T>T		YJM975:T>T		YJM978:T>T		YPS128:T>T		YPS606:T>T	AA:187		UWOPS83_787_3:T>T		UWOPS87_2421:T>T	AA:209		UWOPS03_461_4:P>P		UWOPS05_217_3:P>P	AA:226		SK1:F>F		UWOPS03_461_4:F>F		UWOPS05_217_3:F>F		Y55:F>F		YPS128:F>F		YPS606:F>F	AA:417		DBVPG6044:K>K		NCYC110:K>K		SK1:K>K		Y55:K>K	AA:460		UWOPS03_461_4:I>I		UWOPS83_787_3:I>I		UWOPS87_2421:I>I	AA:514		BC187:V>V		DBVPG1373:V>V		DBVPG1788:V>V		DBVPG6765:V>V		L_1374:V>V		YJM975:V>V		YS9:V>V	AA:524		NCYC110:A>A		SK1:A>A		Y55:A>A	AA:559		BC187:R>R		DBVPG1373:R>R		DBVPG1788:R>R		DBVPG6765:R>R		L_1374:R>R		NCYC110:R>R		S288c:R>R		SK1:R>R		UWOPS03_461_4:R>R		UWOPS83_787_3:R>R		UWOPS87_2421:R>R		Y12:R>R		Y55:R>R		YJM975:R>R		YS2:R>R		YS4:R>R		YS9:R>R	AA:567		NCYC110:L>L		SK1:L>L		Y55:L>L	AA:596		NCYC110:R>R		SK1:R>RID:YBR069C	AA:13		DBVPG1106:S>S	AA:49		SK1:E>E		UWOPS83_787_3:E>E		UWOPS87_2421:E>E		Y55:E>E	AA:67		L_1528:F>F	AA:70		L_1528:F>F	AA:72		L_1528:R>R		SK1:R>R		UWOPS83_787_3:R>R		UWOPS87_2421:R>R		Y55:R>R	AA:80		L_1528:S>S	AA:90		378604X:L>L		DBVPG1106:L>L		DBVPG1373:L>L		DBVPG1853:L>L		DBVPG6765:L>L		L_1374:L>L		YIIc17_E5:L>L		YJM981:L>L		YS9:L>L	AA:92		Y12:K>K	AA:93		L_1528:S>S	AA:96		L_1528:S>S	AA:98		378604X:H>H	AA:99		L_1528:L>L	AA:188		UWOPS87_2421:L>L	AA:189		DBVPG1106:P>P		DBVPG1373:P>P		DBVPG1853:P>P		DBVPG6765:P>P		L_1374:P>P		YJM981:P>P		YS9:P>P	AA:209		DBVPG1106:I>I		DBVPG1373:I>I		DBVPG1853:I>I		DBVPG6765:I>I		L_1374:I>I		YS9:I>I	AA:221		378604X:I>I		DBVPG6044:I>I		SK1:I>I		UWOPS05_227_2:I>I		UWOPS87_2421:I>I		Y55:I>I		YPS128:I>I		YPS606:I>I	AA:223		DBVPG1106:L>L		DBVPG1373:L>L		DBVPG1853:L>L		DBVPG6765:L>L		L_1374:L>L		YS9:L>L	AA:233		378604X:E>E		DBVPG1106:E>E		DBVPG1373:E>E		DBVPG6765:E>E		L_1374:E>E		UWOPS05_227_2:E>E		YIIc17_E5:E>E		YPS128:E>E		YPS606:E>E		YS9:E>E	AA:259		DBVPG1106:A>A		DBVPG6765:A>A		L_1374:A>A		YIIc17_E5:A>A		YJM978:A>A		YS9:A>A	AA:282		378604X:G>G		DBVPG1106:G>G		DBVPG6044:G>G		DBVPG6765:G>G		L_1374:G>G		NCYC110:G>G		SK1:G>G		UWOPS05_227_2:G>G		Y55:G>G		YIIc17_E5:G>G		YJM978:G>G		YPS128:G>G		YPS606:G>G		YS9:G>G	AA:358		S288c:A>A	AA:367		L_1374:S>S	AA:440		UWOPS05_227_2:T>T	AA:457		UWOPS03_461_4:L>L	AA:488		Y9:G>G		YS2:G>G	AA:506		DBVPG1106:I>I		DBVPG1373:I>I		DBVPG1788:I>I		DBVPG1853:I>I		DBVPG6765:I>I		L_1374:I>I		L_1528:I>I		NCYC361:I>I		YIIc17_E5:I>I		YJM981:I>I	AA:512		DBVPG1373:Q>Q		DBVPG1788:Q>Q		DBVPG1853:Q>Q		DBVPG6765:Q>Q		L_1374:Q>Q		L_1528:Q>Q		NCYC361:Q>Q		YIIc17_E5:Q>Q		YJM975:Q>Q		YJM981:Q>Q	AA:547		UWOPS03_461_4:G>G	AA:577		DBVPG1788:S>S		DBVPG6765:S>S		L_1374:S>S		L_1528:S>S		YJM975:S>S		YJM981:S>S		YS2:S>S	AA:589		273614X:D>D		378604X:D>D		K11:D>D		NCYC361:D>D		UWOPS03_461_4:D>D		UWOPS83_787_3:D>D		Y12:D>D		Y9:D>D		YPS128:D>D		YPS606:D>D		YS4:D>D	AA:599		UWOPS83_787_3:S>S		YPS128:S>S		YPS606:S>SID:YBR070C	AA:80		K11:L>L	AA:97		K11:R>R	AA:99		YPS128:R>R	AA:131		YS4:V>V	AA:145		YS4:V>V	AA:155		DBVPG1373:S>S		DBVPG1853:S>S		DBVPG6765:S>S		L_1374:S>S		UWOPS87_2421:S>S		YIIc17_E5:S>S		YJM978:S>S		YPS128:S>S		YPS606:S>S		YS4:S>S	AA:157		DBVPG1373:H>H		DBVPG1853:H>H		DBVPG6044:H>H		DBVPG6765:H>H		L_1374:H>H		SK1:H>H		UWOPS03_461_4:H>H		UWOPS05_227_2:H>H		UWOPS87_2421:H>H		Y55:H>H		YIIc17_E5:H>H		YJM978:H>H		YPS128:H>H		YPS606:H>H		YS4:H>H	AA:176		UWOPS87_2421:I>I		YPS128:I>I		YPS606:I>I	AA:208		DBVPG1788:L>L		DBVPG1853:L>L		DBVPG6765:L>L		L_1374:L>L		YJM978:L>L		YS4:L>LID:YBR071W	AA:12		UWOPS05_227_2:T>T		UWOPS83_787_3:T>T	AA:76		DBVPG1853:S>S		DBVPG6044:S>S		NCYC110:S>S		SK1:S>S		Y55:S>S	AA:167		378604X:D>D		DBVPG1106:D>D		DBVPG1373:D>D		DBVPG1788:D>D		DBVPG1853:D>D		DBVPG6040:D>D		DBVPG6044:D>D		DBVPG6765:D>D		L_1374:D>D		L_1528:D>D		SK1:D>D		UWOPS05_217_3:D>D		UWOPS87_2421:D>D		Y55:D>D		YJM978:D>D		YJM981:D>D		YPS128:D>D		YS9:D>D	AA:174		378604X:V>V		DBVPG1788:V>V		DBVPG1853:V>V		DBVPG6040:V>V		DBVPG6765:V>V		L_1374:V>V		L_1528:V>V		NCYC361:V>V		UWOPS05_217_3:V>V		UWOPS87_2421:V>V		YJM978:V>V		YJM981:V>V		YPS128:V>V		YPS606:V>V		YS9:V>V	AA:187		378604X:I>I		DBVPG1373:I>I		DBVPG1788:I>I		DBVPG1853:I>I		DBVPG6044:I>I		DBVPG6765:I>I		L_1374:I>I		L_1528:I>I		SK1:I>I		UWOPS05_217_3:I>I		UWOPS87_2421:I>I		Y55:I>I		YJM978:I>I		YJM981:I>I		YPS128:I>I		YS9:I>I	AA:201		378604X:G>G		UWOPS05_217_3:G>GID:YBR072W	AA:39		DBVPG6040:L>L	AA:60		Y12:G>G		YS9:G>G	AA:87		DBVPG6044:F>F		SK1:F>F		Y55:F>F	AA:120		DBVPG6044:D>D		SK1:D>D		Y55:D>D	AA:184		273614X:L>L		DBVPG1106:L>L		DBVPG1373:L>L		DBVPG1788:L>L		DBVPG1853:L>L		DBVPG6040:L>L		DBVPG6765:L>L		L_1528:L>L		YJM975:L>L		YS9:L>L	AA:185		YPS606:T>T	AA:205		273614X:E>E		378604X:E>E		DBVPG1106:E>E		DBVPG1373:E>E		DBVPG1788:E>E		DBVPG1853:E>E		DBVPG6044:E>E		DBVPG6765:E>E		L_1528:E>E		SK1:E>E		UWOPS05_217_3:E>E		UWOPS87_2421:E>E		Y55:E>E		YJM975:E>E		YPS606:E>E		YS9:E>EID:YBR073W	AA:12		378604X:K>K		DBVPG6044:K>K		SK1:K>K		UWOPS05_227_2:K>K		UWOPS83_787_3:K>K		YS4:K>K		YS9:K>K	AA:21		378604X:K>K		DBVPG6044:K>K		SK1:K>K		UWOPS05_217_3:K>K		UWOPS05_227_2:K>K		UWOPS83_787_3:K>K		UWOPS87_2421:K>K		YS4:K>K	AA:32		UWOPS83_787_3:V>V		UWOPS87_2421:V>V	AA:35		UWOPS05_217_3:A>A		UWOPS05_227_2:A>A	AA:46		DBVPG1788:L>L	AA:55		378604X:T>T		DBVPG6044:T>T		SK1:T>T		UWOPS05_217_3:T>T		UWOPS05_227_2:T>T		UWOPS83_787_3:T>T		UWOPS87_2421:T>T		YS4:T>T		YS9:T>T	AA:59		DBVPG1106:A>A		DBVPG1373:A>A		DBVPG1788:A>A		DBVPG1853:A>A		DBVPG6044:A>A		DBVPG6765:A>A		L_1528:A>A		SK1:A>A		UWOPS05_217_3:A>A		UWOPS05_227_2:A>A		UWOPS83_787_3:A>A		UWOPS87_2421:A>A		Y12:A>A		YIIc17_E5:A>A		YJM978:A>A		YPS128:A>A		YPS606:A>A		YS4:A>A	AA:170		378604X:S>S		DBVPG6044:S>S		SK1:S>S		UWOPS03_461_4:S>S		UWOPS05_217_3:S>S		Y55:S>S		YS4:S>S	AA:185		378604X:Y>Y		DBVPG1106:Y>Y		DBVPG1373:Y>Y		DBVPG1788:Y>Y		DBVPG1853:Y>Y		DBVPG6044:Y>Y		DBVPG6765:Y>Y		L_1528:Y>Y		SK1:Y>Y		UWOPS03_461_4:Y>Y		UWOPS05_217_3:Y>Y		UWOPS83_787_3:Y>Y		UWOPS87_2421:Y>Y		Y55:Y>Y		YIIc17_E5:Y>Y		YJM975:Y>Y		YJM978:Y>Y		YPS128:Y>Y	AA:249		DBVPG6044:E>E		NCYC110:E>E		SK1:E>E		Y55:E>E	AA:331		YJM975:R>R		YJM978:R>R	AA:352		YPS128:C>C	AA:415		DBVPG1373:Y>Y		DBVPG1788:Y>Y		DBVPG1853:Y>Y		DBVPG6765:Y>Y		L_1374:Y>Y		YJM975:Y>Y		YJM978:Y>Y		YPS128:Y>Y	AA:417		DBVPG1788:K>K	AA:455		DBVPG1373:K>K		DBVPG1788:K>K		DBVPG1853:K>K		DBVPG6765:K>K		L_1374:K>K		UWOPS03_461_4:K>K		UWOPS83_787_3:K>K		UWOPS87_2421:K>K		YJM975:K>K		YJM978:K>K		YPS128:K>K		YPS606:K>K	AA:493		DBVPG1373:A>A		DBVPG1788:A>A		DBVPG1853:A>A		DBVPG6765:A>A		UWOPS03_461_4:A>A		UWOPS83_787_3:A>A		UWOPS87_2421:A>A		YJM978:A>A		YPS128:A>A		YS4:A>A	AA:550		UWOPS87_2421:P>P	AA:557		273614X:F>F		DBVPG1106:F>F		DBVPG1853:F>F		DBVPG6765:F>F		L_1528:F>F		YJM978:F>F		YS4:F>F	AA:582		UWOPS87_2421:L>L	AA:590		YPS128:L>L	AA:633		UWOPS03_461_4:K>K		UWOPS05_227_2:K>K		UWOPS83_787_3:K>K	AA:673		YPS128:S>S	AA:684		273614X:K>K		L_1374:K>K	AA:730		DBVPG1106:P>P		DBVPG1373:P>P		DBVPG1853:P>P		DBVPG6765:P>P		L_1528:P>P	AA:744		273614X:G>G		DBVPG1373:G>G		DBVPG1853:G>G		DBVPG6765:G>G		L_1374:G>G		L_1528:G>G		UWOPS03_461_4:G>G		UWOPS05_227_2:G>G		UWOPS83_787_3:G>G		YPS128:G>G	AA:782		273614X:S>S		DBVPG1373:S>S		DBVPG1788:S>S		DBVPG1853:S>S		DBVPG6765:S>S		L_1374:S>S		L_1528:S>S		UWOPS03_461_4:S>S		UWOPS05_227_2:S>S		YPS128:S>S	AA:803		DBVPG1106:L>L	AA:834		UWOPS03_461_4:N>N		UWOPS05_227_2:N>N		UWOPS83_787_3:N>N	AA:839		UWOPS03_461_4:T>T		UWOPS05_227_2:T>T		UWOPS83_787_3:T>T	AA:847		UWOPS03_461_4:T>T		UWOPS05_217_3:T>T		UWOPS05_227_2:T>T		UWOPS83_787_3:T>T	AA:851		DBVPG1106:T>T		DBVPG1373:T>T		DBVPG1788:T>T		DBVPG1853:T>T		DBVPG6765:T>T		L_1528:T>T		UWOPS03_461_4:T>T		UWOPS05_227_2:T>T		UWOPS83_787_3:T>T		YJM978:T>T		YPS128:T>T	AA:872		DBVPG1106:S>S		DBVPG1373:S>S		DBVPG1788:S>S		DBVPG1853:S>S		DBVPG6765:S>S		L_1528:S>S		Y55:S>S		YJM978:S>S	AA:887		DBVPG1853:P>PID:YBR077C	AA:11		273614X:L>L		DBVPG1373:L>L		DBVPG1853:L>L		DBVPG6765:L>L		L_1374:L>L		L_1528:L>L		Y55:L>L		YJM975:L>L		YS4:G>G	AA:30		UWOPS05_217_3:S>S	AA:53		DBVPG1373:N>N		DBVPG1788:N>N		DBVPG6765:N>N		NCYC361:N>N		UWOPS05_217_3:N>N		UWOPS05_227_2:N>N		YJM975:N>N		YPS128:N>N	AA:68		DBVPG1373:N>N		DBVPG1788:N>N		DBVPG6765:N>N		L_1528:N>N		NCYC361:N>N		UWOPS05_227_2:N>N		YJM975:N>N	AA:88		NCYC361:E>E	AA:117		UWOPS05_227_2:T>T	AA:133		273614X:A>A		DBVPG1373:A>A		DBVPG6765:A>A		L_1374:A>A		L_1528:A>A		UWOPS87_2421:A>A		Y55:A>A		YJM975:A>AID:YBR082C	AA:8		273614X:A>A		322134S:A>A		DBVPG1788:A>A		DBVPG6040:A>A		DBVPG6765:A>A		L_1374:A>A		L_1528:A>A		Y55:A>A		YIIc17_E5:A>A		YJM975:A>A		YS4:A>A	AA:62		YJM975:P>P		YJM978:P>P	AA:96		322134S:P>P		378604X:P>P		DBVPG6044:P>P		DBVPG6765:P>P		L_1374:P>P		L_1528:P>P		NCYC110:P>P		SK1:P>P		UWOPS05_217_3:P>P		UWOPS05_227_2:P>P		UWOPS87_2421:P>P		Y12:P>P		Y55:P>P		YJM975:P>P		YJM978:P>P		YPS128:P>P		YS4:P>P	AA:101		Y12:S>S	AA:121		YS9:V>V	AA:129		YJM975:K>KID:YBR084W	AA:33		273614X:S>S		322134S:S>S		378604X:S>S		DBVPG1373:S>S		DBVPG1788:S>S		DBVPG1853:S>S		DBVPG6044:S>S		DBVPG6765:S>S		K11:S>S		L_1374:S>S		NCYC110:S>S		SK1:S>S		UWOPS03_461_4:S>S		UWOPS05_227_2:S>S		UWOPS83_787_3:S>S		Y12:S>S		Y55:S>S		Y9:S>S		YJM975:S>S		YJM981:S>S		YPS128:S>S		YS2:S>S		YS9:S>S	AA:38		378604X:L>L		K11:L>L		Y12:L>L		Y9:L>L		YPS128:L>L	AA:60		378604X:L>L		DBVPG6044:L>L		K11:L>L		NCYC110:L>L		SK1:L>L		UWOPS83_787_3:L>L		Y12:L>L		Y9:L>L		YPS128:L>L	AA:151		YPS128:K>K		YPS606:K>K	AA:168		UWOPS03_461_4:G>G	AA:219		UWOPS03_461_4:A>A	AA:290		UWOPS03_461_4:V>V	AA:394		273614X:R>R		BC187:R>R		DBVPG1373:R>R		DBVPG1853:R>R		DBVPG6040:R>R		L_1374:R>R		Y55:R>R		YIIc17_E5:R>R		YJM978:R>R		YS9:R>R	AA:420		DBVPG6044:G>G		NCYC110:G>G		UWOPS03_461_4:G>G		UWOPS05_217_3:G>G	AA:433		UWOPS03_461_4:S>S		UWOPS05_217_3:S>S	AA:450		273614X:G>G		322134S:G>G		BC187:G>G		DBVPG1373:G>G		DBVPG6040:G>G		DBVPG6044:G>G		L_1374:G>G		NCYC110:G>G		UWOPS03_461_4:G>G		UWOPS05_217_3:G>G		Y55:G>G		YIIc17_E5:G>G		YJM975:G>G		YJM978:G>G		YPS128:G>G		YPS606:G>G		YS9:G>G	AA:478		378604X:S>S	AA:539		UWOPS03_461_4:L>L		UWOPS05_217_3:L>L	AA:559		YIIc17_E5:I>I	AA:587		Y9:T>	AA:608		DBVPG6044:L>L	AA:682		273614X:L>L		DBVPG1853:L>L		DBVPG6040:L>L		DBVPG6765:L>L		L_1374:L>L		L_1528:L>L		NCYC361:L>L		UWOPS83_787_3:L>L		Y55:L>L		YJM975:L>L		YJM978:L>L		YJM981:L>L	AA:686		273614X:K>K		DBVPG1853:K>K		DBVPG6040:K>K		DBVPG6765:K>K		L_1374:K>K		L_1528:K>K		NCYC361:K>K		UWOPS83_787_3:K>K		Y55:K>K		YJM975:K>K		YJM978:K>K		YJM981:K>K	AA:688		273614X:V>V		DBVPG1853:V>V		DBVPG6040:V>V		DBVPG6765:V>V		L_1374:V>V		L_1528:V>V		NCYC361:V>V		UWOPS83_787_3:V>V		Y55:V>V		YJM975:V>V		YJM978:V>V		YJM981:V>V		YS9:V>V	AA:732		SK1:V>V	AA:737		273614X:V>V		DBVPG1853:V>V		DBVPG6040:V>V		DBVPG6765:V>V		L_1374:V>V		L_1528:V>V		NCYC361:V>V		UWOPS83_787_3:V>V		Y55:V>V		YJM978:V>V		YS9:V>V	AA:739		DBVPG1853:V>V		DBVPG6040:V>V		DBVPG6765:V>V		L_1374:V>V		L_1528:V>V		NCYC361:V>V		UWOPS83_787_3:V>V		Y55:V>V		YJM978:V>V		YS9:V>V	AA:756		DBVPG1853:P>P		DBVPG6040:P>P		L_1374:P>P		L_1528:P>P		NCYC361:P>P		SK1:P>P		UWOPS03_461_4:P>P		UWOPS83_787_3:P>P		Y55:P>P		YJM978:P>P		YS9:P>P	AA:768		UWOPS03_461_4:N>N	AA:908		273614X:T>T		BC187:T>T		DBVPG1788:T>T		DBVPG1853:T>T		DBVPG6040:T>T		DBVPG6765:T>T		L_1374:T>T		L_1528:T>T		Y55:T>T		YIIc17_E5:T>T		YJM975:T>TID:YBR085C-A	AA:12		UWOPS05_227_2:F>F	AA:18		UWOPS83_787_3:S>S	AA:27		DBVPG1373:L>L		DBVPG1788:L>L		DBVPG6040:L>L		DBVPG6765:L>L		L_1374:L>L		UWOPS83_787_3:L>L		UWOPS87_2421:L>L		Y55:L>L		YIIc17_E5:L>L		YJM975:L>L		YJM978:L>LID:YBR085W	AA:5		273614X:A>A		BC187:A>A		DBVPG1373:A>A		DBVPG1788:A>A		DBVPG1853:A>A		DBVPG6765:A>A		L_1374:A>A		Y55:A>A		YIIc17_E5:A>A		YJM978:A>A		YJM981:A>A		YS4:A>A	AA:87		273614X:F>F		DBVPG1373:F>F		DBVPG1788:F>F		DBVPG1853:F>F		DBVPG6765:F>F		L_1528:F>F		Y55:F>F		YJM978:F>F		YJM981:F>F		YPS128:F>F		YPS606:F>F		YS4:F>F	AA:105		DBVPG6044:G>G		NCYC110:G>G		SK1:G>G	AA:111		Y12:G>G	AA:130		273614X:S>S	AA:132		K11:L>L		NCYC110:L>L		SK1:L>L		UWOPS03_461_4:L>L		UWOPS05_217_3:L>L		UWOPS83_787_3:L>L		Y12:L>L		YPS128:L>L		YPS606:L>L		YS2:L>L		YS9:L>L	AA:148		273614X:A>A		DBVPG1106:A>A		DBVPG1373:A>A		DBVPG1788:A>A		DBVPG1853:A>A		DBVPG6044:A>A		DBVPG6765:A>A		L_1528:A>A		NCYC110:A>A		SK1:A>A		Y12:A>A		Y55:A>A		YJM978:A>A		YJM981:A>A		YPS128:A>A		YPS606:A>A		YS4:A>A	AA:193		273614X:R>R		DBVPG1788:R>R		Y55:R>R	AA:290		L_1374:V>V		YJM978:V>V		YPS128:V>VID:YBR087W	AA:3		YIIc17_E5:L>L	AA:12		DBVPG1373:S>S		DBVPG1788:S>S		DBVPG6765:S>S		L_1528:S>S		UWOPS87_2421:S>S		Y55:S>S		YIIc17_E5:S>S		YS4:S>S	AA:62		DBVPG1106:G>G		DBVPG1373:G>G		DBVPG1788:G>G		DBVPG6765:G>G		UWOPS83_787_3:G>G		UWOPS87_2421:G>G		Y55:G>G		YIIc17_E5:G>G		YS4:G>G	AA:78		UWOPS83_787_3:A>A		UWOPS87_2421:A>A	AA:89		DBVPG1106:S>S		DBVPG1373:S>S		DBVPG1788:S>S		DBVPG1853:S>S		DBVPG6765:S>S		UWOPS83_787_3:S>S		UWOPS87_2421:S>S		Y55:S>S		YIIc17_E5:S>S		YS4:S>S	AA:112		YS4:L>L	AA:130		DBVPG6044:G>G		K11:G>G		SK1:G>G		UWOPS03_461_4:G>G	AA:143		DBVPG6765:A>A		Y55:A>A	AA:145		DBVPG1106:S>S		DBVPG1373:S>S		DBVPG1788:S>S		DBVPG1853:S>S		DBVPG6765:S>S		UWOPS83_787_3:S>S		UWOPS87_2421:S>S		Y55:S>S		YIIc17_E5:S>S		YS4:S>S	AA:167		UWOPS05_217_3:L>L	AA:194		DBVPG1106:S>S		DBVPG1373:S>S		DBVPG1788:S>S		DBVPG1853:S>S		DBVPG6765:S>S		UWOPS83_787_3:S>S		UWOPS87_2421:S>S		Y55:S>S		YIIc17_E5:S>S		YJM975:S>S	AA:201		DBVPG1106:I>I		DBVPG1373:I>I		DBVPG1788:I>I		DBVPG1853:I>I		DBVPG6044:I>I		DBVPG6765:I>I		NCYC110:I>I		SK1:I>I		UWOPS03_461_4:I>I		UWOPS05_217_3:I>I		UWOPS83_787_3:I>I		UWOPS87_2421:I>I		Y55:I>I		YIIc17_E5:I>I		YJM975:I>I		YPS606:I>I	AA:219		DBVPG1106:L>L		DBVPG1373:L>L		DBVPG1788:L>L		DBVPG6044:L>L		DBVPG6765:L>L		NCYC110:L>L		SK1:L>L		UWOPS83_787_3:L>L		UWOPS87_2421:L>L		Y55:L>L		YJM975:L>L	AA:242		DBVPG1106:L>L		DBVPG1373:L>L		DBVPG1788:L>L		DBVPG6044:L>L		DBVPG6765:L>L		NCYC110:L>L		SK1:L>L		UWOPS03_461_4:L>L		UWOPS05_217_3:L>L		Y55:L>L		YJM975:L>L		YPS606:L>L	AA:263		DBVPG6044:I>I		NCYC110:I>I		SK1:I>I	AA:281		DBVPG1106:E>E		DBVPG1373:E>E		DBVPG1788:E>E		DBVPG6044:E>E		DBVPG6765:E>E		NCYC110:E>E		SK1:E>E		UWOPS03_461_4:E>E		UWOPS05_217_3:E>E		UWOPS05_227_2:E>E		UWOPS83_787_3:E>E		UWOPS87_2421:E>E		Y55:E>E		YJM975:E>E		YPS606:E>E	AA:317		YS9:N>N	AA:349		DBVPG1106:V>V		DBVPG1373:V>V		DBVPG1788:V>V		DBVPG6044:V>V		DBVPG6765:V>V		NCYC110:V>V		SK1:V>V		UWOPS03_461_4:V>V		UWOPS05_217_3:V>V		UWOPS05_227_2:V>V		UWOPS87_2421:V>V		Y55:V>V		YJM975:V>V		YPS128:V>V		YPS606:V>V	AA:353		DBVPG6044:L>L		NCYC110:L>L		SK1:L>L		UWOPS03_461_4:L>L		UWOPS05_217_3:L>L		UWOPS05_227_2:L>LID:YBR088C	AA:54		DBVPG6040:V>V	AA:57		K11:F>F		Y9:F>F		YPS128:F>F	AA:63		NCYC110:D>D		SK1:D>D		UWOPS05_227_2:D>D	AA:89		K11:T>T		Y9:T>T		YPS128:T>T	AA:100		DBVPG1788:I>I		L_1374:I>I		YJM978:I>I	AA:106		DBVPG6044:T>T		NCYC110:T>T		SK1:T>T		YPS128:T>T		YPS606:T>T	AA:143		DBVPG6044:E>E		NCYC110:E>E		SK1:E>E	AA:181		UWOPS03_461_4:I>I	AA:193		DBVPG1106:T>T	AA:252		DBVPG6044:P>P		NCYC110:P>P		SK1:P>PID:YBR089C-A	AA:34		322134S:A>A		378604X:A>A		DBVPG6044:Y>Y		SK1:Y>Y		Y55:Y>Y		Y9:A>A		YPS606:A>A	AA:43		DBVPG6044:D>D		SK1:D>D		Y55:D>D	AA:100		DBVPG6044:->-		SK1:->-		Y55:->-ID:YBR091C	AA:2		DBVPG6040:S>S	AA:31		DBVPG6040:S>S	AA:36		DBVPG1373:I>I		DBVPG1788:I>I		DBVPG1853:I>I		DBVPG6765:I>I		YJM978:I>I		YS9:I>I	AA:39		DBVPG6040:T>T	AA:43		DBVPG6040:K>KID:YBR092C	AA:6		DBVPG6044:V>V		NCYC110:V>V		SK1:V>V		Y55:V>V	AA:32		DBVPG6044:G>G		NCYC110:G>G		SK1:G>G		Y55:G>G		YPS128:G>G		YPS606:G>G	AA:40		DBVPG1373:F>F		DBVPG1788:F>F		DBVPG6040:F>F		DBVPG6044:F>F		DBVPG6765:F>F		NCYC110:F>F		SK1:F>F		Y55:F>F		YJM975:F>F		YJM978:F>F		YPS128:F>F		YPS606:F>F		YS4:F>F	AA:56		DBVPG1788:I>I		YJM978:I>I	AA:72		273614X:L>L		DBVPG1373:L>L		DBVPG1788:L>L		DBVPG6040:L>L		DBVPG6765:L>L		YJM975:L>L		YJM978:L>L		YS4:L>L	AA:80		273614X:P>P		DBVPG1373:P>P		DBVPG1788:P>P		DBVPG6040:P>P		DBVPG6765:P>P		YJM975:P>P		YJM978:P>P		YS4:P>P	AA:99		DBVPG1788:T>T		DBVPG6040:T>T		DBVPG6765:T>T		YJM975:T>T		YJM978:T>T		YS4:T>T	AA:117		273614X:I>I		DBVPG1373:I>I		DBVPG1788:I>I		DBVPG6040:I>I		DBVPG6765:I>I		L_1374:I>I		YJM975:I>I		YJM978:I>I		YS4:I>I	AA:136		DBVPG1373:L>L		DBVPG1788:L>L		DBVPG6040:L>L		DBVPG6765:L>L		L_1374:L>L		YJM975:L>L		YJM978:L>L		YS4:L>L	AA:167		UWOPS03_461_4:P>P	AA:206		BC187:A>A		DBVPG1106:A>A		DBVPG1373:A>A		DBVPG1788:A>A		DBVPG6040:A>A		DBVPG6765:A>A		L_1374:A>A		YIIc17_E5:A>A		YJM975:A>A		YJM978:A>A		YS4:A>A	AA:208		BC187:T>T		DBVPG1106:T>T		DBVPG1373:T>T		DBVPG1788:T>T		DBVPG6040:T>T		DBVPG6765:T>T		L_1374:T>T		YIIc17_E5:T>T		YJM975:T>T		YJM978:T>T		YS4:T>T	AA:223		BC187:N>N		DBVPG1106:N>N		DBVPG1373:N>N		DBVPG1788:N>N		DBVPG6040:N>N		DBVPG6765:N>N		L_1374:N>N		YIIc17_E5:N>N		YJM975:N>N		YJM978:N>N		YS4:N>N	AA:331		YIIc17_E5:V>V	AA:354		273614X:K>K		322134S:K>K		378604X:K>K		DBVPG1106:K>K		DBVPG1853:K>K		DBVPG6765:K>K		SK1:K>K		UWOPS03_461_4:K>K		UWOPS05_217_3:K>K		Y55:K>K		YIIc17_E5:K>K		YJM975:K>K		YJM978:K>K		YPS606:K>K	AA:368		273614X:T>T		BC187:T>T		DBVPG1106:T>T		DBVPG1853:T>T		DBVPG6040:T>T		DBVPG6765:T>T		SK1:T>T		UWOPS05_217_3:T>T		Y55:T>T		YIIc17_E5:T>T		YJM975:T>T		YJM978:T>T		YPS606:T>T	AA:410		273614X:T>T		DBVPG1106:T>T		DBVPG1853:T>T		DBVPG6765:T>T		YJM975:T>T		YJM978:T>T		YPS128:D>D		YPS606:D>D	AA:411		SK1:G>G		Y55:G>G		YPS606:G>G	AA:430		273614X:A>A		BC187:A>A		DBVPG1106:A>A		DBVPG1788:A>A		DBVPG1853:A>A		DBVPG6040:A>A		DBVPG6765:A>A		SK1:A>A		Y55:A>A		YIIc17_E5:A>A		YJM975:A>A		YJM978:A>A		YPS606:A>A		YS4:A>A		YS9:A>A	AA:439		DBVPG6040:N>N	AA:445		DBVPG6040:N>N	AA:459		DBVPG6040:H>H	AA:461		273614X:N>N		DBVPG1106:N>N		DBVPG1373:N>N		DBVPG1788:N>N		DBVPG1853:N>N		DBVPG6765:N>N		YJM975:N>N		YJM978:N>N		YS9:N>N	AA:464		DBVPG6040:L>L	AA:468		DBVPG6040:->-ID:YBR093C	AA:19		UWOPS83_787_3:T>T	AA:45		DBVPG1788:G>G		UWOPS83_787_3:G>G	AA:56		DBVPG1788:I>I	AA:59		DBVPG1788:D>D		UWOPS83_787_3:D>D	AA:99		DBVPG1788:T>T		UWOPS83_787_3:T>T	AA:105		322134S:S>S	AA:137		UWOPS83_787_3:N>N	AA:162		DBVPG1788:N>N		UWOPS83_787_3:N>N		YS2:N>N	AA:195		UWOPS05_227_2:L>L	AA:217		DBVPG1373:A>A		DBVPG6765:A>A		L_1528:A>A		UWOPS05_227_2:A>A		YJM975:A>A		YJM978:A>A	AA:242		NCYC361:L>L		YS2:L>L	AA:244		NCYC361:K>K		YS2:K>K	AA:250		NCYC361:N>N		YS2:N>N	AA:269		DBVPG1373:A>A		DBVPG6765:A>A		L_1528:A>A		YJM975:A>A		YJM978:A>A	AA:275		NCYC361:V>V		YS2:V>V	AA:278		NCYC361:I>I		YS2:I>I	AA:281		YS2:K>K	AA:295		378604X:T>T		DBVPG1106:T>T		DBVPG1373:T>T		DBVPG6044:T>T		DBVPG6765:T>T		L_1528:T>T		UWOPS05_227_2:T>T		Y55:T>T		YJM975:T>T		YJM978:T>T		YS9:T>T	AA:299		DBVPG1106:E>E		DBVPG1373:E>E		DBVPG6044:E>E		DBVPG6765:E>E		L_1528:E>E		Y55:E>E		YJM975:E>E		YJM978:E>E	AA:304		DBVPG1106:D>D		DBVPG6765:D>D		L_1528:D>D		Y55:D>D		YJM975:D>D		YJM978:D>D	AA:385		YPS606:K>K	AA:402		DBVPG1106:V>V		DBVPG1788:V>V		DBVPG6765:V>V		L_1528:V>V		SK1:V>V		UWOPS87_2421:V>V		Y55:V>V		YJM975:V>V		YJM978:V>V		YS9:V>VID:YBR094W	AA:50		BC187:K>K		DBVPG1106:K>K		DBVPG1788:K>K		DBVPG6765:K>K		L_1374:K>K		L_1528:K>K		YIIc17_E5:K>K		YJM981:K>K		YS4:K>K	AA:81		UWOPS03_461_4:P>P		YPS606:P>P	AA:83		273614X:I>I		BC187:I>I		DBVPG1106:I>I		DBVPG1788:I>I		DBVPG6040:I>I		DBVPG6765:I>I		L_1374:I>I		L_1528:I>I		YJM981:I>I		YS4:I>I	AA:98		273614X:I>I		BC187:I>I		DBVPG1106:I>I		DBVPG1788:I>I		DBVPG6040:I>I		DBVPG6765:I>I		L_1374:I>I		L_1528:I>I		Y55:I>I		YJM981:I>I		YS4:I>I	AA:107		273614X:L>L		BC187:L>L		DBVPG1106:L>L		DBVPG1373:L>L		DBVPG1788:L>L		DBVPG6040:L>L		DBVPG6765:L>L		L_1374:L>L		L_1528:L>L		UWOPS03_461_4:L>L		Y55:L>L		YJM981:L>L		YPS128:L>L		YPS606:L>L		YS4:L>L	AA:117		273614X:N>N		BC187:N>N		DBVPG1106:N>N		DBVPG1373:N>N		DBVPG1788:N>N		DBVPG6040:N>N		DBVPG6765:N>N		L_1374:N>N		L_1528:N>N		S288c:N>N		UWOPS03_461_4:N>N		W303:N>N		YGPM:N>N		YJM981:N>N		YPS128:N>N		YPS606:N>N		YS4:N>N		YS9:N>N	AA:154		273614X:G>G		DBVPG1373:G>G		DBVPG1788:G>G		DBVPG6040:G>G		DBVPG6765:G>G		L_1374:G>G		L_1528:G>G		SK1:G>G		UWOPS03_461_4:G>G		UWOPS05_217_3:G>G		Y55:G>G		YJM981:G>G		YS4:G>G	AA:208		SK1:D>D		UWOPS03_461_4:D>D		UWOPS05_217_3:D>D		UWOPS05_227_2:D>D		Y55:D>D		YPS128:D>D		YPS606:D>D	AA:244		SK1:H>H		UWOPS03_461_4:H>H		UWOPS05_217_3:H>H		UWOPS05_227_2:H>H		YPS128:H>H		YPS606:H>H	AA:260		DBVPG6040:S>S	AA:262		SK1:S>S		UWOPS05_217_3:S>S		UWOPS05_227_2:S>S		YPS128:S>S		YPS606:S>S	AA:313		273614X:V>V		DBVPG1106:V>V		DBVPG1373:V>V		DBVPG1788:V>V		DBVPG6040:V>V		DBVPG6765:V>V		L_1528:V>V		SK1:V>V		UWOPS05_217_3:V>V		UWOPS05_227_2:V>V		Y55:V>V		YPS128:V>V		YPS606:V>V		YS4:V>V	AA:350		UWOPS05_227_2:L>L	AA:354		DBVPG6044:L>L		SK1:L>L		Y55:L>L	AA:384		DBVPG1106:Y>Y		DBVPG1373:Y>Y		DBVPG1788:Y>Y		DBVPG1853:Y>Y		DBVPG6765:Y>Y		L_1528:Y>Y		YS4:Y>Y	AA:387		DBVPG1106:Y>Y		DBVPG1373:Y>Y		DBVPG1788:Y>Y		DBVPG1853:Y>Y		DBVPG6044:Y>Y		DBVPG6765:Y>Y		L_1528:Y>Y		SK1:Y>Y		UWOPS05_227_2:Y>Y		UWOPS83_787_3:Y>Y		Y55:Y>Y		YPS606:Y>Y		YS4:Y>Y	AA:395		UWOPS83_787_3:L>L	AA:397		UWOPS83_787_3:E>E	AA:399		DBVPG1106:P>P		DBVPG1373:P>P		DBVPG1788:P>P		DBVPG1853:P>P		DBVPG6044:P>P		DBVPG6765:P>P		L_1528:P>P		SK1:P>P		UWOPS05_227_2:P>P		UWOPS83_787_3:P>P		Y55:P>P		YPS606:P>P		YS4:P>P	AA:413		DBVPG6044:L>L		SK1:L>L		Y55:L>L	AA:535		DBVPG1373:N>N		DBVPG1788:N>N		DBVPG1853:N>N		L_1528:N>N		UWOPS87_2421:N>N		YJM975:N>N		YS4:N>N	AA:552		DBVPG1373:L>L	AA:607		DBVPG6044:L>L		DBVPG6765:L>L		K11:L>L		SK1:L>L		UWOPS03_461_4:L>L		UWOPS83_787_3:L>L		Y55:L>L		YPS128:L>L		YPS606:L>L	AA:685		YJM975:S>S	AA:725		SK1:P>P		Y55:P>PID:YBR095C	AA:10		DBVPG6044:N>N		NCYC361:N>N		SK1:N>N		UWOPS83_787_3:N>N		Y55:N>N		YPS128:N>N		YPS606:N>N	AA:59		322134S:G>G	AA:98		YS2:H>H	AA:146		DBVPG1373:E>E		DBVPG6765:E>E		L_1374:E>E		L_1528:E>E		UWOPS83_787_3:E>E		UWOPS87_2421:E>E		YIIc17_E5:E>E		YJM975:E>E		YJM978:E>E		YS2:E>E		YS9:E>E	AA:166		DBVPG6765:L>L		L_1374:L>L		UWOPS83_787_3:L>L		UWOPS87_2421:L>L		YS9:L>L	AA:241		DBVPG1373:T>T		DBVPG6765:T>T		L_1374:T>T		UWOPS83_787_3:T>T		YIIc17_E5:T>T		YJM975:T>T		YJM978:T>T	AA:354		378604X:K>K	AA:405		378604X:F>F		DBVPG6044:F>F		NCYC110:F>F		SK1:F>F		UWOPS05_227_2:F>F		UWOPS83_787_3:F>F		Y55:F>F		YPS128:F>F		YPS606:F>F	AA:423		378604X:P>P		NCYC110:P>P		SK1:P>P		UWOPS05_227_2:P>P		Y55:P>P		YPS128:P>P		YPS606:P>PID:YBR096W	AA:33		273614X:Y>Y		BC187:Y>Y		DBVPG1788:Y>Y		DBVPG6765:Y>Y		L_1374:Y>Y		L_1528:Y>Y		SK1:Y>Y		UWOPS03_461_4:Y>Y		UWOPS05_227_2:Y>Y		UWOPS83_787_3:Y>Y		UWOPS87_2421:Y>Y		Y55:Y>Y		YIIc17_E5:Y>Y		YPS128:Y>Y		YPS606:Y>Y		YS4:Y>Y	AA:72		SK1:S>S		Y55:S>S		YPS128:S>S		YPS606:S>S	AA:180		322134S:I>I	AA:209		UWOPS03_461_4:E>E		UWOPS05_227_2:E>E		YPS128:E>E		YPS606:E>E	AA:212		Y9:G>GID:YBR097W	AA:21		273614X:I>	AA:68		DBVPG1106:F>F		DBVPG1373:F>F		DBVPG1788:F>F		DBVPG1853:F>F		DBVPG6044:F>F		DBVPG6765:F>F		L_1374:F>F		L_1528:F>F		UWOPS03_461_4:F>F		UWOPS05_227_2:F>F		UWOPS83_787_3:F>F		Y55:F>F		YJM975:F>F		YJM978:F>F		YJM981:F>F		YPS128:F>F		YPS606:F>F		YS2:F>F		YS4:F>F		YS9:F>F	AA:78		UWOPS03_461_4:K>K		UWOPS05_227_2:K>K	AA:79		DBVPG1106:L>L		DBVPG1373:L>L		DBVPG1788:L>L		DBVPG1853:L>L		DBVPG6765:L>L		L_1374:L>L		L_1528:L>L		YJM975:L>L		YJM978:L>L		YJM981:L>L		YS2:L>L		YS4:L>L		YS9:L>L	AA:115		DBVPG1373:L>L		DBVPG1788:L>L		DBVPG1853:L>L		DBVPG6765:L>L		L_1374:L>L		L_1528:L>L		UWOPS03_461_4:L>L		UWOPS05_227_2:L>L		UWOPS87_2421:L>L		YJM975:L>L		YJM978:L>L		YPS128:L>L		YPS606:L>L		YS2:L>L		YS4:L>L		YS9:L>L	AA:119		DBVPG1373:L>L		DBVPG1788:L>L		DBVPG1853:L>L		DBVPG6765:L>L		L_1374:L>L		L_1528:L>L		UWOPS03_461_4:L>L		UWOPS05_227_2:L>L		UWOPS87_2421:L>L		YJM975:L>L		YJM978:L>L		YPS128:L>L		YPS606:L>L		YS2:L>L		YS4:L>L		YS9:L>L	AA:212		DBVPG6044:S>S		NCYC110:S>S		SK1:S>S		Y55:S>S	AA:218		YS9:T>T	AA:251		DBVPG6044:S>S		NCYC110:S>S		SK1:S>S		Y55:S>S	AA:264		DBVPG6044:E>E		NCYC110:E>E		SK1:E>E		Y55:E>E	AA:268		273614X:T>T		DBVPG1373:T>T		DBVPG1788:T>T		DBVPG1853:T>T		DBVPG6044:T>T		DBVPG6765:T>T		L_1374:T>T		L_1528:T>T		NCYC110:T>T		SK1:T>T		UWOPS05_227_2:T>T		UWOPS83_787_3:T>T		UWOPS87_2421:T>T		Y12:T>T		Y55:T>T		Y9:T>T		YPS128:T>T		YS9:T>T	AA:392		273614X:T>T		BC187:T>T		DBVPG1373:T>T		DBVPG1788:T>T		DBVPG1853:T>T		DBVPG6765:T>T		L_1374:T>T		L_1528:T>T		YJM975:T>T		YJM978:T>T	AA:450		273614X:A>A		BC187:A>A		DBVPG1373:A>A		DBVPG1788:A>A		DBVPG1853:A>A		DBVPG6765:A>A		L_1374:A>A		L_1528:A>A		YIIc17_E5:A>A		YJM975:A>A		YJM978:A>A		YPS128:A>A	AA:491		Y12:V>V		Y9:V>V	AA:514		DBVPG6040:L>L		Y12:L>L		Y9:L>L	AA:544		DBVPG1373:N>N		DBVPG1788:N>N		DBVPG6044:N>N		DBVPG6765:N>N		L_1374:N>N		L_1528:N>N		NCYC110:N>N		SK1:N>N		UWOPS05_227_2:N>N		Y55:N>N		YIIc17_E5:N>N		YJM975:N>N		YJM978:N>N		YPS128:N>N		YPS606:N>N		YS4:N>N	AA:625		UWOPS05_227_2:I>I	AA:640		DBVPG1373:I>I		DBVPG1788:I>I		DBVPG6765:I>I		UWOPS87_2421:I>I		YIIc17_E5:I>I		YJM975:I>I		YS4:I>I	AA:668		322134S:T>T		DBVPG1373:T>T		DBVPG1788:T>T		DBVPG6040:T>T		DBVPG6044:T>T		DBVPG6765:T>T		NCYC110:T>T		SK1:T>T		UWOPS05_227_2:T>T		UWOPS87_2421:T>T		Y12:T>T		Y55:T>T		Y9:T>T		YIIc17_E5:T>T		YJM975:T>T		YPS128:T>T		YPS606:T>T		YS2:T>T		YS4:T>T	AA:670		Y9:S>S	AA:695		DBVPG6044:Y>Y		NCYC110:Y>Y		SK1:Y>Y		Y55:Y>Y	AA:710		DBVPG6044:P>P		NCYC110:P>P		SK1:P>P		Y55:P>P	AA:747		322134S:E>E		BC187:E>E		DBVPG1373:E>E		DBVPG6044:E>E		DBVPG6765:E>E		NCYC110:E>E		SK1:E>E		UWOPS03_461_4:E>E		UWOPS05_227_2:E>E		UWOPS87_2421:E>E		Y55:E>E		YJM975:E>E		YPS128:E>E		YPS606:E>E		YS4:E>E	AA:846		BC187:L>L		DBVPG1106:L>L		DBVPG1373:L>L		DBVPG6044:L>L		DBVPG6765:L>L		NCYC110:L>L		SK1:L>L		UWOPS03_461_4:L>L		UWOPS05_227_2:L>L		Y55:L>L		YIIc17_E5:L>L		YJM975:L>L		YS4:L>L	AA:877		UWOPS03_461_4:V>V		UWOPS05_227_2:V>V	AA:879		UWOPS87_2421:R>R	AA:962		DBVPG1788:Q>Q		UWOPS87_2421:Q>Q	AA:979		Y12:S>S		Y9:S>S	AA:1081		DBVPG1788:N>N		UWOPS87_2421:N>N	AA:1090		DBVPG1788:P>P		DBVPG1853:P>P		DBVPG6040:P>P		DBVPG6765:P>P		L_1528:P>P		UWOPS87_2421:P>P		YJM975:P>P		YJM978:P>P		YS2:P>P		YS4:P>P		YS9:P>P	AA:1129		NCYC110:S>S		SK1:S>S		Y55:S>S	AA:1133		BC187:Q>Q		DBVPG1788:Q>Q		DBVPG1853:Q>Q		DBVPG6040:Q>Q		DBVPG6765:Q>Q		UWOPS87_2421:Q>Q		YJM975:Q>Q		YJM978:Q>Q		YS4:Q>Q		YS9:Q>Q	AA:1135		UWOPS03_461_4:T>T	AA:1144		Y12:A>A		Y9:A>A	AA:1287		DBVPG6044:G>G		SK1:G>G		Y55:G>G	AA:1301		UWOPS03_461_4:F>F		UWOPS05_227_2:F>FID:YBR098W	AA:2		DBVPG1788:S>S		L_1374:S>S	AA:17		DBVPG6044:A>A		SK1:A>A		Y55:A>A	AA:28		Y9:V>V	AA:31		DBVPG6040:I>I		K11:I>I		Y9:I>I	AA:40		Y9:D>D	AA:56		DBVPG1788:S>S	AA:86		273614X:S>S		BC187:S>S		DBVPG1373:S>S		DBVPG1788:S>S		DBVPG1853:S>S		DBVPG6765:S>S		L_1528:S>S		YIIc17_E5:S>S		YS4:S>S	AA:153		DBVPG1788:G>G	AA:185		UWOPS05_217_3:P>P		UWOPS05_227_2:P>P	AA:233		YJM975:Q>Q	AA:245		BC187:R>R		DBVPG1373:R>R		DBVPG1788:R>R		DBVPG1853:R>R		DBVPG6044:R>R		DBVPG6765:R>R		L_1528:R>R		NCYC110:R>R		UWOPS03_461_4:R>R		UWOPS05_217_3:R>R		UWOPS05_227_2:R>R		Y12:R>R		Y55:R>R		Y9:R>R		YIIc17_E5:R>R		YJM975:R>R		YJM978:R>R		YPS128:R>R		YS4:R>R	AA:302		NCYC361:T>T	AA:399		DBVPG6044:I>I		NCYC110:I>I		SK1:I>I	AA:407		UWOPS03_461_4:L>L		UWOPS05_227_2:L>L	AA:417		YJM978:L>L	AA:435		273614X:P>P		322134S:P>P		BC187:P>P		DBVPG1373:P>P		DBVPG1788:P>P		DBVPG6044:P>P		DBVPG6765:P>P		L_1528:P>P		NCYC110:P>P		SK1:P>P		UWOPS05_227_2:P>P		YIIc17_E5:P>P		YJM975:P>P		YS9:P>P	AA:438		UWOPS05_227_2:R>R	AA:455		DBVPG6044:Y>Y		SK1:Y>Y	AA:551		DBVPG6040:L>L	AA:552		UWOPS03_461_4:G>G		UWOPS05_227_2:G>G	AA:677		378604X:L>L		DBVPG6040:L>L		Y12:L>L		Y9:L>L		YS2:L>LID:YBR099C	AA:46		UWOPS03_461_4:I>I		UWOPS05_227_2:I>IID:YBR101C	AA:10		DBVPG6044:A>A		K11:A>A		NCYC110:A>A		SK1:A>A		UWOPS05_217_3:A>A		Y12:A>A		Y55:A>A		YPS606:A>A	AA:44		DBVPG6044:L>L		K11:L>L		NCYC110:L>L		SK1:L>L		UWOPS05_217_3:L>L		Y12:L>L		Y55:L>L		YPS606:L>L	AA:112		K11:G>G		NCYC110:G>G		SK1:G>G		UWOPS03_461_4:G>G		UWOPS05_217_3:G>G		UWOPS05_227_2:G>G		Y12:G>G		Y55:G>G		YPS606:G>G	AA:117		K11:N>N		NCYC110:N>N		SK1:N>N		UWOPS03_461_4:N>N		UWOPS05_217_3:N>N		UWOPS05_227_2:N>N		Y12:N>N		Y55:N>N		YPS606:N>N	AA:163		K11:H>H		SK1:H>H		UWOPS03_461_4:H>H		UWOPS05_217_3:H>H		UWOPS05_227_2:H>H		Y12:H>H		Y55:H>H		YPS606:H>H	AA:171		K11:F>F		SK1:F>F		UWOPS03_461_4:F>F		UWOPS05_217_3:F>F		UWOPS05_227_2:F>F		Y12:F>F		Y55:F>F		YPS606:F>F	AA:188		UWOPS03_461_4:A>A		UWOPS05_217_3:A>A		UWOPS05_227_2:A>A	AA:216		UWOPS03_461_4:V>V		UWOPS05_217_3:V>V		UWOPS05_227_2:V>VID:YBR104W	AA:7		UWOPS05_217_3:T>T		UWOPS83_787_3:T>T	AA:74		YPS128:V>V	AA:237		273614X:I>I		BC187:I>I		DBVPG1106:I>I		DBVPG1373:I>I		DBVPG1788:I>I		DBVPG6040:I>I		DBVPG6765:I>I		L_1374:I>I		Y55:I>I		YJM975:I>I		YJM978:I>I	AA:278		K11:K>K		Y9:K>KID:YBR105C	AA:33		DBVPG1788:A>A	AA:117		UWOPS05_217_3:T>T		UWOPS05_227_2:T>T	AA:146		YPS128:L>L		YPS606:L>L	AA:156		DBVPG6044:C>C		SK1:C>C		UWOPS87_2421:C>C		Y55:C>C	AA:172		273614X:L>L		322134S:L>L		DBVPG1106:L>L		DBVPG1373:L>L		DBVPG6765:L>L	AA:176		DBVPG6765:H>H	AA:189		DBVPG6044:N>N		SK1:N>N		Y55:N>N	AA:199		273614X:S>S		322134S:S>S		DBVPG1106:S>S		DBVPG1373:S>S		DBVPG6044:S>S		DBVPG6765:S>S		K11:S>S		NCYC110:S>S		SK1:S>S		YPS128:S>S		YPS606:S>S	AA:209		DBVPG6044:F>F		NCYC110:F>F		YPS128:F>F		YPS606:F>F	AA:231		DBVPG6044:L>L		NCYC110:L>L	AA:265		DBVPG6044:D>D		NCYC110:D>D		SK1:D>D	AA:282		273614X:Y>Y		322134S:Y>Y		DBVPG1106:Y>Y		DBVPG1373:Y>Y		DBVPG6044:Y>Y		DBVPG6765:Y>Y		NCYC110:Y>Y		SK1:Y>Y		UWOPS05_217_3:Y>Y		YJM975:Y>Y		YPS128:Y>Y		YPS606:Y>Y		YS9:Y>Y	AA:344		273614X:L>L		322134S:L>L		DBVPG1106:L>L		DBVPG1373:L>L		DBVPG6044:L>L		DBVPG6765:L>L		NCYC110:L>L		SK1:L>L		UWOPS05_217_3:L>L		YJM975:L>L		YPS606:L>L		YS9:L>LID:YBR106W	AA:48		378604X:Q>Q	AA:78		273614X:K>K		378604X:K>K		DBVPG1106:K>K		DBVPG1788:K>K		DBVPG6765:K>K		K11:K>K		L_1374:K>K		NCYC361:K>K		SK1:K>K		UWOPS03_461_4:K>K		UWOPS05_227_2:K>K		UWOPS87_2421:K>K		YJM978:K>K		YJM981:K>K		YPS128:K>K		YPS606:K>K		YS9:K>K	AA:146		SK1:T>T		Y55:T>T	AA:169		378604X:T>T	AA:179		DBVPG1373:R>R		DBVPG1788:R>R		DBVPG6765:R>R		L_1374:R>R		YJM978:R>R		YJM981:R>R		YS9:R>RID:YBR107C	AA:4		273614X:T>T	AA:65		273614X:L>L		BC187:L>L		DBVPG6044:L>L		DBVPG6765:L>L		L_1374:L>L		L_1528:L>L		NCYC110:L>L		SK1:L>L		Y55:L>L		YJM981:L>L		YPS128:L>L		YPS606:L>L		YS4:L>L	AA:87		273614X:L>L		BC187:L>L		DBVPG6044:L>L		DBVPG6765:L>L		L_1374:L>L		L_1528:L>L		NCYC110:L>L		SK1:L>L		UWOPS03_461_4:L>L		UWOPS05_217_3:L>L		UWOPS05_227_2:L>L		UWOPS83_787_3:L>L		Y55:L>L		YJM975:L>L		YJM981:L>L		YPS128:L>L		YPS606:L>L		YS4:L>L	AA:90		273614X:C>C		BC187:C>C		DBVPG6765:C>C		L_1374:C>C		L_1528:C>C		YJM975:C>C		YJM981:C>C		YS4:C>C	AA:108		DBVPG6044:G>G		SK1:G>G		UWOPS03_461_4:G>G		UWOPS05_227_2:G>G		UWOPS83_787_3:G>G		Y55:G>G		YPS128:G>G		YPS606:G>G	AA:132		273614X:V>V		BC187:V>V		DBVPG1373:V>V		DBVPG1853:V>V		DBVPG6765:V>V		L_1374:V>V		L_1528:V>V		YJM975:V>V		YJM981:V>V		YS4:V>V		YS9:V>V	AA:161		UWOPS83_787_3:F>F		UWOPS87_2421:F>F	AA:168		BC187:E>E		DBVPG1853:E>E		DBVPG6765:E>E		L_1374:E>E		L_1528:E>E	AA:170		273614X:C>C		BC187:C>C		DBVPG1106:C>C		DBVPG1373:C>C		DBVPG1853:C>C		DBVPG6765:C>C		L_1374:C>C		L_1528:C>C		YJM975:C>C		YS4:C>CID:YBR109C	AA:47		UWOPS87_2421:A>A	AA:87		273614X:L>L		322134S:L>L		BC187:L>L		DBVPG1373:L>L		DBVPG1788:L>L		DBVPG6044:L>L		DBVPG6765:L>L		L_1374:L>L		L_1528:L>L		SK1:L>L		Y55:L>L		YJM981:L>L		YPS128:L>L		YPS606:L>L		YS9:L>L	AA:123		YS9:D>DID:YBR110W	AA:16		273614X:Y>Y		322134S:Y>Y		BC187:Y>Y		DBVPG1853:Y>Y		DBVPG6044:Y>Y		DBVPG6765:Y>Y		L_1528:Y>Y		SK1:Y>Y		UWOPS83_787_3:Y>Y		UWOPS87_2421:Y>Y		Y55:Y>Y	AA:29		DBVPG1853:Y>Y	AA:45		UWOPS83_787_3:V>V		UWOPS87_2421:V>V	AA:59		322134S:A>A		BC187:A>A		DBVPG1106:A>A		DBVPG1853:A>A		DBVPG6044:A>A		DBVPG6765:A>A		L_1528:A>A		SK1:A>A		UWOPS05_217_3:A>A		UWOPS83_787_3:A>A		UWOPS87_2421:A>A		Y55:A>A		YJM981:A>A		YPS128:A>A		YPS606:A>A		YS4:A>A	AA:74		BC187:Y>Y		DBVPG1106:Y>Y		DBVPG1853:Y>Y		DBVPG6044:Y>Y		DBVPG6765:Y>Y		L_1528:Y>Y		SK1:Y>Y		UWOPS83_787_3:Y>Y		Y55:Y>Y		YJM981:Y>Y		YS4:Y>Y	AA:146		YS9:I>I	AA:225		DBVPG1373:Y>Y	AA:227		YPS128:R>R		YPS606:R>R	AA:238		DBVPG6044:D>D		SK1:D>D		Y55:D>D	AA:267		BC187:I>I		DBVPG1106:I>I		DBVPG1373:I>I		DBVPG1853:I>I		DBVPG6765:I>I		UWOPS87_2421:I>I		YJM981:I>I	AA:332		UWOPS87_2421:I>	AA:364		SK1:L>L		Y55:L>L	AA:444		YJM975:D>D		YJM981:D>D	AA:450		DBVPG1373:->-ID:YBR111C	AA:19		273614X:T>T		322134S:T>T		BC187:T>T		DBVPG1853:T>T		DBVPG6765:T>T		L_1374:T>T		L_1528:T>T		YJM981:T>T		YS4:T>T		YS9:T>T	AA:24		DBVPG6044:K>K		Y55:K>K	AA:52		273614X:I>I		322134S:I>I		BC187:I>I		DBVPG1853:I>I		DBVPG6765:I>I		L_1374:I>I		UWOPS03_461_4:I>I		UWOPS05_217_3:I>I		UWOPS05_227_2:I>I		UWOPS87_2421:I>I		Y55:I>I		YJM978:I>I		YJM981:I>I		YPS128:I>I		YPS606:I>I		YS4:I>I		YS9:I>I	AA:78		UWOPS03_461_4:D>D		UWOPS05_217_3:D>D		UWOPS05_227_2:D>D	AA:84		YPS128:T>T		YPS606:T>T	AA:105		SK1:P>P		Y55:P>P	AA:163		UWOPS03_461_4:V>V		UWOPS05_217_3:V>V	AA:192		DBVPG1106:V>V		DBVPG1373:V>V		DBVPG1788:V>V		DBVPG6765:V>V		K11:V>V		L_1374:V>V		SK1:V>V		UWOPS03_461_4:V>V		UWOPS05_217_3:V>V		UWOPS87_2421:V>V		Y12:V>V		Y55:V>V		YJM978:V>V		YJM981:V>V		YPS606:V>V	AA:196		UWOPS87_2421:K>KID:YBR115C	AA:131		K11:L>L	AA:166		322134S:T>T		DBVPG1106:T>T		DBVPG1373:T>T		DBVPG6044:T>T		DBVPG6765:T>T		K11:T>T		L_1528:T>T		SK1:T>T		UWOPS03_461_4:T>T		UWOPS05_217_3:T>T		UWOPS83_787_3:T>T		UWOPS87_2421:T>T		Y55:T>T		YJM978:T>T		YJM981:T>T	AA:197		Y9:Y>Y	AA:278		378604X:T>T		UWOPS05_217_3:T>T		Y12:T>T		Y9:T>T		YPS606:T>T	AA:283		378604X:A>A		UWOPS05_217_3:A>A		Y12:A>A		Y9:A>A		YPS128:A>A		YPS606:A>A	AA:289		378604X:T>T		SK1:T>T		UWOPS05_217_3:T>T		Y12:T>T		Y55:T>T		Y9:T>T		YPS128:T>T		YPS606:T>T	AA:358		378604X:V>V		DBVPG1853:V>V		DBVPG6765:V>V		L_1528:V>V		SK1:V>V		UWOPS05_217_3:V>V		UWOPS05_227_2:V>V		Y55:V>V		Y9:V>V		YIIc17_E5:V>V		YPS128:V>V		YPS606:V>V		YS2:V>V	AA:364		UWOPS83_787_3:D>D	AA:403		DBVPG1853:D>D		DBVPG6765:D>D	AA:507		UWOPS83_787_3:V>V	AA:536		DBVPG1853:G>G		DBVPG6765:G>G		UWOPS05_227_2:G>G		YIIc17_E5:G>G	AA:580		YPS128:D>D	AA:630		DBVPG6044:A>A		SK1:A>A		UWOPS03_461_4:A>A		UWOPS05_217_3:A>A		Y55:A>A	AA:680		273614X:T>T		DBVPG1853:T>T		DBVPG6044:T>T		DBVPG6765:T>T		SK1:T>T		UWOPS03_461_4:T>T		UWOPS05_217_3:T>T		Y55:T>T	AA:819		378604X:P>P	AA:827		BC187:K>K	AA:840		378604X:T>T		DBVPG6044:T>T		NCYC110:T>T		SK1:T>T	AA:975		273614X:V>V		BC187:V>V		DBVPG1106:V>V		DBVPG1373:V>V		DBVPG1788:V>V		DBVPG1853:V>V		DBVPG6765:V>V		YJM978:V>V		YS4:V>V	AA:1008		378604X:A>A		NCYC110:A>A		SK1:A>A		Y55:A>A		YIIc17_E5:A>A		YS2:A>A	AA:1017		378604X:R>R		YS2:R>R	AA:1018		DBVPG1106:L>L		DBVPG1373:L>L		DBVPG1788:L>L		DBVPG1853:L>L		DBVPG6765:L>L		UWOPS03_461_4:L>L		YS4:L>L	AA:1046		YIIc17_E5:S>S	AA:1067		UWOPS03_461_4:H>H	AA:1081		YJM975:L>L	AA:1106		DBVPG1373:F>F		DBVPG1853:F>F		DBVPG6044:F>F		DBVPG6765:F>F		SK1:F>F		UWOPS03_461_4:F>F		Y12:F>F		Y55:F>F		YIIc17_E5:F>F		YJM975:F>F		YPS128:F>F		YPS606:F>F		YS2:F>F		YS4:F>F	AA:1115		DBVPG1373:T>T		DBVPG1853:T>T		DBVPG6765:T>T		YJM975:T>T		YS2:T>T		YS4:T>T	AA:1130		378604X:K>K		DBVPG1373:K>K		DBVPG1853:K>K		DBVPG6044:K>K		DBVPG6765:K>K		SK1:K>K		UWOPS03_461_4:K>K		Y12:K>K		Y55:K>K		YIIc17_E5:K>K		YJM975:K>K		YPS606:K>K		YS2:K>K		YS4:K>K		YS9:K>K	AA:1163		UWOPS83_787_3:R>R	AA:1232		UWOPS83_787_3:N>N	AA:1259		378604X:L>L		DBVPG1373:L>L		DBVPG1788:L>L		DBVPG6044:L>L		DBVPG6765:L>L		SK1:L>L		UWOPS03_461_4:L>L		Y12:L>L		Y55:L>L		YJM975:L>L		YS4:L>L		YS9:L>L	AA:1277		YIIc17_E5:S>S	AA:1291		DBVPG1373:L>L		DBVPG1788:L>L		DBVPG6765:L>L		UWOPS03_461_4:L>L		YJM975:L>L		YS4:L>L		YS9:L>L	AA:1306		YS4:T>T	AA:1371		DBVPG1373:L>L		DBVPG1788:L>L		DBVPG6765:L>L		YS4:L>L		YS9:L>L	AA:1387		BC187:G>G		DBVPG1373:G>G		DBVPG1788:G>G		DBVPG6765:G>G		YJM978:G>G	AA:1389		YPS128:S>SID:YBR119W	AA:30		DBVPG6044:N>N		SK1:N>N	AA:86		273614X:E>E		378604X:E>E		BC187:E>E		DBVPG1788:E>E		DBVPG1853:E>E		DBVPG6765:E>E		L_1374:E>E		L_1528:E>E		YJM978:E>E	AA:106		273614X:V>V		BC187:V>V		DBVPG1788:V>V		DBVPG1853:V>V		DBVPG6765:V>V		L_1374:V>V		L_1528:V>V		UWOPS03_461_4:V>V		YS4:V>V	AA:139		DBVPG1853:K>K	AA:168		YS4:R>R	AA:237		BC187:G>G		DBVPG1853:G>G		DBVPG6765:G>G	AA:239		273614X:T>T		BC187:T>T		DBVPG1853:T>T		DBVPG6765:T>T		YIIc17_E5:T>T	AA:242		273614X:L>L		BC187:L>L		DBVPG1853:L>L		DBVPG6765:L>L		YIIc17_E5:L>LID:YBR120C	AA:77		SK1:L>L		Y55:L>L	AA:84		BC187:L>L		DBVPG1373:L>L		DBVPG1788:L>L		DBVPG1853:L>L		L_1528:L>L		YJM981:L>L	AA:105		YPS606:I>I	AA:135		UWOPS03_461_4:Y>Y		UWOPS05_217_3:Y>Y	AA:138		YPS606:R>RID:YBR121C	AA:128		BC187:A>A		DBVPG1106:A>A		DBVPG1853:A>A		DBVPG6044:A>A		DBVPG6765:A>A		L_1374:A>A		L_1528:A>A		NCYC110:A>A		SK1:A>A		UWOPS03_461_4:A>A		UWOPS05_227_2:A>A		Y12:A>A		Y55:A>A		YIIc17_E5:A>A		YJM981:A>A	AA:205		UWOPS03_461_4:R>R	AA:216		NCYC110:G>G		SK1:G>G		UWOPS03_461_4:G>G	AA:224		UWOPS03_461_4:Y>Y		YS9:Y>Y	AA:244		378604X:N>N		Y12:N>N	AA:249		378604X:P>P	AA:257		UWOPS05_227_2:K>K	AA:383		DBVPG1788:Y>Y		DBVPG1853:Y>Y		DBVPG6765:Y>Y	AA:400		UWOPS83_787_3:T>T		UWOPS87_2421:T>T	AA:430		Y9:T>T	AA:468		DBVPG1106:G>G		DBVPG1373:G>G		YIIc17_E5:G>G	AA:473		Y55:K>K	AA:478		UWOPS05_217_3:D>D		UWOPS05_227_2:D>D	AA:553		DBVPG1106:L>L		DBVPG1373:L>L		YIIc17_E5:L>L	AA:566		273614X:V>V		378604X:V>V		DBVPG1106:V>V		DBVPG1373:V>V		DBVPG6765:V>V		K11:V>V		SK1:V>V		UWOPS03_461_4:V>V		UWOPS05_217_3:V>V		Y12:V>V		Y55:V>V		Y9:V>V		YIIc17_E5:V>V		YPS128:V>V		YPS606:V>V		YS2:V>V	AA:569		DBVPG1106:I>I		DBVPG1373:I>I		YIIc17_E5:I>I	AA:636		K11:I>IID:YBR122C	AA:91		273614X:D>D		BC187:D>D		DBVPG1373:D>D		DBVPG1788:D>D		DBVPG1853:D>D		DBVPG6765:D>D		L_1528:D>D		YIIc17_E5:D>D		YJM981:D>D	AA:97		UWOPS03_461_4:S>S	AA:115		SK1:T>T		Y55:T>T	AA:147		DBVPG1853:A>A	AA:178		378604X:->-		K11:->-		UWOPS03_461_4:->-		UWOPS05_217_3:->-		UWOPS05_227_2:->-		YS2:->-ID:YBR123C	AA:4		BC187:E>E		YJM975:E>E	AA:92		DBVPG6044:D>D		NCYC110:D>D		Y55:D>D	AA:185		K11:S>S	AA:329		UWOPS87_2421:R>R		Y9:R>R	AA:353		DBVPG1373:L>L		DBVPG1853:L>L		DBVPG6765:L>L		L_1374:L>L		YJM975:L>L	AA:363		YJM975:G>G	AA:369		273614X:Y>Y		DBVPG1373:Y>Y		DBVPG1853:Y>Y		DBVPG6765:Y>Y		L_1374:Y>Y		YJM975:Y>Y		YS9:Y>Y	AA:375		SK1:D>D		Y55:D>D	AA:432		273614X:R>R		DBVPG1373:R>R		DBVPG1788:R>R		DBVPG1853:R>R		DBVPG6765:R>R		L_1528:R>R		YS9:R>R	AA:462		UWOPS03_461_4:K>K		UWOPS05_217_3:K>K		UWOPS05_227_2:K>K	AA:479		273614X:R>R		DBVPG1373:R>R		DBVPG1788:R>R		DBVPG1853:R>R		DBVPG6765:R>R		L_1528:R>R		YJM981:R>R		YS9:R>R	AA:500		YIIc17_E5:K>K	AA:620		DBVPG1853:D>DID:YBR125C	AA:8		Y12:P>P		Y9:P>P	AA:11		K11:E>E	AA:161		DBVPG1373:I>I	AA:200		DBVPG6044:R>R		NCYC110:R>R		SK1:R>R		Y55:R>R	AA:240		BC187:V>V		DBVPG1788:V>V		DBVPG1853:V>V		DBVPG6044:V>V		DBVPG6765:V>V		K11:V>V		L_1528:V>V		SK1:V>V		UWOPS03_461_4:V>V		UWOPS05_217_3:V>V		UWOPS87_2421:V>V		Y55:V>V		Y9:V>V		YJM981:V>V		YS4:V>V		YS9:V>V	AA:293		UWOPS03_461_4:L>L		UWOPS05_217_3:L>L	AA:309		378604X:C>C		BC187:C>C		DBVPG1788:C>C		DBVPG6044:C>C		DBVPG6765:C>C		K11:C>C		NCYC110:C>C		SK1:C>C		UWOPS03_461_4:C>C		UWOPS05_217_3:C>C		UWOPS87_2421:C>C		Y55:C>C		YJM981:C>C		YS4:C>C		YS9:C>C	AA:327		UWOPS03_461_4:Y>Y		UWOPS05_217_3:Y>YID:YBR126C	AA:43		DBVPG1373:S>S		DBVPG6765:S>S		L_1528:S>S		YJM975:S>S		YJM981:S>S		YS4:S>S	AA:166		DBVPG6044:L>L		NCYC110:L>L		SK1:L>L		Y55:L>L	AA:196		DBVPG1106:I>I		DBVPG1373:I>I		DBVPG6765:I>I		L_1374:I>I		L_1528:I>I		YJM981:I>I		YS2:I>I		YS9:I>I	AA:205		273614X:K>K		378604X:K>K		DBVPG1106:K>K		DBVPG1373:K>K		DBVPG1853:K>K		DBVPG6044:K>K		DBVPG6765:K>K		L_1374:K>K		L_1528:K>K		NCYC110:K>K		SK1:K>K		UWOPS03_461_4:K>K		UWOPS05_217_3:K>K		UWOPS05_227_2:K>K		UWOPS87_2421:K>K		Y55:K>K		YJM981:K>K		YS2:K>K		YS9:K>K	AA:227		273614X:S>S		DBVPG1106:S>S		DBVPG1373:S>S		DBVPG6765:S>S		L_1374:S>S		L_1528:S>S		YJM981:S>S	AA:230		273614X:R>R		378604X:R>R		DBVPG1106:R>R		DBVPG1373:R>R		DBVPG6044:R>R		DBVPG6765:R>R		L_1374:R>R		L_1528:R>R		NCYC110:R>R		SK1:R>R		UWOPS87_2421:R>R		Y55:R>R		YJM981:R>R		YS2:R>R		YS9:R>R	AA:235		273614X:N>N		378604X:N>N		DBVPG1106:N>N		DBVPG1373:N>N		DBVPG1853:N>N		DBVPG6765:N>N		L_1374:N>N		L_1528:N>N		UWOPS87_2421:N>N		YJM981:N>N		YS2:N>N		YS9:N>N	AA:257		DBVPG6044:I>I		SK1:I>I		Y55:I>I	AA:271		DBVPG6044:V>V		SK1:V>V		UWOPS03_461_4:V>V		UWOPS05_217_3:V>V		UWOPS05_227_2:V>V		Y55:V>V	AA:276		UWOPS87_2421:Q>Q		YS2:Q>Q		YS9:Q>Q	AA:314		UWOPS03_461_4:H>H		UWOPS05_217_3:H>H	AA:320		YPS128:K>K	AA:378		UWOPS05_217_3:L>L	AA:380		UWOPS05_217_3:A>A		YPS128:A>A	AA:384		SK1:V>V		Y12:V>V		Y55:V>V	AA:390		273614X:T>T		UWOPS05_217_3:T>T		UWOPS87_2421:T>T		YIIc17_E5:T>T		YJM981:T>T		YS9:T>T	AA:421		UWOPS83_787_3:A>A	AA:483		378604X:T>TID:YBR128C	AA:19		UWOPS87_2421:I>I	AA:39		UWOPS03_461_4:E>E		UWOPS05_217_3:E>E		UWOPS05_227_2:E>E		UWOPS87_2421:E>E		Y9:E>E		YIIc17_E5:E>E		YS4:E>E		YS9:E>E	AA:58		DBVPG6040:D>D		SK1:D>D		UWOPS03_461_4:D>D		UWOPS05_217_3:D>D		UWOPS05_227_2:D>D		Y55:D>D		Y9:D>D		YIIc17_E5:D>D		YPS128:D>D		YS4:D>D	AA:62		UWOPS03_461_4:I>I		UWOPS05_217_3:I>I		UWOPS05_227_2:I>I		Y9:I>I		YIIc17_E5:I>I		YS4:I>I	AA:79		DBVPG6040:L>L		SK1:L>L		UWOPS03_461_4:L>L		UWOPS05_217_3:L>L		UWOPS05_227_2:L>L		Y55:L>L		Y9:L>L		YIIc17_E5:L>L		YPS128:L>L		YS4:L>L	AA:86		SK1:K>K		Y55:K>K		YPS128:K>K	AA:101		YIIc17_E5:R>R	AA:324		YS2:A>AID:YBR129C	AA:70		DBVPG1853:Q>Q	AA:85		378604X:T>T		DBVPG1853:T>T		DBVPG6044:T>T		K11:T>T		SK1:T>T		UWOPS03_461_4:T>T		UWOPS05_217_3:T>T		UWOPS83_787_3:T>T		Y55:T>T		YPS606:T>T	AA:116		322134S:T>T		378604X:T>T		DBVPG1853:T>T		DBVPG6040:T>T		DBVPG6044:T>T		SK1:T>T		UWOPS03_461_4:T>T		UWOPS05_217_3:T>T		UWOPS83_787_3:T>T		Y55:T>T		YS2:T>T	AA:136		DBVPG1853:E>E		YS2:E>E	AA:197		UWOPS83_787_3:L>L	AA:217		YS9:V>V	AA:248		SK1:S>S		Y55:S>SID:YBR130C	AA:102		UWOPS03_461_4:F>F		UWOPS05_217_3:F>F		UWOPS83_787_3:F>F	AA:110		273614X:K>K		322134S:K>K		378604X:K>K		DBVPG6040:K>K		DBVPG6044:K>K		SK1:K>K		UWOPS03_461_4:K>K		UWOPS05_217_3:K>K		UWOPS05_227_2:K>K		UWOPS83_787_3:K>K		YPS128:K>K		YPS606:K>K		YS2:K>K	AA:167		DBVPG6040:L>L		YPS128:L>L	AA:179		378604X:L>L	AA:183		UWOPS05_217_3:I>I		UWOPS05_227_2:I>I	AA:199		DBVPG6040:S>S		YPS128:S>S	AA:274		378604X:R>R		DBVPG6044:R>R		SK1:R>R		UWOPS05_217_3:R>R		UWOPS05_227_2:R>R		UWOPS83_787_3:R>R		Y55:R>R		YS2:R>R	AA:289		DBVPG6040:L>L	AA:297		378604X:V>V		DBVPG6040:V>V		DBVPG6044:V>V		SK1:V>V		UWOPS05_217_3:V>V		UWOPS05_227_2:V>V		UWOPS83_787_3:V>V		Y55:V>V		YS2:V>V	AA:362		YS9:P>P	AA:389		DBVPG6044:N>N		SK1:N>N		Y55:N>NID:YBR132C	AA:7		378604X:T>T		Y12:T>T		Y9:T>T		YPS606:T>T	AA:124		DBVPG1853:L>L		DBVPG6044:L>L		SK1:L>L		UWOPS03_461_4:L>L		Y12:L>L		Y55:L>L		Y9:L>L	AA:152		UWOPS03_461_4:P>P		UWOPS05_227_2:P>P	AA:176		378604X:L>L		DBVPG1788:L>L		DBVPG1853:L>L		DBVPG6044:L>L		DBVPG6765:L>L		L_1374:L>L		SK1:L>L		UWOPS03_461_4:L>L		UWOPS05_227_2:L>L		Y12:L>L		Y55:L>L		YJM975:L>L		YJM978:L>L		YS9:L>L	AA:200		378604X:S>S		DBVPG1788:S>S		DBVPG1853:S>S		DBVPG6044:S>S		DBVPG6765:S>S		L_1374:S>S		SK1:S>S		UWOPS03_461_4:S>S		UWOPS05_227_2:S>S		Y12:S>S		Y55:S>S		YJM975:S>S		YJM978:S>S		YS9:S>S	AA:216		378604X:S>S		DBVPG1788:S>S		DBVPG1853:S>S		DBVPG6044:S>S		DBVPG6765:S>S		L_1374:S>S		SK1:S>S		UWOPS05_227_2:S>S		Y12:S>S		Y55:S>S		YJM975:S>S		YJM978:S>S		YS9:S>S	AA:315		273614X:R>R		322134S:R>R		DBVPG1788:R>R		DBVPG1853:R>R		DBVPG6044:R>R		DBVPG6765:R>R		L_1374:R>R		SK1:R>R		UWOPS03_461_4:R>R		UWOPS05_227_2:R>R		UWOPS83_787_3:R>R		Y12:R>R		Y55:R>R		YIIc17_E5:R>R		YJM975:R>R		YJM978:R>R		YPS128:R>R	AA:342		UWOPS03_461_4:V>V		UWOPS05_227_2:V>V		UWOPS83_787_3:V>V		Y12:V>V		YPS128:V>V	AA:360		YS2:A>A	AA:389		273614X:L>L		BC187:L>L		L_1374:L>L		L_1528:L>L		UWOPS05_217_3:L>L	AA:486		322134S:H>H		DBVPG1106:H>H		DBVPG1853:H>H		DBVPG6044:H>H		DBVPG6765:H>H		L_1374:H>H		L_1528:H>H		NCYC361:H>H		SK1:H>H		UWOPS03_461_4:H>H		UWOPS05_217_3:H>H		UWOPS05_227_2:H>H		UWOPS83_787_3:H>H		Y55:H>H		Y9:H>H		YIIc17_E5:H>H		YJM975:H>H		YPS128:H>H		YPS606:H>H		YS2:H>H		YS9:H>H	AA:502		322134S:Q>Q		DBVPG1106:Q>Q		DBVPG6765:Q>Q		L_1374:Q>Q		L_1528:Q>Q		NCYC361:Q>Q		UWOPS05_217_3:Q>Q		YIIc17_E5:Q>Q		YJM975:Q>Q		YS9:Q>Q	AA:506		Y9:A>A	AA:536		Y9:L>L	AA:549		DBVPG1853:Y>Y		UWOPS03_461_4:Y>Y		UWOPS05_217_3:Y>Y		UWOPS05_227_2:Y>Y		UWOPS83_787_3:Y>Y	AA:589		Y9:K>KID:YBR133C	AA:107		273614X:S>S		322134S:S>S		DBVPG1373:S>S		DBVPG1788:S>S		DBVPG6765:S>S		L_1528:S>S		YJM975:S>S		YJM978:S>S		YJM981:S>S		YS9:S>S	AA:134		YPS606:R>R	AA:147		UWOPS03_461_4:R>R		UWOPS05_217_3:R>R		UWOPS05_227_2:R>R	AA:181		YS9:P>P	AA:218		YPS606:R>R	AA:317		YPS606:S>S	AA:364		273614X:E>E		DBVPG6044:E>E		DBVPG6765:E>E		L_1374:E>E		L_1528:E>E		SK1:E>E		UWOPS05_217_3:E>E		UWOPS05_227_2:E>E		Y55:E>E		YIIc17_E5:E>E		YJM978:E>E		YPS606:E>E		YS4:E>E	AA:443		YPS128:T>T		YPS606:T>T	AA:487		YS4:H>H	AA:569		SK1:L>L		UWOPS05_217_3:L>L		Y55:L>L	AA:659		BC187:L>L		DBVPG1106:L>L		DBVPG1373:L>L		DBVPG6765:L>L		YJM975:L>L		YJM981:L>L	AA:661		378604X:S>S		BC187:S>S		DBVPG1106:S>S		DBVPG1373:S>S		DBVPG6044:S>S		DBVPG6765:S>S		SK1:S>S		UWOPS05_217_3:S>S		UWOPS05_227_2:S>S		UWOPS87_2421:S>S		Y12:S>S		Y55:S>S		YJM975:S>S		YJM981:S>S		YPS128:S>S	AA:688		322134S:Y>Y		BC187:Y>Y		DBVPG1106:Y>Y		DBVPG1373:Y>Y		DBVPG6765:Y>Y		YJM975:Y>Y		YJM981:Y>Y	AA:744		322134S:L>L		BC187:L>L		DBVPG1373:L>L		DBVPG6765:L>L		L_1528:L>L		UWOPS87_2421:L>L		YJM975:L>L		YJM978:L>L	AA:750		UWOPS87_2421:G>G	AA:753		UWOPS87_2421:S>S	AA:766		322134S:P>P		BC187:P>P		DBVPG1373:P>P		DBVPG1788:P>P		DBVPG6765:P>P		L_1528:P>P		UWOPS87_2421:P>P		YJM975:P>P		YJM978:P>P	AA:797		DBVPG6044:L>L		SK1:L>L		Y55:L>L	AA:819		UWOPS87_2421:C>CID:YBR135W	AA:32		DBVPG1373:P>P	AA:40		378604X:E>E		SK1:E>E		Y55:E>E		YPS128:E>E	AA:47		378604X:P>P		SK1:P>P		Y55:P>P		YPS128:P>P	AA:92		378604X:A>A		SK1:A>A		UWOPS05_227_2:A>A		Y55:A>A		YPS128:A>A		YS4:A>A	AA:124		YPS128:Q>Q		YPS606:Q>QID:YBR136W	AA:8		273614X:L>L		BC187:L>L		DBVPG1106:L>L		DBVPG1373:L>L		DBVPG6765:L>L		L_1374:L>L		YJM975:L>L	AA:63		273614X:I>I		322134S:I>I		BC187:I>I		DBVPG1106:I>I		DBVPG1853:I>I		DBVPG6765:I>I		L_1374:I>I		NCYC361:I>I		UWOPS03_461_4:I>I		UWOPS05_217_3:I>I		UWOPS83_787_3:I>I		UWOPS87_2421:I>I		Y12:I>I		Y9:I>I		YJM975:I>I	AA:129		YPS606:S>S	AA:148		NCYC361:A>A	AA:157		SK1:Y>Y	AA:190		DBVPG1853:S>S		UWOPS05_217_3:S>S		UWOPS05_227_2:S>S		Y9:S>S		YPS606:S>S	AA:225		273614X:V>V		322134S:V>V		BC187:V>V		DBVPG1373:V>V		DBVPG1788:V>V		DBVPG6765:V>V		L_1374:V>V		YJM975:V>V		YJM978:V>V		YS4:V>V	AA:237		UWOPS05_217_3:A>A		UWOPS05_227_2:A>A	AA:343		273614X:L>L		BC187:L>L		DBVPG1373:L>L		DBVPG1788:L>L		DBVPG6765:L>L		L_1374:L>L		L_1528:L>L		YJM975:L>L		YJM978:L>L		YJM981:L>L	AA:350		DBVPG6044:L>L		NCYC110:L>L		NCYC361:L>L		UWOPS05_227_2:L>L	AA:375		378604X:P>P		Y9:P>P		YS4:P>P	AA:416		322134S:R>R		BC187:R>R		DBVPG1373:R>R		DBVPG1788:R>R		DBVPG1853:R>R		DBVPG6044:R>R		DBVPG6765:R>R		L_1374:R>R		L_1528:R>R		NCYC110:R>R		UWOPS05_227_2:R>R		YJM975:R>R		YJM981:R>R	AA:552		322134S:S>S		378604X:S>S		DBVPG1106:S>S		DBVPG1788:S>S		DBVPG1853:S>S		DBVPG6044:S>S		DBVPG6765:S>S		L_1374:S>S		L_1528:S>S		NCYC110:S>S		UWOPS05_217_3:S>S		UWOPS05_227_2:S>S		Y55:S>S		YJM975:S>S		YJM981:S>S	AA:602		322134S:N>N		DBVPG1106:N>N		DBVPG1788:N>N		DBVPG6765:N>N		L_1374:N>N		L_1528:N>N		YJM975:N>N	AA:608		L_1528:T>T	AA:622		322134S:V>V		DBVPG1106:V>V		DBVPG1788:V>V		DBVPG6765:V>V		L_1374:V>V		L_1528:V>V		NCYC361:V>V		YJM975:V>V	AA:677		UWOPS05_217_3:K>K		UWOPS05_227_2:K>K	AA:690		DBVPG6044:V>V		SK1:V>V		Y55:V>V	AA:699		DBVPG6044:L>L		SK1:L>L		Y55:L>L	AA:757		322134S:N>N		378604X:N>N		DBVPG1106:N>N		DBVPG1788:N>N		DBVPG6044:N>N		DBVPG6765:N>N		L_1374:N>N		L_1528:N>N		NCYC361:N>N		SK1:N>N		UWOPS05_227_2:N>N		Y55:N>N		Y9:N>N		YIIc17_E5:N>N		YJM975:N>N	AA:821		273614X:K>K		BC187:K>K		DBVPG1788:K>K		DBVPG6765:K>K		L_1528:K>K		NCYC361:K>K		SK1:K>K		Y9:K>K		YIIc17_E5:K>K		YJM975:K>K		YS9:K>K	AA:828		YPS606:A>A	AA:853		273614X:R>R		BC187:R>R		DBVPG1106:R>R		DBVPG1373:R>R		DBVPG1788:R>R		DBVPG6765:R>R		L_1528:R>R		NCYC361:R>R		YIIc17_E5:R>R		YJM975:R>R	AA:906		273614X:V>V		BC187:V>V		DBVPG1373:V>V		DBVPG1788:V>V		DBVPG1853:V>V		DBVPG6765:V>V		L_1374:V>V		L_1528:V>V		SK1:V>V		UWOPS03_461_4:V>V		UWOPS87_2421:V>V		Y55:V>V		Y9:V>V		YIIc17_E5:V>V		YS9:V>V	AA:918		DBVPG6044:P>P		SK1:P>P		Y55:P>P	AA:988		273614X:V>V		BC187:V>V		DBVPG1373:V>V		DBVPG1788:V>V		DBVPG6765:V>V		L_1374:V>V		NCYC361:V>V		YIIc17_E5:V>V		YJM975:V>V		YJM978:V>V	AA:1015		273614X:V>V		BC187:V>V		DBVPG1373:V>V		DBVPG1853:V>V		DBVPG6044:V>V		DBVPG6765:V>V		L_1374:V>V		NCYC110:V>V		NCYC361:V>V		SK1:V>V		UWOPS03_461_4:V>V		Y55:V>V		YJM975:V>V		YJM978:V>V	AA:1039		273614X:T>T		BC187:T>T		DBVPG1373:T>T		DBVPG1788:T>T		DBVPG6765:T>T		L_1374:T>T		NCYC361:T>T		YJM975:T>T		YJM978:T>T	AA:1070		273614X:R>R		DBVPG1373:R>R		DBVPG1788:R>R		DBVPG6765:R>R		L_1374:R>R		NCYC361:R>R		YJM975:R>R		YJM978:R>R	AA:1082		UWOPS03_461_4:P>P		UWOPS05_217_3:P>P	AA:1093		273614X:L>L		DBVPG1373:L>L		DBVPG1788:L>L		DBVPG6765:L>L		L_1374:L>L		NCYC361:L>L		YJM978:L>L	AA:1147		DBVPG1853:N>N		DBVPG6044:N>N		NCYC110:N>N		UWOPS05_217_3:N>N		Y55:N>N	AA:1185		BC187:Q>Q		DBVPG1106:Q>Q		DBVPG1373:Q>Q		L_1374:Q>Q		YJM978:Q>Q	AA:1208		DBVPG6044:N>N		NCYC110:N>N		SK1:N>N		Y55:N>N	AA:1218		DBVPG1106:S>S		DBVPG1373:S>S		L_1374:S>S		YIIc17_E5:S>S		YJM978:S>S	AA:1231		BC187:L>L		DBVPG1106:L>L		DBVPG1373:L>L		DBVPG6044:L>L		L_1374:L>L		NCYC110:L>L		SK1:L>L		Y55:L>L		YIIc17_E5:L>L		YJM978:L>L	AA:1243		BC187:L>L		DBVPG1106:L>L		DBVPG1373:L>L		YIIc17_E5:L>L		YJM978:L>L	AA:1280		BC187:S>S		DBVPG1373:S>S		DBVPG6044:S>S		NCYC361:S>S		SK1:S>S		UWOPS05_217_3:S>S		UWOPS05_227_2:S>S		Y55:S>S		YIIc17_E5:S>S	AA:1302		YPS128:I>I		YPS606:I>I	AA:1320		BC187:I>I		DBVPG6044:I>I		NCYC361:I>I		SK1:I>I		Y55:I>I		YIIc17_E5:I>I	AA:1330		UWOPS03_461_4:N>N		UWOPS05_227_2:N>N	AA:1341		SK1:S>S		Y55:S>S	AA:1351		SK1:R>R		Y55:R>R	AA:1399		UWOPS03_461_4:L>L		UWOPS05_227_2:L>L	AA:1406		322134S:E>E		DBVPG1373:E>E		L_1374:E>E		NCYC361:E>E		YJM978:E>E	AA:1442		L_1374:Y>Y	AA:1450		YJM978:S>S	AA:1498		YPS128:R>R	AA:1607		Y9:T>T	AA:1844		378604X:V>V		Y12:V>V	AA:1916		L_1528:A>A	AA:1923		K11:L>L		UWOPS83_787_3:L>L		YPS128:L>L		YS4:L>L	AA:1941		YS4:L>L	AA:2029		UWOPS83_787_3:L>L		UWOPS87_2421:L>L		YPS128:L>L	AA:2042		UWOPS83_787_3:P>P		YPS128:P>P	AA:2055		K11:V>V		UWOPS83_787_3:V>V		UWOPS87_2421:V>V		YPS128:V>V	AA:2072		K11:G>G		UWOPS83_787_3:G>G		UWOPS87_2421:G>G		YPS128:G>G	AA:2093		BC187:F>F		DBVPG1788:F>F		DBVPG6765:F>F		K11:F>F		L_1374:F>F		L_1528:F>F		UWOPS05_227_2:F>F		UWOPS83_787_3:F>F		UWOPS87_2421:F>F		YIIc17_E5:F>F		YPS128:F>F	AA:2185		K11:L>L		UWOPS05_227_2:L>L		UWOPS87_2421:L>L		YPS128:L>L	AA:2206		BC187:Y>Y		DBVPG1373:Y>Y		DBVPG1788:Y>Y		DBVPG1853:Y>Y		DBVPG6765:Y>Y		K11:Y>Y		L_1374:Y>Y		L_1528:Y>Y		NCYC361:Y>Y		UWOPS05_227_2:Y>Y		UWOPS87_2421:Y>Y		YPS128:Y>Y		YS4:Y>Y	AA:2226		BC187:H>H		DBVPG1373:H>H		DBVPG1788:H>H		DBVPG1853:H>H		DBVPG6765:H>H		K11:H>H		L_1374:H>H		L_1528:H>H		NCYC361:H>H		UWOPS05_227_2:H>H		UWOPS87_2421:H>H		YPS128:H>H		YS2:H>H		YS4:H>H	AA:2272		378604X:L>L	AA:2339		SK1:S>S		Y55:S>SID:YBR137W	AA:8		378604X:L>L		NCYC361:L>L		Y12:L>L		YPS128:L>L		YPS606:L>L	AA:53		322134S:N>N		378604X:N>N		BC187:N>N		DBVPG6044:N>N		DBVPG6765:N>N		NCYC361:N>N		SK1:N>N		UWOPS05_217_3:N>N		UWOPS05_227_2:N>N		Y12:N>N		Y55:N>N		YJM975:N>N		YJM981:N>N		YPS128:N>N		YPS606:N>N	AA:58		YIIc17_E5:P>P	AA:94		322134S:T>T		NCYC361:T>T	AA:98		DBVPG6044:F>F		SK1:F>F		Y55:F>F	AA:136		322134S:S>S		BC187:S>S		DBVPG6765:S>S		UWOPS05_217_3:S>S		YJM975:S>S		YPS128:S>S	AA:164		YPS128:L>LID:YBR138C	AA:9		378604X:R>R	AA:42		BC187:A>A		DBVPG1373:A>A		DBVPG6044:A>A		DBVPG6765:A>A		L_1374:A>A		L_1528:A>A		NCYC361:A>A		SK1:A>A		Y55:A>A		YJM975:A>A	AA:60		378604X:V>V		BC187:V>V		DBVPG1373:V>V		DBVPG1853:V>V		DBVPG6044:V>V		DBVPG6765:V>V		L_1374:V>V		L_1528:V>V		NCYC361:V>V		SK1:V>V		UWOPS83_787_3:V>V		UWOPS87_2421:V>V		Y55:V>V	AA:69		BC187:L>L		DBVPG1373:L>L		DBVPG1788:L>L		DBVPG6044:L>L		DBVPG6765:L>L		L_1374:L>L		L_1528:L>L		NCYC361:L>L		SK1:L>L		Y55:L>L	AA:80		378604X:F>F	AA:96		DBVPG6044:N>N		SK1:N>N		Y55:N>N	AA:134		BC187:Y>Y		DBVPG1373:Y>Y		DBVPG1788:Y>Y		DBVPG6765:Y>Y		L_1374:Y>Y		L_1528:Y>Y	AA:148		DBVPG6044:F>F		NCYC110:F>F		SK1:F>F		Y55:F>F	AA:165		BC187:D>D		DBVPG1106:D>D		DBVPG1373:D>D		DBVPG1788:D>D		DBVPG6765:D>D		L_1374:D>D		L_1528:D>D	AA:177		BC187:R>R		DBVPG1106:R>R		DBVPG1373:R>R		DBVPG1788:R>R		DBVPG6765:R>R		L_1528:R>R	AA:197		DBVPG6044:V>V		NCYC110:V>V		SK1:V>V		Y55:V>V	AA:222		DBVPG6044:V>V		NCYC110:V>V		SK1:V>V		Y55:V>V	AA:416		BC187:L>L		DBVPG1373:L>L		DBVPG1788:L>L		DBVPG6765:L>L		L_1374:L>L		L_1528:L>L		YJM975:L>L		YJM978:L>L		YS4:L>L		YS9:L>L	AA:420		BC187:A>A		DBVPG1373:A>A		DBVPG1788:A>A		DBVPG6044:A>A		DBVPG6765:A>A		L_1374:A>A		L_1528:A>A		SK1:A>A		UWOPS05_217_3:A>A		Y55:A>A		YJM975:A>A		YJM978:A>A		YPS128:A>A		YPS606:A>A		YS4:A>A		YS9:A>A	AA:455		YPS128:F>F		YPS606:F>F	AA:520		BC187:L>L		DBVPG1373:L>L		DBVPG6765:L>L		L_1374:L>L		L_1528:L>L		YJM975:L>L		YJM978:L>L		YS9:L>LID:YBR139W	AA:33		DBVPG1106:T>T		DBVPG1373:T>T		DBVPG6044:T>T		DBVPG6765:T>T		L_1374:T>T		L_1528:T>T		NCYC361:T>T		SK1:T>T		Y55:T>T		YJM975:T>T	AA:43		DBVPG6044:L>L		SK1:L>L		Y55:L>L	AA:75		YPS128:S>S		YPS606:S>S	AA:91		DBVPG1106:V>V		DBVPG1788:V>V		DBVPG1853:V>V		DBVPG6044:V>V		DBVPG6765:V>V		L_1374:V>V		L_1528:V>V		SK1:V>V		Y55:V>V		YJM975:V>V		YJM978:V>V	AA:108		DBVPG1106:Y>Y		DBVPG1788:Y>Y		DBVPG6044:Y>Y		DBVPG6765:Y>Y		L_1374:Y>Y		L_1528:Y>Y		SK1:Y>Y		Y55:Y>Y		YJM975:Y>Y		YJM978:Y>Y	AA:111		DBVPG1106:F>F		DBVPG1788:F>F		DBVPG6044:F>F		DBVPG6765:F>F		L_1374:F>F		L_1528:F>F		NCYC110:F>F		SK1:F>F		Y55:F>F		YJM975:F>F		YJM978:F>F	AA:227		378604X:Q>Q		DBVPG6044:Q>Q		NCYC110:Q>Q		SK1:Q>Q		UWOPS87_2421:Q>Q		Y12:Q>Q		Y55:Q>Q	AA:309		DBVPG6044:V>V		NCYC110:V>V		SK1:V>V		Y55:V>V	AA:381		378604X:T>T		Y12:T>T	AA:411		DBVPG6044:A>A		NCYC110:A>A		SK1:A>A		Y55:A>A	AA:421		DBVPG1106:L>L		DBVPG1373:L>L		DBVPG6765:L>L		L_1374:L>L		L_1528:L>L	AA:441		UWOPS05_227_2:R>R		YPS128:R>R		YPS606:R>R	AA:499		DBVPG1106:S>S		DBVPG1373:S>S		DBVPG1788:S>S		DBVPG6765:S>S		L_1374:S>S		L_1528:S>S		YS4:S>SID:YBR141C	AA:58		DBVPG6044:D>D		NCYC110:D>D		SK1:D>D		Y55:D>D	AA:68		DBVPG1853:I>I	AA:112		DBVPG6044:K>K		SK1:K>K		Y55:K>K	AA:146		322134S:N>N		DBVPG1373:N>N		DBVPG6044:N>N		DBVPG6765:N>N		SK1:N>N		Y55:N>N		YJM975:N>N		YJM978:N>N		YPS128:N>N	AA:182		322134S:L>L		DBVPG1106:L>L		DBVPG1373:L>L		DBVPG6765:L>L		YJM975:L>L		YJM978:L>L		YPS128:L>L	AA:209		322134S:I>I		DBVPG1106:I>I		DBVPG1373:I>I		DBVPG6765:I>I		YJM975:I>I		YJM978:I>I		YPS128:I>I	AA:218		DBVPG1106:L>L	AA:276		DBVPG1373:C>C		DBVPG6765:C>C		L_1374:C>C		L_1528:C>C		YJM975:C>C		YJM978:C>C	AA:330		UWOPS03_461_4:L>L		UWOPS05_227_2:L>LID:YBR145W	AA:2		DBVPG1373:P>P		DBVPG1788:P>P		DBVPG6765:P>P		L_1528:P>P		YJM975:P>P		YS2:P>P	AA:56		DBVPG1853:G>G	AA:66		NCYC110:L>L		SK1:L>L		Y55:L>L	AA:109		UWOPS87_2421:V>V	AA:141		DBVPG1853:A>A		UWOPS05_227_2:A>A	AA:174		DBVPG1853:G>G		UWOPS05_227_2:G>G		UWOPS83_787_3:G>G	AA:222		NCYC110:F>F		SK1:F>F		Y55:F>F	AA:233		DBVPG1373:G>G		DBVPG1788:G>G		DBVPG6040:G>G		DBVPG6765:G>G		L_1528:G>G		YJM975:G>G	AA:247		UWOPS05_227_2:I>I		UWOPS83_787_3:I>I	AA:278		NCYC110:A>A		SK1:A>A		Y55:A>A	AA:292		DBVPG1373:I>I		DBVPG6765:I>I		L_1528:I>I		UWOPS05_227_2:I>I		UWOPS83_787_3:I>I		YJM975:I>I		YPS128:I>I		YPS606:I>I		YS9:I>I	AA:301		NCYC110:N>N		SK1:N>N		Y55:N>N	AA:328		273614X:D>D		DBVPG1373:D>D		DBVPG6040:D>D		DBVPG6765:D>D		L_1528:D>D		NCYC110:D>D		SK1:D>D		UWOPS05_227_2:D>D		UWOPS83_787_3:D>D		Y55:D>D		Y9:D>D		YJM978:D>D		YPS128:D>D		YPS606:D>D		YS2:D>D		YS9:D>DID:YBR146W	AA:60		DBVPG6044:R>R		SK1:R>R		Y55:R>R	AA:86		273614X:F>F		378604X:F>F		BC187:F>F		DBVPG1106:F>F		DBVPG1788:F>F		DBVPG1853:F>F		DBVPG6040:F>F		DBVPG6044:F>F		DBVPG6765:F>F		K11:F>F		L_1528:F>F		SK1:F>F		UWOPS03_461_4:F>F		UWOPS05_227_2:F>F		UWOPS83_787_3:F>F		Y55:F>F		YJM975:F>F		YPS128:F>F		YPS606:F>F		YS4:F>F	AA:92		BC187:I>I		DBVPG1106:I>I		DBVPG1373:I>I		DBVPG1788:I>I		DBVPG1853:I>I		DBVPG6040:I>I		DBVPG6044:I>I		DBVPG6765:I>I		L_1528:I>I		SK1:I>I		UWOPS83_787_3:I>I		Y55:I>I		YJM975:I>I		YS4:I>I	AA:99		UWOPS83_787_3:K>K	AA:107		DBVPG6044:L>L		SK1:L>L		Y55:L>L	AA:120		378604X:L>L	AA:159		322134S:K>K		BC187:K>K		DBVPG1106:K>K		DBVPG1373:K>K		DBVPG1788:K>K		DBVPG1853:K>K		DBVPG6044:K>K		DBVPG6765:K>K		L_1374:K>K		L_1528:K>K		SK1:K>K		UWOPS03_461_4:K>K		UWOPS05_227_2:K>K		UWOPS83_787_3:K>K		UWOPS87_2421:K>K		Y55:K>K		YJM975:K>K		YPS128:K>K		YPS606:K>K		YS4:K>K		YS9:K>K	AA:172		322134S:G>G		BC187:G>G		DBVPG1106:G>G		DBVPG1373:G>G		DBVPG1788:G>G		DBVPG1853:G>G		DBVPG6044:G>G		DBVPG6765:G>G		L_1374:G>G		L_1528:G>G		SK1:G>G		UWOPS03_461_4:G>G		UWOPS05_227_2:G>G		UWOPS83_787_3:G>G		UWOPS87_2421:G>G		Y55:G>G		YJM975:G>G		YJM978:G>G		YS4:G>G		YS9:G>G	AA:208		322134S:K>K		BC187:K>K		DBVPG1373:K>K		DBVPG1788:K>K		DBVPG1853:K>K		DBVPG6765:K>K		L_1374:K>K		L_1528:K>K		YJM975:K>K		YJM978:K>K		YS9:K>K	AA:229		DBVPG6044:H>H		SK1:H>H		Y55:H>H	AA:242		K11:L>L		Y12:L>L		YIIc17_E5:L>L		YPS128:L>L		YPS606:L>L		YS2:L>L	AA:244		UWOPS03_461_4:S>S		UWOPS05_227_2:S>S		UWOPS87_2421:S>SID:YBR147W	AA:21		DBVPG1106:S>S		DBVPG1373:S>S		DBVPG1788:S>S		DBVPG1853:S>S		DBVPG6044:S>S		DBVPG6765:S>S		K11:S>S		L_1374:S>S		L_1528:S>S		NCYC361:S>S		SK1:S>S		UWOPS03_461_4:S>S		UWOPS05_227_2:S>S		UWOPS83_787_3:S>S		Y12:S>S		Y55:S>S		YJM975:S>S		YPS128:S>S		YPS606:S>S		YS4:S>S	AA:29		DBVPG1106:F>F		DBVPG1373:F>F		DBVPG1788:F>F		DBVPG1853:F>F		DBVPG6044:F>F		DBVPG6765:F>F		K11:F>F		L_1374:F>F		L_1528:F>F		SK1:F>F		UWOPS03_461_4:F>F		UWOPS05_227_2:F>F		UWOPS83_787_3:F>F		Y12:F>F		Y55:F>F		YJM975:F>F		YPS128:F>F		YPS606:F>F		YS4:F>F	AA:33		DBVPG6765:I>I		L_1528:I>I		YJM975:I>I		YS4:I>I	AA:36		Y12:N>N	AA:42		DBVPG1373:A>A		DBVPG1788:A>A		DBVPG1853:A>A		DBVPG6044:A>A		DBVPG6765:A>A		K11:A>A		L_1374:A>A		L_1528:A>A		NCYC361:A>A		SK1:A>A		UWOPS03_461_4:A>A		UWOPS05_227_2:A>A		UWOPS83_787_3:A>A		Y12:A>A		Y55:A>A		YJM975:A>A		YPS128:A>A		YS4:A>A	AA:59		DBVPG1373:F>F		DBVPG1788:F>F		DBVPG6765:F>F		L_1374:F>F		L_1528:F>F		Y12:F>F		YS4:F>F	AA:60		DBVPG6044:N>N		SK1:N>N		Y55:N>N	AA:70		DBVPG1853:L>L		UWOPS83_787_3:L>L		YPS128:L>L	AA:78		DBVPG1106:A>A		DBVPG1788:A>A		DBVPG6765:A>A		L_1374:A>A		L_1528:A>A		UWOPS03_461_4:A>A		UWOPS05_227_2:A>A		Y12:A>A		YS4:A>A	AA:95		UWOPS83_787_3:D>D		YPS128:D>D	AA:155		UWOPS03_461_4:T>T		YPS128:T>T	AA:200		DBVPG1788:A>A		DBVPG6044:A>A		DBVPG6765:A>A		L_1374:A>A		SK1:A>A		Y55:A>A		YS9:A>A	AA:212		DBVPG6044:G>G		DBVPG6765:G>G		L_1374:G>G		SK1:G>G		Y55:G>G		YS9:G>G	AA:231		SK1:F>F	AA:236		DBVPG6765:C>C		L_1374:C>C		YS9:C>CID:YBR148W	AA:29		BC187:I>I		DBVPG1788:I>I		DBVPG6040:I>I		DBVPG6765:I>I		L_1374:I>I		UWOPS03_461_4:I>I		YJM975:I>I		YJM978:I>I		YS4:I>I	AA:36		DBVPG1788:L>L		DBVPG6040:L>L		DBVPG6765:L>L		L_1374:L>L		YJM975:L>L		YJM978:L>L		YS4:L>L	AA:47		BC187:L>L		DBVPG1788:L>L		DBVPG6040:L>L		DBVPG6765:L>L		L_1374:L>L		YJM975:L>L		YJM978:L>L		YS4:L>L		YS9:L>L	AA:64		273614X:L>L		Y9:L>L	AA:76		DBVPG6044:R>R		SK1:R>R		Y55:R>R		YPS128:R>R		YPS606:R>R	AA:82		273614X:V>V		DBVPG6044:V>V		SK1:V>V		UWOPS83_787_3:V>V		Y55:V>V		Y9:V>V		YPS128:V>V		YPS606:V>V	AA:86		YS2:F>F	AA:120		UWOPS83_787_3:S>S	AA:141		DBVPG6044:N>N		NCYC110:N>N		SK1:N>N		Y55:N>N	AA:178		DBVPG6044:S>S		NCYC110:S>S		SK1:S>S		Y55:S>S	AA:277		DBVPG6044:I>I		NCYC110:I>I		SK1:I>I		Y55:I>I	AA:306		UWOPS05_227_2:N>N	AA:328		UWOPS05_227_2:E>E	AA:331		YJM978:N>N	AA:348		273614X:S>S		322134S:S>S		378604X:S>S		DBVPG1373:S>S		DBVPG6040:S>S		DBVPG6044:S>S		DBVPG6765:S>S		K11:S>S		L_1528:S>S		SK1:S>S		UWOPS05_227_2:S>S		Y55:S>S		YJM978:S>S		YPS128:S>S		YPS606:S>S		YS9:S>S	AA:380		YS9:N>N	AA:385		DBVPG6044:T>T		SK1:T>T		Y55:T>T	AA:410		322134S:T>T		BC187:T>T		DBVPG1373:T>T		DBVPG6040:T>T		DBVPG6765:T>T		L_1528:T>T		YJM978:T>T		YS9:T>T	AA:536		DBVPG6044:P>P		K11:P>P		SK1:P>P		UWOPS83_787_3:P>P		Y55:P>P		YPS128:P>P		YPS606:P>P	AA:588		273614X:L>L		BC187:L>L		DBVPG1788:L>L		DBVPG6765:L>L		K11:L>L		UWOPS03_461_4:L>L		UWOPS05_227_2:L>L		UWOPS83_787_3:L>L		YIIc17_E5:L>L		YJM975:L>L		YPS128:L>L		YPS606:L>L		YS2:L>LID:YBR149W	AA:67		DBVPG1853:T>T	AA:84		K11:L>L		Y12:L>L		Y9:L>L		YPS606:L>L	AA:93		DBVPG6044:E>E		SK1:E>E		Y55:E>E	AA:127		DBVPG6044:L>L		SK1:L>L		Y55:L>L	AA:131		YPS606:H>H	AA:134		DBVPG1853:L>L	AA:162		DBVPG1853:A>A	AA:194		SK1:F>F		Y55:F>F	AA:284		322134S:P>P		378604X:P>P		BC187:P>P		DBVPG1106:P>P		DBVPG6040:P>P		DBVPG6765:P>P		L_1528:P>P		NCYC110:P>P		SK1:P>P		UWOPS83_787_3:P>P		Y12:P>P		Y55:P>P		Y9:P>P		YJM981:P>P		YPS606:P>P	AA:298		UWOPS83_787_3:F>F	AA:307		UWOPS83_787_3:Q>Q	AA:316		UWOPS83_787_3:Y>Y	AA:339		K11:L>LID:YBR151W	AA:17		UWOPS87_2421:Q>Q	AA:58		273614X:A>A		DBVPG6044:A>A		DBVPG6765:A>A		L_1374:A>A		L_1528:A>A		SK1:A>A		UWOPS87_2421:A>A		Y55:A>A		YIIc17_E5:A>A		YS4:A>A	AA:83		UWOPS87_2421:V>V	AA:137		DBVPG1373:I>I		DBVPG6044:I>I		DBVPG6765:I>I		L_1374:I>I		L_1528:I>I		UWOPS87_2421:I>I		Y55:I>I		YJM978:I>I	AA:166		DBVPG6044:D>D		SK1:D>D		Y55:D>D	AA:177		DBVPG1373:D>D		DBVPG6765:D>D		L_1374:D>D		L_1528:D>D		UWOPS87_2421:D>D		YJM978:D>D	AA:193		L_1528:P>P	AA:241		L_1528:S>S	AA:255		UWOPS03_461_4:H>H	AA:269		L_1528:L>L	AA:279		L_1528:G>G	AA:284		L_1528:T>T	AA:293		L_1528:L>L	AA:304		L_1528:P>P	AA:313		L_1528:Y>YID:YBR154C	AA:15		L_1528:A>A	AA:18		L_1528:T>T	AA:35		L_1528:V>V	AA:105		L_1528:F>F	AA:124		L_1528:V>V	AA:137		UWOPS87_2421:E>E	AA:189		322134S:G>G		DBVPG1788:G>G		DBVPG1853:G>G		DBVPG6044:G>G		DBVPG6765:G>G		L_1374:G>G		L_1528:G>G		NCYC110:G>G		SK1:G>G		UWOPS83_787_3:G>G		Y55:G>G		Y9:G>G		YIIc17_E5:G>G		YS9:G>G	AA:195		DBVPG1788:V>V		DBVPG1853:V>V		DBVPG6044:V>V		DBVPG6765:V>V		L_1374:V>V		L_1528:V>V		NCYC110:V>V		SK1:V>V		UWOPS83_787_3:V>V		Y55:V>V		YIIc17_E5:V>V		YS9:V>VID:YBR155W	AA:13		L_1528:P>P	AA:15		DBVPG1373:K>K		L_1528:K>K		YS4:K>K	AA:19		L_1528:G>G	AA:23		DBVPG6044:P>P		NCYC110:P>P		SK1:P>P	AA:36		UWOPS03_461_4:T>T		UWOPS05_227_2:T>T	AA:39		L_1528:E>E	AA:48		L_1528:P>P	AA:50		L_1528:F>F	AA:60		L_1528:A>A	AA:118		322134S:S>S		DBVPG6044:S>S		DBVPG6765:S>S		L_1374:S>S		L_1528:S>S		NCYC110:S>S		SK1:S>S		UWOPS03_461_4:S>S		UWOPS05_227_2:S>S		YJM978:S>S		YPS128:S>S		YPS606:S>S		YS4:S>S	AA:121		322134S:E>E		DBVPG1106:E>E		DBVPG1373:E>E		DBVPG6765:E>E		L_1374:E>E		L_1528:E>E		YJM978:E>E		YS4:E>E	AA:124		322134S:Y>Y		DBVPG6044:Y>Y		DBVPG6765:Y>Y		L_1374:Y>Y		L_1528:Y>Y		NCYC110:Y>Y		SK1:Y>Y		UWOPS03_461_4:Y>Y		UWOPS05_227_2:Y>Y		YJM978:Y>Y		YPS128:Y>Y		YPS606:Y>Y		YS4:Y>Y	AA:150		YPS128:I>I		YPS606:I>I	AA:157		322134S:C>C		DBVPG1106:C>C		DBVPG6044:C>C		DBVPG6765:C>C		L_1374:C>C		L_1528:C>C		NCYC110:C>C		SK1:C>C		UWOPS03_461_4:C>C		UWOPS05_227_2:C>C		YPS128:C>C		YPS606:C>C		YS4:C>C	AA:166		322134S:F>F		DBVPG1106:F>F		DBVPG6044:F>F		DBVPG6765:F>F		L_1374:F>F		L_1528:F>F		NCYC110:F>F		SK1:F>F		UWOPS03_461_4:F>F		UWOPS05_227_2:F>F		Y55:F>F		YPS128:F>F		YPS606:F>F		YS4:F>F	AA:188		UWOPS03_461_4:E>E		UWOPS05_227_2:E>E	AA:303		YPS606:Q>Q	AA:322		BC187:F>F		DBVPG1106:F>F		DBVPG1373:F>F		DBVPG1853:F>F		DBVPG6765:F>F		L_1374:F>F	AA:359		BC187:I>I		DBVPG1106:I>I		DBVPG1373:I>I		DBVPG1853:I>I		DBVPG6765:I>I		L_1374:I>I		SK1:I>I		Y55:I>I		YJM978:I>I		YPS606:I>I		YS4:I>I	AA:379		BC187:A>A		DBVPG1106:A>A		DBVPG1373:A>A		DBVPG1853:A>A		DBVPG6765:A>A		L_1374:A>A		YJM978:A>AID:YBR156C	AA:61		YPS606:K>K	AA:104		BC187:D>D		DBVPG1373:D>D		DBVPG1788:D>D		DBVPG1853:D>D		DBVPG6044:D>D		DBVPG6765:D>D		L_1528:D>D		SK1:D>D		UWOPS03_461_4:D>D		UWOPS87_2421:D>D		Y55:D>D		YJM978:D>D		YPS606:D>D	AA:130		YS9:T>T	AA:145		DBVPG1788:P>P	AA:192		DBVPG6044:N>N		SK1:N>N	AA:247		DBVPG1853:I>I	AA:257		L_1528:G>G	AA:274		L_1528:K>K	AA:280		L_1528:S>S	AA:297		DBVPG6765:A>A		YJM981:A>A	AA:304		L_1528:L>L	AA:311		L_1528:S>S		UWOPS87_2421:K>K	AA:324		L_1528:K>K	AA:332		L_1528:L>L	AA:362		L_1528:F>F	AA:385		DBVPG1373:P>P		DBVPG6765:P>P		L_1374:P>P		L_1528:P>P		YJM978:P>P		YJM981:P>P	AA:406		L_1528:Q>Q	AA:449		YIIc17_E5:P>P	AA:474		DBVPG1853:T>T	AA:499		DBVPG1373:E>E		L_1528:E>E		YJM975:E>E		YJM978:E>E	AA:519		UWOPS87_2421:A>A	AA:525		UWOPS87_2421:K>K	AA:532		SK1:L>L		Y55:L>L	AA:595		DBVPG1853:A>A		DBVPG6765:A>A	AA:602		DBVPG1373:L>L		L_1374:L>L		L_1528:L>L		YJM975:L>L		YJM978:L>L	AA:608		DBVPG1373:V>V		L_1374:V>V		L_1528:V>V		YJM975:V>V		YJM978:V>V	AA:618		DBVPG1373:T>T		DBVPG1853:T>T		DBVPG6765:T>T		L_1374:T>T		L_1528:T>T		SK1:T>T		UWOPS87_2421:T>T		Y55:T>T		YJM975:T>T		YJM978:T>T	AA:627		DBVPG1853:S>S		DBVPG6765:S>S	AA:633		DBVPG1373:T>T		L_1374:T>T		L_1528:T>T		YJM975:T>T		YJM978:T>TID:YBR157C	AA:12		UWOPS87_2421:I>I	AA:69		UWOPS87_2421:G>G	AA:72		UWOPS05_227_2:L>L	AA:88		YPS606:V>V	AA:111		UWOPS83_787_3:S>S		UWOPS87_2421:S>S	AA:175		UWOPS83_787_3:S>S		UWOPS87_2421:S>S	AA:177		UWOPS83_787_3:F>F		UWOPS87_2421:F>F	AA:195		273614X:S>S		BC187:S>S		DBVPG1788:S>S		DBVPG1853:S>S		DBVPG6765:S>S		L_1374:S>S		YJM975:S>S	AA:200		UWOPS87_2421:I>I	AA:210		DBVPG1853:N>N	AA:216		273614X:G>G		BC187:G>G		DBVPG1106:G>G		DBVPG1788:G>G		DBVPG1853:G>G		DBVPG6765:G>G		L_1374:G>G		UWOPS05_227_2:G>G		UWOPS87_2421:G>G		YJM975:G>GID:YBR159W	AA:62		DBVPG6044:G>G		SK1:G>G		Y55:G>G	AA:80		322134S:R>R	AA:106		273614X:L>L		322134S:L>L		DBVPG1788:L>L		DBVPG6765:L>L		L_1528:L>L		NCYC361:L>L		UWOPS83_787_3:L>L		UWOPS87_2421:L>L		YJM975:L>L		YS9:L>L	AA:149		322134S:G>G		DBVPG1788:G>G		DBVPG6044:G>G		DBVPG6765:G>G		NCYC361:G>G		SK1:G>G		UWOPS83_787_3:G>G		UWOPS87_2421:G>G		Y55:G>G		YS9:G>G	AA:175		UWOPS87_2421:T>T	AA:186		Y9:P>P	AA:225		322134S:G>G		DBVPG1853:G>G		NCYC361:G>G		UWOPS83_787_3:G>G		YS2:G>G	AA:264		DBVPG1853:R>R	AA:276		DBVPG6044:F>F		SK1:F>F		Y55:F>F	AA:277		322134S:V>V		DBVPG1853:V>V		NCYC361:V>V		UWOPS83_787_3:V>V		YS2:V>V	AA:287		YS9:R>	AA:322		DBVPG1853:N>N		DBVPG6765:N>N		L_1374:N>N		NCYC361:N>N		UWOPS83_787_3:N>N		YJM981:N>N		YS2:N>N		YS4:N>N	AA:337		DBVPG1853:L>L		DBVPG6765:L>L		L_1374:L>L		NCYC361:L>L		UWOPS83_787_3:L>L		YJM981:L>L		YS2:L>L		YS4:L>LID:YBR160W	AA:30		322134S:P>P		DBVPG1106:P>P		DBVPG1373:P>P		DBVPG1788:P>P		DBVPG1853:P>P		DBVPG6765:P>P		L_1374:P>P		L_1528:P>P		NCYC110:P>P		NCYC361:P>P		SK1:P>P		UWOPS83_787_3:P>P		Y55:P>P		YJM975:P>P		YJM981:P>P		YS4:P>P	AA:44		L_1528:L>L	AA:86		NCYC361:L>L		UWOPS83_787_3:L>L	AA:115		DBVPG1853:K>K	AA:126		322134S:Y>Y	AA:139		DBVPG1106:P>P		DBVPG1373:P>P		DBVPG1788:P>P		DBVPG6765:P>P		L_1528:P>P		YJM975:P>P		YJM978:P>P	AA:141		UWOPS83_787_3:N>N	AA:195		UWOPS05_227_2:T>T	AA:213		UWOPS87_2421:S>SID:YBR162C	AA:105		DBVPG1853:N>N	AA:112		UWOPS87_2421:E>E		YPS128:E>E		YPS606:E>E	AA:229		W303:F>F	AA:292		273614X:S>S		DBVPG1106:S>S		DBVPG1373:S>S		DBVPG1788:S>S		UWOPS03_461_4:S>S		YJM978:S>S		YS9:S>S	AA:304		Y12:G>G	AA:309		273614X:G>G		DBVPG1106:G>G		DBVPG1373:G>G		DBVPG1788:G>G		DBVPG6765:G>G		UWOPS03_461_4:G>G		YJM978:G>G		YS9:G>G	AA:312		273614X:A>A		DBVPG1106:A>A		DBVPG1373:A>A		DBVPG1788:A>A		DBVPG6765:A>A		L_1374:A>A		UWOPS03_461_4:A>A		YJM978:A>A		YS9:A>A	AA:348		DBVPG1106:L>L		DBVPG1373:L>L		DBVPG1788:L>L		DBVPG6765:L>L		L_1528:L>L		UWOPS03_461_4:L>L		YJM978:L>L		YS9:L>L	AA:387		UWOPS03_461_4:I>I	AA:418		DBVPG1106:S>S		DBVPG1788:S>S		DBVPG6765:S>S		L_1374:S>S		L_1528:S>S		YJM978:S>S		YS9:S>S	AA:432		YPS128:F>FID:YBR162W-A	AA:2		273614X:A>A		322134S:A>A		DBVPG6040:A>A	AA:53		DBVPG6044:V>V		SK1:V>V		Y55:V>V	AA:60		DBVPG6044:L>L		SK1:L>L		Y55:L>LID:YBR163W	AA:47		UWOPS03_461_4:D>D		UWOPS05_227_2:D>D		YPS128:D>D		YPS606:D>D	AA:69		SK1:T>T		Y55:T>T	AA:102		UWOPS87_2421:S>S	AA:112		Y12:L>L		YPS128:L>L		YPS606:L>L		YS2:L>L	AA:146		DBVPG1853:F>F	AA:165		DBVPG1373:V>V		L_1528:V>V	AA:181		DBVPG6044:P>P		SK1:P>P		Y55:P>P	AA:249		273614X:V>V		DBVPG1788:V>V		DBVPG6765:V>V		L_1528:V>V		YJM978:V>V	AA:252		273614X:L>L		DBVPG1788:L>L		DBVPG6765:L>L		L_1528:L>L		YJM978:L>L	AA:284		DBVPG1853:K>K	AA:316		273614X:I>I		322134S:I>I		BC187:I>I		DBVPG1788:I>I		DBVPG1853:I>I		DBVPG6765:I>I		L_1528:I>I		YJM975:I>I		YJM978:I>I	AA:372		322134S:G>G		BC187:G>G		DBVPG1373:G>G		DBVPG1788:G>G		DBVPG1853:G>G		DBVPG6765:G>G		L_1528:G>G		YJM975:G>G		YJM978:G>G		YS2:G>G	AA:440		YPS128:V>V		YPS606:V>V	AA:525		UWOPS83_787_3:S>S		UWOPS87_2421:S>S	AA:586		273614X:->-		BC187:->-		DBVPG1373:->-		DBVPG1788:->-		DBVPG1853:->-		DBVPG6044:->-		DBVPG6765:->-		L_1374:->-		L_1528:->-		SK1:->-		UWOPS83_787_3:->-		UWOPS87_2421:->-		Y55:->-		YJM975:->-		YJM978:->-		YPS128:->-		YS2:->-		YS9:->-ID:YBR164C	AA:7		273614X:S>S		322134S:S>S		BC187:S>S		DBVPG1106:S>S		DBVPG1373:S>S		DBVPG1788:S>S		DBVPG6040:S>S		DBVPG6044:S>S		DBVPG6765:S>S		SK1:S>S		UWOPS05_227_2:S>S		UWOPS83_787_3:S>S		UWOPS87_2421:S>S		Y55:S>S		YJM978:S>S		YPS606:S>S	AA:24		273614X:L>L		322134S:L>L		DBVPG1106:L>L		DBVPG1373:L>L		DBVPG6040:L>L		DBVPG6044:L>L		DBVPG6765:L>L		SK1:L>L		UWOPS05_227_2:L>L		UWOPS83_787_3:L>L		UWOPS87_2421:L>L		Y55:L>L		YIIc17_E5:L>L		YJM978:L>L		YPS606:L>L	AA:175		DBVPG6044:I>I		SK1:I>I		Y55:I>IID:YBR165W	AA:71		273614X:C>C		BC187:C>C		DBVPG1106:C>C		DBVPG1788:C>C		DBVPG1853:C>C		DBVPG6765:C>C		UWOPS83_787_3:C>C		UWOPS87_2421:C>C		YJM975:C>C		YJM981:C>C		YS9:C>C	AA:128		SK1:F>F	AA:144		UWOPS05_217_3:F>F	AA:165		UWOPS83_787_3:N>N	AA:203		273614X:S>S		BC187:S>S		DBVPG1106:S>S		DBVPG1373:S>S		DBVPG1853:S>S		DBVPG6765:S>S		L_1374:S>S		L_1528:S>S		UWOPS03_461_4:S>S		UWOPS05_217_3:S>S		YJM975:S>S		YS9:S>S	AA:215		Y55:N>N	AA:255		UWOPS03_461_4:T>T		UWOPS05_217_3:T>T	AA:262		SK1:T>T		Y55:T>T	AA:278		SK1:->-		Y55:->-ID:YBR166C	AA:51		DBVPG1373:L>L		DBVPG1788:L>L		DBVPG6040:L>L		DBVPG6044:L>L		DBVPG6765:L>L		L_1374:L>L		L_1528:L>L		SK1:L>L		UWOPS03_461_4:L>L		UWOPS05_227_2:L>L		UWOPS83_787_3:L>L		Y55:L>L		YPS128:L>L		YPS606:L>L	AA:149		SK1:H>H		UWOPS03_461_4:H>H		UWOPS83_787_3:H>H		Y55:H>H	AA:231		273614X:I>I		DBVPG1373:I>I		DBVPG1788:I>I		DBVPG6040:I>I		DBVPG6765:I>I		L_1374:I>I		L_1528:I>I		YJM975:I>I		YJM978:I>I		YS4:I>I		YS9:I>I	AA:300		273614X:I>I		322134S:I>I		DBVPG1373:I>I		DBVPG6044:I>I		L_1374:I>I		L_1528:I>I		NCYC361:I>I		SK1:I>I		UWOPS03_461_4:I>I		UWOPS83_787_3:I>I		YJM975:I>I		YJM978:I>I	AA:315		273614X:N>N		322134S:N>N		DBVPG1373:N>N		L_1374:N>N		L_1528:N>N		YJM975:N>NID:YBR167C	AA:7		322134S:T>T		DBVPG1373:T>T		DBVPG1788:T>T		DBVPG6765:T>T		L_1528:T>T		SK1:T>T		UWOPS05_227_2:T>T		UWOPS83_787_3:T>T		UWOPS87_2421:T>T		Y55:T>T		YJM975:T>T		YJM978:T>T		YPS606:T>TID:YBR168W	AA:65		DBVPG1106:D>D		DBVPG1373:D>D		DBVPG1788:D>D		DBVPG6765:D>D		YJM978:D>D	AA:139		YPS128:V>V	AA:247		UWOPS87_2421:F>F	AA:343		273614X:K>K		322134S:K>K		BC187:K>K		DBVPG1788:K>K		DBVPG1853:K>K		DBVPG6040:K>K		DBVPG6044:K>K		DBVPG6765:K>K		NCYC110:K>K		SK1:K>K		UWOPS87_2421:K>K		Y55:K>K		YJM975:K>K		YJM978:K>K		YS2:K>K	AA:408		UWOPS87_2421:R>RID:YBR169C	AA:16		322134S:L>L		BC187:L>L		DBVPG1373:L>L		DBVPG1788:L>L		DBVPG6040:L>L		L_1374:L>L		L_1528:L>L		UWOPS05_227_2:L>L		YJM978:L>L	AA:21		YPS606:N>N	AA:40		322134S:V>V		BC187:V>V		DBVPG1373:V>V		DBVPG1788:V>V		DBVPG6040:V>V		L_1374:V>V		L_1528:V>V		YJM978:V>V	AA:65		DBVPG6044:V>V		Y55:V>V	AA:69		UWOPS05_227_2:K>K	AA:71		BC187:I>I		DBVPG1106:I>I		DBVPG1373:I>I		DBVPG1788:I>I		DBVPG1853:I>I		DBVPG6040:I>I		DBVPG6765:I>I		L_1374:I>I		L_1528:I>I		YJM978:I>I	AA:104		BC187:V>V		DBVPG1106:V>V		DBVPG1373:V>V		DBVPG1788:V>V		DBVPG1853:V>V		DBVPG6040:V>V		DBVPG6044:V>V		DBVPG6765:V>V		L_1374:V>V		L_1528:V>V		UWOPS05_227_2:V>V		UWOPS87_2421:V>V		Y55:V>V		YJM978:V>V	AA:118		UWOPS05_227_2:L>L	AA:126		YPS606:V>V	AA:158		BC187:A>A		DBVPG1106:A>A		DBVPG1373:A>A		DBVPG1788:A>A		DBVPG1853:A>A		DBVPG6040:A>A		DBVPG6044:A>A		DBVPG6765:A>A		L_1374:A>A		L_1528:A>A		UWOPS87_2421:A>A		Y55:A>A		YJM975:A>A		YJM978:A>A	AA:168		UWOPS87_2421:P>P	AA:179		BC187:A>A		DBVPG1106:A>A		DBVPG1788:A>A		DBVPG1853:A>A		DBVPG6040:A>A		DBVPG6765:A>A		L_1374:A>A		L_1528:A>A		YJM975:A>A		YJM978:A>A	AA:209		UWOPS87_2421:Y>Y	AA:271		UWOPS87_2421:S>S		YPS128:A>A		YPS606:A>A	AA:277		DBVPG6044:V>V		Y55:V>V	AA:345		322134S:T>T		378604X:T>T		DBVPG1106:T>T		DBVPG1373:T>T		DBVPG6044:T>T		DBVPG6765:T>T		L_1374:T>T		NCYC361:T>T		UWOPS87_2421:T>T		Y55:T>T		YIIc17_E5:T>T		YJM975:T>T		YJM978:T>T		YPS128:T>T		YPS606:T>T		YS4:T>T		YS9:T>T	AA:390		UWOPS87_2421:R>R	AA:414		322134S:D>D		DBVPG1373:D>D		DBVPG1788:D>D		DBVPG1853:D>D		DBVPG6044:D>D		DBVPG6765:D>D		NCYC361:D>D		UWOPS87_2421:D>D		Y55:D>D		YJM978:D>D	AA:419		UWOPS87_2421:F>F	AA:438		DBVPG1853:D>D		DBVPG6044:D>D		Y55:D>D	AA:488		DBVPG6044:G>G		Y55:G>G	AA:522		Y9:K>K	AA:542		322134S:L>L		BC187:L>L		DBVPG1373:L>L		DBVPG1788:L>L		DBVPG1853:L>L		DBVPG6044:L>L		DBVPG6765:L>L		L_1374:L>L		L_1528:L>L		UWOPS03_461_4:L>L		UWOPS83_787_3:L>L		Y55:L>L	AA:562		DBVPG6044:K>K		Y55:K>K	AA:565		DBVPG6044:A>A		Y55:A>A	AA:580		DBVPG6044:T>T		Y55:T>T	AA:608		DBVPG6044:T>T		Y55:T>T	AA:644		UWOPS83_787_3:A>A	AA:669		UWOPS83_787_3:L>LID:YBR170C	AA:111		DBVPG1853:L>L	AA:115		DBVPG1853:E>E	AA:125		YPS128:E>E		YPS606:E>E	AA:136		UWOPS83_787_3:L>L	AA:223		DBVPG1106:A>A		DBVPG1373:A>A		DBVPG1788:A>A		DBVPG6765:A>A		L_1374:A>A		YJM975:A>A	AA:372		DBVPG1106:S>S		DBVPG1373:S>S		DBVPG1788:S>S		DBVPG6765:S>S		L_1528:S>S		YJM975:S>S		YPS128:S>S	AA:375		UWOPS03_461_4:G>G	AA:397		UWOPS03_461_4:S>S	AA:423		DBVPG6044:A>A		SK1:A>A		Y55:A>A	AA:458		DBVPG1853:P>P	AA:463		YPS606:L>L	AA:551		322134S:A>A		BC187:A>A		DBVPG1106:A>A		DBVPG1373:A>A		DBVPG1788:A>A		DBVPG1853:A>A		DBVPG6765:A>A		L_1528:A>A		YJM978:A>AID:YBR171W	AA:13		UWOPS87_2421:G>G	AA:168		YPS606:K>K	AA:175		DBVPG6044:A>A		NCYC110:A>A		SK1:A>A		Y55:A>A	AA:190		YPS606:C>C	AA:197		322134S:K>K		DBVPG1106:K>K		DBVPG1373:K>K		DBVPG1853:K>K		DBVPG6765:K>K		UWOPS87_2421:K>KID:YBR172C	AA:6		BC187:S>S		DBVPG1106:S>S		DBVPG1373:S>S		DBVPG1788:S>S		DBVPG1853:S>S		DBVPG6044:S>S		DBVPG6765:S>S		L_1528:S>S		UWOPS87_2421:S>S		Y55:S>S		YJM978:S>S		YS2:S>S	AA:17		BC187:F>F		DBVPG1106:F>F		DBVPG1373:F>F		DBVPG1788:F>F		DBVPG1853:F>F		DBVPG6044:F>F		DBVPG6765:F>F		L_1528:F>F		UWOPS87_2421:F>F		Y55:F>F		YJM978:F>F		YS2:F>F	AA:64		BC187:S>S		DBVPG1106:S>S		DBVPG1788:S>S		DBVPG6765:S>S		UWOPS87_2421:S>S		Y55:S>S		YJM978:S>S		YPS128:S>S		YS2:S>S	AA:88		Y55:S>S	AA:94		BC187:A>A		DBVPG1106:A>A		DBVPG1788:A>A		DBVPG1853:A>A		DBVPG6765:A>A		L_1374:A>A		SK1:A>A		UWOPS87_2421:A>A		Y55:A>A	AA:129		DBVPG1853:T>T	AA:150		DBVPG1106:A>A		DBVPG1853:A>A		DBVPG6765:A>A		L_1374:A>A		YJM975:A>A		YS9:A>A	AA:188		DBVPG1106:P>P		DBVPG1853:P>P		DBVPG6765:P>P		L_1374:P>P		L_1528:P>P		SK1:P>P		UWOPS87_2421:P>P		Y55:P>P		YJM975:P>P		YS9:P>P	AA:209		DBVPG1373:R>R		DBVPG1853:R>R		DBVPG6765:R>R		L_1374:R>R		L_1528:R>R		SK1:R>R		UWOPS87_2421:R>R		Y55:R>R		YJM975:R>R		YPS606:R>R		YS9:R>R	AA:217		BC187:I>I		DBVPG1106:I>I		DBVPG1373:I>I		DBVPG1853:I>I		DBVPG6765:I>I		L_1374:I>I		L_1528:I>I		NCYC110:I>I		SK1:I>I		UWOPS87_2421:I>I		Y55:I>I		YJM975:I>I		YPS606:I>I		YS9:I>I	AA:244		DBVPG1853:L>L	AA:266		BC187:K>K		DBVPG1373:K>K		DBVPG1853:K>K		DBVPG6044:K>K		DBVPG6765:K>K		L_1374:K>K		L_1528:K>K		NCYC110:K>K		SK1:K>K		UWOPS03_461_4:K>K		UWOPS87_2421:K>K		Y55:K>K		YJM975:K>K		YPS606:K>K		YS9:K>K	AA:272		DBVPG6044:T>T		NCYC110:T>T		SK1:T>T		Y55:T>T		YPS606:T>T	AA:334		BC187:K>K		DBVPG1373:K>K		DBVPG1853:K>K		DBVPG6765:K>K		L_1374:K>K		L_1528:K>K		UWOPS87_2421:K>K		YJM975:K>K		YS9:K>K	AA:434		DBVPG6044:K>K		NCYC110:K>K		SK1:K>K		Y55:K>K	AA:472		273614X:N>N		322134S:N>N		BC187:N>N		DBVPG1373:N>N		DBVPG1788:N>N		DBVPG1853:N>N		DBVPG6044:N>N		DBVPG6765:N>N		NCYC110:N>N		SK1:N>N		Y55:N>N		YJM975:N>N		YPS128:N>N		YPS606:N>N	AA:479		273614X:I>I		322134S:I>I		BC187:I>I		DBVPG1373:I>I		DBVPG1788:I>I		DBVPG6765:I>I		YJM975:I>I	AA:578		DBVPG6044:S>S		SK1:S>S		Y55:S>S	AA:624		322134S:T>T		BC187:T>T		DBVPG1106:T>T		DBVPG1373:T>T		DBVPG1788:T>T		DBVPG6765:T>T		UWOPS87_2421:T>T		YJM975:T>T	AA:655		322134S:T>T		DBVPG1106:T>T		DBVPG1373:T>T		DBVPG1788:T>T		DBVPG1853:T>T		DBVPG6765:T>T		SK1:T>T		UWOPS83_787_3:T>T		UWOPS87_2421:T>T		Y55:T>T		YJM975:T>T	AA:706		NCYC110:Q>Q		SK1:Q>Q		Y55:Q>QID:YBR173C	AA:77		378604X:G>G	AA:97		YJM978:N>N	AA:113		DBVPG1853:K>K	AA:117		BC187:I>I		DBVPG1106:I>I		DBVPG1373:I>I		DBVPG1788:I>I		DBVPG1853:I>I		DBVPG6765:I>I		L_1374:I>I		L_1528:I>I		UWOPS87_2421:I>I		YJM975:I>I		YJM978:I>IID:YBR175W	AA:17		378604X:T>T		DBVPG1373:T>T		DBVPG1788:T>T		DBVPG6765:T>T		UWOPS87_2421:T>T		YS9:T>T	AA:19		378604X:A>A	AA:21		378604X:I>I		DBVPG1373:I>I		DBVPG1788:I>I		DBVPG6765:I>I		NCYC110:I>I		SK1:I>I		UWOPS05_227_2:I>I		UWOPS87_2421:I>I		Y55:I>I		YPS128:I>I		YPS606:I>I	AA:91		NCYC110:T>T		SK1:T>T		Y55:T>T	AA:107		378604X:K>K	AA:133		378604X:T>T		DBVPG1106:T>T		DBVPG1373:T>T		DBVPG1788:T>T		YS9:T>T	AA:157		UWOPS87_2421:G>G	AA:185		UWOPS03_461_4:R>R	AA:208		NCYC110:S>S		SK1:S>S		Y55:S>S	AA:217		UWOPS03_461_4:D>D	AA:231		378604X:P>P		DBVPG1106:P>P		DBVPG1373:P>P		DBVPG1788:P>P		DBVPG6765:P>P		YS9:P>P	AA:236		UWOPS87_2421:V>V	AA:259		UWOPS87_2421:G>G	AA:285		NCYC361:H>H		SK1:H>H		UWOPS03_461_4:H>H		UWOPS87_2421:H>H		Y55:H>H		YPS128:H>H		YPS606:H>HID:YBR176W	AA:7		UWOPS87_2421:Q>Q	AA:27		DBVPG1788:T>T	AA:42		DBVPG1853:S>S		UWOPS87_2421:S>S	AA:110		DBVPG1853:G>G	AA:156		UWOPS83_787_3:L>L		UWOPS87_2421:L>L	AA:171		K11:P>P		UWOPS83_787_3:P>P		UWOPS87_2421:P>P	AA:226		273614X:L>L		322134S:L>L		BC187:L>L		DBVPG1373:L>L		DBVPG1788:L>L		DBVPG1853:L>L		DBVPG6040:L>L		DBVPG6044:L>L		DBVPG6765:L>L		L_1374:L>L		L_1528:L>L		NCYC110:L>L		SK1:L>L		UWOPS03_461_4:L>L		UWOPS83_787_3:L>L		UWOPS87_2421:L>L		Y55:L>L		YJM975:L>L		YJM978:L>L		YPS128:L>L		YPS606:L>L		YS2:L>L		YS9:L>L	AA:305		BC187:F>F		DBVPG1373:F>F		DBVPG1788:F>F		DBVPG1853:F>F		DBVPG6040:F>F		DBVPG6044:F>F		DBVPG6765:F>F		L_1528:F>F		NCYC110:F>F		NCYC361:F>F		SK1:F>F		UWOPS03_461_4:F>F		UWOPS83_787_3:F>F		Y55:F>F		YJM975:F>F		YS2:F>FID:YBR177C	AA:3		L_1528:E>E	AA:10		DBVPG1106:I>I		DBVPG1373:I>I		DBVPG1788:I>I		L_1374:I>I		L_1528:I>I		NCYC361:I>I		YJM978:I>I		YS2:I>I	AA:14		L_1528:H>H	AA:67		YIIc17_E5:L>L	AA:89		UWOPS83_787_3:F>F		UWOPS87_2421:F>F		YIIc17_E5:F>F	AA:114		UWOPS87_2421:K>K	AA:198		DBVPG1106:V>V		DBVPG1373:V>V		DBVPG1788:V>V		DBVPG6765:V>V		L_1528:V>V		YJM978:V>V	AA:205		DBVPG6044:G>G	AA:305		DBVPG6044:R>R		Y55:R>R	AA:322		DBVPG6044:P>P		Y55:P>P	AA:357		DBVPG1373:Y>Y		DBVPG1788:Y>Y		DBVPG6765:Y>Y		L_1528:Y>Y		NCYC361:Y>Y		YS2:Y>Y	AA:363		YPS128:G>G		YPS606:G>G	AA:373		DBVPG6044:A>A		Y55:A>A	AA:414		DBVPG6044:L>L		Y55:L>L		YPS128:L>L		YPS606:L>LID:YBR179C	AA:58		Y12:F>F		Y9:F>F	AA:91		DBVPG1853:N>N		DBVPG6044:N>N		NCYC110:N>N		SK1:N>N		Y55:N>N	AA:99		L_1528:L>L	AA:111		DBVPG1853:D>D	AA:113		UWOPS83_787_3:L>L		UWOPS87_2421:L>L	AA:121		DBVPG1373:P>P	AA:135		L_1528:V>V	AA:138		L_1528:L>L	AA:143		L_1528:D>D	AA:148		DBVPG1853:T>T		NCYC110:T>T		SK1:T>T		Y12:T>T		Y55:T>T		Y9:T>T	AA:155		L_1528:N>N	AA:158		L_1528:K>K	AA:172		L_1528:V>V	AA:190		L_1528:V>V	AA:196		L_1528:V>V	AA:202		DBVPG6044:A>A		SK1:A>A		Y55:A>A	AA:212		L_1528:L>L	AA:214		L_1528:P>P	AA:217		L_1528:Q>Q	AA:249		DBVPG1106:T>T		DBVPG1788:T>T		DBVPG6765:T>T		L_1374:T>T		YJM978:T>T	AA:258		DBVPG1373:S>S	AA:266		DBVPG1373:E>E	AA:267		L_1528:I>I	AA:287		322134S:K>K		BC187:A>A		DBVPG1373:A>A		DBVPG1788:A>A		DBVPG6040:A>A		DBVPG6765:A>A		L_1374:A>A		SK1:A>A		UWOPS83_787_3:A>A		Y55:A>A		YJM975:A>A		YPS128:A>A		YPS606:A>A		YS9:A>A	AA:311		DBVPG1373:L>L		DBVPG1788:L>L		DBVPG1853:L>L		DBVPG6044:L>L		DBVPG6765:L>L		L_1374:L>L		L_1528:L>L		NCYC110:L>L		SK1:L>L		Y55:L>L		YJM978:L>L		YPS128:L>L		YPS606:L>L	AA:342		DBVPG1373:N>N		DBVPG1853:N>N		DBVPG6040:N>N		DBVPG6044:N>N		DBVPG6765:N>N		L_1374:N>N		L_1528:N>N		NCYC110:N>N		SK1:N>N		UWOPS05_227_2:N>N		Y55:N>N		YJM978:N>N		YPS128:N>N		YPS606:N>N	AA:449		W303:E>E	AA:465		W303:L>L	AA:504		YS9:L>L	AA:545		YPS128:L>L		YPS606:L>L	AA:561		BC187:I>I		DBVPG1373:I>I		DBVPG1788:I>I		DBVPG6040:I>I		DBVPG6765:I>I		L_1374:I>I		L_1528:I>I		UWOPS83_787_3:I>I		YS9:I>I	AA:581		SK1:G>G		YPS128:G>G		YPS606:G>G	AA:602		NCYC110:G>G		SK1:G>G	AA:718		UWOPS87_2421:V>V	AA:754		273614X:I>I		BC187:I>I		DBVPG1373:I>I		DBVPG1788:I>I		DBVPG6040:I>I		DBVPG6765:I>I		L_1528:I>I		YJM975:I>I		YS9:I>I	AA:786		L_1528:T>T	AA:821		UWOPS83_787_3:N>N		UWOPS87_2421:N>N	AA:847		YPS128:V>V		YPS606:V>VID:YBR180W	AA:33		DBVPG1373:L>L		DBVPG6765:L>L		L_1374:L>L		YJM975:L>L		YJM978:L>L		YS2:L>L	AA:34		YIIc17_E5:G>G	AA:67		322134S:L>L		DBVPG1373:L>L		DBVPG6040:L>L		DBVPG6044:L>L		DBVPG6765:L>L		L_1374:L>L		SK1:L>L		UWOPS03_461_4:L>L		UWOPS05_227_2:L>L		UWOPS83_787_3:L>L		Y12:L>L		Y55:L>L		Y9:L>L		YIIc17_E5:L>L		YJM975:L>L		YJM978:L>L		YPS128:L>L		YPS606:L>L		YS2:L>L		YS4:L>L	AA:70		UWOPS83_787_3:Q>Q	AA:124		273614X:P>P		BC187:P>P		DBVPG6765:P>P		L_1374:P>P		UWOPS83_787_3:P>P		YJM975:P>P		YJM978:P>P		YS2:P>P	AA:126		L_1528:S>S	AA:128		L_1528:N>N	AA:144		L_1528:S>S	AA:147		L_1528:T>T	AA:157		L_1528:A>A	AA:160		L_1528:S>S	AA:161		YS4:V>V	AA:167		L_1528:G>G	AA:177		L_1528:F>F	AA:187		UWOPS83_787_3:L>L	AA:192		L_1528:L>L	AA:202		L_1528:A>A	AA:212		L_1528:F>F	AA:217		L_1528:V>V	AA:234		L_1528:R>R	AA:237		L_1528:A>A	AA:239		L_1528:A>A	AA:248		L_1528:G>G		UWOPS83_787_3:G>G	AA:263		L_1528:G>G	AA:282		L_1528:I>I	AA:284		L_1528:V>V	AA:286		L_1528:A>A	AA:297		L_1528:G>G	AA:300		L_1528:D>D	AA:307		L_1528:K>K	AA:316		NCYC110:P>P		SK1:P>P		Y55:P>P	AA:330		L_1528:P>P	AA:334		273614X:I>I		BC187:I>I		DBVPG6765:I>I		L_1374:I>I		L_1528:I>I		NCYC110:I>I		SK1:I>I		Y55:I>I		Y9:I>I		YJM975:I>I		YPS606:I>I	AA:336		NCYC110:K>K		SK1:K>K		Y55:K>K	AA:352		NCYC110:P>P		SK1:P>P		Y55:P>P	AA:371		273614X:T>T		BC187:T>T		DBVPG1106:T>T		DBVPG6765:T>T		L_1374:T>T		L_1528:T>T		YJM975:T>T		YS2:T>T	AA:380		NCYC110:S>S		SK1:S>S		Y55:S>S	AA:386		273614X:S>S		BC187:S>S		DBVPG1106:S>S		DBVPG6040:S>S		DBVPG6765:S>S		L_1374:S>S		L_1528:S>S		SK1:S>S		UWOPS03_461_4:S>S		UWOPS05_227_2:S>S		UWOPS87_2421:S>S		Y55:S>S		Y9:S>S		YJM975:S>S		YPS606:S>S		YS2:S>S	AA:408		SK1:Y>Y		Y55:Y>Y	AA:414		273614X:A>A		322134S:A>A		BC187:A>A		DBVPG1106:A>A		DBVPG6040:A>A		DBVPG6765:A>A		L_1374:A>A		L_1528:A>A		SK1:A>A		UWOPS05_227_2:A>A		UWOPS87_2421:A>A		Y55:A>A		Y9:A>A		YIIc17_E5:A>A		YJM975:A>A		YJM978:A>A		YPS606:A>A		YS2:A>A	AA:488		K11:G>G		Y9:G>G		YIIc17_E5:G>G		YPS606:G>G		YS4:G>G	AA:508		L_1528:A>A	AA:515		322134S:S>S		BC187:S>S		DBVPG1106:S>S		DBVPG1788:S>S		DBVPG1853:S>S		DBVPG6040:S>S		DBVPG6765:S>S		L_1374:S>S		L_1528:S>S		YJM975:S>S		YJM978:S>S		YS2:S>S	AA:546		322134S:L>L		BC187:L>L		DBVPG1106:L>L		DBVPG1788:L>L		DBVPG1853:L>L		DBVPG6040:L>L		DBVPG6765:L>L		K11:L>L		L_1374:L>L		L_1528:L>L		SK1:L>L		UWOPS03_461_4:L>L		UWOPS87_2421:L>L		Y55:L>L		YIIc17_E5:L>L		YJM975:L>L		YJM978:L>L		YPS128:L>L		YPS606:L>L		YS2:L>L		YS4:L>L	AA:548		DBVPG1853:L>L	AA:560		L_1528:L>L	AA:563		L_1528:F>F	AA:569		L_1528:A>AID:YBR181C	AA:82		BC187:S>S		DBVPG1106:S>S		DBVPG1373:S>S		DBVPG1788:S>S		DBVPG6765:S>S		L_1374:S>S		L_1528:S>S		YJM975:S>S		YJM978:S>S	AA:182		322134S:Q>Q		YIIc17_E5:Q>Q	AA:195		L_1528:V>V	AA:216		L_1528:L>L	AA:224		L_1528:A>A	AA:232		L_1528:S>S	AA:233		UWOPS87_2421:S>SID:YBR182C	AA:28		DBVPG1853:L>L		DBVPG6044:L>L		K11:L>L		SK1:L>L		UWOPS03_461_4:L>L		UWOPS83_787_3:L>L		Y55:L>L		Y9:L>L		YPS128:L>L		YPS606:L>L	AA:45		K11:V>V	AA:96		UWOPS83_787_3:L>L	AA:113		UWOPS03_461_4:S>S		UWOPS05_227_2:S>S		Y12:S>S		Y9:S>S		YPS606:S>S	AA:189		DBVPG6044:F>F		Y55:F>F	AA:206		UWOPS83_787_3:E>E	AA:216		DBVPG1853:R>R	AA:219		UWOPS03_461_4:P>P		UWOPS05_227_2:P>P		YPS128:P>P		YPS606:P>P	AA:239		DBVPG1373:S>S		DBVPG6765:S>S		L_1374:S>S		L_1528:S>S		YJM975:S>S		YJM978:S>S	AA:244		UWOPS83_787_3:L>L	AA:281		DBVPG1373:G>G		DBVPG6765:G>G		L_1374:G>G		YJM975:G>G		YJM978:G>G	AA:286		DBVPG1373:E>E		DBVPG6765:E>E		L_1374:E>E		YJM975:E>E		YJM978:E>E	AA:299		DBVPG1106:A>A		DBVPG1373:A>A		DBVPG6765:A>A		L_1374:A>A		YJM975:A>A		YJM978:A>A	AA:355		DBVPG1106:T>T		DBVPG1373:T>T		DBVPG6765:T>T		L_1374:T>T		YJM975:T>T		YJM978:T>T	AA:382		DBVPG1106:K>K		DBVPG1373:K>K		DBVPG6765:K>K		L_1374:K>K		UWOPS03_461_4:K>K		UWOPS05_227_2:K>K		UWOPS83_787_3:K>K		UWOPS87_2421:K>K		YJM975:K>K		YJM978:K>K		YPS606:K>KID:YBR183W	AA:35		273614X:P>P		322134S:P>P		BC187:P>P		DBVPG1106:P>P		DBVPG1373:P>P		DBVPG1788:P>P		DBVPG6044:P>P		DBVPG6765:P>P		L_1374:P>P		L_1528:P>P		SK1:P>P		UWOPS03_461_4:P>P		UWOPS05_227_2:P>P		UWOPS83_787_3:P>P		UWOPS87_2421:P>P		Y55:P>P		YJM978:P>P	AA:48		YS9:V>V	AA:51		DBVPG6044:L>L		SK1:L>L		UWOPS03_461_4:L>L		UWOPS05_227_2:L>L		UWOPS83_787_3:L>L		Y55:L>L	AA:81		UWOPS87_2421:V>V	AA:96		DBVPG1373:L>L	AA:113		273614X:L>L	AA:139		322134S:G>G	AA:180		273614X:L>L		322134S:L>L		BC187:L>L		DBVPG1106:L>L		DBVPG1373:L>L		DBVPG1788:L>L		DBVPG1853:L>L		DBVPG6044:L>L		DBVPG6765:L>L		L_1528:L>L		SK1:L>L		UWOPS03_461_4:L>L		UWOPS05_217_3:L>L		UWOPS83_787_3:L>L		UWOPS87_2421:L>L		Y55:L>L		YJM978:L>L		YPS128:L>L		YPS606:L>L	AA:193		UWOPS05_217_3:N>N	AA:228		YS4:S>S		YS9:S>S	AA:238		YS4:L>L		YS9:L>L	AA:248		DBVPG1853:T>T		UWOPS05_217_3:T>T		UWOPS05_227_2:T>T		UWOPS83_787_3:T>T		YPS128:T>T		YPS606:T>T	AA:263		NCYC361:R>R		UWOPS83_787_3:R>R		UWOPS87_2421:R>R	AA:282		DBVPG1853:F>F		UWOPS05_217_3:F>F		UWOPS05_227_2:F>F		YPS128:F>FID:YBR185C	AA:19		DBVPG1106:S>S		DBVPG1788:S>S		DBVPG1853:S>S		DBVPG6765:S>S		L_1374:S>S		L_1528:S>S	AA:31		DBVPG1106:L>L		DBVPG1788:L>L		DBVPG1853:L>L		DBVPG6765:L>L		L_1374:L>L		L_1528:L>L	AA:65		L_1374:P>P	AA:76		322134S:A>A		DBVPG1106:A>A		DBVPG1788:A>A		DBVPG1853:A>A		DBVPG6044:A>A		DBVPG6765:A>A		L_1374:A>A		SK1:A>A		UWOPS03_461_4:A>A		UWOPS05_217_3:A>A		UWOPS83_787_3:A>A		UWOPS87_2421:A>A		Y55:A>A	AA:85		UWOPS87_2421:L>L	AA:91		322134S:T>T		DBVPG1106:T>T		DBVPG1788:T>T		DBVPG1853:T>T		DBVPG6044:T>T		DBVPG6765:T>T		L_1374:T>T		L_1528:T>T		SK1:T>T		UWOPS03_461_4:T>T		UWOPS05_217_3:T>T		UWOPS87_2421:T>T		Y55:T>T		YPS128:T>T		YS9:T>T	AA:129		DBVPG1788:S>S		DBVPG1853:S>S		DBVPG6044:S>S		L_1374:S>S		L_1528:S>S		NCYC361:S>S		SK1:S>S		UWOPS87_2421:S>S		Y55:S>S	AA:131		UWOPS87_2421:A>A	AA:135		UWOPS03_461_4:L>L		UWOPS05_217_3:L>L	AA:192		UWOPS87_2421:L>L	AA:252		UWOPS87_2421:P>P	AA:270		273614X:G>G		BC187:G>G		DBVPG1373:G>G		DBVPG1788:G>G		DBVPG6765:G>G		L_1374:G>G		L_1528:G>G		NCYC361:G>G		UWOPS05_217_3:G>G		UWOPS05_227_2:G>G		UWOPS83_787_3:G>G		UWOPS87_2421:G>G		YJM975:G>G		YJM978:G>GID:YBR186W	AA:6		322134S:D>D		DBVPG1373:D>D		DBVPG1788:D>D		DBVPG6765:D>D		L_1528:D>D		NCYC110:D>D		NCYC361:D>D		SK1:D>D		UWOPS03_461_4:D>D		UWOPS05_217_3:D>D		UWOPS05_227_2:D>D		Y55:D>D		Y9:D>D		YJM978:D>D		YJM981:D>D		YPS128:D>D		YPS606:D>D	AA:174		273614X:S>S		DBVPG1373:S>S		DBVPG6040:S>S		DBVPG6765:S>S		L_1528:S>S		NCYC361:S>S		YJM978:S>S		YS9:S>S	AA:184		273614X:E>E		DBVPG1373:E>E		DBVPG1788:E>E		DBVPG1853:E>E		DBVPG6040:E>E		DBVPG6765:E>E		L_1528:E>E		NCYC361:E>E		UWOPS87_2421:E>E		YJM978:E>E		YS9:E>E	AA:233		DBVPG1373:S>S		DBVPG1853:S>S		DBVPG6040:S>S		DBVPG6765:S>S		L_1528:S>S		UWOPS87_2421:S>S		YJM975:S>S		YJM978:S>S		YS9:S>S	AA:259		YS9:F>F	AA:265		DBVPG1373:S>S	AA:309		UWOPS87_2421:L>L	AA:313		DBVPG1373:H>H	AA:337		YS2:F>F	AA:365		NCYC110:F>F		SK1:F>F		Y55:F>F	AA:376		DBVPG1853:F>F	AA:398		UWOPS83_787_3:D>D	AA:509		UWOPS83_787_3:L>L	AA:513		UWOPS83_787_3:A>A	AA:515		UWOPS83_787_3:K>K	AA:517		UWOPS83_787_3:S>S	AA:522		UWOPS03_461_4:S>S		UWOPS05_217_3:S>S		UWOPS05_227_2:S>S	AA:553		UWOPS83_787_3:A>AID:YBR191W	AA:72		UWOPS83_787_3:V>V	AA:77		DBVPG6044:N>N		SK1:N>N		UWOPS05_217_3:N>N		UWOPS05_227_2:N>N		Y55:N>N	AA:86		NCYC361:E>E	AA:109		YS2:V>V	AA:120		378604X:K>K	AA:126		BC187:V>V		DBVPG1106:V>V		DBVPG1373:V>V		DBVPG1788:V>V		DBVPG6765:V>V		L_1374:V>V		L_1528:V>V		YJM978:V>V		YPS606:V>VID:YBR193C	AA:69		322134S:L>L		DBVPG1373:L>L		DBVPG6044:L>L		DBVPG6765:L>L		L_1374:L>L		SK1:L>L		UWOPS05_217_3:L>L		UWOPS05_227_2:L>L		UWOPS87_2421:L>L		Y55:L>L		YIIc17_E5:L>L		YJM975:L>L		YJM981:L>L		YPS606:L>L	AA:77		322134S:V>V	AA:119		BC187:I>I		DBVPG1373:I>I		DBVPG6040:I>I		DBVPG6765:I>I		L_1374:I>I		UWOPS87_2421:I>I		YIIc17_E5:I>I		YJM975:I>I		YJM978:I>I		YJM981:I>I	AA:153		SK1:R>R		Y55:R>RID:YBR195C	AA:146		Y9:A>A	AA:190		YIIc17_E5:V>V	AA:217		BC187:L>L		DBVPG1106:L>L		DBVPG1788:L>L		DBVPG6044:L>L		DBVPG6765:L>L		K11:L>L		NCYC361:L>L		SK1:L>L		Y55:L>L		Y9:L>L		YJM978:L>L		YPS128:L>L	AA:277		BC187:S>S		DBVPG1788:S>S		DBVPG1853:S>S		DBVPG6040:S>S		DBVPG6044:S>S		DBVPG6765:S>S		K11:S>S		SK1:S>S		Y12:S>S		Y55:S>S		Y9:S>S		YJM975:S>S		YJM978:S>S		YPS128:S>S	AA:362		BC187:E>E		DBVPG1788:E>E		DBVPG1853:E>E		DBVPG6040:E>E		DBVPG6765:E>E		K11:E>E		SK1:E>E		UWOPS03_461_4:E>E		Y12:E>E		Y55:E>E		Y9:E>E		YJM978:E>E		YPS128:E>E		YS2:E>EID:YBR197C	AA:9		UWOPS05_227_2:V>V	AA:48		UWOPS05_227_2:S>S	AA:53		BC187:A>A		DBVPG1106:A>A		DBVPG1373:A>A		DBVPG1853:A>A		DBVPG6040:A>A		DBVPG6044:A>A		DBVPG6765:A>A		L_1528:A>A		SK1:A>A		UWOPS05_227_2:A>A		Y55:A>A		YJM978:A>A		YJM981:A>A		YS9:A>A	AA:71		BC187:P>P		DBVPG1106:P>P		DBVPG1373:P>P		DBVPG6765:P>P		K11:P>P		L_1528:P>P		YJM978:P>P		YJM981:P>P		YS9:P>P	AA:97		BC187:T>T		DBVPG1106:T>T		DBVPG1373:T>T		DBVPG1853:T>T		DBVPG6040:T>T		DBVPG6765:T>T		L_1528:T>T		YJM978:T>T		YJM981:T>T		YS9:T>T	AA:102		W303:L>L	AA:167		UWOPS05_227_2:K>K	AA:180		YS4:A>AID:YBR199W	AA:11		273614X:P>P		322134S:P>P		DBVPG1106:P>P		DBVPG1373:P>P		DBVPG1853:P>P		DBVPG6040:P>P		DBVPG6765:P>P		L_1528:P>P		Y9:P>P		YJM978:P>P		YPS128:P>P		YPS606:P>P		YS4:P>P		YS9:P>P	AA:40		273614X:V>V		322134S:V>V		DBVPG1106:V>V		DBVPG1373:V>V		DBVPG1853:V>V		DBVPG6040:V>V		DBVPG6765:V>V		L_1374:V>V		L_1528:V>V		YJM978:V>V		YS2:V>V	AA:64		YS4:S>S		YS9:S>S	AA:74		DBVPG1106:E>E	AA:85		378604X:E>E	AA:99		YS9:L>L	AA:116		273614X:E>E		322134S:E>E		BC187:E>E		DBVPG1106:E>E		DBVPG1373:E>E		DBVPG1853:E>E		DBVPG6040:E>E		DBVPG6765:E>E		L_1374:E>E		L_1528:E>E		YS2:E>E	AA:169		YS9:T>T	AA:187		YS4:I>I	AA:243		Y9:S>S	AA:252		Y9:L>L	AA:398		322134S:K>K		DBVPG1373:K>K		DBVPG1788:K>K		DBVPG6765:K>K		L_1528:K>K		NCYC361:K>K		YJM975:K>K	AA:411		Y12:H>H		Y9:H>H	AA:465		322134S:->-		DBVPG1373:->-		DBVPG1788:->-		DBVPG6765:->-		L_1528:->-		Y12:->-		Y9:->-		YJM978:->-		YPS606:->-		YS2:->-ID:YBR201W	AA:70		BC187:S>S		DBVPG1106:S>S		DBVPG1853:S>S		DBVPG6765:S>S		L_1528:S>S		W303:S>S		YJM975:S>S		YJM978:S>S		YJM981:S>S		YS2:S>S	AA:81		BC187:L>L		DBVPG1106:L>L		DBVPG1853:L>L		DBVPG6044:L>L		DBVPG6765:L>L		L_1528:L>L		NCYC110:L>L		NCYC361:L>L		SK1:L>L		UWOPS03_461_4:L>L		UWOPS83_787_3:L>L		UWOPS87_2421:L>L		W303:L>L		Y55:L>L		YJM975:L>L		YJM978:L>L		YJM981:L>L		YPS606:L>L		YS2:L>L	AA:115		NCYC361:A>A	AA:131		322134S:L>L		BC187:L>L		DBVPG1106:L>L		DBVPG1853:L>L		DBVPG6765:L>L		W303:L>L		YJM975:L>L		YJM978:L>L		YJM981:L>L		YS2:L>L	AA:155		Y9:I>I	AA:159		322134S:A>A		BC187:A>A		DBVPG1106:A>A		DBVPG1853:A>A		DBVPG6765:A>A		W303:A>A		YJM975:A>A		YJM978:A>A		YJM981:A>A	AA:167		322134S:R>R		BC187:R>R		DBVPG1106:R>R		DBVPG1853:R>R		DBVPG6765:R>R		W303:R>R		YJM975:R>R		YJM978:R>R		YJM981:R>R	AA:180		UWOPS03_461_4:H>H		UWOPS05_217_3:H>H		UWOPS05_227_2:H>H		UWOPS83_787_3:H>HID:YBR202W	AA:24		322134S:F>F		BC187:F>F		DBVPG1788:F>F		DBVPG1853:F>F		L_1374:F>F		L_1528:F>F		YJM975:F>F		YJM978:F>F	AA:35		322134S:S>S		BC187:S>S		DBVPG1788:S>S		DBVPG1853:S>S		L_1374:S>S		L_1528:S>S		YJM975:S>S		YJM978:S>S	AA:52		DBVPG1853:I>I	AA:107		322134S:Q>Q		BC187:Q>Q		DBVPG1788:Q>Q		DBVPG1853:Q>Q		DBVPG6044:Q>Q		DBVPG6765:Q>Q		K11:Q>Q		L_1374:Q>Q		L_1528:Q>Q		NCYC110:Q>Q		UWOPS03_461_4:Q>Q		UWOPS05_227_2:Q>Q		W303:Q>Q		Y12:Q>Q		Y55:Q>Q		YJM975:Q>Q		YPS606:Q>Q	AA:169		322134S:T>T		BC187:T>T		DBVPG1788:T>T		DBVPG1853:T>T		DBVPG6765:T>T		L_1374:T>T		L_1528:T>T		W303:T>T		YJM975:T>T	AA:220		UWOPS05_227_2:I>I	AA:228		273614X:R>R		322134S:R>R		DBVPG1373:R>R		DBVPG1788:R>R		DBVPG6044:R>R		DBVPG6765:R>R		L_1374:R>R		L_1528:R>R		UWOPS05_227_2:R>R		UWOPS87_2421:R>R		W303:R>R		Y55:R>R		YJM975:R>R		YPS606:R>R		YS4:R>R	AA:233		273614X:D>D		322134S:D>D		DBVPG1373:D>D		DBVPG1788:D>D		DBVPG6765:D>D		L_1374:D>D		L_1528:D>D		W303:D>D		YJM975:D>D	AA:269		DBVPG6044:V>V		UWOPS87_2421:V>V		Y55:V>V		YPS128:V>V		YPS606:V>V		YS4:V>V	AA:279		273614X:T>T		322134S:T>T		DBVPG1106:T>T		DBVPG1373:T>T		DBVPG1788:T>T		DBVPG1853:T>T		DBVPG6765:T>T		L_1374:T>T		L_1528:T>T		W303:T>T		YJM975:T>T	AA:286		DBVPG6044:S>S		Y55:S>S	AA:295		273614X:K>K		DBVPG1106:K>K		DBVPG1373:K>K		DBVPG1788:K>K		DBVPG1853:K>K		DBVPG6040:K>K		DBVPG6765:K>K		L_1528:K>K	AA:303		273614X:R>R		DBVPG1106:R>R		DBVPG1373:R>R		DBVPG1788:R>R		DBVPG6040:R>R		DBVPG6765:R>R		L_1528:R>R	AA:305		UWOPS87_2421:S>S	AA:358		378604X:A>A	AA:408		YS9:G>G	AA:410		DBVPG1106:V>V		DBVPG1373:V>V		DBVPG1853:V>V		DBVPG6040:V>V		DBVPG6044:V>V		DBVPG6765:V>V		L_1528:V>V		NCYC110:V>V		UWOPS83_787_3:V>V		UWOPS87_2421:V>V		W303:V>V		Y55:V>V		YJM975:V>V		YJM978:V>V		YJM981:V>V		YPS128:V>V	AA:420		378604X:P>P	AA:428		378604X:V>V		DBVPG1106:V>V		DBVPG1373:V>V		DBVPG1853:V>V		DBVPG6040:V>V		DBVPG6044:V>V		DBVPG6765:V>V		L_1528:V>V		NCYC110:V>V		SK1:V>V		UWOPS03_461_4:V>V		UWOPS05_227_2:V>V		UWOPS83_787_3:V>V		UWOPS87_2421:V>V		W303:V>V		Y12:V>V		Y55:V>V		Y9:V>V		YIIc17_E5:V>V		YJM975:V>V		YJM978:V>V		YJM981:V>V		YPS128:V>V	AA:469		UWOPS83_787_3:L>L		UWOPS87_2421:L>L		YPS128:L>L		YPS606:L>L	AA:477		YS9:S>S	AA:488		UWOPS03_461_4:S>S		UWOPS05_227_2:S>S	AA:496		378604X:A>A		DBVPG6044:A>A		NCYC110:A>A		SK1:A>A		UWOPS03_461_4:A>A		UWOPS05_227_2:A>A		UWOPS87_2421:A>A		Y55:A>A		YPS128:A>A		YPS606:A>A	AA:540		UWOPS83_787_3:V>V	AA:569		322134S:P>P	AA:570		DBVPG6044:L>L		NCYC110:L>L		SK1:L>L		Y55:L>L	AA:586		L_1374:L>L	AA:623		UWOPS83_787_3:N>N	AA:631		YPS128:T>T		YPS606:T>T	AA:643		UWOPS83_787_3:A>A	AA:709		UWOPS87_2421:D>D	AA:743		DBVPG1853:K>K	AA:773		YPS128:G>G	AA:832		UWOPS83_787_3:N>N		UWOPS87_2421:N>N	AA:835		UWOPS83_787_3:A>A		UWOPS87_2421:A>A	AA:845		DBVPG1853:A>AID:YBR203W	AA:10		322134S:V>V	AA:52		YJM975:I>I		YJM978:I>I	AA:53		DBVPG1853:S>S	AA:66		K11:C>C	AA:138		YPS606:F>F	AA:141		UWOPS05_217_3:K>K		UWOPS05_227_2:K>K	AA:157		DBVPG6044:L>L		SK1:L>L		Y55:L>L		Y9:L>L		YPS606:L>L	AA:173		K11:L>L		NCYC361:L>L		UWOPS05_217_3:L>L		YPS606:L>L	AA:175		K11:K>K		NCYC361:K>K		UWOPS05_217_3:K>K		YPS606:K>K	AA:178		K11:Y>Y		NCYC361:Y>Y		UWOPS05_217_3:Y>Y		YPS606:Y>Y	AA:188		K11:N>N		NCYC361:N>N		UWOPS05_217_3:N>N		YPS606:N>N	AA:205		322134S:G>G		DBVPG1373:G>G		DBVPG6044:G>G		K11:G>G		L_1374:G>G		L_1528:G>G		NCYC361:G>G		SK1:G>G		UWOPS05_217_3:G>G		Y55:G>G		YJM975:G>G		YJM978:G>G		YPS606:G>G	AA:224		YPS606:A>A	AA:295		322134S:R>R		DBVPG1373:R>R		DBVPG6044:R>R		DBVPG6765:R>R		L_1374:R>R		L_1528:R>R		NCYC110:R>R		NCYC361:R>R		Y55:R>R		YJM975:R>R		YPS606:R>R		YS2:R>R	AA:306		YS4:P>P	AA:321		UWOPS05_217_3:S>S	AA:434		378604X:S>S		DBVPG6044:S>S		NCYC110:S>S		Y55:S>S		YPS128:S>S		YPS606:S>S	AA:539		DBVPG1373:Y>Y		DBVPG6765:Y>Y		L_1374:Y>Y		YJM975:Y>Y		YJM981:Y>Y		YS2:Y>Y		YS4:Y>Y	AA:556		378604X:L>L		DBVPG1373:L>L		DBVPG6765:L>L		L_1374:L>L		YJM975:L>L		YJM981:L>L		YS2:L>L		YS4:L>L	AA:561		UWOPS83_787_3:V>V	AA:664		322134S:E>E	AA:692		322134S:C>C		378604X:C>C		BC187:C>C		DBVPG1106:C>C		DBVPG1373:C>C		DBVPG1788:C>C		DBVPG1853:C>C		L_1374:C>C		SK1:C>C		UWOPS03_461_4:C>C		UWOPS05_227_2:C>C		UWOPS83_787_3:C>C		Y12:C>C		Y55:C>C		Y9:C>C		YJM975:C>C		YJM978:C>C		YJM981:C>C		YPS128:C>C		YS4:C>C		YS9:C>C	AA:718		322134S:Y>Y		378604X:Y>Y		BC187:Y>Y		DBVPG1106:Y>Y		DBVPG1373:Y>Y		DBVPG1788:Y>Y		DBVPG1853:Y>Y		L_1374:Y>Y		L_1528:Y>Y		SK1:Y>Y		UWOPS03_461_4:Y>Y		UWOPS05_227_2:Y>Y		Y55:Y>Y		YJM975:Y>Y		YJM978:Y>Y		YJM981:Y>Y		YPS128:Y>Y		YS9:Y>Y	AA:733		SK1:D>D		Y55:D>D	AA:791		YS4:N>N	AA:815		UWOPS05_217_3:L>L		UWOPS05_227_2:L>L	AA:867		DBVPG6044:V>V		SK1:V>V		UWOPS83_787_3:V>V		Y55:V>V	AA:899		DBVPG1106:S>S		DBVPG1373:S>S		DBVPG1788:S>S		DBVPG6040:S>S		DBVPG6765:S>S		L_1528:S>S		W303:S>S		YJM975:S>S		YS9:S>S	AA:901		DBVPG1106:L>L		DBVPG1373:L>L		DBVPG1788:L>L		DBVPG1853:L>L		DBVPG6040:L>L		DBVPG6765:L>L		L_1528:L>L		W303:L>L		YJM975:L>L		YS9:L>LID:YBR204C	AA:8		DBVPG1106:E>E		DBVPG1373:E>E		DBVPG1788:E>E		DBVPG6044:E>E		L_1528:E>E		SK1:E>E		UWOPS05_217_3:E>E		UWOPS05_227_2:E>E		Y55:E>E		YJM975:E>E		YJM978:E>E	AA:34		DBVPG6044:A>A		SK1:A>A		Y55:A>A	AA:56		DBVPG6044:E>E		SK1:E>E		Y55:E>E	AA:105		DBVPG6044:L>L		SK1:L>L		UWOPS05_217_3:L>L		Y55:L>L	AA:122		322134S:P>P	AA:134		DBVPG1106:P>P		DBVPG1373:P>P		DBVPG1788:P>P		YJM975:P>P		YJM978:P>P	AA:148		322134S:D>D		DBVPG1106:D>D		DBVPG1373:D>D		DBVPG1788:D>D		YJM975:D>D		YJM978:D>D	AA:155		DBVPG6044:T>T		SK1:T>T		Y55:T>T	AA:158		322134S:P>P		DBVPG1106:P>P		DBVPG6040:P>P		DBVPG6044:P>P		SK1:P>P		Y55:P>P		YJM975:P>P		YJM978:P>P		YS4:P>P	AA:162		DBVPG1106:N>N		DBVPG1373:N>N		DBVPG1788:N>N		DBVPG1853:N>N		DBVPG6040:N>N		DBVPG6044:N>N		SK1:N>N		Y55:N>N		YJM975:N>N		YJM978:N>N		YS4:N>N		YS9:N>N	AA:175		DBVPG1106:G>G		DBVPG1788:G>G		DBVPG6040:G>G		YJM975:G>G		YJM978:G>G		YS4:G>G		YS9:G>G	AA:190		DBVPG1106:H>H		DBVPG1788:H>H		DBVPG6040:H>H		YJM975:H>H		YJM978:H>H		YS9:H>H	AA:233		K11:L>L	AA:303		378604X:L>L	AA:360		UWOPS87_2421:L>LID:YBR205W	AA:78		322134S:H>H		DBVPG1373:H>H		DBVPG1788:H>H		DBVPG6040:H>H		L_1374:H>H		UWOPS83_787_3:H>H		YJM978:H>H	AA:85		322134S:K>K		DBVPG1373:K>K		DBVPG1788:K>K		DBVPG6040:K>K		DBVPG6044:K>K		L_1374:K>K		NCYC110:K>K		SK1:K>K		UWOPS83_787_3:K>K		Y55:K>K		YJM978:K>K	AA:124		L_1374:L>L		L_1528:L>L	AA:202		DBVPG1106:L>L		DBVPG1373:L>L		DBVPG6040:L>L		L_1374:L>L		L_1528:L>L		W303:L>L		YPS128:L>L		YPS606:L>L	AA:217		UWOPS05_217_3:T>T	AA:249		YPS128:T>T		YPS606:T>T	AA:284		K11:T>T		UWOPS03_461_4:T>T		UWOPS05_217_3:T>T	AA:326		DBVPG1106:G>G		DBVPG6040:G>G		DBVPG6765:G>G		L_1528:G>G		W303:G>G		YJM981:G>GID:YBR207W	AA:27		UWOPS05_227_2:S>S	AA:53		UWOPS05_227_2:G>G	AA:100		BC187:E>E		DBVPG1373:E>E		DBVPG1788:E>E		DBVPG6044:E>E		L_1374:E>E		SK1:E>E		UWOPS05_217_3:E>E		UWOPS05_227_2:E>E		Y55:E>E		YJM975:E>E		YJM978:E>E		YJM981:E>E		YS4:E>E		YS9:E>E	AA:111		DBVPG1373:D>D		DBVPG1788:D>D		DBVPG6044:D>D		SK1:D>D		UWOPS05_217_3:D>D		UWOPS05_227_2:D>D		Y55:D>D		YJM975:D>D		YJM981:D>D		YS4:D>D		YS9:D>D	AA:144		322134S:L>L		UWOPS83_787_3:L>L		UWOPS87_2421:L>L	AA:157		322134S:Y>Y		UWOPS83_787_3:Y>Y		UWOPS87_2421:Y>Y	AA:175		DBVPG6044:L>L		SK1:L>L	AA:245		DBVPG6044:L>L		SK1:L>L	AA:261		DBVPG1106:S>S		DBVPG1373:S>S		DBVPG6765:S>S		L_1374:S>S		UWOPS05_217_3:S>S		W303:S>S		Y55:S>S		YJM975:S>S		YS9:S>S	AA:304		322134S:I>I	AA:316		DBVPG1373:L>L		L_1374:L>L	AA:341		DBVPG1373:D>D		DBVPG6765:D>D		L_1374:D>D		UWOPS05_227_2:D>D		UWOPS87_2421:D>D		W303:D>D		Y55:D>D		YS9:D>D	AA:374		378604X:T>T		DBVPG1373:T>T		DBVPG6044:T>T		DBVPG6765:T>T		L_1374:T>T		SK1:T>T		UWOPS05_217_3:T>T		UWOPS05_227_2:T>T		UWOPS87_2421:T>T		W303:T>T		Y55:T>T		YPS128:T>T		YPS606:T>T		YS9:T>T	AA:409		378604X:I>I		DBVPG1373:I>I		DBVPG1788:I>I		DBVPG6040:I>I		DBVPG6044:I>I		DBVPG6765:I>I		L_1374:I>I		SK1:I>I		UWOPS05_217_3:I>I		UWOPS05_227_2:I>I		UWOPS83_787_3:I>I		UWOPS87_2421:I>I		W303:I>I		Y55:I>I		YPS128:I>I		YPS606:I>I		YS4:I>I		YS9:I>I	AA:456		378604X:L>L	AA:464		DBVPG1373:A>A		DBVPG1788:A>A		DBVPG6765:A>A		UWOPS05_217_3:A>A		UWOPS05_227_2:A>A		W303:A>A		Y55:A>A		YS9:A>AID:YBR210W	AA:15		273614X:L>L		DBVPG1373:L>L		DBVPG1853:L>L		DBVPG6040:L>L		DBVPG6765:L>L		L_1528:L>L		UWOPS87_2421:L>L		W303:L>L		Y55:L>L		YJM975:L>L		YS2:L>L		YS9:L>L	AA:27		DBVPG6040:T>T	AA:43		DBVPG6044:L>L		SK1:L>L	AA:50		UWOPS05_227_2:L>L		UWOPS87_2421:L>L	AA:63		273614X:A>A		DBVPG1373:A>A		DBVPG1853:A>A		DBVPG6040:A>A		DBVPG6765:A>A		UWOPS03_461_4:A>A		UWOPS05_227_2:A>A		W303:A>A		Y55:A>A		YJM975:A>A		YJM978:A>A		YS2:A>A		YS9:A>A	AA:68		YS4:N>N	AA:70		UWOPS87_2421:Y>Y	AA:86		273614X:S>S		378604X:S>S		DBVPG1373:S>S		DBVPG6765:S>S		Y55:S>S		YJM978:S>S		YS2:S>S		YS9:S>SID:YBR212W	AA:13		273614X:E>E		322134S:E>E		378604X:E>E		BC187:E>E		DBVPG6765:E>E		W303:E>E		Y55:E>E		YJM975:E>E		YS2:E>E		YS4:E>E		YS9:E>E	AA:98		DBVPG6044:S>S		NCYC110:S>S		SK1:S>S		Y9:S>S		YPS606:S>S		YS4:S>S	AA:122		UWOPS05_217_3:T>T	AA:130		273614X:Y>Y		322134S:Y>Y		DBVPG6765:Y>Y		YJM975:Y>Y		YS2:Y>Y	AA:167		DBVPG6044:T>T		NCYC110:T>T		NCYC361:T>T		SK1:T>T		UWOPS05_217_3:T>T		UWOPS83_787_3:T>T		Y9:T>T		YPS606:T>T		YS4:T>T	AA:181		NCYC361:T>T		UWOPS83_787_3:T>T	AA:223		273614X:R>R		DBVPG1788:R>R		DBVPG1853:R>R		DBVPG6765:R>R		L_1528:R>R		NCYC361:R>R		UWOPS83_787_3:R>R		W303:R>R		YJM975:R>R		YJM978:R>R		YJM981:R>R	AA:248		UWOPS05_217_3:R>R	AA:257		UWOPS83_787_3:K>K	AA:261		273614X:G>G		DBVPG1788:G>G		DBVPG6765:G>G		L_1528:G>G		YJM975:G>G		YJM978:G>G		YJM981:G>G	AA:263		W303:A>A	AA:317		YIIc17_E5:N>N	AA:346		DBVPG1853:D>D	AA:416		Y12:Q>Q		YS4:Q>Q	AA:437		DBVPG6044:A>A		NCYC110:A>A		SK1:A>A		Y12:A>A		YPS128:A>A		YPS606:A>A		YS4:A>A	AA:442		DBVPG1373:T>T	AA:455		DBVPG6044:L>L		NCYC110:L>L		SK1:L>L	AA:469		378604X:Q>Q		W303:Q>Q	AA:542		DBVPG1373:D>D		L_1374:D>D		L_1528:D>D		YJM978:D>D		YS2:D>D		YS9:D>D	AA:544		378604X:A>A		DBVPG1373:A>A		DBVPG1788:A>A		DBVPG6765:A>A		L_1374:A>A		L_1528:A>A		UWOPS05_217_3:A>A		UWOPS05_227_2:A>A		UWOPS87_2421:A>A		W303:A>A		Y55:A>A		YJM978:A>A		YPS128:A>A		YPS606:A>A		YS2:A>A		YS9:A>A	AA:570		UWOPS03_461_4:N>N		UWOPS05_217_3:N>N		UWOPS05_227_2:N>N		UWOPS87_2421:N>N	AA:585		YS4:P>P	AA:631		UWOPS03_461_4:D>D		UWOPS05_227_2:D>D		UWOPS87_2421:D>DID:YBR213W	AA:32		DBVPG6044:L>L		UWOPS83_787_3:L>L		UWOPS87_2421:L>L	AA:45		DBVPG1788:D>D		Y55:D>D		YS2:D>D		YS9:D>D	AA:53		DBVPG1373:K>K		DBVPG1788:K>K		DBVPG1853:K>K		DBVPG6040:K>K		L_1374:K>K		L_1528:K>K		NCYC361:K>K		Y55:K>K		YJM975:K>K		YJM978:K>K		YS2:K>K		YS9:K>K	AA:84		DBVPG1373:E>E		DBVPG1788:E>E		DBVPG6044:E>E		L_1374:E>E		L_1528:E>E		SK1:E>E		UWOPS87_2421:E>E		Y55:E>E		YJM975:E>E		YS2:E>E		YS4:E>E		YS9:E>E	AA:88		273614X:Y>Y		DBVPG1373:Y>Y		DBVPG1788:Y>Y		L_1374:Y>Y		L_1528:Y>Y		NCYC361:Y>Y		UWOPS87_2421:Y>Y		Y55:Y>Y		YJM975:Y>Y		YS2:Y>Y		YS9:Y>Y	AA:201		YPS606:G>G	AA:203		273614X:L>L		DBVPG1373:L>L		DBVPG1788:L>L		DBVPG6765:L>L		L_1528:L>L		Y55:L>L		YJM978:L>L		YS2:L>L		YS9:L>L	AA:212		UWOPS05_217_3:P>P	AA:241		YS2:V>V	AA:266		Y12:R>R		Y9:R>RID:YBR214W	AA:65		UWOPS83_787_3:G>G	AA:149		DBVPG6044:P>P		NCYC110:P>P		UWOPS83_787_3:P>P	AA:155		DBVPG6044:F>F		NCYC110:F>F	AA:161		NCYC361:D>D	AA:162		UWOPS83_787_3:L>L	AA:179		273614X:Q>Q		322134S:Q>Q		BC187:Q>Q		DBVPG1106:Q>Q		DBVPG1373:Q>Q		DBVPG1788:Q>Q		DBVPG6040:Q>Q		L_1374:Q>Q		L_1528:Q>Q		NCYC361:Q>Q		Y55:Q>Q		YJM975:Q>Q		YJM978:Q>Q		YS2:Q>Q		YS9:Q>Q	AA:203		DBVPG6044:P>P		NCYC110:P>P	AA:211		DBVPG6044:E>E		NCYC110:E>E	AA:224		273614X:V>V		BC187:V>V		DBVPG1106:V>V		DBVPG1373:V>V		DBVPG1788:V>V		DBVPG6040:V>V		L_1374:V>V		L_1528:V>V		NCYC361:V>V		Y55:V>V		YJM975:V>V		YJM978:V>V		YJM981:V>V	AA:227		DBVPG6044:V>V		NCYC110:V>V	AA:233		273614X:E>E		BC187:E>E		DBVPG1106:E>E		DBVPG1373:E>E		DBVPG1788:E>E		DBVPG6040:E>E		L_1374:E>E		L_1528:E>E		NCYC361:E>E		Y55:E>E		YJM975:E>E		YJM978:E>E		YJM981:E>E	AA:316		378604X:I>I		BC187:I>I		DBVPG1106:I>I		DBVPG1373:I>I		DBVPG1788:I>I		DBVPG6040:I>I		DBVPG6765:I>I		L_1374:I>I		L_1528:I>I		NCYC361:I>I		SK1:I>I		Y12:I>I		Y55:I>I		Y9:I>I		YJM978:I>I		YJM981:I>I		YPS128:I>I		YPS606:I>I		YS2:I>I	AA:351		SK1:I>I		UWOPS05_217_3:I>I		UWOPS05_227_2:I>I		Y12:I>I		Y9:I>I		YPS128:I>I		YPS606:I>I	AA:382		DBVPG1106:L>L	AA:391		273614X:H>H		378604X:H>H		BC187:H>H		DBVPG1106:H>H		DBVPG1373:H>H		DBVPG6040:H>H		DBVPG6765:H>H		K11:H>H		L_1374:H>H		NCYC110:H>H		NCYC361:H>H		SK1:H>H		UWOPS05_217_3:H>H		UWOPS05_227_2:H>H		W303:H>H		Y12:H>H		Y55:H>H		Y9:H>H		YJM975:H>H		YJM978:H>H		YJM981:H>H		YPS606:H>H		YS2:H>H		YS4:H>H		YS9:H>H	AA:424		K11:A>A		SK1:A>A		W303:A>A		Y12:A>A		Y9:A>A		YPS606:A>A		YS4:A>A	AA:456		DBVPG1373:P>P		Y55:P>P	AA:457		NCYC110:P>P	AA:472		273614X:A>A		378604X:A>A		DBVPG1373:A>A		DBVPG1788:A>A		DBVPG6044:A>A		DBVPG6765:A>A		K11:A>A		L_1374:A>A		NCYC110:A>A		NCYC361:A>A		SK1:A>A		UWOPS05_217_3:A>A		UWOPS05_227_2:A>A		W303:A>A		Y12:A>A		Y55:A>A		Y9:A>A		YJM975:A>A		YS2:A>A		YS4:A>A		YS9:A>A	AA:496		DBVPG6044:L>L		NCYC110:L>L	AA:520		K11:R>R		SK1:R>R		Y12:R>R		Y9:R>RID:YBR217W	AA:4		UWOPS05_217_3:I>I		UWOPS05_227_2:I>I	AA:7		SK1:S>S		Y12:S>S		Y9:S>S	AA:55		SK1:S>S		Y12:S>S		Y9:S>S	AA:57		YS9:L>L	AA:62		UWOPS83_787_3:D>D	AA:134		UWOPS05_227_2:I>I		UWOPS83_787_3:I>I	AA:142		DBVPG6044:K>K		NCYC110:K>K		UWOPS05_227_2:K>K		UWOPS83_787_3:K>K	AA:184		UWOPS05_217_3:A>A		UWOPS05_227_2:A>AID:YBR220C	AA:76		273614X:D>D	AA:108		273614X:I>I		DBVPG1853:I>I		DBVPG6765:I>I		Y55:I>I		YJM975:I>I		YJM981:I>I		YS2:I>I		YS9:I>I	AA:168		UWOPS05_227_2:L>L	AA:171		273614X:A>A		DBVPG6765:A>A		K11:A>A		UWOPS05_227_2:A>A		W303:A>A		YJM975:A>A		YJM978:A>A		YJM981:A>A		YPS128:A>A		YS2:A>A	AA:173		Y12:T>T	AA:202		UWOPS05_227_2:Y>Y	AA:220		K11:K>K		W303:K>K	AA:259		DBVPG1788:S>S		DBVPG6765:S>S		YJM975:S>S		YJM978:S>S		YJM981:S>S		YS2:S>S	AA:260		UWOPS03_461_4:K>K	AA:319		YPS606:L>L	AA:331		K11:L>L		UWOPS03_461_4:L>L		UWOPS05_227_2:L>L		UWOPS83_787_3:L>L	AA:372		273614X:T>T		DBVPG1373:T>T		DBVPG1788:T>T		DBVPG6040:T>T		DBVPG6765:T>T		L_1374:T>T		L_1528:T>T		UWOPS03_461_4:T>T		UWOPS05_227_2:T>T		UWOPS83_787_3:T>T		YJM975:T>T		YJM978:T>T		YJM981:T>T		YS2:T>T	AA:377		UWOPS03_461_4:K>K		UWOPS05_227_2:K>K	AA:390		322134S:L>L		YS4:L>L	AA:400		273614X:A>A		322134S:A>A		DBVPG1373:A>A		DBVPG1788:A>A		DBVPG6040:A>A		DBVPG6044:A>A		DBVPG6765:A>A		K11:A>A		L_1374:A>A		L_1528:A>A		NCYC110:A>A		UWOPS03_461_4:A>A		UWOPS05_227_2:A>A		UWOPS83_787_3:A>A		W303:A>A		YJM975:A>A		YJM978:A>A		YJM981:A>A		YPS606:A>A		YS2:A>A		YS4:A>A	AA:410		322134S:F>F		W303:F>F		YS4:F>F	AA:422		K11:C>C		YPS606:C>C	AA:426		UWOPS03_461_4:F>F		UWOPS05_227_2:F>F		UWOPS83_787_3:F>F	AA:436		322134S:T>T		DBVPG6044:T>T		NCYC110:T>T		UWOPS83_787_3:T>T		W303:T>T		YS4:T>T	AA:461		322134S:L>L		K11:L>L		YS4:L>L	AA:484		DBVPG1373:F>F		DBVPG1788:F>F		DBVPG1853:F>F		DBVPG6040:F>F		DBVPG6044:F>F		DBVPG6765:F>F		L_1374:F>F		L_1528:F>F		NCYC110:F>F		UWOPS03_461_4:F>F		UWOPS05_227_2:F>F		UWOPS83_787_3:F>F		Y55:F>F		YJM975:F>F		YJM981:F>F		YS2:F>FID:YBR221C	AA:153		273614X:P>P		378604X:P>P		BC187:P>P		DBVPG1373:P>P		DBVPG1788:P>P		DBVPG6765:P>P		L_1374:P>P		NCYC361:P>P		UWOPS05_227_2:P>P		UWOPS87_2421:P>P		Y55:P>P		YS2:P>P		YS4:P>P	AA:196		DBVPG6044:A>A		NCYC110:A>A	AA:266		UWOPS87_2421:G>G		YPS128:G>G	AA:279		DBVPG6044:P>P		NCYC110:P>P	AA:355		UWOPS05_217_3:I>I		UWOPS05_227_2:I>IID:YBR223C	AA:110		YS2:G>G	AA:114		SK1:L>L	AA:165		UWOPS05_227_2:L>L		UWOPS83_787_3:L>L	AA:181		UWOPS05_227_2:H>H		UWOPS83_787_3:H>H	AA:210		322134S:N>N		378604X:N>N		DBVPG1373:N>N		DBVPG1788:N>N		DBVPG6040:N>N		DBVPG6765:N>N		L_1374:N>N		L_1528:N>N		NCYC361:N>N		SK1:N>N		UWOPS05_227_2:N>N		UWOPS83_787_3:N>N		UWOPS87_2421:N>N		W303:N>N		Y55:N>N		YIIc17_E5:N>N		YJM981:N>N		YPS128:N>N		YS2:N>N		YS4:N>N	AA:220		UWOPS05_227_2:P>P	AA:252		UWOPS05_227_2:L>L	AA:314		YPS128:G>G		YPS606:G>G	AA:317		YS9:L>L	AA:380		DBVPG6044:F>F	AA:397		DBVPG6044:Y>Y	AA:405		DBVPG6044:E>E		UWOPS03_461_4:E>E	AA:438		273614X:H>H		322134S:H>H		378604X:H>H		DBVPG1106:H>H		DBVPG6040:H>H		DBVPG6765:H>H		L_1374:H>H		W303:H>H		Y55:H>H		YJM975:H>H		YJM981:H>H		YS9:H>H	AA:477		DBVPG6040:K>K		DBVPG6044:K>K		UWOPS03_461_4:K>K		UWOPS05_227_2:K>K		UWOPS87_2421:K>K		W303:K>K		YPS606:K>K	AA:503		UWOPS03_461_4:F>F		UWOPS05_227_2:F>F		YPS606:F>F	AA:506		YPS606:D>D	AA:510		L_1374:C>C		L_1528:C>C	AA:519		UWOPS03_461_4:V>V		UWOPS05_227_2:V>V		YPS606:V>V	AA:524		L_1528:P>PID:YBR227C	AA:39		DBVPG6044:P>P		NCYC110:P>P	AA:46		UWOPS03_461_4:L>L		UWOPS05_217_3:L>L	AA:57		DBVPG6044:K>K		NCYC110:K>K		UWOPS03_461_4:K>K		UWOPS05_217_3:K>K	AA:97		DBVPG6044:D>D		NCYC110:D>D	AA:137		UWOPS03_461_4:L>L		UWOPS05_217_3:L>L		UWOPS05_227_2:L>L	AA:162		UWOPS87_2421:A>A	AA:218		SK1:G>G		UWOPS87_2421:G>G		YIIc17_E5:G>G	AA:223		DBVPG6044:S>S		NCYC110:S>S	AA:252		DBVPG6044:I>I		SK1:I>I		UWOPS87_2421:I>I		YPS128:I>I		YPS606:I>I	AA:280		322134S:G>G		DBVPG1853:G>G		DBVPG6765:G>G		L_1374:G>G		L_1528:G>G		NCYC361:G>G		SK1:G>G		UWOPS05_217_3:G>G		UWOPS87_2421:G>G		W303:G>G		Y55:G>G		Y9:G>G		YJM978:G>G		YJM981:G>G		YPS128:G>G		YPS606:G>G		YS4:G>G		YS9:G>G	AA:320		DBVPG6044:K>K		SK1:K>K		UWOPS87_2421:K>K		YPS128:K>K		YPS606:K>K	AA:346		YPS128:T>T		YPS606:T>T	AA:353		Y12:F>F		Y9:F>F	AA:372		DBVPG6044:L>L		SK1:L>L		UWOPS87_2421:L>L	AA:435		YS9:L>L	AA:443		DBVPG6040:P>P		W303:P>P	AA:450		SK1:V>V	AA:457		K11:Y>Y		UWOPS03_461_4:V>V		UWOPS05_227_2:V>V		UWOPS83_787_3:V>V		Y12:Y>Y		YIIc17_E5:Y>Y	AA:482		DBVPG6044:D>D	AA:506		UWOPS03_461_4:S>S		UWOPS05_217_3:S>S		UWOPS05_227_2:S>S		UWOPS83_787_3:S>SID:YBR228W	AA:8		DBVPG1106:H>H		L_1528:H>H	AA:100		273614X:V>V		DBVPG1106:V>V		DBVPG1373:V>V		DBVPG6765:V>V		L_1528:V>V		Y55:V>V		YJM975:V>V		YJM978:V>V		YS2:V>V		YS4:V>V		YS9:V>V	AA:164		273614X:E>E		DBVPG1373:E>E		DBVPG6765:E>E		L_1374:E>E		L_1528:E>E		Y55:E>E		YJM978:E>E		YS2:E>E		YS4:E>E		YS9:E>E	AA:189		273614X:V>V		DBVPG1373:V>V		DBVPG6040:V>V		DBVPG6765:V>V		L_1374:V>V		L_1528:V>V		Y55:V>V		YJM978:V>V		YS2:V>V		YS4:V>V		YS9:V>V	AA:271		273614X:N>N		322134S:N>N		DBVPG1373:N>N		DBVPG6040:N>N		DBVPG6765:N>N		K11:N>N		L_1374:N>N		L_1528:N>N		SK1:N>N		UWOPS05_227_2:N>N		UWOPS83_787_3:N>N		UWOPS87_2421:N>N		W303:N>N		Y12:N>N		Y55:N>N		Y9:N>N		YJM975:N>N		YJM978:N>N		YPS606:N>N		YS2:N>N	AA:282		UWOPS87_2421:C>CID:YBR230C	AA:114		UWOPS03_461_4:L>L		UWOPS05_227_2:L>LID:YBR231C	AA:41		DBVPG1106:D>D		DBVPG1373:D>D		DBVPG1788:D>D		DBVPG6765:D>D		L_1374:D>D		UWOPS03_461_4:D>D		UWOPS05_217_3:D>D		W303:D>D		Y12:D>D		Y55:D>D		YIIc17_E5:D>D		YJM975:D>D	AA:104		YPS128:I>I		YPS606:I>I	AA:121		YPS128:N>N	AA:180		BC187:E>E		DBVPG1106:E>E		DBVPG1788:E>E		DBVPG6765:E>E		L_1374:E>E		Y55:E>E		YJM975:E>E		YJM978:E>E	AA:187		DBVPG6040:F>F	AA:209		DBVPG6040:S>S	AA:214		BC187:R>R		DBVPG1106:R>R		DBVPG1788:R>R		DBVPG6040:R>R		DBVPG6044:R>R		DBVPG6765:R>R		L_1374:R>R		NCYC361:R>R		SK1:R>R		UWOPS05_217_3:R>R		W303:R>R		Y55:R>R		YJM975:R>R		YJM978:R>R		YPS128:R>R	AA:221		DBVPG6040:P>P	AA:237		DBVPG6040:T>T	AA:253		DBVPG6044:G>G		YPS128:G>G		YPS606:G>G	AA:284		YJM975:Y>YID:YBR233W	AA:41		UWOPS05_217_3:I>I	AA:53		BC187:S>S		DBVPG1106:S>S		DBVPG1373:S>S		DBVPG1788:S>S		DBVPG1853:S>S		DBVPG6765:S>S		L_1374:S>S		W303:S>S		Y55:S>S		YJM975:S>S		YJM981:S>S		YS9:S>S	AA:55		322134S:N>N		DBVPG6044:N>N		SK1:N>N		UWOPS83_787_3:N>N		YIIc17_E5:N>N	AA:126		322134S:A>A		SK1:A>A	AA:131		UWOPS83_787_3:V>V	AA:201		DBVPG1788:G>G	AA:203		UWOPS05_217_3:P>P	AA:282		DBVPG6040:L>L		SK1:L>L		YIIc17_E5:L>L	AA:386		BC187:A>A		DBVPG1788:A>A		DBVPG1853:A>A		DBVPG6765:A>A		L_1374:A>A		UWOPS05_227_2:A>A		W303:A>A		Y55:A>A		YJM978:A>A		YJM981:A>A		YPS128:A>A		YS2:A>A	AA:413		Y9:L>LID:YBR233W-A	AA:4		K11:N>N		SK1:N>N		YIIc17_E5:N>N	AA:63		322134S:K>K		378604X:K>K		BC187:K>K		DBVPG1788:K>K		DBVPG1853:K>K		DBVPG6044:K>K		DBVPG6765:K>K		L_1374:K>K		L_1528:K>K		SK1:K>K		UWOPS05_227_2:K>K		UWOPS87_2421:K>K		Y55:K>K		YIIc17_E5:K>K		YJM978:K>K		YS9:K>KID:YBR236C	AA:15		378604X:D>D		DBVPG1106:D>D		DBVPG1788:D>D		DBVPG1853:D>D		DBVPG6765:D>D		L_1528:D>D		Y55:D>D		YS2:D>D		YS4:D>D		YS9:D>D	AA:88		DBVPG6044:E>E	AA:109		378604X:E>E		BC187:E>E		DBVPG1106:E>E		DBVPG1788:E>E		DBVPG1853:E>E		DBVPG6765:E>E		L_1528:E>E		Y55:E>E		YS2:E>E		YS4:E>E		YS9:E>E	AA:162		378604X:T>T		BC187:T>T		DBVPG1106:T>T		DBVPG1853:T>T		DBVPG6040:T>T		DBVPG6765:T>T		L_1528:T>T		Y55:T>T		YS2:T>T		YS4:T>T		YS9:T>T	AA:266		UWOPS05_227_2:L>L	AA:277		DBVPG6040:G>G	AA:286		UWOPS05_227_2:S>S	AA:321		SK1:K>K		Y12:K>K		Y9:K>K	AA:358		YS4:L>L	AA:426		DBVPG6044:Q>Q		NCYC110:Q>QID:YBR237W	AA:81		UWOPS05_217_3:S>S		UWOPS05_227_2:S>S	AA:224		DBVPG6044:T>T	AA:262		DBVPG6044:L>L	AA:294		S288c:R>R	AA:302		DBVPG6044:T>T	AA:305		DBVPG1373:G>G		DBVPG6040:G>G		DBVPG6044:G>G		DBVPG6765:G>G		L_1374:G>G		W303:G>G		Y55:G>G	AA:307		DBVPG1373:T>T		DBVPG6040:T>T		DBVPG6765:T>T		L_1374:T>T		W303:T>T		Y55:T>T	AA:340		DBVPG6044:R>R	AA:356		Y9:A>A	AA:367		DBVPG1373:G>G		DBVPG6040:G>G		DBVPG6044:G>G		DBVPG6765:G>G		L_1374:G>G		W303:G>G		Y55:G>G	AA:381		DBVPG6044:G>G	AA:412		322134S:V>V		YS4:V>V	AA:425		K11:F>F		UWOPS03_461_4:F>F		UWOPS05_217_3:F>F	AA:435		YPS606:T>T	AA:535		UWOPS03_461_4:Q>Q		UWOPS05_227_2:Q>Q	AA:539		UWOPS05_227_2:D>D	AA:561		378604X:Y>Y		DBVPG1373:Y>Y		DBVPG1788:Y>Y		DBVPG6765:Y>Y		L_1374:Y>Y		L_1528:Y>Y		W303:Y>Y		Y55:Y>Y	AA:619		378604X:G>G		DBVPG1373:G>G		DBVPG1788:G>G		DBVPG6765:G>G		L_1528:G>G		W303:G>G		Y55:G>G		YJM978:G>G	AA:764		SK1:Q>Q		Y12:Q>Q	AA:810		SK1:Y>Y		Y12:Y>Y		Y9:Y>Y	AA:815		UWOPS05_227_2:G>G	AA:825		273614X:I>I		BC187:I>I		DBVPG1788:I>I		DBVPG6765:I>I		UWOPS05_227_2:I>I		YJM975:I>I		YJM978:I>I		YS9:I>I	AA:848		273614X:K>K		BC187:K>K		DBVPG1788:K>K		DBVPG6765:K>K		SK1:K>K		UWOPS05_227_2:K>K		Y12:K>K		Y9:K>K		YIIc17_E5:K>K		YJM975:K>K		YJM978:K>K		YS9:K>KID:YBR239C	AA:22		273614X:N>N		DBVPG1106:N>N		DBVPG1788:N>N		DBVPG1853:N>N		DBVPG6040:N>N		DBVPG6044:N>N		DBVPG6765:N>N		K11:N>N		L_1374:N>N		L_1528:N>N		SK1:N>N		UWOPS03_461_4:N>N		UWOPS05_227_2:N>N		UWOPS83_787_3:N>N		UWOPS87_2421:N>N		W303:N>N		Y55:N>N		Y9:N>N		YIIc17_E5:N>N		YJM975:N>N		YPS128:N>N		YPS606:N>N	AA:37		DBVPG6044:N>N	AA:67		UWOPS03_461_4:T>T		UWOPS05_227_2:T>T	AA:69		YPS128:V>V		YPS606:V>V	AA:128		273614X:Q>Q		DBVPG1788:Q>Q		DBVPG1853:Q>Q		DBVPG6040:Q>Q		DBVPG6044:Q>Q		DBVPG6765:Q>Q		L_1374:Q>Q		L_1528:Q>Q		SK1:Q>Q		UWOPS05_217_3:Q>Q		UWOPS05_227_2:Q>Q		Y55:Q>Q		YIIc17_E5:Q>Q		YJM975:Q>Q		YPS128:Q>Q	AA:161		SK1:I>I		YIIc17_E5:I>I		YPS128:I>I	AA:166		DBVPG6044:L>L	AA:173		DBVPG6044:T>T	AA:184		UWOPS05_217_3:N>N		UWOPS05_227_2:N>N	AA:188		273614X:G>G		DBVPG1373:G>G		DBVPG1853:G>G		DBVPG6040:G>G		DBVPG6765:G>G		L_1374:G>G		L_1528:G>G		Y55:G>G		YJM975:G>G		YPS128:G>G	AA:200		YPS128:K>K	AA:275		273614X:S>S	AA:282		DBVPG1373:S>S	AA:292		YJM981:T>T	AA:348		UWOPS05_217_3:A>A		UWOPS05_227_2:A>A	AA:408		UWOPS05_217_3:T>T		UWOPS05_227_2:T>T	AA:422		YIIc17_E5:P>P	AA:432		BC187:I>I		DBVPG1106:I>I		DBVPG1373:I>I		DBVPG6765:I>I		L_1528:I>I		NCYC361:I>I		UWOPS05_217_3:I>I		UWOPS05_227_2:I>I		UWOPS83_787_3:I>I		W303:I>I		Y55:I>I		YIIc17_E5:I>I		YJM978:I>I		YJM981:I>I		YPS128:I>I		YS2:I>IID:YBR240C	AA:25		DBVPG6044:R>R	AA:67		DBVPG6044:E>E	AA:142		UWOPS05_227_2:R>R	AA:163		K11:T>T		NCYC361:T>T		SK1:T>T		UWOPS05_227_2:T>T		UWOPS83_787_3:T>T		UWOPS87_2421:T>T		YIIc17_E5:T>T		YPS128:T>T		YPS606:T>T	AA:180		SK1:N>N		UWOPS05_227_2:N>N		UWOPS83_787_3:N>N		UWOPS87_2421:N>N		YIIc17_E5:N>N		YPS128:N>N		YPS606:N>N	AA:212		SK1:Q>Q	AA:216		SK1:E>E		UWOPS05_227_2:P>P		UWOPS83_787_3:E>E		UWOPS87_2421:E>E		YIIc17_E5:P>P		YJM975:P>P		YPS128:P>P		YPS606:E>E		YS4:P>P	AA:222		YIIc17_E5:S>S	AA:238		UWOPS87_2421:S>S	AA:344		YIIc17_E5:L>L	AA:355		UWOPS87_2421:P>P	AA:358		NCYC110:N>N	AA:367		NCYC110:L>L	AA:369		DBVPG6040:V>V		DBVPG6765:V>V		W303:V>V		Y55:V>V		YJM975:V>V		YJM981:V>V		YS4:V>V	AA:371		UWOPS03_461_4:Y>Y		UWOPS05_217_3:Y>Y		UWOPS05_227_2:Y>Y	AA:376		DBVPG6040:L>L		DBVPG6765:L>L		W303:L>L		Y55:L>L		YJM975:L>L		YJM981:L>L		YS4:L>L	AA:377		NCYC110:L>L	AA:387		DBVPG6040:Y>Y		DBVPG6765:Y>Y		W303:Y>Y		Y55:Y>Y		YJM975:Y>Y		YJM981:Y>Y		YS4:Y>Y	AA:447		NCYC110:A>AID:YBR242W	AA:90		K11:L>L		SK1:L>L		UWOPS83_787_3:L>L		YS4:L>L	AA:145		YIIc17_E5:I>I	AA:202		K11:F>F		SK1:F>F		Y12:F>F	AA:211		DBVPG6044:T>T		K11:T>T		SK1:T>T		UWOPS05_217_3:T>T		UWOPS05_227_2:T>T		Y12:T>T		YIIc17_E5:T>T		YPS128:T>T		YPS606:T>T	AA:225		DBVPG6044:R>R		K11:R>R		SK1:R>R		UWOPS03_461_4:R>R		UWOPS05_217_3:R>R		UWOPS05_227_2:R>R		Y12:R>R		YPS128:R>R		YPS606:R>RID:YBR243C	AA:22		YPS606:P>P	AA:55		UWOPS83_787_3:L>L	AA:91		NCYC110:I>I	AA:115		NCYC110:G>G		SK1:G>G		Y12:G>G		YPS606:G>G		YS4:G>G	AA:133		UWOPS83_787_3:I>I	AA:165		UWOPS03_461_4:I>I		UWOPS05_227_2:I>I	AA:178		UWOPS83_787_3:T>T	AA:209		Y12:A>A	AA:216		Y12:I>I	AA:218		UWOPS03_461_4:I>I	AA:243		BC187:L>L		DBVPG6765:L>L		L_1374:L>L		L_1528:L>L		Y55:L>L		YJM978:L>L	AA:249		Y12:P>P	AA:277		YPS606:N>N	AA:355		UWOPS03_461_4:N>N		UWOPS05_217_3:N>N		UWOPS05_227_2:N>N	AA:379		Y12:D>D	AA:387		DBVPG6044:E>E		SK1:E>E		UWOPS03_461_4:E>E		UWOPS05_217_3:E>E		UWOPS05_227_2:E>E		Y12:E>E		YPS606:E>E	AA:389		DBVPG6044:I>I		SK1:I>I	AA:436		DBVPG1788:A>AID:YBR244W	AA:16		YIIc17_E5:E>E	AA:17		DBVPG6044:S>S		NCYC110:S>S		UWOPS03_461_4:S>S		UWOPS05_217_3:S>S		UWOPS83_787_3:S>S		UWOPS87_2421:S>S		YPS606:S>S	AA:23		YS9:L>L	AA:87		DBVPG6040:Y>Y		YIIc17_E5:Y>Y	AA:107		YJM978:V>V		YS9:V>V	AA:124		DBVPG6044:I>IID:YBR246W	AA:44		DBVPG1373:R>R		DBVPG1853:R>R		DBVPG6044:R>R		K11:R>R		NCYC110:R>R		SK1:R>R		UWOPS03_461_4:R>R		UWOPS05_217_3:R>R		UWOPS05_227_2:R>R		UWOPS87_2421:R>R		YIIc17_E5:R>R		YPS606:R>R	AA:68		DBVPG1373:L>L		DBVPG1853:L>L		DBVPG6044:L>L		K11:L>L		NCYC110:L>L		SK1:L>L		UWOPS03_461_4:L>L		UWOPS05_217_3:L>L		UWOPS05_227_2:L>L		Y12:L>L		YIIc17_E5:L>L		YPS128:L>L		YPS606:L>L	AA:72		DBVPG1373:L>L		DBVPG1853:L>L		DBVPG6044:L>L		K11:L>L		NCYC110:L>L		SK1:L>L		UWOPS03_461_4:L>L		UWOPS05_217_3:L>L		UWOPS05_227_2:L>L		Y12:L>L		YIIc17_E5:L>L		YPS606:L>L	AA:124		SK1:I>I	AA:148		DBVPG6044:T>T		NCYC110:T>T	AA:164		322134S:A>A		DBVPG6044:A>A		K11:A>A		NCYC110:A>A		SK1:A>A		UWOPS03_461_4:A>A		UWOPS05_217_3:A>A		UWOPS05_227_2:A>A		UWOPS83_787_3:A>A		Y12:A>A		YIIc17_E5:A>A	AA:185		322134S:I>I		DBVPG6044:I>I		K11:I>I		NCYC110:I>I		SK1:I>I		UWOPS03_461_4:I>I		UWOPS05_217_3:I>I		UWOPS05_227_2:I>I		UWOPS83_787_3:I>I		Y12:I>I		Y9:I>I		YIIc17_E5:I>I		YPS606:I>I	AA:203		DBVPG6044:A>A		NCYC110:A>A	AA:213		K11:D>D	AA:223		322134S:R>R		DBVPG6044:R>R		K11:R>R		NCYC110:R>R		SK1:R>R		UWOPS03_461_4:R>R		UWOPS05_227_2:R>R		UWOPS83_787_3:R>R		UWOPS87_2421:R>R		Y12:R>R		Y9:R>R	AA:292		322134S:P>P		K11:P>P		NCYC110:P>P		SK1:P>P		UWOPS05_227_2:P>P		UWOPS83_787_3:P>P		UWOPS87_2421:P>P		Y12:P>P		Y9:P>P	AA:294		NCYC361:V>V	AA:316		NCYC361:E>E	AA:344		322134S:S>S		K11:S>S		SK1:S>S		UWOPS83_787_3:S>S		Y12:S>S		Y9:S>SID:YBR247C	AA:221		UWOPS03_461_4:L>L	AA:225		SK1:T>T		Y12:T>T		YIIc17_E5:T>T		YPS128:T>T		YPS606:T>T	AA:248		378604X:V>V	AA:280		UWOPS03_461_4:N>N	AA:335		SK1:A>A		Y12:A>A		Y9:A>A		YIIc17_E5:A>A	AA:380		SK1:Y>Y		Y12:Y>Y		Y9:Y>Y		YIIc17_E5:Y>Y		YPS128:Y>Y	AA:400		DBVPG6040:L>L	AA:410		DBVPG6040:T>T	AA:434		YS4:Q>Q	AA:436		DBVPG1853:Q>Q	AA:440		DBVPG6040:L>L	AA:468		DBVPG6040:F>F		NCYC361:F>F	AA:483		DBVPG6040:N>N		NCYC361:N>NID:YBR248C	AA:25		L_1374:G>G	AA:46		378604X:I>I		SK1:I>I		UWOPS05_227_2:I>I		Y12:I>I		YIIc17_E5:I>I	AA:68		378604X:P>P	AA:116		378604X:E>E		YPS128:E>E		YPS606:E>E	AA:156		DBVPG6765:E>E	AA:178		378604X:F>F		DBVPG6044:F>F		SK1:F>F		UWOPS87_2421:F>F		YIIc17_E5:F>F		YPS128:F>F		YPS606:F>F		YS4:F>F	AA:179		DBVPG6765:I>I		UWOPS05_227_2:I>I	AA:329		UWOPS03_461_4:V>V		UWOPS87_2421:V>V	AA:330		DBVPG6044:G>G		UWOPS03_461_4:G>G		UWOPS05_227_2:G>G	AA:365		UWOPS03_461_4:T>T		UWOPS05_227_2:T>T	AA:372		378604X:E>E	AA:378		322134S:G>G		378604X:G>G		SK1:G>G		YPS128:G>G		YPS606:G>G	AA:403		UWOPS05_217_3:V>V	AA:404		BC187:D>D		DBVPG1373:D>D		DBVPG1788:D>D		DBVPG6765:D>D		L_1374:D>D		L_1528:D>D		Y55:D>D		YJM978:D>D		YS2:D>DID:YBR249C	AA:28		273614X:Y>Y	AA:54		YJM978:G>G		YJM981:G>G	AA:81		378604X:L>L		K11:L>L		SK1:L>L		Y12:L>L		Y9:L>L	AA:207		378604X:T>T		DBVPG6044:T>T		SK1:T>T		UWOPS83_787_3:T>T		YPS128:T>T		YPS606:T>T	AA:326		273614X:I>I	AA:356		322134S:K>K		DBVPG1373:K>K		DBVPG1788:K>K		DBVPG1853:K>K		DBVPG6040:K>K		DBVPG6765:K>K		SK1:K>K		Y12:K>K		Y55:K>K		Y9:K>K		YIIc17_E5:K>K		YPS128:K>K		YPS606:K>K		YS2:K>K		YS4:K>KID:YBR251W	AA:29		DBVPG6044:L>L		NCYC110:L>L	AA:41		UWOPS05_227_2:P>P	AA:49		K11:S>S		UWOPS83_787_3:S>S	AA:59		DBVPG6044:L>L		NCYC110:L>L		SK1:L>L		UWOPS05_227_2:L>L		Y12:L>L		Y9:L>L		YPS128:L>L		YPS606:L>L	AA:103		UWOPS03_461_4:G>G		UWOPS05_227_2:G>G	AA:158		378604X:T>T		DBVPG6044:T>T		K11:T>T		NCYC110:T>T		SK1:T>T		UWOPS03_461_4:T>T		UWOPS05_227_2:T>T		UWOPS83_787_3:T>T		YPS128:T>T		YPS606:T>T		YS4:T>T	AA:183		378604X:E>E		DBVPG6044:E>E		K11:E>E		SK1:E>E		UWOPS03_461_4:E>E		UWOPS05_227_2:E>E		UWOPS83_787_3:E>E		YPS128:E>E		YS4:E>E	AA:191		K11:S>S		SK1:S>S	AA:260		DBVPG1788:G>G		DBVPG6765:G>G		L_1374:G>GID:YBR252W	AA:9		DBVPG6040:L>L		K11:L>L		SK1:L>L		UWOPS05_217_3:L>L		Y12:L>L		YS4:L>L	AA:57		NCYC110:V>V	AA:88		Y12:Y>Y	AA:94		UWOPS05_217_3:V>V	AA:112		DBVPG6044:V>V		NCYC110:V>V	AA:132		DBVPG6044:L>L		NCYC110:L>L	AA:145		YIIc17_E5:T>TID:YBR253W	AA:18		YS4:S>S	AA:44		K11:E>E		SK1:E>E	AA:50		K11:V>V		SK1:V>V	AA:62		UWOPS83_787_3:T>T	AA:101		DBVPG6044:E>E	AA:113		YS4:E>EID:YBR254C	AA:14		Y12:P>P		Y9:P>P	AA:56		378604X:N>N		DBVPG6040:N>N		K11:N>N		SK1:N>N		Y12:N>N		Y9:N>N		YPS128:N>N		YPS606:N>N	AA:85		378604X:D>D		K11:D>D		SK1:D>D	AA:105		378604X:S>S		K11:S>S		SK1:S>S	AA:133		378604X:F>F		DBVPG6040:F>F		K11:F>F		SK1:F>F		UWOPS05_227_2:F>F		UWOPS83_787_3:F>F		YPS128:F>F		YPS606:F>FID:YBR256C	AA:11		378604X:T>T		SK1:T>T		UWOPS87_2421:T>T	AA:20		378604X:D>D		DBVPG6040:D>D		K11:D>D		SK1:D>D		UWOPS05_227_2:D>D	AA:52		DBVPG6044:G>G		NCYC110:G>G	AA:55		378604X:L>L		DBVPG6040:L>L		SK1:L>L		UWOPS05_227_2:L>L		UWOPS83_787_3:L>L	AA:79		378604X:V>V		DBVPG6040:V>V		SK1:V>V		UWOPS05_227_2:V>V	AA:116		378604X:V>V		DBVPG6040:V>V		SK1:V>V		UWOPS05_227_2:V>V	AA:129		378604X:F>F		DBVPG6040:F>F		SK1:F>F		UWOPS05_227_2:F>F	AA:160		UWOPS83_787_3:P>P	AA:169		UWOPS87_2421:I>I	AA:174		DBVPG6040:H>H		DBVPG6044:H>H		NCYC110:H>H		SK1:H>H		UWOPS05_227_2:H>H		UWOPS83_787_3:H>H		UWOPS87_2421:H>H		YPS128:H>H		YPS606:H>HID:YBR257W	AA:16		DBVPG6040:C>C		SK1:C>C		Y12:C>C	AA:36		DBVPG6040:L>L		SK1:L>L		UWOPS83_787_3:L>L		Y12:L>L		YIIc17_E5:L>L		YPS128:L>L		YPS606:L>L	AA:59		DBVPG6044:N>N		UWOPS05_217_3:N>N	AA:221		SK1:K>K		UWOPS03_461_4:K>K		UWOPS05_227_2:K>K		UWOPS83_787_3:K>K		UWOPS87_2421:K>K		YPS606:K>K	AA:228		UWOPS03_461_4:T>T		UWOPS05_227_2:T>T	AA:252		Y12:R>R		YPS606:R>RID:YBR258C	AA:44		DBVPG6044:E>E		SK1:E>E		UWOPS05_227_2:E>E		UWOPS83_787_3:E>E		UWOPS87_2421:E>E		Y12:E>E		YIIc17_E5:E>E	AA:86		BC187:Y>YID:YBR259W	AA:2		K11:S>S		SK1:S>S		UWOPS83_787_3:S>S		YIIc17_E5:S>S		YPS128:S>S		YS2:S>S	AA:43		YPS128:V>V	AA:92		SK1:Q>Q		YIIc17_E5:Q>Q		YS4:Q>Q	AA:144		UWOPS83_787_3:F>F	AA:193		UWOPS83_787_3:C>C		YPS128:C>C		YPS606:C>C	AA:233		UWOPS83_787_3:E>E	AA:265		DBVPG6044:Q>Q		NCYC110:Q>Q		UWOPS05_227_2:Q>Q		YPS128:Q>Q		YPS606:Q>Q	AA:302		DBVPG6044:R>R		K11:R>R		SK1:R>R		UWOPS05_227_2:R>R		UWOPS83_787_3:R>R		Y9:R>R		YPS128:R>R		YPS606:R>R		YS4:R>R	AA:353		UWOPS05_227_2:L>L	AA:385		NCYC110:D>D		NCYC361:D>D		SK1:D>D		Y9:D>D		YIIc17_E5:D>D		YS4:D>D	AA:498		Y9:E>E	AA:502		Y9:V>V	AA:507		NCYC110:N>N	AA:516		NCYC110:L>L		SK1:L>L		UWOPS05_217_3:L>L		UWOPS05_227_2:L>L		Y12:L>L		Y9:L>L		YIIc17_E5:L>L		YPS128:L>L		YPS606:L>L	AA:528		DBVPG6040:S>S		NCYC110:S>S		SK1:S>S		UWOPS05_217_3:S>S		UWOPS05_227_2:S>S		Y12:S>S		YIIc17_E5:S>S		YPS128:S>S		YPS606:S>S	AA:566		NCYC110:F>F	AA:579		BC187:D>D		DBVPG1373:D>D		DBVPG1788:D>D		DBVPG6040:D>D		DBVPG6765:D>D		L_1374:D>D		SK1:D>D		UWOPS05_217_3:D>D		UWOPS05_227_2:D>D		Y12:D>D		Y55:D>D		YIIc17_E5:D>D		YPS128:D>D		YPS606:D>D		YS9:D>D	AA:651		DBVPG6044:L>L		UWOPS05_217_3:L>L		UWOPS05_227_2:L>L		YPS128:L>L		YPS606:L>L	AA:687		YPS128:L>L		YPS606:L>LID:YBR260C	AA:18		DBVPG1853:S>S	AA:50		378604X:S>S		DBVPG6765:S>S		L_1528:S>S		Y55:S>S		YJM975:S>S		YJM978:S>S	AA:65		BC187:F>F		DBVPG1853:F>F		DBVPG6765:F>F		L_1528:F>F		Y55:F>F		YJM975:F>F		YJM978:F>F	AA:77		SK1:V>V		YS4:V>V	AA:121		273614X:T>T		DBVPG6044:T>T		UWOPS03_461_4:T>T		UWOPS05_217_3:T>T		UWOPS05_227_2:T>T	AA:148		273614X:S>S		DBVPG6044:S>S		UWOPS03_461_4:S>S		UWOPS05_217_3:S>S		UWOPS05_227_2:S>S		UWOPS83_787_3:S>S	AA:198		273614X:F>F		DBVPG6044:F>F		NCYC110:F>F		SK1:K>K		UWOPS03_461_4:K>K		UWOPS05_217_3:K>K		UWOPS05_227_2:F>F		UWOPS83_787_3:F>F		UWOPS87_2421:F>F		YPS128:F>F		YPS606:F>F	AA:229		SK1:F>F		Y9:F>F		YPS128:F>F	AA:251		SK1:A>A		Y9:A>A		YIIc17_E5:A>A		YS9:A>A	AA:277		273614X:L>L		NCYC110:L>L		SK1:L>L		UWOPS03_461_4:L>L		UWOPS05_227_2:L>L		Y9:L>L		YIIc17_E5:L>L		YPS128:L>L		YPS606:L>L		YS9:L>L	AA:282		YPS128:S>S		YPS606:S>S	AA:311		SK1:L>L		Y9:L>L		YIIc17_E5:L>L		YS9:L>L	AA:337		NCYC110:P>P		SK1:P>P		UWOPS05_227_2:P>P		UWOPS87_2421:P>P		Y9:P>P		YIIc17_E5:P>P		YPS128:P>P		YPS606:P>P		YS4:P>P		YS9:P>P	AA:360		UWOPS05_227_2:S>S	AA:369		UWOPS05_227_2:T>T	AA:398		NCYC110:I>I		YPS128:I>I		YPS606:I>I	AA:406		NCYC110:G>G		YPS128:G>G		YPS606:G>G	AA:436		NCYC110:I>I		SK1:I>I		Y9:I>I		YIIc17_E5:I>I		YPS128:I>I		YPS606:I>I		YS9:I>I	AA:440		NCYC110:L>L		SK1:L>L		Y9:L>L		YIIc17_E5:L>L		YPS128:L>L		YPS606:L>L		YS9:L>L	AA:477		273614X:L>L		UWOPS05_227_2:L>L		UWOPS83_787_3:L>L		UWOPS87_2421:L>L	AA:496		273614X:I>I		UWOPS05_227_2:I>I		UWOPS83_787_3:I>I		UWOPS87_2421:I>I	AA:527		273614X:P>P		UWOPS05_227_2:P>P		UWOPS83_787_3:P>P		UWOPS87_2421:P>P	AA:542		SK1:S>S	AA:570		DBVPG6044:E>E		NCYC110:E>E		UWOPS83_787_3:E>E		UWOPS87_2421:E>E		YPS606:E>E	AA:590		UWOPS87_2421:L>L	AA:596		YPS606:D>D	AA:640		DBVPG6044:D>D	AA:654		SK1:V>VID:YBR261C	AA:44		K11:L>L		NCYC110:L>L		SK1:L>L		UWOPS83_787_3:L>L		Y9:L>L		YPS128:L>L		YPS606:L>L		YS9:L>L	AA:53		K11:L>L		Y9:L>L	AA:56		SK1:R>R		UWOPS83_787_3:R>R		YPS128:R>R		YPS606:R>R		YS9:R>R	AA:63		NCYC110:N>N	AA:74		UWOPS83_787_3:I>I	AA:97		NCYC110:P>P		SK1:P>P		UWOPS03_461_4:P>P		UWOPS83_787_3:P>P		UWOPS87_2421:P>P		Y9:P>P		YIIc17_E5:P>P		YPS128:P>P		YPS606:P>P	AA:100		NCYC110:E>E		YPS128:E>E		YPS606:E>E	AA:108		UWOPS03_461_4:E>E		UWOPS87_2421:E>E	AA:115		UWOPS03_461_4:I>I		UWOPS87_2421:I>I	AA:142		UWOPS05_227_2:V>V	AA:145		DBVPG6044:L>L		NCYC110:L>L		SK1:L>L		UWOPS03_461_4:L>L		UWOPS83_787_3:L>L		UWOPS87_2421:L>L		Y9:L>L		YIIc17_E5:L>L		YPS128:L>L		YPS606:L>L	AA:206		UWOPS87_2421:L>LID:YBR262C	AA:53		YS2:V>V		YS9:V>V	AA:78		SK1:S>SID:YBR263W	AA:17		SK1:R>R		Y9:R>R	AA:38		DBVPG6044:P>P	AA:63		378604X:F>F	AA:112		378604X:G>G		UWOPS05_217_3:G>G		YPS128:G>G		YPS606:G>G	AA:301		273614X:F>F	AA:323		378604X:L>L		SK1:L>L		Y9:L>L	AA:356		378604X:L>L		SK1:L>L		Y9:L>L	AA:362		UWOPS05_227_2:D>D	AA:378		DBVPG6044:A>A		NCYC110:A>A		SK1:A>A		UWOPS05_227_2:A>A		Y9:A>A	AA:430		273614X:K>K	AA:436		BC187:V>V		Y55:V>V		YJM981:V>V		YS4:V>VID:YBR264C	AA:43		SK1:T>T		UWOPS05_217_3:T>T		Y12:T>T	AA:75		378604X:L>L		UWOPS05_217_3:L>L		YIIc17_E5:L>L		YPS128:L>L		YPS606:L>L	AA:143		DBVPG6044:G>G		NCYC110:G>G	AA:144		YS4:L>L	AA:159		YPS606:T>T	AA:181		SK1:E>EID:YBR265W	AA:13		UWOPS05_217_3:T>T	AA:31		322134S:A>A	AA:111		378604X:T>T		YIIc17_E5:T>T		YS4:T>T	AA:120		322134S:A>A		378604X:A>A		DBVPG1788:A>A		DBVPG1853:A>A		DBVPG6040:A>A		K11:A>A		SK1:A>A		UWOPS03_461_4:A>A		UWOPS87_2421:A>A		Y9:A>A		YIIc17_E5:A>A		YJM975:A>A		YPS128:A>A		YS4:A>A	AA:122		K11:P>P		SK1:P>P		Y9:P>P	AA:126		K11:R>R		SK1:R>R		Y9:R>R	AA:148		YS4:A>A	AA:152		K11:A>A		SK1:A>A		UWOPS03_461_4:A>A		Y9:A>A	AA:157		322134S:T>T	AA:170		YPS128:A>A	AA:233		Y12:P>P	AA:251		Y12:A>A		YIIc17_E5:A>A	AA:290		DBVPG6040:L>L		Y12:L>LID:YBR267W	AA:14		378604X:L>L	AA:49		UWOPS03_461_4:T>T		UWOPS05_217_3:T>T		UWOPS05_227_2:T>T		UWOPS83_787_3:T>T	AA:62		378604X:S>S		DBVPG6040:S>S		DBVPG6044:S>S		UWOPS03_461_4:S>S		UWOPS05_217_3:S>S		UWOPS05_227_2:S>S		UWOPS83_787_3:S>S		Y12:S>S		Y9:S>S		YPS128:S>S	AA:65		DBVPG6044:A>A	AA:170		378604X:N>N		DBVPG6044:N>N		SK1:N>N		UWOPS03_461_4:N>N		UWOPS05_217_3:N>N		UWOPS05_227_2:N>N		UWOPS87_2421:N>N		Y12:N>N		Y9:N>N		YPS128:N>N	AA:174		DBVPG6044:K>K		SK1:K>K		UWOPS03_461_4:K>K		UWOPS05_217_3:K>K		UWOPS05_227_2:K>K		UWOPS87_2421:K>K		YPS128:K>K	AA:197		SK1:L>L	AA:226		SK1:T>T	AA:251		378604X:L>L		UWOPS05_227_2:L>L		Y12:L>L		Y9:L>L	AA:329		273614X:E>E		322134S:E>E		DBVPG1106:E>E		DBVPG1373:E>E		DBVPG6765:E>E		L_1374:E>E		Y55:E>E		YJM978:E>E		YJM981:E>E		YS2:E>E	AA:345		DBVPG6044:R>R	AA:367		DBVPG6044:R>RID:YBR268W	AA:38		SK1:L>LID:YBR269C	AA:35		DBVPG6044:T>T		K11:T>T		SK1:T>T		UWOPS05_227_2:T>T		UWOPS87_2421:T>T		YIIc17_E5:T>T		YPS128:T>T	AA:61		K11:S>S	AA:119		SK1:P>PID:YBR270C	AA:19		322134S:T>T		378604X:T>T		DBVPG1373:T>T		DBVPG1853:T>T		DBVPG6044:T>T		DBVPG6765:T>T		L_1374:T>T		L_1528:T>T		Y55:T>T		YJM978:T>T		YJM981:T>T		YS2:T>T		YS4:T>T	AA:49		378604X:D>D		DBVPG1853:D>D		L_1374:D>D		L_1528:D>D		YJM978:D>D		YJM981:D>D		YS2:D>D	AA:84		DBVPG6765:R>R		Y55:R>R		YS2:R>R		YS4:R>R	AA:112		322134S:N>N		378604X:N>N		DBVPG1853:N>N		DBVPG6044:N>N		DBVPG6765:N>N		K11:N>N		L_1374:N>N		NCYC361:N>N		SK1:N>N		Y55:N>N		YJM978:N>N		YJM981:N>N		YS2:N>N		YS4:N>N	AA:218		DBVPG6044:P>P	AA:242		K11:S>S		SK1:S>S		W303:S>S		Y9:S>S		YPS128:S>S		YPS606:S>S	AA:244		YS4:G>G	AA:248		K11:S>S		SK1:S>S		Y9:S>S		YPS128:S>S		YPS606:S>S	AA:259		UWOPS03_461_4:G>G		UWOPS05_227_2:G>G	AA:283		UWOPS03_461_4:L>L		UWOPS05_227_2:L>L	AA:322		YS4:F>F	AA:338		UWOPS87_2421:E>E	AA:398		DBVPG1373:V>V	AA:441		YPS128:Q>Q		YPS606:Q>Q	AA:466		UWOPS87_2421:L>L	AA:483		DBVPG6044:L>L		NCYC110:L>LID:YBR271W	AA:6		DBVPG6044:D>D	AA:78		Y12:K>K		Y9:K>K	AA:143		273614X:S>S		378604X:S>S		DBVPG1106:S>S		DBVPG1373:S>S		DBVPG1853:S>S		DBVPG6044:S>S		DBVPG6765:S>S		L_1374:S>S		L_1528:S>S		SK1:S>S		UWOPS05_227_2:S>S		UWOPS87_2421:S>S		Y12:S>S		Y55:S>S		Y9:S>S		YJM978:S>S		YJM981:S>S		YPS128:S>S		YS2:S>S	AA:149		SK1:G>G		UWOPS05_227_2:G>G		Y12:G>G		Y9:G>G	AA:176		UWOPS05_227_2:L>L	AA:179		378604X:R>R		SK1:R>R		UWOPS05_227_2:R>R		Y12:R>R		Y9:R>R	AA:186		UWOPS87_2421:R>R	AA:198		378604X:L>L		SK1:L>L		UWOPS05_227_2:L>L		Y12:L>L		Y9:L>L	AA:216		DBVPG6044:N>N		K11:N>N		NCYC110:N>N		YPS128:N>N	AA:314		273614X:E>E		378604X:E>E		BC187:E>E		DBVPG1373:E>E		DBVPG1853:E>E		DBVPG6040:E>E		DBVPG6044:E>E		DBVPG6765:E>E		K11:E>E		L_1374:E>E		L_1528:E>E		NCYC110:E>E		SK1:E>E		UWOPS05_227_2:E>E		UWOPS87_2421:E>E		Y55:E>E		YJM975:E>E		YJM978:E>E		YPS128:E>E	AA:317		DBVPG6044:D>D		K11:D>D		NCYC110:D>D		UWOPS87_2421:D>D		YPS128:D>D	AA:346		K11:P>P	AA:348		DBVPG6044:H>H		K11:H>H		NCYC110:H>H		SK1:H>H		UWOPS87_2421:H>H		YPS128:H>HID:YBR272C	AA:92		DBVPG6044:V>V	AA:102		DBVPG6044:V>V	AA:209		273614X:G>G		322134S:G>G		378604X:G>G		DBVPG1373:G>G		DBVPG1788:G>G		DBVPG6765:G>G		UWOPS03_461_4:G>G		UWOPS05_227_2:G>G		UWOPS83_787_3:G>G		UWOPS87_2421:G>G		Y55:G>G		YJM978:G>G		YPS128:G>G		YPS606:G>G	AA:238		W303:V>V	AA:291		322134S:L>L	AA:304		W303:S>S	AA:395		273614X:T>T		322134S:T>T		378604X:T>T		DBVPG1373:T>T		DBVPG1788:T>T		DBVPG6765:T>T		L_1528:T>T		UWOPS03_461_4:T>T		UWOPS05_227_2:T>T		UWOPS83_787_3:T>T		UWOPS87_2421:T>T		Y55:T>T		YJM978:T>T		YS2:T>T		YS9:T>T	AA:407		322134S:E>E		378604X:E>E		BC187:E>E		DBVPG1373:E>E		DBVPG1788:E>E		DBVPG6765:E>E		L_1528:E>E		UWOPS03_461_4:E>E		UWOPS05_227_2:E>E		UWOPS83_787_3:E>E		UWOPS87_2421:E>E		Y55:E>E		YJM978:E>E		YS2:E>E		YS9:E>E	AA:420		273614X:S>S	AA:425		UWOPS03_461_4:I>I		UWOPS05_227_2:I>I		UWOPS87_2421:I>I	AA:430		322134S:V>V		378604X:V>V		BC187:V>V		DBVPG1373:V>V		DBVPG1788:V>V		DBVPG6765:V>V		L_1528:V>V		UWOPS03_461_4:V>V		UWOPS05_227_2:V>V		UWOPS83_787_3:V>V		UWOPS87_2421:V>V		Y55:V>V		YJM978:V>V		YS2:V>V		YS9:V>V	AA:442		322134S:D>D		378604X:D>D		BC187:D>D		DBVPG1373:D>D		DBVPG1788:D>D		DBVPG6765:D>D		L_1528:D>D		UWOPS03_461_4:D>D		UWOPS05_227_2:D>D		UWOPS83_787_3:D>D		UWOPS87_2421:D>D		Y55:D>D		YJM978:D>D		YS2:D>D		YS9:D>D	AA:468		YIIc17_E5:Y>YID:YBR273C	AA:57		Y12:L>L	AA:62		YPS606:D>D	AA:77		YPS606:V>V	AA:119		YJM978:S>S	AA:286		322134S:S>S		378604X:S>S		DBVPG1373:S>S		DBVPG6765:S>S		K11:S>S		L_1528:S>S		UWOPS05_227_2:S>S		UWOPS83_787_3:S>S		UWOPS87_2421:S>S		Y55:S>S		YJM975:S>S		YJM978:S>S	AA:327		SK1:D>D	AA:344		UWOPS87_2421:E>EID:YBR274W	AA:25		YJM975:A>A	AA:133		273614X:H>H		378604X:H>H		DBVPG1106:H>H		DBVPG1853:H>H		DBVPG6044:H>H		DBVPG6765:H>H		L_1374:H>H		L_1528:H>H		Y55:H>H		YPS606:H>H	AA:158		BC187:L>L		DBVPG1106:L>L		DBVPG1853:L>L		UWOPS83_787_3:L>L		Y55:L>L	AA:165		273614X:S>S		378604X:S>S		BC187:S>S		DBVPG1106:S>S		DBVPG6044:S>S		DBVPG6765:S>S		L_1374:S>S		L_1528:S>S		UWOPS03_461_4:S>S		UWOPS05_217_3:S>S		UWOPS83_787_3:S>S		UWOPS87_2421:S>S		Y55:S>S		YPS606:S>S	AA:188		273614X:A>A		BC187:A>A		DBVPG1106:A>A		DBVPG6044:A>A		DBVPG6765:A>A		L_1374:A>A		L_1528:A>A		UWOPS83_787_3:A>A		Y55:A>A		YPS606:A>A	AA:215		378604X:L>L		UWOPS87_2421:L>L	AA:291		UWOPS87_2421:D>D	AA:312		YS9:L>L	AA:352		273614X:T>T		322134S:T>T		BC187:T>T		DBVPG6765:T>T		L_1374:T>T		L_1528:T>T		UWOPS83_787_3:T>T		Y55:T>T		YS9:T>T	AA:430		322134S:P>P		BC187:P>P		DBVPG6765:P>P		L_1374:P>P		SK1:P>P		UWOPS05_217_3:P>P		UWOPS83_787_3:P>P		UWOPS87_2421:P>P		Y12:P>P		YIIc17_E5:P>P		YS9:P>P	AA:459		322134S:G>G		BC187:G>G		DBVPG1373:G>G		DBVPG6765:G>G		L_1374:G>G		UWOPS05_217_3:G>G		UWOPS83_787_3:G>G		UWOPS87_2421:G>G	AA:481		UWOPS87_2421:C>CID:YBR276C	AA:41		W303:G>G	AA:71		273614X:V>V	AA:103		378604X:P>P		DBVPG6040:P>P		NCYC110:P>P	AA:106		SK1:T>T		UWOPS05_227_2:T>T		W303:T>T		YIIc17_E5:T>T		YPS606:T>T	AA:158		UWOPS05_227_2:R>R	AA:229		K11:P>P		SK1:P>P		W303:P>P		YIIc17_E5:P>P		YPS606:P>P	AA:285		UWOPS05_227_2:N>N	AA:302		322134S:E>E	AA:332		YPS128:E>E	AA:338		322134S:G>G	AA:352		UWOPS87_2421:N>N	AA:492		378604X:Y>Y		SK1:Y>Y		W303:Y>Y		Y12:Y>Y		Y9:Y>Y		YIIc17_E5:Y>Y		YPS128:Y>Y		YPS606:Y>Y	AA:517		DBVPG6044:P>P	AA:531		Y12:R>R		Y9:R>R	AA:663		YS4:D>D	AA:665		YPS128:V>V		YPS606:V>V	AA:673		YIIc17_E5:G>G	AA:701		UWOPS05_227_2:F>F	AA:712		SK1:S>S		W303:S>S	AA:730		DBVPG6040:S>S	AA:737		YPS128:I>I		YPS606:I>I	AA:766		Y12:P>P		Y9:P>PID:YBR278W	AA:3		K11:N>N		SK1:N>N		W303:N>N		YIIc17_E5:N>N		YPS128:N>N	AA:24		K11:D>D		SK1:D>D		UWOPS05_227_2:D>D		W303:D>D		Y12:D>D		Y9:D>D		YIIc17_E5:D>D		YPS128:D>D	AA:26		UWOPS05_227_2:E>E	AA:102		K11:K>K		SK1:K>K		UWOPS03_461_4:K>K		UWOPS05_217_3:K>K		UWOPS05_227_2:K>K		W303:K>K		Y12:K>K		Y9:K>K		YIIc17_E5:K>K		YPS128:K>K	AA:109		Y9:Q>Q	AA:158		SK1:Q>Q		UWOPS03_461_4:Q>Q		UWOPS05_217_3:Q>Q		UWOPS87_2421:Q>Q		W303:Q>Q		Y12:Q>Q		YPS128:Q>Q	AA:178		UWOPS87_2421:S>S	AA:185		SK1:H>H		W303:H>HID:YBR279W	AA:50		YJM975:T>T	AA:57		UWOPS03_461_4:Q>Q		UWOPS05_217_3:Q>Q		UWOPS05_227_2:Q>Q	AA:60		SK1:E>E		UWOPS03_461_4:E>E		UWOPS05_217_3:E>E		UWOPS05_227_2:E>E		W303:E>E		Y12:E>E		YIIc17_E5:E>E	AA:66		SK1:V>V		UWOPS03_461_4:V>V		UWOPS05_217_3:V>V		UWOPS05_227_2:V>V		W303:V>V		Y12:V>V		YIIc17_E5:V>V	AA:91		DBVPG6044:L>L		SK1:L>L		UWOPS03_461_4:L>L		UWOPS05_217_3:L>L		UWOPS05_227_2:L>L		UWOPS87_2421:L>L		W303:L>L		Y12:L>L		YIIc17_E5:L>L		YPS128:L>L	AA:143		YPS128:D>D	AA:177		DBVPG6044:K>K		SK1:K>K		UWOPS03_461_4:K>K		UWOPS05_217_3:K>K		UWOPS05_227_2:K>K		UWOPS87_2421:K>K		W303:K>K		Y12:K>K		YPS128:K>K	AA:186		DBVPG6044:D>D		SK1:D>D		UWOPS03_461_4:D>D		UWOPS05_217_3:D>D		UWOPS05_227_2:D>D		UWOPS87_2421:D>D		W303:D>D		Y12:D>D		YIIc17_E5:D>D		YPS128:D>D	AA:191		YPS128:T>T	AA:192		DBVPG6044:A>A		NCYC110:A>A		SK1:A>A		UWOPS03_461_4:A>A		UWOPS05_217_3:A>A		UWOPS05_227_2:A>A		W303:A>A		Y12:A>A		Y9:A>A	AA:199		DBVPG6044:F>F		SK1:F>F		UWOPS03_461_4:F>F		UWOPS05_217_3:F>F		UWOPS05_227_2:F>F		W303:F>F		Y12:F>F		YPS128:F>F	AA:208		DBVPG6044:S>S		NCYC110:S>S		SK1:S>S		UWOPS03_461_4:S>S		UWOPS05_217_3:S>S		UWOPS05_227_2:S>S		UWOPS87_2421:S>S		W303:S>S		Y12:S>S		Y9:S>S		YPS128:S>S	AA:211		Y55:T>T	AA:217		378604X:L>L	AA:229		NCYC110:E>E		SK1:E>E		UWOPS03_461_4:E>E		UWOPS05_227_2:E>E		UWOPS87_2421:E>E		W303:E>E		Y12:E>E		Y9:E>E		YPS128:E>E	AA:238		SK1:T>T		W303:T>T		Y12:T>T		Y9:T>T		YPS128:T>T	AA:337		BC187:N>N	AA:360		SK1:T>T		W303:T>T		Y12:T>T	AA:379		SK1:S>SID:YBR280C	AA:3		DBVPG6765:E>E		L_1528:E>E		Y55:E>E	AA:129		DBVPG6044:N>N		Y9:N>N	AA:153		K11:E>E		W303:E>E		YIIc17_E5:E>E		YPS128:E>E		YPS606:E>E	AA:196		K11:F>F		UWOPS05_227_2:F>F		UWOPS87_2421:F>F		W303:F>F		YPS128:F>F		YPS606:F>F	AA:216		UWOPS05_227_2:L>L		W303:L>L		YPS128:L>L		YPS606:L>L	AA:263		SK1:R>R		UWOPS05_227_2:R>R		UWOPS87_2421:R>R		W303:R>R		YPS128:R>R		YPS606:R>R	AA:314		UWOPS05_227_2:P>P	AA:377		UWOPS05_227_2:L>L		UWOPS87_2421:L>L	AA:390		SK1:S>S		W303:S>S	AA:404		SK1:L>L		W303:L>L	AA:420		SK1:N>N		W303:N>N	AA:447		UWOPS05_227_2:A>A		UWOPS87_2421:A>A		Y12:A>A		YPS128:A>A	AA:466		DBVPG6044:I>I		K11:I>I		SK1:I>I		UWOPS87_2421:I>I		W303:I>I		Y12:I>I		YIIc17_E5:I>I		YPS128:I>I	AA:470		DBVPG6044:A>A		K11:A>A		UWOPS05_227_2:A>A		UWOPS87_2421:A>A		Y12:A>A		YIIc17_E5:A>A		YPS128:A>A	AA:480		K11:I>I		YIIc17_E5:I>I	AA:494		SK1:L>L		W303:L>L	AA:499		DBVPG6044:D>D		UWOPS83_787_3:D>D	AA:527		K11:L>L		SK1:L>L		UWOPS05_227_2:L>L		UWOPS87_2421:L>L		W303:L>L		Y12:L>L		YIIc17_E5:L>L		YPS128:L>L	AA:529		K11:V>V		SK1:V>V		UWOPS05_227_2:V>V		UWOPS87_2421:V>V		W303:V>V		Y12:V>V		YIIc17_E5:V>V		YPS128:V>V	AA:632		Y12:L>L		YPS128:L>L		YPS606:L>LID:YBR281C	AA:3		DBVPG6044:D>D	AA:58		YPS128:I>I		YPS606:I>I	AA:59		SK1:R>R		Y12:R>R	AA:60		DBVPG1853:L>L	AA:93		K11:L>L	AA:100		YIIc17_E5:G>G	AA:103		DBVPG6044:T>T		SK1:T>T		UWOPS03_461_4:T>T		UWOPS05_217_3:T>T		UWOPS05_227_2:T>T		UWOPS83_787_3:T>T		UWOPS87_2421:T>T		Y12:T>T		YIIc17_E5:T>T		YPS128:T>T		YPS606:T>T	AA:183		DBVPG6044:L>L		SK1:L>L		UWOPS05_217_3:L>L		UWOPS05_227_2:L>L		UWOPS83_787_3:L>L		UWOPS87_2421:L>L		W303:L>L		YPS128:L>L		YPS606:L>L	AA:213		UWOPS05_217_3:P>P		UWOPS05_227_2:P>P	AA:219		DBVPG6044:K>K		UWOPS05_217_3:K>K		UWOPS05_227_2:K>K	AA:221		SK1:A>A		Y12:A>A		Y9:A>A	AA:223		UWOPS87_2421:Y>Y		YPS128:Y>Y		YPS606:Y>Y	AA:227		DBVPG6044:Y>Y		UWOPS05_217_3:Y>Y		UWOPS05_227_2:Y>Y		UWOPS83_787_3:Y>Y	AA:254		SK1:S>S		Y12:S>S		Y9:S>S	AA:320		UWOPS03_461_4:I>I		UWOPS05_217_3:I>I	AA:350		YPS128:T>T		YPS606:T>T	AA:377		SK1:N>N		Y12:N>N		Y9:N>N	AA:398		SK1:T>T		Y12:T>T		Y9:T>T	AA:469		SK1:L>L		W303:L>L		YPS128:L>L		YPS606:L>L	AA:477		SK1:G>G		W303:G>G	AA:490		SK1:G>G		W303:G>G		YPS128:G>G	AA:493		SK1:V>V	AA:498		SK1:F>F		YPS128:F>F	AA:524		YPS128:I>I	AA:550		DBVPG6044:G>G		SK1:G>G		UWOPS03_461_4:G>G	AA:571		UWOPS87_2421:Q>Q		YPS128:Q>Q	AA:646		K11:D>D		SK1:D>D		Y9:D>D	AA:654		SK1:G>G		Y9:G>G	AA:677		K11:I>I		SK1:I>I		Y12:I>I		Y9:I>I	AA:689		UWOPS03_461_4:L>L		UWOPS05_217_3:L>L		UWOPS05_227_2:L>L	AA:690		DBVPG6044:T>T		UWOPS83_787_3:T>T	AA:701		UWOPS83_787_3:T>T	AA:769		UWOPS03_461_4:L>L		UWOPS05_217_3:L>L		UWOPS05_227_2:L>L	AA:786		DBVPG6765:V>V	AA:811		YIIc17_E5:T>TID:YBR282W	AA:7		DBVPG1373:S>S		DBVPG1788:S>S		DBVPG1853:S>S		DBVPG6040:S>S		DBVPG6765:S>S		UWOPS83_787_3:S>S		Y55:S>S		YJM975:S>S		YJM978:S>S		YS9:S>S	AA:45		378604X:T>T		DBVPG6765:T>T		Y55:T>T		YJM975:T>T		YJM978:T>T		YS9:T>T	AA:51		UWOPS87_2421:T>T	AA:70		UWOPS87_2421:Y>Y		W303:Y>Y		YPS128:Y>Y	AA:103		UWOPS05_217_3:H>H		UWOPS05_227_2:H>H	AA:130		UWOPS03_461_4:R>R		UWOPS05_217_3:R>R		UWOPS05_227_2:R>RID:YBR283C	AA:27		DBVPG6044:L>L		SK1:L>L	AA:72		378604X:F>F	AA:148		Y55:V>V	AA:171		DBVPG1853:I>I		DBVPG6044:I>I		DBVPG6765:I>I		SK1:I>I		Y55:I>I		YS9:I>I	AA:209		UWOPS87_2421:D>D		YPS128:D>D	AA:239		DBVPG6044:F>F		SK1:F>F	AA:371		BC187:S>S		DBVPG1106:S>S		DBVPG1373:S>S		DBVPG6765:S>S		L_1528:S>S		Y55:S>S		YS4:S>S	AA:372		K11:A>A	AA:386		BC187:Q>Q		DBVPG1106:Q>Q		DBVPG1373:Q>Q		DBVPG6765:Q>Q		L_1528:Q>Q		Y55:Q>Q		YS9:Q>Q	AA:422		BC187:V>V		DBVPG1106:V>V		DBVPG1373:V>V		DBVPG6765:V>V		L_1528:V>V		Y55:V>V		YS9:V>V	AA:431		K11:A>A		UWOPS03_461_4:A>A		UWOPS05_227_2:A>A		W303:A>A		Y9:A>A		YIIc17_E5:A>A		YPS128:A>A	AA:471		378604X:Y>Y		BC187:Y>Y		DBVPG1106:Y>Y		DBVPG1373:Y>Y		DBVPG6765:Y>Y		L_1528:Y>Y		Y55:Y>Y		YS9:Y>YID:YBR284W	AA:102		DBVPG6044:T>T		NCYC110:T>T		SK1:T>T	AA:178		DBVPG6044:K>K		NCYC110:K>K		SK1:K>K	AA:197		UWOPS03_461_4:L>L		UWOPS05_227_2:L>L	AA:200		322134S:G>G		DBVPG1788:G>G		DBVPG1853:G>G		DBVPG6040:G>G		DBVPG6765:G>G		UWOPS83_787_3:G>G		Y55:G>G		YJM975:G>G		YJM981:G>G		YS9:G>G	AA:256		DBVPG6044:L>L		NCYC110:L>L		SK1:L>L	AA:297		322134S:F>F		DBVPG1788:F>F		DBVPG1853:F>F		DBVPG6040:F>F		DBVPG6044:F>F		DBVPG6765:F>F		K11:F>F		NCYC110:F>F		SK1:F>F		UWOPS03_461_4:F>F		UWOPS05_227_2:F>F		UWOPS83_787_3:F>F		YJM975:F>F		YJM978:F>F		YJM981:F>F		YS2:F>F		YS9:F>F	AA:368		DBVPG6044:H>H		NCYC110:H>H		SK1:H>H	AA:405		322134S:L>L	AA:407		322134S:K>K	AA:420		DBVPG1853:S>S		DBVPG6765:S>S		L_1374:S>S		UWOPS83_787_3:S>S		Y55:S>S		YJM975:S>S		YJM978:S>S		YJM981:S>S		YS2:S>S	AA:432		DBVPG1853:S>S		DBVPG6765:S>S		L_1374:S>S		UWOPS83_787_3:S>S		Y55:S>S		YJM975:S>S		YJM978:S>S		YS2:S>S	AA:483		DBVPG6765:L>L		L_1374:L>L		L_1528:L>L		SK1:L>L		Y55:L>L		YJM975:L>L		YS2:L>L	AA:487		DBVPG6765:P>P		YJM975:P>P	AA:506		DBVPG6044:F>F		SK1:F>F		UWOPS05_217_3:F>F		YPS128:F>F		YPS606:F>F	AA:593		K11:T>T		Y9:T>T	AA:610		K11:A>A		Y9:A>A	AA:617		DBVPG6044:A>A		NCYC110:A>A		SK1:A>A	AA:654		322134S:L>L		BC187:L>L		DBVPG1106:L>L		DBVPG6765:L>L		K11:L>L		L_1528:L>L		UWOPS05_217_3:L>L		Y12:L>L		Y55:L>L		Y9:L>L		YJM975:L>L		YJM981:L>L		YS2:L>L	AA:692		UWOPS03_461_4:I>I	AA:698		UWOPS83_787_3:Y>Y	AA:759		UWOPS87_2421:F>F	AA:797		BC187:A>AID:YBR285W	AA:114		YPS128:P>PID:YBR286W	AA:14		L_1374:Y>Y	AA:85		322134S:N>N		DBVPG1853:N>N		DBVPG6044:N>N		DBVPG6765:N>N		SK1:N>N		UWOPS05_217_3:N>N		UWOPS83_787_3:N>N		Y55:N>N		YJM975:N>N		YJM978:N>N		YPS128:N>N		YPS606:N>N		YS2:N>N	AA:178		SK1:K>K		YPS128:K>K		YPS606:K>K	AA:215		UWOPS05_217_3:F>F		YPS128:F>F		YPS606:F>F	AA:318		Y55:V>V	AA:454		K11:T>T	AA:484		378604X:D>D		YIIc17_E5:D>D	AA:510		DBVPG6044:R>R	AA:528		UWOPS03_461_4:F>FID:YBR287W	AA:46		K11:S>S	AA:66		UWOPS87_2421:L>L	AA:116		YPS606:G>G	AA:127		378604X:L>L		K11:L>L		UWOPS03_461_4:L>L		UWOPS87_2421:L>L		Y12:L>L		Y9:L>L	AA:167		378604X:L>L		K11:L>L		UWOPS87_2421:L>L		Y12:L>L		Y9:L>L		YPS606:L>L	AA:205		378604X:E>E		K11:E>E		UWOPS87_2421:E>E		Y12:E>E		Y9:E>E	AA:210		378604X:E>E		K11:E>E		UWOPS87_2421:E>E		Y12:E>E		Y9:E>E	AA:257		378604X:P>P		K11:P>P		Y12:P>P		Y9:P>P		YIIc17_E5:P>P	AA:302		378604X:L>L		UWOPS03_461_4:L>L		YIIc17_E5:L>L	AA:307		378604X:L>L		UWOPS03_461_4:L>L		YIIc17_E5:L>L		YPS128:L>L		YPS606:L>LID:YBR288C	AA:3		YPS128:L>L	AA:23		L_1374:T>T	AA:26		UWOPS05_227_2:S>S		UWOPS83_787_3:S>S		Y12:S>S		YIIc17_E5:S>S		YPS128:S>S		YPS606:S>S	AA:29		Y12:H>H	AA:145		BC187:I>I	AA:230		YIIc17_E5:K>K	AA:319		YIIc17_E5:S>S		YS4:S>S	AA:337		YS4:E>E	AA:393		DBVPG6044:G>G		NCYC110:G>G		SK1:G>G		UWOPS05_227_2:G>G		UWOPS83_787_3:G>G		Y12:G>G		YIIc17_E5:G>G		YPS128:G>G		YPS606:G>G		YS4:G>G	AA:413		UWOPS05_227_2:G>G		Y12:G>G		YIIc17_E5:G>G		YPS128:G>G		YPS606:G>G	AA:435		YPS128:L>L		YPS606:L>L	AA:438		DBVPG6044:S>S		SK1:S>S	AA:448		YS4:Y>Y	AA:483		322134S:L>LID:YBR290W	AA:32		378604X:T>T		Y12:T>T		YIIc17_E5:T>T	AA:46		DBVPG6040:G>G	AA:184		378604X:I>I		DBVPG6044:I>I		K11:I>I		SK1:I>I		UWOPS03_461_4:I>I		UWOPS05_227_2:I>I		Y12:I>I		YIIc17_E5:I>I		YPS606:I>I	AA:200		UWOPS83_787_3:L>L	AA:227		YS4:P>P	AA:234		DBVPG1788:V>V		DBVPG6044:V>V		DBVPG6765:V>V		K11:V>V		SK1:V>V		UWOPS03_461_4:V>V		UWOPS05_227_2:V>V		UWOPS83_787_3:V>V		YIIc17_E5:V>V		YPS606:V>V	AA:239		DBVPG6765:S>S	AA:266		YPS606:F>F	AA:281		UWOPS83_787_3:I>IID:YBR291C	AA:34		Y9:E>E	AA:47		UWOPS05_217_3:S>S	AA:66		YJM978:I>I		YJM981:I>I	AA:79		SK1:G>G		UWOPS03_461_4:G>G		UWOPS05_217_3:G>G		Y9:G>G		YIIc17_E5:G>G		YPS606:G>G	AA:120		SK1:L>L		UWOPS83_787_3:L>L		YPS606:L>L	AA:227		DBVPG6044:V>V		SK1:V>V	AA:229		UWOPS83_787_3:V>V	AA:265		DBVPG6044:G>G		K11:G>G		SK1:G>G		UWOPS03_461_4:G>G		UWOPS87_2421:G>G		YIIc17_E5:G>G		YPS128:G>G		YPS606:G>G	AA:294		K11:V>VID:YBR293W	AA:2		YPS606:S>S	AA:59		L_1374:S>S		YJM978:S>S	AA:108		DBVPG6044:A>A		NCYC110:A>A		SK1:A>A		YPS606:A>A	AA:110		378604X:F>F	AA:113		378604X:T>T		DBVPG6044:T>T		DBVPG6765:T>T		NCYC110:T>T		SK1:T>T		UWOPS05_217_3:T>T		YIIc17_E5:T>T		YPS606:T>T	AA:143		378604X:P>P		YIIc17_E5:P>P	AA:197		DBVPG6044:T>T		NCYC110:T>T		SK1:T>T	AA:200		YIIc17_E5:S>S	AA:205		DBVPG6044:L>L		NCYC110:L>L		SK1:L>L		UWOPS03_461_4:L>L		UWOPS05_217_3:L>L		YIIc17_E5:L>L		YPS606:L>L	AA:266		DBVPG6044:V>V		NCYC110:V>V		SK1:V>V		YIIc17_E5:V>V	AA:272		DBVPG6040:A>A	AA:312		DBVPG6765:L>L		K11:L>L		UWOPS03_461_4:L>L		UWOPS05_217_3:L>L		UWOPS87_2421:L>L		Y12:L>L		YIIc17_E5:L>L		YPS128:L>L		YPS606:L>L	AA:350		UWOPS83_787_3:T>T	AA:395		DBVPG6765:G>G		K11:G>G		UWOPS87_2421:G>G		Y12:G>G		YPS128:G>G		YPS606:G>G	AA:425		K11:G>G		Y12:G>G	AA:458		K11:L>L		NCYC110:L>L		SK1:L>L		UWOPS03_461_4:L>L		UWOPS05_217_3:L>L		UWOPS87_2421:L>L		Y12:L>L		YIIc17_E5:L>L		YPS128:L>L		YPS606:L>LID:YBR296C	AA:15		YPS128:L>L	AA:36		YS2:S>S	AA:58		378604X:G>G	AA:98		UWOPS87_2421:S>S	AA:155		Y12:L>L	AA:167		UWOPS05_217_3:S>S		YPS128:S>S		YPS606:S>S	AA:177		378604X:V>V		DBVPG6044:V>V		SK1:V>V		UWOPS05_217_3:L>L		Y12:V>V		YIIc17_E5:V>V		YPS128:L>L		YPS606:L>L	AA:200		UWOPS05_217_3:T>T		YPS128:T>T		YPS606:T>T	AA:208		YPS128:S>S		YPS606:S>S	AA:210		DBVPG6044:N>N		SK1:N>N		Y12:N>N	AA:222		DBVPG6040:A>A	AA:255		DBVPG6040:I>I		DBVPG6044:L>L		SK1:L>L		UWOPS05_217_3:L>L		UWOPS87_2421:L>L		Y12:L>L		YPS128:L>L		YPS606:L>L	AA:264		Y12:P>P	AA:290		DBVPG6044:G>G		SK1:G>G		UWOPS05_217_3:G>G		UWOPS87_2421:G>G		Y12:G>G		YPS128:G>G		YPS606:G>G	AA:308		273614X:A>A		DBVPG1373:A>A		DBVPG6040:A>A		DBVPG6044:A>A		L_1528:A>A		SK1:A>A		UWOPS87_2421:A>A		Y12:A>A		YJM975:A>A		YPS128:A>A		YPS606:A>A		YS4:A>A	AA:310		Y12:N>N		YPS128:N>N		YPS606:N>N	AA:318		DBVPG6044:K>K		SK1:K>K		UWOPS87_2421:K>K		Y12:K>K	AA:326		378604X:L>L		UWOPS87_2421:L>L	AA:370		378604X:V>V		DBVPG1788:V>V		DBVPG6044:V>V		DBVPG6765:V>V		SK1:V>V		UWOPS83_787_3:V>V		UWOPS87_2421:V>V		Y12:V>V		YIIc17_E5:V>V		YPS606:V>V	AA:392		378604X:S>S		UWOPS83_787_3:S>S		UWOPS87_2421:S>S		YIIc17_E5:S>S	AA:425		378604X:N>N		DBVPG6044:N>N		K11:N>N		NCYC361:N>N		SK1:N>N		UWOPS05_227_2:N>N		UWOPS83_787_3:N>N		UWOPS87_2421:N>N		Y12:N>N		YIIc17_E5:N>N		YPS606:N>N	AA:450		378604X:V>V		YIIc17_E5:V>V	AA:453		378604X:L>L		DBVPG1788:L>L		DBVPG6044:L>L		DBVPG6765:L>L		K11:L>L		SK1:L>L		UWOPS05_227_2:L>L		UWOPS83_787_3:L>L		UWOPS87_2421:L>L		Y12:L>L		YIIc17_E5:L>L		YPS606:L>L		YS4:L>L	AA:489		378604X:L>L		K11:S>S		NCYC361:S>S		SK1:S>S		UWOPS05_217_3:L>L		UWOPS05_227_2:L>L		UWOPS87_2421:L>L		Y12:L>L		YIIc17_E5:L>L		YPS128:L>L		YPS606:S>S	AA:514		K11:A>A		NCYC361:A>A		SK1:A>A		Y12:A>A	AA:537		YPS606:A>A	AA:542		UWOPS05_217_3:G>G		UWOPS05_227_2:G>GID:YBR298C	AA:3		322134S:G>G		DBVPG6044:G>G		K11:G>G		NCYC110:G>G		UWOPS03_461_4:G>G		UWOPS05_227_2:N>N		UWOPS87_2421:G>G		YPS606:G>G		YS2:G>G	AA:7		UWOPS03_461_4:L>L		UWOPS05_227_2:L>L		UWOPS83_787_3:L>L	AA:27		UWOPS03_461_4:N>N		UWOPS05_227_2:N>N		UWOPS83_787_3:N>N	AA:53		UWOPS03_461_4:G>G		UWOPS05_227_2:G>G		UWOPS83_787_3:G>G	AA:55		YPS606:G>G	AA:66		322134S:V>V		DBVPG6044:V>V		K11:V>V		NCYC110:V>V		Y55:V>V	AA:68		322134S:D>D		DBVPG6044:D>D		NCYC110:D>D		UWOPS03_461_4:D>D		UWOPS05_227_2:D>D		UWOPS83_787_3:D>D		UWOPS87_2421:D>D		Y55:D>D	AA:88		322134S:P>P		UWOPS87_2421:P>P	AA:89		378604X:L>L		DBVPG6044:L>L		K11:L>L		NCYC110:L>L		SK1:L>L		YPS606:L>L		YS2:L>L	AA:103		DBVPG6044:S>S		NCYC110:S>S		SK1:S>S		UWOPS87_2421:S>S		Y55:S>S	AA:113		UWOPS87_2421:E>E		YPS606:E>E	AA:124		DBVPG6044:Y>Y		NCYC110:Y>Y		UWOPS87_2421:Y>Y		YPS606:Y>Y	AA:127		322134S:P>P		UWOPS03_461_4:P>P		UWOPS05_217_3:P>P		UWOPS05_227_2:P>P		YPS606:P>P		YS2:P>P		YS4:P>P	AA:129		UWOPS87_2421:F>F	AA:136		UWOPS87_2421:L>L	AA:155		322134S:L>L		UWOPS03_461_4:L>L		UWOPS05_217_3:L>L		UWOPS05_227_2:L>L		UWOPS87_2421:L>L		YPS606:L>L	AA:159		322134S:A>A		K11:G>G		SK1:G>G		UWOPS83_787_3:G>G		UWOPS87_2421:A>A		YPS128:A>A		YPS606:G>G		YS2:A>A	AA:161		322134S:E>E		BC187:E>E		DBVPG1788:E>E		DBVPG1853:E>E		DBVPG6044:E>E		NCYC110:E>E		SK1:E>E		UWOPS03_461_4:E>E		UWOPS05_217_3:E>E		UWOPS05_227_2:E>E		UWOPS87_2421:E>E		YPS128:E>E		YPS606:E>E	AA:163		322134S:V>V		DBVPG6044:V>V		NCYC110:V>V		SK1:V>V		UWOPS03_461_4:V>V		UWOPS05_217_3:V>V		UWOPS05_227_2:V>V		UWOPS87_2421:V>V		YPS128:V>V		YPS606:G>G	AA:174		322134S:Y>Y		DBVPG6044:Y>Y		NCYC110:Y>Y		SK1:Y>Y		UWOPS83_787_3:Y>Y		UWOPS87_2421:Y>Y		YPS128:Y>Y		YPS606:Y>Y		YS2:Y>Y	AA:176		322134S:G>G		UWOPS03_461_4:G>G		UWOPS05_217_3:G>G		UWOPS05_227_2:G>G		UWOPS83_787_3:G>G		UWOPS87_2421:G>G		YPS128:G>G		YPS606:G>G		YS2:G>G	AA:181		322134S:L>L		UWOPS03_461_4:L>L		UWOPS05_217_3:L>L		UWOPS05_227_2:L>L		UWOPS87_2421:L>L		YPS128:L>L		YPS606:L>L		YS2:L>L	AA:205		322134S:A>A		DBVPG6040:A>A		DBVPG6044:A>A		K11:A>A		NCYC110:A>A		SK1:A>A		UWOPS03_461_4:A>A		UWOPS05_217_3:A>A		UWOPS05_227_2:A>A		UWOPS83_787_3:A>A		UWOPS87_2421:A>A		Y12:A>A		YPS128:A>A		YPS606:A>A	AA:259		322134S:N>N		DBVPG1373:G>G		UWOPS03_461_4:N>N		UWOPS05_217_3:N>N		UWOPS05_227_2:N>N		UWOPS83_787_3:N>N		UWOPS87_2421:N>N		YPS128:N>N		YPS606:N>N	AA:277		UWOPS03_461_4:L>L		UWOPS05_217_3:L>L		UWOPS05_227_2:L>L	AA:282		UWOPS03_461_4:P>P		UWOPS05_217_3:P>P		UWOPS05_227_2:P>P	AA:302		UWOPS83_787_3:K>K		UWOPS87_2421:K>K		YPS128:K>K		YPS606:K>K	AA:306		UWOPS03_461_4:D>D		UWOPS05_217_3:D>D		UWOPS05_227_2:D>D		UWOPS83_787_3:D>D		UWOPS87_2421:D>D		YPS128:D>D		YPS606:D>D	AA:307		Y9:Q>Q	AA:316		322134S:L>L		DBVPG6040:L>L		K11:L>L		NCYC110:L>L		UWOPS03_461_4:L>L		UWOPS05_217_3:L>L		UWOPS05_227_2:L>L		UWOPS83_787_3:L>L		UWOPS87_2421:L>L		Y12:L>L		YPS128:L>L		YPS606:L>L	AA:323		322134S:K>K		DBVPG6040:K>K		K11:K>K		NCYC110:K>K		UWOPS03_461_4:K>K		UWOPS05_217_3:K>K		UWOPS05_227_2:K>K		UWOPS83_787_3:K>K		UWOPS87_2421:K>K		Y12:K>K		YPS128:K>K		YPS606:K>K	AA:327		UWOPS83_787_3:V>V		UWOPS87_2421:V>V		YPS128:V>V		YPS606:V>V	AA:360		UWOPS03_461_4:R>R		UWOPS05_217_3:R>R		UWOPS05_227_2:R>R		UWOPS87_2421:R>R		YPS128:R>R	AA:387		Y12:F>F	AA:390		DBVPG6040:K>K		K11:K>K		SK1:K>K		UWOPS83_787_3:K>K		Y12:K>K	AA:397		Y12:T>T	AA:399		DBVPG6040:F>F		K11:F>F		SK1:F>F		UWOPS03_461_4:F>F		UWOPS05_217_3:F>F		UWOPS05_227_2:F>F		UWOPS83_787_3:F>F		Y12:F>F		Y9:F>F	AA:401		UWOPS87_2421:F>F		YPS128:F>F	AA:405		UWOPS03_461_4:Q>Q		UWOPS05_217_3:Q>Q		UWOPS05_227_2:Q>Q		UWOPS87_2421:Q>Q		YPS128:Q>Q	AA:432		UWOPS87_2421:G>G		YPS128:G>G		YPS606:G>G	AA:443		UWOPS03_461_4:I>I		UWOPS05_217_3:I>I		UWOPS05_227_2:I>I	AA:446		K11:L>L		SK1:L>L		UWOPS03_461_4:L>L		UWOPS05_217_3:L>L		UWOPS05_227_2:L>L		UWOPS83_787_3:L>L		UWOPS87_2421:L>L		Y12:L>L		YPS128:L>L		YPS606:L>L	AA:462		K11:L>L		SK1:L>L		UWOPS03_461_4:L>L		UWOPS05_217_3:L>L		UWOPS05_227_2:L>L		UWOPS83_787_3:L>L		YPS606:L>L	AA:467		378604X:F>F	AA:468		K11:F>F		SK1:F>F		UWOPS03_461_4:F>F		UWOPS05_217_3:F>F		UWOPS05_227_2:F>F		UWOPS83_787_3:F>F		Y9:F>F		YPS606:F>F	AA:477		K11:V>V		SK1:V>V		UWOPS83_787_3:V>V		YPS606:V>V	AA:481		UWOPS87_2421:V>V		YPS128:V>V	AA:482		K11:S>S		SK1:S>S		UWOPS05_217_3:S>S		UWOPS05_227_2:S>S		UWOPS83_787_3:S>S	AA:491		UWOPS05_217_3:T>T		UWOPS05_227_2:T>T		UWOPS87_2421:T>T		YPS128:T>T	AA:494		YPS606:I>I	AA:496		K11:L>L		SK1:L>L		UWOPS83_787_3:L>L	AA:508		UWOPS87_2421:V>V		YPS128:V>V		YPS606:V>V	AA:512		322134S:I>I		SK1:I>I	AA:514		UWOPS05_217_3:Y>Y		UWOPS05_227_2:Y>Y	AA:519		SK1:E>E		UWOPS05_217_3:E>E		UWOPS05_227_2:E>E		UWOPS83_787_3:E>E		YPS606:E>E	AA:522		SK1:N>N		UWOPS05_217_3:N>N		UWOPS05_227_2:N>N		UWOPS87_2421:N>N		Y9:N>N		YPS128:N>N		YPS606:N>N	AA:526		UWOPS87_2421:K>K		YPS128:K>K		YPS606:K>K	AA:542		UWOPS05_227_2:A>A	AA:549		UWOPS05_227_2:T>T		YPS606:T>T	AA:552		Y12:R>R	AA:556		UWOPS03_461_4:N>N		UWOPS05_227_2:N>N		UWOPS83_787_3:N>N		Y9:E>E	AA:567		UWOPS05_227_2:A>A	AA:572		UWOPS05_227_2:S>S		Y9:S>S	AA:575		SK1:V>V		UWOPS05_227_2:V>V		Y9:V>V		YPS128:V>V		YPS606:V>V	AA:585		UWOPS05_227_2:A>A	AA:593		UWOPS05_227_2:P>P		Y12:P>P	AA:614		YPS128:K>K		YPS606:K>KID:YBR301W	AA:4		DBVPG1853:L>L		L_1374:L>L		YJM975:L>L	AA:19		DBVPG1853:S>S		L_1374:S>S		YJM975:S>S		YJM981:S>S	AA:34		322134S:V>V		DBVPG1853:V>V		L_1374:V>V		YJM975:V>V		YJM981:V>V	AA:59		Y55:T>T		YS4:T>T	AA:69		K11:A>A	AA:76		SK1:F>F	AA:78		SK1:T>T	AA:81		DBVPG1853:T>T		SK1:T>T		Y55:T>T		YJM975:T>T		YJM978:T>T		YJM981:T>T		YS2:T>T		YS4:T>T	AA:88		K11:V>V		UWOPS83_787_3:V>V		Y9:V>V		YPS128:V>V		YPS606:V>V	AA:95		K11:V>V		SK1:V>V		UWOPS87_2421:V>V		YS2:V>V	AA:100		Y9:S>S	AA:105		DBVPG1853:A>A		Y55:A>A		YS4:A>A	AA:107		SK1:S>S		YS2:S>S	AA:119		Y55:A>AID:YCL001W	AA:15		DBVPG1373:N>N		DBVPG1788:N>N		DBVPG6765:N>N		L_1374:N>N		Y55:N>N		YJM975:N>N	AA:62		273614X:G>G		DBVPG6044:G>G		UWOPS87_2421:G>G		Y12:G>G	AA:136		Y55:L>L	AA:149		SK1:L>LID:YCL004W	AA:29		UWOPS05_217_3:Q>Q		UWOPS05_227_2:Q>Q	AA:39		DBVPG6040:G>G	AA:72		322134S:Q>Q		378604X:Q>Q		DBVPG1853:Q>Q		DBVPG6044:Q>Q		K11:Q>Q		SK1:Q>Q		UWOPS83_787_3:Q>Q		Y12:Q>Q		YPS128:Q>Q		YS4:Q>Q		YS9:Q>Q	AA:144		DBVPG1853:V>V		UWOPS05_227_2:V>V		UWOPS83_787_3:V>V	AA:159		BC187:T>T	AA:164		273614X:G>G		322134S:G>G		DBVPG6044:G>G		SK1:G>G		UWOPS87_2421:G>G		Y12:G>G		YPS128:G>G		YS9:G>G	AA:180		DBVPG1853:L>L	AA:200		273614X:S>S		322134S:S>S		DBVPG1853:S>S		DBVPG6044:S>S		K11:S>S		SK1:S>S		UWOPS05_227_2:S>S		UWOPS83_787_3:S>S		UWOPS87_2421:S>S		YPS128:S>S	AA:224		322134S:F>F		K11:F>F		SK1:F>F		UWOPS87_2421:F>F		YPS128:F>F	AA:249		322134S:I>I		K11:I>I		SK1:I>I	AA:273		UWOPS03_461_4:L>L	AA:358		YPS128:E>E		YPS606:E>E	AA:431		YS9:S>SID:YCL005W	AA:25		DBVPG6044:L>L	AA:35		YPS128:F>F	AA:91		273614X:G>G		322134S:G>G		378604X:G>G		SK1:G>G		UWOPS05_217_3:G>G		Y9:G>G		YPS128:G>G		YPS606:G>G		YS2:G>G		YS4:G>G	AA:161		273614X:H>H		322134S:H>H		378604X:H>H		SK1:H>H		UWOPS05_217_3:H>H		Y9:H>H		YPS128:H>H		YPS606:H>H		YS2:H>H		YS4:H>H	AA:219		SK1:N>N		UWOPS05_217_3:N>N		UWOPS05_227_2:N>N		YS2:N>N		YS4:N>NID:YCL010C	AA:8		L_1528:V>V	AA:18		DBVPG6765:A>A	AA:67		L_1528:V>V	AA:72		DBVPG6044:S>S		NCYC110:S>S	AA:129		DBVPG6044:A>A		L_1528:S>S		NCYC110:A>A		SK1:A>A		Y9:A>A	AA:137		322134S:R>R		DBVPG6040:R>R	AA:138		DBVPG6044:G>G		NCYC110:G>G	AA:156		378604X:G>G		UWOPS05_217_3:G>G	AA:171		L_1528:N>N	AA:198		378604X:G>G		UWOPS05_217_3:G>G		YPS128:G>G	AA:234		378604X:E>E		DBVPG6044:E>E		NCYC110:E>E		UWOPS05_217_3:E>E		Y9:E>E		YPS128:E>EID:YCL011C	AA:6		YPS128:G>G	AA:149		NCYC110:V>V	AA:158		DBVPG6044:G>G		NCYC110:G>G	AA:180		DBVPG6044:K>K		UWOPS03_461_4:K>K		UWOPS05_227_2:K>K		YIIc17_E5:K>K		YPS128:K>K		YS4:K>K	AA:294		DBVPG6044:G>G		YIIc17_E5:G>G		YPS128:G>G	AA:333		322134S:T>T		DBVPG1853:T>T		DBVPG6044:T>T		NCYC110:T>T		UWOPS05_227_2:T>T		YIIc17_E5:T>T		YPS128:T>T		YPS606:T>TID:YCL016C	AA:26		K11:D>D		YPS606:D>D	AA:59		UWOPS83_787_3:V>V	AA:79		273614X:Q>Q		BC187:Q>Q		DBVPG6765:Q>Q		L_1374:Q>Q		SK1:Q>Q		Y55:Q>Q		YJM978:Q>Q	AA:91		YJM978:L>L	AA:101		DBVPG6040:F>F		UWOPS83_787_3:F>F		YIIc17_E5:F>F	AA:205		BC187:H>H		DBVPG6765:H>H		SK1:H>H		Y55:H>H		YJM978:H>H		YS4:H>H	AA:231		BC187:I>I		DBVPG6765:I>I		SK1:I>I		Y55:I>I		YJM978:I>I		YS4:I>I	AA:234		DBVPG6044:V>V		Y12:V>V		YPS606:V>V	AA:245		BC187:A>A		DBVPG6765:A>A		SK1:A>A		Y55:A>A		YJM978:A>A		YS4:A>A	AA:265		Y12:A>A	AA:283		UWOPS03_461_4:K>K		UWOPS05_217_3:K>K		Y12:K>K		YPS606:K>K	AA:289		Y12:P>P		YPS606:P>P	AA:313		YS4:V>V	AA:350		UWOPS03_461_4:E>E		UWOPS05_217_3:E>E		YPS606:E>E	AA:371		YPS606:L>LID:YCL017C	AA:38		UWOPS05_217_3:A>A	AA:272		DBVPG1373:T>T		DBVPG6044:T>T		DBVPG6765:T>T		L_1528:T>T		SK1:T>T		UWOPS03_461_4:T>T		UWOPS05_217_3:T>T		Y55:T>T		YJM975:T>T		YPS128:T>T		YPS606:T>T	AA:332		DBVPG1373:L>L		L_1528:L>L		UWOPS03_461_4:L>L		UWOPS05_217_3:L>L		UWOPS05_227_2:L>L		Y55:L>L		YJM975:L>L		YJM978:L>L	AA:347		DBVPG1373:E>E		L_1528:E>E		SK1:E>E		Y55:E>E		YJM975:E>E		YJM978:E>E	AA:359		UWOPS03_461_4:D>D		UWOPS05_217_3:D>D		UWOPS05_227_2:D>DID:YCL021W-A	AA:4		273614X:T>T		BC187:T>T		DBVPG1373:T>T		DBVPG1788:T>T		DBVPG6765:T>T		L_1374:T>T		L_1528:T>T		Y55:T>T		YJM975:T>T		YJM978:T>T		YJM981:T>T	AA:29		UWOPS05_217_3:S>S		Y12:S>S		YIIc17_E5:S>S		YPS128:S>S		YPS606:S>S	AA:49		L_1528:S>S	AA:59		UWOPS05_217_3:K>K	AA:79		Y12:F>F	AA:109		322134S:V>V		DBVPG6040:V>V		YPS128:V>V		YPS606:V>V	AA:122		Y12:F>FID:YCL025C	AA:18		DBVPG1853:T>T		DBVPG6044:T>T		K11:T>T		SK1:T>T		UWOPS03_461_4:T>T		UWOPS05_217_3:T>T		UWOPS05_227_2:T>T		UWOPS83_787_3:T>T		YPS606:T>T		YS4:T>T	AA:34		UWOPS03_461_4:T>T		UWOPS05_227_2:T>T	AA:77		NCYC110:T>T	AA:108		DBVPG1853:N>N		K11:N>N		UWOPS83_787_3:N>N		Y9:N>N	AA:121		DBVPG1853:Q>Q	AA:139		322134S:L>L		DBVPG1373:L>L		DBVPG1853:L>L		DBVPG6765:L>L		K11:L>L		NCYC110:L>L		NCYC361:L>L		SK1:L>L		UWOPS05_217_3:L>L		UWOPS05_227_2:L>L		UWOPS83_787_3:L>L		Y55:L>L		Y9:L>L		YJM975:L>L		YJM978:L>L		YJM981:L>L		YS4:L>L	AA:190		NCYC110:P>P		Y9:P>P	AA:201		378604X:A>A		NCYC110:A>A		UWOPS03_461_4:A>A		UWOPS05_217_3:A>A		UWOPS05_227_2:A>A		UWOPS83_787_3:A>A		Y9:A>A		YIIc17_E5:A>A	AA:327		SK1:G>G	AA:393		DBVPG6044:V>V		NCYC110:V>V	AA:445		DBVPG6044:S>S		NCYC110:S>S		UWOPS05_217_3:S>S	AA:466		K11:I>I		UWOPS83_787_3:I>I		YIIc17_E5:I>I	AA:498		UWOPS87_2421:C>C		YPS128:C>C		YPS606:C>C	AA:574		378604X:L>L		K11:L>L		NCYC361:L>L		UWOPS87_2421:L>L		Y12:L>L	AA:596		DBVPG1373:->-		DBVPG1788:->-		DBVPG6765:->-		Y55:->-		YJM978:->-		YJM981:->-ID:YCL026C-A	AA:8		DBVPG6765:L>L		YJM975:L>L	AA:17		DBVPG6044:I>I		NCYC110:I>I	AA:28		273614X:V>V		322134S:V>V		BC187:V>V		DBVPG1106:V>V		DBVPG6044:V>V		DBVPG6765:V>V		L_1528:V>V		NCYC110:V>V		SK1:V>V		UWOPS05_227_2:V>V		Y55:V>V		YJM975:V>V		YJM978:V>V		YJM981:V>V	AA:52		273614X:V>V		322134S:V>V		BC187:V>V		DBVPG1106:V>V		DBVPG6765:V>V		L_1528:V>V		Y55:V>V		YJM975:V>V		YJM978:V>V		YJM981:V>V	AA:66		273614X:I>I		BC187:I>I		DBVPG1106:I>I		DBVPG1373:I>I		DBVPG6044:I>I		DBVPG6765:I>I		L_1528:I>I		NCYC110:I>I		UWOPS05_227_2:I>I		Y55:I>I		YJM975:I>I		YJM978:I>I		YJM981:I>I	AA:87		273614X:R>R		BC187:R>R		DBVPG1106:R>R		DBVPG1373:R>R		DBVPG6044:R>R		DBVPG6765:R>R		L_1528:R>R		NCYC110:R>R		UWOPS03_461_4:R>R		UWOPS05_227_2:R>R		Y55:R>R		YJM975:R>R	AA:107		UWOPS03_461_4:Q>Q		UWOPS05_227_2:Q>Q	AA:135		YPS128:A>A		YPS606:A>AID:YCL028W	AA:3		273614X:T>T		DBVPG1106:T>T		DBVPG1373:T>T		DBVPG1853:T>T		DBVPG6040:T>T		DBVPG6044:T>T		DBVPG6765:T>T		K11:T>T		L_1374:T>T		L_1528:T>T		NCYC110:T>T		SK1:T>T		UWOPS05_227_2:T>T		UWOPS87_2421:T>T		Y55:T>T		YJM975:T>T		YJM978:T>T		YJM981:T>T		YS9:T>T	AA:18		K11:N>N	AA:22		DBVPG1853:A>A	AA:44		NCYC110:S>S	AA:62		Y12:D>D		Y9:D>D	AA:92		273614X:G>G		DBVPG1106:G>G		DBVPG1373:G>G		DBVPG1853:G>G		DBVPG6765:G>G		L_1374:G>G		L_1528:G>G		NCYC110:G>G		YJM975:G>G		YJM978:G>G		YJM981:G>G		YS9:G>G	AA:114		K11:F>F		SK1:F>F		YPS128:F>F		YPS606:F>F		YS2:F>F	AA:140		YJM978:A>A		YJM981:A>A	AA:207		SK1:L>L		Y9:L>L		YIIc17_E5:L>L		YPS606:L>L		YS4:L>L	AA:212		SK1:S>S		UWOPS87_2421:S>S		Y9:S>S		YIIc17_E5:S>S		YPS606:S>S		YS4:S>S	AA:229		YIIc17_E5:G>G	AA:287		UWOPS87_2421:Q>Q	AA:298		YPS606:Q>Q	AA:306		YS4:Q>Q	AA:310		273614X:H>H		322134S:H>H		DBVPG1373:H>H		DBVPG1788:H>H		DBVPG1853:H>H		DBVPG6044:H>H		DBVPG6765:H>H		L_1374:H>H		NCYC110:H>H		Y9:H>H		YIIc17_E5:H>H		YJM978:H>H		YJM981:H>H		YPS606:H>H		YS2:H>H		YS4:H>H		YS9:H>H	AA:358		378604X:P>P		DBVPG1853:P>P		DBVPG6044:P>P		NCYC110:P>P		Y9:P>P		YPS606:P>P		YS4:P>P	AA:364		378604X:Q>Q		Y9:Q>Q		YS4:Q>Q	AA:366		378604X:S>S		DBVPG6044:S>S		NCYC110:S>S		Y9:S>S		YPS606:S>S		YS4:S>S	AA:377		DBVPG1373:N>N	AA:387		378604X:F>F		Y9:F>F		YS4:F>FID:YCL029C	AA:26		UWOPS05_227_2:G>G	AA:59		K11:T>T		SK1:T>T		Y12:T>T		YIIc17_E5:T>T		YS4:T>T	AA:68		DBVPG6044:I>I		K11:I>I		SK1:I>I		UWOPS05_227_2:I>I		Y12:I>I		YIIc17_E5:I>I		YPS606:I>I		YS4:I>I	AA:90		DBVPG1853:T>T		DBVPG6044:T>T		K11:T>T		SK1:T>T		Y12:T>T		YIIc17_E5:T>T		YPS606:T>T		YS4:T>T	AA:122		DBVPG6765:R>R	AA:209		UWOPS05_227_2:Q>Q	AA:214		Y12:H>H		Y9:H>H	AA:277		DBVPG6044:A>A		K11:A>A		UWOPS05_227_2:A>A		UWOPS83_787_3:A>A		UWOPS87_2421:A>A		Y12:A>A		Y9:A>A		YIIc17_E5:A>A		YPS606:A>A	AA:300		DBVPG6040:T>T		UWOPS05_227_2:T>T	AA:322		SK1:Q>Q		Y12:Q>Q		Y9:Q>Q		YPS606:Q>Q	AA:364		Y12:S>S		Y9:S>S	AA:421		UWOPS03_461_4:T>T		UWOPS05_217_3:T>T		UWOPS05_227_2:T>T	AA:427		UWOPS05_217_3:A>A		UWOPS05_227_2:A>A	AA:437		Y12:Q>QID:YCL030C	AA:40		NCYC361:I>I		UWOPS83_787_3:L>L	AA:52		YJM975:V>V		YJM978:V>V	AA:92		DBVPG6044:E>E		NCYC361:E>E		SK1:E>E		UWOPS03_461_4:E>E		UWOPS83_787_3:E>E		Y12:E>E		YIIc17_E5:E>E		YPS128:E>E		YPS606:E>E		YS4:E>E	AA:96		322134S:V>V		DBVPG1373:V>V		DBVPG1788:V>V		DBVPG6044:V>V		NCYC361:V>V		SK1:V>V		UWOPS03_461_4:V>V		UWOPS83_787_3:V>V		Y12:V>V		YIIc17_E5:V>V		YPS128:V>V		YPS606:V>V		YS4:V>V	AA:150		DBVPG6044:G>G		SK1:G>G		YIIc17_E5:G>G		YS4:G>G	AA:154		DBVPG6044:T>T		NCYC361:T>T		SK1:T>T		UWOPS83_787_3:T>T		YIIc17_E5:T>T		YPS128:T>T		YPS606:T>T	AA:160		DBVPG6044:Y>Y		NCYC361:Y>Y		SK1:Y>Y		UWOPS83_787_3:Y>Y		YIIc17_E5:Y>Y		YPS128:Y>Y		YPS606:Y>Y	AA:164		DBVPG6044:L>L		NCYC361:L>L		SK1:L>L		UWOPS83_787_3:L>L		YIIc17_E5:L>L		YPS128:L>L		YPS606:L>L	AA:190		DBVPG6044:R>R		SK1:R>R		YIIc17_E5:R>R		YS4:R>R	AA:222		DBVPG6044:V>V		NCYC361:V>V		SK1:V>V		UWOPS05_217_3:V>V		UWOPS83_787_3:V>V		YPS606:V>V		YS2:V>V		YS4:V>V	AA:260		DBVPG6044:P>P	AA:293		DBVPG6044:K>K	AA:306		DBVPG6044:A>A	AA:315		UWOPS03_461_4:V>V		UWOPS05_217_3:V>V	AA:329		DBVPG6044:L>L		UWOPS83_787_3:L>L	AA:353		DBVPG6044:L>L		DBVPG6765:L>L		L_1528:L>L		NCYC361:L>L		SK1:L>L		UWOPS03_461_4:L>L		UWOPS05_217_3:L>L		UWOPS83_787_3:L>L		YJM975:L>L		YS2:L>L		YS4:L>L	AA:394		YS2:N>N		YS4:N>N	AA:397		UWOPS03_461_4:D>D		UWOPS05_217_3:D>D	AA:418		UWOPS03_461_4:P>P		UWOPS05_217_3:P>P		UWOPS83_787_3:P>P	AA:420		SK1:L>L		YS4:L>L	AA:468		K11:V>V		Y9:V>V	AA:476		K11:P>P		SK1:P>P		Y9:P>P	AA:523		K11:V>V		SK1:V>V		Y9:V>V	AA:535		DBVPG6044:G>G		K11:G>G		SK1:G>G		UWOPS05_217_3:G>G		Y9:G>G	AA:600		UWOPS05_217_3:V>V		YPS128:V>V	AA:606		UWOPS05_217_3:A>A		UWOPS83_787_3:A>A		YPS128:A>A	AA:617		UWOPS83_787_3:S>S		UWOPS87_2421:S>S		YPS128:S>S	AA:619		DBVPG6044:A>A		NCYC110:A>A		UWOPS05_217_3:A>A		UWOPS83_787_3:A>A		UWOPS87_2421:A>A		YPS128:A>A	AA:653		DBVPG1853:L>L		K11:L>L		SK1:L>L		Y9:L>L	AA:711		UWOPS05_217_3:V>V		UWOPS05_227_2:V>V	AA:726		DBVPG1853:T>T		DBVPG6044:T>T		K11:T>T		NCYC110:T>T		SK1:T>T		UWOPS05_217_3:T>T		UWOPS05_227_2:T>T		UWOPS83_787_3:T>T		Y9:T>T		YPS128:T>T		YS2:T>T		YS4:T>T	AA:732		DBVPG1853:T>T		DBVPG6044:T>T		K11:T>T		NCYC110:T>T		SK1:T>T		UWOPS05_217_3:T>T		UWOPS05_227_2:T>T		UWOPS83_787_3:T>T		Y9:T>T		YPS128:T>T		YPS606:T>T		YS2:T>T		YS4:T>T	AA:778		UWOPS83_787_3:D>DID:YCL031C	AA:57		UWOPS03_461_4:N>N		UWOPS05_217_3:N>N		UWOPS87_2421:N>N		YPS606:N>N	AA:71		YS4:V>V	AA:101		K11:S>S		SK1:S>S		UWOPS03_461_4:S>S		UWOPS05_217_3:S>S		UWOPS83_787_3:S>S		UWOPS87_2421:S>S		Y12:S>S		Y9:S>S		YIIc17_E5:S>S		YPS128:S>S		YPS606:S>S		YS4:S>S	AA:118		DBVPG6044:Y>Y	AA:142		DBVPG6044:K>K	AA:145		DBVPG6044:S>S	AA:150		DBVPG6044:K>K		UWOPS05_217_3:K>K		UWOPS05_227_2:K>K		UWOPS83_787_3:K>K		UWOPS87_2421:K>K		Y12:K>K		Y9:K>K		YIIc17_E5:K>K		YPS128:K>K		YPS606:K>K		YS4:K>K	AA:159		L_1528:T>T	AA:161		L_1528:T>T	AA:175		L_1528:L>L	AA:210		L_1528:D>D	AA:220		L_1528:T>T	AA:222		L_1528:S>S	AA:230		L_1528:I>I	AA:243		L_1528:A>A	AA:280		DBVPG6044:D>DID:YCL032W	AA:7		SK1:A>A		UWOPS05_217_3:A>A		UWOPS83_787_3:A>A		Y12:A>A		YS2:A>A	AA:16		K11:S>S		UWOPS05_217_3:S>S		Y12:S>S	AA:46		Y55:S>S	AA:57		273614X:C>C		BC187:C>C		DBVPG6040:C>C		DBVPG6044:C>C		DBVPG6765:C>C		K11:C>C		NCYC361:C>C		SK1:C>C		UWOPS05_217_3:C>C		UWOPS05_227_2:C>C		UWOPS83_787_3:C>C		Y12:C>C		Y55:C>C		YJM975:C>C		YJM978:C>C		YJM981:C>C		YPS606:C>C		YS2:C>C		YS4:C>C	AA:75		UWOPS03_461_4:L>L		UWOPS05_227_2:L>L	AA:135		SK1:S>S		YPS606:S>S	AA:138		UWOPS03_461_4:T>T		UWOPS05_227_2:T>T	AA:163		BC187:S>S		DBVPG6044:S>S		K11:S>S		SK1:S>S		UWOPS03_461_4:S>S		UWOPS05_227_2:S>S		UWOPS83_787_3:S>S		YIIc17_E5:S>S		YJM975:S>S		YJM978:S>S		YJM981:S>S		YPS606:S>S		YS2:S>S	AA:243		DBVPG6044:N>N	AA:264		DBVPG6044:C>C	AA:288		DBVPG6040:V>V		DBVPG6044:V>V		L_1374:V>V		L_1528:V>V		SK1:V>V		UWOPS03_461_4:V>V		UWOPS05_227_2:V>V		UWOPS83_787_3:V>V		Y55:V>V		YJM981:V>V		YPS606:V>V		YS2:V>V		YS4:V>V	AA:297		UWOPS03_461_4:L>L		UWOPS05_227_2:L>L		UWOPS83_787_3:L>L	AA:300		UWOPS03_461_4:L>L		UWOPS05_227_2:L>L		UWOPS83_787_3:L>L	AA:317		378604X:H>H		SK1:H>H		YS2:H>H	AA:323		YS9:R>RID:YCL033C	AA:3		UWOPS83_787_3:K>K	AA:35		UWOPS83_787_3:S>S	AA:67		273614X:A>A	AA:77		SK1:Y>Y	AA:143		L_1374:G>G	AA:148		UWOPS03_461_4:L>L	AA:156		UWOPS03_461_4:H>HID:YCL034W	AA:66		K11:N>N		SK1:N>N	AA:72		UWOPS05_217_3:R>R		UWOPS05_227_2:R>R	AA:79		K11:L>L		SK1:L>L		UWOPS03_461_4:L>L		UWOPS05_217_3:L>L		UWOPS05_227_2:L>L		UWOPS83_787_3:L>L	AA:99		DBVPG6044:R>R		K11:R>R		NCYC110:R>R		SK1:R>R		UWOPS03_461_4:R>R		UWOPS05_217_3:R>R		UWOPS05_227_2:R>R		UWOPS83_787_3:R>R	AA:126		SK1:A>A		YS9:A>A	AA:205		UWOPS03_461_4:Q>Q		UWOPS05_217_3:Q>Q		UWOPS05_227_2:Q>Q		UWOPS83_787_3:Q>Q	AA:235		SK1:G>G	AA:238		322134S:K>K	AA:243		DBVPG6044:T>T		NCYC110:T>T		SK1:T>T		UWOPS03_461_4:T>T		UWOPS05_217_3:T>T		UWOPS83_787_3:T>T		YPS606:T>T	AA:259		DBVPG6765:R>R		Y55:R>R	AA:347		322134S:P>P		DBVPG6044:P>P		NCYC110:P>P		SK1:P>P		UWOPS03_461_4:P>P		YPS128:P>P		YPS606:P>PID:YCL035C	AA:59		K11:G>G		SK1:G>G		YS2:G>G	AA:82		322134S:G>G	AA:90		K11:D>D	AA:98		DBVPG6044:G>G		K11:G>G		UWOPS03_461_4:G>G		UWOPS05_217_3:G>G		UWOPS05_227_2:G>G		YPS128:G>G		YPS606:G>GID:YCL036W	AA:30		YS9:G>G	AA:70		DBVPG1853:C>C	AA:137		DBVPG6040:S>S	AA:188		YPS606:S>S	AA:250		273614X:T>T		DBVPG1788:T>T		DBVPG6765:T>T		Y55:T>T		YJM981:T>T	AA:300		273614X:G>G		322134S:G>G		DBVPG1853:G>G		DBVPG6765:G>G		L_1374:G>G		SK1:G>G		UWOPS05_227_2:G>G		Y55:G>G		YJM981:G>G		YS4:G>G	AA:331		YPS606:L>L	AA:333		273614X:N>N		DBVPG1853:N>N		DBVPG6765:N>N		L_1374:N>N		Y55:N>N		YJM981:N>N		YS4:N>N	AA:345		273614X:L>L		DBVPG1106:L>L		DBVPG6765:L>L		L_1374:L>L		Y55:L>L		YJM981:L>L	AA:361		UWOPS05_227_2:I>I	AA:391		SK1:D>D		YPS128:D>D		YPS606:D>D		YS9:D>D	AA:443		322134S:G>G		DBVPG1106:G>G		DBVPG6765:G>G		L_1374:G>G		SK1:G>G		UWOPS03_461_4:G>G		UWOPS05_227_2:G>G		Y55:G>G		YPS128:G>G		YPS606:G>G		YS2:G>G		YS4:G>G		YS9:G>G	AA:445		YS2:S>S	AA:531		UWOPS03_461_4:K>K		UWOPS05_227_2:K>K	AA:536		SK1:S>S	AA:547		YS2:G>G	AA:554		K11:T>T		SK1:T>T		UWOPS03_461_4:T>T		UWOPS05_227_2:T>T		YPS128:T>T		YPS606:T>TID:YCL038C	AA:32		SK1:S>S		UWOPS83_787_3:S>S		YPS128:S>S		YPS606:S>S		YS2:S>S		YS4:S>S	AA:39		SK1:V>V		UWOPS83_787_3:V>V		YPS606:V>V		YS2:V>V		YS4:V>V	AA:51		YPS128:L>L		YPS606:L>L	AA:73		Y9:T>T	AA:106		322134S:L>L		DBVPG1106:L>L		DBVPG1373:L>L		DBVPG1788:L>L		DBVPG6765:L>L		K11:L>L		L_1374:L>L		L_1528:L>L		SK1:L>L		Y55:L>L		YIIc17_E5:L>L		YJM981:L>L		YS4:L>L		YS9:L>L	AA:435		DBVPG6044:S>S		UWOPS05_217_3:S>S		UWOPS83_787_3:S>S	AA:463		Y12:K>K		Y9:K>K		YS9:K>K	AA:465		DBVPG6044:S>S		NCYC110:S>S		Y12:S>S		Y9:S>S		YS9:S>SID:YCL039W	AA:24		NCYC361:V>V		UWOPS05_217_3:V>V		UWOPS83_787_3:V>V		UWOPS87_2421:V>V		YIIc17_E5:V>V		YPS128:V>V		YPS606:V>V	AA:35		NCYC361:L>L		SK1:L>L		UWOPS05_217_3:L>L		UWOPS83_787_3:L>L		UWOPS87_2421:L>L		YIIc17_E5:L>L		YPS128:L>L		YPS606:L>L		YS2:L>L	AA:40		DBVPG1853:L>L		NCYC361:L>L		SK1:L>L		UWOPS05_217_3:L>L		UWOPS05_227_2:L>L		UWOPS83_787_3:L>L		UWOPS87_2421:L>L		Y9:L>L		YIIc17_E5:L>L		YPS128:L>L		YPS606:L>L		YS2:L>L	AA:53		NCYC361:L>L		SK1:L>L		UWOPS05_217_3:L>L		UWOPS83_787_3:L>L		UWOPS87_2421:L>L		Y9:L>L		YS2:L>L	AA:88		UWOPS87_2421:S>S	AA:98		NCYC110:R>R	AA:140		NCYC110:C>C	AA:145		YIIc17_E5:I>I	AA:214		YPS128:N>N		YPS606:N>N	AA:217		UWOPS87_2421:N>N		Y9:N>N	AA:224		SK1:E>E	AA:354		YPS128:V>V		YPS606:V>V	AA:360		L_1528:D>D	AA:391		YPS128:F>F		YPS606:F>F	AA:397		YS9:I>I	AA:404		Y55:S>S	AA:406		YS9:P>P	AA:424		YPS128:P>P		YPS606:P>P	AA:476		DBVPG1853:V>V		SK1:V>V	AA:492		YS4:E>E	AA:503		DBVPG6044:I>I	AA:594		322134S:R>R		BC187:R>R		DBVPG6040:R>R		DBVPG6765:R>R		L_1528:R>R		SK1:R>R		Y55:R>R		YIIc17_E5:R>R		YJM975:R>R		YJM981:R>R		YS4:R>R	AA:599		322134S:D>D		BC187:D>D		DBVPG1853:D>D		DBVPG6040:D>D		DBVPG6765:D>D		L_1528:D>D		NCYC361:D>D		SK1:D>D		Y55:D>D		YIIc17_E5:D>D		YJM975:D>D		YJM981:D>D		YS4:D>D	AA:631		322134S:L>L		BC187:L>L		DBVPG1788:L>L		DBVPG1853:L>L		DBVPG6040:L>L		DBVPG6765:L>L		L_1528:L>L		NCYC110:L>L		NCYC361:L>L		SK1:L>L		UWOPS05_217_3:L>L		Y12:L>L		YIIc17_E5:L>L		YJM975:L>L		YJM981:L>L		YS9:L>L	AA:648		BC187:I>I		DBVPG1788:I>I		DBVPG1853:I>I		DBVPG6040:I>I		DBVPG6765:I>I		L_1528:I>I		NCYC110:I>I		NCYC361:I>I		SK1:I>I		UWOPS05_217_3:I>I		Y12:I>I		YIIc17_E5:I>I		YJM975:I>I		YJM978:I>I		YJM981:I>I		YS9:I>I	AA:655		BC187:G>G		DBVPG1788:G>G		DBVPG1853:G>G		DBVPG6040:G>G		DBVPG6765:G>G		L_1374:G>G		L_1528:G>G		NCYC110:G>G		NCYC361:G>G		SK1:G>G		UWOPS05_217_3:G>G		Y12:G>G		YIIc17_E5:G>G		YJM975:G>G		YJM978:G>G		YJM981:G>G		YS9:G>G	AA:693		BC187:L>L		DBVPG1788:L>L		DBVPG6040:L>L		DBVPG6765:L>L		L_1374:L>L		L_1528:L>L		NCYC110:L>L		NCYC361:L>L		SK1:L>L		UWOPS05_217_3:L>L		Y12:L>L		Y55:L>L		YIIc17_E5:L>L		YJM975:L>L		YJM978:L>L		YJM981:L>L		YS9:L>L	AA:698		YIIc17_E5:S>S	AA:729		NCYC361:A>A	AA:734		DBVPG6044:D>D		NCYC110:D>DID:YCL043C	AA:24		UWOPS83_787_3:Q>Q		YIIc17_E5:Q>Q	AA:27		UWOPS83_787_3:V>V		YIIc17_E5:V>V	AA:31		322134S:D>D	AA:41		273614X:S>S		322134S:S>S		378604X:S>S		DBVPG1788:S>S		DBVPG1853:S>S		DBVPG6040:S>S		DBVPG6044:S>S		DBVPG6765:S>S		L_1374:S>S		NCYC110:S>S		SK1:S>S		UWOPS05_217_3:S>S		UWOPS05_227_2:S>S		UWOPS83_787_3:S>S		Y12:S>S		Y55:S>S		YIIc17_E5:S>S		YJM975:S>S		YJM978:S>S		YJM981:S>S		YS2:S>S		YS4:S>S	AA:44		Y12:E>E	AA:46		UWOPS05_217_3:I>I		UWOPS05_227_2:I>I	AA:78		378604X:L>L	AA:143		UWOPS05_217_3:V>V		UWOPS05_227_2:V>V	AA:189		273614X:D>D		322134S:D>D		DBVPG1106:D>D		DBVPG1788:D>D		DBVPG1853:D>D		DBVPG6040:D>D		DBVPG6044:D>D		DBVPG6765:D>D		L_1374:D>D		L_1528:D>D		NCYC110:D>D		NCYC361:D>D		UWOPS05_217_3:D>D		UWOPS05_227_2:D>D		Y55:D>D		YJM981:D>D		YS4:D>D	AA:217		322134S:N>N		DBVPG1106:N>N		DBVPG1788:N>N		DBVPG1853:N>N		L_1374:N>N		L_1528:N>N		NCYC361:N>N		UWOPS05_217_3:N>N		UWOPS05_227_2:N>N		Y55:N>N		YJM978:N>N		YJM981:N>N		YS4:N>N	AA:274		322134S:K>K		DBVPG1106:K>K		DBVPG1788:K>K		DBVPG1853:K>K		L_1374:K>K		L_1528:K>K		NCYC110:K>K		NCYC361:K>K		UWOPS83_787_3:K>K		Y55:K>K		YJM978:K>K		YJM981:K>K		YS4:K>K	AA:303		NCYC110:G>G	AA:345		DBVPG1106:E>E		DBVPG1853:E>E		YJM975:E>E		YJM978:E>E		YJM981:E>EID:YCL044C	AA:22		DBVPG6765:D>D		L_1528:D>D		Y55:D>D		YJM975:D>D		YJM981:D>D		YS9:D>D	AA:92		UWOPS05_217_3:H>H	AA:110		NCYC361:T>T	AA:112		378604X:I>I		NCYC110:I>I		UWOPS05_217_3:I>I		UWOPS83_787_3:I>I		Y9:I>I		YPS606:I>I		YS9:I>I	AA:203		378604X:G>G	AA:213		378604X:D>D		UWOPS05_217_3:D>D		UWOPS83_787_3:D>D	AA:225		378604X:P>P		UWOPS83_787_3:P>P	AA:288		DBVPG6044:L>L		UWOPS05_217_3:L>L		Y55:L>L		YPS128:L>L		YPS606:L>L	AA:345		Y9:R>R	AA:353		DBVPG6044:T>T		SK1:T>T		UWOPS83_787_3:T>T		Y55:T>T		YPS128:T>T		YPS606:T>TID:YCL045C	AA:38		K11:A>A	AA:67		NCYC110:C>C		SK1:C>C		UWOPS03_461_4:C>C		UWOPS05_227_2:C>C	AA:117		DBVPG1853:L>L	AA:201		YJM978:A>A	AA:208		DBVPG1853:P>P		DBVPG6040:P>P	AA:216		DBVPG1853:L>L		DBVPG6040:L>L		K11:L>L		NCYC110:L>L		NCYC361:L>L		SK1:L>L		UWOPS03_461_4:L>L		UWOPS05_217_3:L>L		UWOPS05_227_2:L>L		UWOPS83_787_3:L>L		Y12:L>L		YIIc17_E5:L>L		YPS606:L>L		YS4:L>L	AA:227		UWOPS03_461_4:V>V		UWOPS05_217_3:V>V		UWOPS05_227_2:V>V	AA:258		K11:T>T		UWOPS03_461_4:T>T		UWOPS05_217_3:T>T		UWOPS05_227_2:T>T	AA:269		DBVPG6040:E>E	AA:365		DBVPG6044:P>P		NCYC110:P>P		SK1:P>P		UWOPS03_461_4:P>P		UWOPS05_217_3:P>P	AA:405		YIIc17_E5:K>K	AA:509		NCYC361:L>L	AA:572		YS9:L>L	AA:587		YPS128:H>H		YPS606:H>H	AA:599		YS2:I>I	AA:616		NCYC110:I>I		SK1:I>I		UWOPS05_217_3:I>I	AA:692		378604X:T>TID:YCL049C	AA:68		UWOPS83_787_3:I>I	AA:105		Y9:N>N		YPS128:N>N		YPS606:N>N	AA:189		YPS128:V>V		YPS606:V>V	AA:212		273614X:V>V		DBVPG1373:V>V		DBVPG6765:V>V		K11:V>V		L_1528:V>V		UWOPS03_461_4:V>V		UWOPS05_217_3:V>V		Y9:V>V		YIIc17_E5:V>V		YJM975:V>V		YJM978:V>V		YPS128:V>V		YPS606:V>V		YS9:V>V	AA:239		Y9:P>P		YIIc17_E5:P>P		YS9:P>P	AA:250		YS9:T>T	AA:267		YPS128:T>T		YPS606:T>T	AA:288		DBVPG6044:E>EID:YCL050C	AA:32		DBVPG6044:T>T		NCYC110:T>T		SK1:T>T		YS4:T>T	AA:46		322134S:S>S		DBVPG1373:S>S		DBVPG6044:S>S		DBVPG6765:S>S		K11:S>S		L_1528:S>S		NCYC110:S>S		NCYC361:S>S		UWOPS03_461_4:S>S		UWOPS05_227_2:S>S		UWOPS83_787_3:S>S		UWOPS87_2421:S>S		Y12:S>S		Y55:S>S		YIIc17_E5:S>S		YJM975:S>S		YJM978:S>S		YPS128:S>S		YPS606:S>S		YS4:S>S	AA:61		DBVPG6044:P>P	AA:77		NCYC361:I>I		UWOPS83_787_3:I>I	AA:162		322134S:L>L		DBVPG6044:L>L		K11:L>L		NCYC110:L>L		UWOPS03_461_4:L>L		UWOPS05_227_2:L>L		Y9:L>L		YIIc17_E5:L>L		YPS128:L>L		YPS606:L>L	AA:208		DBVPG6044:S>S		K11:S>S		NCYC110:S>S		UWOPS03_461_4:S>S		UWOPS05_227_2:S>S		UWOPS83_787_3:S>S		Y9:S>S		YIIc17_E5:S>S		YPS128:S>S		YPS606:S>S	AA:260		DBVPG6040:R>R		DBVPG6044:R>R		K11:R>R		NCYC110:R>R		UWOPS03_461_4:R>R		UWOPS05_227_2:R>R		UWOPS83_787_3:R>R		Y9:R>R		YIIc17_E5:R>R		YPS128:R>R		YPS606:R>R	AA:264		DBVPG6040:F>F		UWOPS83_787_3:F>F	AA:275		DBVPG6040:T>T	AA:299		UWOPS87_2421:I>IID:YCL051W	AA:34		DBVPG6040:L>L	AA:54		DBVPG1373:P>P	AA:214		322134S:S>S		DBVPG1853:S>S		DBVPG6040:S>S		Y12:S>S		YS4:S>S	AA:263		322134S:T>T		DBVPG1853:T>T		DBVPG6040:T>T		DBVPG6044:T>T		K11:T>T		UWOPS03_461_4:T>T		UWOPS05_227_2:T>T		UWOPS83_787_3:T>T		UWOPS87_2421:T>T		YPS606:T>T		YS4:T>T	AA:279		322134S:E>E		DBVPG1853:E>E		DBVPG6040:E>E		DBVPG6044:E>E		UWOPS83_787_3:E>E		UWOPS87_2421:E>E		YIIc17_E5:E>E		YPS606:E>E	AA:330		K11:L>L		UWOPS03_461_4:L>L		UWOPS05_227_2:L>L	AA:350		322134S:L>L		DBVPG1853:L>L		DBVPG6040:L>L		DBVPG6044:L>L		K11:L>L		UWOPS83_787_3:L>L		YIIc17_E5:L>L		YPS606:L>L	AA:362		DBVPG6040:F>F	AA:371		DBVPG6044:S>S		SK1:S>S	AA:430		DBVPG6044:N>N		SK1:N>N	AA:497		UWOPS03_461_4:G>G		UWOPS05_217_3:G>G	AA:540		322134S:N>N		DBVPG6040:N>N		Y12:N>N		YIIc17_E5:N>NID:YCL052C	AA:47		YS4:T>T	AA:76		DBVPG6044:L>L		NCYC110:L>L		SK1:L>L	AA:84		Y55:S>S	AA:90		DBVPG6044:P>P		NCYC110:P>P		SK1:P>P	AA:111		YS4:Q>Q	AA:163		322134S:L>L		DBVPG1853:L>L		K11:L>L		NCYC110:L>L		SK1:L>L		UWOPS03_461_4:L>L		UWOPS05_217_3:L>L		UWOPS05_227_2:L>L		UWOPS87_2421:L>L		Y12:L>L		Y9:L>L		YIIc17_E5:L>L		YPS128:L>L		YS4:L>L	AA:213		322134S:H>H		DBVPG1853:H>H		K11:H>H		UWOPS87_2421:H>H		Y9:H>H		YIIc17_E5:H>H		YPS128:H>H		YS4:H>H	AA:215		322134S:T>T		DBVPG1853:T>T		DBVPG6044:T>T		K11:T>T		NCYC110:T>T		SK1:T>T		UWOPS03_461_4:T>T		UWOPS05_217_3:T>T		UWOPS05_227_2:T>T		UWOPS87_2421:T>T		Y12:T>T		Y9:T>T		YIIc17_E5:T>T		YPS128:T>T		YS4:T>T	AA:253		322134S:L>L		DBVPG1853:L>L		K11:L>L		Y12:L>L		Y9:L>L		YIIc17_E5:L>L		YPS128:L>L		YS4:L>L	AA:268		322134S:L>L		DBVPG1853:L>L		K11:L>L		Y12:L>L		Y9:L>L		YIIc17_E5:L>L		YPS128:L>L		YS4:L>L	AA:320		DBVPG1373:L>L		DBVPG1788:L>L		DBVPG6765:L>L		UWOPS05_227_2:L>L		Y55:L>L		YJM975:L>L		YJM978:L>L		YPS128:L>L	AA:399		DBVPG6044:I>I		NCYC110:I>I		UWOPS83_787_3:I>I		YPS128:I>I		YPS606:I>IID:YCL055W	AA:48		YPS128:D>D		YPS606:D>D	AA:165		L_1528:L>L	AA:183		322134S:E>E		DBVPG6044:E>E		NCYC110:E>E		UWOPS05_227_2:E>E		Y12:E>E		Y9:E>E		YPS128:E>E		YPS606:E>E	AA:205		322134S:T>T		DBVPG6044:T>T		NCYC110:T>T		UWOPS05_227_2:T>T		UWOPS83_787_3:T>T		Y12:T>T		Y9:T>T		YPS128:T>T		YPS606:T>T	AA:223		322134S:R>R		DBVPG6044:R>R		NCYC110:R>R		UWOPS05_227_2:R>R		UWOPS83_787_3:R>R		Y12:R>R		Y9:R>R		YPS128:R>R		YPS606:R>R	AA:264		YPS128:T>T		YPS606:T>T	AA:297		DBVPG6044:L>L		NCYC110:L>L	AA:300		UWOPS03_461_4:F>F		UWOPS05_227_2:F>FID:YCL056C	AA:37		UWOPS05_217_3:G>G		UWOPS05_227_2:G>G	AA:99		YJM975:K>K		YJM978:K>KID:YCL057C-A	AA:51		378604X:V>V		DBVPG6040:V>V		DBVPG6044:V>V		UWOPS83_787_3:V>V		YIIc17_E5:V>V		YPS128:V>V	AA:69		DBVPG6044:L>L	AA:78		YJM975:G>G	AA:89		378604X:S>S		DBVPG6040:S>S		DBVPG6044:S>S		UWOPS83_787_3:S>S		YIIc17_E5:S>S		YPS128:S>SID:YCL057W	AA:22		378604X:R>R		DBVPG6040:R>R		DBVPG6044:R>R		NCYC110:R>R		UWOPS03_461_4:R>R		UWOPS83_787_3:R>R		Y9:R>R		YPS128:R>R		YPS606:R>R	AA:75		YPS128:S>S		YPS606:S>S	AA:104		YIIc17_E5:S>S	AA:120		322134S:L>L		DBVPG1853:L>L		DBVPG6044:L>L		K11:L>L		NCYC110:L>L		UWOPS03_461_4:L>L		UWOPS05_227_2:L>L		UWOPS83_787_3:L>L		YIIc17_E5:L>L		YPS128:L>L	AA:134		K11:F>F	AA:178		UWOPS05_227_2:R>R	AA:184		322134S:I>I		DBVPG1853:I>I		DBVPG6040:I>I		K11:I>I		UWOPS05_227_2:I>I		YIIc17_E5:I>I	AA:196		322134S:S>S		DBVPG1853:S>S		DBVPG6040:S>S		DBVPG6044:S>S		K11:S>S		NCYC110:S>S		UWOPS05_227_2:S>S		UWOPS83_787_3:S>S		YIIc17_E5:S>S		YPS128:S>S	AA:208		YS4:F>F	AA:275		UWOPS83_787_3:E>E	AA:288		UWOPS83_787_3:L>L	AA:347		273614X:N>N		322134S:N>N		BC187:N>N		DBVPG1373:N>N		DBVPG6040:N>N		DBVPG6765:N>N		K11:N>N		L_1374:N>N		NCYC110:N>N		UWOPS05_227_2:N>N		UWOPS83_787_3:N>N		Y12:N>N		Y9:N>N		YIIc17_E5:N>N		YPS606:N>N		YS4:N>N		YS9:N>N	AA:376		NCYC110:E>E	AA:475		322134S:A>A		K11:A>A		Y12:A>A		Y9:A>A		YIIc17_E5:A>A	AA:552		DBVPG1853:S>S		DBVPG6044:S>S		K11:S>S		NCYC110:S>S		Y12:S>S		Y9:S>S		YIIc17_E5:S>S	AA:567		DBVPG1853:S>S	AA:677		322134S:I>I		378604X:I>I		DBVPG1853:I>I		DBVPG6040:I>I		DBVPG6044:I>I		NCYC110:I>I		UWOPS87_2421:I>I		Y9:I>I		YIIc17_E5:I>I		YS9:I>I	AA:680		322134S:A>A		378604X:A>A		DBVPG1853:A>A		DBVPG6040:A>A		DBVPG6044:A>A		NCYC110:A>A		UWOPS87_2421:A>A		Y9:A>A		YIIc17_E5:A>A		YPS128:A>A		YS4:A>A		YS9:A>AID:YCL059C	AA:36		DBVPG6044:A>A		NCYC110:A>A	AA:77		BC187:E>E		DBVPG1373:E>E		DBVPG1788:E>E		DBVPG1853:E>E		DBVPG6040:E>E		DBVPG6044:E>E		DBVPG6765:E>E		K11:E>E		L_1374:E>E		L_1528:E>E		NCYC110:E>E		SK1:E>E		UWOPS05_227_2:E>E		Y55:E>E		Y9:E>E		YJM978:E>E		YPS128:E>E		YPS606:E>E		YS4:E>E		YS9:E>E	AA:116		DBVPG1853:I>I		DBVPG6040:I>I		K11:I>I		YPS128:I>I		YPS606:I>I		YS9:I>I	AA:126		DBVPG1853:I>I		DBVPG6040:I>I		DBVPG6044:I>I		K11:I>I		NCYC110:I>I		YPS606:I>I		YS9:I>IID:YCL063W	AA:48		DBVPG6040:L>L	AA:59		DBVPG6040:L>L	AA:62		YS2:L>L		YS9:L>L	AA:64		L_1528:V>V	AA:80		DBVPG6040:R>R	AA:95		DBVPG6040:V>V	AA:106		DBVPG6040:N>N	AA:125		DBVPG6040:T>T	AA:139		DBVPG6040:I>I	AA:145		DBVPG6040:L>L		YS9:L>L	AA:148		DBVPG6040:V>V	AA:154		DBVPG1373:P>P	AA:166		378604X:V>V		DBVPG6040:V>V		K11:V>V		Y12:V>V	AA:180		DBVPG6040:K>K	AA:182		UWOPS03_461_4:Q>Q	AA:189		DBVPG6040:L>L	AA:217		UWOPS83_787_3:N>N	AA:236		YPS128:S>S		YPS606:S>S	AA:243		DBVPG6044:L>L		UWOPS87_2421:L>L	AA:254		UWOPS87_2421:V>V	AA:283		DBVPG6044:S>S		UWOPS87_2421:S>S	AA:327		UWOPS87_2421:P>P	AA:351		378604X:N>N		DBVPG1853:N>N		DBVPG6044:N>N		K11:N>N		UWOPS87_2421:N>N		Y12:N>N		YPS128:N>N		YPS606:N>N	AA:368		YPS128:L>L		YPS606:L>L	AA:408		DBVPG6044:V>V	AA:414		DBVPG1853:Q>QID:YCL064C	AA:16		DBVPG1853:P>P		DBVPG6040:P>P		DBVPG6044:P>P		NCYC110:P>P		Y12:P>P		YIIc17_E5:P>P		YPS606:P>P	AA:75		UWOPS05_227_2:A>A	AA:98		YPS128:V>V		YPS606:V>V	AA:101		DBVPG1853:I>I		DBVPG6044:I>I		NCYC110:I>I		UWOPS03_461_4:I>I		UWOPS05_217_3:I>I		Y12:I>I		YIIc17_E5:I>I		YPS128:I>I		YPS606:I>I	AA:112		DBVPG1853:G>G		Y12:G>G		YIIc17_E5:G>G	AA:135		DBVPG1853:I>I		NCYC361:I>I		UWOPS87_2421:I>I		Y12:I>I		YIIc17_E5:I>I	AA:175		DBVPG1106:G>G		DBVPG1373:G>G		DBVPG1853:G>G		DBVPG6044:G>G		DBVPG6765:G>G		L_1374:G>G		L_1528:G>G		NCYC361:G>G		SK1:G>G		UWOPS03_461_4:G>G		UWOPS87_2421:G>G		Y12:G>G		Y55:G>G		YIIc17_E5:G>G		YJM975:G>G		YJM978:G>G		YPS128:G>G		YPS606:G>G	AA:182		UWOPS87_2421:G>G	AA:193		UWOPS03_461_4:L>L	AA:210		NCYC361:N>N	AA:279		SK1:V>V	AA:307		378604X:A>A		DBVPG6044:A>A		NCYC361:A>A		UWOPS03_461_4:A>A		UWOPS05_217_3:A>A		UWOPS87_2421:A>A		Y12:A>A		YPS128:A>A		YPS606:A>A	AA:345		378604X:D>D		NCYC361:D>D		UWOPS03_461_4:D>D		UWOPS05_217_3:D>D	AA:355		DBVPG6044:V>V		YPS128:V>VID:YCL066W	AA:5		UWOPS03_461_4:K>K		UWOPS05_227_2:K>K	AA:40		UWOPS87_2421:C>C	AA:45		YJM981:R>R	AA:55		378604X:V>V		DBVPG1853:V>V		DBVPG6044:V>V		K11:V>V		UWOPS03_461_4:V>V		UWOPS05_227_2:V>V		UWOPS83_787_3:V>V		UWOPS87_2421:V>V		YIIc17_E5:V>V		YPS128:V>V		YPS606:V>V	AA:63		378604X:K>K		DBVPG1853:K>K		DBVPG6044:K>K		K11:K>K		UWOPS03_461_4:K>K		UWOPS83_787_3:K>K		UWOPS87_2421:K>K		YIIc17_E5:K>K		YPS128:K>K		YPS606:K>K	AA:104		DBVPG1373:S>S		YJM978:S>SID:YCL068C	AA:17		DBVPG6040:D>D	AA:30		378604X:I>I		DBVPG1373:I>I		DBVPG1853:I>I		DBVPG6040:I>I		DBVPG6044:I>I		DBVPG6765:I>I		SK1:I>I		UWOPS03_461_4:I>I		UWOPS05_217_3:I>I		Y12:I>I		Y55:I>I		YIIc17_E5:I>I		YJM978:I>I		YPS128:I>I		YPS606:I>I	AA:35		UWOPS03_461_4:V>V		UWOPS05_217_3:V>V	AA:47		378604X:L>L		DBVPG1853:L>L		DBVPG6044:L>L		UWOPS03_461_4:L>L		UWOPS05_217_3:L>L		Y12:L>L		YIIc17_E5:L>L		YPS128:L>L		YPS606:L>L	AA:106		L_1528:L>L	AA:168		DBVPG6044:T>T		UWOPS05_217_3:T>T		UWOPS05_227_2:T>T		UWOPS83_787_3:T>T		Y12:T>T		YIIc17_E5:T>T		YPS128:T>T		YPS606:T>TID:YCR002C	AA:51		273614X:T>T		K11:T>T	AA:119		273614X:K>K		322134S:K>K		DBVPG6044:K>K		K11:K>K		SK1:K>K		UWOPS03_461_4:K>K		UWOPS83_787_3:K>K		YIIc17_E5:K>K	AA:291		322134S:H>H		DBVPG6044:H>H		SK1:H>H		UWOPS03_461_4:H>H		UWOPS05_217_3:H>H		YIIc17_E5:H>H		YPS606:H>H		YS4:H>HID:YCR004C	AA:62		322134S:A>A		378604X:A>A		DBVPG6044:A>A		K11:A>A		SK1:A>A		UWOPS03_461_4:A>A		UWOPS05_217_3:A>A		UWOPS05_227_2:A>A		UWOPS83_787_3:A>A		YIIc17_E5:A>A		YPS128:A>A		YPS606:A>A	AA:66		YIIc17_E5:T>T	AA:166		DBVPG6040:A>A	AA:180		378604X:P>P		DBVPG6044:P>P		K11:P>P		SK1:P>P		UWOPS05_217_3:P>P		UWOPS05_227_2:P>P		UWOPS87_2421:P>P		YIIc17_E5:P>P		YPS128:P>P		YPS606:P>P		YS4:P>P	AA:207		YJM978:K>K	AA:227		378604X:A>A		K11:A>A		SK1:A>A		UWOPS87_2421:A>A		YS4:A>A	AA:244		378604X:C>C		DBVPG1373:C>C		DBVPG1788:C>C		DBVPG6040:C>C		DBVPG6765:C>C		K11:C>C		NCYC361:C>C		SK1:C>C		UWOPS05_227_2:C>C		UWOPS87_2421:C>C		Y55:C>C		YJM978:C>C		YJM981:C>C		YPS128:C>C		YS4:C>C		YS9:C>CID:YCR005C	AA:26		YPS606:L>L	AA:45		322134S:V>V		378604X:V>V		DBVPG6044:V>V		K11:V>V		SK1:V>V		UWOPS83_787_3:V>V		UWOPS87_2421:V>V		Y12:V>V		Y9:V>V		YS2:V>V	AA:49		378604X:G>G		DBVPG6044:G>G		K11:G>G		SK1:G>G		UWOPS83_787_3:G>G		UWOPS87_2421:G>G		Y12:G>G		Y9:G>G		YS2:G>G	AA:71		DBVPG6044:S>S	AA:162		DBVPG6044:S>S		UWOPS83_787_3:S>S		UWOPS87_2421:S>S		Y12:S>S		Y9:S>S	AA:282		DBVPG6044:A>A	AA:307		K11:L>L	AA:312		DBVPG1106:N>N	AA:373		273614X:Y>Y		DBVPG6044:Y>Y		K11:Y>Y		SK1:Y>Y		UWOPS83_787_3:Y>Y		UWOPS87_2421:Y>Y		Y9:Y>Y		YIIc17_E5:Y>Y		YPS128:Y>Y	AA:374		YS4:E>E	AA:393		273614X:V>V		DBVPG6044:V>V		K11:V>V		SK1:V>V		UWOPS83_787_3:V>V		UWOPS87_2421:V>V		YIIc17_E5:V>V		YPS128:V>VID:YCR008W	AA:23		DBVPG1853:S>S		SK1:S>S		UWOPS87_2421:S>S		YPS128:S>S		YPS606:S>S		YS2:S>S		YS4:S>S	AA:45		UWOPS03_461_4:I>I		UWOPS05_227_2:I>I	AA:66		DBVPG1853:L>L		SK1:L>L		YIIc17_E5:L>L		YPS128:L>L		YPS606:L>L		YS2:L>L		YS4:L>L	AA:75		DBVPG1853:R>R		SK1:R>R		YIIc17_E5:R>R		YPS128:R>R		YPS606:R>R		YS2:R>R		YS4:R>R	AA:99		DBVPG1853:S>S		SK1:S>S		YIIc17_E5:S>S		YPS128:S>S		YPS606:S>S		YS2:S>S		YS4:S>S	AA:164		Y12:I>I		Y9:I>I	AA:170		322134S:P>P		378604X:P>P		DBVPG1853:P>P		SK1:P>P		Y12:P>P		Y9:P>P		YIIc17_E5:P>P		YPS128:P>P		YS2:P>P		YS4:P>P	AA:189		322134S:C>C		378604X:C>C		DBVPG1853:C>C		SK1:C>C		UWOPS05_217_3:C>C		UWOPS05_227_2:C>C		UWOPS83_787_3:C>C		Y12:C>C		Y9:C>C		YIIc17_E5:C>C		YS2:C>C		YS4:C>C	AA:195		322134S:H>H		378604X:H>H		DBVPG1853:H>H		SK1:H>H		Y12:H>H		Y9:H>H		YIIc17_E5:H>H		YS2:H>H		YS4:H>H	AA:277		378604X:G>G	AA:289		322134S:S>S		378604X:S>S		DBVPG1853:S>S		SK1:S>S		UWOPS83_787_3:S>S		UWOPS87_2421:S>S		Y12:S>S		Y9:S>S		YIIc17_E5:S>S		YPS128:S>S		YS4:S>S	AA:316		L_1528:G>G	AA:318		L_1528:C>C	AA:331		L_1528:R>R	AA:350		L_1528:Y>Y	AA:355		L_1528:F>F	AA:378		378604X:S>S		DBVPG1853:S>S		SK1:S>S		UWOPS83_787_3:S>S		UWOPS87_2421:S>S		Y12:S>S		YIIc17_E5:S>S		YPS128:S>S	AA:414		L_1528:L>L	AA:438		DBVPG1853:V>V	AA:460		L_1528:D>D	AA:525		UWOPS05_217_3:G>G	AA:591		273614X:R>R		BC187:R>R		DBVPG1106:R>R		DBVPG1373:R>R		DBVPG1788:R>R		DBVPG6765:R>R		Y55:R>R		YJM975:R>R		YJM978:R>R		YS4:R>RID:YCR009C	AA:8		378604X:K>K		DBVPG6044:K>K		NCYC361:K>K		SK1:K>K		UWOPS03_461_4:K>K		UWOPS83_787_3:K>K		Y12:K>K		YIIc17_E5:K>K		YS4:K>K	AA:10		378604X:I>I		DBVPG6044:I>I		NCYC361:I>I		SK1:I>I		UWOPS03_461_4:I>I		UWOPS83_787_3:I>I		Y12:I>I		YIIc17_E5:I>I		YS4:I>I	AA:58		322134S:L>L		378604X:L>L		DBVPG6044:L>L		NCYC361:L>L		SK1:L>L		UWOPS03_461_4:L>L		Y12:L>L		YS4:L>L	AA:143		NCYC361:D>D	AA:172		DBVPG6044:L>L		SK1:L>L		YS4:L>L	AA:201		273614X:F>F		378604X:T>T		DBVPG1373:F>F		DBVPG1853:F>F		DBVPG6044:F>F		DBVPG6765:F>F		L_1374:F>F		L_1528:F>F		NCYC361:F>F		SK1:T>T		Y55:F>F		YPS606:F>F		YS4:T>T	AA:220		DBVPG6044:Y>Y	AA:238		322134S:Y>Y		DBVPG6044:Y>Y		SK1:Y>Y		YPS606:Y>YID:YCR010C	AA:11		L_1528:T>T	AA:14		L_1528:E>E	AA:16		L_1528:A>A	AA:46		L_1528:Y>Y	AA:70		YIIc17_E5:A>A	AA:110		DBVPG1853:A>A	AA:115		DBVPG6040:I>IID:YCR011C	AA:52		322134S:L>L		K11:T>T		SK1:L>L		Y12:T>T	AA:135		DBVPG1788:F>F	AA:137		BC187:C>C	AA:142		K11:P>P	AA:185		UWOPS05_217_3:A>A		UWOPS05_227_2:A>A	AA:214		Y12:F>F		Y9:F>F	AA:270		322134S:R>R	AA:288		YIIc17_E5:G>G	AA:323		YIIc17_E5:G>G	AA:350		K11:L>L		Y12:L>L		Y9:L>L	AA:354		YS4:G>G	AA:369		DBVPG6044:A>A	AA:393		DBVPG6044:V>V	AA:403		DBVPG6044:E>E		K11:E>E		UWOPS83_787_3:E>E		Y9:E>E		YIIc17_E5:E>E	AA:462		DBVPG6044:S>S	AA:498		K11:F>F		Y9:F>F	AA:515		SK1:I>I	AA:519		DBVPG6044:D>D	AA:565		DBVPG6044:A>A	AA:580		L_1374:N>N	AA:610		YPS606:V>V	AA:650		Y55:G>G	AA:742		322134S:L>L		SK1:L>L	AA:753		Y9:G>G	AA:778		UWOPS03_461_4:S>S	AA:802		DBVPG6044:T>T		UWOPS87_2421:T>T	AA:834		DBVPG6765:L>L		L_1528:L>L	AA:835		YPS128:T>T		YPS606:T>T	AA:926		378604X:T>T	AA:987		322134S:V>V		378604X:V>V		DBVPG6044:V>V		SK1:V>V		UWOPS83_787_3:V>V		UWOPS87_2421:V>V		Y12:V>V		Y9:V>V		YIIc17_E5:V>V		YPS128:V>V		YPS606:V>VID:YCR015C	AA:131		K11:G>G	AA:182		UWOPS03_461_4:S>S		UWOPS05_227_2:S>S	AA:247		K11:G>G		UWOPS03_461_4:G>G		UWOPS05_227_2:G>GID:YCR016W	AA:20		UWOPS03_461_4:S>S		UWOPS05_217_3:S>S	AA:70		322134S:G>G		K11:G>G		SK1:G>G		Y12:G>G	AA:75		YIIc17_E5:K>K	AA:139		UWOPS03_461_4:P>P		UWOPS05_217_3:P>P	AA:164		322134S:N>N		K11:N>N		SK1:N>N		UWOPS03_461_4:N>N		UWOPS05_217_3:N>N		Y12:N>N		YPS128:N>N		YPS606:N>N	AA:183		DBVPG1788:L>L		L_1374:L>L	AA:212		YPS128:K>K		YPS606:K>K	AA:267		K11:L>L		SK1:L>LID:YCR017C	AA:16		DBVPG6044:C>C		K11:C>C		SK1:C>C		UWOPS83_787_3:C>C		YPS128:C>C		YS4:C>C	AA:74		YS2:A>A	AA:389		322134S:L>L		378604X:L>L		DBVPG1853:L>L		DBVPG6044:L>L		SK1:L>L		UWOPS83_787_3:L>L		Y12:L>L		YIIc17_E5:L>L		YPS128:L>L	AA:405		322134S:N>N		378604X:N>N		DBVPG1853:N>N		DBVPG6044:N>N		SK1:N>N		UWOPS83_787_3:N>N		Y12:N>N		YIIc17_E5:N>N		YJM978:N>N		YPS128:N>N		YPS606:N>N		YS4:N>N	AA:439		UWOPS83_787_3:I>I	AA:455		K11:I>I		Y12:I>I	AA:549		322134S:F>F		DBVPG6044:F>F		K11:F>F		SK1:F>F		UWOPS05_217_3:F>F		UWOPS05_227_2:F>F		UWOPS83_787_3:F>F		UWOPS87_2421:F>F		Y12:F>F		YIIc17_E5:F>F		YPS128:F>F		YPS606:F>F	AA:651		378604X:A>A	AA:654		273614X:L>L		322134S:L>L		378604X:L>L		SK1:L>L		UWOPS05_217_3:L>L		UWOPS05_227_2:L>L		UWOPS87_2421:L>L		Y9:L>L		YIIc17_E5:L>L		YJM978:L>L		YPS128:L>L		YS4:L>L	AA:728		K11:G>G		Y9:G>G	AA:860		DBVPG6044:P>P		K11:P>P		NCYC110:P>P		NCYC361:P>P		SK1:P>P		Y9:P>P		YPS606:P>PID:YCR018C	AA:24		YIIc17_E5:E>E		YPS128:E>E	AA:70		BC187:V>V		DBVPG1373:V>V		DBVPG1788:V>V		DBVPG6765:V>V		L_1374:V>V		L_1528:V>V		Y55:V>V		YJM975:V>V		YJM978:V>V	AA:85		BC187:R>R		DBVPG1373:R>R		DBVPG1788:R>R		DBVPG6765:R>R		L_1374:R>R		L_1528:R>R		Y55:R>R		YJM975:R>R		YJM978:R>R	AA:126		UWOPS03_461_4:Y>Y		UWOPS05_217_3:Y>Y	AA:211		322134S:A>A		K11:A>A		SK1:A>A		UWOPS03_461_4:A>A		UWOPS05_217_3:A>A		UWOPS87_2421:A>A		YIIc17_E5:A>A		YPS606:A>A	AA:222		SK1:->-ID:YCR020C	AA:5		SK1:T>T		UWOPS05_217_3:T>T		Y9:T>T		YIIc17_E5:T>T	AA:26		NCYC361:K>K		UWOPS83_787_3:K>K	AA:38		UWOPS05_227_2:L>L	AA:142		SK1:A>A		YIIc17_E5:A>A	AA:181		YPS128:F>F		YPS606:F>F	AA:193		YIIc17_E5:E>EID:YCR020C-A	AA:30		UWOPS05_227_2:A>A	AA:62		UWOPS05_217_3:S>S		YIIc17_E5:S>S	AA:65		DBVPG1853:T>T		DBVPG6044:T>T		K11:T>T		SK1:T>T		UWOPS05_217_3:T>T		UWOPS05_227_2:T>T		UWOPS83_787_3:T>T		Y9:T>T		YIIc17_E5:T>T		YPS128:T>TID:YCR021C	AA:80		378604X:F>F		DBVPG1853:F>F		DBVPG6040:F>F		DBVPG6044:F>F		DBVPG6765:F>F		K11:F>F		SK1:F>F		UWOPS05_217_3:F>F		UWOPS05_227_2:F>F		UWOPS83_787_3:F>F		Y12:F>F		Y55:F>F		Y9:F>F		YIIc17_E5:F>F		YPS128:F>F		YPS606:F>F		YS4:F>F	AA:87		UWOPS83_787_3:S>S	AA:112		UWOPS05_217_3:P>P		UWOPS05_227_2:P>P	AA:195		NCYC361:L>L	AA:197		378604X:T>T		DBVPG6044:T>T		K11:T>T		NCYC361:T>T		SK1:T>T		UWOPS05_217_3:T>T		UWOPS05_227_2:T>T		UWOPS83_787_3:T>T		UWOPS87_2421:T>T		Y12:T>T		Y9:T>T		YIIc17_E5:T>T		YPS128:T>T		YPS606:T>T		YS2:T>T	AA:217		UWOPS05_217_3:L>L		UWOPS05_227_2:L>L	AA:259		UWOPS03_461_4:F>F		UWOPS05_227_2:F>F	AA:265		YJM978:Y>Y	AA:267		K11:L>L	AA:273		322134S:D>D		378604X:D>D		DBVPG6044:D>D		K11:D>D		NCYC361:D>D		SK1:D>D		UWOPS03_461_4:D>D		UWOPS05_217_3:D>D		Y12:D>D		Y9:D>D		YIIc17_E5:D>D		YPS128:D>D		YPS606:D>D		YS2:D>D		YS4:D>D	AA:297		UWOPS03_461_4:A>AID:YCR023C	AA:2		UWOPS83_787_3:A>A	AA:29		UWOPS83_787_3:S>S	AA:83		DBVPG1853:S>S		UWOPS83_787_3:S>S	AA:85		SK1:K>K	AA:106		DBVPG1853:L>L	AA:134		DBVPG1853:T>T	AA:160		DBVPG6040:G>G		DBVPG6765:G>G		L_1374:G>G		NCYC361:G>G		Y55:G>G	AA:218		378604X:L>L		YPS128:L>L		YPS606:L>L	AA:250		UWOPS83_787_3:P>P	AA:350		DBVPG6044:F>F	AA:496		K11:I>I		UWOPS05_217_3:I>I		YPS128:I>I		YPS606:I>I		YS4:I>IID:YCR024C	AA:40		273614X:I>I	AA:70		273614X:T>T	AA:114		UWOPS05_217_3:S>S	AA:124		UWOPS83_787_3:N>N	AA:140		DBVPG6044:L>L	AA:146		DBVPG6044:R>R		K11:R>R		SK1:R>R		UWOPS83_787_3:R>R		Y12:R>R		Y9:R>R		YIIc17_E5:R>R		YPS606:R>R	AA:158		DBVPG1853:S>S		DBVPG6040:S>S		DBVPG6044:S>S		DBVPG6765:S>S		K11:S>S		L_1374:S>S		L_1528:S>S		SK1:S>S		UWOPS05_217_3:S>S		UWOPS05_227_2:S>S		UWOPS83_787_3:S>S		Y12:S>S		Y55:S>S		YIIc17_E5:S>S		YJM975:S>S		YJM978:S>S		YJM981:S>S		YPS606:S>S	AA:186		Y12:C>C		Y9:C>C	AA:228		SK1:L>L	AA:267		UWOPS83_787_3:E>E	AA:298		UWOPS05_227_2:S>S	AA:301		DBVPG6044:N>N		NCYC110:N>N	AA:321		UWOPS83_787_3:E>E	AA:332		UWOPS83_787_3:T>T	AA:341		UWOPS83_787_3:K>K	AA:350		UWOPS05_227_2:F>F	AA:456		273614X:H>H	AA:472		DBVPG6044:G>G		NCYC110:G>G		UWOPS83_787_3:G>G	AA:491		UWOPS87_2421:D>DID:YCR026C	AA:12		NCYC361:N>N	AA:26		378604X:F>F		UWOPS87_2421:F>F		Y55:F>F	AA:31		378604X:D>D		NCYC361:D>D		UWOPS87_2421:D>D		Y12:D>D		Y55:D>D		YS9:D>D	AA:35		UWOPS03_461_4:S>S		UWOPS05_227_2:S>S	AA:249		K11:E>E		SK1:E>E		Y12:E>E		Y9:E>E	AA:259		UWOPS05_217_3:R>R	AA:284		DBVPG6044:K>K		Y55:K>K	AA:293		DBVPG6044:S>S		Y55:S>S	AA:324		YIIc17_E5:E>E	AA:353		273614X:L>L		BC187:L>L		DBVPG1853:L>L		DBVPG6044:L>L		K11:L>L		L_1528:L>L		SK1:L>L		UWOPS05_217_3:L>L		UWOPS83_787_3:L>L		Y12:L>L		Y55:L>L		Y9:L>L		YIIc17_E5:L>L		YJM975:L>L		YPS606:L>L		YS2:L>L		YS4:L>L	AA:411		UWOPS05_217_3:V>V	AA:463		UWOPS05_217_3:I>I	AA:477		UWOPS83_787_3:D>D	AA:486		UWOPS05_217_3:N>N	AA:548		DBVPG6044:A>A		Y55:A>A		YPS606:A>A	AA:563		DBVPG6044:P>P		UWOPS05_217_3:P>P		UWOPS83_787_3:P>P		Y12:P>P		Y9:P>P		YPS606:P>P	AA:573		UWOPS05_217_3:T>T	AA:628		L_1374:F>F	AA:688		DBVPG6044:L>LID:YCR027C	AA:10		YPS128:N>N		YPS606:N>N	AA:76		NCYC361:E>E		UWOPS83_787_3:E>E	AA:79		NCYC361:L>L	AA:105		UWOPS05_217_3:L>L	AA:107		DBVPG6044:P>P		Y55:P>P	AA:140		YPS128:V>V		YPS606:V>V	AA:153		378604X:A>A		K11:A>A		NCYC361:A>A		SK1:A>A		UWOPS05_217_3:A>A		UWOPS87_2421:A>A		Y12:A>A	AA:162		273614X:R>RID:YCR028C	AA:21		322134S:K>K		378604X:K>K		K11:K>K		UWOPS83_787_3:K>K		UWOPS87_2421:K>K		Y12:K>K		Y55:K>K		YPS128:K>K		YPS606:K>K	AA:100		S288c:V>V		UWOPS03_461_4:P>P	AA:111		322134S:A>A	AA:210		L_1528:P>P	AA:214		L_1528:Y>Y		S288c:L>L	AA:231		NCYC361:K>K		S288c:L>L	AA:233		L_1528:S>S	AA:235		L_1528:T>T	AA:237		L_1528:F>F		S288c:F>F	AA:263		L_1528:S>S		S288c:S>S	AA:265		S288c:I>I	AA:268		S288c:V>V	AA:269		378604X:L>L		K11:L>L		NCYC361:L>L		UWOPS05_217_3:L>L		UWOPS87_2421:L>L		YPS128:L>L		YPS606:L>L		YS4:L>L	AA:287		378604X:N>N		DBVPG6044:N>N		K11:N>N		NCYC361:N>N		UWOPS05_217_3:N>N		UWOPS87_2421:N>N		Y55:N>N		YPS128:N>N		YPS606:N>N		YS4:N>N	AA:298		S288c:L>L	AA:302		S288c:N>N	AA:312		S288c:N>N	AA:315		S288c:S>S	AA:316		YJM978:G>G	AA:319		S288c:A>A	AA:328		S288c:S>S	AA:348		S288c:S>S	AA:352		S288c:V>V	AA:363		S288c:L>L	AA:371		S288c:A>A	AA:375		S288c:G>G	AA:379		S288c:Y>Y	AA:383		S288c:A>A	AA:386		S288c:F>F	AA:389		322134S:A>A		S288c:A>A	AA:397		S288c:L>L	AA:399		UWOPS87_2421:E>E	AA:406		273614X:S>S		NCYC361:S>S		UWOPS03_461_4:S>S		UWOPS05_217_3:S>S		UWOPS87_2421:S>S		Y12:S>S		Y55:S>S		YPS128:S>S		YPS606:S>S	AA:419		S288c:S>S	AA:428		S288c:V>V	AA:430		S288c:K>K	AA:432		S288c:E>E	AA:435		S288c:C>C	AA:437		S288c:A>A	AA:445		S288c:S>S	AA:465		S288c:Q>Q	AA:471		322134S:A>A	AA:476		SK1:G>G		UWOPS03_461_4:G>G		UWOPS05_227_2:G>G		Y12:G>G	AA:479		273614X:D>D	AA:513		K11:->-		NCYC361:->-		SK1:->-		UWOPS03_461_4:->-		UWOPS05_227_2:->-		UWOPS83_787_3:->-		UWOPS87_2421:->-		Y55:->-		YPS606:->-		YS2:->-ID:YCR033W	AA:14		NCYC110:K>K		Y55:K>K	AA:50		YS4:A>A	AA:56		NCYC110:P>P		Y55:P>P	AA:116		Y55:S>S	AA:174		DBVPG6044:P>P		UWOPS05_217_3:P>P		UWOPS05_227_2:P>P		UWOPS83_787_3:P>P		Y12:P>P		Y55:P>P		YPS128:P>P		YPS606:P>P		YS9:P>P	AA:201		YS9:T>T	AA:280		K11:F>F		UWOPS05_217_3:F>F		Y12:F>F		YS2:F>F		YS9:F>F	AA:302		YS9:E>E	AA:334		YS4:G>G	AA:353		DBVPG1853:K>K	AA:389		322134S:T>T		BC187:T>T		DBVPG1106:T>T		DBVPG1373:T>T		DBVPG1788:T>T		DBVPG1853:T>T		DBVPG6044:T>T		DBVPG6765:T>T		K11:T>T		NCYC361:T>T		SK1:T>T		UWOPS03_461_4:T>T		Y12:T>T		Y55:T>T		YJM975:T>T		YJM981:T>T		YPS128:T>T		YPS606:T>T		YS2:T>T		YS9:T>T	AA:416		DBVPG6044:S>S		K11:S>S		UWOPS03_461_4:S>S		Y12:S>S		Y55:S>S		YS9:S>S	AA:432		NCYC361:K>K		UWOPS87_2421:K>K		YPS128:K>K		YPS606:K>K	AA:455		DBVPG1853:E>E		K11:E>E		NCYC361:E>E		UWOPS03_461_4:E>E		Y12:E>E		Y55:E>E		YPS128:E>E		YPS606:E>E	AA:538		UWOPS03_461_4:F>F		Y12:F>F		YS2:F>F	AA:572		NCYC361:L>L		UWOPS03_461_4:L>L		UWOPS83_787_3:L>L		UWOPS87_2421:L>L		Y12:L>L		Y55:L>L		YPS128:L>L		YPS606:L>L		YS2:L>L	AA:663		L_1528:S>S	AA:692		UWOPS83_787_3:F>F		Y55:F>F	AA:708		Y9:C>C	AA:738		UWOPS83_787_3:A>A		Y55:A>A	AA:822		273614X:N>N		322134S:N>N		BC187:N>N		DBVPG1106:N>N		DBVPG1788:N>N		DBVPG6765:N>N		L_1528:N>N		NCYC110:N>N		SK1:N>N		Y12:N>N		Y55:N>N		Y9:N>N		YJM978:N>N		YPS128:N>N		YPS606:N>N		YS4:N>N		YS9:N>N	AA:841		UWOPS05_227_2:I>I	AA:890		DBVPG1373:S>S		YJM975:S>S	AA:901		Y12:P>P		Y9:P>P	AA:903		378604X:L>L	AA:938		DBVPG1853:G>G	AA:966		DBVPG1853:Q>Q	AA:968		378604X:E>E		UWOPS03_461_4:E>E	AA:991		YS4:G>G	AA:1002		YPS128:S>S		YPS606:S>S	AA:1028		YS9:T>	AA:1114		DBVPG1853:H>H	AA:1129		273614X:V>V		DBVPG1853:V>V		DBVPG6044:V>V		Y55:V>V		YPS128:V>V		YPS606:V>V	AA:1148		DBVPG6044:L>L		NCYC110:L>L		Y55:L>L	AA:1171		UWOPS83_787_3:I>I	AA:1217		UWOPS83_787_3:S>S	AA:1221		YPS606:K>KID:YCR035C	AA:63		YS2:A>A	AA:216		K11:K>K		Y9:K>K		YS4:K>K		YS9:K>K	AA:242		K11:K>K		UWOPS03_461_4:K>K		UWOPS05_227_2:K>K		Y55:K>K		YPS128:K>K		YPS606:K>K		YS4:K>K		YS9:K>K	AA:260		YS9:R>R	AA:338		K11:A>A		Y12:A>A		YS4:A>A		YS9:A>AID:YCR037C	AA:34		DBVPG6040:T>T	AA:43		SK1:T>T	AA:55		UWOPS03_461_4:P>P	AA:57		DBVPG6040:P>P	AA:148		DBVPG6044:E>E		K11:E>E		UWOPS03_461_4:E>E		Y55:E>E	AA:151		DBVPG1788:T>T		YIIc17_E5:T>T	AA:180		DBVPG6044:K>K		K11:K>K		Y55:K>K		Y9:K>K	AA:188		UWOPS03_461_4:L>L	AA:212		378604X:L>L	AA:280		DBVPG1788:I>I	AA:285		DBVPG1106:K>K		DBVPG1788:K>K		DBVPG1853:K>K		DBVPG6040:K>K		DBVPG6765:K>K		SK1:K>K		YIIc17_E5:K>K	AA:314		UWOPS03_461_4:S>S		UWOPS05_217_3:S>S	AA:318		UWOPS87_2421:K>K		YPS128:K>K		YPS606:K>K	AA:373		K11:N>N		Y12:N>N		Y9:N>N	AA:385		K11:L>L		UWOPS03_461_4:L>L		UWOPS05_217_3:L>L		Y12:L>L		Y9:L>L	AA:402		Y12:G>G		Y9:G>G	AA:448		UWOPS05_217_3:L>L	AA:463		UWOPS05_217_3:G>G	AA:469		UWOPS05_217_3:T>T		Y12:T>T	AA:533		273614X:A>A		378604X:A>A		UWOPS83_787_3:A>A		Y12:A>A		Y55:A>A		YPS128:A>A		YPS606:A>A	AA:615		273614X:L>L		378604X:L>L		Y55:L>L	AA:682		273614X:C>C		378604X:C>C		DBVPG6040:C>C		UWOPS03_461_4:C>C		UWOPS05_227_2:C>C		Y12:C>C		Y9:C>C		YPS128:C>C		YPS606:C>C	AA:712		273614X:Q>Q		378604X:Q>Q		DBVPG6040:Q>Q		UWOPS03_461_4:Q>Q		UWOPS05_227_2:Q>Q		Y12:Q>Q		Y9:Q>Q		YPS606:Q>Q	AA:716		273614X:I>I		378604X:I>I		DBVPG6040:I>I		UWOPS03_461_4:I>I		UWOPS05_227_2:I>I		Y12:I>I		Y9:I>I		YPS606:I>I	AA:722		273614X:T>T		378604X:T>T		Y12:T>T		Y9:T>T	AA:818		DBVPG6044:V>V		UWOPS03_461_4:V>V		UWOPS05_227_2:V>V		UWOPS83_787_3:V>V		Y55:V>V		Y9:V>V		YPS606:V>V		YS9:V>V	AA:822		273614X:V>V	AA:907		378604X:V>V		DBVPG6044:V>V		UWOPS03_461_4:V>V		UWOPS05_227_2:V>V		UWOPS83_787_3:V>V		Y55:V>V		Y9:V>V		YPS606:V>V		YS9:V>VID:YCR038C	AA:32		DBVPG1373:T>T	AA:64		W303:I>I	AA:69		UWOPS03_461_4:V>V		UWOPS05_227_2:V>V	AA:81		378604X:L>L		DBVPG6044:L>L		NCYC110:L>L		UWOPS03_461_4:L>L		UWOPS05_227_2:L>L		Y12:L>L		Y55:L>L		YIIc17_E5:L>L		YPS128:L>L		YPS606:L>L	AA:157		DBVPG6040:L>L	AA:202		378604X:T>T		DBVPG6044:T>T		K11:T>T		UWOPS03_461_4:T>T		UWOPS05_227_2:T>T		Y12:T>T		Y55:T>T		YIIc17_E5:T>T		YPS606:T>T	AA:318		DBVPG6044:L>L		YPS606:L>L	AA:356		UWOPS05_227_2:I>I	AA:378		DBVPG1373:N>N	AA:385		UWOPS05_227_2:G>G	AA:506		UWOPS03_461_4:L>L		UWOPS05_227_2:L>L	AA:507		YPS606:S>S	AA:618		UWOPS03_461_4:K>K		UWOPS05_227_2:K>KID:YCR039C	AA:172		BC187:S>SID:YCR042C	AA:20		DBVPG6044:L>L		NCYC110:L>L		UWOPS05_217_3:L>L		UWOPS05_227_2:L>L		Y12:L>L		Y55:L>L		YPS128:L>L		YS9:L>L	AA:111		Y12:I>I		Y9:I>I	AA:202		UWOPS03_461_4:K>K		UWOPS05_227_2:K>K		Y9:K>K	AA:216		BC187:C>C		DBVPG1788:C>C		DBVPG1853:C>C		DBVPG6044:C>C		DBVPG6765:C>C		L_1528:C>C		NCYC110:C>C		SK1:C>C		UWOPS03_461_4:C>C		UWOPS05_227_2:C>C		Y55:C>C		Y9:C>C		YIIc17_E5:C>C		YPS128:C>C		YPS606:C>C	AA:239		YPS128:F>F		YPS606:F>F	AA:408		DBVPG6044:E>E		Y12:E>E		Y55:E>E		Y9:E>E		YS9:E>E	AA:464		YS9:S>S	AA:469		K11:S>S		YS2:S>S	AA:473		UWOPS05_217_3:T>T		UWOPS05_227_2:T>T		UWOPS83_787_3:T>T		Y12:T>T	AA:476		YS2:S>S	AA:569		K11:N>N	AA:572		K11:A>A	AA:578		DBVPG6044:F>F		Y55:F>F	AA:584		K11:G>G		Y12:G>G	AA:601		DBVPG6044:L>L		Y55:L>L	AA:635		UWOPS05_217_3:I>I		UWOPS05_227_2:I>I		YPS128:I>I		YPS606:I>I	AA:645		DBVPG6044:P>P		K11:P>P		L_1528:P>P		SK1:P>P		UWOPS05_217_3:P>P		UWOPS05_227_2:P>P		UWOPS83_787_3:P>P		Y12:P>P		Y55:P>P		YPS128:P>P		YPS606:P>P	AA:662		DBVPG6044:N>N		K11:N>N		UWOPS05_217_3:N>N		UWOPS05_227_2:N>N		UWOPS83_787_3:N>N		Y12:N>N		Y55:N>N		YPS128:N>N		YPS606:N>N	AA:722		YPS128:A>A	AA:758		DBVPG6044:E>E		K11:E>E		UWOPS05_227_2:E>E		UWOPS83_787_3:E>E		Y12:E>E		Y55:E>E		Y9:E>E		YPS128:E>E	AA:822		DBVPG6044:T>T		K11:T>T		UWOPS05_227_2:T>T		UWOPS83_787_3:T>T		Y55:T>T		Y9:T>T		YPS128:T>T		YPS606:T>T	AA:887		DBVPG6044:V>V		UWOPS05_227_2:V>V		UWOPS83_787_3:V>V		Y55:V>V	AA:898		K11:Y>Y		UWOPS05_227_2:Y>Y		UWOPS83_787_3:Y>Y		Y55:Y>Y		Y9:Y>Y		YPS128:Y>Y		YPS606:Y>Y	AA:914		UWOPS83_787_3:G>G	AA:949		K11:L>L		UWOPS05_227_2:L>L		UWOPS83_787_3:L>L		Y9:L>L		YPS128:L>L		YPS606:L>L	AA:967		K11:F>F		UWOPS83_787_3:F>F		Y9:F>F	AA:970		DBVPG6044:C>C	AA:973		UWOPS05_217_3:P>P		UWOPS05_227_2:P>P	AA:976		DBVPG6044:L>L	AA:987		YIIc17_E5:P>P	AA:1017		UWOPS03_461_4:L>L		UWOPS05_217_3:L>L		UWOPS05_227_2:L>L	AA:1042		UWOPS83_787_3:L>L	AA:1111		K11:L>L		UWOPS03_461_4:L>L		UWOPS05_217_3:L>L		UWOPS05_227_2:L>L		Y12:L>L		Y9:L>L	AA:1137		NCYC361:E>E	AA:1140		273614X:Y>Y		DBVPG1106:Y>Y		DBVPG1373:Y>Y		DBVPG1788:Y>Y		DBVPG1853:Y>Y		DBVPG6044:Y>Y		K11:Y>Y		L_1528:Y>Y		NCYC361:Y>Y		SK1:Y>Y		UWOPS03_461_4:Y>Y		UWOPS05_217_3:Y>Y		UWOPS05_227_2:Y>Y		UWOPS83_787_3:Y>Y		Y12:Y>Y		Y55:Y>Y		Y9:Y>Y		YIIc17_E5:Y>Y		YJM975:Y>Y		YJM981:Y>Y	AA:1166		L_1528:S>S	AA:1195		UWOPS03_461_4:G>G		UWOPS05_217_3:G>G		UWOPS05_227_2:G>G	AA:1227		K11:Y>Y		Y9:Y>Y	AA:1241		UWOPS03_461_4:P>P		UWOPS05_227_2:P>P	AA:1248		Y55:R>R	AA:1253		YS4:D>D	AA:1257		YPS128:V>V	AA:1259		Y55:Y>Y	AA:1317		Y9:A>A	AA:1340		Y55:P>P	AA:1359		Y55:L>L		Y9:L>L	AA:1380		DBVPG1106:P>P		DBVPG1853:P>P		SK1:P>P		UWOPS05_217_3:P>P		UWOPS05_227_2:P>P		Y55:P>P		Y9:P>P		YIIc17_E5:P>P		YS4:P>P	AA:1391		UWOPS05_217_3:T>T		UWOPS05_227_2:T>T	AA:1401		Y55:T>TID:YCR045C	AA:49		322134S:I>I		378604X:I>I		DBVPG6044:I>I		K11:I>I		NCYC110:I>I		SK1:I>I		Y12:I>I	AA:77		322134S:P>P		DBVPG6044:P>P		K11:P>P		NCYC110:P>P		SK1:P>P		UWOPS03_461_4:P>P		UWOPS83_787_3:P>P		UWOPS87_2421:P>P		Y12:P>P		YPS128:P>P	AA:121		DBVPG6044:N>N		K11:N>N		NCYC110:N>N		UWOPS03_461_4:N>N		UWOPS83_787_3:N>N		UWOPS87_2421:N>N		Y9:N>N		YPS128:N>N	AA:156		DBVPG6044:N>N		NCYC110:N>N		UWOPS83_787_3:N>N		UWOPS87_2421:N>N		Y55:N>N		Y9:N>N		YPS128:N>N		YPS606:N>N	AA:185		K11:E>E	AA:219		DBVPG6044:G>G		K11:G>G		SK1:G>G		UWOPS05_227_2:G>G		UWOPS83_787_3:G>G		UWOPS87_2421:G>G		Y55:G>G		Y9:G>G		YPS606:G>G	AA:272		SK1:G>G		UWOPS83_787_3:G>G		Y9:G>G	AA:296		YPS606:A>A	AA:303		273614X:D>D		BC187:D>D		DBVPG6040:D>D		Y12:D>D		Y55:D>D		YPS606:D>D	AA:305		BC187:Y>Y		DBVPG6040:Y>Y	AA:313		273614X:E>E		Y12:E>E		Y55:E>E	AA:317		BC187:T>T		DBVPG6040:T>T	AA:374		SK1:V>V		UWOPS83_787_3:V>V		Y9:V>V		YPS606:V>V	AA:377		SK1:I>I		UWOPS83_787_3:I>I		Y9:I>I		YPS606:I>I	AA:459		273614X:N>N		BC187:N>N		K11:N>N		SK1:N>N		UWOPS05_217_3:N>N		UWOPS05_227_2:N>N		UWOPS83_787_3:N>N		Y12:N>N		Y55:N>N		Y9:N>N		YPS606:N>NID:YCR046C	AA:56		UWOPS05_217_3:A>A		UWOPS05_227_2:A>A	AA:95		K11:L>L	AA:161		378604X:L>L		DBVPG1106:L>L		DBVPG1373:L>L		DBVPG1853:L>L		DBVPG6765:L>L		K11:L>L		SK1:L>L		UWOPS03_461_4:L>L		UWOPS05_217_3:L>L		UWOPS05_227_2:L>L		UWOPS83_787_3:L>L		Y12:L>L		Y9:L>L		YJM975:L>L		YJM978:L>L		YJM981:L>L		YS4:L>L		YS9:L>LID:YCR047C	AA:14		378604X:Y>Y		DBVPG1106:D>D		K11:Y>Y		L_1528:D>D		UWOPS05_217_3:D>D	AA:66		322134S:T>T		BC187:T>T		DBVPG1853:T>T		DBVPG6765:T>T		K11:T>T		SK1:T>T		UWOPS05_217_3:T>T		Y55:T>T		YJM978:T>T		YPS606:T>T		YS2:T>T		YS4:T>T		YS9:T>T	AA:114		NCYC361:A>A		SK1:A>A		Y12:A>A		Y55:A>A	AA:129		322134S:S>S		DBVPG1853:S>S		NCYC361:S>S		SK1:S>S		Y12:S>S		Y55:S>S		YPS606:S>S		YS2:S>S	AA:194		YPS606:K>K	AA:197		DBVPG6044:L>L		NCYC361:L>L		Y55:L>L	AA:259		378604X:K>K		DBVPG1853:K>KID:YCR048W	AA:45		378604X:S>S		DBVPG6044:S>S		L_1528:S>S		SK1:S>S		UWOPS83_787_3:S>S		Y12:S>S		Y55:S>S		YPS606:S>S		YS2:S>S	AA:78		UWOPS83_787_3:E>E	AA:84		K11:D>D	AA:112		SK1:S>S		Y12:S>S		YS2:S>S	AA:114		UWOPS05_217_3:F>F	AA:131		DBVPG6044:N>N		K11:N>N		SK1:N>N		UWOPS05_217_3:N>N		UWOPS05_227_2:N>N		Y12:N>N		Y55:N>N		YS2:N>N	AA:192		DBVPG6044:F>F	AA:200		273614X:I>I		322134S:I>I		DBVPG6044:I>I		K11:I>I		SK1:I>I		UWOPS05_227_2:I>I		UWOPS83_787_3:I>I		YPS606:I>I		YS2:I>I	AA:237		273614X:L>L		K11:L>L		SK1:L>L		UWOPS83_787_3:L>L		YS2:L>L		YS9:L>L	AA:312		L_1528:A>A	AA:323		L_1528:Q>Q	AA:338		SK1:S>S		UWOPS83_787_3:S>S		UWOPS87_2421:S>S		Y12:S>S		YS2:S>S		YS4:S>S		YS9:S>S	AA:344		L_1528:R>R	AA:347		L_1528:L>L	AA:350		L_1528:S>S	AA:354		L_1528:C>C	AA:356		322134S:F>F		L_1528:F>F	AA:361		NCYC110:Q>Q		Y55:Q>Q	AA:363		L_1528:K>K	AA:365		L_1528:N>N	AA:368		L_1528:P>P	AA:381		DBVPG1853:L>L		NCYC110:L>L		UWOPS83_787_3:L>L		UWOPS87_2421:L>L		Y12:L>L		Y55:L>L		Y9:L>L		YPS606:L>L		YS2:L>L		YS4:L>L		YS9:L>L	AA:387		L_1528:Y>Y	AA:391		L_1528:Y>Y	AA:406		Y12:V>V		Y9:V>V	AA:408		L_1528:A>A	AA:419		322134S:T>T		DBVPG1853:T>T		K11:T>T		NCYC110:T>T		SK1:T>T		UWOPS83_787_3:T>T		UWOPS87_2421:T>T		Y12:T>T		Y55:T>T		Y9:T>T		YPS606:T>T		YS2:T>T		YS4:T>T		YS9:T>T	AA:423		L_1528:F>F	AA:438		L_1528:P>P	AA:447		L_1528:T>T	AA:454		L_1528:L>L	AA:459		L_1528:P>P	AA:468		L_1528:T>T	AA:483		L_1528:L>L	AA:486		L_1528:F>F	AA:488		YS4:D>D	AA:489		L_1528:R>R	AA:492		L_1528:Y>Y	AA:521		L_1528:Y>Y	AA:524		L_1528:S>S	AA:526		L_1528:G>G	AA:538		378604X:F>F		DBVPG6040:F>F		K11:F>F		NCYC110:F>F		SK1:F>F		UWOPS83_787_3:F>F		UWOPS87_2421:F>F		Y12:F>F		Y55:F>F		Y9:F>F		YPS606:F>F	AA:552		378604X:F>F	AA:591		DBVPG6044:V>V		NCYC110:V>V		Y55:V>V	AA:601		L_1528:S>SID:YCR050C	AA:24		DBVPG6044:K>K		NCYC110:K>K		Y55:K>K	AA:51		DBVPG6044:N>N		NCYC110:N>N		Y55:N>N	AA:63		378604X:A>A	AA:77		378604X:V>V		DBVPG6040:V>V		K11:V>V		NCYC110:V>V		SK1:V>V		UWOPS83_787_3:V>V		UWOPS87_2421:V>V		Y12:V>V		Y55:V>V		Y9:V>V		YPS606:V>V	AA:89		L_1528:A>AID:YCR051W	AA:80		DBVPG6044:D>D		UWOPS87_2421:D>D		Y55:D>D	AA:111		UWOPS03_461_4:E>E		UWOPS05_217_3:E>E		UWOPS05_227_2:E>E	AA:168		DBVPG6044:D>D		UWOPS03_461_4:D>D		UWOPS05_217_3:D>D		UWOPS05_227_2:D>D		UWOPS83_787_3:D>D		UWOPS87_2421:D>D		Y55:D>D		Y9:D>D		YPS128:D>D		YS4:D>D	AA:187		YS9:A>A	AA:197		YS4:A>A		YS9:A>A	AA:216		YPS128:P>PID:YCR052W	AA:4		322134S:Q>Q		BC187:Q>Q		DBVPG1373:Q>Q		DBVPG1788:Q>Q		DBVPG6040:Q>Q		DBVPG6044:Q>Q		DBVPG6765:Q>Q		SK1:Q>Q		UWOPS05_217_3:Q>Q		UWOPS83_787_3:Q>Q		Y55:Q>Q		Y9:Q>Q		YJM975:Q>Q		YJM978:Q>Q		YPS128:Q>Q		YS4:Q>Q	AA:9		DBVPG6040:P>P		DBVPG6044:P>P		SK1:P>P		Y55:P>P		Y9:P>P		YPS128:P>P	AA:12		UWOPS83_787_3:Y>Y		YS4:Y>Y	AA:16		UWOPS05_217_3:A>A		UWOPS05_227_2:A>A	AA:171		YS2:D>D	AA:181		378604X:A>A	AA:187		378604X:I>I	AA:191		YJM978:L>L	AA:226		DBVPG6044:G>G		Y55:G>G		YS2:G>G	AA:261		378604X:L>L	AA:279		DBVPG6044:D>D		Y55:D>D		YPS606:D>D	AA:285		378604X:N>N		DBVPG1373:N>N		DBVPG6040:N>N		DBVPG6044:N>N		L_1374:N>N		L_1528:N>N		SK1:N>N		UWOPS05_217_3:N>N		UWOPS05_227_2:N>N		UWOPS87_2421:N>N		Y55:N>N		YIIc17_E5:N>N		YJM975:N>N		YJM978:N>N		YPS606:N>N		YS2:N>N	AA:314		378604X:K>K		DBVPG6044:K>K		K11:K>K		Y55:K>K		YPS606:K>K		YS2:K>K	AA:325		K11:T>T	AA:327		378604X:A>A		DBVPG1853:A>A		DBVPG6040:A>A		K11:A>A		UWOPS05_217_3:A>A		UWOPS05_227_2:A>A	AA:376		Y12:L>	AA:389		UWOPS05_217_3:E>E	AA:422		322134S:L>L		DBVPG1373:L>L		DBVPG1788:L>L		DBVPG1853:L>L		DBVPG6040:L>L		DBVPG6044:L>L		L_1374:L>L		UWOPS05_217_3:L>L		UWOPS05_227_2:L>L		UWOPS87_2421:L>L		YIIc17_E5:L>L		YJM975:L>L		YJM978:L>L		YPS128:L>L		YPS606:L>L	AA:425		DBVPG6044:H>H	AA:453		DBVPG1853:D>D		DBVPG6040:D>D		DBVPG6044:D>D		UWOPS03_461_4:D>D		UWOPS05_227_2:D>D		UWOPS87_2421:D>D		Y55:D>D		YPS128:D>D		YPS606:D>D	AA:461		UWOPS87_2421:Y>Y	AA:469		UWOPS03_461_4:S>S		UWOPS05_227_2:S>S		YPS128:S>S		YPS606:S>S	AA:477		322134S:L>L		DBVPG1853:L>L		SK1:L>L		UWOPS03_461_4:L>L		UWOPS05_227_2:L>L		UWOPS87_2421:L>L		Y55:L>L		YPS128:L>L		YPS606:L>LID:YCR053W	AA:10		L_1528:S>S	AA:11		UWOPS83_787_3:T>	AA:13		L_1528:S>S	AA:18		L_1528:T>T		UWOPS03_461_4:T>T		UWOPS05_227_2:T>T		UWOPS87_2421:T>T		Y55:T>T		Y9:T>T		YPS606:T>T	AA:37		L_1528:I>I	AA:44		L_1528:V>V	AA:47		UWOPS87_2421:A>A	AA:59		L_1528:Q>Q	AA:62		UWOPS03_461_4:A>A		UWOPS05_227_2:A>A		UWOPS83_787_3:A>A	AA:64		L_1528:A>A	AA:119		UWOPS87_2421:P>P		YPS606:P>P	AA:131		DBVPG6044:V>V		UWOPS03_461_4:V>V		UWOPS83_787_3:V>V		UWOPS87_2421:V>V		Y55:V>V		YPS606:V>V	AA:156		UWOPS87_2421:V>V	AA:190		273614X:S>S		DBVPG6044:S>S		UWOPS83_787_3:S>S		UWOPS87_2421:S>S		Y12:S>S		Y55:S>S		Y9:S>S		YPS606:S>S	AA:199		YPS606:T>T	AA:225		322134S:F>F		SK1:F>F	AA:249		273614X:A>A		UWOPS03_461_4:A>A		UWOPS87_2421:A>A		Y12:A>A		YPS606:A>A		YS2:A>A		YS4:A>A	AA:253		273614X:Y>Y		UWOPS03_461_4:Y>Y		UWOPS87_2421:Y>Y		Y12:Y>Y		Y9:Y>Y		YPS606:Y>Y		YS2:Y>Y		YS4:Y>Y	AA:257		273614X:S>S		DBVPG6044:S>S		UWOPS03_461_4:S>S		UWOPS83_787_3:S>S		UWOPS87_2421:S>S		Y12:S>S		Y55:S>S		YPS606:S>S		YS2:S>S		YS4:S>S	AA:272		Y12:F>F	AA:317		DBVPG1373:Y>Y		YJM975:Y>Y	AA:379		YS4:D>D	AA:399		UWOPS83_787_3:E>E	AA:441		UWOPS87_2421:S>S	AA:483		378604X:L>L		DBVPG6044:L>L		NCYC110:L>L		SK1:L>L		UWOPS03_461_4:L>L		UWOPS83_787_3:L>L		UWOPS87_2421:L>L		Y55:L>L		Y9:L>L		YPS128:L>L		YS4:L>L		YS9:L>LID:YCR057C	AA:151		378604X:S>S		Y9:S>S	AA:161		DBVPG6044:I>I		NCYC110:I>I		Y55:I>I		YPS128:I>I	AA:189		378604X:G>G		DBVPG1788:G>G		DBVPG6044:G>G		NCYC110:G>G		Y55:G>G		YPS128:G>G	AA:244		DBVPG6044:S>S		NCYC110:S>S		Y55:S>S	AA:256		UWOPS83_787_3:F>F	AA:262		378604X:G>G		DBVPG6040:G>G		DBVPG6044:G>G		NCYC110:K>K		UWOPS03_461_4:G>G		UWOPS05_217_3:G>G		UWOPS05_227_2:G>G		UWOPS83_787_3:G>G		Y55:G>G		Y9:G>G		YPS128:G>G		YS4:G>G	AA:283		UWOPS83_787_3:E>E	AA:294		DBVPG6044:L>L		K11:L>L		NCYC110:L>L		UWOPS05_217_3:L>L		UWOPS05_227_2:L>L		UWOPS83_787_3:L>L		Y55:L>L	AA:334		K11:S>S		UWOPS83_787_3:S>S		YPS128:S>S	AA:344		YPS128:H>H	AA:391		DBVPG6044:T>T		UWOPS05_217_3:T>T		UWOPS05_227_2:T>T		UWOPS83_787_3:T>T		Y55:T>T	AA:446		DBVPG1788:C>C	AA:503		DBVPG6044:F>F		Y55:F>F	AA:508		DBVPG6044:Q>Q		UWOPS83_787_3:Q>Q		Y55:Q>Q		Y9:Q>Q		YS4:Q>Q	AA:526		SK1:D>D	AA:580		378604X:F>F		UWOPS83_787_3:F>F		Y9:F>F	AA:670		DBVPG6044:L>L		Y55:L>L	AA:689		378604X:T>T		UWOPS03_461_4:T>T		UWOPS05_217_3:T>T		UWOPS05_227_2:T>T		Y12:T>T	AA:717		Y9:L>L	AA:754		DBVPG6040:V>V	AA:763		Y9:I>I	AA:775		DBVPG6044:L>L		NCYC110:L>L	AA:802		K11:L>L		Y9:L>L	AA:863		K11:D>D		UWOPS03_461_4:D>D		UWOPS05_217_3:D>D		UWOPS05_227_2:D>D		Y12:D>D		Y9:D>D		YPS606:D>D	AA:905		Y12:P>PID:YCR059C	AA:74		273614X:D>D		322134S:D>D		DBVPG6044:D>D		K11:D>D		NCYC110:D>D		SK1:D>D		UWOPS05_217_3:D>D		YPS606:D>D		YS4:D>D	AA:121		UWOPS05_217_3:P>P	AA:129		UWOPS05_217_3:P>P	AA:137		DBVPG6040:A>A	AA:140		YIIc17_E5:P>P	AA:185		322134S:R>R		DBVPG1853:R>R		DBVPG6044:R>R		K11:R>R		NCYC110:R>R		SK1:R>R		UWOPS05_217_3:R>R		UWOPS83_787_3:R>R		Y12:R>R		YPS128:R>R		YS2:R>R		YS4:R>R	AA:236		DBVPG1853:G>G		DBVPG6044:G>G		K11:G>G		NCYC110:G>G		UWOPS05_217_3:G>G		Y12:G>G		YPS128:G>GID:YCR060W	AA:13		378604X:S>S		DBVPG6044:S>S		K11:S>S		NCYC110:S>S		SK1:S>S		UWOPS05_217_3:S>S		UWOPS05_227_2:S>S		Y55:S>S		Y9:S>S		YPS606:S>S		YS2:S>S	AA:92		273614X:G>G		DBVPG1853:G>G		DBVPG6040:G>G		DBVPG6044:G>G		K11:G>G		NCYC110:G>G		SK1:G>G		Y55:G>G		Y9:G>G		YPS128:G>G		YPS606:G>GID:YCR061W	AA:25		UWOPS05_217_3:D>D		UWOPS05_227_2:D>D	AA:37		322134S:N>N		NCYC361:N>N		SK1:N>N	AA:52		K11:A>A		NCYC110:A>A		UWOPS87_2421:A>A		Y12:A>A		Y55:A>A		Y9:A>A		YS4:A>A	AA:144		L_1528:S>S	AA:165		DBVPG6040:N>N		K11:N>N		UWOPS05_217_3:N>N		UWOPS05_227_2:N>N		YS4:N>N	AA:189		L_1528:V>V	AA:204		L_1528:T>T	AA:214		L_1528:P>P	AA:218		L_1528:N>N	AA:225		L_1528:S>S	AA:241		L_1528:T>T	AA:244		YJM975:T>T		YJM981:T>T	AA:249		L_1528:N>N	AA:256		L_1528:G>G	AA:270		L_1528:R>R	AA:282		L_1528:A>A	AA:288		UWOPS83_787_3:P>P	AA:293		L_1528:D>D	AA:295		L_1528:P>P	AA:316		L_1374:V>V	AA:333		UWOPS83_787_3:F>F	AA:356		YS4:L>L	AA:386		K11:A>A	AA:397		K11:T>T		UWOPS83_787_3:T>T		Y12:T>T		Y55:T>T		Y9:T>T	AA:404		K11:L>L		UWOPS05_217_3:L>L		UWOPS83_787_3:L>L		Y12:L>L		Y55:L>L		Y9:L>L	AA:408		DBVPG1853:F>F	AA:451		Y55:H>H	AA:472		Y9:L>L	AA:518		UWOPS05_217_3:S>S	AA:559		UWOPS05_217_3:K>K	AA:562		UWOPS05_217_3:T>T	AA:577		UWOPS83_787_3:F>F	AA:582		UWOPS05_217_3:D>DID:YCR063W	AA:7		YPS128:R>R	AA:79		K11:D>D		UWOPS87_2421:D>D		Y55:D>D		YPS128:D>D		YS4:D>D	AA:105		YPS128:C>C		YS4:C>CID:YCR065W	AA:18		Y55:E>E	AA:62		UWOPS03_461_4:P>P		UWOPS05_227_2:P>P	AA:69		DBVPG6040:A>A	AA:104		UWOPS87_2421:G>G	AA:106		DBVPG6040:L>L	AA:155		UWOPS83_787_3:N>N	AA:246		DBVPG6040:F>F		K11:F>F		NCYC110:F>F		Y55:F>F		YPS606:F>F	AA:252		UWOPS83_787_3:Q>Q	AA:275		NCYC110:S>S		Y55:S>S	AA:340		K11:F>F		UWOPS83_787_3:F>F	AA:417		K11:P>P	AA:443		Y55:R>R	AA:488		YS4:G>G	AA:560		378604X:G>GID:YCR066W	AA:98		273614X:L>L		SK1:L>L	AA:133		Y55:N>N		Y9:N>N	AA:177		Y55:I>I	AA:234		K11:N>N		YS4:N>N	AA:346		K11:P>P		UWOPS03_461_4:P>P		UWOPS87_2421:P>P		YPS128:P>P		YS2:P>P		YS4:P>P	AA:478		K11:E>E		NCYC110:E>E		Y55:E>E		YPS128:E>E		YS4:E>EID:YCR068W	AA:36		378604X:P>P		BC187:P>P		DBVPG1373:P>P		DBVPG1788:P>P		DBVPG6765:P>P		L_1374:P>P		L_1528:P>P		UWOPS03_461_4:P>P		UWOPS05_217_3:P>P		Y55:P>P		YIIc17_E5:P>P		YJM975:P>P	AA:113		L_1528:A>A	AA:144		378604X:F>F		BC187:F>F		DBVPG1106:F>F		DBVPG1788:F>F		DBVPG6765:F>F		L_1374:F>F		L_1528:F>F		Y55:F>F		YIIc17_E5:F>F		YJM975:F>F	AA:173		378604X:N>N		BC187:N>N		DBVPG1106:N>N		DBVPG1788:N>N		DBVPG6765:N>N		L_1374:N>N		L_1528:N>N		YIIc17_E5:N>N		YJM975:N>N	AA:249		273614X:G>G		BC187:G>G		DBVPG1106:G>G		DBVPG1788:G>G		DBVPG6765:G>G		L_1374:G>G		L_1528:G>G		Y55:G>G		YJM975:G>G	AA:349		273614X:A>A		DBVPG1106:A>A		DBVPG1788:A>A		DBVPG6044:A>A		DBVPG6765:A>A		L_1374:A>A		L_1528:A>A		NCYC110:A>A		Y55:A>A		YJM975:A>A	AA:379		273614X:I>I		DBVPG6044:I>I		DBVPG6765:I>I		L_1374:I>I		L_1528:I>I		NCYC110:I>I		UWOPS05_217_3:I>I		Y55:I>I		YIIc17_E5:I>I		YJM975:I>I		YS2:I>I	AA:382		273614X:F>F		DBVPG1788:F>F		DBVPG6044:F>F		DBVPG6765:F>F		L_1528:F>F		NCYC110:F>F		Y55:F>F		YJM975:F>F		YS2:F>F	AA:504		SK1:S>S		UWOPS83_787_3:S>S	AA:506		YPS128:C>CID:YCR069W	AA:38		UWOPS05_227_2:K>K	AA:54		378604X:E>E	AA:60		NCYC110:S>S	AA:72		UWOPS05_217_3:L>L		UWOPS05_227_2:L>L	AA:201		YPS606:D>D	AA:206		K11:T>T		UWOPS83_787_3:T>T	AA:209		UWOPS05_217_3:D>D		UWOPS05_227_2:D>D	AA:218		YPS128:L>L		YPS606:L>L	AA:219		L_1374:R>R	AA:221		YPS128:L>L		YPS606:L>L	AA:264		UWOPS05_217_3:N>N		UWOPS05_227_2:N>N	AA:301		DBVPG6044:I>I		DBVPG6765:I>I		L_1374:I>I		NCYC110:I>I		Y55:I>I		YIIc17_E5:I>I		YJM978:I>IID:YCR071C	AA:69		273614X:E>E		378604X:E>E		DBVPG1373:E>E		DBVPG6044:E>E		DBVPG6765:E>E		L_1374:E>E		UWOPS03_461_4:E>E		UWOPS05_217_3:E>E		UWOPS05_227_2:E>E		Y55:E>E		YJM975:E>E		YJM978:E>E		YPS128:E>E		YS4:E>E	AA:88		273614X:K>K		378604X:K>K		DBVPG1373:K>K		DBVPG6044:K>K		DBVPG6765:K>K		L_1374:K>K		Y55:K>K		YJM975:K>K		YJM978:K>K		YS4:K>K	AA:125		Y9:S>SID:YCR073W-A	AA:57		K11:A>A		Y12:A>A		Y9:A>A	AA:124		DBVPG6044:R>R	AA:152		DBVPG6044:G>G		K11:G>G		UWOPS05_227_2:G>G		Y12:G>G		YPS128:G>G		YPS606:G>G	AA:170		322134S:C>C		DBVPG1853:C>C		L_1374:C>C		SK1:C>C		Y55:C>C		YJM978:C>C	AA:203		YIIc17_E5:D>D	AA:212		378604X:N>N		DBVPG6044:N>N		NCYC110:N>N	AA:226		UWOPS05_217_3:P>P	AA:265		NCYC110:K>K	AA:309		YPS128:F>F		YPS606:F>FID:YCR076C	AA:16		UWOPS05_217_3:S>S	AA:25		YS2:R>R	AA:41		YPS128:D>D		YPS606:D>D	AA:53		UWOPS05_217_3:S>S		YS2:S>S		YS4:S>S	AA:83		YS2:V>V		YS4:V>V	AA:208		YIIc17_E5:P>P	AA:219		L_1528:P>P	AA:247		YPS128:S>S		YPS606:S>SID:YCR077C	AA:10		NCYC110:G>G	AA:50		UWOPS05_217_3:T>T		UWOPS05_227_2:T>T	AA:68		273614X:G>G		BC187:G>G		DBVPG6044:G>G		DBVPG6765:G>G		NCYC361:G>G		SK1:G>G		UWOPS05_217_3:G>G		UWOPS05_227_2:G>G		UWOPS83_787_3:G>G		Y55:G>G		YJM975:G>G		YJM978:G>G		YJM981:G>G		YPS128:G>G	AA:84		273614X:A>A		BC187:A>A		L_1374:A>A		NCYC361:A>A		SK1:A>A		Y55:A>A		YJM975:A>A		YJM978:A>A		YJM981:A>A	AA:93		DBVPG6044:D>D		NCYC110:D>D	AA:120		273614X:P>P		BC187:P>P		DBVPG6044:P>P		L_1374:P>P		NCYC110:P>P		NCYC361:P>P		SK1:P>P		UWOPS05_227_2:P>P		UWOPS83_787_3:P>P		Y55:P>P		YJM975:P>P		YJM978:P>P		YJM981:P>P		YPS128:P>P		YPS606:P>P	AA:130		UWOPS05_227_2:P>P	AA:167		273614X:P>P		DBVPG1788:P>P		DBVPG6765:P>P		L_1374:P>P		NCYC361:P>P		SK1:P>P		Y55:P>P		YJM978:P>P		YJM981:P>P	AA:207		UWOPS05_227_2:N>N		UWOPS87_2421:N>N	AA:228		DBVPG1373:S>S		DBVPG1788:S>S		DBVPG6765:S>S		L_1374:S>S		L_1528:S>S		NCYC361:S>S		SK1:S>S		UWOPS83_787_3:S>S		UWOPS87_2421:S>S		Y55:S>S		YIIc17_E5:S>S		YJM978:S>S		YJM981:S>S		YPS128:S>S		YPS606:S>S	AA:230		DBVPG1373:T>T		DBVPG1788:T>T		DBVPG6765:T>T		L_1374:T>T		NCYC361:T>T		SK1:T>T		Y55:T>T		YIIc17_E5:T>T		YJM978:T>T		YJM981:T>T	AA:324		DBVPG1373:K>K		DBVPG1788:K>K		DBVPG6040:K>K		DBVPG6044:K>K		DBVPG6765:K>K		L_1528:K>K		NCYC110:K>K		SK1:K>K		UWOPS87_2421:K>K		Y12:K>K		Y55:K>K		Y9:K>K		YIIc17_E5:K>K		YJM981:K>K		YPS128:K>K		YPS606:K>K	AA:395		NCYC110:I>I	AA:410		NCYC110:I>I	AA:417		NCYC110:D>D	AA:420		NCYC110:A>A	AA:433		UWOPS87_2421:L>L	AA:445		DBVPG6040:A>A	AA:458		YJM975:Y>Y		YJM981:Y>Y	AA:564		NCYC110:I>I	AA:577		Y55:T>T	AA:592		DBVPG1106:L>L		DBVPG1788:L>L		DBVPG1853:L>L		DBVPG6040:L>L		DBVPG6765:L>L		L_1374:L>L		L_1528:L>L		NCYC110:L>L		NCYC361:L>L		SK1:L>L		UWOPS05_217_3:L>L		UWOPS05_227_2:L>L		Y55:L>L		YIIc17_E5:L>L		YJM975:L>L		YJM981:L>L		YS9:L>L	AA:596		NCYC110:V>V	AA:643		NCYC110:L>L	AA:660		K11:S>S	AA:731		DBVPG6040:I>I	AA:733		378604X:A>A	AA:762		UWOPS83_787_3:L>L		UWOPS87_2421:L>L	AA:782		UWOPS05_217_3:V>V		UWOPS05_227_2:V>V	AA:792		273614X:I>I		378604X:I>I		DBVPG1106:I>I		DBVPG1788:I>I		DBVPG1853:I>I		DBVPG6040:I>I		DBVPG6044:I>I		DBVPG6765:I>I		K11:I>I		L_1374:I>I		NCYC361:I>I		SK1:I>I		UWOPS05_217_3:I>I		UWOPS05_227_2:I>I		UWOPS83_787_3:I>I		UWOPS87_2421:I>I		Y55:I>I		YIIc17_E5:I>I		YJM981:I>I		YPS606:I>IID:YCR082W	AA:11		273614X:V>V		322134S:V>V		DBVPG1373:V>V		DBVPG6040:V>V		DBVPG6765:V>V		SK1:V>V		UWOPS03_461_4:V>V		UWOPS05_217_3:V>V		W303:V>V		Y55:V>V		YJM975:V>V		YPS606:V>V		YS9:V>V	AA:46		YJM975:V>V	AA:56		UWOPS83_787_3:I>I	AA:106		L_1528:A>AID:YCR083W	AA:18		YPS606:I>I	AA:29		L_1528:K>K	AA:32		L_1528:N>N	AA:48		L_1528:I>I	AA:49		273614X:D>D		322134S:D>D		BC187:D>D		DBVPG1373:D>D		DBVPG1788:D>D		DBVPG6765:D>D		L_1374:D>D		SK1:D>D		UWOPS05_217_3:D>D		UWOPS05_227_2:D>D		W303:D>D		Y55:D>D		YJM975:D>D		YPS606:D>D		YS2:D>D		YS4:D>D		YS9:D>D	AA:70		L_1528:Q>Q	AA:83		L_1528:D>D	AA:89		L_1528:A>A	AA:104		UWOPS05_217_3:K>K		UWOPS05_227_2:K>K	AA:106		L_1528:G>G	AA:111		SK1:K>K	AA:112		L_1528:I>I	AA:120		L_1528:L>L	AA:125		L_1528:K>KID:YCR086W	AA:122		BC187:F>F	AA:129		UWOPS83_787_3:V>V	AA:133		273614X:I>I		BC187:I>I		DBVPG1853:I>I		DBVPG6040:I>I		DBVPG6765:I>I		UWOPS03_461_4:I>I		W303:I>I		Y55:I>I		YJM975:I>IID:YCR087C-A	AA:9		273614X:C>C		378604X:C>C		BC187:C>C		DBVPG1373:C>C		DBVPG6040:C>C		DBVPG6765:C>C		K11:C>C		L_1374:C>C		SK1:C>C		UWOPS05_217_3:C>C		UWOPS05_227_2:C>C		W303:C>C		Y55:C>C		YJM975:C>C		YJM981:C>C		YPS606:C>C	AA:62		273614X:L>L		DBVPG1373:L>L		DBVPG6040:L>L		DBVPG6765:L>L		L_1374:L>L		W303:L>L		Y55:L>L		YJM975:L>L	AA:87		UWOPS03_461_4:V>V		UWOPS05_217_3:V>V		UWOPS05_227_2:V>V	AA:108		BC187:T>T	AA:111		273614X:G>G		DBVPG1853:G>G		DBVPG6040:G>G		DBVPG6765:G>G		L_1374:G>G		W303:G>G		Y55:G>G		YJM975:G>G	AA:142		UWOPS83_787_3:V>VID:YCR088W	AA:8		DBVPG1106:Y>Y		DBVPG1853:Y>Y		DBVPG6765:Y>Y		L_1374:Y>Y		UWOPS05_227_2:Y>Y		W303:Y>Y		Y55:Y>Y		YJM981:Y>Y	AA:72		DBVPG6044:S>S	AA:91		UWOPS05_227_2:A>A	AA:112		DBVPG1853:K>K	AA:131		DBVPG6765:L>L	AA:163		DBVPG6044:S>S		SK1:S>S		UWOPS05_227_2:S>S		Y9:S>S		YPS128:S>S	AA:218		DBVPG6044:L>L		YPS128:L>L	AA:221		L_1528:N>N	AA:269		UWOPS03_461_4:K>K	AA:279		UWOPS03_461_4:E>E	AA:319		Y9:A>A	AA:327		273614X:S>S		322134S:S>S		DBVPG1373:S>S		DBVPG1788:S>S		DBVPG6044:S>S		DBVPG6765:S>S		L_1374:S>S		L_1528:S>S		UWOPS05_217_3:S>S		UWOPS05_227_2:S>S		W303:S>S		Y55:S>S		YIIc17_E5:S>S		YPS128:S>S	AA:443		UWOPS05_217_3:E>E		UWOPS05_227_2:E>E	AA:466		DBVPG6040:E>E	AA:467		DBVPG6044:P>P	AA:505		DBVPG6044:E>E	AA:544		378604X:D>D		DBVPG6044:D>D		SK1:D>D	AA:578		DBVPG1853:G>GID:YCR090C	AA:56		L_1528:A>A	AA:69		YPS128:K>K		YPS606:K>K	AA:140		UWOPS83_787_3:E>E	AA:172		DBVPG6044:T>T		YPS128:T>T		YPS606:T>TID:YCR091W	AA:10		SK1:S>S		UWOPS83_787_3:S>S		Y9:S>S		YPS128:S>S		YPS606:S>S	AA:92		378604X:L>L		DBVPG6040:L>L		DBVPG6044:L>L		SK1:L>L		UWOPS05_227_2:L>L		UWOPS83_787_3:L>L		UWOPS87_2421:L>L		YPS128:L>L		YPS606:L>L		YS4:L>L	AA:205		378604X:G>G		DBVPG6040:G>G		NCYC110:G>G		SK1:G>G		UWOPS05_227_2:G>G		UWOPS83_787_3:G>G		UWOPS87_2421:G>G		Y12:G>G		YPS606:G>G		YS4:G>G	AA:271		YPS606:P>P	AA:338		YS4:V>V	AA:354		UWOPS87_2421:V>V	AA:361		YPS606:I>I	AA:362		UWOPS03_461_4:K>K		UWOPS05_227_2:K>K	AA:382		Y12:P>P		Y9:P>P	AA:454		DBVPG1106:N>N		DBVPG1373:N>N		DBVPG1853:N>N		DBVPG6044:N>N		DBVPG6765:N>N		K11:N>N		L_1374:N>N		NCYC110:N>N		SK1:N>N		UWOPS87_2421:N>N		Y12:N>N		Y9:N>N		YIIc17_E5:N>N		YJM975:N>N		YPS128:N>N		YPS606:N>N	AA:682		NCYC361:I>I		SK1:I>I		Y12:I>I	AA:710		DBVPG1853:R>R	AA:716		SK1:I>IID:YCR095C	AA:6		YS4:A>A	AA:19		DBVPG6040:S>S	AA:36		W303:T>T	AA:90		DBVPG6040:N>N	AA:93		378604X:L>L		Y12:L>L	AA:117		UWOPS05_227_2:I>I	AA:161		UWOPS05_227_2:A>A	AA:268		DBVPG1853:T>T		SK1:T>T		UWOPS83_787_3:T>T		YPS606:T>T		YS4:T>T	AA:293		DBVPG1853:L>L		SK1:L>L		YPS128:L>L		YPS606:L>L		YS4:L>L	AA:295		DBVPG1853:I>I		SK1:I>I		YS4:I>I	AA:320		UWOPS83_787_3:K>K	AA:354		378604X:H>H		K11:H>HID:YDL001W	AA:64		273614X:G>G		378604X:G>G		BC187:G>G		DBVPG1373:G>G		DBVPG1853:G>G		DBVPG6044:G>G		DBVPG6765:G>G		K11:G>G		L_1528:G>G		UWOPS05_227_2:G>G		UWOPS87_2421:G>G		Y12:G>G		YJM978:G>G		YPS606:G>G		YS4:G>G		YS9:G>G	AA:135		273614X:T>T		378604X:T>T		BC187:T>T		DBVPG1373:T>T		DBVPG1853:T>T		DBVPG6044:T>T		DBVPG6765:T>T		K11:T>T		NCYC110:T>T		SK1:T>T		UWOPS87_2421:T>T		Y12:T>T		Y55:T>T		YJM975:T>T		YJM978:T>T		YPS606:T>T		YS2:T>T		YS4:T>T	AA:217		273614X:G>G		378604X:G>G		BC187:G>G		DBVPG1373:G>G		DBVPG1853:G>G		DBVPG6044:G>G		DBVPG6765:G>G		K11:G>G		NCYC110:G>G		SK1:G>G		UWOPS83_787_3:G>G		UWOPS87_2421:G>G		Y12:G>G		Y55:G>G		YJM975:G>G		YJM978:G>G		YPS128:G>G		YPS606:G>G		YS2:G>G		YS4:G>G	AA:347		273614X:P>P		DBVPG1373:P>P		DBVPG1788:P>P		DBVPG1853:P>P		DBVPG6044:P>P		DBVPG6765:P>P		K11:P>P		NCYC110:P>P		SK1:P>P		UWOPS83_787_3:P>P		UWOPS87_2421:P>P		Y55:P>P		Y9:P>P		YIIc17_E5:P>P		YJM978:P>P		YPS128:P>P		YPS606:P>P		YS2:P>P		YS4:P>P		YS9:P>P	AA:394		UWOPS03_461_4:L>L	AA:402		UWOPS03_461_4:L>LID:YDL002C	AA:24		UWOPS83_787_3:I>I	AA:74		DBVPG6765:K>K		K11:K>K		L_1374:K>K		SK1:K>K		UWOPS03_461_4:K>K		UWOPS05_217_3:K>K		UWOPS83_787_3:K>K		UWOPS87_2421:K>K		YIIc17_E5:K>K		YJM978:K>K		YJM981:K>K		YPS128:K>KID:YDL003W	AA:6		UWOPS05_217_3:P>P	AA:44		322134S:A>A		DBVPG6040:A>A		NCYC361:A>A	AA:113		YPS128:T>T		YPS606:T>T	AA:137		UWOPS03_461_4:V>V	AA:189		273614X:E>E		322134S:E>E		BC187:E>E		DBVPG1788:E>E		DBVPG1853:E>E		DBVPG6040:E>E		DBVPG6044:E>E		DBVPG6765:E>E		K11:E>E		L_1528:E>E		NCYC361:E>E		SK1:E>E		UWOPS05_227_2:E>E		UWOPS83_787_3:E>E		Y12:E>E		Y55:E>E		YJM975:E>E		YPS606:E>E		YS2:E>E		YS4:E>E		YS9:E>E	AA:201		273614X:D>D		K11:D>D		Y12:D>D		YS4:D>D	AA:246		322134S:D>D	AA:247		UWOPS05_217_3:L>L		UWOPS05_227_2:L>L	AA:265		322134S:E>E	AA:280		K11:T>T		YS4:T>T	AA:313		UWOPS83_787_3:G>	AA:437		378604X:S>S		DBVPG1373:S>S		DBVPG1788:S>S		DBVPG1853:S>S		DBVPG6040:S>S		DBVPG6044:S>S		DBVPG6765:S>S		K11:S>S		L_1374:S>S		L_1528:S>S		NCYC110:S>S		SK1:S>S		UWOPS05_217_3:S>S		UWOPS83_787_3:S>S		UWOPS87_2421:S>S		Y55:S>S		Y9:S>S		YIIc17_E5:S>S		YS2:S>S		YS4:S>S	AA:502		BC187:V>V		DBVPG1373:V>V		DBVPG1788:V>V		DBVPG1853:V>V		DBVPG6765:V>V		L_1528:V>V		YJM978:V>V		YS4:V>VID:YDL004W	AA:53		YIIc17_E5:N>N	AA:64		273614X:L>L		DBVPG1788:L>L		DBVPG1853:L>L		DBVPG6040:L>L		DBVPG6044:L>L		DBVPG6765:L>L		K11:L>L		L_1374:L>L		L_1528:L>L		SK1:L>L		UWOPS03_461_4:L>L		UWOPS87_2421:L>L		Y12:L>L		Y55:L>L		Y9:L>L		YIIc17_E5:L>L		YPS606:L>L		YS4:L>L	AA:88		273614X:K>K		DBVPG6044:K>K		K11:K>K		SK1:K>K		UWOPS03_461_4:K>K		UWOPS87_2421:K>K		Y12:K>K		Y55:K>K		YPS606:K>K	AA:125		UWOPS03_461_4:N>N	AA:155		273614X:L>L		378604X:L>L		DBVPG6044:L>L		SK1:L>L		UWOPS03_461_4:L>L		UWOPS87_2421:L>L		Y55:L>L		Y9:L>LID:YDL005C	AA:113		273614X:E>E		BC187:E>E		DBVPG1788:E>E		DBVPG1853:E>E		DBVPG6040:E>E		DBVPG6044:E>E		DBVPG6765:E>E		K11:E>E		L_1374:E>E		SK1:E>E		UWOPS83_787_3:E>E		Y55:E>E		Y9:E>E		YIIc17_E5:E>E		YJM975:E>E		YJM981:E>E		YPS606:E>E		YS4:E>E	AA:201		273614X:A>A		DBVPG6040:A>A		UWOPS05_227_2:A>A		UWOPS83_787_3:A>A	AA:206		273614X:F>F		BC187:F>F		DBVPG1788:F>F		DBVPG1853:F>F		DBVPG6040:F>F		DBVPG6044:F>F		DBVPG6765:F>F		L_1374:F>F		SK1:F>F		UWOPS05_227_2:F>F		UWOPS83_787_3:F>F		Y55:F>F		YJM975:F>F		YPS606:F>F		YS4:F>F		YS9:F>F	AA:220		SK1:A>A		Y55:A>A	AA:335		DBVPG6044:N>N		SK1:N>N		Y55:N>N	AA:368		DBVPG6044:T>T		SK1:T>T		Y55:T>T	AA:397		YJM978:D>D	AA:413		322134S:I>I		BC187:I>I		DBVPG1788:I>I		DBVPG6040:I>I		DBVPG6044:I>I		DBVPG6765:I>I		K11:I>I		L_1374:I>I		NCYC361:I>I		SK1:I>I		UWOPS05_217_3:I>I		YJM975:I>I		YJM978:I>I		YPS128:I>I		YS2:I>I		YS4:I>I	AA:426		UWOPS03_461_4:L>L		UWOPS05_217_3:L>LID:YDL006W	AA:5		K11:S>S	AA:31		322134S:F>F		NCYC361:F>F	AA:99		DBVPG6044:A>A		SK1:A>A		Y55:A>A		YS9:A>A	AA:128		YS9:S>S	AA:149		YS9:G>G	AA:196		DBVPG6040:A>A		Y9:A>A		YS2:A>A		YS9:A>A	AA:198		DBVPG1853:T>T	AA:216		DBVPG6044:T>T		SK1:T>T		UWOPS03_461_4:T>T		UWOPS05_217_3:T>T		Y55:T>T	AA:261		DBVPG6044:L>L		SK1:L>L		Y55:L>LID:YDL007W	AA:87		UWOPS05_217_3:K>K		UWOPS05_227_2:K>K	AA:91		NCYC110:E>E		NCYC361:E>E		SK1:E>E		Y55:E>E	AA:166		UWOPS05_227_2:P>P	AA:191		UWOPS05_227_2:I>I	AA:212		NCYC110:G>G		SK1:G>G		Y55:G>G	AA:224		K11:A>A	AA:237		YS4:A>A	AA:247		UWOPS03_461_4:I>I	AA:270		273614X:V>V		DBVPG6040:V>V		NCYC110:V>V		SK1:V>V		UWOPS03_461_4:V>V		UWOPS87_2421:V>V		Y55:V>V		YPS606:V>V	AA:274		DBVPG1106:N>N		DBVPG1788:N>N		DBVPG6765:N>N		L_1528:N>N		YJM981:N>N	AA:300		UWOPS03_461_4:R>R	AA:309		UWOPS03_461_4:L>L	AA:339		SK1:I>I		Y55:I>I	AA:382		UWOPS03_461_4:T>T		UWOPS05_217_3:T>T		UWOPS05_227_2:T>T	AA:429		UWOPS87_2421:E>EID:YDL008W	AA:56		UWOPS83_787_3:K>K	AA:69		322134S:C>C		BC187:C>C		DBVPG1373:C>C		DBVPG1788:C>C		DBVPG6044:C>C		DBVPG6765:C>C		K11:C>C		L_1374:C>C		NCYC110:C>C		SK1:C>C		UWOPS05_217_3:C>C		UWOPS05_227_2:C>C		UWOPS83_787_3:C>C		YIIc17_E5:C>C		YJM978:C>C		YPS128:C>C		YPS606:C>C	AA:82		K11:L>L		YPS128:L>L		YPS606:L>L	AA:95		DBVPG1788:R>R	AA:131		DBVPG6044:A>A		K11:A>A		NCYC110:A>A		SK1:A>A		UWOPS05_217_3:A>A		UWOPS05_227_2:A>A		Y55:A>A		YPS128:A>A		YPS606:A>A	AA:159		DBVPG1853:D>DID:YDL010W	AA:12		UWOPS03_461_4:L>L		UWOPS05_227_2:L>L	AA:66		UWOPS83_787_3:T>T	AA:87		UWOPS03_461_4:L>L		UWOPS05_217_3:L>L		UWOPS05_227_2:L>L	AA:150		UWOPS03_461_4:Y>Y		UWOPS05_217_3:Y>Y		UWOPS05_227_2:Y>Y	AA:158		273614X:I>I		DBVPG1853:I>I		DBVPG6044:I>I		NCYC110:I>I		NCYC361:I>I		SK1:I>I		UWOPS03_461_4:I>I		UWOPS05_217_3:I>I		UWOPS05_227_2:I>I		UWOPS83_787_3:I>I		UWOPS87_2421:I>I		Y55:I>I		YPS128:I>I		YPS606:I>I	AA:178		273614X:T>T	AA:199		273614X:I>I		DBVPG1853:I>I		DBVPG6044:I>I		NCYC110:I>I		NCYC361:I>I		SK1:I>I		UWOPS03_461_4:I>I		UWOPS05_217_3:I>I		UWOPS05_227_2:I>I		UWOPS83_787_3:I>I		UWOPS87_2421:I>I		Y55:I>I		YPS606:I>I		YS2:I>I	AA:221		273614X:S>S		DBVPG6044:S>S		NCYC110:S>S		NCYC361:S>S		SK1:S>S		UWOPS83_787_3:S>S		UWOPS87_2421:S>S		Y55:S>S		YPS606:S>S		YS2:S>SID:YDL013W	AA:94		NCYC361:T>T	AA:99		K11:V>V	AA:171		K11:L>L	AA:205		UWOPS83_787_3:L>L		UWOPS87_2421:L>L	AA:232		UWOPS83_787_3:T>T		UWOPS87_2421:T>T	AA:301		DBVPG6040:S>S		DBVPG6044:S>S		SK1:S>S		Y55:S>S	AA:323		DBVPG1788:E>E	AA:405		DBVPG6044:D>D		Y55:D>D		Y9:D>D		YPS128:D>D	AA:483		273614X:D>D	AA:511		273614X:G>G		DBVPG6044:G>G		K11:G>G		SK1:G>G		UWOPS03_461_4:G>G		UWOPS05_217_3:G>G		Y55:G>G		Y9:G>G		YPS128:G>G	AA:532		UWOPS03_461_4:C>C		UWOPS05_217_3:C>C	AA:533		DBVPG6044:P>P		K11:P>P		SK1:P>P		Y55:P>P		Y9:P>P	AA:611		DBVPG6044:R>R		SK1:R>R		Y55:R>RID:YDL014W	AA:53		YS2:G>G	AA:146		DBVPG6040:P>P		NCYC110:P>P		SK1:P>P		Y55:P>P	AA:163		NCYC110:F>F		SK1:F>F	AA:167		UWOPS03_461_4:G>G		UWOPS05_217_3:G>G	AA:196		L_1374:A>A	AA:211		273614X:A>A		NCYC110:A>A		SK1:A>A		UWOPS03_461_4:A>A		UWOPS05_217_3:A>A		UWOPS83_787_3:A>A		Y55:A>A		Y9:A>A		YS2:A>A	AA:219		BC187:P>P		DBVPG1106:P>P	AA:220		UWOPS03_461_4:I>I		UWOPS05_217_3:I>I	AA:235		YS2:G>GID:YDL015C	AA:49		K11:K>K		UWOPS87_2421:K>K		YPS128:K>K	AA:130		273614X:A>A		K11:A>A		UWOPS87_2421:A>A		YPS128:A>A		YPS606:A>A	AA:173		YS9:G>G	AA:191		DBVPG6044:L>L		K11:L>L		SK1:L>L		UWOPS03_461_4:L>L		UWOPS05_217_3:L>L		UWOPS87_2421:L>L		Y55:L>L		YPS128:L>L		YPS606:L>L	AA:201		YPS128:D>D		YPS606:D>D	AA:203		DBVPG1373:L>L		DBVPG1853:L>L		DBVPG6044:L>L		DBVPG6765:L>L		L_1374:L>L		L_1528:L>L		NCYC361:L>L		SK1:L>L		UWOPS03_461_4:L>L		UWOPS05_217_3:L>L		UWOPS87_2421:L>L		Y55:L>L		YIIc17_E5:L>L		YJM978:L>L		YPS128:L>L		YPS606:L>L		YS9:L>L	AA:206		YPS128:L>L		YPS606:L>L	AA:232		YPS128:K>K		YPS606:K>K	AA:268		SK1:F>F		Y55:F>F	AA:288		L_1528:Y>Y	AA:309		L_1528:V>VID:YDL017W	AA:6		378604X:K>K		DBVPG1853:K>K		DBVPG6040:K>K		DBVPG6044:K>K		NCYC110:K>K		SK1:K>K		UWOPS03_461_4:K>K		UWOPS05_217_3:K>K		UWOPS83_787_3:K>K		UWOPS87_2421:K>K		Y12:K>K		Y55:K>K		Y9:K>K		YIIc17_E5:K>K		YPS128:K>K		YPS606:K>K		YS4:K>K	AA:39		YPS606:I>I	AA:65		L_1528:F>F	AA:84		L_1528:P>P	AA:99		L_1528:G>G	AA:103		DBVPG6040:V>V		DBVPG6044:V>V		K11:V>V		NCYC110:V>V		SK1:V>V		UWOPS83_787_3:V>V		UWOPS87_2421:V>V		Y12:V>V		Y55:V>V		YPS606:V>V		YS2:V>V	AA:105		L_1528:P>P	AA:106		DBVPG6040:L>L		DBVPG6044:L>L		NCYC110:L>L		SK1:L>L		Y55:L>L	AA:124		L_1528:P>P	AA:142		UWOPS87_2421:K>K	AA:248		UWOPS03_461_4:G>G		UWOPS05_217_3:G>G		UWOPS05_227_2:G>G	AA:276		378604X:A>A		DBVPG6040:A>A		DBVPG6044:A>A		K11:A>A		NCYC110:A>A		SK1:A>A		UWOPS03_461_4:A>A		UWOPS05_217_3:A>A		UWOPS05_227_2:A>A		UWOPS83_787_3:A>A		UWOPS87_2421:A>A		Y55:A>A		Y9:A>A		YPS128:A>A		YPS606:A>A		YS2:A>A	AA:344		L_1528:C>C	AA:357		L_1528:L>L	AA:371		L_1528:E>E	AA:379		L_1528:K>K	AA:382		L_1528:T>T	AA:384		L_1528:G>G	AA:387		L_1528:P>P	AA:389		L_1528:Y>Y	AA:396		L_1528:F>F	AA:399		L_1528:L>L	AA:410		L_1528:E>E	AA:416		L_1528:P>P		UWOPS03_461_4:P>P		UWOPS05_217_3:P>P	AA:418		L_1528:T>T	AA:425		L_1528:A>A	AA:456		L_1528:K>K	AA:460		L_1528:A>A	AA:482		L_1528:E>E	AA:484		L_1528:T>T	AA:500		L_1528:K>K	AA:504		YPS128:L>L		YPS606:L>LID:YDL018C	AA:27		DBVPG6040:E>E		DBVPG6044:E>E		K11:E>E		NCYC110:E>E		SK1:E>E		UWOPS03_461_4:E>E		UWOPS05_217_3:E>E		UWOPS83_787_3:E>E		Y55:E>E		Y9:E>E		YPS128:E>E		YPS606:E>E	AA:63		DBVPG6040:V>V		DBVPG6044:V>V		NCYC110:V>V		SK1:V>V		UWOPS03_461_4:V>V		UWOPS05_217_3:V>V		UWOPS83_787_3:V>V		Y12:V>V		Y55:V>V		Y9:V>V		YPS128:V>V		YPS606:V>V	AA:81		DBVPG6040:S>S		DBVPG6044:S>S		NCYC110:S>S		SK1:S>S		Y55:S>S	AA:103		DBVPG6044:Y>Y		NCYC110:Y>Y		SK1:Y>Y		UWOPS03_461_4:Y>Y		UWOPS05_217_3:Y>Y		UWOPS83_787_3:Y>Y		Y12:Y>Y		Y55:Y>Y		Y9:Y>Y		YPS128:Y>Y		YPS606:Y>Y	AA:122		UWOPS03_461_4:R>R		UWOPS05_217_3:R>R	AA:131		DBVPG6044:R>R		NCYC110:R>R		SK1:R>R		UWOPS03_461_4:R>R		UWOPS05_217_3:R>R		UWOPS87_2421:R>R		Y12:R>R		Y55:R>R		Y9:R>R		YPS128:R>R		YPS606:R>R	AA:150		DBVPG6044:D>D		NCYC110:D>D		SK1:D>D		UWOPS03_461_4:D>D		UWOPS05_217_3:D>D		UWOPS87_2421:D>D		Y12:D>D		Y55:D>D		Y9:D>D		YPS606:D>D	AA:155		DBVPG6044:S>S		NCYC110:S>S		SK1:S>S		UWOPS03_461_4:S>S		UWOPS05_217_3:S>S		UWOPS87_2421:S>S		Y12:S>S		Y55:S>S		Y9:S>S		YPS128:S>S		YPS606:S>S	AA:218		UWOPS03_461_4:R>RID:YDL020C	AA:4		YS2:T>T	AA:12		DBVPG1853:L>L		DBVPG6044:L>L		SK1:L>L		UWOPS87_2421:L>L		Y12:L>L		Y55:L>L		Y9:L>L		YPS128:L>L	AA:27		DBVPG1853:H>H		DBVPG6044:H>H		K11:H>H		SK1:H>H		UWOPS03_461_4:H>H		UWOPS05_217_3:H>H		UWOPS87_2421:H>H		Y12:H>H		Y55:H>H		Y9:H>H		YPS128:H>H	AA:45		DBVPG1853:P>P		DBVPG6044:P>P		K11:P>P		SK1:P>P		UWOPS03_461_4:P>P		UWOPS05_217_3:P>P		UWOPS87_2421:P>P		Y12:P>P		Y55:P>P		Y9:P>P		YPS128:P>P	AA:55		DBVPG1853:N>N		DBVPG6044:N>N		K11:N>N		SK1:N>N		UWOPS03_461_4:N>N		UWOPS05_217_3:N>N		UWOPS87_2421:N>N		Y12:N>N		Y55:N>N		Y9:N>N		YPS128:N>N	AA:66		DBVPG6044:H>H		SK1:H>H		Y55:H>H	AA:75		UWOPS03_461_4:I>I		UWOPS05_217_3:I>I	AA:130		UWOPS03_461_4:L>L		UWOPS05_217_3:L>L	AA:135		DBVPG1853:N>N		DBVPG6044:N>N		SK1:N>N		UWOPS03_461_4:L>L		UWOPS05_217_3:L>L		UWOPS87_2421:N>N		Y55:N>N		YPS128:N>N	AA:200		K11:N>N		UWOPS03_461_4:N>N		UWOPS05_217_3:N>N		UWOPS87_2421:N>N		Y9:N>N		YPS606:N>N	AA:222		K11:D>D		UWOPS03_461_4:D>D		UWOPS05_217_3:D>D		UWOPS87_2421:D>D		Y9:D>D		YPS606:D>D	AA:318		NCYC361:L>L		UWOPS87_2421:L>L		Y12:L>L		Y9:L>L		YPS606:L>L		YS2:L>L		YS4:L>L	AA:327		NCYC361:F>F		UWOPS05_217_3:F>F		UWOPS87_2421:F>F		Y12:F>F		Y9:F>F		YPS606:F>F		YS2:F>F		YS4:F>F	AA:375		DBVPG6040:N>N	AA:432		NCYC361:G>G		UWOPS03_461_4:G>G		UWOPS05_217_3:G>G		UWOPS83_787_3:G>G		UWOPS87_2421:G>G		Y12:G>G		Y9:G>G		YPS128:G>G		YPS606:G>G		YS2:G>G	AA:452		NCYC361:A>A		UWOPS87_2421:A>A		Y12:A>A		Y9:A>A		YPS128:A>A		YPS606:A>A		YS2:A>A	AA:468		UWOPS03_461_4:H>H		UWOPS05_217_3:H>H	AA:491		UWOPS03_461_4:K>K		UWOPS05_217_3:K>KID:YDL021W	AA:15		DBVPG6044:L>L		SK1:L>L		Y55:L>L	AA:37		BC187:T>T	AA:77		UWOPS87_2421:T>T		YPS128:T>T		YPS606:T>T		YS9:T>T	AA:97		DBVPG6765:F>F		UWOPS87_2421:F>F		YPS128:F>F		YPS606:F>F		YS4:F>F	AA:143		DBVPG6044:E>E		SK1:E>E		Y55:E>E	AA:198		378604X:S>S		BC187:S>S		DBVPG1106:S>S		DBVPG1788:S>S		DBVPG6765:S>S		L_1374:S>S		YJM978:S>S		YS9:S>S	AA:204		UWOPS87_2421:L>L		Y9:L>L		YPS128:L>L	AA:258		NCYC110:D>D		SK1:D>D		UWOPS03_461_4:D>D		UWOPS05_217_3:D>D		UWOPS05_227_2:D>D		UWOPS83_787_3:D>D		UWOPS87_2421:D>D		Y55:D>D		Y9:D>D		YPS128:D>D		YPS606:D>D	AA:291		UWOPS03_461_4:D>D		UWOPS05_217_3:D>D		UWOPS05_227_2:D>DID:YDL022W	AA:12		Y12:S>S	AA:65		YS2:A>A	AA:85		YPS128:I>I		YPS606:I>I	AA:97		UWOPS87_2421:P>P	AA:186		SK1:T>T		UWOPS83_787_3:T>T		UWOPS87_2421:T>T		W303:T>T		Y55:T>T		YPS128:T>T	AA:188		SK1:V>V		UWOPS87_2421:V>V		W303:V>V		Y55:V>V	AA:231		UWOPS83_787_3:V>V	AA:250		SK1:L>L		Y55:L>L	AA:287		UWOPS83_787_3:S>S	AA:316		UWOPS87_2421:R>RID:YDL027C	AA:25		K11:N>N	AA:76		K11:S>S		SK1:S>S		UWOPS03_461_4:S>S		UWOPS05_217_3:S>S		UWOPS83_787_3:S>S		UWOPS87_2421:S>S		W303:S>S		Y55:S>S		YPS128:S>S		YPS606:S>S		YS9:S>S	AA:121		DBVPG6040:P>P	AA:139		L_1528:S>S	AA:182		L_1528:K>K	AA:184		L_1528:V>V	AA:185		UWOPS83_787_3:L>L	AA:209		L_1528:K>K	AA:215		L_1528:I>I	AA:218		273614X:R>R		DBVPG6040:R>R		DBVPG6044:R>R		L_1528:R>R		SK1:R>R		UWOPS03_461_4:R>R		UWOPS05_217_3:R>R		UWOPS05_227_2:R>R		UWOPS83_787_3:R>R		W303:R>R		Y12:R>R		Y55:R>R		YPS128:R>R		YPS606:R>R		YS9:R>R	AA:223		L_1528:N>N	AA:227		L_1528:K>K	AA:234		L_1528:V>V	AA:246		L_1528:F>F	AA:248		L_1528:Q>Q	AA:255		L_1528:F>F	AA:264		L_1528:S>S	AA:271		L_1528:V>V	AA:275		UWOPS83_787_3:I>I	AA:331		UWOPS83_787_3:D>D	AA:376		DBVPG6044:I>I		NCYC110:I>I		W303:I>IID:YDL028C	AA:77		K11:P>P		YS2:P>P		YS4:P>P	AA:278		W303:S>S	AA:307		273614X:T>T		378604X:T>T		BC187:T>T		DBVPG1106:T>T		DBVPG1373:T>T		DBVPG1788:T>T		DBVPG6765:T>T		K11:T>T		L_1528:T>T		SK1:T>T		UWOPS83_787_3:T>T		Y55:T>T		YIIc17_E5:T>T		YJM975:T>T		YJM978:T>T		YPS606:T>T		YS2:T>T		YS9:T>T	AA:365		K11:N>N		UWOPS83_787_3:N>N	AA:414		273614X:K>K		DBVPG6044:K>K		SK1:K>K		Y55:K>K		Y9:K>K	AA:522		273614X:D>D		DBVPG6044:D>D		UWOPS87_2421:D>D		W303:D>D		Y55:D>D	AA:532		273614X:G>G		DBVPG6044:G>G		SK1:G>G		UWOPS87_2421:G>G		W303:G>G		Y55:G>G	AA:552		UWOPS83_787_3:K>K		Y12:K>K		Y9:K>K		YS4:K>K		YS9:K>K	AA:619		UWOPS03_461_4:N>N		UWOPS05_217_3:N>N	AA:718		DBVPG6040:P>P	AA:719		UWOPS87_2421:F>F	AA:725		DBVPG6040:I>I		DBVPG6044:I>I		SK1:I>I		UWOPS83_787_3:I>I		UWOPS87_2421:I>I		Y55:I>I		Y9:I>I		YPS128:I>I		YS4:I>I		YS9:I>IID:YDL029W	AA:36		SK1:R>R		W303:R>R		Y55:R>R	AA:76		YS9:G>G	AA:146		322134S:Y>Y		Y12:Y>Y		YIIc17_E5:Y>Y	AA:153		YIIc17_E5:G>G	AA:199		YS9:R>R	AA:245		273614X:L>L		DBVPG6044:L>L		Y55:L>L		YPS606:L>L	AA:260		273614X:A>A		DBVPG6044:A>A		K11:A>A		UWOPS03_461_4:A>A		UWOPS05_227_2:A>A		W303:A>A		Y12:A>A		Y55:A>A		YPS606:A>A	AA:276		DBVPG6044:G>G		W303:G>G		Y55:G>G	AA:295		UWOPS03_461_4:S>S		UWOPS05_217_3:S>S	AA:311		273614X:G>G		DBVPG6044:G>G		K11:G>G		NCYC361:G>G		UWOPS03_461_4:G>G		UWOPS05_217_3:G>G		UWOPS83_787_3:G>G		W303:G>G		Y12:G>G		Y55:G>G		YPS606:G>G	AA:314		273614X:S>S		DBVPG6044:S>S		K11:S>S		W303:S>S		Y12:S>S		Y55:S>S		YPS606:S>S	AA:343		273614X:I>I		DBVPG6044:I>I		K11:I>I		NCYC361:I>I		UWOPS83_787_3:I>I		W303:I>I		Y12:I>I		Y55:I>I		YIIc17_E5:I>I		YPS606:I>IID:YDL030W	AA:52		DBVPG6765:S>S		L_1374:S>S	AA:53		Y12:L>L		YPS128:L>L		YS4:L>L		YS9:L>L	AA:71		Y12:Q>Q		YPS128:Q>Q		YS9:Q>Q	AA:89		Y12:K>K		Y9:K>K		YPS128:K>K		YPS606:K>K		YS9:K>K	AA:115		K11:K>K		UWOPS83_787_3:K>K	AA:122		K11:E>E		UWOPS83_787_3:E>E		UWOPS87_2421:E>E		Y9:E>E		YPS606:E>E		YS9:E>E	AA:209		UWOPS03_461_4:L>L	AA:269		UWOPS83_787_3:C>C	AA:316		DBVPG1853:Y>Y	AA:356		UWOPS03_461_4:I>I		UWOPS05_227_2:I>I	AA:373		273614X:G>G		322134S:G>G		UWOPS03_461_4:G>G		UWOPS05_227_2:G>G		UWOPS83_787_3:G>G		UWOPS87_2421:G>G		Y12:G>G		YPS606:G>G	AA:387		322134S:Q>Q		UWOPS87_2421:Q>Q	AA:400		273614X:L>L		322134S:L>L		UWOPS87_2421:L>L		Y12:L>L		YPS606:L>L	AA:421		DBVPG1106:Y>Y		DBVPG1853:Y>Y		DBVPG6044:Y>Y		DBVPG6765:Y>Y		L_1528:Y>Y		SK1:Y>Y		W303:Y>Y		Y55:Y>Y		YS9:Y>Y	AA:442		273614X:N>N		UWOPS03_461_4:N>N		UWOPS05_227_2:N>N		UWOPS87_2421:N>N		Y12:N>N		YPS606:N>N	AA:457		273614X:P>P		322134S:P>P		UWOPS03_461_4:P>P		UWOPS05_227_2:P>P		UWOPS83_787_3:P>P		UWOPS87_2421:P>P		Y12:P>P		YPS606:P>P	AA:487		UWOPS03_461_4:A>A		UWOPS05_227_2:A>AID:YDL033C	AA:5		UWOPS03_461_4:Y>Y		UWOPS05_227_2:Y>Y	AA:54		YS4:R>R	AA:98		NCYC110:N>N		SK1:N>N	AA:113		NCYC110:R>R		SK1:R>R	AA:156		NCYC110:Y>Y		SK1:Y>Y	AA:204		UWOPS05_227_2:T>T	AA:247		YS4:L>L	AA:318		UWOPS83_787_3:P>P		UWOPS87_2421:P>P		Y9:P>P		YPS606:P>P		YS9:P>PID:YDL036C	AA:29		DBVPG6044:G>G		NCYC110:G>G		SK1:G>G		Y55:G>G	AA:78		273614X:F>F		322134S:F>F		DBVPG1853:F>F		K11:F>F		UWOPS03_461_4:F>F		UWOPS05_217_3:F>F		UWOPS05_227_2:F>F		Y9:F>F	AA:105		DBVPG6044:L>L		NCYC110:L>L		SK1:L>L		Y55:L>L	AA:120		YJM978:K>K	AA:127		K11:K>K	AA:151		NCYC110:G>G		SK1:G>G		W303:G>G		Y55:G>G	AA:154		BC187:H>H		L_1374:H>H		L_1528:H>H		YIIc17_E5:H>H		YJM978:H>H		YJM981:H>H	AA:188		322134S:I>I		DBVPG1853:I>I		K11:I>I		NCYC110:I>I		SK1:I>I		UWOPS05_227_2:I>I		W303:I>I		Y12:I>I		Y55:I>I		Y9:I>I		YPS606:I>I	AA:199		322134S:V>V		BC187:V>V		DBVPG1853:V>V		K11:V>V		L_1528:V>V		NCYC110:V>V		SK1:V>V		UWOPS05_227_2:V>V		W303:V>V		Y12:V>V		Y55:V>V		Y9:V>V		YIIc17_E5:V>V		YJM978:V>V		YJM981:V>V		YPS606:V>V	AA:227		322134S:L>L		BC187:L>L		DBVPG1853:L>L		K11:L>L		L_1528:L>L		NCYC110:L>L		SK1:L>L		UWOPS05_227_2:L>L		W303:L>L		Y12:L>L		Y55:L>L		Y9:L>L		YIIc17_E5:L>L		YJM978:L>L		YJM981:L>L		YPS606:L>L	AA:347		DBVPG1853:L>L	AA:358		SK1:S>S		Y55:S>SID:YDL042C	AA:25		DBVPG1853:T>T		DBVPG6044:T>T		SK1:T>T		UWOPS05_217_3:T>T		UWOPS83_787_3:T>T		W303:T>T		Y55:T>T		YPS128:T>T	AA:120		DBVPG6040:P>P		DBVPG6044:P>P		SK1:P>P		W303:P>P		Y55:P>P		YPS128:P>P		YPS606:P>P		YS9:P>P	AA:170		DBVPG6044:L>L		SK1:L>L		UWOPS03_461_4:L>L		W303:L>L		Y55:L>L		YPS128:L>L		YPS606:L>L		YS9:L>L	AA:271		YS9:I>I	AA:306		DBVPG6765:S>S		L_1374:S>S	AA:316		L_1374:L>L	AA:365		YPS128:G>G		YPS606:G>G	AA:380		DBVPG1853:P>P		DBVPG6044:P>P		W303:P>P		Y55:P>P		YPS128:P>P		YPS606:P>P		YS4:P>P	AA:396		DBVPG1853:C>C	AA:441		273614X:D>D		322134S:D>D		K11:D>D		Y9:D>D		YS9:D>DID:YDL044C	AA:17		273614X:H>H		322134S:H>H		DBVPG6044:H>H		NCYC110:H>H		SK1:H>H		UWOPS03_461_4:H>H		UWOPS05_227_2:H>H		UWOPS83_787_3:H>H		W303:H>H		Y55:H>H		Y9:H>H		YPS128:H>H		YPS606:H>H	AA:69		273614X:N>N		322134S:N>N	AA:71		DBVPG6044:R>R		NCYC110:R>R		SK1:R>R		Y55:R>R		Y9:R>R		YPS128:R>R		YPS606:R>R	AA:86		273614X:L>L		322134S:L>L		DBVPG6044:L>L		NCYC110:L>L		SK1:L>L		UWOPS03_461_4:L>L		UWOPS05_227_2:L>L		UWOPS83_787_3:L>L		Y55:L>L		Y9:L>L		YPS128:L>L		YPS606:L>L	AA:110		Y9:R>R	AA:127		273614X:D>D		322134S:D>D		DBVPG6044:D>D		NCYC110:D>D		SK1:D>D		UWOPS03_461_4:D>D		UWOPS83_787_3:D>D		Y55:D>D		Y9:D>D		YPS128:D>D		YPS606:D>D	AA:129		273614X:V>V		322134S:V>V		DBVPG6044:V>V		NCYC110:V>V		SK1:V>V		UWOPS03_461_4:V>V		UWOPS83_787_3:V>V		Y55:V>V		Y9:V>V		YPS128:V>V		YPS606:V>V	AA:137		273614X:A>A		322134S:A>A		DBVPG6044:A>A		NCYC110:A>A		SK1:A>A		UWOPS03_461_4:A>A		UWOPS83_787_3:A>A		Y55:A>A		YPS128:A>A		YPS606:A>A	AA:166		DBVPG6044:R>R		NCYC110:R>R		SK1:R>R		Y55:R>R	AA:211		273614X:L>L	AA:219		DBVPG6044:I>I		NCYC110:I>I		SK1:I>I		Y55:I>I	AA:250		YS9:L>L	AA:256		UWOPS05_217_3:A>A	AA:274		YJM981:A>A	AA:285		YJM981:P>P	AA:336		DBVPG6044:L>L		NCYC110:L>L		SK1:L>L		Y55:L>L	AA:348		DBVPG1853:R>R	AA:350		DBVPG6044:L>L		NCYC110:L>L		SK1:L>L		Y55:L>LID:YDL045C	AA:48		K11:R>R	AA:52		273614X:L>L		DBVPG1853:L>L		K11:L>L		YPS606:L>L	AA:70		DBVPG1853:L>L		YPS606:L>L	AA:73		DBVPG1853:Y>Y		YPS606:Y>Y	AA:82		DBVPG1853:F>F		DBVPG6044:F>F		NCYC110:F>F		SK1:F>F		UWOPS03_461_4:F>F		UWOPS05_217_3:F>F		UWOPS05_227_2:F>F		Y55:F>F		YPS606:F>F	AA:84		DBVPG1853:K>K		YPS606:K>K	AA:192		YPS606:Q>Q	AA:216		YS9:L>L	AA:221		UWOPS03_461_4:F>F		UWOPS05_217_3:F>F	AA:284		DBVPG6044:H>H		SK1:H>H		UWOPS05_227_2:H>H		Y55:H>H	AA:305		UWOPS05_227_2:K>KID:YDL045W-A	AA:14		NCYC110:P>P		SK1:P>P		Y55:P>P	AA:16		273614X:L>L		DBVPG6040:L>L		SK1:L>L		Y55:L>L	AA:37		K11:L>L		NCYC110:L>L		SK1:L>L		UWOPS05_227_2:L>L		Y55:L>L		YPS128:L>L		YPS606:L>L	AA:62		273614X:T>T		K11:T>T		NCYC110:T>T		SK1:T>T		UWOPS05_227_2:T>T		UWOPS83_787_3:T>T		Y55:T>T		Y9:T>T		YPS128:T>T		YPS606:T>T	AA:66		DBVPG1106:A>A		NCYC110:A>A		SK1:A>A		Y55:A>AID:YDL046W	AA:8		DBVPG1853:L>L		DBVPG6044:L>L		NCYC110:L>L		SK1:L>L		UWOPS03_461_4:L>L		UWOPS05_217_3:L>L		UWOPS05_227_2:L>L		UWOPS83_787_3:L>L		UWOPS87_2421:L>L		Y55:L>L		YPS606:L>L	AA:23		DBVPG1106:V>V		DBVPG1788:V>V		DBVPG6765:V>V		L_1374:V>V		L_1528:V>V		NCYC361:V>V		YJM975:V>V		YJM978:V>V		YJM981:V>V	AA:30		UWOPS87_2421:L>L	AA:48		NCYC110:D>D		SK1:D>D		Y55:D>D	AA:66		BC187:P>P		DBVPG1106:P>P		DBVPG1788:P>P		DBVPG1853:P>P		DBVPG6765:P>P		L_1374:P>P		L_1528:P>P		NCYC361:P>P		UWOPS83_787_3:P>P		YJM975:P>P		YJM978:P>P		YJM981:P>P		YPS128:P>P		YPS606:P>P	AA:74		BC187:T>T		DBVPG1106:T>T		DBVPG1788:T>T		DBVPG1853:T>T		DBVPG6765:T>T		L_1374:T>T		L_1528:T>T		NCYC110:T>T		NCYC361:T>T		UWOPS03_461_4:T>T		UWOPS05_217_3:T>T		UWOPS05_227_2:T>T		UWOPS83_787_3:T>T		UWOPS87_2421:T>T		Y55:T>T		YJM975:T>T		YJM978:T>T		YJM981:T>T	AA:127		BC187:P>P		DBVPG1788:P>P		L_1374:P>P		L_1528:P>P		NCYC361:P>P		YJM978:P>P		YJM981:P>P	AA:128		NCYC110:G>G		SK1:G>G		Y55:G>G	AA:140		K11:G>G	AA:142		BC187:V>V		DBVPG1788:V>V		L_1374:V>V		L_1528:V>V		NCYC110:V>V		SK1:V>V		UWOPS03_461_4:V>V		UWOPS05_217_3:V>V		UWOPS05_227_2:V>V		UWOPS83_787_3:V>V		Y55:V>V		YJM978:V>V		YJM981:V>V	AA:151		NCYC110:A>A		SK1:A>A		Y55:A>A	AA:163		UWOPS87_2421:C>CID:YDL047W	AA:55		UWOPS87_2421:H>H	AA:67		273614X:T>T		322134S:T>T		DBVPG1788:T>T		DBVPG1853:T>T		DBVPG6044:T>T		DBVPG6765:T>T		K11:T>T		L_1374:T>T		L_1528:T>T		NCYC110:T>T		SK1:T>T		UWOPS05_217_3:T>T		UWOPS05_227_2:T>T		UWOPS83_787_3:T>T		UWOPS87_2421:T>T		YJM978:T>T		YS2:T>T	AA:107		YS9:K>K	AA:124		YS9:Y>Y	AA:126		273614X:F>F		322134S:F>F		DBVPG1373:F>F		DBVPG1853:F>F		DBVPG6765:F>F		L_1374:F>F		L_1528:F>F		UWOPS05_217_3:F>F		UWOPS05_227_2:F>F		UWOPS83_787_3:F>F		UWOPS87_2421:F>F		YJM978:F>F		YS2:F>F	AA:238		DBVPG1853:H>H		UWOPS83_787_3:H>H	AA:243		DBVPG1106:E>E		DBVPG1373:E>E		DBVPG1788:E>E		DBVPG1853:E>E		DBVPG6044:E>E		DBVPG6765:E>E		L_1528:E>E		NCYC361:E>E		SK1:E>E		UWOPS05_217_3:E>E		UWOPS87_2421:E>E		Y12:E>E		YIIc17_E5:E>E		YJM978:E>E		YPS128:E>E	AA:259		UWOPS05_217_3:S>S	AA:268		YS9:G>G	AA:270		DBVPG1853:V>V	AA:288		DBVPG1106:S>S		DBVPG1373:S>S		DBVPG1853:S>S		DBVPG6044:S>S		DBVPG6765:S>S		L_1528:S>S		NCYC110:S>S		NCYC361:S>S		SK1:S>S		UWOPS87_2421:S>S		Y12:S>S		YIIc17_E5:S>S		YJM978:S>S		YPS128:S>SID:YDL048C	AA:40		DBVPG6040:A>A		UWOPS05_217_3:A>A		UWOPS05_227_2:A>A		YPS606:A>A	AA:58		BC187:S>S		DBVPG1373:S>S		DBVPG6765:S>S		L_1374:S>S		L_1528:S>S		NCYC361:S>S		YJM975:S>S		YJM978:S>S		YJM981:S>S	AA:66		Y9:S>S	AA:109		DBVPG6765:S>S		L_1374:S>S		L_1528:S>S		NCYC361:S>S		YJM975:S>S		YJM978:S>S		YJM981:S>S	AA:198		378604X:H>H		BC187:H>H		DBVPG1373:H>H		DBVPG1788:H>H		DBVPG6765:H>H		L_1374:H>H		L_1528:H>H		NCYC361:H>H		UWOPS03_461_4:H>H		UWOPS05_217_3:H>H		UWOPS83_787_3:H>H		W303:H>H		YJM975:H>H		YJM978:H>H		YJM981:H>H		YPS128:H>H		YS9:H>H	AA:216		378604X:S>S		BC187:S>S		DBVPG1373:S>S		DBVPG1788:S>S		DBVPG6040:S>S		DBVPG6044:S>S		DBVPG6765:S>S		L_1374:S>S		L_1528:S>S		NCYC110:S>S		NCYC361:S>S		SK1:S>S		UWOPS03_461_4:S>S		UWOPS05_217_3:S>S		UWOPS83_787_3:S>S		W303:S>S		Y55:S>S		YJM975:S>S		YJM978:S>S		YJM981:S>S		YPS128:S>S		YS2:S>S		YS9:S>S	AA:250		UWOPS03_461_4:S>S		UWOPS05_217_3:S>S	AA:261		YS4:S>S	AA:292		W303:H>H		YS9:H>H	AA:300		DBVPG6044:E>E		NCYC110:E>E		SK1:E>E		Y55:E>E	AA:309		UWOPS87_2421:C>C	AA:333		DBVPG1106:Q>Q		DBVPG1373:Q>Q		DBVPG1788:Q>Q		DBVPG1853:Q>Q		DBVPG6040:Q>Q		DBVPG6044:Q>Q		DBVPG6765:Q>Q		L_1374:Q>Q		L_1528:Q>Q		NCYC110:Q>Q		NCYC361:Q>Q		SK1:Q>Q		UWOPS03_461_4:Q>Q		UWOPS05_217_3:Q>Q		UWOPS83_787_3:Q>Q		UWOPS87_2421:Q>Q		W303:Q>Q		Y55:Q>Q		YJM975:Q>Q		YJM978:Q>Q		YS2:Q>Q		YS4:Q>Q		YS9:Q>Q	AA:350		W303:D>D		YS9:D>D	AA:355		DBVPG6040:S>S		DBVPG6044:S>S		NCYC110:S>S		SK1:S>S		UWOPS03_461_4:S>S		UWOPS05_217_3:S>S		Y55:S>S	AA:387		UWOPS83_787_3:L>L	AA:390		DBVPG6044:I>I		K11:I>I		SK1:I>I		UWOPS03_461_4:I>I		UWOPS05_217_3:I>I		Y12:I>I		Y55:I>I	AA:433		DBVPG6044:C>C		SK1:C>C		Y55:C>C	AA:438		BC187:L>L		DBVPG1373:L>L		DBVPG6765:L>L		K11:N>N		L_1374:L>L		L_1528:L>L		NCYC361:L>L		YJM975:L>L		YJM978:L>L		YJM981:L>L	AA:450		DBVPG1106:K>K		DBVPG1373:K>K		DBVPG1788:K>K		DBVPG1853:K>K		DBVPG6040:K>K		L_1374:K>K		NCYC361:K>K		UWOPS87_2421:K>K		W303:K>K		YJM978:K>K		YPS128:K>K		YPS606:K>K		YS2:K>K		YS4:K>KID:YDL049C	AA:18		322134S:A>A		DBVPG6044:A>A		NCYC110:A>A		SK1:A>A		UWOPS03_461_4:A>A		UWOPS05_217_3:A>A		UWOPS05_227_2:A>A		UWOPS87_2421:A>A		W303:A>A		Y55:A>A		YPS128:A>A	AA:21		DBVPG6044:D>D		NCYC110:D>D		SK1:D>D		Y55:D>D	AA:29		YPS128:P>P	AA:45		273614X:V>V		322134S:V>V		DBVPG6044:V>V		NCYC110:V>V		SK1:V>V		UWOPS03_461_4:V>V		UWOPS05_217_3:V>V		UWOPS05_227_2:V>V		W303:V>V		Y55:V>V	AA:53		273614X:T>T		DBVPG6044:T>T		NCYC110:T>T		SK1:T>T		Y55:T>T	AA:147		DBVPG6044:P>P		NCYC110:P>P		SK1:P>P		UWOPS87_2421:P>P		YPS128:P>P		YPS606:P>P	AA:257		273614X:L>L		UWOPS83_787_3:L>L		UWOPS87_2421:L>L		YPS128:L>L		YPS606:L>L	AA:264		UWOPS83_787_3:A>AID:YDL051W	AA:56		YS9:A>A	AA:100		YPS128:E>E		YPS606:E>E		YS4:E>E		YS9:E>E	AA:126		UWOPS03_461_4:A>A		UWOPS05_217_3:A>A	AA:264		Y9:S>SID:YDL052C	AA:60		YS9:G>G	AA:97		DBVPG1853:P>P	AA:197		273614X:V>V		DBVPG1106:V>V		DBVPG6040:V>V		DBVPG6765:V>V		L_1374:V>V		L_1528:V>V		SK1:V>V		UWOPS03_461_4:V>V		UWOPS05_227_2:V>V		Y55:V>V		YIIc17_E5:V>V		YJM975:V>V		YS9:V>V	AA:250		273614X:A>A		DBVPG1106:A>A		DBVPG6040:A>A		DBVPG6765:A>A		L_1374:A>A		L_1528:A>A		YIIc17_E5:A>A		YJM975:A>A		YS9:A>AID:YDL053C	AA:62		378604X:Q>Q	AA:75		YIIc17_E5:N>N		YJM975:N>N	AA:105		322134S:F>F		DBVPG1106:F>F		DBVPG1373:F>F		DBVPG1853:F>F		DBVPG6044:F>F		DBVPG6765:F>F		L_1374:F>F		L_1528:F>F		SK1:F>F		UWOPS87_2421:F>F		Y55:F>F		YIIc17_E5:F>F		YJM975:F>F		YS4:F>F	AA:182		322134S:L>L		DBVPG1106:L>L		DBVPG1373:L>L		DBVPG6044:L>L		DBVPG6765:L>L		L_1374:L>L		L_1528:L>L		SK1:L>L		Y55:L>L		YIIc17_E5:L>L		YJM975:L>L		YS4:L>LID:YDL056W	AA:83		K11:A>A		Y12:A>A		YPS128:A>A		YPS606:A>A	AA:86		YPS128:L>L		YPS606:L>L	AA:178		378604X:S>S		DBVPG1106:S>S		DBVPG6765:S>S		L_1374:S>S		L_1528:S>S		NCYC110:S>S		SK1:S>S		Y55:S>S		YIIc17_E5:S>S		YJM978:S>S		YJM981:S>S		YPS606:S>S	AA:244		DBVPG1106:S>S		DBVPG1373:S>S	AA:295		DBVPG1373:T>T		YPS128:T>T		YPS606:T>T	AA:306		DBVPG1373:S>S		YPS128:S>S		YPS606:S>S	AA:308		DBVPG1373:G>G		YPS128:G>G		YPS606:G>G	AA:334		UWOPS03_461_4:R>R	AA:380		DBVPG6044:P>P		SK1:P>P		Y55:P>P	AA:382		DBVPG1373:H>H	AA:390		DBVPG1106:P>P	AA:392		DBVPG6044:D>D		SK1:D>D		Y55:D>D		Y9:D>D	AA:418		K11:G>G		Y9:G>G	AA:454		DBVPG1373:L>L		DBVPG6044:L>L		K11:L>L		UWOPS03_461_4:L>L		Y55:L>L		Y9:L>L	AA:470		DBVPG1373:H>H		DBVPG6044:H>H		K11:H>H		SK1:H>H		UWOPS03_461_4:H>H		Y55:H>H		Y9:H>H		YPS128:H>H		YPS606:H>H	AA:542		DBVPG1373:S>S	AA:566		YS4:T>T	AA:671		DBVPG1373:L>L		DBVPG6040:L>L		K11:L>L		NCYC110:L>L		SK1:L>L		Y55:L>L		YPS128:L>L		YPS606:L>L		YS4:L>L	AA:688		YPS128:F>F		YPS606:F>F	AA:705		NCYC110:R>R		SK1:R>R		Y55:R>R		YPS128:R>R		YPS606:R>R	AA:728		DBVPG6040:L>L	AA:736		DBVPG6040:T>T	AA:762		SK1:L>L		Y55:L>L	AA:819		Y12:G>G	AA:829		UWOPS05_227_2:A>AID:YDL057W	AA:6		SK1:V>V		UWOPS05_227_2:V>V		Y55:V>V		YS2:V>V		YS4:V>V	AA:14		DBVPG6040:P>P	AA:70		DBVPG6040:A>A	AA:81		UWOPS83_787_3:N>N		YPS606:N>N	AA:138		UWOPS05_227_2:D>D	AA:143		273614X:V>V		DBVPG1106:V>V		DBVPG6040:V>V		SK1:V>V		UWOPS05_227_2:V>V		UWOPS83_787_3:V>V		Y55:V>V		YPS606:V>V		YS4:V>V	AA:185		273614X:I>I		DBVPG6040:I>I		SK1:I>I		UWOPS05_227_2:I>I		UWOPS83_787_3:I>I		Y55:I>I		YPS606:I>I	AA:188		273614X:A>A		DBVPG6040:A>A	AA:205		273614X:P>P		DBVPG6040:P>P		SK1:P>P		UWOPS05_227_2:P>P		UWOPS83_787_3:P>P		Y55:P>P		YPS606:P>P	AA:249		DBVPG6044:Q>Q		SK1:Q>Q		Y55:Q>Q	AA:276		UWOPS05_227_2:F>F	AA:309		UWOPS05_227_2:G>GID:YDL058W	AA:34		Y9:S>S	AA:61		DBVPG1853:P>P	AA:113		DBVPG6040:P>P	AA:144		DBVPG6044:H>H		SK1:H>H		UWOPS83_787_3:H>H		Y55:H>H		YPS128:H>H		YPS606:H>H	AA:149		DBVPG6044:F>F		SK1:F>F		UWOPS03_461_4:F>F		UWOPS83_787_3:F>F		Y12:F>F		Y55:F>F		Y9:F>F		YPS128:F>F		YPS606:F>F	AA:232		273614X:L>L		DBVPG6040:L>L		DBVPG6044:L>L		SK1:L>L		UWOPS03_461_4:L>L		UWOPS83_787_3:L>L		Y12:L>L		Y55:L>L		YPS128:L>L		YPS606:L>L		YS9:L>L	AA:234		273614X:S>S		DBVPG6040:S>S		DBVPG6044:S>S		SK1:S>S		UWOPS03_461_4:S>S		UWOPS83_787_3:S>S		Y12:S>S		Y55:S>S		YPS128:S>S		YPS606:S>S		YS9:S>S	AA:239		UWOPS03_461_4:E>E	AA:269		UWOPS03_461_4:F>F	AA:271		273614X:E>E		DBVPG6040:E>E		DBVPG6044:E>E		SK1:E>E		UWOPS03_461_4:E>E		UWOPS83_787_3:E>E		Y12:E>E		Y55:E>E		YPS128:E>E		YPS606:E>E	AA:298		DBVPG6040:R>R		UWOPS83_787_3:R>R		Y12:R>R		Y55:R>R		YPS128:R>R	AA:303		Y12:N>N	AA:323		273614X:H>H	AA:393		YS2:P>P	AA:394		DBVPG6044:I>I		SK1:I>I	AA:413		YPS128:V>V	AA:425		YS2:L>L		YS9:L>L	AA:465		DBVPG6044:S>S		SK1:S>S		UWOPS05_227_2:S>S		UWOPS87_2421:S>S		Y55:S>S		YPS128:S>S		YS2:S>S		YS4:S>S		YS9:S>S	AA:496		L_1528:E>E	AA:627		DBVPG6044:L>L		NCYC110:L>L		SK1:L>L		Y55:L>L	AA:722		DBVPG6040:E>E		YS4:E>E	AA:729		DBVPG6040:F>F		DBVPG6044:F>F		K11:F>F		NCYC110:F>F		SK1:F>F		UWOPS05_227_2:F>F		UWOPS83_787_3:F>F		Y55:F>F		YPS128:F>F		YS4:F>F	AA:736		DBVPG6040:Q>Q		YS4:Q>Q	AA:896		Y12:N>N		YS4:N>N	AA:942		DBVPG6040:E>E		UWOPS03_461_4:E>E		UWOPS05_217_3:E>E		UWOPS05_227_2:E>E		UWOPS83_787_3:E>E		Y12:E>E		YPS128:E>E		YPS606:E>E		YS4:E>E	AA:999		DBVPG6040:L>L		Y12:L>L		YS4:L>L	AA:1011		DBVPG6044:E>E		SK1:E>E		Y55:E>E	AA:1038		DBVPG6040:K>K		DBVPG6044:K>K		NCYC110:K>K		SK1:K>K		UWOPS03_461_4:K>K		UWOPS05_217_3:K>K		UWOPS83_787_3:K>K		Y55:K>K		YPS606:K>K		YS4:K>K	AA:1043		DBVPG6040:S>S		UWOPS83_787_3:S>S		YS4:S>S	AA:1089		DBVPG6044:A>A		NCYC110:A>A		SK1:A>A		Y55:A>A	AA:1129		DBVPG6040:L>L		DBVPG6044:L>L		NCYC110:L>L		SK1:L>L		UWOPS83_787_3:L>L		Y55:L>L		YS4:L>L	AA:1144		L_1528:R>R	AA:1150		K11:L>L		Y12:L>L	AA:1167		YPS606:Q>Q	AA:1291		DBVPG6040:Y>Y		K11:Y>Y		Y12:Y>Y		Y9:Y>Y		YS4:Y>Y		YS9:Y>Y	AA:1309		DBVPG6044:T>T		NCYC110:T>T		SK1:T>T		Y55:T>T	AA:1330		DBVPG6040:E>E		K11:E>E		Y12:E>E		Y9:E>E		YS9:E>E	AA:1380		DBVPG1373:T>T	AA:1471		DBVPG6044:Q>Q		SK1:Q>Q		UWOPS03_461_4:Q>Q		UWOPS05_227_2:Q>Q		Y12:Q>Q		Y55:Q>Q		Y9:Q>Q		YS4:Q>Q	AA:1706		DBVPG1853:T>T		DBVPG6044:T>T		SK1:T>T		UWOPS83_787_3:T>T		Y55:T>T		YPS128:T>T		YS2:T>T	AA:1733		YS2:E>E	AA:1741		DBVPG1853:I>I		UWOPS83_787_3:I>I	AA:1766		UWOPS83_787_3:G>G	AA:1788		DBVPG6044:Q>Q		SK1:Q>Q		UWOPS05_217_3:Q>Q		UWOPS05_227_2:Q>Q		UWOPS83_787_3:Q>Q		Y55:Q>Q		YPS128:Q>Q		YS2:Q>QID:YDL059C	AA:3		DBVPG6044:I>I		SK1:I>I		Y55:I>I	AA:79		DBVPG6044:G>G		SK1:G>G		Y55:G>G	AA:158		SK1:G>G		UWOPS87_2421:G>G		Y55:G>G	AA:197		UWOPS03_461_4:V>V		UWOPS87_2421:V>V	AA:211		UWOPS03_461_4:N>N		UWOPS87_2421:N>NID:YDL060W	AA:120		DBVPG6044:S>S		SK1:S>S		UWOPS03_461_4:S>S		UWOPS05_227_2:S>S		UWOPS83_787_3:S>S		Y12:S>S		Y55:S>S		YPS128:S>S		YS4:S>S	AA:163		UWOPS87_2421:S>S	AA:303		NCYC110:L>L		SK1:L>L		Y12:L>L		Y55:L>L		YPS128:L>L		YPS606:L>L	AA:332		UWOPS87_2421:D>D	AA:336		UWOPS87_2421:V>V	AA:344		273614X:D>D		NCYC110:D>D		SK1:D>D		UWOPS83_787_3:D>D		UWOPS87_2421:D>D		Y12:D>D		Y55:D>D		Y9:D>D		YPS128:D>D		YPS606:D>D		YS4:D>D	AA:393		NCYC110:P>P		SK1:P>P		Y55:P>P	AA:401		UWOPS03_461_4:A>A	AA:417		K11:A>A		UWOPS03_461_4:A>A		Y12:A>A		Y9:A>A		YS9:A>A	AA:449		UWOPS87_2421:Y>Y	AA:493		Y9:S>S		YPS128:S>S		YPS606:S>S	AA:494		273614X:A>A	AA:558		DBVPG6044:R>R		NCYC110:R>R		SK1:R>R		UWOPS03_461_4:R>R		UWOPS05_227_2:R>R		UWOPS83_787_3:R>R		UWOPS87_2421:R>R		Y55:R>R		YPS128:R>R		YPS606:R>R		YS9:R>R	AA:583		UWOPS03_461_4:G>G		UWOPS05_227_2:G>G	AA:593		UWOPS03_461_4:V>V		UWOPS05_227_2:V>V	AA:598		DBVPG6044:L>L		NCYC110:L>L		SK1:L>L		UWOPS03_461_4:L>L		UWOPS05_227_2:L>L		UWOPS83_787_3:L>L		Y55:L>L		YPS606:L>L	AA:610		DBVPG6044:S>S		NCYC110:S>S		SK1:S>S		UWOPS05_227_2:S>S		UWOPS83_787_3:S>S		Y55:S>S		YPS606:S>S		YS9:S>S	AA:627		YPS606:P>P	AA:635		UWOPS05_227_2:S>S	AA:654		YS9:A>A	AA:734		Y12:P>PID:YDL063C	AA:2		UWOPS03_461_4:G>G	AA:37		DBVPG1853:L>L	AA:49		322134S:D>D		DBVPG6044:P>P		DBVPG6765:D>D		SK1:P>P		UWOPS83_787_3:P>P		Y55:P>P		YS4:P>P		YS9:P>P	AA:85		UWOPS83_787_3:L>L		YS9:L>L	AA:95		YS4:V>V	AA:139		DBVPG1853:L>L	AA:148		L_1374:L>L	AA:203		DBVPG1853:L>L		DBVPG6044:L>L		NCYC110:L>L		SK1:L>L		UWOPS05_227_2:L>L		UWOPS83_787_3:L>L		Y12:L>L		Y55:L>L		Y9:L>L	AA:207		DBVPG1853:G>G	AA:238		DBVPG6044:S>S		NCYC110:S>S		SK1:S>S		UWOPS83_787_3:S>S		UWOPS87_2421:S>S		Y12:S>S		Y55:S>S		Y9:S>S	AA:248		DBVPG1788:N>N		DBVPG1853:N>N		DBVPG6044:N>N		DBVPG6765:N>N		L_1374:N>N		L_1528:N>N		NCYC110:N>N		SK1:N>N		UWOPS03_461_4:N>N		UWOPS83_787_3:N>N		UWOPS87_2421:N>N		Y12:N>N		Y55:N>N		Y9:N>N		YJM975:N>N		YJM978:N>N		YS9:N>N	AA:293		Y12:S>S		Y9:S>S	AA:313		UWOPS03_461_4:G>G	AA:385		K11:L>L		Y12:L>L		YS4:L>L	AA:461		UWOPS87_2421:G>G		YPS128:G>G		YPS606:G>G	AA:470		UWOPS83_787_3:L>L	AA:489		UWOPS83_787_3:F>F	AA:496		YS2:E>E	AA:497		SK1:L>L		Y55:L>L	AA:504		K11:V>V	AA:521		UWOPS87_2421:I>I		YPS128:I>I		YPS606:I>I	AA:569		K11:S>S	AA:589		DBVPG6044:N>N		K11:N>N		NCYC110:N>N		SK1:N>N		UWOPS83_787_3:N>N		UWOPS87_2421:N>N		Y55:N>N		YPS128:N>N		YPS606:N>N	AA:598		DBVPG6044:C>C		K11:C>C		NCYC110:C>C		SK1:C>C		UWOPS83_787_3:C>C		UWOPS87_2421:C>C		Y55:C>C		YPS128:C>C		YPS606:C>CID:YDL064W	AA:61		DBVPG6044:T>T		NCYC110:T>T		SK1:T>T		Y55:T>T		Y9:T>T	AA:71		DBVPG6044:K>K		NCYC110:K>K		SK1:K>K		UWOPS03_461_4:K>K		UWOPS05_217_3:K>K		UWOPS05
[truncated: 1,200,000 more chars]
