# Supplementary material for: Proteome-wide evidence for enhanced positive Darwinian selection within intrinsically disordered regions in proteins
Source: Genome Biol. 2011 Jul 19;12(7):R65. doi: 10.1186/gb-2011-12-7-r65 (PMC3218827; doi:10.1186/gb-2011-12-7-r65)
Supplement: Additional file 5 — Synonymous SNPs in S. paradoxus genes studied. The identity of each affected amino acid in each affected strain is shown for each of 3,756 genes. [file gb-2011-12-7-r65-S5.RTF]

ID:YAL001C	AA:20		A12:T>T		DBVPG6304:T>T		YPS138:T>T	AA:24		UWOPS91_917_1:V>V	AA:28		UWOPS91_917_1:L>L	AA:35		A12:S>S		DBVPG6304:S>S		UWOPS91_917_1:S>S		YPS138:S>S	AA:61		A12:E>E		DBVPG6304:E>E		UFRJ50816:E>E		UWOPS91_917_1:E>E		YPS138:E>E	AA:134		A12:R>R		DBVPG6304:R>R		UFRJ50816:R>R	AA:140		DBVPG4650:D>D	AA:150		A12:D>D	AA:155		N_43:S>S		N_44:S>S		N_45:S>S	AA:190		UWOPS91_917_1:N>N	AA:201		IFO1804:D>D	AA:249		A12:S>S		UFRJ50816:S>S		YPS138:S>S	AA:251		Q89_8:I>I		UWOPS91_917_1:I>I	AA:269		A12:G>G		UFRJ50816:G>G		YPS138:G>G	AA:270		UWOPS91_917_1:S>S	AA:275		UWOPS91_917_1:Y>Y	AA:292		IFO1804:L>L		N_44:L>L		N_45:L>L	AA:296		A12:S>S		A4:S>S		UFRJ50816:S>S		UWOPS91_917_1:S>S		YPS138:S>S	AA:309		A12:K>K		A4:K>K		UFRJ50816:K>K		UWOPS91_917_1:K>K		YPS138:K>K	AA:324		UWOPS91_917_1:I>I	AA:327		A12:E>E		A4:E>E		IFO1804:E>E		N_44:E>E		N_45:E>E		YPS138:E>E	AA:345		A12:D>D		A4:D>D		UWOPS91_917_1:D>D		YPS138:D>D	AA:350		A12:R>R		A4:R>R		UWOPS91_917_1:R>R		YPS138:R>R	AA:379		A12:A>A		A4:A>A	AA:391		IFO1804:N>N		N_44:N>N		N_45:N>N	AA:404		IFO1804:N>N		N_45:N>N	AA:412		UWOPS91_917_1:V>V	AA:443		DBVPG6304:S>S	AA:469		A12:G>G		A4:G>G		DBVPG6304:G>G		UFRJ50791:G>G		UWOPS91_917_1:G>G	AA:471		DBVPG6304:S>S	AA:480		UWOPS91_917_1:R>R	AA:515		A12:T>T		A4:T>T		DBVPG6304:T>T		N_44:T>T		N_45:T>T		UFRJ50791:T>T		UWOPS91_917_1:T>T		YPS138:T>T	AA:539		A4:L>L		DBVPG6304:L>L		UFRJ50791:L>L		UWOPS91_917_1:L>L		YPS138:L>L	AA:573		DBVPG6304:T>T		UFRJ50791:T>T		UWOPS91_917_1:T>T		YPS138:T>T	AA:585		UWOPS91_917_1:Y>Y	AA:591		UWOPS91_917_1:Y>Y	AA:602		UFRJ50791:L>L		YPS138:L>L	AA:605		N_44:R>R		N_45:R>R		UWOPS91_917_1:R>R	AA:610		DBVPG6304:S>S		UFRJ50791:S>S		UWOPS91_917_1:S>S		YPS138:S>S	AA:612		UWOPS91_917_1:L>L	AA:615		N_43:A>A		N_44:A>A		N_45:A>A		UFRJ50791:A>A		UWOPS91_917_1:A>A		YPS138:A>A	AA:647		UFRJ50791:G>G	AA:654		A4:I>I		UFRJ50791:I>I		YPS138:I>I	AA:659		UWOPS91_917_1:S>S	AA:677		UWOPS91_917_1:K>K	AA:685		A4:S>S		UFRJ50791:S>S		UWOPS91_917_1:S>S		YPS138:S>S	AA:688		UWOPS91_917_1:P>P	AA:689		A12:I>I		A4:I>I		UFRJ50791:I>I		YPS138:I>I	AA:710		A12:T>T		A4:T>T		N_43:T>T		N_44:T>T		N_45:T>T		UFRJ50816:T>T		UWOPS91_917_1:T>T		YPS138:T>T	AA:716		UWOPS91_917_1:F>F	AA:726		A12:T>T		A4:T>T		UFRJ50816:T>T		YPS138:T>T	AA:735		A12:G>G		UFRJ50816:G>G		YPS138:G>G	AA:755		YPS138:F>F	AA:812		A12:I>I		A4:I>I		N_43:I>I		N_44:I>I		N_45:I>I		UFRJ50816:I>I		YPS138:I>I	AA:824		A12:T>T		A4:T>T	AA:831		A12:F>F		A4:F>F		UFRJ50816:F>F		YPS138:F>F	AA:835		A12:L>L		A4:L>L		UFRJ50816:L>L		YPS138:L>L	AA:845		A12:D>D		A4:D>D		UFRJ50816:D>D		YPS138:D>D	AA:882		N_43:E>E	AA:887		N_43:S>S		N_45:S>S	AA:901		N_43:D>D		N_45:D>D	AA:904		A12:Y>Y		A4:Y>Y		DBVPG6304:Y>Y		N_43:Y>Y		N_45:Y>Y		UFRJ50816:Y>Y		YPS138:Y>Y	AA:916		A12:P>P		A4:P>P		DBVPG6304:P>P		UFRJ50816:P>P		UWOPS91_917_1:P>P		YPS138:P>P	AA:925		UWOPS91_917_1:Y>Y	AA:927		A12:K>K		A4:K>K		DBVPG6304:K>K		UFRJ50816:K>K		UWOPS91_917_1:K>K		YPS138:K>K	AA:936		UWOPS91_917_1:I>I	AA:958		DBVPG6304:R>R	AA:993		UWOPS91_917_1:L>L	AA:1007		A12:S>S		A4:S>S		DBVPG6304:S>S		N_43:S>S		N_45:S>S		UFRJ50816:S>S		Y7:S>S	AA:1011		A12:L>L		A4:L>L		DBVPG6304:L>L		UFRJ50816:L>L		UWOPS91_917_1:L>L	AA:1021		A12:V>V		A4:V>V		DBVPG6304:V>V		UFRJ50816:V>V		UWOPS91_917_1:V>V	AA:1022		N_43:S>S		N_45:S>S	AA:1024		A12:P>P		A4:P>P		DBVPG6304:P>P		UFRJ50816:P>P		UWOPS91_917_1:P>P	AA:1055		UWOPS91_917_1:G>G	AA:1063		A12:Y>Y		A4:Y>Y		DBVPG6304:Y>Y		UFRJ50816:Y>Y	AA:1077		A12:V>V		A4:V>V		DBVPG6304:V>V		Q89_8:V>V		UFRJ50816:V>V		UWOPS91_917_1:V>VID:YAL005C	AA:13		A4:Y>Y		N_43:P>P		N_44:P>P		N_45:P>P		UFRJ50791:Y>Y		YPS138:Y>Y	AA:35		A12:G>G		UFRJ50791:G>G	AA:41		A12:S>S	AA:45		A12:N>N		N_43:N>N		N_44:N>N		N_45:N>N		UFRJ50791:N>N	AA:72		A12:T>T		DBVPG6304:T>T		UFRJ50791:T>T	AA:140		A12:K>K		DBVPG6304:K>K		UWOPS91_917_1:K>K	AA:149		UWOPS91_917_1:S>S	AA:156		UWOPS91_917_1:V>V	AA:162		A12:I>I	AA:176		UWOPS91_917_1:V>V	AA:180		DBVPG6304:A>A		UWOPS91_917_1:A>A	AA:199		N_43:A>A		N_44:A>A		N_45:A>A	AA:212		UWOPS91_917_1:Q>Q	AA:238		DBVPG6304:V>V	AA:256		N_43:L>L		N_44:L>L		N_45:L>L		UWOPS91_917_1:L>L	AA:267		N_43:L>L		N_44:L>L		N_45:L>L		UWOPS91_917_1:L>L	AA:285		A12:F>F		DBVPG6304:S>S	AA:288		DBVPG6304:P>P	AA:289		UWOPS91_917_1:E>E	AA:307		UWOPS91_917_1:S>S	AA:310		UWOPS91_917_1:V>V	AA:312		N_43:V>V		N_44:V>V		N_45:V>V		UWOPS91_917_1:V>V	AA:342		DBVPG6304:L>L		UWOPS91_917_1:L>L	AA:369		A12:A>A		YPS138:A>A	AA:373		A12:L>L		UWOPS91_917_1:L>L		YPS138:L>L	AA:379		N_43:E>E		N_44:E>E	AA:407		CBS432:H>H		CBS5829:H>H		DBVPG4650:H>H		N_43:H>H		N_44:H>H		Q59_1:H>H		UWOPS91_917_1:H>H		YPS138:H>H	AA:447		N_43:L>L		N_44:L>L		UWOPS91_917_1:L>L	AA:473		CBS432:I>I		CBS5829:I>I		DBVPG4650:I>I		Q59_1:I>I	AA:483		YPS138:T>T	AA:500		UWOPS91_917_1:V>V	AA:501		DBVPG4650:T>T	AA:511		A4:L>L		UWOPS91_917_1:L>L		YPS138:L>L	AA:516		A4:T>T		YPS138:T>T	AA:522		A4:L>L		YPS138:L>L	AA:531		A4:T>T		UWOPS91_917_1:T>T		YPS138:T>T	AA:554		N_44:F>F		N_45:F>F	AA:566		A4:D>D		YPS138:D>D	AA:583		N_44:P>P	AA:606		A4:S>S		UFRJ50791:S>S		YPS138:S>S	AA:608		CBS432:T>T		CBS5829:T>T		N_44:T>T		N_45:T>T		Q32_3:T>T		Q95_3:T>T		S36_7:T>TID:YAL007C	AA:3		DBVPG6304:Y>Y		UFRJ50816:Y>Y		YPS138:Y>Y	AA:96		A12:L>L		DBVPG6304:L>L		UFRJ50816:L>L		YPS138:L>L	AA:100		DBVPG6304:V>V		N_45:V>V		UFRJ50816:V>V		YPS138:V>V	AA:113		N_45:F>F	AA:127		YPS138:S>S	AA:129		A12:K>K		DBVPG6304:K>K		UFRJ50816:K>K		YPS138:K>K	AA:158		A12:Y>Y		DBVPG6304:Y>Y		UFRJ50791:Y>Y		UFRJ50816:Y>Y		YPS138:Y>Y	AA:166		N_45:E>E	AA:173		N_45:L>L	AA:174		A12:C>C		DBVPG6304:C>C		UFRJ50816:C>C		YPS138:C>C	AA:192		N_44:A>A		N_45:A>A	AA:208		A12:P>P		DBVPG6304:P>P		UFRJ50791:P>P		YPS138:P>PID:YAL008W	AA:1		DBVPG6304:H>H		UFRJ50791:H>H		UFRJ50816:H>H	AA:24		A12:V>V		DBVPG6304:V>V		UFRJ50791:V>V		UFRJ50816:V>V		UWOPS91_917_1:V>V	AA:27		A12:Q>Q		DBVPG6304:Q>Q		UFRJ50791:Q>Q		UFRJ50816:Q>Q		UWOPS91_917_1:Q>Q	AA:47		A12:S>S		DBVPG6304:S>S		N_44:S>S		N_45:S>S		UFRJ50791:S>S		UFRJ50816:S>S		UWOPS91_917_1:S>S	AA:58		A12:Q>Q		DBVPG6304:Q>Q		UFRJ50791:Q>Q		UFRJ50816:Q>Q		UWOPS91_917_1:Q>Q	AA:91		A12:A>A		DBVPG6304:A>A		UFRJ50791:A>A		UFRJ50816:A>A		UWOPS91_917_1:A>A	AA:107		A12:L>L		DBVPG6304:L>L		N_44:L>L		N_45:L>L		UWOPS91_917_1:L>L	AA:113		A12:->-		A4:->-		DBVPG6304:->-		UWOPS91_917_1:->-	AA:121		A12:N>N		A4:N>N		DBVPG6304:N>N		UWOPS91_917_1:N>N	AA:149		A12:P>P		A4:P>P		DBVPG6304:P>P		UWOPS91_917_1:P>P	AA:156		N_43:V>V		N_44:V>V		N_45:V>V	AA:160		A12:N>N		A4:N>N		UWOPS91_917_1:N>NID:YAL009W	AA:9		A12:H>H		A4:H>H		UFRJ50816:H>H	AA:17		A4:T>T	AA:42		A12:T>T		A4:T>T		UFRJ50816:T>T	AA:91		UWOPS91_917_1:V>V	AA:108		CBS5829:Q>Q		DBVPG4650:Q>Q		Q62_5:Q>Q		Y6_5:Q>Q		Y7:Q>Q		Z1_1:Q>Q	AA:116		A12:G>G		DBVPG6304:G>G		N_43:G>G		N_44:G>G		N_45:G>G		UFRJ50816:G>G	AA:130		UWOPS91_917_1:S>S	AA:143		A12:I>I		DBVPG6304:I>I		N_44:I>I		N_45:I>I		UFRJ50816:I>I		UWOPS91_917_1:I>I	AA:152		N_44:L>L		N_45:L>L	AA:162		A4:L>L	AA:169		A12:S>S		A4:S>S		DBVPG6304:S>S		UFRJ50816:S>S		UWOPS91_917_1:S>S	AA:207		Y7:L>L	AA:216		UFRJ50816:F>F	AA:221		UFRJ50816:G>G	AA:232		UFRJ50816:P>P	AA:248		A12:A>A		A4:A>A		DBVPG6304:A>A		UWOPS91_917_1:A>A	AA:251		A12:R>R		A4:R>R		DBVPG6304:R>R		UFRJ50816:R>RID:YAL010C	AA:20		UFRJ50816:T>T		UWOPS91_917_1:N>N	AA:28		A12:S>S		DBVPG6304:S>S		UFRJ50791:S>S		UFRJ50816:S>S		YPS138:S>S	AA:54		A12:S>S		DBVPG6304:S>S		UFRJ50791:S>S		UFRJ50816:S>S		UWOPS91_917_1:S>S		YPS138:S>S	AA:56		A12:L>L		DBVPG6304:L>L		UFRJ50791:L>L		UFRJ50816:L>L		UWOPS91_917_1:L>L		YPS138:L>L	AA:62		A12:G>G		DBVPG6304:G>G		UFRJ50791:G>G		UFRJ50816:G>G		UWOPS91_917_1:G>G		YPS138:G>G	AA:68		A12:L>L		DBVPG6304:L>L		UFRJ50791:L>L		UFRJ50816:L>L		YPS138:L>L	AA:77		A12:L>L		DBVPG6304:L>L		UFRJ50791:L>L		UFRJ50816:L>L		UWOPS91_917_1:L>L		YPS138:L>L	AA:108		A12:S>S		DBVPG6304:S>S		UFRJ50791:S>S		UFRJ50816:S>S		UWOPS91_917_1:S>S		YPS138:S>S	AA:137		CBS5829:L>L		IFO1804:L>L		KPN3829:L>L		N_43:L>L		N_44:L>L		N_45:L>L		Q59_1:L>L		S36_7:L>L		T21_4:L>L		Z1_1:L>L	AA:149		IFO1804:R>R		N_43:R>R		N_44:R>R		N_45:R>R	AA:156		A12:V>V		DBVPG6304:V>V		UFRJ50791:V>V		UFRJ50816:V>V		UWOPS91_917_1:V>V		YPS138:V>V	AA:161		A12:I>I		DBVPG6304:I>I		UFRJ50791:I>I		UFRJ50816:I>I		YPS138:I>I	AA:166		A12:Q>Q		DBVPG6304:Q>Q		KPN3829:D>D		N_43:D>D		N_44:D>D		N_45:D>D		UFRJ50791:D>D		UFRJ50816:Q>Q		UWOPS91_917_1:Q>Q		YPS138:D>D	AA:172		A12:T>T		DBVPG6304:T>T		UFRJ50791:T>T		UFRJ50816:T>T		UWOPS91_917_1:T>T		YPS138:T>T	AA:173		N_43:E>E		N_45:E>E	AA:177		N_43:H>H		N_44:H>H		N_45:H>H	AA:179		A12:K>K		DBVPG6304:K>K		N_43:K>K		N_44:K>K		N_45:K>K		UFRJ50791:K>K		UFRJ50816:K>K		UWOPS91_917_1:K>K		YPS138:K>K	AA:184		A12:C>C		DBVPG6304:C>C		UFRJ50816:C>C		YPS138:C>C	AA:193		A12:S>S		DBVPG6304:S>S		UFRJ50816:S>S		UWOPS91_917_1:S>S		YPS138:S>S	AA:203		A12:F>F		CBS5829:F>F		DBVPG4650:F>F		DBVPG6304:F>F		KPN3829:F>F		N_17:F>F		N_43:F>F		N_44:F>F		Q59_1:F>F		T21_4:F>F		UFRJ50816:F>F		UWOPS91_917_1:F>F		Y6_5:F>F		YPS138:F>F		Z1_1:F>F	AA:205		A12:S>S	AA:215		A12:S>S		DBVPG6304:S>S		UFRJ50816:S>S		UWOPS91_917_1:S>S		YPS138:S>S	AA:227		A12:T>T		DBVPG6304:T>T		YPS138:T>T	AA:233		DBVPG6304:L>L		UFRJ50816:G>G		YPS138:L>L	AA:234		A12:T>T	AA:242		A12:R>R		DBVPG6304:R>R		N_43:R>R		N_45:R>R		UWOPS91_917_1:R>R	AA:246		DBVPG6304:S>S		YPS138:S>S	AA:255		A12:G>G		DBVPG6304:G>G		UFRJ50816:G>G		UWOPS91_917_1:G>G		YPS138:G>G	AA:259		A12:E>E		DBVPG6304:E>E		UWOPS91_917_1:E>E		YPS138:E>E	AA:270		A12:L>L		DBVPG6304:L>L		UFRJ50816:L>L		UWOPS91_917_1:L>L		YPS138:L>L	AA:278		A12:T>T		DBVPG6304:T>T		UFRJ50816:T>T		UWOPS91_917_1:T>T	AA:285		DBVPG6304:I>I		UFRJ50816:I>I		YPS138:I>I	AA:288		A12:G>G	AA:294		A12:T>T		DBVPG6304:T>T		UFRJ50816:T>T	AA:299		A12:E>E		DBVPG6304:E>E		UFRJ50816:E>E	AA:315		A12:N>N		DBVPG6304:N>N		N_44:N>N		UFRJ50816:N>N		UWOPS91_917_1:N>N	AA:361		UWOPS91_917_1:S>S	AA:362		DBVPG6304:D>D		UFRJ50816:D>D	AA:367		UWOPS91_917_1:K>K	AA:399		A12:A>A		A4:A>A		DBVPG6304:A>A		N_44:A>A		UFRJ50816:A>A		UWOPS91_917_1:A>A	AA:407		A12:S>S		A4:S>S		DBVPG6304:S>S		UFRJ50816:S>S		UWOPS91_917_1:S>S	AA:415		N_44:Q>Q	AA:436		A12:L>L		A4:L>L		DBVPG6304:L>L		UFRJ50816:L>L	AA:440		N_44:T>T		N_45:T>T	AA:443		UFRJ50816:T>T		UWOPS91_917_1:T>T	AA:447		UWOPS91_917_1:S>S	AA:464		N_44:S>S		N_45:S>S	AA:468		A12:I>I		A4:I>I		DBVPG6304:I>I	AA:486		A12:L>L		A4:L>L		DBVPG6304:L>L		UFRJ50816:L>L		UWOPS91_917_1:L>L	AA:493		UWOPS91_917_1:L>LID:YAL011W	AA:3		KPN3829:S>S		N_17:S>S	AA:24		A4:R>R		DBVPG6304:R>R		UFRJ50791:R>R		UFRJ50816:R>R		UWOPS91_917_1:R>R		YPS138:R>R	AA:27		A12:S>S		A4:S>S		DBVPG6304:S>S		IFO1804:S>S		KPN3829:S>S		N_17:S>S		N_45:S>S		UFRJ50791:S>S		UFRJ50816:S>S		UWOPS91_917_1:S>S		YPS138:S>S	AA:33		A12:T>T		A4:T>T		DBVPG6304:T>T		UFRJ50791:T>T		UFRJ50816:T>T		UWOPS91_917_1:T>T		YPS138:T>T	AA:37		A12:I>I		A4:I>I		DBVPG6304:I>I		UFRJ50791:I>I		UFRJ50816:I>I		UWOPS91_917_1:I>I		YPS138:I>I	AA:42		A12:I>I		A4:I>I		DBVPG6304:I>I		UFRJ50791:I>I		UFRJ50816:I>I		UWOPS91_917_1:I>I		YPS138:I>I	AA:67		UWOPS91_917_1:G>G	AA:90		A12:K>K		UFRJ50791:K>K		UFRJ50816:K>K		UWOPS91_917_1:K>K		YPS138:K>K	AA:108		A12:T>T		A4:T>T		N_43:T>T		N_45:T>T		UFRJ50791:T>T		YPS138:T>T	AA:112		A12:G>G		A4:G>G		UFRJ50791:G>G		UWOPS91_917_1:G>G		YPS138:G>G	AA:124		N_43:S>S		N_45:S>S	AA:126		A12:G>G		A4:G>G		UFRJ50791:G>G		YPS138:G>G	AA:134		A12:R>R		A4:R>R		UFRJ50791:R>R		UFRJ50816:R>R		UWOPS91_917_1:R>R		YPS138:R>R	AA:161		N_43:H>H		N_45:H>H	AA:163		A12:S>S		A4:S>S		UFRJ50791:S>S		UFRJ50816:S>S		YPS138:S>S	AA:171		A12:S>S		A4:S>S		UFRJ50791:S>S		UFRJ50816:S>S		YPS138:S>S	AA:176		A12:F>F		A4:F>F		UFRJ50791:F>F		UFRJ50816:F>F		UWOPS91_917_1:F>F		YPS138:F>F	AA:215		UFRJ50791:L>L		UFRJ50816:L>L	AA:216		A12:L>L	AA:220		A12:L>L		UFRJ50791:L>L		UFRJ50816:L>L	AA:229		A12:L>L		UFRJ50791:L>L		UFRJ50816:L>L	AA:264		N_43:L>L		N_45:L>L	AA:267		A12:R>R		UFRJ50791:R>R		UFRJ50816:R>R	AA:269		A12:G>G		UFRJ50791:G>G		UFRJ50816:G>G	AA:275		N_43:S>S		N_45:S>S	AA:283		N_43:H>H		N_44:H>H		N_45:H>H		UFRJ50791:H>H		UFRJ50816:H>H	AA:309		A12:F>F		UFRJ50791:F>F		UFRJ50816:F>F	AA:322		A12:Q>Q		UFRJ50791:Q>Q		UFRJ50816:Q>Q	AA:350		A12:C>C		N_44:C>C		N_45:C>C		UFRJ50791:C>C		UFRJ50816:C>C	AA:352		N_44:P>P		N_45:P>P	AA:353		A12:->-		UFRJ50791:->-		UFRJ50816:->-	AA:395		YPS138:L>L	AA:432		DBVPG6304:Y>Y		UFRJ50816:Y>Y		UWOPS91_917_1:Y>Y		YPS138:Y>Y	AA:470		DBVPG6304:F>F		UFRJ50816:F>F		YPS138:F>F	AA:480		UWOPS91_917_1:L>L	AA:487		UWOPS91_917_1:Q>Q	AA:539		A12:G>G		DBVPG6304:G>G		UFRJ50816:G>G		UWOPS91_917_1:G>G		YPS138:G>G	AA:547		A12:I>I		DBVPG6304:I>I		UFRJ50816:I>I	AA:563		A12:A>A		DBVPG6304:A>A		UFRJ50816:A>A	AA:582		UWOPS91_917_1:N>N	AA:621		A12:->-		DBVPG6304:->-		UFRJ50816:->-		UWOPS91_917_1:->-	AA:625		A12:F>F	AA:634		A12:F>F		DBVPG6304:F>F		UFRJ50816:F>F		UWOPS91_917_1:F>FID:YAL013W	AA:5		UFRJ50816:C>C	AA:35		DBVPG6304:V>V		YPS138:V>V	AA:47		UWOPS91_917_1:T>T	AA:50		UWOPS91_917_1:S>S	AA:76		IFO1804:T>T	AA:113		CBS5829:L>L	AA:131		YPS138:L>L	AA:149		CBS5829:F>F		DBVPG4650:F>F		IFO1804:F>F		N_44:F>F		N_45:F>F		Q62_5:F>F		S36_7:F>F		UFRJ50816:F>F		Y6_5:F>F		YPS138:F>F	AA:157		N_44:V>V		N_45:V>V	AA:184		Q62_5:L>L	AA:188		UFRJ50816:V>V	AA:197		UFRJ50816:A>A	AA:226		UWOPS91_917_1:V>V	AA:228		DBVPG6304:R>R		UFRJ50791:R>R		UFRJ50816:R>R		UWOPS91_917_1:R>R	AA:239		N_44:V>V	AA:261		DBVPG6304:G>G		N_43:G>G		N_44:G>G		UFRJ50791:G>G		UFRJ50816:G>G		UWOPS91_917_1:G>G	AA:266		UWOPS91_917_1:V>V	AA:281		UWOPS91_917_1:S>S	AA:286		DBVPG6304:P>P		UWOPS91_917_1:P>P	AA:297		DBVPG6304:V>V		UFRJ50791:V>V		UFRJ50816:V>V	AA:306		DBVPG6304:V>V		UFRJ50791:V>V		UFRJ50816:V>V		UWOPS91_917_1:V>V	AA:325		UWOPS91_917_1:C>C	AA:327		UWOPS91_917_1:L>L	AA:332		N_43:A>A		N_45:A>A	AA:338		DBVPG6304:V>V		UFRJ50791:V>V		UFRJ50816:V>V		UWOPS91_917_1:V>V	AA:339		N_43:E>E	AA:349		UWOPS91_917_1:S>S	AA:364		DBVPG6304:P>P		UFRJ50791:P>P		UFRJ50816:P>P	AA:398		DBVPG6304:V>V		UFRJ50791:V>V		UFRJ50816:V>VID:YAL016W	AA:37		UWOPS91_917_1:S>S	AA:83		DBVPG6304:L>L		UFRJ50791:L>L		UWOPS91_917_1:L>L	AA:87		DBVPG6304:V>V		UFRJ50791:V>V		UWOPS91_917_1:V>V	AA:95		N_44:F>F		N_45:F>F	AA:100		DBVPG6304:K>K		UFRJ50791:K>K	AA:129		DBVPG6304:S>S		UFRJ50791:S>S		UFRJ50816:S>S		UWOPS91_917_1:S>S	AA:195		A4:V>V		UFRJ50791:V>V		UFRJ50816:V>V		YPS138:V>V	AA:218		A4:D>D		UFRJ50791:D>D		UFRJ50816:D>D		YPS138:D>D	AA:227		DBVPG6304:->-	AA:256		CBS5829:H>H		T21_4:H>H		Z1_1:H>H	AA:300		CBS5829:S>S		T21_4:S>S		Z1_1:S>S	AA:301		A4:K>K		YPS138:K>K	AA:391		YPS138:V>V	AA:418		N_43:G>G		N_45:G>G	AA:421		N_43:L>L		N_45:L>L	AA:458		Y6_5:V>V	AA:507		N_43:->-		N_45:->-	AA:527		N_43:F>F		N_45:F>F	AA:563		DBVPG6304:G>G		UFRJ50816:G>G		UWOPS91_917_1:G>G	AA:575		DBVPG6304:G>G		UFRJ50816:G>G		UWOPS91_917_1:G>G	AA:583		DBVPG6304:L>L		N_45:L>L		UFRJ50816:L>L		UWOPS91_917_1:L>L	AA:589		DBVPG6304:L>L		UFRJ50816:L>L	AA:617		UWOPS91_917_1:V>VID:YAL018C	AA:6		UFRJ50816:E>E		UWOPS91_917_1:E>E	AA:22		A4:V>V		UFRJ50816:V>V		UWOPS91_917_1:V>V	AA:25		A4:P>P		UFRJ50816:P>P		UWOPS91_917_1:P>P	AA:29		A4:H>H		UFRJ50816:H>H		UWOPS91_917_1:H>H	AA:46		A4:G>G		UFRJ50816:G>G		UWOPS91_917_1:G>G		YPS138:G>G	AA:54		A4:I>I		UFRJ50816:I>I		UWOPS91_917_1:I>I		YPS138:I>I	AA:62		UWOPS91_917_1:S>S	AA:66		A4:G>G		N_43:G>G		N_45:G>G		UFRJ50816:G>G		UWOPS91_917_1:G>G		YPS138:G>G	AA:69		N_43:L>L		N_45:L>L	AA:71		UWOPS91_917_1:I>I	AA:73		A4:E>E		N_43:E>E		N_45:E>E		UFRJ50816:E>E		UWOPS91_917_1:E>E		YPS138:E>E	AA:94		UWOPS91_917_1:I>I	AA:96		UWOPS91_917_1:P>P	AA:102		UWOPS91_917_1:F>F	AA:106		A4:G>G		UFRJ50816:G>G		YPS138:G>G	AA:110		UWOPS91_917_1:F>F	AA:121		A4:A>A		UFRJ50816:A>A		UWOPS91_917_1:A>A		YPS138:A>A	AA:124		UWOPS91_917_1:L>L	AA:130		UWOPS91_917_1:K>K	AA:135		UWOPS91_917_1:Y>Y	AA:136		N_43:A>A		N_45:A>A	AA:139		A4:Y>Y		N_43:Y>Y		N_45:Y>Y		UFRJ50816:Y>Y		UWOPS91_917_1:L>L		YPS138:Y>Y	AA:150		A4:P>P		UFRJ50816:P>P		YPS138:P>P	AA:153		A4:D>D		N_43:D>D		N_45:D>D		UFRJ50816:D>D		YPS138:D>D	AA:163		A4:E>E		UFRJ50816:E>E		UWOPS91_917_1:E>E		YPS138:E>E	AA:165		YPS138:C>C	AA:167		UWOPS91_917_1:E>E	AA:183		A4:F>F		N_43:F>F		N_45:F>F		UFRJ50816:F>F		UWOPS91_917_1:F>F		YPS138:F>F	AA:190		UFRJ50816:S>S		UWOPS91_917_1:S>S	AA:194		UWOPS91_917_1:A>A	AA:212		UWOPS91_917_1:T>T	AA:214		UWOPS91_917_1:T>T	AA:218		A4:V>V		UFRJ50816:V>V		YPS138:V>V	AA:228		N_45:Y>Y		UWOPS91_917_1:L>L	AA:230		A4:S>S		UFRJ50816:S>S		YPS138:S>S	AA:237		UWOPS91_917_1:T>T	AA:239		A4:T>T		N_43:T>T		N_45:T>T		UFRJ50816:T>T		UWOPS91_917_1:T>T		YPS138:T>T	AA:240		KPN3828:S>S	AA:243		N_43:N>N		N_45:N>N	AA:248		N_43:D>D		N_45:D>D		UWOPS91_917_1:L>L	AA:257		UWOPS91_917_1:A>A	AA:269		A4:L>L		N_43:L>L		N_45:L>L		UFRJ50816:L>L		UWOPS91_917_1:L>L		YPS138:L>L	AA:277		A4:V>V		UFRJ50816:V>V		YPS138:V>V	AA:283		UFRJ50816:K>K	AA:288		UWOPS91_917_1:L>L	AA:293		A4:P>P		UFRJ50816:P>P	AA:296		A4:V>V		UFRJ50816:V>V	AA:306		UWOPS91_917_1:G>G	AA:319		UWOPS91_917_1:A>AID:YAL019W	AA:36		A12:C>C	AA:47		A12:T>T	AA:50		A12:S>S	AA:65		A12:R>R		UWOPS91_917_1:R>R	AA:78		Q32_3:Q>Q	AA:81		UWOPS91_917_1:L>L	AA:98		UWOPS91_917_1:R>R	AA:107		UWOPS91_917_1:T>T	AA:135		UWOPS91_917_1:N>N	AA:164		A12:S>S	AA:174		UWOPS91_917_1:V>V	AA:195		UWOPS91_917_1:R>R	AA:199		A12:A>A		DBVPG6304:A>A	AA:205		A12:V>V		DBVPG6304:V>V	AA:209		DBVPG4650:S>S		N_17:S>S		Q62_5:S>S		Y6_5:S>S		Z1_1:S>S	AA:223		N_44:T>T		N_45:T>T	AA:231		DBVPG6304:T>T		UWOPS91_917_1:T>T	AA:286		DBVPG6304:E>E		UWOPS91_917_1:E>E	AA:307		T21_4:L>L	AA:329		Y7:L>L	AA:356		N_44:L>L		N_45:L>L		UWOPS91_917_1:L>L	AA:358		DBVPG6304:S>S	AA:363		UWOPS91_917_1:T>T	AA:433		N_44:L>L		N_45:L>L	AA:439		A4:L>L		N_44:L>L		N_45:L>L		UWOPS91_917_1:L>L	AA:455		A4:C>C	AA:605		A4:C>C		YPS138:C>C	AA:607		N_44:Q>Q		N_45:Q>Q	AA:625		A4:->-		N_44:->-		N_45:->-		YPS138:->-	AA:628		A4:P>P		YPS138:P>P	AA:696		A4:I>I		N_45:I>I		UWOPS91_917_1:I>I		YPS138:I>I	AA:702		CBS432:F>F		DBVPG4650:F>F		KPN3828:F>F		KPN3829:F>F		N_17:F>F		N_45:F>F		Q59_1:F>F		T21_4:F>F		Z1_1:F>F	AA:708		UWOPS91_917_1:P>P	AA:711		A4:F>F		YPS138:F>F	AA:713		N_45:H>H		UWOPS91_917_1:H>H	AA:716		A4:T>T		UWOPS91_917_1:T>T		YPS138:T>T	AA:726		YPS138:C>C	AA:762		UWOPS91_917_1:G>G		YPS138:G>G	AA:768		A4:V>V		N_45:V>V		UWOPS91_917_1:V>V		YPS138:V>V	AA:773		N_45:R>R		UWOPS91_917_1:R>R		YPS138:R>R	AA:790		UWOPS91_917_1:K>K		YPS138:K>K	AA:842		UWOPS91_917_1:Q>Q	AA:872		DBVPG6304:K>K		UWOPS91_917_1:K>K		YPS138:K>K	AA:876		UWOPS91_917_1:I>I	AA:879		DBVPG6304:I>I		YPS138:I>I	AA:882		DBVPG6304:G>G		YPS138:G>G	AA:892		DBVPG6304:I>I		UWOPS91_917_1:I>I		YPS138:I>I	AA:931		DBVPG6304:I>I	AA:935		IFO1804:I>I	AA:1004		DBVPG6304:L>L	AA:1007		DBVPG6304:F>F	AA:1065		UWOPS91_917_1:C>C	AA:1070		UWOPS91_917_1:I>I	AA:1083		UWOPS91_917_1:F>F	AA:1107		UWOPS91_917_1:H>HID:YAL020C	AA:78		N_44:E>E		UFRJ50791:E>E	AA:81		N_43:F>F		N_44:F>F	AA:109		N_17:T>T		Q62_5:T>T	AA:116		A12:E>E		UFRJ50791:E>E		UFRJ50816:E>E	AA:127		UFRJ50791:L>L		UFRJ50816:L>L	AA:154		A12:P>P		UFRJ50791:P>P		UFRJ50816:P>P		UWOPS91_917_1:P>P	AA:157		A12:G>G		CBS432:G>G		UFRJ50791:G>G		UFRJ50816:G>G	AA:165		N_43:E>E		N_44:E>E	AA:205		IFO1804:E>E		N_43:E>E		N_44:E>E		N_45:E>E	AA:214		A12:R>R		UFRJ50791:R>R		UFRJ50816:R>R		UWOPS91_917_1:R>R	AA:220		A12:C>C		UFRJ50791:C>C		UFRJ50816:C>C		UWOPS91_917_1:C>C	AA:247		UWOPS91_917_1:P>P	AA:285		UWOPS91_917_1:V>V	AA:290		UWOPS91_917_1:G>G	AA:297		Q62_5:V>V	AA:302		A12:G>G	AA:327		A12:G>G		IFO1804:G>G		N_43:G>G		N_44:G>G		N_45:G>G		UWOPS91_917_1:G>G	AA:330		A12:Y>YID:YAL022C	AA:5		A4:V>V		UFRJ50816:V>V		UWOPS91_917_1:V>V		YPS138:V>V	AA:12		A12:V>V	AA:18		A4:G>G		DBVPG6304:G>G		UFRJ50816:G>G		UWOPS91_917_1:G>G		YPS138:G>G	AA:53		A4:I>I		DBVPG6304:I>I		UFRJ50816:I>I		UWOPS91_917_1:I>I		YPS138:I>I	AA:58		UWOPS91_917_1:T>T	AA:95		A4:L>L		DBVPG6304:L>L		UWOPS91_917_1:L>L		YPS138:L>L	AA:101		A4:R>R		DBVPG6304:R>R		YPS138:R>R	AA:106		DBVPG6304:I>I		YPS138:I>I	AA:128		A4:Y>Y		DBVPG6304:Y>Y		YPS138:Y>Y	AA:147		A4:L>L		DBVPG6304:L>L		Q32_3:L>L		Q62_5:L>L		Q89_8:L>L		UFRJ50816:L>L		Y6_5:L>L		YPS138:L>L	AA:150		CBS432:G>G	AA:155		N_44:A>A		N_45:A>A	AA:159		A4:V>V		DBVPG6304:V>V		UFRJ50816:V>V		YPS138:V>V	AA:185		A4:F>F		DBVPG6304:F>F		UFRJ50791:F>F		UFRJ50816:F>F		YPS138:F>F	AA:187		DBVPG6304:V>V		UFRJ50791:V>V		UFRJ50816:V>V		YPS138:V>V	AA:214		A4:D>D		DBVPG6304:D>D		UFRJ50791:D>D		UFRJ50816:D>D	AA:254		DBVPG6304:V>V		UFRJ50791:V>V		UFRJ50816:V>V	AA:289		DBVPG6304:A>A		IFO1804:A>A		N_43:A>A		N_44:A>A		N_45:A>A		UFRJ50791:A>A		UFRJ50816:A>A	AA:291		DBVPG6304:L>L		UFRJ50791:L>L		UFRJ50816:L>L	AA:293		DBVPG4650:L>L		Q95_3:L>L		S36_7:L>L	AA:295		DBVPG6304:P>P		UFRJ50791:P>P		UFRJ50816:P>P	AA:300		DBVPG6304:V>V		UFRJ50791:V>V		UFRJ50816:V>V	AA:310		DBVPG6304:Y>Y		UFRJ50791:Y>Y		UFRJ50816:Y>Y	AA:329		UFRJ50791:G>G		UFRJ50816:G>G	AA:369		DBVPG4650:G>G		IFO1804:G>G		N_43:G>G		N_45:G>G		Q95_3:G>G		S36_7:G>G	AA:377		DBVPG6304:K>K	AA:379		DBVPG6304:Q>Q		UFRJ50791:Q>Q		UFRJ50816:Q>Q		UWOPS91_917_1:Q>Q	AA:404		DBVPG6304:K>K		UFRJ50816:K>K	AA:420		DBVPG6304:S>S		UWOPS91_917_1:S>S	AA:426		UWOPS91_917_1:N>N	AA:434		A4:S>S		DBVPG6304:S>S		YPS138:S>S	AA:456		DBVPG6304:P>P		UWOPS91_917_1:P>P	AA:498		CBS432:L>L		CBS5829:L>L		N_17:L>L	AA:506		DBVPG6304:I>I		UFRJ50816:I>I		UWOPS91_917_1:I>IID:YAL023C	AA:20		CBS5829:N>N		Q32_3:N>N		Q62_5:N>N		Q89_8:N>N		Q95_3:N>N	AA:66		A4:A>A		DBVPG6304:A>A		IFO1804:A>A		N_44:A>A		N_45:A>A		UFRJ50816:A>A		YPS138:A>A	AA:74		A12:I>I		A4:I>I		DBVPG6304:I>I		UFRJ50816:I>I		YPS138:I>I	AA:88		IFO1804:T>T	AA:122		A12:L>L		A4:L>L		DBVPG6304:L>L		UFRJ50791:L>L		UFRJ50816:L>L		YPS138:L>L	AA:132		A12:L>L		A4:L>L		DBVPG6304:L>L		UFRJ50791:L>L		UFRJ50816:L>L		YPS138:L>L	AA:142		A12:L>L		A4:L>L		DBVPG6304:L>L		IFO1804:L>L		N_43:L>L		N_44:L>L		UFRJ50791:L>L		UFRJ50816:L>L		YPS138:L>L	AA:145		A12:V>V		A4:V>V		DBVPG6304:V>V		UFRJ50791:V>V		UFRJ50816:V>V		YPS138:V>V	AA:151		A12:S>S		A4:S>S		DBVPG6304:S>S		UFRJ50791:S>S		UFRJ50816:S>S		YPS138:S>S	AA:167		A12:G>G		A4:G>G		DBVPG6304:G>G		UFRJ50791:G>G		UFRJ50816:G>G		YPS138:G>G	AA:220		A12:F>F		DBVPG6304:F>F		UFRJ50791:F>F		YPS138:F>F	AA:227		A12:L>L		DBVPG6304:L>L		UFRJ50791:L>L		YPS138:L>L	AA:242		A12:D>D		DBVPG6304:D>D		UFRJ50791:D>D		UWOPS91_917_1:D>D		YPS138:D>D	AA:272		UWOPS91_917_1:G>G	AA:297		A12:D>D		DBVPG6304:D>D		UFRJ50791:D>D		UWOPS91_917_1:D>D		YPS138:D>D	AA:299		A12:I>I		DBVPG6304:I>I		UFRJ50791:I>I		YPS138:I>I	AA:319		A12:E>E		DBVPG6304:E>E		UFRJ50791:E>E		UWOPS91_917_1:E>E		YPS138:E>E	AA:322		A12:T>T		DBVPG6304:T>T		N_43:T>T		N_44:T>T		N_45:T>T		UFRJ50791:T>T		UWOPS91_917_1:T>T		YPS138:T>T	AA:328		N_43:A>A		N_44:A>A		N_45:A>A		UWOPS91_917_1:A>A	AA:332		A12:T>T		DBVPG6304:T>T		UFRJ50791:T>T		UWOPS91_917_1:T>T		YPS138:T>T	AA:348		A12:G>G		DBVPG6304:G>G		UFRJ50791:G>G		YPS138:G>G	AA:351		N_43:L>L		N_44:L>L		N_45:L>L	AA:358		A12:N>N	AA:370		A12:F>F		DBVPG6304:F>F		UWOPS91_917_1:F>F		YPS138:F>F	AA:400		DBVPG6304:L>L		UFRJ50791:L>L		UWOPS91_917_1:L>L		YPS138:L>L	AA:408		UWOPS91_917_1:N>N	AA:411		DBVPG6304:S>S		UFRJ50791:S>S		UWOPS91_917_1:S>S		YPS138:S>S	AA:414		DBVPG6304:S>S		UFRJ50791:S>S		UWOPS91_917_1:S>S		YPS138:S>S	AA:420		DBVPG6304:D>D		UFRJ50791:D>D		YPS138:D>D	AA:430		DBVPG6304:V>V		UFRJ50791:V>V		UFRJ50816:V>V		UWOPS91_917_1:V>V		YPS138:V>V	AA:449		DBVPG6304:L>L		UFRJ50791:L>L		UFRJ50816:L>L		UWOPS91_917_1:L>L		YPS138:L>L	AA:467		DBVPG6304:I>I		UFRJ50791:I>I		UFRJ50816:I>I		YPS138:I>I	AA:493		UFRJ50791:L>L		UFRJ50816:L>L		UWOPS91_917_1:L>L		YPS138:L>L	AA:500		DBVPG6304:G>G	AA:515		UFRJ50816:T>T		UWOPS91_917_1:T>T		YPS138:T>T	AA:550		UFRJ50816:V>V		UWOPS91_917_1:V>V	AA:562		UFRJ50816:F>F	AA:577		A12:T>T		UFRJ50816:T>T		UWOPS91_917_1:T>T	AA:591		IFO1804:A>A		N_44:A>A		N_45:A>A	AA:592		A12:T>T		UFRJ50816:T>T		UWOPS91_917_1:T>T	AA:602		A12:S>S		UFRJ50816:S>S		UWOPS91_917_1:S>S	AA:635		A12:Y>Y		IFO1804:Y>Y		N_44:Y>Y		N_45:Y>Y		UFRJ50816:Y>Y		UWOPS91_917_1:Y>Y	AA:657		Q62_5:L>L	AA:664		DBVPG6304:G>G	AA:666		A12:H>H		DBVPG6304:H>H		UFRJ50816:H>H		UWOPS91_917_1:H>H	AA:671		A12:V>V		DBVPG6304:V>V		UFRJ50816:V>V	AA:682		A12:R>R		IFO1804:R>R		N_44:R>R		N_45:R>R		UFRJ50816:R>R	AA:685		A12:L>L		CBS432:L>L		CBS5829:L>L		IFO1804:L>L		N_44:L>L		N_45:L>L		Q59_1:L>L		Q62_5:L>L		Q95_3:L>L		T21_4:L>L		UFRJ50816:L>L	AA:696		IFO1804:F>F		N_44:F>F		N_45:F>F	AA:706		IFO1804:A>A		N_44:A>A		N_45:A>AID:YAL025C	AA:32		A4:K>K		UWOPS91_917_1:K>K	AA:38		A4:N>N		KPN3828:N>N		KPN3829:N>N		N_17:N>N		N_43:N>N		N_45:N>N		Q95_3:N>N		Y7:N>N	AA:94		N_43:D>D		N_45:D>D	AA:107		A12:D>D		UWOPS91_917_1:D>D	AA:126		A12:V>V		A4:V>V		UFRJ50791:V>V	AA:129		UWOPS91_917_1:P>P	AA:136		A12:G>G		A4:G>G		N_43:G>G		N_45:G>G		UFRJ50791:G>G		UWOPS91_917_1:G>G	AA:155		A12:L>L		A4:L>L		N_43:L>L		N_45:L>L		UFRJ50791:L>L		UWOPS91_917_1:L>L	AA:177		A12:E>E		A4:E>E		UFRJ50791:E>E	AA:181		A12:L>L		A4:L>L		N_43:L>L		N_45:L>L		UFRJ50791:L>L		UWOPS91_917_1:L>L	AA:202		A12:H>H		A4:H>H		UFRJ50791:H>H		UWOPS91_917_1:H>H	AA:204		N_43:F>F		N_45:F>F	AA:216		A12:Q>Q		A4:Q>Q		UFRJ50791:Q>Q		UWOPS91_917_1:Q>Q	AA:226		A12:L>L		A4:L>L		UFRJ50791:L>L	AA:232		N_43:L>L		N_45:L>L	AA:238		DBVPG4650:A>A		N_45:A>A		Q32_3:A>A		Q62_5:A>A		Q89_8:A>A		Q95_3:A>A		T21_4:A>A	AA:242		A12:D>D		A4:D>D		N_43:D>D		N_45:D>D		UFRJ50791:T>T		UWOPS91_917_1:D>D	AA:256		A12:T>T		A4:T>T		UFRJ50791:T>T	AA:257		N_43:A>A		N_45:A>A	AA:267		A12:Q>Q		A4:Q>Q		UFRJ50791:Q>Q		UWOPS91_917_1:Q>Q		YPS138:Q>Q	AA:297		DBVPG4650:N>NID:YAL027W	AA:4		A12:T>T		UWOPS91_917_1:T>T		YPS138:T>T	AA:47		N_43:->-		N_44:->-		N_45:->-		UWOPS91_917_1:->-	AA:101		A12:Y>Y		A4:Y>Y		UFRJ50791:Y>Y		YPS138:Y>Y	AA:149		A12:L>L		A4:L>L		N_43:L>L		N_44:L>L		N_45:L>L		UFRJ50791:L>L		YPS138:L>L	AA:154		N_43:Y>Y		N_44:Y>Y		N_45:Y>Y	AA:159		A12:N>N		A4:N>N		UFRJ50791:N>N		YPS138:N>N	AA:168		A12:V>V		A4:V>V		UFRJ50791:V>V		YPS138:V>V	AA:178		A12:T>T		A4:T>T		UFRJ50791:T>T		YPS138:T>T	AA:204		A4:C>C		UFRJ50791:C>C		UFRJ50816:C>C		YPS138:C>C	AA:233		CBS432:I>I	AA:251		A12:Y>Y		A4:Y>Y		UFRJ50791:Y>Y		UFRJ50816:Y>Y		YPS138:Y>YID:YAL028W	AA:1		A12:H>H		A4:H>H		UFRJ50816:H>H		YPS138:H>H	AA:7		A12:P>P		A4:P>P		UFRJ50816:P>P		YPS138:P>P	AA:12		CBS5829:V>V		Q59_1:V>V		Q62_5:V>V		T21_4:V>V		Z1_1:V>V	AA:39		A12:I>I		UFRJ50816:I>I	AA:43		A12:S>S		UFRJ50816:S>S	AA:67		A12:I>I		DBVPG6304:I>I		UFRJ50816:I>I	AA:76		UWOPS91_917_1:P>P	AA:91		A12:A>A		DBVPG6304:A>A		UFRJ50816:A>A	AA:108		A12:T>T		DBVPG6304:T>T		UFRJ50816:T>T	AA:118		UFRJ50816:C>C	AA:120		N_43:F>F		N_45:F>F	AA:133		A12:N>N		DBVPG6304:N>N		UFRJ50816:N>N		UWOPS91_917_1:N>N	AA:147		N_43:R>R		N_45:R>R	AA:160		DBVPG6304:K>K	AA:170		UWOPS91_917_1:L>L	AA:202		A4:P>P		DBVPG6304:P>P		UFRJ50791:P>P		UFRJ50816:P>P	AA:222		A4:R>R		DBVPG6304:R>R		UFRJ50791:R>R		UFRJ50816:R>R	AA:227		A4:W>W		DBVPG6304:W>W		UFRJ50791:W>W		UFRJ50816:W>W	AA:231		N_45:I>I	AA:243		UWOPS91_917_1:V>V	AA:247		N_43:C>C		N_45:C>C	AA:265		N_43:I>I		N_45:I>I	AA:279		A4:Q>Q		DBVPG6304:Q>Q		UFRJ50791:Q>Q		UFRJ50816:Q>Q	AA:284		A4:V>V		DBVPG6304:V>V		UFRJ50791:V>V		UFRJ50816:V>V		UWOPS91_917_1:V>V	AA:287		UWOPS91_917_1:R>R	AA:291		DBVPG6304:N>N	AA:315		A4:R>R		DBVPG6304:R>R		UFRJ50791:R>R		UFRJ50816:R>R	AA:321		N_43:->-		N_45:->-	AA:344		A4:F>F		DBVPG6304:F>F		UFRJ50791:F>F		UFRJ50816:F>F	AA:353		A4:T>T		DBVPG6304:T>T		UFRJ50791:T>T		UFRJ50816:T>T	AA:355		A4:I>I		DBVPG6304:I>I		UFRJ50791:I>I		UFRJ50816:I>I	AA:367		A4:L>L		DBVPG6304:L>L		UFRJ50791:L>L		UFRJ50816:L>L	AA:392		A4:I>I		DBVPG6304:I>I		UFRJ50791:I>I		UFRJ50816:I>I	AA:410		UWOPS91_917_1:->-	AA:417		A4:G>G		DBVPG6304:G>G		UFRJ50816:G>G	AA:419		A4:S>S		DBVPG6304:S>S		UFRJ50816:S>S		UWOPS91_917_1:S>S	AA:423		A4:E>E	AA:428		UWOPS91_917_1:V>V	AA:430		UWOPS91_917_1:P>P	AA:433		UWOPS91_917_1:F>F	AA:446		UFRJ50816:->-	AA:452		N_43:Y>Y		N_45:Y>Y	AA:469		DBVPG6304:A>A		N_43:A>A		N_45:A>A		UFRJ50816:A>A		UWOPS91_917_1:A>A	AA:475		N_43:R>R		N_45:R>R		UWOPS91_917_1:R>R	AA:479		A12:R>R		DBVPG6304:R>R		UFRJ50816:R>R	AA:483		DBVPG6304:H>H	AA:495		UWOPS91_917_1:Y>Y	AA:504		A12:R>R		A4:R>R		DBVPG6304:R>R		UFRJ50816:R>R	AA:522		A12:Y>Y		A4:Y>Y		DBVPG6304:Y>Y		UFRJ50791:Y>Y		UFRJ50816:Y>Y		UWOPS91_917_1:Y>YID:YAL032C	AA:13		UFRJ50816:S>S	AA:24		DBVPG6304:R>R		UFRJ50816:R>R		YPS138:R>R	AA:27		DBVPG6304:P>P		UFRJ50816:P>P		YPS138:P>P	AA:35		N_45:A>A		UFRJ50816:K>K	AA:51		UFRJ50791:S>S	AA:56		UFRJ50791:Q>Q	AA:59		UFRJ50816:L>L	AA:73		A4:A>A		DBVPG6304:A>A		N_43:A>A		N_45:A>A		UFRJ50791:A>A		YPS138:A>A	AA:90		UFRJ50816:E>E		UWOPS91_917_1:E>E	AA:96		A4:G>G		DBVPG6304:G>G		UFRJ50791:G>G		YPS138:G>G	AA:97		UWOPS91_917_1:L>L	AA:99		N_43:V>V		N_44:V>V		N_45:V>V	AA:105		UFRJ50816:R>R		UWOPS91_917_1:R>R	AA:110		A4:A>A		DBVPG6304:A>A		UFRJ50791:A>A		YPS138:A>A	AA:111		UFRJ50816:L>L		UWOPS91_917_1:L>L	AA:114		A4:L>L		DBVPG6304:L>L		N_44:V>V		UFRJ50791:L>L		YPS138:L>L	AA:119		N_44:S>S		N_45:S>S	AA:144		UWOPS91_917_1:E>E	AA:145		A4:K>K		DBVPG6304:K>K		UFRJ50791:K>K		UFRJ50816:K>K		YPS138:K>K	AA:161		A4:K>K		DBVPG6304:K>K		UFRJ50791:K>K		UFRJ50816:K>K	AA:169		A4:E>E		DBVPG6304:E>E		UFRJ50791:E>E		UFRJ50816:E>E	AA:174		A4:K>K		DBVPG6304:K>K		UFRJ50791:K>K		UFRJ50816:K>K		UWOPS91_917_1:K>K	AA:180		A4:A>A		DBVPG6304:A>A		UFRJ50791:A>A		UFRJ50816:A>A		YPS138:A>A	AA:192		A4:N>N		DBVPG6304:N>N		UFRJ50791:N>N		UFRJ50816:N>N		UWOPS91_917_1:N>N		YPS138:N>N	AA:197		DBVPG6304:A>A	AA:201		A4:R>R		DBVPG6304:R>R		UFRJ50791:R>R		UFRJ50816:R>R		YPS138:R>R	AA:205		A4:A>A		DBVPG6304:A>A		UFRJ50791:A>A		UFRJ50816:A>A		YPS138:A>A	AA:210		A4:N>N		DBVPG6304:N>N		UFRJ50791:N>N		UFRJ50816:N>N		YPS138:N>N	AA:213		A4:K>K		DBVPG6304:K>K		UFRJ50791:K>K		UFRJ50816:K>K		YPS138:K>K	AA:215		A4:N>N		DBVPG6304:N>N		UFRJ50791:N>N		UFRJ50816:N>N		YPS138:N>N	AA:220		A4:P>P		DBVPG6304:P>P		UFRJ50791:P>P		UFRJ50816:P>P		YPS138:P>P	AA:237		N_44:T>T		N_45:T>T	AA:291		DBVPG6304:T>T		UFRJ50816:T>T		YPS138:T>T	AA:299		N_44:N>N	AA:306		DBVPG6304:A>A		N_44:A>A		UFRJ50816:A>A		YPS138:A>A	AA:308		DBVPG6304:V>V		UFRJ50816:V>V		YPS138:V>V	AA:317		DBVPG6304:R>R		UFRJ50816:R>R		YPS138:R>R	AA:322		UWOPS91_917_1:Q>Q	AA:326		DBVPG6304:Q>Q		UFRJ50816:Q>Q		YPS138:Q>Q	AA:332		DBVPG6304:S>S		UFRJ50816:S>S		YPS138:S>S	AA:343		DBVPG6304:F>F		N_44:F>F		N_45:F>F		UFRJ50816:F>F		YPS138:F>FID:YAL033W	AA:38		A12:C>C		A4:C>C		DBVPG6304:C>C		UFRJ50791:C>C		YPS138:C>C	AA:51		KPN3828:L>L		KPN3829:L>L		UFRJ50816:L>L	AA:62		UFRJ50816:I>I	AA:73		UFRJ50816:L>L	AA:80		A12:P>P		A4:P>P		DBVPG6304:P>P		UFRJ50791:P>P	AA:85		A12:Y>Y		A4:Y>Y		DBVPG6304:Y>Y		N_44:Y>Y		N_45:Y>Y		UFRJ50791:Y>Y		YPS138:Y>Y	AA:108		A12:I>I		A4:I>I		DBVPG6304:I>I	AA:118		UFRJ50816:L>L	AA:132		A12:S>S		A4:S>S		DBVPG6304:S>S		UFRJ50791:S>S	AA:147		A12:R>RID:YAL034C	AA:10		A4:Q>Q		UFRJ50791:Q>Q		YPS138:Q>Q	AA:17		A4:F>F		YPS138:F>F	AA:68		UFRJ50791:L>L		YPS138:L>L	AA:76		IFO1804:A>A		N_44:A>A		N_45:A>A	AA:77		UFRJ50791:P>P		UFRJ50816:P>P		UWOPS91_917_1:P>P		YPS138:P>P	AA:91		IFO1804:P>P		N_44:P>P		N_45:P>P	AA:99		UFRJ50791:L>L		UFRJ50816:L>L		YPS138:L>L	AA:105		N_44:A>A	AA:111		IFO1804:P>P		N_44:P>P		N_45:P>P		UFRJ50791:P>P		UFRJ50816:P>P		YPS138:P>P	AA:146		UFRJ50791:S>S		UFRJ50816:S>S		UWOPS91_917_1:S>S		YPS138:S>S	AA:148		UFRJ50791:S>S		UFRJ50816:S>S		UWOPS91_917_1:S>S		YPS138:S>S	AA:154		UFRJ50791:H>H		UFRJ50816:H>H		YPS138:H>H	AA:163		UFRJ50791:Q>Q		UFRJ50816:Q>Q		YPS138:Q>Q	AA:185		IFO1804:N>N		N_45:N>N	AA:187		UFRJ50791:Y>Y		UFRJ50816:Y>Y		UWOPS91_917_1:Y>Y		YPS138:Y>Y	AA:212		UFRJ50791:S>S		UFRJ50816:S>S		YPS138:S>S	AA:238		IFO1804:R>R		N_45:R>R		UFRJ50816:R>R		UWOPS91_917_1:R>R		YPS138:R>R	AA:241		YPS138:I>I	AA:248		UFRJ50816:A>A		UWOPS91_917_1:A>A		YPS138:A>A	AA:261		A4:Y>Y		UFRJ50816:Y>Y		UWOPS91_917_1:Y>Y		YPS138:Y>Y	AA:265		IFO1804:R>R	AA:270		UFRJ50816:S>S		UWOPS91_917_1:S>S		YPS138:S>S	AA:303		DBVPG6304:G>G		UFRJ50816:G>G		YPS138:G>G	AA:332		A4:S>S		DBVPG6304:S>S		UFRJ50816:S>S		UWOPS91_917_1:S>S		YPS138:S>S	AA:357		A4:N>N		DBVPG6304:N>N		UFRJ50816:N>N		YPS138:N>N	AA:386		UWOPS91_917_1:I>IID:YAL034W-A	AA:5		DBVPG4650:G>G	AA:6		N_44:H>H		N_45:H>H	AA:18		N_44:I>I		N_45:I>I	AA:41		N_44:G>G		N_45:G>G	AA:46		A4:Q>Q	AA:57		A4:V>V		DBVPG6304:V>V	AA:66		A4:G>G		DBVPG6304:G>G	AA:68		A4:Q>Q		DBVPG6304:Q>Q	AA:73		A4:L>L		DBVPG6304:L>L	AA:130		A4:F>F		DBVPG6304:F>F		IFO1804:F>F		N_43:F>F		N_44:F>F		YPS138:F>F	AA:136		A12:Q>Q		YPS138:Q>Q	AA:161		IFO1804:E>E		N_44:E>E	AA:172		UWOPS91_917_1:L>L	AA:174		IFO1804:I>I		N_44:I>I		UWOPS91_917_1:I>I	AA:176		A12:L>L		A4:L>L		DBVPG6304:L>L		IFO1804:L>L		N_43:L>L		N_44:L>L		N_45:L>L		UWOPS91_917_1:L>L		YPS138:L>L	AA:178		UWOPS91_917_1:L>L	AA:196		UWOPS91_917_1:I>I	AA:206		A12:F>F		A4:F>F		DBVPG6304:F>F		UWOPS91_917_1:F>F		YPS138:F>F	AA:211		IFO1804:C>C		N_43:C>C		N_44:C>C		N_45:C>C	AA:231		UWOPS91_917_1:I>I	AA:236		A12:I>I		A4:I>I		CBS432:I>I		DBVPG4650:I>I		DBVPG6304:I>I		IFO1804:I>I		N_43:I>I		N_44:I>I		N_45:I>I		Q59_1:I>I		Q62_5:I>I		Q95_3:I>I		UWOPS91_917_1:I>I		Y7:I>I		YPS138:I>I	AA:245		A12:->-		A4:->-		DBVPG6304:->-		UWOPS91_917_1:->-		YPS138:->-	AA:275		A12:->-		A4:->-		DBVPG6304:->-		UFRJ50816:->-		UWOPS91_917_1:->-		YPS138:->-ID:YAL035W	AA:17		UWOPS91_917_1:E>E	AA:19		KPN3828:F>F		Q95_3:F>F	AA:32		A12:S>S		CBS432:S>S		DBVPG6304:S>S		KPN3828:S>S		N_44:S>S		N_45:S>S		Q62_5:S>S		Q95_3:S>S		UFRJ50791:S>S		UFRJ50816:S>S		Y7:S>S		YPS138:S>S	AA:43		A12:I>I		DBVPG6304:I>I		UFRJ50791:I>I		UFRJ50816:I>I		YPS138:I>I	AA:63		A12:H>H		DBVPG6304:H>H		UFRJ50791:H>H		UFRJ50816:H>H		YPS138:H>H	AA:67		DBVPG6304:L>L		YPS138:L>L	AA:74		UFRJ50816:L>L	AA:85		N_44:L>L		N_45:L>L	AA:90		UFRJ50816:G>G	AA:94		DBVPG6304:L>L		UFRJ50791:L>L		UFRJ50816:L>L		YPS138:L>L	AA:104		DBVPG6304:L>L		UFRJ50816:L>L		YPS138:L>L	AA:124		Q59_1:V>V	AA:131		T21_4:I>I	AA:155		DBVPG6304:A>A		UFRJ50816:A>A		YPS138:A>A	AA:171		DBVPG6304:S>S		UFRJ50816:S>S		YPS138:S>S	AA:177		DBVPG6304:L>L		UFRJ50816:L>L		YPS138:L>L	AA:185		DBVPG6304:L>L		UFRJ50816:L>L		YPS138:L>L	AA:195		N_43:F>F		N_44:F>F		N_45:F>F	AA:222		DBVPG6304:C>C		IFO1804:C>C		N_43:C>C		N_44:C>C		N_45:C>C		UFRJ50816:C>C		YPS138:C>C	AA:244		DBVPG6304:->-		UFRJ50791:->-		UFRJ50816:->-		YPS138:->-	AA:256		DBVPG6304:A>A		UFRJ50791:A>A		UFRJ50816:A>A		YPS138:A>A	AA:264		DBVPG6304:S>S		UFRJ50791:S>S		UFRJ50816:S>S		YPS138:S>S	AA:267		N_43:I>I		N_44:I>I		N_45:I>I	AA:277		A4:D>D		DBVPG6304:D>D		UFRJ50791:D>D		UFRJ50816:D>D		YPS138:D>D	AA:307		A4:V>V		DBVPG6304:V>V		IFO1804:V>V		N_43:V>V		N_45:V>V		UFRJ50791:V>V		YPS138:V>V	AA:322		UWOPS91_917_1:V>V	AA:327		A4:I>I		DBVPG6304:I>I		UFRJ50791:I>I		YPS138:I>I	AA:365		A4:T>T		DBVPG6304:T>T		UFRJ50791:T>T		UFRJ50816:T>T		UWOPS91_917_1:T>T		YPS138:T>T	AA:393		DBVPG6304:G>G	AA:406		A12:L>L		A4:L>L		DBVPG6304:L>L		UFRJ50791:L>L		UFRJ50816:L>L		YPS138:L>L	AA:456		A12:I>I		A4:I>I		DBVPG6304:I>I		YPS138:I>I	AA:478		A12:T>T		A4:T>T		DBVPG6304:T>T		IFO1804:T>T		N_43:T>T		N_44:T>T		N_45:T>T		UFRJ50816:T>T		UWOPS91_917_1:T>T		YPS138:T>T	AA:508		A12:I>I		A4:I>I		DBVPG6304:I>I		UFRJ50816:I>I		UWOPS91_917_1:I>I		YPS138:I>I	AA:518		A12:V>V		DBVPG6304:V>V		UFRJ50816:V>V		UWOPS91_917_1:V>V		YPS138:V>V	AA:559		A12:F>F		A4:F>F		DBVPG6304:F>F		UFRJ50816:F>F	AA:570		A12:I>I		A4:I>I		DBVPG6304:I>I		UFRJ50816:I>I		YPS138:I>I	AA:576		IFO1804:H>H		N_43:H>H		N_44:H>H		N_45:H>H	AA:589		IFO1804:S>S		N_43:S>S		N_44:S>S		N_45:S>S	AA:627		A4:D>D		DBVPG6304:D>D		YPS138:D>D	AA:643		A4:V>V		DBVPG6304:V>V		YPS138:V>V	AA:659		A4:D>D		DBVPG6304:D>D		N_43:D>D		N_45:D>D		YPS138:D>D	AA:673		A4:T>T		DBVPG6304:T>T		YPS138:T>T	AA:713		A4:T>T		DBVPG6304:T>T		YPS138:T>T	AA:732		Z1_1:Q>Q	AA:734		YPS138:V>V	AA:736		A4:N>N		DBVPG6304:N>N		YPS138:N>N	AA:742		A4:T>T		DBVPG6304:T>T		UFRJ50791:T>T		YPS138:T>T	AA:780		A4:N>N		DBVPG6304:N>N		IFO1804:N>N		N_43:N>N		N_44:N>N		N_45:N>N		UFRJ50791:N>N		UFRJ50816:N>N		YPS138:N>N	AA:791		A4:W>W		DBVPG6304:W>W		UFRJ50791:W>W		UFRJ50816:W>W		YPS138:W>W	AA:804		DBVPG6304:I>I		UFRJ50791:I>I		UFRJ50816:I>I		YPS138:I>I	AA:848		DBVPG6304:V>V		UFRJ50791:V>V		UFRJ50816:V>V		YPS138:V>V	AA:884		DBVPG6304:R>R	AA:949		A12:I>I		A4:I>I		DBVPG6304:I>I		IFO1804:I>I		N_44:I>I		N_45:I>I		UFRJ50791:I>I		UFRJ50816:I>I	AA:965		UFRJ50791:I>I		UFRJ50816:I>I	AA:982		IFO1804:L>L		N_44:L>L		N_45:L>LID:YAL036C	AA:10		IFO1804:E>E		N_43:E>E		N_45:E>E	AA:11		A12:D>D		YPS138:D>D	AA:16		A12:N>N		DBVPG6304:N>N		UFRJ50816:N>N		UWOPS91_917_1:N>N		YPS138:N>N	AA:18		A12:G>G		DBVPG6304:G>G		UFRJ50816:G>G		UWOPS91_917_1:G>G		YPS138:G>G	AA:28		A12:S>S		DBVPG6304:S>S		UFRJ50816:S>S		UWOPS91_917_1:S>S		YPS138:S>S	AA:36		A12:F>F		DBVPG6304:F>F		UFRJ50816:F>F		UWOPS91_917_1:F>F		YPS138:F>F	AA:108		IFO1804:L>L		N_43:L>L		N_44:L>L		N_45:L>L	AA:120		UWOPS91_917_1:K>K	AA:150		DBVPG6304:A>A		IFO1804:A>A		N_43:A>A		N_44:A>A		N_45:A>A		UWOPS91_917_1:A>A		YPS138:A>A	AA:163		DBVPG6304:A>A		UFRJ50816:A>A		UWOPS91_917_1:A>A		YPS138:A>A	AA:203		DBVPG6304:G>G		UWOPS91_917_1:G>G	AA:217		DBVPG6304:K>K	AA:219		DBVPG6304:V>V		UWOPS91_917_1:V>V	AA:226		DBVPG6304:L>L	AA:245		UWOPS91_917_1:A>A	AA:252		DBVPG6304:L>L	AA:258		DBVPG6304:K>K	AA:265		DBVPG6304:V>V	AA:278		UWOPS91_917_1:A>A	AA:288		A12:S>S		A4:S>S	AA:295		A12:V>V		A4:V>V		IFO1804:V>V		N_43:V>V		N_44:V>V		UWOPS91_917_1:V>V	AA:297		IFO1804:P>P		N_43:P>P		N_44:P>P	AA:313		A12:F>F		A4:F>F		IFO1804:F>F		N_43:F>F		N_44:F>F		UWOPS91_917_1:F>F	AA:315		A4:I>I	AA:322		A12:G>G		A4:G>G		UWOPS91_917_1:G>G	AA:338		A12:A>A		A4:A>A	AA:345		A12:F>F		A4:F>F		IFO1804:F>F		N_43:F>F		N_44:F>FID:YAL037W	AA:65		KPN3828:L>L	AA:124		YPS138:R>R	AA:141		N_17:V>V		N_44:V>V		N_45:V>V	AA:147		A12:V>V		A4:V>V		DBVPG6304:V>V		UWOPS91_917_1:V>V		YPS138:V>V	AA:159		A4:->-		CBS432:->-		CBS5829:->-		DBVPG4650:->-		DBVPG6304:->-		KPN3828:->-		N_17:->-		N_44:->-		N_45:->-		Q59_1:->-		Q62_5:->-		Q89_8:->-		S36_7:->-		T21_4:->-		UWOPS91_917_1:->-		YPS138:->-		Z1_1:->-	AA:180		DBVPG6304:F>F		UWOPS91_917_1:F>F		YPS138:F>F	AA:188		A4:G>G		DBVPG6304:G>G		YPS138:G>G	AA:216		A4:K>K		YPS138:K>K	AA:230		DBVPG6304:G>G		YPS138:G>G	AA:247		N_17:A>A		N_44:A>A	AA:254		DBVPG6304:S>S		YPS138:S>S	AA:266		KPN3828:S>S	AA:270		CBS432:L>L		DBVPG4650:L>L		KPN3828:L>L		N_17:L>L		N_44:L>L		Q59_1:L>L		S36_7:L>L		T21_4:L>L		Y7:L>L		Z1_1:L>LID:YAL038W	AA:20		UFRJ50816:S>S		UWOPS91_917_1:S>S	AA:48		UFRJ50816:N>N	AA:59		UFRJ50816:V>V	AA:84		UWOPS91_917_1:V>V	AA:120		UFRJ50816:S>S	AA:123		UFRJ50816:V>V	AA:124		A4:L>L		YPS138:L>L	AA:143		UFRJ50816:D>D	AA:159		A4:V>V		YPS138:V>V	AA:170		UFRJ50816:G>G	AA:207		UWOPS91_917_1:D>D	AA:233		A4:L>L		UFRJ50791:L>L	AA:239		UWOPS91_917_1:D>D	AA:252		A4:V>V		UFRJ50791:V>V	AA:272		UWOPS91_917_1:W>W	AA:322		UWOPS91_917_1:D>D	AA:328		IFO1804:T>T		N_43:T>T		N_45:T>T	AA:335		A4:G>G		DBVPG6304:G>G		IFO1804:G>G		N_43:G>G		N_45:G>G		UFRJ50791:G>G		UWOPS91_917_1:G>G		YPS138:G>G	AA:360		IFO1804:S>S		N_43:S>S		N_45:S>S	AA:439		UWOPS91_917_1:D>DID:YAL039C	AA:22		UFRJ50791:D>D	AA:54		A4:I>I		DBVPG6304:I>I		IFO1804:I>I		N_45:I>I		UFRJ50791:I>I		UWOPS91_917_1:I>I	AA:57		A4:V>V		DBVPG6304:V>V		UFRJ50791:V>V	AA:58		KPN3828:E>E	AA:59		Q95_3:K>K	AA:65		A4:Q>Q		DBVPG6304:Q>Q		UFRJ50791:Q>Q	AA:92		A4:L>L		DBVPG6304:L>L		UFRJ50791:L>L		UWOPS91_917_1:L>L		YPS138:L>L	AA:101		Q59_1:A>A	AA:106		UWOPS91_917_1:V>V	AA:110		A4:G>G		DBVPG6304:G>G		UFRJ50791:G>G		UWOPS91_917_1:G>G		YPS138:G>G	AA:112		A4:F>F		DBVPG6304:F>F		UFRJ50791:F>F		YPS138:F>F	AA:114		UWOPS91_917_1:L>L	AA:140		A4:L>L		DBVPG6304:L>L		UFRJ50791:L>L		UWOPS91_917_1:L>L		YPS138:L>L	AA:150		A4:V>V		DBVPG6304:V>V		UFRJ50791:V>V		UWOPS91_917_1:V>V		YPS138:V>V	AA:153		UWOPS91_917_1:E>E	AA:154		A4:A>A		DBVPG6304:A>A		UFRJ50791:A>A		YPS138:A>A	AA:155		DBVPG4650:V>V		Q89_8:V>V	AA:183		A4:S>S		DBVPG6304:S>S		UFRJ50791:S>S		YPS138:S>S	AA:185		A4:F>F		DBVPG6304:F>F		IFO1804:F>F		N_44:P>P		N_45:F>F		UFRJ50791:F>F		YPS138:F>F	AA:192		UWOPS91_917_1:I>I	AA:212		A4:P>P		DBVPG6304:P>P		UFRJ50791:P>P		UWOPS91_917_1:P>P		YPS138:P>P	AA:221		N_44:D>D		N_45:D>D	AA:225		A4:Q>Q		DBVPG6304:Q>Q		UFRJ50791:Q>Q		UWOPS91_917_1:Q>Q		YPS138:Q>Q	AA:231		A4:S>S		DBVPG6304:S>S		YPS138:S>S	AA:237		UWOPS91_917_1:S>S	AA:239		N_17:S>S		N_44:S>S		N_45:S>S		Q95_3:S>S	AA:258		A4:N>N		DBVPG6304:N>N		YPS138:N>NID:YAL040C	AA:20		A4:S>S		UFRJ50816:S>S		UWOPS91_917_1:S>S		YPS138:S>S	AA:32		UWOPS91_917_1:H>H	AA:41		UWOPS91_917_1:F>F	AA:60		IFO1804:S>S		N_43:S>S		N_45:S>S	AA:67		A12:P>P		A4:P>P		UFRJ50816:P>P		YPS138:P>P	AA:68		CBS432:S>S		KPN3829:S>S		Q59_1:S>S		Q89_8:S>S	AA:75		A12:N>N		A4:N>N		UFRJ50816:N>N		UWOPS91_917_1:N>N		YPS138:N>N	AA:114		A12:S>S	AA:118		A12:S>S		IFO1804:S>S		N_43:S>S		N_45:S>S		UFRJ50816:S>S		UWOPS91_917_1:S>S		YPS138:S>S	AA:121		A12:S>S		DBVPG6304:S>S		UFRJ50816:S>S		UWOPS91_917_1:S>S		YPS138:S>S	AA:131		UWOPS91_917_1:P>P	AA:153		A12:A>A		DBVPG6304:A>A		UFRJ50816:A>A		YPS138:A>A	AA:157		IFO1804:T>T		N_43:T>T		N_45:T>T	AA:168		A12:V>V		DBVPG6304:V>V		UFRJ50816:V>V		YPS138:V>V	AA:177		N_45:S>S	AA:190		A12:Q>Q		DBVPG6304:Q>Q		UFRJ50816:Q>Q		UWOPS91_917_1:Q>Q		YPS138:Q>Q	AA:212		A12:K>K		DBVPG6304:K>K		N_43:K>K		N_45:K>K		UWOPS91_917_1:K>K		YPS138:K>K	AA:244		N_43:I>I		N_45:A>A		UWOPS91_917_1:I>I	AA:249		N_43:I>I		N_45:I>I	AA:277		N_43:I>I		N_45:I>I	AA:278		A12:L>L		DBVPG6304:L>L		UWOPS91_917_1:L>L		YPS138:L>L	AA:284		A12:A>A		DBVPG6304:A>A		N_43:A>A		N_45:A>A		UWOPS91_917_1:A>A		YPS138:A>A	AA:308		CBS5829:G>G		Q32_3:G>G		Q95_3:G>G		T21_4:G>G	AA:313		UWOPS91_917_1:N>N	AA:316		N_43:F>F		N_45:F>F		UWOPS91_917_1:F>F		YPS138:F>F	AA:360		N_43:S>S		N_45:S>S		UWOPS91_917_1:S>S	AA:364		UWOPS91_917_1:F>F	AA:375		N_43:Q>Q		N_45:Q>Q		UWOPS91_917_1:Q>Q	AA:378		UWOPS91_917_1:S>S	AA:381		DBVPG6304:L>L		UWOPS91_917_1:L>L	AA:419		DBVPG6304:K>K	AA:421		DBVPG6304:S>S	AA:437		DBVPG6304:I>I	AA:441		UWOPS91_917_1:S>S	AA:448		DBVPG6304:T>T	AA:461		UWOPS91_917_1:R>R	AA:465		CBS5829:Y>Y		N_17:Y>Y		Q32_3:Y>Y		Q95_3:Y>Y		T21_4:Y>Y	AA:473		DBVPG6304:F>F		UWOPS91_917_1:F>F		YPS138:F>F	AA:489		DBVPG6304:N>N		YPS138:N>N	AA:497		DBVPG6304:S>S		YPS138:S>S	AA:499		DBVPG6304:R>R		IFO1804:R>R		N_43:R>R		N_45:R>R		YPS138:R>R	AA:539		DBVPG6304:Q>Q		YPS138:Q>Q	AA:555		YPS138:T>T	AA:558		DBVPG6304:S>S		YPS138:S>S	AA:560		DBVPG6304:G>G		YPS138:G>G	AA:574		DBVPG6304:T>T		YPS138:T>TID:YAL041W	AA:1		UWOPS91_917_1:H>H	AA:6		A4:T>T		UFRJ50791:T>T		UFRJ50816:T>T	AA:71		YPS138:A>A	AA:82		A4:K>K		UFRJ50791:K>K		UFRJ50816:K>K		YPS138:K>K	AA:93		A4:R>R		UFRJ50791:R>R		UFRJ50816:R>R		YPS138:R>R	AA:109		UFRJ50791:R>R		UFRJ50816:R>R		YPS138:R>R	AA:110		IFO1804:T>T		N_43:T>T		N_44:T>T		N_45:T>T	AA:116		A4:G>G		UFRJ50791:G>G		UFRJ50816:G>G		YPS138:G>G	AA:145		A4:T>T		UFRJ50816:T>T		YPS138:T>T	AA:172		A4:T>T		UFRJ50816:T>T		YPS138:T>T	AA:226		A4:V>V		UFRJ50816:V>V		YPS138:V>V	AA:229		A4:->-		UFRJ50816:->-		YPS138:->-	AA:360		IFO1804:L>L		N_43:L>L		N_44:L>L	AA:374		IFO1804:I>I		N_43:I>I		N_44:I>I	AA:384		DBVPG6304:->-	AA:386		IFO1804:L>L		N_43:L>L		N_44:L>L	AA:393		DBVPG6304:L>L	AA:398		YPS138:V>V	AA:431		DBVPG6304:I>I		YPS138:I>I	AA:446		DBVPG6304:I>I	AA:459		DBVPG6304:F>F	AA:470		DBVPG6304:P>P	AA:513		IFO1804:F>F		N_43:F>F		N_44:F>F	AA:525		DBVPG6304:F>F	AA:529		CBS5829:R>R		N_17:R>R		Q95_3:R>R	AA:533		DBVPG6304:L>L	AA:546		CBS432:I>I		CBS5829:I>I		DBVPG6304:I>I		IFO1804:I>I		N_17:I>I		N_43:I>I		N_44:I>I		N_45:I>I		Q32_3:I>I		Q95_3:I>I		T21_4:I>I	AA:551		DBVPG6304:I>I		IFO1804:I>I		N_43:I>I		N_44:I>I		N_45:I>I	AA:567		DBVPG6304:G>G	AA:588		N_45:A>A	AA:627		N_43:R>R		N_45:R>R	AA:652		N_43:I>I		N_45:I>I	AA:655		DBVPG6304:C>C	AA:713		CBS5829:C>C		Q95_3:C>C		Y7:C>C	AA:733		A4:R>R	AA:740		A4:R>R	AA:760		IFO1804:R>R		N_45:R>R	AA:769		A4:I>I	AA:799		A4:I>I	AA:808		A4:V>VID:YAL042W	AA:2		IFO1804:L>L		N_43:L>L		N_44:L>L		N_45:L>L	AA:6		CBS432:Q>Q	AA:32		A4:R>R		YPS138:R>R	AA:63		DBVPG6304:S>S		YPS138:S>S	AA:79		DBVPG6304:R>R		UWOPS91_917_1:R>R		YPS138:R>R	AA:102		DBVPG6304:P>P		YPS138:P>P	AA:112		DBVPG6304:A>A		UWOPS91_917_1:A>A		YPS138:A>A	AA:123		UWOPS91_917_1:H>H	AA:148		N_43:I>I		N_44:I>I		N_45:I>I	AA:152		N_43:I>I		N_44:I>I		N_45:I>I	AA:183		UWOPS91_917_1:P>P	AA:192		UWOPS91_917_1:L>L	AA:203		DBVPG6304:I>I		N_43:I>I		N_45:I>I		UFRJ50816:I>I		UWOPS91_917_1:I>I		YPS138:I>I	AA:205		DBVPG6304:A>A		UFRJ50816:A>A		UWOPS91_917_1:A>A		YPS138:A>A	AA:213		DBVPG6304:L>L		UFRJ50816:L>L		UWOPS91_917_1:L>L		YPS138:L>L	AA:233		UWOPS91_917_1:I>I	AA:248		DBVPG6304:Q>Q		UFRJ50816:Q>Q		YPS138:Q>Q	AA:282		A4:G>G		DBVPG6304:G>G		UFRJ50816:G>G		YPS138:G>G	AA:287		N_43:V>V	AA:294		DBVPG6304:V>V		UFRJ50816:V>V		YPS138:V>V	AA:330		A4:I>I		DBVPG6304:I>I		UFRJ50816:I>I		YPS138:I>I	AA:336		A4:D>D		DBVPG6304:D>D		UFRJ50816:D>D		YPS138:D>D	AA:350		A12:P>P	AA:354		A4:R>R		DBVPG6304:R>R		YPS138:R>R	AA:363		A12:R>R		A4:R>R		DBVPG6304:R>R		UFRJ50791:R>R		UFRJ50816:R>R		YPS138:R>R	AA:368		A12:D>D		A4:D>D		DBVPG6304:D>D		UFRJ50791:D>D		UFRJ50816:D>D		YPS138:D>D	AA:373		N_43:V>V	AA:383		A12:I>I		A4:I>I		DBVPG6304:I>I		UFRJ50791:I>I		UFRJ50816:I>I		YPS138:I>IID:YAL043C	AA:6		UWOPS91_917_1:L>L	AA:13		Q89_8:H>H	AA:14		UWOPS91_917_1:L>L	AA:28		UWOPS91_917_1:V>V	AA:34		UWOPS91_917_1:P>P	AA:40		Q32_3:F>F	AA:41		A12:K>K		DBVPG6304:K>K		UFRJ50816:K>K	AA:46		A12:G>G		UFRJ50816:G>G	AA:52		N_43:P>P		N_45:P>P	AA:53		UWOPS91_917_1:D>D	AA:76		A12:I>I		DBVPG6304:I>I		UFRJ50816:I>I	AA:82		UWOPS91_917_1:K>K	AA:85		A12:L>L		UFRJ50816:L>L	AA:90		UWOPS91_917_1:S>S	AA:95		CBS432:L>L	AA:96		UWOPS91_917_1:S>S	AA:111		UWOPS91_917_1:F>F	AA:115		A12:E>E		DBVPG6304:E>E		UFRJ50816:E>E	AA:120		UWOPS91_917_1:I>I	AA:122		UWOPS91_917_1:L>L	AA:125		UWOPS91_917_1:R>R		YPS138:L>L	AA:127		UWOPS91_917_1:E>E	AA:130		UWOPS91_917_1:Q>Q	AA:141		UWOPS91_917_1:L>L	AA:143		A12:S>S		DBVPG6304:S>S		UFRJ50816:S>S	AA:155		UWOPS91_917_1:R>R	AA:171		A12:I>I		DBVPG6304:I>I		UFRJ50816:I>I	AA:181		A12:T>T		DBVPG6304:T>T		IFO1804:T>T		N_45:T>T		UFRJ50816:T>T	AA:191		UWOPS91_917_1:L>L	AA:217		UWOPS91_917_1:E>E	AA:220		IFO1804:A>A		N_45:A>A	AA:228		UWOPS91_917_1:G>G	AA:234		IFO1804:S>S		N_45:S>S	AA:242		DBVPG6304:Q>Q		UFRJ50816:Q>Q		UWOPS91_917_1:V>V	AA:245		A4:D>D		DBVPG6304:D>D		UFRJ50816:D>D		UWOPS91_917_1:D>D	AA:252		UFRJ50816:P>P	AA:258		A4:E>E		DBVPG6304:E>E		IFO1804:E>E		N_45:E>E		UFRJ50816:E>E		UWOPS91_917_1:E>E	AA:278		A4:D>D		UFRJ50816:D>D	AA:281		A4:S>S		UFRJ50816:S>S	AA:283		A4:D>D		UFRJ50816:D>D		UWOPS91_917_1:D>D	AA:287		N_45:S>S	AA:303		A4:N>N		N_17:N>N		N_45:N>N		UWOPS91_917_1:N>N	AA:307		A4:T>T		UWOPS91_917_1:T>T	AA:319		A4:L>L		UWOPS91_917_1:L>L	AA:327		N_17:I>I	AA:340		A4:L>L	AA:344		UWOPS91_917_1:D>D	AA:345		A4:Q>Q	AA:353		A4:L>L	AA:364		UWOPS91_917_1:P>P	AA:407		A4:T>T	AA:418		A4:Q>Q	AA:422		N_45:L>L	AA:459		A4:E>E	AA:471		A4:L>L	AA:479		A4:S>S		DBVPG6304:S>S	AA:490		A4:N>N		DBVPG6304:N>N	AA:492		A4:L>L		DBVPG6304:L>L	AA:501		CBS432:A>A		CBS5829:A>A		Q62_5:A>A		T21_4:A>A		Y7:A>A	AA:510		CBS432:K>K		CBS5829:K>K		Q62_5:K>K		T21_4:K>K		Y7:K>K	AA:517		DBVPG6304:G>G		UFRJ50791:G>G		UWOPS91_917_1:G>G	AA:519		UWOPS91_917_1:L>L	AA:528		DBVPG6304:R>R		UWOPS91_917_1:R>R	AA:551		UWOPS91_917_1:I>I	AA:555		UWOPS91_917_1:F>F	AA:558		DBVPG6304:S>S		UFRJ50791:S>S		YPS138:S>S	AA:603		UWOPS91_917_1:S>S	AA:622		DBVPG6304:V>V		UFRJ50791:V>V		YPS138:V>V	AA:626		YPS138:Q>Q	AA:638		UWOPS91_917_1:E>E	AA:640		DBVPG6304:D>D		UFRJ50791:D>D		YPS138:D>D	AA:732		A12:L>L		DBVPG6304:L>L		YPS138:L>L	AA:743		A12:L>L		DBVPG6304:L>L		UFRJ50791:L>L		YPS138:L>L	AA:756		CBS432:T>T		KPN3828:T>T		KPN3829:T>T		Q32_3:T>T	AA:768		A12:N>N		DBVPG6304:N>N		IFO1804:N>N		N_43:N>N		N_44:N>N		N_45:N>N		UFRJ50791:N>N		YPS138:N>N	AA:784		A12:S>S		DBVPG6304:S>S		UFRJ50791:S>S		YPS138:S>SID:YAL044C	AA:27		DBVPG6304:G>G	AA:48		DBVPG6304:E>E	AA:54		DBVPG6304:I>I	AA:72		CBS5829:S>S		DBVPG6304:S>S		Q95_3:S>S	AA:81		DBVPG6304:Q>Q	AA:90		DBVPG6304:L>L	AA:114		DBVPG6304:T>T	AA:153		DBVPG6304:F>F	AA:161		DBVPG6304:R>R	AA:165		DBVPG6304:L>L		UWOPS91_917_1:L>L	AA:167		N_43:T>TID:YAL044W-A	AA:19		DBVPG6304:S>S	AA:42		DBVPG6304:L>L	AA:79		CBS432:I>I		CBS5829:I>I		DBVPG6304:I>I		IFO1804:I>I		N_17:I>I		N_44:I>I		N_45:I>I		Q32_3:I>I		Q59_1:I>I		Q89_8:I>I		Q95_3:I>I	AA:81		DBVPG6304:E>EID:YAL046C	AA:27		DBVPG4650:L>L	AA:36		IFO1804:K>K		N_44:K>K		N_45:K>K	AA:49		UFRJ50791:N>N	AA:59		UFRJ50791:S>S	AA:99		UFRJ50791:I>I	AA:114		UFRJ50791:T>T	AA:116		UFRJ50791:S>SID:YAL047C	AA:1		A4:->-		IFO1804:->-		N_45:->-		YPS138:->-	AA:23		UWOPS91_917_1:K>K	AA:25		CBS432:R>R		Q32_3:R>R		Q95_3:R>R		Y6_5:R>R		Y7:R>R	AA:33		UFRJ50791:L>L		YPS138:L>L	AA:34		UWOPS91_917_1:K>K	AA:35		CBS432:R>R		CBS5829:R>R		Q32_3:R>R		Q62_5:R>R		Q95_3:R>R		UWOPS91_917_1:S>S		Y6_5:R>R		Y7:R>R	AA:38		A4:R>R		UFRJ50791:R>R		UWOPS91_917_1:R>R		YPS138:R>R	AA:52		A4:S>S		UFRJ50791:S>S		UWOPS91_917_1:S>S		YPS138:S>S	AA:59		A4:A>A		UFRJ50791:A>A		YPS138:A>A	AA:87		A4:E>E		UFRJ50791:E>E		UWOPS91_917_1:E>E		YPS138:E>E	AA:97		A4:S>S		UFRJ50791:S>S		UFRJ50816:S>S		YPS138:S>S	AA:124		UFRJ50791:Q>Q		UFRJ50816:Q>Q		UWOPS91_917_1:Q>Q		YPS138:Q>Q	AA:148		UFRJ50791:Q>Q		UFRJ50816:Q>Q		UWOPS91_917_1:Q>Q	AA:151		UWOPS91_917_1:K>K	AA:156		IFO1804:E>E		N_43:E>E	AA:167		N_17:L>L	AA:172		UFRJ50816:N>N		UWOPS91_917_1:N>N	AA:178		IFO1804:E>E		N_43:E>E		N_45:E>E	AA:179		CBS432:L>L		CBS5829:L>L		Q95_3:L>L		Y6_5:L>L		Y7:L>L	AA:197		UWOPS91_917_1:I>I	AA:218		UFRJ50816:L>L	AA:224		A12:Q>Q	AA:230		UWOPS91_917_1:T>T	AA:246		N_43:K>K		N_45:K>K		UWOPS91_917_1:K>K	AA:256		UWOPS91_917_1:S>S	AA:259		DBVPG6304:K>K	AA:269		UWOPS91_917_1:Q>Q	AA:277		A12:T>T		DBVPG6304:T>T		UWOPS91_917_1:N>N	AA:286		N_43:L>L		N_45:L>L	AA:289		UWOPS91_917_1:Q>Q	AA:319		UWOPS91_917_1:K>K	AA:321		UWOPS91_917_1:L>L	AA:328		UWOPS91_917_1:L>L	AA:332		A12:L>L		DBVPG6304:L>L		N_43:L>L		N_45:L>L		UWOPS91_917_1:L>L	AA:344		UWOPS91_917_1:P>P	AA:354		N_43:E>E		N_45:E>E		UWOPS91_917_1:E>E	AA:366		UWOPS91_917_1:D>D	AA:385		CBS5829:E>E	AA:392		A12:S>S		DBVPG6304:S>S	AA:395		UWOPS91_917_1:A>A	AA:397		CBS5829:S>S		Q62_5:S>S	AA:409		N_45:Q>Q	AA:416		UWOPS91_917_1:D>D	AA:424		UWOPS91_917_1:K>K	AA:426		UWOPS91_917_1:L>L	AA:436		UWOPS91_917_1:S>S	AA:444		A12:L>L		A4:L>L		DBVPG6304:L>L		N_43:L>L		N_45:L>L	AA:449		UWOPS91_917_1:E>E	AA:455		UWOPS91_917_1:A>A	AA:472		A4:N>N		DBVPG6304:N>N		YPS138:N>N	AA:474		UWOPS91_917_1:T>T	AA:475		A4:N>N		DBVPG6304:N>N		YPS138:N>N	AA:480		UWOPS91_917_1:S>S	AA:483		UWOPS91_917_1:L>L	AA:486		A4:Q>Q		DBVPG6304:Q>Q		UWOPS91_917_1:Q>Q		YPS138:Q>Q	AA:488		UWOPS91_917_1:F>F	AA:491		UWOPS91_917_1:T>T	AA:510		A4:L>L		DBVPG6304:L>L		UWOPS91_917_1:L>L		YPS138:L>L	AA:514		UWOPS91_917_1:E>E	AA:515		A4:I>I		DBVPG6304:I>I		YPS138:I>I	AA:544		N_43:V>V	AA:546		UWOPS91_917_1:I>I	AA:562		A4:N>N		DBVPG6304:N>N		YPS138:N>N	AA:597		UWOPS91_917_1:T>T	AA:600		UWOPS91_917_1:S>S	AA:609		A4:N>N		DBVPG6304:N>N		UWOPS91_917_1:N>N		YPS138:N>N	AA:611		A4:R>R		DBVPG6304:R>R		YPS138:R>RID:YAL048C	AA:9		A4:F>F	AA:19		IFO1804:A>A		N_45:A>A	AA:40		A12:A>A		A4:A>A	AA:47		A12:P>P		A4:P>P		IFO1804:P>P		N_45:P>P		YPS138:P>P	AA:71		A12:R>R		A4:R>R		YPS138:R>R	AA:102		A12:F>F		IFO1804:A>A		N_17:A>A		N_45:A>A		S36_7:A>A		UWOPS91_917_1:F>F		YPS138:A>A	AA:106		IFO1804:V>V		N_45:V>V	AA:122		A12:S>S		YPS138:S>S	AA:141		IFO1804:D>D		N_44:D>D		N_45:D>D		Q95_3:D>D		S36_7:D>D	AA:148		A12:K>K		IFO1804:K>K		N_45:K>K		YPS138:K>K	AA:166		A12:Y>Y		DBVPG6304:Y>Y		UWOPS91_917_1:Y>Y		YPS138:Y>Y	AA:201		IFO1804:S>S		N_44:S>S	AA:203		A12:K>K		DBVPG6304:K>K		UWOPS91_917_1:K>K		YPS138:K>K	AA:213		A12:C>C		DBVPG6304:C>C		YPS138:C>C	AA:217		A12:K>K		DBVPG6304:K>K		UWOPS91_917_1:K>K		YPS138:K>K	AA:220		A12:N>N		DBVPG6304:N>N		YPS138:N>N	AA:223		A12:S>S		DBVPG6304:S>S		UWOPS91_917_1:S>S		YPS138:S>S	AA:229		A12:S>S		DBVPG6304:S>S		UWOPS91_917_1:S>S		YPS138:S>S	AA:247		A12:E>E		DBVPG6304:E>E		UWOPS91_917_1:E>E		YPS138:E>E	AA:272		UWOPS91_917_1:L>L	AA:281		A12:K>K		DBVPG6304:K>K		N_43:K>K		N_44:K>K	AA:300		A12:G>G		DBVPG6304:G>G	AA:310		A12:E>E		DBVPG6304:E>E	AA:320		A12:I>I		DBVPG6304:I>I	AA:325		A12:F>F		DBVPG6304:F>F		N_43:F>F		N_44:F>F	AA:356		A12:I>I		DBVPG6304:I>I	AA:385		A12:V>V	AA:400		A12:K>K		DBVPG6304:K>K	AA:423		N_43:N>N		N_44:N>N	AA:425		Z1_1:D>D	AA:433		A12:K>K		N_43:K>K	AA:452		A12:L>L		UWOPS91_917_1:L>L	AA:473		A12:F>F		IFO1804:F>F		N_43:F>F		UWOPS91_917_1:F>F	AA:475		IFO1804:P>P		N_43:P>P	AA:483		A12:R>R		UWOPS91_917_1:R>R	AA:489		UWOPS91_917_1:A>A	AA:495		A12:Q>Q	AA:513		A12:I>I		IFO1804:I>I		N_43:I>I		UWOPS91_917_1:I>I	AA:521		A12:V>V		UWOPS91_917_1:V>V	AA:556		UWOPS91_917_1:N>N	AA:565		A12:L>L		UWOPS91_917_1:L>L	AA:572		IFO1804:E>E		N_43:E>E		N_45:E>E	AA:587		A12:P>P		UWOPS91_917_1:S>S		YPS138:P>P	AA:592		A12:D>D	AA:598		A12:S>S		UWOPS91_917_1:S>S	AA:608		UWOPS91_917_1:T>T	AA:619		A12:R>R		A4:R>R	AA:626		A12:L>L	AA:647		A12:G>G		A4:G>GID:YAL049C	AA:26		A12:I>I		A4:I>I		DBVPG6304:I>I		YPS138:I>I	AA:38		A12:V>V		A4:V>V		DBVPG6304:V>V		YPS138:V>V	AA:49		UWOPS91_917_1:H>H	AA:55		DBVPG6304:E>E	AA:77		A4:P>P		DBVPG6304:P>P		UFRJ50791:P>P		UWOPS91_917_1:P>P	AA:83		A4:A>A		CBS432:A>A		DBVPG6304:A>A		N_17:A>A		N_43:A>A		N_45:A>A		Q89_8:A>A		Q95_3:A>A		UFRJ50791:A>A		UWOPS91_917_1:A>A	AA:85		A4:I>I		DBVPG6304:I>I		UFRJ50791:I>I		UWOPS91_917_1:I>I	AA:89		A4:S>S		DBVPG6304:S>S		UFRJ50791:S>S		UWOPS91_917_1:S>S	AA:96		A4:I>I		DBVPG6304:I>I		N_43:I>I		N_45:I>I		UFRJ50791:I>I		UWOPS91_917_1:I>I	AA:98		N_43:A>A	AA:100		A4:N>N		DBVPG6304:N>N		UFRJ50791:N>N		UWOPS91_917_1:N>N	AA:102		N_43:L>L		N_45:L>L		UWOPS91_917_1:L>L	AA:110		N_43:Q>Q		N_45:Q>Q	AA:111		A4:V>V		DBVPG6304:V>V		UFRJ50791:V>V		UWOPS91_917_1:V>V	AA:125		A4:F>F		DBVPG6304:F>F		UFRJ50791:F>F		UWOPS91_917_1:F>F	AA:133		N_43:L>L		N_45:L>L	AA:140		N_43:V>V		N_45:V>V	AA:173		A4:V>V		DBVPG6304:V>V		N_45:V>V		UFRJ50791:V>V		UWOPS91_917_1:V>V	AA:175		A4:V>V		DBVPG6304:V>V		UFRJ50791:V>V	AA:178		A4:G>G		DBVPG6304:G>G		UFRJ50791:G>G	AA:184		A4:D>D		DBVPG6304:D>D		UFRJ50791:D>D	AA:186		DBVPG6304:T>T	AA:188		A4:L>L		DBVPG6304:L>L		UFRJ50791:L>L		UWOPS91_917_1:L>L	AA:197		UWOPS91_917_1:V>V	AA:203		UWOPS91_917_1:I>I	AA:221		N_45:I>I	AA:241		Q89_8:G>G		Q95_3:G>G	AA:245		A12:S>S		A4:S>S		UFRJ50791:S>SID:YAL054C	AA:7		YPS138:I>I	AA:62		A4:I>I		DBVPG6304:I>I		YPS138:I>I	AA:66		DBVPG6304:V>V		YPS138:V>V	AA:81		DBVPG6304:T>T		YPS138:T>T	AA:90		DBVPG6304:K>K		YPS138:K>K	AA:100		A4:G>G		N_43:N>N	AA:121		A4:A>A		UWOPS91_917_1:Q>Q	AA:141		A4:R>R		DBVPG6304:R>R		UWOPS91_917_1:R>R		YPS138:R>R	AA:151		A4:D>D		DBVPG6304:D>D		UWOPS91_917_1:D>D		YPS138:D>D	AA:158		A4:T>T		DBVPG6304:T>T		UWOPS91_917_1:T>T	AA:182		UWOPS91_917_1:A>A	AA:188		A4:A>A		DBVPG6304:A>A		UWOPS91_917_1:A>A	AA:195		UWOPS91_917_1:E>E	AA:198		A4:S>S		DBVPG6304:S>S	AA:211		A4:V>V		DBVPG6304:V>V	AA:217		CBS432:F>F	AA:222		A4:G>G		DBVPG6304:G>G		UWOPS91_917_1:G>G	AA:239		A4:S>S		UFRJ50791:S>S	AA:243		A4:T>T		DBVPG6304:T>T		N_43:T>T		UFRJ50791:T>T	AA:261		A4:D>D		DBVPG6304:D>D		UFRJ50791:D>D		UWOPS91_917_1:Y>Y	AA:263		A4:E>E		UFRJ50791:E>E	AA:267		UWOPS91_917_1:A>A	AA:271		UWOPS91_917_1:E>E	AA:278		UWOPS91_917_1:R>R	AA:289		A4:S>S		UFRJ50791:S>S	AA:295		A4:L>L		UFRJ50791:L>L	AA:306		A4:C>C		DBVPG6304:C>C		UFRJ50791:C>C		UWOPS91_917_1:C>C	AA:333		A4:A>A		UFRJ50791:A>A		UWOPS91_917_1:A>A	AA:337		A4:L>L		UFRJ50791:L>L	AA:363		CBS432:H>H		IFO1804:H>H		N_43:H>H		N_45:H>H	AA:381		A4:T>T		DBVPG6304:T>T		UFRJ50791:T>T	AA:389		A4:A>A		DBVPG6304:A>A		UFRJ50791:A>A	AA:392		A4:S>S		DBVPG6304:S>S		UFRJ50791:S>S	AA:394		A4:S>S		UFRJ50791:S>S	AA:401		A4:P>P		DBVPG6304:P>P		UFRJ50791:P>P		UWOPS91_917_1:P>P	AA:404		N_44:S>S		N_45:S>S	AA:411		UWOPS91_917_1:Y>Y	AA:419		A4:E>E		DBVPG6304:E>E		UFRJ50791:E>E		UWOPS91_917_1:E>E	AA:427		A4:P>P		DBVPG6304:P>P		IFO1804:P>P		N_43:P>P		N_45:P>P		UFRJ50791:P>P		UWOPS91_917_1:P>P	AA:474		UWOPS91_917_1:I>I	AA:477		CBS432:K>K	AA:492		IFO1804:F>F		N_43:F>F		N_45:F>F	AA:494		N_45:A>A	AA:538		DBVPG6304:A>A	AA:542		DBVPG6304:V>V		UWOPS91_917_1:V>V	AA:546		DBVPG6304:L>L		UWOPS91_917_1:L>L	AA:551		UWOPS91_917_1:I>I	AA:575		DBVPG6304:R>R		UWOPS91_917_1:R>R	AA:585		DBVPG6304:Q>Q	AA:591		DBVPG6304:A>A		UWOPS91_917_1:A>A	AA:627		IFO1804:A>A		N_43:A>A		N_44:A>A		N_45:A>A	AA:642		A4:L>L		IFO1804:L>L		N_43:L>L		N_44:L>L		N_45:L>L		UWOPS91_917_1:L>L	AA:645		A4:L>L	AA:647		A4:P>P		IFO1804:P>P		N_43:P>P		N_44:P>P		N_45:P>P	AA:655		Y7:P>P	AA:673		A4:H>H		IFO1804:H>H		N_43:H>H		N_44:H>H		N_45:H>H		UWOPS91_917_1:H>H	AA:679		UWOPS91_917_1:K>K	AA:688		UWOPS91_917_1:Q>Q	AA:694		A4:L>L	AA:704		N_43:P>P		N_44:P>P		N_45:P>P	AA:713		A4:S>S		UWOPS91_917_1:S>SID:YAL055W	AA:4		IFO1804:W>W	AA:14		A4:C>C		DBVPG6304:C>C		YPS138:C>C	AA:43		IFO1804:H>H		N_45:H>H	AA:48		A4:L>L		DBVPG6304:L>L	AA:50		A4:N>N		DBVPG6304:N>N	AA:65		A4:Y>Y		DBVPG6304:Y>Y		IFO1804:Y>Y		N_45:Y>Y	AA:84		A4:L>L		DBVPG6304:L>L	AA:101		A4:S>S		DBVPG6304:S>S	AA:132		IFO1804:A>A		N_44:A>A		N_45:A>A	AA:143		A4:K>K		DBVPG6304:K>KID:YAL059W	AA:16		DBVPG6304:I>I		UFRJ50791:I>I		UFRJ50816:I>I		YPS138:I>I	AA:22		DBVPG6304:Y>Y	AA:35		DBVPG6304:I>I		UFRJ50791:I>I		UFRJ50816:I>I		YPS138:I>I	AA:46		DBVPG6304:L>L		UFRJ50791:L>L		UFRJ50816:L>L		YPS138:L>L	AA:66		A12:L>L		DBVPG6304:L>L		IFO1804:L>L		N_43:L>L		N_44:L>L		N_45:L>L		UFRJ50791:L>L		UFRJ50816:L>L		YPS138:L>L	AA:84		A12:H>H		DBVPG6304:H>H		IFO1804:H>H		N_43:H>H		N_44:H>H		N_45:H>H		UFRJ50791:H>H		UFRJ50816:H>H		YPS138:H>HID:YAL060W	AA:2		UFRJ50816:S>S	AA:24		UWOPS91_917_1:L>L	AA:46		N_43:V>V		N_44:V>V		N_45:V>V	AA:49		A12:T>T		YPS138:T>T	AA:50		N_43:R>R		N_44:R>R		N_45:R>R	AA:52		A12:E>E		YPS138:E>E	AA:77		A12:S>S		UWOPS91_917_1:S>S		YPS138:S>S	AA:92		A12:A>A		YPS138:A>A	AA:101		A12:A>A		YPS138:A>A	AA:123		A12:A>A		A4:A>A		DBVPG6304:A>A		YPS138:A>A	AA:126		A12:A>A		A4:A>A		DBVPG6304:A>A		UWOPS91_917_1:A>A		YPS138:A>A	AA:134		A12:S>S		A4:S>S		DBVPG6304:S>S		N_43:S>S		N_44:S>S		N_45:S>S		UWOPS91_917_1:S>S		YPS138:S>S	AA:143		A12:A>A		A4:A>A		DBVPG6304:A>A		YPS138:A>A	AA:147		A12:S>S		A4:S>S		DBVPG6304:S>S		YPS138:S>S	AA:185		UWOPS91_917_1:G>G	AA:209		A12:K>K		A4:K>K		DBVPG6304:K>K		YPS138:K>K	AA:230		UWOPS91_917_1:G>G	AA:237		N_43:D>D		N_45:D>D	AA:250		N_43:D>D		N_45:D>D	AA:266		A4:I>I		DBVPG6304:I>I		YPS138:I>I	AA:292		UWOPS91_917_1:N>N	AA:294		UWOPS91_917_1:A>A	AA:301		IFO1804:L>L	AA:311		A4:N>N		DBVPG6304:N>N		UWOPS91_917_1:N>N		YPS138:N>N	AA:316		A4:D>D		DBVPG6304:D>D		UWOPS91_917_1:D>D		YPS138:D>D	AA:319		A4:N>N		N_43:N>N		YPS138:N>N	AA:329		A4:G>G		DBVPG6304:G>G		UWOPS91_917_1:G>G		YPS138:G>G	AA:335		N_43:D>D	AA:340		A4:T>T		UWOPS91_917_1:T>T		YPS138:T>T	AA:347		N_43:P>P		UWOPS91_917_1:P>P		YPS138:P>P	AA:350		N_43:N>N	AA:353		N_43:T>T	AA:365		YPS138:L>LID:YAL061W	AA:6		DBVPG6304:I>I		IFO1804:I>I		N_43:I>I		N_44:I>I		N_45:I>I		UWOPS91_917_1:I>I		YPS138:I>I	AA:31		UWOPS91_917_1:N>N	AA:43		UWOPS91_917_1:Q>Q	AA:45		DBVPG6304:L>L		IFO1804:L>L		N_43:L>L		N_44:L>L		N_45:L>L		UWOPS91_917_1:L>L	AA:48		DBVPG6304:I>I		UWOPS91_917_1:I>I		YPS138:I>I	AA:50		UWOPS91_917_1:R>R	AA:72		DBVPG6304:A>A	AA:85		UWOPS91_917_1:T>T	AA:94		UWOPS91_917_1:L>L	AA:98		UWOPS91_917_1:L>L	AA:100		UWOPS91_917_1:R>R	AA:103		UWOPS91_917_1:A>A	AA:105		DBVPG6304:V>V		UWOPS91_917_1:V>V	AA:116		UWOPS91_917_1:V>V	AA:122		DBVPG6304:S>S		IFO1804:S>S		N_44:S>S		N_45:S>S		UWOPS91_917_1:S>S	AA:124		UWOPS91_917_1:F>F	AA:139		UWOPS91_917_1:G>G	AA:141		UWOPS91_917_1:H>H	AA:143		UWOPS91_917_1:T>T	AA:146		N_44:K>K		N_45:K>K	AA:153		UWOPS91_917_1:V>V	AA:166		N_45:Q>Q		UWOPS91_917_1:Q>Q	AA:169		UWOPS91_917_1:R>R	AA:182		UWOPS91_917_1:N>N	AA:190		N_45:A>A	AA:200		UWOPS91_917_1:G>G	AA:202		UWOPS91_917_1:S>S	AA:207		UWOPS91_917_1:->-	AA:214		UWOPS91_917_1:A>A	AA:225		UWOPS91_917_1:Y>Y	AA:258		UWOPS91_917_1:S>S	AA:264		UWOPS91_917_1:V>V	AA:284		YPS138:K>K	AA:293		UWOPS91_917_1:H>H	AA:319		DBVPG6304:I>I		IFO1804:I>I		N_44:I>I		N_45:I>I		UWOPS91_917_1:I>I		YPS138:I>I	AA:324		DBVPG6304:K>K		UWOPS91_917_1:K>K		YPS138:K>K	AA:329		DBVPG6304:L>L		YPS138:L>L	AA:352		DBVPG6304:L>L		YPS138:L>L	AA:375		DBVPG6304:S>S		UWOPS91_917_1:S>S		YPS138:S>S	AA:401		DBVPG6304:I>I		IFO1804:I>I		N_43:I>I		N_44:I>I		N_45:I>I		UWOPS91_917_1:I>I		YPS138:I>I	AA:403		A4:F>F		DBVPG6304:F>F		UFRJ50791:F>F		UWOPS91_917_1:F>F		YPS138:F>F	AA:407		UWOPS91_917_1:L>L	AA:410		A4:V>V		DBVPG6304:V>V		YPS138:V>VID:YAL062W	AA:6		A4:L>L		DBVPG6304:L>L		UFRJ50791:L>L		YPS138:L>L	AA:8		A4:L>L		DBVPG6304:L>L		IFO1804:L>L		N_44:L>L		N_45:L>L		UFRJ50791:L>L		YPS138:L>L	AA:11		A4:V>V		DBVPG6304:V>V		UFRJ50791:V>V		YPS138:V>V	AA:13		A4:L>L		DBVPG6304:L>L		UFRJ50791:L>L		YPS138:L>L	AA:32		UFRJ50791:F>F	AA:48		A4:N>N		DBVPG6304:N>N		YPS138:N>N	AA:50		A4:P>P		DBVPG6304:P>P		YPS138:P>P	AA:62		A4:S>S		DBVPG6304:S>S		YPS138:S>S	AA:71		DBVPG6304:L>L		YPS138:L>L	AA:75		A4:L>L		DBVPG6304:L>L		YPS138:L>L	AA:77		DBVPG6304:P>P		YPS138:P>P	AA:93		DBVPG6304:T>T		YPS138:T>T	AA:96		YPS138:L>L	AA:111		IFO1804:L>L		N_45:L>L	AA:122		IFO1804:G>G		N_45:G>G		YPS138:G>G	AA:126		UWOPS91_917_1:D>D	AA:128		IFO1804:P>P		N_45:P>P		UWOPS91_917_1:P>P	AA:151		UWOPS91_917_1:I>I	AA:155		UWOPS91_917_1:T>T	AA:159		YPS138:N>N	AA:172		UWOPS91_917_1:G>G	AA:178		IFO1804:G>G		N_45:G>G	AA:186		YPS138:A>A	AA:189		UWOPS91_917_1:D>D		YPS138:D>D	AA:192		IFO1804:L>L		N_45:L>L	AA:197		UWOPS91_917_1:A>A	AA:199		UWOPS91_917_1:N>N	AA:205		UWOPS91_917_1:H>H	AA:209		YPS138:R>R	AA:214		IFO1804:L>L		N_45:L>L	AA:224		IFO1804:G>G		N_45:G>G		UWOPS91_917_1:G>G	AA:227		YPS138:T>T	AA:238		YPS138:N>N	AA:250		A4:R>R		UWOPS91_917_1:R>R		YPS138:R>R	AA:258		IFO1804:R>R		N_44:R>R	AA:281		A4:N>N		YPS138:N>N	AA:298		A4:P>P		YPS138:P>P	AA:303		A4:R>R		YPS138:R>R	AA:315		A4:S>S		YPS138:S>S	AA:332		A4:A>A		YPS138:A>A	AA:351		A4:N>N		UWOPS91_917_1:N>N		YPS138:N>N	AA:355		A4:R>R		UWOPS91_917_1:R>R		YPS138:R>R	AA:378		A4:A>A		UWOPS91_917_1:A>A		YPS138:A>A	AA:389		A4:V>V		UWOPS91_917_1:V>V		YPS138:V>V	AA:395		UWOPS91_917_1:P>P	AA:415		IFO1804:T>T		N_44:T>T	AA:430		A12:G>G	AA:438		A12:G>G		A4:G>G		UWOPS91_917_1:G>G	AA:457		A12:K>K		A4:K>K		UWOPS91_917_1:K>KID:YAR002C-A	AA:5		A12:Q>Q		A4:Q>Q		YPS138:Q>Q	AA:11		A12:G>G		A4:G>G		DBVPG6304:G>G		UWOPS91_917_1:G>G		YPS138:G>G	AA:25		YPS138:I>I	AA:26		UWOPS91_917_1:L>L	AA:50		A12:D>D		A4:D>D	AA:78		A12:A>A		A4:A>A		UFRJ50816:A>A		UWOPS91_917_1:A>A	AA:82		UWOPS91_917_1:S>S	AA:98		A12:T>T		A4:T>T		UFRJ50816:T>T		UWOPS91_917_1:T>T	AA:100		A12:A>A		A4:A>A		N_43:A>A		UFRJ50816:A>A		UWOPS91_917_1:A>A	AA:107		N_43:A>A	AA:113		N_43:I>I		UWOPS91_917_1:I>I	AA:135		A12:V>V		A4:V>V		UFRJ50816:V>V		UWOPS91_917_1:V>V	AA:145		A12:E>E		A4:E>E		CBS432:E>E		DBVPG4650:E>E		KPN3828:E>E		N_17:E>E		Q32_3:E>E		Q62_5:E>E		Q95_3:E>E		UFRJ50816:E>E		UWOPS91_917_1:E>E		Y6_5:E>E		Z1_1:E>E	AA:151		A12:G>G		A4:G>G		UFRJ50816:G>G	AA:158		A12:K>K		A4:K>K		UFRJ50816:K>K	AA:160		A12:Y>Y		A4:Y>Y		N_43:Y>Y		N_45:Y>Y		UFRJ50816:Y>Y		UWOPS91_917_1:Y>Y	AA:173		A12:T>T		A4:T>T		UFRJ50816:T>T		UWOPS91_917_1:T>T	AA:175		A12:Q>Q		A4:Q>Q		DBVPG6304:Q>Q		UFRJ50816:Q>Q		UWOPS91_917_1:Q>Q	AA:178		A12:T>T		A4:T>T		DBVPG6304:T>T		UFRJ50816:T>T		UWOPS91_917_1:T>T	AA:193		UWOPS91_917_1:S>S	AA:194		A12:T>T		A4:T>T		UFRJ50816:T>T	AA:204		A12:P>P		A4:P>P		DBVPG6304:P>P		UFRJ50816:P>P		UWOPS91_917_1:P>P	AA:211		A12:F>F		A4:F>F		DBVPG6304:F>F		UFRJ50816:F>F		UWOPS91_917_1:F>F	AA:214		A12:L>L		A4:L>L		DBVPG6304:L>L		UFRJ50816:L>LID:YAR002W	AA:16		A12:S>S		A4:S>S		DBVPG6304:S>S		UFRJ50791:S>S		UFRJ50816:S>S		YPS138:S>S	AA:52		UWOPS91_917_1:I>I	AA:56		UWOPS91_917_1:I>I	AA:69		A12:->-		A4:->-		DBVPG6304:->-		UFRJ50791:->-		UFRJ50816:->-		UWOPS91_917_1:->-		YPS138:->-	AA:75		N_43:I>I	AA:101		A12:A>A		A4:A>A		DBVPG6304:A>A		UFRJ50791:A>A		UFRJ50816:A>A		YPS138:A>A	AA:108		N_43:P>P	AA:121		N_43:L>L		N_45:L>L	AA:135		UFRJ50791:R>R		UFRJ50816:R>R	AA:142		A12:H>H		A4:H>H		DBVPG6304:H>H		UFRJ50791:H>H		UFRJ50816:H>H		UWOPS91_917_1:H>H	AA:165		UWOPS91_917_1:T>T	AA:210		A12:->-		A4:->-		DBVPG6304:->-		N_44:->-		N_45:->-		UFRJ50791:->-		UFRJ50816:->-		UWOPS91_917_1:->-		YPS138:->-	AA:217		A12:I>I		A4:I>I		DBVPG6304:I>I		UFRJ50791:I>I		UFRJ50816:I>I		UWOPS91_917_1:I>I		YPS138:I>I	AA:229		Y7:I>I	AA:272		A12:R>R		A4:R>R		DBVPG6304:R>R		UFRJ50791:R>R		UFRJ50816:R>R		YPS138:R>R	AA:303		A12:A>A		DBVPG6304:A>A		UFRJ50791:A>A		UFRJ50816:A>A		YPS138:A>A	AA:319		A12:L>L		DBVPG6304:L>L		UFRJ50791:L>L		UFRJ50816:L>L		YPS138:L>L	AA:358		UFRJ50791:I>I		UFRJ50816:I>I		YPS138:I>I	AA:374		A12:I>I		UFRJ50791:I>I		UFRJ50816:I>I		YPS138:I>I	AA:412		CBS5829:S>S	AA:429		N_17:G>G	AA:450		N_17:S>S	AA:451		A12:R>R		UFRJ50816:R>R		YPS138:R>R	AA:462		N_17:L>L	AA:478		N_17:->-	AA:483		IFO1804:R>R		N_44:R>R		N_45:R>R	AA:495		IFO1804:G>G		N_44:G>G		N_45:G>G		Y7:G>G	AA:500		N_17:V>V	AA:503		A12:F>F		A4:F>F		IFO1804:F>F		N_44:F>F		N_45:F>F		YPS138:F>F	AA:505		N_17:L>L	AA:506		A12:R>R		A4:R>R		YPS138:R>R	AA:507		N_17:F>F	AA:509		A12:K>K		A4:K>K		YPS138:K>K	AA:513		N_17:N>N	AA:517		N_17:R>R	AA:526		N_17:I>I	AA:533		N_17:E>EID:YAR003W	AA:38		A4:T>T		DBVPG6304:T>T		IFO1804:T>T		N_45:T>T		UFRJ50791:T>T		UFRJ50816:T>T		UWOPS91_917_1:T>T		YPS138:T>T	AA:57		A12:C>C		A4:C>C		DBVPG6304:C>C		UFRJ50791:C>C		UFRJ50816:C>C		UWOPS91_917_1:C>C		YPS138:C>C	AA:117		A12:N>N		A4:N>N		DBVPG6304:N>N		IFO1804:N>N		N_44:N>N		N_45:N>N		Q95_3:N>N		UFRJ50816:N>N		Y7:N>N	AA:130		A12:T>T		DBVPG6304:T>T		UFRJ50816:T>T	AA:173		A12:N>N		A4:N>N		DBVPG6304:N>N		IFO1804:N>N		N_44:N>N		N_45:N>N		UFRJ50816:N>N		YPS138:N>N	AA:200		IFO1804:R>R		N_44:R>R		N_45:R>R	AA:203		UFRJ50816:L>L	AA:222		A12:N>N	AA:223		A12:R>R		A4:R>R		DBVPG6304:R>R		UFRJ50816:R>R		YPS138:R>R	AA:243		N_44:Y>Y		N_45:Y>Y	AA:276		A12:I>I	AA:288		A12:T>T	AA:309		A12:F>F	AA:337		A12:Y>Y		A4:Y>Y		DBVPG6304:Y>Y		YPS138:Y>Y	AA:344		A12:N>N		A4:N>N		DBVPG6304:N>N		N_44:N>N		N_45:N>N		YPS138:N>N	AA:409		A12:L>L		DBVPG6304:L>L		UWOPS91_917_1:L>L		YPS138:L>L	AA:418		A12:R>R		DBVPG6304:R>R		UFRJ50816:R>R		UWOPS91_917_1:R>R		YPS138:R>RID:YAR007C	AA:1		A12:->-		A4:->-		DBVPG6304:->-		UFRJ50816:->-		UWOPS91_917_1:->-		YPS138:->-	AA:4		IFO1804:L>L		N_43:L>L		N_44:L>L		N_45:L>L		UWOPS91_917_1:L>L	AA:16		A12:E>E		A4:E>E		DBVPG6304:E>E		UFRJ50816:E>E		UWOPS91_917_1:E>E		YPS138:E>E	AA:24		A12:L>L		A4:L>L		DBVPG6304:L>L		UFRJ50816:L>L		YPS138:L>L	AA:41		A12:R>R		A4:R>R		DBVPG6304:R>R		UFRJ50816:R>R		UWOPS91_917_1:R>R		YPS138:R>R	AA:48		UWOPS91_917_1:Y>Y	AA:54		IFO1804:S>S		N_43:S>S		N_44:S>S		N_45:S>S	AA:62		A12:N>N		A4:N>N		DBVPG6304:N>N		UFRJ50816:N>N		YPS138:N>N	AA:77		UWOPS91_917_1:G>G	AA:81		IFO1804:K>K		N_43:K>K		N_44:K>K		N_45:K>K	AA:109		A12:S>S		A4:S>S		DBVPG6304:S>S		IFO1804:S>S		N_43:S>S		N_44:S>S		N_45:S>S		UFRJ50816:S>S		UWOPS91_917_1:S>S		YPS138:S>S	AA:125		A12:Q>Q		A4:Q>Q		DBVPG6304:Q>Q		UFRJ50816:Q>Q		UWOPS91_917_1:Q>Q		YPS138:Q>Q	AA:131		N_43:N>N	AA:140		UWOPS91_917_1:Y>Y	AA:166		A12:N>N		A4:N>N		DBVPG6304:N>N		UFRJ50816:N>N		UWOPS91_917_1:N>N		YPS138:N>N	AA:170		A12:A>A		A4:A>A		DBVPG6304:A>A		UFRJ50816:A>A		UWOPS91_917_1:A>A		YPS138:A>A	AA:184		A12:N>N		A4:N>N		IFO1804:N>N		N_43:N>N		N_44:N>N		N_45:N>N		UFRJ50816:N>N		YPS138:N>N	AA:187		A12:S>S		A4:S>S		DBVPG6304:S>S		IFO1804:S>S		N_43:S>S		N_44:S>S		N_45:S>S		UFRJ50816:S>S		YPS138:S>S	AA:197		A12:L>L		A4:L>L		DBVPG6304:L>L		UFRJ50816:L>L		YPS138:L>L	AA:201		A12:N>N		A4:N>N		DBVPG6304:N>N		UFRJ50816:N>N	AA:234		IFO1804:S>S		N_43:S>S		N_44:S>S		N_45:S>S	AA:258		A12:L>L		A4:L>L		UFRJ50816:L>L	AA:264		A12:L>L		A4:L>L		UFRJ50816:L>L	AA:268		A12:I>I		A4:I>I		UFRJ50816:I>I	AA:288		A12:R>R		A4:R>R		UFRJ50816:R>R	AA:293		A12:F>F		A4:F>F		UFRJ50816:F>F	AA:322		A12:N>N		A4:N>N		UFRJ50816:N>N		UWOPS91_917_1:N>N	AA:327		A12:P>P		A4:P>P		UFRJ50816:P>P	AA:360		A12:Q>Q		IFO1804:Q>Q		N_43:Q>Q		N_44:Q>Q		UWOPS91_917_1:Q>Q	AA:368		A12:Y>Y		UWOPS91_917_1:Y>Y	AA:393		A12:S>S		A4:S>S		UWOPS91_917_1:S>S	AA:406		IFO1804:G>G		N_43:G>G		N_44:G>G		N_45:G>G	AA:419		A12:S>S		A4:S>S		UWOPS91_917_1:S>S	AA:424		A12:I>I		A4:I>I		UWOPS91_917_1:I>I	AA:432		N_43:S>S		N_45:S>S	AA:444		A12:Q>Q		A4:Q>Q		UWOPS91_917_1:Q>Q	AA:448		A12:N>N		A4:N>N		N_43:N>N		N_45:N>N		UWOPS91_917_1:N>N	AA:496		A12:H>H		IFO1804:H>H		N_43:H>H		N_45:H>H		UFRJ50816:H>H		UWOPS91_917_1:H>H	AA:499		A12:F>F		IFO1804:F>F		N_43:F>F		N_45:F>F		UFRJ50816:F>F		UWOPS91_917_1:F>F	AA:508		IFO1804:Q>Q		N_43:Q>Q		N_45:Q>Q		UWOPS91_917_1:Q>Q	AA:521		UWOPS91_917_1:D>D	AA:522		A12:D>D		UFRJ50816:D>D	AA:525		A12:L>L		IFO1804:L>L		N_43:L>L		N_45:L>L		UFRJ50816:L>L		UWOPS91_917_1:L>L	AA:533		A12:V>V		IFO1804:V>V		N_43:V>V		N_45:V>V		UWOPS91_917_1:V>V	AA:538		A12:A>A		DBVPG6304:A>A		UFRJ50816:A>A	AA:563		A12:L>L		DBVPG6304:L>L		IFO1804:L>L		N_43:L>L		N_45:L>L		UFRJ50816:L>L		UWOPS91_917_1:L>L	AA:577		UWOPS91_917_1:N>NID:YAR008W	AA:57		DBVPG6304:L>L		UFRJ50816:L>L	AA:75		DBVPG6304:P>P		UFRJ50816:P>P		YPS138:P>P	AA:93		IFO1804:L>L		N_43:L>L		N_44:L>L		N_45:L>L	AA:105		DBVPG6304:F>F		UFRJ50816:F>F		UWOPS91_917_1:F>F		YPS138:F>F	AA:127		DBVPG6304:I>I		IFO1804:I>I		N_43:I>I		N_44:I>I		N_45:I>I		UFRJ50816:I>I		YPS138:I>I	AA:150		DBVPG6304:P>P		YPS138:P>P	AA:155		IFO1804:V>V		N_43:V>V		N_44:V>V		N_45:V>V	AA:157		DBVPG6304:I>I		UFRJ50816:I>I		UWOPS91_917_1:I>I		YPS138:I>I	AA:170		DBVPG6304:C>C		IFO1804:C>C		N_43:C>C		N_44:C>C		UWOPS91_917_1:C>C		YPS138:C>C	AA:173		IFO1804:G>G		N_43:G>G		N_44:G>G	AA:180		UWOPS91_917_1:T>T	AA:201		DBVPG6304:S>S		UFRJ50816:S>S		UWOPS91_917_1:S>S		YPS138:S>S	AA:223		N_43:S>S		N_44:S>S	AA:227		DBVPG6304:P>P		UFRJ50816:P>P		UWOPS91_917_1:P>P		YPS138:P>P	AA:244		UWOPS91_917_1:G>G	AA:248		DBVPG6304:R>R		UFRJ50816:R>R		YPS138:R>R	AA:264		DBVPG6304:T>T		UFRJ50816:T>T		UWOPS91_917_1:T>T		YPS138:T>T	AA:268		YPS138:P>PID:YAR014C	AA:1		A12:->-		A4:->-		DBVPG6304:->-		UFRJ50816:->-		UWOPS91_917_1:->-		YPS138:->-	AA:8		UWOPS91_917_1:L>L	AA:22		DBVPG4650:Y>Y		Q95_3:Y>Y		Y7:Y>Y	AA:47		A12:S>S		A4:S>S		DBVPG6304:S>S		N_43:S>S		N_44:S>S		N_45:S>S		UFRJ50816:S>S		UWOPS91_917_1:S>S		YPS138:S>S	AA:62		A12:D>D		A4:D>D		DBVPG6304:D>D		UFRJ50816:D>D		UWOPS91_917_1:D>D		YPS138:D>D	AA:75		A12:L>L		A4:L>L		DBVPG6304:L>L		UFRJ50816:L>L		YPS138:L>L	AA:78		A12:Q>Q		A4:Q>Q		DBVPG6304:Q>Q		N_43:Q>Q		N_45:Q>Q		UFRJ50816:Q>Q		UWOPS91_917_1:Q>Q	AA:94		A4:P>P		UFRJ50816:P>P	AA:96		UWOPS91_917_1:E>E	AA:126		A4:I>I	AA:140		N_43:A>A		N_45:A>A	AA:154		UWOPS91_917_1:T>T		YPS138:T>T	AA:157		A12:S>S		DBVPG6304:S>S		UFRJ50816:S>S		UWOPS91_917_1:S>S		YPS138:S>S	AA:160		A12:S>S		A4:S>S		DBVPG6304:S>S		N_43:T>T		N_44:T>T		N_45:T>T		UFRJ50791:S>S	AA:166		UWOPS91_917_1:P>P		YPS138:P>P	AA:167		N_43:S>S		N_45:S>S	AA:170		UWOPS91_917_1:G>G	AA:176		UWOPS91_917_1:N>N	AA:178		N_43:S>S		N_45:S>S	AA:181		A12:K>K		DBVPG6304:K>K		UFRJ50816:K>K		YPS138:K>K	AA:184		A12:G>G		UFRJ50816:G>G		YPS138:G>G	AA:192		UWOPS91_917_1:A>A	AA:193		A12:E>E		DBVPG6304:E>E		UFRJ50816:E>E		YPS138:E>E	AA:196		A12:K>K		UFRJ50816:K>K		YPS138:K>K	AA:199		A12:D>D		DBVPG6304:D>D		UFRJ50816:D>D		YPS138:D>D	AA:205		UWOPS91_917_1:D>D	AA:207		A12:P>P		UFRJ50816:P>P		YPS138:P>P	AA:213		A12:G>G		UFRJ50816:G>G		YPS138:G>G	AA:215		A12:D>D	AA:216		YPS138:R>R	AA:218		A12:D>D		A4:D>D		DBVPG6304:D>D		UFRJ50816:D>D		UWOPS91_917_1:A>A	AA:233		N_43:A>A		N_44:A>A		N_45:A>A	AA:235		N_43:E>E		N_44:E>E		N_45:E>E	AA:253		A4:S>S		DBVPG6304:S>S		UFRJ50816:S>S		YPS138:S>S	AA:257		CBS432:S>S		KPN3829:S>S		N_17:S>S		Q62_5:S>S		Z1_1:S>S	AA:259		N_43:D>D		N_44:D>D		N_45:D>D	AA:260		UWOPS91_917_1:S>S	AA:271		UWOPS91_917_1:K>K	AA:272		UWOPS91_917_1:G>G	AA:275		UWOPS91_917_1:L>L	AA:307		UWOPS91_917_1:D>D	AA:312		UWOPS91_917_1:L>L	AA:315		A4:D>D		DBVPG6304:D>D		YPS138:D>D	AA:326		A4:Y>Y		DBVPG6304:Y>Y		UFRJ50816:Y>Y		YPS138:Y>Y	AA:343		A4:N>N		DBVPG6304:N>N		UFRJ50816:N>N		UWOPS91_917_1:N>N		YPS138:N>N	AA:366		A4:D>D		DBVPG6304:D>D		UFRJ50816:D>D		UWOPS91_917_1:D>D		YPS138:D>D	AA:397		A4:I>I		DBVPG6304:I>I		IFO1804:I>I		N_44:I>I		UFRJ50816:I>I		UWOPS91_917_1:I>I		YPS138:I>I	AA:400		A4:P>P		DBVPG6304:P>P		IFO1804:P>P		N_44:P>P		UFRJ50816:P>P		UWOPS91_917_1:P>P	AA:408		A4:T>T		DBVPG6304:T>T		UFRJ50816:T>T	AA:429		A4:Q>Q		DBVPG6304:Q>Q		IFO1804:Q>Q		N_45:Q>Q		UFRJ50816:Q>Q	AA:431		UFRJ50816:L>L	AA:433		IFO1804:C>C	AA:444		A4:L>L		DBVPG6304:L>L		UFRJ50816:L>L	AA:463		A4:D>D	AA:494		A12:E>E		A4:E>E		DBVPG6304:E>E		UFRJ50816:E>E	AA:502		A12:Y>Y		A4:Y>Y		DBVPG6304:Y>Y		UFRJ50816:Y>Y	AA:542		N_45:L>L	AA:551		A4:E>E		DBVPG6304:E>E		UFRJ50791:E>E	AA:565		A12:N>N		A4:N>N		DBVPG6304:N>N		N_45:N>N		UFRJ50791:N>N		YPS138:N>N	AA:582		N_45:E>E	AA:587		A12:E>E		A4:E>E		DBVPG6304:E>E		UFRJ50791:E>E	AA:613		N_44:S>S		N_45:S>S	AA:651		A12:I>I		A4:I>I		DBVPG6304:I>I		UFRJ50791:I>I		YPS138:I>I	AA:665		N_43:L>L		N_44:L>L		N_45:L>L	AA:669		A12:Q>Q		A4:Q>Q		DBVPG6304:Q>Q		UFRJ50791:Q>Q		YPS138:Q>Q	AA:676		A4:T>T		DBVPG6304:T>T		UFRJ50791:T>T		UFRJ50816:T>T		YPS138:T>T	AA:678		A12:T>T		A4:T>T		DBVPG6304:T>T		UFRJ50791:T>T		YPS138:T>T	AA:685		N_43:G>G		N_44:G>G		N_45:G>G	AA:700		Q59_1:S>S		Q95_3:S>SID:YAR015W	AA:10		N_43:T>T		N_44:T>T		N_45:T>T	AA:48		CBS432:V>V		CBS5829:V>V		DBVPG4650:V>V		KPN3829:V>V		N_17:V>V		N_44:V>V		N_45:V>V		Q32_3:V>V		Q59_1:V>V		Q62_5:V>V		Q89_8:V>V		Q95_3:V>V		T21_4:V>V	AA:62		DBVPG6304:L>L		UFRJ50816:L>L		YPS138:L>L	AA:73		DBVPG6304:T>T		UFRJ50816:T>T		YPS138:T>T	AA:78		N_45:V>V	AA:85		DBVPG6304:N>N		N_45:N>N		UFRJ50816:N>N		YPS138:N>N	AA:88		N_45:I>I	AA:110		A4:V>V		DBVPG6304:V>V		UFRJ50816:V>V		YPS138:V>V	AA:138		A4:G>G		DBVPG6304:G>G		UFRJ50816:G>G		YPS138:G>G	AA:144		A4:L>L		DBVPG6304:L>L		N_45:L>L		UFRJ50816:L>L		YPS138:L>L	AA:148		N_45:E>E	AA:164		A12:L>L		A4:L>L		DBVPG6304:L>L		UFRJ50816:L>L	AA:184		A12:T>T		A4:T>T		DBVPG6304:T>T		UFRJ50816:T>T	AA:191		KPN3828:H>H		N_17:H>H	AA:193		N_43:F>F		N_45:F>F	AA:204		A12:I>I		A4:I>I		DBVPG6304:I>I	AA:208		A12:L>L		A4:L>L		DBVPG6304:L>L	AA:222		A12:N>N		A4:N>N		DBVPG6304:N>N	AA:225		A12:L>L		A4:L>L		DBVPG6304:L>L	AA:285		N_43:D>D		N_45:D>DID:YAR018C	AA:9		UWOPS91_917_1:Y>Y	AA:18		A4:Y>Y		CBS5829:Y>Y		DBVPG6304:Y>Y		Q32_3:Y>Y		Q59_1:Y>Y		T21_4:Y>Y		UFRJ50816:Y>Y		UWOPS91_917_1:Y>Y		YPS138:Y>Y	AA:57		UWOPS91_917_1:Y>Y	AA:68		A4:I>I		DBVPG6304:I>I		UFRJ50816:I>I		YPS138:I>I	AA:78		A4:L>L		DBVPG6304:L>L		UFRJ50816:L>L		YPS138:L>L	AA:87		A4:L>L		DBVPG6304:L>L		UFRJ50816:L>L		UWOPS91_917_1:L>L		YPS138:L>L	AA:104		UWOPS91_917_1:S>S	AA:106		A4:R>R		UFRJ50816:R>R		YPS138:R>R	AA:119		A4:A>A		UFRJ50816:A>A		UWOPS91_917_1:A>A		YPS138:A>A	AA:120		CBS432:N>N		DBVPG4650:N>N	AA:121		IFO1804:L>L		N_43:L>L		N_44:L>L		N_45:L>L	AA:135		A4:N>N		IFO1804:N>N		N_43:N>N		N_44:N>N		N_45:N>N		UFRJ50816:N>N		YPS138:N>N	AA:170		A4:P>P		UFRJ50816:P>P		YPS138:P>P	AA:171		N_45:S>S	AA:172		A4:Y>Y	AA:174		UWOPS91_917_1:Q>Q	AA:191		CBS432:L>L		DBVPG4650:L>L		Q32_3:L>L		Q59_1:L>L		Q62_5:L>L		T21_4:L>L		UWOPS91_917_1:T>T		Y7:L>L		Z1_1:L>L	AA:198		A4:E>E		DBVPG6304:E>E		UFRJ50816:E>E		UWOPS91_917_1:E>E		YPS138:E>E	AA:199		UWOPS91_917_1:L>L	AA:201		UWOPS91_917_1:K>K	AA:202		A4:A>A		DBVPG6304:A>A		UFRJ50816:A>A		UWOPS91_917_1:A>A		YPS138:A>A	AA:203		UWOPS91_917_1:L>L	AA:213		IFO1804:Q>Q		N_43:Q>Q		N_45:Q>Q	AA:229		A4:D>D		DBVPG6304:D>D		UFRJ50816:D>D		UWOPS91_917_1:D>D		YPS138:D>D	AA:231		UWOPS91_917_1:Y>Y	AA:271		IFO1804:G>G		N_43:G>G		N_45:G>G	AA:278		A4:R>R		DBVPG6304:R>R		UFRJ50816:R>R		YPS138:R>R	AA:326		A4:L>L		DBVPG6304:L>L		YPS138:L>L	AA:332		A4:Y>Y		DBVPG6304:Y>Y		UFRJ50816:Y>Y		YPS138:Y>Y	AA:351		N_43:E>E		N_45:E>E	AA:361		A4:L>L		DBVPG6304:L>L		N_43:L>L		N_45:L>L		YPS138:L>L	AA:368		N_43:L>L		N_45:L>L	AA:372		A4:E>E		DBVPG6304:E>E		YPS138:E>E	AA:386		A4:L>L		DBVPG6304:L>L		YPS138:L>L	AA:390		N_43:T>T		N_45:T>T	AA:404		A4:R>R		DBVPG6304:R>R		YPS138:R>RID:YAR019C	AA:9		UWOPS91_917_1:V>V	AA:17		N_43:K>K		N_44:K>K		N_45:K>K	AA:19		UWOPS91_917_1:S>S	AA:30		CBS432:S>S		DBVPG4650:S>S		N_17:S>S		Q89_8:S>S		UWOPS91_917_1:S>S	AA:36		UWOPS91_917_1:T>T	AA:39		A12:S>S		DBVPG6304:S>S		UFRJ50791:S>S		UFRJ50816:S>S		YPS138:S>S	AA:46		A12:K>K		DBVPG6304:K>K		UFRJ50791:K>K		UFRJ50816:K>K		UWOPS91_917_1:K>K		YPS138:K>K	AA:48		UWOPS91_917_1:S>S	AA:52		N_43:A>A		N_45:A>A		UWOPS91_917_1:I>I	AA:55		UWOPS91_917_1:C>C	AA:57		A12:Q>Q		DBVPG6304:Q>Q		N_43:Q>Q		N_44:Q>Q		N_45:Q>Q		UFRJ50791:Q>Q		UFRJ50816:Q>Q		YPS138:Q>Q	AA:62		UWOPS91_917_1:S>S	AA:77		UWOPS91_917_1:N>N	AA:83		UWOPS91_917_1:F>F	AA:92		N_43:R>R		N_44:R>R		N_45:R>R	AA:99		UWOPS91_917_1:K>K	AA:108		UWOPS91_917_1:K>K	AA:110		A12:P>P		UWOPS91_917_1:P>P	AA:117		UWOPS91_917_1:D>D	AA:123		A12:S>S		DBVPG6304:S>S		N_43:S>S		N_44:S>S		N_45:S>S		UFRJ50791:S>S		UFRJ50816:S>S		YPS138:S>S	AA:124		UWOPS91_917_1:K>K	AA:128		N_43:T>T		N_44:T>T		N_45:T>T		UWOPS91_917_1:L>L	AA:131		A12:F>F		A4:L>L		DBVPG6304:L>L		N_43:F>F		N_44:F>F		N_45:F>F		UFRJ50791:F>F		UFRJ50816:L>L		UWOPS91_917_1:F>F		YPS138:L>L	AA:134		A12:L>L		DBVPG6304:L>L		UFRJ50791:L>L		UFRJ50816:L>L		UWOPS91_917_1:L>L		YPS138:L>L	AA:156		UWOPS91_917_1:N>N	AA:176		UWOPS91_917_1:T>T		Z1_1:L>L	AA:185		A12:N>N		DBVPG6304:N>N		IFO1804:N>N		N_43:N>N		N_44:N>N		N_45:N>N		UFRJ50791:N>N		UFRJ50816:N>N		YPS138:N>N	AA:186		UWOPS91_917_1:V>V	AA:188		IFO1804:D>D		N_43:D>D		N_44:D>D		N_45:D>D	AA:189		A4:I>I		UFRJ50816:I>I		UWOPS91_917_1:I>I		YPS138:I>I	AA:191		IFO1804:S>S		N_43:S>S		N_44:S>S		N_45:S>S	AA:203		UWOPS91_917_1:L>L	AA:212		IFO1804:K>K		N_43:K>K		N_44:K>K		N_45:K>K	AA:215		UWOPS91_917_1:F>F	AA:216		A12:V>V		DBVPG6304:V>V		UFRJ50791:V>V		UFRJ50816:V>V		YPS138:V>V	AA:228		UWOPS91_917_1:V>V	AA:234		A12:R>R		DBVPG6304:R>R		UFRJ50791:R>R		UFRJ50816:R>R		YPS138:R>R	AA:237		A4:P>P		UFRJ50816:P>P		UWOPS91_917_1:F>F	AA:244		A12:I>I		DBVPG6304:I>I		UFRJ50791:I>I		UFRJ50816:I>I		YPS138:I>I	AA:246		UWOPS91_917_1:L>L	AA:250		IFO1804:S>S		N_43:S>S		N_44:S>S		N_45:S>S		UWOPS91_917_1:S>S	AA:253		UWOPS91_917_1:N>N	AA:257		A12:I>I		DBVPG6304:I>I		IFO1804:I>I		N_43:I>I		N_44:I>I		N_45:I>I		UFRJ50791:I>I		UFRJ50816:I>I		YPS138:I>I	AA:265		UWOPS91_917_1:L>L	AA:281		A12:T>T		DBVPG6304:T>T		UFRJ50791:T>T		UFRJ50816:T>T		YPS138:T>T	AA:284		UWOPS91_917_1:Q>Q	AA:287		UWOPS91_917_1:I>I	AA:289		UWOPS91_917_1:A>A	AA:309		IFO1804:F>F		N_43:F>F		N_44:F>F		N_45:F>F	AA:311		A4:S>S		UWOPS91_917_1:S>S	AA:327		A12:T>T		A4:T>T		DBVPG6304:T>T		IFO1804:T>T		N_43:T>T		N_44:T>T		N_45:T>T		UFRJ50791:T>T		UFRJ50816:T>T		YPS138:T>T	AA:338		UWOPS91_917_1:V>V	AA:340		UWOPS91_917_1:L>L	AA:351		A12:S>S		A4:S>S		DBVPG6304:S>S		UFRJ50791:S>S		UFRJ50816:S>S		YPS138:S>S	AA:352		UWOPS91_917_1:T>T	AA:358		IFO1804:G>G		N_43:G>G		N_44:G>G		N_45:G>G	AA:373		A12:L>L		A4:L>L		UFRJ50816:L>L		YPS138:L>L	AA:375		UWOPS91_917_1:P>P	AA:388		UWOPS91_917_1:P>P	AA:398		A12:S>S		A4:S>S		IFO1804:S>S		N_43:S>S		N_44:S>S		N_45:S>S		UFRJ50816:S>S		YPS138:S>S	AA:399		UWOPS91_917_1:R>R	AA:404		IFO1804:V>V		N_43:V>V		N_44:V>V		N_45:V>V	AA:412		A12:T>T		A4:T>T		IFO1804:T>T		N_43:T>T		N_44:T>T		N_45:T>T		UFRJ50816:T>T		YPS138:T>T	AA:417		IFO1804:L>L		UWOPS91_917_1:L>L	AA:420		A12:S>S		A4:S>S		UFRJ50816:S>S		YPS138:S>S	AA:425		A12:I>I		A4:I>I		UFRJ50816:I>I		UWOPS91_917_1:I>I		YPS138:I>I	AA:427		UWOPS91_917_1:S>S	AA:433		N_43:D>D		N_44:D>D		N_45:D>D	AA:440		A12:N>N		A4:N>N		UFRJ50816:N>N		YPS138:N>N	AA:449		N_43:I>I		N_44:I>I		N_45:I>I		UWOPS91_917_1:I>I	AA:451		N_43:P>P		N_44:P>P		N_45:P>P	AA:452		UWOPS91_917_1:S>S	AA:494		N_43:L>L		N_44:L>L		N_45:L>L	AA:497		N_43:L>L		N_44:L>L		N_45:L>L	AA:512		N_43:S>S		N_44:S>S		N_45:S>S	AA:548		A4:Y>Y		UFRJ50816:Y>Y		YPS138:Y>Y	AA:550		A12:I>I		A4:I>I		IFO1804:I>I		N_44:I>I		N_45:I>I		UFRJ50816:I>I		YPS138:I>I	AA:575		A12:L>L		A4:L>L		YPS138:L>L	AA:579		IFO1804:N>N		N_44:N>N		N_45:N>N	AA:581		IFO1804:Q>Q		N_44:Q>Q		N_45:Q>Q		Q62_5:Q>Q		UWOPS91_917_1:Q>Q		Z1_1:Q>Q	AA:584		A4:Y>Y		IFO1804:Y>Y		N_44:Y>Y		N_45:Y>Y		UWOPS91_917_1:Y>Y		YPS138:Y>Y	AA:607		UWOPS91_917_1:I>I	AA:612		A4:N>N		YPS138:N>N	AA:627		N_45:P>P	AA:628		A4:K>K	AA:641		IFO1804:T>T		N_44:T>T		N_45:T>T	AA:643		A4:P>P		IFO1804:P>P		N_44:P>P		N_45:P>P		UWOPS91_917_1:P>P	AA:645		A4:L>L		IFO1804:L>L		N_44:L>L		N_45:L>L		Q62_5:L>L		UWOPS91_917_1:L>L		Z1_1:L>L	AA:657		IFO1804:P>P		N_44:P>P		N_45:P>P		Q62_5:P>P		UWOPS91_917_1:P>P		Z1_1:P>P	AA:683		A4:A>A		YPS138:A>A	AA:703		A4:N>N		Q62_5:N>N		UFRJ50816:N>N		UWOPS91_917_1:N>N		YPS138:N>N		Z1_1:N>N	AA:709		Q62_5:L>L		UWOPS91_917_1:L>L	AA:715		A4:P>P		UFRJ50816:P>P		UWOPS91_917_1:P>P		YPS138:P>P	AA:744		IFO1804:N>N		N_43:N>N		N_44:N>N		N_45:N>N	AA:758		A4:Y>Y		UFRJ50816:Y>Y		YPS138:Y>Y	AA:799		UWOPS91_917_1:T>T	AA:803		N_43:S>S		N_44:S>S		N_45:S>S	AA:804		A4:N>N		DBVPG6304:N>N		UFRJ50816:N>N		YPS138:N>N	AA:812		A4:D>D		DBVPG6304:D>D		UFRJ50816:D>D		YPS138:D>D	AA:815		A4:K>K		DBVPG6304:K>K		N_43:K>K		N_44:K>K		N_45:K>K		UFRJ50816:K>K		UWOPS91_917_1:K>K		YPS138:K>K	AA:821		A4:S>S		DBVPG6304:S>S		UFRJ50816:S>S		UWOPS91_917_1:S>S		YPS138:S>S	AA:831		UWOPS91_917_1:R>R	AA:833		N_43:I>I		N_44:I>I		N_45:I>I	AA:835		UWOPS91_917_1:G>G	AA:839		A4:L>L		DBVPG6304:L>L		UFRJ50816:L>L		YPS138:L>L	AA:849		UWOPS91_917_1:V>V	AA:858		UWOPS91_917_1:L>L	AA:860		N_43:T>T		N_44:T>T		N_45:T>T		UWOPS91_917_1:T>T	AA:863		DBVPG6304:R>R		UFRJ50816:R>R	AA:865		UWOPS91_917_1:I>I	AA:867		DBVPG6304:R>R		UFRJ50816:R>R		YPS138:R>R	AA:871		UWOPS91_917_1:G>G	AA:885		A4:K>K		DBVPG6304:K>K		N_43:K>K		N_44:K>K		N_45:K>K		UFRJ50816:K>K		UWOPS91_917_1:K>K		YPS138:K>K		Z1_1:K>K	AA:911		UFRJ50816:N>N		UWOPS91_917_1:N>N	AA:913		UWOPS91_917_1:E>E	AA:917		UWOPS91_917_1:E>E	AA:923		UWOPS91_917_1:I>I	AA:926		A4:V>V		DBVPG6304:V>V		UFRJ50816:V>V		YPS138:V>V	AA:936		UWOPS91_917_1:Y>Y	AA:941		N_43:S>S		N_45:S>S	AA:960		A12:Q>Q		A4:Q>Q		DBVPG6304:Q>Q		N_43:Q>Q		N_45:Q>Q		UFRJ50816:Q>Q		YPS138:Q>Q	AA:962		A12:P>P		A4:P>P		DBVPG6304:P>P		UFRJ50816:P>P		YPS138:P>P	AA:966		A12:V>V		A4:V>V		DBVPG6304:V>V		N_43:V>V		N_45:V>V		UFRJ50816:V>V		YPS138:V>V	AA:971		A12:A>A		A4:A>A		DBVPG6304:A>A		UFRJ50816:A>A		YPS138:A>A	AA:973		UWOPS91_917_1:S>SID:YAR035W	AA:10		A4:R>R		UFRJ50816:R>R		UWOPS91_917_1:R>R		YPS138:R>R	AA:28		A4:V>V		UFRJ50816:V>V		UWOPS91_917_1:V>V		YPS138:V>V	AA:78		UFRJ50816:I>I		YPS138:I>I	AA:87		N_45:D>D	AA:95		UFRJ50816:E>E		YPS138:E>E	AA:116		Q32_3:A>A	AA:145		UFRJ50791:A>A		UFRJ50816:A>A	AA:151		N_45:A>A		UFRJ50791:A>A		UFRJ50816:A>A		YPS138:A>A	AA:158		UFRJ50791:V>V		UFRJ50816:V>V		YPS138:V>V	AA:164		UFRJ50791:E>E		UFRJ50816:E>E		YPS138:E>E	AA:178		UFRJ50791:A>A		UFRJ50816:A>A		YPS138:A>A	AA:189		UFRJ50791:H>H		UFRJ50816:H>H		YPS138:H>H	AA:196		UFRJ50791:L>L		UFRJ50816:L>L		YPS138:L>L	AA:201		UFRJ50791:V>V		UFRJ50816:V>V		YPS138:V>V	AA:216		UFRJ50791:L>L		UFRJ50816:L>L		YPS138:L>L	AA:324		A4:V>V		DBVPG6304:V>V		UFRJ50791:V>V		UFRJ50816:V>V		UWOPS91_917_1:V>V		YPS138:V>V	AA:326		A4:P>P		DBVPG6304:P>P		UFRJ50791:P>P		UFRJ50816:P>P		UWOPS91_917_1:P>P		YPS138:P>P	AA:333		N_44:D>D		N_45:D>D	AA:340		A4:R>R		DBVPG6304:R>R		N_44:R>R		N_45:R>R		UFRJ50791:R>R		UFRJ50816:R>R		UWOPS91_917_1:R>R		YPS138:R>R	AA:365		A4:R>R		DBVPG6304:R>R		UFRJ50816:R>R		UWOPS91_917_1:R>R		YPS138:R>R	AA:368		A4:A>A		DBVPG6304:A>A		UFRJ50816:A>A		UWOPS91_917_1:A>A		YPS138:A>A	AA:379		A4:V>V		DBVPG6304:V>V		UFRJ50816:V>V		UWOPS91_917_1:V>V		YPS138:V>V	AA:384		A4:V>V		DBVPG6304:V>V		UFRJ50816:V>V		UWOPS91_917_1:V>V		YPS138:V>V	AA:389		UWOPS91_917_1:V>V	AA:393		A4:I>I		DBVPG6304:I>I		UFRJ50816:I>I		UWOPS91_917_1:I>I		YPS138:I>I	AA:447		A4:V>V		DBVPG6304:V>V		YPS138:V>V	AA:453		A4:G>G		DBVPG6304:G>G		IFO1804:G>G		N_44:G>G		N_45:G>G		UWOPS91_917_1:G>G		YPS138:G>G	AA:487		UWOPS91_917_1:V>V	AA:540		A12:L>L		A4:L>L		YPS138:L>L	AA:554		A12:V>V		IFO1804:V>V		N_43:V>V		N_45:V>V	AA:565		IFO1804:P>P		N_43:P>P		N_45:P>P	AA:585		A12:L>L		A4:L>L	AA:599		A12:E>E		A4:E>E		IFO1804:E>E		N_17:E>E		N_43:E>E		N_45:E>E		S36_7:E>E		Y6_5:E>E		Y7:E>E	AA:606		A12:G>G		A4:G>G		IFO1804:G>G		N_43:G>G		N_45:G>G	AA:609		A12:G>G		A4:G>G		UWOPS91_917_1:G>G	AA:621		A12:V>V		A4:V>V		UWOPS91_917_1:V>V	AA:632		A12:A>A		A4:A>A		UWOPS91_917_1:A>A	AA:634		Q89_8:R>R	AA:680		A12:Q>Q		A4:Q>Q		UWOPS91_917_1:Q>Q	AA:690		A12:A>A		A4:A>AID:YAR062W	AA:168		UWOPS91_917_1:T>T	AA:170		UWOPS91_917_1:->-ID:YAR066W	AA:46		N_45:C>C	AA:70		N_45:T>T	AA:186		N_45:T>T	AA:214		A12:V>V		N_45:V>V	AA:221		N_45:V>VID:YBL003C	AA:22		A12:V>V		A4:V>V		DBVPG6304:V>V		YPS138:V>V	AA:29		A12:G>G		A4:G>G		DBVPG6304:G>G		YPS138:G>G	AA:33		A12:V>V		A4:V>V		DBVPG6304:V>V		YPS138:V>V	AA:39		A12:Q>Q		A4:Q>Q		DBVPG6304:Q>Q		YPS138:Q>Q	AA:40		UWOPS91_917_1:F>F	AA:64		A12:I>I		A4:I>I		DBVPG6304:I>I	AA:68		UWOPS91_917_1:F>F		YPS138:F>F	AA:78		N_43:D>D		N_44:D>D		N_45:D>D	AA:93		UWOPS91_917_1:V>V		YPS138:V>V	AA:97		YPS138:S>S	AA:100		UWOPS91_917_1:S>S		YPS138:S>S	AA:104		UWOPS91_917_1:T>T		YPS138:T>T	AA:127		A12:F>F		A4:F>F		DBVPG6304:F>F		N_43:F>F		N_44:F>F		N_45:F>F		UWOPS91_917_1:A>A		YPS138:F>FID:YBL005W	AA:9		UWOPS91_917_1:S>S	AA:13		UWOPS91_917_1:T>T	AA:21		UWOPS91_917_1:R>R	AA:24		A12:K>K		A4:K>K		DBVPG6304:K>K		UFRJ50816:K>K		YPS138:K>K	AA:28		N_44:K>K	AA:32		A12:T>T		A4:T>T		DBVPG6304:T>T		UFRJ50816:T>T		UWOPS91_917_1:T>T		YPS138:T>T	AA:36		A12:S>S		A4:S>S		DBVPG6304:S>S		UFRJ50816:S>S		YPS138:S>S	AA:39		A12:C>C		A4:C>C		DBVPG6304:C>C		UFRJ50816:C>C		UWOPS91_917_1:C>C		YPS138:C>C	AA:48		DBVPG6304:L>L	AA:70		A4:P>P		DBVPG6304:P>P		UFRJ50816:P>P		YPS138:P>P	AA:74		CBS432:A>A		N_17:A>A		Q62_5:A>A		Q89_8:A>A		Q95_3:A>A		Y6_5:A>A	AA:83		IFO1804:T>T		N_44:T>T		N_45:T>T	AA:86		A4:K>K		DBVPG6304:K>K		IFO1804:K>K		N_44:K>K		N_45:K>K		UFRJ50791:K>K		UFRJ50816:K>K		UWOPS91_917_1:K>K		YPS138:K>K	AA:112		UWOPS91_917_1:S>S	AA:126		DBVPG4650:F>F	AA:130		UWOPS91_917_1:S>S	AA:146		A12:L>L		A4:L>L		DBVPG6304:L>L		UFRJ50791:L>L		UFRJ50816:L>L		YPS138:L>L	AA:149		UWOPS91_917_1:L>L	AA:154		UWOPS91_917_1:D>D	AA:166		UWOPS91_917_1:L>L	AA:168		UWOPS91_917_1:N>N	AA:187		A12:P>P		A4:P>P		UWOPS91_917_1:P>P		YPS138:P>P	AA:195		UWOPS91_917_1:T>T	AA:201		A12:K>K		A4:K>K		DBVPG6304:K>K		IFO1804:K>K		N_43:K>K		N_44:K>K		N_45:K>K		UFRJ50791:K>K		UFRJ50816:K>K		YPS138:K>K	AA:211		IFO1804:F>F		N_43:F>F		N_44:F>F		N_45:F>F	AA:219		A12:V>V		A4:V>V		DBVPG6304:V>V		IFO1804:V>V		N_43:V>V		N_44:V>V		N_45:V>V		UFRJ50791:V>V		UWOPS91_917_1:V>V		YPS138:V>V	AA:228		UWOPS91_917_1:L>L	AA:233		IFO1804:L>L		N_43:L>L		N_44:L>L		N_45:L>L	AA:258		A12:K>K		A4:K>K		UFRJ50791:K>K		UWOPS91_917_1:K>K	AA:262		UWOPS91_917_1:N>N	AA:290		A12:I>I		A4:I>I		DBVPG6304:I>I		UFRJ50791:I>I		UFRJ50816:I>I		UWOPS91_917_1:I>I	AA:294		A12:I>I		A4:I>I		DBVPG6304:I>I		UFRJ50791:I>I		UWOPS91_917_1:I>I	AA:299		A12:P>P		A4:P>P		DBVPG6304:P>P		N_43:P>P		N_44:P>P		N_45:P>P		UFRJ50791:P>P		UFRJ50816:P>P		UWOPS91_917_1:P>P	AA:304		UWOPS91_917_1:L>L	AA:350		A12:T>T		A4:T>T		DBVPG6304:T>T		UFRJ50816:T>T		UWOPS91_917_1:T>T	AA:379		A12:F>F		A4:F>F		UFRJ50816:F>F		UWOPS91_917_1:F>F	AA:382		A12:L>L		A4:L>L		UFRJ50816:L>L	AA:386		A12:A>A		A4:A>A		DBVPG6304:A>A		UFRJ50816:A>A	AA:418		A12:V>V		A4:V>V		UFRJ50816:V>V		UWOPS91_917_1:V>V	AA:431		UWOPS91_917_1:Y>Y	AA:436		A12:V>V		A4:V>V		DBVPG6304:V>V		UFRJ50816:V>V		UWOPS91_917_1:V>V	AA:453		A12:K>K		A4:K>K		DBVPG6304:K>K		UFRJ50816:K>K		UWOPS91_917_1:K>K	AA:457		A12:V>V		A4:V>V		DBVPG6304:V>V		UFRJ50816:V>V		UWOPS91_917_1:V>V	AA:466		UWOPS91_917_1:G>G	AA:471		UWOPS91_917_1:E>E	AA:494		A12:L>L		A4:L>L		DBVPG6304:L>L		UFRJ50791:L>L		UFRJ50816:L>L		UWOPS91_917_1:L>L	AA:508		A12:I>I		A4:I>I		DBVPG6304:I>I		UFRJ50816:I>I		UWOPS91_917_1:I>I	AA:510		A12:E>E		A4:E>E		DBVPG6304:E>E		IFO1804:E>E		N_44:E>E		N_45:E>E		UFRJ50816:E>E		UWOPS91_917_1:E>E	AA:536		IFO1804:I>I		N_44:I>I		N_45:I>I	AA:541		A12:L>L		A4:L>L		DBVPG6304:L>L		UFRJ50791:L>L		UFRJ50816:L>L		UWOPS91_917_1:L>L		YPS138:L>L	AA:545		A12:S>S		A4:S>S		UFRJ50791:S>S		UFRJ50816:S>S		UWOPS91_917_1:S>S		YPS138:S>S	AA:579		A12:E>E		A4:E>E		DBVPG6304:E>E		UFRJ50791:E>E		UFRJ50816:E>E		UWOPS91_917_1:E>E		YPS138:E>E	AA:583		UWOPS91_917_1:S>S	AA:584		N_44:L>L		N_45:L>L	AA:591		A12:T>T		A4:T>T		DBVPG6304:T>T		UFRJ50791:T>T		UFRJ50816:T>T		UWOPS91_917_1:T>T		YPS138:T>T	AA:597		A12:L>L		A4:L>L		DBVPG6304:L>L		UFRJ50791:L>L		UFRJ50816:L>L		YPS138:L>L	AA:619		A12:T>T		A4:T>T		DBVPG6304:T>T		N_44:T>T		N_45:T>T		UFRJ50791:T>T		UWOPS91_917_1:T>T		YPS138:T>T	AA:626		A12:A>A		A4:A>A		DBVPG6304:A>A		UFRJ50791:A>A		UFRJ50816:A>A		UWOPS91_917_1:A>A		YPS138:A>A	AA:628		A12:S>S		CBS432:S>S		CBS5829:S>S		DBVPG4650:S>S		DBVPG6304:S>S		KPN3828:S>S		N_17:S>S		N_45:S>S		Q32_3:S>S		Q89_8:S>S		T21_4:S>S		UFRJ50791:S>S		UFRJ50816:S>S		YPS138:S>S		Z1_1:S>S	AA:638		A12:L>L		DBVPG6304:L>L		UFRJ50791:L>L		UWOPS91_917_1:L>L		YPS138:L>L	AA:640		A12:E>E		DBVPG6304:E>E		UFRJ50791:E>E		UWOPS91_917_1:E>E		YPS138:E>E	AA:664		N_43:A>A		N_44:A>A	AA:670		A12:P>P		DBVPG6304:P>P		N_44:P>P		UFRJ50791:P>P		UWOPS91_917_1:P>P		YPS138:P>P	AA:700		A12:T>T		UFRJ50791:T>T		YPS138:T>T	AA:720		A12:S>S		N_43:S>S		UFRJ50791:S>S		YPS138:S>S	AA:733		A12:D>D		UFRJ50791:D>D		UWOPS91_917_1:D>D		YPS138:D>D	AA:735		A12:I>I		UFRJ50791:I>I		YPS138:I>I	AA:773		A12:S>S		UFRJ50791:S>S		UFRJ50816:S>S		YPS138:S>S	AA:779		N_43:I>I		N_44:I>I	AA:805		A12:E>E		A4:E>E		UFRJ50791:E>E		UFRJ50816:E>E		UWOPS91_917_1:E>E		YPS138:E>E	AA:815		A12:F>F		A4:F>F		UFRJ50791:F>F		UFRJ50816:F>F		UWOPS91_917_1:F>F		YPS138:F>F	AA:834		UFRJ50791:G>G		UFRJ50816:G>G		YPS138:G>G	AA:837		UFRJ50791:L>L		UFRJ50816:L>L		YPS138:L>L	AA:858		UFRJ50791:G>G		UFRJ50816:G>G		UWOPS91_917_1:G>G		YPS138:G>G	AA:868		UFRJ50791:Q>Q		UFRJ50816:Q>Q	AA:877		CBS432:V>V		N_17:V>V		Q32_3:V>V		Q95_3:V>V		T21_4:V>V		Y6_5:V>V	AA:879		UFRJ50791:A>A		UFRJ50816:A>A	AA:892		UWOPS91_917_1:L>L	AA:904		UFRJ50791:S>S		UFRJ50816:S>S	AA:910		UFRJ50791:S>S		UFRJ50816:S>S		UWOPS91_917_1:S>S	AA:926		A12:Q>Q		UFRJ50791:Q>Q		UFRJ50816:Q>Q	AA:936		A12:N>N		A4:N>N		UFRJ50791:N>N		UFRJ50816:N>N		UWOPS91_917_1:N>N	AA:944		IFO1804:G>G		N_43:G>G		N_44:G>G		N_45:G>G	AA:957		A12:D>D		A4:D>D		UFRJ50791:D>D		UFRJ50816:D>D		UWOPS91_917_1:D>D	AA:958		IFO1804:L>L		N_43:L>L		N_44:L>L		N_45:L>LID:YBL006C	AA:4		UWOPS91_917_1:A>A	AA:7		N_45:F>F	AA:22		A12:T>T		A4:T>T		DBVPG6304:T>T		UFRJ50791:T>T	AA:49		A12:G>G		A4:G>G		DBVPG6304:G>G		UFRJ50791:G>G	AA:55		UWOPS91_917_1:S>S	AA:58		A12:N>N		A4:N>N		DBVPG6304:N>N		UFRJ50791:N>N	AA:68		A12:F>F		A4:F>F		DBVPG6304:F>F		UFRJ50791:F>F		UWOPS91_917_1:F>F	AA:87		N_45:L>L	AA:93		A12:A>A		A4:A>A		DBVPG6304:A>A		UFRJ50791:A>A		UWOPS91_917_1:A>A	AA:116		N_45:V>V	AA:118		A4:V>V		DBVPG6304:V>V		UFRJ50791:V>V		UWOPS91_917_1:V>V	AA:136		A4:L>L		DBVPG6304:L>L		UFRJ50791:L>L		UWOPS91_917_1:L>L	AA:140		UWOPS91_917_1:V>V	AA:147		A4:R>R		DBVPG6304:R>R		UFRJ50791:R>R	AA:151		A4:Q>Q		DBVPG6304:Q>Q	AA:179		DBVPG6304:S>SID:YBL007C	AA:16		A4:L>L		UFRJ50791:L>L		UFRJ50816:L>L	AA:17		N_45:P>P	AA:63		A4:S>S		UFRJ50816:S>S	AA:80		CBS432:H>H		DBVPG4650:H>H		N_17:H>H		Q32_3:H>H		Q59_1:H>H		Q62_5:H>H		Q95_3:H>H		T21_4:H>H		Y6_5:H>H		Z1_1:H>H	AA:82		A4:L>L		UFRJ50816:L>L	AA:141		A4:T>T		UFRJ50816:T>T		UWOPS91_917_1:T>T	AA:166		A4:E>E		UFRJ50816:E>E	AA:174		DBVPG4650:T>T	AA:175		UWOPS91_917_1:A>A	AA:181		CBS432:Q>Q		DBVPG4650:Q>Q		KPN3828:Q>Q		Q32_3:Q>Q		Q59_1:Q>Q		Q62_5:Q>Q		Q95_3:Q>Q		T21_4:Q>Q		Y6_5:Q>Q		Z1_1:Q>Q	AA:182		A4:S>S		UFRJ50816:S>S	AA:196		A12:N>N		A4:N>N		N_43:N>N		N_44:N>N		N_45:N>N		UFRJ50816:N>N	AA:245		A12:S>S		A4:S>S		UFRJ50816:S>S		UWOPS91_917_1:S>S	AA:248		A12:S>S		A4:S>S		N_45:S>S		UFRJ50791:S>S		UFRJ50816:S>S		UWOPS91_917_1:S>S	AA:282		A12:E>E		A4:E>E		UFRJ50791:E>E		UFRJ50816:E>E	AA:291		A12:S>S		A4:S>S		UFRJ50791:S>S		UFRJ50816:S>S	AA:329		A12:N>N		A4:N>N		IFO1804:N>N		N_44:N>N		N_45:N>N		UFRJ50791:N>N		UFRJ50816:N>N	AA:331		A12:P>P		A4:P>P		UFRJ50791:P>P		UFRJ50816:P>P	AA:347		IFO1804:L>L		N_44:L>L		N_45:L>L	AA:350		IFO1804:K>K		N_44:K>K		N_45:K>K	AA:352		IFO1804:C>C		N_44:C>C		N_45:C>C	AA:356		UWOPS91_917_1:D>D	AA:404		CBS432:G>G		CBS5829:G>G		DBVPG4650:G>G		KPN3829:G>G		N_17:G>G		Q89_8:G>G		Q95_3:G>G		T21_4:G>G		Y6_5:G>G	AA:405		UWOPS91_917_1:R>R	AA:417		A12:I>I		A4:I>I		DBVPG6304:I>I		IFO1804:I>I		N_17:I>I		N_44:I>I		N_45:I>I		UFRJ50791:I>I		UFRJ50816:I>I		UWOPS91_917_1:I>I	AA:424		A12:C>C		A4:C>C		DBVPG6304:C>C		UFRJ50791:C>C		UFRJ50816:C>C	AA:443		A12:G>G		A4:G>G		DBVPG6304:G>G		UFRJ50791:G>G		UWOPS91_917_1:G>G	AA:477		A12:C>C		A4:C>C		DBVPG6304:C>C		UFRJ50791:C>C	AA:484		UWOPS91_917_1:L>L	AA:493		A12:G>G		A4:G>G		DBVPG6304:G>G		IFO1804:G>G		N_44:G>G		N_45:G>G		UFRJ50791:G>G		UWOPS91_917_1:G>G	AA:507		A12:->-		A4:->-		DBVPG6304:->-		UWOPS91_917_1:->-	AA:517		A12:N>N		A4:N>N		DBVPG6304:N>N		UWOPS91_917_1:N>N	AA:523		A12:P>P		A4:P>P		UWOPS91_917_1:L>L	AA:527		UWOPS91_917_1:L>L	AA:533		DBVPG6304:Y>Y		UWOPS91_917_1:Y>Y	AA:552		A12:V>V		A4:V>V		DBVPG6304:V>V	AA:566		A12:Y>Y		A4:Y>Y		DBVPG6304:Y>Y		YPS138:Y>Y	AA:577		IFO1804:A>A		N_44:A>A	AA:582		UWOPS91_917_1:E>E	AA:587		UWOPS91_917_1:I>I	AA:593		UWOPS91_917_1:V>V	AA:597		UWOPS91_917_1:F>F	AA:602		UWOPS91_917_1:G>G	AA:605		A4:I>I		DBVPG6304:I>I		YPS138:I>I	AA:612		A4:S>S		DBVPG6304:S>S		N_43:S>S		N_44:S>S		N_45:S>S		UWOPS91_917_1:L>L		YPS138:S>S	AA:620		A4:R>R		DBVPG6304:R>R		UFRJ50816:R>R		UWOPS91_917_1:R>R		YPS138:R>R	AA:638		A4:F>F		UFRJ50816:F>F		YPS138:F>F	AA:645		A4:T>T		DBVPG6304:T>T		YPS138:T>T	AA:653		UWOPS91_917_1:F>F	AA:659		A4:L>L	AA:663		UWOPS91_917_1:S>S	AA:680		UWOPS91_917_1:I>I	AA:683		UWOPS91_917_1:L>L	AA:686		A4:L>L		DBVPG6304:L>L		UFRJ50816:L>L		YPS138:L>L	AA:690		A4:A>A		DBVPG6304:A>A		UFRJ50816:A>A		UWOPS91_917_1:A>A		YPS138:A>A	AA:695		DBVPG6304:H>H		UFRJ50816:H>H		YPS138:H>H	AA:709		UWOPS91_917_1:S>S	AA:711		DBVPG6304:L>L		UFRJ50816:L>L		UWOPS91_917_1:L>L		YPS138:L>L	AA:739		DBVPG6304:I>I		N_43:I>I		N_44:I>I		UFRJ50816:I>I		YPS138:I>I	AA:744		UWOPS91_917_1:R>R	AA:754		DBVPG6304:R>R		UFRJ50816:R>R		YPS138:R>R	AA:761		UWOPS91_917_1:R>R	AA:763		DBVPG6304:Q>Q		UFRJ50816:Q>Q		YPS138:Q>Q	AA:770		UWOPS91_917_1:L>L	AA:782		UWOPS91_917_1:F>F	AA:785		UWOPS91_917_1:F>F	AA:787		UFRJ50816:T>T		UWOPS91_917_1:T>T	AA:792		UFRJ50816:G>G		YPS138:G>G	AA:800		DBVPG6304:R>R		UFRJ50816:R>R	AA:803		UFRJ50816:W>W		YPS138:W>W	AA:831		UFRJ50816:N>N	AA:877		UFRJ50816:E>E		UWOPS91_917_1:E>E		YPS138:E>E	AA:900		A4:A>A		UFRJ50816:A>A		UWOPS91_917_1:A>A		YPS138:A>A	AA:916		UWOPS91_917_1:R>R	AA:918		A4:G>G		CBS432:G>G		CBS5829:G>G		DBVPG4650:G>G		N_17:G>G		N_43:G>G		N_44:G>G		N_45:G>G		Q32_3:G>G		Q59_1:G>G		Q95_3:G>G		T21_4:G>G		UFRJ50816:G>G		UWOPS91_917_1:G>G		YPS138:G>G	AA:937		CBS5829:V>V	AA:973		A4:L>L		UFRJ50791:L>L		UFRJ50816:L>L		UWOPS91_917_1:L>L		YPS138:L>L	AA:987		UWOPS91_917_1:V>V	AA:989		A4:L>L		DBVPG6304:L>L		UFRJ50791:L>L		UFRJ50816:L>L		UWOPS91_917_1:L>L	AA:992		Q32_3:V>V	AA:1003		UWOPS91_917_1:I>I	AA:1029		A4:S>S		DBVPG6304:S>S		N_43:S>S		N_44:S>S		N_45:S>S		UFRJ50791:S>S		UFRJ50816:S>S		UWOPS91_917_1:S>S	AA:1082		UWOPS91_917_1:R>R	AA:1092		A4:R>R		UFRJ50791:R>R		UFRJ50816:R>R	AA:1113		A4:V>V	AA:1118		N_43:E>E		N_44:E>E		UFRJ50791:E>E		UWOPS91_917_1:E>E	AA:1124		N_43:N>N		N_44:N>N		N_45:N>N	AA:1137		IFO1804:I>I		UFRJ50791:I>I	AA:1193		UFRJ50791:F>FID:YBL009W	AA:5		A4:A>A		CBS432:A>A		CBS5829:A>A		DBVPG4650:A>A		DBVPG6304:A>A		KPN3828:A>A		N_17:A>A		Q59_1:A>A		Q95_3:A>A		T21_4:A>A		UFRJ50816:A>A	AA:19		A4:L>L		DBVPG6304:L>L		UFRJ50816:L>L	AA:22		UWOPS91_917_1:A>A	AA:32		A4:N>N		DBVPG6304:N>N		IFO1804:N>N		N_43:N>N		N_44:N>N		N_45:N>N		UFRJ50816:N>N		UWOPS91_917_1:N>N	AA:63		A4:S>S		DBVPG6304:S>S		UFRJ50816:S>S		UWOPS91_917_1:S>S	AA:73		A4:S>S		DBVPG6304:S>S		UFRJ50816:S>S		UWOPS91_917_1:S>S	AA:81		UWOPS91_917_1:T>T	AA:95		A4:G>G		DBVPG6304:G>G		UFRJ50816:G>G		UWOPS91_917_1:G>G	AA:107		A4:I>I		DBVPG6304:I>I		UFRJ50816:I>I	AA:121		A4:Q>Q		DBVPG6304:Q>Q		UFRJ50816:Q>Q	AA:127		A4:S>S		UFRJ50816:S>S		UWOPS91_917_1:S>S	AA:135		A4:R>R		UFRJ50816:R>R	AA:143		IFO1804:K>K		N_43:K>K		N_45:K>K	AA:144		A4:L>L		UFRJ50816:L>L	AA:163		UWOPS91_917_1:S>S	AA:167		A4:A>A		UFRJ50816:A>A		UWOPS91_917_1:A>A	AA:173		A4:S>S		N_43:S>S		N_45:S>S		UFRJ50816:S>S		UWOPS91_917_1:S>S	AA:175		N_43:K>K		N_45:K>K	AA:208		A4:T>T		UFRJ50816:T>T	AA:223		UFRJ50816:L>L	AA:239		CBS432:V>V		KPN3828:V>V		KPN3829:V>V	AA:240		UFRJ50816:E>E	AA:245		UFRJ50816:N>N	AA:276		N_44:I>I	AA:279		IFO1804:D>D		N_44:D>D		N_45:D>D		UFRJ50816:D>D		UWOPS91_917_1:D>D		YPS138:D>D	AA:294		UWOPS91_917_1:N>N	AA:299		IFO1804:S>S		N_44:S>S		N_45:S>S		UFRJ50816:S>S		UWOPS91_917_1:S>S		YPS138:S>S	AA:328		UWOPS91_917_1:K>K		YPS138:K>K	AA:335		UFRJ50816:L>L		UWOPS91_917_1:L>L		YPS138:L>L	AA:341		UWOPS91_917_1:V>V	AA:344		UFRJ50816:D>D		YPS138:D>D	AA:348		YPS138:L>L	AA:369		UWOPS91_917_1:L>L	AA:383		UWOPS91_917_1:C>C	AA:388		UWOPS91_917_1:I>I	AA:411		A12:S>S		DBVPG6304:S>S		UFRJ50816:S>S		UWOPS91_917_1:S>S		YPS138:S>S	AA:423		A12:Q>Q		DBVPG6304:Q>Q		UFRJ50816:Q>Q		UWOPS91_917_1:Q>Q		YPS138:Q>Q	AA:427		A12:I>I		DBVPG6304:I>I		UFRJ50816:I>I		YPS138:I>I	AA:434		A12:G>G		DBVPG6304:G>G		UFRJ50816:G>G		UWOPS91_917_1:G>G		YPS138:G>G	AA:443		IFO1804:N>N		N_43:N>N		N_44:N>N		N_45:N>N	AA:444		UWOPS91_917_1:L>L	AA:447		UWOPS91_917_1:L>L	AA:450		UWOPS91_917_1:L>L	AA:454		UWOPS91_917_1:R>R	AA:458		A12:G>G		DBVPG6304:G>G		UFRJ50816:G>G		UWOPS91_917_1:G>G		YPS138:G>G	AA:460		A12:S>S		DBVPG6304:S>S		UFRJ50791:S>S		UFRJ50816:S>S		UWOPS91_917_1:S>S		YPS138:S>S	AA:466		UWOPS91_917_1:L>L	AA:470		A12:V>V		DBVPG6304:V>V		UFRJ50791:V>V		UFRJ50816:V>V		UWOPS91_917_1:V>V		YPS138:V>V	AA:506		A12:A>A		DBVPG6304:A>A		UFRJ50791:A>A		UFRJ50816:A>A		UWOPS91_917_1:A>A		YPS138:A>A	AA:519		A12:V>V		DBVPG6304:V>V		UFRJ50791:V>V		UFRJ50816:V>V		YPS138:V>V	AA:531		A12:L>L		DBVPG6304:L>L		UFRJ50791:L>L		UFRJ50816:L>L		YPS138:L>L	AA:559		A12:I>I		DBVPG6304:I>I		UFRJ50791:I>I		UFRJ50816:I>I		YPS138:I>I	AA:565		N_43:T>T		N_44:T>T		N_45:T>T	AA:569		A12:H>H		DBVPG6304:H>H		UFRJ50791:H>H		UFRJ50816:H>H		UWOPS91_917_1:H>H		YPS138:H>H	AA:581		A12:F>F		DBVPG6304:F>F		UFRJ50791:F>F		UFRJ50816:F>F		YPS138:F>F	AA:586		A12:L>L		DBVPG6304:L>L		UFRJ50791:L>L		UFRJ50816:L>L		YPS138:L>L	AA:591		N_43:L>L		N_44:L>L		N_45:L>L	AA:592		A12:P>P		DBVPG6304:P>P		UFRJ50791:P>P		UFRJ50816:P>P		UWOPS91_917_1:P>P		YPS138:P>P	AA:594		N_43:P>P		N_44:P>P		N_45:P>P	AA:609		A12:L>L		DBVPG6304:L>L		UFRJ50791:L>L		UFRJ50816:L>L		UWOPS91_917_1:L>L		YPS138:L>L	AA:626		A12:T>T		DBVPG6304:T>T		UFRJ50791:T>T		UFRJ50816:T>T		YPS138:T>T	AA:635		UWOPS91_917_1:I>I	AA:647		A12:R>R		DBVPG6304:R>R		UFRJ50791:R>R		UFRJ50816:R>R		UWOPS91_917_1:R>R		YPS138:R>R	AA:651		A12:K>K		DBVPG6304:K>K		UFRJ50791:K>K		UFRJ50816:K>K		UWOPS91_917_1:K>K		YPS138:K>K	AA:658		A12:L>L		DBVPG6304:L>L		UFRJ50791:L>L		UFRJ50816:L>L		YPS138:L>L	AA:667		DBVPG6304:L>L		UFRJ50791:L>L		UFRJ50816:L>L		YPS138:L>LID:YBL010C	AA:10		IFO1804:L>L		N_43:L>L		N_44:L>L		N_45:L>L	AA:42		CBS5829:L>L		N_17:L>L		Q32_3:L>L		Q89_8:L>L		Q95_3:L>L		T21_4:L>L	AA:63		A12:N>N		A4:N>N		DBVPG6304:N>N		UFRJ50816:N>N		YPS138:N>N	AA:97		N_44:L>L		N_45:L>L	AA:102		A12:K>K		A4:K>K	AA:124		A12:S>S		A4:S>S		YPS138:S>S	AA:150		N_43:S>S		N_44:S>S		N_45:S>S	AA:243		A12:L>L		A4:L>L		N_43:L>L		N_44:L>L		N_45:L>L		YPS138:L>L	AA:245		A12:I>I		A4:I>I	AA:257		A12:S>S		A4:S>S		YPS138:S>S	AA:276		A4:L>L	AA:281		A12:H>H		A4:H>H		UFRJ50816:H>HID:YBL011W	AA:11		UWOPS91_917_1:A>A	AA:23		A12:N>N		A4:N>N		DBVPG6304:N>N	AA:33		A12:S>S		A4:S>S		DBVPG6304:S>S		UWOPS91_917_1:S>S	AA:43		UWOPS91_917_1:P>P	AA:50		A12:L>L		A4:L>L		DBVPG6304:L>L		UWOPS91_917_1:L>L	AA:61		UWOPS91_917_1:I>I	AA:72		A4:R>R		DBVPG6304:R>R		N_43:R>R		N_45:R>R		UWOPS91_917_1:R>R	AA:74		N_45:S>S	AA:84		UWOPS91_917_1:I>I	AA:92		A12:N>N		A4:N>N		DBVPG6304:N>N		UWOPS91_917_1:N>N	AA:97		A4:P>P		DBVPG6304:P>P		UWOPS91_917_1:P>P	AA:130		UWOPS91_917_1:A>A	AA:133		A4:F>F		DBVPG6304:F>F		N_43:F>F		N_45:F>F		UWOPS91_917_1:F>F	AA:135		A4:A>A		DBVPG6304:A>A	AA:143		A4:D>D		DBVPG6304:D>D		UWOPS91_917_1:D>D	AA:147		UWOPS91_917_1:P>P	AA:165		A4:H>H		DBVPG6304:H>H		UWOPS91_917_1:H>H	AA:203		A4:K>K		DBVPG6304:K>K	AA:210		A4:P>P		DBVPG6304:P>P	AA:212		A4:V>V		DBVPG6304:V>V	AA:214		A4:T>T		DBVPG6304:T>T	AA:226		A12:A>A		A4:A>A		DBVPG6304:A>A		UWOPS91_917_1:A>A		YPS138:A>A	AA:240		A12:H>H		A4:H>H		DBVPG6304:H>H	AA:242		A12:A>A		A4:A>A		DBVPG6304:A>A		UFRJ50816:A>A		UWOPS91_917_1:A>A	AA:251		A4:P>P		DBVPG6304:P>P		UFRJ50816:P>P		UWOPS91_917_1:P>P	AA:255		DBVPG4650:S>S	AA:256		A12:H>H		A4:H>H		DBVPG6304:H>H		UFRJ50816:H>H		UWOPS91_917_1:H>H	AA:267		A12:G>G		DBVPG6304:G>G		UFRJ50816:G>G		UWOPS91_917_1:G>G		YPS138:G>G	AA:296		A12:P>P		UFRJ50816:P>P		UWOPS91_917_1:P>P		YPS138:P>P	AA:305		A12:V>V		UFRJ50816:V>V		YPS138:V>V	AA:321		A12:Y>Y		UFRJ50816:Y>Y		YPS138:Y>Y	AA:324		A12:P>P		UFRJ50816:P>P		UWOPS91_917_1:P>P		YPS138:P>P	AA:334		A12:L>L		IFO1804:L>L		N_43:L>L		N_44:L>L		N_45:L>L		UFRJ50816:L>L		UWOPS91_917_1:L>L		YPS138:L>L	AA:344		A12:S>S		IFO1804:S>S		N_43:S>S		N_44:S>S		N_45:S>S		UFRJ50816:S>S		UWOPS91_917_1:S>S		YPS138:S>S	AA:358		IFO1804:V>V		N_43:V>V		N_44:V>V		N_45:V>V	AA:369		N_45:F>F	AA:411		A12:A>A		A4:A>A		DBVPG6304:A>A		UFRJ50816:A>A		YPS138:A>A	AA:414		A12:H>H		A4:H>H		DBVPG6304:H>H		UFRJ50816:H>H		YPS138:H>H	AA:430		A12:A>A		A4:A>A		DBVPG6304:A>A		UFRJ50816:A>A		UWOPS91_917_1:A>A		YPS138:A>A	AA:439		A12:R>R		A4:R>R		DBVPG6304:R>R		UFRJ50816:R>R		YPS138:R>R	AA:442		A12:G>G		A4:G>G		DBVPG6304:G>G		IFO1804:G>G		N_43:G>G		N_45:G>G		UFRJ50816:G>G		UWOPS91_917_1:G>G		YPS138:G>G	AA:444		UWOPS91_917_1:C>C	AA:452		A12:P>P		A4:P>P		DBVPG6304:P>P		UFRJ50816:P>P		UWOPS91_917_1:P>P		YPS138:P>P	AA:458		A12:S>S		A4:S>S		DBVPG6304:S>S		UFRJ50816:S>S		UWOPS91_917_1:S>S		YPS138:S>S	AA:472		A12:A>A		A4:A>A		DBVPG6304:A>A		UFRJ50816:A>A		UWOPS91_917_1:A>A		YPS138:A>A	AA:482		A12:K>K		A4:K>K		DBVPG6304:K>K		UFRJ50816:K>K		UWOPS91_917_1:K>K		YPS138:K>K	AA:496		UWOPS91_917_1:I>I	AA:516		A12:L>L		A4:L>L		DBVPG6304:L>L		UFRJ50816:L>L		YPS138:L>L	AA:520		UWOPS91_917_1:P>P	AA:522		A12:N>N		A4:N>N		DBVPG6304:N>N		UFRJ50816:N>N		YPS138:N>N	AA:526		A12:I>I		A4:I>I		DBVPG6304:I>I		UFRJ50816:I>I		UWOPS91_917_1:I>I		YPS138:I>I	AA:533		A4:S>S	AA:558		A12:L>L		A4:L>L		DBVPG6304:L>L		UFRJ50791:L>L		YPS138:L>L	AA:564		A12:S>S		A4:S>S		DBVPG6304:S>S		UFRJ50791:S>S		YPS138:S>S	AA:568		A12:L>L		A4:L>L	AA:579		UWOPS91_917_1:A>A	AA:593		A4:L>L		DBVPG6304:L>L		IFO1804:L>L		N_43:L>L		UFRJ50791:L>L		UFRJ50816:L>L		YPS138:L>L	AA:601		DBVPG4650:A>A		Q62_5:A>A		Q95_3:A>A	AA:611		IFO1804:E>E		N_43:E>E	AA:616		A4:R>R		UFRJ50791:R>R		UFRJ50816:R>R		YPS138:R>R	AA:618		A4:T>T		UFRJ50791:T>T		UFRJ50816:T>T		YPS138:T>T	AA:624		A4:R>R		UFRJ50791:R>R		UFRJ50816:R>R		YPS138:R>R	AA:649		A4:N>N		UFRJ50791:N>N		UFRJ50816:N>N		YPS138:N>N	AA:667		A4:N>N		UFRJ50791:N>N		UFRJ50816:N>N		YPS138:N>N	AA:691		A4:A>A		UFRJ50791:A>A		UFRJ50816:A>A		YPS138:A>A	AA:729		IFO1804:I>I		N_43:I>IID:YBL013W	AA:32		A12:V>V		A4:V>V		DBVPG6304:V>V	AA:43		A12:L>L		A4:L>L		DBVPG6304:L>L		N_43:L>L		N_44:L>L	AA:66		A12:V>V		A4:V>V		DBVPG6304:V>V	AA:75		A4:G>G		DBVPG6304:G>G	AA:77		A4:Q>Q		DBVPG6304:Q>Q	AA:80		A4:V>V		DBVPG6304:V>V	AA:111		N_44:S>S	AA:135		DBVPG6304:A>A	AA:159		DBVPG6304:S>S		IFO1804:S>S		N_44:S>S	AA:172		DBVPG6304:R>R	AA:181		A12:T>T		DBVPG6304:T>T	AA:184		A12:T>T		DBVPG6304:T>T	AA:193		A12:F>F		DBVPG6304:F>F	AA:199		A12:V>V		DBVPG6304:V>V	AA:202		A12:T>T		DBVPG6304:T>T		UWOPS91_917_1:T>T	AA:246		A12:L>L		DBVPG6304:L>L	AA:250		UWOPS91_917_1:T>T	AA:257		A12:L>L		DBVPG6304:L>L		UWOPS91_917_1:L>L	AA:259		UWOPS91_917_1:Q>Q	AA:263		UWOPS91_917_1:Q>Q	AA:269		A12:K>K		DBVPG6304:K>K		UWOPS91_917_1:K>K	AA:274		DBVPG6304:H>H	AA:295		UWOPS91_917_1:N>N	AA:307		A12:K>K		DBVPG6304:K>K		UWOPS91_917_1:K>K	AA:310		UWOPS91_917_1:A>A	AA:372		DBVPG6304:F>F		UFRJ50791:F>F		UWOPS91_917_1:F>F	AA:377		A12:V>V		A4:V>V		DBVPG6304:V>V		UFRJ50791:V>V		UWOPS91_917_1:V>VID:YBL014C	AA:60		A12:L>L		A4:L>L		DBVPG6304:L>L		UWOPS91_917_1:L>L	AA:67		A12:F>F		A4:F>F		DBVPG6304:F>F		UWOPS91_917_1:F>F	AA:97		A4:K>K		DBVPG6304:K>K		UWOPS91_917_1:K>K	AA:107		A4:I>I		DBVPG6304:I>I		UWOPS91_917_1:I>I	AA:140		A4:N>N	AA:143		A4:K>K		DBVPG6304:K>K		UWOPS91_917_1:K>K	AA:157		UWOPS91_917_1:V>V	AA:168		A12:L>L	AA:171		UWOPS91_917_1:Y>Y	AA:179		A12:F>F		A4:F>F		DBVPG6304:F>F		UWOPS91_917_1:F>F	AA:187		A4:R>R		DBVPG6304:R>R		UWOPS91_917_1:R>R	AA:202		A12:N>N	AA:206		A12:I>I	AA:208		A12:I>I	AA:219		A4:->-		DBVPG6304:->-		UWOPS91_917_1:->-	AA:238		A12:F>F	AA:241		UWOPS91_917_1:A>A	AA:250		UWOPS91_917_1:I>I	AA:255		N_43:P>P		N_44:P>P		N_45:P>P	AA:261		A12:I>I	AA:263		A12:S>S	AA:265		A12:G>G		UWOPS91_917_1:R>R	AA:271		A12:I>I	AA:274		A12:V>V	AA:287		A12:I>I		A4:A>A	AA:292		IFO1804:Y>Y		N_43:Y>Y		N_44:Y>Y		N_45:Y>Y	AA:300		A4:N>N		DBVPG6304:D>D		UFRJ50791:D>D	AA:302		A4:R>R		DBVPG6304:R>R		UFRJ50791:R>R	AA:305		A4:C>C		DBVPG6304:C>C		UFRJ50791:C>C		UWOPS91_917_1:C>C	AA:316		A4:V>V		DBVPG6304:V>V		UFRJ50791:V>V		UWOPS91_917_1:V>V	AA:322		A12:P>P	AA:332		A12:F>F		DBVPG6304:F>F		UFRJ50791:F>F		UWOPS91_917_1:F>F	AA:335		A4:T>T		DBVPG6304:T>T		UFRJ50791:T>T		UWOPS91_917_1:T>T	AA:337		A4:F>F		DBVPG6304:F>F		UFRJ50791:F>F		UWOPS91_917_1:F>F	AA:339		A12:L>L		A4:L>L		DBVPG6304:G>G		IFO1804:G>G		N_43:G>G		N_44:G>G		N_45:G>G		UFRJ50791:G>G		UWOPS91_917_1:G>G	AA:342		A12:I>I	AA:350		DBVPG6304:L>L		UFRJ50791:L>L	AA:352		A12:R>R	AA:358		A12:->-		DBVPG6304:->-		UFRJ50791:->-		UWOPS91_917_1:->-	AA:362		A12:N>N		DBVPG6304:N>N		UFRJ50791:N>N		UWOPS91_917_1:N>N	AA:365		KPN3828:N>N	AA:377		A12:I>I		UWOPS91_917_1:I>I	AA:383		A12:C>C		DBVPG6304:C>C		UFRJ50791:C>C		UWOPS91_917_1:C>C	AA:400		A12:C>C		DBVPG6304:C>C		UFRJ50791:C>C		UWOPS91_917_1:C>C	AA:406		A12:L>L		DBVPG6304:L>L		UFRJ50791:L>L	AA:412		UWOPS91_917_1:P>P	AA:428		UWOPS91_917_1:Y>Y	AA:438		UWOPS91_917_1:L>L	AA:445		UWOPS91_917_1:D>D	AA:448		UWOPS91_917_1:R>R	AA:454		UWOPS91_917_1:Q>Q	AA:455		A12:I>I		A4:I>I		DBVPG6304:I>I	AA:487		UWOPS91_917_1:V>V	AA:496		A12:->-		A4:->-	AA:499		A12:L>L		A4:L>L		IFO1804:L>L		N_43:L>L		N_45:L>L		UWOPS91_917_1:L>L	AA:508		A12:I>I		A4:I>I	AA:519		YPS138:P>P	AA:521		A12:K>K		A4:K>K		YPS138:K>K	AA:528		A12:L>L	AA:542		A12:W>W		A4:W>W	AA:620		A4:F>F	AA:651		IFO1804:T>T		N_44:T>T		N_45:T>T	AA:696		UWOPS91_917_1:S>S	AA:730		A12:R>R		A4:R>R	AA:734		A12:T>T		A4:T>T	AA:736		IFO1804:L>L		N_45:L>L	AA:743		A12:A>A		A4:A>A		UWOPS91_917_1:A>A	AA:768		A12:T>T		A4:T>T		UFRJ50816:T>T	AA:771		A12:N>N		A4:N>N		UFRJ50816:N>N		UWOPS91_917_1:N>N	AA:805		DBVPG4650:S>S		Q62_5:S>S	AA:810		A12:V>V		A4:V>V		IFO1804:V>V		N_45:V>V		UFRJ50816:V>V		UWOPS91_917_1:V>V	AA:823		IFO1804:I>I		N_45:I>I	AA:831		A12:Q>Q		A4:Q>Q		IFO1804:Q>Q		N_45:Q>Q		UFRJ50816:Q>Q		UWOPS91_917_1:Q>Q	AA:834		IFO1804:D>D		N_45:D>D	AA:857		A12:P>P		A4:P>P		UFRJ50816:P>P	AA:891		Q59_1:S>SID:YBL015W	AA:19		A4:F>F		DBVPG6304:F>F		UWOPS91_917_1:F>F	AA:21		UWOPS91_917_1:F>F	AA:82		IFO1804:V>V	AA:85		A4:S>S		DBVPG6304:S>S		UFRJ50816:S>S		UWOPS91_917_1:S>S	AA:94		Q62_5:G>G		Y7:G>G		Z1_1:G>G	AA:99		A4:P>P		DBVPG6304:P>P		IFO1804:P>P		N_43:P>P		N_44:P>P		N_45:P>P		UFRJ50816:P>P		UWOPS91_917_1:P>P	AA:103		UWOPS91_917_1:R>R	AA:132		IFO1804:P>P		N_43:P>P		N_44:P>P		N_45:P>P		UWOPS91_917_1:P>P	AA:151		A4:F>F		DBVPG6304:F>F		UFRJ50816:F>F		YPS138:F>F	AA:176		A4:D>D		DBVPG6304:D>D		UFRJ50816:D>D		UWOPS91_917_1:D>D		YPS138:D>D	AA:210		A4:V>V		DBVPG6304:V>V		UFRJ50816:V>V		UWOPS91_917_1:V>V		YPS138:V>V	AA:240		A4:I>I		UFRJ50816:I>I		UWOPS91_917_1:I>I		YPS138:I>I	AA:246		N_17:H>H	AA:277		A4:T>T		UFRJ50816:T>T		YPS138:T>T	AA:281		A4:N>N		UFRJ50816:N>N		UWOPS91_917_1:N>N		YPS138:N>N	AA:284		A4:S>S		UFRJ50816:S>S		UWOPS91_917_1:S>S		YPS138:S>S	AA:286		IFO1804:N>N		N_44:N>N		N_45:N>N	AA:294		A4:E>E		UFRJ50816:E>E		YPS138:E>E	AA:306		A4:V>V		UWOPS91_917_1:V>V		YPS138:V>V	AA:309		A4:Q>Q		UWOPS91_917_1:Q>Q		YPS138:Q>Q	AA:325		A4:H>H		YPS138:H>H	AA:352		IFO1804:D>D		N_43:D>D		N_44:D>D		N_45:D>D	AA:356		IFO1804:I>I		N_43:I>I		N_44:I>I		N_45:I>I	AA:369		IFO1804:I>I		N_43:I>I		N_44:I>I		N_45:I>I		YPS138:I>I	AA:373		YPS138:F>F	AA:375		YPS138:V>V	AA:385		YPS138:N>N	AA:399		YPS138:V>V	AA:415		A12:S>S		YPS138:S>S	AA:440		A12:I>I		DBVPG6304:I>I		UWOPS91_917_1:I>I		YPS138:I>I	AA:514		UWOPS91_917_1:L>LID:YBL016W	AA:9		N_43:Y>Y		N_44:Y>Y		N_45:Y>Y	AA:12		N_43:V>V		N_45:V>V		Q62_5:V>V		S36_7:V>V		Y6_5:V>V		Z1_1:V>V	AA:36		Q62_5:S>S		S36_7:S>S		Y6_5:S>S		Z1_1:S>S	AA:41		CBS5829:N>N		N_43:N>N		N_44:N>N		N_45:N>N		Q62_5:N>N		S36_7:N>N		Y6_5:N>N		Z1_1:N>N	AA:58		Q89_8:T>T		S36_7:T>T		Y6_5:T>T		Z1_1:T>T	AA:91		N_44:D>D		N_45:D>D		UFRJ50816:D>D		YPS138:D>D	AA:118		DBVPG6304:Y>Y		UFRJ50816:Y>Y		YPS138:Y>Y	AA:122		YPS138:->-	AA:147		N_44:G>G		N_45:G>G	AA:150		A4:V>V		DBVPG6304:V>V		UFRJ50816:V>V		YPS138:V>V	AA:172		N_44:G>G		N_45:G>G	AA:180		A4:G>G		DBVPG6304:G>G		N_44:G>G		N_45:G>G		UFRJ50816:G>G		UWOPS91_917_1:G>G		YPS138:G>G	AA:189		A4:P>P		DBVPG6304:P>P		N_44:P>P		N_45:P>P		UFRJ50816:P>P		UWOPS91_917_1:P>P		YPS138:P>P	AA:195		A4:->-		DBVPG6304:->-		UFRJ50816:->-		UWOPS91_917_1:->-		YPS138:->-	AA:205		UWOPS91_917_1:I>I	AA:227		A4:V>V		DBVPG6304:V>V		UFRJ50816:V>V		YPS138:V>V	AA:254		A4:G>G		DBVPG6304:G>G		N_44:G>G		N_45:G>G		UFRJ50816:G>G		YPS138:G>G	AA:256		A4:S>S		DBVPG6304:S>S		UFRJ50816:S>S		YPS138:S>S	AA:266		N_44:V>V		N_45:V>V	AA:274		A4:H>H		DBVPG6304:H>H		UFRJ50816:H>H		YPS138:H>H	AA:288		A4:L>L		UFRJ50816:L>L		YPS138:L>L	AA:294		N_45:R>R	AA:312		A4:V>V		UFRJ50816:V>V		UWOPS91_917_1:V>V		YPS138:V>V	AA:341		N_44:L>L	AA:347		DBVPG6304:N>N		UFRJ50816:N>N		YPS138:N>NID:YBL019W	AA:7		A4:Y>Y		DBVPG6304:Y>Y		UFRJ50791:Y>Y		UFRJ50816:Y>Y		UWOPS91_917_1:Y>Y		YPS138:Y>Y	AA:15		UWOPS91_917_1:T>T	AA:34		A4:L>L		DBVPG6304:L>L		YPS138:L>L	AA:36		A4:E>E		DBVPG6304:E>E		UFRJ50791:E>E		UFRJ50816:E>E		UWOPS91_917_1:E>E		YPS138:E>E	AA:43		A4:K>K		DBVPG6304:K>K		UFRJ50791:K>K		UFRJ50816:K>K		YPS138:K>K	AA:61		CBS432:F>F		N_17:F>F	AA:66		UWOPS91_917_1:G>G	AA:70		A4:P>P		DBVPG6304:P>P		UFRJ50791:P>P		UFRJ50816:P>P		UWOPS91_917_1:P>P		YPS138:P>P	AA:74		DBVPG6304:V>V		UFRJ50791:V>V		UFRJ50816:V>V		UWOPS91_917_1:V>V		YPS138:V>V	AA:92		DBVPG6304:N>N		UFRJ50791:N>N		UFRJ50816:N>N		UWOPS91_917_1:N>N		YPS138:N>N	AA:98		DBVPG6304:N>N		UFRJ50791:N>N		UFRJ50816:N>N	AA:106		N_44:I>I		UWOPS91_917_1:I>I	AA:109		DBVPG6304:Q>Q		UFRJ50791:Q>Q		UFRJ50816:Q>Q	AA:127		DBVPG6304:T>T		IFO1804:T>T		N_43:T>T		N_44:T>T		N_45:T>T		UFRJ50791:T>T		UWOPS91_917_1:T>T		YPS138:T>T	AA:136		CBS432:V>V		CBS5829:V>V		IFO1804:V>V		N_17:V>V		N_43:V>V		N_44:V>V		N_45:V>V		Q59_1:V>V		Q62_5:V>V		Q89_8:V>V		T21_4:V>V		Y6_5:V>V		Y7:V>V		Z1_1:V>V	AA:137		DBVPG6304:V>V		UFRJ50791:V>V		UFRJ50816:V>V		UWOPS91_917_1:V>V		YPS138:V>V	AA:141		DBVPG6304:P>P		UFRJ50791:P>P		YPS138:P>P	AA:146		IFO1804:I>I		N_43:I>I		N_45:I>I	AA:148		A12:->-		DBVPG6304:->-		UFRJ50791:->-		UFRJ50816:->-		UWOPS91_917_1:->-		YPS138:->-	AA:151		CBS5829:I>I	AA:152		A12:->-		DBVPG6304:->-		UFRJ50791:->-		YPS138:->-	AA:178		A12:D>D		DBVPG6304:D>D		UFRJ50791:D>D		UWOPS91_917_1:D>D		YPS138:D>D	AA:185		A12:I>I		DBVPG6304:I>I		UWOPS91_917_1:I>I		YPS138:I>I	AA:191		UWOPS91_917_1:L>L	AA:203		A12:->-		DBVPG6304:->-		UWOPS91_917_1:->-		YPS138:->-	AA:240		CBS432:->-	AA:247		N_43:H>H		N_44:H>H		N_45:H>H	AA:305		A12:S>S		A4:S>S		DBVPG6304:S>S		UFRJ50816:S>S		YPS138:S>S	AA:383		UWOPS91_917_1:S>S	AA:395		UWOPS91_917_1:F>F	AA:402		N_43:V>V		N_44:V>V		N_45:V>V	AA:414		A12:R>R		A4:R>R		UFRJ50816:R>R		YPS138:R>R	AA:423		UWOPS91_917_1:T>T	AA:442		A12:I>I		A4:I>I		UFRJ50816:I>I		UWOPS91_917_1:I>I	AA:450		Y6_5:F>F	AA:455		A12:S>S		A4:S>S		CBS432:S>S		N_43:S>S		N_44:S>S		N_45:S>S		UFRJ50816:S>S		UWOPS91_917_1:S>S		YPS138:S>S	AA:484		A12:Q>Q		A4:Q>Q		UWOPS91_917_1:Q>Q		YPS138:Q>QID:YBL020W	AA:8		A12:Q>Q		DBVPG6304:Q>Q		YPS138:Q>Q	AA:21		A12:F>F		DBVPG6304:F>F		UWOPS91_917_1:F>F		YPS138:F>F	AA:45		A4:D>D		UFRJ50816:D>D		UWOPS91_917_1:D>D	AA:46		A12:P>P		A4:P>P		UFRJ50816:P>P	AA:64		A12:A>A		A4:A>A		DBVPG6304:A>A		UFRJ50816:A>A		YPS138:A>A	AA:71		IFO1804:S>S		N_45:S>S	AA:91		A12:V>V		A4:V>V		DBVPG6304:V>V		UFRJ50816:V>V		UWOPS91_917_1:V>V		YPS138:V>V	AA:102		UWOPS91_917_1:V>V	AA:108		A12:L>L		A4:L>L		DBVPG6304:L>L		UFRJ50816:L>L		YPS138:L>L	AA:111		UWOPS91_917_1:N>N	AA:113		A12:K>K		A4:K>K		DBVPG6304:K>K		UFRJ50816:K>K		UWOPS91_917_1:K>K		YPS138:K>K	AA:127		N_45:T>T	AA:159		UWOPS91_917_1:D>D	AA:174		A12:Q>Q		A4:Q>Q		DBVPG6304:Q>Q		UFRJ50816:Q>Q		YPS138:Q>Q	AA:187		A12:H>H		A4:H>H		DBVPG6304:H>H		UFRJ50816:H>H		YPS138:H>H	AA:194		A12:I>I		A4:I>I		DBVPG6304:I>I		UFRJ50816:I>I		UWOPS91_917_1:I>I		YPS138:I>I	AA:203		A12:L>L		A4:L>L		DBVPG6304:L>L		UFRJ50816:L>L		UWOPS91_917_1:L>L		YPS138:L>L	AA:217		A4:F>F		DBVPG6304:F>F		UFRJ50816:F>F		UWOPS91_917_1:F>F		YPS138:F>F	AA:224		A4:R>R		DBVPG6304:R>R		UFRJ50816:R>R		YPS138:R>R	AA:227		A4:Q>Q		DBVPG6304:Q>Q		IFO1804:Q>Q		N_45:Q>Q		UFRJ50816:Q>Q		UWOPS91_917_1:Q>Q		YPS138:Q>Q	AA:229		A4:L>L		DBVPG6304:L>L		IFO1804:L>L		N_45:L>L		UFRJ50816:L>L		UWOPS91_917_1:L>L		YPS138:L>L	AA:248		A4:E>E		DBVPG6304:E>E		UFRJ50816:E>E		UWOPS91_917_1:E>E		YPS138:E>E	AA:273		UWOPS91_917_1:I>I	AA:298		A4:E>E		DBVPG6304:E>E		UFRJ50816:E>E		UWOPS91_917_1:E>E		YPS138:E>E	AA:331		DBVPG6304:K>K		YPS138:K>K	AA:332		A4:Q>Q		UFRJ50816:Q>Q	AA:347		YPS138:K>K	AA:349		UWOPS91_917_1:G>G	AA:353		Q95_3:R>R		Z1_1:R>R	AA:399		UWOPS91_917_1:->-	AA:416		DBVPG6304:V>V		UFRJ50816:V>V		YPS138:V>V	AA:423		DBVPG6304:I>I		UFRJ50816:I>I		YPS138:I>I	AA:437		A4:V>V		DBVPG6304:V>V		UFRJ50791:V>V		UFRJ50816:V>V		YPS138:V>V	AA:447		A4:H>H		DBVPG6304:H>H		UFRJ50791:H>H		UFRJ50816:H>H		UWOPS91_917_1:H>H		YPS138:H>H	AA:468		N_43:D>D		N_45:D>D	AA:481		A4:->-		DBVPG6304:->-		UFRJ50791:->-		UFRJ50816:->-		UWOPS91_917_1:->-		YPS138:->-	AA:491		A4:V>V		DBVPG6304:V>V		N_43:V>V		N_45:V>V		UFRJ50791:V>V		UFRJ50816:V>V		UWOPS91_917_1:V>V		YPS138:V>V	AA:496		N_45:F>F	AA:502		UWOPS91_917_1:R>R	AA:548		A4:Q>Q		DBVPG6304:Q>Q		UFRJ50791:Q>Q		UFRJ50816:Q>QID:YBL021C	AA:4		DBVPG6304:E>E		YPS138:E>E	AA:10		DBVPG6304:Y>Y		YPS138:Y>Y	AA:28		DBVPG6304:K>K		YPS138:K>K	AA:74		CBS432:C>C		CBS5829:C>C		DBVPG4650:C>C		N_17:C>C		Q95_3:C>C		S36_7:C>C		Y6_5:C>C		Y7:C>C	AA:90		DBVPG6304:P>P		UFRJ50816:P>P		YPS138:P>P	AA:92		YPS138:T>T	AA:94		DBVPG6304:K>K		IFO1804:K>K		N_43:K>K		N_44:K>K		N_45:K>K		UFRJ50816:K>K		YPS138:K>K	AA:100		DBVPG6304:N>N		UFRJ50816:N>N	AA:107		DBVPG6304:D>D		UFRJ50816:D>D		YPS138:D>D	AA:112		DBVPG6304:T>T		UFRJ50816:T>T	AA:113		IFO1804:S>S		N_43:S>S		N_44:S>S		N_45:S>S	AA:115		DBVPG6304:Q>Q		UFRJ50816:Q>Q		YPS138:Q>Q	AA:117		DBVPG6304:L>L		UFRJ50816:L>L		YPS138:L>L	AA:126		DBVPG6304:G>G		UFRJ50816:G>G		YPS138:G>G	AA:131		DBVPG6304:D>D		UFRJ50816:D>D		YPS138:D>DID:YBL023C	AA:3		A12:G>G		DBVPG6304:G>G		IFO1804:G>G		N_44:G>G		N_45:G>G		UFRJ50816:G>G	AA:24		A12:V>V		DBVPG6304:V>V		UFRJ50816:V>V	AA:29		A12:V>V		DBVPG6304:V>V		UFRJ50816:V>V	AA:38		A12:Y>Y		DBVPG6304:Y>Y		UFRJ50791:Y>Y		UFRJ50816:Y>Y		UWOPS91_917_1:S>S	AA:52		A12:E>E		DBVPG6304:E>E		UFRJ50791:E>E		UFRJ50816:E>E	AA:75		DBVPG6304:R>R	AA:78		UWOPS91_917_1:D>D	AA:85		A12:K>K		DBVPG6304:K>K		UFRJ50791:K>K		UFRJ50816:K>K		UWOPS91_917_1:K>K	AA:91		A12:H>H		DBVPG6304:H>H		UFRJ50791:H>H		UFRJ50816:H>H		UWOPS91_917_1:H>H	AA:107		DBVPG6304:S>S		UWOPS91_917_1:L>L	AA:125		IFO1804:L>L		N_44:L>L		N_45:L>L	AA:145		UWOPS91_917_1:S>S	AA:151		A12:L>L		CBS5829:L>L		DBVPG6304:L>L		IFO1804:L>L		N_44:L>L		N_45:L>L		UFRJ50791:L>L		UWOPS91_917_1:L>L	AA:160		A12:N>N		A4:N>N		DBVPG6304:N>N		UFRJ50791:N>N	AA:187		A4:V>V		DBVPG6304:V>V		UFRJ50791:V>V		UWOPS91_917_1:V>V	AA:200		A4:T>T		DBVPG6304:T>T		UFRJ50791:T>T		UWOPS91_917_1:T>T	AA:227		A4:R>R		DBVPG6304:R>R		IFO1804:R>R		N_43:R>R		N_44:R>R		N_45:R>R		UFRJ50791:R>R	AA:238		CBS432:S>S		CBS5829:S>S	AA:256		A4:D>D		DBVPG6304:D>D		UFRJ50791:D>D	AA:262		A4:E>E		DBVPG6304:E>E		UFRJ50791:E>E	AA:308		A12:E>E		A4:E>E		DBVPG6304:E>E		UFRJ50791:E>E	AA:310		A4:A>A		DBVPG6304:A>A		IFO1804:A>A		N_43:A>A		N_44:A>A		N_45:A>A		UFRJ50791:A>A	AA:318		A4:I>I		DBVPG6304:I>I		UFRJ50791:I>I	AA:360		A4:D>D		DBVPG6304:D>D	AA:366		A4:S>S		DBVPG6304:S>S	AA:376		IFO1804:I>I		N_43:I>I		N_44:I>I		N_45:I>I	AA:406		CBS5829:A>A	AA:407		IFO1804:A>A		N_43:A>A		N_44:A>A		N_45:A>A	AA:418		A4:E>E	AA:440		A12:Y>Y		A4:Y>Y		DBVPG6304:Y>Y	AA:445		A12:E>E		A4:E>E		DBVPG6304:E>E	AA:458		A12:A>A		A4:A>A		DBVPG6304:A>A	AA:459		IFO1804:L>L		N_44:L>L		N_45:L>L	AA:460		A12:L>L		A4:L>L		DBVPG6304:L>L	AA:472		A12:P>P		A4:P>P		DBVPG6304:P>P	AA:481		A12:T>T	AA:490		A12:T>T		A4:T>T		DBVPG6304:T>T		UFRJ50791:T>T	AA:493		A12:G>G		A4:G>G		DBVPG6304:G>G		UFRJ50791:G>G	AA:506		A12:C>C		A4:C>C		DBVPG6304:C>C		UFRJ50791:C>C	AA:519		A12:F>F		A4:F>F		DBVPG6304:F>F		UFRJ50791:F>F	AA:539		A12:F>F		A4:F>F		DBVPG6304:F>F		UFRJ50791:F>F	AA:546		A12:V>V		A4:V>V		DBVPG6304:V>V		UFRJ50791:V>V	AA:551		A12:R>R		A4:R>R		DBVPG6304:R>R		UFRJ50791:R>R	AA:556		A12:L>L		A4:L>L		DBVPG6304:L>L		IFO1804:L>L		N_44:L>L		N_45:L>L		UFRJ50791:L>L	AA:591		A12:T>T		A4:T>T		DBVPG6304:T>T		IFO1804:T>T		N_45:T>T		UFRJ50791:T>T	AA:602		A4:L>L		DBVPG6304:L>L		UFRJ50791:L>L	AA:606		A4:P>P		DBVPG6304:P>P		UFRJ50791:P>P	AA:607		N_44:C>C		N_45:C>C	AA:610		A4:L>L		DBVPG6304:L>L		UFRJ50791:L>L	AA:615		A4:I>I		DBVPG6304:I>I		UFRJ50791:I>I	AA:618		A4:S>S		DBVPG6304:S>S		UFRJ50791:S>S	AA:631		DBVPG6304:S>S	AA:644		IFO1804:V>V	AA:652		A4:Y>Y		DBVPG6304:Y>Y		UFRJ50791:Y>Y	AA:654		A4:L>L		DBVPG6304:L>L		UFRJ50791:L>L	AA:656		DBVPG6304:F>F		UFRJ50791:F>F	AA:684		IFO1804:L>L		N_44:L>L		N_45:L>L	AA:691		A4:E>E		DBVPG6304:E>E		IFO1804:E>E		N_44:E>E		N_45:E>E		UFRJ50791:E>E	AA:694		IFO1804:P>P		N_44:P>P		N_45:P>P	AA:702		DBVPG6304:L>L		UFRJ50791:L>L	AA:712		DBVPG6304:Y>Y		UFRJ50791:Y>Y	AA:718		DBVPG6304:R>R		UFRJ50791:R>R	AA:723		DBVPG6304:L>L		UFRJ50791:L>L	AA:764		IFO1804:L>L		N_44:L>L		N_45:L>L	AA:782		IFO1804:R>R		N_44:R>R		N_45:R>R	AA:812		DBVPG6304:V>V	AA:819		DBVPG6304:D>D		UFRJ50816:D>D	AA:845		DBVPG6304:P>P		IFO1804:P>P		N_43:P>P		N_45:P>P		UWOPS91_917_1:P>P	AA:851		DBVPG6304:E>EID:YBL024W	AA:10		A12:P>P		DBVPG6304:P>P		UFRJ50816:P>P		UWOPS91_917_1:P>P		YPS138:P>P	AA:33		A12:F>F		DBVPG6304:F>F		UFRJ50816:F>F		YPS138:F>F	AA:37		N_45:P>P	AA:39		A12:F>F		DBVPG6304:F>F		UFRJ50816:F>F		UWOPS91_917_1:F>F	AA:42		N_43:L>L		N_44:L>L		N_45:L>L	AA:49		A12:S>S		DBVPG6304:S>S		UFRJ50816:S>S		YPS138:S>S	AA:62		A12:R>R		DBVPG6304:R>R		YPS138:R>R	AA:65		A12:->-		DBVPG6304:->-		N_43:->-		N_45:->-		UFRJ50816:->-		YPS138:->-	AA:93		A12:I>I		DBVPG6304:I>I		UFRJ50816:I>I		YPS138:I>I	AA:157		A12:R>R		DBVPG6304:R>R		UFRJ50816:R>R		YPS138:R>R	AA:177		A12:T>T		DBVPG6304:T>T		UFRJ50816:T>T		YPS138:T>T	AA:199		A12:H>H		DBVPG6304:H>H		UFRJ50816:H>H		YPS138:H>H	AA:215		A12:Q>Q		DBVPG6304:Q>Q		UFRJ50816:Q>Q		YPS138:Q>Q	AA:243		A12:I>I		DBVPG6304:I>I		UFRJ50791:I>I		UFRJ50816:I>I		YPS138:I>I	AA:256		A12:T>T		DBVPG6304:T>T		UFRJ50791:T>T		UFRJ50816:T>T		YPS138:T>T	AA:259		YPS138:W>W	AA:277		A12:V>V		DBVPG6304:V>V		UFRJ50791:V>V		UFRJ50816:V>V		YPS138:V>V	AA:292		A12:->-		DBVPG6304:->-		UFRJ50791:->-		UFRJ50816:->-		YPS138:->-	AA:302		A12:V>V		DBVPG6304:V>V		UFRJ50791:V>V		UFRJ50816:V>V		YPS138:V>V	AA:307		A12:V>V		DBVPG6304:V>V		N_43:V>V		N_44:V>V		N_45:V>V		UFRJ50791:V>V		UFRJ50816:V>V		YPS138:V>V	AA:322		N_43:S>S		N_44:S>S		N_45:S>S	AA:340		A12:L>L		DBVPG6304:L>L		UFRJ50791:L>L		UFRJ50816:L>L		YPS138:L>L	AA:344		KPN3828:->-	AA:352		N_43:F>F		N_44:F>F		N_45:F>F	AA:354		DBVPG6304:G>G		UFRJ50791:G>G		UFRJ50816:G>G	AA:364		N_43:G>G		N_44:G>G		N_45:G>G	AA:366		N_43:S>S		N_44:S>S		N_45:S>S	AA:448		A12:F>F		DBVPG6304:F>F		UFRJ50791:F>F		UWOPS91_917_1:F>F	AA:454		A12:R>R		DBVPG6304:R>R		UFRJ50791:R>R	AA:463		A12:V>V		DBVPG6304:V>V	AA:476		A12:I>I		DBVPG6304:I>I		N_43:I>I		N_45:I>I		UFRJ50791:I>I		UWOPS91_917_1:I>I	AA:489		A12:Y>Y		DBVPG6304:Y>Y	AA:498		UWOPS91_917_1:S>S	AA:509		DBVPG6304:V>V		UWOPS91_917_1:V>V	AA:514		A12:V>V		DBVPG6304:V>V		UFRJ50816:V>V		UWOPS91_917_1:V>V	AA:546		A12:Q>Q		DBVPG6304:Q>Q		UWOPS91_917_1:Q>Q	AA:559		UWOPS91_917_1:N>N	AA:562		UWOPS91_917_1:V>V	AA:565		UWOPS91_917_1:H>H	AA:588		A12:I>I		DBVPG6304:I>I	AA:592		UWOPS91_917_1:L>L	AA:601		A12:C>C		A4:C>C		DBVPG6304:C>C	AA:610		A12:R>R		A4:R>R		DBVPG6304:R>R	AA:623		A12:V>V		A4:V>V		DBVPG6304:V>V	AA:631		A12:T>T		A4:T>T		DBVPG6304:T>T	AA:655		A12:R>R		A4:R>R		DBVPG6304:R>R		UWOPS91_917_1:R>R	AA:682		A12:I>I		A4:I>I		DBVPG6304:I>I		UWOPS91_917_1:I>IID:YBL025W	AA:6		A4:I>I		DBVPG6304:I>I		UFRJ50791:I>I		YPS138:I>I	AA:15		A12:F>F		A4:F>F		DBVPG6304:F>F		UFRJ50791:F>F		YPS138:F>F	AA:23		A12:G>G	AA:34		A12:S>S		A4:S>S		DBVPG6304:S>S		IFO1804:S>S		N_43:S>S		N_44:S>S		N_45:S>S		UFRJ50791:S>S		UFRJ50816:S>S		YPS138:S>S	AA:39		A12:E>E		A4:E>E		DBVPG6304:E>E		UFRJ50791:E>E		YPS138:E>E	AA:44		A12:I>I		A4:I>I		DBVPG6304:I>I		IFO1804:I>I		N_43:I>I		N_44:I>I		N_45:I>I		UFRJ50791:I>I		YPS138:I>I	AA:47		Q62_5:G>G		Z1_1:G>G	AA:64		A12:G>G		A4:G>G		DBVPG6304:G>G		UFRJ50791:G>G		UFRJ50816:G>G		YPS138:G>G	AA:66		A12:D>D		A4:D>D		DBVPG6304:D>D		UFRJ50791:D>D		UFRJ50816:D>D		YPS138:D>D	AA:99		A12:T>T		A4:T>T		DBVPG6304:T>T		UFRJ50791:T>T		UFRJ50816:T>T		YPS138:T>T	AA:129		Q59_1:A>AID:YBL028C	AA:4		A12:T>T		A4:T>T		DBVPG6304:T>T		UWOPS91_917_1:T>T		YPS138:T>T	AA:9		UWOPS91_917_1:K>K	AA:24		A12:R>R		YPS138:R>R	AA:31		A12:T>T		A4:T>T		DBVPG6304:T>T		UWOPS91_917_1:T>T		YPS138:T>T	AA:35		A12:K>K		A4:K>K		DBVPG6304:K>K		YPS138:K>K	AA:47		UWOPS91_917_1:V>V	AA:56		UWOPS91_917_1:K>K	AA:60		A12:D>D		A4:D>D		DBVPG6304:D>D		YPS138:D>D	AA:64		A12:Q>Q		A4:Q>Q		DBVPG6304:Q>Q		UWOPS91_917_1:Q>Q		YPS138:Q>Q	AA:66		N_43:L>L		N_44:L>L		N_45:L>L	AA:71		CBS5829:L>L		Q32_3:L>L		Q59_1:L>L		Y7:L>L		Z1_1:L>L	AA:79		A12:R>R		A4:R>R		DBVPG6304:R>R		UWOPS91_917_1:R>R		YPS138:R>R	AA:93		A4:S>S		DBVPG6304:S>S		UWOPS91_917_1:S>S		YPS138:S>S	AA:94		N_45:K>KID:YBL029W	AA:17		A12:->-		DBVPG6304:->-		UFRJ50816:->-		UWOPS91_917_1:->-		YPS138:->-	AA:27		A12:->-		DBVPG6304:->-		UFRJ50816:->-		UWOPS91_917_1:->-		YPS138:->-	AA:33		A12:R>R		DBVPG6304:R>R		IFO1804:R>R		N_43:R>R		N_44:R>R		N_45:R>R		UFRJ50816:R>R		UWOPS91_917_1:R>R		YPS138:R>R	AA:40		UWOPS91_917_1:V>V	AA:48		UWOPS91_917_1:I>I	AA:49		A12:T>T		DBVPG6304:T>T		UFRJ50816:T>T		YPS138:T>T	AA:68		A12:S>S		DBVPG6304:S>S		UFRJ50816:S>S		UWOPS91_917_1:S>S		YPS138:S>S	AA:71		A12:I>I		DBVPG6304:I>I		UFRJ50816:I>I		YPS138:I>I	AA:76		A12:N>N		DBVPG6304:N>N		IFO1804:N>N		N_43:N>N		N_44:N>N		N_45:N>N		UFRJ50816:N>N		UWOPS91_917_1:N>N		YPS138:N>N	AA:98		A12:E>E		DBVPG6304:E>E		IFO1804:E>E		N_43:E>E		N_44:E>E		N_45:E>E		UFRJ50816:E>E		UWOPS91_917_1:E>E		YPS138:E>E	AA:100		DBVPG6304:I>I		IFO1804:I>I		N_43:I>I		N_44:I>I		N_45:I>I		UFRJ50816:I>I		UWOPS91_917_1:I>I		YPS138:I>I	AA:108		DBVPG6304:Q>Q		UFRJ50816:Q>Q		UWOPS91_917_1:Q>Q		YPS138:Q>Q	AA:118		A12:R>R		DBVPG6304:R>R		UFRJ50816:R>R		UWOPS91_917_1:R>R		YPS138:R>R	AA:123		A12:I>I		DBVPG6304:I>I		UFRJ50816:I>I		UWOPS91_917_1:I>I		YPS138:I>I	AA:128		IFO1804:V>V		N_43:V>V		N_44:V>V		N_45:V>V	AA:136		A12:R>R		DBVPG6304:R>R		UFRJ50816:R>R		UWOPS91_917_1:R>R		YPS138:R>R	AA:158		A12:W>W		DBVPG6304:W>W		UFRJ50816:W>W		UWOPS91_917_1:W>W		YPS138:W>W	AA:181		A12:Y>Y		DBVPG6304:Y>Y		UFRJ50816:Y>Y		YPS138:Y>Y	AA:198		A12:F>F		DBVPG6304:F>F		UFRJ50816:F>F		UWOPS91_917_1:F>F		YPS138:F>F	AA:251		A12:I>I		A4:I>I		DBVPG6304:I>I		UFRJ50816:I>I		UWOPS91_917_1:I>I		YPS138:I>I	AA:275		A12:T>T		A4:T>T		DBVPG6304:T>T		UFRJ50816:T>T		UWOPS91_917_1:T>T	AA:309		IFO1804:V>V		N_43:V>V		N_45:V>V	AA:323		UFRJ50816:V>V	AA:339		A12:V>V		A4:V>V		DBVPG6304:V>V		UFRJ50816:V>V		YPS138:V>V	AA:341		A12:L>L		A4:L>L		DBVPG6304:L>L		UFRJ50816:L>L		YPS138:L>L	AA:365		A12:S>S		A4:S>S		DBVPG6304:S>S		UFRJ50816:S>S		YPS138:S>S	AA:372		IFO1804:F>FID:YBL030C	AA:4		A12:K>K	AA:9		IFO1804:I>I		N_43:I>I		N_45:I>I	AA:42		A4:A>A		UWOPS91_917_1:A>A	AA:52		A4:A>A		UWOPS91_917_1:A>A	AA:92		IFO1804:L>L		N_43:L>L		N_44:L>L		N_45:L>L	AA:100		A4:T>T		IFO1804:T>T		N_43:T>T		N_44:T>T		N_45:T>T		UWOPS91_917_1:T>T	AA:122		A4:V>V		DBVPG6304:V>V	AA:159		A4:S>S		DBVPG6304:S>S		UWOPS91_917_1:S>S	AA:167		A4:T>T		DBVPG6304:T>T		UWOPS91_917_1:T>T	AA:178		A4:L>L		DBVPG6304:L>L	AA:188		A4:A>A		DBVPG6304:A>A		UWOPS91_917_1:A>A	AA:199		UWOPS91_917_1:E>E	AA:211		DBVPG4650:D>D		Y6_5:D>D	AA:226		A4:S>S		UWOPS91_917_1:S>S	AA:236		A4:I>I		DBVPG6304:I>I		UWOPS91_917_1:I>I	AA:242		A4:A>A		DBVPG6304:A>A		UWOPS91_917_1:A>A	AA:252		A4:A>A		DBVPG6304:A>A	AA:296		DBVPG6304:L>L	AA:307		DBVPG6304:P>PID:YBL031W	AA:51		A12:Y>Y		DBVPG6304:Y>Y		N_43:Y>Y		N_44:Y>Y		UFRJ50816:Y>Y		UWOPS91_917_1:Y>Y		YPS138:Y>Y	AA:68		A12:I>I		DBVPG6304:I>I		UFRJ50816:I>I		UWOPS91_917_1:I>I		YPS138:I>I	AA:84		UWOPS91_917_1:R>R	AA:90		UWOPS91_917_1:V>V	AA:95		A12:C>C		DBVPG6304:C>C		UFRJ50816:C>C		UWOPS91_917_1:C>C		YPS138:C>C	AA:127		A12:L>L		DBVPG6304:L>L		N_43:L>L		N_44:L>L		UFRJ50816:L>L		UWOPS91_917_1:L>L		YPS138:L>L	AA:132		DBVPG6304:R>R		UFRJ50816:R>R		UWOPS91_917_1:R>R		YPS138:R>R	AA:188		UWOPS91_917_1:L>L	AA:196		A12:L>L		DBVPG6304:L>L		UFRJ50816:L>L		YPS138:L>L	AA:209		N_43:G>G		N_44:G>G		N_45:G>G	AA:215		A12:D>D		DBVPG6304:D>D		UFRJ50816:D>D		YPS138:D>D	AA:224		A12:G>G		DBVPG6304:G>G		UFRJ50816:G>G		UWOPS91_917_1:G>G		YPS138:G>G	AA:231		UFRJ50816:D>D	AA:238		A12:S>S		DBVPG6304:S>S		N_43:S>S		N_44:S>S		N_45:S>S		UFRJ50816:S>S		UWOPS91_917_1:S>S		YPS138:S>S	AA:263		UFRJ50816:R>R	AA:268		N_43:->-		N_44:->-		N_45:->-	AA:270		KPN3828:G>G	AA:275		A12:K>K		DBVPG6304:K>K		N_43:K>K		N_44:K>K		N_45:K>K		UFRJ50816:K>K		YPS138:K>K	AA:284		A12:R>R		DBVPG6304:R>R		UFRJ50816:R>R		YPS138:R>RID:YBL032W	AA:68		A12:A>A		A4:A>A		DBVPG6304:A>A		UFRJ50791:A>A		UWOPS91_917_1:A>A		YPS138:A>A	AA:117		CBS5829:L>L		DBVPG4650:L>L		Q32_3:L>L		Q59_1:L>L		Q62_5:L>L		Z1_1:L>L	AA:127		IFO1804:L>L		N_43:L>L		N_45:L>L		UWOPS91_917_1:L>L	AA:130		UWOPS91_917_1:I>I	AA:140		CBS5829:W>W		DBVPG4650:W>W		Q32_3:W>W		Q59_1:W>W		Q62_5:W>W		Y7:W>W		Z1_1:W>W	AA:143		A4:R>R		YPS138:R>R	AA:156		UWOPS91_917_1:N>N	AA:162		UWOPS91_917_1:N>N	AA:165		UWOPS91_917_1:I>I	AA:184		UWOPS91_917_1:I>I	AA:190		A4:N>N		YPS138:N>N	AA:198		A4:R>R		DBVPG6304:R>R		UWOPS91_917_1:R>R		YPS138:R>R	AA:210		UWOPS91_917_1:E>E	AA:219		UWOPS91_917_1:D>D	AA:222		UWOPS91_917_1:A>A	AA:225		UWOPS91_917_1:N>N	AA:259		A4:S>S		YPS138:S>S	AA:267		A4:F>F		YPS138:F>F	AA:275		IFO1804:A>A		N_43:A>A		N_45:A>A	AA:281		A4:D>D		UWOPS91_917_1:D>D		YPS138:D>D	AA:318		A12:I>I		YPS138:I>I	AA:348		A12:F>F		IFO1804:F>F		N_43:F>F		N_45:F>F		UWOPS91_917_1:F>F		YPS138:F>F	AA:365		UWOPS91_917_1:F>F	AA:373		UWOPS91_917_1:F>FID:YBL033C	AA:67		UWOPS91_917_1:I>I	AA:73		UWOPS91_917_1:R>R	AA:78		UWOPS91_917_1:L>L	AA:80		UWOPS91_917_1:P>P	AA:91		UWOPS91_917_1:N>N	AA:92		DBVPG6304:N>N	AA:96		UWOPS91_917_1:R>R	AA:97		DBVPG6304:V>V	AA:100		DBVPG6304:L>L		UFRJ50816:L>L		UWOPS91_917_1:L>L	AA:102		UWOPS91_917_1:L>L	AA:111		DBVPG6304:S>S		UWOPS91_917_1:S>S	AA:118		UWOPS91_917_1:P>P	AA:123		UWOPS91_917_1:L>L	AA:135		DBVPG6304:L>L		UWOPS91_917_1:L>L	AA:146		DBVPG6304:I>I		UWOPS91_917_1:I>I	AA:151		DBVPG6304:Q>Q	AA:153		CBS432:L>L	AA:161		DBVPG6304:N>N		UWOPS91_917_1:C>C	AA:169		DBVPG6304:G>G		N_44:P>P		N_45:P>P		UWOPS91_917_1:G>G	AA:188		DBVPG6304:C>C		UWOPS91_917_1:C>C	AA:199		UWOPS91_917_1:C>C	AA:220		N_43:L>L		N_45:L>L	AA:223		UWOPS91_917_1:G>G	AA:235		UWOPS91_917_1:R>R	AA:238		UWOPS91_917_1:E>E	AA:239		Q32_3:D>D	AA:241		DBVPG6304:V>V		UFRJ50816:V>V		UWOPS91_917_1:V>V	AA:244		DBVPG6304:G>G		UFRJ50816:G>G		UWOPS91_917_1:G>G	AA:259		DBVPG6304:Q>Q		UFRJ50816:Q>Q	AA:275		UWOPS91_917_1:D>D	AA:276		IFO1804:E>E		N_43:E>E		N_45:E>E		UWOPS91_917_1:P>P	AA:291		UWOPS91_917_1:S>S	AA:293		UWOPS91_917_1:L>L	AA:297		DBVPG6304:I>I		UWOPS91_917_1:I>I	AA:301		UWOPS91_917_1:Q>Q	AA:304		UWOPS91_917_1:P>P	AA:314		CBS432:L>L		CBS5829:L>L	AA:315		UWOPS91_917_1:P>P	AA:333		A12:S>S	AA:341		IFO1804:Y>Y		N_43:Y>Y		N_45:Y>YID:YBL036C	AA:3		A12:I>I		UFRJ50816:I>I		UWOPS91_917_1:I>I		YPS138:I>I	AA:19		A12:I>I		UFRJ50816:I>I		YPS138:I>I	AA:33		A12:D>D		UFRJ50816:D>D		UWOPS91_917_1:D>D		YPS138:D>D	AA:35		A12:S>S		UFRJ50816:S>S		UWOPS91_917_1:S>S		YPS138:S>S	AA:40		A12:L>L		DBVPG6304:L>L		YPS138:L>L	AA:43		YPS138:S>S	AA:67		A12:D>D		DBVPG6304:D>D		UFRJ50816:D>D		UWOPS91_917_1:D>D		YPS138:D>D	AA:75		CBS432:G>G		DBVPG4650:G>G		IFO1804:G>G		N_17:G>G		N_43:G>G		N_45:G>G		Q32_3:G>G	AA:79		A12:L>L		DBVPG6304:L>L		UFRJ50816:L>L		YPS138:L>L	AA:82		A12:L>L		DBVPG6304:L>L		UFRJ50816:L>L		UWOPS91_917_1:L>L		YPS138:L>L	AA:84		UFRJ50816:I>I	AA:110		A12:S>S		DBVPG6304:S>S		N_43:S>S		N_44:S>S		N_45:S>S		UFRJ50816:S>S		UWOPS91_917_1:S>S		YPS138:S>S	AA:124		A12:N>N		DBVPG6304:N>N		UFRJ50816:N>N		UWOPS91_917_1:N>N		YPS138:N>N	AA:128		IFO1804:Q>Q		N_43:Q>Q		N_44:Q>Q		N_45:Q>Q	AA:139		A12:A>A		DBVPG6304:A>A		UFRJ50816:A>A		UWOPS91_917_1:A>A		YPS138:A>A	AA:154		A12:V>V		UFRJ50816:V>V		UWOPS91_917_1:V>V		YPS138:V>V	AA:162		UWOPS91_917_1:N>N	AA:167		UFRJ50816:G>G	AA:178		A12:L>L		DBVPG6304:L>L		N_43:L>L		N_44:L>L		N_45:L>L		UFRJ50816:L>L		UWOPS91_917_1:L>L	AA:209		A12:I>I		DBVPG6304:L>L		UFRJ50816:I>I		UWOPS91_917_1:L>L		YPS138:I>I	AA:213		UWOPS91_917_1:V>VID:YBL038W	AA:11		A12:R>R		A4:R>R		UFRJ50816:R>R		YPS138:R>R	AA:27		A12:->-		A4:->-		UFRJ50816:->-		YPS138:->-	AA:65		A12:L>L		A4:L>L		UFRJ50816:L>L		YPS138:L>L	AA:71		UWOPS91_917_1:K>K	AA:77		KPN3829:S>S	AA:87		A4:R>R		UFRJ50816:R>R		YPS138:R>R	AA:89		UWOPS91_917_1:L>L	AA:99		A4:S>S		UFRJ50816:S>S		YPS138:S>S	AA:127		Q59_1:S>S	AA:153		A4:T>T		UFRJ50816:T>T		YPS138:T>T	AA:170		N_43:G>G		N_44:G>G		N_45:G>G	AA:186		UWOPS91_917_1:S>S		YPS138:S>S	AA:205		UWOPS91_917_1:I>I	AA:218		N_43:N>N		N_44:N>N		N_45:N>NID:YBL041W	AA:48		N_45:I>I	AA:63		DBVPG6304:K>K		UFRJ50791:K>K		UFRJ50816:K>K	AA:75		DBVPG6304:S>S		N_45:S>S		UFRJ50791:S>S		UFRJ50816:S>S	AA:104		DBVPG6304:R>R		UFRJ50791:R>R		UFRJ50816:R>R	AA:118		UFRJ50791:P>P		UFRJ50816:P>P	AA:125		DBVPG6304:V>V		UFRJ50791:V>V		UFRJ50816:V>V	AA:137		DBVPG6304:T>T		N_45:T>T		UFRJ50791:T>T		UFRJ50816:T>T	AA:152		DBVPG6304:S>S		UFRJ50791:S>S		UFRJ50816:S>S	AA:159		UFRJ50791:T>T	AA:161		DBVPG6304:R>R		UFRJ50791:R>R	AA:176		DBVPG6304:L>L		UFRJ50791:L>L	AA:183		DBVPG6304:C>C		UFRJ50791:C>C	AA:185		DBVPG6304:T>T		UFRJ50791:T>T	AA:202		DBVPG6304:Q>Q	AA:222		DBVPG6304:F>F		UFRJ50791:F>F	AA:228		DBVPG6304:L>L	AA:238		UWOPS91_917_1:->-ID:YBL045C	AA:16		N_43:L>L		N_44:L>L		N_45:L>L	AA:45		UWOPS91_917_1:T>T	AA:49		DBVPG6304:I>I		YPS138:I>I	AA:61		DBVPG6304:G>G		UWOPS91_917_1:G>G		YPS138:G>G	AA:67		DBVPG6304:A>A		UWOPS91_917_1:A>A		YPS138:A>A	AA:85		N_45:L>L	AA:104		DBVPG6304:T>T		UWOPS91_917_1:T>T		YPS138:T>T	AA:117		DBVPG6304:D>D		YPS138:D>D	AA:160		CBS432:G>G		CBS5829:G>G		DBVPG4650:G>G		N_17:G>G		N_45:G>G		Q32_3:G>G		UFRJ50791:G>G		Y6_5:G>G		Y7:G>G		YPS138:G>G	AA:172		CBS432:S>S		CBS5829:S>S		N_17:S>S		Q32_3:S>S		Y6_5:S>S		Y7:S>S	AA:232		UFRJ50791:N>N		UWOPS91_917_1:N>N		YPS138:N>N	AA:246		DBVPG6304:N>N		UFRJ50791:N>N		UWOPS91_917_1:N>N		YPS138:N>N	AA:248		DBVPG6304:T>T		UFRJ50791:T>T		UWOPS91_917_1:T>T		YPS138:T>T	AA:265		DBVPG6304:E>E		UFRJ50791:E>E		UFRJ50816:E>E		UWOPS91_917_1:E>E		YPS138:E>E	AA:270		DBVPG6304:V>V		UFRJ50791:V>V		UFRJ50816:V>V		UWOPS91_917_1:V>V		YPS138:V>V	AA:291		N_43:A>A		UWOPS91_917_1:A>A	AA:295		A12:L>L		A4:L>L		DBVPG6304:L>L		UFRJ50791:L>L		UFRJ50816:L>L		UWOPS91_917_1:L>L		YPS138:L>L	AA:298		A12:L>L		A4:L>L		DBVPG6304:L>L		UFRJ50791:L>L		UFRJ50816:L>L		UWOPS91_917_1:L>L		YPS138:L>L	AA:300		N_43:R>R	AA:306		UFRJ50791:E>E		UFRJ50816:E>E	AA:319		A12:T>T		DBVPG6304:T>T		N_43:T>T		UFRJ50791:T>T		UFRJ50816:T>T		UWOPS91_917_1:T>T		YPS138:T>T	AA:323		A12:N>N		A4:N>N		DBVPG6304:N>N		YPS138:N>N	AA:328		A12:L>L		A4:L>L		DBVPG6304:L>L		UFRJ50791:L>L		UFRJ50816:L>L		UWOPS91_917_1:L>L		YPS138:L>L	AA:339		UWOPS91_917_1:L>L	AA:350		A12:L>L		A4:L>L		DBVPG6304:L>L		UFRJ50791:L>L		UFRJ50816:L>L		UWOPS91_917_1:L>L		YPS138:L>L	AA:359		A12:D>D		A4:D>D		DBVPG6304:D>D		UFRJ50791:D>D		UFRJ50816:D>D		YPS138:D>D	AA:362		A12:V>V		A4:V>V		DBVPG6304:V>V		UFRJ50791:V>V		UFRJ50816:V>V		YPS138:V>V	AA:371		A12:K>K		A4:K>K		DBVPG6304:K>K		UFRJ50816:K>K		UWOPS91_917_1:K>K		YPS138:K>K	AA:393		A12:N>N		A4:N>N		DBVPG6304:N>N		UFRJ50816:N>N		UWOPS91_917_1:N>N		YPS138:N>N	AA:434		A12:P>P		A4:P>P		DBVPG6304:P>P		YPS138:P>P	AA:436		N_43:A>A		N_44:A>A		N_45:A>A	AA:449		A12:V>V		A4:V>V		DBVPG6304:V>V		UWOPS91_917_1:V>VID:YBL049W	AA:27		IFO1804:->-		N_43:->-		N_45:->-	AA:36		A12:F>F		A4:F>F		DBVPG6304:F>F		UFRJ50791:F>F		YPS138:F>F	AA:58		A12:N>N		A4:N>N		DBVPG6304:N>N		UFRJ50791:N>N		YPS138:N>N	AA:66		A4:P>P		DBVPG6304:P>P		UFRJ50791:P>P		UFRJ50816:P>P		YPS138:P>P	AA:71		DBVPG6304:I>I		UFRJ50791:I>I		UFRJ50816:I>I		YPS138:I>I	AA:73		A4:H>H		DBVPG6304:H>H		IFO1804:H>H		N_43:H>H		N_45:H>H		UFRJ50791:H>H		UFRJ50816:H>H		YPS138:H>H	AA:115		DBVPG6304:A>A		YPS138:A>AID:YBL050W	AA:11		DBVPG6304:L>L		UFRJ50791:L>L		YPS138:L>L	AA:12		UWOPS91_917_1:L>L	AA:14		DBVPG6304:N>N		UFRJ50791:N>N		UWOPS91_917_1:N>N		YPS138:N>N	AA:30		UWOPS91_917_1:L>L	AA:38		UWOPS91_917_1:T>T	AA:41		UWOPS91_917_1:C>C	AA:69		UWOPS91_917_1:T>T	AA:78		UWOPS91_917_1:V>V	AA:83		UWOPS91_917_1:F>F	AA:91		UWOPS91_917_1:R>R	AA:100		UWOPS91_917_1:V>V	AA:104		UWOPS91_917_1:N>N	AA:109		UWOPS91_917_1:P>P	AA:112		UWOPS91_917_1:P>P	AA:114		UWOPS91_917_1:T>T	AA:121		UWOPS91_917_1:->-	AA:123		DBVPG6304:F>F		UFRJ50791:F>F		UFRJ50816:F>F		YPS138:F>F	AA:124		CBS5829:N>N		KPN3828:N>N		Q62_5:N>N	AA:132		DBVPG6304:I>I		UFRJ50791:I>I		UFRJ50816:I>I		YPS138:I>I	AA:136		UWOPS91_917_1:Y>Y	AA:150		DBVPG6304:L>L		UFRJ50791:L>L		UFRJ50816:L>L		UWOPS91_917_1:L>L	AA:170		IFO1804:T>T		N_44:T>T		N_45:T>T	AA:171		DBVPG6304:L>L		UFRJ50791:L>L		UFRJ50816:L>L		UWOPS91_917_1:L>L	AA:194		DBVPG6304:P>P		UFRJ50791:P>P		UFRJ50816:P>P		UWOPS91_917_1:P>P	AA:213		UFRJ50791:C>C		UFRJ50816:C>C	AA:225		DBVPG6304:T>T		UFRJ50791:T>T		UFRJ50816:T>T		UWOPS91_917_1:T>T		YPS138:T>T	AA:248		UWOPS91_917_1:F>F	AA:283		A4:L>L		DBVPG6304:L>L		UFRJ50816:L>L		UWOPS91_917_1:L>L		YPS138:L>L	AA:290		UWOPS91_917_1:->-ID:YBL051C	AA:13		A4:L>L		DBVPG6304:L>L		UFRJ50816:L>L		UWOPS91_917_1:L>L	AA:26		A4:D>D		DBVPG6304:D>D		UFRJ50816:D>D		UWOPS91_917_1:D>D	AA:31		A4:H>H		DBVPG6304:H>H		UFRJ50816:H>H	AA:36		A4:Y>Y		DBVPG6304:Y>Y		UFRJ50816:Y>Y		UWOPS91_917_1:Y>Y	AA:40		A4:A>A		DBVPG6304:A>A		UFRJ50816:A>A	AA:54		A4:Y>Y		DBVPG6304:Y>Y		UFRJ50816:Y>Y	AA:63		A4:V>V		DBVPG6304:V>V		UFRJ50816:V>V		UWOPS91_917_1:V>V	AA:102		A4:V>V		DBVPG6304:V>V		UFRJ50791:V>V		UFRJ50816:V>V		UWOPS91_917_1:V>V		YPS138:V>V	AA:106		A4:T>T		DBVPG6304:T>T		UFRJ50791:T>T		UFRJ50816:T>T		UWOPS91_917_1:T>T		YPS138:T>T	AA:120		A4:F>F		DBVPG6304:F>F		IFO1804:S>S		N_44:S>S		N_45:S>S		UFRJ50791:S>S		UFRJ50816:F>F		UWOPS91_917_1:S>S		YPS138:F>F	AA:123		IFO1804:P>P		N_44:P>P		N_45:P>P	AA:126		UWOPS91_917_1:S>S	AA:139		A4:N>N		DBVPG6304:N>N		UFRJ50791:N>N		UFRJ50816:N>N		YPS138:N>N	AA:141		A4:S>S		DBVPG6304:S>S		UFRJ50791:S>S		UFRJ50816:S>S		YPS138:S>S	AA:145		A4:Y>Y		DBVPG6304:Y>Y		UFRJ50791:Y>Y		UFRJ50816:Y>Y		UWOPS91_917_1:Y>Y		YPS138:Y>Y	AA:153		A4:G>G		DBVPG6304:G>G		N_43:G>G		N_44:G>G		N_45:G>G		UFRJ50791:P>P		UFRJ50816:P>P		UWOPS91_917_1:G>G		YPS138:G>G	AA:159		A4:T>T		DBVPG6304:T>T		UFRJ50791:T>T		UFRJ50816:T>T		YPS138:T>T	AA:164		A4:Q>Q		DBVPG6304:Q>Q		UFRJ50791:Q>Q		UFRJ50816:Q>Q		YPS138:Q>Q	AA:170		A4:Q>Q		DBVPG6304:Q>Q		UFRJ50791:Q>Q		UFRJ50816:Q>Q		UWOPS91_917_1:Q>Q		YPS138:Q>Q	AA:176		N_43:K>K		N_44:K>K		N_45:Q>Q		UWOPS91_917_1:K>K	AA:179		A4:Q>Q		DBVPG6304:Q>Q		UFRJ50791:Q>Q		UFRJ50816:Q>Q		YPS138:Q>Q	AA:191		A4:A>A		UFRJ50791:A>A		UFRJ50816:A>A		UWOPS91_917_1:A>A		YPS138:A>A	AA:204		UWOPS91_917_1:P>P	AA:226		UWOPS91_917_1:S>S	AA:228		A4:N>N		DBVPG6304:N>N		UFRJ50791:N>N		UFRJ50816:N>N		UWOPS91_917_1:N>N		YPS138:N>N	AA:231		A12:I>I		A4:I>I		DBVPG6304:I>I		UFRJ50791:I>I		UFRJ50816:I>I		YPS138:I>I	AA:251		A12:N>N		A4:N>N		DBVPG6304:N>N		UFRJ50791:N>N		UFRJ50816:N>N		YPS138:N>N	AA:291		A12:T>T		A4:T>T		DBVPG6304:S>S		UFRJ50791:S>S		UFRJ50816:S>S		UWOPS91_917_1:T>T		YPS138:T>T	AA:305		A12:A>A		A4:A>A		DBVPG6304:A>A		UFRJ50791:A>A		UFRJ50816:A>A		YPS138:A>A	AA:310		UWOPS91_917_1:K>K	AA:314		UWOPS91_917_1:I>I	AA:317		A12:R>R		A4:R>R		DBVPG6304:R>R		UFRJ50791:R>R		UFRJ50816:R>R		UWOPS91_917_1:R>R		YPS138:R>R	AA:323		A12:V>V		A4:V>V		DBVPG6304:V>V		N_43:V>V		UFRJ50791:V>V		UFRJ50816:V>V		UWOPS91_917_1:V>V		YPS138:V>V	AA:325		UWOPS91_917_1:G>G	AA:329		A12:C>C		A4:C>C		DBVPG6304:C>C		UFRJ50791:C>C		UFRJ50816:C>C		UWOPS91_917_1:C>C		YPS138:C>C	AA:331		UWOPS91_917_1:V>V	AA:333		UWOPS91_917_1:I>I	AA:338		UWOPS91_917_1:S>S	AA:345		UWOPS91_917_1:Y>Y	AA:349		UWOPS91_917_1:Y>Y	AA:353		UWOPS91_917_1:E>E	AA:360		A12:L>L		A4:L>L		UFRJ50816:L>L		UWOPS91_917_1:L>L		YPS138:L>L	AA:362		UWOPS91_917_1:S>S	AA:365		A12:E>E		A4:E>E		N_43:E>E		UWOPS91_917_1:E>E		YPS138:E>E	AA:374		A12:L>L		A4:L>L		UWOPS91_917_1:L>L		YPS138:L>L	AA:389		CBS432:A>A		CBS5829:A>A		KPN3828:A>A		N_17:A>A		Q62_5:A>A		Q89_8:A>A	AA:406		A12:Q>Q		A4:Q>Q		UWOPS91_917_1:Q>Q		YPS138:Q>Q	AA:426		UWOPS91_917_1:G>G	AA:438		UWOPS91_917_1:T>T	AA:440		UWOPS91_917_1:N>N	AA:451		A12:I>I		YPS138:I>I	AA:453		A12:N>N		YPS138:N>N	AA:462		CBS432:N>N		CBS5829:N>N		KPN3828:N>N		N_17:N>N		Q62_5:N>N		Q89_8:N>N	AA:465		UWOPS91_917_1:N>N		YPS138:N>N	AA:484		UWOPS91_917_1:S>S	AA:517		A4:L>L		DBVPG6304:L>L		UWOPS91_917_1:L>L		YPS138:L>L	AA:535		A4:E>E		DBVPG6304:E>E		UWOPS91_917_1:E>E		YPS138:E>E	AA:538		A4:T>T		DBVPG6304:T>T		UFRJ50816:T>T		YPS138:T>T	AA:546		A4:G>G		DBVPG6304:G>G		UFRJ50816:G>G		YPS138:G>G	AA:561		N_43:L>L		N_44:L>L		N_45:L>L	AA:570		A4:D>D		DBVPG6304:D>D		UFRJ50816:D>D		YPS138:D>D	AA:581		A4:I>I		DBVPG6304:I>I		UFRJ50816:I>I		YPS138:I>I	AA:599		KPN3829:T>T		N_17:T>T		N_43:T>T		N_44:T>T		N_45:T>T		Q32_3:T>T		Q62_5:T>T		Z1_1:T>T	AA:617		UWOPS91_917_1:S>S	AA:655		A12:L>L		A4:L>L		DBVPG6304:L>L		UFRJ50816:L>L		YPS138:L>LID:YBL052C	AA:1		DBVPG6304:->-		IFO1804:->-		N_44:->-		N_45:->-		UFRJ50791:->-		UFRJ50816:->-		YPS138:->-	AA:5		IFO1804:D>D		N_44:D>D		N_45:D>D	AA:51		DBVPG6304:E>E		UFRJ50791:E>E		UFRJ50816:E>E		YPS138:E>E	AA:80		UFRJ50791:K>K		UFRJ50816:K>K		YPS138:K>K	AA:110		DBVPG6304:T>T		UFRJ50791:T>T		UFRJ50816:T>T		UWOPS91_917_1:E>E		YPS138:T>T	AA:129		DBVPG6304:F>F		UWOPS91_917_1:F>F	AA:139		DBVPG6304:F>F		UFRJ50791:F>F		UFRJ50816:F>F		YPS138:F>F	AA:142		A12:R>R		DBVPG6304:R>R		UFRJ50791:R>R		UFRJ50816:R>R		UWOPS91_917_1:R>R		YPS138:R>R	AA:153		DBVPG6304:L>L		UFRJ50791:L>L		UFRJ50816:L>L		UWOPS91_917_1:L>L		YPS138:L>L	AA:158		UWOPS91_917_1:A>A	AA:160		A12:Y>Y		DBVPG6304:Y>Y		UFRJ50791:Y>Y		UFRJ50816:Y>Y		UWOPS91_917_1:Y>Y		YPS138:Y>Y	AA:165		DBVPG6304:Y>Y		IFO1804:Y>Y		N_43:Y>Y		N_44:Y>Y		UFRJ50791:Y>Y		UFRJ50816:Y>Y		YPS138:Y>Y	AA:208		A12:S>S		A4:S>S		DBVPG6304:S>S		UFRJ50816:S>S		UWOPS91_917_1:S>S		YPS138:S>S	AA:214		A12:R>R		A4:R>R		DBVPG6304:R>R		UFRJ50816:R>R		UWOPS91_917_1:R>R		YPS138:R>R	AA:225		UWOPS91_917_1:E>E	AA:234		A12:P>P		A4:P>P		DBVPG6304:P>P		UFRJ50816:P>P		YPS138:P>P	AA:251		A12:L>L		A4:L>L		DBVPG6304:L>L		UFRJ50816:L>L		UWOPS91_917_1:L>L		YPS138:L>L	AA:269		UWOPS91_917_1:L>L	AA:272		A12:D>D		A4:D>D		DBVPG6304:D>D		UFRJ50816:D>D		YPS138:D>D	AA:298		A12:I>I		A4:I>I		DBVPG6304:I>I		UFRJ50816:I>I		UWOPS91_917_1:I>I		YPS138:I>I	AA:325		A12:V>V		A4:V>V		DBVPG6304:V>V		UFRJ50816:V>V		YPS138:V>V	AA:350		IFO1804:K>K		N_43:K>K		N_45:K>K	AA:365		A12:V>V		A4:V>V		DBVPG6304:V>V		UWOPS91_917_1:V>V		YPS138:V>V	AA:367		A12:A>A		A4:A>A		DBVPG6304:A>A		UWOPS91_917_1:A>A	AA:378		A12:L>L		A4:L>L		DBVPG6304:L>L		YPS138:L>L	AA:394		A12:E>E		A4:E>E		DBVPG6304:E>E		UFRJ50816:E>E		UWOPS91_917_1:E>E		YPS138:E>E	AA:414		IFO1804:Y>Y		N_43:Y>Y		N_44:Y>Y		N_45:Y>Y	AA:423		A4:L>L		DBVPG6304:L>L		IFO1804:L>L		N_43:L>L		N_45:L>L		UFRJ50816:L>L		UWOPS91_917_1:L>L		YPS138:L>L	AA:430		A4:F>F		DBVPG6304:F>F		UFRJ50816:F>F		UWOPS91_917_1:F>F		YPS138:F>F	AA:441		A4:F>F		DBVPG6304:F>F		UFRJ50816:F>F		UWOPS91_917_1:F>F		YPS138:F>F	AA:443		A4:A>A		DBVPG6304:A>A		IFO1804:A>A		N_43:A>A		N_44:A>A		N_45:A>A		UFRJ50816:A>A		UWOPS91_917_1:A>A		YPS138:A>A	AA:455		A4:R>R		DBVPG6304:R>R	AA:456		UWOPS91_917_1:E>E	AA:458		A4:L>L		DBVPG6304:L>L		IFO1804:L>L		N_43:L>L		N_44:L>L		N_45:L>L		UFRJ50816:L>L		UWOPS91_917_1:L>L		YPS138:L>L	AA:466		A4:V>V		DBVPG6304:V>V		UFRJ50816:V>V		YPS138:V>V	AA:476		A4:F>F		DBVPG6304:F>F		UFRJ50816:F>F		UWOPS91_917_1:F>F		YPS138:F>F	AA:489		A4:V>V		DBVPG6304:V>V		UFRJ50816:V>V		UWOPS91_917_1:V>V		YPS138:V>V	AA:510		A4:P>P		DBVPG6304:P>P		UFRJ50816:P>P		UWOPS91_917_1:P>P		YPS138:P>P	AA:531		UWOPS91_917_1:K>K	AA:538		A4:F>F		DBVPG6304:F>F		UFRJ50816:F>F		UWOPS91_917_1:F>F		YPS138:F>F	AA:572		A4:V>V		DBVPG6304:V>V		UFRJ50791:V>V		UFRJ50816:V>V		YPS138:V>V	AA:578		UWOPS91_917_1:L>L	AA:584		N_43:R>R		N_44:R>R		N_45:R>R	AA:586		A4:R>R		DBVPG6304:R>R		UFRJ50791:R>R		UFRJ50816:R>R		UWOPS91_917_1:R>R		YPS138:R>R	AA:609		A4:S>S		DBVPG6304:S>S		UFRJ50791:S>S		UFRJ50816:S>S		YPS138:S>S	AA:619		A4:K>K		DBVPG6304:K>K		UFRJ50791:K>K		UFRJ50816:K>K		YPS138:K>K	AA:623		A4:E>E		DBVPG6304:E>E		UFRJ50791:E>E		UFRJ50816:E>E		UWOPS91_917_1:E>E		YPS138:E>E	AA:625		UWOPS91_917_1:T>T	AA:630		A4:K>K		DBVPG6304:K>K		UFRJ50791:K>K		UFRJ50816:K>K		YPS138:K>K	AA:633		A4:F>F		DBVPG6304:F>F		N_43:F>F		N_44:F>F		N_45:F>F		UFRJ50791:F>F		UFRJ50816:F>F		UWOPS91_917_1:F>F		YPS138:F>F	AA:642		A4:G>G		DBVPG6304:G>G		UFRJ50791:G>G		UFRJ50816:G>G		YPS138:G>G	AA:648		N_43:Q>Q		N_44:Q>Q		N_45:Q>Q	AA:650		UWOPS91_917_1:S>S	AA:660		UWOPS91_917_1:S>S	AA:662		UWOPS91_917_1:Y>Y	AA:699		UWOPS91_917_1:P>P	AA:706		UWOPS91_917_1:S>S	AA:713		UWOPS91_917_1:F>F	AA:722		A4:R>R		DBVPG6304:R>R		UFRJ50791:R>R		UFRJ50816:R>R		UWOPS91_917_1:R>R		YPS138:R>R	AA:724		N_43:E>E		N_44:E>E		N_45:E>E	AA:726		A4:K>K		DBVPG6304:K>K		UFRJ50791:K>K		UFRJ50816:K>K		YPS138:K>K	AA:735		UWOPS91_917_1:I>I	AA:737		UWOPS91_917_1:P>P	AA:766		A4:E>E		DBVPG6304:E>E		UFRJ50791:E>E		UFRJ50816:E>E		YPS138:E>E	AA:779		N_44:R>R		N_45:R>R	AA:814		UWOPS91_917_1:L>L	AA:818		UWOPS91_917_1:E>E	AA:827		DBVPG6304:K>K		UFRJ50816:K>K		UWOPS91_917_1:K>K		YPS138:K>K	AA:837		UWOPS91_917_1:L>LID:YBL054W	AA:1		A12:H>H		DBVPG6304:H>H		IFO1804:H>H		N_44:H>H		N_45:H>H		UFRJ50791:H>H		UFRJ50816:H>H		YPS138:H>H	AA:30		A12:R>R		A4:R>R		DBVPG6304:R>R		UFRJ50791:R>R		UFRJ50816:R>R		YPS138:R>R	AA:44		CBS432:C>C	AA:47		A12:L>L		DBVPG6304:L>L		UFRJ50791:L>L		UFRJ50816:L>L		UWOPS91_917_1:L>L		YPS138:L>L	AA:50		A12:Q>Q		A4:Q>Q		DBVPG6304:Q>Q		UFRJ50791:Q>Q		UFRJ50816:Q>Q		UWOPS91_917_1:Q>Q		YPS138:Q>Q	AA:60		UWOPS91_917_1:A>A	AA:74		A12:V>V		A4:V>V		DBVPG6304:V>V		UFRJ50791:V>V		UFRJ50816:V>V		UWOPS91_917_1:V>V		YPS138:V>V	AA:77		A4:R>R	AA:79		IFO1804:I>I		N_43:I>I		N_45:I>I	AA:82		UWOPS91_917_1:V>V	AA:96		A12:T>T		A4:T>T		DBVPG6304:T>T		UFRJ50791:T>T		UFRJ50816:T>T		UWOPS91_917_1:T>T		YPS138:T>T	AA:147		A12:G>G		A4:G>G		DBVPG6304:G>G		UFRJ50791:G>G		UFRJ50816:G>G		YPS138:G>G	AA:166		DBVPG6304:V>V		UFRJ50791:V>V		UFRJ50816:V>V	AA:172		A12:T>T		A4:T>T		DBVPG6304:T>T		UFRJ50791:T>T		UFRJ50816:T>T		UWOPS91_917_1:T>T		YPS138:T>T	AA:178		UWOPS91_917_1:I>I	AA:190		UFRJ50791:F>F		UFRJ50816:F>F	AA:232		N_44:I>I	AA:234		UWOPS91_917_1:G>G	AA:236		A4:Y>Y		DBVPG6304:Y>Y		UFRJ50791:Y>Y		YPS138:Y>Y	AA:240		A4:L>L		DBVPG6304:L>L		YPS138:L>L	AA:266		UWOPS91_917_1:N>N	AA:270		UWOPS91_917_1:R>R	AA:279		A4:V>V		DBVPG6304:V>V		UFRJ50791:V>V		YPS138:V>V	AA:296		A4:L>L		DBVPG6304:L>L		UFRJ50791:L>L		UWOPS91_917_1:L>L		YPS138:L>L	AA:331		UFRJ50791:S>S	AA:340		UFRJ50791:H>H	AA:346		UFRJ50791:R>R	AA:377		N_45:F>F	AA:380		UFRJ50791:V>V		YPS138:V>V	AA:405		A12:K>K		UFRJ50791:K>K	AA:443		A12:F>F		UFRJ50791:F>F		YPS138:F>F	AA:464		A12:N>N		IFO1804:N>N		N_43:N>N		N_44:N>N		Q59_1:N>N		Q89_8:N>N		UFRJ50791:N>N	AA:468		A12:V>V		IFO1804:V>V		N_43:V>V		N_44:V>V		UFRJ50791:V>V		YPS138:V>V	AA:491		A12:I>I		UFRJ50791:I>I		YPS138:I>I	AA:494		A12:F>F		IFO1804:F>F		N_43:F>F		Q89_8:F>F		UFRJ50791:F>F		YPS138:F>FID:YBL056W	AA:28		A4:G>G		DBVPG6304:G>G		UFRJ50791:G>G	AA:31		UFRJ50816:T>T	AA:41		A4:N>N		DBVPG6304:N>N		UFRJ50791:N>N		UFRJ50816:N>N		UWOPS91_917_1:N>N		YPS138:N>N	AA:44		UWOPS91_917_1:R>R	AA:119		A4:T>T		DBVPG6304:T>T		UFRJ50791:T>T		UFRJ50816:T>T		UWOPS91_917_1:T>T		YPS138:T>T	AA:127		IFO1804:Y>Y		N_43:Y>Y		N_44:Y>Y	AA:131		A4:F>F		DBVPG6304:F>F		UFRJ50791:F>F		UFRJ50816:F>F		YPS138:F>F	AA:146		A4:Q>Q		DBVPG6304:Q>Q		UFRJ50791:Q>Q		UFRJ50816:Q>Q		UWOPS91_917_1:Q>Q		YPS138:Q>Q	AA:151		A4:V>V		DBVPG6304:V>V		UFRJ50791:V>V		UFRJ50816:V>V		YPS138:V>V	AA:168		DBVPG6304:R>R		UFRJ50791:R>R		UFRJ50816:R>R	AA:206		A4:M>M		DBVPG6304:M>M		UFRJ50791:M>M		UFRJ50816:M>M		YPS138:M>M	AA:220		A12:I>I		A4:I>I		DBVPG6304:I>I		UFRJ50791:I>I		UFRJ50816:I>I		UWOPS91_917_1:I>I		YPS138:I>I	AA:245		A12:T>T		A4:T>T		DBVPG6304:T>T		UFRJ50791:T>T		UFRJ50816:T>T	AA:258		IFO1804:H>H		N_43:H>H		N_44:H>H		N_45:H>H	AA:383		A12:S>S		A4:S>S		YPS138:S>S	AA:412		A12:I>I		A4:I>I		DBVPG6304:I>I		UWOPS91_917_1:I>I		YPS138:I>I	AA:415		UWOPS91_917_1:I>I	AA:451		UWOPS91_917_1:F>F	AA:459		UWOPS91_917_1:F>FID:YBL057C	AA:17		UFRJ50791:A>A		UFRJ50816:A>A	AA:23		A4:V>V		UFRJ50791:V>V		UFRJ50816:V>V	AA:72		A4:L>L		DBVPG6304:L>L		UFRJ50791:L>L	AA:85		N_43:A>A	AA:109		IFO1804:T>T		N_43:T>T		N_44:T>T		N_45:T>T	AA:115		A4:R>R		DBVPG6304:R>R		IFO1804:R>R		N_44:R>R		N_45:R>R		UFRJ50791:R>R		UFRJ50816:R>R	AA:121		A4:R>R		DBVPG6304:R>R		UFRJ50791:R>R		UFRJ50816:R>R	AA:125		A4:P>P		DBVPG6304:P>P		UFRJ50791:P>P		UFRJ50816:P>P	AA:137		A4:D>D		DBVPG6304:D>D		IFO1804:D>D		N_44:D>D		N_45:D>D		UFRJ50791:D>D		UFRJ50816:D>D	AA:165		A12:L>L		A4:L>L		DBVPG6304:L>L		IFO1804:L>L		N_44:L>L		N_45:L>L		UFRJ50791:L>L		UFRJ50816:L>L		YPS138:L>L	AA:181		IFO1804:L>L		N_44:L>L		N_45:L>L	AA:205		A12:T>T		A4:T>T		DBVPG6304:T>T		UFRJ50791:T>T		UFRJ50816:T>T		YPS138:T>TID:YBL058W	AA:12		IFO1804:K>K		N_43:K>K		N_44:K>K		N_45:K>K	AA:40		A4:I>I	AA:53		IFO1804:L>L		N_43:L>L		N_44:L>L		N_45:L>L		UFRJ50816:L>L	AA:58		IFO1804:V>V		N_43:V>V		N_44:V>V		N_45:V>V		UFRJ50816:V>V	AA:68		A4:L>L		UFRJ50816:L>L	AA:90		A4:R>R		UFRJ50816:R>R	AA:100		A4:L>L		UFRJ50816:L>L	AA:175		A4:F>F		UFRJ50816:F>F	AA:189		A4:G>G		IFO1804:G>G		N_43:G>G		N_44:G>G		N_45:G>G		UFRJ50816:G>G	AA:207		N_43:K>K		N_44:K>K		N_45:K>K	AA:214		A4:V>V		DBVPG6304:V>V		UFRJ50816:V>V	AA:244		UFRJ50816:F>F	AA:253		A4:E>E		DBVPG6304:E>E		UFRJ50816:E>E	AA:258		N_43:V>V		N_44:V>V	AA:259		A4:R>R		DBVPG6304:R>R		UFRJ50816:R>R	AA:279		N_43:I>I		N_44:I>I	AA:338		A4:L>L	AA:352		A4:G>G		DBVPG6304:G>G	AA:363		N_43:S>S		N_44:S>S		N_45:S>S	AA:368		A4:A>A		DBVPG6304:A>A	AA:372		N_43:I>I		N_44:I>I		N_45:I>I	AA:378		DBVPG6304:I>I	AA:395		UWOPS91_917_1:Q>Q	AA:407		UWOPS91_917_1:I>I	AA:422		A4:H>H		CBS432:H>H		DBVPG6304:H>H		N_17:H>H		N_43:H>H		N_44:H>H		N_45:H>H		UWOPS91_917_1:H>H	AA:425		A4:P>P		DBVPG6304:P>P		UWOPS91_917_1:P>PID:YBL060W	AA:29		A12:L>L		A4:L>L		DBVPG6304:L>L		UFRJ50791:L>L		UFRJ50816:L>L		YPS138:L>L	AA:41		A12:T>T		A4:T>T		DBVPG6304:T>T		UWOPS91_917_1:T>T		YPS138:T>T	AA:43		N_43:F>F		N_45:F>F	AA:51		A12:H>H	AA:56		UWOPS91_917_1:I>I	AA:68		UWOPS91_917_1:F>F	AA:73		A12:V>V		A4:V>V		CBS432:V>V		DBVPG6304:V>V		N_17:V>V		N_43:V>V		N_45:V>V		UFRJ50791:V>V		UFRJ50816:V>V		UWOPS91_917_1:V>V		YPS138:V>V	AA:79		A12:R>R		A4:R>R		UFRJ50791:R>R		UFRJ50816:R>R		YPS138:R>R	AA:94		A12:T>T		A4:T>T		DBVPG6304:T>T		UFRJ50791:T>T		UFRJ50816:T>T		YPS138:T>T	AA:97		YPS138:P>P	AA:101		A12:N>N		A4:N>N		CBS432:N>N		N_17:N>N		N_43:N>N		N_44:N>N		N_45:N>N		UFRJ50791:N>N		UFRJ50816:N>N		YPS138:N>N	AA:104		N_43:L>L		N_44:L>L		N_45:L>L	AA:106		A12:Q>Q		A4:Q>Q		N_43:Q>Q		N_44:Q>Q		N_45:Q>Q		UFRJ50791:Q>Q		UFRJ50816:Q>Q		YPS138:Q>Q	AA:121		UWOPS91_917_1:E>E	AA:125		UWOPS91_917_1:V>V	AA:139		UWOPS91_917_1:T>T	AA:141		A12:Q>Q		A4:Q>Q		N_43:Q>Q		N_45:Q>Q		UFRJ50791:Q>Q		UFRJ50816:Q>Q		UWOPS91_917_1:Q>Q		YPS138:Q>Q	AA:147		UWOPS91_917_1:L>L	AA:166		UWOPS91_917_1:V>V	AA:168		A12:Y>Y		A4:Y>Y		UFRJ50791:Y>Y		UFRJ50816:Y>Y		YPS138:Y>Y	AA:173		A12:T>T		A4:T>T	AA:176		A12:P>P		A4:P>P		UFRJ50791:P>P		UFRJ50816:P>P		YPS138:P>P	AA:182		UWOPS91_917_1:V>V	AA:189		A12:V>V		A4:V>V		UFRJ50791:V>V		UFRJ50816:V>V		YPS138:V>V	AA:197		N_43:K>K		N_44:K>K		N_45:K>K	AA:208		UWOPS91_917_1:V>V	AA:240		UWOPS91_917_1:F>F	AA:251		UWOPS91_917_1:D>D	AA:257		A12:V>V		A4:V>V		DBVPG6304:V>V		UFRJ50791:V>V		UWOPS91_917_1:V>V		YPS138:V>V	AA:265		A12:G>G		A4:G>G		DBVPG6304:G>G		UFRJ50791:G>G		YPS138:G>G	AA:269		A12:A>A		A4:A>A		DBVPG6304:A>A		UFRJ50791:A>A	AA:271		Q32_3:F>F	AA:280		A12:I>I		A4:I>I		DBVPG6304:I>I		UFRJ50791:I>I	AA:283		UWOPS91_917_1:R>R	AA:287		A12:P>P		A4:P>P		DBVPG6304:P>P		UFRJ50791:P>P	AA:298		A12:C>C		A4:C>C		DBVPG6304:C>C		UFRJ50791:C>C		YPS138:C>C	AA:308		N_43:R>R		N_45:R>R	AA:322		A12:F>F		A4:F>F		DBVPG6304:F>F		UFRJ50791:F>F		YPS138:F>F	AA:334		A12:I>I		A4:I>I		DBVPG6304:I>I		UFRJ50791:I>I		YPS138:I>I	AA:335		N_43:V>V		N_45:V>V	AA:355		A4:A>A		DBVPG6304:A>A		UFRJ50791:A>A		YPS138:A>A	AA:365		A12:E>E		A4:E>E		DBVPG6304:E>E		UFRJ50791:E>E		YPS138:E>E	AA:368		A12:L>L		A4:L>L		DBVPG6304:L>L		UFRJ50791:L>L		YPS138:L>L	AA:379		A4:V>V		DBVPG6304:V>V		UFRJ50791:V>V		YPS138:V>V	AA:405		A4:G>G		DBVPG6304:G>G		UFRJ50791:G>G		YPS138:G>G	AA:419		A4:R>R		DBVPG6304:R>R		UFRJ50791:R>R		YPS138:R>R	AA:450		A12:V>V		CBS432:V>V		DBVPG6304:V>V		KPN3828:V>V		KPN3829:V>V		N_17:V>V		N_43:V>V		N_44:V>V		N_45:V>V		Q32_3:V>V		UFRJ50791:V>V		UWOPS91_917_1:V>V		YPS138:V>V	AA:461		A12:S>S		N_43:S>S		N_44:S>S		N_45:S>S		UFRJ50791:S>S		UFRJ50816:S>S		YPS138:S>S	AA:488		UWOPS91_917_1:I>I	AA:490		A12:N>N		UFRJ50791:N>N		UFRJ50816:N>N		YPS138:N>N	AA:493		A12:T>T		N_43:T>T		N_44:T>T		N_45:T>T		UFRJ50791:T>T		UFRJ50816:T>T		UWOPS91_917_1:T>T		YPS138:T>T	AA:497		UWOPS91_917_1:F>F	AA:528		A12:E>E		UFRJ50791:E>E		UFRJ50816:E>E		UWOPS91_917_1:E>E		YPS138:E>E	AA:541		CBS432:I>I		KPN3828:I>I		KPN3829:I>I		N_17:I>I		N_43:I>I		N_44:I>I		N_45:I>I		Q32_3:I>I	AA:561		CBS432:Y>Y		KPN3828:Y>Y		KPN3829:Y>Y		N_17:Y>Y	AA:563		A12:I>I	AA:569		A12:T>T		UFRJ50791:T>T		UFRJ50816:T>T		UWOPS91_917_1:T>T	AA:573		N_44:Q>Q	AA:580		UWOPS91_917_1:I>I	AA:583		A12:I>I		UFRJ50791:I>I		UFRJ50816:I>I	AA:599		UWOPS91_917_1:V>V	AA:603		N_43:F>F		N_44:F>F	AA:614		N_43:->-		N_45:->-	AA:636		N_45:I>I	AA:648		A12:T>T		DBVPG6304:T>T		UFRJ50791:T>T		UFRJ50816:T>T	AA:661		A12:Q>Q		DBVPG6304:Q>Q		UFRJ50791:Q>Q		UFRJ50816:Q>Q	AA:681		A12:H>H		CBS432:H>H		DBVPG6304:H>H		IFO1804:H>H		KPN3828:H>H		KPN3829:H>H		N_17:H>H		N_43:H>H		N_45:H>H		Q59_1:H>H		UFRJ50816:H>H		UWOPS91_917_1:H>HID:YBL061C	AA:18		A12:K>K		DBVPG6304:K>K		UFRJ50816:K>K	AA:29		A12:V>V		DBVPG6304:V>V		UFRJ50816:V>V	AA:44		IFO1804:L>L		N_43:L>L		N_44:L>L		N_45:L>L	AA:54		A12:S>S		DBVPG6304:S>S		UFRJ50816:S>S	AA:72		A12:S>S		DBVPG6304:S>S		UFRJ50791:S>S		UFRJ50816:S>S	AA:84		IFO1804:T>T		N_43:T>T		N_44:T>T		N_45:T>T	AA:104		A12:T>T		DBVPG6304:T>T		UFRJ50791:T>T		UFRJ50816:T>T	AA:121		A12:S>S		DBVPG6304:S>S		IFO1804:S>S		N_43:S>S		N_44:S>S		N_45:S>S		UFRJ50791:S>S		UFRJ50816:S>S	AA:124		A12:S>S		DBVPG6304:S>S		UFRJ50791:S>S		UFRJ50816:S>S	AA:128		UWOPS91_917_1:S>S	AA:134		A12:S>S		DBVPG6304:S>S		UFRJ50791:S>S		UFRJ50816:S>S		UWOPS91_917_1:S>S	AA:149		IFO1804:L>L		N_43:L>L		N_44:L>L		N_45:L>L		UWOPS91_917_1:L>L	AA:152		A12:D>D		DBVPG6304:D>D		UFRJ50791:D>D		UFRJ50816:D>D	AA:160		UWOPS91_917_1:A>A	AA:173		IFO1804:K>K		N_43:K>K		N_44:K>K		N_45:K>K	AA:192		A12:L>L		DBVPG6304:L>L		UFRJ50791:L>L		UFRJ50816:L>L		UWOPS91_917_1:L>L	AA:197		A12:K>K		DBVPG6304:K>K		UFRJ50791:K>K		UFRJ50816:K>K	AA:211		A12:A>A		DBVPG6304:A>A		UFRJ50791:A>A		UFRJ50816:A>A	AA:219		A12:A>A		DBVPG6304:A>A		UFRJ50791:A>A		UFRJ50816:A>A		UWOPS91_917_1:A>A	AA:222		A12:A>A		DBVPG6304:A>A		UFRJ50791:A>A		UFRJ50816:A>A		UWOPS91_917_1:A>A	AA:231		A12:G>G		DBVPG6304:G>G		UFRJ50791:G>G		UFRJ50816:G>G	AA:250		A12:L>L		UFRJ50791:L>L		UFRJ50816:L>L		YPS138:L>L	AA:286		N_43:A>A		N_44:A>A		N_45:A>A	AA:299		A12:E>E		UFRJ50791:E>E		UWOPS91_917_1:E>E		YPS138:E>E	AA:306		A12:E>E		YPS138:E>E	AA:310		UWOPS91_917_1:A>A	AA:311		A12:A>A		YPS138:A>A	AA:314		A12:E>E		DBVPG6304:E>E		UFRJ50791:E>E		YPS138:E>E	AA:326		UWOPS91_917_1:V>V	AA:328		A12:N>N		DBVPG6304:N>N		UFRJ50791:N>N		YPS138:N>N	AA:394		A12:K>K		DBVPG6304:K>K		YPS138:K>K	AA:410		N_44:G>G		N_45:G>G	AA:419		A12:A>A		DBVPG6304:A>A		YPS138:A>A	AA:421		A12:L>L		DBVPG6304:L>L		YPS138:L>L	AA:423		A12:Q>Q		DBVPG6304:Q>Q		YPS138:Q>Q	AA:431		A12:S>S		DBVPG6304:S>S		YPS138:S>S	AA:435		A12:L>L		DBVPG6304:L>L		YPS138:L>L	AA:470		DBVPG6304:L>L		YPS138:L>L	AA:507		A12:T>T		A4:T>T		DBVPG6304:T>T		YPS138:T>T	AA:529		A12:T>T		A4:T>T		DBVPG6304:T>T		YPS138:T>T	AA:556		A12:Q>Q		A4:Q>Q		DBVPG6304:Q>Q		YPS138:Q>Q	AA:558		IFO1804:K>K		N_44:K>K		N_45:K>K	AA:559		DBVPG6304:T>T	AA:578		A12:P>P		A4:P>P		DBVPG6304:P>P		YPS138:P>P	AA:580		A12:S>S		A4:S>S		DBVPG6304:S>S		YPS138:S>S	AA:598		CBS432:P>P		N_17:P>P	AA:634		A4:S>S		DBVPG6304:S>S		UFRJ50816:S>S		YPS138:S>S	AA:648		A4:K>K		DBVPG6304:K>K		UFRJ50816:K>K		YPS138:K>K	AA:651		CBS432:R>R		KPN3829:R>R		N_17:R>R		N_43:R>R		N_44:R>R		N_45:R>R	AA:664		A4:L>L		DBVPG6304:L>L		YPS138:L>LID:YBL066C	AA:3		UFRJ50791:G>G		YPS138:G>G	AA:10		A12:F>F		UFRJ50791:F>F		YPS138:F>F	AA:30		A12:Q>Q		UFRJ50791:Q>Q		YPS138:Q>Q	AA:34		IFO1804:T>T		N_43:T>T	AA:52		A12:N>N		UFRJ50791:N>N		YPS138:N>N	AA:83		N_43:P>P	AA:90		A12:S>S		UFRJ50791:S>S		UFRJ50816:S>S	AA:112		A12:R>R		IFO1804:R>R		N_43:R>R		N_45:R>R		Q32_3:R>R		UFRJ50791:R>R		UFRJ50816:R>R	AA:145		A12:R>R		IFO1804:R>R		N_43:R>R		N_45:R>R		UFRJ50791:R>R		UFRJ50816:R>R		UWOPS91_917_1:R>R	AA:154		A12:T>T		UFRJ50791:T>T		UFRJ50816:T>T		UWOPS91_917_1:T>T	AA:163		A12:P>P		IFO1804:P>P		N_43:P>P		N_45:P>P		UFRJ50791:P>P		UFRJ50816:P>P		UWOPS91_917_1:P>P	AA:178		A12:T>T		UFRJ50791:T>T		UFRJ50816:T>T		UWOPS91_917_1:T>T	AA:185		A12:S>S		IFO1804:S>S		N_43:S>S		N_45:S>S		UFRJ50791:S>S		UFRJ50816:S>S		UWOPS91_917_1:S>S	AA:201		Q32_3:T>T	AA:203		UWOPS91_917_1:Q>Q	AA:221		UFRJ50791:P>P		UFRJ50816:P>P		UWOPS91_917_1:P>P	AA:226		A12:N>N		UFRJ50791:N>N		UFRJ50816:N>N	AA:230		A12:P>P		A4:P>P		DBVPG6304:P>P		UFRJ50791:L>L		UFRJ50816:P>P		YPS138:P>P	AA:232		UWOPS91_917_1:T>T	AA:252		A12:V>V		IFO1804:V>V		N_45:V>V		UFRJ50791:V>V		UFRJ50816:V>V		UWOPS91_917_1:V>V	AA:258		A12:L>L		UFRJ50791:L>L		UFRJ50816:L>L	AA:281		A12:L>L		UFRJ50791:L>L		UFRJ50816:L>L		UWOPS91_917_1:L>L	AA:288		IFO1804:A>A		N_44:A>A	AA:292		A12:P>P		UFRJ50791:P>P		UFRJ50816:P>P	AA:363		A12:E>E		A4:E>E	AA:376		A12:Y>Y		A4:Y>Y	AA:385		A12:R>R		A4:R>R	AA:389		A12:I>I		A4:I>I		UWOPS91_917_1:I>I	AA:402		A12:I>I		A4:I>I		IFO1804:I>I		N_43:I>I		N_44:I>I		UWOPS91_917_1:I>I	AA:410		A12:L>L		A4:L>L		IFO1804:L>L		N_43:L>L		N_44:L>L		N_45:L>L	AA:436		A12:I>I		A4:I>I		UFRJ50816:I>I		UWOPS91_917_1:I>I		YPS138:I>I	AA:446		A12:F>F		A4:F>F		UFRJ50816:F>F		UWOPS91_917_1:F>F		YPS138:F>F	AA:454		UWOPS91_917_1:T>T	AA:456		IFO1804:Q>Q		N_43:Q>Q		N_44:Q>Q		N_45:Q>Q	AA:464		A4:S>S		UFRJ50816:S>S		UWOPS91_917_1:S>S		YPS138:S>S	AA:466		UWOPS91_917_1:T>T	AA:467		A4:A>A		UFRJ50816:A>A		YPS138:A>A	AA:474		UWOPS91_917_1:P>P	AA:478		IFO1804:L>L		N_43:L>L		N_44:L>L		N_45:L>L	AA:489		A4:T>T		DBVPG6304:T>T		UFRJ50816:T>T		YPS138:T>T	AA:491		Q32_3:I>I		UWOPS91_917_1:I>I	AA:493		UWOPS91_917_1:T>T	AA:496		IFO1804:L>L		N_43:L>L		N_44:L>L		N_45:L>L	AA:503		UWOPS91_917_1:P>P	AA:507		UWOPS91_917_1:T>T	AA:508		N_45:P>P	AA:509		A4:P>P		DBVPG6304:P>P		UWOPS91_917_1:P>P		YPS138:P>P	AA:512		DBVPG6304:P>P	AA:519		UWOPS91_917_1:V>V	AA:521		IFO1804:L>L		N_43:L>L		N_45:L>L		UWOPS91_917_1:L>L	AA:525		CBS432:L>L	AA:528		A4:I>I		DBVPG6304:I>I		UFRJ50816:I>I		UWOPS91_917_1:I>I		YPS138:I>I	AA:529		IFO1804:N>N		N_43:N>N		N_45:N>N	AA:530		UWOPS91_917_1:V>V	AA:531		A4:T>T		DBVPG6304:T>T		UFRJ50816:T>T		YPS138:T>T	AA:533		UWOPS91_917_1:D>D	AA:548		UWOPS91_917_1:G>G	AA:555		UWOPS91_917_1:A>A	AA:558		UWOPS91_917_1:K>K	AA:561		A4:L>L		DBVPG6304:L>L		UFRJ50816:L>L		YPS138:L>L	AA:565		A4:P>P		DBVPG6304:P>P		UFRJ50816:P>P		UWOPS91_917_1:P>P		YPS138:P>P	AA:569		UWOPS91_917_1:S>S	AA:571		A4:G>G		DBVPG6304:G>G		UFRJ50816:G>G		UWOPS91_917_1:G>G		YPS138:G>G	AA:573		UWOPS91_917_1:I>I	AA:576		IFO1804:L>L		N_43:L>L		N_45:L>L	AA:616		DBVPG6304:N>N	AA:630		A4:A>A		DBVPG6304:A>A		UFRJ50816:A>A		YPS138:A>A	AA:646		A4:I>I		DBVPG6304:I>I		N_43:I>I		N_45:I>I		UFRJ50816:I>I		UWOPS91_917_1:I>I		YPS138:I>I	AA:658		A4:L>L		DBVPG6304:L>L		UFRJ50816:L>L		UWOPS91_917_1:L>L		YPS138:L>L	AA:662		UWOPS91_917_1:T>T	AA:667		UWOPS91_917_1:A>A	AA:680		Q32_3:I>I	AA:697		A4:G>G		DBVPG6304:G>G		N_43:G>G		N_44:G>G		N_45:G>G		UFRJ50816:G>G		UWOPS91_917_1:G>G		YPS138:G>G	AA:711		A4:P>P		DBVPG6304:P>P		UWOPS91_917_1:P>P		YPS138:P>P	AA:719		YPS138:L>L	AA:723		A12:S>S		A4:S>S		DBVPG6304:S>S		UWOPS91_917_1:S>S		YPS138:S>S	AA:726		A12:T>T		A4:T>T		DBVPG6304:T>T		YPS138:T>T	AA:776		A12:T>T		A4:T>T		DBVPG6304:T>T		UFRJ50816:T>T		YPS138:T>T	AA:780		UWOPS91_917_1:S>S	AA:791		A12:T>T		A4:T>T		DBVPG6304:T>T		UFRJ50816:T>T		YPS138:T>T	AA:804		A12:S>S		A4:S>S		DBVPG6304:S>S		UFRJ50816:S>S		YPS138:S>S	AA:810		A12:L>L		A4:L>L		DBVPG6304:L>L		UFRJ50816:L>L		YPS138:L>L	AA:815		A12:V>V		DBVPG6304:V>V		UFRJ50816:V>V		YPS138:V>V	AA:819		N_43:P>P		N_44:P>P		N_45:P>P		UWOPS91_917_1:P>P	AA:831		A12:V>V		DBVPG6304:V>V		UFRJ50816:V>V		UWOPS91_917_1:V>V	AA:834		CBS432:T>T		DBVPG4650:T>T		N_43:T>T		N_44:T>T		N_45:T>T		Q32_3:T>T		UWOPS91_917_1:T>T	AA:852		A12:P>P		DBVPG6304:P>P		UFRJ50816:P>P		YPS138:P>P	AA:876		N_43:F>F		N_44:F>F		N_45:F>F	AA:905		A12:P>P		DBVPG6304:P>P		UFRJ50816:P>P		UWOPS91_917_1:P>P		YPS138:P>P	AA:909		A12:D>D		DBVPG6304:D>D		UFRJ50816:D>D		UWOPS91_917_1:D>D		YPS138:D>D	AA:912		N_43:L>L		N_44:L>L		N_45:L>L		UWOPS91_917_1:L>L	AA:930		A12:L>L		A4:L>L		DBVPG6304:L>L		UFRJ50816:L>L		YPS138:L>L	AA:938		A12:H>H		DBVPG6304:H>H		UFRJ50816:H>H		YPS138:H>H	AA:951		A12:N>N		A4:N>N		DBVPG6304:N>N		UFRJ50791:N>N		UFRJ50816:N>N		YPS138:N>N	AA:955		A12:S>S		A4:S>S	AA:958		A12:L>L		A4:L>L		DBVPG6304:L>L		UFRJ50791:L>L		UFRJ50816:L>L		UWOPS91_917_1:L>L		YPS138:L>L	AA:966		A12:Y>Y		A4:Y>Y		DBVPG6304:Y>Y		UFRJ50791:Y>Y		UFRJ50816:Y>Y		UWOPS91_917_1:Y>Y		YPS138:Y>Y	AA:995		A12:I>I		A4:I>I		DBVPG6304:I>I		N_43:I>I		N_44:I>I		N_45:I>I		UFRJ50791:I>I		UFRJ50816:I>I		YPS138:I>I	AA:998		A12:P>P		A4:P>P		DBVPG6304:P>P		UFRJ50791:P>P		UFRJ50816:P>P		YPS138:P>P	AA:1029		N_43:L>L		N_44:L>L		N_45:L>L	AA:1037		A12:I>I		A4:I>I		DBVPG6304:I>I		UFRJ50791:I>I		YPS138:I>I	AA:1040		A12:V>V		A4:V>V		DBVPG6304:V>V		UFRJ50791:V>V		YPS138:V>V	AA:1044		N_43:L>L		N_44:L>L		N_45:L>L	AA:1071		A12:C>C	AA:1083		A12:I>I		A4:I>I		DBVPG6304:I>I		UFRJ50791:I>I		UFRJ50816:I>I		UWOPS91_917_1:I>I		YPS138:I>I	AA:1090		A12:T>T		A4:T>T		DBVPG6304:T>T		UFRJ50816:T>T		UWOPS91_917_1:T>T		YPS138:T>T	AA:1102		A12:S>S		A4:S>S		DBVPG6304:S>S		UFRJ50816:S>S		UWOPS91_917_1:S>S		YPS138:S>S	AA:1112		A12:K>K		A4:K>K		DBVPG4650:K>K		DBVPG6304:K>K		N_43:K>K		N_45:K>K		UFRJ50816:K>K		UWOPS91_917_1:K>K		YPS138:K>K	AA:1120		A12:L>L		A4:L>L		DBVPG6304:L>L		UFRJ50816:L>L		YPS138:L>L	AA:1126		UWOPS91_917_1:Q>QID:YBL068W	AA:7		N_43:L>L		N_45:L>L	AA:25		N_43:Q>Q		N_45:Q>Q	AA:43		A12:V>V		A4:V>V		UFRJ50816:V>V	AA:46		A12:G>G		A4:G>G		UFRJ50816:G>G		YPS138:G>G	AA:80		A12:H>H		A4:H>H		DBVPG6304:H>H		UFRJ50816:H>H		UWOPS91_917_1:H>H		YPS138:H>H	AA:89		A12:A>A		A4:A>A		DBVPG6304:A>A		UFRJ50816:A>A		YPS138:A>A	AA:123		A12:Q>Q		A4:Q>Q		DBVPG6304:Q>Q		UFRJ50816:Q>Q		UWOPS91_917_1:Q>Q		YPS138:Q>Q	AA:127		A12:->-		A4:->-		DBVPG6304:->-		UFRJ50816:->-		YPS138:->-	AA:133		CBS5829:G>G		Q95_3:G>G		S36_7:G>G		T21_4:G>G		Y6_5:G>G		Y7:G>G		Z1_1:G>G	AA:143		UWOPS91_917_1:G>G	AA:155		UWOPS91_917_1:V>V	AA:157		A12:->-		A4:->-		DBVPG6304:->-		UFRJ50791:->-		UFRJ50816:->-		UWOPS91_917_1:->-		YPS138:->-	AA:166		A12:I>I		A4:I>I		DBVPG6304:I>I		UFRJ50791:I>I		UFRJ50816:I>I		YPS138:I>I	AA:174		A12:V>V		A4:V>V		DBVPG6304:V>V		UFRJ50791:V>V		UFRJ50816:V>V		YPS138:V>V	AA:185		UWOPS91_917_1:A>A	AA:191		A12:S>S		A4:S>S		DBVPG6304:S>S		UFRJ50791:S>S		UFRJ50816:S>S		UWOPS91_917_1:S>S		YPS138:S>S	AA:211		CBS432:V>V	AA:215		A12:F>F		A4:F>F		DBVPG6304:F>F		UFRJ50791:F>F		UFRJ50816:F>F		UWOPS91_917_1:F>F		YPS138:F>F	AA:224		A12:I>I		A4:I>I		DBVPG6304:I>I		UFRJ50791:I>I		UFRJ50816:I>I		YPS138:I>I	AA:229		UWOPS91_917_1:Q>Q	AA:232		A12:I>I		A4:I>I		DBVPG6304:I>I		UFRJ50791:I>I		UFRJ50816:I>I		YPS138:I>I	AA:235		N_45:I>I	AA:262		UFRJ50791:T>T		UFRJ50816:T>T	AA:301		A12:T>T		A4:T>T		DBVPG6304:T>T		UFRJ50791:T>T		UFRJ50816:T>T		UWOPS91_917_1:T>T		YPS138:T>TID:YBL069W	AA:8		A12:I>I		A4:I>I		DBVPG6304:I>I	AA:19		A12:L>L		DBVPG6304:L>L		UFRJ50791:L>L	AA:46		A12:L>L		DBVPG6304:L>L		UFRJ50791:L>L	AA:69		N_44:L>L		N_45:L>L	AA:111		UFRJ50791:S>S	AA:125		N_44:V>V		N_45:V>V	AA:128		Y7:P>P	AA:132		A12:I>I		DBVPG6304:I>I		UFRJ50791:I>I		YPS138:I>I	AA:201		A12:I>I		DBVPG6304:I>I		IFO1804:I>I		N_44:I>I		N_45:I>I		UFRJ50791:I>I	AA:205		IFO1804:I>I		N_43:I>I		N_44:I>I		N_45:I>I	AA:216		A12:Y>Y		DBVPG6304:Y>Y		UFRJ50791:Y>Y		YPS138:Y>Y	AA:221		IFO1804:T>T		N_44:T>T	AA:289		DBVPG6304:Y>Y		UFRJ50816:Y>Y		YPS138:Y>Y	AA:291		IFO1804:A>A		N_43:A>A		N_44:A>A	AA:311		UWOPS91_917_1:T>T	AA:321		DBVPG6304:T>T		UFRJ50816:T>T		UWOPS91_917_1:T>T		YPS138:T>T	AA:324		UWOPS91_917_1:Y>Y	AA:334		DBVPG6304:I>I		UFRJ50816:I>I		YPS138:I>I	AA:340		UWOPS91_917_1:I>I	AA:355		DBVPG6304:R>R		YPS138:R>R	AA:365		DBVPG6304:V>V		UFRJ50816:V>V		UWOPS91_917_1:V>V		YPS138:V>V	AA:368		N_43:I>I		N_44:I>I		N_45:I>I	AA:373		N_43:R>R		N_44:R>R		N_45:R>R		UWOPS91_917_1:R>R		YPS138:R>R	AA:389		UWOPS91_917_1:L>L	AA:410		UWOPS91_917_1:K>K	AA:412		UWOPS91_917_1:I>I	AA:418		UWOPS91_917_1:C>C	AA:421		N_43:L>L		N_44:L>L		N_45:L>LID:YBL072C	AA:40		UWOPS91_917_1:A>A	AA:47		N_43:S>S		N_45:S>S	AA:66		UWOPS91_917_1:S>S	AA:86		UWOPS91_917_1:H>H	AA:89		UWOPS91_917_1:F>F	AA:100		N_43:V>V		N_44:V>V		N_45:V>V	AA:130		A12:I>I		A4:I>I		DBVPG6304:I>I		N_43:I>I		N_44:I>I		N_45:I>I		UFRJ50791:I>I		UWOPS91_917_1:I>I		YPS138:I>I	AA:141		A12:E>E		A4:E>E		DBVPG6304:E>E		N_43:E>E		N_44:E>E		N_45:E>E		UFRJ50791:E>E		UWOPS91_917_1:E>E		YPS138:E>E	AA:175		UWOPS91_917_1:F>FID:YBL074C	AA:2		A4:N>N		DBVPG6304:N>N		UFRJ50816:N>N		UWOPS91_917_1:R>R		YPS138:N>N	AA:22		A4:E>E		DBVPG6304:E>E		UFRJ50816:E>E		UWOPS91_917_1:E>E		YPS138:E>E	AA:28		A4:H>H		DBVPG6304:H>H		UFRJ50816:H>H		UWOPS91_917_1:H>H		YPS138:H>H	AA:76		N_17:S>S		Q62_5:S>S		Q89_8:S>S		S36_7:S>S		Y7:S>S		Z1_1:S>S	AA:98		A4:K>K		DBVPG6304:K>K		UWOPS91_917_1:K>K		YPS138:K>K	AA:107		A4:L>L		DBVPG6304:L>L		UWOPS91_917_1:L>L		YPS138:L>L	AA:111		A4:A>A		DBVPG6304:A>A		UWOPS91_917_1:A>A		YPS138:A>A	AA:121		A4:V>V		DBVPG6304:G>G		IFO1804:G>G		N_43:G>G		N_44:G>G		N_45:G>G		UFRJ50816:V>V		UWOPS91_917_1:V>V		YPS138:V>V	AA:125		N_43:A>A		N_44:A>A		N_45:A>A	AA:134		IFO1804:G>G		N_45:G>G	AA:152		A4:L>L		DBVPG6304:L>L		UWOPS91_917_1:L>L		YPS138:L>L	AA:154		KPN3828:Y>Y	AA:155		N_43:S>S		N_44:S>S		N_45:S>S	AA:165		CBS5829:G>G		DBVPG4650:G>G		KPN3828:G>G		N_43:G>G		N_44:G>G		N_45:G>G		Z1_1:G>G	AA:184		CBS5829:A>A		DBVPG4650:A>A		KPN3828:A>A		Q62_5:A>A		Z1_1:A>A	AA:211		UFRJ50816:T>T	AA:217		A4:V>V		UFRJ50816:V>V		UWOPS91_917_1:V>V	AA:227		A4:V>V		UFRJ50816:V>V		UWOPS91_917_1:V>V		YPS138:V>V	AA:231		UWOPS91_917_1:I>I	AA:253		N_43:S>S		N_44:S>S		N_45:S>S	AA:263		A4:H>H		UFRJ50816:H>H		UWOPS91_917_1:H>H		YPS138:H>H	AA:268		UWOPS91_917_1:F>F	AA:271		A4:D>D		IFO1804:F>F		N_17:F>F		N_43:F>F		N_44:F>F		N_45:F>F		Q62_5:F>F		Q89_8:F>F		S36_7:F>F		UFRJ50816:D>D		UWOPS91_917_1:F>F		Y7:F>F		YPS138:D>D		Z1_1:F>F	AA:279		A4:Y>Y		UFRJ50816:Y>Y		YPS138:Y>Y	AA:310		A4:Q>Q		UFRJ50816:Q>Q		YPS138:Q>Q	AA:316		UWOPS91_917_1:V>V	AA:319		UWOPS91_917_1:I>I	AA:322		UWOPS91_917_1:D>D	AA:325		UWOPS91_917_1:G>G	AA:328		UWOPS91_917_1:P>P	AA:333		A4:V>V		UFRJ50816:V>V		UWOPS91_917_1:V>V		YPS138:V>V	AA:336		UWOPS91_917_1:S>S	AA:351		UWOPS91_917_1:F>FID:YBL075C	AA:4		A12:E>E		A4:E>E		UFRJ50791:E>E		UFRJ50816:E>E	AA:15		A12:G>G		A4:G>G		UFRJ50791:G>G		UFRJ50816:G>G		YPS138:G>G	AA:16		N_44:A>A	AA:37		A12:D>D		UFRJ50791:D>D		UFRJ50816:D>D		YPS138:D>D	AA:45		A12:T>T		A4:T>T		UFRJ50791:T>T		UFRJ50816:T>T		YPS138:T>T	AA:67		A12:A>A		A4:A>A		UFRJ50791:A>A		UFRJ50816:A>A		YPS138:A>A	AA:70		A12:A>A		A4:A>A		N_44:A>A		UFRJ50791:A>A		UFRJ50816:A>A		YPS138:A>A	AA:89		A12:E>E		A4:E>E		UFRJ50791:E>E		UFRJ50816:E>E		UWOPS91_917_1:E>E		YPS138:E>E	AA:92		A12:K>K		A4:K>K		UFRJ50791:K>K		UFRJ50816:K>K		UWOPS91_917_1:K>K		YPS138:K>K	AA:99		N_44:N>N		UWOPS91_917_1:N>N	AA:103		UWOPS91_917_1:K>K	AA:105		A12:T>T		A4:T>T		N_44:T>T		UFRJ50791:T>T		UFRJ50816:T>T		UWOPS91_917_1:T>T		YPS138:T>T	AA:115		UWOPS91_917_1:A>A	AA:118		A4:S>S		DBVPG6304:S>S		UWOPS91_917_1:R>R	AA:122		UWOPS91_917_1:R>R	AA:124		A12:D>D		UWOPS91_917_1:D>D	AA:128		UWOPS91_917_1:Y>Y	AA:138		N_44:I>I	AA:146		UWOPS91_917_1:K>K	AA:153		UWOPS91_917_1:K>K	AA:156		A12:K>K		A4:K>K		UFRJ50791:K>K		UFRJ50816:K>K		YPS138:K>K	AA:160		UWOPS91_917_1:K>K	AA:164		UWOPS91_917_1:S>S	AA:170		A12:N>N		A4:N>N		UFRJ50791:N>N		UFRJ50816:N>N		UWOPS91_917_1:N>N		YPS138:N>N	AA:172		A4:D>D	AA:181		A12:P>P		A4:P>P		UFRJ50816:P>P		UWOPS91_917_1:P>P		YPS138:P>P	AA:207		A12:E>E		A4:E>E		UFRJ50816:E>E		UWOPS91_917_1:E>E		YPS138:E>E	AA:224		A12:S>S		A4:S>S		UFRJ50816:S>S		UWOPS91_917_1:S>S		YPS138:S>S	AA:237		UFRJ50791:R>R		UFRJ50816:R>R		YPS138:R>R	AA:242		A4:T>T		IFO1804:T>T		N_44:T>T		UWOPS91_917_1:T>T	AA:245		UWOPS91_917_1:G>G	AA:246		UWOPS91_917_1:G>G	AA:250		KPN3828:I>I		Q32_3:I>I		Q89_8:I>I		Q95_3:I>I		T21_4:I>I	AA:276		A12:A>A		A4:A>A		IFO1804:S>S		N_43:S>S		N_44:S>S		N_45:S>S		UFRJ50791:A>A		UFRJ50816:A>A		UWOPS91_917_1:A>A		YPS138:A>A	AA:278		A12:V>V		A4:V>V		DBVPG6304:V>V		UFRJ50791:V>V		UFRJ50816:V>V		YPS138:V>V	AA:280		UWOPS91_917_1:A>A	AA:293		UWOPS91_917_1:N>N	AA:295		A12:E>E		A4:E>E		UFRJ50791:E>E		UFRJ50816:E>E		YPS138:E>E	AA:317		DBVPG6304:L>L	AA:341		A12:S>S		A4:S>S		DBVPG6304:S>S		IFO1804:S>S		N_43:S>S		N_44:S>S		UFRJ50791:S>S		UFRJ50816:S>S		UWOPS91_917_1:S>S		YPS138:S>S	AA:359		A12:Y>Y		DBVPG6304:Y>Y		UFRJ50791:Y>Y		UFRJ50816:Y>Y		UWOPS91_917_1:Y>Y		YPS138:Y>Y	AA:386		A12:A>A		DBVPG6304:A>A		UFRJ50791:A>A		UFRJ50816:A>A		UWOPS91_917_1:A>A		YPS138:A>A	AA:394		A12:S>S	AA:404		A12:T>T		A4:T>T		DBVPG6304:T>T		UFRJ50791:T>T		UFRJ50816:T>T		UWOPS91_917_1:T>T		YPS138:T>T	AA:408		IFO1804:F>F	AA:411		A12:A>A		A4:A>A		DBVPG6304:A>A		UFRJ50816:A>A		UWOPS91_917_1:A>A		YPS138:A>A	AA:422		IFO1804:E>E		N_43:E>E		N_44:E>E		N_45:E>E	AA:425		N_45:L>L	AA:441		UWOPS91_917_1:I>I	AA:460		IFO1804:H>H		N_43:H>H		N_44:H>H		N_45:H>H	AA:464		A4:G>G		DBVPG6304:G>G		UFRJ50816:G>G		UWOPS91_917_1:G>G		YPS138:G>G	AA:478		CBS432:E>E		CBS5829:E>E	AA:484		UWOPS91_917_1:V>V	AA:490		A4:T>T		DBVPG6304:T>T		UFRJ50816:T>T		UWOPS91_917_1:T>T		YPS138:T>T	AA:504		A4:Y>Y		DBVPG6304:Y>Y		UFRJ50816:Y>Y		UWOPS91_917_1:Y>Y		YPS138:Y>Y	AA:512		Q62_5:D>D		Q95_3:D>D	AA:537		N_43:P>P		N_44:P>P		N_45:P>P	AA:553		A4:K>K		DBVPG6304:K>K		Q62_5:E>E		Q89_8:E>E		Q95_3:E>E		S36_7:E>E		UFRJ50816:K>K		Y7:E>E		YPS138:K>K	AA:556		A4:R>R		DBVPG6304:R>R		UFRJ50816:R>R		UWOPS91_917_1:R>R		YPS138:R>R	AA:569		A4:T>T		DBVPG6304:T>T		N_43:T>T		N_44:T>T		N_45:T>T		UFRJ50816:T>T		YPS138:T>T	AA:575		A12:T>T		A4:T>T		CBS432:F>F		CBS5829:F>F		DBVPG6304:F>F		N_44:T>T		N_45:F>F		Q32_3:F>F		Q59_1:F>F		Q62_5:F>F		UFRJ50791:T>T		UFRJ50816:T>T		UWOPS91_917_1:T>T		Y7:F>F		YPS138:T>T	AA:578		A4:G>G		DBVPG6304:G>G		UFRJ50816:G>G		YPS138:G>G	AA:587		A4:T>T		N_43:T>T		N_44:T>T		N_45:T>T		UFRJ50816:T>T	AA:596		N_44:N>N		N_45:N>N	AA:613		A4:S>S		UFRJ50816:S>S		YPS138:S>S	AA:643		A12:P>P		A4:P>P		IFO1804:D>D		N_45:D>D		UFRJ50791:P>P		UFRJ50816:P>P		YPS138:P>PID:YBL078C	AA:3		A12:G>G		A4:G>G		DBVPG6304:G>G		UFRJ50816:G>G		UWOPS91_917_1:G>G	AA:7		A12:E>E		A4:E>E		DBVPG6304:E>E		UFRJ50816:E>E		UWOPS91_917_1:E>E		YPS138:E>E	AA:21		N_43:H>H	AA:49		A4:L>L		DBVPG6304:L>L		UFRJ50791:L>L		UFRJ50816:L>L		UWOPS91_917_1:L>L		YPS138:L>L	AA:54		CBS432:D>D		CBS5829:D>D		N_43:R>R		N_45:R>R		Q32_3:D>D		Q59_1:D>D	AA:57		A4:Y>Y		DBVPG6304:Y>Y		UFRJ50791:Y>Y		UFRJ50816:Y>Y		UWOPS91_917_1:Y>Y		YPS138:Y>Y	AA:92		A4:N>N		DBVPG6304:N>N		UFRJ50791:N>N		UFRJ50816:N>N		UWOPS91_917_1:N>N		YPS138:N>N	AA:114		N_45:F>FID:YBL080C	AA:13		UWOPS91_917_1:E>E	AA:16		UWOPS91_917_1:N>N	AA:17		UWOPS91_917_1:A>A	AA:31		A4:L>L		DBVPG6304:L>L		N_43:L>L		N_44:L>L		N_45:L>L		UFRJ50816:L>L		UWOPS91_917_1:L>L	AA:38		A4:K>K		DBVPG6304:K>K		IFO1804:K>K		N_43:K>K		N_44:K>K		N_45:K>K		UFRJ50816:K>K		UWOPS91_917_1:K>K	AA:44		UWOPS91_917_1:N>N	AA:46		UWOPS91_917_1:I>I	AA:87		IFO1804:S>S		N_43:S>S		N_44:S>S		N_45:S>S	AA:94		A12:Q>Q		A4:Q>Q		DBVPG6304:Q>Q		UFRJ50791:Q>Q		UFRJ50816:Q>Q	AA:97		UWOPS91_917_1:T>T	AA:103		A12:L>L		A4:L>L		DBVPG6304:L>L		UWOPS91_917_1:L>L	AA:107		UWOPS91_917_1:G>G	AA:117		A12:L>L		A4:L>L		DBVPG6304:L>L		UFRJ50791:L>L		UFRJ50816:L>L		UWOPS91_917_1:L>L	AA:133		A12:A>A		A4:A>A		DBVPG6304:A>A		UFRJ50816:A>A		UWOPS91_917_1:A>A	AA:135		A12:T>T		A4:T>T		DBVPG6304:T>T		UFRJ50791:T>T		UFRJ50816:T>T	AA:174		A12:L>L		A4:L>L		DBVPG6304:L>L		IFO1804:L>L		N_43:L>L		N_44:L>L		N_45:L>L		UFRJ50791:L>L		UFRJ50816:L>L	AA:183		UWOPS91_917_1:N>N	AA:205		A12:P>P		A4:P>P		DBVPG6304:P>P		UFRJ50791:P>P		UFRJ50816:P>P		UWOPS91_917_1:P>P	AA:241		A12:P>P		A4:P>P		UFRJ50791:P>P		UFRJ50816:P>P		UWOPS91_917_1:P>P	AA:247		IFO1804:I>I		N_43:I>I		N_44:I>I		N_45:I>I		UWOPS91_917_1:I>I	AA:249		A12:T>T		A4:T>T		UFRJ50791:T>T		UFRJ50816:T>T		UWOPS91_917_1:T>T	AA:256		UWOPS91_917_1:V>V	AA:259		A12:S>S		A4:Q>Q		UFRJ50791:S>S		UFRJ50816:S>S	AA:266		A12:S>S		A4:S>S		UFRJ50791:S>S		UFRJ50816:S>S	AA:276		Q32_3:G>G	AA:283		UWOPS91_917_1:R>R	AA:292		A12:I>I		A4:I>I		UFRJ50791:I>I		UFRJ50816:I>I		UWOPS91_917_1:I>I	AA:299		A4:L>L		UFRJ50791:L>L		UFRJ50816:L>L		UWOPS91_917_1:L>L	AA:306		A12:A>A		UFRJ50791:A>A		UFRJ50816:A>A		UWOPS91_917_1:A>A	AA:312		UWOPS91_917_1:L>L	AA:319		Q62_5:A>A		Y6_5:A>A		Y7:A>A	AA:320		UWOPS91_917_1:G>G	AA:327		IFO1804:S>S		N_43:S>S		N_44:S>S		N_45:S>S	AA:355		A12:V>V		UWOPS91_917_1:V>V	AA:362		A12:N>N	AA:373		A12:D>D		UWOPS91_917_1:D>D	AA:378		IFO1804:T>T		N_43:T>T		N_44:T>T		N_45:T>T	AA:380		A12:H>H	AA:383		A12:G>G		UWOPS91_917_1:G>G	AA:415		UWOPS91_917_1:R>R	AA:423		UWOPS91_917_1:T>T	AA:434		A12:F>F		UWOPS91_917_1:F>F	AA:438		A12:R>R		UFRJ50791:R>R		UWOPS91_917_1:R>R	AA:444		UWOPS91_917_1:S>S	AA:454		A12:L>L		UWOPS91_917_1:L>L	AA:460		A12:I>I		UFRJ50791:I>I	AA:463		A12:L>L		UFRJ50791:L>L		UWOPS91_917_1:L>L	AA:472		A12:L>L		UFRJ50791:L>L		UWOPS91_917_1:L>L	AA:489		UWOPS91_917_1:T>T	AA:494		UWOPS91_917_1:S>S	AA:513		UWOPS91_917_1:K>K	AA:525		UWOPS91_917_1:N>N	AA:528		A12:V>V	AA:531		A12:R>R		UFRJ50791:R>R		UWOPS91_917_1:R>R	AA:537		A12:R>R		UFRJ50791:R>RID:YBL081W	AA:17		N_43:H>H		N_45:H>H	AA:26		UWOPS91_917_1:T>T	AA:28		A4:L>L		CBS432:L>L		CBS5829:L>L		DBVPG6304:L>L		N_17:L>L		N_43:L>L		N_44:L>L		N_45:L>L		Q32_3:L>L		Q62_5:L>L		Q89_8:L>L		Q95_3:L>L		T21_4:L>L		UFRJ50816:L>L		UWOPS91_917_1:L>L		Y6_5:L>L		Z1_1:L>L	AA:34		UWOPS91_917_1:T>T	AA:38		N_45:L>L	AA:58		A4:V>V		DBVPG6304:V>V		UFRJ50816:V>V		UWOPS91_917_1:V>V	AA:59		N_44:V>V		N_45:V>V	AA:67		A4:V>V		DBVPG6304:V>V		UFRJ50816:V>V	AA:87		UWOPS91_917_1:R>R	AA:100		A4:V>V		DBVPG6304:V>V		UFRJ50816:V>V		UWOPS91_917_1:V>V	AA:107		N_44:V>V		N_45:V>V		UWOPS91_917_1:V>V	AA:147		A4:Q>Q		DBVPG6304:Q>Q		UFRJ50816:Q>Q	AA:156		A4:V>V		DBVPG6304:V>V		UFRJ50816:V>V	AA:167		N_17:V>V	AA:195		N_44:G>G		N_45:G>G	AA:219		Y7:->-	AA:220		A4:R>R		DBVPG6304:R>R		N_44:R>R		N_45:R>R		UFRJ50816:R>R		UWOPS91_917_1:R>R		YPS138:R>R	AA:224		N_44:R>R		N_45:R>R	AA:226		A4:->-		DBVPG6304:->-		UFRJ50816:->-		YPS138:->-	AA:234		N_44:G>G		N_45:G>G	AA:244		A4:G>G		DBVPG6304:G>G		UFRJ50791:G>G		UFRJ50816:G>G		UWOPS91_917_1:G>G		YPS138:G>G	AA:249		UWOPS91_917_1:I>I	AA:269		A4:H>H		DBVPG6304:H>H		UFRJ50791:H>H		UWOPS91_917_1:H>H		YPS138:H>H	AA:286		A4:G>G		DBVPG6304:G>G		UFRJ50791:G>G		UWOPS91_917_1:G>G		YPS138:G>G	AA:325		A4:R>R		UFRJ50791:R>R		UFRJ50816:R>R		YPS138:R>R	AA:326		N_43:R>R		N_44:R>R		N_45:R>R	AA:338		A4:->-		UFRJ50791:->-		UFRJ50816:->-		UWOPS91_917_1:->-		YPS138:->-	AA:347		Q62_5:G>G		UWOPS91_917_1:G>GTable S5. Synonymous SNPs in S. paradoxus genes studied. The identity of each affected amino acid in each affected strain is shown for each of 3756 genes.ID:YBL082C	AA:3		YPS138:L>L	AA:13		UWOPS91_917_1:L>L	AA:22		A4:G>G		UFRJ50816:G>G		YPS138:G>G	AA:24		A4:L>L		UFRJ50816:L>L	AA:49		CBS5829:P>P		Q32_3:P>P		Q59_1:P>P		Q62_5:P>P		Q95_3:P>P		T21_4:P>P		Z1_1:P>P	AA:71		A4:S>S		UFRJ50816:S>S		UWOPS91_917_1:S>S		YPS138:S>S	AA:89		A4:H>H		UFRJ50816:H>H		YPS138:H>H	AA:90		N_45:L>L	AA:94		A4:F>F		UFRJ50816:F>F		UWOPS91_917_1:F>F		YPS138:F>F	AA:104		A4:L>L		N_43:L>L		N_45:L>L		UFRJ50816:L>L		UWOPS91_917_1:L>L		YPS138:L>L	AA:117		A4:V>V		YPS138:V>V	AA:133		A4:L>L		UWOPS91_917_1:L>L		YPS138:L>L	AA:140		A4:V>V		UWOPS91_917_1:V>V	AA:143		IFO1804:T>T		N_45:T>T	AA:154		A4:A>A	AA:157		UWOPS91_917_1:F>F	AA:166		UWOPS91_917_1:D>D	AA:228		A4:I>I		DBVPG6304:I>I	AA:236		DBVPG6304:L>L	AA:243		IFO1804:V>V		N_43:V>V		N_45:V>V	AA:267		N_43:S>S	AA:276		Q32_3:T>T		Q95_3:T>T		Y7:T>T	AA:282		A12:T>T		A4:T>T		DBVPG6304:T>T		UWOPS91_917_1:T>T	AA:295		A12:H>H		A4:H>H		DBVPG6304:H>H		UWOPS91_917_1:H>H	AA:300		UWOPS91_917_1:L>L	AA:304		A12:V>V		A4:V>V		DBVPG6304:V>V		UWOPS91_917_1:V>V	AA:310		UWOPS91_917_1:L>L	AA:316		A12:C>C		A4:C>C		DBVPG6304:C>C		UWOPS91_917_1:C>C	AA:335		A12:R>R		DBVPG6304:R>R	AA:341		UWOPS91_917_1:G>G	AA:342		CBS432:E>E	AA:352		A12:L>L		A4:L>L		DBVPG6304:L>L		IFO1804:L>L		N_45:L>L		UWOPS91_917_1:L>L		YPS138:L>L	AA:356		UWOPS91_917_1:A>A	AA:363		A12:T>T		A4:T>T		DBVPG6304:T>T		Q59_1:T>T		UWOPS91_917_1:T>T	AA:372		UWOPS91_917_1:R>R	AA:386		A12:A>A		DBVPG6304:A>A		YPS138:A>A	AA:405		A12:S>S		A4:S>S		DBVPG6304:S>S		UWOPS91_917_1:S>S		YPS138:S>S	AA:422		UWOPS91_917_1:F>F	AA:424		IFO1804:L>L		N_44:L>L		N_45:L>L	AA:462		A12:T>T		A4:T>T		DBVPG6304:T>T		UWOPS91_917_1:T>T		YPS138:T>TID:YBL084C	AA:3		A4:Q>Q		DBVPG6304:Q>Q		IFO1804:Q>Q		N_43:Q>Q		N_45:Q>Q		UFRJ50791:Q>Q		UFRJ50816:Q>Q		YPS138:Q>Q	AA:26		A4:T>T		DBVPG6304:T>T		UFRJ50791:T>T		UFRJ50816:T>T		YPS138:T>T	AA:45		IFO1804:L>L		N_43:L>L		N_45:L>L	AA:47		A4:H>H		YPS138:H>H	AA:81		A4:K>K		DBVPG6304:K>K		YPS138:K>K	AA:84		A4:S>S		DBVPG6304:S>S		YPS138:S>S	AA:97		A4:Q>Q		DBVPG6304:Q>Q		UWOPS91_917_1:Q>Q		YPS138:Q>Q	AA:98		CBS5829:L>L		IFO1804:L>L		N_44:L>L		N_45:L>L	AA:136		UWOPS91_917_1:Y>Y	AA:155		UWOPS91_917_1:P>P	AA:163		UWOPS91_917_1:Y>Y	AA:167		UWOPS91_917_1:A>A	AA:175		A4:S>S		DBVPG6304:S>S		UWOPS91_917_1:S>S		YPS138:S>S	AA:182		A4:A>A		DBVPG6304:T>T		IFO1804:A>A		N_43:A>A		N_44:A>A		N_45:A>A		UFRJ50791:A>A		YPS138:T>T	AA:203		A4:D>D		DBVPG6304:D>D		YPS138:D>D	AA:220		UWOPS91_917_1:P>P	AA:222		A4:N>N		DBVPG6304:N>N		UWOPS91_917_1:N>N	AA:234		A4:F>F		IFO1804:S>S		N_43:S>S		N_44:S>S		N_45:S>S	AA:239		A4:D>D		DBVPG6304:D>D		IFO1804:D>D		N_43:D>D		N_44:D>D		N_45:D>D	AA:259		UWOPS91_917_1:L>L	AA:263		A4:R>R		DBVPG6304:R>R		IFO1804:R>R		N_43:R>R		N_44:R>R		N_45:R>R		UWOPS91_917_1:R>R	AA:267		A4:K>K		DBVPG6304:K>K		UWOPS91_917_1:K>K	AA:276		UWOPS91_917_1:E>E	AA:283		UWOPS91_917_1:Q>Q	AA:291		UWOPS91_917_1:D>D	AA:297		UWOPS91_917_1:I>I	AA:300		UWOPS91_917_1:E>E	AA:302		UWOPS91_917_1:L>L	AA:306		UWOPS91_917_1:K>K	AA:310		A4:Y>Y		DBVPG6304:Y>Y	AA:314		A4:S>S	AA:324		UWOPS91_917_1:I>I	AA:327		DBVPG6304:L>L		UWOPS91_917_1:L>L	AA:335		A4:L>L		DBVPG6304:L>L		IFO1804:L>L		N_43:L>L		N_45:L>L		UFRJ50791:L>L		UWOPS91_917_1:L>L	AA:353		UFRJ50791:P>P		UWOPS91_917_1:P>P	AA:379		UFRJ50791:N>N	AA:388		UWOPS91_917_1:D>D	AA:399		UFRJ50791:L>L		UWOPS91_917_1:L>L	AA:403		IFO1804:R>R		N_43:R>R		N_44:R>R		N_45:R>R		UFRJ50791:R>R		UWOPS91_917_1:R>R	AA:409		DBVPG6304:L>L		UFRJ50791:L>L		UWOPS91_917_1:L>L	AA:430		UFRJ50791:L>L		UWOPS91_917_1:L>L	AA:444		UFRJ50791:T>T		UWOPS91_917_1:T>T	AA:450		UFRJ50791:Q>Q	AA:489		A4:T>T		UFRJ50791:T>T		UWOPS91_917_1:T>T	AA:504		A4:K>K		UWOPS91_917_1:K>K	AA:525		A4:S>S		UFRJ50791:S>S		UWOPS91_917_1:S>S	AA:531		A4:H>H		CBS5829:H>H		IFO1804:H>H		N_43:H>H		N_44:H>H		N_45:H>H		Q32_3:H>H		Q89_8:H>H		Q95_3:H>H		T21_4:H>H		UFRJ50791:H>H		Y6_5:H>H	AA:534		A4:S>S		UFRJ50791:S>S		UWOPS91_917_1:S>S	AA:541		N_43:F>F		N_44:F>F		N_45:F>F	AA:572		A4:H>H		UFRJ50791:H>H	AA:580		A4:H>H		UFRJ50791:H>H		UWOPS91_917_1:H>H	AA:602		A4:L>L		UFRJ50791:L>L		UWOPS91_917_1:L>L	AA:604		A4:S>S		UFRJ50791:S>S		UWOPS91_917_1:S>S	AA:630		A4:A>A		UFRJ50791:A>A		UWOPS91_917_1:A>A	AA:639		A4:L>L		UFRJ50791:L>L		UWOPS91_917_1:L>L	AA:646		A4:Y>Y		UFRJ50791:Y>Y		UWOPS91_917_1:Y>Y	AA:656		N_45:E>E	AA:676		A4:L>L		UFRJ50791:L>L	AA:694		A4:A>A		UFRJ50791:A>A		UFRJ50816:A>A		UWOPS91_917_1:A>A	AA:698		A4:E>E		UFRJ50791:E>E	AA:706		UWOPS91_917_1:Y>Y	AA:739		UWOPS91_917_1:F>FID:YBL086C	AA:15		A12:T>T		A4:T>T		CBS432:T>T		CBS5829:T>T		DBVPG4650:T>T		DBVPG6304:T>T		N_17:T>T		N_45:T>T		Q32_3:T>T		Q62_5:T>T		Q89_8:T>T		Q95_3:T>T		T21_4:T>T		UFRJ50791:T>T		UFRJ50816:T>T		Y7:T>T	AA:44		A12:D>D		A4:D>D		DBVPG6304:D>D		UFRJ50816:D>D	AA:83		DBVPG4650:P>P	AA:91		A12:P>P		A4:P>P		DBVPG6304:P>P		UFRJ50816:P>P	AA:107		A12:L>L		A4:L>L	AA:134		A12:V>V		A4:V>V		DBVPG6304:V>V		UFRJ50816:V>V		UWOPS91_917_1:V>V	AA:138		A12:P>P		A4:P>P		DBVPG6304:P>P		UFRJ50816:P>P		UWOPS91_917_1:P>P	AA:141		A12:E>E		A4:E>E		DBVPG6304:E>E		UFRJ50816:E>E		UWOPS91_917_1:E>E	AA:153		CBS5829:K>K		DBVPG4650:K>K		N_17:K>K		N_43:K>K		Q32_3:K>K		Q59_1:K>K		Q62_5:K>K		Q89_8:K>K		T21_4:K>K		UWOPS91_917_1:K>K		Y6_5:K>K		Y7:K>K	AA:179		A12:S>S		A4:S>S		DBVPG6304:S>S		UFRJ50816:S>S		UWOPS91_917_1:S>S		YPS138:S>S	AA:187		A12:P>P		A4:P>P		DBVPG6304:P>P		UFRJ50816:P>P		UWOPS91_917_1:V>V		YPS138:P>P	AA:216		YPS138:N>N	AA:218		A12:P>P		A4:P>P		DBVPG6304:P>P		UFRJ50816:P>P		UWOPS91_917_1:P>P		YPS138:P>P	AA:223		A12:T>T		A4:T>T		CBS432:T>T		CBS5829:T>T		DBVPG6304:T>T		N_43:T>T		N_44:T>T		N_45:T>T		Q59_1:T>T		Q62_5:T>T		Q89_8:T>T		UFRJ50816:T>T		UWOPS91_917_1:T>T		Y6_5:T>T		YPS138:T>T	AA:233		A12:T>T		A4:T>T		DBVPG6304:T>T		UFRJ50816:T>T		YPS138:T>T	AA:254		A12:K>K		A4:K>K		DBVPG6304:K>K		UFRJ50816:K>K		UWOPS91_917_1:K>K		YPS138:K>K	AA:259		A12:D>D		A4:D>D		DBVPG6304:D>D		N_43:D>D		N_44:D>D		N_45:D>D		UFRJ50816:D>D		UWOPS91_917_1:D>D		YPS138:D>D	AA:273		A12:N>N		A4:N>N		DBVPG6304:N>N		UFRJ50816:N>N		YPS138:N>N	AA:296		A12:V>V		A4:V>V		DBVPG6304:V>V		IFO1804:V>V		N_43:V>V		N_44:V>V		N_45:V>V		UFRJ50816:V>V		UWOPS91_917_1:V>V		YPS138:V>V	AA:309		UWOPS91_917_1:L>L	AA:314		UWOPS91_917_1:K>K	AA:318		A12:S>S		A4:S>S		UFRJ50816:S>S		YPS138:S>S	AA:320		A12:S>S		A4:S>S		UFRJ50816:S>S		YPS138:S>S	AA:325		N_45:L>L	AA:332		A12:T>T		UFRJ50816:T>T		YPS138:T>T	AA:376		A12:V>V		A4:V>V		UFRJ50816:V>V		YPS138:V>V	AA:388		IFO1804:A>A		N_43:A>A		N_44:A>A		N_45:A>A	AA:406		A12:T>T		A4:T>T		UFRJ50816:T>T		YPS138:T>T	AA:415		A12:I>I		A4:I>I		UFRJ50816:I>I		UWOPS91_917_1:I>I		YPS138:I>I	AA:419		A12:S>S		A4:S>S		UFRJ50816:S>S		UWOPS91_917_1:S>S		YPS138:S>S	AA:422		A12:T>T		A4:T>T		UFRJ50816:T>T		UWOPS91_917_1:T>T		YPS138:T>T	AA:426		YPS138:L>L	AA:455		A12:K>K		A4:K>K		UFRJ50816:K>K		YPS138:K>KID:YBL087C	AA:40		N_43:A>A		N_44:A>A	AA:56		A12:K>K		YPS138:K>K	AA:60		A12:V>V		DBVPG6304:V>V		UFRJ50791:V>V		UFRJ50816:V>V		YPS138:V>V	AA:66		A12:V>V		DBVPG6304:V>V		UFRJ50791:V>V		UFRJ50816:V>V		YPS138:V>V	AA:70		A12:L>L		DBVPG6304:L>L		UFRJ50816:L>L		YPS138:L>L	AA:75		A12:K>K		DBVPG6304:K>K		UFRJ50816:K>K		YPS138:K>K	AA:79		A12:A>A		A4:A>A		DBVPG6304:A>A		UFRJ50816:A>A		YPS138:A>A	AA:85		A12:L>L		A4:L>L		DBVPG6304:L>L		UFRJ50816:L>L		YPS138:L>L	AA:88		A12:A>A		A4:A>A		UFRJ50816:A>A		YPS138:A>A	AA:90		A12:L>L		A4:L>L		UFRJ50816:L>L		YPS138:L>LID:YBL089W	AA:15		A12:C>C		A4:C>C		DBVPG6304:C>C		UFRJ50791:C>C	AA:19		A12:C>C		A4:C>C		DBVPG6304:C>C		IFO1804:C>C		N_43:C>C		N_44:C>C		N_45:C>C		UFRJ50791:C>C		YPS138:C>C	AA:43		A12:A>A		A4:A>A		DBVPG6304:A>A		UFRJ50791:A>A	AA:77		A12:V>V		A4:V>V		DBVPG6304:V>V	AA:116		A12:I>I		A4:I>I		DBVPG6304:I>I		UFRJ50816:I>I	AA:138		A12:R>R		A4:R>R		UFRJ50816:R>R	AA:171		N_45:->-	AA:194		A12:H>H		A4:H>H		DBVPG6304:H>H		IFO1804:H>H		N_43:H>H		N_44:H>H		N_45:H>H		UFRJ50791:H>H		UFRJ50816:H>H	AA:203		A12:R>R		A4:R>R		DBVPG6304:R>R		UFRJ50791:R>R		UFRJ50816:R>R	AA:220		Q59_1:I>I	AA:221		A12:H>H		A4:H>H		DBVPG6304:H>H		IFO1804:H>H		N_43:H>H		N_44:H>H		N_45:H>H		UFRJ50791:H>H		UFRJ50816:H>H	AA:227		A12:L>L		DBVPG6304:L>L		UFRJ50791:L>L		UFRJ50816:L>L	AA:229		A12:R>R		A4:R>R		DBVPG6304:R>R		UFRJ50791:R>R		UFRJ50816:R>R	AA:265		A12:I>I		A4:I>I		DBVPG6304:I>I		UFRJ50791:I>I		UFRJ50816:I>I	AA:277		IFO1804:G>G		N_44:G>G		N_45:G>G	AA:284		A12:S>S		A4:S>S	AA:336		IFO1804:I>I		N_44:I>I		N_45:I>I	AA:342		A4:S>S		IFO1804:S>S		N_44:S>S		N_45:S>S	AA:347		A12:V>V		A4:V>V		UFRJ50791:V>V		UFRJ50816:V>V	AA:349		A12:F>F		A4:F>F		IFO1804:F>F		N_44:F>F		N_45:F>F		UFRJ50791:F>F		UFRJ50816:F>F	AA:354		A4:T>T	AA:364		A12:D>D		A4:D>D		UFRJ50791:D>D		UFRJ50816:D>D	AA:372		A12:R>R		A4:R>R		UFRJ50791:R>R		UFRJ50816:R>R	AA:435		A12:->-		A4:->-		N_45:->-		UFRJ50791:->-		UFRJ50816:->-	AA:453		N_45:->-ID:YBL090W	AA:9		A12:->-		A4:->-		UFRJ50791:->-		UFRJ50816:->-	AA:12		A12:G>G		A4:G>G		UFRJ50791:G>G		UFRJ50816:G>G	AA:56		A12:P>P		A4:P>P		UFRJ50791:P>P		UFRJ50816:P>P		YPS138:P>P	AA:76		A12:S>S		A4:S>S		N_43:S>S		N_44:S>S		N_45:S>S		UFRJ50791:S>S		YPS138:S>S	AA:87		A12:L>L		A4:L>L		N_43:L>L		N_44:L>L		N_45:L>L		UFRJ50791:L>L		UFRJ50816:L>L		YPS138:L>L	AA:111		UWOPS91_917_1:V>V	AA:114		Q95_3:S>S		Y7:S>S	AA:125		A12:K>K		A4:K>K		UFRJ50791:K>K		YPS138:K>K	AA:146		UWOPS91_917_1:G>G	AA:173		A12:L>L		A4:L>L		UFRJ50791:L>L		YPS138:L>LID:YBL091C	AA:14		A12:A>A		A4:A>A		DBVPG6304:A>A		UFRJ50791:A>A		UWOPS91_917_1:A>A	AA:17		UWOPS91_917_1:L>L	AA:32		IFO1804:N>N		N_45:N>N	AA:57		A4:L>L	AA:70		A12:F>F		A4:F>F		DBVPG6304:F>F		UFRJ50791:F>F		YPS138:F>F	AA:93		A12:A>A		DBVPG6304:A>A		YPS138:A>A	AA:99		IFO1804:S>S		N_45:S>S	AA:103		A12:G>G		A4:G>G		DBVPG6304:G>G	AA:105		A12:T>T	AA:107		A12:Y>Y		A4:Y>Y		DBVPG6304:Y>Y		YPS138:Y>Y	AA:112		A4:S>S		YPS138:S>S	AA:123		IFO1804:E>E		N_45:E>E	AA:138		A4:K>K		YPS138:K>K	AA:150		A12:G>G		DBVPG6304:G>G	AA:165		A12:I>I		DBVPG6304:I>I		YPS138:I>I	AA:170		A12:S>S		DBVPG6304:S>S		IFO1804:S>S		N_45:S>S		UWOPS91_917_1:H>H		YPS138:S>S	AA:175		A12:Q>Q		DBVPG6304:Q>Q		YPS138:Q>Q	AA:192		N_17:I>I	AA:194		A12:T>T		A4:T>T		DBVPG6304:T>T		IFO1804:T>T		N_45:T>T		YPS138:T>T	AA:209		A12:P>P		DBVPG6304:P>P		YPS138:P>P	AA:211		DBVPG6304:F>F		YPS138:F>F	AA:223		A4:N>N		DBVPG6304:N>N		YPS138:N>N	AA:225		A4:Q>Q		DBVPG6304:Q>Q		YPS138:Q>Q	AA:283		A4:T>T		DBVPG6304:T>T		YPS138:T>T	AA:291		DBVPG4650:I>I	AA:299		A4:V>V		UWOPS91_917_1:V>V		YPS138:V>V	AA:310		UWOPS91_917_1:R>R	AA:331		A4:L>L		YPS138:L>L	AA:357		A12:P>P		A4:P>P		DBVPG6304:P>P		UFRJ50791:P>P		UWOPS91_917_1:K>K		YPS138:P>P	AA:359		A4:D>D		YPS138:D>D	AA:365		N_44:I>I		N_45:I>I	AA:369		A4:N>N		YPS138:N>N	AA:378		A12:L>L		A4:L>L		DBVPG6304:L>L		UFRJ50791:L>L		UWOPS91_917_1:N>N	AA:380		A4:K>K		UWOPS91_917_1:K>K		YPS138:K>K	AA:383		A12:L>L		A4:L>L		DBVPG6304:L>L		UFRJ50791:L>L		UWOPS91_917_1:S>S		YPS138:S>SID:YBL092W	AA:13		N_43:V>V		N_44:V>V		N_45:V>V	AA:15		CBS432:L>L		UWOPS91_917_1:L>L	AA:30		A12:F>F	AA:32		A12:P>P		A4:P>P		CBS432:P>P		CBS5829:P>P		DBVPG6304:P>P		IFO1804:P>P		N_17:P>P		N_43:P>P		N_44:P>P		N_45:P>P		Q32_3:P>P		Q62_5:P>P		Q89_8:P>P		S36_7:P>P		UFRJ50791:P>P		UFRJ50816:P>P		Y6_5:P>P		Y7:P>P		Z1_1:P>P	AA:43		UWOPS91_917_1:S>S	AA:54		UWOPS91_917_1:L>L	AA:78		UWOPS91_917_1:V>V	AA:103		A12:L>L		A4:L>L		CBS432:L>L		CBS5829:L>L		DBVPG6304:L>L		IFO1804:L>L		N_17:L>L		N_43:L>L		N_45:L>L		Q62_5:L>L		Q89_8:L>L		S36_7:L>L		T21_4:L>L		UFRJ50816:L>L		UWOPS91_917_1:L>L		Y6_5:L>L		Z1_1:L>L	AA:122		N_44:W>WID:YBL093C	AA:12		UWOPS91_917_1:S>S	AA:47		A12:D>D		A4:D>D		YPS138:D>D	AA:51		UWOPS91_917_1:A>A	AA:65		A4:N>N	AA:94		A12:F>F		A4:F>F		DBVPG6304:F>F		UWOPS91_917_1:F>F	AA:118		A12:Q>Q		A4:Q>Q		UFRJ50791:Q>Q	AA:144		A12:T>T		A4:T>T		DBVPG6304:T>T		UFRJ50791:T>T		UWOPS91_917_1:T>T	AA:148		A12:S>S		A4:S>S		DBVPG6304:S>S		UFRJ50791:S>S		UWOPS91_917_1:S>S	AA:153		A12:L>L		A4:L>L		DBVPG6304:L>L		UFRJ50791:L>L		UWOPS91_917_1:L>L	AA:162		A4:K>K	AA:164		A12:L>L		A4:L>L		DBVPG6304:L>L		UFRJ50791:L>L		UWOPS91_917_1:L>L	AA:168		A12:K>K		A4:K>K		DBVPG6304:K>K		UFRJ50791:K>K	AA:169		UWOPS91_917_1:T>T	AA:170		CBS5829:G>G	AA:183		A12:D>D		A4:D>D		DBVPG6304:D>D		UFRJ50791:D>D		UWOPS91_917_1:D>DID:YBL095W	AA:10		A12:I>I		A4:I>I		N_43:I>I		N_44:I>I		N_45:I>I	AA:13		N_43:H>H		N_44:H>H		N_45:H>H	AA:15		A12:S>S		A4:S>S	AA:34		A12:T>T		A4:T>T		DBVPG6304:T>T		UFRJ50816:T>T	AA:67		A12:C>C		A4:C>C		DBVPG6304:C>C		UFRJ50791:C>C		UFRJ50816:C>C	AA:85		A12:D>D		A4:D>D		DBVPG6304:D>D		UFRJ50791:D>D		UFRJ50816:D>D	AA:88		N_43:A>A		N_44:A>A		N_45:A>A	AA:101		A12:V>V		A4:V>V		DBVPG6304:V>V		UFRJ50791:V>V		UFRJ50816:V>V	AA:121		A12:A>A		A4:A>A		DBVPG6304:A>A		UFRJ50791:A>A		UFRJ50816:A>A	AA:140		A12:I>I		A4:I>I		DBVPG6304:I>I		UFRJ50791:I>I		UFRJ50816:I>I		UWOPS91_917_1:I>I	AA:143		UWOPS91_917_1:F>F	AA:145		A12:G>G		A4:G>G		DBVPG6304:G>G		UFRJ50791:G>G		UFRJ50816:G>G		UWOPS91_917_1:G>G	AA:149		UWOPS91_917_1:M>M	AA:171		A12:I>I		A4:I>I		DBVPG6304:I>I		UFRJ50791:I>I		UFRJ50816:I>I	AA:174		UWOPS91_917_1:->-	AA:177		A12:T>T		A4:T>T		DBVPG6304:T>T		UFRJ50791:T>T		UFRJ50816:T>T	AA:186		UWOPS91_917_1:S>S	AA:208		UWOPS91_917_1:->-	AA:213		UWOPS91_917_1:L>L	AA:228		UWOPS91_917_1:L>L	AA:233		A12:T>T		DBVPG6304:T>T		UFRJ50791:T>T		UFRJ50816:T>T		YPS138:T>T	AA:237		A12:I>I		DBVPG6304:I>I		UFRJ50791:I>I		UFRJ50816:I>I		UWOPS91_917_1:I>I		YPS138:I>I	AA:243		A12:V>V		DBVPG6304:V>V		UFRJ50791:V>V		UFRJ50816:V>V		YPS138:V>V	AA:268		UWOPS91_917_1:N>N	AA:280		A12:I>I		DBVPG6304:I>I		UFRJ50791:I>I		UFRJ50816:I>I		YPS138:I>IID:YBL098W	AA:19		UWOPS91_917_1:F>F	AA:32		UWOPS91_917_1:K>K	AA:47		A12:I>I		UFRJ50791:I>I		UFRJ50816:I>I		UWOPS91_917_1:I>I	AA:53		A12:R>R		UFRJ50791:R>R		UFRJ50816:R>R		UWOPS91_917_1:R>R		YPS138:R>R	AA:82		A12:I>I		A4:I>I		DBVPG6304:I>I		UFRJ50791:I>I		UFRJ50816:I>I		YPS138:I>I	AA:100		A12:H>H		A4:H>H		DBVPG6304:H>H		UFRJ50791:H>H		UFRJ50816:H>H		UWOPS91_917_1:H>H		YPS138:H>H	AA:119		A12:S>S		A4:S>S		DBVPG6304:S>S		IFO1804:S>S		N_43:S>S		N_44:S>S		N_45:S>S		UFRJ50791:S>S		UFRJ50816:S>S		UWOPS91_917_1:S>S		YPS138:S>S	AA:157		A12:I>I		A4:I>I		DBVPG6304:I>I		UFRJ50791:I>I		UFRJ50816:I>I		UWOPS91_917_1:I>I		YPS138:I>I	AA:261		A12:K>K		A4:K>K		DBVPG6304:K>K		YPS138:K>K	AA:275		A12:L>L		A4:L>L		DBVPG6304:L>L		YPS138:L>L	AA:281		CBS5829:E>E		S36_7:E>E	AA:297		A12:V>V		A4:V>V		DBVPG6304:V>V		UFRJ50816:V>V	AA:302		N_17:K>K		N_43:K>K		N_45:K>K	AA:312		A12:T>T		A4:T>T		DBVPG6304:T>T		N_17:T>T		N_43:T>T		N_45:T>T		UFRJ50816:T>T	AA:326		A12:D>D		A4:D>D		DBVPG6304:D>D		UFRJ50816:D>D	AA:349		UWOPS91_917_1:I>I	AA:399		UWOPS91_917_1:R>R	AA:453		UWOPS91_917_1:F>F	AA:466		A12:L>L		A4:L>L		DBVPG6304:L>L		UFRJ50816:L>L		YPS138:L>LID:YBL099W	AA:12		N_43:R>R		N_45:R>R	AA:47		N_43:G>G		N_44:G>G		N_45:G>G	AA:63		A12:I>I		DBVPG6304:I>I		UFRJ50816:I>I		YPS138:I>I	AA:132		YPS138:N>N	AA:142		A12:T>T	AA:149		A12:T>T		DBVPG6304:T>T		UFRJ50816:T>T		UWOPS91_917_1:T>T		YPS138:T>T	AA:183		UFRJ50816:D>D	AA:185		N_43:G>G		N_44:G>G		N_45:G>G	AA:192		DBVPG6304:G>G		UFRJ50816:G>G		YPS138:G>G	AA:235		UWOPS91_917_1:L>L	AA:242		DBVPG6304:G>G		UFRJ50816:G>G		YPS138:G>G	AA:271		A12:S>S		DBVPG6304:S>S		UFRJ50816:S>S		YPS138:S>S	AA:286		A12:G>G		DBVPG6304:G>G		UFRJ50816:G>G		UWOPS91_917_1:G>G	AA:291		Z1_1:T>T	AA:323		UWOPS91_917_1:S>S	AA:334		A12:I>I		DBVPG6304:I>I		UWOPS91_917_1:I>I	AA:337		A12:V>V		DBVPG6304:V>V		UWOPS91_917_1:V>V	AA:341		A12:S>S		DBVPG6304:S>S		UWOPS91_917_1:S>S	AA:351		UWOPS91_917_1:F>F	AA:356		A12:T>T		DBVPG6304:T>T		UFRJ50791:T>T	AA:366		IFO1804:G>G		N_44:G>G		N_45:G>G	AA:381		A12:G>G		DBVPG6304:G>G	AA:403		A12:I>I		CBS432:I>I		CBS5829:I>I		DBVPG4650:I>I		DBVPG6304:I>I		IFO1804:I>I		KPN3828:I>I		KPN3829:I>I		N_17:I>I		N_43:I>I		N_44:I>I		N_45:I>I		Q59_1:I>I		Q95_3:I>I		T21_4:I>I		UFRJ50791:I>I		Y6_5:I>I	AA:411		A12:D>D		DBVPG6304:D>D		UFRJ50791:D>D	AA:419		IFO1804:S>S		N_43:S>S		N_44:S>S		N_45:S>S	AA:432		A12:S>S		DBVPG6304:S>S		UFRJ50791:S>S	AA:444		IFO1804:T>T	AA:450		DBVPG6304:G>G	AA:458		A12:T>T		DBVPG6304:T>T		UFRJ50791:T>T	AA:487		A12:I>I	AA:506		IFO1804:Q>Q		N_43:Q>Q		N_44:Q>Q		N_45:Q>QID:YBL102W	AA:7		A12:G>G		A4:G>G		DBVPG6304:G>G		UFRJ50791:G>G		YPS138:G>G	AA:9		UWOPS91_917_1:L>L	AA:16		A12:->-		A4:->-		DBVPG6304:->-		IFO1804:->-		N_43:->-		N_45:->-		UFRJ50791:->-		YPS138:->-	AA:32		UWOPS91_917_1:V>V	AA:41		A12:A>A		A4:A>A		DBVPG6304:A>A		UFRJ50791:A>A		YPS138:A>A	AA:47		A12:I>I		A4:I>I		DBVPG6304:I>I		UFRJ50791:I>I		UWOPS91_917_1:I>I		YPS138:I>I	AA:50		A12:G>G		A4:G>G		CBS432:G>G		CBS5829:G>G		DBVPG4650:G>G		DBVPG6304:G>G		IFO1804:G>G		KPN3829:G>G		N_17:G>G		N_43:G>G		N_45:G>G		UFRJ50791:G>G		UWOPS91_917_1:G>G		YPS138:G>G	AA:135		Y7:R>R	AA:147		A12:R>R		DBVPG6304:R>R		YPS138:R>R	AA:149		A12:R>R		DBVPG6304:R>R		YPS138:R>R	AA:168		CBS432:V>V		CBS5829:V>V		N_17:V>V		N_43:V>V		N_44:V>V		N_45:V>V		Q59_1:V>V		Y6_5:V>V	AA:190		DBVPG6304:I>I		UFRJ50816:I>I		YPS138:I>I	AA:196		DBVPG6304:C>C		UFRJ50816:C>C		UWOPS91_917_1:C>C		YPS138:C>C	AA:215		UWOPS91_917_1:N>NID:YBL103C	AA:1		UWOPS91_917_1:->-	AA:16		A12:D>D		A4:D>D		DBVPG6304:D>D		UFRJ50791:D>D		UWOPS91_917_1:D>D	AA:18		A12:G>G		A4:G>G		DBVPG6304:G>G		UFRJ50791:G>G	AA:21		A12:L>L		A4:L>L		DBVPG6304:L>L		UFRJ50791:L>L	AA:24		A12:N>N		A4:N>N		DBVPG6304:N>N		UFRJ50791:N>N		UWOPS91_917_1:N>N	AA:30		A12:L>L		A4:L>L		DBVPG6304:L>L		IFO1804:L>L		N_43:L>L		N_44:L>L		N_45:L>L		UFRJ50791:L>L		UWOPS91_917_1:L>L	AA:33		A12:S>S	AA:37		UFRJ50791:K>K	AA:39		A12:E>E		A4:E>E		DBVPG6304:E>E		UFRJ50791:E>E	AA:42		UWOPS91_917_1:I>I	AA:46		A12:P>P		A4:P>P		DBVPG6304:P>P		UFRJ50791:P>P	AA:48		IFO1804:T>T		N_44:T>T		N_45:T>T		UWOPS91_917_1:T>T	AA:55		UWOPS91_917_1:N>N	AA:74		UWOPS91_917_1:I>I	AA:87		N_43:I>I		N_44:I>I		N_45:I>I	AA:134		A12:K>K		A4:K>K		DBVPG6304:K>K		N_43:K>K		N_44:K>K		N_45:K>K		YPS138:K>K	AA:140		A12:L>L		A4:L>L		DBVPG6304:L>L		N_43:L>L		N_44:L>L		N_45:L>L		UFRJ50816:L>L		YPS138:L>L	AA:151		A12:T>T	AA:186		A12:K>K	AA:188		A12:L>L	AA:196		A12:A>A	AA:229		A12:S>S	AA:239		A12:T>T	AA:243		N_45:G>G	AA:248		A12:S>S	AA:253		DBVPG6304:G>G		UFRJ50816:G>G	AA:255		A12:R>R	AA:263		A12:V>V		A4:V>V		CBS432:V>V		CBS5829:V>V		DBVPG6304:V>V		N_17:V>V		N_43:V>V		N_44:V>V		N_45:V>V		Q32_3:V>V		Q59_1:V>V		Q89_8:V>V		T21_4:V>V		UFRJ50816:V>V		YPS138:V>V	AA:275		A12:G>G	AA:276		A4:L>L		DBVPG6304:L>L		UFRJ50816:L>L		YPS138:L>L	AA:282		A4:S>S		DBVPG6304:S>S		UFRJ50816:S>S		YPS138:S>S	AA:286		A4:S>S		DBVPG6304:S>S		UFRJ50816:S>S		YPS138:S>S	AA:287		Q32_3:Y>Y	AA:307		N_43:D>D		N_44:D>D	AA:309		N_43:L>L		N_45:L>L	AA:312		A4:T>T		DBVPG6304:T>T		UFRJ50816:T>T		YPS138:T>T	AA:316		A12:T>T		A4:T>T		DBVPG6304:T>T		N_43:T>T		N_44:T>T		N_45:T>T		UFRJ50816:T>T		YPS138:T>T	AA:320		A12:F>F	AA:336		DBVPG6304:L>L		UFRJ50816:L>L		YPS138:L>L	AA:338		A12:T>T	AA:355		A12:R>R	AA:371		DBVPG6304:R>R	AA:398		A4:P>P		DBVPG6304:P>P		UFRJ50791:P>P		YPS138:P>P	AA:403		A4:S>S		DBVPG6304:S>S		UFRJ50791:S>S		YPS138:S>S	AA:421		A4:I>I		DBVPG6304:I>I		UFRJ50791:I>I		UWOPS91_917_1:I>I	AA:434		A12:G>G		A4:G>G		DBVPG6304:G>G		UFRJ50791:G>G		UWOPS91_917_1:G>G		YPS138:G>G	AA:459		A12:L>L		A4:L>L		DBVPG6304:L>L		N_44:L>L		N_45:L>L		UFRJ50791:L>L		UWOPS91_917_1:L>L		YPS138:L>LID:YBL104C	AA:1		N_43:->-		N_44:->-		N_45:->-	AA:10		A12:P>P		A4:P>P		DBVPG6304:P>P		UFRJ50816:P>P		YPS138:P>P	AA:25		N_43:G>G		N_44:G>G		N_45:G>G	AA:47		A12:S>S		YPS138:S>S	AA:54		N_45:G>G	AA:79		A12:G>G		A4:G>G		DBVPG6304:G>G		UFRJ50791:G>G		UFRJ50816:G>G		YPS138:G>G	AA:89		A12:P>P		A4:P>P		DBVPG6304:P>P		N_43:P>P		N_45:P>P		UFRJ50791:P>P		UFRJ50816:P>P		YPS138:P>P	AA:95		A12:H>H		A4:H>H		DBVPG6304:H>H		UFRJ50791:H>H		UFRJ50816:H>H		YPS138:H>H	AA:102		CBS432:R>R		CBS5829:R>R		KPN3829:R>R		N_17:R>R		Q32_3:R>R		Q59_1:R>R		Q62_5:R>R		T21_4:R>R		Y6_5:R>R		Z1_1:R>R	AA:130		A12:G>G		A4:G>G		DBVPG6304:G>G		UFRJ50791:G>G		UFRJ50816:G>G		YPS138:G>G	AA:140		A12:V>V		A4:V>V		DBVPG6304:V>V		N_43:V>V		N_44:V>V		N_45:V>V		UFRJ50791:V>V		UFRJ50816:V>V		YPS138:V>V	AA:146		A12:S>S		A4:S>S		DBVPG6304:S>S		UFRJ50791:S>S		UFRJ50816:S>S	AA:187		A12:F>F		A4:F>F		DBVPG6304:F>F		UFRJ50791:F>F		UFRJ50816:F>F	AA:197		Q59_1:S>S	AA:210		N_43:V>V		N_44:V>V		N_45:V>V	AA:227		A12:A>A		A4:A>A		DBVPG6304:A>A		UFRJ50791:A>A		UFRJ50816:A>A		UWOPS91_917_1:A>A	AA:238		DBVPG6304:T>T		N_43:S>S		N_44:S>S		N_45:S>S	AA:246		N_43:P>P		N_44:P>P	AA:256		UWOPS91_917_1:L>L	AA:258		UFRJ50791:G>G		UFRJ50816:G>G	AA:261		A12:G>G		DBVPG6304:G>G		IFO1804:G>G		N_44:G>G		N_45:G>G		UFRJ50791:G>G		UFRJ50816:G>G		UWOPS91_917_1:G>G		YPS138:G>G	AA:281		A12:L>L		A4:L>L		DBVPG6304:L>L		UFRJ50791:L>L		UFRJ50816:L>L		UWOPS91_917_1:L>L	AA:283		CBS432:V>V		KPN3829:V>V		N_17:V>V		Q32_3:V>V		Q59_1:V>V		Q62_5:V>V		Y6_5:V>V		Z1_1:V>V	AA:312		UWOPS91_917_1:L>L	AA:319		A4:S>S		DBVPG6304:S>S		UFRJ50791:S>S		UFRJ50816:S>S		YPS138:S>S	AA:329		UWOPS91_917_1:A>A	AA:338		N_43:A>A		N_44:A>A		N_45:A>A	AA:339		UWOPS91_917_1:L>L	AA:346		A4:A>A		DBVPG6304:A>A		UFRJ50791:A>A		UFRJ50816:A>A		UWOPS91_917_1:A>A		YPS138:A>A	AA:367		A12:G>G		A4:G>G		DBVPG6304:G>G		UFRJ50791:G>G		UFRJ50816:G>G		UWOPS91_917_1:G>G		YPS138:G>G	AA:375		UWOPS91_917_1:A>A	AA:376		A4:K>K		DBVPG6304:K>K		UFRJ50791:K>K		UFRJ50816:K>K		YPS138:K>K	AA:415		A4:A>A	AA:440		A12:L>L		A4:L>L		UFRJ50791:L>L		UFRJ50816:L>L		YPS138:L>L	AA:456		N_45:S>S	AA:474		A12:L>L		UFRJ50791:L>L		UFRJ50816:L>L		UWOPS91_917_1:L>L		YPS138:L>L	AA:504		N_43:L>L		N_45:L>L	AA:510		A12:I>I		UFRJ50791:I>I		UFRJ50816:I>I		YPS138:I>I	AA:518		UWOPS91_917_1:D>D	AA:535		A12:K>K		UFRJ50791:K>K		UFRJ50816:K>K		UWOPS91_917_1:K>K		YPS138:K>K	AA:537		UWOPS91_917_1:L>L	AA:541		A12:P>P		N_43:P>P		N_45:P>P		UFRJ50791:P>P		UFRJ50816:P>P		UWOPS91_917_1:P>P		YPS138:P>P	AA:637		UFRJ50791:S>S		UFRJ50816:S>S		UWOPS91_917_1:S>S		YPS138:S>S	AA:671		DBVPG6304:V>V		N_45:V>V		UFRJ50791:V>V		UFRJ50816:V>V		UWOPS91_917_1:V>V		YPS138:V>V	AA:678		DBVPG6304:T>T		UFRJ50791:T>T		UFRJ50816:T>T		YPS138:T>T	AA:683		UWOPS91_917_1:S>S	AA:699		DBVPG6304:S>S		UFRJ50791:S>S		UFRJ50816:S>S		YPS138:S>S	AA:710		DBVPG6304:R>R		UFRJ50791:R>R		UFRJ50816:R>R		YPS138:R>R	AA:725		DBVPG6304:N>N		UFRJ50791:N>N		UFRJ50816:N>N		UWOPS91_917_1:N>N		YPS138:N>N	AA:742		IFO1804:A>A	AA:768		A12:S>S		DBVPG6304:S>S		IFO1804:S>S		N_44:S>S		N_45:S>S		UFRJ50791:S>S		UFRJ50816:S>S		UWOPS91_917_1:S>S		YPS138:S>S	AA:783		A12:Y>Y		DBVPG6304:Y>Y		IFO1804:Y>Y		N_44:Y>Y		N_45:Y>Y		UFRJ50791:Y>Y		UFRJ50816:Y>Y		UWOPS91_917_1:Y>Y		YPS138:Y>Y	AA:792		A12:P>P		DBVPG6304:P>P		UFRJ50791:P>P		UFRJ50816:P>P		YPS138:P>P	AA:804		A12:H>H		CBS432:H>H		CBS5829:H>H		DBVPG4650:H>H		DBVPG6304:H>H		IFO1804:H>H		KPN3828:H>H		N_17:H>H		N_44:H>H		N_45:H>H		Q32_3:H>H		Q59_1:H>H		Q62_5:H>H		S36_7:H>H		T21_4:H>H		UFRJ50791:H>H		UFRJ50816:H>H		Y6_5:H>H		YPS138:H>H	AA:834		A12:L>L		DBVPG6304:L>L		UFRJ50791:L>L		UFRJ50816:L>L		YPS138:L>L	AA:872		A12:D>D		DBVPG6304:D>D		UFRJ50791:D>D		UFRJ50816:D>D		UWOPS91_917_1:D>D		YPS138:D>D	AA:885		UWOPS91_917_1:L>L	AA:909		A12:Q>Q		DBVPG6304:Q>Q		UFRJ50791:Q>Q		UFRJ50816:Q>Q		UWOPS91_917_1:Q>Q		YPS138:Q>Q	AA:919		A12:I>I		DBVPG6304:I>I		IFO1804:I>I		N_44:I>I		N_45:I>I		UFRJ50791:I>I		UFRJ50816:I>I		UWOPS91_917_1:I>I		YPS138:I>I	AA:953		UFRJ50816:F>F	AA:967		A12:K>K		DBVPG6304:K>K		UFRJ50816:K>K		YPS138:K>K	AA:979		UWOPS91_917_1:S>S	AA:991		A12:V>V		DBVPG6304:V>V		UFRJ50816:V>V		YPS138:V>V	AA:1012		N_44:V>V		N_45:V>V		UWOPS91_917_1:V>V	AA:1016		A12:T>T		DBVPG6304:T>T		UFRJ50816:T>T		YPS138:T>T	AA:1019		UWOPS91_917_1:V>V	AA:1035		A12:K>K		DBVPG6304:K>K		UFRJ50816:K>K		UWOPS91_917_1:K>K		YPS138:K>KID:YBL107C	AA:1		A12:->-		A4:->-		DBVPG6304:->-		UFRJ50816:->-		UWOPS91_917_1:->-	AA:41		A12:A>A		A4:A>A		DBVPG6304:A>A		UWOPS91_917_1:A>A	AA:45		A12:V>V		A4:V>V		DBVPG6304:V>V		UFRJ50791:V>V		UFRJ50816:V>V	AA:56		A12:Q>Q		A4:Q>Q		DBVPG6304:Q>Q		UFRJ50791:Q>Q		UFRJ50816:Q>Q	AA:58		A12:E>E		A4:E>E		DBVPG6304:E>E		UFRJ50791:E>E		UFRJ50816:E>E		UWOPS91_917_1:E>E	AA:73		N_43:I>I		N_44:I>I	AA:113		A12:L>L		A4:L>L		DBVPG6304:L>L		UFRJ50791:L>L		UFRJ50816:L>L		UWOPS91_917_1:L>L		YPS138:L>L	AA:120		UWOPS91_917_1:K>K	AA:133		N_43:N>N		N_44:N>N		N_45:N>N	AA:147		A12:L>L		A4:L>L		DBVPG6304:L>L		N_43:L>L		N_44:L>L		N_45:L>L		UFRJ50791:L>L		UFRJ50816:L>L		UWOPS91_917_1:L>L		YPS138:L>L	AA:163		N_45:R>R	AA:165		UWOPS91_917_1:V>V	AA:183		A12:L>L		A4:L>L		DBVPG6304:L>L		N_43:L>L		N_44:L>L		N_45:L>L		UFRJ50791:L>L		UFRJ50816:L>L		UWOPS91_917_1:L>L		YPS138:L>LID:YBR002C	AA:8		A12:Q>Q		A4:Q>Q		DBVPG6304:Q>Q		UFRJ50791:Q>Q		UWOPS91_917_1:Q>Q		YPS138:Q>Q	AA:13		A12:P>P		A4:P>P		DBVPG6304:P>P		N_43:P>P		N_44:P>P		N_45:P>P		UFRJ50791:P>P		UWOPS91_917_1:P>P		YPS138:P>P	AA:36		DBVPG6304:R>R	AA:47		A4:R>R		DBVPG6304:R>R		UFRJ50791:R>R		YPS138:R>R	AA:52		A4:P>P		DBVPG6304:P>P		N_43:P>P		N_44:P>P		N_45:P>P		UFRJ50791:P>P		UWOPS91_917_1:P>P		YPS138:P>P	AA:56		A4:Q>Q		DBVPG6304:Q>Q		UFRJ50791:Q>Q		UWOPS91_917_1:Q>Q		YPS138:Q>Q	AA:76		A4:G>G		DBVPG6304:G>G		UFRJ50791:G>G		UWOPS91_917_1:G>G		YPS138:G>G	AA:152		A4:R>R		DBVPG6304:R>R		UFRJ50791:R>R		UFRJ50816:R>R		UWOPS91_917_1:R>R	AA:178		IFO1804:E>E		N_43:E>E		N_44:E>E		N_45:E>E	AA:197		A4:P>P		DBVPG6304:P>P		UFRJ50791:P>P		UFRJ50816:P>P		UWOPS91_917_1:P>P	AA:201		UWOPS91_917_1:F>F	AA:209		Q59_1:S>S		Q89_8:S>S		S36_7:S>S		Y7:S>S	AA:217		A4:T>T		DBVPG6304:T>T		UFRJ50791:T>T		UFRJ50816:T>T		UWOPS91_917_1:T>T	AA:222		A4:S>S		DBVPG6304:S>S		IFO1804:S>S		N_43:S>S		N_44:S>S		N_45:S>S		UFRJ50816:S>S		UWOPS91_917_1:S>S	AA:230		Q89_8:L>L		S36_7:L>L		Y7:L>L	AA:253		IFO1804:N>N		N_43:N>N		N_44:N>N		N_45:N>N	AA:279		UWOPS91_917_1:T>T	AA:286		UFRJ50816:F>FID:YBR003W	AA:15		A12:S>S		A4:S>S		DBVPG6304:S>S		UFRJ50791:S>S		YPS138:S>S	AA:30		A12:N>N		A4:N>N		DBVPG6304:N>N		UFRJ50791:N>N		UWOPS91_917_1:N>N		YPS138:N>N	AA:35		A12:L>L		A4:L>L		DBVPG6304:L>L		UFRJ50791:L>L		UWOPS91_917_1:L>L		YPS138:L>L	AA:54		N_43:N>N		N_44:N>N		N_45:N>N	AA:65		A12:G>G		A4:G>G		DBVPG6304:G>G		UFRJ50791:G>G		YPS138:G>G	AA:67		A12:G>G		A4:G>G		DBVPG6304:G>G		UWOPS91_917_1:G>G		YPS138:G>G	AA:71		A12:L>L		A4:L>L		DBVPG6304:L>L		UFRJ50791:L>L		UWOPS91_917_1:L>L		YPS138:L>L	AA:99		A12:S>S		A4:S>S		DBVPG6304:S>S		YPS138:S>S	AA:110		A12:K>K		A4:K>K		DBVPG6304:K>K		UFRJ50816:K>K		UWOPS91_917_1:K>K		YPS138:K>K	AA:119		UWOPS91_917_1:D>D	AA:127		A12:Q>Q		A4:Q>Q		DBVPG6304:Q>Q		UFRJ50816:Q>Q		YPS138:Q>Q	AA:131		A12:F>F		A4:F>F		DBVPG6304:F>F		UFRJ50816:F>F		UWOPS91_917_1:F>F	AA:138		A12:I>I		A4:I>I		DBVPG6304:I>I		UFRJ50816:I>I		UWOPS91_917_1:I>I		YPS138:I>I	AA:145		A12:H>H		A4:H>H		DBVPG6304:H>H		UFRJ50816:H>H		YPS138:H>H	AA:147		A12:I>I		A4:I>I		DBVPG6304:I>I		UFRJ50816:I>I		UWOPS91_917_1:I>I		YPS138:I>I	AA:152		A12:P>P		A4:P>P		DBVPG6304:P>P		N_43:P>P		N_44:P>P		N_45:P>P		UFRJ50816:P>P		UWOPS91_917_1:P>P		YPS138:P>P	AA:171		N_44:I>I		N_45:I>I	AA:176		A12:R>R		DBVPG6304:R>R		UFRJ50816:R>R	AA:207		A12:S>S		DBVPG6304:S>S		UFRJ50816:S>S	AA:213		A12:T>T		DBVPG6304:T>T		UFRJ50816:T>T		UWOPS91_917_1:T>T	AA:221		UWOPS91_917_1:G>G	AA:235		A12:L>L		DBVPG6304:L>L		UFRJ50816:L>L		UWOPS91_917_1:L>L	AA:237		UWOPS91_917_1:N>N	AA:240		N_43:V>V		N_44:V>V		N_45:V>V		UWOPS91_917_1:V>V	AA:270		A12:T>T		UFRJ50816:T>T	AA:280		A12:I>I		IFO1804:I>I		N_43:I>I		N_44:I>I		UFRJ50816:I>I		UWOPS91_917_1:I>I	AA:282		N_17:S>S	AA:311		A12:T>T		IFO1804:T>T		N_43:T>T		N_44:T>T		UFRJ50816:T>T		UWOPS91_917_1:T>T	AA:316		IFO1804:Y>Y		N_43:Y>Y		N_44:Y>Y	AA:322		UWOPS91_917_1:L>L	AA:327		IFO1804:L>L		N_43:L>L		N_44:L>L	AA:333		UWOPS91_917_1:R>R	AA:357		IFO1804:G>G		N_17:G>G		N_43:G>G		N_44:G>G		Q62_5:G>G		Q89_8:G>G		Y7:G>G		Z1_1:G>G	AA:360		A12:F>F		IFO1804:F>F		N_43:F>F		N_44:F>F		UFRJ50816:F>F	AA:379		A12:P>P		A4:P>P		UFRJ50816:P>P		UWOPS91_917_1:P>P	AA:385		IFO1804:K>K		N_43:K>K		N_44:K>K		N_45:K>K	AA:389		A12:A>A		A4:A>A		IFO1804:A>A		N_43:A>A		N_44:A>A		N_45:A>A		UFRJ50816:A>A		UWOPS91_917_1:A>A	AA:406		A4:L>L		UFRJ50816:L>L		UWOPS91_917_1:L>L	AA:413		A4:E>E		UFRJ50816:E>E	AA:420		UWOPS91_917_1:T>T	AA:426		A4:L>L		IFO1804:L>L		N_43:L>L		N_44:L>L		N_45:L>L		UFRJ50816:L>L	AA:431		UWOPS91_917_1:A>A	AA:437		A4:A>A		UFRJ50816:A>A	AA:449		A4:R>R		UFRJ50816:R>R		UWOPS91_917_1:R>R	AA:470		A4:T>T		UFRJ50816:T>T		UWOPS91_917_1:T>T		YPS138:T>TID:YBR004C	AA:18		UWOPS91_917_1:K>K	AA:23		A4:Y>Y		DBVPG6304:Y>Y		UFRJ50791:Y>Y		UFRJ50816:Y>Y		UWOPS91_917_1:Y>Y		YPS138:Y>Y	AA:24		UWOPS91_917_1:V>V	AA:28		A4:Y>Y		DBVPG6304:Y>Y		UFRJ50791:Y>Y		UFRJ50816:Y>Y		UWOPS91_917_1:Y>Y		YPS138:Y>Y	AA:32		CBS432:T>T		DBVPG4650:T>T		UWOPS91_917_1:T>T	AA:42		A4:->-		DBVPG6304:->-		N_43:->-		N_45:->-		UFRJ50816:->-		UWOPS91_917_1:->-		YPS138:->-	AA:57		YPS138:->-	AA:65		UWOPS91_917_1:Y>Y	AA:91		UWOPS91_917_1:G>G	AA:105		DBVPG6304:D>D		N_43:D>D		N_45:D>D		UFRJ50816:D>D		UWOPS91_917_1:I>I		YPS138:D>D	AA:121		DBVPG6304:P>P	AA:136		A4:R>R		DBVPG6304:R>R		UFRJ50816:R>R		YPS138:R>R	AA:146		A12:A>A		A4:A>A		DBVPG6304:A>A		UFRJ50816:A>A		YPS138:A>A	AA:154		UWOPS91_917_1:P>P	AA:156		A4:T>T		DBVPG6304:T>T		IFO1804:T>T		N_43:T>T		N_45:T>T		UFRJ50816:T>T		YPS138:T>T	AA:160		A12:T>T		A4:T>T		DBVPG6304:T>T		UFRJ50816:T>T		YPS138:T>T	AA:163		UWOPS91_917_1:L>L	AA:191		A12:R>R		A4:R>R		DBVPG6304:R>R		IFO1804:Y>Y		N_17:Y>Y		N_43:Y>Y		N_44:Y>Y		N_45:Y>Y		UWOPS91_917_1:Y>Y		YPS138:R>R	AA:193		UWOPS91_917_1:S>S	AA:199		UWOPS91_917_1:K>K	AA:215		A12:I>I		A4:I>I		DBVPG6304:I>I		UFRJ50816:I>I		YPS138:I>I	AA:219		UWOPS91_917_1:->-	AA:227		A12:E>E		A4:E>E		DBVPG6304:E>E		UFRJ50816:E>E		YPS138:E>E	AA:232		IFO1804:P>P		N_43:P>P		N_44:P>P		N_45:P>P	AA:240		A12:L>L		A4:L>L		DBVPG6304:L>L		YPS138:L>L	AA:269		UWOPS91_917_1:A>A	AA:309		A12:->-		A4:->-		DBVPG6304:->-		YPS138:->-	AA:318		A12:N>N		A4:N>N		DBVPG6304:N>N		IFO1804:N>N		N_17:N>N		N_43:N>N		N_44:N>N		N_45:N>N		Q62_5:N>N		UWOPS91_917_1:N>N		Y7:N>N		YPS138:N>N		Z1_1:N>N	AA:326		UWOPS91_917_1:L>L	AA:336		UWOPS91_917_1:S>S	AA:349		A12:R>R		A4:R>R		DBVPG6304:R>R		UFRJ50816:R>R		YPS138:R>R	AA:358		UWOPS91_917_1:R>R	AA:372		UWOPS91_917_1:S>S	AA:379		UWOPS91_917_1:I>I	AA:386		A12:I>I		A4:I>I		DBVPG6304:I>I		UFRJ50816:I>I		YPS138:I>I	AA:390		UWOPS91_917_1:F>F	AA:409		UWOPS91_917_1:P>P	AA:422		UWOPS91_917_1:A>AID:YBR005W	AA:9		UWOPS91_917_1:E>E	AA:14		UWOPS91_917_1:V>V	AA:21		A4:H>H		DBVPG6304:H>H		UFRJ50791:H>H		UFRJ50816:H>H		UWOPS91_917_1:H>H		YPS138:H>H	AA:29		A4:Y>Y		DBVPG6304:Y>Y		UFRJ50791:Y>Y		UFRJ50816:Y>Y		UWOPS91_917_1:Y>Y		YPS138:Y>Y	AA:32		A4:S>S		DBVPG6304:S>S		UFRJ50791:S>S		UFRJ50816:S>S		YPS138:S>S	AA:39		UWOPS91_917_1:S>S	AA:47		UWOPS91_917_1:L>L	AA:70		A4:G>G		DBVPG6304:G>G		UFRJ50791:G>G		UFRJ50816:G>G		UWOPS91_917_1:G>G		YPS138:G>G	AA:83		A4:P>P		DBVPG6304:P>P		UFRJ50791:P>P		UFRJ50816:P>P		UWOPS91_917_1:P>P		YPS138:P>P	AA:94		A4:G>G		DBVPG6304:G>G		UFRJ50791:G>G		UFRJ50816:G>G		UWOPS91_917_1:G>G		YPS138:G>G	AA:108		Q95_3:T>T	AA:112		UWOPS91_917_1:N>N	AA:116		A4:L>L		DBVPG6304:L>L		UFRJ50791:L>L		UFRJ50816:L>L		UWOPS91_917_1:L>L		YPS138:L>L	AA:118		A4:Y>Y		DBVPG6304:Y>Y		UFRJ50791:Y>Y		UFRJ50816:Y>Y		UWOPS91_917_1:Y>Y		YPS138:Y>Y	AA:129		A4:D>D		DBVPG6304:D>D		UFRJ50791:D>D		UFRJ50816:D>D		UWOPS91_917_1:D>D		YPS138:D>D	AA:136		A4:P>P		DBVPG6304:P>P		UFRJ50791:P>P		UFRJ50816:P>P		YPS138:P>P	AA:138		A4:P>P		DBVPG6304:P>P		UFRJ50791:P>P		UFRJ50816:P>P		UWOPS91_917_1:P>P		YPS138:P>P	AA:174		A4:P>P		DBVPG6304:P>P		UFRJ50791:P>P		UFRJ50816:P>P		YPS138:P>P	AA:201		A4:I>I		DBVPG6304:I>I		UFRJ50791:I>I		YPS138:I>I	AA:209		IFO1804:V>V		N_43:V>V		N_44:V>V		N_45:V>VID:YBR006W	AA:9		A12:P>P		UFRJ50791:P>P		UWOPS91_917_1:P>P		YPS138:P>P	AA:15		A12:N>N		A4:N>N		UFRJ50791:N>N		UWOPS91_917_1:N>N		YPS138:N>N	AA:21		UWOPS91_917_1:G>G	AA:30		A12:G>G		A4:G>G		UFRJ50791:G>G		YPS138:G>G	AA:40		N_43:P>P		N_44:P>P		N_45:P>P	AA:66		UWOPS91_917_1:E>E	AA:70		A12:T>T		A4:T>T		DBVPG6304:T>T		N_43:T>T		N_44:T>T		N_45:T>T		UFRJ50791:T>T		UWOPS91_917_1:T>T		YPS138:T>T	AA:87		A12:Y>Y		A4:Y>Y		DBVPG6304:Y>Y		N_43:Y>Y		N_44:Y>Y		N_45:Y>Y		UFRJ50791:Y>Y		UWOPS91_917_1:Y>Y		YPS138:Y>Y	AA:109		KPN3828:L>L	AA:111		A12:E>E		A4:E>E		DBVPG6304:E>E		UFRJ50791:E>E		UWOPS91_917_1:E>E		YPS138:E>E	AA:120		A12:A>A		A4:A>A		DBVPG6304:A>A		YPS138:A>A	AA:136		A12:A>A		A4:A>A		DBVPG6304:A>A		YPS138:A>A	AA:140		A12:P>P		A4:P>P		DBVPG6304:P>P		UWOPS91_917_1:P>P		YPS138:P>P	AA:173		A12:K>K		A4:K>K		DBVPG6304:K>K		UFRJ50791:K>K		UFRJ50816:K>K		UWOPS91_917_1:K>K	AA:207		UWOPS91_917_1:G>G	AA:210		A12:K>K		A4:K>K		DBVPG6304:K>K		N_43:K>K		N_44:K>K		N_45:K>K		UFRJ50791:K>K		UFRJ50816:K>K		UWOPS91_917_1:K>K		YPS138:K>K	AA:235		A12:V>V		DBVPG6304:V>V		UFRJ50791:V>V		UFRJ50816:V>V		YPS138:V>V	AA:261		UWOPS91_917_1:L>L	AA:290		A12:K>K		A4:K>K		DBVPG6304:K>K		UFRJ50791:K>K		UFRJ50816:K>K		YPS138:K>K	AA:297		UWOPS91_917_1:T>T	AA:300		UWOPS91_917_1:C>C	AA:331		UFRJ50791:L>L		UFRJ50816:L>L		UWOPS91_917_1:L>L		YPS138:L>L	AA:342		N_17:N>N	AA:344		A12:S>S		DBVPG6304:S>S		UFRJ50791:S>S		UFRJ50816:S>S		UWOPS91_917_1:S>S		YPS138:S>S	AA:355		A12:D>D		A4:D>D		DBVPG6304:D>D		UFRJ50791:D>D		UFRJ50816:D>D		UWOPS91_917_1:D>D		YPS138:D>D	AA:373		UWOPS91_917_1:L>L	AA:378		UWOPS91_917_1:Y>Y	AA:383		A12:L>L		A4:L>L		DBVPG6304:L>L		N_43:L>L		N_45:L>L		UFRJ50791:L>L		UFRJ50816:L>L		UWOPS91_917_1:L>L	AA:399		A4:G>G		DBVPG6304:G>G		UFRJ50791:G>G		UFRJ50816:G>G	AA:428		UWOPS91_917_1:V>V	AA:429		A4:F>F		DBVPG6304:F>F		UFRJ50791:F>F		UFRJ50816:F>F	AA:442		CBS432:A>A	AA:449		UWOPS91_917_1:S>S	AA:452		UWOPS91_917_1:T>T	AA:455		A4:F>F		UFRJ50791:F>F		UFRJ50816:F>F	AA:471		A4:G>G	AA:475		A4:S>S		N_43:S>S		N_45:S>S		UFRJ50791:S>S		UFRJ50816:S>S	AA:484		A4:V>V		UFRJ50791:V>V		UFRJ50816:V>VID:YBR007C	AA:1		A4:L>L		DBVPG6304:L>L		IFO1804:L>L		N_44:L>L		N_45:L>L		UFRJ50791:L>L		UWOPS91_917_1:L>L	AA:4		A4:T>T		DBVPG6304:T>T		YPS138:T>T	AA:37		IFO1804:I>I		N_44:I>I		N_45:I>I	AA:57		UWOPS91_917_1:Y>Y	AA:94		UFRJ50791:R>R		YPS138:R>R	AA:123		UWOPS91_917_1:K>K	AA:132		UFRJ50791:Q>Q		YPS138:Q>Q	AA:136		UFRJ50791:->-		UWOPS91_917_1:->-		YPS138:->-	AA:144		UFRJ50791:S>S		UWOPS91_917_1:S>S		YPS138:S>S	AA:151		UWOPS91_917_1:->-	AA:170		UWOPS91_917_1:L>L	AA:206		UWOPS91_917_1:P>P	AA:213		N_44:F>F		N_45:F>F	AA:227		UWOPS91_917_1:I>I	AA:254		UFRJ50791:T>T		YPS138:T>T	AA:261		UFRJ50791:I>I		UFRJ50816:I>I		YPS138:I>I	AA:263		N_44:F>F		N_45:F>F	AA:283		UWOPS91_917_1:V>V	AA:288		UWOPS91_917_1:S>S	AA:303		A4:T>T		UFRJ50791:T>T		UFRJ50816:T>T		UWOPS91_917_1:T>T	AA:305		UFRJ50791:R>R		UFRJ50816:R>R		YPS138:R>R	AA:317		UFRJ50791:I>I		UFRJ50816:I>I		YPS138:I>I	AA:322		UWOPS91_917_1:G>G	AA:330		UFRJ50791:T>T		UFRJ50816:T>T	AA:332		UFRJ50791:K>K		UFRJ50816:K>K		YPS138:K>K	AA:336		UWOPS91_917_1:I>I	AA:354		UFRJ50791:F>F		UFRJ50816:F>F		YPS138:F>F	AA:360		UWOPS91_917_1:R>R	AA:364		UFRJ50791:F>F		UFRJ50816:F>F		YPS138:F>F	AA:400		UFRJ50791:R>R		UFRJ50816:R>R		YPS138:R>R	AA:406		A4:R>R		UFRJ50791:R>R		UFRJ50816:R>R		UWOPS91_917_1:R>R		YPS138:R>R	AA:419		N_44:F>F		N_45:F>F	AA:439		A4:->-		UFRJ50791:->-		UFRJ50816:->-		YPS138:->-	AA:457		A4:I>I		N_44:I>I		N_45:I>I		UFRJ50791:I>I		UWOPS91_917_1:I>I	AA:464		A4:R>R		UFRJ50791:R>R		UFRJ50816:R>R	AA:467		A4:E>E		CBS432:E>E		N_44:E>E		N_45:E>E		UFRJ50791:E>E		UFRJ50816:E>E		UWOPS91_917_1:E>E	AA:469		A4:I>I		UFRJ50791:I>I		UFRJ50816:I>I	AA:479		A4:V>V		N_44:V>V		N_45:V>V		UFRJ50791:V>V		UFRJ50816:V>V		UWOPS91_917_1:V>V	AA:486		UWOPS91_917_1:D>D	AA:489		CBS432:T>T		UWOPS91_917_1:T>T	AA:524		A4:P>P		UFRJ50791:P>P		UWOPS91_917_1:P>P	AA:533		A4:R>R		UFRJ50791:R>R		UWOPS91_917_1:R>R	AA:537		N_43:A>A		N_44:A>A		N_45:A>A	AA:556		A4:L>L		UFRJ50791:L>L	AA:570		A4:F>F		UFRJ50791:F>F		UWOPS91_917_1:F>F	AA:576		A4:S>S		UFRJ50791:S>S	AA:588		A4:G>G	AA:597		N_43:I>I		N_44:I>I		N_45:I>I	AA:616		A4:W>W		N_43:W>W		N_44:W>W		N_45:W>W		UFRJ50791:W>W		UFRJ50816:W>W		UWOPS91_917_1:W>W		YPS138:W>W	AA:619		A4:I>I		UFRJ50816:I>I		UWOPS91_917_1:I>I		YPS138:I>I	AA:645		UFRJ50816:I>I		YPS138:I>I	AA:653		A4:->-		UFRJ50816:->-		UWOPS91_917_1:->-		YPS138:->-	AA:658		A4:Q>Q		N_43:Q>Q		N_44:Q>Q		N_45:Q>Q		UFRJ50816:Q>Q		UWOPS91_917_1:Q>Q		YPS138:Q>Q	AA:665		UWOPS91_917_1:A>A	AA:670		UWOPS91_917_1:V>V	AA:683		UWOPS91_917_1:N>N	AA:698		UFRJ50816:R>R		UWOPS91_917_1:R>R		YPS138:R>R	AA:727		UWOPS91_917_1:I>I	AA:741		UWOPS91_917_1:F>FID:YBR008C	AA:9		A4:C>C		DBVPG6304:C>C		UFRJ50791:C>C		YPS138:C>C	AA:51		A12:C>C		A4:C>C		DBVPG6304:C>C		UFRJ50791:C>C		UFRJ50816:C>C		YPS138:C>C	AA:55		A4:->-		DBVPG6304:->-		UFRJ50791:->-		UFRJ50816:->-		UWOPS91_917_1:->-		YPS138:->-	AA:64		A4:S>S		DBVPG6304:S>S		UFRJ50791:S>S		UFRJ50816:S>S		YPS138:S>S	AA:71		UFRJ50791:K>K		UFRJ50816:K>K	AA:83		A4:->-		DBVPG6304:->-		UFRJ50791:->-		UFRJ50816:->-		UWOPS91_917_1:->-	AA:100		A4:N>N		DBVPG6304:N>N		UFRJ50791:N>N		UFRJ50816:N>N		UWOPS91_917_1:N>N	AA:107		A12:N>N		A4:N>N		DBVPG6304:N>N		UFRJ50791:N>N		UFRJ50816:N>N		UWOPS91_917_1:N>N		YPS138:N>N	AA:176		UWOPS91_917_1:N>N	AA:199		A4:V>V		DBVPG6304:V>V		N_43:V>V		N_45:V>V		UFRJ50816:V>V		UWOPS91_917_1:V>V	AA:204		A4:T>T		DBVPG6304:I>I		UFRJ50816:T>T		UWOPS91_917_1:T>T		YPS138:T>T	AA:313		A4:G>G		UFRJ50816:G>G		UWOPS91_917_1:G>G	AA:330		A4:C>C		UFRJ50816:C>C		UWOPS91_917_1:C>C		YPS138:C>C	AA:342		A4:A>A		UFRJ50816:A>A		UWOPS91_917_1:A>A	AA:358		UWOPS91_917_1:V>V	AA:380		A12:T>T		A4:T>T		YPS138:T>T	AA:406		N_43:I>I	AA:429		A12:G>G		A4:G>G		YPS138:G>G	AA:502		A12:F>F		A4:F>F		DBVPG6304:F>F		YPS138:F>F	AA:519		A12:G>G		A4:G>G		DBVPG6304:G>G		N_43:G>G		N_45:G>G		YPS138:G>GID:YBR009C	AA:16		A12:V>V		DBVPG6304:S>S		UFRJ50791:S>S		YPS138:V>V	AA:39		N_44:D>D		N_45:D>D		UFRJ50791:D>D		YPS138:D>D	AA:61		UFRJ50791:D>D		YPS138:D>D	AA:65		N_44:S>S		N_45:S>S		UFRJ50791:S>S		YPS138:S>SID:YBR010W	AA:2		UWOPS91_917_1:A>A	AA:11		UFRJ50816:S>S		UWOPS91_917_1:S>S	AA:30		A4:A>A		N_43:A>A		N_44:A>A		N_45:A>A		UFRJ50816:A>A		UWOPS91_917_1:A>A		YPS138:A>A	AA:37		N_43:K>K		N_44:K>K		N_45:K>K	AA:48		UWOPS91_917_1:A>A	AA:59		N_45:T>T	AA:61		UWOPS91_917_1:L>L	AA:72		A4:V>V		N_43:V>V		N_44:V>V		N_45:V>V		UFRJ50816:V>V		UWOPS91_917_1:V>V		YPS138:V>V	AA:75		UWOPS91_917_1:I>I	AA:79		A4:F>F		UFRJ50816:F>F		YPS138:F>F	AA:93		A4:L>L		UFRJ50816:L>L		YPS138:L>L	AA:104		UFRJ50816:L>L	AA:116		A4:K>K		UFRJ50816:K>K		UWOPS91_917_1:K>K		YPS138:K>K	AA:131		A4:L>L		UFRJ50816:L>L		YPS138:L>LID:YBR011C	AA:31		UWOPS91_917_1:G>G	AA:40		DBVPG6304:R>R		UFRJ50816:R>R	AA:49		DBVPG6304:T>T		UFRJ50816:T>T		UWOPS91_917_1:T>T	AA:65		UWOPS91_917_1:V>V	AA:67		DBVPG6304:F>F		UFRJ50816:F>F		UWOPS91_917_1:F>F	AA:94		DBVPG6304:N>N		UFRJ50816:N>N		UWOPS91_917_1:N>N	AA:114		UWOPS91_917_1:N>N	AA:157		IFO1804:S>S		N_43:S>S		N_45:S>S	AA:163		A12:T>T		UWOPS91_917_1:T>T	AA:179		UWOPS91_917_1:F>F	AA:225		A12:F>F		UWOPS91_917_1:F>F	AA:234		IFO1804:V>V		N_43:V>V		N_45:V>V	AA:257		IFO1804:V>V		N_43:V>V		N_45:V>V	AA:275		A12:Q>Q	AA:279		A12:G>G		UWOPS91_917_1:G>G	AA:284		UWOPS91_917_1:G>GID:YBR014C	AA:3		UFRJ50816:T>T	AA:9		A4:S>S		CBS432:S>S		CBS5829:S>S		DBVPG4650:S>S		DBVPG6304:S>S		N_43:S>S		N_44:S>S		N_45:S>S		Q32_3:S>S		Q59_1:S>S		Q62_5:S>S		Q95_3:S>S		T21_4:S>S		UFRJ50791:S>S		UFRJ50816:S>S		YPS138:S>S	AA:12		A4:S>S		DBVPG6304:S>S		N_43:S>S		N_44:S>S		N_45:S>S		UFRJ50816:S>S		YPS138:S>S	AA:14		A4:R>R		DBVPG6304:R>R		UFRJ50816:R>R		YPS138:R>R	AA:33		UFRJ50816:C>C	AA:43		A4:T>T		DBVPG6304:T>T		N_43:T>T		N_44:T>T		N_45:T>T		UFRJ50791:T>T		YPS138:T>T	AA:48		IFO1804:V>V		N_43:V>V		N_44:V>V		N_45:V>V	AA:51		A12:C>C		A4:C>C		DBVPG6304:C>C		IFO1804:C>C		N_43:C>C		N_44:C>C		N_45:C>C		YPS138:C>C	AA:54		A4:T>T		DBVPG6304:T>T		IFO1804:T>T		N_43:T>T		N_44:T>T		N_45:T>T		UFRJ50791:T>T		YPS138:T>T	AA:57		UFRJ50816:F>F	AA:61		Q62_5:V>V	AA:72		CBS432:Q>Q		CBS5829:Q>Q		Q62_5:Q>Q	AA:85		A12:I>I		A4:I>I		DBVPG6304:I>I		IFO1804:I>I		N_43:I>I		N_44:I>I		N_45:I>I		UFRJ50791:I>I		YPS138:I>I	AA:108		A12:L>L		A4:L>L		DBVPG6304:L>L		IFO1804:L>L		N_43:L>L		N_44:L>L		N_45:L>L		UFRJ50791:L>L		YPS138:L>L	AA:123		A12:V>V		A4:V>V		DBVPG6304:V>V		UFRJ50791:V>V		YPS138:V>V	AA:140		A12:C>C		A4:C>C		DBVPG6304:C>C		IFO1804:C>C		N_43:C>C		N_44:C>C		N_45:C>C		UFRJ50791:C>C		YPS138:C>C	AA:145		UFRJ50816:L>L	AA:186		A12:->-		A4:->-		IFO1804:->-		N_43:->-		N_44:->-		N_45:->-		UFRJ50791:->-		YPS138:->-	AA:188		A12:I>I		A4:I>I		DBVPG6304:I>I		IFO1804:I>I		N_43:I>I		N_44:I>I		N_45:I>I		UFRJ50791:I>I		YPS138:I>I	AA:190		A12:Y>Y		A4:Y>Y		DBVPG6304:Y>Y		UFRJ50791:Y>Y		YPS138:Y>Y	AA:198		A12:F>F		A4:F>F		DBVPG6304:F>F		UFRJ50791:F>F		YPS138:F>FID:YBR016W	AA:7		A12:Y>Y		A4:Y>Y		DBVPG6304:Y>Y		UFRJ50791:Y>Y		YPS138:Y>Y	AA:10		A12:T>T		A4:T>T		DBVPG6304:T>T		UFRJ50791:T>T		YPS138:T>T	AA:74		A12:Q>Q		A4:Q>Q		UFRJ50791:Q>Q		YPS138:Q>Q	AA:79		A12:Q>Q		A4:Q>Q		UFRJ50791:Q>Q		YPS138:Q>Q	AA:84		A12:Q>Q		A4:Q>Q		UFRJ50791:Q>Q		YPS138:Q>Q	AA:92		A12:Y>Y		A4:Y>Y		UFRJ50791:Y>Y		YPS138:Y>Y	AA:100		CBS432:R>R	AA:110		A12:L>L		DBVPG6304:L>L		UFRJ50791:L>LID:YBR017C	AA:3		UWOPS91_917_1:G>G	AA:6		UWOPS91_917_1:L>L	AA:32		UWOPS91_917_1:L>L	AA:38		UWOPS91_917_1:R>R	AA:41		A4:I>I		DBVPG6304:I>I	AA:42		UWOPS91_917_1:V>V	AA:48		A4:N>N		DBVPG6304:N>N		UWOPS91_917_1:N>N	AA:57		A4:T>T		DBVPG6304:T>T		UFRJ50791:T>T		UFRJ50816:T>T		UWOPS91_917_1:T>T		YPS138:T>T	AA:59		DBVPG4650:L>L		N_17:L>L		T21_4:L>L		UWOPS91_917_1:L>L	AA:66		UWOPS91_917_1:N>N	AA:76		A4:L>L	AA:78		A12:L>L		A4:L>L		DBVPG6304:L>L		IFO1804:L>L		N_44:L>L		N_45:L>L		UFRJ50791:L>L	AA:100		UWOPS91_917_1:A>A	AA:106		UWOPS91_917_1:R>R	AA:116		UWOPS91_917_1:H>H	AA:119		UWOPS91_917_1:I>I	AA:122		A12:H>H		A4:H>H		DBVPG6304:H>H		UFRJ50791:H>H		UFRJ50816:H>H	AA:130		IFO1804:V>V		N_43:V>V	AA:149		IFO1804:I>I		N_43:I>I		N_44:I>I		N_45:I>I	AA:150		UWOPS91_917_1:D>D	AA:168		UWOPS91_917_1:T>T	AA:184		A12:E>E		A4:E>E		DBVPG6304:E>E		UFRJ50791:E>E		UFRJ50816:E>E		UWOPS91_917_1:E>E	AA:191		UWOPS91_917_1:Y>Y	AA:205		A12:G>G		A4:G>G		DBVPG6304:G>G		UFRJ50791:G>G		UFRJ50816:G>G		UWOPS91_917_1:G>G	AA:227		UWOPS91_917_1:R>R	AA:262		A12:L>L		A4:L>L		DBVPG6304:L>L		UFRJ50791:L>L		UFRJ50816:L>L	AA:331		UFRJ50791:R>R		UFRJ50816:R>R	AA:340		A4:V>V		UFRJ50791:V>V		UFRJ50816:V>V	AA:342		A12:C>C		A4:C>C		DBVPG6304:C>C		UFRJ50791:C>C		UFRJ50816:C>C		UWOPS91_917_1:C>C	AA:363		A12:I>I		A4:I>I		DBVPG6304:I>I		UFRJ50816:I>I		UWOPS91_917_1:I>I	AA:379		A12:S>S		A4:S>S		DBVPG6304:S>S		UFRJ50816:S>S	AA:405		A12:L>L		A4:L>L		DBVPG6304:L>L		UFRJ50816:L>L	AA:461		A12:S>S		A4:S>S		UFRJ50791:S>S		UFRJ50816:S>S		YPS138:S>S	AA:463		UWOPS91_917_1:V>V	AA:470		A4:F>F		UFRJ50791:F>F		UFRJ50816:F>F	AA:485		N_17:P>P		Q62_5:P>P		T21_4:P>P		Z1_1:P>P	AA:486		UFRJ50791:S>S		UFRJ50816:S>S	AA:503		A12:L>L		A4:L>L		DBVPG6304:L>L		UFRJ50791:L>L		UFRJ50816:L>L		UWOPS91_917_1:L>L		YPS138:L>L	AA:522		A12:I>I		A4:I>I		DBVPG6304:I>I		UFRJ50791:I>I		UFRJ50816:I>I		UWOPS91_917_1:I>I		YPS138:I>I	AA:526		A12:I>I		A4:I>I		DBVPG6304:I>I		UFRJ50791:I>I		UFRJ50816:I>I		UWOPS91_917_1:I>I		YPS138:I>I	AA:528		UWOPS91_917_1:I>I	AA:531		N_17:F>F		Q62_5:F>F		T21_4:F>F		Z1_1:F>F	AA:551		A12:L>L		A4:L>L		DBVPG6304:L>L		N_43:L>L		N_44:L>L		N_45:L>L		UFRJ50791:L>L		UFRJ50816:L>L		UWOPS91_917_1:L>L		YPS138:L>L	AA:557		UWOPS91_917_1:G>G	AA:565		UWOPS91_917_1:F>F	AA:589		A4:L>L		DBVPG6304:L>L		UFRJ50791:L>L		UFRJ50816:L>L		YPS138:L>L	AA:613		UWOPS91_917_1:R>R	AA:615		Q59_1:G>G	AA:620		UWOPS91_917_1:A>A	AA:626		A4:L>L		DBVPG6304:L>L		N_43:L>L		N_44:L>L		UFRJ50791:L>L		UFRJ50816:L>L		UWOPS91_917_1:L>L		YPS138:L>L	AA:632		A4:G>G		DBVPG6304:G>G		UFRJ50791:G>G		UWOPS91_917_1:G>G		YPS138:G>G	AA:640		A4:Y>Y		DBVPG6304:Y>Y		UFRJ50791:Y>Y		UWOPS91_917_1:Y>Y		YPS138:Y>Y	AA:648		UWOPS91_917_1:Q>Q	AA:651		N_43:W>W		N_44:W>W		N_45:W>W	AA:662		A4:A>A		DBVPG6304:A>A		UFRJ50791:A>A		UWOPS91_917_1:A>A		YPS138:A>A	AA:686		A4:->-		DBVPG6304:->-		UFRJ50791:->-		YPS138:->-	AA:690		YPS138:K>K	AA:703		A4:L>L		DBVPG6304:L>L		UFRJ50791:L>L		UFRJ50816:L>L		YPS138:L>L	AA:724		UWOPS91_917_1:I>I	AA:758		A4:I>I		DBVPG6304:I>I		UFRJ50791:I>I		UFRJ50816:I>I		UWOPS91_917_1:I>I		YPS138:I>I	AA:778		A4:P>P		DBVPG6304:P>P		UFRJ50791:P>P		UFRJ50816:P>P		UWOPS91_917_1:P>P		YPS138:P>P	AA:816		UWOPS91_917_1:S>S	AA:831		A4:Q>Q		DBVPG6304:Q>Q		UFRJ50791:Q>Q		UFRJ50816:Q>Q		UWOPS91_917_1:Q>Q		YPS138:Q>Q	AA:837		UWOPS91_917_1:H>H	AA:843		UWOPS91_917_1:I>I	AA:878		UWOPS91_917_1:E>E	AA:895		IFO1804:H>H		N_43:H>H		N_44:H>H		N_45:H>H	AA:900		IFO1804:K>K	AA:908		UWOPS91_917_1:V>V	AA:916		UWOPS91_917_1:C>CID:YBR018C	AA:10		A4:T>T		UFRJ50816:T>T		UWOPS91_917_1:T>T		YPS138:T>T	AA:17		A4:F>F		UFRJ50816:F>F		YPS138:F>F	AA:20		N_43:L>L		N_44:L>L		N_45:L>L	AA:29		A12:L>L		A4:L>L		UFRJ50816:L>L		YPS138:L>L	AA:45		A4:S>S		DBVPG6304:S>S		UFRJ50816:S>S		UWOPS91_917_1:S>S		YPS138:S>S	AA:57		UWOPS91_917_1:T>T	AA:74		UWOPS91_917_1:H>H	AA:84		A4:V>V		DBVPG6304:V>V		UFRJ50791:V>V		UFRJ50816:V>V		UWOPS91_917_1:V>V		YPS138:V>V	AA:86		A4:I>I		DBVPG6304:I>I		UFRJ50791:I>I		UFRJ50816:I>I		YPS138:I>I	AA:92		A4:L>L		DBVPG6304:L>L		UFRJ50791:L>L		UFRJ50816:L>L		UWOPS91_917_1:L>L		YPS138:L>L	AA:98		UWOPS91_917_1:V>V	AA:106		A4:L>L		DBVPG6304:L>L		UFRJ50791:L>L		UFRJ50816:L>L		UWOPS91_917_1:L>L		YPS138:L>L	AA:127		DBVPG6304:V>V		UFRJ50791:V>V		UFRJ50816:V>V		UWOPS91_917_1:V>V		YPS138:V>V	AA:145		DBVPG6304:F>F		UFRJ50791:F>F		UFRJ50816:F>F		YPS138:F>F	AA:154		DBVPG6304:K>K		IFO1804:K>K		N_44:K>K		N_45:K>K		UFRJ50791:K>K		UFRJ50816:K>K		UWOPS91_917_1:K>K		YPS138:K>K	AA:190		DBVPG6304:V>V		UFRJ50791:V>V		UFRJ50816:V>V		YPS138:V>V	AA:220		A12:V>V		DBVPG6304:V>V		UFRJ50791:V>V		UFRJ50816:V>V		YPS138:V>V	AA:222		UFRJ50791:Q>Q		UFRJ50816:Q>Q	AA:228		A12:N>N		DBVPG6304:N>N		UFRJ50791:N>N		UFRJ50816:N>N		YPS138:N>N	AA:251		A12:N>N		DBVPG6304:N>N		UFRJ50791:N>N		UFRJ50816:N>N		YPS138:N>N	AA:264		A12:P>P		DBVPG6304:P>P		UFRJ50791:P>P		UFRJ50816:P>P		YPS138:P>P	AA:293		A12:I>I		DBVPG6304:I>I		UFRJ50791:I>I		UFRJ50816:I>I		YPS138:I>I	AA:301		A12:L>L		DBVPG6304:L>L		UFRJ50791:L>L		UFRJ50816:L>L		YPS138:L>L	AA:320		A12:R>R		DBVPG6304:R>R		UFRJ50791:R>R		UFRJ50816:R>R		YPS138:R>R	AA:336		A12:L>L		DBVPG6304:L>L		UFRJ50816:L>L		YPS138:L>L	AA:363		A12:L>L		DBVPG6304:L>L		N_43:L>L		N_45:L>L		UFRJ50816:L>L		YPS138:L>LID:YBR019C	AA:25		A12:G>G		A4:G>G		DBVPG6304:G>G		UFRJ50791:G>G		UFRJ50816:G>G		UWOPS91_917_1:G>G		YPS138:G>G	AA:31		A12:N>N		A4:N>N	AA:41		A12:L>L		A4:L>L		N_43:L>L		N_44:L>L		N_45:L>L		UWOPS91_917_1:L>L	AA:53		N_43:S>S		N_44:S>S		N_45:S>S	AA:62		UWOPS91_917_1:A>A	AA:83		UWOPS91_917_1:I>I	AA:100		A12:T>T		A4:T>T		UFRJ50816:T>T		UWOPS91_917_1:T>T	AA:106		A12:I>I		A4:I>I		DBVPG6304:I>I		N_17:I>I		N_43:I>I		N_44:I>I		N_45:I>I		UFRJ50816:I>I		UWOPS91_917_1:I>I	AA:112		A4:D>D		DBVPG6304:D>D		N_43:S>S		N_44:S>S		N_45:S>S		UFRJ50791:D>D		UWOPS91_917_1:D>D		YPS138:D>D	AA:115		A12:C>C		A4:C>C		DBVPG6304:C>C	AA:138		A12:N>N		A4:N>N		DBVPG6304:N>N		UFRJ50816:N>N	AA:141		A12:F>F		A4:F>F		DBVPG6304:F>F		UFRJ50816:F>F		UWOPS91_917_1:F>F	AA:148		UWOPS91_917_1:R>R	AA:206		A12:G>G		A4:G>G		DBVPG6304:G>G		UFRJ50816:G>G	AA:208		A12:P>P		A4:P>P		DBVPG6304:P>P		IFO1804:P>P		N_43:P>P		N_44:P>P		N_45:P>P		UFRJ50816:P>P		UWOPS91_917_1:P>P	AA:220		UFRJ50816:N>N		UWOPS91_917_1:N>N		YPS138:N>N	AA:223		A12:R>R		A4:R>R		DBVPG6304:R>R		UFRJ50816:R>R		UWOPS91_917_1:R>R		YPS138:R>R	AA:235		UWOPS91_917_1:P>P	AA:239		A12:T>T		A4:T>T		DBVPG6304:T>T		UFRJ50816:T>T		YPS138:T>T	AA:245		A12:R>R		A4:R>R		DBVPG6304:R>R		UFRJ50816:R>R		YPS138:R>R	AA:247		UWOPS91_917_1:N>N	AA:273		A12:T>T		A4:T>T		DBVPG6304:T>T		IFO1804:T>T		N_43:T>T		N_44:T>T		N_45:T>T		UFRJ50816:T>T		UWOPS91_917_1:T>T		YPS138:T>T	AA:285		UWOPS91_917_1:T>T	AA:292		IFO1804:V>V		N_17:V>V		N_43:V>V		N_44:V>V		N_45:V>V	AA:298		YPS138:L>L	AA:305		A12:N>N		A4:N>N		DBVPG6304:N>N		UWOPS91_917_1:N>N		YPS138:N>N	AA:309		A12:A>A		A4:A>A		UFRJ50816:A>A		UWOPS91_917_1:A>A		YPS138:A>A	AA:319		A12:G>G		A4:G>G		DBVPG6304:G>G		UFRJ50816:G>G		UWOPS91_917_1:G>G		YPS138:G>G	AA:418		A4:T>T		UFRJ50791:T>T		UFRJ50816:T>T		UWOPS91_917_1:T>T		YPS138:T>T	AA:437		UFRJ50791:V>V		UFRJ50816:V>V		UWOPS91_917_1:V>V		YPS138:V>V	AA:450		UWOPS91_917_1:Y>Y	AA:464		A4:V>V		IFO1804:V>V		N_43:V>V		N_44:V>V		N_45:V>V		UFRJ50791:V>V		UFRJ50816:V>V		UWOPS91_917_1:V>V		YPS138:V>V	AA:476		IFO1804:T>T		N_43:T>T		N_44:T>T		N_45:T>T	AA:488		UWOPS91_917_1:V>V	AA:504		A4:A>A		UFRJ50791:A>A		UFRJ50816:A>A		YPS138:A>A	AA:507		UWOPS91_917_1:V>V	AA:513		A4:G>G	AA:517		UWOPS91_917_1:T>T	AA:522		A4:A>A		UFRJ50791:A>A		UFRJ50816:A>A		UWOPS91_917_1:A>A		YPS138:A>A	AA:541		N_17:S>S	AA:545		DBVPG6304:G>G		UFRJ50791:G>G	AA:554		A4:I>I		DBVPG6304:I>I		UFRJ50791:I>I		YPS138:I>I	AA:559		A4:S>S		DBVPG6304:S>S		UFRJ50791:S>S		YPS138:S>S	AA:581		A4:L>L		DBVPG6304:L>L		IFO1804:L>L		N_44:L>L		N_45:L>L		UFRJ50791:L>L		UWOPS91_917_1:L>L		YPS138:L>L	AA:599		A4:L>L		DBVPG6304:L>L		UFRJ50791:L>L		UFRJ50816:L>L		UWOPS91_917_1:L>L		YPS138:L>L	AA:608		A4:T>T		DBVPG6304:T>T		UFRJ50791:T>T		UFRJ50816:T>T		YPS138:T>T	AA:615		UWOPS91_917_1:I>I	AA:623		A4:L>L		DBVPG6304:L>L		UFRJ50791:L>L		UFRJ50816:L>L		UWOPS91_917_1:L>L		YPS138:L>L	AA:628		A12:I>I		A4:I>I		DBVPG6304:P>P		UFRJ50791:P>P		UFRJ50816:I>I		UWOPS91_917_1:I>I		YPS138:P>P	AA:630		UWOPS91_917_1:T>T	AA:634		A4:L>L		DBVPG6304:L>L		UFRJ50791:L>L		UFRJ50816:L>L		UWOPS91_917_1:L>L		YPS138:L>L	AA:657		A12:Q>Q		A4:Q>Q		DBVPG6304:Q>Q		UFRJ50791:Q>Q		UFRJ50816:Q>Q		UWOPS91_917_1:Q>Q		YPS138:Q>Q	AA:661		A12:N>N		A4:N>N		DBVPG6304:N>N		UFRJ50791:N>N		UFRJ50816:N>N		YPS138:N>N	AA:664		A12:F>F		A4:F>F		DBVPG6304:F>F		UFRJ50791:F>F		UFRJ50816:F>F		UWOPS91_917_1:F>F		YPS138:F>F	AA:689		A4:F>FID:YBR020W	AA:12		UWOPS91_917_1:P>P	AA:15		A4:N>N		DBVPG6304:N>N		UFRJ50791:N>N		UFRJ50816:N>N		YPS138:N>N	AA:18		UWOPS91_917_1:A>A	AA:20		A4:E>E		DBVPG6304:E>E		UFRJ50791:E>E		UFRJ50816:E>E		YPS138:E>E	AA:30		A4:P>P		DBVPG6304:P>P		UFRJ50791:P>P		UFRJ50816:P>P		YPS138:P>P	AA:44		IFO1804:P>P		N_17:P>P		N_43:P>P		N_44:P>P		N_45:P>P	AA:53		A4:R>R		DBVPG6304:R>R		IFO1804:R>R		N_17:R>R		N_43:R>R		N_44:R>R		N_45:R>R		UFRJ50791:R>R		UFRJ50816:R>R		YPS138:R>R	AA:69		IFO1804:L>L	AA:79		A4:C>C		DBVPG6304:C>C		UFRJ50791:C>C		UFRJ50816:C>C		UWOPS91_917_1:C>C		YPS138:C>C	AA:98		A4:P>P		DBVPG6304:P>P		UFRJ50791:P>P		UFRJ50816:P>P		UWOPS91_917_1:P>P		YPS138:P>P	AA:103		A4:R>R		DBVPG6304:R>R		IFO1804:R>R		N_43:R>R		N_44:R>R		N_45:R>R		UFRJ50791:R>R		UFRJ50816:R>R		UWOPS91_917_1:R>R	AA:108		UWOPS91_917_1:P>P	AA:120		A4:V>V		DBVPG6304:V>V		UFRJ50791:V>V		UFRJ50816:V>V		YPS138:V>V	AA:133		A4:V>V		DBVPG6304:V>V		IFO1804:V>V		N_43:V>V		N_44:V>V		N_45:V>V		UFRJ50791:V>V		UFRJ50816:V>V		UWOPS91_917_1:V>V		YPS138:V>V	AA:136		IFO1804:S>S		N_43:S>S		N_44:S>S		N_45:S>S	AA:152		A4:A>A		DBVPG6304:A>A		UFRJ50791:A>A		YPS138:A>A	AA:191		IFO1804:Y>Y		N_43:Y>Y		N_45:Y>Y		UWOPS91_917_1:Y>Y	AA:224		IFO1804:G>G	AA:251		A4:R>R		DBVPG6304:R>R	AA:252		UWOPS91_917_1:E>E	AA:262		A4:V>V		DBVPG6304:V>V		IFO1804:V>V		N_43:V>V		N_45:V>V		UFRJ50816:V>V		UWOPS91_917_1:V>V		YPS138:V>V	AA:265		N_45:N>N	AA:269		UWOPS91_917_1:T>T	AA:274		UWOPS91_917_1:Y>Y	AA:285		IFO1804:A>A		N_43:A>A		N_44:A>A		N_45:A>A	AA:287		A4:V>V	AA:298		A4:S>S		UFRJ50816:S>S		YPS138:S>S	AA:307		A4:K>K		UFRJ50816:K>K		UWOPS91_917_1:K>K		YPS138:K>K	AA:321		A12:Y>Y		A4:Y>Y		UFRJ50816:Y>Y		UWOPS91_917_1:Y>Y		YPS138:Y>Y	AA:336		A12:I>I		A4:I>I		UFRJ50816:I>I	AA:343		N_43:L>L		N_44:L>L		N_45:L>L	AA:353		A12:K>K		A4:K>K		N_43:K>K		N_44:K>K		N_45:K>K		UFRJ50816:K>K		UWOPS91_917_1:K>K	AA:365		N_43:A>A	AA:386		A12:Q>Q		A4:Q>Q		N_43:Q>Q		N_44:Q>Q		N_45:Q>Q		UFRJ50816:Q>Q	AA:397		A12:V>V		A4:V>V		UFRJ50816:V>V	AA:410		A12:L>L		A4:L>L		DBVPG6304:L>L		UFRJ50816:L>L	AA:414		A12:A>A		A4:A>A		DBVPG6304:A>A		N_43:A>A		N_44:A>A		N_45:A>A		UFRJ50816:A>A	AA:424		A12:K>K		A4:K>K		DBVPG6304:K>K		UFRJ50816:K>K	AA:449		A12:D>D		A4:D>D		DBVPG6304:D>D		UFRJ50816:D>D	AA:453		UWOPS91_917_1:S>S	AA:455		N_43:A>A		N_44:A>A		N_45:A>A	AA:463		A12:S>S		A4:S>S		DBVPG6304:S>S		UFRJ50816:S>S		UWOPS91_917_1:S>S	AA:469		A12:G>G		A4:G>G		DBVPG6304:G>G		UFRJ50816:G>G	AA:474		A12:T>T		DBVPG6304:T>T		UFRJ50816:T>T	AA:488		CBS5829:K>K	AA:497		N_43:Y>Y		N_44:Y>Y		N_45:Y>Y	AA:504		A12:K>K		DBVPG6304:K>K		UFRJ50816:K>K	AA:529		IFO1804:->-		N_43:->-		N_44:->-		N_45:->-ID:YBR021W	AA:18		UFRJ50816:S>S		YPS138:S>S	AA:54		IFO1804:A>A	AA:65		YPS138:K>K	AA:73		N_43:K>K		N_44:K>K		N_45:K>K	AA:80		YPS138:V>V	AA:94		YPS138:S>S	AA:102		N_44:K>K		N_45:K>K	AA:125		YPS138:F>F	AA:136		N_43:G>G		N_44:G>G		N_45:G>G	AA:139		YPS138:L>L	AA:168		UWOPS91_917_1:G>G		YPS138:G>G	AA:179		UWOPS91_917_1:S>S		YPS138:S>S	AA:203		UWOPS91_917_1:Y>Y	AA:206		UWOPS91_917_1:Q>Q	AA:208		A12:Y>Y	AA:218		A12:L>L		UWOPS91_917_1:L>L	AA:231		A12:P>P		N_44:P>P		N_45:P>P		UWOPS91_917_1:P>P	AA:244		A12:F>F	AA:258		N_45:L>L	AA:259		UWOPS91_917_1:L>L	AA:288		IFO1804:I>I		N_44:I>I		N_45:I>I	AA:291		YPS138:A>A	AA:294		UWOPS91_917_1:R>R		YPS138:R>R	AA:301		N_44:T>T	AA:320		A12:G>G		YPS138:G>G	AA:337		UWOPS91_917_1:R>R	AA:344		A12:S>S		YPS138:S>S	AA:365		A12:I>I		UWOPS91_917_1:I>I		YPS138:I>I	AA:378		A12:N>N		UWOPS91_917_1:N>N		YPS138:N>N	AA:382		A12:P>P		YPS138:P>P	AA:389		UWOPS91_917_1:F>F	AA:415		UWOPS91_917_1:G>G	AA:423		A12:L>L		DBVPG6304:L>L		UWOPS91_917_1:L>L		YPS138:L>L	AA:436		IFO1804:Y>Y	AA:437		A12:I>I		DBVPG6304:I>I		UFRJ50816:I>I		YPS138:I>I	AA:439		A12:I>I		DBVPG6304:I>I		UFRJ50816:I>I		YPS138:I>I	AA:441		UWOPS91_917_1:R>R	AA:445		UWOPS91_917_1:F>F	AA:451		A12:L>L		DBVPG6304:L>L		UFRJ50816:L>L		YPS138:L>L	AA:462		UWOPS91_917_1:S>S	AA:466		YPS138:T>T	AA:468		A12:A>A		DBVPG6304:A>A		UFRJ50816:A>A		UWOPS91_917_1:A>A		YPS138:A>A	AA:476		A12:L>L		DBVPG6304:L>L		UFRJ50816:L>L		UWOPS91_917_1:L>L		YPS138:L>L	AA:486		UWOPS91_917_1:D>D	AA:488		A12:F>F		DBVPG6304:F>F		UFRJ50816:F>F		YPS138:F>F	AA:493		UWOPS91_917_1:G>G	AA:501		UWOPS91_917_1:Y>Y	AA:504		UWOPS91_917_1:Q>Q	AA:508		UWOPS91_917_1:F>F	AA:518		UWOPS91_917_1:N>N	AA:524		A12:A>A		DBVPG6304:A>A		YPS138:A>A	AA:534		UWOPS91_917_1:P>P	AA:537		A12:I>I		DBVPG6304:I>I	AA:543		UWOPS91_917_1:P>P	AA:550		UWOPS91_917_1:G>G	AA:554		UWOPS91_917_1:L>L	AA:557		UWOPS91_917_1:L>L	AA:561		A12:V>V		DBVPG6304:V>V	AA:563		UWOPS91_917_1:Y>Y	AA:566		UWOPS91_917_1:S>S	AA:573		UWOPS91_917_1:L>L	AA:577		UWOPS91_917_1:F>F	AA:581		UWOPS91_917_1:G>G	AA:583		UWOPS91_917_1:P>P	AA:588		DBVPG6304:I>I		UWOPS91_917_1:I>I	AA:609		DBVPG6304:D>D		N_43:D>D		N_44:D>D		N_45:D>D	AA:611		UWOPS91_917_1:I>I	AA:626		DBVPG6304:H>H	AA:631		DBVPG6304:T>TID:YBR022W	AA:19		UWOPS91_917_1:R>R	AA:20		A12:I>I		DBVPG6304:I>I		UFRJ50791:I>I		UFRJ50816:I>I	AA:32		IFO1804:G>G		N_45:G>G		UWOPS91_917_1:G>G	AA:45		A12:K>K		DBVPG6304:K>K		IFO1804:K>K		N_45:K>K		UFRJ50791:K>K		UFRJ50816:K>K		UWOPS91_917_1:K>K	AA:47		A12:E>E		DBVPG6304:E>E		UFRJ50791:E>E		UFRJ50816:E>E		UWOPS91_917_1:E>E	AA:101		UWOPS91_917_1:T>T	AA:109		UWOPS91_917_1:K>K	AA:125		A12:I>I		IFO1804:I>I		N_45:I>I		UFRJ50791:I>I		UFRJ50816:I>I		UWOPS91_917_1:I>I	AA:138		UWOPS91_917_1:G>G	AA:142		A12:L>L		UWOPS91_917_1:L>L	AA:147		UWOPS91_917_1:I>I	AA:152		UWOPS91_917_1:F>F	AA:153		IFO1804:G>G		N_45:G>G	AA:154		UWOPS91_917_1:V>V	AA:157		UWOPS91_917_1:K>K	AA:160		UWOPS91_917_1:E>E	AA:174		A12:V>V		UFRJ50791:V>V		UFRJ50816:V>V	AA:176		UWOPS91_917_1:Q>QID:YBR024W	AA:86		A4:L>L		DBVPG6304:L>L		N_43:L>L		N_44:L>L		N_45:L>L		UFRJ50791:L>L		UFRJ50816:L>L		UWOPS91_917_1:L>L		YPS138:L>L	AA:114		UFRJ50791:R>R		UFRJ50816:R>R	AA:122		A4:G>G		DBVPG6304:G>G		UFRJ50791:G>G		UFRJ50816:G>G		YPS138:G>G	AA:136		A4:T>T		DBVPG6304:T>T		UFRJ50791:T>T		UFRJ50816:T>T		YPS138:T>T	AA:165		A4:L>L		DBVPG6304:L>L		UFRJ50791:L>L		UFRJ50816:L>L		UWOPS91_917_1:L>L		YPS138:L>L	AA:177		A4:H>H		DBVPG6304:H>H		UFRJ50791:H>H		UFRJ50816:H>H		YPS138:H>H	AA:182		UWOPS91_917_1:P>P	AA:189		IFO1804:P>P		N_43:P>P		N_44:P>P		N_45:P>P	AA:199		UFRJ50791:E>E		UFRJ50816:E>E	AA:212		IFO1804:T>T	AA:228		UWOPS91_917_1:Y>Y	AA:233		A4:R>R		DBVPG6304:R>R		IFO1804:R>R		N_43:R>R		N_44:R>R		N_45:R>R		UFRJ50791:R>R		UFRJ50816:R>R		UWOPS91_917_1:R>R		YPS138:R>R	AA:272		A4:L>L		DBVPG6304:L>L		UFRJ50791:L>L		UFRJ50816:L>L		YPS138:L>L	AA:281		UWOPS91_917_1:A>A	AA:286		A12:E>E		A4:E>E		DBVPG6304:E>E		UFRJ50791:E>E		UFRJ50816:E>E		YPS138:E>EID:YBR025C	AA:10		DBVPG6304:V>V	AA:53		DBVPG6304:G>G		UFRJ50791:G>G	AA:110		IFO1804:L>L	AA:124		IFO1804:S>S		N_43:S>S		N_44:S>S		N_45:S>S	AA:158		A12:V>V		A4:V>V		DBVPG6304:V>V		UFRJ50791:V>V		YPS138:V>V	AA:179		A12:V>V		A4:V>V		DBVPG6304:V>V		UFRJ50791:V>V		YPS138:V>V	AA:189		N_43:R>R		N_45:R>R	AA:222		A12:T>T		A4:T>T		DBVPG6304:T>T		UFRJ50791:T>T		YPS138:T>T	AA:229		A12:S>S		A4:S>S		DBVPG6304:S>S		N_43:S>S		N_44:S>S		N_45:S>S		UFRJ50791:S>S		YPS138:S>S	AA:240		N_43:I>I		N_44:I>I		N_45:I>I	AA:243		A12:S>S		A4:S>S		N_43:S>S		N_44:S>S		N_45:S>S		UFRJ50791:S>S		YPS138:S>S	AA:293		A12:T>T		A4:T>T		DBVPG6304:T>T		UFRJ50791:T>T		YPS138:T>T	AA:299		A12:S>S		A4:S>S		DBVPG6304:S>S		UFRJ50791:S>S		UWOPS91_917_1:S>S		YPS138:S>S	AA:316		A12:C>C		A4:C>C		DBVPG6304:C>C		N_43:C>C		N_44:C>C		UWOPS91_917_1:C>C		YPS138:C>C	AA:327		A12:S>S		A4:S>S		DBVPG6304:S>S		UWOPS91_917_1:S>S		YPS138:S>S	AA:347		A12:G>G		A4:G>G		DBVPG6304:G>G		YPS138:G>G	AA:375		CBS432:Q>Q		N_17:Q>Q		N_45:Q>Q	AA:381		UWOPS91_917_1:T>TID:YBR026C	AA:29		A12:G>G		DBVPG6304:G>G		UFRJ50816:G>G		UWOPS91_917_1:F>F		YPS138:G>G	AA:31		A12:L>L		A4:L>L		DBVPG6304:L>L		YPS138:G>G	AA:34		N_43:N>N		N_45:N>N	AA:73		A12:P>P		DBVPG6304:P>P		YPS138:P>P	AA:87		DBVPG6304:C>C		UFRJ50816:C>C		YPS138:C>C	AA:99		DBVPG6304:I>I		YPS138:I>I	AA:106		N_43:I>I		N_45:I>I	AA:110		DBVPG4650:T>T	AA:145		DBVPG6304:V>V		UFRJ50816:V>V		YPS138:V>V	AA:160		UWOPS91_917_1:I>I	AA:170		DBVPG6304:V>V		UFRJ50816:V>V		UWOPS91_917_1:V>V		YPS138:V>V	AA:173		DBVPG6304:V>V		UFRJ50816:V>V		YPS138:V>V	AA:176		A12:I>I		A4:I>I		DBVPG6304:I>I		N_45:I>I		UFRJ50816:I>I		UWOPS91_917_1:I>I	AA:193		A12:->-	AA:221		A12:T>T		A4:T>T		DBVPG6304:T>T		UFRJ50816:T>T	AA:223		UWOPS91_917_1:A>A	AA:252		UWOPS91_917_1:I>I	AA:256		UWOPS91_917_1:P>P	AA:264		A12:N>N		A4:N>N		DBVPG6304:N>N		UFRJ50816:N>N	AA:302		A12:C>C		A4:C>C		UFRJ50816:C>C		UWOPS91_917_1:C>C	AA:313		A12:Q>Q		A4:Q>Q		N_43:Q>Q		UFRJ50816:Q>Q	AA:318		UFRJ50816:R>R	AA:329		A12:H>H		A4:H>H		UFRJ50816:H>H	AA:335		A12:V>V		A4:V>V		DBVPG6304:V>V		UFRJ50816:V>V		UWOPS91_917_1:V>V	AA:365		A12:G>G		A4:G>G		DBVPG6304:G>G		UWOPS91_917_1:G>GID:YBR028C	AA:26		A12:R>R		DBVPG6304:R>R		UFRJ50791:R>R		UFRJ50816:R>R		UWOPS91_917_1:R>R		YPS138:R>R	AA:33		A12:A>A		A4:A>A		DBVPG6304:A>A		N_43:A>A		N_44:A>A		N_45:A>A		UFRJ50791:A>A		UFRJ50816:A>A		YPS138:A>A	AA:51		N_17:F>F	AA:60		KPN3828:W>W	AA:87		KPN3828:L>L	AA:96		A12:P>P		A4:P>P		DBVPG6304:P>P		UFRJ50791:P>P		YPS138:P>P	AA:113		A12:G>G		A4:G>G		DBVPG6304:G>G		UFRJ50791:G>G		YPS138:G>G	AA:119		A12:I>I		A4:I>I		DBVPG6304:I>I		UFRJ50791:I>I		UWOPS91_917_1:I>I		YPS138:I>I	AA:125		A12:F>F		A4:F>F		DBVPG6304:F>F		UFRJ50791:F>F		YPS138:F>F	AA:135		A12:F>F		A4:F>F		DBVPG6304:F>F		UFRJ50791:F>F		UWOPS91_917_1:F>F		YPS138:F>F	AA:163		A12:T>T		A4:T>T		DBVPG6304:T>T		UFRJ50791:T>T	AA:177		UWOPS91_917_1:A>A	AA:185		UWOPS91_917_1:I>I	AA:192		A4:->-		DBVPG6304:->-		UFRJ50791:->-	AA:203		UWOPS91_917_1:T>T	AA:207		A4:V>V		DBVPG6304:V>V		IFO1804:V>V		N_43:V>V		N_44:V>V		N_45:V>V	AA:244		IFO1804:T>T		KPN3828:T>T		KPN3829:T>T		N_17:T>T		N_43:T>T		N_45:T>T		UWOPS91_917_1:T>T	AA:257		UWOPS91_917_1:C>C	AA:265		UWOPS91_917_1:T>T	AA:287		UWOPS91_917_1:K>K	AA:289		A4:F>F		DBVPG6304:F>F		UFRJ50791:F>F		YPS138:F>F	AA:291		A4:T>T		DBVPG6304:T>T		UFRJ50791:T>T		UWOPS91_917_1:T>T		YPS138:T>T	AA:312		A4:D>D		DBVPG6304:D>D		UFRJ50791:D>D		YPS138:D>D	AA:332		T21_4:P>P	AA:341		UWOPS91_917_1:V>V	AA:347		A4:V>V		DBVPG6304:V>V		UFRJ50791:V>V		YPS138:V>V	AA:365		A4:C>C		DBVPG6304:C>C		UFRJ50791:C>C		YPS138:C>C	AA:370		A4:L>L		DBVPG6304:L>L		IFO1804:L>L		N_44:L>L		N_45:L>L		UFRJ50791:L>L		UWOPS91_917_1:L>L		YPS138:L>L	AA:388		A4:V>V		DBVPG6304:V>V		IFO1804:V>V		N_44:V>V		N_45:V>V		UFRJ50791:V>V		UWOPS91_917_1:V>V		YPS138:V>V	AA:455		A12:R>R		A4:R>R		DBVPG6304:R>R		UFRJ50816:R>R		YPS138:R>R	AA:466		A12:S>S		A4:S>S		DBVPG6304:S>S		UFRJ50816:S>S		YPS138:S>S	AA:468		UWOPS91_917_1:E>E	AA:497		A12:V>V		A4:V>V		DBVPG6304:V>V		UFRJ50816:V>V		UWOPS91_917_1:V>V	AA:500		A12:->-		A4:->-		DBVPG6304:->-		IFO1804:->-		N_44:->-		N_45:->-		UFRJ50816:->-		UWOPS91_917_1:->-	AA:508		A12:I>I		A4:I>I		DBVPG6304:I>I		UFRJ50816:I>I	AA:519		A12:F>F		A4:F>F		DBVPG6304:F>F		UFRJ50816:F>F		UWOPS91_917_1:F>FID:YBR030W	AA:33		A4:S>S		DBVPG6304:S>S		UFRJ50816:S>S		YPS138:S>S	AA:42		UWOPS91_917_1:A>A	AA:44		A4:T>T		DBVPG6304:T>T	AA:46		A4:I>I		DBVPG6304:I>I		UFRJ50816:I>I		YPS138:I>I	AA:50		A4:E>E		DBVPG6304:E>E		N_44:E>E		N_45:E>E		UFRJ50816:E>E		UWOPS91_917_1:E>E		YPS138:E>E	AA:55		N_44:L>L		N_45:L>L	AA:57		UWOPS91_917_1:K>K	AA:67		A4:S>S		DBVPG6304:S>S		N_44:S>S		N_45:S>S		UWOPS91_917_1:S>S		YPS138:S>S	AA:69		A4:A>A		DBVPG6304:A>A		UFRJ50816:A>A		UWOPS91_917_1:A>A		YPS138:A>A	AA:82		UWOPS91_917_1:A>A	AA:86		A4:A>A		DBVPG6304:A>A		UFRJ50816:A>A		YPS138:A>A	AA:92		UWOPS91_917_1:T>T	AA:106		A4:T>T		UFRJ50816:T>T		UWOPS91_917_1:T>T		YPS138:T>T	AA:108		UWOPS91_917_1:R>R	AA:110		UWOPS91_917_1:R>R	AA:120		A4:P>P		UFRJ50816:P>P		UWOPS91_917_1:P>P		YPS138:P>P	AA:130		A12:L>L		A4:L>L		UFRJ50816:L>L		YPS138:L>L	AA:156		N_44:A>A		N_45:A>A	AA:174		UWOPS91_917_1:G>G	AA:182		A4:N>N		DBVPG6304:N>N		UFRJ50816:N>N	AA:187		UWOPS91_917_1:Y>Y	AA:219		A4:I>I		DBVPG6304:I>I		IFO1804:I>I		N_44:I>I		N_45:I>I		UFRJ50791:I>I		UFRJ50816:I>I		UWOPS91_917_1:I>I	AA:221		A4:D>D		DBVPG6304:D>D		IFO1804:D>D		N_44:D>D		N_45:D>D		UFRJ50791:D>D		UFRJ50816:D>D	AA:234		A4:F>F		DBVPG6304:F>F		UFRJ50791:F>F	AA:236		A4:S>S		DBVPG6304:S>S		IFO1804:S>S		N_44:S>S		N_45:S>S		UFRJ50791:S>S		UFRJ50816:S>S	AA:239		A4:D>D		DBVPG6304:D>D		UFRJ50791:D>D		UFRJ50816:D>D	AA:248		UWOPS91_917_1:G>G	AA:250		UWOPS91_917_1:C>C	AA:253		UWOPS91_917_1:L>L	AA:255		UWOPS91_917_1:A>A	AA:258		CBS432:Y>Y		DBVPG6304:Y>Y		IFO1804:Y>Y		KPN3828:Y>Y		KPN3829:Y>Y		N_17:Y>Y		N_44:Y>Y		N_45:Y>Y		Q32_3:Y>Y		Q62_5:Y>Y		Q89_8:Y>Y		Q95_3:Y>Y		UFRJ50791:Y>Y		UWOPS91_917_1:Y>Y		Y7:Y>Y	AA:274		DBVPG6304:T>T		UFRJ50791:T>T	AA:291		DBVPG6304:L>L		UFRJ50791:L>L	AA:308		CBS432:G>G		DBVPG6304:G>G		IFO1804:G>G		KPN3828:G>G		KPN3829:G>G		N_17:G>G		N_44:G>G		N_45:G>G		Q62_5:G>G		Q89_8:G>G		Q95_3:G>G		UFRJ50791:G>G	AA:311		UWOPS91_917_1:N>N	AA:330		DBVPG6304:I>I		UFRJ50791:I>I	AA:333		DBVPG6304:S>S		UFRJ50791:S>S	AA:349		CBS432:T>T		DBVPG6304:T>T		IFO1804:T>T		KPN3828:T>T		KPN3829:T>T		N_17:T>T		N_43:T>T		N_44:T>T		N_45:T>T		Q62_5:T>T		Q89_8:T>T		UFRJ50791:T>T	AA:355		IFO1804:Y>Y		N_43:Y>Y		N_44:Y>Y		N_45:Y>Y	AA:382		DBVPG6304:S>S		UFRJ50791:S>S	AA:399		DBVPG6304:E>E		UFRJ50791:E>E	AA:453		DBVPG6304:S>S		UWOPS91_917_1:S>S	AA:463		DBVPG6304:L>L		IFO1804:L>L		N_43:L>L		N_44:L>L		N_45:L>L		UWOPS91_917_1:L>L	AA:502		DBVPG6304:K>K		IFO1804:K>K		N_43:K>K		N_44:K>K		N_45:K>K		UFRJ50791:K>K		UWOPS91_917_1:K>K	AA:514		DBVPG6304:L>L		UFRJ50791:L>L	AA:540		UWOPS91_917_1:V>V	AA:555		IFO1804:S>S		N_43:S>S		N_44:S>S		N_45:S>SID:YBR031W	AA:42		IFO1804:V>V		N_43:V>V		N_44:V>V		N_45:V>V	AA:45		A4:N>N		DBVPG6304:N>N		UFRJ50791:N>N		UWOPS91_917_1:N>N		YPS138:N>N	AA:60		A4:T>T		DBVPG6304:T>T		IFO1804:T>T		N_43:T>T		N_44:T>T		N_45:T>T		UFRJ50791:T>T		UWOPS91_917_1:T>T		YPS138:T>T	AA:63		DBVPG6304:E>E	AA:72		A4:A>A		DBVPG6304:A>A		IFO1804:A>A		N_43:A>A		N_44:A>A		N_45:A>A		UFRJ50791:A>A		UFRJ50816:A>A		UWOPS91_917_1:A>A		YPS138:A>A	AA:116		A4:N>N		DBVPG6304:N>N		UFRJ50791:N>N		UFRJ50816:N>N		YPS138:N>N	AA:123		UWOPS91_917_1:A>A	AA:125		IFO1804:A>A		N_43:A>A		N_44:A>A		N_45:A>A	AA:126		A4:I>I		DBVPG6304:I>I		UFRJ50791:I>I		UFRJ50816:I>I		UWOPS91_917_1:I>I	AA:132		A4:A>A		DBVPG6304:A>A		UFRJ50791:A>A		UFRJ50816:A>A		UWOPS91_917_1:A>A	AA:141		A4:R>R		DBVPG6304:R>R		IFO1804:R>R		N_43:R>R		N_45:R>R		UFRJ50791:R>R		UFRJ50816:R>R		UWOPS91_917_1:R>R	AA:148		A4:I>I		DBVPG6304:I>I		UFRJ50791:I>I		UFRJ50816:I>I		UWOPS91_917_1:I>I		YPS138:I>I	AA:152		UWOPS91_917_1:V>V	AA:178		A4:L>L		DBVPG6304:L>L		UFRJ50791:L>L		UFRJ50816:L>L		UWOPS91_917_1:L>L	AA:182		A4:L>L		DBVPG6304:L>L		UFRJ50791:L>L		UFRJ50816:L>L	AA:187		UFRJ50791:L>L	AA:203		UWOPS91_917_1:R>R	AA:217		UFRJ50816:K>K	AA:222		A4:V>V		UFRJ50816:V>V		UWOPS91_917_1:V>V	AA:232		UWOPS91_917_1:S>S	AA:266		UWOPS91_917_1:T>T	AA:275		UWOPS91_917_1:T>T	AA:355		UWOPS91_917_1:F>F	AA:358		CBS432:T>T		Q59_1:T>T		Q89_8:T>T		Q95_3:T>T		T21_4:T>T		Y7:T>T	AA:361		UFRJ50816:H>HID:YBR033W	AA:10		A12:G>G		A4:G>G		DBVPG6304:G>G		IFO1804:G>G		N_43:G>G		N_44:G>G		N_45:G>G		UFRJ50816:G>G		UWOPS91_917_1:G>G		YPS138:G>G	AA:14		A12:P>P		A4:P>P		DBVPG6304:P>P		UFRJ50816:P>P		UWOPS91_917_1:P>P		YPS138:P>P	AA:49		IFO1804:R>R		N_43:R>R		N_44:R>R		N_45:R>R	AA:56		A12:C>C		A4:C>C		DBVPG6304:C>C		UFRJ50816:C>C		UWOPS91_917_1:C>C		YPS138:C>C	AA:90		A12:V>V		DBVPG6304:V>V		UWOPS91_917_1:V>V		YPS138:V>V	AA:132		A12:N>N		DBVPG6304:N>N		YPS138:N>N	AA:186		A12:T>T		DBVPG6304:T>T		IFO1804:T>T		N_43:T>T		N_44:T>T		N_45:T>T		UFRJ50791:T>T		YPS138:T>T	AA:195		A4:D>D		DBVPG6304:D>D		UFRJ50791:D>D		UWOPS91_917_1:D>D		YPS138:D>D	AA:204		A12:G>G		A4:G>G		DBVPG6304:G>G		UFRJ50791:G>G		UWOPS91_917_1:G>G		YPS138:G>G	AA:207		IFO1804:I>I		N_44:I>I		N_45:I>I	AA:208		A4:A>A		DBVPG6304:A>A		UFRJ50791:A>A		YPS138:A>A	AA:222		A12:C>C		A4:C>C		DBVPG6304:C>C		UFRJ50791:C>C		UWOPS91_917_1:C>C		YPS138:C>C	AA:225		UWOPS91_917_1:N>N	AA:227		A4:R>R		DBVPG6304:R>R		UFRJ50791:R>R		YPS138:R>R	AA:231		UWOPS91_917_1:S>S	AA:240		UFRJ50791:R>R		YPS138:R>R	AA:242		A4:N>N		DBVPG6304:N>N		UFRJ50791:N>N		UWOPS91_917_1:N>N		YPS138:N>N	AA:264		A4:A>A		DBVPG6304:A>A		UFRJ50791:A>A		UWOPS91_917_1:A>A		YPS138:A>A	AA:273		UWOPS91_917_1:S>S	AA:289		UWOPS91_917_1:K>K	AA:312		UWOPS91_917_1:K>K	AA:359		UWOPS91_917_1:I>I	AA:374		A4:I>I		UFRJ50791:I>I		UWOPS91_917_1:I>I		YPS138:I>I	AA:392		A4:P>P	AA:406		A4:N>N	AA:421		UFRJ50816:F>F	AA:422		UWOPS91_917_1:C>C	AA:430		UWOPS91_917_1:S>S	AA:435		DBVPG6304:S>S		IFO1804:S>S		N_43:S>S		N_45:S>S		UWOPS91_917_1:S>S	AA:444		DBVPG6304:F>F		UFRJ50816:F>F	AA:466		DBVPG6304:I>I		UFRJ50816:I>I	AA:470		DBVPG6304:S>S		UFRJ50816:S>S	AA:482		DBVPG6304:R>R		N_44:R>R		UFRJ50816:R>R	AA:484		DBVPG6304:K>K		UFRJ50816:K>K	AA:488		DBVPG6304:F>F		UFRJ50816:F>F	AA:494		DBVPG6304:L>L		UFRJ50816:L>L		YPS138:L>L	AA:516		A4:F>F		DBVPG6304:F>F		UFRJ50816:F>F		YPS138:F>F	AA:519		A4:F>F		DBVPG6304:F>F		UFRJ50816:F>F		YPS138:F>F	AA:524		IFO1804:L>L		N_43:L>L		N_44:L>L		N_45:L>L	AA:551		IFO1804:A>A		N_43:A>A		N_44:A>A		N_45:A>A	AA:561		A12:L>L		A4:L>L		DBVPG6304:L>L		UFRJ50816:L>L		YPS138:L>L	AA:567		N_44:L>L		N_45:L>L	AA:572		UFRJ50816:L>L		YPS138:L>L	AA:599		A12:I>I		A4:I>I		DBVPG6304:I>I		UFRJ50816:I>I		YPS138:I>I	AA:606		A12:I>I		A4:I>I		DBVPG6304:I>I		UFRJ50816:I>I		YPS138:I>I	AA:641		A12:T>T		A4:T>T		DBVPG6304:T>T		UFRJ50816:T>T		YPS138:T>T	AA:649		N_43:F>F		N_44:F>F		N_45:F>F	AA:682		A12:Q>Q	AA:718		IFO1804:S>S		N_43:S>S		N_44:S>S		N_45:S>S	AA:723		A12:N>N		A4:N>N		DBVPG6304:N>N		IFO1804:N>N		N_43:N>N		N_44:N>N		N_45:N>N		UFRJ50816:N>N		YPS138:N>N	AA:725		A12:E>E		A4:E>E		DBVPG6304:E>E		UFRJ50816:E>E		YPS138:E>E	AA:730		IFO1804:L>L		N_43:L>L		N_44:L>L		N_45:L>L	AA:734		A12:P>P		A4:P>P		DBVPG6304:P>P		IFO1804:P>P		N_43:P>P		N_44:P>P		N_45:P>P		UFRJ50791:P>P		UFRJ50816:P>P		UWOPS91_917_1:P>P		YPS138:P>P	AA:751		A12:P>P		A4:P>P		DBVPG6304:P>P		UFRJ50791:P>P		UFRJ50816:P>P		YPS138:P>P	AA:770		A4:K>K		DBVPG6304:K>K		UFRJ50791:K>K		UFRJ50816:K>K		UWOPS91_917_1:K>K		YPS138:K>K	AA:784		UWOPS91_917_1:V>V	AA:833		UWOPS91_917_1:T>T	AA:835		A4:L>L		DBVPG6304:L>L		UFRJ50791:L>L		UFRJ50816:L>L		YPS138:L>L	AA:846		IFO1804:V>V		N_43:V>V		N_45:V>V		UWOPS91_917_1:V>V	AA:866		UWOPS91_917_1:S>SID:YBR034C	AA:17		A12:V>V		A4:V>V		DBVPG6304:V>V		IFO1804:G>G		N_43:G>G		N_45:G>G		UFRJ50816:V>V		UWOPS91_917_1:V>V	AA:25		A12:V>V		A4:V>V		DBVPG6304:V>V		IFO1804:V>V		N_43:V>V		N_45:V>V		UFRJ50816:V>V		UWOPS91_917_1:V>V	AA:32		A12:V>V		A4:V>V		DBVPG6304:V>V		UFRJ50816:V>V	AA:44		A12:F>F		A4:F>F		DBVPG6304:F>F		IFO1804:F>F		N_45:F>F		UFRJ50816:F>F		UWOPS91_917_1:F>F	AA:49		IFO1804:V>V		N_43:V>V		N_45:V>V	AA:69		A12:V>V		A4:V>V		DBVPG6304:V>V		UFRJ50816:V>V		UWOPS91_917_1:V>V	AA:82		A12:L>L		A4:L>L		DBVPG6304:L>L		UFRJ50816:L>L	AA:102		DBVPG6304:L>L	AA:105		DBVPG6304:G>G	AA:112		A12:L>L		A4:L>L		IFO1804:L>L		N_43:L>L		UFRJ50816:L>L	AA:156		IFO1804:I>I		N_43:I>I		N_45:I>I	AA:166		A12:I>I		A4:I>I		DBVPG6304:I>I		YPS138:I>I	AA:214		A12:V>V		A4:V>V		UFRJ50791:V>V		UFRJ50816:V>V	AA:217		A12:P>P		A4:P>P		DBVPG6304:P>P		UFRJ50791:P>P		YPS138:P>P	AA:230		A12:M>M		A4:M>M		DBVPG6304:M>M		UFRJ50791:M>M		UFRJ50816:M>M		YPS138:M>M	AA:237		UWOPS91_917_1:P>P	AA:255		A12:H>H		A4:H>H		DBVPG6304:H>H		UFRJ50791:H>H		UFRJ50816:H>H		UWOPS91_917_1:H>H		YPS138:H>H	AA:258		A12:N>N		A4:N>N	AA:342		A12:I>I		A4:I>I		DBVPG6304:I>I		UFRJ50816:I>I		UWOPS91_917_1:I>I		YPS138:I>I	AA:345		A12:G>G		A4:G>G		DBVPG6304:G>G		YPS138:G>GID:YBR035C	AA:10		A4:W>W		DBVPG6304:W>W		UFRJ50816:W>W		UWOPS91_917_1:W>W		YPS138:W>W	AA:16		A4:S>S		DBVPG6304:S>S		UFRJ50816:S>S		YPS138:S>S	AA:21		A4:S>S		DBVPG6304:S>S		UFRJ50816:S>S		UWOPS91_917_1:S>S		YPS138:S>S	AA:29		DBVPG6304:T>T		UWOPS91_917_1:T>T	AA:32		A4:E>E		DBVPG6304:E>E		UFRJ50791:E>E		UFRJ50816:E>E		UWOPS91_917_1:E>E		YPS138:E>E	AA:35		A4:L>L		DBVPG6304:L>L		UFRJ50816:L>L		UWOPS91_917_1:L>L		YPS138:L>L	AA:71		A4:V>V		DBVPG6304:V>V		UFRJ50816:V>V		UWOPS91_917_1:V>V		YPS138:V>V	AA:87		A4:P>P		DBVPG6304:P>P		UFRJ50816:P>P		YPS138:P>P	AA:100		A4:P>P		DBVPG6304:P>P		IFO1804:P>P		N_43:P>P		N_44:P>P		N_45:P>P		UFRJ50816:P>P		UWOPS91_917_1:P>P		YPS138:P>P	AA:120		A4:E>E		DBVPG6304:E>E		UFRJ50816:E>E		UWOPS91_917_1:E>E	AA:135		A4:S>S		CBS432:S>S		CBS5829:S>S		DBVPG6304:S>S		IFO1804:S>S		KPN3829:S>S		N_17:S>S		N_43:S>S		N_44:S>S		N_45:S>S		UFRJ50816:S>S		UWOPS91_917_1:S>S	AA:153		A4:K>K		DBVPG6304:K>K		UFRJ50816:K>K		UWOPS91_917_1:K>K	AA:187		UWOPS91_917_1:A>A	AA:196		UFRJ50816:I>I	AA:215		IFO1804:G>G		N_43:G>G		N_45:G>G	AA:226		DBVPG6304:L>L		UFRJ50816:L>L		UWOPS91_917_1:L>LID:YBR037C	AA:16		UWOPS91_917_1:L>L	AA:20		A4:H>H		DBVPG6304:H>H		YPS138:H>H	AA:32		KPN3829:A>A	AA:41		KPN3829:Q>Q	AA:42		A12:G>G		A4:G>G		DBVPG6304:G>G		YPS138:G>G	AA:50		A12:V>V		A4:V>V		DBVPG6304:V>V		UWOPS91_917_1:V>V		YPS138:V>V	AA:57		A12:G>G		A4:G>G		DBVPG6304:G>G		UWOPS91_917_1:G>G		YPS138:G>G	AA:75		A12:V>V		A4:V>V		DBVPG6304:V>V		YPS138:V>V	AA:80		A12:L>L		A4:L>L		DBVPG6304:L>L		YPS138:L>L	AA:104		A12:L>L		A4:L>L		DBVPG6304:L>L		N_43:L>L		N_44:L>L		N_45:L>L		YPS138:L>L	AA:125		N_43:N>N		N_45:N>N	AA:130		N_43:R>R		N_45:R>R	AA:152		A12:K>K		UFRJ50816:K>K	AA:154		A4:K>K		DBVPG6304:K>K		YPS138:K>K	AA:187		A12:V>V		DBVPG6304:V>V		UFRJ50816:V>V	AA:195		A12:L>L		A4:L>L		DBVPG6304:L>L		N_43:L>L		N_44:L>L		N_45:L>L		UFRJ50816:L>L		UWOPS91_917_1:L>L	AA:213		A12:T>T		A4:T>T		DBVPG6304:T>T		UFRJ50816:T>T	AA:219		A12:S>S		A4:S>S		DBVPG6304:S>S		UFRJ50816:S>S	AA:223		A12:P>P		A4:P>P		DBVPG6304:P>P		N_43:P>P		N_44:P>P		N_45:P>P		UFRJ50816:P>P		UWOPS91_917_1:P>P	AA:233		A12:H>H		A4:H>H		DBVPG6304:H>H		UFRJ50816:H>H		UWOPS91_917_1:H>H		YPS138:H>H	AA:264		A12:E>E		A4:E>E		DBVPG6304:E>E		UFRJ50816:E>E		YPS138:E>E	AA:307		A12:R>R		A4:R>R		DBVPG6304:R>R		N_43:R>R		N_44:R>R		N_45:R>R		UFRJ50791:R>R		UFRJ50816:R>R		UWOPS91_917_1:S>S		YPS138:R>RID:YBR039W	AA:2		UWOPS91_917_1:L>L	AA:19		A4:A>A		IFO1804:A>A		N_43:A>A		N_44:A>A		N_45:A>A		UFRJ50816:A>A		UWOPS91_917_1:A>A	AA:25		A4:Y>Y	AA:47		UWOPS91_917_1:K>K	AA:64		UWOPS91_917_1:L>L	AA:73		UWOPS91_917_1:S>S	AA:94		A4:D>D		YPS138:D>D	AA:100		A4:A>A		YPS138:A>A	AA:103		IFO1804:P>P	AA:107		A4:I>I		YPS138:I>I	AA:112		A4:S>S		YPS138:S>S	AA:133		UWOPS91_917_1:N>N	AA:181		UFRJ50816:D>D		UWOPS91_917_1:D>D		YPS138:D>D	AA:184		UWOPS91_917_1:L>L	AA:189		UFRJ50816:A>A		YPS138:A>A	AA:191		IFO1804:T>T		N_43:T>T		N_45:T>T	AA:192		UWOPS91_917_1:Y>Y	AA:198		UFRJ50816:F>F		UWOPS91_917_1:F>F		YPS138:F>F	AA:217		IFO1804:N>N		N_43:N>N		N_45:N>N	AA:231		IFO1804:E>E		N_43:E>E		N_45:E>E		UFRJ50816:E>E		UWOPS91_917_1:E>E		YPS138:E>E	AA:235		UFRJ50816:D>D		YPS138:D>D	AA:242		UWOPS91_917_1:L>L	AA:263		UFRJ50816:I>I		YPS138:I>I	AA:271		N_43:D>D		N_45:D>D		UFRJ50816:D>D		UWOPS91_917_1:D>D		YPS138:D>D	AA:277		UFRJ50816:A>A		UWOPS91_917_1:A>A		YPS138:A>A	AA:287		N_43:L>L		N_45:L>L	AA:295		UFRJ50816:V>V		YPS138:V>V	AA:305		A12:T>T		UFRJ50791:T>T		UFRJ50816:T>T		UWOPS91_917_1:T>TID:YBR040W	AA:5		UWOPS91_917_1:S>S	AA:12		UWOPS91_917_1:K>K	AA:22		A12:F>F		A4:F>F		DBVPG6304:F>F		UFRJ50791:F>F		UFRJ50816:F>F		UWOPS91_917_1:F>F		YPS138:F>F	AA:26		A12:S>S		A4:S>S		DBVPG6304:S>S		UFRJ50791:S>S		UFRJ50816:S>S		YPS138:S>S	AA:33		IFO1804:L>L		N_45:L>L	AA:37		A12:C>C		A4:C>C		DBVPG6304:C>C		UWOPS91_917_1:C>C		YPS138:C>C	AA:40		IFO1804:P>P		N_45:P>P	AA:41		UWOPS91_917_1:S>S	AA:42		A12:N>N		A4:N>N		DBVPG6304:N>N		UFRJ50791:N>N		UFRJ50816:N>N		YPS138:N>N	AA:45		DBVPG6304:T>T	AA:48		A12:V>V		A4:V>V		DBVPG6304:V>V		IFO1804:V>V		N_45:V>V		UFRJ50791:V>V		UFRJ50816:V>V		UWOPS91_917_1:V>V		YPS138:V>V	AA:57		A12:P>P		A4:P>P		DBVPG6304:P>P		UFRJ50791:P>P		UFRJ50816:P>P		UWOPS91_917_1:P>P		YPS138:P>P	AA:65		A12:S>S		A4:S>S		DBVPG6304:S>S		UFRJ50791:S>S		UWOPS91_917_1:S>S		YPS138:S>S	AA:69		UWOPS91_917_1:S>S	AA:70		CBS432:N>N	AA:71		A12:T>T		A4:T>T		DBVPG6304:T>T		UFRJ50791:T>T		UFRJ50816:T>T		YPS138:T>T	AA:78		A12:V>V		A4:V>V		DBVPG6304:V>V		UFRJ50791:V>V	AA:105		A12:F>F		A4:F>F		DBVPG6304:F>F		UWOPS91_917_1:F>F		YPS138:F>F	AA:114		A4:K>K		DBVPG6304:K>K	AA:116		YPS138:L>L	AA:118		A4:S>S		DBVPG6304:S>S		YPS138:S>S	AA:128		UWOPS91_917_1:Q>Q	AA:134		YPS138:S>S	AA:151		A4:A>A		DBVPG6304:A>A		YPS138:A>A	AA:158		A4:V>V		DBVPG6304:V>V		YPS138:V>V	AA:190		A4:L>L		DBVPG6304:L>L		UWOPS91_917_1:L>L		YPS138:L>L	AA:196		A4:L>L		DBVPG6304:L>L		UWOPS91_917_1:L>L		YPS138:L>L	AA:202		A4:T>T		DBVPG6304:T>T		UWOPS91_917_1:T>T		YPS138:T>T	AA:220		A4:A>A		DBVPG6304:A>A		UWOPS91_917_1:A>A		YPS138:A>A	AA:222		A4:Y>Y		DBVPG6304:Y>Y		YPS138:Y>Y	AA:227		A4:S>S		DBVPG6304:S>S		UWOPS91_917_1:S>S		YPS138:S>S	AA:229		A4:S>S		DBVPG6304:S>S		UWOPS91_917_1:S>S		YPS138:S>S	AA:239		A4:K>K		DBVPG6304:K>K		UWOPS91_917_1:K>K		YPS138:K>K	AA:245		A4:A>A		DBVPG6304:A>A		UWOPS91_917_1:A>A		YPS138:A>A	AA:254		A4:L>L		DBVPG6304:L>L		YPS138:L>L	AA:261		UWOPS91_917_1:L>L	AA:273		IFO1804:E>E		N_43:E>E		N_44:E>E		N_45:E>E	AA:277		UWOPS91_917_1:V>V	AA:281		A4:Q>Q		DBVPG6304:Q>Q		UWOPS91_917_1:Q>Q		YPS138:Q>Q	AA:292		A4:S>S		DBVPG6304:S>S		UWOPS91_917_1:S>S	AA:296		UWOPS91_917_1:S>SID:YBR041W	AA:3		DBVPG6304:P>P		IFO1804:P>P		N_43:P>P		N_45:P>P		UWOPS91_917_1:P>P		YPS138:P>P	AA:9		UWOPS91_917_1:F>F	AA:14		DBVPG6304:I>I		YPS138:I>I	AA:44		DBVPG6304:L>L		YPS138:L>L	AA:57		DBVPG6304:I>I		UWOPS91_917_1:I>I		YPS138:I>I	AA:99		UWOPS91_917_1:R>R	AA:123		A12:R>R		UFRJ50791:R>R		UWOPS91_917_1:R>R		YPS138:R>R	AA:127		A12:I>I		UFRJ50791:I>I		YPS138:I>I	AA:135		A12:Q>Q		UFRJ50791:Q>Q		YPS138:Q>Q	AA:161		A12:G>G		UFRJ50791:G>G		UWOPS91_917_1:G>G		YPS138:G>G	AA:173		A12:G>G		UFRJ50791:G>G		UWOPS91_917_1:G>G		YPS138:G>G	AA:177		A12:V>V		DBVPG6304:V>V		UFRJ50791:V>V		UWOPS91_917_1:V>V		YPS138:V>V	AA:180		A12:L>L		UWOPS91_917_1:L>L		YPS138:L>L	AA:191		A12:D>D		DBVPG6304:D>D		UFRJ50791:D>D		YPS138:D>D	AA:241		A12:R>R		A4:R>R		DBVPG6304:R>R		N_43:R>R		N_44:R>R		N_45:R>R		UFRJ50791:R>R		UWOPS91_917_1:R>R		YPS138:R>R	AA:254		UWOPS91_917_1:L>L	AA:268		Q59_1:I>I	AA:280		A12:V>V		A4:V>V		DBVPG6304:V>V		UFRJ50791:V>V		UFRJ50816:V>V		YPS138:V>V	AA:288		A12:T>T		A4:T>T		DBVPG6304:T>T		UFRJ50791:T>T		UFRJ50816:T>T		UWOPS91_917_1:T>T		YPS138:T>T	AA:298		UWOPS91_917_1:P>P	AA:302		A12:S>S		A4:S>S		DBVPG6304:S>S		UFRJ50791:S>S		UFRJ50816:S>S		YPS138:S>S	AA:309		Q59_1:A>A	AA:313		UWOPS91_917_1:L>L	AA:316		IFO1804:G>G		N_43:G>G		N_44:G>G		N_45:G>G	AA:320		A12:A>A		A4:A>A		DBVPG6304:A>A		UFRJ50791:A>A		UFRJ50816:A>A		UWOPS91_917_1:A>A		YPS138:A>A	AA:327		A12:A>A		A4:A>A		CBS432:A>A		DBVPG4650:A>A		DBVPG6304:A>A		IFO1804:A>A		KPN3828:A>A		N_17:A>A		N_44:A>A		N_45:A>A		Q32_3:A>A		Q59_1:A>A		Q95_3:A>A		S36_7:A>A		UFRJ50791:A>A		UFRJ50816:A>A		UWOPS91_917_1:A>A		Y6_5:A>A		Y7:A>A		YPS138:A>A	AA:329		A12:T>T		A4:T>T		DBVPG6304:T>T		UFRJ50791:T>T		UFRJ50816:T>T		UWOPS91_917_1:T>T		YPS138:T>T	AA:350		A12:R>R		A4:R>R		DBVPG6304:R>R		UFRJ50791:R>R		UFRJ50816:R>R		UWOPS91_917_1:R>R		YPS138:R>R	AA:356		UWOPS91_917_1:P>P	AA:366		A12:V>V		A4:V>V		DBVPG6304:V>V		UFRJ50816:V>V		UWOPS91_917_1:V>V	AA:403		DBVPG6304:A>A	AA:405		A12:T>T		A4:T>T		DBVPG6304:T>T		UWOPS91_917_1:T>T	AA:417		A12:C>C		A4:C>C		DBVPG6304:C>C		UFRJ50816:C>C		UWOPS91_917_1:C>C	AA:455		IFO1804:V>V		N_45:V>V	AA:456		A12:A>A		A4:A>A		DBVPG6304:A>A		UFRJ50816:A>A		UWOPS91_917_1:A>A	AA:465		A12:L>L		A4:L>L		DBVPG6304:L>L		UFRJ50816:L>L	AA:468		UWOPS91_917_1:I>I	AA:506		Q59_1:C>C	AA:520		A12:F>F		UFRJ50816:F>F		UWOPS91_917_1:F>F	AA:566		A12:E>E		UFRJ50816:E>E		UWOPS91_917_1:E>E	AA:586		N_44:K>K		N_45:K>K	AA:599		UWOPS91_917_1:L>L	AA:605		A12:P>P		A4:P>P		UWOPS91_917_1:P>P	AA:633		A12:P>P		A4:P>P		UWOPS91_917_1:P>P	AA:654		IFO1804:L>L		N_43:L>L		N_44:L>L		N_45:L>L	AA:669		A12:L>L		A4:L>LID:YBR043C	AA:3		N_43:N>N		N_44:N>N		N_45:N>N	AA:31		A12:T>T		A4:T>T		UWOPS91_917_1:T>T	AA:41		A12:G>G		A4:G>G	AA:62		UWOPS91_917_1:D>D	AA:78		IFO1804:R>R		KPN3828:R>R		N_17:R>R		N_43:R>R		N_44:R>R		N_45:R>R		Q89_8:R>R		Q95_3:R>R		T21_4:R>R		Y6_5:R>R		Y7:R>R	AA:121		A12:->-		A4:->-	AA:123		A12:D>D		A4:D>D	AA:125		A12:E>E		A4:E>E		UWOPS91_917_1:E>E	AA:145		UWOPS91_917_1:I>I	AA:159		A12:E>E		A4:E>E		DBVPG6304:E>E		UWOPS91_917_1:E>E		YPS138:E>E	AA:190		N_43:I>I		N_44:I>I		N_45:I>I	AA:193		IFO1804:F>F		N_43:F>F		N_44:F>F		N_45:F>F	AA:211		A12:Y>Y		A4:Y>Y		UWOPS91_917_1:Y>Y	AA:215		A12:T>T		A4:T>T		UWOPS91_917_1:H>H	AA:217		UWOPS91_917_1:R>R	AA:245		UWOPS91_917_1:R>R	AA:276		A4:D>D	AA:316		UWOPS91_917_1:P>P	AA:324		IFO1804:S>S		N_43:S>S		N_44:S>S		N_45:S>S	AA:336		A12:->-		UWOPS91_917_1:->-	AA:339		A12:R>R		N_17:R>R		N_43:R>R		N_44:R>R		N_45:R>R		Q59_1:R>R		Q89_8:R>R		UWOPS91_917_1:R>R	AA:360		N_43:V>V		N_44:V>V		N_45:V>V	AA:366		UWOPS91_917_1:P>P	AA:369		A12:S>S	AA:398		N_43:F>F		N_44:F>F		N_45:F>F	AA:403		A12:S>S		UWOPS91_917_1:S>S	AA:417		UWOPS91_917_1:T>T	AA:440		A12:K>K		UWOPS91_917_1:K>K	AA:461		UWOPS91_917_1:S>S	AA:484		A12:P>P	AA:496		A12:I>I		A4:I>I		UWOPS91_917_1:I>I	AA:541		UFRJ50816:T>T	AA:591		A12:I>I		A4:I>I		DBVPG6304:I>I		IFO1804:I>I		N_43:I>I		N_44:I>I		N_45:I>I		UFRJ50816:I>I		UWOPS91_917_1:I>I		YPS138:I>I	AA:617		UWOPS91_917_1:F>F	AA:633		UWOPS91_917_1:I>I	AA:635		A4:C>C	AA:639		A12:Q>Q		YPS138:Q>Q	AA:654		A12:A>A		A4:A>A		DBVPG6304:A>A		YPS138:A>A	AA:669		A4:->-		DBVPG6304:->-		N_43:->-		N_45:->-		UFRJ50816:->-		UWOPS91_917_1:->-		YPS138:->-	AA:676		A4:T>T		CBS432:T>T		DBVPG6304:T>T		IFO1804:T>T		N_17:T>T		N_43:T>T		N_44:T>T		N_45:T>T		UFRJ50816:T>T		UWOPS91_917_1:T>T		YPS138:T>TID:YBR045C	AA:56		A12:L>L		A4:L>L		DBVPG6304:L>L		N_43:L>L		N_45:L>L		YPS138:L>L	AA:86		N_43:V>V		N_45:V>V	AA:89		A12:L>L		A4:L>L		DBVPG6304:L>L		UFRJ50816:L>L		YPS138:L>L	AA:102		A12:R>R		A4:R>R		DBVPG6304:R>R		UFRJ50816:R>R		YPS138:R>R	AA:128		A12:P>P		A4:P>P		DBVPG6304:P>P		UFRJ50816:P>P		YPS138:P>P	AA:130		A12:F>F		A4:F>F		DBVPG6304:F>F		UFRJ50816:F>F		YPS138:F>F	AA:146		A12:I>I		A4:I>I		DBVPG6304:I>I		N_43:I>I		N_45:I>I		YPS138:I>I	AA:154		A4:P>P		YPS138:P>P	AA:190		A4:F>F		YPS138:F>F	AA:233		A4:T>T		DBVPG6304:T>T	AA:238		UWOPS91_917_1:D>D	AA:266		A4:L>L		DBVPG6304:L>L		N_43:L>L		N_45:L>L	AA:274		A4:A>A		DBVPG6304:A>A		UWOPS91_917_1:A>A	AA:309		DBVPG6304:D>D		UWOPS91_917_1:D>D	AA:325		UWOPS91_917_1:I>I	AA:347		UWOPS91_917_1:G>G	AA:351		A4:F>F		DBVPG6304:F>F	AA:352		N_43:F>F		N_45:F>F	AA:363		A4:F>F		DBVPG6304:F>F	AA:389		UWOPS91_917_1:->-	AA:392		UWOPS91_917_1:F>F	AA:394		UWOPS91_917_1:A>A	AA:398		A4:F>F		DBVPG6304:F>F		YPS138:F>F	AA:405		UWOPS91_917_1:I>I	AA:411		A4:F>F		DBVPG6304:F>F		YPS138:F>F	AA:431		UWOPS91_917_1:S>S	AA:445		A4:Q>Q		DBVPG6304:Q>Q		YPS138:Q>Q	AA:451		N_43:I>I		N_44:I>I		N_45:I>I	AA:464		A4:R>R		DBVPG6304:R>R		N_43:R>R		N_44:R>R		N_45:R>R		YPS138:R>R	AA:467		A4:V>V		DBVPG6304:V>V		YPS138:V>V	AA:486		UWOPS91_917_1:K>K	AA:491		UWOPS91_917_1:R>R	AA:493		A4:K>K		DBVPG6304:K>K		UFRJ50816:K>K		YPS138:K>K	AA:498		UWOPS91_917_1:G>G	AA:500		UWOPS91_917_1:I>I	AA:524		UWOPS91_917_1:V>V	AA:533		A4:Y>Y		DBVPG6304:Y>Y		UFRJ50816:Y>Y		UWOPS91_917_1:Y>Y		YPS138:Y>Y	AA:541		UWOPS91_917_1:I>I	AA:570		A4:A>A		DBVPG6304:A>A		UFRJ50816:A>A		UWOPS91_917_1:A>A		YPS138:A>A	AA:594		A4:I>I		DBVPG6304:I>I		UFRJ50816:I>I		YPS138:I>I	AA:611		UWOPS91_917_1:S>S	AA:617		UWOPS91_917_1:P>P	AA:625		N_43:P>P		N_44:P>P		N_45:P>PID:YBR046C	AA:10		A12:T>T		A4:T>T		DBVPG6304:T>T		UWOPS91_917_1:T>T	AA:60		A12:I>I		A4:I>I		DBVPG6304:I>I	AA:64		CBS432:L>L		CBS5829:L>L		KPN3829:L>L	AA:90		DBVPG6304:K>K	AA:100		A12:R>R		A4:R>R		DBVPG6304:R>R		IFO1804:R>R		N_44:R>R		N_45:R>R		UWOPS91_917_1:R>R	AA:153		DBVPG6304:I>I	AA:156		UWOPS91_917_1:G>G	AA:161		UWOPS91_917_1:M>M	AA:163		UWOPS91_917_1:T>T	AA:164		A12:F>F		DBVPG6304:F>F		Q89_8:N>N		T21_4:N>N	AA:174		A12:S>S		DBVPG6304:S>S	AA:181		UWOPS91_917_1:K>K	AA:187		A12:A>A		CBS432:A>A		CBS5829:A>A		DBVPG6304:A>A		IFO1804:A>A		KPN3829:A>A		N_43:A>A		N_44:A>A		N_45:A>A		UWOPS91_917_1:A>A	AA:196		UWOPS91_917_1:C>C	AA:201		CBS432:S>S	AA:230		UWOPS91_917_1:R>R	AA:233		DBVPG6304:C>C		YPS138:C>C	AA:243		UWOPS91_917_1:I>I	AA:247		IFO1804:S>S		N_44:S>S		N_45:S>S		UWOPS91_917_1:V>V	AA:264		A12:L>L		A4:L>L		DBVPG6304:L>L		IFO1804:R>R		N_43:R>R		UWOPS91_917_1:R>R	AA:267		DBVPG6304:P>P		YPS138:P>P	AA:298		UWOPS91_917_1:->-	AA:300		IFO1804:F>F		N_43:F>F	AA:301		UWOPS91_917_1:L>L	AA:316		A12:V>V		DBVPG6304:V>V		IFO1804:V>V		N_43:V>V		YPS138:V>V	AA:318		UWOPS91_917_1:T>T	AA:325		UWOPS91_917_1:D>D	AA:327		UWOPS91_917_1:L>L	AA:333		UWOPS91_917_1:T>TID:YBR047W	AA:47		A4:S>S		UFRJ50816:S>S		UWOPS91_917_1:S>S		YPS138:S>S	AA:50		A4:P>P		UFRJ50816:P>P		YPS138:P>P	AA:52		UFRJ50816:H>H	AA:89		A4:D>D		DBVPG6304:D>D		UFRJ50816:D>D		UWOPS91_917_1:D>D		YPS138:D>D	AA:108		A4:E>E		DBVPG6304:E>E		UFRJ50816:E>E		UWOPS91_917_1:E>E		YPS138:E>E	AA:117		IFO1804:S>S		N_43:S>S		N_45:S>S	AA:124		A4:G>G		DBVPG6304:G>G		UFRJ50816:G>G		UWOPS91_917_1:G>G		YPS138:G>G	AA:132		UWOPS91_917_1:R>R	AA:148		A4:T>T		DBVPG6304:T>T		UFRJ50816:T>T		YPS138:T>T	AA:167		A4:E>E		DBVPG6304:E>E		UFRJ50816:E>E	AA:173		A4:Q>Q		DBVPG6304:Q>Q		UFRJ50816:Q>Q		UWOPS91_917_1:Q>QID:YBR050C	AA:5		UWOPS91_917_1:V>V	AA:24		N_45:F>F	AA:56		A4:R>R		UFRJ50816:R>R		UWOPS91_917_1:R>R	AA:73		UWOPS91_917_1:R>R	AA:82		A4:I>I		UFRJ50816:I>I		UWOPS91_917_1:I>I	AA:154		CBS432:F>F	AA:156		A12:L>L		A4:L>L		UFRJ50816:L>L	AA:164		UWOPS91_917_1:S>S	AA:168		A12:S>S		A4:S>S	AA:171		IFO1804:D>D		N_45:D>D	AA:181		IFO1804:Y>Y	AA:187		A12:V>V		A4:V>V		UWOPS91_917_1:V>V	AA:190		A12:K>K		A4:K>K		UWOPS91_917_1:K>K	AA:206		UWOPS91_917_1:A>A	AA:242		KPN3828:Q>Q	AA:261		IFO1804:E>E		N_43:E>E		N_45:E>E	AA:268		A12:I>I		UWOPS91_917_1:I>I	AA:285		DBVPG4650:L>L		Q59_1:L>L		S36_7:L>L		T21_4:L>L	AA:286		A12:D>D	AA:305		A12:R>R		UWOPS91_917_1:R>R	AA:306		IFO1804:L>L		N_43:L>L	AA:327		Q59_1:T>T	AA:346		A12:V>VID:YBR052C	AA:7		A12:V>V		DBVPG6304:V>V		UFRJ50791:V>V		UFRJ50816:V>V		YPS138:V>V	AA:26		A12:S>S		DBVPG6304:S>S		UFRJ50791:S>S		UFRJ50816:S>S		YPS138:S>S	AA:28		UWOPS91_917_1:T>T	AA:36		A12:C>C		DBVPG6304:C>C		UFRJ50791:C>C		UFRJ50816:C>C		UWOPS91_917_1:C>C		YPS138:C>C	AA:58		UWOPS91_917_1:P>P	AA:67		IFO1804:V>V		N_44:V>V		N_45:V>V		UWOPS91_917_1:V>V	AA:76		UFRJ50816:D>D	AA:90		UFRJ50816:D>D	AA:104		A12:P>P		DBVPG6304:P>P		UFRJ50816:P>P		YPS138:P>P	AA:109		A12:V>V		DBVPG6304:V>V		UFRJ50816:V>V		UWOPS91_917_1:V>V		YPS138:V>V	AA:111		A12:V>V		DBVPG6304:V>V		UFRJ50816:V>V		UWOPS91_917_1:V>V		YPS138:V>V	AA:112		IFO1804:P>P		N_44:P>P		N_45:P>P	AA:128		A12:S>S		DBVPG6304:S>S		UFRJ50816:S>S		UWOPS91_917_1:S>S		YPS138:S>S	AA:132		DBVPG6304:V>V	AA:149		A12:R>R		DBVPG6304:R>R		UFRJ50816:R>R		UWOPS91_917_1:R>R		YPS138:R>R	AA:163		A12:S>S		DBVPG6304:S>S		UFRJ50816:S>S		YPS138:S>S	AA:169		A12:V>V		DBVPG6304:V>V		N_43:V>V		N_44:V>V		N_45:V>V		UFRJ50816:V>V		UWOPS91_917_1:V>V		YPS138:V>V	AA:190		A12:L>L		DBVPG6304:L>L		UFRJ50816:L>L		UWOPS91_917_1:L>L		YPS138:L>LID:YBR053C	AA:13		A12:D>D		A4:D>D		UFRJ50791:D>D		UFRJ50816:D>D	AA:22		N_43:S>S	AA:47		A12:K>K		A4:K>K		UFRJ50791:K>K		UFRJ50816:K>K		UWOPS91_917_1:K>K	AA:58		A12:N>N		A4:N>N		N_43:N>N		UFRJ50791:N>N		UFRJ50816:N>N	AA:69		UFRJ50791:L>L		UFRJ50816:L>L	AA:82		UWOPS91_917_1:F>F	AA:131		A12:I>I	AA:132		Q59_1:A>A	AA:156		UWOPS91_917_1:P>P	AA:165		Q59_1:T>T	AA:177		IFO1804:I>I		N_43:I>I		N_45:I>I	AA:188		CBS432:F>F	AA:200		A12:I>I		A4:I>I		IFO1804:I>I		N_43:I>I		N_45:I>I		UFRJ50791:I>I		UWOPS91_917_1:I>I		YPS138:I>I	AA:208		UWOPS91_917_1:P>P	AA:215		A12:I>I		A4:I>I		UFRJ50791:I>I		UWOPS91_917_1:I>I		YPS138:I>I	AA:233		A12:P>P		A4:P>P		DBVPG6304:P>P		UWOPS91_917_1:P>P		YPS138:P>P	AA:242		A12:T>T	AA:249		UWOPS91_917_1:K>K	AA:252		UWOPS91_917_1:L>L	AA:253		A4:F>F	AA:293		A12:N>N		DBVPG6304:N>N		IFO1804:N>N		N_43:N>N		N_44:N>N		N_45:N>N		UFRJ50816:N>N		UWOPS91_917_1:N>N		YPS138:N>N	AA:299		A12:T>T		A4:T>T		DBVPG6304:T>T		UFRJ50816:T>T		UWOPS91_917_1:T>T		YPS138:T>T	AA:306		A12:T>T		A4:T>T		DBVPG6304:I>I		UFRJ50791:T>T		UFRJ50816:T>T		UWOPS91_917_1:T>T		YPS138:I>I	AA:323		A12:I>I		A4:I>I		DBVPG6304:I>I		N_43:I>I		N_44:I>I		N_45:I>I		UFRJ50816:I>I		UWOPS91_917_1:I>I		YPS138:I>I	AA:343		A12:R>R		A4:R>R		DBVPG6304:R>R		UFRJ50791:R>R		UFRJ50816:R>R		UWOPS91_917_1:R>R		YPS138:R>R	AA:356		A12:N>N		A4:N>N		DBVPG6304:N>N		UFRJ50791:N>N		UFRJ50816:N>N		UWOPS91_917_1:N>N		YPS138:N>NID:YBR054W	AA:5		A12:V>V		A4:V>V		DBVPG6304:V>V		UFRJ50816:V>V		YPS138:V>V	AA:14		N_43:E>E		N_44:E>E		N_45:E>E	AA:20		UFRJ50816:P>P	AA:33		UWOPS91_917_1:S>S	AA:39		UFRJ50816:V>V	AA:45		A12:L>L		A4:L>L		DBVPG6304:L>L		UWOPS91_917_1:L>L		YPS138:L>L	AA:52		UFRJ50791:P>P	AA:61		UFRJ50816:D>D	AA:65		UFRJ50816:Y>Y	AA:74		UFRJ50816:F>F	AA:85		CBS432:N>N		DBVPG6304:N>N		IFO1804:N>N		N_43:N>N		N_44:N>N		N_45:N>N		Q95_3:N>N		UFRJ50816:N>N		UWOPS91_917_1:N>N		Y6_5:N>N		Y7:N>N		YPS138:N>N	AA:91		UFRJ50816:V>V	AA:92		YPS138:R>R	AA:105		UFRJ50816:E>E	AA:110		UFRJ50816:R>R	AA:117		UFRJ50816:Y>Y	AA:122		UFRJ50816:L>L	AA:127		IFO1804:P>P		UFRJ50816:P>P	AA:133		UFRJ50816:L>L		UWOPS91_917_1:L>L	AA:143		IFO1804:A>A		N_43:A>A		N_44:A>A		N_45:A>A	AA:149		DBVPG6304:T>T	AA:155		UFRJ50816:C>C	AA:161		UFRJ50816:C>C	AA:165		DBVPG6304:T>T		UWOPS91_917_1:T>T	AA:170		DBVPG6304:Y>Y	AA:173		DBVPG6304:I>I	AA:181		DBVPG6304:V>V	AA:194		UWOPS91_917_1:K>K	AA:200		IFO1804:V>V		N_43:V>V		N_44:V>V		N_45:V>V	AA:211		A12:I>I		A4:I>I		DBVPG6304:I>I		UWOPS91_917_1:I>I	AA:238		CBS432:T>T		CBS5829:T>T		KPN3829:T>T		N_17:T>T		Q32_3:T>T		Q89_8:T>T		Q95_3:T>T		T21_4:T>T		Y7:T>T	AA:252		UWOPS91_917_1:L>L	AA:265		A12:I>I		A4:I>I		DBVPG6304:I>I	AA:294		A12:F>F		A4:F>F		DBVPG6304:F>F		YPS138:F>F	AA:306		UWOPS91_917_1:K>K	AA:326		IFO1804:A>A		N_43:A>A		N_44:A>A		N_45:A>A	AA:340		N_43:A>A	AA:341		DBVPG6304:T>TID:YBR055C	AA:10		A12:N>N		A4:N>N		DBVPG6304:N>N		UFRJ50816:N>N	AA:33		A12:R>R	AA:34		IFO1804:F>F		N_44:F>F		N_45:F>F	AA:74		UWOPS91_917_1:P>P	AA:90		A4:D>D		DBVPG6304:D>D		UFRJ50791:D>D		UFRJ50816:D>D	AA:129		A4:->-		DBVPG6304:->-		IFO1804:->-		N_44:->-		N_45:->-		UFRJ50791:->-		UFRJ50816:->-	AA:171		A4:->-		DBVPG6304:->-		UFRJ50791:->-		UFRJ50816:->-		UWOPS91_917_1:->-		YPS138:->-	AA:176		A4:N>N		DBVPG6304:N>N		UFRJ50791:N>N		UFRJ50816:N>N		UWOPS91_917_1:N>N		YPS138:N>N	AA:178		A4:S>S		DBVPG6304:S>S		UFRJ50791:S>S		UFRJ50816:S>S		YPS138:S>S	AA:188		A4:L>L		DBVPG6304:L>L		UFRJ50791:L>L		UFRJ50816:L>L		UWOPS91_917_1:L>L		YPS138:L>L	AA:194		UWOPS91_917_1:R>R	AA:200		A4:A>A		DBVPG6304:A>A		IFO1804:A>A		N_43:A>A		N_44:A>A		N_45:A>A		UFRJ50791:A>A		UFRJ50816:A>A		UWOPS91_917_1:A>A		YPS138:A>A	AA:203		UWOPS91_917_1:H>H	AA:223		A12:I>I		A4:I>I		DBVPG6304:I>I		UFRJ50791:I>I		UFRJ50816:I>I		UWOPS91_917_1:I>I		YPS138:I>I	AA:241		A4:T>T		DBVPG6304:T>T		UFRJ50816:T>T		YPS138:T>T	AA:248		A12:Y>Y		A4:Y>Y		DBVPG6304:Y>Y		UFRJ50816:Y>Y		YPS138:Y>Y	AA:257		A12:P>P		A4:P>P		DBVPG6304:P>P		UFRJ50816:P>P		UWOPS91_917_1:P>P		YPS138:P>P	AA:270		A4:A>A		DBVPG6304:A>A		UFRJ50816:A>A		UWOPS91_917_1:A>A		YPS138:A>A	AA:271		N_17:I>I	AA:274		A12:G>G		A4:G>G		DBVPG6304:G>G		UFRJ50816:G>G		UWOPS91_917_1:G>G		YPS138:G>G	AA:286		CBS432:N>N		UWOPS91_917_1:N>N	AA:287		A12:S>S		A4:S>S		DBVPG6304:S>S		UFRJ50816:S>S		YPS138:S>S	AA:296		A12:T>T		A4:T>T		DBVPG6304:T>T		UFRJ50816:T>T		YPS138:T>T	AA:306		IFO1804:V>V		N_43:V>V		N_44:V>V		N_45:V>V	AA:314		N_43:N>N	AA:316		A12:L>L		A4:L>L		DBVPG6304:L>L		UFRJ50816:L>L		UWOPS91_917_1:L>L		YPS138:L>L	AA:326		A12:I>I		A4:I>I		DBVPG6304:I>I		UFRJ50816:I>I		UWOPS91_917_1:I>I		YPS138:I>I	AA:344		UWOPS91_917_1:A>A	AA:362		A12:Q>Q		A4:Q>Q		DBVPG6304:Q>Q		UFRJ50816:Q>Q		UWOPS91_917_1:Q>Q		YPS138:Q>Q	AA:373		IFO1804:S>S		N_43:S>S		N_44:S>S		N_45:S>S	AA:397		A12:L>L		A4:L>L		DBVPG6304:L>L		UFRJ50816:L>L	AA:415		UWOPS91_917_1:I>I	AA:425		A12:A>A		A4:A>A		DBVPG6304:A>A		UFRJ50816:A>A		UWOPS91_917_1:A>A	AA:435		UWOPS91_917_1:V>V	AA:442		A12:->-		A4:->-		DBVPG6304:->-		UFRJ50816:->-	AA:452		N_44:F>F		N_45:F>F		UWOPS91_917_1:F>F	AA:472		A12:->-		A4:->-		DBVPG6304:->-		UFRJ50816:->-		UWOPS91_917_1:->-	AA:477		A12:Q>Q		A4:Q>Q		DBVPG6304:Q>Q		N_44:Q>Q		N_45:Q>Q		UFRJ50816:Q>Q	AA:482		N_17:I>I	AA:498		A12:N>N		A4:N>N		DBVPG6304:N>N		UFRJ50816:N>N	AA:521		A12:A>A		A4:A>A		DBVPG6304:A>A		UFRJ50791:A>A		UFRJ50816:A>A	AA:524		N_44:V>V	AA:534		A12:L>L		A4:L>L		DBVPG6304:L>L		UFRJ50791:L>L		UFRJ50816:L>L	AA:551		A4:V>V		DBVPG6304:V>V		UFRJ50791:V>V		UFRJ50816:V>V		UWOPS91_917_1:V>V	AA:564		A12:I>I		A4:I>I		DBVPG6304:I>I		UFRJ50791:I>I		UFRJ50816:I>I	AA:580		A4:N>N	AA:625		N_43:V>V		N_44:V>V	AA:652		A12:P>P		A4:P>P		DBVPG6304:P>P		UFRJ50791:P>P		UFRJ50816:P>P		UWOPS91_917_1:P>P		YPS138:P>P	AA:664		A12:I>I		A4:I>I		DBVPG6304:I>I		UFRJ50791:I>I		UFRJ50816:I>I		UWOPS91_917_1:I>I		YPS138:I>I	AA:683		A12:C>C		A4:C>C		DBVPG6304:C>C		UFRJ50791:C>C		UFRJ50816:C>C		UWOPS91_917_1:C>C		YPS138:C>C	AA:690		A12:P>P		A4:P>P		DBVPG6304:P>P		N_43:P>P		N_45:P>P		UFRJ50791:P>P		UFRJ50816:P>P		UWOPS91_917_1:P>P		YPS138:P>P	AA:697		YPS138:->-	AA:714		UFRJ50791:T>T		UFRJ50816:T>T	AA:733		A12:L>L		A4:L>L		DBVPG6304:L>L		N_43:L>L		N_45:L>L		UFRJ50791:L>L		UFRJ50816:L>L		UWOPS91_917_1:L>L		YPS138:L>L	AA:740		UWOPS91_917_1:F>F	AA:759		UWOPS91_917_1:V>V	AA:763		A12:L>L		A4:L>L		DBVPG6304:L>L		N_43:L>L		N_45:L>L		UFRJ50791:L>L		UFRJ50816:L>L		UWOPS91_917_1:L>L		YPS138:L>L	AA:767		A12:S>S		A4:S>S		DBVPG6304:S>S		UFRJ50791:S>S		UFRJ50816:S>S		UWOPS91_917_1:S>S		YPS138:S>S	AA:798		UWOPS91_917_1:L>L	AA:805		N_43:I>I		N_45:I>I	AA:809		A12:V>V		A4:V>V		DBVPG6304:V>V		UFRJ50791:V>V		UFRJ50816:V>V	AA:832		A12:V>V		A4:V>V		DBVPG6304:V>V		UFRJ50791:V>V		UFRJ50816:V>V		UWOPS91_917_1:V>V		YPS138:V>V	AA:835		A12:F>F		A4:F>F		DBVPG6304:F>F		UFRJ50791:F>F		UFRJ50816:F>F	AA:856		A12:F>F		A4:F>F		DBVPG6304:F>F		IFO1804:F>F		N_43:F>F		N_45:F>F		UFRJ50791:F>F		UFRJ50816:F>F		UWOPS91_917_1:F>F		YPS138:F>F	AA:879		CBS432:T>T		CBS5829:T>T		N_17:T>T		Q95_3:T>T		T21_4:T>T		Y7:T>T	AA:883		A12:T>T		A4:T>T		DBVPG6304:T>T		UFRJ50816:T>T		UWOPS91_917_1:T>T		YPS138:T>T	AA:897		A12:R>R		A4:R>R		DBVPG6304:R>R		UFRJ50816:R>R		YPS138:R>RID:YBR056W	AA:6		A12:R>R		DBVPG6304:R>R		UFRJ50791:R>R	AA:10		UWOPS91_917_1:E>E	AA:13		UWOPS91_917_1:K>K	AA:26		UWOPS91_917_1:L>L	AA:35		UWOPS91_917_1:L>L	AA:37		UFRJ50816:R>R		UWOPS91_917_1:R>R	AA:42		UWOPS91_917_1:K>K	AA:49		UFRJ50816:V>V		UWOPS91_917_1:V>V	AA:56		A12:V>V		DBVPG6304:V>V		UFRJ50791:V>V	AA:77		UFRJ50816:S>S		UWOPS91_917_1:S>S	AA:83		UWOPS91_917_1:S>S	AA:87		UFRJ50816:K>K	AA:89		A12:H>H		DBVPG6304:H>H		UFRJ50791:H>H	AA:95		UFRJ50816:A>A	AA:98		UFRJ50816:L>L		UWOPS91_917_1:L>L	AA:101		A12:H>H		DBVPG6304:H>H		UFRJ50791:H>H	AA:112		A12:E>E		DBVPG6304:E>E		UFRJ50791:E>E	AA:132		A12:V>V		DBVPG6304:V>V		UFRJ50791:V>V		UFRJ50816:V>V		UWOPS91_917_1:V>V	AA:136		A12:K>K		DBVPG6304:K>K		UFRJ50791:K>K		UFRJ50816:K>K	AA:142		A12:P>P		DBVPG6304:P>P		UFRJ50791:P>P	AA:144		A12:A>A		DBVPG6304:A>A		UFRJ50791:A>A	AA:146		UFRJ50816:L>L	AA:148		UFRJ50816:K>K	AA:155		A12:P>P		DBVPG6304:P>P		UFRJ50816:P>P	AA:179		UFRJ50816:G>G		UWOPS91_917_1:G>G	AA:183		A12:G>G		DBVPG6304:G>G	AA:184		UFRJ50816:A>A		UWOPS91_917_1:A>A	AA:186		UFRJ50816:C>C		UWOPS91_917_1:C>C	AA:188		UFRJ50816:S>S		UWOPS91_917_1:S>S	AA:192		UFRJ50816:S>S		UWOPS91_917_1:S>S	AA:205		UFRJ50816:Y>Y		UWOPS91_917_1:Y>Y	AA:207		UFRJ50816:T>T		UWOPS91_917_1:T>T	AA:216		A12:A>A		DBVPG6304:A>A	AA:230		UFRJ50816:G>G		UWOPS91_917_1:G>G	AA:243		A12:P>P		DBVPG6304:P>P		UFRJ50816:P>P		UWOPS91_917_1:P>P	AA:246		A12:Q>Q		CBS432:Q>Q		CBS5829:Q>Q		DBVPG6304:Q>Q		KPN3829:Q>Q		N_17:Q>Q		Q32_3:Q>Q		Q89_8:Q>Q		Q95_3:Q>Q		Y7:Q>Q	AA:252		A12:E>E		DBVPG6304:E>E		UWOPS91_917_1:E>E	AA:255		A12:N>N		DBVPG6304:N>N		UWOPS91_917_1:N>N	AA:262		UWOPS91_917_1:P>P	AA:271		UFRJ50816:G>G	AA:274		A12:P>P		DBVPG6304:P>P		UFRJ50816:P>P		UWOPS91_917_1:P>P	AA:278		N_43:A>A	AA:296		A12:V>V		DBVPG6304:V>V		UFRJ50791:V>V		UFRJ50816:V>V	AA:307		UFRJ50816:K>K	AA:310		DBVPG6304:N>N	AA:313		UFRJ50816:I>I	AA:316		N_43:L>L		N_44:L>L	AA:320		A12:V>V	AA:323		A12:P>P		CBS432:P>P		CBS5829:P>P		DBVPG6304:P>P		KPN3829:P>P		N_43:P>P		N_44:P>P		Q32_3:P>P		Q95_3:P>P		UFRJ50791:P>P	AA:327		N_43:A>A		N_44:A>A	AA:330		A12:T>T		DBVPG6304:T>T		UFRJ50791:T>T	AA:349		A12:D>D		DBVPG6304:D>D		N_43:D>D		UFRJ50791:D>D		UFRJ50816:D>D	AA:361		A12:Q>Q		DBVPG6304:Q>Q		UFRJ50791:Q>Q		UFRJ50816:Q>Q	AA:379		N_17:Q>Q	AA:386		A12:G>G		DBVPG6304:G>G		UFRJ50791:G>G		UFRJ50816:G>G		UWOPS91_917_1:G>G	AA:397		DBVPG6304:G>G		UFRJ50791:G>G		UFRJ50816:G>G		UWOPS91_917_1:G>G	AA:400		A12:P>P		DBVPG6304:P>P		UFRJ50791:P>P		UFRJ50816:P>P		UWOPS91_917_1:P>P	AA:417		DBVPG6304:S>S		UFRJ50791:S>S		UFRJ50816:S>S		UWOPS91_917_1:S>S	AA:427		DBVPG6304:N>N		UFRJ50791:N>N		UFRJ50816:N>N	AA:445		DBVPG6304:A>A		UFRJ50791:A>A		UFRJ50816:A>A		UWOPS91_917_1:A>A	AA:455		DBVPG6304:F>F		UFRJ50791:F>F		UFRJ50816:F>F		UWOPS91_917_1:F>F		YPS138:F>F	AA:467		DBVPG6304:K>K		YPS138:K>K	AA:490		UWOPS91_917_1:G>G	AA:495		DBVPG6304:L>L		UFRJ50791:L>L		UFRJ50816:L>L		UWOPS91_917_1:L>L		YPS138:L>LID:YBR057C	AA:16		N_43:G>G	AA:25		A4:->-		DBVPG6304:->-		UFRJ50816:->-	AA:27		A12:L>L		DBVPG6304:L>L		UFRJ50816:L>L		UWOPS91_917_1:L>L		YPS138:L>L	AA:41		A4:F>F		DBVPG6304:F>F		UFRJ50816:F>F		YPS138:F>F	AA:54		A4:V>V		DBVPG6304:V>V		IFO1804:V>V		N_43:V>V		N_45:V>V		UFRJ50816:V>V		UWOPS91_917_1:V>V	AA:90		A4:S>S		DBVPG6304:S>S		UFRJ50816:S>S		UWOPS91_917_1:S>S		YPS138:S>S	AA:101		UWOPS91_917_1:C>C	AA:106		A4:L>L		DBVPG6304:L>L		UWOPS91_917_1:L>L	AA:113		UFRJ50791:->-	AA:177		A4:F>F		DBVPG6304:F>F		IFO1804:F>F		N_43:F>F		N_44:F>F		N_45:F>F		UFRJ50791:F>F		UFRJ50816:F>F		UWOPS91_917_1:F>F	AA:198		N_44:C>C	AA:201		IFO1804:I>I		N_43:I>I		N_44:I>I		N_45:I>I	AA:270		UWOPS91_917_1:W>W	AA:275		A12:I>I		DBVPG6304:I>I		IFO1804:I>I		N_43:I>I		N_44:I>I		N_45:I>I		UFRJ50816:I>I		UWOPS91_917_1:I>I		YPS138:I>I	AA:297		A12:->-		DBVPG6304:->-		UFRJ50816:->-		YPS138:->-	AA:303		UWOPS91_917_1:T>T	AA:309		A12:I>I		DBVPG6304:I>I		UFRJ50816:I>I		YPS138:I>IID:YBR058C-A	AA:34		IFO1804:K>K		N_43:K>K		N_44:K>K		N_45:K>K	AA:62		IFO1804:T>T		N_43:T>T		N_44:T>T		N_45:T>T	AA:73		DBVPG6304:H>H		UFRJ50791:H>H		UFRJ50816:H>H		UWOPS91_917_1:H>H	AA:76		A4:T>T		DBVPG6304:T>T		UFRJ50791:T>T		UFRJ50816:T>TID:YBR060C	AA:14		CBS432:F>F		CBS5829:F>F		KPN3829:F>F	AA:28		UWOPS91_917_1:I>I	AA:57		A12:S>S		A4:S>S		CBS432:S>S		CBS5829:S>S		DBVPG4650:S>S		KPN3829:S>S		N_17:S>S		N_44:S>S		N_45:S>S		UFRJ50791:S>S		UFRJ50816:S>S		UWOPS91_917_1:S>S		Y6_5:S>S		YPS138:S>S	AA:95		UWOPS91_917_1:Q>Q	AA:97		N_44:I>I		N_45:I>I	AA:99		A12:F>F		A4:F>F		UFRJ50791:F>F		UFRJ50816:F>F		YPS138:F>F	AA:141		A12:R>R		A4:R>R		UFRJ50791:R>R		UFRJ50816:R>R		YPS138:R>R	AA:146		A12:D>D		A4:D>D		UFRJ50791:D>D		UFRJ50816:D>D		YPS138:D>D	AA:148		A12:V>V		A4:V>V		UFRJ50791:V>V		UFRJ50816:V>V		YPS138:V>V	AA:153		A12:L>L		A4:L>L		UFRJ50791:L>L		UFRJ50816:L>L		UWOPS91_917_1:L>L		YPS138:L>L	AA:164		A12:M>M		A4:M>M		N_43:M>M		N_44:M>M		N_45:M>M		UFRJ50791:M>M		UFRJ50816:M>M		YPS138:M>M	AA:187		A12:V>V		A4:V>V		N_43:V>V		N_44:V>V		N_45:V>V		UFRJ50791:V>V		UFRJ50816:V>V		UWOPS91_917_1:V>V		YPS138:V>V	AA:189		CBS432:S>S		CBS5829:S>S	AA:271		A12:L>L		A4:L>L		DBVPG6304:L>L		N_43:L>L		N_44:L>L		UFRJ50791:L>L		UWOPS91_917_1:L>L		YPS138:L>L	AA:285		A4:F>F		DBVPG6304:F>F		UFRJ50791:F>F		YPS138:F>F	AA:293		DBVPG6304:K>K		N_43:K>K		N_44:K>K		N_45:K>K		UFRJ50791:K>K		UWOPS91_917_1:K>K	AA:310		A4:E>E		DBVPG6304:E>E		UFRJ50791:E>E		UWOPS91_917_1:E>E		YPS138:E>E	AA:328		IFO1804:K>K	AA:332		UFRJ50791:S>S	AA:338		A4:I>I		DBVPG6304:I>I		UFRJ50791:I>I		UWOPS91_917_1:I>I		YPS138:I>I	AA:355		UWOPS91_917_1:R>R	AA:357		A4:H>H		DBVPG6304:H>H		YPS138:H>H	AA:361		IFO1804:V>V		N_43:V>V		N_45:V>V	AA:372		UFRJ50791:S>S		UWOPS91_917_1:S>S	AA:439		A4:I>I		DBVPG6304:I>I		UFRJ50791:I>I		YPS138:I>I	AA:518		A4:L>L		DBVPG6304:L>L		IFO1804:L>L		N_44:L>L		N_45:L>L		UWOPS91_917_1:L>L		YPS138:L>L	AA:538		A4:Y>Y		DBVPG6304:Y>Y		UFRJ50816:Y>Y		UWOPS91_917_1:Y>Y		YPS138:Y>Y	AA:548		A4:S>S		DBVPG6304:S>S		UFRJ50816:S>S		YPS138:S>S	AA:554		UWOPS91_917_1:G>G	AA:568		A4:V>V		DBVPG6304:V>V		UFRJ50816:V>V		UWOPS91_917_1:V>VID:YBR061C	AA:8		DBVPG6304:P>P	AA:14		DBVPG6304:Q>Q		IFO1804:Q>Q		N_43:Q>Q		N_44:Q>Q		N_45:Q>Q		UWOPS91_917_1:Q>Q	AA:45		IFO1804:R>R		N_44:R>R		N_45:R>R	AA:67		N_45:I>I	AA:73		A12:V>V		DBVPG6304:V>V		UFRJ50791:V>V		UWOPS91_917_1:V>V	AA:76		UWOPS91_917_1:T>T	AA:86		A12:K>K		DBVPG6304:K>K		UFRJ50791:K>K		UWOPS91_917_1:K>K	AA:88		DBVPG6304:I>I		IFO1804:I>I		N_44:I>I		N_45:I>I		UFRJ50791:I>I		UWOPS91_917_1:I>I	AA:95		A12:Q>Q		DBVPG6304:Q>Q		UFRJ50791:Q>Q		UWOPS91_917_1:Q>Q	AA:106		A12:A>A		DBVPG6304:A>A		UFRJ50791:A>A	AA:117		A12:T>T		DBVPG6304:T>T		UFRJ50791:T>T	AA:131		N_45:->-	AA:133		IFO1804:A>A		N_44:A>A		N_45:A>A	AA:190		A12:T>T		DBVPG6304:T>T		IFO1804:T>T		N_44:T>T		N_45:T>T		UFRJ50791:T>T		UFRJ50816:T>T		UWOPS91_917_1:T>T		YPS138:T>T	AA:208		A12:F>F		DBVPG6304:F>F		UFRJ50791:F>F		UFRJ50816:F>F		YPS138:F>F	AA:246		UWOPS91_917_1:K>K	AA:253		A12:L>L		DBVPG6304:L>L		UFRJ50791:L>L		UFRJ50816:L>L		UWOPS91_917_1:L>L		YPS138:L>L	AA:266		A12:S>S		DBVPG6304:S>S		UFRJ50816:S>S		YPS138:S>S	AA:280		UWOPS91_917_1:E>E	AA:288		A12:S>S		CBS432:S>S		CBS5829:S>S		DBVPG6304:S>S		KPN3829:S>S		N_17:S>S		N_43:S>S		N_44:S>S		N_45:S>S		UFRJ50791:S>S		UFRJ50816:S>S		UWOPS91_917_1:S>S		YPS138:S>SID:YBR065C	AA:14		A12:->-		A4:->-		DBVPG6304:->-		UFRJ50816:->-		UWOPS91_917_1:->-		YPS138:->-	AA:17		A12:L>L		DBVPG6304:L>L		UFRJ50816:L>L		UWOPS91_917_1:L>L		YPS138:L>L	AA:22		YPS138:->-	AA:31		UWOPS91_917_1:V>V	AA:35		A12:L>L		DBVPG6304:L>L		UFRJ50816:L>L		UWOPS91_917_1:L>L		YPS138:L>L	AA:41		A12:P>P		CBS432:P>P		CBS5829:P>P		DBVPG6304:P>P		KPN3828:P>P		KPN3829:P>P		N_17:P>P		N_43:P>P		N_45:P>P		Q89_8:P>P		Q95_3:P>P		UFRJ50816:P>P		UWOPS91_917_1:P>P		YPS138:P>P	AA:55		UWOPS91_917_1:F>F	AA:61		A12:V>V		DBVPG6304:V>V		UFRJ50816:V>V		UWOPS91_917_1:V>V		YPS138:V>V	AA:83		A12:D>D		DBVPG6304:D>D		UFRJ50816:D>D		UWOPS91_917_1:D>D		YPS138:D>D	AA:95		UWOPS91_917_1:G>G	AA:99		A12:T>T		DBVPG6304:T>T		N_43:T>T		N_44:T>T		N_45:T>T		UFRJ50816:T>T		UWOPS91_917_1:T>T		YPS138:T>T	AA:105		A12:P>P		DBVPG6304:P>P		UFRJ50816:P>P		YPS138:P>P	AA:114		UWOPS91_917_1:S>S	AA:169		N_43:->-		N_44:->-		N_45:->-	AA:197		A12:H>H		DBVPG6304:H>H		UFRJ50816:H>H		YPS138:H>H	AA:208		A12:V>V		UFRJ50816:V>V		YPS138:V>V	AA:241		A12:R>R		A4:R>R		IFO1804:R>R		N_43:R>R		N_44:R>R		N_45:R>R		UFRJ50791:R>R		UFRJ50816:R>R		YPS138:R>R	AA:247		IFO1804:Q>Q		N_43:Q>Q		N_44:Q>Q		N_45:Q>Q	AA:288		A12:P>P		A4:P>P		DBVPG6304:P>P		UFRJ50791:P>P		UFRJ50816:P>P		YPS138:P>P	AA:302		A12:->-		DBVPG6304:->-		IFO1804:G>G		N_44:G>G		N_45:G>G		UFRJ50816:->-		UWOPS91_917_1:->-		YPS138:->-	AA:316		A12:T>T		A4:S>S		DBVPG6304:T>T		UFRJ50791:S>S		UFRJ50816:T>T		YPS138:T>T	AA:324		A12:->-		A4:->-		IFO1804:->-		N_44:->-		N_45:->-		UFRJ50791:->-		UFRJ50816:->-		YPS138:->-	AA:333		A12:T>T		A4:T>T		DBVPG6304:T>T		UFRJ50791:T>T		UFRJ50816:T>T		YPS138:T>T	AA:338		A12:G>G		A4:G>G		DBVPG6304:G>G		UFRJ50791:G>G		UFRJ50816:G>G		YPS138:G>G	AA:340		A12:L>L		A4:L>L		DBVPG6304:L>L		UFRJ50791:L>L		UFRJ50816:L>L		YPS138:L>LID:YBR066C	AA:9		A12:P>P		A4:P>P		DBVPG6304:P>P		UFRJ50791:P>P		UFRJ50816:P>P	AA:11		A12:S>S		A4:S>S		DBVPG6304:S>S		UFRJ50791:S>S		UFRJ50816:S>S		UWOPS91_917_1:S>S	AA:33		A12:S>S		A4:S>S		UFRJ50791:S>S		UFRJ50816:S>S	AA:59		A12:G>G		A4:G>G		DBVPG6304:G>G		UFRJ50791:G>G		UFRJ50816:G>G		UWOPS91_917_1:G>G	AA:67		A12:T>T		A4:T>T		DBVPG6304:T>T		UFRJ50791:T>T		UFRJ50816:T>T		UWOPS91_917_1:T>T	AA:136		A12:T>T		A4:T>T		DBVPG6304:T>T		UFRJ50816:T>T		UWOPS91_917_1:T>T	AA:139		DBVPG4650:T>T	AA:146		A12:K>K		A4:K>K		DBVPG6304:K>K		UFRJ50816:K>K	AA:157		UFRJ50816:Q>Q	AA:160		A12:I>I		A4:I>I		CBS432:I>I		CBS5829:I>I		DBVPG6304:I>I		KPN3828:I>I		N_17:I>I		N_43:I>I		N_45:I>I		UFRJ50816:I>I		UWOPS91_917_1:I>I	AA:166		A12:F>F		A4:F>F		DBVPG6304:F>F		UFRJ50816:F>F	AA:173		A12:L>L		A4:L>L		DBVPG6304:L>L		UFRJ50816:L>L	AA:174		IFO1804:I>I		N_43:I>I		N_45:I>I		UWOPS91_917_1:I>I	AA:177		UWOPS91_917_1:F>F	AA:190		A12:L>L		A4:L>L		DBVPG6304:L>L		UFRJ50816:L>L		UWOPS91_917_1:L>LID:YBR067C	AA:11		A12:N>N		A4:N>N		DBVPG6304:N>N		UFRJ50791:N>N		UFRJ50816:N>N		UWOPS91_917_1:N>N	AA:16		UWOPS91_917_1:G>G	AA:18		IFO1804:A>A		N_44:A>A		N_45:A>A		UWOPS91_917_1:A>A	AA:44		IFO1804:R>R		N_44:R>R		N_45:R>R	AA:48		A12:G>G		A4:G>G		DBVPG6304:G>G		UFRJ50791:G>G		UFRJ50816:G>G	AA:55		DBVPG6304:S>S		UFRJ50816:S>S	AA:75		A12:G>G		A4:G>G		DBVPG6304:G>G		UFRJ50791:G>G	AA:79		A12:S>S		A4:S>S		DBVPG6304:S>S		UFRJ50791:S>S	AA:81		UFRJ50816:R>R		UWOPS91_917_1:R>R	AA:110		IFO1804:D>D		N_44:D>D		N_45:D>D		UFRJ50816:D>D		UWOPS91_917_1:D>D	AA:127		N_45:G>G	AA:129		UFRJ50791:G>G	AA:132		DBVPG4650:V>V		Q59_1:V>V		Q95_3:V>V		T21_4:V>V		Y7:V>V		Z1_1:V>V	AA:138		A12:G>G		UFRJ50791:G>G	AA:141		A4:S>S		DBVPG6304:S>S		IFO1804:S>S		N_44:S>S		UFRJ50816:S>S		UWOPS91_917_1:S>S	AA:144		Y7:G>G	AA:146		A12:S>S		DBVPG6304:S>S		UFRJ50791:S>S		UFRJ50816:S>S		UWOPS91_917_1:S>S	AA:148		UFRJ50816:D>D		UWOPS91_917_1:D>D	AA:152		A12:D>D		A4:D>D		UFRJ50791:D>D		UFRJ50816:G>G		UWOPS91_917_1:D>D	AA:162		A12:S>S		A4:S>S		DBVPG6304:S>S		UFRJ50791:S>S	AA:171		UFRJ50816:V>V		UWOPS91_917_1:V>V	AA:183		DBVPG6304:S>S		UFRJ50816:S>S		UWOPS91_917_1:S>SID:YBR068C	AA:2		A12:T>T		DBVPG6304:T>T		UFRJ50791:T>T	AA:17		UWOPS91_917_1:F>F	AA:43		Q62_5:V>V	AA:53		A12:P>P		A4:P>P		DBVPG6304:P>P		UFRJ50791:P>P		UFRJ50816:P>P		UWOPS91_917_1:P>P		YPS138:P>P	AA:68		A12:S>S		DBVPG6304:S>S		UFRJ50791:S>S		UWOPS91_917_1:S>S	AA:82		N_43:P>P		N_45:P>P	AA:89		N_45:C>C	AA:111		A12:A>A		UWOPS91_917_1:A>A	AA:123		CBS432:P>P		CBS5829:P>P		UWOPS91_917_1:P>P	AA:140		A12:G>G		DBVPG6304:G>G		UWOPS91_917_1:G>G	AA:155		A12:P>P		DBVPG6304:P>P		UFRJ50791:P>P		UWOPS91_917_1:P>P	AA:219		A12:N>N		DBVPG6304:N>N		UFRJ50791:N>N		UWOPS91_917_1:N>N	AA:262		A12:V>V		DBVPG6304:V>V		UFRJ50791:V>V		UFRJ50816:V>V		YPS138:V>V	AA:267		A12:P>P		DBVPG6304:P>P		UFRJ50791:P>P		UFRJ50816:P>P		YPS138:P>P	AA:280		A12:T>T		DBVPG6304:T>T		UFRJ50791:T>T		UFRJ50816:T>T		YPS138:T>T	AA:285		A12:L>L		DBVPG6304:L>L		UFRJ50791:L>L		UFRJ50816:L>L		YPS138:L>L	AA:312		A12:E>E		IFO1804:E>E		N_43:E>E		N_45:E>E		UFRJ50791:E>E		UFRJ50816:E>E		YPS138:E>E	AA:315		A12:S>S		DBVPG6304:S>S		UFRJ50791:S>S		UFRJ50816:S>S		YPS138:S>S	AA:356		UWOPS91_917_1:N>N	AA:360		UWOPS91_917_1:N>N	AA:363		A12:T>T		DBVPG6304:T>T		UFRJ50791:T>T		UFRJ50816:T>T		YPS138:T>T	AA:372		A12:I>I		DBVPG6304:I>I		UFRJ50791:I>I		UFRJ50816:I>I		UWOPS91_917_1:I>I		YPS138:I>I	AA:402		A12:R>R		A4:R>R		DBVPG6304:R>R		UFRJ50791:R>R		UFRJ50816:R>R		UWOPS91_917_1:R>R		YPS138:R>R	AA:431		UWOPS91_917_1:K>K	AA:442		UWOPS91_917_1:N>N	AA:445		A12:R>R		A4:R>R		DBVPG6304:R>R		IFO1804:R>R		N_43:R>R		N_44:R>R		N_45:R>R		UFRJ50791:R>R		UFRJ50816:R>R		UWOPS91_917_1:R>R		YPS138:R>R	AA:464		A12:S>S		A4:S>S		DBVPG6304:S>S		UFRJ50791:S>S		UFRJ50816:S>S		UWOPS91_917_1:S>S		YPS138:S>S	AA:474		A12:R>R		A4:R>R		DBVPG6304:R>R		UFRJ50791:R>R		UFRJ50816:R>R		YPS138:R>R	AA:478		UWOPS91_917_1:V>V	AA:491		A12:T>T		A4:T>T		DBVPG6304:T>T		UFRJ50791:T>T		UFRJ50816:T>T		YPS138:T>T	AA:514		UWOPS91_917_1:R>R	AA:519		UWOPS91_917_1:F>F	AA:521		UWOPS91_917_1:L>L	AA:524		A12:R>R		A4:R>R		DBVPG6304:R>R		UFRJ50791:R>R		UFRJ50816:R>R		YPS138:R>R	AA:530		A12:A>A		A4:A>A		CBS432:A>A		CBS5829:A>A		DBVPG4650:A>A		DBVPG6304:A>A		IFO1804:A>A		N_17:A>A		N_43:A>A		N_44:A>A		N_45:A>A		Q32_3:A>A		Q59_1:A>A		Q89_8:A>A		Q95_3:A>A		T21_4:A>A		UFRJ50791:A>A		UFRJ50816:A>A		UWOPS91_917_1:A>A		Y6_5:A>A		Y7:A>A		YPS138:A>A		Z1_1:A>A	AA:532		UWOPS91_917_1:L>L	AA:535		UWOPS91_917_1:R>R	AA:538		UWOPS91_917_1:F>F	AA:544		UWOPS91_917_1:F>F	AA:550		A12:C>C		A4:C>C		DBVPG6304:C>C		UFRJ50791:C>C		UFRJ50816:C>C		UWOPS91_917_1:C>C		YPS138:C>C	AA:552		UWOPS91_917_1:F>F	AA:565		A12:A>A		A4:A>A		DBVPG6304:A>A		IFO1804:A>A		N_44:A>A		N_45:A>A		YPS138:A>A	AA:569		A12:V>V		A4:V>V		DBVPG6304:V>V		UFRJ50791:V>V		UFRJ50816:V>V		YPS138:V>V	AA:576		A12:->-		A4:->-		DBVPG6304:->-		IFO1804:->-		N_43:->-		N_44:->-		N_45:->-		UFRJ50791:->-		UFRJ50816:->-		UWOPS91_917_1:->-		YPS138:->-	AA:581		DBVPG6304:V>V	AA:587		UWOPS91_917_1:G>G	AA:591		CBS432:Y>Y		CBS5829:Y>YID:YBR069C	AA:23		A12:T>T		A4:T>T		DBVPG6304:T>T		UFRJ50791:T>T		UFRJ50816:R>R		UWOPS91_917_1:T>T		YPS138:T>T	AA:44		A12:R>R		UFRJ50816:R>R		YPS138:R>R	AA:64		A12:K>K		A4:K>K		IFO1804:K>K		N_43:K>K		N_44:K>K		UFRJ50816:K>K		YPS138:K>K	AA:83		A12:H>H		A4:H>H		UFRJ50816:H>H		YPS138:H>H	AA:106		A12:Y>Y		A4:Y>Y		UFRJ50816:Y>Y		YPS138:Y>Y	AA:127		A12:A>A		A4:A>A		UFRJ50816:A>A		YPS138:A>A	AA:150		A12:N>N		A4:N>N		UFRJ50816:N>N		YPS138:N>N	AA:155		A12:H>H		A4:H>H		UFRJ50816:H>H		YPS138:H>H	AA:160		IFO1804:K>K		N_44:K>K		N_45:K>K	AA:198		A12:Q>Q		A4:Q>Q		IFO1804:Q>Q		N_43:Q>Q		N_44:Q>Q		N_45:Q>Q		UWOPS91_917_1:Q>Q		YPS138:Q>Q	AA:225		A12:S>S		A4:S>S		IFO1804:S>S		N_44:S>S		N_45:S>S		YPS138:S>S	AA:232		A12:S>S		A4:V>V		IFO1804:S>S		N_44:S>S		N_45:S>S		UFRJ50816:V>V		UWOPS91_917_1:V>V		YPS138:V>V	AA:256		UWOPS91_917_1:N>N	AA:262		A12:G>G		A4:G>G		UFRJ50816:G>G		UWOPS91_917_1:G>G		YPS138:G>G	AA:266		IFO1804:S>S		N_45:S>S	AA:269		A12:V>V		A4:V>V		UFRJ50816:V>V		YPS138:V>V	AA:271		UWOPS91_917_1:R>R	AA:286		A12:->-		A4:->-		IFO1804:->-		N_45:->-		UFRJ50816:->-		UWOPS91_917_1:->-		YPS138:->-	AA:297		A4:T>T		UFRJ50816:T>T		YPS138:T>T	AA:298		UWOPS91_917_1:V>V	AA:365		A4:T>T		UFRJ50816:T>T		YPS138:T>T	AA:385		UWOPS91_917_1:E>E	AA:407		A12:N>N	AA:410		A4:N>N		UFRJ50816:N>N		UWOPS91_917_1:N>N		YPS138:N>N	AA:428		A12:D>D		A4:D>D		UFRJ50816:D>D		YPS138:D>D	AA:434		CBS432:N>N		CBS5829:N>N		N_17:N>N		N_45:N>N	AA:442		A12:N>N		A4:N>N		UFRJ50816:N>N		UWOPS91_917_1:N>N		YPS138:N>N	AA:488		IFO1804:T>T		N_43:T>T		N_44:T>T		N_45:T>T		UWOPS91_917_1:T>T	AA:515		A12:G>G		A4:G>G		DBVPG6304:G>G		UFRJ50791:G>G		UWOPS91_917_1:G>G		YPS138:G>G	AA:524		A12:T>T		A4:T>T		DBVPG6304:T>T		UFRJ50791:T>T		YPS138:T>T	AA:529		A12:L>L		A4:L>L		DBVPG6304:L>L		UFRJ50791:L>L		YPS138:L>L	AA:532		DBVPG6304:V>V		IFO1804:V>V		N_43:V>V		N_44:V>V		N_45:V>V		UWOPS91_917_1:V>V	AA:548		A12:L>L		A4:L>L		DBVPG6304:L>L		UFRJ50791:L>L		YPS138:L>L	AA:550		UFRJ50816:F>F	AA:559		UWOPS91_917_1:F>F	AA:567		UFRJ50816:I>I	AA:574		A12:W>W		A4:W>W		DBVPG6304:W>W		UFRJ50791:W>W		YPS138:W>W	AA:592		A12:F>F		A4:F>F		DBVPG6304:F>F		IFO1804:F>F		N_43:F>F		N_44:F>F		N_45:F>F		UFRJ50791:F>F		UWOPS91_917_1:F>F		YPS138:F>F	AA:603		UFRJ50816:->-	AA:607		UWOPS91_917_1:->-	AA:610		YPS138:L>L	AA:612		UFRJ50816:N>NID:YBR070C	AA:10		N_43:S>S	AA:37		A12:T>T		A4:T>T		DBVPG6304:T>T		UFRJ50816:T>T		UWOPS91_917_1:T>T		YPS138:T>T	AA:51		A12:M>M		A4:M>M		DBVPG6304:M>M		UFRJ50816:M>M		YPS138:M>M	AA:52		CBS5829:G>G	AA:65		A12:->-		A4:->-		DBVPG6304:->-		UFRJ50816:->-		UWOPS91_917_1:->-		YPS138:->-	AA:86		UWOPS91_917_1:T>T	AA:91		UWOPS91_917_1:D>D	AA:106		N_17:G>G	AA:127		A12:H>H		A4:H>H		DBVPG6304:H>H		N_43:H>H		N_45:H>H		UFRJ50816:H>H	AA:136		A12:K>K		A4:K>K		DBVPG6304:K>K		UFRJ50816:K>K	AA:139		UWOPS91_917_1:E>E	AA:140		A12:S>S		A4:S>S		DBVPG6304:S>S		UFRJ50816:S>S	AA:143		A12:G>G		A4:G>G		DBVPG6304:G>G	AA:146		A12:I>I		A4:I>I		DBVPG6304:I>I	AA:151		UWOPS91_917_1:V>V	AA:155		UWOPS91_917_1:L>L	AA:164		UWOPS91_917_1:F>F	AA:177		UWOPS91_917_1:S>S	AA:180		N_43:Y>Y		N_45:Y>Y	AA:183		A12:E>E		A4:E>E		DBVPG6304:E>E		UFRJ50816:E>E		UWOPS91_917_1:E>E		YPS138:E>E	AA:198		UWOPS91_917_1:L>L	AA:208		UWOPS91_917_1:L>L	AA:209		A12:G>G		A4:G>G		DBVPG6304:G>G	AA:228		UWOPS91_917_1:E>E	AA:234		UWOPS91_917_1:V>VID:YBR071W	AA:8		UWOPS91_917_1:S>S	AA:32		A12:S>S		A4:S>S		DBVPG6304:S>S		UFRJ50816:S>S	AA:38		IFO1804:G>G		N_43:G>G		N_44:G>G		N_45:G>G		UWOPS91_917_1:G>G	AA:57		A12:S>S		A4:S>S		DBVPG6304:S>S	AA:61		A12:K>K		A4:K>K		DBVPG6304:K>K	AA:71		IFO1804:K>K		N_43:K>K		N_44:K>K		N_45:K>K	AA:77		A12:L>L		A4:L>L		DBVPG6304:L>L	AA:85		A12:Y>Y		A4:Y>Y		DBVPG6304:Y>Y	AA:95		A12:N>N		A4:N>N		DBVPG6304:N>N		UFRJ50816:N>N		UWOPS91_917_1:N>N	AA:98		A12:K>K		A4:K>K		DBVPG6304:K>K		UFRJ50816:K>K		UWOPS91_917_1:K>K	AA:101		A12:K>K		A4:K>K		DBVPG6304:K>K		UFRJ50816:K>K	AA:103		IFO1804:R>R		N_43:R>R		N_44:R>R		N_45:R>R	AA:107		A12:S>S		A4:S>S		DBVPG6304:S>S		UFRJ50816:S>S		UWOPS91_917_1:S>S	AA:115		A12:E>E		DBVPG6304:E>E		UFRJ50816:E>E		UWOPS91_917_1:E>E	AA:144		A12:Y>Y		DBVPG6304:Y>Y		UFRJ50816:Y>Y		YPS138:Y>Y	AA:156		IFO1804:A>A		N_43:A>A		N_45:A>A	AA:169		IFO1804:K>K		N_43:K>K		N_45:K>K	AA:205		UWOPS91_917_1:T>TID:YBR072W	AA:20		UWOPS91_917_1:A>A	AA:29		UWOPS91_917_1:G>G	AA:30		UWOPS91_917_1:L>L	AA:33		UWOPS91_917_1:Y>Y	AA:44		A4:A>A		YPS138:A>A	AA:49		UWOPS91_917_1:G>G	AA:54		IFO1804:R>R		N_45:R>R	AA:57		UWOPS91_917_1:N>N	AA:63		A12:Y>Y		A4:Y>Y		UWOPS91_917_1:Y>Y		YPS138:Y>Y	AA:76		UWOPS91_917_1:N>N	AA:84		UWOPS91_917_1:G>G	AA:88		A12:S>S		A4:S>S		YPS138:S>S	AA:96		UWOPS91_917_1:D>D	AA:122		UWOPS91_917_1:E>E	AA:123		A12:Y>Y		A4:Y>Y		UWOPS91_917_1:Y>Y		YPS138:Y>Y	AA:127		IFO1804:K>K		N_43:K>K		N_45:K>K	AA:136		A12:I>I		A4:I>I		YPS138:I>I	AA:147		A12:K>K		A4:K>K		UWOPS91_917_1:K>K		YPS138:K>K	AA:155		A12:S>S		A4:S>S		IFO1804:S>S		N_43:S>S		N_45:S>S		UWOPS91_917_1:S>S		Y6_5:S>S		YPS138:S>S	AA:161		UWOPS91_917_1:V>V	AA:167		A12:Y>Y		A4:Y>Y		IFO1804:Y>Y		N_43:Y>Y		N_45:Y>Y		UWOPS91_917_1:Y>Y		YPS138:Y>Y	AA:170		A12:V>V		A4:V>V		YPS138:V>V	AA:175		UWOPS91_917_1:I>I	AA:186		A12:L>L		A4:L>L		YPS138:L>L	AA:188		A12:V>V		A4:V>V		IFO1804:V>V		N_43:V>V		N_45:V>V		UWOPS91_917_1:V>V		YPS138:V>V	AA:203		A12:K>K		A4:K>K		YPS138:K>KID:YBR073W	AA:12		UWOPS91_917_1:R>R	AA:95		A4:A>A	AA:119		A4:L>L		UFRJ50816:L>L		UWOPS91_917_1:L>L		YPS138:L>L	AA:139		UWOPS91_917_1:Q>Q	AA:152		A4:S>S		DBVPG6304:S>S		UFRJ50816:S>S		YPS138:S>S	AA:157		A4:L>L		DBVPG6304:L>L		UFRJ50816:L>L		UWOPS91_917_1:L>L		YPS138:L>L	AA:160		A4:A>A		DBVPG6304:A>A		UFRJ50816:A>A	AA:193		UWOPS91_917_1:F>F	AA:201		A4:F>F		DBVPG6304:F>F		UFRJ50816:F>F	AA:248		N_43:A>A		N_44:A>A		N_45:A>A	AA:263		DBVPG6304:R>R		UFRJ50816:R>R		UWOPS91_917_1:R>R	AA:287		UWOPS91_917_1:N>N	AA:299		DBVPG6304:L>L		IFO1804:L>L		N_43:L>L		N_44:L>L		N_45:L>L		UFRJ50816:L>L		UWOPS91_917_1:L>L	AA:302		DBVPG6304:D>D		UFRJ50816:D>D		UWOPS91_917_1:D>D	AA:306		UWOPS91_917_1:S>S	AA:310		CBS432:L>L		DBVPG6304:L>L		IFO1804:L>L		N_43:L>L		N_44:L>L		N_45:L>L		Q32_3:L>L		Q62_5:L>L		Q89_8:L>L		Q95_3:L>L		T21_4:L>L		UFRJ50816:L>L		UWOPS91_917_1:L>L		Z1_1:L>L	AA:313		Q59_1:D>D		Q62_5:D>D		Q89_8:D>D		Q95_3:D>D		T21_4:D>D		Z1_1:D>D	AA:319		DBVPG6304:T>T		UFRJ50816:T>T		UWOPS91_917_1:T>T	AA:325		DBVPG6304:L>L		UFRJ50816:L>L		UWOPS91_917_1:L>L	AA:329		UWOPS91_917_1:L>L	AA:345		DBVPG6304:G>G		UFRJ50816:G>G		UWOPS91_917_1:G>G	AA:349		DBVPG6304:S>S		IFO1804:S>S		N_43:S>S		N_44:S>S		UFRJ50816:S>S		UWOPS91_917_1:S>S	AA:352		DBVPG6304:C>C		UFRJ50816:C>C		UWOPS91_917_1:C>C	AA:377		DBVPG6304:L>L		UFRJ50816:L>L		UWOPS91_917_1:L>L	AA:399		DBVPG6304:R>R		UFRJ50816:R>R		UWOPS91_917_1:R>R	AA:408		DBVPG6304:Y>Y		UFRJ50816:Y>Y		UWOPS91_917_1:Y>Y	AA:425		DBVPG6304:L>L		UFRJ50816:L>L		UWOPS91_917_1:L>L	AA:436		IFO1804:V>V		N_43:V>V		N_44:V>V		N_45:V>V	AA:461		DBVPG6304:K>K		UFRJ50816:K>K	AA:464		DBVPG6304:L>L		UFRJ50816:L>L		UWOPS91_917_1:L>L	AA:478		DBVPG6304:F>F		UFRJ50816:F>F	AA:491		DBVPG6304:S>S		UFRJ50816:S>S	AA:544		DBVPG6304:L>L		UFRJ50816:L>L	AA:552		DBVPG6304:T>T		UFRJ50816:T>T	AA:557		DBVPG6304:F>F		UFRJ50816:F>F	AA:583		DBVPG6304:T>T	AA:585		DBVPG6304:S>S	AA:596		DBVPG6304:K>K		UFRJ50816:K>K	AA:616		IFO1804:D>D		N_44:D>D		N_45:D>D	AA:621		DBVPG6304:G>G		UFRJ50816:G>G		YPS138:G>G	AA:626		DBVPG6304:S>S		UFRJ50816:S>S		YPS138:S>S	AA:635		Q62_5:L>L		Q89_8:L>L		Q95_3:L>L		T21_4:L>L	AA:639		DBVPG6304:L>L		UFRJ50816:L>L		YPS138:L>L	AA:648		DBVPG6304:E>E		UFRJ50816:E>E		YPS138:E>E	AA:657		DBVPG6304:T>T		UFRJ50816:T>T		YPS138:T>T	AA:660		DBVPG6304:L>L		UFRJ50816:L>L		YPS138:L>L	AA:664		DBVPG6304:E>E		UFRJ50816:E>E		YPS138:E>E	AA:679		IFO1804:G>G		N_44:G>G		N_45:G>G	AA:688		DBVPG6304:S>S		UFRJ50816:S>S		YPS138:S>S	AA:691		DBVPG6304:T>T		UFRJ50816:T>T		YPS138:T>T	AA:697		DBVPG6304:P>P		UFRJ50816:P>P		YPS138:P>P	AA:731		DBVPG6304:S>S		UFRJ50816:S>S		YPS138:S>S	AA:739		YPS138:R>R	AA:752		A12:Y>Y	AA:779		YPS138:L>L	AA:788		A12:G>G		IFO1804:G>G		N_44:G>G		N_45:G>G		YPS138:G>G	AA:801		A12:K>K		DBVPG6304:K>K		YPS138:K>K	AA:830		N_44:Y>Y		N_45:Y>Y	AA:839		N_44:A>A		N_45:A>A	AA:863		N_43:A>A		N_44:A>A		N_45:A>A	AA:878		A12:C>C		DBVPG6304:C>C		YPS138:C>C	AA:901		A12:S>S		DBVPG6304:S>S	AA:912		A12:A>A		DBVPG6304:A>AID:YBR077C	AA:30		A12:L>L		DBVPG6304:L>L		UWOPS91_917_1:L>L	AA:79		N_43:L>L		N_44:L>L		N_45:L>L		UWOPS91_917_1:I>I	AA:100		UWOPS91_917_1:V>V	AA:103		N_43:V>V		N_44:V>V		N_45:V>V	AA:110		A12:I>I		DBVPG6304:I>I		UFRJ50816:I>I	AA:125		A12:G>G		DBVPG6304:G>G		UFRJ50816:G>G		UWOPS91_917_1:G>G		YPS138:G>G	AA:145		A12:R>R		DBVPG6304:R>R		UFRJ50816:R>R		UWOPS91_917_1:R>R		YPS138:R>R	AA:153		N_43:R>R		N_44:R>R		N_45:R>RID:YBR082C	AA:42		UWOPS91_917_1:A>A	AA:49		A12:K>K		A4:K>K		DBVPG6304:K>K		UFRJ50816:K>K		UWOPS91_917_1:R>R		YPS138:K>K	AA:58		UWOPS91_917_1:I>I	AA:79		A12:C>C		A4:C>C		DBVPG6304:C>C		UFRJ50816:C>C		YPS138:C>C	AA:99		A12:E>E		A4:E>E		DBVPG6304:E>E		UFRJ50816:E>E		YPS138:E>EID:YBR084W	AA:5		A12:L>L		UFRJ50816:L>L		UWOPS91_917_1:L>L		YPS138:L>L	AA:11		N_43:S>S		N_44:S>S		N_45:S>S	AA:24		UFRJ50816:L>L		YPS138:L>L	AA:44		N_44:A>A		N_45:A>A		UWOPS91_917_1:A>A	AA:49		A12:E>E		UFRJ50816:E>E	AA:52		A12:N>N		UFRJ50816:N>N	AA:58		UFRJ50816:I>I	AA:69		A12:L>L		UFRJ50816:L>L	AA:102		CBS432:K>K		DBVPG4650:K>K		KPN3828:K>K		KPN3829:K>K		N_17:K>K		N_44:K>K		Q32_3:K>K		UWOPS91_917_1:K>K	AA:107		A12:I>I		UFRJ50816:I>I		UWOPS91_917_1:I>I	AA:112		A12:L>L		UFRJ50816:L>L		UWOPS91_917_1:L>L	AA:124		A12:S>S		UFRJ50816:S>S	AA:144		A12:T>T		UFRJ50816:T>T	AA:173		A12:F>F		UFRJ50816:F>F		UWOPS91_917_1:F>F	AA:194		A12:G>G		UFRJ50816:G>G		UWOPS91_917_1:G>G		YPS138:G>G	AA:197		A12:A>A		UFRJ50816:A>A		UWOPS91_917_1:A>A		YPS138:A>A	AA:200		A12:L>L		UFRJ50816:L>L		UWOPS91_917_1:L>L		YPS138:L>L	AA:206		UWOPS91_917_1:V>V	AA:277		N_45:S>S	AA:290		UWOPS91_917_1:V>V	AA:298		A12:T>T		A4:T>T		UFRJ50816:T>T		YPS138:T>T	AA:301		N_45:P>P	AA:304		A12:V>V		A4:V>V		DBVPG6304:V>V		UFRJ50816:V>V		YPS138:V>V	AA:314		A12:S>S		A4:S>S		DBVPG6304:S>S		UFRJ50816:S>S		YPS138:S>S	AA:336		CBS5829:L>L	AA:338		A12:L>L		A4:L>L		DBVPG6304:L>L		N_45:L>L		UFRJ50816:L>L		YPS138:L>L	AA:377		A12:G>G		A4:G>G		DBVPG6304:G>G		UFRJ50816:G>G		YPS138:G>G	AA:386		A12:K>K		A4:K>K		DBVPG6304:K>K		UFRJ50816:K>K		YPS138:K>K	AA:402		N_43:V>V	AA:403		A12:A>A		A4:A>A		DBVPG6304:A>A		UFRJ50816:A>A		UWOPS91_917_1:A>A		YPS138:A>A	AA:411		DBVPG6304:G>G	AA:415		A12:S>S		A4:S>S		DBVPG6304:S>S		UFRJ50816:S>S		UWOPS91_917_1:S>S		YPS138:S>S	AA:421		A12:L>L		A4:L>L		DBVPG6304:L>L		UFRJ50816:L>L		UWOPS91_917_1:L>L		YPS138:L>L	AA:442		A12:L>L		DBVPG6304:L>L	AA:465		A12:E>E		A4:E>E		DBVPG6304:E>E		UFRJ50816:E>E		UWOPS91_917_1:E>E		YPS138:E>E	AA:470		IFO1804:L>L	AA:474		UWOPS91_917_1:I>I	AA:477		A12:I>I		A4:I>I		DBVPG6304:I>I		UFRJ50816:I>I		UWOPS91_917_1:I>I		YPS138:I>I	AA:480		A12:A>A		A4:A>A		DBVPG6304:A>A		UWOPS91_917_1:A>A		YPS138:A>A	AA:485		A4:A>A		YPS138:A>A	AA:496		UWOPS91_917_1:A>A	AA:509		A12:V>V		DBVPG6304:V>V		IFO1804:V>V		N_43:V>V		N_44:V>V		UWOPS91_917_1:V>V		YPS138:V>V	AA:541		A12:P>P		DBVPG6304:P>P		UFRJ50816:P>P		UWOPS91_917_1:P>P		YPS138:P>P	AA:575		A12:I>I		IFO1804:I>I		N_43:I>I		N_44:I>I		N_45:I>I		UFRJ50816:I>I		UWOPS91_917_1:I>I		YPS138:I>I	AA:578		A12:A>A		UFRJ50816:A>A		UWOPS91_917_1:A>A		YPS138:A>A	AA:584		A12:F>F		UFRJ50816:F>F		YPS138:F>F	AA:589		IFO1804:G>G		N_43:G>G		N_44:G>G		N_45:G>G	AA:595		A12:A>A		UFRJ50816:A>A		YPS138:A>A	AA:602		A12:L>L		UFRJ50816:L>L		YPS138:L>L	AA:615		IFO1804:I>I		N_43:I>I		N_44:I>I		N_45:I>I	AA:635		A12:G>G		UFRJ50816:G>G		UWOPS91_917_1:G>G		YPS138:G>G	AA:641		A12:A>A		UFRJ50816:A>A		YPS138:A>A	AA:650		A12:P>P		UFRJ50816:P>P		YPS138:P>P	AA:657		IFO1804:E>E		N_43:E>E		N_45:E>E	AA:669		UWOPS91_917_1:A>A	AA:685		A12:L>L		UFRJ50816:L>L		UWOPS91_917_1:L>L		YPS138:L>L	AA:705		A12:Y>Y		UFRJ50816:Y>Y		UWOPS91_917_1:Y>Y		YPS138:Y>Y	AA:709		N_43:E>E		N_45:E>E	AA:711		CBS432:G>G	AA:714		UWOPS91_917_1:F>F	AA:736		A12:V>V		UWOPS91_917_1:V>V		YPS138:V>V	AA:739		A12:V>V		UWOPS91_917_1:V>V		YPS138:V>V	AA:741		UWOPS91_917_1:T>T	AA:748		A12:H>H		UWOPS91_917_1:H>H		YPS138:H>H	AA:756		A12:P>P		UWOPS91_917_1:P>P		YPS138:P>P	AA:785		A12:N>N		UWOPS91_917_1:N>N	AA:798		A12:N>N		UWOPS91_917_1:N>N		YPS138:N>N	AA:806		N_45:A>A	AA:819		N_43:G>G	AA:826		A12:S>S		YPS138:S>S	AA:836		A12:A>A		DBVPG6304:A>A		UWOPS91_917_1:A>A		YPS138:A>A	AA:840		A12:A>A		DBVPG6304:A>A		UWOPS91_917_1:A>A		YPS138:A>A	AA:844		A12:V>V		DBVPG6304:V>V		UWOPS91_917_1:V>V		YPS138:V>V	AA:852		N_43:S>S		N_45:S>S	AA:855		A12:F>F		YPS138:F>F	AA:858		YPS138:D>D	AA:892		A12:T>T		DBVPG6304:T>T		YPS138:T>T	AA:894		A12:R>R		DBVPG6304:R>R		YPS138:R>R	AA:905		YPS138:I>I	AA:906		UWOPS91_917_1:A>A	AA:914		UWOPS91_917_1:H>H	AA:920		UWOPS91_917_1:G>G	AA:926		A12:T>T		DBVPG6304:T>T		YPS138:T>T	AA:933		A12:R>R		DBVPG6304:R>R		N_45:R>R		UWOPS91_917_1:R>R		YPS138:R>R	AA:935		A12:S>S		DBVPG6304:S>S		UWOPS91_917_1:S>S		YPS138:S>S	AA:937		A12:G>G		DBVPG6304:G>G		UWOPS91_917_1:G>G		YPS138:G>G	AA:968		UWOPS91_917_1:D>D	AA:973		UWOPS91_917_1:G>GID:YBR085C-A	AA:18		CBS5829:I>I	AA:35		A12:A>A		A4:A>A		DBVPG6304:A>A		UFRJ50816:A>A		UWOPS91_917_1:A>A	AA:61		UWOPS91_917_1:F>FID:YBR085W	AA:30		A4:A>A		UFRJ50816:A>A		UWOPS91_917_1:A>A		YPS138:A>A	AA:74		UWOPS91_917_1:S>S	AA:81		A4:A>A		UFRJ50816:A>A		UWOPS91_917_1:A>A		YPS138:A>A	AA:90		A4:Q>Q		UWOPS91_917_1:Q>Q		YPS138:Q>Q	AA:106		A4:F>F		UFRJ50816:F>F		UWOPS91_917_1:F>F		YPS138:F>F	AA:113		A12:G>G		A4:G>G		IFO1804:G>G		N_43:G>G		N_45:G>G		UFRJ50816:G>G		UWOPS91_917_1:G>G		YPS138:G>G	AA:121		IFO1804:A>A		N_43:A>A		N_45:A>A	AA:128		A4:A>A		UFRJ50816:A>A		UWOPS91_917_1:A>A		YPS138:A>A	AA:141		A12:R>R		A4:R>R		UWOPS91_917_1:R>R		YPS138:R>R	AA:144		IFO1804:L>L		N_43:L>L		N_45:L>L	AA:145		A12:A>A		A4:A>A		UWOPS91_917_1:A>A		YPS138:A>A	AA:187		A12:V>V		A4:V>V		YPS138:V>V	AA:189		A12:I>I		A4:I>I		UWOPS91_917_1:I>I		YPS138:I>I	AA:211		A12:S>S		UWOPS91_917_1:S>S		YPS138:S>S	AA:217		IFO1804:L>L		N_43:L>L		N_45:L>L	AA:228		A12:T>T		A4:T>T		IFO1804:T>T		KPN3829:T>T		N_43:T>T		N_45:T>T		UWOPS91_917_1:T>T		YPS138:T>T	AA:252		A12:V>V		A4:V>V		YPS138:V>V	AA:277		A12:C>C		A4:C>C		YPS138:C>C	AA:286		A12:A>A		A4:A>A		YPS138:A>AID:YBR087W	AA:5		N_43:V>V		N_44:V>V		N_45:V>V	AA:21		UWOPS91_917_1:G>G	AA:23		A4:T>T		DBVPG6304:T>T		UWOPS91_917_1:T>T	AA:63		A4:S>S		DBVPG6304:S>S	AA:69		A4:K>K	AA:72		A4:V>V		DBVPG6304:V>V		UWOPS91_917_1:V>V	AA:74		A4:Q>Q		DBVPG6304:Q>Q		UWOPS91_917_1:Q>Q	AA:78		UWOPS91_917_1:A>A	AA:83		UWOPS91_917_1:L>L	AA:91		A4:P>P		CBS432:P>P		DBVPG6304:P>P		KPN3828:P>P		KPN3829:P>P		N_43:P>P		N_44:P>P		N_45:P>P		UWOPS91_917_1:P>P	AA:93		A4:H>H		DBVPG6304:H>H		N_43:H>H		N_44:H>H		UWOPS91_917_1:H>H	AA:108		A4:V>V		DBVPG6304:V>V	AA:112		N_43:L>L		N_44:L>L		N_45:L>L	AA:116		A4:V>V		DBVPG6304:V>V		UWOPS91_917_1:V>V	AA:122		A4:V>V		DBVPG6304:V>V		UWOPS91_917_1:V>V	AA:129		A12:D>D	AA:137		A12:C>C		A4:C>C		DBVPG6304:C>C	AA:165		A12:I>I		A4:I>I		DBVPG6304:I>I		UWOPS91_917_1:I>I		YPS138:I>I	AA:173		A12:S>S		A4:S>S		DBVPG6304:S>S		UFRJ50816:S>S		UWOPS91_917_1:S>S		YPS138:S>S	AA:175		UWOPS91_917_1:S>S	AA:186		A12:L>L		A4:L>L		DBVPG6304:L>L		UFRJ50816:L>L		UWOPS91_917_1:L>L		YPS138:L>L	AA:205		A12:V>V		A4:V>V		DBVPG6304:V>V		UFRJ50791:V>V		UFRJ50816:V>V		YPS138:V>V	AA:207		A12:T>T		A4:T>T		UFRJ50791:T>T		UFRJ50816:T>T		UWOPS91_917_1:T>T		YPS138:T>T	AA:219		A12:L>L		A4:L>L		UFRJ50791:L>L		UFRJ50816:L>L		UWOPS91_917_1:L>L		YPS138:L>L	AA:226		A12:S>S		A4:S>S		UFRJ50791:S>S		UFRJ50816:S>S		UWOPS91_917_1:S>S		YPS138:S>S	AA:228		A12:G>G		A4:G>G		UFRJ50791:G>G		UFRJ50816:G>G		YPS138:G>G	AA:230		N_43:L>L		N_44:L>L		N_45:L>L	AA:231		UWOPS91_917_1:R>R	AA:242		YPS138:L>L	AA:246		A12:L>L		A4:L>L		UFRJ50791:L>L		UFRJ50816:L>L		UWOPS91_917_1:L>L		YPS138:L>L	AA:256		A12:K>K		A4:K>K		UFRJ50791:K>K		UFRJ50816:K>K		YPS138:K>K	AA:271		A12:V>V		A4:V>V		N_43:V>V		N_44:V>V		N_45:V>V		UFRJ50791:V>V		UFRJ50816:V>V		UWOPS91_917_1:V>V		YPS138:V>V	AA:274		UWOPS91_917_1:R>R	AA:281		UWOPS91_917_1:E>E	AA:286		A12:L>L		A4:L>L		UFRJ50791:L>L		UFRJ50816:L>L		UWOPS91_917_1:L>L		YPS138:L>L	AA:299		A4:I>I		N_43:I>I		N_44:I>I		N_45:I>I		UFRJ50816:I>I		UWOPS91_917_1:I>I		YPS138:I>IID:YBR088C	AA:46		A12:I>I		DBVPG6304:I>I		UFRJ50791:I>I	AA:90		A12:Y>Y		DBVPG6304:Y>Y		UFRJ50791:Y>Y		UFRJ50816:Y>Y		UWOPS91_917_1:Y>Y	AA:110		UWOPS91_917_1:I>I	AA:112		A12:N>N		DBVPG6304:N>N		UFRJ50791:N>N		UFRJ50816:N>N		UWOPS91_917_1:N>N		YPS138:N>N	AA:126		A12:V>V		A4:V>V		DBVPG6304:V>V		UFRJ50791:V>V		UFRJ50816:V>V		YPS138:V>V	AA:166		A4:V>V		DBVPG6304:V>V		UFRJ50791:V>V		UFRJ50816:V>V		UWOPS91_917_1:V>V		YPS138:V>V	AA:200		A4:I>I		DBVPG6304:I>I		UFRJ50816:I>I		UWOPS91_917_1:I>I		YPS138:I>I	AA:225		N_44:D>D		N_45:D>D	AA:227		A4:V>V		DBVPG6304:V>V		UFRJ50816:V>V		YPS138:V>V	AA:229		UWOPS91_917_1:F>F	AA:236		A4:L>L		UFRJ50816:L>L		UWOPS91_917_1:L>L		YPS138:L>L	AA:243		A4:I>I		DBVPG6304:I>I		UFRJ50816:I>I		YPS138:I>I	AA:245		UWOPS91_917_1:D>DID:YBR089C-A	AA:14		N_43:V>V		N_44:V>V		N_45:V>V	AA:23		A12:G>G		A4:G>G		DBVPG6304:G>G		UWOPS91_917_1:G>G	AA:34		UWOPS91_917_1:S>S	AA:41		A12:Y>Y		A4:Y>Y		DBVPG6304:Y>Y		N_43:Y>Y		N_44:Y>Y		N_45:Y>Y		UWOPS91_917_1:Y>Y	AA:54		A12:G>G		A4:G>G		DBVPG6304:G>G		N_43:G>G		N_44:G>G		N_45:G>G		UWOPS91_917_1:G>G	AA:76		A12:V>V		A4:V>V		DBVPG6304:V>V	AA:82		N_43:P>P		N_44:P>P		N_45:P>P		UWOPS91_917_1:P>P	AA:99		UWOPS91_917_1:G>GID:YBR091C	AA:2		UWOPS91_917_1:V>V	AA:14		UWOPS91_917_1:S>S	AA:19		A4:->-		UFRJ50816:->-		UWOPS91_917_1:->-	AA:46		A4:S>S		UFRJ50816:S>S		UWOPS91_917_1:S>S		YPS138:S>S	AA:59		A4:L>L		UWOPS91_917_1:L>L		YPS138:L>L	AA:75		A4:N>N		UFRJ50816:N>N		UWOPS91_917_1:N>N		YPS138:N>N	AA:78		A4:E>E		UFRJ50816:E>E		YPS138:E>E	AA:101		UWOPS91_917_1:A>AID:YBR092C	AA:2		A4:C>C		DBVPG6304:C>C		UFRJ50816:C>C	AA:5		UWOPS91_917_1:G>G	AA:8		A4:V>V		DBVPG6304:V>V		UWOPS91_917_1:V>V	AA:18		A4:T>T		DBVPG6304:T>T		UWOPS91_917_1:T>T	AA:24		A4:T>T		DBVPG6304:T>T		UFRJ50816:T>T		UWOPS91_917_1:T>T	AA:41		A4:E>E		DBVPG6304:E>E		UFRJ50816:E>E		UWOPS91_917_1:E>E	AA:52		A4:S>S		DBVPG6304:S>S		UFRJ50816:S>S		UWOPS91_917_1:S>S	AA:59		A4:N>N		DBVPG6304:N>N		UFRJ50816:N>N		UWOPS91_917_1:N>N	AA:62		N_43:I>I	AA:97		A4:H>H		DBVPG6304:H>H		UFRJ50816:H>H	AA:108		A4:L>L		DBVPG6304:L>L		UFRJ50816:L>L		UWOPS91_917_1:L>L	AA:130		A4:P>P		DBVPG6304:P>P		IFO1804:P>P		KPN3828:P>P		KPN3829:P>P		N_43:P>P		N_44:P>P		N_45:P>P		UFRJ50816:P>P		UWOPS91_917_1:P>P	AA:165		A4:V>V		DBVPG6304:V>V		UWOPS91_917_1:V>V	AA:171		A4:L>L		DBVPG6304:L>L		UFRJ50816:L>L	AA:176		DBVPG6304:T>T	AA:180		DBVPG6304:V>V	AA:185		UWOPS91_917_1:I>I	AA:194		DBVPG6304:V>V		UFRJ50816:V>V		UWOPS91_917_1:V>V	AA:198		DBVPG6304:C>C		UFRJ50816:C>C		UWOPS91_917_1:C>C	AA:204		UWOPS91_917_1:S>S	AA:208		UWOPS91_917_1:R>R	AA:234		UWOPS91_917_1:V>V	AA:237		KPN3828:A>A		N_43:A>A		N_44:A>A		N_45:A>A	AA:244		DBVPG6304:P>P		YPS138:P>P	AA:263		DBVPG6304:T>T		UWOPS91_917_1:T>T		YPS138:T>T	AA:275		UWOPS91_917_1:Q>Q	AA:334		N_43:C>C		N_44:C>C	AA:337		UWOPS91_917_1:H>H	AA:364		N_43:V>V	AA:370		A4:P>P		YPS138:P>P	AA:382		UWOPS91_917_1:G>G	AA:399		A4:T>T		YPS138:T>T	AA:415		A4:V>V		UWOPS91_917_1:V>V		YPS138:V>V	AA:420		N_43:T>T	AA:428		CBS432:L>L		CBS5829:L>L		DBVPG4650:L>L		KPN3829:L>L		N_17:L>L		N_43:L>L		T21_4:L>L		Y6_5:L>L		Y7:L>L	AA:431		UWOPS91_917_1:N>N	AA:439		A4:S>S		UWOPS91_917_1:S>S		YPS138:S>S	AA:442		UWOPS91_917_1:N>N	AA:445		A4:C>C		N_43:C>C		YPS138:C>C	AA:456		UWOPS91_917_1:N>NID:YBR093C	AA:37		DBVPG6304:S>S		N_43:S>S		N_44:S>S	AA:51		DBVPG6304:D>D	AA:60		DBVPG6304:G>G	AA:82		N_43:L>L		N_44:L>L		N_45:L>L	AA:91		DBVPG6304:T>T		UWOPS91_917_1:T>T	AA:95		N_43:V>V		N_44:V>V		N_45:V>V	AA:108		DBVPG6304:I>I		UWOPS91_917_1:I>I	AA:116		N_17:V>V	AA:121		DBVPG6304:S>S		UWOPS91_917_1:S>S	AA:157		UWOPS91_917_1:V>V	AA:170		UWOPS91_917_1:L>L	AA:181		UFRJ50816:G>G		UWOPS91_917_1:G>G	AA:209		UWOPS91_917_1:E>E	AA:217		UFRJ50816:G>G		UWOPS91_917_1:G>G	AA:223		UWOPS91_917_1:V>V	AA:226		UWOPS91_917_1:V>V	AA:228		UWOPS91_917_1:S>S	AA:237		UWOPS91_917_1:S>S	AA:244		UFRJ50816:V>V	AA:251		UWOPS91_917_1:P>P	AA:272		UWOPS91_917_1:S>S	AA:280		UFRJ50816:V>V	AA:304		IFO1804:T>T		N_43:T>T		N_45:T>T	AA:321		UFRJ50816:M>M		YPS138:M>M	AA:344		UWOPS91_917_1:H>H	AA:382		DBVPG6304:V>V		UWOPS91_917_1:S>S	AA:391		UFRJ50816:S>S		YPS138:S>S	AA:392		N_43:F>F		N_44:F>F		N_45:F>F	AA:415		UFRJ50816:I>I		YPS138:I>I	AA:419		T21_4:E>E	AA:428		YPS138:Q>QID:YBR094W	AA:26		DBVPG6304:H>H		UFRJ50816:H>H		UWOPS91_917_1:H>H		YPS138:H>H	AA:45		UFRJ50816:K>K	AA:48		UWOPS91_917_1:V>V	AA:65		N_43:S>S		UWOPS91_917_1:S>S	AA:73		CBS432:T>T		CBS5829:T>T		DBVPG4650:T>T		DBVPG6304:T>T		KPN3829:T>T		N_43:T>T		N_44:T>T		N_45:T>T		Q32_3:T>T		Q62_5:T>T		Q89_8:T>T		UFRJ50816:T>T		UWOPS91_917_1:T>T		YPS138:T>T	AA:80		DBVPG6304:Q>Q		UFRJ50816:Q>Q		YPS138:Q>Q	AA:83		N_44:I>I		N_45:I>I	AA:90		UWOPS91_917_1:L>L	AA:91		DBVPG6304:P>P		UFRJ50816:P>P		YPS138:P>P	AA:94		DBVPG6304:L>L		UFRJ50816:L>L		UWOPS91_917_1:L>L		YPS138:L>L	AA:96		DBVPG6304:A>A		UFRJ50816:A>A		UWOPS91_917_1:A>A		YPS138:A>A	AA:109		DBVPG6304:D>D		UFRJ50816:D>D		YPS138:D>D	AA:133		UWOPS91_917_1:S>S	AA:146		DBVPG6304:I>I		UFRJ50816:I>I		YPS138:I>I	AA:151		DBVPG6304:T>T		UFRJ50816:T>T		YPS138:T>T	AA:152		UWOPS91_917_1:V>V	AA:161		UWOPS91_917_1:T>T	AA:172		UWOPS91_917_1:A>A	AA:174		UWOPS91_917_1:F>F	AA:183		UWOPS91_917_1:L>L	AA:188		N_43:A>A		N_45:A>A	AA:193		UWOPS91_917_1:L>L	AA:229		UWOPS91_917_1:A>A	AA:258		A4:N>N		UFRJ50816:N>N		UWOPS91_917_1:N>N		YPS138:N>N	AA:268		A4:A>A		UFRJ50816:A>A		YPS138:A>A	AA:313		A4:V>V		UFRJ50816:V>V		UWOPS91_917_1:V>V		YPS138:V>V	AA:316		A4:L>L		UFRJ50816:L>L		UWOPS91_917_1:L>L		YPS138:L>L	AA:323		A4:T>T		IFO1804:T>T		N_44:T>T		N_45:T>T		UFRJ50816:T>T		UWOPS91_917_1:T>T		YPS138:T>T	AA:332		A4:T>T		UFRJ50816:T>T		UWOPS91_917_1:T>T		YPS138:T>T	AA:334		UWOPS91_917_1:N>N	AA:374		A4:R>R		UFRJ50816:R>R		UWOPS91_917_1:R>R		YPS138:R>R	AA:398		A4:L>L		DBVPG6304:L>L		UFRJ50816:L>L		UWOPS91_917_1:L>L		YPS138:L>L	AA:421		A4:H>H		DBVPG6304:H>H		UFRJ50816:H>H		YPS138:H>H	AA:442		A4:S>S		DBVPG6304:S>S		IFO1804:S>S		N_43:S>S		N_44:S>S		N_45:S>S		UFRJ50816:S>S		UWOPS91_917_1:S>S		YPS138:S>S	AA:456		IFO1804:A>A		N_43:A>A		N_44:A>A		N_45:A>A	AA:468		A4:E>E		DBVPG6304:E>E		UFRJ50816:E>E		YPS138:E>E	AA:481		IFO1804:S>S		N_43:S>S		N_44:S>S	AA:512		IFO1804:A>A		N_43:A>A		N_44:A>A	AA:526		IFO1804:G>G		N_43:G>G		N_44:G>G		N_45:G>G	AA:537		A4:S>S		DBVPG6304:S>S		UFRJ50816:S>S		UWOPS91_917_1:S>S		YPS138:S>S	AA:540		N_43:R>R		N_44:R>R		N_45:R>R	AA:559		DBVPG6304:N>N		YPS138:N>N	AA:589		N_43:K>K		N_44:K>K		N_45:K>K	AA:590		A4:P>P		DBVPG6304:P>P		UFRJ50816:P>P		UWOPS91_917_1:P>P		YPS138:P>P	AA:612		A4:T>T		DBVPG6304:T>T		UFRJ50816:T>T		UWOPS91_917_1:T>T		YPS138:T>T	AA:616		A4:L>L		DBVPG6304:L>L		UFRJ50816:L>L		UWOPS91_917_1:L>L		YPS138:L>L	AA:634		CBS432:E>E		CBS5829:E>E		KPN3828:E>E		KPN3829:E>E		N_17:E>E		N_43:E>E		N_44:E>E		N_45:E>E		Q32_3:E>E		Q59_1:E>E		Q89_8:E>E		Y6_5:E>E	AA:636		UWOPS91_917_1:I>I	AA:641		UWOPS91_917_1:K>K	AA:672		A4:N>N		DBVPG6304:N>N		N_43:N>N		N_44:N>N		N_45:N>N		UFRJ50816:N>N		UWOPS91_917_1:N>N		YPS138:N>N	AA:687		A4:Y>Y		DBVPG6304:Y>Y		UFRJ50816:Y>Y		YPS138:Y>Y	AA:703		A4:T>T		DBVPG6304:T>T		UFRJ50816:T>T		UWOPS91_917_1:T>T		YPS138:T>T	AA:710		A4:L>L		DBVPG6304:L>L		UFRJ50816:L>L		UWOPS91_917_1:L>L		YPS138:L>L	AA:712		IFO1804:D>D		N_43:D>D		N_45:D>D	AA:734		A4:V>V		DBVPG6304:V>V		UFRJ50816:V>V		YPS138:V>V	AA:746		UWOPS91_917_1:I>I	AA:752		DBVPG6304:G>G		YPS138:G>GID:YBR095C	AA:17		A4:T>T		DBVPG6304:T>T		UFRJ50791:L>L		UFRJ50816:L>L		YPS138:T>T	AA:26		UWOPS91_917_1:L>L	AA:43		UFRJ50791:D>D		UFRJ50816:D>D	AA:54		DBVPG6304:V>V		UFRJ50791:V>V	AA:62		A12:T>T		DBVPG6304:T>T		UFRJ50791:T>T	AA:69		UWOPS91_917_1:S>S	AA:96		A12:L>L		DBVPG6304:L>L		UFRJ50791:L>L		UWOPS91_917_1:L>L	AA:107		IFO1804:I>I		N_44:I>I	AA:154		A12:V>V		DBVPG6304:V>V		UFRJ50791:V>V		UWOPS91_917_1:V>V		YPS138:V>V	AA:166		A12:T>T		DBVPG6304:T>T		UFRJ50791:T>T		UWOPS91_917_1:T>T		YPS138:T>T	AA:169		A12:D>D		A4:D>D		DBVPG6304:D>D		UFRJ50791:I>I		YPS138:D>D	AA:172		A12:V>V		DBVPG6304:V>V		UFRJ50791:V>V		UWOPS91_917_1:V>V		YPS138:V>V	AA:210		A12:P>P		A4:P>P		DBVPG6304:P>P		UFRJ50791:P>P		YPS138:P>P	AA:212		A12:I>I		DBVPG6304:I>I		UFRJ50791:I>I		YPS138:I>I	AA:236		A4:Y>Y	AA:241		A12:I>I		A4:I>I		UFRJ50791:I>I		YPS138:I>I	AA:260		A12:P>P		A4:P>P		DBVPG6304:P>P		YPS138:P>P	AA:296		N_44:L>L		N_45:L>L	AA:314		A4:V>V		DBVPG6304:V>V		N_44:V>V		N_45:V>V		YPS138:V>V	AA:320		S36_7:S>S	AA:335		A4:V>V		DBVPG6304:V>V		YPS138:V>V	AA:354		A12:P>P		A4:V>V		DBVPG6304:P>P		UFRJ50791:P>P		UWOPS91_917_1:P>P		YPS138:V>V	AA:384		A4:I>I		DBVPG6304:I>I		YPS138:I>I	AA:404		A4:K>K		DBVPG6304:K>K		YPS138:K>K	AA:406		A4:A>A		DBVPG6304:A>A		YPS138:A>AID:YBR096W	AA:11		UWOPS91_917_1:F>F	AA:21		DBVPG6304:S>S		UFRJ50816:S>S	AA:43		DBVPG6304:L>L		UFRJ50816:L>L	AA:65		A4:T>T	AA:68		A4:D>D		DBVPG6304:D>D		UFRJ50816:D>D	AA:74		UFRJ50816:F>F	AA:115		UWOPS91_917_1:Y>Y	AA:119		UWOPS91_917_1:A>A	AA:129		A4:I>I		DBVPG6304:I>I		UFRJ50816:I>I		UWOPS91_917_1:I>I		YPS138:I>I	AA:140		DBVPG6304:R>R	AA:143		A4:C>C		DBVPG6304:C>C		UFRJ50816:C>C		YPS138:C>C	AA:166		UWOPS91_917_1:L>L	AA:172		A4:V>V		DBVPG6304:V>V		UFRJ50816:V>V		UWOPS91_917_1:V>V		YPS138:V>V	AA:183		UWOPS91_917_1:K>K	AA:219		N_17:L>L		Q59_1:L>L	AA:225		UWOPS91_917_1:D>DID:YBR097W	AA:5		A4:L>L		DBVPG6304:L>L		YPS138:L>L	AA:24		A4:L>L		DBVPG6304:L>L		YPS138:L>L	AA:33		A4:L>L		DBVPG6304:L>L		UFRJ50791:L>L		UWOPS91_917_1:L>L		YPS138:L>L	AA:41		A4:T>T		DBVPG6304:T>T		UFRJ50791:T>T		UWOPS91_917_1:T>T		YPS138:T>T	AA:51		N_43:I>I		N_44:I>I		N_45:I>I	AA:65		A4:L>L		DBVPG6304:L>L		UFRJ50791:L>L		UWOPS91_917_1:L>L	AA:69		A4:L>L		DBVPG6304:L>L		UFRJ50791:L>L	AA:71		A4:R>R		DBVPG6304:R>R		UFRJ50791:R>R	AA:86		N_43:L>L		N_44:L>L		N_45:L>L	AA:109		A4:L>L		DBVPG6304:L>L		UFRJ50791:L>L		YPS138:L>L	AA:119		IFO1804:L>L		N_43:L>L		N_44:L>L		N_45:L>L	AA:134		UWOPS91_917_1:A>A	AA:137		A12:D>D		A4:D>D		DBVPG6304:D>D		UFRJ50791:D>D		UWOPS91_917_1:D>D		YPS138:D>D	AA:154		A12:L>L		A4:L>L		DBVPG6304:L>L		UFRJ50791:L>L		UWOPS91_917_1:L>L		YPS138:L>L	AA:168		A12:A>A		A4:A>A		DBVPG6304:A>A		YPS138:A>A	AA:179		DBVPG4650:N>N		KPN3828:N>N	AA:181		UWOPS91_917_1:G>G	AA:185		UWOPS91_917_1:F>F	AA:200		IFO1804:E>E		N_43:E>E		N_44:E>E		N_45:E>E	AA:226		UFRJ50791:L>L	AA:235		IFO1804:A>A		N_43:A>A		N_44:A>A		N_45:A>A	AA:261		A4:L>L		DBVPG6304:L>L		UFRJ50791:L>L		UWOPS91_917_1:L>L		YPS138:L>L	AA:268		UWOPS91_917_1:T>T	AA:280		A12:L>L		A4:L>L		DBVPG6304:L>L		YPS138:L>L	AA:306		UFRJ50791:T>T	AA:310		DBVPG6304:D>D	AA:313		A12:R>R		A4:R>R		DBVPG6304:R>R		YPS138:R>R	AA:341		UWOPS91_917_1:L>L	AA:359		UWOPS91_917_1:L>L	AA:363		A12:L>L		DBVPG6304:L>L		YPS138:L>L	AA:378		A12:A>A		DBVPG6304:A>A		YPS138:A>A	AA:384		A12:V>V		DBVPG6304:V>V		YPS138:V>V	AA:388		A12:R>R		DBVPG6304:R>R		YPS138:R>R	AA:390		UWOPS91_917_1:L>L	AA:394		A12:L>L		DBVPG6304:L>L		N_43:L>L		N_44:L>L		N_45:L>L		YPS138:L>L	AA:405		A12:Q>Q		DBVPG6304:Q>Q		YPS138:Q>Q	AA:419		UWOPS91_917_1:E>E	AA:423		A12:L>L		DBVPG6304:L>L		UFRJ50791:L>L		YPS138:L>L	AA:429		UWOPS91_917_1:L>L	AA:446		UWOPS91_917_1:L>L	AA:450		UWOPS91_917_1:A>A	AA:456		A12:V>V		DBVPG6304:V>V		UFRJ50791:V>V		YPS138:V>V	AA:459		UWOPS91_917_1:E>E	AA:466		DBVPG6304:V>V	AA:471		A12:C>C		DBVPG6304:C>C		UFRJ50791:C>C		UFRJ50816:C>C		YPS138:C>C	AA:479		UWOPS91_917_1:D>D	AA:486		A12:L>L		DBVPG6304:L>L		UFRJ50791:L>L		UFRJ50816:L>L		YPS138:L>L	AA:490		UWOPS91_917_1:Q>Q	AA:493		UWOPS91_917_1:T>T	AA:495		A12:V>V		DBVPG6304:V>V		N_44:V>V		N_45:V>V		UFRJ50791:V>V		UFRJ50816:V>V		YPS138:V>V	AA:506		UWOPS91_917_1:F>F	AA:512		UWOPS91_917_1:P>P	AA:546		A12:F>F		A4:F>F		DBVPG6304:F>F		UWOPS91_917_1:F>F		YPS138:F>F	AA:550		A12:T>T		A4:T>T		DBVPG6304:T>T		UFRJ50791:T>T		UFRJ50816:T>T		YPS138:T>T	AA:557		N_44:D>D		N_45:D>D	AA:597		A12:K>K		A4:K>K		DBVPG6304:K>K		UFRJ50791:K>K		UFRJ50816:K>K		UWOPS91_917_1:K>K		YPS138:K>K	AA:622		A12:S>S		A4:S>S		DBVPG6304:S>S		UFRJ50791:S>S		UFRJ50816:S>S		UWOPS91_917_1:S>S		YPS138:S>S	AA:629		A12:N>N		A4:N>N		DBVPG6304:N>N		UFRJ50791:N>N		UFRJ50816:N>N		UWOPS91_917_1:N>N		YPS138:N>N	AA:680		A4:Q>Q		DBVPG6304:Q>Q		N_43:Q>Q		N_44:Q>Q		N_45:Q>Q		UFRJ50791:Q>Q		UFRJ50816:Q>Q		YPS138:Q>Q	AA:685		UWOPS91_917_1:L>L	AA:689		A4:G>G		DBVPG6304:G>G		UFRJ50791:G>G		UFRJ50816:G>G		YPS138:G>G	AA:704		A4:S>S		DBVPG6304:S>S		UFRJ50791:S>S		UFRJ50816:S>S		UWOPS91_917_1:S>S		YPS138:S>S	AA:710		N_43:P>P		N_44:P>P	AA:729		UFRJ50791:L>L		UFRJ50816:L>L	AA:737		A4:I>I		DBVPG6304:I>I		UFRJ50791:I>I		UFRJ50816:I>I		YPS138:I>I	AA:741		A4:I>I		DBVPG6304:I>I		UFRJ50791:I>I		UFRJ50816:I>I		YPS138:I>I	AA:754		N_43:F>F		N_44:F>F		N_45:F>F	AA:755		A4:K>K		DBVPG6304:K>K		UFRJ50791:K>K		UFRJ50816:K>K		UWOPS91_917_1:K>K		YPS138:K>K	AA:768		A4:S>S		DBVPG6304:S>S		UFRJ50791:S>S		UFRJ50816:S>S		YPS138:S>S	AA:773		A4:L>L		DBVPG6304:L>L		UFRJ50791:L>L		YPS138:L>L	AA:777		A4:S>S		DBVPG6304:S>S		UFRJ50791:S>S		UFRJ50816:S>S		YPS138:S>S	AA:784		UWOPS91_917_1:L>L	AA:797		UWOPS91_917_1:F>F	AA:802		A4:I>I		DBVPG6304:I>I		UFRJ50791:I>I		YPS138:I>I	AA:825		A4:S>S		DBVPG6304:S>S		UFRJ50791:S>S		UWOPS91_917_1:S>S		YPS138:S>S	AA:832		A4:T>T		DBVPG6304:T>T		UFRJ50791:T>T		YPS138:T>T	AA:836		UWOPS91_917_1:V>V	AA:850		A4:D>D		DBVPG6304:D>D		UFRJ50791:D>D		UWOPS91_917_1:D>D		YPS138:D>D	AA:857		A4:F>F		DBVPG6304:F>F		UFRJ50791:F>F		YPS138:F>F	AA:867		UWOPS91_917_1:I>I	AA:879		UWOPS91_917_1:R>R	AA:883		A4:V>V		N_45:V>V		UFRJ50791:V>V		UWOPS91_917_1:V>V		YPS138:V>V	AA:891		A4:S>S		DBVPG6304:S>S		UFRJ50791:S>S		UWOPS91_917_1:S>S		YPS138:S>S	AA:896		A4:N>N		DBVPG6304:N>N		UFRJ50791:N>N		UWOPS91_917_1:N>N		YPS138:N>N	AA:901		A4:V>V		DBVPG6304:V>V		YPS138:V>V	AA:909		A4:N>N		DBVPG6304:N>N		N_45:N>N		UWOPS91_917_1:N>N		YPS138:N>N	AA:919		Y7:F>F	AA:939		A4:N>N		DBVPG6304:N>N		YPS138:N>N	AA:954		UWOPS91_917_1:K>K	AA:960		UWOPS91_917_1:S>S	AA:964		A4:S>S		DBVPG6304:S>S		YPS138:S>S	AA:997		A4:T>T		DBVPG6304:T>T		YPS138:T>T	AA:1001		A4:K>K		DBVPG6304:K>K		UWOPS91_917_1:K>K		YPS138:K>K	AA:1007		UWOPS91_917_1:G>G	AA:1011		A4:N>N		DBVPG6304:N>N		YPS138:N>N	AA:1022		A4:V>V		DBVPG6304:V>V		YPS138:V>V	AA:1025		UWOPS91_917_1:S>S	AA:1039		A4:T>T		DBVPG6304:T>T		UWOPS91_917_1:T>T		YPS138:T>T	AA:1051		N_43:E>E		N_45:E>E	AA:1062		UFRJ50791:P>P	AA:1074		N_43:T>T		N_45:T>T	AA:1089		A4:S>S		DBVPG6304:S>S		UWOPS91_917_1:S>S		YPS138:S>S	AA:1098		UWOPS91_917_1:T>T	AA:1103		IFO1804:G>G		N_43:G>G		N_45:G>G	AA:1111		IFO1804:K>K		N_43:K>K		N_45:K>K	AA:1112		UWOPS91_917_1:E>E	AA:1122		UWOPS91_917_1:S>S	AA:1123		UFRJ50791:L>L	AA:1125		A4:Y>Y		DBVPG6304:Y>Y		YPS138:Y>Y	AA:1133		A4:Q>Q		DBVPG6304:Q>Q		YPS138:Q>Q	AA:1139		UWOPS91_917_1:N>N	AA:1144		UWOPS91_917_1:A>A	AA:1150		UWOPS91_917_1:G>G	AA:1152		A4:I>I		DBVPG6304:I>I		UWOPS91_917_1:I>I		YPS138:I>I	AA:1156		A4:K>K		DBVPG6304:K>K		YPS138:K>K	AA:1168		KPN3828:F>F	AA:1169		UWOPS91_917_1:L>L	AA:1173		UWOPS91_917_1:C>C	AA:1184		IFO1804:K>K		N_43:K>K		N_44:K>K		N_45:K>K	AA:1185		UWOPS91_917_1:N>N	AA:1187		UWOPS91_917_1:Y>Y	AA:1215		UWOPS91_917_1:D>D	AA:1219		A4:L>L		DBVPG6304:L>L		UWOPS91_917_1:L>L	AA:1221		IFO1804:R>R		N_45:R>R	AA:1233		UWOPS91_917_1:A>A	AA:1239		UWOPS91_917_1:I>I	AA:1250		UWOPS91_917_1:T>T	AA:1253		IFO1804:G>G		N_43:G>G		N_44:G>G		N_45:G>G	AA:1260		UWOPS91_917_1:I>I	AA:1263		UWOPS91_917_1:N>N	AA:1270		UWOPS91_917_1:S>S	AA:1272		A4:G>G		DBVPG6304:G>G		UFRJ50791:G>G		YPS138:G>G	AA:1283		A4:C>C		DBVPG6304:C>C		UFRJ50791:C>C		YPS138:C>C	AA:1302		DBVPG6304:L>L		UFRJ50791:L>L		YPS138:L>L	AA:1350		DBVPG6304:S>S		UFRJ50791:S>S		YPS138:S>S	AA:1352		DBVPG6304:I>I		UFRJ50791:I>I		YPS138:I>I	AA:1377		UFRJ50791:L>L		YPS138:L>L	AA:1401		UFRJ50791:N>N		YPS138:N>N	AA:1406		DBVPG6304:L>L		IFO1804:L>L		N_43:L>L		N_44:L>L		UFRJ50791:L>L		YPS138:L>L	AA:1417		DBVPG6304:V>V		UFRJ50791:V>V		YPS138:V>V	AA:1421		DBVPG6304:L>L		UFRJ50791:L>L		YPS138:L>L	AA:1432		DBVPG4650:T>T		DBVPG6304:T>T		IFO1804:T>T		KPN3828:T>T		KPN3829:T>T		N_17:T>T		N_43:T>T		N_44:T>T		N_45:T>T		UFRJ50791:T>T		YPS138:T>T	AA:1451		DBVPG6304:G>G		UFRJ50791:G>G		YPS138:G>G	AA:1454		DBVPG6304:Q>Q		UFRJ50791:Q>Q		YPS138:Q>QID:YBR098W	AA:18		N_44:S>S		N_45:S>S	AA:21		A12:I>I		A4:I>I		DBVPG6304:I>I		UFRJ50816:I>I	AA:72		A12:L>L		A4:L>L		DBVPG6304:L>L		UFRJ50816:L>L		YPS138:L>L	AA:73		N_44:N>N		N_45:N>N	AA:115		A12:N>N		A4:N>N		DBVPG6304:N>N		UFRJ50791:N>N		UFRJ50816:N>N		YPS138:N>N	AA:123		A12:L>L		A4:L>L		DBVPG6304:L>L		UFRJ50791:L>L		UFRJ50816:L>L		YPS138:L>L	AA:138		A12:K>K		A4:K>K		DBVPG6304:K>K		UFRJ50791:K>K		UFRJ50816:K>K		YPS138:K>K	AA:148		A12:N>N		A4:N>N		YPS138:N>N	AA:189		A12:Q>Q		A4:Q>Q		UFRJ50791:Q>Q		UFRJ50816:Q>Q		YPS138:Q>Q	AA:217		A12:V>V		A4:V>V		UFRJ50791:V>V		UFRJ50816:V>V		YPS138:V>V	AA:220		A12:H>H		A4:H>H		UFRJ50791:H>H		UFRJ50816:H>H		YPS138:H>H	AA:253		IFO1804:D>D		N_44:D>D		N_45:D>D	AA:265		IFO1804:T>T		N_44:T>T		N_45:T>T	AA:267		A12:A>A		A4:A>A		UFRJ50791:A>A		UFRJ50816:A>A		YPS138:A>A	AA:271		A12:T>T		A4:T>T		UFRJ50791:T>T		UFRJ50816:T>T		YPS138:T>T	AA:284		A12:L>L		A4:L>L		UFRJ50791:L>L		UFRJ50816:L>L		YPS138:L>L	AA:303		A12:T>T		A4:T>T		DBVPG6304:T>T		UFRJ50791:T>T		UFRJ50816:T>T		YPS138:T>T	AA:328		A12:A>A		A4:A>A		UFRJ50791:A>A		UFRJ50816:A>A		YPS138:A>A	AA:344		IFO1804:N>N		N_43:N>N		N_45:N>N	AA:382		A4:F>F		UFRJ50791:F>F		UFRJ50816:F>F		YPS138:F>F	AA:420		A4:Q>Q		DBVPG6304:Q>Q		UFRJ50816:Q>Q		YPS138:Q>Q	AA:481		A12:Y>Y		A4:Y>Y		DBVPG6304:Y>Y		UFRJ50816:Y>Y	AA:536		A12:E>E		A4:E>E		DBVPG6304:E>E		UFRJ50816:E>E	AA:537		N_43:E>E		N_44:E>E	AA:546		A12:S>S		A4:S>S		DBVPG6304:S>S		N_43:S>S		N_44:S>S		N_45:S>S		UFRJ50816:S>S	AA:562		UWOPS91_917_1:R>R	AA:574		N_43:V>V		N_44:V>V	AA:575		A12:H>H		A4:H>H		DBVPG6304:H>H		UFRJ50816:H>H	AA:577		A12:V>V		A4:V>V		DBVPG6304:V>V		UFRJ50816:V>V		UWOPS91_917_1:V>V	AA:586		UWOPS91_917_1:S>S	AA:596		A12:K>K		A4:K>K		DBVPG6304:K>K		UFRJ50816:K>K		UWOPS91_917_1:K>K	AA:598		A12:R>R		A4:R>R		DBVPG6304:R>R		UFRJ50791:R>R		UFRJ50816:R>R		UWOPS91_917_1:R>R	AA:611		A12:L>L		A4:L>L		DBVPG6304:L>L		UFRJ50791:L>L		UFRJ50816:L>L	AA:637		A12:K>K		A4:K>K		DBVPG6304:K>K		UFRJ50791:K>K		UFRJ50816:K>K		YPS138:K>K	AA:641		A12:V>V		A4:V>V		DBVPG6304:V>V		UFRJ50791:V>V		UFRJ50816:V>V		UWOPS91_917_1:V>V		YPS138:V>V	AA:643		A12:S>S		A4:S>S		DBVPG6304:S>S		N_43:S>S		N_44:S>S		N_45:S>S		UFRJ50791:S>S		UFRJ50816:S>S		UWOPS91_917_1:S>S		YPS138:S>S	AA:646		UWOPS91_917_1:P>P	AA:663		A12:D>D		A4:D>D		DBVPG6304:D>D		YPS138:D>D	AA:683		A12:T>T		A4:T>T		DBVPG6304:T>T		UFRJ50791:T>T		UFRJ50816:T>T		YPS138:T>T	AA:690		A12:A>A		A4:A>A		DBVPG6304:A>A		UFRJ50791:A>A		UFRJ50816:A>A		YPS138:A>AID:YBR099C	AA:1		A12:L>L		A4:L>L		DBVPG6304:L>L		UFRJ50791:L>L		UFRJ50816:L>L		UWOPS91_917_1:L>L	AA:3		A12:C>C		A4:C>C		DBVPG6304:C>C		UFRJ50816:C>C		UWOPS91_917_1:C>C	AA:13		UWOPS91_917_1:T>T	AA:22		A12:T>T		A4:T>T		DBVPG6304:T>T		UFRJ50816:T>T		UWOPS91_917_1:T>T	AA:25		N_43:F>F		N_44:F>F	AA:37		UWOPS91_917_1:R>R	AA:53		A12:V>V		A4:V>V		DBVPG6304:V>V		N_43:V>V		N_44:V>V		N_45:V>V		UFRJ50816:V>V	AA:62		N_43:V>V		N_44:V>V	AA:83		A12:D>D		A4:D>D		DBVPG6304:D>D		UFRJ50816:D>D	AA:118		A12:K>K		A4:K>K		DBVPG6304:K>K		UFRJ50816:K>KID:YBR101C	AA:10		UWOPS91_917_1:I>I	AA:41		UWOPS91_917_1:R>R	AA:57		DBVPG6304:V>V	AA:96		N_44:G>G		N_45:G>G	AA:114		UWOPS91_917_1:A>A	AA:124		DBVPG6304:I>I		UWOPS91_917_1:I>I	AA:129		DBVPG6304:V>V		UWOPS91_917_1:V>V	AA:130		UWOPS91_917_1:V>V	AA:145		DBVPG6304:V>V		UFRJ50816:V>V	AA:150		DBVPG6304:L>L		UFRJ50816:L>L	AA:152		UWOPS91_917_1:A>A	AA:166		UWOPS91_917_1:H>H	AA:171		N_43:R>R		N_44:R>R		N_45:R>R	AA:172		UWOPS91_917_1:V>V	AA:180		UWOPS91_917_1:T>T	AA:188		DBVPG6304:T>T		UFRJ50791:T>T		UFRJ50816:T>T		UWOPS91_917_1:T>T		YPS138:T>T	AA:214		CBS432:I>I		CBS5829:I>I		DBVPG6304:I>I		KPN3828:I>I		N_17:I>I		N_43:I>I		N_45:I>I		Q32_3:I>I		Q59_1:I>I		UFRJ50791:I>I		UFRJ50816:I>I		YPS138:I>I	AA:234		DBVPG6304:V>V		UFRJ50791:V>V		UFRJ50816:V>V		YPS138:V>V	AA:246		UWOPS91_917_1:F>F	AA:259		UWOPS91_917_1:L>L	AA:273		DBVPG6304:H>H		UFRJ50791:H>H		UFRJ50816:H>H		YPS138:H>HID:YBR104W	AA:36		A12:K>K		YPS138:K>K	AA:44		CBS432:G>G		UWOPS91_917_1:G>G	AA:47		A12:A>A		DBVPG6304:A>A		UFRJ50791:A>A		UWOPS91_917_1:A>A		YPS138:A>A	AA:62		DBVPG6304:L>L	AA:72		UWOPS91_917_1:L>L	AA:75		UWOPS91_917_1:L>L	AA:132		A4:V>V		DBVPG6304:V>V		UFRJ50791:V>V		YPS138:V>V	AA:140		A4:L>L		DBVPG6304:L>L		UFRJ50791:L>L		YPS138:L>L	AA:141		UWOPS91_917_1:P>P	AA:143		UFRJ50791:S>S	AA:151		A4:T>T		DBVPG6304:T>T		UFRJ50791:T>T		UWOPS91_917_1:T>T		YPS138:T>T	AA:161		A4:S>S		DBVPG6304:S>S		UFRJ50791:S>S		UWOPS91_917_1:S>S		YPS138:S>S	AA:182		UWOPS91_917_1:F>F	AA:197		A12:G>G		A4:G>G		UFRJ50791:G>G		YPS138:G>G	AA:215		UWOPS91_917_1:T>T	AA:217		IFO1804:F>F		N_43:F>F		N_44:F>F		N_45:F>F	AA:222		A12:A>A		A4:A>A		UFRJ50791:A>A		YPS138:A>A	AA:262		A12:V>V		A4:V>V		UFRJ50791:V>V		UWOPS91_917_1:V>V	AA:272		A12:L>L		A4:L>L		UWOPS91_917_1:L>L		YPS138:L>L	AA:287		A12:T>T		A4:T>T		UWOPS91_917_1:T>T		YPS138:T>T	AA:307		UWOPS91_917_1:R>RID:YBR105C	AA:29		A12:I>I		A4:I>I		DBVPG6304:I>I		UWOPS91_917_1:I>I		YPS138:I>I	AA:45		A12:D>D		YPS138:D>D	AA:50		A4:S>S		DBVPG6304:S>S		UFRJ50816:S>S		UWOPS91_917_1:S>S		YPS138:S>S	AA:52		A4:V>V		DBVPG6304:V>V		UFRJ50816:V>V		UWOPS91_917_1:V>V		YPS138:V>V	AA:62		A12:Q>Q		A4:Q>Q		DBVPG6304:Q>Q		IFO1804:Q>Q		N_43:Q>Q		N_44:Q>Q		N_45:Q>Q		UFRJ50816:Q>Q		UWOPS91_917_1:Q>Q		YPS138:Q>Q	AA:64		A12:I>I		A4:I>I		DBVPG6304:I>I		UFRJ50816:I>I		YPS138:I>I	AA:71		A12:L>L		A4:L>L		DBVPG6304:L>L		UFRJ50816:L>L		UWOPS91_917_1:L>L		YPS138:L>L	AA:89		UWOPS91_917_1:L>L	AA:93		UWOPS91_917_1:S>S		YPS138:S>S	AA:101		A12:A>A		A4:A>A		DBVPG6304:A>A		UFRJ50816:A>A		UWOPS91_917_1:A>A		YPS138:R>R	AA:141		A4:G>G		DBVPG6304:G>G		UWOPS91_917_1:G>G		YPS138:G>G	AA:155		A4:F>F		DBVPG6304:F>F		IFO1804:F>F		N_44:F>F		UFRJ50816:F>F		UWOPS91_917_1:F>F		YPS138:F>F	AA:157		A4:F>F		DBVPG6304:F>F		UFRJ50816:F>F		UWOPS91_917_1:F>F		YPS138:F>F	AA:160		A4:Y>Y		DBVPG6304:Y>Y		UFRJ50816:Y>Y		UWOPS91_917_1:Y>Y		YPS138:Y>Y	AA:173		DBVPG6304:F>F		UWOPS91_917_1:F>F		YPS138:F>F	AA:177		UWOPS91_917_1:Y>Y	AA:209		UWOPS91_917_1:A>A	AA:211		A4:F>F		DBVPG6304:F>F		IFO1804:F>F		N_43:F>F		N_44:F>F		N_45:F>F		UFRJ50816:F>F		UWOPS91_917_1:F>F		YPS138:F>F	AA:241		UWOPS91_917_1:S>S	AA:246		T21_4:A>A		UWOPS91_917_1:A>A	AA:267		UWOPS91_917_1:I>I	AA:282		A4:G>G		N_45:I>I		YPS138:G>G	AA:296		IFO1804:L>L		N_43:L>L		N_44:L>L		N_45:L>L	AA:304		A4:V>V		IFO1804:V>V		N_43:V>V		N_44:V>V		N_45:V>V		YPS138:V>V	AA:308		YPS138:V>V	AA:314		A4:C>C		CBS432:C>C		DBVPG4650:C>C		IFO1804:C>C		KPN3828:C>C		N_17:C>C		N_43:C>C		N_44:C>C		N_45:C>C		Q32_3:C>C		YPS138:C>C	AA:348		A12:H>H		A4:H>H		YPS138:H>H	AA:353		A12:L>L		A4:L>L		YPS138:L>LID:YBR106W	AA:2		UWOPS91_917_1:N>N	AA:40		UWOPS91_917_1:I>I	AA:43		UWOPS91_917_1:S>S	AA:55		UWOPS91_917_1:V>V	AA:59		N_43:D>D	AA:61		N_43:T>T		N_44:T>T		N_45:T>T	AA:73		A12:S>S		A4:S>S		YPS138:S>S	AA:81		A12:V>V		A4:V>V		UFRJ50816:V>V		UWOPS91_917_1:V>V		YPS138:V>V	AA:86		A12:D>D		A4:D>D		UFRJ50816:D>D		YPS138:D>D	AA:99		A12:I>I		A4:I>I		UWOPS91_917_1:I>I		YPS138:I>I	AA:120		A12:F>F		A4:F>F		DBVPG6304:F>F		UWOPS91_917_1:F>F		YPS138:F>F	AA:122		A12:Q>Q		A4:Q>Q		UFRJ50816:Q>Q		UWOPS91_917_1:Q>Q		YPS138:Q>Q	AA:128		A12:K>K		A4:K>K		DBVPG6304:K>K		N_43:K>K		N_44:K>K		N_45:K>K		UWOPS91_917_1:K>K		YPS138:K>K	AA:140		A12:L>L		A4:L>L		DBVPG6304:L>L		N_43:L>L		N_44:L>L		N_45:L>L		UFRJ50791:L>L		UFRJ50816:L>L		UWOPS91_917_1:L>L		YPS138:L>L	AA:146		A12:T>T		A4:T>T		DBVPG6304:T>T		UFRJ50791:T>T		UFRJ50816:T>T		UWOPS91_917_1:T>T		YPS138:T>TID:YBR107C	AA:19		A12:K>K		A4:K>K		DBVPG6304:K>K		UFRJ50816:K>K	AA:52		A12:L>L		A4:L>L		DBVPG6304:L>L		UFRJ50816:L>L		YPS138:L>L	AA:54		UWOPS91_917_1:I>I	AA:59		UWOPS91_917_1:N>N	AA:103		DBVPG6304:A>A		YPS138:A>A	AA:119		CBS432:A>A		CBS5829:A>A		DBVPG4650:A>A		KPN3828:A>A		KPN3829:A>A		N_43:A>A		N_45:A>A		Q32_3:A>A		Q62_5:A>A		Q95_3:A>A		T21_4:A>A		Y6_5:A>A	AA:125		A12:Y>Y		A4:Y>Y		DBVPG6304:Y>Y		UFRJ50816:Y>Y		UWOPS91_917_1:Y>Y		YPS138:Y>Y	AA:149		A12:H>H		A4:H>H		DBVPG6304:H>H		UFRJ50816:H>H		UWOPS91_917_1:H>H		YPS138:H>H	AA:152		A12:T>T		A4:T>T		DBVPG6304:T>T		UFRJ50816:T>T		UWOPS91_917_1:T>T		YPS138:T>T	AA:156		UWOPS91_917_1:P>P	AA:160		A12:Q>Q		A4:Q>Q		UFRJ50816:Q>Q		YPS138:Q>Q	AA:180		UWOPS91_917_1:L>L	AA:185		UWOPS91_917_1:V>V	AA:196		UWOPS91_917_1:V>V	AA:207		A12:D>D		A4:D>D		UFRJ50816:D>D		UWOPS91_917_1:D>D		YPS138:D>D	AA:218		A12:->-		A4:->-		UFRJ50791:->-		UFRJ50816:->-		UWOPS91_917_1:S>S		YPS138:->-	AA:231		A12:Q>Q		A4:Q>Q		UFRJ50816:Q>Q		YPS138:Q>Q	AA:243		A12:S>S		A4:S>S		DBVPG6304:S>S		IFO1804:S>S		N_43:S>S		N_45:S>S		UFRJ50791:S>S		UFRJ50816:S>S		UWOPS91_917_1:S>S		YPS138:S>SID:YBR109C	AA:11		UWOPS91_917_1:N>N	AA:26		A12:I>I		A4:I>I		DBVPG6304:I>I		UFRJ50791:I>I		UWOPS91_917_1:I>I	AA:31		A12:S>S		A4:S>S		DBVPG6304:S>S		UFRJ50791:S>S		UWOPS91_917_1:S>S		YPS138:S>S	AA:51		UWOPS91_917_1:V>V	AA:104		A12:T>T		A4:T>T		DBVPG6304:T>T		UFRJ50791:T>T		UWOPS91_917_1:T>T	AA:107		A12:K>K		A4:K>K		UFRJ50791:K>K		UWOPS91_917_1:L>L	AA:128		A12:I>I		A4:I>I		DBVPG6304:I>I		UFRJ50791:I>I	AA:132		IFO1804:K>K		N_43:K>K		N_45:K>K	AA:141		A12:F>F		A4:F>F		DBVPG6304:F>F		UFRJ50791:F>F		UWOPS91_917_1:F>FID:YBR110W	AA:4		UWOPS91_917_1:E>E	AA:11		A4:A>A		DBVPG6304:A>A		UWOPS91_917_1:A>A		YPS138:A>A	AA:13		UWOPS91_917_1:I>I	AA:16		A4:Y>Y		DBVPG6304:Y>Y		N_43:Y>Y		N_44:Y>Y		N_45:Y>Y		YPS138:Y>Y	AA:62		A12:F>F		A4:F>F		UWOPS91_917_1:F>F		YPS138:F>F	AA:91		A12:V>V		A4:V>V		IFO1804:V>V		N_43:V>V		N_44:V>V		N_45:V>V		UWOPS91_917_1:V>V		YPS138:V>V	AA:97		A12:L>L		A4:L>L		UWOPS91_917_1:L>L		YPS138:L>L	AA:134		UWOPS91_917_1:L>L	AA:140		A12:S>S		A4:S>S		YPS138:S>S	AA:144		IFO1804:L>L	AA:157		A12:L>L		A4:L>L		UWOPS91_917_1:L>L		YPS138:L>L	AA:165		A12:A>A		A4:A>A		UWOPS91_917_1:A>A		YPS138:A>A	AA:171		A12:L>L		A4:L>L		UWOPS91_917_1:L>L		YPS138:L>L	AA:189		A12:E>E		A4:E>E		UWOPS91_917_1:E>E		YPS138:E>E	AA:193		UWOPS91_917_1:S>S	AA:205		UWOPS91_917_1:A>A	AA:208		N_44:K>K	AA:215		A12:R>R		A4:R>R		UWOPS91_917_1:R>R		YPS138:R>R	AA:224		A12:L>L		A4:L>L		UWOPS91_917_1:L>L		YPS138:L>L	AA:238		A12:D>D		A4:D>D		YPS138:D>D	AA:247		A12:T>T		A4:T>T		UWOPS91_917_1:T>T		YPS138:T>T	AA:251		UWOPS91_917_1:I>I	AA:256		UWOPS91_917_1:R>R	AA:262		A12:E>E		A4:E>E		UWOPS91_917_1:E>E		YPS138:E>E	AA:275		A12:T>T		A4:T>T		YPS138:T>T	AA:294		UWOPS91_917_1:Y>Y	AA:302		N_43:P>P		N_45:P>P	AA:311		UWOPS91_917_1:K>K	AA:314		A4:L>L		UWOPS91_917_1:L>L		YPS138:L>L	AA:338		A4:S>S		UWOPS91_917_1:S>S		YPS138:S>S	AA:341		UWOPS91_917_1:D>D	AA:342		A4:Y>Y		YPS138:Y>Y	AA:346		KPN3828:L>L	AA:354		A4:S>S		YPS138:S>S	AA:358		N_43:S>S	AA:372		UWOPS91_917_1:F>F	AA:382		UWOPS91_917_1:N>N	AA:394		A12:V>V		A4:V>V		UWOPS91_917_1:V>V		YPS138:V>V	AA:397		A12:L>L		A4:L>L		UWOPS91_917_1:L>L		YPS138:L>L	AA:403		A12:R>R		A4:R>R		N_43:R>R		N_45:R>R		UWOPS91_917_1:R>R		YPS138:R>R	AA:422		A12:L>L		A4:L>L		N_43:L>L		N_45:L>L		UWOPS91_917_1:L>L		YPS138:L>L	AA:428		A12:Q>Q		A4:Q>Q		UWOPS91_917_1:Q>Q	AA:436		A12:S>S		A4:S>S		UWOPS91_917_1:S>S	AA:448		A12:I>I		A4:I>I		UWOPS91_917_1:I>IID:YBR111C	AA:85		A12:S>S		UFRJ50791:S>S	AA:101		A12:->-		CBS432:->-		IFO1804:->-		N_43:->-		N_45:->-		UFRJ50791:->-		UWOPS91_917_1:->-	AA:107		A12:G>G		UFRJ50791:G>G		UWOPS91_917_1:G>G	AA:143		A12:I>I		IFO1804:I>I		N_43:I>I		N_45:I>I		UFRJ50791:I>I		UWOPS91_917_1:N>N		YPS138:I>I	AA:154		A12:A>A		UFRJ50791:A>A		YPS138:A>A	AA:157		A12:T>T		IFO1804:T>T		N_45:T>T		UFRJ50791:T>T		UWOPS91_917_1:T>T		YPS138:T>T	AA:159		A12:G>G		UFRJ50791:G>G		UWOPS91_917_1:G>G		YPS138:G>G	AA:168		A12:I>I		UFRJ50791:I>I		UWOPS91_917_1:I>I		YPS138:I>I	AA:174		A12:V>V		UFRJ50791:V>V		YPS138:A>A	AA:206		IFO1804:W>W		N_43:W>W		N_45:W>W	AA:223		A12:G>G		YPS138:G>GID:YBR115C	AA:7		A4:S>S		UFRJ50816:S>S	AA:8		IFO1804:T>T		N_43:T>T		N_44:T>T		N_45:T>T	AA:58		N_17:S>S	AA:61		UWOPS91_917_1:I>I	AA:113		A4:R>R		UWOPS91_917_1:H>H	AA:120		IFO1804:P>P		N_43:P>P		N_44:P>P		N_45:P>P	AA:124		UWOPS91_917_1:A>A	AA:136		UWOPS91_917_1:S>S	AA:140		A12:V>V		A4:V>V		IFO1804:V>V		N_43:V>V		N_44:V>V		N_45:V>V		UFRJ50816:V>V		UWOPS91_917_1:V>V		YPS138:V>V	AA:153		A12:N>N		A4:N>N		DBVPG6304:N>N		UFRJ50816:N>N		UWOPS91_917_1:N>N		YPS138:N>N	AA:180		A12:H>H		A4:H>H		DBVPG6304:H>H		IFO1804:H>H		N_43:H>H		N_45:H>H		UFRJ50816:H>H		UWOPS91_917_1:H>H		YPS138:H>H	AA:197		UWOPS91_917_1:K>K	AA:205		IFO1804:V>V		N_43:V>V		N_45:V>V	AA:232		A12:S>S		A4:S>S		DBVPG6304:S>S		UWOPS91_917_1:S>S		YPS138:S>S	AA:235		IFO1804:I>I		N_43:I>I		N_45:I>I	AA:259		A12:F>F		A4:F>F		DBVPG6304:F>F		UWOPS91_917_1:F>F		YPS138:F>F	AA:276		IFO1804:V>V		N_43:V>V		N_44:V>V		N_45:V>V	AA:308		UFRJ50816:N>N	AA:310		A4:P>P		UFRJ50816:P>P		UWOPS91_917_1:P>P		YPS138:P>P	AA:376		A12:->-		DBVPG6304:->-		UFRJ50816:->-		YPS138:->-	AA:381		A12:S>S		A4:S>S		DBVPG6304:S>S		UFRJ50816:S>S		UWOPS91_917_1:S>S		YPS138:S>S	AA:385		UWOPS91_917_1:L>L	AA:389		A12:V>V		A4:V>V		DBVPG6304:V>V		UFRJ50816:V>V		UWOPS91_917_1:V>V		YPS138:V>V	AA:414		A12:S>S		A4:S>S		DBVPG6304:S>S		UFRJ50816:S>S		UWOPS91_917_1:S>S		YPS138:S>S	AA:426		A12:L>L		A4:L>L		DBVPG6304:L>L		UFRJ50816:L>L		UWOPS91_917_1:L>L		YPS138:L>L	AA:438		A4:R>R		DBVPG6304:R>R		N_44:A>A		N_45:A>A		UFRJ50791:R>R		UFRJ50816:R>R		UWOPS91_917_1:A>A		YPS138:R>R	AA:445		A4:->-		DBVPG6304:->-		UFRJ50816:->-		UWOPS91_917_1:->-		YPS138:->-	AA:453		UWOPS91_917_1:I>I	AA:455		A4:R>R		DBVPG6304:R>R		UFRJ50816:R>R		YPS138:R>R	AA:461		A4:S>S		DBVPG6304:S>S		N_44:S>S		N_45:S>S		UFRJ50816:S>S		UWOPS91_917_1:S>S		YPS138:S>S	AA:477		A4:A>A		DBVPG6304:A>A		UFRJ50791:A>A		UFRJ50816:A>A		UWOPS91_917_1:V>V		YPS138:A>A	AA:505		A4:D>D		DBVPG6304:D>D		UFRJ50791:D>D		UFRJ50816:D>D		UWOPS91_917_1:G>G		YPS138:D>D	AA:510		A4:G>G		N_45:G>G		UFRJ50791:G>G		UFRJ50816:G>G		UWOPS91_917_1:G>G		YPS138:G>G	AA:521		N_45:K>K		UWOPS91_917_1:K>K	AA:528		YPS138:R>R	AA:530		UWOPS91_917_1:W>W	AA:536		A4:T>T		DBVPG6304:T>T		UFRJ50791:T>T		UFRJ50816:T>T		UWOPS91_917_1:T>T		YPS138:T>T	AA:568		A4:G>G		DBVPG6304:G>G		UFRJ50791:G>G		UFRJ50816:G>G		UWOPS91_917_1:G>G		YPS138:G>G	AA:604		A4:L>L		DBVPG6304:L>L		UFRJ50791:L>L		UFRJ50816:L>L		UWOPS91_917_1:L>L		YPS138:L>L	AA:641		UWOPS91_917_1:F>F	AA:643		A4:L>L		DBVPG6304:L>L		UFRJ50791:L>L		UFRJ50816:L>L		UWOPS91_917_1:L>L		YPS138:L>L	AA:654		UFRJ50791:G>G		UFRJ50816:G>G	AA:673		UWOPS91_917_1:M>M	AA:688		A4:P>P		DBVPG6304:P>P		UFRJ50791:P>P		UFRJ50816:P>P		UWOPS91_917_1:P>P		YPS138:P>P	AA:700		A4:T>T		DBVPG6304:T>T		UFRJ50791:T>T		UFRJ50816:T>T		UWOPS91_917_1:T>T		YPS138:T>T	AA:723		CBS432:->-	AA:751		UWOPS91_917_1:F>F	AA:764		A4:S>S		UFRJ50816:S>S	AA:766		A4:T>T		DBVPG6304:T>T		N_44:T>T		N_45:T>T		UFRJ50816:T>T	AA:816		YPS138:I>I	AA:825		YPS138:N>N	AA:839		YPS138:T>T	AA:847		UFRJ50816:->-		YPS138:->-	AA:878		UFRJ50816:L>L		YPS138:L>L	AA:898		UFRJ50791:->-		UFRJ50816:->-		YPS138:->-	AA:904		UFRJ50791:N>N		UFRJ50816:N>N		YPS138:N>N	AA:956		UWOPS91_917_1:Q>Q	AA:981		UWOPS91_917_1:I>I	AA:991		UWOPS91_917_1:V>V	AA:1003		UWOPS91_917_1:A>A	AA:1008		UFRJ50791:T>T		UFRJ50816:T>T		UWOPS91_917_1:T>T	AA:1013		UFRJ50791:A>A		UFRJ50816:A>A		UWOPS91_917_1:A>A		YPS138:A>A	AA:1020		N_43:R>R		N_45:R>R		UFRJ50791:R>R		UFRJ50816:R>R		YPS138:R>R	AA:1032		UFRJ50791:D>D		UFRJ50816:D>D		UWOPS91_917_1:D>D		YPS138:D>D	AA:1042		A4:T>T		UFRJ50791:T>T		UFRJ50816:T>T		UWOPS91_917_1:T>T	AA:1052		N_43:W>W		N_45:W>W	AA:1060		A4:C>C		UFRJ50791:C>C		UFRJ50816:C>C		YPS138:C>C	AA:1067		UWOPS91_917_1:C>C	AA:1076		A4:A>A		UFRJ50791:A>A		UFRJ50816:A>A		UWOPS91_917_1:A>A		YPS138:A>A	AA:1081		UWOPS91_917_1:T>TID:YBR119W	AA:2		UWOPS91_917_1:T>T	AA:7		A4:L>L		DBVPG6304:L>L		YPS138:L>L	AA:71		DBVPG6304:V>V		UFRJ50816:V>V		YPS138:V>V	AA:80		DBVPG6304:S>S		UFRJ50816:S>S		YPS138:S>S	AA:86		N_43:T>T		N_44:T>T		N_45:T>T	AA:94		DBVPG6304:N>N		UFRJ50816:N>N		YPS138:N>N	AA:97		N_43:L>L		N_44:L>L		N_45:L>L	AA:118		N_43:R>R	AA:122		DBVPG6304:L>L		UFRJ50791:L>L		UFRJ50816:L>L		YPS138:L>L	AA:146		N_43:V>V		N_44:V>V		N_45:V>V	AA:169		DBVPG6304:K>K		N_43:K>K		N_44:K>K		N_45:K>K		UFRJ50791:K>K		UFRJ50816:K>K		UWOPS91_917_1:K>K		YPS138:K>K	AA:172		DBVPG6304:V>V		UFRJ50791:V>V		UFRJ50816:V>V		UWOPS91_917_1:V>V		YPS138:V>V	AA:175		UWOPS91_917_1:L>L	AA:188		DBVPG6304:E>E		UFRJ50791:E>E		UFRJ50816:E>E	AA:189		N_43:I>I		N_44:I>I		N_45:I>I	AA:209		DBVPG6304:E>E		UFRJ50791:E>E		UFRJ50816:E>E		YPS138:E>E	AA:232		DBVPG6304:N>N		UFRJ50791:N>N		UFRJ50816:N>N		YPS138:N>N	AA:234		DBVPG6304:V>V		UFRJ50791:V>V		UFRJ50816:V>V		UWOPS91_917_1:V>V		YPS138:V>V	AA:243		A12:G>G		DBVPG6304:G>G		UFRJ50791:G>G		UFRJ50816:G>G		UWOPS91_917_1:G>G		YPS138:G>G	AA:254		A12:G>G		DBVPG6304:G>G		UFRJ50791:G>G		UFRJ50816:G>G		UWOPS91_917_1:G>G		YPS138:G>G	AA:272		A12:V>V		DBVPG6304:V>V		UFRJ50791:V>V		UFRJ50816:V>V		YPS138:V>V	AA:275		A12:R>R		DBVPG6304:R>R		UFRJ50791:R>R		UFRJ50816:R>R		YPS138:R>R	AA:289		A12:S>S		DBVPG6304:S>S		UFRJ50791:S>S		UWOPS91_917_1:S>S		YPS138:S>S	AA:293		A12:L>L		DBVPG6304:L>L		UFRJ50791:L>L		UWOPS91_917_1:L>L		YPS138:L>LID:YBR120C	AA:3		N_43:T>T		N_44:T>T		N_45:T>T	AA:19		UWOPS91_917_1:T>T	AA:40		A12:I>I		A4:I>I		N_43:I>I		N_44:I>I		N_45:I>I		UFRJ50816:I>I		UWOPS91_917_1:I>I		YPS138:I>I	AA:60		N_43:L>L		N_44:L>L		N_45:L>L	AA:62		UWOPS91_917_1:V>V	AA:67		A12:L>L		DBVPG6304:L>L		UFRJ50816:L>L		UWOPS91_917_1:R>R		YPS138:L>L	AA:69		UWOPS91_917_1:L>L	AA:71		A12:I>I		DBVPG6304:I>I		UWOPS91_917_1:I>I		YPS138:I>I	AA:74		UWOPS91_917_1:F>F	AA:92		UWOPS91_917_1:S>S	AA:100		UWOPS91_917_1:I>I	AA:109		UWOPS91_917_1:R>R	AA:117		A12:G>G		DBVPG6304:G>G		YPS138:G>G	AA:121		A12:Y>Y		DBVPG6304:Y>Y		YPS138:Y>Y	AA:126		UWOPS91_917_1:C>C	AA:130		UWOPS91_917_1:I>I	AA:144		A12:F>F		DBVPG6304:F>F		YPS138:F>F	AA:156		A12:L>L		DBVPG6304:L>L		UFRJ50791:L>L		YPS138:L>L	AA:159		A12:L>L		DBVPG6304:L>L		UFRJ50791:L>L		UWOPS91_917_1:L>L		YPS138:L>L	AA:162		CBS432:R>R		Q62_5:R>R		Q95_3:R>R		Y6_5:R>R		Z1_1:R>RID:YBR121C	AA:67		UWOPS91_917_1:K>K	AA:87		UWOPS91_917_1:I>I	AA:96		UWOPS91_917_1:S>S	AA:137		UWOPS91_917_1:F>F	AA:156		UWOPS91_917_1:K>K	AA:168		A12:N>N		A4:N>N		DBVPG6304:N>N		UFRJ50791:N>N		UFRJ50816:N>N		YPS138:N>N	AA:173		A12:C>C		A4:C>C		DBVPG6304:C>C		UFRJ50791:C>C		UWOPS91_917_1:C>C		YPS138:C>C	AA:179		A12:N>N		A4:N>N		DBVPG6304:N>N		UFRJ50791:N>N		UFRJ50816:N>N		UWOPS91_917_1:N>N		YPS138:N>N	AA:185		A12:L>L		A4:L>L		DBVPG6304:L>L		IFO1804:L>L		UFRJ50791:L>L		UFRJ50816:L>L		YPS138:L>L	AA:194		UWOPS91_917_1:L>L	AA:197		A12:S>S		DBVPG6304:S>S		UWOPS91_917_1:S>S		YPS138:S>S	AA:211		A12:I>I		A4:I>I	AA:213		UWOPS91_917_1:L>L	AA:216		UWOPS91_917_1:I>I	AA:232		A4:T>T		DBVPG6304:T>T		UFRJ50791:T>T		YPS138:T>T	AA:236		UWOPS91_917_1:L>L	AA:255		UWOPS91_917_1:N>N	AA:267		UWOPS91_917_1:S>S	AA:276		UWOPS91_917_1:T>T	AA:278		A4:D>D		DBVPG6304:D>D		UFRJ50791:D>D		YPS138:D>D	AA:279		UWOPS91_917_1:T>T	AA:294		UWOPS91_917_1:V>V	AA:296		UWOPS91_917_1:G>G	AA:304		A12:H>H		A4:H>H		DBVPG6304:H>H		UFRJ50791:H>H		YPS138:H>H	AA:305		UWOPS91_917_1:M>M	AA:309		DBVPG4650:S>S		Q32_3:S>S		Q89_8:S>S		Y7:S>S	AA:322		A12:L>L		A4:L>L		DBVPG6304:L>L		UFRJ50791:L>L		YPS138:L>L	AA:327		N_43:N>N		N_44:N>N		N_45:N>N	AA:352		Q59_1:S>S	AA:364		A12:G>G		A4:G>G		DBVPG6304:G>G		UWOPS91_917_1:G>G		YPS138:G>G	AA:372		A12:I>I		A4:I>I		DBVPG6304:I>I		YPS138:I>I	AA:380		A12:->-		A4:->-		DBVPG6304:->-		YPS138:->-	AA:396		A12:S>S		A4:S>S		DBVPG6304:S>S		N_44:S>S		N_45:S>S		UWOPS91_917_1:S>S		YPS138:S>S	AA:413		A12:R>R		DBVPG6304:R>R		UWOPS91_917_1:R>R		YPS138:R>R	AA:486		A12:W>W		DBVPG6304:W>W		UWOPS91_917_1:W>W		YPS138:W>W	AA:537		A12:T>T		DBVPG6304:T>T		UFRJ50791:T>T		YPS138:T>T	AA:544		A12:F>F		DBVPG6304:F>F		UFRJ50791:F>F		UWOPS91_917_1:F>F		YPS138:F>F	AA:546		UWOPS91_917_1:A>A	AA:564		N_43:L>L	AA:566		A12:I>I		DBVPG6304:I>I		UFRJ50791:I>I		YPS138:I>I	AA:585		A12:I>I		DBVPG6304:I>I		UFRJ50791:I>I		UWOPS91_917_1:I>I		YPS138:I>I	AA:606		A12:P>P		UFRJ50791:P>P		YPS138:P>P	AA:615		A12:C>C		UFRJ50791:C>C		YPS138:C>C	AA:632		A12:V>V		A4:V>V		UFRJ50791:V>V		YPS138:V>V	AA:662		A12:I>I		YPS138:I>I	AA:665		A12:T>T		A4:T>T		UFRJ50791:T>T		UFRJ50816:T>T		YPS138:T>TID:YBR122C	AA:6		UWOPS91_917_1:L>L	AA:12		UWOPS91_917_1:T>T	AA:19		A4:T>T		N_43:T>T		N_44:T>T		N_45:T>T		UFRJ50791:T>T		UWOPS91_917_1:T>T		YPS138:T>T	AA:43		DBVPG4650:F>F	AA:46		N_17:C>C		N_43:C>C		N_44:C>C		N_45:C>C	AA:68		A12:D>D		A4:D>D		YPS138:D>D	AA:76		A12:L>L		A4:L>L		UFRJ50791:L>L		YPS138:L>L	AA:78		N_44:E>E		N_45:E>E	AA:93		A12:V>V		DBVPG6304:V>V		YPS138:V>V	AA:95		A12:P>P		DBVPG6304:P>P		N_17:P>P		N_43:P>P		N_44:P>P		N_45:P>P		YPS138:P>P	AA:102		N_17:I>I		N_43:I>I		N_44:I>I		N_45:I>I		YPS138:I>I	AA:130		A12:H>H		DBVPG6304:H>H		UFRJ50816:H>H		YPS138:H>H	AA:137		UWOPS91_917_1:I>I	AA:140		A12:R>R		DBVPG6304:R>R		UFRJ50816:R>R		UWOPS91_917_1:R>R		YPS138:R>R	AA:158		A12:S>S		DBVPG6304:S>S		UFRJ50816:S>S		UWOPS91_917_1:S>S		YPS138:S>SID:YBR123C	AA:2		A12:Q>Q		A4:Q>Q		DBVPG6304:Q>Q		UFRJ50791:Q>Q		UFRJ50816:Q>Q	AA:13		Q32_3:A>A	AA:17		UWOPS91_917_1:F>F	AA:26		IFO1804:Y>Y		N_44:Y>Y		N_45:Y>Y	AA:33		A12:K>K		A4:K>K		DBVPG6304:K>K		N_17:K>K		Q95_3:K>K		UFRJ50791:K>K		UFRJ50816:K>K		UWOPS91_917_1:K>K		YPS138:K>K		Z1_1:K>K	AA:49		IFO1804:I>I		N_44:I>I		N_45:I>I	AA:52		A12:I>I		A4:I>I		DBVPG6304:I>I		UFRJ50791:I>I		UFRJ50816:I>I		UWOPS91_917_1:I>I		YPS138:I>I	AA:67		UWOPS91_917_1:S>S	AA:69		UWOPS91_917_1:Y>Y	AA:77		A12:V>V		A4:V>V		DBVPG6304:V>V		YPS138:V>V	AA:80		IFO1804:L>L		N_44:L>L		N_45:L>L	AA:88		A12:I>I		A4:I>I		DBVPG6304:I>I		UFRJ50791:I>I		UFRJ50816:I>I		YPS138:I>I	AA:125		A12:T>T		A4:T>T		DBVPG6304:T>T		UFRJ50791:T>T		UFRJ50816:T>T		YPS138:T>T	AA:130		UWOPS91_917_1:C>C	AA:132		A12:L>L		A4:L>L		DBVPG6304:L>L		UFRJ50791:L>L		UFRJ50816:L>L		YPS138:L>L	AA:139		A12:F>F		A4:F>F		DBVPG6304:F>F		UFRJ50791:F>F		UFRJ50816:F>F		YPS138:F>F	AA:143		A12:->-		A4:->-		DBVPG6304:->-		UFRJ50791:->-		UFRJ50816:->-		YPS138:->-	AA:148		UWOPS91_917_1:V>V	AA:154		IFO1804:I>I	AA:159		A12:S>S		A4:S>S		DBVPG6304:S>S		UFRJ50791:S>S		UFRJ50816:S>S		UWOPS91_917_1:S>S		YPS138:S>S	AA:173		A12:Y>Y		A4:Y>Y		DBVPG6304:Y>Y		UFRJ50791:Y>Y		UFRJ50816:Y>Y		YPS138:Y>Y	AA:209		A12:I>I		A4:I>I		DBVPG6304:I>I		UFRJ50791:I>I		UFRJ50816:I>I		YPS138:I>I	AA:213		A12:H>H		A4:H>H		DBVPG6304:H>H		UFRJ50791:H>H		UFRJ50816:H>H		YPS138:H>H	AA:221		CBS432:S>S		IFO1804:S>S		KPN3828:S>S		N_45:S>S		Q89_8:S>S	AA:240		A12:G>G		A4:G>G		DBVPG6304:G>G		N_45:G>G		UFRJ50791:G>G		UFRJ50816:G>G		YPS138:G>G	AA:274		A12:S>S		A4:S>S		DBVPG6304:S>S		UFRJ50791:S>S		UFRJ50816:S>S		YPS138:S>S	AA:303		A12:S>S		A4:S>S		DBVPG6304:S>S		UFRJ50791:S>S		UFRJ50816:S>S		YPS138:S>S	AA:320		A12:F>F		A4:F>F		DBVPG6304:F>F		UFRJ50791:F>F		UFRJ50816:F>F		YPS138:F>F	AA:342		A12:G>G		A4:G>G		N_45:G>G		Q62_5:G>G		Q95_3:G>G		T21_4:G>G		UFRJ50791:G>G		Y6_5:G>G	AA:361		A12:Q>Q		A4:Q>Q		UFRJ50791:Q>Q	AA:375		A12:A>A		A4:A>A		DBVPG6304:A>A		N_45:A>A		UFRJ50791:A>A	AA:385		N_17:F>F	AA:395		A12:T>T		A4:T>T		DBVPG6304:T>T	AA:423		A12:R>R		A4:R>R		DBVPG6304:R>R		YPS138:R>R	AA:427		N_43:I>I		N_45:I>I	AA:439		A12:Q>Q		A4:Q>Q		DBVPG6304:Q>Q		N_43:Q>Q		N_45:Q>Q		YPS138:Q>Q	AA:446		A12:G>G		A4:G>G		DBVPG6304:G>G		YPS138:G>G	AA:452		A12:W>W		A4:W>W		DBVPG6304:W>W		YPS138:W>W	AA:472		A12:K>K		A4:K>K		DBVPG6304:K>K		YPS138:K>K	AA:511		A12:->-		A4:->-		DBVPG6304:->-		IFO1804:->-		N_43:->-		N_45:->-		UWOPS91_917_1:->-		YPS138:->-	AA:533		UWOPS91_917_1:F>F	AA:536		UWOPS91_917_1:Y>Y	AA:542		UWOPS91_917_1:R>R	AA:570		UWOPS91_917_1:->-	AA:573		UWOPS91_917_1:L>L	AA:576		IFO1804:D>D		N_43:D>D		N_44:D>D		N_45:D>D	AA:578		A12:A>A		A4:A>A		DBVPG6304:A>A		YPS138:A>A	AA:597		UWOPS91_917_1:L>L	AA:600		DBVPG4650:T>T		IFO1804:T>T		N_17:T>T		N_43:T>T		N_44:T>T		N_45:T>T		Q32_3:T>T		Q59_1:T>T		Y6_5:T>T		Y7:T>T	AA:603		A12:F>F		A4:F>F		DBVPG6304:F>F		YPS138:F>F	AA:613		A12:T>T		A4:T>T		DBVPG6304:T>T		YPS138:T>TID:YBR125C	AA:15		UFRJ50816:I>I	AA:21		UFRJ50816:R>R	AA:24		UWOPS91_917_1:F>F	AA:37		UWOPS91_917_1:K>K	AA:45		A12:R>R		UFRJ50816:R>R		YPS138:R>R	AA:69		A12:L>L		UWOPS91_917_1:L>L		YPS138:L>L	AA:73		A12:N>N		IFO1804:N>N		N_43:N>N		N_44:N>N		N_45:N>N		UWOPS91_917_1:N>N		YPS138:N>N	AA:108		UWOPS91_917_1:S>S		YPS138:S>S	AA:118		IFO1804:V>V		N_43:V>V		N_44:V>V		N_45:V>V		UWOPS91_917_1:V>V		YPS138:V>V	AA:147		UWOPS91_917_1:S>S		YPS138:S>S	AA:155		UWOPS91_917_1:H>H	AA:163		IFO1804:T>T	AA:168		UWOPS91_917_1:T>T		YPS138:T>T	AA:181		N_44:R>R	AA:185		A4:Y>Y		DBVPG6304:Y>Y		UWOPS91_917_1:Y>Y		YPS138:Y>Y	AA:191		A4:G>G		DBVPG6304:G>G		UWOPS91_917_1:G>G		YPS138:G>G	AA:216		UWOPS91_917_1:C>C	AA:229		UWOPS91_917_1:L>L	AA:234		DBVPG6304:Y>Y		UFRJ50816:Y>Y		YPS138:Y>Y	AA:246		DBVPG6304:F>F		UFRJ50816:F>F		YPS138:F>F	AA:324		N_43:V>VID:YBR126C	AA:6		UWOPS91_917_1:R>R	AA:48		A4:G>G		DBVPG6304:G>G		UFRJ50791:G>G	AA:56		A4:G>G		DBVPG6304:G>G		UFRJ50791:G>G	AA:72		UWOPS91_917_1:V>V	AA:76		UWOPS91_917_1:C>C	AA:107		UWOPS91_917_1:S>S	AA:135		UFRJ50791:G>G	AA:138		A4:F>F		UFRJ50791:F>F		UWOPS91_917_1:F>F	AA:149		A4:D>D		UFRJ50791:D>D		UFRJ50816:D>D		UWOPS91_917_1:D>D	AA:161		A4:L>L		UFRJ50791:L>L		UFRJ50816:L>L	AA:166		UFRJ50791:T>T		UFRJ50816:T>T	AA:180		A4:P>P		UFRJ50791:P>P		UFRJ50816:P>P	AA:202		Y6_5:I>I	AA:207		A4:T>T		UFRJ50791:T>T		UFRJ50816:T>T	AA:209		UFRJ50791:Y>Y		UFRJ50816:Y>Y	AA:229		N_45:L>L	AA:231		N_45:Q>Q	AA:248		UFRJ50791:I>I		UFRJ50816:I>I	AA:272		UFRJ50791:Q>Q		UFRJ50816:Q>Q	AA:275		UFRJ50791:S>S		UFRJ50816:S>S	AA:338		DBVPG6304:Q>Q		UFRJ50816:Q>Q		YPS138:Q>Q	AA:377		A4:L>L		DBVPG6304:L>L		UFRJ50816:L>L		YPS138:L>L	AA:385		UFRJ50816:R>R	AA:422		A4:L>L		DBVPG6304:L>L		UFRJ50816:L>L		YPS138:L>L	AA:428		N_44:L>L		N_45:L>L	AA:454		A4:R>R		DBVPG6304:R>R		YPS138:R>R	AA:486		CBS432:G>G		KPN3828:G>G		N_17:G>GID:YBR128C	AA:10		A12:R>R		DBVPG6304:R>R		N_44:R>R		N_45:R>R		UFRJ50816:R>R		UWOPS91_917_1:R>R		YPS138:R>R	AA:16		A12:R>R		DBVPG6304:R>R		UFRJ50816:R>R		YPS138:R>R	AA:28		N_44:R>R		N_45:R>R	AA:42		T21_4:K>K	AA:65		A4:I>I		DBVPG6304:I>I		UFRJ50816:I>I		YPS138:I>I	AA:70		A4:V>V		DBVPG6304:V>V		UFRJ50816:V>V		YPS138:V>V	AA:90		A4:L>L		DBVPG6304:L>L		N_44:L>L		N_45:L>L		Q59_1:L>L		Q62_5:L>L		S36_7:L>L		T21_4:L>L		UFRJ50791:L>L		UFRJ50816:L>L		UWOPS91_917_1:L>L		Y6_5:L>L		Y7:L>L		YPS138:L>L		Z1_1:L>L	AA:103		A12:I>I		A4:I>I		DBVPG6304:I>I		UFRJ50791:I>I		UFRJ50816:I>I		UWOPS91_917_1:I>I		YPS138:Y>Y	AA:112		N_43:K>K		N_44:K>K		N_45:K>K		Q59_1:K>K		Q62_5:K>K		Q95_3:K>K		S36_7:K>K		T21_4:K>K		Y6_5:K>K		Y7:K>K		Z1_1:K>K	AA:116		N_43:S>S		N_44:S>S		N_45:S>S	AA:121		UWOPS91_917_1:G>G	AA:126		A4:N>N		DBVPG6304:N>N		UFRJ50791:N>N		UFRJ50816:N>N		UWOPS91_917_1:N>N		YPS138:N>N	AA:131		UWOPS91_917_1:G>G	AA:136		A4:I>I		DBVPG6304:I>I		UFRJ50791:I>I		UFRJ50816:I>I		UWOPS91_917_1:I>I		YPS138:I>I	AA:150		UWOPS91_917_1:R>R	AA:162		A4:L>L		DBVPG6304:L>L		N_43:L>L		N_44:L>L		N_45:L>L		UFRJ50791:L>L		UFRJ50816:L>L		UWOPS91_917_1:L>L		YPS138:L>L	AA:167		A12:E>E		A4:E>E		DBVPG6304:E>E		UFRJ50791:E>E		UFRJ50816:E>E		UWOPS91_917_1:H>H		YPS138:E>E	AA:204		A12:V>V		A4:V>V		DBVPG6304:V>V		UFRJ50791:V>V		UFRJ50816:V>V		UWOPS91_917_1:V>V		YPS138:V>V	AA:213		UWOPS91_917_1:L>L	AA:272		A12:I>I		A4:I>I		DBVPG6304:I>I		UFRJ50791:I>I		UFRJ50816:I>I		UWOPS91_917_1:I>IID:YBR129C	AA:27		UWOPS91_917_1:G>G	AA:79		A12:R>R		DBVPG6304:R>R		YPS138:R>R	AA:84		UWOPS91_917_1:F>F	AA:87		A12:E>E		DBVPG6304:E>E		UWOPS91_917_1:E>E		YPS138:E>E	AA:99		UWOPS91_917_1:L>L	AA:105		A12:K>K		DBVPG6304:K>K		YPS138:K>K	AA:114		A12:R>R		DBVPG6304:R>R		UWOPS91_917_1:R>R		YPS138:R>R	AA:163		A12:I>I		DBVPG6304:I>I		IFO1804:I>I		N_44:I>I		N_45:I>I		UWOPS91_917_1:I>I		YPS138:I>I	AA:170		A12:T>T		DBVPG6304:T>T		YPS138:T>T	AA:171		UWOPS91_917_1:F>F	AA:193		A12:A>A		DBVPG6304:A>A		YPS138:A>A	AA:204		UWOPS91_917_1:E>E	AA:209		N_44:G>G		N_45:G>G	AA:220		UWOPS91_917_1:L>L	AA:225		UWOPS91_917_1:I>I	AA:231		Q62_5:R>R		Q95_3:R>R		Z1_1:R>R	AA:236		A12:R>R		DBVPG6304:R>R		UWOPS91_917_1:R>R	AA:246		Q59_1:S>S		Q62_5:S>S		Q95_3:S>S		UWOPS91_917_1:S>S		Z1_1:S>S	AA:279		A12:L>L		DBVPG6304:L>L		N_44:L>L		N_45:L>L		UWOPS91_917_1:L>L		YPS138:L>L	AA:286		A12:S>S		DBVPG6304:S>S		N_44:S>S		N_45:S>S		UWOPS91_917_1:S>S		YPS138:S>S	AA:294		A12:C>C		DBVPG6304:C>C		UWOPS91_917_1:V>V		YPS138:C>CID:YBR130C	AA:10		A12:T>T		A4:T>T		DBVPG6304:T>T		UFRJ50816:T>T		UWOPS91_917_1:G>G		YPS138:T>T	AA:55		UFRJ50816:G>G	AA:98		A12:I>I		A4:I>I		UFRJ50816:I>I		UWOPS91_917_1:I>I		YPS138:I>I	AA:134		UWOPS91_917_1:H>H	AA:137		N_43:L>L		N_44:L>L		N_45:L>L	AA:190		UWOPS91_917_1:R>R	AA:196		A4:F>F		UFRJ50816:F>F		UWOPS91_917_1:F>F		YPS138:F>F	AA:200		UWOPS91_917_1:F>F	AA:215		A4:I>I		YPS138:I>I	AA:236		UWOPS91_917_1:T>T	AA:240		A4:L>L		DBVPG6304:L>L		UWOPS91_917_1:L>L		YPS138:L>L	AA:243		UWOPS91_917_1:T>T	AA:244		UFRJ50816:D>D	AA:254		A4:R>R		DBVPG6304:R>R		N_43:R>R		N_45:R>R		UWOPS91_917_1:R>R		YPS138:R>R	AA:270		A4:V>V		DBVPG6304:V>V		UWOPS91_917_1:V>V		YPS138:V>V	AA:283		UWOPS91_917_1:F>F	AA:285		A4:G>G		DBVPG6304:G>G		YPS138:G>G	AA:289		A4:F>F		DBVPG6304:F>F		YPS138:F>F	AA:299		UWOPS91_917_1:L>L	AA:309		A4:F>F		DBVPG6304:F>F		UFRJ50791:F>F		YPS138:F>F	AA:316		A4:F>F		DBVPG6304:F>F		YPS138:F>F	AA:322		A4:L>L		DBVPG6304:L>L		YPS138:L>L	AA:327		UWOPS91_917_1:R>R	AA:348		A4:G>G		DBVPG6304:G>G		UFRJ50791:G>G		UWOPS91_917_1:G>G		YPS138:G>G	AA:354		A12:A>A		A4:A>A		DBVPG6304:L>L		UFRJ50791:L>L		UFRJ50816:A>A		UWOPS91_917_1:A>A		YPS138:A>A	AA:357		UWOPS91_917_1:->-	AA:364		A4:Q>Q		DBVPG6304:Q>Q		UFRJ50791:Q>Q		YPS138:Q>Q	AA:367		UWOPS91_917_1:I>I	AA:376		N_43:V>V		N_44:V>V		N_45:V>V		UWOPS91_917_1:V>V	AA:378		UWOPS91_917_1:K>K	AA:381		UWOPS91_917_1:N>N	AA:384		UWOPS91_917_1:G>G	AA:410		UWOPS91_917_1:V>V	AA:420		A12:D>D		A4:D>D		DBVPG6304:D>D		UFRJ50791:S>S		UFRJ50816:D>D		UWOPS91_917_1:D>D		YPS138:D>DID:YBR132C	AA:18		A12:I>I		A4:I>I		DBVPG6304:I>I		UFRJ50791:V>V		UFRJ50816:I>I		UWOPS91_917_1:I>I		YPS138:I>I	AA:21		A12:F>F		A4:F>F		UFRJ50791:F>F		UFRJ50816:F>F		UWOPS91_917_1:F>F		YPS138:F>F	AA:48		A12:H>H		A4:H>H		UFRJ50791:H>H		UFRJ50816:A>A		YPS138:H>H	AA:55		A12:E>E		A4:E>E		IFO1804:E>E		UWOPS91_917_1:E>E		YPS138:E>E	AA:67		UWOPS91_917_1:V>V	AA:77		IFO1804:A>A		N_43:A>A		N_45:A>A	AA:93		A4:S>S		DBVPG6304:S>S		UFRJ50791:S>S		UFRJ50816:S>S		UWOPS91_917_1:S>S		YPS138:S>S	AA:107		YPS138:G>G	AA:147		IFO1804:I>I		N_43:I>I		N_45:I>I		UWOPS91_917_1:I>I	AA:150		N_43:Q>Q		N_45:Q>Q		UWOPS91_917_1:Q>Q	AA:152		A4:Q>Q		DBVPG6304:Q>Q		UFRJ50791:Q>Q		UFRJ50816:Q>Q		UWOPS91_917_1:Q>Q		YPS138:Q>Q	AA:170		A4:T>T		DBVPG6304:T>T		IFO1804:T>T		N_43:T>T		N_45:T>T		UFRJ50791:T>T		UFRJ50816:T>T		UWOPS91_917_1:T>T		YPS138:T>T	AA:204		UWOPS91_917_1:K>K	AA:218		A12:Y>Y		A4:Y>Y		DBVPG6304:Y>Y		UFRJ50791:Y>Y		UFRJ50816:Y>Y		UWOPS91_917_1:Y>Y		YPS138:Y>Y	AA:235		A12:T>T		A4:T>T		DBVPG6304:T>T		UFRJ50816:T>T		YPS138:T>T	AA:237		A12:P>P		A4:P>P		DBVPG6304:P>P		IFO1804:P>P		N_43:P>P		N_45:P>P		UFRJ50816:P>P		YPS138:P>P	AA:254		UWOPS91_917_1:N>N	AA:286		A12:F>F		A4:F>F		DBVPG6304:F>F		UFRJ50816:F>F		UWOPS91_917_1:F>F		YPS138:F>F	AA:290		A12:H>H		A4:H>H		DBVPG6304:H>H		UFRJ50816:H>H		UWOPS91_917_1:H>H		YPS138:H>H	AA:297		UWOPS91_917_1:S>S	AA:306		UWOPS91_917_1:A>A	AA:395		N_43:N>N		N_45:N>N	AA:396		A12:T>T		A4:T>T		DBVPG6304:T>T		UFRJ50816:T>T		UWOPS91_917_1:T>T		YPS138:T>T	AA:404		A12:V>V		A4:V>V		DBVPG6304:V>V		N_43:V>V		N_45:V>V		UFRJ50816:V>V		UWOPS91_917_1:V>V		YPS138:V>V	AA:417		UWOPS91_917_1:N>N	AA:420		UWOPS91_917_1:A>A	AA:434		A12:->-		A4:->-		DBVPG6304:->-		UFRJ50816:->-		UWOPS91_917_1:->-		YPS138:->-	AA:459		A12:R>R		A4:R>R		DBVPG6304:R>R		UFRJ50816:R>R		YPS138:R>R	AA:471		A12:S>S		A4:S>S		DBVPG6304:S>S		UFRJ50816:S>S		UWOPS91_917_1:S>S		YPS138:S>S	AA:473		A12:C>C		A4:C>C		DBVPG6304:C>C		UFRJ50816:C>C		UWOPS91_917_1:C>C		YPS138:C>C	AA:497		UWOPS91_917_1:D>D	AA:513		A12:S>S		A4:S>S		DBVPG6304:S>S		UFRJ50816:S>S		YPS138:S>S	AA:515		N_43:F>F		N_45:F>F	AA:518		A12:K>K		A4:K>K		DBVPG6304:K>K		N_43:K>K		N_45:K>K		UFRJ50816:K>K		YPS138:K>K	AA:523		UWOPS91_917_1:P>P	AA:529		A12:R>R		A4:R>R		DBVPG6304:R>R		UFRJ50816:R>R		UWOPS91_917_1:R>R		YPS138:R>R	AA:569		A12:S>S		A4:S>S		DBVPG6304:S>S		UFRJ50816:S>S		UWOPS91_917_1:S>S		YPS138:S>S	AA:576		N_43:F>F		N_45:F>F	AA:593		A12:T>T		A4:T>T		DBVPG6304:T>T		UFRJ50816:T>T		YPS138:T>TID:YBR133C	AA:26		CBS432:F>F		CBS5829:F>F		N_17:F>F	AA:28		A12:Y>Y		A4:Y>Y		DBVPG6304:Y>Y		UFRJ50791:Y>Y		UFRJ50816:Y>Y		UWOPS91_917_1:S>S		YPS138:S>S	AA:32		A12:E>E		DBVPG6304:E>E		UWOPS91_917_1:E>E	AA:36		A12:S>S		DBVPG6304:S>S		UWOPS91_917_1:S>S		YPS138:S>S	AA:61		A12:V>V		A4:V>V		DBVPG6304:V>V		IFO1804:V>V		N_43:V>V		N_44:V>V		N_45:V>V		UWOPS91_917_1:V>V		YPS138:V>V	AA:66		UWOPS91_917_1:S>S	AA:76		A12:T>T		A4:T>T		DBVPG6304:T>T		UWOPS91_917_1:T>T		YPS138:T>T	AA:88		UWOPS91_917_1:L>L	AA:99		A12:I>I		A4:I>I		DBVPG6304:I>I		YPS138:I>I	AA:104		UWOPS91_917_1:->-	AA:141		A12:V>V		A4:V>V		DBVPG6304:V>V		UFRJ50816:V>V		UWOPS91_917_1:V>V		YPS138:V>V	AA:147		UWOPS91_917_1:I>I	AA:175		A12:G>G		A4:G>G		DBVPG6304:G>G		UFRJ50816:G>G		YPS138:G>G	AA:204		A12:D>D		A4:D>D		DBVPG6304:D>D		N_43:V>V		N_44:V>V		N_45:V>V		UFRJ50816:D>D		YPS138:D>D	AA:234		A12:Y>Y		A4:Y>Y		DBVPG6304:Y>Y		UFRJ50816:Y>Y		UWOPS91_917_1:Y>Y	AA:251		N_43:T>T		N_45:T>T		UWOPS91_917_1:D>D	AA:277		N_45:I>I	AA:287		A12:P>P		A4:P>P		DBVPG6304:P>P		UFRJ50816:P>P	AA:295		A12:->-		A4:->-		DBVPG6304:->-		UFRJ50816:->-	AA:302		A12:V>V		A4:V>V		DBVPG6304:V>V		UFRJ50816:V>V		UWOPS91_917_1:V>V	AA:311		A12:A>A		A4:A>A		DBVPG6304:A>A		UFRJ50816:A>A		UWOPS91_917_1:A>A	AA:335		A12:R>R		A4:R>R		DBVPG6304:R>R		UFRJ50816:R>R	AA:344		UWOPS91_917_1:M>M	AA:345		A12:I>I		A4:I>I		DBVPG6304:I>I		UFRJ50816:I>I	AA:359		Q59_1:T>T	AA:383		A12:L>L		A4:L>L		DBVPG6304:L>L		IFO1804:L>L		N_43:L>L		N_45:L>L		UFRJ50816:L>L	AA:389		UFRJ50816:L>L	AA:393		UWOPS91_917_1:F>F	AA:402		A12:V>V		A4:V>V		DBVPG6304:V>V		UFRJ50816:V>V	AA:407		UWOPS91_917_1:I>I	AA:474		A12:V>V		A4:V>V		DBVPG6304:V>V		UFRJ50816:V>V	AA:501		UWOPS91_917_1:P>P	AA:505		A12:L>L		DBVPG6304:L>L		IFO1804:L>L		N_43:L>L		N_44:L>L		N_45:L>L		UFRJ50816:L>L		UWOPS91_917_1:L>L	AA:507		UWOPS91_917_1:Q>Q	AA:513		A12:I>I		DBVPG6304:I>I		UFRJ50816:I>I		UWOPS91_917_1:I>I	AA:523		A12:Q>Q		DBVPG6304:Q>Q		YPS138:Q>Q	AA:532		IFO1804:G>G		N_43:G>G		N_44:G>G		N_45:G>G	AA:536		UWOPS91_917_1:A>A	AA:540		A12:V>V		DBVPG6304:V>V		UFRJ50791:V>V		UWOPS91_917_1:V>V		YPS138:V>V	AA:544		UWOPS91_917_1:L>L	AA:555		A12:T>T		DBVPG6304:T>T		UFRJ50791:T>T		YPS138:T>T	AA:556		UWOPS91_917_1:Q>Q	AA:562		A12:I>I		DBVPG6304:I>I		IFO1804:I>I		N_43:I>I		N_44:I>I		N_45:I>I		UFRJ50791:I>I		UWOPS91_917_1:I>I		YPS138:I>I	AA:565		IFO1804:L>L		N_43:L>L		N_44:L>L		N_45:L>L	AA:572		A12:L>L		A4:L>L		DBVPG6304:L>L		UFRJ50791:L>L		YPS138:L>L	AA:580		UFRJ50791:I>I		UWOPS91_917_1:I>I	AA:589		A12:G>G		A4:G>G		YPS138:G>G	AA:627		UFRJ50791:A>A		UWOPS91_917_1:A>A	AA:629		UFRJ50791:F>F		UWOPS91_917_1:F>F	AA:707		A12:A>A		A4:A>A		UFRJ50791:A>A		UFRJ50816:A>A		UWOPS91_917_1:A>A		YPS138:A>A	AA:710		UFRJ50791:S>S		UFRJ50816:S>S		UWOPS91_917_1:S>S	AA:715		UWOPS91_917_1:T>T	AA:737		Q59_1:L>L	AA:742		A12:R>R		A4:R>R		DBVPG6304:R>R		YPS138:R>R	AA:748		IFO1804:L>L		N_43:L>L		N_44:L>L		N_45:L>L	AA:761		UWOPS91_917_1:L>L	AA:796		A12:R>R		A4:R>R		DBVPG6304:R>R	AA:822		A12:N>N		A4:N>N		DBVPG6304:N>N		UFRJ50791:N>N		UFRJ50816:N>N		YPS138:N>NID:YBR135W	AA:16		A4:Q>Q		DBVPG6304:Q>Q		UFRJ50816:Q>Q		UWOPS91_917_1:Q>Q	AA:22		A4:L>L		DBVPG6304:L>L		UFRJ50816:L>L		UWOPS91_917_1:L>L	AA:27		A4:S>S		DBVPG6304:S>S		UFRJ50816:S>S		UWOPS91_917_1:S>S	AA:32		UWOPS91_917_1:P>P	AA:36		UWOPS91_917_1:D>D	AA:46		A4:L>L		IFO1804:L>L		N_43:L>L		N_44:L>L		N_45:L>L		UFRJ50816:L>L		UWOPS91_917_1:L>L	AA:51		UWOPS91_917_1:L>L	AA:56		A4:S>S		UFRJ50816:S>S		UWOPS91_917_1:S>S	AA:63		UWOPS91_917_1:V>V	AA:69		A4:L>L		UFRJ50816:L>L		UWOPS91_917_1:L>L	AA:84		A4:G>G		UFRJ50816:G>G	AA:86		A4:E>E		UFRJ50816:E>E	AA:93		A4:P>P		UFRJ50816:P>P	AA:98		A4:L>L		UFRJ50816:L>L		UWOPS91_917_1:L>L	AA:105		UFRJ50816:N>N	AA:108		UFRJ50816:A>A	AA:112		UFRJ50816:A>A	AA:123		N_43:Q>Q		N_44:Q>Q		N_45:Q>QID:YBR136W	AA:26		A12:V>V		A4:V>V		DBVPG6304:V>V		YPS138:V>V	AA:31		Q59_1:V>V	AA:33		IFO1804:T>T		N_43:T>T		N_44:T>T		N_45:T>T	AA:51		A12:L>L		A4:L>L		DBVPG6304:L>L		UFRJ50816:L>L		UWOPS91_917_1:L>L		YPS138:L>L	AA:75		A12:S>S		CBS432:S>S		DBVPG6304:S>S		UFRJ50816:S>S		YPS138:S>S	AA:80		A12:L>L		DBVPG6304:L>L		UFRJ50816:L>L		YPS138:L>L	AA:81		CBS432:L>L		CBS5829:L>L		KPN3829:L>L		N_17:L>L		Q59_1:L>L		T21_4:L>L	AA:95		A12:G>G		DBVPG6304:G>G		IFO1804:G>G		N_43:G>G		N_44:G>G		N_45:G>G		UFRJ50816:G>G		UWOPS91_917_1:G>G		YPS138:G>G	AA:104		UFRJ50816:D>D	AA:106		A12:F>F	AA:122		A12:F>F		DBVPG6304:F>F		UFRJ50816:F>F		UWOPS91_917_1:F>F		YPS138:F>F	AA:125		A12:R>R		DBVPG6304:R>R		UFRJ50816:R>R		UWOPS91_917_1:R>R		YPS138:R>R	AA:148		A12:A>A		DBVPG6304:A>A		UFRJ50816:A>A		UWOPS91_917_1:A>A		YPS138:A>A	AA:171		A12:L>L		DBVPG6304:L>L		YPS138:L>L	AA:180		A12:S>S		DBVPG6304:S>S		UWOPS91_917_1:S>S		YPS138:S>S	AA:206		N_43:L>L		N_44:L>L		N_45:L>L	AA:209		A12:T>T		UWOPS91_917_1:T>T		YPS138:T>T	AA:231		A12:L>L		A4:L>L		UWOPS91_917_1:L>L		YPS138:L>L	AA:237		UWOPS91_917_1:A>A	AA:246		A12:L>L		UWOPS91_917_1:L>L		YPS138:L>L	AA:262		UWOPS91_917_1:T>T	AA:268		Y6_5:C>C	AA:285		UWOPS91_917_1:K>K	AA:291		A12:N>N		A4:N>N		DBVPG6304:N>N		YPS138:N>N	AA:308		A12:S>S		A4:S>S		DBVPG6304:S>S		UFRJ50816:S>S		YPS138:S>S	AA:311		A12:F>F		A4:F>F		DBVPG6304:F>F		UFRJ50816:F>F		YPS138:F>F	AA:319		UWOPS91_917_1:F>F	AA:320		A12:P>P		A4:P>P		DBVPG6304:P>P		UFRJ50816:P>P		YPS138:P>P	AA:326		A12:D>D		A4:D>D		DBVPG6304:D>D		UFRJ50816:D>D		UWOPS91_917_1:D>D		YPS138:D>D	AA:335		A12:F>F		A4:F>F		DBVPG6304:F>F		UFRJ50816:F>F		UWOPS91_917_1:F>F		YPS138:F>F	AA:339		A12:T>T		A4:T>T		DBVPG6304:T>T		UFRJ50816:T>T		UWOPS91_917_1:T>T		YPS138:T>T	AA:377		A4:S>S		DBVPG6304:S>S		UFRJ50816:S>S		UWOPS91_917_1:S>S	AA:379		A4:L>L		DBVPG6304:L>L		UFRJ50816:L>L		YPS138:L>L	AA:390		N_45:Y>Y	AA:417		UFRJ50816:V>V	AA:436		UWOPS91_917_1:L>L	AA:441		A4:V>V		DBVPG6304:V>V		UFRJ50816:V>V		UWOPS91_917_1:V>V	AA:464		A4:R>R		DBVPG6304:R>R		UFRJ50816:R>R		UWOPS91_917_1:R>R	AA:482		A4:K>K	AA:496		A4:E>E		UFRJ50816:E>E	AA:498		UWOPS91_917_1:T>T	AA:503		A4:E>E		DBVPG6304:E>E		UFRJ50816:E>E		UWOPS91_917_1:E>E	AA:509		A4:R>R	AA:516		A4:S>S		DBVPG6304:S>S	AA:520		IFO1804:K>K		N_44:K>K		N_45:K>K	AA:528		A4:S>S		UFRJ50816:S>S		UWOPS91_917_1:S>S	AA:542		A4:G>G		UFRJ50816:G>G		UWOPS91_917_1:G>G	AA:547		A4:L>L		UFRJ50816:L>L	AA:564		A4:G>G		UFRJ50816:G>G	AA:572		IFO1804:L>L		N_44:L>L		N_45:L>L	AA:586		A12:L>L		A4:L>L		IFO1804:L>L		N_44:L>L		N_45:L>L		UFRJ50816:L>L		UWOPS91_917_1:L>L	AA:630		UWOPS91_917_1:A>A	AA:656		A12:S>S		A4:S>S		UWOPS91_917_1:S>S	AA:669		UWOPS91_917_1:I>I	AA:675		A12:V>V		A4:V>V		UWOPS91_917_1:V>V	AA:685		A12:S>S		A4:S>S	AA:694		UWOPS91_917_1:Q>Q	AA:695		A12:L>L		A4:L>L	AA:700		IFO1804:V>V		N_45:V>V		UWOPS91_917_1:V>V	AA:725		A12:Q>Q		A4:Q>Q	AA:729		A12:I>I		A4:I>I		UWOPS91_917_1:I>I		YPS138:I>I	AA:767		A12:N>N		A4:N>N		YPS138:N>N	AA:800		A12:K>K		A4:K>K		DBVPG6304:K>K		YPS138:K>K	AA:818		A12:K>K		A4:K>K		DBVPG6304:K>K		YPS138:K>K	AA:841		A12:L>L		A4:L>L		DBVPG6304:L>L		IFO1804:L>L		N_43:L>L		N_45:L>L		UWOPS91_917_1:L>L		YPS138:L>L	AA:862		A12:N>N		A4:N>N		DBVPG6304:N>N		UFRJ50816:N>N		YPS138:N>N	AA:868		IFO1804:Q>Q		N_43:Q>Q		N_45:Q>Q		UWOPS91_917_1:Q>Q	AA:871		UWOPS91_917_1:A>A	AA:884		A4:F>F		DBVPG6304:F>F		UFRJ50816:F>F		YPS138:F>F	AA:892		A12:H>H	AA:896		A12:G>G	AA:897		A4:R>R		DBVPG6304:R>R		UFRJ50816:R>R		UWOPS91_917_1:R>R		YPS138:R>R	AA:911		A12:S>S	AA:914		A12:I>I		A4:I>I		DBVPG6304:I>I		IFO1804:I>I		N_43:I>I		N_45:I>I		UFRJ50816:I>I		UWOPS91_917_1:I>I		YPS138:I>I	AA:921		A12:S>S	AA:927		A12:A>A	AA:933		A12:L>L	AA:938		A12:G>G	AA:942		A12:V>V	AA:957		A12:L>L	AA:961		A12:E>E	AA:964		A12:T>T	AA:982		A4:G>G		DBVPG6304:G>G		UFRJ50816:G>G		YPS138:G>G	AA:997		A4:K>K		DBVPG6304:K>K		UFRJ50816:K>K		YPS138:K>K	AA:1000		A4:S>S		DBVPG6304:S>S		UFRJ50816:S>S		YPS138:S>S	AA:1005		A4:K>K		DBVPG6304:K>K		IFO1804:K>K		N_43:K>K		N_44:K>K		N_45:K>K		UFRJ50816:K>K		YPS138:K>K	AA:1013		A4:A>A		DBVPG6304:A>A		UFRJ50816:A>A		YPS138:A>A	AA:1018		A4:P>P		DBVPG6304:P>P		UFRJ50816:P>P		YPS138:P>P	AA:1022		IFO1804:I>I		N_43:I>I		N_44:I>I		N_45:I>I	AA:1029		A4:F>F		DBVPG6304:F>F		UFRJ50816:F>F		YPS138:F>F	AA:1030		IFO1804:A>A		N_43:A>A		N_44:A>A		N_45:A>A	AA:1036		IFO1804:I>I		N_43:I>I		N_44:I>I		N_45:I>I	AA:1039		A4:T>T		DBVPG6304:T>T		UFRJ50816:T>T		YPS138:T>T	AA:1047		A4:A>A		DBVPG6304:A>A		UFRJ50816:A>A		YPS138:A>A	AA:1052		A4:S>S		DBVPG6304:S>S		UFRJ50816:S>S		YPS138:S>S	AA:1059		A4:N>N		DBVPG6304:N>N		UFRJ50816:N>N		YPS138:N>N	AA:1083		IFO1804:K>K		N_44:K>K		N_45:K>K	AA:1095		IFO1804:L>L		N_43:L>L		N_44:L>L	AA:1096		A4:G>G		DBVPG6304:G>G		UFRJ50816:G>G		YPS138:G>G	AA:1120		A12:I>I		A4:I>I		DBVPG6304:I>I		YPS138:I>I	AA:1130		A12:K>K		A4:K>K		DBVPG6304:K>K		UFRJ50816:K>K		YPS138:K>K	AA:1141		UWOPS91_917_1:N>N	AA:1159		CBS432:V>V		CBS5829:V>V	AA:1164		UWOPS91_917_1:L>L	AA:1166		IFO1804:P>P	AA:1170		A12:Q>Q		A4:Q>Q		DBVPG6304:Q>Q		UFRJ50816:Q>Q		YPS138:Q>Q	AA:1172		IFO1804:E>E		N_43:E>E		N_44:E>E		N_45:E>E	AA:1176		A12:K>K		A4:K>K		DBVPG6304:K>K		UFRJ50816:K>K		YPS138:K>K	AA:1188		A12:L>L		A4:L>L		DBVPG6304:L>L		YPS138:L>L	AA:1189		UWOPS91_917_1:K>K	AA:1201		A12:N>N		DBVPG6304:N>N		UFRJ50816:N>N		YPS138:N>N	AA:1218		UWOPS91_917_1:S>S	AA:1234		A12:A>A		A4:A>A		DBVPG6304:A>A		YPS138:A>A	AA:1247		UWOPS91_917_1:S>S	AA:1263		UWOPS91_917_1:L>L	AA:1276		A12:L>L		A4:L>L		YPS138:L>L	AA:1281		A12:S>S		A4:S>S		YPS138:S>S	AA:1313		A12:A>A		A4:A>A		UWOPS91_917_1:A>A		YPS138:A>A	AA:1328		IFO1804:T>T		N_44:T>T		N_45:T>T	AA:1352		A12:V>V		A4:V>V		IFO1804:V>V		N_44:V>V		N_45:V>V		UFRJ50816:V>V		UWOPS91_917_1:V>V		YPS138:V>V	AA:1377		UWOPS91_917_1:K>K	AA:1379		UWOPS91_917_1:T>T	AA:1395		UWOPS91_917_1:T>T	AA:1397		UWOPS91_917_1:S>S	AA:1400		A12:L>L		A4:L>L		DBVPG6304:L>L		UFRJ50816:L>L		YPS138:L>L	AA:1407		IFO1804:T>T		N_43:T>T		N_44:T>T		N_45:T>T		UWOPS91_917_1:T>T	AA:1412		UWOPS91_917_1:R>R	AA:1421		UWOPS91_917_1:Y>Y	AA:1426		UWOPS91_917_1:H>H	AA:1430		A12:Q>Q		A4:Q>Q	AA:1439		IFO1804:Q>Q		N_43:Q>Q		N_45:Q>Q	AA:1441		UWOPS91_917_1:T>T	AA:1445		UWOPS91_917_1:I>I	AA:1455		UWOPS91_917_1:L>L	AA:1460		A12:T>T		A4:T>T		DBVPG6304:T>T		UFRJ50816:T>T		UWOPS91_917_1:T>T		YPS138:T>T	AA:1470		A12:L>L		A4:L>L		DBVPG6304:L>L		UFRJ50816:L>L		YPS138:L>L	AA:1483		UWOPS91_917_1:F>F	AA:1489		A4:F>F		DBVPG6304:F>F		UFRJ50816:F>F		YPS138:F>F	AA:1496		UWOPS91_917_1:T>T	AA:1498		UWOPS91_917_1:R>R	AA:1508		A4:L>L		DBVPG6304:L>L		UFRJ50816:L>L		YPS138:L>L	AA:1517		UWOPS91_917_1:S>S	AA:1522		UWOPS91_917_1:D>D	AA:1526		A4:S>S		DBVPG6304:S>S		UFRJ50816:S>S		YPS138:S>S	AA:1535		DBVPG6304:Y>Y	AA:1539		A4:L>L		DBVPG6304:L>L		UFRJ50816:L>L	AA:1563		IFO1804:D>D		N_43:D>D		UFRJ50816:D>D	AA:1565		UWOPS91_917_1:D>D	AA:1568		A4:E>E		DBVPG6304:E>E		UFRJ50816:E>E	AA:1569		UWOPS91_917_1:V>V	AA:1572		UWOPS91_917_1:Q>Q	AA:1574		UWOPS91_917_1:N>N	AA:1576		UWOPS91_917_1:A>A	AA:1582		IFO1804:I>I		N_43:I>I		N_45:I>I	AA:1588		UWOPS91_917_1:L>L	AA:1608		UWOPS91_917_1:S>S	AA:1619		IFO1804:N>N	AA:1628		DBVPG6304:Y>Y	AA:1629		IFO1804:D>D		N_43:D>D	AA:1630		DBVPG6304:L>L	AA:1641		DBVPG6304:E>E	AA:1660		N_44:F>F		N_45:F>F	AA:1696		IFO1804:L>L		N_44:L>L	AA:1703		DBVPG6304:L>L	AA:1713		UWOPS91_917_1:C>C	AA:1715		DBVPG6304:E>E		UWOPS91_917_1:E>E	AA:1719		DBVPG6304:P>P	AA:1722		DBVPG6304:E>E		IFO1804:E>E		N_44:E>E	AA:1746		DBVPG6304:H>H		UWOPS91_917_1:H>H	AA:1758		DBVPG6304:R>R		IFO1804:R>R		UWOPS91_917_1:R>R	AA:1766		DBVPG6304:K>K		UWOPS91_917_1:K>K	AA:1782		DBVPG6304:I>I		UWOPS91_917_1:I>I	AA:1789		DBVPG6304:I>I		UWOPS91_917_1:I>I	AA:1801		DBVPG6304:Y>YID:YBR137W	AA:50		A12:V>V		A4:V>V		DBVPG6304:V>V	AA:55		A12:P>P		A4:P>P		DBVPG6304:P>P	AA:70		A12:C>C		A4:C>C		DBVPG6304:C>C	AA:72		A12:F>F		A4:F>F		DBVPG6304:F>F	AA:75		A12:V>V		A4:V>V		DBVPG6304:V>V	AA:76		KPN3828:T>T	AA:77		A12:Y>Y		A4:Y>Y		DBVPG6304:Y>Y	AA:80		A12:S>S		A4:S>S		DBVPG6304:S>S	AA:119		A12:F>F		A4:F>F		DBVPG6304:F>F	AA:130		A12:G>G		A4:G>G		DBVPG6304:G>G		N_43:G>G		N_44:G>G		N_45:G>G	AA:132		A12:V>V		A4:V>V		DBVPG6304:V>V	AA:150		A12:G>G		DBVPG6304:G>G	AA:152		A12:K>K		DBVPG6304:K>K	AA:160		A12:A>A		DBVPG6304:A>A	AA:172		A12:L>L		DBVPG6304:L>L		N_43:L>L		N_44:L>L		N_45:L>L	AA:174		A12:D>D		DBVPG6304:D>DID:YBR138C	AA:24		UWOPS91_917_1:P>P	AA:63		IFO1804:->-		N_43:->-		N_45:->-	AA:64		A12:S>S		A4:S>S		UWOPS91_917_1:S>S		YPS138:S>S	AA:69		A12:F>F		A4:F>F		UWOPS91_917_1:F>F		YPS138:F>F	AA:122		A4:I>I		UFRJ50816:I>I		YPS138:I>I	AA:128		N_17:L>L		N_43:L>L		N_44:L>L		N_45:L>L		UWOPS91_917_1:L>L	AA:150		A12:F>F		A4:F>F		N_17:F>F		N_43:F>F		N_44:F>F		N_45:F>F		UFRJ50816:F>F		UWOPS91_917_1:F>F		YPS138:F>F	AA:153		N_17:A>A		N_43:A>A		N_44:A>A		N_45:A>A	AA:170		A12:D>D		A4:D>D		UFRJ50816:D>D		YPS138:D>D	AA:179		A12:I>I		A4:I>I		UFRJ50816:I>I		YPS138:I>I	AA:214		A12:C>C		A4:C>C		DBVPG6304:C>C	AA:221		A12:K>K		A4:K>K		DBVPG6304:K>K		UFRJ50816:K>K		YPS138:K>K	AA:272		A12:I>I		A4:I>I		DBVPG6304:I>I		UFRJ50816:I>I		YPS138:I>I	AA:274		A12:S>S		A4:S>S		DBVPG6304:S>S		UFRJ50816:S>S		YPS138:S>S	AA:296		UFRJ50816:I>I	AA:372		A12:R>R		A4:R>R		DBVPG6304:R>R		YPS138:R>R	AA:397		A12:N>N		A4:N>N		DBVPG6304:N>N		YPS138:N>N	AA:426		A12:L>L		A4:L>L		DBVPG6304:L>L		YPS138:L>L	AA:438		A12:L>L		A4:L>L		DBVPG6304:L>L		N_43:L>L	AA:495		DBVPG6304:I>I	AA:498		N_43:->-		N_44:->-		N_45:->-ID:YBR139W	AA:4		UWOPS91_917_1:L>L	AA:7		A12:V>V		A4:V>V		DBVPG6304:V>V		UFRJ50816:V>V	AA:14		YPS138:I>I	AA:51		UWOPS91_917_1:L>L	AA:55		A12:R>R		DBVPG6304:R>R		UFRJ50816:R>R		YPS138:R>R	AA:62		A12:L>L		DBVPG6304:L>L		UFRJ50816:L>L		UWOPS91_917_1:L>L		YPS138:L>L	AA:72		A12:A>A		DBVPG6304:A>A		UFRJ50816:A>A		UWOPS91_917_1:A>A		YPS138:A>A	AA:76		A12:L>L		DBVPG6304:L>L		YPS138:L>L	AA:79		A12:R>R		CBS5829:R>R		DBVPG6304:R>R		N_44:R>R		N_45:R>R		T21_4:R>R		Y6_5:R>R		YPS138:R>R	AA:96		A12:G>G		DBVPG6304:G>G		YPS138:G>G	AA:121		A12:P>P		DBVPG6304:P>P		N_44:P>P		UFRJ50791:P>P		UWOPS91_917_1:P>P		YPS138:P>P	AA:124		A12:L>L		DBVPG6304:L>L		UWOPS91_917_1:L>L		YPS138:L>L	AA:130		A12:P>P		DBVPG6304:P>P		UFRJ50791:P>P		UFRJ50816:P>P		UWOPS91_917_1:P>P		YPS138:P>P	AA:135		A12:F>F		UFRJ50791:F>F		UWOPS91_917_1:F>F		YPS138:F>F	AA:139		A12:L>L		UWOPS91_917_1:L>L		YPS138:L>L	AA:143		UWOPS91_917_1:G>G	AA:148		A12:G>G		UFRJ50791:G>G		UFRJ50816:G>G		YPS138:G>G	AA:175		UFRJ50791:V>V		UFRJ50816:V>V		UWOPS91_917_1:V>V		YPS138:V>V	AA:185		UWOPS91_917_1:S>S	AA:195		YPS138:Y>Y	AA:204		UFRJ50791:A>A		UFRJ50816:A>A		YPS138:A>A	AA:208		UFRJ50791:L>L		UFRJ50816:L>L		YPS138:L>L	AA:215		UWOPS91_917_1:I>I	AA:222		UFRJ50791:G>G		UFRJ50816:G>G		UWOPS91_917_1:G>G		YPS138:G>G	AA:253		UWOPS91_917_1:T>T	AA:271		UFRJ50791:G>G		UFRJ50816:G>G		UWOPS91_917_1:G>G		YPS138:G>G	AA:297		DBVPG6304:L>L		UFRJ50791:L>L		UFRJ50816:L>L	AA:352		DBVPG6304:V>V		UFRJ50791:V>V		UFRJ50816:V>V		UWOPS91_917_1:V>V	AA:392		A12:P>P		DBVPG6304:P>P	AA:394		Q62_5:Q>Q		Z1_1:Q>Q	AA:398		N_43:A>A		N_44:A>A		N_45:A>A		UWOPS91_917_1:A>A	AA:403		UWOPS91_917_1:H>H	AA:407		N_43:V>V		N_44:V>V		N_45:V>V	AA:412		Q59_1:G>G		Q89_8:G>G	AA:447		A12:V>V		DBVPG6304:V>V		UFRJ50816:V>V	AA:451		CBS432:T>T		CBS5829:T>T	AA:458		A12:V>V		DBVPG6304:V>V		UFRJ50816:V>V	AA:467		A12:L>L		DBVPG6304:L>L		UFRJ50816:L>L	AA:470		A12:Y>Y		DBVPG6304:Y>Y		UFRJ50816:Y>Y	AA:477		UFRJ50816:P>P	AA:479		A12:D>D		A4:D>D		DBVPG6304:D>D		N_43:D>D		N_44:D>D		N_45:D>D		UFRJ50816:D>DID:YBR141C	AA:12		UWOPS91_917_1:A>A	AA:31		UWOPS91_917_1:Q>Q	AA:39		N_43:L>L		N_44:L>L	AA:45		A12:V>V		DBVPG6304:V>V		UFRJ50791:V>V		UFRJ50816:V>V		UWOPS91_917_1:V>V		YPS138:V>V	AA:54		A12:E>E		A4:E>E		DBVPG6304:E>E		N_43:E>E		N_44:E>E		UFRJ50791:E>E		UFRJ50816:E>E		UWOPS91_917_1:E>E		YPS138:E>E	AA:59		A12:Q>Q		A4:Q>Q		DBVPG6304:Q>Q		UFRJ50791:Q>Q		UFRJ50816:Q>Q		UWOPS91_917_1:Q>Q		YPS138:Q>Q	AA:68		UWOPS91_917_1:R>R	AA:75		N_43:N>N		N_44:N>N	AA:79		A12:I>I		A4:I>I		DBVPG6304:I>I		UFRJ50791:I>I		UFRJ50816:I>I		UWOPS91_917_1:I>I		YPS138:I>I	AA:89		A12:A>A		A4:A>A		DBVPG6304:A>A		IFO1804:A>A		N_43:A>A		N_44:A>A		UFRJ50791:A>A		UFRJ50816:A>A		YPS138:A>A	AA:105		A12:H>H		A4:H>H		DBVPG6304:H>H		UFRJ50791:H>H		UFRJ50816:H>H		YPS138:H>H	AA:107		A12:I>I		A4:I>I		DBVPG6304:G>G		UFRJ50791:I>I		UFRJ50816:I>I		YPS138:I>I	AA:115		A12:T>T		A4:T>T		UFRJ50791:T>T		UFRJ50816:T>T		YPS138:T>T	AA:132		A12:A>A		A4:A>A		IFO1804:I>I		N_44:I>I		N_45:I>I		UFRJ50791:A>A		UFRJ50816:A>A		YPS138:A>A	AA:134		A12:V>V		A4:V>V		IFO1804:V>V		N_43:V>V		N_44:V>V		N_45:V>V		UFRJ50791:V>V		UFRJ50816:V>V		YPS138:V>V	AA:223		N_44:V>V		N_45:V>V	AA:235		A12:->-		A4:->-		UFRJ50791:->-		UFRJ50816:->-		YPS138:->-	AA:243		A12:V>V		A4:V>V		DBVPG6304:V>V		IFO1804:R>R		N_44:R>R		N_45:R>R		UFRJ50791:V>V		UFRJ50816:V>V		YPS138:V>V	AA:255		A12:Q>Q		A4:Q>Q		DBVPG6304:Q>Q		UFRJ50791:Q>Q		UFRJ50816:Q>Q		UWOPS91_917_1:Q>Q		YPS138:Q>Q	AA:270		UFRJ50791:S>S		UFRJ50816:S>S	AA:281		A12:L>L		A4:L>L		UFRJ50791:L>L		UFRJ50816:L>L		YPS138:L>L	AA:293		A12:Y>Y		A4:Y>Y		N_44:Y>Y		N_45:Y>Y		UFRJ50791:Y>Y		UFRJ50816:Y>Y		YPS138:Y>Y	AA:295		A12:F>F		A4:F>F		UFRJ50791:F>F		UFRJ50816:F>F		YPS138:F>F	AA:302		A12:V>V		A4:V>V		UFRJ50816:V>V		YPS138:V>V	AA:314		IFO1804:L>L		N_44:L>L		N_45:L>LID:YBR145W	AA:15		UFRJ50816:F>F	AA:20		A12:G>G		A4:G>G		UFRJ50816:G>G		YPS138:G>G	AA:27		A12:V>V		A4:V>V		UFRJ50816:V>V		YPS138:V>V	AA:30		N_43:P>P		N_44:P>P		N_45:P>P	AA:38		A12:L>L		A4:L>L		YPS138:L>L	AA:68		A12:G>G		A4:G>G		YPS138:G>G	AA:76		A12:V>V		A4:V>V		N_43:V>V		N_44:V>V		N_45:V>V		YPS138:V>V	AA:128		IFO1804:T>T		N_43:T>T		N_44:T>T		N_45:T>T	AA:129		A12:F>F		A4:F>F		KPN3828:F>F		N_17:F>F		YPS138:F>F	AA:131		A12:E>E		A4:E>E		YPS138:E>E	AA:135		A12:A>A		A4:A>A		YPS138:A>A	AA:150		A12:A>A		A4:A>A		YPS138:A>A	AA:158		A12:A>A		YPS138:A>A	AA:161		A12:T>T		A4:T>T		YPS138:T>T	AA:165		A12:A>A		A4:A>A		YPS138:A>A	AA:166		KPN3828:L>L		N_17:L>L		Q32_3:L>L	AA:175		A12:Q>Q		A4:Q>Q		IFO1804:Q>Q		N_43:Q>Q		N_44:Q>Q		YPS138:Q>Q	AA:180		DBVPG4650:S>S	AA:204		IFO1804:I>I		N_43:I>I		N_44:I>I	AA:222		A12:F>F		A4:F>F		DBVPG6304:F>F	AA:225		UWOPS91_917_1:F>F	AA:230		DBVPG6304:D>D	AA:237		A12:K>K		A4:K>K		DBVPG6304:K>K		UWOPS91_917_1:K>K	AA:239		A12:T>T		A4:T>T		DBVPG6304:T>T		IFO1804:T>T		N_43:T>T		UWOPS91_917_1:T>T	AA:246		A12:V>V		A4:V>V		DBVPG6304:V>V		IFO1804:V>V		UWOPS91_917_1:V>V	AA:249		A12:V>V		A4:V>V		DBVPG6304:V>V	AA:256		UWOPS91_917_1:I>I	AA:258		A12:A>A		A4:A>A		DBVPG6304:A>A		UWOPS91_917_1:A>A	AA:270		A12:V>V		A4:V>V		DBVPG6304:V>V		UFRJ50816:V>V		UWOPS91_917_1:V>V	AA:294		A12:I>I		A4:I>I		DBVPG6304:I>I		UFRJ50791:I>I		UFRJ50816:I>I		UWOPS91_917_1:I>I	AA:309		N_43:L>L		N_45:L>L	AA:321		A4:I>I		DBVPG6304:I>I		UFRJ50791:I>I		UFRJ50816:I>I	AA:344		A4:R>R		DBVPG6304:R>R		UFRJ50791:R>R		UFRJ50816:R>R	AA:349		N_43:T>T		N_45:T>TID:YBR146W	AA:16		A12:A>A		A4:A>A		DBVPG6304:A>A		UFRJ50816:A>A		YPS138:A>A	AA:35		A12:K>K		A4:K>K		DBVPG6304:K>K		UFRJ50816:K>K		YPS138:K>K	AA:38		IFO1804:V>V		N_43:V>V		N_44:V>V		N_45:V>V	AA:55		A12:I>I		A4:I>I		DBVPG6304:I>I		UFRJ50816:I>I		YPS138:I>I	AA:117		A12:D>D		A4:D>D		DBVPG6304:D>D		IFO1804:D>D		N_44:D>D		N_45:D>D		UFRJ50816:D>D		UWOPS91_917_1:D>D		YPS138:D>D	AA:120		A12:L>L		A4:L>L		DBVPG6304:L>L		UFRJ50816:L>L		YPS138:L>L	AA:141		UWOPS91_917_1:D>D	AA:142		IFO1804:N>N		N_44:N>N		N_45:N>N	AA:152		UWOPS91_917_1:G>G	AA:156		A12:A>A		A4:A>A		DBVPG6304:A>A		UFRJ50816:A>A		YPS138:A>A	AA:161		UWOPS91_917_1:K>K	AA:168		A12:F>F		A4:F>F		DBVPG6304:F>F		UFRJ50816:F>F		YPS138:F>F	AA:170		UWOPS91_917_1:V>V	AA:174		IFO1804:G>G		N_45:G>G	AA:186		A12:Y>Y		A4:Y>Y		DBVPG6304:Y>Y		UFRJ50816:Y>Y		YPS138:Y>Y	AA:189		UWOPS91_917_1:K>K	AA:192		UWOPS91_917_1:D>D	AA:215		UWOPS91_917_1:T>T	AA:218		UWOPS91_917_1:G>G	AA:222		A12:G>G		A4:G>G		DBVPG6304:G>G		UFRJ50791:G>G		UFRJ50816:G>G		YPS138:G>G	AA:223		UWOPS91_917_1:Q>Q	AA:229		A12:H>H		A4:H>H		DBVPG6304:H>H		UFRJ50791:H>H		UFRJ50816:H>H		UWOPS91_917_1:H>H		YPS138:H>H	AA:233		UWOPS91_917_1:K>K	AA:237		UWOPS91_917_1:V>V	AA:251		UWOPS91_917_1:V>V	AA:256		UWOPS91_917_1:Y>Y	AA:264		A4:P>P		DBVPG6304:P>P		UFRJ50791:P>P		UFRJ50816:P>P		YPS138:P>PID:YBR147W	AA:23		N_43:S>S		N_44:S>S		N_45:S>S	AA:46		N_43:S>S		N_44:S>S		N_45:S>S	AA:116		A12:P>P		DBVPG6304:P>P		N_43:P>P		N_44:P>P		N_45:P>P		UFRJ50791:P>P		UWOPS91_917_1:P>P		YPS138:P>P	AA:128		UWOPS91_917_1:V>V	AA:148		UWOPS91_917_1:S>S	AA:151		A12:E>E		UFRJ50791:E>E		YPS138:E>E	AA:173		UWOPS91_917_1:S>S	AA:178		A12:A>A		UFRJ50791:A>A		UWOPS91_917_1:A>A		YPS138:A>A	AA:190		A12:G>G		UFRJ50791:G>G		UWOPS91_917_1:G>G	AA:205		A12:I>I		A4:I>I		N_43:I>I		N_44:I>I		N_45:I>I		UFRJ50791:I>I		UWOPS91_917_1:I>I	AA:221		UWOPS91_917_1:L>L	AA:240		A12:V>V		A4:V>V		UFRJ50791:V>V		UWOPS91_917_1:V>V		YPS138:V>V	AA:265		A12:D>D		A4:D>D		N_43:D>D		N_44:D>D		N_45:D>D		UFRJ50791:D>D		UWOPS91_917_1:D>D		YPS138:D>D	AA:270		A12:V>V		A4:V>V		UFRJ50791:V>V		UWOPS91_917_1:V>V		YPS138:V>V	AA:290		UWOPS91_917_1:F>F	AA:294		A12:F>F		A4:F>F		DBVPG6304:F>F		UFRJ50791:F>F		YPS138:F>F	AA:302		UWOPS91_917_1:E>EID:YBR148W	AA:24		N_43:T>T		N_44:T>T		N_45:T>T	AA:43		A12:P>P		A4:P>P		DBVPG6304:P>P		UFRJ50816:P>P		YPS138:P>P	AA:45		A12:A>A		A4:A>A		DBVPG6304:A>A		UFRJ50816:A>A		YPS138:A>A	AA:66		A12:D>D		A4:D>D		DBVPG6304:D>D		N_43:D>D		N_44:D>D		N_45:D>D		UFRJ50816:D>D		YPS138:D>D	AA:84		A12:S>S		A4:S>S	AA:125		A12:D>D		A4:D>D		DBVPG6304:D>D		UFRJ50816:D>D	AA:128		N_44:K>K		N_45:K>K	AA:132		A12:T>T		A4:T>T		DBVPG6304:T>T		UFRJ50816:T>T	AA:135		A12:I>I		A4:I>I		DBVPG6304:I>I		UFRJ50816:I>I	AA:137		A12:N>N		A4:N>N		DBVPG6304:N>N		UFRJ50816:N>N	AA:171		A12:F>F		A4:F>F		UFRJ50816:F>F	AA:173		N_44:D>D		N_45:D>D	AA:180		A4:Q>Q		UFRJ50816:Q>Q		YPS138:Q>Q	AA:195		N_45:T>T	AA:197		UFRJ50816:S>S		YPS138:S>S	AA:232		A4:L>L		UFRJ50816:L>L		YPS138:L>L	AA:240		A4:F>F		UFRJ50816:F>F		YPS138:F>F	AA:264		A4:Y>Y		UFRJ50816:Y>Y		YPS138:Y>Y	AA:266		N_44:I>I		N_45:I>I	AA:277		A4:N>N		UFRJ50816:N>N		YPS138:N>N	AA:292		A4:S>S		UFRJ50816:S>S		YPS138:S>S	AA:325		A4:L>L		UFRJ50816:L>L		YPS138:L>L	AA:330		N_17:L>L	AA:359		A4:I>I		UFRJ50816:I>I		YPS138:I>I	AA:365		N_44:D>D		N_45:D>D	AA:366		A4:L>L		UFRJ50816:L>L	AA:377		IFO1804:S>S		N_44:S>S		N_45:S>S	AA:378		A12:A>A		A4:A>A		UFRJ50816:A>A		YPS138:A>A	AA:419		A12:Q>Q		A4:Q>Q		UFRJ50816:Q>Q		YPS138:Q>Q	AA:425		A4:S>S		YPS138:S>S	AA:428		A4:L>L		YPS138:L>L	AA:449		A12:L>L		A4:L>L		IFO1804:L>L		N_45:L>L		UFRJ50816:L>L		UWOPS91_917_1:L>L		YPS138:L>L	AA:457		UWOPS91_917_1:K>K	AA:459		A12:Q>Q		A4:Q>Q		UFRJ50816:Q>Q		UWOPS91_917_1:Q>Q		YPS138:Q>Q	AA:476		IFO1804:R>R		N_45:R>R	AA:480		A12:L>L		A4:L>L		UFRJ50816:L>L		UWOPS91_917_1:L>L		YPS138:L>L	AA:494		YPS138:Y>Y	AA:502		IFO1804:E>E		N_43:E>E		N_45:E>E	AA:519		A12:T>T		A4:T>T		UFRJ50816:T>T		YPS138:T>T	AA:528		A12:L>L		A4:L>L		UFRJ50816:L>L		YPS138:L>L	AA:533		A12:F>F		A4:F>F		UFRJ50816:F>F		UWOPS91_917_1:F>F		YPS138:F>F	AA:536		UWOPS91_917_1:Q>Q	AA:537		IFO1804:L>L		N_43:L>L		N_45:L>L	AA:558		N_43:F>F	AA:560		A12:E>E		A4:E>E		UFRJ50816:E>E		UWOPS91_917_1:E>E	AA:565		UWOPS91_917_1:F>F	AA:568		A12:E>E		A4:E>E		UFRJ50816:E>E	AA:584		IFO1804:I>I		N_43:I>I		N_45:I>I	AA:592		UWOPS91_917_1:K>K	AA:607		UWOPS91_917_1:D>DID:YBR149W	AA:6		A12:T>T		A4:T>T		DBVPG6304:T>T		N_43:T>T		N_44:T>T		N_45:T>T		UFRJ50791:T>T		UWOPS91_917_1:T>T	AA:11		N_43:I>I		N_44:I>I		N_45:I>I	AA:12		A12:V>V		A4:V>V		DBVPG6304:V>V		UFRJ50791:V>V		UFRJ50816:V>V	AA:19		UWOPS91_917_1:K>K	AA:21		UWOPS91_917_1:T>T	AA:36		N_43:L>L		N_44:L>L		N_45:L>L	AA:49		UWOPS91_917_1:E>E	AA:58		A12:I>I		A4:I>I		DBVPG6304:I>I		UFRJ50791:I>I		UFRJ50816:I>I		UWOPS91_917_1:I>I		YPS138:I>I	AA:61		UWOPS91_917_1:G>G	AA:68		A12:A>A		A4:A>A		DBVPG6304:A>A		N_43:A>A		N_44:A>A		N_45:A>A		UFRJ50791:A>A		UWOPS91_917_1:A>A		YPS138:A>A	AA:75		DBVPG6304:P>P		YPS138:P>P	AA:98		UWOPS91_917_1:T>T	AA:109		A12:V>V		A4:V>V		DBVPG6304:V>V		UFRJ50816:V>V		UWOPS91_917_1:V>V		YPS138:V>V	AA:122		UWOPS91_917_1:L>L	AA:125		A4:V>V		DBVPG6304:V>V		UFRJ50816:V>V		UWOPS91_917_1:V>V		YPS138:V>V	AA:148		N_44:L>L		N_45:L>L	AA:157		A4:G>G		DBVPG6304:G>G		N_43:G>G		N_44:G>G		N_45:G>G		UFRJ50816:G>G		UWOPS91_917_1:G>G		YPS138:G>G	AA:161		A4:Y>Y		DBVPG6304:Y>Y		UFRJ50816:Y>Y		UWOPS91_917_1:Y>Y		YPS138:Y>Y	AA:166		A4:D>D		DBVPG6304:D>D		UFRJ50816:D>D		YPS138:D>D	AA:171		A4:Y>Y		DBVPG6304:Y>Y		UFRJ50816:Y>Y		UWOPS91_917_1:Y>Y		YPS138:Y>Y	AA:185		UWOPS91_917_1:R>R	AA:192		N_43:S>S		N_44:S>S		N_45:S>S	AA:204		A4:K>K		DBVPG6304:K>K		UFRJ50816:K>K		UWOPS91_917_1:K>K		YPS138:K>K	AA:217		A4:T>T		DBVPG6304:T>T		UFRJ50816:T>T		UWOPS91_917_1:T>T		YPS138:T>T	AA:237		A4:L>L		DBVPG6304:L>L		UFRJ50816:L>L		YPS138:L>L	AA:244		A4:G>G		UFRJ50816:G>G		YPS138:G>G	AA:245		IFO1804:S>S		UWOPS91_917_1:S>S	AA:251		A4:L>L		UFRJ50816:L>L		UWOPS91_917_1:L>L		YPS138:L>L	AA:255		UWOPS91_917_1:L>L	AA:267		N_43:G>G	AA:270		N_43:L>L		N_45:L>L	AA:294		A4:S>S		UFRJ50816:S>S		YPS138:S>S	AA:300		A12:S>S		A4:S>S		UFRJ50816:S>S		UWOPS91_917_1:S>S		YPS138:S>S	AA:311		IFO1804:V>V	AA:319		N_17:R>R		Y6_5:R>R	AA:320		A12:F>F		A4:F>F		UWOPS91_917_1:F>F		YPS138:F>F	AA:329		IFO1804:L>L	AA:335		A12:N>N		A4:N>NID:YBR151W	AA:13		A4:D>D		DBVPG6304:D>D		UWOPS91_917_1:D>D		YPS138:D>D	AA:21		YPS138:K>K	AA:31		A4:K>K		DBVPG6304:K>K		YPS138:K>K	AA:35		DBVPG4650:R>R	AA:49		A4:R>R		DBVPG4650:R>R		DBVPG6304:R>R		UWOPS91_917_1:R>R		YPS138:R>R	AA:54		UWOPS91_917_1:E>E	AA:61		A4:K>K		UWOPS91_917_1:K>K		YPS138:K>K	AA:64		A12:I>I		A4:I>I		UWOPS91_917_1:I>I		YPS138:I>I	AA:70		UWOPS91_917_1:L>L	AA:73		A4:S>S		CBS5829:S>S		KPN3828:S>S		N_17:S>S		N_43:S>S		N_44:S>S		N_45:S>S		Q62_5:S>S		Q95_3:S>S		UWOPS91_917_1:S>S		YPS138:S>S	AA:78		A12:K>K		A4:K>K		UWOPS91_917_1:K>K		YPS138:K>K	AA:81		YPS138:F>F	AA:96		N_43:L>L		N_44:L>L		N_45:L>L	AA:119		N_43:E>E		N_44:E>E		N_45:E>E	AA:133		A12:L>L		A4:L>L		DBVPG6304:L>L		UWOPS91_917_1:L>L		YPS138:L>L	AA:159		A12:L>L		A4:L>L		CBS5829:L>L		DBVPG6304:L>L		KPN3828:L>L		N_17:L>L		N_43:L>L		N_44:L>L		N_45:L>L		Q62_5:L>L		Q95_3:L>L		UFRJ50816:L>L		UWOPS91_917_1:L>L		Y7:L>L		YPS138:L>L		Z1_1:L>L	AA:164		A12:S>S		A4:S>S		DBVPG6304:S>S		UFRJ50816:S>S		UWOPS91_917_1:S>S		YPS138:S>S	AA:171		UWOPS91_917_1:L>L	AA:177		A12:D>D		A4:D>D		DBVPG6304:D>D		N_43:D>D		N_44:D>D		N_45:D>D		UFRJ50816:D>D		UWOPS91_917_1:D>D		YPS138:D>D	AA:184		A12:S>S		A4:S>S		DBVPG6304:S>S		YPS138:S>S	AA:202		A12:A>A		A4:A>A		DBVPG6304:A>A		UFRJ50816:A>A		YPS138:A>A	AA:208		A12:S>S		A4:S>S		DBVPG6304:S>S		N_43:S>S		N_45:S>S		UFRJ50816:S>S		YPS138:S>S	AA:231		A12:L>L		A4:L>L		DBVPG6304:L>L		UFRJ50816:L>L		YPS138:L>L	AA:241		A12:S>S		A4:S>S		DBVPG6304:S>S		UFRJ50816:S>S		YPS138:S>S	AA:244		A12:R>R		A4:R>R		DBVPG6304:R>R		UFRJ50816:R>R		YPS138:R>R	AA:269		A12:L>L		A4:L>L		DBVPG6304:L>L		UFRJ50816:L>L		YPS138:L>L	AA:275		A12:L>L		A4:L>L		DBVPG6304:L>L		UFRJ50816:L>L		YPS138:L>L	AA:292		A12:H>H		A4:H>H		DBVPG6304:H>H		N_43:H>H		UFRJ50816:H>H		UWOPS91_917_1:H>H		YPS138:H>H	AA:294		A12:I>I		A4:I>I		DBVPG6304:I>I		UFRJ50816:I>I		UWOPS91_917_1:I>I		YPS138:I>I	AA:296		A12:P>P		A4:P>P		DBVPG6304:P>P		N_43:P>P		UFRJ50816:P>P		UWOPS91_917_1:P>P		YPS138:P>P	AA:299		A12:P>P		A4:P>P		DBVPG6304:P>P		N_43:P>P		UFRJ50816:P>P		UWOPS91_917_1:P>P		YPS138:P>P	AA:304		UWOPS91_917_1:P>P	AA:305		A12:E>E		A4:E>E		DBVPG6304:E>E		UFRJ50816:E>E		YPS138:E>E	AA:308		A12:R>R		A4:R>R		DBVPG6304:R>R		N_43:R>R		UFRJ50816:R>R		YPS138:R>R	AA:313		A12:Y>Y		A4:Y>Y		DBVPG6304:Y>Y		UFRJ50816:Y>Y		UWOPS91_917_1:Y>Y		YPS138:Y>YID:YBR154C	AA:11		N_43:T>T		N_45:T>T	AA:15		A4:T>T		DBVPG6304:T>T		UFRJ50791:T>T		UFRJ50816:T>T		UWOPS91_917_1:T>T		YPS138:T>T	AA:25		A12:S>S		A4:S>S		DBVPG6304:S>S		UFRJ50791:S>S		UFRJ50816:S>S		YPS138:S>S	AA:38		UWOPS91_917_1:L>L	AA:65		A12:L>L		A4:L>L		DBVPG6304:L>L		N_43:L>L		N_45:L>L		UFRJ50791:L>L		UFRJ50816:L>L		UWOPS91_917_1:L>L		YPS138:L>L	AA:109		UWOPS91_917_1:T>T	AA:113		N_43:I>I		N_44:I>I		N_45:I>I	AA:130		UWOPS91_917_1:G>G	AA:141		A12:A>A		A4:A>A		DBVPG6304:A>A		UWOPS91_917_1:A>A	AA:143		A12:F>F		A4:F>F		DBVPG6304:F>F		N_43:F>F		N_44:F>F		N_45:F>F		UWOPS91_917_1:F>F	AA:146		A12:L>L		A4:L>L		DBVPG6304:L>L		UWOPS91_917_1:L>L	AA:149		A12:R>R		A4:R>R		DBVPG6304:R>R		N_43:R>R		N_44:R>R		N_45:R>R		UFRJ50791:R>R	AA:173		A12:G>G		A4:G>G		DBVPG6304:G>G		UFRJ50791:G>GID:YBR155W	AA:27		A12:P>P		DBVPG6304:P>P		N_43:P>P		N_44:P>P		N_45:P>P		UWOPS91_917_1:P>P		YPS138:P>P	AA:29		UWOPS91_917_1:L>L	AA:40		UWOPS91_917_1:I>I	AA:42		N_43:K>K		N_44:K>K		N_45:K>K		UWOPS91_917_1:K>K	AA:48		A12:P>P		DBVPG6304:P>P		UWOPS91_917_1:P>P		YPS138:P>P	AA:54		N_43:L>L		N_44:L>L		N_45:L>L	AA:65		A12:V>V		DBVPG6304:V>V		UWOPS91_917_1:V>V		YPS138:V>V	AA:70		A12:L>L		DBVPG6304:L>L		UWOPS91_917_1:L>L		YPS138:L>L	AA:83		N_43:A>A		N_44:A>A		N_45:A>A	AA:104		UWOPS91_917_1:E>E	AA:123		A12:L>L		DBVPG6304:L>L		UWOPS91_917_1:L>L		YPS138:L>L	AA:125		A12:A>A		DBVPG6304:A>A		YPS138:A>A	AA:134		Y7:L>L	AA:139		A12:R>R		A4:R>R		DBVPG6304:R>R		YPS138:R>R	AA:140		UWOPS91_917_1:C>C	AA:142		UWOPS91_917_1:E>E	AA:152		A12:P>P		DBVPG6304:P>P		UWOPS91_917_1:P>P		YPS138:P>P	AA:157		IFO1804:C>C		N_43:C>C		N_44:C>C	AA:164		IFO1804:A>A		N_43:A>A		N_44:A>A	AA:206		UWOPS91_917_1:L>L	AA:210		UWOPS91_917_1:E>E	AA:223		IFO1804:K>K		N_43:K>K		N_44:K>K	AA:225		UWOPS91_917_1:I>I	AA:238		UWOPS91_917_1:N>N	AA:245		UWOPS91_917_1:V>V	AA:248		A12:L>L		A4:L>L		UFRJ50791:L>L		UWOPS91_917_1:L>L		YPS138:L>L	AA:252		UWOPS91_917_1:K>K	AA:260		UWOPS91_917_1:D>D	AA:269		UWOPS91_917_1:A>A	AA:270		A12:L>L		A4:L>L		YPS138:L>L	AA:271		UWOPS91_917_1:I>I	AA:275		UWOPS91_917_1:T>T	AA:284		UWOPS91_917_1:E>E	AA:297		UWOPS91_917_1:L>L	AA:310		A12:G>G		A4:G>G		DBVPG6304:G>G		UFRJ50791:G>G		YPS138:G>G	AA:315		A12:T>T		A4:T>T		UFRJ50791:T>T		UWOPS91_917_1:T>T		YPS138:T>T	AA:318		A12:K>K		A4:K>K		UFRJ50791:K>K		UWOPS91_917_1:K>K		YPS138:K>K	AA:322		A12:F>F		A4:F>F		DBVPG6304:F>F		UFRJ50791:F>F		UWOPS91_917_1:F>F		YPS138:F>F	AA:328		A12:G>G		A4:G>G		DBVPG6304:G>G		UFRJ50791:G>G		YPS138:G>G	AA:344		A12:K>K		A4:K>K		DBVPG6304:K>K		UFRJ50791:K>K		UWOPS91_917_1:K>K		YPS138:K>K	AA:357		IFO1804:L>L		N_43:L>L		N_45:L>LID:YBR156C	AA:14		A4:S>S		DBVPG6304:S>S		UFRJ50791:S>S		UWOPS91_917_1:S>S		YPS138:S>S	AA:18		A4:I>I		DBVPG6304:I>I		UWOPS91_917_1:I>I	AA:25		UFRJ50816:V>V		UWOPS91_917_1:S>S	AA:28		IFO1804:R>R		N_43:R>R		N_45:R>R	AA:38		UFRJ50816:Y>Y	AA:66		A4:I>I		DBVPG6304:I>I		UWOPS91_917_1:I>I	AA:74		UFRJ50816:I>I		UWOPS91_917_1:I>I	AA:78		A4:G>G		DBVPG6304:G>G	AA:82		UFRJ50816:S>S	AA:106		A4:S>S		DBVPG4650:S>S		DBVPG6304:S>S		Q32_3:S>S	AA:109		UFRJ50816:V>V		UWOPS91_917_1:V>V	AA:114		A4:H>H		DBVPG6304:H>H	AA:120		A4:W>W		DBVPG6304:W>W		N_43:W>W		N_45:W>W	AA:130		UFRJ50816:V>V		UWOPS91_917_1:V>V	AA:138		UFRJ50816:L>L		UWOPS91_917_1:L>L	AA:170		UWOPS91_917_1:F>F	AA:185		A4:W>W		DBVPG6304:W>W		UFRJ50816:W>W	AA:199		A4:T>T		UFRJ50816:T>T	AA:209		A12:I>I		A4:I>I		UFRJ50816:I>I	AA:222		A12:Q>Q		A4:Q>Q		DBVPG6304:Q>Q		UFRJ50816:Q>Q	AA:229		UWOPS91_917_1:S>S	AA:249		A12:S>S		A4:S>S	AA:254		A12:F>F		A4:F>F		CBS5829:V>V		DBVPG6304:F>F		YPS138:F>F	AA:255		A12:T>T		A4:T>T		UFRJ50816:T>T		YPS138:T>T	AA:271		A12:C>C		A4:C>C		UFRJ50816:C>C		YPS138:C>C	AA:279		A12:G>G		A4:G>G		DBVPG6304:G>G		UFRJ50816:G>G		YPS138:G>G	AA:316		A12:N>N		A4:N>N		DBVPG6304:N>N		UFRJ50816:N>N		YPS138:N>N	AA:324		IFO1804:S>S		N_43:S>S		N_44:S>S		N_45:S>S	AA:347		IFO1804:I>I		N_43:I>I		N_44:I>I		N_45:I>I		UFRJ50816:I>I	AA:360		YPS138:N>N	AA:382		A12:T>T		A4:T>T		DBVPG6304:T>T		UFRJ50816:T>T		YPS138:T>T	AA:387		IFO1804:F>F		N_43:F>F		N_44:F>F		N_45:F>F	AA:403		A12:C>C		A4:C>C		DBVPG6304:C>C		UFRJ50816:C>C		UWOPS91_917_1:C>C		YPS138:C>C	AA:406		A12:R>R		A4:R>R		DBVPG6304:R>R		UFRJ50816:R>R		YPS138:R>R	AA:408		IFO1804:R>R		N_43:R>R		N_44:R>R		N_45:R>R	AA:410		A12:V>V		A4:V>V		DBVPG6304:V>V		UWOPS91_917_1:V>V		YPS138:V>V	AA:420		UWOPS91_917_1:A>A	AA:426		A12:L>L		A4:L>L		DBVPG6304:L>L		IFO1804:L>L		N_43:L>L		N_44:L>L		N_45:L>L		UWOPS91_917_1:L>L		YPS138:L>L	AA:433		UWOPS91_917_1:F>F	AA:456		A12:R>R		A4:R>R		DBVPG6304:R>R		IFO1804:R>R		N_43:R>R		N_44:R>R		N_45:R>R		UWOPS91_917_1:R>R		YPS138:R>R	AA:479		UWOPS91_917_1:W>W	AA:500		IFO1804:L>L		N_43:L>L		N_44:L>L		N_45:L>L	AA:525		UWOPS91_917_1:L>L	AA:542		UWOPS91_917_1:V>V	AA:560		A12:I>I		A4:I>I		YPS138:I>I	AA:567		UWOPS91_917_1:H>H	AA:585		A12:I>I		A4:I>I		YPS138:I>I	AA:602		A12:L>L		A4:L>L		DBVPG6304:L>L		UFRJ50791:L>L		UWOPS91_917_1:L>L		YPS138:L>L	AA:613		A12:F>F		A4:F>F		UWOPS91_917_1:F>F		YPS138:F>F	AA:644		UWOPS91_917_1:I>I	AA:655		A4:L>L		DBVPG6304:L>L		UFRJ50791:L>L		YPS138:L>L	AA:657		UWOPS91_917_1:P>P	AA:664		N_43:C>C		N_44:C>C		N_45:C>C	AA:677		A4:Y>Y		DBVPG6304:Y>Y		N_43:Y>Y		N_44:Y>Y		N_45:Y>Y		UFRJ50791:Y>Y		UWOPS91_917_1:Y>Y		YPS138:Y>Y	AA:681		A4:G>G		DBVPG6304:G>G		N_43:G>G		N_44:G>G		N_45:G>G		UFRJ50791:G>G		UWOPS91_917_1:G>G		YPS138:G>GID:YBR157C	AA:17		IFO1804:S>S		N_43:S>S		N_44:S>S		N_45:S>S	AA:24		A12:N>N		CBS432:N>N		CBS5829:N>N		DBVPG6304:N>N		IFO1804:N>N		N_17:N>N		N_43:N>N		N_44:N>N		N_45:N>N		UFRJ50791:N>N		YPS138:N>N	AA:32		A12:I>I		DBVPG6304:I>I		UFRJ50791:I>I		YPS138:I>I	AA:50		UWOPS91_917_1:->-	AA:78		A12:V>V		DBVPG6304:V>V		UFRJ50791:V>V		UWOPS91_917_1:V>V		YPS138:V>V	AA:85		A12:->-		DBVPG6304:->-		UFRJ50791:->-		UFRJ50816:->-		YPS138:->-	AA:102		A12:N>N		DBVPG6304:N>N		UFRJ50791:N>N		UFRJ50816:N>N		UWOPS91_917_1:N>N		YPS138:N>N	AA:160		IFO1804:T>T		N_43:T>T		N_44:T>T		N_45:T>T	AA:193		IFO1804:K>K		N_43:K>K		N_45:K>K	AA:196		A12:G>G		DBVPG6304:G>G		UFRJ50791:G>G		UFRJ50816:G>G		UWOPS91_917_1:G>G		YPS138:G>G	AA:201		A12:M>M		DBVPG6304:M>M		UWOPS91_917_1:M>M		YPS138:M>M	AA:219		DBVPG6304:T>T		YPS138:T>T	AA:229		DBVPG6304:N>N		UFRJ50816:N>N		UWOPS91_917_1:N>N		YPS138:N>N	AA:249		A4:F>F		DBVPG6304:F>F		YPS138:F>FID:YBR159W	AA:14		A4:F>F		UFRJ50816:F>F		YPS138:F>F	AA:19		CBS432:G>G		CBS5829:G>G		KPN3828:G>G		Q95_3:G>G		Y6_5:G>G		Z1_1:G>G	AA:29		UWOPS91_917_1:V>V	AA:33		UFRJ50816:T>T		YPS138:T>T	AA:38		UWOPS91_917_1:R>R	AA:46		A4:L>L		UFRJ50816:L>L		UWOPS91_917_1:L>L		YPS138:L>L	AA:59		A4:A>A		DBVPG6304:A>A		UFRJ50816:A>A		UWOPS91_917_1:A>A		YPS138:A>A	AA:61		UWOPS91_917_1:T>T	AA:69		A4:G>G		DBVPG6304:G>G		UFRJ50816:G>G		YPS138:G>G	AA:76		N_45:K>K	AA:79		A4:A>A		DBVPG6304:A>A		UFRJ50816:A>A		YPS138:A>A	AA:114		N_45:V>V	AA:133		N_45:K>K	AA:141		N_45:I>I	AA:171		DBVPG6304:T>T		UFRJ50816:T>T		YPS138:T>T	AA:177		CBS432:T>T		CBS5829:T>T		N_17:T>T		Q32_3:T>T		Q59_1:T>T		Q95_3:T>T		T21_4:T>T	AA:178		UWOPS91_917_1:L>L	AA:180		UWOPS91_917_1:I>I	AA:189		UWOPS91_917_1:V>V	AA:191		UWOPS91_917_1:T>T	AA:192		DBVPG6304:V>V		UFRJ50816:V>V		YPS138:V>V	AA:203		DBVPG6304:G>G		N_45:G>G		UFRJ50816:G>G		YPS138:G>G	AA:209		UWOPS91_917_1:G>G	AA:214		UWOPS91_917_1:L>L	AA:218		DBVPG6304:P>P		UFRJ50816:P>P		YPS138:P>P	AA:222		CBS432:T>T		CBS5829:T>T		DBVPG6304:T>T		N_17:T>T		Q32_3:T>T		Q59_1:T>T		Q95_3:T>T		T21_4:T>T		UFRJ50816:T>T		Y7:T>T		YPS138:T>T	AA:231		N_45:Q>Q	AA:232		UWOPS91_917_1:S>S	AA:237		UWOPS91_917_1:L>L	AA:239		UWOPS91_917_1:G>G	AA:247		UWOPS91_917_1:D>D	AA:250		DBVPG6304:L>L		UWOPS91_917_1:L>L	AA:259		UWOPS91_917_1:S>S	AA:263		N_43:I>I		N_45:I>I		UWOPS91_917_1:I>I	AA:266		UWOPS91_917_1:S>S	AA:275		DBVPG6304:Q>Q		UFRJ50816:Q>Q		YPS138:Q>Q	AA:277		UWOPS91_917_1:V>V	AA:281		DBVPG6304:L>L		UFRJ50816:L>L		YPS138:L>L	AA:285		UWOPS91_917_1:G>G	AA:293		CBS432:R>R	AA:296		DBVPG6304:T>T		UFRJ50816:T>T		YPS138:T>T	AA:298		DBVPG6304:T>T		UFRJ50816:T>T		YPS138:T>T	AA:299		UWOPS91_917_1:P>P	AA:302		UWOPS91_917_1:A>A	AA:309		UFRJ50816:V>V		YPS138:V>V	AA:313		UWOPS91_917_1:T>T	AA:317		CBS432:Y>Y		CBS5829:Y>Y		KPN3829:Y>Y		N_17:Y>Y		Q32_3:Y>Y		Q59_1:Y>Y		Q62_5:Y>Y		Q89_8:Y>Y		Q95_3:Y>Y		T21_4:Y>Y		Y7:Y>Y		Z1_1:Y>Y	AA:318		UWOPS91_917_1:S>S	AA:331		UWOPS91_917_1:S>S	AA:332		A12:I>I		IFO1804:I>I		N_43:I>I		N_45:I>I		UFRJ50816:I>I		YPS138:I>I	AA:335		UWOPS91_917_1:R>R	AA:337		A12:L>L		UFRJ50816:L>L		YPS138:L>L	AA:346		IFO1804:K>K		N_43:K>K		N_45:K>KID:YBR160W	AA:14		A12:V>V		A4:V>V		UFRJ50816:V>V		YPS138:V>V	AA:18		CBS432:T>T		CBS5829:T>T		DBVPG4650:T>T		IFO1804:T>T		KPN3829:T>T		N_17:T>T		N_43:T>T		N_45:T>T		Q62_5:T>T		Q95_3:T>T		T21_4:T>T		Z1_1:T>T	AA:26		A12:L>L		A4:L>L		UFRJ50816:L>L		YPS138:L>L	AA:29		A12:R>R		A4:R>R		DBVPG6304:R>R		IFO1804:R>R		N_43:R>R		N_45:R>R		UFRJ50816:R>R		YPS138:R>R	AA:42		UFRJ50816:I>I	AA:67		A12:D>D		A4:D>D		DBVPG6304:D>D		UFRJ50816:D>D		YPS138:D>D	AA:79		A12:S>S		A4:S>S		DBVPG6304:S>S		UFRJ50816:S>S		UWOPS91_917_1:S>S		YPS138:S>S	AA:95		A4:L>L		DBVPG6304:L>L		UFRJ50816:L>L		UWOPS91_917_1:L>L		YPS138:L>L	AA:98		A4:Y>Y		DBVPG6304:Y>Y		UFRJ50816:Y>Y		UWOPS91_917_1:Y>Y		YPS138:Y>Y	AA:113		A4:V>V		DBVPG6304:V>V		UFRJ50816:V>V		UWOPS91_917_1:V>V		YPS138:V>V	AA:115		A4:K>K		DBVPG6304:K>K		UFRJ50816:K>K		UWOPS91_917_1:K>K		YPS138:K>K	AA:129		A12:S>S		A4:S>S		DBVPG6304:S>S		N_44:S>S		N_45:S>S		UFRJ50816:S>S		UWOPS91_917_1:S>S		YPS138:S>S	AA:133		A4:L>L		DBVPG6304:L>L		UFRJ50816:L>L		UWOPS91_917_1:L>L		YPS138:L>L	AA:137		UWOPS91_917_1:L>L	AA:139		A4:P>P		UFRJ50816:P>P		UWOPS91_917_1:P>P		YPS138:P>P	AA:148		UWOPS91_917_1:G>G	AA:163		UWOPS91_917_1:V>V	AA:183		N_44:L>L		N_45:L>L	AA:185		UFRJ50816:G>G	AA:206		UFRJ50816:C>C		YPS138:C>C	AA:228		A12:L>L		UFRJ50816:L>L		YPS138:L>L	AA:237		UWOPS91_917_1:P>P	AA:254		A12:R>R		N_44:R>R		N_45:R>R		UFRJ50816:R>R		UWOPS91_917_1:R>R		YPS138:R>R	AA:263		UWOPS91_917_1:S>S	AA:271		A12:L>L		UFRJ50816:L>L		YPS138:L>L	AA:273		A12:D>D		UFRJ50816:D>D		UWOPS91_917_1:D>D		YPS138:D>D	AA:285		A12:S>S		UFRJ50816:S>S		YPS138:S>SID:YBR162C	AA:4		UWOPS91_917_1:G>G	AA:7		UWOPS91_917_1:R>R	AA:29		A12:A>A		A4:A>A		DBVPG6304:A>A		UFRJ50816:A>A		UWOPS91_917_1:A>A		YPS138:A>A	AA:34		UWOPS91_917_1:N>N	AA:51		UWOPS91_917_1:S>S	AA:67		A4:D>D		DBVPG6304:D>D		UFRJ50816:D>D		UWOPS91_917_1:D>D		YPS138:D>D	AA:76		UWOPS91_917_1:S>S	AA:77		A12:S>S		A4:S>S		DBVPG6304:S>S		UFRJ50791:S>S		YPS138:S>S	AA:103		A12:S>S		A4:S>S		UFRJ50791:S>S		UWOPS91_917_1:S>S		YPS138:S>S	AA:109		A12:Q>Q		A4:Q>Q		UFRJ50791:Q>Q		YPS138:Q>Q	AA:115		N_43:H>H		N_45:H>H	AA:123		A12:A>A		A4:A>A		CBS432:A>A		CBS5829:A>A		KPN3829:A>A		Q59_1:A>A		Q95_3:A>A		T21_4:A>A		UFRJ50791:A>A		UWOPS91_917_1:A>A		Y7:A>A		YPS138:A>A	AA:148		CBS5829:T>T		DBVPG4650:T>T		KPN3829:T>T		Q59_1:T>T		Q95_3:T>T		T21_4:T>T		Y7:T>T	AA:167		N_43:N>N		N_45:N>N	AA:174		A12:F>F		A4:F>F		UFRJ50791:F>F		UWOPS91_917_1:F>F		YPS138:F>F	AA:194		A12:G>G		A4:G>G		UFRJ50791:G>G		YPS138:G>G	AA:195		N_43:K>K	AA:223		UWOPS91_917_1:G>G	AA:235		A12:V>V		A4:V>V		UFRJ50791:V>V		UWOPS91_917_1:V>V		YPS138:V>V	AA:238		UWOPS91_917_1:S>S	AA:250		A12:S>S		A4:S>S		UFRJ50791:S>S		UWOPS91_917_1:S>S		YPS138:S>S	AA:268		YPS138:A>A	AA:298		A12:R>R		A4:R>R		UFRJ50791:R>R		UWOPS91_917_1:R>R		YPS138:R>R	AA:303		A12:C>C		A4:C>C		DBVPG6304:C>C		UFRJ50791:C>C		YPS138:C>C	AA:353		UWOPS91_917_1:Q>Q	AA:355		UWOPS91_917_1:I>I	AA:377		UWOPS91_917_1:G>G	AA:389		A12:R>R		A4:R>R		DBVPG6304:R>R		UWOPS91_917_1:R>R	AA:398		A4:T>T		DBVPG6304:T>T		YPS138:T>T	AA:413		A12:->-		A4:->-		DBVPG6304:->-		YPS138:->-	AA:416		A12:V>V		A4:V>V		DBVPG6304:V>V		YPS138:V>VID:YBR162W-A	AA:10		A12:L>L		UWOPS91_917_1:L>L	AA:16		A4:N>N		DBVPG6304:N>N	AA:19		IFO1804:N>N		N_43:N>N		N_44:N>N		N_45:N>N	AA:22		A12:Y>Y	AA:24		A4:K>K		DBVPG6304:K>K		UFRJ50791:K>K	AA:46		A4:G>G		UFRJ50791:G>G	AA:53		A4:V>V		UFRJ50791:V>V	AA:55		A4:G>G		UFRJ50791:G>G	AA:58		UWOPS91_917_1:L>LID:YBR163W	AA:4		UWOPS91_917_1:R>R	AA:12		A12:F>F		A4:F>F		UWOPS91_917_1:F>F		YPS138:F>F	AA:18		A12:R>R		A4:R>R		N_43:R>R		N_44:R>R		N_45:R>R		UWOPS91_917_1:R>R		YPS138:R>R	AA:44		A12:P>P		A4:P>P		YPS138:P>P	AA:79		IFO1804:E>E		N_43:E>E		N_44:E>E		N_45:E>E	AA:84		A12:S>S		A4:S>S		UWOPS91_917_1:S>S		YPS138:S>S	AA:108		A12:F>F		A4:F>F		YPS138:F>F	AA:114		A12:N>N		A4:N>N		UWOPS91_917_1:N>N		YPS138:N>N	AA:121		A12:L>L		A4:L>L		YPS138:L>L	AA:137		A12:T>T		A4:T>T		UWOPS91_917_1:T>T		YPS138:T>T	AA:173		IFO1804:K>K		N_43:K>K		N_44:K>K		N_45:K>K		UWOPS91_917_1:K>K	AA:179		IFO1804:T>T		N_43:T>T		N_44:T>T		N_45:T>T	AA:190		IFO1804:T>T		N_43:T>T		N_44:T>T		N_45:T>T		UWOPS91_917_1:T>T	AA:195		A12:A>A		A4:A>A		UFRJ50791:A>A		UFRJ50816:A>A		YPS138:A>A	AA:198		A12:K>K		A4:K>K		IFO1804:K>K		N_43:K>K		N_45:K>K		UFRJ50791:K>K		UFRJ50816:K>K		UWOPS91_917_1:K>K		YPS138:K>K	AA:225		A12:F>F		A4:F>F		DBVPG6304:F>F		UFRJ50791:F>F		UFRJ50816:F>F		YPS138:F>F	AA:230		A12:G>G		A4:G>G		DBVPG6304:G>G		UFRJ50791:G>G		UFRJ50816:G>G		UWOPS91_917_1:G>G		YPS138:G>G	AA:239		UWOPS91_917_1:G>G	AA:254		Q59_1:N>N		Q62_5:N>N		Q89_8:N>N		Q95_3:N>N		Y7:N>N		Z1_1:N>N	AA:278		A12:H>H		A4:H>H		DBVPG6304:H>H		UFRJ50791:H>H	AA:307		UWOPS91_917_1:L>L	AA:317		UWOPS91_917_1:L>L	AA:321		A12:I>I		A4:I>I		DBVPG6304:I>I		UFRJ50791:I>I		UWOPS91_917_1:I>I	AA:323		A12:T>T		A4:T>T		DBVPG6304:T>T		UFRJ50791:T>T		UWOPS91_917_1:T>T		YPS138:T>T	AA:334		A12:S>S		A4:S>S		DBVPG6304:S>S		UFRJ50791:S>S	AA:340		A12:K>K		A4:K>K		DBVPG6304:K>K		UFRJ50791:K>K		UWOPS91_917_1:K>K		YPS138:K>K	AA:342		A12:Q>Q		A4:Q>Q		DBVPG6304:Q>Q		UFRJ50791:Q>Q		YPS138:Q>Q	AA:360		N_43:T>T		N_45:T>T	AA:367		N_43:N>N		N_44:N>N		N_45:N>N	AA:372		UWOPS91_917_1:G>G	AA:374		UWOPS91_917_1:D>D	AA:382		UWOPS91_917_1:T>T	AA:387		A12:L>L		A4:L>L		DBVPG6304:L>L		N_43:L>L		N_44:L>L		N_45:L>L		UFRJ50791:L>L		UWOPS91_917_1:L>L		YPS138:L>L	AA:392		UWOPS91_917_1:P>P	AA:394		UWOPS91_917_1:F>F	AA:401		YPS138:L>L	AA:408		A12:N>N		A4:N>N		DBVPG6304:N>N		UFRJ50791:N>N		UWOPS91_917_1:N>N		YPS138:N>N	AA:422		UWOPS91_917_1:F>F	AA:438		UWOPS91_917_1:F>F	AA:442		A12:I>I		A4:I>I		DBVPG6304:I>I		N_43:I>I		N_44:I>I		N_45:I>I		UFRJ50791:I>I		UWOPS91_917_1:I>I		YPS138:I>I	AA:448		UWOPS91_917_1:S>S	AA:469		UWOPS91_917_1:T>T	AA:476		A12:F>F		A4:F>F		DBVPG6304:F>F		UFRJ50791:F>F		UFRJ50816:F>F		YPS138:F>F	AA:479		UWOPS91_917_1:R>R	AA:492		UFRJ50791:S>S		UFRJ50816:S>S		YPS138:S>S	AA:495		A12:L>L		A4:L>L		DBVPG6304:L>L		UFRJ50791:L>L		UFRJ50816:L>L		YPS138:L>L	AA:497		UWOPS91_917_1:I>I	AA:498		A12:E>E		A4:E>E		DBVPG6304:E>E		UFRJ50791:E>E		UFRJ50816:E>E		YPS138:E>E	AA:505		UWOPS91_917_1:N>N	AA:513		UWOPS91_917_1:Y>Y	AA:516		A12:V>V		A4:V>V		UFRJ50791:V>V		UFRJ50816:V>V		UWOPS91_917_1:V>V		YPS138:V>V	AA:539		UWOPS91_917_1:E>E	AA:541		UFRJ50791:T>T		UFRJ50816:T>T		UWOPS91_917_1:T>T	AA:550		UWOPS91_917_1:K>K	AA:551		A4:F>F		UFRJ50791:F>F		UFRJ50816:F>F		YPS138:F>F	AA:553		UWOPS91_917_1:D>D	AA:555		UWOPS91_917_1:R>R	AA:557		A12:V>V		A4:V>V		UFRJ50791:V>V		UFRJ50816:V>V		YPS138:V>V	AA:559		UWOPS91_917_1:S>S	AA:569		A4:V>V		UFRJ50791:V>V		UFRJ50816:V>V		YPS138:V>VID:YBR164C	AA:40		A4:V>V		UFRJ50816:V>V		YPS138:V>V	AA:55		A12:I>I		A4:I>I		UFRJ50816:I>I	AA:57		IFO1804:F>F		N_44:F>F		N_45:F>F	AA:63		A12:Q>Q		A4:Q>Q		UFRJ50816:Q>Q	AA:83		A12:G>G		A4:G>G		IFO1804:G>G		N_45:G>G		UFRJ50816:G>G		UWOPS91_917_1:G>G	AA:85		A12:T>T		A4:T>T		IFO1804:T>T		N_45:T>T		UFRJ50816:T>T		YPS138:T>T	AA:116		A12:E>E		A4:E>E		UFRJ50816:E>E		UWOPS91_917_1:E>E		YPS138:E>E	AA:120		A12:V>V		A4:V>V		IFO1804:V>V		N_43:V>V		N_45:V>V		UFRJ50816:V>V		UWOPS91_917_1:V>V		YPS138:V>V	AA:177		A12:H>H		A4:H>H		UFRJ50816:H>H		YPS138:H>HID:YBR165W	AA:12		A12:D>D		DBVPG6304:D>D		UFRJ50816:D>D		UWOPS91_917_1:D>D	AA:24		A12:Q>Q		DBVPG6304:Q>Q		UFRJ50816:Q>Q		UWOPS91_917_1:Q>Q	AA:59		UWOPS91_917_1:G>G	AA:64		A12:D>D		DBVPG6304:D>D		UFRJ50816:D>D		UWOPS91_917_1:D>D	AA:67		A12:I>I		DBVPG6304:I>I		N_44:I>I		N_45:I>I		UFRJ50816:I>I		UWOPS91_917_1:I>I	AA:73		A12:S>S		DBVPG6304:S>S		UFRJ50816:S>S		UWOPS91_917_1:S>S	AA:87		A12:T>T		A4:T>T		DBVPG6304:T>T		UFRJ50816:T>T	AA:99		A12:L>L		DBVPG6304:L>L		UFRJ50816:L>L		UWOPS91_917_1:L>L	AA:128		A4:F>F		DBVPG6304:F>F		UFRJ50816:F>F	AA:144		Y6_5:F>F	AA:147		A4:L>L		DBVPG6304:L>L		UFRJ50816:L>L	AA:151		A4:L>L	AA:155		A4:D>D		DBVPG6304:D>D		UFRJ50816:D>D	AA:197		N_43:K>K		N_44:K>K		N_45:K>K	AA:201		CBS5829:I>I	AA:221		A12:K>K		A4:K>K	AA:276		A12:R>R		A4:R>R		DBVPG6304:R>RID:YBR166C	AA:6		A12:I>I		DBVPG6304:I>I		UFRJ50816:I>I	AA:21		Q59_1:G>G		Q62_5:G>G		Q89_8:G>G		Q95_3:G>G		Y6_5:G>G		Z1_1:G>G	AA:49		N_43:V>V		N_44:V>V		N_45:V>V	AA:53		N_43:I>I		N_44:I>I		N_45:I>I	AA:58		A12:N>N		DBVPG6304:N>N	AA:60		A12:T>T		DBVPG6304:T>T		UWOPS91_917_1:T>T	AA:62		N_43:F>F		N_44:F>F		N_45:F>F	AA:73		A12:F>F		DBVPG6304:F>F		N_43:F>F		N_44:F>F		N_45:F>F		UFRJ50791:F>F		UWOPS91_917_1:F>F	AA:123		A12:P>P		A4:P>P		DBVPG6304:P>P		UFRJ50791:P>P		UFRJ50816:P>P	AA:129		A12:->-		A4:->-		DBVPG6304:->-		UFRJ50791:->-		UFRJ50816:->-	AA:136		A12:L>L		A4:L>L		DBVPG6304:R>R		UFRJ50791:R>R		UFRJ50816:L>L	AA:143		A12:A>A		A4:A>A		DBVPG6304:A>A		N_17:R>R		N_43:R>R		N_45:R>R		UFRJ50791:A>A		UFRJ50816:A>A	AA:160		N_43:I>I		N_45:I>I	AA:179		A12:P>P		A4:P>P		DBVPG6304:P>P		UFRJ50791:P>P		UFRJ50816:P>P	AA:219		A12:N>N		A4:N>N		DBVPG6304:N>N		UFRJ50791:N>N		UFRJ50816:N>N	AA:249		A12:R>R		A4:R>R		UFRJ50791:R>R		UFRJ50816:R>R	AA:259		A12:T>T		A4:T>T		UFRJ50791:T>T		UFRJ50816:T>T	AA:291		A12:T>T		A4:T>T		N_17:T>T		N_43:T>T		N_45:T>T		UFRJ50791:T>T		UFRJ50816:T>T	AA:304		A12:I>I		A4:I>I		UFRJ50791:I>I		UFRJ50816:I>I	AA:326		A12:R>R		A4:R>R		UFRJ50791:R>R	AA:344		A12:D>D		A4:D>D		UFRJ50816:D>D	AA:349		A12:T>T		A4:T>T		UFRJ50816:T>T	AA:367		N_17:C>C		N_43:C>C		N_45:C>C	AA:380		S36_7:D>D	AA:401		A12:S>S		A4:S>S	AA:405		A12:F>F		A4:F>F	AA:410		A12:I>I		A4:I>I	AA:437		A12:T>T		A4:T>T	AA:456		A12:H>H		A4:H>HID:YBR167C	AA:6		A12:D>D		A4:D>D		YPS138:D>D	AA:31		A12:D>D		A4:D>D	AA:63		A12:T>T		A4:T>T		N_43:N>N		N_44:N>N		N_45:N>N		UFRJ50816:T>T	AA:69		UWOPS91_917_1:F>F	AA:80		A12:I>I		A4:I>I		UFRJ50816:I>I		UWOPS91_917_1:I>I	AA:85		A12:P>P		A4:P>P		N_43:P>P		N_44:P>P		N_45:P>P		UFRJ50816:P>P		UWOPS91_917_1:P>P	AA:107		A12:N>N		A4:N>N		N_43:N>N		N_44:N>N		N_45:N>N		UFRJ50816:N>N		UWOPS91_917_1:N>N	AA:109		UWOPS91_917_1:D>D	AA:120		UWOPS91_917_1:L>LID:YBR168W	AA:6		UWOPS91_917_1:K>K	AA:33		UWOPS91_917_1:I>I	AA:40		A12:S>S		UFRJ50816:S>S		YPS138:S>S	AA:41		UWOPS91_917_1:T>T	AA:43		UWOPS91_917_1:F>F	AA:47		A12:P>P		A4:P>P		UFRJ50816:P>P		UWOPS91_917_1:P>P		YPS138:P>P	AA:49		UWOPS91_917_1:L>L	AA:59		A12:I>I		A4:I>I		UFRJ50816:I>I		UWOPS91_917_1:I>I		YPS138:I>I	AA:74		A12:T>T		A4:T>T		UWOPS91_917_1:T>T		YPS138:T>T	AA:78		A12:L>L		A4:L>L		DBVPG6304:L>L		UFRJ50816:L>L		UWOPS91_917_1:L>L		YPS138:L>L	AA:90		UWOPS91_917_1:V>V	AA:102		A4:L>L		DBVPG6304:L>L		UFRJ50816:L>L		UWOPS91_917_1:L>L	AA:105		A12:G>G		A4:G>G		DBVPG6304:G>G		UFRJ50816:G>G		UWOPS91_917_1:G>G	AA:127		A4:L>L		DBVPG6304:L>L	AA:142		N_44:L>L	AA:150		A4:E>E		DBVPG6304:E>E	AA:157		A12:N>N		A4:N>N		DBVPG6304:N>N	AA:175		A12:N>N		A4:N>N		DBVPG6304:N>N	AA:196		CBS432:Y>Y		CBS5829:Y>Y		KPN3828:Y>Y		KPN3829:Y>Y	AA:203		N_44:T>T	AA:212		A12:V>V		A4:V>V		DBVPG6304:V>V	AA:213		N_43:Y>Y		N_44:Y>Y	AA:220		A12:A>A		A4:A>A		DBVPG6304:A>A	AA:225		A12:L>L		A4:L>L		DBVPG6304:L>L	AA:239		A12:I>I		A4:I>I		DBVPG6304:I>I	AA:350		UFRJ50816:T>T		YPS138:T>T	AA:377		UFRJ50816:D>D	AA:413		A12:L>L		A4:L>L		UFRJ50816:L>L		YPS138:L>LID:YBR169C	AA:2		A12:I>I		A4:I>I		UFRJ50816:I>I		UWOPS91_917_1:I>I		YPS138:I>I	AA:4		N_43:V>V		N_44:V>V		N_45:V>V	AA:10		A12:A>A		A4:A>A		N_43:V>V		N_44:V>V		N_45:V>V		UFRJ50816:A>A		YPS138:A>A	AA:13		A12:V>V		A4:V>V		UFRJ50816:V>V		YPS138:V>V	AA:16		A12:T>T		A4:T>T		UFRJ50816:T>T		YPS138:T>T	AA:53		UWOPS91_917_1:I>I	AA:110		UWOPS91_917_1:E>E	AA:112		N_43:G>G		N_44:G>G		N_45:G>G	AA:113		A12:T>T		A4:T>T		UFRJ50791:T>T		UWOPS91_917_1:T>T		YPS138:T>T	AA:118		A12:V>V		A4:V>V		UFRJ50791:V>V		YPS138:V>V	AA:126		UWOPS91_917_1:I>I	AA:134		A12:I>I		A4:I>I		UFRJ50791:I>I		YPS138:I>I	AA:135		UWOPS91_917_1:L>L	AA:146		A12:I>I		IFO1804:I>I		N_43:I>I		N_44:I>I		N_45:I>I		UFRJ50791:I>I		UWOPS91_917_1:I>I		YPS138:I>I	AA:166		A12:L>L		IFO1804:L>L		N_44:L>L		N_45:L>L		UFRJ50791:L>L		UWOPS91_917_1:L>L		YPS138:L>L	AA:168		A12:R>R		UFRJ50791:R>R		UFRJ50816:R>R		UWOPS91_917_1:R>R		YPS138:R>R	AA:173		UWOPS91_917_1:I>I	AA:182		A12:C>C	AA:192		T21_4:N>N		Y6_5:N>N	AA:220		A12:K>K		UFRJ50791:K>K		YPS138:K>K	AA:238		A12:C>C	AA:245		A12:F>F		UFRJ50791:F>F		YPS138:F>F	AA:250		A12:V>V		UWOPS91_917_1:V>V		YPS138:V>V	AA:305		T21_4:S>S	AA:333		A12:->-		DBVPG6304:->-	AA:336		IFO1804:T>T		N_44:T>T		N_45:T>T	AA:341		A12:Y>Y		DBVPG6304:Y>Y		UWOPS91_917_1:Y>Y	AA:392		UWOPS91_917_1:A>A	AA:413		A12:R>R		DBVPG6304:R>R	AA:434		UWOPS91_917_1:R>R	AA:446		A12:K>K		DBVPG6304:K>K		UFRJ50816:K>K	AA:467		A12:I>I		DBVPG6304:I>I		N_43:I>I		N_44:I>I		N_45:I>I		UFRJ50816:I>I	AA:514		UWOPS91_917_1:R>R	AA:530		A12:S>S		UFRJ50816:S>S		UWOPS91_917_1:S>S	AA:536		UWOPS91_917_1:I>I	AA:538		UWOPS91_917_1:Y>Y	AA:552		A4:K>K		UFRJ50816:K>K		UWOPS91_917_1:K>K	AA:579		A4:S>S		UFRJ50816:S>S	AA:603		A4:->-		UFRJ50816:->-	AA:617		A12:V>V		A4:V>V		YPS138:V>V	AA:622		A12:T>T		A4:T>T		UFRJ50816:T>T		YPS138:T>T	AA:640		A12:S>S		A4:S>S		N_43:S>S		N_44:S>S		N_45:S>S		UFRJ50816:S>S		YPS138:S>S	AA:662		A12:V>V		A4:V>V		UFRJ50816:V>V		YPS138:V>V	AA:666		A12:I>I		A4:I>I		UFRJ50816:I>I		YPS138:I>IID:YBR170C	AA:34		N_43:Y>Y		N_45:Y>Y	AA:48		A4:I>I		DBVPG6304:I>I		UFRJ50816:I>I		UWOPS91_917_1:I>I		YPS138:I>I	AA:64		A4:I>I		DBVPG6304:I>I		UFRJ50816:I>I		UWOPS91_917_1:I>I		YPS138:I>I	AA:70		A4:S>S		DBVPG6304:S>S		N_43:S>S		N_45:S>S		UFRJ50816:S>S		UWOPS91_917_1:S>S		YPS138:S>S	AA:85		UWOPS91_917_1:L>L	AA:97		UWOPS91_917_1:D>D	AA:125		A4:K>K		DBVPG6304:K>K	AA:152		N_43:I>I		N_45:I>I	AA:156		UWOPS91_917_1:V>V	AA:169		A4:H>H		DBVPG6304:H>H		UWOPS91_917_1:H>H		YPS138:H>H	AA:180		A4:G>G		DBVPG6304:G>G		UWOPS91_917_1:G>G		YPS138:G>G	AA:185		A12:V>V		A4:V>V		DBVPG6304:V>V		UWOPS91_917_1:R>R	AA:225		A4:H>H		DBVPG6304:H>H		UWOPS91_917_1:H>H		YPS138:H>H	AA:234		DBVPG6304:K>K	AA:241		UWOPS91_917_1:F>F	AA:283		UWOPS91_917_1:V>V	AA:285		N_43:V>V		N_44:V>V		N_45:V>V	AA:286		UWOPS91_917_1:Q>Q	AA:289		UWOPS91_917_1:L>L	AA:300		UWOPS91_917_1:L>L	AA:315		UWOPS91_917_1:V>V	AA:326		UWOPS91_917_1:T>T	AA:327		N_43:C>C		N_44:C>C		N_45:C>C	AA:328		UWOPS91_917_1:V>V	AA:329		A12:P>P		A4:P>P	AA:358		UWOPS91_917_1:N>N	AA:369		A12:P>P		A4:P>P		DBVPG6304:P>P	AA:374		A12:V>V		A4:V>V		DBVPG6304:V>V	AA:375		UWOPS91_917_1:A>A	AA:385		A12:I>I		A4:I>I		DBVPG6304:I>I		UWOPS91_917_1:I>I	AA:401		UWOPS91_917_1:I>I	AA:435		A12:V>V		A4:V>V		DBVPG6304:V>V		UFRJ50816:V>V	AA:465		N_45:T>T	AA:483		A12:A>A		A4:A>A		DBVPG6304:A>A		UFRJ50816:A>A	AA:509		A12:H>H		DBVPG6304:H>H		UFRJ50816:H>H		UWOPS91_917_1:H>H		YPS138:H>H	AA:510		N_43:V>V	AA:515		UWOPS91_917_1:P>P	AA:536		DBVPG6304:L>L		N_43:L>L		N_45:L>L		UFRJ50816:L>L		UWOPS91_917_1:L>L		YPS138:L>L	AA:562		A4:V>V		DBVPG6304:V>V		N_43:V>V		N_45:V>V		UFRJ50816:V>V		UWOPS91_917_1:V>V		YPS138:V>V	AA:565		UWOPS91_917_1:L>L	AA:572		N_43:T>T		N_45:T>T	AA:578		A4:S>S		DBVPG6304:S>S		N_43:S>S		N_45:S>S		UFRJ50816:S>S		UWOPS91_917_1:S>S		YPS138:S>SID:YBR171W	AA:9		A12:F>F		A4:F>F		DBVPG6304:F>F		UFRJ50816:F>F		UWOPS91_917_1:F>F		YPS138:F>F	AA:14		UWOPS91_917_1:T>T	AA:20		A12:E>E		A4:E>E		DBVPG6304:E>E		UFRJ50816:E>E		UWOPS91_917_1:E>E		YPS138:E>E	AA:25		UWOPS91_917_1:T>T	AA:38		A12:F>F		A4:F>F		DBVPG6304:F>F		UFRJ50816:F>F		YPS138:F>F	AA:64		A12:S>S		A4:S>S		DBVPG6304:S>S		N_43:S>S		N_44:S>S		N_45:S>S		UFRJ50816:S>S		UWOPS91_917_1:S>S		YPS138:S>S	AA:77		A12:Q>Q		A4:Q>Q		DBVPG6304:Q>Q		UFRJ50816:Q>Q		YPS138:Q>Q	AA:82		A12:S>S		A4:S>S		DBVPG6304:S>S		UFRJ50816:S>S		UWOPS91_917_1:S>S		YPS138:S>S	AA:89		A12:E>E		A4:E>E		DBVPG6304:E>E		N_43:E>E		N_44:E>E		N_45:E>E		UFRJ50816:E>E		UWOPS91_917_1:E>E		YPS138:E>E	AA:94		UWOPS91_917_1:A>A	AA:108		CBS5829:L>L	AA:127		A12:G>G		A4:G>G		DBVPG6304:G>G		UFRJ50816:G>G		UWOPS91_917_1:G>G		YPS138:G>G	AA:143		A12:L>L		A4:L>L		DBVPG6304:L>L		UFRJ50816:L>L		UWOPS91_917_1:L>L		YPS138:L>L	AA:148		A12:T>T		A4:T>T		DBVPG6304:T>T	AA:150		A12:Q>Q		A4:Q>Q		DBVPG6304:Q>Q		UFRJ50816:Q>Q		UWOPS91_917_1:Q>Q		YPS138:Q>Q	AA:160		A12:V>V		A4:V>V		DBVPG6304:V>V		IFO1804:V>V		N_43:V>V		N_44:V>V		N_45:V>V		UFRJ50816:V>V		UWOPS91_917_1:V>V		YPS138:V>V	AA:170		A12:I>I		A4:I>I		DBVPG6304:I>I		IFO1804:I>I		N_43:I>I		N_44:I>I		N_45:I>I		UFRJ50816:I>I		UWOPS91_917_1:I>I		YPS138:I>I	AA:176		A12:L>L		A4:L>L		DBVPG6304:L>L		UFRJ50816:L>L		UWOPS91_917_1:L>L		YPS138:L>L	AA:181		DBVPG4650:Q>QID:YBR172C	AA:16		A4:V>V		IFO1804:V>V		N_43:V>V		N_45:V>V		UWOPS91_917_1:V>V		YPS138:V>V	AA:19		UWOPS91_917_1:I>I	AA:34		A12:T>T		A4:T>T		IFO1804:T>T		N_17:T>T		N_43:T>T		N_45:T>T		YPS138:T>T	AA:41		UWOPS91_917_1:S>S	AA:49		A12:S>S		A4:S>S		CBS432:S>S		CBS5829:S>S		DBVPG4650:S>S		IFO1804:S>S		N_17:S>S		N_43:S>S		N_45:S>S		S36_7:S>S		UWOPS91_917_1:S>S		YPS138:S>S	AA:68		A12:L>L		UWOPS91_917_1:L>L		YPS138:L>L	AA:86		IFO1804:I>I		N_17:I>I		N_43:I>I		N_44:I>I		N_45:I>I		UWOPS91_917_1:I>I	AA:129		A12:R>R		A4:C>C		UFRJ50791:C>C		UFRJ50816:C>C		UWOPS91_917_1:C>C		YPS138:C>C	AA:138		A12:I>I		UWOPS91_917_1:I>I		YPS138:I>I	AA:142		A12:N>N		YPS138:N>N	AA:144		A12:A>A		UWOPS91_917_1:A>A		YPS138:A>A	AA:156		UWOPS91_917_1:P>P	AA:162		A12:L>L		A4:L>L		UWOPS91_917_1:L>L		YPS138:L>L	AA:174		A12:L>L		A4:L>L		N_43:L>L		N_44:L>L		UWOPS91_917_1:L>L		YPS138:L>L	AA:181		A12:G>G		YPS138:G>G	AA:188		A12:->-		A4:->-		YPS138:->-	AA:193		A12:H>H		A4:H>H		N_43:H>H		N_44:H>H		N_45:H>H		UWOPS91_917_1:H>H		YPS138:H>H	AA:195		A12:L>L		A4:L>L		CBS432:L>L		CBS5829:L>L		DBVPG4650:L>L		KPN3828:L>L		N_17:L>L		N_43:L>L		N_44:L>L		N_45:L>L		S36_7:L>L		UWOPS91_917_1:L>L		YPS138:L>L	AA:213		A12:V>V		A4:S>S		DBVPG6304:S>S		UFRJ50791:S>S		UWOPS91_917_1:S>S		YPS138:S>S	AA:219		A12:V>V		A4:V>V		CBS432:V>V		CBS5829:V>V		KPN3828:V>V		N_17:V>V		N_43:V>V		N_44:V>V		UWOPS91_917_1:V>V	AA:233		A12:A>A		A4:A>A		N_43:A>A		N_44:A>A		N_45:A>A		UWOPS91_917_1:A>A	AA:240		A12:->-		A4:->-		UWOPS91_917_1:->-		YPS138:->-	AA:269		A12:P>P		A4:P>P		YPS138:P>P	AA:272		A12:->-	AA:308		A12:C>C		A4:C>C		DBVPG6304:C>C		YPS138:C>C	AA:313		A12:F>F		A4:F>F		CBS432:F>F		CBS5829:F>F		DBVPG4650:F>F		DBVPG6304:F>F		KPN3829:F>F		N_17:F>F		N_43:F>F		N_44:F>F		N_45:F>F		UWOPS91_917_1:F>F		YPS138:F>F	AA:318		A12:F>F		A4:F>F		DBVPG6304:F>F		YPS138:F>F	AA:324		A12:F>F		UWOPS91_917_1:F>F	AA:349		UWOPS91_917_1:T>T	AA:355		IFO1804:F>F		N_43:F>F		N_44:F>F		N_45:F>F	AA:373		A12:L>L		A4:L>L		DBVPG6304:L>L		IFO1804:L>L		N_43:L>L		N_44:L>L		N_45:L>L		UWOPS91_917_1:L>L		YPS138:L>L	AA:401		UWOPS91_917_1:S>S	AA:407		UWOPS91_917_1:S>S	AA:427		A12:F>F		A4:F>F		DBVPG6304:F>F		UWOPS91_917_1:F>F		YPS138:F>F	AA:439		A12:C>C		A4:C>C		DBVPG6304:C>C		UWOPS91_917_1:C>C		YPS138:C>C	AA:441		A12:W>W		A4:W>W		DBVPG6304:W>W		UWOPS91_917_1:W>W		YPS138:W>W	AA:465		A12:I>I		A4:I>I		DBVPG6304:I>I		UFRJ50791:I>I		YPS138:I>I	AA:474		A12:H>H		A4:H>H		DBVPG6304:H>H		N_43:H>H		N_44:H>H		N_45:H>H		UFRJ50791:H>H		UWOPS91_917_1:H>H		YPS138:H>H	AA:477		A12:A>A		A4:A>A		DBVPG6304:A>A		UFRJ50791:A>A		YPS138:A>A	AA:489		UWOPS91_917_1:F>F	AA:501		A12:->-		A4:->-		DBVPG6304:->-		UFRJ50791:->-		UWOPS91_917_1:->-		YPS138:->-	AA:538		N_43:C>C		N_44:C>C		N_45:C>C	AA:541		UWOPS91_917_1:L>L	AA:542		UFRJ50791:C>C	AA:544		A4:S>S		UFRJ50791:S>S		YPS138:S>S	AA:559		A4:V>V		N_43:V>V		N_45:V>V		UFRJ50791:V>V		UWOPS91_917_1:V>V		YPS138:V>V	AA:599		A4:S>S		UFRJ50791:S>S		YPS138:S>S	AA:620		A4:P>P		UFRJ50791:P>P		UFRJ50816:P>P		UWOPS91_917_1:P>P		YPS138:P>P	AA:627		A4:S>S		IFO1804:S>S		N_43:S>S		N_44:S>S		N_45:S>S		UFRJ50791:S>S		UFRJ50816:S>S		UWOPS91_917_1:S>S		YPS138:S>S	AA:629		A4:A>A		UFRJ50791:A>A		UFRJ50816:A>A		YPS138:A>A	AA:673		UWOPS91_917_1:R>R	AA:678		A12:S>S		A4:S>S		UFRJ50791:S>S		UFRJ50816:S>S		UWOPS91_917_1:S>S		YPS138:S>S	AA:710		A12:F>F		A4:F>F		UFRJ50791:F>F		UFRJ50816:F>F		UWOPS91_917_1:F>F		YPS138:F>F	AA:720		A12:L>L		A4:L>L		UFRJ50791:L>L		UFRJ50816:L>L		UWOPS91_917_1:L>L		YPS138:L>L	AA:726		A12:G>G		UFRJ50791:G>G		UFRJ50816:G>G		YPS138:G>G	AA:731		A12:L>L		A4:L>L		UFRJ50791:L>L		UFRJ50816:L>L		UWOPS91_917_1:L>L		YPS138:L>LID:YBR173C	AA:7		IFO1804:F>F		N_43:F>F		N_44:F>F		N_45:F>F	AA:15		A12:A>A		A4:A>A		DBVPG6304:A>A		IFO1804:A>A		N_43:A>A		N_44:A>A		N_45:A>A		UFRJ50816:A>A		UWOPS91_917_1:A>A		YPS138:A>A	AA:20		UWOPS91_917_1:G>G	AA:23		A12:A>A		A4:A>A		DBVPG6304:A>A		UFRJ50816:A>A		UWOPS91_917_1:A>A		YPS138:A>A	AA:32		A12:I>I		DBVPG6304:I>I		IFO1804:I>I		N_43:I>I		N_44:I>I		N_45:I>I		UFRJ50816:I>I		UWOPS91_917_1:I>I		YPS138:I>I	AA:47		DBVPG6304:T>T		UFRJ50816:T>T		YPS138:T>T	AA:83		CBS432:S>S		DBVPG4650:S>S		N_17:S>S		N_43:S>S		N_45:S>S		Q62_5:S>S		Q89_8:S>S		UFRJ50816:S>S		Y7:S>S		YPS138:S>S	AA:88		UWOPS91_917_1:G>G	AA:113		Z1_1:F>F	AA:123		A4:G>G		DBVPG6304:G>G		UFRJ50816:G>G	AA:145		A4:W>W		DBVPG6304:W>W		UFRJ50816:W>W	AA:147		CBS432:D>D		DBVPG4650:D>D		N_17:D>D		N_43:D>D		N_45:D>DID:YBR175W	AA:11		A12:Q>Q		A4:Q>Q		DBVPG6304:Q>Q	AA:18		A12:C>C		A4:C>C		DBVPG6304:C>C	AA:19		IFO1804:A>A		N_44:A>A		N_45:A>A	AA:33		IFO1804:G>G		N_43:G>G		N_44:G>G	AA:50		A12:L>L		A4:L>L		DBVPG6304:L>L	AA:55		IFO1804:A>A		N_43:A>A		N_44:A>A		N_45:A>A	AA:79		A12:V>V		A4:V>V		CBS432:V>V		DBVPG6304:V>V		N_43:V>V		N_44:V>V		N_45:V>V		YPS138:V>V	AA:84		N_43:L>L		N_44:L>L		N_45:L>L	AA:94		A12:G>G		A4:G>G		DBVPG6304:G>G		N_43:G>G		N_44:G>G		N_45:G>G		YPS138:G>G	AA:103		A12:T>T		A4:T>T		DBVPG6304:T>T		YPS138:T>T	AA:105		N_43:N>N		N_44:N>N		N_45:N>N	AA:111		A12:L>L		A4:L>L		DBVPG6304:L>L		N_43:L>L		N_44:L>L		N_45:L>L		YPS138:L>L	AA:119		A12:S>S		A4:S>S		CBS432:S>S		CBS5829:S>S		DBVPG6304:S>S		N_43:S>S		N_44:S>S		N_45:S>S		Q32_3:S>S		S36_7:S>S		T21_4:S>S		YPS138:S>S	AA:143		A12:S>S		A4:S>S		DBVPG6304:S>S		YPS138:S>S	AA:152		A12:S>S		A4:S>S		DBVPG6304:S>S		YPS138:S>S	AA:176		N_43:T>T		N_44:T>T		N_45:T>T	AA:193		A12:S>S		A4:S>S		DBVPG6304:S>S		N_43:S>S		N_44:S>S		N_45:S>S		YPS138:S>S	AA:209		N_43:L>L		N_44:L>L		N_45:L>L	AA:212		A12:V>V		A4:V>V		DBVPG6304:V>V		N_43:V>V		N_44:V>V		N_45:V>V		YPS138:V>V	AA:237		A12:L>L		A4:L>L		DBVPG6304:L>L		YPS138:L>L	AA:248		A12:P>P		A4:P>P		DBVPG6304:P>P		YPS138:P>P	AA:263		N_43:G>G		N_45:G>G	AA:270		A12:S>S		A4:S>S		DBVPG6304:S>S		UWOPS91_917_1:S>S		YPS138:S>S	AA:292		A12:S>S		DBVPG6304:S>S		N_43:S>S		N_45:S>S		UWOPS91_917_1:S>S		YPS138:S>S	AA:302		A12:S>S		DBVPG6304:S>S		UFRJ50816:S>S		YPS138:S>SID:YBR176W	AA:25		A12:Y>Y		A4:Y>Y		DBVPG6304:Y>Y		UFRJ50816:Y>Y		UWOPS91_917_1:Y>Y	AA:42		UWOPS91_917_1:S>S	AA:46		UWOPS91_917_1:A>A	AA:76		UWOPS91_917_1:Y>Y	AA:88		IFO1804:K>K		N_43:K>K	AA:120		A12:L>L		A4:L>L		DBVPG6304:L>L		IFO1804:L>L		N_43:L>L		UFRJ50791:L>L		UFRJ50816:L>L	AA:131		CBS5829:S>S	AA:132		IFO1804:K>K		N_43:K>K	AA:147		IFO1804:K>K		N_43:K>K	AA:151		A12:K>K		A4:K>K		DBVPG6304:K>K		UFRJ50791:K>K		UFRJ50816:K>K	AA:155		A4:E>E		DBVPG6304:E>E		UFRJ50791:E>E	AA:157		A12:C>C		A4:C>C		DBVPG6304:C>C		UFRJ50791:C>C	AA:160		A12:G>G		A4:G>G		DBVPG6304:G>G		UFRJ50791:G>G	AA:167		A12:I>I		DBVPG6304:I>I		UFRJ50791:I>I	AA:176		A12:S>S		DBVPG6304:S>S		UFRJ50791:S>S		YPS138:S>S	AA:182		A12:V>V		DBVPG6304:V>V		UFRJ50791:V>V		YPS138:V>V	AA:206		IFO1804:C>C		N_43:C>C		N_45:C>C	AA:212		DBVPG6304:E>E		UFRJ50791:E>E		YPS138:E>E	AA:214		IFO1804:V>V		N_43:V>V		N_45:V>V	AA:224		DBVPG6304:S>S		UFRJ50791:S>S		UWOPS91_917_1:S>S		YPS138:S>S	AA:239		A12:T>T		DBVPG6304:T>T		UFRJ50791:T>T		YPS138:T>T	AA:242		A12:Q>Q		DBVPG6304:Q>Q		UFRJ50791:Q>Q		UWOPS91_917_1:Q>Q		YPS138:Q>Q	AA:248		A12:D>D		DBVPG4650:D>D		DBVPG6304:D>D		UFRJ50791:D>D		YPS138:D>D	AA:264		A12:T>T		DBVPG6304:T>T		UFRJ50791:T>T		YPS138:T>T	AA:266		A12:N>N		DBVPG6304:N>N		UFRJ50791:N>N		YPS138:N>N	AA:275		UFRJ50791:L>L	AA:279		UWOPS91_917_1:I>I	AA:301		IFO1804:L>L		N_43:L>L		N_45:L>LID:YBR177C	AA:23		A12:V>V		DBVPG6304:V>V		UFRJ50816:V>V		UWOPS91_917_1:M>M	AA:41		A12:T>T		DBVPG6304:T>T		N_17:T>T		N_43:T>T		N_44:T>T		N_45:T>T		UFRJ50816:T>T	AA:52		A12:I>I		DBVPG6304:I>I		UWOPS91_917_1:I>I	AA:64		A12:N>N		DBVPG4650:N>N		DBVPG6304:N>N		UFRJ50816:N>N		UWOPS91_917_1:N>N	AA:86		DBVPG4650:S>S	AA:113		A12:I>I		DBVPG6304:I>I		N_17:I>I		N_44:I>I		N_45:I>I		UFRJ50816:I>I		UWOPS91_917_1:I>I		YPS138:I>I	AA:117		UWOPS91_917_1:E>E	AA:171		UWOPS91_917_1:C>C	AA:176		UWOPS91_917_1:I>I	AA:192		A12:A>A		DBVPG6304:A>A		UFRJ50816:A>A		UWOPS91_917_1:A>A		YPS138:A>A	AA:199		IFO1804:S>S		N_17:S>S		N_44:S>S		N_45:S>S	AA:233		IFO1804:V>V		N_43:V>V		N_44:V>V		N_45:V>V	AA:237		DBVPG6304:->-		UFRJ50816:->-		UWOPS91_917_1:->-		YPS138:->-	AA:247		DBVPG6304:T>T		IFO1804:T>T		N_43:T>T		N_44:T>T		N_45:T>T		UFRJ50816:T>T		UWOPS91_917_1:T>T		YPS138:T>T	AA:268		DBVPG6304:R>R		UFRJ50816:R>R		YPS138:R>R	AA:287		A4:G>G		DBVPG6304:G>G		YPS138:G>G	AA:298		A4:I>I		DBVPG6304:I>I		UWOPS91_917_1:I>I		YPS138:I>I	AA:314		UWOPS91_917_1:W>W	AA:320		A4:V>V		DBVPG6304:V>V		N_43:V>V		N_44:V>V		N_45:V>V		UWOPS91_917_1:V>V		YPS138:V>V	AA:345		A4:E>E		DBVPG6304:E>E		UWOPS91_917_1:E>E		YPS138:E>E	AA:356		IFO1804:E>E		N_43:E>E		N_44:E>E		N_45:E>E	AA:373		A12:S>S		A4:S>S		DBVPG6304:S>S		UFRJ50816:S>S		UWOPS91_917_1:S>S		YPS138:S>S	AA:378		A12:A>A		A4:Q>Q		DBVPG6304:A>A		IFO1804:Q>Q		N_43:Q>Q		N_44:Q>Q		N_45:Q>Q		UFRJ50816:A>A		UWOPS91_917_1:Q>Q		YPS138:Q>Q	AA:387		UWOPS91_917_1:V>V	AA:390		UWOPS91_917_1:Q>Q	AA:397		A12:L>L		A4:L>L		DBVPG6304:L>L		UFRJ50816:L>L		YPS138:L>L	AA:406		IFO1804:E>E		N_43:E>E		N_44:E>E		N_45:E>E	AA:414		A12:L>L		A4:L>L		DBVPG6304:L>L		UFRJ50816:L>L		UWOPS91_917_1:L>L		YPS138:L>L	AA:420		A12:->-		A4:->-		DBVPG6304:->-		UFRJ50791:->-		UFRJ50816:->-		YPS138:->-	AA:444		A12:S>S		A4:S>S		DBVPG6304:S>S		UFRJ50791:S>S		UFRJ50816:S>S		UWOPS91_917_1:S>S		YPS138:S>SID:YBR179C	AA:2		DBVPG6304:I>I		IFO1804:I>I		N_44:I>I		N_45:I>I		UFRJ50791:I>I		UWOPS91_917_1:I>I		YPS138:I>I	AA:19		UWOPS91_917_1:F>F	AA:38		UWOPS91_917_1:Q>Q	AA:42		A4:S>S	AA:53		A4:R>R		DBVPG6304:R>R		UFRJ50791:R>R		YPS138:R>R	AA:70		IFO1804:->-		N_44:->-		N_45:->-	AA:73		CBS432:L>L		IFO1804:L>L		N_44:L>L		N_45:L>L	AA:98		IFO1804:T>T		N_44:T>T		N_45:T>T	AA:112		IFO1804:A>A		N_44:A>A		N_45:A>A	AA:121		UWOPS91_917_1:L>L	AA:128		N_44:->-		N_45:->-	AA:138		N_44:T>T		N_45:T>T	AA:152		N_44:R>R		N_45:R>R		UWOPS91_917_1:R>R	AA:161		DBVPG6304:W>W	AA:166		DBVPG6304:L>L		YPS138:L>L	AA:186		DBVPG6304:R>R		YPS138:R>R	AA:195		DBVPG6304:C>C		YPS138:C>C	AA:240		A4:F>F		DBVPG6304:F>F		YPS138:F>F	AA:250		A4:T>T		DBVPG6304:T>T		YPS138:T>T	AA:263		N_45:->-	AA:278		A12:->-		A4:->-		DBVPG6304:->-		YPS138:->-	AA:313		A12:R>R		A4:R>R		DBVPG6304:R>R		YPS138:R>R	AA:341		A12:I>I		A4:I>I		DBVPG6304:I>I		IFO1804:I>I		N_45:I>I		YPS138:I>I	AA:350		A12:F>F		A4:F>F		YPS138:F>F	AA:360		A12:T>T		IFO1804:D>D		N_45:D>D	AA:399		A12:L>L		A4:L>L		DBVPG6304:L>L		YPS138:L>L	AA:416		IFO1804:W>W		N_45:W>W	AA:426		A12:R>R		A4:R>R		DBVPG6304:R>R	AA:435		A12:V>V		A4:V>V		DBVPG6304:V>V	AA:455		IFO1804:I>I		N_44:I>I		N_45:I>I	AA:536		A12:R>R	AA:566		DBVPG6304:L>L	AA:589		A4:V>V		DBVPG6304:V>V		YPS138:V>V	AA:648		UFRJ50816:L>L		YPS138:L>L	AA:651		UWOPS91_917_1:R>R	AA:659		A4:S>S		DBVPG6304:S>S		UFRJ50816:S>S		UWOPS91_917_1:S>S		YPS138:S>S	AA:677		A4:F>F	AA:679		UWOPS91_917_1:R>R	AA:695		A4:->-		DBVPG6304:->-		N_17:->-		N_43:->-		N_45:->-		UFRJ50816:->-		UWOPS91_917_1:->-		YPS138:->-	AA:717		A4:D>D		DBVPG6304:D>D		N_17:D>D		N_43:D>D		N_45:D>D		UFRJ50816:D>D		UWOPS91_917_1:D>D		YPS138:D>D	AA:737		N_43:A>A		N_45:A>A	AA:744		UWOPS91_917_1:K>K	AA:758		N_43:Q>Q		N_45:Q>Q	AA:760		A12:Y>Y		A4:Y>Y		DBVPG6304:Y>Y		UFRJ50816:Y>Y		UWOPS91_917_1:Y>Y		YPS138:Y>Y	AA:762		A12:I>I		A4:I>I		DBVPG6304:I>I		UFRJ50816:I>I		YPS138:I>I	AA:766		UWOPS91_917_1:I>I	AA:777		N_43:S>S		N_45:S>S	AA:781		A12:G>G		A4:G>G		DBVPG6304:G>G		UFRJ50816:G>G		UWOPS91_917_1:G>G		YPS138:G>G	AA:786		A12:G>G		A4:G>G		DBVPG6304:G>G		UFRJ50816:G>G		UWOPS91_917_1:G>G		YPS138:G>G	AA:811		A12:I>I		A4:I>I		DBVPG6304:I>I		UFRJ50816:I>I		UWOPS91_917_1:I>I		YPS138:I>I	AA:831		N_43:G>G		N_45:G>G	AA:836		A12:L>L		N_43:L>L		N_45:L>L		UFRJ50816:L>L		UWOPS91_917_1:L>L		YPS138:L>L	AA:840		A12:A>A		UFRJ50816:A>A		UWOPS91_917_1:A>A		YPS138:A>A	AA:853		YPS138:S>SID:YBR180W	AA:18		IFO1804:Q>Q		N_43:Q>Q		N_44:Q>Q		N_45:Q>Q		T21_4:Q>Q	AA:20		A12:S>S		A4:S>S		DBVPG6304:S>S		UFRJ50791:S>S		UFRJ50816:S>S		UWOPS91_917_1:S>S	AA:32		A12:D>D		A4:D>D		DBVPG6304:D>D		UFRJ50791:D>D		UFRJ50816:D>D	AA:45		UWOPS91_917_1:V>V	AA:102		IFO1804:T>T		N_43:T>T		N_44:T>T		N_45:T>T	AA:120		A12:G>G		A4:G>G		DBVPG6304:G>G		UFRJ50791:G>G	AA:133		A12:A>A		A4:A>A		DBVPG6304:A>A		IFO1804:A>A		N_43:A>A		N_44:A>A		N_45:A>A	AA:136		IFO1804:L>L		N_43:L>L		N_44:L>L		N_45:L>L	AA:157		IFO1804:A>A		N_43:A>A		N_44:A>A		N_45:A>A	AA:160		A4:S>S		DBVPG6304:S>S	AA:167		A12:G>G		A4:G>G		DBVPG6304:G>G	AA:174		IFO1804:G>G		N_43:G>G		N_44:G>G		N_45:G>G	AA:178		A12:L>L		A4:L>L		DBVPG6304:L>L	AA:183		A4:L>L		DBVPG6304:L>L	AA:193		A4:L>L		DBVPG6304:L>L		IFO1804:L>L		N_43:L>L		N_44:L>L		N_45:L>L	AA:196		A4:V>V		DBVPG6304:V>V		IFO1804:V>V		N_43:V>V		N_44:V>V		N_45:V>V	AA:199		IFO1804:N>N		N_43:N>N		N_44:N>N		N_45:N>N	AA:201		A4:A>A		DBVPG6304:A>A	AA:212		A4:F>F		DBVPG6304:F>F		YPS138:F>F	AA:217		A4:V>V		DBVPG6304:V>V		YPS138:V>V	AA:230		IFO1804:P>P		N_43:P>P		N_44:P>P		N_45:P>P	AA:234		A4:R>R		DBVPG6304:R>R		IFO1804:R>R		N_43:R>R		N_44:R>R		N_45:R>R		YPS138:R>R	AA:259		A4:I>I		DBVPG6304:I>I		YPS138:I>I	AA:263		Q59_1:G>G	AA:300		N_43:D>D		N_44:D>D		N_45:D>D	AA:305		Q95_3:E>E	AA:328		N_43:L>L		N_44:L>L		N_45:L>L	AA:343		UWOPS91_917_1:F>F	AA:344		A4:F>F		DBVPG6304:F>F		YPS138:F>F	AA:346		A4:Q>Q		DBVPG6304:Q>Q		N_43:Q>Q		N_44:Q>Q		N_45:Q>Q		UWOPS91_917_1:Q>Q		YPS138:Q>Q	AA:349		A4:P>P		DBVPG6304:P>P		N_43:P>P		N_44:P>P		N_45:P>P		UWOPS91_917_1:P>P		YPS138:P>P	AA:353		A4:K>K		DBVPG6304:K>K		YPS138:K>K	AA:357		N_43:T>T		N_44:T>T		N_45:T>T	AA:378		N_43:L>L		N_44:L>L		N_45:L>L	AA:390		A4:S>S		DBVPG6304:S>S		UWOPS91_917_1:S>S		YPS138:S>S	AA:398		A4:N>N		DBVPG6304:N>N		N_44:N>N		N_45:N>N		YPS138:N>N	AA:399		UWOPS91_917_1:F>F	AA:412		A4:G>G		YPS138:G>G	AA:414		YPS138:A>A	AA:416		UWOPS91_917_1:L>L	AA:452		A4:L>L		N_44:L>L		N_45:L>L		YPS138:L>L	AA:457		N_44:L>L		N_45:L>L	AA:462		A4:T>T		YPS138:T>T	AA:464		N_44:G>G		N_45:G>G		UWOPS91_917_1:G>G	AA:485		A4:T>T		N_44:T>T		N_45:T>T		UWOPS91_917_1:T>T		YPS138:T>T	AA:492		N_44:C>C		N_45:C>C	AA:494		A4:N>N		N_44:N>N		N_45:N>N		YPS138:N>N	AA:499		N_44:Y>Y		N_45:Y>Y	AA:503		N_44:L>L		N_45:L>L	AA:505		UWOPS91_917_1:P>P	AA:508		UWOPS91_917_1:A>A	AA:510		UWOPS91_917_1:G>G	AA:512		UWOPS91_917_1:V>V	AA:516		N_44:S>S		N_45:S>S	AA:519		DBVPG4650:R>R		Q95_3:R>R		T21_4:R>R	AA:521		UWOPS91_917_1:V>V	AA:531		A4:L>L		YPS138:L>L	AA:544		YPS138:T>T	AA:548		N_44:I>I	AA:560		UWOPS91_917_1:L>L	AA:563		UWOPS91_917_1:F>F	AA:569		UWOPS91_917_1:A>AID:YBR181C	AA:15		A12:L>L		A4:L>L		DBVPG6304:L>L		YPS138:L>L	AA:32		N_43:G>G		N_45:G>G	AA:34		A12:S>S		A4:S>S		DBVPG6304:S>S		N_43:S>S		N_45:S>S		YPS138:S>S	AA:47		A
[truncated: 1,200,000 more chars]
