# Supplementary material for: Proteome-wide evidence for enhanced positive Darwinian selection within intrinsically disordered regions in proteins
Source: Genome Biol. 2011 Jul 19;12(7):R65. doi: 10.1186/gb-2011-12-7-r65 (PMC3218827; doi:10.1186/gb-2011-12-7-r65)
Supplement: Additional file 6 — Chromosomal location of protein-coding region SNPs in the different strains of S. cerevisiae. The direction of each gene as well as the base change in each SNP and its affect on codons and encoded amino acids is shown. [file gb-2011-12-7-r65-S6.ZIP › Additional_data_file_6.rtf]

PROTEIN  DIRECTION  PHASE  STRAIN	GENOMIC POSITION      SNP	  CODON		AA==========================================================================================YAL001C      -      1      273614X       chr01:147600         G>A         ACA>ATA      T>IYAL001C      -      1      378604X       chr01:147600         G>A         ACA>ATA      T>IYAL001C      -      1      DBVPG1853     chr01:147600         G>A         ACA>ATA      T>IYAL001C      -      1      K11           chr01:147600         G>A         ACA>ATA      T>IYAL001C      -      1      SK1           chr01:147600         G>A         ACA>ATA      T>IYAL001C      -      1      Y12           chr01:147600         G>A         ACA>ATA      T>IYAL001C      -      1      273614X       chr01:147609         T>A         TAT>TTT      Y>FYAL001C      -      1      378604X       chr01:147609         T>A         TAT>TTT      Y>FYAL001C      -      1      DBVPG1853     chr01:147609         T>A         TAT>TTT      Y>FYAL001C      -      1      SK1           chr01:147609         T>A         TAT>TTT      Y>FYAL001C      -      1      Y12           chr01:147609         T>A         TAT>TTT      Y>FYAL001C      -      1      Y55           chr01:147609         T>A         TAT>TTT      Y>FYAL001C      -      1      YPS128        chr01:147609         T>A         TAT>TTT      Y>FYAL001C      -      1      YPS606        chr01:147609         T>A         TAT>TTT      Y>FYAL001C      -      1      273614X       chr01:147657         G>C         ACT>AGT      T>SYAL001C      -      1      378604X       chr01:147657         G>C         ACT>AGT      T>SYAL001C      -      1      SK1           chr01:147657         G>C         ACT>AGT      T>SYAL001C      -      1      UWOPS05_217_3 chr01:147657         G>C         ACT>AGT      T>SYAL001C      -      1      UWOPS05_227_2 chr01:147657         G>C         ACT>AGT      T>SYAL001C      -      1      Y12           chr01:147657         G>C         ACT>AGT      T>SYAL001C      -      1      Y55           chr01:147657         G>C         ACT>AGT      T>SYAL001C      -      1      YPS128        chr01:147657         G>C         ACT>AGT      T>SYAL001C      -      1      YPS606        chr01:147657         G>C         ACT>AGT      T>SYAL001C      -      1      UWOPS05_217_3 chr01:147699         G>A         TCA>TTA      S>LYAL001C      -      1      UWOPS05_227_2 chr01:147699         G>A         TCA>TTA      S>LYAL001C      -      1      273614X       chr01:147719         T>A         GTA>GTT      V>VYAL001C      -      1      378604X       chr01:147719         T>A         GTA>GTT      V>VYAL001C      -      1      SK1           chr01:147719         T>A         GTA>GTT      V>VYAL001C      -      1      Y12           chr01:147719         T>A         GTA>GTT      V>VYAL001C      -      1      273614X       chr01:147728         A>G         TGT>TGC      C>CYAL001C      -      1      378604X       chr01:147728         A>G         TGT>TGC      C>CYAL001C      -      1      DBVPG1106     chr01:147728         A>G         TGT>TGC      C>CYAL001C      -      1      DBVPG6765     chr01:147728         A>G         TGT>TGC      C>CYAL001C      -      1      L_1528        chr01:147728         A>G         TGT>TGC      C>CYAL001C      -      1      SK1           chr01:147728         A>G         TGT>TGC      C>CYAL001C      -      1      UWOPS83_787_3 chr01:147728         A>G         TGT>TGC      C>CYAL001C      -      1      UWOPS87_2421  chr01:147728         A>G         TGT>TGC      C>CYAL001C      -      1      Y12           chr01:147728         A>G         TGT>TGC      C>CYAL001C      -      1      Y55           chr01:147728         A>G         TGT>TGC      C>CYAL001C      -      1      YIIc17_E5     chr01:147728         A>G         TGT>TGC      C>CYAL001C      -      1      YJM975        chr01:147728         A>G         TGT>TGC      C>CYAL001C      -      1      YJM981        chr01:147728         A>G         TGT>TGC      C>CYAL001C      -      1      YPS128        chr01:147728         A>G         TGT>TGC      C>CYAL001C      -      1      L_1528        chr01:147740         C>T         TTG>TTA      L>LYAL001C      -      1      273614X       chr01:147761         T>A         CCA>CCT      P>PYAL001C      -      1      378604X       chr01:147761         T>A         CCA>CCT      P>PYAL001C      -      1      DBVPG6044     chr01:147761         T>A         CCA>CCT      P>PYAL001C      -      1      SK1           chr01:147761         T>A         CCA>CCT      P>PYAL001C      -      1      UWOPS05_217_3 chr01:147761         T>A         CCA>CCT      P>PYAL001C      -      1      UWOPS05_227_2 chr01:147761         T>A         CCA>CCT      P>PYAL001C      -      1      UWOPS83_787_3 chr01:147761         T>A         CCA>CCT      P>PYAL001C      -      1      Y12           chr01:147761         T>A         CCA>CCT      P>PYAL001C      -      1      Y55           chr01:147761         T>A         CCA>CCT      P>PYAL001C      -      1      YPS128        chr01:147761         T>A         CCA>CCT      P>PYAL001C      -      1      DBVPG1106     chr01:147787         C>G         GTC>CTC      V>LYAL001C      -      1      DBVPG6765     chr01:147787         C>G         GTC>CTC      V>LYAL001C      -      1      L_1528        chr01:147787         C>G         GTC>CTC      V>LYAL001C      -      1      YIIc17_E5     chr01:147787         C>G         GTC>CTC      V>LYAL001C      -      1      YJM975        chr01:147787         C>G         GTC>CTC      V>LYAL001C      -      1      YJM981        chr01:147787         C>G         GTC>CTC      V>LYAL001C      -      1      DBVPG6044     chr01:147797         T>C         GTA>GTG      V>VYAL001C      -      1      Y55           chr01:147797         T>C         GTA>GTG      V>VYAL001C      -      1      DBVPG6044     chr01:147887         C>T         AAG>AAA      K>KYAL001C      -      1      Y55           chr01:147887         C>T         AAG>AAA      K>KYAL001C      -      1      273614X       chr01:148148         A>T         GGT>GGA      G>GYAL001C      -      1      DBVPG1853     chr01:148148         A>T         GGT>GGA      G>GYAL001C      -      1      DBVPG6040     chr01:148148         A>T         GGT>GGA      G>GYAL001C      -      1      SK1           chr01:148148         A>T         GGT>GGA      G>GYAL001C      -      1      UWOPS05_217_3 chr01:148148         A>T         GGT>GGA      G>GYAL001C      -      1      UWOPS05_227_2 chr01:148148         A>T         GGT>GGA      G>GYAL001C      -      1      UWOPS83_787_3 chr01:148148         A>T         GGT>GGA      G>GYAL001C      -      1      Y12           chr01:148148         A>T         GGT>GGA      G>GYAL001C      -      1      Y55           chr01:148148         A>T         GGT>GGA      G>GYAL001C      -      1      YPS128        chr01:148148         A>T         GGT>GGA      G>GYAL001C      -      1      SK1           chr01:148262         G>A         AAC>AAT      N>NYAL001C      -      1      Y55           chr01:148598         A>G         CTT>CTC      L>LYAL001C      -      1      UWOPS05_217_3 chr01:148612         G>C         CTT>GTT      L>VYAL001C      -      1      UWOPS05_227_2 chr01:148612         G>C         CTT>GTT      L>VYAL001C      -      1      UWOPS05_217_3 chr01:148751         G>A         AAC>AAT      N>NYAL001C      -      1      UWOPS05_227_2 chr01:148751         G>A         AAC>AAT      N>NYAL001C      -      1      DBVPG6044     chr01:148820         A>G         ACT>ACC      T>TYAL001C      -      1      Y55           chr01:148820         A>G         ACT>ACC      T>TYAL001C      -      1      273614X       chr01:148888         C>T         GAC>AAC      D>NYAL001C      -      1      DBVPG1853     chr01:148888         C>T         GAC>AAC      D>NYAL001C      -      1      DBVPG6044     chr01:148888         C>T         GAC>AAC      D>NYAL001C      -      1      SK1           chr01:148888         C>T         GAC>AAC      D>NYAL001C      -      1      UWOPS05_217_3 chr01:148888         C>T         GAC>AAC      D>NYAL001C      -      1      UWOPS05_227_2 chr01:148888         C>T         GAC>AAC      D>NYAL001C      -      1      Y55           chr01:148888         C>T         GAC>AAC      D>NYAL001C      -      1      YPS128        chr01:148888         C>T         GAC>AAC      D>NYAL001C      -      1      YS4           chr01:148895         A>T         ACT>ACA      T>TYAL001C      -      1      NCYC110       chr01:148951         T>G         AAA>CAA      K>QYAL001C      -      1      273614X       chr01:148976         C>G         TCG>TCC      S>SYAL001C      -      1      322134S       chr01:149127         T>C         CAT>CGT      H>RYAL001C      -      1      DBVPG6044     chr01:149189         A>G         GCT>GCC      A>AYAL001C      -      1      NCYC110       chr01:149189         A>G         GCT>GCC      A>AYAL001C      -      1      Y55           chr01:149189         A>G         GCT>GCC      A>AYAL001C      -      1      322134S       chr01:149228         T>C         GGA>GGG      G>GYAL001C      -      1      273614X       chr01:149270         T>C         GTA>GTG      V>VYAL001C      -      1      DBVPG1853     chr01:149270         T>C         GTA>GTG      V>VYAL001C      -      1      SK1           chr01:149270         T>C         GTA>GTG      V>VYAL001C      -      1      273614X       chr01:149309         T>C         TTA>TTG      L>LYAL001C      -      1      322134S       chr01:149309         T>C         TTA>TTG      L>LYAL001C      -      1      DBVPG1373     chr01:149309         T>C         TTA>TTG      L>LYAL001C      -      1      DBVPG1853     chr01:149309         T>C         TTA>TTG      L>LYAL001C      -      1      DBVPG6044     chr01:149309         T>C         TTA>TTG      L>LYAL001C      -      1      DBVPG6765     chr01:149309         T>C         TTA>TTG      L>LYAL001C      -      1      NCYC110       chr01:149309         T>C         TTA>TTG      L>LYAL001C      -      1      SK1           chr01:149309         T>C         TTA>TTG      L>LYAL001C      -      1      UWOPS83_787_3 chr01:149309         T>C         TTA>TTG      L>LYAL001C      -      1      Y55           chr01:149309         T>C         TTA>TTG      L>LYAL001C      -      1      YJM978        chr01:149309         T>C         TTA>TTG      L>LYAL001C      -      1      YPS606        chr01:149309         T>C         TTA>TTG      L>LYAL001C      -      1      YS4           chr01:149309         T>C         TTA>TTG      L>LYAL001C      -      1      YS9           chr01:149309         T>C         TTA>TTG      L>LYAL001C      -      1      SK1           chr01:149456         G>A         AAC>AAT      N>NYAL001C      -      1      Y12           chr01:149456         G>A         AAC>AAT      N>NYAL001C      -      1      322134S       chr01:149469         C>T         GGC>GAC      G>DYAL001C      -      1      DBVPG1106     chr01:149469         C>T         GGC>GAC      G>DYAL001C      -      1      DBVPG1373     chr01:149469         C>T         GGC>GAC      G>DYAL001C      -      1      DBVPG1788     chr01:149469         C>T         GGC>GAC      G>DYAL001C      -      1      DBVPG6765     chr01:149469         C>T         GGC>GAC      G>DYAL001C      -      1      L_1374        chr01:149469         C>T         GGC>GAC      G>DYAL001C      -      1      L_1528        chr01:149469         C>T         GGC>GAC      G>DYAL001C      -      1      YJM975        chr01:149469         C>T         GGC>GAC      G>DYAL001C      -      1      YJM978        chr01:149469         C>T         GGC>GAC      G>DYAL001C      -      1      YS4           chr01:149469         C>T         GGC>GAC      G>DYAL001C      -      1      273614X       chr01:149596         T>C         AAT>GAT      N>DYAL001C      -      1      K11           chr01:149596         T>C         AAT>GAT      N>DYAL001C      -      1      NCYC110       chr01:149596         T>C         AAT>GAT      N>DYAL001C      -      1      SK1           chr01:149596         T>C         AAT>GAT      N>DYAL001C      -      1      UWOPS03_461_4 chr01:149596         T>C         AAT>GAT      N>DYAL001C      -      1      UWOPS05_217_3 chr01:149596         T>C         AAT>GAT      N>DYAL001C      -      1      UWOPS83_787_3 chr01:149596         T>C         AAT>GAT      N>DYAL001C      -      1      Y12           chr01:149596         T>C         AAT>GAT      N>DYAL001C      -      1      Y55           chr01:149596         T>C         AAT>GAT      N>DYAL001C      -      1      YPS606        chr01:149596         T>C         AAT>GAT      N>DYAL001C      -      1      YS9           chr01:149596         T>C         AAT>GAT      N>DYAL001C      -      1      273614X       chr01:149775         C>T         CGT>CAT      R>HYAL001C      -      1      322134S       chr01:149775         C>T         CGT>CAT      R>HYAL001C      -      1      DBVPG1106     chr01:149775         C>T         CGT>CAT      R>HYAL001C      -      1      DBVPG1373     chr01:149775         C>T         CGT>CAT      R>HYAL001C      -      1      DBVPG1788     chr01:149775         C>T         CGT>CAT      R>HYAL001C      -      1      DBVPG6765     chr01:149775         C>T         CGT>CAT      R>HYAL001C      -      1      K11           chr01:149775         C>T         CGT>CAT      R>HYAL001C      -      1      L_1374        chr01:149775         C>T         CGT>CAT      R>HYAL001C      -      1      L_1528        chr01:149775         C>T         CGT>CAT      R>HYAL001C      -      1      SK1           chr01:149775         C>T         CGT>CAT      R>HYAL001C      -      1      UWOPS03_461_4 chr01:149775         C>T         CGT>CAT      R>HYAL001C      -      1      UWOPS05_217_3 chr01:149775         C>T         CGT>CAT      R>HYAL001C      -      1      UWOPS83_787_3 chr01:149775         C>T         CGT>CAT      R>HYAL001C      -      1      UWOPS87_2421  chr01:149775         C>T         CGT>CAT      R>HYAL001C      -      1      Y12           chr01:149775         C>T         CGT>CAT      R>HYAL001C      -      1      Y55           chr01:149775         C>T         CGT>CAT      R>HYAL001C      -      1      YIIc17_E5     chr01:149775         C>T         CGT>CAT      R>HYAL001C      -      1      YJM975        chr01:149775         C>T         CGT>CAT      R>HYAL001C      -      1      YJM978        chr01:149775         C>T         CGT>CAT      R>HYAL001C      -      1      YS4           chr01:149775         C>T         CGT>CAT      R>HYAL001C      -      1      YS9           chr01:149775         C>T         CGT>CAT      R>HYAL001C      -      1      273614X       chr01:149798         C>T         GGG>GGA      G>GYAL001C      -      1      UWOPS83_787_3 chr01:149798         C>T         GGG>GGA      G>GYAL001C      -      1      YS9           chr01:149798         C>T         GGG>GGA      G>GYAL001C      -      1      273614X       chr01:149805         G>A         CCA>CTA      P>LYAL001C      -      1      YS9           chr01:149805         G>A         CCA>CTA      P>LYAL001C      -      1      273614X       chr01:149915         C>A         GGG>GGT      G>GYAL001C      -      1      K11           chr01:149915         C>A         GGG>GGT      G>GYAL001C      -      1      SK1           chr01:149915         C>A         GGG>GGT      G>GYAL001C      -      1      UWOPS05_217_3 chr01:149915         C>A         GGG>GGT      G>GYAL001C      -      1      UWOPS83_787_3 chr01:149915         C>A         GGG>GGT      G>GYAL001C      -      1      Y12           chr01:149915         C>A         GGG>GGT      G>GYAL001C      -      1      Y55           chr01:149915         C>A         GGG>GGT      G>GYAL001C      -      1      YS9           chr01:149915         C>A         GGG>GGT      G>GYAL001C      -      1      322134S       chr01:149921         T>C         ACA>ACG      T>TYAL001C      -      1      322134S       chr01:149940         T>C         GAT>GGT      D>GYAL001C      -      1      DBVPG1853     chr01:149975         A>G         TTT>TTC      F>FYAL001C      -      1      K11           chr01:149975         A>G         TTT>TTC      F>FYAL001C      -      1      SK1           chr01:149975         A>G         TTT>TTC      F>FYAL001C      -      1      UWOPS05_217_3 chr01:149975         A>G         TTT>TTC      F>FYAL001C      -      1      UWOPS83_787_3 chr01:149975         A>G         TTT>TTC      F>FYAL001C      -      1      Y55           chr01:149975         A>G         TTT>TTC      F>FYAL001C      -      1      YS9           chr01:149975         A>G         TTT>TTC      F>FYAL001C      -      1      DBVPG1788     chr01:150047         C>T         AAG>AAA      K>KYAL001C      -      1      322134S       chr01:150305         G>A         TAC>TAT      Y>YYAL001C      -      1      DBVPG1853     chr01:150305         G>A         TAC>TAT      Y>YYAL001C      -      1      DBVPG6765     chr01:150305         G>A         TAC>TAT      Y>YYAL001C      -      1      K11           chr01:150305         G>A         TAC>TAT      Y>YYAL001C      -      1      L_1374        chr01:150305         G>A         TAC>TAT      Y>YYAL001C      -      1      L_1528        chr01:150305         G>A         TAC>TAT      Y>YYAL001C      -      1      NCYC110       chr01:150305         G>A         TAC>TAT      Y>YYAL001C      -      1      SK1           chr01:150305         G>A         TAC>TAT      Y>YYAL001C      -      1      UWOPS05_217_3 chr01:150305         G>A         TAC>TAT      Y>YYAL001C      -      1      UWOPS05_227_2 chr01:150305         G>A         TAC>TAT      Y>YYAL001C      -      1      UWOPS83_787_3 chr01:150305         G>A         TAC>TAT      Y>YYAL001C      -      1      Y12           chr01:150305         G>A         TAC>TAT      Y>YYAL001C      -      1      Y55           chr01:150305         G>A         TAC>TAT      Y>YYAL001C      -      1      YIIc17_E5     chr01:150305         G>A         TAC>TAT      Y>YYAL001C      -      1      YJM978        chr01:150305         G>A         TAC>TAT      Y>YYAL001C      -      1      YPS606        chr01:150305         G>A         TAC>TAT      Y>YYAL001C      -      1      YS9           chr01:150305         G>A         TAC>TAT      Y>YYAL001C      -      1      UWOPS83_787_3 chr01:150360         T>G         AAA>ACA      K>TYAL001C      -      1      K11           chr01:150464         C>T         CAG>CAA      Q>QYAL001C      -      1      SK1           chr01:150464         C>T         CAG>CAA      Q>QYAL001C      -      1      Y12           chr01:150464         C>T         CAG>CAA      Q>QYAL001C      -      1      UWOPS05_217_3 chr01:150628         G>A         CTG>TTG      L>LYAL001C      -      1      UWOPS05_227_2 chr01:150628         G>A         CTG>TTG      L>LYAL001C      -      1      322134S       chr01:150646         T>G         ATC>CTC      I>LYAL001C      -      1      NCYC110       chr01:150755         A>G         AAT>AAC      N>NYAL001C      -      1      Y55           chr01:150755         A>G         AAT>AAC      N>NYAL001C      -      1      YPS606        chr01:150805         G>A         CTA>TTA      L>LYAL001C      -      1      UWOPS03_461_4 chr01:150806         G>A         AGC>AGT      S>SYAL001C      -      1      UWOPS05_217_3 chr01:150806         G>A         AGC>AGT      S>SYAL001C      -      1      UWOPS05_227_2 chr01:150806         G>A         AGC>AGT      S>SYAL001C      -      1      DBVPG1373     chr01:150923         G>A         TGC>TGT      C>CYAL001C      -      1      DBVPG1788     chr01:150923         G>A         TGC>TGT      C>CYAL001C      -      1      DBVPG6044     chr01:150923         G>A         TGC>TGT      C>CYAL001C      -      1      DBVPG6765     chr01:150923         G>A         TGC>TGT      C>CYAL001C      -      1      L_1374        chr01:150923         G>A         TGC>TGT      C>CYAL001C      -      1      UWOPS03_461_4 chr01:150923         G>A         TGC>TGT      C>CYAL001C      -      1      UWOPS05_217_3 chr01:150923         G>A         TGC>TGT      C>CYAL001C      -      1      UWOPS05_227_2 chr01:150923         G>A         TGC>TGT      C>CYAL001C      -      1      Y55           chr01:150923         G>A         TGC>TGT      C>CYAL001C      -      1      YIIc17_E5     chr01:150923         G>A         TGC>TGT      C>CYAL001C      -      1      YJM975        chr01:150923         G>A         TGC>TGT      C>CYAL001C      -      1      YS9           chr01:150923         G>A         TGC>TGT      C>CYAL001C      -      0      DBVPG6044     chr01:151124         A>G         TCT>TCC      S>SYAL001C      -      0      Y55           chr01:151124         A>G         TCT>TCC      S>SYAL005C      -      0      322134S       chr01:139588         C>a         GCA>TCA      A>SYAL005C      -      0      YJM978        chr01:139688         G>A         GCC>GCT      A>AYAL005C      -      0      YJM981        chr01:139688         G>A         GCC>GCT      A>AYAL005C      -      0      DBVPG1373     chr01:139694         G>A         ACC>ACT      T>TYAL005C      -      0      DBVPG1853     chr01:139694         G>A         ACC>ACT      T>TYAL005C      -      0      DBVPG6765     chr01:139694         G>A         ACC>ACT      T>TYAL005C      -      0      NCYC361       chr01:139694         G>A         ACC>ACT      T>TYAL005C      -      0      UWOPS03_461_4 chr01:139694         G>A         ACC>ACT      T>TYAL005C      -      0      UWOPS05_217_3 chr01:139694         G>A         ACC>ACT      T>TYAL005C      -      0      Y55           chr01:139694         G>A         ACC>ACT      T>TYAL005C      -      0      YIIc17_E5     chr01:139694         G>A         ACC>ACT      T>TYAL005C      -      0      YJM978        chr01:139694         G>A         ACC>ACT      T>TYAL005C      -      0      YJM981        chr01:139694         G>A         ACC>ACT      T>TYAL005C      -      0      YPS128        chr01:139694         G>A         ACC>ACT      T>TYAL005C      -      0      YPS606        chr01:139694         G>A         ACC>ACT      T>TYAL005C      -      0      YS9           chr01:139694         G>A         ACC>ACT      T>TYAL005C      -      0      UWOPS03_461_4 chr01:139829         G>A         TCC>TCT      S>SYAL005C      -      0      UWOPS05_217_3 chr01:139829         G>A         TCC>TCT      S>SYAL005C      -      0      DBVPG1373     chr01:139958         A>G         ACT>ACC      T>TYAL005C      -      0      DBVPG1373     chr01:139982         A>G         AAT>AAC      N>NYAL005C      -      0      DBVPG1373     chr01:139988         A>G         ATT>ATC      I>IYAL005C      -      0      DBVPG1373     chr01:140099         G>A         GCC>GCT      A>AYAL005C      -      0      DBVPG1373     chr01:140144         A>G         GAT>GAC      D>DYAL005C      -      0      322134S       chr01:140169         A>G         TTC>TCC      F>SYAL005C      -      0      378604X       chr01:140169         A>G         TTC>TCC      F>SYAL005C      -      0      DBVPG6040     chr01:140169         A>G         TTC>TCC      F>SYAL005C      -      0      DBVPG6044     chr01:140169         A>G         TTC>TCC      F>SYAL005C      -      0      DBVPG6765     chr01:140169         A>G         TTC>TCC      F>SYAL005C      -      0      NCYC110       chr01:140169         A>G         TTC>TCC      F>SYAL005C      -      0      NCYC361       chr01:140169         A>G         TTC>TCC      F>SYAL005C      -      0      SK1           chr01:140169         A>G         TTC>TCC      F>SYAL005C      -      0      UWOPS05_217_3 chr01:140169         A>G         TTC>TCC      F>SYAL005C      -      0      UWOPS83_787_3 chr01:140169         A>G         TTC>TCC      F>SYAL005C      -      0      UWOPS87_2421  chr01:140169         A>G         TTC>TCC      F>SYAL005C      -      0      Y55           chr01:140169         A>G         TTC>TCC      F>SYAL005C      -      0      Y9            chr01:140169         A>G         TTC>TCC      F>SYAL005C      -      0      YGPM          chr01:140169         A>G         TTC>TCC      F>SYAL005C      -      0      YIIc17_E5     chr01:140169         A>G         TTC>TCC      F>SYAL005C      -      0      YJM978        chr01:140169         A>G         TTC>TCC      F>SYAL005C      -      0      YPS606        chr01:140169         A>G         TTC>TCC      F>SYAL005C      -      0      322134S       chr01:140182         A>G         TCA>CCA      S>PYAL005C      -      0      378604X       chr01:140182         A>G         TCA>CCA      S>PYAL005C      -      0      DBVPG6040     chr01:140182         A>G         TCA>CCA      S>PYAL005C      -      0      DBVPG6044     chr01:140182         A>G         TCA>CCA      S>PYAL005C      -      0      DBVPG6765     chr01:140182         A>G         TCA>CCA      S>PYAL005C      -      0      NCYC110       chr01:140182         A>G         TCA>CCA      S>PYAL005C      -      0      NCYC361       chr01:140182         A>G         TCA>CCA      S>PYAL005C      -      0      SK1           chr01:140182         A>G         TCA>CCA      S>PYAL005C      -      0      UWOPS05_217_3 chr01:140182         A>G         TCA>CCA      S>PYAL005C      -      0      UWOPS83_787_3 chr01:140182         A>G         TCA>CCA      S>PYAL005C      -      0      UWOPS87_2421  chr01:140182         A>G         TCA>CCA      S>PYAL005C      -      0      Y55           chr01:140182         A>G         TCA>CCA      S>PYAL005C      -      0      Y9            chr01:140182         A>G         TCA>CCA      S>PYAL005C      -      0      YGPM          chr01:140182         A>G         TCA>CCA      S>PYAL005C      -      0      YIIc17_E5     chr01:140182         A>G         TCA>CCA      S>PYAL005C      -      0      YJM978        chr01:140182         A>G         TCA>CCA      S>PYAL005C      -      0      YPS606        chr01:140182         A>G         TCA>CCA      S>PYAL005C      -      0      UWOPS83_787_3 chr01:140285         G>C         TCC>TCG      S>SYAL005C      -      0      DBVPG1373     chr01:140288         A>G         TCT>TCC      S>SYAL005C      -      0      DBVPG1373     chr01:140348         T>A         CCA>CCT      P>PYAL005C      -      0      DBVPG6044     chr01:140348         T>A         CCA>CCT      P>PYAL005C      -      0      K11           chr01:140348         T>A         CCA>CCT      P>PYAL005C      -      0      NCYC110       chr01:140348         T>A         CCA>CCT      P>PYAL005C      -      0      Y55           chr01:140348         T>A         CCA>CCT      P>PYAL005C      -      0      DBVPG1106     chr01:140376         C>a         GGT>GTT      G>VYAL005C      -      0      UWOPS05_217_3 chr01:140387         G>A         GAC>GAT      D>DYAL005C      -      0      UWOPS05_227_2 chr01:140387         G>A         GAC>GAT      D>DYAL005C      -      0      YPS128        chr01:140387         G>A         GAC>GAT      D>DYAL005C      -      0      YPS606        chr01:140387         G>A         GAC>GAT      D>DYAL005C      -      0      DBVPG1373     chr01:140399         T>C         AAA>AAG      K>KYAL005C      -      0      K11           chr01:140399         T>C         AAA>AAG      K>KYAL005C      -      0      UWOPS83_787_3 chr01:140402         T>C         CAA>CAG      Q>QYAL005C      -      0      DBVPG6044     chr01:140420         G>A         ACC>ACT      T>TYAL005C      -      0      K11           chr01:140420         G>A         ACC>ACT      T>TYAL005C      -      0      NCYC110       chr01:140420         G>A         ACC>ACT      T>TYAL005C      -      0      Y55           chr01:140420         G>A         ACC>ACT      T>TYAL005C      -      0      Y9            chr01:140420         G>A         ACC>ACT      T>TYAL005C      -      0      322134S       chr01:140453         T>C         CAA>CAG      Q>QYAL005C      -      0      DBVPG6040     chr01:140453         T>C         CAA>CAG      Q>QYAL005C      -      0      DBVPG6765     chr01:140453         T>C         CAA>CAG      Q>QYAL005C      -      0      L_1528        chr01:140453         T>C         CAA>CAG      Q>QYAL005C      -      0      UWOPS87_2421  chr01:140462         G>A         GAC>GAT      D>DYAL005C      -      0      YPS128        chr01:140477         T>C         AGA>AGG      R>RYAL005C      -      0      YPS606        chr01:140477         T>C         AGA>AGG      R>RYAL005C      -      0      K11           chr01:140528         C>T         TTG>TTA      L>LYAL005C      -      0      DBVPG1106     chr01:140639         T>A         GAA>GAT      E>DYAL005C      -      0      322134S       chr01:140648         G>A         ACC>ACT      T>TYAL005C      -      0      DBVPG1106     chr01:140648         G>A         ACC>ACT      T>TYAL005C      -      0      DBVPG6040     chr01:140648         G>A         ACC>ACT      T>TYAL005C      -      0      DBVPG6044     chr01:140648         G>A         ACC>ACT      T>TYAL005C      -      0      DBVPG6765     chr01:140648         G>A         ACC>ACT      T>TYAL005C      -      0      K11           chr01:140648         G>A         ACC>ACT      T>TYAL005C      -      0      L_1528        chr01:140648         G>A         ACC>ACT      T>TYAL005C      -      0      NCYC110       chr01:140648         G>A         ACC>ACT      T>TYAL005C      -      0      UWOPS05_217_3 chr01:140648         G>A         ACC>ACT      T>TYAL005C      -      0      UWOPS05_227_2 chr01:140648         G>A         ACC>ACT      T>TYAL005C      -      0      UWOPS83_787_3 chr01:140648         G>A         ACC>ACT      T>TYAL005C      -      0      YJM975        chr01:140648         G>A         ACC>ACT      T>TYAL005C      -      0      YPS128        chr01:140648         G>A         ACC>ACT      T>TYAL005C      -      0      YPS606        chr01:140648         G>A         ACC>ACT      T>TYAL005C      -      0      322134S       chr01:140811         A>G         TTC>TCC      F>SYAL005C      -      0      BC187         chr01:140811         A>G         TTC>TCC      F>SYAL005C      -      0      DBVPG1106     chr01:140811         A>G         TTC>TCC      F>SYAL005C      -      0      DBVPG1373     chr01:140811         A>G         TTC>TCC      F>SYAL005C      -      0      DBVPG6044     chr01:140811         A>G         TTC>TCC      F>SYAL005C      -      0      DBVPG6765     chr01:140811         A>G         TTC>TCC      F>SYAL005C      -      0      K11           chr01:140811         A>G         TTC>TCC      F>SYAL005C      -      0      L_1528        chr01:140811         A>G         TTC>TCC      F>SYAL005C      -      0      NCYC110       chr01:140811         A>G         TTC>TCC      F>SYAL005C      -      0      S288c         chr01:140811         A>G         TTC>TCC      F>SYAL005C      -      0      SK1           chr01:140811         A>G         TTC>TCC      F>SYAL005C      -      0      UWOPS05_217_3 chr01:140811         A>G         TTC>TCC      F>SYAL005C      -      0      UWOPS05_227_2 chr01:140811         A>G         TTC>TCC      F>SYAL005C      -      0      UWOPS83_787_3 chr01:140811         A>G         TTC>TCC      F>SYAL005C      -      0      YGPM          chr01:140811         A>G         TTC>TCC      F>SYAL005C      -      0      YJM975        chr01:140811         A>G         TTC>TCC      F>SYAL005C      -      0      YPS128        chr01:140811         A>G         TTC>TCC      F>SYAL005C      -      0      YPS606        chr01:140811         A>G         TTC>TCC      F>SYAL005C      -      0      322134S       chr01:141032         T>A         GGA>GGT      G>GYAL005C      -      0      BC187         chr01:141032         T>A         GGA>GGT      G>GYAL005C      -      0      DBVPG1106     chr01:141032         T>A         GGA>GGT      G>GYAL005C      -      0      DBVPG1373     chr01:141032         T>A         GGA>GGT      G>GYAL005C      -      0      DBVPG6044     chr01:141032         T>A         GGA>GGT      G>GYAL005C      -      0      K11           chr01:141032         T>A         GGA>GGT      G>GYAL005C      -      0      L_1528        chr01:141032         T>A         GGA>GGT      G>GYAL005C      -      0      SK1           chr01:141032         T>A         GGA>GGT      G>GYAL005C      -      0      UWOPS05_217_3 chr01:141032         T>A         GGA>GGT      G>GYAL005C      -      0      UWOPS05_227_2 chr01:141032         T>A         GGA>GGT      G>GYAL005C      -      0      Y55           chr01:141032         T>A         GGA>GGT      G>GYAL005C      -      0      YGPM          chr01:141032         T>A         GGA>GGT      G>GYAL005C      -      0      YJM978        chr01:141032         T>A         GGA>GGT      G>GYAL005C      -      0      YPS128        chr01:141032         T>A         GGA>GGT      G>GYAL005C      -      0      YPS606        chr01:141032         T>A         GGA>GGT      G>GYAL005C      -      0      322134S       chr01:141137         A>T         CCT>CCA      P>PYAL005C      -      0      BC187         chr01:141137         A>T         CCT>CCA      P>PYAL005C      -      0      DBVPG1106     chr01:141137         A>T         CCT>CCA      P>PYAL005C      -      0      DBVPG1373     chr01:141137         A>T         CCT>CCA      P>PYAL005C      -      0      DBVPG6044     chr01:141137         A>T         CCT>CCA      P>PYAL005C      -      0      SK1           chr01:141137         A>T         CCT>CCA      P>PYAL005C      -      0      UWOPS05_227_2 chr01:141137         A>T         CCT>CCA      P>PYAL005C      -      0      UWOPS87_2421  chr01:141137         A>T         CCT>CCA      P>PYAL005C      -      0      Y55           chr01:141137         A>T         CCT>CCA      P>PYAL005C      -      0      YGPM          chr01:141137         A>T         CCT>CCA      P>PYAL005C      -      0      YJM978        chr01:141137         A>T         CCT>CCA      P>PYAL005C      -      0      YPS128        chr01:141137         A>T         CCT>CCA      P>PYAL005C      -      0      YPS606        chr01:141137         A>T         CCT>CCA      P>PYAL005C      -      0      DBVPG6044     chr01:141186         G>C         GCT>GGT      A>GYAL005C      -      0      SK1           chr01:141186         G>C         GCT>GGT      A>GYAL005C      -      0      UWOPS05_227_2 chr01:141186         G>C         GCT>GGT      A>GYAL005C      -      0      Y55           chr01:141186         G>C         GCT>GGT      A>GYAL005C      -      0      UWOPS87_2421  chr01:141221         C>T         TTG>TTA      L>LYAL005C      -      0      BC187         chr01:141344         A>G         GAT>GAC      D>DYAL005C      -      0      DBVPG1106     chr01:141344         A>G         GAT>GAC      D>DYAL005C      -      0      DBVPG1373     chr01:141344         A>G         GAT>GAC      D>DYAL005C      -      0      UWOPS87_2421  chr01:141344         A>G         GAT>GAC      D>DYAL005C      -      0      YIIc17_E5     chr01:141344         A>G         GAT>GAC      D>DYAL005C      -      0      YJM978        chr01:141344         A>G         GAT>GAC      D>DYAL005C      -      0      YPS606        chr01:141344         A>G         GAT>GAC      D>DYAL005C      -      0      DBVPG1373     chr01:141365         A>G         CGT>CGC      R>RYAL007C      -      0      DBVPG1853     chr01:137754         G>A         GCC>GCT      A>AYAL007C      -      0      DBVPG6040     chr01:137754         G>A         GCC>GCT      A>AYAL007C      -      0      NCYC110       chr01:137796         T>C         TTA>TTG      L>LYAL007C      -      0      Y55           chr01:137796         T>C         TTA>TTG      L>LYAL007C      -      0      DBVPG1853     chr01:138064         T>C         AAA>AGA      K>RYAL007C      -      0      NCYC361       chr01:138064         T>C         AAA>AGA      K>RYAL007C      -      0      YS2           chr01:138064         T>C         AAA>AGA      K>RYAL007C      -      0      NCYC110       chr01:138077         C>T         GAC>AAC      D>NYAL007C      -      0      Y55           chr01:138077         C>T         GAC>AAC      D>NYAL007C      -      0      UWOPS83_787_3 chr01:138222         T>C         GAA>GAG      E>EYAL007C      -      0      322134S       chr01:138259         G>A         GCA>GTA      A>VYAL007C      -      0      DBVPG1788     chr01:138259         G>A         GCA>GTA      A>VYAL007C      -      0      YJM975        chr01:138259         G>A         GCA>GTA      A>VYAL007C      -      0      322134S       chr01:138265         C>A         AGT>ATT      S>IYAL007C      -      0      K11           chr01:138337         G>C         TCT>TGT      S>CYAL007C      -      0      NCYC110       chr01:138342         G>A         ATC>ATT      I>IYAL007C      -      0      Y55           chr01:138342         G>A         ATC>ATT      I>IYAL008W      +      0      K11           chr01:136960         G>A         TTG>TTA      L>LYAL008W      +      0      SK1           chr01:136960         G>A         TTG>TTA      L>LYAL008W      +      0      BC187         chr01:136963         T>C         AAT>AAC      N>NYAL008W      +      0      DBVPG1106     chr01:136963         T>C         AAT>AAC      N>NYAL008W      +      0      DBVPG1788     chr01:136963         T>C         AAT>AAC      N>NYAL008W      +      0      DBVPG6765     chr01:136963         T>C         AAT>AAC      N>NYAL008W      +      0      L_1374        chr01:136963         T>C         AAT>AAC      N>NYAL008W      +      0      L_1528        chr01:136963         T>C         AAT>AAC      N>NYAL008W      +      0      UWOPS05_227_2 chr01:136963         T>C         AAT>AAC      N>NYAL008W      +      0      Y55           chr01:136963         T>C         AAT>AAC      N>NYAL008W      +      0      YPS606        chr01:136963         T>C         AAT>AAC      N>NYAL008W      +      0      K11           chr01:136971         A>T         AAG>ATG      K>MYAL008W      +      0      SK1           chr01:136971         A>T         AAG>ATG      K>MYAL008W      +      0      L_1374        chr01:137049         G>A         GGG>GAG      G>EYAL008W      +      0      K11           chr01:137065         C>T         GGC>GGT      G>GYAL008W      +      0      SK1           chr01:137065         C>T         GGC>GGT      G>GYAL008W      +      0      Y12           chr01:137065         C>T         GGC>GGT      G>GYAL008W      +      0      YS9           chr01:137065         C>T         GGC>GGT      G>GYAL008W      +      0      DBVPG6040     chr01:137089         G>A         TTG>TTA      L>LYAL008W      +      0      UWOPS05_217_3 chr01:137134         G>T         GGG>GGT      G>GYAL008W      +      0      YPS606        chr01:137134         G>T         GGG>GGT      G>GYAL008W      +      0      BC187         chr01:137150         C>G         CAG>GAG      Q>EYAL008W      +      0      DBVPG1106     chr01:137150         C>G         CAG>GAG      Q>EYAL008W      +      0      DBVPG1788     chr01:137150         C>G         CAG>GAG      Q>EYAL008W      +      0      DBVPG6765     chr01:137150         C>G         CAG>GAG      Q>EYAL008W      +      0      L_1528        chr01:137150         C>G         CAG>GAG      Q>EYAL008W      +      0      UWOPS05_217_3 chr01:137150         C>G         CAG>GAG      Q>EYAL008W      +      0      YPS606        chr01:137150         C>G         CAG>GAG      Q>EYAL008W      +      0      BC187         chr01:137169         C>A         ACT>AAT      T>NYAL008W      +      0      DBVPG1106     chr01:137169         C>A         ACT>AAT      T>NYAL008W      +      0      DBVPG1788     chr01:137169         C>A         ACT>AAT      T>NYAL008W      +      0      DBVPG6765     chr01:137169         C>A         ACT>AAT      T>NYAL008W      +      0      L_1528        chr01:137169         C>A         ACT>AAT      T>NYAL008W      +      0      UWOPS05_217_3 chr01:137169         C>A         ACT>AAT      T>NYAL008W      +      0      UWOPS87_2421  chr01:137169         C>A         ACT>AAT      T>NYAL008W      +      0      Y55           chr01:137169         C>A         ACT>AAT      T>NYAL008W      +      0      YPS606        chr01:137169         C>A         ACT>AAT      T>NYAL008W      +      0      BC187         chr01:137239         G>A         TTG>TTA      L>LYAL008W      +      0      DBVPG1106     chr01:137239         G>A         TTG>TTA      L>LYAL008W      +      0      DBVPG1788     chr01:137239         G>A         TTG>TTA      L>LYAL008W      +      0      DBVPG6040     chr01:137239         G>A         TTG>TTA      L>LYAL008W      +      0      DBVPG6765     chr01:137239         G>A         TTG>TTA      L>LYAL008W      +      0      L_1528        chr01:137239         G>A         TTG>TTA      L>LYAL008W      +      0      UWOPS05_217_3 chr01:137239         G>A         TTG>TTA      L>LYAL008W      +      0      UWOPS87_2421  chr01:137239         G>A         TTG>TTA      L>LYAL008W      +      0      Y55           chr01:137239         G>A         TTG>TTA      L>LYAL008W      +      0      YPS606        chr01:137239         G>A         TTG>TTA      L>LYAL008W      +      0      DBVPG6040     chr01:137258         G>A         GTT>ATT      V>IYAL008W      +      0      322134S       chr01:137266         A>G         GGA>GGG      G>GYAL008W      +      0      BC187         chr01:137266         A>G         GGA>GGG      G>GYAL008W      +      0      DBVPG1106     chr01:137266         A>G         GGA>GGG      G>GYAL008W      +      0      DBVPG1788     chr01:137266         A>G         GGA>GGG      G>GYAL008W      +      0      DBVPG6765     chr01:137266         A>G         GGA>GGG      G>GYAL008W      +      0      L_1528        chr01:137266         A>G         GGA>GGG      G>GYAL008W      +      0      UWOPS05_217_3 chr01:137266         A>G         GGA>GGG      G>GYAL008W      +      0      UWOPS87_2421  chr01:137266         A>G         GGA>GGG      G>GYAL008W      +      0      Y55           chr01:137266         A>G         GGA>GGG      G>GYAL008W      +      0      YPS606        chr01:137266         A>G         GGA>GGG      G>GYAL008W      +      0      322134S       chr01:137338         A>G         TTA>TTG      L>LYAL008W      +      0      BC187         chr01:137338         A>G         TTA>TTG      L>LYAL008W      +      0      DBVPG1106     chr01:137338         A>G         TTA>TTG      L>LYAL008W      +      0      DBVPG1788     chr01:137338         A>G         TTA>TTG      L>LYAL008W      +      0      DBVPG6040     chr01:137338         A>G         TTA>TTG      L>LYAL008W      +      0      DBVPG6765     chr01:137338         A>G         TTA>TTG      L>LYAL008W      +      0      L_1374        chr01:137338         A>G         TTA>TTG      L>LYAL008W      +      0      L_1528        chr01:137338         A>G         TTA>TTG      L>LYAL008W      +      0      SK1           chr01:137338         A>G         TTA>TTG      L>LYAL008W      +      0      UWOPS05_217_3 chr01:137338         A>G         TTA>TTG      L>LYAL008W      +      0      UWOPS87_2421  chr01:137338         A>G         TTA>TTG      L>LYAL008W      +      0      Y12           chr01:137338         A>G         TTA>TTG      L>LYAL008W      +      0      Y55           chr01:137338         A>G         TTA>TTG      L>LYAL008W      +      0      YPS606        chr01:137338         A>G         TTA>TTG      L>LYAL008W      +      0      YS9           chr01:137338         A>G         TTA>TTG      L>LYAL009W      +      0      273614X       chr01:135954         C>T         TCC>TCT      S>SYAL009W      +      0      322134S       chr01:135954         C>T         TCC>TCT      S>SYAL009W      +      0      378604X       chr01:135954         C>T         TCC>TCT      S>SYAL009W      +      0      DBVPG1106     chr01:135954         C>T         TCC>TCT      S>SYAL009W      +      0      DBVPG1373     chr01:135954         C>T         TCC>TCT      S>SYAL009W      +      0      DBVPG1788     chr01:135954         C>T         TCC>TCT      S>SYAL009W      +      0      DBVPG1853     chr01:135954         C>T         TCC>TCT      S>SYAL009W      +      0      DBVPG6040     chr01:135954         C>T         TCC>TCT      S>SYAL009W      +      0      DBVPG6044     chr01:135954         C>T         TCC>TCT      S>SYAL009W      +      0      DBVPG6765     chr01:135954         C>T         TCC>TCT      S>SYAL009W      +      0      L_1374        chr01:135954         C>T         TCC>TCT      S>SYAL009W      +      0      NCYC361       chr01:135954         C>T         TCC>TCT      S>SYAL009W      +      0      UWOPS03_461_4 chr01:135954         C>T         TCC>TCT      S>SYAL009W      +      0      UWOPS05_217_3 chr01:135954         C>T         TCC>TCT      S>SYAL009W      +      0      Y55           chr01:135954         C>T         TCC>TCT      S>SYAL009W      +      0      YJM975        chr01:135954         C>T         TCC>TCT      S>SYAL009W      +      0      YPS128        chr01:135954         C>T         TCC>TCT      S>SYAL009W      +      0      YPS606        chr01:135954         C>T         TCC>TCT      S>SYAL009W      +      0      YS2           chr01:135954         C>T         TCC>TCT      S>SYAL009W      +      0      YS4           chr01:135954         C>T         TCC>TCT      S>SYAL009W      +      0      YS9           chr01:135954         C>T         TCC>TCT      S>SYAL009W      +      0      DBVPG1106     chr01:135972         G>A         CGG>CGA      R>RYAL009W      +      0      322134S       chr01:136043         G>A         CGC>CAC      R>HYAL009W      +      0      378604X       chr01:136043         G>A         CGC>CAC      R>HYAL009W      +      0      DBVPG1106     chr01:136043         G>A         CGC>CAC      R>HYAL009W      +      0      DBVPG1373     chr01:136043         G>A         CGC>CAC      R>HYAL009W      +      0      DBVPG1788     chr01:136043         G>A         CGC>CAC      R>HYAL009W      +      0      DBVPG1853     chr01:136043         G>A         CGC>CAC      R>HYAL009W      +      0      DBVPG6765     chr01:136043         G>A         CGC>CAC      R>HYAL009W      +      0      L_1374        chr01:136043         G>A         CGC>CAC      R>HYAL009W      +      0      UWOPS87_2421  chr01:136043         G>A         CGC>CAC      R>HYAL009W      +      0      YJM975        chr01:136043         G>A         CGC>CAC      R>HYAL009W      +      0      YS2           chr01:136043         G>A         CGC>CAC      R>HYAL009W      +      0      YS4           chr01:136043         G>A         CGC>CAC      R>HYAL009W      +      0      273614X       chr01:136049         C>T         GCT>GTT      A>VYAL009W      +      0      322134S       chr01:136059         A>G         CAA>CAG      Q>QYAL009W      +      0      378604X       chr01:136059         A>G         CAA>CAG      Q>QYAL009W      +      0      DBVPG1106     chr01:136059         A>G         CAA>CAG      Q>QYAL009W      +      0      DBVPG1373     chr01:136059         A>G         CAA>CAG      Q>QYAL009W      +      0      DBVPG1788     chr01:136059         A>G         CAA>CAG      Q>QYAL009W      +      0      DBVPG1853     chr01:136059         A>G         CAA>CAG      Q>QYAL009W      +      0      DBVPG6765     chr01:136059         A>G         CAA>CAG      Q>QYAL009W      +      0      L_1374        chr01:136059         A>G         CAA>CAG      Q>QYAL009W      +      0      UWOPS87_2421  chr01:136059         A>G         CAA>CAG      Q>QYAL009W      +      0      YJM975        chr01:136059         A>G         CAA>CAG      Q>QYAL009W      +      0      YS4           chr01:136059         A>G         CAA>CAG      Q>QYAL009W      +      0      322134S       chr01:136155         C>G         GTC>GTG      V>VYAL009W      +      0      DBVPG1106     chr01:136155         C>G         GTC>GTG      V>VYAL009W      +      0      DBVPG1373     chr01:136155         C>G         GTC>GTG      V>VYAL009W      +      0      DBVPG1788     chr01:136155         C>G         GTC>GTG      V>VYAL009W      +      0      DBVPG1853     chr01:136155         C>G         GTC>GTG      V>VYAL009W      +      0      DBVPG6040     chr01:136155         C>G         GTC>GTG      V>VYAL009W      +      0      DBVPG6765     chr01:136155         C>G         GTC>GTG      V>VYAL009W      +      0      L_1374        chr01:136155         C>G         GTC>GTG      V>VYAL009W      +      0      NCYC361       chr01:136155         C>G         GTC>GTG      V>VYAL009W      +      0      UWOPS87_2421  chr01:136155         C>G         GTC>GTG      V>VYAL009W      +      0      YIIc17_E5     chr01:136155         C>G         GTC>GTG      V>VYAL009W      +      0      YS2           chr01:136155         C>G         GTC>GTG      V>VYAL009W      +      0      YS4           chr01:136155         C>G         GTC>GTG      V>VYAL009W      +      0      322134S       chr01:136162         C>T         CTC>TTC      L>FYAL009W      +      0      DBVPG1106     chr01:136162         C>T         CTC>TTC      L>FYAL009W      +      0      DBVPG1373     chr01:136162         C>T         CTC>TTC      L>FYAL009W      +      0      DBVPG1788     chr01:136162         C>T         CTC>TTC      L>FYAL009W      +      0      DBVPG1853     chr01:136162         C>T         CTC>TTC      L>FYAL009W      +      0      DBVPG6040     chr01:136162         C>T         CTC>TTC      L>FYAL009W      +      0      DBVPG6044     chr01:136162         C>T         CTC>TTC      L>FYAL009W      +      0      DBVPG6765     chr01:136162         C>T         CTC>TTC      L>FYAL009W      +      0      L_1374        chr01:136162         C>T         CTC>TTC      L>FYAL009W      +      0      NCYC110       chr01:136162         C>T         CTC>TTC      L>FYAL009W      +      0      NCYC361       chr01:136162         C>T         CTC>TTC      L>FYAL009W      +      0      UWOPS03_461_4 chr01:136162         C>T         CTC>TTC      L>FYAL009W      +      0      UWOPS87_2421  chr01:136162         C>T         CTC>TTC      L>FYAL009W      +      0      Y55           chr01:136162         C>T         CTC>TTC      L>FYAL009W      +      0      YIIc17_E5     chr01:136162         C>T         CTC>TTC      L>FYAL009W      +      0      YJM975        chr01:136162         C>T         CTC>TTC      L>FYAL009W      +      0      YPS128        chr01:136162         C>T         CTC>TTC      L>FYAL009W      +      0      YPS606        chr01:136162         C>T         CTC>TTC      L>FYAL009W      +      0      YS2           chr01:136162         C>T         CTC>TTC      L>FYAL009W      +      0      YS4           chr01:136162         C>T         CTC>TTC      L>FYAL009W      +      0      YS9           chr01:136162         C>T         CTC>TTC      L>FYAL009W      +      0      322134S       chr01:136197         C>A         TTC>TTA      F>LYAL009W      +      0      DBVPG1106     chr01:136197         C>T         TTC>TTT      F>FYAL009W      +      0      DBVPG1373     chr01:136197         C>T         TTC>TTT      F>FYAL009W      +      0      DBVPG1788     chr01:136197         C>T         TTC>TTT      F>FYAL009W      +      0      DBVPG1853     chr01:136197         C>T         TTC>TTT      F>FYAL009W      +      0      DBVPG6765     chr01:136197         C>T         TTC>TTT      F>FYAL009W      +      0      L_1374        chr01:136197         C>T         TTC>TTT      F>FYAL009W      +      0      UWOPS87_2421  chr01:136197         C>T         TTC>TTT      F>FYAL009W      +      0      YIIc17_E5     chr01:136197         C>T         TTC>TTT      F>FYAL009W      +      0      YJM975        chr01:136197         C>T         TTC>TTT      F>FYAL009W      +      0      YS4           chr01:136197         C>T         TTC>TTT      F>FYAL009W      +      0      W303          chr01:136274         C>a         ACC>AaC      T>NYAL009W      +      0      YS9           chr01:136326         G>A         CAG>CAA      Q>QYAL009W      +      0      UWOPS87_2421  chr01:136550         G>A         TGG>TAG      W>*YAL009W      +      0      DBVPG6040     chr01:136578         C>T         GCC>GCT      A>AYAL009W      +      0      DBVPG6044     chr01:136578         C>T         GCC>GCT      A>AYAL009W      +      0      NCYC110       chr01:136578         C>T         GCC>GCT      A>AYAL009W      +      0      NCYC361       chr01:136578         C>T         GCC>GCT      A>AYAL009W      +      0      Y55           chr01:136578         C>T         GCC>GCT      A>AYAL010C      -      0      DBVPG6044     chr01:134187         C>T         TGA>TAA      *>*YAL010C      -      0      NCYC110       chr01:134187         C>T         TGA>TAA      *>*YAL010C      -      0      Y55           chr01:134187         C>T         TGA>TAA      *>*YAL010C      -      0      K11           chr01:134192         G>A         TCC>TCT      S>SYAL010C      -      0      Y9            chr01:134192         G>A         TCC>TCT      S>SYAL010C      -      0      YPS128        chr01:134192         G>A         TCC>TCT      S>SYAL010C      -      0      YPS606        chr01:134192         G>A         TCC>TCT      S>SYAL010C      -      0      378604X       chr01:134221         C>T         GCA>ACA      A>TYAL010C      -      0      DBVPG1106     chr01:134221         C>T         GCA>ACA      A>TYAL010C      -      0      DBVPG1373     chr01:134221         C>T         GCA>ACA      A>TYAL010C      -      0      DBVPG1853     chr01:134221         C>T         GCA>ACA      A>TYAL010C      -      0      DBVPG6765     chr01:134221         C>T         GCA>ACA      A>TYAL010C      -      0      L_1374        chr01:134221         C>T         GCA>ACA      A>TYAL010C      -      0      L_1528        chr01:134221         C>T         GCA>ACA      A>TYAL010C      -      0      YJM981        chr01:134221         C>T         GCA>ACA      A>TYAL010C      -      0      Y9            chr01:134258         T>G         GCA>GCC      A>AYAL010C      -      0      Y9            chr01:134330         C>A         CTG>CTT      L>LYAL010C      -      0      DBVPG6044     chr01:134416         A>G         TTA>CTA      L>LYAL010C      -      0      NCYC110       chr01:134416         A>G         TTA>CTA      L>LYAL010C      -      0      Y55           chr01:134416         A>G         TTA>CTA      L>LYAL010C      -      0      Y9            chr01:134416         A>G         TTA>CTA      L>LYAL010C      -      0      322134S       chr01:134477         T>C         AGA>AGG      R>RYAL010C      -      0      378604X       chr01:134477         T>C         AGA>AGG      R>RYAL010C      -      0      DBVPG1106     chr01:134477         T>C         AGA>AGG      R>RYAL010C      -      0      DBVPG1373     chr01:134477         T>C         AGA>AGG      R>RYAL010C      -      0      DBVPG6044     chr01:134477         T>C         AGA>AGG      R>RYAL010C      -      0      DBVPG6765     chr01:134477         T>C         AGA>AGG      R>RYAL010C      -      0      L_1374        chr01:134477         T>C         AGA>AGG      R>RYAL010C      -      0      L_1528        chr01:134477         T>C         AGA>AGG      R>RYAL010C      -      0      Y55           chr01:134477         T>C         AGA>AGG      R>RYAL010C      -      0      YPS128        chr01:134477         T>C         AGA>AGG      R>RYAL010C      -      0      YPS606        chr01:134477         T>C         AGA>AGG      R>RYAL010C      -      0      UWOPS05_227_2 chr01:134483         T>A         GAA>GAT      E>DYAL010C      -      0      DBVPG6044     chr01:134522         T>C         TTA>TTG      L>LYAL010C      -      0      Y55           chr01:134522         T>C         TTA>TTG      L>LYAL010C      -      0      DBVPG6044     chr01:134533         A>G         TTA>CTA      L>LYAL010C      -      0      NCYC110       chr01:134533         A>G         TTA>CTA      L>LYAL010C      -      0      Y55           chr01:134533         A>G         TTA>CTA      L>LYAL010C      -      0      322134S       chr01:134589         G>A         TCA>TTA      S>LYAL010C      -      0      378604X       chr01:134589         G>A         TCA>TTA      S>LYAL010C      -      0      DBVPG1106     chr01:134589         G>A         TCA>TTA      S>LYAL010C      -      0      DBVPG1373     chr01:134589         G>A         TCA>TTA      S>LYAL010C      -      0      DBVPG1788     chr01:134589         G>A         TCA>TTA      S>LYAL010C      -      0      DBVPG6040     chr01:134589         G>A         TCA>TTA      S>LYAL010C      -      0      DBVPG6044     chr01:134589         G>A         TCA>TTA      S>LYAL010C      -      0      DBVPG6765     chr01:134589         G>A         TCA>TTA      S>LYAL010C      -      0      L_1528        chr01:134589         G>A         TCA>TTA      S>LYAL010C      -      0      Y55           chr01:134589         G>A         TCA>TTA      S>LYAL010C      -      0      YIIc17_E5     chr01:134589         G>A         TCA>TTA      S>LYAL010C      -      0      YJM978        chr01:134589         G>A         TCA>TTA      S>LYAL010C      -      0      322134S       chr01:134606         A>G         AAT>AAC      N>NYAL010C      -      0      378604X       chr01:134606         A>G         AAT>AAC      N>NYAL010C      -      0      DBVPG1106     chr01:134606         A>G         AAT>AAC      N>NYAL010C      -      0      DBVPG1373     chr01:134606         A>G         AAT>AAC      N>NYAL010C      -      0      DBVPG1788     chr01:134606         A>G         AAT>AAC      N>NYAL010C      -      0      DBVPG6040     chr01:134606         A>G         AAT>AAC      N>NYAL010C      -      0      DBVPG6044     chr01:134606         A>G         AAT>AAC      N>NYAL010C      -      0      DBVPG6765     chr01:134606         A>G         AAT>AAC      N>NYAL010C      -      0      Y55           chr01:134606         A>G         AAT>AAC      N>NYAL010C      -      0      YIIc17_E5     chr01:134606         A>G         AAT>AAC      N>NYAL010C      -      0      YJM978        chr01:134606         A>G         AAT>AAC      N>NYAL010C      -      0      YPS128        chr01:134606         A>G         AAT>AAC      N>NYAL010C      -      0      YPS606        chr01:134606         A>G         AAT>AAC      N>NYAL010C      -      0      322134S       chr01:134641         G>A         CCA>TCA      P>SYAL010C      -      0      378604X       chr01:134641         G>A         CCA>TCA      P>SYAL010C      -      0      DBVPG1106     chr01:134641         G>A         CCA>TCA      P>SYAL010C      -      0      DBVPG1373     chr01:134641         G>A         CCA>TCA      P>SYAL010C      -      0      DBVPG1788     chr01:134641         G>A         CCA>TCA      P>SYAL010C      -      0      DBVPG1853     chr01:134641         G>A         CCA>TCA      P>SYAL010C      -      0      DBVPG6040     chr01:134641         G>A         CCA>TCA      P>SYAL010C      -      0      DBVPG6765     chr01:134641         G>A         CCA>TCA      P>SYAL010C      -      0      YIIc17_E5     chr01:134641         G>A         CCA>TCA      P>SYAL010C      -      0      YJM978        chr01:134641         G>A         CCA>TCA      P>SYAL010C      -      0      322134S       chr01:134663         G>A         TCC>TCT      S>SYAL010C      -      0      378604X       chr01:134663         G>A         TCC>TCT      S>SYAL010C      -      0      DBVPG1106     chr01:134663         G>A         TCC>TCT      S>SYAL010C      -      0      DBVPG1373     chr01:134663         G>A         TCC>TCT      S>SYAL010C      -      0      DBVPG1788     chr01:134663         G>A         TCC>TCT      S>SYAL010C      -      0      DBVPG6765     chr01:134663         G>A         TCC>TCT      S>SYAL010C      -      0      YIIc17_E5     chr01:134663         G>A         TCC>TCT      S>SYAL010C      -      0      YJM978        chr01:134663         G>A         TCC>TCT      S>SYAL010C      -      0      YS2           chr01:134663         G>A         TCC>TCT      S>SYAL010C      -      0      UWOPS05_227_2 chr01:134669         T>G         CCA>CCC      P>PYAL010C      -      0      UWOPS05_217_3 chr01:134687         A>G         AAT>AAC      N>NYAL010C      -      0      DBVPG6040     chr01:134819         C>A         TCG>TCT      S>SYAL010C      -      0      DBVPG6044     chr01:135158         C>T         AAG>AAA      K>KYAL010C      -      0      NCYC110       chr01:135158         C>T         AAG>AAA      K>KYAL010C      -      0      Y55           chr01:135158         C>T         AAG>AAA      K>KYAL010C      -      0      YJM978        chr01:135171         T>G         CAA>CCA      Q>PYAL010C      -      0      322134S       chr01:135183         C>T         AGT>AAT      S>NYAL010C      -      0      378604X       chr01:135183         C>T         AGT>AAT      S>NYAL010C      -      0      DBVPG1373     chr01:135183         C>T         AGT>AAT      S>NYAL010C      -      0      DBVPG1788     chr01:135183         C>T         AGT>AAT      S>NYAL010C      -      0      DBVPG6765     chr01:135183         C>T         AGT>AAT      S>NYAL010C      -      0      L_1374        chr01:135183         C>T         AGT>AAT      S>NYAL010C      -      0      L_1528        chr01:135183         C>T         AGT>AAT      S>NYAL010C      -      0      YIIc17_E5     chr01:135183         C>T         AGT>AAT      S>NYAL010C      -      0      YJM978        chr01:135183         C>T         AGT>AAT      S>NYAL010C      -      0      YJM981        chr01:135183         C>T         AGT>AAT      S>NYAL010C      -      0      YS2           chr01:135183         C>T         AGT>AAT      S>NYAL010C      -      0      322134S       chr01:135224         G>A         TAC>TAT      Y>YYAL010C      -      0      378604X       chr01:135224         G>A         TAC>TAT      Y>YYAL010C      -      0      DBVPG1373     chr01:135224         G>A         TAC>TAT      Y>YYAL010C      -      0      DBVPG1788     chr01:135224         G>A         TAC>TAT      Y>YYAL010C      -      0      DBVPG6044     chr01:135224         G>A         TAC>TAT      Y>YYAL010C      -      0      DBVPG6765     chr01:135224         G>A         TAC>TAT      Y>YYAL010C      -      0      L_1374        chr01:135224         G>A         TAC>TAT      Y>YYAL010C      -      0      L_1528        chr01:135224         G>A         TAC>TAT      Y>YYAL010C      -      0      NCYC110       chr01:135224         G>A         TAC>TAT      Y>YYAL010C      -      0      Y55           chr01:135224         G>A         TAC>TAT      Y>YYAL010C      -      0      YJM975        chr01:135224         G>A         TAC>TAT      Y>YYAL010C      -      0      YJM981        chr01:135224         G>A         TAC>TAT      Y>YYAL010C      -      0      YPS606        chr01:135224         G>A         TAC>TAT      Y>YYAL010C      -      0      YS2           chr01:135224         G>A         TAC>TAT      Y>YYAL010C      -      0      SK1           chr01:135321         G>A         ACG>ATG      T>MYAL010C      -      0      273614X       chr01:135327         G>C         GCG>GGG      A>GYAL010C      -      0      322134S       chr01:135327         G>C         GCG>GGG      A>GYAL010C      -      0      DBVPG6040     chr01:135327         G>C         GCG>GGG      A>GYAL010C      -      0      DBVPG6765     chr01:135327         G>C         GCG>GGG      A>GYAL010C      -      0      L_1374        chr01:135327         G>C         GCG>GGG      A>GYAL010C      -      0      L_1528        chr01:135327         G>C         GCG>GGG      A>GYAL010C      -      0      NCYC110       chr01:135327         G>C         GCG>GGG      A>GYAL010C      -      0      SK1           chr01:135327         G>C         GCG>GGG      A>GYAL010C      -      0      UWOPS03_461_4 chr01:135327         G>C         GCG>GGG      A>GYAL010C      -      0      Y55           chr01:135327         G>C         GCG>GGG      A>GYAL010C      -      0      YJM975        chr01:135327         G>C         GCG>GGG      A>GYAL010C      -      0      YJM981        chr01:135327         G>C         GCG>GGG      A>GYAL010C      -      0      YPS606        chr01:135327         G>C         GCG>GGG      A>GYAL010C      -      0      YS2           chr01:135327         G>C         GCG>GGG      A>GYAL010C      -      0      273614X       chr01:135342         A>T         TTC>TAC      F>YYAL010C      -      0      L_1528        chr01:135355         G>A         CCA>TCA      P>SYAL010C      -      0      322134S       chr01:135419         T>A         AAA>AAT      K>NYAL010C      -      0      378604X       chr01:135419         T>A         AAA>AAT      K>NYAL010C      -      0      DBVPG6040     chr01:135419         T>A         AAA>AAT      K>NYAL010C      -      0      DBVPG6765     chr01:135419         T>A         AAA>AAT      K>NYAL010C      -      0      L_1374        chr01:135419         T>A         AAA>AAT      K>NYAL010C      -      0      L_1528        chr01:135419         T>A         AAA>AAT      K>NYAL010C      -      0      NCYC110       chr01:135419         T>A         AAA>AAT      K>NYAL010C      -      0      UWOPS03_461_4 chr01:135419         T>A         AAA>AAT      K>NYAL010C      -      0      UWOPS05_217_3 chr01:135419         T>A         AAA>AAT      K>NYAL010C      -      0      Y55           chr01:135419         T>A         AAA>AAT      K>NYAL010C      -      0      YJM975        chr01:135419         T>A         AAA>AAT      K>NYAL010C      -      0      YJM981        chr01:135419         T>A         AAA>AAT      K>NYAL010C      -      0      YPS606        chr01:135419         T>A         AAA>AAT      K>NYAL010C      -      0      YS2           chr01:135419         T>A         AAA>AAT      K>NYAL010C      -      0      DBVPG6040     chr01:135511         G>A         CCC>TCC      P>SYAL011W      +      0      L_1528        chr01:132246         G>A         GAG>GAA      E>EYAL011W      +      0      UWOPS05_217_3 chr01:132305         A>G         GAT>GGT      D>GYAL011W      +      0      UWOPS05_227_2 chr01:132305         A>G         GAT>GGT      D>GYAL011W      +      0      322134S       chr01:132426         A>G         GGA>GGG      G>GYAL011W      +      0      BC187         chr01:132426         A>G         GGA>GGG      G>GYAL011W      +      0      DBVPG1373     chr01:132426         A>G         GGA>GGG      G>GYAL011W      +      0      DBVPG1788     chr01:132426         A>G         GGA>GGG      G>GYAL011W      +      0      DBVPG1853     chr01:132426         A>G         GGA>GGG      G>GYAL011W      +      0      DBVPG6765     chr01:132426         A>G         GGA>GGG      G>GYAL011W      +      0      L_1374        chr01:132426         A>G         GGA>GGG      G>GYAL011W      +      0      L_1528        chr01:132426         A>G         GGA>GGG      G>GYAL011W      +      0      UWOPS87_2421  chr01:132426         A>G         GGA>GGG      G>GYAL011W      +      0      YIIc17_E5     chr01:132426         A>G         GGA>GGG      G>GYAL011W      +      0      YJM981        chr01:132426         A>G         GGA>GGG      G>GYAL011W      +      0      YS2           chr01:132426         A>G         GGA>GGG      G>GYAL011W      +      0      YS9           chr01:132426         A>G         GGA>GGG      G>GYAL011W      +      0      DBVPG6044     chr01:132429         G>A         CTG>CTA      L>LYAL011W      +      0      Y55           chr01:132429         G>A         CTG>CTA      L>LYAL011W      +      0      UWOPS87_2421  chr01:132457         A>G         AAC>GAC      N>DYAL011W      +      0      322134S       chr01:132471         T>C         ACT>ACC      T>TYAL011W      +      0      BC187         chr01:132471         T>C         ACT>ACC      T>TYAL011W      +      0      DBVPG1373     chr01:132471         T>C         ACT>ACC      T>TYAL011W      +      0      DBVPG1788     chr01:132471         T>C         ACT>ACC      T>TYAL011W      +      0      DBVPG1853     chr01:132471         T>C         ACT>ACC      T>TYAL011W      +      0      DBVPG6044     chr01:132471         T>C         ACT>ACC      T>TYAL011W      +      0      DBVPG6765     chr01:132471         T>C         ACT>ACC      T>TYAL011W      +      0      L_1374        chr01:132471         T>C         ACT>ACC      T>TYAL011W      +      0      L_1528        chr01:132471         T>C         ACT>ACC      T>TYAL011W      +      0      UWOPS05_217_3 chr01:132471         T>C         ACT>ACC      T>TYAL011W      +      0      UWOPS05_227_2 chr01:132471         T>C         ACT>ACC      T>TYAL011W      +      0      UWOPS87_2421  chr01:132471         T>C         ACT>ACC      T>TYAL011W      +      0      Y55           chr01:132471         T>C         ACT>ACC      T>TYAL011W      +      0      YIIc17_E5     chr01:132471         T>C         ACT>ACC      T>TYAL011W      +      0      YJM981        chr01:132471         T>C         ACT>ACC      T>TYAL011W      +      0      YPS128        chr01:132471         T>C         ACT>ACC      T>TYAL011W      +      0      YS9           chr01:132471         T>C         ACT>ACC      T>TYAL011W      +      0      YPS128        chr01:132525         C>T         TCC>TCT      S>SYAL011W      +      0      DBVPG6044     chr01:132555         T>C         TTT>TTC      F>FYAL011W      +      0      K11           chr01:132555         T>C         TTT>TTC      F>FYAL011W      +      0      UWOPS05_217_3 chr01:132555         T>C         TTT>TTC      F>FYAL011W      +      0      UWOPS05_227_2 chr01:132555         T>C         TTT>TTC      F>FYAL011W      +      0      Y55           chr01:132555         T>C         TTT>TTC      F>FYAL011W      +      0      322134S       chr01:132621         A>G         CCA>CCG      P>PYAL011W      +      0      DBVPG1373     chr01:132621         A>G         CCA>CCG      P>PYAL011W      +      0      DBVPG1788     chr01:132621         A>G         CCA>CCG      P>PYAL011W      +      0      DBVPG1853     chr01:132621         A>G         CCA>CCG      P>PYAL011W      +      0      DBVPG6765     chr01:132621         A>G         CCA>CCG      P>PYAL011W      +      0      L_1374        chr01:132621         A>G         CCA>CCG      P>PYAL011W      +      0      L_1528        chr01:132621         A>G         CCA>CCG      P>PYAL011W      +      0      YJM981        chr01:132621         A>G         CCA>CCG      P>PYAL011W      +      0      YS9           chr01:132621         A>G         CCA>CCG      P>PYAL011W      +      0      Y55           chr01:132717         C>T         TTC>TTT      F>FYAL011W      +      0      322134S       chr01:132726         G>A         AAG>AAA      K>KYAL011W      +      0      DBVPG1373     chr01:132726         G>A         AAG>AAA      K>KYAL011W      +      0      DBVPG1788     chr01:132726         G>A         AAG>AAA      K>KYAL011W      +      0      DBVPG1853     chr01:132726         G>A         AAG>AAA      K>KYAL011W      +      0      DBVPG6765     chr01:132726         G>A         AAG>AAA      K>KYAL011W      +      0      L_1374        chr01:132726         G>A         AAG>AAA      K>KYAL011W      +      0      L_1528        chr01:132726         G>A         AAG>AAA      K>KYAL011W      +      0      Y55           chr01:132726         G>A         AAG>AAA      K>KYAL011W      +      0      YJM981        chr01:132726         G>A         AAG>AAA      K>KYAL011W      +      0      YS9           chr01:132726         G>A         AAG>AAA      K>KYAL011W      +      0      UWOPS05_217_3 chr01:132738         C>T         ATC>ATT      I>IYAL011W      +      0      UWOPS05_227_2 chr01:132738         C>T         ATC>ATT      I>IYAL011W      +      0      322134S       chr01:132834         A>G         GAA>GAG      E>EYAL011W      +      0      BC187         chr01:132834         A>G         GAA>GAG      E>EYAL011W      +      0      DBVPG1373     chr01:132834         A>G         GAA>GAG      E>EYAL011W      +      0      DBVPG1788     chr01:132834         A>G         GAA>GAG      E>EYAL011W      +      0      DBVPG1853     chr01:132834         A>G         GAA>GAG      E>EYAL011W      +      0      DBVPG6765     chr01:132834         A>G         GAA>GAG      E>EYAL011W      +      0      L_1374        chr01:132834         A>G         GAA>GAG      E>EYAL011W      +      0      L_1528        chr01:132834         A>G         GAA>GAG      E>EYAL011W      +      0      Y55           chr01:132834         A>G         GAA>GAG      E>EYAL011W      +      0      YJM981        chr01:132834         A>G         GAA>GAG      E>EYAL011W      +      0      YS9           chr01:132834         A>G         GAA>GAG      E>EYAL011W      +      0      273614X       chr01:132931         C>T         CTG>TTG      L>LYAL011W      +      0      K11           chr01:132931         C>T         CTG>TTG      L>LYAL011W      +      0      273614X       chr01:132981         A>G         AAA>AAG      K>KYAL011W      +      0      DBVPG6040     chr01:132997         C>T         CCT>TCT      P>SYAL011W      +      0      322134S       chr01:133083         A>G         GAA>GAG      E>EYAL011W      +      0      BC187         chr01:133083         A>G         GAA>GAG      E>EYAL011W      +      0      DBVPG1373     chr01:133083         A>G         GAA>GAG      E>EYAL011W      +      0      DBVPG1788     chr01:133083         A>G         GAA>GAG      E>EYAL011W      +      0      DBVPG6765     chr01:133083         A>G         GAA>GAG      E>EYAL011W      +      0      L_1374        chr01:133083         A>G         GAA>GAG      E>EYAL011W      +      0      L_1528        chr01:133083         A>G         GAA>GAG      E>EYAL011W      +      0      UWOPS05_217_3 chr01:133083         A>G         GAA>GAG      E>EYAL011W      +      0      YJM975        chr01:133083         A>G         GAA>GAG      E>EYAL011W      +      0      YS9           chr01:133083         A>G         GAA>GAG      E>EYAL011W      +      0      273614X       chr01:133137         C>T         TTC>TTT      F>FYAL011W      +      0      BC187         chr01:133137         C>T         TTC>TTT      F>FYAL011W      +      0      DBVPG1373     chr01:133137         C>T         TTC>TTT      F>FYAL011W      +      0      DBVPG1788     chr01:133137         C>T         TTC>TTT      F>FYAL011W      +      0      DBVPG6765     chr01:133137         C>T         TTC>TTT      F>FYAL011W      +      0      L_1374        chr01:133137         C>T         TTC>TTT      F>FYAL011W      +      0      L_1528        chr01:133137         C>T         TTC>TTT      F>FYAL011W      +      0      UWOPS05_217_3 chr01:133137         C>T         TTC>TTT      F>FYAL011W      +      0      Y55           chr01:133137         C>T         TTC>TTT      F>FYAL011W      +      0      YJM975        chr01:133137         C>T         TTC>TTT      F>FYAL011W      +      0      YJM981        chr01:133137         C>T         TTC>TTT      F>FYAL011W      +      0      YS9           chr01:133137         C>T         TTC>TTT      F>FYAL011W      +      0      BC187         chr01:133177         G>A         GTT>ATT      V>IYAL011W      +      0      DBVPG1373     chr01:133177         G>A         GTT>ATT      V>IYAL011W      +      0      DBVPG1788     chr01:133177         G>A         GTT>ATT      V>IYAL011W      +      0      DBVPG6765     chr01:133177         G>A         GTT>ATT      V>IYAL011W      +      0      L_1374        chr01:133177         G>A         GTT>ATT      V>IYAL011W      +      0      L_1528        chr01:133177         G>A         GTT>ATT      V>IYAL011W      +      0      YJM975        chr01:133177         G>A         GTT>ATT      V>IYAL011W      +      0      YJM981        chr01:133177         G>A         GTT>ATT      V>IYAL011W      +      0      YS9           chr01:133177         G>A         GTT>ATT      V>IYAL011W      +      0      YPS128        chr01:133201         A>G         ACA>GCA      T>AYAL011W      +      0      YPS606        chr01:133201         A>G         ACA>GCA      T>AYAL011W      +      0      UWOPS05_217_3 chr01:133214         C>A         GCA>GAA      A>EYAL011W      +      0      YJM975        chr01:133234         G>T         GAC>TAC      D>YYAL011W      +      0      YJM981        chr01:133234         G>T         GAC>TAC      D>YYAL011W      +      0      YS9           chr01:133234         G>T         GAC>TAC      D>YYAL011W      +      0      DBVPG6040     chr01:133363         A>G         ACA>GCA      T>AYAL011W      +      0      NCYC361       chr01:133363         A>G         ACA>GCA      T>AYAL011W      +      0      YS9           chr01:133363         A>G         ACA>GCA      T>AYAL011W      +      0      YPS606        chr01:133364         C>T         ACA>ATA      T>IYAL011W      +      0      YPS606        chr01:133371         C>T         GCC>GCT      A>AYAL011W      +      0      DBVPG1373     chr01:133377         G>A         TCG>TCA      S>SYAL011W      +      0      DBVPG1788     chr01:133377         G>A         TCG>TCA      S>SYAL011W      +      0      DBVPG6765     chr01:133377         G>A         TCG>TCA      S>SYAL011W      +      0      UWOPS87_2421  chr01:133377         G>A         TCG>TCA      S>SYAL011W      +      0      YJM975        chr01:133377         G>A         TCG>TCA      S>SYAL011W      +      0      YJM981        chr01:133377         G>A         TCG>TCA      S>SYAL011W      +      0      UWOPS05_217_3 chr01:133423         G>A         GGT>AGT      G>SYAL011W      +      0      DBVPG6040     chr01:133432         T>G         TCA>GCA      S>AYAL011W      +      0      UWOPS05_217_3 chr01:133432         T>G         TCA>GCA      S>AYAL011W      +      0      YS9           chr01:133432         T>G         TCA>GCA      S>AYAL011W      +      0      UWOPS05_217_3 chr01:133446         A>G         CAA>CAG      Q>QYAL011W      +      0      BC187         chr01:133473         G>A         CCG>CCA      P>PYAL011W      +      0      L_1374        chr01:133473         G>A         CCG>CCA      P>PYAL011W      +      0      L_1528        chr01:133473         G>A         CCG>CCA      P>PYAL011W      +      0      322134S       chr01:133618         G>A         GTA>ATA      V>IYAL011W      +      0      DBVPG1106     chr01:133618         G>A         GTA>ATA      V>IYAL011W      +      0      DBVPG1788     chr01:133618         G>A         GTA>ATA      V>IYAL011W      +      0      DBVPG6765     chr01:133618         G>A         GTA>ATA      V>IYAL011W      +      0      L_1374        chr01:133618         G>A         GTA>ATA      V>IYAL011W      +      0      L_1528        chr01:133618         G>A         GTA>ATA      V>IYAL011W      +      0      UWOPS87_2421  chr01:133618         G>A         GTA>ATA      V>IYAL011W      +      0      YJM975        chr01:133618         G>A         GTA>ATA      V>IYAL011W      +      0      YJM981        chr01:133618         G>A         GTA>ATA      V>IYAL011W      +      0      322134S       chr01:133643         A>G         GAT>GGT      D>GYAL011W      +      0      DBVPG1106     chr01:133643         A>G         GAT>GGT      D>GYAL011W      +      0      DBVPG1788     chr01:133643         A>G         GAT>GGT      D>GYAL011W      +      0      DBVPG6040     chr01:133643         A>G         GAT>GGT      D>GYAL011W      +      0      DBVPG6765     chr01:133643         A>G         GAT>GGT      D>GYAL011W      +      0      K11           chr01:133643         A>G         GAT>GGT      D>GYAL011W      +      0      L_1528        chr01:133643         A>G         GAT>GGT      D>GYAL011W      +      0      UWOPS05_217_3 chr01:133643         A>G         GAT>GGT      D>GYAL011W      +      0      UWOPS87_2421  chr01:133643         A>G         GAT>GGT      D>GYAL011W      +      0      Y55           chr01:133643         A>G         GAT>GGT      D>GYAL011W      +      0      YJM975        chr01:133643         A>G         GAT>GGT      D>GYAL011W      +      0      YJM981        chr01:133643         A>G         GAT>GGT      D>GYAL011W      +      0      YS9           chr01:133643         A>G         GAT>GGT      D>GYAL011W      +      0      322134S       chr01:133752         G>A         GAG>GAA      E>EYAL011W      +      0      DBVPG1106     chr01:133752         G>A         GAG>GAA      E>EYAL011W      +      0      DBVPG1788     chr01:133752         G>A         GAG>GAA      E>EYAL011W      +      0      DBVPG1853     chr01:133752         G>A         GAG>GAA      E>EYAL011W      +      0      DBVPG6765     chr01:133752         G>A         GAG>GAA      E>EYAL011W      +      0      L_1374        chr01:133752         G>A         GAG>GAA      E>EYAL011W      +      0      L_1528        chr01:133752         G>A         GAG>GAA      E>EYAL011W      +      0      UWOPS87_2421  chr01:133752         G>A         GAG>GAA      E>EYAL011W      +      0      Y55           chr01:133752         G>A         GAG>GAA      E>EYAL011W      +      0      YJM975        chr01:133752         G>A         GAG>GAA      E>EYAL011W      +      0      YJM981        chr01:133752         G>A         GAG>GAA      E>EYAL011W      +      0      YS9           chr01:133752         G>A         GAG>GAA      E>EYAL011W      +      0      322134S       chr01:133860         G>A         GGG>GGA      G>GYAL011W      +      0      DBVPG1106     chr01:133860         G>A         GGG>GGA      G>GYAL011W      +      0      DBVPG1788     chr01:133860         G>A         GGG>GGA      G>GYAL011W      +      0      DBVPG1853     chr01:133860         G>A         GGG>GGA      G>GYAL011W      +      0      DBVPG6765     chr01:133860         G>A         GGG>GGA      G>GYAL011W      +      0      L_1374        chr01:133860         G>A         GGG>GGA      G>GYAL011W      +      0      L_1528        chr01:133860         G>A         GGG>GGA      G>GYAL011W      +      0      NCYC110       chr01:133860         G>A         GGG>GGA      G>GYAL011W      +      0      UWOPS05_217_3 chr01:133860         G>A         GGG>GGA      G>GYAL011W      +      0      UWOPS87_2421  chr01:133860         G>A         GGG>GGA      G>GYAL011W      +      0      Y55           chr01:133860         G>A         GGG>GGA      G>GYAL011W      +      0      YJM975        chr01:133860         G>A         GGG>GGA      G>GYAL011W      +      0      YJM981        chr01:133860         G>A         GGG>GGA      G>GYAL011W      +      0      YS9           chr01:133860         G>A         GGG>GGA      G>GYAL011W      +      0      322134S       chr01:134007         T>C         GCT>GCC      A>AYAL013W      +      0      DBVPG6765     chr01:129408         C>T         TCC>TCT      S>SYAL013W      +      0      273614X       chr01:129477         A>G         AAA>AAG      K>KYAL013W      +      0      DBVPG1373     chr01:129477         A>G         AAA>AAG      K>KYAL013W      +      0      DBVPG1788     chr01:129477         A>G         AAA>AAG      K>KYAL013W      +      0      DBVPG6040     chr01:129477         A>G         AAA>AAG      K>KYAL013W      +      0      DBVPG6765     chr01:129477         A>G         AAA>AAG      K>KYAL013W      +      0      L_1374        chr01:129477         A>G         AAA>AAG      K>KYAL013W      +      0      YJM975        chr01:129477         A>G         AAA>AAG      K>KYAL013W      +      0      YJM981        chr01:129477         A>G         AAA>AAG      K>KYAL013W      +      0      273614X       chr01:129518         T>C         CTC>CCC      L>PYAL013W      +      0      DBVPG1373     chr01:129518         T>C         CTC>CCC      L>PYAL013W      +      0      DBVPG1788     chr01:129518         T>C         CTC>CCC      L>PYAL013W      +      0      DBVPG6040     chr01:129518         T>C         CTC>CCC      L>PYAL013W      +      0      DBVPG6765     chr01:129518         T>C         CTC>CCC      L>PYAL013W      +      0      L_1374        chr01:129518         T>C         CTC>CCC      L>PYAL013W      +      0      YJM975        chr01:129518         T>C         CTC>CCC      L>PYAL013W      +      0      YJM981        chr01:129518         T>C         CTC>CCC      L>PYAL013W      +      0      DBVPG6765     chr01:129786         C>A         CCC>CCA      P>PYAL013W      +      0      DBVPG6765     chr01:129817         C>T         CTG>TTG      L>LYAL013W      +      0      322134S       chr01:130255         T>C         TGC>CGC      C>RYAL013W      +      0      DBVPG1373     chr01:130255         T>C         TGC>CGC      C>RYAL013W      +      0      DBVPG1788     chr01:130255         T>C         TGC>CGC      C>RYAL013W      +      0      DBVPG6765     chr01:130255         T>C         TGC>CGC      C>RYAL013W      +      0      L_1374        chr01:130255         T>C         TGC>CGC      C>RYAL013W      +      0      L_1528        chr01:130255         T>C         TGC>CGC      C>RYAL013W      +      0      SK1           chr01:130255         T>C         TGC>CGC      C>RYAL013W      +      0      UWOPS87_2421  chr01:130255         T>C         TGC>CGC      C>RYAL013W      +      0      Y55           chr01:130255         T>C         TGC>CGC      C>RYAL013W      +      0      YIIc17_E5     chr01:130255         T>C         TGC>CGC      C>RYAL013W      +      0      YJM978        chr01:130255         T>C         TGC>CGC      C>RYAL013W      +      0      YPS128        chr01:130255         T>C         TGC>CGC      C>RYAL013W      +      0      YPS606        chr01:130255         T>C         TGC>CGC      C>RYAL013W      +      0      YJM978        chr01:130268         A>G         GAG>GGG      E>GYAL013W      +      0      L_1374        chr01:130272         C>T         CCC>CCT      P>PYAL013W      +      0      L_1528        chr01:130272         C>T         CCC>CCT      P>PYAL013W      +      0      YIIc17_E5     chr01:130272         C>T         CCC>CCT      P>PYAL013W      +      0      DBVPG1373     chr01:130293         C>T         TGC>TGT      C>CYAL013W      +      0      DBVPG1788     chr01:130293         C>T         TGC>TGT      C>CYAL013W      +      0      DBVPG6765     chr01:130293         C>T         TGC>TGT      C>CYAL013W      +      0      L_1374        chr01:130293         C>T         TGC>TGT      C>CYAL013W      +      0      L_1528        chr01:130293         C>T         TGC>TGT      C>CYAL013W      +      0      SK1           chr01:130293         C>T         TGC>TGT      C>CYAL013W      +      0      YIIc17_E5     chr01:130293         C>T         TGC>TGT      C>CYAL013W      +      0      YJM978        chr01:130293         C>T         TGC>TGT      C>CYAL013W      +      0      YS9           chr01:130293         C>T         TGC>TGT      C>CYAL013W      +      0      DBVPG1373     chr01:130363         C>T         CTC>TTC      L>FYAL013W      +      0      DBVPG1788     chr01:130363         C>T         CTC>TTC      L>FYAL013W      +      0      DBVPG6765     chr01:130363         C>T         CTC>TTC      L>FYAL013W      +      0      L_1374        chr01:130363         C>T         CTC>TTC      L>FYAL013W      +      0      L_1528        chr01:130363         C>T         CTC>TTC      L>FYAL013W      +      0      SK1           chr01:130363         C>T         CTC>TTC      L>FYAL013W      +      0      UWOPS87_2421  chr01:130363         C>T         CTC>TTC      L>FYAL013W      +      0      YIIc17_E5     chr01:130363         C>T         CTC>TTC      L>FYAL013W      +      0      YS9           chr01:130363         C>T         CTC>TTC      L>FYAL013W      +      0      Y55           chr01:130455         A>G         GAA>GAG      E>EYAL013W      +      0      273614X       chr01:130471         C>a         CAC>aAC      H>NYAL016W      +      0      NCYC110       chr01:124973         T>C         TCT>CCT      S>PYAL016W      +      0      Y55           chr01:124973         T>C         TCT>CCT      S>PYAL016W      +      0      NCYC110       chr01:125134         A>G         GCA>GCG      A>AYAL016W      +      0      Y55           chr01:125134         A>G         GCA>GCG      A>AYAL016W      +      0      Y9            chr01:125134         A>G         GCA>GCG      A>AYAL016W      +      0      Y9            chr01:125152         G>A         GAG>GAA      E>EYAL016W      +      0      DBVPG6044     chr01:125159         G>T         GCC>TCC      A>SYAL016W      +      0      NCYC110       chr01:125159         G>T         GCC>TCC      A>SYAL016W      +      0      Y55           chr01:125159         G>T         GCC>TCC      A>SYAL016W      +      0      DBVPG6044     chr01:125278         A>G         GAA>GAG      E>EYAL016W      +      0      Y55           chr01:125278         A>G         GAA>GAG      E>EYAL016W      +      0      DBVPG6044     chr01:125287         A>G         GTA>GTG      V>VYAL016W      +      0      Y55           chr01:125287         A>G         GTA>GTG      V>VYAL016W      +      0      DBVPG6044     chr01:125420         G>A         GTT>ATT      V>IYAL016W      +      0      Y55           chr01:125420         G>A         GTT>ATT      V>IYAL016W      +      0      YS9           chr01:125549         T>C         TTG>CTG      L>LYAL016W      +      0      YS9           chr01:125601         A>T         AAC>ATC      N>IYAL016W      +      0      DBVPG6044     chr01:125611         G>A         CAG>CAA      Q>QYAL016W      +      0      Y55           chr01:125611         G>A         CAG>CAA      Q>QYAL016W      +      0      Y9            chr01:125611         G>A         CAG>CAA      Q>QYAL016W      +      0      YPS128        chr01:125611         G>A         CAG>CAA      Q>QYAL016W      +      0      YPS606        chr01:125611         G>A         CAG>CAA      Q>QYAL016W      +      0      YS9           chr01:125675         T>C         TTG>CTG      L>LYAL016W      +      0      Y55           chr01:125794         A>T         TCA>TCT      S>SYAL016W      +      0      Y9            chr01:125794         A>T         TCA>TCT      S>SYAL016W      +      0      YPS128        chr01:125794         A>T         TCA>TCT      S>SYAL016W      +      0      YPS606        chr01:125794         A>T         TCA>TCT      S>SYAL016W      +      0      YS9           chr01:125794         A>T         TCA>TCT      S>SYAL016W      +      0      L_1528        chr01:125813         A>G         AGT>GGT      S>GYAL016W      +      0      Y55           chr01:125896         T>C         GCT>GCC      A>AYAL016W      +      0      UWOPS87_2421  chr01:125938         T>C         GAT>GAC      D>DYAL016W      +      0      L_1528        chr01:125944         A>C         TCA>TCC      S>SYAL016W      +      0      UWOPS87_2421  chr01:126259         G>A         GAG>GAA      E>EYAL016W      +      0      Y55           chr01:126259         G>A         GAG>GAA      E>EYAL016W      +      0      YPS128        chr01:126259         G>A         GAG>GAA      E>EYAL016W      +      0      YPS606        chr01:126259         G>A         GAG>GAA      E>EYAL016W      +      0      Y55           chr01:126268         T>A         CCT>CCA      P>PYAL016W      +      0      NCYC361       chr01:126349         T>A         GTT>GTA      V>VYAL016W      +      0      273614X       chr01:126359         A>T         AGA>TGA      R>*YAL016W      +      0      YS9           chr01:126421         T>A         CGT>CGA      R>RYAL016W      +      0      UWOPS87_2421  chr01:126463         A>G         TTA>TTG      L>LYAL016W      +      0      Y12           chr01:126463         A>G         TTA>TTG      L>LYAL016W      +      0      Y55           chr01:126463         A>G         TTA>TTG      L>LYAL016W      +      0      YPS128        chr01:126463         A>G         TTA>TTG      L>LYAL016W      +      0      UWOPS87_2421  chr01:126469         A>G         GAA>GAG      E>EYAL016W      +      0      Y12           chr01:126469         A>G         GAA>GAG      E>EYAL016W      +      0      Y55           chr01:126469         A>G         GAA>GAG      E>EYAL016W      +      0      YPS128        chr01:126469         A>G         GAA>GAG      E>EYAL016W      +      0      UWOPS87_2421  chr01:126484         G>A         AGG>AGA      R>RYAL016W      +      0      Y12           chr01:126484         G>A         AGG>AGA      R>RYAL016W      +      0      Y55           chr01:126484         G>A         AGG>AGA      R>RYAL016W      +      0      YPS128        chr01:126484         G>A         AGG>AGA      R>RYAL016W      +      0      Y12           chr01:126502         T>C         GCT>GCC      A>AYAL016W      +      0      Y55           chr01:126502         T>C         GCT>GCC      A>AYAL016W      +      0      YPS128        chr01:126502         T>C         GCT>GCC      A>AYAL016W      +      0      YS9           chr01:126516         T>A         GTG>GAG      V>EYAL016W      +      0      DBVPG6044     chr01:126554         T>C         TTA>CTA      L>LYAL016W      +      0      UWOPS05_217_3 chr01:126554         T>C         TTA>CTA      L>LYAL016W      +      0      UWOPS05_227_2 chr01:126554         T>C         TTA>CTA      L>LYAL016W      +      0      Y55           chr01:126554         T>C         TTA>CTA      L>LYAL016W      +      0      DBVPG6044     chr01:126601         G>A         AGG>AGA      R>RYAL016W      +      0      L_1374        chr01:126601         G>A         AGG>AGA      R>RYAL016W      +      0      NCYC361       chr01:126601         G>A         AGG>AGA      R>RYAL016W      +      0      UWOPS05_217_3 chr01:126601         G>A         AGG>AGA      R>RYAL016W      +      0      UWOPS05_227_2 chr01:126601         G>A         AGG>AGA      R>RYAL016W      +      0      UWOPS87_2421  chr01:126601         G>A         AGG>AGA      R>RYAL016W      +      0      Y12           chr01:126601         G>A         AGG>AGA      R>RYAL016W      +      0      Y55           chr01:126601         G>A         AGG>AGA      R>RYAL016W      +      0      YPS128        chr01:126601         G>A         AGG>AGA      R>RYAL016W      +      0      YPS128        chr01:126657         C>T         GCC>GTC      A>VYAL016W      +      0      YIIc17_E5     chr01:126777         T>A         TTA>TAA      L>*YAL018C      -      0      UWOPS05_217_3 chr01:118576         G>C         CCG>GCG      P>AYAL018C      -      0      DBVPG6044     chr01:118607         A>G         ATT>ATC      I>IYAL018C      -      0      NCYC110       chr01:118607         A>G         ATT>ATC      I>IYAL018C      -      0      Y55           chr01:118607         A>G         ATT>ATC      I>IYAL018C      -      0      YPS606        chr01:118607         A>G         ATT>ATC      I>IYAL018C      -      0      DBVPG6044     chr01:118654         C>T         GAT>AAT      D>NYAL018C      -      0      Y55           chr01:118654         C>T         GAT>AAT      D>NYAL018C      -      0      YPS606        chr01:118724         A>G         ATT>ATC      I>IYAL018C      -      0      YS4           chr01:118724         A>G         ATT>ATC      I>IYAL018C      -      0      K11           chr01:118766         G>A         CAC>CAT      H>HYAL018C      -      0      Y12           chr01:118766         G>A         CAC>CAT      H>HYAL018C      -      0      YS9           chr01:118766         G>A         CAC>CAT      H>HYAL018C      -      0      DBVPG6044     chr01:118823         T>C         AAA>AAG      K>KYAL018C      -      0      K11           chr01:118823         T>C         AAA>AAG      K>KYAL018C      -      0      Y12           chr01:118823         T>C         AAA>AAG      K>KYAL018C      -      0      Y55           chr01:118823         T>C         AAA>AAG      K>KYAL018C      -      0      YPS606        chr01:118823         T>C         AAA>AAG      K>KYAL018C      -      0      YS4           chr01:118823         T>C         AAA>AAG      K>KYAL018C      -      0      YS9           chr01:118823         T>C         AAA>AAG      K>KYAL018C      -      0      K11           chr01:118998         G>A         GCA>GTA      A>VYAL018C      -      0      SK1           chr01:118998         G>A         GCA>GTA      A>VYAL018C      -      0      Y12           chr01:118998         G>A         GCA>GTA      A>VYAL018C      -      0      Y55           chr01:118998         G>A         GCA>GTA      A>VYAL018C      -      0      YPS606        chr01:118998         G>A         GCA>GTA      A>VYAL018C      -      0      YS4           chr01:118998         G>A         GCA>GTA      A>VYAL018C      -      0      YS9           chr01:118998         G>A         GCA>GTA      A>VYAL018C      -      0      YPS606        chr01:119020         C>T         GTC>ATC      V>IYAL018C      -      0      K11           chr01:119051         C>T         GAG>GAA      E>EYAL018C      -      0      Y12           chr01:119051         C>T         GAG>GAA      E>EYAL018C      -      0      YS9           chr01:119051         C>T         GAG>GAA      E>EYAL018C      -      0      Y55           chr01:119150         G>A         TTC>TTT      F>FYAL018C      -      0      K11           chr01:119168         C>G         CTG>CTC      L>LYAL018C      -      0      YS9           chr01:119168         C>G         CTG>CTC      L>LYAL018C      -      0      YS9           chr01:119243         C>T         TTG>TTA      L>LYAL018C      -      0      273614X       chr01:119345         G>A         TTC>TTT      F>FYAL018C      -      0      378604X       chr01:119345         G>A         TTC>TTT      F>FYAL018C      -      0      BC187         chr01:119345         G>A         TTC>TTT      F>FYAL018C      -      0      DBVPG1788     chr01:119345         G>A         TTC>TTT      F>FYAL018C      -      0      DBVPG6765     chr01:119345         G>A         TTC>TTT      F>FYAL018C      -      0      L_1374        chr01:119345         G>A         TTC>TTT      F>FYAL018C      -      0      YJM981        chr01:119345         G>A         TTC>TTT      F>FYAL018C      -      0      273614X       chr01:119351         A>G         GAT>GAC      D>DYAL018C      -      0      378604X       chr01:119351         A>G         GAT>GAC      D>DYAL018C      -      0      BC187         chr01:119351         A>G         GAT>GAC      D>DYAL018C      -      0      DBVPG1788     chr01:119351         A>G         GAT>GAC      D>DYAL018C      -      0      DBVPG6765     chr01:119351         A>G         GAT>GAC      D>DYAL018C      -      0      L_1374        chr01:119351         A>G         GAT>GAC      D>DYAL018C      -      0      NCYC110       chr01:119351         A>G         GAT>GAC      D>DYAL018C      -      0      Y55           chr01:119351         A>G         GAT>GAC      D>DYAL018C      -      0      YJM981        chr01:119351         A>G         GAT>GAC      D>DYAL018C      -      0      YPS606        chr01:119351         A>G         GAT>GAC      D>DYAL018C      -      0      NCYC110       chr01:119382         G>A         ACC>ATC      T>IYAL018C      -      0      Y55           chr01:119382         G>A         ACC>ATC      T>IYAL018C      -      0      YS9           chr01:119388         C>T         AGC>AAC      S>NYAL018C      -      0      273614X       chr01:119395         C>T         GTT>ATT      V>IYAL018C      -      0      273614X       chr01:119405         G>C         CAC>CAG      H>QYAL018C      -      0      BC187         chr01:119405         G>C         CAC>CAG      H>QYAL018C      -      0      DBVPG1788     chr01:119405         G>C         CAC>CAG      H>QYAL018C      -      0      DBVPG6765     chr01:119405         G>C         CAC>CAG      H>QYAL018C      -      0      YJM981        chr01:119405         G>C         CAC>CAG      H>QYAL018C      -      0      273614X       chr01:119461         G>A         CTG>TTG      L>LYAL018C      -      0      378604X       chr01:119461         G>A         CTG>TTG      L>LYAL018C      -      0      BC187         chr01:119461         G>A         CTG>TTG      L>LYAL018C      -      0      DBVPG1788     chr01:119461         G>A         CTG>TTG      L>LYAL018C      -      0      DBVPG6765     chr01:119461         G>A         CTG>TTG      L>LYAL018C      -      0      L_1374        chr01:119461         G>A         CTG>TTG      L>LYAL018C      -      0      YIIc17_E5     chr01:119461         G>A         CTG>TTG      L>LYAL018C      -      0      YJM981        chr01:119461         G>A         CTG>TTG      L>LYAL019W      +      0      DBVPG6044     chr01:114929         T>C         TCG>CCG      S>PYAL019W      +      0      K11           chr01:114929         T>C         TCG>CCG      S>PYAL019W      +      0      Y12           chr01:114929         T>C         TCG>CCG      S>PYAL019W      +      0      Y55           chr01:114929         T>C         TCG>CCG      S>PYAL019W      +      0      YPS128        chr01:114929         T>C         TCG>CCG      S>PYAL019W      +      0      YPS606        chr01:114929         T>C         TCG>CCG      S>PYAL019W      +      0      K11           chr01:115063         G>A         AGG>AGA      R>RYAL019W      +      0      Y12           chr01:115063         G>A         AGG>AGA      R>RYAL019W      +      0      DBVPG6040     chr01:115110         A>C         AAG>ACG      K>TYAL019W      +      0      YPS606        chr01:115135         G>A         GAG>GAA      E>EYAL019W      +      0      DBVPG6044     chr01:115168         C>T         CCC>CCT      P>PYAL019W      +      0      K11           chr01:115168         C>T         CCC>CCT      P>PYAL019W      +      0      NCYC110       chr01:115168         C>T         CCC>CCT      P>PYAL019W      +      0      Y12           chr01:115168         C>T         CCC>CCT      P>PYAL019W      +      0      Y55           chr01:115168         C>T         CCC>CCT      P>PYAL019W      +      0      K11           chr01:115183         T>C         ACT>ACC      T>TYAL019W      +      0      YPS606        chr01:115184         C>T         CTG>TTG      L>LYAL019W      +      0      DBVPG6044     chr01:115336         A>G         TTA>TTG      L>LYAL019W      +      0      NCYC110       chr01:115336         A>G         TTA>TTG      L>LYAL019W      +      0      Y55           chr01:115336         A>G         TTA>TTG      L>LYAL019W      +      0      YPS606        chr01:115459         G>A         CAG>CAA      Q>QYAL019W      +      0      DBVPG6044     chr01:115495         T>C         AAT>AAC      N>NYAL019W      +      0      K11           chr01:115495         T>C         AAT>AAC      N>NYAL019W      +      0      NCYC110       chr01:115495         T>C         AAT>AAC      N>NYAL019W      +      0      Y12           chr01:115495         T>C         AAT>AAC      N>NYAL019W      +      0      Y55           chr01:115495         T>C         AAT>AAC      N>NYAL019W      +      0      YPS606        chr01:115495         T>C         AAT>AAC      N>NYAL019W      +      0      DBVPG6044     chr01:115513         G>C         CTG>CTC      L>LYAL019W      +      0      K11           chr01:115513         G>C         CTG>CTC      L>LYAL019W      +      0      NCYC110       chr01:115513         G>C         CTG>CTC      L>LYAL019W      +      0      Y12           chr01:115513         G>C         CTG>CTC      L>LYAL019W      +      0      Y55           chr01:115513         G>C         CTG>CTC      L>LYAL019W      +      0      YPS606        chr01:115513         G>C         CTG>CTC      L>LYAL019W      +      0      DBVPG6044     chr01:115591         G>C         GAG>GAC      E>DYAL019W      +      0      NCYC110       chr01:115591         G>C         GAG>GAC      E>DYAL019W      +      0      Y55           chr01:115591         G>C         GAG>GAC      E>DYAL019W      +      0      DBVPG6044     chr01:115607         T>C         TTA>CTA      L>LYAL019W      +      0      K11           chr01:115607         T>C         TTA>CTA      L>LYAL019W      +      0      NCYC110       chr01:115607         T>C         TTA>CTA      L>LYAL019W      +      0      Y55           chr01:115607         T>C         TTA>CTA      L>LYAL019W      +      0      YS2           chr01:115607         T>C         TTA>CTA      L>LYAL019W      +      0      SK1           chr01:115630         A>G         CTA>CTG      L>LYAL019W      +      0      YS9           chr01:115630         A>G         CTA>CTG      L>LYAL019W      +      0      DBVPG6044     chr01:115660         A>C         GCA>GCC      A>AYAL019W      +      0      Y55           chr01:115660         A>C         GCA>GCC      A>AYAL019W      +      0      SK1           chr01:115666         C>T         GAC>GAT      D>DYAL019W      +      0      YS9           chr01:115666         C>T         GAC>GAT      D>DYAL019W      +      0      DBVPG6044     chr01:115786         C>T         GAC>GAT      D>DYAL019W      +      0      UWOPS05_227_2 chr01:115786         C>T         GAC>GAT      D>DYAL019W      +      0      Y55           chr01:115786         C>T         GAC>GAT      D>DYAL019W      +      0      YPS606        chr01:115786         C>T         GAC>GAT      D>DYAL019W      +      0      YS2           chr01:115786         C>T         GAC>GAT      D>DYAL019W      +      0      YPS606        chr01:115795         T>C         ATT>ATC      I>IYAL019W      +      0      YS2           chr01:115801         T>C         GAT>GAC      D>DYAL019W      +      0      DBVPG6044     chr01:115907         G>A         GAC>AAC      D>NYAL019W      +      0      Y55           chr01:115907         G>A         GAC>AAC      D>NYAL019W      +      0      DBVPG6044     chr01:115922         G>A         GCA>ACA      A>TYAL019W      +      0      Y55           chr01:115922         G>A         GCA>ACA      A>TYAL019W      +      0      DBVPG6765     chr01:115975         G>A         AGG>AGA      R>RYAL019W      +      0      UWOPS05_227_2 chr01:115978         G>A         CTG>CTA      L>LYAL019W      +      0      Y12           chr01:115978         G>A         CTG>CTA      L>LYAL019W      +      0      YS2           chr01:115978         G>A         CTG>CTA      L>LYAL019W      +      0      YS4           chr01:115978         G>A         CTG>CTA      L>LYAL019W      +      0      YS9           chr01:115978         G>A         CTG>CTA      L>LYAL019W      +      0      Y12           chr01:115999         T>C         AGT>AGC      S>SYAL019W      +      0      YS2           chr01:115999         T>C         AGT>AGC      S>SYAL019W      +      0      YS4           chr01:115999         T>C         AGT>AGC      S>SYAL019W      +      0      Y12           chr01:116038         A>G         AAA>AAG      K>KYAL019W      +      0      YS2           chr01:116038         A>G         AAA>AAG      K>KYAL019W      +      0      YS4           chr01:116038         A>G         AAA>AAG      K>KYAL019W      +      0      YS9           chr01:116038         A>G         AAA>AAG      K>KYAL019W      +      0      YS2           chr01:116134         G>C         GGG>GGC      G>GYAL019W      +      0      DBVPG1853     chr01:116176         T>G         AAT>AAG      N>KYAL019W      +      0      DBVPG6044     chr01:116179         T>A         AAT>AAA      N>KYAL019W      +      0      Y55           chr01:116179         T>A         AAT>AAA      N>KYAL019W      +      0      UWOPS03_461_4 chr01:116207         G>A         GCA>ACA      A>TYAL019W      +      0      UWOPS05_227_2 chr01:116207         G>A         GCA>ACA      A>TYAL019W      +      0      YS2           chr01:116207         G>A         GCA>ACA      A>TYAL019W      +      0      UWOPS03_461_4 chr01:116239         A>T         ACA>ACT      T>TYAL019W      +      0      UWOPS05_227_2 chr01:116239         A>T         ACA>ACT      T>TYAL019W      +      0      378604X       chr01:116328         T>C         ATT>ACT      I>TYAL019W      +      0      BC187         chr01:116328         T>C         ATT>ACT      I>TYAL019W      +      0      DBVPG1373     chr01:116328         T>C         ATT>ACT      I>TYAL019W      +      0      DBVPG1788     chr01:116328         T>C         ATT>ACT      I>TYAL019W      +      0      DBVPG6040     chr01:116328         T>C         ATT>ACT      I>TYAL019W      +      0      DBVPG6044     chr01:116328         T>C         ATT>ACT      I>TYAL019W      +      0      DBVPG6765     chr01:116328         T>C         ATT>ACT      I>TYAL019W      +      0      L_1374        chr01:116328         T>C         ATT>ACT      I>TYAL019W      +      0      NCYC361       chr01:116328         T>C         ATT>ACT      I>TYAL019W      +      0      UWOPS03_461_4 chr01:116328         T>C         ATT>ACT      I>TYAL019W      +      0      Y12           chr01:116328         T>C         ATT>ACT      I>TYAL019W      +      0      Y55           chr01:116328         T>C         ATT>ACT      I>TYAL019W      +      0      YPS128        chr01:116328         T>C         ATT>ACT      I>TYAL019W      +      0      YPS606        chr01:116328         T>C         ATT>ACT      I>TYAL019W      +      0      YS4           chr01:116328         T>C         ATT>ACT      I>TYAL019W      +      0      YS9           chr01:116328         T>C         ATT>ACT      I>TYAL019W      +      0      DBVPG6044     chr01:116335         C>T         AAC>AAT      N>NYAL019W      +      0      UWOPS03_461_4 chr01:116335         C>T         AAC>AAT      N>NYAL019W      +      0      Y12           chr01:116335         C>T         AAC>AAT      N>NYAL019W      +      0      Y55           chr01:116335         C>T         AAC>AAT      N>NYAL019W      +      0      YPS128        chr01:116335         C>T         AAC>AAT      N>NYAL019W      +      0      YPS606        chr01:116335         C>T         AAC>AAT      N>NYAL019W      +      0      YS4           chr01:116335         C>T         AAC>AAT      N>NYAL019W      +      0      YS9           chr01:116335         C>T         AAC>AAT      N>NYAL019W      +      0      UWOPS87_2421  chr01:116341         T>C         ACT>ACC      T>TYAL019W      +      0      Y12           chr01:116341         T>C         ACT>ACC      T>TYAL019W      +      0      YPS128        chr01:116341         T>C         ACT>ACC      T>TYAL019W      +      0      YPS606        chr01:116341         T>C         ACT>ACC      T>TYAL019W      +      0      YS4           chr01:116341         T>C         ACT>ACC      T>TYAL019W      +      0      YS9           chr01:116341         T>C         ACT>ACC      T>TYAL019W      +      0      DBVPG6044     chr01:116458         C>A         AAC>AAA      N>KYAL019W      +      0      Y55           chr01:116458         C>A         AAC>AAA      N>KYAL019W      +      0      UWOPS03_461_4 chr01:116512         C>T         GAC>GAT      D>DYAL019W      +      0      UWOPS05_217_3 chr01:116512         C>T         GAC>GAT      D>DYAL019W      +      0      UWOPS87_2421  chr01:116512         C>T         GAC>GAT      D>DYAL019W      +      0      Y12           chr01:116512         C>T         GAC>GAT      D>DYAL019W      +      0      YPS128        chr01:116512         C>T         GAC>GAT      D>DYAL019W      +      0      YPS606        chr01:116512         C>T         GAC>GAT      D>DYAL019W      +      0      YS4           chr01:116512         C>T         GAC>GAT      D>DYAL019W      +      0      YS9           chr01:116512         C>T         GAC>GAT      D>DYAL019W      +      0      UWOPS03_461_4 chr01:116521         T>G         TTT>TTG      F>LYAL019W      +      0      UWOPS05_217_3 chr01:116521         T>G         TTT>TTG      F>LYAL019W      +      0      DBVPG6044     chr01:116527         T>C         GCT>GCC      A>AYAL019W      +      0      Y12           chr01:116527         T>C         GCT>GCC      A>AYAL019W      +      0      Y55           chr01:116527         T>C         GCT>GCC      A>AYAL019W      +      0      YPS128        chr01:116527         T>C         GCT>GCC      A>AYAL019W      +      0      YPS606        chr01:116527         T>C         GCT>GCC      A>AYAL019W      +      0      YS9           chr01:116527         T>C         GCT>GCC      A>AYAL019W      +      0      DBVPG6044     chr01:116549         A>G         ATC>GTC      I>VYAL019W      +      0      UWOPS87_2421  chr01:116549         A>G         ATC>GTC      I>VYAL019W      +      0      Y12           chr01:116549         A>G         ATC>GTC      I>VYAL019W      +      0      Y55           chr01:116549         A>G         ATC>GTC      I>VYAL019W      +      0      YPS128        chr01:116549         A>G         ATC>GTC      I>VYAL019W      +      0      YPS606        chr01:116549         A>G         ATC>GTC      I>VYAL019W      +      0      YS4           chr01:116549         A>G         ATC>GTC      I>VYAL019W      +      0      YS9           chr01:116549         A>G         ATC>GTC      I>VYAL019W      +      0      UWOPS87_2421  chr01:116611         A>G         AGA>AGG      R>RYAL019W      +      0      YS4           chr01:116714         A>G         ATG>GTG      M>VYAL019W      +      0      UWOPS87_2421  chr01:116778         C>T         CCG>CTG      P>LYAL019W      +      0      Y12           chr01:116851         T>A         CCT>CCA      P>PYAL019W      +      0      Y9            chr01:116851         T>A         CCT>CCA      P>PYAL019W      +      0      Y9            chr01:116872         C>G         TAC>TAG      Y>*YAL019W      +      0      SK1           chr01:116906         G>C         GAT>CAT      D>HYAL019W      +      0      UWOPS03_461_4 chr01:116950         C>T         ACC>ACT      T>TYAL019W      +      0      UWOPS05_217_3 chr01:116950         C>T         ACC>ACT      T>TYAL019W      +      0      K11           chr01:117104         T>C         TTA>CTA      L>LYAL019W      +      0      UWOPS03_461_4 chr01:117104         T>C         TTA>CTA      L>LYAL019W      +      0      UWOPS05_217_3 chr01:117104         T>C         TTA>CTA      L>LYAL019W      +      0      Y12           chr01:117104         T>C         TTA>CTA      L>LYAL019W      +      0      Y55           chr01:117394         A>T         ATA>ATT      I>IYAL019W      +      0      322134S       chr01:117400         A>G         AAA>AAG      K>KYAL019W      +      0      SK1           chr01:117400         A>G         AAA>AAG      K>KYAL019W      +      0      DBVPG1853     chr01:117500         T>C         TCT>CCT      S>PYAL019W      +      0      K11           chr01:117505         A>C         TCA>TCC      S>SYAL019W      +      0      UWOPS03_461_4 chr01:117505         A>C         TCA>TCC      S>SYAL019W      +      0      UWOPS05_217_3 chr01:117505         A>C         TCA>TCC      S>SYAL019W      +      0      UWOPS83_787_3 chr01:117505         A>C         TCA>TCC      S>SYAL019W      +      0      UWOPS87_2421  chr01:117505         A>C         TCA>TCC      S>SYAL019W      +      0      Y12           chr01:117505         A>C         TCA>TCC      S>SYAL019W      +      0      Y55           chr01:117505         A>C         TCA>TCC      S>SYAL019W      +      0      YS2           chr01:117505         A>C         TCA>TCC      S>SYAL019W      +      0      YS9           chr01:117505         A>C         TCA>TCC      S>SYAL019W      +      0      K11           chr01:117511         C>T         TCC>TCT      S>SYAL019W      +      0      UWOPS83_787_3 chr01:117511         C>T         TCC>TCT      S>SYAL019W      +      0      UWOPS87_2421  chr01:117511         C>T         TCC>TCT      S>SYAL019W      +      0      Y12           chr01:117511         C>T         TCC>TCT      S>SYAL019W      +      0      YS2           chr01:117511         C>T         TCC>TCT      S>SYAL019W      +      0      YS9           chr01:117511         C>T         TCC>TCT      S>SYAL019W      +      0      Y55           chr01:117529         A>G         GCA>GCG      A>AYAL019W      +      0      K11           chr01:117670         C>T         AGC>AGT      S>SYAL019W      +      0      UWOPS83_787_3 chr01:117670         C>T         AGC>AGT      S>SYAL019W      +      0      Y12           chr01:117670         C>T         AGC>AGT      S>SYAL019W      +      0      YS2           chr01:117670         C>T         AGC>AGT      S>SYAL019W      +      0      YS9           chr01:117670         C>T         AGC>AGT      S>SYAL019W      +      0      K11           chr01:117679         G>A         ACG>ACA      T>TYAL019W      +      0      UWOPS83_787_3 chr01:117679         G>A         ACG>ACA      T>TYAL019W      +      0      Y12           chr01:117679         G>A         ACG>ACA      T>TYAL019W      +      0      YS2           chr01:117679         G>A         ACG>ACA      T>TYAL019W      +      0      YS9           chr01:117679         G>A         ACG>ACA      T>TYAL019W      +      0      UWOPS87_2421  chr01:117700         A>G         CTA>CTG      L>LYAL019W      +      0      Y12           chr01:117709         T>C         AAT>AAC      N>NYAL019W      +      0      UWOPS83_787_3 chr01:117826         G>C         GTG>GTC      V>VYAL019W      +      0      322134S       chr01:117941         C>T         CTA>TTA      L>LYAL019W      +      0      DBVPG6044     chr01:117941         C>T         CTA>TTA      L>LYAL019W      +      0      K11           chr01:117941         C>T         CTA>TTA      L>LYAL019W      +      0      NCYC110       chr01:117941         C>T         CTA>TTA      L>LYAL019W      +      0      UWOPS05_217_3 chr01:117941         C>T         CTA>TTA      L>LYAL019W      +      0      UWOPS83_787_3 chr01:117941         C>T         CTA>TTA      L>LYAL019W      +      0      YPS606        chr01:117941         C>T         CTA>TTA      L>LYAL019W      +      0      YS4           chr01:117941         C>T         CTA>TTA      L>LYAL019W      +      0      DBVPG6044     chr01:118093         G>A         AGG>AGA      R>RYAL019W      +      0      NCYC110       chr01:118093         G>A         AGG>AGA      R>RYAL019W      +      0      Y55           chr01:118093         G>A         AGG>AGA      R>RYAL019W      +      0      UWOPS05_217_3 chr01:118117         G>A         AAG>AAA      K>KYAL019W      +      0      YPS606        chr01:118117         G>A         AAG>AAA      K>KYAL019W      +      0      DBVPG6044     chr01:118123         T>C         GTT>GTC      V>VYAL019W      +      0      NCYC110       chr01:118123         T>C         GTT>GTC      V>VYAL019W      +      0      Y55           chr01:118123         T>C         GTT>GTC      V>VYAL019W      +      0      UWOPS05_217_3 chr01:118132         C>T         ACC>ACT      T>TYAL019W      +      0      YPS606        chr01:118132         C>T         ACC>ACT      T>TYAL019W      +      0      DBVPG6044     chr01:118144         T>C         ACT>ACC      T>TYAL019W      +      0      K11           chr01:118144         T>C         ACT>ACC      T>TYAL019W      +      0      NCYC110       chr01:118144         T>C         ACT>ACC      T>TYAL019W      +      0      Y55           chr01:118144         T>C         ACT>ACC      T>TYAL019W      +      0      YS4           chr01:118144         T>C         ACT>ACC      T>TYAL020C      -      0      UWOPS83_787_3 chr01:113694         G>C         CCT>CGT      P>RYAL020C      -      0      UWOPS83_787_3 chr01:113732         T>C         GGA>GGG      G>GYAL020C      -      0      K11           chr01:113790         G>A         ACT>ATT      T>IYAL020C      -      0      UWOPS03_461_4 chr01:113790         G>A         ACT>ATT      T>IYAL020C      -      0      UWOPS05_227_2 chr01:113790         G>A         ACT>ATT      T>IYAL020C      -      0      UWOPS87_2421  chr01:113790         G>A         ACT>ATT      T>IYAL020C      -      0      YPS606        chr01:113790         G>A         ACT>ATT      T>IYAL020C      -      0      YS4           chr01:113790         G>A         ACT>ATT      T>IYAL020C      -      0      YS9           chr01:113790         G>A         ACT>ATT      T>IYAL020C      -      0      UWOPS83_787_3 chr01:113807         A>G         AGT>AGC      S>SYAL020C      -      0      Y55           chr01:113813         G>T         ACC>ACA      T>TYAL020C      -      0      K11           chr01:113858         G>C         CTC>CTG      L>LYAL020C      -      0      UWOPS03_461_4 chr01:113858         G>C         CTC>CTG      L>LYAL020C      -      0      UWOPS05_227_2 chr01:113858         G>C         CTC>CTG      L>LYAL020C      -      0      UWOPS83_787_3 chr01:113858         G>C         CTC>CTG      L>LYAL020C      -      0      UWOPS87_2421  chr01:113858         G>C         CTC>CTG      L>LYAL020C      -      0      Y55           chr01:113858         G>C         CTC>CTG      L>LYAL020C      -      0      YPS606        chr01:113858         G>C         CTC>CTG      L>LYAL020C      -      0      YS4           chr01:113858         G>C         CTC>CTG      L>LYAL020C      -      0      YS9           chr01:113858         G>C         CTC>CTG      L>LYAL020C      -      0      UWOPS05_217_3 chr01:113875         C>a         GGC>TGC      G>CYAL020C      -      0      UWOPS83_787_3 chr01:113885         C>T         TCG>TCA      S>SYAL020C      -      0      K11           chr01:113891         T>C         GTA>GTG      V>VYAL020C      -      0      YPS606        chr01:113891         T>C         GTA>GTG      V>VYAL020C      -      0      YS4           chr01:113891         T>C         GTA>GTG      V>VYAL020C      -      0      UWOPS83_787_3 chr01:113894         C>A         ACG>ACT      T>TYAL020C      -      0      K11           chr01:113908         C>T         GCG>ACG      A>TYAL020C      -      0      UWOPS05_227_2 chr01:113908         C>T         GCG>ACG      A>TYAL020C      -      0      YPS606        chr01:113908         C>T         GCG>ACG      A>TYAL020C      -      0      YS4           chr01:113908         C>T         GCG>ACG      A>TYAL020C      -      0      YS9           chr01:113908         C>T         GCG>ACG      A>TYAL020C      -      0      UWOPS05_227_2 chr01:113940         A>G         CTG>CCG      L>PYAL020C      -      0      UWOPS05_227_2 chr01:113946         A>G         GTG>GCG      V>AYAL020C      -      0      YS9           chr01:113981         G>A         TTC>TTT      F>FYAL020C      -      0      UWOPS05_227_2 chr01:113987         A>G         ACT>ACC      T>TYAL020C      -      0      UWOPS05_227_2 chr01:113994         A>G         CTG>CCG      L>PYAL020C      -      0      UWOPS83_787_3 chr01:113996         G>A         CGC>CGT      R>RYAL020C      -      0      UWOPS83_787_3 chr01:114002         G>A         TCC>TCT      S>SYAL020C      -      0      UWOPS83_787_3 chr01:114011         C>T         GTG>GTA      V>VYAL020C      -      0      UWOPS83_787_3 chr01:114023         G>A         GGC>GGT      G>GYAL020C      -      0      UWOPS05_227_2 chr01:114042         A>G         ATG>ACG      M>TYAL020C      -      0      UWOPS05_217_3 chr01:114071         T>C         GTA>GTG      V>VYAL020C      -      0      UWOPS05_227_2 chr01:114071         T>C         GTA>GTG      V>VYAL020C      -      0      Y55           chr01:114071         T>C         GTA>GTG      V>VYAL020C      -      0      UWOPS83_787_3 chr01:114076         C>G         GCC>CCC      A>PYAL020C      -      0      YJM981        chr01:114077         C>T         GTG>GTA      V>VYAL020C      -      0      K11           chr01:114103         C>T         GTA>ATA      V>IYAL020C      -      0      UWOPS05_217_3 chr01:114103         C>T         GTA>ATA      V>IYAL020C      -      0      UWOPS87_2421  chr01:114103         C>T         GTA>ATA      V>IYAL020C      -      0      Y12           chr01:114103         C>T         GTA>ATA      V>IYAL020C      -      0      Y55           chr01:114103         C>T         GTA>ATA      V>IYAL020C      -      0      YPS606        chr01:114103         C>T         GTA>ATA      V>IYAL020C      -      0      YS4           chr01:114103         C>T         GTA>ATA      V>IYAL020C      -      0      Y12           chr01:114113         C>T         CTG>CTA      L>LYAL020C      -      0      YS4           chr01:114113         C>T         CTG>CTA      L>LYAL020C      -      0      K11           chr01:114134         T>C         CAA>CAG      Q>QYAL020C      -      0      UWOPS05_217_3 chr01:114134         T>C         CAA>CAG      Q>QYAL020C      -      0      Y12           chr01:114134         T>C         CAA>CAG      Q>QYAL020C      -      0      Y55           chr01:114134         T>C         CAA>CAG      Q>QYAL020C      -      0      YPS606        chr01:114134         T>C         CAA>CAG      Q>QYAL020C      -      0      YS4           chr01:114134         T>C         CAA>CAG      Q>QYAL020C      -      0      K11           chr01:114149         T>G         ACA>ACC      T>TYAL020C      -      0      NCYC110       chr01:114149         T>G         ACA>ACC      T>TYAL020C      -      0      Y12           chr01:114149         T>G         ACA>ACC      T>TYAL020C      -      0      Y55           chr01:114149         T>G         ACA>ACC      T>TYAL020C      -      0      YPS606        chr01:114149         T>G         ACA>ACC      T>TYAL020C      -      0      YS4           chr01:114149         T>G         ACA>ACC      T>TYAL020C      -      0      K11           chr01:114228         T>C         GAG>GGG      E>GYAL020C      -      0      Y12           chr01:114228         T>C         GAG>GGG      E>GYAL020C      -      0      Y55           chr01:114228         T>C         GAG>GGG      E>GYAL020C      -      0      YPS606        chr01:114228         T>C         GAG>GGG      E>GYAL020C      -      0      YS4           chr01:114228         T>C         GAG>GGG      E>GYAL020C      -      0      NCYC110       chr01:114259         G>C         CAG>GAG      Q>EYAL020C      -      0      Y55           chr01:114259         G>C         CAG>GAG      Q>EYAL020C      -      0      YPS606        chr01:114259         G>C         CAG>GAG      Q>EYAL020C      -      0      YS4           chr01:114259         G>C         CAG>GAG      Q>EYAL020C      -      0      SK1           chr01:114284         T>C         AGA>AGG      R>RYAL020C      -      0      Y12           chr01:114358         C>A         GCA>TCA      A>SYAL020C      -      0      YPS128        chr01:114358         C>A         GCA>TCA      A>SYAL020C      -      0      YPS606        chr01:114358         C>A         GCA>TCA      A>SYAL020C      -      0      UWOPS05_217_3 chr01:114466         G>A         CTG>TTG      L>LYAL020C      -      0      UWOPS05_217_3 chr01:114556         C>A         GAG>TAG      E>*YAL020C      -      0      K11           chr01:114590         A>G         TCT>TCC      S>SYAL020C      -      0      Y12           chr01:114590         A>G         TCT>TCC      S>SYAL020C      -      0      YPS128        chr01:114590         A>G         TCT>TCC      S>SYAL020C      -      0      K11           chr01:113666         A>G         TCT>TCC      S>SYAL020C      -      0      YS4           chr01:113666         A>G         TCT>TCC      S>SYAL020C      -      0      K11           chr01:113689         G>A         CTG>TTG      L>LYAL022C      -      0      Y9            chr01:108881         C>T         AGG>AGA      R>RYAL022C      -      0      273614X       chr01:108882         C>G         AGG>ACG      R>TYAL022C      -      0      UWOPS03_461_4 chr01:108908         G>A         TTC>TTT      F>FYAL022C      -      0      UWOPS05_217_3 chr01:108908         G>A         TTC>TTT      F>FYAL022C      -      0      SK1           chr01:108929         G>A         GGC>GGT      G>GYAL022C      -      0      Y55           chr01:108944         T>G         ACA>ACC      T>TYAL022C      -      0      Y55           chr01:108968         T>C         GGA>GGG      G>GYAL022C      -      0      BC187         chr01:109001         C>T         CTG>CTA      L>LYAL022C      -      0      YJM975        chr01:109001         C>T         CTG>CTA      L>LYAL022C      -      0      YJM981        chr01:109001         C>T         CTG>CTA      L>LYAL022C      -      0      273614X       chr01:109151         C>G         ATG>ATC      M>IYAL022C      -      0      UWOPS03_461_4 chr01:109444         G>A         CTA>TTA      L>LYAL022C      -      0      UWOPS05_217_3 chr01:109444         G>A         CTA>TTA      L>LYAL022C      -      0      DBVPG6040     chr01:109448         G>C         CTC>CTG      L>LYAL022C      -      0      UWOPS05_217_3 chr01:109625         A>G         AGT>AGC      S>SYAL022C      -      0      UWOPS05_227_2 chr01:109625         A>G         AGT>AGC      S>SYAL022C      -      0      UWOPS05_217_3 chr01:109640         G>A         AGC>AGT      S>SYAL022C      -      0      UWOPS05_227_2 chr01:109640         G>A         AGC>AGT      S>SYAL022C      -      0      DBVPG1853     chr01:109669         C>T         GTG>ATG      V>MYAL022C      -      0      322134S       chr01:109703         G>A         GGC>GGT      G>GYAL022C      -      0      DBVPG1853     chr01:109721         A>C         TCT>TCG      S>SYAL022C      -      0      DBVPG6044     chr01:109721         A>C         TCT>TCG      S>SYAL022C      -      0      UWOPS05_227_2 chr01:109721         A>C         TCT>TCG      S>SYAL022C      -      0      UWOPS87_2421  chr01:109721         A>C         TCT>TCG      S>SYAL022C      -      0      Y55           chr01:109721         A>C         TCT>TCG      S>SYAL022C      -      0      273614X       chr01:109736         A>G         GCT>GCC      A>AYAL022C      -      0      322134S       chr01:109736         A>G         GCT>GCC      A>AYAL022C      -      0      DBVPG1373     chr01:109736         A>G         GCT>GCC      A>AYAL022C      -      0      DBVPG1788     chr01:109736         A>G         GCT>GCC      A>AYAL022C      -      0      DBVPG1853     chr01:109736         A>G         GCT>GCC      A>AYAL022C      -      0      DBVPG6040     chr01:109736         A>G         GCT>GCC      A>AYAL022C      -      0      DBVPG6044     chr01:109736         A>G         GCT>GCC      A>AYAL022C      -      0      DBVPG6765     chr01:109736         A>G         GCT>GCC      A>AYAL022C      -      0      L_1374        chr01:109736         A>G         GCT>GCC      A>AYAL022C      -      0      L_1528        chr01:109736         A>G         GCT>GCC      A>AYAL022C      -      0      SK1           chr01:109736         A>G         GCT>GCC      A>AYAL022C      -      0      UWOPS05_227_2 chr01:109736         A>G         GCT>GCC      A>AYAL022C      -      0      UWOPS87_2421  chr01:109736         A>G         GCT>GCC      A>AYAL022C      -      0      Y55           chr01:109736         A>G         GCT>GCC      A>AYAL022C      -      0      YIIc17_E5     chr01:109736         A>G         GCT>GCC      A>AYAL022C      -      0      YJM981        chr01:109736         A>G         GCT>GCC      A>AYAL022C      -      0      YS9           chr01:109736         A>G         GCT>GCC      A>AYAL022C      -      0      YPS606        chr01:109784         C>T         GGG>GGA      G>GYAL022C      -      0      DBVPG1853     chr01:109796         A>G         GGT>GGC      G>GYAL022C      -      0      DBVPG6044     chr01:109796         A>G         GGT>GGC      G>GYAL022C      -      0      UWOPS05_227_2 chr01:109796         A>G         GGT>GGC      G>GYAL022C      -      0      UWOPS87_2421  chr01:109796         A>G         GGT>GGC      G>GYAL022C      -      0      Y55           chr01:109796         A>G         GGT>GGC      G>GYAL022C      -      0      YPS606        chr01:109796         A>G         GGT>GGC      G>GYAL022C      -      0      273614X       chr01:109850         T>C         ACA>ACG      T>TYAL022C      -      0      UWOPS87_2421  chr01:109863         C>A         GGG>GTG      G>VYAL022C      -      0      DBVPG6044     chr01:109888         T>C         ATG>GTG      M>VYAL022C      -      0      UWOPS05_227_2 chr01:109888         T>C         ATG>GTG      M>VYAL022C      -      0      UWOPS87_2421  chr01:109888         T>C         ATG>GTG      M>VYAL022C      -      0      Y55           chr01:109888         T>C         ATG>GTG      M>VYAL022C      -      0      YPS606        chr01:109888         T>C         ATG>GTG      M>VYAL022C      -      0      DBVPG6044     chr01:109985         G>A         AAC>AAT      N>NYAL022C      -      0      Y55           chr01:109985         G>A         AAC>AAT      N>NYAL022C      -      0      DBVPG6044     chr01:110015         T>C         AGA>AGG      R>RYAL022C      -      0      NCYC110       chr01:110015         T>C         AGA>AGG      R>RYAL022C      -      0      UWOPS05_227_2 chr01:110015         T>C         AGA>AGG      R>RYAL022C      -      0      Y55           chr01:110015         T>C         AGA>AGG      R>RYAL022C      -      0      YPS606        chr01:110015         T>C         AGA>AGG      R>RYAL022C      -      0      DBVPG1788     chr01:110102         G>A         ACC>ACT      T>TYAL022C      -      0      DBVPG1853     chr01:110102         G>A         ACC>ACT      T>TYAL022C      -      0      DBVPG6044     chr01:110102         G>A         ACC>ACT      T>TYAL022C      -      0      DBVPG6765     chr01:110102         G>A         ACC>ACT      T>TYAL022C      -      0      NCYC110       chr01:110102         G>A         ACC>ACT      T>TYAL022C      -      0      UWOPS05_227_2 chr01:110102         G>A         ACC>ACT      T>TYAL022C      -      0      Y55           chr01:110102         G>A         ACC>ACT      T>TYAL022C      -      0      YJM978        chr01:110102         G>T         ACC>ACA      T>TYAL022C      -      0      YPS606        chr01:110102         G>A         ACC>ACT      T>TYAL022C      -      0      YS4           chr01:110102         G>A         ACC>ACT      T>TYAL022C      -      0      DBVPG1853     chr01:110132         T>C         CAA>CAG      Q>QYAL022C      -      0      DBVPG6044     chr01:110132         T>C         CAA>CAG      Q>QYAL022C      -      0      DBVPG6765     chr01:110132         T>C         CAA>CAG      Q>QYAL022C      -      0      NCYC110       chr01:110132         T>C         CAA>CAG      Q>QYAL022C      -      0      UWOPS05_227_2 chr01:110132         T>C         CAA>CAG      Q>QYAL022C      -      0      Y55           chr01:110132         T>C         CAA>CAG      Q>QYAL022C      -      0      YPS606        chr01:110132         T>C         CAA>CAG      Q>QYAL022C      -      0      DBVPG1853     chr01:110147         G>A         ATC>ATT      I>IYAL022C      -      0      DBVPG6044     chr01:110147         G>A         ATC>ATT      I>IYAL022C      -      0      DBVPG6765     chr01:110147         G>A         ATC>ATT      I>IYAL022C      -      0      NCYC110       chr01:110147         G>A         ATC>ATT      I>IYAL022C      -      0      UWOPS05_227_2 chr01:110147         G>A         ATC>ATT      I>IYAL022C      -      0      Y55           chr01:110147         G>A         ATC>ATT      I>IYAL022C      -      0      DBVPG6044     chr01:110201         T>G         TCA>TCC      S>SYAL022C      -      0      NCYC110       chr01:110201         T>G         TCA>TCC      S>SYAL022C      -      0      Y55           chr01:110201         T>G         TCA>TCC      S>SYAL022C      -      0      UWOPS05_217_3 chr01:110294         A>T         GAT>GAA      D>EYAL022C      -      0      UWOPS05_227_2 chr01:110294         A>T         GAT>GAA      D>EYAL022C      -      0      DBVPG1853     chr01:110381         C>T         GTG>GTA      V>VYAL023C      -      0      322134S       chr01:106296         T>G         ATT>CTT      I>LYAL023C      -      0      YS2           chr01:106354         A>G         TTT>TTC      F>FYAL023C      -      0      UWOPS03_461_4 chr01:106531         G>A         TAC>TAT      Y>YYAL023C      -      0      UWOPS05_217_3 chr01:106531         G>A         TAC>TAT      Y>YYAL023C      -      0      DBVPG6044     chr01:106582         T>A         CTA>CTT      L>LYAL023C      -      0      NCYC110       chr01:106582         T>A         CTA>CTT      L>LYAL023C      -      0      UWOPS03_461_4 chr01:106582         T>A         CTA>CTT      L>LYAL023C      -      0      UWOPS05_217_3 chr01:106582         T>A         CTA>CTT      L>LYAL023C      -      0      Y55           chr01:106582         T>A         CTA>CTT      L>LYAL023C      -      0      YPS128        chr01:106582         T>A         CTA>CTT      L>LYAL023C      -      0      L_1528        chr01:106665         T>C         ATC>GTC      I>VYAL023C      -      0      UWOPS87_2421  chr01:106681         C>T         ACG>ACA      T>TYAL023C      -      0      L_1374        chr01:106801         T>C         CCA>CCG      P>PYAL023C      -      0      YJM975        chr01:106801         T>C         CCA>CCG      P>PYAL023C      -      0      YJM981        chr01:106801         T>C         CCA>CCG      P>PYAL023C      -      0      UWOPS03_461_4 chr01:106890         A>G         TTA>CTA      L>LYAL023C      -      0      UWOPS05_217_3 chr01:106890         A>G         TTA>CTA      L>LYAL023C      -      0      273614X       chr01:106945         T>C         CCA>CCG      P>PYAL023C      -      0      DBVPG6040     chr01:106954         C>T         AGG>AGA      R>RYAL023C      -      0      NCYC110       chr01:106954         C>T         AGG>AGA      R>RYAL023C      -      0      Y55           chr01:106954         C>T         AGG>AGA      R>RYAL023C      -      0      YPS606        chr01:106954         C>T         AGG>AGA      R>RYAL023C      -      0      DBVPG6040     chr01:107002         C>T         AAG>AAA      K>KYAL023C      -      0      UWOPS03_461_4 chr01:107002         C>T         AAG>AAA      K>KYAL023C      -      0      UWOPS05_217_3 chr01:107002         C>T         AAG>AAA      K>KYAL023C      -      0      Y55           chr01:107002         C>T         AAG>AAA      K>KYAL023C      -      0      Y9            chr01:107002         C>T         AAG>AAA      K>KYAL023C      -      0      YPS128        chr01:107002         C>T         AAG>AAA      K>KYAL023C      -      0      YPS606        chr01:107002         C>T         AAG>AAA      K>KYAL023C      -      0      273614X       chr01:107029         C>T         GAG>GAA      E>EYAL023C      -      0      DBVPG6040     chr01:107029         C>T         GAG>GAA      E>EYAL023C      -      0      UWOPS03_461_4 chr01:107029         C>T         GAG>GAA      E>EYAL023C      -      0      UWOPS05_217_3 chr01:107029         C>T         GAG>GAA      E>EYAL023C      -      0      Y55           chr01:107029         C>T         GAG>GAA      E>EYAL023C      -      0      Y9            chr01:107029         C>T         GAG>GAA      E>EYAL023C      -      0      YPS128        chr01:107029         C>T         GAG>GAA      E>EYAL023C      -      0      YPS606        chr01:107029         C>T         GAG>GAA      E>EYAL023C      -      0      UWOPS03_461_4 chr01:107056         C>T         TTG>TTA      L>LYAL023C      -      0      UWOPS05_217_3 chr01:107056         C>T         TTG>TTA      L>LYAL023C      -      0      322134S       chr01:107061         T>A         AGT>TGT      S>CYAL023C      -      0      UWOPS05_217_3 chr01:107077         C>T         TTG>TTA      L>LYAL023C      -      0      Y9            chr01:107079         A>G         TTG>CTG      L>LYAL023C      -      0      YS4           chr01:107088         C>T         GGC>AGC      G>SYAL023C      -      0      Y9            chr01:107191         G>A         AAC>AAT      N>NYAL023C      -      0      SK1           chr01:107246         A>C         GTG>GGG      V>GYAL023C      -      0      UWOPS05_217_3 chr01:107413         A>G         TAT>TAC      Y>YYAL023C      -      0      UWOPS05_217_3 chr01:107464         T>C         TCA>TCG      S>SYAL023C      -      0      UWOPS05_217_3 chr01:107485         A>G         CTT>CTC      L>LYAL023C      -      0      DBVPG6040     chr01:107506         A>G         GTT>GTC      V>VYAL023C      -      0      UWOPS05_217_3 chr01:107506         A>G         GTT>GTC      V>VYAL023C      -      0      YS9           chr01:107506         A>G         GTT>GTC      V>VYAL023C      -      0      273614X       chr01:107539         G>A         GGC>GGT      G>GYAL023C      -      0      322134S       chr01:107539         G>A         GGC>GGT      G>GYAL023C      -      0      BC187         chr01:107539         G>A         GGC>GGT      G>GYAL023C      -      0      DBVPG1373     chr01:107539         G>A         GGC>GGT      G>GYAL023C      -      0      DBVPG1788     chr01:107539         G>A         GGC>GGT      G>GYAL023C      -      0      DBVPG6040     chr01:107539         G>A         GGC>GGT      G>GYAL023C      -      0      DBVPG6765     chr01:107539         G>A         GGC>GGT      G>GYAL023C      -      0      L_1374        chr01:107539         G>A         GGC>GGT      G>GYAL023C      -      0      SK1           chr01:107539         G>A         GGC>GGT      G>GYAL023C      -      0      UWOPS05_217_3 chr01:107539         G>A         GGC>GGT      G>GYAL023C      -      0      YJM975        chr01:107539         G>A         GGC>GGT      G>GYAL023C      -      0      YPS128        chr01:107539         G>A         GGC>GGT      G>GYAL023C      -      0      YPS606        chr01:107539         G>A         GGC>GGT      G>GYAL023C      -      0      YS2           chr01:107539         G>A         GGC>GGT      G>GYAL023C      -      0      YS9           chr01:107539         G>A         GGC>GGT      G>GYAL023C      -      0      378604X       chr01:107889         T>A         AAC>TAC      N>YYAL023C      -      0      322134S       chr01:107971         A>G         AGT>AGC      S>SYAL023C      -      0      DBVPG1788     chr01:107971         A>G         AGT>AGC      S>SYAL023C      -      0      SK1           chr01:107971         A>G         AGT>AGC      S>SYAL023C      -      0      Y55           chr01:107971         A>G         AGT>AGC      S>SYAL023C      -      0      YIIc17_E5     chr01:107971         A>G         AGT>AGC      S>SYAL023C      -      0      YJM975        chr01:107971         A>G         AGT>AGC      S>SYAL023C      -      0      YJM978        chr01:107971         A>G         AGT>AGC      S>SYAL023C      -      0      YPS128        chr01:107971         A>G         AGT>AGC      S>SYAL023C      -      0      YPS606        chr01:107971         A>G         AGT>AGC      S>SYAL023C      -      0      YS9           chr01:107971         A>G         AGT>AGC      S>SYAL023C      -      0      YPS128        chr01:108085         C>T         GCG>GCA      A>AYAL023C      -      0      YPS606        chr01:108085         C>T         GCG>GCA      A>AYAL023C      -      0      Y55           chr01:108109         A>G         TAT>TAC      Y>YYAL023C      -      0      YJM978        chr01:108109         A>G         TAT>TAC      Y>YYAL023C      -      0      YPS128        chr01:108109         A>G         TAT>TAC      Y>YYAL023C      -      0      YPS606        chr01:108109         A>G         TAT>TAC      Y>YYAL023C      -      0      YS4           chr01:108109         A>G         TAT>TAC      Y>YYAL023C      -      0      L_1374        chr01:108325         C>A         TTG>TTT      L>FYAL023C      -      0      Y55           chr01:108392         G>A         GCT>GTT      A>VYAL023C      -      0      UWOPS05_217_3 chr01:108427         C>T         GAG>GAA      E>EYAL023C      -      0      YPS128        chr01:108427         C>T         GAG>GAA      E>EYAL023C      -      0      SK1           chr01:108458         C>T         AGC>AAC      S>NYAL023C      -      0      Y55           chr01:108502         A>G         ATT>ATC      I>IYAL025C      -      0      DBVPG6040     chr01:100274         G>A         TAC>TAT      Y>YYAL025C      -      0      SK1           chr01:100274         G>A         TAC>TAT      Y>YYAL025C      -      0      Y9            chr01:100274         G>A         TAC>TAT      Y>YYAL025C      -      0      YS4           chr01:100274         G>A         TAC>TAT      Y>YYAL025C      -      0      273614X       chr01:100399         G>C         CAA>GAA      Q>EYAL025C      -      0      322134S       chr01:100399         G>C         CAA>GAA      Q>EYAL025C      -      0      378604X       chr01:100399         G>C         CAA>GAA      Q>EYAL025C      -      0      BC187         chr01:100399         G>C         CAA>GAA      Q>EYAL025C      -      0      DBVPG1373     chr01:100399         G>C         CAA>GAA      Q>EYAL025C      -      0      DBVPG1788     chr01:100399         G>C         CAA>GAA      Q>EYAL025C      -      0      DBVPG6040     chr01:100399         G>C         CAA>GAA      Q>EYAL025C      -      0      DBVPG6765     chr01:100399         G>C         CAA>GAA      Q>EYAL025C      -      0      L_1374        chr01:100399         G>C         CAA>GAA      Q>EYAL025C      -      0      L_1528        chr01:100399         G>C         CAA>GAA      Q>EYAL025C      -      0      UWOPS87_2421  chr01:100399         G>C         CAA>GAA      Q>EYAL025C      -      0      Y55           chr01:100399         G>C         CAA>GAA      Q>EYAL025C      -      0      Y9            chr01:100399         G>C         CAA>GAA      Q>EYAL025C      -      0      YJM975        chr01:100399         G>C         CAA>GAA      Q>EYAL025C      -      0      YPS128        chr01:100399         G>C         CAA>GAA      Q>EYAL025C      -      0      YPS606        chr01:100399         G>C         CAA>GAA      Q>EYAL025C      -      0      YS4           chr01:100399         G>C         CAA>GAA      Q>EYAL025C      -      0      YS9           chr01:100399         G>C         CAA>GAA      Q>EYAL025C      -      0      UWOPS87_2421  chr01:100517         C>T         GAG>GAA      E>EYAL025C      -      0      DBVPG6044     chr01:100688         C>T         TTG>TTA      L>LYAL025C      -      0      UWOPS83_787_3 chr01:100688         C>T         TTG>TTA      L>LYAL025C      -      0      Y55           chr01:100688         C>T         TTG>TTA      L>LYAL025C      -      0      YS9           chr01:100698         C>a         AGA>ATA      R>IYAL025C      -      0      DBVPG1853     chr01:100796         C>T         CAG>CAA      Q>QYAL025C      -      0      DBVPG6044     chr01:100841         C>T         AAG>AAA      K>KYAL025C      -      0      UWOPS83_787_3 chr01:100841         C>T         AAG>AAA      K>KYAL025C      -      0      Y55           chr01:100841         C>T         AAG>AAA      K>KYAL025C      -      0      DBVPG1853     chr01:101027         C>T         AGG>AGA      R>RYAL027W      +      0      W303          chr01:94749          T>C         TTT>TCT      F>SYAL027W      +      0      SK1           chr01:94768          C>G         ATC>ATG      I>MYAL027W      +      0      UWOPS87_2421  chr01:94768          C>G         ATC>ATG      I>MYAL027W      +      0      YPS606        chr01:94768          C>G         ATC>ATG      I>MYAL027W      +      0      YS4           chr01:94768          C>G         ATC>ATG      I>MYAL027W      +      0      DBVPG6044     chr01:94788          C>A         ACT>AAT      T>NYAL027W      +      0      UWOPS83_787_3 chr01:94788          C>A         ACT>AAT      T>NYAL027W      +      0      Y55           chr01:94788          C>A         ACT>AAT      T>NYAL027W      +      0      DBVPG6040     chr01:94864          A>G         CCA>CCG      P>PYAL027W      +      0      DBVPG6044     chr01:94864          A>G         CCA>CCG      P>PYAL027W      +      0      K11           chr01:94864          A>G         CCA>CCG      P>PYAL027W      +      0      SK1           chr01:94864          A>G         CCA>CCG      P>PYAL027W      +      0      UWOPS83_787_3 chr01:94864          A>G         CCA>CCG      P>PYAL027W      +      0      Y55           chr01:94864          A>G         CCA>CCG      P>PYAL027W      +      0      YPS606        chr01:94864          A>G         CCA>CCG      P>PYAL027W      +      0      YS4           chr01:94864          A>G         CCA>CCG      P>PYAL027W      +      0      DBVPG6040     chr01:94916          A>G         ATC>GTC      I>VYAL027W      +      0      DBVPG6044     chr01:94916          A>G         ATC>GTC      I>VYAL027W      +      0      K11           chr01:94916          A>G         ATC>GTC      I>VYAL027W      +      0      SK1           chr01:94916          A>G         ATC>GTC      I>VYAL027W      +      0      UWOPS83_787_3 chr01:94916          A>G         ATC>GTC      I>VYAL027W      +      0      UWOPS87_2421  chr01:94916          A>G         ATC>GTC      I>VYAL027W      +      0      Y55           chr01:94916          A>G         ATC>GTC      I>VYAL027W      +      0      YPS606        chr01:94916          A>G         ATC>GTC      I>VYAL027W      +      0      YS4           chr01:94916          A>G         ATC>GTC      I>VYAL027W      +      0      YIIc17_E5     chr01:94975          C>T         TCC>TCT      S>SYAL027W      +      0      DBVPG6040     chr01:95019          T>C         ATC>ACC      I>TYAL027W      +      0      DBVPG6044     chr01:95019          T>C         ATC>ACC      I>TYAL027W      +      0      K11           chr01:95019          T>C         ATC>ACC      I>TYAL027W      +      0      SK1           chr01:95019          T>C         ATC>ACC      I>TYAL027W      +      0      UWOPS83_787_3 chr01:95019          T>C         ATC>ACC      I>TYAL027W      +      0      UWOPS87_2421  chr01:95019          T>C         ATC>ACC      I>TYAL027W      +      0      Y55           chr01:95019          T>C         ATC>ACC      I>TYAL027W      +      0      YPS606        chr01:95019          T>C         ATC>ACC      I>TYAL027W      +      0      YS4           chr01:95019          T>C         ATC>ACC      I>TYAL027W      +      0      DBVPG6765     chr01:95020          C>G         ATC>ATG      I>MYAL027W      +      0      DBVPG6040     chr01:95044          A>T         GCA>GCT      A>AYAL027W      +      0      DBVPG6044     chr01:95044          A>T         GCA>GCT      A>AYAL027W      +      0      K11           chr01:95044          A>T         GCA>GCT      A>AYAL027W      +      0      SK1           chr01:95044          A>T         GCA>GCT      A>AYAL027W      +      0      UWOPS83_787_3 chr01:95044          A>T         GCA>GCT      A>AYAL027W      +      0      Y55           chr01:95044          A>T         GCA>GCT      A>AYAL027W      +      0      YPS606        chr01:95044          A>T         GCA>GCT      A>AYAL027W      +      0      YS4           chr01:95044          A>T         GCA>GCT      A>AYAL027W      +      0      DBVPG6040     chr01:95056          G>A         ACG>ACA      T>TYAL027W      +      0      DBVPG6044     chr01:95056          G>A         ACG>ACA      T>TYAL027W      +      0      K11           chr01:95056          G>A         ACG>ACA      T>TYAL027W      +      0      SK1           chr01:95056          G>A         ACG>ACA      T>TYAL027W      +      0      UWOPS03_461_4 chr01:95056          G>A         ACG>ACA      T>TYAL027W      +      0      UWOPS83_787_3 chr01:95056          G>A         ACG>ACA      T>TYAL027W      +      0      UWOPS87_2421  chr01:95056          G>A         ACG>ACA      T>TYAL027W      +      0      Y55           chr01:95056          G>A         ACG>ACA      T>TYAL027W      +      0      Y9            chr01:95056          G>A         ACG>ACA      T>TYAL027W      +      0      YPS606        chr01:95056          G>A         ACG>ACA      T>TYAL027W      +      0      DBVPG6040     chr01:95074          T>A         TCT>TCA      S>SYAL027W      +      0      DBVPG6044     chr01:95074          T>A         TCT>TCA      S>SYAL027W      +      0      K11           chr01:95074          T>A         TCT>TCA      S>SYAL027W      +      0      SK1           chr01:95074          T>A         TCT>TCA      S>SYAL027W      +      0      UWOPS83_787_3 chr01:95074          T>A         TCT>TCA      S>SYAL027W      +      0      Y55           chr01:95074          T>A         TCT>TCA      S>SYAL027W      +      0      YPS606        chr01:95074          T>A         TCT>TCA      S>SYAL027W      +      0      YS4           chr01:95074          T>A         TCT>TCA      S>SYAL027W      +      0      YS9           chr01:95074          T>A         TCT>TCA      S>SYAL027W      +      0      DBVPG1853     chr01:95117          T>C         TTG>CTG      L>LYAL027W      +      0      DBVPG6040     chr01:95117          T>C         TTG>CTG      L>LYAL027W      +      0      DBVPG6044     chr01:95117          T>C         TTG>CTG      L>LYAL027W      +      0      K11           chr01:95117          T>C         TTG>CTG      L>LYAL027W      +      0      SK1           chr01:95117          T>C         TTG>CTG      L>LYAL027W      +      0      UWOPS03_461_4 chr01:95117          T>C         TTG>CTG      L>LYAL027W      +      0      UWOPS83_787_3 chr01:95117          T>C         TTG>CTG      L>LYAL027W      +      0      Y55           chr01:95117          T>C         TTG>CTG      L>LYAL027W      +      0      Y9            chr01:95117          T>C         TTG>CTG      L>LYAL027W      +      0      YPS128        chr01:95117          T>C         TTG>CTG      L>LYAL027W      +      0      YPS606        chr01:95117          T>C         TTG>CTG      L>LYAL027W      +      0      YS4           chr01:95117          T>C         TTG>CTG      L>LYAL027W      +      0      YS9           chr01:95117          T>C         TTG>CTG      L>LYAL027W      +      0      YIIc17_E5     chr01:95124          A>C         TAC>TCC      Y>SYAL027W      +      0      DBVPG1853     chr01:95140          G>A         GAG>GAA      E>EYAL027W      +      0      DBVPG6040     chr01:95140          G>A         GAG>GAA      E>EYAL027W      +      0      DBVPG6044     chr01:95140          G>A         GAG>GAA      E>EYAL027W      +      0      K11           chr01:95140          G>A         GAG>GAA      E>EYAL027W      +      0      SK1           chr01:95140          G>A         GAG>GAA      E>EYAL027W      +      0      UWOPS03_461_4 chr01:95140          G>A         GAG>GAA      E>EYAL027W      +      0      UWOPS83_787_3 chr01:95140          G>A         GAG>GAA      E>EYAL027W      +      0      UWOPS87_2421  chr01:95140          G>A         GAG>GAA      E>EYAL027W      +      0      Y55           chr01:95140          G>A         GAG>GAA      E>EYAL027W      +      0      Y9            chr01:95140          G>A         GAG>GAA      E>EYAL027W      +      0      YPS606        chr01:95140          G>A         GAG>GAA      E>EYAL027W      +      0      YS4           chr01:95140          G>A         GAG>GAA      E>EYAL027W      +      0      YS9           chr01:95140          G>A         GAG>GAA      E>EYAL027W      +      0      DBVPG6044     chr01:95156          A>G         AAC>GAC      N>DYAL027W      +      0      Y55           chr01:95156          A>G         AAC>GAC      N>DYAL027W      +      0      DBVPG1853     chr01:95170          G>A         AAG>AAA      K>KYAL027W      +      0      DBVPG6040     chr01:95170          G>A         AAG>AAA      K>KYAL027W      +      0      DBVPG6044     chr01:95170          G>A         AAG>AAA      K>KYAL027W      +      0      K11           chr01:95170          G>A         AAG>AAA      K>KYAL027W      +      0      SK1           chr01:95170          G>A         AAG>AAA      K>KYAL027W      +      0      UWOPS03_461_4 chr01:95170          G>A         AAG>AAA      K>KYAL027W      +      0      UWOPS83_787_3 chr01:95170          G>A         AAG>AAA      K>KYAL027W      +      0      UWOPS87_2421  chr01:95170          G>A         AAG>AAA      K>KYAL027W      +      0      Y55           chr01:95170          G>A         AAG>AAA      K>KYAL027W      +      0      Y9            chr01:95170          G>A         AAG>AAA      K>KYAL027W      +      0      YPS128        chr01:95170          G>A         AAG>AAA      K>KYAL027W      +      0      YPS606        chr01:95170          G>A         AAG>AAA      K>KYAL027W      +      0      YS9           chr01:95170          G>A         AAG>AAA      K>KYAL027W      +      0      DBVPG1853     chr01:95191          T>C         TAT>TAC      Y>YYAL027W      +      0      DBVPG6040     chr01:95191          T>C         TAT>TAC      Y>YYAL027W      +      0      DBVPG6044     chr01:95191          T>C         TAT>TAC      Y>YYAL027W      +      0      K11           chr01:95191          T>C         TAT>TAC      Y>YYAL027W      +      0      SK1           chr01:95191          T>C         TAT>TAC      Y>YYAL027W      +      0      UWOPS03_461_4 chr01:95191          T>C         TAT>TAC      Y>YYAL027W      +      0      UWOPS83_787_3 chr01:95191          T>C         TAT>TAC      Y>YYAL027W      +      0      UWOPS87_2421  chr01:95191          T>C         TAT>TAC      Y>YYAL027W      +      0      Y55           chr01:95191          T>C         TAT>TAC      Y>YYAL027W      +      0      YPS128        chr01:95191          T>C         TAT>TAC      Y>YYAL027W      +      0      YPS606        chr01:95191          T>C         TAT>TAC      Y>YYAL027W      +      0      YS9           chr01:95191          T>C         TAT>TAC      Y>YYAL027W      +      0      DBVPG1853     chr01:95239          A>T         GGA>GGT      G>GYAL027W      +      0      DBVPG6040     chr01:95239          A>T         GGA>GGT      G>GYAL027W      +      0      DBVPG6044     chr01:95239          A>T         GGA>GGT      G>GYAL027W      +      0      K11           chr01:95239          A>T         GGA>GGT      G>GYAL027W      +      0      SK1           chr01:95239          A>T         GGA>GGT      G>GYAL027W      +      0      UWOPS83_787_3 chr01:95239          A>T         GGA>GGT      G>GYAL027W      +      0      UWOPS87_2421  chr01:95239          A>T         GGA>GGT      G>GYAL027W      +      0      Y55           chr01:95239          A>T         GGA>GGT      G>GYAL027W      +      0      Y9            chr01:95239          A>T         GGA>GGT      G>GYAL027W      +      0      YPS128        chr01:95239          A>T         GGA>GGT      G>GYAL027W      +      0      YPS606        chr01:95239          A>T         GGA>GGT      G>GYAL027W      +      0      YS9           chr01:95239          A>T         GGA>GGT      G>GYAL027W      +      0      UWOPS03_461_4 chr01:95282          G>A         GTA>ATA      V>IYAL027W      +      0      UWOPS87_2421  chr01:95282          G>A         GTA>ATA      V>IYAL027W      +      0      Y55           chr01:95306          G>T         GGG>TGG      G>WYAL027W      +      0      DBVPG1853     chr01:95328          C>T         ACA>ATA      T>IYAL027W      +      0      DBVPG6040     chr01:95328          C>T         ACA>ATA      T>IYAL027W      +      0      DBVPG6044     chr01:95328          C>T         ACA>ATA      T>IYAL027W      +      0      K11           chr01:95328          C>T         ACA>ATA      T>IYAL027W      +      0      SK1           chr01:95328          C>T         ACA>ATA      T>IYAL027W      +      0      Y55           chr01:95328          C>T         ACA>ATA      T>IYAL027W      +      0      Y9            chr01:95328          C>T         ACA>ATA      T>IYAL027W      +      0      YPS128        chr01:95328          C>T         ACA>ATA      T>IYAL027W      +      0      YPS606        chr01:95328          C>T         ACA>ATA      T>IYAL027W      +      0      YS4           chr01:95328          C>T         ACA>ATA      T>IYAL027W      +      0      UWOPS83_787_3 chr01:95332          G>A         CCG>CCA      P>PYAL027W      +      0      UWOPS03_461_4 chr01:95354          G>T         GTA>TTA      V>LYAL027W      +      0      DBVPG1853     chr01:95413          G>C         GTG>GTC      V>VYAL027W      +      0      DBVPG6040     chr01:95413          G>C         GTG>GTC      V>VYAL027W      +      0      DBVPG6044     chr01:95413          G>C         GTG>GTC      V>VYAL027W      +      0      K11           chr01:95413          G>C         GTG>GTC      V>VYAL027W      +      0      NCYC110       chr01:95413          G>C         GTG>GTC      V>VYAL027W      +      0      SK1           chr01:95413          G>C         GTG>GTC      V>VYAL027W      +      0      UWOPS03_461_4 chr01:95413          G>C         GTG>GTC      V>VYAL027W      +      0      UWOPS87_2421  chr01:95413          G>C         GTG>GTC      V>VYAL027W      +      0      Y55           chr01:95413          G>C         GTG>GTC      V>VYAL027W      +      0      Y9            chr01:95413          G>C         GTG>GTC      V>VYAL027W      +      0      YPS128        chr01:95413          G>C         GTG>GTC      V>VYAL027W      +      0      YPS606        chr01:95413          G>C         GTG>GTC      V>VYAL027W      +      0      YS4           chr01:95413          G>C         GTG>GTC      V>VYAL027W      +      0      DBVPG1853     chr01:95449          G>A         CAG>CAA      Q>QYAL027W      +      0      DBVPG6040     chr01:95449          G>A         CAG>CAA      Q>QYAL027W      +      0      K11           chr01:95449          G>A         CAG>CAA      Q>QYAL027W      +      0      NCYC110       chr01:95449          G>A         CAG>CAA      Q>QYAL027W      +      0      SK1           chr01:95449          G>A         CAG>CAA      Q>QYAL027W      +      0      UWOPS03_461_4 chr01:95449          G>A         CAG>CAA      Q>QYAL027W      +      0      UWOPS87_2421  chr01:95449          G>A         CAG>CAA      Q>QYAL027W      +      0      Y55           chr01:95449          G>A         CAG>CAA      Q>QYAL027W      +      0      Y9            chr01:95449          G>A         CAG>CAA      Q>QYAL027W      +      0      YPS128        chr01:95449          G>A         CAG>CAA      Q>QYAL027W      +      0      YS4           chr01:95449          G>A         CAG>CAA      Q>QYAL028W      +      0      DBVPG1853     chr01:92907          A>T         AAT>TAT      N>YYAL028W      +      0      DBVPG6044     chr01:92935          G>A         GGC>GAC      G>DYAL028W      +      0      K11           chr01:92935          G>A         GGC>GAC      G>DYAL028W      +      0      UWOPS05_217_3 chr01:92935          G>A         GGC>GAC      G>DYAL028W      +      0      UWOPS05_227_2 chr01:92935          G>A         GGC>GAC      G>DYAL028W      +      0      Y12           chr01:92935          G>A         GGC>GAC      G>DYAL028W      +      0      Y55           chr01:92935          G>A         GGC>GAC      G>DYAL028W      +      0      YPS606        chr01:92935          G>A         GGC>GAC      G>DYAL028W      +      0      DBVPG6044     chr01:92942          A>G         GGA>GGG      G>GYAL028W      +      0      NCYC110       chr01:92942          A>G         GGA>GGG      G>GYAL028W      +      0      Y12           chr01:92942          A>G         GGA>GGG      G>GYAL028W      +      0      Y55           chr01:92942          A>G         GGA>GGG      G>GYAL028W      +      0      YPS606        chr01:92942          A>G         GGA>GGG      G>GYAL028W      +      0      DBVPG6044     chr01:92956          A>G         GAT>GGT      D>GYAL028W      +      0      K11           chr01:92956          A>G         GAT>GGT      D>GYAL028W      +      0      NCYC110       chr01:92956          A>G         GAT>GGT      D>GYAL028W      +      0      Y12           chr01:92956          A>G         GAT>GGT      D>GYAL028W      +      0      Y55           chr01:92956          A>G         GAT>GGT      D>GYAL028W      +      0      YPS606        chr01:92956          A>G         GAT>GGT      D>GYAL028W      +      0      DBVPG6044     chr01:92984          G>A         AAG>AAA      K>KYAL028W      +      0      NCYC110       chr01:92984          G>A         AAG>AAA      K>KYAL028W      +      0      UWOPS05_217_3 chr01:92984          G>A         AAG>AAA      K>KYAL028W      +      0      UWOPS05_227_2 chr01:92984          G>A         AAG>AAA      K>KYAL028W      +      0      Y12           chr01:92984          G>A         AAG>AAA      K>KYAL028W      +      0      Y55           chr01:92984          G>A         AAG>AAA      K>KYAL028W      +      0      UWOPS03_461_4 chr01:93041          C>G         CAC>CAG      H>QYAL028W      +      0      UWOPS05_217_3 chr01:93041          C>G         CAC>CAG      H>QYAL028W      +      0      UWOPS05_227_2 chr01:93041          C>G         CAC>CAG      H>QYAL028W      +      0      Y9            chr01:93108          C>a         CAC>aAC      H>NYAL028W      +      0      Y12           chr01:93113          A>G         GAA>GAG      E>EYAL028W      +      0      Y12           chr01:93137          G>A         GAG>GAA      E>EYAL028W      +      0      DBVPG6044     chr01:93185          C>T         TAC>TAT      Y>YYAL028W      +      0      NCYC110       chr01:93185          C>T         TAC>TAT      Y>YYAL028W      +      0      UWOPS03_461_4 chr01:93185          C>T         TAC>TAT      Y>YYAL028W      +      0      UWOPS87_2421  chr01:93185          C>T         TAC>TAT      Y>YYAL028W      +      0      Y12           chr01:93185          C>T         TAC>TAT      Y>YYAL028W      +      0      Y55           chr01:93185          C>T         TAC>TAT      Y>YYAL028W      +      0      YPS606        chr01:93185          C>T         TAC>TAT      Y>YYAL028W      +      0      YS9           chr01:93282          C>T         CCA>TCA      P>SYAL028W      +      0      Y12           chr01:93311          C>G         ACC>ACG      T>TYAL028W      +      0      YPS606        chr01:93311          C>G         ACC>ACG      T>TYAL028W      +      0      NCYC361       chr01:93320          A>G         GGA>GGG      G>GYAL028W      +      0      Y12           chr01:93320          A>G         GGA>GGG      G>GYAL028W      +      0      YPS606        chr01:93320          A>G         GGA>GGG      G>GYAL028W      +      0      DBVPG6044     chr01:93323          A>G         CCA>CCG      P>PYAL028W      +      0      NCYC110       chr01:93323          A>G         CCA>CCG      P>PYAL028W      +      0      Y55           chr01:93323          A>G         CCA>CCG      P>PYAL028W      +      0      Y12           chr01:93326          C>T         TTC>TTT      F>FYAL028W      +      0      YPS606        chr01:93326          C>T         TTC>TTT      F>FYAL028W      +      0      YS9           chr01:93342          G>A         GAA>AAA      E>KYAL028W      +      0      NCYC361       chr01:93379          A>T         GAC>GTC      D>VYAL028W      +      0      NCYC361       chr01:93387          C>A         CCC>ACC      P>TYAL028W      +      0      DBVPG6044     chr01:93404          G>A         TTG>TTA      L>LYAL028W      +      0      NCYC110       chr01:93404          G>A         TTG>TTA      L>LYAL028W      +      0      Y55           chr01:93404          G>A         TTG>TTA      L>LYAL028W      +      0      DBVPG1373     chr01:93408          G>T         GCC>TCC      A>SYAL028W      +      0      UWOPS03_461_4 chr01:93425          C>T         GCC>GCT      A>AYAL028W      +      0      UWOPS87_2421  chr01:93425          C>T         GCC>GCT      A>AYAL028W      +      0      YPS606        chr01:93425          C>T         GCC>GCT      A>AYAL028W      +      0      UWOPS03_461_4 chr01:93444          A>G         AAC>GAC      N>DYAL028W      +      0      UWOPS03_461_4 chr01:93450          A>G         AAG>GAG      K>EYAL028W      +      0      UWOPS87_2421  chr01:93461          T>C         CTT>CTC      L>LYAL028W      +      0      L_1528        chr01:93465          T>A         TCA>ACA      S>TYAL028W      +      0      NCYC110       chr01:93467          A>G         TCA>TCG      S>SYAL028W      +      0      UWOPS03_461_4 chr01:93467          A>G         TCA>TCG      S>SYAL028W      +      0      UWOPS87_2421  chr01:93467          A>G         TCA>TCG      S>SYAL028W      +      0      Y12           chr01:93467          A>G         TCA>TCG      S>SYAL028W      +      0      Y55           chr01:93467          A>G         TCA>TCG      S>SYAL028W      +      0      YPS606        chr01:93467          A>G         TCA>TCG      S>SYAL028W      +      0      UWOPS87_2421  chr01:93554          G>A         AAG>AAA      K>KYAL028W      +      0      DBVPG1373     chr01:93564          G>A         GCA>ACA      A>TYAL028W      +      0      DBVPG1853     chr01:93564          G>A         GCA>ACA      A>TYAL028W      +      0      DBVPG6040     chr01:93564          G>A         GCA>ACA      A>TYAL028W      +      0      NCYC110       chr01:93564          G>A         GCA>ACA      A>TYAL028W      +      0      SK1           chr01:93564          G>A         GCA>ACA      A>TYAL028W      +      0      UWOPS03_461_4 chr01:93564          G>A         GCA>ACA      A>TYAL028W      +      0      UWOPS87_2421  chr01:93564          G>A         GCA>ACA      A>TYAL028W      +      0      Y55           chr01:93564          G>A         GCA>ACA      A>TYAL028W      +      0      Y9            chr01:93564          G>A         GCA>ACA      A>TYAL028W      +      0      YPS606        chr01:93564          G>A         GCA>ACA      A>TYAL028W      +      0      YS9           chr01:93564          G>A         GCA>ACA      A>TYAL028W      +      0      Y9            chr01:93574          C>T         CCG>CTG      P>LYAL028W      +      0      Y9            chr01:93603          A>G         ATA>GTA      I>VYAL028W      +      0      DBVPG6040     chr01:93611          G>A         AAG>AAA      K>KYAL028W      +      0      DBVPG1853     chr01:93675          A>G         ATA>GTA      I>VYAL028W      +      0      NCYC110       chr01:93734          G>A         TCG>TCA      S>SYAL028W      +      0      NCYC110       chr01:93746          G>A         CCG>CCA      P>PYAL028W      +      0      UWOPS87_2421  chr01:93752          T>A         TCT>TCA      S>SYAL028W      +      0      DBVPG6040     chr01:93757          C>T         GCT>GTT      A>VYAL028W      +      0      Y9            chr01:93757          C>T         GCT>GTT      A>VYAL028W      +      0      DBVPG1788     chr01:93790          T>A         ATT>AAT      I>NYAL028W      +      0      DBVPG1853     chr01:93790          T>A         ATT>AAT      I>NYAL028W      +      0      DBVPG6040     chr01:93790          T>A         ATT>AAT      I>NYAL028W      +      0      DBVPG6765     chr01:93790          T>A         ATT>AAT      I>NYAL028W      +      0      L_1528        chr01:93790          T>A         ATT>AAT      I>NYAL028W      +      0      UWOPS83_787_3 chr01:93790          T>A         ATT>AAT      I>NYAL028W      +      0      Y9            chr01:93790          T>A         ATT>AAT      I>NYAL028W      +      0      YIIc17_E5     chr01:93790          T>A         ATT>AAT      I>NYAL028W      +      0      YPS606        chr01:93790          T>A         ATT>AAT      I>NYAL028W      +      0      UWOPS83_787_3 chr01:93866          G>T         TCG>TCT      S>SYAL028W      +      0      Y55           chr01:93866          G>T         TCG>TCT      S>SYAL028W      +      0      DBVPG6040     chr01:93881          G>C         ACG>ACC      T>TYAL028W      +      0      DBVPG1373     chr01:93918          C>T         CCT>TCT      P>SYAL028W      +      0      YS9           chr01:93934          C>a         ACA>AaA      T>KYAL028W      +      0      Y55           chr01:94236          A>G         ACA>GCA      T>AYAL028W      +      0      YS4           chr01:94286          T>C         GCT>GCC      A>AYAL028W      +      0      DBVPG1373     chr01:94387          C>T         ACA>ATA      T>IYAL028W      +      0      YPS128        chr01:94415          C>G         GCC>GCG      A>AYAL028W      +      0      BC187         chr01:94473          G>A         GTA>ATA      V>IYAL028W      +      0      DBVPG1373     chr01:94473          G>A         GTA>ATA      V>IYAL028W      +      0      DBVPG1788     chr01:94473          G>A         GTA>ATA      V>IYAL028W      +      0      DBVPG6765     chr01:94473          G>A         GTA>ATA      V>IYAL028W      +      0      SK1           chr01:94473          G>A         GTA>ATA      V>IYAL028W      +      0      UWOPS83_787_3 chr01:94473          G>A         GTA>ATA      V>IYAL028W      +      0      UWOPS87_2421  chr01:94473          G>A         GTA>ATA      V>IYAL028W      +      0      Y55           chr01:94473          G>A         GTA>ATA      V>IYAL028W      +      0      YJM975        chr01:94473          G>A         GTA>ATA      V>IYAL028W      +      0      YPS128        chr01:94473          G>A         GTA>ATA      V>IYAL028W      +      0      YS4           chr01:94473          G>A         GTA>ATA      V>IYAL028W      +      0      YS9           chr01:94473          G>A         GTA>ATA      V>IYAL032C      -      0      DBVPG1853     chr01:83338          A>G         TAG>CAG      *>QYAL032C      -      0      DBVPG6044     chr01:83338          A>G         TAG>CAG      *>QYAL032C      -      0      NCYC110       chr01:83338          A>G         TAG>CAG      *>QYAL032C      -      0      UWOPS03_461_4 chr01:83338          A>G         TAG>CAG      *>QYAL032C      -      0      UWOPS05_217_3 chr01:83338          A>G         TAG>CAG      *>QYAL032C      -      0      UWOPS83_787_3 chr01:83338          A>G         TAG>CAG      *>QYAL032C      -      0      Y55           chr01:83338          A>G         TAG>CAG      *>QYAL032C      -      0      Y9            chr01:83338          A>G         TAG>CAG      *>QYAL032C      -      0      YPS128        chr01:83338          A>G         TAG>CAG      *>QYAL032C      -      0      YPS606        chr01:83338          A>G         TAG>CAG      *>QYAL032C      -      0      YS4           chr01:83338          A>G         TAG>CAG      *>QYAL032C      -      0      UWOPS03_461_4 chr01:83357          T>C         AAA>AAG      K>KYAL032C      -      0      DBVPG1853     chr01:83410          C>G         GCC>CCC      A>PYAL032C      -      0      DBVPG6044     chr01:83453          G>A         AAC>AAT      N>NYAL032C      -      0      NCYC110       chr01:83453          G>A         AAC>AAT      N>NYAL032C      -      0      Y55           chr01:83453          G>A         AAC>AAT      N>NYAL032C      -      0      DBVPG6044     chr01:83576          A>G         GAT>GAC      D>DYAL032C      -      0      UWOPS03_461_4 chr01:83576          A>G         GAT>GAC      D>DYAL032C      -      0      UWOPS05_217_3 chr01:83576          A>G         GAT>GAC      D>DYAL032C      -      0      Y55           chr01:83576          A>G         GAT>GAC      D>DYAL032C      -      0      Y9            chr01:83576          A>G         GAT>GAC      D>DYAL032C      -      0      DBVPG6044     chr01:83605          C>T         GGC>AGC      G>SYAL032C      -      0      Y55           chr01:83605          C>T         GGC>AGC      G>SYAL032C      -      0      273614X       chr01:83614          T>C         ATA>GTA      I>VYAL032C      -      0      322134S       chr01:83614          T>C         ATA>GTA      I>VYAL032C      -      0      DBVPG6044     chr01:83614          T>C         ATA>GTA      I>VYAL032C      -      0      SK1           chr01:83614          T>C         ATA>GTA      I>VYAL032C      -      0      UWOPS03_461_4 chr01:83614          T>C         ATA>GTA      I>VYAL032C      -      0      UWOPS05_217_3 chr01:83614          T>C         ATA>GTA      I>VYAL032C      -      0      UWOPS87_2421  chr01:83614          T>C         ATA>GTA      I>VYAL032C      -      0      Y12           chr01:83614          T>C         ATA>GTA      I>VYAL032C      -      0      Y55           chr01:83614          T>C         ATA>GTA      I>VYAL032C      -      0      Y9            chr01:83614          T>C         ATA>GTA      I>VYAL032C      -      0      YPS128        chr01:83614          T>C         ATA>GTA      I>VYAL032C      -      0      YS2           chr01:83614          T>C         ATA>GTA      I>VYAL032C      -      0      UWOPS03_461_4 chr01:83630          T>C         AGA>AGG      R>RYAL032C      -      0      UWOPS05_217_3 chr01:83630          T>C         AGA>AGG      R>RYAL032C      -      0      Y9            chr01:83721          T>C         AAC>AGC      N>SYAL032C      -      0      DBVPG6040     chr01:83791          C>a         GCT>TCT      A>SYAL032C      -      0      Y55           chr01:83798          C>T         AAG>AAA      K>KYAL032C      -      0      DBVPG6044     chr01:83904          T>C         GAC>GGC      D>GYAL032C      -      0      K11           chr01:83904          T>C         GAC>GGC      D>GYAL032C      -      0      Y55           chr01:83904          T>C         GAC>GGC      D>GYAL032C      -      0      Y9            chr01:83904          T>C         GAC>GGC      D>GYAL032C      -      0      K11           chr01:84047          G>A         GTC>GTT      V>VYAL032C      -      0      SK1           chr01:84047          G>A         GTC>GTT      V>VYAL032C      -      0      YS2           chr01:84077          C>T         AAG>AAA      K>KYAL032C      -      0      DBVPG6044     chr01:84109          A>G         TTG>CTG      L>LYAL032C      -      0      K11           chr01:84109          A>G         TTG>CTG      L>LYAL032C      -      0      UWOPS05_217_3 chr01:84109          A>G         TTG>CTG      L>LYAL032C      -      0      UWOPS05_227_2 chr01:84109          A>G         TTG>CTG      L>LYAL032C      -      0      UWOPS87_2421  chr01:84109          A>G         TTG>CTG      L>LYAL032C      -      0      Y55           chr01:84109          A>G         TTG>CTG      L>LYAL032C      -      0      YPS128        chr01:84109          A>G         TTG>CTG      L>LYAL032C      -      0      YS2           chr01:84109          A>G         TTG>CTG      L>LYAL032C      -      0      DBVPG6044     chr01:84168          T>C         AAT>AGT      N>SYAL032C      -      0      K11           chr01:84168          T>C         AAT>AGT      N>SYAL032C      -      0      Y12           chr01:84168          T>C         AAT>AGT      N>SYAL032C      -      0      Y55           chr01:84168          T>C         AAT>AGT      N>SYAL032C      -      0      YPS128        chr01:84168          T>C         AAT>AGT      N>SYAL032C      -      0      Y12           chr01:84320          T>C         GAA>GAG      E>EYAL032C      -      0      DBVPG6044     chr01:84350          A>G         GAT>GAC      D>DYAL032C      -      0      NCYC110       chr01:84350          A>G         GAT>GAC      D>DYAL032C      -      0      Y12           chr01:84350          A>G         GAT>GAC      D>DYAL032C      -      0      Y55           chr01:84350          A>G         GAT>GAC      D>DYAL032C      -      0      YPS128        chr01:84350          A>G         GAT>GAC      D>DYAL032C      -      0      YPS606        chr01:84350          A>G         GAT>GAC      D>DYAL032C      -      0      UWOPS05_227_2 chr01:84412          A>G         TCA>CCA      S>PYAL032C      -      0      UWOPS87_2421  chr01:84412          A>G         TCA>CCA      S>PYAL032C      -      0      DBVPG1853     chr01:84439          G>C         CAA>GAA      Q>EYAL032C      -      0      NCYC110       chr01:84439          G>C         CAA>GAA      Q>EYAL032C      -      0      UWOPS05_227_2 chr01:84439          G>C         CAA>GAA      Q>EYAL032C      -      0      UWOPS87_2421  chr01:84439          G>C         CAA>GAA      Q>EYAL032C      -      0      Y12           chr01:84439          G>C         CAA>GAA      Q>EYAL032C      -      0      Y55           chr01:84439          G>C         CAA>GAA      Q>EYAL032C      -      0      YPS128        chr01:84439          G>C         CAA>GAA      Q>EYAL032C      -      0      YPS606        chr01:84439          G>C         CAA>GAA      Q>EYAL033W      +      0      UWOPS83_787_3 chr01:82712          A>G         GTA>GTG      V>VYAL033W      +      0      Y55           chr01:82712          A>G         GTA>GTG      V>VYAL033W      +      0      YPS606        chr01:82712          A>G         GTA>GTG      V>VYAL033W      +      0      UWOPS03_461_4 chr01:82730          T>C         TAT>TAC      Y>YYAL033W      +      0      UWOPS05_227_2 chr01:82730          T>C         TAT>TAC      Y>YYAL033W      +      0      Y9            chr01:82970          C>T         TGC>TGT      C>CYAL033W      +      0      DBVPG6044     chr01:83030          C>T         GAC>GAT      D>DYAL033W      +      0      NCYC110       chr01:83030          C>T         GAC>GAT      D>DYAL033W      +      0      Y55           chr01:83030          C>T         GAC>GAT      D>DYAL033W      +      0      YPS606        chr01:83030          C>T         GAC>GAT      D>DYAL033W      +      0      DBVPG1853     chr01:83170          T>C         ATT>ACT      I>TYAL034C      -      0      SK1           chr01:80716          A>T         TTA>ATA      L>IYAL034C      -      0      SK1           chr01:80749          C>G         GGC>CGC      G>RYAL034C      -      0      322134S       chr01:80848          G>A         CTA>TTA      L>LYAL034C      -      0      DBVPG6044     chr01:80932          G>A         CTA>TTA      L>LYAL034C      -      0      NCYC110       chr01:80932          G>A         CTA>TTA      L>LYAL034C      -      0      Y55           chr01:80932          G>A         CTA>TTA      L>LYAL034C      -      0      DBVPG6044     chr01:80947          G>A         CTA>TTA      L>LYAL034C      -      0      K11           chr01:80947          G>A         CTA>TTA      L>LYAL034C      -      0      NCYC110       chr01:80947          G>A         CTA>TTA      L>LYAL034C      -      0      SK1           chr01:80947          G>A         CTA>TTA      L>LYAL034C      -      0      UWOPS03_461_4 chr01:80947          G>A         CTA>TTA      L>LYAL034C      -      0      Y55           chr01:80947          G>A         CTA>TTA      L>LYAL034C      -      0      322134S       chr01:81011          C>A         AAG>AAT      K>NYAL034C      -      0      SK1           chr01:81254          T>C         GGA>GGG      G>GYAL034C      -      0      SK1           chr01:81307          T>C         AGA>GGA      R>GYAL034C      -      0      DBVPG6044     chr01:81328          A>G         TTA>CTA      L>LYAL034C      -      0      NCYC110       chr01:81328          A>G         TTA>CTA      L>LYAL034C      -      0      Y55           chr01:81328          A>G         TTA>CTA      L>LYAL034C      -      0      UWOPS05_227_2 chr01:81342          C>A         AGA>ATA      R>IYAL034C      -      0      DBVPG6765     chr01:81366          G>A         CCT>CTT      P>LYAL034C      -      0      BC187         chr01:81443          G>A         AAC>AAT      N>NYAL034C      -      0      DBVPG1373     chr01:81443          G>A         AAC>AAT      N>NYAL034C      -      0      DBVPG1788     chr01:81443          G>A         AAC>AAT      N>NYAL034C      -      0      DBVPG6040     chr01:81443          G>A         AAC>AAT      N>NYAL034C      -      0      DBVPG6765     chr01:81443          G>A         AAC>AAT      N>NYAL034C      -      0      L_1374        chr01:81443          G>A         AAC>AAT      N>NYAL034C      -      0      YIIc17_E5     chr01:81443          G>A         AAC>AAT      N>NYAL034C      -      0      YJM981        chr01:81443          G>A         AAC>AAT      N>NYAL034C      -      0      YS9           chr01:81443          G>A         AAC>AAT      N>NYAL034C      -      0      K11           chr01:81605          T>C         TCA>TCG      S>SYAL034C      -      0      SK1           chr01:81605          T>C         TCA>TCG      S>SYAL034C      -      0      322134S       chr01:81613          C>T         GCC>ACC      A>TYAL034C      -      0      322134S       chr01:81685          G>T         CAT>AAT      H>NYAL034C      -      0      K11           chr01:81692          A>G         GTT>GTC      V>VYAL034C      -      0      SK1           chr01:81692          A>G         GTT>GTC      V>VYAL034C      -      0      K11           chr01:81732          T>C         AAT>AGT      N>SYAL034C      -      0      SK1           chr01:81732          T>C         AAT>AGT      N>SYAL034C      -      0      UWOPS83_787_3 chr01:81732          T>C         AAT>AGT      N>SYAL034C      -      0      Y55           chr01:81732          T>C         AAT>AGT      N>SYAL034C      -      0      YPS606        chr01:81732          T>C         AAT>AGT      N>SYAL034C      -      0      K11           chr01:81842          A>G         AGT>AGC      S>SYAL034C      -      0      SK1           chr01:81842          A>G         AGT>AGC      S>SYAL034C      -      0      UWOPS05_227_2 chr01:81842          A>G         AGT>AGC      S>SYAL034C      -      0      UWOPS83_787_3 chr01:81842          A>G         AGT>AGC      S>SYAL034C      -      0      UWOPS05_227_2 chr01:81867          C>T         CGT>CAT      R>HYAL034W-A    +      0      L_1374        chr01:79817          A>G         GAA>GAG      E>EYAL034W-A    +      0      UWOPS03_461_4 chr01:79861          C>T         TCC>TTC      S>FYAL034W-A    +      0      UWOPS05_217_3 chr01:79861          C>T         TCC>TTC      S>FYAL034W-A    +      0      UWOPS05_227_2 chr01:79861          C>T         TCC>TTC      S>FYAL034W-A    +      0      DBVPG6040     chr01:79925          A>G         GAA>GAG      E>EYAL034W-A    +      0      DBVPG6044     chr01:79925          A>G         GAA>GAG      E>EYAL034W-A    +      0      SK1           chr01:79925          A>G         GAA>GAG      E>EYAL034W-A    +      0      UWOPS03_461_4 chr01:79925          A>G         GAA>GAG      E>EYAL034W-A    +      0      UWOPS05_217_3 chr01:79925          A>G         GAA>GAG      E>EYAL034W-A    +      0      UWOPS05_227_2 chr01:79925          A>G         GAA>GAG      E>EYAL034W-A    +      0      Y55           chr01:79925          A>G         GAA>GAG      E>EYAL034W-A    +      0      Y9            chr01:79925          A>G         GAA>GAG      E>EYAL034W-A    +      0      YPS606        chr01:79925          A>G         GAA>GAG      E>EYAL034W-A    +      0      SK1           chr01:80051          T>C         GAT>GAC      D>DYAL034W-A    +      0      378604X       chr01:80070          A>G         AAT>GAT      N>DYAL034W-A    +      0      SK1           chr01:80078          G>A         TTG>TTA      L>LYAL034W-A    +      0      DBVPG6040     chr01:80086          G>C         AGT>ACT      S>TYAL034W-A    +      0      DBVPG6765     chr01:80109          G>C         GTG>CTG      V>LYAL034W-A    +      0      UWOPS05_227_2 chr01:80177          A>G         AGA>AGG      R>RYAL034W-A    +      0      NCYC361       chr01:80263          A>G         GAG>GGG      E>GYAL034W-A    +      0      378604X       chr01:80306          T>G         GAT>GAG      D>EYAL034W-A    +      0      DBVPG6044     chr01:80327          T>C         GTT>GTC      V>VYAL034W-A    +      0      UWOPS03_461_4 chr01:80327          T>C         GTT>GTC      V>VYAL034W-A    +      0      UWOPS05_227_2 chr01:80327          T>C         GTT>GTC      V>VYAL034W-A    +      0      Y55           chr01:80327          T>C         GTT>GTC      V>VYAL034W-A    +      0      DBVPG1373     chr01:80369          A>G         CTA>CTG      L>LYAL034W-A    +      0      K11           chr01:80555          G>A         GAG>GAA      E>EYAL034W-A    +      0      SK1           chr01:80555          G>A         GAG>GAA      E>EYAL034W-A    +      0      UWOPS03_461_4 chr01:80555          G>A         GAG>GAA      E>EYAL034W-A    +      0      Y55           chr01:80555          G>A         GAG>GAA      E>EYAL035W      +      0      273614X       chr01:76457          G>A         CAG>CAA      Q>QYAL035W      +      0      DBVPG6765     chr01:76457          G>A         CAG>CAA      Q>QYAL035W      +      0      L_1374        chr01:76457          G>A         CAG>CAA      Q>QYAL035W      +      0      L_1528        chr01:76457          G>A         CAG>CAA      Q>QYAL035W      +      0      YJM981        chr01:76457          G>A         CAG>CAA      Q>QYAL035W      +      0      DBVPG6044     chr01:76529          T>C         AAT>AAC      N>NYAL035W      +      0      Y55           chr01:76529          T>C         AAT>AAC      N>NYAL035W      +      0      YS4           chr01:76763          G>A         AGG>AGA      R>RYAL035W      +      0      YS9           chr01:76763          G>A         AGG>AGA      R>RYAL035W      +      0      K11           chr01:76841          G>A         GAG>GAA      E>EYAL035W      +      0      YPS606        chr01:76841          G>A         GAG>GAA      E>EYAL035W      +      0      K11           chr01:76862          C>T         TCC>TCT      S>SYAL035W      +      0      322134S       chr01:76934          T>C         GGT>GGC      G>GYAL035W      +      0      322134S       chr01:77000          A>G         AGA>AGG      R>RYAL035W      +      0      322134S       chr01:77074          A>G         AAA>AGA      K>RYAL035W      +      0      DBVPG6044     chr01:77074          A>G         AAA>AGA      K>RYAL035W      +      0      K11           chr01:77074          A>G         AAA>AGA      K>RYAL035W      +      0      NCYC110       chr01:77074          A>G         AAA>AGA      K>RYAL035W      +      0      Y55           chr01:77074          A>G         AAA>AGA      K>RYAL035W      +      0      YPS606        chr01:77074          A>G         AAA>AGA      K>RYAL035W      +      0      YS4           chr01:77074          A>G         AAA>AGA      K>RYAL035W      +      0      YS9           chr01:77074          A>G         AAA>AGA      K>RYAL035W      +      0      DBVPG1106     chr01:77116          G>T         CGA>CTA      R>LYAL035W      +      0      DBVPG1373     chr01:77116          G>T         CGA>CTA      R>LYAL035W      +      0      DBVPG6044     chr01:77116          G>T         CGA>CTA      R>LYAL035W      +      0      DBVPG6765     chr01:77116          G>T         CGA>CTA      R>LYAL035W      +      0      K11           chr01:77116          G>T         CGA>CTA      R>LYAL035W      +      0      L_1374        chr01:77116          G>T         CGA>CTA      R>LYAL035W      +      0      NCYC110       chr01:77116          G>T         CGA>CTA      R>LYAL035W      +      0      SK1           chr01:77116          G>T         CGA>CTA      R>LYAL035W      +      0      Y55           chr01:77116          G>T         CGA>CTA      R>LYAL035W      +      0      YIIc17_E5     chr01:77116          G>T         CGA>CTA      R>LYAL035W      +      0      YPS606        chr01:77116          G>T         CGA>CTA      R>LYAL035W      +      0      YS4           chr01:77116          G>T         CGA>CTA      R>LYAL035W      +      0      YS9           chr01:77116          G>T         CGA>CTA      R>LYAL035W      +      0      K11           chr01:77153          A>G         AAA>AAG      K>KYAL035W      +      0      YPS606        chr01:77153          A>G         AAA>AAG      K>KYAL035W      +      0      YS9           chr01:77227          C>T         GCC>GTC      A>VYAL035W      +      0      DBVPG6044     chr01:77434          G>A         GGA>GAA      G>EYAL035W      +      0      NCYC110       chr01:77434          G>A         GGA>GAA      G>EYAL035W      +      0      UWOPS03_461_4 chr01:77434          G>A         GGA>GAA      G>EYAL035W      +      0      Y55           chr01:77434          G>A         GGA>GAA      G>EYAL035W      +      0      Y9            chr01:77434          G>A         GGA>GAA      G>EYAL035W      +      0      YPS606        chr01:77434          G>A         GGA>GAA      G>EYAL035W      +      0      DBVPG6044     chr01:77555          T>G         CAT>CAG      H>QYAL035W      +      0      K11           chr01:77555          T>G         CAT>CAG      H>QYAL035W      +      0      NCYC110       chr01:77555          T>G         CAT>CAG      H>QYAL035W      +      0      UWOPS03_461_4 chr01:77555          T>G         CAT>CAG      H>QYAL035W      +      0      UWOPS05_227_2 chr01:77555          T>G         CAT>CAG      H>QYAL035W      +      0      Y55           chr01:77555          T>G         CAT>CAG      H>QYAL035W      +      0      Y9            chr01:77555          T>G         CAT>CAG      H>QYAL035W      +      0      YPS606        chr01:77555          T>G         CAT>CAG      H>QYAL035W      +      0      DBVPG6044     chr01:77582          T>A         GCT>GCA      A>AYAL035W      +      0      NCYC110       chr01:77582          T>A         GCT>GCA      A>AYAL035W      +      0      Y55           chr01:77582          T>A         GCT>GCA      A>AYAL035W      +      0      Y9            chr01:77582          T>A         GCT>GCA      A>AYAL035W      +      0      YPS606        chr01:77582          T>A         GCT>GCA      A>AYAL035W      +      0      UWOPS03_461_4 chr01:77609          C>T         TCC>TCT      S>SYAL035W      +      0      UWOPS05_227_2 chr01:77609          C>T         TCC>TCT      S>SYAL035W      +      0      DBVPG6044     chr01:77675          C>T         ACC>ACT      T>TYAL035W      +      0      NCYC110       chr01:77675          C>T         ACC>ACT      T>TYAL035W      +      0      Y55           chr01:77675          C>T         ACC>ACT      T>TYAL035W      +      0      YS9           chr01:77683          C>A         ACT>AAT      T>NYAL035W      +      0      DBVPG1853     chr01:77723          T>C         GGT>GGC      G>GYAL035W      +      0      DBVPG1853     chr01:77738          C>T         GGC>GGT      G>GYAL035W      +      0      DBVPG6044     chr01:77738          C>T         GGC>GGT      G>GYAL035W      +      0      K11           chr01:77738          C>T         GGC>GGT      G>GYAL035W      +      0      NCYC110       chr01:77738          C>T         GGC>GGT      G>GYAL035W      +      0      SK1           chr01:77738          C>T         GGC>GGT      G>GYAL035W      +      0      UWOPS03_461_4 chr01:77738          C>T         GGC>GGT      G>GYAL035W      +      0      UWOPS05_227_2 chr01:77738          C>T         GGC>GGT      G>GYAL035W      +      0      Y55           chr01:77738          C>T         GGC>GGT      G>GYAL035W      +      0      Y9            chr01:77738          C>T         GGC>GGT      G>GYAL035W      +      0      YPS606        chr01:77738          C>T         GGC>GGT      G>GYAL035W      +      0      YS2           chr01:77867          C>a         CAC>CAa      H>QYAL035W      +      0      Y9            chr01:77885          A>G         TTA>TTG      L>LYAL035W      +      0      YS2           chr01:77886          C>a         CGT>aGT      R>SYAL035W      +      0      322134S       chr01:77913          A>C         ATC>CTC      I>LYAL035W      +      0      K11           chr01:78188          T>C         AAT>AAC      N>NYAL035W      +      0      SK1           chr01:78188          T>C         AAT>AAC      N>NYAL035W      +      0      UWOPS03_461_4 chr01:78188          T>C         AAT>AAC      N>NYAL035W      +      0      UWOPS05_227_2 chr01:78188          T>C         AAT>AAC      N>NYAL035W      +      0      YPS128        chr01:78188          T>C         AAT>AAC      N>NYAL035W      +      0      YPS606        chr01:78188          T>C         AAT>AAC      N>NYAL035W      +      0      YS4           chr01:78188          T>C         AAT>AAC      N>NYAL035W      +      0      YPS128        chr01:78272          G>A         TTG>TTA      L>LYAL035W      +      0      YPS606        chr01:78272          G>A         TTG>TTA      L>LYAL035W      +      0      YPS128        chr01:78290          C>T         TCC>TCT      S>SYAL035W      +      0      YPS606        chr01:78290          C>T         TCC>TCT      S>SYAL035W      +      0      SK1           chr01:78347          A>G         GTA>GTG      V>VYAL035W      +      0      YPS128        chr01:78347          A>G         GTA>GTG      V>VYAL035W      +      0      YPS606        chr01:78347          A>G         GTA>GTG      V>VYAL035W      +      0      YS4           chr01:78347          A>G         GTA>GTG      V>VYAL035W      +      0      SK1           chr01:78393          T>C         TTG>CTG      L>LYAL035W      +      0      YS4           chr01:78393          T>C         TTG>CTG      L>LYAL035W      +      0      DBVPG6044     chr01:78462          C>T         CTA>TTA      L>LYAL035W      +      0      K11           chr01:78462          C>T         CTA>TTA      L>LYAL035W      +      0      NCYC110       chr01:78462          C>T         CTA>TTA      L>LYAL035W      +      0      Y55           chr01:78462          C>T         CTA>TTA      L>LYAL035W      +      0      Y9            chr01:78462          C>T         CTA>TTA      L>LYAL035W      +      0      YPS128        chr01:78462          C>T         CTA>TTA      L>LYAL035W      +      0      YPS606        chr01:78462          C>T         CTA>TTA      L>LYAL035W      +      0      YS4           chr01:78462          C>T         CTA>TTA      L>LYAL035W      +      0      DBVPG6044     chr01:78561          T>C         TTA>CTA      L>LYAL035W      +      0      K11           chr01:78561          T>C         TTA>CTA      L>LYAL035W      +      0      NCYC110       chr01:78561          T>C         TTA>CTA      L>LYAL035W      +      0      Y55           chr01:78561          T>C         TTA>CTA      L>LYAL035W      +      0      Y9            chr01:78561          T>C         TTA>CTA      L>LYAL035W      +      0      YPS128        chr01:78561          T>C         TTA>CTA      L>LYAL035W      +      0      YPS606        chr01:78561          T>C         TTA>CTA      L>LYAL035W      +      0      YS4           chr01:78561          T>C         TTA>CTA      L>LYAL035W      +      0      UWOPS03_461_4 chr01:78572          C>T         GCC>GCT      A>AYAL035W      +      0      UWOPS05_217_3 chr01:78572          C>T         GCC>GCT      A>AYAL035W      +      0      UWOPS03_461_4 chr01:78584          T>C         TCT>TCC      S>SYAL035W      +      0      UWOPS05_217_3 chr01:78584          T>C         TCT>TCC      S>SYAL035W      +      0      K11           chr01:78701          A>G         CAA>CAG      Q>QYAL035W      +      0      UWOPS03_461_4 chr01:78701          A>G         CAA>CAG      Q>QYAL035W      +      0      UWOPS05_217_3 chr01:78701          A>G         CAA>CAG      Q>QYAL035W      +      0      Y9            chr01:78701          A>G         CAA>CAG      Q>QYAL035W      +      0      YPS128        chr01:78701          A>G         CAA>CAG      Q>QYAL035W      +      0      YPS606        chr01:78701          A>G         CAA>CAG      Q>QYAL035W      +      0      DBVPG6044     chr01:78788          G>A         GTG>GTA      V>VYAL035W      +      0      NCYC110       chr01:78788          G>A         GTG>GTA      V>VYAL035W      +      0      Y55           chr01:78788          G>A         GTG>GTA      V>VYAL035W      +      0      DBVPG1853     chr01:78802          T>C         GTT>GCT      V>AYAL035W      +      0      UWOPS05_217_3 chr01:78812          C>T         GCC>GCT      A>AYAL035W      +      0      DBVPG1373     chr01:78884          T>A         GCT>GCA      A>AYAL035W      +      0      DBVPG6044     chr01:78911          T>C         ATT>ATC      I>IYAL035W      +      0      K11           chr01:78911          T>C         ATT>ATC      I>IYAL035W      +      0      Y55           chr01:78911          T>C         ATT>ATC      I>IYAL035W      +      0      Y9            chr01:78911          T>C         ATT>ATC      I>IYAL035W      +      0      YPS128        chr01:78911          T>C         ATT>ATC      I>IYAL035W      +      0      YS2           chr01:78911          T>C         ATT>ATC      I>IYAL035W      +      0      K11           chr01:78930          G>A         GTC>ATC      V>IYAL035W      +      0      Y9            chr01:78930          G>A         GTC>ATC      V>IYAL035W      +      0      YPS128        chr01:78930          G>A         GTC>ATC      V>IYAL035W      +      0      YS2           chr01:78995          A>G         AAA>AAG      K>KYAL035W      +      0      378604X       chr01:79013          T>C         GCT>GCC      A>AYAL035W      +      0      K11           chr01:79013          T>C         GCT>GCC      A>AYAL035W      +      0      Y9            chr01:79013          T>C         GCT>GCC      A>AYAL035W      +      0      YPS128        chr01:79013          T>C         GCT>GCC      A>AYAL035W      +      0      YS2           chr01:79013          T>C         GCT>GCC      A>AYAL035W      +      0      378604X       chr01:79086          C>T         CTG>TTG      L>LYAL035W      +      0      K11           chr01:79086          C>T         CTG>TTG      L>LYAL035W      +      0      Y9            chr01:79086          C>T         CTG>TTG      L>LYAL035W      +      0      YPS128        chr01:79086          C>T         CTG>TTG      L>LYAL035W      +      0      YS2           chr01:79086          C>T         CTG>TTG      L>LYAL035W      +      0      DBVPG6044     chr01:79178          C>T         GTC>GTT      V>VYAL036C      -      0      K11           chr01:75074          G>A         GAC>GAT      D>DYAL036C      -      0      SK1           chr01:75074          G>A         GAC>GAT      D>DYAL036C      -      0      UWOPS03_461_4 chr01:75074          G>A         GAC>GAT      D>DYAL036C      -      0      Y9            chr01:75074          G>A         GAC>GAT      D>DYAL036C      -      0      YPS606        chr01:75074          G>A         GAC>GAT      D>DYAL036C      -      0      W303          chr01:75092          C>a         TTG>TTT      L>FYAL036C      -      0      YS4           chr01:75139          G>A         CTG>TTG      L>LYAL036C      -      0      378604X       chr01:75212          T>C         AGA>AGG      R>RYAL036C      -      0      BC187         chr01:75212          T>C         AGA>AGG      R>RYAL036C      -      0      DBVPG1853     chr01:75212          T>C         AGA>AGG      R>RYAL036C      -      0      DBVPG6040     chr01:75212          T>C         AGA>AGG      R>RYAL036C      -      0      DBVPG6044     chr01:75212          T>C         AGA>AGG      R>RYAL036C      -      0      DBVPG6765     chr01:75212          T>C         AGA>AGG      R>RYAL036C      -      0      L_1528        chr01:75212          T>C         AGA>AGG      R>RYAL036C      -      0      UWOPS03_461_4 chr01:75212          T>C         AGA>AGG      R>RYAL036C      -      0      UWOPS05_227_2 chr01:75212          T>C         AGA>AGG      R>RYAL036C      -      0      Y55           chr01:75212          T>C         AGA>AGG      R>RYAL036C      -      0      Y9            chr01:75212          T>C         AGA>AGG      R>RYAL036C      -      0      YPS606        chr01:75212          T>C         AGA>AGG      R>RYAL036C      -      0      YS9           chr01:75212          T>C         AGA>AGG      R>RYAL036C      -      0      YS4           chr01:75233          A>G         TTT>TTC      F>FYAL036C      -      0      UWOPS03_461_4 chr01:75278          A>G         AAT>AAC      N>NYAL036C      -      0      UWOPS05_227_2 chr01:75278          A>G         AAT>AAC      N>NYAL036C      -      0      Y9            chr01:75278          A>G         AAT>AAC      N>NYAL036C      -      0      YPS606        chr01:75278          A>G         AAT>AAC      N>NYAL036C      -      0      YS4           chr01:75287          A>G         GAT>GAC      D>DYAL036C      -      0      YS4           chr01:75308          C>T         GAG>GAA      E>EYAL036C      -      0      DBVPG6044     chr01:75389          C>T         CTG>CTA      L>LYAL036C      -      0      Y55           chr01:75389          C>T         CTG>CTA      L>LYAL036C      -      0      DBVPG6765     chr01:75490          A>G         TTC>CTC      F>LYAL036C      -      0      SK1           chr01:75521          G>A         AGC>AGT      S>SYAL036C      -      0      DBVPG6044     chr01:75536          G>A         ATC>ATT      I>IYAL036C      -      0      Y55           chr01:75536          G>A         ATC>ATT      I>IYAL036C      -      0      UWOPS03_461_4 chr01:75548          C>T         GGG>GGA      G>GYAL036C      -      0      UWOPS05_227_2 chr01:75548          C>T         GGG>GGA      G>GYAL036C      -      0      Y9            chr01:75551          C>T         CTG>CTA      L>LYAL036C      -      0      DBVPG6044     chr01:75599          T>C         AAA>AAG      K>KYAL036C      -      0      Y55           chr01:75599          T>C         AAA>AAG      K>KYAL036C      -      0      DBVPG6044     chr01:75848          G>A         ACC>ACT      T>TYAL036C      -      0      UWOPS05_227_2 chr01:75857          G>A         ACC>ACT      T>TYAL036C      -      0      UWOPS05_227_2 chr01:75913          A>G         TTA>CTA      L>LYAL036C      -      0      DBVPG6044     chr01:76031          T>C         GAA>GAG      E>EYAL036C      -      0      Y55           chr01:76031          T>C         GAA>GAG      E>EYAL036C      -      0      DBVPG6044     chr01:76052          C>T         AAG>AAA      K>KYAL036C      -      0      Y55           chr01:76052          C>T         AAG>AAA      K>KYAL036C      -      0      YPS606        chr01:76052          C>T         AAG>AAA      K>KYAL036C      -      0      YPS606        chr01:76097          T>C         CAA>CAG      Q>QYAL036C      -      0      DBVPG6044     chr01:76118          T>C         GAA>GAG      E>EYAL036C      -      0      Y55           chr01:76118          T>C         GAA>GAG      E>EYAL036C      -      0      YPS606        chr01:76118          T>C         GAA>GAG      E>EYAL037W      +      0      273614X       chr01:74096          G>A         GGA>AGA      G>RYAL037W      +      0      273614X       chr01:74107          G>C         GAG>GAC      E>DYAL037W      +      0      273614X       chr01:74114          A>T         ACA>TCA      T>SYAL037W      +      0      DBVPG6040     chr01:74137          T>C         TAT>TAC      Y>YYAL037W      +      0      DBVPG1373     chr01:74140          A>G         GAA>GAG      E>EYAL037W      +      0      DBVPG1788     chr01:74140          A>G         GAA>GAG      E>EYAL037W      +      0      BC187         chr01:74157          T>G         ATT>AGT      I>SYAL037W      +      0      DBVPG1373     chr01:74157          T>G         ATT>AGT      I>SYAL037W      +      0      DBVPG1788     chr01:74157          T>G         ATT>AGT      I>SYAL037W      +      0      SK1           chr01:74191          G>C         TCG>TCC      S>SYAL037W      +      0      DBVPG6040     chr01:74228          G>A         GTC>ATC      V>IYAL037W      +      0      DBVPG6044     chr01:74228          G>A         GTC>ATC      V>IYAL037W      +      0      NCYC361       chr01:74228          G>A         GTC>ATC      V>IYAL037W      +      0      UWOPS05_227_2 chr01:74228          G>A         GTC>ATC      V>IYAL037W      +      0      UWOPS83_787_3 chr01:74228          G>A         GTC>ATC      V>IYAL037W      +      0      Y12           chr01:74228          G>A         GTC>ATC      V>IYAL037W      +      0      Y55           chr01:74228          G>A         GTC>ATC      V>IYAL037W      +      0      Y9            chr01:74228          G>A         GTC>ATC      V>IYAL037W      +      0      YIIc17_E5     chr01:74228          G>A         GTC>ATC      V>IYAL037W      +      0      YPS128        chr01:74228          G>A         GTC>ATC      V>IYAL037W      +      0      YJM981        chr01:74240          T>A         TGC>AGC      C>SYAL037W      +      0      DBVPG6040     chr01:74242          C>T         TGC>TGT      C>CYAL037W      +      0      NCYC361       chr01:74242          C>T         TGC>TGT      C>CYAL037W      +      0      UWOPS05_227_2 chr01:74242          C>T         TGC>TGT      C>CYAL037W      +      0      YIIc17_E5     chr01:74242          C>T         TGC>TGT      C>CYAL037W      +      0      DBVPG6044     chr01:74243          T>C         TCA>CCA      S>PYAL037W      +      0      Y12           chr01:74243          T>C         TCA>CCA      S>PYAL037W      +      0      Y55           chr01:74243          T>C         TCA>CCA      S>PYAL037W      +      0      Y9            chr01:74243          T>C         TCA>CCA      S>PYAL037W      +      0      YPS128        chr01:74243          T>C         TCA>CCA      S>PYAL037W      +      0      NCYC361       chr01:74305          T>C         AAT>AAC      N>NYAL037W      +      0      UWOPS05_227_2 chr01:74326          C>T         TTC>TTT      F>FYAL037W      +      0      DBVPG1106     chr01:74328          T>C         GTT>GCT      V>AYAL037W      +      0      L_1374        chr01:74328          T>C         GTT>GCT      V>AYAL037W      +      0      DBVPG6040     chr01:74335          G>A         CAG>CAA      Q>QYAL037W      +      0      NCYC361       chr01:74402          C>T         CTG>TTG      L>LYAL037W      +      0      BC187         chr01:74452          G>A         CAG>CAA      Q>QYAL037W      +      0      UWOPS05_227_2 chr01:74455          C>T         TAC>TAT      Y>YYAL037W      +      0      UWOPS05_227_2 chr01:74467          C>T         AAC>AAT      N>NYAL037W      +      0      UWOPS05_227_2 chr01:74491          A>G         TCA>TCG      S>SYAL037W      +      0      YPS128        chr01:74523          C>G         TCA>TGA      S>*YAL037W      +      0      K11           chr01:74539          T>C         GAT>GAC      D>DYAL037W      +      0      NCYC361       chr01:74539          T>C         GAT>GAC      D>DYAL037W      +      0      SK1           chr01:74539          T>C         GAT>GAC      D>DYAL037W      +      0      UWOPS05_227_2 chr01:74542          C>T         TGC>TGT      C>CYAL037W      +      0      DBVPG1106     chr01:74573          A>G         ATG>GTG      M>VYAL037W      +      0      YS9           chr01:74581          C>T         TAC>TAT      Y>YYAL037W      +      0      K11           chr01:74613          A>C         CAA>CCA      Q>PYAL037W      +      0      SK1           chr01:74646          G>A         AGT>AAT      S>NYAL037W      +      0      YS2           chr01:74675          G>A         GAT>AAT      D>NYAL037W      +      0      YS4           chr01:74675          G>A         GAT>AAT      D>NYAL037W      +      0      DBVPG1853     chr01:74787          G>A         AGA>AAA      R>KYAL037W      +      0      DBVPG6040     chr01:74787          G>A         AGA>AAA      R>KYAL037W      +      0      K11           chr01:74787          G>A         AGA>AAA      R>KYAL037W      +      0      SK1           chr01:74787          G>A         AGA>AAA      R>KYAL037W      +      0      UWOPS83_787_3 chr01:74787          G>A         AGA>AAA      R>KYAL037W      +      0      Y9            chr01:74787          G>A         AGA>AAA      R>KYAL037W      +      0      YPS606        chr01:74787          G>A         AGA>AAA      R>KYAL037W      +      0      YS2           chr01:74787          G>A         AGA>AAA      R>KYAL037W      +      0      YS4           chr01:74787          G>A         AGA>AAA      R>KYAL037W      +      0      DBVPG6044     chr01:74791          G>A         ATG>ATA      M>IYAL037W      +      0      Y55           chr01:74791          G>A         ATG>ATA      M>IYAL037W      +      0      UWOPS83_787_3 chr01:74815          T>C         GGT>GGC      G>GYAL038W      +      0      YS9           chr01:71841          A>G         AGA>GGA      R>GYAL038W      +      0      YJM978        chr01:72808          C>a         CCA>CaA      P>QYAL038W      +      0      YPS128        chr01:72863          C>T         ATC>ATT      I>IYAL038W      +      0      YPS606        chr01:72863          C>T         ATC>ATT      I>IYAL038W      +      0      SK1           chr01:72953          T>C         GCT>GCC      A>AYAL038W      +      0      YPS128        chr01:72953          T>C         GCT>GCC      A>AYAL038W      +      0      YPS606        chr01:72953          T>C         GCT>GCC      A>AYAL039C      -      0      DBVPG6044     chr01:68726          G>C         TCC>TCG      S>SYAL039C      -      0      SK1           chr01:68726          G>C         TCC>TCG      S>SYAL039C      -      0      UWOPS03_461_4 chr01:68726          G>C         TCC>TCG      S>SYAL039C      -      0      Y12           chr01:68726          G>C         TCC>TCG      S>SYAL039C      -      0      Y55           chr01:68726          G>C         TCC>TCG      S>SYAL039C      -      0      YPS606        chr01:68726          G>C         TCC>TCG      S>SYAL039C      -      0      YS4           chr01:68726          G>C         TCC>TCG      S>SYAL039C      -      0      DBVPG6044     chr01:68740          A>G         TCC>CCC      S>PYAL039C      -      0      SK1           chr01:68740          A>G         TCC>CCC      S>PYAL039C      -      0      UWOPS03_461_4 chr01:68740          A>G         TCC>CCC      S>PYAL039C      -      0      Y12           chr01:68740          A>G         TCC>CCC      S>PYAL039C      -      0      Y55           chr01:68740          A>G         TCC>CCC      S>PYAL039C      -      0      YPS606        chr01:68740          A>G         TCC>CCC      S>PYAL039C      -      0      YS4           chr01:68740          A>G         TCC>CCC      S>PYAL039C      -      0      YPS606        chr01:68761          C>T         GAC>AAC      D>NYAL039C      -      0      YJM978        chr01:68786          A>C         GCT>GCG      A>AYAL039C      -      0      DBVPG6044     chr01:68870          A>G         GAT>GAC      D>DYAL039C      -      0      SK1           chr01:68870          A>G         GAT>GAC      D>DYAL039C      -      0      Y12           chr01:68870          A>G         GAT>GAC      D>DYAL039C      -      0      Y55           chr01:68870          A>G         GAT>GAC      D>DYAL039C      -      0      YPS128        chr01:68870          A>G         GAT>GAC      D>DYAL039C      -      0      YPS606        chr01:68870          A>G         GAT>GAC      D>DYAL039C      -      0      YS4           chr01:68870          A>G         GAT>GAC      D>DYAL039C      -      0      DBVPG6044     chr01:68876          G>C         GTC>GTG      V>VYAL039C      -      0      Y55           chr01:68876          G>C         GTC>GTG      V>VYAL039C      -      0      SK1           chr01:68960          T>C         CCA>CCG      P>PYAL039C      -      0      YPS128        chr01:68960          T>C         CCA>CCG      P>PYAL039C      -      0      273614X       chr01:69065          A>G         CCT>CCC      P>PYAL039C      -      0      273614X       chr01:69113          T>C         GAA>GAG      E>EYAL039C      -      0      SK1           chr01:69113          T>C         GAA>GAG      E>EYAL039C      -      0      Y12           chr01:69113          T>C         GAA>GAG      E>EYAL039C      -      0      YPS128        chr01:69113          T>C         GAA>GAG      E>EYAL039C      -      0      YS4           chr01:69113          T>C         GAA>GAG      E>EYAL039C      -      0      UWOPS03_461_4 chr01:69125          C>G         GGG>GGC      G>GYAL039C      -      0      UWOPS05_217_3 chr01:69125          C>G         GGG>GGC      G>GYAL039C      -      0      SK1           chr01:69281          G>A         ATC>ATT      I>IYAL039C      -      0      Y12           chr01:69281          G>A         ATC>ATT      I>IYAL039C      -      0      YPS128        chr01:69281          G>A         ATC>ATT      I>IYAL039C      -      0      Y55           chr01:69290          G>T         ATC>ATA      I>IYAL039C      -      0      SK1           chr01:69404          C>T         GAG>GAA      E>EYAL039C      -      0      YJM978        chr01:69418          G>T         CCA>ACA      P>TYAL040C      -      0      322134S       chr01:65786          G>A         ACT>ATT      T>IYAL040C      -      0      UWOPS05_217_3 chr01:65801          C>T         AGC>AAC      S>NYAL040C      -      0      322134S       chr01:65872          G>A         CAC>CAT      H>HYAL040C      -      0      DBVPG1373     chr01:65872          G>A         CAC>CAT      H>HYAL040C      -      0      NCYC361       chr01:65872          G>A         CAC>CAT      H>HYAL040C      -      0      SK1           chr01:65872          G>A         CAC>CAT      H>HYAL040C      -      0      YJM981        chr01:65872          G>A         CAC>CAT      H>HYAL040C      -      0      322134S       chr01:65893          A>C         GTT>GTG      V>VYAL040C      -      0      DBVPG1373     chr01:65893          A>C         GTT>GTG      V>VYAL040C      -      0      NCYC361       chr01:65893          A>C         GTT>GTG      V>VYAL040C      -      0      SK1           chr01:65893          A>C         GTT>GTG      V>VYAL040C      -      0      YJM981        chr01:65893          A>C         GTT>GTG      V>VYAL040C      -      0      322134S       chr01:65911          T>C         TCA>TCG      S>SYAL040C      -      0      DBVPG1373     chr01:65911          T>C         TCA>TCG      S>SYAL040C      -      0      NCYC361       chr01:65911          T>C         TCA>TCG      S>SYAL040C      -      0      SK1           chr01:65911          T>C         TCA>TCG      S>SYAL040C      -      0      YJM981        chr01:65911          T>C         TCA>TCG      S>SYAL040C      -      0      322134S       chr01:65992          G>A         ATC>ATT      I>IYAL040C      -      0      DBVPG1373     chr01:65992          G>A         ATC>ATT      I>IYAL040C      -      0      DBVPG6765     chr01:65992          G>A         ATC>ATT      I>IYAL040C      -      0      NCYC361       chr01:65992          G>A         ATC>ATT      I>IYAL040C      -      0      SK1           chr01:65992          G>A         ATC>ATT      I>IYAL040C      -      0      UWOPS87_2421  chr01:65992          G>A         ATC>ATT      I>IYAL040C      -      0      YJM978        chr01:65992          G>A         ATC>ATT      I>IYAL040C      -      0      YJM981        chr01:65992          G>A         ATC>ATT      I>IYAL040C      -      0      YS4           chr01:65992          G>A         ATC>ATT      I>IYAL040C      -      0      322134S       chr01:66004          T>C         CAA>CAG      Q>QYAL040C      -      0      DBVPG1373     chr01:66004          T>C         CAA>CAG      Q>QYAL040C      -      0      DBVPG6044     chr01:66004          T>C         CAA>CAG      Q>QYAL040C      -      0      DBVPG6765     chr01:66004          T>C         CAA>CAG      Q>QYAL040C      -      0      NCYC361       chr01:66004          T>C         CAA>CAG      Q>QYAL040C      -      0      SK1           chr01:66004          T>C         CAA>CAG      Q>QYAL040C      -      0      UWOPS05_217_3 chr01:66004          T>C         CAA>CAG      Q>QYAL040C      -      0      UWOPS87_2421  chr01:66004          T>C         CAA>CAG      Q>QYAL040C      -      0      Y55           chr01:66004          T>C         CAA>CAG      Q>QYAL040C      -      0      Y9            chr01:66004          T>C         CAA>CAG      Q>QYAL040C      -      0      YJM978        chr01:66004          T>C         CAA>CAG      Q>QYAL040C      -      0      YJM981        chr01:66004          T>C         CAA>CAG      Q>QYAL040C      -      0      YPS128        chr01:66004          T>C         CAA>CAG      Q>QYAL040C      -      0      YPS606        chr01:66004          T>C         CAA>CAG      Q>QYAL040C      -      0      322134S       chr01:66034          A>G         AAT>AAC      N>NYAL040C      -      0      DBVPG1373     chr01:66034          A>G         AAT>AAC      N>NYAL040C      -      0      DBVPG6765     chr01:66034          A>G         AAT>AAC      N>NYAL040C      -      0      NCYC361       chr01:66034          A>G         AAT>AAC      N>NYAL040C      -      0      UWOPS87_2421  chr01:66034          A>G         AAT>AAC      N>NYAL040C      -      0      YJM978        chr01:66034          A>G         AAT>AAC      N>NYAL040C      -      0      YJM981        chr01:66034          A>G         AAT>AAC      N>NYAL040C      -      0      YS4           chr01:66034          A>G         AAT>AAC      N>NYAL040C      -      0      Y9            chr01:66044          G>C         TCT>TGT      S>CYAL040C      -      0      DBVPG6044     chr01:66130          A>G         TCT>TCC      S>SYAL040C      -      0      UWOPS05_217_3 chr01:66130          A>G         TCT>TCC      S>SYAL040C      -      0      Y55           chr01:66130          A>G         TCT>TCC      S>SYAL040C      -      0      Y9            chr01:66130          A>G         TCT>TCC      S>SYAL040C      -      0      YPS128        chr01:66130          A>G         TCT>TCC      S>SYAL040C      -      0      YPS606        chr01:66130          A>G         TCT>TCC      S>SYAL040C      -      0      DBVPG6044     chr01:66169          T>G         CCA>CCC      P>PYAL040C      -      0      UWOPS05_217_3 chr01:66169          T>G         CCA>CCC      P>PYAL040C      -      0      Y55           chr01:66169          T>G         CCA>CCC      P>PYAL040C      -      0      YPS128        chr01:66169          T>G         CCA>CCC      P>PYAL040C      -      0      YPS606        chr01:66169          T>G         CCA>CCC      P>PYAL040C      -      0      UWOPS05_217_3 chr01:66350          G>A         TCA>TTA      S>LYAL040C      -      0      YS4           chr01:66414          T>G         AAA>CAA      K>QYAL040C      -      0      YS9           chr01:66414          T>G         AAA>CAA      K>QYAL040C      -      0      NCYC361       chr01:66447          T>A         AAA>TAA      K>*YAL040C      -      0      YS4           chr01:66489          T>C         ATA>GTA      I>VYAL040C      -      0      YS9           chr01:66489          T>C         ATA>GTA      I>VYAL040C      -      0      YPS128        chr01:66547          C>T         GAG>GAA      E>EYAL040C      -      0      DBVPG6044     chr01:66576          G>C         CTT>GTT      L>VYAL040C      -      0      Y55           chr01:66576          G>C         CTT>GTT      L>VYAL040C      -      0      YS4           chr01:66691          C>T         ATG>ATA      M>IYAL040C      -      0      YS9           chr01:66691          C>T         ATG>ATA      M>IYAL040C      -      0      YJM981        chr01:66842          G>A         TCG>TTG      S>LYAL040C      -      0      DBVPG1373     chr01:66949          C>T         ACG>ACA      T>TYAL040C      -      0      YS2           chr01:66985          G>A         AAC>AAT      N>NYAL040C      -      0      YS4           chr01:66985          G>A         AAC>AAT      N>NYAL040C      -      0      YS9           chr01:66985          G>A         AAC>AAT      N>NYAL040C      -      0      DBVPG6044     chr01:67033          T>C         AAA>AAG      K>KYAL040C      -      0      NCYC110       chr01:67033          T>C         AAA>AAG      K>KYAL040C      -      0      Y55           chr01:67033          T>C         AAA>AAG      K>KYAL040C      -      0      YJM975        chr01:67129          A>C         CTT>CTG      L>LYAL040C      -      0      DBVPG6044     chr01:67263          T>C         ACA>GCA      T>AYAL040C      -      0      Y55           chr01:67263          T>C         ACA>GCA      T>AYAL040C      -      0      DBVPG1106     chr01:67270          A>G         CTT>CTC      L>LYAL040C      -      0      DBVPG1373     chr01:67270          A>G         CTT>CTC      L>LYAL040C      -      0      DBVPG1788     chr01:67270          A>G         CTT>CTC      L>LYAL040C      -      0      DBVPG6040     chr01:67270          A>G         CTT>CTC      L>LYAL040C      -      0      DBVPG6044     chr01:67270          A>G         CTT>CTC      L>LYAL040C      -      0      DBVPG6765     chr01:67270          A>G         CTT>CTC      L>LYAL040C      -      0      L_1528        chr01:67270          A>G         CTT>CTC      L>LYAL040C      -      0      NCYC110       chr01:67270          A>G         CTT>CTC      L>LYAL040C      -      0      UWOPS05_227_2 chr01:67270          A>G         CTT>CTC      L>LYAL040C      -      0      Y55           chr01:67270          A>G         CTT>CTC      L>LYAL040C      -      0      YJM981        chr01:67270          A>G         CTT>CTC      L>LYAL040C      -      0      YPS606        chr01:67270          A>G         CTT>CTC      L>LYAL040C      -      0      YS2           chr01:67270          A>G         CTT>CTC      L>LYAL040C      -      0      YS4           chr01:67270          A>G         CTT>CTC      L>LYAL040C      -      0      DBVPG1106     chr01:67276          G>A         TTC>TTT      F>FYAL040C      -      0      DBVPG1373     chr01:67276          G>A         TTC>TTT      F>FYAL040C      -      0      DBVPG1788     chr01:67276          G>A         TTC>TTT      F>FYAL040C      -      0      DBVPG6040     chr01:67276          G>A         TTC>TTT      F>FYAL040C      -      0      DBVPG6044     chr01:67276          G>A         TTC>TTT      F>FYAL040C      -      0      DBVPG6765     chr01:67276          G>A         TTC>TTT      F>FYAL040C      -      0      L_1528        chr01:67276          G>A         TTC>TTT      F>FYAL040C      -      0      NCYC110       chr01:67276          G>A         TTC>TTT      F>FYAL040C      -      0      UWOPS05_217_3 chr01:67276          G>A         TTC>TTT      F>FYAL040C      -      0      UWOPS05_227_2 chr01:67276          G>A         TTC>TTT      F>FYAL040C      -      0      Y55           chr01:67276          G>A         TTC>TTT      F>FYAL040C      -      0      YJM975        chr01:67276          G>A         TTC>TTT      F>FYAL040C      -      0      YJM981        chr01:67276          G>A         TTC>TTT      F>FYAL040C      -      0      YPS606        chr01:67276          G>A         TTC>TTT      F>FYAL040C      -      0      YS2           chr01:67276          G>A         TTC>TTT      F>FYAL040C      -      0      YS4           chr01:67276          G>A         TTC>TTT      F>FYAL040C      -      0      YPS606        chr01:67297          A>G         AAT>AAC      N>NYAL040C      -      0      UWOPS05_227_2 chr01:67342          T>C         AAA>AAG      K>KYAL040C      -      0      YS2           chr01:67370          G>T         GCA>GAA      A>EYAL040C      -      0      YS4           chr01:67370          G>T         GCA>GAA      A>EYAL040C      -      0      DBVPG6044     chr01:67374          T>C         AGT>GGT      S>GYAL040C      -      0      Y55           chr01:67374          T>C         AGT>GGT      S>GYAL040C      -      0      DBVPG1106     chr01:67422          A>C         TCA>GCA      S>AYAL040C      -      0      DBVPG1373     chr01:67422          A>C         TCA>GCA      S>AYAL040C      -      0      DBVPG1788     chr01:67422          A>C         TCA>GCA      S>AYAL040C      -      0      DBVPG6040     chr01:67422          A>C         TCA>GCA      S>AYAL040C      -      0      L_1528        chr01:67422          A>C         TCA>GCA      S>AYAL040C      -      0      YJM975        chr01:67422          A>C         TCA>GCA      S>AYAL040C      -      0      YJM981        chr01:67422          A>C         TCA>GCA      S>AYAL040C      -      0      DBVPG6044     chr01:67438          G>A         GCC>GCT      A>AYAL040C      -      0      Y55           chr01:67438          G>A         GCC>GCT      A>AYAL040C      -      0      DBVPG1106     chr01:67442          G>A         GCC>GTC      A>VYAL040C      -      0      DBVPG1373     chr01:67442          G>A         GCC>GTC      A>VYAL040C      -      0      DBVPG1788     chr01:67442          G>A         GCC>GTC      A>VYAL040C      -      0      DBVPG6040     chr01:67442          G>A         GCC>GTC      A>VYAL040C      -      0      DBVPG6765     chr01:67442          G>A         GCC>GTC      A>VYAL040C      -      0      L_1528        chr01:67442          G>A         GCC>GTC      A>VYAL040C      -      0      YJM975        chr01:67442          G>A         GCC>GTC      A>VYAL040C      -      0      YJM981        chr01:67442          G>A         GCC>GTC      A>VYAL040C      -      0      DBVPG1373     chr01:67477          C>G         AGG>AGC      R>SYAL040C      -      0      DBVPG1788     chr01:67477          C>G         AGG>AGC      R>SYAL040C      -      0      DBVPG6040     chr01:67477          C>G         AGG>AGC      R>SYAL040C      -      0      DBVPG6765     chr01:67477          C>G         AGG>AGC      R>SYAL040C      -      0      L_1528        chr01:67477          C>G         AGG>AGC      R>SYAL040C      -      0      SK1           chr01:67477          C>G         AGG>AGC      R>SYAL040C      -      0      UWOPS05_227_2 chr01:67477          C>G         AGG>AGC      R>SYAL040C      -      0      UWOPS83_787_3 chr01:67477          C>G         AGG>AGC      R>SYAL040C      -      0      YJM975        chr01:67477          C>G         AGG>AGC      R>SYAL040C      -      0      YPS606        chr01:67477          C>G         AGG>AGC      R>SYAL040C      -      0      YS2           chr01:67477          C>G         AGG>AGC      R>SYAL040C      -      0      YS4           chr01:67477          C>G         AGG>AGC      R>SYAL040C      -      0      DBVPG6044     chr01:67507          C>T         AAG>AAA      K>KYAL040C      -      0      UWOPS05_227_2 chr01:67507          C>T         AAG>AAA      K>KYAL040C      -      0      UWOPS83_787_3 chr01:67507          C>T         AAG>AAA      K>KYAL040C      -      0      Y55           chr01:67507          C>T         AAG>AAA      K>KYAL040C      -      0      YPS606        chr01:67507          C>T         AAG>AAA      K>KYAL041W      +      0      UWOPS03_461_4 chr01:62870          C>T         GGC>GGT      G>GYAL041W      +      0      273614X       chr01:62873          A>T         ACA>ACT      T>TYAL041W      +      0      273614X       chr01:62885          T>C         GAT>GAC      D>DYAL041W      +      0      Y55           chr01:62894          A>T         CCA>CCT      P>PYAL041W      +      0      YPS606        chr01:62894          A>T         CCA>CCT      P>PYAL041W      +      0      Y12           chr01:62951          C>T         GTC>GTT      V>VYAL041W      +      0      Y9            chr01:62951          C>T         GTC>GTT      V>VYAL041W      +      0      YS4           chr01:62951          C>T         GTC>GTT      V>VYAL041W      +      0      W303          chr01:62963          C>a         GAC>GAa      D>EYAL041W      +      0      273614X       chr01:62991          C>T         CGG>TGG      R>WYAL041W      +      0      SK1           chr01:62991          C>T         CGG>TGG      R>WYAL041W      +      0      YS4           chr01:63013          C>G         CCT>CGT      P>RYAL041W      +      0      Y12           chr01:63026          T>C         CCT>CCC      P>PYAL041W      +      0      Y55           chr01:63026          T>C         CCT>CCC      P>PYAL041W      +      0      Y9            chr01:63026          T>C         CCT>CCC      P>PYAL041W      +      0      YPS606        chr01:63026          T>C         CCT>CCC      P>PYAL041W      +      0      YS4           chr01:63026          T>C         CCT>CCC      P>PYAL041W      +      0      UWOPS03_461_4 chr01:63086          A>T         CTA>CTT      L>LYAL041W      +      0      DBVPG1373     chr01:63116          G>C         AAG>AAC      K>NYAL041W      +      0      YJM981        chr01:63118          C>T         TCC>TTC      S>FYAL041W      +      0      YJM981        chr01:63130          A>G         AAC>AGC      N>SYAL041W      +      0      273614X       chr01:63262          T>A         TTA>TAA      L>*YAL041W      +      0      UWOPS03_461_4 chr01:63308          C>T         GAC>GAT      D>DYAL041W      +      0      UWOPS05_217_3 chr01:63308          C>T         GAC>GAT      D>DYAL041W      +      0      378604X       chr01:63386          T>C         TTT>TTC      F>FYAL041W      +      0      DBVPG1853     chr01:63386          T>C         TTT>TTC      F>FYAL041W      +      0      DBVPG6044     chr01:63386          T>C         TTT>TTC      F>FYAL041W      +      0      UWOPS05_217_3 chr01:63386          T>C         TTT>TTC      F>FYAL041W      +      0      Y55           chr01:63386          T>C         TTT>TTC      F>FYAL041W      +      0      YPS128        chr01:63386          T>C         TTT>TTC      F>FYAL041W      +      0      YPS606        chr01:63386          T>C         TTT>TTC      F>FYAL041W      +      0      YS2           chr01:63386          T>C         TTT>TTC      F>FYAL041W      +      0      YS9           chr01:63386          T>C         TTT>TTC      F>FYAL041W      +      0      DBVPG1853     chr01:63473          A>C         GCA>GCC      A>AYAL041W      +      0      UWOPS05_217_3 chr01:63485          G>A         GAG>GAA      E>EYAL041W      +      0      322134S       chr01:63521          T>C         TCT>TCC      S>SYAL041W      +      0      378604X       chr01:63521          T>A         TCT>TCA      S>SYAL041W      +      0      DBVPG1373     chr01:63521          T>C         TCT>TCC      S>SYAL041W      +      0      DBVPG1853     chr01:63521          T>A         TCT>TCA      S>SYAL041W      +      0      DBVPG6040     chr01:63521          T>C         TCT>TCC      S>SYAL041W      +      0      DBVPG6044     chr01:63521          T>C         TCT>TCC      S>SYAL041W      +      0      UWOPS05_217_3 chr01:63521          T>C         TCT>TCC      S>SYAL041W      +      0      UWOPS83_787_3 chr01:63521          T>C         TCT>TCC      S>SYAL041W      +      0      Y55           chr01:63521          T>C         TCT>TCC      S>SYAL041W      +      0      YJM975        chr01:63521          T>C         TCT>TCC      S>SYAL041W      +      0      YJM978        chr01:63521          T>C         TCT>TCC      S>SYAL041W      +      0      YPS128        chr01:63521          T>C         TCT>TCC      S>SYAL041W      +      0      YPS606        chr01:63521          T>C         TCT>TCC      S>SYAL041W      +      0      YS2           chr01:63521          T>A         TCT>TCA      S>SYAL041W      +      0      YS9           chr01:63521          T>A         TCT>TCA      S>SYAL041W      +      0      L_1528        chr01:63530          G>A         CAG>CAA      Q>QYAL041W      +      0      UWOPS03_461_4 chr01:63575          C>T         AGC>AGT      S>SYAL041W      +      0      UWOPS05_217_3 chr01:63575          C>T         AGC>AGT      S>SYAL041W      +      0      DBVPG6044     chr01:63647          T>C         CCT>CCC      P>PYAL041W      +      0      Y55           chr01:63647          T>C         CCT>CCC      P>PYAL041W      +      0      378604X       chr01:63659          G>A         TCG>TCA      S>SYAL041W      +      0      DBVPG1853     chr01:63659          G>A         TCG>TCA      S>SYAL041W      +      0      DBVPG6044     chr01:63659          G>A         TCG>TCA      S>SYAL041W      +      0      UWOPS03_461_4 chr01:63659          G>A         TCG>TCA      S>SYAL041W      +      0      UWOPS05_217_3 chr01:63659          G>A         TCG>TCA      S>SYAL041W      +      0      Y55           chr01:63659          G>A         TCG>TCA      S>SYAL041W      +      0      Y9            chr01:63659          G>A         TCG>TCA      S>SYAL041W      +      0      YPS128        chr01:63659          G>A         TCG>TCA      S>SYAL041W      +      0      YPS606        chr01:63659          G>A         TCG>TCA      S>SYAL041W      +      0      YS9           chr01:63659          G>A         TCG>TCA      S>SYAL041W      +      0      UWOPS03_461_4 chr01:63692          G>A         AAG>AAA      K>KYAL041W      +      0      UWOPS05_217_3 chr01:63692          G>A         AAG>AAA      K>KYAL041W      +      0      UWOPS03_461_4 chr01:63710          A>T         GAA>GAT      E>DYAL041W      +      0      UWOPS05_217_3 chr01:63710          A>T         GAA>GAT      E>DYAL041W      +      0      DBVPG1373     chr01:63729          T>C         TTG>CTG      L>LYAL041W      +      0      UWOPS03_461_4 chr01:63761          A>G         TTA>TTG      L>LYAL041W      +      0      UWOPS05_217_3 chr01:63761          A>G         TTA>TTG      L>LYAL041W      +      0      Y9            chr01:63781          C>T         ACG>ATG      T>MYAL041W      +      0      YPS606        chr01:63821          T>C         GAT>GAC      D>DYAL041W      +      0      DBVPG6044     chr01:63870          T>C         TTA>CTA      L>LYAL041W      +      0      UWOPS03_461_4 chr01:63870          T>C         TTA>CTA      L>LYAL041W      +      0      UWOPS05_217_3 chr01:63870          T>C         TTA>CTA      L>LYAL041W      +      0      Y55           chr01:63870          T>C         TTA>CTA      L>LYAL041W      +      0      322134S       chr01:63936          T>A         TAT>AAT      Y>NYAL041W      +      0      SK1           chr01:63993          C>T         CAC>TAC      H>YYAL041W      +      0      UWOPS03_461_4 chr01:64067          A>G         AAA>AAG      K>KYAL041W      +      0      UWOPS05_217_3 chr01:64067          A>G         AAA>AAG      K>KYAL041W      +      0      Y9            chr01:64095          C>T         CTG>TTG      L>LYAL041W      +      0      DBVPG6044     chr01:64259          C>T         AAC>AAT      N>NYAL041W      +      0      Y55           chr01:64259          C>T         AAC>AAT      N>NYAL041W      +      0      YS2           chr01:64316          C>T         TTC>TTT      F>FYAL041W      +      0      K11           chr01:64478          C>T         AAC>AAT      N>NYAL041W      +      0      YS2           chr01:64478          C>T         AAC>AAT      N>NYAL041W      +      0      BC187         chr01:64484          G>A         ACG>ACA      T>TYAL041W      +      0      DBVPG1373     chr01:64484          G>A         ACG>ACA      T>TYAL041W      +      0      YJM981        chr01:64484          G>A         ACG>ACA      T>TYAL041W      +      0      YS9           chr01:64496          C>a         GGC>GGa      G>GYAL041W      +      0      SK1           chr01:64500          C>T         CCT>TCT      P>SYAL041W      +      0      DBVPG6044     chr01:64664          A>G         GAA>GAG      E>EYAL041W      +      0      K11           chr01:64664          A>G         GAA>GAG      E>EYAL041W      +      0      Y55           chr01:64664          A>G         GAA>GAG      E>EYAL041W      +      0      YS2           chr01:64664          A>G         GAA>GAG      E>EYAL041W      +      0      DBVPG6044     chr01:64677          C>T         CTA>TTA      L>LYAL041W      +      0      K11           chr01:64677          C>T         CTA>TTA      L>LYAL041W      +      0      UWOPS03_461_4 chr01:64677          C>T         CTA>TTA      L>LYAL041W      +      0      UWOPS05_217_3 chr01:64677          C>T         CTA>TTA      L>LYAL041W      +      0      UWOPS05_227_2 chr01:64677          C>T         CTA>TTA      L>LYAL041W      +      0      Y55           chr01:64677          C>T         CTA>TTA      L>LYAL041W      +      0      YPS606        chr01:64677          C>T         CTA>TTA      L>LYAL041W      +      0      YS2           chr01:64677          C>T         CTA>TTA      L>LYAL041W      +      0      YS4           chr01:64677          C>T         CTA>TTA      L>LYAL041W      +      0      UWOPS03_461_4 chr01:64718          A>C         ATA>ATC      I>IYAL041W      +      0      UWOPS05_217_3 chr01:64718          A>C         ATA>ATC      I>IYAL041W      +      0      UWOPS05_227_2 chr01:64718          A>C         ATA>ATC      I>IYAL041W      +      0      DBVPG6044     chr01:64782          T>C         TTG>CTG      L>LYAL041W      +      0      K11           chr01:64782          T>C         TTG>CTG      L>LYAL041W      +      0      UWOPS05_227_2 chr01:64782          T>C         TTG>CTG      L>LYAL041W      +      0      Y55           chr01:64782          T>C         TTG>CTG      L>LYAL041W      +      0      YPS606        chr01:64782          T>C         TTG>CTG      L>LYAL041W      +      0      YS2           chr01:64782          T>C         TTG>CTG      L>LYAL041W      +      0      YS4           chr01:64782          T>C         TTG>CTG      L>LYAL041W      +      0      BC187         chr01:64796          T>C         AAT>AAC      N>NYAL041W      +      0      YS4           chr01:64894          C>G         ACG>AGG      T>RYAL041W      +      0      UWOPS05_217_3 chr01:64907          A>C         ACA>ACC      T>TYAL041W      +      0      UWOPS05_227_2 chr01:64907          A>C         ACA>ACC      T>TYAL041W      +      0      YIIc17_E5     chr01:64908          G>A         GCC>ACC      A>TYAL041W      +      0      YS2           chr01:64908          G>A         GCC>ACC      A>TYAL041W      +      0      YS4           chr01:64908          G>A         GCC>ACC      A>TYAL041W      +      0      DBVPG6044     chr01:64916          A>C         TCA>TCC      S>SYAL041W      +      0      Y55           chr01:64916          A>C         TCA>TCC      S>SYAL041W      +      0      UWOPS05_217_3 chr01:64934          C>T         CCC>CCT      P>PYAL041W      +      0      UWOPS05_227_2 chr01:64934          C>T         CCC>CCT      P>PYAL041W      +      0      BC187         chr01:64937          C>G         ACC>ACG      T>TYAL041W      +      0      DBVPG1373     chr01:65032          T>C         GTC>GCC      V>AYAL041W      +      0      YIIc17_E5     chr01:65037          C>T         CCT>TCT      P>SYAL041W      +      0      YS2           chr01:65037          C>T         CCT>TCT      P>SYAL041W      +      0      DBVPG6044     chr01:65135          G>A         AGG>AGA      R>RYAL041W      +      0      Y55           chr01:65135          G>A         AGG>AGA      R>RYAL041W      +      0      UWOPS05_227_2 chr01:65159          T>C         AAT>AAC      N>NYAL041W      +      0      DBVPG6044     chr01:65173          G>A         AGC>AAC      S>NYAL041W      +      0      Y55           chr01:65173          G>A         AGC>AAC      S>NYAL041W      +      0      UWOPS05_227_2 chr01:65345          T>A         AAT>AAA      N>KYAL041W      +      0      273614X       chr01:65366          G>A         GCG>GCA      A>AYAL041W      +      0      322134S       chr01:65366          G>A         GCG>GCA      A>AYAL041W      +      0      SK1           chr01:65366          G>A         GCG>GCA      A>AYAL041W      +      0      UWOPS05_227_2 chr01:65366          G>A         GCG>GCA      A>AYAL041W      +      0      Y55           chr01:65366          G>A         GCG>GCA      A>AYAL041W      +      0      YJM975        chr01:65366          G>A         GCG>GCA      A>AYAL041W      +      0      YPS128        chr01:65366          G>A         GCG>GCA      A>AYAL041W      +      0      YS9           chr01:65366          G>A         GCG>GCA      A>AYAL042W      +      0      SK1           chr01:61415          C>T         TGC>TGT      C>CYAL042W      +      0      322134S       chr01:61428          T>C         TTA>CTA      L>LYAL042W      +      0      DBVPG6044     chr01:61452          G>A         GGA>AGA      G>RYAL042W      +      0      SK1           chr01:61452          G>A         GGA>AGA      G>RYAL042W      +      0      UWOPS03_461_4 chr01:61452          G>A         GGA>AGA      G>RYAL042W      +      0      UWOPS05_217_3 chr01:61452          G>A         GGA>AGA      G>RYAL042W      +      0      UWOPS05_227_2 chr01:61452          G>A         GGA>AGA      G>RYAL042W      +      0      Y55           chr01:61452          G>A         GGA>AGA      G>RYAL042W      +      0      YPS128        chr01:61452          G>A         GGA>AGA      G>RYAL042W      +      0      YPS606        chr01:61452          G>A         GGA>AGA      G>RYAL042W      +      0      YS4           chr01:61452          G>A         GGA>AGA      G>RYAL042W      +      0      YS4           chr01:61459          T>A         TTC>TAC      F>YYAL042W      +      0      DBVPG6044     chr01:61586          C>T         GAC>GAT      D>DYAL042W      +      0      UWOPS03_461_4 chr01:61586          C>T         GAC>GAT      D>DYAL042W      +      0      UWOPS05_217_3 chr01:61586          C>T         GAC>GAT      D>DYAL042W      +      0      UWOPS05_227_2 chr01:61586          C>T         GAC>GAT      D>DYAL042W      +      0      UWOPS83_787_3 chr01:61586          C>T         GAC>GAT      D>DYAL042W      +      0      Y55           chr01:61586          C>T         GAC>GAT      D>DYAL042W      +      0      YPS128        chr01:61586          C>T         GAC>GAT      D>DYAL042W      +      0      YPS606        chr01:61586          C>T         GAC>GAT      D>DYAL042W      +      0      YS9           chr01:61646          C>a         AGC>AGa      S>RYAL042W      +      0      322134S       chr01:61681          A>G         CAT>CGT      H>RYAL042W      +      0      YS4           chr01:61709          G>T         GCG>GCT      A>AYAL042W      +      0      UWOPS83_787_3 chr01:61754          C>G         GCC>GCG      A>AYAL042W      +      0      UWOPS03_461_4 chr01:61769          G>A         CAG>CAA      Q>QYAL042W      +      0      UWOPS05_217_3 chr01:61769          G>A         CAG>CAA      Q>QYAL042W      +      0      UWOPS05_227_2 chr01:61769          G>A         CAG>CAA      Q>QYAL042W      +      0      UWOPS83_787_3 chr01:61811          C>T         GAC>GAT      D>DYAL042W      +      0      DBVPG6765     chr01:61901          C>T         GTC>GTT      V>VYAL042W      +      0      K11           chr01:61991          T>C         CCT>CCC      P>PYAL042W      +      0      UWOPS05_217_3 chr01:61991          T>C         CCT>CCC      P>PYAL042W      +      0      UWOPS05_227_2 chr01:61991          T>C         CCT>CCC      P>PYAL042W      +      0      UWOPS83_787_3 chr01:61991          T>C         CCT>CCC      P>PYAL042W      +      0      SK1           chr01:62000          C>G         CCC>CCG      P>PYAL042W      +      0      K11           chr01:62054          G>T         TCG>TCT      S>SYAL042W      +      0      DBVPG6044     chr01:62111          C>T         TCC>TCT      S>SYAL042W      +      0      Y55           chr01:62111          C>T         TCC>TCT      S>SYAL042W      +      0      Y9            chr01:62204          C>a         AAC>AAa      N>KYAL042W      +      0      Y9            chr01:62206          C>a         ACA>AaA      T>KYAL042W      +      0      DBVPG6044     chr01:62309          T>C         CAT>CAC      H>HYAL042W      +      0      K11           chr01:62309          T>C         CAT>CAC      H>HYAL042W      +      0      SK1           chr01:62309          T>C         CAT>CAC      H>HYAL042W      +      0      UWOPS03_461_4 chr01:62309          T>C         CAT>CAC      H>HYAL042W      +      0      UWOPS05_217_3 chr01:62309          T>C         CAT>CAC      H>HYAL042W      +      0      Y55           chr01:62309          T>C         CAT>CAC      H>HYAL042W      +      0      YIIc17_E5     chr01:62309          T>C         CAT>CAC      H>HYAL042W      +      0      YPS606        chr01:62309          T>C         CAT>CAC      H>HYAL042W      +      0      YS4           chr01:62309          T>C         CAT>CAC      H>HYAL042W      +      0      322134S       chr01:62354          A>G         ACA>ACG      T>TYAL042W      +      0      322134S       chr01:62362          T>C         GTT>GCT      V>AYAL042W      +      0      378604X       chr01:62362          T>C         GTT>GCT      V>AYAL042W      +      0      DBVPG1373     chr01:62362          T>C         GTT>GCT      V>AYAL042W      +      0      DBVPG1788     chr01:62362          T>C         GTT>GCT      V>AYAL042W      +      0      DBVPG1853     chr01:62362          T>C         GTT>GCT      V>AYAL042W      +      0      DBVPG6044     chr01:62362          T>C         GTT>GCT      V>AYAL042W      +      0      DBVPG6765     chr01:62362          T>C         GTT>GCT      V>AYAL042W      +      0      K11           chr01:62362          T>C         GTT>GCT      V>AYAL042W      +      0      SK1           chr01:62362          T>C         GTT>GCT      V>AYAL042W      +      0      UWOPS03_461_4 chr01:62362          T>C         GTT>GCT      V>AYAL042W      +      0      UWOPS05_217_3 chr01:62362          T>C         GTT>GCT      V>AYAL042W      +      0      Y55           chr01:62362          T>C         GTT>GCT      V>AYAL042W      +      0      Y9            chr01:62362          T>C         GTT>GCT      V>AYAL042W      +      0      YIIc17_E5     chr01:62362          T>C         GTT>GCT      V>AYAL042W      +      0      YPS606        chr01:62362          T>C         GTT>GCT      V>AYAL042W      +      0      YS4           chr01:62362          T>C         GTT>GCT      V>AYAL042W      +      0      YS4           chr01:62387          C>T         TTC>TTT      F>FYAL042W      +      0      UWOPS03_461_4 chr01:62549          C>T         GGC>GGT      G>GYAL042W      +      0      UWOPS05_217_3 chr01:62549          C>T         GGC>GGT      G>GYAL043C      -      0      NCYC361       chr01:58764          G>A         CAT>TAT      H>YYAL043C      -      0      322134S       chr01:58795          G>A         CCC>CCT      P>PYAL043C      -      0      DBVPG1106     chr01:58795          G>A         CCC>CCT      P>PYAL043C      -      0      DBVPG1373     chr01:58795          G>A         CCC>CCT      P>PYAL043C      -      0      DBVPG1853     chr01:58795          G>A         CCC>CCT      P>PYAL043C      -      0      DBVPG6044     chr01:58795          G>A         CCC>CCT      P>PYAL043C      -      0      DBVPG6765     chr01:58795          G>A         CCC>CCT      P>PYAL043C      -      0      L_1374        chr01:58795          G>A         CCC>CCT      P>PYAL043C      -      0      L_1528        chr01:58795          G>A         CCC>CCT      P>PYAL043C      -      0      NCYC361       chr01:58795          G>A         CCC>CCT      P>PYAL043C      -      0      YJM978        chr01:58795          G>A         CCC>CCT      P>PYAL043C      -      0      YJM981        chr01:58795          G>A         CCC>CCT      P>PYAL043C      -      0      YPS606        chr01:58795          G>A         CCC>CCT      P>PYAL043C      -      0      YS4           chr01:58795          G>A         CCC>CCT      P>PYAL043C      -      0      322134S       chr01:58828          G>A         TTC>TTT      F>FYAL043C      -      0      DBVPG1106     chr01:58828          G>A         TTC>TTT      F>FYAL043C      -      0      DBVPG1373     chr01:58828          G>A         TTC>TTT      F>FYAL043C      -      0      DBVPG1853     chr01:58828          G>A         TTC>TTT      F>FYAL043C      -      0      DBVPG6765     chr01:58828          G>A         TTC>TTT      F>FYAL043C      -      0      L_1374        chr01:58828          G>A         TTC>TTT      F>FYAL043C      -      0      L_1528        chr01:58828          G>A         TTC>TTT      F>FYAL043C      -      0      YJM978        chr01:58828          G>A         TTC>TTT      F>FYAL043C      -      0      YJM981        chr01:58828          G>A         TTC>TTT      F>FYAL043C      -      0      YS4           chr01:58828          G>A         TTC>TTT      F>FYAL043C      -      0      Y9            chr01:58998          T>G         AAC>CAC      N>HYAL043C      -      0      322134S       chr01:59074          C>T         GAG>GAA      E>EYAL043C      -      0      DBVPG1106     chr01:59074          C>T         GAG>GAA      E>EYAL043C      -      0      DBVPG6044     chr01:59074          C>T         GAG>GAA      E>EYAL043C      -      0      DBVPG6765     chr01:59074          C>T         GAG>GAA      E>EYAL043C      -      0      L_1374        chr01:59074          C>T         GAG>GAA      E>EYAL043C      -      0      L_1528        chr01:59074          C>T         GAG>GAA      E>EYAL043C      -      0      NCYC110       chr01:59074          C>T         GAG>GAA      E>EYAL043C      -      0      UWOPS05_217_3 chr01:59074          C>T         GAG>GAA      E>EYAL043C      -      0      UWOPS05_227_2 chr01:59074          C>T         GAG>GAA      E>EYAL043C      -      0      Y55           chr01:59074          C>T         GAG>GAA      E>EYAL043C      -      0      YJM978        chr01:59074          C>T         GAG>GAA      E>EYAL043C      -      0      YJM981        chr01:59074          C>T         GAG>GAA      E>EYAL043C      -      0      YPS606        chr01:59074          C>T         GAG>GAA      E>EYAL043C      -      0      YS4           chr01:59074          C>T         GAG>GAA      E>EYAL043C      -      0      UWOPS05_217_3 chr01:59100          G>C         CTT>GTT      L>VYAL043C      -      0      UWOPS05_227_2 chr01:59100          G>C         CTT>GTT      L>VYAL043C      -      0      YPS606        chr01:59146          T>C         AGA>AGG      R>RYAL043C      -      0      YIIc17_E5     chr01:59164          T>C         TTA>TTG      L>LYAL043C      -      0      322134S       chr01:59230          G>T         GCC>GCA      A>AYAL043C      -      0      DBVPG1106     chr01:59230          G>T         GCC>GCA      A>AYAL043C      -      0      DBVPG1373     chr01:59230          G>T         GCC>GCA      A>AYAL043C      -      0      DBVPG6044     chr01:59230          G>T         GCC>GCA      A>AYAL043C      -      0      DBVPG6765     chr01:59230          G>T         GCC>GCA      A>AYAL043C      -      0      L_1374        chr01:59230          G>T         GCC>GCA      A>AYAL043C      -      0      L_1528        chr01:59230          G>T         GCC>GCA      A>AYAL043C      -      0      NCYC110       chr01:59230          G>T         GCC>GCA      A>AYAL043C      -      0      NCYC361       chr01:59230          G>T         GCC>GCA      A>AYAL043C      -      0      UWOPS05_217_3 chr01:59230          G>T         GCC>GCA      A>AYAL043C      -      0      UWOPS05_227_2 chr01:59230          G>T         GCC>GCA      A>AYAL043C      -      0      Y55           chr01:59230          G>T         GCC>GCA      A>AYAL043C      -      0      YJM981        chr01:59230          G>T         GCC>GCA      A>AYAL043C      -      0      YPS606        chr01:59230          G>T         GCC>GCA      A>AYAL043C      -      0      YS4           chr01:59230          G>T         GCC>GCA      A>AYAL043C      -      0      DBVPG1373     chr01:59266          C>A         CTG>CTT      L>LYAL043C      -      0      322134S       chr01:59350          A>C         ATT>ATG      I>MYAL043C      -      0      DBVPG1373     chr01:59350          A>C         ATT>ATG      I>MYAL043C      -      0      DBVPG1853     chr01:59350          A>C         ATT>ATG      I>MYAL043C      -      0      DBVPG6044     chr01:59350          A>C         ATT>ATG      I>MYAL043C      -      0      DBVPG6765     chr01:59350          A>C         ATT>ATG      I>MYAL043C      -      0      L_1374        chr01:59350          A>C         ATT>ATG      I>MYAL043C      -      0      L_1528        chr01:59350          A>C         ATT>ATG      I>MYAL043C      -      0      NCYC110       chr01:59350          A>C         ATT>ATG      I>MYAL043C      -      0      NCYC361       chr01:59350          A>C         ATT>ATG      I>MYAL043C      -      0      SK1           chr01:59350          A>C         ATT>ATG      I>MYAL043C      -      0      UWOPS05_217_3 chr01:59350          A>C         ATT>ATG      I>MYAL043C      -      0      UWOPS05_227_2 chr01:59350          A>C         ATT>ATG      I>MYAL043C      -      0      Y55           chr01:59350          A>C         ATT>ATG      I>MYAL043C      -      0      YJM981        chr01:59350          A>C         ATT>ATG      I>MYAL043C      -      0      YPS606        chr01:59350          A>C         ATT>ATG      I>MYAL043C      -      0      NCYC361       chr01:59371          G>A         GCC>GCT      A>AYAL043C      -      0      322134S       chr01:59395          G>A         AGC>AGT      S>SYAL043C      -      0      DBVPG1106     chr01:59395          G>A         AGC>AGT      S>SYAL043C      -      0      DBVPG1373     chr01:59395          G>A         AGC>AGT      S>SYAL043C      -      0      DBVPG1853     chr01:59395          G>A         AGC>AGT      S>SYAL043C      -      0      DBVPG6044     chr01:59395          G>A         AGC>AGT      S>SYAL043C      -      0      L_1528        chr01:59395          G>A         AGC>AGT      S>SYAL043C      -      0      NCYC110       chr01:59395          G>A         AGC>AGT      S>SYAL043C      -      0      NCYC361       chr01:59395          G>A         AGC>AGT      S>SYAL043C      -      0      Y55           chr01:59395          G>A         AGC>AGT      S>SYAL043C      -      0      YJM981        chr01:59395          G>A         AGC>AGT      S>SYAL043C      -      0      YPS606        chr01:59395          G>A         AGC>AGT      S>SYAL043C      -      0      UWOPS05_227_2 chr01:59401          C>A         GAG>GAT      E>DYAL043C      -      0      322134S       chr01:59476          C>T         ATG>ATA      M>IYAL043C      -      0      DBVPG1373     chr01:59476          C>T         ATG>ATA      M>IYAL043C      -      0      DBVPG1853     chr01:59476          C>T         ATG>ATA      M>IYAL043C      -      0      DBVPG6765     chr01:59476          C>T         ATG>ATA      M>IYAL043C      -      0      L_1528        chr01:59476          C>T         ATG>ATA      M>IYAL043C      -      0      YJM981        chr01:59476          C>T         ATG>ATA      M>IYAL043C      -      0      322134S       chr01:59497          A>G         GCT>GCC      A>AYAL043C      -      0      DBVPG1373     chr01:59497          A>G         GCT>GCC      A>AYAL043C      -      0      DBVPG1853     chr01:59497          A>G         GCT>GCC      A>AYAL043C      -      0      DBVPG6765     chr01:59497          A>G         GCT>GCC      A>AYAL043C      -      0      L_1528        chr01:59497          A>G         GCT>GCC      A>AYAL043C      -      0      YJM981        chr01:59497          A>G         GCT>GCC      A>AYAL043C      -      0      UWOPS05_217_3 chr01:59641          T>C         GGA>GGG      G>GYAL043C      -      0      322134S       chr01:59660          C>G         AGC>ACC      S>TYAL043C      -      0      DBVPG1373     chr01:59660          C>G         AGC>ACC      S>TYAL043C      -      0      DBVPG1853     chr01:59660          C>G         AGC>ACC      S>TYAL043C      -      0      DBVPG6044     chr01:59660          C>G         AGC>ACC      S>TYAL043C      -      0      DBVPG6765     chr01:59660          C>G         AGC>ACC      S>TYAL043C      -      0      L_1528        chr01:59660          C>G         AGC>ACC      S>TYAL043C      -      0      NCYC110       chr01:59660          C>G         AGC>ACC      S>TYAL043C      -      0      NCYC361       chr01:59660          C>G         AGC>ACC      S>TYAL043C      -      0      SK1           chr01:59660          C>G         AGC>ACC      S>TYAL043C      -      0      UWOPS05_217_3 chr01:59660          C>G         AGC>ACC      S>TYAL043C      -      0      Y55           chr01:59660          C>G         AGC>ACC      S>TYAL043C      -      0      YIIc17_E5     chr01:59660          C>G         AGC>ACC      S>TYAL043C      -      0      YJM981        chr01:59660          C>G         AGC>ACC      S>TYAL043C      -      0      YPS128        chr01:59660          C>G         AGC>ACC      S>TYAL043C      -      0      YS9           chr01:59660          C>G         AGC>ACC      S>TYAL043C      -      0      YS9           chr01:59758          G>A         TAC>TAT      Y>YYAL043C      -      0      DBVPG6044     chr01:59860          C>T         CTG>CTA      L>LYAL043C      -      0      Y55           chr01:59860          C>T         CTG>CTA      L>LYAL043C      -      0      DBVPG1853     chr01:59864          T>A         CAA>CTA      Q>LYAL043C      -      0      322134S       chr01:59977          T>C         TCA>TCG      S>SYAL043C      -      0      DBVPG1106     chr01:59977          T>C         TCA>TCG      S>SYAL043C      -      0      DBVPG1373     chr01:59977          T>C         TCA>TCG      S>SYAL043C      -      0      DBVPG1788     chr01:59977          T>C         TCA>TCG      S>SYAL043C      -      0      DBVPG1853     chr01:59977          T>C         TCA>TCG      S>SYAL043C      -      0      DBVPG6044     chr01:59977          T>C         TCA>TCG      S>SYAL043C      -      0      DBVPG6765     chr01:59977          T>C         TCA>TCG      S>SYAL043C      -      0      L_1374        chr01:59977          T>C         TCA>TCG      S>SYAL043C      -      0      NCYC361       chr01:59977          T>C         TCA>TCG      S>SYAL043C      -      0      UWOPS05_217_3 chr01:59977          T>C         TCA>TCG      S>SYAL043C      -      0      Y55           chr01:59977          T>C         TCA>TCG      S>SYAL043C      -      0      YJM981        chr01:59977          T>C         TCA>TCG      S>SYAL043C      -      0      YS9           chr01:59977          T>C         TCA>TCG      S>SYAL043C      -      0      322134S       chr01:59986          T>C         AAA>AAG      K>KYAL043C      -      0      DBVPG1106     chr01:59986          T>C         AAA>AAG      K>KYAL043C      -      0      DBVPG1373     chr01:59986          T>C         AAA>AAG      K>KYAL043C      -      0      DBVPG1788     chr01:59986          T>C         AAA>AAG      K>KYAL043C      -      0      DBVPG1853     chr01:59986          T>C         AAA>AAG      K>KYAL043C      -      0      DBVPG6044     chr01:59986          T>C         AAA>AAG      K>KYAL043C      -      0      DBVPG6765     chr01:59986          T>C         AAA>AAG      K>KYAL043C      -      0      L_1374        chr01:59986          T>C         AAA>AAG      K>KYAL043C      -      0      NCYC361       chr01:59986          T>C         AAA>AAG      K>KYAL043C      -      0      UWOPS05_217_3 chr01:59986          T>C         AAA>AAG      K>KYAL043C      -      0      Y55           chr01:59986          T>C         AAA>AAG      K>KYAL043C      -      0      YJM981        chr01:59986          T>C         AAA>AAG      K>KYAL043C      -      0      YPS128        chr01:59986          T>C         AAA>AAG      K>KYAL043C      -      0      YS9           chr01:59986          T>C         AAA>AAG      K>KYAL043C      -      0      UWOPS05_217_3 chr01:60024          T>A         AAT>TAT      N>YYAL043C      -      0      DBVPG6044     chr01:60109          C>T         AAG>AAA      K>KYAL043C      -      0      Y55           chr01:60109          C>T         AAG>AAA      K>KYAL043C      -      0      322134S       chr01:60166          T>C         AAA>AAG      K>KYAL043C      -      0      DBVPG1106     chr01:60166          T>C         AAA>AAG      K>KYAL043C      -      0      DBVPG1788     chr01:60166          T>C         AAA>AAG      K>KYAL043C      -      0      DBVPG6765     chr01:60166          T>C         AAA>AAG      K>KYAL043C      -      0      NCYC361       chr01:60166          T>C         AAA>AAG      K>KYAL043C      -      0      322134S       chr01:60199          C>T         AGG>AGA      R>RYAL043C      -      0      DBVPG1106     chr01:60199          C>T         AGG>AGA      R>RYAL043C      -      0      DBVPG1788     chr01:60199          C>T         AGG>AGA      R>RYAL043C      -      0      DBVPG6044     chr01:60199          C>T         AGG>AGA      R>RYAL043C      -      0      DBVPG6765     chr01:60199          C>T         AGG>AGA      R>RYAL043C      -      0      L_1374        chr01:60199          C>T         AGG>AGA      R>RYAL043C      -      0      NCYC361       chr01:60199          C>T         AGG>AGA      R>RYAL043C      -      0      SK1           chr01:60199          C>T         AGG>AGA      R>RYAL043C      -      0      UWOPS03_461_4 chr01:60199          C>T         AGG>AGA      R>RYAL043C      -      0      Y55           chr01:60199          C>T         AGG>AGA      R>RYAL043C      -      0      YJM981        chr01:60199          C>T         AGG>AGA      R>RYAL043C      -      0      YPS128        chr01:60199          C>T         AGG>AGA      R>RYAL043C      -      0      YS9           chr01:60199          C>T         AGG>AGA      R>RYAL043C      -      0      DBVPG1106     chr01:60247          C>T         GAG>GAA      E>EYAL043C      -      0      DBVPG1788     chr01:60247          C>T         GAG>GAA      E>EYAL043C      -      0      DBVPG6765     chr01:60247          C>T         GAG>GAA      E>EYAL043C      -      0      L_1374        chr01:60247          C>T         GAG>GAA      E>EYAL043C      -      0      L_1528        chr01:60247          C>T         GAG>GAA      E>EYAL043C      -      0      NCYC361       chr01:60247          C>T         GAG>GAA      E>EYAL043C      -      0      YJM981        chr01:60247          C>T         GAG>GAA      E>EYAL043C      -      0      YS9           chr01:60247          C>T         GAG>GAA      E>EYAL043C      -      0      SK1           chr01:60268          G>C         GTC>GTG      V>VYAL043C      -      0      YPS128        chr01:60268          G>C         GTC>GTG      V>VYAL043C      -      0      DBVPG1106     chr01:60289          G>T         TCC>TCA      S>SYAL043C      -      0      BC187         chr01:60292          A>T         CTT>CTA      L>LYAL043C      -      0      BC187         chr01:60385          C>T         GAG>GAA      E>EYAL043C      -      0      DBVPG1106     chr01:60385          C>T         GAG>GAA      E>EYAL043C      -      0      DBVPG1373     chr01:60385          C>T         GAG>GAA      E>EYAL043C      -      0      DBVPG1788     chr01:60385          C>T         GAG>GAA      E>EYAL043C      -      0      DBVPG6765     chr01:60385          C>T         GAG>GAA      E>EYAL043C      -      0      L_1374        chr01:60385          C>T         GAG>GAA      E>EYAL043C      -      0      L_1528        chr01:60385          C>T         GAG>GAA      E>EYAL043C      -      0      NCYC361       chr01:60385          C>T         GAG>GAA      E>EYAL043C      -      0      UWOPS03_461_4 chr01:60385          C>T         GAG>GAA      E>EYAL043C      -      0      YJM981        chr01:60385          C>T         GAG>GAA      E>EYAL043C      -      0      YS2           chr01:60385          C>T         GAG>GAA      E>EYAL043C      -      0      YS9           chr01:60385          C>T         GAG>GAA      E>EYAL043C      -      0      BC187         chr01:60418          C>T         AGG>AGA      R>RYAL043C      -      0      DBVPG1106     chr01:60418          C>T         AGG>AGA      R>RYAL043C      -      0      DBVPG1373     chr01:60418          C>T         AGG>AGA      R>RYAL043C      -      0      DBVPG1788     chr01:60418          C>T         AGG>AGA      R>RYAL043C      -      0      DBVPG6765     chr01:60418          C>T         AGG>AGA      R>RYAL043C      -      0      L_1374        chr01:60418          C>T         AGG>AGA      R>RYAL043C      -      0      L_1528        chr01:60418          C>T         AGG>AGA      R>RYAL043C      -      0      NCYC361       chr01:60418          C>T         AGG>AGA      R>RYAL043C      -      0      SK1           chr01:60418          C>T         AGG>AGA      R>RYAL043C      -      0      UWOPS03_461_4 chr01:60418          C>T         AGG>AGA      R>RYAL043C      -      0      Y55           chr01:60418          C>T         AGG>AGA      R>RYAL043C      -      0      YJM981        chr01:60418          C>T         AGG>AGA      R>RYAL043C      -      0      YPS128        chr01:60418          C>T         AGG>AGA      R>RYAL043C      -      0      YPS606        chr01:60418          C>T         AGG>AGA      R>RYAL043C      -      0      YS2           chr01:60418          C>T         AGG>AGA      R>RYAL043C      -      0      YS9           chr01:60418          C>T         AGG>AGA      R>RYAL043C      -      0      Y9            chr01:60419          C>T         AGG>AAG      R>KYAL043C      -      0      UWOPS03_461_4 chr01:60439          A>T         GCT>GCA      A>AYAL043C      -      0      SK1           chr01:60446          T>C         AAA>AGA      K>RYAL043C      -      0      L_1374        chr01:60508          T>G         AAA>AAC      K>NYAL043C      -      0      BC187         chr01:60527          A>G         GTC>GCC      V>AYAL043C      -      0      DBVPG1106     chr01:60527          A>G         GTC>GCC      V>AYAL043C      -      0      DBVPG1373     chr01:60527          A>G         GTC>GCC      V>AYAL043C      -      0      DBVPG1788     chr01:60527          A>G         GTC>GCC      V>AYAL043C      -      0      DBVPG6765     chr01:60527          A>G         GTC>GCC      V>AYAL043C      -      0      L_1374        chr01:60527          A>G         GTC>GCC      V>AYAL043C      -      0      L_1528        chr01:60527          A>G         GTC>GCC      V>AYAL043C      -      0      NCYC361       chr01:60527          A>G         GTC>GCC      V>AYAL043C      -      0      YJM981        chr01:60527          A>G         GTC>GCC      V>AYAL043C      -      0      DBVPG1106     chr01:60559          C>T         GTG>GTA      V>VYAL043C      -      0      UWOPS03_461_4 chr01:60565          G>A         ATC>ATT      I>IYAL043C      -      0      UWOPS05_217_3 chr01:60565          G>A         ATC>ATT      I>IYAL043C      -      0      BC187         chr01:60775          T>C         CTA>CTG      L>LYAL043C      -      0      DBVPG1373     chr01:60775          T>C         CTA>CTG      L>LYAL043C      -      0      DBVPG1788     chr01:60775          T>C         CTA>CTG      L>LYAL043C      -      0      DBVPG6765     chr01:60775          T>C         CTA>CTG      L>LYAL043C      -      0      L_1374        chr01:60775          T>C         CTA>CTG      L>LYAL043C      -      0      L_1528        chr01:60775          T>C         CTA>CTG      L>LYAL043C      -      0      NCYC361       chr01:60775          T>C         CTA>CTG      L>LYAL043C      -      0      SK1           chr01:60775          T>C         CTA>CTG      L>LYAL043C      -      0      Y9            chr01:60775          T>C         CTA>CTG      L>LYAL043C      -      0      YJM975        chr01:60775          T>C         CTA>CTG      L>LYAL043C      -      0      YJM981        chr01:60775          T>C         CTA>CTG      L>LYAL043C      -      0      DBVPG6044     chr01:60877          C>T         GTG>GTA      V>VYAL043C      -      0      Y55           chr01:60877          C>T         GTG>GTA      V>VYAL043C      -      0      BC187         chr01:60913          A>G         CCT>CCC      P>PYAL043C      -      0      DBVPG1373     chr01:60913          A>G         CCT>CCC      P>PYAL043C      -      0      DBVPG1788     chr01:60913          A>G         CCT>CCC      P>PYAL043C      -      0      DBVPG6765     chr01:60913          A>G         CCT>CCC      P>PYAL043C      -      0      L_1374        chr01:60913          A>G         CCT>CCC      P>PYAL043C      -      0      UWOPS03_461_4 chr01:60913          A>G         CCT>CCC      P>PYAL043C      -      0      UWOPS05_217_3 chr01:60913          A>G         CCT>CCC      P>PYAL043C      -      0      YJM975        chr01:60913          A>G         CCT>CCC      P>PYAL043C      -      0      YPS606        chr01:60913          A>G         CCT>CCC      P>PYAL043C      -      0      SK1           chr01:60916          C>T         TTG>TTA      L>LYAL043C      -      0      K11           chr01:60925          C>T         AAG>AAA      K>KYAL043C      -      0      YS4           chr01:60986          T>A         GAG>GTG      E>VYAL043C      -      0      K11           chr01:61012          T>C         ACA>ACG      T>TYAL043C      -      0      DBVPG1373     chr01:61033          T>C         GAA>GAG      E>EYAL043C      -      0      YS9           chr01:61046          G>A         TCT>TTT      S>FYAL044C      -      0      322134S       chr01:58074          G>A         AAC>AAT      N>NYAL044C      -      0      378604X       chr01:58074          G>A         AAC>AAT      N>NYAL044C      -      0      DBVPG1373     chr01:58074          G>A         AAC>AAT      N>NYAL044C      -      0      DBVPG1788     chr01:58074          G>A         AAC>AAT      N>NYAL044C      -      0      DBVPG1853     chr01:58074          G>A         AAC>AAT      N>NYAL044C      -      0      DBVPG6765     chr01:58074          G>A         AAC>AAT      N>NYAL044C      -      0      L_1374        chr01:58074          G>A         AAC>AAT      N>NYAL044C      -      0      NCYC361       chr01:58074          G>A         AAC>AAT      N>NYAL044C      -      0      UWOPS87_2421  chr01:58074          G>A         AAC>AAT      N>NYAL044C      -      0      YS4           chr01:58074          G>A         AAC>AAT      N>NYAL044C      -      0      DBVPG6044     chr01:58080          C>A         GTG>GTT      V>VYAL044C      -      0      NCYC110       chr01:58080          C>A         GTG>GTT      V>VYAL044C      -      0      Y55           chr01:58080          C>A         GTG>GTT      V>VYAL044C      -      0      378604X       chr01:58167          C>T         GAG>GAA      E>EYAL044C      -      0      UWOPS87_2421  chr01:58167          C>T         GAG>GAA      E>EYAL044C      -      0      273614X       chr01:58196          A>C         TCC>GCC      S>AYAL044C      -      0      322134S       chr01:58196          A>C         TCC>GCC      S>AYAL044C      -      0      378604X       chr01:58196          A>C         TCC>GCC      S>AYAL044C      -      0      DBVPG1853     chr01:58196          A>C         TCC>GCC      S>AYAL044C      -      0      DBVPG6044     chr01:58196          A>C         TCC>GCC      S>AYAL044C      -      0      DBVPG6765     chr01:58196          A>C         TCC>GCC      S>AYAL044C      -      0      K11           chr01:58196          A>C         TCC>GCC      S>AYAL044C      -      0      L_1374        chr01:58196          A>C         TCC>GCC      S>AYAL044C      -      0      NCYC361       chr01:58196          A>C         TCC>GCC      S>AYAL044C      -      0      UWOPS87_2421  chr01:58196          A>C         TCC>GCC      S>AYAL044C      -      0      Y12           chr01:58196          A>C         TCC>GCC      S>AYAL044C      -      0      YGPM          chr01:58196          A>C         TCC>GCC      S>AYAL044C      -      0      YIIc17_E5     chr01:58196          A>C         TCC>GCC      S>AYAL044C      -      0      YPS606        chr01:58196          A>C         TCC>GCC      S>AYAL044C      -      0      YS4           chr01:58196          A>C         TCC>GCC      S>AYAL044C      -      0      YS9           chr01:58196          A>C         TCC>GCC      S>AYAL044C      -      0      273614X       chr01:58247          A>C         TCC>GCC      S>AYAL044C      -      0      322134S       chr01:58247          A>C         TCC>GCC      S>AYAL044C      -      0      378604X       chr01:58247          A>C         TCC>GCC      S>AYAL044C      -      0      DBVPG1373     chr01:58247          A>C         TCC>GCC      S>AYAL044C      -      0      DBVPG1853     chr01:58247          A>C         TCC>GCC      S>AYAL044C      -      0      DBVPG6044     chr01:58247          A>C         TCC>GCC      S>AYAL044C      -      0      DBVPG6765     chr01:58247          A>C         TCC>GCC      S>AYAL044C      -      0      K11           chr01:58247          A>C         TCC>GCC      S>AYAL044C      -      0      L_1374        chr01:58247          A>C         TCC>GCC      S>AYAL044C      -      0      NCYC361       chr01:58247          A>C         TCC>GCC      S>AYAL044C      -      0      UWOPS05_217_3 chr01:58247          A>C         TCC>GCC      S>AYAL044C      -      0      UWOPS87_2421  chr01:58247          A>C         TCC>GCC      S>AYAL044C      -      0      Y12           chr01:58247          A>C         TCC>GCC      S>AYAL044C      -      0      Y9            chr01:58247          A>C         TCC>GCC      S>AYAL044C      -      0      YGPM          chr01:58247          A>C         TCC>GCC      S>AYAL044C      -      0      YIIc17_E5     chr01:58247          A>C         TCC>GCC      S>AYAL044C      -      0      YPS606        chr01:58247          A>C         TCC>GCC      S>AYAL044C      -      0      YS4           chr01:58247          A>C         TCC>GCC      S>AYAL044C      -      0      273614X       chr01:58424          C>T         GCT>ACT      A>TYAL044C      -      0      322134S       chr01:58424          C>T         GCT>ACT      A>TYAL044C      -      0      378604X       chr01:58424          C>T         GCT>ACT      A>TYAL044C      -      0      DBVPG1106     chr01:58424          C>T         GCT>ACT      A>TYAL044C      -      0      DBVPG1373     chr01:58424          C>T         GCT>ACT      A>TYAL044C      -      0      DBVPG1853     chr01:58424          C>T         GCT>ACT      A>TYAL044C      -      0      DBVPG6044     chr01:58424          C>T         GCT>ACT      A>TYAL044C      -      0      DBVPG6765     chr01:58424          C>T         GCT>ACT      A>TYAL044C      -      0      K11           chr01:58424          C>T         GCT>ACT      A>TYAL044C      -      0      NCYC361       chr01:58424          C>T         GCT>ACT      A>TYAL044C      -      0      S288c         chr01:58424          C>T         GCT>ACT      A>TYAL044C      -      0      UWOPS03_461_4 chr01:58424          C>T         GCT>ACT      A>TYAL044C      -      0      UWOPS05_217_3 chr01:58424          C>T         GCT>ACT      A>TYAL044C      -      0      UWOPS87_2421  chr01:58424          C>T         GCT>ACT      A>TYAL044C      -      0      Y12           chr01:58424          C>T         GCT>ACT      A>TYAL044C      -      0      Y9            chr01:58424          C>T         GCT>ACT      A>TYAL044C      -      0      YGPM          chr01:58424          C>T         GCT>ACT      A>TYAL044C      -      0      YPS606        chr01:58424          C>T         GCT>ACT      A>TYAL044C      -      0      YS4           chr01:58424          C>T         GCT>ACT      A>TYAL044W-A    +      0      K11           chr01:57611          G>A         GCG>GCA      A>AYAL044W-A    +      0      K11           chr01:57623          C>T         TAC>TAT      Y>YYAL044W-A    +      0      DBVPG6044     chr01:57626          C>T         AAC>AAT      N>NYAL044W-A    +      0      NCYC110       chr01:57626          C>T         AAC>AAT      N>NYAL044W-A    +      0      Y55           chr01:57626          C>T         AAC>AAT      N>NYAL044W-A    +      0      K11           chr01:57629          T>C         GAT>GAC      D>DYAL044W-A    +      0      Y9            chr01:57635          C>T         CAC>CAT      H>HYAL044W-A    +      0      YIIc17_E5     chr01:57635          C>T         CAC>CAT      H>HYAL044W-A    +      0      K11           chr01:57647          C>T         GGC>GGT      G>GYAL044W-A    +      0      UWOPS03_461_4 chr01:57668          T>C         AAT>AAC      N>NYAL044W-A    +      0      UWOPS05_217_3 chr01:57668          T>C         AAT>AAC      N>NYAL044W-A    +      0      322134S       chr01:57670          T>C         GTC>GCC      V>AYAL044W-A    +      0      378604X       chr01:57670          T>C         GTC>GCC      V>AYAL044W-A    +      0      DBVPG1373     chr01:57670          T>C         GTC>GCC      V>AYAL044W-A    +      0      DBVPG1788     chr01:57670          T>C         GTC>GCC      V>AYAL044W-A    +      0      DBVPG1853     chr01:57670          T>C         GTC>GCC      V>AYAL044W-A    +      0      DBVPG6765     chr01:57670          T>C         GTC>GCC      V>AYAL044W-A    +      0      L_1374        chr01:57670          T>C         GTC>GCC      V>AYAL044W-A    +      0      L_1528        chr01:57670          T>C         GTC>GCC      V>AYAL044W-A    +      0      NCYC361       chr01:57670          T>C         GTC>GCC      V>AYAL044W-A    +      0      YS4           chr01:57670          T>C         GTC>GCC      V>AYAL044W-A    +      0      YS9           chr01:57670          T>C         GTC>GCC      V>AYAL044W-A    +      0      K11           chr01:57758          C>T         TCC>TCT      S>SYAL044W-A    +      0      YIIc17_E5     chr01:57804          C>a         CAA>aAA      Q>KYAL044W-A    +      0      K11           chr01:57809          G>A         TTG>TTA      L>LYAL046C      -      0      DBVPG1788     chr01:57042          C>T         CCG>CCA      P>PYAL046C      -      0      DBVPG1853     chr01:57042          C>T         CCG>CCA      P>PYAL046C      -      0      DBVPG6765     chr01:57042          C>T         CCG>CCA      P>PYAL046C      -      0      YJM975        chr01:57042          C>T         CCG>CCA      P>PYAL046C      -      0      DBVPG1853     chr01:57156          G>A         TTC>TTT      F>FYAL046C      -      0      DBVPG6765     chr01:57156          G>A         TTC>TTT      F>FYAL046C      -      0      L_1528        chr01:57156          G>A         TTC>TTT      F>FYAL046C      -      0      YIIc17_E5     chr01:57207          A>C         GTT>GTG      V>VYAL046C      -      0      Y9            chr01:57319          G>C         ACG>AGG      T>RYAL046C      -      0      YIIc17_E5     chr01:57319          G>C         ACG>AGG      T>RYAL046C      -      0      DBVPG1373     chr01:57321          C>T         CTG>CTA      L>LYAL046C      -      0      DBVPG1788     chr01:57321          C>T         CTG>CTA      L>LYAL046C      -      0      DBVPG1853     chr01:57321          C>T         CTG>CTA      L>LYAL046C      -      0      DBVPG6765     chr01:57321          C>T         CTG>CTA      L>LYAL046C      -      0      L_1528        chr01:57321          C>T         CTG>CTA      L>LYAL046C      -      0      NCYC361       chr01:57321          C>T         CTG>CTA      L>LYAL046C      -      0      YS9           chr01:57321          C>T         CTG>CTA      L>LYAL046C      -      0      DBVPG6044     chr01:57352          G>T         TCT>TAT      S>YYAL046C      -      0      Y55           chr01:57352          G>T         TCT>TAT      S>YYAL047C      -      0      UWOPS05_217_3 chr01:55010          G>A         CTA>TTA      L>LYAL047C      -      0      UWOPS05_227_2 chr01:55010          G>A         CTA>TTA      L>LYAL047C      -      0      YS4           chr01:55011          G>A         AAC>AAT      N>NYAL047C      -      0      322134S       chr01:55146          C>T         CTG>CTA      L>LYAL047C      -      0      378604X       chr01:55146          C>T         CTG>CTA      L>LYAL047C      -      0      DBVPG1106     chr01:55146          C>T         CTG>CTA      L>LYAL047C      -      0      DBVPG1373     chr01:55146          C>T         CTG>CTA      L>LYAL047C      -      0      DBVPG1788     chr01:55146          C>T         CTG>CTA      L>LYAL047C      -      0      DBVPG1853     chr01:55146          C>T         CTG>CTA      L>LYAL047C      -      0      DBVPG6040     chr01:55146          C>T         CTG>CTA      L>LYAL047C      -      0      DBVPG6765     chr01:55146          C>T         CTG>CTA      L>LYAL047C      -      0      L_1374        chr01:55146          C>T         CTG>CTA      L>LYAL047C      -      0      L_1528        chr01:55146          C>T         CTG>CTA      L>LYAL047C      -      0      SK1           chr01:55146          C>T         CTG>CTA      L>LYAL047C      -      0      YJM978        chr01:55146          C>T         CTG>CTA      L>LYAL047C      -      0      YS4           chr01:55146          C>T         CTG>CTA      L>LYAL047C      -      0      L_1374        chr01:55161          A>G         ACT>ACC      T>TYAL047C      -      0      322134S       chr01:55172          A>G         TTA>CTA      L>LYAL047C      -      0      378604X       chr01:55172          A>G         TTA>CTA      L>LYAL047C      -      0      DBVPG1106     chr01:55172          A>G         TTA>CTA      L>LYAL047C      -      0      DBVPG1373     chr01:55172          A>G         TTA>CTA      L>LYAL047C      -      0      DBVPG1788     chr01:55172          A>G         TTA>CTA      L>LYAL047C      -      0      DBVPG1853     chr01:55172          A>G         TTA>CTA      L>LYAL047C      -      0      DBVPG6040     chr01:55172          A>G         TTA>CTA      L>LYAL047C      -      0      DBVPG6765     chr01:55172          A>G         TTA>CTA      L>LYAL047C      -      0      L_1374        chr01:55172          A>G         TTA>CTA      L>LYAL047C      -      0      L_1528        chr01:55172          A>G         TTA>CTA      L>LYAL047C      -      0      SK1           chr01:55172          A>G         TTA>CTA      L>LYAL047C      -      0      UWOPS05_217_3 chr01:55172          A>G         TTA>CTA      L>LYAL047C      -      0      UWOPS05_227_2 chr01:55172          A>G         TTA>CTA      L>LYAL047C      -      0      Y12           chr01:55172          A>G         TTA>CTA      L>LYAL047C      -      0      YIIc17_E5     chr01:55172          A>G         TTA>CTA      L>LYAL047C      -      0      YJM978        chr01:55172          A>G         TTA>CTA      L>LYAL047C      -      0      YS4           chr01:55172          A>G         TTA>CTA      L>LYAL047C      -      0      Y12           chr01:55181          C>T         GGC>AGC      G>SYAL047C      -      0      YIIc17_E5     chr01:55181          C>T         GGC>AGC      G>SYAL047C      -      0      YJM978        chr01:55200          T>A         CCA>CCT      P>PYAL047C      -      0      322134S       chr01:55214          A>C         TCC>GCC      S>AYAL047C      -      0      378604X       chr01:55214          A>C         TCC>GCC      S>AYAL047C      -      0      DBVPG1106     chr01:55214          A>C         TCC>GCC      S>AYAL047C      -      0      DBVPG1788     chr01:55214          A>C         TCC>GCC      S>AYAL047C      -      0      DBVPG1853     chr01:55214          A>C         TCC>GCC      S>AYAL047C      -      0      DBVPG6040     chr01:55214          A>C         TCC>GCC      S>AYAL047C      -      0      DBVPG6765     chr01:55214          A>C         TCC>GCC      S>AYAL047C      -      0      L_1374        chr01:55214          A>C         TCC>GCC      S>AYAL047C      -      0      L_1528        chr01:55214          A>C         TCC>GCC      S>AYAL047C      -      0      SK1           chr01:55214          A>C         TCC>GCC      S>AYAL047C      -      0      YJM978        chr01:55214          A>C         TCC>GCC      S>AYAL047C      -      0      YS4           chr01:55214          A>C         TCC>GCC      S>AYAL047C      -      0      322134S       chr01:55257          A>G         ACT>ACC      T>TYAL047C      -      0      378604X       chr01:55257          A>G         ACT>ACC      T>TYAL047C      -      0      DBVPG1106     chr01:55257          A>G         ACT>ACC      T>TYAL047C      -      0      DBVPG1373     chr01:55257          A>G         ACT>ACC      T>TYAL047C      -      0      DBVPG1788     chr01:55257          A>G         ACT>ACC      T>TYAL047C      -      0      DBVPG1853     chr01:55257          A>G         ACT>ACC      T>TYAL047C      -      0      DBVPG6040     chr01:55257          A>G         ACT>ACC      T>TYAL047C      -      0      DBVPG6765     chr01:55257          A>G         ACT>ACC      T>TYAL047C      -      0      L_1374        chr01:55257          A>G         ACT>ACC      T>TYAL047C      -      0      L_1528        chr01:55257          A>G         ACT>ACC      T>TYAL047C      -      0      SK1           chr01:55257          A>G         ACT>ACC      T>TYAL047C      -      0      YS4           chr01:55257          A>G         ACT>ACC      T>TYAL047C      -      0      378604X       chr01:55328          C>A         GTA>TTA      V>LYAL047C      -      0      DBVPG1106     chr01:55328          C>A         GTA>TTA      V>LYAL047C      -      0      DBVPG1373     chr01:55328          C>A         GTA>TTA      V>LYAL047C      -      0      DBVPG1788     chr01:55328          C>A         GTA>TTA      V>LYAL047C      -      0      DBVPG1853     chr01:55328          C>A         GTA>TTA      V>LYAL047C      -      0      DBVPG6040     chr01:55328          C>A         GTA>TTA      V>LYAL047C      -      0      DBVPG6765     chr01:55328          C>A         GTA>TTA      V>LYAL047C      -      0      L_1374        chr01:55328          C>A         GTA>TTA      V>LYAL047C      -      0      L_1528        chr01:55328          C>A         GTA>TTA      V>LYAL047C      -      0      SK1           chr01:55328          C>A         GTA>TTA      V>LYAL047C      -      0      YS4           chr01:55328          C>A         GTA>TTA      V>LYAL047C      -      0      322134S       chr01:55376          C>A         GAA>TAA      E>*YAL047C      -      0      DBVPG6044     chr01:55427          G>A         CTT>TTT      L>FYAL047C      -      0      Y55           chr01:55427          G>A         CTT>TTT      L>FYAL047C      -      0      YIIc17_E5     chr01:55453          C>T         AGC>AAC      S>NYAL047C      -      0      DBVPG1106     chr01:55509          T>C         TTA>TTG      L>LYAL047C      -      0      DBVPG1373     chr01:55509          T>C         TTA>TTG      L>LYAL047C      -      0      DBVPG1788     chr01:55509          T>C         TTA>TTG      L>LYAL047C      -      0      DBVPG1853     chr01:55509          T>C         TTA>TTG      L>LYAL047C      -      0      DBVPG6040     chr01:55509          T>C         TTA>TTG      L>LYAL047C      -      0      DBVPG6765     chr01:55509          T>C         TTA>TTG      L>LYAL047C      -      0      L_1374        chr01:55509          T>C         TTA>TTG      L>LYAL047C      -      0      L_1528        chr01:55509          T>C         TTA>TTG      L>LYAL047C      -      0      SK1           chr01:55509          T>C         TTA>TTG      L>LYAL047C      -      0      UWOPS05_217_3 chr01:55509          T>C         TTA>TTG      L>LYAL047C      -      0      YIIc17_E5     chr01:55509          T>C         TTA>TTG      L>LYAL047C      -      0      YPS606        chr01:55509          T>C         TTA>TTG      L>LYAL047C      -      0      YS4           chr01:55509          T>C         TTA>TTG      L>LYAL047C      -      0      UWOPS05_217_3 chr01:55521          C>T         GAG>GAA      E>EYAL047C      -      0      YIIc17_E5     chr01:55521          C>T         GAG>GAA      E>EYAL047C      -      0      SK1           chr01:55526          A>G         TTG>CTG      L>LYAL047C      -      0      YS4           chr01:55526          A>G         TTG>CTG      L>LYAL047C      -      0      UWOPS05_217_3 chr01:55545          A>G         AAT>AAC      N>NYAL047C      -      0      YIIc17_E5     chr01:55545          A>G         AAT>AAC      N>NYAL047C      -      0      YIIc17_E5     chr01:55556          C>A         GTA>TTA      V>LYAL047C      -      0      YS4           chr01:55584          A>T         CTT>CTA      L>LYAL047C      -      0      DBVPG1373     chr01:55689          T>C         GAA>GAG      E>EYAL047C      -      0      DBVPG1788     chr01:55689          T>C         GAA>GAG      E>EYAL047C      -      0      DBVPG1853     chr01:55689          T>C         GAA>GAG      E>EYAL047C      -      0      DBVPG6040     chr01:55689          T>C         GAA>GAG      E>EYAL047C      -      0      DBVPG6765     chr01:55689          T>C         GAA>GAG      E>EYAL047C      -      0      L_1374        chr01:55689          T>C         GAA>GAG      E>EYAL047C      -      0      L_1528        chr01:55689          T>C         GAA>GAG      E>EYAL047C      -      0      SK1           chr01:55689          T>C         GAA>GAG      E>EYAL047C      -      0      YPS128        chr01:55689          T>C         GAA>GAG      E>EYAL047C      -      0      YS4           chr01:55689          T>C         GAA>GAG      E>EYAL047C      -      0      YS4           chr01:55722          C>T         TTG>TTA      L>LYAL047C      -      0      DBVPG1373     chr01:55746          A>G         GTT>GTC      V>VYAL047C      -      0      DBVPG1788     chr01:55746          A>G         GTT>GTC      V>VYAL047C      -      0      DBVPG1853     chr01:55746          A>G         GTT>GTC      V>VYAL047C      -      0      DBVPG6040     chr01:55746          A>G         GTT>GTC      V>VYAL047C      -      0      DBVPG6044     chr01:55746          A>G         GTT>GTC      V>VYAL047C      -      0      DBVPG6765     chr01:55746          A>G         GTT>GTC      V>VYAL047C      -      0      L_1374        chr01:55746          A>G         GTT>GTC      V>VYAL047C      -      0      L_1528        chr01:55746          A>G         GTT>GTC      V>VYAL047C      -      0      S288c         chr01:55746          A>G         GTT>GTC      V>VYAL047C      -      0      SK1           chr01:55746          A>G         GTT>GTC      V>VYAL047C      -      0      W303          chr01:55746          A>G         GTT>GTC      V>VYAL047C      -      0      Y55           chr01:55746          A>G         GTT>GTC      V>VYAL047C      -      0      Y9            chr01:55746          A>G         GTT>GTC      V>VYAL047C      -      0      YPS128        chr01:55746          A>G         GTT>GTC      V>VYAL047C      -      0      YS4           chr01:55746          A>G         GTT>GTC      V>VYAL047C      -      0      DBVPG1373     chr01:55794          T>C         CAA>CAG      Q>QYAL047C      -      0      DBVPG1788     chr01:55794          T>C         CAA>CAG      Q>QYAL047C      -      0      DBVPG1853     chr01:55794          T>C         CAA>CAG      Q>QYAL047C      -      0      DBVPG6040     chr01:55794          T>C         CAA>CAG      Q>QYAL047C      -      0      DBVPG6765     chr01:55794          T>C         CAA>CAG      Q>QYAL047C      -      0      L_1374        chr01:55794          T>C         CAA>CAG      Q>QYAL047C      -      0      L_1528        chr01:55794          T>C         CAA>CAG      Q>QYAL047C      -      0      SK1           chr01:55794          T>C         CAA>CAG      Q>QYAL047C      -      0      Y9            chr01:55794          T>C         CAA>CAG      Q>QYAL047C      -      0      YPS128        chr01:55794          T>C         CAA>CAG      Q>QYAL047C      -      0      YS4           chr01:55794          T>C         CAA>CAG      Q>QYAL047C      -      0      DBVPG6044     chr01:55900          T>C         AAA>AGA      K>RYAL047C      -      0      Y55           chr01:55900          T>C         AAA>AGA      K>RYAL047C      -      0      YPS128        chr01:55902          C>T         GAG>GAA      E>EYAL047C      -      0      YPS128        chr01:55911          C>T         TTG>TTA      L>LYAL047C      -      0      SK1           chr01:55915          C>T         AGA>AAA      R>KYAL047C      -      0      Y9            chr01:55915          C>G         AGA>ACA      R>TYAL047C      -      0      BC187         chr01:55954          A>T         ATT>AAT      I>NYAL047C      -      0      DBVPG1373     chr01:55954          A>T         ATT>AAT      I>NYAL047C      -      0      DBVPG1788     chr01:55954          A>T         ATT>AAT      I>NYAL047C      -      0      DBVPG1853     chr01:55954          A>T         ATT>AAT      I>NYAL047C      -      0      DBVPG6765     chr01:55954          A>T         ATT>AAT      I>NYAL047C      -      0      L_1374        chr01:55954          A>T         ATT>AAT      I>NYAL047C      -      0      L_1528        chr01:55954          A>T         ATT>AAT      I>NYAL047C      -      0      NCYC361       chr01:55954          A>T         ATT>AAT      I>NYAL047C      -      0      S288c         chr01:55954          A>T         ATT>AAT      I>NYAL047C      -      0      SK1           chr01:55954          A>T         ATT>AAT      I>NYAL047C      -      0      UWOPS03_461_4 chr01:55954          A>T         ATT>AAT      I>NYAL047C      -      0      W303          chr01:55954          A>T         ATT>AAT      I>NYAL047C      -      0      Y55           chr01:55954          A>T         ATT>AAT      I>NYAL047C      -      0      Y9            chr01:55954          A>T         ATT>AAT      I>NYAL047C      -      0      YPS128        chr01:55954          A>T         ATT>AAT      I>NYAL047C      -      0      YS4           chr01:55954          A>T         ATT>AAT      I>NYAL047C      -      0      BC187         chr01:56008          A>G         GTC>GCC      V>AYAL047C      -      0      DBVPG1373     chr01:56008          A>G         GTC>GCC      V>AYAL047C      -      0      DBVPG1788     chr01:56008          A>G         GTC>GCC      V>AYAL047C      -      0      DBVPG1853     chr01:56008          A>G         GTC>GCC      V>AYAL047C      -      0      DBVPG6765     chr01:56008          A>G         GTC>GCC      V>AYAL047C      -      0      L_1528        chr01:56008          A>G         GTC>GCC      V>AYAL047C      -      0      NCYC361       chr01:56008          A>G         GTC>GCC      V>AYAL047C      -      0      SK1           chr01:56008          A>G         GTC>GCC      V>AYAL047C      -      0      Y9            chr01:56008          A>G         GTC>GCC      V>AYAL047C      -      0      DBVPG6765     chr01:56059          A>C         ATT>AGT      I>SYAL047C      -      0      L_1374        chr01:56059          A>C         ATT>AGT      I>SYAL047C      -      0      DBVPG1853     chr01:56083          T>C         GAA>GGA      E>GYAL047C      -      0      BC187         chr01:56108          T>C         ACT>GCT      T>AYAL047C      -      0      DBVPG1373     chr01:56108          T>C         ACT>GCT      T>AYAL047C      -      0      DBVPG1788     chr01:56108          T>C         ACT>GCT      T>AYAL047C      -      0      DBVPG1853     chr01:56108          T>C         ACT>GCT      T>AYAL047C      -      0      DBVPG6765     chr01:56108          T>C         ACT>GCT      T>AYAL047C      -      0      L_1374        chr01:56108          T>C         ACT>GCT      T>AYAL047C      -      0      NCYC361       chr01:56108          T>C         ACT>GCT      T>AYAL047C      -      0      SK1           chr01:56108          T>C         ACT>GCT      T>AYAL047C      -      0      UWOPS03_461_4 chr01:56108          T>C         ACT>GCT      T>AYAL047C      -      0      Y55           chr01:56108          T>C         ACT>GCT      T>AYAL047C      -      0      Y9            chr01:56108          T>C         ACT>GCT      T>AYAL047C      -      0      YPS128        chr01:56108          T>C         ACT>GCT      T>AYAL047C      -      0      YS4           chr01:56108          T>C         ACT>GCT      T>AYAL047C      -      0      BC187         chr01:56130          T>C         TCA>TCG      S>SYAL047C      -      0      DBVPG1373     chr01:56130          T>C         TCA>TCG      S>SYAL047C      -      0      DBVPG1788     chr01:56130          T>C         TCA>TCG      S>SYAL047C      -      0      DBVPG1853     chr01:56130          T>C         TCA>TCG      S>SYAL047C      -      0      DBVPG6765     chr01:56130          T>C         TCA>TCG      S>SYAL047C      -      0      L_1374        chr01:56130          T>C         TCA>TCG      S>SYAL047C      -      0      NCYC361       chr01:56130          T>C         TCA>TCG      S>SYAL047C      -      0      SK1           chr01:56130          T>C         TCA>TCG      S>SYAL047C      -      0      BC187         chr01:56145          T>G         TTA>TTC      L>FYAL047C      -      0      DBVPG1373     chr01:56145          T>G         TTA>TTC      L>FYAL047C      -      0      DBVPG1788     chr01:56145          T>G         TTA>TTC      L>FYAL047C      -      0      DBVPG1853     chr01:56145          T>G         TTA>TTC      L>FYAL047C      -      0      DBVPG6765     chr01:56145          T>G         TTA>TTC      L>FYAL047C      -      0      L_1374        chr01:56145          T>G         TTA>TTC      L>FYAL047C      -      0      NCYC361       chr01:56145          T>G         TTA>TTC      L>FYAL047C      -      0      SK1           chr01:56145          T>G         TTA>TTC      L>FYAL047C      -      0      UWOPS03_461_4 chr01:56145          T>G         TTA>TTC      L>FYAL047C      -      0      Y9            chr01:56145          T>G         TTA>TTC      L>FYAL047C      -      0      YPS128        chr01:56145          T>G         TTA>TTC      L>FYAL047C      -      0      YS4           chr01:56145          T>G         TTA>TTC      L>FYAL047C      -      0      UWOPS03_461_4 chr01:56160          T>A         CCA>CCT      P>PYAL047C      -      0      Y9            chr01:56160          T>A         CCA>CCT      P>PYAL047C      -      0      BC187         chr01:56196          A>G         GAT>GAC      D>DYAL047C      -      0      DBVPG1373     chr01:56196          A>G         GAT>GAC      D>DYAL047C      -      0      DBVPG1788     chr01:56196          A>G         GAT>GAC      D>DYAL047C      -      0      DBVPG6765     chr01:56196          A>G         GAT>GAC      D>DYAL047C      -      0      L_1374        chr01:56196          A>G         GAT>GAC      D>DYAL047C      -      0      NCYC361       chr01:56196          A>G         GAT>GAC      D>DYAL047C      -      0      SK1           chr01:56196          A>G         GAT>GAC      D>DYAL047C      -      0      YJM981        chr01:56196          A>G         GAT>GAC      D>DYAL047C      -      0      YPS128        chr01:56212          G>A         GCG>GTG      A>VYAL047C      -      0      YS4           chr01:56250          G>T         ATC>ATA      I>IYAL047C      -      0      YPS128        chr01:56286          A>G         GTT>GTC      V>VYAL047C      -      0      BC187         chr01:56352          G>A         GCC>GCT      A>AYAL047C      -      0      DBVPG1373     chr01:56352          G>A         GCC>GCT      A>AYAL047C      -      0      DBVPG6765     chr01:56352          G>A         GCC>GCT      A>AYAL047C      -      0      L_1374        chr01:56352          G>A         GCC>GCT      A>AYAL047C      -      0      NCYC361       chr01:56352          G>A         GCC>GCT      A>AYAL047C      -      0      YJM975        chr01:56352          G>A         GCC>GCT      A>AYAL047C      -      0      YJM981        chr01:56352          G>A         GCC>GCT      A>AYAL047C      -      0      YPS128        chr01:56369          C>T         GAG>AAG      E>KYAL047C      -      0      SK1           chr01:56400          G>A         CAC>CAT      H>HYAL047C      -      0      Y9            chr01:56476          A>G         GTC>GCC      V>AYAL047C      -      0      YPS128        chr01:56481          C>T         AAG>AAA      K>KYAL047C      -      0      UWOPS03_461_4 chr01:56598          T>C         AGA>AGG      R>RYAL047C      -      0      UWOPS05_217_3 chr01:56598          T>C         AGA>AGG      R>RYAL047C      -      0      UWOPS03_461_4 chr01:56619          T>C         GTA>GTG      V>VYAL047C      -      0      UWOPS05_217_3 chr01:56619          T>C         GTA>GTG      V>VYAL047C      -      0      DBVPG1788     chr01:56787          G>A         AGC>AGT      S>SYAL047C      -      0      DBVPG1853     chr01:56787          G>A         AGC>AGT      S>SYAL047C      -      0      DBVPG6765     chr01:56787          G>A         AGC>AGT      S>SYAL047C      -      0      L_1374        chr01:56787          G>A         AGC>AGT      S>SYAL047C      -      0      NCYC361       chr01:56787          G>A         AGC>AGT      S>SYAL047C      -      0      SK1           chr01:56787          G>A         AGC>AGT      S>SYAL047C      -      0      UWOPS05_217_3 chr01:56787          G>A         AGC>AGT      S>SYAL047C      -      0      YJM975        chr01:56787          G>A         AGC>AGT      S>SYAL047C      -      0      NCYC361       chr01:56844          C>G         TGG>TGC      W>CYAL048C      -      0      DBVPG1373     chr01:52817          T>C         TCA>TCG      S>SYAL048C      -      0      NCYC361       chr01:52850          A>G         TGT>TGC      C>CYAL048C      -      0      YIIc17_E5     chr01:52850          A>G         TGT>TGC      C>CYAL048C      -      0      YPS128        chr01:52850          A>G         TGT>TGC      C>CYAL048C      -      0      DBVPG1788     chr01:52859          T>C         GTA>GTG      V>VYAL048C      -      0      DBVPG6765     chr01:52859          T>C         GTA>GTG      V>VYAL048C      -      0      L_1374        chr01:52859          T>C         GTA>GTG      V>VYAL048C      -      0      L_1528        chr01:52859          T>C         GTA>GTG      V>VYAL048C      -      0      SK1           chr01:52859          T>C         GTA>GTG      V>VYAL048C      -      0      UWOPS05_217_3 chr01:52859          T>C         GTA>GTG      V>VYAL048C      -      0      YJM978        chr01:52859          T>C         GTA>GTG      V>VYAL048C      -      0      YIIc17_E5     chr01:52877          C>T         GGG>GGA      G>GYAL048C      -      0      DBVPG1788     chr01:52940          C>T         CCG>CCA      P>PYAL048C      -      0      DBVPG6765     chr01:52940          C>T         CCG>CCA      P>PYAL048C      -      0      L_1374        chr01:52940          C>T         CCG>CCA      P>PYAL048C      -      0      L_1528        chr01:52940          C>T         CCG>CCA      P>PYAL048C      -      0      NCYC361       chr01:52940          C>T         CCG>CCA      P>PYAL048C      -      0      SK1           chr01:52940          C>T         CCG>CCA      P>PYAL048C      -      0      YJM978        chr01:52940          C>T         CCG>CCA      P>PYAL048C      -      0      DBVPG1788     chr01:52952          G>T         GGC>GGA      G>GYAL048C      -      0      DBVPG6765     chr01:52952          G>T         GGC>GGA      G>GYAL048C      -      0      L_1374        chr01:52952          G>T         GGC>GGA      G>GYAL048C      -      0      L_1528        chr01:52952          G>T         GGC>GGA      G>GYAL048C      -      0      SK1           chr01:52952          G>T         GGC>GGA      G>GYAL048C      -      0      YJM978        chr01:52952          G>T         GGC>GGA      G>GYAL048C      -      0      DBVPG1788     chr01:52985          A>G         TTT>TTC      F>FYAL048C      -      0      DBVPG6765     chr01:52985          A>G         TTT>TTC      F>FYAL048C      -      0      L_1374        chr01:52985          A>G         TTT>TTC      F>FYAL048C      -      0      L_1528        chr01:52985          A>G         TTT>TTC      F>FYAL048C      -      0      YJM978        chr01:52985          A>G         TTT>TTC      F>FYAL048C      -      0      DBVPG1788     chr01:52997          A>G         CTT>CTC      L>LYAL048C      -      0      DBVPG6765     chr01:52997          A>G         CTT>CTC      L>LYAL048C      -      0      L_1374        chr01:52997          A>G         CTT>CTC      L>LYAL048C      -      0      L_1528        chr01:52997          A>G         CTT>CTC      L>LYAL048C      -      0      SK1           chr01:52997          A>G         CTT>CTC      L>LYAL048C      -      0      YJM978        chr01:52997          A>G         CTT>CTC      L>LYAL048C      -      0      DBVPG1788     chr01:53147          T>G         ACA>ACC      T>TYAL048C      -      0      DBVPG1853     chr01:53147          T>G         ACA>ACC      T>TYAL048C      -      0      DBVPG6040     chr01:53147          T>G         ACA>ACC      T>TYAL048C      -      0      DBVPG6765     chr01:53147          T>G         ACA>ACC      T>TYAL048C      -      0      L_1374        chr01:53147          T>G         ACA>ACC      T>TYAL048C      -      0      L_1528        chr01:53147          T>G         ACA>ACC      T>TYAL048C      -      0      NCYC361       chr01:53147          T>G         ACA>ACC      T>TYAL048C      -      0      SK1           chr01:53147          T>G         ACA>ACC      T>TYAL048C      -      0      UWOPS05_227_2 chr01:53147          T>G         ACA>ACC      T>TYAL048C      -      0      YJM978        chr01:53147          T>G         ACA>ACC      T>TYAL048C      -      0      YPS128        chr01:53147          T>G         ACA>ACC      T>TYAL048C      -      0      DBVPG1788     chr01:53168          A>G         GTT>GTC      V>VYAL048C      -      0      DBVPG1853     chr01:53168          A>G         GTT>GTC      V>VYAL048C      -      0      DBVPG6040     chr01:53168          A>G         GTT>GTC      V>VYAL048C      -      0      DBVPG6765     chr01:53168          A>G         GTT>GTC      V>VYAL048C      -      0      L_1374        chr01:53168          A>G         GTT>GTC      V>VYAL048C      -      0      L_1528        chr01:53168          A>G         GTT>GTC      V>VYAL048C      -      0      NCYC361       chr01:53168          A>G         GTT>GTC      V>VYAL048C      -      0      SK1           chr01:53168          A>G         GTT>GTC      V>VYAL048C      -      0      YJM978        chr01:53168          A>G         GTT>GTC      V>VYAL048C      -      0      378604X       chr01:53267          C>T         CAG>CAA      Q>QYAL048C      -      0      DBVPG1788     chr01:53267          C>T         CAG>CAA      Q>QYAL048C      -      0      DBVPG1853     chr01:53267          C>T         CAG>CAA      Q>QYAL048C      -      0      DBVPG6040     chr01:53267          C>T         CAG>CAA      Q>QYAL048C      -      0      DBVPG6765     chr01:53267          C>T         CAG>CAA      Q>QYAL048C      -      0      L_1374        chr01:53267          C>T         CAG>CAA      Q>QYAL048C      -      0      L_1528        chr01:53267          C>T         CAG>CAA      Q>QYAL048C      -      0      NCYC361       chr01:53267          C>T         CAG>CAA      Q>QYAL048C      -      0      SK1           chr01:53267          C>T         CAG>CAA      Q>QYAL048C      -      0      YJM978        chr01:53267          C>T         CAG>CAA      Q>QYAL048C      -      0      YS2           chr01:53514          G>A         ACC>ATC      T>IYAL048C      -      0      DBVPG6040     chr01:53520          T>C         CAA>CGA      Q>RYAL048C      -      0      378604X       chr01:53543          C>T         CAG>CAA      Q>QYAL048C      -      0      DBVPG1788     chr01:53543          C>T         CAG>CAA      Q>QYAL048C      -      0      DBVPG1853     chr01:53543          C>T         CAG>CAA      Q>QYAL048C      -      0      DBVPG6040     chr01:53543          C>T         CAG>CAA      Q>QYAL048C      -      0      DBVPG6765     chr01:53543          C>T         CAG>CAA      Q>QYAL048C      -      0      L_1528        chr01:53543          C>T         CAG>CAA      Q>QYAL048C      -      0      NCYC361       chr01:53543          C>T         CAG>CAA      Q>QYAL048C      -      0      SK1           chr01:53543          C>T         CAG>CAA      Q>QYAL048C      -      0      YS2           chr01:53543          C>T         CAG>CAA      Q>QYAL048C      -      0      YS2           chr01:53786          A>C         CTT>CTG      L>LYAL048C      -      0      378604X       chr01:53837          A>G         GTT>GTC      V>VYAL048C      -      0      DBVPG1788     chr01:53837          A>G         GTT>GTC      V>VYAL048C      -      0      DBVPG6765     chr01:53837          A>G         GTT>GTC      V>VYAL048C      -      0      L_1528        chr01:53837          A>G         GTT>GTC      V>VYAL048C      -      0      NCYC361       chr01:53837          A>G         GTT>GTC      V>VYAL048C      -      0      SK1           chr01:53837          A>G         GTT>GTC      V>VYAL048C      -      0      273614X       chr01:53844          C>T         AGG>AAG      R>KYAL048C      -      0      378604X       chr01:53844          C>T         AGG>AAG      R>KYAL048C      -      0      DBVPG1788     chr01:53844          C>T         AGG>AAG      R>KYAL048C      -      0      DBVPG1853     chr01:53844          C>T         AGG>AAG      R>KYAL048C      -      0      DBVPG6765     chr01:53844          C>T         AGG>AAG      R>KYAL048C      -      0      K11           chr01:53844          C>T         AGG>AAG      R>KYAL048C      -      0      L_1528        chr01:53844          C>T         AGG>AAG      R>KYAL048C      -      0      NCYC361       chr01:53844          C>T         AGG>AAG      R>KYAL048C      -      0      SK1           chr01:53844          C>T         AGG>AAG      R>KYAL048C      -      0      YS2           chr01:53844          C>T         AGG>AAG      R>KYAL048C      -      0      378604X       chr01:53852          G>A         CTC>CTT      L>LYAL048C      -      0      DBVPG1788     chr01:53852          G>A         CTC>CTT      L>LYAL048C      -      0      DBVPG1853     chr01:53852          G>A         CTC>CTT      L>LYAL048C      -      0      DBVPG6765     chr01:53852          G>A         CTC>CTT      L>LYAL048C      -      0      L_1528        chr01:53852          G>A         CTC>CTT      L>LYAL048C      -      0      SK1           chr01:53852          G>A         CTC>CTT      L>LYAL048C      -      0      YS2           chr01:53852          G>A         CTC>CTT      L>LYAL048C      -      0      378604X       chr01:53936          A>T         GCT>GCA      A>AYAL048C      -      0      DBVPG1788     chr01:53936          A>T         GCT>GCA      A>AYAL048C      -      0      DBVPG1853     chr01:53936          A>T         GCT>GCA      A>AYAL048C      -      0      DBVPG6040     chr01:53936          A>T         GCT>GCA      A>AYAL048C      -      0      DBVPG6765     chr01:53936          A>T         GCT>GCA      A>AYAL048C      -      0      L_1528        chr01:53936          A>T         GCT>GCA      A>AYAL048C      -      0      SK1           chr01:53936          A>T         GCT>GCA      A>AYAL048C      -      0      273614X       chr01:54137          G>A         GAC>GAT      D>DYAL048C      -      0      K11           chr01:54137          G>A         GAC>GAT      D>DYAL048C      -      0      YS9           chr01:54137          G>A         GAC>GAT      D>DYAL048C      -      0      273614X       chr01:54176          A>G         GCT>GCC      A>AYAL048C      -      0      378604X       chr01:54176          A>G         GCT>GCC      A>AYAL048C      -      0      DBVPG1373     chr01:54176          A>G         GCT>GCC      A>AYAL048C      -      0      DBVPG1853     chr01:54176          A>G         GCT>GCC      A>AYAL048C      -      0      DBVPG6040     chr01:54176          A>G         GCT>GCC      A>AYAL048C      -      0      DBVPG6765     chr01:54176          A>G         GCT>GCC      A>AYAL048C      -      0      K11           chr01:54176          A>G         GCT>GCC      A>AYAL048C      -      0      L_1528        chr01:54176          A>G         GCT>GCC      A>AYAL048C      -      0      SK1           chr01:54176          A>G         GCT>GCC      A>AYAL048C      -      0      Y9            chr01:54176          A>G         GCT>GCC      A>AYAL048C      -      0      YPS606        chr01:54176          A>G         GCT>GCC      A>AYAL048C      -      0      YS9           chr01:54176          A>G         GCT>GCC      A>AYAL048C      -      0      378604X       chr01:54199          G>A         CTA>TTA      L>LYAL048C      -      0      DBVPG1373     chr01:54199          G>A         CTA>TTA      L>LYAL048C      -      0      DBVPG1853     chr01:54199          G>A         CTA>TTA      L>LYAL048C      -      0      DBVPG6040     chr01:54199          G>A         CTA>TTA      L>LYAL048C      -      0      DBVPG6765     chr01:54199          G>A         CTA>TTA      L>LYAL048C      -      0      L_1528        chr01:54199          G>A         CTA>TTA      L>LYAL048C      -      0      SK1           chr01:54199          G>A         CTA>TTA      L>LYAL048C      -      0      YJM975        chr01:54199          G>A         CTA>TTA      L>LYAL048C      -      0      YPS606        chr01:54199          G>A         CTA>TTA      L>LYAL048C      -      0      273614X       chr01:54245          A>G         GCT>GCC      A>AYAL048C      -      0      378604X       chr01:54245          A>G         GCT>GCC      A>AYAL048C      -      0      DBVPG1373     chr01:54245          A>G         GCT>GCC      A>AYAL048C      -      0      DBVPG1853     chr01:54245          A>G         GCT>GCC      A>AYAL048C      -      0      DBVPG6040     chr01:54245          A>G         GCT>GCC      A>AYAL048C      -      0      DBVPG6765     chr01:54245          A>G         GCT>GCC      A>AYAL048C      -      0      K11           chr01:54245          A>G         GCT>GCC      A>AYAL048C      -      0      L_1528        chr01:54245          A>G         GCT>GCC      A>AYAL048C      -      0      SK1           chr01:54245          A>G         GCT>GCC      A>AYAL048C      -      0      Y9            chr01:54245          A>G         GCT>GCC      A>AYAL048C      -      0      YPS128        chr01:54245          A>G         GCT>GCC      A>AYAL048C      -      0      YPS606        chr01:54245          A>G         GCT>GCC      A>AYAL048C      -      0      YS9           chr01:54273          T>C         AAC>AGC      N>SYAL048C      -      0      DBVPG1373     chr01:54320          T>C         GAA>GAG      E>EYAL048C      -      0      DBVPG1853     chr01:54320          T>C         GAA>GAG      E>EYAL048C      -      0      DBVPG6040     chr01:54320          T>C         GAA>GAG      E>EYAL048C      -      0      DBVPG6765     chr01:54320          T>C         GAA>GAG      E>EYAL048C      -      0      L_1528        chr01:54320          T>C         GAA>GAG      E>EYAL048C      -      0      SK1           chr01:54320          T>C         GAA>GAG      E>EYAL048C      -      0      YJM975        chr01:54320          T>C         GAA>GAG      E>EYAL048C      -      0      YJM978        chr01:54320          T>C         GAA>GAG      E>EYAL048C      -      0      UWOPS05_227_2 chr01:54386          A>G         AGT>AGC      S>SYAL048C      -      0      UWOPS05_227_2 chr01:54392          C>T         GAG>GAA      E>EYAL048C      -      0      UWOPS05_227_2 chr01:54458          G>A         GTC>GTT      V>VYAL048C      -      0      DBVPG1106     chr01:54509          A>G         GTT>GTC      V>VYAL048C      -      0      DBVPG1373     chr01:54509          A>G         GTT>GTC      V>VYAL048C      -      0      DBVPG1853     chr01:54509          A>G         GTT>GTC      V>VYAL048C      -      0      DBVPG6040     chr01:54509          A>G         GTT>GTC      V>VYAL048C      -      0      DBVPG6765     chr01:54509          A>G         GTT>GTC      V>VYAL048C      -      0      L_1528        chr01:54509          A>G         GTT>GTC      V>VYAL048C      -      0      SK1           chr01:54509          A>G         GTT>GTC      V>VYAL048C      -      0      UWOPS05_227_2 chr01:54509          A>G         GTT>GTC      V>VYAL048C      -      0      Y12           chr01:54509          A>G         GTT>GTC      V>VYAL048C      -      0      Y9            chr01:54509          A>G         GTT>GTC      V>VYAL048C      -      0      YJM975        chr01:54509          A>G         GTT>GTC      V>VYAL048C      -      0      YJM978        chr01:54509          A>G         GTT>GTC      V>VYAL048C      -      0      YPS128        chr01:54509          A>G         GTT>GTC      V>VYAL048C      -      0      YPS606        chr01:54509          A>G         GTT>GTC      V>VYAL048C      -      0      YS4           chr01:54509          A>G         GTT>GTC      V>VYAL048C      -      0      UWOPS05_227_2 chr01:54626          A>C         CCT>CCG      P>PYAL048C      -      0      DBVPG1106     chr01:54662          G>T         ATC>ATA      I>IYAL048C      -      0      DBVPG1853     chr01:54662          G>T         ATC>ATA      I>IYAL048C      -      0      DBVPG6040     chr01:54662          G>T         ATC>ATA      I>IYAL048C      -      0      DBVPG6765     chr01:54662          G>T         ATC>ATA      I>IYAL048C      -      0      L_1528        chr01:54662          G>T         ATC>ATA      I>IYAL048C      -      0      SK1           chr01:54662          G>T         ATC>ATA      I>IYAL048C      -      0      YJM975        chr01:54662          G>T         ATC>ATA      I>IYAL048C      -      0      YJM978        chr01:54662          G>T         ATC>ATA      I>IYAL048C      -      0      YJM981        chr01:54662          G>T         ATC>ATA      I>IYAL048C      -      0      YS9           chr01:54662          G>T         ATC>ATA      I>IYAL048C      -      0      UWOPS05_227_2 chr01:54676          G>A         CCA>TCA      P>SYAL048C      -      0      378604X       chr01:54680          C>T         GTG>GTA      V>VYAL048C      -      0      DBVPG1106     chr01:54680          C>T         GTG>GTA      V>VYAL048C      -      0      DBVPG1853     chr01:54680          C>T         GTG>GTA      V>VYAL048C      -      0      DBVPG6040     chr01:54680          C>T         GTG>GTA      V>VYAL048C      -      0      DBVPG6765     chr01:54680          C>T         GTG>GTA      V>VYAL048C      -      0      L_1528        chr01:54680          C>T         GTG>GTA      V>VYAL048C      -      0      SK1           chr01:54680          C>T         GTG>GTA      V>VYAL048C      -      0      YJM975        chr01:54680          C>T         GTG>GTA      V>VYAL048C      -      0      YJM978        chr01:54680          C>T         GTG>GTA      V>VYAL048C      -      0      YJM981        chr01:54680          C>T         GTG>GTA      V>VYAL048C      -      0      322134S       chr01:54743          A>C         GTT>GTG      V>VYAL048C      -      0      378604X       chr01:54743          A>C         GTT>GTG      V>VYAL048C      -      0      DBVPG1106     chr01:54743          A>C         GTT>GTG      V>VYAL048C      -      0      DBVPG1373     chr01:54743          A>C         GTT>GTG      V>VYAL048C      -      0      DBVPG1853     chr01:54743          A>C         GTT>GTG      V>VYAL048C      -      0      DBVPG6040     chr01:54743          A>C         GTT>GTG      V>VYAL048C      -      0      DBVPG6765     chr01:54743          A>C         GTT>GTG      V>VYAL048C      -      0      L_1528        chr01:54743          A>C         GTT>GTG      V>VYAL048C      -      0      SK1           chr01:54743          A>C         GTT>GTG      V>VYAL048C      -      0      YJM975        chr01:54743          A>C         GTT>GTG      V>VYAL048C      -      0      YJM978        chr01:54743          A>C         GTT>GTG      V>VYAL048C      -      0      YJM981        chr01:54743          A>C         GTT>GTG      V>VYAL048C      -      0      322134S       chr01:54785          A>G         ACT>ACC      T>TYAL048C      -      0      378604X       chr01:54785          A>G         ACT>ACC      T>TYAL048C      -      0      DBVPG1106     chr01:54785          A>G         ACT>ACC      T>TYAL048C      -      0      DBVPG1373     chr01:54785          A>G         ACT>ACC      T>TYAL048C      -      0      DBVPG1853     chr01:54785          A>G         ACT>ACC      T>TYAL048C      -      0      DBVPG6765     chr01:54785          A>G         ACT>ACC      T>TYAL048C      -      0      L_1528        chr01:54785          A>G         ACT>ACC      T>TYAL048C      -      0      SK1           chr01:54785          A>G         ACT>ACC      T>TYAL048C      -      0      YJM975        chr01:54785          A>G         ACT>ACC      T>TYAL048C      -      0      YJM978        chr01:54785          A>G         ACT>ACC      T>TYAL048C      -      0      YJM981        chr01:54785          A>G         ACT>ACC      T>TYAL049C      -      0      UWOPS87_2421  chr01:51859          A>G         GTT>GTC      V>VYAL049C      -      0      273614X       chr01:51952          T>G         GCA>GCC      A>AYAL049C      -      0      322134S       chr01:51952          T>G         GCA>GCC      A>AYAL049C      -      0      BC187         chr01:51952          T>G         GCA>GCC      A>AYAL049C      -      0      DBVPG1373     chr01:51952          T>G         GCA>GCC      A>AYAL049C      -      0      DBVPG1788     chr01:51952          T>G         GCA>GCC      A>AYAL049C      -      0      DBVPG1853     chr01:51952          T>G         GCA>GCC      A>AYAL049C      -      0      DBVPG6765     chr01:51952          T>G         GCA>GCC      A>AYAL049C      -      0      SK1           chr01:51952          T>G         GCA>GCC      A>AYAL049C      -      0      UWOPS87_2421  chr01:51952          T>A         GCA>GCT      A>AYAL049C      -      0      Y12           chr01:51952          T>G         GCA>GCC      A>AYAL049C      -      0      Y9            chr01:51952          T>G         GCA>GCC      A>AYAL049C      -      0      YIIc17_E5     chr01:51952          T>G         GCA>GCC      A>AYAL049C      -      0      YJM975        chr01:51952          T>G         GCA>GCC      A>AYAL049C      -      0      YS4           chr01:51952          T>G         GCA>GCC      A>AYAL049C      -      0      273614X       chr01:52102          T>G         GCA>GCC      A>AYAL049C      -      0      NCYC361       chr01:52164          C>a         GGT>TGT      G>CYAL049C      -      0      DBVPG1373     chr01:52165          C>T         GGG>GGA      G>GYAL049C      -      0      DBVPG1788     chr01:52165          C>T         GGG>GGA      G>GYAL049C      -      0      DBVPG1853     chr01:52165          C>T         GGG>GGA      G>GYAL049C      -      0      DBVPG6765     chr01:52165          C>T         GGG>GGA      G>GYAL049C      -      0      SK1           chr01:52165          C>T         GGG>GGA      G>GYAL049C      -      0      YJM975        chr01:52165          C>T         GGG>GGA      G>GYAL049C      -      0      YJM981        chr01:52165          C>T         GGG>GGA      G>GYAL049C      -      0      K11           chr01:52218          C>G         GTG>CTG      V>LYAL049C      -      0      UWOPS05_217_3 chr01:52287          T>C         ACC>GCC      T>AYAL049C      -      0      DBVPG1373     chr01:52311          A>G         TTT>CTT      F>LYAL049C      -      0      DBVPG1788     chr01:52311          A>G         TTT>CTT      F>LYAL049C      -      0      DBVPG6765     chr01:52311          A>G         TTT>CTT      F>LYAL049C      -      0      YJM975        chr01:52311          A>G         TTT>CTT      F>LYAL049C      -      0      YJM981        chr01:52311          A>G         TTT>CTT      F>LYAL049C      -      0      DBVPG6765     chr01:52461          C>T         GTT>ATT      V>IYAL054C      -      0      322134S       chr01:43005          G>A         TCC>TCT      S>SYAL054C      -      0      378604X       chr01:43005          G>A         TCC>TCT      S>SYAL054C      -      0      DBVPG1373     chr01:43005          G>A         TCC>TCT      S>SYAL054C      -      0      DBVPG1853     chr01:43005          G>A         TCC>TCT      S>SYAL054C      -      0      DBVPG6040     chr01:43005          G>A         TCC>TCT      S>SYAL054C      -      0      DBVPG6765     chr01:43005          G>A         TCC>TCT      S>SYAL054C      -      0      K11           chr01:43005          G>A         TCC>TCT      S>SYAL054C      -      0      L_1374        chr01:43005          G>A         TCC>TCT      S>SYAL054C      -      0      NCYC361       chr01:43005          G>A         TCC>TCT      S>SYAL054C      -      0      SK1           chr01:43005          G>A         TCC>TCT      S>SYAL054C      -      0      Y9            chr01:43005          G>A         TCC>TCT      S>SYAL054C      -      0      YJM975        chr01:43005          G>A         TCC>TCT      S>SYAL054C      -      0      YPS128        chr01:43005          G>A         TCC>TCT      S>SYAL054C      -      0      YS4           chr01:43005          G>A         TCC>TCT      S>SYAL054C      -      0      YS9           chr01:43005          G>A         TCC>TCT      S>SYAL054C      -      0      YPS606        chr01:43021          A>T         TTG>TAG      L>*YAL054C      -      0      Y9            chr01:43116          A>G         GAT>GAC      D>DYAL054C      -      0      322134S       chr01:43138          C>T         AGT>AAT      S>NYAL054C      -      0      DBVPG1373     chr01:43138          C>T         AGT>AAT      S>NYAL054C      -      0      DBVPG6040     chr01:43138          C>T         AGT>AAT      S>NYAL054C      -      0      DBVPG6765     chr01:43138          C>T         AGT>AAT      S>NYAL054C      -      0      L_1374        chr01:43138          C>T         AGT>AAT      S>NYAL054C      -      0      NCYC361       chr01:43138          C>T         AGT>AAT      S>NYAL054C      -      0      SK1           chr01:43138          C>T         AGT>AAT      S>NYAL054C      -      0      YJM975        chr01:43138          C>T         AGT>AAT      S>NYAL054C      -      0      YS9           chr01:43138          C>T         AGT>AAT      S>NYAL054C      -      0      UWOPS83_787_3 chr01:43239          A>G         ATT>ATC      I>IYAL054C      -      0      DBVPG1853     chr01:43317          G>A         ATC>ATT      I>IYAL054C      -      0      YPS606        chr01:43317          G>A         ATC>ATT      I>IYAL054C      -      0      YS4           chr01:43317          G>A         ATC>ATT      I>IYAL054C      -      0      NCYC110       chr01:43341          A>G         GCT>GCC      A>AYAL054C      -      0      DBVPG1853     chr01:43383          A>G         TAT>TAC      Y>YYAL054C      -      0      YPS606        chr01:43383          A>G         TAT>TAC      Y>YYAL054C      -      0      YS4           chr01:43383          A>G         TAT>TAC      Y>YYAL054C      -      0      YPS606        chr01:43449          G>C         GTC>GTG      V>VYAL054C      -      0      DBVPG1373     chr01:43479          G>A         AAC>AAT      N>NYAL054C      -      0      DBVPG1788     chr01:43479          G>A         AAC>AAT      N>NYAL054C      -      0      DBVPG6765     chr01:43479          G>A         AAC>AAT      N>NYAL054C      -      0      SK1           chr01:43479          G>A         AAC>AAT      N>NYAL054C      -      0      UWOPS03_461_4 chr01:43479          G>A         AAC>AAT      N>NYAL054C      -      0      UWOPS05_217_3 chr01:43479          G>A         AAC>AAT      N>NYAL054C      -      0      UWOPS83_787_3 chr01:43479          G>A         AAC>AAT      N>NYAL054C      -      0      YJM975        chr01:43479          G>A         AAC>AAT      N>NYAL054C      -      0      YJM978        chr01:43479          G>A         AAC>AAT      N>NYAL054C      -      0      YJM981        chr01:43479          G>A         AAC>AAT      N>NYAL054C      -      0      UWOPS83_787_3 chr01:43536          G>A         TTC>TTT      F>FYAL054C      -      0      273614X       chr01:43566          A>G         GTT>GTC      V>VYAL054C      -      0      DBVPG1373     chr01:43566          A>G         GTT>GTC      V>VYAL054C      -      0      DBVPG1788     chr01:43566          A>G         GTT>GTC      V>VYAL054C      -      0      DBVPG1853     chr01:43566          A>G         GTT>GTC      V>VYAL054C      -      0      DBVPG6765     chr01:43566          A>G         GTT>GTC      V>VYAL054C      -      0      SK1           chr01:43566          A>G         GTT>GTC      V>VYAL054C      -      0      Y9            chr01:43566          A>G         GTT>GTC      V>VYAL054C      -      0      YJM975        chr01:43566          A>G         GTT>GTC      V>VYAL054C      -      0      YJM978        chr01:43566          A>G         GTT>GTC      V>VYAL054C      -      0      YJM981        chr01:43566          A>G         GTT>GTC      V>VYAL054C      -      0      YPS606        chr01:43566          A>G         GTT>GTC      V>VYAL054C      -      0      YS2           chr01:43566          A>G         GTT>GTC      V>VYAL054C      -      0      YS4           chr01:43566          A>G         GTT>GTC      V>VYAL054C      -      0      YS9           chr01:43566          A>G         GTT>GTC      V>VYAL054C      -      0      YS4           chr01:43667          A>G         TGG>CGG      W>RYAL054C      -      0      DBVPG1788     chr01:43692          C>T         GAG>GAA      E>EYAL054C      -      0      DBVPG6765     chr01:43692          C>T         GAG>GAA      E>EYAL054C      -      0      SK1           chr01:43692          C>T         GAG>GAA      E>EYAL054C      -      0      YJM975        chr01:43692          C>T         GAG>GAA      E>EYAL054C      -      0      YJM978        chr01:43692          C>T         GAG>GAA      E>EYAL054C      -      0      YJM981        chr01:43692          C>T         GAG>GAA      E>EYAL054C      -      0      YS9           chr01:43692          C>T         GAG>GAA      E>EYAL054C      -      0      378604X       chr01:43785          C>G         GCG>GCC      A>AYAL054C      -      0      DBVPG1788     chr01:43785          C>G         GCG>GCC      A>AYAL054C      -      0      DBVPG1853     chr01:43785          C>G         GCG>GCC      A>AYAL054C      -      0      DBVPG6765     chr01:43785          C>G         GCG>GCC      A>AYAL054C      -      0      SK1           chr01:43785          C>G         GCG>GCC      A>AYAL054C      -      0      UWOPS03_461_4 chr01:43785          C>G         GCG>GCC      A>AYAL054C      -      0      UWOPS05_217_3 chr01:43785          C>G         GCG>GCC      A>AYAL054C      -      0      YJM978        chr01:43785          C>G         GCG>GCC      A>AYAL054C      -      0      YJM981        chr01:43785          C>G         GCG>GCC      A>AYAL054C      -      0      YPS128        chr01:43785          C>G         GCG>GCC      A>AYAL054C      -      0      YPS606        chr01:43785          C>G         GCG>GCC      A>AYAL054C      -      0      YS2           chr01:43785          C>G         GCG>GCC      A>AYAL054C      -      0      YS4           chr01:43785          C>G         GCG>GCC      A>AYAL054C      -      0      YS9           chr01:43785          C>G         GCG>GCC      A>AYAL054C      -      0      UWOPS03_461_4 chr01:43814          C>T         GAA>AAA      E>KYAL054C      -      0      UWOPS05_217_3 chr01:43814          C>T         GAA>AAA      E>KYAL054C      -      0      UWOPS05_227_2 chr01:43814          C>T         GAA>AAA      E>KYAL054C      -      0      273614X       chr01:43851          C>T         GCG>GCA      A>AYAL054C      -      0      DBVPG1853     chr01:43851          C>T         GCG>GCA      A>AYAL054C      -      0      K11           chr01:43851          C>T         GCG>GCA      A>AYAL054C      -      0      Y9            chr01:43851          C>T         GCG>GCA      A>AYAL054C      -      0      YIIc17_E5     chr01:43851          C>T         GCG>GCA      A>AYAL054C      -      0      YPS128        chr01:43851          C>T         GCG>GCA      A>AYAL054C      -      0      YS2           chr01:43851          C>T         GCG>GCA      A>AYAL054C      -      0      273614X       chr01:43857          A>C         ACT>ACG      T>TYAL054C      -      0      K11           chr01:43857          A>C         ACT>ACG      T>TYAL054C      -      0      Y9            chr01:43857          A>C         ACT>ACG      T>TYAL054C      -      0      YIIc17_E5     chr01:43857          A>C         ACT>ACG      T>TYAL054C      -      0      YPS128        chr01:43857          A>C         ACT>ACG      T>TYAL054C      -      0      UWOPS03_461_4 chr01:44121          C>T         AAG>AAA      K>KYAL054C      -      0      UWOPS83_787_3 chr01:44121          C>T         AAG>AAA      K>KYAL054C      -      0      SK1           chr01:44151          C>T         TTG>TTA      L>LYAL054C      -      0      DBVPG6765     chr01:44193          C>A         AAG>AAT      K>NYAL054C      -      0      273614X       chr01:44210          C>T         GTC>ATC      V>IYAL054C      -      0      YS4           chr01:44277          A>G         GGT>GGC      G>GYAL054C      -      0      273614X       chr01:44279          C>T         GGT>AGT      G>SYAL054C      -      0      378604X       chr01:44385          A>G         GGT>GGC      G>GYAL054C      -      0      DBVPG6765     chr01:44385          A>G         GGT>GGC      G>GYAL054C      -      0      NCYC361       chr01:44385          A>G         GGT>GGC      G>GYAL054C      -      0      SK1           chr01:44385          A>G         GGT>GGC      G>GYAL054C      -      0      UWOPS05_217_3 chr01:44385          A>G         GGT>GGC      G>GYAL054C      -      0      YJM975        chr01:44385          A>G         GGT>GGC      G>GYAL054C      -      0      YJM978        chr01:44385          A>G         GGT>GGC      G>GYAL054C      -      0      YJM981        chr01:44385          A>G         GGT>GGC      G>GYAL054C      -      0      YS9           chr01:44385          A>G         GGT>GGC      G>GYAL054C      -      0      UWOPS03_461_4 chr01:44421          T>C         GAA>GAG      E>EYAL054C      -      0      UWOPS83_787_3 chr01:44421          T>C         GAA>GAG      E>EYAL054C      -      0      UWOPS83_787_3 chr01:44470          C>A         GGC>GTC      G>VYAL054C      -      0      UWOPS03_461_4 chr01:44529          G>A         ACC>ACT      T>TYAL054C      -      0      Y9            chr01:44547          G>A         GGC>GGT      G>GYAL054C      -      0      273614X       chr01:44589          A>G         ACT>ACC      T>TYAL054C      -      0      322134S       chr01:44589          A>G         ACT>ACC      T>TYAL054C      -      0      378604X       chr01:44589          A>G         ACT>ACC      T>TYAL054C      -      0      DBVPG1853     chr01:44589          A>G         ACT>ACC      T>TYAL054C      -      0      DBVPG6765     chr01:44589          A>G         ACT>ACC      T>TYAL054C      -      0      L_1374        chr01:44589          A>G         ACT>ACC      T>TYAL054C      -      0      NCYC361       chr01:44589          A>G         ACT>ACC      T>TYAL054C      -      0      SK1           chr01:44589          A>G         ACT>ACC      T>TYAL054C      -      0      UWOPS05_217_3 chr01:44589          A>G         ACT>ACC      T>TYAL054C      -      0      Y9            chr01:44589          A>G         ACT>ACC      T>TYAL054C      -      0      YJM975        chr01:44589          A>G         ACT>ACC      T>TYAL054C      -      0      YPS606        chr01:44589          A>G         ACT>ACC      T>TYAL054C      -      0      YS4           chr01:44589          A>G         ACT>ACC      T>TYAL054C      -      0      YS9           chr01:44667          G>T         TCC>TCA      S>SYAL054C      -      0      322134S       chr01:44676          G>A         GGC>GGT      G>GYAL054C      -      0      DBVPG6765     chr01:44676          G>A         GGC>GGT      G>GYAL054C      -      0      L_1374        chr01:44676          G>A         GGC>GGT      G>GYAL054C      -      0      NCYC361       chr01:44676          G>A         GGC>GGT      G>GYAL054C      -      0      SK1           chr01:44676          G>A         GGC>GGT      G>GYAL054C      -      0      YJM975        chr01:44676          G>A         GGC>GGT      G>GYAL054C      -      0      UWOPS03_461_4 chr01:44684          T>C         AAA>GAA      K>EYAL054C      -      0      SK1           chr01:44687          G>A         CCT>TCT      P>SYAL054C      -      0      NCYC361       chr01:44799          G>C         GAC>GAG      D>EYAL054C      -      0      322134S       chr01:44892          A>G         ACT>ACC      T>TYAL054C      -      0      378604X       chr01:44892          A>G         ACT>ACC      T>TYAL054C      -      0      DBVPG1373     chr01:44892          A>G         ACT>ACC      T>TYAL054C      -      0      DBVPG1853     chr01:44892          A>G         ACT>ACC      T>TYAL054C      -      0      DBVPG6765     chr01:44892          A>G         ACT>ACC      T>TYAL054C      -      0      L_1374        chr01:44892          A>G         ACT>ACC      T>TYAL054C      -      0      NCYC361       chr01:44892          A>G         ACT>ACC      T>TYAL054C      -      0      SK1           chr01:44892          A>G         ACT>ACC      T>TYAL054C      -      0      UWOPS05_217_3 chr01:44892          A>G         ACT>ACC      T>TYAL054C      -      0      Y9            chr01:44892          A>G         ACT>ACC      T>TYAL054C      -      0      YJM975        chr01:44892          A>G         ACT>ACC      T>TYAL054C      -      0      YPS606        chr01:44892          A>G         ACT>ACC      T>TYAL054C      -      0      322134S       chr01:44936          C>A         GCC>TCC      A>SYAL054C      -      0      378604X       chr01:44936          C>A         GCC>TCC      A>SYAL054C      -      0      DBVPG1373     chr01:44936          C>A         GCC>TCC      A>SYAL054C      -      0      DBVPG1853     chr01:44936          C>A         GCC>TCC      A>SYAL054C      -      0      DBVPG6765     chr01:44936          C>A         GCC>TCC      A>SYAL054C      -      0      L_1374        chr01:44936          C>A         GCC>TCC      A>SYAL054C      -      0      NCYC361       chr01:44936          C>A         GCC>TCC      A>SYAL054C      -      0      SK1           chr01:44936          C>A         GCC>TCC      A>SYAL054C      -      0      UWOPS05_217_3 chr01:44936          C>A         GCC>TCC      A>SYAL054C      -      0      Y9            chr01:44936          C>A         GCC>TCC      A>SYAL054C      -      0      YIIc17_E5     chr01:44936          C>A         GCC>TCC      A>SYAL054C      -      0      YJM975        chr01:44936          C>A         GCC>TCC      A>SYAL054C      -      0      DBVPG6044     chr01:44993          G>A         CTA>TTA      L>LYAL054C      -      0      Y55           chr01:44993          G>A         CTA>TTA      L>LYAL055W      +      0      UWOPS05_217_3 chr01:42181          C>T         CCA>TCA      P>SYAL055W      +      0      DBVPG6044     chr01:42187          C>T         CCA>TCA      P>SYAL055W      +      0      Y55           chr01:42187          C>T         CCA>TCA      P>SYAL055W      +      0      UWOPS83_787_3 chr01:42211          A>T         ACA>TCA      T>SYAL055W      +      0      DBVPG6040     chr01:42234          T>A         GGT>GGA      G>GYAL055W      +      0      UWOPS05_217_3 chr01:42244          G>A         GCA>ACA      A>TYAL055W      +      0      UWOPS05_227_2 chr01:42244          G>A         GCA>ACA      A>TYAL055W      +      0      378604X       chr01:42258          G>A         ACG>ACA      T>TYAL055W      +      0      DBVPG1106     chr01:42258          G>A         ACG>ACA      T>TYAL055W      +      0      DBVPG1788     chr01:42258          G>A         ACG>ACA      T>TYAL055W      +      0      DBVPG1853     chr01:42258          G>A         ACG>ACA      T>TYAL055W      +      0      DBVPG6040     chr01:42258          G>A         ACG>ACA      T>TYAL055W      +      0      DBVPG6044     chr01:42258          G>A         ACG>ACA      T>TYAL055W      +      0      DBVPG6765     chr01:42258          G>A         ACG>ACA      T>TYAL055W      +      0      K11           chr01:42258          G>A         ACG>ACA      T>TYAL055W      +      0      L_1374        chr01:42258          G>A         ACG>ACA      T>TYAL055W      +      0      L_1528        chr01:42258          G>A         ACG>ACA      T>TYAL055W      +      0      SK1           chr01:42258          G>A         ACG>ACA      T>TYAL055W      +      0      UWOPS05_217_3 chr01:42258          G>A         ACG>ACA      T>TYAL055W      +      0      UWOPS05_227_2 chr01:42258          G>A         ACG>ACA      T>TYAL055W      +      0      UWOPS83_787_3 chr01:42258          G>A         ACG>ACA      T>TYAL055W      +      0      UWOPS87_2421  chr01:42258          G>A         ACG>ACA      T>TYAL055W      +      0      Y55           chr01:42258          G>A         ACG>ACA      T>TYAL055W      +      0      YIIc17_E5     chr01:42258          G>A         ACG>ACA      T>TYAL055W      +      0      YPS128        chr01:42258          G>A         ACG>ACA      T>TYAL055W      +      0      YPS606        chr01:42258          G>A         ACG>ACA      T>TYAL055W      +      0      YS4           chr01:42258          G>A         ACG>ACA      T>TYAL055W      +      0      YS9           chr01:42258          G>A         ACG>ACA      T>TYAL055W      +      0      UWOPS87_2421  chr01:42272          A>T         TAT>TTT      Y>FYAL055W      +      0      UWOPS05_217_3 chr01:42285          A>G         ACA>ACG      T>TYAL055W      +      0      UWOPS05_227_2 chr01:42285          A>G         ACA>ACG      T>TYAL055W      +      0      DBVPG1853     chr01:42291          A>T         GCA>GCT      A>AYAL055W      +      0      DBVPG6044     chr01:42291          A>T         GCA>GCT      A>AYAL055W      +      0      K11           chr01:42291          A>T         GCA>GCT      A>AYAL055W      +      0      Y55           chr01:42291          A>T         GCA>GCT      A>AYAL055W      +      0      YIIc17_E5     chr01:42291          A>T         GCA>GCT      A>AYAL055W      +      0      YPS128        chr01:42291          A>T         GCA>GCT      A>AYAL055W      +      0      YPS606        chr01:42291          A>T         GCA>GCT      A>AYAL055W      +      0      YS4           chr01:42291          A>T         GCA>GCT      A>AYAL055W      +      0      DBVPG6044     chr01:42307          G>A         GCA>ACA      A>TYAL055W      +      0      Y55           chr01:42307          G>A         GCA>ACA      A>TYAL055W      +      0      DBVPG1853     chr01:42316          C>T         CCA>TCA      P>SYAL055W      +      0      DBVPG6044     chr01:42316          C>T         CCA>TCA      P>SYAL055W      +      0      K11           chr01:42316          C>T         CCA>TCA      P>SYAL055W      +      0      Y55           chr01:42316          C>T         CCA>TCA      P>SYAL055W      +      0      YIIc17_E5     chr01:42316          C>T         CCA>TCA      P>SYAL055W      +      0      YPS128        chr01:42316          C>T         CCA>TCA      P>SYAL055W      +      0      YPS606        chr01:42316          C>T         CCA>TCA      P>SYAL055W      +      0      YS4           chr01:42316          C>T         CCA>TCA      P>SYAL055W      +      0      UWOPS05_227_2 chr01:42323          G>A         GGT>GAT      G>DYAL055W      +      0      UWOPS87_2421  chr01:42378          C>T         AGC>AGT      S>SYAL055W      +      0      UWOPS05_227_2 chr01:42405          G>A         AAG>AAA      K>KYAL055W      +      0      UWOPS05_227_2 chr01:42422          C>T         GCT>GTT      A>VYAL055W      +      0      UWOPS87_2421  chr01:42471          G>A         ATG>ATA      M>IYAL055W      +      0      378604X       chr01:42485          G>A         GGG>GAG      G>EYAL055W      +      0      DBVPG1106     chr01:42485          G>A         GGG>GAG      G>EYAL055W      +      0      DBVPG1788     chr01:42485          G>A         GGG>GAG      G>EYAL055W      +      0      DBVPG6040     chr01:42485          G>A         GGG>GAG      G>EYAL055W      +      0      DBVPG6765     chr01:42485          G>A         GGG>GAG      G>EYAL055W      +      0      L_1374        chr01:42485          G>A         GGG>GAG      G>EYAL055W      +      0      L_1528        chr01:42485          G>A         GGG>GAG      G>EYAL055W      +      0      SK1           chr01:42485          G>A         GGG>GAG      G>EYAL055W      +      0      Y55           chr01:42485          G>A         GGG>GAG      G>EYAL055W      +      0      YS9           chr01:42485          G>A         GGG>GAG      G>EYAL055W      +      0      378604X       chr01:42528          T>C         TAT>TAC      Y>YYAL055W      +      0      DBVPG1106     chr01:42528          T>C         TAT>TAC      Y>YYAL055W      +      0      DBVPG1788     chr01:42528          T>C         TAT>TAC      Y>YYAL055W      +      0      DBVPG6040     chr01:42528          T>C         TAT>TAC      Y>YYAL055W      +      0      DBVPG6765     chr01:42528          T>C         TAT>TAC      Y>YYAL055W      +      0      L_1374        chr01:42528          T>C         TAT>TAC      Y>YYAL055W      +      0      L_1528        chr01:42528          T>C         TAT>TAC      Y>YYAL055W      +      0      SK1           chr01:42528          T>C         TAT>TAC      Y>YYAL055W      +      0      UWOPS05_227_2 chr01:42528          T>C         TAT>TAC      Y>YYAL055W      +      0      UWOPS83_787_3 chr01:42528          T>C         TAT>TAC      Y>YYAL055W      +      0      YS9           chr01:42528          T>C         TAT>TAC      Y>YYAL055W      +      0      322134S       chr01:42555          A>T         TCA>TCT      S>SYAL055W      +      0      378604X       chr01:42555          A>T         TCA>TCT      S>SYAL055W      +      0      DBVPG1106     chr01:42555          A>T         TCA>TCT      S>SYAL055W      +      0      DBVPG1788     chr01:42555          A>T         TCA>TCT      S>SYAL055W      +      0      DBVPG6040     chr01:42555          A>T         TCA>TCT      S>SYAL055W      +      0      DBVPG6765     chr01:42555          A>T         TCA>TCT      S>SYAL055W      +      0      L_1374        chr01:42555          A>T         TCA>TCT      S>SYAL055W      +      0      L_1528        chr01:42555          A>T         TCA>TCT      S>SYAL055W      +      0      SK1           chr01:42555          A>T         TCA>TCT      S>SYAL055W      +      0      YS9           chr01:42555          A>T         TCA>TCT      S>SYAL055W      +      0      378604X       chr01:42563          G>A         AGA>AAA      R>KYAL055W      +      0      DBVPG1106     chr01:42563          G>A         AGA>AAA      R>KYAL055W      +      0      DBVPG1788     chr01:42563          G>A         AGA>AAA      R>KYAL055W      +      0      DBVPG6040     chr01:42563          G>A         AGA>AAA      R>KYAL055W      +      0      DBVPG6765     chr01:42563          G>A         AGA>AAA      R>KYAL055W      +      0      L_1374        chr01:42563          G>A         AGA>AAA      R>KYAL055W      +      0      L_1528        chr01:42563          G>A         AGA>AAA      R>KYAL055W      +      0      SK1           chr01:42563          G>A         AGA>AAA      R>KYAL055W      +      0      YS9           chr01:42563          G>A         AGA>AAA      R>KYAL055W      +      0      322134S       chr01:42591          G>A         TTG>TTA      L>LYAL055W      +      0      378604X       chr01:42591          G>A         TTG>TTA      L>LYAL055W      +      0      DBVPG1106     chr01:42591          G>A         TTG>TTA      L>LYAL055W      +      0      DBVPG1788     chr01:42591          G>A         TTG>TTA      L>LYAL055W      +      0      DBVPG6040     chr01:42591          G>A         TTG>TTA      L>LYAL055W      +      0      DBVPG6765     chr01:42591          G>A         TTG>TTA      L>LYAL055W      +      0      L_1374        chr01:42591          G>A         TTG>TTA      L>LYAL055W      +      0      NCYC361       chr01:42591          G>A         TTG>TTA      L>LYAL055W      +      0      SK1           chr01:42591          G>A         TTG>TTA      L>LYAL055W      +      0      UWOPS05_227_2 chr01:42591          G>A         TTG>TTA      L>LYAL055W      +      0      YS9           chr01:42591          G>A         TTG>TTA      L>LYAL055W      +      0      322134S       chr01:42630          A>G         GCA>GCG      A>AYAL055W      +      0      378604X       chr01:42630          A>G         GCA>GCG      A>AYAL055W      +      0      DBVPG1106     chr01:42630          A>G         GCA>GCG      A>AYAL055W      +      0      DBVPG1788     chr01:42630          A>G         GCA>GCG      A>AYAL055W      +      0      DBVPG6040     chr01:42630          A>G         GCA>GCG      A>AYAL055W      +      0      DBVPG6765     chr01:42630          A>G         GCA>GCG      A>AYAL055W      +      0      L_1374        chr01:42630          A>G         GCA>GCG      A>AYAL055W      +      0      NCYC361       chr01:42630          A>G         GCA>GCG      A>AYAL055W      +      0      SK1           chr01:42630          A>G         GCA>GCG      A>AYAL055W      +      0      YS9           chr01:42630          A>G         GCA>GCG      A>AYAL055W      +      0      322134S       chr01:42636          C>T         GTC>GTT      V>VYAL055W      +      0      378604X       chr01:42636          C>T         GTC>GTT      V>VYAL055W      +      0      DBVPG1106     chr01:42636          C>T         GTC>GTT      V>VYAL055W      +      0      DBVPG1788     chr01:42636          C>T         GTC>GTT      V>VYAL055W      +      0      DBVPG1853     chr01:42636          C>T         GTC>GTT      V>VYAL055W      +      0      DBVPG6040     chr01:42636          C>T         GTC>GTT      V>VYAL055W      +      0      DBVPG6765     chr01:42636          C>T         GTC>GTT      V>VYAL055W      +      0      K11           chr01:42636          C>T         GTC>GTT      V>VYAL055W      +      0      L_1374        chr01:42636          C>T         GTC>GTT      V>VYAL055W      +      0      NCYC361       chr01:42636          C>T         GTC>GTT      V>VYAL055W      +      0      SK1           chr01:42636          C>T         GTC>GTT      V>VYAL055W      +      0      Y12           chr01:42636          C>T         GTC>GTT      V>VYAL055W      +      0      Y9            chr01:42636          C>T         GTC>GTT      V>VYAL055W      +      0      YIIc17_E5     chr01:42636          C>T         GTC>GTT      V>VYAL055W      +      0      YPS128        chr01:42636          C>T         GTC>GTT      V>VYAL055W      +      0      YPS606        chr01:42636          C>T         GTC>GTT      V>VYAL055W      +      0      YS4           chr01:42636          C>T         GTC>GTT      V>VYAL055W      +      0      YS9           chr01:42636          C>T         GTC>GTT      V>VYAL055W      +      0      378604X       chr01:42663          C>G         GTC>GTG      V>VYAL055W      +      0      322134S       chr01:42685          G>A         GTA>ATA      V>IYAL055W      +      0      378604X       chr01:42685          G>A         GTA>ATA      V>IYAL055W      +      0      DBVPG1106     chr01:42685          G>A         GTA>ATA      V>IYAL055W      +      0      DBVPG6040     chr01:42685          G>A         GTA>ATA      V>IYAL055W      +      0      DBVPG6765     chr01:42685          G>A         GTA>ATA      V>IYAL055W      +      0      L_1374        chr01:42685          G>A         GTA>ATA      V>IYAL055W      +      0      NCYC361       chr01:42685          G>A         GTA>ATA      V>IYAL055W      +      0      SK1           chr01:42685          G>A         GTA>ATA      V>IYAL055W      +      0      UWOPS05_227_2 chr01:42685          G>A         GTA>ATA      V>IYAL055W      +      0      YS9           chr01:42685          G>A         GTA>ATA      V>IYAL055W      +      0      UWOPS87_2421  chr01:42691          G>A         GAT>AAT      D>NYAL059W      +      0      DBVPG1373     chr01:36525          C>T         CGA>TGA      R>*YAL059W      +      0      DBVPG1853     chr01:36525          C>T         CGA>TGA      R>*YAL059W      +      0      DBVPG6040     chr01:36525          C>T         CGA>TGA      R>*YAL059W      +      0      DBVPG6765     chr01:36525          C>T         CGA>TGA      R>*YAL059W      +      0      L_1528        chr01:36525          C>T         CGA>TGA      R>*YAL059W      +      0      NCYC110       chr01:36525          C>T         CGA>TGA      R>*YAL059W      +      0      SK1           chr01:36525          C>T         CGA>TGA      R>*YAL059W      +      0      UWOPS03_461_4 chr01:36525          C>T         CGA>TGA      R>*YAL059W      +      0      Y55           chr01:36525          C>T         CGA>TGA      R>*YAL059W      +      0      YPS128        chr01:36525          C>T         CGA>TGA      R>*YAL059W      +      0      YPS606        chr01:36525          C>T         CGA>TGA      R>*YAL059W      +      0      YS4           chr01:36525          C>T         CGA>TGA      R>*YAL059W      +      0      YS9           chr01:36525          C>T         CGA>TGA      R>*YAL059W      +      0      YPS128        chr01:36551          T>A         ACT>ACA      T>TYAL059W      +      0      YPS606        chr01:36551          T>A         ACT>ACA      T>TYAL059W      +      0      UWOPS03_461_4 chr01:36553          T>C         ATA>ACA      I>TYAL059W      +      0      DBVPG6765     chr01:36556          T>C         CTG>CCG      L>PYAL059W      +      0      DBVPG1106     chr01:36575          A>G         AAA>AAG      K>KYAL059W      +      0      DBVPG1373     chr01:36575          A>G         AAA>AAG      K>KYAL059W      +      0      DBVPG1853     chr01:36575          A>G         AAA>AAG      K>KYAL059W      +      0      DBVPG6040     chr01:36575          A>G         AAA>AAG      K>KYAL059W      +      0      DBVPG6765     chr01:36575          A>G         AAA>AAG      K>KYAL059W      +      0      L_1528        chr01:36575          A>G         AAA>AAG      K>KYAL059W      +      0      NCYC110       chr01:36575          A>G         AAA>AAG      K>KYAL059W      +      0      Y55           chr01:36575          A>G         AAA>AAG      K>KYAL059W      +      0      YPS128        chr01:36575          A>G         AAA>AAG      K>KYAL059W      +      0      YPS606        chr01:36575          A>G         AAA>AAG      K>KYAL059W      +      0      YS9           chr01:36575          A>G         AAA>AAG      K>KYAL059W      +      0      UWOPS03_461_4 chr01:36596          A>G         ATA>ATG      I>MYAL059W      +      0      DBVPG1106     chr01:36638          A>G         GAA>GAG      E>EYAL059W      +      0      DBVPG1373     chr01:36638          A>G         GAA>GAG      E>EYAL059W      +      0      DBVPG1853     chr01:36638          A>G         GAA>GAG      E>EYAL059W      +      0      DBVPG6040     chr01:36638          A>G         GAA>GAG      E>EYAL059W      +      0      DBVPG6765     chr01:36638          A>G         GAA>GAG      E>EYAL059W      +      0      L_1528        chr01:36638          A>G         GAA>GAG      E>EYAL059W      +      0      NCYC110       chr01:36638          A>G         GAA>GAG      E>EYAL059W      +      0      Y55           chr01:36638          A>G         GAA>GAG      E>EYAL059W      +      0      YPS128        chr01:36638          A>G         GAA>GAG      E>EYAL059W      +      0      YPS606        chr01:36638          A>G         GAA>GAG      E>EYAL059W      +      0      YS9           chr01:36638          A>G         GAA>GAG      E>EYAL059W      +      0      UWOPS03_461_4 chr01:36757          C>T         GCC>GTC      A>VYAL059W      +      0      YS4           chr01:36770          G>T         AAG>AAT      K>NYAL059W      +      0      BC187         chr01:36801          G>A         GAA>AAA      E>KYAL059W      +      0      BC187         chr01:36814          A>C         GAC>GCC      D>AYAL059W      +      0      DBVPG1106     chr01:36814          A>C         GAC>GCC      D>AYAL059W      +      0      DBVPG1373     chr01:36814          A>C         GAC>GCC      D>AYAL059W      +      0      DBVPG1853     chr01:36814          A>C         GAC>GCC      D>AYAL059W      +      0      DBVPG6040     chr01:36814          A>C         GAC>GCC      D>AYAL059W      +      0      DBVPG6044     chr01:36814          A>C         GAC>GCC      D>AYAL059W      +      0      DBVPG6765     chr01:36814          A>C         GAC>GCC      D>AYAL059W      +      0      K11           chr01:36814          A>C         GAC>GCC      D>AYAL059W      +      0      S288c         chr01:36814          A>C         GAC>GCC      D>AYAL059W      +      0      SK1           chr01:36814          A>C         GAC>GCC      D>AYAL059W      +      0      UWOPS03_461_4 chr01:36814          A>C         GAC>GCC      D>AYAL059W      +      0      W303          chr01:36814          A>C         GAC>GCC      D>AYAL059W      +      0      Y55           chr01:36814          A>C         GAC>GCC      D>AYAL059W      +      0      YJM978        chr01:36814          A>C         GAC>GCC      D>AYAL059W      +      0      YS4           chr01:36814          A>C         GAC>GCC      D>AYAL059W      +      0      YS9           chr01:36814          A>C         GAC>GCC      D>AYAL059W      +      0      273614X       chr01:36899          C>T         TCC>TCT      S>SYAL059W      +      0      BC187         chr01:36899          C>T         TCC>TCT      S>SYAL059W      +      0      DBVPG1106     chr01:36899          C>T         TCC>TCT      S>SYAL059W      +      0      DBVPG1373     chr01:36899          C>T         TCC>TCT      S>SYAL059W      +      0      DBVPG1853     chr01:36899          C>T         TCC>TCT      S>SYAL059W      +      0      DBVPG6040     chr01:36899          C>T         TCC>TCT      S>SYAL059W      +      0      DBVPG6765     chr01:36899          C>T         TCC>TCT      S>SYAL059W      +      0      K11           chr01:36899          C>T         TCC>TCT      S>SYAL059W      +      0      SK1           chr01:36899          C>T         TCC>TCT      S>SYAL059W      +      0      UWOPS03_461_4 chr01:36899          C>T         TCC>TCT      S>SYAL059W      +      0      YJM978        chr01:36899          C>T         TCC>TCT      S>SYAL059W      +      0      YPS128        chr01:36899          C>T         TCC>TCT      S>SYAL059W      +      0      YS4           chr01:36899          C>T         TCC>TCT      S>SYAL059W      +      0      YS9           chr01:36899          C>T         TCC>TCT      S>SYAL059W      +      0      UWOPS03_461_4 chr01:36926          T>C         AAT>AAC      N>NYAL059W      +      0      DBVPG1853     chr01:36941          C>T         GGC>GGT      G>GYAL059W      +      0      UWOPS03_461_4 chr01:36941          C>T         GGC>GGT      G>GYAL059W      +      0      273614X       chr01:37009          C>A         GCA>GAA      A>EYAL059W      +      0      BC187         chr01:37009          C>A         GCA>GAA      A>EYAL059W      +      0      DBVPG1106     chr01:37009          C>A         GCA>GAA      A>EYAL059W      +      0      DBVPG1373     chr01:37009          C>A         GCA>GAA      A>EYAL059W      +      0      DBVPG6040     chr01:37009          C>A         GCA>GAA      A>EYAL059W      +      0      DBVPG6765     chr01:37009          C>A         GCA>GAA      A>EYAL059W      +      0      L_1528        chr01:37009          C>A         GCA>GAA      A>EYAL059W      +      0      YJM978        chr01:37009          C>A         GCA>GAA      A>EYAL059W      +      0      273614X       chr01:37020          A>G         AGT>GGT      S>GYAL059W      +      0      BC187         chr01:37020          A>G         AGT>GGT      S>GYAL059W      +      0      DBVPG1106     chr01:37020          A>G         AGT>GGT      S>GYAL059W      +      0      DBVPG1373     chr01:37020          A>G         AGT>GGT      S>GYAL059W      +      0      DBVPG1853     chr01:37020          A>G         AGT>GGT      S>GYAL059W      +      0      DBVPG6040     chr01:37020          A>G         AGT>GGT      S>GYAL059W      +      0      DBVPG6765     chr01:37020          A>G         AGT>GGT      S>GYAL059W      +      0      L_1528        chr01:37020          A>G         AGT>GGT      S>GYAL059W      +      0      SK1           chr01:37020          A>G         AGT>GGT      S>GYAL059W      +      0      YJM978        chr01:37020          A>G         AGT>GGT      S>GYAL059W      +      0      UWOPS03_461_4 chr01:37029          A>G         AAT>GAT      N>DYAL059W      +      0      Y9            chr01:37029          A>G         AAT>GAT      N>DYAL059W      +      0      DBVPG1853     chr01:37040          G>T         AAG>AAT      K>NYAL059W      +      0      273614X       chr01:37070          C>T         GAC>GAT      D>DYAL059W      +      0      BC187         chr01:37070          C>T         GAC>GAT      D>DYAL059W      +      0      DBVPG1106     chr01:37070          C>T         GAC>GAT      D>DYAL059W      +      0      DBVPG1373     chr01:37070          C>T         GAC>GAT      D>DYAL059W      +      0      DBVPG6040     chr01:37070          C>T         GAC>GAT      D>DYAL059W      +      0      DBVPG6765     chr01:37070          C>T         GAC>GAT      D>DYAL059W      +      0      L_1528        chr01:37070          C>T         GAC>GAT      D>DYAL059W      +      0      YJM978        chr01:37070          C>T         GAC>GAT      D>DYAL059W      +      0      YPS128        chr01:37093          T>C         GTG>GCG      V>AYAL060W      +      0      Y55           chr01:35224          C>T         ATC>ATT      I>IYAL060W      +      0      K11           chr01:35338          A>T         AAA>AAT      K>NYAL060W      +      0      Y55           chr01:35344          C>T         TCC>TCT      S>SYAL060W      +      0      DBVPG1853     chr01:35422          G>A         GTG>GTA      V>VYAL060W      +      0      K11           chr01:35422          G>A         GTG>GTA      V>VYAL060W      +      0      UWOPS03_461_4 chr01:35422          G>A         GTG>GTA      V>VYAL060W      +      0      UWOPS05_227_2 chr01:35422          G>A         GTG>GTA      V>VYAL060W      +      0      UWOPS87_2421  chr01:35422          G>A         GTG>GTA      V>VYAL060W      +      0      YS9           chr01:35422          G>A         GTG>GTA      V>VYAL060W      +      0      273614X       chr01:35467          G>A         GCG>GCA      A>AYAL060W      +      0      322134S       chr01:35467          G>A         GCG>GCA      A>AYAL060W      +      0      DBVPG1106     chr01:35467          G>A         GCG>GCA      A>AYAL060W      +      0      DBVPG6040     chr01:35467          G>A         GCG>GCA      A>AYAL060W      +      0      DBVPG6044     chr01:35467          G>A         GCG>GCA      A>AYAL060W      +      0      DBVPG6765     chr01:35467          G>A         GCG>GCA      A>AYAL060W      +      0      L_1374        chr01:35467          G>A         GCG>GCA      A>AYAL060W      +      0      L_1528        chr01:35467          G>A         GCG>GCA      A>AYAL060W      +      0      NCYC361       chr01:35467          G>A         GCG>GCA      A>AYAL060W      +      0      SK1           chr01:35467          G>A         GCG>GCA      A>AYAL060W      +      0      Y55           chr01:35467          G>A         GCG>GCA      A>AYAL060W      +      0      YJM975        chr01:35467          G>A         GCG>GCA      A>AYAL060W      +      0      YJM978        chr01:35467          G>A         GCG>GCA      A>AYAL060W      +      0      273614X       chr01:35509          A>G         AAA>AAG      K>KYAL060W      +      0      322134S       chr01:35509          A>G         AAA>AAG      K>KYAL060W      +      0      DBVPG1106     chr01:35509          A>G         AAA>AAG      K>KYAL060W      +      0      DBVPG6040     chr01:35509          A>G         AAA>AAG      K>KYAL060W      +      0      DBVPG6044     chr01:35509          A>G         AAA>AAG      K>KYAL060W      +      0      DBVPG6765     chr01:35509          A>G         AAA>AAG      K>KYAL060W      +      0      L_1374        chr01:35509          A>G         AAA>AAG      K>KYAL060W      +      0      L_1528        chr01:35509          A>G         AAA>AAG      K>KYAL060W      +      0      NCYC361       chr01:35509          A>G         AAA>AAG      K>KYAL060W      +      0      SK1           chr01:35509          A>G         AAA>AAG      K>KYAL060W      +      0      Y55           chr01:35509          A>G         AAA>AAG      K>KYAL060W      +      0      YJM975        chr01:35509          A>G         AAA>AAG      K>KYAL060W      +      0      YJM978        chr01:35509          A>G         AAA>AAG      K>KYAL060W      +      0      273614X       chr01:35515          T>C         TGT>TGC      C>CYAL060W      +      0      322134S       chr01:35515          T>C         TGT>TGC      C>CYAL060W      +      0      DBVPG1106     chr01:35515          T>C         TGT>TGC      C>CYAL060W      +      0      DBVPG6040     chr01:35515          T>C         TGT>TGC      C>CYAL060W      +      0      DBVPG6765     chr01:35515          T>C         TGT>TGC      C>CYAL060W      +      0      L_1374        chr01:35515          T>C         TGT>TGC      C>CYAL060W      +      0      L_1528        chr01:35515          T>C         TGT>TGC      C>CYAL060W      +      0      NCYC361       chr01:35515          T>C         TGT>TGC      C>CYAL060W      +      0      SK1           chr01:35515          T>C         TGT>TGC      C>CYAL060W      +      0      YJM975        chr01:35515          T>C         TGT>TGC      C>CYAL060W      +      0      YJM978        chr01:35515          T>C         TGT>TGC      C>CYAL060W      +      0      DBVPG6044     chr01:35608          A>G         GTA>GTG      V>VYAL060W      +      0      Y55           chr01:35608          A>G         GTA>GTG      V>VYAL060W      +      0      273614X       chr01:35632          G>C         CCG>CCC      P>PYAL060W      +      0      322134S       chr01:35632          G>C         CCG>CCC      P>PYAL060W      +      0      378604X       chr01:35632          G>C         CCG>CCC      P>PYAL060W      +      0      DBVPG1106     chr01:35632          G>C         CCG>CCC      P>PYAL060W      +      0      DBVPG1853     chr01:35632          G>C         CCG>CCC      P>PYAL060W      +      0      DBVPG6040     chr01:35632          G>C         CCG>CCC      P>PYAL060W      +      0      DBVPG6765     chr01:35632          G>C         CCG>CCC      P>PYAL060W      +      0      K11           chr01:35632          G>C         CCG>CCC      P>PYAL060W      +      0      L_1374        chr01:35632          G>C         CCG>CCC      P>PYAL060W      +      0      L_1528        chr01:35632          G>C         CCG>CCC      P>PYAL060W      +      0      SK1           chr01:35632          G>C         CCG>CCC      P>PYAL060W      +      0      YJM978        chr01:35632          G>C         CCG>CCC      P>PYAL060W      +      0      YS9           chr01:35632          G>C         CCG>CCC      P>PYAL060W      +      0      YS9           chr01:35642          G>C         GAA>CAA      E>QYAL060W      +      0      273614X       chr01:35669          G>A         GTT>ATT      V>IYAL060W      +      0      322134S       chr01:35669          G>A         GTT>ATT      V>IYAL060W      +      0      DBVPG1106     chr01:35669          G>A         GTT>ATT      V>IYAL060W      +      0      DBVPG6040     chr01:35669          G>A         GTT>ATT      V>IYAL060W      +      0      DBVPG6765     chr01:35669          G>A         GTT>ATT      V>IYAL060W      +      0      L_1374        chr01:35669          G>A         GTT>ATT      V>IYAL060W      +      0      L_1528        chr01:35669          G>A         GTT>ATT      V>IYAL060W      +      0      SK1           chr01:35669          G>A         GTT>ATT      V>IYAL060W      +      0      YJM978        chr01:35669          G>A         GTT>ATT      V>IYAL060W      +      0      322134S       chr01:35737          G>A         TTG>TTA      L>LYAL060W      +      0      DBVPG1106     chr01:35737          G>A         TTG>TTA      L>LYAL060W      +      0      DBVPG1853     chr01:35737          G>A         TTG>TTA      L>LYAL060W      +      0      DBVPG6040     chr01:35737          G>A         TTG>TTA      L>LYAL060W      +      0      DBVPG6765     chr01:35737          G>A         TTG>TTA      L>LYAL060W      +      0      L_1374        chr01:35737          G>A         TTG>TTA      L>LYAL060W      +      0      L_1528        chr01:35737          G>A         TTG>TTA      L>LYAL060W      +      0      SK1           chr01:35737          G>A         TTG>TTA      L>LYAL060W      +      0      YJM978        chr01:35737          G>A         TTG>TTA      L>LYAL060W      +      0      322134S       chr01:35752          T>C         GGT>GGC      G>GYAL060W      +      0      378604X       chr01:35752          T>C         GGT>GGC      G>GYAL060W      +      0      DBVPG1106     chr01:35752          T>C         GGT>GGC      G>GYAL060W      +      0      DBVPG1853     chr01:35752          T>C         GGT>GGC      G>GYAL060W      +      0      DBVPG6040     chr01:35752          T>C         GGT>GGC      G>GYAL060W      +      0      DBVPG6044     chr01:35752          T>C         GGT>GGC      G>GYAL060W      +      0      DBVPG6765     chr01:35752          T>C         GGT>GGC      G>GYAL060W      +      0      K11           chr01:35752          T>C         GGT>GGC      G>GYAL060W      +      0      L_1374        chr01:35752          T>C         GGT>GGC      G>GYAL060W      +      0      L_1528        chr01:35752          T>C         GGT>GGC      G>GYAL060W      +      0      SK1           chr01:35752          T>C         GGT>GGC      G>GYAL060W      +      0      UWOPS05_227_2 chr01:35752          T>C         GGT>GGC      G>GYAL060W      +      0      UWOPS87_2421  chr01:35752          T>C         GGT>GGC      G>GYAL060W      +      0      Y55           chr01:35752          T>C         GGT>GGC      G>GYAL060W      +      0      YJM978        chr01:35752          T>C         GGT>GGC      G>GYAL060W      +      0      YS9           chr01:35752          T>C         GGT>GGC      G>GYAL060W      +      0      DBVPG1106     chr01:35766          G>C         TGT>TCT      C>SYAL060W      +      0      322134S       chr01:35797          T>C         GCT>GCC      A>AYAL060W      +      0      DBVPG1106     chr01:35797          T>C         GCT>GCC      A>AYAL060W      +      0      DBVPG1853     chr01:35797          T>C         GCT>GCC      A>AYAL060W      +      0      DBVPG6040     chr01:35797          T>C         GCT>GCC      A>AYAL060W      +      0      DBVPG6765     chr01:35797          T>C         GCT>GCC      A>AYAL060W      +      0      L_1374        chr01:35797          T>C         GCT>GCC      A>AYAL060W      +      0      L_1528        chr01:35797          T>C         GCT>GCC      A>AYAL060W      +      0      SK1           chr01:35797          T>C         GCT>GCC      A>AYAL060W      +      0      YJM978        chr01:35797          T>C         GCT>GCC      A>AYAL060W      +      0      YS9           chr01:35797          T>C         GCT>GCC      A>AYAL060W      +      0      322134S       chr01:35819          A>G         ATT>GTT      I>VYAL060W      +      0      378604X       chr01:35819          A>G         ATT>GTT      I>VYAL060W      +      0      DBVPG1106     chr01:35819          A>G         ATT>GTT      I>VYAL060W      +      0      DBVPG1853     chr01:35819          A>G         ATT>GTT      I>VYAL060W      +      0      DBVPG6040     chr01:35819          A>G         ATT>GTT      I>VYAL060W      +      0      DBVPG6765     chr01:35819          A>G         ATT>GTT      I>VYAL060W      +      0      K11           chr01:35819          A>G         ATT>GTT      I>VYAL060W      +      0      L_1374        chr01:35819          A>G         ATT>GTT      I>VYAL060W      +      0      L_1528        chr01:35819          A>G         ATT>GTT      I>VYAL060W      +      0      SK1           chr01:35819          A>G         ATT>GTT      I>VYAL060W      +      0      UWOPS05_227_2 chr01:35819          A>G         ATT>GTT      I>VYAL060W      +      0      UWOPS87_2421  chr01:35819          A>G         ATT>GTT      I>VYAL060W      +      0      YJM978        chr01:35819          A>G         ATT>GTT      I>VYAL060W      +      0      YS9           chr01:35819          A>G         ATT>GTT      I>VYAL060W      +      0      378604X       chr01:35890          T>A         CAT>CAA      H>QYAL060W      +      0      UWOPS05_227_2 chr01:35890          T>A         CAT>CAA      H>QYAL060W      +      0      UWOPS83_787_3 chr01:35906          C>T         CTA>TTA      L>LYAL060W      +      0      YJM978        chr01:35941          T>A         GAT>GAA      D>EYAL060W      +      0      322134S       chr01:35971          T>C         GTT>GTC      V>VYAL060W      +      0      DBVPG1106     chr01:35971          T>C         GTT>GTC      V>VYAL060W      +      0      DBVPG1853     chr01:35971          T>C         GTT>GTC      V>VYAL060W      +      0      DBVPG6040     chr01:35971          T>C         GTT>GTC      V>VYAL060W      +      0      DBVPG6765     chr01:35971          T>C         GTT>GTC      V>VYAL060W      +      0      L_1374        chr01:35971          T>C         GTT>GTC      V>VYAL060W      +      0      L_1528        chr01:35971          T>C         GTT>GTC      V>VYAL060W      +      0      SK1           chr01:35971          T>C         GTT>GTC      V>VYAL060W      +      0      UWOPS83_787_3 chr01:35971          T>C         GTT>GTC      V>VYAL060W      +      0      YPS128        chr01:35971          T>C         GTT>GTC      V>VYAL060W      +      0      YS9           chr01:35971          T>C         GTT>GTC      V>VYAL060W      +      0      378604X       chr01:35977          C>T         TTC>TTT      F>FYAL060W      +      0      378604X       chr01:36006          A>G         AAG>AGG      K>RYAL060W      +      0      DBVPG1106     chr01:36006          A>G         AAG>AGG      K>RYAL060W      +      0      DBVPG1373     chr01:36006          A>G         AAG>AGG      K>RYAL060W      +      0      DBVPG1853     chr01:36006          A>G         AAG>AGG      K>RYAL060W      +      0      DBVPG6040     chr01:36006          A>G         AAG>AGG      K>RYAL060W      +      0      DBVPG6765     chr01:36006          A>G         AAG>AGG      K>RYAL060W      +      0      L_1374        chr01:36006          A>G         AAG>AGG      K>RYAL060W      +      0      L_1528        chr01:36006          A>G         AAG>AGG      K>RYAL060W      +      0      SK1           chr01:36006          A>G         AAG>AGG      K>RYAL060W      +      0      UWOPS83_787_3 chr01:36006          A>G         AAG>AGG      K>RYAL060W      +      0      YPS606        chr01:36006          A>G         AAG>AGG      K>RYAL060W      +      0      YS9           chr01:36006          A>G         AAG>AGG      K>RYAL060W      +      0      322134S       chr01:36049          C>G         GTC>GTG      V>VYAL060W      +      0      378604X       chr01:36049          C>G         GTC>GTG      V>VYAL060W      +      0      DBVPG1106     chr01:36049          C>G         GTC>GTG      V>VYAL060W      +      0      DBVPG1853     chr01:36049          C>G         GTC>GTG      V>VYAL060W      +      0      DBVPG6040     chr01:36049          C>G         GTC>GTG      V>VYAL060W      +      0      DBVPG6765     chr01:36049          C>G         GTC>GTG      V>VYAL060W      +      0      L_1528        chr01:36049          C>G         GTC>GTG      V>VYAL060W      +      0      SK1           chr01:36049          C>G         GTC>GTG      V>VYAL060W      +      0      UWOPS83_787_3 chr01:36049          C>G         GTC>GTG      V>VYAL060W      +      0      YPS128        chr01:36049          C>G         GTC>GTG      V>VYAL060W      +      0      YPS606        chr01:36049          C>G         GTC>GTG      V>VYAL060W      +      0      YS9           chr01:36049          C>G         GTC>GTG      V>VYAL060W      +      0      DBVPG6044     chr01:36120          C>A         GCC>GAC      A>DYAL060W      +      0      K11           chr01:36120          C>A         GCC>GAC      A>DYAL060W      +      0      NCYC110       chr01:36120          C>A         GCC>GAC      A>DYAL060W      +      0      W303          chr01:36120          C>A         GCC>GAC      A>DYAL060W      +      0      Y55           chr01:36120          C>A         GCC>GAC      A>DYAL060W      +      0      YGPM          chr01:36120          C>A         GCC>GAC      A>DYAL060W      +      0      YS4           chr01:36120          C>A         GCC>GAC      A>DYAL060W      +      0      378604X       chr01:36161          G>A         GCC>ACC      A>TYAL060W      +      0      DBVPG1373     chr01:36161          G>A         GCC>ACC      A>TYAL060W      +      0      DBVPG1853     chr01:36161          G>A         GCC>ACC      A>TYAL060W      +      0      DBVPG6044     chr01:36161          G>A         GCC>ACC      A>TYAL060W      +      0      DBVPG6765     chr01:36161          G>A         GCC>ACC      A>TYAL060W      +      0      L_1528        chr01:36161          G>A         GCC>ACC      A>TYAL060W      +      0      NCYC110       chr01:36161          G>A         GCC>ACC      A>TYAL060W      +      0      SK1           chr01:36161          G>A         GCC>ACC      A>TYAL060W      +      0      UWOPS83_787_3 chr01:36161          G>A         GCC>ACC      A>TYAL060W      +      0      Y55           chr01:36161          G>A         GCC>ACC      A>TYAL060W      +      0      YPS128        chr01:36161          G>A         GCC>ACC      A>TYAL060W      +      0      YPS606        chr01:36161          G>A         GCC>ACC      A>TYAL060W      +      0      YS9           chr01:36161          G>A         GCC>ACC      A>TYAL060W      +      0      DBVPG1853     chr01:36233          G>C         GAG>CAG      E>QYAL061W      +      0      YS4           chr01:33462          C>A         GCG>GAG      A>EYAL061W      +      0      273614X       chr01:33503          A>G         AAG>GAG      K>EYAL061W      +      0      DBVPG1373     chr01:33503          A>G         AAG>GAG      K>EYAL061W      +      0      DBVPG1788     chr01:33503          A>G         AAG>GAG      K>EYAL061W      +      0      DBVPG6765     chr01:33503          A>G         AAG>GAG      K>EYAL061W      +      0      L_1374        chr01:33503          A>G         AAG>GAG      K>EYAL061W      +      0      YJM975        chr01:33503          A>G         AAG>GAG      K>EYAL061W      +      0      YJM978        chr01:33503          A>G         AAG>GAG      K>EYAL061W      +      0      YJM981        chr01:33503          A>G         AAG>GAG      K>EYAL061W      +      0      DBVPG1853     chr01:33512          C>T         CAT>TAT      H>YYAL061W      +      0      DBVPG6044     chr01:33513          A>G         CAT>CGT      H>RYAL061W      +      0      NCYC110       chr01:33513          A>G         CAT>CGT      H>RYAL061W      +      0      UWOPS03_461_4 chr01:33513          A>G         CAT>CGT      H>RYAL061W      +      0      UWOPS05_217_3 chr01:33513          A>G         CAT>CGT      H>RYAL061W      +      0      Y55           chr01:33513          A>G         CAT>CGT      H>RYAL061W      +      0      YIIc17_E5     chr01:33513          A>G         CAT>CGT      H>RYAL061W      +      0      YPS606        chr01:33513          A>G         CAT>CGT      H>RYAL061W      +      0      YS4           chr01:33513          A>G         CAT>CGT      H>RYAL061W      +      0      273614X       chr01:33549          A>C         GAA>GCA      E>AYAL061W      +      0      DBVPG1373     chr01:33549          A>C         GAA>GCA      E>AYAL061W      +      0      DBVPG1788     chr01:33549          A>C         GAA>GCA      E>AYAL061W      +      0      DBVPG1853     chr01:33549          A>C         GAA>GCA      E>AYAL061W      +      0      DBVPG6044     chr01:33549          A>C         GAA>GCA      E>AYAL061W      +      0      DBVPG6765     chr01:33549          A>C         GAA>GCA      E>AYAL061W      +      0      L_1528        chr01:33549          A>C         GAA>GCA      E>AYAL061W      +      0      NCYC110       chr01:33549          A>C         GAA>GCA      E>AYAL061W      +      0      SK1           chr01:33549          A>C         GAA>GCA      E>AYAL061W      +      0      UWOPS03_461_4 chr01:33549          A>C         GAA>GCA      E>AYAL061W      +      0      UWOPS05_217_3 chr01:33549          A>C         GAA>GCA      E>AYAL061W      +      0      Y55           chr01:33549          A>C         GAA>GCA      E>AYAL061W      +      0      Y9            chr01:33549          A>C         GAA>GCA      E>AYAL061W      +      0      YIIc17_E5     chr01:33549          A>C         GAA>GCA      E>AYAL061W      +      0      YJM975        chr01:33549          A>C         GAA>GCA      E>AYAL061W      +      0      YJM978        chr01:33549          A>C         GAA>GCA      E>AYAL061W      +      0      YJM981        chr01:33549          A>C         GAA>GCA      E>AYAL061W      +      0      YPS606        chr01:33549          A>C         GAA>GCA      E>AYAL061W      +      0      YS4           chr01:33549          A>C         GAA>GCA      E>AYAL061W      +      0      YS9           chr01:33549          A>C         GAA>GCA      E>AYAL061W      +      0      378604X       chr01:33613          A>G         GAA>GAG      E>EYAL061W      +      0      DBVPG1373     chr01:33613          A>G         GAA>GAG      E>EYAL061W      +      0      DBVPG1788     chr01:33613          A>G         GAA>GAG      E>EYAL061W      +      0      DBVPG1853     chr01:33613          A>G         GAA>GAG      E>EYAL061W      +      0      DBVPG6765     chr01:33613          A>G         GAA>GAG      E>EYAL061W      +      0      L_1528        chr01:33613          A>G         GAA>GAG      E>EYAL061W      +      0      NCYC110       chr01:33613          A>G         GAA>GAG      E>EYAL061W      +      0      SK1           chr01:33613          A>G         GAA>GAG      E>EYAL061W      +      0      UWOPS03_461_4 chr01:33613          A>G         GAA>GAG      E>EYAL061W      +      0      UWOPS05_217_3 chr01:33613          A>G         GAA>GAG      E>EYAL061W      +      0      Y55           chr01:33613          A>G         GAA>GAG      E>EYAL061W      +      0      YIIc17_E5     chr01:33613          A>G         GAA>GAG      E>EYAL061W      +      0      YJM975        chr01:33613          A>G         GAA>GAG      E>EYAL061W      +      0      YJM978        chr01:33613          A>G         GAA>GAG      E>EYAL061W      +      0      YPS606        chr01:33613          A>G         GAA>GAG      E>EYAL061W      +      0      YS4           chr01:33613          A>G         GAA>GAG      E>EYAL061W      +      0      YS9           chr01:33613          A>G         GAA>GAG      E>EYAL061W      +      0      DBVPG1853     chr01:33691          G>A         GAG>GAA      E>EYAL061W      +      0      UWOPS03_461_4 chr01:33691          G>A         GAG>GAA      E>EYAL061W      +      0      UWOPS05_217_3 chr01:33691          G>A         GAG>GAA      E>EYAL061W      +      0      UWOPS83_787_3 chr01:33691          G>A         GAG>GAA      E>EYAL061W      +      0      YPS606        chr01:33691          G>A         GAG>GAA      E>EYAL061W      +      0      YS4           chr01:33691          G>A         GAG>GAA      E>EYAL061W      +      0      SK1           chr01:33701          G>A         GGT>AGT      G>SYAL061W      +      0      YPS606        chr01:33742          G>A         GAG>GAA      E>EYAL061W      +      0      UWOPS03_461_4 chr01:33748          A>T         ACA>ACT      T>TYAL061W      +      0      378604X       chr01:33757          C>T         TGC>TGT      C>CYAL061W      +      0      NCYC110       chr01:33757          C>T         TGC>TGT      C>CYAL061W      +      0      Y55           chr01:33757          C>T         TGC>TGT      C>CYAL061W      +      0      YIIc17_E5     chr01:33757          C>T         TGC>TGT      C>CYAL061W      +      0      DBVPG1788     chr01:33763          C>T         GAC>GAT      D>DYAL061W      +      0      YS9           chr01:33763          C>T         GAC>GAT      D>DYAL061W      +      0      DBVPG1853     chr01:33772          T>C         CGT>CGC      R>RYAL061W      +      0      YS4           chr01:33772          T>C         CGT>CGC      R>RYAL061W      +      0      SK1           chr01:33790          C>G         AAC>AAG      N>KYAL061W      +      0      YIIc17_E5     chr01:33865          G>C         GCG>GCC      A>AYAL061W      +      0      YPS606        chr01:33865          G>C         GCG>GCC      A>AYAL061W      +      0      273614X       chr01:33893          C>G         CGT>GGT      R>GYAL061W      +      0      BC187         chr01:33893          C>G         CGT>GGT      R>GYAL061W      +      0      DBVPG1373     chr01:33893          C>G         CGT>GGT      R>GYAL061W      +      0      DBVPG1788     chr01:33893          C>G         CGT>GGT      R>GYAL061W      +      0      DBVPG6765     chr01:33893          C>G         CGT>GGT      R>GYAL061W      +      0      L_1374        chr01:33893          C>G         CGT>GGT      R>GYAL061W      +      0      L_1528        chr01:33893          C>G         CGT>GGT      R>GYAL061W      +      0      SK1           chr01:33893          C>G         CGT>GGT      R>GYAL061W      +      0      YJM975        chr01:33893          C>G         CGT>GGT      R>GYAL061W      +      0      YJM978        chr01:33893          C>G         CGT>GGT      R>GYAL061W      +      0      YJM981        chr01:33893          C>G         CGT>GGT      R>GYAL061W      +      0      UWOPS03_461_4 chr01:33901          G>A         GTG>GTA      V>VYAL061W      +      0      UWOPS83_787_3 chr01:33904          G>A         ATG>ATA      M>IYAL061W      +      0      YS9           chr01:33927          T>C         GTA>GCA      V>AYAL061W      +      0      YS4           chr01:33942          C>T         CCC>CTC      P>LYAL061W      +      0      273614X       chr01:33997          A>G         AGA>AGG      R>RYAL061W      +      0      BC187         chr01:33997          A>G         AGA>AGG      R>RYAL061W      +      0      DBVPG1373     chr01:33997          A>G         AGA>AGG      R>RYAL061W      +      0      DBVPG1788     chr01:33997          A>G         AGA>AGG      R>RYAL061W      +      0      DBVPG6765     chr01:33997          A>G         AGA>AGG      R>RYAL061W      +      0      L_1374        chr01:33997          A>G         AGA>AGG      R>RYAL061W      +      0      L_1528        chr01:33997          A>G         AGA>AGG      R>RYAL061W      +      0      SK1           chr01:33997          A>G         AGA>AGG      R>RYAL061W      +      0      YJM975        chr01:33997          A>G         AGA>AGG      R>RYAL061W      +      0      YJM978        chr01:33997          A>G         AGA>AGG      R>RYAL061W      +      0      YJM981        chr01:33997          A>G         AGA>AGG      R>RYAL061W      +      0      UWOPS83_787_3 chr01:34018          C>T         GGC>GGT      G>GYAL061W      +      0      273614X       chr01:34171          T>A         ACT>ACA      T>TYAL061W      +      0      BC187         chr01:34171          T>A         ACT>ACA      T>TYAL061W      +      0      DBVPG1373     chr01:34171          T>A         ACT>ACA      T>TYAL061W      +      0      DBVPG1788     chr01:34171          T>A         ACT>ACA      T>TYAL061W      +      0      DBVPG6765     chr01:34171          T>A         ACT>ACA      T>TYAL061W      +      0      L_1374        chr01:34171          T>A         ACT>ACA      T>TYAL061W      +      0      L_1528        chr01:34171          T>A         ACT>ACA      T>TYAL061W      +      0      SK1           chr01:34171          T>A         ACT>ACA      T>TYAL061W      +      0      YJM981        chr01:34171          T>A         ACT>ACA      T>TYAL061W      +      0      YS4           chr01:34171          T>A         ACT>ACA      T>TYAL061W      +      0      YIIc17_E5     chr01:34183          C>A         GCC>GCA      A>AYAL061W      +      0      DBVPG6044     chr01:34246          T>C         TTT>TTC      F>FYAL061W      +      0      NCYC110       chr01:34246          T>C         TTT>TTC      F>FYAL061W      +      0      Y55           chr01:34246          T>C         TTT>TTC      F>FYAL061W      +      0      UWOPS87_2421  chr01:34258          G>A         GGG>GGA      G>GYAL061W      +      0      UWOPS87_2421  chr01:34297          T>G         ACT>ACG      T>TYAL061W      +      0      273614X       chr01:34309          C>T         ACC>ACT      T>TYAL061W      +      0      BC187         chr01:34309          C>T         ACC>ACT      T>TYAL061W      +      0      DBVPG1373     chr01:34309          C>T         ACC>ACT      T>TYAL061W      +      0      DBVPG6040     chr01:34309          C>T         ACC>ACT      T>TYAL061W      +      0      DBVPG6044     chr01:34309          C>T         ACC>ACT      T>TYAL061W      +      0      DBVPG6765     chr01:34309          C>T         ACC>ACT      T>TYAL061W      +      0      L_1374        chr01:34309          C>T         ACC>ACT      T>TYAL061W      +      0      SK1           chr01:34309          C>T         ACC>ACT      T>TYAL061W      +      0      UWOPS05_217_3 chr01:34309          C>T         ACC>ACT      T>TYAL061W      +      0      UWOPS83_787_3 chr01:34309          C>T         ACC>ACT      T>TYAL061W      +      0      UWOPS87_2421  chr01:34309          C>T         ACC>ACT      T>TYAL061W      +      0      Y55           chr01:34309          C>T         ACC>ACT      T>TYAL061W      +      0      YJM981        chr01:34309          C>T         ACC>ACT      T>TYAL061W      +      0      YS4           chr01:34309          C>T         ACC>ACT      T>TYAL061W      +      0      YS9           chr01:34309          C>T         ACC>ACT      T>TYAL061W      +      0      DBVPG6040     chr01:34315          G>A         GTG>GTA      V>VYAL061W      +      0      DBVPG6044     chr01:34315          G>A         GTG>GTA      V>VYAL061W      +      0      Y55           chr01:34315          G>A         GTG>GTA      V>VYAL061W      +      0      UWOPS87_2421  chr01:34333          C>T         GGC>GGT      G>GYAL061W      +      0      UWOPS83_787_3 chr01:34335          A>G         CAT>CGT      H>RYAL061W      +      0      UWOPS87_2421  chr01:34370          T>C         TTG>CTG      L>LYAL061W      +      0      UWOPS05_217_3 chr01:34372          G>A         TTG>TTA      L>LYAL061W      +      0      SK1           chr01:34424          G>A         GCA>ACA      A>TYAL061W      +      0      SK1           chr01:34498          C>T         AAC>AAT      N>NYAL061W      +      0      UWOPS83_787_3 chr01:34505          G>A         GAC>AAC      D>NYAL061W      +      0      DBVPG1373     chr01:34516          T>C         GAT>GAC      D>DYAL061W      +      0      L_1528        chr01:34516          T>C         GAT>GAC      D>DYAL061W      +      0      SK1           chr01:34516          T>C         GAT>GAC      D>DYAL061W      +      0      UWOPS83_787_3 chr01:34516          T>C         GAT>GAC      D>DYAL061W      +      0      UWOPS87_2421  chr01:34516          T>C         GAT>GAC      D>DYAL061W      +      0      YJM978        chr01:34516          T>C         GAT>GAC      D>DYAL061W      +      0      YJM981        chr01:34516          T>C         GAT>GAC      D>DYAL061W      +      0      DBVPG1373     chr01:34555          A>G         ACA>ACG      T>TYAL061W      +      0      L_1528        chr01:34555          A>G         ACA>ACG      T>TYAL061W      +      0      SK1           chr01:34555          A>G         ACA>ACG      T>TYAL061W      +      0      UWOPS87_2421  chr01:34555          A>G         ACA>ACG      T>TYAL061W      +      0      YJM978        chr01:34555          A>G         ACA>ACG      T>TYAL061W      +      0      YJM981        chr01:34555          A>G         ACA>ACG      T>TYAL061W      +      0      DBVPG6044     chr01:34574          C>T         CCA>TCA      P>SYAL061W      +      0      UWOPS83_787_3 chr01:34598          A>G         AGG>GGG      R>GYAL062W      +      0      K11           chr01:31609          C>T         ATC>ATT      I>IYAL062W      +      0      Y12           chr01:31609          C>T         ATC>ATT      I>IYAL062W      +      0      YS2           chr01:31609          C>T         ATC>ATT      I>IYAL062W      +      0      YS9           chr01:31609          C>T         ATC>ATT      I>IYAL062W      +      0      UWOPS03_461_4 chr01:31621          G>A         GTG>GTA      V>VYAL062W      +      0      UWOPS05_227_2 chr01:31621          G>A         GTG>GTA      V>VYAL062W      +      0      UWOPS87_2421  chr01:31621          G>A         GTG>GTA      V>VYAL062W      +      0      322134S       chr01:31636          T>C         ATT>ATC      I>IYAL062W      +      0      DBVPG1373     chr01:31636          T>C         ATT>ATC      I>IYAL062W      +      0      DBVPG6765     chr01:31636          T>C         ATT>ATC      I>IYAL062W      +      0      L_1374        chr01:31636          T>C         ATT>ATC      I>IYAL062W      +      0      NCYC361       chr01:31636          T>C         ATT>ATC      I>IYAL062W      +      0      SK1           chr01:31636          T>C         ATT>ATC      I>IYAL062W      +      0      YJM975        chr01:31636          T>C         ATT>ATC      I>IYAL062W      +      0      UWOPS03_461_4 chr01:31675          T>C         ATT>ATC      I>IYAL062W      +      0      UWOPS05_227_2 chr01:31675          T>C         ATT>ATC      I>IYAL062W      +      0      UWOPS87_2421  chr01:31684          C>A         GTC>GTA      V>VYAL062W      +      0      UWOPS05_227_2 chr01:31692          G>A         AGG>AAG      R>KYAL062W      +      0      UWOPS87_2421  chr01:31693          G>A         AGG>AGA      R>RYAL062W      +      0      322134S       chr01:31756          C>T         TAC>TAT      Y>YYAL062W      +      0      DBVPG6765     chr01:31756          C>T         TAC>TAT      Y>YYAL062W      +      0      L_1374        chr01:31756          C>T         TAC>TAT      Y>YYAL062W      +      0      NCYC361       chr01:31756          C>T         TAC>TAT      Y>YYAL062W      +      0      SK1           chr01:31756          C>T         TAC>TAT      Y>YYAL062W      +      0      UWOPS03_461_4 chr01:31756          C>T         TAC>TAT      Y>YYAL062W      +      0      UWOPS87_2421  chr01:31756          C>T         TAC>TAT      Y>YYAL062W      +      0      Y12           chr01:31756          C>T         TAC>TAT      Y>YYAL062W      +      0      YJM975        chr01:31756          C>T         TAC>TAT      Y>YYAL062W      +      0      YS2           chr01:31756          C>T         TAC>TAT      Y>YYAL062W      +      0      YS2           chr01:31795          T>C         GGT>GGC      G>GYAL062W      +      0      322134S       chr01:31822          C>T         AAC>AAT      N>NYAL062W      +      0      DBVPG6765     chr01:31822          C>T         AAC>AAT      N>NYAL062W      +      0      L_1374        chr01:31822          C>T         AAC>AAT      N>NYAL062W      +      0      NCYC361       chr01:31822          C>T         AAC>AAT      N>NYAL062W      +      0      SK1           chr01:31822          C>T         AAC>AAT      N>NYAL062W      +      0      UWOPS03_461_4 chr01:31822          C>T         AAC>AAT      N>NYAL062W      +      0      UWOPS87_2421  chr01:31822          C>T         AAC>AAT      N>NYAL062W      +      0      Y12           chr01:31822          C>T         AAC>AAT      N>NYAL062W      +      0      YJM975        chr01:31822          C>T         AAC>AAT      N>NYAL062W      +      0      YS2           chr01:31822          C>T         AAC>AAT      N>NYAL062W      +      0      273614X       chr01:31849          T>C         TTT>TTC      F>FYAL062W      +      0      322134S       chr01:31849          T>C         TTT>TTC      F>FYAL062W      +      0      L_1374        chr01:31849          T>C         TTT>TTC      F>FYAL062W      +      0      NCYC361       chr01:31849          T>C         TTT>TTC      F>FYAL062W      +      0      SK1           chr01:31849          T>C         TTT>TTC      F>FYAL062W      +      0      YJM975        chr01:31849          T>C         TTT>TTC      F>FYAL062W      +      0      YJM978        chr01:31849          T>C         TTT>TTC      F>FYAL062W      +      0      Y12           chr01:31918          C>T         GAC>GAT      D>DYAL062W      +      0      YS2           chr01:31918          C>T         GAC>GAT      D>DYAL062W      +      0      273614X       chr01:31976          C>T         CTG>TTG      L>LYAL062W      +      0      322134S       chr01:31976          C>T         CTG>TTG      L>LYAL062W      +      0      DBVPG1788     chr01:31976          C>T         CTG>TTG      L>LYAL062W      +      0      DBVPG6765     chr01:31976          C>T         CTG>TTG      L>LYAL062W      +      0      L_1374        chr01:31976          C>T         CTG>TTG      L>LYAL062W      +      0      NCYC361       chr01:31976          C>T         CTG>TTG      L>LYAL062W      +      0      SK1           chr01:31976          C>T         CTG>TTG      L>LYAL062W      +      0      YJM975        chr01:31976          C>T         CTG>TTG      L>LYAL062W      +      0      YJM978        chr01:31976          C>T         CTG>TTG      L>LYAL062W      +      0      273614X       chr01:31990          T>C         ATT>ATC      I>IYAL062W      +      0      322134S       chr01:31990          T>C         ATT>ATC      I>IYAL062W      +      0      DBVPG1788     chr01:31990          T>C         ATT>ATC      I>IYAL062W      +      0      DBVPG6765     chr01:31990          T>C         ATT>ATC      I>IYAL062W      +      0      L_1374        chr01:31990          T>C         ATT>ATC      I>IYAL062W      +      0      NCYC361       chr01:31990          T>C         ATT>ATC      I>IYAL062W      +      0      SK1           chr01:31990          T>C         ATT>ATC      I>IYAL062W      +      0      YJM975        chr01:31990          T>C         ATT>ATC      I>IYAL062W      +      0      YJM978        chr01:31990          T>C         ATT>ATC      I>IYAL062W      +      0      YPS128        chr01:32140          G>A         CCG>CCA      P>PYAL062W      +      0      273614X       chr01:32146          C>T         GCC>GCT      A>AYAL062W      +      0      322134S       chr01:32146          C>T         GCC>GCT      A>AYAL062W      +      0      DBVPG1788     chr01:32146          C>T         GCC>GCT      A>AYAL062W      +      0      DBVPG6765     chr01:32146          C>T         GCC>GCT      A>AYAL062W      +      0      SK1           chr01:32146          C>T         GCC>GCT      A>AYAL062W      +      0      YJM975        chr01:32146          C>T         GCC>GCT      A>AYAL062W      +      0      YJM978        chr01:32146          C>T         GCC>GCT      A>AYAL062W      +      0      273614X       chr01:32159          T>C         TTA>CTA      L>LYAL062W      +      0      322134S       chr01:32159          T>C         TTA>CTA      L>LYAL062W      +      0      DBVPG1788     chr01:32159          T>C         TTA>CTA      L>LYAL062W      +      0      DBVPG6765     chr01:32159          T>C         TTA>CTA      L>LYAL062W      +      0      NCYC361       chr01:32159          T>C         TTA>CTA      L>LYAL062W      +      0      SK1           chr01:32159          T>C         TTA>CTA      L>LYAL062W      +      0      UWOPS03_461_4 chr01:32159          T>C         TTA>CTA      L>LYAL062W      +      0      YJM975        chr01:32159          T>C         TTA>CTA      L>LYAL062W      +      0      YJM978        chr01:32159          T>C         TTA>CTA      L>LYAL062W      +      0      YS9           chr01:32179          A>G         GCA>GCG      A>AYAL062W      +      0      UWOPS03_461_4 chr01:32270          T>G         TTG>GTG      L>VYAL062W      +      0      273614X       chr01:32272          G>A         TTG>TTA      L>LYAL062W      +      0      DBVPG1788     chr01:32272          G>A         TTG>TTA      L>LYAL062W      +      0      DBVPG6765     chr01:32272          G>A         TTG>TTA      L>LYAL062W      +      0      K11           chr01:32272          G>A         TTG>TTA      L>LYAL062W      +      0      SK1           chr01:32272          G>A         TTG>TTA      L>LYAL062W      +      0      UWOPS83_787_3 chr01:32272          G>A         TTG>TTA      L>LYAL062W      +      0      Y12           chr01:32272          G>A         TTG>TTA      L>LYAL062W      +      0      YJM978        chr01:32272          G>A         TTG>TTA      L>LYAL062W      +      0      YPS128        chr01:32272          G>A         TTG>TTA      L>LYAL062W      +      0      YS9           chr01:32272          G>A         TTG>TTA      L>LYAL062W      +      0      K11           chr01:32281          C>T         ATC>ATT      I>IYAL062W      +      0      Y12           chr01:32281          C>T         ATC>ATT      I>IYAL062W      +      0      YS9           chr01:32281          C>T         ATC>ATT      I>IYAL062W      +      0      UWOPS03_461_4 chr01:32302          G>A         GTG>GTA      V>VYAL062W      +      0      UWOPS83_787_3 chr01:32302          G>A         GTG>GTA      V>VYAL062W      +      0      YPS128        chr01:32305          T>C         TCT>TCC      S>SYAL062W      +      0      NCYC110       chr01:32494          C>T         GAC>GAT      D>DYAL062W      +      0      Y55           chr01:32494          C>T         GAC>GAT      D>DYAL062W      +      0      273614X       chr01:32512
[truncated: 1,200,000 more chars]
