# Supplementary material for: Proteome-wide evidence for enhanced positive Darwinian selection within intrinsically disordered regions in proteins
Source: Genome Biol. 2011 Jul 19;12(7):R65. doi: 10.1186/gb-2011-12-7-r65 (PMC3218827; doi:10.1186/gb-2011-12-7-r65)
Supplement: Additional file 7 — Chromosomal location of protein-coding region SNPs in the different strains of S. paradoxus. The direction of each gene as well as the base change in each SNP and its affect on codons and encoded amino acids is shown. [file gb-2011-12-7-r65-S7.ZIP › Additional_data_file_7.rtf]

PROTEIN  DIRECTION  PHASE  STRAIN       GENOMIC POSITION      SNP         CODON         AA==========================================================================================YAL001C      +      0      A12           c218:25212           G>C         AAG>AAC      K>NYAL001C      +      0      A4            c218:25212           G>C         AAG>AAC      K>NYAL001C      +      0      DBVPG6304     c218:25212           G>C         AAG>AAC      K>NYAL001C      +      0      UFRJ50816     c218:25212           G>C         AAG>AAC      K>NYAL001C      +      0      A12           c218:25228           T>C         TGT>CGT      C>RYAL001C      +      0      A4            c218:25228           T>C         TGT>CGT      C>RYAL001C      +      0      UFRJ50816     c218:25228           T>C         TGT>CGT      C>RYAL001C      +      0      UWOPS91_917_1 c218:25228           T>A         TGT>AGT      C>SYAL001C      +      0      N_43          c218:25231           G>A         GAT>AAT      D>NYAL001C      +      0      N_45          c218:25231           G>A         GAT>AAT      D>NYAL001C      +      0      A12           c218:25234           A>G         AGC>GGC      S>GYAL001C      +      0      A4            c218:25234           A>G         AGC>GGC      S>GYAL001C      +      0      UFRJ50816     c218:25234           A>G         AGC>GGC      S>GYAL001C      +      0      N_43          c218:25259           C>A         ACA>AAA      T>KYAL001C      +      0      N_45          c218:25259           C>A         ACA>AAA      T>KYAL001C      +      0      UWOPS91_917_1 c218:25270           G>A         GAC>AAC      D>NYAL001C      +      0      A12           c218:25280           G>A         AGT>AAT      S>NYAL001C      +      0      A4            c218:25280           G>A         AGT>AAT      S>NYAL001C      +      0      DBVPG6304     c218:25280           G>A         AGT>AAT      S>NYAL001C      +      0      UFRJ50816     c218:25280           G>A         AGT>AAT      S>NYAL001C      +      0      UWOPS91_917_1 c218:25280           G>A         AGT>AAT      S>NYAL001C      +      0      A12           c218:25296           A>T         GTA>GTT      V>VYAL001C      +      0      A4            c218:25296           A>T         GTA>GTT      V>VYAL001C      +      0      DBVPG6304     c218:25296           A>T         GTA>GTT      V>VYAL001C      +      0      Q89_8         c218:25296           A>T         GTA>GTT      V>VYAL001C      +      0      UFRJ50816     c218:25296           A>T         GTA>GTT      V>VYAL001C      +      0      UWOPS91_917_1 c218:25296           A>T         GTA>GTT      V>VYAL001C      +      0      A12           c218:25338           C>T         TAC>TAT      Y>YYAL001C      +      0      A4            c218:25338           C>T         TAC>TAT      Y>YYAL001C      +      0      DBVPG6304     c218:25338           C>T         TAC>TAT      Y>YYAL001C      +      0      UFRJ50816     c218:25338           C>T         TAC>TAT      Y>YYAL001C      +      0      UWOPS91_917_1 c218:25362           G>A         GGG>GGA      G>GYAL001C      +      0      UWOPS91_917_1 c218:25408           A>G         AAG>GAG      K>EYAL001C      +      0      A12           c218:25438           A>G         ATA>GTA      I>VYAL001C      +      0      A4            c218:25438           A>G         ATA>GTA      I>VYAL001C      +      0      DBVPG6304     c218:25438           A>G         ATA>GTA      I>VYAL001C      +      0      UFRJ50816     c218:25438           A>G         ATA>GTA      I>VYAL001C      +      0      UWOPS91_917_1 c218:25438           A>G         ATA>GTA      I>VYAL001C      +      0      A12           c218:25455           A>T         CCA>CCT      P>PYAL001C      +      0      A4            c218:25455           A>T         CCA>CCT      P>PYAL001C      +      0      DBVPG6304     c218:25455           A>T         CCA>CCT      P>PYAL001C      +      0      UFRJ50816     c218:25455           A>T         CCA>CCT      P>PYAL001C      +      0      UWOPS91_917_1 c218:25455           A>T         CCA>CCT      P>PYAL001C      +      0      N_43          c218:25461           T>C         AGT>AGC      S>SYAL001C      +      0      N_45          c218:25461           T>C         AGT>AGC      S>SYAL001C      +      0      A12           c218:25464           A>G         GTA>GTG      V>VYAL001C      +      0      A4            c218:25464           A>G         GTA>GTG      V>VYAL001C      +      0      DBVPG6304     c218:25464           A>G         GTA>GTG      V>VYAL001C      +      0      UFRJ50816     c218:25464           A>G         GTA>GTG      V>VYAL001C      +      0      UWOPS91_917_1 c218:25464           A>G         GTA>GTG      V>VYAL001C      +      0      N_43          c218:25487           G>A         TGC>TAC      C>YYAL001C      +      0      N_45          c218:25487           G>A         TGC>TAC      C>YYAL001C      +      0      UWOPS91_917_1 c218:25487           G>A         TGC>TAC      C>YYAL001C      +      0      A12           c218:25494           C>G         CTC>CTG      L>LYAL001C      +      0      A4            c218:25494           C>G         CTC>CTG      L>LYAL001C      +      0      DBVPG6304     c218:25494           C>G         CTC>CTG      L>LYAL001C      +      0      UFRJ50816     c218:25494           C>G         CTC>CTG      L>LYAL001C      +      0      UWOPS91_917_1 c218:25494           C>G         CTC>CTG      L>LYAL001C      +      0      A12           c218:25506           G>A         TCG>TCA      S>SYAL001C      +      0      A4            c218:25506           G>A         TCG>TCA      S>SYAL001C      +      0      DBVPG6304     c218:25506           G>A         TCG>TCA      S>SYAL001C      +      0      N_43          c218:25506           G>A         TCG>TCA      S>SYAL001C      +      0      N_45          c218:25506           G>A         TCG>TCA      S>SYAL001C      +      0      UFRJ50816     c218:25506           G>A         TCG>TCA      S>SYAL001C      +      0      Y7            c218:25506           G>A         TCG>TCA      S>SYAL001C      +      0      UWOPS91_917_1 c218:25513           A>C         ATT>CTT      I>LYAL001C      +      0      UWOPS91_917_1 c218:25546           C>T         CTA>TTA      L>LYAL001C      +      0      Q62_5         c218:25565           A>C         GAC>GCC      D>AYAL001C      +      0      T21_4         c218:25565           A>C         GAC>GCC      D>AYAL001C      +      0      A12           c218:25568           G>A         GGG>GAG      G>EYAL001C      +      0      A4            c218:25568           G>A         GGG>GAG      G>EYAL001C      +      0      DBVPG6304     c218:25568           G>A         GGG>GAG      G>EYAL001C      +      0      UFRJ50816     c218:25568           G>A         GGG>GAG      G>EYAL001C      +      0      UWOPS91_917_1 c218:25568           G>A         GGG>GAG      G>EYAL001C      +      0      YPS138        c218:25568           G>A         GGG>GAG      G>EYAL001C      +      0      DBVPG6304     c218:25653           C>T         CGC>CGT      R>RYAL001C      +      0      UWOPS91_917_1 c218:25719           T>C         ATT>ATC      I>IYAL001C      +      0      UWOPS91_917_1 c218:25729           G>A         GCA>ACA      A>TYAL001C      +      0      A12           c218:25746           A>G         AAA>AAG      K>KYAL001C      +      0      A4            c218:25746           A>G         AAA>AAG      K>KYAL001C      +      0      DBVPG6304     c218:25746           A>G         AAA>AAG      K>KYAL001C      +      0      UFRJ50816     c218:25746           A>G         AAA>AAG      K>KYAL001C      +      0      UWOPS91_917_1 c218:25746           A>G         AAA>AAG      K>KYAL001C      +      0      YPS138        c218:25746           A>G         AAA>AAG      K>KYAL001C      +      0      UWOPS91_917_1 c218:25752           C>T         TAC>TAT      Y>YYAL001C      +      0      A12           c218:25779           C>T         CCC>CCT      P>PYAL001C      +      0      A4            c218:25779           C>T         CCC>CCT      P>PYAL001C      +      0      DBVPG6304     c218:25779           C>T         CCC>CCT      P>PYAL001C      +      0      UFRJ50816     c218:25779           C>T         CCC>CCT      P>PYAL001C      +      0      UWOPS91_917_1 c218:25779           C>T         CCC>CCT      P>PYAL001C      +      0      YPS138        c218:25779           C>T         CCC>CCT      P>PYAL001C      +      0      N_45          c218:25786           C>T         CCT>TCT      P>SYAL001C      +      0      A12           c218:25815           T>C         TAT>TAC      Y>YYAL001C      +      0      A4            c218:25815           T>C         TAT>TAC      Y>YYAL001C      +      0      DBVPG6304     c218:25815           T>C         TAT>TAC      Y>YYAL001C      +      0      N_43          c218:25815           T>C         TAT>TAC      Y>YYAL001C      +      0      N_45          c218:25815           T>C         TAT>TAC      Y>YYAL001C      +      0      UFRJ50816     c218:25815           T>C         TAT>TAC      Y>YYAL001C      +      0      YPS138        c218:25815           T>C         TAT>TAC      Y>YYAL001C      +      0      N_43          c218:25824           C>T         GAC>GAT      D>DYAL001C      +      0      N_45          c218:25824           C>T         GAC>GAT      D>DYAL001C      +      0      N_43          c218:25866           T>C         AGT>AGC      S>SYAL001C      +      0      N_45          c218:25866           T>C         AGT>AGC      S>SYAL001C      +      0      N_43          c218:25881           G>A         GAG>GAA      E>EYAL001C      +      0      A12           c218:25898           G>A         AGT>AAT      S>NYAL001C      +      0      A4            c218:25898           G>A         AGT>AAT      S>NYAL001C      +      0      UFRJ50816     c218:25898           G>A         AGT>AAT      S>NYAL001C      +      0      YPS138        c218:25898           G>A         AGT>AAT      S>NYAL001C      +      0      A12           c218:25920           T>A         GAT>GAA      D>EYAL001C      +      0      A4            c218:25920           T>A         GAT>GAA      D>EYAL001C      +      0      N_43          c218:25920           T>A         GAT>GAA      D>EYAL001C      +      0      N_45          c218:25920           T>A         GAT>GAA      D>EYAL001C      +      0      UFRJ50816     c218:25920           T>A         GAT>GAA      D>EYAL001C      +      0      YPS138        c218:25920           T>A         GAT>GAA      D>EYAL001C      +      0      A12           c218:25992           C>T         GAC>GAT      D>DYAL001C      +      0      A4            c218:25992           C>T         GAC>GAT      D>DYAL001C      +      0      UFRJ50816     c218:25992           C>T         GAC>GAT      D>DYAL001C      +      0      YPS138        c218:25992           C>T         GAC>GAT      D>DYAL001C      +      0      A12           c218:26022           T>C         CTT>CTC      L>LYAL001C      +      0      A4            c218:26022           T>C         CTT>CTC      L>LYAL001C      +      0      UFRJ50816     c218:26022           T>C         CTT>CTC      L>LYAL001C      +      0      YPS138        c218:26022           T>C         CTT>CTC      L>LYAL001C      +      0      A12           c218:26034           C>T         TTC>TTT      F>FYAL001C      +      0      A4            c218:26034           C>T         TTC>TTT      F>FYAL001C      +      0      UFRJ50816     c218:26034           C>T         TTC>TTT      F>FYAL001C      +      0      YPS138        c218:26034           C>T         TTC>TTT      F>FYAL001C      +      0      A12           c218:26055           T>A         ACT>ACA      T>TYAL001C      +      0      A4            c218:26055           T>A         ACT>ACA      T>TYAL001C      +      0      A12           c218:26091           A>T         ATA>ATT      I>IYAL001C      +      0      A4            c218:26091           A>T         ATA>ATT      I>IYAL001C      +      0      N_43          c218:26091           A>T         ATA>ATT      I>IYAL001C      +      0      N_44          c218:26091           A>T         ATA>ATT      I>IYAL001C      +      0      N_45          c218:26091           A>T         ATA>ATT      I>IYAL001C      +      0      UFRJ50816     c218:26091           A>T         ATA>ATT      I>IYAL001C      +      0      YPS138        c218:26091           A>T         ATA>ATT      I>IYAL001C      +      0      A12           c218:26183           G>A         AGG>AAG      R>KYAL001C      +      0      A4            c218:26183           G>A         AGG>AAG      R>KYAL001C      +      0      UFRJ50816     c218:26183           G>A         AGG>AAG      R>KYAL001C      +      0      YPS138        c218:26183           G>A         AGG>AAG      R>KYAL001C      +      0      YPS138        c218:26262           C>T         TTC>TTT      F>FYAL001C      +      0      A12           c218:26322           A>G         GGA>GGG      G>GYAL001C      +      0      UFRJ50816     c218:26322           A>G         GGA>GGG      G>GYAL001C      +      0      YPS138        c218:26322           A>G         GGA>GGG      G>GYAL001C      +      0      UWOPS91_917_1 c218:26342           C>a         TCT>TaT      S>YYAL001C      +      0      A12           c218:26349           T>C         ACT>ACC      T>TYAL001C      +      0      A4            c218:26349           T>C         ACT>ACC      T>TYAL001C      +      0      UFRJ50816     c218:26349           T>C         ACT>ACC      T>TYAL001C      +      0      YPS138        c218:26349           T>C         ACT>ACC      T>TYAL001C      +      0      UWOPS91_917_1 c218:26379           T>C         TTT>TTC      F>FYAL001C      +      0      A12           c218:26397           C>T         ACC>ACT      T>TYAL001C      +      0      A4            c218:26397           C>T         ACC>ACT      T>TYAL001C      +      0      N_43          c218:26397           C>T         ACC>ACT      T>TYAL001C      +      0      N_44          c218:26397           C>T         ACC>ACT      T>TYAL001C      +      0      N_45          c218:26397           C>T         ACC>ACT      T>TYAL001C      +      0      UFRJ50816     c218:26397           C>T         ACC>ACT      T>TYAL001C      +      0      UWOPS91_917_1 c218:26397           C>T         ACC>ACT      T>TYAL001C      +      0      YPS138        c218:26397           C>T         ACC>ACT      T>TYAL001C      +      0      A12           c218:26418           A>C         AGA>AGC      R>SYAL001C      +      0      A4            c218:26418           A>C         AGA>AGC      R>SYAL001C      +      0      UFRJ50816     c218:26418           A>C         AGA>AGC      R>SYAL001C      +      0      UWOPS91_917_1 c218:26418           A>C         AGA>AGC      R>SYAL001C      +      0      YPS138        c218:26418           A>C         AGA>AGC      R>SYAL001C      +      0      A12           c218:26460           T>C         ATT>ATC      I>IYAL001C      +      0      A4            c218:26460           T>C         ATT>ATC      I>IYAL001C      +      0      UFRJ50791     c218:26460           T>C         ATT>ATC      I>IYAL001C      +      0      YPS138        c218:26460           T>C         ATT>ATC      I>IYAL001C      +      0      UWOPS91_917_1 c218:26463           T>C         CCT>CCC      P>PYAL001C      +      0      A4            c218:26472           C>A         TCC>TCA      S>SYAL001C      +      0      UFRJ50791     c218:26472           C>A         TCC>TCA      S>SYAL001C      +      0      UWOPS91_917_1 c218:26472           C>A         TCC>TCA      S>SYAL001C      +      0      YPS138        c218:26472           C>A         TCC>TCA      S>SYAL001C      +      0      UWOPS91_917_1 c218:26496           G>A         AAG>AAA      K>KYAL001C      +      0      A4            c218:26537           A>C         AAG>ACG      K>TYAL001C      +      0      N_43          c218:26537           A>C         AAG>ACG      K>TYAL001C      +      0      N_44          c218:26537           A>C         AAG>ACG      K>TYAL001C      +      0      N_45          c218:26537           A>C         AAG>ACG      K>TYAL001C      +      0      UFRJ50791     c218:26537           A>C         AAG>ACG      K>TYAL001C      +      0      YPS138        c218:26537           A>C         AAG>ACG      K>TYAL001C      +      0      UWOPS91_917_1 c218:26545           G>A         GTT>ATT      V>IYAL001C      +      0      UWOPS91_917_1 c218:26550           G>A         TCG>TCA      S>SYAL001C      +      0      UWOPS91_917_1 c218:26552           C>A         ACT>AAT      T>NYAL001C      +      0      A4            c218:26565           T>A         ATT>ATA      I>IYAL001C      +      0      UFRJ50791     c218:26565           T>A         ATT>ATA      I>IYAL001C      +      0      YPS138        c218:26565           T>A         ATT>ATA      I>IYAL001C      +      0      DBVPG4650     c218:26584           G>A         GGT>AGT      G>SYAL001C      +      0      UFRJ50791     c218:26586           T>C         GGT>GGC      G>GYAL001C      +      0      CBS432        c218:26587           G>T         GCC>TCC      A>SYAL001C      +      0      UWOPS91_917_1 c218:26600           C>T         TCA>TTA      S>LYAL001C      +      0      N_43          c218:26613           A>C         GAA>GAC      E>DYAL001C      +      0      N_44          c218:26613           A>C         GAA>GAC      E>DYAL001C      +      0      N_45          c218:26613           A>C         GAA>GAC      E>DYAL001C      +      0      CBS432        c218:26620           C>T         CCA>TCA      P>SYAL001C      +      0      N_43          c218:26682           T>C         GCT>GCC      A>AYAL001C      +      0      N_44          c218:26682           T>C         GCT>GCC      A>AYAL001C      +      0      N_45          c218:26682           T>C         GCT>GCC      A>AYAL001C      +      0      UFRJ50791     c218:26682           T>C         GCT>GCC      A>AYAL001C      +      0      UWOPS91_917_1 c218:26682           T>C         GCT>GCC      A>AYAL001C      +      0      YPS138        c218:26682           T>C         GCT>GCC      A>AYAL001C      +      0      UWOPS91_917_1 c218:26691           A>G         TTA>TTG      L>LYAL001C      +      0      DBVPG6304     c218:26697           G>A         TCG>TCA      S>SYAL001C      +      0      UFRJ50791     c218:26697           G>A         TCG>TCA      S>SYAL001C      +      0      UWOPS91_917_1 c218:26697           G>A         TCG>TCA      S>SYAL001C      +      0      YPS138        c218:26697           G>A         TCG>TCA      S>SYAL001C      +      0      N_44          c218:26710           C>A         CGA>AGA      R>RYAL001C      +      0      N_45          c218:26710           C>A         CGA>AGA      R>RYAL001C      +      0      UWOPS91_917_1 c218:26710           C>A         CGA>AGA      R>RYAL001C      +      0      UFRJ50791     c218:26721           G>A         TTG>TTA      L>LYAL001C      +      0      YPS138        c218:26721           G>A         TTG>TTA      L>LYAL001C      +      0      UWOPS91_917_1 c218:26754           T>C         TAT>TAC      Y>YYAL001C      +      0      UWOPS91_917_1 c218:26772           T>C         TAT>TAC      Y>YYAL001C      +      0      DBVPG6304     c218:26808           A>G         ACA>ACG      T>TYAL001C      +      0      UFRJ50791     c218:26808           A>G         ACA>ACG      T>TYAL001C      +      0      UWOPS91_917_1 c218:26808           A>G         ACA>ACG      T>TYAL001C      +      0      YPS138        c218:26808           A>G         ACA>ACG      T>TYAL001C      +      0      DBVPG6304     c218:26839           A>G         AAC>GAC      N>DYAL001C      +      0      UFRJ50791     c218:26839           A>G         AAC>GAC      N>DYAL001C      +      0      UWOPS91_917_1 c218:26839           A>G         AAC>GAC      N>DYAL001C      +      0      YPS138        c218:26839           A>G         AAC>GAC      N>DYAL001C      +      0      T21_4         c218:26861           A>T         AAA>ATA      K>IYAL001C      +      0      A4            c218:26908           C>T         CTG>TTG      L>LYAL001C      +      0      DBVPG6304     c218:26908           C>T         CTG>TTG      L>LYAL001C      +      0      UFRJ50791     c218:26908           C>T         CTG>TTG      L>LYAL001C      +      0      UWOPS91_917_1 c218:26908           C>T         CTG>TTG      L>LYAL001C      +      0      YPS138        c218:26908           C>T         CTG>TTG      L>LYAL001C      +      0      DBVPG6304     c218:26926           G>A         GAC>AAC      D>NYAL001C      +      0      UFRJ50791     c218:26926           G>A         GAC>AAC      D>NYAL001C      +      0      UWOPS91_917_1 c218:26940           C>G         AGC>AGG      S>RYAL001C      +      0      A12           c218:26982           T>C         ACT>ACC      T>TYAL001C      +      0      A4            c218:26982           T>C         ACT>ACC      T>TYAL001C      +      0      DBVPG6304     c218:26982           T>C         ACT>ACC      T>TYAL001C      +      0      N_44          c218:26982           T>C         ACT>ACC      T>TYAL001C      +      0      N_45          c218:26982           T>C         ACT>ACC      T>TYAL001C      +      0      UFRJ50791     c218:26982           T>C         ACT>ACC      T>TYAL001C      +      0      UWOPS91_917_1 c218:26982           T>C         ACT>ACC      T>TYAL001C      +      0      YPS138        c218:26982           T>C         ACT>ACC      T>TYAL001C      +      0      A4            c218:27008           T>A         TTC>TAC      F>YYAL001C      +      0      DBVPG6304     c218:27008           T>A         TTC>TAC      F>YYAL001C      +      0      UFRJ50791     c218:27008           T>A         TTC>TAC      F>YYAL001C      +      0      UWOPS91_917_1 c218:27008           T>A         TTC>TAC      F>YYAL001C      +      0      UWOPS91_917_1 c218:27087           C>T         CGC>CGT      R>RYAL001C      +      0      DBVPG6304     c218:27114           A>G         TCA>TCG      S>SYAL001C      +      0      A12           c218:27120           G>A         GGG>GGA      G>GYAL001C      +      0      A4            c218:27120           G>A         GGG>GGA      G>GYAL001C      +      0      DBVPG6304     c218:27120           G>A         GGG>GGA      G>GYAL001C      +      0      UFRJ50791     c218:27120           G>A         GGG>GGA      G>GYAL001C      +      0      UWOPS91_917_1 c218:27120           G>A         GGG>GGA      G>GYAL001C      +      0      UWOPS91_917_1 c218:27127           T>C         TCT>CCT      S>PYAL001C      +      0      N_44          c218:27176           G>A         AGA>AAA      R>KYAL001C      +      0      N_45          c218:27176           G>A         AGA>AAA      R>KYAL001C      +      0      UWOPS91_917_1 c218:27197           G>A         AGC>AAC      S>NYAL001C      +      0      DBVPG6304     c218:27198           C>T         AGC>AGT      S>SYAL001C      +      0      UWOPS91_917_1 c218:27291           T>C         GTT>GTC      V>VYAL001C      +      0      IFO1804       c218:27315           C>T         AAC>AAT      N>NYAL001C      +      0      N_45          c218:27315           C>T         AAC>AAT      N>NYAL001C      +      0      A12           c218:27322           T>A         TTG>ATG      L>MYAL001C      +      0      A4            c218:27322           T>A         TTG>ATG      L>MYAL001C      +      0      DBVPG6304     c218:27322           T>A         TTG>ATG      L>MYAL001C      +      0      YPS138        c218:27322           T>A         TTG>ATG      L>MYAL001C      +      0      UWOPS91_917_1 c218:27323           T>C         TTG>TCG      L>SYAL001C      +      0      IFO1804       c218:27354           T>C         AAT>AAC      N>NYAL001C      +      0      N_44          c218:27354           T>C         AAT>AAC      N>NYAL001C      +      0      N_45          c218:27354           T>C         AAT>AAC      N>NYAL001C      +      0      A12           c218:27390           A>G         GCA>GCG      A>AYAL001C      +      0      A4            c218:27390           A>G         GCA>GCG      A>AYAL001C      +      0      A12           c218:27477           A>G         AGA>AGG      R>RYAL001C      +      0      A4            c218:27477           A>G         AGA>AGG      R>RYAL001C      +      0      UWOPS91_917_1 c218:27477           A>G         AGA>AGG      R>RYAL001C      +      0      YPS138        c218:27477           A>G         AGA>AGG      R>RYAL001C      +      0      A12           c218:27492           T>C         GAT>GAC      D>DYAL001C      +      0      A4            c218:27492           T>C         GAT>GAC      D>DYAL001C      +      0      UWOPS91_917_1 c218:27492           T>C         GAT>GAC      D>DYAL001C      +      0      YPS138        c218:27492           T>C         GAT>GAC      D>DYAL001C      +      0      A12           c218:27511           A>G         ATT>GTT      I>VYAL001C      +      0      A4            c218:27511           A>G         ATT>GTT      I>VYAL001C      +      0      YPS138        c218:27511           A>G         ATT>GTT      I>VYAL001C      +      0      A12           c218:27546           G>A         GAG>GAA      E>EYAL001C      +      0      A4            c218:27546           G>A         GAG>GAA      E>EYAL001C      +      0      IFO1804       c218:27546           G>A         GAG>GAA      E>EYAL001C      +      0      N_44          c218:27546           G>A         GAG>GAA      E>EYAL001C      +      0      N_45          c218:27546           G>A         GAG>GAA      E>EYAL001C      +      0      YPS138        c218:27546           G>A         GAG>GAA      E>EYAL001C      +      0      UWOPS91_917_1 c218:27555           T>C         ATT>ATC      I>IYAL001C      +      0      A12           c218:27600           A>G         AAA>AAG      K>KYAL001C      +      0      A4            c218:27600           A>G         AAA>AAG      K>KYAL001C      +      0      UFRJ50816     c218:27600           A>G         AAA>AAG      K>KYAL001C      +      0      UWOPS91_917_1 c218:27600           A>G         AAA>AAG      K>KYAL001C      +      0      YPS138        c218:27600           A>G         AAA>AAG      K>KYAL001C      +      0      A12           c218:27631           C>T         CCA>TCA      P>SYAL001C      +      0      A4            c218:27631           C>T         CCA>TCA      P>SYAL001C      +      0      UFRJ50816     c218:27631           C>T         CCA>TCA      P>SYAL001C      +      0      UWOPS91_917_1 c218:27631           C>T         CCA>TCA      P>SYAL001C      +      0      YPS138        c218:27631           C>T         CCA>TCA      P>SYAL001C      +      0      A12           c218:27639           T>C         TCT>TCC      S>SYAL001C      +      0      A4            c218:27639           T>C         TCT>TCC      S>SYAL001C      +      0      UFRJ50816     c218:27639           T>C         TCT>TCC      S>SYAL001C      +      0      UWOPS91_917_1 c218:27639           T>C         TCT>TCC      S>SYAL001C      +      0      YPS138        c218:27639           T>C         TCT>TCC      S>SYAL001C      +      0      IFO1804       c218:27651           G>C         CTG>CTC      L>LYAL001C      +      0      N_44          c218:27651           G>C         CTG>CTC      L>LYAL001C      +      0      N_45          c218:27651           G>C         CTG>CTC      L>LYAL001C      +      0      UWOPS91_917_1 c218:27702           C>T         TAC>TAT      Y>YYAL001C      +      0      IFO1804       c218:27710           G>T         AGG>ATG      R>MYAL001C      +      0      N_44          c218:27710           G>T         AGG>ATG      R>MYAL001C      +      0      N_45          c218:27710           G>T         AGG>ATG      R>MYAL001C      +      0      UWOPS91_917_1 c218:27717           C>T         TCC>TCT      S>SYAL001C      +      0      A12           c218:27720           T>A         GGT>GGA      G>GYAL001C      +      0      UFRJ50816     c218:27720           T>A         GGT>GGA      G>GYAL001C      +      0      YPS138        c218:27720           T>A         GGT>GGA      G>GYAL001C      +      0      Q89_8         c218:27774           C>T         ATC>ATT      I>IYAL001C      +      0      UWOPS91_917_1 c218:27774           C>T         ATC>ATT      I>IYAL001C      +      0      A12           c218:27780           T>C         TCT>TCC      S>SYAL001C      +      0      UFRJ50816     c218:27780           T>C         TCT>TCC      S>SYAL001C      +      0      YPS138        c218:27780           T>C         TCT>TCC      S>SYAL001C      +      0      UWOPS91_917_1 c218:27830           A>G         AAG>AGG      K>RYAL001C      +      0      UWOPS91_917_1 c218:27839           G>A         GGA>GAA      G>EYAL001C      +      0      IFO1804       c218:27924           T>C         GAT>GAC      D>DYAL001C      +      0      UWOPS91_917_1 c218:27957           C>T         AAC>AAT      N>NYAL001C      +      0      N_43          c218:28062           C>T         TCC>TCT      S>SYAL001C      +      0      N_44          c218:28062           C>T         TCC>TCT      S>SYAL001C      +      0      N_45          c218:28062           C>T         TCC>TCT      S>SYAL001C      +      0      A12           c218:28077           T>C         GAT>GAC      D>DYAL001C      +      0      DBVPG4650     c218:28107           T>C         GAT>GAC      D>DYAL001C      +      0      A12           c218:28125           G>A         AGG>AGA      R>RYAL001C      +      0      DBVPG6304     c218:28125           G>A         AGG>AGA      R>RYAL001C      +      0      UFRJ50816     c218:28125           G>A         AGG>AGA      R>RYAL001C      +      0      N_43          c218:28210           A>G         AGA>GGA      R>GYAL001C      +      0      A12           c218:28303           T>C         TCA>CCA      S>PYAL001C      +      0      DBVPG6304     c218:28303           T>C         TCA>CCA      S>PYAL001C      +      0      N_43          c218:28303           T>C         TCA>CCA      S>PYAL001C      +      0      N_44          c218:28303           T>C         TCA>CCA      S>PYAL001C      +      0      N_45          c218:28303           T>C         TCA>CCA      S>PYAL001C      +      0      UFRJ50816     c218:28303           T>C         TCA>CCA      S>PYAL001C      +      0      YPS138        c218:28303           T>C         TCA>CCA      S>PYAL001C      +      0      A12           c218:28344           G>A         GAG>GAA      E>EYAL001C      +      0      DBVPG6304     c218:28344           G>A         GAG>GAA      E>EYAL001C      +      0      UFRJ50816     c218:28344           G>A         GAG>GAA      E>EYAL001C      +      0      UWOPS91_917_1 c218:28344           G>A         GAG>GAA      E>EYAL001C      +      0      YPS138        c218:28344           G>A         GAG>GAA      E>EYAL001C      +      0      UWOPS91_917_1 c218:28383           A>C         CAA>CAC      Q>HYAL001C      +      0      A12           c218:28385           T>C         TTT>TCT      F>SYAL001C      +      0      DBVPG6304     c218:28385           T>C         TTT>TCT      F>SYAL001C      +      0      YPS138        c218:28385           T>C         TTT>TCT      F>SYAL001C      +      0      N_43          c218:28386           T>G         TTT>TTG      F>LYAL001C      +      0      N_44          c218:28386           T>G         TTT>TTG      F>LYAL001C      +      0      N_45          c218:28386           T>G         TTT>TTG      F>LYAL001C      +      0      A12           c218:28393           C>T         CAT>TAT      H>YYAL001C      +      0      DBVPG6304     c218:28393           C>T         CAT>TAT      H>YYAL001C      +      0      N_43          c218:28393           C>T         CAT>TAT      H>YYAL001C      +      0      N_44          c218:28393           C>T         CAT>TAT      H>YYAL001C      +      0      N_45          c218:28393           C>T         CAT>TAT      H>YYAL001C      +      0      UWOPS91_917_1 c218:28393           C>T         CAT>TAT      H>YYAL001C      +      0      Y7            c218:28393           C>T         CAT>TAT      H>YYAL001C      +      0      YPS138        c218:28393           C>T         CAT>TAT      H>YYAL001C      +      0      A12           c218:28422           G>A         TCG>TCA      S>SYAL001C      +      0      DBVPG6304     c218:28422           G>A         TCG>TCA      S>SYAL001C      +      0      UWOPS91_917_1 c218:28422           G>A         TCG>TCA      S>SYAL001C      +      0      YPS138        c218:28422           G>A         TCG>TCA      S>SYAL001C      +      0      UWOPS91_917_1 c218:28443           A>T         CTA>CTT      L>LYAL001C      +      0      UWOPS91_917_1 c218:28455           A>G         GTA>GTG      V>VYAL001C      +      0      A12           c218:28467           G>C         ACG>ACC      T>TYAL001C      +      0      DBVPG6304     c218:28467           G>C         ACG>ACC      T>TYAL001C      +      0      YPS138        c218:28467           G>C         ACG>ACC      T>TYAL001C      +      0      A12           c218:28474           A>G         AAT>GAT      N>DYAL001C      +      0      DBVPG6304     c218:28474           A>G         AAT>GAT      N>DYAL001C      +      0      UWOPS91_917_1 c218:28474           A>G         AAT>GAT      N>DYAL001C      +      0      YPS138        c218:28474           A>G         AAT>GAT      N>DYAL001C      +      0      UWOPS91_917_1 c218:28487           T>C         GTT>GCT      V>AYAL005C      +      0      DBVPG6304     c218:34699           T>G         ATT>ATG      I>MYAL005C      +      0      A4            c218:34717           C>T         TAC>TAT      Y>YYAL005C      +      0      UFRJ50791     c218:34717           C>T         TAC>TAT      Y>YYAL005C      +      0      YPS138        c218:34717           C>T         TAC>TAT      Y>YYAL005C      +      0      CBS432        c218:34786           G>A         ACG>ACA      T>TYAL005C      +      0      CBS5829       c218:34786           G>A         ACG>ACA      T>TYAL005C      +      0      N_44          c218:34786           G>A         ACG>ACA      T>TYAL005C      +      0      N_45          c218:34786           G>A         ACG>ACA      T>TYAL005C      +      0      Q32_3         c218:34786           G>A         ACG>ACA      T>TYAL005C      +      0      Q95_3         c218:34786           G>A         ACG>ACA      T>TYAL005C      +      0      S36_7         c218:34786           G>A         ACG>ACA      T>TYAL005C      +      0      A4            c218:34792           T>A         TCT>TCA      S>SYAL005C      +      0      UFRJ50791     c218:34792           T>A         TCT>TCA      S>SYAL005C      +      0      YPS138        c218:34792           T>A         TCT>TCA      S>SYAL005C      +      0      N_44          c218:34861           T>G         CCT>CCG      P>PYAL005C      +      0      A4            c218:34912           C>T         GAC>GAT      D>DYAL005C      +      0      YPS138        c218:34912           C>T         GAC>GAT      D>DYAL005C      +      0      N_44          c218:34948           C>T         TTC>TTT      F>FYAL005C      +      0      N_45          c218:34948           C>T         TTC>TTT      F>FYAL005C      +      0      A4            c218:35017           C>T         ACC>ACT      T>TYAL005C      +      0      UWOPS91_917_1 c218:35017           C>T         ACC>ACT      T>TYAL005C      +      0      YPS138        c218:35017           C>T         ACC>ACT      T>TYAL005C      +      0      A4            c218:35044           A>G         TTA>TTG      L>LYAL005C      +      0      YPS138        c218:35044           A>G         TTA>TTG      L>LYAL005C      +      0      A4            c218:35062           C>T         ACC>ACT      T>TYAL005C      +      0      YPS138        c218:35062           C>T         ACC>ACT      T>TYAL005C      +      0      A4            c218:35075           T>C         TTG>CTG      L>LYAL005C      +      0      UWOPS91_917_1 c218:35075           T>C         TTG>CTG      L>LYAL005C      +      0      YPS138        c218:35075           T>C         TTG>CTG      L>LYAL005C      +      0      DBVPG4650     c218:35107           T>C         ACT>ACC      T>TYAL005C      +      0      UWOPS91_917_1 c218:35110           C>G         GTC>GTG      V>VYAL005C      +      0      YPS138        c218:35161           C>T         ACC>ACT      T>TYAL005C      +      0      CBS432        c218:35191           T>C         ATT>ATC      I>IYAL005C      +      0      CBS5829       c218:35191           T>C         ATT>ATC      I>IYAL005C      +      0      DBVPG4650     c218:35191           T>C         ATT>ATC      I>IYAL005C      +      0      Q59_1         c218:35191           T>C         ATT>ATC      I>IYAL005C      +      0      N_43          c218:35267           T>C         TTG>CTG      L>LYAL005C      +      0      N_44          c218:35267           T>C         TTG>CTG      L>LYAL005C      +      0      UWOPS91_917_1 c218:35267           T>C         TTG>CTG      L>LYAL005C      +      0      CBS432        c218:35389           T>C         CAT>CAC      H>HYAL005C      +      0      CBS5829       c218:35389           T>C         CAT>CAC      H>HYAL005C      +      0      DBVPG4650     c218:35389           T>C         CAT>CAC      H>HYAL005C      +      0      N_43          c218:35389           T>C         CAT>CAC      H>HYAL005C      +      0      N_44          c218:35389           T>C         CAT>CAC      H>HYAL005C      +      0      Q59_1         c218:35389           T>C         CAT>CAC      H>HYAL005C      +      0      UWOPS91_917_1 c218:35389           T>C         CAT>CAC      H>HYAL005C      +      0      YPS138        c218:35389           T>C         CAT>CAC      H>HYAL005C      +      0      N_43          c218:35432           T>A         TCC>ACC      S>TYAL005C      +      0      N_44          c218:35432           T>A         TCC>ACC      S>TYAL005C      +      0      UWOPS91_917_1 c218:35432           T>A         TCC>ACC      S>TYAL005C      +      0      YPS138        c218:35432           T>A         TCC>ACC      S>TYAL005C      +      0      N_43          c218:35473           A>G         GAA>GAG      E>EYAL005C      +      0      N_44          c218:35473           A>G         GAA>GAG      E>EYAL005C      +      0      A12           c218:35491           A>G         TTA>TTG      L>LYAL005C      +      0      UWOPS91_917_1 c218:35491           A>G         TTA>TTG      L>LYAL005C      +      0      YPS138        c218:35491           A>G         TTA>TTG      L>LYAL005C      +      0      A12           c218:35503           T>C         GCT>GCC      A>AYAL005C      +      0      YPS138        c218:35503           T>C         GCT>GCC      A>AYAL005C      +      0      A12           c218:35533           T>C         TTT>TTC      F>FYAL005C      +      0      DBVPG6304     c218:35584           G>A         TTG>TTA      L>LYAL005C      +      0      UWOPS91_917_1 c218:35584           G>A         TTG>TTA      L>LYAL005C      +      0      N_43          c218:35674           T>C         GTT>GTC      V>VYAL005C      +      0      N_44          c218:35674           T>C         GTT>GTC      V>VYAL005C      +      0      N_45          c218:35674           T>C         GTT>GTC      V>VYAL005C      +      0      UWOPS91_917_1 c218:35674           T>C         GTT>GTC      V>VYAL005C      +      0      UWOPS91_917_1 c218:35680           C>T         GTC>GTT      V>VYAL005C      +      0      UWOPS91_917_1 c218:35689           T>C         TCT>TCC      S>SYAL005C      +      0      UWOPS91_917_1 c218:35743           G>A         GAG>GAA      E>EYAL005C      +      0      DBVPG6304     c218:35746           A>T         CCA>CCT      P>PYAL005C      +      0      DBVPG6304     c218:35755           T>C         TCT>TCC      S>SYAL005C      +      0      N_43          c218:35809           G>A         TTG>TTA      L>LYAL005C      +      0      N_44          c218:35809           G>A         TTG>TTA      L>LYAL005C      +      0      N_45          c218:35809           G>A         TTG>TTA      L>LYAL005C      +      0      UWOPS91_917_1 c218:35809           G>A         TTG>TTA      L>LYAL005C      +      0      N_43          c218:35840           T>C         TTA>CTA      L>LYAL005C      +      0      N_44          c218:35840           T>C         TTA>CTA      L>LYAL005C      +      0      N_45          c218:35840           T>C         TTA>CTA      L>LYAL005C      +      0      UWOPS91_917_1 c218:35840           T>C         TTA>CTA      L>LYAL005C      +      0      DBVPG6304     c218:35896           C>T         GTC>GTT      V>VYAL005C      +      0      UWOPS91_917_1 c218:35974           G>A         CAG>CAA      Q>QYAL005C      +      0      N_43          c218:36013           C>T         GCC>GCT      A>AYAL005C      +      0      N_44          c218:36013           C>T         GCC>GCT      A>AYAL005C      +      0      N_45          c218:36013           C>T         GCC>GCT      A>AYAL005C      +      0      DBVPG6304     c218:36070           T>C         GCT>GCC      A>AYAL005C      +      0      UWOPS91_917_1 c218:36070           T>C         GCT>GCC      A>AYAL005C      +      0      UWOPS91_917_1 c218:36082           C>T         GTC>GTT      V>VYAL005C      +      0      A12           c218:36124           C>T         ATC>ATT      I>IYAL005C      +      0      UWOPS91_917_1 c218:36142           T>C         GTT>GTC      V>VYAL005C      +      0      UWOPS91_917_1 c218:36163           C>T         TCC>TCT      S>SYAL005C      +      0      A12           c218:36190           G>A         AAG>AAA      K>KYAL005C      +      0      DBVPG6304     c218:36190           G>A         AAG>AAA      K>KYAL005C      +      0      UWOPS91_917_1 c218:36190           G>A         AAG>AAA      K>KYAL005C      +      0      N_43          c218:36375           C>G         ACT>AGT      T>SYAL005C      +      0      N_44          c218:36375           C>G         ACT>AGT      T>SYAL005C      +      0      N_45          c218:36375           C>G         ACT>AGT      T>SYAL005C      +      0      A12           c218:36394           T>C         ACT>ACC      T>TYAL005C      +      0      DBVPG6304     c218:36394           T>C         ACT>ACC      T>TYAL005C      +      0      UFRJ50791     c218:36394           T>C         ACT>ACC      T>TYAL005C      +      0      A12           c218:36475           T>C         AAT>AAC      N>NYAL005C      +      0      N_43          c218:36475           T>C         AAT>AAC      N>NYAL005C      +      0      N_44          c218:36475           T>C         AAT>AAC      N>NYAL005C      +      0      N_45          c218:36475           T>C         AAT>AAC      N>NYAL005C      +      0      UFRJ50791     c218:36475           T>C         AAT>AAC      N>NYAL005C      +      0      A12           c218:36487           T>C         TCT>TCC      S>SYAL005C      +      0      A12           c218:36505           T>C         GGT>GGC      G>GYAL005C      +      0      UFRJ50791     c218:36505           T>C         GGT>GGC      G>GYAL005C      +      0      A12           c218:36516           A>G         GAC>GGC      D>GYAL005C      +      0      N_43          c218:36516           A>G         GAC>GGC      D>GYAL005C      +      0      N_44          c218:36516           A>G         GAC>GGC      D>GYAL005C      +      0      N_45          c218:36516           A>G         GAC>GGC      D>GYAL005C      +      0      UFRJ50791     c218:36516           A>G         GAC>GGC      D>GYAL005C      +      0      N_43          c218:36571           A>T         CCA>CCT      P>PYAL005C      +      0      N_44          c218:36571           A>T         CCA>CCT      P>PYAL005C      +      0      N_45          c218:36571           A>T         CCA>CCT      P>PYAL007C      +      0      A12           c218:37829           C>T         CCC>CCT      P>PYAL007C      +      0      DBVPG6304     c218:37829           C>T         CCC>CCT      P>PYAL007C      +      0      UFRJ50791     c218:37829           C>T         CCC>CCT      P>PYAL007C      +      0      YPS138        c218:37829           C>T         CCC>CCT      P>PYAL007C      +      0      N_44          c218:37833           T>G         TTC>GTC      F>VYAL007C      +      0      N_45          c218:37833           T>G         TTC>GTC      F>VYAL007C      +      0      A12           c218:37860           G>A         GTC>ATC      V>IYAL007C      +      0      DBVPG6304     c218:37860           G>A         GTC>ATC      V>IYAL007C      +      0      UFRJ50791     c218:37860           G>A         GTC>ATC      V>IYAL007C      +      0      YPS138        c218:37860           G>A         GTC>ATC      V>IYAL007C      +      0      N_44          c218:37877           A>G         GCA>GCG      A>AYAL007C      +      0      N_45          c218:37877           A>G         GCA>GCG      A>AYAL007C      +      0      A12           c218:37931           T>C         TGT>TGC      C>CYAL007C      +      0      DBVPG6304     c218:37931           T>C         TGT>TGC      C>CYAL007C      +      0      UFRJ50816     c218:37931           T>C         TGT>TGC      C>CYAL007C      +      0      YPS138        c218:37931           T>C         TGT>TGC      C>CYAL007C      +      0      N_45          c218:37932           C>T         CTG>TTG      L>LYAL007C      +      0      N_45          c218:37955           G>A         GAG>GAA      E>EYAL007C      +      0      A12           c218:37979           T>C         TAT>TAC      Y>YYAL007C      +      0      DBVPG6304     c218:37979           T>C         TAT>TAC      Y>YYAL007C      +      0      UFRJ50791     c218:37979           T>C         TAT>TAC      Y>YYAL007C      +      0      UFRJ50816     c218:37979           T>C         TAT>TAC      Y>YYAL007C      +      0      YPS138        c218:37979           T>C         TAT>TAC      Y>YYAL007C      +      0      A12           c218:38066           A>G         AAA>AAG      K>KYAL007C      +      0      DBVPG6304     c218:38066           A>G         AAA>AAG      K>KYAL007C      +      0      UFRJ50816     c218:38066           A>G         AAA>AAG      K>KYAL007C      +      0      YPS138        c218:38066           A>G         AAA>AAG      K>KYAL007C      +      0      YPS138        c218:38072           C>G         TCC>TCG      S>SYAL007C      +      0      N_45          c218:38114           C>T         TTC>TTT      F>FYAL007C      +      0      DBVPG6304     c218:38153           A>G         GTA>GTG      V>VYAL007C      +      0      N_45          c218:38153           A>G         GTA>GTG      V>VYAL007C      +      0      UFRJ50816     c218:38153           A>G         GTA>GTG      V>VYAL007C      +      0      YPS138        c218:38153           A>G         GTA>GTG      V>VYAL007C      +      0      A12           c218:38165           A>G         TTA>TTG      L>LYAL007C      +      0      DBVPG6304     c218:38165           A>G         TTA>TTG      L>LYAL007C      +      0      UFRJ50816     c218:38165           A>G         TTA>TTG      L>LYAL007C      +      0      YPS138        c218:38165           A>G         TTA>TTG      L>LYAL007C      +      0      DBVPG6304     c218:38444           C>T         TAC>TAT      Y>YYAL007C      +      0      UFRJ50816     c218:38444           C>T         TAC>TAT      Y>YYAL007C      +      0      YPS138        c218:38444           C>T         TAC>TAT      Y>YYAL008W      -      0      DBVPG6304     c218:38640           C>T         CAT>CAT      H>HYAL008W      -      0      UFRJ50791     c218:38640           C>T         CAT>CAT      H>HYAL008W      -      0      UFRJ50816     c218:38640           C>T         CAT>CAT      H>HYAL008W      -      0      A12           c218:38672           G>A         AAA>AAT      K>NYAL008W      -      0      DBVPG6304     c218:38672           G>A         AAA>AAT      K>NYAL008W      -      0      N_44          c218:38672           G>A         AAA>AAT      K>NYAL008W      -      0      N_45          c218:38672           G>A         AAA>AAT      K>NYAL008W      -      0      UFRJ50791     c218:38672           G>A         AAA>AAT      K>NYAL008W      -      0      N_44          c218:38702           G>A         GAA>GAT      E>DYAL008W      -      0      N_45          c218:38702           G>A         GAA>GAT      E>DYAL008W      -      0      A12           c218:38708           G>A         GTT>GTT      V>VYAL008W      -      0      DBVPG6304     c218:38708           G>A         GTT>GTT      V>VYAL008W      -      0      UFRJ50791     c218:38708           G>A         GTT>GTT      V>VYAL008W      -      0      UFRJ50816     c218:38708           G>A         GTT>GTT      V>VYAL008W      -      0      UWOPS91_917_1 c218:38708           G>A         GTT>GTT      V>VYAL008W      -      0      A12           c218:38719           A>G         CAA>CAA      Q>QYAL008W      -      0      DBVPG6304     c218:38719           A>G         CAA>CAA      Q>QYAL008W      -      0      UFRJ50791     c218:38719           A>G         CAA>CAA      Q>QYAL008W      -      0      UFRJ50816     c218:38719           A>G         CAA>CAA      Q>QYAL008W      -      0      UWOPS91_917_1 c218:38719           A>G         CAA>CAA      Q>QYAL008W      -      0      N_44          c218:38726           A>G         AGG>AGC      R>SYAL008W      -      0      N_45          c218:38726           A>G         AGG>AGC      R>SYAL008W      -      0      A12           c218:38777           G>A         AGC>AGT      S>SYAL008W      -      0      DBVPG6304     c218:38777           G>A         AGC>AGT      S>SYAL008W      -      0      N_44          c218:38777           G>A         AGC>AGT      S>SYAL008W      -      0      N_45          c218:38777           G>A         AGC>AGT      S>SYAL008W      -      0      UFRJ50791     c218:38777           G>A         AGC>AGT      S>SYAL008W      -      0      UFRJ50816     c218:38777           G>A         AGC>AGT      S>SYAL008W      -      0      UWOPS91_917_1 c218:38777           G>A         AGC>AGT      S>SYAL008W      -      0      A12           c218:38810           T>C         CAG>CAG      Q>QYAL008W      -      0      DBVPG6304     c218:38810           T>C         CAG>CAG      Q>QYAL008W      -      0      UFRJ50791     c218:38810           T>C         CAG>CAG      Q>QYAL008W      -      0      UFRJ50816     c218:38810           T>C         CAG>CAG      Q>QYAL008W      -      0      UWOPS91_917_1 c218:38810           T>C         CAG>CAG      Q>QYAL008W      -      0      A12           c218:38822           A>G         TTG>TTC      L>FYAL008W      -      0      DBVPG6304     c218:38822           A>G         TTG>TTC      L>FYAL008W      -      0      UFRJ50791     c218:38822           A>G         TTG>TTC      L>FYAL008W      -      0      UFRJ50816     c218:38822           A>G         TTG>TTC      L>FYAL008W      -      0      A12           c218:38909           T>A         GCT>GCT      A>AYAL008W      -      0      DBVPG6304     c218:38909           T>A         GCT>GCT      A>AYAL008W      -      0      UFRJ50791     c218:38909           T>A         GCT>GCT      A>AYAL008W      -      0      UFRJ50816     c218:38909           T>A         GCT>GCT      A>AYAL008W      -      0      UWOPS91_917_1 c218:38909           T>A         GCT>GCT      A>AYAL008W      -      0      A12           c218:38957           T>G         CTT>CTC      L>LYAL008W      -      0      DBVPG6304     c218:38957           T>G         CTT>CTC      L>LYAL008W      -      0      N_44          c218:38957           T>G         CTT>CTC      L>LYAL008W      -      0      N_45          c218:38957           T>G         CTT>CTC      L>LYAL008W      -      0      UWOPS91_917_1 c218:38957           T>G         CTT>CTC      L>LYAL008W      -      0      A12           c218:38975           G>T         TGA>TGA      *>*YAL008W      -      0      A4            c218:38975           G>T         TGA>TGA      *>*YAL008W      -      0      DBVPG6304     c218:38975           G>T         TGA>TGA      *>*YAL008W      -      0      UWOPS91_917_1 c218:38975           G>T         TGA>TGA      *>*YAL008W      -      0      N_44          c218:38995           G>C         TAA>GAA      *>EYAL008W      -      0      N_45          c218:38995           G>C         TAA>GAA      *>EYAL008W      -      0      A12           c218:39000           C>T         AAC>AAC      N>NYAL008W      -      0      A4            c218:39000           C>T         AAC>AAC      N>NYAL008W      -      0      DBVPG6304     c218:39000           C>T         AAC>AAC      N>NYAL008W      -      0      UWOPS91_917_1 c218:39000           C>T         AAC>AAC      N>NYAL008W      -      0      N_44          c218:39018           G>A         GGA>GTA      G>VYAL008W      -      0      N_45          c218:39018           G>A         GGA>GTA      G>VYAL008W      -      0      A12           c218:39083           A>G         CCA>CCC      P>PYAL008W      -      0      A4            c218:39083           A>G         CCA>CCC      P>PYAL008W      -      0      DBVPG6304     c218:39083           A>G         CCA>CCC      P>PYAL008W      -      0      UWOPS91_917_1 c218:39083           A>G         CCA>CCC      P>PYAL008W      -      0      N_43          c218:39104           A>G         GTT>GTC      V>VYAL008W      -      0      N_44          c218:39104           A>G         GTT>GTC      V>VYAL008W      -      0      N_45          c218:39104           A>G         GTT>GTC      V>VYAL008W      -      0      A12           c218:39116           A>G         AAC>AAC      N>NYAL008W      -      0      A4            c218:39116           A>G         AAC>AAC      N>NYAL008W      -      0      UWOPS91_917_1 c218:39116           A>G         AAC>AAC      N>NYAL008W      -      0      A12           c218:39147           C>T         TTT>TAT      F>YYAL008W      -      0      A4            c218:39147           C>T         TTT>TAT      F>YYAL008W      -      0      DBVPG6304     c218:39147           C>T         TTT>TAT      F>YYAL008W      -      0      UWOPS91_917_1 c218:39147           C>T         TTT>TAT      F>YYAL008W      -      0      A12           c218:39157           G>A         AAT>TAT      N>YYAL008W      -      0      DBVPG6304     c218:39157           G>A         AAT>TAT      N>YYAL008W      -      0      N_43          c218:39157           G>A         AAT>TAT      N>YYAL008W      -      0      N_44          c218:39157           G>A         AAT>TAT      N>YYAL008W      -      0      N_45          c218:39157           G>A         AAT>TAT      N>YYAL008W      -      0      UWOPS91_917_1 c218:39157           G>A         AAT>TAT      N>YYAL008W      -      0      UWOPS91_917_1 c218:39187           A>G         GCC>CCC      A>PYAL009W      -      0      A12           c218:39548           A>G         CAC>CAC      H>HYAL009W      -      0      A4            c218:39548           A>G         CAC>CAC      H>HYAL009W      -      0      UFRJ50816     c218:39548           A>G         CAC>CAC      H>HYAL009W      -      0      A4            c218:39572           A>G         ACT>ACC      T>TYAL009W      -      0      A12           c218:39647           G>A         ACT>ACT      T>TYAL009W      -      0      A4            c218:39647           G>A         ACT>ACT      T>TYAL009W      -      0      UFRJ50816     c218:39647           G>A         ACT>ACT      T>TYAL009W      -      0      N_43          c218:39671           C>T         AAT>AAA      N>KYAL009W      -      0      N_45          c218:39671           C>T         AAT>AAA      N>KYAL009W      -      0      UWOPS91_917_1 c218:39794           A>T         GTA>GTA      V>VYAL009W      -      0      CBS5829       c218:39845           C>T         CAA>CAA      Q>QYAL009W      -      0      DBVPG4650     c218:39845           C>T         CAA>CAA      Q>QYAL009W      -      0      Q62_5         c218:39845           C>T         CAA>CAA      Q>QYAL009W      -      0      Y6_5          c218:39845           C>T         CAA>CAA      Q>QYAL009W      -      0      Y7            c218:39845           C>T         CAA>CAA      Q>QYAL009W      -      0      Z1_1          c218:39845           C>T         CAA>CAA      Q>QYAL009W      -      0      A12           c218:39869           T>C         GGA>GGG      G>GYAL009W      -      0      DBVPG6304     c218:39869           T>C         GGA>GGG      G>GYAL009W      -      0      N_43          c218:39869           T>C         GGA>GGG      G>GYAL009W      -      0      N_44          c218:39869           T>C         GGA>GGG      G>GYAL009W      -      0      N_45          c218:39869           T>C         GGA>GGG      G>GYAL009W      -      0      UFRJ50816     c218:39869           T>C         GGA>GGG      G>GYAL009W      -      0      UWOPS91_917_1 c218:39911           G>A         TCT>TCT      S>SYAL009W      -      0      A12           c218:39950           G>A         ATT>ATT      I>IYAL009W      -      0      DBVPG6304     c218:39950           G>A         ATT>ATT      I>IYAL009W      -      0      N_44          c218:39950           G>A         ATT>ATT      I>IYAL009W      -      0      N_45          c218:39950           G>A         ATT>ATT      I>IYAL009W      -      0      UFRJ50816     c218:39950           G>A         ATT>ATT      I>IYAL009W      -      0      UWOPS91_917_1 c218:39950           G>A         ATT>ATT      I>IYAL009W      -      0      N_44          c218:39977           T>C         CTT>CTG      L>LYAL009W      -      0      N_45          c218:39977           T>C         CTT>CTG      L>LYAL009W      -      0      A4            c218:40007           A>G         CTC>CTC      L>LYAL009W      -      0      A12           c218:40028           C>T         TCT>TCA      S>SYAL009W      -      0      A4            c218:40028           C>T         TCT>TCA      S>SYAL009W      -      0      DBVPG6304     c218:40028           C>T         TCT>TCA      S>SYAL009W      -      0      UFRJ50816     c218:40028           C>T         TCT>TCA      S>SYAL009W      -      0      UWOPS91_917_1 c218:40028           C>T         TCT>TCA      S>SYAL009W      -      0      A12           c218:40049           T>C         AGC>AGG      S>RYAL009W      -      0      A4            c218:40049           T>C         AGC>AGG      S>RYAL009W      -      0      DBVPG6304     c218:40049           T>C         AGC>AGG      S>RYAL009W      -      0      UWOPS91_917_1 c218:40049           T>C         AGC>AGG      S>RYAL009W      -      0      UFRJ50816     c218:40073           T>C         TTT>TTG      F>LYAL009W      -      0      A12           c218:40079           G>A         GAA>GAT      E>DYAL009W      -      0      A4            c218:40079           G>A         GAA>GAT      E>DYAL009W      -      0      DBVPG6304     c218:40079           G>A         GAA>GAT      E>DYAL009W      -      0      UFRJ50816     c218:40133           A>C         TAT>TAG      Y>*YAL009W      -      0      Y7            c218:40142           C>T         TTG>TTA      L>LYAL009W      -      0      UFRJ50816     c218:40169           G>A         TTT>TTT      F>FYAL009W      -      0      UFRJ50816     c218:40184           A>G         GGG>GGC      G>GYAL009W      -      0      UFRJ50816     c218:40217           T>A         CCA>CCT      P>PYAL009W      -      0      A12           c218:40227           T>C         ATA>AGA      I>RYAL009W      -      0      A4            c218:40227           T>C         ATA>AGA      I>RYAL009W      -      0      DBVPG6304     c218:40227           T>C         ATA>AGA      I>RYAL009W      -      0      UFRJ50816     c218:40227           T>C         ATA>AGA      I>RYAL009W      -      0      UWOPS91_917_1 c218:40245           A>G         GGC>GCC      G>AYAL009W      -      0      UFRJ50816     c218:40251           C>G         TTC>TCC      F>SYAL009W      -      0      A12           c218:40265           A>G         GCG>GCC      A>AYAL009W      -      0      A4            c218:40265           A>G         GCG>GCC      A>AYAL009W      -      0      DBVPG6304     c218:40265           A>G         GCG>GCC      A>AYAL009W      -      0      UWOPS91_917_1 c218:40265           A>G         GCG>GCC      A>AYAL009W      -      0      A12           c218:40275           T>C         CGC>CGC      R>RYAL009W      -      0      A4            c218:40275           T>C         CGC>CGC      R>RYAL009W      -      0      DBVPG6304     c218:40275           T>C         CGC>CGC      R>RYAL009W      -      0      UFRJ50816     c218:40275           T>C         CGC>CGC      R>RYAL010C      +      0      UWOPS91_917_1 c218:40497           A>G         CTA>CTG      L>LYAL010C      +      0      A12           c218:40518           A>G         CTA>CTG      L>LYAL010C      +      0      A4            c218:40518           A>G         CTA>CTG      L>LYAL010C      +      0      DBVPG6304     c218:40518           A>G         CTA>CTG      L>LYAL010C      +      0      UFRJ50816     c218:40518           A>G         CTA>CTG      L>LYAL010C      +      0      UWOPS91_917_1 c218:40518           A>G         CTA>CTG      L>LYAL010C      +      0      N_44          c218:40531           C>A         CAG>AAG      Q>KYAL010C      +      0      N_45          c218:40531           C>A         CAG>AAG      Q>KYAL010C      +      0      UFRJ50816     c218:40551           A>G         ACA>ACG      T>TYAL010C      +      0      A12           c218:40572           A>C         ATA>ATC      I>IYAL010C      +      0      A4            c218:40572           A>C         ATA>ATC      I>IYAL010C      +      0      DBVPG6304     c218:40572           A>C         ATA>ATC      I>IYAL010C      +      0      N_44          c218:40584           G>A         TCG>TCA      S>SYAL010C      +      0      N_45          c218:40584           G>A         TCG>TCA      S>SYAL010C      +      0      A12           c218:40606           A>G         ATT>GTT      I>VYAL010C      +      0      A4            c218:40606           A>G         ATT>GTT      I>VYAL010C      +      0      DBVPG6304     c218:40606           A>G         ATT>GTT      I>VYAL010C      +      0      UFRJ50816     c218:40606           A>G         ATT>GTT      I>VYAL010C      +      0      UWOPS91_917_1 c218:40635           T>C         TCT>TCC      S>SYAL010C      +      0      UFRJ50816     c218:40647           C>T         ACC>ACT      T>TYAL010C      +      0      UWOPS91_917_1 c218:40647           C>T         ACC>ACT      T>TYAL010C      +      0      N_44          c218:40656           A>T         ACA>ACT      T>TYAL010C      +      0      N_45          c218:40656           A>T         ACA>ACT      T>TYAL010C      +      0      A12           c218:40666           C>T         CTA>TTA      L>LYAL010C      +      0      A4            c218:40666           C>T         CTA>TTA      L>LYAL010C      +      0      DBVPG6304     c218:40666           C>T         CTA>TTA      L>LYAL010C      +      0      UFRJ50816     c218:40666           C>T         CTA>TTA      L>LYAL010C      +      0      UWOPS91_917_1 c218:40715           A>G         TAT>TGT      Y>CYAL010C      +      0      N_44          c218:40731           G>A         CAG>CAA      Q>QYAL010C      +      0      A12           c218:40755           T>A         TCT>TCA      S>SYAL010C      +      0      A4            c218:40755           T>A         TCT>TCA      S>SYAL010C      +      0      DBVPG6304     c218:40755           T>A         TCT>TCA      S>SYAL010C      +      0      UFRJ50816     c218:40755           T>A         TCT>TCA      S>SYAL010C      +      0      UWOPS91_917_1 c218:40755           T>A         TCT>TCA      S>SYAL010C      +      0      A12           c218:40779           G>C         GCG>GCC      A>AYAL010C      +      0      A4            c218:40779           G>C         GCG>GCC      A>AYAL010C      +      0      DBVPG6304     c218:40779           G>C         GCG>GCC      A>AYAL010C      +      0      N_44          c218:40779           G>C         GCG>GCC      A>AYAL010C      +      0      UFRJ50816     c218:40779           G>C         GCG>GCC      A>AYAL010C      +      0      UWOPS91_917_1 c218:40779           G>C         GCG>GCC      A>AYAL010C      +      0      A12           c218:40819           A>G         AGT>GGT      S>GYAL010C      +      0      DBVPG6304     c218:40819           A>G         AGT>GGT      S>GYAL010C      +      0      UFRJ50816     c218:40819           A>G         AGT>GGT      S>GYAL010C      +      0      UWOPS91_917_1 c218:40819           A>G         AGT>GGT      S>GYAL010C      +      0      A12           c218:40826           G>A         AGT>AAT      S>NYAL010C      +      0      N_44          c218:40841           T>C         CTG>CCG      L>PYAL010C      +      0      A12           c218:40852           C>G         CAA>GAA      Q>EYAL010C      +      0      A4            c218:40852           C>G         CAA>GAA      Q>EYAL010C      +      0      DBVPG6304     c218:40852           C>G         CAA>GAA      Q>EYAL010C      +      0      N_44          c218:40852           C>G         CAA>GAA      Q>EYAL010C      +      0      UFRJ50816     c218:40852           C>G         CAA>GAA      Q>EYAL010C      +      0      UWOPS91_917_1 c218:40852           C>G         CAA>GAA      Q>EYAL010C      +      0      UWOPS91_917_1 c218:40859           C>T         ACA>ATA      T>IYAL010C      +      0      A12           c218:40867           G>A         GAT>AAT      D>NYAL010C      +      0      DBVPG6304     c218:40867           G>A         GAT>AAT      D>NYAL010C      +      0      UFRJ50816     c218:40867           G>A         GAT>AAT      D>NYAL010C      +      0      UWOPS91_917_1 c218:40867           G>A         GAT>AAT      D>NYAL010C      +      0      UWOPS91_917_1 c218:40875           G>A         AAG>AAA      K>KYAL010C      +      0      DBVPG6304     c218:40890           C>T         GAC>GAT      D>DYAL010C      +      0      UFRJ50816     c218:40890           C>T         GAC>GAT      D>DYAL010C      +      0      UWOPS91_917_1 c218:40893           A>G         TCA>TCG      S>SYAL010C      +      0      A12           c218:40978           T>C         TCA>CCA      S>PYAL010C      +      0      DBVPG6304     c218:40978           T>C         TCA>CCA      S>PYAL010C      +      0      N_44          c218:40978           T>C         TCA>CCA      S>PYAL010C      +      0      UFRJ50816     c218:40978           T>C         TCA>CCA      S>PYAL010C      +      0      UWOPS91_917_1 c218:40978           T>C         TCA>CCA      S>PYAL010C      +      0      A12           c218:40989           G>A         CAG>CAA      Q>QYAL010C      +      0      DBVPG6304     c218:40989           G>A         CAG>CAA      Q>QYAL010C      +      0      UFRJ50816     c218:40989           G>A         CAG>CAA      Q>QYAL010C      +      0      UWOPS91_917_1 c218:40989           G>A         CAG>CAA      Q>QYAL010C      +      0      A12           c218:41005           A>G         ATC>GTC      I>VYAL010C      +      0      DBVPG6304     c218:41005           A>G         ATC>GTC      I>VYAL010C      +      0      N_44          c218:41005           A>G         ATC>GTC      I>VYAL010C      +      0      UFRJ50816     c218:41005           A>G         ATC>GTC      I>VYAL010C      +      0      UWOPS91_917_1 c218:41005           A>G         ATC>GTC      I>VYAL010C      +      0      A12           c218:41018           G>A         AGA>AAA      R>KYAL010C      +      0      DBVPG6304     c218:41018           G>A         AGA>AAA      R>KYAL010C      +      0      UFRJ50816     c218:41018           G>A         AGA>AAA      R>KYAL010C      +      0      A12           c218:41031           T>C         AAT>AAC      N>NYAL010C      +      0      DBVPG6304     c218:41031           T>C         AAT>AAC      N>NYAL010C      +      0      N_44          c218:41031           T>C         AAT>AAC      N>NYAL010C      +      0      UFRJ50816     c218:41031           T>C         AAT>AAC      N>NYAL010C      +      0      UWOPS91_917_1 c218:41031           T>C         AAT>AAC      N>NYAL010C      +      0      A12           c218:41079           G>A         GAG>GAA      E>EYAL010C      +      0      DBVPG6304     c218:41079           G>A         GAG>GAA      E>EYAL010C      +      0      UFRJ50816     c218:41079           G>A         GAG>GAA      E>EYAL010C      +      0      A12           c218:41094           C>T         ACC>ACT      T>TYAL010C      +      0      DBVPG6304     c218:41094           C>T         ACC>ACT      T>TYAL010C      +      0      UFRJ50816     c218:41094           C>T         ACC>ACT      T>TYAL010C      +      0      A12           c218:41112           A>T         GGA>GGT      G>GYAL010C      +      0      A12           c218:41119           A>G         ATA>GTA      I>VYAL010C      +      0      DBVPG6304     c218:41121           A>T         ATA>ATT      I>IYAL010C      +      0      UFRJ50816     c218:41121           A>T         ATA>ATT      I>IYAL010C      +      0      YPS138        c218:41121           A>T         ATA>ATT      I>IYAL010C      +      0      A12           c218:41142           A>G         ACA>ACG      T>TYAL010C      +      0      DBVPG6304     c218:41142           A>G         ACA>ACG      T>TYAL010C      +      0      UFRJ50816     c218:41142           A>G         ACA>ACG      T>TYAL010C      +      0      UWOPS91_917_1 c218:41142           A>G         ACA>ACG      T>TYAL010C      +      0      A12           c218:41164           T>C         TTA>CTA      L>LYAL010C      +      0      DBVPG6304     c218:41164           T>C         TTA>CTA      L>LYAL010C      +      0      UFRJ50816     c218:41164           T>C         TTA>CTA      L>LYAL010C      +      0      UWOPS91_917_1 c218:41164           T>C         TTA>CTA      L>LYAL010C      +      0      YPS138        c218:41164           T>C         TTA>CTA      L>LYAL010C      +      0      DBVPG6304     c218:41190           T>C         CTT>CTC      L>LYAL010C      +      0      YPS138        c218:41190           T>C         CTT>CTC      L>LYAL010C      +      0      A12           c218:41199           G>A         GAG>GAA      E>EYAL010C      +      0      DBVPG6304     c218:41199           G>A         GAG>GAA      E>EYAL010C      +      0      UWOPS91_917_1 c218:41199           G>A         GAG>GAA      E>EYAL010C      +      0      YPS138        c218:41199           G>A         GAG>GAA      E>EYAL010C      +      0      A12           c218:41211           A>G         GGA>GGG      G>GYAL010C      +      0      DBVPG6304     c218:41211           A>G         GGA>GGG      G>GYAL010C      +      0      UFRJ50816     c218:41211           A>G         GGA>GGG      G>GYAL010C      +      0      UWOPS91_917_1 c218:41211           A>G         GGA>GGG      G>GYAL010C      +      0      YPS138        c218:41211           A>G         GGA>GGG      G>GYAL010C      +      0      DBVPG6304     c218:41238           G>A         TCG>TCA      S>SYAL010C      +      0      YPS138        c218:41238           G>A         TCG>TCA      S>SYAL010C      +      0      A12           c218:41250           G>A         AGG>AGA      R>RYAL010C      +      0      DBVPG6304     c218:41250           G>A         AGG>AGA      R>RYAL010C      +      0      N_43          c218:41250           G>A         AGG>AGA      R>RYAL010C      +      0      N_45          c218:41250           G>A         AGG>AGA      R>RYAL010C      +      0      UWOPS91_917_1 c218:41250           G>A         AGG>AGA      R>RYAL010C      +      0      A12           c218:41274           G>A         ACG>ACA      T>TYAL010C      +      0      UFRJ50816     c218:41277           A>C         GGA>GGC      G>GYAL010C      +      0      A12           c218:41295           G>A         ACG>ACA      T>TYAL010C      +      0      DBVPG6304     c218:41295           G>A         ACG>ACA      T>TYAL010C      +      0      YPS138        c218:41295           G>A         ACG>ACA      T>TYAL010C      +      0      A12           c218:41331           C>T         TCC>TCT      S>SYAL010C      +      0      DBVPG6304     c218:41331           C>T         TCC>TCT      S>SYAL010C      +      0      UFRJ50816     c218:41331           C>T         TCC>TCT      S>SYAL010C      +      0      UWOPS91_917_1 c218:41331           C>T         TCC>TCT      S>SYAL010C      +      0      YPS138        c218:41331           C>T         TCC>TCT      S>SYAL010C      +      0      A12           c218:41361           C>T         TCC>TCT      S>SYAL010C      +      0      A12           c218:41367           C>T         TTC>TTT      F>FYAL010C      +      0      CBS5829       c218:41367           C>T         TTC>TTT      F>FYAL010C      +      0      DBVPG4650     c218:41367           C>T         TTC>TTT      F>FYAL010C      +      0      DBVPG6304     c218:41367           C>T         TTC>TTT      F>FYAL010C      +      0      KPN3829       c218:41367           C>T         TTC>TTT      F>FYAL010C      +      0      N_17          c218:41367           C>T         TTC>TTT      F>FYAL010C      +      0      N_43          c218:41367           C>T         TTC>TTT      F>FYAL010C      +      0      N_44          c218:41367           C>T         TTC>TTT      F>FYAL010C      +      0      Q59_1         c218:41367           C>T         TTC>TTT      F>FYAL010C      +      0      T21_4         c218:41367           C>T         TTC>TTT      F>FYAL010C      +      0      UFRJ50816     c218:41367           C>T         TTC>TTT      F>FYAL010C      +      0      UWOPS91_917_1 c218:41367           C>T         TTC>TTT      F>FYAL010C      +      0      Y6_5          c218:41367           C>T         TTC>TTT      F>FYAL010C      +      0      YPS138        c218:41367           C>T         TTC>TTT      F>FYAL010C      +      0      Z1_1          c218:41367           C>T         TTC>TTT      F>FYAL010C      +      0      A12           c218:41397           A>G         TCA>TCG      S>SYAL010C      +      0      DBVPG6304     c218:41397           A>G         TCA>TCG      S>SYAL010C      +      0      UFRJ50816     c218:41397           A>G         TCA>TCG      S>SYAL010C      +      0      UWOPS91_917_1 c218:41397           A>G         TCA>TCG      S>SYAL010C      +      0      YPS138        c218:41397           A>G         TCA>TCG      S>SYAL010C      +      0      A12           c218:41424           C>T         TGC>TGT      C>CYAL010C      +      0      DBVPG6304     c218:41424           C>T         TGC>TGT      C>CYAL010C      +      0      UFRJ50816     c218:41424           C>T         TGC>TGT      C>CYAL010C      +      0      YPS138        c218:41424           C>T         TGC>TGT      C>CYAL010C      +      0      A12           c218:41439           G>A         AAG>AAA      K>KYAL010C      +      0      DBVPG6304     c218:41439           G>A         AAG>AAA      K>KYAL010C      +      0      N_43          c218:41439           G>A         AAG>AAA      K>KYAL010C      +      0      N_44          c218:41439           G>A         AAG>AAA      K>KYAL010C      +      0      N_45          c218:41439           G>A         AAG>AAA      K>KYAL010C      +      0      UFRJ50791     c218:41439           G>A         AAG>AAA      K>KYAL010C      +      0      UFRJ50816     c218:41439           G>A         AAG>AAA      K>KYAL010C      +      0      UWOPS91_917_1 c218:41439           G>A         AAG>AAA      K>KYAL010C      +      0      YPS138        c218:41439           G>A         AAG>AAA      K>KYAL010C      +      0      N_43          c218:41445           T>C         CAT>CAC      H>HYAL010C      +      0      N_44          c218:41445           T>C         CAT>CAC      H>HYAL010C      +      0      N_45          c218:41445           T>C         CAT>CAC      H>HYAL010C      +      0      N_43          c218:41457           G>A         GAG>GAA      E>EYAL010C      +      0      N_45          c218:41457           G>A         GAG>GAA      E>EYAL010C      +      0      A12           c218:41460           C>T         ACC>ACT      T>TYAL010C      +      0      DBVPG6304     c218:41460           C>T         ACC>ACT      T>TYAL010C      +      0      UFRJ50791     c218:41460           C>T         ACC>ACT      T>TYAL010C      +      0      UFRJ50816     c218:41460           C>T         ACC>ACT      T>TYAL010C      +      0      UWOPS91_917_1 c218:41460           C>T         ACC>ACT      T>TYAL010C      +      0      YPS138        c218:41460           C>T         ACC>ACT      T>TYAL010C      +      0      A12           c218:41472           T>A         AAT>AAA      N>KYAL010C      +      0      DBVPG6304     c218:41472           T>A         AAT>AAA      N>KYAL010C      +      0      UFRJ50791     c218:41472           T>A         AAT>AAA      N>KYAL010C      +      0      UFRJ50816     c218:41472           T>A         AAT>AAA      N>KYAL010C      +      0      YPS138        c218:41472           T>A         AAT>AAA      N>KYAL010C      +      0      A12           c218:41478           C>T         GAC>GAT      D>DYAL010C      +      0      DBVPG6304     c218:41478           C>T         GAC>GAT      D>DYAL010C      +      0      KPN3829       c218:41478           C>T         GAC>GAT      D>DYAL010C      +      0      N_43          c218:41478           C>T         GAC>GAT      D>DYAL010C      +      0      N_44          c218:41478           C>T         GAC>GAT      D>DYAL010C      +      0      N_45          c218:41478           C>T         GAC>GAT      D>DYAL010C      +      0      UFRJ50791     c218:41478           C>T         GAC>GAT      D>DYAL010C      +      0      UFRJ50816     c218:41478           C>T         GAC>GAT      D>DYAL010C      +      0      UWOPS91_917_1 c218:41478           C>T         GAC>GAT      D>DYAL010C      +      0      YPS138        c218:41478           C>T         GAC>GAT      D>DYAL010C      +      0      N_43          c218:41486           A>G         GAA>GGA      E>GYAL010C      +      0      N_44          c218:41486           A>G         GAA>GGA      E>GYAL010C      +      0      N_45          c218:41486           A>G         GAA>GGA      E>GYAL010C      +      0      A12           c218:41493           C>T         ATC>ATT      I>IYAL010C      +      0      DBVPG6304     c218:41493           C>T         ATC>ATT      I>IYAL010C      +      0      UFRJ50791     c218:41493           C>T         ATC>ATT      I>IYAL010C      +      0      UFRJ50816     c218:41493           C>T         ATC>ATT      I>IYAL010C      +      0      YPS138        c218:41493           C>T         ATC>ATT      I>IYAL010C      +      0      A12           c218:41508           G>A         GTG>GTA      V>VYAL010C      +      0      DBVPG6304     c218:41508           G>A         GTG>GTA      V>VYAL010C      +      0      UFRJ50791     c218:41508           G>A         GTG>GTA      V>VYAL010C      +      0      UFRJ50816     c218:41508           G>A         GTG>GTA      V>VYAL010C      +      0      UWOPS91_917_1 c218:41508           G>A         GTG>GTA      V>VYAL010C      +      0      YPS138        c218:41508           G>A         GTG>GTA      V>VYAL010C      +      0      IFO1804       c218:41518           T>C         TCA>CCA      S>PYAL010C      +      0      N_43          c218:41518           T>C         TCA>CCA      S>PYAL010C      +      0      N_44          c218:41518           T>C         TCA>CCA      S>PYAL010C      +      0      N_45          c218:41518           T>C         TCA>CCA      S>PYAL010C      +      0      IFO1804       c218:41529           A>G         AGA>AGG      R>RYAL010C      +      0      N_43          c218:41529           A>G         AGA>AGG      R>RYAL010C      +      0      N_44          c218:41529           A>G         AGA>AGG      R>RYAL010C      +      0      N_45          c218:41529           A>G         AGA>AGG      R>RYAL010C      +      0      CBS5829       c218:41563           T>C         TTG>CTG      L>LYAL010C      +      0      IFO1804       c218:41563           T>C         TTG>CTG      L>LYAL010C      +      0      KPN3829       c218:41563           T>C         TTG>CTG      L>LYAL010C      +      0      N_43          c218:41563           T>C         TTG>CTG      L>LYAL010C      +      0      N_44          c218:41563           T>C         TTG>CTG      L>LYAL010C      +      0      N_45          c218:41563           T>C         TTG>CTG      L>LYAL010C      +      0      Q59_1         c218:41563           T>C         TTG>CTG      L>LYAL010C      +      0      S36_7         c218:41563           T>C         TTG>CTG      L>LYAL010C      +      0      T21_4         c218:41563           T>C         TTG>CTG      L>LYAL010C      +      0      Z1_1          c218:41563           T>C         TTG>CTG      L>LYAL010C      +      0      A12           c218:41584           A>G         ATT>GTT      I>VYAL010C      +      0      DBVPG6304     c218:41584           A>G         ATT>GTT      I>VYAL010C      +      0      UFRJ50791     c218:41584           A>G         ATT>GTT      I>VYAL010C      +      0      UFRJ50816     c218:41584           A>G         ATT>GTT      I>VYAL010C      +      0      UWOPS91_917_1 c218:41584           A>G         ATT>GTT      I>VYAL010C      +      0      YPS138        c218:41584           A>G         ATT>GTT      I>VYAL010C      +      0      A12           c218:41593           C>T         CCA>TCA      P>SYAL010C      +      0      DBVPG6304     c218:41593           C>T         CCA>TCA      P>SYAL010C      +      0      IFO1804       c218:41593           C>T         CCA>TCA      P>SYAL010C      +      0      N_43          c218:41593           C>T         CCA>TCA      P>SYAL010C      +      0      N_44          c218:41593           C>T         CCA>TCA      P>SYAL010C      +      0      N_45          c218:41593           C>T         CCA>TCA      P>SYAL010C      +      0      UFRJ50791     c218:41593           C>T         CCA>TCA      P>SYAL010C      +      0      UFRJ50816     c218:41593           C>T         CCA>TCA      P>SYAL010C      +      0      UWOPS91_917_1 c218:41593           C>T         CCA>TCA      P>SYAL010C      +      0      YPS138        c218:41593           C>T         CCA>TCA      P>SYAL010C      +      0      A12           c218:41652           G>A         TCG>TCA      S>SYAL010C      +      0      DBVPG6304     c218:41652           G>A         TCG>TCA      S>SYAL010C      +      0      UFRJ50791     c218:41652           G>A         TCG>TCA      S>SYAL010C      +      0      UFRJ50816     c218:41652           G>A         TCG>TCA      S>SYAL010C      +      0      UWOPS91_917_1 c218:41652           G>A         TCG>TCA      S>SYAL010C      +      0      YPS138        c218:41652           G>A         TCG>TCA      S>SYAL010C      +      0      A12           c218:41745           G>A         CTG>CTA      L>LYAL010C      +      0      DBVPG6304     c218:41745           G>A         CTG>CTA      L>LYAL010C      +      0      UFRJ50791     c218:41745           G>A         CTG>CTA      L>LYAL010C      +      0      UFRJ50816     c218:41745           G>A         CTG>CTA      L>LYAL010C      +      0      UWOPS91_917_1 c218:41745           G>A         CTG>CTA      L>LYAL010C      +      0      YPS138        c218:41745           G>A         CTG>CTA      L>LYAL010C      +      0      A12           c218:41772           G>A         TTG>TTA      L>LYAL010C      +      0      DBVPG6304     c218:41772           G>A         TTG>TTA      L>LYAL010C      +      0      UFRJ50791     c218:41772           G>A         TTG>TTA      L>LYAL010C      +      0      UFRJ50816     c218:41772           G>A         TTG>TTA      L>LYAL010C      +      0      YPS138        c218:41772           G>A         TTG>TTA      L>LYAL010C      +      0      A12           c218:41790           C>A         GGC>GGA      G>GYAL010C      +      0      DBVPG6304     c218:41790           C>A         GGC>GGA      G>GYAL010C      +      0      UFRJ50791     c218:41790           C>A         GGC>GGA      G>GYAL010C      +      0      UFRJ50816     c218:41790           C>A         GGC>GGA      G>GYAL010C      +      0      UWOPS91_917_1 c218:41790           C>A         GGC>GGA      G>GYAL010C      +      0      YPS138        c218:41790           C>A         GGC>GGA      G>GYAL010C      +      0      A12           c218:41806           C>T         CTA>TTA      L>LYAL010C      +      0      DBVPG6304     c218:41806           C>T         CTA>TTA      L>LYAL010C      +      0      UFRJ50791     c218:41806           C>T         CTA>TTA      L>LYAL010C      +      0      UFRJ50816     c218:41806           C>T         CTA>TTA      L>LYAL010C      +      0      UWOPS91_917_1 c218:41806           C>T         CTA>TTA      L>LYAL010C      +      0      YPS138        c218:41806           C>T         CTA>TTA      L>LYAL010C      +      0      A12           c218:41814           T>A         TCT>TCA      S>SYAL010C      +      0      DBVPG6304     c218:41814           T>A         TCT>TCA      S>SYAL010C      +      0      UFRJ50791     c218:41814           T>A         TCT>TCA      S>SYAL010C      +      0      UFRJ50816     c218:41814           T>A         TCT>TCA      S>SYAL010C      +      0      UWOPS91_917_1 c218:41814           T>A         TCT>TCA      S>SYAL010C      +      0      YPS138        c218:41814           T>A         TCT>TCA      S>SYAL010C      +      0      KPN3829       c218:41848           C>A         CAA>AAA      Q>KYAL010C      +      0      N_17          c218:41848           C>A         CAA>AAA      Q>KYAL010C      +      0      A12           c218:41853           A>T         GAA>GAT      E>DYAL010C      +      0      DBVPG6304     c218:41853           A>T         GAA>GAT      E>DYAL010C      +      0      UFRJ50791     c218:41853           A>T         GAA>GAT      E>DYAL010C      +      0      UFRJ50816     c218:41853           A>T         GAA>GAT      E>DYAL010C      +      0      UWOPS91_917_1 c218:41853           A>T         GAA>GAT      E>DYAL010C      +      0      YPS138        c218:41853           A>T         GAA>GAT      E>DYAL010C      +      0      A12           c218:41892           G>A         TCG>TCA      S>SYAL010C      +      0      DBVPG6304     c218:41892           G>A         TCG>TCA      S>SYAL010C      +      0      UFRJ50791     c218:41892           G>A         TCG>TCA      S>SYAL010C      +      0      UFRJ50816     c218:41892           G>A         TCG>TCA      S>SYAL010C      +      0      YPS138        c218:41892           G>A         TCG>TCA      S>SYAL010C      +      0      UWOPS91_917_1 c218:41916           C>T         AAC>AAT      N>NYAL010C      +      0      DBVPG6304     c218:41938           G>A         GCA>ACA      A>TYAL010C      +      0      UFRJ50791     c218:41938           G>A         GCA>ACA      A>TYAL010C      +      0      UFRJ50816     c218:41938           G>A         GCA>ACA      A>TYAL010C      +      0      UWOPS91_917_1 c218:41938           G>A         GCA>ACA      A>TYAL010C      +      0      YPS138        c218:41938           G>A         GCA>ACA      A>TYAL010C      +      0      DBVPG6304     c218:41956           C>T         CTC>TTC      L>FYAL010C      +      0      IFO1804       c218:41956           C>T         CTC>TTC      L>FYAL010C      +      0      N_44          c218:41956           C>T         CTC>TTC      L>FYAL010C      +      0      N_45          c218:41956           C>T         CTC>TTC      L>FYAL010C      +      0      UFRJ50791     c218:41956           C>T         CTC>TTC      L>FYAL010C      +      0      UFRJ50816     c218:41956           C>T         CTC>TTC      L>FYAL010C      +      0      UWOPS91_917_1 c218:41956           C>T         CTC>TTC      L>FYAL010C      +      0      YPS138        c218:41956           C>T         CTC>TTC      L>FYAL011W      -      0      KPN3829       c218:42090           T>C         AGC>AGC      S>SYAL011W      -      0      N_17          c218:42090           T>C         AGC>AGC      S>SYAL011W      -      0      A4            c218:42152           C>T         CGT>CGA      R>RYAL011W      -      0      DBVPG6304     c218:42152           C>T         CGT>CGA      R>RYAL011W      -      0      UFRJ50791     c218:42152           C>T         CGT>CGA      R>RYAL011W      -      0      UFRJ50816     c218:42152           C>T         CGT>CGA      R>RYAL011W      -      0      UWOPS91_917_1 c218:42152           C>T         CGT>CGA      R>RYAL011W      -      0      YPS138        c218:42152           C>T         CGT>CGA      R>RYAL011W      -      0      A12           c218:42161           G>A         TCT>TCT      S>SYAL011W      -      0      A4            c218:42161           G>A         TCT>TCT      S>SYAL011W      -      0      DBVPG6304     c218:42161           G>A         TCT>TCT      S>SYAL011W      -      0      IFO1804       c218:42161           G>A         TCT>TCT      S>SYAL011W      -      0      KPN3829       c218:42161           G>A         TCT>TCT      S>SYAL011W      -      0      N_17          c218:42161           G>A         TCT>TCT      S>SYAL011W      -      0      N_45          c218:42161           G>A         TCT>TCT      S>SYAL011W      -      0      UFRJ50791     c218:42161           G>A         TCT>TCT      S>SYAL011W      -      0      UFRJ50816     c218:42161           G>A         TCT>TCT      S>SYAL011W      -      0      UWOPS91_917_1 c218:42161           G>A         TCT>TCT      S>SYAL011W      -      0      YPS138        c218:42161           G>A         TCT>TCT      S>SYAL011W      -      0      A12           c218:42179           C>T         ACG>ACA      T>TYAL011W      -      0      A4            c218:42179           C>T         ACG>ACA      T>TYAL011W      -      0      DBVPG6304     c218:42179           C>T         ACG>ACA      T>TYAL011W      -      0      UFRJ50791     c218:42179           C>T         ACG>ACA      T>TYAL011W      -      0      UFRJ50816     c218:42179           C>T         ACG>ACA      T>TYAL011W      -      0      UWOPS91_917_1 c218:42179           C>T         ACG>ACA      T>TYAL011W      -      0      YPS138        c218:42179           C>T         ACG>ACA      T>TYAL011W      -      0      A12           c218:42191           A>G         ATC>ATC      I>IYAL011W      -      0      A4            c218:42191           A>G         ATC>ATC      I>IYAL011W      -      0      DBVPG6304     c218:42191           A>G         ATC>ATC      I>IYAL011W      -      0      UFRJ50791     c218:42191           A>G         ATC>ATC      I>IYAL011W      -      0      UFRJ50816     c218:42191           A>G         ATC>ATC      I>IYAL011W      -      0      UWOPS91_917_1 c218:42191           A>G         ATC>ATC      I>IYAL011W      -      0      YPS138        c218:42191           A>G         ATC>ATC      I>IYAL011W      -      0      A12           c218:42206           C>T         ATC>ATA      I>IYAL011W      -      0      A4            c218:42206           C>T         ATC>ATA      I>IYAL011W      -      0      DBVPG6304     c218:42206           C>T         ATC>ATA      I>IYAL011W      -      0      UFRJ50791     c218:42206           C>T         ATC>ATA      I>IYAL011W      -      0      UFRJ50816     c218:42206           C>T         ATC>ATA      I>IYAL011W      -      0      UWOPS91_917_1 c218:42206           C>T         ATC>ATA      I>IYAL011W      -      0      YPS138        c218:42206           C>T         ATC>ATA      I>IYAL011W      -      0      UWOPS91_917_1 c218:42281           T>C         GGT>GGG      G>GYAL011W      -      0      UWOPS91_917_1 c218:42330           T>A         CAG>CTG      Q>LYAL011W      -      0      A12           c218:42352           C>T         AAG>AAG      K>KYAL011W      -      0      UFRJ50791     c218:42352           C>T         AAG>AAG      K>KYAL011W      -      0      UFRJ50816     c218:42352           C>T         AAG>AAG      K>KYAL011W      -      0      UWOPS91_917_1 c218:42352           C>T         AAG>AAG      K>KYAL011W      -      0      YPS138        c218:42352           C>T         AAG>AAG      K>KYAL011W      -      0      N_43          c218:42372           T>C         TTT>TGT      F>CYAL011W      -      0      N_45          c218:42372           T>C         TTT>TGT      F>CYAL011W      -      0      UWOPS91_917_1 c218:42372           T>C         TTT>TGT      F>CYAL011W      -      0      UWOPS91_917_1 c218:42387           A>G         AAG>ACG      K>TYAL011W      -      0      A12           c218:42393           C>G         GTC>GCC      V>AYAL011W      -      0      A4            c218:42393           C>G         GTC>GCC      V>AYAL011W      -      0      UFRJ50791     c218:42393           C>G         GTC>GCC      V>AYAL011W      -      0      UWOPS91_917_1 c218:42393           C>G         GTC>GCC      V>AYAL011W      -      0      YPS138        c218:42393           C>G         GTC>GCC      V>AYAL011W      -      0      A12           c218:42406           A>T         ACT>ACT      T>TYAL011W      -      0      A4            c218:42406           A>T         ACT>ACT      T>TYAL011W      -      0      N_43          c218:42406           A>T         ACT>ACT      T>TYAL011W      -      0      N_45          c218:42406           A>T         ACT>ACT      T>TYAL011W      -      0      UFRJ50791     c218:42406           A>T         ACT>ACT      T>TYAL011W      -      0      YPS138        c218:42406           A>T         ACT>ACT      T>TYAL011W      -      0      A12           c218:42416           G>A         GGT>GGT      G>GYAL011W      -      0      A4            c218:42416           G>A         GGT>GGT      G>GYAL011W      -      0      UFRJ50791     c218:42416           G>A         GGT>GGT      G>GYAL011W      -      0      UWOPS91_917_1 c218:42416           G>A         GGT>GGT      G>GYAL011W      -      0      YPS138        c218:42416           G>A         GGT>GGT      G>GYAL011W      -      0      A12           c218:42424           C>T         GAT>AAT      D>NYAL011W      -      0      UFRJ50791     c218:42424           C>T         GAT>AAT      D>NYAL011W      -      0      UWOPS91_917_1 c218:42424           C>T         GAT>AAT      D>NYAL011W      -      0      YPS138        c218:42424           C>T         GAT>AAT      D>NYAL011W      -      0      A12           c218:42432           A>G         CTC>CCC      L>PYAL011W      -      0      N_43          c218:42432           A>G         CTC>CCC      L>PYAL011W      -      0      N_45          c218:42432           A>G         CTC>CCC      L>PYAL011W      -      0      UFRJ50791     c218:42432           A>G         CTC>CCC      L>PYAL011W      -      0      UWOPS91_917_1 c218:42432           A>G         CTC>CCC      L>PYAL011W      -      0      YPS138        c218:42432           A>G         CTC>CCC      L>PYAL011W      -      0      A12           c218:42440           C>T         TTC>TTA      F>LYAL011W      -      0      A4            c218:42440           C>T         TTC>TTA      F>LYAL011W      -      0      UFRJ50791     c218:42440           C>T         TTC>TTA      F>LYAL011W      -      0      UWOPS91_917_1 c218:42440           C>T         TTC>TTA      F>LYAL011W      -      0      YPS138        c218:42440           C>T         TTC>TTA      F>LYAL011W      -      0      N_43          c218:42452           T>A         TCG>TCT      S>SYAL011W      -      0      N_45          c218:42452           T>A         TCG>TCT      S>SYAL011W      -      0      A12           c218:42458           C>G         GGG>GGC      G>GYAL011W      -      0      A4            c218:42458           C>G         GGG>GGC      G>GYAL011W      -      0      UFRJ50791     c218:42458           C>G         GGG>GGC      G>GYAL011W      -      0      YPS138        c218:42458           C>G         GGG>GGC      G>GYAL011W      -      0      A12           c218:42482           G>A         CGT>CGT      R>RYAL011W      -      0      A4            c218:42482           G>A         CGT>CGT      R>RYAL011W      -      0      UFRJ50791     c218:42482           G>A         CGT>CGT      R>RYAL011W      -      0      UFRJ50816     c218:42482           G>A         CGT>CGT      R>RYAL011W      -      0      UWOPS91_917_1 c218:42482           G>A         CGT>CGT      R>RYAL011W      -      0      YPS138        c218:42482           G>A         CGT>CGT      R>RYAL011W      -      0      A12           c218:42539           T>C         CAT>CAG      H>QYAL011W      -      0      A4            c218:42539           T>C         CAT>CAG      H>QYAL011W      -      0      N_43          c218:42539           T>C         CAT>CAG      H>QYAL011W      -      0      N_45          c218:42539           T>C         CAT>CAG      H>QYAL011W      -      0      UFRJ50791     c218:42539           T>C         CAT>CAG      H>QYAL011W      -      0      UFRJ50816     c218:42539           T>C         CAT>CAG      H>QYAL011W      -      0      UWOPS91_917_1 c218:42539           T>C         CAT>CAG      H>QYAL011W      -      0      YPS138        c218:42539           T>C         CAT>CAG      H>QYAL011W      -      0      N_43          c218:42563           A>G         CAT>CAC      H>HYAL011W      -      0      N_45          c218:42563           A>G         CAT>CAC      H>HYAL011W      -      0      A12           c218:42569           C>G         TCC>TCC      S>SYAL011W      -      0      A4            c218:42569           C>G         TCC>TCC      S>SYAL011W      -      0      UFRJ50791     c218:42569           C>G         TCC>TCC      S>SYAL011W      -      0      UFRJ50816     c218:42569           C>G         TCC>TCC      S>SYAL011W      -      0      YPS138        c218:42569           C>G         TCC>TCC      S>SYAL011W      -      0      N_43          c218:42575           G>A         AGG>AGT      R>SYAL011W      -      0      N_45          c218:42575           G>A         AGG>AGT      R>SYAL011W      -      0      A12           c218:42595           G>A         TCT>TCT      S>SYAL011W      -      0      A4            c218:42595           G>A         TCT>TCT      S>SYAL011W      -      0      UFRJ50791     c218:42595           G>A         TCT>TCT      S>SYAL011W      -      0      UFRJ50816     c218:42595           G>A         TCT>TCT      S>SYAL011W      -      0      YPS138        c218:42595           G>A         TCT>TCT      S>SYAL011W      -      0      A12           c218:42608           A>G         TTT>TTC      F>FYAL011W      -      0      A4            c218:42608           A>G         TTT>TTC      F>FYAL011W      -      0      UFRJ50791     c218:42608           A>G         TTT>TTC      F>FYAL011W      -      0      UFRJ50816     c218:42608           A>G         TTT>TTC      F>FYAL011W      -      0      UWOPS91_917_1 c218:42608           A>G         TTT>TTC      F>FYAL011W      -      0      YPS138        c218:42608           A>G         TTT>TTC      F>FYAL011W      -      0      UWOPS91_917_1 c218:42694           C>T         CTC>ATC      L>IYAL011W      -      0      UFRJ50791     c218:42725           C>A         CTT>CTT      L>LYAL011W      -      0      UFRJ50816     c218:42725           C>A         CTT>CTT      L>LYAL011W      -      0      A12           c218:42730           C>A         TTG>TTG      L>LYAL011W      -      0      A12           c218:42740           T>C         CTT>CTG      L>LYAL011W      -      0      UFRJ50791     c218:42740           T>C         CTT>CTG      L>LYAL011W      -      0      UFRJ50816     c218:42740           T>C         CTT>CTG      L>LYAL011W      -      0      A12           c218:42767           A>T         TTG>TTA      L>LYAL011W      -      0      UFRJ50791     c218:42767           A>T         TTG>TTA      L>LYAL011W      -      0      UFRJ50816     c218:42767           A>T         TTG>TTA      L>LYAL011W      -      0      N_43          c218:42768           C>T         TTG>TAG      L>*YAL011W      -      0      N_45          c218:42768           C>T         TTG>TAG      L>*YAL011W      -      0      N_43          c218:42822           A>G         CAA>CCA      Q>PYAL011W      -      0      N_45          c218:42822           A>G         CAA>CCA      Q>PYAL011W      -      0      A12           c218:42838           T>C         TTG>GTG      L>VYAL011W      -      0      UFRJ50791     c218:42838           T>C         TTG>GTG      L>VYAL011W      -      0      UFRJ50816     c218:42838           T>C         TTG>GTG      L>VYAL011W      -      0      N_43          c218:42872           T>C         CTG>CTG      L>LYAL011W      -      0      N_45          c218:42872           T>C         CTG>CTG      L>LYAL011W      -      0      A12           c218:42881           G>T         AGG>AGA      R>RYAL011W      -      0      UFRJ50791     c218:42881           G>T         AGG>AGA      R>RYAL011W      -      0      UFRJ50816     c218:42881           G>T         AGG>AGA      R>RYAL011W      -      0      A12           c218:42887           T>C         GGA>GGG      G>GYAL011W      -      0      UFRJ50791     c218:42887           T>C         GGA>GGG      G>GYAL011W      -      0      UFRJ50816     c218:42887           T>C         GGA>GGG      G>GYAL011W      -      0      N_43          c218:42905           C>G         TCT>TCC      S>SYAL011W      -      0      N_45          c218:42905           C>G         TCT>TCC      S>SYAL011W      -      0      N_43          c218:42916           C>T         CAT>AAT      H>NYAL011W      -      0      N_45          c218:42916           C>T         CAT>AAT      H>NYAL011W      -      0      UFRJ50791     c218:42920           G>A         CAA>CAT      Q>HYAL011W      -      0      UFRJ50816     c218:42920           G>A         CAA>CAT      Q>HYAL011W      -      0      N_43          c218:42929           A>G         CAT>CAC      H>HYAL011W      -      0      N_44          c218:42929           A>G         CAT>CAC      H>HYAL011W      -      0      N_45          c218:42929           A>G         CAT>CAC      H>HYAL011W      -      0      UFRJ50791     c218:42929           A>G         CAT>CAC      H>HYAL011W      -      0      UFRJ50816     c218:42929           A>G         CAT>CAC      H>HYAL011W      -      0      UFRJ50791     c218:42944           G>A         TGG>TGT      W>CYAL011W      -      0      UFRJ50816     c218:42944           G>A         TGG>TGT      W>CYAL011W      -      0      A12           c218:43007           A>G         TTC>TTC      F>FYAL011W      -      0      UFRJ50791     c218:43007           A>G         TTC>TTC      F>FYAL011W      -      0      UFRJ50816     c218:43007           A>G         TTC>TTC      F>FYAL011W      -      0      A12           c218:43046           C>T         CAG>CAA      Q>QYAL011W      -      0      UFRJ50791     c218:43046           C>T         CAG>CAA      Q>QYAL011W      -      0      UFRJ50816     c218:43046           C>T         CAG>CAA      Q>QYAL011W      -      0      A12           c218:43101           A>G         AGG>ACG      R>TYAL011W      -      0      A12           c218:43130           G>A         TGC>TGT      C>CYAL011W      -      0      N_44          c218:43130           G>A         TGC>TGT      C>CYAL011W      -      0      N_45          c218:43130           G>A         TGC>TGT      C>CYAL011W      -      0      UFRJ50791     c218:43130           G>A         TGC>TGT      C>CYAL011W      -      0      UFRJ50816     c218:43130           G>A         TGC>TGT      C>CYAL011W      -      0      N_44          c218:43136           T>C         CCG>CCG      P>PYAL011W      -      0      N_45          c218:43136           T>C         CCG>CCG      P>PYAL011W      -      0      A12           c218:43139           C>T         TAA>TAA      *>*YAL011W      -      0      UFRJ50791     c218:43139           C>T         TAA>TAA      *>*YAL011W      -      0      UFRJ50816     c218:43139           C>T         TAA>TAA      *>*YAL011W      -      0      UFRJ50816     c218:43237           G>C         TTT>GTT      F>VYAL011W      -      0      UWOPS91_917_1 c218:43237           G>C         TTT>GTT      F>VYAL011W      -      0      YPS138        c218:43237           G>C         TTT>GTT      F>VYAL011W      -      0      IFO1804       c218:43241           C>T         TTC>TTA      F>LYAL011W      -      0      N_44          c218:43241           C>T         TTC>TTA      F>LYAL011W      -      0      YPS138        c218:43265           C>T         TTG>TTA      L>LYAL011W      -      0      DBVPG4650     c218:43322           G>T         TAC>TAA      Y>*YAL011W      -      0      DBVPG6304     c218:43376           G>A         TAC>TAT      Y>YYAL011W      -      0      UFRJ50816     c218:43376           G>A         TAC>TAT      Y>YYAL011W      -      0      UWOPS91_917_1 c218:43376           G>A         TAC>TAT      Y>YYAL011W      -      0      YPS138        c218:43376           G>A         TAC>TAT      Y>YYAL011W      -      0      DBVPG6304     c218:43468           G>A         CGC>TGC      R>CYAL011W      -      0      UFRJ50816     c218:43468           G>A         CGC>TGC      R>CYAL011W      -      0      UWOPS91_917_1 c218:43468           G>A         CGC>TGC      R>CYAL011W      -      0      YPS138        c218:43468           G>A         CGC>TGC      R>CYAL011W      -      0      DBVPG6304     c218:43490           C>G         TTC>TTC      F>FYAL011W      -      0      UFRJ50816     c218:43490           C>G         TTC>TTC      F>FYAL011W      -      0      YPS138        c218:43490           C>G         TTC>TTC      F>FYAL011W      -      0      A12           c218:43499           A>G         ATG>ATC      M>IYAL011W      -      0      DBVPG6304     c218:43499           A>G         ATG>ATC      M>IYAL011W      -      0      UFRJ50816     c218:43499           A>G         ATG>ATC      M>IYAL011W      -      0      YPS138        c218:43499           A>G         ATG>ATC      M>IYAL011W      -      0      UWOPS91_917_1 c218:43520           G>A         CTT>CTT      L>LYAL011W      -      0      UWOPS91_917_1 c218:43541           T>C         CAA>CAG      Q>QYAL011W      -      0      A12           c218:43562           T>G         CAG>CAC      Q>HYAL011W      -      0      DBVPG6304     c218:43562           T>G         CAG>CAC      Q>HYAL011W      -      0      UFRJ50816     c218:43562           T>G         CAG>CAC      Q>HYAL011W      -      0      UWOPS91_917_1 c218:43562           T>G         CAG>CAC      Q>HYAL011W      -      0      YPS138        c218:43562           T>G         CAG>CAC      Q>HYAL011W      -      0      IFO1804       c218:43579           C>T         GAC>AAC      D>NYAL011W      -      0      N_43          c218:43579           C>T         GAC>AAC      D>NYAL011W      -      0      N_44          c218:43579           C>T         GAC>AAC      D>NYAL011W      -      0      UWOPS91_917_1 c218:43579           C>T         GAC>AAC      D>NYAL011W      -      0      A12           c218:43588           G>A         ATT>TTT      I>FYAL011W      -      0      DBVPG6304     c218:43588           G>A         ATT>TTT      I>FYAL011W      -      0      UFRJ50816     c218:43588           G>A         ATT>TTT      I>FYAL011W      -      0      UWOPS91_917_1 c218:43588           G>A         ATT>TTT      I>FYAL011W      -      0      A12           c218:43602           A>G         CTC>CCC      L>PYAL011W      -      0      DBVPG6304     c218:43602           A>G         CTC>CCC      L>PYAL011W      -      0      UFRJ50816     c218:43602           A>G         CTC>CCC      L>PYAL011W      -      0      YPS138        c218:43602           A>G         CTC>CCC      L>PYAL011W      -      0      UWOPS91_917_1 c218:43619           T>C         TTT>TTG      F>LYAL011W      -      0      A12           c218:43697           G>A         GGG>GGT      G>GYAL011W      -      0      DBVPG6304     c218:43697           G>A         GGG>GGT      G>GYAL011W      -      0      UFRJ50816     c218:43697           G>A         GGG>GGT      G>GYAL011W      -      0      UWOPS91_917_1 c218:43697           G>A         GGG>GGT      G>GYAL011W      -      0      YPS138        c218:43697           G>A         GGG>GGT      G>GYAL011W      -      0      A12           c218:43715           A>G         AAA>AAC      K>NYAL011W      -      0      DBVPG6304     c218:43715           A>G         AAA>AAC      K>NYAL011W      -      0      UFRJ50816     c218:43715           A>G         AAA>AAC      K>NYAL011W      -      0      YPS138        c218:43715           A>G         AAA>AAC      K>NYAL011W      -      0      A12           c218:43721           A>G         ATC>ATC      I>IYAL011W      -      0      DBVPG6304     c218:43721           A>G         ATC>ATC      I>IYAL011W      -      0      UFRJ50816     c218:43721           A>G         ATC>ATC      I>IYAL011W      -      0      A12           c218:43750           C>T         GGG>AGG      G>RYAL011W      -      0      DBVPG6304     c218:43750           C>T         GGG>AGG      G>RYAL011W      -      0      UFRJ50816     c218:43750           C>T         GGG>AGG      G>RYAL011W      -      0      A12           c218:43769           G>A         GCC>GCT      A>AYAL011W      -      0      DBVPG6304     c218:43769           G>A         GCC>GCT      A>AYAL011W      -      0      UFRJ50816     c218:43769           G>A         GCC>GCT      A>AYAL011W      -      0      UWOPS91_917_1 c218:43826           G>A         AAC>AAT      N>NYAL011W      -      0      DBVPG6304     c218:43897           T>C         CAA>GAA      Q>EYAL011W      -      0      IFO1804       c218:43897           T>C         CAA>GAA      Q>EYAL011W      -      0      N_43          c218:43897           T>C         CAA>GAA      Q>EYAL011W      -      0      UFRJ50816     c218:43897           T>C         CAA>GAA      Q>EYAL011W      -      0      UWOPS91_917_1 c218:43897           T>C         CAA>GAA      Q>EYAL011W      -      0      A12           c218:43905           T>C         ATA>AGA      I>RYAL011W      -      0      DBVPG6304     c218:43905           T>C         ATA>AGA      I>RYAL011W      -      0      IFO1804       c218:43905           T>C         ATA>AGA      I>RYAL011W      -      0      N_43          c218:43905           T>C         ATA>AGA      I>RYAL011W      -      0      UFRJ50816     c218:43905           T>C         ATA>AGA      I>RYAL011W      -      0      UWOPS91_917_1 c218:43905           T>C         ATA>AGA      I>RYAL011W      -      0      IFO1804       c218:43911           C>T         GTC>GAC      V>DYAL011W      -      0      N_43          c218:43911           C>T         GTC>GAC      V>DYAL011W      -      0      A12           c218:43925           T>C         TTC>TTG      F>LYAL011W      -      0      DBVPG6304     c218:43925           T>C         TTC>TTG      F>LYAL011W      -      0      UFRJ50816     c218:43925           T>C         TTC>TTG      F>LYAL011W      -      0      UWOPS91_917_1 c218:43925           T>C         TTC>TTG      F>LYAL011W      -      0      A12           c218:43943           T>C         TAA>TAG      *>*YAL011W      -      0      DBVPG6304     c218:43943           T>C         TAA>TAG      *>*YAL011W      -      0      UFRJ50816     c218:43943           T>C         TAA>TAG      *>*YAL011W      -      0      UWOPS91_917_1 c218:43943           T>C         TAA>TAG      *>*YAL011W      -      0      A12           c218:43956           G>A         TTC>TTC      F>FYAL011W      -      0      T21_4         c218:43968           C>a         TCT>TTT      S>FYAL011W      -      0      A12           c218:43982           A>G         TTT>TTC      F>FYAL011W      -      0      DBVPG6304     c218:43982           A>G         TTT>TTC      F>FYAL011W      -      0      UFRJ50816     c218:43982           A>G         TTT>TTC      F>FYAL011W      -      0      UWOPS91_917_1 c218:43982           A>G         TTT>TTC      F>FYAL013W      -      0      UFRJ50816     c218:45731           T>C         TGT>TGT      C>CYAL013W      -      0      DBVPG6304     c218:45820           A>G         GTT>GTC      V>VYAL013W      -      0      YPS138        c218:45820           A>G         GTT>GTC      V>VYAL013W      -      0      UWOPS91_917_1 c218:45856           C>T         ACT>ACA      T>TYAL013W      -      0      UWOPS91_917_1 c218:45865           T>C         TCC>TCG      S>SYAL013W      -      0      UWOPS91_917_1 c218:45897           G>A         CTT>TTT      L>FYAL013W      -      0      UWOPS91_917_1 c218:45919           G>A         CAA>CAT      Q>HYAL013W      -      0      IFO1804       c218:45945           G>T         ACT>ACT      T>TYAL013W      -      0      CBS5829       c218:46054           G>A         CTT>CTT      L>LYAL013W      -      0      YPS138        c218:46108           G>A         CTC>CTT      L>LYAL013W      -      0      CBS5829       c218:46162           G>A         TTC>TTT      F>FYAL013W      -      0      DBVPG4650     c218:46162           G>A         TTC>TTT      F>FYAL013W      -      0      IFO1804       c218:46162           G>A         TTC>TTT      F>FYAL013W      -      0      N_44          c218:46162           G>A         TTC>TTT      F>FYAL013W      -      0      N_45          c218:46162           G>A         TTC>TTT      F>FYAL013W      -      0      Q62_5         c218:46162           G>A         TTC>TTT      F>FYAL013W      -      0      S36_7         c218:46162           G>A         TTC>TTT      F>FYAL013W      -      0      UFRJ50816     c218:46162           G>A         TTC>TTT      F>FYAL013W      -      0      Y6_5          c218:46162           G>A         TTC>TTT      F>FYAL013W      -      0      YPS138        c218:46162           G>A         TTC>TTT      F>FYAL013W      -      0      N_44          c218:46186           T>C         GTT>GTG      V>VYAL013W      -      0      N_45          c218:46186           T>C         GTT>GTG      V>VYAL013W      -      0      UFRJ50816     c218:46231           A>G         TGG>TGC      W>CYAL013W      -      0      Q62_5         c218:46269           A>G         CTT>CTT      L>LYAL013W      -      0      UFRJ50816     c218:46279           T>C         GTC>GTG      V>VYAL013W      -      0      UFRJ50816     c218:46308           T>C         GCG>GCG      A>AYAL013W      -      0      UFRJ50791     c218:46315           C>G         TAG>TAC      *>YYAL013W      -      0      UFRJ50816     c218:46315           C>G         TAG>TAC      *>YYAL013W      -      0      UFRJ50791     c218:46323           A>G         ATC>CTC      I>LYAL013W      -      0      UFRJ50816     c218:46323           A>G         ATC>CTC      I>LYAL013W      -      0      N_43          c218:46350           G>A         CTC>TTC      L>FYAL013W      -      0      N_44          c218:46350           G>A         CTC>TTC      L>FYAL013W      -      0      UFRJ50816     c218:46350           G>A         CTC>TTC      L>FYAL013W      -      0      UWOPS91_917_1 c218:46350           G>A         CTC>TTC      L>FYAL013W      -      0      UWOPS91_917_1 c218:46366           C>T         TTC>TTA      F>LYAL013W      -      0      UFRJ50791     c218:46372           G>A         TGA>TGT      *>CYAL013W      -      0      UFRJ50816     c218:46372           G>A         TGA>TGT      *>CYAL013W      -      0      UWOPS91_917_1 c218:46393           A>G         GTA>GTC      V>VYAL013W      -      0      DBVPG6304     c218:46399           T>C         CGA>CGG      R>RYAL013W      -      0      UFRJ50791     c218:46399           T>C         CGA>CGG      R>RYAL013W      -      0      UFRJ50816     c218:46399           T>C         CGA>CGG      R>RYAL013W      -      0      UWOPS91_917_1 c218:46399           T>C         CGA>CGG      R>RYAL013W      -      0      N_44          c218:46432           C>G         GTG>GTC      V>VYAL013W      -      0      DBVPG6304     c218:46498           G>A         GGT>GGT      G>GYAL013W      -      0      N_43          c218:46498           G>A         GGT>GGT      G>GYAL013W      -      0      N_44          c218:46498           G>A         GGT>GGT      G>GYAL013W      -      0      UFRJ50791     c218:46498           G>A         GGT>GGT      G>GYAL013W      -      0      UFRJ50816     c218:46498           G>A         GGT>GGT      G>GYAL013W      -      0      UWOPS91_917_1 c218:46498           G>A         GGT>GGT      G>GYAL013W      -      0      UWOPS91_917_1 c218:46513           C>T         GTC>GTA      V>VYAL013W      -      0      DBVPG6304     c218:46553           T>A         CAG>CTG      Q>LYAL013W      -      0      UFRJ50791     c218:46553           T>A         CAG>CTG      Q>LYAL013W      -      0      UFRJ50816     c218:46553           T>A         CAG>CTG      Q>LYAL013W      -      0      UWOPS91_917_1 c218:46558           T>C         TCT>TCG      S>SYAL013W      -      0      DBVPG6304     c218:46573           T>C         CCA>CCG      P>PYAL013W      -      0      UWOPS91_917_1 c218:46573           T>C         CCA>CCG      P>PYAL013W      -      0      DBVPG6304     c218:46608           T>C         GTC>GTC      V>VYAL013W      -      0      UFRJ50791     c218:46608           T>C         GTC>GTC      V>VYAL013W      -      0      UFRJ50816     c218:46608           T>C         GTC>GTC      V>VYAL013W      -      0      N_43          c218:46611           C>T         GAT>AAT      D>NYAL013W      -      0      DBVPG6304     c218:46621           T>C         ATC>ATG      I>MYAL013W      -      0      UFRJ50791     c218:46621           T>C         ATC>ATG      I>MYAL013W      -      0      UFRJ50816     c218:46621           T>C         ATC>ATG      I>MYAL013W      -      0      UWOPS91_917_1 c218:46621           T>C         ATC>ATG      I>MYAL013W      -      0      DBVPG6304     c218:46633           G>A         GTG>GTT      V>VYAL013W      -      0      UFRJ50791     c218:46633           G>A         GTG>GTT      V>VYAL013W      -      0      UFRJ50816     c218:46633           G>A         GTG>GTT      V>VYAL013W      -      0      UWOPS91_917_1 c218:46633           G>A         GTG>GTT      V>VYAL013W      -      0      UWOPS91_917_1 c218:46690           A>G         TGC>TGC      C>CYAL013W      -      0      UWOPS91_917_1 c218:46696           T>C         CTG>CTG      L>LYAL013W      -      0      N_43          c218:46711           G>A         GCC>GCT      A>AYAL013W      -      0      N_45          c218:46711           G>A         GCC>GCT      A>AYAL013W      -      0      DBVPG6304     c218:46723           C>T         TGC>TGA      C>*YAL013W      -      0      N_43          c218:46723           C>T         TGC>TGA      C>*YAL013W      -      0      N_45          c218:46723           C>T         TGC>TGA      C>*YAL013W      -      0      UFRJ50791     c218:46723           C>T         TGC>TGA      C>*YAL013W      -      0      UFRJ50816     c218:46723           C>T         TGC>TGA      C>*YAL013W      -      0      UWOPS91_917_1 c218:46723           C>T         TGC>TGA      C>*YAL013W      -      0      DBVPG6304     c218:46729           G>C         GTC>GTG      V>VYAL013W      -      0      UFRJ50791     c218:46729           G>C         GTC>GTG      V>VYAL013W      -      0      UFRJ50816     c218:46729           G>C         GTC>GTG      V>VYAL013W      -      0      UWOPS91_917_1 c218:46729           G>C         GTC>GTG      V>VYAL013W      -      0      N_43          c218:46732           C>T         GAG>GAA      E>EYAL013W      -      0      N_43          c218:46743           A>G         TTC>CTC      F>LYAL013W      -      0      N_45          c218:46743           A>G         TTC>CTC      F>LYAL013W      -      0      UWOPS91_917_1 c218:46762           C>A         TCT>TCT      S>SYAL013W      -      0      DBVPG6304     c218:46807           G>A         CCT>CCT      P>PYAL013W      -      0      UFRJ50791     c218:46807           G>A         CCT>CCT      P>PYAL013W      -      0      UFRJ50816     c218:46807           G>A         CCT>CCT      P>PYAL013W      -      0      N_43          c218:46834           C>T         TTC>TTA      F>LYAL013W      -      0      N_45          c218:46834           C>T         TTC>TTA      F>LYAL013W      -      0      DBVPG6304     c218:46901           A>G         TTC>TCC      F>SYAL013W      -      0      UFRJ50791     c218:46901           A>G         TTC>TCC      F>SYAL013W      -      0      UFRJ50816     c218:46901           A>G         TTC>TCC      F>SYAL013W      -      0      UWOPS91_917_1 c218:46901           A>G         TTC>TCC      F>SYAL013W      -      0      DBVPG6304     c218:46909           G>A         GTC>GTT      V>VYAL013W      -      0      UFRJ50791     c218:46909           G>A         GTC>GTT      V>VYAL013W      -      0      UFRJ50816     c218:46909           G>A         GTC>GTT      V>VYAL016W      -      0      DBVPG6304     c219:1052            G>T         TGA>AGA      *>RYAL016W      -      0      UFRJ50791     c219:1052            G>T         TGA>AGA      *>RYAL016W      -      0      UWOPS91_917_1 c219:1052            G>C         TGA>GGA      *>GYAL016W      -      0      DBVPG6304     c219:1061            C>G         TGC>CGC      C>RYAL016W      -      0      UFRJ50791     c219:1061            C>G         TGC>CGC      C>RYAL016W      -      0      UFRJ50791     c219:1128            A>G         AGA>AGC      R>SYAL016W      -      0      UWOPS91_917_1 c219:1144            T>C         AGC>AGC      S>SYAL016W      -      0      DBVPG6304     c219:1281            A>C         TTG>TTG      L>LYAL016W      -      0      UFRJ50791     c219:1281            A>C         TTG>TTG      L>LYAL016W      -      0      UWOPS91_917_1 c219:1281            A>C         TTG>TTG      L>LYAL016W      -      0      DBVPG6304     c219:1293            C>T         GTC>GTA      V>VYAL016W      -      0      UFRJ50791     c219:1293            C>T         GTC>GTA      V>VYAL016W      -      0      UWOPS91_917_1 c219:1293            C>T         GTC>GTA      V>VYAL016W      -      0      DBVPG6304     c219:1302            A>G         AAA>AAC      K>NYAL016W      -      0      N_44          c219:1302            A>G         AAA>AAC      K>NYAL016W      -      0      N_45          c219:1302            A>G         AAA>AAC      K>NYAL016W      -      0      UFRJ50791     c219:1302            A>G         AAA>AAC      K>NYAL016W      -      0      UWOPS91_917_1 c219:1302            A>G         AAA>AAC      K>NYAL016W      -      0      N_44          c219:1319            G>A         TTC>TTC      F>FYAL016W      -      0      N_45          c219:1319            G>A         TTC>TTC      F>FYAL016W      -      0      DBVPG6304     c219:1332            T>C         AAA>AAG      K>KYAL016W      -      0      UFRJ50791     c219:1332            T>C         AAA>AAG      K>KYAL016W      -      0      DBVPG6304     c219:1419            A>G         TCT>TCC      S>SYAL016W      -      0      UFRJ50791     c219:1419            A>G         TCT>TCC      S>SYAL016W      -      0      UFRJ50816     c219:1419            A>G         TCT>TCC      S>SYAL016W      -      0      UWOPS91_917_1 c219:1419            A>G         TCT>TCC      S>SYAL016W      -      0      DBVPG6304     c219:1479            A>G         AAA>AAC      K>NYAL016W      -      0      UFRJ50791     c219:1479            A>G         AAA>AAC      K>NYAL016W      -      0      UFRJ50816     c219:1479            A>G         AAA>AAC      K>NYAL016W      -      0      UWOPS91_917_1 c219:1479            A>G         AAA>AAC      K>NYAL016W      -      0      YPS138        c219:1479            A>G         AAA>AAC      K>NYAL016W      -      0      A4            c219:1500            C>T         AAT>AAA      N>KYAL016W      -      0      UFRJ50791     c219:1500            C>T         AAT>AAA      N>KYAL016W      -      0      UFRJ50816     c219:1500            C>T         AAT>AAA      N>KYAL016W      -      0      UWOPS91_917_1 c219:1500            C>T         AAT>AAA      N>KYAL016W      -      0      YPS138        c219:1500            C>T         AAT>AAA      N>KYAL016W      -      0      A4            c219:1557            A>G         GAA>GAC      E>DYAL016W      -      0      UFRJ50791     c219:1557            A>G         GAA>GAC      E>DYAL016W      -      0      UFRJ50816     c219:1557            A>G         GAA>GAC      E>DYAL016W      -      0      YPS138        c219:1557            A>G         GAA>GAC      E>DYAL016W      -      0      A4            c219:1617            G>T         GTA>GTA      V>VYAL016W      -      0      UFRJ50791     c219:1617            G>T         GTA>GTA      V>VYAL016W      -      0      UFRJ50816     c219:1617            G>T         GTA>GTA      V>VYAL016W      -      0      YPS138        c219:1617            G>T         GTA>GTA      V>VYAL016W      -      0      A4            c219:1632            G>A         TTG>TTT      L>FYAL016W      -      0      UFRJ50791     c219:1632            G>A         TTG>TTT      L>FYAL016W      -      0      UFRJ50816     c219:1632            G>A         TTG>TTT      L>FYAL016W      -      0      YPS138        c219:1632            G>A         TTG>TTT      L>FYAL016W      -      0      A4            c219:1686            G>A         GAT>GAT      D>DYAL016W      -      0      UFRJ50791     c219:1686            G>A         GAT>GAT      D>DYAL016W      -      0      UFRJ50816     c219:1686            G>A         GAT>GAT      D>DYAL016W      -      0      YPS138        c219:1686            G>A         GAT>GAT      D>DYAL016W      -      0      DBVPG6304     c219:1713            A>C         TAG>TAG      *>*YAL016W      -      0      A4            c219:1721            A>G         TGT>CGT      C>RYAL016W      -      0      DBVPG6304     c219:1721            A>G         TGT>CGT      C>RYAL016W      -      0      UFRJ50791     c219:1721            A>G         TGT>CGT      C>RYAL016W      -      0      UFRJ50816     c219:1721            A>G         TGT>CGT      C>RYAL016W      -      0      YPS138        c219:1721            A>G         TGT>CGT      C>RYAL016W      -      0      A4            c219:1730            G>A         ATC>TTC      I>FYAL016W      -      0      UFRJ50791     c219:1730            G>A         ATC>TTC      I>FYAL016W      -      0      UFRJ50816     c219:1730            G>A         ATC>TTC      I>FYAL016W      -      0      YPS138        c219:1730            G>A         ATC>TTC      I>FYAL016W      -      0      A4            c219:1752            A>G         GAA>GAC      E>DYAL016W      -      0      UFRJ50791     c219:1752            A>G         GAA>GAC      E>DYAL016W      -      0      UFRJ50816     c219:1752            A>G         GAA>GAC      E>DYAL016W      -      0      YPS138        c219:1752            A>G         GAA>GAC      E>DYAL016W      -      0      CBS5829       c219:1800            G>A         CAC>CAT      H>HYAL016W      -      0      T21_4         c219:1800            G>A         CAC>CAT      H>HYAL016W      -      0      Z1_1          c219:1800            G>A         CAC>CAT      H>HYAL016W      -      0      A4            c219:1851            A>G         AGA>AGC      R>SYAL016W      -      0      UFRJ50816     c219:1851            A>G         AGA>AGC      R>SYAL016W      -      0      YPS138        c219:1851            A>G         AGA>AGC      R>SYAL016W      -      0      CBS5829       c219:1932            T>C         TCT>TCG      S>SYAL016W      -      0      T21_4         c219:1932            T>C         TCT>TCG      S>SYAL016W      -      0      Z1_1          c219:1932            T>C         TCT>TCG      S>SYAL016W      -      0      A4            c219:1935            C>T         AAA>AAA      K>KYAL016W      -      0      YPS138        c219:1935            C>T         AAA>AAA      K>KYAL016W      -      0      UFRJ50816     c219:2109            A>G         TAA>TAC      *>YYAL016W      -      0      YPS138        c219:2205            G>A         GTC>GTT      V>VYAL016W      -      0      N_43          c219:2253            T>C         ATC>ATG      I>MYAL016W      -      0      N_45          c219:2253            T>C         ATC>ATG      I>MYAL016W      -      0      N_43          c219:2286            G>A         GGC>GGT      G>GYAL016W      -      0      N_45          c219:2286            G>A         GGC>GGT      G>GYAL016W      -      0      N_43          c219:2295            G>A         CTT>CTT      L>LYAL016W      -      0      N_45          c219:2295            G>A         CTT>CTT      L>LYAL016W      -      0      Y6_5          c219:2406            C>a         GTA>GTT      V>VYAL016W      -      0      N_43          c219:2553            C>T         TGA>TGA      *>*YAL016W      -      0      N_45          c219:2553            C>T         TGA>TGA      *>*YAL016W      -      0      DBVPG6304     c219:2594            A>G         TTT>CTT      F>LYAL016W      -      0      UFRJ50816     c219:2594            A>G         TTT>CTT      F>LYAL016W      -      0      N_43          c219:2615            G>A         TTC>TTC      F>FYAL016W      -      0      N_45          c219:2615            G>A         TTC>TTC      F>FYAL016W      -      0      DBVPG6304     c219:2645            G>A         AGA>TGA      R>*YAL016W      -      0      UFRJ50816     c219:2645            G>A         AGA>TGA      R>*YAL016W      -      0      UWOPS91_917_1 c219:2645            G>A         AGA>TGA      R>*YAL016W      -      0      UWOPS91_917_1 c219:2706            G>A         GAA>GAT      E>DYAL016W      -      0      DBVPG6304     c219:2721            T>C         GGC>GGG      G>GYAL016W      -      0      UFRJ50816     c219:2721            T>C         GGC>GGG      G>GYAL016W      -      0      UWOPS91_917_1 c219:2721            T>C         GGC>GGG      G>GYAL016W      -      0      UWOPS91_917_1 c219:2736            A>T         TGG>TGA      W>*YAL016W      -      0      DBVPG6304     c219:2757            A>G         GGC>GGC      G>GYAL016W      -      0      UFRJ50816     c219:2757            A>G         GGC>GGC      G>GYAL016W      -      0      UWOPS91_917_1 c219:2757            A>G         GGC>GGC      G>GYAL016W      -      0      DBVPG6304     c219:2781            T>C         CTT>CTG      L>LYAL016W      -      0      N_45          c219:2781            T>C         CTT>CTG      L>LYAL016W      -      0      UFRJ50816     c219:2781            T>C         CTT>CTG      L>LYAL016W      -      0      UWOPS91_917_1 c219:2781            T>C         CTT>CTG      L>LYAL016W      -      0      DBVPG6304     c219:2799            G>A         CTC>CTT      L>LYAL016W      -      0      UFRJ50816     c219:2799            G>A         CTC>CTT      L>LYAL016W      -      0      DBVPG6304     c219:2805            C>T         TTT>TTA      F>LYAL016W      -      0      N_45          c219:2805            C>T         TTT>TTA      F>LYAL016W      -      0      UFRJ50816     c219:2805            C>T         TTT>TTA      F>LYAL016W      -      0      UWOPS91_917_1 c219:2805            C>T         TTT>TTA      F>LYAL016W      -      0      DBVPG6304     c219:2844            G>A         CAA>CAT      Q>HYAL016W      -      0      UFRJ50816     c219:2844            G>A         CAA>CAT      Q>HYAL016W      -      0      UWOPS91_917_1 c219:2885            T>C         GTA>GTA      V>VYAL016W      -      0      DBVPG6304     c219:2897            G>T         TTC>ATC      F>IYAL016W      -      0      A12           c219:2915            T>C         TTG>GTG      L>VYAL016W      -      0      DBVPG6304     c219:2915            T>C         TTG>GTG      L>VYAL018C      +      0      UWOPS91_917_1 c219:8274            G>A         GCG>GCA      A>AYAL018C      +      0      UWOPS91_917_1 c219:8313            G>A         GGG>GGA      G>GYAL018C      +      0      A4            c219:8343            A>G         GTA>GTG      V>VYAL018C      +      0      UFRJ50816     c219:8343            A>G         GTA>GTG      V>VYAL018C      +      0      A4            c219:8352            A>G         CCA>CCG      P>PYAL018C      +      0      UFRJ50816     c219:8352            A>G         CCA>CCG      P>PYAL018C      +      0      UWOPS91_917_1 c219:8367            G>A         TTG>TTA      L>LYAL018C      +      0      UFRJ50816     c219:8382            A>G         AAA>AAG      K>KYAL018C      +      0      N_45          c219:8389            C>A         CTC>ATC      L>IYAL018C      +      0      A4            c219:8400            T>C         GTT>GTC      V>VYAL018C      +      0      UFRJ50816     c219:8400            T>C         GTT>GTC      V>VYAL018C      +      0      YPS138        c219:8400            T>C         GTT>GTC      V>VYAL018C      +      0      A4            c219:8424            A>T         CTA>CTT      L>LYAL018C      +      0      UFRJ50816     c219:8424            A>T         CTA>CTT      L>LYAL018C      +      0      YPS138        c219:8424            A>T         CTA>CTT      L>LYAL018C      +      0      KPN3828       c219:8434            T>A         TTC>ATC      F>IYAL018C      +      0      UWOPS91_917_1 c219:8460            G>A         GCG>GCA      A>AYAL018C      +      0      KPN3828       c219:8484            T>A         TTT>TTA      F>LYAL018C      +      0      UWOPS91_917_1 c219:8487            G>A         TTG>TTA      L>LYAL018C      +      0      N_43          c219:8502            T>C         AAT>AAC      N>NYAL018C      +      0      N_45          c219:8502            T>C         AAT>AAC      N>NYAL018C      +      0      KPN3828       c219:8511            T>C         AGT>AGC      S>SYAL018C      +      0      UWOPS91_917_1 c219:8514            T>C         TAT>TAC      Y>YYAL018C      +      0      UWOPS91_917_1 c219:8520            C>G         ACC>ACG      T>TYAL018C      +      0      A4            c219:8533            A>G         ATA>GTA      I>VYAL018C      +      0      UFRJ50816     c219:8533            A>G         ATA>GTA      I>VYAL018C      +      0      YPS138        c219:8533            A>G         ATA>GTA      I>VYAL018C      +      0      A4            c219:8541            G>A         TCG>TCA      S>SYAL018C      +      0      UFRJ50816     c219:8541            G>A         TCG>TCA      S>SYAL018C      +      0      YPS138        c219:8541            G>A         TCG>TCA      S>SYAL018C      +      0      UWOPS91_917_1 c219:8542            G>A         GGT>AGT      G>SYAL018C      +      0      N_45          c219:8547            C>T         TAC>TAT      Y>YYAL018C      +      0      A4            c219:8577            C>T         GTC>GTT      V>VYAL018C      +      0      UFRJ50816     c219:8577            C>T         GTC>GTT      V>VYAL018C      +      0      YPS138        c219:8577            C>T         GTC>GTT      V>VYAL018C      +      0      UWOPS91_917_1 c219:8589            G>A         ACG>ACA      T>TYAL018C      +      0      UWOPS91_917_1 c219:8595            A>G         ACA>ACG      T>TYAL018C      +      0      A4            c219:8632            A>G         ATT>GTT      I>VYAL018C      +      0      UFRJ50816     c219:8632            A>G         ATT>GTT      I>VYAL018C      +      0      YPS138        c219:8632            A>G         ATT>GTT      I>VYAL018C      +      0      UWOPS91_917_1 c219:8649            T>C         GCT>GCC      A>AYAL018C      +      0      UFRJ50816     c219:8661            A>G         TCA>TCG      S>SYAL018C      +      0      UWOPS91_917_1 c219:8661            A>C         TCA>TCC      S>SYAL018C      +      0      UWOPS91_917_1 c219:8667            A>G         TTA>TTG      L>LYAL018C      +      0      T21_4         c219:8668            C>a         CAA>aAA      Q>KYAL018C      +      0      A4            c219:8682            T>C         TTT>TTC      F>FYAL018C      +      0      N_43          c219:8682            T>C         TTT>TTC      F>FYAL018C      +      0      N_45          c219:8682            T>C         TTT>TTC      F>FYAL018C      +      0      UFRJ50816     c219:8682            T>C         TTT>TTC      F>FYAL018C      +      0      UWOPS91_917_1 c219:8682            T>C         TTT>TTC      F>FYAL018C      +      0      YPS138        c219:8682            T>C         TTT>TTC      F>FYAL018C      +      0      DBVPG4650     c219:8707            A>C         AAT>CAT      N>HYAL018C      +      0      N_17          c219:8707            A>C         AAT>CAT      N>HYAL018C      +      0      N_43          c219:8707            A>C         AAT>CAT      N>HYAL018C      +      0      N_45          c219:8707            A>C         AAT>CAT      N>HYAL018C      +      0      Q59_1         c219:8707            A>C         AAT>CAT      N>HYAL018C      +      0      Q62_5         c219:8707            A>C         AAT>CAT      N>HYAL018C      +      0      Q89_8         c219:8707            A>C         AAT>CAT      N>HYAL018C      +      0      S36_7         c219:8707            A>C         AAT>CAT      N>HYAL018C      +      0      T21_4         c219:8707            A>C         AAT>CAT      N>HYAL018C      +      0      UWOPS91_917_1 c219:8707            A>C         AAT>CAT      N>HYAL018C      +      0      Y6_5          c219:8707            A>C         AAT>CAT      N>HYAL018C      +      0      UWOPS91_917_1 c219:8730            G>A         GAG>GAA      E>EYAL018C      +      0      YPS138        c219:8736            C>T         TGC>TGT      C>CYAL018C      +      0      A4            c219:8742            A>G         GAA>GAG      E>EYAL018C      +      0      UFRJ50816     c219:8742            A>G         GAA>GAG      E>EYAL018C      +      0      UWOPS91_917_1 c219:8742            A>G         GAA>GAG      E>EYAL018C      +      0      YPS138        c219:8742            A>G         GAA>GAG      E>EYAL018C      +      0      A4            c219:8749            G>A         GGT>AGT      G>SYAL018C      +      0      UFRJ50816     c219:8749            G>A         GGT>AGT      G>SYAL018C      +      0      YPS138        c219:8749            G>A         GGT>AGT      G>SYAL018C      +      0      A4            c219:8772            T>C         GAT>GAC      D>DYAL018C      +      0      N_43          c219:8772            T>C         GAT>GAC      D>DYAL018C      +      0      N_45          c219:8772            T>C         GAT>GAC      D>DYAL018C      +      0      UFRJ50816     c219:8772            T>C         GAT>GAC      D>DYAL018C      +      0      YPS138        c219:8772            T>C         GAT>GAC      D>DYAL018C      +      0      A4            c219:8781            A>G         CCA>CCG      P>PYAL018C      +      0      UFRJ50816     c219:8781            A>G         CCA>CCG      P>PYAL018C      +      0      YPS138        c219:8781            A>G         CCA>CCG      P>PYAL018C      +      0      A4            c219:8814            C>T         TAC>TAT      Y>YYAL018C      +      0      N_43          c219:8814            C>T         TAC>TAT      Y>YYAL018C      +      0      N_45          c219:8814            C>T         TAC>TAT      Y>YYAL018C      +      0      UFRJ50816     c219:8814            C>T         TAC>TAT      Y>YYAL018C      +      0      YPS138        c219:8814            C>T         TAC>TAT      Y>YYAL018C      +      0      N_43          c219:8823            T>C         GCT>GCC      A>AYAL018C      +      0      N_45          c219:8823            T>C         GCT>GCC      A>AYAL018C      +      0      UWOPS91_917_1 c219:8826            T>C         TAT>TAC      Y>YYAL018C      +      0      UWOPS91_917_1 c219:8841            A>G         AAA>AAG      K>KYAL018C      +      0      A4            c219:8845            G>A         GTC>ATC      V>IYAL018C      +      0      UFRJ50816     c219:8845            G>A         GTC>ATC      V>IYAL018C      +      0      YPS138        c219:8845            G>A         GTC>ATC      V>IYAL018C      +      0      UWOPS91_917_1 c219:8859            G>A         TTG>TTA      L>LYAL018C      +      0      A4            c219:8868            A>T         GCA>GCT      A>AYAL018C      +      0      UFRJ50816     c219:8868            A>T         GCA>GCT      A>AYAL018C      +      0      UWOPS91_917_1 c219:8868            A>T         GCA>GCT      A>AYAL018C      +      0      YPS138        c219:8868            A>T         GCA>GCT      A>AYAL018C      +      0      UWOPS91_917_1 c219:8901            T>C         TTT>TTC      F>FYAL018C      +      0      A4            c219:8913            A>G         GGA>GGG      G>GYAL018C      +      0      UFRJ50816     c219:8913            A>G         GGA>GGG      G>GYAL018C      +      0      YPS138        c219:8913            A>G         GGA>GGG      G>GYAL018C      +      0      UWOPS91_917_1 c219:8925            C>T         TTC>TTT      F>FYAL018C      +      0      UWOPS91_917_1 c219:8934            G>A         TTG>TTA      L>LYAL018C      +      0      UWOPS91_917_1 c219:8943            C>T         CCC>CCT      P>PYAL018C      +      0      UWOPS91_917_1 c219:8949            T>C         ATT>ATC      I>IYAL018C      +      0      A4            c219:8967            C>T         ACC>ACT      T>TYAL018C      +      0      N_43          c219:8967            C>T         ACC>ACT      T>TYAL018C      +      0      N_45          c219:8967            C>T         ACC>ACT      T>TYAL018C      +      0      UFRJ50816     c219:8967            C>T         ACC>ACT      T>TYAL018C      +      0      UWOPS91_917_1 c219:8967            C>T         ACC>ACT      T>TYAL018C      +      0      YPS138        c219:8967            C>T         ACC>ACT      T>TYAL018C      +      0      N_43          c219:8994            C>T         GAC>GAT      D>DYAL018C      +      0      N_45          c219:8994            C>T         GAC>GAT      D>DYAL018C      +      0      A4            c219:9012            G>A         GAG>GAA      E>EYAL018C      +      0      N_43          c219:9012            G>A         GAG>GAA      E>EYAL018C      +      0      N_45          c219:9012            G>A         GAG>GAA      E>EYAL018C      +      0      UFRJ50816     c219:9012            G>A         GAG>GAA      E>EYAL018C      +      0      UWOPS91_917_1 c219:9012            G>A         GAG>GAA      E>EYAL018C      +      0      YPS138        c219:9012            G>A         GAG>GAA      E>EYAL018C      +      0      UWOPS91_917_1 c219:9018            C>T         ATC>ATT      I>IYAL018C      +      0      N_43          c219:9024            C>A         CTC>CTA      L>LYAL018C      +      0      N_45          c219:9024            C>A         CTC>CTA      L>LYAL018C      +      0      A4            c219:9033            T>C         GGT>GGC      G>GYAL018C      +      0      N_43          c219:9033            T>C         GGT>GGC      G>GYAL018C      +      0      N_45          c219:9033            T>C         GGT>GGC      G>GYAL018C      +      0      UFRJ50816     c219:9033            T>C         GGT>GGC      G>GYAL018C      +      0      UWOPS91_917_1 c219:9033            T>C         GGT>GGC      G>GYAL018C      +      0      YPS138        c219:9033            T>C         GGT>GGC      G>GYAL018C      +      0      UWOPS91_917_1 c219:9045            G>A         TCG>TCA      S>SYAL018C      +      0      A4            c219:9055            C>T         CTG>TTG      L>LYAL018C      +      0      N_43          c219:9055            C>T         CTG>TTG      L>LYAL018C      +      0      N_45          c219:9055            C>T         CTG>TTG      L>LYAL018C      +      0      UFRJ50816     c219:9055            C>T         CTG>TTG      L>LYAL018C      +      0      UWOPS91_917_1 c219:9055            C>T         CTG>TTG      L>LYAL018C      +      0      YPS138        c219:9055            C>T         CTG>TTG      L>LYAL018C      +      0      A4            c219:9069            C>T         ATC>ATT      I>IYAL018C      +      0      UFRJ50816     c219:9069            C>T         ATC>ATT      I>IYAL018C      +      0      UWOPS91_917_1 c219:9069            C>T         ATC>ATT      I>IYAL018C      +      0      YPS138        c219:9069            C>T         ATC>ATT      I>IYAL018C      +      0      A4            c219:9093            G>A         GGG>GGA      G>GYAL018C      +      0      UFRJ50816     c219:9093            G>A         GGG>GGA      G>GYAL018C      +      0      UWOPS91_917_1 c219:9093            G>A         GGG>GGA      G>GYAL018C      +      0      YPS138        c219:9093            G>A         GGG>GGA      G>GYAL018C      +      0      A4            c219:9144            T>C         CAT>CAC      H>HYAL018C      +      0      UFRJ50816     c219:9144            T>C         CAT>CAC      H>HYAL018C      +      0      UWOPS91_917_1 c219:9144            T>C         CAT>CAC      H>HYAL018C      +      0      A4            c219:9156            C>T         CCC>CCT      P>PYAL018C      +      0      UFRJ50816     c219:9156            C>T         CCC>CCT      P>PYAL018C      +      0      UWOPS91_917_1 c219:9156            C>T         CCC>CCT      P>PYAL018C      +      0      A4            c219:9165            G>A         GTG>GTA      V>VYAL018C      +      0      UFRJ50816     c219:9165            G>A         GTG>GTA      V>VYAL018C      +      0      UWOPS91_917_1 c219:9165            G>A         GTG>GTA      V>VYAL018C      +      0      UWOPS91_917_1 c219:9171            A>T         GAA>GAT      E>DYAL018C      +      0      UFRJ50816     c219:9184            A>C         ATT>CTT      I>LYAL018C      +      0      N_43          c219:9190            G>A         GCA>ACA      A>TYAL018C      +      0      N_45          c219:9190            G>A         GCA>ACA      A>TYAL018C      +      0      UFRJ50816     c219:9190            G>A         GCA>ACA      A>TYAL018C      +      0      UWOPS91_917_1 c219:9190            G>A         GCA>ACA      A>TYAL018C      +      0      A4            c219:9196            C>T         CCA>TCA      P>SYAL018C      +      0      UFRJ50816     c219:9196            C>T         CCA>TCA      P>SYAL018C      +      0      UWOPS91_917_1 c219:9205            A>G         ACC>GCC      T>AYAL018C      +      0      UFRJ50816     c219:9213            G>A         GAG>GAA      E>EYAL018C      +      0      UWOPS91_917_1 c219:9213            G>A         GAG>GAA      E>EYAL019W      -      0      N_45          c219:9491            T>C         TGA>TGG      *>WYAL019W      -      0      UWOPS91_917_1 c219:9491            T>C         TGA>TGG      *>WYAL019W      -      0      UWOPS91_917_1 c219:9503            G>C         ATC>ATG      I>MYAL019W      -      0      A12           c219:9581            G>A         TGT>TGT      C>CYAL019W      -      0      UWOPS91_917_1 c219:9593            C>T         TGC>TGA      C>*YAL019W      -      0      A12           c219:9614            C>T         ACT>ACA      T>TYAL019W      -      0      A12           c219:9623            A>G         AGT>AGC      S>SYAL019W      -      0      Y7            c219:9658            T>A         GTG>TTG      V>LYAL019W      -      0      A12           c219:9659            G>T         AGC>AGA      S>RYAL019W      -      0      A12           c219:9668            C>A         CGG>CGT      R>RYAL019W      -      0      UWOPS91_917_1 c219:9668            C>A         CGG>CGT      R>RYAL019W      -      0      UWOPS91_917_1 c219:9674            C>T         TGG>TGA      W>*YAL019W      -      0      Q32_3         c219:9707            A>T         CAG>CAA      Q>QYAL019W      -      0      UWOPS91_917_1 c219:9716            A>G         CTC>CTC      L>LYAL019W      -      0      UWOPS91_917_1 c219:9731            G>A         AGA>AGT      R>SYAL019W      -      0      UWOPS91_917_1 c219:9767            G>C         CGA>CGG      R>RYAL019W      -      0      UWOPS91_917_1 c219:9794            T>C         ACG>ACG      T>TYAL019W      -      0      UWOPS91_917_1 c219:9878            C>A         AAC>AAT      N>NYAL019W      -      0      A12           c219:9884            T>C         TTT>TTG      F>LYAL019W      -      0      N_44          c219:9887            T>C         TGT>TGG      C>WYAL019W      -      0      N_45          c219:9887            T>C         TGT>TGG      C>WYAL019W      -      0      A12           c219:9892            A>G         TAG>CAG      *>QYAL019W      -      0      UWOPS91_917_1 c219:9905            C>T         TGT>TGA      C>*YAL019W      -      0      UWOPS91_917_1 c219:9923            T>C         GAT>GAG      D>EYAL019W      -      0      A12           c219:9947            G>A         AGA>AGT      R>SYAL019W      -      0      DBVPG6304     c219:9947            G>A         AGA>AGT      R>SYAL019W      -      0      A12           c219:9965            C>G         AGC>AGC      S>SYAL019W      -      0      UWOPS91_917_1 c219:9968            T>C         TTT>TTG      F>LYAL019W      -      0      A12           c219:9974            C>T         TTT>TTA      F>LYAL019W      -      0      A12           c219:9986            G>A         AAA>AAT      K>NYAL019W      -      0      DBVPG6304     c219:9986            G>A         AAA>AAT      K>NYAL019W      -      0      UWOPS91_917_1 c219:9995            C>T         GTA>GTA      V>VYAL019W      -      0      UWOPS91_917_1 c219:10058           A>G         CGA>CGC      R>RYAL019W      -      0      A12           c219:10070           C>A         GCT>GCT      A>AYAL019W      -      0      DBVPG6304     c219:10070           C>A         GCT>GCT      A>AYAL019W      -      0      A12           c219:10088           G>A         GTA>GTT      V>VYAL019W      -      0      DBVPG6304     c219:10088           G>A         GTA>GTT      V>VYAL019W      -      0      DBVPG4650     c219:10100           C>T         TCT>TCA      S>SYAL019W      -      0      N_17          c219:10100           C>T         TCT>TCA      S>SYAL019W      -      0      Q62_5         c219:10100           C>T         TCT>TCA      S>SYAL019W      -      0      Y6_5          c219:10100           C>T         TCT>TCA      S>SYAL019W      -      0      Z1_1          c219:10100           C>T         TCT>TCA      S>SYAL019W      -      0      N_44          c219:10142           C>T         ACG>ACA      T>TYAL019W      -      0      N_45          c219:10142           C>T         ACG>ACA      T>TYAL019W      -      0      DBVPG6304     c219:10166           G>T         ACC>ACA      T>TYAL019W      -      0      UWOPS91_917_1 c219:10166           G>T         ACC>ACA      T>TYAL019W      -      0      DBVPG6304     c219:10206           G>A         ACC>ATC      T>IYAL019W      -      0      UWOPS91_917_1 c219:10206           G>A         ACC>ATC      T>IYAL019W      -      0      DBVPG6304     c219:10250           A>T         CAT>CAA      H>QYAL019W      -      0      UWOPS91_917_1 c219:10250           A>T         CAT>CAA      H>QYAL019W      -      0      UWOPS91_917_1 c219:10273           A>G         ACT>CCT      T>PYAL019W      -      0      N_44          c219:10317           T>C         TTC>TGC      F>CYAL019W      -      0      N_45          c219:10317           T>C         TTC>TGC      F>CYAL019W      -      0      N_44          c219:10323           G>A         AAC>ATC      N>IYAL019W      -      0      N_45          c219:10323           G>A         AAC>ATC      N>IYAL019W      -      0      DBVPG6304     c219:10331           C>T         GAA>GAA      E>EYAL019W      -      0      UWOPS91_917_1 c219:10331           C>T         GAA>GAA      E>EYAL019W      -      0      T21_4         c219:10395           T>A         TTG>TTG      L>LYAL019W      -      0      DBVPG6304     c219:10421           G>A         AGA>AGT      R>SYAL019W      -      0      UWOPS91_917_1 c219:10421           G>A         AGA>AGT      R>SYAL019W      -      0      UWOPS91_917_1 c219:10439           T>C         TAC>TAG      Y>*YAL019W      -      0      Y7            c219:10460           G>A         CTT>CTT      L>LYAL019W      -      0      N_44          c219:10535           A>G         GAG>GAC      E>DYAL019W      -      0      N_45          c219:10535           A>G         GAG>GAC      E>DYAL019W      -      0      UWOPS91_917_1 c219:10535           A>G         GAG>GAC      E>DYAL019W      -      0      N_44          c219:10541           G>A         CTT>CTT      L>LYAL019W      -      0      N_45          c219:10541           G>A         CTT>CTT      L>LYAL019W      -      0      UWOPS91_917_1 c219:10541           G>A         CTT>CTT      L>LYAL019W      -      0      DBVPG6304     c219:10547           G>A         AGT>AGT      S>SYAL019W      -      0      UWOPS91_917_1 c219:10562           C>T         ACC>ACA      T>TYAL019W      -      0      A4            c219:10639           G>A         CCA>TCA      P>SYAL019W      -      0      DBVPG6304     c219:10639           G>A         CCA>TCA      P>SYAL019W      -      0      UWOPS91_917_1 c219:10639           G>A         CCA>TCA      P>SYAL019W      -      0      UWOPS91_917_1 c219:10646           T>A         TTG>TTT      L>FYAL019W      -      0      A4            c219:10717           G>A         ATC>TTC      I>FYAL019W      -      0      DBVPG6304     c219:10717           G>A         ATC>TTC      I>FYAL019W      -      0      A4            c219:10733           T>C         ATT>ATG      I>MYAL019W      -      0      DBVPG6304     c219:10733           T>C         ATT>ATG      I>MYAL019W      -      0      UWOPS91_917_1 c219:10733           T>C         ATT>ATG      I>MYAL019W      -      0      A4            c219:10739           A>G         ATG>ATC      M>IYAL019W      -      0      DBVPG6304     c219:10739           A>G         ATG>ATC      M>IYAL019W      -      0      UWOPS91_917_1 c219:10739           A>G         ATG>ATC      M>IYAL019W      -      0      A4            c219:10748           C>T         AGC>AGA      S>RYAL019W      -      0      DBVPG6304     c219:10748           C>T         AGC>AGA      S>RYAL019W      -      0      N_44          c219:10772           C>T         CTT>CTA      L>LYAL019W      -      0      N_45          c219:10772           C>T         CTT>CTA      L>LYAL019W      -      0      A4            c219:10778           A>G         TTG>TTC      L>FYAL019W      -      0      DBVPG6304     c219:10778           A>G         TTG>TTC      L>FYAL019W      -      0      A4            c219:10790           G>A         CTT>CTT      L>LYAL019W      -      0      N_44          c219:10790           G>A         CTT>CTT      L>LYAL019W      -      0      N_45          c219:10790           G>A         CTT>CTT      L>LYAL019W      -      0      UWOPS91_917_1 c219:10790           G>A         CTT>CTT      L>LYAL019W      -      0      A4            c219:10838           C>A         TGC>TGT      C>CYAL019W      -      0      UWOPS91_917_1 c219:10904           T>C         TTC>TTG      F>LYAL019W      -      0      A4            c219:10982           C>T         TTC>TTA      F>LYAL019W      -      0      A4            c219:11183           T>G         TTG>TTC      L>FYAL019W      -      0      A4            c219:11288           G>A         TGC>TGT      C>CYAL019W      -      0      YPS138        c219:11288           G>A         TGC>TGT      C>CYAL019W      -      0      N_44          c219:11294           G>C         CAA>CAG      Q>QYAL019W      -      0      N_45          c219:11294           G>C         CAA>CAG      Q>QYAL019W      -      0      A4            c219:11301           T>C         CTG>CGG      L>RYAL019W      -      0      DBVPG4650     c219:11301           T>C         CTG>CGG      L>RYAL019W      -      0      N_44          c219:11301           T>C         CTG>CGG      L>RYAL019W      -      0      N_45          c219:11301           T>C         CTG>CGG      L>RYAL019W      -      0      T21_4         c219:11301           T>C         CTG>CGG      L>RYAL019W      -      0      YPS138        c219:11301           T>C         CTG>CGG      L>RYAL019W      -      0      Z1_1          c219:11301           T>C         CTG>CGG      L>RYAL019W      -      0      A4            c219:11318           G>A         AGG>AGT      R>SYAL019W      -      0      UWOPS91_917_1 c219:11318           G>A         AGG>AGT      R>SYAL019W      -      0      YPS138        c219:11318           G>A         AGG>AGT      R>SYAL019W      -      0      A4            c219:11348           T>C         TAA>TAG      *>*YAL019W      -      0      N_44          c219:11348           T>C         TAA>TAG      *>*YAL019W      -      0      N_45          c219:11348           T>C         TAA>TAG      *>*YAL019W      -      0      YPS138        c219:11348           T>C         TAA>TAG      *>*YAL019W      -      0      A4            c219:11357           A>G         CCA>CCC      P>PYAL019W      -      0      YPS138        c219:11357           A>G         CCA>CCC      P>PYAL019W      -      0      N_45          c219:11445           T>C         GAT>GGT      D>GYAL019W      -      0      UWOPS91_917_1 c219:11538           G>C         TCT>TGT      S>CYAL019W      -      0      A4            c219:11561           C>T         ATC>ATA      I>IYAL019W      -      0      N_45          c219:11561           C>T         ATC>ATA      I>IYAL019W      -      0      UWOPS91_917_1 c219:11561           C>T         ATC>ATA      I>IYAL019W      -      0      YPS138        c219:11561           C>T         ATC>ATA      I>IYAL019W      -      0      A4            c219:11568           G>A         ACC>ATC      T>IYAL019W      -      0      YPS138        c219:11568           G>A         ACC>ATC      T>IYAL019W      -      0      CBS432        c219:11579           G>A         TTT>TTT      F>FYAL019W      -      0      DBVPG4650     c219:11579           G>A         TTT>TTT      F>FYAL019W      -      0      KPN3828       c219:11579           G>A         TTT>TTT      F>FYAL019W      -      0      KPN3829       c219:11579           G>A         TTT>TTT      F>FYAL019W      -      0      N_17          c219:11579           G>A         TTT>TTT      F>FYAL019W      -      0      N_45          c219:11579           G>A         TTT>TTT      F>FYAL019W      -      0      Q59_1         c219:11579           G>A         TTT>TTT      F>FYAL019W      -      0      T21_4         c219:11579           G>A         TTT>TTT      F>FYAL019W      -      0      Z1_1          c219:11579           G>A         TTT>TTT      F>FYAL019W      -      0      UWOPS91_917_1 c219:11597           G>C         CCT>CCG      P>PYAL019W      -      0      A4            c219:11606           A>G         TTT>TTC      F>FYAL019W      -      0      YPS138        c219:11606           A>G         TTT>TTC      F>FYAL019W      -      0      N_45          c219:11612           C>G         CAT>CAC      H>HYAL019W      -      0      UWOPS91_917_1 c219:11612           C>G         CAT>CAC      H>HYAL019W      -      0      A4            c219:11621           G>A         ACG>ACT      T>TYAL019W      -      0      UWOPS91_917_1 c219:11621           G>A         ACG>ACT      T>TYAL019W      -      0      YPS138        c219:11621           G>A         ACG>ACT      T>TYAL019W      -      0      A4            c219:11630           G>A         GAA>GAT      E>DYAL019W      -      0      UWOPS91_917_1 c219:11630           G>A         GAA>GAT      E>DYAL019W      -      0      YPS138        c219:11630           G>A         GAA>GAT      E>DYAL019W      -      0      A4            c219:11636           C>A         AAG>AAT      K>NYAL019W      -      0      UWOPS91_917_1 c219:11636           C>A         AAG>AAT      K>NYAL019W      -      0      YPS138        c219:11636           C>A         AAG>AAT      K>NYAL019W      -      0      YPS138        c219:11651           G>A         TGT>TGT      C>CYAL019W      -      0      UWOPS91_917_1 c219:11759           A>G         GGC>GGC      G>GYAL019W      -      0      YPS138        c219:11759           A>G         GGC>GGC      G>GYAL019W      -      0      A4            c219:11777           A>G         GTT>GTC      V>VYAL019W      -      0      N_45          c219:11777           A>G         GTT>GTC      V>VYAL019W      -      0      UWOPS91_917_1 c219:11777           A>G         GTT>GTC      V>VYAL019W      -      0      YPS138        c219:11777           A>G         GTT>GTC      V>VYAL019W      -      0      N_45          c219:11792           T>C         CGG>CGG      R>RYAL019W      -      0      UWOPS91_917_1 c219:11792           T>C         CGG>CGG      R>RYAL019W      -      0      YPS138        c219:11792           T>C         CGG>CGG      R>RYAL019W      -      0      DBVPG4650     c219:11816           T>C         TGT>TGG      C>WYAL019W      -      0      Q89_8         c219:11816           T>C         TGT>TGG      C>WYAL019W      -      0      Z1_1          c219:11816           T>C         TGT>TGG      C>WYAL019W      -      0      A4            c219:11824           C>G         TGC>CGC      C>RYAL019W      -      0      UWOPS91_917_1 c219:11824           C>G         TGC>CGC      C>RYAL019W      -      0      YPS138        c219:11824           C>G         TGC>CGC      C>RYAL019W      -      0      UWOPS91_917_1 c219:11843           C>T         AAA>AAA      K>KYAL019W      -      0      YPS138        c219:11843           C>T         AAA>AAA      K>KYAL019W      -      0      UWOPS91_917_1 c219:11895           C>T         GTG>GAG      V>EYAL019W      -      0      YPS138        c219:11895           C>T         GTG>GAG      V>EYAL019W      -      0      UWOPS91_917_1 c219:12001           A>G         CAA>CAA      Q>QYAL019W      -      0      DBVPG6304     c219:12029           T>C         TAT>TAG      Y>*YAL019W      -      0      UWOPS91_917_1 c219:12029           T>C         TAT>TAG      Y>*YAL019W      -      0      YPS138        c219:12029           T>C         TAT>TAG      Y>*YAL019W      -      0      DBVPG6304     c219:12065           G>A         CAA>CAT      Q>HYAL019W      -      0      YPS138        c219:12065           G>A         CAA>CAT      Q>HYAL019W      -      0      DBVPG6304     c219:12089           C>T         AAG>AAA      K>KYAL019W      -      0      UWOPS91_917_1 c219:12089           C>T         AAG>AAA      K>KYAL019W      -      0      YPS138        c219:12089           C>T         AAG>AAA      K>KYAL019W      -      0      UWOPS91_917_1 c219:12101           G>A         ATT>ATT      I>IYAL019W      -      0      DBVPG6304     c219:12110           G>A         ATT>ATT      I>IYAL019W      -      0      YPS138        c219:12110           G>A         ATT>ATT      I>IYAL019W      -      0      DBVPG6304     c219:12119           A>G         GGT>GGC      G>GYAL019W      -      0      YPS138        c219:12119           A>G         GGT>GGC      G>GYAL019W      -      0      IFO1804       c219:12126           C>T         GGT>GAT      G>DYAL019W      -      0      UWOPS91_917_1 c219:12139           G>A         GTC>TTC      V>FYAL019W      -      0      DBVPG6304     c219:12149           C>T         ATC>ATA      I>IYAL019W      -      0      UWOPS91_917_1 c219:12149           C>T         ATC>ATA      I>IYAL019W      -      0      YPS138        c219:12149           C>T         ATC>ATA      I>IYAL019W      -      0      DBVPG6304     c219:12198           A>G         ATC>ACC      I>TYAL019W      -      0      IFO1804       c219:12198           A>G         ATC>ACC      I>TYAL019W      -      0      UWOPS91_917_1 c219:12198           A>G         ATC>ACC      I>TYAL019W      -      0      YPS138        c219:12198           A>G         ATC>ACC      I>TYAL019W      -      0      UWOPS91_917_1 c219:12220           G>A         AAA>TAA      K>*YAL019W      -      0      DBVPG6304     c219:12245           C>G         GAA>GAC      E>DYAL019W      -      0      UWOPS91_917_1 c219:12245           C>G         GAA>GAC      E>DYAL019W      -      0      DBVPG6304     c219:12266           C>A         ATA>ATT      I>IYAL019W      -      0      IFO1804       c219:12278           C>T         ATT>ATA      I>IYAL019W      -      0      DBVPG6304     c219:12305           T>C         AAT>AAG      N>KYAL019W      -      0      UWOPS91_917_1 c219:12305           T>C         AAT>AAG      N>KYAL019W      -      0      DBVPG6304     c219:12383           G>A         TTG>TTT      L>FYAL019W      -      0      IFO1804       c219:12430           A>G         TTT>CTT      F>LYAL019W      -      0      N_43          c219:12430           A>G         TTT>CTT      F>LYAL019W      -      0      DBVPG6304     c219:12485           T>C         CTT>CTG      L>LYAL019W      -      0      DBVPG6304     c219:12494           T>A         TTC>TTT      F>FYAL019W      -      0      UWOPS91_917_1 c219:12668           G>A         TGT>TGT      C>CYAL019W      -      0      UWOPS91_917_1 c219:12683           G>A         ATC>ATT      I>IYAL019W      -      0      UWOPS91_917_1 c219:12689           T>C         TAT>TAG      Y>*YAL019W      -      0      DBVPG6304     c219:12695           T>C         TTC>TTG      F>LYAL019W      -      0      N_43          c219:12695           T>C         TTC>TTG      F>LYAL019W      -      0      N_44          c219:12695           T>C         TTC>TTG      F>LYAL019W      -      0      N_45          c219:12695           T>C         TTC>TTG      F>LYAL019W      -      0      UWOPS91_917_1 c219:12695           T>C         TTC>TTG      F>LYAL019W      -      0      UWOPS91_917_1 c219:12722           G>A         TTT>TTT      F>FYAL019W      -      0      KPN3829       c219:12729           C>T         AGC>AAC      S>NYAL019W      -      0      N_17          c219:12729           C>T         AGC>AAC      S>NYAL019W      -      0      Y6_5          c219:12729           C>T         AGC>AAC      S>NYAL019W      -      0      DBVPG6304     c219:12730           T>C         AGC>GGC      S>GYAL019W      -      0      UWOPS91_917_1 c219:12730           T>C         AGC>GGC      S>GYAL019W      -      0      N_43          c219:12743           T>C         GAT>GAG      D>EYAL019W      -      0      N_44          c219:12743           T>C         GAT>GAG      D>EYAL019W      -      0      N_45          c219:12743           T>C         GAT>GAG      D>EYAL019W      -      0      CBS5829       c219:12764           G>A         TTG>TTT      L>FYAL019W      -      0      UWOPS91_917_1 c219:12794           A>G         CAT>CAC      H>HYAL020C      +      0      A12           c219:13172           T>C         TAT>TAC      Y>YYAL020C      +      0      A12           c219:13181           C>G         GGC>GGG      G>GYAL020C      +      0      IFO1804       c219:13181           C>G         GGC>GGG      G>GYAL020C      +      0      N_43          c219:13181           C>G         GGC>GGG      G>GYAL020C      +      0      N_44          c219:13181           C>G         GGC>GGG      G>GYAL020C      +      0      N_45          c219:13181           C>G         GGC>GGG      G>GYAL020C      +      0      UWOPS91_917_1 c219:13181           C>A         GGC>GGA      G>GYAL020C      +      0      A12           c219:13215           G>A         GAT>AAT      D>NYAL020C      +      0      A12           c219:13246           C>T         GCG>GTG      A>VYAL020C      +      0      UWOPS91_917_1 c219:13246           C>T         GCG>GTG      A>VYAL020C      +      0      A12           c219:13256           T>C         GGT>GGC      G>GYAL020C      +      0      A12           c219:13267           T>A         GTA>GAA      V>EYAL020C      +      0      UWOPS91_917_1 c219:13267           T>A         GTA>GAA      V>EYAL020C      +      0      Q62_5         c219:13271           C>A         GTC>GTA      V>VYAL020C      +      0      UWOPS91_917_1 c219:13292           G>A         GGG>GGA      G>GYAL020C      +      0      UWOPS91_917_1 c219:13307           A>G         GTA>GTG      V>VYAL020C      +      0      A12           c219:13369           C>A         GCA>GAA      A>EYAL020C      +      0      UFRJ50791     c219:13369           C>A         GCA>GAA      A>EYAL020C      +      0      N_17          c219:13389           C>T         CGT>TGT      R>CYAL020C      +      0      Q89_8         c219:13389           C>T         CGT>TGT      R>CYAL020C      +      0      Q95_3         c219:13389           C>T         CGT>TGT      R>CYAL020C      +      0      A12           c219:13392           G>C         GGC>CGC      G>RYAL020C      +      0      UFRJ50791     c219:13392           G>C         GGC>CGC      G>RYAL020C      +      0      UFRJ50816     c219:13392           G>C         GGC>CGC      G>RYAL020C      +      0      UWOPS91_917_1 c219:13392           G>C         GGC>CGC      G>RYAL020C      +      0      A12           c219:13399           G>T         AGG>ATG      R>MYAL020C      +      0      UFRJ50791     c219:13399           G>T         AGG>ATG      R>MYAL020C      +      0      UFRJ50816     c219:13399           G>T         AGG>ATG      R>MYAL020C      +      0      UFRJ50791     c219:13419           C>T         CCA>TCA      P>SYAL020C      +      0      UFRJ50816     c219:13419           C>T         CCA>TCA      P>SYAL020C      +      0      UWOPS91_917_1 c219:13421           A>T         CCA>CCT      P>PYAL020C      +      0      A12           c219:13471           C>T         GCT>GTT      A>VYAL020C      +      0      UFRJ50791     c219:13471           C>T         GCT>GTT      A>VYAL020C      +      0      UFRJ50816     c219:13471           C>T         GCT>GTT      A>VYAL020C      +      0      UWOPS91_917_1 c219:13471           C>T         GCT>GTT      A>VYAL020C      +      0      A12           c219:13502           T>C         TGT>TGC      C>CYAL020C      +      0      UFRJ50791     c219:13502           T>C         TGT>TGC      C>CYAL020C      +      0      UFRJ50816     c219:13502           T>C         TGT>TGC      C>CYAL020C      +      0      UWOPS91_917_1 c219:13502           T>C         TGT>TGC      C>CYAL020C      +      0      A12           c219:13509           T>C         TTC>CTC      F>LYAL020C      +      0      UFRJ50791     c219:13509           T>C         TTC>CTC      F>LYAL020C      +      0      UFRJ50816     c219:13509           T>C         TTC>CTC      F>LYAL020C      +      0      UWOPS91_917_1 c219:13509           T>C         TTC>CTC      F>LYAL020C      +      0      A12           c219:13520           G>C         CGG>CGC      R>RYAL020C      +      0      UFRJ50791     c219:13520           G>C         CGG>CGC      R>RYAL020C      +      0      UFRJ50816     c219:13520           G>C         CGG>CGC      R>RYAL020C      +      0      UWOPS91_917_1 c219:13520           G>C         CGG>CGC      R>RYAL020C      +      0      IFO1804       c219:13547           A>G         GAA>GAG      E>EYAL020C      +      0      N_43          c219:13547           A>G         GAA>GAG      E>EYAL020C      +      0      N_44          c219:13547           A>G         GAA>GAG      E>EYAL020C      +      0      N_45          c219:13547           A>G         GAA>GAG      E>EYAL020C      +      0      CBS432        c219:13641           A>G         AGG>GGG      R>GYAL020C      +      0      A12           c219:13659           G>A         GTA>ATA      V>IYAL020C      +      0      UFRJ50791     c219:13659           G>A         GTA>ATA      V>IYAL020C      +      0      UFRJ50816     c219:13659           G>A         GTA>ATA      V>IYAL020C      +      0      N_43          c219:13667           A>G         GAA>GAG      E>EYAL020C      +      0      N_44          c219:13667           A>G         GAA>GAG      E>EYAL020C      +      0      A12           c219:13691           G>A         GGG>GGA      G>GYAL020C      +      0      CBS432        c219:13691           G>T         GGG>GGT      G>GYAL020C      +      0      UFRJ50791     c219:13691           G>A         GGG>GGA      G>GYAL020C      +      0      UFRJ50816     c219:13691           G>A         GGG>GGA      G>GYAL020C      +      0      A12           c219:13700           G>C         CCG>CCC      P>PYAL020C      +      0      UFRJ50791     c219:13700           G>C         CCG>CCC      P>PYAL020C      +      0      UFRJ50816     c219:13700           G>C         CCG>CCC      P>PYAL020C      +      0      UWOPS91_917_1 c219:13700           G>C         CCG>CCC      P>PYAL020C      +      0      UWOPS91_917_1 c219:13706           C>G         GAC>GAG      D>EYAL020C      +      0      UWOPS91_917_1 c219:13713           G>C         GCC>CCC      A>PYAL020C      +      0      UWOPS91_917_1 c219:13720           G>C         GGT>GCT      G>AYAL020C      +      0      A12           c219:13734           A>G         ATC>GTC      I>VYAL020C      +      0      N_43          c219:13734           A>G         ATC>GTC      I>VYAL020C      +      0      N_44          c219:13734           A>G         ATC>GTC      I>VYAL020C      +      0      UFRJ50791     c219:13734           A>G         ATC>GTC      I>VYAL020C      +      0      UFRJ50816     c219:13734           A>G         ATC>GTC      I>VYAL020C      +      0      UWOPS91_917_1 c219:13752           G>A         GGC>AGC      G>SYAL020C      +      0      UFRJ50791     c219:13779           C>T         CTG>TTG      L>LYAL020C      +      0      UFRJ50816     c219:13779           C>T         CTG>TTG      L>LYAL020C      +      0      A12           c219:13814           G>A         GAG>GAA      E>EYAL020C      +      0      UFRJ50791     c219:13814           G>A         GAG>GAA      E>EYAL020C      +      0      UFRJ50816     c219:13814           G>A         GAG>GAA      E>EYAL020C      +      0      N_17          c219:13835           G>A         ACG>ACA      T>TYAL020C      +      0      Q62_5         c219:13835           G>A         ACG>ACA      T>TYAL020C      +      0      A12           c219:13867           G>A         AGG>AAG      R>KYAL020C      +      0      UFRJ50791     c219:13867           G>A         AGG>AAG      R>KYAL020C      +      0      N_43          c219:13919           C>T         TTC>TTT      F>FYAL020C      +      0      N_44          c219:13919           C>T         TTC>TTT      F>FYAL020C      +      0      N_44          c219:13928           A>G         GAA>GAG      E>EYAL020C      +      0      UFRJ50791     c219:13928           A>G         GAA>GAG      E>EYAL020C      +      0      N_44          c219:13989           A>G         ACT>GCT      T>AYAL020C      +      0      UFRJ50791     c219:13989           A>G         ACT>GCT      T>AYAL020C      +      0      N_44          c219:14098           G>A         GGC>GAC      G>DYAL020C      +      0      A12           c219:14154           T>C         TTC>CTC      F>LYAL022C      +      0      DBVPG6304     c219:17342           C>T         ACC>ATC      T>IYAL022C      +      0      UFRJ50816     c219:17342           C>T         ACC>ATC      T>IYAL022C      +      0      DBVPG6304     c219:17355           A>T         ATA>ATT      I>IYAL022C      +      0      UFRJ50816     c219:17355           A>T         ATA>ATT      I>IYAL022C      +      0      UWOPS91_917_1 c219:17355           A>T         ATA>ATT      I>IYAL022C      +      0      CBS432        c219:17379           G>A         CTG>CTA      L>LYAL022C      +      0      CBS5829       c219:17379           G>A         CTG>CTA      L>LYAL022C      +      0      N_17          c219:17379           G>A         CTG>CTA      L>LYAL022C      +      0      DBVPG6304     c219:17418           T>A         GAT>GAA      D>EYAL022C      +      0      UWOPS91_917_1 c219:17418           T>A         GAT>GAA      D>EYAL022C      +      0      DBVPG6304     c219:17434           A>G         AAC>GAC      N>DYAL022C      +      0      UFRJ50816     c219:17434           A>G         AAC>GAC      N>DYAL022C      +      0      DBVPG6304     c219:17451           C>A         GAC>GAA      D>EYAL022C      +      0      Q32_3         c219:17467           A>G         AAT>GAT      N>DYAL022C      +      0      N_45          c219:17494           T>C         TCA>CCA      S>PYAL022C      +      0      DBVPG6304     c219:17505           A>G         CCA>CCG      P>PYAL022C      +      0      UWOPS91_917_1 c219:17505           A>G         CCA>CCG      P>PYAL022C      +      0      DBVPG6304     c219:17513           C>G         ACA>AGA      T>RYAL022C      +      0      UWOPS91_917_1 c219:17513           C>G         ACA>AGA      T>RYAL022C      +      0      DBVPG6304     c219:17571           G>A         GGG>GGA      G>GYAL022C      +      0      UWOPS91_917_1 c219:17595           C>T         AAC>AAT      N>NYAL022C      +      0      DBVPG6304     c219:17613           G>A         TCG>TCA      S>SYAL022C      +      0      UWOPS91_917_1 c219:17613           G>A         TCG>TCA      S>SYAL022C      +      0      DBVPG6304     c219:17661           G>A         AAG>AAA      K>KYAL022C      +      0      UFRJ50816     c219:17661           G>A         AAG>AAA      K>KYAL022C      +      0      DBVPG6304     c219:17736           G>A         CAG>CAA      Q>QYAL022C      +      0      UFRJ50791     c219:17736           G>A         CAG>CAA      Q>QYAL022C      +      0      UFRJ50816     c219:17736           G>A         CAG>CAA      Q>QYAL022C      +      0      UWOPS91_917_1 c219:17736           G>A         CAG>CAA      Q>QYAL022C      +      0      DBVPG6304     c219:17742           G>A         AAG>AAA      K>KYAL022C      +      0      DBVPG4650     c219:17766           C>G         GGC>GGG      G>GYAL022C      +      0      IFO1804       c219:17766           C>G         GGC>GGG      G>GYAL022C      +      0      N_43          c219:17766           C>G         GGC>GGG      G>GYAL022C      +      0      N_45          c219:17766           C>G         GGC>GGG      G>GYAL022C      +      0      Q95_3         c219:17766           C>G         GGC>GGG      G>GYAL022C      +      0      S36_7         c219:17766           C>G         GGC>GGG      G>GYAL022C      +      0      UFRJ50791     c219:17886           A>G         GGA>GGG      G>GYAL022C      +      0      UFRJ50816     c219:17886           A>G         GGA>GGG      G>GYAL022C      +      0      DBVPG6304     c219:17943           C>T         TAC>TAT      Y>YYAL022C      +      0      UFRJ50791     c219:17943           C>T         TAC>TAT      Y>YYAL022C      +      0      UFRJ50816     c219:17943           C>T         TAC>TAT      Y>YYAL022C      +      0      DBVPG6304     c219:17973           C>G         GTC>GTG      V>VYAL022C      +      0      UFRJ50791     c219:17973           C>G         GTC>GTG      V>VYAL022C      +      0      UFRJ50816     c219:17973           C>G         GTC>GTG      V>VYAL022C      +      0      DBVPG6304     c219:17988           C>T         CCC>CCT      P>PYAL022C      +      0      UFRJ50791     c219:17988           C>T         CCC>CCT      P>PYAL022C      +      0      UFRJ50816     c219:17988           C>T         CCC>CCT      P>PYAL022C      +      0      DBVPG4650     c219:17992           C>T         CTA>TTA      L>LYAL022C      +      0      Q95_3         c219:17992           C>T         CTA>TTA      L>LYAL022C      +      0      S36_7         c219:17992           C>T         CTA>TTA      L>LYAL022C      +      0      DBVPG6304     c219:18000           G>T         CTG>CTT      L>LYAL022C      +      0      UFRJ50791     c219:18000           G>T         CTG>CTT      L>LYAL022C      +      0      UFRJ50816     c219:18000           G>T         CTG>CTT      L>LYAL022C      +      0      DBVPG6304     c219:18006           T>C         GCT>GCC      A>AYAL022C      +      0      IFO1804       c219:18006           T>C         GCT>GCC      A>AYAL022C      +      0      N_43          c219:18006           T>C         GCT>GCC      A>AYAL022C      +      0      N_44          c219:18006           T>C         GCT>GCC      A>AYAL022C      +      0      N_45          c219:18006           T>C         GCT>GCC      A>AYAL022C      +      0      UFRJ50791     c219:18006           T>C         GCT>GCC      A>AYAL022C      +      0      UFRJ50816     c219:18006           T>C         GCT>GCC      A>AYAL022C      +      0      DBVPG6304     c219:18111           T>A         GTT>GTA      V>VYAL022C      +      0      UFRJ50791     c219:18111           T>A         GTT>GTA      V>VYAL022C      +      0      UFRJ50816     c219:18111           T>A         GTT>GTA      V>VYAL022C      +      0      CBS432        c219:18212           G>A         CGT>CAT      R>HYAL022C      +      0      A4            c219:18231           T>C         GAT>GAC      D>DYAL022C      +      0      DBVPG6304     c219:18231           T>C         GAT>GAC      D>DYAL022C      +      0      UFRJ50791     c219:18231           T>C         GAT>GAC      D>DYAL022C      +      0      UFRJ50816     c219:18231           T>C         GAT>GAC      D>DYAL022C      +      0      A4            c219:18259           G>A         GCC>ACC      A>TYAL022C      +      0      DBVPG6304     c219:18259           G>A         GCC>ACC      A>TYAL022C      +      0      UFRJ50791     c219:18259           G>A         GCC>ACC      A>TYAL022C      +      0      UFRJ50816     c219:18259           G>A         GCC>ACC      A>TYAL022C      +      0      A4            c219:18266           A>G         GAC>GGC      D>GYAL022C      +      0      DBVPG6304     c219:18266           A>G         GAC>GGC      D>GYAL022C      +      0      UFRJ50791     c219:18266           A>G         GAC>GGC      D>GYAL022C      +      0      UFRJ50816     c219:18266           A>G         GAC>GGC      D>GYAL022C      +      0      DBVPG6304     c219:18312           G>A         GTG>GTA      V>VYAL022C      +      0      UFRJ50791     c219:18312           G>A         GTG>GTA      V>VYAL022C      +      0      UFRJ50816     c219:18312           G>A         GTG>GTA      V>VYAL022C      +      0      YPS138        c219:18312           G>A         GTG>GTA      V>VYAL022C      +      0      A4            c219:18318           C>T         TTC>TTT      F>FYAL022C      +      0      DBVPG6304     c219:18318           C>T         TTC>TTT      F>FYAL022C      +      0      UFRJ50791     c219:18318           C>T         TTC>TTT      F>FYAL022C      +      0      UFRJ50816     c219:18318           C>T         TTC>TTT      F>FYAL022C      +      0      YPS138        c219:18318           C>T         TTC>TTT      F>FYAL022C      +      0      A4            c219:18396           A>G         GTA>GTG      V>VYAL022C      +      0      DBVPG6304     c219:18396           A>G         GTA>GTG      V>VYAL022C      +      0      UFRJ50816     c219:18396           A>G         GTA>GTG      V>VYAL022C      +      0      YPS138        c219:18396           A>G         GTA>GTG      V>VYAL022C      +      0      N_44          c219:18408           C>G         GCC>GCG      A>AYAL022C      +      0      N_45          c219:18408           C>G         GCC>GCG      A>AYAL022C      +      0      CBS432        c219:18423           G>A         GGG>GGA      G>GYAL022C      +      0      A4            c219:18430           T>C         TTG>CTG      L>LYAL022C      +      0      DBVPG6304     c219:18430           T>C         TTG>CTG      L>LYAL022C      +      0      Q32_3         c219:18430           T>C         TTG>CTG      L>LYAL022C      +      0      Q62_5         c219:18430           T>C         TTG>CTG      L>LYAL022C      +      0      Q89_8         c219:18430           T>C         TTG>CTG      L>LYAL022C      +      0      UFRJ50816     c219:18430           T>C         TTG>CTG      L>LYAL022C      +      0      Y6_5          c219:18430           T>C         TTG>CTG      L>LYAL022C      +      0      YPS138        c219:18430           T>C         TTG>CTG      L>LYAL022C      +      0      A4            c219:18489           T>C         TAT>TAC      Y>YYAL022C      +      0      DBVPG6304     c219:18489           T>C         TAT>TAC      Y>YYAL022C      +      0      YPS138        c219:18489           T>C         TAT>TAC      Y>YYAL022C      +      0      N_44          c219:18553           A>G         ATT>GTT      I>VYAL022C      +      0      N_45          c219:18553           A>G         ATT>GTT      I>VYAL022C      +      0      DBVPG6304     c219:18555           T>A         ATT>ATA      I>IYAL022C      +      0      YPS138        c219:18555           T>A         ATT>ATA      I>IYAL022C      +      0      A4            c219:18570           A>G         CGA>CGG      R>RYAL022C      +      0      DBVPG6304     c219:18570           A>G         CGA>CGG      R>RYAL022C      +      0      YPS138        c219:18570           A>G         CGA>CGG      R>RYAL022C      +      0      A4            c219:18586           T>C         TTG>CTG      L>LYAL022C      +      0      DBVPG6304     c219:18586           T>C         TTG>CTG      L>LYAL022C      +      0      UWOPS91_917_1 c219:18586           T>C         TTG>CTG      L>LYAL022C      +      0      YPS138        c219:18586           T>C         TTG>CTG      L>LYAL022C      +      0      A4            c219:18609           C>G         ATC>ATG      I>MYAL022C      +      0      DBVPG6304     c219:18609           C>G         ATC>ATG      I>MYAL022C      +      0      YPS138        c219:18609           C>G         ATC>ATG      I>MYAL022C      +      0      A4            c219:18615           C>A         TCC>TCA      S>SYAL022C      +      0      DBVPG6304     c219:18615           C>A         TCC>TCA      S>SYAL022C      +      0      YPS138        c219:18615           C>A         TCC>TCA      S>SYAL022C      +      0      UWOPS91_917_1 c219:18699           T>C         ACT>ACC      T>TYAL022C      +      0      A4            c219:18714           C>T         ATC>ATT      I>IYAL022C      +      0      DBVPG6304     c219:18714           C>T         ATC>ATT      I>IYAL022C      +      0      UFRJ50816     c219:18714           C>T         ATC>ATT      I>IYAL022C      +      0      UWOPS91_917_1 c219:18714           C>T         ATC>ATT      I>IYAL022C      +      0      YPS138        c219:18714           C>T         ATC>ATT      I>IYAL022C      +      0      A4            c219:18819           C>T         GGC>GGT      G>GYAL022C      +      0      DBVPG6304     c219:18819           C>T         GGC>GGT      G>GYAL022C      +      0      UFRJ50816     c219:18819           C>T         GGC>GGT      G>GYAL022C      +      0      UWOPS91_917_1 c219:18819           C>T         GGC>GGT      G>GYAL022C      +      0      YPS138        c219:18819           C>T         GGC>GGT      G>GYAL022C      +      0      A12           c219:18837           C>A         GTC>GTA      V>VYAL022C      +      0      A4            c219:18858           T>C         GTT>GTC      V>VYAL022C      +      0      UFRJ50816     c219:18858           T>C         GTT>GTC      V>VYAL022C      +      0      UWOPS91_917_1 c219:18858           T>C         GTT>GTC      V>VYAL022C      +      0      YPS138        c219:18858           T>C         GTT>GTC      V>VYAL023C      +      0      DBVPG6304     c219:19246           A>T         GAG>GTG      E>VYAL023C      +      0      A12           c219:19339           A>G         GAG>GGG      E>GYAL023C      +      0      DBVPG6304     c219:19339           A>G         GAG>GGG      E>GYAL023C      +      0      UFRJ50816     c219:19339           A>G         GAG>GGG      E>GYAL023C      +      0      IFO1804       c219:19352           A>G         GCA>GCG      A>AYAL023C      +      0      N_44          c219:19352           A>G         GCA>GCG      A>AYAL023C      +      0      N_45          c219:19352           A>G         GCA>GCG      A>AYAL023C      +      0      IFO1804       c219:19382           T>C         TTT>TTC      F>FYAL023C      +      0      N_44          c219:19382           T>C         TTT>TTC      F>FYAL023C      +      0      N_45          c219:19382           T>C         TTT>TTC      F>FYAL023C      +      0      A12           c219:19415           A>G         TTA>TTG      L>LYAL023C      +      0      CBS432        c219:19415           A>G         TTA>TTG      L>LYAL023C      +      0      CBS5829       c219:19415           A>G         TTA>TTG      L>LYAL023C      +      0      IFO1804       c219:19415           A>G         TTA>TTG      L>LYAL023C      +      0      N_44          c219:19415           A>G         TTA>TTG      L>LYAL023C      +      0      N_45          c219:19415           A>G         TTA>TTG      L>LYAL023C      +      0      Q59_1         c219:19415           A>G         TTA>TTG      L>LYAL023C      +      0      Q62_5         c219:19415           A>G         TTA>TTG      L>LYAL023C      +      0      Q95_3         c219:19415           A>G         TTA>TTG      L>LYAL023C      +      0      T21_4         c219:19415           A>G         TTA>TTG      L>LYAL023C      +      0      UFRJ50816     c219:19415           A>G         TTA>TTG      L>LYAL023C      +      0      A12           c219:19424           G>A         AGG>AGA      R>RYAL023C      +      0      IFO1804       c219:19424           G>A         AGG>AGA      R>RYAL023C      +      0      N_44          c219:19424           G>A         AGG>AGA      R>RYAL023C      +      0      N_45          c219:19424           G>A         AGG>AGA      R>RYAL023C      +      0      UFRJ50816     c219:19424           G>A         AGG>AGA      R>RYAL023C      +      0      A12           c219:19457           T>G         GTT>GTG      V>VYAL023C      +      0      DBVPG6304     c219:19457           T>G         GTT>GTG      V>VYAL023C      +      0      UFRJ50816     c219:19457           T>G         GTT>GTG      V>VYAL023C      +      0      A12           c219:19472           T>C         CAT>CAC      H>HYAL023C      +      0      DBVPG6304     c219:19472           T>C         CAT>CAC      H>HYAL023C      +      0      UFRJ50816     c219:19472           T>C         CAT>CAC      H>HYAL023C      +      0      UWOPS91_917_1 c219:19472           T>C         CAT>CAC      H>HYAL023C      +      0      DBVPG6304     c219:19478           T>C         GGT>GGC      G>GYAL023C      +      0      DBVPG6304     c219:19484           T>G         TTT>TTG      F>LYAL023C      +      0      Q62_5         c219:19499           G>A         CTG>CTA      L>LYAL023C      +      0      DBVPG6304     c219:19543           T>G         ATG>AGG      M>RYAL023C      +      0      A12           c219:19565           C>T         TAC>TAT      Y>YYAL023C      +      0      IFO1804       c219:19565           C>T         TAC>TAT      Y>YYAL023C      +      0      N_44          c219:19565           C>T         TAC>TAT      Y>YYAL023C      +      0      N_45          c219:19565           C>T         TAC>TAT      Y>YYAL023C      +      0      UFRJ50816     c219:19565           C>T         TAC>TAT      Y>YYAL023C      +      0      UWOPS91_917_1 c219:19565           C>T         TAC>TAT      Y>YYAL023C      +      0      UWOPS91_917_1 c219:19620           T>C         TAT>CAT      Y>HYAL023C      +      0      A12           c219:19664           C>T         TCC>TCT      S>SYAL023C      +      0      UFRJ50816     c219:19664           C>T         TCC>TCT      S>SYAL023C      +      0      UWOPS91_917_1 c219:19664           C>T         TCC>TCT      S>SYAL023C      +      0      A12           c219:19694           C>T         ACC>ACT      T>TYAL023C      +      0      UFRJ50816     c219:19694           C>T         ACC>ACT      T>TYAL023C      +      0      UWOPS91_917_1 c219:19694           C>T         ACC>ACT      T>TYAL023C      +      0      IFO1804       c219:19697           G>A         GCG>GCA      A>AYAL023C      +      0      N_44          c219:19697           G>A         GCG>GCA      A>AYAL023C      +      0      N_45          c219:19697           G>A         GCG>GCA      A>AYAL023C      +      0      A12           c219:19739           C>G         ACC>ACG      T>TYAL023C      +      0      UFRJ50816     c219:19739           C>G         ACC>ACG      T>TYAL023C      +      0      UWOPS91_917_1 c219:19739           C>G         ACC>ACG      T>TYAL023C      +      0      UFRJ50816     c219:19784           C>T         TTC>TTT      F>FYAL023C      +      0      UFRJ50816     c219:19820           G>A         GTG>GTA      V>VYAL023C      +      0      UWOPS91_917_1 c219:19820           G>A         GTG>GTA      V>VYAL023C      +      0      UWOPS91_917_1 c219:19848           C>T         CAC>TAC      H>YYAL023C      +      0      UFRJ50816     c219:19925           A>C         ACA>ACC      T>TYAL023C      +      0      UWOPS91_917_1 c219:19925           A>C         ACA>ACC      T>TYAL023C      +      0      YPS138        c219:19925           A>C         ACA>ACC      T>TYAL023C      +      0      DBVPG6304     c219:19970           T>C         GGT>GGC      G>GYAL023C      +      0      UFRJ50791     c219:19989           T>C         TTA>CTA      L>LYAL023C      +      0      UFRJ50816     c219:19989           T>C         TTA>CTA      L>LYAL023C      +      0      UWOPS91_917_1 c219:19989           T>C         TTA>CTA      L>LYAL023C      +      0      YPS138        c219:19989           T>C         TTA>CTA      L>LYAL023C      +      0      DBVPG6304     c219:20069           T>C         ATT>ATC      I>IYAL023C      +      0      UFRJ50791     c219:20069           T>C         ATT>ATC      I>IYAL023C      +      0      UFRJ50816     c219:20069           T>C         ATT>ATC      I>IYAL023C      +      0      YPS138        c219:20069           T>C         ATT>ATC      I>IYAL023C      +      0      N_43          c219:20070           A>T         ATC>TTC      I>FYAL023C      +      0      N_44          c219:20070           A>T         ATC>TTC      I>FYAL023C      +      0      N_45          c219:20070           A>T         ATC>TTC      I>FYAL023C      +      0      DBVPG6304     c219:20121           C>T         CTA>TTA      L>LYAL023C      +      0      UFRJ50791     c219:20121           C>T         CTA>TTA      L>LYAL023C      +      0      UFRJ50816     c219:20121           C>T         CTA>TTA      L>LYAL023C      +      0      UWOPS91_917_1 c219:20121           C>T         CTA>TTA      L>LYAL023C      +      0      YPS138        c219:20121           C>T         CTA>TTA      L>LYAL023C      +      0      DBVPG6304     c219:20180           T>G         GTT>GTG      V>VYAL023C      +      0      UFRJ50791     c219:20180           T>G         GTT>GTG      V>VYAL023C      +      0      UFRJ50816     c219:20180           T>G         GTT>GTG      V>VYAL023C      +      0      UWOPS91_917_1 c219:20180           T>G         GTT>GTG      V>VYAL023C      +      0      YPS138        c219:20180           T>G         GTT>GTG      V>VYAL023C      +      0      DBVPG6304     c219:20210           C>T         GAC>GAT      D>DYAL023C      +      0      UFRJ50791     c219:20210           C>T         GAC>GAT      D>DYAL023C      +      0      YPS138        c219:20210           C>T         GAC>GAT      D>DYAL023C      +      0      DBVPG6304     c219:20228           C>T         TCC>TCT      S>SYAL023C      +      0      UFRJ50791     c219:20228           C>T         TCC>TCT      S>SYAL023C      +      0      UWOPS91_917_1 c219:20228           C>T         TCC>TCT      S>SYAL023C      +      0      YPS138        c219:20228           C>T         TCC>TCT      S>SYAL023C      +      0      DBVPG6304     c219:20237           C>T         TCC>TCT      S>SYAL023C      +      0      UFRJ50791     c219:20237           C>T         TCC>TCT      S>SYAL023C      +      0      UWOPS91_917_1 c219:20237           C>T         TCC>TCT      S>SYAL023C      +      0      YPS138        c219:20237           C>T         TCC>TCT      S>SYAL023C      +      0      UWOPS91_917_1 c219:20246           T>C         AAT>AAC      N>NYAL023C      +      0      DBVPG6304     c219:20268           C>T         CTG>TTG      L>LYAL023C      +      0      UFRJ50791     c219:20268           C>T         CTG>TTG      L>LYAL023C      +      0      UWOPS91_917_1 c219:20268           C>T         CTG>TTG      L>LYAL023C      +      0      YPS138        c219:20268           C>T         CTG>TTG      L>LYAL023C      +      0      Q32_3         c219:20269           T>A         CTG>CAG      L>QYAL023C      +      0      A12           c219:20360           T>C         TTT>TTC      F>FYAL023C      +      0      DBVPG6304     c219:20360           T>C         TTT>TTC      F>FYAL023C      +      0      UWOPS91_917_1 c219:20360           T>C         TTT>TTC      F>FYAL023C      +      0      YPS138        c219:20360           T>C         TTT>TTC      F>FYAL023C      +      0      A12           c219:20396           C>T         AAC>AAT      N>NYAL023C      +      0      N_43          c219:20417           G>A         TTG>TTA      L>LYAL023C      +      0      N_44          c219:20417           G>A         TTG>TTA      L>LYAL023C      +      0      N_45          c219:20417           G>A         TTG>TTA      L>LYAL023C      +      0      A12           c219:20426           C>A         GGC>GGA      G>GYAL023C      +      0      DBVPG6304     c219:20426           C>A         GGC>GGA      G>GYAL023C      +      0      UFRJ50791     c219:20426           C>A         GGC>GGA      G>GYAL023C      +      0      YPS138        c219:20426           C>A         GGC>GGA      G>GYAL023C      +      0      A12           c219:20474           T>C         ACT>ACC      T>TYAL023C      +      0      DBVPG6304     c219:20474           T>C         ACT>ACC      T>TYAL023C      +      0      UFRJ50791     c219:20474           T>C         ACT>ACC      T>TYAL023C      +      0      UWOPS91_917_1 c219:20474           T>C         ACT>ACC      T>TYAL023C      +      0      YPS138        c219:20474           T>C         ACT>ACC      T>TYAL023C      +      0      N_43          c219:20486           T>C         GCT>GCC      A>AYAL023C      +      0      N_44          c219:20486           T>C         GCT>GCC      A>AYAL023C      +      0      N_45          c219:20486           T>C         GCT>GCC      A>AYAL023C      +      0      UWOPS91_917_1 c219:20486           T>C         GCT>GCC      A>AYAL023C      +      0      A12           c219:20504           G>A         ACG>ACA      T>TYAL023C      +      0      DBVPG6304     c219:20504           G>A         ACG>ACA      T>TYAL023C      +      0      N_43          c219:20504           G>A         ACG>ACA      T>TYAL023C      +      0      N_44          c219:20504           G>A         ACG>ACA      T>TYAL023C      +      0      N_45          c219:20504           G>A         ACG>ACA      T>TYAL023C      +      0      UFRJ50791     c219:20504           G>A         ACG>ACA      T>TYAL023C      +      0      UWOPS91_917_1 c219:20504           G>A         ACG>ACA      T>TYAL023C      +      0      YPS138        c219:20504           G>A         ACG>ACA      T>TYAL023C      +      0      A12           c219:20513           A>G         GAA>GAG      E>EYAL023C      +      0      DBVPG6304     c219:20513           A>G         GAA>GAG      E>EYAL023C      +      0      UFRJ50791     c219:20513           A>G         GAA>GAG      E>EYAL023C      +      0      UWOPS91_917_1 c219:20513           A>G         GAA>GAG      E>EYAL023C      +      0      YPS138        c219:20513           A>G         GAA>GAG      E>EYAL023C      +      0      N_43          c219:20535           A>G         ATT>GTT      I>VYAL023C      +      0      N_44          c219:20535           A>G         ATT>GTT      I>VYAL023C      +      0      N_45          c219:20535           A>G         ATT>GTT      I>VYAL023C      +      0      A12           c219:20573           C>T         ATC>ATT      I>IYAL023C      +      0      DBVPG6304     c219:20573           C>T         ATC>ATT      I>IYAL023C      +      0      UFRJ50791     c219:20573           C>T         ATC>ATT      I>IYAL023C      +      0      YPS138        c219:20573           C>T         ATC>ATT      I>IYAL023C      +      0      A12           c219:20579           T>C         GAT>GAC      D>DYAL023C      +      0      DBVPG6304     c219:20579           T>C         GAT>GAC      D>DYAL023C      +      0      UFRJ50791     c219:20579           T>C         GAT>GAC      D>DYAL023C      +      0      UWOPS91_917_1 c219:20579           T>C         GAT>GAC      D>DYAL023C      +      0      YPS138        c219:20579           T>C         GAT>GAC      D>DYAL023C      +      0      A12           c219:20644           C>T         TCG>TTG      S>LYAL023C      +      0      DBVPG6304     c219:20644           C>T         TCG>TTG      S>LYAL023C      +      0      UFRJ50791     c219:20644           C>T         TCG>TTG      S>LYAL023C      +      0      UWOPS91_917_1 c219:20644           C>T         TCG>TTG      S>LYAL023C      +      0      YPS138        c219:20644           C>T         TCG>TTG      S>LYAL023C      +      0      UWOPS91_917_1 c219:20654           C>T         GGC>GGT      G>GYAL023C      +      0      A12           c219:20744           T>C         GAT>GAC      D>DYAL023C      +      0      DBVPG6304     c219:20744           T>C         GAT>GAC      D>DYAL023C      +      0      UFRJ50791     c219:20744           T>C         GAT>GAC      D>DYAL023C      +      0      UWOPS91_917_1 c219:20744           T>C         GAT>GAC      D>DYAL023C      +      0      YPS138        c219:20744           T>C         GAT>GAC      D>DYAL023C      +      0      A12           c219:20789           G>A         TTG>TTA      L>LYAL023C      +      0      DBVPG6304     c219:20789           G>A         TTG>TTA      L>LYAL023C      +      0      UFRJ50791     c219:20789           G>A         TTG>TTA      L>LYAL023C      +      0      YPS138        c219:20789           G>A         TTG>TTA      L>LYAL023C      +      0      A12           c219:20810           C>T         TTC>TTT      F>FYAL023C      +      0      DBVPG6304     c219:20810           C>T         TTC>TTT      F>FYAL023C      +      0      UFRJ50791     c219:20810           C>T         TTC>TTT      F>FYAL023C      +      0      YPS138        c219:20810           C>T         TTC>TTT      F>FYAL023C      +      0      YPS138        c219:20821           A>G         AAG>AGG      K>RYAL023C      +      0      A12           c219:20969           C>T         GGC>GGT      G>GYAL023C      +      0      A4            c219:20969           C>T         GGC>GGT      G>GYAL023C      +      0      DBVPG6304     c219:20969           C>T         GGC>GGT      G>GYAL023C      +      0      UFRJ50791     c219:20969           C>T         GGC>GGT      G>GYAL023C      +      0      UFRJ50816     c219:20969           C>T         GGC>GGT      G>GYAL023C      +      0      YPS138        c219:20969           C>T         GGC>GGT      G>GYAL023C      +      0      A12           c219:21017           G>C         TCG>TCC      S>SYAL023C      +      0      A4            c219:21017           G>C         TCG>TCC      S>SYAL023C      +      0      DBVPG6304     c219:21017           G>C         TCG>TCC      S>SYAL023C      +      0      UFRJ50791     c219:21017           G>C         TCG>TCC      S>SYAL023C      +      0      UFRJ50816     c219:21017           G>C         TCG>TCC      S>SYAL023C      +      0      YPS138        c219:21017           G>C         TCG>TCC      S>SYAL023C      +      0      A12           c219:21035           C>T         GTC>GTT      V>VYAL023C      +      0      A4            c219:21035           C>T         GTC>GTT      V>VYAL023C      +      0      DBVPG6304     c219:21035           C>T         GTC>GTT      V>VYAL023C      +      0      UFRJ50791     c219:21035           C>T         GTC>GTT      V>VYAL023C      +      0      UFRJ50816     c219:21035           C>T         GTC>GTT      V>VYAL023C      +      0      YPS138        c219:21035           C>T         GTC>GTT      V>VYAL023C      +      0      A12           c219:21044           C>T         CTC>CTT      L>LYAL023C      +      0      A4            c219:21044           C>T         CTC>CTT      L>LYAL023C      +      0      DBVPG6304     c219:21044           C>T         CTC>CTT      L>LYAL023C      +      0      IFO1804       c219:21044           C>T         CTC>CTT      L>LYAL023C      +      0      N_43          c219:21044           C>T         CTC>CTT      L>LYAL023C      +      0      N_44          c219:21044           C>T         CTC>CTT      L>LYAL023C      +      0      UFRJ50791     c219:21044           C>T         CTC>CTT      L>LYAL023C      +      0      UFRJ50816     c219:21044           C>T         CTC>CTT      L>LYAL023C      +      0      YPS138        c219:21044           C>T         CTC>CTT      L>LYAL023C      +      0      A12           c219:21072           T>C         TTG>CTG      L>LYAL023C      +      0      A4            c219:21072           T>C         TTG>CTG      L>LYAL023C      +      0      DBVPG6304     c219:21072           T>C         TTG>CTG      L>LYAL023C      +      0      UFRJ50791     c219:21072           T>C         TTG>CTG      L>LYAL023C      +      0      UFRJ50816     c219:21072           T>C         TTG>CTG      L>LYAL023C      +      0      YPS138        c219:21072           T>C         TTG>CTG      L>LYAL023C      +      0      A12           c219:21102           T>C         TTA>CTA      L>LYAL023C      +      0      A4            c219:21102           T>C         TTA>CTA      L>LYAL023C      +      0      DBVPG6304     c219:21102           T>C         TTA>CTA      L>LYAL023C      +      0      UFRJ50791     c219:21102           T>C         TTA>CTA      L>LYAL023C      +      0      UFRJ50816     c219:21102           T>C         TTA>CTA      L>LYAL023C      +      0      YPS138        c219:21102           T>C         TTA>CTA      L>LYAL023C      +      0      IFO1804       c219:21206           C>A         ACC>ACA      T>TYAL023C      +      0      A12           c219:21248           C>A         ATC>ATA      I>IYAL023C      +      0      A4            c219:21248           C>A         ATC>ATA      I>IYAL023C      +      0      DBVPG6304     c219:21248           C>A         ATC>ATA      I>IYAL023C      +      0      UFRJ50816     c219:21248           C>A         ATC>ATA      I>IYAL023C      +      0      YPS138        c219:21248           C>A         ATC>ATA      I>IYAL023C      +      0      A4            c219:21272           C>T         GCC>GCT      A>AYAL023C      +      0      DBVPG6304     c219:21272           C>T         GCC>GCT      A>AYAL023C      +      0      IFO1804       c219:21272           C>G         GCC>GCG      A>AYAL023C      +      0      N_44          c219:21272           C>G         GCC>GCG      A>AYAL023C      +      0      N_45          c219:21272           C>G         GCC>GCG      A>AYAL023C      +      0      UFRJ50816     c219:21272           C>T         GCC>GCT      A>AYAL023C      +      0      YPS138        c219:21272           C>T         GCC>GCT      A>AYAL023C      +      0      Q89_8         c219:21298           A>G         AAA>AGA      K>RYAL023C      +      0      CBS5829       c219:21410           C>T         AAC>AAT      N>NYAL023C      +      0      Q32_3         c219:21410           C>T         AAC>AAT      N>NYAL023C      +      0      Q62_5         c219:21410           C>T         AAC>AAT      N>NYAL023C      +      0      Q89_8         c219:21410           C>T         AAC>AAT      N>NYAL023C      +      0      Q95_3         c219:21410           C>T         AAC>AAT      N>NYAL025C      +      0      DBVPG4650     c219:26593           C>T         AAC>AAT      N>NYAL025C      +      0      A12           c219:26683           A>G         CAA>CAG      Q>QYAL025C      +      0      A4            c219:26683           A>G         CAA>CAG      Q>QYAL025C      +      0      UFRJ50791     c219:26683           A>G         CAA>CAG      Q>QYAL025C      +      0      UWOPS91_917_1 c219:26683           A>G         CAA>CAG      Q>QYAL025C      +      0      YPS138        c219:26683           A>G         CAA>CAG      Q>QYAL025C      +      0      N_43          c219:26713           A>G         GCA>GCG      A>AYAL025C      +      0      N_45          c219:26713           A>G         GCA>GCG      A>AYAL025C      +      0      A12           c219:26716           A>G         ACA>ACG      T>TYAL025C      +      0      A4            c219:26716           A>G         ACA>ACG      T>TYAL025C      +      0      UFRJ50791     c219:26716           A>G         ACA>ACG      T>TYAL025C      +      0      N_44          c219:26745           T>A         TTG>TAG      L>*YAL025C      +      0      A12           c219:26758           G>A         ACG>ACA      T>TYAL025C      +      0      A4            c219:26758           G>A         ACG>ACA      T>TYAL025C      +      0      UFRJ50791     c219:26758           G>A         ACG>ACA      T>TYAL025C      +      0      DBVPG4650     c219:26770           A>G         GCA>GCG      A>AYAL025C      +      0      N_45          c219:26770           A>G         GCA>GCG      A>AYAL025C      +      0      Q32_3         c219:26770           A>G         GCA>GCG      A>AYAL025C      +      0      Q62_5         c219:26770           A>G         GCA>GCG      A>AYAL025C      +      0      Q89_8         c219:26770           A>G         GCA>GCG      A>AYAL025C      +      0      Q95_3         c219:26770           A>G         GCA>GCG      A>AYAL025C      +      0      T21_4         c219:26770           A>G         GCA>GCG      A>AYAL025C      +      0      N_43          c219:26788           A>G         TTA>TTG      L>LYAL025C      +      0      N_45          c219:26788           A>G         TTA>TTG      L>LYAL025C      +      0      A12           c219:26806           G>A         CTG>CTA      L>LYAL025C      +      0      A4            c219:26806           G>A         CTG>CTA      L>LYAL025C      +      0      UFRJ50791     c219:26806           G>A         CTG>CTA      L>LYAL025C      +      0      A12           c219:26836           G>A         CAG>CAA      Q>QYAL025C      +      0      A4            c219:26836           G>A         CAG>CAA      Q>QYAL025C      +      0      UFRJ50791     c219:26836           G>A         CAG>CAA      Q>QYAL025C      +      0      UWOPS91_917_1 c219:26836           G>A         CAG>CAA      Q>QYAL025C      +      0      N_43          c219:26872           C>T         TTC>TTT      F>FYAL025C      +      0      N_45          c219:26872           C>T         TTC>TTT      F>FYAL025C      +      0      A12           c219:26878           C>T         CAC>CAT      H>HYAL025C      +      0      A4            c219:26878           C>T         CAC>CAT      H>HYAL025C      +      0      UFRJ50791     c219:26878           C>T         CAC>CAT      H>HYAL025C      +      0      UWOPS91_917_1 c219:26878           C>T         CAC>CAT      H>HYAL025C      +      0      A12           c219:26941           G>A         TTG>TTA      L>LYAL025C      +      0      A4            c219:26941           G>A         TTG>TTA      L>LYAL025C      +      0      N_43          c219:26941           G>A         TTG>TTA      L>LYAL025C      +      0      N_45          c219:26941           G>A         TTG>TTA      L>LYAL025C      +      0      UFRJ50791     c219:26941           G>A         TTG>TTA      L>LYAL025C      +      0      UWOPS91_917_1 c219:26941           G>A         TTG>TTA      L>LYAL025C      +      0      A12           c219:26953           G>A         GAG>GAA      E>EYAL025C      +      0      A4            c219:26953           G>A         GAG>GAA      E>EYAL025C      +      0      UFRJ50791     c219:26953           G>A         GAG>GAA      E>EYAL025C      +      0      A12           c219:26968           A>T         GGA>GGT      G>GYAL025C      +      0      A4            c219:26968           A>T         GGA>GGT      G>GYAL025C      +      0      N_43          c219:26968           A>T         GGA>GGT      G>GYAL025C      +      0      N_45          c219:26968           A>T         GGA>GGT      G>GYAL025C      +      0      UFRJ50791     c219:26968           A>T         GGA>GGT      G>GYAL025C      +      0      UWOPS91_917_1 c219:26968           A>T         GGA>GGT      G>GYAL025C      +      0      UFRJ50791     c219:26991           G>A         AGA>AAA      R>KYAL025C      +      0      A12           c219:27017           C>T         CTG>TTG      L>LYAL025C      +      0      A4            c219:27017           C>T         CTG>TTG      L>LYAL025C      +      0      N_43          c219:27017           C>T         CTG>TTG      L>LYAL025C      +      0      N_45          c219:27017           C>T         CTG>TTG      L>LYAL025C      +      0      UFRJ50791     c219:27017           C>T         CTG>TTG      L>LYAL025C      +      0      UWOPS91_917_1 c219:27017           C>T         CTG>TTG      L>LYAL025C      +      0      A12           c219:27076           C>T         AGC>AGT      S>SYAL025C      +      0      A4            c219:27076           C>T         AGC>AGT      S>SYAL025C      +      0      UWOPS91_917_1 c219:27097           A>T         CCA>CCT      P>PYAL025C      +      0      A12           c219:27106           T>G         GTT>GTG      V>VYAL025C      +      0      A4            c219:27106           T>G         GTT>GTG      V>VYAL025C      +      0      UFRJ50791     c219:27106           T>G         GTT>GTG      V>VYAL025C      +      0      N_45          c219:27114           A>G         AAG>AGG      K>RYAL025C      +      0      A12           c219:27163           C>T         GAC>GAT      D>DYAL025C      +      0      UWOPS91_917_1 c219:27163           C>T         GAC>GAT      D>DYAL025C      +      0      N_43          c219:27202           C>T         GAC>GAT      D>DYAL025C      +      0      N_45          c219:27202           C>T         GAC>GAT      D>DYAL025C      +      0      UWOPS91_917_1 c219:27202           C>A         GAC>GAA      D>EYAL025C      +      0      A12           c219:27286           C>T         GAC>GAT      D>DYAL025C      +      0      A4            c219:27286           C>T         GAC>GAT      D>DYAL025C      +      0      N_43          c219:27286           C>T         GAC>GAT      D>DYAL025C      +      0      N_45          c219:27286           C>T         GAC>GAT      D>DYAL025C      +      0      UWOPS91_917_1 c219:27286           C>T         GAC>GAT      D>DYAL025C      +      0      A4            c219:27370           T>C         AAT>AAC      N>NYAL025C      +      0      KPN3828       c219:27370           T>C         AAT>AAC      N>NYAL025C      +      0      KPN3829       c219:27370           T>C         AAT>AAC      N>NYAL025C      +      0      N_17          c219:27370           T>C         AAT>AAC      N>NYAL025C      +      0      N_43          c219:27370           T>C         AAT>AAC      N>NYAL025C      +      0      N_45          c219:27370           T>C         AAT>AAC      N>NYAL025C      +      0      Q95_3         c219:27370           T>C         AAT>AAC      N>NYAL025C      +      0      Y7            c219:27370           T>C         AAT>AAC      N>NYAL025C      +      0      A4            c219:27388           A>G         AAA>AAG      K>KYAL025C      +      0      UWOPS91_917_1 c219:27388           A>G         AAA>AAG      K>KYAL025C      +      0      N_43          c219:27470           G>A         GTG>ATG      V>MYAL025C      +      0      N_45          c219:27470           G>A         GTG>ATG      V>MYAL027W      -      0      A12           c219:32217           T>C         ACT>ACG      T>TYAL027W      -      0      UWOPS91_917_1 c219:32217           T>C         ACT>ACG      T>TYAL027W      -      0      YPS138        c219:32217           T>C         ACT>ACG      T>TYAL027W      -      0      A12           c219:32340           C>T         TGC>TGA      C>*YAL027W      -      0      UWOPS91_917_1 c219:32340           C>T         TGC>TGA      C>*YAL027W      -      0      YPS138        c219:32340           C>T         TGC>TGA      C>*YAL027W      -      0      N_43          c219:32346           T>C         TAA>TAG      *>*YAL027W      -      0      N_44          c219:32346           T>C         TAA>TAG      *>*YAL027W      -      0      N_45          c219:32346           T>C         TAA>TAG      *>*YAL027W      -      0      UWOPS91_917_1 c219:32346           T>C         TAA>TAG      *>*YAL027W      -      0      A12           c219:32460           A>C         CAC>CAG      H>QYAL027W      -      0      A4            c219:32460           A>C         CAC>CAG      H>QYAL027W      -      0      UFRJ50816     c219:32460           A>C         CAC>CAG      H>QYAL027W      -      0      YPS138        c219:32460           A>C         CAC>CAG      H>QYAL027W      -      0      A12           c219:32466           T>C         TTC>TTG      F>LYAL027W      -      0      A4            c219:32466           T>C         TTC>TTG      F>LYAL027W      -      0      UFRJ50791     c219:32466           T>C         TTC>TTG      F>LYAL027W      -      0      UFRJ50816     c219:32466           T>C         TTC>TTG      F>LYAL027W      -      0      A12           c219:32508           A>G         TAT>TAC      Y>YYAL027W      -      0      A4            c219:32508           A>G         TAT>TAC      Y>YYAL027W      -      0      UFRJ50791     c219:32508           A>G         TAT>TAC      Y>YYAL027W      -      0      YPS138        c219:32508           A>G         TAT>TAC      Y>YYAL027W      -      0      A12           c219:32589           T>C         AGT>AGG      S>RYAL027W      -      0      A4            c219:32589           T>C         AGT>AGG      S>RYAL027W      -      0      UFRJ50791     c219:32589           T>C         AGT>AGG      S>RYAL027W      -      0      UFRJ50816     c219:32589           T>C         AGT>AGG      S>RYAL027W      -      0      YPS138        c219:32589           T>C         AGT>AGG      S>RYAL027W      -      0      A12           c219:32652           A>C         CTT>CTG      L>LYAL027W      -      0      A4            c219:32652           A>C         CTT>CTG      L>LYAL027W      -      0      N_43          c219:32652           A>C         CTT>CTG      L>LYAL027W      -      0      N_44          c219:32652           A>C         CTT>CTG      L>LYAL027W      -      0      N_45          c219:32652           A>C         CTT>CTG      L>LYAL027W      -      0      UFRJ50791     c219:32652           A>C         CTT>CTG      L>LYAL027W      -      0      YPS138        c219:32652           A>C         CTT>CTG      L>LYAL027W      -      0      N_43          c219:32667           G>A         TAT>TAT      Y>YYAL027W      -      0      N_44          c219:32667           G>A         TAT>TAT      Y>YYAL027W      -      0      N_45          c219:32667           G>A         TAT>TAT      Y>YYAL027W      -      0      A12           c219:32682           T>A         AAT>AAT      N>NYAL027W      -      0      A4            c219:32682           T>A         AAT>AAT      N>NYAL027W      -      0      UFRJ50791     c219:32682           T>A         AAT>AAT      N>NYAL027W      -      0      YPS138        c219:32682           T>A         AAT>AAT      N>NYAL027W      -      0      A12           c219:32700           G>A         TAA>TAT      *>YYAL027W      -      0      YPS138        c219:32700           G>A         TAA>TAT      *>YYAL027W      -      0      A12           c219:32709           G>A         GTA>GTT      V>VYAL027W      -      0      A4            c219:32709           G>A         GTA>GTT      V>VYAL027W      -      0      UFRJ50791     c219:32709           G>A         GTA>GTT      V>VYAL027W      -      0      YPS138        c219:32709           G>A         GTA>GTT      V>VYAL027W      -      0      A12           c219:32739           C>A         ACC>ACT      T>TYAL027W      -      0      A4            c219:32739           C>A         ACC>ACT      T>TYAL027W      -      0      UFRJ50791     c219:32739           C>A         ACC>ACT      T>TYAL027W      -      0      YPS138        c219:32739           C>A         ACC>ACT      T>TYAL027W      -      0      A4            c219:32808           T>C         ATT>ATG      I>MYAL027W      -      0      UFRJ50791     c219:32808           T>C         ATT>ATG      I>MYAL027W      -      0      UFRJ50816     c219:32808           T>C         ATT>ATG      I>MYAL027W      -      0      YPS138        c219:32808           T>C         ATT>ATG      I>MYAL027W      -      0      A4            c219:32817           C>G         TGT>TGC      C>CYAL027W      -      0      UFRJ50791     c219:32817           C>G         TGT>TGC      C>CYAL027W      -      0      UFRJ50816     c219:32817           C>G         TGT>TGC      C>CYAL027W      -      0      YPS138        c219:32817           C>G         TGT>TGC      C>CYAL027W      -      0      A4            c219:32891           C>T         GTA>ATA      V>IYAL027W      -      0      N_43          c219:32891           C>T         GTA>ATA      V>IYAL027W      -      0      N_45          c219:32891           C>T         GTA>ATA      V>IYAL027W      -      0      UFRJ50791     c219:32891           C>T         GTA>ATA      V>IYAL027W      -      0      UFRJ50816     c219:32891           C>T         GTA>ATA      V>IYAL027W      -      0      YPS138        c219:32891           C>T         GTA>ATA      V>IYAL027W      -      0      CBS432        c219:32905           C>a         ATT>ATT      I>IYAL027W      -      0      A12           c219:32959           C>T         TAT>TAT      Y>YYAL027W      -      0      A4            c219:32959           C>T         TAT>TAT      Y>YYAL027W      -      0      UFRJ50791     c219:32959           C>T         TAT>TAT      Y>YYAL027W      -      0      UFRJ50816     c219:32959           C>T         TAT>TAT      Y>YYAL027W      -      0      YPS138        c219:32959           C>T         TAT>TAT      Y>YYAL027W      -      0      N_43          c219:32960           T>C         TAT>GAT      Y>DYAL027W      -      0      N_45          c219:32960           T>C         TAT>GAT      Y>DYAL028W      -      0      A12           c219:33222           C>T         CAT>CAT      H>HYAL028W      -      0      A4            c219:33222           C>T         CAT>CAT      H>HYAL028W      -      0      UFRJ50816     c219:33222           C>T         CAT>CAT      H>HYAL028W      -      0      YPS138        c219:33222           C>T         CAT>CAT      H>HYAL028W      -      0      A12           c219:33241           A>G         CCT>CCT      P>PYAL028W      -      0      A4            c219:33241           A>G         CCT>CCT      P>PYAL028W      -      0      UFRJ50816     c219:33241           A>G         CCT>CCT      P>PYAL028W      -      0      YPS138        c219:33241           A>G         CCT>CCT      P>PYAL028W      -      0      CBS5829       c219:33254           G>A         GTC>GTT      V>VYAL028W      -      0      Q59_1         c219:33254           G>A         GTC>GTT      V>VYAL028W      -      0      Q62_5         c219:33254           G>A         GTC>GTT      V>VYAL028W      -      0      T21_4         c219:33254           G>A         GTC>GTT      V>VYAL028W      -      0      Z1_1          c219:33254           G>A         GTC>GTT      V>VYAL028W      -      0      A12           c219:33314           G>C         TGT>TGG      C>WYAL028W      -      0      UFRJ50816     c219:33314           G>C         TGT>TGG      C>WYAL028W      -      0      A12           c219:33335           A>G         ATC>ATC      I>IYAL028W      -      0      UFRJ50816     c219:33335           A>G         ATC>ATC      I>IYAL028W      -      0      A12           c219:33347           G>A         AGC>AGT      S>SYAL028W      -      0      UFRJ50816     c219:33347           G>A         AGC>AGT      S>SYAL028W      -      0      A12           c219:33358           A>G         TGC>CGC      C>RYAL028W      -      0      N_43          c219:33358           A>G         TGC>CGC      C>RYAL028W      -      0      N_45          c219:33358           A>G         TGC>CGC      C>RYAL028W      -      0      UFRJ50816     c219:33358           A>G         TGC>CGC      C>RYAL028W      -      0      A12           c219:33377           T>C         AAC>AAG      N>KYAL028W      -      0      UFRJ50816     c219:33377           T>C         AAC>AAG      N>KYAL028W      -      0      A12           c219:33419           G>A         ATT>ATT      I>IYAL028W      -      0      DBVPG6304     c219:33419           G>A         ATT>ATT      I>IYAL028W      -      0      UFRJ50816     c219:33419           G>A         ATT>ATT      I>IYAL028W      -      0      A12           c219:33445           G>C         CTC>GTC      L>VYAL028W      -      0      DBVPG6304     c219:33445           G>C         CTC>GTC      L>VYAL028W      -      0      UFRJ50816     c219:33445           G>C         CTC>GTC      L>VYAL028W      -      0      UWOPS91_917_1 c219:33448           A>G         CCG>CCG      P>PYAL028W      -      0      A12           c219:33461           C>T         TTC>TTA      F>LYAL028W      -      0      DBVPG6304     c219:33461           C>T         TTC>TTA      F>LYAL028W      -      0      UFRJ50816     c219:33461           C>T         TTC>TTA      F>LYAL028W      -      0      UWOPS91_917_1 c219:33461           C>T         TTC>TTA      F>LYAL028W      -      0      KPN3828       c219:33483           C>T         TTT>TAT      F>YYAL028W      -      0      A12           c219:33491           G>A         GCG>GCT      A>AYAL028W      -      0      DBVPG6304     c219:33491           G>A         GCG>GCT      A>AYAL028W      -      0      UFRJ50816     c219:33491           G>A         GCG>GCT      A>AYAL028W      -      0      A12           c219:33542           G>A         ACA>ACT      T>TYAL028W      -      0      DBVPG6304     c219:33542           G>A         ACA>ACT      T>TYAL028W      -      0      UFRJ50816     c219:33542           G>A         ACA>ACT      T>TYAL028W      -      0      UFRJ50816     c219:33574           T>A         TGT>TGT      C>CYAL028W      -      0      N_43          c219:33580           G>A         TTT>TTT      F>FYAL028W      -      0      N_45          c219:33580           G>A         TTT>TTT      F>FYAL028W      -      0      A12           c219:33618           C>T         AAC>AAC      N>NYAL028W      -      0      DBVPG6304     c219:33618           C>T         AAC>AAC      N>NYAL028W      -      0      UFRJ50816     c219:33618           C>T         AAC>AAC      N>NYAL028W      -      0      UWOPS91_917_1 c219:33618           C>T         AAC>AAC      N>NYAL028W      -      0      DBVPG6304     c219:33626           G>A         AAA>AAT      K>NYAL028W      -      0      UFRJ50816     c219:33626           G>A         AAA>AAT      K>NYAL028W      -      0      A12           c219:33638           C>G         GAA>GAC      E>DYAL028W      -      0      DBVPG6304     c219:33638           C>G         GAA>GAC      E>DYAL028W      -      0      N_43          c219:33638           C>G         GAA>GAC      E>DYAL028W      -      0      N_45          c219:33638           C>G         GAA>GAC      E>DYAL028W      -      0      UFRJ50816     c219:33638           C>G         GAA>GAC      E>DYAL028W      -      0      UWOPS91_917_1 c219:33638           C>G         GAA>GAC      E>DYAL028W      -      0      N_43          c219:33659           C>T         CGA>CGA      R>RYAL028W      -      0      N_45          c219:33659           C>T         CGA>CGA      R>RYAL028W      -      0      UFRJ50816     c219:33682           G>A         GTA>TTA      V>LYAL028W      -      0      DBVPG6304     c219:33698           C>T         AAA>AAA      K>KYAL028W      -      0      UWOPS91_917_1 c219:33728           G>A         CTG>CTT      L>LYAL028W      -      0      A4            c219:33797           A>C         CAC>CAG      H>QYAL028W      -      0      DBVPG6304     c219:33797           A>C         CAC>CAG      H>QYAL028W      -      0      UFRJ50816     c219:33797           A>C         CAC>CAG      H>QYAL028W      -      0      UWOPS91_917_1 c219:33797           A>C         CAC>CAG      H>QYAL028W      -      0      DBVPG6304     c219:33811           C>T         TTT>ATT      F>IYAL028W      -      0      UFRJ50816     c219:33811           C>T         TTT>ATT      F>IYAL028W      -      0      UWOPS91_917_1 c219:33811           C>T         TTT>ATT      F>IYAL028W      -      0      A4            c219:33824           A>C         CCG>CCG      P>PYAL028W      -      0      DBVPG6304     c219:33824           A>C         CCG>CCG      P>PYAL028W      -      0      UFRJ50791     c219:33824           A>C         CCG>CCG      P>PYAL028W      -      0      UFRJ50816     c219:33824           A>C         CCG>CCG      P>PYAL028W      -      0      A4            c219:33884           C>T         CGC>CGA      R>RYAL028W      -      0      DBVPG6304     c219:33884           C>T         CGC>CGA      R>RYAL028W      -      0      UFRJ50791     c219:33884           C>T         CGC>CGA      R>RYAL028W      -      0      UFRJ50816     c219:33884           C>T         CGC>CGA      R>RYAL028W      -      0      A4            c219:33900           T>C         TGG>TGG      W>WYAL028W      -      0      DBVPG6304     c219:33900           T>C         TGG>TGG      W>WYAL028W      -      0      UFRJ50791     c219:33900           T>C         TGG>TGG      W>WYAL028W      -      0      UFRJ50816     c219:33900           T>C         TGG>TGG      W>WYAL028W      -      0      N_45          c219:33911           A>G         ATT>ATC      I>IYAL028W      -      0      A4            c219:33949           A>G         GTG>CTG      V>LYAL028W      -      0      DBVPG6304     c219:33949           A>G         GTG>CTG      V>LYAL028W      -      0      UFRJ50791     c219:33949           A>G         GTG>CTG      V>LYAL028W      -      0      UFRJ50816     c219:33949           A>G         GTG>CTG      V>LYAL028W      -      0      UWOPS91_917_1 c219:33949           A>C         GTG>GTG      V>VYAL028W      -      0      N_43          c219:33955           C>A         CGA>TGA      R>*YAL028W      -      0      N_45          c219:33955           C>A         CGA>TGA      R>*YAL028W      -      0      N_43          c219:33961           G>A         TGT>TGT      C>CYAL028W      -      0      N_45          c219:33961           G>A         TGT>TGT      C>CYAL028W      -      0      A4            c219:33999           T>C         TAG>TGG      *>WYAL028W      -      0      DBVPG6304     c219:33999           T>C         TAG>TGG      *>WYAL028W      -      0      UFRJ50791     c219:33999           T>C         TAG>TGG      *>WYAL028W      -      0      UFRJ50816     c219:33999           T>C         TAG>TGG      *>WYAL028W      -      0      A4            c219:34010           G>A         AAG>AAT      K>NYAL028W      -      0      DBVPG6304     c219:34010           G>A         AAG>AAT      K>NYAL028W      -      0      UFRJ50791     c219:34010           G>A         AAG>AAT      K>NYAL028W      -      0      UFRJ50816     c219:34010           G>A         AAG>AAT      K>NYAL028W      -      0      N_43          c219:34013           G>A         ATA>ATT      I>IYAL028W      -      0      N_45          c219:34013           G>A         ATA>ATT      I>IYAL028W      -      0      DBVPG6304     c219:34046           C>T         AAC>AAA      N>KYAL028W      -      0      A4            c219:34055           T>C         CAA>CAG      Q>QYAL028W      -      0      DBVPG6304     c219:34055           T>C         CAA>CAG      Q>QYAL028W      -      0      UFRJ50791     c219:34055           T>C         CAA>CAG      Q>QYAL028W      -      0      UFRJ50816     c219:34055           T>C         CAA>CAG      Q>QYAL028W      -      0      A4            c219:34070           A>G         GTT>GTC      V>VYAL028W      -      0      DBVPG6304     c219:34070           A>G         GTT>GTC      V>VYAL028W      -      0      UFRJ50791     c219:34070           A>G         GTT>GTC      V>VYAL028W      -      0      UFRJ50816     c219:34070           A>G         GTT>GTC      V>VYAL028W      -      0      UWOPS91_917_1 c219:34070           A>G         GTT>GTC      V>VYAL028W      -      0      UWOPS91_917_1 c219:34081           A>G         CGG>CGG      R>RYAL028W      -      0      DBVPG6304     c219:34092           C>T         AAC>AAC      N>NYAL028W      -      0      N_43          c219:34117           G>A         ACC>TCC      T>SYAL028W      -      0      N_45          c219:34117           G>A         ACC>TCC      T>SYAL028W      -      0      A4            c219:34163           C>T         AGA>AGA      R>RYAL028W      -      0      DBVPG6304     c219:34163           C>T         AGA>AGA      R>RYAL028W      -      0      UFRJ50791     c219:34163           C>T         AGA>AGA      R>RYAL028W      -      0      UFRJ50816     c219:34163           C>T         AGA>AGA      R>RYAL028W      -      0      A4            c219:34170           G>A         CGA>CTA      R>LYAL028W      -      0      DBVPG6304     c219:34170           G>A         CGA>CTA      R>LYAL028W      -      0      UFRJ50791     c219:34170           G>A         CGA>CTA      R>LYAL028W      -      0      UFRJ50816     c219:34170           G>A         CGA>CTA      R>LYAL028W      -      0      N_43          c219:34181           A>T         TGA>TGA      *>*YAL028W      -      0      N_45          c219:34181           A>T         TGA>TGA      *>*YAL028W      -      0      N_43          c219:34193           A>G         AGA>AGC      R>SYAL028W      -      0      N_45          c219:34193           A>G         AGA>AGC      R>SYAL028W      -      0      A4            c219:34243           T>G         GTC>CTC      V>LYAL028W      -      0      DBVPG6304     c219:34243           T>G         GTC>CTC      V>LYAL028W      -      0      UFRJ50791     c219:34243           T>G         GTC>CTC      V>LYAL028W      -      0      UFRJ50816     c219:34243           T>G         GTC>CTC      V>LYAL028W      -      0      A4            c219:34250           G>A         TTC>TTT      F>FYAL028W      -      0      DBVPG6304     c219:34250           G>A         TTC>TTT      F>FYAL028W      -      0      UFRJ50791     c219:34250           G>A         TTC>TTT      F>FYAL028W      -      0      UFRJ50816     c219:34250           G>A         TTC>TTT      F>FYAL028W      -      0      N_43          c219:34262           T>C         ATT>ATG      I>MYAL028W      -      0      N_45          c219:34262           T>C         ATT>ATG      I>MYAL028W      -      0      A4            c219:34277           A>C         ACT>ACG      T>TYAL028W      -      0      DBVPG6304     c219:34277           A>C         ACT>ACG      T>TYAL028W      -      0      UFRJ50791     c219:34277           A>C         ACT>ACG      T>TYAL028W      -      0      UFRJ50816     c219:34277           A>C         ACT>ACG      T>TYAL028W      -      0      A4            c219:34283           G>A         ATT>ATT      I>IYAL028W      -      0      DBVPG6304     c219:34283           G>A         ATT>ATT      I>IYAL028W      -      0      UFRJ50791     c219:34283           G>A         ATT>ATT      I>IYAL028W      -      0      UFRJ50816     c219:34283           G>A         ATT>ATT      I>IYAL028W      -      0      A4            c219:34312           G>A         GTC>TTC      V>FYAL028W      -      0      A4            c219:34319           G>A         CTG>CTT      L>LYAL028W      -      0      DBVPG6304     c219:34319           G>A         CTG>CTT      L>LYAL028W      -      0      UFRJ50791     c219:34319           G>A         CTG>CTT      L>LYAL028W      -      0      UFRJ50816     c219:34319           G>A         CTG>CTT      L>LYAL028W      -      0      A4            c219:34347           G>T         CTG>CAG      L>QYAL028W      -      0      DBVPG6304     c219:34347           G>T         CTG>CAG      L>QYAL028W      -      0      N_43          c219:34347           G>T         CTG>CAG      L>QYAL028W      -      0      N_45          c219:34347           G>T         CTG>CAG      L>QYAL028W      -      0      UFRJ50816     c219:34347           G>T         CTG>CAG      L>QYAL028W      -      0      A4            c219:34356           G>A         AGC>ATC      S>IYAL028W      -      0      DBVPG6304     c219:34356           G>A         AGC>ATC      S>IYAL028W      -      0      UFRJ50816     c219:34356           G>A         AGC>ATC      S>IYAL028W      -      0      UWOPS91_917_1 c219:34356           G>A         AGC>ATC      S>IYAL028W      -      0      A4            c219:34368           C>T         GTG>GAG      V>EYAL028W      -      0      DBVPG6304     c219:34368           C>T         GTG>GAG      V>EYAL028W      -      0      UFRJ50791     c219:34368           C>T         GTG>GAG      V>EYAL028W      -      0      UFRJ50816     c219:34368           C>T         GTG>GAG      V>EYAL028W      -      0      N_43          c219:34387           T>C         AAC>GAC      N>DYAL028W      -      0      N_45          c219:34387           T>C         AAC>GAC      N>DYAL028W      -      0      A4            c219:34396           G>T         ATC>ATC      I>IYAL028W      -      0      DBVPG6304     c219:34396           G>T         ATC>ATC      I>IYAL028W      -      0      UFRJ50791     c219:34396           G>T         ATC>ATC      I>IYAL028W      -      0      UFRJ50816     c219:34396           G>T         ATC>ATC      I>IYAL028W      -      0      A4            c219:34421           G>T         TGC>TGA      C>*YAL028W      -      0      DBVPG6304     c219:34421           G>T         TGC>TGA      C>*YAL028W      -      0      UFRJ50791     c219:34421           G>T         TGC>TGA      C>*YAL028W      -      0      UFRJ50816     c219:34421           G>T         TGC>TGA      C>*YAL028W      -      0      UWOPS91_917_1 c219:34421           G>T         TGC>TGA      C>*YAL028W      -      0      UWOPS91_917_1 c219:34448           C>T         TAA>TAA      *>*YAL028W      -      0      A4            c219:34469           T>C         GGT>GGG      G>GYAL028W      -      0      DBVPG6304     c219:34469           T>C         GGT>GGG      G>GYAL028W      -      0      UFRJ50816     c219:34469           T>C         GGT>GGG      G>GYAL028W      -      0      UWOPS91_917_1 c219:34475           G>A         TCC>TCT      S>SYAL028W      -      0      A4            c219:34477           G>A         TCC>TCC      S>SYAL028W      -      0      DBVPG6304     c219:34477           G>A         TCC>TCC      S>SYAL028W      -      0      N_43          c219:34477           G>C         TCC>GCC      S>AYAL028W      -      0      N_45          c219:34477           G>C         TCC>GCC      S>AYAL028W      -      0      UFRJ50816     c219:34477           G>A         TCC>TCC      S>SYAL028W      -      0      DBVPG6304     c219:34484           T>C         TTC>TTG      F>LYAL028W      -      0      A4            c219:34489           T>C         GAG>GAG      E>EYAL028W      -      0      UWOPS91_917_1 c219:34502           G>C         GTA>GTG      V>VYAL028W      -      0      UWOPS91_917_1 c219:34508           G>A         CCT>CCT      P>PYAL028W      -      0      UWOPS91_917_1 c219:34517           G>A         TTT>TTT      F>FYAL028W      -      0      UFRJ50816     c219:34556           C>T         TGA>TGA      *>*YAL028W      -      0      N_43          c219:34574           G>A         TAT>TAT      Y>YYAL028W      -      0      N_45          c219:34574           G>A         TAT>TAT      Y>YYAL028W      -      0      UWOPS91_917_1 c219:34586           T>C         CAC>CAG      H>QYAL028W      -      0      DBVPG6304     c219:34625           G>A         GCA>GCT      A>AYAL028W      -      0      N_43          c219:34625           G>A         GCA>GCT      A>AYAL028W      -      0      N_45          c219:34625           G>A         GCA>GCT      A>AYAL028W      -      0      UFRJ50816     c219:34625           G>A         GCA>GCT      A>AYAL028W      -      0      UWOPS91_917_1 c219:34625           G>A         GCA>GCT      A>AYAL028W      -      0      N_43          c219:34643           C>T         AGA>AGA      R>RYAL028W      -      0      N_45          c219:34643           C>T         AGA>AGA      R>RYAL028W      -      0      UWOPS91_917_1 c219:34643           C>T         AGA>AGA      R>RYAL028W      -      0      A12           c219:34655           T>C         CGT>CGG      R>RYAL028W      -      0      DBVPG6304     c219:34655           T>C         CGT>CGG      R>RYAL028W      -      0      UFRJ50816     c219:34655           T>C         CGT>CGG      R>RYAL028W      -      0      DBVPG6304     c219:34667           A>G         CAT>CAC      H>HYAL028W      -      0      N_43          c219:34687           C>T         CAA>AAA      Q>KYAL028W      -      0      N_45          c219:34687           C>T         CAA>AAA      Q>KYAL028W      -      0      UWOPS91_917_1 c219:34703           A>G         TAT>TAC      Y>YYAL028W      -      0      A12           c219:34730           C>T         AGA>AGA      R>RYAL028W      -      0      A4            c219:34730           C>T         AGA>AGA      R>RYAL028W      -      0      DBVPG6304     c219:34730           C>T         AGA>AGA      R>RYAL028W      -      0      UFRJ50816     c219:34730           C>T         AGA>AGA      R>RYAL028W      -      0      A12           c219:34763           A>G         AAA>AAC      K>NYAL028W      -      0      A4            c219:34763           A>G         AAA>AAC      K>NYAL028W      -      0      DBVPG6304     c219:34763           A>G         AAA>AAC      K>NYAL028W      -      0      UFRJ50791     c219:34763           A>G         AAA>AAC      K>NYAL028W      -      0      UFRJ50816     c219:34763           A>G         AAA>AAC      K>NYAL028W      -      0      UWOPS91_917_1 c219:34763           A>G         AAA>AAC      K>NYAL028W      -      0      A12           c219:34770           A>G         GAT>GCT      D>AYAL028W      -      0      A4            c219:34770           A>G         GAT>GCT      D>AYAL028W      -      0      DBVPG6304     c219:34770           A>G         GAT>GCT      D>AYAL028W      -      0      UFRJ50791     c219:34770           A>G         GAT>GCT      D>AYAL028W      -      0      UFRJ50816     c219:34770           A>G         GAT>GCT      D>AYAL028W      -      0      UWOPS91_917_1 c219:34770           A>G         GAT>GCT      D>AYAL028W      -      0      A12           c219:34784           G>A         TAC>TAT      Y>YYAL028W      -      0      A4            c219:34784           G>A         TAC>TAT      Y>YYAL028W      -      0      DBVPG6304     c219:34784           G>A         TAC>TAT      Y>YYAL028W      -      0      UFRJ50791     c219:34784           G>A         TAC>TAT      Y>YYAL028W      -      0      UFRJ50816     c219:34784           G>A         TAC>TAT      Y>YYAL028W      -      0      UWOPS91_917_1 c219:34784           G>A         TAC>TAT      Y>YYAL028W      -      0      N_43          c219:34806           G>A         TAG>TTG      *>LYAL028W      -      0      N_45          c219:34806           G>A         TAG>TTG      *>LYAL032C      +      0      DBVPG4650     c219:43303           A>G         AAA>AGA      K>RYAL032C      +      0      N_44          c219:43333           C>T         TCA>TTA      S>LYAL032C      +      0      N_45          c219:43333           C>T         TCA>TTA      S>LYAL032C      +      0      DBVPG6304     c219:43346           C>T         CGC>CGT      R>RYAL032C      +      0      UFRJ50816     c219:43346           C>T         CGC>CGT      R>RYAL032C      +      0      YPS138        c219:43346           C>T         CGC>CGT      R>RYAL032C      +      0      DBVPG6304     c219:43355           C>G         CCC>CCG      P>PYAL032C      +      0      UFRJ50816     c219:43355           C>G         CCC>CCG      P>PYAL032C      +      0      YPS138        c219:43355           C>G         CCC>CCG      P>PYAL032C      +      0      N_45          c219:43379           T>C         GCT>GCC      A>AYAL032C      +      0      DBVPG6304     c219:43403           C>T         TTC>TTT      F>FYAL032C      +      0      N_44          c219:43403           C>T         TTC>TTT      F>FYAL032C      +      0      N_45          c219:43403           C>T         TTC>TTT      F>FYAL032C      +      0      UFRJ50816     c219:43403           C>T         TTC>TTT      F>FYAL032C      +      0      YPS138        c219:43403           C>T         TTC>TTT      F>FYAL032C      +      0      DBVPG6304     c219:43436           C>A         TCC>TCA      S>SYAL032C      +      0      UFRJ50816     c219:43436           C>A         TCC>TCA      S>SYAL032C      +      0      YPS138        c219:43436           C>A         TCC>TCA      S>SYAL032C      +      0      UWOPS91_917_1 c219:43452           C>A         CAA>AAA      Q>KYAL032C      +      0      DBVPG6304     c219:43454           A>G         CAA>CAG      Q>QYAL032C      +      0      UFRJ50816     c219:43454           A>G         CAA>CAG      Q>QYAL032C      +      0      YPS138        c219:43454           A>G         CAA>CAG      Q>QYAL032C      +      0      UWOPS91_917_1 c219:43460           C>A         GAC>GAA      D>EYAL032C      +      0      UWOPS91_917_1 c219:43466           G>A         CAG>CAA      Q>QYAL032C      +      0      DBVPG6304     c219:43481           A>G         AGA>AGG      R>RYAL032C      +      0      UFRJ50816     c219:43481           A>G         AGA>AGG      R>RYAL032C      +      0      YPS138        c219:43481           A>G         AGA>AGG      R>RYAL032C      +      0      KPN3829       c219:43486           A>G         AAG>AGG      K>RYAL032C      +      0      DBVPG6304     c219:43508           C>T         GTC>GTT      V>VYAL032C      +      0      UFRJ50816     c219:43508           C>T         GTC>GTT      V>VYAL032C      +      0      YPS138        c219:43508           C>T         GTC>GTT      V>VYAL032C      +      0      DBVPG6304     c219:43514           A>G         GCA>GCG      A>AYAL032C      +      0      N_44          c219:43514           A>G         GCA>GCG      A>AYAL032C      +      0      UFRJ50816     c219:43514           A>G         GCA>GCG      A>AYAL032C      +      0      YPS138        c219:43514           A>G         GCA>GCG      A>AYAL032C      +      0      DBVPG6304     c219:43525           A>G         AAT>AGT      N>SYAL032C      +      0      N_44          c219:43535           T>C         AAT>AAC      N>NYAL032C      +      0      DBVPG6304     c219:43559           C>T         ACC>ACT      T>TYAL032C      +      0      UFRJ50816     c219:43559           C>T         ACC>ACT      T>TYAL032C      +      0      YPS138        c219:43559           C>T         ACC>ACT      T>TYAL032C      +      0      DBVPG6304     c219:43579           C>T         ACT>ATT      T>IYAL032C      +      0      N_44          c219:43608           A>G         ATT>GTT      I>VYAL032C      +      0      N_44          c219:43616           A>G         GTA>GTG      V>VYAL032C      +      0      DBVPG6304     c219:43686           A>T         ACG>TCG      T>SYAL032C      +      0      N_44          c219:43687           C>T         ACG>ATG      T>MYAL032C      +      0      N_44          c219:43721           C>G         ACC>ACG      T>TYAL032C      +      0      N_45          c219:43721           C>G         ACC>ACG      T>TYAL032C      +      0      A4            c219:43772           T>C         CCT>CCC      P>PYAL032C      +      0      DBVPG6304     c219:43772           T>C         CCT>CCC      P>PYAL032C      +      0      UFRJ50791     c219:43772           T>C         CCT>CCC      P>PYAL032C      +      0      UFRJ50816     c219:43772           T>C         CCT>CCC      P>PYAL032C      +      0      YPS138        c219:43772           T>C         CCT>CCC      P>PYAL032C      +      0      A4            c219:43787           C>T         AAC>AAT      N>NYAL032C      +      0      DBVPG6304     c219:43787           C>T         AAC>AAT      N>NYAL032C      +      0      UFRJ50791     c219:43787           C>T         AAC>AAT      N>NYAL032C      +      0      UFRJ50816     c219:43787           C>T         AAC>AAT      N>NYAL032C      +      0      YPS138        c219:43787           C>T         AAC>AAT      N>NYAL032C      +      0      A4            c219:43793           A>G         AAA>AAG      K>KYAL032C      +      0      DBVPG6304     c219:43793           A>G         AAA>AAG      K>KYAL032C      +      0      UFRJ50791     c219:43793           A>G         AAA>AAG      K>KYAL032C      +      0      UFRJ50816     c219:43793           A>G         AAA>AAG      K>KYAL032C      +      0      YPS138        c219:43793           A>G         AAA>AAG      K>KYAL032C      +      0      A4            c219:43802           T>C         AAT>AAC      N>NYAL032C      +      0      DBVPG6304     c219:43802           T>C         AAT>AAC      N>NYAL032C      +      0      UFRJ50791     c219:43802           T>C         AAT>AAC      N>NYAL032C      +      0      UFRJ50816     c219:43802           T>C         AAT>AAC      N>NYAL032C      +      0      YPS138        c219:43802           T>C         AAT>AAC      N>NYAL032C      +      0      A4            c219:43817           C>T         GCC>GCT      A>AYAL032C      +      0      DBVPG6304     c219:43817           C>T         GCC>GCT      A>AYAL032C      +      0      UFRJ50791     c219:43817           C>T         GCC>GCT      A>AYAL032C      +      0      UFRJ50816     c219:43817           C>T         GCC>GCT      A>AYAL032C      +      0      YPS138        c219:43817           C>T         GCC>GCT      A>AYAL032C      +      0      A4            c219:43829           C>G         CGC>CGG      R>RYAL032C      +      0      DBVPG6304     c219:43829           C>G         CGC>CGG      R>RYAL032C      +      0      UFRJ50791     c219:43829           C>G         CGC>CGG      R>RYAL032C      +      0      UFRJ50816     c219:43829           C>G         CGC>CGG      R>RYAL032C      +      0      YPS138        c219:43829           C>G         CGC>CGG      R>RYAL032C      +      0      DBVPG6304     c219:43841           T>C         GCT>GCC      A>AYAL032C      +      0      UWOPS91_917_1 c219:43850           T>C         AAT>AAC      N>NYAL032C      +      0      A4            c219:43856           C>T         AAC>AAT      N>NYAL032C      +      0      DBVPG6304     c219:43856           C>T         AAC>AAT      N>NYAL032C      +      0      UFRJ50791     c219:43856           C>T         AAC>AAT      N>NYAL032C      +      0      UFRJ50816     c219:43856           C>T         AAC>AAT      N>NYAL032C      +      0      YPS138        c219:43856           C>T         AAC>AAT      N>NYAL032C      +      0      A4            c219:43892           G>A         GCG>GCA      A>AYAL032C      +      0      DBVPG6304     c219:43892           G>A         GCG>GCA      A>AYAL032C      +      0      UFRJ50791     c219:43892           G>A         GCG>GCA      A>AYAL032C      +      0      UFRJ50816     c219:43892           G>A         GCG>GCA      A>AYAL032C      +      0      YPS138        c219:43892           G>A         GCG>GCA      A>AYAL032C      +      0      A4            c219:43910           A>G         AAA>AAG      K>KYAL032C      +      0      DBVPG6304     c219:43910           A>G         AAA>AAG      K>KYAL032C      +      0      UFRJ50791     c219:43910           A>G         AAA>AAG      K>KYAL032C      +      0      UFRJ50816     c219:43910           A>G         AAA>AAG      K>KYAL032C      +      0      UWOPS91_917_1 c219:43910           A>G         AAA>AAG      K>KYAL032C      +      0      A4            c219:43925           A>G         GAA>GAG      E>EYAL032C      +      0      DBVPG6304     c219:43925           A>G         GAA>GAG      E>EYAL032C      +      0      UFRJ50791     c219:43925           A>G         GAA>GAG      E>EYAL032C      +      0      UFRJ50816     c219:43925           A>G         GAA>GAG      E>EYAL032C      +      0      A4            c219:43949           G>A         AAG>AAA      K>KYAL032C      +      0      DBVPG6304     c219:43949           G>A         AAG>AAA      K>KYAL032C      +      0      UFRJ50791     c219:43949           G>A         AAG>AAA      K>KYAL032C      +      0      UFRJ50816     c219:43949           G>A         AAG>AAA      K>KYAL032C      +      0      A4            c219:43972           C>T         ACG>ATG      T>MYAL032C      +      0      DBVPG6304     c219:43972           C>T         ACG>ATG      T>MYAL032C      +      0      UFRJ50791     c219:43972           C>T         ACG>ATG      T>MYAL032C      +      0      UFRJ50816     c219:43972           C>T         ACG>ATG      T>MYAL032C      +      0      A4            c219:43997           A>G         AAA>AAG      K>KYAL032C      +      0      DBVPG6304     c219:43997           A>G         AAA>AAG      K>KYAL032C      +      0      UFRJ50791     c219:43997           A>G         AAA>AAG      K>KYAL032C      +      0      UFRJ50816     c219:43997           A>G         AAA>AAG      K>KYAL032C      +      0      YPS138        c219:43997           A>G         AAA>AAG      K>KYAL032C      +      0      UWOPS91_917_1 c219:44000           G>A         GAG>GAA      E>EYAL032C      +      0      UFRJ50791     c219:44005           G>C         AGC>ACC      S>TYAL032C      +      0      UFRJ50816     c219:44005           G>C         AGC>ACC      S>TYAL032C      +      0      N_44          c219:44038           A>T         CAA>CTA      Q>LYAL032C      +      0      N_45          c219:44038           A>T         CAA>CTA      Q>LYAL032C      +      0      N_44          c219:44075           C>T         AGC>AGT      S>SYAL032C      +      0      N_45          c219:44075           C>T         AGC>AGT      S>SYAL032C      +      0      A4            c219:44090           A>G         CTA>CTG      L>LYAL032C      +      0      DBVPG6304     c219:44090           A>G         CTA>CTG      L>LYAL032C      +      0      UFRJ50791     c219:44090           A>G         CTA>CTG      L>LYAL032C      +      0      YPS138        c219:44090           A>G         CTA>CTG      L>LYAL032C      +      0      UFRJ50816     c219:44099           A>G         CTA>CTG      L>LYAL032C      +      0      UWOPS91_917_1 c219:44099           A>G         CTA>CTG      L>LYAL032C      +      0      A4            c219:44102           G>A         GCG>GCA      A>AYAL032C      +      0      DBVPG6304     c219:44102           G>A         GCG>GCA      A>AYAL032C      +      0      UFRJ50791     c219:44102           G>A         GCG>GCA      A>AYAL032C      +      0      YPS138        c219:44102           G>A         GCG>GCA      A>AYAL032C      +      0      UFRJ50816     c219:44117           G>A         AGG>AGA      R>RYAL032C      +      0      UWOPS91_917_1 c219:44117           G>A         AGG>AGA      R>RYAL032C      +      0      N_43          c219:44135           A>C         GTA>GTC      V>VYAL032C      +      0      N_44          c219:44135           A>C         GTA>GTC      V>VYAL032C      +      0      N_45          c219:44135           A>C         GTA>GTC      V>VYAL032C      +      0      UWOPS91_917_1 c219:44139           T>C         TTG>CTG      L>LYAL032C      +      0      A4            c219:44144           C>T         GGC>GGT      G>GYAL032C      +      0      DBVPG6304     c219:44144           C>T         GGC>GGT      G>GYAL032C      +      0      UFRJ50791     c219:44144           C>T         GGC>GGT      G>GYAL032C      +      0      YPS138        c219:44144           C>T         GGC>GGT      G>GYAL032C      +      0      UFRJ50816     c219:44162           G>A         GAG>GAA      E>EYAL032C      +      0      UWOPS91_917_1 c219:44162           G>A         GAG>GAA      E>EYAL032C      +      0      A4            c219:44213           T>C         GCT>GCC      A>AYAL032C      +      0      DBVPG6304     c219:44213           T>C         GCT>GCC      A>AYAL032C      +      0      N_43          c219:44213           T>C         GCT>GCC      A>AYAL032C      +      0      N_45          c219:44213           T>C         GCT>GCC      A>AYAL032C      +      0      UFRJ50791     c219:44213           T>C         GCT>GCC      A>AYAL032C      +      0      YPS138        c219:44213           T>C         GCT>GCC      A>AYAL032C      +      0      UFRJ50816     c219:44253           T>C         TTG>CTG      L>LYAL032C      +      0      UFRJ50791     c219:44264           A>G         CAA>CAG      Q>QYAL032C      +      0      UFRJ50791     c219:44279           C>T         AGC>AGT      S>SYAL032C      +      0      A12           c219:44323           T>C         GTT>GCT      V>AYAL032C      +      0      A4            c219:44323           T>C         GTT>GCT      V>AYAL032C      +      0      DBVPG6304     c219:44323           T>C         GTT>GCT      V>AYAL032C      +      0      UFRJ50791     c219:44323           T>C         GTT>GCT      V>AYAL032C      +      0      YPS138        c219:44323           T>C         GTT>GCT      V>AYAL032C      +      0      UFRJ50816     c219:44327           A>G         AAA>AAG      K>KYAL032C      +      0      UFRJ50816     c219:44351           T>C         CAT>CAC      H>HYAL032C      +      0      UFRJ50816     c219:44360           C>T         ATC>ATT      I>IYAL032C      +      0      N_43          c219:44377           A>T         GAA>GTA      E>VYAL032C      +      0      N_44          c219:44377           A>T         GAA>GTA      E>VYAL032C      +      0      N_45          c219:44377           A>T         GAA>GTA      E>VYAL032C      +      0      UFRJ50816     c219:44393           A>G         TCA>TCG      S>SYAL032C      +      0      UFRJ50816     c219:44426           G>C         CAG>CAC      Q>HYAL033W      -      0      A12           c219:44533           C>T         CAT>CAA      H>QYAL033W      -      0      A12           c219:44586           C>T         TGT>AGT      C>SYAL033W      -      0      A4            c219:44586           C>T         TGT>AGT      C>SYAL033W      -      0      DBVPG6304     c219:44586           C>T         TGT>AGT      C>SYAL033W      -      0      UFRJ50791     c219:44586           C>T         TGT>AGT      C>SYAL033W      -      0      UFRJ50816     c219:44586           C>T         TGT>AGT      C>SYAL033W      -      0      YPS138        c219:44586           C>T         TGT>AGT      C>SYAL033W      -      0      A12           c219:44607           G>A         AGA>TGA      R>*YAL033W      -      0      UFRJ50816     c219:44608           C>T         CAC>CAA      H>QYAL033W      -      0      A12           c219:44644           A>G         TGC>TGC      C>CYAL033W      -      0      A4            c219:44644           A>G         TGC>TGC      C>CYAL033W      -      0      DBVPG6304     c219:44644           A>G         TGC>TGC      C>CYAL033W      -      0      UFRJ50791     c219:44644           A>G         TGC>TGC      C>CYAL033W      -      0      YPS138        c219:44644           A>G         TGC>TGC      C>CYAL033W      -      0      KPN3828       c219:44683           A>G         CTC>CTC      L>LYAL033W      -      0      KPN3829       c219:44683           A>G         CTC>CTC      L>LYAL033W      -      0      UFRJ50816     c219:44683           A>G         CTC>CTC      L>LYAL033W      -      0      N_44          c219:44686           C>T         TAT>TAA      Y>*YAL033W      -      0      Y7            c219:44710           G>A         CAG>CAT      Q>HYAL033W      -      0      UFRJ50816     c219:44716           C>T         ATC>ATA      I>IYAL033W      -      0      UFRJ50816     c219:44745           G>A         GAG>TAG      E>*YAL033W      -      0      UFRJ50816     c219:44751           G>A         CTG>TTG      L>LYAL033W      -      0      A12           c219:44770           G>A         CCT>CCT      P>PYAL033W      -      0      A4            c219:44770           G>A         CCT>CCT      P>PYAL033W      -      0      DBVPG6304     c219:44770           G>A         CCT>CCT      P>PYAL033W      -      0      UFRJ50791     c219:44770           G>A         CCT>CCT      P>PYAL033W      -      0      A12           c219:44785           G>A         TAT>TAT      Y>YYAL033W      -      0      A4            c219:44785           G>A         TAT>TAT      Y>YYAL033W      -      0      DBVPG6304     c219:44785           G>A         TAT>TAT      Y>YYAL033W      -      0      N_44          c219:44785           G>A         TAT>TAT      Y>YYAL033W      -      0      N_45          c219:44785           G>A         TAT>TAT      Y>YYAL033W      -      0      UFRJ50791     c219:44785           G>A         TAT>TAT      Y>YYAL033W      -      0      YPS138        c219:44785           G>A         TAT>TAT      Y>YYAL033W      -      0      UFRJ50816     c219:44835           G>A         CAT>TAT      H>YYAL033W      -      0      A12           c219:44854           G>A         ATC>ATT      I>IYAL033W      -      0      A4            c219:44854           G>A         ATC>ATT      I>IYAL033W      -      0      DBVPG6304     c219:44854           G>A         ATC>ATT      I>IYAL033W      -      0      A12           c219:44881           C>T         GAC>GAA      D>EYAL033W      -      0      A4            c219:44881           C>T         GAC>GAA      D>EYAL033W      -      0      DBVPG6304     c219:44881           C>T         GAC>GAA      D>EYAL033W      -      0      UFRJ50791     c219:44881           C>T         GAC>GAA      D>EYAL033W      -      0      UFRJ50816     c219:44884           A>C         CTT>CTG      L>LYAL033W      -      0      N_43          c219:44920           C>T         AGC>AGA      S>RYAL033W      -      0      N_44          c219:44920           C>T         AGC>AGA      S>RYAL033W      -      0      N_45          c219:44920           C>T         AGC>AGA      S>RYAL033W      -      0      A12           c219:44926           T>C         TCT>TCG      S>SYAL033W      -      0      A4            c219:44926           T>C         TCT>TCG      S>SYAL033W      -      0      DBVPG6304     c219:44926           T>C         TCT>TCG      S>SYAL033W      -      0      UFRJ50791     c219:44926           T>C         TCT>TCG      S>SYAL033W      -      0      A12           c219:44973           C>T         CGA>AGA      R>RYAL034C      +      0      A4            c219:45844           G>A         GAT>AAT      D>NYAL034C      +      0      UWOPS91_917_1 c219:45857           A>C         AAG>ACG      K>TYAL034C      +      0      UWOPS91_917_1 c219:45876           T>A         ATT>ATA      I>IYAL034C      +      0      A4            c219:45893           G>A         CGG>CAG      R>QYAL034C      +      0      DBVPG6304     c219:45893           G>A         CGG>CAG      R>QYAL034C      +      0      UFRJ50816     c219:45893           G>A         CGG>CAG      R>QYAL034C      +      0      UWOPS91_917_1 c219:45893           G>A         CGG>CAG      R>QYAL034C      +      0      YPS138        c219:45893           G>A         CGG>CAG      R>QYAL034C      +      0      UWOPS91_917_1 c219:45926           A>G         AAT>AGT      N>SYAL034C      +      0      A4            c219:45963           T>C         AAT>AAC      N>NYAL034C      +      0      DBVPG6304     c219:45963           T>C         AAT>AAC      N>NYAL034C      +      0      UFRJ50816     c219:45963           T>C         AAT>AAC      N>NYAL034C      +      0      YPS138        c219:45963           T>C         AAT>AAC      N>NYAL034C      +      0      UWOPS91_917_1 c219:46001           A>G         AAC>AGC      N>SYAL034C      +      0      A4            c219:46038           T>C         TCT>TCC      S>SYAL034C      +      0      DBVPG6304     c219:46038           T>C         TCT>TCC      S>SYAL034C      +      0      UFRJ50816     c219:46038           T>C         TCT>TCC      S>SYAL034C      +      0      UWOPS91_917_1 c219:46038           T>C         TCT>TCC      S>SYAL034C      +      0      YPS138        c219:46038           T>C         TCT>TCC      S>SYAL034C      +      0      DBVPG6304     c219:46125           C>T         GGC>GGT      G>GYAL034C      +      0      UFRJ50816     c219:46125           C>T         GGC>GGT      G>GYAL034C      +      0      YPS138        c219:46125           C>T         GGC>GGT      G>GYAL034C      +      0      DBVPG6304     c219:46148           C>A         GCA>GAA      A>EYAL034C      +      0      UFRJ50816     c219:46148           C>A         GCA>GAA      A>EYAL034C      +      0      UWOPS91_917_1 c219:46148           C>A         GCA>GAA      A>EYAL034C      +      0      YPS138        c219:46148           C>A         GCA>GAA      A>EYAL034C      +      0      UWOPS91_917_1 c219:46210           C>G         CCC>GCC      P>AYAL034C      +      0      UFRJ50816     c219:46224           A>G         TCA>TCG      S>SYAL034C      +      0      UWOPS91_917_1 c219:46224           A>G         TCA>TCG      S>SYAL034C      +      0      YPS138        c219:46224           A>G         TCA>TCG      S>SYAL034C      +      0      IFO1804       c219:46239           C>T         CGC>CGT      R>RYAL034C      +      0      A4            c219:46251           C>T         TAC>TAT      Y>YYAL034C      +      0      UFRJ50816     c219:46251           C>T         TAC>TAT      Y>YYAL034C      +      0      UWOPS91_917_1 c219:46251           C>T         TAC>TAT      Y>YYAL034C      +      0      YPS138        c219:46251           C>T         TAC>TAT      Y>YYAL034C      +      0      UFRJ50816     c219:46290           G>C         GCG>GCC      A>AYAL034C      +      0      UWOPS91_917_1 c219:46290           G>C         GCG>GCC      A>AYAL034C      +      0      YPS138        c219:46290           G>C         GCG>GCC      A>AYAL034C      +      0      YPS138        c219:46311           T>C         ATT>ATC      I>IYAL034C      +      0      IFO1804       c219:46320           A>G         AGA>AGG      R>RYAL034C      +      0      N_45          c219:46320           A>G         AGA>AGG      R>RYAL034C      +      0      UFRJ50816     c219:46320           A>G         AGA>AGG      R>RYAL034C      +      0      UWOPS91_917_1 c219:46320           A>G         AGA>AGG      R>RYAL034C      +      0      YPS138        c219:46320           A>G         AGA>AGG      R>RYAL034C      +      0      UWOPS91_917_1 c219:46370           A>G         GAT>GGT      D>GYAL034C      +      0      UFRJ50791     c219:46398           T>C         TCT>TCC      S>SYAL034C      +      0      UFRJ50816     c219:46398           T>C         TCT>TCC      S>SYAL034C      +      0      YPS138        c219:46398           T>C         TCT>TCC      S>SYAL034C      +      0      UWOPS91_917_1 c219:46466           C>T         GCG>GTG      A>VYAL034C      +      0      UFRJ50791     c219:46473           C>T         TAC>TAT      Y>YYAL034C      +      0      UFRJ50816     c219:46473           C>T         TAC>TAT      Y>YYAL034C      +      0      UWOPS91_917_1 c219:46473           C>T         TAC>TAT      Y>YYAL034C      +      0      YPS138        c219:46473           C>T         TAC>TAT      Y>YYAL034C      +      0      IFO1804       c219:46479           T>C         AAT>AAC      N>NYAL034C      +      0      N_45          c219:46479           T>C         AAT>AAC      N>NYAL034C      +      0      UFRJ50791     c219:46526           G>A         CGC>CAC      R>HYAL034C      +      0      UFRJ50816     c219:46526           G>A         CGC>CAC      R>HYAL034C      +      0      UWOPS91_917_1 c219:46526           G>A         CGC>CAC      R>HYAL034C      +      0      YPS138        c219:46526           G>A         CGC>CAC      R>HYAL034C      +      0      UFRJ50791     c219:46545           A>G         CAA>CAG      Q>QYAL034C      +      0      UFRJ50816     c219:46545           A>G         CAA>CAG      Q>QYAL034C      +      0      YPS138        c219:46545           A>G         CAA>CAG      Q>QYAL034C      +      0      UFRJ50791     c219:46572           T>C         CAT>CAC      H>HYAL034C      +      0      UFRJ50816     c219:46572           T>C         CAT>CAC      H>HYAL034C      +      0      YPS138        c219:46572           T>C         CAT>CAC      H>HYAL034C      +      0      UFRJ50791     c219:46590           A>G         TCA>TCG      S>SYAL034C      +      0      UFRJ50816     c219:46590           A>G         TCA>TCG      S>SYAL034C      +      0      UWOPS91_917_1 c219:46590           A>G         TCA>TCG      S>SYAL034C      +      0      YPS138        c219:46590           A>G         TCA>TCG      S>SYAL034C      +      0      UFRJ50791     c219:46596           T>A         TCT>TCA      S>SYAL034C      +      0      UFRJ50816     c219:46596           T>A         TCT>TCA      S>SYAL034C      +      0      UWOPS91_917_1 c219:46596           T>A         TCT>TCA      S>SYAL034C      +      0      YPS138        c219:46596           T>A         TCT>TCA      S>SYAL034C      +      0      Q95_3         c219:46627           A>C         ATC>CTC      I>LYAL034C      +      0      UWOPS91_917_1 c219:46636           A>G         ATT>GTT      I>VYAL034C      +      0      UFRJ50791     c219:46693           A>T         ACT>TCT      T>SYAL034C      +      0      UFRJ50816     c219:46693           A>T         ACT>TCT      T>SYAL034C      +      0      YPS138        c219:46693           A>T         ACT>TCT      T>SYAL034C      +      0      IFO1804       c219:46701           A>C         CCA>CCC      P>PYAL034C      +      0      N_44          c219:46701           A>C         CCA>CCC      P>PYAL034C      +      0      N_45          c219:46701           A>C         CCA>CCC      P>PYAL034C      +      0      UFRJ50791     c219:46701           A>C         CCA>CCC      P>PYAL034C      +      0      UFRJ50816     c219:46701           A>C         CCA>CCC      P>PYAL034C      +      0      YPS138        c219:46701           A>C         CCA>CCC      P>PYAL034C      +      0      N_44          c219:46719           T>C         GCT>GCC      A>AYAL034C      +      0      UFRJ50791     c219:46737           A>G         TTA>TTG      L>LYAL034C      +      0      UFRJ50816     c219:46737           A>G         TTA>TTG      L>LYAL034C      +      0      YPS138        c219:46737           A>G         TTA>TTG      L>LYAL034C      +      0      IFO1804       c219:46761           A>G         CCA>CCG      P>PYAL034C      +      0      N_44          c219:46761           A>G         CCA>CCG      P>PYAL034C      +      0      N_45          c219:46761           A>G         CCA>CCG      P>PYAL034C      +      0      UFRJ50791     c219:46803           C>T         CCC>CCT      P>PYAL034C      +      0      UFRJ50816     c219:46803           C>T         CCC>CCT      P>PYAL034C      +      0      UWOPS91_917_1 c219:46803           C>T         CCC>CCT      P>PYAL034C      +      0      YPS138        c219:46803           C>T         CCC>CCT      P>PYAL034C      +      0      IFO1804       c219:46806           C>A         GCC>GCA      A>AYAL034C      +      0      N_44          c219:46806           C>A         GCC>GCA      A>AYAL034C      +      0      N_45          c219:46806           C>A         GCC>GCA      A>AYAL034C      +      0      UFRJ50791     c219:46828           T>C         TTA>CTA      L>LYAL034C      +      0      YPS138        c219:46828           T>C         TTA>CTA      L>LYAL034C      +      0      A4            c219:46983           C>T         TTC>TTT      F>FYAL034C      +      0      YPS138        c219:46983           C>T         TTC>TTT      F>FYAL034C      +      0      A4            c219:47004           A>G         CAA>CAG      Q>QYAL034C      +      0      UFRJ50791     c219:47004           A>G         CAA>CAG      Q>QYAL034C      +      0      YPS138        c219:47004           A>G         CAA>CAG      Q>QYAL034C      +      0      A4            c219:47024           T>A         TTC>TAC      F>YYAL034C      +      0      N_44          c219:47024           T>A         TTC>TAC      F>YYAL034C      +      0      N_45          c219:47024           T>A         TTC>TAC      F>YYAL034C      +      0      UFRJ50791     c219:47024           T>A         TTC>TAC      F>YYAL034C      +      0      YPS138        c219:47024           T>A         TTC>TAC      F>YYAL034W-A    -      0      A4            c219:47157           T>C         AGC>AGG      S>RYAL034W-A    -      0      UFRJ50791     c219:47157           T>C         AGC>AGG      S>RYAL034W-A    -      0      DBVPG4650     c219:47163           A>C         GGT>GGG      G>GYAL034W-A    -      0      N_44          c219:47166           G>A         CAT>CAT      H>HYAL034W-A    -      0      N_45          c219:47166           G>A         CAT>CAT      H>HYAL034W-A    -      0      N_44          c219:47204           C>T         ATA>ATA      I>IYAL034W-A    -      0      N_45          c219:47204           C>T         ATA>ATA      I>IYAL034W-A    -      0      N_44          c219:47271           C>T         GGC>GGA      G>GYAL034W-A    -      0      N_45          c219:47271           C>T         GGC>GGA      G>GYAL034W-A    -      0      A4            c219:47288           A>G         CAG>CAG      Q>QYAL034W-A    -      0      A4            c219:47319           C>T         GTA>GTA      V>VYAL034W-A    -      0      DBVPG6304     c219:47319           C>T         GTA>GTA      V>VYAL034W-A    -      0      N_44          c219:47343           T>C         AAC>AAG      N>KYAL034W-A    -      0      N_45          c219:47343           T>C         AAC>AAG      N>KYAL034W-A    -      0      A4            c219:47346           G>C         GGC>GGG      G>GYAL034W-A    -      0      DBVPG6304     c219:47346           G>C         GGC>GGG      G>GYAL034W-A    -      0      A4            c219:47353           G>T         CAA>CAA      Q>QYAL034W-A    -      0      DBVPG6304     c219:47353           G>T         CAA>CAA      Q>QYAL034W-A    -      0      A4            c219:47367           C>T         CTC>CTA      L>LYAL034W-A    -      0      DBVPG6304     c219:47367           C>T         CTC>CTA      L>LYAL034W-A    -      0      DBVPG4650     c219:47429           A>T         TCG>ACG      S>TYAL034W-A    -      0      N_17          c219:47429           A>T         TCG>ACG      S>TYAL034W-A    -      0      YPS138        c219:47526           C>T         TTC>TTA      F>LYAL034W-A    -      0      A4            c219:47540           G>A         TTT>TTT      F>FYAL034W-A    -      0      DBVPG6304     c219:47540           G>A         TTT>TTT      F>FYAL034W-A    -      0      IFO1804       c219:47540           G>A         TTT>TTT      F>FYAL034W-A    -      0      N_43          c219:47540           G>A         TTT>TTT      F>FYAL034W-A    -      0      N_44          c219:47540           G>A         TTT>TTT      F>FYAL034W-A    -      0      YPS138        c219:47540           G>A         TTT>TTT      F>FYAL034W-A    -      0      A12           c219:47556           T>C         CAA>CAG      Q>QYAL034W-A    -      0      YPS138        c219:47556           T>C         CAA>CAG      Q>QYAL034W-A    -      0      IFO1804       c219:47559           C>T         TGC>TGA      C>*YAL034W-A    -      0      N_43          c219:47559           C>T         TGC>TGA      C>*YAL034W-A    -      0      N_44          c219:47559           C>T         TGC>TGA      C>*YAL034W-A    -      0      N_45          c219:47559           C>T         TGC>TGA      C>*YAL034W-A    -      0      DBVPG6304     c219:47601           C>T         CAC>CAA      H>QYAL034W-A    -      0      A12           c219:47610           T>A         CAG>CAT      Q>HYAL034W-A    -      0      A4            c219:47610           T>A         CAG>CAT      Q>HYAL034W-A    -      0      DBVPG6304     c219:47610           T>A         CAG>CAT      Q>HYAL034W-A    -      0      YPS138        c219:47610           T>A         CAG>CAT      Q>HYAL034W-A    -      0      A12           c219:47621           T>C         CAT>GAT      H>DYAL034W-A    -      0      A4            c219:47621           T>C         CAT>GAT      H>DYAL034W-A    -      0      CBS432        c219:47621           T>C         CAT>GAT      H>DYAL034W-A    -      0      CBS5829       c219:47621           T>C         CAT>GAT      H>DYAL034W-A    -      0      DBVPG4650     c219:47621           T>C         CAT>GAT      H>DYAL034W-A    -      0      DBVPG6304     c219:47621           T>C         CAT>GAT      H>DYAL034W-A    -      0      IFO1804       c219:47621           T>C         CAT>GAT      H>DYAL034W-A    -      0      N_43          c219:47621           T>C         CAT>GAT      H>DYAL034W-A    -      0      N_44          c219:47621           T>C         CAT>GAT      H>DYAL034W-A    -      0      N_45          c219:47621           T>C         CAT>GAT      H>DYAL034W-A    -      0      Q32_3         c219:47621           T>C         CAT>GAT      H>DYAL034W-A    -      0      Q59_1         c219:47621           T>C         CAT>GAT      H>DYAL034W-A    -      0      Q95_3         c219:47621           T>C         CAT>GAT      H>DYAL034W-A    -      0      Y6_5          c219:47621           T>C         CAT>GAT      H>DYAL034W-A    -      0      YPS138        c219:47621           T>C         CAT>GAT      H>DYAL034W-A    -      0      IFO1804       c219:47631           T>C         GAG>GAG      E>EYAL034W-A    -      0      N_44          c219:47631           T>C         GAG>GAG      E>EYAL034W-A    -      0      A12           c219:47658           T>C         TTT>TTG      F>LYAL034W-A    -      0      A4            c219:47658           T>C         TTT>TTG      F>LYAL034W-A    -      0      DBVPG6304     c219:47658           T>C         TTT>TTG      F>LYAL034W-A    -      0      IFO1804       c219:47658           T>C         TTT>TTG      F>LYAL034W-A    -      0      N_43          c219:47658           T>C         TTT>TTG      F>LYAL034W-A    -      0      N_44          c219:47658           T>C         TTT>TTG      F>LYAL034W-A    -      0      N_45          c219:47658           T>C         TTT>TTG      F>LYAL034W-A    -      0      UWOPS91_917_1 c219:47658           T>C         TTT>TTG      F>LYAL034W-A    -      0      YPS138        c219:47658           T>C         TTT>TTG      F>LYAL034W-A    -      0      UWOPS91_917_1 c219:47664           C>T         CTT>CTA      L>LYAL034W-A    -      0      IFO1804       c219:47670           C>T         ATC>ATA      I>IYAL034W-A    -      0      N_44          c219:47670           C>T         ATC>ATA      I>IYAL034W-A    -      0      UWOPS91_917_1 c219:47670           C>G         ATC>ATC      I>IYAL034W-A    -      0      A12           c219:47676           C>T         CTG>CTA      L>LYAL034W-A    -      0      A4            c219:47676           C>T         CTG>CTA      L>LYAL034W-A    -      0      DBVPG6304     c219:47676           C>T         CTG>CTA      L>LYAL034W-A    -      0      IFO1804       c219:47676           C>T         CTG>CTA      L>LYAL034W-A    -      0      N_43          c219:47676           C>T         CTG>CTA      L>LYAL034W-A    -      0      N_44          c219:47676           C>T         CTG>CTA      L>LYAL034W-A    -      0      N_45          c219:47676           C>T         CTG>CTA      L>LYAL034W-A    -      0      UWOPS91_917_1 c219:47676           C>T         CTG>CTA      L>LYAL034W-A    -      0      YPS138        c219:47676           C>T         CTG>CTA      L>LYAL034W-A    -      0      UWOPS91_917_1 c219:47682           G>A         CTG>CTT      L>LYAL034W-A    -      0      UWOPS91_917_1 c219:47736           G>A         ATT>ATT      I>IYAL034W-A    -      0      A12           c219:47768           G>A         TTC>TTC      F>FYAL034W-A    -      0      A4            c219:47768           G>A         TTC>TTC      F>FYAL034W-A    -      0      DBVPG6304     c219:47768           G>A         TTC>TTC      F>FYAL034W-A    -      0      UWOPS91_917_1 c219:47768           G>A         TTC>TTC      F>FYAL034W-A    -      0      YPS138        c219:47768           G>A         TTC>TTC      F>FYAL034W-A    -      0      IFO1804       c219:47781           A>G         TGT>TGC      C>CYAL034W-A    -      0      N_43          c219:47781           A>G         TGT>TGC      C>CYAL034W-A    -      0      N_44          c219:47781           A>G         TGT>TGC      C>CYAL034W-A    -      0      N_45          c219:47781           A>G         TGT>TGC      C>CYAL034W-A    -      0      UWOPS91_917_1 c219:47841           C>T         ATC>ATA      I>IYAL034W-A    -      0      A12           c219:47856           C>T         ATA>ATA      I>IYAL034W-A    -      0      A4            c219:47856           C>T         ATA>ATA      I>IYAL034W-A    -      0      CBS432        c219:47856           C>T         ATA>ATA      I>IYAL034W-A    -      0      DBVPG4650     c219:47856           C>T         ATA>ATA      I>IYAL034W-A    -      0      DBVPG6304     c219:47856           C>T         ATA>ATA      I>IYAL034W-A    -      0      IFO1804       c219:47856           C>T         ATA>ATA      I>IYAL034W-A    -      0      N_43          c219:47856           C>T         ATA>ATA      I>IYAL034W-A    -      0      N_44          c219:47856           C>T         ATA>ATA      I>IYAL034W-A    -      0      N_45          c219:47856           C>T         ATA>ATA      I>IYAL034W-A    -      0      Q59_1         c219:47856           C>T         ATA>ATA      I>IYAL034W-A    -      0      Q62_5         c219:47856           C>T         ATA>ATA      I>IYAL034W-A    -      0      Q95_3         c219:47856           C>T         ATA>ATA      I>IYAL034W-A    -      0      UWOPS91_917_1 c219:47856           C>T         ATA>ATA      I>IYAL034W-A    -      0      Y7            c219:47856           C>T         ATA>ATA      I>IYAL034W-A    -      0      YPS138        c219:47856           C>T         ATA>ATA      I>IYAL034W-A    -      0      A12           c219:47883           C>T         TAA>TAA      *>*YAL034W-A    -      0      A4            c219:47883           C>T         TAA>TAA      *>*YAL034W-A    -      0      DBVPG6304     c219:47883           C>T         TAA>TAA      *>*YAL034W-A    -      0      UWOPS91_917_1 c219:47883           C>T         TAA>TAA      *>*YAL034W-A    -      0      YPS138        c219:47883           C>T         TAA>TAA      *>*YAL034W-A    -      0      IFO1804       c219:47889           T>C         TTT>TTG      F>LYAL034W-A    -      0      N_43          c219:47889           T>C         TTT>TTG      F>LYAL034W-A    -      0      N_44          c219:47889           T>C         TTT>TTG      F>LYAL034W-A    -      0      N_45          c219:47889           T>C         TTT>TTG      F>LYAL034W-A    -      0      A12           c219:47975           G>A         TAG>TAG      *>*YAL034W-A    -      0      A4            c219:47975           G>A         TAG>TAG      *>*YAL034W-A    -      0      DBVPG6304     c219:47975           G>A         TAG>TAG      *>*YAL034W-A    -      0      UFRJ50816     c219:47975           G>A         TAG>TAG      *>*YAL034W-A    -      0      UWOPS91_917_1 c219:47975           G>A         TAG>TAG      *>*YAL034W-A    -      0      YPS138        c219:47975           G>A         TAG>TAG      *>*YAL035W      -      0      A12           c219:48349           A>G         ATC>CTC      I>LYAL035W      -      0      CBS432        c219:48349           A>G         ATC>CTC      I>LYAL035W      -      0      DBVPG6304     c219:48349           A>G         ATC>CTC      I>LYAL035W      -      0      KPN3828       c219:48349           A>G         ATC>CTC      I>LYAL035W      -      0      N_44          c219:48349           A>G         ATC>CTC      I>LYAL035W      -      0      N_45          c219:48349           A>G         ATC>CTC      I>LYAL035W      -      0      Q62_5         c219:48349           A>G         ATC>CTC      I>LYAL035W      -      0      Q95_3         c219:48349           A>G         ATC>CTC      I>LYAL035W      -      0      UFRJ50791     c219:48349           A>G         ATC>CTC      I>LYAL035W      -      0      UFRJ50816     c219:48349           A>G         ATC>CTC      I>LYAL035W      -      0      UWOPS91_917_1 c219:48349           A>G         ATC>CTC      I>LYAL035W      -      0      Y7            c219:48349           A>G         ATC>CTC      I>LYAL035W      -      0      YPS138        c219:48349           A>G         ATC>CTC      I>LYAL035W      -      0      UWOPS91_917_1 c219:48356           G>T         GAA>GAA      E>EYAL035W      -      0      KPN3828       c219:48362           A>G         TTC>TTC      F>FYAL035W      -      0      Q95_3         c219:48362           A>G         TTC>TTC      F>FYAL035W      -      0      UWOPS91_917_1 c219:48362           A>T         TTC>TTA      F>LYAL035W      -      0      A12           c219:48401           G>A         AGT>AGT      S>SYAL035W      -      0      CBS432        c219:48401           G>A         AGT>AGT      S>SYAL035W      -      0      DBVPG6304     c219:48401           G>A         AGT>AGT      S>SYAL035W      -      0      KPN3828       c219:48401           G>A         AGT>AGT      S>SYAL035W      -      0      N_44          c219:48401           G>A         AGT>AGT      S>SYAL035W      -      0      N_45          c219:48401           G>A         AGT>AGT      S>SYAL035W      -      0      Q62_5         c219:48401           G>A         AGT>AGT      S>SYAL035W      -      0      Q95_3         c219:48401           G>A         AGT>AGT      S>SYAL035W      -      0      UFRJ50791     c219:48401           G>A         AGT>AGT      S>SYAL035W      -      0      UFRJ50816     c219:48401           G>A         AGT>AGT      S>SYAL035W      -      0      Y7            c219:48401           G>A         AGT>AGT      S>SYAL035W      -      0      YPS138        c219:48401           G>A         AGT>AGT      S>SYAL035W      -      0      T21_4         c219:48431           T>C         ATC>ATG      I>MYAL035W      -      0      A12           c219:48434           A>G         ATC>ATC      I>IYAL035W      -      0      DBVPG6304     c219:48434           A>G         ATC>ATC      I>IYAL035W      -      0      UFRJ50791     c219:48434           A>G         ATC>ATC      I>IYAL035W      -      0      UFRJ50816     c219:48434           A>G         ATC>ATC      I>IYAL035W      -      0      YPS138        c219:48434           A>G         ATC>ATC      I>IYAL035W      -      0      A12           c219:48494           G>A         CAT>CAT      H>HYAL035W      -      0      DBVPG6304     c219:48494           G>A         CAT>CAT      H>HYAL035W      -      0      UFRJ50791     c219:48494           G>A         CAT>CAT      H>HYAL035W      -      0      UFRJ50816     c219:48494           G>A         CAT>CAT      H>HYAL035W      -      0      YPS138        c219:48494           G>A         CAT>CAT      H>HYAL035W      -      0      DBVPG6304     c219:48506           A>G         CTT>CTC      L>LYAL035W      -      0      YPS138        c219:48506           A>G         CTT>CTC      L>LYAL035W      -      0      UFRJ50816     c219:48527           G>T         CTC>CTA      L>LYAL035W      -      0      N_44          c219:48560           A>C         CTT>CTG      L>LYAL035W      -      0      N_45          c219:48560           A>C         CTT>CTG      L>LYAL035W      -      0      UFRJ50816     c219:48575           C>T         GGA>GGA      G>GYAL035W      -      0      DBVPG6304     c219:48587           T>C         CTT>CTG      L>LYAL035W      -      0      UFRJ50791     c219:48587           T>C         CTT>CTG      L>LYAL035W      -      0      UFRJ50816     c219:48587           T>C         CTT>CTG      L>LYAL035W      -      0      YPS138        c219:48587           T>C         CTT>CTG      L>LYAL035W      -      0      DBVPG6304     c219:48611           G>T         TTC>TTA      F>LYAL035W      -      0      UFRJ50816     c219:48611           G>T         TTC>TTA      F>LYAL035W      -      0      YPS138        c219:48611           G>T         TTC>TTA      F>LYAL035W      -      0      DBVPG6304     c219:48617           T>C         CTG>CTG      L>LYAL035W      -      0      UFRJ50816     c219:48617           T>C         CTG>CTG      L>LYAL035W      -      0      YPS138        c219:48617           T>C         CTG>CTG      L>LYAL035W      -      0      Q59_1         c219:48677           C>A         GTT>GTT      V>VYAL035W      -      0      T21_4         c219:48698           G>A         ATT>ATT      I>IYAL035W      -      0      N_44          c219:48710           C>T         AGC>AGA      S>RYAL035W      -      0      N_45          c219:48710           C>T         AGC>AGA      S>RYAL035W      -      0      DBVPG6304     c219:48770           T>C         GCT>GCG      A>AYAL035W      -      0      UFRJ50816     c219:48770           T>C         GCT>GCG      A>AYAL035W      -      0      YPS138        c219:48770           T>C         GCT>GCG      A>AYAL035W      -      0      DBVPG6304     c219:48818           C>T         TCT>TCA      S>SYAL035W      -      0      UFRJ50816     c219:48818           C>T         TCT>TCA      S>SYAL035W      -      0      YPS138        c219:48818           C>T         TCT>TCA      S>SYAL035W      -      0      DBVPG6304     c219:48836           A>G         CTT>CTC      L>LYAL035W      -      0      UFRJ50816     c219:48836           A>G         CTT>CTC      L>LYAL035W      -      0      YPS138        c219:48836           A>G         CTT>CTC      L>LYAL035W      -      0      DBVPG6304     c219:48860           T>A         CTT>CTT      L>LYAL035W      -      0      UFRJ50816     c219:48860           T>A         CTT>CTT      L>LYAL035W      -      0      YPS138        c219:48860           T>A         CTT>CTT      L>LYAL035W      -      0      DBVPG6304     c219:48887           G>A         TAA>TAT      *>YYAL035W      -      0      UFRJ50816     c219:48887           G>A         TAA>TAT      *>YYAL035W      -      0      YPS138        c219:48887           G>A         TAA>TAT      *>YYAL035W      -      0      N_43          c219:48890           C>G         TTC>TTC      F>FYAL035W      -      0      N_44          c219:48890           C>G         TTC>TTC      F>FYAL035W      -      0      N_45          c219:48890           C>G         TTC>TTC      F>FYAL035W      -      0      DBVPG6304     c219:48914           C>T         AGC>AGA      S>RYAL035W      -      0      UFRJ50816     c219:48914           C>T         AGC>AGA      S>RYAL035W      -      0      YPS138        c219:48914           C>T         AGC>AGA      S>RYAL035W      -      0      DBVPG6304     c219:48971           G>A         TGC>TGT      C>CYAL035W      -      0      IFO1804       c219:48971           G>A         TGC>TGT      C>CYAL035W      -      0      N_43          c219:48971           G>A         TGC>TGT      C>CYAL035W      -      0      N_44          c219:48971           G>A         TGC>TGT      C>CYAL035W      -      0      N_45          c219:48971           G>A         TGC>TGT      C>CYAL035W      -      0      UFRJ50816     c219:48971           G>A         TGC>TGT      C>CYAL035W      -      0      YPS138        c219:48971           G>A         TGC>TGT      C>CYAL035W      -      0      IFO1804       c219:49022           C>T         TTT>TTA      F>LYAL035W      -      0      N_43          c219:49022           C>T         TTT>TTA      F>LYAL035W      -      0      N_44          c219:49022           C>T         TTT>TTA      F>LYAL035W      -      0      N_45          c219:49022           C>T         TTT>TTA      F>LYAL035W      -      0      DBVPG6304     c219:49037           G>T         TAG>TAA      *>*YAL035W      -      0      UFRJ50791     c219:49037           G>T         TAG>TAA      *>*YAL035W      -      0      UFRJ50816     c219:49037           G>T         TAG>TAA      *>*YAL035W      -      0      YPS138        c219:49037           G>T         TAG>TAA      *>*YAL035W      -      0      DBVPG6304     c219:49073           A>G         GCC>GCC      A>AYAL035W      -      0      UFRJ50791     c219:49073           A>G         GCC>GCC      A>AYAL035W      -      0      UFRJ50816     c219:49073           A>G         GCC>GCC      A>AYAL035W      -      0      YPS138        c219:49073           A>G         GCC>GCC      A>AYAL035W      -      0      DBVPG6304     c219:49097           A>G         AGC>AGC      S>SYAL035W      -      0      UFRJ50791     c219:49097           A>G         AGC>AGC      S>SYAL035W      -      0      UFRJ50816     c219:49097           A>G         AGC>AGC      S>SYAL035W      -      0      YPS138        c219:49097           A>G         AGC>AGC      S>SYAL035W      -      0      N_43          c219:49106           G>A         ATC>ATT      I>IYAL035W      -      0      N_44          c219:49106           G>A         ATC>ATT      I>IYAL035W      -      0      N_45          c219:49106           G>A         ATC>ATT      I>IYAL035W      -      0      A4            c219:49136           A>G         GAC>GAC      D>DYAL035W      -      0      DBVPG6304     c219:49136           A>G         GAC>GAC      D>DYAL035W      -      0      UFRJ50791     c219:49136           A>G         GAC>GAC      D>DYAL035W      -      0      UFRJ50816     c219:49136           A>G         GAC>GAC      D>DYAL035W      -      0      YPS138        c219:49136           A>G         GAC>GAC      D>DYAL035W      -      0      A4            c219:49208           T>C         ATC>ATG      I>MYAL035W      -      0      DBVPG6304     c219:49208           T>C         ATC>ATG      I>MYAL035W      -      0      UFRJ50791     c219:49208           T>C         ATC>ATG      I>MYAL035W      -      0      UFRJ50816     c219:49208           T>C         ATC>ATG      I>MYAL035W      -      0      YPS138        c219:49208           T>C         ATC>ATG      I>MYAL035W      -      0      A4            c219:49217           T>C         CAC>CAG      H>QYAL035W      -      0      DBVPG6304     c219:49217           T>C         CAC>CAG      H>QYAL035W      -      0      UFRJ50791     c219:49217           T>C         CAC>CAG      H>QYAL035W      -      0      YPS138        c219:49217           T>C         CAC>CAG      H>QYAL035W      -      0      A4            c219:49226           C>T         GTC>GTA      V>VYAL035W      -      0      DBVPG6304     c219:49226           C>T         GTC>GTA      V>VYAL035W      -      0      IFO1804       c219:49226           C>T         GTC>GTA      V>VYAL035W      -      0      N_43          c219:49226           C>T         GTC>GTA      V>VYAL035W      -      0      N_45          c219:49226           C>T         GTC>GTA      V>VYAL035W      -      0      UFRJ50791     c219:49226           C>T         GTC>GTA      V>VYAL035W      -      0      YPS138        c219:49226           C>T         GTC>GTA      V>VYAL035W      -      0      UWOPS91_917_1 c219:49271           T>C         GTT>GTG      V>VYAL035W      -      0      A4            c219:49286           A>T         ATC>ATA      I>IYAL035W      -      0      DBVPG6304     c219:49286           A>T         ATC>ATA      I>IYAL035W      -      0      UFRJ50791     c219:49286           A>T         ATC>ATA      I>IYAL035W      -      0      YPS138        c219:49286           A>T         ATC>ATA      I>IYAL035W      -      0      IFO1804       c219:49289           T>C         ATC>ATG      I>MYAL035W      -      0      N_43          c219:49289           T>C         ATC>ATG      I>MYAL035W      -      0      N_45          c219:49289           T>C         ATC>ATG      I>MYAL035W      -      0      A4            c219:49400           G>T         ACC>ACA      T>TYAL035W      -      0      DBVPG6304     c219:49400           G>T         ACC>ACA      T>TYAL035W      -      0      UFRJ50791     c219:49400           G>T         ACC>ACA      T>TYAL035W      -      0      UFRJ50816     c219:49400           G>T         ACC>ACA      T>TYAL035W      -      0      UWOPS91_917_1 c219:49400           G>T         ACC>ACA      T>TYAL035W      -      0      YPS138        c219:49400           G>T         ACC>ACA      T>TYAL035W      -      0      DBVPG6304     c219:49484           C>T         GGA>GGA      G>GYAL035W      -      0      UWOPS91_917_1 c219:49499           T>C         AAT>AAG      N>KYAL035W      -      0      A12           c219:49523           G>A         CTT>CTT      L>LYAL035W      -      0      A4            c219:49523           G>A         CTT>CTT      L>LYAL035W      -      0      DBVPG6304     c219:49523           G>A         CTT>CTT      L>LYAL035W      -      0      UFRJ50791     c219:49523           G>A         CTT>CTT      L>LYAL035W      -      0      UFRJ50816     c219:49523           G>A         CTT>CTT      L>LYAL035W      -      0      YPS138        c219:49523           G>A         CTT>CTT      L>LYAL035W      -      0      A4            c219:49532           C>A         TAA>TAT      *>YYAL035W      -      0      DBVPG6304     c219:49532           C>A         TAA>TAT      *>YYAL035W      -      0      UFRJ50791     c219:49532           C>A         TAA>TAT      *>YYAL035W      -      0      UFRJ50816     c219:49532           C>A         TAA>TAT      *>YYAL035W      -      0      UWOPS91_917_1 c219:49532           C>A         TAA>TAT      *>YYAL035W      -      0      YPS138        c219:49532           C>A         TAA>TAT      *>YYAL035W      -      0      UWOPS91_917_1 c219:49604           T>C         TGT>TGG      C>WYAL035W      -      0      A12           c219:49655           G>C         ATA>ATG      I>MYAL035W      -      0      A4            c219:49655           G>C         ATA>ATG      I>MYAL035W      -      0      DBVPG6304     c219:49655           G>C         ATA>ATG      I>MYAL035W      -      0      UFRJ50791     c219:49655           G>C         ATA>ATG      I>MYAL035W      -      0      UFRJ50816     c219:49655           G>C         ATA>ATG      I>MYAL035W      -      0      UWOPS91_917_1 c219:49655           G>C         ATA>ATG      I>MYAL035W      -      0      YPS138        c219:49655           G>C         ATA>ATG      I>MYAL035W      -      0      A12           c219:49673           G>A         ATC>ATT      I>IYAL035W      -      0      A4            c219:49673           G>A         ATC>ATT      I>IYAL035W      -      0      DBVPG6304     c219:49673           G>A         ATC>ATT      I>IYAL035W      -      0      YPS138        c219:49673           G>A         ATC>ATT      I>IYAL035W      -      0      A12           c219:49721           A>G         AGA>AGC      R>SYAL035W      -      0      A4            c219:49721           A>G         AGA>AGC      R>SYAL035W      -      0      DBVPG6304     c219:49721           A>G         AGA>AGC      R>SYAL035W      -      0      UFRJ50791     c219:49721           A>G         AGA>AGC      R>SYAL035W      -      0      UFRJ50816     c219:49721           A>G         AGA>AGC      R>SYAL035W      -      0      YPS138        c219:49721           A>G         AGA>AGC      R>SYAL035W      -      0      A12           c219:49739           A>G         ACC>ACC      T>TYAL035W      -      0      A4            c219:49739           A>G         ACC>ACC      T>TYAL035W      -      0      DBVPG6304     c219:49739           A>G         ACC>ACC      T>TYAL035W      -      0      IFO1804       c219:49739           A>G         ACC>ACC      T>TYAL035W      -      0      N_43          c219:49739           A>G         ACC>ACC      T>TYAL035W      -      0      N_44          c219:49739           A>G         ACC>ACC      T>TYAL035W      -      0      N_45          c219:49739           A>G         ACC>ACC      T>TYAL035W      -      0      UFRJ50816     c219:49739           A>G         ACC>ACC      T>TYAL035W      -      0      UWOPS91_917_1 c219:49739           A>G         ACC>ACC      T>TYAL035W      -      0      YPS138        c219:49739           A>G         ACC>ACC      T>TYAL035W      -      0      A12           c219:49763           C>T         AAT>AAA      N>KYAL035W      -      0      A4            c219:49763           C>T         AAT>AAA      N>KYAL035W      -      0      DBVPG6304     c219:49763           C>T         AAT>AAA      N>KYAL035W      -      0      IFO1804       c219:49763           C>T         AAT>AAA      N>KYAL035W      -      0      N_43          c219:49763           C>T         AAT>AAA      N>KYAL035W      -      0      N_44          c219:49763           C>T         AAT>AAA      N>KYAL035W      -      0      N_45          c219:49763           C>T         AAT>AAA      N>KYAL035W      -      0      UFRJ50816     c219:49763           C>T         AAT>AAA      N>KYAL035W      -      0      UWOPS91_917_1 c219:49763           C>T         AAT>AAA      N>KYAL035W      -      0      YPS138        c219:49763           C>T         AAT>AAA      N>KYAL035W      -      0      A12           c219:49784           G>T         ATG>ATA      M>IYAL035W      -      0      A4            c219:49784           G>T         ATG>ATA      M>IYAL035W      -      0      DBVPG6304     c219:49784           G>T         ATG>ATA      M>IYAL035W      -      0      UFRJ50816     c219:49784           G>T         ATG>ATA      M>IYAL035W      -      0      YPS138        c219:49784           G>T         ATG>ATA      M>IYAL035W      -      0      A12           c219:49829           G>A         ATC>ATT      I>IYAL035W      -      0      A4            c219:49829           G>A         ATC>ATT      I>IYAL035W      -      0      DBVPG6304     c219:49829           G>A         ATC>ATT      I>IYAL035W      -      0      UFRJ50816     c219:49829           G>A         ATC>ATT      I>IYAL035W      -      0      UWOPS91_917_1 c219:49829           G>A         ATC>ATT      I>IYAL035W      -      0      YPS138        c219:49829           G>A         ATC>ATT      I>IYAL035W      -      0      A12           c219:49838           C>T         AGC>AGA      S>RYAL035W      -      0      DBVPG6304     c219:49838           C>T         AGC>AGA      S>RYAL035W      -      0      UFRJ50816     c219:49838           C>T         AGC>AGA      S>RYAL035W      -      0      UWOPS91_917_1 c219:49838           C>T         AGC>AGA      S>RYAL035W      -      0      YPS138        c219:49838           C>T         AGC>AGA      S>RYAL035W      -      0      A12           c219:49859           T>C         GTT>GTG      V>VYAL035W      -      0      DBVPG6304     c219:49859           T>C         GTT>GTG      V>VYAL035W      -      0      UFRJ50816     c219:49859           T>C         GTT>GTG      V>VYAL035W      -      0      UWOPS91_917_1 c219:49859           T>C         GTT>GTG      V>VYAL035W      -      0      YPS138        c219:49859           T>C         GTT>GTG      V>VYAL035W      -      0      A12           c219:49943           A>G         TTG>TTC      L>FYAL035W      -      0      A4            c219:49943           A>G         TTG>TTC      L>FYAL035W      -      0      DBVPG6304     c219:49943           A>G         TTG>TTC      L>FYAL035W      -      0      UFRJ50816     c219:49943           A>G         TTG>TTC      L>FYAL035W      -      0      YPS138        c219:49943           A>G         TTG>TTC      L>FYAL035W      -      0      A12           c219:49967           G>A         AGA>AGT      R>SYAL035W      -      0      A4            c219:49967           G>A         AGA>AGT      R>SYAL035W      -      0      DBVPG6304     c219:49967           G>A         AGA>AGT      R>SYAL035W      -      0      IFO1804       c219:49967           G>A         AGA>AGT      R>SYAL035W      -      0      N_43          c219:49967           G>A         AGA>AGT      R>SYAL035W      -      0      N_44          c219:49967           G>A         AGA>AGT      R>SYAL035W      -      0      N_45          c219:49967           G>A         AGA>AGT      R>SYAL035W      -      0      UFRJ50816     c219:49967           G>A         AGA>AGT      R>SYAL035W      -      0      YPS138        c219:49967           G>A         AGA>AGT      R>SYAL035W      -      0      A12           c219:49982           T>G         TTC>TTC      F>FYAL035W      -      0      A4            c219:49982           T>G         TTC>TTC      F>FYAL035W      -      0      DBVPG6304     c219:49982           T>G         TTC>TTC      F>FYAL035W      -      0      UFRJ50816     c219:49982           T>G         TTC>TTC      F>FYAL035W      -      0      A12           c219:50000           G>A         CAA>CAT      Q>HYAL035W      -      0      A4            c219:50000           G>A         CAA>CAT      Q>HYAL035W      -      0      DBVPG6304     c219:50000           G>A         CAA>CAT      Q>HYAL035W      -      0      UFRJ50816     c219:50000           G>A         CAA>CAT      Q>HYAL035W      -      0      YPS138        c219:50000           G>A         CAA>CAT      Q>HYAL035W      -      0      A12           c219:50015           C>T         ATA>ATA      I>IYAL035W      -      0      A4            c219:50015           C>T         ATA>ATA      I>IYAL035W      -      0      DBVPG6304     c219:50015           C>T         ATA>ATA      I>IYAL035W      -      0      UFRJ50816     c219:50015           C>T         ATA>ATA      I>IYAL035W      -      0      YPS138        c219:50015           C>T         ATA>ATA      I>IYAL035W      -      0      IFO1804       c219:50033           A>G         CAT>CAC      H>HYAL035W      -      0      N_43          c219:50033           A>G         CAT>CAC      H>HYAL035W      -      0      N_44          c219:50033           A>G         CAT>CAC      H>HYAL035W      -      0      N_45          c219:50033           A>G         CAT>CAC      H>HYAL035W      -      0      IFO1804       c219:50072           A>G         AGT>AGC      S>SYAL035W      -      0      N_43          c219:50072           A>G         AGT>AGC      S>SYAL035W      -      0      N_44          c219:50072           A>G         AGT>AGC      S>SYAL035W      -      0      N_45          c219:50072           A>G         AGT>AGC      S>SYAL035W      -      0      N_43          c219:50102           G>A         CAA>CAT      Q>HYAL035W      -      0      N_44          c219:50102           G>A         CAA>CAT      Q>HYAL035W      -      0      N_45          c219:50102           G>A         CAA>CAT      Q>HYAL035W      -      0      A4            c219:50142           G>A         CAA>CTA      Q>LYAL035W      -      0      DBVPG6304     c219:50142           G>A         CAA>CTA      Q>LYAL035W      -      0      YPS138        c219:50142           G>A         CAA>CTA      Q>LYAL035W      -      0      A4            c219:50167           C>T         GGC>AGC      G>SYAL035W      -      0      CBS432        c219:50167           C>T         GGC>AGC      G>SYAL035W      -      0      CBS5829       c219:50167           C>T         GGC>AGC      G>SYAL035W      -      0      DBVPG6304     c219:50167           C>T         GGC>AGC      G>SYAL035W      -      0      N_17          c219:50167           C>T         GGC>AGC      G>SYAL035W      -      0      N_43          c219:50167           C>T         GGC>AGC      G>SYAL035W      -      0      N_45          c219:50167           C>T         GGC>AGC      G>SYAL035W      -      0      Q62_5         c219:50167           C>T         GGC>AGC      G>SYAL035W      -      0      Y6_5          c219:50167           C>T         GGC>AGC      G>SYAL035W      -      0      Y7            c219:50167           C>T         GGC>AGC      G>SYAL035W      -      0      YPS138        c219:50167           C>T         GGC>AGC      G>SYAL035W      -      0      Z1_1          c219:50167           C>T         GGC>AGC      G>SYAL035W      -      0      A4            c219:50186           A>G         GAC>GAC      D>DYAL035W      -      0      DBVPG6304     c219:50186           A>G         GAC>GAC      D>DYAL035W      -      0      YPS138        c219:50186           A>G         GAC>GAC      D>DYAL035W      -      0      Y6_5          c219:50194           C>T         TTC>ATC      F>IYAL035W      -      0      Z1_1          c219:50194           C>T         TTC>ATC      F>IYAL035W      -      0      A4            c219:50219           A>T         GAT>GAA      D>EYAL035W      -      0      DBVPG6304     c219:50219           A>T         GAT>GAA      D>EYAL035W      -      0      YPS138        c219:50219           A>T         GAT>GAA      D>EYAL035W      -      0      A4            c219:50234           G>A         GTA>GTT      V>VYAL035W      -      0      DBVPG6304     c219:50234           G>A         GTA>GTT      V>VYAL035W      -      0      YPS138        c219:50234           G>A         GTA>GTT      V>VYAL035W      -      0      A4            c219:50283           A>T         GAT>GAT      D>DYAL035W      -      0      DBVPG6304     c219:50283           A>T         GAT>GAT      D>DYAL035W      -      0      N_43          c219:50283           A>T         GAT>GAT      D>DYAL035W      -      0      N_45          c219:50283           A>T         GAT>GAT      D>DYAL035W      -      0      YPS138        c219:50283           A>T         GAT>GAT      D>DYAL035W      -      0      A4            c219:50324           A>G         ACG>ACC      T>TYAL035W      -      0      DBVPG6304     c219:50324           A>G         ACG>ACC      T>TYAL035W      -      0      YPS138        c219:50324           A>G         ACG>ACC      T>TYAL035W      -      0      A4            c219:50351           T>C         AAC>AAG      N>KYAL035W      -      0      DBVPG6304     c219:50351           T>C         AAC>AAG      N>KYAL035W      -      0      YPS138        c219:50351           T>C         AAC>AAG      N>KYAL035W      -      0      A4            c219:50365           A>G         TTC>CTC      F>LYAL035W      -      0      DBVPG6304     c219:50365           A>G         TTC>CTC      F>LYAL035W      -      0      YPS138        c219:50365           A>G         TTC>CTC      F>LYAL035W      -      0      A4            c219:50444           A>G         ACC>ACC      T>TYAL035W      -      0      DBVPG6304     c219:50444           A>G         ACC>ACC      T>TYAL035W      -      0      YPS138        c219:50444           A>G         ACC>ACC      T>TYAL035W      -      0      A4            c219:50469           A>G         CAA>CCA      Q>PYAL035W      -      0      DBVPG6304     c219:50469           A>G         CAA>CCA      Q>PYAL035W      -      0      YPS138        c219:50469           A>G         CAA>CCA      Q>PYAL035W      -      0      Z1_1          c219:50501           A>C         CAA>CAG      Q>QYAL035W      -      0      YPS138        c219:50507           G>C         GTC>GTG      V>VYAL035W      -      0      A4            c219:50513           G>A         AAC>AAT      N>NYAL035W      -      0      DBVPG6304     c219:50513           G>A         AAC>AAT      N>NYAL035W      -      0      YPS138        c219:50513           G>A         AAC>AAT      N>NYAL035W      -      0      A4            c219:50522           C>T         AGT>AGA      S>RYAL035W      -      0      DBVPG6304     c219:50522           C>T         AGT>AGA      S>RYAL035W      -      0      UFRJ50791     c219:50522           C>T         AGT>AGA      S>RYAL035W      -      0      YPS138        c219:50522           C>T         AGT>AGA      S>RYAL035W      -      0      A4            c219:50531           G>A         ACC>ACT      T>TYAL035W      -      0      DBVPG6304     c219:50531           G>A         ACC>ACT      T>TYAL035W      -      0      UFRJ50791     c219:50531           G>A         ACC>ACT      T>TYAL035W      -      0      YPS138        c219:50531           G>A         ACC>ACT      T>TYAL035W      -      0      A4            c219:50588           A>C         TTT>TTG      F>LYAL035W      -      0      DBVPG6304     c219:50588           A>C         TTT>TTG      F>LYAL035W      -      0      UFRJ50791     c219:50588           A>C         TTT>TTG      F>LYAL035W      -      0      UFRJ50816     c219:50588           A>C         TTT>TTG      F>LYAL035W      -      0      YPS138        c219:50588           A>C         TTT>TTG      F>LYAL035W      -      0      A4            c219:50645           G>A         AAC>AAT      N>NYAL035W      -      0      DBVPG6304     c219:50645           G>A         AAC>AAT      N>NYAL035W      -      0      IFO1804       c219:50645           G>A         AAC>AAT      N>NYAL035W      -      0      N_43          c219:50645           G>A         AAC>AAT      N>NYAL035W      -      0      N_44          c219:50645           G>A         AAC>AAT      N>NYAL035W      -      0      N_45          c219:50645           G>A         AAC>AAT      N>NYAL035W      -      0      UFRJ50791     c219:50645           G>A         AAC>AAT      N>NYAL035W      -      0      UFRJ50816     c219:50645           G>A         AAC>AAT      N>NYAL035W      -      0      YPS138        c219:50645           G>A         AAC>AAT      N>NYAL035W      -      0      A4            c219:50678           T>C         TGG>TGG      W>WYAL035W      -      0      DBVPG6304     c219:50678           T>C         TGG>TGG      W>WYAL035W      -      0      UFRJ50791     c219:50678           T>C         TGG>TGG      W>WYAL035W      -      0      UFRJ50816     c219:50678           T>C         TGG>TGG      W>WYAL035W      -      0      YPS138        c219:50678           T>C         TGG>TGG      W>WYAL035W      -      0      DBVPG6304     c219:50711           T>C         TTT>TTG      F>LYAL035W      -      0      UFRJ50791     c219:50711           T>C         TTT>TTG      F>LYAL035W      -      0      UFRJ50816     c219:50711           T>C         TTT>TTG      F>LYAL035W      -      0      YPS138        c219:50711           T>C         TTT>TTG      F>LYAL035W      -      0      DBVPG6304     c219:50717           C>T         ATC>ATA      I>IYAL035W      -      0      UFRJ50791     c219:50717           C>T         ATC>ATA      I>IYAL035W      -      0      UFRJ50816     c219:50717           C>T         ATC>ATA      I>IYAL035W      -      0      YPS138        c219:50717           C>T         ATC>ATA      I>IYAL035W      -      0      IFO1804       c219:50726           C>T         TGC>TGA      C>*YAL035W      -      0      DBVPG6304     c219:50849           C>T         GTC>GTA      V>VYAL035W      -      0      UFRJ50791     c219:50849           C>T         GTC>GTA      V>VYAL035W      -      0      UFRJ50816     c219:50849           C>T         GTC>GTA      V>VYAL035W      -      0      YPS138        c219:50849           C>T         GTC>GTA      V>VYAL035W      -      0      DBVPG6304     c219:50957           T>C         AGG>AGG      R>RYAL035W      -      0      A12           c219:51152           A>G         ATC>ATC      I>IYAL035W      -      0      A4            c219:51152           A>G         ATC>ATC      I>IYAL035W      -      0      DBVPG6304     c219:51152           A>G         ATC>ATC      I>IYAL035W      -      0      IFO1804       c219:51152           A>G         ATC>ATC      I>IYAL035W      -      0      N_44          c219:51152           A>G         ATC>ATC      I>IYAL035W      -      0      N_45          c219:51152           A>G         ATC>ATC      I>IYAL035W      -      0      UFRJ50791     c219:51152           A>G         ATC>ATC      I>IYAL035W      -      0      UFRJ50816     c219:51152           A>G         ATC>ATC      I>IYAL035W      -      0      A12           c219:51165           A>G         GGA>GCA      G>AYAL035W      -      0      A4            c219:51165           A>G         GGA>GCA      G>AYAL035W      -      0      DBVPG6304     c219:51165           A>G         GGA>GCA      G>AYAL035W      -      0      UFRJ50791     c219:51165           A>G         GGA>GCA      G>AYAL035W      -      0      UFRJ50816     c219:51165           A>G         GGA>GCA      G>AYAL035W      -      0      A12           c219:51183           T>G         AGA>ACA      R>TYAL035W      -      0      A4            c219:51183           T>G         AGA>ACA      R>TYAL035W      -      0      CBS432        c219:51183           T>G         AGA>ACA      R>TYAL035W      -      0      CBS5829       c219:51183           T>G         AGA>ACA      R>TYAL035W      -      0      DBVPG6304     c219:51183           T>G         AGA>ACA      R>TYAL035W      -      0      IFO1804       c219:51183           T>G         AGA>ACA      R>TYAL035W      -      0      KPN3829       c219:51183           T>G         AGA>ACA      R>TYAL035W      -      0      N_44          c219:51183           T>G         AGA>ACA      R>TYAL035W      -      0      N_45          c219:51183           T>G         AGA>ACA      R>TYAL035W      -      0      Q32_3         c219:51183           T>G         AGA>ACA      R>TYAL035W      -      0      Q62_5         c219:51183           T>G         AGA>ACA      R>TYAL035W      -      0      Q95_3         c219:51183           T>G         AGA>ACA      R>TYAL035W      -      0      S36_7         c219:51183           T>G         AGA>ACA      R>TYAL035W      -      0      UFRJ50791     c219:51183           T>G         AGA>ACA      R>TYAL035W      -      0      UFRJ50816     c219:51183           T>G         AGA>ACA      R>TYAL035W      -      0      Y7            c219:51183           T>G         AGA>ACA      R>TYAL035W      -      0      A12           c219:51194           G>A         CAA>CAT      Q>HYAL035W      -      0      A4            c219:51194           G>A         CAA>CAT      Q>HYAL035W      -      0      DBVPG6304     c219:51194           G>A         CAA>CAT      Q>HYAL035W      -      0      UFRJ50791     c219:51200           G>A         ATC>ATT      I>IYAL035W      -      0      UFRJ50816     c219:51200           G>A         ATC>ATT      I>IYAL035W      -      0      IFO1804       c219:51251           C>T         CTT>CTA      L>LYAL035W      -      0      N_44          c219:51251           C>T         CTT>CTA      L>LYAL035W      -      0      N_45          c219:51251           C>T         CTT>CTA      L>LYAL036C      +      0      A12           c219:51631           T>C         TTT>TTC      F>FYAL036C      +      0      A4            c219:51631           T>C         TTT>TTC      F>FYAL036C      +      0      IFO1804       c219:51631           T>C         TTT>TTC      F>FYAL036C      +      0      N_43          c219:51631           T>C         TTT>TTC      F>FYAL036C      +      0      N_44          c219:51631           T>C         TTT>TTC      F>FYAL036C      +      0      A12           c219:51652           C>G         GCC>GCG      A>AYAL036C      +      0      A4            c219:51652           C>G         GCC>GCG      A>AYAL036C      +      0      A12           c219:51700           G>C         GGG>GGC      G>GYAL036C      +      0      A4            c219:51700           G>C         GGG>GGC      G>GYAL036C      +      0      UWOPS91_917_1 c219:51700           G>C         GGG>GGC      G>GYAL036C      +      0      A4            c219:51721           T>C         ATT>ATC      I>IYAL036C      +      0      A12           c219:51727           T>C         TTT>TTC      F>FYAL036C      +      0      A4            c219:51727           T>C         TTT>TTC      F>FYAL036C      +      0      IFO1804       c219:51727           T>C         TTT>TTC      F>FYAL036C      +      0      N_43          c219:51727           T>C         TTT>TTC      F>FYAL036C      +      0      N_44          c219:51727           T>C         TTT>TTC      F>FYAL036C      +      0      UWOPS91_917_1 c219:51727           T>C         TTT>TTC      F>FYAL036C      +      0      IFO1804       c219:51775           G>A         CCG>CCA      P>PYAL036C      +      0      N_43          c219:51775           G>A         CCG>CCA      P>PYAL036C      +      0      N_44          c219:51775           G>A         CCG>CCA      P>PYAL036C      +      0      A12           c219:51781           A>G         GTA>GTG      V>VYAL036C      +      0      A4            c219:51781           A>G         GTA>GTG      V>VYAL036C      +      0      IFO1804       c219:51781           A>G         GTA>GTG      V>VYAL036C      +      0      N_43          c219:51781           A>G         GTA>GTG      V>VYAL036C      +      0      N_44          c219:51781           A>G         GTA>GTG      V>VYAL036C      +      0      UWOPS91_917_1 c219:51781           A>G         GTA>GTG      V>VYAL036C      +      0      A12           c219:51802           C>T         TCC>TCT      S>SYAL036C      +      0      A4            c219:51802           C>T         TCC>TCT      S>SYAL036C      +      0      UWOPS91_917_1 c219:51832           T>A         GCT>GCA      A>AYAL036C      +      0      DBVPG6304     c219:51871           C>T         GTC>GTT      V>VYAL036C      +      0      DBVPG6304     c219:51892           G>A         AAG>AAA      K>KYAL036C      +      0      DBVPG6304     c219:51908           T>C         TTA>CTA      L>LYAL036C      +      0      UWOPS91_917_1 c219:51931           T>C         GCT>GCC      A>AYAL036C      +      0      DBVPG6304     c219:51986           T>C         TTA>CTA      L>LYAL036C      +      0      DBVPG6304     c219:52009           G>C         GTG>GTC      V>VYAL036C      +      0      UWOPS91_917_1 c219:52009           G>C         GTG>GTC      V>VYAL036C      +      0      DBVPG6304     c219:52015           A>G         AAA>AAG      K>KYAL036C      +      0      DBVPG6304     c219:52057           G>T         GGG>GGT      G>GYAL036C      +      0      UWOPS91_917_1 c219:52057           G>T         GGG>GGT      G>GYAL036C      +      0      DBVPG6304     c219:52177           T>C         GCT>GCC      A>AYAL036C      +      0      UFRJ50816     c219:52177           T>C         GCT>GCC      A>AYAL036C      +      0      UWOPS91_917_1 c219:52177           T>C         GCT>GCC      A>AYAL036C      +      0      YPS138        c219:52177           T>C         GCT>GCC      A>AYAL036C      +      0      DBVPG6304     c219:52216           T>C         GCT>GCC      A>AYAL036C      +      0      IFO1804       c219:52216           T>C         GCT>GCC      A>AYAL036C      +      0      N_43          c219:52216           T>C         GCT>GCC      A>AYAL036C      +      0      N_44          c219:52216           T>C         GCT>GCC      A>AYAL036C      +      0      N_45          c219:52216           T>C         GCT>GCC      A>AYAL036C      +      0      UWOPS91_917_1 c219:52216           T>C         GCT>GCC      A>AYAL036C      +      0      YPS138        c219:52216           T>C         GCT>GCC      A>AYAL036C      +      0      UWOPS91_917_1 c219:52306           G>A         AAG>AAA      K>KYAL036C      +      0      IFO1804       c219:52342           G>T         CTG>CTT      L>LYAL036C      +      0      N_43          c219:52342           G>T         CTG>CTT      L>LYAL036C      +      0      N_44          c219:52342           G>T         CTG>CTT      L>LYAL036C      +      0      N_45          c219:52342           G>T         CTG>CTT      L>LYAL036C      +      0      A12           c219:52558           C>T         TTC>TTT      F>FYAL036C      +      0      DBVPG6304     c219:52558           C>T         TTC>TTT      F>FYAL036C      +      0      UFRJ50816     c219:52558           C>T         TTC>TTT      F>FYAL036C      +      0      UWOPS91_917_1 c219:52558           C>T         TTC>TTT      F>FYAL036C      +      0      YPS138        c219:52558           C>T         TTC>TTT      F>FYAL036C      +      0      A12           c219:52582           C>T         AGC>AGT      S>SYAL036C      +      0      DBVPG6304     c219:52582           C>T         AGC>AGT      S>SYAL036C      +      0      UFRJ50816     c219:52582           C>T         AGC>AGT      S>SYAL036C      +      0      UWOPS91_917_1 c219:52582           C>T         AGC>AGT      S>SYAL036C      +      0      YPS138        c219:52582           C>T         AGC>AGT      S>SYAL036C      +      0      A12           c219:52612           G>A         GGG>GGA      G>GYAL036C      +      0      DBVPG6304     c219:52612           G>A         GGG>GGA      G>GYAL036C      +      0      UFRJ50816     c219:52612           G>A         GGG>GGA      G>GYAL036C      +      0      UWOPS91_917_1 c219:52612           G>C         GGG>GGC      G>GYAL036C      +      0      YPS138        c219:52612           G>A         GGG>GGA      G>GYAL036C      +      0      A12           c219:52618           T>C         AAT>AAC      N>NYAL036C      +      0      DBVPG6304     c219:52618           T>C         AAT>AAC      N>NYAL036C      +      0      UFRJ50816     c219:52618           T>C         AAT>AAC      N>NYAL036C      +      0      UWOPS91_917_1 c219:52618           T>C         AAT>AAC      N>NYAL036C      +      0      YPS138        c219:52618           T>C         AAT>AAC      N>NYAL036C      +      0      A12           c219:52633           C>T         GAC>GAT      D>DYAL036C      +      0      YPS138        c219:52633           C>T         GAC>GAT      D>DYAL036C      +      0      IFO1804       c219:52636           A>G         GAA>GAG      E>EYAL036C      +      0      N_43          c219:52636           A>G         GAA>GAG      E>EYAL036C      +      0      N_45          c219:52636           A>G         GAA>GAG      E>EYAL037W      -      0      A12           c219:52880           T>C         ACC>AGC      T>SYAL037W      -      0      DBVPG6304     c219:52880           T>C         ACC>AGC      T>SYAL037W      -      0      UFRJ50816     c219:52880           T>C         ACC>AGC      T>SYAL037W      -      0      UWOPS91_917_1 c219:52880           T>C         ACC>AGC      T>SYAL037W      -      0      A12           c219:52913           T>C         AAC>AGC      N>SYAL037W      -      0      DBVPG6304     c219:52913           T>C         AAC>AGC      N>SYAL037W      -      0      UFRJ50816     c219:52913           T>C         AAC>AGC      N>SYAL037W      -      0      UWOPS91_917_1 c219:52913           T>C         AAC>AGC      N>SYAL037W      -      0      A12           c219:52934           G>T         CTG>CAG      L>QYAL037W      -      0      DBVPG6304     c219:52934           G>T         CTG>CAG      L>QYAL037W      -      0      UFRJ50816     c219:52934           G>T         CTG>CAG      L>QYAL037W      -      0      UWOPS91_917_1 c219:52934           G>T         CTG>CAG      L>QYAL037W      -      0      UWOPS91_917_1 c219:52978           C>T         TGT>TGA      C>*YAL037W      -      0      A12           c219:52985           T>G         AGG>ACG      R>TYAL037W      -      0      DBVPG6304     c219:52985           T>G         AGG>ACG      R>TYAL037W      -      0      UFRJ50816     c219:52985           T>G         AGG>ACG      R>TYAL037W      -      0      YPS138        c219:52985           T>G         AGG>ACG      R>TYAL037W      -      0      N_43          c219:53005           T>C         ATT>ATG      I>MYAL037W      -      0      N_45          c219:53005           T>C         ATT>ATG      I>MYAL037W      -      0      UWOPS91_917_1 c219:53037           G>C         ACT>GCT      T>AYAL037W      -      0      A12           c219:53047           T>C         TAT>TAG      Y>*YAL037W      -      0      DBVPG6304     c219:53047           T>C         TAT>TAG      Y>*YAL037W      -      0      YPS138        c219:53047           T>C         TAT>TAG      Y>*YAL037W      -      0      KPN3828       c219:53065           C>a         CTG>CTT      L>LYAL037W      -      0      A12           c219:53096           C>G         CAC>CCC      H>PYAL037W      -      0      DBVPG6304     c219:53096           C>G         CAC>CCC      H>PYAL037W      -      0      UFRJ50791     c219:53096           C>G         CAC>CCC      H>PYAL037W      -      0      UWOPS91_917_1 c219:53096           C>G         CAC>CCC      H>PYAL037W      -      0      YPS138        c219:53096           C>G         CAC>CCC      H>PYAL037W      -      0      YPS138        c219:53242           G>A         CGT>CGT      R>RYAL037W      -      0      N_17          c219:53293           C>T         GTC>GTA      V>VYAL037W      -      0      N_44          c219:53293           C>T         GTC>GTA      V>VYAL037W      -      0      N_45          c219:53293           C>T         GTC>GTA      V>VYAL037W      -      0      A12           c219:53311           C>A         GTA>GTT      V>VYAL037W      -      0      A4            c219:53311           C>A         GTA>GTT      V>VYAL037W      -      0      DBVPG6304     c219:53311           C>A         GTA>GTT      V>VYAL037W      -      0      UWOPS91_917_1 c219:53311           C>A         GTA>GTT      V>VYAL037W      -      0      YPS138        c219:53311           C>A         GTA>GTT      V>VYAL037W      -      0      N_17          c219:53342           T>C         AAA>AGA      K>RYAL037W      -      0      N_44          c219:53342           T>C         AAA>AGA      K>RYAL037W      -      0      N_45          c219:53342           T>C         AAA>AGA      K>RYAL037W      -      0      A4            c219:53348           A>C         TGA>TGA      *>*YAL037W      -      0      CBS432        c219:53348           A>C         TGA>TGA      *>*YAL037W      -      0      CBS5829       c219:53348           A>C         TGA>TGA      *>*YAL037W      -      0      DBVPG4650     c219:53348           A>C         TGA>TGA      *>*YAL037W      -      0      DBVPG6304     c219:53348           A>C         TGA>TGA      *>*YAL037W      -      0      KPN3828       c219:53348           A>C         TGA>TGA      *>*YAL037W      -      0      N_17          c219:53348           A>C         TGA>TGA      *>*YAL037W      -      0      N_44          c219:53348           A>C         TGA>TGA      *>*YAL037W      -      0      N_45          c219:53348           A>C         TGA>TGA      *>*YAL037W      -      0      Q59_1         c219:53348           A>C         TGA>TGA      *>*YAL037W      -      0      Q62_5         c219:53348           A>C         TGA>TGA      *>*YAL037W      -      0      Q89_8         c219:53348           A>C         TGA>TGA      *>*YAL037W      -      0      S36_7         c219:53348           A>C         TGA>TGA      *>*YAL037W      -      0      T21_4         c219:53348           A>C         TGA>TGA      *>*YAL037W      -      0      UWOPS91_917_1 c219:53348           A>C         TGA>TGA      *>*YAL037W      -      0      YPS138        c219:53348           A>C         TGA>TGA      *>*YAL037W      -      0      Z1_1          c219:53348           A>C         TGA>TGA      *>*YAL037W      -      0      UWOPS91_917_1 c219:53365           T>C         ATT>ATG      I>MYAL037W      -      0      A4            c219:53377           C>T         TAC>TAA      Y>*YAL037W      -      0      DBVPG6304     c219:53377           C>T         TAC>TAA      Y>*YAL037W      -      0      UWOPS91_917_1 c219:53377           C>T         TAC>TAA      Y>*YAL037W      -      0      YPS138        c219:53377           C>T         TAC>TAA      Y>*YAL037W      -      0      A4            c219:53399           T>C         GCA>GGA      A>GYAL037W      -      0      UWOPS91_917_1 c219:53399           T>C         GCA>GGA      A>GYAL037W      -      0      YPS138        c219:53399           T>C         GCA>GGA      A>GYAL037W      -      0      DBVPG6304     c219:53410           A>G         TTT>TTC      F>FYAL037W      -      0      UWOPS91_917_1 c219:53410           A>G         TTT>TTC      F>FYAL037W      -      0      YPS138        c219:53410           A>G         TTT>TTC      F>FYAL037W      -      0      A4            c219:53423           T>G         AGA>ACA      R>TYAL037W      -      0      DBVPG6304     c219:53423           T>G         AGA>ACA      R>TYAL037W      -      0      YPS138        c219:53423           T>G         AGA>ACA      R>TYAL037W      -      0      A4            c219:53434           T>A         GGT>GGT      G>GYAL037W      -      0      DBVPG6304     c219:53434           T>A         GGT>GGT      G>GYAL037W      -      0      YPS138        c219:53434           T>A         GGT>GGT      G>GYAL037W      -      0      A4            c219:53461           A>C         ATC>ATG      I>MYAL037W      -      0      A4            c219:53473           G>C         ATA>ATG      I>MYAL037W      -      0      DBVPG6304     c219:53473           G>C         ATA>ATG      I>MYAL037W      -      0      YPS138        c219:53473           G>C         ATA>ATG      I>MYAL037W      -      0      A4            c219:53479           C>T         TTT>TTA      F>LYAL037W      -      0      DBVPG6304     c219:53479           C>T         TTT>TTA      F>LYAL037W      -      0      YPS138        c219:53479           C>T         TTT>TTA      F>LYAL037W      -      0      A4            c219:53518           A>T         AAG>AAA      K>KYAL037W      -      0      N_17          c219:53518           A>G         AAG>AAC      K>NYAL037W      -      0      N_44          c219:53518           A>G         AAG>AAC      K>NYAL037W      -      0      YPS138        c219:53518           A>T         AAG>AAA      K>KYAL037W      -      0      DBVPG4650     c219:53520           T>C         AAG>GAG      K>EYAL037W      -      0      T21_4         c219:53535           T>C         ATC>GTC      I>VYAL037W      -      0      T21_4         c219:53544           T>C         CAA>GAA      Q>EYAL037W      -      0      DBVPG6304     c219:53560           T>C         GGT>GGG      G>GYAL037W      -      0      YPS138        c219:53560           T>C         GGT>GGG      G>GYAL037W      -      0      N_17          c219:53592           G>A         GCT>TCT      A>SYAL037W      -      0      N_44          c219:53592           G>A         GCT>TCT      A>SYAL037W      -      0      N_17          c219:53611           A>G         GCC>GCC      A>AYAL037W      -      0      N_44          c219:53611           A>G         GCC>GCC      A>AYAL037W      -      0      N_17          c219:53631           G>A         AGA>TGA      R>*YAL037W      -      0      N_44          c219:53631           G>A         AGA>TGA      R>*YAL037W      -      0      DBVPG6304     c219:53632           G>A         AGT>AGT      S>SYAL037W      -      0      YPS138        c219:53632           G>A         AGT>AGT      S>SYAL037W      -      0      N_17          c219:53642           A>G         GTT>GCT      V>AYAL037W      -      0      N_44          c219:53642           A>G         GTT>GCT      V>AYAL037W      -      0      KPN3828       c219:53669           A>G         TCG>TCG      S>SYAL037W      -      0      CBS432        c219:53680           C>T         TTA>TTA      L>LYAL037W      -      0      DBVPG4650     c219:53680           C>T         TTA>TTA      L>LYAL037W      -      0      KPN3828       c219:53680           C>T         TTA>TTA      L>LYAL037W      -      0      N_17          c219:53680           C>T         TTA>TTA      L>LYAL037W      -      0      N_44          c219:53680           C>T         TTA>TTA      L>LYAL037W      -      0      Q59_1         c219:53680           C>T         TTA>TTA      L>LYAL037W      -      0      S36_7         c219:53680           C>T         TTA>TTA      L>LYAL037W      -      0      T21_4         c219:53680           C>T         TTA>TTA      L>LYAL037W      -      0      Y7            c219:53680           C>T         TTA>TTA      L>LYAL037W      -      0      Z1_1          c219:53680           C>T         TTA>TTA      L>LYAL038W      -      0      UFRJ50816     c219:54478           A>G         TCT>TCC      S>SYAL038W      -      0      UWOPS91_917_1 c219:54478           A>G         TCT>TCC      S>SYAL038W      -      0      UFRJ50816     c219:54499           G>C         GAT>GAG      D>EYAL038W      -      0      UFRJ50816     c219:54562           T>A         AAC>AAT      N>NYAL038W      -      0      A12           c219:54582           C>G         GTG>CTG      V>LYAL038W      -      0      A4            c219:54582           C>G         GTG>CTG      V>LYAL038W      -      0      DBVPG6304     c219:54582           C>G         GTG>CTG      V>LYAL038W      -      0      UFRJ50816     c219:54582           C>G         GTG>CTG      V>LYAL038W      -      0      YPS138        c219:54582           C>G         GTG>CTG      V>LYAL038W      -      0      UFRJ50816     c219:54595           G>A         GTA>GTT      V>VYAL038W      -      0      UWOPS91_917_1 c219:54671           C>a         GTC>GTC      V>VYAL038W      -      0      UFRJ50816     c219:54733           G>A         TGG>TGT      W>CYAL038W      -      0      UFRJ50816     c219:54778           A>G         AGC>AGC      S>SYAL038W      -      0      UFRJ50816     c219:54787           A>G         GTC>GTC      V>VYAL038W      -      0      A4            c219:54790           G>A         CTT>CTT      L>LYAL038W      -      0      YPS138        c219:54790           G>A         CTT>CTT      L>LYAL038W      -      0      UFRJ50816     c219:54847           A>G         GAT>GAC      D>DYAL038W      -      0      UFRJ50816     c219:54883           G>A         CAA>CAT      Q>HYAL038W      -      0      A4            c219:54895           G>A         GTC>GTT      V>VYAL038W      -      0      YPS138        c219:54895           G>A         GTC>GTT      V>VYAL038W      -      0      UFRJ50816     c219:54928           G>A         GGC>GGT      G>GYAL038W      -      0      UWOPS91_917_1 c219:55039           G>A         GAC>GAT      D>DYAL038W      -      0      A4            c219:55117           A>G         CTT>CTC      L>LYAL038W      -      0      UFRJ50791     c219:55117           A>G         CTT>CTC      L>LYAL038W      -      0      UWOPS91_917_1 c219:55135           G>A         GAC>GAT      D>DYAL038W      -      0      A4            c219:55174           A>G         GTC>GTC      V>VYAL038W      -      0      UFRJ50791     c219:55174           A>G         GTC>GTC      V>VYAL038W      -      0      UWOPS91_917_1 c219:55234           T>C         TGG>TGG      W>WYAL038W      -      0      UFRJ50791     c219:55313           C>T         GGT>GAT      G>DYAL038W      -      0      UWOPS91_917_1 c219:55313           C>T         GGT>GAT      G>DYAL038W      -      0      UWOPS91_917_1 c219:55384           G>A         GAT>GAT      D>DYAL038W      -      0      IFO1804       c219:55402           A>G         ACA>ACC      T>TYAL038W      -      0      N_43          c219:55402           A>G         ACA>ACC      T>TYAL038W      -      0      N_45          c219:55402           A>G         ACA>ACC      T>TYAL038W      -      0      A4            c219:55423           G>A         GGT>GGT      G>GYAL038W      -      0      DBVPG6304     c219:55423           G>A         GGT>GGT      G>GYAL038W      -      0      IFO1804       c219:55423           G>A         GGT>GGT      G>GYAL038W      -      0      N_43          c219:55423           G>A         GGT>GGT      G>GYAL038W      -      0      N_45          c219:55423           G>A         GGT>GGT      G>GYAL038W      -      0      UFRJ50791     c219:55423           G>A         GGT>GGT      G>GYAL038W      -      0      UWOPS91_917_1 c219:55423           G>A         GGT>GGT      G>GYAL038W      -      0      YPS138        c219:55423           G>A         GGT>GGT      G>GYAL038W      -      0      IFO1804       c219:55498           G>A         AGC>AGT      S>SYAL038W      -      0      N_43          c219:55498           G>A         AGC>AGT      S>SYAL038W      -      0      N_45          c219:55498           G>A         AGC>AGT      S>SYAL038W      -      0      N_43          c219:55596           T>C         TTC>GTC      F>VYAL038W      -      0      UWOPS91_917_1 c219:55735           G>A         GAC>GAT      D>DYAL039C      +      0      N_44          c219:58143           G>A         GGT>GAT      G>DYAL039C      +      0      A4            c219:58150           T>C         AAT>AAC      N>NYAL039C      +      0      DBVPG6304     c219:58150           T>C         AAT>AAC      N>NYAL039C      +      0      YPS138        c219:58150           T>C         AAT>AAC      N>NYAL039C      +      0      UWOPS91_917_1 c219:58158           G>A         GGG>GAG      G>EYAL039C      +      0      A4            c219:58166           G>A         GCA>ACA      A>TYAL039C      +      0      DBVPG6304     c219:58166           G>A         GCA>ACA      A>TYAL039C      +      0      UWOPS91_917_1 c219:58166           G>A         GCA>ACA      A>TYAL039C      +      0      YPS138        c219:58166           G>A         GCA>ACA      A>TYAL039C      +      0      N_44          c219:58172           T>G         TCC>GCC      S>AYAL039C      +      0      N_45          c219:58172           T>G         TCC>GCC      S>AYAL039C      +      0      N_17          c219:58207           G>T         TCG>TCT      S>SYAL039C      +      0      N_44          c219:58207           G>A         TCG>TCA      S>SYAL039C      +      0      N_45          c219:58207           G>A         TCG>TCA      S>SYAL039C      +      0      Q95_3         c219:58207           G>T         TCG>TCT      S>SYAL039C      +      0      UWOPS91_917_1 c219:58213           G>A         TCG>TCA      S>SYAL039C      +      0      A4            c219:58231           C>T         TCC>TCT      S>SYAL039C      +      0      DBVPG6304     c219:58231           C>T         TCC>TCT      S>SYAL039C      +      0      YPS138        c219:58231           C>T         TCC>TCT      S>SYAL039C      +      0      A4            c219:58249           A>G         CAA>CAG      Q>QYAL039C      +      0      DBVPG6304     c219:58249           A>G         CAA>CAG      Q>QYAL039C      +      0      UFRJ50791     c219:58249           A>G         CAA>CAG      Q>QYAL039C      +      0      UWOPS91_917_1 c219:58249           A>G         CAA>CAG      Q>QYAL039C      +      0      YPS138        c219:58249           A>G         CAA>CAG      Q>QYAL039C      +      0      A4            c219:58256           G>A         GAA>AAA      E>KYAL039C      +      0      DBVPG6304     c219:58256           G>A         GAA>AAA      E>KYAL039C      +      0      UFRJ50791     c219:58256           G>A         GAA>AAA      E>KYAL039C      +      0      UWOPS91_917_1 c219:58256           G>A         GAA>AAA      E>KYAL039C      +      0      YPS138        c219:58256           G>A         GAA>AAA      E>KYAL039C      +      0      N_44          c219:58261           C>T         GAC>GAT      D>DYAL039C      +      0      N_45          c219:58261           C>T         GAC>GAT      D>DYAL039C      +      0      A4            c219:58288           A>T         CCA>CCT      P>PYAL039C      +      0      DBVPG6304     c219:58288           A>T         CCA>CCT      P>PYAL039C      +      0      UFRJ50791     c219:58288           A>T         CCA>CCT      P>PYAL039C      +      0      UWOPS91_917_1 c219:58288           A>G         CCA>CCG      P>PYAL039C      +      0      YPS138        c219:58288           A>T         CCA>CCT      P>PYAL039C      +      0      N_44          c219:58314           G>A         GGC>GAC      G>DYAL039C      +      0      UWOPS91_917_1 c219:58348           C>A         ATC>ATA      I>IYAL039C      +      0      IFO1804       c219:58369           A>G         CCA>CCG      P>PYAL039C      +      0      N_44          c219:58369           A>G         CCA>CCG      P>PYAL039C      +      0      N_45          c219:58369           A>G         CCA>CCG      P>PYAL039C      +      0      A4            c219:58375           T>C         AGT>AGC      S>SYAL039C      +      0      DBVPG6304     c219:58375           T>C         AGT>AGC      S>SYAL039C      +      0      UFRJ50791     c219:58375           T>C         AGT>AGC      S>SYAL039C      +      0      YPS138        c219:58375           T>C         AGT>AGC      S>SYAL039C      +      0      UWOPS91_917_1 c219:58451           G>A         GGC>AGC      G>SYAL039C      +      0      DBVPG4650     c219:58459           C>G         GTC>GTG      V>VYAL039C      +      0      Q89_8         c219:58459           C>G         GTC>GTG      V>VYAL039C      +      0      A4            c219:58462           C>G         GCC>GCG      A>AYAL039C      +      0      DBVPG6304     c219:58462           C>G         GCC>GCG      A>AYAL039C      +      0      UFRJ50791     c219:58462           C>G         GCC>GCG      A>AYAL039C      +      0      YPS138        c219:58462           C>G         GCC>GCG      A>AYAL039C      +      0      UWOPS91_917_1 c219:58465           G>A         GAG>GAA      E>EYAL039C      +      0      A4            c219:58474           C>G         GTC>GTG      V>VYAL039C      +      0      DBVPG6304     c219:58474           C>G         GTC>GTG      V>VYAL039C      +      0      UFRJ50791     c219:58474           C>G         GTC>GTG      V>VYAL039C      +      0      UWOPS91_917_1 c219:58474           C>G         GTC>GTG      V>VYAL039C      +      0      YPS138        c219:58474           C>G         GTC>GTG      V>VYAL039C      +      0      A4            c219:58504           A>G         CTA>CTG      L>LYAL039C      +      0      DBVPG6304     c219:58504           A>G         CTA>CTG      L>LYAL039C      +      0      UFRJ50791     c219:58504           A>G         CTA>CTG      L>LYAL039C      +      0      UWOPS91_917_1 c219:58504           A>G         CTA>CTG      L>LYAL039C      +      0      YPS138        c219:58504           A>G         CTA>CTG      L>LYAL039C      +      0      UWOPS91_917_1 c219:58580           T>C         TTG>CTG      L>LYAL039C      +      0      A4            c219:58588           C>T         TTC>TTT      F>FYAL039C      +      0      DBVPG6304     c219:58588           C>T         TTC>TTT      F>FYAL039C      +      0      UFRJ50791     c219:58588           C>T         TTC>TTT      F>FYAL039C      +      0      YPS138        c219:58588           C>T         TTC>TTT      F>FYAL039C      +      0      A4            c219:58594           A>G         GGA>GGG      G>GYAL039C      +      0      DBVPG6304     c219:58594           A>G         GGA>GGG      G>GYAL039C      +      0      UFRJ50791     c219:58594           A>G         GGA>GGG      G>GYAL039C      +      0      UWOPS91_917_1 c219:58594           A>G         GGA>GGG      G>GYAL039C      +      0      YPS138        c219:58594           A>G         GGA>GGG      G>GYAL039C      +      0      UWOPS91_917_1 c219:58606           C>T         GTC>GTT      V>VYAL039C      +      0      Q59_1         c219:58621           C>A         GCC>GCA      A>AYAL039C      +      0      A4            c219:58646           C>T         CTG>TTG      L>LYAL039C      +      0      DBVPG6304     c219:58646           C>T         CTG>TTG      L>LYAL039C      +      0      UFRJ50791     c219:58646           C>T         CTG>TTG      L>LYAL039C      +      0      UWOPS91_917_1 c219:58646           C>T         CTG>TTG      L>LYAL039C      +      0      YPS138        c219:58646           C>T         CTG>TTG      L>LYAL039C      +      0      A4            c219:58657           C>T         TCC>TCT      S>SYAL039C      +      0      DBVPG6304     c219:58657           C>T         TCC>TCT      S>SYAL039C      +      0      UFRJ50791     c219:58657           C>T         TCC>TCT      S>SYAL039C      +      0      YPS138        c219:58657           C>T         TCC>TCT      S>SYAL039C      +      0      A4            c219:58663           C>T         TTC>TTT      F>FYAL039C      +      0      DBVPG6304     c219:58663           C>T         TTC>TTT      F>FYAL039C      +      0      IFO1804       c219:58663           C>T         TTC>TTT      F>FYAL039C      +      0      N_45          c219:58663           C>T         TTC>TTT      F>FYAL039C      +      0      UFRJ50791     c219:58663           C>T         TTC>TTT      F>FYAL039C      +      0      YPS138        c219:58663           C>T         TTC>TTT      F>FYAL039C      +      0      A4            c219:58729           A>G         CAA>CAG      Q>QYAL039C      +      0      DBVPG6304     c219:58729           A>G         CAA>CAG      Q>QYAL039C      +      0      UFRJ50791     c219:58729           A>G         CAA>CAG      Q>QYAL039C      +      0      Q95_3         c219:58747           A>G         AAA>AAG      K>KYAL039C      +      0      KPN3828       c219:58750           A>G         GAA>GAG      E>EYAL039C      +      0      A4            c219:58753           T>A         GTT>GTA      V>VYAL039C      +      0      DBVPG6304     c219:58753           T>A         GTT>GTA      V>VYAL039C      +      0      UFRJ50791     c219:58753           T>A         GTT>GTA      V>VYAL039C      +      0      A4            c219:58762           A>C         ATA>ATC      I>IYAL039C      +      0      DBVPG6304     c219:58762           A>C         ATA>ATC      I>IYAL039C      +      0      IFO1804       c219:58762           A>C         ATA>ATC      I>IYAL039C      +      0      N_45          c219:58762           A>C         ATA>ATC      I>IYAL039C      +      0      UFRJ50791     c219:58762           A>C         ATA>ATC      I>IYAL039C      +      0      UWOPS91_917_1 c219:58762           A>C         ATA>ATC      I>IYAL039C      +      0      UWOPS91_917_1 c219:58841           G>T         GTA>TTA      V>LYAL039C      +      0      UFRJ50791     c219:58858           C>T         GAC>GAT      D>DYAL039C      +      0      UWOPS91_917_1 c219:58910           T>C         TCG>CCG      S>PYAL040C      +      0      DBVPG6304     c219:60124           C>T         ACC>ACT      T>TYAL040C      +      0      YPS138        c219:60124           C>T         ACC>ACT      T>TYAL040C      +      0      DBVPG6304     c219:60166           C>A         GGC>GGA      G>GYAL040C      +      0      YPS138        c219:60166           C>A         GGC>GGA      G>GYAL040C      +      0      DBVPG6304     c219:60172           C>T         TCC>TCT      S>SYAL040C      +      0      YPS138        c219:60172           C>T         TCC>TCT      S>SYAL040C      +      0      YPS138        c219:60181           T>C         ACT>ACC      T>TYAL040C      +      0      DBVPG6304     c219:60229           A>G         CAA>CAG      Q>QYAL040C      +      0      YPS138        c219:60229           A>G         CAA>CAG      Q>QYAL040C      +      0      DBVPG6304     c219:60349           G>A         CGG>CGA      R>RYAL040C      +      0      IFO1804       c219:60349           G>A         CGG>CGA      R>RYAL040C      +      0      N_43          c219:60349           G>A         CGG>CGA      R>RYAL040C      +      0      N_45          c219:60349           G>A         CGG>CGA      R>RYAL040C      +      0      YPS138        c219:60349           G>A         CGG>CGA      R>RYAL040C      +      0      DBVPG6304     c219:60355           C>T         TCC>TCT      S>SYAL040C      +      0      YPS138        c219:60355           C>T         TCC>TCT      S>SYAL040C      +      0      DBVPG6304     c219:60379           C>T         AAC>AAT      N>NYAL040C      +      0      YPS138        c219:60379           C>T         AAC>AAT      N>NYAL040C      +      0      DBVPG6304     c219:60427           C>T         TTC>TTT      F>FYAL040C      +      0      UWOPS91_917_1 c219:60427           C>T         TTC>TTT      F>FYAL040C      +      0      YPS138        c219:60427           C>T         TTC>TTT      F>FYAL040C      +      0      CBS5829       c219:60451           C>T         TAC>TAT      Y>YYAL040C      +      0      N_17          c219:60451           C>T         TAC>TAT      Y>YYAL040C      +      0      Q32_3         c219:60451           C>T         TAC>TAT      Y>YYAL040C      +      0      Q95_3         c219:60451           C>T         TAC>TAT      Y>YYAL040C      +      0      T21_4         c219:60451           C>T         TAC>TAT      Y>YYAL040C      +      0      UWOPS91_917_1 c219:60463           A>G         AGA>AGG      R>RYAL040C      +      0      IFO1804       c219:60500           A>G         ACT>GCT      T>AYAL040C      +      0      DBVPG6304     c219:60502           T>C         ACT>ACC      T>TYAL040C      +      0      UWOPS91_917_1 c219:60523           G>A         TCG>TCA      S>SYAL040C      +      0      DBVPG6304     c219:60535           C>T         ATC>ATT      I>IYAL040C      +      0      DBVPG6304     c219:60583           G>C         TCG>TCC      S>SYAL040C      +      0      DBVPG6304     c219:60589           A>G         AAA>AAG      K>KYAL040C      +      0      UWOPS91_917_1 c219:60683           A>C         ATG>CTG      M>LYAL040C      +      0      DBVPG6304     c219:60703           C>T         CTC>CTT      L>LYAL040C      +      0      UWOPS91_917_1 c219:60703           C>T         CTC>CTT      L>LYAL040C      +      0      UWOPS91_917_1 c219:60712           C>T         TCC>TCT      S>SYAL040C      +      0      N_43          c219:60721           A>G         CAA>CAG      Q>QYAL040C      +      0      N_45          c219:60721           A>G         CAA>CAG      Q>QYAL040C      +      0      UWOPS91_917_1 c219:60721           A>G         CAA>CAG      Q>QYAL040C      +      0      UWOPS91_917_1 c219:60754           T>C         TTT>TTC      F>FYAL040C      +      0      N_43          c219:60766           C>T         TCC>TCT      S>SYAL040C      +      0      N_45          c219:60766           C>T         TCC>TCT      S>SYAL040C      +      0      UWOPS91_917_1 c219:60766           C>T         TCC>TCT      S>SYAL040C      +      0      N_17          c219:60774           C>T         CCG>CTG      P>LYAL040C      +      0      UWOPS91_917_1 c219:60788           G>A         GTT>ATT      V>IYAL040C      +      0      UWOPS91_917_1 c219:60794           C>T         CTT>TTT      L>FYAL040C      +      0      CBS432        c219:60800           T>G         TCC>GCC      S>AYAL040C      +      0      KPN3828       c219:60800           T>G         TCC>GCC      S>AYAL040C      +      0      N_43          c219:60800           T>G         TCC>GCC      S>AYAL040C      +      0      N_45          c219:60800           T>G         TCC>GCC      S>AYAL040C      +      0      Q59_1         c219:60800           T>G         TCC>GCC      S>AYAL040C      +      0      Q62_5         c219:60800           T>G         TCC>GCC      S>AYAL040C      +      0      UWOPS91_917_1 c219:60800           T>G         TCC>GCC      S>AYAL040C      +      0      Y6_5          c219:60800           T>G         TCC>GCC      S>AYAL040C      +      0      Z1_1          c219:60800           T>G         TCC>GCC      S>AYAL040C      +      0      N_45          c219:60835           C>A         GCC>GCA      A>AYAL040C      +      0      N_43          c219:60898           T>C         TTT>TTC      F>FYAL040C      +      0      N_45          c219:60898           T>C         TTT>TTC      F>FYAL040C      +      0      UWOPS91_917_1 c219:60898           T>C         TTT>TTC      F>FYAL040C      +      0      YPS138        c219:60898           T>C         TTT>TTC      F>FYAL040C      +      0      UWOPS91_917_1 c219:60907           C>T         AAC>AAT      N>NYAL040C      +      0      CBS5829       c219:60922           C>T         GGC>GGT      G>GYAL040C      +      0      Q32_3         c219:60922           C>T         GGC>GGT      G>GYAL040C      +      0      Q95_3         c219:60922           C>T         GGC>GGT      G>GYAL040C      +      0      T21_4         c219:60922           C>T         GGC>GGT      G>GYAL040C      +      0      A12           c219:60994           A>G         GCA>GCG      A>AYAL040C      +      0      DBVPG6304     c219:60994           A>G         GCA>GCG      A>AYAL040C      +      0      N_43          c219:60994           A>G         GCA>GCG      A>AYAL040C      +      0      N_45          c219:60994           A>G         GCA>GCG      A>AYAL040C      +      0      UWOPS91_917_1 c219:60994           A>G         GCA>GCG      A>AYAL040C      +      0      YPS138        c219:60994           A>G         GCA>GCG      A>AYAL040C      +      0      A12           c219:61012           C>T         CTC>CTT      L>LYAL040C      +      0      DBVPG6304     c219:61012           C>T         CTC>CTT      L>LYAL040C      +      0      UWOPS91_917_1 c219:61012           C>T         CTC>CTT      L>LYAL040C      +      0      YPS138        c219:61012           C>T         CTC>CTT      L>LYAL040C      +      0      N_43          c219:61015           C>T         ATC>ATT      I>IYAL040C      +      0      N_45          c219:61015           C>T         ATC>ATT      I>IYAL040C      +      0      CBS5829       c219:61065           T>C         CTG>CCG      L>PYAL040C      +      0      UWOPS91_917_1 c219:61079           G>A         GAC>AAC      D>NYAL040C      +      0      UWOPS91_917_1 c219:61085           G>A         GAA>AAA      E>KYAL040C      +      0      N_43          c219:61099           C>T         ATC>ATT      I>IYAL040C      +      0      N_45          c219:61099           C>T         ATC>ATT      I>IYAL040C      +      0      N_43          c219:61114           T>C         ATT>ATC      I>IYAL040C      +      0      N_45          c219:61114           T>C         ATT>ATC      I>IYAL040C      +      0      UWOPS91_917_1 c219:61114           T>C         ATT>ATC      I>IYAL040C      +      0      UWOPS91_917_1 c219:61197           C>T         ACC>ATC      T>IYAL040C      +      0      A12           c219:61210           G>A         AAG>AAA      K>KYAL040C      +      0      DBVPG6304     c219:61210           G>A         AAG>AAA      K>KYAL040C      +      0      N_43          c219:61210           G>A         AAG>AAA      K>KYAL040C      +      0      N_45          c219:61210           G>A         AAG>AAA      K>KYAL040C      +      0      UWOPS91_917_1 c219:61210           G>A         AAG>AAA      K>KYAL040C      +      0      YPS138        c219:61210           G>A         AAG>AAA      K>KYAL040C      +      0      UWOPS91_917_1 c219:61232           A>C         AAC>CAC      N>HYAL040C      +      0      A12           c219:61276           G>A         CAG>CAA      Q>QYAL040C      +      0      DBVPG6304     c219:61276           G>A         CAG>CAA      Q>QYAL040C      +      0      UFRJ50816     c219:61276           G>A         CAG>CAA      Q>QYAL040C      +      0      UWOPS91_917_1 c219:61276           G>A         CAG>CAA      Q>QYAL040C      +      0      YPS138        c219:61276           G>A         CAG>CAA      Q>QYAL040C      +      0      A12           c219:61296           C>G         ACT>AGT      T>SYAL040C      +      0      DBVPG6304     c219:61296           C>G         ACT>AGT      T>SYAL040C      +      0      IFO1804       c219:61296           C>G         ACT>AGT      T>SYAL040C      +      0      N_43          c219:61296           C>G         ACT>AGT      T>SYAL040C      +      0      N_45          c219:61296           C>G         ACT>AGT      T>SYAL040C      +      0      UFRJ50816     c219:61296           C>G         ACT>AGT      T>SYAL040C      +      0      YPS138        c219:61296           C>G         ACT>AGT      T>SYAL040C      +      0      N_45          c219:61315           C>A         TCC>TCA      S>SYAL040C      +      0      A12           c219:61342           T>C         GTT>GTC      V>VYAL040C      +      0      DBVPG6304     c219:61342           T>C         GTT>GTC      V>VYAL040C      +      0      UFRJ50816     c219:61342           T>C         GTT>GTC      V>VYAL040C      +      0      YPS138        c219:61342           T>C         GTT>GTC      V>VYAL040C      +      0      IFO1804       c219:61375           G>T         ACG>ACT      T>TYAL040C      +      0      N_43          c219:61375           G>T         ACG>ACT      T>TYAL040C      +      0      N_45          c219:61375           G>T         ACG>ACT      T>TYAL040C      +      0      A12           c219:61387           A>T         GCA>GCT      A>AYAL040C      +      0      DBVPG6304     c219:61387           A>T         GCA>GCT      A>AYAL040C      +      0      UFRJ50816     c219:61387           A>T         GCA>GCT      A>AYAL040C      +      0      YPS138        c219:61387           A>T         GCA>GCT      A>AYAL040C      +      0      UWOPS91_917_1 c219:61453           C>T         CCC>CCT      P>PYAL040C      +      0      A12           c219:61483           C>T         TCC>TCT      S>SYAL040C      +      0      DBVPG6304     c219:61483           C>T         TCC>TCT      S>SYAL040C      +      0      UFRJ50816     c219:61483           C>T         TCC>TCT      S>SYAL040C      +      0      UWOPS91_917_1 c219:61483           C>T         TCC>TCT      S>SYAL040C      +      0      YPS138        c219:61483           C>T         TCC>TCT      S>SYAL040C      +      0      A12           c219:61492           T>C         TCT>TCC      S>SYAL040C      +      0      IFO1804       c219:61492           T>C         TCT>TCC      S>SYAL040C      +      0      N_43          c219:61492           T>C         TCT>TCC      S>SYAL040C      +      0      N_45          c219:61492           T>C         TCT>TCC      S>SYAL040C      +      0      UFRJ50816     c219:61492           T>C         TCT>TCC      S>SYAL040C      +      0      UWOPS91_917_1 c219:61492           T>C         TCT>TCC      S>SYAL040C      +      0      YPS138        c219:61492           T>C         TCT>TCC      S>SYAL040C      +      0      A12           c219:61504           T>C         TCT>TCC      S>SYAL040C      +      0      UWOPS91_917_1 c219:61573           C>A         TTC>TTA      F>LYAL040C      +      0      A12           c219:61621           T>C         AAT>AAC      N>NYAL040C      +      0      A4            c219:61621           T>C         AAT>AAC      N>NYAL040C      +      0      UFRJ50816     c219:61621           T>C         AAT>AAC      N>NYAL040C      +      0      UWOPS91_917_1 c219:61621           T>C         AAT>AAC      N>NYAL040C      +      0      YPS138        c219:61621           T>C         AAT>AAC      N>NYAL040C      +      0      CBS432        c219:61642           C>T         AGC>AGT      S>SYAL040C      +      0      KPN3829       c219:61642           C>T         AGC>AGT      S>SYAL040C      +      0      Q59_1         c219:61642           C>T         AGC>AGT      S>SYAL040C      +      0      Q89_8         c219:61642           C>T         AGC>AGT      S>SYAL040C      +      0      A12           c219:61645           A>G         CCA>CCG      P>PYAL040C      +      0      A4            c219:61645           A>G         CCA>CCG      P>PYAL040C      +      0      UFRJ50816     c219:61645           A>G         CCA>CCG      P>PYAL040C      +      0      YPS138        c219:61645           A>G         CCA>CCG      P>PYAL040C      +      0      IFO1804       c219:61666           T>G         TCT>TCG      S>SYAL040C      +      0      N_43          c219:61666           T>A         TCT>TCA      S>SYAL040C      +      0      N_45          c219:61666           T>G         TCT>TCG      S>SYAL040C      +      0      IFO1804       c219:61695           A>G         AAT>AGT      N>SYAL040C      +      0      N_43          c219:61695           A>G         AAT>AGT      N>SYAL040C      +      0      N_45          c219:61695           A>G         AAT>AGT      N>SYAL040C      +      0      UFRJ50816     c219:61718           T>G         TTA>GTA      L>VYAL040C      +      0      UWOPS91_917_1 c219:61723           C>T         TTC>TTT      F>FYAL040C      +      0      UWOPS91_917_1 c219:61750           C>T         CAC>CAT      H>HYAL040C      +      0      A4            c219:61761           C>T         GCC>GTC      A>VYAL040C      +      0      UFRJ50816     c219:61761           C>T         GCC>GTC      A>VYAL040C      +      0      YPS138        c219:61761           C>T         GCC>GTC      A>VYAL040C      +      0      A4            c219:61786           G>T         TCG>TCT      S>SYAL040C      +      0      UFRJ50816     c219:61786           G>T         TCG>TCT      S>SYAL040C      +      0      UWOPS91_917_1 c219:61786           G>C         TCG>TCC      S>SYAL040C      +      0      YPS138        c219:61786           G>T         TCG>TCT      S>SYAL041W      -      0      UWOPS91_917_1 c219:62222           C>T         CAT>CAT      H>HYAL041W      -      0      A4            c219:62236           A>G         ACG>ACC      T>TYAL041W      -      0      UFRJ50791     c219:62236           A>G         ACG>ACC      T>TYAL041W      -      0      UFRJ50816     c219:62236           A>G         ACG>ACC      T>TYAL041W      -      0      A4            c219:62326           A>G         AGG>AGC      R>SYAL041W      -      0      UFRJ50791     c219:62326           A>G         AGG>AGC      R>SYAL041W      -      0      UFRJ50816     c219:62326           A>G         AGG>AGC      R>SYAL041W      -      0      A4            c219:62344           T>A         TGA>TGT      *>CYAL041W      -      0      CBS432        c219:62344           T>G         TGA>TGC      *>CYAL041W      -      0      DBVPG4650     c219:62344           T>G         TGA>TGC      *>CYAL041W      -      0      IFO1804       c219:62344           T>G         TGA>TGC      *>CYAL041W      -      0      KPN3829       c219:62344           T>G         TGA>TGC      *>CYAL041W      -      0      N_44          c219:62344           T>G         TGA>TGC      *>CYAL041W      -      0      N_45          c219:62344           T>G         TGA>TGC      *>CYAL041W      -      0      Q32_3         c219:62344           T>G         TGA>TGC      *>CYAL041W      -      0      UFRJ50791     c219:62344           T>A         TGA>TGT      *>CYAL041W      -      0      UFRJ50816     c219:62344           T>A         TGA>TGT      *>CYAL041W      -      0      YPS138        c219:62344           T>A         TGA>TGT      *>CYAL041W      -      0      UFRJ50791     c219:62358           C>T         TAT>AAT      Y>NYAL041W      -      0      UFRJ50816     c219:62358           C>T         TAT>AAT      Y>NYAL041W      -      0      UWOPS91_917_1 c219:62358           C>T         TAT>AAT      Y>NYAL041W      -      0      YPS138        c219:62358           C>T         TAT>AAT      Y>NYAL041W      -      0      UFRJ50791     c219:62400           C>T         GAG>AAG      E>KYAL041W      -      0      UFRJ50816     c219:62400           C>T         GAG>AAG      E>KYAL041W      -      0      YPS138        c219:62400           C>T         GAG>AAG      E>KYAL041W      -      0      YPS138        c219:62431           T>A         GCT>GCT      A>AYAL041W      -      0      IFO1804       c219:62452           G>A         TTG>TTT      L>FYAL041W      -      0      N_43          c219:62452           G>A         TTG>TTT      L>FYAL041W      -      0      N_44          c219:62452           G>A         TTG>TTT      L>FYAL041W      -      0      N_45          c219:62452           G>A         TTG>TTT      L>FYAL041W      -      0      A4            c219:62465           C>T         AAG>AAG      K>KYAL041W      -      0      UFRJ50791     c219:62465           C>T         AAG>AAG      K>KYAL041W      -      0      UFRJ50816     c219:62465           C>T         AAG>AAG      K>KYAL041W      -      0      YPS138        c219:62465           C>T         AAG>AAG      K>KYAL041W      -      0      A4            c219:62497           G>A         CGA>CGT      R>RYAL041W      -      0      UFRJ50791     c219:62497           G>A         CGA>CGT      R>RYAL041W      -      0      UFRJ50816     c219:62497           G>A         CGA>CGT      R>RYAL041W      -      0      YPS138        c219:62497           G>A         CGA>CGT      R>RYAL041W      -      0      UFRJ50791     c219:62545           T>C         CGA>CGG      R>RYAL041W      -      0      UFRJ50816     c219:62545           T>C         CGA>CGG      R>RYAL041W      -      0      YPS138        c219:62545           T>C         CGA>CGG      R>RYAL041W      -      0      IFO1804       c219:62548           A>G         ACT>ACC      T>TYAL041W      -      0      N_43          c219:62548           A>G         ACT>ACC      T>TYAL041W      -      0      N_44          c219:62548           A>G         ACT>ACC      T>TYAL041W      -      0      N_45          c219:62548           A>G         ACT>ACC      T>TYAL041W      -      0      A4            c219:62566           C>T         GGT>GGA      G>GYAL041W      -      0      UFRJ50791     c219:62566           C>T         GGT>GGA      G>GYAL041W      -      0      UFRJ50816     c219:62566           C>T         GGT>GGA      G>GYAL041W      -      0      YPS138        c219:62566           C>T         GGT>GGA      G>GYAL041W      -      0      A4            c219:62647           T>C         AGT>AGG      S>RYAL041W      -      0      UFRJ50816     c219:62647           T>C         AGT>AGG      S>RYAL041W      -      0      YPS138        c219:62647           T>C         AGT>AGG      S>RYAL041W      -      0      A4            c219:62653           G>A         ACT>ACT      T>TYAL041W      -      0      UFRJ50816     c219:62653           G>A         ACT>ACT      T>TYAL041W      -      0      YPS138        c219:62653           G>A         ACT>ACT      T>TYAL041W      -      0      A4            c219:62725           T>C         TGC>TGG      C>WYAL041W      -      0      UFRJ50816     c219:62725           T>C         TGC>TGG      C>WYAL041W      -      0      YPS138        c219:62725           T>C         TGC>TGG      C>WYAL041W      -      0      A4            c219:62734           C>A         ACA>ACT      T>TYAL041W      -      0      UFRJ50816     c219:62734           C>A         ACA>ACT      T>TYAL041W      -      0      YPS138        c219:62734           C>A         ACA>ACT      T>TYAL041W      -      0      A4            c219:62752           C>T         GAC>GAA      D>EYAL041W      -      0      UFRJ50816     c219:62752           C>T         GAC>GAA      D>EYAL041W      -      0      YPS138        c219:62752           C>T         GAC>GAA      D>EYAL041W      -      0      A4            c219:62767           T>C         TTT>TTG      F>LYAL041W      -      0      UFRJ50816     c219:62767           T>C         TTT>TTG      F>LYAL041W      -      0      YPS138        c219:62767           T>C         TTT>TTG      F>LYAL041W      -      0      IFO1804       c219:62773           C>T         TGG>TGA      W>*YAL041W      -      0      N_43          c219:62773           C>T         TGG>TGA      W>*YAL041W      -      0      N_44          c219:62773           C>T         TGG>TGA      W>*YAL041W      -      0      N_45          c219:62773           C>T         TGG>TGA      W>*YAL041W      -      0      A4            c219:62791           T>C         ATC>ATG      I>MYAL041W      -      0      IFO1804       c219:62791           T>C         ATC>ATG      I>MYAL041W      -      0      N_43          c219:62791           T>C         ATC>ATG      I>MYAL041W      -      0      N_44          c219:62791           T>C         ATC>ATG      I>MYAL041W      -      0      N_45          c219:62791           T>C         ATC>ATG      I>MYAL041W      -      0      UFRJ50816     c219:62791           T>C         ATC>ATG      I>MYAL041W      -      0      YPS138        c219:62791           T>C         ATC>ATG      I>MYAL041W      -      0      A4            c219:62896           G>A         GTT>GTT      V>VYAL041W      -      0      UFRJ50816     c219:62896           G>A         GTT>GTT      V>VYAL041W      -      0      YPS138        c219:62896           G>A         GTT>GTT      V>VYAL041W      -      0      A4            c219:62905           C>T         TGA>TGA      *>*YAL041W      -      0      UFRJ50816     c219:62905           C>T         TGA>TGA      *>*YAL041W      -      0      YPS138        c219:62905           C>T         TGA>TGA      *>*YAL041W      -      0      A4            c219:62911           T>G         TAG>TAC      *>YYAL041W      -      0      IFO1804       c219:62911           T>G         TAG>TAC      *>YYAL041W      -      0      N_43          c219:62911           T>G         TAG>TAC      *>YYAL041W      -      0      N_44          c219:62911           T>G         TAG>TAC      *>YYAL041W      -      0      UFRJ50816     c219:62911           T>G         TAG>TAC      *>YYAL041W      -      0      YPS138        c219:62911           T>G         TAG>TAC      *>YYAL041W      -      0      A4            c219:63029           C>G         CTG>CCG      L>PYAL041W      -      0      UFRJ50816     c219:63029           C>G         CTG>CCG      L>PYAL041W      -      0      YPS138        c219:63029           C>G         CTG>CCG      L>PYAL041W      -      0      A4            c219:63178           A>C         CAT>CAG      H>QYAL041W      -      0      YPS138        c219:63178           A>C         CAT>CAG      H>QYAL041W      -      0      IFO1804       c219:63298           C>T         CTT>CTA      L>LYAL041W      -      0      N_43          c219:63298           C>T         CTT>CTA      L>LYAL041W      -      0      N_44          c219:63298           C>T         CTT>CTA      L>LYAL041W      -      0      IFO1804       c219:63340           G>A         ATT>ATT      I>IYAL041W      -      0      N_43          c219:63340           G>A         ATT>ATT      I>IYAL041W      -      0      N_44          c219:63340           G>A         ATT>ATT      I>IYAL041W      -      0      DBVPG6304     c219:63370           C>T         TAA>TAA      *>*YAL041W      -      0      IFO1804       c219:63376           C>T         CTT>CTA      L>LYAL041W      -      0      N_43          c219:63376           C>T         CTT>CTA      L>LYAL041W      -      0      N_44          c219:63376           C>T         CTT>CTA      L>LYAL041W      -      0      DBVPG6304     c219:63397           C>T         CTG>CTA      L>LYAL041W      -      0      YPS138        c219:63412           T>C         GTT>GTG      V>VYAL041W      -      0      DBVPG6304     c219:63442           T>C         ATA>ATG      I>MYAL041W      -      0      UFRJ50816     c219:63442           T>C         ATA>ATG      I>MYAL041W      -      0      YPS138        c219:63442           T>C         ATA>ATG      I>MYAL041W      -      0      DBVPG6304     c219:63511           C>T         ATC>ATA      I>IYAL041W      -      0      YPS138        c219:63511           C>T         ATC>ATA      I>IYAL041W      -      0      DBVPG6304     c219:63556           G>T         ATT>ATA      I>IYAL041W      -      0      DBVPG6304     c219:63595           A>G         TTC>TTC      F>FYAL041W      -      0      DBVPG6304     c219:63628           C>T         CCT>CCA      P>PYAL041W      -      0      Q62_5         c219:63707           T>A         CGT>CTT      R>LYAL041W      -      0      DBVPG6304     c219:63724           G>A         AGG>AGT      R>SYAL041W      -      0      IFO1804       c219:63757           A>G         TTT>TTC      F>FYAL041W      -      0      N_43          c219:63757           A>G         TTT>TTC      F>FYAL041W      -      0      N_44          c219:63757           A>G         TTT>TTC      F>FYAL041W      -      0      DBVPG6304     c219:63793           G>A         TTT>TTT      F>FYAL041W      -      0      CBS5829       c219:63805           A>G         CGA>CGC      R>RYAL041W      -      0      N_17          c219:63805           A>G         CGA>CGC      R>RYAL041W      -      0      Q95_3         c219:63805           A>G         CGA>CGC      R>RYAL041W      -      0      DBVPG6304     c219:63817           G>C         CTT>CTG      L>LYAL041W      -      0      CBS432        c219:63856           A>G         ATT>ATC      I>IYAL041W      -      0      CBS5829       c219:63856           A>G         ATT>ATC      I>IYAL041W      -      0      DBVPG6304     c219:63856           A>G         ATT>ATC      I>IYAL041W      -      0      IFO1804       c219:63856           A>G         ATT>ATC      I>IYAL041W      -      0      N_17          c219:63856           A>G         ATT>ATC      I>IYAL041W      -      0      N_43          c219:63856           A>G         ATT>ATC      I>IYAL041W      -      0      N_44          c219:63856           A>G         ATT>ATC      I>IYAL041W      -      0      N_45          c219:63856           A>G         ATT>ATC      I>IYAL041W      -      0      Q32_3         c219:63856           A>G         ATT>ATC      I>IYAL041W      -      0      Q95_3         c219:63856           A>G         ATT>ATC      I>IYAL041W      -      0      T21_4         c219:63856           A>G         ATT>ATC      I>IYAL041W      -      0      DBVPG6304     c219:63871           C>T         ATT>ATA      I>IYAL041W      -      0      IFO1804       c219:63871           C>T         ATT>ATA      I>IYAL041W      -      0      N_43          c219:63871           C>T         ATT>ATA      I>IYAL041W      -      0      N_44          c219:63871           C>T         ATT>ATA      I>IYAL041W      -      0      N_45          c219:63871           C>T         ATT>ATA      I>IYAL041W      -      0      DBVPG6304     c219:63919           C>T         GGT>GGA      G>GYAL041W      -      0      N_45          c219:63982           C>T         GCT>GCA      A>AYAL041W      -      0      N_43          c219:64051           G>A         CAG>CAT      Q>HYAL041W      -      0      N_45          c219:64051           G>A         CAG>CAT      Q>HYAL041W      -      0      DBVPG6304     c219:64053           A>T         CAG>AAG      Q>KYAL041W      -      0      DBVPG6304     c219:64075           C>T         TAT>TAA      Y>*YAL041W      -      0      CBS432        c219:64083           A>G         ATT>CTT      I>LYAL041W      -      0      CBS5829       c219:64083           A>G         ATT>CTT      I>LYAL041W      -      0      N_17          c219:64083           A>G         ATT>CTT      I>LYAL041W      -      0      N_43          c219:64083           A>G         ATT>CTT      I>LYAL041W      -      0      N_45          c219:64083           A>G         ATT>CTT      I>LYAL041W      -      0      Q32_3         c219:64083           A>G         ATT>CTT      I>LYAL041W      -      0      Q95_3         c219:64083           A>G         ATT>CTT      I>LYAL041W      -      0      T21_4         c219:64083           A>G         ATT>CTT      I>LYAL041W      -      0      Y7            c219:64083           A>G         ATT>CTT      I>LYAL041W      -      0      N_43          c219:64099           T>G         CGG>CGC      R>RYAL041W      -      0      N_45          c219:64099           T>G         CGG>CGC      R>RYAL041W      -      0      N_43          c219:64119           C>T         TAA>AAA      *>KYAL041W      -      0      N_45          c219:64119           C>T         TAA>AAA      *>KYAL041W      -      0      N_43          c219:64174           C>T         ATT>ATA      I>IYAL041W      -      0      N_45          c219:64174           C>T         ATT>ATA      I>IYAL041W      -      0      DBVPG6304     c219:64183           A>G         TGT>TGC      C>CYAL041W      -      0      DBVPG6304     c219:64300           T>C         TGA>TGG      *>WYAL041W      -      0      N_43          c219:64300           T>C         TGA>TGG      *>WYAL041W      -      0      N_45          c219:64300           T>C         TGA>TGG      *>WYAL041W      -      0      CBS5829       c219:64357           A>G         TGT>TGC      C>CYAL041W      -      0      Q95_3         c219:64357           A>G         TGT>TGC      C>CYAL041W      -      0      Y7            c219:64357           A>G         TGT>TGC      C>CYAL041W      -      0      DBVPG6304     c219:64388           A>G         AGA>ACA      R>TYAL041W      -      0      IFO1804       c219:64388           A>G         AGA>ACA      R>TYAL041W      -      0      N_43          c219:64388           A>G         AGA>ACA      R>TYAL041W      -      0      N_45          c219:64388           A>G         AGA>ACA      R>TYAL041W      -      0      A4            c219:64417           A>T         AGG>AGA      R>RYAL041W      -      0      A4            c219:64438           G>A         CGA>CGT      R>RYAL041W      -      0      IFO1804       c219:64499           G>C         AGA>AGA      R>RYAL041W      -      0      N_45          c219:64499           G>C         AGA>AGA      R>RYAL041W      -      0      A4            c219:64525           A>G         ATT>ATC      I>IYAL041W      -      0      A4            c219:64596           A>G         GTC>CTC      V>LYAL041W      -      0      A4            c219:64615           C>T         ATT>ATA      I>IYAL041W      -      0      A4            c219:64642           G>T         GTT>GTA      V>VYAL041W      -      0      A4            c219:64654           A>G         TGG>TGC      W>CYAL042W      -      0      IFO1804       c219:65055           C>T         CTT>CTA      L>LYAL042W      -      0      N_43          c219:65055           C>T         CTT>CTA      L>LYAL042W      -      0      N_44          c219:65055           C>T         CTT>CTA      L>LYAL042W      -      0      N_45          c219:65055           C>T         CTT>CTA      L>LYAL042W      -      0      CBS432        c219:65067           A>T         CAA>CAA      Q>QYAL042W      -      0      IFO1804       c219:65088           C>T         AGC>AGA      S>RYAL042W      -      0      N_43          c219:65088           C>T         AGC>AGA      S>RYAL042W      -      0      N_44          c219:65088           C>T         AGC>AGA      S>RYAL042W      -      0      N_45          c219:65088           C>T         AGC>AGA      S>RYAL042W      -      0      A4            c219:65145           G>A         CGA>CGT      R>RYAL042W      -      0      YPS138        c219:65145           G>A         CGA>CGT      R>RYAL042W      -      0      A4            c219:65166           G>A         GAA>GAT      E>DYAL042W      -      0      YPS138        c219:65166           G>A         GAA>GAT      E>DYAL042W      -      0      DBVPG6304     c219:65238           A>G         TCG>TCC      S>SYAL042W      -      0      YPS138        c219:65238           A>G         TCG>TCC      S>SYAL042W      -      0      N_43          c219:65280           G>A         TGA>TGT      *>CYAL042W      -      0      N_44          c219:65280           G>A         TGA>TGT      *>CYAL042W      -      0      N_45          c219:65280           G>A         TGA>TGT      *>CYAL042W      -      0      DBVPG6304     c219:65286           T>C         CGG>CGG      R>RYAL042W      -      0      UWOPS91_917_1 c219:65286           T>C         CGG>CGG      R>RYAL042W      -      0      YPS138        c219:65286           T>C         CGG>CGG      R>RYAL042W      -      0      DBVPG6304     c219:65355           G>A         CCC>CCT      P>PYAL042W      -      0      YPS138        c219:65355           G>A         CCC>CCT      P>PYAL042W      -      0      DBVPG6304     c219:65385           G>A         GCC>GCT      A>AYAL042W      -      0      UWOPS91_917_1 c219:65385           G>A         GCC>GCT      A>AYAL042W      -      0      YPS138        c219:65385           G>A         GCC>GCT      A>AYAL042W      -      0      UWOPS91_917_1 c219:65412           A>G         TAA>TAC      *>YYAL042W      -      0      UWOPS91_917_1 c219:65418           G>A         CAC>CAT      H>HYAL042W      -      0      N_43          c219:65493           C>T         ATC>ATA      I>IYAL042W      -      0      N_44          c219:65493           C>T         ATC>ATA      I>IYAL042W      -      0      N_45          c219:65493           C>T         ATC>ATA      I>IYAL042W      -      0      DBVPG6304     c219:65501           G>A         CGA>TGA      R>*YAL042W      -      0      YPS138        c219:65501           G>A         CGA>TGA      R>*YAL042W      -      0      N_43          c219:65505           A>G         ATT>ATC      I>IYAL042W      -      0      N_44          c219:65505           A>G         ATT>ATC      I>IYAL042W      -      0      N_45          c219:65505           A>G         ATT>ATC      I>IYAL042W      -      0      UWOPS91_917_1 c219:65564           G>A         AGA>TGA      R>*YAL042W      -      0      DBVPG6304     c219:65589           A>G         AAA>AAC      K>NYAL042W      -      0      YPS138        c219:65589           A>G         AAA>AAC      K>NYAL042W      -      0      UWOPS91_917_1 c219:65598           T>C         CCC>CCG      P>PYAL042W      -      0      DBVPG6304     c219:65618           G>A         ACA>TCA      T>SYAL042W      -      0      UWOPS91_917_1 c219:65625           A>G         CTC>CTC      L>LYAL042W      -      0      DBVPG6304     c219:65658           A>G         ATT>ATC      I>IYAL042W      -      0      N_43          c219:65658           A>G         ATT>ATC      I>IYAL042W      -      0      N_45          c219:65658           A>G         ATT>ATC      I>IYAL042W      -      0      UFRJ50816     c219:65658           A>G         ATT>ATC      I>IYAL042W      -      0      UWOPS91_917_1 c219:65658           A>G         ATT>ATC      I>IYAL042W      -      0      YPS138        c219:65658           A>G         ATT>ATC      I>IYAL042W      -      0      DBVPG6304     c219:65664           C>T         GCC>GCA      A>AYAL042W      -      0      UFRJ50816     c219:65664           C>T         GCC>GCA      A>AYAL042W      -      0      UWOPS91_917_1 c219:65664           C>T         GCC>GCA      A>AYAL042W      -      0      YPS138        c219:65664           C>T         GCC>GCA      A>AYAL042W      -      0      DBVPG6304     c219:65688           C>T         CTG>CTA      L>LYAL042W      -      0      UFRJ50816     c219:65688           C>T         CTG>CTA      L>LYAL042W      -      0      UWOPS91_917_1 c219:65688           C>T         CTG>CTA      L>LYAL042W      -      0      YPS138        c219:65688           C>T         CTG>CTA      L>LYAL042W      -      0      DBVPG6304     c219:65730           T>C         TTT>TTG      F>LYAL042W      -      0      YPS138        c219:65730           T>C         TTT>TTG      F>LYAL042W      -      0      UWOPS91_917_1 c219:65748           C>T         ATA>ATA      I>IYAL042W      -      0      DBVPG6304     c219:65793           T>C         CAG>CAG      Q>QYAL042W      -      0      UFRJ50816     c219:65793           T>C         CAG>CAG      Q>QYAL042W      -      0      YPS138        c219:65793           T>C         CAG>CAG      Q>QYAL042W      -      0      DBVPG6304     c219:65817           C>A         ATG>ATT      M>IYAL042W      -      0      UFRJ50816     c219:65817           C>A         ATG>ATT      M>IYAL042W      -      0      YPS138        c219:65817           C>A         ATG>ATT      M>IYAL042W      -      0      A4            c219:65895           G>A         GGC>GGT      G>GYAL042W      -      0      DBVPG6304     c219:65895           G>A         GGC>GGT      G>GYAL042W      -      0      UFRJ50816     c219:65895           G>A         GGC>GGT      G>GYAL042W      -      0      YPS138        c219:65895           G>A         GGC>GGT      G>GYAL042W      -      0      N_43          c219:65910           G>A         GTC>GTT      V>VYAL042W      -      0      A4            c219:65921           T>C         TTG>GTG      L>VYAL042W      -      0      DBVPG6304     c219:65921           T>C         TTG>GTG      L>VYAL042W      -      0      UFRJ50816     c219:65921           T>C         TTG>GTG      L>VYAL042W      -      0      UWOPS91_917_1 c219:65921           T>C         TTG>GTG      L>VYAL042W      -      0      YPS138        c219:65921           T>C         TTG>GTG      L>VYAL042W      -      0      DBVPG6304     c219:65931           C>A         GTC>GTT      V>VYAL042W      -      0      UFRJ50816     c219:65931           C>A         GTC>GTT      V>VYAL042W      -      0      YPS138        c219:65931           C>A         GTC>GTT      V>VYAL042W      -      0      A4            c219:65947           A>G         GAA>GCA      E>AYAL042W      -      0      DBVPG4650     c219:65947           A>G         GAA>GCA      E>AYAL042W      -      0      DBVPG6304     c219:65947           A>G         GAA>GCA      E>AYAL042W      -      0      KPN3829       c219:65947           A>G         GAA>GCA      E>AYAL042W      -      0      N_43          c219:65947           A>G         GAA>GCA      E>AYAL042W      -      0      Q32_3         c219:65947           A>G         GAA>GCA      E>AYAL042W      -      0      S36_7         c219:65947           A>G         GAA>GCA      E>AYAL042W      -      0      UFRJ50816     c219:65947           A>G         GAA>GCA      E>AYAL042W      -      0      UWOPS91_917_1 c219:65947           A>G         GAA>GCA      E>AYAL042W      -      0      YPS138        c219:65947           A>G         GAA>GCA      E>AYAL042W      -      0      UFRJ50816     c219:65971           C>T         CTT>CAT      L>HYAL042W      -      0      A4            c219:66039           G>A         ATA>ATT      I>IYAL042W      -      0      DBVPG6304     c219:66039           G>A         ATA>ATT      I>IYAL042W      -      0      UFRJ50816     c219:66039           G>A         ATA>ATT      I>IYAL042W      -      0      YPS138        c219:66039           G>A         ATA>ATT      I>IYAL042W      -      0      A4            c219:66057           G>A         GAT>GAT      D>DYAL042W      -      0      DBVPG6304     c219:66057           G>A         GAT>GAT      D>DYAL042W      -      0      UFRJ50816     c219:66057           G>A         GAT>GAT      D>DYAL042W      -      0      YPS138        c219:66057           G>A         GAT>GAT      D>DYAL042W      -      0      DBVPG6304     c219:66083           C>T         TGG>AGG      W>RYAL042W      -      0      UFRJ50791     c219:66083           C>T         TGG>AGG      W>RYAL042W      -      0      UFRJ50816     c219:66083           C>T         TGG>AGG      W>RYAL042W      -      0      YPS138        c219:66083           C>T         TGG>AGG      W>RYAL042W      -      0      A12           c219:66099           C>T         CCT>CCA      P>PYAL042W      -      0      A4            c219:66113           G>T         AGG>AGG      R>RYAL042W      -      0      DBVPG6304     c219:66113           G>T         AGG>AGG      R>RYAL042W      -      0      YPS138        c219:66113           G>T         AGG>AGG      R>RYAL042W      -      0      CBS432        c219:66123           T>C         CAC>CAG      H>QYAL042W      -      0      CBS5829       c219:66123           T>C         CAC>CAG      H>QYAL042W      -      0      DBVPG4650     c219:66123           T>C         CAC>CAG      H>QYAL042W      -      0      KPN3829       c219:66123           T>C         CAC>CAG      H>QYAL042W      -      0      N_17          c219:66123           T>C         CAC>CAG      H>QYAL042W      -      0      N_43          c219:66123           T>C         CAC>CAG      H>QYAL042W      -      0      N_45          c219:66123           T>C         CAC>CAG      H>QYAL042W      -      0      Q32_3         c219:66123           T>C         CAC>CAG      H>QYAL042W      -      0      Q59_1         c219:66123           T>C         CAC>CAG      H>QYAL042W      -      0      Q89_8         c219:66123           T>C         CAC>CAG      H>QYAL042W      -      0      Q95_3         c219:66123           T>C         CAC>CAG      H>QYAL042W      -      0      S36_7         c219:66123           T>C         CAC>CAG      H>QYAL042W      -      0      Y7            c219:66123           T>C         CAC>CAG      H>QYAL042W      -      0      A12           c219:66138           T>C         AGA>AGG      R>RYAL042W      -      0      A4            c219:66138           T>C         AGA>AGG      R>RYAL042W      -      0      DBVPG6304     c219:66138           T>C         AGA>AGG      R>RYAL042W      -      0      UFRJ50791     c219:66138           T>C         AGA>AGG      R>RYAL042W      -      0      UFRJ50816     c219:66138           T>C         AGA>AGG      R>RYAL042W      -      0      YPS138        c219:66138           T>C         AGA>AGG      R>RYAL042W      -      0      A12           c219:66147           C>T         TTT>TTA      F>LYAL042W      -      0      A4            c219:66147           C>T         TTT>TTA      F>LYAL042W      -      0      DBVPG6304     c219:66147           C>T         TTT>TTA      F>LYAL042W      -      0      UFRJ50791     c219:66147           C>T         TTT>TTA      F>LYAL042W      -      0      UFRJ50816     c219:66147           C>T         TTT>TTA      F>LYAL042W      -      0      YPS138        c219:66147           C>T         TTT>TTA      F>LYAL042W      -      0      A12           c219:66153           G>A         GAT>GAT      D>DYAL042W      -      0      A4            c219:66153           G>A         GAT>GAT      D>DYAL042W      -      0      DBVPG6304     c219:66153           G>A         GAT>GAT      D>DYAL042W      -      0      UFRJ50791     c219:66153           G>A         GAT>GAT      D>DYAL042W      -      0      UFRJ50816     c219:66153           G>A         GAT>GAT      D>DYAL042W      -      0      YPS138        c219:66153           G>A         GAT>GAT      D>DYAL042W      -      0      N_43          c219:66168           C>T         GTG>GTA      V>VYAL042W      -      0      YPS138        c219:66189           C>A         GAA>GAT      E>DYAL042W      -      0      A12           c219:66198           G>A         ATT>ATT      I>IYAL042W      -      0      A4            c219:66198           G>A         ATT>ATT      I>IYAL042W      -      0      DBVPG6304     c219:66198           G>A         ATT>ATT      I>IYAL042W      -      0      UFRJ50791     c219:66198           G>A         ATT>ATT      I>IYAL042W      -      0      UFRJ50816     c219:66198           G>A         ATT>ATT      I>IYAL042W      -      0      YPS138        c219:66198           G>A         ATT>ATT      I>IYAL042W      -      0      A12           c219:66242           G>T         GAC>AAC      D>NYAL042W      -      0      A4            c219:66242           G>T         GAC>AAC      D>NYAL042W      -      0      DBVPG6304     c219:66242           G>T         GAC>AAC      D>NYAL042W      -      0      UFRJ50791     c219:66242           G>T         GAC>AAC      D>NYAL042W      -      0      UFRJ50816     c219:66242           G>T         GAC>AAC      D>NYAL042W      -      0      YPS138        c219:66242           G>T         GAC>AAC      D>NYAL042W      -      0      YPS138        c219:66255           G>A         GAA>GAT      E>DYAL042W      -      0      IFO1804       c219:66281           G>A         CCA>TCA      P>SYAL042W      -      0      N_43          c219:66281           G>A         CCA>TCA      P>SYAL043C      +      0      A12           c219:66568           T>C         TCT>TCC      S>SYAL043C      +      0      DBVPG6304     c219:66568           T>C         TCT>TCC      S>SYAL043C      +      0      UFRJ50791     c219:66568           T>C         TCT>TCC      S>SYAL043C      +      0      YPS138        c219:66568           T>C         TCT>TCC      S>SYAL043C      +      0      A12           c219:66616           T>C         AAT>AAC      N>NYAL043C      +      0      DBVPG6304     c219:66616           T>C         AAT>AAC      N>NYAL043C      +      0      IFO1804       c219:66616           T>C         AAT>AAC      N>NYAL043C      +      0      N_43          c219:66616           T>C         AAT>AAC      N>NYAL043C      +      0      N_44          c219:66616           T>C         AAT>AAC      N>NYAL043C      +      0      N_45          c219:66616           T>C         AAT>AAC      N>NYAL043C      +      0      UFRJ50791     c219:66616           T>C         AAT>AAC      N>NYAL043C      +      0      YPS138        c219:66616           T>C         AAT>AAC      N>NYAL043C      +      0      CBS432        c219:66652           C>T         ACC>ACT      T>TYAL043C      +      0      KPN3828       c219:66652           C>T         ACC>ACT      T>TYAL043C      +      0      KPN3829       c219:66652           C>T         ACC>ACT      T>TYAL043C      +      0      Q32_3         c219:66652           C>T         ACC>ACT      T>TYAL043C      +      0      A12           c219:66689           C>T         CTG>TTG      L>LYAL043C      +      0      DBVPG6304     c219:66689           C>T         CTG>TTG      L>LYAL043C      +      0      UFRJ50791     c219:66689           C>T         CTG>TTG      L>LYAL043C      +      0      YPS138        c219:66689           C>T         CTG>TTG      L>LYAL043C      +      0      UWOPS91_917_1 c219:66698           C>T         CCT>TCT      P>SYAL043C      +      0      A12           c219:66724           G>A         TTG>TTA      L>LYAL043C      +      0      DBVPG6304     c219:66724           G>A         TTG>TTA      L>LYAL043C      +      0      YPS138        c219:66724           G>A         TTG>TTA      L>LYAL043C      +      0      YPS138        c219:66934           G>A         CTG>CTA      L>LYAL043C      +      0      DBVPG6304     c219:66949           G>T         AAG>AAT      K>NYAL043C      +      0      UFRJ50791     c219:66949           G>T         AAG>AAT      K>NYAL043C      +      0      YPS138        c219:66949           G>T         AAG>AAT      K>NYAL043C      +      0      DBVPG6304     c219:67000           C>T         GAC>GAT      D>DYAL043C      +      0      UFRJ50791     c219:67000           C>T         GAC>GAT      D>DYAL043C      +      0      YPS138        c219:67000           C>T         GAC>GAT      D>DYAL043C      +      0      UWOPS91_917_1 c219:67006           A>G         GAA>GAG      E>EYAL043C      +      0      DBVPG6304     c219:67017           A>G         GAG>GGG      E>GYAL043C      +      0      UFRJ50791     c219:67017           A>G         GAG>GGG      E>GYAL043C      +      0      UWOPS91_917_1 c219:67017           A>G         GAG>GGG      E>GYAL043C      +      0      YPS138        c219:67017           A>G         GAG>GGG      E>GYAL043C      +      0      YPS138        c219:67042           G>A         CAG>CAA      Q>QYAL043C      +      0      DBVPG6304     c219:67054           G>A         GTG>GTA      V>VYAL043C      +      0      UFRJ50791     c219:67054           G>A         GTG>GTA      V>VYAL043C      +      0      YPS138        c219:67054           G>A         GTG>GTA      V>VYAL043C      +      0      DBVPG6304     c219:67085           G>A         GTC>ATC      V>IYAL043C      +      0      IFO1804       c219:67085           G>A         GTC>ATC      V>IYAL043C      +      0      N_43          c219:67085           G>A         GTC>ATC      V>IYAL043C      +      0      N_44          c219:67085           G>A         GTC>ATC      V>IYAL043C      +      0      N_45          c219:67085           G>A         GTC>ATC      V>IYAL043C      +      0      UFRJ50791     c219:67085           G>A         GTC>ATC      V>IYAL043C      +      0      UWOPS91_917_1 c219:67085           G>A         GTC>ATC      V>IYAL043C      +      0      YPS138        c219:67085           G>A         GTC>ATC      V>IYAL043C      +      0      DBVPG6304     c219:67102           C>G         ACC>ACG      T>TYAL043C      +      0      UWOPS91_917_1 c219:67111           C>G         TCC>TCG      S>SYAL043C      +      0      UWOPS91_917_1 c219:67172           G>A         GCC>ACC      A>TYAL043C      +      0      DBVPG6304     c219:67194           G>A         AGG>AAG      R>KYAL043C      +      0      UFRJ50791     c219:67194           G>A         AGG>AAG      R>KYAL043C      +      0      YPS138        c219:67194           G>A         AGG>AAG      R>KYAL043C      +      0      DBVPG6304     c219:67246           G>T         TCG>TCT      S>SYAL043C      +      0      UFRJ50791     c219:67246           G>T         TCG>TCT      S>SYAL043C      +      0      YPS138        c219:67246           G>T         TCG>TCT      S>SYAL043C      +      0      UWOPS91_917_1 c219:67255           C>T         TTC>TTT      F>FYAL043C      +      0      UWOPS91_917_1 c219:67267           C>A         ATC>ATA      I>IYAL043C      +      0      UWOPS91_917_1 c219:67285           C>A         GTC>GTA      V>VYAL043C      +      0      UWOPS91_917_1 c219:67302           A>G         CAG>CGG      Q>RYAL043C      +      0      DBVPG6304     c219:67336           T>A         CGT>CGA      R>RYAL043C      +      0      UWOPS91_917_1 c219:67336           T>G         CGT>CGG      R>RYAL043C      +      0      UWOPS91_917_1 c219:67361           C>T         CTA>TTA      L>LYAL043C      +      0      DBVPG6304     c219:67369           A>C         GGA>GGC      G>GYAL043C      +      0      UFRJ50791     c219:67369           A>C         GGA>GGC      G>GYAL043C      +      0      UWOPS91_917_1 c219:67369           A>C         GGA>GGC      G>GYAL043C      +      0      CBS432        c219:67390           A>G         AAA>AAG      K>KYAL043C      +      0      CBS5829       c219:67390           A>G         AAA>AAG      K>KYAL043C      +      0      Q62_5         c219:67390           A>G         AAA>AAG      K>KYAL043C      +      0      T21_4         c219:67390           A>G         AAA>AAG      K>KYAL043C      +      0      Y7            c219:67390           A>G         AAA>AAG      K>KYAL043C      +      0      CBS432        c219:67417           G>C         GCG>GCC      A>AYAL043C      +      0      CBS5829       c219:67417           G>C         GCG>GCC      A>AYAL043C      +      0      Q62_5         c219:67417           G>C         GCG>GCC      A>AYAL043C      +      0      T21_4         c219:67417           G>C         GCG>GCC      A>AYAL043C      +      0      Y7            c219:67417           G>C         GCG>GCC      A>AYAL043C      +      0      A4            c219:67442           T>C         TTG>CTG      L>LYAL043C      +      0      DBVPG6304     c219:67442           T>C         TTG>CTG      L>LYAL043C      +      0      A4            c219:67450           T>C         AAT>AAC      N>NYAL043C      +      0      DBVPG6304     c219:67450           T>C         AAT>AAC      N>NYAL043C      +      0      A4            c219:67483           A>G         TCA>TCG      S>SYAL043C      +      0      DBVPG6304     c219:67483           A>G         TCA>TCG      S>SYAL043C      +      0      A4            c219:67507           C>G         CTC>CTG      L>LYAL043C      +      0      A4            c219:67543           G>A         GAG>GAA      E>EYAL043C      +      0      N_45          c219:67654           A>T         CTA>CTT      L>LYAL043C      +      0      A4            c219:67666           A>G         CAA>CAG      Q>QYAL043C      +      0      A4            c219:67699           G>T         ACG>ACT      T>TYAL043C      +      0      UWOPS91_917_1 c219:67828           G>A         CCG>CCA      P>PYAL043C      +      0      A4            c219:67861           G>A         TTG>TTA      L>LYAL043C      +      0      A4            c219:67885           G>A         CAG>CAA      Q>QYAL043C      +      0      UWOPS91_917_1 c219:67888           T>C         GAT>GAC      D>DYAL043C      +      0      A4            c219:67900           T>C         CTT>CTC      L>LYAL043C      +      0      N_17          c219:67939           C>T         ATC>ATT      I>IYAL043C      +      0      A4
[truncated: 1,200,000 more chars]
