# Supplementary material for: Proteome-wide evidence for enhanced positive Darwinian selection within intrinsically disordered regions in proteins
Source: Genome Biol. 2011 Jul 19;12(7):R65. doi: 10.1186/gb-2011-12-7-r65 (PMC3218827; doi:10.1186/gb-2011-12-7-r65)
Supplement: Additional file 8 — FastA file containing the ORF DNA sequence of all analyzed S. cerevisiae genes. Strand, phase, chromosone and genomic start/finish coordinates are specified in the header line. [file gb-2011-12-7-r65-S8.RTF]

>YAL001C	-:1:chr01:147596:151008AAATCACTTTGAATCAGCTGTGGGATATATCTGGTAAATATTTTGATTTGTCTGATAAAAAAGTTAAACAGTTCGTGCTTTCATGCGTGATATTGAAAAAGGACATTGAGGTGTATTGTGATGGTGCTATAACAACTAAAAATGTGACTGATATTATAGGCGACGCTAATCATTCATACTCGGTTGGGATTACTGAGGACAGCCTATGGACATTATTAACGGGATACACAAAAAAGGAGTCAACTATTGGAAATTCTGCATTTGAACTACTTCTCGAAGTTGCCAAATCAGGAGAAAAAGGGATCAATACTATGGATTTGGCGCAGGTAACTGGGCAAGATCCTAGAAGTGTGACTGGACGTATCAAGAAAATAAACCACCTGTTAACAAGTTCACAACTGATTTATAAGGGACACGTCGTGAAGCAATTGAAGCTAAAAAAATTCAGCCATGACGGGGTGGATAGTAATCCCTATATTAATATTAGGGATCATTTAGCAACAATAGTTGAGGTGGTAAAACGATCAAAAAATGGTATTCGCCAGATAATTGATTTAAAGCGTGAATTGAAATTTGACAAAGAGAAAAGACTTTCTAAAGCTTTTATTGCAGCTATTGCATGGTTAGATGAAAAGGAGTACTTAAAGAAAGTGCTTGTAGTATCACCCAAGAATCCTGCCATTAAAATCAGATGTGTAAAATACGTGAAAGATATTCCAGACTCTAAAGGCTCGCCTTCATTTGAGTATGATAGCAATAGCGCGGATGAAGATTCTGTATCAGATAGCAAGGCAGCTTTCGAAGATGAAGACTTAGTCGAAGGTTTAGATAATTTCAATGCGACTGATTTATTACAAAATCAAGGCCTTGTTATGGAAGAGAAAGAGGATGCTGTAAAGAATGAAGTTCTTCTTAATCGATTTTATCCACTTCAAAATCAGACTTATGACATTGCAGATAAGTCTGGCCTTAAAGGAATTTCAACTATGGATGTTGTAAATCGAATTACCGGAAAAGAATTTCAGCGAGCTTTTACCAAATCAAGCGAATATTATTTAGAAAGTGTGGATAAGCAAAAAGAAAATACAGGGGGGTATAGGCTTTTTCGCATATACGATTTTGAGGGAAAGAAGAAGTTTTTTAGGCTGTTCACAGCTCAGAACTTTCAAAAGTTAACAAATGCGGAAGACGAAATATCCGTTCCAAAAGGGTTTGATGAGCTAGGCAAATCTCGTACCGATTTGAAAACTCTCAACGAGGATAATTTCGTCGCACTCAACAACACTGTTAGATTTACAACGGACAGCGATGGACAGGATATATTCTTCTGGCACGGTGAATTAAAAATTCCCCCAAACTCAAAAAAAACTCCGAATAAAAACAAACGGAAGAGGCAGGTTAAAAACAGTACTAATGCTTCTGTTGCAGGAAACATTTCGAATCCCAAAAGGATTAAGCTAGAGCAGCATGTCAGCACTGCACAGGAGCCGAAATCTGCTGAAGATAGTCCAAGTTCAAACGGAGGCACTGTTGTCAAAGGCAAGGTGGTTAACTTCGGCGGCTTTTCTGCCCGCTCTTTGCGTTCACTACAGAGACAGAGAGCCATTTTGAAAGTTATGAATACGATTGGTGGGGTAGCATACCTGAGAGAACAATTTTACGAAAGCGTTTCTAAATATATGGGCTCCACAACGACATTAGATAAAAAGACTGTCCGTGGTGATGTTGATTTGATGGTAGAAAGCGAAAAATTAGGAGCCAGAACAGAGCCTGTATCAGGAAGAAAAATTATTTTTTTGCCCACTGTTGGAGAGGACGCTATCCAAAGGTACATCCTGAAAGAAAAAGATAGTAAAAAAGCAACCTTTACTGATGTTATACATGATACGGAAATATACTTCTTTGACCAAACGGAAAAAAATAGGTTTCACAGAGGAAAGAAATCAGTTGAAAGAATTCGTAAGTTTCAGAACCGCCAAAAGAATGCTAAGATCAAAGCTTCAGATGACGCTATCTCTAAGAAGAGTACGTCGGTCAACGTATCAGATGGAAAGATCAAAAGGAGAGACAAAAAAGTGTCTGCTGGTAGGACAACGGTGGTCGTGGAAAATACTAAAGAAGACAAAACTGTCTATCATGCAGGCACTAAAGATGGTGTTCAGGCTTTAATCAGAGCTGTTGTAGTTACTAAAAGTATTAAAAATGAAATAATGTGGGACAAAATAACAAAATTATTTCCTAATAATTCTTTAGATAACCTAAAAAAGAAATGGACGGCACGGCGAGTAAGAATGGGTCATAGTGGTTGGAGGGCATATGTCGATAAGTGGAAAAAAATGCTCGTTCTAGCCATTAAAAGTGAAAAGATTTCACTGAGGGATGTTGAAGAACTAGATCTTATCAAATTGCTTGATATTTGGACCTCTTTTGATGAAAAGGAAATAAAAAGGCCGCTCTTTCTTTATAAGAACTACGAAGAGAATAGAAAAAAATTTACTCTGGTACGTGATGACACACTTACACATTCTGGCAACGATCTGGCCATGTCTTCTATGATTCAAAGAGAGATCTCTTCTTTAAAAAAAACTTACACTAGAAAGATTTCCGCTTCTACTAAGGACTTATCGAAGAGTCAAAGCGACGATTATATTCGCACAGTGATCCGGTCCATATTAATAGAAAGTCCTTCGACCACTAGAAATGAAATAGAGGCGTTGAAGAACGTTGGAAACGAATCAATAGATAACGTCATCATGGATATGGCTAAGGAAAAGCAAATTTATCTCCATGGCTCAAAACTTGAATGTACTGATACTTTACCAGACATTTTGGAAAATAGAGGAAATTATAAAGATTTTGGTGTAGCTTTTCAGTATAGATGTAAGGTTAATGAATTATTGGAGGCCGGAAACGCTATTGTTATCAATCAAGAGCCGTCCGATATATCCTCTTGGGTTTTAATTGATTTGATTTCGGGAGAGCTATTGAATATGGATGTAATTCCAATGGTGAGAAATGTTCGACCTTTAACGTATACTTCAAGGAGATTTGAAATACGAACATTAACTCCCCCTCTGATTATATATGCCAATTCTCAGACAAAATTGAATACAGCAAGGAAGTCTGCTGTCAAAGTTCCACTGGGCAAACCATTTTCTCGTTTATGGGTGAATGGATCTGGTTCCATTAGGCCAAACATATGGAAGCAGGTAGTTACTATGGTCGTTAACGAAATAATATTTCATCCAGGGATAACATTGAGTAGATTGCAATCTAGGTGTCGTGAAGTACTTTCGCTTCATGAAATATCAGAAATATGCAAATGGCTCCTAGAAAGACAAGTATTAATAACTACTGATTTTGATGGCTATTGGGTCAATCATAATTGGTATTCTATATATGAATCTACATAA>YAL001C	-:0:chr01:151099:151168ATGGTACTGACGATTTATCCTGACGAACTCGTACAAATAGTGTCTGATAAAATTGCTTCAAATAAGGGAA>YAL005C	-:0:chr01:139505:141433ATGTCAAAAGCTGTCGGTATTGATTTAGGTACAACATACTCGTGTGTTGCTCACTTTGCTAATGATCGTGTGGACATTATTGCCAACGATCAAGGTAACAGAACCACTCCATCTTTTGTCGCTTTCACTGACACTGAAAGATTGATTGGTGATGCTGCTAAGAATCAAGCTGCTATGAATCCTTCGAATACCGTTTTCGACGCTAAGCGTTTGATCGGTAGAAACTTCAACGACCCAGAAGTGCAGGCTGACATGAAGCACTTCCCATTCAAGTTGATCGATGTTGACGGTAAGCCTCAAATTCAAGTTGAATTTAAGGGTGAAACCAAGAACTTTACCCCAGAACAAATCTCCTCCATGGTCTTGGGTAAGATGAAGGAAACTGCCGAATCTTACTTGGGAGCCAAGGTCAATGACGCTGTCGTCACTGTCCCAGCTTACTTCAACGATTCTCAAAGACAAGCTACCAAGGATGCTGGTACCATTGCTGGTTTGAATGTCTTGCGTATTATTAACGAACCTACCGCCGCTGCCATTGCTTACGGTTTGGACAAGAAGGGTAAGGAAGAACACGTCTTGATTTTCGACTTGGGTGGTGGTACTTTCGATGTCTCTTTGTTGTTCATTGAAGACGGTATCTTTGAAGTTAAGGCCACCGCTGGTGACACCCATTTGGGTGGTGAAGATTTTGACAACAGATTGGTCAACCACTTCATCCAAGAATTCAAGAGAAAGAACAAGAAGGACTTGTCTACCAACCAAAGAGCTTTGAGAAGATTAAGAACCGCTTGTGAAAGAGCCAAGAGAACTTTGTCTTCCTCCGCTCAAACTTCCGTTGAAATTGACTCTTTGTTCGAAGGTATCGATTTCTACACTTCCATCACCAGAGCCAGATTCGAAGAATTGTGTGCTGACTTGTTCAGATCTACTTTGGACCCAGTTGAAAAGGTCTTGAGAGATGCTAAATTGGACAAATCTCAAGTCGATGAAATTGTCTTGGTCGGTGGTTCTACCAGAATTCCAAAGGTCCAAAAATTGGTCACTGACTACTTCAACGGTAAGGAACCAAACAGATCTATCAACCCAGATGAAGCTGTTGCTTACGGTGCTGCTGTTCAAGCTGCTATTTTGACTGGTGACGAATCTTCCAAGACTCAAGATCTATTGTTGTTGGATGTCGCTCCATTATCCTTGGGTATTGAAACTGCTGGTGGTGTCATGACCAAGTTGATTCCAAGAAACTCTACCATTTCAACAAAGAAGTTCGAGATCTTTTCCACTTATGCTGATAACCAACCAGGTGTCTTGATTCAAGTCTTTGAAGGTGAAAGAGCCAAGACTAAGGACAACAACTTGTTGGGTAAGTTCGAATTGAGTGGTATTCCACCAGCTCCAAGAGGTGTCCCACAAATTGAAGTCACTTTCGATGTCGACTCTAACGGTATTTTGAATGTTTCCGCCGTCGAAAAGGGTACTGGTAAGTCTAACAAGATCACTATTACCAACGACAAGGGTAGATTGTCCAAGGAAGATATCGAAAAGATGGTTGCTGAAGCCGAAAAATTCAAGGAAGAAGATGAAAAGGAATCTCAAAGAATTGCTTCCAAGAACCAATTGGAATCCATTGCTTACTCTTTGAAGAACACCATTTCTGAAGCTGGTGACAAATTGGAACAAGCTGACAAGGACACCGTCACCAAGAAGGCTGAAGAGACTATTTCTTGGTTAGACAGCAACACCACTGCCAGCAAGGAAGAATTCGATGACAAGTTGAAGGAGTTGCAAGACATTGCCAACCCAATCATGTCTAAGTTGTACCAAGCTGGTGGTGCTCCAGGTGGCGCTGCAGGTGGTGCTCCAGGCGGTTTCCCAGGTGGTGCTCCTCCAGCTCCAGAGGCTGAAGGTCCAACCGTTGAAGAAGTTGATTAA>YAL007C	-:0:chr01:137700:138347ATGATCAAATCTACAATTGCTCTACCCTCTTTCTTCATTGTTTTAATTTTGGCGTTGGTCAATTCAGTGGCTGCATCTTCCAGTTATGCACCAGTTGCTATCAGTTTACCAGCATTCAGCAAAGAATGCCTGTACTACGATATGGTTACTGAGGATGATTCCCTGGCTGTGGGTTACCAAGTTCTAACCGGTGGTAATTTTGAGATTGATTTTGATATTACTGCTCCTGATGGATCTGTGATTACTAGTGAGAAACAAAAGAAGTACTCAGACTTTTTATTAAAATCGTTTGGAGTGGGGAAGTATACCTTTTGTTTTTCCAATAACTATGGTACAGCGTTGAAAAAGGTAGAAATTACACTAGAAAAGGAAAAAACTTTGACTGACGAGCATGAAGCTGATGTCAATAACGATGACATTATTGCCAATAACGCCGTGGAGGAAATAGATAGAAACTTGAACAAAATCACGAAGACTTTGAACTATTTGAGAGCCAGAGAATGGAGAAACATGTCTACCGTCAATTCCACCGAATCAAGGTTGACTTGGTTATCTATATTGATTATCATTATCATTGCCGTTATAAGTATTGCCCAAGTTCTACTTATTCAATTCCTCTTCACTGGTCGTCAAAAAAATTACGTTTAA>YAL008W	+:0:chr01:136916:137512ATGACTTTGGCTTTTAATATGCAACGGTTGGTGTTTCGTAATTTGAATGTTGGGAAGCGCATGTTCAAGAACGTCCCCTTATGGAGGTTTAATGTCGCCAATAAATTAGGAAAGCCCTTAACTCGCTCTGTAGGGTTAGGCGGTGCTGGCATAGTTGCTGGTGGCTTTTACTTGATGAATCGCCAGCCTTCTAAGTTGATATTCAATGATTCTTTAGGGGCAGCTGTCAAACAACAGGGTCCCTTGGAACCAACTGTGGGCAACAGTACGGCAATTACCGAGGAAAGGAGGAACAAAATAAGTAGTCACAAGCAGATGTTTTTGGGATCATTATTCGGTGTTGTTTTAGGAGTTACGGTGGCTAAGATATCAATTTTGTTTATGTATGTCGGTATTACAAGCATGCTTCTTTGTGAATGGTTACGGTACAAGGGATGGATTCGCATTAATTTGAAAAATATCAAATCTGTAATTGTTTTGAAAGATGTAGACTTGAAGAAACTGCTTATTGATGGGTTATTGGGTACAGAATACATGGGTTTTAAAGTATTCTTTACATTGAGTTTCGTATTAGCAAGTTTAAATGCTAACAAATGA>YAL009W	+:0:chr01:135856:136635ATGGAGCCAGAGAGCATAGGCGATGTGGGGAACCATGCCCAGGATGATAGTGCCAGTATAGTGTCCGGGCCTCGCAGGCGTTCTACTAGCAAGACATCCAGTGCGAAGAATATACGGAACTCCAGTAATATCTCTCCAGCATCGATGATTTTCAGGAATTTGTTGATACTGGAGGATGATTTAAGACGCCAAGCTCACGAACAAAAGATACTGAAGTGGCAATTCACTTTGTTCTTAGCGTCTATGGCCGGTGTAGGCGCATTTACCTTCTACGAACTTTATTTCACTTCAGATTATGTCAAGGGCCTCCATAGGGTTATTTTGCAATTCACTCTTTCTTTCATTTCCATTACTGTAGTTCTTTTTCATATCAGTGGACAATATAGAAGAACTATCGTCATTCCAAGAAGATTTTTTACCTCTACTAATAAAGGGATTAGGCAGTTTAATGTGAAGCTAGTTAAAGTACAGTCTACGTGGGACGAGAAATACACAGATTCAGTAAGATTTGTGAGTCGAACAATTGCTTATTGTAATATTTATTGTTTGAAAAAATTTCTGTGGCTTAAAGACGATAATGCCATTGTGAAATTTTGGAAAAGTGTCACGATACAATCCCAACCGAGGATCGGAGCTGTGGATGTGAAATTAGTCCTCAACCCCAGAGCATTTAGTGCAGAGATTAGAGAAGGATGGGAGATTTATAGAGACGAGTTTTGGGCCAGGGAAGGTGCTAGAAGACGCAAACAAGCGCACGAACTCCGACCTAAATCAGAATGA>YAL010C	-:0:chr01:134186:135667ATGCTACCCTATATGGACCAAGTACTAAGGGCATTTTATCAGAGCACCCATTGGAGTACGCAAAATAGCTACGAGGATATAACGGCCACATCGAGAACATTATTAGATTTCCGAATTCCCTCAGCAATACACCTGCAAATTTCCAACAAATCTACTCCCAATACATTCAATTCTTTAGATTTTTCTACGAGGTCCAGGATAAATGGTTCTCTGAGTTATTTATACTCCGATGCACAGCAATTGGAGAAATTCATGCGCAACTCTACTGATATCCCATTACAAGATGCCACCGAAACATACAGACAATTGCAACCAAACCTCAATTTCAGTGTTAGTAGTGCGAATACGTTGAGTAGTGACAACACCACAGTCGACAATGACAAGAAATTACTACATGACTCGAAATTTGTTAAAAAATCCCTTTATTATGGTAGAATGTACTACCCCAGCTCTGATTTAGAAGCAATGATAATAAAACGACTAAGTCCACAAACCCAATTTATGCTTAAGGGTGTCAGTAGTTTCAAAGAAAGCTTAAACGTTTTAACGTGCTATTTTCAAAGAGATTCTCACCGCAATTTACAGGAGTGGATATTTTCCACCAGTGATCTATTATGTGGTTATAGAGTATTACACAATTTCCTTACCACGCCTTCCAAGTTTAACACCTCACTGTACAATAATTCTTCGTTGTCGCTTGGTGCTGAATTTTGGTTAGGGTTAGTAAGTTTAAGCCCCGGTTGTTCGACAACTTTAAGATATTACACACATTCTACAAACACAGGACGACCACTAACTTTGACATTATCTTGGCAACCATTATTCGGCCATATATCCTCCACATATTCGGCCAAGACAGGGACAAATTCTACTTTTTGCGCGAAGTATGATTTTAATCTTTATTCGATTGAATCAAATCTTTCATTTGGGTGCGAATTTTGGCAAAAAAAGCATCATTTGCTTGAAACCAATAAAAACAATAATGATAAATTAGAACCAATCTCCGACGAATTGGTTGATATAAATCCAAACAGCAGAGCGACTAAACTACTGCACGAAAATGTACCGGATCTGAATTCAGCTGTTAACGATATTCCTTCTACACTAGATATACCTGTTCACAAACAAAAGCTATTAAATGATTTAACTTATGCATTCTCGTCGTCATTAAGAAAAATCGATGAAGAAAGATCTACCATCGAAAAATTTGATAACAAAATAAATAGTTCCATTTTTACCAGTGTTTGGAAATTAAGCACGTCATTACGTGACAAGACTTTAAAACTATTATGGGAAGGCAAATGGAGGGGATTTTTAATATCTGCCGGGACAGAGCTGGTATTCACTAGAGGCTTTCAAGAAAGTTTATCCGATGATGAAAAGAATGATAATGCAATATCTATATCAGCAACTGATACAGAAAACGGCAATATACCAGTTTTCCCGGCAAAGTTTGGCATACAATTCCAGTACTCCACATGA>YAL011W	+:0:chr01:132202:134079ATGCCTGCTGTCTTGAGAACCAGGTCCAAAGAATCCTCTATAGAGCAGAAGCCTGCTTCCAGAACTAGAACGAGATCAAGAAGGGGCAAGCGTGGTCGTGACGATGATGATGATGACGACGATGAGGAAAGCGATGATGCATACGATGAAGTAGGTAATGACTATGACGAGTATGCTTCAAGAGCGAAGCTGGCCACCAATAGGCCCTTCGAAATAGTCGCGGGACTGCCTGCTAGTGTGGAGCTGCCCAACTATAACTCTTCGCTTACTCATCCGCAATCAATTAAAAATTCTGGGGTGCTTTACGACTCTCTGGTCAGTTCCAGAAGAACCTGGGTTCAGGGTGAGATGTTTGAACTGTATTGGCGAAGACCTAAGAAAATTGTTAGTGAATCTACCCCAGCAGCGACGGAGAGTCCAACATCTGGAACGATTCCTTTGATTCGAGATAAGATGCAGAAAATGTGCGATTGTGTAATGAGTGGAGGTCCTCACACGTTCAAAGTTAGACTTTTCATACTGAAGAATGACAAAATCGAACAGAAATGGCAAGATGAGCAAGAGTTGAAGAAAAAGGAAAAGGAACTGAAACGAAAGAACGATGCAGAGGCCAAAAGATTGAGGATGGAGGAAAGGAAAAGGCAGCAGATGCAAAAGAAAATAGCCAAGGAACAAAAACTTCAATTGCAGAAGGAAAATAAAGCCAAGCAGAAGTTGGAACAGGAGGCGCTGAAGCTAAAAAGAAAGGAAGAAATGAAAAAACTAAAGGAACAAAATAAAAATAAACAGGGTTCACCTTCTTCCTCCATGCATGACCCAAGAATGATAATGAATTTGAATTTGATGGCACAAGAAGATCCAAAACTAAACACTTTAATGGAAACCGTCGCAAAGGGTCTTGCCAATAATAGTCAACTGGAGGAATTTAAAAAGTTCATTGAAATTGCCAAAAAAAGGTCACTAGAGGAGAACCCAGTTAATAAGCGTCCATCTGTCACAACAACGCGACCTGCACCTCCCTCTAAAGCTAAAGACGTAGCCGAAGATCACCGGTTAAACTCGATAACCTTGGTGAAAAGTTCCAAAACCGCTGCCACGGAACCTGAACCAAAAAAAGCTGATGACGAGAATGCAGAGAAGCAACAGTCCAAAGAGGCAAAGACAACTGCCGAATCGACTCAAGTAGATGTCAAGAAAGAAGAAGAAGATGTGAAGGAAAAGGGTGTGAAATCAGAGGATACACAAAAGAAAGAAGATAATCAAGTGGTACCGAAAAGGAAAAGAAGAAAGAACGCAATAAAGGAAGATAAAGATATGCAATTGACCGCGTTCCAACAGAAATACGTTCAAGGTGCGGAGATCATCCTGGAGTATTTAGAATTCACCCATTCGAGGTATTACCTGCCTAAGAAATCAGTAGTAGAATTTTTGGAGGATACGGATGAGATTATAATATCTTGGATTGTTATACACAATTCTAAAGAAATTGAGAAGTTCAAAACCAAGAAAATAAAAGCTAAACTGAAAGCCGACCAAAAACTAAACAAGGAGGATGCCAAGCCAGGCTCTGATGTGGAGAAGGAAGTCAGCTTTAATCCTCTTTTTGAAGCCGATTGCCCTACCCCTCTCTACACCCCAATGACAATGAAGTTATCGGGGATTCACAAAAGATTTAACCAAATCATCCGAAATAGCGTTTCTCCAATGGAAGAAGTTGTTAAAGAAATGGAAAAAATTCTGCAAATTGGTACTAGATTGTCTGGCTATAATCTGTGGTACCAATTGGATGGATACGATGATGAAGCTTTGAGCGAAAGTTTGCGGTTCGAACTAAATGAGTGGGAGCACGCCATGAGAAGCAGAAGACACAAAAGATAA>YAL013W	+:0:chr01:129271:130533ATGAGTCAGCAAACACCACAGGAAAGTGAACAGACCACAGCGAAAGAACAGGACCTTGATCAAGAGAGCGTGTTGAGCAACATTGACTTCAATACGGATTTGAATCACAATTTGAATTTATCGGAATACTGTATATCCAGTGACGCAGGAACAGAGAAGATGGATAGCGACGAGGAGAAGTCGTTGGCCAATCTGCCGGAGTTGAAATACGCTCCCAAGCTATCCAGCCTGGTGAAGCAAGAGACGCTCACCGAGAGCTTGAAAAGACCACACGAAGATGAGAAAGAGGCGATAGATGAGGCCAAGAAGATGAAAGTGCCGGGAGAGAACGAGGACGAAAGCAAGGAAGAGGAAAAGAGTCAAGAACTGGAAGAGGCAATTGACAGCAAGGAGAAGAGCACCGACGCCAGGGACGAGCAAGGGGACGAAGGTGATAATGAGGAGGAAAACAACGAGGAGGATAATGAAAACGAAAACGAGCATACAGCACCGCCTGCGCTGGTGATGCCCTCCCCCATCGAAATGGAGGAACAGAGGATGACTGCGCTGAAGGAAATCACCGACATCGAGTACAAGTTCGCGCAATTGCGCCAAAAACTATATGACAATCAATTGGTGCGGTTGCAAACGGAGCTGCAGATGTGTCTGGAAGGGTCACACCCGGAATTGCAGGTCTACTACTCGAAGATTGCCGCGATCCGTGACTACAAGCTACACCGAGCGTACCAGCGACAGAAGTACGAGCTTTCATGCATCAACACAGAAACAATCGCTACCAGGACATTCATTCACCAGGACTTCCACAAGAAGGTCACCGACCTGCGAGCCAGGCTGCTGAACAGAACCACGCAGACCTGGTACGATATCAACAAGGAGCGCCGCGATATGGATATAGTCATCCCAGATGTCAATTACCACGTCCCCATCAAACTTGATAACAAGACGCTGAGCTGTATCACGGGCTACGCCAGCGCAGCACAGCTGTGCTATCCCGGCGAGCCCGTGGCAGAGGACCTCGCTTGCGAAAGCATCGAGTACCGCTACAGAGCCAACCCGGTGGACAAACTCGAAGTCATTGTGGACCGAATGAGGCTCAATAACGAGATTAGCGACCTCGAAGGCCTGCGCAAATATTTCCACTCCTTCCCGGGTGCTCCTGAGTTGAACCCGCTTAGAGACTCCGAAATCAACGACGACTTCCACCAGTGGGCCCAGTGTGACCGCCACACTGGACCCCATACCACTTCTTTTTGTTATTCTTAA>YAL016W	+:0:chr01:124880:126787ATGTCTGGAGCAAGATCAACAACGGCAGGTGCCGTGCCCTCGGCAGCAACAACATCAACAACATCAACAACGTCAAACTCTAAGGACTCAGACTCTAACGAGTCATTATATCCCTTGGCTCTGCTTATGGATGAGTTAAAACATGATGATATTGCTAATAGGGTAGAAGCCATGAAAAAACTAGATACCATCGCGTTGGCACTCGGTCCCGAAAGAACAAGAAACGAGTTGATTCCCTTTTTAACGGAAGTTGCACAAGATGATGAAGATGAGGTGTTTGCCGTTTTAGCCGAACAGTTAGGAAAATTTGTCCCCTACATTGGCGGTCCTCAATACGCCACAATCCTATTACCAGTTTTGGAAATTTTGGCATCTGCAGAAGAAACTTTGGTTAGAGAAAAGGCCGTAGATTCTCTGAATAACGTGGCCCAAGAACTTTCTCAAGAACAATTATTTAGTGACTTCGTCCCTTTAATTGAACATTTAGCTACTGCAGATTGGTTTTCTTCAAAAGTTTCTGCTTGTGGTCTTTTCAAGTCTGTTATTGTTAGAATCAAAGATGATTCATTGAGAAAGAATATCCTGGCTTTATACTTACAACTCGCTCAAGATGATACTCCAATGGTGAAAAGGGCCGTCGGTAAAAACCTGCCCATCTTGATCGATCTGTTGACTCAAAATTTGGGATTATCTACAGACGAAGATTGGGATTACATTTCTAACATTTTCCAGAAAATCATTAACGATAATCAAGATTCTGTCAAGTTTCTGGCAGTTGATTGTTTAATTTCCATCTTGAAATTTTTTAACGCTAAAGGTGATGAGTCTCACACTCAAGATTTATTGAACTCTGCTGTCAAATTAATTGGTGACGAAGCGTGGAGGGTACGTTACATGGCTGCCGATAGATTTTCAGATTTAGCCTCGCAATTCAGTTCCAACCAAGCATATATCGATGAATTAGTACAACCATTTTTGAACCTTTGTGAGGACAACGAGGGAGATGTTAGGGAAGCTGTGGCTAAACAAGTTTCTGGGTTTGCCAAGTTCCTAAATGATCCTTCAATTATATTGAATAAGATCTTACCTGCTGTGCAGAATTTGAGTATGGACGAAAGTGAAACAGTGAGATCTGCTTTGGCTTCTAAGATTACAAATATTGTATTACTGTTGAATAAAGATCAAGTCATTAACAATTTTCTTCCGATTTTACTGAATATGCTAAGAGATGAGTTCCCTGACGTTCGTTTAAATATCATTGCCAGCTTGAAGGTTGTCAATGACGTAATAGGAATTGAGCTGCTATCAGACTCTTTGTTACCTGCCATAACAGAATTAGCCAAGGACGTGAATTGGAGAGTTAGAATGGCTATAATTGAGTACATACCTATCTTGGCAGAACAATTAGGTATGCAATTTTTTGACCAACAGTTAAGCGATTTATGTCTTTCATGGTTGTGGGATACTGTTTATTCTATCAGAGAAGCCGCAGTGAATAATTTAAAAAGGTTAACGGAAATATTTGGCTCTGATTGGTGTCGTGATGAAATTATTTCAAGACTGCTCAAATTTGATCTACAATTACTGGAAAATTTTGTCTCGAGGTTCACAATACTCTCTGCTCTAACCACTTTGGTGCCCGTGGTATCGTTAGATGTCGTTACTGAGCAACTATTACCATTCATTTCTCACTTGGCTGATGACGGTGTTCCAAATATTAGGTTTAATGTGGCCAAATCCTACGCTGTGATAGTGAAGGTTTTAATTAAGGACGAGGCCAAATATGATGCATTAATTAAGAACACAATTTTACCCTCATTGCAAACGCTGTGTCAAGACGAAGATGTTGATGTAAAATACTTCGCTAAGAAAAGTTTGGCAGAATGTCAAGAACTTTTAAAAAATTGA>YAL018C	-:0:chr01:118565:119542ATGAGTTTTACAGGTTCATTAGCGCTGGCAGGTATTGGTGGTTTGGTGTACAAGTTTGGCGGAGGCCAATCGTATGAAAAACTGCCATATGTCAACATCCCCTTCAATCAGTACTTGGATAAAGTTTACAAGAAGCACTTCAGCAAAGTTATGAGCCGTACCAGATACGTGCTAATGAACTTTTTCAAAGATGCATTCACAGGAGGTGCATTCATGTACCCGTTCAAAGGGTTTTTAGAGTTCAACACCAATAAGAGCAGTTACTCAACGACTATGTTAGGTATATTATCGAGTTACTTGATAATGTTTGCCCTGGTAAGCTTTGTGTATTGGGCTACAATTACGCCAATGTACACAGCTTTTTTGATTGTGCTGGGCCCTATTGGGCTTTTCATTGCCATTTTTCATTCCTTTTTGCAAGCAAATGTTTTCACTTTATTGTTTATGAGACTCAGTCATTTTAATAATCATTTAGTGGAGGTGTGCTTAGAGAAGAATGGTTTAGAGGAGAATTTGAGTGAAGTCAAACCAATCAAGTACTATGCACCTATTAACTCGATATATTTCTGGGCTTACTATTTCCCATTCAAGTTGGTCAAGTACATGTTAGGCCTGTCGGTTCTTTTTGTGCTGTTGGTTATATCTTTTTTCCCACTTATTGGACCAATCCTTTTTCATATTTTAATTTCTCCTTTCATCACTCAAATTTACTTCACGAAAGTTTTACGTTTACAAAATTTCGACAACATACAAAGACGTGAAAATATTTATCTCCACGCTGGCCAATATGCCTCATTTGGTTTCTTGGCCGGTTTGATTGAGTCCGTGCCCATTTTAGCGGGCTTTGCCATTTCTACAAATACTATTGGTAGCGTTTTATTTAACCTTGATCATCCAATGGTCCCTGAAAATTTAGTAGAAACTCAAGCTGAAATTGAAGCAGCACCACAAGATATCAATCAACAACCGAATCAATAA>YAL019W	+:0:chr01:114920:118315ATGAGTGGTTCGCATTCAAATGATGAGGATGACGTAGTGCAAGTGCCCGAGACGTCCTCTCCCACCAAGGTAGCATCGTCGTCTCCCTTAAAGCCTACTTCGCCAACAGTTCCGGATGCAAGTGTGGCGTCTTTGAGAAGCAGGTTTACTTTCAAGCCTTCAGATCCCAGCGAAGGAGCTCATACTTCGAAGCCGCTCCCATCTGGGAGTCCTGAGGTAGCACTGGTTAACCTTGCGAGAGAGTTCCCCGATTTCTCTCAAACTCTGGTGCAGGCTGTTTTCAAATCTAACTCTTTTAACTTACAGTCTGCCAGGGAACGTCTTACAAGATTGAGGCAGCAAAGACAAAATTGGACATGGAACAAGAACGCATCTCCCAAGAAGTCAGAAACCCCGCCACCTGTCAAGAAGTCATTACCACTGGCAAACACAGGCCGTTTATCATCTATCCATGGCAATATCAACAACAAATCCTCCAAGATTACCGTGGCCAAACAGAAAACGTCCATTTTTGACCGTTACTCAAACGTCATCAACCAGAAACAATACACTTTTGAGCTGCCAACTAACTTGAATATAGACTCGGAGGCACTGAGCAAGTTGCCCGTGAACTACAACAAAAAGAGAAGGCTGGTAAGGGCAGATCAGCATCCAATTGGCAAGTCTTATGAGTCATCCGCTACACAATTAGGTTCTGCAAGAGAGAAACTACTGGCGAACCGCAAATACGGTCGTCATGCAAACGACAACGATGAAGAGGAGGAAGAGAGTATGATGACGGACGATGACGATGCAAGTGGCGACGACTACACAGAATCCACGCCGCAGATAAATCTGGATGAACAAGTTTTACAGTTTATTAATGACTCTGATATTGTCGATCTCTCGGACCTCTCAGATACCACGATGCATAAGGCTCAACTCATAGCCTCACATAGGCCATATTCTTCTTTAAATGCCTTTGTAAACACAAATTTCAATGATAAGGACACTGAGGAGAACGCATCGAACAAGAGAAAAAGACGTGCGGCTGCATCCGCCAATGAGAGTGAGAGGCTGCTCGATAAAATCACCCAAAGTATAAGAGGTTACAATGCAATTGAGTCTGTGATCAAGAAATGTTCTTCCTACGGTGATTTGGTCACTTCGCAAATGAAGAAATGGGGTGTGCAAGTGGAAGGCGATAACTCTGAGTTGGACCTGATGAACCTTGGGGAAGACGATGACGACGACAATGATGATGGCAATAACGATAATAATAATAGCAACAATAACAATACCGCTGGCGCAGACGCCACTAGCAAGGAAAAAGAAGATACAAAGGCCGTAGTGGAAGGTTTTGATGAAACTAGCGCAGAACCTACTCCAGCACCAGCACCAGCACCAGTGGAAAGAGAAACAAAACGAATTAGAAACACAACTAAGCCAAAAGTGGTCGAAGATGAAGATGACGATGTAGATTTGGAGGCAATCGATGACGAATTGCCGCAGTCTGAGCATGAAGATGATGACTATGAAGAGGAGGACGAAGACTATAACGATGAGGAAGAAGATGTGGAATATGATGATGGTGACGATGATGACGATGATGACGATGAATTTGTCGCTACCAGAAAAAACACACACGTGATCTCCACCACGAGCAGAAATGGCCGTAAACCTATTGTCAAGTTCTTCAAGGGCAAACCCAGACTGTTAAGCCCGGAAATTTCACTAAAAGACTACCAACAAACGGGTATAAACTGGTTGAATCTGCTATACCAAAACAAGATGTCATGTATCCTTGCAGACGACATGGGTCTAGGTAAAACATGTCAAGTCATTTCATTTTTCGCATATTTGAAACAAATAAACGAACCGGGTCCTCACTTGGTTGTTGTGCCATCATCGACGCTAGAAAATTGGTTAAGGGAGTTCCAGAAATTCGCACCTGCTTTGAAGATTGAACCCTACTATGGCTCTTTACAAGAAAGGGAAGAATTGCGTGATATCCTGGAAAGGAACGCTGGGAAATATGATGTTATCGTGACCACGTATAACTTGGCTGCAGGTAATAAATACGACGTTTCGTTTTTGAAAAATAGAAACTTCAATGTTGTGGTTTATGATGAAGGTCATATGTTGAAAAATTCCACTTCAGAGAGATTTGCCAAACTGATGAAAATTCGTGCCAATTTCCGCCTTTTATTAACTGGTACGCCATTACAAAATAACTTGAAGGAACTAATGTCGCTGTTGGAATTTATCATGCCAAATCTTTTCATTTCCAAAAAGGAATCATTTGACGCAATCTTCAAACAACGTGCCAAGACCACAGACGATAACAAAAATCACAACCCGCTATTAGCGCAAGAAGCCATTACAAGAGCTAAAACGATGATGAAGCCATTTATTTTGAGAAGACGTAAGGATCAAGTGTTGAAACATTTGCCACCAAAGCACACGCATATTCAGTATTGTGAATTGAACGCAATACAAAAAAAAATATATGATAAGGAAATACAAATCGTGTTAGAACATAAGAGAATGATTAAAGATGGCGAATTGCCAAAAGATGCAAAAGAAAAGTCTAAATTACAATCTTCAAGTTCCAAAAATTTAATAATGGCATTGCGAAAGGCCTCTCTGCATCCACTTTTGTTCAGAAATATCTATAATGATAAAATCATCACTAAAATGAGTGATGCCATATTGGATGAACCTGCTTATGCTGAAAACGGTAACAAAGAGTATATTAAGGAAGATATGAGCTATATGACGGATTTTGAGTTGCACAAACTATGCTGCAATTTCCCGAACACGTTATCCAAATACCAACTTCATAATGACGAGTGGATGCAATCTGGGAAGATAGACGCTTTGAAAAAATTGCTGAAAACAATCATTGTTGACAAACAGGAAAAGGTGCTGATATTTTCCTTATTCACTCAAGTCCTGGATATTCTAGAGATGGTTTTGTCCACCTTAGATTATAAATTTTTAAGATTAGATGGTTCCACGCAAGTGAATGATAGACAACTACTAATAGATAAGTTTTATGAAGATAAGGATATTCCCATTTTCATCTTATCAACAAAGGCAGGTGGATTCGGTATTAATTTGGTGTGCGCAAATAATGTTATTATATTCGATCAAAGTTTTAACCCACATGATGACAGACAAGCTGCTGATAGGGCACATCGTGTGGGACAAACAAAGGAAGTTAATATAACCACTTTAATTACTAAGGATTCCATAGAGGAAAAGATTCATCAACTGGCCAAAAATAAACTAGCTTTAGATTCGTATATCAGTGAAGATAAAAAATCTCAAGATGTGTTGGAAAGTAAAGTTAGTGATATGTTGGAGGATATAATTTATGATGAAAACTCGAAACCGAAGGGAACCAAAGAATAA>YAL020C	-:0:chr01:113615:114616ATGAGTTGTGTGTATGCGTTTGGGTCTAATGGGCAAAGGCAACTGGGACTGGGGCACGATGAGGATATGGATACCCCACAGAGGTCTGTGCCAGGAGATGATGGAGCAATAGTCAGGAAGATAGCGTGCGGTGGGAACCACAGCGTGATGCTGACAAATGACGGGAATCTGGTAGGATGTGGAGATAACAGACGGGGAGAACTGGATAGTGCGCAAGCACTGCGGCAGGTGCATGACTGGAGGCCCGTGGAAGTACCGGCACCCGTGGTGGATGTGGCGTGCGGCTGGGACACGACAGTTATTGTGGATGCTGATGGCCGTGTATGGCAGAGAGGAGGCGGTTGCTACGAGTTCACTCAGCAACATGTGCCATTGAATTCCAACGATGAGCGCATCGCAGTATACGGATGTTTCCAGAACTTTGTGGTGGTGCAAGGCACCCGAGTATACGGCTGGGGCAGCAACACAAAGTGTCAATTGCAAGAGCCCAAATCCCGATCACTGAAAGAGCCCGTATTGGTGTACGATACCGGGTCTGTGGCCGTAGACTACGTGGCCATGGGCAAGGACTTCATGGTCATAGTGGACGAGGGCGGCCGCATAGTGCACGCATCCGGTCGCCTGCCCACTGGGTTCGAGCTCAAACAACAGCAAAAAAGACACAATCTAGTGGTACTGTGCATGTGGACCTCGATCCACCTGTGGAATGCGCGCCTCAATACGGTAGAGTCGTTTGGTAGGGGCACACATTCCCAACTCTTCCCGCAAGAGCGCCTAGACTTCCCTATTGTCGGTGTTGCAACCGGGAGTGAGCACGGTATTCTAACTACTGCTAATCAAGAAGGCAAGTCTCACTGTTACAATGTATACTGCTGGGGCTGGGGAGAGCATGGCAACTGCGGCCCGCAAAAGGCGTCCCAGCCTGGACTGCAGCTCGTGGGCCAATACTCTGGAAAACCTCGCGTGTTTGGCGGATGTGCCACCACGTGGATCGTGCTCTAG>YAL022C	-:0:chr01:108878:110431ATGAGTACTAGTGCGGACACTGATACCATCAAGAAGCCAATCCTTGCGGTGCCAGAGCCTGCACTGGCCGATACGCATTCAGAGGAGATATCACGCTCTGGAGAAGAACATGAATCAGAGAACAACGAGCACTCAGATGAAGAAGGCGATAATTATTCTGAAAGAGAGCAATCTGTGTCAACCGAACCACTGGATACATTGCCGTTAAGAAAAAAGTTGAAAAATCTTTCATATATTACATTTTTCGCCATAGGAATAGGTCTTTTATGGCCGTGGAACTGTATCCTCAGTGCCTCGCAATATTTTAAGCACGATATTTTCAAAGACACCTCCATTTGGGCAAAGATCTTCACAAGCTCTATGATGTCCTTTTCTACCATATCGTCAATGCTGTTCAACATCTACTTGGCCAAAAGACAGTACAAATACTCGAGAAGGGTCATAAACGGGCTTGTGTGGGAGATTATTGTCTTTACTGTCATGTGCTTCTTTACAATTTTGCATTTCCTTTTACCTAAATGGTTCAATTTCATGTTTATAATGATGCTTGTAGTGATCAGTTCCATGGGGACAGCCATGACACAGAATGGTATCATGGCCATAGCCAACGTCTTCGGTTCCGAGTACAGTCAAGGTGTCATGGTGGGGCAAGCCGTTGCTGGTGTCCTGCCCTCCCTAGTGCTTTTTGCCCTAGCTTTCATCGAGAACTCTTCTGTGTCTACTACGGGCGGGATTCTTCTATACTTTTTTACCACAACACTCGTGGTCACCATTTGTGTGGTCATGTTCAGCGTGAGCAAAATCAGTCGGAAAGTGAACGAGAATTGGAATGTGGAAGACGGACATATCACTGATGTGTTGTTAGGGTCTCTGCGCTCCAATGAGGAGGAAATCCGTATTGTTGGCCGCATTGACCAAATGGAGGATGAAGACCACCGCCGCACCAACGGCACTCGCGACGACAACGACGAAGGTGAGGAACTCCAACTAAAAGTGCCTTTCGAGGTCTTATTTGCCAAACTAAAGTACCTGGTTCTTTCCATATTCACCACGTTTGTCGTAACTCTTGTTTTTCCTGTATTTGCGTCTGCCACCTACGTGACAGGGCTTCCTTTAAGCAACGCACAGTACATACCTCTCATATTCACGCTGTGGAACCTAGGCGACCTTTACGGAAGAGTCATTGCCGACTGGCCCATGTTCCGTGACCAGAAATTTACGCCACGCAAAACCTTCATCTACTCATTGTTGCGGGTGGCCGCAATACCACTGTTCTTGATGTTCACAGCCATCACCTCCTCTTCAAGCGGCGATGAAGAGCACAATGGCTCCGTAATCGTTGACTTGTGTTACATGCTACTGCAGTTCCTTTTCGGCGTAACCAACGGGCACGTCATCTCTATGAGTTTCATGAAGGTGCCAGAGCAACTGGACAACGACGACGAGAAGGAAGCGGCCGGTGGATTCACTAATATTTTCGTTTCTACAGGACTAGCTCTGGGCAGCATCATAAGCTACGTATTCGTCTTCATAATTGACTTTATTATCAGGTAG>YAL023C	-:0:chr01:106273:108552ATGTCCTCGTCTTCGTCTACCGGGTACAGCAAAAACAATGCCGCCCACATTAAGCAAGAGAATACACTGAGACAAAGAGAATCGTCTTCCATCAGCGTCAGTGAGGAACTTTCGAGCGCTGATGAGAGAGACGCGGAAGATTTCTCGAAGGAAAAGCCCGCTGCACAAAGCTCACTGTTACGCCTGGAATCCGTTGTAATGCCGGTGATCTTTACTGCATTGGCGTTGTTTACCAGGATGTACAAAATCGGCATCAACAACCATGTTGTTTGGGATGAGGCGCACTTTGGTAAATTTGGTTCTTATTACTTGAGACACGAATTTTACCACGATGTCCATCCTCCCCTAGGAAAAATGCTGGTCGGGTTGTCTGGTTATTTGGCAGGCTACAACGGTTCTTGGGACTTCCCTTCTGGGGAAATTTACCCAGACTATTTGGATTATGTTAAAATGAGACTGTTCAACGCGTCATTTTCCGCGCTCTGTGTGCCATTGGCCTACTTCACTGCCAAAGCTATTGGATTTTCTTTACCAACAGTTTGGCTGATGACCGTGTTGGTTTTGTTTGAAAACTCGTATAGTACTTTGGGCAGGTTCATTCTTTTGGACTCCATGCTACTTTTCTTCACTGTCGCATCGTTCTTTAGTTTTGTTATGTTCCACAACCAGAGGTCCAAGCCGTTCTCTAGAAAGTGGTGGAAATGGCTGTTGATCACTGGTATTTCTTTGGGTTGCACTATTTCCGTCAAAATGGTGGGTCTATTTATCATCACTATGGTCGGTATCTATACTGTGATTGACTTATGGACCTTTTTGGCAGATAAATCCATGTCATGGAAAACCTATATTAACCACTGGTTGGCAAGAATATTTGGTCTTATTATCGTCCCCTTCTGCATTTTCCTATTGTGCTTCAAAATACATTTTGACCTATTATCGCATTCTGGTACAGGTGATGCTAACATGCCATCTCTTTTCCAAGCAAGATTAGTGGGTTCTGACGTCGGACAAGGCCCCCGTGACATTGCTCTAGGTTCCTCCGTTGTTTCCATCAAAAACCAAGCTCTTGGAGGATCTCTATTGCACTCACATATACAAACTTATCCAGATGGGTCCAACCAACAACAAGTAACCTGTTATGGTTACAAAGATGCTAACAACGAATGGTTTTTCAACAGAGAAAGAGGCTTACCATCATGGTCAGAAAACGAAACTGACATCGAGTATTTGAAGCCAGGTACCTCCTATAGATTGGTACACAAAAGCACGGGCAGAAACTTGCACACCCACCCAGTTGCTGCACCAGTGTCAAAGACACAATGGGAGGTTTCTGGTTACGGTGACAATGTTGTTGGTGACAACAAAGACAATTGGGTTATTGAGATCATGGACCAAAGAGGAGATGAAGACCCTGAGAAGTTGCACACATTGACCACCTCTTTCCGTATCAAGAACTTGGAGATGGGCTGTTACTTGGCTCAAACCGGTAACAGTTTGCCCGAATGGGGTTTCAGACAACAAGAGGTTGTCTGCATGAAAAACCCATTCAAGAGGGACAAGAGGACCTGGTGGAACATCGAGACCCACGAAAATGAAAGGTTGCCACCAAGACCCGAAGATTTTCAATACCCAAAGACCAACTTCTTAAAAGACTTCATTCATTTAAATCTAGCCATGATGGCCACTAATAACGCTTTGGTGCCAGATCCAGACAAATTTGATTACTTAGCTTCCTCAGCATGGCAATGGCCAACTTTGAATGTGGGTTTGAGACTATGTGGCTGGGGTGATGATAATCCAAAATACTTCCTATTGGGTACCCCAGCTTCCACGTGGGCTTCTAGTGTTGCCGTCCTCGCATTCATGGCCACGGTCGTTATCTTACTGATCAGATGGCAAAGACAATATGTGGACCTAAGAAATCCATCTAACTGGAACGTTTTCTTAATGGGCGGGTTCTACCCACTACTAGCTTGGGGCCTACACTACATGCCATTCGTTATCATGTCTAGAGTCACCTACGTTCATCATTACTTGCCTGCCTTGTATTTTGCACTGATCATTTTGGCGTACTGTTTCGACGCCGGTTTGCAAAAATGGTCCAGATCTAAGTGCGGCCGTATCATGCGGTTCGTCCTATACGCCGGATTCATGGCACTTGTAATTGGTTGCTTCTGGTACTTCTCCCCAATATCATTTGGTATGGAGGGACCAAGTAGTAACTTCCGCTACTTAAACTGGTTTTCCACTTGGGACATTGCCGACAAGCAAGAAGCATGA>YAL025C	-:0:chr01:100226:101146ATGTCCGACGAAATTGTTTGGCAAGTGATTAATCAAAGTTTCTGCTCTCATAGAATTAAGGCACCTAATGGTCAAAATTTTTGCAGAAATGAGTATAACGTCACTGGGTTGTGTACAAGGCAATCATGCCCACTTGCCAACTCCAAGTATGCAACAGTGAAGTGTGACAATGGGAAACTGTACTTGTATATGAAGACGCCTGAAAGAGCGCACACTCCTGCCAAGTTATGGGAAAGAATCAAACTATCCAAAAATTACACGAAGGCTTTACAACAGATCGACGAACACCTACTACATTGGAGCAAGTTTTTCCGTCATAAGTGTAAACAGAGATTTACAAAATTGACGCAGGTCATGATAACAGAGAGACGTTTAGCATTAAGGGAGGAGGAGAGACACTACGTTGGTGTAGCACCAAAGGTCAAAAGAAGAGAACAAAACAGGGAGAGAAAGGCATTGGTAGCTGCCAAGATTGAAAAAGCCATCGAAAAAGAATTGATGGACAGATTAAAGAGTGGTGCTTACGGTGATAAACCATTAAATGTTGACGAAAAGGTCTGGAAGAAGATAATGGGTCAAATGGAAGAGGAGAATTCTCAGGACGAGGAAGAAGACTGGGATGAAGAAGAGGAAAGTGATGATGGGGAAGTTGAATACGTTGCAGATGATGGCGAAGGTGAATACGTTGACGTGGATGACTTAGAAAAGTGGTTAGCTGACTCTGACAGAGAAGCTTCCAGCGCCAGTCAAAGCGAAAGCGACAGTGAAAGCGAGAGCGACAGCGATAGTGATGAAGAAAATAAGAATTCAGCCAAGAGACGTAAGAAGGGAACTAGTGCCAAAACTAAACGTCCTAAAGTCGAAATCGAATACGAGGAGGAACACGAAGTTCAAAATGCTGAGCAAGAAGTGGCACAATAA>YAL027W	+:0:chr01:94688:95473ATGGCACCAAGTATAGCAACGGTAAAGATAGCCAGGGACATGGTTTTGCCATTACGTATATTTGTCAATAGAAAGCAGATCCTTCAAACCAATGATAAGACTAGCAATAAGTCGAATGCCACTATATTTGAAGCACCATTATTATCAAATAACTCCATAATCTGCTTAAAATCACCAAATACAAGAATATATTTATCGCAACAAGATAAGAAGAATCTTTGTGACGAGATCAAGGAGGACCTGTTATTGATTGTTTACGAACTAGCGTCCCCGGAAATCATCAGTTCCGTACTCAGCAAAATAAGAGTTGGTCATTCTACTGATTTCCAAATCAACGTTCTGCCCAAACTTTTTGCAGGTGCCGATACGGATAATGCGGTAACTTCTCACATCCAGTCTGTGACAAGGCTGGCTAAATTCAAATACAAGTTGCACTACAAACATAAGTGGGAGCTCGACATATTCATCAACAGCATTAAGAAGATCGCCAATTTAAGGCACTATTTGATGTTTCAAACATTAACATTAAACGGTTTCTCATTAAATGCAGGACCCAAAACGTTATTAGCTAGGAAAATAGAAAAACAGCCCCAGGTACCTAATTTGTTAATAGAAAATGGGGACGCTGATGCCCTGGATACACCGGTGGAAGAGGATATAAAACCTGTAATAGAATTTATGTACAAGCCTGTTATTAATTTAGGTGAAATTATTGATGTACATGTGTTGCATAGGCCTAGAAGACATAAGGTACGTACCCAGTCGAAGCAACCCCAGGAGGAATGA>YAL028W	+:0:chr01:92901:94487ATGCAAAATGCTCAAATAAAGAGCTCTTCTAAAGGCAGCGGAATAGATGGTACAGATCGCAATAGCAAAGATGGTGTAGAAAAGAGACCCCTGGAAGATGTAAAGCAAATGATTGACGCTGGAACACCAGATGTTGGCCACAAATCTACTGTTGAAACTAAGCCAAACGTTGGATGGCAAGCCTCTCACAGTAATTTGGCTGCATTACACGAAAAAGAGCAGAAATATGAAATGGAGCACCATCATGCTCGTCATAAACTGCATCGTCAAGTTATTCCGGATTACACGTCTGCCTCGACCGCAATGTTCAGCGATTGTATGTTCAACGCAGCACCAGATAAAGTACGAAGTCTCAGTACGATGAAGTCTTCTGGACTCTCGCCAAAACACCCATTTAACGTAGTCGCCACCTTTAAAGGACCATTCCCGCAGCATAGTGTAGAATCAAAGCCTCTCGATGGTGGATACTCTGCCAAAGACCATTTTCCCTCATTTAAGATGTTGCAAGCCCAGCAGCACCCAGCCCATCGCCATTACAAAGACAACGACAAGTACGGTCTTAAATCACCTTCCCGGTCCTTCGTGAAGGACAAGAAAAGGTTGGTTCACCGGTTTTTGAAATCCATGGAGCCTTCTTCGTCTGGGCAATCTAAGGATTCGTCTGCACTGGCGCCGGCTTTCGATCCAATATTGCCCAATGTTATATCTAAGCCTTCCAAGCGACCCACACATCATTCGCATTCATCAGACGGGAGTTCTAGCACGCAGACAGATATATCGTTACAGAGCTTGCTTTACCATGATCTTGAAAGCTCACCAAAGAAACATGTTTCGCCCTCAAGACCGCCCTCTGTAGCTTCCGAATCCTCTCCTGCCGTTGCTAATCCCATTGGGCTTTCGCCAAAAGACGCCTGCAATGCATCGTTTTCGCAGTCGTCCTCATCTTCGTTGTCTTCTTCTTCATCGTCTTCATCATCGACGTCATTCTCACAGTCAGTGGCTGTTGATCCTCTTGAACCTCCTGGAAATATCACATATAGTAGTTCGAATCTTTCGCTAAATTCAGATGAATTAGACTACTATCAGCGTCATATCGGATTGCAGTTACAGCAGACAGAAGCTTTACTAAAGCACAGTTTGAAAGATGAGGTTCTGAAAGATGAAAATGACCTTGTTAAAAACATTGCAAATTTTGACAAGATCGTTAAAGAGCTAAGGGACTTAAGATCCAGGACCATTGGATGGAAAGAGCTTGTTGAAGAGGATTATTTAATGAATTTGAAACAGGATTTTGACAAGGAAAACCCCGAATCATTTGAGGCACGTTTGAGTGATACAATAAATACAAACGTGGCAAAATTACAAGATTTAGAGAAAAGAATGGCTTCTTGCAAAGACAGGTTGGCCTCTAGGAAGGAAGTAATGAGGAAAATGGAAAGTTTATTGTCTTTGGAGAATTCCTTAATGATATCCAAAAAAAATGTAACATTCGCATCTAAATACCGCAACGAGGCCCTTGATATTGTCTTTTTAATTATCATCATCGTCATATGCTATACCTTCAAGCATCTAGTATCGCATAAATAA>YAL032C	-:0:chr01:83336:84475ATGTTTAGTAACAGACTACCACCTCCAAAACATTCTCAAGGACGAGTTTCGACGGCTTTGAGCTCAGATCGCGTTGAGCCGGCAATATTGACTGACCAAATCGCTAAAAACGTTAAGCTCGATGATTTTATTCCAAAGAGACAGTCTAATTTCGAACTATCGGTTCCTTTGCCAACGAAAGCAGAAATCCAAGAATGTACAGCAAGAACCAAGTCATACATTCAGCGGCTTGTGAATGCGAAACTAGCCAACTCAAATAACAGGGCATCATCAAGGTACGTCACCGAAACACATCAGGCACCCGCGAATCTATTATTGAACAACAGCCACCATATTGAGGTAGTGTCCAAGCAAATGGATCCATTGTTGCCAAGGTTCGTTGGGAAGAAGGCGAGAAAGGTTGTAGCACCCACAGAAAACGACGAAGTCGTGCCTGTTCTCCATATGGATGGCAGCAATGATAGGGGAGAAGCTGATCCAAATGAGTGGAAGATACCTGCAGCTGTGTCAAACTGGAAAAATCCAAATGGTTATACCGTGGCCTTGGAAAGACGTGTAGGTAAAGCTCTTGACAACGAAAATAATACCATCAACGATGGGTTTATGAAGCTCTCCGAAGCGTTAGAAAACGCTGACAAGAAGGCAAGACAAGAGATCAGGTCCAAAATGGAATTGAAGCGGCTTGCTATGGAACAGGAAATGCTTGCTAAAGAATCTAAATTGAAAGAATTGAGCCAACGAGCCAGATACCACAACGGGACTCCGCAGACGGGAGCAATAGTTAAGCCCAAAAAGCAAACGAGCACAGTGGCCAGACTAAAAGAGCTGGCGTACTCTCAAGGAAGAGACGTATCCGAAAAGATAATTCTGGGCGCAGCAAAGCGTTCAGAACAACCGGATCTGCAGTACGATTCAAGATTTTTCACAAGAGGGGCAAATGCCTCCGCCAAAAGGCATGAAGACCAGGTTTATGACAACCCACTGTTCGTCCAACAAGATATTGAAAGCATATACAAGACCAACTACGAAAAGCTGGACGAAGCGGTCAATGTTAAGAGTGAAGGTGCCAGTGGTTCTCACGGCCCCATTCAGTTTACTAAAGCTGAATCCGATGATAAATCGGATAACTATGGCGCCTAG>YAL033W	+:0:chr01:82707:83228ATGGTACGTTTAAAAAGTAGATATATCCTTTTTGAAATTATATTCCCACCTACAGACACCAACGTTGAGGAATCTGTGTCGAAAGCAGACATCTTGCTTTCGCATCACAGAGCATCGCCTGCGGATGTGTCCATAAAGTCGATACTCCAAGAGATACGACGCTCGCTGTCGTTGAATCTGGGCGACTATGGGTCTGCAAAATGTAACTCTCTCTTGCAGTTGAAATACTTTTCAAATAAGACGTCTACGGGGATAATCCGATGCCATCGAGAGGATTGCGACCTTGTTATCATGGCATTGATGTTGATGTCGAAAATTGGCGACGTCGATGGACTGATCGTGAACCCCGTCAAGGTAAGTGGGACCATCAAGAAAATAGAGCAGTTTGCTATGAGAAGGAATTCTAAAATTCTGAACATAATCAAGTGTAGTCAATCATCACACCTCAGCGATAATGACTTTATTATCAATGATTTCAAGAAAATTGGAAGGGAAAACGAAAACGAAAACGAGGACGATTAG>YAL034C	-:0:chr01:80711:81952ATGGGATTATATTCTCCTGAATCTGAAAAGTCTCAATTAAATATGAATTACATTGGTAAGGATGATTCGCAGTCCATTTTCAGACGTCTGAATCAGAATTTGAAAGCAAGTAACAACAATAACGATAGTAATAAAAACGGTTTAAACATGAGTGATTATAGCAATAATTCACCCTATGGGCGCTCGTACGACGTAAGAATTAACCAGAACTCACAAAATAATGGCAATGGATGCTTTTCTGGCAGCATTGACTCCTTGGTTGATGAACATATAATACCATCGCCACCTTTGTCGCCCAAGCTGGAGTCGAAAATTAGCCACAATGGCTCACCCCGCATGGCCTCTTCAGTGCTAGTGGGATCTACGCCTAAAGGCGCTGTAGAGAATGTGCTGTTCGTGAAGCCTGTATGGCCCAATGGGTTATCAAGAAAAAGGTACCGCTACGCCACCTACGGGTTTCTGTCTCAATACAAAATTTTCAGCAATTTGGCCCAACCATATTCTAAGAACATTATCAACCGGTACAACAATCTGGCCTATAATGCTAGACATAAATATTCCAAATACAATGATGATATGACTCCTCCTCCTCTGCCTTCCTCTTCTTCTAGATTACCTTCCCCGTTAGCATCTCCGAATTTGAATAGACAAGCAAGATATAATATGAGGAAACAGGCTCTCTACAATAACAATCTAGGAAAGTTTGAATCCGACACTGAATGGATACCACGGAAACGCAAGGTATACTCACCACAAAGAAGAACGATGACTACCAGTCCACATCGCGCCAAGAAGTTCTCACCCTCTGCATCCACTCCTCACACTAACATTGCATCCATTGAGGCGATTCATGATGCTCCTCAATATATACCAAACGTCTCATGGAAAAAATTACCAGATTACTCTCCGCCCTTATCTACGCTTCCTACAGACAGTAACAAGTCACTCAAGATCGAGTGGAAGGGGTCTCCAATGGACCTGTCCACAGACCCGCTGAGGAACGAGCTACACCCTGCTGAACTAGTTCTCGCTCAAACTCTAAGGTTACCTTGTGATTTGTATCTGGATTCTAAGAGAAGGTTATTTTTGGAAAAAGTTTATAGACTAAAGAAAGGGTTGCCGTTTAGAAGGACCGACGCCCAAAAAGCCTGTAGGATCGACGTTAATAAAGCATCAAGACTATTCCAAGCTTTCGAGAAGGTTGGCTGGCTACAGGATTCGAATTTTACGAAGTACTTATAA>YAL034W-A	+:0:chr01:79719:80588ATGTCTGCTCCCACTATGAGATCCACCTCAATATTGACAGAGCATTTGGGATATCCGCCCATCTCGCTTGTTGATGATATCATTAATGCTGTAAATGAAATTATGTACAAGTGCACTGCTGCCATGGAAAAATATCTGCTATCCAAGAGCAAAATCGGCGAGGAAGATTATGGAGAAGAGATCAAAAGTGGAGTTGCTAAGTTGGAATCACTTTTGGAAAACTCCGTGGATAAGAATTTTGACAAACTAGAACTATATGTTTTGAGGAACGTCCTTCGAATCCCTGAAGAGTATTTGGACGCCAATGTTTTTAGATTGGAGAACCAAAAGGATCTGGTCATTGTAGATGAGAATGAGTTGAAGAAAAGTGAGGAGAAACTTCGAGAGAAAGTGAACGACGTGGAGTTAGCGTTCAAAAAGAATGAAATGCTATTGAAAAGAGTTACAAAAGTGAAAAGACTGTTGTTTACGATAAGAGGATTCAAACAAAAGCTAAACGAGTTACTGAAATGCAAAGACGATGTACAATTGCAGAAAATTTTGGAGTCGTTAAAACCTATAGATGACACAATGACTCTACTGACTGATTCATTACGTAAACTATATGTTGATAGTGAAAGTACCAGTTCAACAGAGGAGGTAGAGGCACTACTGCAGAGATTGAAGACCAACGGGAAGCAAAATAATAAGGATTTCAGAACACGATATATCGATATAAGGACGAATAATGTCCTACGAAAATTGGGGCTACTAGGTGATAAAGAGGACGAAAAACAGTCTGCCAAGCCGGATGCGAGGACGCAAGCAGGGGATATAGTTAGTATAGATATTGAAGAGCCTCAATTGGATTTACTTGATGATGTGTTATAA>YAL035W	+:0:chr01:76428:79436ATGGCGAAAAAGAGTAAAAAGAACCAACAGAACTACTGGGATGAGGAATTCGAAGAAGACGCCGCCCAGAACGAAGAAATCAGTGCCACGCCAACTCCAAATCCAGAAAGCAGCGCAGGTGCAGATGACACTTCCAGAGAAGCAAGTGCAAGTGCTGAAGGTGCTGAGGCCATTGAAGGCGACTTCATGTCTACTTTGAAGCAATCGAAGAAGAAGCAAGAAAAGAAGGTTATTGAAGAGAAGAAGGATGGTAAGCCTATACTAAAGTCCAAGAAGGAAAAGGAGAAGGAAAAAAAGGAAAAGGAGAAGCAGAAGAAGAAAGAACAAGCTGCCAGGAAGAAGGCCCAACAGCAAGCTCAAAAGGAGAAGAACAAGGAGTTGAACAAGCAAAATGTTGAAAAAGCTGCTGCTGAGAAGGCTGCTGCTGAGAAATCCCAAAAATCTAAAGGTGAAAGTGATAAACCAAGTGCTAGTGCTAAGAAGCCAGCCAAGAAAGTACCTGCCGGTTTGGCTGCTTTGAGACGTCAATTAGAATTGAAGAAACAACTTGAAGAACAAGAAAAGTTGGAAAGAGAGGAAGAAGAAAGATTGGAGAAAGAAGAGGAGGAAAGATTGGCCAACGAAGAAAAAATGAAGGAAGAAGCTAAAGCAGCTAAAAAGGAAAAGGAGAAGGCAAAGCGTGAAAAACGAAAGGCTGAAGGTAAGCTATTGACCAGAAAGCAAAAAGAAGAAAAGAAATTATTGGAAAGAAGACGTGCCGCTTTATTGTCTTCCGGTAACGTCAAAGTTGCCGGTCTGGCCAAGAAGGATGGAGAAGAAAACAAACCAAAGAAGGTTGTTTACAGCAAGAAGAAGAAGAGAACAACCCAGGAAAACGCCTCCGAAGCCATTAAATCTGACTCTAAGAAAGACTCGGAAGTTGTACCTGATGACGAACTCAAAGAATCCGAAGATGTTTTGATTGATGATTGGGAAAATTTGGCTCTTGGTGATGATGACGAGGAGGGAACCAACGAAGAAACGCAAGAATCCACCGCAAGCCATGAAAATGAAGACCAAAATCAAGGCGAAGAAGAAGAAGAAGGAGAAGAAGAAGAAGAAGAAGAAGAAGAAAGAGCACATGTGCATGAAGTTGCCAAAAGCACACCAGCAGCTACACCAGCAGCTACTCCAACTCCATCCAGCGCTTCTCCAAACAAAAAAGATCTTCGTTCCCCAATTTGTTGTATTTTGGGTCATGTCGATACCGGTAAGACTAAATTGTTAGACAAAATCAGACAAACCAACGTTCAAGGTGGTGAAGCTGGTGGCATCACCCAACAGATTGGTGCCACTTATTTCCCCATCGACGCTATTAAGGCAAAAACTAAAGTTATGGCTGAATATGAAAAACAAACTTTTGATGTCCCAGGTCTTTTGGTTATTGATACCCCAGGTCACGAATCCTTCTCTAACTTACGTTCAAGAGGTTCTTCATTGTGTAACATCGCAATTTTGGTTATTGACATTATGCATGGTTTGGAACAACAGACTATTGAATCTATCAAACTGTTAAGAGATAGAAAGGCTCCATTTGTCGTTGCCCTAAACAAAATTGATAGATTATATGACTGGAAAGCCATTCCAAACAATTCATTCAGAGACTCCTTTGCAAAGCAATCAAGAGCTGTTCAAGAGGAATTTCAATCTAGGTATTCTAAGATTCAATTGGAATTAGCTGAACAAGGTTTGAATTCGGAATTGTATTTCCAAAACAAAAATATGTCTAAGTATGTCTCCATTGTCCCAACATCTGCCGTCACCGGTGAGGGTGTTCCAGATTTATTGTGGTTGCTATTAGAATTGACCCAAAAGAGGATGTCCAAACAATTGATGTACTTGTCTCACGTGGAAGCAACCATTTTGGAAGTGAAAGTCGTAGAAGGTTTTGGTACCACAATTGATGTTATCTTGTCCAACGGTTACTTGAGAGAAGGTGACCGTATTGTACTGTGTGGTATGAATGGTCCAATTGTAACGAATATCAGAGCATTACTAACACCACAACCATTACGTGAACTACGTTTGAAATCTGAATATGTCCACCACAAAGAAGTCAAGGCTGCTTTAGGTGTCAAGATTGCCGCTAATGATTTAGAAAAAGCCGTTTCTGGTTCTAGGCTGCTAGTTGTCGGTCCTGAAGATGACGAAGATGAATTGATGGACGACGTTATGGATGATTTGACTGGTTTGTTGGACTCCGTTGACACAACTGGTAAAGGTGTTGTGGTCCAAGCATCCACCTTGGGTTCTTTGGAAGCTTTGTTGGATTTCTTGAAAGACATGAAAATCCCTGTGATGTCTATCGGGTTAGGTCCAGTGTACAAGCGTGATGTTATGAAAGCCTCCACTATGTTGGAAAAGGCTCCAGAGTATGCCGTGATGTTATGTTTTGATGTTAAAGTGGATAAGGAAGCTGAACAATACGCTGAACAAGAAGGAATTAAGATCTTTAATGCAGACGTCATCTATCATTTATTTGATTCATTTACAGCATACCAAGAAAAGTTATTGGAAGAACGTCGTAAAGATTTCCTAGATTACGCTATTTTCCCATGTGTCTTACAAACCTTACAAATTATTAACAAACGTGGTCCAATGATTATTGGTGTAGACGTTCTGGAAGGTACTCTACGTGTGGGAACTCCTATTTGCGCTGTGAAAACCGACCCTACTACAAAGGAAAGACAAACTTTGATATTAGGTAAAGTCATCTCTTTAGAAATCAACCATCAACCTGTCCAAGAAGTAAAGAAGGGCCAAACCGCTGCTGGTGTTGCCGTCCGTCTAGAAGATCCCTCCGGTCAACAACCTATCTGGGGTCGTCATGTTGACGAGAATGATACATTATACTCCTTGGTTTCAAGAAGATCTATTGACACTTTGAAGGATAAAGCTTTTAGGGACCAAGTTGCTAGATCCGATTGGCTGCTATTGAAGAAGCTGAAGGTCGTTTTCGGCATCGAATGA>YAL036C	-:0:chr01:75044:76153ATGTCTACTACAGTTGAAAAAATCAAAGCTATCGAAGATGAAATGGCCCGTACCCAAAAGAACAAGGCCACATCTTTCCATTTGGGTCAACTGAAGGCCAAGCTGGCCAAACTGAGAAGAGAATTGTTGACCAGTGCTTCATCCGGCAGCGGTGGTGGTGCTGGTATTGGTTTTGATGTGGCTAGAACTGGTGTGGCCAGTGTGGGGTTTGTCGGGTTCCCGTCGGTGGGGAAATCTACATTACTGTCCAAGTTGACTGGTACTGAGTCTGAAGCAGCTGAGTACGAGTTTACCACCCTGGTTACCGTCCCCGGTGTCATTCGTTATAAAGGTGCCAAGATCCAAATGTTGGATTTACCTGGTATTATCGATGGTGCTAAGGATGGTAGAGGTAGAGGTAAGCAAGTTATTGCCGTGGCAAGAACCTGTAACCTGTTATTTATCATCCTAGATGTGAACAAACCCTTGCATCATAAGCAAATCATTGAGAAGGAACTGGAAGGTGTGGGGATTCGTCTGAATAAAACTCCGCCAGATATCTTGATCAAAAAAAAAGAGAAAGGTGGTATTTCCATCACAAACACAGTCCCATTGACCCATCTGGGGAATGACGAAATCAGAGCCGTTATGAGCGAGTACAGAATAAATAGCGCTGAGATTGCCTTCAGGTGTGATGCCACTGTGGATGATTTGATTGATGTTTTGGAAGCTTCGTCAAGAAGATACATGCCTGCCATCTATGTGTTAAACAAGATTGATTCTCTGTCAATAGAGGAATTGGAATTACTTTACCGAATTCCTAATGCCGTGCCTATTTCGTCTGGTCAAGATTGGAACTTGGACGAGCTGTTGCAAGTCATGTGGGATAGACTAAATCTAGTCCGTATTTACACTAAACCAAAGGGCCAAATACCAGATTTTACCGACCCTGTGGTGCTAAGATCAGACCGTTGCAGTGTCAAGGATTTTTGTAACCAAATTCATAAATCTTTAGTGGACGACTTTAGAAATGCTCTGGTTTACGGTAGCAGTGTCAAACATCAACCTCAATACGTGGGGTTGAGTCACATTTTGGAAGACGAAGATGTTGTTACCATCTTGAAAAAGTGA>YAL037W	+:0:chr01:74021:74824ATGGATATGGAAATCGAAGATTCAAGCCCCATAGATGACCTGAAGTTACAAAAACTGGATACCAATGTTTATTTTGGACCCTGTGAGATATTGACACAACCTATTCTTTTGCAATATGAAAATATTAAGTTCATCATTGGTGTCAATCTAAGTACTGAAAAGATAGCGTCGTTTTATACCCAGTATTTCAGGAACTCTAATTCGGTAGTCGTGAATCTTTGCTCACCAACTACAGCAGCAGTAGCAACAAAGAAGGCCGCAATTGATTTGTATATACGAAACAATACAATACTACTACAGAAATTCGTTGGACAGTACTTGCAGATGGGCAAAAAGATAAAAACATCTTTAACACAGGCACAAACCGATACAATCCAATCACTGCCCCAGTTTTGTAATTCGAATGTCCTCAGTGGTGAGCCCTTGGTACAGTACCAGGCATTCAACGATCTGTTGGCACTCTTTAAGTCATTTAGTCATTTTGGAAATATCTTGGTTATATCATCACATTCCTATGATTGCGCACTTCTCAAATTTCTTATTTCCAGGGTGATGACCTACTATCCACTAGTGACCATCCAGGATTCTTTGCAATATATGAAAGCAACCCTGAACATATCCATCAGTACATCCGATGAGTTCGATATTCTGAATGATAAAGAACTGTGGGAGTTTGGCCAAACCCAGGAAATTCTAAAACGTAGGCAGACGAGCTCAGTCAAGAGGAGATGTGTCAATTTACCAGAAAACTCTACGATCGATAACAGAATGCTTATGGGTACCACAAAGCGAGGTCGCTTTTGA>YAL038W	+:0:chr01:71787:73289ATGTCTAGATTAGAAAGATTGACCTCATTAAACGTTGTTGCTGGTTCTGACTTGAGAAGAACCTCCATCATTGGTACCATCGGTCCAAAGACCAACAACCCAGAAACCTTGGTTGCTTTGAGAAAGGCTGGTTTGAACATTGTCCGTATGAACTTCTCTCACGGTTCTTACGAATACCACAAGTCTGTCATTGACAACGCCAGAAAGTCCGAAGAATTGTACCCAGGTAGACCATTGGCCATTGCTTTGGACACCAAGGGTCCAGAAATCAGAACTGGTACCACCACCAACGATGTTGACTACCCAATCCCACCAAACCACGAAATGATCTTCACCACCGATGACAAGTACGCTAAGGCTTGTGACGACAAGATCATGTACGTTGACTACAAGAACATCACCAAGGTCATCTCCGCTGGTAGAATCATCTACGTTGATGATGGTGTTTTGTCTTTCCAAGTTTTGGAAGTCGTTGACGACAAGACTTTGAAGGTCAAGGCTTTGAACGCCGGTAAGATCTGTTCCCACAAGGGTGTCAACTTACCAGGTACCGATGTCGATTTGCCAGCTTTGTCTGAAAAGGACAAGGAAGATTTGAGATTCGGTGTCAAGAACGGTGTCCACATGGTCTTCGCTTCTTTCATCAGAACCGCCAACGATGTTTTGACCATCAGAGAAGTCTTGGGTGAACAAGGTAAGGACGTCAAGATCATTGTCAAGATTGAAAACCAACAAGGTGTTAACAACTTCGACGAAATCTTGAAGGTCACTGACGGTGTTATGGTTGCCAGAGGTGACTTGGGTATTGAAATCCCAGCCCCAGAAGTCTTGGCTGTCCAAAAGAAATTGATTGCTAAGTCTAACTTGGCTGGTAAGCCAGTTATCTGTGCTACCCAAATGTTGGAATCCATGACTTACAACCCAAGACCAACCAGAGCTGAAGTTTCCGATGTCGGTAACGCTATCTTGGATGGTGCTGACTGTGTTATGTTGTCTGGTGAAACCGCCAAGGGTAACTACCCAATCAACGCCGTTACCACTATGGCTGAAACCGCTGTCATTGCTGAACAAGCTATCGCTTACTTGCCAAACTACGATGACATGAGAAACTGTACTCCAAAGCCAACCTCCACCACCGAAACCGTCGCTGCCTCCGCTGTCGCTGCTGTTTTCGAACAAAAGGCCAAGGCTATCATTGTCTTGTCCACTTCCGGTACCACCCCAAGATTGGTTTCCAAGTACAGACCAAACTGTCCAATCATCTTGGTTACCAGATGCCCAAGAGCTGCTAGATTCTCTCACTTGTACAGAGGTGTCTTCCCATTCGTTTTCGAAAAGGAACCTGTCTCTGACTGGACTGATGATGTTGAAGCCCGTATCAACTTCGGTATTGAAAAGGCTAAGGAATTCGGTATCTTGAAGAAGGGTGACACTTACGTTTCCATCCAAGGTTTCAAGGCCGGTGCTGGTCACTCCAACACTTTGCAAGTCTCTACCGTTTAA>YAL039C	-:0:chr01:68717:69526ATGGGTTGGTTTTGGGCAGATCAAAAAACTACGGGCAAAGATATTGGTGGGGCAGCAGTATCATCCATGTCAGGGTGCCCAGTCATGCACGAGTCGTCGTCGTCGTCGCCACCATCCTCTGAGTGCCCCGTTATGCAGGGAGATAACGATAGAATAAACCCGCTGAACAATATGCCGGAGTTGGCAGCATCCAAACAGCCTGGCCAAAAGATGGACTTGCCCGTTGATCGGACCATCTCCAGCATCCCCAAGAGTCCAGACAGTAACGAGTTCTGGGAGTATCCTTCTCCACAACAGATGTACAATGCTATGGTTAGAAAGGGCAAGATTGGCGGTAGCGGCGAAGTCGCCGAAGATGCAGTGGAGTCCATGGTGCAGGTCCACAACTTTCTAAATGAAGGGTGCTGGCAGGAAGTGCTCGAATGGGAAAAACCGCACACAGATGAAAGCCACGTGCAGCCTAAGTTGCTGAAATTCATGGGGAAACCGGGCGTATTGAGCCCTCGTGCTCGCTGGATGCACCTGTGCGGCCTACTGTTTCCGTCCCATTTTAGCCAAGAACTACCATTCGACAGGCACGACTGGATTGTACTCCGAGGCGAGCGCAAAGCGGAACAACAACCTCCAACCTTCAAGGAAGTTAGATACGTCTTGGATTTCTACGGAGGGCCCGACGACGAAAACGGAATGCCTACTTTCCACGTGGATGTCCGTCCTGCCCTAGATAGTCTAGACAATGCTAAGGACCGGATGACCCGTTTCTTGGACCGGATGATCTCGGGTCCGTCCTCTTCGTCCTCCGCCCCTTAA>YAL040C	-:0:chr01:65779:67521ATGGCCATATTGAAGGATACCATAATTAGATACGCTAATGCAAGGTATGCTACCGCTAGTGGCACTTCCACCGCCACTGCCGCCTCTGTCAGCGCTGCCTCATGTCCTAATTTGCCCTTGCTCTTGCAAAAGAGGCGGGCCATTGCTAGTGCAAAGTCTAAAAACCCTAATCTCGTTAAAAGAGAATTGCAAGCACATCACTCAGCGATCAGCGAATACAATAATGATCAATTGGACCACTATTTCCGTCTTTCCCACACAGAAAGGCCGCTGTACAACCTGACTAACTTCAACTCTCAGCCACAAGTTAATCCGAAGATGCGTTTCTTGATCTTTGACTTCATCATGTACTGTCACACAAGACTCAATCTATCGACCTCGACTTTGTTCCTTACTTTCACTATCTTGGACAAGTATTCCTCGCGGTTCATTATCAAGAGTTACAACTACCAGCTCTTGTCCTTGACCGCGCTTTGGATTTCGTCCAAATTTTGGGACTCCAAGAATAGAATGGCCACTTTGAAAGTCTTGCAAAACTTGTGTTGCAATCAATATTCTATAAAGCAATTCACGACTATGGAAATGCATCTTTTCAAATCACTCGATTGGTCCATCTGTCAGTCGGCAACATTCGACTCCTACATCGACATCTTCTTGTTCCAATCTACGTCCCCGTTATCGCCTGGCGTTGTCCTTTCTGCCCCTTTGGAAGCTTTCATTCAACAGAAACTGGCCTTATTAAATAACGCTGCTGGTACTGCTATTAATAAATCGTCCTCTTCTCAAGGCCCCTCTTTGAACATCAACGAGATCAAATTGGGTGCCATTATGTTGTGCGAGTTAGCTTCCTTCAATCTCGAATTATCATTTAAATATGATCGTTCACTAATTGCGCTGGGTGCAATTAACCTCATCAAATTATCTTTGAACTACTATAATTCAAACCTTTGGGAAAATATCAATCTGGCTTTGGAGGAAAACTGCCAAGACCTAGATATTAAATTGTCAGAAATCTCTAATACTTTATTGGATATAGCAATGGACCAAAATTCTTTCCCCTCCAGTTTCAAATCAAAATATTTGAATAGCAATAAGACATCTTTAGCAAAATCTCTCTTAGACGCATTACAAAACTATTGTATTCAATTGAAACTGGAAGAATTCTACCGTTCACAAGAATTGGAAACCATGTACAATACTATCTTTGCTCAGTCCTTTGACAGCGATTCATTGACTTGTGTTTACTCAAATGCTACTACTCCAAAGAGCGCTACGGTTTCATCTGCGGCCACAGACTATTTCTCGGATCACACTCATTTAAGAAGGTTGACCAAAGATAGCATTTCTCCACCATTTGCCTTCACTCCAACCTCATCTTCATCCTCTCCATCTCCATTCAATTCCCCTTACAAGACTTCAAGTTCAATGACGACCCCAGACTCTGCATCACACCATTCACATTCAGGTTCGTTCTCTTCTACCCAAAATTCTTTTAAAAGGTCACTGAGCATCCCACAAAATTCAAGCATCTTTTGGCCAAGCCCACTAACTCCCACCACCCCATCTCTAATGTCAAATAGAAAATTATTACAAAATTTATCTGTGCGTTCAAAAAGATTATTTCCTGTTAGACCCATGGCCACTGCTCACCCATGCTCTGCCCCCACCCAACTGAAAAAGAGATCAACTTCCTCTGTGGATTGTGATTTTAATGATAGTAGCAACCTCAAGAAAACTCGCTGA>YAL041W	+:0:chr01:62841:65405ATGGCGATCCAAACCCGTTTTGCCTCGGGCACATCTTTATCCGATTTGAAACCAAAACCAAGTGCAACTTCCATCTCCATACCCATGCAAAATGTCATGAACAAGCCTGTCACGGAACAGGACTCACTGTTCCATATATGCGCAAACATCCGGAAAAGACTGGAGGTGTTACCTCAACTCAAACCTTTTTTACAATTGGCCTACCAATCGAGCGAGGTTTTGAGTGAAAGGCAATCTCTTTTGCTATCCCAAAAGCAGCATCAGGAACTGCTCAAGTCCAATGGCGCTAACCGGGACAGTAGCGACTTGGCACCAACTTTAAGGTCTAGCTCTATCTCCACAGCTACCAGTCTCATGTCGATGGAAGGTATATCATACACGAATTCGAATCCCTCGGCCACCCCAAATATGGAGGACACTTTACTGACTTTTAGTATGGGTATTTTGCCCATTACCATGGATTGCGACCCTGTGACACAACTATCACAGCTGTTTCAACAAGGTGCGCCCCTCTGTATACTTTTCAACTCTGTGAAGCCGCAATTTAAATTACCGGTAATAGCATCTGACGATTTGAAAGTCTGTAAAAAATCCATTTATGACTTTATATTGGGCTGCAAGAAACACTTTGCATTTAACGATGAGGAGCTTTTCACTATATCCGACGTTTTTGCCAACTCTACTTCCCAGCTGGTCAAAGTGCTAGAAGTAGTAGAAACGCTAATGAATTCCAGCCCTACTATTTTCCCCTCTAAGAGTAAGACACAGCAAATCATGAACGCAGAAAACCAACACCGACATCAGCCTCAGCAGTCTTCGAAGAAGCATAACGAGTATGTTAAAATTATCAAGGAATTCGTTGCAACGGAAAGAAAATATGTTCACGATTTGGAAATTTTGGATAAATATAGACAGCAGTTATTAGACAGCAATCTAATAACGTCTGAAGAGTTGTACATGTTGTTCCCTAATTTGGGTGATGCTATAGATTTTCAAAGAAGATTTCTAATATCCTTGGAAATAAATGCTTTAGTAGAACCTTCCAAGCAAAGAATCGGGGCTCTTTTCATGCATTCCAAACATTTTTTTAAGTTGTATGAGCCTTGGTCTATTGGCCAAAATGCAGCCATCGAATTTCTCTCTTCAACTTTGCACAAGATGAGGGTTGATGAATCGCAGCGGTTCATAATTAACAATAAACTGGAATTGCAATCCTTCCTTTATAAACCCGTGCAAAGGCTTTGTAGATATCCCCTGTTGGTCAAAGAATTGCTTGCTGAATCGAGTGACGATAATAATACGAAAGAACTTGAAGCTGCTTTAGATATTTCTAAAAATATTGCGAGAAGTATCAACGAAAATCAAAGAAGAACAGAAAATCATCAAGTGGTGAAGAAACTTTATGGTAGAGTGGTCAACTGGAAGGGTTATAGAATTTCCAAGTTCGGTGAGTTATTATATTTCGATAAAGTGTTCATTTCAACAACAAATAGCTCCTCGGAACCTGAAAGAGAATTTGAGGTTTATCTTTTTGAAAAAATCATCATCCTTTTTTCAGAGGTAGTGACTAAGAAATCTGCATCATCACTAATCCTTAAGAAGAAATCCTCAACCTCAGCATCAATCTCCGCCTCGAACATAACGGACAACAATGGCAGCCCTCACCACAGTTACCATAAGAGGCATAGCAATAGTAGTAGCAGTAATAATATCCATTTATCTTCGTCTTCAGCAGCGGCGATAATACATTCCAGTACCAATAGTAGTGACAACAATTCCAACAATTCATCATCATCCTCATTATTCAAGCTGTCCGCTAACGAACCTAAGCTGGATCTAAGAGGTCGAATTATGATAATGAATCTGAATCAAATCATACCGCAAAACAACCGGTCATTAAATATAACATGGGAATCCATAAAAGAGCAAGGTAATTTCCTTTTGAAATTCAAAAATGAGGAAACAAGAGATAATTGGTCATCGTGTTTACAACAGTTGATTCATGATCTGAAAAATGAGCAGTTTAAGGCAAGACATCACTCTTCAACATCGACGACTTCATCGACAGCCAAATCATCTTCAATGATGTCACCCACCACAACTATGAATACACCGAATCATCACAACAGCCGCCAGACACACGATAGTATGGCTTCTTTCTCAAGTTCTCATATGAAAAGGGTTTCGGATGTCCTGCCTAAACGGAGGACCACTTCATCAAGTTTCGAAAGTGAAATTAAATCCATTTCAGAAAATTTCAAGAACTCTATTCCAGAATCTTCCATACTCTTCAGGATATCATATAATAACAACTCTAATAATACCTCTAGTAGCGAGATCTTCACACTTTTGGTAGAAAAAGTTTGGAATTTTGACGACTTGATAATGGCGATCAATTCTAAAATTTCGAATACACATAATAACAACATTTCACCAATCACCAAGATCAAATATCAGGACGAAGATGGGGATTTTGTTGTGTTAGGTAGCGATGAAGATTGGAATGTTGCTAAAGAAATGTTGGCGGAAAACAATGAGAAATTCTTGAACATTCGTCTGTATTGA>YAL042W	+:0:chr01:61317:62564ATGAAAAGGTCCACGTTGCTGTCGCTGGACGCATTCGCTAAGACCGAAGAGGACGTACGAGTCCGCACCAGGGCCGGCGGGCTGATCACTTTATCGTGCATCTTGACCACGTTATTTCTGCTGGTGAACGAGTGGGGACAGTTCAATTCTGTGGTAACAAGGCCACAATTGGTGGTGGACCGTGACCGACACGCAAAGCTGGAGCTTAATATGGATGTGACATTTCCATCGATGCCATGTGACCTGGTGAATCTCGATATTATGGACGACTCTGGAGAGATGCAACTAGACATTCTTGACGCAGGGTTCACGATGTCTAGGTTGAATAGCGAGGGTCGCCCCGTGGGAGATGCTACTGAGTTGCATGTGGGTGGGAACGGCGACGGAACCGCGCCGGTTAATAACGATCCTAACTATTGTGGGCCATGTTACGGTGCCAAAGATCAGTCGCAGAATGAGAATCTAGCACAGGAAGAGAAGGTTTGCTGCCAAGACTGTGATGCAGTGAGATCAGCATACTTGGAGGCAGGCTGGGCTTTTTTCGACGGGAAGAATATCGAGCAGTGTGAAAGAGAGGGCTATGTCAGCAAGATTAACGAGCACTTGAATGAAGGCTGCAGGATCAAAGGTTCTGCACAAATTAACAGAATTCAGGGGAATCTTCACTTTGCCCCTGGAAAACCCTACCAGAATGCATATGGACATTTTCATGATACTTCTTTGTACGACAAGACTTCGAATTTGAACTTCAACCACATCATCAATCATTTGAGCTTTGGGAAGCCGATCCAGTCCCACAGTAAGTTGTTAGGAAACGATAAGCGCCACGGCGGCGCCGTAGTTGCCACTTCTCCCTTGGACGGACGCCAGGTGTTCCCGGACAGGAACACACACTTTCACCAGTTCTCGTATTTTGCCAAGATTGTCCCCACCAGATATGAGTACTTGGATAATGTTGTCATTGAGACCGCGCAGTTCAGCGCCACTTTTCATTCCCGACCTCTTGCCGGTGGAAGGGACAAGGATCATCCAAACACACTTCACGTTAGGGGTGGTATCCCTGGTATGTTCGTCTTTTTCGAAATGTCTCCATTGAAAGTCATCAATAAGGAACAGCACGGGCAGACTTGGTCGGGCTTCATCTTGAATTGTATCACCAGCATTGGTGGTGTCCTAGCTGTGGGCACTGTCATGGACAAGCTATTCTACAAAGCACAGAGATCGATCTGGGGCAAGAAGAGCCAGTAG>YAL043C	-:0:chr01:58696:61053ATGTCATCTGCAGAGATGGAACAATTGTTACAGGCCAAGACACTGGCCATGCACAACAATCCAACGGAGATGCTGCCCAAGGTGCTCGAAACTACGGCATCCATGTACCACAACGGTAATCTCAGCAAGCTGAAGTTGCCTTTGGCCAAGTTTTTTACACAGTTAGTTCTAGACGTGGTGTCGATGGACTCTCCAATTGCGAATACTGAGAGACCGTTTATTGCTGCTCAATATCTGCCACTACTTCTTGCTATGGCGCAATCCACCGCGGACGTACTAGTGTACAAGAATATCGTGCTTATTATGTGCGCTTCATACCCGCTGGTGTTGGATCTGGTTGCTAAGACATCAAACCAGGAAATGTTTGATCAGTTGTGTATGCTGAAGAAGTTCGTGCTCTCGCACTGGAGAACTGCATATCCTTTGCGTGCCACCGTTGACGATGAAACGGATGTCGAACAATGGCTGGCGCAGATTGACCAAAATATCGGCGTGAAATTAGCGACCATCAAGTTCATATCTGAGGTCGTGCTGTCGCAAACTAAATCACCCAGCGGCAACGAGATTAATTCATCTACCATCCCGGATAACCACCCTGTGTTGAACAAACCGGCTTTGGAGAGCGAGGCTAAGAGGCTTCTTGATATGTTGCTAAACTACCTAATTGAGGAACAGTACATGGTCTCGTCCGTTTTCATTGGTATCATCAATTCTTTATCCTTCGTCATCAAAAGAAGGCCGCAGACAACAATAAGAATTCTTTCCGGGCTGTTGCGTTTCAACGTCGACGCCAAGTTTCCCCTAGAGGGCAAGTCTGACTTGAACTACAAACTATCCAAGAGATTTGTTGAAAGGGCGTACAAGAACTTTGTGCAATTTGGGCTAAAAAATCAAATCATTACAAAATCCCTCTCATCCGGATCAGGGTCATCGATCTACTCCAAGCTGACCAAGATTTCTCAAACTTTACACGTTATTGGCGAAGAGACCAAGAGCAAGGGAATTTTGAACTTCGACCCTTCCAAGGGCAATAGCAAGAAAACGTTGTCCAGGCAGGACAAACTAAAATACATCTCACTATGGAAAAGGCAATTATCCGCGTTATTGTCTACTCTAGGGGTGTCCACAAAGACCCCCACGCCTGTGTCCGCACCTGCAACGGGCTCTTCAACCGAAAACATGCTTGATCAACTGAAGATATTGCAAAAATACACCCTCAACAAGGCTTCACACCAGGGCAATACTTTTTTCAACAACTCACCCAAACCAATCAGCAACACCTACTCATCTGTGTACTCATTGATGAACAGTTCGAACTCCAACCAGGATGTGACCCAGCTACCCAATGACATACTTATCAAGCTGTCCACAGAGGCCATCTTGCAAATGGACAGCACGAAACTGATCACCGGATTGTCTATCGTTGCTTCGAGGTACACGGATTTAATGAATACGTACATCAATTCTGTACCGTCCTCGTCATCATCAAAGAGGAAATCCGACGATGATGACGACGGCAACGACAATGAAGAAGTTGGAAACGATGGCCCAACGGCTAATAGCAAGAAAATCAAAATGGAAACAGAACCACTAGCGGAGGAACCAGAGGAGCCCGAAGACGATGACCGAATGCAGAAGATGCTTCAAGAAGAGGAAAGCGCCCAAGAAATCTCAGGAGATGCCAACAAATCAACTTCTGCCATTAAGGAGATCGCACCCCCCTTTGAACCTGACTCATTGACGCAGGATGAAAAACTAAAGTACCTCTCAAAGCTGACCAAGAAACTGTTTGAATTATCCGGTCGCCAGGATACTACCCGGGCCAAATCTTCGTCTTCCTCCTCCATATTACTGGACGATGACGACTCCTCGTCATGGTTACACGTCTTAATCAGATTGGTTACGAGAGGAATCGAAGCACAAGAGGCCAGTGACCTGATTCGTGAAGAACTGCTTGGCTTCTTCATCCAGGATTTCGAGCAACGTGTCAGTCTGATCATTGAATGGCTCAATGAAGAATGGTTCTTCCAAACCTCGCTGCATCAAGATCCCTCTAACTACAAAAAATGGTCCTTAAGAGTTCTCGAGTCTCTGGGTCCATTCCTTGAAAACAAACACAGACGATTCTTCATCAGACTTATGAGCGAACTGCCCAGTCTTCAAAGCGATCATCTTGAGGCACTGAAGCCTATCTGCCTGGATCCGGCAAGAAGTTCCCTTGGTTTCCAAACGCTAAAGTTTCTCATTATGTTTAGACCCCCAGTGCAGGACACTGTTCGCGACCTGCTGCATCAGCTAAAGCAAGAAGATGAAGGCTTACACAAGCAGTGCGATTCACTGCTTGACAGGCTAAAATGA>YAL044C	-:0:chr01:57951:58463ATGTTACGCACTACTAGACTATGGACCACCCGCATGCCCGCTGTGAGCAAATTGTTTTTGAGAAACAGCTCCGGCAATGCCCTAAACAAGAATAAACTACCATTTTTGTACTCATCCCAAGGACCTCAAGCCGTGAGGTACACTTCCCAACATGAGTGGATAGCTGTGCATCAGGACAAGACTGCCTTTGTCGGAATTACAAAATACGCCACTGATTCCTTAGGGGACGCTACCTATGTTGAGTTGCCAGAAGTGGGCACTGAGATTTCCCAAGGTGAGTCGCTAGGGTCCATTGAGTCCGTCAAGTCAGCCTCCGAGATCTACCAGCCTGCCGATGGTACCGTAGAGGAAATTAACACTAATCTTGAGGAAAATCCAGGTGTGGTGAACGAAGATCCTATGGGTGACGGCTGGCTAGTCAAAATGAAGCTTGGTGAGGGCGTTAATGTGGAACAGGTCGAGGGTCTAATGTCCTTAGAACAGTACGAAAAGACACTGGTTCATGATGACTGA>YAL044W-A	+:0:chr01:57519:57851ATGTTCAAGAGAGCAATGAGCACAGATGGTCCCGTGGCACGTACCATCCTGAAGAGACTGGAATGCGGCTTTCCAGATTACAAGAACTTTGCGTTTGGCCTCTACAACGATTCTCACAAGCATAAGGGCCATGCTGGTGTACAGGGAAATGTCTCTGCTGAGACACATTTCCGGATTGAGATGGTCAGTAAAAAGTTCGAAGGCCTGAAACTTCCACAACGCCATCGTATGGTTTATTCCCTCTTGCAAGACGAGATGGCTCAGGCGAACGGTATCCATGCTTTACAATTGTCACTAAAGACCCCACAGGAGTATGAATCCAAAGCGAAATAG>YAL046C	-:0:chr01:57030:57386ATGAAGCTCCCACAGACCATGCTACGTTCTATATCTGTGAAGCATGTCCGGTGGCCAAGGATTCTGACGGGCTCAAAGCTTTGGTACTCAACGCAGATGGCAATGACTCCGGAGGAGAAGATGATCACCGATAAACTACAACAGGAACTGGAACCTGAAGTGTGTAAAGTGCAAGACGTTTCCGGTGGCTGCGGATCCATGTTTGCTATCAACATAACAAGCAAGAAGTTCAACGGACTGAGTCTCATCAAGCAGCACCAGCTGGTGAACAGAATTTTGAGGGACGATATTTCCAGATGGCATGGCCTACAATTGACCACTAAGAAGTCAACTGGGAAGGGTCCGGCATCATCATGA>YAL047C	-:0:chr01:54990:56858ATGGTACGTCGATGGATTCCTAGTGGCAGGCATCTTCGCAATAATGACAACACTGGTGATGATGACGACAGCGAGTTCACAAACTCGATGGATTCTGGGATGTCCATACCATCACTCAGGGACTCCATGACCACGAGGTCATCTCATAACGATCCCATCAAGCCTGCTCTGATGAACGATTCCAACAAAGTCAAAAATTTGGAGAAGGAGTTGACTAATGCCAAAATCAAGATTCAAGTACTCTATGAATACATTCGCAGAATCCCTAATAAAGACGGCAATGCACCATCGCTGGGCAATGACACTGATTTTAGAAATTCGATTATCGAGGGTCTAAATCTTGAAATAAACAAATTGAAACAGGATTTAAAGGCGAAGGAAGTCGAGTACCAAGATACGCTACAATTCGTTCAAGAGAATTTAGAAAATTCGGAAAGTATCGTGAATACGATCAATCACCTATTGTCCTTTATATTGACGCATTTTAATGAGCAAGATGAAAATGCCCATCTTCTTGATAAAGAAGAGAGGGAGACCCTGGAGGAAACTTTAGAGCTGAGCTCGGATTATGTTCTTGAAAAAATGGATACTTTGTCCAAGTTCATCATCCAATTCTTGCAGGACTTTTTGCATTCCAAAAGTCGAGCGGAATCAAAGCAGGATAAGGAAGAATTTCTTTCACTGGCCCAGTCCTCACCAGCAGGATCACAGTTAGAAAGTAGGGACTCACCATCAAGTAAAGAGGAGAATACTGATGGTGGATACCAGAATGACGAAATTCATGACAGTAACAACCACATTGATACAGAAAATGTAATGGCAAACAGTACTTCCTTACCAATTTCAGCCGTCGAGTCTCGTTTTGAGAAAACCTTAGACACTCAATTAGAGATTGTCATAGAAATTTTGCACAAAGAATATGATCAATTTATAAATTCCATTAGATTGAAATTCGAGAAATCGCAAAAATTAGAAAAAATAATAGCGTCCAAACTAAATGAGCAGTCTCATCTACTAGATTCCTTAGAATTGGAAGAAAATTCTAGTTCCGTTATAGAAAAACAAGATCATTTGATTTCCCAACTGAAGGAAAAGATCGAATCGCAATCTGTTTTGATAAACAATTTGGAAAAATTGAAGGAAGACATCATTAAAATGAAACAAAATGAAAAAGTTTTAACCAAAGAACTGGAAACTCAGACCAAAATCAATAAACTAAAGGAAAATAATTGGGACAGCTACATCAATGACTTGGAAAAACAAATCAATGACCTTCAAATCGATAAATCAGAGGAATTCCACGTAATACAAAATCAGTTAGACAAATTAGACTTGGAGAATTACCAATTAAAAAATCAGTTAAATACTTTGGACAACCAAAAGTTAATACTATCCCAATATGAAAGCAATTTTATCAAATTTAATCAGAACCTTTTACTACACCTTGACAGTATTTTCAATATTCTGCAGAAAATCTTACAAGAAAGTTCTATTGCCCAATTTGACAGGAAAATGAAATCCATAAAATCTGTACCAAATGCGTTGAAAAACTTGAATTTGATTCAGCCCAAACTAGAATCGCTCTACACTTTTATAGAGACTGCATTAGAGTCCATAATAAACTCATATATCTCTTCACTGATTTCCATGGAAACCCCAGAGCAGCCACATCAACAGGGCAATGAATTAACGGCAACTCCAAATAAAGAACTGACCTTACGAATAGAGGAGTTACAAAGAAGATGGATATCCGAAAGAGAAAGACGGAAGCTCGATGCTAACGCTTCAGAAGCTCGAATCAAGGCTTTAGAACAGGAAAATGAGTCATTGAGATCGAAACTTTTCAACCTATCAATCAACAATCCCTAA>YAL048C	-:0:chr01:52802:54790ATGACTAAAGAAACGATTCGGGTAGTTATTTGCGGTGATGAAGGGGTTGGTAAATCCAGTCTGATTGTATCATTAACAAAAGCTGAATTCATACCGACCATACAGGACGTGCTGCCACCCATCAGTATCCCAAGAGATTTCTCATCATCACCTACATATTCTCCTAAGAATACAGTACTTATAGACACTTCAGATTCGGACCTCATAGCTTTAGACCATGAGTTGAAGTCCGCCGACGTAATTTGGCTTGTGTATTGCGATCACGAATCGTATGACCATGTTTCTCTCTTTTGGTTGCCTCATTTCAGATCCCTGGGGTTGAATATTCCTGTCATTCTCTGCAAAAATAAATGTGATTCCATATCGAATGTTAATGCCAATGCAATGGTCGTGTCAGAGAACAGTGATGATGATATCGATACCAAAGTGGAGGATGAAGAATTTATCCCAATATTAATGGAGTTTAAAGAAATCGACACTTGCATTAAGACAAGTGCCAAGACACAGTTTGATCTTAACCAAGCGTTTTATCTTTGCCAAAGGGCTATAACACACCCAATATCACCTTTATTTGATGCCATGGTAGGTGAACTAAAGCCATTGGCTGTCATGGCTTTAAAAAGAATTTTTCTTTTAAGCGATTTAAACCAGGACTCATATTTAGATGACAACGAAATCTTGGGCTTACAAAAAAAGTGCTTCAATAAGAGTATCGATGTAAACGAACTGAATTTTATTAAAGATTTGCTTTTGGATATTTCCAAGCACGATCAAGAGTACATTAACCGCAAGCTATACGTACCGGGAAAAGGCATCACCAAAGATGGTTTCCTTGTACTGAACAAAATATACGCTGAAAGGGGGAGACACGAAACTACATGGGCTATCCTAAGAACTTTCCATTACACAGATTCCTTGTGTATTAATGACAAAATTCTCCATCCGAGGTTGGTTGTCCCTGACACTTCCAGTGTGGAATTGAGCCCCAAGGGCTACAGATTTCTTGTAGATATTTTTTTGAAGTTTGATATCGACAATGATGGTGGTTTGAATAATCAAGAATTACATCGTCTATTTAAGTGCACACCAGGGCTGCCTAAACTATGGACCTCAACGAATTTCCCCTTCTCCACTGTCGTAAACAACAAGGGTTGCATCACCTTACAAGGCTGGCTAGCACAATGGAGTATGACGACTTTCTTGAACTATAGCACAACTACCGCTTACTTGGTGTATTTTGGCTTTCAGGAAGATGCAAGACTAGCCCTACAAGTAACCAAGCCAAGGAAAATGAGACGCCGTTCCGGGAAACTTTACAGATCCAATATCAATGACAGAAAAGTGTTCAATTGCTTTGTCATTGGAAAGCCATGTTGCGGCAAAAGCTCTTTGCTAGAGGCCTTCTTGGGCAGATCTTTCTCGGAGGAGTATTCTCCGACAATCAAACCAAGAATTGCAGTCAATAGTTTAGAACTCAAAGGTGGGAAACAGTACTATTTGATTTTGCAAGAACTTGGAGAACAGGAATACGCCATACTAGAGAATAAGGATAAGTTAAAAGAATGTGACGTAATCTGTCTAACATACGATTCCAGCGACCCAGAATCATTCTCCTACTTAGTTTCGCTTCTAGACAAATTCACACATTTACAAGATCTACCGTTGGTATTTGTAGCTTCTAAGGCAGATTTGGATAAGCAGCAACAAAGGTGTCAAATCCAACCAGATGAACTGGCAGACGAGCTATTTGTGAACCACCCACTGCACATATCATCCAGATGGCTAAGTTCCCTTAATGAATTGTTTATTAAAATCACAGAAGCTGCTCTTGATCCTGGCAAAAACACTCCGGGATTGCCAGAAGAAACAGCAGCAAAAGATGTCGACTACAGACAAACGGCTCTCATTTTTGGGTCCACTGTTGGATTCGTAGCACTATGTTCCTTTACGTTAATGAAATTATTCAAATCATCAAAATTCTCAAAATAA>YAL049C	-:0:chr01:51856:52596ATGGCATCTAATCAACCTGGCAAGTGTTGCTTTGAAGGAGTTTGTCACGATGGAACACCCAAGGGTCGTCGTGAAGAAATCTTCGGTTTAGATACTTATGCAGCAGGCTCTACATCTCCCAAGGAAAAAGTTATAGTTATCTTGACAGATGTGTATGGCAATAAATTCAACAATGTTTTATTAACGGCCGACAAATTTGCTAGTGCTGGGTACATGGTCTTTGTTCCCGATATTTTATTCGGCGATGCTATCTCATCGGACAAACCAATTGATCGTGATGCCTGGTTTCAAAGACATTCTCCTGAAGTCACCAAGAAAATTGTTGATGGATTCATGAAGTTGTTAAAACTTGAATATGACCCAAAGTTTATTGGCGTTGTGGGTTACTGTTTTGGTGCAAAGTTTGCCGTCCAACACATTAGTGGCGACGGGGGTCTTGCCAATGCTGCAGCCATTGCACATCCATCTTTCGTCAGCATCGAGGAAATTGAAGCAATTGATAGCAAGAAACCAATATTGATTTCAGCAGCGGAAGAGGATCACATCTTTCCGGCAAACTTAAGACACTTAACGGAGGAAAAATTAAAGGATAATCACGCTACTTACCAGTTAGACCTCTTCAGTGGTGTGGCTCACGGGTTTGCAGCAAGAGGCGATATATCCATACCTGCCGTAAAATATGCGAAGGAGAAAGTCTTGCTCGACCAAATATACTGGTTCAATCATTTTTCGAATGTTTAA>YAL054C	-:0:chr01:42882:45023ATGTCGCCCTCTGCCGTACAATCATCAAAACTAGAAGAACAGTCAAGTGAAATTGACAAGTTGAAAGCAAAAATGTCCCAGTCTGCCGCCACTGCGCAGCAGAAGAAGGAACATGAGTATGAACATTTGACTTCGGTCAAGATCGTGCCACAACGGCCCATCTCAGATAGACTGCAGCCCGCAATTGCTACCCACTATTCTCCACACTTGGACGGGTTGCAGGACTATCAGCGCTTGCACAAGGAGTCTATTGAAGACCCTGCTAAGTTCTTCGGTTCTAAAGCTACCCAATTTTTAAACTGGTCTAAGCCATTCGATAAGGTGTTCATCCCAGACCCTAAAACGGGCAGGCCCTCCTTCCAGAACAATGCATGGTTCCTCAACGGCCAATTAAACGCCTGTTACAACTGTGTTGACAGACATGCCTTGAAGACTCCTAACAAGAAAGCCATTATTTTCGAAGGTGACGAGCCTGGCCAAGGCTATTCCATTACCTACAAGGAACTACTTGAAGAAGTTTGTCAAGTGGCACAAGTGCTGACTTACTCTATGGGCGTTCGCAAGGGCGATACTGTTGCCGTGTACATGCCTATGGTCCCAGAAGCAATCATAACCTTGTTGGCCATTTCCCGTATCGGTGCCATTCACTCCGTAGTCTTTGCCGGGTTTTCTTCCAACTCCTTGAGAGATCGTATCAACGATGGGGACTCTAAAGTTGTCATCACTACAGATGAATCCAACAGAGGTGGTAAAGTCATTGAGACTAAAAGAATTGTTGATGACGCGCTAAGAGAGACCCCAGGCGTGAGACACGTCTTGGTTTATAGAAAGACCAACAATCCATCTGTTGCTTTCCATGCCCCCAGAGATTTGGATTGGGCAACAGAAAAGAAGAAATACAAGACCTACTATCCATGCACACCCGTTGATTCTGAGGATCCATTATTCTTGTTGTATACGTCTGGTTCTACTGGTGCCCCCAAGGGTGTTCAACATTCTACCGCAGGTTACTTGCTGGGAGCTTTGTTGACCATGCGCTACACTTTTGACACTCACCAAGAAGACGTTTTCTTCACAGCTGGAGACATTGGCTGGATTACAGGCCACACTTATGTGGTTTATGGTCCCTTACTATATGGTTGTGCCACTTTGGTCTTTGAAGGGACTCCTGCGTACCCAAATTACTCCCGTTATTGGGATATTATTGATGAACACAAAGTCACCCAATTTTATGTTGCGCCAACTGCTTTGCGTTTGTTGAAAAGAGCTGGTGATTCCTACATCGAAAATCATTCCTTAAAATCTTTGCGTTGCTTGGGTTCGGTCGGTGAGCCAATTGCTGCTGAAGTTTGGGAGTGGTACTCTGAAAAAATAGGTAAAAATGAAATCCCCATTGTAGACACCTACTGGCAAACAGAATCTGGTTCGCATCTGGTCACCCCGCTGGCTGGTGGTGTTACACCAATGAAACCGGGTTCTGCCTCATTCCCCTTCTTCGGTATTGATGCAGTTGTTCTTGACCCTAACACTGGTGAAGAACTTAACACCAGCCACGCAGAGGGTGTCCTTGCCGTCAAAGCTGCATGGCCATCATTTGCAAGAACTATTTGGAAAAATCATGATAGGTATCTAGACACTTATTTGAACCCTTACCCTGGCTACTATTTCACTGGTGATGGTGCTGCAAAGGATAAGGATGGTTATATCTGGATTTTGGGTCGTGTAGACGATGTGGTGAACGTCTCTGGTCACCGTCTGTCTACCGCTGAAATTGAGGCTGCTATTATCGAAGATCCAATTGTGGCCGAGTGTGCTGTTGTCGGATTCAACGATGACTTGACTGGTCAAGCAGTTGCTGCATTTGTGGTGTTGAAAAACAAATCTAGTTGGTCCACCGCAACAGATGATGAATTACAAGATATCAAGAAGCATTTGGTCTTTACTGTTAGAAAAGACATCGGGCCATTTGCCGCACCAAAATTGATCATTTTAGTGGATGACTTGCCCAAGACAAGATCCGGCAAAATTATGAGACGTATTTTAAGAAAAATCCTAGCAGGAGAAAGTGACCAACTAGGCGACGTTTCTACATTGTCAAACCCTGGCATTGTTAGACATCTAATTGATTCGGTCAAGTTGTAA>YAL055W	+:0:chr01:42178:42720ATGCCACCACCATCAAGAAGTAGAATAAACAAAACAAGAACATTAGGAATAGTGGGTACAGCTATAGCAGTGTTGGTCACGTCCTACTATATATATCAAAAGGTGACAAGTGCAAAGGAAGATAATGGGGCACGACCTCCAGAGGGTGATTCAGTAAAAGAGAACAAAAAGGCAAGGAAGAGCAAATGTATTATAATGAGCAAGTCGATACAAGGACTGCCCATAAAGTGGGAGGAGTACGCCGCTGATGAAGTGGTTTTGCTGGTACCTACGAGCCACACTGATGGATCAATGAAACAAGCCATTGGGGATGCCTTTCGCAAGACGAAAAACGAACACAAAATCATATATTGCGATAGCATGGATGGATTATGGTCATGTGTAAGACGGCTAGGTAAATTTCAGTGCATATTGAACTCCAGGGACTTCACAAGTAGTGGTGGTAGCGATGCAGCAGTCGTTCCTGAAGATATAGGCAGGTTTGTCAAATTTGTTGTTGATAGCGATGTAGAGGATGTGCTGATTGACACTTTATGCAATTAA>YAL059W	+:0:chr01:36510:37148ATGTGGGAACAAAGACGACAAAAGGTAGTTTTTTCCTTGACTATACTGGTAAGATATCGTCTAAAACAAAGCATGGCCAAGAAAATATCAAAGAATTCAAGAGCTGCTAGACAATCGGATGCTCTTGAACCAGAGGTAAAGGATTTAAGTGAACTACCTAGAGCTGAAAAAACCGATTTGACTAATATTTTGATTAGAACAGCAGCCAAGAATGAGGCATTGCTGGAAGCAAAGATATCTAAGAAAGCCAATAAAAGTAAGAGGGGCAAGAAGTTAAATAAAAAGGCTCTGGAAGACAAACTGGACAACTCTATTTCATCCATGGACAGGGATCGTTTAGTGAAGGCCTTGAATTTTACCAATCGTCTGGACGGTAAAATTGCCAAGTCCATTTCTCGTGCCAAGTACATTCAAAATACAAGAAAGGCTGGCTGGGATAGCACCAATGAGACTATAAAAAAAGAGCTGGCTTTTTTGAACGGAGGGTTGTCTGTGCAGGCAAAAAGTGCTAGTGAAGGTAATGCTGAAAAGGAAGATGAGGAGATCCCAGAAGTTTTTGACTCTTTAGCAGAGGATAACACAGTGCAGAAGACTCCTACAAATAGATTCGGTGTCCTGCCAGACGATGTTGAAGAATAG>YAL060W	+:0:chr01:35156:36304ATGAGAGCTTTGGCATATTTCAAGAAGGGTGATATTCACTTCACTAATGATATCCCTAGGCCAGAAATCCAAACCGACGATGAGGTTATTATCGACGTCTCTTGGTGTGGGATTTGTGGCTCGGATCTTCACGAGTACTTGGATGGTCCAATCTTCATGCCTAAAGATGGAGAGTGCCATAAATTATCCAACGCTGCTTTACCTCTGGCAATGGGCCATGAGATGTCAGGAATTGTTTCCAAGGTTGGTCCTAAAGTGACAAAGGTGAAGGTTGGCGACCACGTGGTCGTTGATGCTGCCAGCAGTTGTGCGGACCTGCATTGCTGGCCACACTCCAAATTTTACAATTCCAAACCATGTGATGCTTGTCAGAGGGGCAGTGAAAATCTATGTACCCACGCCGGTTTTGTAGGACTAGGTGTGATCAGTGGTGGCTTTGCTGAACAAGTCGTAGTCTCTCAACATCACATTATCCCGGTTCCAAAGGAAATTCCTCTAGATGTGGCTGCTTTAGTTGAGCCTCTTTCTGTCACCTGGCATGCTGTTAAGATTTCTGGTTTCAAAAAAGGCAGTTCAGCCTTGGTTCTTGGTGCAGGTCCCATTGGGTTGTGTACCATTTTGGTACTTAAGGGAATGGGGGCTAGTAAAATTGTAGTGTCTGAAATTGCAGAGAGAAGAATAGAAATGGCCAAGAAACTGGGCGTTGAGGTGTTCAATCCCTCCAAGCACGGTCATAAATCTATAGAGATACTACGTGGTTTGACCAAGAGCCATGATGGGTTTGATTACAGTTATGATTGTTCTGGTATTCAAGTTACTTTCGAAACCTCTTTGAAGGCATTAACATTCAAGGGGACAGCCACCAACATTGCAGTTTGGGGTCCAAAACCTGTCCCATTCCAACCAATGGATGTGACTCTCCAAGAGAAAGTTATGACTGGTTCGATCGGCTATGTTGTCGAAGCCTTCGAAGAAGTTGTTCGTGCCATCCACAACGGAGACATCGCCATGGAAGATTGTAAGCAACTAATCACTGGTAAGCAAAGGATTGAGGACGGTTGGGAAAAGGGATTCCAAGAGTTGATGGATCACAAGGAATCCAACGTTAAGATTCTATTGACGCCTAACAATCACGGTGAAATGAAGTAA>YAL061W	+:0:chr01:33449:34702ATGAGAGCCTTAGCGTATTTCGGTAAAGGTAACATCAGATTCACCAACCATTTAAAGGAGCCACATATTGTGGCGCCCGATGAGCTTGTGATTGATATCGAATGGTGTGGTATTTGCGGTACGGACCTGCATGAGTACACAGATGGTCCTATCTTTTTCCCAGAAGATGGACACACACATGAGATTAGTCATAACCCATTGCCACAGGCGATGGGCCACGAAATGGCTGGTACCGTTTTGGAGGTGGGCCCTGGTGTGAAAAACTTGAAAGTGGGAGACAAGGTAGTTGTCGAGCCCACAGGTACATGCAGAGACCGGTATCGTTGGCCCCTGTCGCCAAACGTTGACAAGGAATGGTGCGCTGCTTGCAAAAAGGGCTACTATAACATTTGTTCATATTTGGGGCTTTGTGGTGCGGGTGTGCAGAGCGGTGGATTTGCAGAACGTGTTGTGATGAACGAATCTCACTGCTACAAAGTACCGGACTTCGTGCCCTTAGACGTTGCAGCTTTGATTCAACCGTTGGCTGTGTGCTGGCATGCAATTAGAGTCTGCGAGTTCAAAGCAGGCTCTACGGCTTTGATCATTGGTGCTGGCCCCATCGGACTGGGCACGATACTGGCGTTGAACGCTGCAGGTTGCAAGGACATCGTCGTTTCAGAGCCTGCCAAGGTAAGAAGAGAACTGGCTGAAAAAATGGGTGCCAGGGTTTACGACCCAACTGCGCACGCTGCCAAGGAGAGCATTGATTATCTGAGGTCGATTGCTGATGGTGGAGACGGCTTCGATTACACATTTGATTGCTCCGGGTTGGAAGTCACATTGAATGCTGCTATTCAGTGTCTCACTTTCAGAGGCACCGCAGTGAACTTGGCCATGTGGGGCCATCACAAGATACAGTTTTCTCCGATGGACATCACATTGCATGAAAGAAAGTACACAGGGTCCATGTGCTACACACACCACGATTTTGAGGCAGTAATAGAAGCTTTGGAAGAAGGCAGGATTGACATTGATAGAGCAAGACATATGATAACGGGCAGAGTCAACATTGAGGACGGCCTTGATGGCGCCATCATGAAGCTGATAAACGAGAAGGAGTCTACAATCAAGATTATTCTGACTCCAAACAATCACGGAGAGTTGAACAGGGAAGCCGATAATGAGAAGAAAGAAATTTCCGAGCTGAGCAGTCGGAAAGATCAAGAAAGACTACGAGAATCAATAAACGAGGCTAAACTGCGTCACACATGA>YAL062W	+:0:chr01:31568:32941ATGACAAGCGAACCAGAGTTTCAGCAGGCTTACGATGAGATCGTTTCTTCTGTGGAGGATTCCAAAATTTTTGAAAAATTCCCACAGTATAAAAAAGTGTTACCTATTGTTTCTGTCCCGGAGAGGATCATTCAATTCAGGGTCACGTGGGAAAATGATAATGGCGAGCAAGAAGTGGCTCAAGGATACAGGGTGCAGTTCAATTCAGCCAAGGGCCCTTACAAGGGTGGCCTACGCTTCCACCCATCAGTGAACCTGTCTATCCTAAAATTTTTGGGTTTTGAACAGATCTTCAAGAATGCGCTCACTGGGCTAGATATGGGCGGTGGTAAGGGTGGCCTGTGTGTGGACTTGAAAGGCAAGTCTGACAACGAGATCAGAAGGATTTGTTATGCGTTCATGAGAGAACTGAGCAGGCATATTGGTAAGGACACAGACGTGCCCGCAGGAGATATTGGTGTCGGTGGCCGTGAAATTGGCTACCTATTCGGCGCTTACAGATCATACAAGAACTCCTGGGAAGGTGTGTTGACTGGTAAGGGTTTAAACTGGGGTGGCTCACTTATCAGGCCGGAGGCCACCGGGTTCGGCTTAGTTTACTATACGCAAGCAATGATCGATTATGCAACAAACGGCAAGGAGTCGTTTGAGGGCAAACGTGTGACAATCTCCGGAAGTGGCAATGTTGCGCAATATGCAGCTTTGAAAGTGATCGAGCTGGGTGGTATTGTGGTGTCTTTATCCGATTCGAAGGGGTGCATCATCTCTGAGACGGGCATTACTTCTGAGCAAATTCACGATATCGCTTCCGCCAAGATCCGTTTCAAGTCGTTAGAGGAAATCGTTGATGAATACTCTACTTTCAGCGAAAGTAAGATGAAGTACGTTGCAGGAGCACGCCCATGGACGCATGTGAGCAACGTCGACATTGCCTTGCCCTGTGCCACCCAAAACGAGGTCAGTGGTGACGAAGCCAAGGCCCTAGTGGCATCTGGCGTTAAGTTCGTTGCCGAAGGTGCTAACATGGGTTCTACACCCGAGGCTATTTCTGTTTTCGAAACAGCGCGTAGCACTGCAACCAATGCAAAGGATGCAGTTTGGTTTGGGCCACCAAAGGCAGCTAACCTGGGCGGCGTGGCAGTATCCGGTCTGGAAATGGCTCAGAATTCTCAAAAAGTAACTTGGACTGCCGAGCGGGTCGATCAAGAACTAAAGAAGATAATGATCAACTGCTTCAACGACTGCATACAGGCCGCACAAGAGTACTCTACGGAAAAAAATACAAACACCTTGCCATCATTGGTCAAGGGGGCCAACATTGCCAGCTTCGTCATGGTGGCTGACGCAATGCTTGACCAGGGAGACGTTTTTTAG>YAR002C-A	-:0:chr01:154067:154726ATGCTTTTAACCTCTCTTTTACAGGTTTTTGCCTGCTGTCTAGTTTTACCAGCTCAAGTTACTGCGTTCTATTATTATACTTCTGGTGCTGAACGTAAGTGCTTCCACAAGGAATTGTCTAAAGGTACTTTGTTCCAAGCAACTTACAAGGCACAAATTTACGATGACCAATTACAAAATTACAGAGACGCTGGTGCGCAAGATTTTGGTGTCTTGATTGATATTGAGGAAACTTTTGATGATAACCACCTGGTTGTTCATCAGAAAGGTTCAGCAAGTGGTGATTTAACTTTCCTTGCGTCTGATTCGGGTGAACATAAAATCTGTATTCAGCCCGAAGCTGGTGGCTGGTTGATTAAGGCTAAAACGAAGATTGACGTTGAATTCCAAGTGGGCTCTGATGAAAAGTTAGATTCTAAGGGTAAAGCCACTATTGACATTCTACATGCCAAGGTTAACGTCCTAAACTCCAAGATCGGCGAAATTAGAAGAGAGCAAAAATTGATGAGAGATCGTGAAGCTACCTTTAGAGACGCCTCTGAAGCTGTTAATTCTCGTGCTATGTGGTGGATTGTCATTCAATTAATTGTTCTCGCTGTTACTTGTGGCTGGCAAATGAAACACCTGGGCAAATTTTTTGTTAAGCAAAAAATTTTATGA>YAR002W	+:0:chr01:152259:153878ATGCATCGTAAATCATTGAGGAGGGCTAGCGCTACTGTGCCTTCCGCTCCCTATCGAAAGCAGATTATTAGCAATGCACACAATAAACCAAGCCTTTTCTCTAAAATTAAAACTTTCTTTACCCAAAAAGATTCAGCCAGAGTGAGTCCAAGGAATAATGTTGCTAATAAACAACCACGCAATGAGTCTTTTAACAGAAGAATCTCAAGTATGCCTGGAGGTTATTTCCATTCTGAGATATCCCCAGATTCTACTGTAAACCGTTCCGTAGTTGTTTCTGCAGTGGGTGAAGCCAGAAACGACATTGAGAATAAAGAAGAGGAGTATGATGAAACACATGAAACTAACATCTCCAATGCAAAGCTTGCAAACTTTTTTAGTAAAAAAGGTAATGAGCCTTTATCAGAAATTGAAATAGAGGGTGTGATGTCATTGTTACAAAAATCAAGCAAATCCATGATAACTTCGGAAGGAGAACAAAAATCAGCCGAAGGTAATAATATCGACCAGTCGCTTATCTTGAAGGAGTCAGGAAGTACACCAATCAGCATATCTAATGCGCCGACCTTCAACCCAAAATATGATACTTCAAATGCGTCAATGAATACGACTTTGGGAAGCATTGGTTCAAGAAAATACAGTTTCAATTATTCTAGCCTGCCCTCACCATACAAAACAACCGTTTATAGATATAGTGCAGCGAAAAAGATCCCCGATACATACACAGCCAACACATCTGCTCAAAGTATAGCATCTGCTAAATCGGTAAGAAGTGGTGTTTCAAAGTCAGCTCCTAGTAAGAAAATAAGTAATACAGCTGCGGCATTGGTCTCACTATTAGATGAAAATGACAGTAAGAAGAATAATGCAGCTTCAGAACTTGCTAATCCATACTCCTCATATGTAAGCCAAATACGCAAACATAAGAGAGTTTCTCCAAATGCTGCACCAAGGCAAGAGATCAGTGAAGAAGAAACTACTGTTAAGCCATTATTTCAAAACGTTCCTGAACAAGGCGAAGAACCAATGAAACAACTGAACGCCACCAAAATTTCACCATCTGCGCCAAGCAAAGATTCTTTTACTAAATACAAACCTGCAAGGTCCTCATCCTTACGCTCAAATGTCGTCGTAGCTGAAACCTCACCTGAAAAGAAGGATGGTGGAGATAAACCTCCATCCTCTGCTTTTAACTTCTCGTTTAATACTTCAAGAAACGTTGAACCTACTGAGAATGCTTATAAGAGCGAGAACGCACCATCTGCATCATCAAAGGAATTCAATTTTACCAACCTACAGGCGAAGCCGTTAGTTGGAAAGCCAAAAACCGAACTTACAAAGGGCGATTCTACTCCCGTCCAACCAGATCTTTCGGTTACTCCTCAAAAAAGTTCATCGAAAGGCTTTGTTTTTAATAGTGTTCAAAAGAAATCACGGTCCAATCTTTCACAAGAAAACGATAATGAAGGTAAACATATCAGCGCCTCAATTGATAACGACTTTTCAGAGGAAAAGGCGGAAGAGTTTGATTTCAATGTTCCCGTGGTGTCTAAGCAGCTAGGAAATGGCTTGGTTGATGAAAATAAAGTTGAGGCTTTCAAGTCCCTATATACCTTTTGA>YAR003W	+:0:chr01:155007:156287ATGAACATCCTTTTACAGGATCCATTCGCTGTTCTTAAGGAACATCCTGAGAAGCTCACACATACGATTGAGAACCCTTTACGCACTGAATGTCTCCAGTTCAGTCCTTGCGGTGATTACCTGGCTCTTGGGTGTGCCAATGGAGCACTTGTTATTTACGATATGGATACGTTCAGGCCTATTTGTGTCCCAGGAAATATGTTGGGAGCACATGTTCGACCCATTACATCTATCGCATGGTCTCCAGATGGTAGATTGTTGCTTACAAGCTCTAGAGACTGGTCAATAAAACTGTGGGATCTTTCAAAGCCAAGTAAGCCTTTGAAAGAAATACGATTCGATTCTCCAATTTGGGGTTGCCAATGGCTGGATGCTAAAAGGCGGCTTTGTGTAGCTACGATATTTGAGGAAAGTGACGCATATGTTATTGACTTCAGCAATGATCCGGTCGCAAGCCTTCTCAGTAAATCAGACGAAAAACAATTGAGTTCGACACCTGATCATGGATATGTTCTTGTTTGTACAGTACATACCAAACATCCAAATATTATTATTGTTGGAACTTCAAAAGGTTGGCTAGACTTCTATAAATTCCATTCTCTATATCAAACAGAATGTATTCATTCCCTTAAAATCACGAGTTCTAATATCAAACATTTAATTGTCTCGCAAAATGGTGAAAGATTAGCTATTAACTGCTCCGATAGAACAATAAGACAATACGAAATAAGTATTGATGATGAAAACTCTGCGGTTGAGTTGACCTTAGAGCATAAGTACCAGGATGTGATTAATAAATTACAGTGGAACTGTATCCTCTTTAGTAATAATACTGCCGAATACTTAGTCGCTTCTACACATGGTTCTTCTGCACATGAACTATACATCTGGGAAACGACTAGTGGAACGTTGGTGAGAGTCCTGGAAGGGGCTGAAGAGGAGTTGATAGATATAAATTGGGACTTCTATAGTATGAGTATAGTGAGTAATGGTTTTGAATCTGGGAACGTGTATGTGTGGTCTGTTGTTATTCCGCCAAAGTGGAGTGCTTTGGCGCCAGATTTTGAAGAAGTAGAAGAGAATGTCGACTATTTGGAGAAGGAAGATGAATTTGATGAGGTCGATGAGGCAGAACAGCAGCAAGGACTAGAACAAGAGGAAGAAATAGCTATCGATCTTCGGACGAGAGAGCAATATGATGTTAGAGGTAATAACTTGCTTGTAGAACGGTTCACAATCCCTACAGATTATACGAGGATAATTAAGATGCAGTCATCATAG>YAR007C	-:0:chr01:156756:158621ATGAGCAGTGTTCAACTTTCGAGGGGCGATTTTCATAGCATCTTCACCAATAAGCAAAGGTACGATAATCCCACCGGTGGCGTTTATCAAGTTTATAACACCAGGAAATCTGATGGGGCTAACAGCAACAGAAAGAATTTGATCATGATTTCCGATGGTATTTACCATATGAAGGCTCTGTTGAGAAACCAAGCTGCATCCAAGTTCCAGTCAATGGAACTACAAAGGGGTGATATCATTCGCGTGATAATTGCAGAACCTGCTATTGTCAGGGAAAGAAAGAAATACGTTCTTTTAGTAGATGACTTTGAGTTGGTCCAGTCGCGTGCTGATATGGTCAACCAAACTAGTACTTTTTTGGATAACTATTTCTCAGAGCATCCAAATGAAACCTTAAAAGACGAAGATATAACTGACAGTGGTAATGTTGCCAATCAAACAAACGCCAGCAATGCTGGTGTCCCTGATATGCTGCATTCAAACTCAAACTTGAATGCAAATGAGAGAAAATTCGCCAATGAAAACCCTAATTCGCAAAAAACCAGACCAATTTTTGCCATCGAACAACTGTCTCCATACCAAAACGTTTGGACTATCAAAGCAAGAGTTTCCTACAAGGGAGAAATTAAAACGTGGCACAATCAAAGAGGTGATGGTAAACTATTCAATGTCAACTTCTTGGATACCTCTGGAGAAATCCGAGCCACGGCGTTTAATGATTTTGCTACAAAATTTAACGAAATTTTACAAGAAGGCAAAGTATACTATGTATCAAAGGCAAAACTCCAACCAGCTAAGCCCCAATTTACTAATCTAACACACCCTTATGAACTGAATTTGGATAGAGACACTGTTATAGAAGAATGTTTCGATGAAAGTAATGTTCCGAAAACCCATTTCAATTTCATCAAACTAGATGCTATTCAGAACCAGGAAGTAAATTCCAACGTAGACGTCCTCGGTATTATCCAAACTATAAACCCACATTTTGAGCTAACTTCAAGGGCTGGGAAGAAATTCGATCGTCGTGACATCACAATTGTTGACGACTCTGGGTTTTCTATCTCTGTTGGCCTATGGAATCAGCAAGCCCTTGATTTCAACCTTCCTGAAGGTTCTGTTGCTGCCATTAAAGGTGTTCGTGTGACGGATTTTGGTGGCAAATCTTTGTCTATGGGATTTTCTAGTACCCTGATTCCGAATCCAGAAATTCCTGAGGCATATGCCTTAAAGGGTTGGTATGATTCCAAGGGCCGCAACGCAAACTTCATCACTTTAAAGCAAGAACCCGGTATGGGTGGTCAATCGGCTGCTAGCTTAACAAAATTCATTGCTCAGCGTATTACTATTGCTAGAGCTCAAGCTGAAAATCTAGGAAGAAGCGAGAAAGGTGACTTTTTTAGTGTTAAAGCTGCTATAAGTTTCTTAAAAGTTGATAATTTTGCATATCCTGCCTGTTCTAATGAGAATTGTAATAAGAAAGTTCTGGAACAGCCTGATGGTACTTGGAGATGTGAGAAGTGCGACACCAATAATGCAAGGCCAAATTGGAGATACATCTTGACAATATCAATTATTGACGAAACCAATCAACTATGGCTCACTTTATTTGACGACCAAGCTAAACAATTATTGGGTGTTGATGCTAATACATTAATGTCTTTGAAGGAAGAAGACCCCAACGAATTCACAAAAATTACTCAAAGTATCCAAATGAACGAATATGACTTTAGGATTAGAGCGCGTGAGGATACATACAATGATCAAAGCAGAATTAGATATACCGTTGCTAACCTACACAGCTTGAATTACAGGGCTGAAGCCGACTATCTTGCCGATGAGTTATCCAAGGCTTTGTTAGCTTAA>YAR008W	+:0:chr01:158967:159794ATGCCACCGCTAGTATTTGACATAGATCACATCAAACTTCTAAGGAAATGGGGTATTTGTGGTGTGTTATCTGGAACTTTGCCTACTGCAGCACAGCAAAATGTATTTTTGTCGGTACCTTTGAGGCTTATGTTAGAAGATGTGCTGTGGCTGCATTTGAACAATCTTGCCGATGTGAAATTAATAAGACAAGAGGGAGATGAGATTATGGAGGGAATAACATTAGAGCGGGGCGCCAAACTATCTAAAATTGTCAACGATCGTTTGAACAAGTCATTTGAATATCAGAGAAAGTTCAAAAAGGATGAACACATTGCAAAATTAAAGAAAATCGGTAGAATCAATGATAAAACCACAGCTGAAGAATTGCAACGGCTTGATAAATCTAGCAATAATGACCAGCTAATTGAATCTTCTTTGTTCATTGACATTGCTAATACCTCTATGATTTTAAGAGACATACGGAGTGATTCAGACAGCTTATCCCGCGATGATATCAGTGATTTGTTATTTAAGCAGTACAGACAGGCAGGAAAAATGCAGACCTATTTCTTATACAAGGCATTGAGAGATCAAGGGTACGTTTTGTCCCCAGGTGGACGTTTTGGTGGGAAGTTTATAGCATACCCTGGTGATCCTCTTCGTTTCCATTCACATCTGACGATACAAGATGCGATTGATTATCATAATGAGCCGATTGACCTAATATCCATGATAAGTGGTGCAAGACTAGGAACGACTGTGAAAAAACTTTGGGTCATAGGCGGTGTTGCGGAAGAGACAAAGGAAACTCATTTCTTCTCAATAGAATGGGCTGGATTTGGTTAA>YAR014C	-:0:chr01:166743:168866ATGAGTAATAAGGAAGAGCATGTTGATGAGACTTCCGCAAGTGGTGTCAAGGAGGTTAGTTCTATAGCTGCTAGACATGACAATGGTTACGCGCCTTCGCTAATCACGTCGACGTCGGGCATGGACTCCTTTCAATCGCACGCGCTACTGAATGATCCAACCCTAATAGAGGATTATTCAGATATCATTAATAATAGGCCTACAAGTGGAAGTAAACTTACTTTAGGAAACGAGGATTCGGAGAGTATGGGCGGAAGTGTTGTGGTGACTCCGACTTCGAATAAGAGTTCGCCCTTCAATTCGAAACTTAATATACTGAGTAATGCGGCAGAAAAGGGCCATGATGTTCTGCGAAATAGAGATGATGACAAAGAGTTAGAGGAGGAGAACGTGGAAAAACATATGCATAGCAACAGTAAAAGGGATCAGCGACATTACAAAGAAAATTCTTCGGAATTGCCCGACTCATATGATTATTCGGATTCTGAATTTGAGGACAATTTGGAAAGAAGGCTACAAGAGATCGAAACTGATTCCGTGGATAGCGCCGATAAGGATGAGGTGCATTTTTCAGTGAATAATACAATGAACCCTGACGTTGACGATTTTAGTGATGGCTTGAAATACGCCATCTCGGAGGACGAGGATGAAGAGGAAAACTACTCTGATGACGATGACTTTGACAGAAAATTTCAAGATTCAGGGTTTCAAGGGGAGAAAGACGATTTGGAGGAGGAAAACGACGATTACCAACCCTTATCGCCGCCAAGAGAACTGGACCCTGATAAACTATATGCTTTATATGCGTTCAATGGCCATGATTCTTCACATTGTCAATTAGGGCAAGACGAACCTTGTATACTTTTGAATGACCAGGATGCCTATTGGTGGCTGGTTAAGAGGATCACTGATGGTAAAATCGGGTTTGCGCCAGCGGAAATTCTAGAAACCTTTCCAGAAAGACTAGCTCGATTGAATTGCTGGAAAAATGAAAACATGTCTTCTCAATCGGTTGCTTCCTCCGATTCGAAAGATGATTCCATCAGCTCTGGTAACAAGAATCAGAGTGATGCAGAGAGTATAATTCCGACACCTGCACTGAATGGATATGGTAAGGGGAACAAATCTGTTAGTTTCAATGATGTTGTCGGTTATGCAGACAGGTTTATAGATGATGCAATTGAAGATACCTCGTTAGATAGTAACGATGATGGCGGTGAAGGCAATGGACAGTCGTACGACGATGATGTTGATAACGACAAGGAAACAAAAGTAACGCACCGGGACGAATACACTGAAGCGAAGTTAAATTTTGCCAAATTCCAGGATGATGATACGAGCGATGTAGTGAGCGACGTCTCCTTTAGTACATCTTTGAATACACCACTAAATGTGAAAAAAGTTCGGAGACAAGACAACAAAAACGAAAGTGAGCCTAAGACATCATCGAGTAAAGACAGAGAAGATGATTATAATGCAAATCGGTATGTGGGGCAAGAAAAGTCTGAACCGGTTGACAGTGATTACGATACCGATCTGAAAAAAGTATTTGAAGCGCCTCGTATGCCATTTGCGAATGGTATGGCAAAATCAGACTCTCAAAATTCCCTTTCAACAATTGGCGAGTTTTCACCGTCGTCGTCAGAATGGACAAACGAATCACCATCCACTCCAATAGTTGAAGAAAGCAGCAGTATTCCATCATCTAGAGCAATAAGGACATTCACATATATCATGCAAAATCGAAAATTGAGAGACAACAAACGTGGAAAACACAGAGGGCAAATTCAAGCCAGCTTGGGGTCTAGTGGAGGAATGCCAAATCAAACTGACGCAGAACAACCTAAAGAGGAGCTAGAAAAACATCACAGTACCCCCGAAGAAGAAAAGCAATCAACCTTATCGTTACACTCATCATCTGAAGAAGATTTTTATATGGATGAACAAAGGGCGGTATCATCAGCAAGCATAAATAGTTCACTTTCCGGATCAAGGGCATTGTCTAATACCAATATGTCCGATCCGGCTTCGAAGCCCAATTCTTTGGTTCAACATCTCTATGCTCCAGTTTTTGACAGAATGGATGTGTTGATGAAACAATTGGATGAAATTATTCGTAAATGA>YAR015W	+:0:chr01:169370:170290ATGTCAATTACGAAGACTGAACTGGACGGTATATTGCCATTGGTGGCCAGAGGTAAAGTTAGAGACATATATGAGGTAGACGCTGGTACGTTGCTGTTTGTTGCTACGGATCGTATCTCTGCATATGACGTTATTATGGAAAACAGCATTCCTGAAAAGGGGATCCTATTGACCAAACTGTCAGAGTTCTGGTTCAAGTTCCTGTCCAACGATGTTCGTAATCATTTGGTCGACATCGCCCCAGGTAAGACTATTTTCGATTATCTACCTGCAAAATTGAGCGAACCAAAGTACAAAACGCAACTAGAAGACCGCTCTCTATTGGTTCACAAACATAAACTAATTCCATTGGAAGTAATTGTCAGAGGCTACATCACCGGATCTGCTTGGAAAGAGTACGTAAAAACAGGTACTGTGCATGGTTTGAAACAACCTCAAGGACTTAAAGAATCTCAAGAGTTCCCAGAACCAATCTTCACCCCATCGACCAAGGCTGAACAAGGTGAACATGACGAAAACATCTCTCCTGCCCAGGCCGCTGAGCTGGTGGGTGAAGATTTGTCACGTAGAGTGGCAGAACTGGCTGTAAAACTGTACTCCAAGTGCAAAGATTATGCTAAGGAGAAGGGCATCATCATCGCAGACACTAAATTCGAATTCGGTATTGACGAAAAGACCAATGAAATTATTCTAGTGGACGAGGTGCTAACGCCAGACTCCTCTAGATTCTGGAACGGTGCCTCTTATAAGGTAGGAGAATCCCAAGATTCTTACGATAAGCAATTTTTAAGAGACTGGCTTACTGCTAATAAGTTGAACGGTGTTAACGGCGTCAAAATGCCCCAAGACATTGTCGACAGGACAAGGGCCAAATATATAGAGGCTTATGAAACATTGACAGGGTCTAAATGGTCTCACTAA>YAR018C	-:0:chr01:170391:171698ATGCATAGACGACAGTTTTTCCAAGAATACCGTAGTCCTCAGCAGCAGCAGGGACACCCACCAAGGTCAGAATACCAAGTTCTCGAAGAAATTGGGAGAGGTTCATTTGGGTCTGTACGAAAAGTCATCCATATACCTACCAAGAAACTTTTGGTTAGAAAGGATATCAAATATGGCCATATGAATAGCAAAGAGAGACAACAGCTGATCGCTGAATGTAGCATTCTATCGCAGTTGAAGCATGAAAATATTGTAGAATTTTATAACTGGGACTTCGATGAACAAAAAGAAGTGTTATACCTTTATATGGAATATTGTTCCAGGGGTGATTTATCCCAGATGATTAAGCACTACAAACAGGAGCATAAATATATACCAGAAAAAATTGTGTGGGGTATCCTGGCCCAATTATTGACTGCGCTCTATAAATGTCATTATGGTGTTGAATTGCCAACTTTGACCACAATATATGACCGGATGAAACCACCGGTAAAAGGCAAAAACATCGTTATCCATCGTGATCTGAAACCAGGAAATATATTCTTAAGCTATGATGATAGCGATTACAATATTAATGAACAAGTAGACGGTCACGAGGAAGTGAATAGTAATTATTACAGAGACCATAGAGTGAATTCAGGGAAAAGGGGGAGCCCTATGGACTATAGTCAAGTTGTGGTAAAGTTAGGTGATTTTGGGTTAGCCAAATCTCTGGAAACTAGTATTCAATTTGCCACAACATACGTCGGTACACCATATTACATGTCGCCTGAAGTGTTGATGGACCAACCATACTCCCCACTATCCGACATCTGGTCACTAGGTTGTGTTATTTTTGAGATGTGTTCGTTGCACCCCCCATTTCAGGCAAAAAATTATCTCGAGCTACAAACTAAGATTAAAAACGGGAAATGTGACACCGTCCCTGAGTATTACTCTAGAGGGCTTAATGCCATAATACATTCAATGATAGATGTGAACTTAAGAACCAGGCCTTCCACTTTTGAATTACTGCAAGATATTCAGATACGAACTGCAAGAAAGTCGTTGCAATTAGAGAGATTTGAAAGGAAGTTACTGGACTATGAAAATGAGCTGACAAACATTGAAAAAATCCTCGAGAAGCAAGCTATTGAATACGAAAGAGAACTGAGTCAGTTGAAGGAACAATTTACCCAGGCAGTGGAGGAGCGAGCCAGGGAAGTAATTAGCGGTAAGAAAGTTGGTAAGGTTCCAGAATCTATAAACGGATATTATGGTAAAAAATTTGCCAAACCTGCATACCACTGGCAAACAAGATATCGATAA>YAR019C	-:0:chr01:172209:175133ATGAACAGTATGGCCGATACCGATAGAGTCAACTTGACTCCCATCCAGAGGGCATCTGAGAAATCCGTGCAATACCACTTGAAGCAGGTCATTGGGAGGGGTTCTTACGGGGTAGTTTACAAAGCCATTAATAAACATACTGACCAAGTCGTGGCAATAAAGGAGGTCGTGTACGAAAATGATGAGGAACTTAATGACATTATGGCAGAAATTAGCTTGTTAAAAAATTTAAACCATAACAATATTGTTAAATACCACGGCTTCATACGAAAAAGCTATGAATTGTATATCCTCCTCGAATACTGCGCTAATGGTTCTTTGAGGAGGCTCATTTCAAGGAGCTCTACCGGATTAAGTGAAAATGAATCGAAAACCTATGTGACACAGACACTATTGGGGCTGAAATATTTACACGGTGAAGGAGTCATCCACAGGGACATCAAGGCGGCTAACATCCTGCTGAGTGCTGATAACACTGTCAAACTTGCTGATTTTGGCGTTTCCACTATTGTGAACTCCAGCGCCTTAACGCTAGCGGGCACACTCAATTGGATGGCTCCAGAGATCCTGGGCAACAGGGGAGCTTCTACGCTCAGCGACATTTGGTCTCTAGGTGCCACTGTAGTTGAAATGCTCACAAAGAATCCACCCTACCACAATTTGACAGACGCCAATATCTACTACGCTGTTGAAAATGATACCTACTACCCACCTAGCTCTTTCTCTGAGCCACTAAAGGATTTCTTATCTAAATGCTTTGTGAAAAACATGTACAAGAGGCCGACAGCCGACCAGTTACTCAAGCATGTGTGGATCAACTCTACCGAAAATGTGAAGGTCGACAAGCTCAACAAGTTCAAGGAGGACTTTACCGACGCTGATTATCATTGGGATGCCGATTTTCAAGAAGAGAAACTAAATATATCACCCTCTAAATTCAGTCTTCGAGCGGCTCCCGCTCCCTGGGCAGAAAACAATCAAGAACTAGATTTAATGCCCCCCACTGAAAGTCAATTGCTGAGCCAATTGAAGAGTTCATCTAAGCCTTTGACGGACTTGCATGTGCTTTTCAGTGTTTGCTCCCTCGAGAACATCGCTGATACAATTATCGAGTGTCTGTCGCGCACAACTGTTGATAAACGATTAATAACTGCATTTGGCTCCATTTTTGTTTACGATACCCAGCATAACCACTCTAGGTTGCGACTGAAATTCATCGCTATGGGAGGAATTCCACTGATCATTAAATTCGAACATTTAGCCAAAGAGTTCGTCATCGACTACCCTCAGACTTTAATTGAATGTGGAATAATGTATCCTCCGAATTTTGCATCGCTGAAAACCCCAAAGTATATTTTAGAACTCGTCTATAGGTTCTACGATTTAACATCCACAGCCTTCTGGTGTCGCTGGTGTTTCAAACACCTCGATATATCACTCCTTCTGAATAACATCCATGAAAGAAGAGCCCAATCCATACTACTAAAGCTATCGTCATATGCACCATGGTCTTTTGAGAAAATTTTGCCCTCTTTAATTGACTCTAAGCTAAAAAAGAAAATTTTAATCAGTCCTCAAATTACTTACGTAGTCTTCAAATCAATAAACTATATGATAACCACGAATGACGATAAAATACACAAGTCCGCTATTCCTTCTTCTTCCTCTCTACCGTTATCCTCCTCACCCACGAGGAACTCACCAGTGAATTCGGTACAGTCTCCATCAAGGTCCCCTGTTCATTCTTTGATGGCAACGCGTCCCTCTTCTCCAATGCGACACAAGAGCATTTCAAACTTTCCCCATCTGACCATATCTTCAAAATCAAGACTACTAATTGAATTACCGGAGGGTTTCTTTACCTGGCTAACATCTTTTTTTGTTGACATGGCCCAAATCAAAGATTTATCTGTTTTAAAGTACTTCACCAAGCTTTGTTACCTTACAGTACATATAAACAGCACTTTTTTAAATGATCTGCTTGACAACGATGCTTTTTTTGCTTTTATCCGGAATATTGATACCATCATTCCCTTTATCGACGACGCAAAGACAGCAGCTTTCATTTGGAAACAAATCACTGCTATATGCGTTGAAATGAGTTTGGATATGGACCAAATGAGTGCTTCTTTATTTTCTACAGCTATGAATTTCATCAGAAAAAAGAATAACACCTCCATAAGTGGACTGGAGATCATACTGAACTGCTTGCATTTTACGTTACGCAATGTAAATGATGATGTGGCTCCTACAGTGGGCTCATCAGAGTCTCATAGTGTTTTCCTCATAAAGGTCAACAATGACGCTGCTATTGAATTACCGATTGATCAATTAGTTGACCTGTTTTATGCATTGAATGACGATGACGTTAACCTCAGTAAACTAATTAGCATTTTCACGAAGATATGCTCATTGCCCGGTTTTGAAAACCTTACAATTAATATCATATTTCACCCGAACTTTTATGAAAAGATTGTTTCTTTCTTTGATACCTATTTCAACAGTTTACTTATTCAAATCGATCTATTGAAATTCATAAAGCTAATATTCTCAAAATCGTTATTGAAGCTATATGACTACACAGGACAGCCGGATCCTATAAAGCAAACCGAACCAAATCGTCGTAATAAGGCCACTGTTTTCAAACTTCGTGCCATTTTAGTACAAATAACGGAGTTTTTAAACAACAACTGGAACAACGGATGTCCAAAAAGGAATTCAAATCAAGTTGGGGGGGACTCAGTTTTGATCTGTCAGCTATGTGAGGATATCCGTTCATTATCAAAAAAAGGAAGCCTGCAAAAAGTTTCAAGCGTCACTGCAGCAATTGGTAGTTCTCCAACAAAAGATGAGCGTAGTAATTTGCGATCCTCCAAAGATAAAAGTGACGGCTTTTCCGTCCCCATTACAACATTTCAAACATAA>YAR035W	+:0:chr01:190187:192250ATGCCAAACTTAAAGAGACTACCCATCCCGCCACTGCAGGACACGCTCAACCGCTACCTGGCACGCGTGGAACCCCTGCAGGACGAGCGCCAAAACCGCCGTACGCGCCGCACTGTGCTCTCCGCAGAAAACCTGGACGCATTGAACACGCTGCACGAGCGGCTGCTAGAATACGACGCACGGCTCGCGGAAAGCAACCCAGAGTCCTCATACATCGAGCAGTTCTGGTATGACGCGTACTTGCTATATGATGCAACTGTCGTTCTCAACGTCAACCCGTACTTCCAACTGCAGGACGACCCAACCATCAAAGACACACCAGAGACGGCGGCACAGGGCCCCTATGGCGCACACACGGTGCAGGTTCGTCGTGCCGCACGACTCACCACCTCTATTCTCAAGTTCATCCGCCAGATTCGCCACGGCACACTCCGCACAGACACTGTGCGCGGCAAAACGCCGCTGTCGATGGACCAGTATGAGCGGCTATTCGGCTCCAGTAGAATCCCTCCGGGTCCCGGCGAGCCCTCTTGCCACTTGCAAACAGACGCCACGTCGCATCACGTGGTGGCGATGTATCGTGGCCAGTTCTACTGGTTCGACGTGCTGGACACACGCAACGAGCCCATCTTCGCCACCCCAGAACAACTGGAGTGGAACCTCTACTCGATCATCATGGACGCGGAATCCGCCGGAAGCGGATCCGCGCCCTTTGGCGTGTTCACCACAGAGTCGCGCCGGGTGTGGTCCAACATCAGGGACTATCTGTTCCATGCGGACGACTGCACCAACTGGCGCAATCTCAAGCTGATCGACTCCGCGCTGTTCGTGGTCTGTCTCGACGACGTGGCGTTTGCCGCCGATCAGCAGGACGAGCTCACGCGTTCGATGCTGTGCGGGACTTCTACCATCAATCTCGACCCGCACCAACACCAGCCGCCATTGAACGTGCAGACAGGCACCTGTCTCAACCGCTGGTACGACAAGTTACAACTGATCGTGACCAAGAACGGTAAGGCGGGCATCAACTTCGAACACACCGGTGTGGACGGCCACACTGTGCTGCGGCTCGCCACAGACATCTACACAGACTCGATCCTGAGCTTCGCACGCGGTGTCACCAAGAACGTCGTCGACATCTTTAGCGACGACGATGGAAAACCATCGTCGTCGTCGTTGGCCTCGGCGGCTCACTCCGCCAACTTGATCACCATCCCTCGTAAACTGGAATGGCGCACTGACAATTTCCTGCAATCGTCGCTGCACTTTGCCGAGACGCGCATCTCGGACTTGATCTCGCAATACGAGTTTGTTAATCTTGACTTCTCCAACTACGGCGCGTCCCATATCAAGACAGTGTTCAAGTGCTCGCCAGACGCCTTCGTGCAGCAGGTGTTCCAGGTCGCATACTTCGCGTTGTACGGTCGCTTCGAGACCGTGTACGAGCCTGCCATGACCAAGGCGTTCCAAAACGGCCGCACAGAGGCCATCCGCTCCGTCACGGGCCAATCGAAGCTCTTTGTCAAGTCACTACTGGACCAGGATGCCTCGGACGCCACCAAAATTCAGCTCTTGCACGACGCCTGTACGGCGCACTCGCAAATCACAAGGGAATGCTCCCAGGGGCTCGGCCAGGACCGTCACTTGTATGCGCTCTACTGCCTCTGGAACCAATGGTACAAGGACAAGTTGGAGCTCCCACCCATCTTCCGCGACAAGTCCTGGACTACCATGCAGAACAACGTCTTGAGCACCTCCAACTGCGGTAACCCCTGCCTCAAGAGCTTCGGGTTCGGGCCTGTCACCGCCAACGGCTTCGGCATCGGCTACATCATCAGAGACCACTCCGTCTCTGTGGTGGTGTCCTCAAGGCATCGCCAGACTGCTCGGTTTGCGTCGCTCATGGAAAAGTCGCTGCTGGAGATCGACCGCATCTTCAAACGGCAGCAAGCTCGCGCAGCAAAACCCGCTGCCAGGACCACTGCTAGCGCCAACACCAAATCAGAAGACATGAAATACCTGTTGTCCGGCTACGATTACTTCGACGTGAGCGTGTCCGGTTGA>YAR062W	+:0:chr01:218540:219136ATGACAGGTTACTTTTTACCACCACAAACAAGTTCTTACACGTTCAGGTTTGCTAAGGTCGATGACTCTGCAATTCTATCAGTCGGTGGTAACGTTGCGTTCGAATGTTGTGCACAAGAACAACCTCCAATTACATCGACGGATTTTACAATCAATGGTATTAAGCCATGGCAAGGAAGTTTGCCTGATAACATCGGAGGGACTGTCTACATGTATGCAGGCTACTATTATCCGCTGAAGGTTGTTTACTCCAATGCCGTTTCCTGGGGCACGCTTCCAATTAGCGTGGAATTGCCTGATGGTACTACTGTTAGTGATGACTTTGAAGGGTACGTTTACTCTTTTGACGATGATTTAAGTCAGTCAAATTGTACTATCCCTGATCCTTCAAAACATACTACTAGCATCGTCACAACTACTACCGAACTGTGGACTGGTACTTTTACTTCTACATCTACTGAAATGACCACCGTCACCGGTACTAATGGTCAACCAACTGACGAAACCGTTATTGTTGCCAAAGCTCCAACCACTGCCACCTCATCCAGTTTGTCATCATCTTCTTCAGAACAAATCACCAGCTCTATCACGTCTTAG>YAR066W	+:0:chr01:221040:221651ATGTTCAATCGTTTTAACAAATTCCAAGCTGCTGTCGCTTTGGCCCTACTCTCTCGCGGCGCTCTCGGTGACTCTTACACCAATAGCACCTCCTCCGCAGACTTGAGTTCTATCACTTCCGTCTCGTCAGCTAGTGCAAGTGCCACCGCTTCCGACTCACTTTCTTCCAGTGACGGTACCGTTTATTTGCCATCCACAACAATTAGCGGTGATCTCACAGTTACTGGTAAAGTAATTGCAACCGAGGCCGTGGAAGTCGCTGCCGGTGGTAAGTTGACTTTACTTGACGGTGAAAAATACGTCTTCTCATCTGATCTAAAAGTTCACGGTGATTTGGTTGTCGAAAAGTCTGAAGCAAGCTACGAAGGTACCGCGTTCGACGTTTCTGGTGAGACTTTTGAAGTTTCCGGTAACTTCAGTGCTGAAGAAACTGGCGCTGTCTCCGCATCTATCTATTCATTCACACCTAGCTCGTTCAAGAGCAGCGGTGACATTTCTTTGAGTTTGTCAAAGGCCAAGAAGGGTGAAGTCACCTTTTCTCCATACTCTAACGCTGGTACCTTTTCTTTGTCAAATGCTATTCTCAACGGTGGTTCTGTTTCCGGTTTGTAA>YBL003C	-:0:chr02:235397:235795ATGTCCGGTGGTAAAGGTGGTAAAGCTGGTTCAGCTGCTAAAGCTTCTCAATCTAGATCTGCTAAAGCTGGTTTAACATTCCCAGTTGGTAGAGTGCACAGATTGCTAAGAAGAGGTAACTACGCCCAGAGAATTGGTTCTGGTGCTCCAGTCTATCTAACTGCTGTCTTAGAATATTTGGCTGCTGAAATTTTAGAATTGGCTGGTAATGCTGCTAGAGATAACAAAAAAACCAGAATTATTCCAAGACATTTACAATTGGCCATCAGAAATGATGATGAATTGAACAAGCTATTGGGTAATGTTACCATCGCCCAAGGTGGTGTTTTGCCAAACATTCACCAAAACTTGTTGCCAAAGAAGTCTGCCAAGACTGCCAAAGCTTCTCAAGAACTGTAA>YBL005W	+:0:chr02:217473:220403ATGAAAGTGAAGAAATCAACTAGATCAAAAGTTTCGACAGCATGTGTCAATTGCAGAAAAAGGAAAATCAAATGCACAGGTAAATATCCATGTACCAACTGCATTTCTTACGATTGTACGTGTGTATTCCTAAAAAAACATTTACCGCAGAAGGAGGATAGTTCCCAGTCTTTGCCTACTACAGCTGTTGCTCCACCCTCTTCCCACGCCAATGTAGAGGCTTCAGCAGATGTACAGCATCTGGACACTGCGATTAAGCTAGATAATCAATATTACTTCAAACTGATGAACGACCTGATACAGACTCCAGTCTCTCCGAGTGCGACGCATGCTCCTGATACTTCCAATAATCCTACTAATGATAATAATATTCTCTTTAAAGATGATTCCAAATATCAAAATCAACTGGTTACGTATCAAAATATTCTGACAAATTTGTACGCTCTGCCGCCTTGTGATGACACTCAGCTCTTGATTGATAAAACGAAGTCGCAGTTGAATAACCTGATTAACAGTTGGAATCCCGAAATAAACTACCCCAAGCTTTCCAGTTTCTCTCCTCGCCCACAAAGATCGATAGAAACGTATCTTTTAACCAACAAGTATAGAAATAAAATACACATGACGAGGTTCTCCTTTTGGACAGACCAAATGGTTAAATCACAAAGTCCAGATTCATTTCTAGCCACCACTCCACTAGTAGATGAAGTATTTGGTCTTTTCTCTCCAATACAGGCTTTTTCACTAAGAGGTATAGGATATTTAATTAAAAAAAATATCGAAAACACGGGTTCATCGATGTTAATAGATACAAAGGAAACTATTTATCTAATATTAAGATTGTTTGATTTGTGTTATGAACATTTGATCCAAGGTTGCATCTCTATTTCTAATCCATTAGAGAACTATCTTCAAAAAATAAAGCAAACTCCTACTACGACGGCATCTGCTAGTTTGCCTACTTCCCCAGCACCTTTATCTAACGATTTAGTCATTTCTGTTATTCATCAACTACCTCAGCCATTTATACAATCGATTACCGGGTTTACGACTACTCAATTGATAGAAAATTTACATGATTCATTTTCGATGTTTCGAATAGTTACTCAAATGTATGCTCAACATAGGAAGCGCTTTGCGGAATTTTTAAACCAAGCTTTCTCCTTGCCCCATCAAGAAAAGAGTGTTTTATTCTCGTCATTCTGCTCATCAGAATATCTTCTATCTACTCTTTGTTACGCATACTACAATGTTACCCTATATCACATGTTGGACATAAACACTTTAGATTACCTAGAGATTTTAGTGTCATTGCTAGAAATCCAAAATGAAATTGATGAGCGTTTTGGATTTGAAAAAATGCTAGAAGTTGCGGTTACATGCTCCACTAAGATGGGATTGTCTCGTTGGGAGTATTATGTTGGAATAGACGAAAATACTGCCGAACGGAGAAGAAAAATATGGTGGAAAATATACAGTCTGGAAAAGCGTTTTTTAACTGATCTTGGTGATTTATCCTTAATAAATGAACATCAAATGAATTGTCTCTTGCCGAAGGATTTCAGGGACATGGGATTCATTAACCATAAAGAATTTTTAACGAAAATTGGTACGTCCTCTTTATCACCGTCATCGCCCAAGCTAAAAAACTTGTCATTGTCCAGGCTTATTGAATATGGTGAGTTAGCGATAGCCCAAATTGTTGGAGATTTTTTTTCAGAGACTCTTTATAATGAGAAATTCACGTCTTTAGAAGTATCCGTTAAACCCACAATTATCAGACAAAAGTTATTGGAGAAAGTTTTTGAGGACATTGAATCTTTTAGGTTAAAATTGGCCAAAATAAAGCTTCACACCTCAAGAGTTTTTCAAGTAGCTCACTGCAAATATCCAGAATATCCAAAAAACGATCTAATTGAAGCAGCTAAATTTGTAAGTTACCATAAAAATACATGGTTCTCCATCTTGGGTGCTGTTAACAATCTTATTGCTAGGCTATCTGAAGATCCAGAGGTGATAACTGAGCAAAGCATGAAATATGCGAATGAAATGTTTCAAGAATGGAGGGAAATTAATCAATTCTTAATACAGGTTGATACTGATTTTATTGTTTGGGCATGTTTGGACTTTTATGAACTGATATTTTTCGTGATGGCTTCAAAATTTTATGTGGAAGACCCGCACATCACTTTAGAGGATGTTATCAACACTTTGAAAGTTTTTAAGAGAATAACTAACATTATTTCTTTTTTTAATAATAATTTGGACGAGAAGGATTATGATTGTCAAACTTTCAGGGAGTTTTCGAGAAGTTCGAGTTTGGTTGCCATATCCATAAGAATCATATTTTTAAAATACTGCTATGCCGAACAAATTGATAGAGCCGAATTCATCGAACGTTTGAAAGAAGTTGAACCGGGTCTAAGTGACCTTTTGCGTGAGTTTTTTGATACCCGCTCTTTTATTTACAGGTACATGTTGAAATCCGTTGAAAAATCAGGCTTTCATTTAATAATTAGAAAAATGTTAGAAAGCGACTATAAATTTTTGTATAGAGACAAATTGGCCACTGGTAATATTCCAGACCAAGGAAATTCAAGCCAAATTTCTCAGTTGTATGACAGTACTGCTCCTTCATACAACAATGCTTCTGCCTCAGCAGCAAACTCACCGTTGAAGTTATCGTCTTTGTTGAACTCTGGAGAGGAATCGTACACTCAAGACGCATCAGAAAATGTTCCATGTAATCTGCGGCATCAAGATCGATCGTTACAACAGACAAAAAGACAACATTCTGCGCCTAGCCAAATAAGCGCTAATGAGAATAATATATACAACTTGGGTACTTTAGAGGAGTTTGTCAGCAGTGGTGACCTGACTGATTTATATCATACTCTGTGGAATGACAATACTTCATATCCCTTCTTATGA>YBL006C	-:0:chr02:216590:217132ATGAGTGGAAGTAATATGGGATACTATGACGTACTCGCAGGGCTTTCAGCGCTGGAAAAGTCATCCCAGGTAGTGTTCAGTGCGACTGAGCTTCAACAGCTTACGCAACAATCTCATGCTACCGACAAGGGCATAGAGGGCAGTGAGAATAGTAAAGCGAAGGTATCCAAGCCCAAAAGAGTAGCTGTACATGGTTACCTAGGCGGTAAAGTGTCGCTCGCCGATGCGGCACAGGTGGAGTATGAAGTAGGCCATTCACTGCTGGGCAGTTATGTGCCACGCCAGCAGTTGGAAGCCCTGTCAAGTGTCGACTTTTCGCACCATTTCCACCGCACATTAGAATGCAAAGCTGCTCTAGAGACACACGATGTTTTTCTTGCCGGCGCAGGACAATTGTCTCTACCCTTCCAATCACACATAGAGAGCCCCAGGAATAGCGAGGCTAAAAGGAAAAGAAAAGTGATAATATGCAAACGGTGTCAATCACGATTTATAGGTTCCCATAGGCGGTCTCAACTTAGAGAACATGCCTGCGTAGATTAA>YBL007C	-:0:chr02:212635:216369ATGACTGTGTTTCTGGGCATCTATAGGGCCGTCTATGCCTATGAGCCGCAGACACCAGAAGAACTGGCCATCCAAGAAGACGATCTGTTGTACCTCTTACAGAAGTCAGACATTGACGATTGGTGGACAGTAAAGAAAAGAGTCATTGGTTCCGATAGCGAAGAACCGGTGGGTCTAGTGCCCTCCACTTACATTGAAGAAGCTCCTGTTTTGAAGAAGGTAAGAGCCATTTATGATTATGAACAGGTGCAAAATGCTGATGAAGAATTGACGTTTCATGAAAATGACGTTTTTGATGTATTCGATGATAAAGATGCTGATTGGCTGTTGGTTAAGTCTACCGTTTCCAATGAATTCGGCTTCATTCCAGGCAATTACGTCGAACCAGAGAATGGGTCCACTTCCAAGCAGGAACAGGCTCCCGCTGCCGCTGAAGCTCCTGCAGCTACTCCTGCAGCCGCTCCTGCTTCTGCTGCCGTACTACCCACCAACTTTTTACCACCTCCTCAACACAATGATAGAGCTCGTATGATGCAAAGTAAAGAAGACCAAGCTCCAGATGAGGATGAAGAGGGCCCTCCTCCTGCCATGCCTGCAAGACCAACGGCTACTACCGAAACTACTGATGCCACCGCTGCGGCTGTTCGATCTCGTACAAGACTATCATACAGCGATAACGATAATGACGATGAAGAGGATGATTACTATTACAACAGCAACAGCAACAACGTTGGAAATCACGAATACAATACAGAGTATCACTCTTGGAACGTTACCGAAATCGAGGGAAGGAAAAAGAAGAAGGCCAAGCTATCAATCGGTAACAACAAGATAAACTTCATACCCCAAAAGGGGACTCCTCATGAGTGGTCAATTGATAAATTGGTCTCTTATGATAACGAAAAGAAACACATGTTCTTGGAATTTGTTGATCCATATAGAAGCCTTGAATTACACACTGGTAATACCACCACATGTGAAGAGATCATGAACATTATTGGTGAATATAAAGGTGCTTCCCGTGATCCTGGGTTGAGAGAAGTCGAAATGGCTTCCAAATCCAAAAAGAGAGGTATTGTTCAATATGACTTCATGGCTGAATCACAGGACGAATTAACCATAAAATCAGGCGATAAAGTCTACATTTTAGATGATAAAAAATCTAAGGACTGGTGGATGTGCCAACTGGTTGATTCAGGGAAAAGCGGTCTCGTTCCTGCACAGTTTATTGAGCCTGTTCGTGACAAAAAACATACTGAATCTACAGCAAGCGGTATCATCAAGTCTATCAAGAAAAACTTCACCAAATCTCCATCTAGGTCGAGATCTAGATCAAGATCTAAATCCAATGCCAATGCCAGTTGGAAAGATGATGAACTACAAAACGATGTTGTAGGTAGTGCTGCCGGTAAAAGGTCAAGAAAAAGTTCATTGTCCTCTCACAAAAAAAACTCTTCGGCGACCAAAGATTTCCCCAATCCAAAAAAATCGCGTCTATGGGTTGATAGAAGTGGTACTTTCAAAGTGGATGCAGAATTCATTGGATGTGCTAAGGGGAAAATCCACTTGCACAAGGCTAATGGTGTCAAGATTGCTGTCGCCGCTGATAAACTATCCAATGAAGATTTAGCGTATGTGGAAAAAATTACTGGGTTCTCCTTAGAGAAATTTAAGGCGAATGATGGGTCTAGTTCCCGTGGCACTGATTCAAGAGACTCTGAAAGAGAAAGAAGAAGGAGATTGAAGGAGCAGGAGGAAAAAGAACGCGATAGGAGATTGAAAGAGCGTGAATTATACGAATTGAAAAAAGCCAGAGAACTCCTAGATGAAGAAAGATCAAGGTTGCAAGAAAAGGAATTGCCTCCAATAAAACCACCAAGACCAACCTCTACTACCTCCGTTCCAAATACGACGTCCGTACCACCAGCCGAAAGCAGCAATAACAACAACTCCAGTAACAAATATGATTGGTTCGAATTTTTCTTGAATTGCGGCGTTGATGTAAGCAATTGTCAAAGATATACAATAAATTTTGATAGAGAACAGCTTACTGAAGACATGATGCCTGACATCAATAACTCAATGTTAAGAACCTTGGGTCTGCGCGAAGGTGATATTGTTAGAGTAATGAAACATCTTGACAAGAAATTTGGAAGAGAAAATATTGCATCAATTCCTACAAATGCCACTGGAAATATGTTTTCACAACCTGATGGTTCATTGAACGTTGCTACAAGTCCGGAAACCTCACTGCCTCAGCAATTGTTGCCACAAACAACAAGTCCTGCTCAAACAGCTCCTTCCACATCAGCTGAGACAGATGATGCTTGGACTGTTAAACCGGCTTCTAAGTCTGAATCTAATTTACTCTCTAAGAAATCAGAATTTACTGGTTCCATGCAGGATTTATTAGACTTGCAACCTTTAGAACCTAAAAAGGCAGCTGCCTCGACACCTGAACCAAACCTAAAAGATTTGGAACCCGTGAAAACTGGCGGCACCACTGTTCCTGCTGCTCCGGTTTCATCTGCTCCGGTTTCATCTGCTCCCGCTCCATTGGATCCATTCAAAACCGGTGGCAACAATATCTTACCCTTATCTACTGGCTTTGTGATGATGCCAATGATTACTGGGGGCGATATGCTGCCTATGCAAAGAACCGGCGGCTTCGTTGTACCACAAACAACTTTTGGTATGCAATCGCAAGTTACTGGTGGTATCCTACCTGTTCAAAAGACAGGAAATGGGTTAATTCCTATCTCTAACACTGGCGGCGCAATGATGCCACAAACTACGTTCGGCGCAGCAGCTACCGTCTTACCTCTGCAAAAAACTGGAGGTGGCCTAATACCAATTGCCACCACAGGAGGTGCGCAATTCCCGCAAACTTCATTCAATGTTCAGGGGCAACAACAACTTCCAACAGGTTCAATATTACCTGTCCAAAAAACTGCTAATGGATTAATTTCAGCTAATACTGGTGTCTCAATGCCAACTGTACAAAGAACAGGCGGCACTATGATTCCACAAACATCATTTGGCGTTTCACAGCAATTAACAGGTGGTGCAATGATGACCCAACCTCAAAATACAGGGAGTGCCATGATGCCTCAAACATCCTTCAATGCTGTACCACAAATTACTGGAGGAGCAATGATGCCTCAAACGTCCTTCAATGCTTTACCACAAGTTACAGGGGGAGCAATGATGCCTTTGCAGAGAACTGGTGGTGCTTTGAATACATTTAATACTGGAGGCGCCATGATTCCCCAGACTTCGTTCAGCTCTCAAGCTCAAAACACTGGCGGGTTCCGGCCGCAATCCCAATTCGGTTTGACTTTACAAAAAACCGGAGGTATTGCGCCATTAAACCAGAACCAGTTCACTGGTGGAGCCATGAATACTTTAAGTACAGGGGGCGTTCTTCAACAACAGCAACCACAAACGATGAATACCTTCAATACTGGTGGTGTTATGCAAGAGCTCCAAATGATGACTACTTTCAATACTGGTGGTGCTATGCAACAACCACAGATGATGAACACATTCAACACCGATGGAATTATGCAACAACCACAGATGATGAACACTTTTAACACTGGGGGTGCTATGCAACAACCACAACAACAAGCATTACAGAACCAACCTACTGGATTTGGGTTTGGTAATGGACCTCAACAATCAAGGCAAGCCAACATATTCAATGCTACTGCATCAAATCCGTTTGGATTCTAG>YBL009W	+:0:chr02:207197:209227ATGAATTTTGATGCGGTAGCAGATCAACAGATGACTGACAGAAGGTATTTTGCTCTCGAAGTAGCAGAAAGCGATGATGCCGACAGCTCATTAAATTCTTCATCCATGGGAAGCCCCGCAGTGGATGTAGGAAGAAAAGTTTACAAAATCACTTCGCACAAGGGTTCAGCAGAAGATGAGAGTCAATCCTTTTTTACTTCTTCAGATTCTCCAACATCCAAAACAAGGCCTGTAGGTAAAACCATCGAAAACGATGATTACTATGGTAAAAGATCTTCTACAGGTTCATCGCTCAAACAACTCTTCAATAAAATTAATATTAATGATACCGCTCACTCTTCAAACAAAGAAAATGTATCTCAGTCGGTGCTATCGGAAAACAAGCTGCTCTCTCCATCTAAAAGGTTGTCGAAGCAAGGTCTTACAAAGGTGACTAACTCCAAGTTTCGTACACCCTTGAGACCTATTTCAAACCAATCGACTTTATCAAGGGATGAGCCTGTTAAAGATTTTAGATCACTTAAGTTTCGGAGCGGTAGTGATTTCAAATGCTGGGGTGACGAGAAGACAAGTTCTCATGTTCATTCATCCAGTGTGAACTCAGTTAATTCCTTTACTTCTACCACCTCTTCTTCAAAGTGGAAGTTCTGGAAAAATGATAACCTATTGTCGAGGTCGCTATCTTCCAGATCTGTGAATGACCAAGATCCGAACTTTGTTCAGCCAAAACCAACCAATTCGTTACAAAAGAAGTCTTCAATTTCAAGTTTTCACAATTCTATTTTTGGTGGTGGCAAACACACAGAGAAGAAGAGGAATTCTGGATTTATTATGCCCGATCATCAGAGCACGAAGGAGCTAAACCACAAACATTCATCATCGAACCTTTCCTTTAGAAGTTTGAAGCATAAAACATCACATTCTTCACTAAATAAGCTCAAAGTAAGGCGTAAAGGAAATACACAAGAACTGAATCATCCGATCAAAAAAACTTGCCAAATATCATTGCCTGTTCCAGACCAAGTCTCAAAGGACAAGATTCAACTGAAATTGAAAAATTCAACATCATTGGCATCCTTATCTTCAGAAGTCACTCCCATAAACACTTTGGACTACAATGATTCAATTTTGCAACAAATATTACAACTTTGTGATGTTAAGTATATATTACATGATCTACGTGAAGCTCAGTCATTAGGCTTGTTTACGTTGAATACTAGATCGGTTCAGCTGTCTCATAACTTTTGGCAAACTTATCATAGCGATATGCAAACTTCACTCATTTGCAAGAAAGTATGTCTAGGGGCTCTAAGTGATTTGACTACTTCAAACTTGATATCCTTACATGAATTGAAATCATTACGGTTAATACAAGGAACTAGCGGTGTCGCCAATTTACTGCAAGCTTATGTTGTGCCCTCAAATCAATGTGAAAATGACCAAAACTTGATACTGTACTTATTTTTCAAATACCAGGGAACTCCTCTATCAAGGTGCTCTAACATTGATTACTCTCAAGCATTGTCTATTTTCTGGCAGTGCAGCAGTATTTTATATGTTGCTGAATCCAAATTTCAGCTCGAACACAGGAATCTAACTTTGGACCATATTTTGATAGACTCTAAAGGGAATGTTACTTTAATTGATATGAAGTGCTGTCGTTTCTTGAATATAGACAACAATAAGGCTTCCTATACAAGATTAGACCATCATTATTTCTTCCAAGGCCGGGGGACTCTTCAATTCGAGATATATGAACTGATGAGAAGCATGCTGCCTCAGCCAATATCCTGGGCTACATTTGAACCAAGAACAAACCTATTATGGTTATATCACCTAAGTAGCAGCCTGCTAAAAATGGCCAAAAAAGCCGTAGTCAGCGGCGCTTTGAACCGGGAAGAAAATATCTTAATCGAGTTGACGCACTTACTCGATCCTGCTCGAAAACATTCCAAAACAATTTTCAAAAAGGAACTTGTTATAAGAACTTGCGGTGATTTGTTATCTTTGAAGGGAGAAATAATGCAGTAA>YBL010C	-:0:chr02:206110:206952ATGAGCGATAGAGATCAGATAGAACCTGTCACAAATGCATTAGATGCAGAAAGTGATTCTAGTGATGATTTTGGGAATTTTTCAGATGCCTCAGTTGAAAATGACCTCTATAACCAGAATTCTACTTTAACGACGTCTTCCGAATCTGTAGTAGATAACTGCTTGAATAAGATACTACCAAAGGGAGAATTTGACCTGGAAGAAGAAACCATAAAGAATGACTGTTTCAAGCTGAGCAAACTTATTGAAGATGAACGACCCCATGTCATTTATGAACAACTCGTACAATTAGATCCTGTGTTACAGCCATTCATATGGAATAAATCTCATATACGCAGAAACTTGCTCCACATTTTGAGATTATCGGATAATAATGGCTCTGAGGGTGTTGGTACGAAGAGGGAGGAAGAACCGTTGAATGATGAGTTGTTCAAAAGGATATGCGATGCAGTGGAGAAAAATGAGCAAACGGCTACTGGTCTCTTCCTGAGGGATAATTTCAAAATTGACTATACACCACCAATGACTTTAAAATCTCTTCAAAAGGAAGAGGAGCGTGAGCAAGAGCAGCATATACCGCAATTGCTTATGGCAGATTTTACTAGTATGGATGAAGAATCTTTACGCCAATACCACGATACATTGTGTCAATCCATTGACTTTTTGGTTAGTAAATCAAGATCATTGAAGAAACAACAGCGAGACCTCCTAAAGGATAAGACAACTTTTGAAAATGTAGTGACGAATTTGACAGGACATACCCAAAGACTACAGCGAGACGAAATTGCATTGTATAACAAAAAGCGAAACAAAAAGAAAAGGTTCAGTTGGGTAGGATACTAA>YBL011W	+:0:chr02:203541:205820ATGCCTGCACCAAAACTCACGGAGAAATTTGCCTCTTCCAAGAGCACACAGAAAACTACGAATTACAGTTCCATCGAGGCCAAAAGCGTCAAGACGTCGGCTGATCAGGCATACATCTACCAAGAGCCTAGCGCTACCAAGAAGATACTTTACTCCATCGCCACATGGCTGTTGTACAACATCTTCCACTGCTTCTTTAGAGAAATCAGAGGCCGGGGCAGTTTCAAGGTACCGCAACAGGGACCGGTGATCTTTGTTGCGGCTCCGCATGCTAACCAGTTCGTCGACCCTGTAATCCTTATGGGCGAGGTGAAGAAATCTGTCAACAGACGTGTGTCCTTCTTGATTGCGGAGAGCTCATTAAAGCAACCCCCCATAGGGTTTTTGGCTAGTTTCTTCATGGCCATAGGCGTGGTAAGGCCGCAGGATAATTTGAAACCGGCAGAAGGTACTATCCGCGTAGATCCAACAGACTACAAGAGAGTTATCGGCCACGACACGCATTTCTTGACTGATTGTATGCCAAAGGGTCTCATCGGGTTACCCAAATCAATGGGATTTGGAGAAATCCAGTCCATAGAAAGTGACACGAGTTTGACCCTAAGAAAAGAGTTCAAAATGGCCAAACCAGAGATTAAAACTGCTTTACTCACCGGCACTACTTATAAATATGCCGCTAAAGTCGACCAATCTTGCGTTTACCATAGAGTTTTTGAGCATTTGGCCCATAACAACTGCATTGGGATCTTTCCTGAAGGTGGGTCCCACGACAGAACAAACTTGTTGCCCCTGAAAGCAGGTGTGGCGATTATGGCTCTTGGTTGCATGGATAAGCATCCTGACGTCAATGTTAAGATTGTTCCCTGCGGTATGAATTATTTCCATCCACATAAGTTCAGGTCGAGAGCGGTTGTTGAATTCGGTGACCCCATTGAAATACCGAAGGAACTAGTCGCCAAGTACCACAACCCGGAAACGAACAGAGATGCAGTGAAAGAATTATTAGATACCATATCGAAGGGTTTACAATCCGTTACCGTTACATGTTCTGATTATGAAACTTTGATGGTGGTTCAAACGATAAGAAGACTATATATGACACAATTTAGCACCAAGTTACCGTTGCCCTTGATTGTGGAAATGAACAGAAGAATGGTCAAAGGTTACGAATTCTATAGAAACGATCCTAAAATAGCGGACTTGACCAAAGATATAATGGCATATAATGCCGCCTTGAGACACTATAATCTTCCTGATCACCTTGTGGAGGAGGCAAAGGTAAATTTCGCAAAAAACCTCGGACTTGTTTTTTTTAGATCCATCGGGCTCTGCATCCTCTTTTCGTTAGCCATGCCAGGTATCATTATGTTCTCACCTGTCTTCATATTAGCCAAGAGAATTTCTCAAGAAAAGGCCCGTACCGCTTTGTCCAAGTCTACAGTTAAAATAAAGGCTAACGATGTCATTGCCACGTGGAAAATCTTGATTGGGATGGGATTTGCGCCCTTGCTTTACATCTTTTGGTCCGTTTTAATCACTTATTACCTCAGACATAAACCATGGAATAAAATATATGTTTTTTCCGGGTCTTACATCTCGTGTGTTATAGTCACGTATTCCGCCTTAATCGTGGGTGATATTGGTATGGATGGTTTCAAATCTTTGAGACCACTGGTTTTATCTCTTACATCTCCAAAGGGCTTGCAAAAGCTACAAAAGGATCGTAGAAATCTGGCAGAAAGAATAATCGAAGTTGTAAATAACTTTGGAAGCGAATTATTCCCCGATTTCGATAGTGCCGCCCTACGTGAAGAATTCGACGTCATCGATGAAGAGGAAGAAGATCGAAAAACCTCAGAATTGAATCGCAGGAAAATGCTAAGAAAACAGAAAATAAAAAGACAAGAAAAAGATTCGTCATCACCTATCATCAGCCAACGTGACAACCACGATGCCTATGAACACCATAACCAAGATTCCGATGGCGTCTCATTGGTCAATAGTGACAATTCCCTCTCTAACATTCCATTATTCTCTTCTACTTTTCATCGTAAGTCAGAGTCTTCCTTAGCTTCGACATCCGTTGCACCTTCTTCTTCCTCCGAATTTGAGGTAGAAAACGAAATCTTGGAGGAAAAAAATGGATTAGCAAGTAAAATCGCACAGGCCGTCTTAAACAAGAGAATTGGTGAAAATACTGCCAGGGAAGAGGAAGAGGAAGAAGAAGAGGAAGAAGAAGAAGAGGAAGAAGAAGAAGAAGGGAAAGAAGGAGATGCGTAG>YBL013W	+:0:chr02:202059:203264ATGGTTAAAATGAGAAGAATAACACCTACACGCCTCCTATTCACATGCAGATATATTTCAAACAATGCTTCTCCACCAGTGCAGCCCTTGAATGTGCTTTTCTTTGGTAGCGACACTTTCAGTAATTTCTCATTGCAAGCACTCAATGAGTTGCGTCAAAATAATGGAAGCTGTGGTATAGTGGACAATATTCAAGTAGTAACTAGGTCGCCGAAGTGGTGCGGTAGACAGAAGTCTATTTTGAAATACCCGCCGATCTTCGATATGGCAGAGAAGCTTCAATTGCCACGCCCAATTACATGCGACACCAAGCAGGAAATGTTGGCGCTAAGCAAACTGACACCCAGTCGCCAAGGAAATCCGGAGAACGACGGCTCCGGTGCTCCGTTCAACGCGATCATTGCGGTTTCTTTTGGGAAGCTCATTCCGGGTGACTTGATCCGCGCGGTGCCATTGGCGCTAAACGTCCATCCTTCGCTACTTCCCAGACATAAAGGCAGTGCACCTATCCAGCGAGCTCTGCTCGAGGGTGACACTTACACCGGTGTAACTATACAGACACTGCATCCGGATCGGTTTGACCATGGTGCAATTGTAGCGCAGACGGAGCCACTGGCGATCGCAACAATGCTGTCAAAAGGGAGAGTCAATGATTCGACGGCAGATTTTAATTCTGAGGGCCTGCCTCGGAGGACTGCCATACTGATGGACCAGTTGGGCGCCCTTGGCGCCCAACTTTTGGGCCAAACGCTTCGTGAGAGGTTATATCTACCACAGAACCGTGTGCAAGCGCCAACAGCTTACAAACCCAGTTACGCCCACCGTATAACAACAGAAGATAAACGCATTCATTGGGCGCGCGATTCTGCCGCCGAACTACTTAACAAACTCGAAACGCTCGGTCCTCTGCACGCGTTCAAAGAAGCGACAGCGGCAAGAAAGGACGCTCAAAATTCAGTATTAAAACGAATATTGTTTCATGAGTGTAAAGTGATGAGAGACGCACGTCTAGATAATGGCAGTAAACCGGGCATGTTCAAATATGATGATATTAAAGACTGTATTTTGGTCACCTGTCGCGGCAACTTACTACTATGTGTGAGCCGCCTCCAGTTCGAAGGTTTCGCCGTAGAACGTGCTGGCCAGTTCATGGCGCGCCTGCGGAAAAGATGCGGCGCCCTGAGTGAAAAGTTAGTTTTCCTGTAA>YBL014C	-:0:chr02:199067:201751ATGAGTGAGGGACAAATTCCAAGCTCAGATGTGTTAGGCTCGCAATTGGGTGTTGGTGTACAAGGCGCCAGTCTTTACTGTCCGCAAGAAAATTACACTACGAAGAAGCAAGAAAACCCACAATGGCTAAGACCAGTAGATGATACATTGGCCGAGGATGCACTAGACCTTCATATAGTGGTCAAAAGTCTGCTTTGTGATACTGCCATAAGATATATTTCCGACGACAAGGTTTTGCAAGAATCAGATGCCGACGATGATCTCATAACTAGCGATATTGATGAAGATACGGACAATCAGGGGGATACATCTATAGTGGTCAATCCAGTGATACCCGTGGTACCGAAAGATGTCCATTTTTTTAAGAAAGTTGACGTTGGTAATGATTCTATGTTTGGTGTGAACTGCGATACGCCAGTTTCATTTCAAGATTACATTCCTTCAGATTTGCTGCGCAACTTAGATGATACATTACAAGAAAGTACTAACTCTTCGAGGCCGATGCAAGACGCCTTCTTTTGGGATCCTACTGTGGCTAATCGACTGGACTCTCAATATATTCAGACAGCATCAGATTTAAGAAATTATAGAGACGGAACCGAAATCATAGCATATGCCTCGGGTAAAACAGGCTCAGTCCTAAATATAGCAGTTCTGACACGACAAAATACATTGCATTTAAACCGACATAACAATGTAACGAGTATCGAATTGCATTCACCTATCAAGAGTATTAAGATACCGGGGGCCTCTGAGTCCATCGGGCGGAGATCAAACCTGGTGGGCATAATCACCGAAAATTCTTTCCAAATCTTTAGAATTGAAAGTGTCCACTCAAGATCATGTGATGTCATGGTTAGTAGTTCAGAACCACTGTATTTTGTTGAAATAGACGACCTTCAGGTGGTAGATTTTGCATTCAATCCTTGGGACTTACAACAATTCGCAATCATTGATATAAAAGGTAACTGGAGTATCGGGAGGATACCAAAGAATTTCAATAATAATAACAAAAGGAAACTACAGTTAATAGATAACCTCCACGGTACGATTTTTGATCCAGAAGAATTATCGTCATGGAAAAGAATAGAATGGTTTTCACACTTCCAAAAAATACTGGTCTTTGATAGGTCCAAAATGATTGAGATCGACTTTATGAACAATTGGCAAACGGAAGTAGTACAAGCGAAAGCATGGTCAAACATACGTGATTATAAGCGGATAGATGATAAAAATGGTATTTTACTCACTTCAAGGGAAATAATCATCGTAGGGGCATCAGAATCAAATGATCCAGTGAGAAGAATATCTTGGAAACACGATCTGGATCCAGATGATACTACGCTCAGAATCACTGTACAAAAGGTTAAAAAGCCTGACCATATTCTTCTGGTGGCCTTTGTATATTCGATGCGGCATAAACGCATCTATATGCATGTATTTTCCCATAGGAAAGCAAACTTGTTTCAATCCTTAGGATGCTCAACAGTTCTTGAAATTCCGGGTGGGACCCCCACCGGTATAGAGACAATCTTAACACTGGATCATATAGACGATGAATCTCGGAGAGAGGAGGATGCAGATGAAAATTTTGAGTTAGTTGTAGATTTTTTAGTCAAACTAAGGAACTCATCCGAGGTTTATTACTATGCTTTATCAAACACTCAAAACTCCGAACCGAACAAACAAGAAACCCCCATCATTGTCGACCATCCCGAATGGGCGTCACTTTTCAATAACGCTGATGAACGTGAAAAGGAGAGTATCGGTGCACTGGTTTCTCAAATTAAGTTGAAGGAAAGAGAGCGCATATCTCGAGTCCAAAATTTAATTGAGCATGAAAACAGTCACGACGAAGATAAATATCTCCAAGATTTGGGATATCGCTTATCTATAGCTACAAACGAATTGCTTGAATCTTGGCAGAAGACGAAAGATGAAAGCATCCTTAGTGGGTCTTTGAGCCATTCAAAATTGAAAAACCTTTTGGAGAATTCAGATTCCTTCGCGAGTATACCTGAATTTTCTTCATTACTAGATCAGTTTTTCCAGTACTATCAGGATCAGGATGTGACATTTATTGGTTTTGAGAAATTGCTACATTTGTTTTTACATGAAGACGTTCCGGGCTTGGATATCTTTTACAACAAATTATTGCAATGTTGGGTTTTGGTATCCCCGCAAGCGGAACTGCTGACAAAGGAGATAGTCAAAGACATTATCTGGAGTTTAGCGAGACTTGAAAAACCATCACTTTTTGAACCGATTCAAAACGAAATTTCACGATCACTAAGTGGTCCTTACCAGGATATTATCTCCTCTTGGGATATGGATGATATCAATGAAGAAGATGAATCTAATGAATTTAATTTTGATAGTCAATTTTCAGCACCCTTTAATGGCAGGCCTCCATTTAATTTAAATTCGCAATCTCAAATTCCAACGATCAAATCATCACAAAGTAGCGGACTGGCAAGGAGGAAAAGAATATTAAAGACACAATCACAAAAAGCTACTCCGTTATCACAATCCACTCAAAACTTAAGTGTCCTGCCAGATTCAATGACACCAGCATTTACTCTAATGCAGCCCCCTTCCTCGCAGATATCATTTGTGAACGATTCACAACCGCGTAATTCTCAAAAAGCCAAAAAGAAGAAGAAAAGGATCCGGGGGTTTGGATAA>YBL015W	+:0:chr02:194125:195705ATGACAATTTCTAATTTGTTAAAGCAGAGAGTTAGGTATGCTCCCTATCTGAAAAAAGTTAAGGAAGCTCACGAGCTTATTCCATTGTTCAAGAATGGTCAGTACCTTGGGTGGTCCGGTTTTACAGGAGTGGGTACTCCCAAGGCAGTGCCGGAGGCACTGATAGATCACGTGGAGAAGAACAATTTACAAGGGAAGTTGAGATTCAACCTTTTTGTTGGAGCTTCTGCTGGTCCAGAGGAAAACCGTTGGGCTGAACACGACATGATCATTAAGAGAGCCCCTCATCAAGTAGGGAAACCCATTGCAAAGGCAATTAACCAGGGTAGAATTGAGTTCTTTGATAAACATCTGTCCATGTTCCCTCAGGATCTGACATACGGGTTCTACACCAGGGAAAGAAAAGACAACAAAATCCTTGATTATACTATAATCGAGGCAACGGCCATTAAAGAGGACGGGTCTATCGTCCCAGGTCCCTCTGTCGGTGGTTCTCCAGAATTCATTACAGTCAGTGATAAAGTGATTATTGAGGTTAACACGGCTACGCCTTCGTTCGAGGGTATTCACGATATAGACATGCCCGTGAACCCACCTTTCAGGAAACCATACCCATATCTGAAAGTGGACGACAAGTGTGGTGTTGACTCCATCCCGGTTGATCCTGAAAAGGTTGTTGCGATTGTGGAGTCCACCATGAGGGACCAGGTCCCACCAAATACGCCCTCTGACGACATGTCCAGGGCTATTGCAGGTCATTTGGTCGAGTTTTTCAGAAACGAGGTAAAACATGGTAGGCTACCTGAAAACCTGCTGCCTTTACAAAGTGGTATAGGTAACATTGCTAACGCTGTCATTGAAGGGCTTGCTGGCGCCCAATTCAAGCACTTGACTGTATGGACGGAAGTGCTGCAGGACTCGTTCTTGGATCTTTTCGAGAACGGATCTTTGGACTACGCCACTGCTACTTCCGTGAGATTGACTGAAAAGGGTTTCGACAGAGCCTTTGCAAACTGGGAAAATTTCAAACACAGATTGTGTTTGAGATCTCAAGTTGTCTCGAACAATCCGGAAATGATCCGTAGATTGGGTGTCATCGCCATGAATACCCCAGTAGAAGTTGACATTTACGCGCACGCCAATTCTACAAATGTGAATGGTTCCCGTATGTTGAACGGGTTGGGTGGATCTGCTGATTTCTTGAGAAATGCAAAGTTGTCCATCATGCATGCCCCCTCTGCAAGACCAACTAAAGTAGACCCTACCGGTATCTCTACCATTGTTCCTATGGCCTCTCATGTAGATCAAACTGAGCATGACCTGGACATCTTGGTCACTGACCAAGGTTTGGCGGATCTAAGAGGTCTATCGCCTAAGGAAAGAGCCCGTGAAATCATCAACAAGTGTGCTCATCCCGATTATCAAGCTTTGTTGACCGATTACTTGGACAGAGCAGAGCATTACGCTAAAAAGCACAATTGCTTGCATGAACCACACATGCTAAAGAATGCTTTCAAGTTCCACACCAACTTAGCTGAAAAGGGTACAATGAAGGTCGACAGCTGGGAACCAGTTGACTAG>YBL016W	+:0:chr02:192454:193515ATGCCAAAGAGAATTGTATACAATATATCCAGTGACTTCCAGTTGAAGTCGTTACTGGGAGAGGGTGCATACGGTGTGGTATGTTCTGCAACGCATAAGCCCACGGGAGAAATCGTGGCAATAAAAAAGATCGAACCATTCGATAAGCCTTTGTTCGCATTACGTACGCTGCGTGAAATAAAGATCCTGAAGCACTTCAAGCACGAAAATATCATAACAATCTTCAACATTCAACGCCCTGACTCGTTCGAAAACTTCAATGAGGTCTACATAATTCAAGAGCTAATGCAGACAGATTTACACCGTGTAATCTCCACCCAGATGCTGAGTGACGATCATATACAATATTTTATATACCAAACCTTGAGAGCAGTGAAAGTGCTGCATGGTTCGAACGTCATCCATCGTGATTTAAAGCCCTCCAACCTTCTCATAAACTCCAACTGTGACTTGAAAGTATGTGATTTCGGTTTAGCAAGAATCATTGACGAGTCAGCCGCGGACAATTCAGAGCCCACAGGTCAGCAAAGCGGCATGACCGAGTATGTGGCCACACGTTGGTACAGGGCGCCAGAGGTGATGTTAACCTCTGCCAAATACTCAAGGGCCATGGACGTGTGGTCCTGCGGATGTATTCTCGCTGAACTTTTCTTAAGACGGCCAATCTTCCCTGGCAGAGATTATCGCCATCAACTACTACTGATATTCGGTATCATCGGTACACCTCACTCAGATAATGATTTGCGGTGTATAGAGTCACCCAGGGCTAGAGAGTACATAAAGTCGCTTCCCATGTACCCTGCCGCGCCACTGGAGAAGATGTTCCCTCGAGTCAACCCGAAAGGCATAGATCTTTTACAGCGTATGCTTGTTTTTGACCCTGCGAAGAGGATTACTGCTAAGGAGGCACTGGAGCATCCGTATTTGCAAACATACCACGATCCAAACGACGAACCTGAAGGCGAACCCATCCCACCCAGCTTCTTCGAGTTTGATCACTACAAGGAGGCACTAACGACGAAAGACCTCAAGAAACTCATTTGGAACGAAATATTTAGTTAG>YBL019W	+:0:chr02:184356:185918ATGTCATCAAGCGAAAACACGTTACTGGATGGAAAGTCAGAGAACACAATACGATTTTTAACTTTCAATGTCAATGGTATAAGAACCTTTTTTCATTATCAACCATTTTCTCAAATGAATCAATCCCTTAGATCTGTTTTCGACTTTTTTCGAGCAGACATAATAACATTCCAAGAGCTCAAGACGGAAAAATTGTCTATCTCTAAGTGGGGGAGAGTTGATGGTTTTTATTCTTTTATTTCTATCCCTCAAACCAGAAAGGGATATTCTGGCGTTGGCTGCTGGATTAGAATTCCGGAAAAGAACCACCCACTATACCATGCATTACAAGTCGTTAAGGCAGAAGAAGGTATAACGGGTTACTTGACAATAAAAAATGGTAAGCATTCAGCAATCTCCTATAGAAACGACGTAAATCAAGGAATTGGTGGTTACGATTCTTTAGATCCCGATTTAGATGAGAAAAGTGCACTGGAACTAGATTCAGAAGGCAGATGTGTTATGGTTGAACTGGCATGTGGAATAGTTATTATCAGTGTATATTGTCCCGCAAATTCGAACTCATCGGAGGAGGGTGAGATGTTTAGATTAAGGTTCTTGAAAGTTTTATTAAGAAGAGTTCGGAATTTGGACAAAATTGGGAAGAAGATTGTGCTAATGGGCGACGTAAATGTTTGCCGGGATCTTATAGACAGTGCCGATACATTAGAACAATTCTCAATTCCAATAACAGATCCCATGGGTGGAACAAAGTTAGAAGCACAATATAGGGATAAAGCAATCCAATTTATTATCAATCCGGACACGCCACATCGGAGGATATTTAATCAAATATTGGCTGATTCACTTTTACCAGACGCGAGTAAAAGGGGGATACTGATAGACACTACGAGGCTAATTCAAACAAGAAATCGACTTAAAATGTATACAGTCTGGAATATGTTAAAAAATTTAAGACCTTCGAATTATGGCTCACGGATAGATTTTATCCTAGTGTCCTTAAAGCTTGAACGATGCATAAAAGCAGCTGACATTCTTCCGGATATATTGGGCTCTGACCATTGTCCTGTGTATTCTGATTTAGATATACTGGACGACAGAATTGAACCTGGTACGACACAAGTTCCCATACCAAAATTCGAAGCAAGGTACAAATATAATTTAAGAAACCATAATGTTTTAGAGATGTTTGCCAAAAAGGATACGAATAAAGAATCTAATAAACAAAAATATTGTGTATCAAAAGTCATGAATACCAAAAAAAACAGCAACATCAAAAACAAATCGCTCGACTCATTTTTCCAGAAGGTAAATGGAGAAAAAGATGACAGGATTAAAGAATCCTCTGAAATTCCACAGCAAGCTAAAAAAAGAATCTCCACGCCAAAGTTGAATTTCAAGGATGTCTTTGGAAAGCCTCCCCTGTGCAGGCATGGGGAGGAATCCATGCTGAAAACATCGAAAACTTCGGCCAATCCAGGTAGAAAGTTCTGGATTTGCAAGAGATCTCGGGGTGATTCAAATAATACAGAATCATCTTGTGGGTTTTTTCAGTGGGTTTAA>YBL020W	+:0:chr02:182404:184128ATGGCGAAAAAAAACTCACAATTGCCCTCTACTAGTGAGCAGATCTTGGAAAGGTCCACAACAGGAGCTACCTTCCTCATGATGGGCCAACTTTTCACCAAACTGGTAACGTTCATACTAAATAATTTGTTGATCAGGTTTCTGTCGCCCAGAATTTTCGGTATCACGGCCTTTCTAGAATTTATACAGGGCACAGTGTTATTTTTTAGCAGAGATGCGATTCGTCTGTCGACGTTGAGAATCTCAGACTCCGGTAATGGAATAATCGATGATGACGACGAGGAGGAGTACCAGGAAACTCATTACAAGTCTAAAGTTTTGCAAACCGCAGTCAATTTTGCTTACATTCCGTTTTGGATCGGGTTTCCACTGTCCATTGGTCTTATCGCCTGGCAGTACAGAAACATCAACGCGTATTTCATCACTCTTCCATTCTTCAGGTGGTCGATTTTTCTTATCTGGCTGAGTATCATCGTGGAGCTGTTAAGCGAGCCATTCTTCATCGTCAACCAGTTTATGTTGAACTATGCCGCAAGGTCAAGATTTGAAAGCATCGCGGTGACTACAGGATGTATTGTCAATTTTATAGTTGTTTATGCCGTTCAGCAATCCCGCTACCCAATGGGGGTTGTCACATCGGACATTGACAAAGAAGGCATCGCCATATTGGCATTTGCCTTGGGAAAGTTAGCACATTCGATCACCCTGCTAGCATGTTACTACTGGGACTATCTCAAGAATTTCAAACCAAAGAAATTGTTCAGTACCAGGCTAACGAAGATAAAAACGCGTGAAAATAACGAATTGAAGAAAGGCTACCCAAAGAGCACATCTTATTTTTTCCAAAACGACATTTTACAGCACTTCAAAAAAGTTTATTTTCAACTATGTTTTAAGCATTTGTTGACAGAGGGTGATAAGTTGATTATCAATTCTTTATGTACTGTGGAAGAACAAGGCATTTACGCTCTATTGTCGAACTATGGATCGCTACTAACAAGATTATTATTTGCGCCGATCGAAGAATCTCTGCGGTTATTTTTGGCCCGTTTATTATCCTCGCATAACCCTAAAAATTTAAAACTATCTATTGAAGTCCTGGTGAATTTAACAAGGTTTTACATATACTTATCGTTAATGATCATTGTATTTGGGCCTGCCAATTCATCCTTTTTATTGCAGTTCTTGATTGGCTCGAAATGGTCCACTACTTCCGTTTTGGACACTATAAGAGTCTACTGCTTTTACATCCCATTTTTATCGCTTAATGGTATTTTTGAAGCTTTTTTCCAGAGTGTAGCCACTGGTGACCAAATTTTGAAACATTCATATTTTATGATGGCCTTTTCTGGTATTTTCCTGCTCAATTCCTGGCTTCTTATTGAAAAACTCAAACTATCAATCGAAGGCTTGATATTGAGTAACATCATTAACATGGTGTTGAGAATATTGTATTGTGGAGTTTTCTTGAATAAATTTCATAGGGAACTGTTTACAGATTCCTCTTTTTTCTTCAATTTTAAGGATTTCAAAACAGTTATTATTGCTGGCTCAACGATCTGTCTACTTGACTGGTGGTTTATTGGGTACGTTAAAAATTTACAACAATTTGTTGTTAACGTATTATTCGCAATGGGATTGTTAGCGTTAATTTTGGTCAAGGAGCGCCAAACCATACAATCTTTTATTAACAAGAGGGCGGTTTCCAATTCTAAAGATGTATAA>YBL021C	-:0:chr02:181663:182097ATGAATACCAACGAGTCCGAACATGTTAGCACAAGCCCAGAGGATACTCAGGAGAACGGTGGAAACGCTAGCTCCAGCGGCAGTTTGCAGCAAATTTCCACGCTAAGAGAGCAGGACAGATGGCTACCCATCAACAATGTAGCGCGACTCATGAAGAATACTCTCCCACCGAGTGCTAAGGTATCGAAAGATGCGAAAGAGTGCATGCAGGAGTGTGTCAGTGAGCTCATTTCTTTTGTGACTAGCGAGGCCAGCGATCGATGCGCTGCTGACAAAAGAAAGACGATAAACGGGGAAGACATTCTCATATCATTGCACGCCTTAGGATTCGAGAACTATGCAGAGGTGTTGAAAATCTACTTGGCTAAATACAGGCAACAACAGGCGCTGAAGAATCAACTAATGTATGAGCAGGACGACGAAGAGGTGCCTTGA>YBL023C	-:0:chr02:174923:177529ATGTCTGATAATAGAAGACGTAGACGTGAGGAAGATGATTCGGACTCAGAAAATGAGCTACCGCCATCCTCACCTCAGCAACATTTTAGAGGGGGGATGAACCCAGTTTCATCACCTATTGGTTCTCCAGATATGATTAACCCTGAAGGTGACGATAATGAAGTTGACGATGTACCAGATATTGATGAAGTTGAAGAACAAATGAACGAAGTAGATTTGATGGACGATAATATGTATGAGGATTATGCGGCTGATCATAATAGAGATAGATATGATCCAGATCAAGTTGACGACAGGGAACAACAAGAACTATCTTTAAGCGAACGTCGCCGCATTGATGCTCAACTAAATGAAAGAGATAGGCTATTGAGAAATGTTGCCTACATAGATGACGAGGACGAAGAACAAGAAGGTGCAGCGCAACTTGATGAAATGGGCCTTCCTGTGCAAAGACGAAGAAGGAGAAGGCAGTATGAGGACTTAGAGAATAGTGATGACGATCTATTGAGTGACATGGACATTGACCCATTAAGAGAAGAACTTACTTTAGAATCTCTGAGTAACGTTAAGGCTAACAGTTACTCGGAATGGATAACACAACCTAATGTCTCAAGAACTATTGCCAGAGAGTTAAAATCATTCCTGCTGGAATATACAGATGAAACGGGTCGTTCTGTATATGGTGCACGTATTAGAACATTAGGTGAAATGAATTCTGAATCTTTGGAGGTTAATTATAGACACTTAGCGGAGTCCAAAGCCATCCTGGCACTATTTTTAGCTAAATGTCCAGAAGAAATGTTGAAAATATTCGATCTCGTGGCTATGGAGGCAACAGAATTGCATTACCCAGATTATGCCCGTATTCACTCCGAAATTCACGTAAGAATCTCTGATTTTCCAACAATATACAGTTTACGTGAATTGCGTGAGTCTAATTTGTCCTCTCTAGTACGCGTCACTGGGGTGGTGACAAGAAGAACAGGAGTCTTCCCTCAATTAAAATATGTCAAATTCAATTGTTTGAAATGTGGCTCCATTTTGGGCCCATTTTTTCAAGATTCTAATGAAGAAATTAGAATCTCATTCTGTACAAACTGCAAATCAAAAGGTCCCTTTAGAGTCAATGGAGAAAAAACTGTGTACCGAAATTATCAAAGGGTTACGCTCCAGGAAGCTCCCGGAACCGTTCCTCCAGGCCGTCTACCAAGACATAGAGAAGTCATTTTGTTGGCGGATTTGGTAGATGTATCCAAGCCAGGTGAAGAGGTAGAAGTTACCGGCATCTACAAAAATAACTACGATGGTAACTTGAATGCAAAGAACGGATTCCCCGTTTTTGCAACAATTATCGAGGCAAATTCTATAAAAAGAAGAGAGGGCAATACAGCTAACGAAGGCGAAGAAGGGTTGGATGTTTTCAGTTGGACTGAAGAAGAAGAACGTGAATTTAGAAAGATTTCTAGGGATCGTGGTATAATTGATAAAATTATATCATCGATGGCACCGTCTATCTATGGTCATAGAGATATTAAAACTGCAGTCGCGTGCTCATTATTTGGAGGTGTTCCAAAAAACGTCAATGGAAAACATTCTATTCGTGGTGATATCAATGTGTTATTATTAGGTGATCCAGGTACTGCCAAATCTCAAATCTTAAAATACGTCGAGAAAACAGCGCATAGAGCGGTCTTTGCAACTGGTCAGGGTGCTTCGGCTGTCGGTCTGACAGCATCCGTCAGAAAAGATCCTATTACTAAAGAATGGACCTTAGAAGGGGGGGCGCTAGTATTGGCTGATAAGGGTGTTTGTTTAATTGATGAATTCGATAAGATGAACGATCAGGATCGTACATCTATTCATGAGGCTATGGAACAGCAAAGTATTTCCATTTCCAAGGCCGGTATTGTTACTACATTACAAGCGCGCTGCTCAATTATTGCTGCGGCAAATCCTAATGGTGGTAGATATAATTCAACCTTGCCTTTAGCTCAGAATGTTAGTTTGACCGAGCCTATTCTGTCTAGATTTGATATTTTATGTGTTGTCAGAGACCTTGTTGATGAAGAGGCAGACGAAAGATTGGCCACATTTGTTGTCGATTCTCATGTAAGATCTCATCCAGAAAACGATGAAGATCGAGAAGGCGAAGAGCTTAAAAATAATGGCGAATCGGCGATAGAACAAGGAGAAGATGAAATAAATGAGCAGCTTAATGCAAGGCAAAGAAGACTTCAAAGACAAAGGAAGAAGGAGGAAGAGATCTCGCCTATTCCACAGGAATTATTGATGAAATATATTCACTATGCGAGGACAAAAATATACCCTAAATTGCATCAGATGGATATGGATAAGGTTAGCAGGGTATATGCGGATTTGAGAAGAGAAAGTATTTCTACAGGTTCGTTTCCAATCACAGTTCGTCATTTAGAATCTATTCTAAGAATTGCAGAATCTTTTGCTAAAATGAGATTATCTGAATTTGTGTCTTCTTATGATTTGGATAGAGCTATCAAAGTTGTAGTTGATTCCTTTGTTGATGCCCAAAAAGTCAGTGTTCGCCGCCAACTCCGCAGGTCTTTCGCAATTTATACCTTGGGTCACTAA>YBL024W	+:0:chr02:172537:174591ATGGCTAGAAGAAAGAATTTCAAAAAAGGGAACAAGAAGACTTTTGGTGCTCGTGATGACTCGAGAGCTCAAAAAAACTGGTCTGAACTGGTAAAGGAAAATGAAAAATGGGAAAAATACTATAAGACTTTAGCTCTTTTCCCAGAAGATCAATGGGAAGAATTTAAAAAGACATGTCAAGCTCCACTTCCTCTAACTTTTAGAATTACAGGTTCTAGAAAGCATGCCGGTGAGGTCCTGAATTTGTTTAAAGAAAGACATCTACCAAACTTGACTAATGTTGAGTTTGAAGGTGAGAAGATTAAGGCCCCTGTAGAATTACCTTGGTATCCAGACCATCTTGCTTGGCAATTGGACGTTCCTAAGACGGTTATTAGAAAGAATGAACAATTCGCAAAAACTCAGAGATTTTTAGTTGTTGAAAATGCCGTTGGTAATATCTCAAGACAAGAAGCCGTTTCAATGATTCCTCCAATCGTTCTAGAAGTAAAACCTCATCACACTGTTTTAGATATGTGTGCTGCTCCTGGCTCCAAAACTGCTCAATTAATCGAAGCCTTGCACAAGGATACAGATGAACCATCTGGTTTCGTTGTAGCTAATGATGCCGATGCCAGAAGATCTCATATGTTGGTTCACCAATTGAAGAGATTGAACAGTGCCAACTTGATGGTTGTCAACCATGACGCCCAATTCTTCCCACGTATCAGATTACATGGCAACTCAAATAACAAGAATGATGTTTTAAAATTTGACAGAATCCTGTGTGACGTTCCATGTTCTGGTGATGGTACCATGAGGAAAAATGTTAATGTTTGGAAAGACTGGAACACACAAGCAGGTCTTGGTTTGCATGCTGTTCAGCTGAATATATTAAACAGGGGTTTGCATCTTCTAAAGAACAACGGTAGATTGGTTTACTCAACCTGTTCTTTAAATCCTATTGAAAATGAAGCGGTTGTTGCCGAAGCGTTAAGAAAGTGGGGTGACAAGATTAGATTAGTTAACTGTGATGATAAGCTTCCTGGCCTAATAAGATCCAAGGGTGTATCCAAATGGCCTGTCTATGACAGAAATTTGACTGAGAAAACCAAAGGAGACGAAGGTACACTAGATAGTTTCTTTTCACCATCTGAAGAAGAGGCATCGAAATTCAATTTACAAAATTGTATGAGGGTTTATCCTCACCAACAAAACACAGGCGGATTTTTCATTACTGTTTTCGAAAAAGTCGAAGATAGCACTGAGGCGGCTACAGAGAAACTATCTTCTGAAACCCCAGCTCTAGAGTCTGAAGGACCTCAAACAAAGAAAATAAAGGTAGAAGAAGTCCAAAAGAAAGAAAGACTACCACGTGACGCGAACGAAGAGCCTTTTGTTTTCGTTGATCCACAGCACGAAGCTTTAAAAGTTTGTTGGGATTTCTACGGCATCGATAATATTTTCGACAGAAACACTTGTTTAGTGCGTAACGCCACTGGTGAACCAACAAGAGTGGTTTACACTGTGTGTCCAGCATTGAAGGATGTTATTCAAGCGAATGACGATAGGTTGAAGATTATTTATTCTGGTGTAAAATTGTTTGTCTCTCAAAGAAGTGATATCGAATGTTCATGGAGAATCCAAAGTGAATCATTGCCAATAATGAAACACCATATGAAATCTAATAGAATTGTTGAAGCTAATTTAGAGATGTTAAAACACTTGTTAATCGAATCTTTCCCTAACTTTGACGACATTCGTTCGAAGAACATCGATAATGATTTTGTTGAAAAGATGACAAAATTAAGCTCTGGTTGCGCCTTTATTGATGTGTCAAGAAATGACCCTGCCAAAGAAAACTTATTCTTGCCTGTGTGGAAAGGCAACAAGTGTATCAATTTGATGGTTTGTAAAGAAGATACTCATGAGCTATTATATAGGATCTTTGGTATTGATGCGAATGCCAAGGCTACTCCAAGCGCTGAAGAAAAAGAAAAAGAAAAAGAAACGACTGAATCTCCCGCAGAAACTACTACCGGAACCTCTACTGAAGCTCCTAGCGCTGCTAATTGA>YBL025W	+:0:chr02:171484:171921ATGGATAGAAATGTATATGAAGCCTGCAGCAATATAATTAAGGAATTTGGAACACATGTAGTGAGCGCCGATGAAGTACTCGCAGAAAAAATAGATAATGCTGTTCCTATACCGTTCAAAACAAGGGAGGAGATAGATGCCGACGTGGAAAAAGATAGAAACGAAGGAGTTTTCGAAGGTAATATCATTCCTGATATCGACCTACGTGTAGTACACTACTACGCCACGCAGTTGTGTCTAAATAAGTATCCTCATTTGATTAACGCCTTTGATGAGACAAGCCTTATAACATTGGGTTTACTCATCGAAAAGTGGGTAAAAGACTATCTAACCAGCATCCAGACAGAACAGGGAAGGCAAAGTAAGGTAATCGGGAAGGGGCCATGCGAATTCATATCAAAACATATTGATTATAGGCATGCGCCAGGTAATATCTGA>YBL028C	-:0:chr02:167521:167841ATGGCCAAATCATTACGTGCTAGCAGTCATCTAAATGCTAAATCTGTCAAAAGACGTGGCGTTTTCCAAAAAGCAGTAGATGCACGTGAACAAAGAATATCCGATAAACTAAAGGAAGACTTACTAAAGCAAAAATTAGAAGATCTAAAAAAGAAAGAAGAGCAAGGAATTGACATGGATGTCGACGAGAAAAAATCCAATGAAGAAGCTCCCAGAAAAAAAATCAGCACCTCTGGTTGGAGAGACGGTAGACATCACACTTATAAAAAGGCTAAGCTGATGAAACAATCTAAGAAAAAAACTTCTTTCACCAGATTCTGA>YBL029W	+:0:chr02:166137:167267ATGTGCGCCAATATCCCCGAATTCGACTCTTTTTACGAAAATGAAAATATAAATTACAACTTGGAATCATTCGCACCTTTAAATTGTGATGTTAATTCGCCCTTTCTCCCCATTAATAACAATGACATCAACGTTAATGCTTATGGTGACGAAAATTTAACGTACTCCAACTTTTTACTGTCTTATAACGATAAGCTGGCTACTACAACTGCTAAAAACAATAGCATTAATAATAGTAATAGTAATAATAATAGTAATAATAATAAAAATAATAATAATAATCATAATAATAATAATCTACTCGGTAATGACATCAGTCAGATGGCCTTTTTACTCGATTACCCTTCTACTCTCAACGAACCGCAATTTGCCGTAAATTGTAAAGACATTTACAGAAAGGATATATCAACGCCTTCGTCATTAGTTTCGAGTCTGCCACCTGCAAAGTTTTCGTTATCCCTATCGAATTCGCCTTCTCCGCCACCACCATCGTCGTCCTCTTTGAAACATGGGGAAGCAATAATTTCTAATACCAGCGAAAGCAGTGACATATTTGCCGATCCTAATTCGTTTGAAAAGGATACTATGCCCCTAACGCAAGAACTGACGCTAGAAAATCTAAATAATCAATTAAACTATCCCGATTTCACGATAAACGCCATCGAGCAGGATCCTGCCCCTTCGTCTTTTTCATCCTCATCTTCGTCTTCGGAGTCAACGGTCTCTTCCAGCAGGAAGAGGAAGCCCTGTCATGATTCCTACACACATTCTTCACCCTCTTCCTCAGAGTCTAAGAAAATTTCCGACTCGAGATTATCCGCCGAAGGTTTAGCCAAAGTGCTTAATTTAGAATCCCCCGAAGAAGCTTTAAAAAGAGAACGCTTTATATTGGGTATTTTCCAGAATGAATTAAATTACCCGCTAGGTTACAAAACGTGGATTAGGGATACTACAAAAGAATATAGGACAAAGTTAATCAATCAACTGCACGAACGAGTAAAGGTAAAGTACCCCGAATACAACCAGTCCATACTGGAAACAATAATTAGACGAGGCACCTACTACATGATGCAGAGTAGGTTAAGAAGAGAGAGGAGAATGAAGCTTAAGGAACGTAAAAGAACAACCTAA>YBL030C	-:0:chr02:163044:164000ATGTCTTCCAACGCCCAAGTCAAAACCCCATTACCTCCAGCCCCAGCTCCAAAGAAGGAATCTAACTTTTTGATTGATTTCTTAATGGGTGGTGTCAGTGCCGCTGTCGCCAAAACTGCTGCATCTCCCATCGAAAGAGTTAAACTTTTGATCCAAAACCAAGATGAAATGTTAAAACAAGGTACTTTGGACAGAAAATACGCAGGTATCTTAGACTGTTTCAAGAGAACCGCTACACAGGAAGGTGTTATCTCATTCTGGAGAGGTAACACTGCTAACGTTATCCGTTATTTCCCCACTCAAGCTTTGAATTTCGCCTTCAAGGACAAGATCAAGGCCATGTTTGGTTTCAAGAAGGAAGAAGGTTACGCCAAATGGTTTGCCGGTAACTTGGCATCTGGTGGTGCTGCTGGTGCCTTGTCATTACTATTTGTTTACTCTTTGGATTATGCAAGAACTAGATTGGCTGCTGACTCCAAGTCCTCTAAAAAGGGTGGTGCTCGTCAATTCAACGGTTTGATCGATGTCTACAAGAAGACCTTAAAATCTGATGGTGTTGCTGGTCTTTACAGAGGTTTCTTACCTTCTGTCGTTGGTATTGTTGTCTACAGAGGTCTATACTTCGGTATGTACGATTCTTTGAAGCCTCTATTGTTGACTGGTTCTTTGGAAGGTTCATTCTTGGCTTCATTCTTGTTGGGTTGGGTTGTTACTACTGGTGCTTCTACATGTTCTTACCCATTGGATACCGTTAGAAGAAGAATGATGATGACCTCCGGTCAAGCTGTTAAGTACGACGGTGCCTTTGACTGTTTGAGGAAGATTGTTGCTGCTGAAGGTGTTGGTTCTCTATTCAAGGGTTGTGGTGCTAACATCTTAAGAGGTGTCGCAGGTGCTGGTGTTATCTCAATGTACGACCAACTGCAAATGATCTTGTTTGGTAAGAAGTTCAAATAA>YBL031W	+:0:chr02:161702:162718ATGAATGATAAACTCCAAGAAGAGCATAACGAAAAAGACACCACTTCACAAATTAATGGTTTCACGCCGCCGCATATGAGTATAGACTTTCATTCAAATAACAATAGCAATATCATCGAGACTATTGGCGTTTCCAAAAGATTAGGAAATTCTGTATTAAGTGAACTGGATTCTAGAGCTAGCTCAAAATTTGAATTTTTAAAAGATCAATCCGAACAACAATACAACGGCGACAAGAACAATGAACCAAAATCGGGCTCGTATAATATTAATGAGTTCTTCCAAGCAAAGCACGACTCTCAATTTGGCCAGATGGAGTCGCTAGACACACATTATACCCTTTTACATACGCCCAAGAGAAAGTCACAACATGCAATCCCACAGGATCGATCGGATTCGATGAAAAGGTCGAGACCGTCCCGCTCAATTCCATACACTACACCAGTGGTAAACGATATCACAAGAAGAATACGAAGATTGAAATTACGGAACTCATTGGTTAATGGTAATGACATAGTGGCTAGAGCGAGATCTATGCAAGCAAATTCTAACATTAATTCAATAAAGAACACACCATTGTCAAAGCCCAAGCCATTTATGCACAAACCAAACTTTCTAATGCCCACTACGAATTCTTTGAACAAGATCAATTCCGCTCACCGTAATACATCCTCCTCTTCGACAGCTTCATCGATACCAAGATCTAAAGTACACAGATCGATATCAATAAGAGATTTACACGCCAAAACCAAACCGGTAGAACGCACGCCAGTTGCGCAAGGAACAAATTCGCAATTGAAGAATTCAGTTTCCGTTTTTGATAGACTTTACAAGCAAACAACGTTCTCTAGGTCGACGTCTATGAACAACTTATCGTCAGGAACCTCTGCAAAATCCAAGGAACACACAAATGTAAAGACTCGGTTGGTTAAAAGTAAGACAAGTGGTTCATTGTCCAGTAACCTTAAGCAAAGTACTGCCACCGGCACAAAGAGTGATAGACCTATTTGGCGGTGA>YBL032W	+:0:chr02:160187:161332ATGTCACAGTTCTTCGAAGCTGCTACTCCCGTTGCAATTCCCACAAACAATACCAACGGCGGCTCCAGTGATGCCGGCAGCGCCGCCACTGGCGGCGCCCCCGTTGTTGGCACCACCGCTCAACCCACCATCAATCACAGGCTTTTGCTGTCATTGAAAGAGGCTGCCAAGATCATTGGCACTAAGGGCTCCACCATCTCACGCATAAGAGCTGCAAACGCCGTCAAGATCGGTATTTCTGAAAAGGTGCCCGGTTGCTCTGACAGGATCCTGTCCTGTGCTGGGAACGTAATCAATGTGGCCAATGCCATTGGTGATATTGTTGACGTGCTTAACAAACGGAATCCCGAAAATGAGGACGCAGCTGAGGGCGAAGCGGAAGAGCACTACTACTTCCACTTTTTGAACCATATTTTACCAGCTCCCTCAAAGGACGAGATCAGAGATCTGCAGCAACTGGAGGACATCGGTTATGTGAGGCTCATTGTGGCCAATTCCCATATCTCATCGATTATCGGGAAAGCAGGCGCCACCATCAAGTCCCTGATCAATAAGCACGGCGTTAAGATCGTGGCTTCCAAGGACTTCTTACCTGCTAGCGACGAGAGAATTATCGAGATCCAGGGTTTCCCAGGATCCATCACCAATGTACTTATCGAAATTAGCGAGATCATCTTGAGTGACGTTGACGTCAGATTCAGCACAGAAAGATCTTATTTCCCTCATCTGAAAAAGTCCTCCGGTGAGCCAACTTCCCCTTCTACCTCATCTAACACTAGGATCGAATTGAAGATTCCAGAACTGTATGTAGGCGCCATTATTGGCCGTGGAATGAACAGAATTAAGAATTTGAAAACTTTCACAAAAACCAATATTGTCGTGGAAAGGAAGGATGACGATGATAAAGACGAAAATTTTAGAAAATTCATAATCACAAGTAAATTTCCTAAGAATGTCAAACTTGCTGAGTCCATGCTTTTGAAGAACCTGAATACTGAAATTGAGAAACGTGAAAACTACAAGAGAAAATTGGAAGCTGCCGAAGGAGATGCCACTGTTGTTACTGAACGCTCTGATTCTGCTTCTTTCTTGGAAGAGAAGGAAGAACCTCAAGAGAATCATGATAACAAAGAGGAGCAGTCGTAG>YBL033C	-:0:chr02:158659:159696ATGACCATAGATAACTACGACAACAGTAAACAGGATAGCAGCAAATACGAGGTTAGTGGTACGGGTGATGGCAGGAACGGCGATGGCGGCTTGCCTCTAGTACAATGTGTCGCAAGAGCTCGTATCCCAACCACACAGGGTCCGGATATCTTTTTACATCTTTACAGTAACAACAGGGACAACAAGGAACATCTAGCCATTGTGTTTGGTGAAGACATACGGTCGCGCTCGCTATTCCGTAGAAGACAGTGCGAGACGCAACAAGATAGAATGATCAGGGGCGCTTATATTGGCAAACTGTATCCCGGCAGAACTGTGGCAGACGAAGACGATAGACTCGGATTAGCTCTGGAGTTTGATGATAGTACAGGTGAGTTATTAGCTTCCAAAGCCACTACATGGGACGCCCATAACGACACGCTGGTACGGATCCATTCTGAATGTTACACCGGTGAAAACGCATGGAGCGCCCGTTGTGATTGTGGTGAACAATTCGATAGGGCCGGTAGGCTTATCGCTTGCGACCACGAACCCACAAGCAACATCAAAGGTGGAAACGGCCATGGTGTTATCGTGTATCTAAGACAAGAGGGTCGTGGCATCGGGTTAGGTGAGAAACTCAAGGCCTACAACCTGCAAGACTTAGGTGCTGACACAGTGCAGGCCAACTTAATGCTGAAACATCCCGTGGATGCTAGGGACTTCTCGCTGGGTAAGGCTATTTTGCTGGATCTTGGCATCGGTAATGTCAGGCTGCTGACAAATAACCCGGAAAAGATAAAACAGGTAGATCATGCGCCTTACCTTAAGTGCGTTGAACGAGTGCCTATGGTACCCATACACTGGACAAACTCCAGTGAAGGCATAGACTCCAAGGAGATAGAAGGTTATCTCAGGACCAAGATAGAAAGAATGGGTCATTTACTAACGGAGCCTCTGAAACTTCATACAAACCCTCAACCTACTGAGACAAGTGAAGCCCAAAACCAAAACCGTATGAACTCTGCGTTGTCATCAACATCGACGCTGGCAATATAA>YBL036C	-:0:chr02:150450:151223ATGTCCACTGGTATTACTTATGATGAAGATAGAAAGACACAATTAATTGCCCAATACGAATCTGTAAGGGAAGTTGTGAATGCAGAGGCAAAAAATGTTCATGTTAATGAAAATGCCTCCAAAATTTTATTATTGGTTGTTTCGAAATTGAAACCAGCTAGCGATATACAAATTCTTTACGACCATGGTGTGAGGGAGTTCGGGGAAAACTATGTTCAAGAGTTGATCGAAAAGGCAAAATTACTACCAGACGATATCAAGTGGCACTTTATTGGCGGTTTGCAAACGAATAAATGTAAAGATTTGGCTAAAGTGCCAAATTTATACTCTGTTGAAACAATCGACTCCTTGAAGAAAGCCAAAAAATTAAACGAATCGAGGGCTAAATTTCAACCAGATTGCAACCCAATATTGTGTAATGTTCAAATTAATACCTCCCATGAGGATCAAAAATCGGGCTTGAATAATGAAGCAGAAATATTTGAAGTCATCGACTTCTTCTTATCCGAAGAATGCAAGTATATTAAGTTGAATGGGTTAATGACTATTGGTTCATGGAACGTCTCTCATGAAGATAGCAAAGAAAACAGAGACTTTGCTACGCTGGTTGAGTGGAAGAAGAAGATTGATGCTAAATTTGGTACATCATTGAAATTGTCTATGGGGATGAGTGCTGATTTCAGGGAGGCTATAAGGCAAGGAACAGCGGAAGTCAGAATTGGTACCGACATTTTTGGTGCTAGACCTCCAAAAAATGAAGCTAGAATCATTTAG>YBL038W	+:0:chr02:146190:146888ATGTTCCCCTATTTAACAAGAATGAATTTATCCATAAAGATGGGAGGGCTAACTTTAAAAGAAAGTTCCCCCAATGCTTTTTTAAATAACACCACTATTGCTAGGAGATTCAAGCATGAATATGCCCCACGTTTCAAAATCGTACAGAAAAAGCAAAAAGGTAGAGTGCCAGTTCGTACAGGTGGTTCAATAAAGGGATCCACTTTGCAATTTGGTAAGTACGGATTACGACTGAAAAGCGAAGGTATTAGGATATCCGCGCAACAGCTGAAGGAAGCAGATAACGCAATTATGAGATATGTCAGGCCTTTAAACAATGGTCATTTGTGGAGGCGTTTGTGTACAAACGTTGCTGTATGTATCAAGGGTAACGAAACAAGAATGGGTAAAGGTAAAGGTGGGTTTGACCACTGGATGGTGAGGGTACCCACAGGGAAGATCCTTTTCGAAATAAATGGTGATGATTTGCATGAAAAAGTCGCACGGGAAGCCTTCAGAAAAGCAGGCACCAAGTTACCTGGGGTATATGAATTTGTATCCTTGGATTCTCTGGTTAGAGTTGGATTACATAGTTTCAAAAATCCTAAAGACGACCCAGTGAAGAATTTTTACGATGAAAATGCAAAGAAGCCATCAAAGAAGTATTTGAATATTTTAAAGTCTCAAGAACCACAGTACAAACTCTTCAGAGGACGTTGA>YBL041W	+:0:chr02:141250:141975ATGGCCACTATTGCATCAGAATACTCTTCGGAGGCGTCAAATACACCCATTGAACATCAATTCAATCCTTACGGTGATAATGGTGGTACAATCCTGGGCATTGCAGGTGAAGATTTCGCAGTGTTAGCAGGCGATACAAGAAATATCACCGATTACTCAATTAATTCTCGTTATGAACCCAAGGTTTTTGATTGTGGTGATAACATAGTCATGTCGGCGAATGGATTTGCAGCAGACGGCGACGCTTTAGTAAAAAGATTCAAAAATAGTGTAAAATGGTACCATTTCGACCACAACGACAAAAAACTATCTATAAACTCTGCAGCAAGGAACATTCAACATCTTCTGTACGGGAAGAGGTTTTTCCCTTACTACGTTCATACGATCATTGCGGGTCTTGACGAAGATGGTAAGGGCGCTGTCTATTCGTTCGACCCAGTTGGCTCCTACGAAAGAGAACAGTGTAGAGCAGGTGGTGCTGCGGCATCATTGATCATGCCATTTTTGGACAATCAGGTTAATTTCAAAAATCAATATGAGCCAGGTACAAACGGTAAAGTCAAAAAGCCTTTGAAATACTTGTCCGTGGAAGAAGTCATCAAACTGGTGAGAGACTCGTTCACTTCTGCTACAGAAAGACATATACAAGTGGGTGATGGGCTGGAAATCCTTATTGTCACCAAGGATGGTGTAAGGAAAGAATTTTATGAGCTAAAAAGAGATTAA>YBL045C	-:0:chr02:134146:135519ATGCTAAGAACAGTAACTTCAAAGACTGTATCTAACCAGTTCAAGAGGTCTTTGGCTACAGCTGTAGCAACCCCCAAGGCCGAAGTAACGCAATTATCTAACGGTATAGTTGTAGCCACTGAGCATAATCCTTCCGCTCACACAGCCTCTGTCGGTGTTGTCTTCGGCTCCGGTGCTGCCAACGAAAACCCTTATAACAACGGGGTTTCCAATTTATGGAAGAACATCTTTCTATCCAAAGAAAACTCTGCTGTTGCCGCCAAGGAAGGTTTGGCATTGTCTTCCAATATCTCCAGAGACTTCCAATCTTACATTGTATCTTCTTTGCCAGGTTCTACCGATAAATCACTAGACTTCTTGAATCAGTCTTTCATCCAACAAAAGGCTAATTTGCTATCTTCTTCTAACTTCGAGGCCACGAAGAAATCTGTCTTGAAGCAAGTTCAAGATTTTGAAGAAAACGACCATCCCAACAGAGTTTTGGAACATTTACACTCCACCGCCTTCCAAAACACTCCATTATCTTTGCCTACTAGAGGTACTTTGGAGTCCTTGGAGAATTTAGTTGTTGCTGATCTGGAATCTTTTGCCAATAACCATTTCTTGAATTCAAATGCTGTTGTTGTTGGTACCGGTAATATCAAACATGAGGATTTAGTAAATTCCATCGAATCCAAAAACCTAAGTTTGCAAACCGGTACCAAGCCCGTACTCAAGAAAAAGGCCGCTTTCTTGGGTTCCGAAGTTAGATTGAGAGACGACACCTTGCCAAAGGCGTGGATTTCGCTGGCTGTGGAAGGTGAACCTGTCAATTCACCAAATTATTTTGTTGCTAAATTAGCCGCCCAAATTTTCGGCTCATACAACGCCTTCGAACCCGCTTCTAGATTACAAGGTATCAAATTGTTAGACAACATACAGGAGTACCAATTATGCGACAATTTCAATCATTTCTCTCTTTCTTACAAAGATTCCGGTTTATGGGGGTTCTCTACGGCCACGAGAAACGTTACCATGATTGACGACCTCATCCATTTCACTTTGAAACAATGGAACAGATTGACCATTTCTGTCACTGATACTGAAGTTGAACGTGCCAAATCGCTGTTGAAATTACAATTAGGGCAGTTATACGAATCTGGTAATCCCGTCAATGACGCTAACTTGTTAGGCGCAGAAGTCCTAATCAAGGGCTCCAAACTGTCTCTGGGTGAGGCTTTCAAGAAAATTGATGCAATCACAGTCAAAGATGTTAAGGCTTGGGCCGGTAAGAGATTATGGGACCAAGATATTGCTATTGCTGGTACAGGCCAAATTGAAGGGTTATTAGATTACATGAGAATCAGAAGTGACATGTCCATGATGAGATGGTAA>YBL049W	+:0:chr02:126831:127247ATGGGATTGCGTTACTCCATATATATTGAAAATCCGTTATCTTCCCCATCATCATCGTATAAATCAATAAACGACCCGTTATTCCACTCTCAGCATCGATCGCAAAAAAACGTGAGCTTCATCACCTACGGTTGTAGACATTGCAAGACACATCTTTCCAGTTCCTTCCAGATTATTTCTAGAGATTATAGGGGTAGGACCGGAACTGCTTATTTAATGAACAAAGTTGTTAATGTCGTTGAAGGAAAGGTCGAGCAACGAAGAATGTTGACTGGCGACTACTTAGTCTGTGATATTCTTTGTCATTGGTGCAAGAGGAACGTAGGTTGGAAATACTTGCAGAGCAGCAATGATGATCAGCAGTATAAGGAAGGAAAGTTTATCTTAGAGCTGAAAAACATTTGTAAATGTACTTGA>YBL050W	+:0:chr02:125128:125157ATGTCAGACCCTGTAGAGTTATTGAAAAGA>YBL050W	+:0:chr02:125274:126122GCTGAGAAGAAGGGTGTTCCTTCATCGGGTTTCATGAAATTGTTTAGCGGTTCTGATTCATACAAGTTTGAGGAGGCTGCTGATCTTTGTGTCCAAGCAGCCACCATTTACCGTCTAAGAAAAGAGTTAAACTTGGCAGGAGACTCGTTTTTGAAAGCTGCTGACTATCAGAAAAAGGCTGGTAATGAAGACGAAGCAGGAAATACCTACGTAGAGGCTTATAAATGCTTTAAAAGCGGTGGAAACTCTGTGAACGCCGTGGATTCATTAGAAAATGCTATTCAAATTTTTACTCATAGGGGGCAGTTCCGGAGAGGTGCTAATTTCAAGTTTGAGCTTGGAGAAATTCTAGAAAATGATTTGCATGACTATGCAAAAGCTATAGATTGCTATGAGCTCGCTGGTGAGTGGTATGCCCAAGACCAGTCGGTAGCATTATCGAATAAGTGTTTTATCAAATGCGCAGATCTAAAGGCTCTTGACGGTCAATATATTGAAGCAAGTGATATATATTCGAAGTTGATCAAGAGCAGCATGGGCAATAGATTGAGCCAATGGAGTTTGAAGGATTACTTCCTAAAAAAAGGGCTTTGTCAGCTAGCTGCTACTGATGCAGTCGCTGCTGCAAGAACTTTACAAGAGGGTCAAAGTGAAGATCCGAATTTTGCGGATTCAAGGGAGTCAAATTTCTTGAAAAGTTTAATCGATGCTGTTAATGAAGGTGATAGCGAGCAGCTAAGCGAACACTGTAAGGAGTTTGACAATTTTATGAGACTAGATAAATGGAAAATTACCATTTTGAATAAAATTAAGGAGTCCATCCAGCAACAAGAAGATGATTTGTTATGA>YBL051C	-:0:chr02:122756:124762ATGGAGACCAGTTCTTTTGAGAATGCTCCTCCTGCAGCCATCAATGATGCTCAGGATAATAATATAAATACGGAGACTAATGACCAGGAAACAAATCAGCAATCTATCGAAACTAGAGATGCAATTGACAAAGAAAACGGTGTGCAAACGGAAACTGGTGAGAACTCTGCAAAAAATGCCGAACAAAACGTTTCTTCTACAAATTTGAATAATGCCCCCACCAATGGTGCTTTGGACGATGATGTTATCCCAAATGCTATTGTTATTAAAAACATTCCGTTTGCTATTAAAAAAGAGCAATTGTTAGACATTATTGAAGAAATGGATCTTCCCCTTCCTTATGCCTTCAATTACCACTTTGATAACGGTATTTTCAGAGGACTAGCCTTTGCGAATTTCACCACTCCTGAAGAAACTACTCAAGTGATAACTTCTTTGAATGGAAAGGAAATCAGCGGGAGGAAATTGAAAGTGGAATATAAAAAAATGCTTCCCCAAGCTGAAAGAGAAAGAATCGAGAGGGAGAAGAGAGAGAAAAGAGGACAATTAGAAGAACAACACAGATCGTCATCTAATCTTTCTTTGGATTCTTTATCTAAAATGAGTGGAAGCGGAAACAATAATACTTCTAACAATCAATTATTCTCGACTCTAATGAACGGCATTAATGCTAATAGCATGATGAACAGTCCAATGAATAATACCATTAACAATAACAGTTCTAATAACAACAATAGTGGTAACATCATTCTGAACCAACCTTCACTTTCTGCCCAACATACTTCTTCATCGTTGTACCAAACAAACGTTAATAATCAAGCCCAGATGTCCACTGAGAGATTTTATGCGCCTTTACCATCAACTTCCACTTTGCCTCTCCCACCCCAACAACTGGACTTCAATGACCCTGACACTTTGGAAATTTATTCCCAATTATTGTTATTTAAGGATAGAGAAAAGTATTATTACGAGTTGGCTTATCCCATGGGTATATCCGCTTCCCACAAGAGAATTATCAATGTTTTGTGCTCGTACTTAGGGCTAGTAGAAGTATATGATCCAAGATTTATTATTATCAGAAGAAAGATTCTGGATCATGCTAATTTACAATCTCATTTGCAACAACAAGGTCAAATGACATCTGCTCATCCTTTGCAGCCAAACTCCACTGGCGGCTCCATGAATAGGTCACAATCTTATACAAGTTTGTTACAGGCCCATGCAGCAGCTGCAGCGAATAGTATTAGCAATCAGGCCGTTAACAATTCTTCCAACAGCAATACTATTAACAGTAATAACGGTAACGGTAACAATGTCATCATTAATAACAATAGCGCCAGCTCAACACCAAAAATTTCTTCACAGGGACAATTCTCCATGCAACCAACACTAACCTCACCTAAAATGAACATACACCATAGTTCTCAATACAATTCCGCAGACCAACCGCAACAACCTCAACCACAAACACAGCAAAATGTTCAGTCAGCTGCGCAACAACAACAATCTTTTTTAAGACAACAAGCTACTTTAACACCATCCTCAAGAATTCCATCCGGTTATTCTGCCAACCATTATCAAATCAATTCCGTTAATCCCTTACTGAGAAATTCTCAAATTTCACCTCCAAATTCACAAATCCCAATCAACAGCCAAACCCTATCCCAAGCGCAACCACCAGCACAGTCCCAAACTCAACAACGGGTACCAGTGGCATACCAAAATGCTTCATTGTCTTCCCAGCAGTTGTACAACCTTAACGGCCCATCTTCAGCAAACTCACAGTCCCAACTGCTTCCACAGCACACAAATGGCTCAGTACATTCTAATTTCTCATATCAGTCTTATCACGATGAGTCCATGTTGTCCGCACACAATTTGAATAGTGCCGACTTGATCTATAAATCTTTGAGTCACTCTGGACTAGATGATGGCTTGGAACAGGGCTTGAATCGTTCTTTAAGCGGACTGGATTTACAAAACCAAAACAAGAAGAATCTATGGTAA>YBL052C	-:0:chr02:119382:121877ATGTCATTAACAGCAAACGACGAATCGCCAAAACCCAAAAAAAATGCATTATTGAAAAACTTAGAGATCGATGATCTGATACATTCTCAATTTGTCAGAAGCGATACAAATGGACATAGAACTACAAGACGACTATTCAACTCCGATGCCAGTATATCACATCGAATAAGAGGAAGTGTTCGGTCTGATAAAGGCCTTAATAAAATAAAAAAAGGGTTGATTTCCCAGCAGTCCAAACTTGCGTCAGAAAATTCTTCTCAAAATATCGTTAATAGGGACAATAAGATGGGAGCAGTAAGTTTCCCCATTATTGAACCTAATATTGAAGTCAGCGAGGAGTTGAAGGTTAGAATTAAGTATGATTCTATCAAATTTTTCAATTTTGAAAGACTAATATCTAAATCTTCAGTCATAGCACCTTTAGTTAACAAAAATATAACATCATCCGGTCCTCTAATCGGGTTTCAAAGAAGAGTTAACAGGTTAAAGCAAACATGGGATCTAGCAACCGAAAACATGGAGTACCCATATTCTTCTGATAATACGCCATTCAGGGATAACGATTCTTGGCAATGGTACGTACCATACGGCGGAACAATAAAAAAAATGAAAGATTTCAGTACAAAAAGAACTTTACCCACCTGGGAAGATAAAATAAAGTTTCTTACATTTTTAGAAAACTCTAAGTCTGCAACGTACATTAATGGTAACGTATCACTTTGCAATCATAATGAAACCGATCAAGAAAACGAAGATAGGAAAAAAAGGAAAGGGAAAGTACCAAGAATCAAAAATAAAGTGTGGTTTTCCCAGATAGAATACATTGTTCTTCGAAATTATGAAATTAAACCTTGGTATACATCTCCTTTTCCGGAACACATCAACCAAAATAAAATGGTTTTTATATGTGAGTTCTGCCTAAAATATATGACTTCTCGATATACTTTTTATAGACACCAACTAAAGTGTCTAACTTTTAAGCCCCCCGGAAATGAAATTTATCGCGACGGTAAGCTGTCTGTTTGGGAAATTGATGGGCGGGAGAATGTCTTGTATTGTCAAAATCTTTGCCTGTTGGCAAAATGTTTTATCAATTCTAAGACTTTGTATTACGATGTTGAACCGTTTATATTCTATATTCTAACGGAGAGAGAGGATACAGAGAACCATCCCTATCAAAACGCAGCCAAATTCCATTTCGTAGGCTATTTCTCCAAGGAAAAATTCAACTCCAATGACTATAACCTAAGTTGTATTTTAACTCTACCCATATACCAGAGGAAAGGATATGGTCAGTTTTTGATGGAATTTTCATATTTATTATCCAGAAAGGAGTCAAAATTTGGAACTCCTGAAAAACCATTGTCGGATTTAGGATTATTGACTTACAGAACGTTTTGGAAGATAAAATGTGCTGAAGTGCTATTAAAATTAAGAGACAGTGCTAGACGTCGATCAAATAATAAAAATGAAGATACTTTTCAGCAGGTTAGCCTAAACGATATCGCTAAACTAACAGGAATGATACCAACAGACGTTGTGTTTGGATTGGAACAACTTCAAGTTTTGTATCGCCATAAAACACGCTCATTATCCAGTTTGGATGATTTCAACTATATTATTAAAATCGATTCTTGGAACAGGATTGAAAATATTTACAAAACTTGGAGCTCAAAAAACTATCCTCGCGTCAAATATGACAAACTATTGTGGGAACCTATTATATTAGGGCCGTCATTTGGTATAAATGGGATGATGAACTTAGAACCCACCGCATTAGCGGACGAAGCTCTTACAAATGAAACTATGGCTCCGGTAATTTCGAATAACACACATATAGAAAACTATAACAACAGTAGAGCACATAATAAACGCAGAAGAAGAAGAAGAAGAAGTAGTGAGCACAAAACATCCAAGCTTCATGTAAACAATATCATAGAACCGGAAGTACCTGCTACTGATTTTTTTGAAGACACTGTTTCCTCCTTAACAGAGTATATGTGTGATTATAAGAACACAAATAATGATAGATTAATCTATCAAGCGGAAAAAAGAGTGCTGGAAAGCATCCATGATCGCAAAGGGATACCAAGATCAAAATTTAGTACAGAAACTCATTGGGAGCTCTGCTTCACTATTAAAAACTCCGAAACACCACTTGGAAATCATGCAGCTAGGAGAAACGATACTGGAATATCAAGTTTAGAGCAGGATGAAGTAGAAAACGATGTAGATACTGAATTATATGTAGGTGAAAACGCCAAAGAAGATGAAGACGAAGACGAAGACTTTACCCTTGATGATGACATTGAGGATGAGCAAATATCAGAAGAAAATGATGAGGAGGAAGACACATATGAAGAAGACAGTGATGATGATGAGGATGGGAAGAGAAAAGGACAAGAGCAGGATGAAAACGATATAGAAAGCCACATAAGGAAGGAGAGAGTCAGAAAAAGAAGAAAAATAACTCTAATAGAGGATGACGAAGAATAA>YBL054W	+:0:chr02:117592:119169ATGACGTTGCCGAAACTCAGTAGCGTTTCTGTTTCATCAGGACATGTTAGTGCCAACTCACATGGTTTCTCAATACTAAGCAAACACCCTCACCCAAATAATCTTGTCCATTCCCACTCACTTTCTCACACAAATGCGAAGAGCCACCTGCCTATCAGTAGCACTAGCACTAAAGAGAACAGCACGAACAAGGAGGAGGCGGAATCACTCAAAAAAAACAACCCCTCTTCTTGGGACCCTAGTGATGATATCAAGCTCCGCCACCTGAAAGAGATCAAGAACTTGGGCTGGAAGGAGATTGCACATCATTTCCCAAATAGAACTCCTAACGCTTGTCAATTCAGATGGAGGAGACTGAAATCAGGCAACTTAAAGTCTAACAAGACCGCAGTTATTGACATCAATAAGCTATTCGGCGTGTATGCGACTGGTGATGCTACCCCATCCGCGGGTACTCCGTCTGCGGAAGAAGCCGTAAAAGAGGAAGCTGTTGAGGATGAAGATATTACTGCAGGTTCTAGTGCTATCGAGGATTCTCCACCGGATTTCAAGCCATTAGTTAAGCCTAAATACATGGACAGAAAACTGATAACTCAAAGATCTACATCAACATTTTCGGACCATGAGCCACAACACACGAAACCAAGGAAACTGTTTGTTAAGCCAAGGTCCTTCTCTCATTCTATAACAACCAACACGCCTAATGTAAAGACTGCTCAGCAGACAAATCTAAGCCTTTATAACACTACTTCAGCAAAGACAAATAAAGCTGTTAATTCCAATGATTATGAGAATATTGGTCTTGTGCCTAAAATTATTATCAGGTCTAGAAGGAACTCCTTTATTCCTTCAACTCAAATCCCTCATTCAACGACGAAGACTAGGAAAAACTCGCATTCCGTAATTTCTTCTAGAAGATCATCTTTTAACATGATGCATTCAAGAAGATCATCCTTCAACTCTCACGCTCCTACGGAGCCTATTTCTAGAAGAGCTTCCTTGGTAGTTAGCCCGTATATGTCACCCAGAAGGTTATCTACATCGCAATCCGTTCATTATCATCCACAGCACCAATACTATCTTAACCCCATAGCGTCTCCCAACTGCAAGACAGACCACGCAAATGACAAAATCACGCACACGAGGACTTTCTTAGATATGCAAAAATTCGCTAATAAACACCCGTGGTCTAGAGAAGATGATGAAGTGCTACTTAACAATACGAAGGACAAACAAAACCATTTGTCGCCGCTAGAAATTTCTATTGTTCTGCCTAATAATAGATCCGAGTTGGAAATCCAACAAAGAATGGATTATTTAAAGAGAAAAGGACGTGTAAGTGGTTTTCATACAAATGAAGGATGCAAAGACGAGGAGGAGGAGGATGACATTGACCCACTGCACAAGGAGAATGGCATTAACACGCCATCGCAGCAATCGCAAAATTACGGTATGTTAGAGGCTAAACATGATAATCCAAAGTCTAGTGAGCTTTCTTCTATGACCAGCGCAAATGACATACGCAATGAGCAAGATGAACTTCCAGGTATAAATTCTATCTTTAAAAATATATTTTAA>YBL056W	+:0:chr02:113765:115171ATGGGTCAAATATTGTCCAATCCAATTATCGACAAAGAGCATCATTCTGGTACGGACTGTTTGACAGCGTTTGGACTATGTGCCATGCAAGGCTGGCGTATGTCCATGGAAGATGCCCATATTGTTGAGCCGAACCTTTTGGCTGAGTCTGACGAGGAACATCTCGCATTTTATGGTATATTTGACGGTCATGGTGGTTCTTCTGTAGCGGAGTTTTGTGGCTCTAAAATGATATCTATACTGAAAAAACAGGAGAGCTTCAAGAGCGGTATGTTAGAGCAGTGCTTAATAGATACCTTTTTAGCTACAGATGTTGAGTTGTTGAAAGATGAAAAATTAAAAGATGACCATAGTGGTTGTACAGCAACTGTGATATTGGTATCTCAATTGAAAAAGCTACTAATTTGCGCCAATTCCGGTGATAGTAGAACAGTTCTATCTACTGGTGGTAATAGTAAAGCAATGTCATTTGATCATAAGCCCACATTGTTAAGTGAAAAATCTCGTATTGTAGCTGCTGATGGTTTTGTTGAGATGGACCGTGTTAATGGAAATTTAGCGTTATCAAGAGCCATAGGTGATTTTGAATTCAAATCTAACACAAAATTGGGACCTCATGAGCAAGTCGTTACATGTGTTCCTGATATCATTTGTCACAATTTGAATTATGATGAGGATGAATTTGTTATTTTAGCATGTGATGGTATTTGGGATTGTTTAACTTCTCAAGAGTGTGTTGACTTGGTTCACTACGGTATAAGTCAAGGTAATATGACGTTGAGCGACATTTCATCTAGAATCGTAGATGTTTGTTGTTCACCCACAACTGAAGGCTCAGGAATTGGCTGTGATAATATGAGTATTTCCATTGTTGCTTTACTAAAGGAAAATGAATCTGAGTCACAATGGTTTGAGCGTATGAGATCAAAAAATTACAATATCCAAACGTCTTTTGTCCAAAGAAGGAAAAGTATTTTTGATTTCCATGATTTTTCGGATGATGATAACGAAGTGTTCGCAATAACCACCAAAAAATTACAAGACCGCTTGAATCGTAGTAAAGATAATGACGACATGGAAATTGATGATCTTGATACCGAATTGGACAGCAGTGCTACTCCCTCAAAGTTATCAGGTGAGGATAGAACTGGCCCTATTGACTTGTTTTCGTTGGAGGCTCTATTAGAAGCCGGGATTCAAATAAGGCAGAGGCCCAGCTCCGACAGTGACGGCAACACTTCTTATTTCCATGGTGCTTCTTTATCAGATATGTTGGCATCTTTAAGCAATGCGGCTGCAGGAGAAACAGAACCTAATGATGCTGATGATAACGATGATAACGACGGCGAAGAAAACGGCAAGAATGAAAATGCGAAGAAGGGTTCCAAGATTGAAGAAATTGAATAA>YBL057C	-:0:chr02:112803:113447ATGATAACGTCCTTTTTAATGGAAAAGATGACAGTTTCTTCGAATTACACCATAGCTTTATGGGCTACTTTCACCGCAATCTCCTTTGCCGTGGGTTACCAGTTGGGTACGTCGAACGCATCATCAACTAAGAAATCCTCAGCTACTTTACTGCGTTCTAAAGAAATGAAGGAAGGAAAGCTACACAATGACACTGATGAGGAGGAAAGTGAAAGCGAAGATGAGAGCGATGAGGACGAAGATATTGAGTCCACTTCATTGAATGATATACCTGGAGAAGTTAGGATGGCATTGGTGATTCGTCAAGATCTTGGCATGACAAAGGGCAAAATAGCGGCACAATGCTGTCACGCAGCATTGTCATGTTTTAGACATATTGCTACAAACCCCGCGCGTGCTTCATACAACCCAATTATGACACAAAGATGGCTAAATGCTGGACAGGCTAAAATAACGTTGAAATGTCCAGATAAGTTTACAATGGACGAGTTGTATGCGAAGGCTATATCACTTGGGGTGAATGCAGCAGTTATTCATGATGCTGGTAGAACACAGATTGCTGCGGGAAGTGCCACCGTATTGGGTTTAGGACCTGCTCCAAAAGCAGTATTGGATCAAATAACAGGTGATTTGAAATTGTATTGA>YBL058W	+:0:chr02:111439:112710ATGGCGGAAATACCTGATGAAACCATCCAGCAGTTCATGGCATTGACCAATGTGTCGCATAACATAGCCGTTCAATATCTCTCTGAATTTGGAGATTTAAATGAAGCACTAAATTCCTATTATGCTTCTCAAACGGATGACCAAAAGGATAGAAGAGAGGAAGCACATTGGAACAGACAGCAGGAGAAGGCCCTCAAGCAAGAAGCCTTCTCCACCAACTCTTCGAATAAAGCCATAAATACGGAGCACGTTGGTGGGTTATGTCCAAAACCAGGATCCTCACAAGGTAGCAACGAGTACTTGAAAAGGAAAGGTTCTACCTCTCCTGAACCAACCAAGGGTAGTAGCCGCTCTGGAAGTGGTAACAACTCCAGGTTTATGAGCTTTTCGGATATGGTAAGAGGTCAAGCTGATGATGACGATGAAGATCAACCGAGAAATACTTTTGCTGGTGGTGAAACATCCGGCTTAGAGGTTACAGATCCTTCAGATCCTAATTCATTACTGAAGGATTTGCTGGAAAAAGCGAGAAGGGGTGGTCAAATGGGCGCTGAAAACGGATTCCGTGATGACGAAGACCATGAAATGGGTGCCAATAGGTTTACTGGAAGAGGTTTTAGATTAGGGTCAACCATCGACGCAGCAGATGAAGTCGTAGAAGACAACACTTCACAATCACAACGTAGACCAGAAAAAGTCACAAGAGAAATTACATTTTGGAAGGAAGGTTTTCAAGTGGCCGATGGTCCGCTTTATCGCTATGATGATCCTGCGAACAGTTTCTATTTGAGCGAGTTAAATCAAGGGAGGGCTCCATTAAAGCTCTTAGATGTGCAATTTGGACAAGAAGTTGAAGTTAATGTATATAAAAAATTAGATGAGTCTTATAAAGCTCCGACGAGAAAACTGGGCGGTTTTTCAGGCCAGGGCCAAAGACTAGGATCTCCTATCCCGGGTGAATCGTCACCTGCGGAGGTTCCAAAGAATGAGACACCCGCTGCTCAGGAACAACCCATGCCGGACAATGAGCCAAAACAAGGCGACACCTCCATCCAAATTAGATACGCAAATGGCAAAAGAGAAGTTTTGCACTGCAATTCCACAGATACAGTAAAGTTTTTGTATGAGCATGTGACATCAAATGCGAACACTGACCCATCGAGGAATTTCACCTTGAATTATGCCTTTCCTATCAAACCAATAAGCAACGATGAGACAACATTGAAGGACGCTGATCTGCTGAACTCCGTTGTCGTGCAAAGATGGGCATGA>YBL060W	+:0:chr02:107934:109997ATGTGCGCCAGTTTAAACGAGGTAAAAAAGAATGACACCTATGGGGTCTCACAAAAGGGCTACAATGACAATTTCAGTGAAAGTGAGGGCGTCCTTCATGGTAGTAAGTCGATGCCCACTAGCATGAAAAATATGCTACAGTCTCCCACGATGGTCAACATGTGTGATATTTTACAAAACAAGGAAGCTGCTAATGACGAAAAACCTGTGATACCTACTACGGATACCGCCACTGCGGGGACTGGTACTGAAGATATTAGCTCCACTCAATCCGAGGAAACTGATCAGAATAGTCATCTTATTGCCTCAGAGATCTTGGAAGGCACTTTCAAAGATGTATCTTACAAGGAATATGCAAATTTCTTGGGAAACGATAACAATAATCAAGTCTTGACTGAGTTTGTAAAGTTATTGAGTCCTTTGCCGTCGTCACTATTAGAAACGCTTTTCAATTTATCGAAAAGTATATATTTCATTGCAGAAGCGCAAAATATCGACCGGATACTAGAGTGCTTGAGCATAGAATGGATAGCTTGCCACCCGAACACACATTGGAAGTCAGGCTATAAGTCATGTCATATAGTCTTATTTTCCCTGTTGATCCTTAATTCGGATTTGCACAACAACTTTCAAGTTGACCATAAAAAGATTAAGTTTTCCATGGTTGCATTTATCAACAATACACTGAGGGCACTAAGAGAGGAAAATGAATACGAAGAATTGAAAATATACTCCCGCGAACATTTGATCATCGAAGAACTTTCCGAATACTATAAAACGTTAAATGAAACGCCTTTACCGTTATGCACAGAATCTAGAACATCAATAAATATATCAGATAACCAATCTTCCTTGAAAAGGTTCTCTACTCTAGGATCACGGGAATTTAGTACATCAAATTTACGTAGTGTTAACTCTAATTCTACTACACTATATTCAAGAGATGGTCAAGTATCTGTACGAGAAATGAGCGCAAAATCAAATAAAAACTTTCACAATAATCACCCCATGGATGCACTCTACCTTAAAGAGTCTTTTGATGACGGTTTAATTACCGAAAACGGCTCCAGTTGGTTCATGGACGATTTAATTCTTATAAGCAAGAAATCTTTACCACGTAAATATTCTAAAAGAGACAAAGATCAAGTGGCGGCACCAAAAATGACCTCTAAGAGAAACAAATCGTTCTTCGGATGGCTAAAACCATCTAAAACGACTACACTTATTGAGCACACATCTAGAAGGACTTCTTTATCGTATTTGAATAAGGATTCTGAATGGGAGAGGGTGAAAATACAGGTCAAGGAGGGCAGAATTTTTATTTTCAAAATTAAACCAGATGTTAAGGATATCATCCAATCAAGTGAAACAGACAGTGCTACCATCGACTATTTCAAAGATATCAGTAGCTCTTATTTTGCTTACTCACTGCTTGAAGCTGAAGCACATGTCGTGCAAGATAATATAATTATAGGTAGTGGAGCAATGAAATCAAATGTGTGTAACAAAAACACCAAGAGGAAAAGTGGCAACTTTACCGTTAGTTTTCCAGAGAATATCAACGGACCAAAGCTTGTTCTGGAGTTCCAGACGAGAAGTGTTGAAGAAGCCCACAAGTTTATGGACTGTATCAACTTCTGGGCAGGTAGGATTTCTCCAGTTCCTTTAACACAATTCGAAGCCGTATCTAACGCAGAATATGGATGGAGTGACAAGATCTTGACAGAGCACGCTTCCCTCAATCTTAAAAATATTGTTGTAAGTGAATGGAAGCCACTATTGGGGCTAGAGCTACTATACGAAGATGCGAAAGATGTAGAGATGGTCGAACTAAAAGAAAGGCTAAAGGAATTGATGAACTTCACCAGACAGCTTGGTATATGGATAGATAAACATAACGAAATAAAGGATAAGCTGGTCGAAATTTGGAGCTTTGACGATAACTATTTTGAAGCAGTCATGAATAATTGGAATTCGAGATATTTGTATATGAATAATCAATATAAGAAACGACTGAGCTACTTGAAAGCTTTGCAAAAAGCCATGGGTTCTGTTCAGTTCTAA>YBL061C	-:0:chr02:105318:107408ATGGCAAGTTCACCGCAGGTACATCCATATAAGAAGCATTTAATGCAATCACAGCATATAAATTTTGATAACAGGGGCCTACAGTTTCAAAACAGTTCGTTGAAGGTAGGTCAAGATTTTTCAGATAATAAAGAGAATAGGGAAAACAGGGACAATGAAGACTTCAGTACCGCAGACCTTCCTAAGCGGTCGGCAAACCAACCGCTTATCAATGAGCATCTTAGAGCTGCGTCAGTACCTCTACTATCAAACGATATAGGCAATAGTCAAGAGGAAGATTTCGTTCCAGTCCCACCCCCACAGCTTCATCTTAATAATTCAAATAATACTTCCTTAAGTTCTTTGGGAAGTACTCCTACAAATTCTCCGTCACCGGGAGCATTAAGACAGACAAATTCGAGTACATCTCTTACGAAGGAGCAAATAAAGAAAAGAACTCGTTCTGTAGATTTGTCTCACATGTACCTGTTGAATGGTAGCAGTGATACCCAATTAACGGCTACTAATGAATCTGTAGCAGATTTGTCACATCAGATGATCAGTCGATATTTGGGTGGTAAAAATAATACTTCCTTAGTTCCAAGGCTAAAAACAATAGAAATGTATAGACAAAACGTTAAAAAATCTAAAGATCCTGAAGTGTTATTCCAGTATGCGCAATATATGTTACAAACCGCACTGACTATTGAATCTTCCAATGCTCTTGTTCAAGATAGTGACAAGGAAGGAAATGTCAGCCAATCTGACTTAAAACTTCAGTTTTTGAAAGAAGCGCAAAGTTATTTGAAGAAACTGAGCATAAAGGGTTATTCAGATGCCCAATACTTATTAGCGGACGGCTACTCATCCGGAGCATTTGGGAAGATTGAAAATAAAGAAGCATTTGTCTTATTTCAAGCAGCTGCAAAGCATGGTCATATAGAAAGTGCTTACAGAGCATCGCATTGCTTAGAAGAAGGACTAGGAACAACAAGAGATTCTCGTAAGTCAGTCAATTTTTTAAAGTTTGCGGCTAGCAGGAATCATCCCTCAGCAATGTATAAATTGGGACTTTATTCATTCTACGGCAGAATGGGTCTTCCAACCGACGTTAATACTAAATTAAATGGGGTAAAATGGTTATCAAGGGCTGCAGCAAGAGCTAATGAGTTGACGGCCGCAGCACCATACGAATTGGCTAAGATTTATCATGAAGGGTTCTTAGATGTTGTCATTCCGGATGAAAAGTATGCAATGGAACTATATATTCAAGCAGCAAGTTTAGGGCATGTTCCTTCAGCAACTTTGTTGGCACAGATTTATGAAACAGGTAATGATACAGTAGGGCAGGATACATCACTTTCTGTACACTATTATACGCAGGCAGCGTTAAAAGGCGATTCTGTGGCAATGTTAGGTTTATGTGCATGGTACTTACTGGGAGCAGAACCTGCTTTTGAAAAAGATGAAAATGAGGCTTTTCAATGGGCCTTGCGTGCAGCTAATGCTGGTTTGCCAAAGGCCCAATTCACTCTGGGCTACTTCTATGAGCATGGCAAGGGCTGTGATCGTAACATGGAATACGCATGGAAATGGTACGAGAAAGCTGCAGGGAACGAGGACAAGAGGGCGATTAATAAGCTACGCTCAAGGGACGGTGGACTAGCCAGCATTGGTAAAAAACAGCATAAAAAAAATAAAAGTATCAGTACTTTGAATTTATTCTCTACTGTGGACAGTCAAACAAGTAACGTAGGGTCAAATTCAAGAGTTTCTTCAAAATCTGAAACATTCTTTACTGGAAATCCAAAACGTGATCGTGAGCCACAAGGTTTGCAAATTAACATGAATTCAAACACGAATAGAAATGGTATTAAAACGGGTTCTGATACAAGCATCAGGAAAAGTTCTTCCTCAGCAAAGGGAATGTCAAGGGAAGTTGCTGAGCAGTCGATGGCAGCAAAGCAAGAAGTTAGTTTATCAAATATGGGCAGTTCAAACATGATTCGTAAAGATTTTCCTGCAGTAAAAACTGAGTCAAAAAAACCTACAAGCTTGAAGAACAAGAAGGATAAACAAGGTAAAAAAAAAAAAGACTGTGTAATTATGTAA>YBL066C	-:0:chr02:96671:100117ATGGTGAAGGATAATCGAGATTCTGACCAAGACCAAGATTTTAGTTCTGCTCACATGAAAAGACAACCGGAGCAGCAACAGTTGCAACAGCACCAGTTCCCAAGTAAGAAACAACGAATATCTCACCATGATGACAGTCATCAAATCAACCATAGACCAGTTACCTCATGTACACATTGTAGACAGCACAAAATCAAATGCGATGCTAGTCAAAATTTCCCTCATCCTTGCTCCAGATGCGAAAAAATTGGTCTCCACTGTGAAATCAATCCTCAATTCAGGCCTAAGAAGGGCTCACAGTTGCAACTACTGAGACAAGATGTGGATGAAATCAAATCTAAACTCGATACTCTTCTGGCCAATGACAGCGTTTTCGTTCATCTTTTACAACAGATTCCCATGGGCAATAGCCTTTTGAATAAGCTCAATCTGCATCCAACTCCAACTCCGGGTACTATTATCCCTAACCCAGATTCTTCTCCTTCCTCAGGTTCTCCAACTTCTTCCGCGGCTCAACGAGATTCTAAGGTTTCAGTTCAAACTTATTTGTCCAGGGAACCCCAACTCTTACAAGCAAATCAGGGCAGCAATACGAATAAATTTAAAGCAAATAATGAAGCATCTTCTCACATGACGTTGCGCGCATCTTCTTTAGCGCAAGATTCGAAAGGCTTGGTTGCAACAGAGCCAAATAAGCTGCCCCCGCTGCTAAATGACTCAGCATTGCCTAATAATTCAAAAGAATCTTTACCTCCTGCTTTGCAAATGGCTTTTTATAAGAACAACTCTGCAGGTAACACTCCGAACGGCCCCTTCTCTCCAATTCAAAAAACATATTCCCCTCATACTACGTCGACCACCGTTACAACGACAACAAATCAACCACCATTTGCAGCAACAAGCCACGTAGCAACAAATAACAATGCAGATAGGACGAAGACGCCGGTAGTAGCCACCACCACGACTATGCCATTATTGCCTTCGCCGCATGCAAATGTAGATGAGTTTGTACTGGGCGATATTAGTATTTCCATTGAAAAAGCGAATAGATTACACCATATTTTCGTGACTAGGTATCTGCCGTATTTTCCTATTATGTATTCCAATAACGCCACCGAATTATACTCCCAATCTCAGTTGCTTTTCTGGACCGTGATGTTGACGGCATGTCTGTCTGATCCTGAACCGACGATGTATTGCAAGCTAAGCTCTTTGATCAAGCAACTTGCCATAGAGACCTGCTGGATAAGAACACCTAGATCCACACATATTTCGCAAGCTTTGTTAATATTGTGCATTTGGCCTTTGCCTAACCAAAAAGTCCTAGATGATTGTTCTTACCGTTTTGTAGGATTAGCAAAGTCACTGTCTTATCAATTAGGTTTGCACAGAGGTGAATTCATTTCTGAATTCACAAGAACTCAAACATCAATGCCAAATGCAGAAAAGTGGAGAACTAGGACTTGGCTGGGAATATTTTTTGCCGAACTTTGTTGGGCGAGTATCCTTGGTTTGCCACCAACTTCACAGACAGACTATTTATTAGAAAAAGCCTTATCCTGTGGTGACGAAGAATCAGAAGAAGATAACAATGACAGTATTGACAATAACAACAATGATAAAAGGAACAAGAAAGACGAGCCGCACGTTGAAAGTAAATACAAACTACCGGGCAGTTTTAGAAGATTGCTCAGCCTGGCGAATTTCCAAGCAAAATTGTCTCATATCATTGGTTCTTCCACTTCCAGTCCTGATGGTTTATTGGAACCAAAGTATCGTGCTGAGACACTGTCCATCTTGGGAAAAGAGTTAGATTTATTAGCAAAAACTTTAAATTTCCAGAGTGACGATACTGTCAACATTTATTTTCTTTATGTTAAATTAACTGTCTGTTGTTTTGCATTCCTACCCGAAACACCTCCTACCGATCAAATTCCATATGTCACAGAGGCCTATCTAACAGCTACTAAAATTGTCACTCTATTGAATAATCTTTTAGAAACACACCAATTAATTGAACTGCCTATTTATATTAGACAAGCTGCTACATTTTCTGCACTGATTCTCTTTAAATTGCAGTTGACTCCTTTACTTCCTGACAAATATTTTGATTCAGCAAGGCAATCCGTGGTCACTATCCATAGACTTTATAGAAATCAGTTAACTGCGTGGGCCACTAGTGTTGAGAATGATATTTCGAGAACTGCAAGTATGTTAGAAAAACTGAACTTCGTACTGATCATGCATCCAGAAGTTTTTGTGGAAGAAGACGGTATTATTTCTAGGATGAGATCACATTTAACAGGGTCTCTATTCTATGATTTGGTTTGGTGTGTTCACGAGGCGAGAAGAAGGGAAATGGATCCCGAATATAACAAGCAAGCCTTAGAGAAAGCCGCTAAGAAAAGAAAATTTTCCTCAAATGGTATCTACAATGGCACTTCGTCTACGGGTGGCATAACGGACAGAAAACTATATCCATTGCCACTATATAACCATATCTCCAGAGATGACTTTGAAACTGTAACAAAAACAACACCAAGTGGAACCACTGTTACCACTTTAGTTCCTACTAAGAATGCCTTAAAGCAGGCAGAAAAGCTAGCCAAGACAAATAACGGAGATTCTGACGGTTCTATAATGGAGATTAACGGGATACCTCTTTCCATGCTCGGGGAAACAGGCAGCGTAAAATTTCAAAGTTTATTCGCTAATACCTCGAATAGTAACGATTATAATAATAATAGGACGTTATTGGATGCGTCTAATGACATATCAATTCCCTCTAATTCAATTTATCCAGTGGCTTCTGTCCCCGCTTCGAATAACAATCCACAAAGTACTAAGGTAGACTATTATAGTAACGGACCTAGTGTAATTCCTGATCTCTCCATGAAAAGATCAGTAAGCACTCCCGTTAATCATTTTCCTGCGTCCGTTCCAGGGTTAAGGAACCACCCCGTTGGCAACTTATCTAATAATGTTACATTGGGAATAGACCACCCTATTCCAAGGGAGCACAGTAATTTACAAAATGTCACCATGAATTATAATAATCAATTCAGCAACGCCAACGCGATTGGAAGATCACAAAGTAGTATGTCCCATTCACGTACACCGATTGCTTCGAAGTCAAATAATATGACAGATTTGCATTCCGTCGTTTCCGACCCCGGCTCCTCTAAAAGCACGGCATATCCACCTCTGAGCTTGTTTTCCAAAAGTAATGATATCAATAGCAACAAAACGAACCAACGATTTTCTACGGGCACTAACACTGTAACTTCTTCTAACTTCCAAACCATCGATAATGAAAACAACGTGAAGACTCCTGGAAACAAACTAACGGATTTTTTTCAGCAACAAAGTGCAGGCTGGATTGAAGGCAACTCCAGTAATGATGACTTTTTTGGTTGGTTCGATATGAACATGGAACAAGGTTTTTAA>YBL068W	+:0:chr02:92412:93395ATGAATTCAGAGTCTCGAGAAGATATGGCTATAAATAGTATCAAATTGCTAGCGGGAAACTCCCATCCTGATTTGGCTGAACAAATATCGAAAAAGTTAGGTATTCCACTTTCCAAAGTTGGTGTGTACCAGTATTCTAATAAAGAAACCTCTGTCACCATAGGTGAGAGCCTTCGCGACGAAGATGTGTATATTATCCAAACTGGAATAGGTGAACAAGAAATTAATGATTTCTTGATGGAATTATTAATTTTAATTCATGCTTGCCAAATTGCATCTGCAAGAAAGATCACTACTGTAATACCCAATTTTCCATATGCAAGACAAGACAAGAAAGATAAATCCCGGGCGCCCATTACCGCAAAGTTGGTTGCCAATTTATTGCAAACTGCTGGTGCTGATCATGTCATCACAATGGATCTCCATGCCTCCCAAATTCAAGGGTTTTTCCATATCCCGGTTGACAACCTATATGCAGAACCAAGTGTTTTAAATTATATTAGAGCCCGGAAAACAGATTTCGACAATGCTATTTTGGTGTCGCCTGATGCAGGTGGTGCTAAGAGAGTAGCTGCTTTGGCTGACAAGTTAGATTTAAATTTTGCTTTGATTCACAAAGAGAGGCAAAAAGCTAACGAGGTTTCAAAAATGGTGCTTGTTGGTGATGTTACCAATAAATCATGTTTATTAGTTGATGATATGGCGGATACTTGTGGTACGTTGGTAAAAGCTTGTGATACGTTGATGGAGCATGGTGCCAAAGAAGTTATAGCTATTGTTACACACGGTATTTTCTCCGGTTCAGCAAGAGAAAAGCTAAGAAATAGTAGATTGTCTAGAATTGTTTGCACAAATACCGTTCCGGTAGATTTGGATTTACCTATTGCTGACCAGATCGATATTAGTCCCACGTTCGCTGAAGCTATAAGAAGACTACACAATGGTGAATCCGTGTCATATTTGTTCACCCATGCTCCAGTATAG>YBL069W	+:0:chr02:90739:92028ATGGCTAAAGATATTTTGAAGAACCAGGACCCTAAATTGCAAGCGATGATCGTTGAACACTCGGCGCCTGCTCCCAAAGAGATACCTATGGACGCTCCTGTTTTGAAAAGAGTCGCTAGACCTTTAAGACATGTAAAGTTCATTCCAATTAAATCGCTGATATTCCATACTAAAACAGGGCCCATGGATTTTTCTTATGAAAAGAAGATCAAGACGCCTATTCCTAAGAACAAAATTGTAGTTCGAGTAAGTAATGTGGGTTTGAACCCTGTAGATATGAAAATCAGGAACGGCTACACATCGTCGATTTACGGTGAGATCGGCTTGGGGAGAGAGTACAGTGGTGTTATCACTGAGGTAGGCGAGAACCTAAACTACGCCTGGCACGTGGGTGATGAAGTATACGGTATATACTACCACCCTCATTTGGCCGTGGGATGTTTGCAAAGTTCAATCTTAGTAGATCCAAAGGTGGACCCAATCCTTTTGAGACCAGAATCGGTTAGCGCCGAAGAAGCTGCAGGCTCCTTATTCTGCCTGGCCACCGGCTATAATATTCTGAATAAATTATCCAAGAACAAGTATTTGAAGCAAGATTCAAACGTTTTGATTAATGGTGGAACTTCGTCAGTGGGGATGTTTGTCATACAATTATTGAAGCGTCATTACAAGTTACAGAAAAAACTAGTGATTGTAACGTCTGCAAATGGGCCCCAAGTTTTACAGGAGAAATTTCCTGATCTGGCTGATGAAATGATTTTCATCGATTACTTAACGTGTAGGGGTAAATCTAGTAAACCACTAAGAAAAATGCTCGAAGAAAAGAAAATTTCTCAGTATGATCCGGTCGAGGATAAGGAAACTATACTTAACTATAATGAGGGGAAGTTCGATGTTGTACTCGATTTTGTCGGTGGTTACGATATTTTGAGTCATTCTAGTTCATTGATTCACGGCGGTGGTGCGTACGTAACCACTGTAGGTGATTACGTTGCCAATTATAAAGAAGACATTTTCGATTCATGGGATAACCCAAGTGCCAATGCAAGAAAAATGTTCGGGTCTATTATCTGGTCATATAACTACACACATTACTATTTTGATCCGAATGCAAAGACGGCTTCCGCCAATAATGATTGGATTGAGCAATGTGGCGACTTTTTGAAGAACGGTACTGTAAAATGTGTCGTCGACAAAGTTTATGACTGGAAAGACCATAAAGAAGCATTTTCTTATATGGCCACTCAACGTGCCCAAGGAAAGTTAATCATGAACGTCGAAAAATTCTAG>YBL072C	-:0:chr02:88521:89123ATGGGTATTTCTCGTGATTCTCGTCACAAAAGATCCGCTACCGGTGCCAAGCGTGCTCAATTCAGAAAGAAGAGAAAGTTCGAATTAGGCCGTCAACCAGCCAACACCAAGATCGGTGCTAAGAGAATTCACTCTGTTAGAACTAGAGGTGGTAACAAGAAATACAGAGCTCTAAGAATTGAAACCGGTAACTTTTCTTGGGCTTCTGAAGGTATCTCCAAGAAGACCAGAATTGCTGGTGTTGTTTACCATCCATCCAACAATGAATTGGTTAGAACTAACACTTTGACCAAGGCTGCCATTGTCCAAATTGATGCTACTCCATTCAGACAATGGTTCGAAGCTCACTACGGTCAAACCTTGGGTAAGAAGAAGAACGTCAAGGAAGAAGAAACTGTTGCCAAGAGCAAGAACGCTGAAAGAAAGTGGGCTGCTAGAGCTGCTTCTGCCAAGATCGAATCTTCCGTTGAATCTCAATTCAGCGCCGGTAGATTATACGCTTGTATCTCTTCCAGACCAGGTCAATCCGGTAGATGTGATGGTTACATCTTGGAAGGTGAAGAATTAGCTTTCTACCTAAGAAGATTGACTGCTAAGAAATAG>YBL074C	-:0:chr02:86720:87787ATGAATACTGTACCATTTACATCTGCTCCGATTGAGGTAACTATAGGGATCGATCAGTATTCCTTTAATGTCAAGGAGAATCAGCCATTTCACGGAATCAAGGACATCCCCATTGGACACGTTCATGTCATTCATTTTCAGCATGCAGATAATTCCAGCATGAGGTATGGCTACTGGTTTGACTGTAGAATGGGAAACTTTTACATTCAGTATGATCCTAAAGATGGCCTTTACAAAATGATGGAAGAAAGGGATGGCGCAAAATTCGAGAATATTGTTCACAACTTCAAGGAACGGCAGATGATGGTTTCTTATCCGAAAATTGACGAAGATGATACCTGGTACAATCTTACCGAGTTTGTGCAGATGGATAAAATCCGAAAGATAGTAAGGAAAGATGAAAACCAGTTCTCTTACGTAGATTCTTCGATGACCACAGTTCAAGAAAATGAGCTGCTAAAATCCAGCTTGCAAAAAGCAGGTTCTAAAATGGAAGCCAAGAATGAAGATGATCCTGCACATTCTTTAAACTATACAGTAATAAACTTCAAATCTAGAGAAGCCATAAGGCCTGGCCATGAAATGGAGGATTTTTTAGACAAGTCTTACTACTTGAACACTGTAATGCTACAAGGAATTTTTAAAAATTCAAGTAATTATTTTGGGGAGTTGCAGTTTGCGTTCTTAAATGCCATGTTTTTTGGTAACTACGGGTCGAGTTTGCAATGGCATGCTATGATCGAACTGATATGTTCAAGCGCTACGGTGCCTAAACATATGCTCGATAAATTAGACGAAATCTTATATTATCAGATAAAGACATTGCCTGAACAATACTCAGACATCTTGTTGAATGAACGAGTTTGGAATATTTGTCTGTATTCGTCATTTCAAAAAAACTCCCTACACAACACAGAAAAGATAATGGAAAACAAATATCCAGAATTGCTTGGTAAAGACAATGAAGACGACGCTCTTATTTACGGTATCAGTGATGAAGAAAGGGATGACGAGGATGATGAGCACAACCCTACCATTGTTGGCGGTCTCTATTACCAAAGGCCATAA>YBL075C	-:0:chr02:84497:86446ATGTCTAGAGCAGTTGGTATTGATTTGGGAACAACTTACTCGTGTGTTGCTCATTTTTCCAATGATAGGGTAGAGATAATTGCAAATGATCAAGGTAATAGGACCACTCCATCGTATGTGGCTTTCACAGACACCGAAAGATTAATTGGTGACGCCGCCAAAAATCAAGCTGCAATCAATCCTCATAATACAGTTTTTGATGCAAAGCGGTTAATTGGTCGTAAATTTGATGATCCTGAAGTGACGACAGATGCCAAGCACTTCCCTTTCAAAGTTATATCCAGAGATGGTAAACCTGTAGTGCAAGTAGAATATAAGGGTGAAACGAAAACATTTACGCCTGAGGAAATTTCTTCCATGGTTTTAAGCAAAATGAAGGAAACTGCTGAGAACTATTTGGGAACTACGGTCAATGATGCTGTTGTAACTGTTCCTGCATATTTCAATGATTCTCAAAGACAAGCCACTAAGGATGCAGGAACTATTGCAGGGATGAACGTTTTACGTATTATCAATGAACCCACTGCAGCAGCAATTGCTTATGGCTTGGATAAGAAAGGCAGGGCTGAGCACAATGTCCTGATTTTTGATTTGGGTGGTGGTACTTTTGACGTCTCTTTACTTTCAATTGATGAGGGTGTTTTTGAGGTTAAGGCTACCGCAGGAGACACTCATTTAGGTGGTGAAGATTTTGATAATAGGTTGGTGAACCATTTAGCCACTGAATTCAAAAGGAAAACGAAAAAGGACATCTCTAATAATCAAAGATCGTTAAGAAGATTGAGAACTGCGGCAGAAAGAGCTAAGAGAGCGCTTTCTTCCTCATCTCAAACCTCGATCGAGATCGATTCTTTATTTGAAGGTATGGATTTCTACACTTCGTTAACAAGGGCAAGGTTTGAAGAGCTATGTGCTGATTTATTCAGATCCACATTGGAACCAGTAGAAAAGGTTCTTAAAGATTCGAAGCTGGACAAGTCCCAAATTGATGAGATTGTGTTAGTCGGTGGATCTACCAGAATCCCAAAGATTCAGAAATTAGTTTCTGACTTCTTCAATGGCAAAGAGCCTAATCGTTCTATCAACCCGGATGAGGCTGTTGCTTATGGTGCAGCCGTTCAAGCTGCCATTTTAACCGGCGATCAATCAACAAAGACACAAGATTTACTATTATTGGATGTTGCGCCATTGTCCCTAGGAATTGAAACTGCAGGCGGCATAATGACTAAGCTAATTCCTAGAAACTCAACGATTCCAACAAAGAAATCGGAAACCTTCTCTACCTATGCAGATAATCAACCTGGTGTTTTAATTCAAGTCTTTGAAGGTGAAAGAACAAGAACAAAGGATAATAACTTACTTGGTAAATTCGAATTAAGTGGCATTCCGCCTGCTCCCAGAGGTGTGCCTCAAATTGATGTTACCTTTGATATCGACGCTAATGGTATTCTTAATGTGTCTGCTTTGGAAAAGGGTACTGGTAAGAGTAACAAAATCACGATCACTAACGATAAAGGTAGGCTCTCGAAGGATGATATTGATAGGATGGTTTCTGAAGCTGAAAAATATAGGGCTGACGATGAAAGGGAGGCAGAACGAGTTCAGGCTAAGAATCAGCTTGAATCGTATGCATTTACTTTGAAGAATACCATAAACGAAGCAAGTTTCAAAGAGAAAGTAGGTGAAGATGATGCAAAGAGATTAGAAACAGCGTCTCAGGAAACCATTGACTGGTTAGATGCATCGCAGGCAGCCTCTACGGACGAATATAAGGATAGACAAAAGGAGTTGGAAGGCATTGCCAATCCAATAATGACGAAATTTTACGGTGCTGGTGCCGGCGCAGGTCCTGGAGCGGGGGAATCCGGTGGATTCCCCGGATCCATGCCCAACTCGGGTGCTACGGGAGGTGGAGAAGATACAGGTCCAACAGTGGAAGAGGTTGATTGA>YBL078C	-:0:chr02:80375:80728ATGAAGTCTACATTTAAGTCTGAATATCCATTTGAAAAAAGGAAGGCGGAGTCGGAGAGGATTGCTGACAGGTTCAAGAATAGGATACCTGTGATTTGCGAAAAAGCTGAAAAGTCAGATATTCCAGAGATTGATAAGCGTAAATATCTAGTTCCTGCTGACCTTACCGTAGGGCAATTTGTTTATGTTATAAGAAAGAGAATTATGCTACCCCCTGAGAAGGCCATCTTCATTTTTGTCAATGATACTTTGCCACCTACTGCGGCGTTGATGTCTGCCATATATCAAGAACACAAGGATAAGGACGGGTTTTTGTATGTCACTTACTCAGGAGAAAATACATTTGGCAGGTAG>YBL080C	-:0:chr02:73067:74692ATGTTGCGGCTTGCACGTTTTTATTCTTTGGCCAGAACCAAAGCCATACATAGTCACGGTGCACCCTTCAGGCCGGAATATGCATTGAAGTGTGGATTGGAGATTCATACGCAATTGAACACCAAAAACAAGCTTTTTTCACAATCAACGAATAGCGCCACATCTCTAGTGGATGCACCAAACCATCATACTAGTTATTATGACATAGCTCTACCGGGAACGCAGCCGGTTTTGAATCTGGAAGCAATCTTGTTTGCCATGAAACTATCCTTGGCACTGGGTTCTCAAGTGAATAGCATATCTCAGTTTGATAGGAAGCATTATTTTTATGGAGATCAACCTCAAGGCTATCAACTGACGCAGCACTACAGGCCGTTTGCTCGGGGTGGGAAGATCAATCTGTCGAAGGAATTGGATGATATCGATGAATCAGCCAAGGAAATCGGTATCTTACAGTTGCAGATAGAGCAGGACACGGGGAAGTCACACTATACAGAGACAGATAAAGACGTTATTACTTTGGTTGATTTAAATAGGTCGAACGTCCCGCTTATCGAATTGGTAACTAAACCAGATTTCAGTGATATTAAACAGGTTAGAGCGTTTATTAAAAAATATCAGAATTTGGTACGTCATTTGCACATTTCCTCTGGGGACCTGGAAACGGGCGCCATGCGAGTGGACGTTAACCTCTCAATTAATGAATATGCACGCGTTGAGTTGAAGAATTTACCCAATACTAGTTCCATCATCAATGCAATCAAGTACGAATATCAGCGGCAAGTTGAGTTGATTTCTGTGGGTGACACTAGCTCACTGATGGAGCCAGAAACAAGGGGCTGGACAGGCTCATCAACGGTGAAGTTGAGAAGCAAAGAAACCACGATCGATTACAGGTACATGCCTGATCCAGAATTGCCGTATATCAACCTCGCACCAGACGTTATCAGTGGTGTGAGAGGGTTGATGCCCCAATTACCGGACGATATAATGAGAATTCTTATGAAGAAACCCTATCAATTATCATTGAAGGACGCCAAGATTCTGACTTATAATAGTAACCAAAACGATATGTACAATCATGAGGCGTTGAGATCGTACTACTTAGACACCTTCCGTGAATTTTCCAAGCTTGCTGGTGAACGTAGTAACGCGAAATTACCTACGAATTGGATAATCCATGAATTCTTGGGAGATTTGAACAAGCTACAAATTCCATTGGCCAAAGCAAAAGAAATTTTGGCTCCTCCGGTTTTCGCCCAGTTTTTGAAGTTATTGCATGAAGAGGTTATATCCGCAACTAGTGGTAAAATGTTACTGTTTCACATATTGGAAAATTTCGAGCAGAGTAACTGCCAAGATTTGTCTATTCCAGATTTTTCAAAACTAATCGAGAAATTTGAATTGCATGCGATTAATCAGGTCGACCCTCAAGAATTGATGGATCTCTGTAATGATGTCATTGCACAGCACACAGATGACACTTTTATACGGAACCTGGTTACTGGTAAAAAGAAGTCCTCTTTAAAATTCCTAATTGGACAAGGAATGAGACGAAGCCAGGGACGTATAAAGGCAAACGAATTCGAGAAAAAATTCAAAGAGATCTTAAATATTCAATGGTAA>YBL081W	+:0:chr02:71863:72969ATGCCAGGCCAGATAATCAGCATTCCGTTTTTGTCGCAGAACGAGGACATGGATAAATACTTGTTGGAGTACCGCAGTTTGAAGCTCCTTCATCAGTCCAGTAATTCCTTCCAGTCTCACAATGCGCCCTCCCACCAGTCGAACTACCACCCCCATTACAATCACATGAAATACAACAACACTGGTAGCTATTACTATTACAACAACAACAATAACAGCAGTGTAAACCCACATAACCAAGCTGGTCTACAATCCATTAACAGATCTATTCCATCGGCCCCGTACGGGGCTTACAACCAGAACAGAGCTAATGACGTACCATATATGAATACCCAAAAGAAACACCACAGATTTAGCGCTAACAATAATTTGAACCAGCAAAAATACAAGCAATATCCCCAGTATACGTCCAATCCAATGGTTACTGCACATCTGAAGCAAACGTACCCTCAACTGTACTACAATAGCAACGTCAATGCTCACAACAACAACAACAACAGCAACAACAACAACAACAACAACAACAACAGCAACAACAACAACAATCTTTACAACCAGACGCAGTTCTCCACGAGGTACTTCAACTCGAACTCCTCTCCCTCGTTGACTTCTTCCACTTCTAACTCATCCTCTCCATACAACCAAAGCACCTTCGAATACATTTTGCCGTCAACTTCGGCAGCTTCCACAAATTTATCGTCGTCATCATCAAACAACTCTATGCACACCAACCCAACCACTGCAACATCGACATCCGCCGATTTAATCAATGATTTACCCGTGGGCCCCACGTCCAGTTCGCTTATCTCGGATCTACATTCTCCACCAACTGTATCTTTCCTACCAGCAAGCCAAACCCTGCTCATGTCCTCCACCACATCTAGCTCTATTGGCACCAACATAAACCCACCGCAACATTCACCATCCCCATCGCAAAGGGAGGATTTTTCGACGGCACCAGTGAACATGTCTTCGTCCGCATCACTCTTGATGAATGATTCTTCTTTAGGATGGGGGTCTAACCACATGAACGTATCTTCATCCTCTCAACCAGCATCATCAAGACCCTTTGGCATTTGGAATACTGACATGAGCGTTTGGAGTTGA>YBL082C	-:0:chr02:69748:71124ATGGAAGGTGAACAGTCTCCGCAAGGTGAAAAGTCTCTGCAAAGGAAGCAATTTGTCAGACCTCCGCTGGATCTGTGGCAGGATCTCAAGGACGGTGTGCGCTACGTGATCTTCGATTGTAGGGCCAATCTTATCGTTATGCCCCTTTTGATTTTGTTCGAAAGCATGCTGTGCAAGATTATCATTAAGAAGGTAGCTTACACAGAGATCGATTACAAGGCGTACATGGAGCAGATCGAGATGATTCAGCTCGATGGCATGCTGGACTACTCTCAGGTGAGTGGTGGAACGGGCCCGCTGGTGTATCCAGCAGGCCACGTCTTGATCTACAAGATGATGTACTGGCTAACAGAGGGAATGGACCACGTTGAGCGCGGGCAAGTGTTTTTCAGATACTTGTATCTCCTTACACTGGCGTTACAAATGGCGTGTTACTACCTTTTACATCTACCACCGTGGTGTGTGGTCTTGGCGTGCCTCTCTAAAAGATTGCACTCTATTTACGTGCTACGGTTATTCAATGATTGCTTCACTACTTTGTTTATGGTCGTCACGGTTTTGGGGGCTATCGTGGCCAGCAGGTGCCATCAGCGCCCCAAATTAAAGAAGTCCCTTGCGCTGGTGATCTCCGCAACATACAGTATGGCTGTGAGCATTAAGATGAATGCGCTGTTGTATTTCCCTGCAATGATGATTTCTCTATTCATCCTTAATGACGCGAACGTAATCCTTACTTTGTTGGATCTCGTTGCGATGATTGCATGGCAAGTCGCAGTTGCAGTGCCCTTCCTGCGCAGCTTTCCGCAACAGTACCTGCATTGCGCTTTTAATTTCGGCAGGAAGTTTATGTACCAATGGAGTATCAATTGGCAAATGATGGATGAAGAGGCTTTCAATGATAAGAGGTTCCACTTGGCCCTTTTAATCAGCCACCTGATAGCGCTCACCACACTGTTCGTCACAAGATACCCTCGCATCCTGCCCGATTTATGGTCTTCCCTGTGCCATCCGCTGAGGAAAAATGCAGTGCTCAATGCCAATCCCGCCAAGACTATTCCATTCGTTCTAATCGCATCCAACTTCATCGGCGTCCTATTTTCAAGGTCCCTCCACTACCAGTTTCTATCCTGGTATCACTGGACTTTGCCTATACTGATCTTTTGGTCGGGAATGCCCTTCTTCGTTGGTCCCATTTGGTACGTCTTGCACGAGTGGTGCTGGAATTCCTATCCACCAAACTCACAAGCAAGCACGCTATTGTTGGCATTGAATACTGTTCTGTTGCTTCTATTGGCCTTGACGCAGCTATCTGGTTCGGTCGCCCTCGCCAAAAGCCATCTTCGTACCACCAGCTCTATGGAAAAAAAGCTCAACTGA>YBL084C	-:0:chr02:67166:69442ATGGCGGTAAATCCTGAGTTAGCACCGTTCACCCTCTCGAGAGGAATCCCCAGCTTTGATGATCAAGCTTTGAGCACCATCATACAGCTTCAGGACTGCATTCAGCAGGCTATTCAGCAGTTGAACTACAGTACCGCAGAGTTTTTGGCCGAACTGCTCTATGCTGAATGCTCCATTCTCGATAAATCAAGTGTTTACTGGTCCGATGCGGTATATTTATATGCACTTTCGCTGTTTCTGAATAAAAGCTACCACACTGCGTTCCAGATATCCAAAGAATTCAAGGAGTATCATCTTGGTATCGCTTACATATTCGGGCGTTGTGCTTTACAGCTTTCGCAGGGAGTTAACGAAGCTATCCTCACTCTTCTCTCAATAATAAATGTATTTTCTTCGAACAGCAGTAATACGCGCATAAATATGGTGTTGAATTCTAATCTCGTCCATATTCCCGATTTGGCCACTTTGAATTGCCTGTTAGGCAATCTTTATATGAAACTGGACCATTCTAAGGAGGGAGCATTTTATCATTCTGAAGCATTAGCCATTAATCCTTACCTTTGGGAATCTTACGAAGCGATTTGTAAAATGAGAGCTACCGTGGATCTCAAGAGGGTTTTTTTCGACATTGCAGGGAAAAAAAGTAATAGTCATAATAATAATGCAGCTTCATCGTTTCCGTCTACATCACTATCGCATTTCGAACCTCGTTCACAACCTAGCTTATATTCAAAAACAAACAAAAACGGCAATAATAATATCAATAATAATGTTAATACACTGTTTCAGTCGTCTAATTCTCCCCCTTCTACATCGGCATCTTCTTTTTCTTCCATTCAGCATTTCTCAAGGTCACAGCAGCAACAAGCGAACACATCAATAAGGACCTGCCAGAACAAAAATACTCAAACTCCTAAAAACCCTGCAATCAACAGTAAGACGTCTTCTGCGCTACCAAATAACATTTCCATGAACTTAGTGTCTCCATCCTCCAAACAGCCTACAATAAGCTCGTTGGCCAAAGTTTATAACAGAAACAAACTTTTAACGACTCCTCCATCGAAACTGTTAAATAACGATAGGAACCACCAAAATAACAATAATAATAATAATAATAATAATAATAATAATAATAATAATAATAATAATAATAATAATAATAACATTATAAATAAAACAACTTTCAAAACTCCAAGAAACCTATATTCCTCAACAGGAAGGTTAACAACTTCCAAGAAAAATCCAAGGTCTTTAATAATCAGTAACTCAATACTAACGAGTGATTATCAAATTACGCTGCCTGAAATCATGTATAATTTCGCTTTAATATTAAGGTCGTCATCACAATACAATTCGTTCAAGGCAATAAGACTGTTCGAGTCTCAAATCCCATCTCATATTAAAGACACAATGCCATGGTGTCTAGTGCAATTAGGAAAACTTCATTTTGAGATCATTAATTATGATATGTCCTTAAAGTATTTCAATAGATTGAAAGACCTACAACCGGCAAGGGTAAAAGATATGGAAATTTTTTCTACTTTGCTGTGGCATTTGCATGACAAGGTTAAATCTTCAAATTTGGCAAATGGGCTAATGGATACAATGCCTAATAAGCCCGAAACATGGTGTTGTATAGGTAATTTGCTATCATTGCAAAAGGATCATGATGCCGCAATAAAAGCCTTCGAAAAAGCTACTCAGTTAGACCCAAATTTTGCATACGCGTATACTTTGCAAGGTCATGAACATTCTTCCAACGATTCTTCGGATTCTGCCAAGACATGCTATAGAAAGGCGCTAGCTTGTGATCCTCAGCATTACAATGCATATTACGGATTGGGTACGAGCGCTATGAAATTAGGTCAATATGAAGAAGCGTTGTTATATTTTGAAAAGGCAAGGTCAATTAATCCCGTCAATGTTGTGTTAATCTGTTGTTGCGGTGGTTCTTTAGAAAAGCTGGGCTATAAGGAAAAGGCTCTACAATATTATGAACTAGCATGTCATTTGCAACCGACTTCCTCGCTATCCAAATATAAGATGGGCCAGTTGCTCTATTCCATGACAAGATATAATGTTGCTTTGCAAACTTTTGAAGAATTGGTGAAACTCGTTCCTGATGATGCCACAGCCCATTATTTGCTGGGTCAAACATATAGAATAGTTGGGAGGAAAAAAGATGCAATCAAGGAGCTAACTGTTGCTATGAATTTGGATCCAAAGGGTAACCAAGTTATCATCGATGAATTACAAAAATGTCATATGCAAGAATAA>YBL086C	-:0:chr02:61199:62599ATGCCATTTAATCATAATAGCAAGGCAAAAAGACCAAAGTTTCTACTGGACCTGCAAATCAAAGAATTGGTAAATATTCCTCAATCTTCTGGCTATTGCTACACGAAATGGCGTTTGAAGGATGGAACAGGTACGTCGGGACACAAAGTGGCTCTGGACGGTGAGCATCAAACCACTTCGACCCAGAGTAGAGGTACAACAAAACACGTGCATGTGCAACATCATAGAGCACAATGGAATTATTCATTAGACAAACCAATCCTGGTGAAGCTCCACTTGGATAAAAACGGCAGATTCTTGAAGAAAATTTTGGTGTTGGATGTTTTTTTTGAGTTTGCTGATGCGAATTCCTCGTTGACTTCTAGTTCTAGTCCCAATGGCAAAGTGAAGAAAACGACCTATGCTAACGCCACTGCACTAACGGCCACAGGAAACAATTCATATTCTCAAAAGATAACGGGTAAACTTCTATTGGGAACAGTAGACATAGATATAACAGAATACGTCAAAGAAGACGAAACTCCCACAACAAACAGATTTCTCCTAAAGCACTCAAAGGTCAATTCTATAATCAACGTCTCATTGCAGTTGAAGCTAGTTAGAGGCTCTTACGAAGATTTCAACATTTCCAAATCGTTTACTAATGGCCAGCTCGCTAATTACAGGCCAGGAATAAATACTATACTGGATAACACGTCTGAACTATCTAGCCCCACATCAACAACAAACCAGATGTCACCCAAGAATACATTCTCCAATTTTAACGGTATTGGAACAACTGTAGCGAAACCCGGTACAAATGCGACAGGAAACAGCACAAGTATTAAATCACCGACTTCTACCAACCACAAGAGTAGCGAAATGACAACAAAGCCAGGGCTGTCAACAACAATATCTTCCTCTATGAGCCCACTGATTGAGAGCTTGTACCAGAAAACGTTTAAATTACCGTGGGATCCTCGACCAGGTGAATTTACGCCAAGAGAATGCGTGGAAGATATTTTACAAGGCGGAAACGGTTGGGCTAAGAACGAGAAGGGCATAAATTTAATCGATTTACAGGCATTGAGATTGAACGAAATGGAAGAAGAGTACTACAATCCAAATTATGGCAATAACCTTGGGAACAAGGCAAGTAGTTGGCCTCCAAATCCAAGCGATGACGGCTACAGCACAATGGGCAAAAGAGAGTATTTGGAGAAAAAGCAGAATTGGAGTCATATGTCACGAGCACAACGTGCAAAACTGAGGACTCATAATGACGAGGATAACGAAAATACGGCTAATGATAAAGGGAGCGACAAGGATAATAACTCTGTCGAAGACAATAACCCCACGGACTTTTTAACAGATAGAATAAGAGAAAACAAAAACTGGTCTATAATAACCCCATCGGGCTAG>YBL087C	-:0:chr02:59818:60189TTAGGTCTACCAGTCGGTGCCATCATGAACTGTGCTGACAACAGTGGTGCCAGAAACTTGTACATTATCGCCGTCAAAGGCTCTGGTTCCAGATTGAACAGATTGCCAGCCGCCTCTCTAGGTGATATGGTTATGGCCACCGTTAAGAAGGGTAAGCCAGAATTGAGAAAGAAGGTTATGCCAGCTATTGTTGTCCGTCAAGCTAAGTCTTGGAGAAGAAGAGACGGTGTCTTTTTGTACTTCGAAGACAATGCTGGTGTCATCGCTAATCCTAAGGGTGAAATGAAGGGTTCCGCCATCACTGGTCCAGTCGGTAAGGAATGTGCCGATTTATGGCCAAGAGTTGCATCTAACTCCGGTGTTGTTGTGTAA>YBL087C	-:0:chr02:60694:60735ATGTCCGGTAACGGTGCTCAAGGTACTAAGTTTAGAATCTCA>YBL089W	+:0:chr02:49571:50950ATGCCGTCAAACGTACGTTCGGGAGTCTTAACTTTGCTCCATACAGCATGTGGAGCAGGCGTACTTGCAATGCCGTTTGCATTCAAGCCATTTGGGTTAATGCCTGGTCTGATAACGCTAACATTTTGCGGAATATGTTCCTTATGTGGGCTGCTATTACAGACTCGAATAGCGAAGTACGTACCTAAATCTGAGAACGCCTCGTTTGCTAAACTCACCCAACTAATCAATCCGTCAATAAGTGTAGTGTTCGATTTTGCCATTGCTGTTAAATGTTTTGGCGTTGGTGTATCTTACTTAATTATTGTTGGTGACTTAGTGCCACAGATAGTGCAGTCAATTTTTTATCGTAACGATGATAACATGAGTGGTTCGCAAGAGCATCACATGTTCTTAGACAGGCGTTTGTATATAACTCTGATCATAGTGTTTGTTATCTCCCCTTTATGCTTTAAAAGAAGTTTGAATTCTCTACGATATGCTTCTATGATTGCCATTGTTAGTGTCGCATATTTATCTGGTTTGATTATTTACCATTTTGTAAATCGGCATCAGCTAGAGAGAGGGCAAGTATATTTTATGGTACCTCACGGAGATTCTCAGTCTCATTCTCCCCTGACTACATTGCCAATTTTTGTGTTTGCTTACACTTGTCACCACAATATGTTCAGTGTAATTAATGAGCAAGTGGATAAGAGCTTCAAGGTAATCAGGAGGATTCCGATTTTTGCCATCGTGTTGGCCTATTTTTTATACATCATAATTGGTGGTACAGGTTATATGACATTTGGTGAGAATATTGTAGGAAATATCCTCACTTTATACCCGAATTCCATCTCCACCACCATCGGGAGGTTAGCAATGCTGCTATTAGTTATGTTAGCATTTCCATTGCAATGCCATCCTTGCAGATCATCGGTAAAAAACATAATTATATTCATTGAAAATTTCAGAAAAGGTAAGTTATACGATAACAGAGCTAGCTTTATTCCATTAGACAACTTTAATAGTGAAGATCCGCAGGAGGCGCCAACCCAACAAAACAACGAAGAGCCAAATCTGCGTAGTGAGTCTTTACGGCATATCAATATTATCACCCTTTGTATCTTACTGTTCTCATATCTACTGGCTATTTCAATTACGTCTCTAGCAAAAGTCCTAGCAATAGTTGGTGCCACGGGATCTACGTCGATTTCTTTCATTTTGCCAGGCCTTTTTGGTTATAAATTAATTGGCTCAGAATTTACGGGCACGAATGAAAGAGTACCGACAAGCATAAAAATATTCAAATACTTAAGTTTATCTCTATTCATCTGGGGGATAGCAGTAATGGTAGCTTCACTATCAGCGATTGTATTTTTGGGCACATCATCACATTGA>YBL090W	+:0:chr02:48822:49355ATGTTGAAGAGCACGCTGAGGCTTTCAAGAATCTCTCTCAGAAGAGGTTTCACAACGATCGACTGTTTACGCCAACAAAATTCGGATATCGATAAAATCATACTAAATCCAATCAAATTAGCTCAGGGAAGCAACAGCGATCGTGGCCAAACCTCTAAAAGCAAAACTGATAATGCAGATATTTTATCAATGGAAATTCCAGTAGATATGATGCAATCTGCTGGGAGAATAAACAAGAGGGAGCTTCTATCCGAGGCGGAAATTGCTAGAAGTAGCGTGGAGAATGCACAAATGAGATTCAATTCTGGAAAATCTATAATCGTGAATAAGAACAACCCTGCAGAATCATTTAAGAGATTAAACAGGATCATGTTTGAGAACAATATTCCCGGAGATAAAAGAAGTCAACGGTTTTACATGAAGCCGGGGAAAGTGGCTGAATTGAAGAGATCTCAAAGGCATAGGAAGGAATTCATGATGGGCTTCAAGAGGTTGATTGAAATTGTTAAAGATGCCAAGAGGAAAGGATACTAA>YBL091C	-:0:chr02:47360:48625ATGACAGACGCTGAAATAGAAAATTCCCCTGCTTCTGATTTAAAAGAATTGAATTTGGAGAATGAAGGCGTTGAACAGCAAGACCAGGCAAAAGCTGACGAGTCAGACCCAGTAGAAAGCAAAAAGAAGAAGAACAAGAAAAAGAAGAAGAAGAAAAGCAATGTGAAGAAGATTGAATTACTGTTTCCAGATGGAAAGTACCCAGAAGGTGCGTGGATGGACTATCATCAAGATTTCAATCTGCAAAGAACCACGGTTGAAGAATCACGTTATTTGAAAAGGGATCTGGAAAGGGCCGAACATTGGAATGATGTCAGAAAGGGTGCTGAGATACATCGTCGTGTGAGAAGGGCCATCAAGGACAGAATCGTTCCTGGGATGAAGTTAATGGATATCGCTGACATGATCGAAAATACTACAAGAAAGTATACAGGTGCCGAAAATTTATTAGCGATGGAGGATCCCAAATCTCAAGGTATTGGGTTTCCAACGGGTCTCTCTCTCAACCATTGTGCTGCACATTTCACACCCAATGCAGGCGACAAAACCGTTCTGAAATACGAAGACGTGATGAAGGTAGATTATGGTGTGCAGGTAAACGGTAACATCATTGATTCTGCCTTTACTGTTTCCTTTGATCCACAATACGATAACCTGCTAGCCGCTGTAAAGGACGCTACTTACACGGGTATTAAAGAAGCGGGTATCGATGTGAGATTAACCGACATCGGTGAAGCCATCCAAGAAGTTATGGAATCCTACGAAGTGGAAATCAATGGTGAGACTTACCAGGTTAAACCTTGTCGTAATCTATGTGGCCACAGTATCGCACCATATCGTATCCACGGCGGTAAATCCGTTCCCATCGTCAAAAATGGGGACACTACAAAAATGGAGGAAGGTGAGCACTTTGCCATTGAAACTTTTGGTTCTACTGGTAGAGGTTATGTTACTGCCGGTGGGGAAGTTTCTCATTATGCCAGATCTGCTGAAGACCATCAGGTAATGCCCACGTTAGACAGCGCCAAGAACTTGTTAAAAACGATAGACCGCAACTTTGGGACTTTACCGTTCTGTCGCCGATACCTAGACAGACTTGGCCAAGAGAAATACTTATTTGCGTTGAATAACTTGGTTAGACACGGTTTAGTACAGGATTATCCACCATTGAACGATATCCCCGGATCCTACACTGCACAATTCGAACACACCATCTTGTTGCATGCTCACAAAAAGGAAGTCGTTTCGAAAGGTGATGACTACTGA>YBL092W	+:0:chr02:45975:46367ATGGCCTCCTTACCTCACCCAAAGATTGTCAAGAAGCACACCAAGAAGTTCAAGCGTCATCACTCTGACCGTTACCACAGAGTTGCTGAAAACTGGAGAAAGCAAAAGGGTATTGACTCTGTTGTTAGAAGAAGATTCAGAGGTAACATCTCTCAACCAAAGATCGGTTACGGTTCTAACAAGAAGACCAAGTTTTTGTCACCATCTGGTCACAAGACTTTCTTAGTCGCTAACGTTAAGGATTTGGAAACCTTGACCATGCACACCAAGACTTACGCCGCTGAAATTGCTCACAACATCTCCGCTAAGAACAGAGTTGTCATTTTGGCTAGAGCTAAGGCTTTGGGTATCAAGGTCACCAACCCAAAGGGTCGTTTGGCTTTGGAAGCTTAA>YBL093C	-:0:chr02:44253:44915ATGGCTTCTAGAGTGGACGAAACTACAGTCCCCTCATACTACTATTACGTGGATCCGGAAACTACATATACGTACCAACAACCAAATCCTCTACAGGACTTGATATCGGTGTATGGCTTGGATGACATCTCCAGGCAAGTGGCAAGAACAAATTTGGACGGCACTAAAGCCGTGAAGCTAAGAAAATCTTACAAGAACCAGATAGCAGATCTTTCAGGTAAATTCTCCACCATACCGACCAGAGAAAATGGTAAAGGTGGTCAAATAGCACATATTCTTTTCCAAAATAACCCAGACATGATGATACAACCACCTCAGCAGGGTCAAAACATGTCAGAGCAACAATGGCGCGAACAGCTGCGCAATAGAGACATAGCATTGTTTCAGCCTCCAAATTTCGATTGGGACCTTTGCTCTTCGGTACTATCGCAGTTTGAAAGGTCATATCCAAGCGAGTTCGCAAACCAGAACCAAGGGGGAGCCCAAGCGCCGTTTGATATAGACGACTTGGCGTTTGATCTAGACGGTACAGGAAAAAGCCAATCCGGCTCAAATTCAGGTAACAATAGTAAGAAAAGGAAGAACAAATCTAGTGGAAGTTCGATGGCTACACCAACACATAGTGACAGTCATGAGGATATGAAAAGAAGGAGGCTGGAGTAG>YBL095W	+:0:chr02:43274:44086ATGTCCAGAACTATTCCATTTCTATTTAAATTAGTCAACAGGGCAGTAATTTTGCCTACGGCAGGTTTTACATTAGGAGTTGGTGCGTTTGTAAAGGCGTGGCCCGATGATGCCGGTGTTCTATCATTGAATGATCCGCAAACGCCAGCGGAGTTGATTAGTGCGACCAAGAGCCGCCAACCTATGGAGCTGCAGAGGGTTGACATCCTCGCTCAAATCGAGAAAAGCGAGGTTTACAACAAGTTGGCCCAGGATGAGAAGATGCACCATGTCTTATTCAGTGAGAAAATACCAAGCGGGCATAGGGAATATCATGTAGGACAAGGCCTCTTGTTCGGCAAGGGGAAGCTTGAAATTGATCCTTTGGTGTTCCATGATGTGAATCACGGTGAATTAACCGTGATTTATCACTTAGGTGCTGAGTTAGGGAATCGAGACGGTAACGTCCATAAGGGCTTGTTGTCATTGTTGCTGGATGAAGCATTGTGCTATTGTGGCTTCCCTTTGTTGCCTAGTAAAAGAGGTGTAACTGCAAGGCTGTCGCTAGAGTTTTTTGAGGACATTCCTGTAGATACTACGATTATACTAAAAGCAAACGTCAAAGAGATTAAAGGCAGAAAGTGTATCATTGAGGGACACTTGGAACAGTTTCCGTTGGAAGTTTCTTCTCGAAATGGAACTAGAAGTTGGAACTTACCACATATTTGGGGTTTCAACCATAAGCAGGAGATGGCAAAAAAATTTGCCAAGGCCAATTGTATTCTCGTTGAGCCTACTTGGTTCAAATATTTTAAATGGCTTGATATGTTTTGA>YBL098W	+:0:chr02:39142:40524ATGTCTGAATCAGTGGCCATTATAGGTGCAGGATTAGTAGGCTGCCTTGCAGCTTTGGCATTCTCCAAAGAAGGCTACAATGTCACACTATATGATTTTAGACAAGATCCTCGATTGGACACCACCAAAAATAAAAATTTGAAATCCATTAATTTGGCTATTTCTGCTCGTGGCATTGATGCTCTGAAATCAATAGATCCGGATGCTTGTGAACATATTCTGCAAGATATGATTCCCATGAAAGGCAGGATGATTCATGACTTGAAAGGCAGACAGGAATCACAATTGTATGGCTTGCATGGAGAAGCTATTAATTCTATCAATAGATCTGTATTAAATAATAGCCTTTTGGACGAATTAGAAAAATCTACAACAGAACTGAAGTTCGGTCACAAATTAGTCAAAATCGAATGGACAGATGATAAACAAATCTGTCATTTTGCCATTGGGGAAGATTTGAAAACCCCACATACTGAAAAGTATGATTTTGTCATAGGTTGTGACGGAGCATACTCTGCGACGAGATCGCAAATGCAACGTAAAGTTGAGATGGATTTTTCACAAGAATATATGAATTTACGTTACATTGAACTTTACATCCCGCCTACTGAGGAATTCAAGCCAAACTATGGCGGAAATTTTGCAATAGCTCCTGACCATTTGCACATTTGGCCTCGTCATAAATTCATGTTAATTGCGCTCGCCAACAGTGACGGCTCGTTCACTTCAACCTTTTTCGGTTCTAAAGATCAAATATCAGATCTGATAACTTCCAAGTCACGTGTGAGGGAATTCTTAATCGAGAACTTTCCCGATATTATTAATATTATGGATTTGGACGATGCTGTCAAAAGGTTTATCACTTATCCAAAGGAAAGTCTTGTCTGTGTAAACTGTAAGCCATACGATGTACCAGGCGGAAAGGCCATCCTACTCGGCGACGCTGCCCATGCAATGGTTCCATTTTACGGCCAAGGTATGAATTGCGGATTTGAAGATGTGAGAATTCTTATGGCGCTATTGAAAAAGCATTCAGGAGATCGTTCAAGAGCCTTTACTGAGTACACTCAAACAAGACATAAGGACCTAGTTTCTATTACTGAGCTGGCAAAAAGGAACTATAAAGAAATGTCACATGACGTTACATCCAAGCGGTTTTTATTAAGGAAAAAGCTAGATGCTCTCTTTAGTATTATAATGAAGGATAAGTGGATACCTTTGTATACAATGATATCTTTCAGATCCGATATCTCGTATTCTAGAGCTTTAGAAAGGGCTGGAAAGCAAACACGTATCTTGAAATTCTTAGAATCTCTGACACTCGGTATGTTATCTATTGGCGGTTACAAGCTTTTCAAATTTTTGACAAGAGAACGTTCCTGA>YBL099W	+:0:chr02:37050:38687ATGTTGGCTCGTACTGCTGCTATTCGTTCTCTATCGAGAACTCTAATTAACTCTACCAAGGCCGCAAGACCTGCCGCTGCTGCTTTGGCTTCCACCAGAAGATTGGCTTCCACCAAGGCACAACCCACAGAAGTTTCCTCCATCTTAGAGGAAAGAATTAAGGGTGTGTCCGACGAGGCCAATTTGAACGAAACTGGTAGAGTTCTTGCAGTCGGTGATGGTATTGCTCGTGTTTTTGGTTTGAACAACATTCAGGCTGAAGAATTGGTCGAGTTCTCCTCTGGTGTTAAAGGTATGGCTTTGAACTTGGAGCCTGGTCAAGTCGGTATCGTTCTTTTCGGTTCCGATAGACTGGTTAAAGAAGGTGAATTGGTCAAGAGAACCGGTAATATTGTTGATGTCCCAGTCGGTCCAGGCCTTTTGGGTAGAGTTGTCGACGCTTTAGGTAACCCTATTGATGGTAAAGGTCCTATTGACGCTGCCGGTCGTTCAAGAGCTCAAGTCAAAGCACCAGGTATTTTGCCAAGAAGATCTGTCCATGAACCAGTTCAAACCGGTTTGAAAGCCGTTGACGCCTTGGTCCCTATCGGTAGAGGTCAAAGAGAGTTGATTATTGGTGATCGTCAAACAGGTAAGACTGCTGTCGCCTTAGACACCATCTTGAATCAAAAGAGATGGAATAACGGTAGTGACGAATCCAAGAAACTTTACTGTGTTTACGTTGCCGTTGGACAAAAAAGATCTACCGTTGCTCAATTGGTCCAAACTTTGGAACAACATGACGCCATGAAGTACTCTATTATTGTTGCAGCTACTGCATCTGAAGCCGCTCCTCTACAATACTTGGCTCCATTTACTGCCGCATCCATTGGTGAATGGTTCAGAGATAATGGAAAGCACGCTTTGATCGTCTATGACGATTTGTCCAAGCAAGCCGTGGCATACCGTCAATTATCTTTGTTGTTGAGACGTCCTCCTGGTCGTGAAGCCTACCCTGGTGATGTCTTTTACTTGCATCCAAGATTGCTAGAAAGAGCCGCTAAGCTTTCTGAAAAGGAAGGTTCTGGTTCTTTAACTGCTTTGCCTGTTATTGAAACCCAAGGTGGTGATGTCTCCGCTTATATTCCAACCAATGTTATTTCCATTACCGATGGTCAAATATTCTTGGAAGCTGAATTATTCTACAAGGGTATCAGACCTGCCATTAACGTTGGTTTGTCCGTTTCTCGTGTCGGTTCCGCTGCTCAAGTTAAGGCTTTGAAGCAAGTCGCTGGTTCCTTGAAATTGTTTTTGGCTCAATACAGAGAAGTCGCTGCTTTTGCTCAATTCGGTTCCGATTTAGATGCCTCCACCAAGCAAACTTTGGTTAGAGGTGAAAGATTGACTCAATTGTTGAAGCAAAACCAATATTCTCCTTTGGCTACAGAAGAACAGGTTCCATTGATTTATGCCGGTGTTAATGGTCATTTGGATGGTATTGAACTATCAAGAATTGGTGAATTTGAGTCCTCCTTTTTGTCCTATCTAAAATCCAATCACAATGAGCTTTTGACCGAAATTAGAGAAAAGGGTGAATTGTCTAAAGAATTGTTGGCATCTCTAAAGAGTGCTACTGAATCATTTGTTGCCACTTTTTAA>YBL102W	+:0:chr02:24098:24745ATGAGCGAGGAACCACCTTCTGACCAGGTCAATAGTCTCCGTGACTCATTGAATCGATGGAATCAAACAAGACAGCAGAACTCGCAGGGTTTTAATGAATCTGCGAAGACATTGTTCTCAAGCTGGGCGGATTCTCTCAATACCAGGGCCCAGGATATATATCAGACGTTGCCTGTATCTAGACAGGACTTGGTGCAAGACCAGGAGCCGTCGTGGTTCCAATTGTCAAGAACGGAAAGAATGGTACTTTTTGTCTGTTTTCTTTTGGGTGCAACAGCCTGTTTCACTCTTTGTACTTTCCTTTTCCCCGTTCTAGCCGCTAAACCAAGAAAGTTTGGTTTACTATGGACAATGGGGTCCCTACTATTTGTTCTTGCGTTTGGGGTACTTATGGGACCACTCGCGTACTTAAAACATTTGACTGCAAGGGAAAGGCTGCCTTTTTCGATGTTCTTTTTCGCCACATGCTTCATGACGATTTATTTCGCAGCCTTTTCCAAGAACACGGTGCTGACTATTACATGTGCTCTTCTTGAATTAGTTGCCGTCATTTATTATGCTATTTCATATTTCCCATTCGGTGCAACAGGTTTGAGGATGTTAAGCTCTGCTGGTGTCAATTCGGCAAGAGGTGTTCTGCGCATCTGA>YBL103C	-:0:chr02:22075:23535ATGATGAACAATAACGAAAGTGAGGCTGAGAACCAACGTCTACTGGACGAATTAATGAACCAGACAAAAGTCCTCCAGGAAACTTTAGATTTTTCGTTAGTGACACCCACTCCACACCACAATGATGATTACAAGATACACGGAAGTGCCTACCCAGGTGGTGAGACTCCTGCCCAACAGCATGAAAAACTCTCATACATCAATACGCACAACTCTAACGATAATAATAACTTAATGGGCAGTCAAGCGAGGTCCAATTCACAAACTCCTACAGCTTCGACCATATATGAGGAAGCAGAATCGCAATCGTCTTACCTGGATGATATGTTTAGAACAAGCCAAGGCGGTAGACCTGTCACTCAAAATTCCATATCTTCCATAGGGCAGGGTCCCTTGAGATCATCTTATTCTATGGCTTACGACTCACCTGTGGATAGAGCAATGAATACTCCATTACAGCAACAAGAAGGCTTAAAAGCTGAGTTACCACATGACTTTTTATTTCAGCATGGCACCGATGACACAATGTATAACTTAACTGATGATTTGAGCTCCTCTTTATCTTCTAGCATCAATTCTGATATGATGACACCAAACACATATTCATCATCATTTTCTTATAATCCACAAAGCTTGGGCCCAGCATCCGTATCTTCCACATATTCCCCAAAGGTAAGATCACCATCATCGTCATTTCGTGCAGGAAGTTTCCTTTCATCTTCTTTTAGGCATGGTAGCATAAACACACCCAGAACAAGACACACTTCAATAAGTAGCAATATGACTGAAAATATAGGACCTGGAAGTGTTCCAAAGATTTTAGGCGGGTTAACTTCTGATGAGAAACTGAGGCGCAAAAGAGAGTTTCATAATGCCGTCGAAAGGAGAAGAAGAGAATTAATAAAGCAAAAGATAAAAGAACTCGGCCAGTTGGTTCCACCATCTTTATTGAATTACGATGACCTAGGTAAACAAATCAAACCAAATAAGGGTATCATTTTGGATAGAACGGTCGAATATTTACAATACCTGGCCGAAATTTTAGAAATACAAGCACGAAAAAAGAAGGCGTTATTGGCGAAGATAAAAGAATTGGAAGAGAAGAAAAGTTCTGTCGCAGCATTATCTCCTTTTACCAATAACCACCATGCCAGTTCGGGGCAAAATAATAGCGAGAATAGTGAGGAAAGAATTATAGATATTAGATCTGTCCCAAATGCTTTGATGAATGAACAAAATAGTAAGGCTGAATTGCACAATTGGGAGCCACCGTTATATGATTCGGTCGGTAACCACAATCATGCTGGCACTATGGAGAGTCATCCACATACAAACATTCATGAAGAATTAAAGGAGTTCTTATCAGGCGATTTGATTGAAGCCGAAGATAATGCAAAATTAATGTTTGGAGATGACAACTCTAATCCAGCTGACTATCTTTTAGAATTTGGTTCGGGGTAG>YBL104C	-:0:chr02:18177:21293ATGGGTCTCATCAAAAAAGTGACCCATTGGTCATATGATAATTTGATTGATTATCTTTCAGTGAACCCTACAAGAGATGAAGTGACCCACTATAAAGTGGATCCGGAAAATGAATCTGATGAATCAATTATTAAATTACACACTGTGAAAGACTTCGGCAGTATCACCTGCTTAGACTATTCAGAATCAGAAATTGGTATGATTGGAGTTGGTGAAAAGAATGGATATTTAAGGATTTTTAATATCTCTGGACAAAATTCCTCATCGCCAGCGAGCCATGCGCCAGTGGGCTTAAATGCCAATAACGAGACTTCTATGACAAATGCCAGCGGTGGTAAGGCAGCTCAGGCGGAAAATATAGTTGGATCTGTCTCAAATTTAAAGGATACCCAAGGGTACCCTGTTTCAGAAACCAATTATGATATACGTGTACGAGCAAAGAAGCAGCGGTGTATAAACTCTCTGGGAATAAATACGAATGGTCTTATAGCAATGGGTCTTGACAGAAATAAGCATGATTCATCTTTACAAATTTGGGACATGAATTATCATGATGATTCGCATGAAACTATCAACCCAATGTTTAGTTATTGCACAAATGAAAGCATAGTATCCCTAAAGTTCCTCAACGATACCAGTGTATTGGCTGCAAGTACGAAATTTTTGAAAGAAATTGACGTCAGATCCCCCAACCCAATTTATCAACATCCGACACGATTGACGTATGATATCAAACTGAACCCGTTTAACGATTGGCAATTTAGTACTTATGGGGATGATGGGACCTTAGCTATTTGGGATAGGAGAAAATTATCTGACCAAGCTTCACTTGGTGATTTAAATGTAGCTTCCCCTTTATTAACTTTTGAAAAATTGGTTGGATCCGGTGCAGCGTCAAGGAAATACATGAACTCTTGCTTTAGGTGGTCATGTGTGAGGAACAATGAATTTGCCACCTTACATAGAGGTGATACCATCAAAAGATGGAGGTTAGGCTATTACTGCGATAGCAATCGTGATATTGCGGCAGATGACGATAACGAGATGAATATTGAGAATTTGTTTGTTTCTTCCGTGCATGACACAAATACGATGTACGATAGAGTTGCTACATTTGATTATATTCCAAGAAGTAATAATGGAACAAGTCTAATCTGTATGAGACAATCGGGAACAATATACAGAATGCCAATTTCAGAGGTATGCTCCAAGGCTATACTCAATAATAGGAACTCTCTCTTATTGTCAAATTTTGAAAATACTGAGATAGATGAAATTAGAGTCAACAATGAGCATGAAAAGTCCAACTTAGAGAATGTAAAAACAATCCTAAAGAATCTATCGTTTGAGGATTTAGATGTTAGCGAAGATTACTTTCCGTCTGGACATGATGAACCTAATAATGAAATTGAGTACTCCGAGTTGAGTGAAGAGGAAAATGAAGGGAGTAATGATGTCCTTGACAGTAAGCGTGGATTTGAACTGTTCTGGAAGCCAGAAAAACTTCTGGAAAAAGATATAAGTGTAATAATGAGGACGCGAGCTTCATTGGGATATGGTTTGGATCCAATGAACACGGTAGAGATGATTGATTCTTCTAAAAATCTACAAAACAATGCTTATATAAGGAACACATGGAGGTGGATCGCGATTGCAAAGGCTTCTGTTGACGATGGTACGATGGTTTCTGGTGATCTTGACTTAGGTTACGAAGGTGTGATAGGTATTTGGAATGGTATAAACGGAATATCGAACCAAGACAGATATAGACAAGAGACAATTCTTTCTGATAAACAACTAAATAAAGAAATGGAAAAAATCATCAAACTAAGAAGGAAAAATAGGGATCGGAACAGTCCTATTGCCAATGCCGCTGGTTCACCAAAGTACGTTCAGAGAAGACTCTGTTTAATTATCTCAGGATGGGACCTTTCCAGATCTGATTATGAAGACAAGTACAACATAATTATGAAAAACGGCCATTATGAAAAAGCTGCCGCTTGGGCGGTATTTTTTGGTGATATCCCAAAGGCTGTAGAAATATTGGGATCTGCAAAAAAGGAAAGATTGAGACTAATTGCCACCGCTATTGCAGGTTACTTGGCGTATAAGGATCTGCCAGGTAACAATGCATGGAGGCAGCAATGCAGAAAAATGTCCTCCGAATTAGATGATCCTTACTTGAGAGTTATTTTCGCCTTTATCGCAGACAATGATTGGTGGGATATTCTTTATGAACCTGCCATATCTTTGAGAGAACGATTAGGGGTAGCTCTAAGGTTTTTGAATGATACAGACCTAACCACTTTCTTAGATAGAACTTCATCTACAGTTATTGAAAACGGCGAATTAGAAGGTTTAATTCTCACCGGAATAACACCTAATGGTATCGATCTACTGCAATCATACGTTAATAAAACCAGCGATGTTCAGAGTGCCGCACTGATATCAATTTTCGGCTCCCCTAGATATTTCCGCGACCAACGAGTAGATGAATGGATACAAACTTATAGAGATATGCTTAAATCGTGGGAATTATTCTCCATGCGAGCCAGGTTCGATGTTTTAAGATCAAAATTATCGAGAACTAAAACTGGTGTATTAACTGCAGACATAAAGCCCCGGCAAATATATATCCAATGTCAAAACTGTAAACAAAACATCAACACTCCGCGCACATCCTCACCTTCTAGCGCCGTCTCTACTAGTGCTGGAAATTACAAAAATGGTGAAGCCTATAGAAGAAATAACGCTGACTACAAGAAATTTAATACTGGAAGTTCTGAAGCACAAGCAGCTGATGAAAAGCCAAGACATAAGTATTGTTGCCCACACTGTGGGTCTTCATTTCCAAGATGTGCCATATGTCTCATGCCTCTAGGAACGTCAAACTTACCTTTTGTAATAAATGGGACGCAATCACGCGATCCAATGCAGACAGAAGACTCTCAAGATGGTGCAAATCGCGAACTCGTAAGTAGAAAACTGAAGTTGAACGAGTGGTTCAGCTTCTGTTTGAGTTGCAACCATGGTATGCATGCCGGTCACGCTGAAGAATGGTTTGACAGACATAATGTTTGTCCCACTCCAGGTTGCACCTGCCAGTGTAATAAGTAG>YBL107C	-:0:chr02:9961:10551ATGGTTGATAATAGGCGTACGTTTACGGCACCCCAAAGCTTACTTGAAACAAATCTTACTTTTCCGAATGATGAACCGTCACTTACTACAATCACAGTTACGCGAGAAAGATGTGTTGATCCAAGCTTGATAGATTCATTTTTGAGGTTTCTAAGGCATGGAAGTGATGATATAATAAGACAAAAATTAAACAACTATAGGAAAGGCTCTATTAATGGAAAAAATAAGTGTAAGGAATTTTTGAAGCAAGAACTATATCCGAACTGGCAAATACGGAATAATATAATTTCATTTTGCGAAAAAGAGGCAGCAGAGATGAAGAACGAGACCGATCAGCAGTGCGGTAATAATAAAAAGACTACTGCGGAACCACTTATAGACGCAAGAATTGATCCTTACGCTGCAAGAGAAAGAGCGGAGAAACAGGAAGCTCAATATAAGGATTGGACGAAGGTGACAGAGTGGGTGGCAAATAATAGAAAAATAGAACAAATTTTAACTTCTACAACAGAGGGCATTTTAAGGCAAAACTGCGAGCAAAATAATGACTACTTGAAGGAGTTTACGCAATTCTGCAAGGACAACAGTTGA>YBR002C	-:0:chr02:241710:242570ATGGAAACGGATAGTGGTATACCTGGTCATTCATTTGTGTTAAAGTGGACAAAAAACATCTTTTCGCGCACATTGCGTGCATCTAACTGTGTACCTAGACATGTTGGGTTCATCATGGATGGGAACAGGAGATTCGCTAGAAAGAAAGAGATGGACGTAAAGGAGGGCCACGAGGCAGGATTTGTTAGTATGAGTAGAATCTTAGAACTGTGTTATGAAGCAGGAGTCGATACGGCTACCGTGTTTGCCTTTTCAATTGAAAATTTCAAGAGGAGCTCACGGGAAGTTGAATCACTGATGACTTTAGCGCGCGAAAGGATACGACAAATCACAGAACGTGGAGAGCTGGCCTGTAAGTATGGGGTACGCATTAAAATTATCGGCGATCTCTCTTTGTTGGATAAGTCTCTATTAGAAGATGTTCGGGTTGCTGTGGAAACTACAAAGAACAACAAAAGGGCCACGTTAAATATCTGCTTTCCATATACAGGCAGGGAAGAAATCTTGCATGCCATGAAAGAAACAATTGTTCAACATAAGAAGGGCGCCGCTATAGACGAAAGCACGTTAGAATCGCATCTCTACACGGCGGGGGTACCCCCTTTAGATTTATTGATTAGGACAAGTGGCGTTTCCAGATTAAGTGACTTTTTGATATGGCAGGCATCGAGTAAGGGCGTACGCATCGAATTGCTGGATTGTTTATGGCCAGAGTTTGGACCTATACGGATGGCATGGATTTTATTAAAATTTTCGTTTCACAAATCCTTTTTAAACAAAGAGTACAGATTAGAGGAAGGTGATTATGACGAGGAAACCAATGGGGACCCCATCGATTTGAAAGAAAAAAAGTTGAATTAA>YBR003W	+:0:chr02:242811:244232ATGTTTCAAAGGTCTGGCGCTGCTCATCACATCAAATTGATTTCATCTCGAAGATGCCGCTTTAAATCCTCCTTTGCAGTTGCTTTAAACGCTGCCAGTAAGTTGGTAACTCCCAAGATTCTTTGGAATAATCCCATATCATTAGTCTCGAAGGAGATGAACACATTGGCCAAAAATATAGTTGCCCTCATCGGGTCTGGCCATCCGGTGCTAAACAAGGTTACTAGTTACTATTTTGAAACAGAAGGCAAAAAAGTACGTCCCTTGTTAGTGTTGTTGTTGTCAAGAGCACTTTCTGAAATTCCCATGACAGAAAGAAATCACTTGAAAATTGACAAGTCGGATGTTCCTGAGGACCCAATTTACTCTAAACCTAGTCAAAATCAACTATTTCAACGTCCTGCAAGTAGCATATCCCCACTACATATTCTTCACGGTATTAAACCACTAAATCCCTTGACAAAAGGTCCTGAGCCTTTGCCAGAAGAAACTTTTGACAAACAAAGAGGGATTTTACCCAAACAGAGAAGATTAGCAGAGATTGTAGAGATGATACACACTGCGTCTTTACTTCATGATGACGTTATTGATCATTCTGATACAAGAAGAGGAAGGCCAAGCGGAAATGCTGCCTTTACCAACAAGATGGCCGTTTTGGCGGGTGATTTTCTCTTAGGGAGAGCAACAGTGTCAATTTCAAGATTACACAACCCCGAAGTCGTAGAACTAATGTCTAATAGTATTGCGAATCTTGTCGAAGGTGAGTTCATGCAACTGAAAAATACTTCCATTGACGCGGATATCGATACCATCGAAAATGGCCATAAGCTACTTCCGGTTCCTTCTAAAAAGCTTGAAGTTAAAGAGCACGACTTTCGTGTTCCAAGCCGCCAACAGGGGCTGCAATTATCTCATGATCAGATTATAGAAACTGCATTTGAATACTACATACACAAGACCTATCTGAAGACTGCTGCTTTGATATCAAAATCTTGCAGATGTGCTGCTATATTATCTGGGGCATCACCTGCCGTTATCGACGAATGCTATGATTTCGGTAGAAATCTAGGTATATGTTTCCAACTCGTAGATGATATGCTTGATTTTACTGTGTCTGGAAAAGATTTAGGGAAGCCATCCGGCGCGGATCTAAAATTAGGTATTGCTACTGCTCCAGTCTTGTTTGCATGGAAAGAAGACCCATCTTTAGGTCCACTAATTTCACGCAATTTTTCAGAGAGGGGTGATGTTGAAAAAACTATTGATTCTGTGAGACTCCATAATGGTATAGCGAAGACGAAAATACTAGCGGAGGAATATAGGGACAAGGCATTACAAAATCTACGGGATTCTCTTCCTGAATCTGATGCTCGTTCTGCCCTAGAGTTTTTAACTAATAGTATACTAACAAGAAGAAAGTAA>YBR004C	-:0:chr02:244368:245669ATGATTGTGGGGTTGACACTTTATTTTGTATTATTCCGTTCAATACAGTATTTACTGGTTTTTTTGACTCCAATTAGGCAGTTTGATACATCAACATCACTTTTATTAAACGAATTATGTTCTTCTCCCTCTGAAATCAACAGTTATTGGAACAAGTATTTTTGGAATAAGCTACTATCATGGGACAGTGTTTTTTTTATCAAGAACATAACTTCCAAAAACGGAAAACCTCAATTTGAGCATGAATACGCGTTTTCTCAGTTGTGGACTTTTTTCGTTAGGCTGTTTATTAAAAGTAATAACGATAGCATCTACCATGCCTTAAGGGTTGGAGTTGCAATAGAAAATGTTTTATTTTACTTGTCAGGTATTGTTTTATATTTTCTAACAAAAAAAATTTTCAGCCAAAATATAAGGCAATCACAGTTTGCTAGAACTATCGCTAAAAAAACATCTCTGTTGTTTTTCTTAACGAGTGCCGCTGGATTTTTAACAAGCATATATTCTGAACCATTATCTTTTTTTTTTGCATTTGTTGGTATTTGGAGTCGTGAATGCACCATTTCCGTGCCCGTATTGGGTCAATTCGATATTTCGTGGAGATATTGGTTTCCTTACTCCTTTATCAGCATGGCTTGCTTTACCTTAGCATCCTTGAATCGTTCAAACTGTGTTTTGTTAGGGATTTACTTTATTTTTGACCTTATTGAACTAACAAAGAACAGGAAGTTTGTAAAAGCAATATGTTTCCCACTATTATCAGGATCATTAATGTTTTCTGCTCTACTATATCAACAATATTACCTACCATATAAGACATTTTGTCCTCAAAGGGGTGAATGGTGTAAATCTCAATTGTTTTCAAGCATTTTTATCACGAAAACTTCTTTATATTCCTACATTCAGAGTCATTATTGGGGAGTTGGGTTATTGAAATACTGGACCCCAAACAACATCCCAAACTTTTTGTTTGCTGTCCCAAATATTATTATTTTAATCTATTCCTCCATATATTTCAGCAAAATTTATCCCTCCTATAACTTGAAGGCTCTCGTATGGATCACCAGAGCACTGGTCGTCATAGTATGCTTTTTTGCCCATGTCCAAATTCTGAATCGTATAGCCTCCTTTTTACCCTTGCACCTTTGGTATTTGGCTGATAGATTGGTTAAAACTTCTGATCCAAAAAAAATGGAAAATCCGAAAGGTGACGATAAGATAGTCAAGTTTTACATATACTGGTTGGCATTCTGGATACCTTTACAGACTATCCTATTTGCAGCTTTTTTACCACCAGCCTGA>YBR005W	+:0:chr02:245908:246549ATGGGACTTATTTCATACGAAAATGAGGCGATAAACGAGGTGAAAAAGGCAGATAACCATCACGTTAGCAAATTTGTGACTAGTTACTATGGGCCATCATCGTCGTCATGGCAGTCAGGAATATGGATTTTGTTTGTGCTGTTTGTTGCCGCAGTAATCCTTATAATACTGTTCACTTTTGTAGCGAACAGAAGGAGACGAAGGATGGGGCGTGCTCCCATTAGAGGTACGGCATGGTTGACACCGCCTTCATACAGACAGTCTCAGCAACAATATACTGGGACCGTTCAGCAACGGACAGATGATTATGTTCCTGAGTATACAGAAACAGCGAACGAACATGATCTTGGATACTATGACCAGCGGGGCGAGTTTCACCCCAACGATAAGGCTGCATACGTGGCCCCCCCGCCATTGGTACAAGAATGTTCATCAGAATCTGTTAATTCTTTAGAAAGACCTCCCGCTGCTGTAGTTCACCAAGCTAACTCTTTAGATACGGATTACGGTTTAACAAGGCCTAGCAATGGGCGCGTTCCAGCTGTAAGTGATACGGTGGAGCAATTGGAAAGGCTTCCGGGCGGGACTACAACGCAGGAAATTAACCCACCGGAGAGGGCAAAGGTAAATGCAAGGTCATGA>YBR006W	+:0:chr02:247012:248505ATGACTTTGAGTAAGTATTCTAAACCAACTCTAAACGACCCTAATTTATTCAGAGAATCTGGTTATATTGACGGAAAATGGGTTAAGGGCACTGACGAAGTTTTTGAGGTGGTAGACCCTGCTTCCGGCGAAATCATAGCAAGAGTTCCCGAACAACCAGTCTCCGTGGTTGAGGAAGCGATTGATGTTGCCTATGAAACTTTCAAGACGTACAAGAATACAACACCAAGAGAGAGGGCAAAGTGGCTCAGAAACATGTATAACTTAATGCTTGAAAATTTGGATGATCTGGCAACCATCATTACTTTAGAAAATGGTAAAGCTCTAGGGGAAGCTAAAGGAGAAATCAAATACGCGGCTTCGTATTTTGAGTGGTACGCCGAGGAAGCACCCCGTTTATATGGTGCTACTATTCAACCCTTGAACCCTCACAACAGAGTATTCACAATTAGGCAACCTGTTGGTGTATGCGGTATAATTTGTCCATGGAATTTTCCGAGCGCCATGATCACGAGAAAGGCCGCCGCTGCTTTAGCTGTGGGCTGCACAGTAGTCATCAAGCCAGACTCTCAAACGCCGCTATCTGCTTTAGCAATGGCATATTTGGCTGAAAAGGCAGGCTTTCCCAAGGGTTCGTTTAATGTTATTCTTTCACATGCCAACACACCAAAGCTTGGTAAAACATTATGTGAATCACCAAAAGTCAAGAAAGTTACTTTTACTGGTTCTACAAACGTCGGTAAAATCTTGATGAAACAATCTTCTTCTACTTTGAAGAAACTGTCTTTTGAGCTGGGTGGTAACGCCCCTTTCATAGTCTTTGAGGATGCCGATTTGGATCAAGCCTTGGAACAAGCCATGGCTTGTAAATTTAGGGGTTTGGGTCAAACATGTGTGTGCGCAAATAGACTTTACGTTCACTCATCCATAATTGATAAATTTGCGAAATTACTCGCGGAGAGGGTCAAAAAATTCGTAATTGGCCATGGTTTGGACCCAAAAACTACACATGGTTGTGTCATTAACTCCAGCGCTATTGAAAAAGTTGAAAGACATAAACAGGATGCCATTGATAAGGGAGCAAAAGTTGTGCTTGAAGGTGGACGTTTAACTGAGTTAGGTCCTAACTTTTATGCTCCAGTAATTTTGTCACACGTTCCCTCAACAGCTATTGTTTCCAAGGAGGAGACTTTTGGTCCATTATGTCCAATCTTTTCTTTTGATACTATGGAAGAAGTTGTCGGATATGCTAATGATACTGAGTTTGGTTTAGCAGCATATGTCTTTTCTAAAAATGTCAACACTTTATACACTGTGTCTGAAGCTTTGGAAACTGGTATGGTTTCATGTAATACAGGTGTTTTTTCGGATTGTTCTATACCATTTGGTGGTGTTAAAGAGTCAGGATTTGGAAGAGAAGGTTCGCTATATGGTATTGAAGATTACACTGTTTTGAAGACCATCACAATTGGGAATTTGCCAAACAGCATTTAA>YBR007C	-:0:chr02:248807:251017ATGAATCAAAATTTAAAAAACACTTCTTGGGCTGATCGAATAGGAAGCGACGATCAAGAAAGAAAAGCTAACTCTTCAGAGGTTTCACAAAGCCCACCACCCAATAATAGCTTCGAGTCATCAATGGATTCACAATTTAGCTATGCTCATTCAAATAAAAGTAGCATTTCATTCGAATCTATTCAAACCACGGAAAGACTTCTTGACAAATTAGACCTCAGCTTGGAAGATGAACTGATTCTGCAAGAAGCTTTATTAGAAGAAGAAAACGCGTCGAGAAATTCGCAACTGTCACAAACAAGTGGTCCAACATTATGTATGCCTGCGTCGGAATTCCCTTCATTAAGGTATAGAACTAATCCCTCTCCAACATATATACAAGCTAGAGATCGTTCTCTTATCATAGACAATTTAAAAGAAAAGGATTCCACTTTGAGAGGCAAATACTCTTCCGGTAAAGTGGAAAGGCATTTGCCCGTGAAGTCGAGATACTCTTATATCGTCGAAGAAGATTATGACTCTGAGACTTTTAGTGGCATGAAACCCCAAATGAATAGAAATGAGAAAGATTATAAGTACCCAAACCTTGAAAATGGCAATAGAAGCACAAATAGGCCTAATCCTTTCAACTTCGAGAAATATAGGATTGAAAATACTAGACTGCACCATCTTTATCCTACGCTGATATCAGACAATAATACTAGCGTTGACAATAATGCTAATAGTAAGAATAACAGAACTACTAGTAATAATATTAATACATCTACAAAAACTGACAGAATATCCGAGAAGCAGAGTTGCCCCAATGAGTTCACAACCACGCAAAAGAGTAATTGTCTTTATCGGAACGGAAGTTCAACAAGCACCAACACCTCCTTTTCTGAAGTTGGACAACTATCAAAGCCGAAAACTCAGTCTTCTTTTGAAAGTGAATCATCCTCATTCTCCAAACTCAAGTTAACTAAGAGTGATACAACAACCATCAAACCATCTCCTAAACGGTCTAATTCCTCTACCTCGACGATAACGAAAACAAATACCATGACAAATGATATTTCATTACCTCCAACGCCACCATATAAAGCTCATAAAAAAAAAACGTCACTAAATTCGCTAAAAAAACTTTTTAAATCTCCTAGGACAAGAGCAAAAAATAAGAAAGACCTTGAGTCTGAAGGATCAAGCCCTATCCGGTCTGCCACCAATTCATTGGACTTCTCTGGGGAGAATATTCAACTTCCATCTACTTCAAGTACCATTAATAATTCCAGTCCACATTTAGCTAGGTATATTTTTCCTCCAAACCCAGTTTTCCATTTTAAAACAGCATCAACACCTCAGTCATCCACGGATAAAAAAAAGAACAGTAAAGCACGTCCAAATAGGACTCATTTGAGAACTTTCTCGGATTTCCATACAACGGAAAAAGATTCAAAAATAGGTGAACTAAGTGCACTAACAGAACAATCAAATAAGCCCTACCACCCGAAAGTTAGAAGAAGAACACTTTCTTTAGATGGTATGTTGCCCAATAATTCGACGCAATGCATGGATTCCTTCAGTCATAAAAAAGAAGGATCGAACGCAACAAGCAAGTGCGGGAAGCTTAAATTTCACCCAGAACCTTATGATAATGATGAATCTTCACACATCGGACAGGCAATTACTATGCGTCATCAAGGCAAACTTGAAGAATCTGCACAACGACTGAAAAAAGCCTGCGCGTGCGGAAACAAAACTGCGTTTCTGTTATATGGACTAGCCCTGCGACATGGCTGCGGAGTGGACAAGAACTTGAAATTATCATTAGGATATTTAATGGCTGCCACTGATATCAAATCCTTTGCTGCTGAGGTTCTTGATTTAGATATAAATCCGTTAAATTTTGCTTCAATGGACGATATCCCTGATATAGCGCCTGAACCAACAGCACCTGCCTTGTACGAATGTGGTATGGCATATTTAAAGGGTCTTGGGATGGATCATCCCGACGAGCGAAAAGGTTTGAAATTTTTGGAAAAAGCTGCTCTATTAGGCCATGTAGACTCAATGTGCTTAAGCGGGACAATTTGGTCCAAAACTTCAAACGTAAAGAAAAGGGATCTCGCCAGAGCTGCTGCATGGTTTAGGATAGCCGATAAAAAAGGTGCAAATCTATTAGGTTCAGATTGGATTTATAAGGAAAAGTACATGAAACAAGGTCCGAAATAA>YBR008C	-:0:chr02:252564:254210ATGGTATACACTTCAACGTACAGACACACTATCGTTGTTGACCTTTTAGAATATTTGGGTATAGTGTCCAACTTAGAAACTTTACAGAGTGCCCGTGAAGATGAAACAAGAAAACCCGAGAATACCGATAAAAAAGAATGTAAACCCGACTATGATATAGAATGCGGTCCTAATAGATCGTGCTCTGAATCCTCTACCGATTCAGACTCTAGTGGTTCACAGATCGAAAAAAATGATCCTTTCAGGGTGGATTGGAACGGCCCCAGTGATCCTGAGAACCCACAAAACTGGCCCCTACTGAAAAAATCATTGGTAGTATTCCAAATAATGTTACTTACTTGCGTCACGTACATGGGATCCTCCATTTACACACCTGGCCAGGAATATATTCAAGAAGAGTTCCACGTTGGTCATGTAGTGGCAACATTAAATCTTTCTTTATATGTTCTTGGTTATGGTCTAGGTCCCATCATTTTTTCACCGCTATCAGAAACTGCACGCTATGGCCGTCTAAATCTGTACATGGTGACTTTATTTTTTTTCATGATCTTTCAAGTTGGTTGTGCTACTGTGCATAACATCGGCGGTTTAATCGTCATGCGTTTCATCAGTGGCATACTGTGCAGCCCATCGTTGGCCACTGGTGGCGGTACAGTGGCTGATATCATTTCACCAGAAATGGTTCCTCTCGTTTTAGGTATGTGGTCAGCCGGTGCTGTTGCTGCGCCAGTCTTGGCTCCCTTACTAGGCGCTGCTATGGTCGATGCTAAAAATTGGCGATTCATATTTTGGTTATTAATGTGGTTAAGTGCTGCCACTTTTATCTTGTTGGCATTTTTCTTCCCTGAAACACAACACCATAATATTTTGTACCGCCGTGCTTTGAAATTGAGAAAAGAAACTGGTGATGACAGGTACTATACTGAACAGGATAAACTCGATAGAGAAGTTGATGCAAGAACTTTTTTGATCAATACTTTGTATAGGCCTCTCAAAATGATTATCAAAGAGCCTGCAATTTTGGCTTTTGATCTCTATATCGCTGTTGCTTATGGTTGTTTCTACTTATTCTTTGAAGCATTCCCTATTGTATTTGTAGGTATATACCACTTCAGCTTAGTTGAAGTTGGCTTGGCCTATATGGGGTTTTGCGTAGGGTGCGTACTTGCTTATGGCTTATTCGGTATTTTAAACATGAGGATTATTGTACCACGTTTTAGAAACGGCACATTCACCCCGGAAGCTTTTTTAATCGTGGCAATGTGTGTCTGCTGGTGCCTGCCTCTGTCTTTGTTCTTATTTGGTTGGACTGCTCGAGTGCATTGGATTTTGCCAGTTATCTCGGAAGTTTTTTTTGTTTTAGCTGTCTTTAACATTTTCCAAGCAACTTTTGCATATTTGGCTACATGCTACCCAAAGTATGTTGCATCCGTTTTTGCAGGCAATGGTTTTTGTCGGGCTTCGTTTGCCTGTGCTTTTCCGTTGTTTGGTAGAGCAATGTATGACAATTTAGCTACTAAGAACTATCCTGTGGCATGGGGTTCGTCCTTAGTGGGGTTCCTAACTTTAGGTCTAGCTATTATCCCGTTTATACTTTATAAGTATGGGCCATCATTACGTACAAGATCTTCGTACACAGAGGAGTAG>YBR009C	-:0:chr02:255371:255682ATGTCCGGTAGAGGTAAAGGTGGTAAAGGTCTAGGTAAAGGTGGTGCCAAGCGTCACAGAAAGATTCTAAGAGATAACATCCAAGGTATTACTAAGCCAGCTATCAGAAGATTAGCTAGAAGAGGTGGTGTCAAGCGTATTTCTGGTTTGATCTACGAAGAAGTCAGAGCTGTCTTGAAATCCTTCTTGGAATCCGTCATCAGAGACTCTGTTACCTACACCGAACACGCCAAGAGAAAGACTGTTACTTCTTTGGATGTTGTTTATGCTTTGAAGAGACAAGGTAGAACCTTATACGGTTTCGGTGGTTAA>YBR010W	+:0:chr02:256329:256739ATGGCCAGAACAAAGCAAACAGCAAGAAAGTCCACTGGTGGTAAGGCCCCAAGAAAGCAATTAGCTTCTAAGGCTGCCAGAAAATCCGCCCCATCTACCGGTGGTGTTAAGAAGCCTCACAGATATAAGCCAGGTACTGTTGCTTTGAGAGAAATCAGAAGATTCCAAAAATCTACTGAACTGTTGATCAGAAAGTTGCCTTTCCAAAGATTGGTCAGAGAAATCGCTCAAGATTTCAAGACCGACTTGAGATTTCAATCTTCTGCCATCGGTGCCTTGCAAGAATCTGTCGAAGCCTACTTAGTCTCTTTATTTGAAGATACCAACTTGGCTGCCATTCACGCCAAGCGTGTCACTATCCAAAAGAAGGATATCAAGTTGGCTAGAAGATTAAGAGGTGAAAGATCATAG>YBR011C	-:0:chr02:257110:257973ATGACCTACACTACCAGACAAATTGGTGCCAAGAACACCTTGGAATACAAAGTTTATATCGAAAAGGATGGTAAGCCAGTTTCTGCCTTCCACGACATTCCCTTGTACGCTGACAAGGAAAACAACATTTTCAACATGGTTGTTGAAATTCCACGTTGGACCAACGCCAAGTTAGAAATCACCAAGGAAGAAACTTTGAACCCAATCATCCAAGACACCAAGAAGGGCAAGTTGAGATTTGTTAGAAACTGTTTCCCTCACCATGGTTACATTCACAACTATGGTGCTTTCCCACAAACTTGGGAAGACCCAAACGTAAGCCACCCAGAAACTAAGGCAGTTGGTGACAACGATCCAATTGATGTGTTGGAAATTGGTGAAACTATTGCTTACACTGGTCAAGTCAAGCAAGTTAAGGCTCTAGGTATCATGGCTTTATTGGATGAAGGTGAGACCGATTGGAAAGTTATTGCCATTGATATTAACGATCCATTAGCCCCAAAATTGAACGACATTGAGGATGTTGAGAAATACTTCCCAGGTCTGTTGAGGGCTACTAACGAATGGTTCAGAATTTACAAAATCCCAGATGGTAAGCCAGAAAACCAATTTGCCTTCTCCGGTGAAGCTAAGAACAAGAAGTACGCTTTGGATATCATCAAGGAAACACATGACTCCTGGAAACAATTAATTGCTGGTAAGTCTTCTGACAGCAAGGGTATTGATTTGACCAATGTTACTTTGCCTGACACCCCAACCTACTCCAAGGCTGCCTCTGATGCCATCCCACCAGCTTCTCCAAAGGCAGATGCTCCAATTGACAAGTCTATTGACAAGTGGTTCTTCATCTCCGGTTCTGTTTAA>YBR014C	-:0:chr02:266725:267336ATGGCTATTGTTATAAACAAAAGAAACGTGAGAGTCTTGGTAATAACTAATTTACTGCTCATTGTTGTGTTTTTTGTGTTAAGGAATTCGAATGCTAGCGTCAACGAAAGTATTACTACTCACCATCCTGATTCATTGGTGACGTTTGACAATTCAGGAAATGCACCTGGCACTCACCAATCTGTCCATGATACAGTAAATACACAAGATAAGGAAGCCGAAGAAGTTGATAAAAATAGTGGGGACGCTGAATTTGATGCCGCTGCAGAATACAACAAAATAATGGAACAGTCACCCATGATTGTATTTAGCAAGACTGGCTGCCCATATAGCAAAAAACTGAAAGCTTTGTTGACTAATTCGTACACGTTTTCTCCATCTTACTACGTTGTAGAATTGGATAGGCACGAACACACAAAAGAACTACAAGACCAGATTGAAAAAGTCACTGGTAGGAGAACAGTCCCAAACGTTATCATCGGTGGTACTTCCAGAGGTGGTTATACTGAGATAGCAGAGTTACATAAAAATGATGAACTTCTAGACTCTTTCAAAAAATGGAGCGATGGGGCGTTTACTGTAAAAGCTAATTCGCAATCTGAGAGTGCCTAG>YBR016W	+:0:chr02:270247:270633ATGTCTGCTAACGATTACTACGGCGGAACCGCCGGCGAGAAGAGCCAATATTCCCGTCCCTCCAACCCGCCACCTTCATCCGCTCATCAGAACAAAACTCAGGAGCGTGGATACCCACCCCAACAACAGCAGCAGTACTACCAGCAGCAGCAGCAGCATCCCGGATACTATAACCAACAAGGATACAATCAGCAAGGGTACAATCAGCAAGGGTACAATCAGCAGGGCTACAATCAGCAGGGATATAACCAGCAGGGATATAACCAACAAGGCCATCAACAACCAGTCTACGTCCAACAACAACCACCCCAGAGGGGTAACGAAGGTTGTCTGGCTGCATGTCTGGCTGCATTATGTATATGCTGCACCATGGATATGCTATTCTAA>YBR017C	-:0:chr02:270947:273703ATGGCATCGACATGGAAGCCCGCCGAAGACTATGTGTTGCAACTAGCAACTCTTTTACAGAACTGTATGTCACCAAATCCAGAGATTCGTAATAACGCAATGGAAGCTATGGAGAACTTCCAGCTGCAACCTGAATTTCTCAATTATTTGTGTTATATTTTAATTGAAGGCGAATCTGATGATGTATTGAAGCAACACTACTCCCTACAGGATCTTCAGAACAATAGAGCTACCGCCGGTATGCTGTTGAAAAATTCAATGCTAGGGGGAAACAATTTAATTAAGAGCAATAGCCACGACTTAGGATACGTCAAATCAAACATTATACATGGCCTTTATAATTCGAACAATAATCTCGTTTCGAACGTGACAGGTATCGTTATTACTACTTTATTTTCCACTTACTATAGGCAGCATAGAGATGATCCAACTGGTCTTCAAATGCTTTACCAGTTGCTAGAGCTAACCTCAAATGGAAATGAGCCAAGTATTAAGGCTTTATCTAAGATCATGGAAGACAGCGCTCAATTTTTCCAATTGGAATGGTCGGGAAATACGAAGCCTATGGAAGCCTTATTGGATAGTTTTTTTAGGTTTATTTCGAATCCAAATTTCTCACCTGTGATTCGCTCAGAATCGGTGAAATGTATAAATACAGTGATCCCGCTACAAACACAAAGTTTTATTGTGAGATTAGATAAATTCTTAGAAATTATTTTTCAGTTGGCACAAAACGACGAAAACGACCTAGTTAGGGCACAGATTTGCATTAGTTTTAGTTTCTTATTGGAATTCAGACCAGATAAGCTGGTTTCCCATTTAGATGGTATTGTACAATTCATGTTGCATTTGATCACCACTGTAAATGAGGAAAAAGTGGCTATTGAAGCCTGCGAGTTTTTGCACGCCTTTGCAACGAGCCCAAATATTCCTGAACATATCTTACAACCATATGTTAAGGATATCGTGCCAATATTATTATCGAAAATGGTCTATAACGAAGAATCCATCGTTCTCCTGGAAGCTTCTAATGATGATGATGCATTCTTGGAGGATAAAGATGAGGACATCAAGCCCATTGCACCCCGTATTGTGAAAAAGAAAGAGGCAGGAAATGGAGAGGATGCAGATGACAACGAAGATGATGATGATGATGATGATGATGAAGATGGCGATGTTGATACGCAATGGAATTTGAGAAAATGTTCCGCGGCAACGCTAGATGTAATGACGAATATTTTACCTCATCAAGTGATGGATATAGCGTTCCCATTTTTAAGAGAACATTTGGGTTCTGATAGGTGGTTTATTAGAGAAGCTACTATATTAGCACTGGGGGCCATGGCAGAAGGTGGAATGAAGTATTTTAATGATGGCTTACCAGCACTAATACCATTTTTAGTGGAACAATTGAACGATAAGTGGGCACCAGTGAGGAAAATGACATGTTGGACATTAAGTAGGTTTTCACCATGGATATTACAAGACCATACTGAGTTTTTAATTCCAGTCTTAGAACCTATAATAAACACATTAATGGACAAGAAAAAGGATGTTCAAGAGGCGGCTATTAGTAGTGTAGCAGTATTTATTGAAAACGCCGACTCCGAATTGGTTGAAACTTTATTTTATAGTCAATTATTGACGAGTTTTGATAAATGTTTGAAATATTACAAGAAAAAGAATTTAATTATATTATATGATGCCATCGGCAGGTTTGCTGAAAAATGTGCATTAGACGAGACAGCGATGCAAATAATTTTGCCGCCCTTAATTGAAAAATGGGCTTTGCTGTCAGACAGTGACAAGGAGCTGTGGCCACTTTTAGAATGTCTTTCCTGCGTGGCATCATCACTGGGGGAAAGATTCATGCCTATGGCACCAGAAGTGTACAACAGAGCCTTTAGAATTCTATGTCATTGTGTCGAATTGGAAGCCAAATCACATCAAGACCCGACAATAGTAGTGCCTGAGAAGGACTTCATCATCACCTCATTAGATTTGATTGATGGATTGGTACAAGGTCTTGGCGCTCACTCGCAGGATCTATTGTTCCCTCAAGGGACGAAGGATTTAACGATATTGAAAATCATGCTAGAATGTTTGCAGGACCCTGTCCATGAAGTAAGACAAAGCTGCTTTGCCCTGTTGGGAGATATTGTATATTTTTTCAATTCGGAACTGGTAATTGGTAATTTGGAGGATTTCTTGAAGTTGATTGGTACGGAAATAATGCATAACGACGATAGTGATGGTACTCCTGCTGTGATAAATGCGATATGGGCGCTTGGTTTGATAAGCGAACGTATCGATTTGAATACTTATATCATTGATATGTCTAGAATCATTCTAGATTTATTTACCACCAACACACAAATCGTAGACAGCTCTGTGATGGAGAACTTGTCTGTGACCATCGGAAAAATGGGGCTAACACACCCTGAAGTTTTCAGTTCTGGCGCATTTGCCAATGATTCCAACTGGAATAAATGGTGTTTGTCCGTTAACGCATTGGACGATGTAGAGGAAAAGAGTAGCGCGTACATGGGTTTCCTGAAAATTATCAATTTGACCAGCACGGAGGTCACAATGAGTAATGATACCATTCATAAAATTGTTACGGGCCTTTCAAGCAATGTAGAGGCGAATGTTTTTGCGCAAGAGATCTACACCTTTTTGATGAACCATTCTGCCCAAATTTCTGCAATAAATTTCACGCCCGATGAAATCTCCTTCTTACAACAGTTCACCAGCTAA>YBR018C	-:0:chr02:274427:275527ATGACTGCTGAAGAATTTGATTTTTCTAGCCATTCCCATAGACGTTACAATCCACTAACCGATTCATGGATCTTAGTTTCTCCACACAGAGCTAAAAGACCTTGGTTAGGTCAACAGGAGGCTGCTTACAAGCCCACAGCTCCATTGTATGATCCAAAATGCTATCTATGTCCTGGTAACAAAAGAGCTACTGGTAACCTAAACCCAAGATATGAATCAACGTATATTTTCCCCAATGATTATGCTGCCGTTAGGCTCGATCAACCTATTTTACCACAGAATGATTCCAATGAGGATAATCTTAAAAATAGGCTGCTTAAAGTGCAATCTGTGAGAGGCAATTGTTTCGTCATATGTTTTAGCCCCAATCATAATCTAACCATTCCACAAATGAAACAATCAGATCTGGTTCATATTGTTAATTCTTGGCAAGCATTGACTGACGATCTCTCCAGAGAAGCAAGAGAAAATCATAAGCCTTTCAAATATGTCCAAATATTTGAAAACAAAGGTACAGCCATGGGTTGTTCCAACTTACATCCACATGGCCAAGCTTGGTGCTTAGAATCCATCCCTAGTGAAGTTTCGCAAGAATTGAAATCTTTTGATAAATATAAACGTGAACACAATACTGATTTGTTTGCCGATTACGTCAAATTAGAATCAAGAGAGAAGTCAAGAGTCGTAGTGGAGAATGAATCCTTTATTGTTGTTGTTCCATACTGGGCCATCTGGCCATTTGAGACCTTGGTCATTTCAAAGAAGAAGCTTGCCTCAATTAGCCAATTTAACCAAATGGTGAAGGAGGACCTCGCCTCGATTTTAAAGCAACTAACTATTAAGTATGATAATTTATTTGAAACGAGTTTCCCATACTCAATGGGTATCCATCAGGCTCCTTTGAATGCGACTGGTGATGAATTGAGTAATAGTTGGTTTCACATGCATTTCTACCCACCTTTACTGAGATCAGCTACTGTTCGGAAATTCTTGGTTGGTTTTGAATTGTTAGGTGAGCCTCAAAGAGATTTAACTTCGGAACAAGCTGCTGAAAAACTAAGAAATTTAGATGGTCAGATTCATTATCTACAAAGACTGTAA>YBR019C	-:0:chr02:276253:278352ATGACAGCTCAGTTACAAAGTGAAAGTACTTCTAAAATTGTTTTGGTTACAGGTGGTGCTGGATACATTGGTTCACACACTGTGGTAGAGCTAATTGAGAATGGATATGACTGTGTTGTTGCTGATAACCTGTCGAATTCAACTTATGATTCTGTAGCCAGGTTAGAGGTCTTGACCAAGCATCACATTCCCTTCTATGAGGTTGATTTGTGTGACCGAAAAGGTCTGGAAAAGGTTTTCAAAGAATATAAAATTGATTCGGTAATTCACTTTGCTGGTTTAAAGGCTGTAGGTGAATCTACACAAATCCCGCTGAGATACTATCACAATAACATTTTGGGAACTGTCGTTTTATTAGAGTTAATGCAACAATACAACGTTTCCAAATTTGTTTTTTCATCTTCTGCTACTGTCTATGGTGATGCTACGAGATTCCCAAATATGATTCCTATCCCAGAAGAATGTCCCTTAGGGCCTACTAATCCGTATGGTCATACGAAATACGCCATTGAGAATATCTTGAATGATCTTTACAATAGCGACAAAAAAAGTTGGAAGTTTGCTATCTTGCGTTATTTTAACCCAATTGGCGCACATCCCTCTGGATTAATCGGAGAAGATCCGCTAGGTATACCAAACAATTTGTTGCCATATATGGCTCAAGTAGCTGTTGGTAGGCGCGAGAAGCTTTACATCTTCGGAGACGATTATGATTCCAGAGATGGTACCCCGATCAGGGATTATATCCACGTAGTTGATCTAGCAAAAGGTCATATTGCAGCCCTGCAATACCTAGAGGCCTACAATGAAAATGAAGGTTTGTGTCGTGAGTGGAACTTGGGTTCCGGTAAAGGTTCTACAGTTTTTGAAGTTTATCATGCATTCTGCAAAGCTTCTGGTATTGATCTTCCATACAAAGTTACGGGCAGAAGAGCAGGTGATGTTTTGAACTTGACGGCTAAACCAGATAGGGCCAAACGCGAACTGAAATGGCAGACCGAGTTGCAGGTTGAAGACTCCTGCAAGGATTTATGGAAATGGACTACTGAGAATCCTTTTGGTTACCAGTTAAGGGGTGTCGAGGCCAGATTTTCCGCTGAAGATATGCGTTATGACGCAAGATTTGTGACTATTGGTGCCGGCACCAGATTTCAAGCCACGTTTGCCAATTTGGGCGCCAGCATTGTTGACCTGAAAGTGAACGGACAATCAGTTGTTCTTGGCTATGAAAATGAGGAAGGGTATTTGAATCCTGATAGTGCTTATATAGGCGCCACGATCGGCAGGTATGCTAATCGTATTTCGAAGGGTAAGTTTAGTTTATGCAACAAAGACTATCAGTTAACCGTTAATAACGGCGTTAATGCGAATCATAGTAGTATCGGTTCTTTCCACAGAAAAAGATTTTTGGGACCCATCATTCAAAATCCTTCAAAGGATGTTTTTACCGCCGAGTACATGCTGATAGATAATGAGAAGGACACCGAATTTCCAGGTGATCTATTGGTAACCATACAGTATACTGTGAACGTTGCCCAAAAAAGTTTGGAAATGGTATATAAAGGTAAATTGACTGCTGGTGAAGCGACGCCAATAAATTTAACAAATCATAGTTATTTCAATCTGAACAAGCCATATGGAGACACTATTGAGGGTACGGAGATTATGGTGCGTTCAAAAAAATCTGTTGATGTCGACAAAAACATGATTCCTACGGGTAATATCGTCGATAGAGAAATTGCTACCTTTAACTCTACAAAGCCAACGGTCTTAGGCCCCAAAAATCCCCAGTTTGATTGTTGTTTTGTGGTGGATGAAAATGCTAAGCCAAGTCAAATCAATACTCTAAACAATGAATTGACGCTTATTGTCAAGGCTTTTCATCCCGATTCCAATATTACATTAGAAGTTTTAAGTACAGAGCCAACTTATCAATTTTATACCGGTGATTTCTTGTCTGCTGGTTACGAAGCAAGACAAGGTTTTGCAATTGAGCCTGGTAGATACATTGATGCTATCAATCAAGAGAACTGGAAAGATTGTGTAACCTTGAAAAACGGTGAAACTTACGGGTCCAAGATTGTCTACAGATTTTCCTGA>YBR020W	+:0:chr02:279021:280607ATGACTAAATCTCATTCAGAAGAAGTGATTGTACCTGAGTTCAATTCTAGCGCAAAGGAATTACCAAGACCATTGGCCGAAAAGTGCCCGAGCATAATTAAGAAATTTATAAGCGCTTATGATGCTAAACCGGATTTTGTTGCTAGATCGCCTGGTAGAGTCAATCTAATTGGTGAACATATTGATTATTGTGACTTCTCGGTTTTACCTTTAGCTATTGATTTTGATATGCTTTGCGCCGTCAAAGTTTTGAACGAGAAAAATCCATCCATTACCTTAATAAATGCTGATCCCAAATTTGCTCAAAGGAAGTTCGATTTGCCGTTGGACGGTTCTTATGTCACAATTGATCCTTCTGTGTCGGACTGGTCTAATTACTTTAAATGTGGTCTCCATGTTGCTCACTCTTTTCTAAAGAAACTTGCACCGGAAAGGTTTGCCAGTGCTCCTCTGGCCGGGCTGCAAGTCTTCTGTGAGGGTGATGTACCAACTGGCAGTGGATTGTCTTCTTCGGCCGCATTCATTTGTGCCGTTGCTTTAGCTGTTGTTAAAGCGAATATGGGCCCTGGTTATCATATGTCCAAGCAAAATTTAATGCGTATTACGGTCGTTGCAGAACATTATGTTGGTGTTAACAATGGCGGTATGGATCAGGCTGCCTCTGTTTGCGGTGAGGAAGATCATGCTCTATACGTTGAGTTCAAACCGCAGTTGAAGGCTACTCCGTTTAAATTTCCGCAATTAAAAAACCATGAAATTAGCTTTGTTATTGCGAACACCCTTGTTGTATCTAACAAGTTTGAAACCGCCCCAACCAACTATAATTTAAGAGTGGTAGAAGTCACTACAGCTGCAAATGTTTTAGCTGCCACGTACGGTGTTGTTTTACTTTCTGGAAAAGAAGGATCGAGCACGAATAAAGGTAATCTAAGAGATTTCATGAACGTTTATTATGCCAGATATCACAACATTTCCACACCCTGGAACGGCGATATTGAATCCGGCATCGAACGGTTAACAAAGATGCTAGTACTAGTTGAAGAGTCTCTCGCCAATAAGAAACAGGGCTTTAGTGTTGACGATGTCGCACAATCCTTGAATTGTTCTCGCGAAGAATTCACAAGAGACTACTTAACAACATCTCCAGTGAGATTTCAAGTCTTAAAGCTATATCAGAGGGCTAAGCATGTGTATTCTGAATCTTTAAGAGTCTTGAAGGCTGTGAAATTAATGACTACAGCGAGCTTTACTGCCGACGAAGACTTTTTCAAGCAATTTGGTGCCTTGATGAACGAGTCTCAAGCTTCTTGCGATAAACTTTACGAATGTTCTTGTCCAGAGATTGACAAAATTTGTTCCATTGCTTTGTCAAATGGATCATATGGTTCCCGTTTGACCGGAGCTGGCTGGGGTGGTTGTACTGTTCACTTGGTTCCAGGGGGCCCAAATGGCAACATAGAAAAGGTAAAAGAAGCCCTTGCCAATGAGTTCTACAAGGTCAAGTACCCTAAGATCACTGATGCTGAGCTAGAAAATGCTATCATCGTCTCTAAACCAGCATTGGGCAGCTGTCTATATGAATTATAA>YBR021W	+:0:chr02:281443:283344ATGCCAGACAATCTATCATTACATTTAAGCGGCTCTTCAAAAAGATTGAACTCTCGCCAACTTATGGAATCTTCCAATGAGACCTTTGCGCCAAATAATGTGGATTTGGAAAAAGAGTATAAGTCATCTCAGAGTAATATAACTACCGAAGTTTATGAGGCATCGAGCTTTGAAGAAAAAGTAAGCTCAGAAAAACCTCAATACAGCTCATTCTGGAAGAAAATCTATTATGAATATGTGGTCGTTGACAAATCAATCTTGGGTGTTTCTATTCTGGATTCATTTATGTACAACCAGGACTTGAAGCCCGTCGAAAAAGAAAGGCGGGTTTGGTCCTGGTACAATTATTGTTACTTCTGGCTTGCTGAATGTTTCAATATCAACACTTGGCAAATTGCAGCTACAGGTCTACAACTGGGTCTAAATTGGTGGCAGTGTTGGATAACAATTTGGATTGGGTACGGTTTCGTTGGTGCTTTTGTTGTTTTGGCCTCTAGAGTTGGATCTGCTTATCATTTGTCATTCCCTATATCATCTAGAGCATCATTCGGTATTTTCTTCTCTTTATGGCCCGTTATTAACAGAGTCGTCATGGCCATCGTTTGGTATAGTGTCCAAGCTTATATTGCGGCAACTCCCGTATCATTAATGCTGAAATCTATCTTTGGAAAAGATTTACAAGACAAAATCCCAGATCACTTTGGATCACCGAATGCTACTACTTACGAGTTCATGTGTTTTTTTATCTTTTGGGCTGCCAGTCTTCCATTTTTACTGGTTCCACCTCACAAAATTAGACACCTGTTTACTGTTAAAGCCGTCTTGGTTCCGTTTGCTTCTTTTGGTTTCTTAATTTGGGCTATCAGAAGAGCTCACGGGCGTATTGCCTTAGGATCTTTAACCGATGTACAACCTCATGGCTCTGCCTTTTCGTGGGCTTTTCTAAGATCACTAATGGGTTGTATGGCTAATTTCTCCACAATGGTAATCAACGCTCCAGATTTCTCTAGATTTTCAAAAAACCCTAACTCCGCTTTATGGTCCCAATTAGTGTGCATTCCATTTTTGTTTTCCATCACTTGTTTAATTGGAATTCTAGTCACCGCAGCTGGTTATGAAATATACGGTATTAATTACTGGTCGCCACTCGATGTGCTAGAAAAATTTTTACAGACTACTTATAATAAGGGCACAAGAGCTGGTGTTTTCTTAATCTCTTTTGTTTTCGCCGTAGCTCAGTTAGGTACTAATATTTCTGCAAACTCATTATCGTGTGGAACTGATATGTCCGCTATTTTCCCCAAGTTTATCAATATCAAGCGTGGTTCATTATTTTGTGCAGCCATGGCGCTGTGTATTTGTCCATGGAATTTAATGGCAACATCAAGTAAATTTACAATGGCTTTGTCCGCATATGCTATCTTTTTGTCCAGTATTGCCGGTGTTGTCTGCTCAGATTACTTTGTTGTTAGAAGAGGATATATCAAGCTAACACACATATATTCCCATCAAAAGGGCTCTTTTTATATGTACGGAAACAGGTTTGGTATCAATTGGAGAGCTTTGGCTGCATATCTATGTGGGGTGGCTCCTTGCTTGCCCGGTTTCATCGCGGAGGTGGGCGCTCCCGCTATAAAGGTTTCTGATGGAGCTATGAAACTATATTACCTAAGTTACTGGGTAGGATATGGCTTGAGTTTTTCTTCTTATACTGCTCTGTGTTACTTCTTCCCTGTACCTGGGTGCCCTGTTAACAACATTATAAAGGACAAGGGCTGGTTCCAAAGATGGGCCAATGTCGATGATTTTGAAGAAGAGTGGAAAGACACAATTGAGAGGGATGACCTGGTAGATGACAATATTAGTGTCTACGAACACGAACACGAAAAGACTTTCATTTAA>YBR022W	+:0:chr02:283738:284271ATGTCGAACATCACTTATGTCAAAGGTAACATCTTAAAACCAAAGTCATATGCTAGGATCCTCATCCATTCTTGCAACTGCAATGGCTCGTGGGGTGGTGGGATCGCTTATCAACTTGCTTTGCGCTACCCAAAAGCAGAGAAAGATTACGTTGAAGTATGCGAAAAGTATGGTTCTAATTTACTAGGCAAATGCATATTACTTCCCAGTTACGAAAACTCAGATCTGCTGATTTGTTGCTTGTTTACATCATCATTTGGAGGTTCAAGTCATGGAGAAAAGCAAAGTATTTTGAATTATACAAAACTTGCTTTAGATAAATTGAAGACCTTTAGAGAAGCAAAAGACAAAACTCGAACAAGTGAAGATTCAATCGGTGACTATTTAAACGGCCACATCAAATACCCAATAGGGGAATATAAATTGGAAATGCCACAGATAAATAGTGGCATATTCGGAGTCCCTTGGAAAGAGACAGAACGCGTGTTAGAAGAATTTAGTGGCGACATGAGTTTTACAGTATATCAGCTGTAA>YBR024W	+:0:chr02:289445:290350ATGTTGAATAGTTCAAGAAAATATGCTTGTCGTTCCCTATTCAGACAAGCGAACGTCTCAATAAAAGGACTCTTTTATAATGGAGGCGCATATCGAAGAGGGTTTTCAACGGGATGTTGTTTGAGGAGTGATAACAAGGAAAGCCCAAGTGCAAGACAACCACTAGATAGGCTACAACTAGGTGATGAAATCAATGAACCAGAGCCTATTAGAACCAGGTTTTTTCAATTTTCCAGATGGAAGGCCACCATTGCTCTATTGTTGCTAAGTGGTGGGACGTATGCCTATTTATCAAGAAAAAGACGCTTGCTAGAAACTGAAAAGGAAGCAGATGCTAACAGAGCTTACGGTTCAGTAGCACTTGGCGGTCCTTTCAATTTAACAGATTTTAATGGTAAGCCTTTCACTGAGGAGAATTTGAAGGGTAAGTTTTCCATTTTATACTTTGGATTCAGTCATTGCCCCGACATTTGTCCAGAAGAGCTTGACAGATTAACGTATTGGATTTCTGAATTAGATGATAAAGACCATATAAAGATACAGCCATTGTTTATCTCATGTGATCCTGCAAGAGATACACCGGATGTCTTGAAAGAGTACTTAAGCGATTTTCACCCAGCTATCATTGGTTTAACCGGTACGTACGACCAAGTGAAAAGCGTATGCAAAAAATACAAGGTATATTTTTCAACTCCACGTGATGTCAAGCCCAACCAGGATTACTTAGTGGACCATTCGATATTTTTCTATTTGATCGACCCTGAAGGACAGTTTATCGATGCGTTGGGAAGAAACTACGATGAGCAATCTGGTCTCGAAAAGATTCGTGAACAAATTCAGGCGTATGTGCCAAAGGAAGAACGGGAGCGTAGGTCAAAAAAATGGTACTCTTTTATCTTCAATTGA>YBR025C	-:0:chr02:290681:291865ATGCCTCCAAAGAAGCAAGTCGAAGAAAAAAAGGTCTTATTGGGTCGTCCAGGTAATAACTTGAAAGCCGGTATTGTCGGTTTGGCCAATGTTGGTAAGTCTACCTTTTTCCAAGCCATCACTAGATGTCCATTGGGTAACCCAGCTAACTATCCATTCGCTACCATTGATCCAGAAGAAGCCCGTGTTATTGTCCCATCTCCAAGATTTGATAAGTTGTGTGAAATCTACAAGAAGACAGCTTCGGAAGTTCCAGCTCATTTGACCGTTTACGATATTGCTGGTTTGACTAAGGGTGCCTCTGCTGGTGAAGGTTTGGGTAATGCTTTCTTGTCTCACATCAGATCAGTCGATTCTATCTACCAAGTCGTTCGTTGTTTCGATGATGCTGAAATTATCCACGTTGAGGGTGACGTTGATCCAGTTCGTGATTTAGAAATTATTAACCAAGAACTAAGATTGAAAGATATTGAATTCGCACAAAAGGCTTTGGAAGGTGCTGAAAAGATTGCCAAAAGAGGTGGTCAATCTTTGGAAGTCAAACAAAAGAAGGAAGAAATGGATTTGATTACGAAAATCATTAAATTGCTAGAGAGTGGTCAAAGAGTTGCTAATCACTCCTGGACTTCAAAAGAAGTTGAAATTATCAACTCCATGTTCTTGTTGACTGCTAAGCCATGTATCTATTTGATTAATTTATCTGAAAGAGATTACATCAGAAAGAAAAACAAGCATCTGCTAAGAATCAAGGAATGGGTAGACAAGTACTCTCCAGGTGACTTGATCATTCCATTCAGTGTTTCTCTAGAAGAAAGACTATCTCATATGTCCCCAGAAGATGCTGAAGAAGAATTGAAGAAACTGCAGACAATATCTGCCTTGCCAAAGATTATCACTACCATGAGACAAAAGTTAGATTTGATTTCCTTTTTCACCTGCGGTCCAGATGAAGTTCGTGAATGGACCATCAGAAGAGGTACTAAAGCTCCACAAGCTGCTGGTGTTATTCATAACGATTTAATGAATACCTTTATTTTGGCTCAAGTTATGAAATGTGAAGATGTCTTCGAATATAAGGACGATTCTGCCATCAAGGCCGCTGGTAAGTTGATGCAAAAGGGTAAAGACTATGTCGTTGAAGACGGTGATATCATTTACTTCAGAGCTGGTGCTGGTAAGAATTGA>YBR026C	-:0:chr02:292877:294019ATGCTTCCCACATTCAAACGTTACATGTCGTCCTCAGCTCATCAGATTCCCAAGCACTTCAAATCGCTCATCTATTCAACTCATGAAGTTGAGGATTGTACCAAGGTTTTGTCAGTGAAAAATTATACGCCTAAACAAGACTTATCTCAATCAATTGTGTTAAAAACTTTGGCCTTTCCCATAAACCCTTCGGATATCAATCAGTTGCAAGGAGTATACCCGTCTCGTCCAGAAAAGACATACGATTACTCCACAGATGAGCCAGCCGCTATCGCCGGTAATGAGGGTGTCTTTGAAGTTGTTTCTTTACCTTCGGGAAGTTCCAAGGGAGATTTGAAATTGGGTGACCGAGTTATCCCATTGCAGGCAAATCAAGGGACTTGGTCCAATTATAGAGTTTTCTCTAGTAGTTCTGATTTAATCAAGGTAAATGATTTGGATCTGTTTTCTGCGGCAACTGTATCTGTTAATGGTTGTACCGGTTTCCAATTAGTATCAGACTATATCGACTGGAACAGTAACGGTAATGAATGGATTATCCAAAATGCCGGTACATCTAGTGTATCAAAAATAGTTACGCAAGTAGCAAAAGCTAAAGGGATCAAAACATTAAGTGTTATACGTGACCGTGATAATTTTGATGAGGTAGCAAAAGTTTTGGAGGATAAGTATGGTGCTACGAAGGTTATTTCCGAATCGCAAAACAACGACAAGACTTTTGCCAAAGAAGTATTGTCCAAGATTTTGGGTGAAAATGCAAGGGTGAGGCTTGCCTTGAATTCTGTTGGAGGTAAATCCAGTGCATCAATAGCACGTAAGTTGGAAAATAATGCTTTGATGCTCACTTATGGAGGAATGTCAAAACAACCTGTAACTTTACCAACATCTCTACACATTTTCAAAGGCTTGACATCCAAAGGGTACTGGGTGACTGAAAAGAACAAAAAAAACCCCCAAAGCAAGATTGACACCATCAGTGATTTTATCAAAATGTATAATTATGGTCACATTATTTCACCAAGAGATGAAATTGAAACTCTTACCTGGAATACTAACACTACTACTGACGAACAGTTACTAGAACTAGTCAAAAAAGGTATAACTGGGAAGGGGAAGAAAAAAATGGTTGTTTTAGAATGGTAA>YBR028C	-:0:chr02:294425:296002ATGATTTTCTCACTAGATGAAGAACTTCATCGTGTGTCACTAGATGATAAAAAGAATGACATTAAAGTAGATTATTCCTCGGCAATATACAATGATATCAATCATGAACAAGGCAGCTCCATCACATATGAGGAGAGTATAAATCACCTCTCGGTTCATTCAAATGCGATTCCGCTAAATGGCATGAGTCCTGCACATAGGATGAGAAGAAGGTCCTCAGCTTATTCCAAATTCCCTATTCTCACTCCACCAAATACGAGAAGATTTTCTATTACAGGTTCGGACGCAATGCGAACTAATACGAATAGACTATCCATAACACCTCAGGACATTATTTCCTCTAATATTGGAGAAAATGAATTATCAAGGAACCTGCACGATTTTAAGCCCGTGAGAGTCCTAGGCCAAGGTGCCTACGGTAAAGTTCTTCTTGTTAAGGACGTCAATACATCCAAGCTGTATGCTATGAAGCAATTACGGAAAGCAGAAATTTTAATTTCTCAAACAGCCACAGATTCCAAGAGAGAAGACGAAGATAAAAATGATGGTAATAACAACGATAATGACGATGGATTATCAAAGAGGCTTGAGAGAACATTTGCCGAACGGTCTATTCTATCTGAAATAGAACATCCAAACATTGTCAAGTTATTTTATTCTTTTCACGATAACTCAAAGTTATATTTACTGCTACAATATATCCCTGGCGGTGAACTATTTTACCACTTGAAGGAACACGGGACCCTAGATGAGACGACAGTTTCGTTTTACGCGGCAGAAATCAGCTGTGCCTTGAGATTTTTGCATACGAAAGGTGTTGTCTATAGAGATTTAAAACCTGAAAACTGTTTATTGAATCAACGTGGACATTTAGTATTGACAGATTTTGGTCTGAGTAAAAAAAGTGCTAATGATTCCGCGGTTGATGAGGAGGATCCAGAGAACGTCAATGCCCTATACTCGATTATCGGTACGCCTGAATACTGTGCGCCTGAAATATTATTAGGTAAAGCTTACAGTCAGAATTGTGATTGGTATTCTTTGGGATGTCTGTTGTATGACATGTTAGTAGGTAAGCCTCCATATACTGGTAGCAATCATAAGGTGATCATTAATAAGATTCAACAAAACAAGCAAGGCCCCAAAATTCCATTTTATCTGAGCGAGGGAATGAAAGATATATTAAACGCGCTGTTGAAGAAGGAAACTGCCAAAAGGTGGAATGTTGACAAATACTGGGCCAAAACCGGGGCTAATAATAAGCCTACTAAGTCTAAGAAGAAGAAATCAGGGGCTGCAAGAACTAGTCTTTTCACTGAACATTTTATCTTTAGAAAAATAGACTGGAAGTTATTAGAATCTGGACAATTGCAAAAAACCACTCTAGGCCCAATTGTTCCAGTTATTACGGATTTGGAACTAGCGGAAAATTTTGACACAGAATTTACTTCTATGTCATATGAGGAAACCTATACGGATAGCAAGCCGATTAATATCAATTCGGTGAGCAAGTCCCCGGATATGTTCAAGGGATTTAGTTATAAAGCAAGTGGTAGCTATTTGGAGAAGTACTTTTGA>YBR030W	+:0:chr02:298292:299950ATGTCCGTAACTTTTAAGGACGATGTTCATCGGATTCTGAAGTTTGTTGCTAATTGCAATGGCAGGTTTGAAGATTCCAAATGTGATATAAGAGAATCGCCTCTCGGTGGATTGGGTGTCTTTGCAAAGACTGATATTGCAGAAGGTGAGTCAATATTGACGTTGAACAAATCCTCGATTTTTTCTGCATCTAACAGCTCCATTGCCAATTTGTTATGTGACAGTAGCATCGATGGAATGTTAGCTCTAAACATCGCATTCATTTACGAAACTACAGTTTTCAGAAATTCTAGTCATTGGTACCCATTTCTGCGAACTATCCGAATTCGTGATGACGAAGGGCACTTAAACCTGCCGCCAAGCTTTTGGCATGCGGATGCTAAGCGACTTTTAAAAGGAACCAGTTTCGATACCTTATTCGATTCGTTGGCACCAGAAGAAGAAATCATGGAAGGATTTGAAATTGCTGTGGATTTGGCACACAAATGGAACGACGAGTTTGGGTTGGAAATTCCAAAAGGCTTCCTAGACGTTAGTGAGGAGAATCATGAAGAAGATTACAATTTGAAATTAGAAAAGTTTATATCGGTGGCATATACTTTATCATCAAGAGGATTTGAAATTGACGCGTACCATGAGACAGCTTTGGTGCCCATCGCAGACCTGTTCAATCATCATGTTTCCGACCCGGATTTAAAGTTTGTATCGCTATATGATGTGTGCGATAAATGTGGTGAACCAGACATGTGTAAACATCTCATAGCAGAGGAATACTTGGAAGCAGAAAATCTAGACAAAAATATGCCCAAAGTCGCAAGTATGGAGACTCGTGTCATCGATGAAGACTTAATCAAAAGTTTAGAAAATGATTTAGAGAAAGAATACTCTAATGTTACGGCAAACATCGAGGATGATGATGGCGGTATTGAAAACCCTGACGAATGTGTCGATTTAGTGTTAAAGAATGACGTCGCACAAGGCCAAGAAATATTTAATTCCTATGGTGAGCTCTCAAACGTCTTTTTGTTGGCCAGATATGGTTTCACCGTACCTGAGAATCAGTATGATATTGTTCATTTAGGACCTGATTTTATGAAAATCTTAAAGAAAGAAGAAAAATATCAAGAAAAAGTCAAGTGGTGGAGCCAAGTCGGTCATGGCTTGTTTTCAGCATGGTATGCTCAAATGCGCCAAGAAGATGAAGAAGACGAAGACGGTCAAGCAAAATCAGACAATCTGTCTGACGATATAGAGAGCGAAGAGGAAGAGGAAGAGGAAGAGGGTGATGATAGCTTGGAATCATGGCTTTCCCAGCTTTACATAGACTCAAGTGGCGAGCCTTCACCCTCAACGTGGGCCTTAGCTAACCTTTTGACTTTAACGGCCGTTCAATGGGAATCATTGTTTTCCAAAAAGGCCACTCCTCACATCAGTGATTCTATAGTTAACGAAGAAAAGCTGCCCTTCCTGGCCAAAAAAGACAATCCCCATTCCAAGAAACTGCTTTCCAACTTACTTAAGGAAAAGCAACTACCTTGCATAAAAGGCGACAACTCGTCAAAGATAACAAGTGCTACTAAAAGCATGCTCCAAAATGCTCGAACACTTGTACAATCGGAGCATAATATCTTAGATAGATGTCTCAAGAGACTATCCTAA>YBR031W	+:0:chr02:300166:301254ATGTCCCGTCCACAAGTTACTGTTCACTCTTTGACTGGTGAAGCTACTGCCAATGCCTTGCCATTGCCAGCTGTCTTCTCCGCTCCTATCCGTCCAGACATTGTCCACACTGTTTTCACCTCTGTGAACAAGAACAAGAGACAAGCTTACGCTGTTTCTGAAAAGGCTGGTCACCAAACCTCCGCTGAATCCTGGGGTACCGGTCGTGCCGTCGCTCGTATTCCAAGAGTTGGTGGTGGTGGTACCGGTAGATCCGGTCAAGGTGCCTTCGGTAACATGTGTCGTGGTGGTCGTATGTTTGCTCCAACTAAGACCTGGAGAAAGTGGAACGTTAAGGTTAACCACAACGAAAAGCGTTACGCCACTGCTTCTGCTATTGCTGCTACTGCTGTTGCCTCTTTGGTCTTGGCCAGAGGTCACAGAGTCGAAAAGATTCCAGAAATCCCATTGGTTGTCTCCACTGACTTGGAATCTATTCAAAAGACCAAGGAAGCTGTTGCTGCTTTGAAGGCTGTTGGTGCTCACTCCGACTTGTTGAAGGTCTTGAAGTCCAAGAAATTGAGAGCCGGTAAGGGTAAGTACAGAAACAGAAGATGGACTCAAAGAAGAGGTCCATTAGTTGTCTACGCTGAAGACAACGGTATCGTCAAGGCCTTGAGAAACGTTCCAGGTGTTGAAACTGCCAACGTTGCTTCTTTGAACTTGTTGCAATTGGCTCCAGGTGCTCACTTGGGTAGATTCGTTATCTGGACCGAAGCTGCTTTCACCAAGTTGGACCAAGTCTGGGGTTCCGAAACCGTTGCCTCCTCCAAGGTCGGCTACACTTTGCCATCCCATATCATCTCCACTTCTGATGTCACCAGAATTATCAACTCTTCCGAAATCCAATCTGCTATCAGACCAGCTGGCCAAGCTACTCAAAAGCGTACTCACGTTTTGAAGAAGAACCCATTGAAGAACAAGCAAGTCTTGTTGAGATTGAACCCTTACGCCAAGGTCTTTGCTGCTGAAAAGCTAGGTTCCAAGAAGGCTGAAAAGACTGGTACCAAGCCAGCTGCTGTTTTCACCGAAACTTTGAAACACGATTAA>YBR033W	+:0:chr02:301944:304703ATGTCACACCATGTTCCTAATTTATATGGTACACCTATTCGCGACCCTCACGAACGCAAACGCAATTCAGCGTCAATGGGCGAAGTAAATCAGTCAGTTTCTTCGAGGAATTGTGAAAGAGGAAGTGAAAAGGGCACGAAGCAAAGGAAGAAAGCCTCTCACGCTTGTGACCAATGTAGAAGGAAAAGAATCAAGTGCAGGTTTGATAAACATACCGGCGTATGTCAAGGGTGCTTAGAAGTCGGTGAAAAGTGTCAATTTATTAGAGTTCCATTGAAACGTGGTCCTGCAAAAAAAAGGGGCAGTGTAGTATCCATTGAAAAATTCAGCTCGGATAATGATCCTCTTCAATACCGACCAAGAACACATTCATACCCAATGAACTCGGGAAATAACTACTTACCATCACTCGCCAGGAATTCCTCTTTTCCTTCCATAAGCAGTCTATTTGTTCCCTCGATAACTGCTCAATCGCAACAATTTGTAAAGGTGCCGTACGATGACATCAAGCGTAGGAGCTCTCTGGCAATATTAGGCAGCGATTCATCGATATCTACCGAATTTGGGGGAAACTATCGCTTAGATGAAAACTTAAATGTCAGGCAAGAGGGGAAGGATATTGTAGCGAAAGGAATGATAACGCCCGTGGAGGAAATGGGCGCATGTTCATCTAACGTTCGTCGTCAGGGTTCACAATCTCTTCCAATTCAAGAACAGCGCGCTAGCCCGTACATTAACCCTTTTATCTCTGGGAGATCCAGGCTAAGCAGCCTTTCCTATACAAGTGAGGCTACAACTTCAGAAGGTAATACGCAAGGTAAAAACCAATGCATGCTTACTCCCAATAGTGTACGTTCCATTGAAAAGGAACGATTAAACTCGTTAACTGCGGGATTTCCGAACAAAAAATTAGGCACAGATGGTAGAAGTGACAAATGGGACAAAAATTCGACTTGGAAGCCTGTATATCGATCGTCGAATCCTTCTCATCCTAGCACTGAAAAAAATGTTTCCTTGAATCAGGAAGCCAGTGCTAAACCACTTATGCTTGGAACCTACAGACAGTTTGATGCAACCTCGTTCTACAAGGTTTTAGGCATCTACTATAACTTTTTCCATATTAATTTTCCTGTAATACCAATTAACAAAAGTAAATTTACTGACATGCTTGATCCTGAAAAGCCAAACGTGATTGATGAGATCAGACAAATTAACAATGAAATTATCCAGTGTTTTAAAACCGCGCTAGAGGTATTGGTATTTTGCAAAATAAAACAAAGAAGGTCATCGAAATCAACAAAATCATGGTCTCGTGACAGTTTATGCGATTTTCAAAAAGGTCTGTATTACATTCAGAATTTCAACAAATGTATAGCCGACTGCTTCCAAAGTTTAATAACTATAAAACCCGTATTAAAACAAAATTCCAGTGTTATTCCTTCAAGAATAAAGTTCATTTACTTTTCGACAATAATAGTGTTAAATTTTATCTTAATATTGGCCGGTGAGGAAAGCTCGCTTTTGCTAGGCCCCTCTGTTGGGGTTTTTAACGAATTTCAAGCTCATAAGTTATTTCTACCTTTTCAAAATACGTCCCCAATGCTATTATTAAATTCAAACGAGGAAAGTGGTGACGAGATACTGGATTATGCTGTGTTGTTCAAAAGGTTGTACATCCTTTTAAACATTCTGGATACGCTACAAAGCTTTCGGCTAGGTCAACCAAAATTAATTAATCTAAATTTTGGTAGCGCCATTGAAACATATTTTAGCGATAAAACAGGTCATAACCAAGTCGTCGAAAAAGCTCCCGTAGCTTTGGATAACATTCTACGAAACCTTAAACTTGGTGAATTTATAACATATTTTGTCCTTAACAGAAAATCATTGCAAGTAAATGTACCTCACCACTTGCTGTTCACAAATCAAACGGATTACGGAGAGTTCGCTGTTGAAAAAGGGGAACATGATAATATAGCTGGCAAATTTGAGACCCTTTTGAAGAAAAAGGAAATTTTAATCAGAAAATTACTAAATATTGAACAGAAAAATGACCATATTCTAGAAAATTGCTGCAATTCGGATGCTGAAATGAAAAATATCGGAGAGCTAGTCTGCTCAATGATCACTCTGGTATCAGGCATATTAGATTCAATTACTAATATGAACGCAGAAAACTCTGTTGATTTGGATTCAAAGCCCCTTCCGAACGCCTATTTTGCTCAGGACAGTGAAGAAGAATTAATGTCGCCAACACAAAGTATTACGTCAAATCTTGCCAGTGAAGAAAATACACGTTGCACAACCAAAGACTTGATGGGAACTGTTTCTATTTTCATGCTGCCAATGGTGGAAGAATGCTATAATATCATTAGTTTGATAGGACCCATACCTACCACATTAATAAGTTTATACATCCGTAATGGAAATATGGCCAAGGGAATAAATGACAGAATTATGACGTTGTCAACTGCGCTGAACGAATTGGTGCAAATAACCGCACTATTTAATACCCTAGAACCTTTTAGGAAGAATGCACATGACCGTGCTAAGCGTTACTATGTAAGTGCTACAAGTAGTACCGGATGCTACGAATCGGTAATGAAGAGCATGTATTCCGGAAAATGTGCAGCTAGTAACGCCTCAAACGTTGCGCCATCGGAGGAAGAAAACAAAAAGATATTGAAAAAGTTTGCCGACATAGGTTGGAAATTGATGGATGATTCAGAATTGGGTTGTTGCTGTTGCTTCTTTAACTAA>YBR034C	-:0:chr02:304930:305976ATGAGCAAGACAGCCGTGAAAGATTCTGCTACAGAAAAAACCAAGCTAAGTGAAAGCGAACAGCACTACTTCAATTCGTACGATCACTATGGTATTCACGAAGAGATGCTTCAAGATACTGTTCGTACCTTATCTTACAGAAACGCAATTATCCAAAATAAGGATCTTTTTAAGGACAAGATTGTTTTAGACGTCGGTTGCGGTACCGGTATTTTATCCATGTTTGCCGCTAAACACGGTGCGAAGCATGTTATCGGTGTTGATATGTCAAGCATTATTGAGATGGCGAAGGAATTGGTAGAGTTGAACGGATTCAGCGACAAGATCACCTTGCTAAGAGGCAAGTTGGAGGACGTTCATTTACCCTTTCCTAAAGTTGACATCATAATTTCTGAATGGATGGGTTACTTTCTACTATACGAGTCCATGATGGACACCGTTCTTTACGCTAGAGACCACTATTTGGTAGAAGGCGGTCTGATCTTTCCCGACAAGTGCTCCATTCATTTGGCCGGTTTGGAAGACTCTCAGTATAAAGACGAGAAGTTGAACTACTGGCAAGACGTTTACGGGTTTGATTATTCGCCATTTGTTCCGTTGGTCTTACACGAGCCCATCGTCGACACCGTGGAAAGAAACAATGTCAACACCACCTCAGACAAATTGATCGAATTTGATTTAAATACAGTAAAAATATCAGATCTAGCGTTTAAGAGTAACTTTAAATTGACGGCCAAGAGACAAGATATGATTAATGGTATAGTCACCTGGTTCGACATTGTTTTCCCTGCACCAAAGGGTAAGAGACCTGTTGAGTTCTCCACTGGTCCTCATGCTCCATACACTCACTGGAAGCAAACAATATTTTATTTCCCTGATGATCTAGATGCTGAAACTGGTGACACCATTGAAGGTGAATTGGTTTGCTCTCCAAACGAGAAGAATAACAGAGATCTAAATATCAAAATTTCTTACAAGTTCGAATCGAATGGCATCGACGGTAATTCAAGAAGCAGAAAAAACGAAGGTTCTTATTTAATGCATTAA>YBR035C	-:0:chr02:306269:306955ATGACTAAACAAGCTGAGGAGACCCAAAAGCCAATCATATTTGCTCCTGAGACGTATCAATATGATAAATTTACTTTGAATGAAAAACAACTTACTGACGACCCAATCGATCTTTTCACCAAATGGTTCAACGAAGCCAAGGAAGACCCAAGGGAAACGTTGCCAGAAGCAATTACTTTTTCATCCGCGGAACTACCTAGTGGGAGGGTGTCGTCCAGGATTCTTCTTTTTAAGGAGCTCGACCATAGAGGTTTTACTATTTATTCTAACTGGGGAACCTCTAGAAAGGCTCATGATATTGCTACCAACCCGAATGCGGCAATCGTATTCTTTTGGAAGGATCTGCAAAGGCAGGTGAGAGTTGAAGGTATCACAGAGCATGTTAACAGAGAAACTTCTGAAAGATACTTTAAGACGAGACCTCGTGGATCCAAGATCGGTGCATGGGCTTCCCGCCAATCGGATGTTATCAAGAACAGAGAAGAACTAGACGAGTTGACCCAAAAAAACACCGAACGTTTCAAGGATGCTGAAGACATCCCATGTCCAGATTATTGGGGTGGCTTGAGAATCGTTCCACTGGAAATTGAGTTCTGGCAAGGTAGACCCTCGAGATTGCATGATAGATTCGTTTACAGAAGAAAAACAGAAAACGATCCATGGAAAGTCGTTAGACTAGCCCCATGA>YBR037C	-:0:chr02:310564:311451ATGCTGAAGTTGTCAAGAAGTGCCAATCTAAGATTGGTCCAATTGCCAGCCGCAAGATTAAGTGGCAATGGCGCTAAATTGCTCACTCAAAGGGGATTCTTTACTGTAACGCGCTTATGGCAGTCAAATGGCAAGAAACCATTAAGCAGAGTACCTGTGGGCGGTACTCCCATTAAGGATAACGGCAAAGTGCGAGAAGGCTCGATCGAGTTTTCCACGGGAAAGGCCATTGCTCTATTCCTAGCAGTCGGTGGGGCACTTTCTTATTTCTTCAACAGGGAGAAACGCAGATTGGAAACACAGAAGGAGGCTGAAGCAAACAGAGGATACGGTAAACCTTCACTTGGGGGACCCTTCCATCTGGAGGATATGTATGGCAATGAGTTTACGGAGAAAAACCTTCTCGGTAAGTTTTCTATAATATACTTTGGGTTTAGTAACTGTCCTGACATCTGTCCTGATGAACTGGATAAGCTAGGTCTATGGCTTAATACACTCTCTTCAAAGTATGGTATTACTCTGCAGCCATTATTTATAACTTGTGATCCAGCAAGAGACTCCCCTGCTGTATTGAAAGAGTATTTGAGCGACTTTCATCCCTCCATCCTGGGTTTGACGGGGACGTTCGATGAGGTGAAGAACGCATGCAAGAAGTACAGAGTATACTTTTCTACGCCTCCAAACGTCAAACCGGGCCAAGATTATTTGGTAGACCATTCCATCTTCTTTTATCTCATGGACCCTGAAGGACAGTTTGTTGATGCTTTGGGTAGAAATTATGATGAAAAAACGGGCGTGGACAAGATCGTGGAACACGTTAAGAGTTATGTGCCTGCAGAGCAGCGCGCCAAGCAGAAGGAGGCATGGTACTCCTTCTTATTCAAATAA>YBR039W	+:0:chr02:315575:316510ATGTTGTCAAGAATTGTATCAAACAATGCAACACGCTCCGTAATGTGCCACCAAGCGCAAGTGGGTATTCTTTATAAGACTAACCCAGTGAGAACTTATGCTACTTTGAAAGAAGTGGAAATGCGTTTGAAATCTATCAAAAATATTGAGAAGATCACAAAAACTATGAAGATTGTTGCATCTACAAGATTGAGTAAAGCTGAAAAGGCTAAAATTTCCGCAAAGAAGATGGATGAAGCAGAGCAGTTGTTTTACAAGAACGCCGAAACCAAAAATTTGGATGTTGAGGCTACTGAAACAGGTGCTCCTAAAGAGTTGATTGTTGCTATCACCTCTGATAAGGGGTTGTGTGGTTCTATCCACTCTCAATTGGCTAAAGCTGTGAGAAGACATTTGAATGATCAACCAAACGCCGATATAGTCACTATTGGTGATAAAATTAAAATGCAGCTATTGAGAACCCATCCTAACAACATTAAATTGTCTATTAATGGAATTGGTAAAGATGCCCCAACTTTCCAAGAATCTGCTTTGATTGCCGATAAGTTATTGAGTGTCATGAAGGCCGGCACTTACCCAAAGATTTCCATTTTCTACAATGACCCAGTGTCTTCCCTATCTTTTGAACCATCTGAAAAACCGATCTTTAACGCCAAGACCATTGAACAATCCCCATCATTCGGCAAATTTGAGATCGACACGGACGCAAACGTTCCAAGAGATTTGTTTGAATATACTTTGGCTAACCAAATGTTGACAGCAATGGCTCAAGGTTATGCTGCTGAAATTTCCGCCAGAAGAAACGCTATGGATAACGCTTCCAAGAATGCCGGTGATATGATCAATCGTTACTCTATCTTGTACAACAGAACAAGACAAGCTGTCATTACTAATGAACTGGTTGATATTATTACTGGTGCTTCCTCTTTGGGATGA>YBR040W	+:0:chr02:316968:317864ATGGTCGCAATCTCAATGATTTGGTTTTTTACCAAGCGTATGCCCAGAATATTTGCATTAGCTTTTAATTTAATTTCAATATTTCTTTTGATTTTTCTTCTCATCGGCTGTTACAACCCGTCAAATCAGTCAACATTTCTAGTGAAGTATAAATTTGATGACAACTCACCTTTTTATACGATCATAGAGAAATCATATGAAAAATCAAATACAACTCTGGGCTTGGAGGAAGTCATTATAAGATCCGGTTACATGGGTGTTTGTATTGATAACATTCCCTCCCAATATAGCTCTTACAATAATATGACTACATTCTCCAATTCAATTTGCTATGCAAGAAAGAATTTAAGCTCGGTTCCCTTATACAGAGACTTGGAAATTCAACTCTCAAATATTGCATCTTCCAGCTCCAAAACCCAATCAAGCGTTGTCTTGAACATTTTGAAGTTAGCCCAATTAACTTCGGTTAATGTTATACATCCATATGTCTTGATGGCAACTGTAATCCTAACAATCCTAATGTTTCTATTTATTCTGTATGTAACTGTCCCTAAGTTACCATTCAAACTAGCGGTTAATAAATTTCTACTGTTGCTAAGTTCAACTATAGTTTTGACATGGGGTATTGGTGCGATGTGGACTCATGTAGGGATAAATGCAAGTTACAGATTGGTTCCATCATCAAGTATGAATATAATCACTGTCAAGAAGGGCAAGAAGGCAGCAGTAATGGCTTGGTTTAGCTTTGCATTCCTCCTTTTAGACAGTGTGGTTTTATGGTTAATATTTTTAAGGGATAGGAAAAGCTTGAAAGATGAAATTGATAATGTTCCATGTGCCCAAAATAGGTACAATAACTACTCTTCGGATTCATCTACATTGCATTCCAAAGTTTAG>YBR041W	+:0:chr02:318266:320275ATGTCTCCCATACAGGTTGTTGTCTTTGCCTTGTCAAGGATTTTCCTGCTATTATTCAGACTTATCAAGCTAATTATAACCCCTATCCAGAAATCACTGGGTTATCTATTTGGTAATTATTTTGATGAATTAGACCGTAAATATAGATACAAGGAGGATTGGTATATTATTCCTTACTTTTTGAAAAGCGTGTTTTGTTATATCATTGATGTGAGAAGACATAGGTTTCAAAACTGGTACTTATTTATTAAACAGGTCCAACAAAATGGTGACCATTTAGCGATTAGTTACACCCGTCCCATGGCCGAAAAGGGAGAATTTCAACTCGAAACCTTTACGTATATTGAAACTTATAACATAGTGTTGAGATTGTCTCATATTTTGCATTTTGATTATAACGTTCAGGCCGGTGACTACGTGGCAATCGATTGTACTAATAAACCTCTTTTCGTATTTTTATGGCTTTCTTTGTGGAACATTGGGGCTATTCCAGCTTTTTTAAACTATAATACTAAAGGCACTCCGCTGGTTCACTCCCTAAAGATTTCCAATATTACGCAGGTATTTATTGACCCTGATGCCAGTAATCCGATCAGAGAATCGGAAGAAGAAATCAAAAACGCACTTCCTGATGTTAAATTAAACTATCTTGAAGAACAAGACTTAATGCATGAACTTTTAAATTCGCAATCACCGGAATTCTTACAACAAGACAACGTTAGGACACCACTAGGCTTGACCGATTTTAAACCCTCTATGTTAATTTATACATCTGGAACCACTGGTTTGCCTAAATCCGCTATTATGTCTTGGAGAAAATCCTCCGTAGGTTGTCAAGTTTTTGGTCATGTTTTACATATGACTAATGAAAGCACTGTGTTCACAGCCATGCCATTGTTCCATTCAACTGCTGCCTTATTAGGTGCGTGCGCCATTCTATCTCACGGTGGTTGCCTTGCGTTATCGCATAAATTTTCTGCCAGTACATTTTGGAAGCAAGTTTATTTAACAGGAGCCACGCACATCCAATATGTCGGAGAAGTCTGTAGATACCTGTTACATACGCCAATTTCTAAGTATGAAAAGATGCATAAGGTGAAGGTTGCTTATGGTAACGGGCTGAGACCTGACATCTGGCAGGACTTCAGGAAGAGGTTCAACATAGAAGTTATTGGTGAATTCTATGCCGCAACTGAAGCTCCTTTTGCTACAACTACCTTCCAGAAAGGTGACTTTGGAATTGGCGCATGTAGGAACTATGGTACTATAATTCAATGGTTTTTGTCATTCCAACAAACATTGGTAAGGATGGACCCAAATGACGATTCCGTTATATATAGAAATTCCAAGGGTTTCTGCGAAGTGGCCCCTGTTGGCGAACCAGGAGAAATGTTAATGAGAATCTTTTTCCCTAAAAAACCAGAAACATCTTTTCAAGGTTATCTTGGTAATGCCAAGGAAACAAAGTCCAAAGTTGTGAGGGATGTCTTCAGACGTGGCGATGCTTGGTATAGATGTGGAGATTTATTAAAAGCGGACGAATATGGATTATGGTATTTCCTTGATAGAATGGGTGATACTTTCAGATGGAAATCTGAAAATGTTTCCACTACTGAAGTAGAAGATCAGTTGACGGCCAGTAACAAAGAACAATATGCACAAGTTCTAGTTGTTGGTATTAAAGTACCTAAATATGAAGGTAGAGCTGGTTTTGCAGTTATTAAACTAACTGACAACTCTCTTGACATCACTGCAAAGACCAAATTATTAAATGATTCCTTGAGCCGGTTAAATCTACCGTCTTATGCTATGCCCCTATTTGTTAAATTTGTTGATGAAATTAAAATGACAGATAATCATAAAATTTTGAAGAAGGTTTATAGAGAGCAAAAATTACCAAAGGGTTTGGATGGAAATGACACTATTTTTTGGCTCAAGAATTACAAGCGCTATGAAGTCTTGACCGCTGCTGATTGGGAAGCCATCGATGCACAAACAATTAAATTATGA>YBR043C	-:0:chr02:321876:323945ATGCAAGCCCAAGGTTCACAATCGAATGTAGGGTCTTTGAGGAGTAATTGCTCTGACAATTCACTACCGAACAATCATGTTATGATGCACTGCGATGAAAGCAGCGGCACGCCGCACAGCGAGCACAACGATTATAGTTACGAAAAGACCAATCTGGAAAGTACGGCATCAAATAGTCGTGAACACAGAGACAACCAGCTAAGTAGGTTGAAGAGTGAGGAATACGTTGTTCCAAAGAATCAACGTAGGGGACTATTGCCTCAACTCGCCATTATACCGGAGTTCAAGGATGCCAGAGATTATCCACCGATGATGAAAAAGATGATTGTCTTCTTGATTGCGTTTTCCTCCATGATGGGCCCCATGGGCACATCTATCATTTTTCCAGCGATCAACTCAATCACAACAGAATTTAAAACATCAGTGATTATGGTAAACGTTTCAATTGGTGTGTACCTTTTAAGTCTTGGTGTTTTCCCATTGTGGTGGTCTTCTCTATCCGAGCTAGAGGGCAGAAGAACTACTTACATAACTTCATTTGCATTATTGTTTGCATTTAATATCGGGTCTGCTCTAGCTCCTGATATCAACTCATTTATTGCCTTGAGAATGCTCTGTGGGGCTGCTTCTGCCAGTGTTCAAAGTGTAGGTGCTGGAACAGTGGCTGATTTATATATTAGCGAAGATAGAGGTAAAAATTTGAGTTATTACTATTTGGGTCCACTACTGGCGCCGCTACTATCTCCAATTTTTGGATCTTTGTTAGTGAATCGCTGGCCCTGGAGATCCACTCAATGGTTTATGGTTATTTTATCCGGATGTAATGTCATTCTTTTGACGGTGTTACTACCTGAAACATTAAGAAAACAAGATTCTAAAGGCGCTATCGCTCAAATTTTGGCTGAAAGACGTATTCAAGTAGACAATAACGAACGTGGAGAGATACAAGAAGATTATCAGAGGGGAGAAGATGAGACAGATCGAATTGAAAACCAAGTTGCCACATTATCTACTGAGAAGCATAACTACGTTGGAGAGGTAAGGGATCAAGACTCGCTAGATTTAGAAAGTCACTCTAGCCCCAATACTTATGATGGTCGAGCTGGAGAAACCCAATTGCAACGGATTTATACAGAGGCGAGTAGAAGTCTGTATGAATATCAGCTAGATGATAGCGGTATCGATGCAACAACAGCACAAGTTACGAGAATAAGATCAACAGATCCAAAGTTAGCGAGATCGATTCGAGAAAATAGTCTGAGAAAATTACAAACCAACCTGGAAGAGCAAGTCAAAAAAGTGCTATCCAGTAATGGAGGTGAAATCGCTCCTAAACAGGTATCAGCGGTGAGGAAGGTCTGGGACACCTTTTTTGTTTATTTTATCAAGCCTTTAAAATCATTGCACTTCCTAGAATATCCACCCGTGGCACTTGCAATAACATTTTCCGCAATTTCCTTTTCCACAGTATACTTTGTTAATATGACAGTTGAATATAAATATTCAAGGCCTCCTTACAACTTTAAACCATTATACATTGGTCTACTGTATATTCCGAATTCTGTAACATACTTTTTCGCCTCAATTTACGGTGGACGTTGGGTGGACATGCTTTTAAAAAGATACAAAGAGAAATATGGAATTCTTGCTCCTGAAGCTCGTATATCGTGGAATGTTGTTACATCTGTAATATCTTTCCCCATTGCGCTATTGATATTTGGCTGGTGCCTAGATAAAAAATGCCACTGGGTAACGCCACTAATTGGAACAGCCCTCTTTGGATATGCAGCTATGATGACAATTGGTGCTACCCTTTCCTATTTAGTCGATTCATTGCCGGGAAAGGGTGCCACCGGTGTTGCTTTGAATAATTTAATAAGGCAAATCTTGGCTGCAACCGCAGTCTTTGTCACCACACCCATGTTAAACGGTATGGGAACTGGGTGGGCTTTCACAATGCTGGCCTTTATCGTCTTGGGTGCTAGCAGTGTACTTATAATACTGAAAAAGCACGGTGATTACTGGAGAGAGAACTACGATTTACAAAAATTGTACGACAAAATTGATTAA>YBR045C	-:0:chr02:328369:330090ATGGAAACTATTTTGCAGCCAAAGGCTAGACCATTTGAGTCTTTGAAAAGAAAACGTTTTAGAGAATGGTTGAGGCCGTCGACTGCGCATGGATCCCTGTTGCATTCTGATACATTAGATTTGCGTGACTTTGCAAAACCTAATCCCGCTGACACATTTTCTAATCTTGATTCTGGTCATTGTCCTTTGGTCACAACTCCAATAAAATATGAGTGCCCAGATGGAAAGAGTTCTTTTTTCCGAGGAGACACTAAATTTGAAACCCTGTTCAGTAATAGAAAATTCTATGAGTTCAAAGATAATTTGAAAAGGGGATTGAAGAAAATACGTCATGGGAGAAACGGACATCAAAGCGAAAAGAGATGTCCAGTTGTTGAAGAAACAAAAAAGTCTGTGTCAGATAATCTGGACAAACCAGACAATAATACGCCCTGTTTCGACAGATTCCACACAAATTCGAAAGAATTTGAAACGCAATTTGATCATTCAAATAGGAGCCAAAATTCCGAGAAGGCTTATCTAGACAATGAATCCTGTTGGAACCTAAGTGAGAAATTTATTCCTTTTAATAATTTAAAATATGAAGATTTGAAACATTTTGAAGAGAATTTGCAAAGCTTAGCGCCTGCAACTTTTACTCCAATTGAATCAAATGAATCGCTTGATAGGTCAGATTCGACACGTGGCACAAAACGAAGCATTCGCAATGATTCCAGTGATACAACATCTGAAAAGAGGCTATGCTTAAAACAATACTCAGATGAACCTGAATCGGATCATTCGATGGAAAGTACACCATCCATTTACATTACCAAAGAAGTTCAAGAAAGAATTGAAGCATTAAGCTCCACGGATTCGTTTTTAATTGAAAAAGTAGATTTTCCCTCTAACAAAATTGGTTCCAGTGCCTCCGATTATGAAAGTGATAACGAATACAGAAATATGGATGAGGATTCAATAAACGATGTTACCACTGAGAAAGAGGGAAATGTGGTCATACCAGACTCTAATACTAGCACGGTGGACGCAATGGAAAAACCAATTGAAGTGAGCTCGGCCTTAAAAGATGATACATTGGATAAAGACATAGATGATGCGAGCAGTAGCTATTCGGATGATGTAGAGACCACATTCGAGCCAGTTGAATCTGAGGAACTTTCCGATTTATCTGATACAAGCTCAAGTGGAAGTAGTAAAATTTATACTATCCCCACATTTCGCGGCCTTACTAATAGGACCAATATATCACAAATTCTTTCAAAAGTTGGTAAAGCTGATTTAAGCCAAGATAACTTAACGCATTTGATCAAAAGTCATCAAAAAAAGAAAAGATGCGTGAATTTTAGAAATAAAAGATTCTATGATGCTTTCAATCCATATGTTGATAACGAAGAAGATGCAGAGTTATCTGACAGCGAAAACATTTCAGAAATGGATACAGATCTTTGTATAAAGGATCGGAGTACCTCAAGTGTTAGGTTTGATGAAAACTCACGTCTTTTAATCTACAAAAAATCTAAAAAGTTAAACAAAGATGAGACTCAAAGTGGTTATTCGACTACTGAAATGAGGTCAATTCTAAAGACAAAGATGAATTCACAGCATGATGAGGAGTCTCAGAGAGCTTCGAAGTGTGACACAGTAGGTGTAGCCCAATTTTTACATTATTTTCAATATACAGAGTATAAAAGGCAGAGAAATGAAGCAGAAATTATAGACTGA>YBR046C	-:0:chr02:330505:331509ATGAAATGTACTATACCAGAACAGCAAAAAGTCATTTTGATTGATGAAATTGGTGGATACGATGTAATCAAGTATGAGGATTATCCTGTACCATCGATTTCGGAGGAAGAGTTACTAATCAAAAATAAGTACACGGGTGTTAATTACATCGAAAGTTACTTTCGGAAGGGTATTTATCCCTGTGAAAAACCATACGTATTGGGCAGGGAAGCGTCGGGGACCGTTGTAGCAAAAGGTAAAGGGGTAACTAATTTTGAAGTTGGAGACCAAGTTGCTTATATATCTAACTCAACTTTTGCACAGTATTCAAAAATTTCGTCCCAAGGCCCAGTCATGAAGCTGCCAAAGGGAACGAGTGACGAAGAGTTGAAACTTTATGCGGCTGGTTTATTACAAGTTCTCACTGCTTTATCATTTACAAACGAAGCGTATCACGTCAAAAAGGGCGACTATGTATTACTTTTTGCCGCAGCGGGTGGTGTGGGATTGATTTTAAATCAGCTACTCAAGATGAAAGGTGCACATACGATTGCAGTTGCCTCAACTGATGAAAAGCTTAAAATAGCGAAGGAATACGGCGCCGAATACTTGATCAACGCTTCGAAAGAGGATATTTTAAGACAAGTTTTAAAATTCACTAATGGTAAGGGCGTTGATGCTAGTTTTGACTCGGTCGGAAAGGATACCTTTGAAATCAGCTTAGCCGCACTAAAAAGAAAAGGTGTATTCGTTTCCTTTGGTAATGCATCTGGTCTTATCCCGCCATTCTCTATTACCAGACTTTCCCCAAAAAATATTACTTTGGTGAGACCTCAACTTTACGGCTATATCGCCGATCCTGAAGAATGGAAATATTACTCTGACGAGTTTTTTGGTCTGGTTAATTCAAAGAAGTTGAACATCAAAATATACAAAACTTATCCATTACGGGATTATAGAACTGCAGCTGCTGACATAGAAAGTAGAAAAACTGTTGGTAAGCTAGTTCTTGAAATACCACAATAG>YBR047W	+:0:chr02:331831:332358ATGCTTATCAACCACTTGAGTAAGATCCGAACCGTAAGGCACTTTTCTAATATTAAACCCGTTTTATCCAAGGAAGTTTCGCGACGAGTAATTGTTGCACCTGCATCACACTTCAAAACCTCCAGTCCAAATGTGAAAAGTAACATCCCTATTCATGAATATAAACAACTACCGGAAGATTCTAACTACATTGAAAAGCATTATAAGGAACTACAAGTATTTTTGAATGAGTTTTTGATTAAGAAATTAAACAAGACCTATGCGGATTTTGAAGGAGATCCAGACGAGTTGGTATTTCAATTGGAAAAATTCATTGAACTAGAAGTTACTCCGAGATACACAAATCATTCAGCACCTGATGGTTGTGAAGAGAGATTCAAATCCATCGGAGACAGGATAGTCGTTGACCGCTATTTAGATTTTGTGAAAGATGTCAGACTAACACTGCTGCTAAATGGAGGACACTCCTTCATATTTGATGTCATGCTACAAGCCAAAGAAGTATTTGACAAAATGCAGAAAGAATGA>YBR050C	-:0:chr02:337181:338197ATGACTTTGAGTAATTGCGACTCTTTGGATAACTTATTCCAGGACCCTCCAGAGGAAGAAGAAAGTAGTAAATTCGTTGAGGCGGTCAGAACTTTGATGAATAGAAACGATATGGGATATCCTCCCGCCGCTGCAAATGGTACGTATTGCTTAAAAAAAATCAAGTCTTTGAATGCCAAACAGTGGAAAATAAACAAGAAAAGAATGTGCATGTTGCCAGCAGTAAAGAAGAAAAATTTCGACTTTCACGAGCAAAGAAGTTTAATCTTGAATTTAAATTTATGGAAATTCATCAAGTTTATCAATTGTAGTAGTAAAAACAATTACAATAAAAATAATAAGCATGTGAGAAGCTCGAACAACACTGTAAAAAATGAAAATGTTTTACCGTTACAAAAACACAAGAAAGTGGACAATGATCAAAGATTGGAGAACCTTTTTTGGAGAAGCTGGTTTAAGGCACGCAAAAGGAGAGATATAATGGGCAAGCCACGAGAGAGGCATATCAAATTTAACGATAACGTTGAACAGTGTATTATAACTGATGAGCATTTCATACAAAGGCTTCCTTCTACACGGTTGAATTCGACTGATGAACAGCGCCCTTGTTCAAAGTCTGAACTAGATCCCTGTATTGGCAACGCAGCAAGTAAGCGAAGTTTCTATGATTATAACAGCGTTTACGTCGCGAGTGACGCAATTATTACGACTGCCGCTGCCACTGCCATTATCAGTAGTAATAGTGGAGACTATCAGCGTGGGCACGATGTTCGCGATGTTCCAAGAAATGTTTTGTTACAGGCAGGAGAAACAGATTTCAGTAGTGTGCTTCGGGTTGACTCCGATCTCAAGTTATCCAACATAAGTCATCATTCCCCCGTAAAACCTTCGTCAACTTCAAGTCATTCGACCTTCATTTTCGAGTCGGAAACTGACACTGATACTGATACTGACGCTGAAACAGAAAATGACATTGACGCTTACATAGACACCAGTATACCCAACCTGCTCCTATAA>YBR052C	-:0:chr02:338718:339350ATGCCGAAAGTCGCTATTTTGATTTATTCAGTGGACGATATAATTGCCACATTAGCAGAAAATGAAAAAAAAGGTATAGAGATAGCTGGTGGTGAGGCTGAGATTTTCCAAGTGCCAGACGTCAGCTATAAAACTGAATATGCGACAGAAGAGGGAAAAGAGGCAGCCAAAGTCGCCAAAACTAATGCAGATTTTTCCTATAAAATCCTAACAAGAGAAACGTTGGTTGAGTACGATTACTACTTATTCGGAATACCTACTAAGTTTGGAAACTTCCCCGCGGAGTGGAAAAGCTTTTGGGACTCGAATACTGGTGGGCTTTGGGCAAAGGGTTCTCTCCATGGCAAAATTGCCGGTTTGTTTGTTTCAGGCGCAATAAGCGGCAAGGGTGATACAGAAATGTGTATCATGAACGCAATGAGTACTCTGGTCCATCATGGTGTCATTTATGTCCCATTGGGGTATAAGAACGCATACAAGGAATTGACCGACGTTGAAGATGTGAACGGGTCTTGCGCCTGGGGCGCAGGATGTGTTTCTGGAATCGACGGTGGCAGACCTCCCAGTTTATCCGAGCTAAGAGTTCACCAACTTCAAGGGAAGGCCTTTTACGACCGTATCAAAGATTTGTAA>YBR053C	-:0:chr02:339673:340749ATGAGTAGTGTTGGCGATTTTGAAGAAATTATTTTGCATGATTTAAAACCTTATTATCACGTTCCCGGTGCCATACATAGTGAAGGAATAACCTTCGTTAAGGAAACCGGTACACTGTTGTGGGTTGATATTTTTAAAGGAGAGGTTCATAAAGTCGAAGACATAGAACAGCCTGAATCCAGTCATTCTTTTTTTTCCATTAGTAGAGCCAATTATGGCAAGAATGCGTCAATTGAGTACCCTCCCAATCCAGATGAATTAAAAGAATCCGTGGGCTGTATATTTCCAATACTTGATGGGGCTTCACAAAATGAAATCAAACAAGTATTATTTGGTAGTAAATTTGGCATAGGAAAATTGGATTTTAGCAAAAGTGAATGGGAATATGTCATTTTGTATTCCGAATGTCCTGAATTGAGTACAGACAGAGCATATAAGTTGAGATCTAATGATGGGAACGTTTCGCCAGATGGAAAATATATTTATGTGGGGCTAATGAGCGATTTTCCATTTGATTTGGAACCTATTGGGTGTTTGCTTCGTGTTGACCTATTAGCTCACAAGATAGAGCTTGTTTGGAATTGCTTGTTAATTCCTAACGCGATCCACTGGGATGAAAGTGATCAAAAGACGATGTACGTGACTGATTCTTTAAACTTCACTATTTGGAAATGTCCTGGTGGCGACTTGCTAAAACGTGACGAGTTAATTGACGTGAAGAACTCCAACAATCAATCTTTTGAGTCTCCTGAACCAGATGGAAGTGCAATTTGGTTTAGCAAAGACGGAAAACATTCTGGGTTTCTGTTTATCACTGTCTGGTCCACCAGTAAGGTTCAAATGTTCGATTTAACTAACGGAAAATTGCTGAAGGAGTTCATTTTGCCGGAGCAGACACCAAGAGTTTCATGTTGTTGTTTTGTTGGTAAAGATCTCTTCGTGACCACTGCCAATGCAGAAATCAACGATGCTGTAAGAACCAATACTGACAAGAATGGTGGCTGCATCTACAAAATTCCAAACGTGCTAGACGGAAATGTTCCTCTGGAATCTACAAAGCGACAACCTCTCCACTAG>YBR054W	+:0:chr02:343099:344133ATGTCTGATTATGTTGAACTATTGAAAAGAGGTGGTAACGAAGCCATCAAAATTAATCCACCAACCGGTGCTGATTTCCACATTACATCTCGTGGTTCAGACTGGCTTTTCACCGTTTTTTGTGTTAACCTACTATTCGGTGTGATTTTAGTCCCACTAATGTTTAGAAAACCAGTTAAGGACAGATTCGTATATTATACGGCCATTGCTCCAAATTTGTTCATGTCAATTGCTTATTTCACCATGGCTTCTAACTTGGGTTGGATCCCAGTCAGAGCAAAGTACAACCATGTACAAACTTCTACCCAAAAGGAGCACCCAGGTTACAGACAAATTTTTTACGCTAGATACGTTGGCTGGTTTTTGGCTTTCCCATGGCCAATTATTCAAATGTCTCTACTTGGTGGTACTCCATTATGGCAAATTGCTTTCAATGTTGGTATGACTGAAATATTTACTGTCTGCTGGTTGATTGCTGCCTGTGTCCACTCTACTTACAAATGGGGTTACTACACCATTGGTATTGGTGCTGCCATTGTTGTTTGTATTAGTTTGATGACAACTACATTCAACCTGGTCAAGGCCAGAGGTAAGGATGTCTCTAACGTTTTCATTACTTTCATGAGTGTCATCATGTTTTTGTGGCTGATTGCTTACCCAACCTGTTTTGGTATCACAGATGGTGGTAACGTTTTGCAACCAGATTCTGCTACCATTTTCTATGGTATTATTGATTTGTTGATCCTATCTATCTTGCCAGTTCTTTTCATGCCATTGGCTAACTATTTGGGGATTGAAAGATTAGGTTTAATCTTCGACGAAGAACCCGCTGAACATGTTGGTCCAGTTGCTGAAAAGAAGATGCCATCTCCAGCTTCTTTCAAGTCATCTGATTCTGACTCTAGTATCAAGGAGAAGTTAAAGCTAAAGAAGAAGCACAAGAAGGACAAGAAGAAGGCTAAGAAGGCTAAGAAGGCCAAGAAGGCCAAGAAAGCCCAAGAAGAGGAGGAGGATGTAGCCACCGACTCTGAGTAA>YBR055C	-:0:chr02:344600:347299ATGGAGAGGCCATCTTTTTTGGATCAAGAACCACCTGCAGGTTACGTACCAGGTATTGGTCGTGGAGCCACTGGATTTTCAACAAAAGAAAAGCAAGTGGTTAGTAATGATGACAAAGGAAGAAGAATACCGAAAAGGTACCGTGAAAATTTGAACAACCATCTTCAAAGCCAACCGAAAGATGATGAAGATGATGAAGCTGCAAATGTATTCAAAACGCTTGAATTGAAATTAGCACAAAAGAAAAAGAAAAGAGCTAATGAAAAGGATGATGACAATTCAGTTGATTCTTCAAACGTGAAACGGCAATTTGCCGATTTGAAAGAATCATTAGCTGCTGTAACGGAGAGTGAGTGGATGGATATTCCGGATGCCACAGATTTTACAAGAAGAAACAAGAGAAATAGAATTCAAGAGCAATTAAACAGAAAAACTTATGCTGCACCGGATTCGCTAATACCTGGGAATGTTGATTTAAATAAATTAACGGAAGAACGAGAAAAATTATTGCAATCTCAAATAGATGAGAATCTTGCACAATTAACGAAGAATGCAAGTAACCCTATACAGGTTAATAAACCGAACGCTGCTACCGATGCCCTAAGTTACTTAAAGGACTTAGAAAACGATAGAGTAAATTCTCTCTCAGACGCAACGTTAGAAGATTTACAGAAAATGCGCACAATTTTAAAGTCATACAGAAAGGCCGATCCAACAAATCCACAGGGTTGGATAGCTTCTGCCAGATTAGAAGAAAAGGCAAGAAAATTTTCAGTAGCAAAAAAAATAATAGAAAATGGTTGCCAAGAGTGCCCTCGAAGCTCCGATATTTGGCTAGAAAACATTAGACTACACGAATCTGATGTTCACTACTGTAAAACATTAGTGGCAACGGCAATAAATTTTAATCCAACGTCTCCGCTTCTTTGGTTCAAAGCTATTGATTTGGAAAGCACAACGGTTAACAAATATAGAGTAGTGAGAAAAGCACTGCAAGAGATTCCTCGAGATGAGGGCCTATGGAAGCTAGCTGTCAGTTTTGAAGCTGACAAAGCGCAAGTTATAAAAATGTTAGAGAAAGCCACACAATTTATTCCACAAAGTATGGATCTCTTGACTGCATATACTAATTTGCAAAGCTATCATAATGCTAAAATGACTTTGAATTCCTTCAGAAAAATCCTTCCGCAAGAACCGGAAATTTGGATTATCTCTACACTCTTGGAAGAACGAAATAACCCAGATATACCTGTAGATAAACTAGTTAGTTTGCTCAAGGAGGGTTTATTGGAACTCTCTAAAAATGGGTACAAAGCGACCTTGTCAGCATGGTTGAAACGTGCAGAGGCTCTAAATGATGCGCCCAATTCAAATTTAACCTGTCAAGCCATCGTTTACGCTATATTAGAATGGTTAAGAGAAAGTGGCGAGTATGAGTCTGAGTTGAATAATGTTGATCAGATATTAGAAAAAATGCCACACTCAAAGGTACAAATTGCTGTCTTAAAAAAGCTTATTCAGTGGGATCCTTGTGATACAGTTCTTTGGTCTAGACTGAAAATGGCCACTGAAAGCTACCATAAAATTGAAGAGTTATTAGCATTTTTCCAGGAGCTGCTATTTCAGACCAAGAATAGTGATGATATACGAGCAAATATGAGGGAGAAAAGCCCTGGCTTGTTAATGATGTATGTAAGCGAATATTGGAAGGCCCAAAAAGGGGATACTAGGCAAACACTAGTTTTGATTGACCAGATTATAGATTTCGCCCCGCATAATTTGGATTTACGCTTTTTCAAGATAAAGTTATTAGGTCGTTCACTACAACTTGATGAATTACGAGATTTTTTTCAGCAAACTTTCTCCTCTTTAGAGGATTTTAAGATCAGTGGCACGGAAAGATTATATTATAAATACGTAAACTTTCTGCGGTACCAAGATCTGAATGAAGAGGCTATAAAATTCTTGAATGAGAGATGTTTGAAATCATTTCCCATCTGCCACAAATTTTTTTTACAGCTGGGTCAAATTTATCATTCCATGGGCAATATTGAAATGAGTAGAGAAACCTATTTGTCTGGTACAAGGTTAGTGCCCAATTGCCCTTTATTATGGGTTTCCCTATCAAAGATTGACGAGATTGATCTAAAAAATCCAGTAAGGGCAAGATCAATTTTAGATAGAGGATTGTTAAAAAATCCTGACGATGTATTATTTTACATTGCTAAAATCCAAATGGAAATAAGACTTGGTAACTTAGATCAGGCGGAGTTACTCGTCACACAGGCATTGCAAAAGTTTCCAAGCAATGCTTTACTTTGGGTGGAGCAAATCAAGCTGTTTAAGCATGGAAACAAAAGTTCGTTAAAAAAAACAATTTTTCAAGATGCTTTAAGAAGGACACAAAACGATCATCGCGTTCTTTTGGAGATTGGAGTATCCTTTTATGCAGAAGCGCAATATGAAACATCATTAAAATGGTTAGAAAGAGCTCTGAAAAAGTGCTCGCGTTACGGAGATACATGGGTTTGGCTATTTAGGACATATGCAAGGTTAGGCAAGGATACTGTTGATCTCTACAATATGTTCGATCAATGTGAGCCTACTTACGGACCCGAATGGATAGCCGCCTCCAAGAACGTAAAAATGCAATACTGCACACCTAGAGAGATTTTATTGCGCTTGATGAATGACAAATAA>YBR056W	+:0:chr02:347877:349382ATGATTGGCTCACTTAGAAACAAATTTGAGCATTTCAAAGTTTCTGAAAAGGGAGGTCAAAATTTATCTACAACACTACCGAAACTACCCCCTGCAAAGGACCTTGATAGATCAACTATCTACAAGTACCGTTACAATTATGGTGTAAACTTGGGTGCGTTATTTGTGCTCGAACCATGGATTTTTTCTAAAGAAACCATTTGTACAATCGATGGGAAAGAATATGATAGCGAATTTGATGCTATTTCCCAACAATTGAAGAAGCATTCTTCTGAAGACGTTGCGAAAATGTTAAGCGATCACTATAAAAAGTACATTGATCGAATTGACTGGGAATGGCTATCTAAAGATGCCCATATTACAGCGTTACGTATTCCAATTGGATATTGGCATGTCGAAGATGGGAAGCATTTAGATTCGCTTCCATTTGCCCCATTGAGAAAAGTTTATGAGTTAGCCAAACCTTGGGAAAAACTTGGCGAATTAATCAATAATGCCAAGAAAATGAGCATTGGTGTATTGATAGATTTGCATGGTCTACCAGGAGGCGCTAATTGCGACTCACACAGTGGCTCGAAGAGTGGTGAAGCTGCGTTTTTCCACAAGGAAAAGTACATGACCAAAGTCTACAAAGATATTTTACCTGCAATTATTAACACAATGACTCTGGGCAACGAAAACATCATTGGTATTCAAGTGGTTAATGAGGCATGTTTTGATAATAACCCAAAGGGCCAAAAATTCTATTATTCAGAAGCCATTAATACCGTTGAAAAACTTCAACCAGGTTTACCTGTCATAATATCCGATGGTTGGTGGCCCCAACAATGGGCAGACTGGGTTAAAGAAAAACATTTTAGTGAAATAGTGGTCATTGATTCTCATGTCTACCGCTGTTTTTCTGATTCTGATAAGTCGAAGGATGCCAATTCCATTATCAAGGATTTGCCAAATACTGTGAATTTTCCTCATGAAGATGCCGATTATACTGTTGGTGAATTTTCTGGTGTTCTTGATGGACAAACTTGGAATAAAACTTCTGGCGACAGAGACGCTATTGTTCAAAAATACGTACAAACCCAAGCAGATGTATTTTCTCATGTAGCTAGTTGGGGTTGGTTTTTTTGGACTTTGCAGTTTGAATACGGTGATGGAGGTGAATGGGGCTTAGCTCCCATGATGCAGAAAGGAAATTTACCAAAACGTCCTCACGGTGATGACTTACAAGTCGACAAGAAGAAAATAGATTCAATAATCCATGAACACGAGGCTTATTGGAACGGTAAAGGTAAAAATTTTGAACACTGGAGGTTTGAGGATGGCATAAAGACAGCTGTTGATGATATAATTGCCTTTAGGAAATTTGACAATTCATTAATTGGTAGATGGCATTCATGGAAGTCGCAAAGAAGAGCTGAATACGTTTCTGCCAAAAAAGATAGCGAATTTATGTGGGAATGGGATCAAGGCTACCAACGTGGGTTAGATGAGTTTAATAAGTATTAA>YBR057C	-:0:chr02:352191:353291ATGAATTACATGGCTTATGATTATGATCCCCAACATTCGTTGGAAACGTCCTTTAACAATTTGGCATTTCATCCCCACCAACAGTCACAGCAACAAGCCCTATACGAATCTGGTGAAAGAAACGATGCCAGGCCGGGATTAATGAACACCTTAGGTCAGGCGAGTAAAATGAATAATTCCATGCTTCCTCAAGGTTCGTCGGCTAGTCCCTTAACGGGTCAACATAGCTTGAACAGTACAACAAATTTCAACATGCCCCCATCAATGAATACCTATAATTACCAGAATGTACCACAAGCATCTATGAGAAATACTCTCAATCATAACAATATCATGAATGGGGCAACTGCTAATGATTATTGGCTGGATCCAATGAATAATATGACAAATAACAAGGATACTAATGGGAATCCTAATGACTCGATGTCATCAATGTCTAATATGACCGCTAAAACCTCAATCAATAGTACGGCATTCAAGAATTCATTTGTTCCTTTTAATCATGTGACAGCGCTCTCAATGAATAATGTCAACAGCAATGAGATGAATTCAAATAAAGATGACAGAATGGAAGCATTGGAAGTAGAACTACAAATAAAGGAAAGTCAAATCGAATCACTCGAGAACGAAATTCAAAGGCTAAAGAAAATTTTCAACGAGGGGCTAAACTACAAGCAGAATGAGCATAAGTATGAGAAGGAAAACTGCCATATACCCCAAACTTTCGAATTGCCAGCTTCCTTGGAAGTGATATTCAGAAAACTGTCATCGTCACTGCATGCAAAGGAAAAGGAGTTAGCAGAGACGAAAGAAAATCTGGAAAGTATATTAACTGCACTGGCGCTAAATCCAACAAATTCAGTGACTAAGTATGGCAGGTATGATGCAGAATCGATTGCTCACAAAATGGTGGTAAGATTAGAAAACTTAACAAACGAGAACAAAGAAATGGCCAAGATGTTAGCATATGGCAGATCAAAAGAGACCCAAATTGAACTGCAGTTGGCCAAAAAAGAAAATCTAGAATTAAGAGAAAAAATCGCATCTTTGGAAGCCCATTTGGCTTCAAAGGAGTCTTCAAAGGAGGACGTTGCTAATTGA>YBR058C-A	-:0:chr02:356322:356564ATGACACAACATAAAAGCTCGATGGTGTACATACCCACCACTAAGGAAGCTAAAAGACGTAATGGGAAATCAGAAGGCATACTAAATACTATTGAAGAAGTGGTGGAAAAGCTTTATTGGACCTACTACATACATTTACCCTTTTATTTAATGGCCTCTTTTGATTCATTCTTCCTCCATGTTTTTTTTCTCACAATTTTCAGTTTGAGTTTCTTCGGTATACTAAAGTATTGCTTCCTTTGA>YBR060C	-:0:chr02:360650:362512ATGCTAAATGGGGAAGACTTTGTAGAGCATAATGATATCCTATCGTCTCCGGCAAAAAGCAGGAATGTAACCCCAAAAAGGGTTGACCCACATGGAGAAAGACAACTGAGAAGAATTCATTCATCAAAGAAGAATTTGTTGGAAAGAATCTCGCTTGTAGGCAACGAAAGGAAAAATACATCTCCAGATCCGGCACTCAAACCTAAAACGCCAAGTAAAGCTCCCCGTAAACGTGGAAGACCAAGAAAGATACAGGAAGAATTAACTGATAGGATCAAGAAGGATGAGAAAGATACAATTTCCTCTAAGAAAAAGAGGAAATTGGACAAAGATACATCAGGTAATGTCAATGAGGAAAGCAAGACTTCTAACAACAAGCAGGTGATGGAAAAGACGGGGATAAAAGAGAAAAGAGAACGCGAAAAAATACAGGTAGCGACCACAACATATGAAGATAATGTGACTCCACAAACTGATGATAATTTTGTATCAAATTCACCCGAGCCACCAGAACCTGCAACACCATCTAAGAAGTCTTTAACCACTAATCATGATTTTACTTCGCCCCTAAAGCAAATTATAATGAATAATTTAAAAGAATATAAAGACTCAACCTCCCCAGGTAAATTAACCTTGAGTAGAAATTTTACTCCAACCCCTGTACCGAAAAATAAAAAGCTCTACCAAACTTCGGAAACCAAGTCAGCAAGCTCGTTTTTGGATACTTTTGAAGGATATTTCGACCAAAGAAAAATTGTCAGAACTAATGCGAAGTCAAGGCACACCATGTCAATGGCACCTGACGTTACCAGAGAAGAGTTTTCCCTAGTATCAAACTTTTTCAACGAAAATTTTCAAAAACGTCCCAGGCAAAAGTTATTTGAAATTCAGAAAAAAATGTTTCCCCAGTATTGGTTTGAATTGACTCAAGGATTCTCCTTATTATTTTATGGTGTAGGTTCGAAACGTAATTTTTTGGAAGAGTTTGCCATTGACTACTTGTCTCCGAAAATCGCGTACTCGCAACTGGCTTATGAGAATGAATTACAACAAAACAAACCTGTAAATTCCATCCCATGCCTTATTTTAAATGGTTACAACCCTAGCTGTAACTATCGTGACGTCTTCAAAGAGATTACCGATCTTTTGGTCCCCGCTGAGTTGACAAGAAGCGAAACTAAGTACTGGGGCAATCATGTGATTTTGCAGATCCAAAAGATGATTGATTTCTACAAAAATCAACCTTTAGATATCAAATTAATACTTGTAGTGCATAATCTGGATGGTCCTAGCATAAGGAAAAACACTTTTCAGACGATGCTAAGCTTCCTCTCCGTCATCAGACAAATCGCCATAGTCGCCTCTACAGACCACATTTACGCTCCGCTCCTCTGGGACAACATGAAGGCCCAAAACTACAACTTTGTCTTTCATGATATTTCGAATTTTGAACCGTCGACAGTCGAGTCTACGTTCCAAGATGTGATGAAGATGGGTAAAAGCGATACCAGCAGTGGTGCTGAAGGTGCGAAATACGTCTTACAATCACTTACTGTGAACTCCAAGAAGATGTATAAGTTGCTTATTGAAACACAAATGCAGAATATGGGGAATCTATCCGCTAACACAGGTCCTAAGCGTGGTACTCAAAGAACTGGAGTAGAACTTAAACTTTTCAACCATCTCTGTGCCGCTGATTTTATTGCTTCTAATGAGATAGCTCTAAGGTCGATGCTTAGAGAATTCATAGAACATAAAATGGCCAACATAACTAAGAACAATTCTGGAATGGAAATTATTTGGGTACCCTACACGTATGCGGAACTTGAAAAACTTCTGAAAACCGTTTTAAATACTCTATAA>YBR061C	-:0:chr02:364785:365717ATGGGTAAGAGCAGCAAAGATAAAAGAGATTTGTACTATAGAAAAGCAAAAGAGCAGGGCTATAGGGCTAGATCTGCTTTCAAACTACTTCAACTCAATGACCAATTTCATTTCCTGGATGATCCAAACTTAAAAAGAGTTGTAGATTTGTGTGCAGCACCAGGTTCATGGTCACAAGTGCTCTCGAGAAAACTGTTTGATGAAAGTCCCAGTTCAGATAAGGAGGACAGGAAAATTGTCTCTGTCGATTTACAACCGATGTCCCCCATACCTCATGTGACAACGTTGCAAGCCGATATCACTCATCCTAAAACATTGGCGAGGATTCTAAAACTGTTTGGCAACGAAAAGGCCGATTTTGTTTGTAGTGATGGTGCACCTGATGTTACTGGGTTACACGATCTTGACGAATACGTGCAACAACAGTTAATTATGAGTGCGCTGCAACTTACTGCATGCATTCTGAAAAAAGGTGGAACTTTTGTGGCAAAGATCTTCAGAGGTCGTGATATAGATATGCTATATTCCCAATTGGGCTATCTATTCGATAAAATCGTTTGCGCAAAGCCAAGATCATCAAGAGGTACATCTCTGGAAGCTTTTATTGTTTGTTTAGGCTATAACCCACCATCCAATTGGACACCAAAATTAGATGTAAATACATCCGTTGATGAATTTTTTCAAGGCTGTTTTTTGAATAAATTGTGTATATCAGACAAATTGTCTCATTGGAATGAAGAGGAGAGAAACATAGCCGAATTTATGGCTTGTGGAAGTCTTCAAAGTTTCGACTCAGATGCCACTTATCATGACCTACCTTCTTCGGTTGCAGGCACTTCATCGTCCTTAGATCCTGTTCAAAGCCCGACGAACCCTCCCTACAAGAAAGCTTTGGAATTAAAAAGGAGCGGGAAACTCACTAGATCAGTTTGA>YBR065C	-:0:chr02:368582:369676ATGAATGATGAAATTAATGAGCCACCGCCCAATATATGTGAGCAGTGCTTAGGTGATGAGGCTAATATACGAATGACTAAAATTCCACAAGGCTCTGAATGTAAGATTTGTACCTTACCGTTCACCTTATACCATTTTAAGACGTCTAAACGAAGCAATAATATCATTAAAACATTAATATGTGTACGGTGCGCCACTCAGAGAAACATTTGTCAATGCTGTATGCTTGATTCAAGATGGCACATCCCTATACAACTGCGGGATCATTTAATATCCCTTGTGAACGAGGAAAACGTTATGACGGAGGAAGCAAAAAACGACATGATGAAAAGGTTTCTGTCACTAAAAAACGTGAAATTAGGAGGAGCTCAGATTACGAGTGATCCGTCAGAAGCAGATAATATCGTTGATAAATTGAAAAATATACTTCTGCGAGCAACATCAGATGGCCCAAGTACTCCGTTAATAAAAAACACAACTGCATTGTATAAAAATGAAAAAGGTGCAAATGAGGTCAAAAACTTGGAAAAGTACGCGTCCGTGGACATTTCTCATATTTTGAAGAAACTACCCTTGAATGAATCGTTTTTAAAAAACCCCTCCACCAAATCATTTTTTCTGTATAATATTGATGCCTCGATCCCTGAATGGAAAATAACTGATACAGTTTCGCAATTATTGGGTATAAAGAAATGGAAGGATGGAAATTCATTGTCATTAATAGTCAACCACAAGGCGAAGTGTGGTGGCCTAAGATTTCAATCTAGTGAATTGGGGGAGCGGTTTGTCAGTAAAATAAGTGAGACACTTGTCACACCAAAGGGCCTAAAGAGAGGAGTTTTACTTATTGACCGTTTTAGAATCTTTATCATTCCGTGGTCGTCTGGCTTTTCAGCTGCGTCATTCGGAACCAACACTGCAGAAAATATAAAACTTAGTTTAAGTTTGAATAAGCTTATTCAGTTGGAGCTAGGGCTTTCTTTTCCTACAAAAAGCACAGATAACGCAAAGAATGACAAGAAAAAGACATCAAAAAAAGTTCATAAGGACAGATCAAAGAAATCGAAACCTCGCGCTAACAAGTTAACAATATAG>YBR066C	-:0:chr02:370035:370697ATGTCCATAGGTTACAAAGACAACTTGATGTCAACTATTCTAGCTAAAGATAGGAAATGTGAATTTCCCATCAATTTCGAATGTTCGCCCTCCCAGATAACTCTTATGCCTGAAATGTTCTCTTTTAACAATGAAAGGAAATACCAAACCCTGATACCCTTGATGAAAACATCACATCTAATTGATGATGACTTGAAAGATAAATTGAATAAGTGCGCTTTCGATTTTTTCTCCGGAAAACAAGCGAACAGAACGAGTGACGGGACTATTTCAAGATTAACAGCAAGTGGAAAAACGTCACCGATACTGCCATTGCAAAATATTAATATTGTAAAAGCAGAGAATACCGGTAACGGTAAATCAGATCCATATAGCTCAATTAAAATAAGCAAACCGACAAAAACCGTGATAAAATTGAAATCTACGAAAACCAATACAGCAGGACAGAGGACTCGTCATTTCTGTAAGATCTGCTCTACTGGGTTCACCACTTCTGGTCATCTTTCAAGACATAACAGAATCCATACAGGTGAAAAAAATCATATCTGTCCGCATGAGGGCTGTGGACAGAGGTTTAGTAGACATGATAACTGTAATCAGCATTATCGAACTCATGCAAACAAGAAGAAAAGAAACTGGAAGAGGAGGGAGGCTAGCAGTTGA>YBR067C	-:0:chr02:372100:372732ATGTCCGTTTCCAAGATTGCTTTCGTTTTAAGTGCCATTGCCTCTTTGGCCGTCGCTGACACCAGCGCCGCCGAAACTGCTGAATTGCAAGCTATTATCGGTGACATCAACTCTCATCTTTCTGACTACTTGGGTCTAGAAACTGGCAACAGTGGATTCCAAATTCCATCTGATGTCTTGAGTGTGTATCAACAAGTCATGACTTACACCGATGACGCTTACACTACCTTGTTTAGTGAATTGGACTTTGATGCTATCACTAAGACAATTGTTAAATTGCCATGGTACACCACAAGATTGAGTTCTGAAATCGCTGCTGCTCTTGCCTCCGTTTCCCCAGCTTCTTCCGAGGCTGCATCTTCTTCCGAGGCTGCATCTTCTTCCAAGGCTGCATCTTCTTCCGAAGCTACATCCTCTGCCGCTCCATCCTCTTCTGCTGCCCCATCTTCTTCTGCTGCCCCATCATCATCTGCCGAATCATCTTCTAAGGCCGTTTCTTCTTCTGTCGCTCCAACTACCTCTTCTGTCAGCACTTCTACAGTCGAAACTGCTTCCAATGCCGGTCAAAGAGTCAATGCAGGCGCTGCCTCTTTCGGTGCTGTTGTTGCAGGTGCAGCTGCTTTATTGTTATAA>YBR068C	-:0:chr02:373858:375687ATGCTATCTTCAGAAGATTTTGGATCTTCTGGGAAAAAGGAAACTTCTCCTGATTCGATATCGATACGTTCCTTTAGTGCCGGGAATAATTTCCAATCATCATCAAGTGAGAAAACTTATTCTAAGCAAAAATCCGGGAGTGACAAACTTATACATAGATTTGCGGATTCATTCAAAAGAGCCGAGGGTAGCACTACAAGAACTAAGCAAATAAATGAAAATACGTCTGATTTAGAGGATGGCGTTGAGTCTATCACGTCGGATTCCAAGTTGAAAAAGTCCATGAAGTCGCGCCATGTTGTCATGATGTCTTTAGGGACAGGTATTGGGACTGGTCTTTTGGTAGCTAATGCAAAAGGTCTACATTACGGTGGTCCTGCTGCGCTAATAATTGGTTACATCTTGGTTTCTTTCGAGACGTACTTCATGATCCAAGCTGCAGGTGAGATGGCGGTAACCTATCCGACTTTACCAGCAAATTTCAACGCATACTCCTCCATATTCATTTCCAAATCATTTGGATTCGCCACAGTATGGCTTTACTGTTTCCAATGGCTAACGGTTTTGCCTTTAGAGTTAATAACCGCGTCTATGACTATTCAATTTGGGAATGATAAAATAAATCCGGACATTTATATTCTTATTTTCTATGTTTTCTTAGTATTCATTCATTTCTTCGGTGTAAAAGCCTATGGTGAAACGGAATTCATCTTCAATTGCTGTAAAATTTTAATGATTGCAGGTTTCATTATTCTTTCTATTGTTATCAACTGTGGTGGGGCCGGAAATGACGGTTATATCGGGGCCACTTATTGGCATAATCCAGGTGCTTTTGCAGGTGACACATCGATTGGTAGGTTCAAAAACGTTTGCTATATTTTAGTTACTGCTTACTTCTCCTTTGGTGGTATGGAATTATTTGCACTAAGTGTTCAGGAACAGTCTAACCCTAGAAAATCTACTCCGGTGGCAGCCAAGAGAAGCATTTATCGTATCGTTGTGATTTATCTTTTGACTATGATCCTCATTGGATTCAATGTTCCATATAATGATGACCAACTAATGGGCGCAGGCGGATCCGCTACACATGCATCTCCCTATGTCTTAGCCGCTTCTATTCACGGTGTGAAAATTGTTCCACATATTATCAACGCTGTTATTTTGATTTCTGTGGTTTCAGTGGCAAATTCCTCTTTGTATGCTGGTCCAAGACTGATTTGCTCTTTGGCCCAACAAGGCTACGCACCCAAGTTTTTAGATTACGTTGACAGAGAGGGCAGGCCCTTGAGAGCTCTTATTGTGTGTTGCGTTTTCGGCGTCATTGCTTTTGTTGCAGCTTCATCAAAGGAAGAGATCGTGTTTACATGGTTAGCAGCTATCGCAGGCTTGAGTGAATTATTCACATGGACTTCCATAATGTTGTCCCATCTGCGATTCAGACAAGCAATGAAAGTACAGGGAAGGTCTCTAGACGAGTTGGGATACAAGGCCACAACAGGGATTTGGGGTTCCATATACGGTGTCTTTTTTAATATTTTAGTCTTTGTTGCCCAATTTTGGGTAGCATTGGCCCCCTTAGGTAATGGGGGCAAATGCGATGCGGAATCCTTCTTTCAAAATTATTTAGCTTTTCCAATATGGTTGGCCTTTTACTTCGGATATATGGTTTACAACCGAGATTTTACGCTATTAAATCCCCTCGACAAGATTGACCTTGACTTCCACAGACGCATTTATGATCCAGAGCTAATGAGACAAGAGGACGAAGAAAATAAAGAAAAACTAAGGAATATGTCTTTGATGAGAAAAGCTTATCATTTCTGGTGTTAA>YBR069C	-:0:chr02:376571:378430ATGGACGATAGTGTCAGTTTCATTGCCAAAGAGGCCAGTCCAGCACAATATTCGCACAGTTTGCATGAAAGAACACACAGTGAAAAACAAAAGAGAGACTTTACAATAACAGAAAAACAAGATGAGGTATCTGGACAAACAGCGGAGCCTCGAAGGACGGACAGCAAATCCATATTACAGAGGAAATGCAAAGAATTCTTCGACTCTTTTAAAAGGCAGCTGCCACCAGACCGTAATTCCGAACTAGAGTCCCAAGAAAAAAACAACCTGACAAAGTCGATCAAATCTCGTCACTTAGTCATGATCAGTCTCGGTACCGGTATAGGTACTGGTTTACTGGTCGGTAATGGTCAGGTGCTGGGAACAGCTGGTCCTGCCGGGTTAGTCCTTGGTTACGGAATAGCATCGATCATGCTTTACTGTATCATCCAAGCGGCAGGCGAGTTAGGTCTCTGTTATGCAGGACTAACCGGCAATTACACCAGATATCCTTCTATTTTAGTCGACCCTTCGTTGGGTTTTGCAGTTTCTGTGGTTTACACCATTCAATGGCTAACTGTTCTGCCCTTACAATTGGTCACTGCGGCAATGACAGTTAAGTACTGGACGAGTGTAAATGCAGACATCTTTGTTGCCGTGGTTTTCGTTTTTGTAATTATCATTAATCTATTTGGGTCCAGAGGCTATGCTGAAGCCGAGTTCATTTTCAATTCCTGTAAGATATTGATGGTAATCGGATTCGTCATTCTGGCGATTATCATCAATTGTGGTGGTGCAGGTGACAGAAGATACATCGGTGCTGAGTACTGGCACAATCCTGGTCCATTTGCCCACGGTTTCAAAGGTGTATGTACGGTTTTCTGTTATGCTGCATTCAGCTACGGTGGAATTGAAGTTCTACTTCTATCTGCCGCTGAGCAGGAAAACCCAACAAAATCTATTCCCAATGCCTGCAAAAAAGTGGTATACCGTATTCTGCTGATTTACATGCTCACCACCATTTTGGTTTGCTTTCTTGTTCCATACAACAGTGACGAATTATTAGGGTCTAGTGACTCCTCTGGATCCCATGCCTCCCCATTTGTCATTGCTGTTGCATCACATGGTGTCAAAGTTGTGCCTCACTTTATAAATGCTGTTATTTTGATTTCTGTCATTTCTGTTGCAAACTCTTCGTTATATTCTGGGCCAAGATTGTTATTATCATTGGCAGAACAAGGCGTCTTACCTAAATGCTTAGCATATGTTGATAGAAACGGCAGGCCTTTACTTTGTTTTTTTGTTTCCCTTGTCTTTGGATGCATTGGGTTTGTAGCCACTAGTGACGCTGAAGAACAGGTTTTCACCTGGTTATTGGCAATTTCTAGTCTTTCGCAATTATTTATCTGGATGTCAATGTCCTTGTCACATATAAGATTTAGAGATGCCATGGCAAAGCAAGGTCGTTCGATGAACGAAGTTGGATACAAAGCACAAACTGGCTATTGGGGTTCATGGCTTGCAGTTTTAATTGCAATCTTCTTCCTTGTGTGCCAGTTCTGGGTAGCCATCGCACCTGTTAATGAACATGGAAAACTTAACGTCAAAGTGTTTTTCCAAAATTATTTGGCAATGCCCATCGTTTTGTTTGCCTATTTTGGTCACAAGATATACTTCAAATCATGGAGCTTTTGGATTCCAGCCGAAAAAATTGATTTGGACTCACATAGAAACATATTTGTGTCTCCATCGCTTACAGAGATCGACAAAGTTGACGATAACGATGATCTTAAAGAGTATGAAAATTCAGAATCGTCGGAAAATCCAAACAGCTCGCGTTCAAGAAAGTTTTTTAAGAGGATGACCAATTTCTGGTGCTAA>YBR070C	-:0:chr02:379218:379931ATGAAAACGGCCTACTTGGCGTCATTGGTGCTCATCGTATCGACAGCATATGTTATTAGGTTGATAGCGATTCTGCCTTTTTTCCACACTCAAGCAGGTACAGAAAAGGATACGAAAGATGGAGTTAACCTACTGAAAATACGAAAATCGTCAAAGAAACCGCTCAAGATTTTTGTATTCTTAGGATCGGGAGGTCATACTGGTGAAATGATCCGTCTTCTAGAAAATTACCAGGATCTTTTACTGGGTAAGTCGATTGTGTACTTGGGTTATTCTGATGAGGCTTCCAGGCAAAGATTCGCCCACTTTATAAAAAAATTTGGTCATTGCAAAGTAAAATACTATGAATTCATGAAAGCTAGGGAAGTTAAAGCGACTCTCCTACAAAGTGTAAAGACCATCATTGGAACGTTGGTACAATCTTTTGTGCACGTGGTTAGAATCAGATTTGCTATGTGTGGTTCCCCTCATCTGTTTTTATTGAATGGGCCTGGAACATGCTGTATAATATCCTTTTGGTTGAAAATTATGGAACTTCTTTTGCCCCTGTTGGGTTCCTCCCATATAGTTTATGTAGAATCGCTGGCAAGGATTAATACTCCTAGTCTGACCGGAAAAATATTATATTGGGTAGTGGATGAATTCATTGTCCAGTGGCAAGAATTGAGGGACAATTATTTACCAAGATCCAAGTGGTTCGGCATCCTTGTTTAA>YBR071W	+:0:chr02:380408:381043ATGCTAAGACGCTCTAAAAATTCCAGCACTAACACAAACGCAGACACCAAGAAAAGACAATCCATGCATTTAGGATCCAAAAGTTCGCTAATCTCGCTGACTAGCGAATTTGGCCATGGACACTCCAAGACGAAGCAGAAAAAGGAGGAGGGCACAGCTCCTTCTCAATTCCTGTCTCCTACGAATAAGAGAAGCACATCTAGTCAGTCAAAGTTAAAAAGGAGCAGCTTGTTACTGGACGAAACACTGCTTAAAGACTACCACTCGGCCATGAGGCACATGCAAACAAACGCTGCAAAGGAAGAAAAGCTGCGGATGGCTCCATCACCAACGCAGTCAACGAGGAGCGAATCAGACGCCAGTCTTTCCTCCACCAAGAGCTCGATCTCTTCCATATTTTCCCAAGATAATGACTATTCCATTCACGATTTGTTGTACGAAGATATTGAAGAGATGGATAAAACAGACGCTTTCAAAATTAACAACACAATAGCAATCGATGATTCTAAAGCTCTCTTTGTCTTCTGTTCAAACGACTCCTCCTCAAGGACAGCGTCTATCGAAACATTGCACGAATCAAATTTGGACAACCTGGATATGGGTTCCAGTAGAAGGACATCGTTGGACTTTTTTTAA>YBR072W	+:0:chr02:382027:382671ATGTCATTTAACAGTCCATTTTTTGATTTCTTTGACAACATCAACAACGAAGTTGATGCCTTTAACAGATTGCTGGGTGAAGGCGGCTTAAGAGGCTACGCACCAAGACGTCAGTTAGCAAACACACCCGCAAAGGATTCTACTGGCAAGGAAGTTGCTAGACCAAATAACTATGCTGGCGCTCTTTATGATCCCAGAGATGAAACCTTAGATGATTGGTTCGACAATGACTTGTCCCTGTTCCCATCTGGTTTCGGTTTCCCTAGAAGTGTCGCAGTTCCAGTTGATATTTTGGACCATGACAACAACTACGAGTTGAAAGTCGTGGTTCCTGGTGTCAAAAGCAAGAAGGACATTGATATTGAGTACCATCAAAACAAGAACCAAATTTTGGTTTCTGGTGAAATTCCATCTACCTTGAATGAAGAGAGTAAAGACAAGGTCAAGGTCAAGGAGAGCAGCTCTGGTAAGTTCAAGAGAGTCATCACTTTGCCAGACTACCCAGGTGTGGATGCAGACAACATTAAAGCAGACTACGCAAATGGTGTTTTGACATTAACAGTTCCAAAATTGAAGCCTCAGAAGGATGGTAAGAACCACGTCAAGAAGATTGAGGTTTCTTCTCAAGAATCGTGGGGTAACTAA>YBR073W	+:0:chr02:383209:385983ATGCAGATACCGAAATATGAGAACAAGCCATTCAAGCCTCCAAGAAGGGTTGGATCAAATAAGTACACACAACTCAAACCAACCGCCACTGCAGTCACAACAGCCCCTATATCTAAAGCCAAAGTTACTGTCAACTTGAAAAGAAGCATTTCGGCGGGACCTACTTTAAATCTTGCCAAGAAGCCGAATAATCTGTCCTCAAATGAAAACACTAGATATTTTACTATCATGTACAGGAAGCCTACTACCAAAAAGCACAAGACTTGGAGTGGTGATGGCTACGCTACCTTAAAAGCCAGTAGCGATAAGTTATGCTTTTATAACGAAGCAGGGAAATTTCTTGGGTCAAGTATGCTACCAAGTGATTCAGATTCTCTCTTCGAAACTCTTTTCAAAGCAGGCTCCAATGAAGTACAATTGGATTACGAATTGAAGGAAAATGCAGAAATACGTAGCGCCAAAGAAGCCTTATCACAAAACATGGGAAATCCCAGCCCACCGACCACAAGCACAACAGAAACAGTGCCTTCTACGAAGAATGACGGTGGCAAATACCAAATGCCTCTGTCTCAGCTGTTTTCACTAAACACTGTGAAAAGATTCAAATCAGTAACAAAGCAAACAAATGAACACATGACCACAGTACCTAAAACCAGTCAAAATTCCAAAGCCAAAAAATATTATCCAGTATTTGATGTCAACAAAATCGATAATCCTATAGTAATGAACAAAAATGCAGCCGCTGAAGTTGACGTAATTGTTGATCCATTACTGGGCAAATTCTTGCGCCCTCATCAGAGGGAAGGGGTGAAGTTCATGTATGATTGCTTAATGGGCTTGGCAAGACCAACTATTGAAAATCCGGATATCGATTGTACTACTAAAAGTTTAGTGTTAGAAAATGACTCAGATATTAGTGGATGCCTTTTGGCTGATGATATGGGTTTAGGTAAAACACTAATGAGTATAACTTTGATTTGGACATTAATTAGGCAAACTCCTTTTGCATCAAAAGTTTCATGTTCGCAATCAGGCATACCATTAACTGGACTTTGTAAGAAGATTTTAGTCGTTTGTCCCGTTACTTTAATAGGAAATTGGAAAAGAGAATTTGGAAAATGGTTAAATTTGTCAAGAATAGGTGTTTTGACATTAAGCTCAAGGAATTCTCCTGATATGGATAAAATGGCTGTCAGAAATTTTTTAAAAGTGCAACGAACTTATCAAGTCTTGATTATTGGCTACGAAAAACTCTTGAGTGTTTCTGAAGAATTAGAGAAAAATAAACATTTGATTGACATGCTGGTGTGTGACGAAGGCCATCGACTAAAAAACGGGGCTTCTAAAATTTTAAATACGCTGAAGAGTTTAGACATAAGAAGGAAGCTTTTGCTTACGGGAACTCCTATACAAAATGATCTTAATGAGTTTTTCACTATTATAGATTTCATAAACCCAGGAATCCTTGGAAGCTTCGCTTCTTTCAAAAGAAGATTCATTATCCCTATAACTAGAGCCAGAGACACTGCAAACAGATACAACGAAGAATTGTTGGAAAAGGGGGAAGAAAGGTCAAAAGAGATGATAGAAATTACGAAAAGATTTATTTTGAGACGAACAAATGCGATTTTAGAAAAGTACCTTCCTCCAAAGACGGATATAATTTTATTCTGTAAACCATACAGCCAACAGATATTGGCATTCAAAGATATTTTGCAGGGCGCACGTTTAGATTTTGGACAATTGACGTTCAGTTCTTCGCTAGGACTAATAACATTACTGAAAAAGGTTTGTAACTCTCCTGGATTGGTTGGCTCAGATCCCTATTACAAATCACATATAAAGGATACCCAATCTCAGGACAGCTATAGTCGTTCTTTGAACTCTGGTAAGTTAAAGGTATTAATGACATTACTAGAAGGTATTAGGAAGGGTACCAAGGAGAAGGTCGTCGTAGTGTCTAACTACACTCAAACATTGGATATAATTGAAAATTTGATGAATATGGCTGGGATGTCACATTGCAGACTCGACGGTTCCATACCTGCTAAACAAAGGGACTCTATCGTCACATCTTTCAATCGGAATCCAGCCATATTTGGATTCTTGTTGAGTGCAAAATCGGGAGGTGTAGGATTGAATCTAGTCGGTCGTTCGCGACTTATTTTATTTGATAATGATTGGAATCCTTCAGTAGATTTGCAAGCGATGTCACGAATTCATAGAGATGGTCAAAAAAAGCCGTGCTTCATATATAGACTTGTCACAACTGGGTGTATCGATGAGAAAATATTGCAAAGGCAATTAATGAAGAACAGTTTGAGCCAAAAATTTCTAGGTGACTCGGAGATGAGAAATAAAGAATCTTCTAATGATGATCTTTTCAATAAAGAGGACTTGAAGGACCTGTTTTCTGTCCATACAGATACCAAGAGTAACACACATGACTTAATTTGTTCTTGCGATGGTTTAGGTGAGGAAATTGAATATCCTGAAACAAATCAACAGCAGAACACCGTAGAGCTGAGAAAGCGTAGCACTACGACATGGACAAGTGCGCTGGATTTACAAAAGAAAATGAATGAAGCAGCCACCAACGATGATGCCAAAAAGTCACAATACATTAGGCAATGTCTCGTTCATTATAAGCATATCGATCCAGCAAGACAAGATGAATTATTTGATGAGGTTATCACAGATTCGTTCACCGAATTGAAAGATAGTATTACCTTTGCGTTTGTAAAGCCCGGCGAGATATGTCTCAGAGAACAATGA>YBR077C	-:0:chr02:391799:392287ATGGTGATGCTCCATTCTAAAAACGTTAAAGGGTTTCTGGAAAACACTTTGAAACCGTATGATTTGCATTCGGTAGACTTCAAGACATCGTCTTTGCAATCGTCTATGATTATAACCGCCACTAATGGGGGTATACTGTCCTATGCGACGTCAAACAACGATGTACCGAAAAATTCCATAAACGAAATAAACTCGGTCAATAACTTGAAAATGATGAGCTTATTGATTAAGGATAAGTGGTCAGAGGATGAAAACGATACTGAGGAACAGCACTCCAATAGCTGTTACCCTGTGGAAATCGACTCCTTTAAGACAAAAATATATACTTACGAAATGGAAGATTTACATACCTGTGTCGCACAGATACCCAATAGCGACCTTTTGCTATTGTTCATTGCGGAAGGTAGCTTCCCTTACGGACTACTGGTAATTAAAATTGAAAGAGCTATGAGAGAGTTGACTGATTTGTTTGGCTACAAGCTAGGTTGA>YBR082C	-:2:chr02:406622:407021AGATCCACCTACTTCATGTTCAGCCGGTCCAGTCGGCGATGATCTATATCACTGGCAAGCATCCATCATGGGACCTGCCGATTCCCCATATGCCGGCGGTGTTTTCTTCTTGTCTATCCATTTCCCAACCGACTACCCATTCAAGCCACCAAAGATCTCCTTCACAACCAAGATATATCATCCAAATATCAATGCCAATGGTAACATCTGTCTGGACATCCTAAAGGATCAATGGTCTCCAGCTCTAACTCTATCGAAGGTCCTATTATCCATCTGTTCTTTGTTAACAGACGCTAATCCTGACGATCCTTTAGTACCAGAAATCGCTCATATCTACAAGACTGACAGACCCAAGTACGAAGCTACAGCCAGAGAATGGACAAAGAAATACGCTGTATAA>YBR082C	-:0:chr02:407117:407163ATGTCTTCTTCTAAACGTATTGCTAAAGAACTAAGTGATCTAGAAAG>YBR084W	+:0:chr02:411048:413975ATGTTGTCGAGACTATCTTTATTGAGTAACTCGAGGGCGTTCCAACAGGCCAGATGGCGCATTTACCGCTTAAAAGTTTCGCCAACTGTGCATGCTTCTCAATACCATATTCTTTCTGGTAGGAAACTTGCTCAATCTATTCGAGAAAAAGCCAATGATGAGATACAAGCCATTAAGCTTAAGCATCCTAATTTCAAGCCTACCTTGAAAATCATTCAAGTCGGGGCCAGACCAGACTCATCTACGTACGTGAGGATGAAATTGAAAGCTTCAAAGGACAGCAATGTAGACTGTATCATAGAGAAGTTACCAGCAGAAATCACTGAAGTTGAGCTTTTGAAGAAAATTAGTGACATCAATGATGATGACTCTATCCATGGGTTGCTAATTCAACTACCTCTACCGCGTCACTTGGATGAAACCACGATTACAAACGCTGTGGACTTTAAAAAGGACGTTGATGGGTTCCACAGATATAATGCTGGTGAATTGGCTAAAAAAGGAGGGAAACCATACTTCATACCATGTACTCCTTATGGTTGCATGAAATTACTTGAAGAAGCTCATGTTAAGTTAGATGGTAAGAACGCCGTCGTGTTAGGCAGATCAAGTATCGTCGGAAATCCAATTGCTTCGTTGTTGAAAAATGCGAATGCCACTGTTACTGTCTGTCATAGTCATACAAGGAACATTGCAGAAGTGGTCTCCCAAGCTGATATAGTTATCGCAGCTTGCGGTATTCCTCAATACGTTAAATCAGACTGGATTAAAGAAGGCGCCGTGGTTATTGATGTAGGTATCAACTACGTACCTGATATCAGTAAGAAAAGTGGGCAAAAATTAGTTGGTGATGTTGATTTTGATTCTGTAAAGGAAAAGACATCTTATATTACCCCTGTTCCTGGTGGAGTGGGTCCAATGACTGTCGCTATGCTTGTTTCCAATGTACTATTAGCTGCTAAAAGGCAATTCGTGGAATCTGAAAAGCTTCCAGTTATCAAACCTCTTCCATTACACTTAGAAAGTCCAGTGCCTTCAGATATTGATATATCAAGAGCTCAGAGTCCTAAGCATATCAAGCAAGTTGCCGAGGAGTTGGGAATCCACTCTCACGAATTAGAATTATACGGCCACTATAAGGCAAAAATTTCTCCAAATATTTTTAAAAGATTAGAATCTAGAGAAAACGGTAAGTACGTCCTTGTTGCAGGTATTACTCCGACTCCATTGGGTGAAGGTAAATCCACTACGACTATGGGGTTGGTGCAGGCTTTATCCGCTCATTTAGGGAAACCATCCATCGCGAACGTTAGACAACCATCTCTTGGCCCAACCCTGGGTGTCAAAGGTGGTGCTGCTGGTGGTGGTTATGCCCAAGTTATTCCTATGGACGAGTTCAATTTACATTTGACCGGGGATATTCATGCTATCAGCGCTGCGAACAATCTTCTTGCAGCAGCTATCGACACTAGAATGTTCCATGAAGCCACTCAGAAGAATGATAGTACATTTTACAAGAGACTAGTTCCAAGAAAAAAAGGCATCAGAAAGTTTACCCCATCCATGCAGAGAAGGCTTAAAAGATTGGATATTGAAAAAGAAGACCCTGATGCTTTAACACCTGAAGAAGTCAAAAGATTTGCTAGATTGAACATAAATCCCGATACTATCACTATCAGAAGAGTTGTCGACATCAATGACAGGATGTTAAGACAAATTACCATTGGCGAAGCCGCTACGGAGAAGGGTTTTACAAGGACCACTGGATTCGATATCACTGTTGCCTCTGAATTAATGGCCATTTTAGCTCTATCTAAAAGCTTACACGAGATGAAGGAACGTATTGGACGCATGGTTATTGGTGCTGATTATGATAACAAACCAGTAACAGTAGAAGATATTGGCTGTACCGGTGCTCTGACTGCATTATTACGTGACGCTATAAAGCCTAACTTAATGCAAACTTTGGAAGGGACCCCCGTAATGGTTCACGCTGGTCCTTTCGCCAACATCTCCATCGGCGCATCATCAGTAATTGCAGACTTAATGGCATTGAAGCTTGTTGGTTCAGAAAAGAACCCGTTAAATGACAAGAACATCCATGAACCTGGTTATGTAGTTACTGAAGCAGGATTCGATTTTGCCATGGGTGGTGAAAGATTCTTTGATATCAAATGTCGTTCCTCTGGATTGGTGCCAGATGCAGTTGTCTTAGTCGCAACCGTAAGAGCTTTGAAATCTCATGGAGGTGCTCCAAATGTTAAGCCCGGACAATCATTACCAAAAGAATACACAGAGGAAAACATCGATTTTGTTGCCAAGGGTGTTAGTAATTTGGTTAAGCAGATTGAAAACATCAAAACGTTTGGAATACCAGTCGTTGTAGCAATCAACAGATTTGAAACAGACTCACAGGCAGAGATTGAGGTAATCAAGAAAGCAGCCTTGAATGCTGGCGCATCTCATGCCGTTACTTCTAATCACTGGATGGAAGGTGGTAAAGGTGCAGTAGAATTAGCACATGCTGTGGTAGATGCAACGAAAGAACCAAAGAACTTTAACTTTTTGTACGACGTCAATAGCTCCATCGAGGACAAGCTTACCAGCATCGTCCAAAAAATGTATGGTGGGGCAAAAATCGAAGTATCACCAGAAGCCCAAAAAAAGATAGACACCTACAAAAAACAAGGCTTCGGTAATCTTCCCATCTGTATTGCTAAGACACAATATTCATTATCCCATGATCCATCATTAAAGGGTGTTCCTAGAGGTTTTACGTTCCCCATCAGGGATGTGAGAGCTTCAATAGGTGCAGGTTATTTATACGCTTTGGCTGCAGAAATTCAAACCATACCGGGTCTATCGACATATGCTGGTTACATGGCAGTAGAAGTCGACGACGACGGTGAAATTGAAGGTCTATTTTAA>YBR085C-A	-:0:chr02:418901:419158ATGTCATCTGCTCTATACAAACAAAGCACAAATTTTACTCATTCTACCGGTTCTTTCTTGCAAAGCGCACCTGTTGAACTGACGACAGTCAGTGGGTACCAGGAGTTTTTGAAGAAGCAAGAGAAAAAGAACTATGAAATTCAAACAGTTTTAAGCGAGGATAAATCACATGGTTATGTGCTAAAGGACGGCGAAGTCATCGCTAATATCATTGGTGAGGCCAAAGATTATCTTTTGGATTTGGCCGGCCAGGCTTGA>YBR085W	+:0:chr02:415977:416900ATGAGTAGCGACGCTAAGCAACAAGAAACAAACTTTGCCATTAATTTCTTAATGGGTGGTGTGAGTGCGGCCATCGCTAAAACTGCTGCCTCACCAATCGAAAGAGTCAAGATCTTGATCCAAAATCAAGATGAAATGATCAAGCAAGGAACTTTAGATAAAAAGTATTCCGGTATCGTGGATTGTTTCAAGAGAACTGCAAAGCAAGAGGGACTAATATCCTTTTGGCGAGGAAATACTGCCAATGTTATTCGTTATTTTCCCACTCAAGCTTTGAACTTCGCCTTCAAAGATAAGATTAAGTTGATGTTTGGTTTCAAGAAAGAGGAAGGCTATGGTAAATGGTTTGCAGGTAATCTGGCTTCTGGTGGTGCAGCTGGTGCTCTTTCGTTATTATTTGTTTATTCTTTAGATTTTGCCAGAACCAGACTTGCTGCTGATGCAAAATCGTCGAAAAAGGGTGGCGCTCGCCAATTCAATGGGTTGACTGATGTTTATAAAAAGACCTTGAAATCGGATGGTATCGCAGGATTATACAGAGGATTCATGCCATCAGTAGTGGGTATCGTGGTTTATAGAGGACTATATTTCGGTATGTTTGATTCTCTCAAGCCACTGGTGCTAACTGGTTCATTAGATGGTTCATTCTTGGCTTCATTTTTATTGGGATGGGTGGTCACTACAGGTGCCTCAACATGTTCTTATCCATTAGACACAGTGAGAAGAAGAATGATGATGACTTCAGGTCAAGCAGTAAAGTACAACGGTGCTATAGATTGTCTCAAAAAAATCGTAGCTTCTGAAGGTGTAGGGTCATTGTTCAAAGGCTGCGGGGCAAATATCTTGAGAAGTGTTGCTGGAGCTGGTGTTATTTCCATGTATGACCAGTTGCAAATGATATTGTTCGGTAAAAAATTCAAATGA>YBR087W	+:0:chr02:423759:424823ATGTCATTGTGGGTAGATAAATACAGACCTAAGTCCTTGAATGCTCTTTCACATAATGAAGAGTTGACAAATTTTCTAAAATCGTTATCTGATCAGCCTCGTGATTTACCTCATCTTTTACTGTATGGACCAAATGGTACAGGTAAGAAAACGCGTTGTATGGCATTATTGGAGTCCATATTTGGACCTGGAGTCTATAGATTGAAAATTGATGTCAGACAATTTGTCACTGCTTCGAACAGAAAACTAGAACTGAATGTGGTCAGCTCGCCATACCATTTAGAGATCACGCCAAGTGATATGGGTAACAATGATAGAATTGTCATCCAAGAACTATTGAAAGAAGTGGCTCAAATGGAACAAGTGGACTTTCAAGATTCTAAGGATGGACTTGCCCATAGATATAAGTGTGTTATTATCAACGAGGCGAACTCGTTAACAAAAGATGCTCAAGCTGCTTTAAGACGTACCATGGAAAAATACTCCAAAAACATTAGGTTGATAATGGTCTGCGATTCGATGTCGCCTATAATTGCTCCTATCAAATCCCGTTGTCTGTTGATTCGTTGTCCTGCACCAAGCGATAGCGAAATTTCAACTATCTTGTCTGATGTGGTGACAAATGAAAGAATACAACTAGAAACAAAGGATATTTTAAAAAGAATTGCTCAGGCATCGAATGGAAACTTGCGAGTCTCCCTATTAATGCTTGAATCTATGGCACTGAACAACGAATTAGCATTGAAAAGCAGTAGCCCTATAATAAAACCAGATTGGATTATAGTGATCCATAAATTAACGAGGAAAATCGTTAAAGAGAGATCTGTCAATTCTTTAATCGAATGCAGAGCTGTCCTATACGATTTACTAGCTCATTGTATACCTGCCAATATCATCTTAAAGGAACTAACGTTTTCTTTGTTGGATGTGGAAACCCTGAATACCACGAATAAATCGTCCATAATTGAATACTCAAGTGTTTTTGACGAAAGATTATCACTTGGAAACAAAGCAATATTCCATTTGGAAGGGTTCATAGCAAAAGTTATGTGCTGTCTAGATTAA>YBR088C	-:0:chr02:424984:425760ATGTTAGAAGCAAAATTTGAAGAAGCATCCCTTTTCAAGAGAATAATTGATGGTTTCAAAGATTGTGTCCAGTTGGTCAATTTCCAATGTAAAGAAGATGGTATCATTGCACAAGCTGTCGATGACTCAAGAGTTCTATTGGTCTCCTTGGAAATAGGTGTCGAAGCCTTCCAAGAATATAGATGTGACCATCCTGTTACGTTAGGTATGGATCTAACCTCACTAAGTAAAATCCTACGTTGTGGTAACAACACCGATACATTAACACTAATTGCTGACAACACACCGGATTCCATCATCTTATTATTTGAGGATACCAAGAAAGACCGTATAGCCGAATACTCTCTGAAATTGATGGATATCGATGCTGATTTCTTAAAGATTGAAGAATTACAGTACGACTCCACCCTGTCATTGCCATCTTCCGAATTCTCTAAAATTGTTCGTGACTTGTCCCAATTGAGTGATTCTATTAATATCATGATCACCAAAGAAACAATAAAGTTTGTAGCTGACGGTGATATCGGATCAGGTTCAGTCATAATAAAACCATTCGTGGATATGGAACATCCTGAAACAAGCATCAAACTTGAAATGGATCAACCTGTCGACTTGACGTTCGGAGCTAAATATTTATTGGACATCATTAAGGGCTCCTCCCTTTCTGATAGAGTTGGTATCAGGCTCTCCAGCGAAGCTCCTGCTTTATTCCAATTTGATTTGAAGAGTGGGTTCCTACAGTTTTTCTTGGCTCCTAAATTTAATGACGAAGAATAA>YBR089C-A	-:0:chr02:426184:426483ATGGCCGCAACTAAAGAAGCAAAGCAACCAAAGGAACCAAAGAAGAGGACCACCAGGAGAAAGAAGGATCCTAACGCCCCTAAGAGGCGGTTGTCAGCTTATATGTTCTTTGCTAATGAAAACAGAGACATTGTCCGTTCCGAGAATCCTGACGTAACTTTTGGCCAAGTAGGCAGAATATTGGGTGAGAGGTGGAAGGCCTTAACTGCTGAAGAAAAGCAACCCTATGAATCTAAGGCTCAAGCAGACAAGAAGAGATACGAATCTGAAAAGGAATTGTACAATGCTACACGTGCTTGA>YBR091C	-:0:chr02:427149:427478ATGTCGTTCTTTTTAAATAGTCTAAGGGGCAACCAGGAGGTCTCGCAAGAGAAGCTAGACGTTGCAGGAGTGCAATTCGATGCGATGTGCTCGACTTTCAACAATATTCTCAGTACGTGTCTTGAGAAATGTATTCCGCATGAGGGCTTTGGTGAGCCTGATCTAACGAAAGGTGAGCAATGCTGCATAGACAGATGTGTGGCAAAGATGCATTACAGCAATCGCCTCATTGGAGGATTTGTACAAACACGAGGTTTCGGGCCAGAGAATCAGCTACGACATTATTCGCGATTTGTGGCCAAAGAAATAGCTGATGATTCCAAAAAATAG>YBR092C	-:0:chr02:427692:429095ATGTTTAAGTCTGTTGTTTATTCGGTTCTAGCCGCTGCTTTAGTTAATGCAGGTACAATTCCCCTCGGAGAGTTAGCCGATGTTGCCAAAATTGGCACTCAGGAAGACATATTCCCATTCCTGGGTGGTGCCGGGCCATACTTCTCTTTCCCTGGCGACTATGGTATTTCTCGTGACTTGCCTGAAGGTTGTGAAATGAAACAATTGCAAATGCTTGCCAGACATGGTGAAAGATACCCAACTTACAGTAAAGGTGCTACCATCATGAAAACATGGTATAAGTTGAGCAATTACACACGTCAATTCAACGGCTCATTGTCATTCTTGAACGATGATTACGAGTTTTTCATCCGTGATGACGATGATTTGGAAATGGAAACCACTTTTGCCAACTCAGACAATGTTTTGAATCCATACACTGGTGAGATGGATGCTAAGAGACATGCTCGTGAGTTTTTAGCGCAATATGGCTACATGTTCGAAAATCAAACCAGTTTCCCAATTTTCGCCGCTAGTTCTGAAAGGGTTCATGACACTGCTCAATATTTCATTGATGGTTTAGGTGACCAATTCAACATCTCCTTGCAGACTGTCAGTGAAGCCATGTCCGCCGGCGCAAACACTTTGAGTGCTGGTAATGCGTGCCCAGGATGGGATGAAGATGCTAACGATGACATTTTGGACAAATACGATACCACATACTTGGATGACATTGCCAAGAGATTAAACAAAGAAAACAAGGGTTTGAATTTGACCTCAAAGGACGCCAACACTTTGTTTGCATGGTGTGCATACGAATTGAACGCTAGAGGCTACAGTGATGTTTGTGATATCTTCACCGAAGATGAATTGGTACGTTACTCATACGGCCAGGACCTGGTATCGTTTTACCAGGATGGACCAGGTTATGATATGATCAGATCCGTCGGTGCCAACTTGTTTAACGCTACTTTGAAGTTGTTAAAGCAAAGTGAAACTCAAGACTTAAAAGTCTGGTTGAGTTTTACCCACGATACCGATATCCTAAACTATTTGACCACCGCTGGTATAATTGACGACAAAAACAACTTAACTGCCGAATACGTTCCATTCATGGGCAACACCTTCCATAAGTCCTGGTACGTTCCTCAAGGTGCTCGTGTCTACACCGAAAAATTCCAATGTTCTAACGACACCTACGTCAGATACGTCATTAACGATGCTGTCGTTCCAATTGAAACCTGTTCCACCGGCCCAGGGTTCTCTTGTGAAATCAATGATTTCTACGACTATGCTGAAAAGAGAGTAGCTGGTACTGACTTCCTAAAGGTCTGTAACGTCAGCAGTGTCAGTAACGTCACCGAATTGACCTTCTACTGGGACTGGAATACTACTCACTACAACGATACCCTATTAAAACAATAA>YBR093C	-:0:chr02:429542:430945ATGTTTAAATCTGTTGTTTATTCAATTTTAGCCGCTTCTTTGGCCAATGCAGGTACCATTCCCTTAGGCAAACTAGCCGATGTCGACAAGATTGGTACCCAAAAAGATATCTTCCCATTTTTGGGTGGTGCCGGACCATACTACTCTTTCCCTGGCGACTATGGTATTTCTCGTGATTTGCCTGAAGGTTGTGAAATGAAGCAACTGCAAATGGTTGGTAGACATGGTGAAAGATACCCTACTGTCAGTCTGGCTAAGACTATCAAGAGTACATGGTATAAGTTGAGCAATTACACTCGTCAATTCAACGGCTCATTGTCATTCTTGAACGATGATTACGAGTTTTTCATCCGTGATGACGATGATTTGGAAATGGAAACCACTTTTGCCAACTCGGACGATGTTTTGAACCCATACACTGGTGAAATGAACGCCAAGAGACATGCTCGTGACTTCTTGGCTCAATACGGTTACATGGTCGAAAACCAAACCAGTTTCGCCGTTTTTACCTCTAATTCTAAGAGATGTCATGACACTGCTCAATATTTCATTGATGGTTTAGGTGACCAATTCAACATCACCTTGCAGACTGTCAGTGAAGCTGAATCCGCTGGTGCCAACACTTTGAGTGCTTGTAACTCATGTCCTGCTTGGGACTACGATGCCAATGATGACATTGTAAATGAATACGACACAACCTACTTGGATGACATTGCCAAGAGATTGAACAAGGAAAACAAGGGTTTGAACTTGACCTCAACTGACGCTAGTACTTTATTCTCGTGGTGTGCATTTGAAGTGAACGCTAAAGGTTACAGTGATGTCTGTGATATTTTCACCAAGGATGAATTAGTCCATTACTCCTACTACCAAGACTTGCACACTTATTACCATGAGGGTCCAGGTTACGACATTATCAAGTCTGTCGGTTCCAACTTGTTCAATGCCTCAGTCAAATTATTAAAGCAAAGTGAGATTCAAGACCAAAAGGTTTGGTTGAGTTTTACCCACGATACCGATATCCTAAACTTTTTGACCACCGCTGGTATAATTGACGACAAAAACAACTTAACTGCCGAATACGTTCCATTCATGGGCAACACTTTCCACAGATCCTGGTACGTTCCTCAAGGTGCTCGTGTCTACACCGAAAAATTCCAATGTTCTAACGACACCTACGTCAGATACGTCATTAACGATGCTGTTGTTCCAATTGAAACCTGTTCCACTGGTCCAGGGTTCTCTTGTGAAATCAATGACTTCTACGACTATGCTGAAAAGAGAGTAGCCGGTACTGACTTCCTAAAGGTCTGTAACGTCAGCAGCGTCAGTAACTCTACTGAATTGACCTTCTACTGGGACTGGAACACTACTCATTACAACGCCAGTCTATTGAGACAATAG>YBR094W	+:0:chr02:432030:434291ATGCGGGTTTTAATAACTAACGATGATGGTCCCTTAAGTGATCAGTTTTCACCATACATTAGACCTTTTATTCAGCACATTAAAAGAAATTATCCTGAATGGAAAATCACGGTTTGTGTACCTCATGTCCAGAAATCATGGGTGGGTAAGGCTCATCTTGCTGGTAAAAATTTGACAGCTCAATTCATATATTCTAAAGTTGACGCTGAAGACAATACTTTTTGGGGCCCATTTATCCAGCCACAAATTAGGTCAGAAAACTCTAAATTACCTTATGTTCTCAATGCTGAAATTCCGAAAGATACAATTGAGTGGATATTAATCGATGGAACTCCAGCATCGTGCGCAAATATCGGGCTGCACCTATTGTCTAATGAACCGTTTGATCTAGTACTGTCGGGTCCAAATGTTGGTAGAAATACATCTGCTGCTTATATTACCTCTTCTGGTACCGTAGGAGGCGCAATGGAATCTGTTATTACTGGAAATACAAAGGCCATTGCTATTTCTTGGGCCTATTTTAATGGATTGAAAAACGTCTCCCCACTTTTAATGGAGAAGGCCTCTAAGAGATCTTTAGATGTTATCAAACATCTTGTTAAGAATTGGGACCCAAAAACAGATCTATATAGTATCAATATTCCTTTAGTGGAGAGTTTAAGTGATGATACAAAGGTTTACTATGCACCAATTTGGGAAAATAGATGGATTCCAATATTCAACGGCCCTCACATTAACCTAGAGAATAGCTTTGCTGAGATCGAAGACGGTAATGAATCATCATCGATTTCCTTTAATTGGGCTCCAAAATTTGGGGCACATAAGGATTCTATTCACTATATGGACGAATACAAGGACAGGACTGTTTTAACTGATGCTGAAGTTATTGAATCCGAAATGATCAGTGTCACTCCCATGAAAGCCACTTTCAAGGGTGTGAACCACCTTCTTGGAGAGTTGAAACTGACTGAGGAAGAAAATAATTTATCAAAAACAAATAACCTCATCGTGGTAAGTATAGACCCAATGGAATATATATACAAACCTCTAACCCACGCCCTGAAAAAATATCTGCCACAAGTGGAGATTGTATCAAACTTGCCAGAATTTGACAATGGAGGGTGTGAAAAAGAAATGAAGGTTTTTCATTACGGTGATTACGAACAGCTAGATATGGACAAACTAATGGAACTGCCCAATAATTACTTCACAAACTCTTATATATATAGGAAGGCATTGATAAGAAAACACTTTCTTTCGCATACCATACAGACTTATACTGCGAAGAACCCAGAGTCCATTTTAAAGAAGGCATATTTGGAATCATTCACTATTGATTTAGATTATCGTGAATTTCTAGACGACGCATTAGATGAAAATTGGGAATTACGCCAAGAGTTGGAGAATGAAAGCCAAGATAAATGGTGGATCGTGAAACCGAGTATGAGCGATAAAGGTCAAGGTATCAGGGTATTTAAGACTATTGAAGATTTACAGGCTATTTTCGATTCCTTTGACGATGAAGACAGCGAAGCAGAAGAGAGTGGAAATGACGACGATGCTGATGATGTAAATGGCGAATTCATGGATAATAACAAGGTTAACATTTCCCAATTGCGCCACTTTATTATACAAGAATATTTAACCAATCCGTTACTATTGGCATCTATGGATAATAGAAAGTTCCACATAAGATGTTACGTCGTCTGTAGGGGAGATTTGCAAGTTTTTGTTTATGATAGAATGCTAGCGCTCTTTGCTGCCAAGCCATTTGTTCCTCTAGATCCATATGCGTATTCCGTAACTGATTTGAAAGATTTGGAATGCCACTTGACTAACACATGTCTGCAGAGCAAAAAGAAGGATAAAGATTCTTCTGTTTTGGAGTTTGACTCCATAGAGGAAATTCCAAATGAGAGAAAGTCCAATATTAAGGAGCAAATCCATAGCATAACGAACGACGTTTTCTTAGCTGCTGTAAATGTAAATAGATTAAACTTCCAACCGTTACCAAACGCATTTGAAACATATGGCGTAGACTTTTTGATTGATTCGAATTACGAAGTTAAATTACTAGAGATCAACGCTTTCCCAGACTTCAAACAAACTGGGAAAGATTTGAAGAACCTCATTGATGAATTATTTGATGACACTGTCAAATACTGTGTTACCCCCATCTTTAACGAGAATAGAAATAAGACAGATGATGAAACAGACCCGAACTTCGTAAAAGTCATTGATTACACTTCAAACGGTTGGTGA>YBR095C	-:0:chr02:434400:435692ATGACTATTAGGAGCAGTATGAAAAACAACGCAGAATTGGAAAGCAAGAGTGTGTTAGCTAATGAATCGAATATAATTAGCACATTTACTAGAAGGATAATAAAGGAAAAAAGCGGTAACTATCAGGTACTGAAAAGATCCCTTGATGGAAAACTAATCTATCCTGAGGCCACTGGCATATCCTCTAACAGAGGTAACAAACTTTTGCAGAGAAGCGAAGTAGTAACAAGAAGAGACTTGAACAATTCTAAGCCAATGATTGAGCAAACTGTGTTCTATAACGGTTCGGAGCATCGCCTTCTGCAAACAAATATTGTCACTGATAGTAGACGCAAAAGAATAAAATTCACACCTGACATCAACGTCGAGCCTGTTTTAGTCGGTGATGAAAACGACATCGATGGTAGTGAGAAGGAAGACGAAAATATCACTGATGAGTATTACGGCGAAGAAGACGATGACGACCTATCAAAACTAGTGAATGTAAAGGAGATTTTGACTCCCATTCTTTCCCTAGGGGATATAATAAATCATAAAACTATCTCAAGGACATTTTCAAGCCCAATTTTGAAAAATTTAGCCTTGCAAATAATTCTAATGATTGAAAAAGAACAGATGTCAGTTGTTAGATATTCCCAGTTTTTGGAGGTTTTTTTAGGAGATCATCCTGAACCAATTTATGAATCTAACTTGAACCTACCATCTTATAATCACAATTTGACCCTTCCAGAGGATAGAGGGGCAAGCGACGAGGATGACATTAACAACAAAAACAATATCAACGAGGTTAACTCGAATAGTTTGTCTACTGAAGCTGGTCATATCAATAATGGCATGGAAGAATTTGGAGAGGAAGATCCTTTTTTTGCATTGCCAAGGTTGGAACAATCTAATGCGCTGTTATCGTTGCTGCCATCTTCTTCCGGTTCTGCTTCTATATCAACATTAACAGCGGCCGAACAACAACAGTTAAATGAGGAAATTGAGTCTGCCCGACAGCTGTCGCAGATTGCACTACAGAGGAATAAAGAATTCATAAGAAACTTACAAAAAATCAGAAAGTCTGTAATAAAAGCCAATAGAATAAGAGGTAGGATTTTAAATTGGAGTCGGGAATATTTGGGTATATCTGACGATGATATTACTATCCCGGTGGCTCTACGTGTTGTGAAGAGAGGTCTGATCAGTGCTACAACAAATAAGACAACAAATTTTGAGGAAGAAATTGAAAATACCATGGAAGACGGTGTTGTAGACGATAATGAACCTGATGAAGAAGCTAATAGAGCCTAA>YBR096W	+:0:chr02:436015:436707ATGGGTGTTTGCACTATTTTTAGGTGGCTCTTTGCTGCTTACTTGCTCTCTTCATATAAGTCGCTTCCTGGGGCATATTTTGTTAGGTTTTATTATTATGTTATTCAAAACTTGTTCTTGCCTATGTTTACAGGGTTTGAAACCGAGAATATTAAGAAACTTGAGAAAAATGAATATGGCTGTTTCTCGTACACCAGCCTAGATACCTATGCCTCTCCATTCGAATGTGACTTCTACTTTCATAAGAGCAATAGCACCTATTTTGCGGAATTGGACATTTCAAGAGGTAATCTCATGTGCAAGATTTTTCAAAAATTGATGCTGAATTCCAAGCATTATCCATATATTCCAGTTGCGAACGTTTTCACTAATTTCTTGAAGGAAATCAAGCCTTTTCAAAAGTACTCGGTTTCATCAAGGATTATCTGTTGGGATGAGAAATGGATTTACGTCATGAGTAGATTCACAATTAAAAAGGGCACCGTCTTATGCTCATTATCATTGACCAAATACGTCCTTAAGGACGGTAGAAAGACCATCAAACCAAAGGATGCTCTTGAATACTGTGGTTTATATAATGAGAAGGTCGCCAAAATTTCTGAGGACAATCTAAAGCTTTTAACCGAGCGTTGTGGTTTCCACGAGACAGTACCATTGGAAAACTTGAGCCAAGAGTACTGTTCAGAAATCTAA>YBR097W	+:0:chr02:436945:441309ATGGGGGCACAATTATCACTAGTGGTCCAAGCATCACCTTCCATAGCCATTTTTTCATATATCGATGTCTTAGAGGAAGTACACTACGTTTCACAGTTAAACTCATCAAGATTCTTAAAAACATGCAAGGCACTGGACCCTAACGGCGAAATTGTTATCAAAGTGTTTATTAAACCAAAAGACCAATATAGCTTACGACCTTTTCTCCAACGTATAAGGGCTCAATCGTTTAAGTTGGGACAACTACCGCACGTTTTAAACTACAGTAAATTGATCGAGACAAATAGAGCCGGCTACATGATACGGCAGCACTTAAAAAATAATTTATATGACAGATTGAGTTTGAGACCTTACTTACAAGACATTGAACTGAAATTCATTGCTTTCCAGTTGTTAAATACATTAAAGGACATTCATAATCTGAATATTGTCCATGGTGATATAAAGACAGAAAATATCCTAGTAACAAGTTGGAATTGGTGTATATTGACAGATTTTGCTGCATTTATCAAACCCGTATATTTGCCTGAAGATAATCCAGGTGAATTTTTATTCTACTTCGACACCTCGAAGAGAAGAACCTGTTATCTAGCCCCGGAGAGGTTTAACTCTAAACTTTACCAAGATGGAAAATCTAACAATGGTAGGCTAACTAAAGAAATGGACATATTTAGTCTTGGATGTGTTATTGCAGAAATATTTGCTGAAGGAAGACCCATCTTCAACTTATCACAGCTATTCAAATATAAAAGTAATTCATATGACGTAAACAGGGAATTTCTCATGGAGGAAATGAATTCTACCGATTTAAGGAACTTGGTTCTAGACATGATTCAACTAGATCCATCCAAAAGACTTTCATGTGATGAACTACTGAATAAATATCGTGGCATTTTCTTCCCCGATTATTTCTACACTTTCATTTATGATTATTTCAGAAATTTGGTTACTATGACAACAAGCACACCGATATCAGATAACACTTGCACCAATAGTACCTTGGAAGACAATGTAAAACTTTTAGATGAAACTACGGAAAAAATATACAGAGATTTTTCCCAAATATGTCATTGTTTGGACTTTCCTTTAATAAAAGACGGGGGTGAGATTGGTTCAGACCCCCCAATTTTGGAATCTTACAAAATAGAGATAGAAATTAGTCGGTTTTTAAACACAAACTTATATTTCCCCCAAAATTACCATTTAGTCTTACAGCAGTTTACCAAAGTATCCGAAAAGATAAAATCAGTTAAAGAGGAATGTGCCTTACTCTTTATCTCTTATTTGTCTCATAGTATAAGAAGTATTGTTTCGACTGCTACGAAACTTAAAAATTTAGAACTATTAGCAGTATTTGCACAATTCGTTTCTGATGAAAATAAAATTGATCGAGTCGTACCTTATTTCGTATGTTGTTTTGAAGATAGTGACCAGGACGTCCAGGCCCTATCTTTGTTAACATTAATCCAGGTACTCACCTCTGTAAGAAAATTGAATCAATTGAACGAGAATATATTTGTGGACTACTTACTTCCGAGACTGAAAAGATTACTTATTTCCAATAGGCAGAATACCAATTATTTAAGGATTGTGTTTGCTAATTGTTTGAGCGACTTGGCCATTATCATTAATAGATTTCAAGAATTTACATTTGCTCAGCACTGCAATGATAACTCAATGGATAACAACACGGAAATCATGGAAAGCAGTACCAAGTATTCAGCAAAATTGATCCAAAGTGTCGAAGATTTAACTGTCTCTTTTTTAACAGATAATGATACTTATGTAAAGATGGCACTTTTGCAAAACATTCTTCCACTTTGTAAATTTTTTGGTAGGGAAAGAACAAACGATATTATACTGAGTCATTTAATAACCTACCTTAATGATAAGGACCCAGCATTGCGAGTTTCCTTAATTCAAACAATATCCGGAATATCAATTCTTTTGGGTACCGTTACATTAGAACAGTATATTTTACCATTGTTAATCCAGACCATCACTGATTCGGAAGAATTAGTAGTGATCAGTGTTTTACAAAGCTTAAAATCTTTGTTCAAGACTGGGTTGATTAGGAAAAAATATTATATTGATATATCAAAAACAACATCTCCCTTGTTGTTGCATCCTAATAATTGGATAAGACAGTTTACTTTGATGATAATTATAGAAATTATTAATAAGTTATCAAAAGCCGAAGTGTACTGCATTCTCTATCCAATAATAAGGCCTTTCTTCGAATTTGACGTTGAGTTCAACTTCAAATCAATGATAAGCTGTTGCAAGCAACCAGTGTCAAGATCGGTTTACAATCTATTGTGTAGTTGGTCTGTTAGAGCGTCAAAATCTTTATTTTGGAAAAAAATCATCACAAATCATGTAGATTCATTCGGAAATAATAGAATCGAATTTATAACAAAAAATTACTCAAGTAAAAATTATGGATTTAATAAAAGAGATACGAAATCAAGTTCCTCGCTGAAGGGTATTAAAACATCATCCACCGTCTATTCACATGACAACAAGGAAATTCCCTTAACTGCTGAAGACATAAATTGGATTGATAAGTTCCACATTATTGGGCTAACAGAAAAAGATATTTGGAAAATTGTGGCTTTGAGGGGTTATGTAATAAGGACAGCGAGAGTTATGGCAGCGAACCCTGATTTTCCATATAATAATAGTAATTACCGTCCATTAGTACAGAACTCACCACCTAACCTAAACCTTACGAATATTATGCCAAGAAACATTTTCTTTGATGTAGAGTTTGCTGAAGAGTCGACAAGTGAGGGACAAGATTCTAACTTAGAGAACCAACAGATATATAAATATGATGAAAGTGAGAAAGATAGCAATAAGCTAAATATTAACGGTAGCAAACAGCTATCTACCGTCATGGACATAAATGGATCACTAATATTCAAAAATAAGTCCATTGCCACTACTACTTCTAATTTGAAGAACGTTTTTGTTCAGTTAGAACCAACGTCCTATCACATGCATTCTCCAAATCATGGCTTGAAAGATAATGCAAATGTTAAACCAGAAAGGAAGGTAGTCGTCAGCAACAGCTATGAAGGCGACGTTGAAAGCATAGAAAAATTCCTATCGACTTTCAAAATTTTACCTCCTCTGAGAGATTATAAGGAGTTTGGGCCTATTCAAGAGATTGTACGGAGTCCAAACATGGGTAATTTGAGGGGCAAGTTGATAGCTACTTTGATGGAAAACGAACCCAATTCTATTACGTCTTCTGCTGTTTCTCCAGGAGAAACACCCTATTTAATAACAGGTTCAGATCAAGGTGTAATCAAGATTTGGAACCTGAAAGAGATTATCGTGGGCGAGGTTTACTCTTCTTCTTTAACTTATGACTGCTCCTCTACCGTAACTCAGATAACCATGATTCCTAACTTTGACGCGTTTGCCGTTTCCAGTAAAGATGGACAAATAATTGTATTAAAGGTTAATCATTACCAACAAGAAAGTGAAGTCAAATTTTTGAATTGCGAATGCATCAGGAAAATTAACTTGAAGAATTTTGGTAAAAATGAATACGCAGTGAGAATGAGAGCATTTGTGAATGAGGAAAAATCTCTACTAGTAGCATTGACGAATTTGTCAAGGGTTATTATATTTGATATTAGAACCCTGGAGAGGTTACAAATTATAGAGAATTCTCCAAGGCATGGTGCCGTTTCAAGCATCTGTATCGATGAAGAGTGTTGTGTCCTAATTTTGGGGACGACTAGAGGTATTATTGATATATGGGATATCCGTTTCAACGTGCTGATAAGGAGTTGGTCCTTTGGGGACCACGCACCAATCACGCATGTGGAGGTTTGTCAGTTTTATGGAAAGAATTCTGTAATTGTTGTAGGAGGTAGTTCAAAAACATTTCTAACAATATGGAATTTTGTTAAGGGGCATTGTCAGTATGCTTTCATAAATTCTGATGAACAGCCATCTATGGAGCACTTTTTACCAATTGAGAAAGGCTTAGAAGAATTAAATTTTTGTGGAATCAGGTCTTTAAACGCACTAAGCACTATCTCAGTATCTAATGATAAAATTCTTCTTACCGATGAAGCAACAAGTTCCATTGTTATGTTTAGCCTAAATGAGCTTTCTTCTTCTAAAGCAGTAATAAGTCCTTCAAGATTCAGTGACGTTTTTATTCCTACACAAGTTACGGCAAATCTCACAATGTTATTGAGAAAAATGAAACGTACTAGCACTCATTCAGTAGATGATTCTCTATATCATCATGATATTATAAATTCTATATCTACATGTGAAGTTGATGAGACACCTTTGCTGGTTGCTTGTGATAACTCAGGGCTTATTGGAATCTTCCAATAA>YBR098W	+:0:chr02:441509:443584ATGAGCCAGATCGTTGATTTTGTTGAGGACAAAGATTCAAGAAACGATGCCAGTATTCAGATCATCGATGGACCCTCAAATGTTGAAATTATCGCTCTCTCGGAATCAATGGATCAAGATGAGTGCAAAAGGGCACATGTAAGCTCTGCAGAGATGATTCCATCATCACCGCAAAGAAAATCTGTTTCGAATGATGTTGAGAATGTTGACCTAAACAAATCTATCGAACTTTCTGCGCCGTTCTTCCAAGATATCAGTATAAGCAAGCTAGATGACTTTTCTACAACAGTAAATTCCATCATAGATTCGTCTCTCAGAAATGAGAATAATGCAAAAGGAAACGCGAAAAAACTTTTGGATGATCTAATAAGTGATGAATGGTCAGCTGATCTTGAGTCGAGTGGAAAGAAGCACAACAAATCACAATACAATTTGAGGGACATCGCAGAGAAATGGGGAGTACAGTCTTTAAAAAATCCAGAGCCTATTGCTGTTGACTGCGAATATAAGACACAAGGAATCGGAAAAACCAATAGTGACATTAGTGATAGTCCGAAATCACAGATAGGAGCAGCTGATATACTGTTCGATTTTCCACTGTCTCCAGTAAAACATGAGAACCCAACTGAAGAAAAGCACAATTCGATTGCTAATGAAAATTCTTCACCAGATAATAGCCTGAAACCAGCAGGAAAACAAAATCATGGTGAAGATGGGACATCCATGGCAAAAAGGGTATACAATAAAGGTGAAGACGAGCAAGAACACCTTCCGAAAGGAAAGAAGAGAACCATAGCGTTATCAAGAACACTAATCAACAGCACCAAACTACCTGATACAGTAGAACTAAATCTTTCTAAATTTCTCGATTCCTCAGATAGTATTACTACTGATGTACTTTCAACCCCTGCAAAGGGGTCTAACATAGTAAGGACAGGTAGTCAACCAATCTTTAGCAACGCTAATTGTTTTCAGGAAGCAAAACGCTCGAAAACATTAACGGCTGAGGATCCCAAATGTACTAAAAATACTGCCAGAGAGGTATCACAACTAGAGAATTATATTGCCTATGGGCAATACTATACTAGAGAAGACTCAAAAAACAAAATACGACACTTGTTAAAAGAAAACAAAAATGCTTTTAAGCGAGTTAACCAGATATATCGAGATAATATAAAAGCACGCTCTCAAATGATTATAGAGTTTTCGCCTAGCCTTCTCCAGTTATTTAAAAAAGGAGACAGTGATCTGCAACAACAATTGGCACCAGCAGTTGTGCAATCAAGCTATAACGATTCTATGCCGCTTTTAAGATTTCTTCGAAAATGTGACAGTATTTACGACTTTAGTAACGATTTCTATTACCCCTGTGATCCCAAAATAGTTGAAGAAAACGTTTTGATTCTATATTATGATGCGCAAGAATTTTTTGAACAATACACTTCACAAAAGAAAGAATTATATAGGAAGATACGATTTTTCTCAAAGAATGGAAAACATGTGATTCTTATACTAAGCGATATAAATAAACTCAAAAGAGCTATTTTCCAATTAGAAAATGAAAAGTACAAAGCTAGGGTAGAACAACGATTGTCAGGAACAGAAGAAGCTTTAAGACCGAGAAGTAAAAAATCAAGCCAAGTTGGAAAATTAGGGATAAAAAAATTTGATTTAGAGCAACGATTGCGCTTCATTGATAGAGAATGGCATGTCAAAATACATACTGTAAATTCACATATGGAGTTTATTAATTCTCTGCCGAACCTAGTGTCATTAATTGGAAAACAGCGCATGGATCCCGCAATTCGGTATATGAAATATGCTCATTTGAATGTAAAATCCGCTCAGGATAGTACAGAAACGCTAAAGAAAACCTTTCATCAGATAGGGAGAATGCCTGAAATGAAGGCGAATAATGTCGTGAGCCTATACCCCAGTTTTCAATCATTACTTGAAGATATTGAAAAGGGAAGACTGCAATCAGACAACGAAGGTAAATACTTGATGACTGAGGCAGTAGAAAAAAGATTGTACAAACTGTTTACTTGTACTGATCCAAATGATACTATTGAATGA>YBR099C	-:0:chr02:442918:443301ATGCGCTGTTTTCCAATTAATGACACTAGGTTCGGCAGAGAATTAATAAACTCCATATGTGAATTTACAGTATGTATTTTGACATGCCATTCTCTATCAATGAAGCGCAATCGTTGCTCTAAATCAAATTTTTTTATCCCTAATTTTCCAACTTGGCTTGATTTTTTACTTCTCGGTCTTAAAGCTTCTTCTGTTCCTGACAATCGTTGTTCTACCCTAGCTTTGTACTTTTCATTTTCTAATTGGAAAATAGCTCTTTTGAGTTTATTTATATCGCTTAGTATAAGAATCACATGTTTTCCATTCTTTGAGAAAAATCGTATCTTCCTATATAATTCTTTCTTTTGTGAAGTGTATTGTTCAAAAAATTCTTGCGCATCATAA>YBR101C	-:0:chr02:443815:444687ATGGAAAAGCTATTACAGTGGTCTATTGCGAATTCTCAAGGGGACAAAGAAGCTATGGCTAGGGCCGGCCAACCTGATCCTAAATTGCTACAGCAGTTATTCGGTGGTGGTGGTCCTGACGATCCAACCTTAATGAAAGAATCCATGGCTGTTATTATGAATCCGGAGGTTGACTTAGAAACAAAACTCGTTGCATTTGACAACTTTGAAATGTTGATTGAGAACTTAGATAATGCTAATAATATCGAAAATTTAAAACTGTGGGAGCCATTGTTGGATGTTCTTGTTCAGACGAAGGATGAAGAACTACGTGCTGCTGCTTTATCCATTATTGGAACGGCTGTGCAAAACAACTTGGATTCGCAAAATAATTTCATGAAATACGACAATGGTCTGCGAAGCCTTATCGAAATAGCTAGTGACAAGACAAAGCCACTCGACGTGAGAACAAAAGCTTTTTACGCACTATCTAATCTAATAAGAAACCACAAAGATATCTCAGAAAAGTTTTTCAAATTAAATGGGCTCGACTGCATAGCACCTGTATTAAGTGATAACACCGCCAAACCAAAACTGAAAATGAGAGCCATTGCCTTATTGACCGCATATTTGTCATCTGTTAAGATTGATGAAAATATAATCAGTGTGCTGAGAAAGGATGGAGTAATTGAAAGTACGATTGAGTGCTTGTCTGACGAGAGTAACTTGAACATCATAGATAGAGTTCTGTCTTTTCTCTCTCACCTGATATCTTCCGGAATAAAATTTAATGAACAGGAATTGCACAAATTGAACGAAGGTTACAAACATATCGAGCCTCTAAAGGACAGACTTAATGAAGACGATTATTTAGCCGTAAAGTATGTATTATGA>YBR104W	+:0:chr02:449661:450650ATGAGTGAAGAATTTCCTACGCCGCAGCTTTTGGATGAATTGGAAGATCAACAGAAAGTTACCACGCCGAATGAGAAGAGAGAGTTAAGTTCTAATCGTGTGTTGAAGGATATTTTTGCGGGTACCATCGGTGGTATTGCACAAGTTCTTGTAGGGCAACCGTTTGATACAACAAAAGTTAGGCTTCAAACAGCCACCACAAGAACAACAACGTTGGAGGTATTGCGTAACTTGGTAAAGAATGAGGGTGTGTTTGCATTCTACAAAGGTGCGTTAACACCGTTATTGGGTGTTGGTATATGTGTCTCAGTACAATTTGGTGTGAATGAGGCGATGAAAAGATTTTTCCAAAATTACAACGCTTCTAAGAACCCGAATATGAGCTCACAGGACGTAGATCTTTCACGTTCAAATACTCTGCCACTATCCCAGTACTACGTTTGTGGGTTGACAGGTGGTGTAGTCAATTCATTCTTGGCATCGCCCATTGAACAAATAAGAATTCGTTTGCAGACGCAAACATCTAATGGTGGAGACAGAGAATTCAAAGGTCCTTGGGACTGTATTAAAAAATTGAAAGCTCAAGGTGGATTGATGAGGGGTTTATTTCCCACAATGATAAGAGCCGGACATGGGCTTGGTACATATTTTCTTGTCTACGAAGCATTGGTTGCCAGAGAAATTGGTACGGGACTAACAAGAAACGAAATTCCTCCTTGGAAATTATGCTTATTTGGTGCATTTTCCGGGACAATGTTGTGGTTGACAGTATACCCATTGGACGTTGTGAAATCCATTATACAAAACGATGATTTACGAAAACCTAAATACAAGAATTCCATTTCTTACGTAGCAAAGACCATTTATGCAAAAGAAGGTATCAGAGCCTTTTTCAAAGGGTTTGGGCCAACAATGGTTAGGTCCGCGCCCGTTAATGGTGCGACATTCCTGACGTTTGAATTAGTCATGAGATTTCTGGGGGAAGAGTAG>YBR105C	-:0:chr02:450875:451963ATGATCAATAATCCTAAGGTAGACAGTGTAGCGGAGAAACCCAAAGCTGTGACATCAAAGCAGTCGGAGCAAGCGGCTTCGCCAGAACCAACACCAGCCCCCCCTGTTTCTAGAAATCAGTATCCGATCACGTTCAACTTGACTTCAACCGCACCCTTTCATCTTCATGACCGTCATCGCTACTTACAAGAGCAAGATCTTTACAAGTGCGCTTCTAGGGATTCGTTGTCTTCCCTGCAGCAACTCGCCCATACACCTAATGGGTCGACAAGGAAGAAATATATTGTTGAGGACCAATCTCCCTATAGTTCGGAAAATCCAGTCATTGTGACCTCTTCCTATAACCATACGGTTTGCACAAACTACTTAAGACCAAGAATGCAGTTTACAGGGTACCAGATATCAGGATACAAACGCTATCAGGTAACAGTTAACTTAAAGACTGTAGATTTGCCAAAGAAAGATTGCACGTCGCTGTCCCCTCATTTATCTGGATTTTTGTCGATAAGAGGACTCACGAACCAACACCCGGAAATCAGTACATATTTTGAAGCCTACGCGGTAAACCACAAGGAATTAGGGTTCTTGTCCTCTAGCTGGAAAGATGAACCTGTTTTAAACGAATTCAAAGCCACAGACCAAACAGACTTAGAACACTGGATAAATTTCCCCTCCTTTAGACAGCTTTTCCTGATGAGCCAAAAAAACGGTCTCAACTCAACTGACGACAATGGCACTACAAATGCAGCCAAGAAGTTGCCTCCACAGCAGCTTCCTACTACACCAAGCGCAGACGCTGGTAACATATCAAGAATTTTTAGCCAAGAGAAACAATTTGACAACTACTTGAACGAACGGTTTATATTTATGAAATGGAAGGAAAAATTTTTGGTACCAGATGCCCTATTAATGGAAGGTGTAGACGGCGCATCTTATGATGGGTTTTATTACATTGTCCATGATCAAGTTACCGGGAACATTCAAGGGTTTTACTATCATCAAGATGCTGAAAAGTTCCAACAGCTGGAATTAGTACCATCTTTGAAAAATAAAGTCGAGTCCAGTGATTGTTCTTTTGAGTTTGCTTGA>YBR106W	+:0:chr02:452652:453218ATGAATCCTCAAGTCAGTAACATCATCATCATGTTGGTCATGATGCAACTCTCCCGTCGCATTGACATGGAGGACCCAACCATCATCATGTACATTAGAATTTTATACTGTTCTTCCATCGGTATCTCTTGGATCATCTACCAAATGGCCAGAAAGAGAATTGTTGCTAAAAACGACATGACTACCATGAAGTACGTCGAACCTGGTAATGCTATGTCCGGCGAAGGTGAGAAGCTGCAAGTTACTACCGTCAGAGACTACGATTTGAAGGAAATAGACAGTGCTATCAAGTCTATCTACACTGGTATGGCTATGATGGGTTTCATGCATTTGTACTTGAAATACACCAACCCATTGTTCATGCAATCCATTTCTCCAGTGAAAAGCGCTTTGGAACACAACGAAGTGAAAATTCACCTCTTCGGTAAGCCTGCAACCGGCGATTTGAAGAGACCATTCAAGGCTCCATCTTTGTTTGGTGGTATGGGTCAAACTGGTCCAAAGACCGACAAGAAATCTATCGAAGAAGCTGAAAGAGCCGGTAACGCTGGTGTTAAGGCTGAATGA>YBR107C	-:0:chr02:453787:454524ATGCCTTATACTTGGAAGTTTTTAGGAATCAGCAAGCAACTTTCACTGGAAAATGGTATAGCAAAGTTGAACCAATTGCTAAACCTGGAAGTAGATTTAGACATTCAAACCATTCGAGTACCCAGTGATCCTGATGGTGGTACCGCCGCTGACGAGTATATCCGTTACGAAATGAGGTTGGACATCTCTAATTTAGATGAGGGCACTTACTCTAAGTTCATTTTCTTGGGGAACTCAAAAATGGAGGTGCCGATGTTTTTGTGCTATTGCGGGACAGATAACCGCAATGAAGTGGTATTGCAGTGGCTGAAGGCAGAGTACGGTGTCATTATGTGGCCGATAAAATTTGAACAGAAGACCATGATAAAGTTAGCTGACGCCAGCATTGTGCACGTAACGAAAGAGAACATCGAACAAATTACCTGGTTTAGCTCAAAGTTATATTTTGAACCAGAAACACAAGACAAAAACTTGCGGCAATTCAGTATAGAAATACCGAGAGAAAGCTGTGAGGGTTTGGCTCTTGGCTATGGTAATACTATGCACCCGTATAACGATGCAATTGTACCCTACATTTATAATGAAACGGGGATGGCGGTGGAAAGATTACCGTTGACTTCCGTCATATTGGCAGGGCACACGAAAATAATGAGAGAATCCATTGTCACTTCAACACGAAGTCTCAGGAACAGAGTTCTAGCAGTTGTACTCCAATCGATTCAGTTTACCAGCGAGTAA>YBR109C	-:0:chr02:457913:458356ATGTCCTCCAATCTTACCGAAGAACAAATTGCTGAATTCAAAGAAGCCTTTGCCCTCTTTGATAAAGATAACAATGGCTCTATCTCATCAAGTGAATTGGCCACTGTGATGAGGTCATTGGGTCTTTCGCCCAGTGAAGCAGAAGTAAATGATTTGATGAACGAAATAGATGTTGATGGTAACCATCAAATCGAATTTAGTGAATTTTTGGCTCTGATGTCTCGTCAACTCAAATCAAATGACTCTGAACAAGAACTACTAGAAGCTTTTAAAGTATTCGATAAGAACGGTGATGGTTTAATCTCCGCCGCTGAGTTGAAACACGTGCTAACATCCATTGGTGAAAAATTGACTGATGCCGAAGTAGATGATATGCTAAGAGAGGTTAGTGATGGATCAGGCGAGATCAACATTCAACAATTCGCTGCTTTGTTATCTAAATAG>YBR110W	+:0:chr02:458866:460215ATGTTTTTGGAAATTCCTCGGTGGTTACTTGCCTTAATAATATTATACCTTTCCATACCGTTAGTGGTTTATTATGTTATACCCTACTTGTTTTATGGCAACAAGTCGACCAAAAAAAGGATCATCATATTTGTGCTGGGTGATGTAGGACACTCTCCAAGGATATGCTATCACGCTATAAGTTTCAGTAAGTTAGGTTGGCAAGTCGAGCTATGCGGTTATGTGGAGGACACTCTACCCAAAATTATTTCCAGTGATCCAAATATCACCGTCCATCATATGTCAAACTTGAAAAGAAAGGGAGGCGGAACATCAGTTATATTTATGGTAAAGAAGGTGCTTTTTCAAGTTTTAAGTATTTTCAAATTACTTTGGGAATTGAGAGGAAGCGATTACATACTAGTTCAAAATCCACCGAGCATACCCATTCTTCCGATTGCTGTGCTATACAAGTTGACCGGTTGTAAACTAATTATTGATTGGCACAATCTAGCATATTCGATATTGCAACTAAAATTTAAAGGAAACTTTTACCATCCTTTAGTGTTGATATCTTACATGGTAGAGATGATATTCAGCAAATTTGCTGATTATAACTTGACTGTTACTGAAGCAATGAGGAAATATTTAATTCAAAGCTTTCACTTGAATCCAAAGAGATGTGCTGTTCTCTACGACCGCCCGGCTTCCCAATTTCAACCTTTGGCAGGTGACATTTCTCGTCAAAAAGCCCTAACTACCAAAGCCTTTATAAAGAATTATATTCGCGATGATTTTGATACAGAAAAAGGCGATAAAATTATTGTGACTTCAACATCATTCACCCCTGATGAAGATATTGGTATTTTATTAGGTGCCCTAAAGATTTACGAAAACTCTTATGTCAAATTTGATTCAAGTTTGCCTAAGATCTTGTGTTTTATAACGGGTAAAGGACCACTAAAGGAGAAATATATGAAGCAAGTAGAAGAATATGACTGGAAGCGCTGTCAAATCGAATTTGTGTGGTTGTCAGCAGAGGATTACCCAAAGTTATTACAATTATGCGATTACGGAGTTTCCCTGCATACTTCAAGTTCAGGGTTGGACCTGCCAATGAAAATTTTAGATATGTTTGGCTCAGGTCTTCCTGTTATTGCAATGAACTATCCAGTGCTTGACGAATTAGTACAACACAATGTAAATGGGTTAAAATTTGTTGATAGAAGGGAGCTTCATGAATCTCTGATTTTTGCTATGAAAGATGCTGATTTATACCAAAAATTGAAGAAAAATGTAACGCAGGAAGCTGAGAACAGATGGCAATCAAATTGGGAACGAACAATGAGAGATTTGAAGCTAATTCATTGA>YBR111C	-:0:chr02:461172:461867ATGTTTTTGAGAAACGTAAGAGTTATTTCCCTCAATTCAAGGAGACTATTTAGAACTATGAGCACCGTCAAGGGTAAACCAGAAGATGCTAAAATAATTGAAGCCCGTCACGTTAAAGAAACATCTGACTGCAAGTGGATCGGTTTACAAAAAATCATCTACAAAGATCCCAACGGGAAGGAAAGGGAATGGGATAGCGCAGTCCGCACCACTAGAAGTTCCGGTGGTGTAGACGGCATTGGTATCTTAACCATTCTAAAATACAAAGATGGAAAACCTGATGAAATTTTGCTACAAAAGCAGTTTCGCCCTCCCGTTGAGGGCGTATGCATTGAGATGCCAGCCGGTTTGATAGATGCGGGTGAAGATATTGACACTGCTGCCTTAAGAGAATTAAAAGAAGAGACTGGTTACAGTGGTAAAATAATTTCTAAAAGCCCAACAGTTTTTAACGATCCTGGTTTCACAAACACCAACCTCTGTCTGGTTACTGTCGAAGTTGATATGAGTTTACCTGAAAACCAAAAACCAGTTACTCAATTGGAAGATAATGAATTTATCGAATGCTTTAGCGTTGAGTTGCACAAGTTTCCTGATGAAATGGTTAAGTTGGATCAACAAGGCTACAAGTTGGATGCACGTGTTCAAAACGTAGCTCAAGGTATCCTCATGGCCAAGCAATACCACATCAAATAG>YBR115C	-:0:chr02:469742:473920ATGACTAACGAAAAGGTCTGGATAGAGAAGTTGGATAATCCAACTCTTTCAGTGTTACCACATGACTTTTTACGCCCACAACAAGAACCTTATACGAAACAAGCTACATATTCGTTACAGCTACCTCAGCTCGATGTGCCTCATGATAGTTTTTCTAACAAATACGCTGTCGCTTTGAGTGTATGGGCTGCATTGATATATAGAGTAACCGGTGACGATGATATTGTTCTTTATATTGCGAATAACAAAATCTTAAGATTCAATATTCAACCAACGTGGTCATTTAATGAGCTGTATTCTACAATTAACAATGAGTTGAACAAGCTCAATTCTATTGAGGCCAATTTTTCCTTTGACGAGCTAGCTGAAAAAATTCAAAGTTGCCAAGATCTGGAAAGGACCCCTCAGTTGTTCCGTTTGGCCTTTTTGGAAAACCAAGATTTCAAATTAGACGAGTTCAAGCATCATTTAGTGGACTTTGCTTTGAATTTGGATACCAGTAATAATGCGCATGTTTTGAACTTAATTTATAACAGCTTACTGTATTCGAATGAAAGAGTAACCATTGTTGCGGACCAATTTACTCAATATTTGACTGCTGCGCTAAGCGATCCATCCAATTGCATAACTAAAATCTCTCTGATCACCGCATCATCCAAGGATAGTTTACCTGATCCAACTAAGAACTTGGGCTGGTGCGATTTCGTGGGGTGTATTCACGACATTTTCCAGGACAATGCTGAAGCCTTCCCAGAGAGAACCTGTGTTGTGGAGACTCCAACACTAAATTCCGACAAGTCCCGTTCTTTCACTTATCGCGACATCAACCGCACTTCTAACATAGTTGCCCATTATTTGATTAAAACAGGTATCAAAAGAGGTGATGTAGTGATGATCTATTCTTCTAGGGGTGTGGATTTGATGGTATGTGTGATGGGTGTCTTGAAAGCCGGCGCAACCTTTTCAGTTATCGACCCTGCATATCCCCCAGCCAGACAAACCATTTACTTAGGTGTTGCTAAACCACGTGGGTTGATTGTTATTAGAGCTGCTGGACAATTGGATCAACTAGTAGAAGATTACATCAATGATGAATTGGAGATTGTTTCAAGAATCAATTCCATCGCTATTCAAGAAAATGGTACCATTGAAGGTGGCAAATTGGACAATGGCGAGGATGTTTTGGCTCCATATGATCACTACAAAGACACCAGAACAGGTGTTGTAGTTGGACCAGATTCCAACCCAACCCTATCTTTCACATCTGGTTCCGAAGGTATTCCTAAGGGTGTTCTTGGTAGACATTTTTCCTTGGCTTATTATTTCAATTGGATGTCCAAAAGGTTCAACTTAACAGAAAATGATAAATTCACAATGCTGAGCGGTATTGCACATGATCCAATTCAAAGAGATATGTTTACACCATTATTTTTAGGTGCCCAATTGTATGTCCCTACTCAAGATGATATTGGTACACCGGGCCGTTTAGCGGAATGGATGAGTAAGTATGGTTGCACAGTTACCCATTTAACACCTGCCATGGGTCAATTACTTACTGCCCAAGCTACTACACCATTCCCTAAGTTACATCATGCGTTCTTTGTGGGTGACATTTTAACAAAACGTGATTGTCTGAGGTTACAAACCTTGGCAGAAAATTGCCGTATTGTTAATATGTACGGTACCACTGAAACACAGCGTGCAGTTTCTTATTTCGAAGTTAAATCAAAAAATGACGATCCAAACTTTTTGAAAAAATTGAAAGATGTCATGCCTGCTGGTAAAGGTATGTTGAACGTTCAGCTACTAGTTGTTAACAGGAACGATCGTACTCAAATATGTGGTATTGGCGAAATAGGTGAGATTTATGTTCGTGCAGGTGGTTTGGCCGAAGGTTATAGAGGATTACCAGAATTGAATAAAGAAAAATTTGTGAACAACTGGTTTGTTGAAAAAGATCACTGGAATTATTTGGATAAGGATAATGGTGAACCTTGGAGACAATTCTGGTTAGGTCCAAGAGATAGATTGTACAGAACGGGTGATTTAGGTCGTTATCTACCAAACGGTGACTGTGAATGTTGCGGTAGGGCTGATGATCAAGTTAAAATTCGTGGGTTCAGAATCGAATTAGGAGAAATAGATACGCACATTTCCCAACATCCATTGGTAAGAGAAAACATTACTTTAGTTCGCAAAAATGCCGACAATGAGCCAACATTGATCACATTTATGGTCCCAAGATTTGACAAGCCAGATGACTTGTCTAAGTTCCAAAGTGATGTTCCAAAGGAGGTTGAAACTGACCCTATAGTTAAGGGCTTAATCGGTTACCATCTTTTATCCAAGGACATCAGGACTTTCTTAAAGAAAAGATTGGCTAGCTATGCTATGCCTTCCTTGATTGTGGTTATGGATAAACTACCATTGAATCCAAATGGTAAAGTTGATAAGCCTAAACTTCAATTCCCAACTCCCAAGCAATTAAATTTGGTAGCTGAAAATACAGTTTCTGAAACTGACGACTCTCAGTTTACCAATGTTGAGCGCGAGGTTAGAGACTTATGGTTAAGTATATTACCTACCAAGCCAGCATCTGTATCACCAGATGATTCGTTTTTCGATTTAGGTGGTCATTCTATCTTGGCTACCAAAATGATTTTTACCTTAAAGAAAAAGCTGCAAGTTGATTTACCATTGGGCACAATTTTCAAGTATCCAACGATAAAGGCCTTTGCCGCGGAAATTGACAGAATTAAATCATCGGGTGGATCATCTCAAGGTGAGGTCGTCGAAAATGTCACTGCAAATTATGCGGAAGACGCCAAGAAATTGGTTGAGACGCTACCAAGTTCGTACCCCTCTCGAGAATATTTTGTTGAACCTAATAGTGCCGAAGGAAAAACAACAATTAATGTGTTTGTTACCGGTGTCACAGGATTTCTGGGCTCCTACATCCTTGCAGATTTGTTAGGACGTTCTCCAAAGAACTACAGTTTCAAAGTGTTTGCCCACGTCAGGGCCAAGGATGAAGAAGCTGCATTTGCAAGATTACAAAAGGCAGGTATCACCTATGGTACTTGGAACGAAAAATTTGCCTCAAATATTAAAGTTGTATTAGGCGATTTATCTAAAAGCCAATTTGGTCTTTCAGATGAGAAGTGGATGGATTTGGCAAACACAGTTGATATAATTATCCATAATGGTGCGTTAGTTCACTGGGTTTATCCATATGCCAAATTGAGGGATCCAAATGTTATTTCAACTATCAATGTTATGAGCTTAGCCGCCGTCGGCAAGCCAAAGTTCTTTGACTTTGTTTCCTCCACTTCTACTCTTGACACTGAATACTACTTTAATTTGTCAGATAAACTTGTTAGCGAAGGGAAGCCAGGCATTTTAGAATCAGACGATTTAATGAACTCTGCAAGCGGGCTCACTGGTGGATATGGTCAGTCCAAATGGGCTGCTGAGTACATCATTAGACGTGCAGGTGAAAGGGGCCTACGTGGGTGTATTGTCAGACCAGGTTACGTAACAGGTGCCTCTGCCAATGGTTCTTCAAACACAGATGATTTCTTATTGAGATTTTTGAAAGGTTCAGTCCAATTAGGTAAGATTCCAGATATCGAAAATTCCGTGAATATGGTTCCAGTAGATCATGTTGCTCGTGTTGTTGTTGCTACGTCTTTGAATCCTCCCAAAGAAAATGAATTGGCCGTTGCTCAAGTAACGGGTCACCCAAGAATATTATTCAAAGACTACTTGTATACTTTACACGATTATGGTTACGATGTCGAAATCGAAAGCTATTCTAAATGGAAGAAATCATTGGAGGCGTCTGTTATTGACAGGAATGAAGAAAATGCGTTGTATCCTTTGCTACACATGGTCTTAGACAACTTACCTGAAAGTACCAAAGCTCCGGAACTAGACGATAGGAACGCCGTGGCATCTTTAAAGAAAGACACCGCATGGACAGGTGTTGATTGGTCTAATGGAATAGGTGTTACTCCAGAAGAGGTTGGTATATATATTGCATTTTTAAACAAGGTTGGATTTTTACCTCCACCAACTCATAATGACAAACTTCCACTGCCAAGTATAGAACTAACTCAAGCGCAAATAAGTCTAGTTGCTTCAGGTGCTGGTGCTCGTGGAAGCTCCGCAGCAGCTTAA>YBR119W	+:0:chr02:479332:479339ATGTCAGC>YBR119W	+:2:chr02:479429:480317ACTGTACTTTCAAAATTTACCCAGTAGGCCAGCAAATAAAGAAAATTATACCAGATTACTTCTGAAACACATTAATCCCAACAACAAGTATGCCATTAATCCGTCGCTACCCCTTCCCCACAATAAATTACAAATATCATCACAACCGCTGATGTTACTAGATGATCAAATGGGCCTTCTTGAAGTTTCTATTTCAAGATCATCAAAGATGACTAACCAAGCATTTTTAACGTTTGTCACTCAAGAAGAAGCAGACCGGTTTCTAGAAAAATACACGACAACAGCATTAAAAGTTCAAGGTCGTAAAGTGAGAATGGGAAAGGCTCGAACAAATTCGTTATTGGGTCTTTCAATAGAAATGCAAAAAAAAAAAGGTAATGACGAAACGTACAACCTTGATATAAAGAAGGTGCTTAAAGCAAGGAAACTTAAACGCAAGTTACGTAGTGATGATATATGCGCCAAAAAGTTCAGGCTTAAAAGGCAAATAAGACGCCTGAAGCATAAGCTGAGATCAAGAAAGGTAGAGGAGGCTGAGATTGATAGGATTGTAAAAGAATTTGAAACCCGTAGATTGGAAAACATGAAGTCTCAGCAAGAAAATCTAAAACAATCGCAGAAACCTCTTAAGCGGGCTAAAGTGTCCAATACAATGGAAAATCCACCGAACAAAGTCCTTCTTATACAAAATTTGCCAAGCGGCACTACCGAGCAATTATTGTCGCAAATACTTGGCAATGAGGCTTTAGTTGAAATCAGATTAGTTAGCGTTCGTAACCTAGCTTTCGTGGAATACGAGACCGTTGCTGATGCTACGAAAATCAAGAATCAGTTAGGCTCCACTTACAAGCTACAAAACAATGACGTTACCATAGGATTTGCTAAGTAG>YBR120C	-:0:chr02:480429:480917ATGTCTTCTTCCCAGGTCGTCAGGGATTCTGCCAAAAAATTAGTTAATTTACTGGAAAAATATCCAAAGGATCGTATACACCACTTGGTCTCATTCAGGGATGTACAAATAGCAAGATTTAGACGTGTAGCGGGTCTGCCAAATGTAGATGACAAAGGAAAATCTATAAAAGAGAAAAAACCCTCATTAGATGAAATAAAAAGTATAATTAACAGAACTTCCGGTCCATTAGGACTGAATAAGGAGATGTTAACCAAAATTCAAAATAAAATGGTAGATGAGAAATTCACGGAAGAAAGCATCAACGAGCAAATTCGTGCCTTGAGCACTATAATGAATAATAAATTCAGAAACTATTACGATATTGGCGATAAGCTCTATAAACCTGCAGGAAATCCCCAATATTATCAACGGTTAATAAATGCCGTTGACGGTAAGAAAAAGGAAAGCTTATTTACTGCAATGAGAACTGTATTATTTGGTAAATAA>YBR121C	-:0:chr02:481358:483361ATGAGTGTAGAAGATATCAAGAAGGCTAGAGCCGCTGTTCCATTTAACAGAGAACAGCTAGAAAGTGTTTTGAGAGGAAGATTTTTTTATGCCCCAGCTTTTGACTTGTACGGTGGGGTTTCTGGTTTATATGACTATGGTCCACCAGGTTGTGCATTCCAAAACAACATCATTGACGCCTGGAGAAAGCATTTTATTTTGGAAGAAGATATGTTGGAAGTTGATTGTACTATGTTAACTCCATATGAGGTTTTGAAGACCTCAGGTCATGTTGACAAATTTTCTGATTGGATGTGTAGAGATTTGAAGACTGGTGAGATTTTCAGAGCTGACCATTTAGTCGAAGAAGTTTTGGAAGCTCGCTTAAAGGGTGACCAAGAAGCCAGAGGTTTAGTCGAGGATGCTAATGCCGCTGCTAAGGACGATGCCGAAAAGAAGAAGAGAAAGAAAAAAGTTAAACAAATTAAAGCAGTCAAATTGGATGACGACGTTGTCAAAGAATATGAAGAAATTTTGGCTAAAATAGATGGTTATTCCGGCCCAGAATTAGGTGAATTGATGGAAAAATACGATATCGGTAACCCAGTCACCGGAGAAACTTTAGAATCTCCAAGGGCTTTTAACCTGATGTTTGAAACGGCTATTGGCCCATCTGGTCAATTAAAGGGTTACTTGAGGCCAGAAACTGCCCAAGGTCAATTCTTGAATTTTAACAAGTTGTTAGAATTTAACAACAGTAAGACCCCTTTTGCCTCAGCCTCTATCGGTAAATCATTTAGAAATGAGATTTCCCCAAGGGCCGGTTTATTAAGAGTCCGTGAATTTTTGATGGCAGAGATTGAACATTTCGTTGACCCATTGGACAAATCTCATCCAAAATTTAATGAAATCAAAGACATCAAGCTTTCCTTTTTGCCTCGTGATGTTCAAGAAGCTGGTTCTACTGAACCAATTGTCAAGACAGTTGGTGAAGCAGTTGCTTCAAGAATGGTCGATAATGAAACTTTGGGTTACTTTATTGCCAGAATTTACCAATTTTTGATGAAAATTGGTGTTGATGAGTCCAAATTGAGATTCCGTCAACATATGGCTAATGAAATGGCACATTATGCTGCTGACTGTTGGGATGGTGAATTGAAGACATCTTACGGGTGGATTGAATGTGTCGGATGTGCTGATAGATCTGCCTATGATTTGACCGTTCATTCCAAAAAGACCAAAGAAAAATTGGTTGTTAGACAAAAATTAGATAATCCAATTGAAGTCACCAAGTGGGAAATTGATTTGACGAAAAAATTGTTCGGTCCAAAATTCAGAAAAGATGCACCAAAAGTTGAATCTCATCTATTGAATATGTCTCAAGATGACTTGGCTTCCAAGGCTGAGTTATTGAAGGCAAATGGCAAGTTTACTATAAAGGTTGATGGTGTTGATGGCGAAGTCGAGTTGGACGATAAATTGGTTAAAATCGAACAAAGAACAAAGGTTGAACACGTTAGAGAATATGTTCCTAGTGTCATTGAACCATCTTTCGGTATTGGCCGTATCATCTACTCCGTTTTTGAGCACTCTTTCTGGAACAGACCAGAAGACAATGCCAGAAGTGTTCTTTCCTTCCCACCTTTGGTTGCTCCAACAAAGGTTCTTTTAGTTCCTTTATCTAACCACAAAGATTTAGTTCCTGTTCATCATGAAGTCGCCAAAATCTTAAGAAAGTCTCAAATCCCATTTAAGATTGATGATTCTGGAGTTTCCATTGGTAAAAGATATGCCCGTAATGACGAATTAGGTACTCCATTCGGTGTCACCATTGACTTTGAATCTGCCAAAGACCATTCTGTCACTTTGAGAGAAAGAGATTCCACCAAGCAAGTTAGAGGTTCCGTCGAAAATGTCATCAAGGCTATCCGCGATATCACTTACAATGGTGCTTCTTGGGAAGAAGGTACCAAGGATTTGACTCCTTTTATTGCCCAAGCTGAAGCTGAAGCTGAAACTGACTAA>YBR122C	-:0:chr02:483964:484497ATGTTGAAGTCTATATTTGCGAAGCGATTTGCTTCCACAGGGTCATATCCTGGTTCTACAAGAATTACTTTGCCAAGAAGGCCTGCAAAAAAAATTCAACTGGGGAAGTCACGACCGGCTATTTATCACCAATTCAATGTCAAAATGGAGTTGAGTGATGGAAGTGTAGTTATCCGGAGATCCCAATATCCAAAGGGTGAAATTAGATTAATTCAAGACCAGAGAAATAATCCGCTGTGGAATCCTAGCAGGGATGACCTAGTCGTTGTGGATGCTAATTCTGGTGGTAGCTTGGACAGATTTAACAAGAGGTATAGCTCTTTGTTTTCTGTCGATTCAACGACCCCAAATTCAAGCTCTGAAACAGTTGAACTATCTGAAGAAAATAAAAAGAAAACACAGATTAAAAAGGAGGAGAAGGAAGACGTTTCTGAAAAAGCGTTCGGTATGGATGACTATCTATCTCTGTTAGACGATAGTGAACAACAGATCAAATCAGGTAAGTTGGCCAGTAAGAAGAGAGACAAGAAGTGA>YBR123C	-:0:chr02:484736:486685ATGCCAGTGGAGGAGCCTCTTGCTACACTTTCATCAATTCCAGATTCTAGCGCGGACCAGGCCCCACCGTTAATAGCAGATGAATTTACGTTAGACCTGCCCAGAATTCCAAGTTTAGAATTGCCACTTAATGTCTCCACAAAACACTCTAGTATACAGAAGGCTATAAAAATGTGTGGGGGCATCGAAAAAGTGAAAGAAGCCTTTAAAGAACATGGACCTATCGAATCTCAGCACGGATTACAACTGTACTTAAACGATGATACTGATAGCGACGGATCCAAGAGCTATTTTAATGAGCATCCTGTAATTGGTAAAAGAGTTCCCTTCAGAGATGAGTCGGTCATATTAAAGGTTACAATGCCTAAGGGAACTTTAAGTAAAAATAATAATAGCGTCAAAGATTCTATCAAATCCTTAAAAGATTCGAATAAATTGAGAGTCACTCCTGTTTCCATAGTAGATAACACTATAAAATTTAGAGAAATGTCAGATTTTCAAATCAAACTGGATAATGTCCCCTCTGCAAGAGAGTTCAAAAGTAGCTTTGGCTCTCTGGAATGGAATAATTTCAAAAGTTTTGTCAATTCTGTACCAGATAATGACTCTCAACCTCAGGAAAACATCGGGAATTTGATCTTAGACCGTAGCGTTAAAATTCCGAGTACAGATTTCCAACTGCCGCCACCTCCTAAGTTATCAATGGTTGGTTTCCCCCTGCTTTATAAATATAAAGCTAATCCATTTGCGAAAAAAAAAAAAAACGGTGTCACAGAAGTGAAGGGAACATATATCAAAAACTATCAGTTGTTTGTTCATGATCTTAGTGATAAGACAGTTATACCGTCCCAAGCTCATGAACAAGTCCTGTACGATTTTGAGGTCGCGAAGAAAACAAAAGTCTATCCAGGCACTAAAAGTGACTCTAAATTTTATGAATCGCTGGAGGAATGCTTGAAAATTTTGAGAGAGCTCTTCGCTAGACGTCCAATTTGGGTTAAAAGACATCTTGATGGGATTGTCCCGAAGAAGATACACCATACTATGAAGATAGCGCTGGCACTCATATCTTATCGTTTCACAATGGGGCCTTGGAGAAATACATATATCAAATTTGGTATAGACCCCAGAAGTTCAGTGGAATACGCCCAATATCAAACAGAATATTTTAAGATTGAAAGGAAATTACTATCTTCTCCCATAGTGAAAAAGAATGTACCAAAGCCACCACCCCTGGTATTCGAATCAGACACTCCAGGCGGTATTGACAGCAGGTTCAAATTTGATGGAAAAAGAATACCGTGGTACCTGATGTTGCAGATTGATTTATTAATTGGTGAGCCAAACATTGCAGAAGTTTTTCACAACGTCGAATATTTAGATAAGGCGAATGAACTAACTGGATGGTTCAAAGAATTAGACTTGGTCAAGATAAGAAGGATAGTAAAATACGAACTTGGTTGCATGGTTCAAGGAAATTATGAGTATAATAAATACAAGTTGAAGTATTTCAAGACAATGTTATTTGTTAAAGAATCCATGGTACCCGAAAACAAGAACTCAGAAGAGGGAATGGGTGTTAACACCAATAAAGATGCTGATGGGGACATAAATATGGATGCAGGTAGTCAAATGTCCTCAAATGCCATAGAGGAGGATAAGGGTATCGCTGCTGGTGATGATTTTGATGATAATGGCGCCATAACTGAAGAGCCAGACGACGCTGCTTTGGAAAATGAAGAGATGGACACTGATCAGAACTTGAAGGTGCCTGCAAGTATAGACGATGACGTGGATGACGTTGATGCAGATGAAGAAGAACAAGAAAGTTTTGATGTTAAAACAGCGAGCTTCCAAGATATCATAAATAAAATAGCTAAACTAGATCCTAAAACCGCGGAAACAATGAAAAGCGAGCTCAAGGGATTTGTTGATGAAGTCGATCTGTAA>YBR125C	-:0:chr02:487193:488374ATGGGTCAATTGCTTTCCCATCCGTTGACGGAGAAGACCATAGAATATAATGAATATAAAAACAACCAGGCGTCAACTGGTATTGTTCCACGATTTTACAATTGTGTGGGGTCCATGCAAGGCTATCGTTTGACTCAGGAAGATGCACATCTAATTAGAAATGAAAACTCTGTTGTGTACGTACGATTTTTCAACCCATTCATAGACAAATACGAAACCCTGTCATTGAACGTCTTCGCAGTATTCGATGGTCATGGTGGAGATGATTGTTCGAAATTTCTAAGCGGCGGCCGCCACCATCGCGATGGTAACGGCAGTAGCAACGGTAATGGTGAACCCAACGCTGGCCTGATCAAATGGATTGCGTATAGTTTCGAAAACCACCATTACACATCTACAACTAATAACGACTCATCAAAATTTAAAAGATCCTTCAATACGTTGGAAGGACTTGTTTCTCAAATTTTTAAAGACGCTTTCATATTGCAAGATGAAGAGTTATATCGACATTTTGCCAACAGTTCGTGTGGATCTACAGCAGTAGTAGCATGCATCATAAACGAGGAATCACTGTATGTGGCTAACTGTGGTGATTCGCGATGTATTCTTTCCTCCAAATCGAATGGCATAAAGACGATGTCTTTCGATCATAAGCCACAACACATAGGAGAATTGATACGTATAAACGATAATGGTGGTACTGTCTCTCTTGGAAGAGTGGGTGGAGTACTGGCTCTCAGTAGGGCATTTAGTGATTTCCAATTCAAGAGAGGTGTCACTTATCCACATAGAAGGACAAAACTAACAAATATTACCCAGAATTTAACTTATGGAACCCCACCACAAGAAGCACAAGTTACCGTTGAACCAGACGTATTGATGCACAAGATTGACTATTCTAAAGATGAGTTTTTAGTACTGGCTTGCGACGGTATATGGGATATTTATAACAACAAACAGTTAATTCACTTTATAAAGTATCACCTGGTATCAGGGACGAAGTTGGATACAATAATAACAAAATTGTTGGACCACGGGATAGCGCAGGCAAATAGCAATACTGGTGTCGGCTTTGACAATATGACTGCCATCATTGTAGTGCTAAACCGGAAAGGCGAAACATTGCAAGACTGGTTTAATAAAATGAAGACCCGTTTGGAAAGAGAAAGGGGCCTAGTGTAA>YBR126C	-:0:chr02:488899:490386ATGACTACGGATAACGCTAAGGCGCAACTGACCTCGTCTTCAGGGGGTAACATTATTGTGGTGTCCAACAGGCTTCCCGTGACAATCACTAAAAACAGCAGTACGGGACAGTACGAGTACGCAATGTCGTCCGGAGGGCTGGTCACGGCGTTGGAAGGGTTGAAGAAGACGTACACTTTCAAGTGGTTCGGATGGCCTGGGCTAGAGATTCCTGACGATGAGAAGGATCAGGTGAGGAAGGACTTGCTGGAAAAGTTTAATGCCGTACCCATCTTCCTGAGCGATGAAATCGCAGACTTACACTACAACGGGTTCAGTAATTCTATTCTATGGCCGTTATTCCATTACCATCCTGGTGAGATCAATTTCGACGAGAATGCGTGGTTGGCATACAACGAGGCAAACCAGACGTTCACCAACGAGATTGCTAAGACTATGAACCATAACGATTTAATCTGGGTGCATGATTACCATTTGATGTTGGTTCCGGAAATGTTGAGAGTCAAGATTCACGAGAAGCAACTGCAAAACGTTAAGGTCGGGTGGTTCCTGCACACACCATTCCCTTCGAGTGAAATTTACAGAATCTTACCTGTCAGACAAGAGATTTTGAAGGGTGTTTTGAGTTGTGATTTAGTCGGGTTCCACACATACGATTATGCAAGACATTTCTTGTCTTCCGTGCAAAGAGTGCTTAACGTGAACACATTGCCTAATGGGGTGGAATACCAGGGCAGATTCGTTAACGTAGGGGCCTTCCCTATCGGTATCGACGTGGACAAGTTCACCGATGGGTTGAAAAAGGAATCCGTACAAAAGAGAATCCAACAATTGAAGGAAACTTTCAAGGGCTGCAAGATCATAGTTGGTGTCGACAGGCTGGATTACATCAAAGGTGTGCCTCAGAAGTTGCACGCCATGGAAGTGTTTCTGAACGAGCATCCAGAATGGAGGGGCAAGGTTGTTCTGGTACAGGTTGCAGTGCCAAGTCGTGGAGATGTGGAAGAGTACCAATATTTAAGATCTGTGGTCAATGAGTTGGTCGGTAGAATCAACGGTCAGTTCGGTACTGTGGAATTCGTCCCCATCCATTTCATGCACAAGTCTATACCATTTGAAGAGCTGATTTCGTTATATGCTGTGAGCGATGTTTGTTTGGTCTCGTCCACCCGTGATGGTATGAACTTGGTTTCCTACGAATATATTGCTTGCCAAGAAGAAAAGAAAGGTTCCTTAATCCTGAGTGAGTTCACAGGTGCCGCACAATCCTTGAATGGTGCTATTATTGTAAATCCTTGGAACACCGATGATCTTTCTGATGCCATCAACGAGGCCTTGACTTTGCCCGATGTAAAGAAAGAAGTTAACTGGGAAAAACTTTACAAATACATCTCTAAATACACTTCTGCCTTCTGGGGTGAAAATTTCGTCCATGAATTATACAGTACATCATCAAGCTCAACAAGCTCCTCTGCCACCAAAAACTGA>YBR128C	-:0:chr02:493075:494109ATGCATTGCCCAATTTGCCACCATAGAGCGCATGTAGTGTACTGTGCACATTGTATCAATACGAGCCCAAGTCTGCTACTGAAGCTAAAACTAGATTTAATTTTATTAAAGGATGAGAATAAAGAACTTAACGGGAAAGTCGAACAAATATTAAACGAGGCCATGAACTATGACCAGTTAGATATCAAGAGGATGGAGAAAAAGAAAGATCCCCTGATGAATAGTCTCATGAAGTTAGATGTTTTACGAATGAAAAAGAATAATAACCTGATTAGGCACAGGATAGAACAGCTAAATGAGCGGATTTATAGTAAAAGGAATCACATCAGCGAATTGAAAGTAGAAATTGACAATTATAAATGTTATAAGGTGGGTACTGGTACGGACAAATTAAGAGAGCAAGTGGAAATTAGTGATGCAAAAAATAAGCTGGCACAAGTATCGAAGATTTGTGAATCGGCTAGAGATTACAAGCTCAATCTACTAAATAATTGGTTTGTGATCCAGAAGCTGCAAGATAATTTCCAAATACCTTTCGCTATAGCTTTTCAGCCACTAATATCTTTGAAAAACTTCCGTATTTTACCTCTTGCTATAACAAACGATTCCATCAACATCATGTGGAAGTATATTAGCTTCTTCTCAGACATTCTTATGATTAAACTTCCCTACACAAATAAAATCTGTGAACAACCCATGTTTGAGTTTTCCGATAGCATACAGACAGTAGTACAAAGGTTGATCAAGCTTATCATAAATATTTTACAGATATGTAGACATTTGAAACTCGTACCTTCAACACCCATGGATATCCCATGGCTACTGGACCAGTACGATGTGGATGGGCTGTTCTATAATATGGTGAAACGGAACAAGATGAAGTGTAGGTCCGTCTCGCTATATTGGACTTTTGGGATGTTGTACTCGATGGTTTTGGATAACATGAATAATCCACAAAGAGGACATCCTGCAAGGCGGACCGCACCACCTCCAACAGTCACAGGACCTCATGACCGATGGTACGTGGTAGGCTAG>YBR129C	-:0:chr02:494347:495333ATGATAGCAGGTGCGACTGCTCCGTCAAGTCAGCATGAAATTTTGATCGCATCGAATCTCATCAAGAAGCCATCAACGTCTCAAAATAAAACGCCGACAGCTCAATCCAGTTCAGGTAACAATGGAGCTGCTGATGGTGCACCGCAGGGTTACCATCATCACCATCATCACCATCGTCATTTATGGTGGCCTCGGACAACAGACCATCAATATTGGTGTGTCTTGAGGAAAAACCAATTTGCCTATTACAAAACTCGGGATGAAAGAGAGGCTATAAGTGTCATACCGAGATTTGACATACTTAATTTTAAGATAAGCGAGCTCGATGGTATATTAACTGTTTATACTCCATCCAAGGATTTAATATTCAAATTTCCACGAGGGCAAAATGAGAAGGTTGGTATGGAACTGATGCATAATTGGAAAATTGCCCTTGAAAAATTCCTCTCTAGTCCTAGTGGCAACGAAAGTGTCACTACAGGTAGTGATTATGATGAAGAAGAGGATGATGATGATTTAATTGTAGTTGACGAAAAGGCGGGTCCTTCTAGCAGCAAGCATTCTTGCAGTTTGACGATGGATGAGCAACTGTCTCGTGAAGATAAAGAATTTTATAGGATGTTCGATCCAAGAAATGCAGAGCACCAGGTTTGTTCTGGGATTCTTTACACAAAAGTGAAAAAGAAGAAACTGTTCAATAGGGCTAAATGGCAAAAGTTCAATGTGGAATTGACTAATACTTCGTTCAATTTATACTCTTTCAAGACTGGGAAGTTAAAGAAAAGCATTAAATTGGATAAAATTATCGATTGTATTGAACTTGATAATAATTCGAAGATGAAAAATGACGACACTAATTTTGCGCTAATTACATTTGATGAAAGGTTATCTTTTAAAGCCGCTAACGATCAAGATATGGTGGATTGGATAATAAATTTCAAGAGTGGAATTTTGATAAGAAAAAAATTAAAGGCCGAAAATATATAA>YBR130C	-:0:chr02:495586:496863ATGTCGGACCAGGATAATACCCAGACTTCTTCAAGCAAGTTGGCACCTCACCATAATATTTTTATGGCAAACTTGGAAAGTTCCCCCACAAAAGATAGAAATACCTCCAGCCAAAATGCCTCATCTTCGAGAGTCATTGAATCACTGCATGACCAGATTGATATGCTGACAAAAACAAATCTACAATTGACTACGCAATCTCAAAATTTATTGAGTAAATTGGAATTAGCACAATCCAAAGAATCAAAACTACTGGAAAATTTAAATCTGCTGAAGAACGAGAACGAAAATCTCAACTCGATATTTGAAAGAAAGAACAAGAAGCTAAAAGAGCTAGAGAAAGATTATAGCGAATTGAGTAACCGTTACAATGAACAAAAGGAGAAAATGGATCAATTGAGTAAATTGGCGAAAAATTCTAGTGCCATTGAACAATCTTGCTCTGAAAAATTGCAAAATATGGAAGTTAACTATAATTCATTATTGGAGTCACAAAATCTTTACAGAGACCACTATTCTGATGAAATTTCCAAATTAAATGAAAAAATCGGTTTGTTGGAGTTAGAGTTAAGTAATCAAAATTTAAATTATGGTTCAGATACTAGTTCAAATTCAGATATAGAATTGAATTTAAATAAATTCAATGACTCTGTGAAAGATTTGAAATCTCTGGAAACAGAAAAGGATTCAAAATTAAGCAAGATAATCACTCACTCTTTAGATGAGTTAAATCTACAAAGCTGGCTAAATCTGTACCAGACGAACGAAAATCTGATATCAACTTTTGCTGAAAAGATGGATTTGAAAGACGTCTTGAAAAGAAACGATGAGAAGATTAGTAATAAAGGCGCGGTAGTACAAACCTTGAAGAAGAATGTACAAACCCAGGTGGAAAGCAACAACGCTGACGCTTTGAGTAGCAATAATGCACAGGATATGCTTCCTATTAAGATGGTCAAATTAAGAAAGACGCCGAACACAAACGATTCATCCTCCAATGGTAATAGCAGTAACAATAAAAGAAGAAGTTTCTATACTGCGTCGCCTTTGCTGTCATCGGGTTCTATTCCAAAATCTGCATCCCCGGTTTTGCCTGGTGTTAAAAGAACTGCCTCAGTAAGAAAACCAAGCTCGAGCAGTAGTAAAACAAATGTAACGCATAATAACGATCCAAGTACATCTCCCACAATCTCAGTGCCTCCCGGTGTTACAAGAACTGTTTCCTCCACTCATAAGAAAAAGGGAAATAGTATGGTTGTTCACGGGGCCCAATCCTAG>YBR132C	-:0:chr02:499646:501436ATGACAAAGGAACGTATGACCATCGACTACGAAAATGACGGTGATTTTGAGTACGATAAGAATAAATACAAGACAATAACCACTCGAATAAAGAGTATCGAACCTAGTGAGGGATGGTTGGAACCTTCTGGGTCAGTGGGTCACATAAACACGATACCCGAAGCGGGCGATGTTCACGTGGATGAACATGAGGATAGAGGGTCTTCTATTGATGATGACTCAAGGACTTACCTGCTATATTTCACAGAAACTCGACGTAAACTAGAAAACAGGCACGTCCAGTTGATTGCTATTTCCGGTGTCATTGGTACGGCGCTATTCGTGGCGATCGGAAAAGCTTTATACCGTGGAGGGCCCGCCTCTTTATTATTGGCATTTGCTCTTTGGTGTGTTCCAATACTTTGCATTACTGTGTCTACAGCGGAAATGGTCTGCTTTTTCCCTGTAAGTTCCCCCTTTTTGAGATTAGCAACGAAGTGCGTTGACGATTCATTGGCTGTCATGGCTAGCTGGAATTTCTGGTTTCTTGAATGCGTACAGATCCCTTTCGAGATTGTTTCTGTTAATACAATTATACATTATTGGAGAGATGATTATTCAGCTGGTATTCCGCTCGCCGTTCAAGTAGTTTTGTATCTGCTTATTTCCATTTGTGCAGTCAAATATTACGGTGAAATGGAATTTTGGTTGGCTTCTTTCAAAATTATCCTTGCACTCGGCCTATTTACATTCACGTTCATTACCATGTTGGGTGGAAATCCTGAACATGATCGTTACGGGTTTCGTAATTATGGTGAAAGTCCATTCAAGAAATACTTTCCCGATGGCAATGATGTGGGGAAGTCTTCGGGCTACTTCCAGGGGTTTCTCGCTTGCTTGATTCAGGCATCGTTTACCATAGCTGGTGGCGAGTATATTTCTATGTTAGCGGGAGAGGTCAAACGACCAAGAAAAGTATTACCCAAGGCGTTTAAGCAGGTGTTTGTGAGATTAACATTTTTGTTTTTAGGGAGTTGTCTGTGTGTTGGGATTGTTTGTTCGCCAAATGATCCTGACTTGACAGCAGCAATTAATGAAGCAAGGCCTGGCGCCGGGTCTTCACCTTATGTCATTGCAATGAATAATCTGAAAATTAGAATATTACCTGACATTGTTAATATAGCTTTGATTACAGCCGCCTTTTCTGCTGGTAACGCTTACACTTATTGCTCATCCAGAACATTTTATGGTATGGCATTAGATGGCTACGCGCCAAAAATCTTCACTAGATGCAATAGGCATGGTGTGCCCATTTACTCTGTGGCCATATCTTTGGTATGGGCTTTAGTGAGCCTTTTGCAACTGAATTCTAATAGTGCGGTCGTATTGAATTGGTTAATTAACTTGATTACTGCCTCTCAATTGATTAATTTTGTCGTCCTTTGTATCGTCTATTTATTTTTCAGAAGGGCTTACCACGTCCAACAAGATTCGTTACCCAAGTTGCCATTCCGTTCGTGGGGTCAACCATACACTGCTATTATCGGCCTTGTTTCATGTTCCGCAATGATTTTAATACAGGGCTACACCGTTTTCTTTCCCAAATTATGGAACACACAAGATTTTTTGTTTTCGTATTTAATGGTGTTTATCAACATCGGTATATATGTGGGCTACAAATTTATTTGGAAACGTGGTAAAGATCACTTCAAAAACCCACATGAAATTGACTTTTCTAAAGAGCTAACAGAAATTGAAAACCATGAGATTGAAAGCTCCTTCGAAAAATTTCAATATTATAGCAAAGCATAA>YBR133C	-:0:chr02:501798:504281ATGCATAGCAACGTATTTGTTGGTGTCAAACCAGGCTTTAATCATAAACAGCACAGCAAAAAGTCACGTTTCCTAGAAAATGTCTCTAGTCATTCACCAGAACTGCCTAGTAACTATGATTACGTTTTGCTTCCTATAACAACGCCAAGATATAAGGAAATAGTTGGGCAAGTTTTCAAAGATTTCCAAAGACAATCCATACAGAACTGGAAACCGCTTCAAATTCCTGAACCGCAGTTGCAGGATATCTGTATACCCCCGTTCAACGTCAAGAAGCTAGACAATGACGATACGCCGTCTTACATAGGGCTGTTATCCTCTTGGCTGGAGCTGGAGAGTCGCGATCCAAATGTAAGAGATCTTGGCTTAAAGGTCCTTCTAAACGAATGTAAGTACGCGAGGTTTGTTGGAATCAATAAGCTAATATTGGCGCCTCCACGGGACCTGTCCAACCTGCAATTGTATGGACAGATGATTTACAGGCTCCTGCAAAATCGCATCGTCTTTGCTGCGCCTGCGTTAACCATATCCATTTCTCTGCCACTTTACGAAGACAGCGATCCATTGGCCACTTGGGAACTGTGGAATACCGTGCGGAAACAATGCGAATATCATCCCTCTTTGACTATCTCTTTGGCTTTGCCAAGAACCAGGACTCCTTCGTATGTGCTGAATAGATGGTTAGCCGAACCCGTCTCGTGTCTTTTGGTATCTTCATCCATCTTTGCCAGTAATCAGTACGATTATCCCGTTTTACACAAGTTTAACCAGAATTTGATTTTAAAGTTCCAAAAGGTTAATGGAGATTCACAAATTTTGGGTAATGAATTATGCGTGATATTGCATGGGATGGAGAAATATGCCAATAATGTTAAGGGCGGAGAATCTGCCTATTTGGAATATATAAACTACTTATTGAAAAAGGGCGACAAAGTATTAAATTCCAATAGTAATCACCAATTTTTGCTCCAAGAGGACTCTCGGATAATGCCGCCTCTGAAACCTCATTCAGATAATTTATTAAATTCCACATATTTGACTTTTGAAAAAGATTTGGTGAAGTACGATCTTTACGAATCTGCCATATTAGAGGCGCTTCAAGATCTTGCTCCTCGAGCGAGTGCCAAGAGACCGTTGGTGATCCTAGTAGCCGGTGCGGGAAGAGGACCTTTAGTGGATCGAACTTTTAAGATAATATCAATGTTGTTTATGGATAGTAAGGTTTCTATAATTGCCATTGAAAAAAATCCACAGGCATATCTGTACTTGCAAAAAAGAAATTTCGACTGTTGGGATAATAGAGTGAAATTAATCAAGGAGGATATGACCAAATGGCAAATCAACGAGCCGTCGGAAAAGCGTATTCAGATAGATCTGTGCATAAGTGAACTGCTGGGTTCGTTCGGTTGCAATGAATTATCACCAGAATGTCTCTGGTCTATTGAAAAATATCATTCCCACAATGACACAATTTTCATACCGAGGTCATACTCTTCATACATAGCACCCATTTCGTCACCATTATTCTACCAAAAACTCTCACAAACAAATCGCTCTTTGGAGGCGCCCTGGATAGTCCATAGAGTGCCATACTGTATATTATCCTCAAGGGTAAATGAAGTGTGGCGGTTCGAGCATCCCATGGCCCAAAAAGATACTGTCCAAGACGAAGATGATTTTACAGTTGAATTTTCTCAAAGTTCATTAAATGAGTTCAAGATAAAGCACCGCGGCGAAATCCATGGCTTTATCGGATTCTTCTCGGCAAACTTATATAACAATATATTCTTGTCAACTTTGCCCAATGACAGCACAGTCCGTTTAAAATTTAGCGAAGAAACGTTGATGAATACCAGACGAGAAGAAAATCTAATCAAGAAATGTGACCATACACCAAATATGACCTCGTGGTCTCCAATTATCTTTCCTTTGAAGCAACCAATATCCTTTATAGATGACTCCGAACTTTCTGTGCTGATGTCTCGGATACACTCCGATACAGAACAAAAAGTTTGGTATGAATGGTCTTTGGAGAGTTTCATATACCTTATGTTGTCAAATTACACTTCGGCGGTAACTGCTGCAAGCATGACTATTCCGAGGTCTATAGTTACAGATGACACTAAAACTTTAGCCCATAATCGACATTATTCAGCGACTACCAATCAAAAGCTAGATAATCAAATTGATCTTGACCAAGACATTGAAAACGAAGAAGAACAGGGATTCCTATCCAATCTAGAAACTGGTTGGCAAAGCGTACAAGATATTCACGGACTCAGCGAAACCGCCAAACCGGACCATTTAGATTCTATCAATAAGCCTATGTTTGATCTCAAATCTACTAAAGCGCTTGAACCCTCTAACGAATTGCCAAGGCACGAAGACCTCGAGGAAGATGTTCCAGAAGTTCATGTCAGAGTCAAGACTAGTGTTTCCACGCTACATAATGTCTGTGGCAGAGCCTTTTCCCTGCCTCTGTGA>YBR135W	+:0:chr02:504848:505300ATGTACCATCACTATCACGCCTTCCAAGGCAGAAAGCTTACTGACCAAGAAAGAGCACGCGTGTTGGAGTTTCAAGATTCCATTCACTATTCTCCGCGGTACTCAGACGATAACTATGAGTACAGGCATGTGATGTTACCTAAGGCCATGCTAAAAGTTATCCCATCTGATTACTTCAATTCGGAAGTGGGGACCCTGCGTATATTAACAGAAGACGAATGGAGAGGCCTCGGCATCACACAGTCTTTGGGGTGGGAACATTATGAATGCCATGCGCCAGAACCACACATTTTGCTATTCAAAAGGCCGCTGAACTACGAGGCCGAGCTGAGGGCAGCGACCGCTGCTGCTCAACAGCAACAGCAACAGCAGCAACAGCAGCAACAACAACAACAGCAACATCAAACACAATCGATTTCGAACGATATGCAAGTTCCACCCCAAATCTCCTAG>YBR136W	+:0:chr02:505662:512768ATGGAATCACACGTCAAATATCTTGACGAATTGATATTGGCAATAAAAGACCTGAACTCGGGGGTGGATTCAAAGGTGCAGATTAAAAAAGTGCCCACGGATCCATCTTCTTCTCAGGAGTACGCCAAGAGTTTAAAGATCCTGAACACCCTCATAAGAAACCTAAAAGATCAAAGAAGGAACAATATCATGAAAAATGATACTATATTTTCGAAAACAGTTTCCGCCCTTGCCTTATTGTTGGAGTACAACCCCTTCTTGCTTGTTATGAAGGATTCCAACGGGAACTTTGAGATACAAAGGCTGATAGATGATTTCCTCAACATATCCGTTCTGAACTATGATAATTACCACAGAATATGGTTTATGAGGCGAAAATTAGGCAGCTGGTGCAAAGCATGTGTCGAATTTTACGGAAAACCTGCTAAGTTTCAGCTTACTGCACATTTTGAGAACACCATGAATCTTTACGAACAGGCCTTGACTGAAGTCTTGTTGGGCAAGACTGAGCTTCTCAAATTTTATGACACCTTGAAGGGTCTATACATTCTTTTATACTGGTTCACTTCGGAGTATAGTACTTTTGGGAACTCTATAGCATTCTTAGATTCTTCTTTGGGGTTCACGAAATTTGACTTTAACTTCCAACGATTAATCAGGATTGTTCTTTACGTCTTTGATTCCTGCGAACTAGCAGCACTAGAATATGCCGAAATCCAACTCAAATATATTTCTCTAGTTGTGGACTATGTTTGCAATAGAACAATTTCCACAGCCCTGGATGCCCCAGCGTTAGTTTGTTGTGAACAATTAAAGTTTGTATTGACTACTATGCATCATTTTTTGGATAACAAGTATGGGCTCTTGGATAATGACCCCACTATGGCCAAAGGAATTCTTCGACTATATTCTCTTTGCATTTCTAACGATTTCTCAAAATGCTTTGTAGACCACTTCCCAATTGACCAGTGGGCAGATTTTTCACAAAGTGAACATTTTCCGTTCACGCAGTTGACTAATAAAGCTCTCTCGATTGTATATTTTGATTTGAAAAGAAGGTCCCTACCTGTTGAAGCTTTAAAGTACGATAATAAGTTCAACATCTGGGTATACCAATCGGAGCCGGACTCGAGCTTGAAAAATGTCACTTCTCCCTTTGATGATCGATATAAGCAGCTGGAAAAGCTAAGGTTGCTAGTACTAAAGAAGTTTAACAAGACAGAAAGAGGAACTTTGCTCAAATACCGCGTGAACCAGCTAAGTCCTGGATTTTTTCAAAGAGCTGGAAACGATTTCAAGCTAATTTTAAATGAAGCATCTGTATCCATTCAAACTTGTTTCAAGACAAACAATATAACAAGGCTAACATCATGGACTGTAATTCTCGGACGTCTAGCCTGTCTAGAATCAGAGAAGTTTTCCGGCACTCTGCCAAATTCCACAAAGGATATGGATAATTGGTATGTTTGTCATTTATGCGATATTGAGAAAACTGGCAACCCTTTCGTGCGAATAAATCCAAATAGACCAGAGGCTGCGGGTAAATCAGAAATCTTCAGGATACTTCATTCAAACTTTCTATCTCACCCAAATATAGATGAATTTAGCGAATCTTTGTTAAGTGGCATCTTATTTTCTCTACATAGGATATTTTCACACTTTCAACCTCCAAAACTTACAGATGGAAACGGTCAAATCAATAAGAGCTTTAAACTGGTACAAAAGTGCTTTATGAATTCTAACAGATACCTACGTTTATTAAGTACTAGAATTATACCTTTATTCAATATATCAGACTCTCATAATTCCGAAGATGAACACACTGCCACGCTGATAAAGTTTCTACAATCTCAAAAATTGCCAGTGGTGAAAGAAAATTTAGTCATTGCTTGGACACAATTAACATTGACGACTTCTAATGATGTATTTGATACACTACTTTTGAAACTGATTGATATTTTCAATTCTGATGATTATAGTTTACGAATAATGATGACTTTGCAAATTAAAAATATGGCCAAAATTTTAAAGAAAACACCATATCAATTACTATCGCCTATTTTACCTGTATTACTAAGACAGTTGGGAAAAAACCTCGTGGAAAGAAAAGTTGGCTTTCAAAATTTAATAGAATTATTGGGATATTCTTCAAAAACAATTCTCGATATTTTCCAGAGATATATCATCCCTTATGCAATTATTCAATATAAGAGCGATGTGCTAAGTGAAATTGCTAAGATTATGTGTGATGGCGATACAAGTTTAATTAACCAAATGAAGGTTAATTTACTGAAAAAAAACAGTAGGCAAATATTTGCCGTAGCTTTGGTAAAACACGGATTATTTTCTTTGGATATCTTGGAAACCCTTTTTTTAAATAGGGCTCCAACTTTTGACAAAGGATATATAACTGCATACCTTCCCGATTATAAAACTTTAGCTGAAATAACGAAGCTCTACAAAAACAGCGTTACTAAAGATGCAAGTGACAGCGAGAATGCTAATATGATTTTATGCTCTTTGCGATTTTTAATCACCAATTTTGAAAAAGACAAAAGGCATGGTTCGAAGTACAAAAATATCAATAACTGGACGGATGATCAGGAACAAGCGTTCCAAAAGAAACTACAGGATAATATCTTAGGTATTTTCCAAGTTTTTTCGAGTGACATACATGATGTTGAAGGCCGCACCACTTACTACGAAAAGTTAAGGGTTATCAATGGCATTTCTTTTCTTATCATATATGCCCCCAAAAAATCAATAATTTCCGCATTAGCCCAGATTAGTATTTGTTTGCAAACAGGACTTGGGCTTAAGGAAGTTCGATACGAGGCCTTTAGATGTTGGCATCTGTTAGTTCGCCATCTAAATGATGAAGAACTCTCTACTGTTATAGATAGCTTAATTGCATTCATACTTCAAAAGTGGTCTGAGTTCAACGGAAAACTTCGAAATATAGTGTACAGTATACTGGATACCTTAATCAAAGAGAAATCGGACCTGATTTTGAAATTAAAACCTTACACTACTTTGGCTTTAGTAGGCAAGCCTGAATTAGGTATTTTAGCTCGTGATGGCCAATTTGCAAGAATGGTGAATAAAATAAGAAGTACCACGGACCTTATACCCATATTTGCTAATAACTTGAAAAGTAGTAACAAGTATGTCATAAACCAAAATTTAGACGATATAGAGGTATATCTTCGGAGAAAGCAGACAGAAAGATCGATTGATTTTACACCAAAGAAGGTTGGGCAAACTTCTGATATAACATTAGTTTTGGGTGCTTTATTAGACACTTCTCATAAGTTTAGAAATTTAGACAAGGACCTATGCGAGAAGTGCGCCAAATGTATCAGTATGATTGGTGTTTTAGACGTTACAAAGCATGAGTTTAAAAGAACAACATATTCAGAAAACGAAGTTTATGATTTGAATGATAGTGTTCAAACTATTAAGTTCTTGATATGGGTCATAAATGATATCCTCGTTCCTGCGTTTTGGCAAAGTGAGAATCCCAGCAAGCAATTGTTTGTTGCCCTTGTCATACAGGAATCATTAAAATATTGCGGGCTAAGTTCAGAGTCATGGGATATGAACCATAAAGAATTATATCCAAATGAAGCCAAACTATGGGAAAAGTTTAACTCTGTCTCCAAGACAACCATCTATCCGCTTTTATCTTCCTTGTATCTTGCGCAATCATGGAAAGAATATGTCCCGCTAAAATATCCTTCTAATAACTTCAAGGAAGGATACAAAATTTGGGTGAAAAGGTTTACATTGGATTTATTGAAAACAGGTACAACAGAAAATCATCCATTACACGTGTTTTCCTCTTTGATTAGGGAAGATGATGGCTCACTATCAAATTTTTTGCTACCTTATATTTCTCTGGACATTATTATCAAGGCAGAAAAAGGAACTCCATACGCTGATATTTTAAACGGGATTATTATTGAATTTGACAGCATTTTCACGTGCAATCTGGAAGGAATGAATAACTTGCAAGTGGATTCGTTAAGAATGTGCTATGAATCCATCTTCAGAGTTTTCGAATATTGCAAAAAATGGGCAACTGAGTTTAAACAAAATTACAGTAAACTACACGGCACTTTTATCATTAAAGATACGAAAACAACTAACATGCTTTTGAGAATAGATGAGTTTTTGCGAACAACCCCTTCAGATTTGCTAGCTCAACGCTCCTTAGAGACGGATTCTTTTGAAAGGTCTGCTCTATACCTTGAACAGTGCTATCGACAGAATCCTCACGATAAGAACCAAAATGGACAACTACTGAAAAATTTACAAATCACATACGAAGAAATAGGAGACATTGACTCACTCGATGGTGTACTGAGAACCTTTGCTACAGGAAACTTGGTTTCTAAAATTGAAGAATTGCAATATTCTGAAAACTGGAAACTCGCACAAGACTGCTTTAATGTCCTCGGCAAATTTTCAGATGACCCCAAAACTACAACCAGGATGCTAAAGTCTATGTATGACCACCAATTGTATTCTCAAATAATATCGAACTCTTCGTTCCATTCTTCAGACGGAAAAATTTCTTTGTCTCCAGATGTGAAGGAATGGTACAGCATAGGTCTTGAAGCTGCAAATCTAGAAGGCAATGTTCAAACTTTGAAAAATTGGGTAGAACAAATAGAGAGTTTAAGAAATATTGACGATAGAGAAGTACTTTTGCAGTACAATATTGCGAAAGCTTTAATTGCCATCTCAAACGAGGATCCATTAAGGACTCAAAAATACATCCACAATTCCTTTAGGCTTATCGGAACAAATTTTATAACGTCATCTAAAGAGACGACGCTGCTAAAGAAACAGAATTTATTGATGAAATTACACAGTTTATATGACCTCAGTTTTTTATCTTCTGCGAAAGATAAGTTTGAATACAAAAGTAACACTACCATACTCGATTATCGAATGGAACGTATTGGGGCTGACTTCGTGCCAAATCATTACATATTGTCAATGAGAAAGTCATTTGACCAATTGAAAATGAATGAACAAGCAGACGCTGACTTAGGAAAAACATTCTTCACTTTAGCCCAATTGGCGAGAAACAACGCTAGGCTAGATATAGCCTCCGAATCATTAATGCATTGTTTGGAAAGGCGGTTGCCTCAGGCAGAGTTGGAGTTTGCTGAAATACTATGGAAGCAAGGTGAGAATGATAGAGCCTTAAAGATAGTGCAAGAAATACATGAAAAGTATCAAGAAAATTCCTCGGTTAATGCTCGCGATCGTGCCGCCGTGCTATTAAAGTTTACTGAATGGTTAGACCTTTCGAACAATTCAGCGTCCGAACAAATTATTAAACAATATCAGGATATTTTTCAGATTGATTCTAAATGGGATAAACCATATTACTCTATTGGCTTATACTATAGTAGACTACTTGAGCGCAAAAAAGCAGAGGGTTATATTACTAATGGTCGTTTTGAGTACAGGGCAATATCTTACTTTTTATTGGCATTTGAAAAGAACACTGCTAAAGTAAGAGAAAATTTGCCCAAAGTTATCACGTTTTGGCTAGATATTGCGGCCGCATCAATTTCTGAAGCTCCTGGAAACAGAAAGGAAATGCTGAGTAAGGCTACGGAAGATATATGTAGTCATGTTGAAGAAGCGCTGCAGCATTGTCCCACTTATATTTGGTACTTTGTTTTGACTCAGTTGTTATCTAGGTTATTACATTCTCATCAATCATCGGCCCAGATAATAATGCACATACTGCTAAGTTTGGCTGTTGAATACCCCTCTCATATTTTATGGTATATCACAGCCCTTGTAAATTCCAATTCTTCAAAAAGAGTTCTTCGTGGTAAGCATATTTTAGAAAAGTATAGACAACATTCGCAAAATCCTCATGATCTAGTTTCTAGTGCATTGGATTTAACGAAAGCATTAACTCGTGTCTGTTTGCAAGATGTCAAAAGCATTACAAGTAGATCAGGCAAATCTTTAGAAAAAGACTTCAAATTTGACATGAACGTGGCCCCATCTGCAATGGTTGTTCCAGTAAGAAAAAATTTAGACATCATTTCACCACTAGAGTCTAACTCAATGAGGGGCTATCAACCATTTAGGCCGGTTGTTTCTATAATTAGATTCGGATCATCTTATAAAGTGTTTTCTTCATTAAAGAAGCCAAAACAATTGAACATAATAGGTTCAGATGGCAACATTTATGGGATCATGTGTAAGAAGGAAGATGTCCGACAAGATAACCAATATATGCAGTTCGCCACAACAATGGATTTTCTTCTGAGTAAGGACATAGCTTCAAGAAAAAGAAGCCTGGGCATAAATATTTACTCCGTACTATCTCTTCGAGAAGACTGTGGGATATTGGAAATGGTACCGAATGTTGTAACTTTAAGATCTATTCTTTCTACAAAGTACGAAAGTCTGAAAATTAAGTATAGCCTGAAAAGTCTACATGATAGGTGGCAGCACACCGCAGTAGATGGAAAACTCGAGTTTTACATGGAACAGGTAGATAAATTTCCTCCAATCTTGTACCAATGGTTTTTAGAAAACTTTCCTGATCCAATCAATTGGTTCAACGCCAGGAATACGTATGCCAGATCTTACGCCGTCATGGCAATGGTTGGCCATATATTAGGTCTAGGTGATAGGCACTGTGAAAACATATTACTAGATATACAGACGGGTAAAGTTCTTCATGTAGACTTCGACTGTTTATTTGAGAAAGGCAAAAGGTTACCTGTCCCAGAAATTGTTCCCTTCAGACTAACACCAAATTTATTGGATGCGTTGGGCATAATTGGGACAGAAGGAACATTTAAGAAGTCTAGTGAAGTCACGTTGGCTTTAATGAGAAAAAATGAAGTAGCGTTGATGAATGTGATCGAAACAATTATGTACGATAGAAACATGGACCACTCAATTCAAAAAGCGCTAAAGGTCTTAAGAAACAAAATCCGCGGTATAGATCCGCAGGATGGCCTGGTATTGAGTGTTGCTGGCCAAACAGAAACATTGATCCAAGAAGCAACATCAGAAGACAATCTAAGCAAGATGTATATTGGTTGGCTTCCATTTTGGTAA>YBR137W	+:0:chr02:513038:513577ATGGTGGTTTTAGACAAGAAGTTATTGGAAAGATTGACTTCTCGTAAGGTTCCCTTAGAAGAGCTCGAAGATATGGAAAAACGATGCTTTTTGTCTACTTTTACATATCAAGATGCCTTTGATTTGGGGACTTACATAAGAAATGCAGTTAAAGAAAATTTCCCCGAAAAACCAGTTGCAATTGATATCTCCTTACCAAATGGGCATTGCTTGTTTCGCACAGTGACCTACGGTGGCAGTGCATTGGACAATGATTTTTGGATCCAGAGAAAGAAGAAGACAGCGCTTCGATTTGGTCATTCAAGTTTCTATATGGGCTGCAAGAAAAGTGACAAAACACCGGAGGAAAAGTTTTTTGTGGACTCAAAAGAATACGCTTTCCATGGAGGTGCTGTCCTAATACAATCGGAGAGAAGTGACTACCCCTATGCCTGCTTAACTATAAGTGGATTGAAGCAGGAAGAAGACCATTTAATGGCAGTCAGCTCTTTAATTGCATTTGCTAATGAAAGTCTAGAAGAAGATTTGAATTTGGATTGA>YBR138C	-:0:chr02:513756:515330ATGGAGAAAGATCAAATCCAGCCTAGGGTGTTGGAGTCAGTAGACACAAACTCATTATCCTTGCTTTCGTCCAATACCAGCTCAAATATGAATAGCAACACCAACAATAAACTCAGTATAATTGCCAGTGATATTTCTACAGGATCCGTACTTTCCAGGCCCTTGACTCCTCCAGTCGTCCAGGATATAGAGAATAACTCCATGTTACAATGGCAATTTGAAAAGAAGGAATTTATCTTCGATAGTAACAGCACTCCTTCGAAGCAGGCAAAACCATTGCAGAGAAATTCTCCATACCAAGGCAATAGTCAAAGTGAAAATCAGAACCAACAGTTATTGAATGTAAGAAAGCGTCGCTCACAATGTATAGGTGCCAAGCCTAAAATTCCATCCAAATTGTACCAATCTGTTTCCAAATTAGATCTAATTGATGACAAGAGCTTTACGTCTTTGCCAATTGCGCCTCCATGCAATATCGAAACTAATGAAGATGACAGTGGAAACAACGAATACAACAACAATAAAAAACGGCCCAGGTTGAATCCTGTAAATGAACTGCGAGTGCATAACAACAAACGTAATAGGTACGTCAGTTATGGGCCGAGTTTAGATACAAAAAACTACGAGCTAACAGAAAATACTTCTCAGGATATACCACCGCTAGTCCTGGTTGAAGACTACATACCGTATACCCAAAGTAAATCAACAAAAAAAATGGTATCTATTTCAGATTTGAAATCAAAACTAAGTAAAAGAAGAGATAATCATATCCCGCTGAGAGTAAAAAATTCATATTCTGAAATTAATAAGGAAACCAATAGAAATAGCTTTGAACCGAATTCACTAACACTGATACCCCATATATTAAGGAATACTGAAGAAAATAGGGACGAAAGTAACAACCCCTTAGATTTTATCAAAGAGGAAATTGAAATAAGTGATATTTCCATACCAAATTCCATTGAAAATATGGTGGTTAACTTGGTTAATATTCCATCTTCCAATAAAAGTTACGATGATCTTTATCTATCGGAATTAAATGTTCATTCCCAACTGAGGAAATGTGTCATCTGCGAAAAGGCCCTATATGAAATTAGCTCGAGACTTTTAAATTCAGGATACTATAAAGAAATTGTATGTGAGCAATGCACAGTTAGATATGAAGAAGCCGCTAAAATCTTTGAGAACTGCGAGTTCGAATCATCTATGGATGAATCAAATCTAAGCAGCGGTACTTTTAGTGATCTAGAGAATTCAGCAGAACCGTTTCATTTATCGACAGATGTTCCAAAAAAAATAAATAGGCACATAGAAGATAACAAGATCGACCTTAAAAAGGAAATATCAAAAAAAAAGGACAGCTTCTCTAAGGAACTAATAGAACGATTGCAGTTGCAATTGCTAGAAAATGATAAGTCAATCAAACATCATTTTAATAAAGATGCAATGGGCTCTAAATCAATGAATTGGTTTTTGGAAGCAAGAAGAAAATTGAAGTGGAAGTGGAGAATAAATGGTTTACTTCCGCATTTCTTACGCAATCAAAATAGTGATCGTTTAAATTTTCAACCTTGA>YBR139W	+:0:chr02:515658:517184ATGAAGTATCTAAACTTAGTTTTCGTGCTTCAGCTTCTTATTAGCATCAAATACGCCTCATTCGGCCGAGCCTTTTCTCTTTTTGAAGATGATACCACCTTTGCCAATTTGGATAAACAGCTAAAGCTTCCACAGAATACACAGCAAACCCTTAAATTGGACCGTTTGAATCACGATGATCCGCTGTTTACAACTTTTATTTCTTCTGTGGACACAGATTACAGTTTGAGACTTAGAACAGTAGATCCTTCTAAACTAGGAATTGACACCGTAAAACAATGGTCGGGTTACATGGACTATAAGGATTCCAAACACTTTTTTTACTGGTTTTTTGAAAGTAGGAACGATCCTGCTAACGACCCAATTATTCTTTGGTTAAATGGTGGACCTGGTTGTTCCTCGTTTACTGGGTTGCTATTTGAACTAGGCCCCTCATCAATTGGCGCCGATATGAAACCAATCCACAATCCCTATTCTTGGAATAATAACGCTTCAATGATCTTCTTAGAACAGCCACTCGGAGTCGGCTTTTCCTATGGTGATGAAAAAGTCTCCTCTACAAAATTAGCAGGCAAAGATGCGTACATTTTCCTGGAATTGTTTTTTGAAGCTTTTCCTCATTTACGCTCCAACGATTTCCACATTGCAGGCGAATCCTATGCAGGACATTATATCCCTCAAATTGCACATGAGATCGTTGTCAAGAACCCTGAAAGAACGTTCAATTTAACTTCAGTTATGATTGGTAATGGTATCACAGACCCTTTGATTCAAGCAGATTATTATGAACCAATGGCATGCGGGAAAGGGGGCTATCACCCTGTTCTCTCATCAGAAGAATGTGAGAAAATGAGTAAAGCTGCAGGTCGTTGTCGTAGGTTGAACAAGTTATGTTATGCTTCTAAATCAAGTTTACCATGCATAGTCGCCACTGCTTACTGTGACTCTGCACTTTTGGAACCGTACATTAACACAGGACTCAACGTCTATGACATTAGAGGGCCCTGTGAAGATAATAGTACTGATGGTATGTGTTATACAGGTCTCCGCTATGTCGACCAGTATATGAATTTTCCTGAAGTTCAAGAAACGCTAGGGTCCGACGTGCATAATTATTCTGGCTGTGATAATGACGTGTTCACCGGATTTTTGTTTACGGGCGATGGAAGTAAACCATTTCAACAATATATTGCTGAATTATTAAATCACAACATTCCGGTATTAATATATGCGGGTGATAAGGATTATATTTGTAATTGGCTGGGAAACCATGCTTGGTCCAATGAGTTGGAATGGATCAATAAACGTAGGTATCAGAGAAGGATGTTAAGACCATGGGTCAGTAAAGAAACAGGTGAAGAGTTGGGACAAGTCAAGAACTATGGCCCTTTCACCTTTTTGAGAATATACGATGCCGGTCATATGGTGCCCTATGATCAACCGGAGGCAAGTTTGGAAATGGTCAACAGTTGGATTTCCGGTAATCGTGCTTTTTCGGATCTTTCCACCTTGGAAAATGCTAGTTAG>YBR141C	-:0:chr02:527019:528032ATGCATTCAAGAAAGTCGAAGAGTATTACGGGCAAGAGGAAGCAAGTAGGAAGTAACGTGACGAGAGTAATCAAGCCACAGAAGACAAGAAGGATAATAAGAAGATTCCACCATTTAATTAATAAGCGTCAATCGATATGCAAGTTTTTATGCCTTAAGGAAAACCTAGATGATTCCAACGAGGAGAAGAATGACAAAATTATCAGGTTAAGTATTAAAGGTAATGTAAGACTGGGCAAGTACTATGAAGACGGCAAATCGCAGTCCTTCAATGATGCTATGGAATCACAACTACTGAGGCTGCACTCATTAATTAAAAATGAATCCAAATCAAAGGATACTTCTGACTTGGCTGTGATGTACACATTACTTGGTTACATAATGAATCAGATTAATAAATTGGGAGGACTAGAAACTTACCAAATTGCCAGTCAAAACGGGCAATTGAAGGAACGAGGAGGAGACACGTCCAAGTTGCTCGAGAAATGGATCAGGTCCTCATTTGAAAATTGTCCTGGAGCAGTGGCATTAGAAATTGGTTCATTGAGCTCCGGAAATCGCATATCCCGTTGTGCACTTTTTAGAAATGTGGTTCGTATAGATTTAGAGGAACATGAAGGCGTTATTAAGCAGGATTTTATGGAAAGACCGCTACCGAGAAATGAAAACGACAAATTCGACCTGATATCATGCTCTCTAGTGCTAAATTTTGTCAAAAATCACAGGGATCGCGGTGCAATGTGCCATCGCATGGTCAAATTTCTCAAGCCGCAAGGCTACATCTTTATTGTTCTGCCACAAGCCTGTGTGACGCACTCAAGATACTGCGACAAAACACTGTTACAAAACCTCCTCGGCTCTATCGGGCTCATAATGCTTAATAGCCATCAAAGTAATAAGTTGTACTATTGTTTGTATCAATTGCAGGTAGTTTCACCGCAGCCGAGTAGCTTTTCCAAAAGAATCAAGGTTAACGACGGCCCTGGGTTGAACAATTTTGGTATTACCCTCTAA>YBR145W	+:0:chr02:533756:534811ATGCCTTCGCAAGTCATTCCTGAAAAACAAAAGGCTATTGTCTTTTATGAGACAGATGGAAAATTGGAATATAAAGACGTCACAGTTCCGGAACCTAAGCCTAACGAAATTTTAGTCCACGTTAAATATTCTGGTGTTTGTCATAGTGACTTGCACGCGTGGCACGGTGATTGGCCATTTCAATTGAAATTTCCATTAATCGGTGGTCACGAAGGTGCTGGTGTTGTTGTTAAGTTGGGATCTAACGTTAAGGGCTGGAAAGTCGGTGATTTTGCAGGTATAAAATGGTTGAATGGGACTTGCATGTCCTGTGAATATTGTGAAGTAGGTAATGAATCTCAATGTCCTTATTTGGATGGTACTGGCTTCACACATGATGGTACTTTTCAAGAATACGCAACTGCCGATGCCGTTCAAGCTGCCCATATTCCACCAAACGTCAATCTTGCTGAAGTTGCCCCAATCTTGTGTGCAGGTATCACTGTTTATAAGGCGTTGAAAAGAGCCAATGTGATACCAGGCCAATGGGTCACTATATCCGGTGCATGCGGTGGCTTGGGTTCTCTGGCAATCCAATACGCCCTTGCTATGGGTTACAGGGTCATTGGTATCGATGGTGGTAATGCCAAGCGAAAGTTATTTGAACAATTAGGCGGAGAAATATTCATCGATTTCACGGAAGAAAAAGACATTGTTGGTGCTATAATAAAGGCCACTAATGGCGGTTCTCATGGAGTTATTAATGTGTCTGTTTCTGAAGCAGCTATCGAGGCTTCTACGAGGTATTGTAGGCCCAATGGTACTGTCGTCCTGGTTGGTATGCCAGCTCATGCTTACTGCAATTCCGATGTTTTCAATCAAGTTGTAAAATCAATCTCCATCGTTGGATCTTGTGTTGGAAATAGAGCTGATACAAGGGAGGCTTTAGATTTCTTCGCCAGAGGTTTGATCAAATCTCCGATCCACTTAGCTGGCCTATCGGATGTTCCTGAAATTTTTGCAAAGATGGAGAAGGGTGAAATTGTTGGTAGATATGTTGTTGAGACTTCTAAATGA>YBR146W	+:0:chr02:535254:536090ATGTTTTCAAGGCTTTCTTTGTTTCGAAGGGCAGCTCTGGCTCCTGCCCCTATGAGAATGTCTTTTAGGACTATTTATCAAAAAACCGAGGATGAATTGCCCAGAAGGATCGTCCCAAAGCTAGCCACATTTTATTCGGCGAATCCTAACCATGAAGATCGTATTAATCGACTCGAAAGACTTCTAAGGAAATACATAAAACTGCCATCTCAGAACAACAATGAAGCACAACAAACAAAAGCTCCTTGGATTTCTTTCGATGAGTACGCTTTAATCGGCGGTGGTACGAAATTAAAGCCTACGCAATATACTCAGCTACTGTATATGTTGAATAAACTTCACAACATTGATCCTCAACTTACTAATGATGAAATCACATCCGAACTATCCCAATATTATAAAAAAAGTTCAATGCTTTCCAATAATATCAAAATCAAAACTTTAGATGAGTTTGGAAGAAGTATAGCCGTTGGAAAAAGGAAAAGTTCCACTGCGAAAGTATTCGTTGTTAGAGGTACAGGTGAGATATTAGTTAATGGTCGACAATTGAATGATTACTTCCTTAAGATGAAAGACAGAGAATCAATCATGTATCCACTTCAAGTGATCGAATCAGTTGGAAAGTACAATATTTTTGCGACAACGTCTGGAGGCGGACCTACGGGTCAGGCTGAATCAATCATGCACGCCATTGCGAAAGCTTTGGTTGTGTTCAATCCATTATTAAAATCCAGACTACATAAAGCTGGCGTCTTAACAAGAGATTACAGGCACGTCGAAAGAAAGAAGCCAGGAAAAAAGAAGGCAAGAAAGATGCCAACATGGGTCAAGAGATAG>YBR147W	+:0:chr02:536569:537459ATGAAGCTGATCCCAATTATTTTGAATGCTAAGAATCTCAGTGGGATGGCTGGATCCATATCAATATGCTGTTGGATTGTTGTGTTTGTACCACAGATTTATGAAAACTTCCGAAGACAGTCTGCGGAGGGATTATCACTGCTTTTCATTGTGTTGTGGCTATTAGGTGATATATTTAACGTCATGGGAGCTATGATGCAAAATTTACTCCCAACCATGATAATTTTAGCTGCTTACTATACATTGGCAGATTTAATTTTGTTAATACAGTGCATGTGGTATGACAAGGAGAAGAAGAGCATTTTACAAGAAGTCAAGAAAAATGTTGATCCTGTGCATTTGCCTCCAGCAAACCCCATAAATGAGACTGTCTTACAAGACGTTTTTAATGAATATGAGCCACTTTTGCCAAGGATAGAAGAAGAAGATAGCCAATCGTATAGCTCGCTTGAGCTTGGCAGAACAATAGTCGTAAAGGAAAGAGAGAATTTTTTCAATGACTTCTTAATTGTTTCAGGTGTACTTATTGCCGGAATTTTGTCATGGTATATATCTTACTGTTCCGGCCTAGATAACGGTATTCCTAAAAAAAAACCTGCGTTCGAACAGATTAATTTACCTGCACAAATTTTAGGGTATTTAAGTGCGATACTGTACTTGGGCTCTAGGATCCCTCAGATTGTTCTTAACTTCAAAAGAAAATCATGCGAAGGAGTCTCATTCCTATTCTTTTTATTTGCATGCTTAGGGAACACTTCTTTCATAATCTCAGTGCTCTCAGCATCTTGGCTTATCGGTAGCGCTGGTACGCTGTTGATGGACTTCACAGTTTTTATTCAATTTTTCCTTTACGCCAAACCTAAATACGAGAAAATACTAATAGATAATTAA>YBR148W	+:0:chr02:537870:539699ATGTCCAGCTTAGCCGATACAGTAGAGGGTAGCGAGGCCAAACGCGGCAGATTTTCCAACAATGCATTAACCTCCGATACTGGTATTTTACAGAAAAATAGTACATTAAGAAACTGGTTTTTAAAACCGACAGCAGACCTAAAAAATAGTTGCGAAGATAGAGTTGAAGATGACGTCAATGATGTCTACTTAAATGACAAAAACTCACAAAAATCTGTTGAAGAAAGAAAACTAGGAAGAAAAGTTAGATCTTTTTTCAAACAAACAAACTCAAATAAGGACGAGTCTGTGTTGGAAGATGAAGATGATGCGTTAGTTTGGAAGAAAACTAGCAATAAGTGTGCAAAAAAGGAAAATTCCCATGACATCCAAAAAGGAAGTTTCACAAAAAAAATCAGAAACAGTATTTTCAAGAGTGCAAATGACGTGAAAGAATTCAGGAATGAAAATAACTTACTATTGCCCGTTGAACTTTCTTCTGATGATGAGAATGAGTCTCATTTCACTGATGCTAACTCACATGTTATGCAATCAAAAAGTCCCGAGAAAATACCTTCCAAGGATCAATGCTTGACTAAAGGCGCTAAGAATAAAGGTCTGAAAAAGGAATATGAAAAAAGTTTTGAAGAGTATAGCGACGACAGTGACGATGAATTTTCTCCTGCTACTCCACCGGAGAATGTTCTTGAAGGTCCTTACAAATTTGTGTTTCAGACACCAAACACATTTACATCTCAGCCTAATATAACAGTAGAAAATGATTTTCACAAAGGTGGGAGACATGTGATTGACTATCTGAACAAAAAATTGGCTACTATGAACATTGATATTGATTTGACTTCAGGGGGGAAACAAAACGTATCTTGGGAGGAAGAGCTCGACCAATTGAGCGACCACGTTATAGAAAGTATTACTAACCACATTTCAAAGGGTAGAATGCACGCACAAGAGAAGCAAGATGAGTTGGAAAAGTTAAAATTGGAGAATTTGAACTTGTCAACATTAAAGCAAGAAAATCTCCAACATAAGCAGGAAATTAACTCATTGAAAGACAACCTTGAATCCATTAGTAAAAAAAACAATGATCTTATATTGGAAATGAACAAATTGAAAAAAAAAAGTACGAATAATAAAACAAATGAGTACATATCTACGGATGAAAACGAAAATGAGGAGATTACCAAATCCAATATGGGTCCTGGCATTTTAGAACTGAACGTCAACGAAACTTCAAAAAAATTACAGCAATCCACATTCAAACCCTCGAAATATCTTCCAAGAGAAACGAGAAACAATGAAAATAGATTAAAACACTTGGAAAAGAGAATATTTGGCTTAGAAAAATCTCTTGAAAAAAAAAAAAAACAGGTAAGGGCTGACAGCGTTAGATTAGACTTGAATAGATATACTATCGACCAGTTTTTGACATTACTGAAAAGCCTCAGCGAGGTTTTGCAATTCCACAATGTTTATGGCAATGATTTGAAGGAAAACGACGATAATATTATAAAGATTGAAACCTGCTGTAGTGCGCTAAACATGAAAAATTGTTTCGAAGACTCCTCATTTCGTTTACAAGAAAACAGCTTCAAGAGGCAATTGGGCCCTCTGTTCGCAAACATCAACTTCTCCTTAATCGACCAACTGACAATGAACTTCAGGTTCTACGAGAGATCTGCCAATTTCCAGAAGGAAACAATAGGTGGGTTAAGAATGATGCTACAAGATAAGGATAACTATATCAAAACACTGATGCAACATTTGAAGAAAAAAGAGAGTACAAAGTTGATAAAAGACAGCAAGAATGGCGCCTCCACCTTAACATCTTAA>YBR149W	+:0:chr02:539981:541015ATGTCTTCTTCAGTAGCCTCAACCGAAAACATAGTCGAAAATATGTTGCATCCAAAGACTACAGAAATATACTTTTCACTCAACAATGGTGTTCGTATCCCAGCACTGGGTTTGGGGACAGCAAATCCTCACGAAAAGTTAGCTGAAACAAAACAAGCCGTAAAAGCTGCAATCAAAGCTGGATACAGGCACATTGATACTGCTTGGGCCTACGAGACAGAGCCATTCGTAGGTGAAGCCATCAAGGAGTTATTAGAAGATGGATCTATCAAAAGGGAGGATCTTTTCATAACCACAAAAGTGTGGCCGGTTCTATGGGACGAAGTGGACAGATCATTGAATGAATCTTTGAAAGCTTTAGGCTTGGAATACGTCGACTTGCTCTTGCAACATTGGCCGCTATGTTTTGAAAAGATTAAGGACCCTAAGGGGATCAGCGGACTGGTGAAGACTCCGGTTGATGATTCTGGAAAAACAATGTATGCTGCCGACGGTGACTATTTAGAAACTTACAAGCAATTGGAAAAAATTTACCTTGATCCTAACGATCATCGTGTGAGAGCCATTGGTGTCTCAAATTTTTCCATTGAGTATTTGGAACGTCTCATTAAGGAATGCAGAGTTAAGCCAACGGTGAACCAAGTGGAAACTCACCCTCACTTACCACAAATGGAACTAAGAAAGTTCTGCTTTATGCACGACATTCTGTTAACAGCATACTCACCATTAGGTTCCCATGGCGCACCAAACTTGAAAATCCCACTAGTGAAAAAGCTTGCCGAAAAGTACAATGTCACAGGAAATGACTTGCTAATTTCTTACCATATTAGACAAGGCACTATCGTAATTCCGAGATCCTTGAATCCAGTTAGGATTTCCTCGAGTATTGAATTCGCATCTTTGACAAAGGATGAATTACAAGAGTTGAACGACTTCGGTGAAAAATACCCAGTGAGATTCATCGATGAGCCATTTGCAGCCATCCTTCCAGAGTTTACTGGTAACGGACCAAACTTGGACAATTTAAAGTATTAA>YBR151W	+:0:chr02:545022:545972ATGGCTTTTTTGAATATTTTCAAGCAAAAACGTGGTGATGAAGCTTCACAACTGAGCGCAAAGGGACGGGAGGAAATTTCTCAATCGATTAAGATATGCAAAAGTGACGATGCTGCTAACGAACATAGCTGCTCTGGTGATTGCAAAACAGAAATTGAAGAAGGAGAGCAGGCCTTTGCGAAACTAAAGATCGAACATGAAACTCCTTTGTTGAACTCTTCTAAAACGCCAAAAATTCACTTCGTTGTCCCCACCTCTCAAATCGATTGGCAGCATGATGCCTGCCTCGAGGACCCAAAGTCAGTACAGTATAAAATTTCCCAGTGGTGTGATAAGAATTCAGCTAAATTTTCCAACGTGGGCACAGGCAAGACACTAAACTGTGCAGTTTCATCTTTACCTAAAGATATCATGGATATTGATGTTATGCGGGGAACCAAGAATAATGTACTTATTTTGCCTTACTTCATTTGGCTGAACGACCTTAGATCAGATGACGTCGAAGCAACGCTGGATGGTTTAGTTCCTGACTTATTAGACGAGAATATTTCAAGAGAGAAATTGCTAGAAACACGACCAAATGTTGCCGTTGCACGTGAACGTGCATTTGTATTTATATGTTCACATACTACGAGGGATAAACGATGCGGGATTACGGCACCCTATTTAAAGAAAGTTTTTGACAGTAAATTACAGGAACATGGGCTGTATAGGGACAATTCGGATTACAGGGCAGAGGGTGTCAAAATTGCATTTGTTAATCATGTTGGTGGTCACAAATTTGCTGCAAATGTTCAAATTTATTTACGAAATCCAAATACCTTAATTTGGTTGGGAAGAGTAACTCCAACCATAGTTCCTTCTATTGTCGAACATCTGATTGTTCCTGAAGAACCAACGTTGCCGTTTCCCGAAAAAGTTCGCTGTATTAAGAAATATCAAAGCTGGTAA>YBR154C	-:0:chr02:548356:549003ATGGACCAAGAAAATGAAAGAAACATCTCAAGATTATGGAGAGCATTCAGAACAGTAAAAGAAATGGTTAAGGACAGGGGTTATTTTATCACTCAAGAGGAAGTCGAATTGCCATTGGAAGATTTCAAGGCCAAGTATTGTGACTCCATGGGCAGACCACAACGTAAAATGATGTCCTTCCAGGCAAATCCAACAGAAGAATCTATATCAAAGTTCCCAGACATGGGCTCCTTATGGGTAGAATTTTGTGATGAGCCTTCCGTTGGTGTAAAGACAATGAAGACTTTTGTTATACATATTCAAGAAAAAAATTTCCAAACAGGTATCTTTGTTTACCAAAATAATATTACACCAAGTGCAATGAAATTGGTGCCTTCTATACCACCAGCCACCATTGAAACTTTTAATGAAGCTGCCTTAGTGGTTAATATTACTCACCACGAATTGGTTCCAAAGCATATCAGATTGAGTAGTGATGAGAAAAGAGAGCTTTTAAAAAGGTATAGATTGAAGGAATCCCAATTGCCAAGAATTCAAAGAGCTGATCCTGTAGCCTTATACTTGGGATTGAAAAGAGGCGAAGTAGTTAAAATCATAAGAAAAAGTGAAACCTCTGGTCGTTATGCCAGTTACAGAATCTGTATGTAG>YBR155W	+:0:chr02:549765:550922ATGAGCTCCGTTAACGCAAATGGAGGATATACCAAACCACAAAAATATGTGCCAGGGCCAGGTGATCCTGAACTTCCACCCCAACTATCCGAATTTAAAGATAAAACATCGGATGAAATCTTGAAAGAAATGAACAGAATGCCTTTTTTCATGACCAAGTTGGATGAAACAGACGGTGCAGGTGGTGAAAACGTGGAGTTAGAAGCTTTAAAGGCATTAGCTTATGAAGGCGAACCACACGAAATCGCTGAAAATTTCAAGAAGCAAGGTAACGAACTATACAAAGCAAAAAGATTCAAGGATGCAAGGGAACTTTACTCAAAGGGCTTGGCTGTAGAATGCGAAGATAAATCAATAAATGAGTCACTATATGCCAATAGAGCGGCATGTGAGTTAGAGCTGAAAAATTACAGGAGGTGTATCGAGGACTGCAGTAAAGCTCTAACTATTAACCCCAAGAATGTTAAGTGCTACTATCGTACAAGCAAGGCTTTTTTCCAATTAAACAAGTTGGAGGAGGCCAAATCAGCCGCAACATTTGCCAATCAAAGGATTGACCCAGAGAACAAATCAATTTTGAATATGTTATCAGTGATTGATAGAAAAGAACAAGAATTGAAAGCAAAAGAAGAAAAACAGCAAAGAGAAGCTCAGGAACGTGAAAACAAGAAAATTATGTTAGAGAGCGCAATGACGCTGAGAAACATAACTAACATCAAAACTCACTCTCCAGTAGAGTTACTTAATGAGGGTAAAATAAGGCTAGAAGACCCAATGGATTTTGAATCTCAATTGATCTATCCCGCATTAATTATGTACCCCACGCAAGATGAATTTGATTTTGTAGGTGAAGTAAGTGAGTTAACTACTGTGCAAGAACTTGTTGACCTAGTTTTGGAAGGGCCGCAAGAACGCTTCAAAAAAGAAGGTAAGGAAAACTTCACACCAAAGAAAGTGTTGGTGTTCATGGAAACAAAGGCAGGTGGTTTGATTAAAGCTGGTAAGAAACTGACATTTCACGATATCTTGAAGAAAGAGTCGCCAGATGTACCATTGTTCGATAACGCTTTGAAAATATATATTGTGCCAAAGGTAGAAAGTGAAGGGTGGATTTCCAAGTGGGATAAGCAAAAAGCCTTAGAAAGAAGATCTGTGTGA>YBR156C	-:0:chr02:551098:553194ATGGACTGGGCAATCAAAGCAGCTAGGAAGAAAACTCAAAGGAAGCCAGGCTCTACCCGTTCAATCATAGAAACCCTCGATGATCTAAATAATCTAACAACAGATGCACATTCAGAAATAAATCAACGATTGTACGAAAGTAGTGAATGGTTAAGAAATAATGTTTATATGAATACACTCAAGTATGAAGACAAAAAGATGGAAGAGTCTTTAATTAGCCCCGAAAATACGCATAACAAAATGGATGTCGAATTTCCTAAAATGAAAGGAGAATATGAACTTTCTAACTCCCAGAATGATGCCGCAAAAGACGTTACTAAGACACCCAGAAATGGATTACATAACGATAAAAGTATCACGCCTAAATCACTCCGCAGGAAGGAAGTCACCGAAGGAATGAATAGATTTAGTATACATGATACCAATAAAAGCCCGGTTGAGCCATTGAATAGCGTTAAAGTCGACGCCAATGAAAGCGAAAAATCCTCACCATGGTCACCTTACAAAGTTGAAAAGGTTCTAAGAGAATCCTCCAAGACTTCGGAGTCTCCGATTAATACAAAACGCTTCGATAATCAAACATGGGCGGCTAAGGAAGAAATGGAGAATGAACCTATACTTCAAGCTTTAAAGAAAGCTGAATCAGTAAAGGTGAAACCACCACCGAATTCTGGGATAGCGAGATCTCAAAGAAGGTCGAATATGTTTGTTCCACTGCCGAATAAAGATCCTCTTATTATTCAACACATTCCACCAACAAAATCTTCAGGATCAATACCAAAAGTACGAACAGTAAAGGAATCACCAATCGCATTTAAAAAAAAATCTACGATAAATAGTCCTGCTATAAGAGCTGTGGAAAACAGTGATACCGCGGGATCTACAAAAGCATCCAGTGTTTTCGATAGGCTATCATCTATTCCAACAAAATCATTTGAAAACAAAATTTCCCGTGGAAACGTTGGTCACAAATACTCCTCTTCATCAATCGATTTAACAGGCTCCCCCATGAAAAAAGTTTCTCAAAAGTTTAAGTCAATAAACTCCACGGATACTGATATGCAAGAAGCGTTAAGAGATATATTCTCAGTAAAAAATAAAATAACCAAAAATAATTCACCCAAAGGAAAAAACTCTCGAAAATCCTCTATTCCGAGGTTTGATAAGACTTCTTTGAAGCTAACGACACACAAAAAGCTAGCAATCATTGCAGAACAGAAAAAGAAGTCCAAACACTCTAGTGATGTTCATAAAACTGGTTCAAGACCGCACAGTATTTCTCCAACAAAAATAAGTGTCGATTCAAGCTCGCCATCTAAAGAAGTTAAAAATTATTACCAAAGCCCTGTAAGAGGTTATTTGAGACCAACAAAAGCGTCTATTTCGCCTAATAAAAATAAAAATTTAACAACGTCGCAAACGCCACATCGTTTGAAAATTAAAGAGAAAACATTAAGAAAACTTTCTCCGAATATTGCTGACATCTCCAAGCCAGAGTCTCGTAAATCCAAAAATTATCGGCTTACAAACCTTCAATTACTTCCACCAGCAGAGGCAGAACGAGATGACTTAAAAAAAAAGTTTGATAAAAGGTTATCAGGAATTATGAGGTCTCAACAGGAACATCATCGGCGTAAACAAGAGAAACAAAAAAGGATGTCGCATTTGGAACAAGATCTAAAAAAGCAAACAAGCTTTAGTAATGACTACAAGGACATACGTCTAAAAGAATCTTTGGCTCCTTTCGATAATCATGTGCGAGATACCATCAACAAAAATACTGCATTTAGTACCGACAATATATTGGCCACAATAAATACAGTTGACCATCGAGAAATAATCGGAAATGTGACCCCAAAGATAGCCTCTGTCAATGATTCTTTACCAGAGATAAATACTGATTCTGAAGATGAGGCTAGCGTAACTTTAGCGGCATGGGCTAAATCCCCGTATTTGCAAGAGCAATTGATAAGACAGCAAGATATAAATCCACAAACTATCTTTGGACCAATTCCGCCTTTGCACACCGATGAAATTTTCCCAAATCCTAGGCTAAACAGGTTGAAACCGCGTCAAATTGTGCCCAAAAGGTCTTGA>YBR157C	-:0:chr02:553537:554304ATGGGCAAATTTGAGCAAAAAGAAAGAGAAAGAATAAGCACATTTAGTTTTCCTACTACGGGCAGTCAATCTAGTACATCCATTAAATCACTTGGCAGCCCACTCTATGGACGTTTCAGCTCTCTCTCTTCAACAGAATCTCAGTTCGATAGCAGCAAACAACCCCATGAGTACGAAAAGAGCTTTTATTTTGAAGAGTCTCAAGGCGAAGCTCTGTTCAACAAGCTGAAGACCTACTCTTTCCCTGGAGACAAGGATGGTGTGAAAACGAGGAGAAATTCCTCCATATGTCCAAGAAAGCCTAATGCGGTATCACCTTTAAGGGTAGAAAGTAATGAATTATCATCACATTCGCATTCACGTTCATTATCACACGAACTTACAAAGCCTTCAGGGCGAAGGAAAAGTTACCACAGAAAAAGTCATGCAATCTCATTCAGCAGGTCTTGTAAGCCAAATTTCATTGACGGGTACGATTCTAATTCAAGCATAGGCGTCAATTCAAGAAAAACTTCATTGGCAAGTTCTTTCTTGGACAAAGAATATCATTCTTCACCGGATACTTCATATACCCACCAAATGTCCCCGAAAAACACAATCATGAATACCAATGAACAATTAAGAAGAAACGCAAGCGGTAGATTCGGGAGTTTGAAAGAATTCGCTGAAAAAAATCAGATAAATATTGAGGGCAAAATTTTTGCTCATAAAGTGGAAACTGGAGACATATTGCAACCACTAATTGACTTAGATATTGACAATAAATGA>YBR159W	+:0:chr02:558679:559722ATGACTTTTATGCAACAGCTTCAAGAGGCTGGGGAAAGATTTAGGTGTATCAATGGTCTTTTATGGGTTGTTTTTGGGCTTGGTGTCTTAAAATGTACAACGTTATCACTTAGATTTTTAGCTCTTATTTTTGATCTTTTTTTACTACCAGCAGTCAATTTCGACAAGTATGGTGCTAAAACTGGTAAATACTGTGCTATCACTGGTGCAAGTGACGGTATTGGTAAAGAATTTGCTAGACAAATGGCGAAACGTGGCTTCAACTTGGTATTGATCTCGAGAACGCAATCTAAATTGGAGGCTTTACAAAAAGAACTAGAAGATCAACATCATGTCGTTGTAAAGATTCTAGCCATTGACATTGCGGAGGATAAAGAATCCAACTATGAATCCATTAAGGAATTGTGTGCGCAGTTACCAATCACCGTTTTGGTCAATAATGTTGGTCAATCACACTCCATTCCTGTTCCATTTTTGGAAACAGAAGAGAAGGAGCTTAGAAATATTATCACTATCAATAACACTGCTACATTATTAATTACACAAATCATTGCACCAAAGATTGTGGAAACTGTGAAAGCTGAAAACAAGAAGTCGGGTACTCGTGGTTTGATCTTAACCATGGGCTCATTTGGTGGTCTGATTCCCACCCCACTTTTGGCTACATACAGTGGTTCGAAATCATTCTTACAAGGTTGGTCTAACTCTTTGGCTGGGGAATTATCTAAAGATGCTATCGATGTTGAATTAATCATTTCATATTTGGTCACTAGCTCGATGTCTAAAATCAGAAGATCATCTTTAATGATCCCAAATCCACAACAGTTTGTAAAATCCACTTTAAGAAGCGTTGGCAGACGCTGCGGCTCTCAAGAAAGATACGCTACTATGACCCCTTACTGGGCGCACGCAGTGTATCAATTTGTAATTACAGAGACCTTTGGCGTTTACTCTAAGATTGTTAACTCCATTAATTATTCCTTCCATAAATCTATCAGAATTAGAGCCTTAAAAAAAGCCGCAAGACAGGTTAAAAAGGAATAG>YBR160W	+:0:chr02:560072:560968ATGAGCGGTGAATTAGCAAATTACAAAAGACTTGAGAAAGTCGGTGAAGGTACATACGGTGTTGTTTATAAAGCGTTAGACTTAAGACCTGGCCAAGGTCAAAGAGTAGTCGCATTGAAGAAAATAAGACTAGAGAGTGAAGACGAGGGTGTTCCCAGTACAGCCATCAGAGAAATCTCATTATTGAAGGAATTAAAAGACGATAATATTGTCAGATTATACGATATTGTTCACTCTGATGCACACAAGCTATATCTTGTTTTTGAGTTCCTCGATTTGGACCTGAAAAGATATATGGAGGGTATTCCAAAGGACCAACCGTTAGGAGCTGATATTGTTAAGAAGTTTATGATGCAACTTTGTAAGGGTATTGCATACTGCCACTCACACCGTATTCTGCATCGTGATTTAAAACCGCAGAACTTATTGATTAACAAAGATGGGAATCTAAAACTAGGTGATTTTGGCTTAGCGCGTGCTTTTGGTGTTCCGTTGAGAGCTTACACACATGAAATTGTTACTCTATGGTATAGAGCTCCGGAGGTATTACTGGGTGGAAAACAATATAGTACAGGTGTCGATACATGGTCCATCGGCTGTATATTTGCCGAAATGTGTAACAGGAAACCAATCTTCAGTGGCGATAGTGAGATCGATCAGATTTTCAAGATATTCAGAGTATTGGGAACGCCGAATGAAGCTATATGGCCAGATATTGTCTACTTGCCTGATTTCAAGCCAAGCTTTCCTCAATGGCGCAGAAAAGACCTATCACAAGTGGTACCAAGTCTAGATCCACGCGGTATTGATTTGTTGGACAAACTCCTCGCGTATGACCCTATTAACCGGATTAGCGCCAGAAGAGCAGCCATCCACCCCTACTTCCAAGAATCATAA>YBR162C	-:0:chr02:563198:564565ATGTTACAAAAGCTTTCCATGACCGCATTAGTCGGTTTGTTTTCTTCAGTTGTGTCACTCGTGAACGCTGATTGTACTTACTCTGGCGGTAATTACTATTGTGCTCAAACCGATGCCATCATTTACTCTAACGTCGGTTTATCTGCTACCTACCAAGATGTCACCAATATGGATGAATCTTCTTGTGCTTGTACCCAAGCTGATTTCACTGCTTCTGGTAGCTTGGCTCCCTTCAACGAGGAACTATCTGTTCATTTCAGGGGTCCGATCGAGTTACTACAATTTGGTGTTTACTACCCAAATGGTGAATCTAATGCTTTGAAGAAAAGATCAGAGAAACAATCAATCGAGTCCTGTAAGGAAGGTGAAGCTGTTGTCTCCAGACATAAGCATCAGCACAAGAGGGATGTTGCTGTTGAATATGTTCAAGTTACTTCCACTGTTTACGTCGACAGTAATGGTCAAACAGTCACAGCCGATTCCACCAATACCGTTGTCGGTCCTGCTGTTCCTTCTTCTTACACTAAAGACTCTACTGTTTTGTCCAGCAGTGCTCAAGCAGTTGAAACGAGTGAAAGCCAGTCTTCCATCTCTTCCTCTAAAACCACCTCTTCTGCTGCTGCTGCTTCATCATCTTCGTCATCATCTTCTAACACTAACGGTGACTGGTCCAGAGGTTCCTACTTTGTCCCAGGCTCTACTAGCAACTGTACATTCATGAACAACCAAGGGGGGACTGCCGGTTCTGGTGTCTGGTCGAGTTGTTTCGGTAACTCCATCTCTTTTGCTGCTTCAGATGGTGTTTCTGGTGCTGCCTCCGCTCAAGCTCTTGGTGATGTCACCATTAAATCTGGTAATGAATTCATGATTTTCTCCGGTGAAGAATGTTCCGGTAATAACGGCGATTGTGGTTATTACAGAGAAGGCATTCCTGCTTATCATGGGTTTGGTGGGGCTGATAAAATTTTCGTCTTCGAATTTTCTATGCCAAGTGACACCAGCGGTTCTGCCTACAATCAAGATATGCCAGCTATTTGGTTATTAAATGCAAAAATCCCAAGAACCTTGCAATATGGTGATGCATCTTGTTCTTGTTGGAAAACTGGCTGTGGTGAAATGGATTTGTTCGAAATTTTGACCGCTGGTTCTGATAAATTAATCTCACACATCCATGACGGCCAAGATGGTGGTACTCAAGATTACTTCGAGAGACCAACTGATGGTACTTTAAAGGCTGCCGTAATCTTTAATTCAAGTGATAAAACTATTCACATTATCGAAGTCGACGAAAGCTTTGATGCTACTTTAAGCGATGATGTCGTTGACCAATGGTTGAGCAAGTCCGGTTCTTCTGCGGCCTTGCCATAG>YBR162W-A	+:0:chr02:565226:565423ATGGCCGTACAGACACCAAGACAAAGACTGGCTAATGCCAAGTTTAACAAGAATAACGAAAAGTATAGAAAATACGGTAAGAAGAAGGAGGGCAAAACTGAGAAAACCGCACCTGTGATATCCAAAACTTGGTTGGGTATTCTTCTGTTTCTTCTCGTAGGTGGTGGTGTTTTGCAACTAATCAGCTATATCCTATGA>YBR163W	+:0:chr02:565718:567475ATGCTGGGCCGGGCACTAATCAACAAGTATGGATTTTTAATTCATCCTAGGAGATTTGTACATTTAAACGATAAGTCCCTTGATGGAACATTTATACTACCTTCGAAGAAAAATCATATGTATGATGTTCCAACTAATGATCCAAGCGGTATCCTGAATGCCTCGGATATCGATAGAATCAATAATTTGCCATTTTTCGATAACACCAGTCCGACAAAAGAGACAAATACTAAGGAAGGAGCATTGTTGAGCGAAAAATTGGCCAGCGTTAAAGAATTATTTGGAGAAGACCCTGAAAATCCTTCGTTTATCAATTACAGATTCCCCAGAGGTTTAGAGAATCCCTATTTTGACATTCAAGTAAATCAGCTAAAGAAAAAACGATTATCCGTCACACAATTATGCACTACGCAGAATTGGTGCGAGTTAAGGAATTTTTACGACTTTTATTCTCAAAATTTGTCTAACCAACTACTGAACTTAAAATTTCAGGTGCAGAAAGGTAAAAAGATTCATAAATCACTAGAGGACGAAACGCATCCTGAATTAAATCAGTATAAAAGCTTTACCCACAATTTCCTTGCACTGACAAAATTATCTATGGATATTGATAATGATATGGATGCCCTGCTAGATAACTGGTTCAACTCTATCAATAGATTGGTCTCTTTATTCACCAAAGGTGACGGTCATGCAAGAGAAATAGTTTGTCATGGCTTCATTAACTTAGAAGATGGCAAACTGGTGGAGCATTTGCTGAACAGTGATAGCAAGACAAAGGAAAATGTGATTATATCAGGTGTTATTGACCACTTAACTCTTAGAAATAGGCACAATCATCAAGTACAGAAAGGTGCAGCTCATCTTGATACGGAATATCAGAGCTGGGGTAATATCCTCACTAATTTACTATCAAATCTAAAAGAACTGAAGTCGAATAACGAGATTGTTATCTCTGATATCAAAACTAGGTCAGTACCAAAAATACCATCTATTGAGTCCGTTATAGAATCCTCTAAATTACAGACAATGTATTATAAGTTTTTTTTTTCCCACCTTAGTCAAGACATGACACAAACCTACCATAGCTTTTTAATCAATGCCCAGAGAAGAGGACTCGACGTTGATGCGCCCATAAATCCTACCAAGATTTTAACATTCATACTAACAAACCCACTCTTCGCTAATGATGTGAAGAACCTCCTGTACGGACTACCGATAAATCATTCTGCTTTCGATAACGACGCCAAAGGCTCTAATACATTTGATATGACCGCGTTCAATGATTTGCTGGATAGAGGTCCCACATCTTTTAACGTTCCGATTGAGCAAGACGAAGATAGTTCGGAGTCAACAAAATGTGTATCTTTGCGAGATTATGGACATTTTTATACGAAATGGAAGACACCTCTAACTTTGAAATACTTTGCGGCAAGGTTATCACAGATCTATTTTATTGTGGGTAATCTCGTTTCGAATGATTTAATGATAGAATATTATTACCATAACGATAATTTCCACAATATCATCTTCCCATATGATCCCTTGAAACTGGGAACCCATGCCCATGATTCTGCAATGGTTTGGTTTGGTGGCAGAGATATGCATCCAATTGAGCCTACTCAAAAAAACTTCAACACATACTGTAAGTTTTGTGACTACAGACACGTTTGTTCTTGGAAAAATAAAAATGAATTGAAACTTATTGATCTAGGGAAAGAATTAAAAAAAATTATTCTTGAATCAAGTATGAAGTAG>YBR164C	-:0:chr02:567870:568421ATGGGTAACATTTTTAGTTCAATGTTTGACAAACTATGGGGTTCAAACAAAGAATTGCGTATATTGATTTTGGGTTTGGATGGTGCAGGTAAAACTACCATCTTATATCGTTTACAAATTGGCGAAGTAGTCACTACAAAGCCAACCATTGGTTTCAATGTAGAGACGCTAAGTTATAAAAACTTAAAATTGAACGTCTGGGATCTTGGTGGTCAAACAAGTATCAGGCCCTACTGGAGGTGTTATTATGCAGACACTGCTGCAGTTATTTTCGTTGTTGATTCGACTGATAAAGATCGTATGTCTACAGCCTCTAAGGAACTTCATTTGATGTTACAGGAAGAAGAATTGCAAGATGCAGCACTGCTGGTTTTTGCAAATAAACAAGACCAACCGGGTGCATTAAGTGCCAGTGAAGTCTCCAAAGAACTGAATCTTGTAGAATTGAAGGACAGAAGTTGGTCTATCGTAGCATCCAGTGCAATTAAAGGCGAAGGTATTACCGAAGGTTTAGATTGGTTGATTGATGTTATAAAAGAGGAACAGTTATAG>YBR165W	+:0:chr02:568847:569680ATGGCTTACTCTTTAACAAGGAAATTGCTGAAGGATTGGAAATATTTTATGCGCCATCCTGAGAAGACCCAAGGTCTTTTTCATGTAAGGCCACATGATTCAGACTTACATCTTTGGCACGTGGTAATGTACGAGCCCAGGACTTCTTTGGAAGTATATCTACTACTTTATATTGGCGGAAACGACCAGGATCCATATATCATCATGAAATGTCTGTCTCCAAATTGTTGCTTTCCAATTAACAGAACGGTTTCGATGACACACTTAAACTATCTTTTATTGAAAGACTTGGGATTACAGGATTTACTTTTCCACATATGGCAACCGCTTTTTCATATTCAAGCAACGGAAGACTTACAGTATTCACCGTCAACGGTAAAATTCAACAGGGCGTGGAATAGGATTATATATAAGGACTTTAAATCTTATTTTCCGGAACTGATAGGTACTTTACAACCAGGTGATTACTCTATAGTAAAGAGCTATTCCAAAAATCATAATATCAGCAATAGTAATGGTGGCAGTGTCAATGAATTTATGTCCTCTTACAATGCTCAAAGCCACACCTTTCACGCACAAGACAACAGCAAGAACCCTTATACTAACAGCAGCATAGGAAAGTCTAGTATGTTATCTACTCTCAACAATAATAACGTTAACAAAAGGACACATGACTACAATGCTATCGACTTTATGACCAAAAACTTACTTGCATGTGATGATGACAGTATTCATCCGGTCGTAAGTTCTAAAAGGTCAAGAACGTTGGCATGTCCAGATGAAACGAACGATAATCGCGGTAGTGAGCATTACACAAAGAGGAAAAAAATCTAA>YBR166C	-:0:chr02:569837:571195ATGGTATCAGAGGATAAGATTGAGCAATGGAAAGCCACAAAAGTCATTGGTATAATTGGTCTGGGTGATATGGGCCTATTATACGCTAATAAATTTACAGATGCTGGATGGGGTGTTATATGTTGTGATAGGGAAGAATATTATGATGAACTGAAAGAAAAATATGCCTCAGCTAAATTCGAACTGGTGAAAAATGGTCATTTGGTATCCAGGCAAAGCGACTATATTATCTATAGTGTTGAAGCATCCAATATTAGTAAGATCGTCGCAACGTATGGACCATCTTCTAAGGTTGGAACAATTGTTGGGGGTCAAACGAGTTGTAAGCTGCCGGAAATCGAGGCTTTCGAAAAGTATTTACCCAAGGACTGCGACATCATTACCGTGCATTCCCTTCATGGGCCTAAAGTTAATACTGAAGGCCAACCACTAGTTATTATCAATCACAGATCACAGTACCCAGAATCTTTTGAGTTCGTTAATTCTGTTATGGCATGTTTGAAAAGTAAGCAAGTTTATTTGACATATGAAGAGCATGACAAGATTACCGCTGATACACAAGCTGTGACACATGCTGCTTTCTTAAGTATGGGATCTGCGTGGGCAAAGATAAAGATTTATCCTTGGACTCTGGGTGTAAACAAATGGTACGGTGGCCTAGAAAATGTGAAAGTTAATATATCACTAAGAATCTATTCGAACAAGTGGCATGTTTACGCAGGATTAGCCATAACAAACCCAAGTGCACATCAGCAAATTCTTCAATATGCAACCAGTGCAACAGAACTATTTAGTTTAATGATAGATAACAAAGAACAAGAACTTACTGATAGACTATTAAAAGCTAAGCAATTTGTATTTGGAAAGCATACTGGTCTCTTACTATTGGATGACACGATTTTAGAGAAATATTCGCTATCAAAAAGCAGCATTGGTAACAGCAACAATTGCAAGCCAGTGCCGAATTCACATTTATCATTGTTGGCGATTGTTGATTCGTGGTTTCAACTTGGTATTGATCCATATGATCATATGATTTGTTCGACGCCATTATTCAGAATATTCCTGGGTGTGTCCGAATATCTTTTTTTAAAACCTGGCTTATTAGAACAGACAATTGATGCAGCTATCCATGATAAATCATTCATAAAAGATGATTTAGAATTTGTTATTTCGGCTAGAGAATGGAGCTCGGTTGTTTCTTTTGCCAATTTTGATATATACAAAAAGCAATTTCAGAGTGTTCAAAAGTTCTTTGAGCCAATGCTTCCAGAGGCTAATCTCATTGGCAACGAGATGATAAAAACCATTCTGAGTCATTCTAGTGACCGTTCGGCCGCTGAAAAAAGAAATACATAA>YBR167C	-:0:chr02:571463:571885ATGGCACTCAAAAAGAATACACACAACAAATCTACCAAACGAGTAACGAAACACCCATCTTTGAAAACTCTAACGCATAAGCAAATACACACAACAATTTTCGTTAAGTCTACAACACCATACGTTAGTGCACTGAAAAGAATAAATAAGTTTCTCGATAGCGTTCATAAGCAAGGATCATCCTATGTTGCGGTTTTGGGTATGGGTAAAGCTGTAGAAAAGACACTGGCATTAGGTTGTCATTTTCAAGATCAGAAGAACAAGAAAATAGAGGTTTACACTAAAACTATAGAAGTTCTTGATGAAGTAATAACTGAAGGCCAAGCTGATATCGACATGGAAAGTGATGTTGAAGATGACGATAAAGAAACCCAACTAAAGAAAAGGGCTGTAAGCGGTGTAGAATTGCGTATTTATGTTTAA>YBR168W	+:0:chr02:572366:573607ATGGACACAAATTCTAAAACCAAAGTACAAACTGAAAATAAAAAAATTAAGGCGAAATTTATACATAATCATGGGCAAAAGCCCTCTCTTATCCAAATTACACCTCCAATGATTTCCAGCACTCTGTTTCACGCATATCCACTACTACTAATATTCGACAATGCCTTAGCGAATATAATGTGGCTGTCCGACGATAAATGTTTAACATTCATTTATCTAACGAGCATATGGCTGACCATAAGTTTTTTTATCCCCGTTGAAACAGAGGCTAGCCATTTTCTGCCATTTACAAAGATTTTGAGACTATGGTTAGGCATTATAAGCGGGGCATTTCTATTCTTATCATTCATGTATTATATTGTTTCATTGATAGCTTCGTTAAGAGACACGGAACCTCCTACATTGGATGAAATTGTCGTGCTACTAGAGTCTGTGTTGGATAAACTGGAAGTACTGAGAAACGAGCTGAATGTTTGGAAAAAATTAAAGCTTTCATTTGATGGCGTCAATAAGGAGTGTTCTGGCAAGAGGTTGTTTTGCAGATTATTCTTATTCGGTACAATTTTTCAAATTATTATCATGAGATACATATCACCGGGAACTTATACTAGATTTTTTATTATTACCGGCTTGATATACAATACGAGTAGTTTTCAAGCTACCTTAAGGCTACTTTGGAGGTTTACTGCTGTTAGGAATTTCTATTATTTGGGGATTGAAAGTTTTAAAATTTCGAGTTTCTTACCAAAACACTTAAAGATGGAACAAATTATTCCTTTATCTCAAGGACGTGCAATCACGGTGCCACTGGTAGAGGTATTGCCTAAACTACTTCGGGACAAGAAGGGTGATGACCACATTCATATTCTACAGCTTCTACTTAATGAGCAAAAAGATAACTTTGGTAATGAAGACCTTAAAATACTAGAAATTGAAGTATACGAAAATCAAAGAAGGTGGTATCAAAATAAAAATTGGAGTACAAAATTACTGCCGTACGAAAGACAAAACTACTGCATAGAAATTAAAAACACTGATGGAACTTTAACCATGAGGAGTTGTTTGCCCCCAGATGGACTTGGTGAAGAAGAATTACCCAATAATTGGCATTGGATCAATGATAATTGGGACGGAACTGATTGGATATATTCAGATTCAGCATGGAAAGAAATTGGACAATACAGTTCTTTGGAAAGTTTTACCAGGTCAAGAAAATGGAAACGACGCCTCTTCCATTTGTAA>YBR169C	-:0:chr02:573910:575991ATGAGCACTCCATTTGGCTTAGATTTAGGTAACAATAACTCAGTACTAGCAGTTGCCAGAAATAGGGGTATTGATGTCGTTGTCAATGAAGTTTCTAATAGGTCTACACCATCCTTGGTCGGCTTTGGCCCCAGAAATAGGTACTTAGGTGAATCTGGTAAAACTAAGCAAACATCGAATGTTAAAAACACTGTGGAAAACTTGAAAAGAATCATTGGACTAAAGTTCAAAGACCCTGAATTTGATATCGAGAATAAGTTCTTCACTTCGAAATTGGTACAGCTAAAAAATGGTAAAGTTGGTGTGGAAGTGGAGTTCGGCGGTAAAACACACGTATTTTCAGCTACTCAACTGACTGCTATGTTCATTGATAAGGTGAAGCACACCGTTCAAGAGGAAACGAAGTCATCAATTACCGATGTCTGCCTCGCAGTTCCTGTATGGTATTCGGAAGAACAACGTTATAACATAGCCGATGCTGCCAGAATTGCAGGATTAAATCCTGTAAGGATTGTCAACGATGTGACTGCAGCCGCCGTTTCGTACGGCGTCTTCAAGAATGATCTGCCAGGTCCTGAAGAAAAGCCAAGAATCATTGGCTTAGTGGACATTGGGCATTCTACCTACACCTGTTCTATTATGGCTTTCCGCAAAGGCGAAATGAAAGTATTAGGTACTGCTTATGACAAGCACTTTGGTGGTAGAGATTTCGATCGCGCAATCACAGAACATTTTGCTGATCAGTTTAAGGACAAGTACAAGATTGACATTAGGAAAAATCCGAAAGCTTATAACAGAATTTTAATCGCTGCTGAAAAATTAAAAAAAGTGCTTTCTGCGAACACTACTGCCCCCTTCTCCGTTGAATCTGTTATGGATGATATCGACGTTTCCTCTCAATTGAGCCGTGAAGAGCTGGAAGAATTAGTAGAGCCCTTGTTGAAGCGTGTGACGTATCCAATCACCAATGCATTGGCTCAAGCTAAATTAACTGTCAATGATATTGACTTCGTAGAAATAATTGGTGGTACAACCCGTATCCCAGTTTTAAAGAAGTCAATTTCTGATGTTTTTGGAAAACCTTTGTCATCTACTTTAAATCAAGACGAAGCTGTGGCCAAGGGGGCCGCTTTCATATGTGCCATTCACTCTCCAACTTTAAGGGTCAGGCCGTTTAAATTTGAAGATATTGATCCGTATTCAGTGTCATACACTTGGGATAAGCAGGTCGATGACGAAGACCGTTTGGAAGTATTCCCTGCTAATTCATCATATCCATCAACTAAACTAATTACTTTACATCGTACTGGAGATTTCAGCATGAAAGCGGTGTACACTCATCCTTCGAAACTGCCAAAAGGTACTTCCACCACTATTGCAAAATGGAGCTTCACTGGGGTCAAGGTTCCTAAAGATCAAGATTTTATTCCTGTAAAGGTCAAGTTAAGATGCGATCCTTCCGGCTTGCATATTATCGAGAACGCTTACACAACGGAAGATATTACGGTTCAAGAGCCAGTGCCTTTACCGGAAGACGCACCAGAAGATGCCGAGCCCCAGTTTAAAGAAGTTACTAAAACAATTAAGAAAGATGTGCTAGGTATGACTGCAAAAACATTCGCGCTAAACCCGGTTGAGTTGAACGATCTAATTGAAAAAGAGAATGAATTAAGAAACCAGGATAAGTTAGTTGCCGAAACCGAGGATCGCAAAAATGCCCTTGAAGAGTATATTTATACCCTTCGTGCCAAACTCGATGATGAATACTCCGATTTTGCGTCTGACGCAGAAAAAGAAAAGCTAAAAAACATGTTAGCCACTACTGAAAATTGGTTATATGGTGATGGTGACGATTCTACCAAGGCAAAATACATTGCTAAATATGAGGAGCTGGCATCGTTGGGGAATATTATTAGAGGTAGATATTTAGCAAAGGAGGAAGAAAAAAGACAAGCACTCAGAGCGAATCAAGAAACTTCTAAAATGAATGATATTGCTGAAAAATTGGCTGAGCAAAGAAGGGCACGCGCTGCAAGTGATGATAGCGATGACAACAATGATGAAAACATGGACCTTGATTAA>YBR170C	-:0:chr02:576339:578081ATGCTTATCAGATTTAGATCAAAAAACGGTACACACAGGGTTTCTTGTCAAGAAAACGACCTTTTCGGAACGGTCATTGAAAAGTTGGTGGGTAATCTGGACCCCAATGCCGATGTCGACACATTTACTGTTTGTGAGAAGCCCGGTCAAGGTATTCATGCTGTTTCTGAACTAGCTGATCGAACAGTGATGGATCTAGGACTAAAGCACGGTGACATGCTGATCCTTAACTATTCGGACAAGCCCGCTAATGAGAAAGATGGTGTCAATGTTGAAATCGGATCCGTAGGTATTGACAGCAAGGGAATTCGTCAACACAGGTACGGTCCACTAAGGATCAAAGAACTCGCTGTAGATGAGGAACTAGAGAAAGAGGACGGATTAATTCCTCGTCAAAAATCAAAACTATGCAAGCACGGTGATAGAGGTATGTGTGAATACTGCTCGCCTTTACCTCCTTGGGACAAAGAATATCATGAGAAGAATAAGATCAAACATATATCGTTTCATTCATATCTTAAAAAGCTAAATGAAAATGCTAATAAGAAGGAGAACGGCAGCTCTTATATTTCCCCTCTTTCAGAACCTGATTTTAGAATCAATAAGCGTTGCCATAATGGCCATGAACCATGGCCTCGGGGGATATGTTCTAAATGTCAACCATCGGCAATTACATTACAACAGCAAGAATTTAGAATGGTTGACCACGTTGAATTTCAGAAGAGTGAAATAATTAATGAATTTATTCAGGCGTGGAGGTACACGGGTATGCAAAGATTTGGCTATATGTACGGATCTTATTCTAAGTATGATAACACACCTTTAGGTATAAAGGCCGTCGTTGAGGCGATATACGAGCCCCCTCAGCATGATGAGCAAGACGGTTTAACCATGGACGTAGAACAGGTCAAGAATGAAATGCTACAGATTGATAGACAGGCTCAAGAAATGGGGCTTTCGCGAATTGGCCTAATATTTACAGATTTATCTGACGCGGGAGCTGGGGATGGGTCTGTTTTTTGCAAAAGACATAAGGATTCGTTTTTTCTTTCATCATTAGAAGTTATTATGGCTGCTAGGCATCAAACAAGGCATCCTAATGTAAGCAAGTATAGCGAACAAGGTTTTTTTTCTTCCAAGTTTGTAACTTGCGTTATATCAGGTAATTTGGAAGGTGAAATTGATATTTCGAGCTACCAAGTATCCACAGAAGCTGAAGCATTGGTTACTGCAGATATGATAAGTGGGTCCACATTTCCTTCAATGGCATATATTAATGACACTACAGATGAAAGATATGTACCTGAGATATTTTACATGAAGTCGAATGAATATGGTATAACAGTGAAGGAAAATGCGAAGCCTGCATTTCCGGTAGACTATCTTTTAGTGACGCTGACTCATGGGTTCCCGAATACCGATACGGAAACCAACTCGAAATTCGTTAGTTCCACCGGATTTCCATGGAGCAATCGACAAGCTATGGGGCAATCTCAAGATTATCAAGAATTAAAAAAGTATTTATTCAATGTGGCTTCAAGTGGAGATTTCAATCTTTTGCATGAAAAAATCTCGAACTTTCATTTACTATTATACATAAATTCTCTGCAGATACTCTCTCCAGACGAATGGAAGTTACTAATAGAATCTGCTGTGAAAAATGAATGGGAAGAATCTCTACTAAAACTTGTCTCATCGGCTGGTTGGCAAACGTTAGTCATGATCCTTCAGGAAAGCGGCTAG>YBR171W	+:0:chr02:578359:578979ATGTCCGAATTTAATGAAACAAAATTCTCCAACAACGGGACGTTTTTTGAAACGGAAGAGCCAATTGTGGAGACGAAATCAATCTCCGTTTATACCCCACTCATATATGTCTTTATTCTGGTGGTGTCCCTTGTGATGTTTGCTTCAAGCTACAGAAAGAAGCAGGCCAAAAAAATTAGTGAGCAACCATCCATATTTGACGAAAACGATGCCCATGATCTGTATTTCCAAATAAAGGAAATGAGTGAAAATGAAAAAATTCACGAGAAGGTGTTGAAGGCCGCTTTATTGAACAGAGGAGCAGAATCTGTTAGACGATCATTAAAGTTAAAAGAGTTGGCTCCTCAGATAAACCTTCTATATAAAAATGGCTCTATTGGGGAGGATTACTGGAAGAGATTTGAAACTGAAGTTAAATTAATTGAATTGGAATTTAAAGATACTTTACAAGAAGCTGAAAGATTGCAACCGGGCTGGGTTCAATTGTTCGTTATGGTTTGTAAAGAAATTTGCTTTAATCAAGCTCTCTCTAGACGTTATCAATCAATCTTGAAACGGAAAGAAGTGTGTATTAAAGAGTGGGAGCTGAAAATAAATAATGATGGAAGATTAGTCAATTAG>YBR172C	-:0:chr02:579145:581367ATGATAGCACCAGACTCGCAAAGATTATTCGGTTCCTTTGATGAACAGTTTAAGGACTTAAAACTAGATTCAGTCGATACAGAAAACAATAACACACACGGTGTTTCTACTATCCTGGATTCCTCCCCTGCGAGCGTTAATAACAATACAAACGGTGCTGTTGCTGCAAGCGTGAATACAGTCCCTGGATCGACGTTTAGATCCAATACACCTCTTTTAGGAGGTCGACATCCACTCAGTAGGACGTCTTCCTTAATCGATTCTATTGGTATACAGCGTGCTGCGTCACCATTTTCTTCTATGAAAGAGCCTTTTATTCCTCAAAGTTCGGGAGTAATGAGTTCCTCTTTTTGGCACGGCGACCATCCTGAATCACGGGTTAGTACGCCTGTTCAGCAGCATCCACTATTGCAAAGAAACGAGTCCTCCTCCTCCTTTAGTTATGCTGCTAATCTTGGAGTGAATCTGAGTACACATTCATTGGCGGTAGATATTACTCCATTAAGTACACCAACTGCTGCCCAATCTCATGTCAATTTGTTTCCGTCCTCTGATATTCCTCCTAATATGAGCATGAACGGAATGTCACAACTCCCAGCACCAGTTTCAGTTGAATCCAGTTGGAGGTACATAGACACGCAGGGACAGATTCATGGTCCATTTACTACCCAAATGATGTCACAATGGTACATAGGTGGCTATTTTGCTTCAACCCTTCAGATTTCAAGATTGGGAAGCACTCCAGAAACGCTAGGAATAAACGATATTTTCATCACACTTGGTGAGTTAATGACTAAACTCGAGAAGTATGATACGGATCCATTCACTACTTTTGACAAACTTCACGTTCAAACAACCAGCTCTGATTCTATCAACTTGAATCTAGCTCCTTACGCAAGCGGTGTTGCCGCTACTGGCACTATTAAAGCTACTGAAAACGATATTTTCAAGCCATTGACACATGACAATATCTGGGATATGGATGGAGGTACCACCTCCAAGGGAGTCGATATTAAATTAGCCTCAGCAACCACCATTAGTCAAACAGATGAATCTCACAAGCAAGAATACAAATCGACCACTATGCTGGAAAAAGGGAAGAAGGAAAAATCCGAATCTGTAGCAAAAGCGTTATTGGATGAACAAGAGAAACGGAACAGAGAACTCAAAAGGAAGGAAGAAGCTCGCTTATCGAAGAAACAAAAGCAAAAAGAGGATGATTTATTGAAGAAACAAAAGGAACAAAAGGAACAAAAGGAAAAAGAAGCTTTGGAAGCAGAAAAGCAAAAAAAATCAGAAAAGACAAAAAAGGACACACAAACACAAACAGAAGGCTTTAAGACATCAAAAGATTTGCCTTCATTAAATTCCTCAAGCGCGAATCCGGCACCATGGGCCTCAAAAGTCAAAGTTAACAATGCAATCGAAACTTCAATTAAAAATGGTGTAAGTAGCACTGGAAAGAAGAAAGGAGAACCCTTAGGTCTACAACAGAGGAACAGCAAAGAAGAAAAGCAAAAGGAGGAACTAAAATCCGTGCTTAACTGGGCTAACAAGTCCAGCCTACCATCGAATCAAACTATTGATATTAAATCTCAGTTTCAAAAAAGCCCAAAAGGGATGAAAGAGTCGTCTCCGTTAAAGGAGTTGGAAGATCCAAATTTCATAGAAGAGCAGAAAAAACTATGGGAAAAGGTTCAGAGTTCTTCCAAACAAGTAAAGTCCACATCCTCTGCATCTACTACCACATCTTCTTGGACTACGGTGACTTCTAAAGGGAAAGCACCTATAGGAACCGTTGTCTCCCCATATTCAAAAACCAATACCAGTTTAAATTCTTCATTAACGGCAAAAACTAGCACAACTTCTACTACCACTACCTTTGCTAGCATGAACAATGTTTCTCCCCGGCAAGAATTTATCAAATGGTGTAAATCACAGATGAAATTAAATTCTGGAATAACCAATAACAATGTCTTAGAACTATTATTGAGTTTACCTACGGGGCCAGAATCTAAAGAATTGATTCAAGAAACAATTTATGCCAATAGTGACGTTATGGATGGTAGAAGATTTGCCACTGAATTTATTAAAAGACGTGTTGCGTGCGAAAAACAAGGTGATGATCCACTAAGTTGGAATGAAGCGCTAGCTCTATCAGGCAATGATGACGACGGCTGGGAATTCCAAGTAGTGAGCAAGAAGAAGGGTAGAAAACACTGA>YBR173C	-:0:chr02:581721:582167ATGAATATCGTCCCACAAGATACCTTTAAATCTCAAGTCTCTACAGATCAAGACAAAAGTGTTCTTTCATCTGCAGTGCCCTCCTTGCCAGACACATTACGCCAGCAAGAAGGCGGTGCAGTACCTCTTTCCACACAATTGAATGACAGACATCCATTAGAATCAACATTGAAGAACTGGGAAACCACACAGCGCCAAAGACAAATGGAACAGTATCGACAAATATTTGGCATTGCCGAACCAATGAAAAGGACGATGGAAATGGAAATCGTCAATCGTACCGACTTCAACCCCCTTTCAACAAACGGTAGTATACACCGTGACATACTACTGAACAAAGAGTGCAGCATCGATTGGGAGGACGTCTACCCTGGTACTGGCCTACAAGCCAGCACCATGGTAGGTGATGACGTCCACAGCAAAATCGAAAAACAATTAGGCATTTAA>YBR175W	+:0:chr02:582403:583350ATGTTCCAGTTTGTTACTCCTGTGGGAACACAGAATGGATTGAAAGCTACTTGTGCGAAAATATCTCCTGATGGGCAGTTTCTTGCTATAACACAAGGACTTAACATCCTGATATACGACATTAACAGACGAACTGTATCACAAACACTAGTCACATCACATGCCAGGCCTTTCTCAGAGTTATGCTGGTCTCCAGATGGACAGTGCATAGCTACTGCATCGGATGATTTTTCTGTGGAAATCATTCATTTATCTTATGGGCTGCTGCACACTTTTATCGGCCATACAGCGCCAGTGATATCTCTGACATTCAATAGAAAGGGGAACTTGCTGTTTACATCCTCAATGGACGAAAGTATCAAAATATGGGACACACTGAACGGGTCTTTGATGAAAACAATATCTGCACACTCAGAAGCAGTTGTGTCGGTGGACGTACCCATGAATGATTCATCCATTCTGAGTTCAGGTTCATATGACGGGCTCATACGGATCTTTGATGCAGAGACGGGCCATTGTTTGAAGACATTGACCTATGACAAGGATTGGAAAAGAGAAAATGGTGTGGTACCCATCTCCCAAGTTAAGTTTTCTGAGAATGCAAGATATCTCCTGGTGAAATCGCTGGACGGTGTAGTGAAAATATGGGATTGCATCGGGGGTTGTGTAGTTCGTACTTTCCAGGTTCAGCCTCTTGAAAAGGGCGTACTGCATCATTCCTGCGGTATGGACTTCTTAAATCCTGAAGATGGATCCACTCCTTTGGTGATCAGCGGTTATGAAAATGGCGATATATATTGTTGGAATTCTGATACAAAAAGCCTCTTACAGTTGCTCGATGGTTCTTTATATCACCACAGCAGCCCTGTAATGAGCATACATTGCTTCGGTAATATAATGTGTTCCCTTGCATTGAATGGAGATTGCTGTTTATGGAGATGGGTTTAA>YBR176W	+:0:chr02:583715:584653ATGAATATAATGAAAAGACAATTATGCACCTCCTCTAAGCGATTTTTTTCTACAGCAAAAAACGTTGTCAAATATAATACGATTCAGGATATCAGGAACAAATATTTTACTGGTACGCCTTTGTCGATGTGTACTGCGTATGACTTCATTACTGCAACATGGGTTAACAAGGCTAATTGTGATTTACTCTTAGTCGGAGATTCTTTGGCGATGACTTCATTGGGGTACGATAGCACAATTACACTATCCTTGAATGAGTTTAAATATCATGTTGCCTCTGTGTGCAGAGCTGAAGGTTCTTCCATGGTAGTGGTAGACATGCCATTCGGTACGTTTGAATCGGGCATATCTGATGGCTTGAAGAATGCCATAGATATCATGAAATTGGACAGTAAAGTCACCTCAGTCAAAGTTGAAGTCGGTTCTTACACCAAAGACAAATATGCAATGAAATTTATTGAAGAACTGTGCTCCCGGGGTATACCTGTTATGGCTCATATAGGACTAACTCCCCAAAAAGTTCATTCACTTGGAGGTTATAAAGTGCAAGGTAGCAAAAGTCTATTACAAATGCAGGAGCTGTATGAAACCGCCATGCAACTACAAAAAATTGGCTGTTGGTCTATCCTGATTGAATGTGTCCCCCATAAGATGGCTCAGTTCATAACATCTAAACTCTCAGTACCAACAATAGGTATCGGTGCAGGTAACGGTACCAGTGGACAAGTTCTAGTTATATCTGACCTTCTCGGGATGCAAGGCGATTCTGTCCCAAAATTTGTGAAGCAAGCTGTAAATATGACAGATATTGCCACTCAGGGCTTGAAGGAGTACATTGCAAGTGTAGAGGATAGGACATTTCCTGAGAGAGGTACTCATACTTTCAAAGTTAAGGAGGACTTATGGAATGAATTCCTCTCTTCAATTAACGAAAAGTAA>YBR177C	-:0:chr02:584802:586157ATGTCAGAAGTTTCCAAATGGCCAGCAATCAACCCATTCCATTGGGGATACAATGGTACAGTTTCGCATATTGTCGGTGAAAATGGTTCCATTAAACTCCATTTAAAAGACAACAAGGAGCAAGTTGATTTTGACGAGTTCGCTAACAAATATGTCCCAACGTTGAAGAATGGTGCCCAATTCAAATTGAGTCCTTACTTGTTCACAGGTATTTTGCAAACTTTGTACTTAGGTGCTGCTGATTTCTCTAAGAAATTTCCTGTATTCTACGGCAGGGAAATTGTCAAATTCTCGGATGGTGGAGTTTGCACCGCTGACTGGCTCATAGATTCATGGAAAAAGGATTATGAATTCGATCAAAGTACTACGAGCTTTGATAAAAAAAAATTTGATAAAGACGAGAAGGCGACACATCCAGAAGGATGGCCTCGTTTACAACCACGTACAAGGTACCTGAAAGATAATGAGTTGGAAGAACTACGGGAGGTTGATCTACCCCTAGTAGTTATTCTACATGGTCTTGCTGGTGGTAGTCATGAGCCGATTATAAGATCTCTTGCTGAAAACCTGTCTCGCAGTGGGAGATTTCAAGTGGTCGTCCTAAATACCAGAGGTTGTGCACGTTCCAAAATTACCACCAGAAATTTATTTACAGCTTATCACACAATGGATATTCGCGAGTTTTTGCAAAGAGAAAAGCAAAGACATCCAGATAGAAAACTATACGCTGTGGGATGCTCTTTTGGTGCTACGATGCTGGCAAACTATCTGGGAGAAGAGGGCGATAAATCACCTTTATCCGCAGCTGCTACTTTGTGCAATCCTTGGGATCTTCTCCTTTCAGCAATTAGGATGAGCCAGGATTGGTGGTCAAGAACTTTATTTTCCAAAAATATTGCGCAATTCTTAACAAGAACCGTTCAGGTTAATATGGGTGAATTAGGAGTTCCAAATGGCTCTCTCCCCGATCATCCTCCCACAGTCAAGAATCCATCTTTCTATATGTTCACGCCTGAAAATCTAATAAAGGCAAAGAGCTTTAAATCGACCCGGGAATTTGATGAAGTGTACACTGCGCCTGCTTTAGGCTTCCCAAATGCTATGGAGTATTATAAAGCGGCCAGCTCAATAAACAGAGTTGATACAATTCGGGTTCCTACCCTTGTTATCAATTCCAGGGATGATCCTGTTGTCGGCCCAGATCAACCATACTCAATCGTGGAAAAGAATCCTCGTATTTTGTATTGTAGAACCGATTTAGGTGGTCATTTAGCTTACCTAGATAAAGACAACAACTCGTGGGCTACCAAGGCAATTGCAGAATTTTTCACTAAGTTTGATGAATTAGTCGTATGA>YBR179C	-:0:chr02:586542:589109ATGTCTGAAGGAAAACAACAATTCAAAGACAGCAATAAACCGCACAAGGACTCCACAGATCAGGACGATGATGCCGCCACAATAGTACCACAAACTCTTACTTACTCACGCAACGAAGGTCATTTTTTAGGCAGTAATTTCCATGGAGTAACTGATGACCGTACCACTTTGTTTGATGGTGAAGAGGGGCGTAGGGAGGATGATTTGTTGCCCTCATTGCGTTCCTCCAACTCAAAAGCACACTTAATTTCTTCACAACTTAGCCAGTGGAATTATAACAACAATAGAGTACTTTTAAAAAGATCTATATTAAAGACACAAGCTTTTATGGATCAACTTCAAGAAGAAAATAATATTCGCCCGATTTTTATTGCTGCAAATGATGAACGGGAAAAATTACATGTTCTACAACTGAATATCAAGCTAGATGGTCAGTACAATACCAAAGAAAAGAACGGTTTCAACATTGAGAAAAAAGCTTTATCAAAATTATTTCACTCTCAGATCGTATCAGTGACAAATCATTTAAACGCTTTGAAAAAAAGAGTGGATGACGTTTCATCTAAAGTATTCATCACAGGTGATGTAAATACTGGTAAATCAGCTCTTTGCAACTCTCTATTAAAGCAGCGTTTGCTACCTGAGGATCAGCTACCATGTACCAATGTATTTTCCGAAATACTAGAGGCTCGGGAAAATGACGGCATAGAGGAGGTTCATGCCATACCACTAAACATTGCTCCGACTCTCAAGGAAGCCATTGATATGTATTCAATACAAAATCCCAAGACTTATGAAATACACACTTTGAAAGAACTTCCCGATTTGGTTCCCCAAAACGGAAAATATGCTTTACTGAAGATTTACATAAAGGACGATAAAAGGCCTGCCTCTACTAGTCTATTAAGGAATGGTACTGTTGATATTTCATTAATTGACTCCCCTGGGTTGAACATGGACTCGTTACAAACCGCCGAAGTAATGTCCCGACAAGAAGAAATCGACTTAGTTATATTTGTTGTCAATGCAGAGAACCAGCTAACACTATCTGCCAAGGAGTTTATTTCTTTAGCATCTCGTGAGAAAAAATTAATGTTTTTTGTTGTGAAAAAATTTGACAAAATCAGGGACAAACAACGTTGTAAGGAGTTGATCCTAAAGCAAATCCGTGACCTGTCCCCAGAAACATATAAACGTGCAGCTGATTTTGTCCACTTTGTTTCCAAAAACGGAGATGAATTGCCACATTATCATAATGAAAATGACAATGAAGATCATGGTGACCGAAAACCTGACGACGACCCATATTCTAGCAGCGATCCCGATCCGGATTTTGACAGTCTGGAAGATTCTCTACGCAATTTTGTTTTGAAGAAAAGATCACTTTCTAAATTGTTACCTGCCAAGACTTATCTATCGAAATTATTAAGCGATATAATAATGATCTCCAAATCTAATATGAAAATGTATAGTGAAGAAGAAATAAAAATTAACGAACAACTGGAAACATTGAGACCTGAAATTTTGAGCGCTAGAGCAAAGTGCAATGATTTAACAACTTCCGTTGATCAAATGGCCGAGCAAACAATTACAATGACTTATAACAATACTAAAGAAGCACTTCTCAATGCGTTGGATGTTCCGCTACACGAATATCCGAAATATCAGGGCCTCGGTCAAATTTATGACTTCATATTTTCAACAGAAGCGTTCATAGCAAATCAGATTGATGAGTCAATAGGATCAAGTGAATTATTTGCCAAGCAAAAGACAGATTTACTGGTAAAAAAAATTTATGAGATTGGAAAAAATGAACTTGGTGATGATTTCATGTGTGAACGTGTTTTCAGAAGCGAACTAATGTTTAGAAAAAGGAAGCATTTGATAGGAAAAAGGCTTAAAGTATCTTTGTCAATAACTGATTTGTTTGCTCCAACTTGGAAAGGCTTTTTGTCTTACTTAAGCTGGCAGAAACCTGTCACAGCACCACTGCCGGATATAGAAGGACAAACTAACGAGGGTCAGATTGGCCTAATGAAGTATTTGGGTTTGAAAAATTATCCTTTGACACAATATTGGTCAAGACCATCATTGCTCTTCACATCAAAAATTCCCACTCTAACGCTGTACTTTCTAGGAAGTACAAAGGTTGTGGGGAACATTATATTAAACGGCATTAAATTATCGTCGTGGAGCTCACTCAAGAAGTTATCAGTTCCGGTTATAGTGGTAGGTTCCTTGTTGGGACTTACGTATTTAATTCATGACCTCCCCCGTGCCTTACCAATGAATCTTTCCATTAAATACAAAAGAAAGTTGCAAGAACTGGATTACATTCATCTTAACGCTCAAAGAACCTCAAATGAAGTACGTGATGTTTTGCGTGTACCTACACGTGAAATTCTAAGGTCGTGTGAAATAATTATGGATAAAAAACAAATCACTAAGAAAGAATTGGAAAACAAAAAAGAAAGTAACTTGTTGTCAATCAAATTCTTCCAATCTCTATACGAAGGAACCGTGGCTCAAAAATTGATGGTGGAAGAAATAAATTTAGACATCGATTAG>YBR180W	+:0:chr02:589736:591454ATGGGAAGCGAACCGTTTCAGAAAAAGAATTTGGGTCTGCAAATTAATTCGCAAGAAAGTGGAACAACCCGCTCAACATTTCATTCGCTAGAAGATCTAGGAGATGATGTAATTAATGAAAGCTGGGATCAGGTGAACCAAAAGAGAGCCAATATAGATCATGATGTCTTTCATGAGCACCCTGACTCTTCCCCATCATTGTCAGCACAGAAAGCAAAAACAAAAGAAGAGGAAGTTGCTGTAAAGTCATCGAACTCCCAGTCAAGAGACCCTTCTCCTGATACTCAAGCACATATTCCATATACTTATTTTTCCAAGGATCAAAGACTAATCATTTTTGGAATTATCATTTTTATAGGATTTTTGGGCCCAATGTCTGGAAACATATATATACCGGCTTTACCATTGCTGCAAAGGGAATATGATGTAAGTGCAACAACAATAAACGCTACAGTTTCTGTATTTATGGCTGTTTTTTCCGTTGGTCCATTGTTTTGGGGCGCACTGGCGGATTTTGGTGGAAGGAAATTCTTATATATGGTGTCGTTATCACTAATGTTAATTGTTAATATACTTTTGGCCGCTGTACCAGTCAATATTGCTGCCCTTTTTGTTTTAAGAATTTTCCAAGCTTTCGCTTCCAGCTCTGTGATTTCTCTGGGAGCTGGCACTGTAACAGACGTTGTTCCTCCAAAACACAGGGGAAAGGCCATAGCGTATTTCATGATGGGTCCAAACATGGGTCCTATTATAGCACCCATTGTTGCTGGGCTTATTTTAATGAAAGGAAATTACTGGAGATGGCTTTTTGGTTTCACTTCTATCATGACAGGAATAGCATTGATCTTGGTTACTGCTTTACTTCCAGAAACGCTACGTTGTATAGTTGGTAATGGAGACCCTAAATGGGGTGACAAAAAAGATGAACGTGAAAATAACGAATCTCCATTCTTCGAAGGTAATAAAATATCACATCGGCGTCTGTTCCCAGACATTGGTATCCGTAAACCAGTCAATAATGATGCTTTCTTCCAAGAAAATTTTCCAAAGCCGCCTAAAGCAGGTTTGACACTATATTGGAAAATGATTAAATGTCCTCCAATAATAATCACGTCCGTCAGTACTGCACTCCTGTTCTCCAGTTACTATGCGTTCAGCGTCACGTTTTCGTATTACCTTGAACATGACTACCGTTTTACTATGTTAGAAATTGGTGCTGCTTATGTCTGCCCAGGTGTAGCTATGTTACTAGGATCTCAATCTGGTGGGCACCTCTCAGATTATCTTCGTTCACGCTGGATCAAAAGTCATCCTAAAAAGAAATTCCCGGCAGAGTTTCGTTTATTACTGAACCTAATTGGAATTTTACTAACAATATGTGGCACAATAGGATACGGATGGGCAATCTTCTTTCATTATCATTTTGTGGTTCTTTTAGTTTTTTCCGCTCTCACTGCCTTTGGTATGACCTGGTGCAGCAACACATCAATGACATATTTAACTGAGTTATTCCCCAAAAGAGCTGCTGGAACTGTTGCTGTTAGTAGTTTCTTTCGAAATGTGGGCGCTGCCATTAGTTCCGCTATCATTTTACAGCTCTGTAACGCAATGGGAATTGGATGGTGTTTTACAGGGCTCGGTCTCTGCAGTTCAATTTCATTGATTGGTATATTATATCTTCTCATTTTTCAAAGAAAATATACTGCCAAAGAATTTTAA>YBR181C	-:0:chr02:591707:592411TTGAACATTTCTTACCCAGTCAACGGGTCTCAAAAGACCTTCGAAATTGATGATGAACACCGTATTCGTGTTTTCTTCGACAAGAGAATCGGTCAAGAAGTCGATGGTGAAGCCGTTGGTGATGAATTCAAGGGCTACGTCTTCAAGATCTCTGGTGGTAACGACAAACAAGGTTTCCCAATGAAGCAAGGTGTTTTGTTGCCAACTAGAATCAAGTTGTTGTTGACCAAGAACGTTTCTTGTTACAGACCAAGACGTGATGGTGAAAGAAAGAGAAAGTCCGTCAGAGGTGCCATTGTTGGTCCAGATTTGGCTGTCTTGGCTTTGGTCATTGTCAAGAAGGGTGAACAAGAATTGGAAGGTCTAACTGACACTACTGTTCCAAAGAGATTGGGTCCAAAGAGAGCTAACAACATCAGAAAGTTCTTCGGTTTGTCCAAGGAAGATGACGTTCGTGATTTCGTCATCAGAAGAGAAGTCACCAAGGGTGAAAAGACTTACACCAAGGCTCCAAAGATCCAAAGATTGGTTACTCCTCAAAGATTGCAAAGAAAGAGACACCAAAGAGCTTTGAAGGTCAGAAACGCTCAAGCTCAAAGAGAAGCTGCTGCCGAATACGCTCAATTGTTGGCTAAGAGATTGTCTGAAAGAAAGGCTGAAAAGGCCGAAATCAGAAAGAGAAGAGCTTCTTCTTTGAAGGCTTAA>YBR181C	-:0:chr02:592764:592769ATGAAG>YBR182C	-:0:chr02:593501:594859ATGGGTAGAAGAAAAATTGAAATTGAACCTATCAAAGATGATAGAAATCGTACAGTTACTTTCATAAAGCGAAAAGCAGGACTATTTAAAAAGGCTCATGAATTGTCAGTACTTTGCCAAGTAGACATTGCTGTCATTATTTTAGGATCCAATAATACATTCTACGAATACTCTTCTGTTGATATGAGTAACCTGCTTAATGTTCATCAAAACAACACTGATCTTCCTCATAATATCATAGAACCATCTGATTATGGTGACTATGTGAAAAAACCACGTGTTGTTCTGAATGAAAGGAAGCGCAGGCGAAGAAGAGCAACCGTGTTGCAACCAGCTTCCCATTCTGGGAGTTGTACAGTTTCGAGTCAAGATTCCTCCAGTGTACAAAACAATGGGAATTTAAGCGCTCCGTTGGCGTCAAATGACGCCGGGAACGCTGGTGTAAGTACACCATTGGTGCATTGCCACGGAGCAATATCACGTAGCGGATCCAATCATTCTGACTGTGCAAGAAATAGTGCAGATTATCAAATGTTGCAAGGCGGTTTAAATTCTGGTGGGAGTTTTCATGCTAATGATTATAAAGAAAGCGTAGACCAACAGCATGTTGCAAACGAGGCTATTCATAGGAATTTTATGAACAAGAGGATTAGGCCGGATACTCATTTACTACTTTCTGAATCTAACCACTCTAATTATCATAATTTTTACCCGTCGCCTTACGAGAATTTGCCAAAGCCTTCATTGCCTGCAAGTTTAGTGGGCAATATTCCATCCTTTCAATCGCAATTTGTACAGGTTATTCCGGCAAATAGTAACCCAATGGGAAAAGGATTTAATGGGACGGGTGACAGCGAGAGCTTTGAAGCAAAGCAAAAGATACACCCGACAGTTGCTATATCAAATACTTTGGAAGGTCCAGCTCCAGTGCAGGCGATGGTCCATCACCTGCACCAACTGAACAGCAATAGAGGAAAGCTCTCAGGGAAGCCATATTTAAAGCTAAATATTCCGAAGGCCACAAATGACGCTTGCCAGAGGTCGCCAGCAATGTATTCAGGAACCGCATCACCGAAAACGGATGTACAAGCCACTCCCAATCAAATGCTCGCCAGCAACATGTCCTCCCCTCTTTCTCGTTCAAAGTTTTTGGGATTCAAGAACAATGATATGGACGACTTATATCATAATGGCCGATGTGGCAGCACTTATGTAAATAACAAAACATTCTTTCTGAAACCGCCAATTGGAAGACCGCCTAAATTTCCGAAAAGCCCGTCTTCATCTATTGTGGTTTTTCCTTCCTCGGTAGCTAGTTCAACTTTGAAATCCACGAGTTCGACAAACTCTCCAGATTAA>YBR183W	+:0:chr02:596110:597060ATGGGAATATTTCGTTGGAACTATCCAGAGAGTTCTGTCCCCGGCGTTTGGGGAGAAACAACTTCCACTATTGACTGGTGTGAGGAGAACTATGTCGTTTCTCCCTATATTGCCGAGTGGTCAAACACTTTAACTAACAGCGTATTCATACTGTCAGCGATTTACACAACTTACTCTGCTTACAAGAATAAATTAGAAAAAAGGTTTTTACTTATTGGCTTCGGGTACGGTTTGGTCGGAGTAGGATCATGGCTATTTCATATGACACTGAAGTATAGATTCCAACTATTGGATGAACTTCCAATGATATACGCCATGTGCATTCCGACATGGAGTTTAGTATGCGAGGCCAAAGAGGCATTACTTAACGGAGATAATCACAAGAAGGTTCCTCTATTTGAACAGATATTCATCGGCGTAATTATCGGCCTGGCCGTTACAACAGCAAGCATACTCTACGTTATTTACAAAAATGTCGATATCCATCAAATTTTGTTTGGCGTACAGATTGTAGTTGTGGCTGCTACTGCAGGAAGTTTGACGTACAGATACGTCCATGATCCACTTGCCAAAAGAAATCTCAAGGCTTCAATGGCGCTCGGCGCAATTTTGTTCTTATCTGGCTACATTTCGTGGCTACTTGATATACACTATTGTTCGTTCTGGGTGCACGTTAGAAGAAGTATTTTGGCTTTACCACTTGGTGTACTGCTTGAACCACACGGATGGTGGCATATATTAACTGGTATGGGGATTTATTTCTACATTGTTTCTTTGGAACATTTAAGGGTCATTACGCTCAACGTCAGCTGCAATTACCAGTTCATCTGGAGATGGAAAGTCTTCCCTGAACTGATATGGAAAGGGCGCAAACCCTCAACAAGATATTCACTTGAACTATTTGGCCCATACGTAGAAGATCAATCAATTGAAGTTAAAAAGGAGAAGTAA>YBR185C	-:0:chr02:599118:599954ATGAGTGTATTAAGATCTACATGCCTTTTCTTCCCTCCAAGATCCTTGTTGATATCATTTAACAAGCGGCGATTATTTTCAACTTCGAGGTTAATTTTGAATAAAGAAAGTGAAACTACAAAGAAGAAAGATAAAAGTAAGCAGCAAGATTTTAATCCTCGGCACCTGGGTGTTGCTGCCGAAATATTTATCCCCTCGGCTTACAAGAACCTTCCAAATGTATTTGCTCATCCTCTCATTGTTGCAAATGCATTGATCAGAAGACTTTACACGTTTGGTTTGAACTCTGTTCAAGTCGCATTGTTTCGTTTTCAATCAGGCATTAAACCTTCTTTTTTACTCTGGAAAAACAAAGCCATAGAGACGTATATCAATGTAAACACGTCATTTGCTCATAAAAATTTGTCGGACATCAAAGGGTTAGTTTCATTATGGGTTCAGGAAGCTCTCGAGGCTAGGTCCCGTCAACTTCCAGGTAATGCAACACTGGACTGGCAGTTGATAAAGTTTAATGCAGTTCCTAAACTAGTCTCAGTGCAACCAATCATGATTCCCGGAATGCCGTTAGAGCATTTACAGTTGGTTTACAAATTTGATACGAAGCAAAGACTAATTAAAGTCAATCAGCAAACTAAGAAGACTGAGACGTTAGACCGTGACGTTGTAGACTATATTGCCTTTTTGTGTGATGCTACAACTAATGATATGATTTTAATGGGATCTTTGTTTGAAAGTAAACCAAATGATAAATTACCGAAAAGCTACGAGGACGATGCTAAAGTTGCCATACACAGAATGAAAGTTAACGGTGATATATATCGTTTACCTCCAAGCTAA>YBR186W	+:0:chr02:600548:602098ATGAGCTACATAGTTGACCTACAAGTGCGAGGATCATCTCTGAGGGTTATCAAATGTATGTTTAGAGAGGATGAGCAAATTTCTTCTCTACATTCTGGTTCTGACTCGAAACAAAACAGCAACAAAAAGCTGGGAGAATTTTTGAATCTGTTAAAAGCAGTGGTCAAGAGGAAGTTGGAAAGTTTCCCAAAAGACCGTTTAAAGACTTCGATTATAACTGGCCAAGAATTAATGAGAGAGGGGCAAGGAAGTATTGAAATTAAGGATCCACCAACGGAGGCGCAACAACATTTGATTAGAAGCCTAGCAAAAGTTTTATTACACCAGTTTTCAAGCATAAATGGCAAAGTTAACACAGTAAATGAAGGACAAGATAATTTGTTTCTATCCTTATTCGTTAAAAAAATTTCTATAGAGCAACAGTCCACATCACACGTCTCTATTAAATTGAACTTTCATGAGAAAATAAATTTGGGTCAGCACATAGATTCTATTTTAGATTCAGAGGAGACCAACGAGTCGGACACTTATCACATGGGATCTGTTGACGAGTTCATTATATATCCCTTTTGCTGTCTGGAAGAGCAAGATGAGCTGAAAAATGGCAGCATACTGTCCACAGAGTTCGACAAGATTGACTTAGAACTAGACGAGGATGATGGTTTTGAGGGAGAAACTTTAAACAATTGTATCAATTCGGTGGGAAATTTTGACATTCCACTAAGTAAGCAAACACTAAACTTAGTAAATATATCATATCTTCCAGGCACAACTTTCGAGGGCCAATGGGAATCGTTGTATTTTGGTAACAATATTAAAGAAAGGTTATATAGCTACGCAACTATATCTCTCAAGATAGCAAGATTCAAACAAACTGGAGACTCCAATCAAGAAGACATTACAACGCTAATCACAAATAATAAATTACTCCTTGTCCATGGTCCTCCGGGAACAGGTAAAACAACGTTATGCAAAGCACTTTGCCAAAAATTATCTGTGAGGCGGGAATTTTCAGACGGTTCCGATACCATTGATACTAATTATAAGGGGATAATAATAGAATTGTCTTGTGCACGCATCTTTTCTAAATGGTTTGGAGAATCGTCAAAAAATATATCAATAGTATTCAAAGACATTGAGGAGCTTTTAAAGGTCAATGAGGGACGAGGGATCTTTATATGCCTTTTGATAGATGAGGTTGAAGCAATAGCAAGTTCAAGAACAAATTTGTCAAGTAGAAATGAGTCAACAGATGGAATCCGAGTTGTGAACACTTTACTTACGCAACTTGACAGGCTGAAAAAATACCATAACTTTTTAGCATTAGCCACGTCTAATTTATTAGATTCATTAGATGATGCTTTTGTTGATCGAGCAGATGGAGTTTTTTATGTTGGAAATCCAACAGCTGAGGGCATCCTCCACATTCTAAAGGTTTGCATAGAGGAGATGATAACTTCAGGTATAATTCTTTTTCACGCAAGATCAACTGGAGTCAAGTTCTTCAATAAATATCAGGACATTTTGCGGAAAATTGCTATCAAATGCTCG>YBR186W	+:0:chr02:602212:602355ACCGTTGATATCAGTGGTAGAACAATAAGGAAATTGCCCTTGATGTGTCTGTCTGAATACTTCCGGACATTTCCTGTAGACGACGATGAGTTTGTTCTGGCATTAGCTATGTCAGCGCGGAAGCTGAGCGCAGCGCGGAAGTGA>YBR191W	+:0:chr02:606265:606275ATGGGTAAATC>YBR191W	+:2:chr02:606664:607135ACACGGTTACAGATCTCGTACTCGTTACATGTTCCAACGTGACTTCAGAAAGCATGGTGCCGTCCATCTTTCTACTTACTTGAAGGTCTACAAGGTTGGTGACATTGTCGACATCAAAGCCAATGGTTCTATCCAAAAGGGTATGCCACACAAGTTTTACCAAGGTAAGACCGGTGTTGTCTACAACGTTACTAAGTCTTCTGTTGGTGTTATCATCAACAAGATGGTCGGTAACAGATATCTAGAAAAAAGATTAAACTTAAGAGTTGAACACATCAAGCACTCCAAGTGTAGACAAGAATTTTTGGAAAGAGTTAAGGCCAATGCTGCTAAGCGTGCTGAAGCTAAGGCTCAAGGTGTTGCTGTTCAATTGAAGAGACAACCAGCTCAACCAAGAGAATCCCGTATCGTTTCTACTGAAGGTAACGTTCCTCAAACTTTGGCCCCAGTTCCATACGAAACTTTCATCTAA>YBR193C	-:0:chr02:609077:609748ATGTCACAATCTACTGCATCACTGGTACCAGAGGGCAATCAAGGCTCCCTCCAAGAAGATGTTAGTTTTGATTTCAATGGTGTACCTGGCCAGGCTTTAGATGCCGTGCGCATGCGTTTGGCTCAATTAACTCATTCATTGAGGAGAATCAGAGACGAAATGTCCAAAGCCGAACTGCCACAATGGTATACACTCCAATCACAACTGAATGTCACATTATCCCAGCTCGTTTCTGTAACCTCCACTCTACAACATTTCCAAGAAACTTTAGACTCTACAGTGGTTTACCCTTTGCCTAAATTCCCAACCACCTCTCATGAAAGTTTGGTCACTACCCTTTTGAGGAAGAAGAATATTCCTGAAGTGGACGAATGGATGAAATATGTTAGGGAAACATCTGGAGTAACTACAGCCTTGCTAAAGGATGAAGAAATAGAAAAGCTGCTGCAACAGGACAGAGAAATAACGAATTGGGCGCGCACTACGTTTAGAAACGAATATGGAAAGCACGATTTCAAAAATGAAGAATCCCTAAGCGAAGAACACGCTTCACTTCTAGTACGAGATAGCAAACCTTCGAAACCTTTCAATGTGGATGATGTTCTAAAGTTCACCTTCACAGGGGAAAAGCCCATTATTACGGGATCTACTTCAACATCATCTAGTAATTGA>YBR195C	-:0:chr02:610609:611877ATGAATCAGTGCGCGAAGGACATAACTCATGAAGCCTCCAGTATACCCATCGATTTGCAAGAAAGATACTCGCACTGGAAGAAAAACACTAAACTACTTTATGATTACCTAAACACGAATTCAACAAAGTGGCCGTCCTTAACGTGCCAGTTCTTTCCTGATTTAGATACCACTTCGGATGAGCATCGCATCTTGTTATCCTCATTTACATCTTCCCAAAAACCTGAAGATGAGACCATATATATTAGCAAAATATCCACGTTGGGTCATATAAAATGGTCATCTTTAAATAATTTCGACATGGACGAAATGGAATTCAAACCGGAGAACTCGACAAGGTTTCCCTCCAAACACTTAGTAAATGACATCAGTATTTTCTTCCCAAACGGGGAATGCAATAGGGCAAGATATTTGCCTCAAAATCCAGATATTATAGCCGGCGCCTCTTCAGATGGTGCAATCTACATATTCGATAGAACAAAACACGGCTCTACTAGAATAAGACAGTCCAAAATTTCACATCCCTTTGAGACAAAGCTGTTTGGTTCACATGGTGTTATTCAAGACGTGGAGGCAATGGATACTTCTTCGGCAGATATAAATGAGGCGACTTCTTTAGCCTGGAACTTGCAGCAGGAGGCCCTTTTACTTTCTTCTCACTCCAACGGCCAAGTTCAAGTTTGGGACATTAAACAATATTCGCATGAGAACCCTATAATAGATTTACCCTTAGTGTCAATAAACAGCGACGGAACAGCGGTGAATGATGTAACTTGGATGCCAACACACGATTCCCTCTTTGCTGCTTGTACTGAAGGAAATGCGGTCTCCCTATTAGATCTGAGGACTAAGAAAGAGAAGCTCCAGAGTAACCGTGAAAAACACGATGGTGGAGTAAACTCCTGTAGATTTAACTATAAGAACTCTTTAATTCTAGCATCTGCAGATTCAAATGGGAGGCTAAATTTATGGGATATTAGAAACATGAACAAAAGCCCAATCGCTACCATGGAGCACGGTACTTCCGTTTCAACTTTAGAATGGAGTCCAAATTTCGATACTGTATTGGCAACGGCTGGCCAAGAAGATGGGTTAGTCAAGCTATGGGATACCTCCTGCGAAGAAACTATATTTACCCATGGTGGTCATATGCTCGGTGTGAACGACATTTCGTGGGACGCTCATGACCCTTGGTTAATGTGCAGTGTGGCAAATGATAATTCAGTTCACATATGGAAACCTGCAGGAAACCTTGTTGGACATTCGTGA>YBR197C	-:0:chr02:615198:615851ATGGGAGTGAAGCAAACCCCACCAGTCCAGGTGAAAGTAAGCGACGCGGATTCCACCAACAGAAGGAAATCTAGTAGCCAGGAAGGAAACCCTCAGCTGGTTCAATTAAAGGCAAAGAGCGACAAAGATAAAAGAAAGGGATCTTCAGATTCCACTGCCTCTATAATGGGCAGTTCCAACGCACTTCCGACCAAAAACCTAACCACGCCTCCAGCACTAAACCCCTTAACGACCAGCATTAGTAGGGGTAATACTGCATACGAAAGAAGCGTAAATGGTAGCCGTATAACAATGCACTCAAACCTGGCACCTACGGAGACGCAAGACGTATCATGGTCTGAAATCGATACACTGGATGATGTGAAGAAGATGGCGAAAGAACCTATCGTCAACGACGGGTTCCCACGAGATTTTGAGAGCAATCTCACGCAAATGCGCAAGTCGCACGCTCAACTACTGCGATTAATGAGGGAAAGAAATCAGCGACTAAAGTATGCCAAGCTGAGGAGCCCTCCTCATAAGGATCAGCATAATTCTGCCACGAATAAAGATCAAGAACCAGACGAGGTATTGCATGACCCGGAAATCGCACTTGACGGTGAGAAGTACGTGAGCCAAGTTGTCGATACTATTAAAGATGTTCACCGATGCTGA>YBR199W	+:0:chr02:618904:620298ATGAGGTTTCTTTCAAAAAGGATACTGAAACCTGTACTTTCAGTGATCATTCTAATATCGATTGCCGTAACCGTTGTTCTTTACTTTTTAACGGCGAATGAGAACTATTTGCAAGCAGTCAAGGATAGTGCCAAGTCCCAGTATGCTTCTCTCCGGGAGAGCTACAAAAGTATTACAGGTAAAACTGAAAGCGCTGATGAACTTCCTGATCATGATGCTGAGGTGTTAGATAGCATAATGGACAGGCTACACGAGCCCCTTTATGAAAAGGACACTTTTGACCCGAATGAAGTCCTTGCTGAAAATAAACAGTTGTACGAGGAGTTTTTACTTCAAGAAATTTCTGAGCCAAAGGTGGACAATTTGGTACGTAGCGGGGATCCGTTAGCCGGCAAGGCAAAAGGAACTATTTTGAGTTTGGTGAGAAATTCTGATTTAGAGGATATAATTTCTTCTATTCAACAGCTAGAAGAGGAGTACAATAAGAATTTTGGATATCCCTATACCTTCCTTAACGATGAGGAATTTACTGACGAATTCAAAGATGGAATAAAAAGTATTCTTCCTAAGGACCGTGTAGTAGAATTTGGCACTATTGGTCCAGATAACTGGAATATGCCTGATAGCATAGATAGGGAGAGATACGACCAAGAAATGGACAAAATGAGTAAAGAGAACATTCAGTATGCAGAGGTGGAATCCTATCATAACATGTGTCGTTTCTATTCTAAAGAATTTTACCATCATCCTCTGCTATCTAAATATAAATATGTGTGGAGATTAGAGCCAAACGTGAATTTCTATTGCAAAATTAACTATGACGTTTTCCAATTTATGAACAAGAATGACAAGATTTATGGGTTTGTTTTGAACCTCTATGATAGTCCGCAAACCATCGAAACGCTATGGACTTCAACTATGGATTTTGTCGAGGAACACCCAAACTATTTGAACGTCAACGGAGCCTTCGCATGGCTAAAAGATAACTCTCAAAATCCAAAAAATTATGATTATACTCAAGGGTATTCAACTTGTCATTTCTGGACCAATTTTGAAATAGTCGATTTAGACTTTTTGAGGTCAGAACCTTATGAAAAATATATGCAATATCTTGAAGAAAAGGGAGGATTCTACTATGAAAGGTGGGGTGATGCCCCTGTAAGAAGTTTGGCACTTGCTTTATTTGCGGATAAGTCAAGCATCCATTGGTTCAGGGATATAGGTTATCACCATACTCCGTACACGAACTGTCCAACTTGTCCAGCAGATTCAGATAGGTGTAATGGAAATTGTGTACCAGGCAAATTCACTCCTTGGAGTGATCTAGACAACCAAAATTGCCAGGCAACCTGGATAAGACACTCTATGTCTGAGGAAGAGTTAGAAATGTATTGA>YBR201W	+:0:chr02:623572:624207ATGGATGCTGTAATACTGAATCTCTTAGGCGACATTCCTTTGGTCACAAGATTATGGACAATTGGCTGTCTTGTACTATCAGGTCTCACAAGTCTCCGGATTGTGGATCCAGGGAAGGTAGTGTACAGTTATGATTTAGTATTCAAAAAGGGACAATATGGAAGACTACTTTATTCGATATTCGATTACGGCGCATTTAATTGGATATCCATGATAAACATCTTTGTCAGCGCTAATCACTTATCAACTTTGGAAAACTCATTCAATCTGAGAAGAAAATTCTGTTGGATAATATTTTTACTGTTGGTGATACTGGTAAAGATGACCAGCATTGAACAACCTGCAGCATCACTCGGTGTGTTATTGCATGAGAATCTCGTGTACTACGAACTGAAAAAGAACGGAAACCAAATGAACGTACGATTCTTCGGTGCCATTGATGTTTCACCATCTATATTCCCAATCTACATGAATGCAGTAATGTATTTTGTATATAAGCGTAGCTGGTTAGAAATTGCCATGAATTTCATGCCAGGTCACGTAATTTACTACATGGATGATATAATAGGGAAGATTTATGGCATCGATTTGTGTAAATCTCCGTACGACTGGTTCCGCAACACTGAAACACCCTAA>YBR202W	+:0:chr02:625767:628304ATGAGTGCGGCACTTCCATCAATTCAGCTTCCTGTCGACTATAATAATCTTTTTAATGAAATCACCGATTTTTTGGTGACTTTCAAGCAGGATACGTTGTCTTCTGATGCAACACGAAATGAGAACGAAGATGAAAATTTGGATGCTGAGAATATCGAACAACATCTATTGGAAAAGGGGCCCAAGTACATGGCGATGCTACAGAAAGTGGCTAATAGGGAACTGAATTCCGTGATTATTGATTTAGATGACATTCTACAGTATCAAAACGAAAAGTTTCTCCAGGGCACACAGGCAGACGACCTTGTGTCTGCCATTCAACAAAACGCTAATCACTTCACTGAATTATTTTGCCGTGCCATTGACAACAACATGCCGCTACCAACAAAAGAAATTGACTACAAGGACGACGTTCTTGATGTTATTTTAAACCAAAGGAGACTGAGGAATGAGAGAATGCTCTCGGACAGGACCAATGAGATCCGAAGCGAAAACCTCATGGATACCACGATGGATCCACCCTCTTCCATGAATGATGCGTTAAGAGAAGTTGTCGAGGATGAAACTGAGTTATTTCCTCCTAATTTAACTAGACGCTATTTCCTTTATTTTAAGCCTCTATCGCAAAATTGTGCTCGTCGTTACAGGAAGAAAGCAATTAGTTCTAAACCATTATCTGTTAGGCAGATTAAAGGTGACTTCCTAGGCCAATTGATTACCGTCAGAGGTATTATCACCAGAGTTTCTGATGTCAAACCAGCTGTGGAAGTTATCGCATATACCTGCGATCAATGTGGGTACGAAGTTTTCCAAGAGGTCAACTCTCGTACTTTTACTCCGTTGTCAGAATGTACTTCCGAAGAATGTTCCCAAAATCAAACAAAGGGTCAATTGTTTATGAGTACACGAGCTTCCAAATTTAGTGCCTTTCAGGAATGTAAAATTCAGGAGTTATCACAACAAGTGCCAGTGGGTCATATTCCTAGGTCGCTAAATATCCACGTTAACGGGACGCTGGTAAGATCTTTATCGCCTGGTGATATCGTCGATGTAACTGGTATATTCTTGCCAGCGCCCTACACCGGTTTCAAAGCTTTGAAAGCTGGCTTACTGACAGAAACTTATTTAGAGGCACAGTTTGTCCGTCAACACAAGAAGAAATTTGCGTCTTTCAGTCTGACTTCTGATGTAGAAGAGAGAGTTATGGAATTAATCACCTCTGGTGATGTTTATAATAGGCTGGCAAAATCTATTGCACCGGAAATCTACGGTAACTTGGACGTAAAGAAAGCGTTGCTGCTATTACTTGTCGGAGGTGTTGATAAAAGGGTAGGTGACGGTATGAAAATCAGAGGTGATATCAATGTTTGTCTGATGGGTGATCCCGGTGTTGCCAAATCTCAACTGCTGAAGGCCATTTGCAAAATATCACCTCGAGGAGTGTATACCACTGGTAAGGGTTCCTCAGGCGTTGGTCTGACCGCTGCCGTCATGAAAGATCCTGTCACGGATGAAATGATTCTAGAAGGTGGTGCCTTAGTACTCGCTGATAACGGTATTTGTTGTATCGATGAATTTGATAAGATGGATGAAAGTGACAGAACGGCAATCCATGAAGTTATGGAACAACAAACAATTTCGATATCCAAGGCGGTTATCAATACAAATCCGGGCGCCAGAACCTCAATCTTAGCGGCAGCAAATCCGTTGTATGGTAGAATTAATCCTAGATTATCACCTCTGGACAATATAAATCTACCAGCTGCTTTACTATCCAGATTTGATATTCTCTTCTTAATGTTAGATATACCAAGTAGAGATGACGACGAAAAATTAGCCGAGCACGTTACATATGTGCATATGCATAATAAACAACCGGATTTGGACTTCACTCCAGTAGAACCCTCTAAAATGAGAGAGTACATTGCCTATGCTAAGACAAAGAGGCCAGTGATGAGTGAAGCTGTAAACGATTATGTGGTGCAAGCTTACATCAGATTAAGACAAGACTCCAAGAGAGAAATGGATTCCAAATTTTCCTTTGGTCAAGCCACTCCGAGAACTTTACTAGGTATTATAAGATTATCTCAAGCGCTAGCAAAGTTAAGGTTGGCTGATATGGTGGATATAGATGATGTGGAGGAAGCCTTGAGGTTGGTCCGAGTCTCCAAGGAATCATTGTATCAAGAAACCAACAAATCTAAAGAAGATGAAAGCCCCACAACGAAAATCTTTACGATCATCAAGAAAATGTTACAAGAAACTGGTAAAAACACGCTATCATATGAAAATATTGTGAAAACTGTAAGACTAAGAGGGTTTACCATGCTACAATTAAGCAACTGTATCCAAGAATATTCTTACTTAAACGTCTGGCATTTAATTAATGAGGGTAACACTTTGAAATTCGTGGATGACGGTACCATGGACACAGACCAGGAAGACTCACTAGTGAGCACGCCTAAACTCGCACCACAAACGACCGCTTCCGCTAATGTGAGCGCCCAAGATTCTGATATCGATCTACAAGACGCTTGA>YBR203W	+:0:chr02:629163:631937ATGTCAAGTGCTTCAAGACTTCAAAACGTCAATATTGTTTCTAACAACTATTCTCGTTACGGAACTTCCGTTTACGATAAGTTGTATCATAGTAACGGCAGTGGTAGCAATAATGCTGGTAAGAATTCTACCACAGTTGGTAAACTGTCATCTATCTCACAAAAGTCCAGGAGCAAACAGCGTCATGGCTCAAATTGCTCCAGATCAATGAGTCAATCACCCTTATCGACGTTCAAATCTCCACTCAGCAATCAAAATCAATCCAGCGCTCCTGATGATTTGGCTTCCATAGGTCAGAGACGCAGTGACGACGTCACATCTTTAGATAATGAAACTATCATAACGATGAATTCAAGGAAATCAAGGATTAAAAAAAAGTATAAATCCTTAATCTCCACCTCTTCAAAAAAATTTATGAATAAATTGTACGATCATGGTGCTTCATCCGACTCATTTTCTATATTTTCTCTGAAGACTTCCCATTCTGGTAAACATGAAAATTCCAGATTTGAAAAGCTGAGGAAGAGAAAATACCATGCTTGGGGCAAATTTGCCGATATCAACGATTTACCAGTTGAAATAATTGCCAAAATACTTTCTGAATTCGAGTTAGGTCGTGATCAAAAGACACTCGTAAGATGTCTATACGTATCGAAGAAGTTTTATAAGGCGACAAAGATCGTCCTTTATAGGCTACCTTACTTCACTTCCACTTACAGAGTGGCGCAGTTTGTCACCTCACTAAGATTACATCCGGATAACGGGGCATACGTGAAAGTATTGGACCTATCACACTTGAAGCCGGGAATTATAGGCCAAGACTCGAAGGATTCTCAAGGCCTAGACGATGATGGCCACTCAAGGCGTCACAGACGCCGTCGCCGCAGATCTACTAACACAAGTTTGAATCTACCTCCAGCTACTCCAACATCTACCATTTCTAACGAGGATGATGCCAATAGCGGATTAATCAAAGACGACGCTTCAAATGGCAGTGAAGTTGAAGACTTGGCATTAGCTGGTTGGAGAGACTGGAGATACAGAAATGAACCCCTTTATAGCTCTCCTCTGTTGAACAGCTTTAAACTGAAAAAAGTGGTTTCTCGTTCTTCTTCTATCACTTCTACATCTTCTGGAAATTCCACCGGTGTTCACTCTACAAGGAGACAACGTTCCAATAGTTCCGTTGCTTCCATAACGACGAGTATCATGTCATCCATCTATAACACCTCCCATGTCTCATTGTCTTCAACCACGTCTAATACCAGCAATGGTAACATCTCTTCTGGTAGTAATCTGTCAAGAGTTTCCACTGCTGGATCATTGAAAAAAGCTTCTGCGAAGTCCACAAGGTCATCTCCTCAGAAAGCAAAACCTATATCTGACATTACGTCATCTTCCTGGTTTAGAATGAGGTTAAGTTCAAGAAATAGAAAGGCAAGGACAGCTAATACGATTAATTTAAAGAACTCGAAGGATAAGTCTGACGATGACTTTAAAGTTTTAAAGCACGATTCGGGGCATCCATCCAATTATCGTTCTTCGACATTAAAATTTAGCATTGAACAACCATTTAGTACGCATCATCCGTATGCCAACAAATTTCTTTTAAAGTACGCTCCTTATAAAGATCTGCCCCTAGGTTATATTTTACATATGCTGAACCTGTGTCCTAACCTTGTTGAACTAAACCTTTCCAATTTAGTTATTTGTACCGATTTCAAATTAATCAACCAAAGATCCGAAAGAAGAAGGATGACATCTTCACTGTTGCCCGCTGTCCAGGAGTCTAGTGTTTCTGCAGGCCCTGAGAAAGATTTAGAAATTGTCTATATGACCGATTCTGGCAAAGGTTATGAATATTATGAGGGGTTAAGCAAGAAACACTCACGTTCTTCAAGCCTAGGTACCAATCCATCTAGTTGGATTGGAGGTCAGGCGAATTGGACAGATTACCCACCTCCAATCGATGCACAAACGAAAACAAGAGAGGAACACAGACGCAACAATACCTTGAACAATAAGAACGTCGTATTAAAGAAGCTAAACCCTTTTGAAATTTTTGAAATGATCTGCAATAGAAATGAAGAAAAAGGTGGCTACTGCTCCTTAACTAAGGTGAAAATGAATGACATTGTGTGGTGCAGGCAATATATGGTAAAGTATTTTGTAATGAGGAGCTGGCGAAACGATTTGGATTATAAATCCATGGAAAACAACTCTTATGAAAGACACTTATTCAGTTTCCGGGACTCTGGCTTGGATAGAAATTTTTCCTGGGCTTGTAATGCCAAATTGCATGAATTTGTCGCATTGATGGTTATGGATCATCTTTCAAATTTAGATGATTTAGGATTAGAAGAGCTTTTTAATATCAAGTCCGAAAAGCTATATATAAAAAACTATTGTTGCCGAGACCCGGATATTCTCGAAATATCAAACTTATTTGATATTAGATATGGGGCCGGATCTGAGGCTGATGCTACTTCTGATTCCAATCTCGAAGCAGAATCTTTACAATTCAGGTTGACAATATTAAAAACAGAAAAGCCAACTTCATTTTGGCTCACAAAAGTTTCCAAAGATTATGTTTCTTTAGTGGTTAAATTATGTGTGGATGATGACATTGATATGGACAAAATGAAAGTTGGAAAACCAACTTTGAGAATAGACAGTATTACACACAATTTGATTAGTAGGTTAAAGGAGTTGAGGAGAGTTGATCTAAGGAGAAATGTTGGCGAGAATAGCTACTATGCTGGAAGCATCATATGA>YBR204C	-:0:chr02:632249:633376ATGAATATGGCAGAACGTGCAGAAGCAACAAAAAGTTGGTCCTGTGAGCCCCTTTCTGGAAAGACGCTAGAAGAGATTGTTCAAAATGCAGAAAATGCAGCTGATTTGGTAGCTTACATTCGCAAACCAGAGGTAGATTTGGACTTTAGGTTGAAGTTTATCGCAGAACATGAGGAGTTTTTTAATGAACAACTATCGGATCGAAATTCAAGAATAAGGACGTGTCACAACTTGAGCGATAAAGGGATACGGGGTGATACTGTTTTCGTCTTCGTGCCTGGGTTGGCTGGCAACTTAGAACAATTTGAACCCTTACTCGAGCTTGTGGACTCTGATCAAAAGGCATTTTTGACGTTAGATCTGCCCGGCTTTGGTCATAGTTCAGAATGGAGCGACTACCCCATGCTGAAAGTGGTGGAACTTATTTTCGTTCTTGTATGTGACGTCTTAAGAAAATGGTCTACAGCGGTGCCGAATAATGATAACGTAAATCCATTTAACGGTCATAAGATAGTGCTTGTAGGGCATTCTATGGGTTGTTTTCTGGCATGTCATCTTTACGAGCAGCACATGGCTGATACAAAAGCGGTACAAACGCTAGTTTTGTTGACGCCCCCCAAAGCACATATCGAGCAGCTTTCTAAGGATAAACATATTATCCAGTGGGCTCTCTATGGTGTGTTTAAATTGCCATGGCTATTTGACGTTTATAGAAACAAATTCGATCAGGTAAAAGGTCTACAAAGTTCCGGCATCAAGCAGTATTTTTATCAACAAGGTGATGATGTCAAGTTGAAATACAGGAAGTTCTGGCAATTTAAAAACAACATAAGCAACAAAAGTAGAACCATCATAGGTTATTTACTAGGATGGGAAACTGTTGATTGGGTTAAATTCAATGGTGTATTGACGCAAACGGATATGAAACAGAAAATTATAATATTTGGCGCTGAAAAAGATCCTATAGCTCCTATTGAAAATTTGGAATTTTACAAACAAACTATAAATAAAGAATGTCTTCGTAAAGTGATTATTTTACCTGACTGTTCTCATAATTTATGCTTCGACCGTCCAGAATTGGTGTGTGAAAACTTTCAACGAGAGGTGATTGATAATTCCAAATTGTAG>YBR205W	+:0:chr02:633617:634831ATGTCTGTGCACCACAAAAAGAAGCTAATGCCCAAATCGGCATTGCTGATTAGAAAATACCAAAAGGGAATCAGATCATCATTCATTGGGCTCATCATAGTGTTATCCTTTTTATTTTTCATGAGTGGCTCAAGGTCCCCAGAGGTACCAATAGCTCAGGGCACCAGTGTCAGTCGTGTAGCCAGTAAGGATTATTTGATGCCGTTTACTGACAAGTCACAAGGAGTAATCCATCCCGTGGACGATGGAAAGAAGGAAAAGGGTGTTATGGTCACATTGGCCAGGAATTCAGATTTATGGAACTTGGTAAAATCTATTAGACATGTTGAAGACAGGTTCAATAACAGATATCACTACGATTGGGTGTTCTTGAATGACCAACCATTTAGTGATGAGTTTAAACGTGTTACCAGTGCATTGGTTTCAGGCAAGGCAAAATATGGGACAATCCCAAAGGACCATTGGTCCATTCCATCTTGGATCGATACTGAAAAATTCGATGAGAAAAGGCTTGCAATGGGAAAATTGGATATTCCATATGGTAGTTCCGTGCCTTACCGTCACATGTGCCGTTTCCAGTCAGGGTTCATATGGAGACACCCACTACTGGAGGAGTACGAATGGTTTTGGAGAGTGGATACCGATATCACTTTATTCTGTGATATTCAGTATGACATATTCAAGTTTTTAAAAGTAAATAACAAGAAATATGGATTCATTCTTTCGGTAAGTGAATATGAGCGCACAATCCCAACCTTGTGGGAAACGACAAAGAAATTCATCAAGAAAAATCCTAAATTCTTACACAAAAATAACCTCATGAAATTCATTTCGAATGATGATGGTGACACTTATAATATGTGTCATTTCTGGACCAACTTCGAAATCGGCTCTTTAGACTTCTTCAGGTCCGATGCATACAGAGAATACTTTGATTACTTAGACAGTTCTGGTGGATTCTTCTACGAAAGATGGGGGGATGCGCCAGTGCACTCCATCGCTGCCTCTTTGTTCTTGGACAAGTCAGAAATCCATTTCTTTGACGGTCTTGGATTCCATCATCCAGATTTCACCTCTTGTCCTATCGAACAGAAGATTAGACTACAAAACAAGTGTATCTGCGAACCTAGCAAGGACGTCACCTGGACTCCTGACTACTTCTGTACTAGAAAGTACTTTTCAGCAGGAAATTACAAGCTTCCACCAGGAATTTAA>YBR207W	+:0:chr02:635141:636538ATGGCGTTTGAGGATTATTTTTCTTTCCAGATATTCTTCATTTTCTTAAGGAAATCCTTAGAAATTGTTGTCATTGTTTCTATCCTGTTGACGATCGTCAAACAAGATCTGTCTGTTGAGGACGACAGCCCGTTTGAAGGAAGTTCCTCTTCTGCTGGTCTTCCAAGTCCAAATACGAACACAAACGCAGATTCGACCACTGCCTTCTTACAAGCAGGGCCTTCAGACGGTAATGCTATTGGAACGTCCGCTACGGCTGCTAATAATAAATCGAGGCCGCTAAATGTGGAGGAAGAAGAAGAAATTTACGAGTACTCTAATGAGCTTAGAGACCAGGACCGCGAGTCCGATGAACATACGGCTGACAACGTTAAGCTATATCAGAAATTAAAAATACAAATTCTAGCGGGCGGCGCATTTGGTTTGTTGCTGTGTATGTTAATCGGGGGTGCGTTTGTTAGTATCTTTTATCATATTGGAACCGACTTATGGACACTGAGTGAACATTATTATGAAGGTGTCTTAAGTCTTGTCGCGTCCGTTATTATTTCTGTCATGGGGTTGTTTTTTTTAAGAATGGGGAAGTTAAGAGAGAAGTTCAGAGTGAAATTAGCTTCTATCATTTATTCAAAGGACAATAATTTGCTGGGAAACAAAACCCAAAAAGGTGTGAAATTCAGTCAAAAGTACTCCTTCTTCATATTACCATTTATAACGACTTTGAGAGAAGGGTTAGAAGCTGTGTATTCAATTGGAGGTATCGGTATTGACCAACCACTATCCTCGATTCCCCTCTCTATGGTCCTTGCTACTGCAATCAGTACGGTGTTCGGTATTTTTTTCTTCAGGTACTCAAGTTCTCTTTCACTTAAGATATGCCTTGTAGTCGCCACCTGCTTCCTCTACTTGATCGCGGCTGGTTTGTTTTCCAAAGGTGTATGGCAACTTGAATTGCAAGACTACGTCAACAAATGTAACGGTCAAGACATGAGTGAAGTAGAAAATGGACCCGGTTCGTACGACATATCTCGTTCTGTTTGGCATGTTAATTGTTGCAATGGTGAAAAAGATGGAGGTTGGATGATTTTTACTGCCATTTTTGGTTGGACGAATAGTGCTACCGTCGGCTCTGTAATTAGCTACAACGCCTACTGGTTGGTATTAAAATACGCTTTGAAGCTTCTAATGATAGAAGGAAAATGCGGATATATCCCATATCTACCTATTAGCTGGCAAAAGAAACGTATTATGAAAAGATTAAGCATTGCCAAGGCATCGCTCGATCTCAAACATCATACTTCTGAACTCAACTCTAGCACATCAGAGCCAGACAGTCAAAGACGGTCCAAAGATAGTTCTGTGCCCCTAATAATTGATAGCAGTGGTTCAGCAAATTGA>YBR210W	+:0:chr02:645545:645973ATGTCAGGAACCGGATTATCGCTATTCGTGACGGGTCTTATACTGAATTGTTTGAATTCCATTTGCCAGATATACTTTACTATATTATATGGTGATCTTGAAGCAGACTATATCAACTCAATCGAACTGTGTAAGAGGGTGAATAGGTTGAGTGTACCAGAGGCAATTCTACAAGCTTTCATTTCTGCATTGTTTCTTTTTAACGGGTACTGGTTCGTATTTCTCTTGAATGTCCCTGTTCTAGCATATAACGCAAGTAAAGTCTACAAGAAAACACATCTACTGGACGCCACGGATATTTTCAGAAAATTGGGTAGATGCAAAATTGAATGCTTTTTAAAATTGGGATTTTACCTTCTCATATTCTTTTTCTACTTCTACAGAATGGTCACAGCGTTACTTGAAAATGATGCAAATTTGATAAGCTAA>YBR212W	+:0:chr02:647881:649899ATGATGTCTAACGTTGCTAACGCCTCCCAAAGACAGGAAAATCCATATATTATTCCATTGCCTCCTTCATCCACAGTGGAAACTTCTACTGAACCCCCAAGAACGTTATGGATGGGAGATTTGGATCCGTCCTTCGACGAGGCCACGATTGAGGAGATATGGTCAAAACTGGATAAAAAAGTTATCGTTAAACTAATTAGAGCCAAAAAAAATTTGTTGATTCCTTGTAGCTCCACCTCAAGTAGTAATAACAACACAAGTGAGGAGAATGCTGAAAACCAGCAATCTGCATCAAACAGCACTGATCAGTTAGATAATTCCCAAATGATTAATATTAATGGAATATCCTTCATTGATCCTTCCACTACTCAATTACATCATGCCGGATACTGTTTTGTCGAATTTGAAACTCAAAAGGACGCTAAATTTGCCCTGTCATTAAATGCTACACCTTTACCTAATTTTTATTCCCCAACAACTAACTCCCAAACTAATCCAACGTTCAAAAGGACTTTCAGATTGAATTGGGCGTCTGGAGCTACTTTACAAAGTTCTATACCATCTACCCCAGAATTTTCCTTGTTTGTTGGTGATCTTTCTCCCACCGCCACTGAAGCTGATTTGTTATCTCTTTTCCAAACGAGGTTCAAATCTGTTAAAACCGTCCGCGTGATGACTGACCCTTTAACAGGATCATCTCGTTGTTTTGGATTTGTCAGATTCGGTGATGAAGATGAGCGCCGCAGGGCACTGATTGAGATGAGTGGGAAATGGTTTCAAGGAAGGGCCTTAAGAGTCGCTTATGCCACGCCAAGAAATAATATGATGCTGCAGTTACAGGAACAACAACAACAGCAACAACAATTGCAGCAACAGCATCAGCAGCTAGATCAGGAGGATAATAATGGCCCTCTCTTGATTAAGACTGCAAATAATCTCATTCAAAACAATAGCAACATGCTTCCTCTCAATGCCTTGCATAACGCGCCTCCAATGCATTTGAATGAGGGCGGCATTTCAAACATGCGTGTAAATGATTCACTACCTTCAAACACCTATAATACAGACCCCACTAATACTACTGTTTTTGTGGGAGGACTGGTACCAAAAACTACCGAATTTCAATTACGTTCACTATTCAAACCCTTTGGACCCATTCTAAATGTCAGGATTCCCAATGGTAAAAACTGTGGGTTTGTTAAATTCGAGAAGAGGATTGATGCTGAAGCTTCAATACAAGGCCTGCAAGGCTTTATTGTGGGCGGCAGTCCAATAAGATTATCATGGGGACGCCCATCCAGTTCAAATGCTAAAACAAATTCGACCATCATGGGGGCAAGCCAATATATGTCCTCAAATGGCCTGAGAGCGCCATCTGCGGCTTCTTCAGTCGATAACTCCAAACAAATTCTTGAACAATATGCGGAAGATAAAAGGCGACTCTTTTTACATCAGCAGCAGCAGCAGCAGCAGCAGCAGCAACAGGATGGTAACTTTTCTATGGAGCAAATGGCGCACAACAACTATTACAACTACAATAATTACGATTATCACAGGAACAAGAATGGCAGCCATAGCGACCTCGTTAATCTGCAGAGATCTAATGTCCCGTATATGCAAGAAGACGGTGCACTGTACCCACACCAATATTCAAGTCCCTCATACTCGCTCCATCCGACGGGCAACCAATTCTCCAATGCTACTAATAACTTACCTCAATTTGGGAACGCGATGTCAATTTCTATGCAACTGCCCAATGGCAACAGCAATAAAACGGCCTCAAGCATGAACACAAACCCTAACACAAACATGATCATGAATTCTAACATGAACATGAACATGAACGTAAATCCAGTACCATACGGAATGGGAAACGGTGCAAATATGTATGACGTGTCCAGGATGATGACTCCTCCCTTAAATATAGCCCCGAATTCCAATAATTCGAAATCGAGCATCATGAACAAGCATCCTAATAGGAACAATGTTCCGCCAATTCATCCTTCTCTTCTACATTGA>YBR213W	+:0:chr02:650363:651187ATGGTCAAATCGCTACAGCTAGCCCATCAATTAAAAGACAAGAAAATACTACTAATCGGAGGCGGAGAAGTCGGCTTGACAAGGTTATATAAGCTGATACCTACAGGTTGCAAGCTGACTTTAGTATCTCCTGACCTACACAAATCCATTATTCCGAAGTTTGGAAAATTCATCCAGAACGAGGATCAGCCCGACTACAGAGAAGACGCTAAACGCTTTATCAATCCGAACTGGGACCCCACGAAGAATGAAATTTACGAGTACATCCGCAGTGACTTCAAAGACGAATACCTCGACCTAGAAGACGAGAACGACGCGTGGTACATAATAATGACCTGTATACCCGACCACCCTGAAAGCGCAAGAATATATCACCTTTGTAAGGAAAGATTCGGTAAGCAGCAGCTGGTCAATGTGGCGGACAAACCTGATCTCTGTGACTTTTATTTCGGCGCCAACCTGGAGATAGGAGACCGTCTACAGATTCTGATCTCTACCAACGGCCTTTCTCCGCGCTTTGGCGCTCTAGTAAGAGATGAGATCCGTAACTTATTCACACAAATGGGGGATCTGGCGCTGGAGGACGCTGTCGTCAAACTAGGTGAGCTGAGAAGAGGAATTAGGCTGCTGGCACCAGACGACAAGGATGTCAAGTACCGCATGGATTGGGCCAGACGCTGCACAGACCTCTTCGGCATTCAGCACTGCCACAACATCGACGTAAAACGTCTGCTGGATCTTTTCAAGGTCATGTTTCAAGAACAGAACTGTTCCTTGCAGTTCCCTCCCAGAGAACGGTTGCTTAGCGAGTACTGCTCGTCTTGA>YBR214W	+:0:chr02:651410:652993ATGGCTTCTACTTCGAACACGTTCCCTCCAAGCCAAAGCAATTCTTCCAACAACCTTCCAACTTCTAGACATGCATCCATTGTGGAGATGCTGTCTACTCCGCCATTGCTGCCCCACGTTCAAGTGAACGATACAGACGACAAGGAACAACCAGAAGAGTCCACGCCGCCAACAGCAACAGCGGCAGCGCCTGGTCCAGGTTGTGCTGCTACACCCGCCCCCTTGCGCGATGAAAAGCCTCAATTCAAACTATCAGCTGTTCCTATGACCCAGACGCCATCGCAGTGTCTGTCATGTGTGCACGCCCAAAAATGGCAACACATACCGTTGTCGCAATTGATCGAACAGAATAAGCTTATTTTTGTTCCAGGTTCCATATCGGTAGAGGAGGCATTCAACACTTTGATCAAGTATCATCTAAACTCTATTCCCGTGGAATCTTTCCCCGGCGACATGAACTGTTTTACTTTCGACTACAATGACCTTAATTCGTACCTTTTGCTGGTTTTGAATAAAATCACCGTCAGCAACAAACAGCTTACTGCAGATTGCCAGAATGGAAAACCCGTACCAGTGGGCGAAATGGTGAAACTAACTCCTAAGAATCCGTTCTATAAGCTGCCGGAGAATGAGAGTTTGTCCACGGTGATGGGGATTCTCGGTTCGGGTGTCCATCGTGTAGCGATAACAAACGAAGAGATGACCAAAGTTAAAGGTATTTTGTCCCAACGTCGTTTGATAAAGTATCTTTGGGACAACGCTAGATCTTTTACAAGCTTAGAACCTCTATTAAACTCCTCATTACAAGATTTGCACATCGGTGTTCTCAATATTCAATCTAAGCCAACTTCAAGACAATCCCGTGTCATCTCTATTCAAGGCGAGGAGCCCTTAATCATGGGCCTTTATAAAATGCATGTGGAAAGAATTTCTTCCATTGCAGTCATCGACAAACAAGGTAATTTGCTAGGTAACATATCGGTAACGGACGTAAAACATGTCACAAGAACCTCCCAATACCCCTTACTACATAAGACATGTCGCCATTTCATTTCTGTCATTTTAAACTCCAGAGGATTAGAAACGGGTAAAGATTCTTTCCCCATTTTTCACGTATACCCCAGTAGTTCACTAGCAAGGACTCTTGCCAAGTTAGTTGCTACAAAGTCTCATAGACTATGGATCGTACAACCGCCGGAGTCGTCAACCTCGGCGTCATCCACAAATTTAACAGCTGCTAATACTGCAGCCAATGCAGTTTCTGCCACCGCTCAATCGTCTGCTAATGGTGCGACTCCAATGTCAAAGTCATCCTCTTCAACATCCCTCAACTCCCACTCTCCGTTGATGACAGCAATGGAAGACCCACCATCCCCACGTTCTTCGGCCATCGCGATCCCACCACCAAGTCCCGCTTCCTCAACAAACACACCAAACCTATTCGAAAAGGAATATAGAACGGGTAAGTTGATCGGTGTGGTCTCATTGACAGACATCATCAACTTATTAGCAAGGAAACAAACAGGGAATAAAGAAGTGGATCCGCAATCCGCAAGAAGACAGAGAGGCAGTATCGCCATGTGA>YBR217W	+:0:chr02:657827:658387ATGAGTAGGATCCTAGAGAGCGAAAATGAAACAGAAAGTGACGAAAGCTCCATCATATCCACAAATAATGGAACGGCAATGGAAAGATCCAGAAATAATCAAGAATTAAGATCATCTCCTCATACCGTTCAAAATAGATTGGAACTTTTTAGCAGGAGATTGTCTCAGCTTGGTTTGGCGAGTGACATTTCTGTCGACCAGCAAGTTGAAGATTCCTCTAGTGGCACTTATGAACAGGAAGAGACAATCAAAACGAATGCACAAACAAGCAAACAAAAAAGCCATAAAGACGAAAAAAACATACAAAAGATACAGATAAAATTTCAGCCCATTGGTTCTATTGGGCAGTTAAAACCATCTGTTTGTAAAATATCAATGTCACAGTCTTTTGCAATGGTTATTTTATTTCTTAAGAGACGGCTGAAAATGGACCATGTTTATTGTTATATAAATAATTCGTTTGCGCCAAGTCCGCAGCAAAATATTGGTGAACTTTGGATGCAATTCAAGACTAATGATGAGCTTATTGTAAGTTATTGTGCATCCGTAGCGTTTGGTTAA>YBR220C	-:0:chr02:662990:664672ATGGAACCTAAGCGAAAGAGCGGGTCACTAGCCAAGCATGATTTGCCGCAATTTTATCTTTTAATTATGTTATATTTGGCTCAAGGCATACCTGTAGGATTGGCCTTCGGTACCGTACCGTTTCTACTGAAATCTTTAGCAAAGGAGACCTCGTTTACATCACTGGGAATTTTCTCTATGGCTACATATCCATATTCTTTAAAGATCATATGGTCACCAATAGTAGACTCACTGTACAACAAGCGCATCGGTAGAAGAAGATCATGGATCATTCCAGTACAATTTGTTAGTGGATTTGTGCTATGGGCATTAGGGTGGTGCATATCACAAGGCATAATCTTCGATGGTGTCGACGATGCGTTCCATAATCGCGGTAATGGCACTTTACACAGTGTCAGTATAAAAAATTTGACGTGGTGGTTTGGCCTGTTAGTTTTTCTGTGTGCCACTCAAGACATCGCAGTTGATGGTTGGGCGTTGACGATTTTGTCCAAAGAATCCCTATCATATGCATCTACCGCGCAAACAATAGGTTTGAATATTGGTTATTTTATGTCATTTACCATTTTCCTGTCGTTGAATTCCTCTGATTTCGCCAATAAGTATTTCAGAAACATCCCACTGGATCACGGGTTCATTAGTCTTGGTGGGTACATGAAATTCTCGGGCATGCTTTACATTGTAATAACCATATATATCATCTTTTGCACCAAGGAAAAACCCTACGTAGAGTATTTGCCCAAAGTGGAGCCCATAAATACAAGTGACGGAGGGTCAAAGCCGATAAGTATTGAGTATGACGACGGTGATGTGGTGTCAACTCAGAATACAAGCAGTATAAAGTACATTTACCGCTGCTTTATAAAAGTGTTGAAATTGAAGTCCGTAAGAAGCCTAGCCTTCATTCACATGATTTCGAAATTTGCCTTTCAATGCAACGAAGCCGCCACAAACCTGAAACTACTAGAGCAAGGCTTCAAAAGAGAAGACTTGGCTGTGACAGTACTCATAGACCTGCCGTTCGAAATCATATTTGGGTACTATGTTGTTAAATGGAGCTCCGACAAGGACCCCATGATTCGTGACAATAGAAGATTAAGAAACAGCACGGGCACCAACAAGGTCATCAAGTTCTTAGTTGGGGATGCCGGCGTTTTAACACCATGGTTGTGGGGCTTTTTGGGCCGTCTGGCAGCCGCGGTCTTGGGAAGTTACGTGGTGAAGCAATTCCCCAAGGATGGTGAAATATCCACGGGTTATTTTTGTCTCGTGATATTCCAGCACCTCTTAGGTTCCTTCATGAATACTGTCCAGTTCATTGGAATATCGGCCTTCCATACAAGAGTTGCAGACCCCGTGCTGGGTGGCACATATATGACATTGTTAAATACCCTCAGCAACTTCGGTGGGACATGGCCGCGGTTAATCATTATGTCCATGATCAACTACTTCACCGTGTATCAGTGCACTATTCCTGGCACAAATAAAGTATACGTAACTCACGGCGGCAGCATGCAAGCGTGCACCGAGCTTTTGAATGGCACCGTGACCATCCTGCGTGACGGCTATTACATCACCAATCTCATATGTATTGTAGTCGGACTTTTCCTATATTTTGGATATTTGAAAAGGAAAATCCTCCATTTACAAAGTCTGCCAATCAGTTCCTGGAGATGTACGTAA>YBR221C	-:0:chr02:665148:666248ATGTTTTCCAGACTGCCAACATCATTGGCCAGAAATGTTGCACGTCGTGCCCCAACTTCTTTTGTAAGACCCTCTGCAGCAGCAGCAGCATTGAGATTCTCATCAACAAAGACGATGACCGTCAGAGAGGCCTTGAATAGTGCCATGGCGGAAGAATTGGACCGTGATGATGATGTCTTCCTTATTGGTGAAGAAGTTGCACAATATAACGGGGCTTATAAGGTGTCAAAGGGTTTATTGGACAGGTTCGGTGAACGTCGTGTGGTTGACACACCTATTACCGAATACGGGTTCACAGGTTTGGCCGTTGGTGCCGCTTTGAAGGGTTTGAAGCCAATTGTAGAGTTTATGTCGTTCAATTTCTCTATGCAAGCTATCGATCATGTTGTCAATTCCGCTGCAAAGACTCACTACATGTCTGGTGGTACTCAAAAATGTCAAATGGTCTTCAGAGGTCCTAATGGTGCTGCAGTGGGTGTTGGTGCTCAACATTCACAGGACTTTTCTCCTTGGTACGGTTCCATTCCAGGGTTAAAGGTCCTTGTCCCTTATTCTGCTGAAGATGCTAGGGGTTTGTTAAAGGCCGCCATCAGAGATCCAAACCCTGTTGTATTTTTAGAGAACGAATTGTTGTACGGTGAATCTTTTGAAATCTCAGAAGAAGCTTTATCCCCTGAGTTCACCTTGCCATACAAGGCTAAGATCGAAAGAGAAGGTACCGATATTTCCATTGTTACGTACACAAGAAACGTTCAGTTTTCTTTGGAAGCCGCTGAAATTCTACAAAAGAAATATGGTGTCTCTGCAGAAGTTATCAACTTGCGTTCTATTAGACCTTTAGATACTGAAGCTATCATCAAAACTGTCAAGAAGACAAACCACTTGATTACTGTTGAATCCACTTTCCCATCATTTGGTGTTGGTGCTGAAATTGTCGCCCAAGTTATGGAGTCTGAAGCCTTTGATTACTTGGATGCTCCAATCCAAAGAGTTACTGGTGCCGATGTTCCAACACCTTACGCTAAAGAATTAGAAGATTTCGCTTTCCCTGATACTCCAACCATCGTTAAAGCTGTCAAAGAAGTCTTGTCAATTGAATAA>YBR223C	-:0:chr02:668658:670292ATGTCACGAGAAACAAATTTCAATGGAACTAAGAGGAAGAGGTCGGATGTTGCCGAGAAAGTAGCACAACGGTGGAAGAGCGTCAGGTATAGTGCTGAAATGGAGAATATGGCTCCGGTCAACAGTAACAATGATAGCGACGACTGCGTCATAGTCAGTGAATCGAAAATCATTGATTTGACTAATCAGGAACAAGATTTGAGTGAGAGAATAGAAACAAACGATACGGCAAAAGGTGCCGTTTTTAAACTAATGAAATCGGACTTCTATGAAAGAGAGGATTTTATGGGAGAAGTAGAGGATATGATTACATTGAAAGATATCTTTGGCACTGAGACACTAAAAAGAAGCATACTCTTCAGTTTCCAATACGAACTTGATTTCTTGTTGAGACAATTCCACCAGAACGTAGAGAACATAACCATCGTCGGCCAAAAGGGTACTATTATGCCTATCGAAGCCCGTGCTATGGACGCGACACTGGCAGTAATATTAAAAAAGGTCAAACTTATTGAAATAACGATGCCCCCATTCGCTTCCCACCATACGAAGCTGATCATAAACTTTTACGATAATGGCGAATGCAAAATATTCTTGCCATCTAACAATTTTACGTCAATGGAGACTAACCTGCCTCAACAGGTGTGTTGGTGCAGTCCCCTTTTGAAAATAGGTAAAGAAGGGCTACCAGTACCGTTTAAGAGAAGCTTGATAGAATACCTCAATTCGTACCACCTGAAAGACATTGACGAATTGATTACAAAAAGCGTAGAGGAAGTTAACTTTGCTCCTTTGAGCGAATTAGAATTTGTATATTCTACGCCCTCCAAATTTCAGTCGTCGGGTTTACTGTCGTTTTACAATAAACTAGAAAAACTTTCTGCTGGCACAAGTGCTAGTGATACTGCAAAACATTATCTATGTCAAACTTCATCCATAGGGACATCTCTATCAAGAGCGCGAGACGAAAACTTATGGACACATCTAATGATTCCTCTGTTTACCGGAATCATGTCCCCTCCAGCAAAGGACACCGCTGGGAGGAAGAAAGCAGAAATACTGCCAACGAATTCATTGATTAATGAATATTCGCAGAGAAAAATCAAGCCGTACATTATTTTCCCCACCGAACAAGAGTTTGTCACCAGTCCCTTAAAGTGGTCCAGTTCCGGGTGGTTTCATTTTCAATATCTTCAGAAAAAGAGCTACTACGAAATGCTGCGAAACAAGTTCAAAGTATTTTACAAGCAAGACCCTGCTATGGTTACTAGAAGACGAGGGACGACGCCCGCGCACTCTAAGTTTTACATGCATTGTGCAACAAACTCCGCAGGGCCCTGTGATGCATCGCAGGTATTTAAAGAACTAGAATGGTGCCTTTATACTTCGGCAAACCTCAGCCAAACAGCATGGGGCACCGTTTCAAGAAAACCACGCAATTATGAAGCAGGAGTGCTTTACCATAGTCGCAGGTTAGCAAATACCAGGAAGGTCACGTGCCGTACTTTTACACGTGACCGTAGAGGCTGCGCGGGTAATCCCACCCATGTGGCCGTGCCATTCACGCTGCCAGTCATACCATACGACTTAGCTGAGGACGAGTGCTTTTGCCTTGCTCGTCATGAGAACGACTAA>YBR227C	-:0:chr02:673567:675129ATGTTGAAATCTGCAAGCCAAAACTTTTTTAGAGCTTATTCTAGTAGAATTGGTCGATATGCTGCTACAGCATCAGGGAAACTGGCACAGTCTCGTTTATCGAATATACCCACTCCAAAAGCATTGAAGAAATTCCTCGATGAGTACATTGTTGGTCAAGAGATTGGTAAGAAGGTTTTAAGCGTGGCAGTTTATAATCACTATTTAAGAATCAATGACAAGCAAAAGAAAGGGGAATTACAAAGGCAGAGAGAATTGATGGAACGAGAGAAAATCGCGGATGATAGGGATGAACCAATTTTCAGTGGAAACAGCGAATCAAAGGCTGGTTGGAGGAATCTTCAAAGACAGTTTAATTTAGCAGGTAGAGAGGTTGACGAAGATTTAGAGTTAAGCAAAAGTAATGTCCTTGTCGTTGGTCCCTCAGGTTCTGGTAAGACTTTGTTGGCAACAACATTGGCGAAAATATTAAACGTTCCAATTGCAATTACAGATTGTACACAATTGACTCAAGCAGGTTACATAGGTGAAGATGTTGAAGTATGCATTGAAAGATTGCTCGTAAATGCTGAATTTGATGTTGCGAGAGCAGAAAAAGGTATTATTGTTCTTGATGAGATTGATAAACTAGCCAAGCCTGCCGCAAGCATTGGCACTAAAGACGTGTCCGGAGAAGGGGTTCAACAGTCATTATTGAAAATCATTGAGGGCCATAAAGTGGAAATTACCGTTAAAAGGCCTGTTAAGCATGATATTGACGGACAAAAAAATCAAACTACAACAAAGAAGGACGAAGTATTTGTTGTAGACACTTCAAATATTCTTTTCATGATTATGGGAGCTTTTGTTGGTTTAGATAAGCATATTGTTAAAAGGATAGAAGATATGAAGAAAATACAGAAGGCAGGAGAGTCTGTTGAATCATCGAATAGTAAAGAAGTTGAAAAAGAAAGAGCGAAGAAGTTCAGATTTAGCAATACGCTTGAGCAGGTTGAATTAGATAACGGAAAAAAAGTGTGTGCTTTAGACTTGACCACCCCCACAGATTTAGTTAGCTTTGGTCTAATTCCTGAATTAATTGGCAGAGTCCCAATTATAACTGCTCTGCAGCCTCTACAAAGAGACGATTTATTTCATATATTAAAAGAACCTAAAAATGCCTTATTGGATCAATATGAATATATTTTTAAGCAATTTGGGGTAAGATTGTGTGTTACACAAAAGGCGCTAAAGAAAGTTGCTCAATTTGCTCTCAAGGAAGGAACTGGCGCCAGAGGGTTGAGAGGGATAATGGAAAGACTTCTGCTAAACGTTAATTATGATTGCCCGGGTTCTAACATAGCATACGTTTTAATCGACGAAGCTACTGTCGACTCGTTACAGGAAACAGAGCACTCTTTGGCTTCCCAAGTGGACGTAAAATACTATTCTGGTGATGAAAAGGACAGTTTAATTCGTGACGTTTCTGAAGAAGATAAAAAATTAGGAGTTATGCTAGAAAAAGAGTTGGGTCATTCTGCTAACATTCATACCCCAACAATTCCCAAGAGAAGCTTAACATAA>YBR228W	+:0:chr02:675308:676222ATGTCGCAGAAAATACAGCAGCATCAGTTTCCTGATTTTTATTGTTGTTATCTACTACAATCGATTAACAAGAGGCAATCATTTTACGTTGGATCAACTCCTAACCCAGTACGACGTTTGAGGCAGCATAATGGGAAATTGGCTGTTGGAGGGGCATATCGAACCAAACGTGACGGTTCAAGGCCCTGGGAAATGATAATGATTGTGCGGGGCTTCCCAAGTAAGATAGCAGCTTTACAGTTTGAACATGCATGGCAACATGGGTACCAAACGCATTATATTGCCGAAAAAGATCGTGTTGTAAAGCATAAAGCAGGTGGAAGAACTTTACATCACAAAGTTGCATTAATGAAGTTACTTTTAAAGCATGAATTTTTTCAAAGGATGAACTTGATAGTCGAGGTTTTTAATATAAAGGCTTGGGAGGTTTGGAAACAGGACAAATTTTTTATTGAAAGAGACAGATTTCCGATTAATATACAAATAAATGAAAATGCGCTTGAAGAACCAAAGGAGAAAACTGTGGATGTTCTCATGGATCATAGTGATGAGAATTTAAAAGTGGTGGAAGCTGTATACACTAAAGTCATTGAAAATGAAAGAAACATATTCGAAACTTTCGAAAAGAAACTAACTACAGGGGTAGTGCGATGTGAAATTTGTGAGAAAGAAATTGACTACACGTCTGAAGAGCAAAATCTGAAGCCGTTTGTAGCGCTTTGCAATAATAAAGACTGTGGGTGTGTTAACCACTTGAAATGTCTTCACAGGTACTTTCTAGATGATGAACAGTTGATGGTAGGGAGAAGAAATTTGATACCTCGAGGTGGGAAATGTCCGAAATGTGATATGTTTTGCGACTGGACGACCCTAGTCAAGTTTTCGACAAGAATGAAGTTAGCTCACGGGAAGTAG>YBR230C	-:2:chr02:679544:679937TGCTAAACACGACAGTAACGCTAGCCCAAATTCTGACTCTGAAGACGGTCACCACCACAACAATAAGAAGGAATGCGCTATCGAATATCTGAAGGCACGGTTAAATAGTGCATCAGCAGTCGCTTGCGGCTACCTCCAAGCTTTTGTCAGTAAGACGCAAGACTTTGCCAAAGTATGCTTTTTAGAACTTCAGAATCCCGTTGTCTTGGTCAACTTGTTGCTGCATTCTTCAGTGGTATGTTATTTATGTAACGGGTATGCGAACCACAACGCCAGATTCTTGAAGGGGAAACCTAACTCTACAGTCTTAGCAACAACCGCCGGCGCTCTGGGTCTTTTGACGCTGGACGGTATAATTTCAAAGAAATACTACTCCAGATACGACAAGAAATAA>YBR230C	-:0:chr02:680035:680045ATGTCTGCAAC>YBR231C	-:0:chr02:682174:683085ATGCCAGAAGTAGAGACAAAGATTATACCAAACGAAAAGGAGGACGAGGACGAGGACGGCTACATAGAAGAGGAGGACGAGGACTTCCAGCCTGAAAAAGATAAATTAGGTGGTGGTAGCGACGATAGTGATGCCAGCGATGGCGGTGACGACTATGATGACGGTGTGAACAGAGATAAGGGTAGAAATAAAGTGGACTATTCGCGTATAGAGAGCGAGAGCGGCGGACTGATCAAGACTAGAAGAGCTCGGCAAGCTGAGGAAGAATATGCGAAAACACATAAGTATGAATCGTTAACTGTTGAATCGATCCCTGCAAAAGTAAACAGCATCTGGGAGGAGCTACAGGAAGCTAGTAAGAACCGTCTTTTGAGCAGTTCAGGGAAAGTCGGCTCTGTTCTCGACGGTTCCAAGGAGGCTAGGTCGACAACGGCCGCGCAACAGGAGGACAAAATCCTCATCGAAAGAAACTACAAGTTTGCTGGTGAAACTGTCCATGAGAAAAAATGGGTTTCACGCAGCAGTGCGGAGGGTCAAGAATATCTAAACAGTTTGAAATTTAAGCAGCAGGCGCCTGCTGCTCCTGTTCAACTGGAAAAAGCAGTTAGGACCAAGTCCAATGAAAGCCGGCAACACTTGCGACGGCCACTGAAAAGGCCTCCATTGTTGGAGCAGATCATTTCTGGCGGGCTACGACCCAAGCTAACGACATTAGAAAAATCTCAGCTGGACTGGGCTAGTTATGTAGACCGTGCTGGACTTAACGACGAATTGGTGCTGCACAACAAGGATGGGTTCTTGGCCCGGCAAGAATTTCTGCAGCGGGTCGGGTCTGCAGAGGACGAGAGATACAAAGAATTGCGCCGGCAACAACTTGCTCAGCAGTTGCAGCAGGATAGCGAAGCTTCATAG>YBR233W	+:0:chr02:683423:684664ATGTCCACAGAGACTACAAAACCATCAATCACTACTACACCAACTACTGTACTCGTTTCTCCCAACACGCTCAAAAGGAAAAAGGGTGAAGACACCAGCGAGGAACAACTGGAAGCAGAAATCAAACGCGTGGCCCTGAAGGATGCAGACAGCCACTCTGACAACGACCACGATTCCCCCGACAATGTTCCCTCTGATGTACATTTACGAATGCTGTGCTTAGTCAAACACGCTTCACTGATAGTAGGACACAAGGGCGCCACTATATCTAGAATAAAATCCGAAACCTCTGCTAGAATAAACATATCAAATAATATCAGAGGTGTTCCTGAAAGAATTGTTTACGTGAGGGGTACTTGCGATGATGTAGCCAAGGCATACGGCATGATAGTAAGAGCGCTGCTTGAAGAACATGGTAACGAAGATAACGGGGAAGACATTGAGATTTCTATCAACCTATTGATCCCTCATCATCTTATGGGATGTATAATTGGCAAACGTGGGTCTCGGTTAAGAGAAATTGAGGATTTGAGTGCAGCAAAATTATTCGCGTCCCCGAATCAATTGCTGCTTTCTAACGATAGAATTTTAACCATTAACGGGGTTCCGGATGCTATCCATATTGCTACTTTCTACATTAGTCAAACATTGCTAAACTTTCAGATGGAATCTCCGCAGAAAAATGTGAAAAGGTCCATATACTATCAGCCAACTCAGTTTAACTCAGTATTAATAGACCATAGCCAACCGAACACCATTTTCCATCAAAGAAACCACCAATATCATCCAAGCGATAAACTACTTTCATACAAACCCAACAAAAATTTACCAATATCATCTACTTTGTTGAGTATGGCCACACCGCAGTATACTACTGCTAGCGTTGCAAACGCAACGGCCTTTCAACCGAACTTCGTTATCCCTAATGTGACAGTCTTAGACGGCCCTGTTATCAGTCCGGCACCCGGCAACCATTTATTAATGAACTTTGTACAACAAGAGATATTCATAGATGAGAAATTTGTCGGAAATGTCATTGGCAAAGATGGCAAGCACATAAATTCGGTCAAGGAATCCACAGGTTGTTCAATTATTATACAAGATCCTGTAGAAGGCTCTTCGGAGAGAAGACTTACCATAAGAGGTACGTTCATGGCGTCCCAAGCTGCTATTATGTTGATTAGCAATAAAATTGAAATTGATAGATCAAATGCTGAACGTAAAAGAAGGTCGCCCCTCTAA>YBR233W-A	+:0:chr02:684972:685256ATGGAACACAATCTTTCACCTTTACAGCAAGAAGTATTGGACAAATACAAACAGCTTTCTTTAGATCTAAAAGCTCTGGATGAAACTATAAAAGAACTAAACTATTCGCAACACAGGCAACAGCATTCACAGCAAGAAACTGTATCGCCAGATGAGATACTACAGGAGATGAGGGACATAGAGGTCAAAATTGGTTTAGTTGGGACACTGCTAAAAGGTAGCGTGTACTCGCTTATCTTGCAAAGAAAGCAAGAACAGGAGTCTTTGGGTAGCAATTCTAAATAA>YBR236C	-:0:chr02:690378:691688ATGTCAACCAAACCAGAGAAGCCAATATGGATGTCACAAGAAGACTATGACCGTCAGTACGGCTCAATTACTGGTGATGAATCGTCCACAGTCTCAAAAAAAGACTCCAAAGTTACTGCCAATGCGCCTGGTGATGGTAATGGATCATTACCAGTTTTGCAAAGCAGCTCTATCCTTACTTCTAAGGTGTCTGATCTACCCATTGAGGCAGAATCCGGGTTTAAAATCCAAAAGAGAAGACATGAAAGGTATGATCAAGAGGAAAGATTACGCAAACAGCGTGCTCAAAAATTGAGAGAAGAGCAACTAAAGAGACATGAAATAGAGATGACTGCAAATAGATCCATAAATGTTGACCAGATTGTCCGAGAGCACTATAATGAACGTACAATAATCGCCAATCGCGCTAAGAGGAATCTATCGCCTATTATTAAGCTTCGTAATTTTAACAATGCGATTAAGTATATGTTAATTGACAAATATACAAAACCTGGAGATGTCGTTTTAGAACTCGGATGTGGCAAAGGTGGTGATTTAAGAAAATATGGTGCTGCAGGTATTTCACAGTTTATTGGTATTGACATTTCCAATGCCTCTATCCAAGAAGCTCATAAGAGATACCGGTCCATGAGAAATTTGGACTACCAAGTTGTGCTAATTACTGGGGATTGCTTTGGCGAATCATTAGGTGTTGCAGTAGAGCCCTTCCCAGACTGTAGATTTCCGTGTGATATCGTTTCGACGCAATTTTGCTTGCATTATGCTTTTGAGACCGAAGAAAAAGCAAGGAGAGCTTTACTGAATGTCGCCAAATCACTCAAAATTGGTGGTCACTTTTTTGGCACGATACCAGATTCAGAATTCATTCGTTATAAACTGAATAAATTTCCCAAAGAAGTGGAAAAGCCATCTTGGGGAAACTCCATTTATAAAGTGACATTTGAGAATAATTCATACCAAAAGAATGATTATGAGTTCACATCGCCTTATGGTCAAATGTATACTTATTGGCTAGAAGATGCTATAGATAATGTTCCTGAATATGTTGTGCCATTCGAAACCTTAAGAAGTTTGGCTGATGAATACGGTTTGGAACTAGTATCGCAAATGCCTTTTAATAAATTTTTTGTCCAAGAAATTCCCAAATGGATTGAGAGGTTTTCGCCTAAAATGAGGGAAGGTTTACAAAGATCTGATGGTAGATATGGTGTTGAAGGGGATGAAAAGGAAGCGGCATCATATTTCTATACCATGTTTGCATTCCGTAAGGTAAAACAGTATATCGAACCGGAAAGCGTAAAGCCCAACTGA>YBR237W	+:0:chr02:691964:694513ATGGAAACTATTGATTCGAAGCAAAATATTAATAGGGAGTCTTTATTGGAGGAAAGGAGGAAAAAACTAGCAAAATGGAAACAAAAAAAAGCACAATTTGATGCTCAGAAAGAGCATCAAACTTCACGCAATGACATTGTTACTAACAGTTTAGAAGGTAAGCAGACTACTGAAAAGTTTACGGAAAGACAGGAACGTGTAAAAGAAGAGCTTCGGAAGAGAAAAAATGAATTTAGGAAATCTGACGAACCTGTATCGGTTAAGCCTTCGAAGAAAAAGTCGAAGAGAAGTAAGGTAAAGAAGAAAATATCCTTCGACTTCAGTGATGACGATGATTCTGAAATAGGAGTTTCTTTTAGATCGAAGGAGCACATTCAAAAAGCTCCAGAGCATGATAATGAAAAAGATCCACTGGATGAGTTTATGACGTCATTGAAGGAGGAGAAAATGAGTAATAGTAAAGGCATGTACGACAGAGGCGACATTCTTGATGTAGAGGATCAATTGTTTGAACTTGGAGGAACCGACGATGAGGATGTTGAAGATAATACGGATAATTCTAATATAGCGAAAATTGCTAAACTCAAAGCAAAAAAACGTGTAAAGCAAATTTATTACTCTCCAGAAGAGCTTGAGCCGTTCCAAAAAAATTTTTATATAGAATCTGAAACAGTTTCCTCGATGTCAGAGATGGAAGTTGAGGAGCTTAGACTTAGTCTGGATAATATCAAAATAAAGGGAACAGGTTGCCCCAAACCAGTCACAAAGTGGTCTCAACTGGGACTATCAACGGACACCATGGTTTTAATTACAGAAAAGTTGCACTTTGGCTCCTTAACGCCTATTCAATCCCAGGCTCTTCCCGCTATTATGTCAGGTCGTGATGTTATAGGAATATCAAAAACTGGCTCCGGCAAGACCATCTCCTATCTTTTACCATTGCTAAGACAAGTAAAAGCTCAACGGCCATTATCAAAACATGAAACAGGGCCCATGGGTTTAATTCTGGCTCCAACTAGAGAGCTAGCTTTACAAATACATGAAGAAGTTACTAAATTCACAGAAGCAGATACATCCATTAGATCAGTATGCTGTACAGGAGGTTCTGAAATGAAAAAGCAGATTACTGATCTTAAAAGAGGCACTGAGATTGTTGTTGCCACACCGGGACGATTTATTGATATATTAACACTAAATGATGGGAAATTACTTAGTACTAAAAGAATAACGTTCGTAGTAATGGATGAGGCAGACAGGCTGTTCGATTTAGGTTTTGAACCTCAAATAACGCAAATCATGAAAACTGTTCGACCGGATAAACAGTGTGTTCTATTTAGTGCAACTTTTCCGAACAAACTACGCAGTTTTGCCGTAAGAGTTTTGCATTCCCCAATATCTATTACGATTAATTCAAAGGGAATGGTTAATGAAAACGTAAAACAAAAGTTTAGAATATGCCATTCAGAAGATGAAAAATTTGACAATCTGGTGCAGCTTATCCATGAGCGCAGTGAATTTTTTGACGAGGTTCAAAGTGAAAATGACGGACAGTCAAGCGATGTTGAAGAAGTTGATGCTAAAGCCATTATATTTGTATCAAGTCAGAATATTTGCGACTTCATTTCAAAAAAGCTGTTAAATGCTGGAATTGTGACCTGTGCTATTCATGCAGGTAAGCCATATCAAGAAAGGCTTATGAACTTAGAAAAGTTCAAACGAGAAAAGAACAGTATTCTTCTCTGTACAGAGGTTCTCTCAAGAGGTTTAAATGTTCCCGAAGTGTCGTTGGTTATTATCTATAACGCCGTCAAAACTTTCGCGCAATATGTTCATACTACTGGAAGAACTGCCAGAGGTAGTCGCTCCGGTACTGCTATTACTCTTCTATTGCATGATGAACTGTCAGGTGCTTATATCTTGAGCAAGGCAATGCGCGATGAAGAAATAAAAGCTTTAGACCCTCTGCAAGCAAAAGAACTTCAGGAAATGAGTGCCAAGTTTGAATCCGGTATGAAAAAGGGTAAGTTTAGGTTGTCGAAAGGGTTCGGCGGCAAAGGACTGGAAAATATCAAAAGTAAAAGAGAGGAAGCTCAAAATAAAGATTTGGAGCTCAAAAAGAATGATAAGAGGAGCGATGACTTGGAAAAGAAAATCAGCAATCCACGCGAAGGGCATGACAGTGTGTCTGAGTCTTCTGCGCTTATTCCGAGACTCAATTATGAACTATTCAAAGAGTCTACAGACGGGTCGATCATCTTTTACGCCAAGGTTTATATTAATGATTTACCCCAGATTGTAAGATGGGAGGCTACAAAGAATACAACCTTATTATTTATCAAGCATGAAACCGGATGTAGTATTACTAACAAGGGTAAATTTTACCCTGAAGGGAAGGAACCAAAGAATGAAAACGATGAGCCTAAACTATACTTATTGATCGAAGGCCAAGATGAGAAAGACATACAATTAAGTATAGAATTGCTGGAGCAAAAAGTTAAAGAGGGGGTCGTAAAGGCTGCAAGCTTGTCTTTGAAGAGTACTAAATACTAG>YBR239C	-:0:chr02:698349:699938ATGTGCACTCCAGATGAAAACGATTACAAAACATCAACTGACCCAGATACATCTGCAAATACAAATCATACTCTCGAAAAAAAGAAGAGAAAAAAGAGAAAAAACACAAACGTGGCATGCGTGAACTGTTCGAGATTGCATGTGTCATGTGAGGCCAAAAGGCCATGCTTACGATGTATCAGCAAGGGCCTCACGGCTACATGCGTTGACGCTCCAAGAAAGAAAAGCAAGTACTTGGCTGGAATACCGAATAGAGAGCTCCCAATGAACATACAACCGGACCTTCCACCCCGCAAGATCATGATACCCATTTATAACAACTCCAGCAATAGTAGCCTCAACGTAAATAACATGGGAGAACAGCAAAAATTCACCAGCCCACAACATATAGTTCACAAAGCTAAATTCCTATCGAATGCTGCAGATTCCGAGTACTCTATTCTGTCGAATATCATCTATCAAGACACATTGTCTAACAAGATCCCTATTGACATCCTGTACTCCAACACCAATTCCACATCCAACTCCACAATAGGCAATAGCAGTAACAATAGCCCTACGGGCACAAATACAAGCCCTGAAGAAACAGAAATGGAGAAAATTCGCCAGTTGTACAGCGAACAGCGAGCAAACATACCCCCACATCCGTATCCATCTTCCAATCAAAACGTTTATTCTATTCTACTGGGACCAAATTCAGCCAAGATTGTAGCTTCGCAAGTTAATCTTTTCGCGAACCATTTCCCCTTAGTGCCCGTTGATTCAGCTGATAATTCCTTAAACTTTAAAAGATTATTGCCACGGGACCCATCTGAAAAGAGCTCCCAAATTAATTGGGACTCAAGCATAAACCAATATTACTTGAACAGCGAAACGGTAACATTTCCCGAGTTGGCTATCCCCTTAAAGCGGAGGAAAAACCATTTGGTCTCTGTATCCCTGGAAAGCTGTTCACCAGACGCTGCCAACATCAAGAGCAATGTAGAATGGGAACATTCATTACGTTATTCTACTCCAATGGAAATATACACCTCCATAAATGCCCCCTTTTCGCATACACCTGGATTCCACCATCTTTTGGTGTACCTAAAGCATCGATTCAACCAGCAGGATCTGGTGAAGATGTGTCGGTCCATTGCCGAATTCCGGCCCATTTTCATTGCGTGTTCTGTAACTTTAACCGAGGAGGACATGATTTTTATGGAGCAATGCTACCAAAGAACTCTATTAGAATATGTAAAATTTATTGCACAAATCGGTACCCCGACATGTATTTGGCGAAGAAATGGTCAAATTTCTTATGTCAACGAGGAATTTGAAATTCTATGCGGATGGACAAGAGAGGAGCTACTAAACAAGATGACTTTTATCGTAGAGATCATGGACGATGAAAGTGTACGCGATTACTTCAAAACGCTCTCTAAAGTTGCATATAGGGACTTCAGAGGATCTGAGAAGATGAAAGTTTGCAGGCTCTTAAGCCCCATCAAGGGCAAGATAATTCATTGTTGCTGTATGTGGACATTAAAAAGAGATGTCTCTGGTTTGCCCCTCATGATCTTGGGTAACTTCATGCCCATACTCAATTGA>YBR240C	-:0:chr02:700485:701837ATGGTCAATAGTAAGAGGCAGCAGAGAAGCAAGAAAGTAGCGTCATCCTCCAAAGTGCCCCCCACCAAGGGGAGGACATTTACTGGGTGCTGGGCATGCAGATTCAAGAAACGCAGATGCGACGAGAATAGACCAATCTGTTCACTGTGTGCCAAACATGGAGATAATTGTAGTTACGATATCAGACTTATGTGGTTAGAGGAGAACATCTACAAAGTACGCAAGCATTCACTGATCAGTTCATTACAGGCTCGCAAATCGAAATCGAAACCATTGTGCCAGAAAATCTCAAAATCAAGGTTTAAACAAATGACCCATTTTAGACAACTATCACCCCCGACAAGTGACTGCGAAGACAGCGTGCACGAGGCAAGTAAGGAAACTACGCTTCCTAACGATAATACATTCACCATAAGCGTACGGAGATTGAAGATATACAATAACGCTGTGGCCTCTGTCTTTGGTAGCATGACAAATAGAGATTATACTCAAAAGCGCATAGACAAGAAGTTGGACGAATTGCTTAATATGGTGGAAAACGACATATCTGTGGTCAACCTCAATTGCTCCAAACACGGGCCTTACTCAGTTTTCAGGGCCAATCCGGCTGCAGTTACCTCTGCTCTTACAGATCAGCTGCCGTCGCCGGGTCATTCGATGTCGTCAGCAGAGGAAACCACTACTGCAGCGCTGTCATCGCCGCCAGAAGACAGCACCTCTTTGATCGATATTATCCAAGGCAAAATTTTCGGGATCCTATGGTTTAATTGCTACGGGAACATGATATTGAACCGACAAGAATACACGACGTGGTTCATCAATAAGATGAGGAACTCGTTGACCACGGAATTCATACGCTTCCTTGGGAAAATAATTGACGACCCGGACATCAATATGGCCTCGTGCCTGTTCAAAGAGTGCATCGCTCGTTGGAGTTGTGTCGATTGGCAATCCATAGCTATAACAATGCTGGTCATCATTCATGGATATACCTGCCCCAACTTGACAAAGCTTCTAAGAGTGTGGTTTCTCCAGCAAAAGCTATTGAGATTTTCGATGTATCCGCTGGTCAACTTTATCATAAACAATACACAAGATTTAGACGTCCTGTACCATTGTAATGGATTGCTAGGGAATGCCGACTTATTCGAGGATCCCTACCAGGACGAGCTTACGTCGGAGTTACATGTCTTGGTCACGGAGCGCCTAGTGAATAGTTGGAAAGATACAATATTGCAACAACTTTGCTCATGTCAAGATACCACACTTTCATGTTCCCAACTGCGGTACTGGCAGCTACAACTGAAGTGTAATCAACAATTTTACAAGGATGTATATGCCATGCAGGACTAG>YBR242W	+:0:chr02:704665:705381ATGACAGCAACGATCACAAATAAGAAATCATGCTCTGGTTCTGTTGAGGCAGGAAAGACACGTTTAACAACTGAATGGAAGCCTGAAAGCCAAGTACCCCAATATGTTAAAAATGAGCTCTCTAAACCCCATCCAAACTATATCCTGGCGTTTTTGAACGTGGTACAGCAATTAAAAATTCAGAGAAGAACCGGTTATCTTGACCTCGGTATTAAAGAATGTGAAAGCATATCAGATCACATGTACAGATTGAGCATAATCACCATGCTGATCAAGGATTCGCGGGTTAACCGCGATAAATGTGTCCGGATCGCATTGGTGCACGATATCGCCGAATCTTTAGTTGGCGATATTACTCCAGTTGACCCTATTGGGAAGGAAGAAAAGCATCGTCGGGAATGGGAAACCATAAAATATCTATGTAATGCTCTGATCAAGCCATACAATGAGATCGCGGCAAAGGAAATTATGGACGATTGGTTGGCTTATGAAAATGTCACCTCATTGGAAGCTAGATACGTGAAAGATATCGATAAATATGAAATGCTTGTACAATGTTTTGAGTACGAGAGGGAATACAAAGGAACGAAAAACTTCGATGATTTCTTTGGGGCTGTAGCTAGCATAAAAACAGACGAAGTAAAGGGCTGGACAAGTGACCTTGTCGTGCAGCGCCAAAAATACTTCGCCGATTTGACTCAATCGATAACTAAATAA>YBR243C	-:0:chr02:705442:706788ATGTTGCGACTTTTTTCACTGGCACTTATCACATGCTTAATCTACTATTCCAAAAATCAGGGCCCATCTGCTCTTGTTGCGGCCGTGGGATTTGGTATAGCAGGCTATTTAGCTACAGATATGTTGATTCCACGTGTGGGCAAATCCTTCATCAAAATAGGCCTATTCGGTAAGGACTTGAGTAAACCTGGCCGTCCGGTGCTTCCAGAAACAATAGGTGCTATCCCTGCTGCAGTTTATTTATTTGTAATGTTCATTTACATTCCCTTCATTTTCTACAAGTATATGGTCATAACCACTTCAGGTGGGGGCCATCGCGATGTCTCAGTGGTTGAAGATAACGGTATGAATTCTAATATTTTTCCTCATGATAAACTATCTGAATATTTGAGCGCTATCCTATGCTTGGAAAGTACGGTCCTCTTGGGTATCGCTGATGATTTATTTGATTTACGTTGGAGACATAAGTTTTTCTTGCCTGCCATTGCAGCCATCCCACTGCTAATGGTTTATTATGTGGATTTTGGAGTTACGCATGTACTTATTCCCGGATTCATGGAACGCTGGTTGAAAAAGACTAGTGTTGATTTGGGGCTGTGGTATTATGTTTATATGGCATCGATGGCAATTTTTTGCCCCAACTCCATCAACATCCTGGCAGGTGTTAATGGTTTAGAGGTTGGCCAATGTATAGTGTTGGCCATCTTAGCGCTATTGAACGATTTGCTGTACTTCTCGATGGGGCCATTAGCCACAAGAGACTCCCATAGGTTTTCCGCTGTTTTGATTATCCCATTTTTGGGTGTATCCTTGGCGCTATGGAAATGGAATCGTTGGCCCGCCACAGTGTTTGTGGGAGATACATATTGTTATTTCGCTGGAATGGTATTCGCAGTCGTTGGTATACTGGGTCATTTTTCAAAGACGATGCTTTTGCTATTCATTCCTCAAATCGTTAATTTTATTTATTCATGTCCTCAGCTATTCAAATTGGTCCCCTGCCCGAGACATAGGTTACCTAAATTTAATGAAAAAGACGGTTTAATGTATCCATCAAGAGCAAACTTAAAAGAAGAACCGCCAAAGAGCATTTTTAAACCGATCTTGAAGTTACTATACTGTCTCCATTTGATTGACCTGGAATTTGATGAAAATAATGAGATTATTAGCACCTCTAATATGACGTTAATAAACTTGACATTAGTATGGTTTGGCCCTATGAGGGAAGACAAATTGTGCAATACAATCTTGAAGTTGCAATTCTGCATTGGAATTTTGGCTTTACTTGGAAGACACGCTATAGGCGCTATCATCTTTGGCCACGACAACCTATGGACAGTACGTTGA>YBR244W	+:0:chr02:707523:708011ATGACCACATCTTTTTATGATTTAGAATGCAAGGACAAGAAAGGCGAATCTTTTAAGTTTGACCAACTGAAAGGCAAAGTAGTTCTCATAGTTAATGTTGCCTCCAAGTGCGGCTTCACGCCGCAGTATAAAGAATTGGAAGAACTATACAAAAAATATCAAGATAAGGGGTTTGTTATTTTGGGGTTCCCATGTAATCAGTTCGGGAAGCAGGAACCGGGCTCTGATGAACAAATTACGGAATTTTGCCAGTTGAATTATGGCGTTACATTCCCAATTATGAAGAAGATTGATGTTAACGGAAGTAATGCTGACTCTGTCTATAATTATTTGAAAAGCCAAAAAGCAGGTTTACTAGGTTTCAAGGGTATCAAATGGAATTTTGAAAAGTTCTTAGTTGATTCCAATGGTAAGGTTGTCCAAAGATTTTCCTCCTTAACAAAACCATCGTCCTTGGACCAAGAAATCCAAAGCCTGTTAAGTAAATGA>YBR246W	+:0:chr02:711586:712749ATGGACTCTATTCAAGAATCAGATGTACTAAATGCAGTGAAAACAAAACTACCGCCGTGTTGTTTGAGAATATTCAGGAATAAGATCATCCTTGTTGGGACGTATGATTTAGATAAGTCAACTGGATACAGGTCAGGCTCACTGGATGTCTTCACAATGGACCTCAAACTTTTATGTTCGAATAATACGTATGGCGCAATCCTAGATTTGAAGTTATCCCCCTTTGATGATACATTGATATGCACGGCCCATTCGACAGGTAATATCATGCTATGGAGGATACGGTGCACAGATAAGGATGATTTCCAATCAAACGAACTAGATATTCATGCAATTGCAAATCTACAACTCTTTGAAAAAGATGTTCTCATTGCCTCGTGCCATTTTTCCCCACTTGATTGTAAGAAATTACTTGTAACAAATACGGCAGGCGAAGCAGCTACCATTGATATAAGAACACTATCTGTACAGTTCACGGCATCAGCAATCGCGCAGGCTTACTCAAAACTAGATAAAATAGATTACGAAGTTCAAGGTGCAACCGAAAAAGTCATTCATGTTGAATCGGGACAATTTTTGAAACCCCATGAACTCGAGTGTTGGACTGCGGAATTTGGATCTCTACAGCCTTTTCAAGACGTTGTATTTACAGGAGGTGATGATTCGAGAATTATGGCCCACGACTTACGTTCCAAAGAATTCATTTGGAGTAATAATCGTATTCATGATGCTGGTGTTGTTAGCATTAAGTGTAGTCAACCTAACTTTCGAAATAATAAACCCACGTCCATAATAACTGGATCCTATGATGATAACATTCGTTCTCTGGATTTAAGGATGATGGGTGAGTCCATATTTCCTGGTGCAAATGTCCCCACAGTTAATAAATTGGCGTGTGATCTTGGTGGCGGTGTTTGGAGATTTGTTGAATCCCCCATTGATCAGGAACAATCTCACCACAACGGCTCCGATCGGCTTTTAGTTTGTTGCATGTACAATGGTGCCAAGGTGGTGACCATGAATGATAATTCCGATGAGTATTTTCAAATTCAACATTATCTGAAGAAGGGCCATGATTCGATGTGTTACGGTGGCGACTGGTCAAATTCTTTGATCGCAACATGCTCATTTTATGATAATTCCTTGCAAACATGGATAGTTTAG>YBR247C	-:0:chr02:712999:714450ATGGCCAGAGCATCCTCTACTAAAGCCAGAAAACAGAGGCATGATCCACTTTTAAAGGATTTAGATGCAGCTCAAGGTACCTTGAAAAAAATCAATAAAAAGAAGCTAGCGCAGAACGATGCTGCAAATCACGATGCTGCAAATGAGGAAGATGGATACATAGACTCCAAAGCATCAAGAAAAATTTTGCAGTTGGCCAAGGAACAACAGGATGAAATTGAAGGTGAGGAACTTGCTGAATCAGAAAGAAACAAGCAATTTGAAGCCAGATTCACCACCATGAGCTATGATGATGAAGACGAAGACGAAGACGAAGACGAAGAAGCTTTTGGAGAAGACATCTCCGATTTTGAACCCGAAGGTGATTATAAAGAAGAGGAAGAGATAGTTGAAATCGATGAAGAAGACGCAGCGATGTTTGAGCAATATTTCAAGAAATCAGATGATTTTAACTCTTTAAGTGGTAGCTATAATCTTGCAGACAAGATTATGGCCTCTATCCGAGAAAAGGAAAGTCAAGTTGAAGATATGCAAGATGATGAACCACTTGCTAATGAACAGAATACCTCAAGAGGCAATATTTCATCCGGTTTGAAGAGTGGAGAGGGTGTTGCACTTCCAGAGAAGGTCATCAAAGCATATACTACGGTAGGTAGTATTTTAAAAACTTGGACGCACGGTAAGTTGCCAAAGTTGTTCAAGGTTATCCCATCCTTAAGAAATTGGCAAGATGTCATTTATGTGACCAACCCGGAAGAATGGTCACCACACGTTGTTTACGAAGCAACGAAATTATTTGTATCCAACCTAACTGCTAAAGAATCTCAAAAATTTATTAACCTAATTTTACTTGAACGTTTCCGTGACAATATCGAGACCAGTGAAGACCACTCTTTAAACTATCATATATACCGTGCGGTGAAAAAATCGCTTTATAAACCAAGTGCCTTCTTCAAAGGTTTCCTATTCCCATTGGTTGAAACAGGGTGTAATGTACGTGAAGCGACAATTGCAGGAAGTGTGCTTGCTAAAGTCTCTGTTCCGGCTTTACATTCTTCAGCTGCCTTGAGTTATCTTTTAAGGCTACCTTTCTCTCCACCTACAACTGTTTTTATCAAAATATTGCTCGATAAAAAATATGCTTTACCGTACCAGACGGTGGACGACTGCGTGTATTACTTTATGAGGTTTAGAATTTTGGATGACGGTAGTAATGGGGAAGATGCTACAAGAGTACTCCCAGTGATATGGCACAAGGCTTTCTTAACATTTGCACAACGTTACAAGAACGATATTACCCAGGATCAGAGAGATTTCTTGTTGGAAACTGTTCGTCAAAGAGGCCATAAAGATATCGGTCCTGAGATTAGAAGAGAACTATTGGCAGGAGCCAGCAGGGAGTTTGTTGATCCACAGGAAGCTAATGATGATTTAATGATTGATGTCAATTGA>YBR248C	-:0:chr02:714802:716460ATGCCGGTCGTTCACGTGATTGACGTTGAAAGTGGTAACCTACAGTCACTAACCAATGCAATTGAGCATTTAGGTTACGAAGTACAACTGGTGAAATCACCAAAGGATTTTAACATATCAGGCACGTCAAGATTGATTTTGCCTGGTGTCGGAAATTATGGCCATTTCGTCGACAATTTATTTAATAGAGGATTCGAAAAGCCGATAAGAGAATACATTGAATCTGGAAAACCAATAATGGGAATTTGCGTCGGGCTACAAGCGCTCTTTGCCGGTTCCGTGGAAAGCCCTAAGAGTACGGGTCTGAACTACATTGATTTTAAGTTGTCCAGGTTCGATGATTCAGAAAAGCCAGTACCAGAAATAGGTTGGAATTCTTGCATTCCCTCGGAAAACCTATTCTTTGGATTGGATCCATACAAGAGGTACTATTTCGTCCATTCTTTTGCTGCCATTCTGAATTCAGAAAAGAAAAAAAACCTAGAAAATGACGGTTGGAAAATTGCAAAAGCTAAGTACGGTTCAGAGGAATTTATTGCGGCAGTCAACAAGAATAATATATTCGCTACTCAGTTCCATCCTGAAAAATCAGGTAAAGCTGGTTTGAACGTCATTGAGAATTTTTTGAAGCAACAAAGTCCTCCGATTCCAAACTATAGTGCGGAAGAGAAGGAACTCTTAATGAATGACTATTCAAATTATGGTCTAACACGCAGAATTATTGCTTGTCTTGATGTACGTACTAATGACCAAGGTGATTTGGTGGTTACTAAAGGTGATCAATACGATGTACGTGAAAAAAGTGATGGTAAAGGTGTTAGAAACCTTGGTAAGCCTGTTCAGTTGGCACAGAAATATTACCAACAGGGTGCGGATGAAGTAACATTTTTGAATATAACTTCTTTTAGAGATTGTCCTTTGAAGGATACTCCGATGCTAGAGGTTCTGAAACAAGCCGCAAAGACAGTCTTTGTTCCATTGACAGTCGGTGGGGGGATCAAGGATATTGTCGATGTTGATGGAACCAAAATACCTGCTTTAGAAGTTGCAAGTCTATACTTCAGATCTGGTGCTGATAAAGTATCGATCGGTACGGATGCAGTCTATGCAGCCGAAAAATACTACGAGTTGGGTAACAGAGGAGATGGAACGTCACCAATAGAGACAATCTCGAAAGCATACGGTGCTCAGGCAGTTGTTATTTCTGTCGACCCTAAGAGAGTATATGTAAATTCACAAGCAGATACGAAGAACAAAGTCTTCGAGACAGAATATCCGGGCCCCAATGGAGAGAAATACTGCTGGTACCAATGTACAATCAAAGGTGGAAGAGAATCTAGAGACCTTGGTGTGTGGGAATTAACAAGGGCATGTGAAGCTCTAGGTGCTGGGGAGATTTTATTGAACTGCATAGACAAGGATGGCTCTAATTCTGGTTATGATCTGGAATTGATAGAACATGTTAAAGATGCGGTCAAGATTCCCGTCATTGCATCCAGTGGCGCCGGTGTACCCGAACATTTCGAAGAGGCCTTCCTAAAGACCCGCGCAGATGCTTGCTTGGGTGCAGGTATGTTCCACAGAGGTGAATTCACTGTTAACGATGTAAAGGAGTATTTACTAGAGCACGGATTAAAGGTTAGAATGGATGAAGAGTAA>YBR249C	-:0:chr02:716877:717989ATGAGTGAATCTCCAATGTTCGCTGCCAACGGCATGCCAAAGGTAAATCAAGGTGCTGAAGAAGATGTCAGAATTTTAGGTTACGACCCATTAGCTTCTCCAGCTCTCCTTCAAGTGCAAATCCCAGCCACACCAACTTCTTTGGAAACTGCCAAGAGAGGTAGAAGAGAAGCTATAGATATTATTACCGGTAAAGACGACAGAGTTCTTGTCATTGTCGGTCCTTGTTCCATCCATGATCTAGAAGCCGCTCAAGAATACGCTTTGAGATTAAAGAAATTGTCAGATGAATTAAAAGGTGATTTATCCATCATTATGAGAGCATACTTGGAGAAGCCAAGAACAACCGTCGGCTGGAAAGGTCTAATTAATGACCCTGATGTTAACAACACTTTCAACATCAACAAGGGTTTGCAATCCGCTAGACAATTGTTTGTCAACTTGACAAATATCGGTTTGCCAATTGGTTCTGAAATGCTTGATACCATTTCTCCTCAATACTTGGCTGATTTGGTCTCCTTCGGTGCCATTGGTGCCAGAACCACCGAATCTCAACTGCACAGAGAATTGGCCTCCGGTTTGTCTTTCCCAGTTGGTTTCAAGAACGGTACCGATGGTACCTTAAATGTTGCTGTGGATGCTTGTCAAGCCGCTGCTCATTCTCACCATTTCATGGGTGTTACTAAGCATGGTGTTGCTGCTATCACCACTACTAAGGGTAACGAACACTGCTTCGTTATTCTAAGAGGTGGTAAAAAGGGTACCAACTACGACGCTAAGTCCGTTGCAGAAGCTAAGGCTCAATTGCCTGCCGGTTCCAACGGTCTAATGATTGACTACTCTCACGGTAACTCCAATAAGGATTTCAGAAACCAACCAAAGGTCAATGACGTTGTTTGTGAGCAAATCGCTAACGGTGAAAACGCCATTACCGGTGTCATGATTGAATCAAACATCAACGAAGGTAACCAAGGCATCCCAGCCGAAGGTAAAGCCGGCTTGAAATATGGTGTTTCCATCACTGATGCTTGTATAGGTTGGGAAACTACTGAAGACGTCTTGAGGAAATTGGCTGCTGCTGTCAGACAAAGAAGAGAAGTTAACAAGAAATAG>YBR251W	+:0:chr02:721385:722308ATGTTCAAGAGGCAATTATCGACTAGTGTTCGTTATCTACAGCATTATGATGAGTCGCTATTATCAAGGTACTATCCTGAAAGTCTTTTAAAATCCATAAAACTTGCTCAACAAACGATACCAGAAGATACTAAATTCAGAGTATCTCGTAACGTGGAATTCGCACCACCATATTTGGATGATTTTACCAAAATACATCCTTTTTGGGATTACAAACCAGGCATGCCCCATCTCCACGCTCAAGAGGAGAATAATAACTTCAGCATCTTTAGATGGGACCAGGTACAACAACCATTACCAGGCGAAGGTAACATTCTGCCTCCAGGAGTCAGCTTACCAAACGATGGTGGTCGGAAATCGAAAAGCGCCGATGTAGCTGCAGGGCTACACAAGCAAACCGGTGTAGATCCGGATTATATCACTAGAAAGTTGACTATGAAGCCGCTGGTGATGAAAAGAGTGTCAAATCAGACTGGGAAGGGTAAAATTGCGTCTTTCTATGCCTTGGTTGTCGTTGGTGACAAAAACGGTATGGTAGGTTTGGGAGAAGGTAAATCTCGTGAAGAAATGTCCAAAGCGATCTTTAAAGCTCATTGGGATGCGGTAAGGAACCTGAAGGAAATACCTAGGTATGAAAACAGAACTATTTATGGTGATATAGATTTTAGGTATCATGGTGTGAAACTACATTTAAGAAGTGCAAAACCAGGGTTTGGATTACGTGTAAACCATGTAATCTTTGAGATTTGTGAATGTGCAGGTATCAAAGATCTGAGTGGGAAAGTATATAAATCCAGAAACGATATGAATATAGCTAAAGGTACCATCGAGGCTTTCACGAAAGCTCAAAAGACATTGGATGAGGTTGCCCTGGGTAGAGGCAAAAAGCTTGTTGATGTCAGGAAAGTTTACTATTCAAGCTGA>YBR252W	+:0:chr02:722606:723049ATGACTGCTACTAGCGACAAAGTACTAAAGATTCAATTGCGCTCAGCAAGCGCTACTGTACCTACCAAAGGTTCTGCCACTGCCGCGGGATACGACATTTATGCATCTCAGGATATTACCATTCCGGCTATGGGTCAAGGTATGGTTTCCACCGACATATCGTTCACCGTACCTGTTGGTACCTACGGTCGTATTGCGCCAAGGTCAGGCCTGGCAGTGAAAAACGGTATCCAAACCGGTGCTGGTGTTGTCGACAGAGATTACACCGGTGAAGTTAAAGTAGTTTTATTCAATCATTCACAGAGGGATTTCGCGATCAAAAAAGGTGATCGCGTAGCCCAATTGATTCTGGAAAAAATTGTCGATGATGCCCAGATCGTTGTTGTAGACTCTCTGGAAGAAAGTGCAAGAGGGGCCGGTGGCTTTGGTAGCACTGGTAACTAA>YBR253W	+:0:chr02:723265:723630ATGAGCAACCAGGCACTATATGAGAAACTCGAACAAACCAGGACGATTCTGTCCGTGAAGCTGGCGGAATTGATAAATATGACTACGATAGCCGATAGAAATGATGATGACGAGGGTTCATTCGCACAAGAAAATTCTGAGCTCGCTGTGGCCACGACCAGTGTGATGATGGTGAATAACCAGACCATGCAATTGATTAAAAATGTTCAAGACTTGTTGATCCTGACCAGATCGATAAAAGAGAAATGGCTACTGAACCAAATTCCTGTAACGGAACACTCAAAAGTGACTCGTTTTGACGAGAAGCAGATAGAGGAATTACTGGATAACTGTATAGAAACGTTCGTGGCGGAAAAAACTACGTAA>YBR254C	-:0:chr02:723731:724258ATGCCTCAGTATTTTGCCATTATTGGTAAGAAGGACAATCCTGTCTATGAAATCGAGTTTACCAATGCAGAAAATCCACAGGGTTTTCCTCAGGATTTAAAAGAATTAAATCCGTTCATACTGCATGCATCACTAGATATCGTAGAAGATTTACAATGGCAAATTAATCCGACTTCACAGTTAAATGGGAACGGTGGAAACGGATCCAATGGCGGTGGTGGGTTCCTTCGATCTAGAGCTGTAAACAATACTGATAATTGTTATCTAGGTAAAGTTGATCATTTTTATGGACTAGCAATAACGGCTTATATTAGCTATAGTGGGATGAAGTTCGTGATGATCCATGGGAACTCAGCCAACAGTAGCGTAGTTATTGACGACAATAATATGAGGTCATTCTACCAAGAGGTACACGAACTATACGTAAAGACACTAATGAACCCGTTTTATAAGATTACAGACCCTATTAGGAGCCCGGCATTCGACTCGAGGGTAAGGACATTAGCACGTAAGCATTTGTCCAAATAA>YBR256C	-:0:chr02:727381:728097ATGTTTACTGGTATTGTAGAATGCATGGGGACTGTTTTGGAAAACAACCCATATGATGACTCTGAAAGTGGAGGTCAAGGAGTTTCTATTACTATTGGCAATGCGGGGAGTATTCTCACCGATTGTCACGTTGGAGATTCAATAGCCGTAAATGGGGTATGCCTTACTGTGACCGAGTTTAATAACGACTCCTTCAAAGTTGGGATATCACCAGAAACTATAAAACGAAGTAATGTCGCTTCCTGGATTCAAGGCACCCAGGTCAACTTGGAGAGAGCGGTATCTCAAGACGTTAGGTTCGGTGGTCATTATGTACAGGGTCACGTAGACACTGTTGCTAATATTGTCTCAAGAAGACCTGAGGGGAATTCAATTATTTTTGGGTTTCAGTTAAGAGATCAAGAGTACTTTAAATACATAGTAGAAAAGGGATTCATTTGTATAGATGGAACTTCCTTGACCATAATCAAGGTTGACCCACTTTCGCAAGGTGGAGCCTTCTATATTAGTATGATAAAGCACACCCAAGACAATGTTATCATGCCTTTGAAGAAAATTGGCGACGAGGTTAATATTGAAGTAGATTTGACTGGGAAGATTATTGAGAAGCAAATTCTATTAACGTTGGAAAACCAAATATCAAAGAAAGATAGTACTTTGAATACTATGATCTCAAACATTATCGAGGAGAAGGTTAGAAACTACCTAAATAAATAA>YBR257W	+:0:chr02:728880:729719ATGGATAGAACTCAAACGTTTATTAAAGACTGTCTTTTCACGAAATGCCTGGAGGACCCTGAAAAGCCCTTTAATGAAAATAGATTTCAAGATACGTTGTTGCTACTGCCAACTGATGGCGGATTGACTTCAAGGCTTCAGAGACAGCAAAGAAAATCAAAGCTAAATCTTGACAATTTACAAAAGGTCTCCCAGTTAGAGAGTGCAGACAAACAGTTGGAAAAAAGAGATTACCAAAGAATCAACAAGAACTCTAAAATCGCATTAAGAGAGTACATCAATAATTGTAAAAAAAATACAAAAAAATGTCTGAAATTGGCATACGAAAATAAAATTACTGATAAGGAAGACCTTTTACACTACATAGAGGAGAAGCATCCAACGATATACGAATCATTACCGCAGTACGTTGATTTTGTACCGATGTATAAGGAACTATGGATTAACTATATTAAAGAACTTTTGAACATTACAAAAAACTTAAAAACATTCAATGGATCATTAGCATTATTAAAACTATCTATGGCAGACTATAATGGTGCGCTGTTACGCGTTACAAAGAGCAAAAATAAGACTTTAATAGGCCTTCAAGGCATTGTGATCTGGGACTCTCAGAAATTTTTTATCATGATTGTCAAGGGCAATATAATAGACGAAATAAAATGCATTCCAAAAAAGGGCACAGTATTTCAATTTGAAATCCCAATATCAGATGACGACGATTCGGCATTGAGATATAGTATACTAGGCGATAGGTTCAAGTACAGAAGTGTGGATCGTGCAGGCAGGAAGTTCAAAAGTCGTCGTTGTGATGATATGCTATATTATATACAGAATTAG>YBR258C	-:0:chr02:729729:730157ATGGCGTATAATCAAGAAGATAGTAAAAGACTATCAGACAAGTATAAGAAGGAGGGACATTTTGACAAGTTGAAAAGAGAAATATTGTCTAACCCATGGAATAATACAGAAGAGAATAGTGAATCTTTTGAACAAGCGCTTCGGAAAAGAGTTGCCAGTACTGTTAAAGAAATGGTTAACGAAGATGAAGAATTAATATTTAAAAACAGAGGGCTAACCAGTGCATTGATTGAATCACAATTGGTCAAGGACAACTACCTAAAGCTGGGTAGTAAAATGGAGGGGGATAATGGTGATGGTGAGAAGAAATTTGACTTGGATGTCTATGTACGGTCTAAGTTACAGGATCCCAAACTATTGGAAATGATAAAGGGACAACTTCAGGAAACACTGAACTCTTATGAAGAGGAAGCAAATGGAAGTACGTAA>YBR259W	+:0:chr02:730382:732448ATGTCCATTGACGAGGCTGTTGCCAGATATAGAGATGTTATAGGCAATCTAGCAACTGGCAATTTAAGGCGGATTGTTATACAGTCAGAAAAACTGGCCCAGATCATTGCATCTTCAAAAGGTACCGTTCGTTTCCACCATAAAACTCGCAGCGGTAAAACAGTAATATACAAATGCATCAAAAAGGCGCTGCTTTCCAGTGTCGCGTCTCTATCGTCAGAGTTTTCCTCAGAAACAGATGTTCAGCAATTCCTCCATTTAAATTACATATATCAATCTCACTTCCAAGCTTTGAGTGGACAAATAAACAAATATTGCGGCATGAAGAAATACTACGAGTTAAAGTTTGCAGCAATCGACTACTTAGAAACCGAAGTCCAAACTACCGGGCTTACACTCTCAAGATTCTGGGTGGCATCTTTAGATGAGTTCATTAAAAAAGAGCGATGGCCAGATAATGGATCTAACTTTCAAATCTTTTACAAACTAATGGCTGAGTATTCGTCTTGGAAATGGGATTCTGATGATAAAAGGCAACTCCAGTTCATGTACGAATTTCGAATGAAACTCAAAGAATGTTTGGTGAAATTTTACGAGAATTTTGATCTTCAAAAATCTAGCGATCCTCTGAAAGAACTCATTATACCTTGGGAAAAGATTGTTTATGTCGCCAACTGTATCGATGCATTCACTGGAGAGCAAGTTAGGATCGACGGTGCTGAATTAATCTGGACATCCAAAAATCTTGTGTTCTCTTCGATTTCTTCTGCCGTTCTTAGATTAAATGACCTTCAAAATATGTTCAGCGCATTCCGGCCTTATGGGGAAGAAGCATTAGTTCAAGACTTTGCGCATATACGATCTTTAAAGTGGGATAGTAATGATAAAGTTGAAAGCTTAATTCGTGCCCTTATTTTTAATGATATGTTTCCTTATTTCAATAAGGAACAGGTAGATACGAAGGCAGATGGTATATTCTTTTTGCGCTTACTGAGAAAAAACTTCAAAGAACACATTAATGACGTTAAAGATTTTCATATTCAAGTGATAAAGTACTTGAATTCCCAATTTAAAAATAATTACAGCACATTGATGACGTCATCGAAAACTCAGGATAGAAGGAAAAGTCATAATATGCCTTCTTCTATCTTAGACGATGGTAACAAAATTGGAATGCATGTTTCGCCTATCGATGAATACTCACATTTTATTGATAATGATGAACCGTTGTGGCGAGACAAAGTATATCCAAAGATTTACACAAATGAGCAAACACCTACACCTGACGCATCAGCAATATTTGATTCTCACAAGATATATGCAATTATATCGCTGCTGCGATATTACTTACCTGAGAAGAGAAAGTTTTTTCGGATTTATTATCTGCCCAGTATTTTTAAAAGGATATTATATTACGGTGCAAAGTTTGCTCAACTATATTTCATGGAAGGTTGCTTGGAACGGCTAGTAATAGAATCTCTCCAAATTCTAGAACCCTCTTTGGTACATGCGATAAATAATTTGATCAAGTCTAGCATAGAATCACTCAAAAATGTAACAGTGACAAGTGATGACAAAACTTCATCTGGTGTCATTATTTTGTCCTACAAAGAATTTAAGTCACTATCTGAAGTTAACAAAGATTTCAATGAACCATTTTGGCCTAACCAATCAATTGCGAATAGTTGGCCAGATTTCGCGAATAAGCAATTAAAGAGGGGTCAAATTTTGCAAGACGCATTTGCATTCCACCTTTTTGAAATTGAACTACCAATTATTATCGATACTACAAGAAATACGCATTTGAAACTCGTTTCCAACATGTGTACTACGAGCATATTGTACTTATATAATGAAGTTGATTCCTTATCATTAACTAGCATACAGGAAAAATTAGCTGTTTTACCGACAAGTAAACGGAATGAAATCTTATTGTACAATTTGAACAGGCTAACAAAATTGAAACTATTGTTGTTGAAAGAGAACGAAAAAGGACAAAAGTTTTACGCATTTAATTTTAAGTACAAAAGGGATGGACAGAAAACATCATTAATAAGGTTAATTTAA>YBR260C	-:0:chr02:732634:734634ATGGAAGAGACTGCGAAGAAACCAGCTAGTGCCACAGTAAGTGCGAAGTCGTCGCACGATGGTGGCACCGACGATTTGGCGCACCTTTTTAGCACGCCAGAGATCAAGAAGGTGCTAAATTCCGATGTGGCAATTAACGCTTTGTTGAGCCGTTTAAAGCAGTCGTTGTTGACTTGTGAAGAGTTTATGAAATTTATTAGAAAGAAATACGCCTTCGAAGAAGAGCACGTTCAGGAGCTCTCCAAGCAATATAAGCACTTTTTCAACATTCAGGGTTCCACAAATTCATCCTTAAAAAAGATGATTCACGAAGTTTTGGGATTCGATGGCAAGATGGCTCAAGTAAAGCAGAGTTATATCACCGCCTTGCAGAAAATGTACTCCGAGATTAGTAGTCTTTTATTAACAATGACAAAATTAAGAAAGTCTGTCAAGGAGAACAGCAAAAGGCTGGAAAAGGATGTTTCTGATGCCATACACAGCGCAGAAAAGGCGCAATCTCGTTACAATTCGTTGTGCCAAGATTGGGACAAATTGAGGATGACTGACCCAACAAAAACAAAGCTGACCTTGAGAGGTTCCAAAACTACGAAGGAACAAGAAGAAGAATTGTTAAGAAAAATCGATAACGCCGATCTGGAATATAAGCAAAAAGTAGATCATTCTAATTCTTTGAGAAACACATTCATAACGAAGGAGAGACCTAGAATTGTTCAAGAATTAAAAGATTTGATTTTAGAAATAGACACAGCGATGACTATCCAATTGCAGAAGTACACGATATGGACGGAAAATTTGGTTCTGAATACAGGTGTTACAATAAGCCCATTGGACTCCACCAAATCTATGAAATCATTTGCTGGATCTGTTTCCAATGAACGTGATCTTTACAGTTTTTTGAACAAGTATAACCAAACAGGAAAACATTCCCTACTGATCAACAAAAACTTGATCCCTGTATCATACAAAAAGCATCCATCAATGAATCATGGTCAGAAAAATAAGAGCCCCCCAAAGTTTGCCGTGGACCCTTCAAGAAACTCCATCCCGAAGAGAATGATATCCACTCATAATGAATCTCCATTTTTGAGCAGTAGTAGTAACACTGCCGCTGTTCCGAATGCTAATCTTAACAGTGCTACACCGTCCTTAAATACCAACAAGCAGTTACCACCTACTATGGCTTCATCTATTTCTTCCACTAGCAATGCGGCTGGCGCTATGTCACCGTCTTCTTCCATTGTAACAAGCGACACCACATCATCCATAACAAAGACCCTGGATCCTGGCAACAATAGTCCACAAATTCCAGAAGAATTGATTAACTCTTTAGACTCGGATCGCCCGATTTCTCACATTCAGACTAACAACAATATGCCACCCGGTGTTCAAAAAAATTTCAAAACATTTGGTGTACCGCTAGAATCACTGATAGAATTCGAACAAGACATGGTCCCTGCGATAGTGCGCCAATGCATATACGTTATTGATAAATTTGGGCTTGATCAAGAAGGAATTTATAGAAAATCGGCAAATGTACTTGATGTCAGCAAGTTGAAGGAGGAAATTGATAAGGATCCCGCAAACATTTCCATGATTCTACCATCAAAGCCACATTCTGATTCGGATATTTATTTAGTAGGATCACTGCTGAAAACTTTTTTTGCGTCGCTGCCTGATAGTGTTTTACCAAAGGCCCTATCATCGGAGATCAAGGTTTGTTTACAAATTGAAGACCCAACAACAAGAAAAAATTTTATGCACGGACTAATATATAACCTCCCCGACGCACAATACTGGACGTTAAGGGCTCTTGTCTTTCATTTGAAAAGAGTGTTGGCACATGAAGCCCAAAACAGAATGAATTTAAGGGCTCTTTGTATAATTTGGGGTCCAACTATTGCGCCTGCAAATCCAGATGACGCTAATGATGTAAATTTTCAAATTATGGCAATGGAAGTATTATTAGAGGTTTCAGATCAAGCTTTTGAGCCTGAATAA>YBR261C	-:0:chr02:734827:735525ATGGACGTGCCTGCAGATTCCCATATCAAATACGAAGATGCCATCGACTATTGGACGGATGTGGACGCCACTGTGGATGGTGTTCTAGGTGGATACGGAGAAGGTACAGTAGTACCCACAATGGATGTCCTGGGATCCAATAACTTCCTGCGTAAACTGAAGTCGAGGATGCTGCCTCAGGAGAACAACGTGAAGTACGCTGTAGACATTGGTGCGGGGATTGGACGTGTAAGCAAGACCATGCTTCACAAGCACGCCGCTAAGATAGACCTTGTAGAACCGGTAAAGCCTTTTATCGAGCAAATGCACGTTGAATTGGCTGAGCTGAAAGACAAAGGCCAAATTGGACAAATATATGAAGTAGGGATGCAGGACTGGACCCCCGATGCCGGCAAGTACTGGCTGATCTGGTGCCAATGGTGCGTGGGACACCTGCCAGATGCAGAACTCGTCGCATTCCTGAAAAGATGTATTGTTGGTTTGCAACCTAATGGGACAATCGTGGTCAAAGAAAATAACACACCTACAGATACCGATGATTTCGACGAAACGGATTCTTCGGTCACAAGGTCAGATGCTAAGTTCAGGCAAATCTTCGAAGAAGCTGGCCTAAAGCTCATCGCCAGTGAAAGACAACGTGGCTTACCAAGAGAATTGTACCCAGTGAGGATGTACGCACTCAAGCCCATGCCAAACTAG>YBR262C	-:0:chr02:735715:736035ATGTCGAAATTGGGTCCGTTAGCAAGATCTGTGAAGTGGACTCTCTCTGTGGGAGTAATCGGTTCTGTTTTTTACCTGTACCGATACAGTAACAATGGCTACTTCTACGATCACGATGCTACATGGTTGAAACAAGATCATCAGGTACAGGACCTGGTGGATAGAAAAGAAGTGGTACCTGGGGAGACCAGGAACCGCAAGCTAGTTGTGACGGATGATGGCACTGCATGGAGCAGGACCATGGGGGAGAGCATAAAAGATATTTGGAATGAACAGATAAGAAACTCTGTCGATTGGATCTACTCCTGGGGTAAGAATTAG>YBR263W	+:0:chr02:736259:737731ATGTTTCCCAGAGCTTCTGCATTGGCCAAATGTATGGCAACTGTTCATCGTCGTGGGCTACTCACCAGTGGTGCACAATCGCTAGTCTCCAAACCAGTCTCGGAGGGAGATCCAGAGATGTTTGACATCTTGCAACAAGAACGTCACAGACAAAAACACTCTATCACCCTTATCCCATCAGAAAACTTCACCTCGAAGGCCGTTATGGATTTGCTAGGTTCTGAGCTCCAAAACAAGTATTCTGAAGGTTATCCAGGTGAAAGATACTACGGAGGTAACGAAATCATCGATAAGTCCGAATCCTTGTGTCAAGCAAGAGCTCTGGAACTGTACGGATTAGACCCTGCCAAGTGGGGAGTTAATGTTCAACCATTAAGCGGGGCACCTGCCAATTTGTATGTTTACTCCGCTATCATGAACGTTGGTGAAAGATTAATGGGATTGGATTTGCCAGATGGTGGTCACTTGTCTCACGGGTACCAACTTAAGTCAGGAACGCCAATTTCTTTCATTTCCAAATACTTCCAAAGTATGCCATACCATGTCGACCACACTACGGGGCTAATCGATTACGATAACTTGCAAGTATTGGCCAAGGCATTCAGACCAAAGGTGATCGTGGCCGGTACTTCCGCGTACTCGAGATTAATAGACTACGCTAGATTCAAGGAAATTTCCCAAGGATGCGGCGCATATTTGATGAGTGATATGGCACACATATCCGGTTTGGTGGCAGCCAATGTTGTCCCATCTCCATTTGAACATTCCGATATAGTTACCACAACCACTCACAAGTCCTTGAGAGGCCCAAGAGGTGCTATGATTTTCTTCAGAAAGGGTATCAAGTCTGTCACCAAAAAGGGCAAGGAAATCCCATATGAGTTAGAAAAGAAAATCAACTTCTCAGTTTTCCCAGGACATCAAGGTGGTCCTCACAACCATACTATCGGTGCAATGGCAGTGGCATTAAAGCAAGCGATGTCTCCAGAATTCAAAGAATACCAACAAAAAATTGTCGACAACAGCAAATGGTTTGCTCAGGAACTAACCAAGATGGGTTATAAGTTGGTTTCCGGCGGTACCGATAACCACTTGATTGTTATTGACTTATCCGGTACTCAGGTAGACGGTGCTCGTGTCGAGACAATTTTAAGTGCCTTGAACATTGCTGCTAACAAGAACACCATCCCAGGTGATAAGAGTGCTCTTTTCCCCTCTGGTCTAAGAATCGGTACTCCAGCAATGACCACGAGAGGATTTGGCCGTGAAGAGTTTTCTCAAGTCGCAAAGTACATTGATTCTGCCGTTAAGCTCGCTGAAAATTTGAAAACTTTGGAACCAACAACGAAACTAGATGCAAGATCAAGACTCAATGAGTTCAAGAAGTTGTGTAATGAATCTAGTGAAGTCGCTGCTTTGTCTGGCGAGATTTCCAAGTGGGTCGGTCAATACCCTGTCCCAGGTGATATCTAA>YBR264C	-:0:chr02:737765:738364ATGGAAGCAACCATCAAAGTGGTACTGCTAGGAGATTCATCAGTGGGGAAAACCAGTATAGTGACTAGGCTCAAATCAGGTAAGTTTCTAGCAAAACATGCGGCTACGATAGGTGCGGCGTTCATCACCAAGACAATCGAGGTTCCTTCTAACGACTCCTCTACGGAGAAACGTATCCATATGGAGATATGGGACACGGCGGGTCAGGAACGGTATAAATCACTGGTGCCAATGTATTATCGAGATGCGAATATTGCTTTGATTGTATTCGAATTGGGAGACGTATCCAGTCTGCAATGTGCAAAGACATGGTTTCAAGATTTACAGGACCGTGCTCAGGGAACGCAGGTAATTATCGTGGGCAATAAGTACGATTTAGTCTGCGAAGAGCATTCAGGGGAAGTGACTATACCGGCGGAGTTACAGGGTCTGCCGTATGTGGCCGTAAGTGCAAAGACAGGGTACAATTTCGATACATTGAATAAAATAATAATCAGTTTGGTTCCCGAAAGTCAATTCAAGACATTGTCAAAGAACAATGAACAGGGAAATATACTGGAAATAAATAAAAAAAAAAGCGGCAGTGGCTGTATATGTTAA>YBR265W	+:0:chr02:738577:739539ATGAAGTTTACGTTAGAAGACCAAGTTGTGTTGATCACTGGTGGTTCACAAGGTCTTGGAAAGGAATTCGCCAAAAAATATTATAATGAGGCTGAAAACACAAAGATTATTATCGTCAGTAGGTCAGAGGCTAGACTGCTGGACACATGCAACGAAATTAGGATTGAAGCTCACCTGAGAAGGGAAACCACTGACGAGGGCCAAGTGCAACATAAGTTGGCTGCGCCCTTGGACCTTGAGCAACGGTTATTTTACTACCCATGCGACTTGTCCTGCTACGAATCCGTGGAATGTTTGTTCAATGCCCTGAGAGACTTGGATTTACTCCCTACACAAACGTTATGCTGTGCAGGGGGGGCTGTTCCTAAGTTATTTCGTGGGCTAAGCGGACATGAGTTGAACTTGGGTATGGACATCAACTATAAAACAACTTTGAACGTGGCACATCAGATTGCCCTTGCAGAGCAAACCAAGGAACACCACCTCATCATCTTTTCTAGTGCCACCGCGCTTTACCCATTTGTGGGCTATTCCCAGTATGCGCCTGCAAAAGCTGCAATCAAATCACTGGTAGCAATCTTAAGACAAGAACTGACGAACTTCCGTATCAGTTGTGTTTATCCTGGTAATTTTGAAAGCGAAGGTTTCACTGTAGAGCAGCTAACGAAACCCGAAATTACAAAGTTGATCGAAGGCCCCTCAGACGCTATCCCATGCAAACAAGCATGTGATATCATTGCCAAGTCGCTGGCCAGAGGTGATGAAGACGTTTTTACAGATTTTGTCGGATGGATGATAATGGGGATGGACCTTGGGCTCACCGCAAAGAAAAGCCGCTTTGTTCCGTTGCAATGGATTTTTGGTGTCCTATCAAACATTCTGGTCGTGCCATTCTACATGGTTGGCTGTTCCTGGTATATCAGGAAATGGTTTCGTGAAAATGACGGCAAGAAGGCCAACTGA>YBR267W	+:0:chr02:739836:741017ATGAGTAGCAGTGGTGTTTATACGTGTAATTCATGTGTCCTGACCTTTGATTCAAGCGACGAGCAGCGGGCCCACATGAAGTCCGATTGGCATCGCTACAATTTGAAAAGACGTGTTGCACAATTGCCACCAATATCATTTGAGACATTTGACTCCAAAGTGTCTGCAGCTGCTGCCAGTACTAGTAAGTCTGCTGAAAAGGAGAAACCTGTTACCAAAAAGGAGTTGAAAAGAAGGGAAAAGCAAGCATTGCTCGAAAAGAAAAAGAAACTGTTGGAAATTGCCAGGGCTAATATGCTTGAAAACATGCAAAAGAGCCAAGAGGGAAATACTCCCGACCTAAGCAAGCTCTCTTTGCAAGAAAATGAGGAGAACAAGGAAAAGGAAGAACCTAAGAAGGAGGAGCCTGAACAGTTGACCGAGGAAGAAATGGCGGAAAGAGTAATGCAAGAAAACGTACGCAACAGAGTCGATATTCCACTGGAACAATGTCTATTTTGTGAGCACAATAAGCACTTCAAAGATGTTGAAGAAAACCTGGAACACATGTTTAGGACCCACGGGTTTTATATCCCAGAACAGAAATATCTAGTCGACAAGATCGGCTTGGTAAAATACATGTCGGAGAAGATTGGTCTTGGGAACATTTGTATTGTTTGTAATTACCAGGGGAGAACGTTGACCGCTGTAAGACAGCACATGTTGGCAAAGAGACACTGTAAAATTCCCTACGAAAGCGAGGATGAAAGGTTGGAGATATCTGAATTCTACGATTTTACAAGCTCATACGCAAACTTTAATAGCAACACAACACCAGACAACGAAGATGACTGGGAAGACGTGGGCAGCGACGAAGCCGGAAGCGACGACGAAGATCTGCCACAAGAGTACTTATATAACGATGGTATAGAGCTGCATCTACCGACAGGCATCAAAGTTGGCCACAGGTCCTTGCAAAGATACTACAAGCAAGACTTAAAGCCCGAGGTGATACTGACCGAAGGCCAAGGTACCCTGGTCGCTGCAGAGACGAGATCGTTCTTACCTGCCTTCGACAAAAAGGGCGTGCAGACTCAACAGCGTGTTTGGCAAACTGAGAGGTTCGACAAGAAAAGGCTCGATAAGAGAAGTGCCAAGTTCGTCAATAACCAACCACACTACAGAGACCAACTTCTGCAGTAA>YBR268W	+:0:chr02:741294:741611ATGTTGGCGCGCAGTTTGGGTTACAGGTTGATATCGACCAGTCGTATCTTATATAATAAACCCACGGTTAAGTCAGTGGTGTCGTCGTGTCCTGCGGGAACATCGCTTAACTTAAACATATGGAAGAGCGGTAAAGACGCAGTGGCACTCGAGGACAAGGAGTATCCAAATTGGTTATGGAGCGTATTGGATAGTGATCACGTTGTCGAACATGCGGCTGAGGACCCAGAGGGACAGGCTCTCTTAAAGAGAAGAAAGAACATAAGGAAGGCGAACAGGCAGCGAATCAAGCAAAACAATTTCTTGAGCCAACTTTAA>YBR269C	-:0:chr02:742155:742571ATGTTGTGCGCCATCAAAAGCACAGGTTATCGGTATCCTCGCACGGGAGCCTTAAATCTACTACGTGGACGACCATTTAATATGGCCACCCGAAAAATAACTACTGAAAGGATCCCCGGCCCTCCCAAGCTGCCAAGAGAAGAGCAGGAAGAGTTCGAAAGACTTCAGAGGATTGCCACATCGCAAGAGGCAATAGATCAGTATAATGCGCAGGCCACCGGCGATCGTACAAAGGAGAGTTTGAATTCACCGCTTTTAACCAAAAACGATATTGGGTCATTCTCGCCTGAATTTTCCAAGACCATACCTGAATTCGAAGGTGATGTTAACCCCAAGACTGGTGAAGTTGGGGGTCCCAAGCAGGACCCGTTGAGACACGGCGATTATTCGTTTAACGGAAGAGTAACGGATTTCTAG>YBR270C	-:0:chr02:742756:744393ATGGCAACAGACCTAAATCGTAAAAGAAGTGCTACCTCAGGCTCACTGAGCGTCACGAACCCGAACATAAAAGCAACTAATCGCAAACCAGCTAGAGTTTATAGTGTTTCTTCTGATATAGTACCGCAAGCGCTGACACATCCCGACGAAGATGTGCATCTAAAAACATCCAAATCTCCTCATGATGCCGCTCCTAGATGGTCACAGGTGGGGTTTCAATCTATATTCCATGATGGTTCCAATGCAAGGCGCTCGACGGATTCGATTGAAGAGGAGTACAGCCAAGGCACGGAGAATAACGATGGTCACTCCGAAATAGGTTCATCAAGTAGTAACCGAATGGAGGGGAACACCACGTCTAATGATAGTTTATTTTCTTCCAACTCAAGGGGCAATAAAAGGCGACTGTCCATCTTCACCAATTCAAAAGATAATATGAGAAATCGAAGCAGGAGGGCGTCAAAAAACTACGGCACTGTTATTACCGGCACAAGTAGTAACAACATAAGTAGGAGTGGCTCTAAATTGTTTCATACGAAATCTAATATGAGTGTGAACAGTTTACAATCTTCACTGTCTACAGGACATTCTCATTCAAACAAAGGCAGTAATGTCTTTAGCAAAATGGCAAAAAAGCTTTTACCCTACAAGCCGCATAATTCCATTGGTAAAGACGACGTTGAACCCGTAGTTCCCAGCCCATTCAGTAAATTTCTGCATTCTTCATATGGTAAACATAGGTCACCCGTACAGTTTATCCATACTTCTACTGGAGGCCTTATTGACTCTGGAAAATCTGTATATTCCTTTAACCCTAGCATTAACAACAACCCTAATGATACAGCTCTATCGCTGATCCAAGATGATGCTTTCGACGCGACTAATGTTTCTTTACTACATGATTTACTGAAAAACTTGCCATCCTTGATAGCAAACTATAAAAGCTTCACCGTACAGGAGTTATTTGTTCTGGAGGGGAATATCTGGGGTATATACTGCAGCATAGTTGTGGAGCTATTTAAAAACAAGAGGGTATGGCAATTGCCAGCAAAAATAGAAGATATTGACCGGCTGCTGGAATTTTATATCACTTTGAAAACACAAACCAAGGCTGCCGTAACGCACTCAAGGTTTTTGGCCGAAATCGAGGAATTCATTACCACTTCACTTTACATTTTAGAGAACCAGATTGTGTTCAATTATGCCAATGAGGATACCGTAAATACGGCTTTGAAAAGAGTCGGCATAATCTGGAAGGTGTTTTACCAACAGGTTTACTACGACATGATGGCAGTGTTACTGCCCTTCGAGAAAAGCTTCCAAAAAAATAGTAACTACTGGCTTGACGGATATCTATCTGAACCTTCAAGGTACGCACCGTCAATTGATGTCCTTTTATTAAAGTGTTTTAGAGACTCCATCATTCTTCCGTATTACGAAAGCTTCCTACATACAAATGACGGTGCCAGTAAGAGTTTTCAAAGATATATTTTCAGCGAGGAAGAACAGAACGGCGTCACAGAGGAAGATAAACTGACGCTGCTGCAGTGTTTTGGGATCCTGAATACCATAAAAGGTAACAGCAGAAACCAAAGAATTATTGGTGAACTTCTTGAGGGCATACGCATGAGTATATAA>YBR271W	+:0:chr02:744847:746106ATGTTTGATCCTCTCGATTTGTACACCCCTGATGACATCCAGGTTGAGGCTTTACAGTTCAATCTAGCAGAGAGAGAGCCAAAAGATCCTTGCTCACCGCAACGTGACGAAATATTAACGGCTGTAGATGAAGAAGAGAGTGACGACGATGACACCATTATCGATAATCTGGATCTGCCCTCCGTGAAGTATGCTCCGCCAGAAGTTATCCTTTGTATACTAATACTTCTGAAGCCCGATAGACAAGTAAACTTCAATCAGGAGACAGGGAAGAACAAATCTGTACTGGAGGTTTGCAAGAGTCATGGGCTGGAACCCGATCTGCTGAAAAGACTTTTGACTTGGTATACAGAAGAATGGCCTAACAAGAGGTTAAACTCGTTGGAAAAGATATGCAATAAAATCCCAATGCTGAGATTTACAGTATCCAAAGAACTACTGTTGGGCTACTATACTAGTGTATTGAAAAAATACAATAATAGTTGCGGATTGAATGAGGAAATCATACAAGAACTTCTTAAGGAATTGAGCTCAAGGATATCCGAAAACTGTGGGAGAACGGCCCAACCATCCATAGTAAGATATTTTGAATTAAGAAATCTAAGCACTTCCATACCGCTTCATGAACCCTCTTTAACGGCCGATAATCTTGGTTGGAAAACATGGGGATCATCTTTAATACTTTCACAATTAGTCGTAGATCACCTGGATTACCTACATACCACTAATGTGAACATGTTAGCGAACAGCGACATCAAACAGATTAAAGTATTAGAATTAGGAGCGGGCACTGGTTTAGTAGGACTTTCATGGGCCTTAAAATGGAAAGAACTTTATGGAACTGAAAATATAGAAATCTTCGTGACGGATTTACCCGAGATTGTGACGAATTTGAAGAAAAATGTGTCATTAAATAATTTAGGAGATTTCGTTCAAGCAGAGATATTAGATTGGACTAATCCTCACGATTTTATAGATAAATTTGGACATGAAAATGAATTTGACGTCATATTGATCGCCGACCCGATATATTCGCCTCAGCATCCGGAATGGGTAGTTAACATGATATCGAAGTTTTTGGCGGCATCAGGGACCTGTCACCTAGAAATACCTTTAAGGGCAAAATACGCCAAAGAACGAGAGGTTTTGAAATTATTACTAAAAGAGAGTGATTTAAAAGTAGTTGAAGAACGGCATTCAGAGGGTGTGGATGATTGGGGTGCCGTTAAGTATTTATATAGGCAAATCGTTCGCAATTAG>YBR272C	-:0:chr02:746356:747798ATGAGTGAGAAAGAAACAAATTACGTGGAAAATCTCCTTACGCAATTAGAAAATGAACTTAATGAAGATAACTTGCCTGAAGATATCAACACACTATTAAGGAAATGTTCTTTGAATCTTGTGACTGTTGTCTCCCTTCCAGATATGGATGTTAAACCACTACTGGCTACGATCAAGCGTTTTCTGACGTCTAACGTATCCTATGATTCATTGAATTACGATTACCTTTTGGACGTTGTGGACAAGCTTGTTCCCATGGCTGATTTTGACGATGTTCTAGAAGTTTATAGCGCGGAGGACTTAGTGAAGGCCCTTAGGTCAGAAATAGATCCATTAAAAGTAGCAGCATGTAGAGTCATTGAGAATTCGCAGCCAAAGGGTTTATTCGCGACCAGTAATATCATCGATATTCTATTGGATATTCTATTTGATGAGAAAGTGGAGAATGATAAGCTCATTACAGCAATCGAAAAAGCGTTGGAAAGGTTGAGTACAGATGAATTGATTAGGCGTCGATTATTTGATAATAATTTGCCATACCTGGTGAGCGTAAAGGGTAGAATGGAAACGGTTTCTTTTGTCAGATTAATTGACTTCTTGACTATTGAGTTTCAATTCATCAGCGGTCCTGAGTTCAAGGATATCATATTTTGCTTTACTAAAGAAGAGATACTGAAATCCGTTGAAGATATCCTCGTTTTCATCGAATTGGTTAATTAT
[truncated: 1,200,000 more chars]
