# Supplementary material for: Proteome-wide evidence for enhanced positive Darwinian selection within intrinsically disordered regions in proteins
Source: Genome Biol. 2011 Jul 19;12(7):R65. doi: 10.1186/gb-2011-12-7-r65 (PMC3218827; doi:10.1186/gb-2011-12-7-r65)
Supplement: Additional file 9 — FastA file containing the protein sequence translated from the ORFs of all analyzed S. cerevisiae genes. [file gb-2011-12-7-r65-S9.RTF]

>YAL001CMVLTIYPDELVQIVSDKIASNKGKITLNQLWDISGKYFDLSDKKVKQFVLSCVILKKDIEVYCDGAITTKNVTDIIGDANHSYSVGITEDSLWTLLTGYTKKESTIGNSAFELLLEVAKSGEKGINTMDLAQVTGQDPRSVTGRIKKINHLLTSSQLIYKGHVVKQLKLKKFSHDGVDSNPYINIRDHLATIVEVVKRSKNGIRQIIDLKRELKFDKEKRLSKAFIAAIAWLDEKEYLKKVLVVSPKNPAIKIRCVKYVKDIPDSKGSPSFEYDSNSADEDSVSDSKAAFEDEDLVEGLDNFNATDLLQNQGLVMEEKEDAVKNEVLLNRFYPLQNQTYDIADKSGLKGISTMDVVNRITGKEFQRAFTKSSEYYLESVDKQKENTGGYRLFRIYDFEGKKKFFRLFTAQNFQKLTNAEDEISVPKGFDELGKSRTDLKTLNEDNFVALNNTVRFTTDSDGQDIFFWHGELKIPPNSKKTPNKNKRKRQVKNSTNASVAGNISNPKRIKLEQHVSTAQEPKSAEDSPSSNGGTVVKGKVVNFGGFSARSLRSLQRQRAILKVMNTIGGVAYLREQFYESVSKYMGSTTTLDKKTVRGDVDLMVESEKLGARTEPVSGRKIIFLPTVGEDAIQRYILKEKDSKKATFTDVIHDTEIYFFDQTEKNRFHRGKKSVERIRKFQNRQKNAKIKASDDAISKKSTSVNVSDGKIKRRDKKVSAGRTTVVVENTKEDKTVYHAGTKDGVQALIRAVVVTKSIKNEIMWDKITKLFPNNSLDNLKKKWTARRVRMGHSGWRAYVDKWKKMLVLAIKSEKISLRDVEELDLIKLLDIWTSFDEKEIKRPLFLYKNYEENRKKFTLVRDDTLTHSGNDLAMSSMIQREISSLKKTYTRKISASTKDLSKSQSDDYIRTVIRSILIESPSTTRNEIEALKNVGNESIDNVIMDMAKEKQIYLHGSKLECTDTLPDILENRGNYKDFGVAFQYRCKVNELLEAGNAIVINQEPSDISSWVLIDLISGELLNMDVIPMVRNVRPLTYTSRRFEIRTLTPPLIIYANSQTKLNTARKSAVKVPLGKPFSRLWVNGSGSIRPNIWKQVVTMVVNEIIFHPGITLSRLQSRCREVLSLHEISEICKWLLERQVLITTDFDGYWVNHNWYSIYEST>YAL005CMSKAVGIDLGTTYSCVAHFANDRVDIIANDQGNRTTPSFVAFTDTERLIGDAAKNQAAMNPSNTVFDAKRLIGRNFNDPEVQADMKHFPFKLIDVDGKPQIQVEFKGETKNFTPEQISSMVLGKMKETAESYLGAKVNDAVVTVPAYFNDSQRQATKDAGTIAGLNVLRIINEPTAAAIAYGLDKKGKEEHVLIFDLGGGTFDVSLLFIEDGIFEVKATAGDTHLGGEDFDNRLVNHFIQEFKRKNKKDLSTNQRALRRLRTACERAKRTLSSSAQTSVEIDSLFEGIDFYTSITRARFEELCADLFRSTLDPVEKVLRDAKLDKSQVDEIVLVGGSTRIPKVQKLVTDYFNGKEPNRSINPDEAVAYGAAVQAAILTGDESSKTQDLLLLDVAPLSLGIETAGGVMTKLIPRNSTISTKKFEIFSTYADNQPGVLIQVFEGERAKTKDNNLLGKFELSGIPPAPRGVPQIEVTFDVDSNGILNVSAVEKGTGKSNKITITNDKGRLSKEDIEKMVAEAEKFKEEDEKESQRIASKNQLESIAYSLKNTISEAGDKLEQADKDTVTKKAEETISWLDSNTTASKEEFDDKLKELQDIANPIMSKLYQAGGAPGGAAGGAPGGFPGGAPPAPEAEGPTVEEVD>YAL007CMIKSTIALPSFFIVLILALVNSVAASSSYAPVAISLPAFSKECLYYDMVTEDDSLAVGYQVLTGGNFEIDFDITAPDGSVITSEKQKKYSDFLLKSFGVGKYTFCFSNNYGTALKKVEITLEKEKTLTDEHEADVNNDDIIANNAVEEIDRNLNKITKTLNYLRAREWRNMSTVNSTESRLTWLSILIIIIIAVISIAQVLLIQFLFTGRQKNYV>YAL008WMTLAFNMQRLVFRNLNVGKRMFKNVPLWRFNVANKLGKPLTRSVGLGGAGIVAGGFYLMNRQPSKLIFNDSLGAAVKQQGPLEPTVGNSTAITEERRNKISSHKQMFLGSLFGVVLGVTVAKISILFMYVGITSMLLCEWLRYKGWIRINLKNIKSVIVLKDVDLKKLLIDGLLGTEYMGFKVFFTLSFVLASLNANK>YAL009WMEPESIGDVGNHAQDDSASIVSGPRRRSTSKTSSAKNIRNSSNISPASMIFRNLLILEDDLRRQAHEQKILKWQFTLFLASMAGVGAFTFYELYFTSDYVKGLHRVILQFTLSFISITVVLFHISGQYRRTIVIPRRFFTSTNKGIRQFNVKLVKVQSTWDEKYTDSVRFVSRTIAYCNIYCLKKFLWLKDDNAIVKFWKSVTIQSQPRIGAVDVKLVLNPRAFSAEIREGWEIYRDEFWAREGARRRKQAHELRPKSE>YAL010CMLPYMDQVLRAFYQSTHWSTQNSYEDITATSRTLLDFRIPSAIHLQISNKSTPNTFNSLDFSTRSRINGSLSYLYSDAQQLEKFMRNSTDIPLQDATETYRQLQPNLNFSVSSANTLSSDNTTVDNDKKLLHDSKFVKKSLYYGRMYYPSSDLEAMIIKRLSPQTQFMLKGVSSFKESLNVLTCYFQRDSHRNLQEWIFSTSDLLCGYRVLHNFLTTPSKFNTSLYNNSSLSLGAEFWLGLVSLSPGCSTTLRYYTHSTNTGRPLTLTLSWQPLFGHISSTYSAKTGTNSTFCAKYDFNLYSIESNLSFGCEFWQKKHHLLETNKNNNDKLEPISDELVDINPNSRATKLLHENVPDLNSAVNDIPSTLDIPVHKQKLLNDLTYAFSSSLRKIDEERSTIEKFDNKINSSIFTSVWKLSTSLRDKTLKLLWEGKWRGFLISAGTELVFTRGFQESLSDDEKNDNAISISATDTENGNIPVFPAKFGIQFQYST>YAL011WMPAVLRTRSKESSIEQKPASRTRTRSRRGKRGRDDDDDDDDEESDDAYDEVGNDYDEYASRAKLATNRPFEIVAGLPASVELPNYNSSLTHPQSIKNSGVLYDSLVSSRRTWVQGEMFELYWRRPKKIVSESTPAATESPTSGTIPLIRDKMQKMCDCVMSGGPHTFKVRLFILKNDKIEQKWQDEQELKKKEKELKRKNDAEAKRLRMEERKRQQMQKKIAKEQKLQLQKENKAKQKLEQEALKLKRKEEMKKLKEQNKNKQGSPSSSMHDPRMIMNLNLMAQEDPKLNTLMETVAKGLANNSQLEEFKKFIEIAKKRSLEENPVNKRPSVTTTRPAPPSKAKDVAEDHRLNSITLVKSSKTAATEPEPKKADDENAEKQQSKEAKTTAESTQVDVKKEEEDVKEKGVKSEDTQKKEDNQVVPKRKRRKNAIKEDKDMQLTAFQQKYVQGAEIILEYLEFTHSRYYLPKKSVVEFLEDTDEIIISWIVIHNSKEIEKFKTKKIKAKLKADQKLNKEDAKPGSDVEKEVSFNPLFEADCPTPLYTPMTMKLSGIHKRFNQIIRNSVSPMEEVVKEMEKILQIGTRLSGYNLWYQLDGYDDEALSESLRFELNEWEHAMRSRRHKR>YAL013WMSQQTPQESEQTTAKEQDLDQESVLSNIDFNTDLNHNLNLSEYCISSDAGTEKMDSDEEKSLANLPELKYAPKLSSLVKQETLTESLKRPHEDEKEAIDEAKKMKVPGENEDESKEEEKSQELEEAIDSKEKSTDARDEQGDEGDNEEENNEEDNENENEHTAPPALVMPSPIEMEEQRMTALKEITDIEYKFAQLRQKLYDNQLVRLQTELQMCLEGSHPELQVYYSKIAAIRDYKLHRAYQRQKYELSCINTETIATRTFIHQDFHKKVTDLRARLLNRTTQTWYDINKERRDMDIVIPDVNYHVPIKLDNKTLSCITGYASAAQLCYPGEPVAEDLACESIEYRYRANPVDKLEVIVDRMRLNNEISDLEGLRKYFHSFPGAPELNPLRDSEINDDFHQWAQCDRHTGPHTTSFCYS>YAL016WMSGARSTTAGAVPSAATTSTTSTTSNSKDSDSNESLYPLALLMDELKHDDIANRVEAMKKLDTIALALGPERTRNELIPFLTEVAQDDEDEVFAVLAEQLGKFVPYIGGPQYATILLPVLEILASAEETLVREKAVDSLNNVAQELSQEQLFSDFVPLIEHLATADWFSSKVSACGLFKSVIVRIKDDSLRKNILALYLQLAQDDTPMVKRAVGKNLPILIDLLTQNLGLSTDEDWDYISNIFQKIINDNQDSVKFLAVDCLISILKFFNAKGDESHTQDLLNSAVKLIGDEAWRVRYMAADRFSDLASQFSSNQAYIDELVQPFLNLCEDNEGDVREAVAKQVSGFAKFLNDPSIILNKILPAVQNLSMDESETVRSALASKITNIVLLLNKDQVINNFLPILLNMLRDEFPDVRLNIIASLKVVNDVIGIELLSDSLLPAITELAKDVNWRVRMAIIEYIPILAEQLGMQFFDQQLSDLCLSWLWDTVYSIREAAVNNLKRLTEIFGSDWCRDEIISRLLKFDLQLLENFVSRFTILSALTTLVPVVSLDVVTEQLLPFISHLADDGVPNIRFNVAKSYAVIVKVLIKDEAKYDALIKNTILPSLQTLCQDEDVDVKYFAKKSLAECQELLKN>YAL018CMSFTGSLALAGIGGLVYKFGGGQSYEKLPYVNIPFNQYLDKVYKKHFSKVMSRTRYVLMNFFKDAFTGGAFMYPFKGFLEFNTNKSSYSTTMLGILSSYLIMFALVSFVYWATITPMYTAFLIVLGPIGLFIAIFHSFLQANVFTLLFMRLSHFNNHLVEVCLEKNGLEENLSEVKPIKYYAPINSIYFWAYYFPFKLVKYMLGLSVLFVLLVISFFPLIGPILFHILISPFITQIYFTKVLRLQNFDNIQRRENIYLHAGQYASFGFLAGLIESVPILAGFAISTNTIGSVLFNLDHPMVPENLVETQAEIEAAPQDINQQPNQ>YAL019WMSGSHSNDEDDVVQVPETSSPTKVASSSPLKPTSPTVPDASVASLRSRFTFKPSDPSEGAHTSKPLPSGSPEVALVNLAREFPDFSQTLVQAVFKSNSFNLQSARERLTRLRQQRQNWTWNKNASPKKSETPPPVKKSLPLANTGRLSSIHGNINNKSSKITVAKQKTSIFDRYSNVINQKQYTFELPTNLNIDSEALSKLPVNYNKKRRLVRADQHPIGKSYESSATQLGSAREKLLANRKYGRHANDNDEEEEESMMTDDDDASGDDYTESTPQINLDEQVLQFINDSDIVDLSDLSDTTMHKAQLIASHRPYSSLNAFVNTNFNDKDTEENASNKRKRRAAASANESERLLDKITQSIRGYNAIESVIKKCSSYGDLVTSQMKKWGVQVEGDNSELDLMNLGEDDDDDNDDGNNDNNNSNNNNTAGADATSKEKEDTKAVVEGFDETSAEPTPAPAPAPVERETKRIRNTTKPKVVEDEDDDVDLEAIDDELPQSEHEDDDYEEEDEDYNDEEEDVEYDDGDDDDDDDDEFVATRKNTHVISTTSRNGRKPIVKFFKGKPRLLSPEISLKDYQQTGINWLNLLYQNKMSCILADDMGLGKTCQVISFFAYLKQINEPGPHLVVVPSSTLENWLREFQKFAPALKIEPYYGSLQEREELRDILERNAGKYDVIVTTYNLAAGNKYDVSFLKNRNFNVVVYDEGHMLKNSTSERFAKLMKIRANFRLLLTGTPLQNNLKELMSLLEFIMPNLFISKKESFDAIFKQRAKTTDDNKNHNPLLAQEAITRAKTMMKPFILRRRKDQVLKHLPPKHTHIQYCELNAIQKKIYDKEIQIVLEHKRMIKDGELPKDAKEKSKLQSSSSKNLIMALRKASLHPLLFRNIYNDKIITKMSDAILDEPAYAENGNKEYIKEDMSYMTDFELHKLCCNFPNTLSKYQLHNDEWMQSGKIDALKKLLKTIIVDKQEKVLIFSLFTQVLDILEMVLSTLDYKFLRLDGSTQVNDRQLLIDKFYEDKDIPIFILSTKAGGFGINLVCANNVIIFDQSFNPHDDRQAADRAHRVGQTKEVNITTLITKDSIEEKIHQLAKNKLALDSYISEDKKSQDVLESKVSDMLEDIIYDENSKPKGTKE>YAL020CMSCVYAFGSNGQRQLGLGHDEDMDTPQRSVPGDDGAIVRKIACGGNHSVMLTNDGNLVGCGDNRRGELDSAQALRQVHDWRPVEVPAPVVDVACGWDTTVIVDADGRVWQRGGGCYEFTQQHVPLNSNDERIAVYGCFQNFVVVQGTRVYGWGSNTKCQLQEPKSRSLKEPVLVYDTGSVAVDYVAMGKDFMVIVDEGGRIVHASGRLPTGFELKQQQKRHNLVVLCMWTSIHLWNARLNTVESFGRGTHSQLFPQERLDFPIVGVATGSEHGILTTANQEGKSHCYNVYCWGWGEHGNCGPQKASQPGLQLVGQYSGKPRVFGGCATTWIVL>YAL022CMSTSADTDTIKKPILAVPEPALADTHSEEISRSGEEHESENNEHSDEEGDNYSEREQSVSTEPLDTLPLRKKLKNLSYITFFAIGIGLLWPWNCILSASQYFKHDIFKDTSIWAKIFTSSMMSFSTISSMLFNIYLAKRQYKYSRRVINGLVWEIIVFTVMCFFTILHFLLPKWFNFMFIMMLVVISSMGTAMTQNGIMAIANVFGSEYSQGVMVGQAVAGVLPSLVLFALAFIENSSVSTTGGILLYFFTTTLVVTICVVMFSVSKISRKVNENWNVEDGHITDVLLGSLRSNEEEIRIVGRIDQMEDEDHRRTNGTRDDNDEGEELQLKVPFEVLFAKLKYLVLSIFTTFVVTLVFPVFASATYVTGLPLSNAQYIPLIFTLWNLGDLYGRVIADWPMFRDQKFTPRKTFIYSLLRVAAIPLFLMFTAITSSSSGDEEHNGSVIVDLCYMLLQFLFGVTNGHVISMSFMKVPEQLDNDDEKEAAGGFTNIFVSTGLALGSIISYVFVFIIDFIIR>YAL023CMSSSSSTGYSKNNAAHIKQENTLRQRESSSISVSEELSSADERDAEDFSKEKPAAQSSLLRLESVVMPVIFTALALFTRMYKIGINNHVVWDEAHFGKFGSYYLRHEFYHDVHPPLGKMLVGLSGYLAGYNGSWDFPSGEIYPDYLDYVKMRLFNASFSALCVPLAYFTAKAIGFSLPTVWLMTVLVLFENSYSTLGRFILLDSMLLFFTVASFFSFVMFHNQRSKPFSRKWWKWLLITGISLGCTISVKMVGLFIITMVGIYTVIDLWTFLADKSMSWKTYINHWLARIFGLIIVPFCIFLLCFKIHFDLLSHSGTGDANMPSLFQARLVGSDVGQGPRDIALGSSVVSIKNQALGGSLLHSHIQTYPDGSNQQQVTCYGYKDANNEWFFNRERGLPSWSENETDIEYLKPGTSYRLVHKSTGRNLHTHPVAAPVSKTQWEVSGYGDNVVGDNKDNWVIEIMDQRGDEDPEKLHTLTTSFRIKNLEMGCYLAQTGNSLPEWGFRQQEVVCMKNPFKRDKRTWWNIETHENERLPPRPEDFQYPKTNFLKDFIHLNLAMMATNNALVPDPDKFDYLASSAWQWPTLNVGLRLCGWGDDNPKYFLLGTPASTWASSVAVLAFMATVVILLIRWQRQYVDLRNPSNWNVFLMGGFYPLLAWGLHYMPFVIMSRVTYVHHYLPALYFALIILAYCFDAGLQKWSRSKCGRIMRFVLYAGFMALVIGCFWYFSPISFGMEGPSSNFRYLNWFSTWDIADKQEA>YAL025CMSDEIVWQVINQSFCSHRIKAPNGQNFCRNEYNVTGLCTRQSCPLANSKYATVKCDNGKLYLYMKTPERAHTPAKLWERIKLSKNYTKALQQIDEHLLHWSKFFRHKCKQRFTKLTQVMITERRLALREEERHYVGVAPKVKRREQNRERKALVAAKIEKAIEKELMDRLKSGAYGDKPLNVDEKVWKKIMGQMEEENSQDEEEDWDEEEESDDGEVEYVADDGEGEYVDVDDLEKWLADSDREASSASQSESDSESESDSDSDEENKNSAKRRKKGTSAKTKRPKVEIEYEEEHEVQNAEQEVAQ>YAL027WMAPSIATVKIARDMVLPLRIFVNRKQILQTNDKTSNKSNATIFEAPLLSNNSIICLKSPNTRIYLSQQDKKNLCDEIKEDLLLIVYELASPEIISSVLSKIRVGHSTDFQINVLPKLFAGADTDNAVTSHIQSVTRLAKFKYKLHYKHKWELDIFINSIKKIANLRHYLMFQTLTLNGFSLNAGPKTLLARKIEKQPQVPNLLIENGDADALDTPVEEDIKPVIEFMYKPVINLGEIIDVHVLHRPRRHKVRTQSKQPQEE>YAL028WMQNAQIKSSSKGSGIDGTDRNSKDGVEKRPLEDVKQMIDAGTPDVGHKSTVETKPNVGWQASHSNLAALHEKEQKYEMEHHHARHKLHRQVIPDYTSASTAMFSDCMFNAAPDKVRSLSTMKSSGLSPKHPFNVVATFKGPFPQHSVESKPLDGGYSAKDHFPSFKMLQAQQHPAHRHYKDNDKYGLKSPSRSFVKDKKRLVHRFLKSMEPSSSGQSKDSSALAPAFDPILPNVISKPSKRPTHHSHSSDGSSSTQTDISLQSLLYHDLESSPKKHVSPSRPPSVASESSPAVANPIGLSPKDACNASFSQSSSSSLSSSSSSSSSTSFSQSVAVDPLEPPGNITYSSSNLSLNSDELDYYQRHIGLQLQQTEALLKHSLKDEVLKDENDLVKNIANFDKIVKELRDLRSRTIGWKELVEEDYLMNLKQDFDKENPESFEARLSDTINTNVAKLQDLEKRMASCKDRLASRKEVMRKMESLLSLENSLMISKKNVTFASKYRNEALDIVFLIIIIVICYTFKHLVSHK>YAL032CMFSNRLPPPKHSQGRVSTALSSDRVEPAILTDQIAKNVKLDDFIPKRQSNFELSVPLPTKAEIQECTARTKSYIQRLVNAKLANSNNRASSRYVTETHQAPANLLLNNSHHIEVVSKQMDPLLPRFVGKKARKVVAPTENDEVVPVLHMDGSNDRGEADPNEWKIPAAVSNWKNPNGYTVALERRVGKALDNENNTINDGFMKLSEALENADKKARQEIRSKMELKRLAMEQEMLAKESKLKELSQRARYHNGTPQTGAIVKPKKQTSTVARLKELAYSQGRDVSEKIILGAAKRSEQPDLQYDSRFFTRGANASAKRHEDQVYDNPLFVQQDIESIYKTNYEKLDEAVNVKSEGASGSHGPIQFTKAESDDKSDNYGA>YAL033WMVRLKSRYILFEIIFPPTDTNVEESVSKADILLSHHRASPADVSIKSILQEIRRSLSLNLGDYGSAKCNSLLQLKYFSNKTSTGIIRCHREDCDLVIMALMLMSKIGDVDGLIVNPVKVSGTIKKIEQFAMRRNSKILNIIKCSQSSHLSDNDFIINDFKKIGRENENENEDD>YAL034CMGLYSPESEKSQLNMNYIGKDDSQSIFRRLNQNLKASNNNNDSNKNGLNMSDYSNNSPYGRSYDVRINQNSQNNGNGCFSGSIDSLVDEHIIPSPPLSPKLESKISHNGSPRMASSVLVGSTPKGAVENVLFVKPVWPNGLSRKRYRYATYGFLSQYKIFSNLAQPYSKNIINRYNNLAYNARHKYSKYNDDMTPPPLPSSSSRLPSPLASPNLNRQARYNMRKQALYNNNLGKFESDTEWIPRKRKVYSPQRRTMTTSPHRAKKFSPSASTPHTNIASIEAIHDAPQYIPNVSWKKLPDYSPPLSTLPTDSNKSLKIEWKGSPMDLSTDPLRNELHPAELVLAQTLRLPCDLYLDSKRRLFLEKVYRLKKGLPFRRTDAQKACRIDVNKASRLFQAFEKVGWLQDSNFTKYL>YAL034W-AMSAPTMRSTSILTEHLGYPPISLVDDIINAVNEIMYKCTAAMEKYLLSKSKIGEEDYGEEIKSGVAKLESLLENSVDKNFDKLELYVLRNVLRIPEEYLDANVFRLENQKDLVIVDENELKKSEEKLREKVNDVELAFKKNEMLLKRVTKVKRLLFTIRGFKQKLNELLKCKDDVQLQKILESLKPIDDTMTLLTDSLRKLYVDSESTSSTEEVEALLQRLKTNGKQNNKDFRTRYIDIRTNNVLRKLGLLGDKEDEKQSAKPDARTQAGDIVSIDIEEPQLDLLDDVL>YAL035WMAKKSKKNQQNYWDEEFEEDAAQNEEISATPTPNPESSAGADDTSREASASAEGAEAIEGDFMSTLKQSKKKQEKKVIEEKKDGKPILKSKKEKEKEKKEKEKQKKKEQAARKKAQQQAQKEKNKELNKQNVEKAAAEKAAAEKSQKSKGESDKPSASAKKPAKKVPAGLAALRRQLELKKQLEEQEKLEREEEERLEKEEEERLANEEKMKEEAKAAKKEKEKAKREKRKAEGKLLTRKQKEEKKLLERRRAALLSSGNVKVAGLAKKDGEENKPKKVVYSKKKKRTTQENASEAIKSDSKKDSEVVPDDELKESEDVLIDDWENLALGDDDEEGTNEETQESTASHENEDQNQGEEEEEGEEEEEEEEERAHVHEVAKSTPAATPAATPTPSSASPNKKDLRSPICCILGHVDTGKTKLLDKIRQTNVQGGEAGGITQQIGATYFPIDAIKAKTKVMAEYEKQTFDVPGLLVIDTPGHESFSNLRSRGSSLCNIAILVIDIMHGLEQQTIESIKLLRDRKAPFVVALNKIDRLYDWKAIPNNSFRDSFAKQSRAVQEEFQSRYSKIQLELAEQGLNSELYFQNKNMSKYVSIVPTSAVTGEGVPDLLWLLLELTQKRMSKQLMYLSHVEATILEVKVVEGFGTTIDVILSNGYLREGDRIVLCGMNGPIVTNIRALLTPQPLRELRLKSEYVHHKEVKAALGVKIAANDLEKAVSGSRLLVVGPEDDEDELMDDVMDDLTGLLDSVDTTGKGVVVQASTLGSLEALLDFLKDMKIPVMSIGLGPVYKRDVMKASTMLEKAPEYAVMLCFDVKVDKEAEQYAEQEGIKIFNADVIYHLFDSFTAYQEKLLEERRKDFLDYAIFPCVLQTLQIINKRGPMIIGVDVLEGTLRVGTPICAVKTDPTTKERQTLILGKVISLEINHQPVQEVKKGQTAAGVAVRLEDPSGQQPIWGRHVDENDTLYSLVSRRSIDTLKDKAFRDQVARSDWLLLKKLKVVFGIE>YAL036CMSTTVEKIKAIEDEMARTQKNKATSFHLGQLKAKLAKLRRELLTSASSGSGGGAGIGFDVARTGVASVGFVGFPSVGKSTLLSKLTGTESEAAEYEFTTLVTVPGVIRYKGAKIQMLDLPGIIDGAKDGRGRGKQVIAVARTCNLLFIILDVNKPLHHKQIIEKELEGVGIRLNKTPPDILIKKKEKGGISITNTVPLTHLGNDEIRAVMSEYRINSAEIAFRCDATVDDLIDVLEASSRRYMPAIYVLNKIDSLSIEELELLYRIPNAVPISSGQDWNLDELLQVMWDRLNLVRIYTKPKGQIPDFTDPVVLRSDRCSVKDFCNQIHKSLVDDFRNALVYGSSVKHQPQYVGLSHILEDEDVVTILKK>YAL037WMDMEIEDSSPIDDLKLQKLDTNVYFGPCEILTQPILLQYENIKFIIGVNLSTEKIASFYTQYFRNSNSVVVNLCSPTTAAVATKKAAIDLYIRNNTILLQKFVGQYLQMGKKIKTSLTQAQTDTIQSLPQFCNSNVLSGEPLVQYQAFNDLLALFKSFSHFGNILVISSHSYDCALLKFLISRVMTYYPLVTIQDSLQYMKATLNISISTSDEFDILNDKELWEFGQTQEILKRRQTSSVKRRCVNLPENSTIDNRMLMGTTKRGRF>YAL038WMSRLERLTSLNVVAGSDLRRTSIIGTIGPKTNNPETLVALRKAGLNIVRMNFSHGSYEYHKSVIDNARKSEELYPGRPLAIALDTKGPEIRTGTTTNDVDYPIPPNHEMIFTTDDKYAKACDDKIMYVDYKNITKVISAGRIIYVDDGVLSFQVLEVVDDKTLKVKALNAGKICSHKGVNLPGTDVDLPALSEKDKEDLRFGVKNGVHMVFASFIRTANDVLTIREVLGEQGKDVKIIVKIENQQGVNNFDEILKVTDGVMVARGDLGIEIPAPEVLAVQKKLIAKSNLAGKPVICATQMLESMTYNPRPTRAEVSDVGNAILDGADCVMLSGETAKGNYPINAVTTMAETAVIAEQAIAYLPNYDDMRNCTPKPTSTTETVAASAVAAVFEQKAKAIIVLSTSGTTPRLVSKYRPNCPIILVTRCPRAARFSHLYRGVFPFVFEKEPVSDWTDDVEARINFGIEKAKEFGILKKGDTYVSIQGFKAGAGHSNTLQVSTV>YAL039CMGWFWADQKTTGKDIGGAAVSSMSGCPVMHESSSSSPPSSECPVMQGDNDRINPLNNMPELAASKQPGQKMDLPVDRTISSIPKSPDSNEFWEYPSPQQMYNAMVRKGKIGGSGEVAEDAVESMVQVHNFLNEGCWQEVLEWEKPHTDESHVQPKLLKFMGKPGVLSPRARWMHLCGLLFPSHFSQELPFDRHDWIVLRGERKAEQQPPTFKEVRYVLDFYGGPDDENGMPTFHVDVRPALDSLDNAKDRMTRFLDRMISGPSSSSSAP>YAL040CMAILKDTIIRYANARYATASGTSTATAASVSAASCPNLPLLLQKRRAIASAKSKNPNLVKRELQAHHSAISEYNNDQLDHYFRLSHTERPLYNLTNFNSQPQVNPKMRFLIFDFIMYCHTRLNLSTSTLFLTFTILDKYSSRFIIKSYNYQLLSLTALWISSKFWDSKNRMATLKVLQNLCCNQYSIKQFTTMEMHLFKSLDWSICQSATFDSYIDIFLFQSTSPLSPGVVLSAPLEAFIQQKLALLNNAAGTAINKSSSSQGPSLNINEIKLGAIMLCELASFNLELSFKYDRSLIALGAINLIKLSLNYYNSNLWENINLALEENCQDLDIKLSEISNTLLDIAMDQNSFPSSFKSKYLNSNKTSLAKSLLDALQNYCIQLKLEEFYRSQELETMYNTIFAQSFDSDSLTCVYSNATTPKSATVSSAATDYFSDHTHLRRLTKDSISPPFAFTPTSSSSSPSPFNSPYKTSSSMTTPDSASHHSHSGSFSSTQNSFKRSLSIPQNSSIFWPSPLTPTTPSLMSNRKLLQNLSVRSKRLFPVRPMATAHPCSAPTQLKKRSTSSVDCDFNDSSNLKKTR>YAL041WMAIQTRFASGTSLSDLKPKPSATSISIPMQNVMNKPVTEQDSLFHICANIRKRLEVLPQLKPFLQLAYQSSEVLSERQSLLLSQKQHQELLKSNGANRDSSDLAPTLRSSSISTATSLMSMEGISYTNSNPSATPNMEDTLLTFSMGILPITMDCDPVTQLSQLFQQGAPLCILFNSVKPQFKLPVIASDDLKVCKKSIYDFILGCKKHFAFNDEELFTISDVFANSTSQLVKVLEVVETLMNSSPTIFPSKSKTQQIMNAENQHRHQPQQSSKKHNEYVKIIKEFVATERKYVHDLEILDKYRQQLLDSNLITSEELYMLFPNLGDAIDFQRRFLISLEINALVEPSKQRIGALFMHSKHFFKLYEPWSIGQNAAIEFLSSTLHKMRVDESQRFIINNKLELQSFLYKPVQRLCRYPLLVKELLAESSDDNNTKELEAALDISKNIARSINENQRRTENHQVVKKLYGRVVNWKGYRISKFGELLYFDKVFISTTNSSSEPEREFEVYLFEKIIILFSEVVTKKSASSLILKKKSSTSASISASNITDNNGSPHHSYHKRHSNSSSSNNIHLSSSSAAAIIHSSTNSSDNNSNNSSSSSLFKLSANEPKLDLRGRIMIMNLNQIIPQNNRSLNITWESIKEQGNFLLKFKNEETRDNWSSCLQQLIHDLKNEQFKARHHSSTSTTSSTAKSSSMMSPTTTMNTPNHHNSRQTHDSMASFSSSHMKRVSDVLPKRRTTSSSFESEIKSISENFKNSIPESSILFRISYNNNSNNTSSSEIFTLLVEKVWNFDDLIMAINSKISNTHNNNISPITKIKYQDEDGDFVVLGSDEDWNVAKEMLAENNEKFLNIRLY>YAL042WMKRSTLLSLDAFAKTEEDVRVRTRAGGLITLSCILTTLFLLVNEWGQFNSVVTRPQLVVDRDRHAKLELNMDVTFPSMPCDLVNLDIMDDSGEMQLDILDAGFTMSRLNSEGRPVGDATELHVGGNGDGTAPVNNDPNYCGPCYGAKDQSQNENLAQEEKVCCQDCDAVRSAYLEAGWAFFDGKNIEQCEREGYVSKINEHLNEGCRIKGSAQINRIQGNLHFAPGKPYQNAYGHFHDTSLYDKTSNLNFNHIINHLSFGKPIQSHSKLLGNDKRHGGAVVATSPLDGRQVFPDRNTHFHQFSYFAKIVPTRYEYLDNVVIETAQFSATFHSRPLAGGRDKDHPNTLHVRGGIPGMFVFFEMSPLKVINKEQHGQTWSGFILNCITSIGGVLAVGTVMDKLFYKAQRSIWGKKSQ>YAL043CMSSAEMEQLLQAKTLAMHNNPTEMLPKVLETTASMYHNGNLSKLKLPLAKFFTQLVLDVVSMDSPIANTERPFIAAQYLPLLLAMAQSTADVLVYKNIVLIMCASYPLVLDLVAKTSNQEMFDQLCMLKKFVLSHWRTAYPLRATVDDETDVEQWLAQIDQNIGVKLATIKFISEVVLSQTKSPSGNEINSSTIPDNHPVLNKPALESEAKRLLDMLLNYLIEEQYMVSSVFIGIINSLSFVIKRRPQTTIRILSGLLRFNVDAKFPLEGKSDLNYKLSKRFVERAYKNFVQFGLKNQIITKSLSSGSGSSIYSKLTKISQTLHVIGEETKSKGILNFDPSKGNSKKTLSRQDKLKYISLWKRQLSALLSTLGVSTKTPTPVSAPATGSSTENMLDQLKILQKYTLNKASHQGNTFFNNSPKPISNTYSSVYSLMNSSNSNQDVTQLPNDILIKLSTEAILQMDSTKLITGLSIVASRYTDLMNTYINSVPSSSSSKRKSDDDDDGNDNEEVGNDGPTANSKKIKMETEPLAEEPEEPEDDDRMQKMLQEEESAQEISGDANKSTSAIKEIAPPFEPDSLTQDEKLKYLSKLTKKLFELSGRQDTTRAKSSSSSSILLDDDDSSSWLHVLIRLVTRGIEAQEASDLIREELLGFFIQDFEQRVSLIIEWLNEEWFFQTSLHQDPSNYKKWSLRVLESLGPFLENKHRRFFIRLMSELPSLQSDHLEALKPICLDPARSSLGFQTLKFLIMFRPPVQDTVRDLLHQLKQEDEGLHKQCDSLLDRLK>YAL044CMLRTTRLWTTRMPAVSKLFLRNSSGNALNKNKLPFLYSSQGPQAVRYTSQHEWIAVHQDKTAFVGITKYATDSLGDATYVELPEVGTEISQGESLGSIESVKSASEIYQPADGTVEEINTNLEENPGVVNEDPMGDGWLVKMKLGEGVNVEQVEGLMSLEQYEKTLVHDD>YAL044W-AMFKRAMSTDGPVARTILKRLECGFPDYKNFAFGLYNDSHKHKGHAGVQGNVSAETHFRIEMVSKKFEGLKLPQRHRMVYSLLQDEMAQANGIHALQLSLKTPQEYESKAK>YAL046CMKLPQTMLRSISVKHVRWPRILTGSKLWYSTQMAMTPEEKMITDKLQQELEPEVCKVQDVSGGCGSMFAINITSKKFNGLSLIKQHQLVNRILRDDISRWHGLQLTTKKSTGKGPASS>YAL047CMVRRWIPSGRHLRNNDNTGDDDDSEFTNSMDSGMSIPSLRDSMTTRSSHNDPIKPALMNDSNKVKNLEKELTNAKIKIQVLYEYIRRIPNKDGNAPSLGNDTDFRNSIIEGLNLEINKLKQDLKAKEVEYQDTLQFVQENLENSESIVNTINHLLSFILTHFNEQDENAHLLDKEERETLEETLELSSDYVLEKMDTLSKFIIQFLQDFLHSKSRAESKQDKEEFLSLAQSSPAGSQLESRDSPSSKEENTDGGYQNDEIHDSNNHIDTENVMANSTSLPISAVESRFEKTLDTQLEIVIEILHKEYDQFINSIRLKFEKSQKLEKIIASKLNEQSHLLDSLELEENSSSVIEKQDHLISQLKEKIESQSVLINNLEKLKEDIIKMKQNEKVLTKELETQTKINKLKENNWDSYINDLEKQINDLQIDKSEEFHVIQNQLDKLDLENYQLKNQLNTLDNQKLILSQYESNFIKFNQNLLLHLDSIFNILQKILQESSIAQFDRKMKSIKSVPNALKNLNLIQPKLESLYTFIETALESIINSYISSLISMETPEQPHQQGNELTATPNKELTLRIEELQRRWISERERRKLDANASEARIKALEQENESLRSKLFNLSINNP>YAL048CMTKETIRVVICGDEGVGKSSLIVSLTKAEFIPTIQDVLPPISIPRDFSSSPTYSPKNTVLIDTSDSDLIALDHELKSADVIWLVYCDHESYDHVSLFWLPHFRSLGLNIPVILCKNKCDSISNVNANAMVVSENSDDDIDTKVEDEEFIPILMEFKEIDTCIKTSAKTQFDLNQAFYLCQRAITHPISPLFDAMVGELKPLAVMALKRIFLLSDLNQDSYLDDNEILGLQKKCFNKSIDVNELNFIKDLLLDISKHDQEYINRKLYVPGKGITKDGFLVLNKIYAERGRHETTWAILRTFHYTDSLCINDKILHPRLVVPDTSSVELSPKGYRFLVDIFLKFDIDNDGGLNNQELHRLFKCTPGLPKLWTSTNFPFSTVVNNKGCITLQGWLAQWSMTTFLNYSTTTAYLVYFGFQEDARLALQVTKPRKMRRRSGKLYRSNINDRKVFNCFVIGKPCCGKSSLLEAFLGRSFSEEYSPTIKPRIAVNSLELKGGKQYYLILQELGEQEYAILENKDKLKECDVICLTYDSSDPESFSYLVSLLDKFTHLQDLPLVFVASKADLDKQQQRCQIQPDELADELFVNHPLHISSRWLSSLNELFIKITEAALDPGKNTPGLPEETAAKDVDYRQTALIFGSTVGFVALCSFTLMKLFKSSKFSK>YAL049CMASNQPGKCCFEGVCHDGTPKGRREEIFGLDTYAAGSTSPKEKVIVILTDVYGNKFNNVLLTADKFASAGYMVFVPDILFGDAISSDKPIDRDAWFQRHSPEVTKKIVDGFMKLLKLEYDPKFIGVVGYCFGAKFAVQHISGDGGLANAAAIAHPSFVSIEEIEAIDSKKPILISAAEEDHIFPANLRHLTEEKLKDNHATYQLDLFSGVAHGFAARGDISIPAVKYAKEKVLLDQIYWFNHFSNV>YAL054CMSPSAVQSSKLEEQSSEIDKLKAKMSQSAATAQQKKEHEYEHLTSVKIVPQRPISDRLQPAIATHYSPHLDGLQDYQRLHKESIEDPAKFFGSKATQFLNWSKPFDKVFIPDPKTGRPSFQNNAWFLNGQLNACYNCVDRHALKTPNKKAIIFEGDEPGQGYSITYKELLEEVCQVAQVLTYSMGVRKGDTVAVYMPMVPEAIITLLAISRIGAIHSVVFAGFSSNSLRDRINDGDSKVVITTDESNRGGKVIETKRIVDDALRETPGVRHVLVYRKTNNPSVAFHAPRDLDWATEKKKYKTYYPCTPVDSEDPLFLLYTSGSTGAPKGVQHSTAGYLLGALLTMRYTFDTHQEDVFFTAGDIGWITGHTYVVYGPLLYGCATLVFEGTPAYPNYSRYWDIIDEHKVTQFYVAPTALRLLKRAGDSYIENHSLKSLRCLGSVGEPIAAEVWEWYSEKIGKNEIPIVDTYWQTESGSHLVTPLAGGVTPMKPGSASFPFFGIDAVVLDPNTGEELNTSHAEGVLAVKAAWPSFARTIWKNHDRYLDTYLNPYPGYYFTGDGAAKDKDGYIWILGRVDDVVNVSGHRLSTAEIEAAIIEDPIVAECAVVGFNDDLTGQAVAAFVVLKNKSSWSTATDDELQDIKKHLVFTVRKDIGPFAAPKLIILVDDLPKTRSGKIMRRILRKILAGESDQLGDVSTLSNPGIVRHLIDSVKL>YAL055WMPPPSRSRINKTRTLGIVGTAIAVLVTSYYIYQKVTSAKEDNGARPPEGDSVKENKKARKSKCIIMSKSIQGLPIKWEEYAADEVVLLVPTSHTDGSMKQAIGDAFRKTKNEHKIIYCDSMDGLWSCVRRLGKFQCILNSRDFTSSGGSDAAVVPEDIGRFVKFVVDSDVEDVLIDTLCN>YAL059WMWEQRRQKVVFSLTILVRYRLKQSMAKKISKNSRAARQSDALEPEVKDLSELPRAEKTDLTNILIRTAAKNEALLEAKISKKANKSKRGKKLNKKALEDKLDNSISSMDRDRLVKALNFTNRLDGKIAKSISRAKYIQNTRKAGWDSTNETIKKELAFLNGGLSVQAKSASEGNAEKEDEEIPEVFDSLAEDNTVQKTPTNRFGVLPDDVEE>YAL060WMRALAYFKKGDIHFTNDIPRPEIQTDDEVIIDVSWCGICGSDLHEYLDGPIFMPKDGECHKLSNAALPLAMGHEMSGIVSKVGPKVTKVKVGDHVVVDAASSCADLHCWPHSKFYNSKPCDACQRGSENLCTHAGFVGLGVISGGFAEQVVVSQHHIIPVPKEIPLDVAALVEPLSVTWHAVKISGFKKGSSALVLGAGPIGLCTILVLKGMGASKIVVSEIAERRIEMAKKLGVEVFNPSKHGHKSIEILRGLTKSHDGFDYSYDCSGIQVTFETSLKALTFKGTATNIAVWGPKPVPFQPMDVTLQEKVMTGSIGYVVEAFEEVVRAIHNGDIAMEDCKQLITGKQRIEDGWEKGFQELMDHKESNVKILLTPNNHGEMK>YAL061WMRALAYFGKGNIRFTNHLKEPHIVAPDELVIDIEWCGICGTDLHEYTDGPIFFPEDGHTHEISHNPLPQAMGHEMAGTVLEVGPGVKNLKVGDKVVVEPTGTCRDRYRWPLSPNVDKEWCAACKKGYYNICSYLGLCGAGVQSGGFAERVVMNESHCYKVPDFVPLDVAALIQPLAVCWHAIRVCEFKAGSTALIIGAGPIGLGTILALNAAGCKDIVVSEPAKVRRELAEKMGARVYDPTAHAAKESIDYLRSIADGGDGFDYTFDCSGLEVTLNAAIQCLTFRGTAVNLAMWGHHKIQFSPMDITLHERKYTGSMCYTHHDFEAVIEALEEGRIDIDRARHMITGRVNIEDGLDGAIMKLINEKESTIKIILTPNNHGELNREADNEKKEISELSSRKDQERLRESINEAKLRHT>YAL062WMTSEPEFQQAYDEIVSSVEDSKIFEKFPQYKKVLPIVSVPERIIQFRVTWENDNGEQEVAQGYRVQFNSAKGPYKGGLRFHPSVNLSILKFLGFEQIFKNALTGLDMGGGKGGLCVDLKGKSDNEIRRICYAFMRELSRHIGKDTDVPAGDIGVGGREIGYLFGAYRSYKNSWEGVLTGKGLNWGGSLIRPEATGFGLVYYTQAMIDYATNGKESFEGKRVTISGSGNVAQYAALKVIELGGIVVSLSDSKGCIISETGITSEQIHDIASAKIRFKSLEEIVDEYSTFSESKMKYVAGARPWTHVSNVDIALPCATQNEVSGDEAKALVASGVKFVAEGANMGSTPEAISVFETARSTATNAKDAVWFGPPKAANLGGVAVSGLEMAQNSQKVTWTAERVDQELKKIMINCFNDCIQAAQEYSTEKNTNTLPSLVKGANIASFVMVADAMLDQGDVF>YAR002C-AMLLTSLLQVFACCLVLPAQVTAFYYYTSGAERKCFHKELSKGTLFQATYKAQIYDDQLQNYRDAGAQDFGVLIDIEETFDDNHLVVHQKGSASGDLTFLASDSGEHKICIQPEAGGWLIKAKTKIDVEFQVGSDEKLDSKGKATIDILHAKVNVLNSKIGEIRREQKLMRDREATFRDASEAVNSRAMWWIVIQLIVLAVTCGWQMKHLGKFFVKQKIL>YAR002WMHRKSLRRASATVPSAPYRKQIISNAHNKPSLFSKIKTFFTQKDSARVSPRNNVANKQPRNESFNRRISSMPGGYFHSEISPDSTVNRSVVVSAVGEARNDIENKEEEYDETHETNISNAKLANFFSKKGNEPLSEIEIEGVMSLLQKSSKSMITSEGEQKSAEGNNIDQSLILKESGSTPISISNAPTFNPKYDTSNASMNTTLGSIGSRKYSFNYSSLPSPYKTTVYRYSAAKKIPDTYTANTSAQSIASAKSVRSGVSKSAPSKKISNTAAALVSLLDENDSKKNNAASELANPYSSYVSQIRKHKRVSPNAAPRQEISEEETTVKPLFQNVPEQGEEPMKQLNATKISPSAPSKDSFTKYKPARSSSLRSNVVVAETSPEKKDGGDKPPSSAFNFSFNTSRNVEPTENAYKSENAPSASSKEFNFTNLQAKPLVGKPKTELTKGDSTPVQPDLSVTPQKSSSKGFVFNSVQKKSRSNLSQENDNEGKHISASIDNDFSEEKAEEFDFNVPVVSKQLGNGLVDENKVEAFKSLYTF>YAR003WMNILLQDPFAVLKEHPEKLTHTIENPLRTECLQFSPCGDYLALGCANGALVIYDMDTFRPICVPGNMLGAHVRPITSIAWSPDGRLLLTSSRDWSIKLWDLSKPSKPLKEIRFDSPIWGCQWLDAKRRLCVATIFEESDAYVIDFSNDPVASLLSKSDEKQLSSTPDHGYVLVCTVHTKHPNIIIVGTSKGWLDFYKFHSLYQTECIHSLKITSSNIKHLIVSQNGERLAINCSDRTIRQYEISIDDENSAVELTLEHKYQDVINKLQWNCILFSNNTAEYLVASTHGSSAHELYIWETTSGTLVRVLEGAEEELIDINWDFYSMSIVSNGFESGNVYVWSVVIPPKWSALAPDFEEVEENVDYLEKEDEFDEVDEAEQQQGLEQEEEIAIDLRTREQYDVRGNNLLVERFTIPTDYTRIIKMQSS>YAR007CMSSVQLSRGDFHSIFTNKQRYDNPTGGVYQVYNTRKSDGANSNRKNLIMISDGIYHMKALLRNQAASKFQSMELQRGDIIRVIIAEPAIVRERKKYVLLVDDFELVQSRADMVNQTSTFLDNYFSEHPNETLKDEDITDSGNVANQTNASNAGVPDMLHSNSNLNANERKFANENPNSQKTRPIFAIEQLSPYQNVWTIKARVSYKGEIKTWHNQRGDGKLFNVNFLDTSGEIRATAFNDFATKFNEILQEGKVYYVSKAKLQPAKPQFTNLTHPYELNLDRDTVIEECFDESNVPKTHFNFIKLDAIQNQEVNSNVDVLGIIQTINPHFELTSRAGKKFDRRDITIVDDSGFSISVGLWNQQALDFNLPEGSVAAIKGVRVTDFGGKSLSMGFSSTLIPNPEIPEAYALKGWYDSKGRNANFITLKQEPGMGGQSAASLTKFIAQRITIARAQAENLGRSEKGDFFSVKAAISFLKVDNFAYPACSNENCNKKVLEQPDGTWRCEKCDTNNARPNWRYILTISIIDETNQLWLTLFDDQAKQLLGVDANTLMSLKEEDPNEFTKITQSIQMNEYDFRIRAREDTYNDQSRIRYTVANLHSLNYRAEADYLADELSKALLA>YAR008WMPPLVFDIDHIKLLRKWGICGVLSGTLPTAAQQNVFLSVPLRLMLEDVLWLHLNNLADVKLIRQEGDEIMEGITLERGAKLSKIVNDRLNKSFEYQRKFKKDEHIAKLKKIGRINDKTTAEELQRLDKSSNNDQLIESSLFIDIANTSMILRDIRSDSDSLSRDDISDLLFKQYRQAGKMQTYFLYKALRDQGYVLSPGGRFGGKFIAYPGDPLRFHSHLTIQDAIDYHNEPIDLISMISGARLGTTVKKLWVIGGVAEETKETHFFSIEWAGFG>YAR014CMSNKEEHVDETSASGVKEVSSIAARHDNGYAPSLITSTSGMDSFQSHALLNDPTLIEDYSDIINNRPTSGSKLTLGNEDSESMGGSVVVTPTSNKSSPFNSKLNILSNAAEKGHDVLRNRDDDKELEEENVEKHMHSNSKRDQRHYKENSSELPDSYDYSDSEFEDNLERRLQEIETDSVDSADKDEVHFSVNNTMNPDVDDFSDGLKYAISEDEDEEENYSDDDDFDRKFQDSGFQGEKDDLEEENDDYQPLSPPRELDPDKLYALYAFNGHDSSHCQLGQDEPCILLNDQDAYWWLVKRITDGKIGFAPAEILETFPERLARLNCWKNENMSSQSVASSDSKDDSISSGNKNQSDAESIIPTPALNGYGKGNKSVSFNDVVGYADRFIDDAIEDTSLDSNDDGGEGNGQSYDDDVDNDKETKVTHRDEYTEAKLNFAKFQDDDTSDVVSDVSFSTSLNTPLNVKKVRRQDNKNESEPKTSSSKDREDDYNANRYVGQEKSEPVDSDYDTDLKKVFEAPRMPFANGMAKSDSQNSLSTIGEFSPSSSEWTNESPSTPIVEESSSIPSSRAIRTFTYIMQNRKLRDNKRGKHRGQIQASLGSSGGMPNQTDAEQPKEELEKHHSTPEEEKQSTLSLHSSSEEDFYMDEQRAVSSASINSSLSGSRALSNTNMSDPASKPNSLVQHLYAPVFDRMDVLMKQLDEIIRK>YAR015WMSITKTELDGILPLVARGKVRDIYEVDAGTLLFVATDRISAYDVIMENSIPEKGILLTKLSEFWFKFLSNDVRNHLVDIAPGKTIFDYLPAKLSEPKYKTQLEDRSLLVHKHKLIPLEVIVRGYITGSAWKEYVKTGTVHGLKQPQGLKESQEFPEPIFTPSTKAEQGEHDENISPAQAAELVGEDLSRRVAELAVKLYSKCKDYAKEKGIIIADTKFEFGIDEKTNEIILVDEVLTPDSSRFWNGASYKVGESQDSYDKQFLRDWLTANKLNGVNGVKMPQDIVDRTRAKYIEAYETLTGSKWSH>YAR018CMHRRQFFQEYRSPQQQQGHPPRSEYQVLEEIGRGSFGSVRKVIHIPTKKLLVRKDIKYGHMNSKERQQLIAECSILSQLKHENIVEFYNWDFDEQKEVLYLYMEYCSRGDLSQMIKHYKQEHKYIPEKIVWGILAQLLTALYKCHYGVELPTLTTIYDRMKPPVKGKNIVIHRDLKPGNIFLSYDDSDYNINEQVDGHEEVNSNYYRDHRVNSGKRGSPMDYSQVVVKLGDFGLAKSLETSIQFATTYVGTPYYMSPEVLMDQPYSPLSDIWSLGCVIFEMCSLHPPFQAKNYLELQTKIKNGKCDTVPEYYSRGLNAIIHSMIDVNLRTRPSTFELLQDIQIRTARKSLQLERFERKLLDYENELTNIEKILEKQAIEYERELSQLKEQFTQAVEERAREVISGKKVGKVPESINGYYGKKFAKPAYHWQTRYR>YAR019CMNSMADTDRVNLTPIQRASEKSVQYHLKQVIGRGSYGVVYKAINKHTDQVVAIKEVVYENDEELNDIMAEISLLKNLNHNNIVKYHGFIRKSYELYILLEYCANGSLRRLISRSSTGLSENESKTYVTQTLLGLKYLHGEGVIHRDIKAANILLSADNTVKLADFGVSTIVNSSALTLAGTLNWMAPEILGNRGASTLSDIWSLGATVVEMLTKNPPYHNLTDANIYYAVENDTYYPPSSFSEPLKDFLSKCFVKNMYKRPTADQLLKHVWINSTENVKVDKLNKFKEDFTDADYHWDADFQEEKLNISPSKFSLRAAPAPWAENNQELDLMPPTESQLLSQLKSSSKPLTDLHVLFSVCSLENIADTIIECLSRTTVDKRLITAFGSIFVYDTQHNHSRLRLKFIAMGGIPLIIKFEHLAKEFVIDYPQTLIECGIMYPPNFASLKTPKYILELVYRFYDLTSTAFWCRWCFKHLDISLLLNNIHERRAQSILLKLSSYAPWSFEKILPSLIDSKLKKKILISPQITYVVFKSINYMITTNDDKIHKSAIPSSSSLPLSSSPTRNSPVNSVQSPSRSPVHSLMATRPSSPMRHKSISNFPHLTISSKSRLLIELPEGFFTWLTSFFVDMAQIKDLSVLKYFTKLCYLTVHINSTFLNDLLDNDAFFAFIRNIDTIIPFIDDAKTAAFIWKQITAICVEMSLDMDQMSASLFSTAMNFIRKKNNTSISGLEIILNCLHFTLRNVNDDVAPTVGSSESHSVFLIKVNNDAAIELPIDQLVDLFYALNDDDVNLSKLISIFTKICSLPGFENLTINIIFHPNFYEKIVSFFDTYFNSLLIQIDLLKFIKLIFSKSLLKLYDYTGQPDPIKQTEPNRRNKATVFKLRAILVQITEFLNNNWNNGCPKRNSNQVGGDSVLICQLCEDIRSLSKKGSLQKVSSVTAAIGSSPTKDERSNLRSSKDKSDGFSVPITTFQT>YAR035WMPNLKRLPIPPLQDTLNRYLARVEPLQDERQNRRTRRTVLSAENLDALNTLHERLLEYDARLAESNPESSYIEQFWYDAYLLYDATVVLNVNPYFQLQDDPTIKDTPETAAQGPYGAHTVQVRRAARLTTSILKFIRQIRHGTLRTDTVRGKTPLSMDQYERLFGSSRIPPGPGEPSCHLQTDATSHHVVAMYRGQFYWFDVLDTRNEPIFATPEQLEWNLYSIIMDAESAGSGSAPFGVFTTESRRVWSNIRDYLFHADDCTNWRNLKLIDSALFVVCLDDVAFAADQQDELTRSMLCGTSTINLDPHQHQPPLNVQTGTCLNRWYDKLQLIVTKNGKAGINFEHTGVDGHTVLRLATDIYTDSILSFARGVTKNVVDIFSDDDGKPSSSSLASAAHSANLITIPRKLEWRTDNFLQSSLHFAETRISDLISQYEFVNLDFSNYGASHIKTVFKCSPDAFVQQVFQVAYFALYGRFETVYEPAMTKAFQNGRTEAIRSVTGQSKLFVKSLLDQDASDATKIQLLHDACTAHSQITRECSQGLGQDRHLYALYCLWNQWYKDKLELPPIFRDKSWTTMQNNVLSTSNCGNPCLKSFGFGPVTANGFGIGYIIRDHSVSVVVSSRHRQTARFASLMEKSLLEIDRIFKRQQARAAKPAARTTASANTKSEDMKYLLSGYDYFDVSVSG>YAR062W>YAR066WMFNRFNKFQAAVALALLSRGALGDSYTNSTSSADLSSITSVSSASASATASDSLSSSDGTVYLPSTTISGDLTVTGKVIATEAVEVAAGGKLTLLDGEKYVFSSDLKVHGDLVVEKSEASYEGTAFDVSGETFEVSGNFSAEETGAVSASIYSFTPSSFKSSGDISLSLSKAKKGEVTFSPYSNAGTFSLSNAILNGGSVSGL>YBL003CMSGGKGGKAGSAAKASQSRSAKAGLTFPVGRVHRLLRRGNYAQRIGSGAPVYLTAVLEYLAAEILELAGNAARDNKKTRIIPRHLQLAIRNDDELNKLLGNVTIAQGGVLPNIHQNLLPKKSAKTAKASQEL>YBL005WMKVKKSTRSKVSTACVNCRKRKIKCTGKYPCTNCISYDCTCVFLKKHLPQKEDSSQSLPTTAVAPPSSHANVEASADVQHLDTAIKLDNQYYFKLMNDLIQTPVSPSATHAPDTSNNPTNDNNILFKDDSKYQNQLVTYQNILTNLYALPPCDDTQLLIDKTKSQLNNLINSWNPEINYPKLSSFSPRPQRSIETYLLTNKYRNKIHMTRFSFWTDQMVKSQSPDSFLATTPLVDEVFGLFSPIQAFSLRGIGYLIKKNIENTGSSMLIDTKETIYLILRLFDLCYEHLIQGCISISNPLENYLQKIKQTPTTTASASLPTSPAPLSNDLVISVIHQLPQPFIQSITGFTTTQLIENLHDSFSMFRIVTQMYAQHRKRFAEFLNQAFSLPHQEKSVLFSSFCSSEYLLSTLCYAYYNVTLYHMLDINTLDYLEILVSLLEIQNEIDERFGFEKMLEVAVTCSTKMGLSRWEYYVGIDENTAERRRKIWWKIYSLEKRFLTDLGDLSLINEHQMNCLLPKDFRDMGFINHKEFLTKIGTSSLSPSSPKLKNLSLSRLIEYGELAIAQIVGDFFSETLYNEKFTSLEVSVKPTIIRQKLLEKVFEDIESFRLKLAKIKLHTSRVFQVAHCKYPEYPKNDLIEAAKFVSYHKNTWFSILGAVNNLIARLSEDPEVITEQSMKYANEMFQEWREINQFLIQVDTDFIVWACLDFYELIFFVMASKFYVEDPHITLEDVINTLKVFKRITNIISFFNNNLDEKDYDCQTFREFSRSSSLVAISIRIIFLKYCYAEQIDRAEFIERLKEVEPGLSDLLREFFDTRSFIYRYMLKSVEKSGFHLIIRKMLESDYKFLYRDKLATGNIPDQGNSSQISQLYDSTAPSYNNASASAANSPLKLSSLLNSGEESYTQDASENVPCNLRHQDRSLQQTKRQHSAPSQISANENNIYNLGTLEEFVSSGDLTDLYHTLWNDNTSYPFL>YBL006CMSGSNMGYYDVLAGLSALEKSSQVVFSATELQQLTQQSHATDKGIEGSENSKAKVSKPKRVAVHGYLGGKVSLADAAQVEYEVGHSLLGSYVPRQQLEALSSVDFSHHFHRTLECKAALETHDVFLAGAGQLSLPFQSHIESPRNSEAKRKRKVIICKRCQSRFIGSHRRSQLREHACVD>YBL007CMTVFLGIYRAVYAYEPQTPEELAIQEDDLLYLLQKSDIDDWWTVKKRVIGSDSEEPVGLVPSTYIEEAPVLKKVRAIYDYEQVQNADEELTFHENDVFDVFDDKDADWLLVKSTVSNEFGFIPGNYVEPENGSTSKQEQAPAAAEAPAATPAAAPASAAVLPTNFLPPPQHNDRARMMQSKEDQAPDEDEEGPPPAMPARPTATTETTDATAAAVRSRTRLSYSDNDNDDEEDDYYYNSNSNNVGNHEYNTEYHSWNVTEIEGRKKKKAKLSIGNNKINFIPQKGTPHEWSIDKLVSYDNEKKHMFLEFVDPYRSLELHTGNTTTCEEIMNIIGEYKGASRDPGLREVEMASKSKKRGIVQYDFMAESQDELTIKSGDKVYILDDKKSKDWWMCQLVDSGKSGLVPAQFIEPVRDKKHTESTASGIIKSIKKNFTKSPSRSRSRSRSKSNANASWKDDELQNDVVGSAAGKRSRKSSLSSHKKNSSATKDFPNPKKSRLWVDRSGTFKVDAEFIGCAKGKIHLHKANGVKIAVAADKLSNEDLAYVEKITGFSLEKFKANDGSSSRGTDSRDSERERRRRLKEQEEKERDRRLKERELYELKKARELLDEERSRLQEKELPPIKPPRPTSTTSVPNTTSVPPAESSNNNNSSNKYDWFEFFLNCGVDVSNCQRYTINFDREQLTEDMMPDINNSMLRTLGLREGDIVRVMKHLDKKFGRENIASIPTNATGNMFSQPDGSLNVATSPETSLPQQLLPQTTSPAQTAPSTSAETDDAWTVKPASKSESNLLSKKSEFTGSMQDLLDLQPLEPKKAAASTPEPNLKDLEPVKTGGTTVPAAPVSSAPVSSAPAPLDPFKTGGNNILPLSTGFVMMPMITGGDMLPMQRTGGFVVPQTTFGMQSQVTGGILPVQKTGNGLIPISNTGGAMMPQTTFGAAATVLPLQKTGGGLIPIATTGGAQFPQTSFNVQGQQQLPTGSILPVQKTANGLISANTGVSMPTVQRTGGTMIPQTSFGVSQQLTGGAMMTQPQNTGSAMMPQTSFNAVPQITGGAMMPQTSFNALPQVTGGAMMPLQRTGGALNTFNTGGAMIPQTSFSSQAQNTGGFRPQSQFGLTLQKTGGIAPLNQNQFTGGAMNTLSTGGVLQQQQPQTMNTFNTGGVMQELQMMTTFNTGGAMQQPQMMNTFNTDGIMQQPQMMNTFNTGGAMQQPQQQALQNQPTGFGFGNGPQQSRQANIFNATASNPFGF>YBL009WMNFDAVADQQMTDRRYFALEVAESDDADSSLNSSSMGSPAVDVGRKVYKITSHKGSAEDESQSFFTSSDSPTSKTRPVGKTIENDDYYGKRSSTGSSLKQLFNKININDTAHSSNKENVSQSVLSENKLLSPSKRLSKQGLTKVTNSKFRTPLRPISNQSTLSRDEPVKDFRSLKFRSGSDFKCWGDEKTSSHVHSSSVNSVNSFTSTTSSSKWKFWKNDNLLSRSLSSRSVNDQDPNFVQPKPTNSLQKKSSISSFHNSIFGGGKHTEKKRNSGFIMPDHQSTKELNHKHSSSNLSFRSLKHKTSHSSLNKLKVRRKGNTQELNHPIKKTCQISLPVPDQVSKDKIQLKLKNSTSLASLSSEVTPINTLDYNDSILQQILQLCDVKYILHDLREAQSLGLFTLNTRSVQLSHNFWQTYHSDMQTSLICKKVCLGALSDLTTSNLISLHELKSLRLIQGTSGVANLLQAYVVPSNQCENDQNLILYLFFKYQGTPLSRCSNIDYSQALSIFWQCSSILYVAESKFQLEHRNLTLDHILIDSKGNVTLIDMKCCRFLNIDNNKASYTRLDHHYFFQGRGTLQFEIYELMRSMLPQPISWATFEPRTNLLWLYHLSSSLLKMAKKAVVSGALNREENILIELTHLLDPARKHSKTIFKKELVIRTCGDLLSLKGEIMQ>YBL010CMSDRDQIEPVTNALDAESDSSDDFGNFSDASVENDLYNQNSTLTTSSESVVDNCLNKILPKGEFDLEEETIKNDCFKLSKLIEDERPHVIYEQLVQLDPVLQPFIWNKSHIRRNLLHILRLSDNNGSEGVGTKREEEPLNDELFKRICDAVEKNEQTATGLFLRDNFKIDYTPPMTLKSLQKEEEREQEQHIPQLLMADFTSMDEESLRQYHDTLCQSIDFLVSKSRSLKKQQRDLLKDKTTFENVVTNLTGHTQRLQRDEIALYNKKRNKKKRFSWVGY>YBL011WMPAPKLTEKFASSKSTQKTTNYSSIEAKSVKTSADQAYIYQEPSATKKILYSIATWLLYNIFHCFFREIRGRGSFKVPQQGPVIFVAAPHANQFVDPVILMGEVKKSVNRRVSFLIAESSLKQPPIGFLASFFMAIGVVRPQDNLKPAEGTIRVDPTDYKRVIGHDTHFLTDCMPKGLIGLPKSMGFGEIQSIESDTSLTLRKEFKMAKPEIKTALLTGTTYKYAAKVDQSCVYHRVFEHLAHNNCIGIFPEGGSHDRTNLLPLKAGVAIMALGCMDKHPDVNVKIVPCGMNYFHPHKFRSRAVVEFGDPIEIPKELVAKYHNPETNRDAVKELLDTISKGLQSVTVTCSDYETLMVVQTIRRLYMTQFSTKLPLPLIVEMNRRMVKGYEFYRNDPKIADLTKDIMAYNAALRHYNLPDHLVEEAKVNFAKNLGLVFFRSIGLCILFSLAMPGIIMFSPVFILAKRISQEKARTALSKSTVKIKANDVIATWKILIGMGFAPLLYIFWSVLITYYLRHKPWNKIYVFSGSYISCVIVTYSALIVGDIGMDGFKSLRPLVLSLTSPKGLQKLQKDRRNLAERIIEVVNNFGSELFPDFDSAALREEFDVIDEEEEDRKTSELNRRKMLRKQKIKRQEKDSSSPIISQRDNHDAYEHHNQDSDGVSLVNSDNSLSNIPLFSSTFHRKSESSLASTSVAPSSSSEFEVENEILEEKNGLASKIAQAVLNKRIGENTAREEEEEEEEEEEEEEEEEEGKEGDA>YBL013WMVKMRRITPTRLLFTCRYISNNASPPVQPLNVLFFGSDTFSNFSLQALNELRQNNGSCGIVDNIQVVTRSPKWCGRQKSILKYPPIFDMAEKLQLPRPITCDTKQEMLALSKLTPSRQGNPENDGSGAPFNAIIAVSFGKLIPGDLIRAVPLALNVHPSLLPRHKGSAPIQRALLEGDTYTGVTIQTLHPDRFDHGAIVAQTEPLAIATMLSKGRVNDSTADFNSEGLPRRTAILMDQLGALGAQLLGQTLRERLYLPQNRVQAPTAYKPSYAHRITTEDKRIHWARDSAAELLNKLETLGPLHAFKEATAARKDAQNSVLKRILFHECKVMRDARLDNGSKPGMFKYDDIKDCILVTCRGNLLLCVSRLQFEGFAVERAGQFMARLRKRCGALSEKLVFL>YBL014CMSEGQIPSSDVLGSQLGVGVQGASLYCPQENYTTKKQENPQWLRPVDDTLAEDALDLHIVVKSLLCDTAIRYISDDKVLQESDADDDLITSDIDEDTDNQGDTSIVVNPVIPVVPKDVHFFKKVDVGNDSMFGVNCDTPVSFQDYIPSDLLRNLDDTLQESTNSSRPMQDAFFWDPTVANRLDSQYIQTASDLRNYRDGTEIIAYASGKTGSVLNIAVLTRQNTLHLNRHNNVTSIELHSPIKSIKIPGASESIGRRSNLVGIITENSFQIFRIESVHSRSCDVMVSSSEPLYFVEIDDLQVVDFAFNPWDLQQFAIIDIKGNWSIGRIPKNFNNNNKRKLQLIDNLHGTIFDPEELSSWKRIEWFSHFQKILVFDRSKMIEIDFMNNWQTEVVQAKAWSNIRDYKRIDDKNGILLTSREIIIVGASESNDPVRRISWKHDLDPDDTTLRITVQKVKKPDHILLVAFVYSMRHKRIYMHVFSHRKANLFQSLGCSTVLEIPGGTPTGIETILTLDHIDDESRREEDADENFELVVDFLVKLRNSSEVYYYALSNTQNSEPNKQETPIIVDHPEWASLFNNADEREKESIGALVSQIKLKERERISRVQNLIEHENSHDEDKYLQDLGYRLSIATNELLESWQKTKDESILSGSLSHSKLKNLLENSDSFASIPEFSSLLDQFFQYYQDQDVTFIGFEKLLHLFLHEDVPGLDIFYNKLLQCWVLVSPQAELLTKEIVKDIIWSLARLEKPSLFEPIQNEISRSLSGPYQDIISSWDMDDINEEDESNEFNFDSQFSAPFNGRPPFNLNSQSQIPTIKSSQSSGLARRKRILKTQSQKATPLSQSTQNLSVLPDSMTPAFTLMQPPSSQISFVNDSQPRNSQKAKKKKKRIRGFG>YBL015WMTISNLLKQRVRYAPYLKKVKEAHELIPLFKNGQYLGWSGFTGVGTPKAVPEALIDHVEKNNLQGKLRFNLFVGASAGPEENRWAEHDMIIKRAPHQVGKPIAKAINQGRIEFFDKHLSMFPQDLTYGFYTRERKDNKILDYTIIEATAIKEDGSIVPGPSVGGSPEFITVSDKVIIEVNTATPSFEGIHDIDMPVNPPFRKPYPYLKVDDKCGVDSIPVDPEKVVAIVESTMRDQVPPNTPSDDMSRAIAGHLVEFFRNEVKHGRLPENLLPLQSGIGNIANAVIEGLAGAQFKHLTVWTEVLQDSFLDLFENGSLDYATATSVRLTEKGFDRAFANWENFKHRLCLRSQVVSNNPEMIRRLGVIAMNTPVEVDIYAHANSTNVNGSRMLNGLGGSADFLRNAKLSIMHAPSARPTKVDPTGISTIVPMASHVDQTEHDLDILVTDQGLADLRGLSPKERAREIINKCAHPDYQALLTDYLDRAEHYAKKHNCLHEPHMLKNAFKFHTNLAEKGTMKVDSWEPVD>YBL016WMPKRIVYNISSDFQLKSLLGEGAYGVVCSATHKPTGEIVAIKKIEPFDKPLFALRTLREIKILKHFKHENIITIFNIQRPDSFENFNEVYIIQELMQTDLHRVISTQMLSDDHIQYFIYQTLRAVKVLHGSNVIHRDLKPSNLLINSNCDLKVCDFGLARIIDESAADNSEPTGQQSGMTEYVATRWYRAPEVMLTSAKYSRAMDVWSCGCILAELFLRRPIFPGRDYRHQLLLIFGIIGTPHSDNDLRCIESPRAREYIKSLPMYPAAPLEKMFPRVNPKGIDLLQRMLVFDPAKRITAKEALEHPYLQTYHDPNDEPEGEPIPPSFFEFDHYKEALTTKDLKKLIWNEIFS>YBL019WMSSSENTLLDGKSENTIRFLTFNVNGIRTFFHYQPFSQMNQSLRSVFDFFRADIITFQELKTEKLSISKWGRVDGFYSFISIPQTRKGYSGVGCWIRIPEKNHPLYHALQVVKAEEGITGYLTIKNGKHSAISYRNDVNQGIGGYDSLDPDLDEKSALELDSEGRCVMVELACGIVIISVYCPANSNSSEEGEMFRLRFLKVLLRRVRNLDKIGKKIVLMGDVNVCRDLIDSADTLEQFSIPITDPMGGTKLEAQYRDKAIQFIINPDTPHRRIFNQILADSLLPDASKRGILIDTTRLIQTRNRLKMYTVWNMLKNLRPSNYGSRIDFILVSLKLERCIKAADILPDILGSDHCPVYSDLDILDDRIEPGTTQVPIPKFEARYKYNLRNHNVLEMFAKKDTNKESNKQKYCVSKVMNTKKNSNIKNKSLDSFFQKVNGEKDDRIKESSEIPQQAKKRISTPKLNFKDVFGKPPLCRHGEESMLKTSKTSANPGRKFWICKRSRGDSNNTESSCGFFQWV>YBL020WMAKKNSQLPSTSEQILERSTTGATFLMMGQLFTKLVTFILNNLLIRFLSPRIFGITAFLEFIQGTVLFFSRDAIRLSTLRISDSGNGIIDDDDEEEYQETHYKSKVLQTAVNFAYIPFWIGFPLSIGLIAWQYRNINAYFITLPFFRWSIFLIWLSIIVELLSEPFFIVNQFMLNYAARSRFESIAVTTGCIVNFIVVYAVQQSRYPMGVVTSDIDKEGIAILAFALGKLAHSITLLACYYWDYLKNFKPKKLFSTRLTKIKTRENNELKKGYPKSTSYFFQNDILQHFKKVYFQLCFKHLLTEGDKLIINSLCTVEEQGIYALLSNYGSLLTRLLFAPIEESLRLFLARLLSSHNPKNLKLSIEVLVNLTRFYIYLSLMIIVFGPANSSFLLQFLIGSKWSTTSVLDTIRVYCFYIPFLSLNGIFEAFFQSVATGDQILKHSYFMMAFSGIFLLNSWLLIEKLKLSIEGLILSNIINMVLRILYCGVFLNKFHRELFTDSSFFFNFKDFKTVIIAGSTICLLDWWFIGYVKNLQQFVVNVLFAMGLLALILVKERQTIQSFINKRAVSNSKDV>YBL021CMNTNESEHVSTSPEDTQENGGNASSSGSLQQISTLREQDRWLPINNVARLMKNTLPPSAKVSKDAKECMQECVSELISFVTSEASDRCAADKRKTINGEDILISLHALGFENYAEVLKIYLAKYRQQQALKNQLMYEQDDEEVP>YBL023CMSDNRRRRREEDDSDSENELPPSSPQQHFRGGMNPVSSPIGSPDMINPEGDDNEVDDVPDIDEVEEQMNEVDLMDDNMYEDYAADHNRDRYDPDQVDDREQQELSLSERRRIDAQLNERDRLLRNVAYIDDEDEEQEGAAQLDEMGLPVQRRRRRRQYEDLENSDDDLLSDMDIDPLREELTLESLSNVKANSYSEWITQPNVSRTIARELKSFLLEYTDETGRSVYGARIRTLGEMNSESLEVNYRHLAESKAILALFLAKCPEEMLKIFDLVAMEATELHYPDYARIHSEIHVRISDFPTIYSLRELRESNLSSLVRVTGVVTRRTGVFPQLKYVKFNCLKCGSILGPFFQDSNEEIRISFCTNCKSKGPFRVNGEKTVYRNYQRVTLQEAPGTVPPGRLPRHREVILLADLVDVSKPGEEVEVTGIYKNNYDGNLNAKNGFPVFATIIEANSIKRREGNTANEGEEGLDVFSWTEEEEREFRKISRDRGIIDKIISSMAPSIYGHRDIKTAVACSLFGGVPKNVNGKHSIRGDINVLLLGDPGTAKSQILKYVEKTAHRAVFATGQGASAVGLTASVRKDPITKEWTLEGGALVLADKGVCLIDEFDKMNDQDRTSIHEAMEQQSISISKAGIVTTLQARCSIIAAANPNGGRYNSTLPLAQNVSLTEPILSRFDILCVVRDLVDEEADERLATFVVDSHVRSHPENDEDREGEELKNNGESAIEQGEDEINEQLNARQRRLQRQRKKEEEISPIPQELLMKYIHYARTKIYPKLHQMDMDKVSRVYADLRRESISTGSFPITVRHLESILRIAESFAKMRLSEFVSSYDLDRAIKVVVDSFVDAQKVSVRRQLRRSFAIYTLGH>YBL024WMARRKNFKKGNKKTFGARDDSRAQKNWSELVKENEKWEKYYKTLALFPEDQWEEFKKTCQAPLPLTFRITGSRKHAGEVLNLFKERHLPNLTNVEFEGEKIKAPVELPWYPDHLAWQLDVPKTVIRKNEQFAKTQRFLVVENAVGNISRQEAVSMIPPIVLEVKPHHTVLDMCAAPGSKTAQLIEALHKDTDEPSGFVVANDADARRSHMLVHQLKRLNSANLMVVNHDAQFFPRIRLHGNSNNKNDVLKFDRILCDVPCSGDGTMRKNVNVWKDWNTQAGLGLHAVQLNILNRGLHLLKNNGRLVYSTCSLNPIENEAVVAEALRKWGDKIRLVNCDDKLPGLIRSKGVSKWPVYDRNLTEKTKGDEGTLDSFFSPSEEEASKFNLQNCMRVYPHQQNTGGFFITVFEKVEDSTEAATEKLSSETPALESEGPQTKKIKVEEVQKKERLPRDANEEPFVFVDPQHEALKVCWDFYGIDNIFDRNTCLVRNATGEPTRVVYTVCPALKDVIQANDDRLKIIYSGVKLFVSQRSDIECSWRIQSESLPIMKHHMKSNRIVEANLEMLKHLLIESFPNFDDIRSKNIDNDFVEKMTKLSSGCAFIDVSRNDPAKENLFLPVWKGNKCINLMVCKEDTHELLYRIFGIDANAKATPSAEEKEKEKETTESPAETTTGTSTEAPSAAN>YBL025WMDRNVYEACSNIIKEFGTHVVSADEVLAEKIDNAVPIPFKTREEIDADVEKDRNEGVFEGNIIPDIDLRVVHYYATQLCLNKYPHLINAFDETSLITLGLLIEKWVKDYLTSIQTEQGRQSKVIGKGPCEFISKHIDYRHAPGNI>YBL028CMAKSLRASSHLNAKSVKRRGVFQKAVDAREQRISDKLKEDLLKQKLEDLKKKEEQGIDMDVDEKKSNEEAPRKKISTSGWRDGRHHTYKKAKLMKQSKKKTSFTRF>YBL029WMCANIPEFDSFYENENINYNLESFAPLNCDVNSPFLPINNNDINVNAYGDENLTYSNFLLSYNDKLATTTAKNNSINNSNSNNNSNNNKNNNNNHNNNNLLGNDISQMAFLLDYPSTLNEPQFAVNCKDIYRKDISTPSSLVSSLPPAKFSLSLSNSPSPPPPSSSSLKHGEAIISNTSESSDIFADPNSFEKDTMPLTQELTLENLNNQLNYPDFTINAIEQDPAPSSFSSSSSSSESTVSSSRKRKPCHDSYTHSSPSSSESKKISDSRLSAEGLAKVLNLESPEEALKRERFILGIFQNELNYPLGYKTWIRDTTKEYRTKLINQLHERVKVKYPEYNQSILETIIRRGTYYMMQSRLRRERRMKLKERKRTT>YBL030CMSSNAQVKTPLPPAPAPKKESNFLIDFLMGGVSAAVAKTAASPIERVKLLIQNQDEMLKQGTLDRKYAGILDCFKRTATQEGVISFWRGNTANVIRYFPTQALNFAFKDKIKAMFGFKKEEGYAKWFAGNLASGGAAGALSLLFVYSLDYARTRLAADSKSSKKGGARQFNGLIDVYKKTLKSDGVAGLYRGFLPSVVGIVVYRGLYFGMYDSLKPLLLTGSLEGSFLASFLLGWVVTTGASTCSYPLDTVRRRMMMTSGQAVKYDGAFDCLRKIVAAEGVGSLFKGCGANILRGVAGAGVISMYDQLQMILFGKKFK>YBL031WMNDKLQEEHNEKDTTSQINGFTPPHMSIDFHSNNNSNIIETIGVSKRLGNSVLSELDSRASSKFEFLKDQSEQQYNGDKNNEPKSGSYNINEFFQAKHDSQFGQMESLDTHYTLLHTPKRKSQHAIPQDRSDSMKRSRPSRSIPYTTPVVNDITRRIRRLKLRNSLVNGNDIVARARSMQANSNINSIKNTPLSKPKPFMHKPNFLMPTTNSLNKINSAHRNTSSSSTASSIPRSKVHRSISIRDLHAKTKPVERTPVAQGTNSQLKNSVSVFDRLYKQTTFSRSTSMNNLSSGTSAKSKEHTNVKTRLVKSKTSGSLSSNLKQSTATGTKSDRPIWR>YBL032WMSQFFEAATPVAIPTNNTNGGSSDAGSAATGGAPVVGTTAQPTINHRLLLSLKEAAKIIGTKGSTISRIRAANAVKIGISEKVPGCSDRILSCAGNVINVANAIGDIVDVLNKRNPENEDAAEGEAEEHYYFHFLNHILPAPSKDEIRDLQQLEDIGYVRLIVANSHISSIIGKAGATIKSLINKHGVKIVASKDFLPASDERIIEIQGFPGSITNVLIEISEIILSDVDVRFSTERSYFPHLKKSSGEPTSPSTSSNTRIELKIPELYVGAIIGRGMNRIKNLKTFTKTNIVVERKDDDDKDENFRKFIITSKFPKNVKLAESMLLKNLNTEIEKRENYKRKLEAAEGDATVVTERSDSASFLEEKEEPQENHDNKEEQS>YBL033CMTIDNYDNSKQDSSKYEVSGTGDGRNGDGGLPLVQCVARARIPTTQGPDIFLHLYSNNRDNKEHLAIVFGEDIRSRSLFRRRQCETQQDRMIRGAYIGKLYPGRTVADEDDRLGLALEFDDSTGELLASKATTWDAHNDTLVRIHSECYTGENAWSARCDCGEQFDRAGRLIACDHEPTSNIKGGNGHGVIVYLRQEGRGIGLGEKLKAYNLQDLGADTVQANLMLKHPVDARDFSLGKAILLDLGIGNVRLLTNNPEKIKQVDHAPYLKCVERVPMVPIHWTNSSEGIDSKEIEGYLRTKIERMGHLLTEPLKLHTNPQPTETSEAQNQNRMNSALSSTSTLAI>YBL036CMSTGITYDEDRKTQLIAQYESVREVVNAEAKNVHVNENASKILLLVVSKLKPASDIQILYDHGVREFGENYVQELIEKAKLLPDDIKWHFIGGLQTNKCKDLAKVPNLYSVETIDSLKKAKKLNESRAKFQPDCNPILCNVQINTSHEDQKSGLNNEAEIFEVIDFFLSEECKYIKLNGLMTIGSWNVSHEDSKENRDFATLVEWKKKIDAKFGTSLKLSMGMSADFREAIRQGTAEVRIGTDIFGARPPKNEARII>YBL038WMFPYLTRMNLSIKMGGLTLKESSPNAFLNNTTIARRFKHEYAPRFKIVQKKQKGRVPVRTGGSIKGSTLQFGKYGLRLKSEGIRISAQQLKEADNAIMRYVRPLNNGHLWRRLCTNVAVCIKGNETRMGKGKGGFDHWMVRVPTGKILFEINGDDLHEKVAREAFRKAGTKLPGVYEFVSLDSLVRVGLHSFKNPKDDPVKNFYDENAKKPSKKYLNILKSQEPQYKLFRGR>YBL041WMATIASEYSSEASNTPIEHQFNPYGDNGGTILGIAGEDFAVLAGDTRNITDYSINSRYEPKVFDCGDNIVMSANGFAADGDALVKRFKNSVKWYHFDHNDKKLSINSAARNIQHLLYGKRFFPYYVHTIIAGLDEDGKGAVYSFDPVGSYEREQCRAGGAAASLIMPFLDNQVNFKNQYEPGTNGKVKKPLKYLSVEEVIKLVRDSFTSATERHIQVGDGLEILIVTKDGVRKEFYELKRD>YBL045CMLRTVTSKTVSNQFKRSLATAVATPKAEVTQLSNGIVVATEHNPSAHTASVGVVFGSGAANENPYNNGVSNLWKNIFLSKENSAVAAKEGLALSSNISRDFQSYIVSSLPGSTDKSLDFLNQSFIQQKANLLSSSNFEATKKSVLKQVQDFEENDHPNRVLEHLHSTAFQNTPLSLPTRGTLESLENLVVADLESFANNHFLNSNAVVVGTGNIKHEDLVNSIESKNLSLQTGTKPVLKKKAAFLGSEVRLRDDTLPKAWISLAVEGEPVNSPNYFVAKLAAQIFGSYNAFEPASRLQGIKLLDNIQEYQLCDNFNHFSLSYKDSGLWGFSTATRNVTMIDDLIHFTLKQWNRLTISVTDTEVERAKSLLKLQLGQLYESGNPVNDANLLGAEVLIKGSKLSLGEAFKKIDAITVKDVKAWAGKRLWDQDIAIAGTGQIEGLLDYMRIRSDMSMMRW>YBL049WMGLRYSIYIENPLSSPSSSYKSINDPLFHSQHRSQKNVSFITYGCRHCKTHLSSSFQIISRDYRGRTGTAYLMNKVVNVVEGKVEQRRMLTGDYLVCDILCHWCKRNVGWKYLQSSNDDQQYKEGKFILELKNICKCT>YBL050WMSDPVELLKRAEKKGVPSSGFMKLFSGSDSYKFEEAADLCVQAATIYRLRKELNLAGDSFLKAADYQKKAGNEDEAGNTYVEAYKCFKSGGNSVNAVDSLENAIQIFTHRGQFRRGANFKFELGEILENDLHDYAKAIDCYELAGEWYAQDQSVALSNKCFIKCADLKALDGQYIEASDIYSKLIKSSMGNRLSQWSLKDYFLKKGLCQLAATDAVAAARTLQEGQSEDPNFADSRESNFLKSLIDAVNEGDSEQLSEHCKEFDNFMRLDKWKITILNKIKESIQQQEDDLL>YBL051CMETSSFENAPPAAINDAQDNNINTETNDQETNQQSIETRDAIDKENGVQTETGENSAKNAEQNVSSTNLNNAPTNGALDDDVIPNAIVIKNIPFAIKKEQLLDIIEEMDLPLPYAFNYHFDNGIFRGLAFANFTTPEETTQVITSLNGKEISGRKLKVEYKKMLPQAERERIEREKREKRGQLEEQHRSSSNLSLDSLSKMSGSGNNNTSNNQLFSTLMNGINANSMMNSPMNNTINNNSSNNNNSGNIILNQPSLSAQHTSSSLYQTNVNNQAQMSTERFYAPLPSTSTLPLPPQQLDFNDPDTLEIYSQLLLFKDREKYYYELAYPMGISASHKRIINVLCSYLGLVEVYDPRFIIIRRKILDHANLQSHLQQQGQMTSAHPLQPNSTGGSMNRSQSYTSLLQAHAAAAANSISNQAVNNSSNSNTINSNNGNGNNVIINNNSASSTPKISSQGQFSMQPTLTSPKMNIHHSSQYNSADQPQQPQPQTQQNVQSAAQQQQSFLRQQATLTPSSRIPSGYSANHYQINSVNPLLRNSQISPPNSQIPINSQTLSQAQPPAQSQTQQRVPVAYQNASLSSQQLYNLNGPSSANSQSQLLPQHTNGSVHSNFSYQSYHDESMLSAHNLNSADLIYKSLSHSGLDDGLEQGLNRSLSGLDLQNQNKKNLW>YBL052CMSLTANDESPKPKKNALLKNLEIDDLIHSQFVRSDTNGHRTTRRLFNSDASISHRIRGSVRSDKGLNKIKKGLISQQSKLASENSSQNIVNRDNKMGAVSFPIIEPNIEVSEELKVRIKYDSIKFFNFERLISKSSVIAPLVNKNITSSGPLIGFQRRVNRLKQTWDLATENMEYPYSSDNTPFRDNDSWQWYVPYGGTIKKMKDFSTKRTLPTWEDKIKFLTFLENSKSATYINGNVSLCNHNETDQENEDRKKRKGKVPRIKNKVWFSQIEYIVLRNYEIKPWYTSPFPEHINQNKMVFICEFCLKYMTSRYTFYRHQLKCLTFKPPGNEIYRDGKLSVWEIDGRENVLYCQNLCLLAKCFINSKTLYYDVEPFIFYILTEREDTENHPYQNAAKFHFVGYFSKEKFNSNDYNLSCILTLPIYQRKGYGQFLMEFSYLLSRKESKFGTPEKPLSDLGLLTYRTFWKIKCAEVLLKLRDSARRRSNNKNEDTFQQVSLNDIAKLTGMIPTDVVFGLEQLQVLYRHKTRSLSSLDDFNYIIKIDSWNRIENIYKTWSSKNYPRVKYDKLLWEPIILGPSFGINGMMNLEPTALADEALTNETMAPVISNNTHIENYNNSRAHNKRRRRRRRSSEHKTSKLHVNNIIEPEVPATDFFEDTVSSLTEYMCDYKNTNNDRLIYQAEKRVLESIHDRKGIPRSKFSTETHWELCFTIKNSETPLGNHAARRNDTGISSLEQDEVENDVDTELYVGENAKEDEDEDEDFTLDDDIEDEQISEENDEEEDTYEEDSDDDEDGKRKGQEQDENDIESHIRKERVRKRRKITLIEDDEE>YBL054WMTLPKLSSVSVSSGHVSANSHGFSILSKHPHPNNLVHSHSLSHTNAKSHLPISSTSTKENSTNKEEAESLKKNNPSSWDPSDDIKLRHLKEIKNLGWKEIAHHFPNRTPNACQFRWRRLKSGNLKSNKTAVIDINKLFGVYATGDATPSAGTPSAEEAVKEEAVEDEDITAGSSAIEDSPPDFKPLVKPKYMDRKLITQRSTSTFSDHEPQHTKPRKLFVKPRSFSHSITTNTPNVKTAQQTNLSLYNTTSAKTNKAVNSNDYENIGLVPKIIIRSRRNSFIPSTQIPHSTTKTRKNSHSVISSRRSSFNMMHSRRSSFNSHAPTEPISRRASLVVSPYMSPRRLSTSQSVHYHPQHQYYLNPIASPNCKTDHANDKITHTRTFLDMQKFANKHPWSREDDEVLLNNTKDKQNHLSPLEISIVLPNNRSELEIQQRMDYLKRKGRVSGFHTNEGCKDEEEEDDIDPLHKENGINTPSQQSQNYGMLEAKHDNPKSSELSSMTSANDIRNEQDELPGINSIFKNIF>YBL056WMGQILSNPIIDKEHHSGTDCLTAFGLCAMQGWRMSMEDAHIVEPNLLAESDEEHLAFYGIFDGHGGSSVAEFCGSKMISILKKQESFKSGMLEQCLIDTFLATDVELLKDEKLKDDHSGCTATVILVSQLKKLLICANSGDSRTVLSTGGNSKAMSFDHKPTLLSEKSRIVAADGFVEMDRVNGNLALSRAIGDFEFKSNTKLGPHEQVVTCVPDIICHNLNYDEDEFVILACDGIWDCLTSQECVDLVHYGISQGNMTLSDISSRIVDVCCSPTTEGSGIGCDNMSISIVALLKENESESQWFERMRSKNYNIQTSFVQRRKSIFDFHDFSDDDNEVFAITTKKLQDRLNRSKDNDDMEIDDLDTELDSSATPSKLSGEDRTGPIDLFSLEALLEAGIQIRQRPSSDSDGNTSYFHGASLSDMLASLSNAAAGETEPNDADDNDDNDGEENGKNENAKKGSKIEEIE>YBL057CMITSFLMEKMTVSSNYTIALWATFTAISFAVGYQLGTSNASSTKKSSATLLRSKEMKEGKLHNDTDEEESESEDESDEDEDIESTSLNDIPGEVRMALVIRQDLGMTKGKIAAQCCHAALSCFRHIATNPARASYNPIMTQRWLNAGQAKITLKCPDKFTMDELYAKAISLGVNAAVIHDAGRTQIAAGSATVLGLGPAPKAVLDQITGDLKLY>YBL058WMAEIPDETIQQFMALTNVSHNIAVQYLSEFGDLNEALNSYYASQTDDQKDRREEAHWNRQQEKALKQEAFSTNSSNKAINTEHVGGLCPKPGSSQGSNEYLKRKGSTSPEPTKGSSRSGSGNNSRFMSFSDMVRGQADDDDEDQPRNTFAGGETSGLEVTDPSDPNSLLKDLLEKARRGGQMGAENGFRDDEDHEMGANRFTGRGFRLGSTIDAADEVVEDNTSQSQRRPEKVTREITFWKEGFQVADGPLYRYDDPANSFYLSELNQGRAPLKLLDVQFGQEVEVNVYKKLDESYKAPTRKLGGFSGQGQRLGSPIPGESSPAEVPKNETPAAQEQPMPDNEPKQGDTSIQIRYANGKREVLHCNSTDTVKFLYEHVTSNANTDPSRNFTLNYAFPIKPISNDETTLKDADLLNSVVVQRWA>YBL060WMCASLNEVKKNDTYGVSQKGYNDNFSESEGVLHGSKSMPTSMKNMLQSPTMVNMCDILQNKEAANDEKPVIPTTDTATAGTGTEDISSTQSEETDQNSHLIASEILEGTFKDVSYKEYANFLGNDNNNQVLTEFVKLLSPLPSSLLETLFNLSKSIYFIAEAQNIDRILECLSIEWIACHPNTHWKSGYKSCHIVLFSLLILNSDLHNNFQVDHKKIKFSMVAFINNTLRALREENEYEELKIYSREHLIIEELSEYYKTLNETPLPLCTESRTSINISDNQSSLKRFSTLGSREFSTSNLRSVNSNSTTLYSRDGQVSVREMSAKSNKNFHNNHPMDALYLKESFDDGLITENGSSWFMDDLILISKKSLPRKYSKRDKDQVAAPKMTSKRNKSFFGWLKPSKTTTLIEHTSRRTSLSYLNKDSEWERVKIQVKEGRIFIFKIKPDVKDIIQSSETDSATIDYFKDISSSYFAYSLLEAEAHVVQDNIIIGSGAMKSNVCNKNTKRKSGNFTVSFPENINGPKLVLEFQTRSVEEAHKFMDCINFWAGRISPVPLTQFEAVSNAEYGWSDKILTEHASLNLKNIVVSEWKPLLGLELLYEDAKDVEMVELKERLKELMNFTRQLGIWIDKHNEIKDKLVEIWSFDDNYFEAVMNNWNSRYLYMNNQYKKRLSYLKALQKAMGSVQF>YBL061CMASSPQVHPYKKHLMQSQHINFDNRGLQFQNSSLKVGQDFSDNKENRENRDNEDFSTADLPKRSANQPLINEHLRAASVPLLSNDIGNSQEEDFVPVPPPQLHLNNSNNTSLSSLGSTPTNSPSPGALRQTNSSTSLTKEQIKKRTRSVDLSHMYLLNGSSDTQLTATNESVADLSHQMISRYLGGKNNTSLVPRLKTIEMYRQNVKKSKDPEVLFQYAQYMLQTALTIESSNALVQDSDKEGNVSQSDLKLQFLKEAQSYLKKLSIKGYSDAQYLLADGYSSGAFGKIENKEAFVLFQAAAKHGHIESAYRASHCLEEGLGTTRDSRKSVNFLKFAASRNHPSAMYKLGLYSFYGRMGLPTDVNTKLNGVKWLSRAAARANELTAAAPYELAKIYHEGFLDVVIPDEKYAMELYIQAASLGHVPSATLLAQIYETGNDTVGQDTSLSVHYYTQAALKGDSVAMLGLCAWYLLGAEPAFEKDENEAFQWALRAANAGLPKAQFTLGYFYEHGKGCDRNMEYAWKWYEKAAGNEDKRAINKLRSRDGGLASIGKKQHKKNKSISTLNLFSTVDSQTSNVGSNSRVSSKSETFFTGNPKRDREPQGLQINMNSNTNRNGIKTGSDTSIRKSSSSAKGMSREVAEQSMAAKQEVSLSNMGSSNMIRKDFPAVKTESKKPTSLKNKKDKQGKKKKDCVIM>YBL066CMVKDNRDSDQDQDFSSAHMKRQPEQQQLQQHQFPSKKQRISHHDDSHQINHRPVTSCTHCRQHKIKCDASQNFPHPCSRCEKIGLHCEINPQFRPKKGSQLQLLRQDVDEIKSKLDTLLANDSVFVHLLQQIPMGNSLLNKLNLHPTPTPGTIIPNPDSSPSSGSPTSSAAQRDSKVSVQTYLSREPQLLQANQGSNTNKFKANNEASSHMTLRASSLAQDSKGLVATEPNKLPPLLNDSALPNNSKESLPPALQMAFYKNNSAGNTPNGPFSPIQKTYSPHTTSTTVTTTTNQPPFAATSHVATNNNADRTKTPVVATTTTMPLLPSPHANVDEFVLGDISISIEKANRLHHIFVTRYLPYFPIMYSNNATELYSQSQLLFWTVMLTACLSDPEPTMYCKLSSLIKQLAIETCWIRTPRSTHISQALLILCIWPLPNQKVLDDCSYRFVGLAKSLSYQLGLHRGEFISEFTRTQTSMPNAEKWRTRTWLGIFFAELCWASILGLPPTSQTDYLLEKALSCGDEESEEDNNDSIDNNNNDKRNKKDEPHVESKYKLPGSFRRLLSLANFQAKLSHIIGSSTSSPDGLLEPKYRAETLSILGKELDLLAKTLNFQSDDTVNIYFLYVKLTVCCFAFLPETPPTDQIPYVTEAYLTATKIVTLLNNLLETHQLIELPIYIRQAATFSALILFKLQLTPLLPDKYFDSARQSVVTIHRLYRNQLTAWATSVENDISRTASMLEKLNFVLIMHPEVFVEEDGIISRMRSHLTGSLFYDLVWCVHEARRREMDPEYNKQALEKAAKKRKFSSNGIYNGTSSTGGITDRKLYPLPLYNHISRDDFETVTKTTPSGTTVTTLVPTKNALKQAEKLAKTNNGDSDGSIMEINGIPLSMLGETGSVKFQSLFANTSNSNDYNNNRTLLDASNDISIPSNSIYPVASVPASNNNPQSTKVDYYSNGPSVIPDLSMKRSVSTPVNHFPASVPGLRNHPVGNLSNNVTLGIDHPIPREHSNLQNVTMNYNNQFSNANAIGRSQSSMSHSRTPIASKSNNMTDLHSVVSDPGSSKSTAYPPLSLFSKSNDINSNKTNQRFSTGTNTVTSSNFQTIDNENNVKTPGNKLTDFFQQQSAGWIEGNSSNDDFFGWFDMNMEQGF>YBL068WMNSESREDMAINSIKLLAGNSHPDLAEQISKKLGIPLSKVGVYQYSNKETSVTIGESLRDEDVYIIQTGIGEQEINDFLMELLILIHACQIASARKITTVIPNFPYARQDKKDKSRAPITAKLVANLLQTAGADHVITMDLHASQIQGFFHIPVDNLYAEPSVLNYIRARKTDFDNAILVSPDAGGAKRVAALADKLDLNFALIHKERQKANEVSKMVLVGDVTNKSCLLVDDMADTCGTLVKACDTLMEHGAKEVIAIVTHGIFSGSAREKLRNSRLSRIVCTNTVPVDLDLPIADQIDISPTFAEAIRRLHNGESVSYLFTHAPV>YBL069WMAKDILKNQDPKLQAMIVEHSAPAPKEIPMDAPVLKRVARPLRHVKFIPIKSLIFHTKTGPMDFSYEKKIKTPIPKNKIVVRVSNVGLNPVDMKIRNGYTSSIYGEIGLGREYSGVITEVGENLNYAWHVGDEVYGIYYHPHLAVGCLQSSILVDPKVDPILLRPESVSAEEAAGSLFCLATGYNILNKLSKNKYLKQDSNVLINGGTSSVGMFVIQLLKRHYKLQKKLVIVTSANGPQVLQEKFPDLADEMIFIDYLTCRGKSSKPLRKMLEEKKISQYDPVEDKETILNYNEGKFDVVLDFVGGYDILSHSSSLIHGGGAYVTTVGDYVANYKEDIFDSWDNPSANARKMFGSIIWSYNYTHYYFDPNAKTASANNDWIEQCGDFLKNGTVKCVVDKVYDWKDHKEAFSYMATQRAQGKLIMNVEKF>YBL072CMGISRDSRHKRSATGAKRAQFRKKRKFELGRQPANTKIGAKRIHSVRTRGGNKKYRALRIETGNFSWASEGISKKTRIAGVVYHPSNNELVRTNTLTKAAIVQIDATPFRQWFEAHYGQTLGKKKNVKEEETVAKSKNAERKWAARAASAKIESSVESQFSAGRLYACISSRPGQSGRCDGYILEGEELAFYLRRLTAKK>YBL074CMNTVPFTSAPIEVTIGIDQYSFNVKENQPFHGIKDIPIGHVHVIHFQHADNSSMRYGYWFDCRMGNFYIQYDPKDGLYKMMEERDGAKFENIVHNFKERQMMVSYPKIDEDDTWYNLTEFVQMDKIRKIVRKDENQFSYVDSSMTTVQENELLKSSLQKAGSKMEAKNEDDPAHSLNYTVINFKSREAIRPGHEMEDFLDKSYYLNTVMLQGIFKNSSNYFGELQFAFLNAMFFGNYGSSLQWHAMIELICSSATVPKHMLDKLDEILYYQIKTLPEQYSDILLNERVWNICLYSSFQKNSLHNTEKIMENKYPELLGKDNEDDALIYGISDEERDDEDDEHNPTIVGGLYYQRP>YBL075CMSRAVGIDLGTTYSCVAHFSNDRVEIIANDQGNRTTPSYVAFTDTERLIGDAAKNQAAINPHNTVFDAKRLIGRKFDDPEVTTDAKHFPFKVISRDGKPVVQVEYKGETKTFTPEEISSMVLSKMKETAENYLGTTVNDAVVTVPAYFNDSQRQATKDAGTIAGMNVLRIINEPTAAAIAYGLDKKGRAEHNVLIFDLGGGTFDVSLLSIDEGVFEVKATAGDTHLGGEDFDNRLVNHLATEFKRKTKKDISNNQRSLRRLRTAAERAKRALSSSSQTSIEIDSLFEGMDFYTSLTRARFEELCADLFRSTLEPVEKVLKDSKLDKSQIDEIVLVGGSTRIPKIQKLVSDFFNGKEPNRSINPDEAVAYGAAVQAAILTGDQSTKTQDLLLLDVAPLSLGIETAGGIMTKLIPRNSTIPTKKSETFSTYADNQPGVLIQVFEGERTRTKDNNLLGKFELSGIPPAPRGVPQIDVTFDIDANGILNVSALEKGTGKSNKITITNDKGRLSKDDIDRMVSEAEKYRADDEREAERVQAKNQLESYAFTLKNTINEASFKEKVGEDDAKRLETASQETIDWLDASQAASTDEYKDRQKELEGIANPIMTKFYGAGAGAGPGAGESGGFPGSMPNSGATGGGEDTGPTVEEVD>YBL078CMKSTFKSEYPFEKRKAESERIADRFKNRIPVICEKAEKSDIPEIDKRKYLVPADLTVGQFVYVIRKRIMLPPEKAIFIFVNDTLPPTAALMSAIYQEHKDKDGFLYVTYSGENTFGR>YBL080CMLRLARFYSLARTKAIHSHGAPFRPEYALKCGLEIHTQLNTKNKLFSQSTNSATSLVDAPNHHTSYYDIALPGTQPVLNLEAILFAMKLSLALGSQVNSISQFDRKHYFYGDQPQGYQLTQHYRPFARGGKINLSKELDDIDESAKEIGILQLQIEQDTGKSHYTETDKDVITLVDLNRSNVPLIELVTKPDFSDIKQVRAFIKKYQNLVRHLHISSGDLETGAMRVDVNLSINEYARVELKNLPNTSSIINAIKYEYQRQVELISVGDTSSLMEPETRGWTGSSTVKLRSKETTIDYRYMPDPELPYINLAPDVISGVRGLMPQLPDDIMRILMKKPYQLSLKDAKILTYNSNQNDMYNHEALRSYYLDTFREFSKLAGERSNAKLPTNWIIHEFLGDLNKLQIPLAKAKEILAPPVFAQFLKLLHEEVISATSGKMLLFHILENFEQSNCQDLSIPDFSKLIEKFELHAINQVDPQELMDLCNDVIAQHTDDTFIRNLVTGKKKSSLKFLIGQGMRRSQGRIKANEFEKKFKEILNIQW>YBL081WMPGQIISIPFLSQNEDMDKYLLEYRSLKLLHQSSNSFQSHNAPSHQSNYHPHYNHMKYNNTGSYYYYNNNNNSSVNPHNQAGLQSINRSIPSAPYGAYNQNRANDVPYMNTQKKHHRFSANNNLNQQKYKQYPQYTSNPMVTAHLKQTYPQLYYNSNVNAHNNNNNSNNNNNNNNNSNNNNNLYNQTQFSTRYFNSNSSPSLTSSTSNSSSPYNQSTFEYILPSTSAASTNLSSSSSNNSMHTNPTTATSTSADLINDLPVGPTSSSLISDLHSPPTVSFLPASQTLLMSSTTSSSIGTNINPPQHSPSPSQREDFSTAPVNMSSSASLLMNDSSLGWGSNHMNVSSSSQPASSRPFGIWNTDMSVWS>YBL082CMEGEQSPQGEKSLQRKQFVRPPLDLWQDLKDGVRYVIFDCRANLIVMPLLILFESMLCKIIIKKVAYTEIDYKAYMEQIEMIQLDGMLDYSQVSGGTGPLVYPAGHVLIYKMMYWLTEGMDHVERGQVFFRYLYLLTLALQMACYYLLHLPPWCVVLACLSKRLHSIYVLRLFNDCFTTLFMVVTVLGAIVASRCHQRPKLKKSLALVISATYSMAVSIKMNALLYFPAMMISLFILNDANVILTLLDLVAMIAWQVAVAVPFLRSFPQQYLHCAFNFGRKFMYQWSINWQMMDEEAFNDKRFHLALLISHLIALTTLFVTRYPRILPDLWSSLCHPLRKNAVLNANPAKTIPFVLIASNFIGVLFSRSLHYQFLSWYHWTLPILIFWSGMPFFVGPIWYVLHEWCWNSYPPNSQASTLLLALNTVLLLLLALTQLSGSVALAKSHLRTTSSMEKKLN>YBL084CMAVNPELAPFTLSRGIPSFDDQALSTIIQLQDCIQQAIQQLNYSTAEFLAELLYAECSILDKSSVYWSDAVYLYALSLFLNKSYHTAFQISKEFKEYHLGIAYIFGRCALQLSQGVNEAILTLLSIINVFSSNSSNTRINMVLNSNLVHIPDLATLNCLLGNLYMKLDHSKEGAFYHSEALAINPYLWESYEAICKMRATVDLKRVFFDIAGKKSNSHNNNAASSFPSTSLSHFEPRSQPSLYSKTNKNGNNNINNNVNTLFQSSNSPPSTSASSFSSIQHFSRSQQQQANTSIRTCQNKNTQTPKNPAINSKTSSALPNNISMNLVSPSSKQPTISSLAKVYNRNKLLTTPPSKLLNNDRNHQNNNNNNNNNNNNNNNNNNNNNNNNIINKTTFKTPRNLYSSTGRLTTSKKNPRSLIISNSILTSDYQITLPEIMYNFALILRSSSQYNSFKAIRLFESQIPSHIKDTMPWCLVQLGKLHFEIINYDMSLKYFNRLKDLQPARVKDMEIFSTLLWHLHDKVKSSNLANGLMDTMPNKPETWCCIGNLLSLQKDHDAAIKAFEKATQLDPNFAYAYTLQGHEHSSNDSSDSAKTCYRKALACDPQHYNAYYGLGTSAMKLGQYEEALLYFEKARSINPVNVVLICCCGGSLEKLGYKEKALQYYELACHLQPTSSLSKYKMGQLLYSMTRYNVALQTFEELVKLVPDDATAHYLLGQTYRIVGRKKDAIKELTVAMNLDPKGNQVIIDELQKCHMQE>YBL086CMPFNHNSKAKRPKFLLDLQIKELVNIPQSSGYCYTKWRLKDGTGTSGHKVALDGEHQTTSTQSRGTTKHVHVQHHRAQWNYSLDKPILVKLHLDKNGRFLKKILVLDVFFEFADANSSLTSSSSPNGKVKKTTYANATALTATGNNSYSQKITGKLLLGTVDIDITEYVKEDETPTTNRFLLKHSKVNSIINVSLQLKLVRGSYEDFNISKSFTNGQLANYRPGINTILDNTSELSSPTSTTNQMSPKNTFSNFNGIGTTVAKPGTNATGNSTSIKSPTSTNHKSSEMTTKPGLSTTISSSMSPLIESLYQKTFKLPWDPRPGEFTPRECVEDILQGGNGWAKNEKGINLIDLQALRLNEMEEEYYNPNYGNNLGNKASSWPPNPSDDGYSTMGKREYLEKKQNWSHMSRAQRAKLRTHNDEDNENTANDKGSDKDNNSVEDNNPTDFLTDRIRENKNWSIITPSG>YBL087CMSGNGAQGTKFRISLGLPVGAIMNCADNSGARNLYIIAVKGSGSRLNRLPAASLGDMVMATVKKGKPELRKKVMPAIVVRQAKSWRRRDGVFLYFEDNAGVIANPKGEMKGSAITGPVGKECADLWPRVASNSGVVV>YBL089WMPSNVRSGVLTLLHTACGAGVLAMPFAFKPFGLMPGLITLTFCGICSLCGLLLQTRIAKYVPKSENASFAKLTQLINPSISVVFDFAIAVKCFGVGVSYLIIVGDLVPQIVQSIFYRNDDNMSGSQEHHMFLDRRLYITLIIVFVISPLCFKRSLNSLRYASMIAIVSVAYLSGLIIYHFVNRHQLERGQVYFMVPHGDSQSHSPLTTLPIFVFAYTCHHNMFSVINEQVDKSFKVIRRIPIFAIVLAYFLYIIIGGTGYMTFGENIVGNILTLYPNSISTTIGRLAMLLLVMLAFPLQCHPCRSSVKNIIIFIENFRKGKLYDNRASFIPLDNFNSEDPQEAPTQQNNEEPNLRSESLRHINIITLCILLFSYLLAISITSLAKVLAIVGATGSTSISFILPGLFGYKLIGSEFTGTNERVPTSIKIFKYLSLSLFIWGIAVMVASLSAIVFLGTSSH>YBL090WMLKSTLRLSRISLRRGFTTIDCLRQQNSDIDKIILNPIKLAQGSNSDRGQTSKSKTDNADILSMEIPVDMMQSAGRINKRELLSEAEIARSSVENAQMRFNSGKSIIVNKNNPAESFKRLNRIMFENNIPGDKRSQRFYMKPGKVAELKRSQRHRKEFMMGFKRLIEIVKDAKRKGY>YBL091CMTDAEIENSPASDLKELNLENEGVEQQDQAKADESDPVESKKKKNKKKKKKKSNVKKIELLFPDGKYPEGAWMDYHQDFNLQRTTVEESRYLKRDLERAEHWNDVRKGAEIHRRVRRAIKDRIVPGMKLMDIADMIENTTRKYTGAENLLAMEDPKSQGIGFPTGLSLNHCAAHFTPNAGDKTVLKYEDVMKVDYGVQVNGNIIDSAFTVSFDPQYDNLLAAVKDATYTGIKEAGIDVRLTDIGEAIQEVMESYEVEINGETYQVKPCRNLCGHSIAPYRIHGGKSVPIVKNGDTTKMEEGEHFAIETFGSTGRGYVTAGGEVSHYARSAEDHQVMPTLDSAKNLLKTIDRNFGTLPFCRRYLDRLGQEKYLFALNNLVRHGLVQDYPPLNDIPGSYTAQFEHTILLHAHKKEVVSKGDDY>YBL092WMASLPHPKIVKKHTKKFKRHHSDRYHRVAENWRKQKGIDSVVRRRFRGNISQPKIGYGSNKKTKFLSPSGHKTFLVANVKDLETLTMHTKTYAAEIAHNISAKNRVVILARAKALGIKVTNPKGRLALEA>YBL093CMASRVDETTVPSYYYYVDPETTYTYQQPNPLQDLISVYGLDDISRQVARTNLDGTKAVKLRKSYKNQIADLSGKFSTIPTRENGKGGQIAHILFQNNPDMMIQPPQQGQNMSEQQWREQLRNRDIALFQPPNFDWDLCSSVLSQFERSYPSEFANQNQGGAQAPFDIDDLAFDLDGTGKSQSGSNSGNNSKKRKNKSSGSSMATPTHSDSHEDMKRRRLE>YBL095WMSRTIPFLFKLVNRAVILPTAGFTLGVGAFVKAWPDDAGVLSLNDPQTPAELISATKSRQPMELQRVDILAQIEKSEVYNKLAQDEKMHHVLFSEKIPSGHREYHVGQGLLFGKGKLEIDPLVFHDVNHGELTVIYHLGAELGNRDGNVHKGLLSLLLDEALCYCGFPLLPSKRGVTARLSLEFFEDIPVDTTIILKANVKEIKGRKCIIEGHLEQFPLEVSSRNGTRSWNLPHIWGFNHKQEMAKKFAKANCILVEPTWFKYFKWLDMF>YBL098WMSESVAIIGAGLVGCLAALAFSKEGYNVTLYDFRQDPRLDTTKNKNLKSINLAISARGIDALKSIDPDACEHILQDMIPMKGRMIHDLKGRQESQLYGLHGEAINSINRSVLNNSLLDELEKSTTELKFGHKLVKIEWTDDKQICHFAIGEDLKTPHTEKYDFVIGCDGAYSATRSQMQRKVEMDFSQEYMNLRYIELYIPPTEEFKPNYGGNFAIAPDHLHIWPRHKFMLIALANSDGSFTSTFFGSKDQISDLITSKSRVREFLIENFPDIINIMDLDDAVKRFITYPKESLVCVNCKPYDVPGGKAILLGDAAHAMVPFYGQGMNCGFEDVRILMALLKKHSGDRSRAFTEYTQTRHKDLVSITELAKRNYKEMSHDVTSKRFLLRKKLDALFSIIMKDKWIPLYTMISFRSDISYSRALERAGKQTRILKFLESLTLGMLSIGGYKLFKFLTRERS>YBL099WMLARTAAIRSLSRTLINSTKAARPAAAALASTRRLASTKAQPTEVSSILEERIKGVSDEANLNETGRVLAVGDGIARVFGLNNIQAEELVEFSSGVKGMALNLEPGQVGIVLFGSDRLVKEGELVKRTGNIVDVPVGPGLLGRVVDALGNPIDGKGPIDAAGRSRAQVKAPGILPRRSVHEPVQTGLKAVDALVPIGRGQRELIIGDRQTGKTAVALDTILNQKRWNNGSDESKKLYCVYVAVGQKRSTVAQLVQTLEQHDAMKYSIIVAATASEAAPLQYLAPFTAASIGEWFRDNGKHALIVYDDLSKQAVAYRQLSLLLRRPPGREAYPGDVFYLHPRLLERAAKLSEKEGSGSLTALPVIETQGGDVSAYIPTNVISITDGQIFLEAELFYKGIRPAINVGLSVSRVGSAAQVKALKQVAGSLKLFLAQYREVAAFAQFGSDLDASTKQTLVRGERLTQLLKQNQYSPLATEEQVPLIYAGVNGHLDGIELSRIGEFESSFLSYLKSNHNELLTEIREKGELSKELLASLKSATESFVATF>YBL102WMSEEPPSDQVNSLRDSLNRWNQTRQQNSQGFNESAKTLFSSWADSLNTRAQDIYQTLPVSRQDLVQDQEPSWFQLSRTERMVLFVCFLLGATACFTLCTFLFPVLAAKPRKFGLLWTMGSLLFVLAFGVLMGPLAYLKHLTARERLPFSMFFFATCFMTIYFAAFSKNTVLTITCALLELVAVIYYAISYFPFGATGLRMLSSAGVNSARGVLRI>YBL103CMMNNNESEAENQRLLDELMNQTKVLQETLDFSLVTPTPHHNDDYKIHGSAYPGGETPAQQHEKLSYINTHNSNDNNNLMGSQARSNSQTPTASTIYEEAESQSSYLDDMFRTSQGGRPVTQNSISSIGQGPLRSSYSMAYDSPVDRAMNTPLQQQEGLKAELPHDFLFQHGTDDTMYNLTDDLSSSLSSSINSDMMTPNTYSSSFSYNPQSLGPASVSSTYSPKVRSPSSSFRAGSFLSSSFRHGSINTPRTRHTSISSNMTENIGPGSVPKILGGLTSDEKLRRKREFHNAVERRRRELIKQKIKELGQLVPPSLLNYDDLGKQIKPNKGIILDRTVEYLQYLAEILEIQARKKKALLAKIKELEEKKSSVAALSPFTNNHHASSGQNNSENSEERIIDIRSVPNALMNEQNSKAELHNWEPPLYDSVGNHNHAGTMESHPHTNIHEELKEFLSGDLIEAEDNAKLMFGDDNSNPADYLLEFGSG>YBL104CMGLIKKVTHWSYDNLIDYLSVNPTRDEVTHYKVDPENESDESIIKLHTVKDFGSITCLDYSESEIGMIGVGEKNGYLRIFNISGQNSSSPASHAPVGLNANNETSMTNASGGKAAQAENIVGSVSNLKDTQGYPVSETNYDIRVRAKKQRCINSLGINTNGLIAMGLDRNKHDSSLQIWDMNYHDDSHETINPMFSYCTNESIVSLKFLNDTSVLAASTKFLKEIDVRSPNPIYQHPTRLTYDIKLNPFNDWQFSTYGDDGTLAIWDRRKLSDQASLGDLNVASPLLTFEKLVGSGAASRKYMNSCFRWSCVRNNEFATLHRGDTIKRWRLGYYCDSNRDIAADDDNEMNIENLFVSSVHDTNTMYDRVATFDYIPRSNNGTSLICMRQSGTIYRMPISEVCSKAILNNRNSLLLSNFENTEIDEIRVNNEHEKSNLENVKTILKNLSFEDLDVSEDYFPSGHDEPNNEIEYSELSEEENEGSNDVLDSKRGFELFWKPEKLLEKDISVIMRTRASLGYGLDPMNTVEMIDSSKNLQNNAYIRNTWRWIAIAKASVDDGTMVSGDLDLGYEGVIGIWNGINGISNQDRYRQETILSDKQLNKEMEKIIKLRRKNRDRNSPIANAAGSPKYVQRRLCLIISGWDLSRSDYEDKYNIIMKNGHYEKAAAWAVFFGDIPKAVEILGSAKKERLRLIATAIAGYLAYKDLPGNNAWRQQCRKMSSELDDPYLRVIFAFIADNDWWDILYEPAISLRERLGVALRFLNDTDLTTFLDRTSSTVIENGELEGLILTGITPNGIDLLQSYVNKTSDVQSAALISIFGSPRYFRDQRVDEWIQTYRDMLKSWELFSMRARFDVLRSKLSRTKTGVLTADIKPRQIYIQCQNCKQNINTPRTSSPSSAVSTSAGNYKNGEAYRRNNADYKKFNTGSSEAQAADEKPRHKYCCPHCGSSFPRCAICLMPLGTSNLPFVINGTQSRDPMQTEDSQDGANRELVSRKLKLNEWFSFCLSCNHGMHAGHAEEWFDRHNVCPTPGCTCQCNK>YBL107CMVDNRRTFTAPQSLLETNLTFPNDEPSLTTITVTRERCVDPSLIDSFLRFLRHGSDDIIRQKLNNYRKGSINGKNKCKEFLKQELYPNWQIRNNIISFCEKEAAEMKNETDQQCGNNKKTTAEPLIDARIDPYAARERAEKQEAQYKDWTKVTEWVANNRKIEQILTSTTEGILRQNCEQNNDYLKEFTQFCKDNS>YBR002CMETDSGIPGHSFVLKWTKNIFSRTLRASNCVPRHVGFIMDGNRRFARKKEMDVKEGHEAGFVSMSRILELCYEAGVDTATVFAFSIENFKRSSREVESLMTLARERIRQITERGELACKYGVRIKIIGDLSLLDKSLLEDVRVAVETTKNNKRATLNICFPYTGREEILHAMKETIVQHKKGAAIDESTLESHLYTAGVPPLDLLIRTSGVSRLSDFLIWQASSKGVRIELLDCLWPEFGPIRMAWILLKFSFHKSFLNKEYRLEEGDYDEETNGDPIDLKEKKLN>YBR003WMFQRSGAAHHIKLISSRRCRFKSSFAVALNAASKLVTPKILWNNPISLVSKEMNTLAKNIVALIGSGHPVLNKVTSYYFETEGKKVRPLLVLLLSRALSEIPMTERNHLKIDKSDVPEDPIYSKPSQNQLFQRPASSISPLHILHGIKPLNPLTKGPEPLPEETFDKQRGILPKQRRLAEIVEMIHTASLLHDDVIDHSDTRRGRPSGNAAFTNKMAVLAGDFLLGRATVSISRLHNPEVVELMSNSIANLVEGEFMQLKNTSIDADIDTIENGHKLLPVPSKKLEVKEHDFRVPSRQQGLQLSHDQIIETAFEYYIHKTYLKTAALISKSCRCAAILSGASPAVIDECYDFGRNLGICFQLVDDMLDFTVSGKDLGKPSGADLKLGIATAPVLFAWKEDPSLGPLISRNFSERGDVEKTIDSVRLHNGIAKTKILAEEYRDKALQNLRDSLPESDARSALEFLTNSILTRRK>YBR004CMIVGLTLYFVLFRSIQYLLVFLTPIRQFDTSTSLLLNELCSSPSEINSYWNKYFWNKLLSWDSVFFIKNITSKNGKPQFEHEYAFSQLWTFFVRLFIKSNNDSIYHALRVGVAIENVLFYLSGIVLYFLTKKIFSQNIRQSQFARTIAKKTSLLFFLTSAAGFLTSIYSEPLSFFFAFVGIWSRECTISVPVLGQFDISWRYWFPYSFISMACFTLASLNRSNCVLLGIYFIFDLIELTKNRKFVKAICFPLLSGSLMFSALLYQQYYLPYKTFCPQRGEWCKSQLFSSIFITKTSLYSYIQSHYWGVGLLKYWTPNNIPNFLFAVPNIIILIYSSIYFSKIYPSYNLKALVWITRALVVIVCFFAHVQILNRIASFLPLHLWYLADRLVKTSDPKKMENPKGDDKIVKFYIYWLAFWIPLQTILFAAFLPPA>YBR005WMGLISYENEAINEVKKADNHHVSKFVTSYYGPSSSSWQSGIWILFVLFVAAVILIILFTFVANRRRRRMGRAPIRGTAWLTPPSYRQSQQQYTGTVQQRTDDYVPEYTETANEHDLGYYDQRGEFHPNDKAAYVAPPPLVQECSSESVNSLERPPAAVVHQANSLDTDYGLTRPSNGRVPAVSDTVEQLERLPGGTTTQEINPPERAKVNARS>YBR006WMTLSKYSKPTLNDPNLFRESGYIDGKWVKGTDEVFEVVDPASGEIIARVPEQPVSVVEEAIDVAYETFKTYKNTTPRERAKWLRNMYNLMLENLDDLATIITLENGKALGEAKGEIKYAASYFEWYAEEAPRLYGATIQPLNPHNRVFTIRQPVGVCGIICPWNFPSAMITRKAAAALAVGCTVVIKPDSQTPLSALAMAYLAEKAGFPKGSFNVILSHANTPKLGKTLCESPKVKKVTFTGSTNVGKILMKQSSSTLKKLSFELGGNAPFIVFEDADLDQALEQAMACKFRGLGQTCVCANRLYVHSSIIDKFAKLLAERVKKFVIGHGLDPKTTHGCVINSSAIEKVERHKQDAIDKGAKVVLEGGRLTELGPNFYAPVILSHVPSTAIVSKEETFGPLCPIFSFDTMEEVVGYANDTEFGLAAYVFSKNVNTLYTVSEALETGMVSCNTGVFSDCSIPFGGVKESGFGREGSLYGIEDYTVLKTITIGNLPNSI>YBR007CMNQNLKNTSWADRIGSDDQERKANSSEVSQSPPPNNSFESSMDSQFSYAHSNKSSISFESIQTTERLLDKLDLSLEDELILQEALLEEENASRNSQLSQTSGPTLCMPASEFPSLRYRTNPSPTYIQARDRSLIIDNLKEKDSTLRGKYSSGKVERHLPVKSRYSYIVEEDYDSETFSGMKPQMNRNEKDYKYPNLENGNRSTNRPNPFNFEKYRIENTRLHHLYPTLISDNNTSVDNNANSKNNRTTSNNINTSTKTDRISEKQSCPNEFTTTQKSNCLYRNGSSTSTNTSFSEVGQLSKPKTQSSFESESSSFSKLKLTKSDTTTIKPSPKRSNSSTSTITKTNTMTNDISLPPTPPYKAHKKKTSLNSLKKLFKSPRTRAKNKKDLESEGSSPIRSATNSLDFSGENIQLPSTSSTINNSSPHLARYIFPPNPVFHFKTASTPQSSTDKKKNSKARPNRTHLRTFSDFHTTEKDSKIGELSALTEQSNKPYHPKVRRRTLSLDGMLPNNSTQCMDSFSHKKEGSNATSKCGKLKFHPEPYDNDESSHIGQAITMRHQGKLEESAQRLKKACACGNKTAFLLYGLALRHGCGVDKNLKLSLGYLMAATDIKSFAAEVLDLDINPLNFASMDDIPDIAPEPTAPALYECGMAYLKGLGMDHPDERKGLKFLEKAALLGHVDSMCLSGTIWSKTSNVKKRDLARAAAWFRIADKKGANLLGSDWIYKEKYMKQGPK>YBR008CMVYTSTYRHTIVVDLLEYLGIVSNLETLQSAREDETRKPENTDKKECKPDYDIECGPNRSCSESSTDSDSSGSQIEKNDPFRVDWNGPSDPENPQNWPLLKKSLVVFQIMLLTCVTYMGSSIYTPGQEYIQEEFHVGHVVATLNLSLYVLGYGLGPIIFSPLSETARYGRLNLYMVTLFFFMIFQVGCATVHNIGGLIVMRFISGILCSPSLATGGGTVADIISPEMVPLVLGMWSAGAVAAPVLAPLLGAAMVDAKNWRFIFWLLMWLSAATFILLAFFFPETQHHNILYRRALKLRKETGDDRYYTEQDKLDREVDARTFLINTLYRPLKMIIKEPAILAFDLYIAVAYGCFYLFFEAFPIVFVGIYHFSLVEVGLAYMGFCVGCVLAYGLFGILNMRIIVPRFRNGTFTPEAFLIVAMCVCWCLPLSLFLFGWTARVHWILPVISEVFFVLAVFNIFQATFAYLATCYPKYVASVFAGNGFCRASFACAFPLFGRAMYDNLATKNYPVAWGSSLVGFLTLGLAIIPFILYKYGPSLRTRSSYTEE>YBR009CMSGRGKGGKGLGKGGAKRHRKILRDNIQGITKPAIRRLARRGGVKRISGLIYEEVRAVLKSFLESVIRDSVTYTEHAKRKTVTSLDVVYALKRQGRTLYGFGG>YBR010WMARTKQTARKSTGGKAPRKQLASKAARKSAPSTGGVKKPHRYKPGTVALREIRRFQKSTELLIRKLPFQRLVREIAQDFKTDLRFQSSAIGALQESVEAYLVSLFEDTNLAAIHAKRVTIQKKDIKLARRLRGERS>YBR011CMTYTTRQIGAKNTLEYKVYIEKDGKPVSAFHDIPLYADKENNIFNMVVEIPRWTNAKLEITKEETLNPIIQDTKKGKLRFVRNCFPHHGYIHNYGAFPQTWEDPNVSHPETKAVGDNDPIDVLEIGETIAYTGQVKQVKALGIMALLDEGETDWKVIAIDINDPLAPKLNDIEDVEKYFPGLLRATNEWFRIYKIPDGKPENQFAFSGEAKNKKYALDIIKETHDSWKQLIAGKSSDSKGIDLTNVTLPDTPTYSKAASDAIPPASPKADAPIDKSIDKWFFISGSV>YBR014CMAIVINKRNVRVLVITNLLLIVVFFVLRNSNASVNESITTHHPDSLVTFDNSGNAPGTHQSVHDTVNTQDKEAEEVDKNSGDAEFDAAAEYNKIMEQSPMIVFSKTGCPYSKKLKALLTNSYTFSPSYYVVELDRHEHTKELQDQIEKVTGRRTVPNVIIGGTSRGGYTEIAELHKNDELLDSFKKWSDGAFTVKANSQSESA>YBR016WMSANDYYGGTAGEKSQYSRPSNPPPSSAHQNKTQERGYPPQQQQQYYQQQQQHPGYYNQQGYNQQGYNQQGYNQQGYNQQGYNQQGYNQQGHQQPVYVQQQPPQRGNEGCLAACLAALCICCTMDMLF>YBR017CMASTWKPAEDYVLQLATLLQNCMSPNPEIRNNAMEAMENFQLQPEFLNYLCYILIEGESDDVLKQHYSLQDLQNNRATAGMLLKNSMLGGNNLIKSNSHDLGYVKSNIIHGLYNSNNNLVSNVTGIVITTLFSTYYRQHRDDPTGLQMLYQLLELTSNGNEPSIKALSKIMEDSAQFFQLEWSGNTKPMEALLDSFFRFISNPNFSPVIRSESVKCINTVIPLQTQSFIVRLDKFLEIIFQLAQNDENDLVRAQICISFSFLLEFRPDKLVSHLDGIVQFMLHLITTVNEEKVAIEACEFLHAFATSPNIPEHILQPYVKDIVPILLSKMVYNEESIVLLEASNDDDAFLEDKDEDIKPIAPRIVKKKEAGNGEDADDNEDDDDDDDDEDGDVDTQWNLRKCSAATLDVMTNILPHQVMDIAFPFLREHLGSDRWFIREATILALGAMAEGGMKYFNDGLPALIPFLVEQLNDKWAPVRKMTCWTLSRFSPWILQDHTEFLIPVLEPIINTLMDKKKDVQEAAISSVAVFIENADSELVETLFYSQLLTSFDKCLKYYKKKNLIILYDAIGRFAEKCALDETAMQIILPPLIEKWALLSDSDKELWPLLECLSCVASSLGERFMPMAPEVYNRAFRILCHCVELEAKSHQDPTIVVPEKDFIITSLDLIDGLVQGLGAHSQDLLFPQGTKDLTILKIMLECLQDPVHEVRQSCFALLGDIVYFFNSELVIGNLEDFLKLIGTEIMHNDDSDGTPAVINAIWALGLISERIDLNTYIIDMSRIILDLFTTNTQIVDSSVMENLSVTIGKMGLTHPEVFSSGAFANDSNWNKWCLSVNALDDVEEKSSAYMGFLKIINLTSTEVTMSNDTIHKIVTGLSSNVEANVFAQEIYTFLMNHSAQISAINFTPDEISFLQQFTS>YBR018CMTAEEFDFSSHSHRRYNPLTDSWILVSPHRAKRPWLGQQEAAYKPTAPLYDPKCYLCPGNKRATGNLNPRYESTYIFPNDYAAVRLDQPILPQNDSNEDNLKNRLLKVQSVRGNCFVICFSPNHNLTIPQMKQSDLVHIVNSWQALTDDLSREARENHKPFKYVQIFENKGTAMGCSNLHPHGQAWCLESIPSEVSQELKSFDKYKREHNTDLFADYVKLESREKSRVVVENESFIVVVPYWAIWPFETLVISKKKLASISQFNQMVKEDLASILKQLTIKYDNLFETSFPYSMGIHQAPLNATGDELSNSWFHMHFYPPLLRSATVRKFLVGFELLGEPQRDLTSEQAAEKLRNLDGQIHYLQRL>YBR019CMTAQLQSESTSKIVLVTGGAGYIGSHTVVELIENGYDCVVADNLSNSTYDSVARLEVLTKHHIPFYEVDLCDRKGLEKVFKEYKIDSVIHFAGLKAVGESTQIPLRYYHNNILGTVVLLELMQQYNVSKFVFSSSATVYGDATRFPNMIPIPEECPLGPTNPYGHTKYAIENILNDLYNSDKKSWKFAILRYFNPIGAHPSGLIGEDPLGIPNNLLPYMAQVAVGRREKLYIFGDDYDSRDGTPIRDYIHVVDLAKGHIAALQYLEAYNENEGLCREWNLGSGKGSTVFEVYHAFCKASGIDLPYKVTGRRAGDVLNLTAKPDRAKRELKWQTELQVEDSCKDLWKWTTENPFGYQLRGVEARFSAEDMRYDARFVTIGAGTRFQATFANLGASIVDLKVNGQSVVLGYENEEGYLNPDSAYIGATIGRYANRISKGKFSLCNKDYQLTVNNGVNANHSSIGSFHRKRFLGPIIQNPSKDVFTAEYMLIDNEKDTEFPGDLLVTIQYTVNVAQKSLEMVYKGKLTAGEATPINLTNHSYFNLNKPYGDTIEGTEIMVRSKKSVDVDKNMIPTGNIVDREIATFNSTKPTVLGPKNPQFDCCFVVDENAKPSQINTLNNELTLIVKAFHPDSNITLEVLSTEPTYQFYTGDFLSAGYEARQGFAIEPGRYIDAINQENWKDCVTLKNGETYGSKIVYRFS>YBR020WMTKSHSEEVIVPEFNSSAKELPRPLAEKCPSIIKKFISAYDAKPDFVARSPGRVNLIGEHIDYCDFSVLPLAIDFDMLCAVKVLNEKNPSITLINADPKFAQRKFDLPLDGSYVTIDPSVSDWSNYFKCGLHVAHSFLKKLAPERFASAPLAGLQVFCEGDVPTGSGLSSSAAFICAVALAVVKANMGPGYHMSKQNLMRITVVAEHYVGVNNGGMDQAASVCGEEDHALYVEFKPQLKATPFKFPQLKNHEISFVIANTLVVSNKFETAPTNYNLRVVEVTTAANVLAATYGVVLLSGKEGSSTNKGNLRDFMNVYYARYHNISTPWNGDIESGIERLTKMLVLVEESLANKKQGFSVDDVAQSLNCSREEFTRDYLTTSPVRFQVLKLYQRAKHVYSESLRVLKAVKLMTTASFTADEDFFKQFGALMNESQASCDKLYECSCPEIDKICSIALSNGSYGSRLTGAGWGGCTVHLVPGGPNGNIEKVKEALANEFYKVKYPKITDAELENAIIVSKPALGSCLYEL>YBR021WMPDNLSLHLSGSSKRLNSRQLMESSNETFAPNNVDLEKEYKSSQSNITTEVYEASSFEEKVSSEKPQYSSFWKKIYYEYVVVDKSILGVSILDSFMYNQDLKPVEKERRVWSWYNYCYFWLAECFNINTWQIAATGLQLGLNWWQCWITIWIGYGFVGAFVVLASRVGSAYHLSFPISSRASFGIFFSLWPVINRVVMAIVWYSVQAYIAATPVSLMLKSIFGKDLQDKIPDHFGSPNATTYEFMCFFIFWAASLPFLLVPPHKIRHLFTVKAVLVPFASFGFLIWAIRRAHGRIALGSLTDVQPHGSAFSWAFLRSLMGCMANFSTMVINAPDFSRFSKNPNSALWSQLVCIPFLFSITCLIGILVTAAGYEIYGINYWSPLDVLEKFLQTTYNKGTRAGVFLISFVFAVAQLGTNISANSLSCGTDMSAIFPKFINIKRGSLFCAAMALCICPWNLMATSSKFTMALSAYAIFLSSIAGVVCSDYFVVRRGYIKLTHIYSHQKGSFYMYGNRFGINWRALAAYLCGVAPCLPGFIAEVGAPAIKVSDGAMKLYYLSYWVGYGLSFSSYTALCYFFPVPGCPVNNIIKDKGWFQRWANVDDFEEEWKDTIERDDLVDDNISVYEHEHEKTFI>YBR022WMSNITYVKGNILKPKSYARILIHSCNCNGSWGGGIAYQLALRYPKAEKDYVEVCEKYGSNLLGKCILLPSYENSDLLICCLFTSSFGGSSHGEKQSILNYTKLALDKLKTFREAKDKTRTSEDSIGDYLNGHIKYPIGEYKLEMPQINSGIFGVPWKETERVLEEFSGDMSFTVYQL>YBR024WMLNSSRKYACRSLFRQANVSIKGLFYNGGAYRRGFSTGCCLRSDNKESPSARQPLDRLQLGDEINEPEPIRTRFFQFSRWKATIALLLLSGGTYAYLSRKRRLLETEKEADANRAYGSVALGGPFNLTDFNGKPFTEENLKGKFSILYFGFSHCPDICPEELDRLTYWISELDDKDHIKIQPLFISCDPARDTPDVLKEYLSDFHPAIIGLTGTYDQVKSVCKKYKVYFSTPRDVKPNQDYLVDHSIFFYLIDPEGQFIDALGRNYDEQSGLEKIREQIQAYVPKEERERRSKKWYSFIFN>YBR025CMPPKKQVEEKKVLLGRPGNNLKAGIVGLANVGKSTFFQAITRCPLGNPANYPFATIDPEEARVIVPSPRFDKLCEIYKKTASEVPAHLTVYDIAGLTKGASAGEGLGNAFLSHIRSVDSIYQVVRCFDDAEIIHVEGDVDPVRDLEIINQELRLKDIEFAQKALEGAEKIAKRGGQSLEVKQKKEEMDLITKIIKLLESGQRVANHSWTSKEVEIINSMFLLTAKPCIYLINLSERDYIRKKNKHLLRIKEWVDKYSPGDLIIPFSVSLEERLSHMSPEDAEEELKKLQTISALPKIITTMRQKLDLISFFTCGPDEVREWTIRRGTKAPQAAGVIHNDLMNTFILAQVMKCEDVFEYKDDSAIKAAGKLMQKGKDYVVEDGDIIYFRAGAGKN>YBR026CMLPTFKRYMSSSAHQIPKHFKSLIYSTHEVEDCTKVLSVKNYTPKQDLSQSIVLKTLAFPINPSDINQLQGVYPSRPEKTYDYSTDEPAAIAGNEGVFEVVSLPSGSSKGDLKLGDRVIPLQANQGTWSNYRVFSSSSDLIKVNDLDLFSAATVSVNGCTGFQLVSDYIDWNSNGNEWIIQNAGTSSVSKIVTQVAKAKGIKTLSVIRDRDNFDEVAKVLEDKYGATKVISESQNNDKTFAKEVLSKILGENARVRLALNSVGGKSSASIARKLENNALMLTYGGMSKQPVTLPTSLHIFKGLTSKGYWVTEKNKKNPQSKIDTISDFIKMYNYGHIISPRDEIETLTWNTNTTTDEQLLELVKKGITGKGKKKMVVLEW>YBR028CMIFSLDEELHRVSLDDKKNDIKVDYSSAIYNDINHEQGSSITYEESINHLSVHSNAIPLNGMSPAHRMRRRSSAYSKFPILTPPNTRRFSITGSDAMRTNTNRLSITPQDIISSNIGENELSRNLHDFKPVRVLGQGAYGKVLLVKDVNTSKLYAMKQLRKAEILISQTATDSKREDEDKNDGNNNDNDDGLSKRLERTFAERSILSEIEHPNIVKLFYSFHDNSKLYLLLQYIPGGELFYHLKEHGTLDETTVSFYAAEISCALRFLHTKGVVYRDLKPENCLLNQRGHLVLTDFGLSKKSANDSAVDEEDPENVNALYSIIGTPEYCAPEILLGKAYSQNCDWYSLGCLLYDMLVGKPPYTGSNHKVIINKIQQNKQGPKIPFYLSEGMKDILNALLKKETAKRWNVDKYWAKTGANNKPTKSKKKKSGAARTSLFTEHFIFRKIDWKLLESGQLQKTTLGPIVPVITDLELAENFDTEFTSMSYEETYTDSKPININSVSKSPDMFKGFSYKASGSYLEKYF>YBR030WMSVTFKDDVHRILKFVANCNGRFEDSKCDIRESPLGGLGVFAKTDIAEGESILTLNKSSIFSASNSSIANLLCDSSIDGMLALNIAFIYETTVFRNSSHWYPFLRTIRIRDDEGHLNLPPSFWHADAKRLLKGTSFDTLFDSLAPEEEIMEGFEIAVDLAHKWNDEFGLEIPKGFLDVSEENHEEDYNLKLEKFISVAYTLSSRGFEIDAYHETALVPIADLFNHHVSDPDLKFVSLYDVCDKCGEPDMCKHLIAEEYLEAENLDKNMPKVASMETRVIDEDLIKSLENDLEKEYSNVTANIEDDDGGIENPDECVDLVLKNDVAQGQEIFNSYGELSNVFLLARYGFTVPENQYDIVHLGPDFMKILKKEEKYQEKVKWWSQVGHGLFSAWYAQMRQEDEEDEDGQAKSDNLSDDIESEEEEEEEEGDDSLESWLSQLYIDSSGEPSPSTWALANLLTLTAVQWESLFSKKATPHISDSIVNEEKLPFLAKKDNPHSKKLLSNLLKEKQLPCIKGDNSSKITSATKSMLQNARTLVQSEHNILDRCLKRLS>YBR031WMSRPQVTVHSLTGEATANALPLPAVFSAPIRPDIVHTVFTSVNKNKRQAYAVSEKAGHQTSAESWGTGRAVARIPRVGGGGTGRSGQGAFGNMCRGGRMFAPTKTWRKWNVKVNHNEKRYATASAIAATAVASLVLARGHRVEKIPEIPLVVSTDLESIQKTKEAVAALKAVGAHSDLLKVLKSKKLRAGKGKYRNRRWTQRRGPLVVYAEDNGIVKALRNVPGVETANVASLNLLQLAPGAHLGRFVIWTEAAFTKLDQVWGSETVASSKVGYTLPSHIISTSDVTRIINSSEIQSAIRPAGQATQKRTHVLKKNPLKNKQVLLRLNPYAKVFAAEKLGSKKAEKTGTKPAAVFTETLKHD>YBR033WMSHHVPNLYGTPIRDPHERKRNSASMGEVNQSVSSRNCERGSEKGTKQRKKASHACDQCRRKRIKCRFDKHTGVCQGCLEVGEKCQFIRVPLKRGPAKKRGSVVSIEKFSSDNDPLQYRPRTHSYPMNSGNNYLPSLARNSSFPSISSLFVPSITAQSQQFVKVPYDDIKRRSSLAILGSDSSISTEFGGNYRLDENLNVRQEGKDIVAKGMITPVEEMGACSSNVRRQGSQSLPIQEQRASPYINPFISGRSRLSSLSYTSEATTSEGNTQGKNQCMLTPNSVRSIEKERLNSLTAGFPNKKLGTDGRSDKWDKNSTWKPVYRSSNPSHPSTEKNVSLNQEASAKPLMLGTYRQFDATSFYKVLGIYYNFFHINFPVIPINKSKFTDMLDPEKPNVIDEIRQINNEIIQCFKTALEVLVFCKIKQRRSSKSTKSWSRDSLCDFQKGLYYIQNFNKCIADCFQSLITIKPVLKQNSSVIPSRIKFIYFSTIIVLNFILILAGEESSLLLGPSVGVFNEFQAHKLFLPFQNTSPMLLLNSNEESGDEILDYAVLFKRLYILLNILDTLQSFRLGQPKLINLNFGSAIETYFSDKTGHNQVVEKAPVALDNILRNLKLGEFITYFVLNRKSLQVNVPHHLLFTNQTDYGEFAVEKGEHDNIAGKFETLLKKKEILIRKLLNIEQKNDHILENCCNSDAEMKNIGELVCSMITLVSGILDSITNMNAENSVDLDSKPLPNAYFAQDSEEELMSPTQSITSNLASEENTRCTTKDLMGTVSIFMLPMVEECYNIISLIGPIPTTLISLYIRNGNMAKGINDRIMTLSTALNELVQITALFNTLEPFRKNAHDRAKRYYVSATSSTGCYESVMKSMYSGKCAASNASNVAPSEEENKKILKKFADIGWKLMDDSELGCCCCFFN>YBR034CMSKTAVKDSATEKTKLSESEQHYFNSYDHYGIHEEMLQDTVRTLSYRNAIIQNKDLFKDKIVLDVGCGTGILSMFAAKHGAKHVIGVDMSSIIEMAKELVELNGFSDKITLLRGKLEDVHLPFPKVDIIISEWMGYFLLYESMMDTVLYARDHYLVEGGLIFPDKCSIHLAGLEDSQYKDEKLNYWQDVYGFDYSPFVPLVLHEPIVDTVERNNVNTTSDKLIEFDLNTVKISDLAFKSNFKLTAKRQDMINGIVTWFDIVFPAPKGKRPVEFSTGPHAPYTHWKQTIFYFPDDLDAETGDTIEGELVCSPNEKNNRDLNIKISYKFESNGIDGNSRSRKNEGSYLMH>YBR035CMTKQAEETQKPIIFAPETYQYDKFTLNEKQLTDDPIDLFTKWFNEAKEDPRETLPEAITFSSAELPSGRVSSRILLFKELDHRGFTIYSNWGTSRKAHDIATNPNAAIVFFWKDLQRQVRVEGITEHVNRETSERYFKTRPRGSKIGAWASRQSDVIKNREELDELTQKNTERFKDAEDIPCPDYWGGLRIVPLEIEFWQGRPSRLHDRFVYRRKTENDPWKVVRLAP>YBR037CMLKLSRSANLRLVQLPAARLSGNGAKLLTQRGFFTVTRLWQSNGKKPLSRVPVGGTPIKDNGKVREGSIEFSTGKAIALFLAVGGALSYFFNREKRRLETQKEAEANRGYGKPSLGGPFHLEDMYGNEFTEKNLLGKFSIIYFGFSNCPDICPDELDKLGLWLNTLSSKYGITLQPLFITCDPARDSPAVLKEYLSDFHPSILGLTGTFDEVKNACKKYRVYFSTPPNVKPGQDYLVDHSIFFYLMDPEGQFVDALGRNYDEKTGVDKIVEHVKSYVPAEQRAKQKEAWYSFLFK>YBR039WMLSRIVSNNATRSVMCHQAQVGILYKTNPVRTYATLKEVEMRLKSIKNIEKITKTMKIVASTRLSKAEKAKISAKKMDEAEQLFYKNAETKNLDVEATETGAPKELIVAITSDKGLCGSIHSQLAKAVRRHLNDQPNADIVTIGDKIKMQLLRTHPNNIKLSINGIGKDAPTFQESALIADKLLSVMKAGTYPKISIFYNDPVSSLSFEPSEKPIFNAKTIEQSPSFGKFEIDTDANVPRDLFEYTLANQMLTAMAQGYAAEISARRNAMDNASKNAGDMINRYSILYNRTRQAVITNELVDIITGASSLG>YBR040WMVAISMIWFFTKRMPRIFALAFNLISIFLLIFLLIGCYNPSNQSTFLVKYKFDDNSPFYTIIEKSYEKSNTTLGLEEVIIRSGYMGVCIDNIPSQYSSYNNMTTFSNSICYARKNLSSVPLYRDLEIQLSNIASSSSKTQSSVVLNILKLAQLTSVNVIHPYVLMATVILTILMFLFILYVTVPKLPFKLAVNKFLLLLSSTIVLTWGIGAMWTHVGINASYRLVPSSSMNIITVKKGKKAAVMAWFSFAFLLLDSVVLWLIFLRDRKSLKDEIDNVPCAQNRYNNYSSDSSTLHSKV>YBR041WMSPIQVVVFALSRIFLLLFRLIKLIITPIQKSLGYLFGNYFDELDRKYRYKEDWYIIPYFLKSVFCYIIDVRRHRFQNWYLFIKQVQQNGDHLAISYTRPMAEKGEFQLETFTYIETYNIVLRLSHILHFDYNVQAGDYVAIDCTNKPLFVFLWLSLWNIGAIPAFLNYNTKGTPLVHSLKISNITQVFIDPDASNPIRESEEEIKNALPDVKLNYLEEQDLMHELLNSQSPEFLQQDNVRTPLGLTDFKPSMLIYTSGTTGLPKSAIMSWRKSSVGCQVFGHVLHMTNESTVFTAMPLFHSTAALLGACAILSHGGCLALSHKFSASTFWKQVYLTGATHIQYVGEVCRYLLHTPISKYEKMHKVKVAYGNGLRPDIWQDFRKRFNIEVIGEFYAATEAPFATTTFQKGDFGIGACRNYGTIIQWFLSFQQTLVRMDPNDDSVIYRNSKGFCEVAPVGEPGEMLMRIFFPKKPETSFQGYLGNAKETKSKVVRDVFRRGDAWYRCGDLLKADEYGLWYFLDRMGDTFRWKSENVSTTEVEDQLTASNKEQYAQVLVVGIKVPKYEGRAGFAVIKLTDNSLDITAKTKLLNDSLSRLNLPSYAMPLFVKFVDEIKMTDNHKILKKVYREQKLPKGLDGNDTIFWLKNYKRYEVLTAADWEAIDAQTIKL>YBR043CMQAQGSQSNVGSLRSNCSDNSLPNNHVMMHCDESSGTPHSEHNDYSYEKTNLESTASNSREHRDNQLSRLKSEEYVVPKNQRRGLLPQLAIIPEFKDARDYPPMMKKMIVFLIAFSSMMGPMGTSIIFPAINSITTEFKTSVIMVNVSIGVYLLSLGVFPLWWSSLSELEGRRTTYITSFALLFAFNIGSALAPDINSFIALRMLCGAASASVQSVGAGTVADLYISEDRGKNLSYYYLGPLLAPLLSPIFGSLLVNRWPWRSTQWFMVILSGCNVILLTVLLPETLRKQDSKGAIAQILAERRIQVDNNERGEIQEDYQRGEDETDRIENQVATLSTEKHNYVGEVRDQDSLDLESHSSPNTYDGRAGETQLQRIYTEASRSLYEYQLDDSGIDATTAQVTRIRSTDPKLARSIRENSLRKLQTNLEEQVKKVLSSNGGEIAPKQVSAVRKVWDTFFVYFIKPLKSLHFLEYPPVALAITFSAISFSTVYFVNMTVEYKYSRPPYNFKPLYIGLLYIPNSVTYFFASIYGGRWVDMLLKRYKEKYGILAPEARISWNVVTSVISFPIALLIFGWCLDKKCHWVTPLIGTALFGYAAMMTIGATLSYLVDSLPGKGATGVALNNLIRQILAATAVFVTTPMLNGMGTGWAFTMLAFIVLGASSVLIILKKHGDYWRENYDLQKLYDKID>YBR045CMETILQPKARPFESLKRKRFREWLRPSTAHGSLLHSDTLDLRDFAKPNPADTFSNLDSGHCPLVTTPIKYECPDGKSSFFRGDTKFETLFSNRKFYEFKDNLKRGLKKIRHGRNGHQSEKRCPVVEETKKSVSDNLDKPDNNTPCFDRFHTNSKEFETQFDHSNRSQNSEKAYLDNESCWNLSEKFIPFNNLKYEDLKHFEENLQSLAPATFTPIESNESLDRSDSTRGTKRSIRNDSSDTTSEKRLCLKQYSDEPESDHSMESTPSIYITKEVQERIEALSSTDSFLIEKVDFPSNKIGSSASDYESDNEYRNMDEDSINDVTTEKEGNVVIPDSNTSTVDAMEKPIEVSSALKDDTLDKDIDDASSSYSDDVETTFEPVESEELSDLSDTSSSGSSKIYTIPTFRGLTNRTNISQILSKVGKADLSQDNLTHLIKSHQKKKRCVNFRNKRFYDAFNPYVDNEEDAELSDSENISEMDTDLCIKDRSTSSVRFDENSRLLIYKKSKKLNKDETQSGYSTTEMRSILKTKMNSQHDEESQRASKCDTVGVAQFLHYFQYTEYKRQRNEAEIID>YBR046CMKCTIPEQQKVILIDEIGGYDVIKYEDYPVPSISEEELLIKNKYTGVNYIESYFRKGIYPCEKPYVLGREASGTVVAKGKGVTNFEVGDQVAYISNSTFAQYSKISSQGPVMKLPKGTSDEELKLYAAGLLQVLTALSFTNEAYHVKKGDYVLLFAAAGGVGLILNQLLKMKGAHTIAVASTDEKLKIAKEYGAEYLINASKEDILRQVLKFTNGKGVDASFDSVGKDTFEISLAALKRKGVFVSFGNASGLIPPFSITRLSPKNITLVRPQLYGYIADPEEWKYYSDEFFGLVNSKKLNIKIYKTYPLRDYRTAAADIESRKTVGKLVLEIPQ>YBR047WMLINHLSKIRTVRHFSNIKPVLSKEVSRRVIVAPASHFKTSSPNVKSNIPIHEYKQLPEDSNYIEKHYKELQVFLNEFLIKKLNKTYADFEGDPDELVFQLEKFIELEVTPRYTNHSAPDGCEERFKSIGDRIVVDRYLDFVKDVRLTLLLNGGHSFIFDVMLQAKEVFDKMQKE>YBR050CMTLSNCDSLDNLFQDPPEEEESSKFVEAVRTLMNRNDMGYPPAAANGTYCLKKIKSLNAKQWKINKKRMCMLPAVKKKNFDFHEQRSLILNLNLWKFIKFINCSSKNNYNKNNKHVRSSNNTVKNENVLPLQKHKKVDNDQRLENLFWRSWFKARKRRDIMGKPRERHIKFNDNVEQCIITDEHFIQRLPSTRLNSTDEQRPCSKSELDPCIGNAASKRSFYDYNSVYVASDAIITTAAATAIISSNSGDYQRGHDVRDVPRNVLLQAGETDFSSVLRVDSDLKLSNISHHSPVKPSSTSSHSTFIFESETDTDTDTDAETENDIDAYIDTSIPNLLL>YBR052CMPKVAILIYSVDDIIATLAENEKKGIEIAGGEAEIFQVPDVSYKTEYATEEGKEAAKVAKTNADFSYKILTRETLVEYDYYLFGIPTKFGNFPAEWKSFWDSNTGGLWAKGSLHGKIAGLFVSGAISGKGDTEMCIMNAMSTLVHHGVIYVPLGYKNAYKELTDVEDVNGSCAWGAGCVSGIDGGRPPSLSELRVHQLQGKAFYDRIKDL>YBR053CMSSVGDFEEIILHDLKPYYHVPGAIHSEGITFVKETGTLLWVDIFKGEVHKVEDIEQPESSHSFFSISRANYGKNASIEYPPNPDELKESVGCIFPILDGASQNEIKQVLFGSKFGIGKLDFSKSEWEYVILYSECPELSTDRAYKLRSNDGNVSPDGKYIYVGLMSDFPFDLEPIGCLLRVDLLAHKIELVWNCLLIPNAIHWDESDQKTMYVTDSLNFTIWKCPGGDLLKRDELIDVKNSNNQSFESPEPDGSAIWFSKDGKHSGFLFITVWSTSKVQMFDLTNGKLLKEFILPEQTPRVSCCCFVGKDLFVTTANAEINDAVRTNTDKNGGCIYKIPNVLDGNVPLESTKRQPLH>YBR054WMSDYVELLKRGGNEAIKINPPTGADFHITSRGSDWLFTVFCVNLLFGVILVPLMFRKPVKDRFVYYTAIAPNLFMSIAYFTMASNLGWIPVRAKYNHVQTSTQKEHPGYRQIFYARYVGWFLAFPWPIIQMSLLGGTPLWQIAFNVGMTEIFTVCWLIAACVHSTYKWGYYTIGIGAAIVVCISLMTTTFNLVKARGKDVSNVFITFMSVIMFLWLIAYPTCFGITDGGNVLQPDSATIFYGIIDLLILSILPVLFMPLANYLGIERLGLIFDEEPAEHVGPVAEKKMPSPASFKSSDSDSSIKEKLKLKKKHKKDKKKAKKAKKAKKAKKAQEEEEDVATDSE>YBR055CMERPSFLDQEPPAGYVPGIGRGATGFSTKEKQVVSNDDKGRRIPKRYRENLNNHLQSQPKDDEDDEAANVFKTLELKLAQKKKKRANEKDDDNSVDSSNVKRQFADLKESLAAVTESEWMDIPDATDFTRRNKRNRIQEQLNRKTYAAPDSLIPGNVDLNKLTEEREKLLQSQIDENLAQLTKNASNPIQVNKPNAATDALSYLKDLENDRVNSLSDATLEDLQKMRTILKSYRKADPTNPQGWIASARLEEKARKFSVAKKIIENGCQECPRSSDIWLENIRLHESDVHYCKTLVATAINFNPTSPLLWFKAIDLESTTVNKYRVVRKALQEIPRDEGLWKLAVSFEADKAQVIKMLEKATQFIPQSMDLLTAYTNLQSYHNAKMTLNSFRKILPQEPEIWIISTLLEERNNPDIPVDKLVSLLKEGLLELSKNGYKATLSAWLKRAEALNDAPNSNLTCQAIVYAILEWLRESGEYESELNNVDQILEKMPHSKVQIAVLKKLIQWDPCDTVLWSRLKMATESYHKIEELLAFFQELLFQTKNSDDIRANMREKSPGLLMMYVSEYWKAQKGDTRQTLVLIDQIIDFAPHNLDLRFFKIKLLGRSLQLDELRDFFQQTFSSLEDFKISGTERLYYKYVNFLRYQDLNEEAIKFLNERCLKSFPICHKFFLQLGQIYHSMGNIEMSRETYLSGTRLVPNCPLLWVSLSKIDEIDLKNPVRARSILDRGLLKNPDDVLFYIAKIQMEIRLGNLDQAELLVTQALQKFPSNALLWVEQIKLFKHGNKSSLKKTIFQDALRRTQNDHRVLLEIGVSFYAEAQYETSLKWLERALKKCSRYGDTWVWLFRTYARLGKDTVDLYNMFDQCEPTYGPEWIAASKNVKMQYCTPREILLRLMNDK>YBR056WMIGSLRNKFEHFKVSEKGGQNLSTTLPKLPPAKDLDRSTIYKYRYNYGVNLGALFVLEPWIFSKETICTIDGKEYDSEFDAISQQLKKHSSEDVAKMLSDHYKKYIDRIDWEWLSKDAHITALRIPIGYWHVEDGKHLDSLPFAPLRKVYELAKPWEKLGELINNAKKMSIGVLIDLHGLPGGANCDSHSGSKSGEAAFFHKEKYMTKVYKDILPAIINTMTLGNENIIGIQVVNEACFDNNPKGQKFYYSEAINTVEKLQPGLPVIISDGWWPQQWADWVKEKHFSEIVVIDSHVYRCFSDSDKSKDANSIIKDLPNTVNFPHEDADYTVGEFSGVLDGQTWNKTSGDRDAIVQKYVQTQADVFSHVASWGWFFWTLQFEYGDGGEWGLAPMMQKGNLPKRPHGDDLQVDKKKIDSIIHEHEAYWNGKGKNFEHWRFEDGIKTAVDDIIAFRKFDNSLIGRWHSWKSQRRAEYVSAKKDSEFMWEWDQGYQRGLDEFNKY>YBR057CMNYMAYDYDPQHSLETSFNNLAFHPHQQSQQQALYESGERNDARPGLMNTLGQASKMNNSMLPQGSSASPLTGQHSLNSTTNFNMPPSMNTYNYQNVPQASMRNTLNHNNIMNGATANDYWLDPMNNMTNNKDTNGNPNDSMSSMSNMTAKTSINSTAFKNSFVPFNHVTALSMNNVNSNEMNSNKDDRMEALEVELQIKESQIESLENEIQRLKKIFNEGLNYKQNEHKYEKENCHIPQTFELPASLEVIFRKLSSSLHAKEKELAETKENLESILTALALNPTNSVTKYGRYDAESIAHKMVVRLENLTNENKEMAKMLAYGRSKETQIELQLAKKENLELREKIASLEAHLASKESSKEDVAN>YBR058C-AMTQHKSSMVYIPTTKEAKRRNGKSEGILNTIEEVVEKLYWTYYIHLPFYLMASFDSFFLHVFFLTIFSLSFFGILKYCFL>YBR060CMLNGEDFVEHNDILSSPAKSRNVTPKRVDPHGERQLRRIHSSKKNLLERISLVGNERKNTSPDPALKPKTPSKAPRKRGRPRKIQEELTDRIKKDEKDTISSKKKRKLDKDTSGNVNEESKTSNNKQVMEKTGIKEKREREKIQVATTTYEDNVTPQTDDNFVSNSPEPPEPATPSKKSLTTNHDFTSPLKQIIMNNLKEYKDSTSPGKLTLSRNFTPTPVPKNKKLYQTSETKSASSFLDTFEGYFDQRKIVRTNAKSRHTMSMAPDVTREEFSLVSNFFNENFQKRPRQKLFEIQKKMFPQYWFELTQGFSLLFYGVGSKRNFLEEFAIDYLSPKIAYSQLAYENELQQNKPVNSIPCLILNGYNPSCNYRDVFKEITDLLVPAELTRSETKYWGNHVILQIQKMIDFYKNQPLDIKLILVVHNLDGPSIRKNTFQTMLSFLSVIRQIAIVASTDHIYAPLLWDNMKAQNYNFVFHDISNFEPSTVESTFQDVMKMGKSDTSSGAEGAKYVLQSLTVNSKKMYKLLIETQMQNMGNLSANTGPKRGTQRTGVELKLFNHLCAADFIASNEIALRSMLREFIEHKMANITKNNSGMEIIWVPYTYAELEKLLKTVLNTL>YBR061CMGKSSKDKRDLYYRKAKEQGYRARSAFKLLQLNDQFHFLDDPNLKRVVDLCAAPGSWSQVLSRKLFDESPSSDKEDRKIVSVDLQPMSPIPHVTTLQADITHPKTLARILKLFGNEKADFVCSDGAPDVTGLHDLDEYVQQQLIMSALQLTACILKKGGTFVAKIFRGRDIDMLYSQLGYLFDKIVCAKPRSSRGTSLEAFIVCLGYNPPSNWTPKLDVNTSVDEFFQGCFLNKLCISDKLSHWNEEERNIAEFMACGSLQSFDSDATYHDLPSSVAGTSSSLDPVQSPTNPPYKKALELKRSGKLTRSV>YBR065CMNDEINEPPPNICEQCLGDEANIRMTKIPQGSECKICTLPFTLYHFKTSKRSNNIIKTLICVRCATQRNICQCCMLDSRWHIPIQLRDHLISLVNEENVMTEEAKNDMMKRFLSLKNVKLGGAQITSDPSEADNIVDKLKNILLRATSDGPSTPLIKNTTALYKNEKGANEVKNLEKYASVDISHILKKLPLNESFLKNPSTKSFFLYNIDASIPEWKITDTVSQLLGIKKWKDGNSLSLIVNHKAKCGGLRFQSSELGERFVSKISETLVTPKGLKRGVLLIDRFRIFIIPWSSGFSAASFGTNTAENIKLSLSLNKLIQLELGLSFPTKSTDNAKNDKKKTSKKVHKDRSKKSKPRANKLTI>YBR066CMSIGYKDNLMSTILAKDRKCEFPINFECSPSQITLMPEMFSFNNERKYQTLIPLMKTSHLIDDDLKDKLNKCAFDFFSGKQANRTSDGTISRLTASGKTSPILPLQNINIVKAENTGNGKSDPYSSIKISKPTKTVIKLKSTKTNTAGQRTRHFCKICSTGFTTSGHLSRHNRIHTGEKNHICPHEGCGQRFSRHDNCNQHYRTHANKKKRNWKRREASS>YBR067CMSVSKIAFVLSAIASLAVADTSAAETAELQAIIGDINSHLSDYLGLETGNSGFQIPSDVLSVYQQVMTYTDDAYTTLFSELDFDAITKTIVKLPWYTTRLSSEIAAALASVSPASSEAASSSEAASSSKAASSSEATSSAAPSSSAAPSSSAAPSSSAESSSKAVSSSVAPTTSSVSTSTVETASNAGQRVNAGAASFGAVVAGAAALLL>YBR068CMLSSEDFGSSGKKETSPDSISIRSFSAGNNFQSSSSEKTYSKQKSGSDKLIHRFADSFKRAEGSTTRTKQINENTSDLEDGVESITSDSKLKKSMKSRHVVMMSLGTGIGTGLLVANAKGLHYGGPAALIIGYILVSFETYFMIQAAGEMAVTYPTLPANFNAYSSIFISKSFGFATVWLYCFQWLTVLPLELITASMTIQFGNDKINPDIYILIFYVFLVFIHFFGVKAYGETEFIFNCCKILMIAGFIILSIVINCGGAGNDGYIGATYWHNPGAFAGDTSIGRFKNVCYILVTAYFSFGGMELFALSVQEQSNPRKSTPVAAKRSIYRIVVIYLLTMILIGFNVPYNDDQLMGAGGSATHASPYVLAASIHGVKIVPHIINAVILISVVSVANSSLYAGPRLICSLAQQGYAPKFLDYVDREGRPLRALIVCCVFGVIAFVAASSKEEIVFTWLAAIAGLSELFTWTSIMLSHLRFRQAMKVQGRSLDELGYKATTGIWGSIYGVFFNILVFVAQFWVALAPLGNGGKCDAESFFQNYLAFPIWLAFYFGYMVYNRDFTLLNPLDKIDLDFHRRIYDPELMRQEDEENKEKLRNMSLMRKAYHFWC>YBR069CMDDSVSFIAKEASPAQYSHSLHERTHSEKQKRDFTITEKQDEVSGQTAEPRRTDSKSILQRKCKEFFDSFKRQLPPDRNSELESQEKNNLTKSIKSRHLVMISLGTGIGTGLLVGNGQVLGTAGPAGLVLGYGIASIMLYCIIQAAGELGLCYAGLTGNYTRYPSILVDPSLGFAVSVVYTIQWLTVLPLQLVTAAMTVKYWTSVNADIFVAVVFVFVIIINLFGSRGYAEAEFIFNSCKILMVIGFVILAIIINCGGAGDRRYIGAEYWHNPGPFAHGFKGVCTVFCYAAFSYGGIEVLLLSAAEQENPTKSIPNACKKVVYRILLIYMLTTILVCFLVPYNSDELLGSSDSSGSHASPFVIAVASHGVKVVPHFINAVILISVISVANSSLYSGPRLLLSLAEQGVLPKCLAYVDRNGRPLLCFFVSLVFGCIGFVATSDAEEQVFTWLLAISSLSQLFIWMSMSLSHIRFRDAMAKQGRSMNEVGYKAQTGYWGSWLAVLIAIFFLVCQFWVAIAPVNEHGKLNVKVFFQNYLAMPIVLFAYFGHKIYFKSWSFWIPAEKIDLDSHRNIFVSPSLTEIDKVDDNDDLKEYENSESSENPNSSRSRKFFKRMTNFWC>YBR070CMKTAYLASLVLIVSTAYVIRLIAILPFFHTQAGTEKDTKDGVNLLKIRKSSKKPLKIFVFLGSGGHTGEMIRLLENYQDLLLGKSIVYLGYSDEASRQRFAHFIKKFGHCKVKYYEFMKAREVKATLLQSVKTIIGTLVQSFVHVVRIRFAMCGSPHLFLLNGPGTCCIISFWLKIMELLLPLLGSSHIVYVESLARINTPSLTGKILYWVVDEFIVQWQELRDNYLPRSKWFGILV>YBR071WMLRRSKNSSTNTNADTKKRQSMHLGSKSSLISLTSEFGHGHSKTKQKKEEGTAPSQFLSPTNKRSTSSQSKLKRSSLLLDETLLKDYHSAMRHMQTNAAKEEKLRMAPSPTQSTRSESDASLSSTKSSISSIFSQDNDYSIHDLLYEDIEEMDKTDAFKINNTIAIDDSKALFVFCSNDSSSRTASIETLHESNLDNLDMGSSRRTSLDFF>YBR072WMSFNSPFFDFFDNINNEVDAFNRLLGEGGLRGYAPRRQLANTPAKDSTGKEVARPNNYAGALYDPRDETLDDWFDNDLSLFPSGFGFPRSVAVPVDILDHDNNYELKVVVPGVKSKKDIDIEYHQNKNQILVSGEIPSTLNEESKDKVKVKESSSGKFKRVITLPDYPGVDADNIKADYANGVLTLTVPKLKPQKDGKNHVKKIEVSSQESWGN>YBR073WMQIPKYENKPFKPPRRVGSNKYTQLKPTATAVTTAPISKAKVTVNLKRSISAGPTLNLAKKPNNLSSNENTRYFTIMYRKPTTKKHKTWSGDGYATLKASSDKLCFYNEAGKFLGSSMLPSDSDSLFETLFKAGSNEVQLDYELKENAEIRSAKEALSQNMGNPSPPTTSTTETVPSTKNDGGKYQMPLSQLFSLNTVKRFKSVTKQTNEHMTTVPKTSQNSKAKKYYPVFDVNKIDNPIVMNKNAAAEVDVIVDPLLGKFLRPHQREGVKFMYDCLMGLARPTIENPDIDCTTKSLVLENDSDISGCLLADDMGLGKTLMSITLIWTLIRQTPFASKVSCSQSGIPLTGLCKKILVVCPVTLIGNWKREFGKWLNLSRIGVLTLSSRNSPDMDKMAVRNFLKVQRTYQVLIIGYEKLLSVSEELEKNKHLIDMLVCDEGHRLKNGASKILNTLKSLDIRRKLLLTGTPIQNDLNEFFTIIDFINPGILGSFASFKRRFIIPITRARDTANRYNEELLEKGEERSKEMIEITKRFILRRTNAILEKYLPPKTDIILFCKPYSQQILAFKDILQGARLDFGQLTFSSSLGLITLLKKVCNSPGLVGSDPYYKSHIKDTQSQDSYSRSLNSGKLKVLMTLLEGIRKGTKEKVVVVSNYTQTLDIIENLMNMAGMSHCRLDGSIPAKQRDSIVTSFNRNPAIFGFLLSAKSGGVGLNLVGRSRLILFDNDWNPSVDLQAMSRIHRDGQKKPCFIYRLVTTGCIDEKILQRQLMKNSLSQKFLGDSEMRNKESSNDDLFNKEDLKDLFSVHTDTKSNTHDLICSCDGLGEEIEYPETNQQQNTVELRKRSTTTWTSALDLQKKMNEAATNDDAKKSQYIRQCLVHYKHIDPARQDELFDEVITDSFTELKDSITFAFVKPGEICLREQ>YBR077CMVMLHSKNVKGFLENTLKPYDLHSVDFKTSSLQSSMIITATNGGILSYATSNNDVPKNSINEINSVNNLKMMSLLIKDKWSEDENDTEEQHSNSCYPVEIDSFKTKIYTYEMEDLHTCVAQIPNSDLLLLFIAEGSFPYGLLVIKIERAMRELTDLFGYKLG>YBR082CMSSSKRIAKELSDLERDPPTSCSAGPVGDDLYHWQASIMGPADSPYAGGVFFLSIHFPTDYPFKPPKISFTTKIYHPNINANGNICLDILKDQWSPALTLSKVLLSICSLLTDANPDDPLVPEIAHIYKTDRPKYEATAREWTKKYAV>YBR084WMLSRLSLLSNSRAFQQARWRIYRLKVSPTVHASQYHILSGRKLAQSIREKANDEIQAIKLKHPNFKPTLKIIQVGARPDSSTYVRMKLKASKDSNVDCIIEKLPAEITEVELLKKISDINDDDSIHGLLIQLPLPRHLDETTITNAVDFKKDVDGFHRYNAGELAKKGGKPYFIPCTPYGCMKLLEEAHVKLDGKNAVVLGRSSIVGNPIASLLKNANATVTVCHSHTRNIAEVVSQADIVIAACGIPQYVKSDWIKEGAVVIDVGINYVPDISKKSGQKLVGDVDFDSVKEKTSYITPVPGGVGPMTVAMLVSNVLLAAKRQFVESEKLPVIKPLPLHLESPVPSDIDISRAQSPKHIKQVAEELGIHSHELELYGHYKAKISPNIFKRLESRENGKYVLVAGITPTPLGEGKSTTTMGLVQALSAHLGKPSIANVRQPSLGPTLGVKGGAAGGGYAQVIPMDEFNLHLTGDIHAISAANNLLAAAIDTRMFHEATQKNDSTFYKRLVPRKKGIRKFTPSMQRRLKRLDIEKEDPDALTPEEVKRFARLNINPDTITIRRVVDINDRMLRQITIGEAATEKGFTRTTGFDITVASELMAILALSKSLHEMKERIGRMVIGADYDNKPVTVEDIGCTGALTALLRDAIKPNLMQTLEGTPVMVHAGPFANISIGASSVIADLMALKLVGSEKNPLNDKNIHEPGYVVTEAGFDFAMGGERFFDIKCRSSGLVPDAVVLVATVRALKSHGGAPNVKPGQSLPKEYTEENIDFVAKGVSNLVKQIENIKTFGIPVVVAINRFETDSQAEIEVIKKAALNAGASHAVTSNHWMEGGKGAVELAHAVVDATKEPKNFNFLYDVNSSIEDKLTSIVQKMYGGAKIEVSPEAQKKIDTYKKQGFGNLPICIAKTQYSLSHDPSLKGVPRGFTFPIRDVRASIGAGYLYALAAEIQTIPGLSTYAGYMAVEVDDDGEIEGLF>YBR085C-AMSSALYKQSTNFTHSTGSFLQSAPVELTTVSGYQEFLKKQEKKNYEIQTVLSEDKSHGYVLKDGEVIANIIGEAKDYLLDLAGQA>YBR085WMSSDAKQQETNFAINFLMGGVSAAIAKTAASPIERVKILIQNQDEMIKQGTLDKKYSGIVDCFKRTAKQEGLISFWRGNTANVIRYFPTQALNFAFKDKIKLMFGFKKEEGYGKWFAGNLASGGAAGALSLLFVYSLDFARTRLAADAKSSKKGGARQFNGLTDVYKKTLKSDGIAGLYRGFMPSVVGIVVYRGLYFGMFDSLKPLVLTGSLDGSFLASFLLGWVVTTGASTCSYPLDTVRRRMMMTSGQAVKYNGAIDCLKKIVASEGVGSLFKGCGANILRSVAGAGVISMYDQLQMILFGKKFK>YBR087WMSLWVDKYRPKSLNALSHNEELTNFLKSLSDQPRDLPHLLLYGPNGTGKKTRCMALLESIFGPGVYRLKIDVRQFVTASNRKLELNVVSSPYHLEITPSDMGNNDRIVIQELLKEVAQMEQVDFQDSKDGLAHRYKCVIINEANSLTKDAQAALRRTMEKYSKNIRLIMVCDSMSPIIAPIKSRCLLIRCPAPSDSEISTILSDVVTNERIQLETKDILKRIAQASNGNLRVSLLMLESMALNNELALKSSSPIIKPDWIIVIHKLTRKIVKERSVNSLIECRAVLYDLLAHCIPANIILKELTFSLLDVETLNTTNKSSIIEYSSVFDERLSLGNKAIFHLEGFIAKVMCCLD>YBR088CMLEAKFEEASLFKRIIDGFKDCVQLVNFQCKEDGIIAQAVDDSRVLLVSLEIGVEAFQEYRCDHPVTLGMDLTSLSKILRCGNNTDTLTLIADNTPDSIILLFEDTKKDRIAEYSLKLMDIDADFLKIEELQYDSTLSLPSSEFSKIVRDLSQLSDSINIMITKETIKFVADGDIGSGSVIIKPFVDMEHPETSIKLEMDQPVDLTFGAKYLLDIIKGSSLSDRVGIRLSSEAPALFQFDLKSGFLQFFLAPKFNDEE>YBR089C-AMAATKEAKQPKEPKKRTTRRKKDPNAPKRRLSAYMFFANENRDIVRSENPDVTFGQVGRILGERWKALTAEEKQPYESKAQADKKRYESEKELYNATRA>YBR091CMSFFLNSLRGNQEVSQEKLDVAGVQFDAMCSTFNNILSTCLEKCIPHEGFGEPDLTKGEQCCIDRCVAKMHYSNRLIGGFVQTRGFGPENQLRHYSRFVAKEIADDSKK>YBR092CMFKSVVYSVLAAALVNAGTIPLGELADVAKIGTQEDIFPFLGGAGPYFSFPGDYGISRDLPEGCEMKQLQMLARHGERYPTYSKGATIMKTWYKLSNYTRQFNGSLSFLNDDYEFFIRDDDDLEMETTFANSDNVLNPYTGEMDAKRHAREFLAQYGYMFENQTSFPIFAASSERVHDTAQYFIDGLGDQFNISLQTVSEAMSAGANTLSAGNACPGWDEDANDDILDKYDTTYLDDIAKRLNKENKGLNLTSKDANTLFAWCAYELNARGYSDVCDIFTEDELVRYSYGQDLVSFYQDGPGYDMIRSVGANLFNATLKLLKQSETQDLKVWLSFTHDTDILNYLTTAGIIDDKNNLTAEYVPFMGNTFHKSWYVPQGARVYTEKFQCSNDTYVRYVINDAVVPIETCSTGPGFSCEINDFYDYAEKRVAGTDFLKVCNVSSVSNVTELTFYWDWNTTHYNDTLLKQ>YBR093CMFKSVVYSILAASLANAGTIPLGKLADVDKIGTQKDIFPFLGGAGPYYSFPGDYGISRDLPEGCEMKQLQMVGRHGERYPTVSLAKTIKSTWYKLSNYTRQFNGSLSFLNDDYEFFIRDDDDLEMETTFANSDDVLNPYTGEMNAKRHARDFLAQYGYMVENQTSFAVFTSNSKRCHDTAQYFIDGLGDQFNITLQTVSEAESAGANTLSACNSCPAWDYDANDDIVNEYDTTYLDDIAKRLNKENKGLNLTSTDASTLFSWCAFEVNAKGYSDVCDIFTKDELVHYSYYQDLHTYYHEGPGYDIIKSVGSNLFNASVKLLKQSEIQDQKVWLSFTHDTDILNFLTTAGIIDDKNNLTAEYVPFMGNTFHRSWYVPQGARVYTEKFQCSNDTYVRYVINDAVVPIETCSTGPGFSCEINDFYDYAEKRVAGTDFLKVCNVSSVSNSTELTFYWDWNTTHYNASLLRQ>YBR094WMRVLITNDDGPLSDQFSPYIRPFIQHIKRNYPEWKITVCVPHVQKSWVGKAHLAGKNLTAQFIYSKVDAEDNTFWGPFIQPQIRSENSKLPYVLNAEIPKDTIEWILIDGTPASCANIGLHLLSNEPFDLVLSGPNVGRNTSAAYITSSGTVGGAMESVITGNTKAIAISWAYFNGLKNVSPLLMEKASKRSLDVIKHLVKNWDPKTDLYSINIPLVESLSDDTKVYYAPIWENRWIPIFNGPHINLENSFAEIEDGNESSSISFNWAPKFGAHKDSIHYMDEYKDRTVLTDAEVIESEMISVTPMKATFKGVNHLLGELKLTEEENNLSKTNNLIVVSIDPMEYIYKPLTHALKKYLPQVEIVSNLPEFDNGGCEKEMKVFHYGDYEQLDMDKLMELPNNYFTNSYIYRKALIRKHFLSHTIQTYTAKNPESILKKAYLESFTIDLDYREFLDDALDENWELRQELENESQDKWWIVKPSMSDKGQGIRVFKTIEDLQAIFDSFDDEDSEAEESGNDDDADDVNGEFMDNNKVNISQLRHFIIQEYLTNPLLLASMDNRKFHIRCYVVCRGDLQVFVYDRMLALFAAKPFVPLDPYAYSVTDLKDLECHLTNTCLQSKKKDKDSSVLEFDSIEEIPNERKSNIKEQIHSITNDVFLAAVNVNRLNFQPLPNAFETYGVDFLIDSNYEVKLLEINAFPDFKQTGKDLKNLIDELFDDTVKYCVTPIFNENRNKTDDETDPNFVKVIDYTSNGW>YBR095CMTIRSSMKNNAELESKSVLANESNIISTFTRRIIKEKSGNYQVLKRSLDGKLIYPEATGISSNRGNKLLQRSEVVTRRDLNNSKPMIEQTVFYNGSEHRLLQTNIVTDSRRKRIKFTPDINVEPVLVGDENDIDGSEKEDENITDEYYGEEDDDDLSKLVNVKEILTPILSLGDIINHKTISRTFSSPILKNLALQIILMIEKEQMSVVRYSQFLEVFLGDHPEPIYESNLNLPSYNHNLTLPEDRGASDEDDINNKNNINEVNSNSLSTEAGHINNGMEEFGEEDPFFALPRLEQSNALLSLLPSSSGSASISTLTAAEQQQLNEEIESARQLSQIALQRNKEFIRNLQKIRKSVIKANRIRGRILNWSREYLGISDDDITIPVALRVVKRGLISATTNKTTNFEEEIENTMEDGVVDDNEPDEEANRA>YBR096WMGVCTIFRWLFAAYLLSSYKSLPGAYFVRFYYYVIQNLFLPMFTGFETENIKKLEKNEYGCFSYTSLDTYASPFECDFYFHKSNSTYFAELDISRGNLMCKIFQKLMLNSKHYPYIPVANVFTNFLKEIKPFQKYSVSSRIICWDEKWIYVMSRFTIKKGTVLCSLSLTKYVLKDGRKTIKPKDALEYCGLYNEKVAKISEDNLKLLTERCGFHETVPLENLSQEYCSEI>YBR097WMGAQLSLVVQASPSIAIFSYIDVLEEVHYVSQLNSSRFLKTCKALDPNGEIVIKVFIKPKDQYSLRPFLQRIRAQSFKLGQLPHVLNYSKLIETNRAGYMIRQHLKNNLYDRLSLRPYLQDIELKFIAFQLLNTLKDIHNLNIVHGDIKTENILVTSWNWCILTDFAAFIKPVYLPEDNPGEFLFYFDTSKRRTCYLAPERFNSKLYQDGKSNNGRLTKEMDIFSLGCVIAEIFAEGRPIFNLSQLFKYKSNSYDVNREFLMEEMNSTDLRNLVLDMIQLDPSKRLSCDELLNKYRGIFFPDYFYTFIYDYFRNLVTMTTSTPISDNTCTNSTLEDNVKLLDETTEKIYRDFSQICHCLDFPLIKDGGEIGSDPPILESYKIEIEISRFLNTNLYFPQNYHLVLQQFTKVSEKIKSVKEECALLFISYLSHSIRSIVSTATKLKNLELLAVFAQFVSDENKIDRVVPYFVCCFEDSDQDVQALSLLTLIQVLTSVRKLNQLNENIFVDYLLPRLKRLLISNRQNTNYLRIVFANCLSDLAIIINRFQEFTFAQHCNDNSMDNNTEIMESSTKYSAKLIQSVEDLTVSFLTDNDTYVKMALLQNILPLCKFFGRERTNDIILSHLITYLNDKDPALRVSLIQTISGISILLGTVTLEQYILPLLIQTITDSEELVVISVLQSLKSLFKTGLIRKKYYIDISKTTSPLLLHPNNWIRQFTLMIIIEIINKLSKAEVYCILYPIIRPFFEFDVEFNFKSMISCCKQPVSRSVYNLLCSWSVRASKSLFWKKIITNHVDSFGNNRIEFITKNYSSKNYGFNKRDTKSSSSLKGIKTSSTVYSHDNKEIPLTAEDINWIDKFHIIGLTEKDIWKIVALRGYVIRTARVMAANPDFPYNNSNYRPLVQNSPPNLNLTNIMPRNIFFDVEFAEESTSEGQDSNLENQQIYKYDESEKDSNKLNINGSKQLSTVMDINGSLIFKNKSIATTTSNLKNVFVQLEPTSYHMHSPNHGLKDNANVKPERKVVVSNSYEGDVESIEKFLSTFKILPPLRDYKEFGPIQEIVRSPNMGNLRGKLIATLMENEPNSITSSAVSPGETPYLITGSDQGVIKIWNLKEIIVGEVYSSSLTYDCSSTVTQITMIPNFDAFAVSSKDGQIIVLKVNHYQQESEVKFLNCECIRKINLKNFGKNEYAVRMRAFVNEEKSLLVALTNLSRVIIFDIRTLERLQIIENSPRHGAVSSICIDEECCVLILGTTRGIIDIWDIRFNVLIRSWSFGDHAPITHVEVCQFYGKNSVIVVGGSSKTFLTIWNFVKGHCQYAFINSDEQPSMEHFLPIEKGLEELNFCGIRSLNALSTISVSNDKILLTDEATSSIVMFSLNELSSSKAVISPSRFSDVFIPTQVTANLTMLLRKMKRTSTHSVDDSLYHHDIINSISTCEVDETPLLVACDNSGLIGIFQ>YBR098WMSQIVDFVEDKDSRNDASIQIIDGPSNVEIIALSESMDQDECKRAHVSSAEMIPSSPQRKSVSNDVENVDLNKSIELSAPFFQDISISKLDDFSTTVNSIIDSSLRNENNAKGNAKKLLDDLISDEWSADLESSGKKHNKSQYNLRDIAEKWGVQSLKNPEPIAVDCEYKTQGIGKTNSDISDSPKSQIGAADILFDFPLSPVKHENPTEEKHNSIANENSSPDNSLKPAGKQNHGEDGTSMAKRVYNKGEDEQEHLPKGKKRTIALSRTLINSTKLPDTVELNLSKFLDSSDSITTDVLSTPAKGSNIVRTGSQPIFSNANCFQEAKRSKTLTAEDPKCTKNTAREVSQLENYIAYGQYYTREDSKNKIRHLLKENKNAFKRVNQIYRDNIKARSQMIIEFSPSLLQLFKKGDSDLQQQLAPAVVQSSYNDSMPLLRFLRKCDSIYDFSNDFYYPCDPKIVEENVLILYYDAQEFFEQYTSQKKELYRKIRFFSKNGKHVILILSDINKLKRAIFQLENEKYKARVEQRLSGTEEALRPRSKKSSQVGKLGIKKFDLEQRLRFIDREWHVKIHTVNSHMEFINSLPNLVSLIGKQRMDPAIRYMKYAHLNVKSAQDSTETLKKTFHQIGRMPEMKANNVVSLYPSFQSLLEDIEKGRLQSDNEGKYLMTEAVEKRLYKLFTCTDPNDTIE>YBR099CMRCFPINDTRFGRELINSICEFTVCILTCHSLSMKRNRCSKSNFFIPNFPTWLDFLLLGLKASSVPDNRCSTLALYFSFSNWKIALLSLFISLSIRITCFPFFEKNRIFLYNSFFCEVYCSKNSCAS>YBR101CMEKLLQWSIANSQGDKEAMARAGQPDPKLLQQLFGGGGPDDPTLMKESMAVIMNPEVDLETKLVAFDNFEMLIENLDNANNIENLKLWEPLLDVLVQTKDEELRAAALSIIGTAVQNNLDSQNNFMKYDNGLRSLIEIASDKTKPLDVRTKAFYALSNLIRNHKDISEKFFKLNGLDCIAPVLSDNTAKPKLKMRAIALLTAYLSSVKIDENIISVLRKDGVIESTIECLSDESNLNIIDRVLSFLSHLISSGIKFNEQELHKLNEGYKHIEPLKDRLNEDDYLAVKYVL>YBR104WMSEEFPTPQLLDELEDQQKVTTPNEKRELSSNRVLKDIFAGTIGGIAQVLVGQPFDTTKVRLQTATTRTTTLEVLRNLVKNEGVFAFYKGALTPLLGVGICVSVQFGVNEAMKRFFQNYNASKNPNMSSQDVDLSRSNTLPLSQYYVCGLTGGVVNSFLASPIEQIRIRLQTQTSNGGDREFKGPWDCIKKLKAQGGLMRGLFPTMIRAGHGLGTYFLVYEALVAREIGTGLTRNEIPPWKLCLFGAFSGTMLWLTVYPLDVVKSIIQNDDLRKPKYKNSISYVAKTIYAKEGIRAFFKGFGPTMVRSAPVNGATFLTFELVMRFLGEE>YBR105CMINNPKVDSVAEKPKAVTSKQSEQAASPEPTPAPPVSRNQYPITFNLTSTAPFHLHDRHRYLQEQDLYKCASRDSLSSLQQLAHTPNGSTRKKYIVEDQSPYSSENPVIVTSSYNHTVCTNYLRPRMQFTGYQISGYKRYQVTVNLKTVDLPKKDCTSLSPHLSGFLSIRGLTNQHPEISTYFEAYAVNHKELGFLSSSWKDEPVLNEFKATDQTDLEHWINFPSFRQLFLMSQKNGLNSTDDNGTTNAAKKLPPQQLPTTPSADAGNISRIFSQEKQFDNYLNERFIFMKWKEKFLVPDALLMEGVDGASYDGFYYIVHDQVTGNIQGFYYHQDAEKFQQLELVPSLKNKVESSDCSFEFA>YBR106WMNPQVSNIIIMLVMMQLSRRIDMEDPTIIMYIRILYCSSIGISWIIYQMARKRIVAKNDMTTMKYVEPGNAMSGEGEKLQVTTVRDYDLKEIDSAIKSIYTGMAMMGFMHLYLKYTNPLFMQSISPVKSALEHNEVKIHLFGKPATGDLKRPFKAPSLFGGMGQTGPKTDKKSIEEAERAGNAGVKAE>YBR107CMPYTWKFLGISKQLSLENGIAKLNQLLNLEVDLDIQTIRVPSDPDGGTAADEYIRYEMRLDISNLDEGTYSKFIFLGNSKMEVPMFLCYCGTDNRNEVVLQWLKAEYGVIMWPIKFEQKTMIKLADASIVHVTKENIEQITWFSSKLYFEPETQDKNLRQFSIEIPRESCEGLALGYGNTMHPYNDAIVPYIYNETGMAVERLPLTSVILAGHTKIMRESIVTSTRSLRNRVLAVVLQSIQFTSE>YBR109CMSSNLTEEQIAEFKEAFALFDKDNNGSISSSELATVMRSLGLSPSEAEVNDLMNEIDVDGNHQIEFSEFLALMSRQLKSNDSEQELLEAFKVFDKNGDGLISAAELKHVLTSIGEKLTDAEVDDMLREVSDGSGEINIQQFAALLSK>YBR110WMFLEIPRWLLALIILYLSIPLVVYYVIPYLFYGNKSTKKRIIIFVLGDVGHSPRICYHAISFSKLGWQVELCGYVEDTLPKIISSDPNITVHHMSNLKRKGGGTSVIFMVKKVLFQVLSIFKLLWELRGSDYILVQNPPSIPILPIAVLYKLTGCKLIIDWHNLAYSILQLKFKGNFYHPLVLISYMVEMIFSKFADYNLTVTEAMRKYLIQSFHLNPKRCAVLYDRPASQFQPLAGDISRQKALTTKAFIKNYIRDDFDTEKGDKIIVTSTSFTPDEDIGILLGALKIYENSYVKFDSSLPKILCFITGKGPLKEKYMKQVEEYDWKRCQIEFVWLSAEDYPKLLQLCDYGVSLHTSSSGLDLPMKILDMFGSGLPVIAMNYPVLDELVQHNVNGLKFVDRRELHESLIFAMKDADLYQKLKKNVTQEAENRWQSNWERTMRDLKLIH>YBR111CMFLRNVRVISLNSRRLFRTMSTVKGKPEDAKIIEARHVKETSDCKWIGLQKIIYKDPNGKEREWDSAVRTTRSSGGVDGIGILTILKYKDGKPDEILLQKQFRPPVEGVCIEMPAGLIDAGEDIDTAALRELKEETGYSGKIISKSPTVFNDPGFTNTNLCLVTVEVDMSLPENQKPVTQLEDNEFIECFSVELHKFPDEMVKLDQQGYKLDARVQNVAQGILMAKQYHIK>YBR115CMTNEKVWIEKLDNPTLSVLPHDFLRPQQEPYTKQATYSLQLPQLDVPHDSFSNKYAVALSVWAALIYRVTGDDDIVLYIANNKILRFNIQPTWSFNELYSTINNELNKLNSIEANFSFDELAEKIQSCQDLERTPQLFRLAFLENQDFKLDEFKHHLVDFALNLDTSNNAHVLNLIYNSLLYSNERVTIVADQFTQYLTAALSDPSNCITKISLITASSKDSLPDPTKNLGWCDFVGCIHDIFQDNAEAFPERTCVVETPTLNSDKSRSFTYRDINRTSNIVAHYLIKTGIKRGDVVMIYSSRGVDLMVCVMGVLKAGATFSVIDPAYPPARQTIYLGVAKPRGLIVIRAAGQLDQLVEDYINDELEIVSRINSIAIQENGTIEGGKLDNGEDVLAPYDHYKDTRTGVVVGPDSNPTLSFTSGSEGIPKGVLGRHFSLAYYFNWMSKRFNLTENDKFTMLSGIAHDPIQRDMFTPLFLGAQLYVPTQDDIGTPGRLAEWMSKYGCTVTHLTPAMGQLLTAQATTPFPKLHHAFFVGDILTKRDCLRLQTLAENCRIVNMYGTTETQRAVSYFEVKSKNDDPNFLKKLKDVMPAGKGMLNVQLLVVNRNDRTQICGIGEIGEIYVRAGGLAEGYRGLPELNKEKFVNNWFVEKDHWNYLDKDNGEPWRQFWLGPRDRLYRTGDLGRYLPNGDCECCGRADDQVKIRGFRIELGEIDTHISQHPLVRENITLVRKNADNEPTLITFMVPRFDKPDDLSKFQSDVPKEVETDPIVKGLIGYHLLSKDIRTFLKKRLASYAMPSLIVVMDKLPLNPNGKVDKPKLQFPTPKQLNLVAENTVSETDDSQFTNVEREVRDLWLSILPTKPASVSPDDSFFDLGGHSILATKMIFTLKKKLQVDLPLGTIFKYPTIKAFAAEIDRIKSSGGSSQGEVVENVTANYAEDAKKLVETLPSSYPSREYFVEPNSAEGKTTINVFVTGVTGFLGSYILADLLGRSPKNYSFKVFAHVRAKDEEAAFARLQKAGITYGTWNEKFASNIKVVLGDLSKSQFGLSDEKWMDLANTVDIIIHNGALVHWVYPYAKLRDPNVISTINVMSLAAVGKPKFFDFVSSTSTLDTEYYFNLSDKLVSEGKPGILESDDLMNSASGLTGGYGQSKWAAEYIIRRAGERGLRGCIVRPGYVTGASANGSSNTDDFLLRFLKGSVQLGKIPDIENSVNMVPVDHVARVVVATSLNPPKENELAVAQVTGHPRILFKDYLYTLHDYGYDVEIESYSKWKKSLEASVIDRNEENALYPLLHMVLDNLPESTKAPELDDRNAVASLKKDTAWTGVDWSNGIGVTPEEVGIYIAFLNKVGFLPPPTHNDKLPLPSIELTQAQISLVASGAGARGSSAAA>YBR119WMSALYFQNLPSRPANKENYTRLLLKHINPNNKYAINPSLPLPHNKLQISSQPLMLLDDQMGLLEVSISRSSKMTNQAFLTFVTQEEADRFLEKYTTTALKVQGRKVRMGKARTNSLLGLSIEMQKKKGNDETYNLDIKKVLKARKLKRKLRSDDICAKKFRLKRQIRRLKHKLRSRKVEEAEIDRIVKEFETRRLENMKSQQENLKQSQKPLKRAKVSNTMENPPNKVLLIQNLPSGTTEQLLSQILGNEALVEIRLVSVRNLAFVEYETVADATKIKNQLGSTYKLQNNDVTIGFAK>YBR120CMSSSQVVRDSAKKLVNLLEKYPKDRIHHLVSFRDVQIARFRRVAGLPNVDDKGKSIKEKKPSLDEIKSIINRTSGPLGLNKEMLTKIQNKMVDEKFTEESINEQIRALSTIMNNKFRNYYDIGDKLYKPAGNPQYYQRLINAVDGKKKESLFTAMRTVLFGK>YBR121CMSVEDIKKARAAVPFNREQLESVLRGRFFYAPAFDLYGGVSGLYDYGPPGCAFQNNIIDAWRKHFILEEDMLEVDCTMLTPYEVLKTSGHVDKFSDWMCRDLKTGEIFRADHLVEEVLEARLKGDQEARGLVEDANAAAKDDAEKKKRKKKVKQIKAVKLDDDVVKEYEEILAKIDGYSGPELGELMEKYDIGNPVTGETLESPRAFNLMFETAIGPSGQLKGYLRPETAQGQFLNFNKLLEFNNSKTPFASASIGKSFRNEISPRAGLLRVREFLMAEIEHFVDPLDKSHPKFNEIKDIKLSFLPRDVQEAGSTEPIVKTVGEAVASRMVDNETLGYFIARIYQFLMKIGVDESKLRFRQHMANEMAHYAADCWDGELKTSYGWIECVGCADRSAYDLTVHSKKTKEKLVVRQKLDNPIEVTKWEIDLTKKLFGPKFRKDAPKVESHLLNMSQDDLASKAELLKANGKFTIKVDGVDGEVELDDKLVKIEQRTKVEHVREYVPSVIEPSFGIGRIIYSVFEHSFWNRPEDNARSVLSFPPLVAPTKVLLVPLSNHKDLVPVHHEVAKILRKSQIPFKIDDSGVSIGKRYARNDELGTPFGVTIDFESAKDHSVTLRERDSTKQVRGSVENVIKAIRDITYNGASWEEGTKDLTPFIAQAEAEAETD>YBR122CMLKSIFAKRFASTGSYPGSTRITLPRRPAKKIQLGKSRPAIYHQFNVKMELSDGSVVIRRSQYPKGEIRLIQDQRNNPLWNPSRDDLVVVDANSGGSLDRFNKRYSSLFSVDSTTPNSSSETVELSEENKKKTQIKKEEKEDVSEKAFGMDDYLSLLDDSEQQIKSGKLASKKRDKK>YBR123CMPVEEPLATLSSIPDSSADQAPPLIADEFTLDLPRIPSLELPLNVSTKHSSIQKAIKMCGGIEKVKEAFKEHGPIESQHGLQLYLNDDTDSDGSKSYFNEHPVIGKRVPFRDESVILKVTMPKGTLSKNNNSVKDSIKSLKDSNKLRVTPVSIVDNTIKFREMSDFQIKLDNVPSAREFKSSFGSLEWNNFKSFVNSVPDNDSQPQENIGNLILDRSVKIPSTDFQLPPPPKLSMVGFPLLYKYKANPFAKKKKNGVTEVKGTYIKNYQLFVHDLSDKTVIPSQAHEQVLYDFEVAKKTKVYPGTKSDSKFYESLEECLKILRELFARRPIWVKRHLDGIVPKKIHHTMKIALALISYRFTMGPWRNTYIKFGIDPRSSVEYAQYQTEYFKIERKLLSSPIVKKNVPKPPPLVFESDTPGGIDSRFKFDGKRIPWYLMLQIDLLIGEPNIAEVFHNVEYLDKANELTGWFKELDLVKIRRIVKYELGCMVQGNYEYNKYKLKYFKTMLFVKESMVPENKNSEEGMGVNTNKDADGDINMDAGSQMSSNAIEEDKGIAAGDDFDDNGAITEEPDDAALENEEMDTDQNLKVPASIDDDVDDVDADEEEQESFDVKTASFQDIINKIAKLDPKTAETMKSELKGFVDEVDL>YBR125CMGQLLSHPLTEKTIEYNEYKNNQASTGIVPRFYNCVGSMQGYRLTQEDAHLIRNENSVVYVRFFNPFIDKYETLSLNVFAVFDGHGGDDCSKFLSGGRHHRDGNGSSNGNGEPNAGLIKWIAYSFENHHYTSTTNNDSSKFKRSFNTLEGLVSQIFKDAFILQDEELYRHFANSSCGSTAVVACIINEESLYVANCGDSRCILSSKSNGIKTMSFDHKPQHIGELIRINDNGGTVSLGRVGGVLALSRAFSDFQFKRGVTYPHRRTKLTNITQNLTYGTPPQEAQVTVEPDVLMHKIDYSKDEFLVLACDGIWDIYNNKQLIHFIKYHLVSGTKLDTIITKLLDHGIAQANSNTGVGFDNMTAIIVVLNRKGETLQDWFNKMKTRLERERGLV>YBR126CMTTDNAKAQLTSSSGGNIIVVSNRLPVTITKNSSTGQYEYAMSSGGLVTALEGLKKTYTFKWFGWPGLEIPDDEKDQVRKDLLEKFNAVPIFLSDEIADLHYNGFSNSILWPLFHYHPGEINFDENAWLAYNEANQTFTNEIAKTMNHNDLIWVHDYHLMLVPEMLRVKIHEKQLQNVKVGWFLHTPFPSSEIYRILPVRQEILKGVLSCDLVGFHTYDYARHFLSSVQRVLNVNTLPNGVEYQGRFVNVGAFPIGIDVDKFTDGLKKESVQKRIQQLKETFKGCKIIVGVDRLDYIKGVPQKLHAMEVFLNEHPEWRGKVVLVQVAVPSRGDVEEYQYLRSVVNELVGRINGQFGTVEFVPIHFMHKSIPFEELISLYAVSDVCLVSSTRDGMNLVSYEYIACQEEKKGSLILSEFTGAAQSLNGAIIVNPWNTDDLSDAINEALTLPDVKKEVNWEKLYKYISKYTSAFWGENFVHELYSTSSSSTSSSATKN>YBR128CMHCPICHHRAHVVYCAHCINTSPSLLLKLKLDLILLKDENKELNGKVEQILNEAMNYDQLDIKRMEKKKDPLMNSLMKLDVLRMKKNNNLIRHRIEQLNERIYSKRNHISELKVEIDNYKCYKVGTGTDKLREQVEISDAKNKLAQVSKICESARDYKLNLLNNWFVIQKLQDNFQIPFAIAFQPLISLKNFRILPLAITNDSINIMWKYISFFSDILMIKLPYTNKICEQPMFEFSDSIQTVVQRLIKLIINILQICRHLKLVPSTPMDIPWLLDQYDVDGLFYNMVKRNKMKCRSVSLYWTFGMLYSMVLDNMNNPQRGHPARRTAPPPTVTGPHDRWYVVG>YBR129CMIAGATAPSSQHEILIASNLIKKPSTSQNKTPTAQSSSGNNGAADGAPQGYHHHHHHHRHLWWPRTTDHQYWCVLRKNQFAYYKTRDEREAISVIPRFDILNFKISELDGILTVYTPSKDLIFKFPRGQNEKVGMELMHNWKIALEKFLSSPSGNESVTTGSDYDEEEDDDDLIVVDEKAGPSSSKHSCSLTMDEQLSREDKEFYRMFDPRNAEHQVCSGILYTKVKKKKLFNRAKWQKFNVELTNTSFNLYSFKTGKLKKSIKLDKIIDCIELDNNSKMKNDDTNFALITFDERLSFKAANDQDMVDWIINFKSGILIRKKLKAENI>YBR130CMSDQDNTQTSSSKLAPHHNIFMANLESSPTKDRNTSSQNASSSRVIESLHDQIDMLTKTNLQLTTQSQNLLSKLELAQSKESKLLENLNLLKNENENLNSIFERKNKKLKELEKDYSELSNRYNEQKEKMDQLSKLAKNSSAIEQSCSEKLQNMEVNYNSLLESQNLYRDHYSDEISKLNEKIGLLELELSNQNLNYGSDTSSNSDIELNLNKFNDSVKDLKSLETEKDSKLSKIITHSLDELNLQSWLNLYQTNENLISTFAEKMDLKDVLKRNDEKISNKGAVVQTLKKNVQTQVESNNADALSSNNAQDMLPIKMVKLRKTPNTNDSSSNGNSSNNKRRSFYTASPLLSSGSIPKSASPVLPGVKRTASVRKPSSSSSKTNVTHNNDPSTSPTISVPPGVTRTVSSTHKKKGNSMVVHGAQS>YBR132CMTKERMTIDYENDGDFEYDKNKYKTITTRIKSIEPSEGWLEPSGSVGHINTIPEAGDVHVDEHEDRGSSIDDDSRTYLLYFTETRRKLENRHVQLIAISGVIGTALFVAIGKALYRGGPASLLLAFALWCVPILCITVSTAEMVCFFPVSSPFLRLATKCVDDSLAVMASWNFWFLECVQIPFEIVSVNTIIHYWRDDYSAGIPLAVQVVLYLLISICAVKYYGEMEFWLASFKIILALGLFTFTFITMLGGNPEHDRYGFRNYGESPFKKYFPDGNDVGKSSGYFQGFLACLIQASFTIAGGEYISMLAGEVKRPRKVLPKAFKQVFVRLTFLFLGSCLCVGIVCSPNDPDLTAAINEARPGAGSSPYVIAMNNLKIRILPDIVNIALITAAFSAGNAYTYCSSRTFYGMALDGYAPKIFTRCNRHGVPIYSVAISLVWALVSLLQLNSNSAVVLNWLINLITASQLINFVVLCIVYLFFRRAYHVQQDSLPKLPFRSWGQPYTAIIGLVSCSAMILIQGYTVFFPKLWNTQDFLFSYLMVFINIGIYVGYKFIWKRGKDHFKNPHEIDFSKELTEIENHEIESSFEKFQYYSKA>YBR133CMHSNVFVGVKPGFNHKQHSKKSRFLENVSSHSPELPSNYDYVLLPITTPRYKEIVGQVFKDFQRQSIQNWKPLQIPEPQLQDICIPPFNVKKLDNDDTPSYIGLLSSWLELESRDPNVRDLGLKVLLNECKYARFVGINKLILAPPRDLSNLQLYGQMIYRLLQNRIVFAAPALTISISLPLYEDSDPLATWELWNTVRKQCEYHPSLTISLALPRTRTPSYVLNRWLAEPVSCLLVSSSIFASNQYDYPVLHKFNQNLILKFQKVNGDSQILGNELCVILHGMEKYANNVKGGESAYLEYINYLLKKGDKVLNSNSNHQFLLQEDSRIMPPLKPHSDNLLNSTYLTFEKDLVKYDLYESAILEALQDLAPRASAKRPLVILVAGAGRGPLVDRTFKIISMLFMDSKVSIIAIEKNPQAYLYLQKRNFDCWDNRVKLIKEDMTKWQINEPSEKRIQIDLCISELLGSFGCNELSPECLWSIEKYHSHNDTIFIPRSYSSYIAPISSPLFYQKLSQTNRSLEAPWIVHRVPYCILSSRVNEVWRFEHPMAQKDTVQDEDDFTVEFSQSSLNEFKIKHRGEIHGFIGFFSANLYNNIFLSTLPNDSTVRLKFSEETLMNTRREENLIKKCDHTPNMTSWSPIIFPLKQPISFIDDSELSVLMSRIHSDTEQKVWYEWSLESFIYLMLSNYTSAVTAASMTIPRSIVTDDTKTLAHNRHYSATTNQKLDNQIDLDQDIENEEEQGFLSNLETGWQSVQDIHGLSETAKPDHLDSINKPMFDLKSTKALEPSNELPRHEDLEEDVPEVHVRVKTSVSTLHNVCGRAFSLPL>YBR135WMYHHYHAFQGRKLTDQERARVLEFQDSIHYSPRYSDDNYEYRHVMLPKAMLKVIPSDYFNSEVGTLRILTEDEWRGLGITQSLGWEHYECHAPEPHILLFKRPLNYEAELRAATAAAQQQQQQQQQQQQQQQQHQTQSISNDMQVPPQIS>YBR136WMESHVKYLDELILAIKDLNSGVDSKVQIKKVPTDPSSSQEYAKSLKILNTLIRNLKDQRRNNIMKNDTIFSKTVSALALLLEYNPFLLVMKDSNGNFEIQRLIDDFLNISVLNYDNYHRIWFMRRKLGSWCKACVEFYGKPAKFQLTAHFENTMNLYEQALTEVLLGKTELLKFYDTLKGLYILLYWFTSEYSTFGNSIAFLDSSLGFTKFDFNFQRLIRIVLYVFDSCELAALEYAEIQLKYISLVVDYVCNRTISTALDAPALVCCEQLKFVLTTMHHFLDNKYGLLDNDPTMAKGILRLYSLCISNDFSKCFVDHFPIDQWADFSQSEHFPFTQLTNKALSIVYFDLKRRSLPVEALKYDNKFNIWVYQSEPDSSLKNVTSPFDDRYKQLEKLRLLVLKKFNKTERGTLLKYRVNQLSPGFFQRAGNDFKLILNEASVSIQTCFKTNNITRLTSWTVILGRLACLESEKFSGTLPNSTKDMDNWYVCHLCDIEKTGNPFVRINPNRPEAAGKSEIFRILHSNFLSHPNIDEFSESLLSGILFSLHRIFSHFQPPKLTDGNGQINKSFKLVQKCFMNSNRYLRLLSTRIIPLFNISDSHNSEDEHTATLIKFLQSQKLPVVKENLVIAWTQLTLTTSNDVFDTLLLKLIDIFNSDDYSLRIMMTLQIKNMAKILKKTPYQLLSPILPVLLRQLGKNLVERKVGFQNLIELLGYSSKTILDIFQRYIIPYAIIQYKSDVLSEIAKIMCDGDTSLINQMKVNLLKKNSRQIFAVALVKHGLFSLDILETLFLNRAPTFDKGYITAYLPDYKTLAEITKLYKNSVTKDASDSENANMILCSLRFLITNFEKDKRHGSKYKNINNWTDDQEQAFQKKLQDNILGIFQVFSSDIHDVEGRTTYYEKLRVINGISFLIIYAPKKSIISALAQISICLQTGLGLKEVRYEAFRCWHLLVRHLNDEELSTVIDSLIAFILQKWSEFNGKLRNIVYSILDTLIKEKSDLILKLKPYTTLALVGKPELGILARDGQFARMVNKIRSTTDLIPIFANNLKSSNKYVINQNLDDIEVYLRRKQTERSIDFTPKKVGQTSDITLVLGALLDTSHKFRNLDKDLCEKCAKCISMIGVLDVTKHEFKRTTYSENEVYDLNDSVQTIKFLIWVINDILVPAFWQSENPSKQLFVALVIQESLKYCGLSSESWDMNHKELYPNEAKLWEKFNSVSKTTIYPLLSSLYLAQSWKEYVPLKYPSNNFKEGYKIWVKRFTLDLLKTGTTENHPLHVFSSLIREDDGSLSNFLLPYISLDIIIKAEKGTPYADILNGIIIEFDSIFTCNLEGMNNLQVDSLRMCYESIFRVFEYCKKWATEFKQNYSKLHGTFIIKDTKTTNMLLRIDEFLRTTPSDLLAQRSLETDSFERSALYLEQCYRQNPHDKNQNGQLLKNLQITYEEIGDIDSLDGVLRTFATGNLVSKIEELQYSENWKLAQDCFNVLGKFSDDPKTTTRMLKSMYDHQLYSQIISNSSFHSSDGKISLSPDVKEWYSIGLEAANLEGNVQTLKNWVEQIESLRNIDDREVLLQYNIAKALIAISNEDPLRTQKYIHNSFRLIGTNFITSSKETTLLKKQNLLMKLHSLYDLSFLSSAKDKFEYKSNTTILDYRMERIGADFVPNHYILSMRKSFDQLKMNEQADADLGKTFFTLAQLARNNARLDIASESLMHCLERRLPQAELEFAEILWKQGENDRALKIVQEIHEKYQENSSVNARDRAAVLLKFTEWLDLSNNSASEQIIKQYQDIFQIDSKWDKPYYSIGLYYSRLLERKKAEGYITNGRFEYRAISYFLLAFEKNTAKVRENLPKVITFWLDIAAASISEAPGNRKEMLSKATEDICSHVEEALQHCPTYIWYFVLTQLLSRLLHSHQSSAQIIMHILLSLAVEYPSHILWYITALVNSNSSKRVLRGKHILEKYRQHSQNPHDLVSSALDLTKALTRVCLQDVKSITSRSGKSLEKDFKFDMNVAPSAMVVPVRKNLDIISPLESNSMRGYQPFRPVVSIIRFGSSYKVFSSLKKPKQLNIIGSDGNIYGIMCKKEDVRQDNQYMQFATTMDFLLSKDIASRKRSLGINIYSVLSLREDCGILEMVPNVVTLRSILSTKYESLKIKYSLKSLHDRWQHTAVDGKLEFYMEQVDKFPPILYQWFLENFPDPINWFNARNTYARSYAVMAMVGHILGLGDRHCENILLDIQTGKVLHVDFDCLFEKGKRLPVPEIVPFRLTPNLLDALGIIGTEGTFKKSSEVTLALMRKNEVALMNVIETIMYDRNMDHSIQKALKVLRNKIRGIDPQDGLVLSVAGQTETLIQEATSEDNLSKMYIGWLPFW>YBR137WMVVLDKKLLERLTSRKVPLEELEDMEKRCFLSTFTYQDAFDLGTYIRNAVKENFPEKPVAIDISLPNGHCLFRTVTYGGSALDNDFWIQRKKKTALRFGHSSFYMGCKKSDKTPEEKFFVDSKEYAFHGGAVLIQSERSDYPYACLTISGLKQEEDHLMAVSSLIAFANESLEEDLNLD>YBR138CMEKDQIQPRVLESVDTNSLSLLSSNTSSNMNSNTNNKLSIIASDISTGSVLSRPLTPPVVQDIENNSMLQWQFEKKEFIFDSNSTPSKQAKPLQRNSPYQGNSQSENQNQQLLNVRKRRSQCIGAKPKIPSKLYQSVSKLDLIDDKSFTSLPIAPPCNIETNEDDSGNNEYNNNKKRPRLNPVNELRVHNNKRNRYVSYGPSLDTKNYELTENTSQDIPPLVLVEDYIPYTQSKSTKKMVSISDLKSKLSKRRDNHIPLRVKNSYSEINKETNRNSFEPNSLTLIPHILRNTEENRDESNNPLDFIKEEIEISDISIPNSIENMVVNLVNIPSSNKSYDDLYLSELNVHSQLRKCVICEKALYEISSRLLNSGYYKEIVCEQCTVRYEEAAKIFENCEFESSMDESNLSSGTFSDLENSAEPFHLSTDVPKKINRHIEDNKIDLKKEISKKKDSFSKELIERLQLQLLENDKSIKHHFNKDAMGSKSMNWFLEARRKLKWKWRINGLLPHFLRNQNSDRLNFQP>YBR139WMKYLNLVFVLQLLISIKYASFGRAFSLFEDDTTFANLDKQLKLPQNTQQTLKLDRLNHDDPLFTTFISSVDTDYSLRLRTVDPSKLGIDTVKQWSGYMDYKDSKHFFYWFFESRNDPANDPIILWLNGGPGCSSFTGLLFELGPSSIGADMKPIHNPYSWNNNASMIFLEQPLGVGFSYGDEKVSSTKLAGKDAYIFLELFFEAFPHLRSNDFHIAGESYAGHYIPQIAHEIVVKNPERTFNLTSVMIGNGITDPLIQADYYEPMACGKGGYHPVLSSEECEKMSKAAGRCRRLNKLCYASKSSLPCIVATAYCDSALLEPYINTGLNVYDIRGPCEDNSTDGMCYTGLRYVDQYMNFPEVQETLGSDVHNYSGCDNDVFTGFLFTGDGSKPFQQYIAELLNHNIPVLIYAGDKDYICNWLGNHAWSNELEWINKRRYQRRMLRPWVSKETGEELGQVKNYGPFTFLRIYDAGHMVPYDQPEASLEMVNSWISGNRAFSDLSTLENAS>YBR141CMHSRKSKSITGKRKQVGSNVTRVIKPQKTRRIIRRFHHLINKRQSICKFLCLKENLDDSNEEKNDKIIRLSIKGNVRLGKYYEDGKSQSFNDAMESQLLRLHSLIKNESKSKDTSDLAVMYTLLGYIMNQINKLGGLETYQIASQNGQLKERGGDTSKLLEKWIRSSFENCPGAVALEIGSLSSGNRISRCALFRNVVRIDLEEHEGVIKQDFMERPLPRNENDKFDLISCSLVLNFVKNHRDRGAMCHRMVKFLKPQGYIFIVLPQACVTHSRYCDKTLLQNLLGSIGLIMLNSHQSNKLYYCLYQLQVVSPQPSSFSKRIKVNDGPGLNNFGITL>YBR145WMPSQVIPEKQKAIVFYETDGKLEYKDVTVPEPKPNEILVHVKYSGVCHSDLHAWHGDWPFQLKFPLIGGHEGAGVVVKLGSNVKGWKVGDFAGIKWLNGTCMSCEYCEVGNESQCPYLDGTGFTHDGTFQEYATADAVQAAHIPPNVNLAEVAPILCAGITVYKALKRANVIPGQWVTISGACGGLGSLAIQYALAMGYRVIGIDGGNAKRKLFEQLGGEIFIDFTEEKDIVGAIIKATNGGSHGVINVSVSEAAIEASTRYCRPNGTVVLVGMPAHAYCNSDVFNQVVKSISIVGSCVGNRADTREALDFFARGLIKSPIHLAGLSDVPEIFAKMEKGEIVGRYVVETSK>YBR146WMFSRLSLFRRAALAPAPMRMSFRTIYQKTEDELPRRIVPKLATFYSANPNHEDRINRLERLLRKYIKLPSQNNNEAQQTKAPWISFDEYALIGGGTKLKPTQYTQLLYMLNKLHNIDPQLTNDEITSELSQYYKKSSMLSNNIKIKTLDEFGRSIAVGKRKSSTAKVFVVRGTGEILVNGRQLNDYFLKMKDRESIMYPLQVIESVGKYNIFATTSGGGPTGQAESIMHAIAKALVVFNPLLKSRLHKAGVLTRDYRHVERKKPGKKKARKMPTWVKR>YBR147WMKLIPIILNAKNLSGMAGSISICCWIVVFVPQIYENFRRQSAEGLSLLFIVLWLLGDIFNVMGAMMQNLLPTMIILAAYYTLADLILLIQCMWYDKEKKSILQEVKKNVDPVHLPPANPINETVLQDVFNEYEPLLPRIEEEDSQSYSSLELGRTIVVKERENFFNDFLIVSGVLIAGILSWYISYCSGLDNGIPKKKPAFEQINLPAQILGYLSAILYLGSRIPQIVLNFKRKSCEGVSFLFFLFACLGNTSFIISVLSASWLIGSAGTLLMDFTVFIQFFLYAKPKYEKILIDN>YBR148WMSSLADTVEGSEAKRGRFSNNALTSDTGILQKNSTLRNWFLKPTADLKNSCEDRVEDDVNDVYLNDKNSQKSVEERKLGRKVRSFFKQTNSNKDESVLEDEDDALVWKKTSNKCAKKENSHDIQKGSFTKKIRNSIFKSANDVKEFRNENNLLLPVELSSDDENESHFTDANSHVMQSKSPEKIPSKDQCLTKGAKNKGLKKEYEKSFEEYSDDSDDEFSPATPPENVLEGPYKFVFQTPNTFTSQPNITVENDFHKGGRHVIDYLNKKLATMNIDIDLTSGGKQNVSWEEELDQLSDHVIESITNHISKGRMHAQEKQDELEKLKLENLNLSTLKQENLQHKQEINSLKDNLESISKKNNDLILEMNKLKKKSTNNKTNEYISTDENENEEITKSNMGPGILELNVNETSKKLQQSTFKPSKYLPRETRNNENRLKHLEKRIFGLEKSLEKKKKQVRADSVRLDLNRYTIDQFLTLLKSLSEVLQFHNVYGNDLKENDDNIIKIETCCSALNMKNCFEDSSFRLQENSFKRQLGPLFANINFSLIDQLTMNFRFYERSANFQKETIGGLRMMLQDKDNYIKTLMQHLKKKESTKLIKDSKNGASTLTS>YBR149WMSSSVASTENIVENMLHPKTTEIYFSLNNGVRIPALGLGTANPHEKLAETKQAVKAAIKAGYRHIDTAWAYETEPFVGEAIKELLEDGSIKREDLFITTKVWPVLWDEVDRSLNESLKALGLEYVDLLLQHWPLCFEKIKDPKGISGLVKTPVDDSGKTMYAADGDYLETYKQLEKIYLDPNDHRVRAIGVSNFSIEYLERLIKECRVKPTVNQVETHPHLPQMELRKFCFMHDILLTAYSPLGSHGAPNLKIPLVKKLAEKYNVTGNDLLISYHIRQGTIVIPRSLNPVRISSSIEFASLTKDELQELNDFGEKYPVRFIDEPFAAILPEFTGNGPNLDNLKY>YBR151WMAFLNIFKQKRGDEASQLSAKGREEISQSIKICKSDDAANEHSCSGDCKTEIEEGEQAFAKLKIEHETPLLNSSKTPKIHFVVPTSQIDWQHDACLEDPKSVQYKISQWCDKNSAKFSNVGTGKTLNCAVSSLPKDIMDIDVMRGTKNNVLILPYFIWLNDLRSDDVEATLDGLVPDLLDENISREKLLETRPNVAVARERAFVFICSHTTRDKRCGITAPYLKKVFDSKLQEHGLYRDNSDYRAEGVKIAFVNHVGGHKFAANVQIYLRNPNTLIWLGRVTPTIVPSIVEHLIVPEEPTLPFPEKVRCIKKYQSW>YBR154CMDQENERNISRLWRAFRTVKEMVKDRGYFITQEEVELPLEDFKAKYCDSMGRPQRKMMSFQANPTEESISKFPDMGSLWVEFCDEPSVGVKTMKTFVIHIQEKNFQTGIFVYQNNITPSAMKLVPSIPPATIETFNEAALVVNITHHELVPKHIRLSSDEKRELLKRYRLKESQLPRIQRADPVALYLGLKRGEVVKIIRKSETSGRYASYRICM>YBR155WMSSVNANGGYTKPQKYVPGPGDPELPPQLSEFKDKTSDEILKEMNRMPFFMTKLDETDGAGGENVELEALKALAYEGEPHEIAENFKKQGNELYKAKRFKDARELYSKGLAVECEDKSINESLYANRAACELELKNYRRCIEDCSKALTINPKNVKCYYRTSKAFFQLNKLEEAKSAATFANQRIDPENKSILNMLSVIDRKEQELKAKEEKQQREAQERENKKIMLESAMTLRNITNIKTHSPVELLNEGKIRLEDPMDFESQLIYPALIMYPTQDEFDFVGEVSELTTVQELVDLVLEGPQERFKKEGKENFTPKKVLVFMETKAGGLIKAGKKLTFHDILKKESPDVPLFDNALKIYIVPKVESEGWISKWDKQKALERRSV>YBR156CMDWAIKAARKKTQRKPGSTRSIIETLDDLNNLTTDAHSEINQRLYESSEWLRNNVYMNTLKYEDKKMEESLISPENTHNKMDVEFPKMKGEYELSNSQNDAAKDVTKTPRNGLHNDKSITPKSLRRKEVTEGMNRFSIHDTNKSPVEPLNSVKVDANESEKSSPWSPYKVEKVLRESSKTSESPINTKRFDNQTWAAKEEMENEPILQALKKAESVKVKPPPNSGIARSQRRSNMFVPLPNKDPLIIQHIPPTKSSGSIPKVRTVKESPIAFKKKSTINSPAIRAVENSDTAGSTKASSVFDRLSSIPTKSFENKISRGNVGHKYSSSSIDLTGSPMKKVSQKFKSINSTDTDMQEALRDIFSVKNKITKNNSPKGKNSRKSSIPRFDKTSLKLTTHKKLAIIAEQKKKSKHSSDVHKTGSRPHSISPTKISVDSSSPSKEVKNYYQSPVRGYLRPTKASISPNKNKNLTTSQTPHRLKIKEKTLRKLSPNIADISKPESRKSKNYRLTNLQLLPPAEAERDDLKKKFDKRLSGIMRSQQEHHRRKQEKQKRMSHLEQDLKKQTSFSNDYKDIRLKESLAPFDNHVRDTINKNTAFSTDNILATINTVDHREIIGNVTPKIASVNDSLPEINTDSEDEASVTLAAWAKSPYLQEQLIRQQDINPQTIFGPIPPLHTDEIFPNPRLNRLKPRQIVPKRS>YBR157CMGKFEQKERERISTFSFPTTGSQSSTSIKSLGSPLYGRFSSLSSTESQFDSSKQPHEYEKSFYFEESQGEALFNKLKTYSFPGDKDGVKTRRNSSICPRKPNAVSPLRVESNELSSHSHSRSLSHELTKPSGRRKSYHRKSHAISFSRSCKPNFIDGYDSNSSIGVNSRKTSLASSFLDKEYHSSPDTSYTHQMSPKNTIMNTNEQLRRNASGRFGSLKEFAEKNQINIEGKIFAHKVETGDILQPLIDLDIDNK>YBR159WMTFMQQLQEAGERFRCINGLLWVVFGLGVLKCTTLSLRFLALIFDLFLLPAVNFDKYGAKTGKYCAITGASDGIGKEFARQMAKRGFNLVLISRTQSKLEALQKELEDQHHVVVKILAIDIAEDKESNYESIKELCAQLPITVLVNNVGQSHSIPVPFLETEEKELRNIITINNTATLLITQIIAPKIVETVKAENKKSGTRGLILTMGSFGGLIPTPLLATYSGSKSFLQGWSNSLAGELSKDAIDVELIISYLVTSSMSKIRRSSLMIPNPQQFVKSTLRSVGRRCGSQERYATMTPYWAHAVYQFVITETFGVYSKIVNSINYSFHKSIRIRALKKAARQVKKE>YBR160WMSGELANYKRLEKVGEGTYGVVYKALDLRPGQGQRVVALKKIRLESEDEGVPSTAIREISLLKELKDDNIVRLYDIVHSDAHKLYLVFEFLDLDLKRYMEGIPKDQPLGADIVKKFMMQLCKGIAYCHSHRILHRDLKPQNLLINKDGNLKLGDFGLARAFGVPLRAYTHEIVTLWYRAPEVLLGGKQYSTGVDTWSIGCIFAEMCNRKPIFSGDSEIDQIFKIFRVLGTPNEAIWPDIVYLPDFKPSFPQWRRKDLSQVVPSLDPRGIDLLDKLLAYDPINRISARRAAIHPYFQES>YBR162CMLQKLSMTALVGLFSSVVSLVNADCTYSGGNYYCAQTDAIIYSNVGLSATYQDVTNMDESSCACTQADFTASGSLAPFNEELSVHFRGPIELLQFGVYYPNGESNALKKRSEKQSIESCKEGEAVVSRHKHQHKRDVAVEYVQVTSTVYVDSNGQTVTADSTNTVVGPAVPSSYTKDSTVLSSSAQAVETSESQSSISSSKTTSSAAAASSSSSSSSNTNGDWSRGSYFVPGSTSNCTFMNNQGGTAGSGVWSSCFGNSISFAASDGVSGAASAQALGDVTIKSGNEFMIFSGEECSGNNGDCGYYREGIPAYHGFGGADKIFVFEFSMPSDTSGSAYNQDMPAIWLLNAKIPRTLQYGDASCSCWKTGCGEMDLFEILTAGSDKLISHIHDGQDGGTQDYFERPTDGTLKAAVIFNSSDKTIHIIEVDESFDATLSDDVVDQWLSKSGSSAALP>YBR162W-AMAVQTPRQRLANAKFNKNNEKYRKYGKKKEGKTEKTAPVISKTWLGILLFLLVGGGVLQLISYIL>YBR163WMLGRALINKYGFLIHPRRFVHLNDKSLDGTFILPSKKNHMYDVPTNDPSGILNASDIDRINNLPFFDNTSPTKETNTKEGALLSEKLASVKELFGEDPENPSFINYRFPRGLENPYFDIQVNQLKKKRLSVTQLCTTQNWCELRNFYDFYSQNLSNQLLNLKFQVQKGKKIHKSLEDETHPELNQYKSFTHNFLALTKLSMDIDNDMDALLDNWFNSINRLVSLFTKGDGHAREIVCHGFINLEDGKLVEHLLNSDSKTKENVIISGVIDHLTLRNRHNHQVQKGAAHLDTEYQSWGNILTNLLSNLKELKSNNEIVISDIKTRSVPKIPSIESVIESSKLQTMYYKFFFSHLSQDMTQTYHSFLINAQRRGLDVDAPINPTKILTFILTNPLFANDVKNLLYGLPINHSAFDNDAKGSNTFDMTAFNDLLDRGPTSFNVPIEQDEDSSESTKCVSLRDYGHFYTKWKTPLTLKYFAARLSQIYFIVGNLVSNDLMIEYYYHNDNFHNIIFPYDPLKLGTHAHDSAMVWFGGRDMHPIEPTQKNFNTYCKFCDYRHVCSWKNKNELKLIDLGKELKKIILESSMK>YBR164CMGNIFSSMFDKLWGSNKELRILILGLDGAGKTTILYRLQIGEVVTTKPTIGFNVETLSYKNLKLNVWDLGGQTSIRPYWRCYYADTAAVIFVVDSTDKDRMSTASKELHLMLQEEELQDAALLVFANKQDQPGALSASEVSKELNLVELKDRSWSIVASSAIKGEGITEGLDWLIDVIKEEQL>YBR165WMAYSLTRKLLKDWKYFMRHPEKTQGLFHVRPHDSDLHLWHVVMYEPRTSLEVYLLLYIGGNDQDPYIIMKCLSPNCCFPINRTVSMTHLNYLLLKDLGLQDLLFHIWQPLFHIQATEDLQYSPSTVKFNRAWNRIIYKDFKSYFPELIGTLQPGDYSIVKSYSKNHNISNSNGGSVNEFMSSYNAQSHTFHAQDNSKNPYTNSSIGKSSMLSTLNNNNVNKRTHDYNAIDFMTKNLLACDDDSIHPVVSSKRSRTLACPDETNDNRGSEHYTKRKKI>YBR166CMVSEDKIEQWKATKVIGIIGLGDMGLLYANKFTDAGWGVICCDREEYYDELKEKYASAKFELVKNGHLVSRQSDYIIYSVEASNISKIVATYGPSSKVGTIVGGQTSCKLPEIEAFEKYLPKDCDIITVHSLHGPKVNTEGQPLVIINHRSQYPESFEFVNSVMACLKSKQVYLTYEEHDKITADTQAVTHAAFLSMGSAWAKIKIYPWTLGVNKWYGGLENVKVNISLRIYSNKWHVYAGLAITNPSAHQQILQYATSATELFSLMIDNKEQELTDRLLKAKQFVFGKHTGLLLLDDTILEKYSLSKSSIGNSNNCKPVPNSHLSLLAIVDSWFQLGIDPYDHMICSTPLFRIFLGVSEYLFLKPGLLEQTIDAAIHDKSFIKDDLEFVISAREWSSVVSFANFDIYKKQFQSVQKFFEPMLPEANLIGNEMIKTILSHSSDRSAAEKRNT>YBR167CMALKKNTHNKSTKRVTKHPSLKTLTHKQIHTTIFVKSTTPYVSALKRINKFLDSVHKQGSSYVAVLGMGKAVEKTLALGCHFQDQKNKKIEVYTKTIEVLDEVITEGQADIDMESDVEDDDKETQLKKRAVSGVELRIYV>YBR168WMDTNSKTKVQTENKKIKAKFIHNHGQKPSLIQITPPMISSTLFHAYPLLLIFDNALANIMWLSDDKCLTFIYLTSIWLTISFFIPVETEASHFLPFTKILRLWLGIISGAFLFLSFMYYIVSLIASLRDTEPPTLDEIVVLLESVLDKLEVLRNELNVWKKLKLSFDGVNKECSGKRLFCRLFLFGTIFQIIIMRYISPGTYTRFFIITGLIYNTSSFQATLRLLWRFTAVRNFYYLGIESFKISSFLPKHLKMEQIIPLSQGRAITVPLVEVLPKLLRDKKGDDHIHILQLLLNEQKDNFGNEDLKILEIEVYENQRRWYQNKNWSTKLLPYERQNYCIEIKNTDGTLTMRSCLPPDGLGEEELPNNWHWINDNWDGTDWIYSDSAWKEIGQYSSLESFTRSRKWKRRLFHL>YBR169CMSTPFGLDLGNNNSVLAVARNRGIDVVVNEVSNRSTPSLVGFGPRNRYLGESGKTKQTSNVKNTVENLKRIIGLKFKDPEFDIENKFFTSKLVQLKNGKVGVEVEFGGKTHVFSATQLTAMFIDKVKHTVQEETKSSITDVCLAVPVWYSEEQRYNIADAARIAGLNPVRIVNDVTAAAVSYGVFKNDLPGPEEKPRIIGLVDIGHSTYTCSIMAFRKGEMKVLGTAYDKHFGGRDFDRAITEHFADQFKDKYKIDIRKNPKAYNRILIAAEKLKKVLSANTTAPFSVESVMDDIDVSSQLSREELEELVEPLLKRVTYPITNALAQAKLTVNDIDFVEIIGGTTRIPVLKKSISDVFGKPLSSTLNQDEAVAKGAAFICAIHSPTLRVRPFKFEDIDPYSVSYTWDKQVDDEDRLEVFPANSSYPSTKLITLHRTGDFSMKAVYTHPSKLPKGTSTTIAKWSFTGVKVPKDQDFIPVKVKLRCDPSGLHIIENAYTTEDITVQEPVPLPEDAPEDAEPQFKEVTKTIKKDVLGMTAKTFALNPVELNDLIEKENELRNQDKLVAETEDRKNALEEYIYTLRAKLDDEYSDFASDAEKEKLKNMLATTENWLYGDGDDSTKAKYIAKYEELASLGNIIRGRYLAKEEEKRQALRANQETSKMNDIAEKLAEQRRARAASDDSDDNNDENMDLD>YBR170CMLIRFRSKNGTHRVSCQENDLFGTVIEKLVGNLDPNADVDTFTVCEKPGQGIHAVSELADRTVMDLGLKHGDMLILNYSDKPANEKDGVNVEIGSVGIDSKGIRQHRYGPLRIKELAVDEELEKEDGLIPRQKSKLCKHGDRGMCEYCSPLPPWDKEYHEKNKIKHISFHSYLKKLNENANKKENGSSYISPLSEPDFRINKRCHNGHEPWPRGICSKCQPSAITLQQQEFRMVDHVEFQKSEIINEFIQAWRYTGMQRFGYMYGSYSKYDNTPLGIKAVVEAIYEPPQHDEQDGLTMDVEQVKNEMLQIDRQAQEMGLSRIGLIFTDLSDAGAGDGSVFCKRHKDSFFLSSLEVIMAARHQTRHPNVSKYSEQGFFSSKFVTCVISGNLEGEIDISSYQVSTEAEALVTADMISGSTFPSMAYINDTTDERYVPEIFYMKSNEYGITVKENAKPAFPVDYLLVTLTHGFPNTDTETNSKFVSSTGFPWSNRQAMGQSQDYQELKKYLFNVASSGDFNLLHEKISNFHLLLYINSLQILSPDEWKLLIESAVKNEWEESLLKLVSSAGWQTLVMILQESG>YBR171WMSEFNETKFSNNGTFFETEEPIVETKSISVYTPLIYVFILVVSLVMFASSYRKKQAKKISEQPSIFDENDAHDLYFQIKEMSENEKIHEKVLKAALLNRGAESVRRSLKLKELAPQINLLYKNGSIGEDYWKRFETEVKLIELEFKDTLQEAERLQPGWVQLFVMVCKEICFNQALSRRYQSILKRKEVCIKEWELKINNDGRLVN>YBR172CMIAPDSQRLFGSFDEQFKDLKLDSVDTENNNTHGVSTILDSSPASVNNNTNGAVAASVNTVPGSTFRSNTPLLGGRHPLSRTSSLIDSIGIQRAASPFSSMKEPFIPQSSGVMSSSFWHGDHPESRVSTPVQQHPLLQRNESSSSFSYAANLGVNLSTHSLAVDITPLSTPTAAQSHVNLFPSSDIPPNMSMNGMSQLPAPVSVESSWRYIDTQGQIHGPFTTQMMSQWYIGGYFASTLQISRLGSTPETLGINDIFITLGELMTKLEKYDTDPFTTFDKLHVQTTSSDSINLNLAPYASGVAATGTIKATENDIFKPLTHDNIWDMDGGTTSKGVDIKLASATTISQTDESHKQEYKSTTMLEKGKKEKSESVAKALLDEQEKRNRELKRKEEARLSKKQKQKEDDLLKKQKEQKEQKEKEALEAEKQKKSEKTKKDTQTQTEGFKTSKDLPSLNSSSANPAPWASKVKVNNAIETSIKNGVSSTGKKKGEPLGLQQRNSKEEKQKEELKSVLNWANKSSLPSNQTIDIKSQFQKSPKGMKESSPLKELEDPNFIEEQKKLWEKVQSSSKQVKSTSSASTTTSSWTTVTSKGKAPIGTVVSPYSKTNTSLNSSLTAKTSTTSTTTTFASMNNVSPRQEFIKWCKSQMKLNSGITNNNVLELLLSLPTGPESKELIQETIYANSDVMDGRRFATEFIKRRVACEKQGDDPLSWNEALALSGNDDDGWEFQVVSKKKGRKH>YBR173CMNIVPQDTFKSQVSTDQDKSVLSSAVPSLPDTLRQQEGGAVPLSTQLNDRHPLESTLKNWETTQRQRQMEQYRQIFGIAEPMKRTMEMEIVNRTDFNPLSTNGSIHRDILLNKECSIDWEDVYPGTGLQASTMVGDDVHSKIEKQLGI>YBR175WMFQFVTPVGTQNGLKATCAKISPDGQFLAITQGLNILIYDINRRTVSQTLVTSHARPFSELCWSPDGQCIATASDDFSVEIIHLSYGLLHTFIGHTAPVISLTFNRKGNLLFTSSMDESIKIWDTLNGSLMKTISAHSEAVVSVDVPMNDSSILSSGSYDGLIRIFDAETGHCLKTLTYDKDWKRENGVVPISQVKFSENARYLLVKSLDGVVKIWDCIGGCVVRTFQVQPLEKGVLHHSCGMDFLNPEDGSTPLVISGYENGDIYCWNSDTKSLLQLLDGSLYHHSSPVMSIHCFGNIMCSLALNGDCCLWRWV>YBR176WMNIMKRQLCTSSKRFFSTAKNVVKYNTIQDIRNKYFTGTPLSMCTAYDFITATWVNKANCDLLLVGDSLAMTSLGYDSTITLSLNEFKYHVASVCRAEGSSMVVVDMPFGTFESGISDGLKNAIDIMKLDSKVTSVKVEVGSYTKDKYAMKFIEELCSRGIPVMAHIGLTPQKVHSLGGYKVQGSKSLLQMQELYETAMQLQKIGCWSILIECVPHKMAQFITSKLSVPTIGIGAGNGTSGQVLVISDLLGMQGDSVPKFVKQAVNMTDIATQGLKEYIASVEDRTFPERGTHTFKVKEDLWNEFLSSINEK>YBR177CMSEVSKWPAINPFHWGYNGTVSHIVGENGSIKLHLKDNKEQVDFDEFANKYVPTLKNGAQFKLSPYLFTGILQTLYLGAADFSKKFPVFYGREIVKFSDGGVCTADWLIDSWKKDYEFDQSTTSFDKKKFDKDEKATHPEGWPRLQPRTRYLKDNELEELREVDLPLVVILHGLAGGSHEPIIRSLAENLSRSGRFQVVVLNTRGCARSKITTRNLFTAYHTMDIREFLQREKQRHPDRKLYAVGCSFGATMLANYLGEEGDKSPLSAAATLCNPWDLLLSAIRMSQDWWSRTLFSKNIAQFLTRTVQVNMGELGVPNGSLPDHPPTVKNPSFYMFTPENLIKAKSFKSTREFDEVYTAPALGFPNAMEYYKAASSINRVDTIRVPTLVINSRDDPVVGPDQPYSIVEKNPRILYCRTDLGGHLAYLDKDNNSWATKAIAEFFTKFDELVV>YBR179CMSEGKQQFKDSNKPHKDSTDQDDDAATIVPQTLTYSRNEGHFLGSNFHGVTDDRTTLFDGEEGRREDDLLPSLRSSNSKAHLISSQLSQWNYNNNRVLLKRSILKTQAFMDQLQEENNIRPIFIAANDEREKLHVLQLNIKLDGQYNTKEKNGFNIEKKALSKLFHSQIVSVTNHLNALKKRVDDVSSKVFITGDVNTGKSALCNSLLKQRLLPEDQLPCTNVFSEILEARENDGIEEVHAIPLNIAPTLKEAIDMYSIQNPKTYEIHTLKELPDLVPQNGKYALLKIYIKDDKRPASTSLLRNGTVDISLIDSPGLNMDSLQTAEVMSRQEEIDLVIFVVNAENQLTLSAKEFISLASREKKLMFFVVKKFDKIRDKQRCKELILKQIRDLSPETYKRAADFVHFVSKNGDELPHYHNENDNEDHGDRKPDDDPYSSSDPDPDFDSLEDSLRNFVLKKRSLSKLLPAKTYLSKLLSDIIMISKSNMKMYSEEEIKINEQLETLRPEILSARAKCNDLTTSVDQMAEQTITMTYNNTKEALLNALDVPLHEYPKYQGLGQIYDFIFSTEAFIANQIDESIGSSELFAKQKTDLLVKKIYEIGKNELGDDFMCERVFRSELMFRKRKHLIGKRLKVSLSITDLFAPTWKGFLSYLSWQKPVTAPLPDIEGQTNEGQIGLMKYLGLKNYPLTQYWSRPSLLFTSKIPTLTLYFLGSTKVVGNIILNGIKLSSWSSLKKLSVPVIVVGSLLGLTYLIHDLPRALPMNLSIKYKRKLQELDYIHLNAQRTSNEVRDVLRVPTREILRSCEIIMDKKQITKKELENKKESNLLSIKFFQSLYEGTVAQKLMVEEINLDID>YBR180WMGSEPFQKKNLGLQINSQESGTTRSTFHSLEDLGDDVINESWDQVNQKRANIDHDVFHEHPDSSPSLSAQKAKTKEEEVAVKSSNSQSRDPSPDTQAHIPYTYFSKDQRLIIFGIIIFIGFLGPMSGNIYIPALPLLQREYDVSATTINATVSVFMAVFSVGPLFWGALADFGGRKFLYMVSLSLMLIVNILLAAVPVNIAALFVLRIFQAFASSSVISLGAGTVTDVVPPKHRGKAIAYFMMGPNMGPIIAPIVAGLILMKGNYWRWLFGFTSIMTGIALILVTALLPETLRCIVGNGDPKWGDKKDERENNESPFFEGNKISHRRLFPDIGIRKPVNNDAFFQENFPKPPKAGLTLYWKMIKCPPIIITSVSTALLFSSYYAFSVTFSYYLEHDYRFTMLEIGAAYVCPGVAMLLGSQSGGHLSDYLRSRWIKSHPKKKFPAEFRLLLNLIGILLTICGTIGYGWAIFFHYHFVVLLVFSALTAFGMTWCSNTSMTYLTELFPKRAAGTVAVSSFFRNVGAAISSAIILQLCNAMGIGWCFTGLGLCSSISLIGILYLLIFQRKYTAKEF>YBR181CMKLNISYPVNGSQKTFEIDDEHRIRVFFDKRIGQEVDGEAVGDEFKGYVFKISGGNDKQGFPMKQGVLLPTRIKLLLTKNVSCYRPRRDGERKRKSVRGAIVGPDLAVLALVIVKKGEQELEGLTDTTVPKRLGPKRANNIRKFFGLSKEDDVRDFVIRREVTKGEKTYTKAPKIQRLVTPQRLQRKRHQRALKVRNAQAQREAAAEYAQLLAKRLSERKAEKAEIRKRRASSLKA>YBR182CMGRRKIEIEPIKDDRNRTVTFIKRKAGLFKKAHELSVLCQVDIAVIILGSNNTFYEYSSVDMSNLLNVHQNNTDLPHNIIEPSDYGDYVKKPRVVLNERKRRRRRATVLQPASHSGSCTVSSQDSSSVQNNGNLSAPLASNDAGNAGVSTPLVHCHGAISRSGSNHSDCARNSADYQMLQGGLNSGGSFHANDYKESVDQQHVANEAIHRNFMNKRIRPDTHLLLSESNHSNYHNFYPSPYENLPKPSLPASLVGNIPSFQSQFVQVIPANSNPMGKGFNGTGDSESFEAKQKIHPTVAISNTLEGPAPVQAMVHHLHQLNSNRGKLSGKPYLKLNIPKATNDACQRSPAMYSGTASPKTDVQATPNQMLASNMSSPLSRSKFLGFKNNDMDDLYHNGRCGSTYVNNKTFFLKPPIGRPPKFPKSPSSSIVVFPSSVASSTLKSTSSTNSPD>YBR183WMGIFRWNYPESSVPGVWGETTSTIDWCEENYVVSPYIAEWSNTLTNSVFILSAIYTTYSAYKNKLEKRFLLIGFGYGLVGVGSWLFHMTLKYRFQLLDELPMIYAMCIPTWSLVCEAKEALLNGDNHKKVPLFEQIFIGVIIGLAVTTASILYVIYKNVDIHQILFGVQIVVVAATAGSLTYRYVHDPLAKRNLKASMALGAILFLSGYISWLLDIHYCSFWVHVRRSILALPLGVLLEPHGWWHILTGMGIYFYIVSLEHLRVITLNVSCNYQFIWRWKVFPELIWKGRKPSTRYSLELFGPYVEDQSIEVKKEK>YBR185CMSVLRSTCLFFPPRSLLISFNKRRLFSTSRLILNKESETTKKKDKSKQQDFNPRHLGVAAEIFIPSAYKNLPNVFAHPLIVANALIRRLYTFGLNSVQVALFRFQSGIKPSFLLWKNKAIETYINVNTSFAHKNLSDIKGLVSLWVQEALEARSRQLPGNATLDWQLIKFNAVPKLVSVQPIMIPGMPLEHLQLVYKFDTKQRLIKVNQQTKKTETLDRDVVDYIAFLCDATTNDMILMGSLFESKPNDKLPKSYEDDAKVAIHRMKVNGDIYRLPPS>YBR186WMSYIVDLQVRGSSLRVIKCMFREDEQISSLHSGSDSKQNSNKKLGEFLNLLKAVVKRKLESFPKDRLKTSIITGQELMREGQGSIEIKDPPTEAQQHLIRSLAKVLLHQFSSINGKVNTVNEGQDNLFLSLFVKKISIEQQSTSHVSIKLNFHEKINLGQHIDSILDSEETNESDTYHMGSVDEFIIYPFCCLEEQDELKNGSILSTEFDKIDLELDEDDGFEGETLNNCINSVGNFDIPLSKQTLNLVNISYLPGTTFEGQWESLYFGNNIKERLYSYATISLKIARFKQTGDSNQEDITTLITNNKLLLVHGPPGTGKTTLCKALCQKLSVRREFSDGSDTIDTNYKGIIIELSCARIFSKWFGESSKNISIVFKDIEELLKVNEGRGIFICLLIDEVEAIASSRTNLSSRNESTDGIRVVNTLLTQLDRLKKYHNFLALATSNLLDSLDDAFVDRADGVFYVGNPTAEGILHILKVCIEEMITSGIILFHARSTGVKFFNKYQDILRKIAIKCSTVDISGRTIRKLPLMCLSEYFRTFPVDDDEFVLALAMSARKLSAARK>YBR191WMGKSHGYRSRTRYMFQRDFRKHGAVHLSTYLKVYKVGDIVDIKANGSIQKGMPHKFYQGKTGVVYNVTKSSVGVIINKMVGNRYLEKRLNLRVEHIKHSKCRQEFLERVKANAAKRAEAKAQGVAVQLKRQPAQPRESRIVSTEGNVPQTLAPVPYETFI>YBR193CMSQSTASLVPEGNQGSLQEDVSFDFNGVPGQALDAVRMRLAQLTHSLRRIRDEMSKAELPQWYTLQSQLNVTLSQLVSVTSTLQHFQETLDSTVVYPLPKFPTTSHESLVTTLLRKKNIPEVDEWMKYVRETSGVTTALLKDEEIEKLLQQDREITNWARTTFRNEYGKHDFKNEESLSEEHASLLVRDSKPSKPFNVDDVLKFTFTGEKPIITGSTSTSSSN>YBR195CMNQCAKDITHEASSIPIDLQERYSHWKKNTKLLYDYLNTNSTKWPSLTCQFFPDLDTTSDEHRILLSSFTSSQKPEDETIYISKISTLGHIKWSSLNNFDMDEMEFKPENSTRFPSKHLVNDISIFFPNGECNRARYLPQNPDIIAGASSDGAIYIFDRTKHGSTRIRQSKISHPFETKLFGSHGVIQDVEAMDTSSADINEATSLAWNLQQEALLLSSHSNGQVQVWDIKQYSHENPIIDLPLVSINSDGTAVNDVTWMPTHDSLFAACTEGNAVSLLDLRTKKEKLQSNREKHDGGVNSCRFNYKNSLILASADSNGRLNLWDIRNMNKSPIATMEHGTSVSTLEWSPNFDTVLATAGQEDGLVKLWDTSCEETIFTHGGHMLGVNDISWDAHDPWLMCSVANDNSVHIWKPAGNLVGHS>YBR197CMGVKQTPPVQVKVSDADSTNRRKSSSQEGNPQLVQLKAKSDKDKRKGSSDSTASIMGSSNALPTKNLTTPPALNPLTTSISRGNTAYERSVNGSRITMHSNLAPTETQDVSWSEIDTLDDVKKMAKEPIVNDGFPRDFESNLTQMRKSHAQLLRLMRERNQRLKYAKLRSPPHKDQHNSATNKDQEPDEVLHDPEIALDGEKYVSQVVDTIKDVHRC>YBR199WMRFLSKRILKPVLSVIILISIAVTVVLYFLTANENYLQAVKDSAKSQYASLRESYKSITGKTESADELPDHDAEVLDSIMDRLHEPLYEKDTFDPNEVLAENKQLYEEFLLQEISEPKVDNLVRSGDPLAGKAKGTILSLVRNSDLEDIISSIQQLEEEYNKNFGYPYTFLNDEEFTDEFKDGIKSILPKDRVVEFGTIGPDNWNMPDSIDRERYDQEMDKMSKENIQYAEVESYHNMCRFYSKEFYHHPLLSKYKYVWRLEPNVNFYCKINYDVFQFMNKNDKIYGFVLNLYDSPQTIETLWTSTMDFVEEHPNYLNVNGAFAWLKDNSQNPKNYDYTQGYSTCHFWTNFEIVDLDFLRSEPYEKYMQYLEEKGGFYYERWGDAPVRSLALALFADKSSIHWFRDIGYHHTPYTNCPTCPADSDRCNGNCVPGKFTPWSDLDNQNCQATWIRHSMSEEELEMY>YBR201WMDAVILNLLGDIPLVTRLWTIGCLVLSGLTSLRIVDPGKVVYSYDLVFKKGQYGRLLYSIFDYGAFNWISMINIFVSANHLSTLENSFNLRRKFCWIIFLLLVILVKMTSIEQPAASLGVLLHENLVYYELKKNGNQMNVRFFGAIDVSPSIFPIYMNAVMYFVYKRSWLEIAMNFMPGHVIYYMDDIIGKIYGIDLCKSPYDWFRNTETP>YBR202WMSAALPSIQLPVDYNNLFNEITDFLVTFKQDTLSSDATRNENEDENLDAENIEQHLLEKGPKYMAMLQKVANRELNSVIIDLDDILQYQNEKFLQGTQADDLVSAIQQNANHFTELFCRAIDNNMPLPTKEIDYKDDVLDVILNQRRLRNERMLSDRTNEIRSENLMDTTMDPPSSMNDALREVVEDETELFPPNLTRRYFLYFKPLSQNCARRYRKKAISSKPLSVRQIKGDFLGQLITVRGIITRVSDVKPAVEVIAYTCDQCGYEVFQEVNSRTFTPLSECTSEECSQNQTKGQLFMSTRASKFSAFQECKIQELSQQVPVGHIPRSLNIHVNGTLVRSLSPGDIVDVTGIFLPAPYTGFKALKAGLLTETYLEAQFVRQHKKKFASFSLTSDVEERVMELITSGDVYNRLAKSIAPEIYGNLDVKKALLLLLVGGVDKRVGDGMKIRGDINVCLMGDPGVAKSQLLKAICKISPRGVYTTGKGSSGVGLTAAVMKDPVTDEMILEGGALVLADNGICCIDEFDKMDESDRTAIHEVMEQQTISISKAVINTNPGARTSILAAANPLYGRINPRLSPLDNINLPAALLSRFDILFLMLDIPSRDDDEKLAEHVTYVHMHNKQPDLDFTPVEPSKMREYIAYAKTKRPVMSEAVNDYVVQAYIRLRQDSKREMDSKFSFGQATPRTLLGIIRLSQALAKLRLADMVDIDDVEEALRLVRVSKESLYQETNKSKEDESPTTKIFTIIKKMLQETGKNTLSYENIVKTVRLRGFTMLQLSNCIQEYSYLNVWHLINEGNTLKFVDDGTMDTDQEDSLVSTPKLAPQTTASANVSAQDSDIDLQDA>YBR203WMSSASRLQNVNIVSNNYSRYGTSVYDKLYHSNGSGSNNAGKNSTTVGKLSSISQKSRSKQRHGSNCSRSMSQSPLSTFKSPLSNQNQSSAPDDLASIGQRRSDDVTSLDNETIITMNSRKSRIKKKYKSLISTSSKKFMNKLYDHGASSDSFSIFSLKTSHSGKHENSRFEKLRKRKYHAWGKFADINDLPVEIIAKILSEFELGRDQKTLVRCLYVSKKFYKATKIVLYRLPYFTSTYRVAQFVTSLRLHPDNGAYVKVLDLSHLKPGIIGQDSKDSQGLDDDGHSRRHRRRRRRSTNTSLNLPPATPTSTISNEDDANSGLIKDDASNGSEVEDLALAGWRDWRYRNEPLYSSPLLNSFKLKKVVSRSSSITSTSSGNSTGVHSTRRQRSNSSVASITTSIMSSIYNTSHVSLSSTTSNTSNGNISSGSNLSRVSTAGSLKKASAKSTRSSPQKAKPISDITSSSWFRMRLSSRNRKARTANTINLKNSKDKSDDDFKVLKHDSGHPSNYRSSTLKFSIEQPFSTHHPYANKFLLKYAPYKDLPLGYILHMLNLCPNLVELNLSNLVICTDFKLINQRSERRRMTSSLLPAVQESSVSAGPEKDLEIVYMTDSGKGYEYYEGLSKKHSRSSSLGTNPSSWIGGQANWTDYPPPIDAQTKTREEHRRNNTLNNKNVVLKKLNPFEIFEMICNRNEEKGGYCSLTKVKMNDIVWCRQYMVKYFVMRSWRNDLDYKSMENNSYERHLFSFRDSGLDRNFSWACNAKLHEFVALMVMDHLSNLDDLGLEELFNIKSEKLYIKNYCCRDPDILEISNLFDIRYGAGSEADATSDSNLEAESLQFRLTILKTEKPTSFWLTKVSKDYVSLVVKLCVDDDIDMDKMKVGKPTLRIDSITHNLISRLKELRRVDLRRNVGENSYYAGSII>YBR204CMNMAERAEATKSWSCEPLSGKTLEEIVQNAENAADLVAYIRKPEVDLDFRLKFIAEHEEFFNEQLSDRNSRIRTCHNLSDKGIRGDTVFVFVPGLAGNLEQFEPLLELVDSDQKAFLTLDLPGFGHSSEWSDYPMLKVVELIFVLVCDVLRKWSTAVPNNDNVNPFNGHKIVLVGHSMGCFLACHLYEQHMADTKAVQTLVLLTPPKAHIEQLSKDKHIIQWALYGVFKLPWLFDVYRNKFDQVKGLQSSGIKQYFYQQGDDVKLKYRKFWQFKNNISNKSRTIIGYLLGWETVDWVKFNGVLTQTDMKQKIIIFGAEKDPIAPIENLEFYKQTINKECLRKVIILPDCSHNLCFDRPELVCENFQREVIDNSKL>YBR205WMSVHHKKKLMPKSALLIRKYQKGIRSSFIGLIIVLSFLFFMSGSRSPEVPIAQGTSVSRVASKDYLMPFTDKSQGVIHPVDDGKKEKGVMVTLARNSDLWNLVKSIRHVEDRFNNRYHYDWVFLNDQPFSDEFKRVTSALVSGKAKYGTIPKDHWSIPSWIDTEKFDEKRLAMGKLDIPYGSSVPYRHMCRFQSGFIWRHPLLEEYEWFWRVDTDITLFCDIQYDIFKFLKVNNKKYGFILSVSEYERTIPTLWETTKKFIKKNPKFLHKNNLMKFISNDDGDTYNMCHFWTNFEIGSLDFFRSDAYREYFDYLDSSGGFFYERWGDAPVHSIAASLFLDKSEIHFFDGLGFHHPDFTSCPIEQKIRLQNKCICEPSKDVTWTPDYFCTRKYFSAGNYKLPPGI>YBR207WMAFEDYFSFQIFFIFLRKSLEIVVIVSILLTIVKQDLSVEDDSPFEGSSSSAGLPSPNTNTNADSTTAFLQAGPSDGNAIGTSATAANNKSRPLNVEEEEEIYEYSNELRDQDRESDEHTADNVKLYQKLKIQILAGGAFGLLLCMLIGGAFVSIFYHIGTDLWTLSEHYYEGVLSLVASVIISVMGLFFLRMGKLREKFRVKLASIIYSKDNNLLGNKTQKGVKFSQKYSFFILPFITTLREGLEAVYSIGGIGIDQPLSSIPLSMVLATAISTVFGIFFFRYSSSLSLKICLVVATCFLYLIAAGLFSKGVWQLELQDYVNKCNGQDMSEVENGPGSYDISRSVWHVNCCNGEKDGGWMIFTAIFGWTNSATVGSVISYNAYWLVLKYALKLLMIEGKCGYIPYLPISWQKKRIMKRLSIAKASLDLKHHTSELNSSTSEPDSQRRSKDSSVPLIIDSSGSAN>YBR210WMSGTGLSLFVTGLILNCLNSICQIYFTILYGDLEADYINSIELCKRVNRLSVPEAILQAFISALFLFNGYWFVFLLNVPVLAYNASKVYKKTHLLDATDIFRKLGRCKIECFLKLGFYLLIFFFYFYRMVTALLENDANLIS>YBR212WMMSNVANASQRQENPYIIPLPPSSTVETSTEPPRTLWMGDLDPSFDEATIEEIWSKLDKKVIVKLIRAKKNLLIPCSSTSSSNNNTSEENAENQQSASNSTDQLDNSQMININGISFIDPSTTQLHHAGYCFVEFETQKDAKFALSLNATPLPNFYSPTTNSQTNPTFKRTFRLNWASGATLQSSIPSTPEFSLFVGDLSPTATEADLLSLFQTRFKSVKTVRVMTDPLTGSSRCFGFVRFGDEDERRRALIEMSGKWFQGRALRVAYATPRNNMMLQLQEQQQQQQQLQQQHQQLDQEDNNGPLLIKTANNLIQNNSNMLPLNALHNAPPMHLNEGGISNMRVNDSLPSNTYNTDPTNTTVFVGGLVPKTTEFQLRSLFKPFGPILNVRIPNGKNCGFVKFEKRIDAEASIQGLQGFIVGGSPIRLSWGRPSSSNAKTNSTIMGASQYMSSNGLRAPSAASSVDNSKQILEQYAEDKRRLFLHQQQQQQQQQQQDGNFSMEQMAHNNYYNYNNYDYHRNKNGSHSDLVNLQRSNVPYMQEDGALYPHQYSSPSYSLHPTGNQFSNATNNLPQFGNAMSISMQLPNGNSNKTASSMNTNPNTNMIMNSNMNMNMNVNPVPYGMGNGANMYDVSRMMTPPLNIAPNSNNSKSSIMNKHPNRNNVPPIHPSLLH>YBR213WMVKSLQLAHQLKDKKILLIGGGEVGLTRLYKLIPTGCKLTLVSPDLHKSIIPKFGKFIQNEDQPDYREDAKRFINPNWDPTKNEIYEYIRSDFKDEYLDLEDENDAWYIIMTCIPDHPESARIYHLCKERFGKQQLVNVADKPDLCDFYFGANLEIGDRLQILISTNGLSPRFGALVRDEIRNLFTQMGDLALEDAVVKLGELRRGIRLLAPDDKDVKYRMDWARRCTDLFGIQHCHNIDVKRLLDLFKVMFQEQNCSLQFPPRERLLSEYCSS>YBR214WMASTSNTFPPSQSNSSNNLPTSRHASIVEMLSTPPLLPHVQVNDTDDKEQPEESTPPTATAAAPGPGCAATPAPLRDEKPQFKLSAVPMTQTPSQCLSCVHAQKWQHIPLSQLIEQNKLIFVPGSISVEEAFNTLIKYHLNSIPVESFPGDMNCFTFDYNDLNSYLLLVLNKITVSNKQLTADCQNGKPVPVGEMVKLTPKNPFYKLPENESLSTVMGILGSGVHRVAITNEEMTKVKGILSQRRLIKYLWDNARSFTSLEPLLNSSLQDLHIGVLNIQSKPTSRQSRVISIQGEEPLIMGLYKMHVERISSIAVIDKQGNLLGNISVTDVKHVTRTSQYPLLHKTCRHFISVILNSRGLETGKDSFPIFHVYPSSSLARTLAKLVATKSHRLWIVQPPESSTSASSTNLTAANTAANAVSATAQSSANGATPMSKSSSSTSLNSHSPLMTAMEDPPSPRSSAIAIPPPSPASSTNTPNLFEKEYRTGKLIGVVSLTDIINLLARKQTGNKEVDPQSARRQRGSIAM>YBR217WMSRILESENETESDESSIISTNNGTAMERSRNNQELRSSPHTVQNRLELFSRRLSQLGLASDISVDQQVEDSSSGTYEQEETIKTNAQTSKQKSHKDEKNIQKIQIKFQPIGSIGQLKPSVCKISMSQSFAMVILFLKRRLKMDHVYCYINNSFAPSPQQNIGELWMQFKTNDELIVSYCASVAFG>YBR220CMEPKRKSGSLAKHDLPQFYLLIMLYLAQGIPVGLAFGTVPFLLKSLAKETSFTSLGIFSMATYPYSLKIIWSPIVDSLYNKRIGRRRSWIIPVQFVSGFVLWALGWCISQGIIFDGVDDAFHNRGNGTLHSVSIKNLTWWFGLLVFLCATQDIAVDGWALTILSKESLSYASTAQTIGLNIGYFMSFTIFLSLNSSDFANKYFRNIPLDHGFISLGGYMKFSGMLYIVITIYIIFCTKEKPYVEYLPKVEPINTSDGGSKPISIEYDDGDVVSTQNTSSIKYIYRCFIKVLKLKSVRSLAFIHMISKFAFQCNEAATNLKLLEQGFKREDLAVTVLIDLPFEIIFGYYVVKWSSDKDPMIRDNRRLRNSTGTNKVIKFLVGDAGVLTPWLWGFLGRLAAAVLGSYVVKQFPKDGEISTGYFCLVIFQHLLGSFMNTVQFIGISAFHTRVADPVLGGTYMTLLNTLSNFGGTWPRLIIMSMINYFTVYQCTIPGTNKVYVTHGGSMQACTELLNGTVTILRDGYYITNLICIVVGLFLYFGYLKRKILHLQSLPISSWRCT>YBR221CMFSRLPTSLARNVARRAPTSFVRPSAAAAALRFSSTKTMTVREALNSAMAEELDRDDDVFLIGEEVAQYNGAYKVSKGLLDRFGERRVVDTPITEYGFTGLAVGAALKGLKPIVEFMSFNFSMQAIDHVVNSAAKTHYMSGGTQKCQMVFRGPNGAAVGVGAQHSQDFSPWYGSIPGLKVLVPYSAEDARGLLKAAIRDPNPVVFLENELLYGESFEISEEALSPEFTLPYKAKIEREGTDISIVTYTRNVQFSLEAAEILQKKYGVSAEVINLRSIRPLDTEAIIKTVKKTNHLITVESTFPSFGVGAEIVAQVMESEAFDYLDAPIQRVTGADVPTPYAKELEDFAFPDTPTIVKAVKEVLSIE>YBR223CMSRETNFNGTKRKRSDVAEKVAQRWKSVRYSAEMENMAPVNSNNDSDDCVIVSESKIIDLTNQEQDLSERIETNDTAKGAVFKLMKSDFYEREDFMGEVEDMITLKDIFGTETLKRSILFSFQYELDFLLRQFHQNVENITIVGQKGTIMPIEARAMDATLAVILKKVKLIEITMPPFASHHTKLIINFYDNGECKIFLPSNNFTSMETNLPQQVCWCSPLLKIGKEGLPVPFKRSLIEYLNSYHLKDIDELITKSVEEVNFAPLSELEFVYSTPSKFQSSGLLSFYNKLEKLSAGTSASDTAKHYLCQTSSIGTSLSRARDENLWTHLMIPLFTGIMSPPAKDTAGRKKAEILPTNSLINEYSQRKIKPYIIFPTEQEFVTSPLKWSSSGWFHFQYLQKKSYYEMLRNKFKVFYKQDPAMVTRRRGTTPAHSKFYMHCATNSAGPCDASQVFKELEWCLYTSANLSQTAWGTVSRKPRNYEAGVLYHSRRLANTRKVTCRTFTRDRRGCAGNPTHVAVPFTLPVIPYDLAEDECFCLARHEND>YBR227CMLKSASQNFFRAYSSRIGRYAATASGKLAQSRLSNIPTPKALKKFLDEYIVGQEIGKKVLSVAVYNHYLRINDKQKKGELQRQRELMEREKIADDRDEPIFSGNSESKAGWRNLQRQFNLAGREVDEDLELSKSNVLVVGPSGSGKTLLATTLAKILNVPIAITDCTQLTQAGYIGEDVEVCIERLLVNAEFDVARAEKGIIVLDEIDKLAKPAASIGTKDVSGEGVQQSLLKIIEGHKVEITVKRPVKHDIDGQKNQTTTKKDEVFVVDTSNILFMIMGAFVGLDKHIVKRIEDMKKIQKAGESVESSNSKEVEKERAKKFRFSNTLEQVELDNGKKVCALDLTTPTDLVSFGLIPELIGRVPIITALQPLQRDDLFHILKEPKNALLDQYEYIFKQFGVRLCVTQKALKKVAQFALKEGTGARGLRGIMERLLLNVNYDCPGSNIAYVLIDEATVDSLQETEHSLASQVDVKYYSGDEKDSLIRDVSEEDKKLGVMLEKELGHSANIHTPTIPKRSLT>YBR228WMSQKIQQHQFPDFYCCYLLQSINKRQSFYVGSTPNPVRRLRQHNGKLAVGGAYRTKRDGSRPWEMIMIVRGFPSKIAALQFEHAWQHGYQTHYIAEKDRVVKHKAGGRTLHHKVALMKLLLKHEFFQRMNLIVEVFNIKAWEVWKQDKFFIERDRFPINIQINENALEEPKEKTVDVLMDHSDENLKVVEAVYTKVIENERNIFETFEKKLTTGVVRCEICEKEIDYTSEEQNLKPFVALCNNKDCGCVNHLKCLHRYFLDDEQLMVGRRNLIPRGGKCPKCDMFCDWTTLVKFSTRMKLAHGK>YBR230CMSATAKHDSNASPNSDSEDGHHHNNKKECAIEYLKARLNSASAVACGYLQAFVSKTQDFAKVCFLELQNPVVLVNLLLHSSVVCYLCNGYANHNARFLKGKPNSTVLATTAGALGLLTLDGIISKKYYSRYDKK>YBR231CMPEVETKIIPNEKEDEDEDGYIEEEDEDFQPEKDKLGGGSDDSDASDGGDDYDDGVNRDKGRNKVDYSRIESESGGLIKTRRARQAEEEYAKTHKYESLTVESIPAKVNSIWEELQEASKNRLLSSSGKVGSVLDGSKEARSTTAAQQEDKILIERNYKFAGETVHEKKWVSRSSAEGQEYLNSLKFKQQAPAAPVQLEKAVRTKSNESRQHLRRPLKRPPLLEQIISGGLRPKLTTLEKSQLDWASYVDRAGLNDELVLHNKDGFLARQEFLQRVGSAEDERYKELRRQQLAQQLQQDSEAS>YBR233W-AMEHNLSPLQQEVLDKYKQLSLDLKALDETIKELNYSQHRQQHSQQETVSPDEILQEMRDIEVKIGLVGTLLKGSVYSLILQRKQEQESLGSNSK>YBR233WMSTETTKPSITTTPTTVLVSPNTLKRKKGEDTSEEQLEAEIKRVALKDADSHSDNDHDSPDNVPSDVHLRMLCLVKHASLIVGHKGATISRIKSETSARINISNNIRGVPERIVYVRGTCDDVAKAYGMIVRALLEEHGNEDNGEDIEISINLLIPHHLMGCIIGKRGSRLREIEDLSAAKLFASPNQLLLSNDRILTINGVPDAIHIATFYISQTLLNFQMESPQKNVKRSIYYQPTQFNSVLIDHSQPNTIFHQRNHQYHPSDKLLSYKPNKNLPISSTLLSMATPQYTTASVANATAFQPNFVIPNVTVLDGPVISPAPGNHLLMNFVQQEIFIDEKFVGNVIGKDGKHINSVKESTGCSIIIQDPVEGSSERRLTIRGTFMASQAAIMLISNKIEIDRSNAERKRRSPL>YBR236CMSTKPEKPIWMSQEDYDRQYGSITGDESSTVSKKDSKVTANAPGDGNGSLPVLQSSSILTSKVSDLPIEAESGFKIQKRRHERYDQEERLRKQRAQKLREEQLKRHEIEMTANRSINVDQIVREHYNERTIIANRAKRNLSPIIKLRNFNNAIKYMLIDKYTKPGDVVLELGCGKGGDLRKYGAAGISQFIGIDISNASIQEAHKRYRSMRNLDYQVVLITGDCFGESLGVAVEPFPDCRFPCDIVSTQFCLHYAFETEEKARRALLNVAKSLKIGGHFFGTIPDSEFIRYKLNKFPKEVEKPSWGNSIYKVTFENNSYQKNDYEFTSPYGQMYTYWLEDAIDNVPEYVVPFETLRSLADEYGLELVSQMPFNKFFVQEIPKWIERFSPKMREGLQRSDGRYGVEGDEKEAASYFYTMFAFRKVKQYIEPESVKPN>YBR237WMETIDSKQNINRESLLEERRKKLAKWKQKKAQFDAQKEHQTSRNDIVTNSLEGKQTTEKFTERQERVKEELRKRKNEFRKSDEPVSVKPSKKKSKRSKVKKKISFDFSDDDDSEIGVSFRSKEHIQKAPEHDNEKDPLDEFMTSLKEEKMSNSKGMYDRGDILDVEDQLFELGGTDDEDVEDNTDNSNIAKIAKLKAKKRVKQIYYSPEELEPFQKNFYIESETVSSMSEMEVEELRLSLDNIKIKGTGCPKPVTKWSQLGLSTDTMVLITEKLHFGSLTPIQSQALPAIMSGRDVIGISKTGSGKTISYLLPLLRQVKAQRPLSKHETGPMGLILAPTRELALQIHEEVTKFTEADTSIRSVCCTGGSEMKKQITDLKRGTEIVVATPGRFIDILTLNDGKLLSTKRITFVVMDEADRLFDLGFEPQITQIMKTVRPDKQCVLFSATFPNKLRSFAVRVLHSPISITINSKGMVNENVKQKFRICHSEDEKFDNLVQLIHERSEFFDEVQSENDGQSSDVEEVDAKAIIFVSSQNICDFISKKLLNAGIVTCAIHAGKPYQERLMNLEKFKREKNSILLCTEVLSRGLNVPEVSLVIIYNAVKTFAQYVHTTGRTARGSRSGTAITLLLHDELSGAYILSKAMRDEEIKALDPLQAKELQEMSAKFESGMKKGKFRLSKGFGGKGLENIKSKREEAQNKDLELKKNDKRSDDLEKKISNPREGHDSVSESSALIPRLNYELFKESTDGSIIFYAKVYINDLPQIVRWEATKNTTLLFIKHETGCSITNKGKFYPEGKEPKNENDEPKLYLLIEGQDEKDIQLSIELLEQKVKEGVVKAASLSLKSTKY>YBR239CMCTPDENDYKTSTDPDTSANTNHTLEKKKRKKRKNTNVACVNCSRLHVSCEAKRPCLRCISKGLTATCVDAPRKKSKYLAGIPNRELPMNIQPDLPPRKIMIPIYNNSSNSSLNVNNMGEQQKFTSPQHIVHKAKFLSNAADSEYSILSNIIYQDTLSNKIPIDILYSNTNSTSNSTIGNSSNNSPTGTNTSPEETEMEKIRQLYSEQRANIPPHPYPSSNQNVYSILLGPNSAKIVASQVNLFANHFPLVPVDSADNSLNFKRLLPRDPSEKSSQINWDSSINQYYLNSETVTFPELAIPLKRRKNHLVSVSLESCSPDAANIKSNVEWEHSLRYSTPMEIYTSINAPFSHTPGFHHLLVYLKHRFNQQDLVKMCRSIAEFRPIFIACSVTLTEEDMIFMEQCYQRTLLEYVKFIAQIGTPTCIWRRNGQISYVNEEFEILCGWTREELLNKMTFIVEIMDDESVRDYFKTLSKVAYRDFRGSEKMKVCRLLSPIKGKIIHCCCMWTLKRDVSGLPLMILGNFMPILN>YBR240CMVNSKRQQRSKKVASSSKVPPTKGRTFTGCWACRFKKRRCDENRPICSLCAKHGDNCSYDIRLMWLEENIYKVRKHSLISSLQARKSKSKPLCQKISKSRFKQMTHFRQLSPPTSDCEDSVHEASKETTLPNDNTFTISVRRLKIYNNAVASVFGSMTNRDYTQKRIDKKLDELLNMVENDISVVNLNCSKHGPYSVFRANPAAVTSALTDQLPSPGHSMSSAEETTTAALSSPPEDSTSLIDIIQGKIFGILWFNCYGNMILNRQEYTTWFINKMRNSLTTEFIRFLGKIIDDPDINMASCLFKECIARWSCVDWQSIAITMLVIIHGYTCPNLTKLLRVWFLQQKLLRFSMYPLVNFIINNTQDLDVLYHCNGLLGNADLFEDPYQDELTSELHVLVTERLVNSWKDTILQQLCSCQDTTLSCSQLRYWQLQLKCNQQFYKDVYAMQD>YBR242WMTATITNKKSCSGSVEAGKTRLTTEWKPESQVPQYVKNELSKPHPNYILAFLNVVQQLKIQRRTGYLDLGIKECESISDHMYRLSIITMLIKDSRVNRDKCVRIALVHDIAESLVGDITPVDPIGKEEKHRREWETIKYLCNALIKPYNEIAAKEIMDDWLAYENVTSLEARYVKDIDKYEMLVQCFEYEREYKGTKNFDDFFGAVASIKTDEVKGWTSDLVVQRQKYFADLTQSITK>YBR243CMLRLFSLALITCLIYYSKNQGPSALVAAVGFGIAGYLATDMLIPRVGKSFIKIGLFGKDLSKPGRPVLPETIGAIPAAVYLFVMFIYIPFIFYKYMVITTSGGGHRDVSVVEDNGMNSNIFPHDKLSEYLSAILCLESTVLLGIADDLFDLRWRHKFFLPAIAAIPLLMVYYVDFGVTHVLIPGFMERWLKKTSVDLGLWYYVYMASMAIFCPNSINILAGVNGLEVGQCIVLAILALLNDLLYFSMGPLATRDSHRFSAVLIIPFLGVSLALWKWNRWPATVFVGDTYCYFAGMVFAVVGILGHFSKTMLLLFIPQIVNFIYSCPQLFKLVPCPRHRLPKFNEKDGLMYPSRANLKEEPPKSIFKPILKLLYCLHLIDLEFDENNEIISTSNMTLINLTLVWFGPMREDKLCNTILKLQFCIGILALLGRHAIGAIIFGHDNLWTVR>YBR244WMTTSFYDLECKDKKGESFKFDQLKGKVVLIVNVASKCGFTPQYKELEELYKKYQDKGFVILGFPCNQFGKQEPGSDEQITEFCQLNYGVTFPIMKKIDVNGSNADSVYNYLKSQKAGLLGFKGIKWNFEKFLVDSNGKVVQRFSSLTKPSSLDQEIQSLLSK>YBR246WMDSIQESDVLNAVKTKLPPCCLRIFRNKIILVGTYDLDKSTGYRSGSLDVFTMDLKLLCSNNTYGAILDLKLSPFDDTLICTAHSTGNIMLWRIRCTDKDDFQSNELDIHAIANLQLFEKDVLIASCHFSPLDCKKLLVTNTAGEAATIDIRTLSVQFTASAIAQAYSKLDKIDYEVQGATEKVIHVESGQFLKPHELECWTAEFGSLQPFQDVVFTGGDDSRIMAHDLRSKEFIWSNNRIHDAGVVSIKCSQPNFRNNKPTSIITGSYDDNIRSLDLRMMGESIFPGANVPTVNKLACDLGGGVWRFVESPIDQEQSHHNGSDRLLVCCMYNGAKVVTMNDNSDEYFQIQHYLKKGHDSMCYGGDWSNSLIATCSFYDNSLQTWIV>YBR247CMARASSTKARKQRHDPLLKDLDAAQGTLKKINKKKLAQNDAANHDAANEEDGYIDSKASRKILQLAKEQQDEIEGEELAESERNKQFEARFTTMSYDDEDEDEDEDEEAFGEDISDFEPEGDYKEEEEIVEIDEEDAAMFEQYFKKSDDFNSLSGSYNLADKIMASIREKESQVEDMQDDEPLANEQNTSRGNISSGLKSGEGVALPEKVIKAYTTVGSILKTWTHGKLPKLFKVIPSLRNWQDVIYVTNPEEWSPHVVYEATKLFVSNLTAKESQKFINLILLERFRDNIETSEDHSLNYHIYRAVKKSLYKPSAFFKGFLFPLVETGCNVREATIAGSVLAKVSVPALHSSAALSYLLRLPFSPPTTVFIKILLDKKYALPYQTVDDCVYYFMRFRILDDGSNGEDATRVLPVIWHKAFLTFAQRYKNDITQDQRDFLLETVRQRGHKDIGPEIRRELLAGASREFVDPQEANDDLMIDVN>YBR248CMPVVHVIDVESGNLQSLTNAIEHLGYEVQLVKSPKDFNISGTSRLILPGVGNYGHFVDNLFNRGFEKPIREYIESGKPIMGICVGLQALFAGSVESPKSTGLNYIDFKLSRFDDSEKPVPEIGWNSCIPSENLFFGLDPYKRYYFVHSFAAILNSEKKKNLENDGWKIAKAKYGSEEFIAAVNKNNIFATQFHPEKSGKAGLNVIENFLKQQSPPIPNYSAEEKELLMNDYSNYGLTRRIIACLDVRTNDQGDLVVTKGDQYDVREKSDGKGVRNLGKPVQLAQKYYQQGADEVTFLNITSFRDCPLKDTPMLEVLKQAAKTVFVPLTVGGGIKDIVDVDGTKIPALEVASLYFRSGADKVSIGTDAVYAAEKYYELGNRGDGTSPIETISKAYGAQAVVISVDPKRVYVNSQADTKNKVFETEYPGPNGEKYCWYQCTIKGGRESRDLGVWELTRACEALGAGEILLNCIDKDGSNSGYDLELIEHVKDAVKIPVIASSGAGVPEHFEEAFLKTRADACLGAGMFHRGEFTVNDVKEYLLEHGLKVRMDEE>YBR249CMSESPMFAANGMPKVNQGAEEDVRILGYDPLASPALLQVQIPATPTSLETAKRGRREAIDIITGKDDRVLVIVGPCSIHDLEAAQEYALRLKKLSDELKGDLSIIMRAYLEKPRTTVGWKGLINDPDVNNTFNINKGLQSARQLFVNLTNIGLPIGSEMLDTISPQYLADLVSFGAIGARTTESQLHRELASGLSFPVGFKNGTDGTLNVAVDACQAAAHSHHFMGVTKHGVAAITTTKGNEHCFVILRGGKKGTNYDAKSVAEAKAQLPAGSNGLMIDYSHGNSNKDFRNQPKVNDVVCEQIANGENAITGVMIESNINEGNQGIPAEGKAGLKYGVSITDACIGWETTEDVLRKLAAAVRQRREVNKK>YBR251WMFKRQLSTSVRYLQHYDESLLSRYYPESLLKSIKLAQQTIPEDTKFRVSRNVEFAPPYLDDFTKIHPFWDYKPGMPHLHAQEENNNFSIFRWDQVQQPLPGEGNILPPGVSLPNDGGRKSKSADVAAGLHKQTGVDPDYITRKLTMKPLVMKRVSNQTGKGKIASFYALVVVGDKNGMVGLGEGKSREEMSKAIFKAHWDAVRNLKEIPRYENRTIYGDIDFRYHGVKLHLRSAKPGFGLRVNHVIFEICECAGIKDLSGKVYKSRNDMNIAKGTIEAFTKAQKTLDEVALGRGKKLVDVRKVYYSS>YBR252WMTATSDKVLKIQLRSASATVPTKGSATAAGYDIYASQDITIPAMGQGMVSTDISFTVPVGTYGRIAPRSGLAVKNGIQTGAGVVDRDYTGEVKVVLFNHSQRDFAIKKGDRVAQLILEKIVDDAQIVVVDSLEESARGAGGFGSTGN>YBR253WMSNQALYEKLEQTRTILSVKLAELINMTTIADRNDDDEGSFAQENSELAVATTSVMMVNNQTMQLIKNVQDLLILTRSIKEKWLLNQIPVTEHSKVTRFDEKQIEELLDNCIETFVAEKTT>YBR254CMPQYFAIIGKKDNPVYEIEFTNAENPQGFPQDLKELNPFILHASLDIVEDLQWQINPTSQLNGNGGNGSNGGGGFLRSRAVNNTDNCYLGKVDHFYGLAITAYISYSGMKFVMIHGNSANSSVVIDDNNMRSFYQEVHELYVKTLMNPFYKITDPIRSPAFDSRVRTLARKHLSK>YBR256CMFTGIVECMGTVLENNPYDDSESGGQGVSITIGNAGSILTDCHVGDSIAVNGVCLTVTEFNNDSFKVGISPETIKRSNVASWIQGTQVNLERAVSQDVRFGGHYVQGHVDTVANIVSRRPEGNSIIFGFQLRDQEYFKYIVEKGFICIDGTSLTIIKVDPLSQGGAFYISMIKHTQDNVIMPLKKIGDEVNIEVDLTGKIIEKQILLTLENQISKKDSTLNTMISNIIEEKVRNYLNK>YBR257WMDRTQTFIKDCLFTKCLEDPEKPFNENRFQDTLLLLPTDGGLTSRLQRQQRKSKLNLDNLQKVSQLESADKQLEKRDYQRINKNSKIALREYINNCKKNTKKCLKLAYENKITDKEDLLHYIEEKHPTIYESLPQYVDFVPMYKELWINYIKELLNITKNLKTFNGSLALLKLSMADYNGALLRVTKSKNKTLIGLQGIVIWDSQKFFIMIVKGNIIDEIKCIPKKGTVFQFEIPISDDDDSALRYSILGDRFKYRSVDRAGRKFKSRRCDDMLYYIQN>YBR258CMAYNQEDSKRLSDKYKKEGHFDKLKREILSNPWNNTEENSESFEQALRKRVASTVKEMVNEDEELIFKNRGLTSALIESQLVKDNYLKLGSKMEGDNGDGEKKFDLDVYVRSKLQDPKLLEMIKGQLQETLNSYEEEANGST>YBR259WMSIDEAVARYRDVIGNLATGNLRRIVIQSEKLAQIIASSKGTVRFHHKTRSGKTVIYKCIKKALLSSVASLSSEFSSETDVQQFLHLNYIYQSHFQALSGQINKYCGMKKYYELKFAAIDYLETEVQTTGLTLSRFWVASLDEFIKKERWPDNGSNFQIFYKLMAEYSSWKWDSDDKRQLQFMYEFRMKLKECLVKFYENFDLQKSSDPLKELIIPWEKIVYVANCIDAFTGEQVRIDGAELIWTSKNLVFSSISSAVLRLNDLQNMFSAFRPYGEEALVQDFAHIRSLKWDSNDKVESLIRALIFNDMFPYFNKEQVDTKADGIFFLRLLRKNFKEHINDVKDFHIQVIKYLNSQFKNNYSTLMTSSKTQDRRKSHNMPSSILDDGNKIGMHVSPIDEYSHFIDNDEPLWRDKVYPKIYTNEQTPTPDASAIFDSHKIYAIISLLRYYLPEKRKFFRIYYLPSIFKRILYYGAKFAQLYFMEGCLERLVIESLQILEPSLVHAINNLIKSSIESLKNVTVTSDDKTSSGVIILSYKEFKSLSEVNKDFNEPFWPNQSIANSWPDFANKQLKRGQILQDAFAFHLFEIELPIIIDTTRNTHLKLVSNMCTTSILYLYNEVDSLSLTSIQEKLAVLPTSKRNEILLYNLNRLTKLKLLLLKENEKGQKFYAFNFKYKRDGQKTSLIRLI>YBR260CMEETAKKPASATVSAKSSHDGGTDDLAHLFSTPEIKKVLNSDVAINALLSRLKQSLLTCEEFMKFIRKKYAFEEEHVQELSKQYKHFFNIQGSTNSSLKKMIHEVLGFDGKMAQVKQSYITALQKMYSEISSLLLTMTKLRKSVKENSKRLEKDVSDAIHSAEKAQSRYNSLCQDWDKLRMTDPTKTKLTLRGSKTTKEQEEELLRKIDNADLEYKQKVDHSNSLRNTFITKERPRIVQELKDLILEIDTAMTIQLQKYTIWTENLVLNTGVTISPLDSTKSMKSFAGSVSNERDLYSFLNKYNQTGKHSLLINKNLIPVSYKKHPSMNHGQKNKSPPKFAVDPSRNSIPKRMISTHNESPFLSSSSNTAAVPNANLNSATPSLNTNKQLPPTMASSISSTSNAAGAMSPSSSIVTSDTTSSITKTLDPGNNSPQIPEELINSLDSDRPISHIQTNNNMPPGVQKNFKTFGVPLESLIEFEQDMVPAIVRQCIYVIDKFGLDQEGIYRKSANVLDVSKLKEEIDKDPANISMILPSKPHSDSDIYLVGSLLKTFFASLPDSVLPKALSSEIKVCLQIEDPTTRKNFMHGLIYNLPDAQYWTLRALVFHLKRVLAHEAQNRMNLRALCIIWGPTIAPANPDDANDVNFQIMAMEVLLEVSDQAFEPE>YBR261CMDVPADSHIKYEDAIDYWTDVDATVDGVLGGYGEGTVVPTMDVLGSNNFLRKLKSRMLPQENNVKYAVDIGAGIGRVSKTMLHKHAAKIDLVEPVKPFIEQMHVELAELKDKGQIGQIYEVGMQDWTPDAGKYWLIWCQWCVGHLPDAELVAFLKRCIVGLQPNGTIVVKENNTPTDTDDFDETDSSVTRSDAKFRQIFEEAGLKLIASERQRGLPRELYPVRMYALKPMPN>YBR262CMSKLGPLARSVKWTLSVGVIGSVFYLYRYSNNGYFYDHDATWLKQDHQVQDLVDRKEVVPGETRNRKLVVTDDGTAWSRTMGESIKDIWNEQIRNSVDWIYSWGKN>YBR263WMFPRASALAKCMATVHRRGLLTSGAQSLVSKPVSEGDPEMFDILQQERHRQKHSITLIPSENFTSKAVMDLLGSELQNKYSEGYPGERYYGGNEIIDKSESLCQARALELYGLDPAKWGVNVQPLSGAPANLYVYSAIMNVGERLMGLDLPDGGHLSHGYQLKSGTPISFISKYFQSMPYHVDHTTGLIDYDNLQVLAKAFRPKVIVAGTSAYSRLIDYARFKEISQGCGAYLMSDMAHISGLVAANVVPSPFEHSDIVTTTTHKSLRGPRGAMIFFRKGIKSVTKKGKEIPYELEKKINFSVFPGHQGGPHNHTIGAMAVALKQAMSPEFKEYQQKIVDNSKWFAQELTKMGYKLVSGGTDNHLIVIDLSGTQVDGARVETILSALNIAANKNTIPGDKSALFPSGLRIGTPAMTTRGFGREEFSQVAKYIDSAVKLAENLKTLEPTTKLDARSRLNEFKKLCNESSEVAALSGEISKWVGQYPVPGDI>YBR264CMEATIKVVLLGDSSVGKTSIVTRLKSGKFLAKHAATIGAAFITKTIEVPSNDSSTEKRIHMEIWDTAGQERYKSLVPMYYRDANIALIVFELGDVSSLQCAKTWFQDLQDRAQGTQVIIVGNKYDLVCEEHSGEVTIPAELQGLPYVAVSAKTGYNFDTLNKIIISLVPESQFKTLSKNNEQGNILEINKKKSGSGCIC>YBR265WMKFTLEDQVVLITGGSQGLGKEFAKKYYNEAENTKIIIVSRSEARLLDTCNEIRIEAHLRRETTDEGQVQHKLAAPLDLEQRLFYYPCDLSCYESVECLFNALRDLDLLPTQTLCCAGGAVPKLFRGLSGHELNLGMDINYKTTLNVAHQIALAEQTKEHHLIIFSSATALYPFVGYSQYAPAKAAIKSLVAILRQELTNFRISCVYPGNFESEGFTVEQLTKPEITKLIEGPSDAIPCKQACDIIAKSLARGDEDVFTDFVGWMIMGMDLGLTAKKSRFVPLQWIFGVLSNILVVPFYMVGCSWYIRKWFRENDGKKAN>YBR267WMSSSGVYTCNSCVLTFDSSDEQRAHMKSDWHRYNLKRRVAQLPPISFETFDSKVSAAAASTSKSAEKEKPVTKKELKRREKQALLEKKKKLLEIARANMLENMQKSQEGNTPDLSKLSLQENEENKEKEEPKKEEPEQLTEEEMAERVMQENVRNRVDIPLEQCLFCEHNKHFKDVEENLEHMFRTHGFYIPEQKYLVDKIGLVKYMSEKIGLGNICIVCNYQGRTLTAVRQHMLAKRHCKIPYESEDERLEISEFYDFTSSYANFNSNTTPDNEDDWEDVGSDEAGSDDEDLPQEYLYNDGIELHLPTGIKVGHRSLQRYYKQDLKPEVILTEGQGTLVAAETRSFLPAFDKKGVQTQQRVWQTERFDKKRLDKRSAKFVNNQPHYRDQLLQ>YBR268WMLARSLGYRLISTSRILYNKPTVKSVVSSCPAGTSLNLNIWKSGKDAVALEDKEYPNWLWSVLDSDHVVEHAAEDPEGQALLKRRKNIRKANRQRIKQNNFLSQL>YBR269CMLCAIKSTGYRYPRTGALNLLRGRPFNMATRKITTERIPGPPKLPREEQEEFERLQRIATSQEAIDQYNAQATGDRTKESLNSPLLTKNDIGSFSPEFSKTIPEFEGDVNPKTGEVGGPKQDPLRHGDYSFNGRVTDF>YBR270CMATDLNRKRSATSGSLSVTNPNIKATNRKPARVYSVSSDIVPQALTHPDEDVHLKTSKSPHDAAPRWSQVGFQSIFHDGSNARRSTDSIEEEYSQGTENNDGHSEIGSSSSNRMEGNTTSNDSLFSSNSRGNKRRLSIFTNSKDNMRNRSRRASKNYGTVITGTSSNNISRSGSKLFHTKSNMSVNSLQSSLSTGHSHSNKGSNVFSKMAKKLLPYKPHNSIGKDDVEPVVPSPFSKFLHSSYGKHRSPVQFIHTSTGGLIDSGKSVYSFNPSINNNPNDTALSLIQDDAFDATNVSLLHDLLKNLPSLIANYKSFTVQELFVLEGNIWGIYCSIVVELFKNKRVWQLPAKIEDIDRLLEFYITLKTQTKAAVTHSRFLAEIEEFITTSLYILENQIVFNYANEDTVNTALKRVGIIWKVFYQQVYYDMMAVLLPFEKSFQKNSNYWLDGYLSEPSRYAPSIDVLLLKCFRDSIILPYYESFLHTNDGASKSFQRYIFSEEEQNGVTEEDKLTLLQCFGILNTIKGNSRNQRIIGELLEGIRMSI>YBR271WMFDPLDLYTPDDIQVEALQFNLAEREPKDPCSPQRDEILTAVDEEESDDDDTIIDNLDLPSVKYAPPEVILCILILLKPDRQVNFNQETGKNKSVLEVCKSHGLEPDLLKRLLTWYTEEWPNKRLNSLEKICNKIPMLRFTVSKELLLGYYTSVLKKYNNSCGLNEEIIQELLKELSSRISENCGRTAQPSIVRYFELRNLSTSIPLHEPSLTADNLGWKTWGSSLILSQLVVDHLDYLHTTNVNMLANSDIKQIKVLELGAGTGLVGLSWALKWKELYGTENIEIFVTDLPEIVTNLKKNVSLNNLGDFVQAEILDWTNPHDFIDKFGHENEFDVILIADPIYSPQHPEWVVNMISKFLAASGTCHLEIPLRAKYAKEREVLKLLLKESDLKVVEERHSEGVDDWGAVKYLYRQIVRN>YBR272CMSEKETNYVENLLTQLENELNEDNLPEDINTLLRKCSLNLVTVVSLPDMDVKPLLATIKRFLTSNVSYDSLNYDYLLDVVDKLVPMADFDDVLEVYSAEDLVKALRSEIDPLKVAACRVIENSQPKGLFATSNIIDILLDILFDEKVENDKLITAIEKALERLSTDELIRRRLFDNNLPYLVSVKGRMETVSFVRLIDFLTIEFQFISGPEFKDIIFCFTKEEILKSVEDILVFIELVNYYTKFLLEIRNQDKYWALRHVKKILPVFAQLFEDTENYPDVRAFSTNCLLQLFAEVSRIEEDEYSLFKTMDKDSLKIGSEAKLITEWLELINPQYLVKYHKDVVENYFHVSGYSIGMLRNLSADEECFNAIRNKFSAEIVLRLPYLEQMQVVETLTRYEYTSKFLLNEMPKVMGSLIGDGSAGAIIDLETVHYRNSALRNLLDKGEEKLSVWYEPLLREYSKAVNGKNYSTGSETKIADCR>YBR273CMLEALFRDSVEEAINDSIKEGVVLAVYNTARDDQWLKSWFKGDDVSLDTLAEHSIWLRLVKDTEQFQLFEQVFPNVVVPSIYLIRAGKIELIIQGEDDRHWEKLLACIGIKDKKAGESSSRETNPGLAREEKSSRDVHRKNARERIAETTLEIQRREQLKQRKLAEEERERIIRLVRADRAERKALDETHHRTLDDDKPLDVHDYIKDAQKLHSSKCVLQIRMTDGKTLKHEFNSSETLNDVRKWVDVNRTDGDCPYSFHRGIPRVTFKDSDELKTLETLELTPRSALLLKPLETQNSGLSVTGMEGPSLLGRLYKGFSTWWHNDKDPEVTSQREETSKPNRHEVRSSTPLSGAASSSCFQYNNVREPVQSSAHASPMLTPSGTRYPSETNLTTSRSVSPNVFQFVNNDHQEDPEDPTTFNGNNVHLEKKKDEDKK>YBR274WMSLSQVSPLPHIKDVVLGDTVGQGAFACVKNAHLQMDPSIILAVKFIHVPTCKKMGLSDKDITKEVVLQSKCSKHPNVLRLIDCNVSKEYMWIILEMADGGDLFDKIEPDVGVDSDVAQFYFQQLVSAINYLHVECGVAHRDIKPENILLDKNGNLKLADFGLASQFRRKDGTLRVSMDQRGSPPYMAPEVLYSEEGYYADRTDIWSIGILLFVLLTGQTPWELPSLENEDFVFFIENDGNLNWGPWSKIEFTHLNLLRKILQPDPNKRVTLKALKLHPWVLRRASFSGDDGLCNDPELLAKKLFSHLKVSLSNENYLKFTQDTNSNNRYISTQPIGNELAELEHDSMHFQTVSNTQRAFTSYDSNTNYNSGTGMTQEAKWTQFISYDIAALQFHSDENDCNELVKRHLQFNPNKLTKFYTLQPMDVLLPILEKALNLSQIRVKPDLFANFERLCELLGYDNVFPLIINIKTKSNGGYQLCGSISIIKIEEELKSVGFERKTGDPLEWRRLFKKISTICRDIILIPN>YBR276CMVLEVPSITPGELHDLMRLHQDAEWPECKKMFPWAHDISFGQPPDFPHSLAIVKSQSDANNSALLRNSLEVNDIFQSWKVRTSFHREGDTCETGNDSNGFQYPNNTKELLNLLKFQIRQLELQVDDVALENAATYCHNHSILPFLKVDPRGLSLELKRYSRNKVGSNTTLKRSGQDVWGRRGLFRRFDLQCAKMIEMVDNIVIYCSRTGGSTDMQTESAPACSHEGNCPNCTTLALLLQICLMFVQKGYVGSGGSLYKTNLFICTYQNFNTDIPQTLIGTPLLDNEFFKNNTPLNLCSSPSEIVCFNNVDKNMVLCEKLELNKLTSATRLEETGLICGNTTDWHNYQIIKKNNISLTHRFEENTSIVNLKSLNYDTDNPTTSISQLYNIPNTKEVWKLIIKCTSNSQMPSLTKIRTYLDLLLDDDASKSQEHLHLTFPASGSIGLGNLNIQSVEILLNVCYLIFQVSQVQELLTFMYCEDGYTETSLLLTAYIIFHFNIPLQDALLRIHPRPFFLFPSDLQILGHLQPVLREFSPQNGSNLKLYANALKFRDKSFQLHISSELFSSIFFMKIPLESNFVNLKGPLPSRILRHLYLGSLDHAQNPALLKSLGITHIVSVGEVVSWTLNKDKIAHPVRPHRAITMTNTNEVAGNTTCNKSRNRADTVVSDKQENGSNVVISENSGFQICQIENLDDNGKDPLFHQIDKVLDFISNSEATGGKVLVHCMVGVSRSATVCIAECMRYLQCDLASAYLFVRVRRLNVIIQPNLFFVYELFKWWKKHYNREKDKTMDWHIICRGIAEVNMKYT>YBR278WMSNLVKEKAPVFPISKVKKIAKCDPEYVITSNVAISATAFAAELFVQNLVEESLVLAQLNSKGKTSLRLSLNSIEECVEKRDNFRFLEDAIKQLKKNSALDKKRELNMQPGRSDQEVVIEEPELHEDDGVEEEEEEDEVSEEEEPVHNEELLDDSKDQQNDKSTRSVASLLSRFQYKSALDVGEHSDSSDIEVDHTKSTDP>YBR279WMSKKQEYIAPIKYQNSLPVPQLPPKLLVYPESPETNADSSQLINSLYIKTNVTNLIQQDEDLGMPVDLMKFPGLLNKLDSKLLYGFDNVKLDKDDRILLRDPRIDRLTKTDISKVTFLRRTEYVSNTIAAHDNTSLKRKRRLDDGDSDDENLDVNHIISRVEGTFNKTDKWQHPVKKGVKMVKKWDLLPDTASMDQVYFILKFMGSASLDTKEKKSLNTGIFRPVELEEDEWISMYATDHKDSAILENELEKGMDEMDDDSHEGKIYKFKRIRDYDMKQVAEKPMTELAIRLNDKDGIAYYKPLRSKIELRRRRVNDIIKPLVKEHDIDQLNVTLRNPSTKEANIRDKLRMKFDPINFATVDEEDDEDEEQPEDVKKESEGDSKTEGSEQEGENEKDEEIKQEKENEQDEENKQDENRAADTPETSDAVHTEQKPEEEKETLQEE>YBR280CMSEVESREKEPDAGLSPDIVQATLPFLSSDDIKNLSQTNKYYNTLLDFDHSKILWHELFHKAFGTLKTNDEPFQGRNSAEFKTCTETILREAFPTLSWQEVYQLRAYDAKFYSWGYLKHGRLGYTASSNNELVATSLNGPSPRFKYGVNTPTEVPWFNSRTTSRTSNFTPSEDPLSAIKKDGDEIIAQVSSGGFSFQILTESGNLYSSGSTFSGGLKGPGPSGSQHDYNPFREMIHNMERSYPRITSRSNGSTVNTTGTFSGRRMSGSHPSTAYEPGNATTAQHITIDSGGAPAASPGGSHSGVPRTTMPSMGPHENIYSQIEMLERSANKAVPGNNHIRRMFARNSFPLYSGRDENLGSFNDIQFVAVSSGRSHFLAMDTDNNIYSWDSTESDQGVKIEFANLPSRATNPILKIASGWNFNCCYIYKVGLVAWKERDAIQKGESFAFAKYEIVPNTNDVNGDSRIVDFACLQDNCVFFINNNGDKLWKYHNGLNQIVDLNIVGKLCKINACFASLVLFTDTHCYTLKVTNGDVDKDSLTELDINENVISVASGDYHTVALTERGHLYSWGIESQDCGCLGLGPSEKIVNELHIGNWEGQRNIRVVKPTKIELPEDYICVSVTAGGWQTGALIIKKH>YBR281CMYDSRGVALHSELIHRWNHAFSILSIVAFPKKRLLFAGSQDSKILVFDLPTYNLIHTIRLGESQEETHTRSSVLCLTGSEDENFLFSGGADSLVRIWSIGEKTIRDDFLPVTEIATVYSVTDIGDIFSLAYLDSLETIVFGCQNASLLYVENLIQKIEKKSSDGVENINKLPHRRYDKFFDSLGPTGYSSNSLSQTSLTSLQENCGAAIIEVPSENIIKYAHYGFIYSINKLCPRFNQLLEKSSRTSGAEHIISSAGDGISKLWEFSKDKGQNTVKISLINDKIDNEDSVISQTIEFPFLYCGLTDGIIKIWDLNTQQIISTLKTKHESDVISISVYMDHVFAIDESGITHFYQNQVNHWNPQQGKILSSEIFSKSNAGSVSLLTGGSDGSLTLWDITSLLSAVPLSSNSPINASSTLQTTNLWAAYQSASLNNEEMLNTLRELISFQTVSQSKDTTNTLSLRRCAIYLQQLFLKFGATNSQLFPLPDGGNPVVFAYFQGNGKVSQVKGAKKKRILWYGHYDVISSGNTFNWNTDPFTLTCENGYLKGRGVSDNKGPLVSAIHSVAYLFQQGELVNDVVFLVEGSEEIGSASLKQVCEKYHDIIGKDIDWILLSNSTWVDQEHPCLNYGLRGVINAQIKVWSDKPDGHSGLNGGVYDEPMVNLVKIVSKLQNEQNEIMIPNFYSPLKDLTEEEYQRFQKITELANIDENTTVQDLITNWTKPSLSMTTVKFSGPGNITVIPKSVTMGISIRLVPEQSVEQVKRDLKAYLEESFKQLKSQNHLEIKVLNEAEGWLGDPTNHAYQILKDEITTAWDVEPLLVREGGSISCLRMLERIFDAPAVQIPCGQSTDNGHLANENLRIKNWSNLTEILSKVFNRL>YBR282WMKGSPISQFSKTSINALTRPWKKYRDGELFYGLSKVGNKRVPLTTKQGNKTMYKGTRASGIGRHTKFGGYVINWKKVRTYVTPDMVNFELKPYVNANVPPLKHEFKGFSGGPLDPRLQLLKIKEYIVNGRVQSEGATDTSCYKERG>YBR283CMSGFRLIDIVKPILPILPEVELPFEKLPFDDKIVYTIFAGLIYLFAQFPLVGLPKATTPNVNDPIYFLRGVFGCEPRTLLEFGLFPNISSGLILQLLAGLKVIKVNFKIQSDRELFQSLTKVFAIVQYVILTNIFIFAGYFGDDLSVVQIGLINFQLVGAGIFTTLLAEVIDKGFGFSSGAMIINTVVIATNLVADTFGVSQIKVGEDDQTEAQGALINLIQGLRSKHKTFIGGIISAFNRDYLPNLTTTIIVLAIAIIVCYLQSVRVELPIRSTRARGTNNVYPIKLLYTGCLSVLFSYTILFYIHIFAFVLIQLVAKNEPTHIICKIMGHYENANNLLAVPTFPLSLLAPPTSFFKGVTQQPLTFITYSAFILVTGIWFADKWQAISGSSARDVALEFKDQGITLMGRREQNVAKELNKVIPIAAVTGASVLSLITVIGESLGLKGKAAGIVVGIAGGFSLLEVITIEYQQSGGQSALNQVLGVPGAM>YBR284WMVQNNESVFFVECDSYKESPSTSPIRLDDLDGNDAVSDQGLAFDGDVGITSQARVRNPRAQIFEDSNTDVVLHLDDLDMVPLNTKFDMQMEMGSPMAMPAETPPPVEPLKTKDLAYSSLAHLPSYFFEQTHFRIDRKCLLEMSKLRRNYLTISKQDALSCPQLHSRVAGGYLKPVKEKLFGIRHFLDLEESNTVNLLQDGNYMTELFNSQINIPTFKEFREDFEWCLKIIRDRSLSRFSEKRLQYLVNKFPVFQHLHSKEEMRQSKKVPHKDFYNCRKIDLNLLLSGCFSQWQLTEFIWTKLRKEPDRVIHQAFNGSHITLSQLFKVNFEETGQFFNGLKIIDDSFLEWYKVIYLAKYHLVNDEMEIHTGSHGKQLRYYLIAKTFLEFDNYINGEYLAELLKTFLIKPQEESKYQLCQLSVDFQFYLHYDNSDVDNWWMVFANWLNHYNIFSNNIRWNIRISRIYPELYHTGKVKNFQEYLNLIFKPLFNAENYLHKSLGPILLKFLSQVSSIDLCIQDTDNYIWKNFTAVSCLPKDWTSGGDNPTISQYMYYVYVNLTKLNHIRQALHQNTFTLRSSCSPTSMNRTSQFSNTLNFTEHTEAILNNFLLACGGFLNAENLWNAPPSLVYLFYLSQIPMVVAPLNSIVDSKPTMLQEQAPTGLVLEPSKPYKKNPFMKFFEMGFKISLSSESILYNNSYTKEPIIEEYSVAASIYRLHSADLCELLRNSVITSGFSSTLKNKWLGVSLASHDYFVENTGFVDKWYDCKPNTSLEHNVPIIRRQYRSSTLAGEWRLIIA>YBR285WMTIFSRFSYFDSLFSFKKQEPSPIEIIYCNENNGFINIKSLESPTDDSMEADISHREMATILTRNRNNLGKVAIDKKGVNNHCIDLNELKKGLVANEHKLDNDNSTRHQNTYSPEDSVEFDRFDDKQSRILKCSTRRSYLRYKK>YBR286WMHFSLKQLAVAAFYATNLGSAYVIPQFFQEAFQQEEPIENYLPQLNDDDSSAVAANIPKPHIPYFMKPHVESEKLQDKIKVDDLNATAWDLYRLANYSTPDYGHPTRVIGSKGHNKTMEYILNVFDDMQDYYDVSLQEFEALSGKIISFNLSDAETGKSFANTTAFALSPPVDGFVGKLVEIPNLGCEEKDYASVVPPRHNEKQIALIERGKCPFGDKSNLAGKFGFTAVVIYDNEPKSKEGLHGTLGEPTKHTVATVGVPYKVGKKLIANIALNIDYSLYFAMDSYVEFIKTQNIIADTKHGDPDNIVALGAHSDSVEEGPGINDDGSGTISLLNVAKQLTHFKINNKVRFAWWAAEEEGLLGSNFYAYNLTKEENSKIRVFMDYDMMASPNYEYEIYDANNKENPKGSEELKNLYVDYYKAHHLNYTLVPFDGRSDYVGFINNGIPAGGIATGAEKNNVNNGKVLDRCYHQLCDDVSNLSWDAFITNTKLIAHSVATYADSFEGFPKRETQKHKEVDILNAQQPQFKYRADFLII>YBR287WMVETFSFAHLAYLVFESVLQVVIIALAGFWSASSGLLPKQSQKIISLLNVDLFTPCLIFSKLAKSLSMAKIFEIAIIPIFFGLTTGISFISGKIMSRILDLDKDETNFVVANSVFGNSNSLPVSLTLSLAYTLPNLTWDQIPNDNRDNVASRGILYLLIFQQIGQMLRWSWGYNKLMKWSGENTQHMPPSQVQSLLERTPNIDNEELVNEEQEEQELLEEENNRMNSSFLSSSSIGDKIWQKSCTVFERIRANLNPPLYSMIFAVVVAAIGPLQRELFMEDGFINNTFAEAVTQLGSVSIPLILVVLGSNLYPSAEVFPKTVHHSKLLIGSIIGRMILPSCFLLPIIAIAVKYINVSILDDPIFLVVGFLLTVSPPAIQLTQITQLNEFFEAEMADILFWGYAVLSLPVSIIVVSGAIYVLQWANPT>YBR288CMYLSFYITDTKNKLIFQYLLGATAPSFKHLWTRVRTTCPQLLEDSSSDDYLDHSMVGRDLEVYKYFSVINKLNYWCLASTSKSKGPLDCFTFLETIDRILLEYFDKDKLSIKKIVNNYDRISLIFNCCVEAGEPNVSDMLYVNKIKEAVPERSDLSKFISSTAHNLQQAVQLPQQRQQQLQQNQISRGSNSLIENEEIVPWRTSRASKHENNELYVDLLETFHVVFEKKKSHLRLLTGSIHGIVDVRSYLNDNPLVAVKLNTMGNDIGIPSLHDCVEINDGVFSPSNITFIPPDGKFRLLEYSVDLSSQVKQSGVRMNSIGLMSLHFQNGLGKDSDEFELSLNIENFKKVSQVDDLKIDLQFNVENADPNEIAYKIKILRNTHGRFENSIIMGQGQWIFDKSTATGTVPVLRGCIEYENTGPNFTKKVDLQTVSLEYSYIGQSASGIYVEAIDIVSGLTIGKNTKLYKGAKYKTQTGNFQVRL>YBR290WMPEQELLIGQEMNTLHAGSSTDGINVGNAGRTRDTQTGVEGETEIGSDEEDSIEDEGSSSGGNSTTERLVPHQLREQAARHIGKIGRHFNILDRLFKKRTQQSSDIQQGAMFDGVFSNLSAKPDTTETEGNNEQDIPPTYDEAAADMAPSYYGMDLNNSDIYYDEICIEGLPVGNIANLLWNIIVSTSFQFIGFLITYILHTSHAAKQGSRFGLGLTFIGYGYSMIPNDVTSKVGKNKSLNRMELEDPNEFDDVRLNSQSTTQDKFESHLNHGLDEEKQNIPWLAVFVAFLGLFITLKSIYDYIQVKKLEKKYLNQSQNQA>YBR291CMSSKATKSDVDPLHSFLAGSLAGAAEACITYPFEFAKTRLQLIDKASKASRNPLVLIYKTAKTQGIGSIYVGCPAFIIGNTAKAGIRFLGFDTIKDMLRDSETGELSGTRGVIAGLGAGLLESVAAVTPFEAIKTALIDDKQSATPKYHNNGRGVVRNYSSLVRDKGFSGLYRGVLPVSMRQAANQAVRLGCYNKIKTLIQDYTDSPKDKPLSSGLTFLVGAFSGIVTVYSTMPLDTVKTRMQSLDSTKYSSTMNCFATIFKEEGLKTFWKGATPRLGRLVLSGGIVFTIYEKVLVMLA>YBR293WMSISNWITTAYLITSTSFQPLYGSFSDALGRRNCLFFANGAFTIGCLACGFSKNIYMLSFMRALTGIGGGGLITLSTIVNSDVIPSSKRGIFQAFQNLLLGFGAICGASFGGTIASSIGWRWCFLIQVPISVISSILMNYYVPNQKEYNRQNSSIFQNPGKILRDIDVMGSILIITGLTLQLLYLSLGCSTSKLSWTSPSVLLLLVGSVIILLLFILHERKTSARAIIPMELVNSSYSVVVLSISILVGFASYAYLFTLPLFFQIVLGDSTAKAGLRLTIPSLFTPVGSLITGFSMSKYNCLRLLLYIGISLMFLGNFLFLFIEKTSPNWLIGLFLIPANLGQGITFPTTLFTFIFMFSKSDQATATSTLYLFRSIGSVWGVAISAGVIQLSFAGLLRSNLKGLLDENKIKKLIVQLSANSSYIGSLHGEVKNTVIKSFDEATKRAHLMSTLLSSLALILCILKDNLAKPKTRR>YBR296CMALHQFDYIFAIAMLFAFLDAFNIGANDVANSFASSISSRSLKYWQAMVLAGLCEFLGAVLAGARVSGTIKNNIIDSSIFTNDPAVLMLTMTSALIGSSCWLTFATAIGMPVSTTHSIVGGTIGAGIAAGGANGVVWGWSGVSQIIASWFIAPILAGAIAAIVFSISRFSVLEVKSLERSIKNALLLVGVLVFATFSILTMLIVWKGSPNLHLDDLSETETAVSIVLTGAIASIVYFIFFYPFYRRKVLDQDWTLKLIDIFRGPSFYFKSTDDIPPMPEGHQLTIDYYEGRRNLGTTVSVEDEENKAASNSNDSVKNKEDIQEVDLVRTETEPETKLSTKQYWWSLLKQGPKKWPLLFWLVISHGWTQDVIHAQVNDRDMLSGDLKGMYERSKFYDNRVEYIYSVLQAITAATMSFAHGANDVANATGPLSAVYVIWKTNTIGAKSEVPVWVLAYGGVALVIGCWTYGYNIIKNLGNKMILQSPSRGFSIELAVAITTVMATQLGIPTSTTQIAVGGIVAVGLCNKDLKSVNWRMVAWCYSGWFLTLPIAGLIAGIINGIILNAPRFGVEYQMT>YBR298CMKGLSSLINRKKDRNDSHLDEIENGVNATEFNSIEMEEQGKKSDFDLSHLEYGPGSLIPNDNNEEVPDLLDEAMQDAKEADESERGMPLMTALKTYPKAAAWSLLVSTTLIQEGYDTAILGAFYALPVFQKKYGSLNSNTGDYEISVSWQIGLCLCYMAGEIVGLQMTGPSVDYMGNRYTLIMALFFLAAFIFILYFCKSLGMIAVGQALCGMPWGCFQCLTVSYASEICPLALRYYLTTYSNLCWAFGQLFAAGIMKNSQNKYANSELGYKLPFALQWIWPLPLAVGIFFAPESPWWLVKKGRIDQARRSLERTLSGKGPEKELLVSMELDKIKTTIEKEQKMSDEGTYWDCVKDGINRRRTRIACLCWIGQCSCGASLIGYSTYFYEKAGVSTDTAFTFSIIQYCLGIAATFISWWASKYCGRFDLYAFGLAFQAIMFFIIGGLGCSDTHGAKMGSGALLMVVAFFYNLGIAPVVFCLVSEIPSSRLRTKTIILARNAYNVIQVVVTVLIMYQLNSEKWNWGAKSGFFWGGFCLATLAWAVVDLPETAGRTFIEINELFRLGVPARKFKSTKVDPFAAAKAAAAEINVKDPKEDLETSVVDEGRNTSSVVNK>YBR301WMVKLTSIAAGVAAIAATASATTTLAQSDERVNLVELGVYVSDIRAHLAQYYSFQVAHPTETYPVEIAEAVFNYGDFTTMLTGIAPDQVTRMITGVPWYSSRLKPAISSALSKDGIYTIAN>YCL001WMDYDSSDTMNGGSSNPLITKMNTMKLLYQHYLDKVTPHAKERWAVLGGLLCLFMVRITMAEGWYVICYGLGLFLLNQFLAFLTPKFDMSLQQDEENNELEAGEKSEEFRPFIRRLPEFKFWYNSIRATVISLLLSLFSIFDIPVFWPILLMYFILLFFLTMRRQIQHMIKYRYIPLDIGKKKYSHSSN>YCL004WMTTRLLQLTRPHYRLLSLPLQKPFNIKRQMSAANPSPFGNYLNTITKSLQQNLQTCFHFQAKEIDIIESPSQFYDLLKTKILNSQNRIFIASLYLGKSETELVDCISQALTKNPKLKVSFLLDGLRGTRELPSACSATLLSSLVAKYGSERVDCRLYKTPAYHGWKKVLVPKRFNEGLGLQHMKIYGFDNEVILSGANLSNDYFTNRQDRYYLFKSRNFSNYYFKLHQLISSFSYQIIKPMVDGSINIIWPDSNPTVEPTKNKRLFLREASQLLDGFLKSSKQSLPITAVGQFSTLVYPISQFTPLFPKYNDKSTEKRTILSLLSTITSNAISWTFTAGYFNILPDIKAKLLATPVAEANVITASPFANGFYQSKGVSSNLPGAYLYLSKKFLQDVCRYRQDHAITLREWQRGVVNKPNGWSYHAKGIWLSARDKNDANNWKPFITVIGSSNYTRRAYSLDLESNALIITRDEELRKKMKAELDNLLQYTKPVTLEDFQSDPERHVGTGVKIATSILGKKL>YCL005WMFVVDWSVQLCMGVISPLFRALVQLPLSIFVWNGFQLVALPINIPLRLFLGTSLSRLVAQTSTLDFYVVLTLFQYFAVLCAFGSIIGLIFGFILGVFHSICGVPSVYISLEWKRWFAPIRTVLERASTSIVNIMRGQTIAPIPMPKPNPTHISKPNMKKFHDEPGADDMTITHDVNCYITPCQTPTNEKIQHYNNDSFNTTTTDDEPTDIWDRSDTYQNSFVTNETLMSLSNRAKLRRNASDADIVNIKILRRNSR>YCL010CMDGYWDVVVSSLQDIYNANEVIPFDDELQTKKLNFLNMSKDQLQLHLNTFQEHMENVNRVHRILDNVRSNLSLMLNQSREEKSEENTEDAEEGEGTRMALSQGKKAVGKVGRSYWTSEYNPNAPILVGSEVAYKPRRGSADGEWIQCEVLKVVADGTRFEVRDPEPDELGNSGKVYKCNRKELLLIPPGFPTKNYPPGTKVLARYPETTTFYPAIVIGTKRDGTCRLRFDGEEEVDKETEVTRRLVLPSPTALANLARK>YCL011CMERELGMYGNDRSRSRSPVRRRLSDDRDRYDDYNDSSSNNGNGSRRQRRDRGSRFNDRYDQSYGGSRYHDDRNWPPRRGGRGRGGSRSFRGGRGGGRGRTLGPIVERDLERQFDATKRNFENSIFVRNLTFDCTPEDLKELFGTVGEVVEADIITSKGHHRGMGTVEFTKNESVQDAISKFDGALFMDRKLMVRQDNPPPEAAKEFSKKATREEIDNGFEVFIINLPYSMNWQSLKDMFKECGHVLRADVELDFNGFSRGFGSVIYPTEDEMIRAIDTFNGMEVEGRVLEVREGRFNKRKNNDRYNQRREDLEDTRGTEPGLAQDAAVHIDETAAKFTEGVNPGGDRNCFIYCSNLPFSTARSDLFDLFGPIGKINNAELKPQENGQPTGVAVVEYENLVDADFCIQKLNNYNYGGCSLQISYARRD>YCL016CMSINLHSAPEYDPSYKLIQLTPELLDIIQDPVQNHQLRFKSLDKDKSEVVLCSHDKTWVLKQRKHSNTVLLMREFVPEQPITFDETLLFGLSKPYMDVVGFAKTESEFETRETHGELNLNSVPIYNGELDFSDKIMKRSSTKVIGTLEELLENSPCSALEGISKWHKIGGSVKDGVLCILSQDFLFKALHVLLMSAMAESLDLQHLNVEDTHHAVGKDIEDEFNPYTREIIETVLNKFAVQEQEAENNTWRLRIPFIAQWYGIQALRKYVSGISMPIDEFLIKWKSLFPPFFPCDIDIDMLRGYHFKPTDKTVQYIAKSTLPMDPKERFKVLFRLQSQWDLEDIKPLIEELNSRGMKIDSFIMKYARRKRLGKKTVVTSR>YCL017CMLKSTATRSITRLSQVYNVPAATYRACLVSRRFYSPPAAGVKLDDNFSLETHTDIQAAAKAQASARASASGTTPDAVVASGSTAMSHAYQENTGFGTRPIYLDMQATTPTDPRVLDTMLKFYTGLYGNPHSNTHSYGWETNTAVENARAHVAKMINADPKEIIFTSGATESNNMVLKGVPRFYKKTKKHIITTRTEHKCVLEAARAMMKEGFEVTFLNVDDQGLIDLKELEDAIRPDTCLVSVMAVNNEIGVIQPIKEIGAICRKNKIYFHTDAAQAYGKIHIDVNEMNIDLLSISSHKIYGPKGIGAIYVRRRPRVRLEPLLSGGGQERGLRSGTLAPPLVAGFGEAARLMKKEFDNDQAHIKRLSDKLVKGLLSAEHTTLNGSPDHRYPGCVNVSFAYVEGESLLMALRDIALSSGSACTSASLEPSYVLHALGKDDALAHSSIRFGIGRFSTEEEVDYVVKAVSDRVKFLRELSPLWEMVQEGIDLNSIKWSGH>YCL021W-AMVLTDAEELRSPVITSDMSFFDLESNHSSDSVHLLCEKYTHKLPIESESQTTFRLAPTKQRLYRQSTLYVPLSLKQRVFLFTERVKSIWAGLPRCKPNKYFKVAFALAVLTPLAIWIFYIDFRVH>YCL025CMSSSKSLYELKDLKNSSTEIHATGQDNEIEYFETGSNDRPSSQPHLGYEQHNTSAVRRFFDSFKRADQGPQDEVEATQMNDLTSAISPSSRQAQELEKNESSDNIGANTGHKSDSLKKTIQPRHVLMIALGTGIGTGLLVGNGTALVHAGPAGLLIGYAIMGSILYCIIQACGEMALVYSNLTGGYNAYPSFLVDDGFGFAVAWVYCLQWLCVCPLELVTASMTIKYWTTSVNPDVFVIIFYVLVITINIFGARGYAEAEFFFNCCKILMMTGFFILGIIIDVGGAGNDGFIGGKYWHDPGAFNGKHAIDRFKGVAATLVTAAFAFGGSEFIAITTAEQSNPRKAIPGAAKQMIYRILFLFLATIILLGFLVPYNSDQLLGSTGGGTKASPYVIAVASHGVRVVPHFINAVILLSVLSMANSSFYSSARLFLTLSEQGYAPKVFSYIDRAGRPLIAMGVSALFAVIAFCAASPKEEQVFTWLLAISGLSQLFTWTAICLSHLRFRRAMKVQGRSLGELGFKSQTGVWGSAYACIMMILILIAQFWVAIAPIGEGKLDAQAFFENYLAMPILIALYVGYKVWHKDWKLFIRADKIDLDSHRQIFDEELIKQEDEEYRERLRNGPYWKRVVAFWC>YCL026C-AMSPTGNYLNAITNRRTIYNLKPELPQGVGLDDVKRTVHVILKNTPTAFNSQVNRAVIIVGDTHKRIWDAVASAMPTAEAKKRPESCRDEAYGSVIFFTDEGPTEKLQRDFPALAAAFPTCAAHTTGAVQIQSWTALELLGLGANLQHYNDYVKSALPQDVPIAWTVQSQLVFGVPTALPEEKTFINNVINVYH>YCL028WMDTDKLISEAESHFSQGNHAEAVAKLTSAAQSNPNDEQMSTIESLIQKIAGYVMDNRSGGSDASQDRAAGGGSSFMNTLMADSKGSSQTQLGKLALLATVMTHSSNKGSSNRGFDVGTVMSMLSGSGGGSQSMGASGLAALASQFFKSGNNSQGQGQGQGQGQGQGQGQGQGSFTALASLASSFMNSNNNNQQGQNQSSGGSSFGALASMASSFMHSNNNQNSNNSQQGYNQSYQNGNQNSQGYNNQQYQGGNGGYQQQQGQSGGAFSSLASMAQSYLGGGQTQSNQQQYNQQGQNNQQQYQQQGQNYQHQQQGQQQQQGHSSSFSALASMASSYLGNNSNSNSSYGGQQQANEYGRPQQNGQQQSNEYGRPQYGGNQNSNGQHESFNFSGNFSQQNNNGNQNRY>YCL029CMDRYQRKIGCFIQIPNLGRGQLKYVGPVDTKAGMFAGVDLLANIGKNDGSFMGKKYFQTEYPQSGLFIQLQKVASLIEKASISQTSRRTTMEPLSIPKNRSIVRLTNQFSPMDDPKSPTPMRSFRITSRHSGNQQSMDQEASDHHQQQEFGYDNREDRMEVDSILSSDRKANHNTTSDWKPDNGHMNDLNSSEVTIELREAQLTIEKLQRKQLHYKRLLDDQRMVLEEVQPTFDRYEATIQEREKEIDHLKQQLELERRQQAKQKQFFDAENEQLLAVVSQLHEEIKENEERNLSHNQPTGANEDVELLKKQLEQLRNIEDQFELHKTKWAKEREQLKMHNDSLSKEYQNLSKELFLTKPQDSSSEEVASLTKKLEEANEKIKQLEQAQAQTAVESLPIFDPPAPVDTTAGRQQWCEHCDTMGHNTAECPHHNPDNQQFF>YCL030CMVLPILPLIDDLASWNSKKEYVSLVGQVLLDGSSLSNEEILQFSKEEEVPLVALSLPSGKFSDDEIIAFLNNGVSSLFIASQDAKTAEHLVEQLNVPKERVVVEENGVFSNQFMVKQKFSQDKIVSIKKLSKDMLTKEVLGEVRTDRPDGLYTTLVVDQYERCLGLVYSSKKSIAKAIDLGRGVYYSRSRNEIWIKGETSGNGQKLLQISTDCDSDALKFIVEQENVGFCHLETMSCFGEFKHGLVGLESLLKQRLQDAPEESYTRRLFNDSALLDAKIKEEAEELTEAKGKKELSWEAADLFYFALAKLVANDVSLKDVENNLNMKHLKVTRRKGDAKPKFVGQPKAEEEKLTGPIHLDVVKASDKVGVQKALSRPIQKTSEIMHLVNPIIENVRDKGNSALLEYTEKFDGVKLSNPVLNAPFPEEYFEGLTEEMKEALDLSIENVRKFHAAQLPTETLEVETQPGVLCSRFPRPIEKVGLYIPGGTAILPSTALMLGVPAQVAQCKEIVFASPPRKSDGKVSPEVVYVAEKVGASKIVLAGGAQAVAAMAYGTETIPKVDKILGPGNQFVTAAKMYVQNDTQALCSIDMPAGPSEVLVIADEDADVDFVASDLLSQAEHGIDSQVILVGVNLSEKKIQEIQDAVHNQALQLPRVDIVRKCIAHSTIVLCDGYEEALEMSNQYAPEHLILQIANANDYVKLVDNAGSVFVGAYTPESCGDYSSGTNHTLPTYGYARQYSGANTATFQKFITAQNITPEGLENIGRAVMCVAKKEGLDGHRNAVKIRMSKLGLIPKDFQ>YCL031CMGIEDISAMKNGFIVVPFKLPDHKALPKSQEASLHFMFAKRHQSSNSNESDCLFLVNLPLLSNIEHMKKFVGQLCGKYDTVSHVEELLYNDEFGLHEVDLSALTSDLMSSTDVNEKRYTPRNTALLKFVDAASINNCWNALKKYSNLHAKHPNELFEWTYTTPSFTTFVNFYKPLDIDYLKEDIHTHMAIFEQREAQAQEDVQSSIVDEDGFTLVVGKNTKSLNSIRKKILNKNPLSKHENKAKPISNIDKKAKKDFYRFQVRERKKQEINQLLSKFKEDQERIKVMKAKRKFNPYT>YCL032WMEDGKQAINEGSNDASPDLDVNGTILMNNEDFSQWSVDDVITWCISTLEVEETDPLCQRLRENDIVGDLLPELCLQDCQDLCDGDLNKAIKFKILINKMRDSKLEWKDDKTQEDMITVLKNLYTTTSAKLQEFQSQYTRLRMDVLDVMKTSSSSSPINTHGVSTTVPSSNNTIIPSSDGVSLSQTDYFDTVHNRQSPSRRESPVTVFRQPSLSHSKSLHKDSKNKVPQISTNQSHPSAVSTANTPGPSPNEALKQLRASKEDSCERILKNAMKRHNLADQDWRQYVLVICYGDQERLLELNEKPVIIFKNLKQQGLHPAIMLRRRGDFEEVAMMNGSDNVTPGGRL>YCL033CMNKWSRLYVITVRRTFPGRRNIVLTQYWNKSKKMSDESNDVKWNDALTPLQLMVLRDKATERPNTGAYLHTNESGVYHCANCDRPLYSSKAKFDARCGWPAFYEEVSPGAITYHRDNSLMPARVEICCARCGGHLGHVFEGEGWKQLLNLPKDTRHCVNSASLNLKKD>YCL034WMGFLSDHPHTAITETIFRIVSSRDYTLEVELAPLIQLIKADHNDYNYTVNQEEAARALRKKIKYGNRLQQSRTLDLLDLFISQGVKFTVMYNDDKLLQRLRGMATNSENSGSGEKYEPRIIKKCAAYAISWLNYITQNNLENARAYSGLYQLGQTVKQRYSKSSRSRRSGGGSGGRSNFMDDSADDTLYQSNSLTSADRLYRIPQINMNKEAPRIRLIISDALASAVSLQNSLIGLPKGKFSTDDEEATSKFIQARAIRRKVLRYLQLVTEGEFLGSLIHANDELVAALTAYDDRSAQDDSSDESDHGSYDDGIYDENEQDNSRYIDSESSEEESLSSYQPSTISNPFGDHNKI>YCL035CMVSQETIKHVKDLIAENEIFVASKTYCPYCHAALNTLFEKLKVPRSKVLVLQLNDMKEGADIQAALYEINGQRTVPNIYINGKHIGGNDDLQELRETGELEELLEPILAN>YCL036WMQVQKMVRDNSNNGSDKSVHWERRNNNGAGPRYRSRSGNTGALATKLSNGTLSVRGLVKDRTGSGKIAGCVEAFLDARTQLNTPWDRAKCNWLDQIDYYVQLRKTAFSKELDQLRKPMIDAYVAEMRQKFDASYGQSRAQLEAKLAQVDSEWHMVHGDVHAKLEKLVEERRFLKRLSDTIVPPRSKRSQRLSPLTKEDRANCICPQPKGMSDTAWFEAIQKKMLGMNGTIKLLETEQKLLADEKNSVRKTFWPMVEAHSRSNEFAYLEKCIRLMASQRAICFCLDIEAFETNQNVITEIGISIYDPRENMVPSMVPITKNYHLIIEESLELRNQKWVCDYKDCYLLGESYVLSLKECVHFIQSLINYYLVPVTEEDKTWSRAFVGHHVSGDLKWLETIGVKFPGRGYEGHLDHTLLLAETPGDLDVFILDTEQFYRKSYGEKGSSLGKILRLFEIPHAFLHNAGNDAYYTLHLFMKFCDVNFRKISGMDDVLKVMGQVKVWGERDVREPKVVPMSYAISIEEAVKNRTYRKGVKSSRKERVCQTEFGGLTYFGTAKDAFTSTLPTH>YCL038CMSYGTINDMNESVTNYRIKKAQNNIKGWYAYSFSSEPFVVSAVSTYIPLLLQQFASINGVKVHDHSIPCLSETGSDSDKCVLGLFNNRIFVDTSSFALYVFSLSVLFQTIIVISVSGIVDLWGSVKFKGRILVWFGIVGALSTVAISKLNDTQIYSLAGLYIVANGCFGVINVVGNSLLPIFVKDSLKCQSQGAYEPDKVDSLTTVISGRGASLGYSSALIVQIVSMFLVASKKGSKQDVQVAVLFVGIWWFVWQLPMIWLIDDVTIPIRVDDSTLASARSPYPGEQDALGQLNWKNYLSYGWVSLFESFKHARLLKDVMIFLIAWFIISDSITTINSTAVLFSKAELHMSTLNLIMISVLTVVNAMLGAFMIPQFLATKFRWTSSQTLMYIIIWASFIPFYGILGFFFNAFGLKHKFEMFLLAIWYGLSLGGLSAVSRSVFSLIVPPGKESTFFSMFSITDKGSSILGPFLVGLLTDKTHNIRYSFYFFFLLLMLSLPVLNCLDVKRGRREAEELSQVLPESERRLD>YCL039WMSHTNKIAYVLNNDTEETASPSSVGCFDKKQLTKLLIHTLKELGYDSAANQLLLESGGYQNESNHIQTFFKLIKTGQFHLINWQIVCSLPLAHSSPLRSEWLQRLLIPTPTPATTSLFDHMLLQLQYLQQLMSSVNSSTCSDAEIATLRNYVEIMILVNRQIFLEFFHPVTNSASHKGPHTALPVLYLRKILKNFIEIWDSLLVSNDQFLNEENIFNPETTLRELSTYLTNPKLTAQLNLERDHLIDAISKYIDPNELVPKGRLLHLLKQAIKYQQSQDIFNIIDPDDDASFSSPPHRINLLQDNFSHDLTVTFQEWKTIQDTTDEIWFLTFSPNGKYLASATSESSRGYFITVYDVEQDFKIYKTCVSLSQSVLYLMFSPDSRYLVACPFSEDVTIYDMNATSLPDASATDSFLLYPSTRLSPMDSFKLDTTTYPDDTESSASSSSRPANANSNQSRVWCCDAFHTAERAGWMVVGSPDREAIVHSLTTKESLFSLKGRTCIALGHDENISGRKSIDPAKVLYKPTSSNGNWQYVEDDETFPRVHDVKISYDDKYVLLMTHQGVIDVYDFSGFPSKEELSKQTVDPKNFLIPRIARLDVGKNMTCISLPLNTTHQGFHRQQISESQHLVLVSLQDNELQMWDYKENILIQKYFGQKQQHFIIRSCFAYGNKLVMSGSEDGKIYIWDRIRGNLVSVLSGHSTVMSNSTKPMGKNCNVVASNPADKEMFASGGDDGKIKIWKISRN>YCL043CMKFSAGAVLSWSSLLLASSVFAQQEAVAPEDSAVVKLATDSFNEYIQSHDLVLAEFFAPWCGHCKNMAPEYVKAAETLVEKNITLAQIDCTENQDLCMEHNIPGFPSLKIFKNSDVNNSIDYEGPRTAEAIVQFMIKQSQPAVAVVADLPAYLANETFVTPVIVQSGKIDADFNATFYSMANKHFNDYDFVSAENADDDFKLSIYLPSAMDEPVVYNGKKADIADADVFEKWLQVEALPYFGEIDGSVFAQYVESGLPLGYLFYNDEEELEEYKPLFTELAKKNRGLMNFVSIDARKFGRHAGNLNMKEQFPLFAIHDMTEDLKYGLPQLSEEAFDELSDKIVLESKAIESLVKDFLKGDASPIVKSQEIFENQDSSVFQLVGKNHDEIVNDPKKDVLVLYYAPWCGHCKRLAPTYQELADTYANATSDVLIAKLDHTENDVRGVVIEGYPTIVLYPGGKKSESVVYQGSRSLDSLFDFIKENGHFDVDGKALYEEAQEKAAEEADADAELADEEDAIHDEL>YCL044CMAVFTPPSGNSNSTDHTHTQDDHDKDDNDIKKFYIRPSLGLKLWGPLVPAPDNLPGLYTLITIQSAVGFFALWRLRRLYKLPPPRRIATGTHSDLSFGELPSEMIVNGKTKIKKDIADFPTLNRFSTTHGDIVLAPPPIIPRQSRFVSVRKLLWGLFGSLLLSQSLLELTRLNFLKYDPWCDEMKSVRDKKFFNNIVKYYHEGIDPTKIKVKDAMNGTPLSTNIPEVKQSVALARAQVEAQNPIIKWFGPLEYKPMSFNEYLNRMEFHLDMFEFFQNKRNIRENSIELINSISHNPQSSSTGLEGLSESKKLHLQNVEKRLHFLASSGDSISAPVKKRSSTTLSRGVILPHDTKGPQDIDLDTIRSLYDPWMTLALETSLSIKFIPTTMPSHTKTPTSTDQPLPGPTPKALTNEKTH>YCL045CMKITCTDLVYVFILLFLNTSCVQAVFSDDAFITDWQLANLGPWEKVIPDSRDRNRVLILSNPTETSCLVSSFNVSSGQILFRNVLPFTIDEIQLDSNDHNAMVCVNSSSNHWQKYDLHDWFLLEEGVDNAPSTTILPQSSYLNDQVSIKNNELHILDEQSKLAEWKLELPQGFNKVEYFHREDPLALVLNVNDTQYMGFSANGTELIPVWQRDEWLTNVVDYAVLDVFDSRDVELNKDMKAELDSNSLWNAYWLRLTTNWNRLINLLKENQFSPGRVFTKLLALDAKDTTVSDLKFGFAKILIVLTHDGFIGGLDMVNKGQLIWKLDLEIDQGVKMFWTDKNHDELVVFSHDGHYLTIEVTKDQPIIKSRSPLSERKTVDSVIRLNEHDHQYLIKFEDKDHLLFKLNPGKNTDVPIVANNHSSSHIFVTEHDTNGIYGYIIENDTVKQTWKKAVNSKEKMVAYSKRETTNLNTLGITLGDKSVLYKYLYPNLAAYLIANEEHHTITFNLIDTITGEILITQEHKDSPDFRFPMDIVFGEYWVVYSYFSSEPVPEQKLVVVELYESLTPDERLSNSSDNFSYDPLTGHINKPQFQTKQFIFPEIIKTMSISKTTDDITTKAIVMELENGQITYIPKLLLNARGKPAEEMAKDKKKEFMATPYTPVIPINDNFIITHFRNLLPGSDSQLISIPTNLESTSIICDLGLDVFCTRITPSGQFDLMSPTFEKGKLLITIFVLLVITYFIRPSVSNKKLKSQWLIK>YCL049CMFSKYLVTASSLFVALTSAASTVDLDALLLLPGVESHDGVDTVFSTKDFYQVSFVKSIAPAIVNSSVIFHDVSRGVAMGNVKSRASIFNPEETYYDWEQYQVVNNGDWRTEWAPASDCIWREEKDNSDETPDRFPISVPYNWTSQYSIVDYDTDANEDNLDFRFIKSLLDKKNWLKKINQTVSQSSIMVAPMIRPYNVVQLWYSKDMVWANVQRQYCSGVYPGGTQCSAWSRYYHVDAPTCDEPVASYMTKMLENEVQCPNERNATTLEPLRLNKQGDSDFSLTFEEEEEEETGSKSLWSTLKKIFSKRSIS>YCL050CMSIPADIASLISDKYKSAFDNGNLKFIQTETTKTKDPKTSMPYLISHMPSLIEKPERGQTPEGEDPLGKPEEELTVIPEFGGADNKAYKLLLNKFPVIPEHTLLVTNEYQHQTDALTPTDLLTAYKLLCALDNEESDKRHMVFYNSGPASGSSLDHKHLQILQMPEKFVTFQDRLCNGKEHFLPTFNTEPLQDAKVSFAHFVLPMPESEETVDEDLLAMCYISILQRALTFFQDWLNENPELKKSYNLMLTKEWICVVPRSKAFSDEMKIGFNSTGYCGMILTKNDEVFSKITEKPELINDILLECGFPNTSGQKPNEYNY>YCL051WMPNTHTQHVQISEPNPVNTLSTPSKRGHRHRRSLAISGDFDFLKQPAAIVNLPPPQAAENCPSTAPTAVSSTLSPIRYNRFPCKTNEDAGTLDLPEPRFYPLSPKNNLQTPSPRFFISEEPSFSSPVKGVPDAIINLDDALKTRPRSFKSHRRSESAPPDLEVMVDKGNCAAGSNSMIKEEEDSLIEPESKNEYYEQKLPTALLSPLRPSLCVSEQAIDVDDSALNGSPTHHNHGMQNANARNSNTFNSLKIKGQKQRYYHYTKQLPLTVGCDSQSPKEQRSAASMTINQAMTPSSLAYTPSKLASTPATPVSFYDSNADINLESDNFPLKDNPRYAKDGYPKKCGNSQLNRVLDSDKRQDFSGESRRRRSGSPISHMQHRNLIDNMKGRRNSNTINSIFNYKSQHYEMPYDDMMKNENINAQSMPFSVNGVNNENSIGGVITRADDAPLQHSVVKSCTPDGKEEMNRLKSNDSNEYSKSEGQIRTNSQLSKDILMGEPGDMVDLSSFVNAQRKASNETGDLVFSLSQDDDALKTFHASNSAATSNESWCISDDALGKQAQDSEVRRKRKSKLGLFRHIFSRK>YCL052CMVTRHRVTVLYNAPEDIGNHMRQNDTHLTVRGGSGVVLQQRWLLERTGSLDKSFTRITWRPRADLARSLSVIENELSAGFSVYSNSSDVPERFITNPVYNSFHSEKFDIEQYLPPEVDLNLSWNPEDFTYDISVEPTQIQIVEYRLLKQGEEFTIARVKDEKLEVGVFFVDASDESDVDIGGIRCNWRMDDGKMERCQKTSLLYKQGHIAYNHSTTTTSLYLNEPIGLHPKIMIDLTDFEERPKCMYLMHLQLPLELFIDKFQSSPLLLFGEDDLELPEYSLRDKAWGSESIFELKAGTMNEVTLHTRYIEPSNNKGDKLEVSFDPEVILACDTGDNKVSRNPFYKKGLGYESLFTDDTTFRHLNSTTLLVPIPRPDTKDYSKIKNGTLLCLLISIIYIFSKVFGNNKKKRSVKRE>YCL055WMAFQDPTYDQNKSRHINNSHLQGPNQETIEMKSKHVSFKPSRDFHTNDYSNNYIHGKSLPQQHVTNIENRVDGYPKLQKLFQAKAKQINQFATTPFGCKIGIDSIVPTLNHWIQNENLTFDVVMIGCLTENQFIYPILTQLPLDRLISKPGFLFIWANSQKINELTKLLNNEIWAKKFRRSEELVFVPIDKKSPFYPGLDQDDETLMEKMQWHCWMCITGTVRRSTDGHLIHCNVDTDLSIETKDTTNGAVPSHLYRIAENFSTATRRLHIIPARTGYETPVKVRPGWVIVSPDVMLDNFSPKRYKEEIANLGSNIPLKNEIELLRPRSPVQKAQ>YCL056CMVSKKNTAEISAKDIWENIWSGVSSLLDFFAVLENLGVVNDKLYVSGLLRKVWLCYSCISVIKCVWKLIKLCKVKFKIDQRLDGEGNGLVKDKLINFKKKYNEHIRHITAALLQDLSYLMVLIYPGTRLFKRLSNIITLCRIIV>YCL057C-AMSEQAQTQQPAKSTPSKDSNKNGSSVSTILDTKWDIVLSNMLVKTAMGFGVGVFTSVLFFKRRAFPVWLGIGFGVGRGYAEGDAIFRSSAGLRSSKV>YCL057WMRLLLCKNWFASPVISPLLYTRSLYSMANTTSFPIAPQAPPNWSFTPSDISGKTNEIINNSNNFYDSMSKVESPSVSNFVEPFMKFENELGPIINQLTFLQHVSSDKEIRDASVNSSMKLDELNIDLSLRHDIFLQFARVWQDVQSKADSVERETFKYVEKSYKDYIHSGLELDEGNRLKIKEIKKKISVNSINFSKNLGEQKEYITFTKEQLEGVPDSILTQFETIKSDKDSNETLYKVTFKYPDIFPVMKLASSAQTRKQAFLADQNKVPENEAILLDTLKLRDELASLLGYDTYANYNLYDKMAEDSTTVMNFLNDLKDKLIPLGRKELQVLQDMKAEDVKKLNQGADPNYYIWDHRYYDNKYLLENFNVDLEKISEYFPLEATITGMLEIYETLFNLKFIETKDSQNKSVWHDDVKQIAVWNMDDPKSPNFVGWIYFDLHPRDGKYGHAANFGLSSSFMIDDTTRSYPVTALVCNFSKSTKDKPSLLKHNEIVTFFHELGHGIHDLVGQNKESRFNGPGSVPWDFVEAPSQMLEFWTWNKNELINLSSHYKTGEKIPESLINSLIKTKHVNGALFTLRQLHFGLFDMKVHTCKDLQNLSICDTWNQLRQDISLISNGGTLSKGYDSFGHIMSDSYSAGYYGYLWAEVFATDMYHTKFAKDPLNAKNGIQYRDIVLARGGLYDINDNLKEFLGREPSKDAFLKELGLQN>YCL059CMVSTHNRDKPWDTDDIDKWKIEEFKEEDNASGQPFAEESSFMTLFPKYRESYLKTIWNDVTRALDKHNIACVLDLVEGSMTVKTTRKTYDPAIILKARDLIKLLARSVPFPQAVKILQDDMACDVIKIGNFVTNKERFVKRRQRLVGPNGNTLKALELLTKCYILVQGNTVSAMGPFKGLKEVRRVVEDCMKNIHPIYHIKELMIKRELAKRPELANEDWSRFLPMFKKRNVARKKPKKIRNVEKKVYTPFPPAQLPRKVDLEIESGEYFLSKREKQMKKLNEQKEKQMEREIERQEERAKDFIAPEEEAYKPNQN>YCL063WMATQALEDITERLLIRSQEAILQLDLWIQRQQRSSICQTTDQESLDKLSQQYNQYMSQLNSLYVRSESVRDKLSKEQQRRLITEDNEHQRIEDLVREFQDITLRLNELATVPNEAPNDSPQSQSTRSSLGSFQPRPLKIIERQRLCMVTPSKPPKKSVGFNPINEVDCPSKTNSLPCSPKKQPARNRTLRAAKSHDTGLNKSKKPSSSDTYESFFKNRQRLSLTFFDEMDDEDFDSDQDTIILPNISTPPHVGVTAKGAEFEPLRRYNSHESILSNKPAPSKSLNLGSFSASFFRPSNPTFGTSISNVQVNCHPTVAATMAPSRNGPRISSSKALLSSFIARSDTHTVKENNTNLKHASFMDKFNSSLSTISESFQSKRGRKNKGMNEERISNHNVAQEQKNNMDISVSIEELQDALNTELLF>YCL064CMSIVYNKTPLLRQFFPGKASAQFFLKYECLQPSGSFKSRGIGNLIMKSAIRIQKDGKRSPQVFASSGGNAGFAAATACQRLSLPCTVVVPTATKKRMVDKIRNTGAQVIVSGAYWKEADTFLKTNVMNKIDSQVIEPIYVHPFDNPDIWEGHSSMIDEIVQDLKSQHISVNKVKGIVCSVGGGGLYNGIIQGLERYGLADRIPIVGVETNGCHVFNTSLKIGQPVQFKKITSIATSLGTAVISNQTFEYARKYNTRSVVIEDKDVIETCLKYTHQFNMVIEPACGAALHLGYNTKILENALGSKLAADDIVIIIACGGSSNTIKDLEEALDSMRKKDTPVIEVADNFIFPEKNIVNLKSA>YCL066WMFTSKPAFKIKNKASKSYRNTAVSKKLKEKRLAEHVRPSCFNIIRPLKKDIQIPVPSSRFLNKIQIHRIASGSQNTQFRQFNKTSIKSSKKYLNSFMAFRAYYSQFGSGVKQNVLSSLLAEEWHADKMQHGIWDYFAQQYNFINPGFGFVEWLTNNYAEVRGDGYWEDVFVHLAL>YCL068CMFVLIDNVLAYLLEQDDLFVTARFAIQGQIVSRRVNKIHISNITDVLLQQFISHTLPYNDNIVPKKILDSMRTAVRQLLEATACVSRECPLVKRSQDIKRARKRLLSDWYRLGADANMDAVLLVVNSAWRFLAVWRPFVNSIQHATQELYQNIAHYLLHGNVNIQRVTALLQLVMGQDDLLFSMDDVLQEVFRIQLYLNKMLPHNSHKWQKPSPFDSANLLLNFRDWTTDNALLQELLLSYPTINKNKHKNHSVPRLIQV>YCR002CMDPLSSVQPASYVGFDTITNQIEHRLLKKGFQFNIMVVGQSGLGKSTLINTLFASHLIDSATGDDISALPVTKTTEMKISTHTLVEDRVRLNINVIDTPGFGDFIDNSKAWEPIVKYIKEQHSQYLRKELTAQRERFITDTRVHAILYFLQPNGKELSRLDVEALKRLTEIANVIPVIGKSDTLTLDERTEFRELIQNEFEKYNFKIYPYDSEELTDEELELNRSVRSIIPFAVVGSENEIEINGETFRGRKTRWSAINVEDINQCDFVYLREFLIRTHLQDLIETTSYIHYEGFRARQLIALKENANSRSSAHMSSNAIQR>YCR004CMVKIAIITYSTYGHIDVLAQAVKKGVEAAGGKADIYRVEETLPDEVLTKMNAPQKPEDIPVATEKTLLEYDAFLFGVPTRFGNLPAQWSAFWDKTGGLWAKGSLNGKAAGIFVSTSSYGGGQESTVKACLSYLAHHGIIFLPLGYKNSFAELASIEEVHGGSPWGAGTLAGPDGSRTASPLELRIAEIQGKTFYETAKKLFPAKEAKPSTEKKTTTSDAAKRQTKPAAATTAEKKEDKGLLSCCTVM>YCR005CMTVPYLNSNRNVASYLQSNSSQEKTLKERFSEIYPIHAQDVRQFVKEHGKTKISDVLLEQVYGGMRGIPGSVWEGSVLDPEDGIRFRGRTIADIQKDLPKAKGSSQPLPEALFWLLLTGEVPTQAQVENLSADLMSRSELPSHVVQLLDNLPKDLHPMAQFSIAVTALESESKFAKAYAQGISKQDYWSYTFEDSLDLLGKLPVIAAKIYRNVFKDGKMGEVDPNADYAKNLVNLIGSKDEDFVDLMRLYLTIHSDHEGGNVSAHTSHLVGSALSSPYLSLASGLNGLAGPLHGRANQEVLEWLFALKEEVNDDYSKDTIEKYLWDTLNSGRVIPGYGHAVLRKTDPRYMAQRKFAMDHFPDYELFKLVSSIYEVAPGVLTEHGKTKNPWPNVDAHSGVLLQYYGLKESSFYTVLFGVSRAFGILAQLITDRAIGASIERPKSYSTEKYKELVKNIESKL>YCR008WMTGMNDNNAAIPQQTPRKHALSSKVMQLFRSGSRSSRQGKASSNIQPPSNINTNVPSASKSAKFGLHTPTTATPRVVSNPSNTAGVSKPGMYMPEYYQSASPSHSSSSASLNNHIDINTSKSSSAASLTSSVSALSLSPTSAINISSKSLSPKFSHHSNSNTAITPAPTPTASNINNVNKITNTSAPICGRFLVHKDGTHEHHLKNAKRQEKLSTMIKNMVGASKLRGEAKSAVPDIIMDPKTTLKSNKNPPTLFAGFMKQVVDMDDKYPEGAPTSGALNCPERDIYRSDQKDSKNNTHNITTTKKDRQCFAEKYGRCQEVLGKGAFGVVRICQKKNVSSQDGNKSEKLYAVKEFKRRTSESAEKYSKRLTSEFCISSSLHHTNIVTTLDLFQDAKGEYCEVMEYCAGGDLFTLVVAAGKLEYMEADCFFKQLIRGVVYMHEMGVCHRDLKPENLLLTHDGVLKITDFGNSECFKMAWEKNIHLSGGVCGSSPYIAPEEYIKEEFDPRPVDIWACGVIYMAMRTGRQLWSSAEKDDPFYMNYLKGRKEKGGYEPIESLKRARCRNVIYSMLDPVPYRRINGKQILNSEWGREIKCCHNGRALK>YCR009CMSWEGFKKAINRAGHSVIIKNVDKTIDKEYDMEERRYKVLQRAGEALQKEAKGFLDSLRAVTASQTTIAEVISNLYDDSKYVAGGGYNVGNYYLQCVQDFDSETVKQLDGPLRETVLDPITKFSTYFKEIEEAIKKRDHKKQDFDAAKAKVRRLVDKPAKDASKLPRAEKELSLAKDIFENLNNQLKTELPQLVSLRVPYFDPSFEALIKIQLRFCTDGYTRLAQIQQYLDQQSRDDYANGLLDTKIEELLGQMTSLDICALGIK>YCR010CMSDKEQTSGNTDLENAPAGYYSSHDNDVNGVAEDERPSHDSLGKIYTGGDNNEYIYIGRQKFLKSDLYQAFGGTLNPGLAPAPVHKFANPAPLGLSAFALTTFVLSMFNARAQGITVPNVVVGCAMFYGGLVQLIAGIWEIALENTFGGTALCSYGGFWLSFAAIYIPWFGILEAYEDNESDLNNALGFYLLGWAIFTFGLTVCTMKSTVMFFLLFFLLALTFLLLSIGHFANRLGVTRAGGVLGVVVAFIAWYNAYAGVATKQNSYVLARPFPLPSTERVIF>YCR011CMGSHRRYLYYSILSFLLLSCSVVLAKQDKTPFFEGTSSKNSRLTAQDKGNDTCPPCFNCMLPIFECKQFSECNSYTGRCECIEGFAGDDCSLPLCGGLSPDESGNKDRPIRAQNDTCHCDNGWGGINCDVCQEDFVCDAFMPDPSIKGTCYKNGMIVDKVFSGCNVTNEKILQILNGKIPQITFACDKPNQECNFQFWIDQLESFYCGLSDCAFEYDLEQNTSHYKCNDVQCKCVPDTVLCGAKGSIDISDFLTETIKGPGDFSCDLETRQCKFSEPSMNDLILTVFGDPYITLKCESGECVHYSEIPGYKSPSKDPTVSWQGKLVLALTAVMVLALFTFATFYISKSPLFRNGLGSSKSPIRLPDEDAVNNFLQNEDDTLATLSFENITYSVPSINSDGVEETVLNEISGIVKPGQILAIMGGSGAGKTTLLDILAMKRKTGHVSGSIKVNGISMDRKSFSKIIGFVDQDDFLLPTLTVFETVLNSALLRLPKALSFEAKKARVYKVLEELRIIDIKDRIIGNEFDRGISGGEKRRVSIACELVTSPLVLFLDEPTSGLDASNANNVIECLVRLSSDYNRTLVLSIHQPRSNIFYLFDKLVLLSKGEMVYSGNAKKVSEFLRNEGYICPDNYNIADYLIDITFEAGPQGKRRRIRNISDLEAGTDTNDIDNTIHQTTFTSSDGTTQREWAHLAAHRDEIRSLLRDEEDVEGTDGRRGATEIDLNTKLLHDKYKDSVYYAELSQEIEEVLSEGDEESNVLNGDLPTGQQSAGFLQQLSILNSRSFKNMYRNPKLLLGNYLLTILLSLFLGTLYYNVSNDISGFQNRMGLFFFILTYFGFVTFTGLSSFALERIIFIKERSNNYYSPLAYYISKIMSEVVPLRVVPPILLSLIVYPMTGLNMKDNAFFKCIGILILFNLGISLEILTIGIIFEDLNNSIILSVLVLLGSLLFSGLFINTKNITNVAFKYLKNFSVFYYAYESLLINEVKTLMLKERKYGLNIEVPGATILSTFGFVVQNLVFDIKILALFNVVFLIMGYLALKWIVVEQK>YCR015CMKTIIISDFDETITRVDTICTIAKLPYLLNPRLKPEWGHFTKTYMDGYHKYKYNGTRSLPLLSSGVPTIISQSNFNKLFADELKYQNHNRVVELNSVNEITKQQIFKSISLDQMKTFARDQNHEDCLLRDGFKTFCSSVVKNFESDFYVLSINWSKEFIHEVIGDRRLKNSHIFCNDLKKVSDKCSQSYNGEFDCRLLTGSDKVKILGEILDKIDSGCNKEGNSCSYWYIGDSETDLLSILHPSTNGVLLINPQENPSKFIKITEKIIGIPKDKISSFEADNGPAWLQFCEKEGGKGAYLVKSWDSLKDLIMQVTKM>YCR016WMSENHVPAWKRIALKRQTISSGDESKEKGQSNLIDDDPLNITTHLSTGNLTKKEKKRIINGESKSSTKKGKRVSKPGTKKKEKLSKDEKNSKKNKILKDQLRYLIEFFRTKSESKFPTGILELESVKENYGDSLIKDEPSESGVVEVWKFSKQKQNWLIKHFFNLDEIPSVYNDLLLLYFRDLQGKSKEELISKCKGKLKQWNDYVEDQETKIKALIAEDKASEPINGEEKEEGEKDGNAEQGKQKEVQDEQEEVQMPNKELVQRSLKLLEIWKNDDSEQIELKNFFVDV>YCR017CMLIINGKIIPIAHTICAFSAFFAALVTGYSLHFHKIVTNAHYTYPDEWFPSVSATIGDRYPERSIFQILIALTAFPRFLLLLGHYYLNQSKVCFLVGVLRTVSCGGWVYITSTDDHDIHDIFMITYIVLTLPWDIMITRYSSPLTSKNKGLTATIFFGTLFPMIYWYIQHSVQQRAGAYSIYAYFEWSLILLDIAFDAFAYADFKKIDIVLAFNEKPGNTSFFQIRDSSPINYGEEKSSELQKSGEKKVEKEKPVARSATGSYFRFDSFFYLLTNIFNGFLFWSNVTSLLCSIWHFPLWYMGISGYEAAILGYLGPIFLYLPFVSEAFTQYGVLLGGIIAIGAYIVQMPELRLISVAVGTSITVATFVQNLRYITNAETSFSFALTWLLGLVASVILKMGFYTNNPTWVILDERNGGYNKTALVLTVLFGMLSPYVNSINFEGKRNAQAKSASLIGKLFLAVGFGSLLFGIHQLLTDSSTTIYWAWEGYNESHGPLPWPWGALTCTVMLFASLSSVKFMGKPLVPCLLLLISTAVLSARSITQWPKYIFGGLLYAIAMLWLVPSYFSALGQVQNIWVYVLSFSVYIIFVLAHVWVVAYAFVPMGWVLREKIETVLAFSSTFIIIGALTCKNLNIQLVTMGKKFFIYVFFFAVALLSLTARFVYDIRPTGIPQPYHPDSQLITAGIWTIHFGLDNDMWASEDRMINLIKDMELDVVGLLETDTQRITMGNRDLTSKLAHDLNMYADFGPGPNKHTWGCVLLSKFPIVNSTHHLLPSPVGELAPAIHATLQTYNDTLVDVFVFHSGQEEDEEDRRLQSNYMAKLMGNTTRPAILLSYLVVDPGEGNYNTYVSETSGMHDIDPSDDDRWCEYILYRGLRRTGYARVARGTITDTELQVGKFQVLSEQALVEHSDSMYEYGHMSEPEYEDMKFPDKFLGEGERGHFYHVFDEPRYYL>YCR018CMRYNNYDNSGSSFLTRVVKKSDMEKTLLLNREIDDWKSNDKKKAYKERGRVYASCSFIEVSFSQIRAVDVEKKIENAEQLRDLTRNIVKNKTSSLNEITPSKNRVTSACNSERRTTSQEANNLEGYHSCAQGTSRSASITKKYSKKTTSRPKREKRQTILPNGEIKECSKCKDTWTIQWRSGPDQNRELCSPCGLAYGKRLKKENEKKRQAADKRIDRNNP>YCR020C-AMDILKLSDFIGNTLIVSLTEDRILVGSLVAVDAQMNLLLDHVEERMGSSSRMMGLVSVPRRSVKTIMIDKPVLQELTANKVELMANIV>YCR020CMSCTTDKLIQKYDALVRKTTEHKFAKELCAGTLKDRSLYIYLSQDLQFFETSLRLICKTTSLAPTTHALITLAKKIGFFSNDENSYFHDCLELLAPSLTKEERDNFDNKAIPGVDAYINFLDELRKDASITWPSLVTSLWVAEELYWRWARDTPRAPGLHWKYQKWIDLHDGEHFQTWCEFLKAEVDKFPVEEVESIFVKVSQFEFEFFESCYNA>YCR021CMNDTLSSFLNRNEALGLNPPHGLDMHITKRGSDWLWAVFAVFGFILLCYVVMFFIAENKGSRLTRYALAPAFLITFFEFFAFFTYASDLGWTGVQAEFNHVKVSKSITGEVPGIRQIFYSKYIAWFLSWPCLLFLIELAASTTGENDDISALDMVHSLLIQIVGTLFWVVSLLVGSLIKSTYKWGYYTIGAVAMLVTQGVICQRQFFNLKTRGFNALMLCTCMVIVWLYFICWGLSDGGNRIQPDGEAIFYGVLDLCVFAIYPCYLLIAVSRDGKLPRLSLTGGFSHHHATDDVEDAAPETKEAVPESPRASGETAIHEPEPEAEQAVEDTA>YCR023CMARQKLTFKEQMDGFPWVQLVVVSLVRFSEPIAFSSLFPYVYFMVRDFNIAPNDAQVSKYSGYLSSSFALCQVISAYHWGRFSEKHGRKITLTCGLIGTSVSLLILGFSRNFYQALVARSLMGLLNGNVGVIRTIIGEIATERKHQALAFSTMPLLFQFGAVVGPMIGGFLVFRDGTMNEVPLWFPHFAKRIIRSYPYALPNVVVCMFLMFGLTNATLFLEETHPAFKDRRDYGLEVGDFIKKNIFGIQPKRRPWQKRIQDDSENIHHRNENVNSIRGQDSEEDENSPLVNTTNDDDTESIQSIDPILTRRQSVGLIRTYSLHEPTDAVHANIDTAPDGCKESSIFHHVFHTKVFYPISVNFIMALHLIVYNEFLPVFLAYDLAVDPENPKKLASKFPWKISGGIGYEPEQTGTLLSTTGIFGCFVVIFIFPIVDRNFDCLTIFRTLVKLYPIMYVMVPYVVFLQNERIPSWYTVVYLYIITGIKTFCGALTSPQIMLLIHNSSPLSCRSVINGATISISASARFIGPLVWGYIMSWSQQNDVAWVSWWSLSLFCMVALYQSYKIAPIDDNENELHGQGSEDAYNSQSQSSDLRMAHRSSLSSLSNQRCTT>YCR024CMFHAFTFLKGGRFYSSLTVKSLYEQVHHTSHDPISINGWIKSIRLLKRIAFLDLQDGTSVNPLRIVIPLTNTDEVQFLKILKTGQTLSISNATWQSTPNRKQPFELQIKNPVKSIKLVGPVSENYPLQKKYQTLRYLRSLPTLKYRTAYLSAILRLRSFVEFQFMLYFQKNHFTKVSPPILTSNDCEGAGELFQVSTNTSPTASSYFGKPTYLTVSTQLHLEILALSLSRCWTLSPCFRAEKSDTPRHLSEFWMLEVEMCFVNSVNELTSFVETTIKHIIKACIDNQQELLPKQFISSQENNASSELSINQETQQIKTRWEDLINEKWHNITYTNAIEILKKRHNEVSHFKYEPKWGQPLQTEHEKFLAGEYFKSPVFVTDYPRLCKPFYMKQNSTPDDTVGCFDLLVPGMGEIIGGSLREDDYDKLCREMKARGMNRSGELDWYVSLRKEGSAPHGGFGLGFERFISYLYGNHNIKDAIPFYRTSAESIDF>YCR026CMELQNDLESLDNELNDFSEDPFRDDFITDEDAVRSGWRSAWTRMKYWFYKNRLKWTNNPIVIGDAKDSRDGSNFRRGIPLYELDANGQPIDTELVDENELSFGTGFHSKVPFKIIFRTLFGSLVFAIFLILMINIAKPHHSTRVLSHFGSPEFDPYVKYFNGTHEFFPLTIVISLDGFHPSLISKRNTPFLHDLYELKYDGGMNITSTPFMVPSFPTETFPNHWTLVTGQYPIHHGIVSNVFWDPDLNEEFHPGVLDPRIWNNNDTEPIWQTVQSAFDGDIPFKAATHMWPGSDVNYTKYNEEKLQPEHKNPIARERTPFYFDEFNAKEPLSQKLSKIIEYVDMSTLNERPQLILGYVPNVDAFGHKHGYPSESEYYYEDFTETLGEVDTFLKQLVESLQERNLTSFTNLVIVSDHGMSDIVVPSNVIIWEDLLDEKLRKDYVSHAYLEGPMMAISLKDSGNINEVYHNLKTSIDEDKYTVYVNGNFPKEWNFNDGKNHHMASIWIVPEPGYAVMKKEQLKKVAKGDHKDKNEDNVFTIGSHGYDNNAIDMRSVFIGMGPYFPQGYIEPFQNTEIYNLLCDICGVAEKDRNSNDGTGMLMNQLREPQSSEEVEIEDDFDYLVSKFGEFSTYNIIWGGYPEETEQDNVDNDNDDNDDGNTDEIAAMPSSSLTIKLEMTTSIPSATETLLGETSPSSRSSSSSSIQASATASTVGDWLQDIINDAKDLIDDIIDSIDDLVDSDT>YCR027CMEYATMSSSNSTHNFQRKIALIGARNVGKTTLTVRFVESRFVESYYPTIENEFTRIIPYKSHDCTLEILDTAGQDEVSLLNIKSLTGVRGIILCYSIINRASFDLIPILWDKLVDQLGKDNLPVILVGTKADLGRSTKGVKRCVTKAEGEKLASTIGSQDKRNQAAFIECSAELDYNVEETFMLLLKQMERVEGTLGLDAENNNKCSIM>YCR028CMMKESKSITQHEVERESVSSKRAIKKRLLLFKIDLFVLSFVCLQYWINYVDRVGFTNAYISGMKEDLKMVGNDLTVSNTVFMIGYIVGMVPNNLMLLCVPPRIWLSFCTFAWGLLTLGMYKVTSFKHICAIRFFQALFESCTFSGTHFVLGSWYKEDELPIRSAIFTGSGLVGSMFSGFMQTSIFTHLNGRNGLAGWRWLFIIDFCITLPIAIYGFIFFPGLPDQTSAVSKFSMTRYIFNEQELHYARRRLPARDESTRLDWSTIPRVLKRWHWWMFSLVWVLGGENLGFASNSTFALWLQNQKYTLAQRNNYPSGIFAVGIVSTLCSAVYMSKIPRARHWHVSVFISLVMVIVAVLIRADPLNPKVVFSAQYLGGVAYAGQAVFFSWANIICHADLQERAIVLASMNMFSGAVNAWWSILFFASDMVPKFERGCYALLATAISSGIVSVVIRSLQIKENLSKKQVPYIDANDMPGEDDDDDNQDNENDGDDESMEVELHNEEMAEISNPFR>YCR033WMGYPPPTRRLGDKKRYHYSNNPNRRHPSAVYSKNSFPKSSNNGFVSSPTADNSTNPSVTPSTASVPLPTAAPGSTFGIEAPRPSRYDPSSVSRPSSSSYSSTRKIGSRYNPDVERSSSTTSSTPESMNTSTITHTNTDIGNSRYSRKTMSRYNPQSTSSTNVTHFPSALSNAPPFYVANGSSRRPRSMDDYSPDVTNKLETNNVSSVNNNSPHSYYSRSNKWRSIGTPSRPPFDNHVGNMTTTSNTNSIHQREPFWKANSTTILKSTHSQSSPSLHTKKFHDANKLDKPEASVKVETPSKDETKAISYHDNNFPPRKSVSKPNAPLEPDNIKVGEEDALGKKEVHKSGREIAKEHPTPVKMKEHDELEARAKKVSKINIDGKQDEIWTTAKTVASAVEVSKESQKELTRSVERKESPEIRDYERAYDPKALKTDVTKLTVDNDNKSYEEPLEKVEGCIFPLPKAETRLWELKNQKRNKIISEQKYLLKKAIRNFSEYPFYAQNKLIHQQATGLILTKIISKIKKEEHLKKINLKHDYFDLQKKYEKECEILTKLSENLRKEEIENKRKEHELMEQKRREEGIETEKEKSLRHPSSSSSSRRRNRADFVDDAEMENVLLQIDPNYKHYQAAATIPPLILDPIRKHSYKFCDVNNLVTDKKLWASRILKDASDNFTDHEHSLFLEGYLIHPKKFGKISHYMGGLRSPEECVLHYYRTKKTVNYKQLLIDKNKKRKMSAAAKRRKRKERSNDEEVEVDESKEESTNTIEKEEKSENNAEENVQPVLVQGSEVKGDPLGTPEKVENMIEQRGEEFAGELENAERVNDLKRAHDEVGEESNKSSVIETNNGVQIMDPKGAVQNGYYPEETKELDFSLENALQRKKHKSAPEHKTSYWSVRESQLFPELLKEFGSQWSLISEKLGTKSTTMVRNYYQRNAARNGWKLLVDETDLKRDGTSSESVQQSQILIQPERPNINAYSNIPPQQRPALGYFVGQPTHGHNTSISSIDGSIRPFGPDFHRDTFSKISAPLTTLPPPRLPSIQFPRSEMAEPTVTDLRNRPLDHIDTLADAASSVTNNQNFSNERNAIDIGRKSTTISNLLNNSDRSMKSSFQSASRHEAQLEDTPSMNNIVVQEIKPNITTPRSSSISALLNPVNGNGQSNPDGRPLLPFQHAISQGTPTFPLPAPRTSPISRAPPKFNFSNDPLAALAAVASAPDAMSSFLSKKENNN>YCR035CMAESTTLETIEIHPITFPPEVLARISPELSLQRHLSLGIRPCLRKYEEFRDVAIENNTLSRYADAGNIDTKNNILGSNVLKSGKTIVITSITGGIIEETSAAIKDLDDFGEEELFEVTKEEDIIANYASVYPVVEVERGRVGACTDEEMTISQKLHDSILHSRILPKKALKVKAGVRSANEDGTFSVLYPDELEDDTLNETNLKMKRKWSYVLYAKIVVLSRTGPVFDLCWNSLMYALQSVKLPRAFIDERASDLRMTIRTRGRSATIRETYEIICDQTKSVPLMINAKNIAFASNYGIVELDPECQLQNSDNSEEEEVDIDMDKLNTVLIADLDTEAEETSIHSTISILAAPSGNYKQLTLVGGGAKITPEMIKRSLLLSRVRADDLSTRFNI>YCR037CMRFSHFLKYNAVPEWQNHYLDYNELKNLIYTLQTDELKQETPTGDLNDDADSQTPGPIADIESNIAAGEPSSSKRRFTHKLKRKLFGSKTPSGSKRGDSDEKAIDGNNINEETIELDELSPQGKTTSFNKNFIRKKFFESRSSSVSSEGKTLFSSYDTFVTNLSDEKLKVDDFYKRMEAKFYERFDHLINDLEKEGIVTRLNETFNPEIQALPPLREIISGTSETHSSNNPFEIHSSNIDSELRNRFDYSEEEMDEDDDVDVFADTTDNTALLNYSQFNIKSQKKSLLKQTIINLYIDLCQLKSFIELNRMGFSKITKKSDKVLHMNTRQELIESEEFFKDTYIFQHETLSSLNSKIAQLIEFYAVLMGQPGNVDSCKQELKSYLHDHIVWERSNTWKDMLGLSSQNNDIITIEDEAEKLMQEKLQIEYFKYPLPKPINLKFTKIENLAVPKLFFGKRAMKIGFIIIVTGVLLGVKTFNDPVEHRCMALVECCAFLWASEAIPLHITGLLVPLLTVLFRVLKDDDGKVMGAAAASTEILGTMWSSTIMILLAGFTLGEALSQYNVAKVLASWLLALAGTKPRNVLLMAMSVVFFLSMWISNVASPVLTYSLLTPLLDPLDYTSPFAKALVMGVALSADIGGMASPISSPQNIISMQYLKPYGIGWGQFFAVALPTGILSMLCSWALMILTFKIGKTKLEKFKPIRTRFTIKQYFIIIVTIATILLWCVESQIESAFGSSGEIAVIPIVLFFGTGLLSTKDFNTFPWSIVVLAMGGIALGKAVSSSGLLVTIARALQKKIQNDGVFAILCIFGILMLVVGTFVSHTVSAIIIIPLVQEVGDKLSDPKAAPILVFGCALLASCGMGLASSGFPNVTAISMTDKKGNRWLTVGAFISRGVPASLLAFVCVITLGYGISSSVLKGST>YCR038CMSPKNKYVYICVEYIYIYFAKIHKQSTLSSDTTKMFVLIDNVLAYLLEQDDLFVTARFAIQGQIVSRRVNKIHISNITDVLLQQFISHTLPYNDNIVPKKILDSMRTAVRQLLEATACVSRECPLVKRSQDIKRARKRLLSDWYRLGADANMDAVLLVVNSAWRFLAVWRPFVNSIQHATQELYQNIAHYLLHGNVNIQRVTALIQLVMGQDDLLFSMDDVLQEVFRIQLYLNKMLPHNSHKWQKPSPFDSANLLLNFRDWTTDNALLQELLLSYPTINKNKHKNHSVPRLIQIWVESYWQDSETTLKDILNFWYSHLAEYYEYQELFADIVQLFINKKRTRQLKIHYIGLTDKEIEENKPPLDYENLFLQYEIDKTNANDELCGATDLSDLLFQWKQGELLEVEAFALNVSPWSLAKTLTLLESSLYLDIETIEFTRHFKHNDTTIDSVFTLSNQLSSYVLETTLQQTHTISYWLQVALSCLYLRNLNSLASIITSLQNHSIERLSLPIDVKSDHLFQRLKVVVHPNNNYNVYRRTIKHIFHSQLPCVPFTSLLIRDITFIRDGNDTFTKDGNNVNMQKFNQITKIVAFAQYLQQKQYEDIHCSNTTARSLLGAMIKVHTLYNDNKDRAYQVSIAKVPRLT>YCR039CMNKIPIKDLLNPQITDEFKSSILDINKKLFSICCNLPKLPESVTTEEEVELRDILGFLSRANKNRKISDEEKKLLQTTSQLTTTITVLLKEMRSIENDRSNYQLTQKNKSADGLVFNVVTQDMINKSTKPYRGHRFTKENVRILESWFAKNIENPYLDTKGLENLMKNTSLSRIQIKNWVSNRRRKEKTITIAPELADLLSGEPLAKKKE>YCR042CMMSFSKNATPRAIVSESSTLHEMKFRNFRVAHEKISLDIDLATHCITGSATIIIIPLIQNLEYVTFDCKEMTIKDVLVENRRCDQFIHDDPLQTNLNGLTSQNVLYSDNSIEQSHFLRSKFASLNEYPETDSKSQLTIKIPSSIKISLEDANALSNYTPITPSIKTTPGFQESVFTPITLQIEYEIRNPKSGIKFDTVYADKPWLWNVYTSNGEICSSASYWVPCVDLLDEKSTWELEFSVPRLVKNIGTSKLIGQNGEESEKEKEDTPEHDEEEEGKPARVIKDEDKDSNLKNDEEGKNSKSKDAQDNDEEEEEGESDEEEEEGEEERRNIEESNNPSLRDVIVCCSEYSNIKELPHPIDLTKKKCIFQIINPVAPHHIGWAIGAFNSWSLPLISPPSVDAEDEVEEDKLRENVVDNVNDTMDDDIGSDIIPIQIFTLPTQETDELTVINSTVVCQKIIDFYSKEFGSYPFTCYSMVFLPTAPSKHMDFAALGICNTRLLYPLEVIDKAFSTTNELAWALANQWSCVNITPLDMNDYWCCLGIAGYMVFQVTKKLMGNNTYKYQLKRNSEAIVEQDFEKPPIGSTFTGSSRPISWSSKDLSFIQLKAPMILHILDRRMTKTERSFGMSRVLPKIFLQAMSGDLPNNSLTSSHFQHVCERVNKSKLENFFNEWVYGSGVPILRVTQRFNRKRMVIELGIRQVQDEELGHEKVVGEEGFFKSALDHLEHPDLNRTECFTGSMTIRIHEHDGTPYEHIVEIKDTFTKIDIQYNTKYRRLRKRGGGANDENGVENNNEEKPIVVDVNCLGNVYMSPEECSRFSLTEFNRTSESNELLKQNEAFEWIRIDSDLEWICQMHINQPDYMFSSQLRQDGDIEAQLEAIRYYEDVVVNGGVKSLVYSSILFRTAIDERYFFGIRLAACEALSKYVYDPDFTGGVKHLIQIFQILFCLEDSNIPKSNNFENPKLYFLQCNIPKYLAKVKNENGKCPKLVKQFLLDILVYNENGENKYSDDAYVRSLIENVVKVALNEYKDKAYMEKVKTQLLRYENLVNWLSSYESLIKTTIMYAKYKLHKVGAYDFTELTGMIMHTLTLGINNGDISRESFQNEFLMVLKIMLLEGGLKNKDALVLFTEILCFHEDSYIRDKSVDVLSECVNLVVMDGSLDTISDDIKSSVQSVHNEVKNIKSEDDIELFLSGHYVDDMKIKIEKIGRQNISGLIQICRDMFKGYSPLKILLWDVLNLPVLSLYQRKQIHDLVRVMYTLINSFVVRLETPRERRLVAKMNSNEEGKLDIVIKRESILKVHIKKEVTSTVEAPKKANKIKISLKGDKPVRKVEKQIVKPKVTSKQRKVKSHVNRMGSLPLRFVKIQQQPRVMVHLSSVPYSQFVQITKVTSRSFMVKIRTKNDAKN>YCR043CMIPAPLDASLLREHAFQGTNDLSTVLSPSTFTDEGGYKPVLKYGLGYFNYGLVIDDEVYDYSVCDIIRGHVYDHFWCYFCCFMILFTIWLISLNWCPSSKKSKFDWSKKKDDFKMEGGDLEYQHVKI>YCR045CMKPQCILISLLVNLAYAEEYLVRFKNPTAFQQFTSNSNRSWRQFIDNKIEKKFSIGSFRGVTMNLSKNLVNKLKKSPLVADIVPNFRFEAFEGDSVNSAESSYTFNATAKYSYEDVEEEQNITYQPDAPRHLARISRHYQLPFDVGDKDRYKSWFNYYYEHDYQGQDVNAYIMDTGIFADHPEFEDRVIQGIDLTKEGFGDQNGHGTHVAGLVGSKTYGAAKRVNLVEVKVLGKDGSGEASNVLSGLEFIVEHCTKVSRPQGKKCVANLSLGSFRSPIINMAVEGAIEEGIVFVAAAGNFNLDAYWASPASAENVITVGAFDDHIDTIAKFSNWGPCVNIFAPGVEIESLSHLNYNDTLILSGTSMSTPIVTGVAAILLSKGIEPEMIAQEIEYLSTRNVFHRRTLFFKPSTPNQILYNGVDKLDDPYDDETFPRLNIEAIAKELEEYNATLQTPMSENLQSGSKLWGWNNDVTLPLGEIRLKRRDFMKNL>YCR046CMWSRNVRLLGSWTRSYMVPATKRKTIPVYPPVQRIASSQIMKQVALSEIESLDPGAVKRKLISKKNKDRLKAGDVVRIVYDSSKCSYDTFVGYILSIDRKQLVQDASLLLRNQIAKTAVEIRVPLFSPLIERIDLLTPHVSSRQRNKHYYIRGTRLDVGDLEAGLRRKK>YCR047CMSRPEELAPPEIFYNDSEAHKYTGSTRVQHIQAKMTLRALELLNLQPCSFILDIGCGSGLSGEILTQEGDHVWCGLDISPSMLATGLSRELEGDLMLQDMGTGIPFRAGSFDAAISISAIQWLCNADTSYNDPKQRLMRFFNTLYAALKKGGKFVAQFYPKNDDQVDDILQSAKVAGFSGGLVVDDPESKKNKKYYLVLSSGAPPQGEEQVNLDGVTMDEENVNLKKQLRQRLKGGKDKESAKSFILRKKELMKRRGRKVAKDSKFTGRKRRHRF>YCR048WMTETKDLLQDEEFLKIRRLNSAEANKRHSVTYDNVILPQESMEVSPRSSTTSLVEPVESTEGVESTEAERVAGKQEQEEEYPVDAHMQKYLSHLKSKSRSRFHRKDASKYVSFFGDVSFDPRPTLLDSAINVPFQTTFKGPVLEKQLKNLQLTKTKTKATVKTTVKTTEKTDKADAPPGEKLESNFSGIYVFAWMFLGWIAIRCCTDYYASYGSAWNKLEIVQYMTTDLFTIAMLDLAMFLCTFFVVFVHWLVKKRIINWKWTGFVAVSIFELAFIPVTFPIYVYYFDFNWVTRIFLFLHSVVFVMKSHSFAFYNGYLWDIKQELEYSSKQLQKYKESLSPETREILQKSCDFCLFELNYQTKDNDFPNNISCSNFFMFCLFPVLVYQINYPRTSRIRWRYVLEKVCAIIGTIFLMMVTAQFFMHPVAMRCIQFHNTPTFGGWIPATQEWFHLLFDMIPGFTVLYMLTFYMIWDALLNCVAELTRFADRYFYGDWWNCVSFEEFSRIWNVPVHKFLLRHVYHSSMGALHLSKSQATLFTFFLSAVFHEMAMFAIFRRVRGYLFMFQLSQFVWTALSNTKFLRARPQLSNVVFSFGVCSGPSIIMTLYLTL>YCR050CMVAVHKVRYNVIMILGPEQTPNEKTTLDNCGLARRNLVLLKAVHTNCDSWNMNRYPLTLLKMANMAISWNTALKKKVNNVAWLLLKCNAPMELWYTCLSKNL>YCR051WMNANIWVAASDGNLDRVEHILRESKGAMTPQSKDINGYTPMHAAAAYGHLDLLKKMCNEYNGDINVLDNDGDTPLHHVEDVATARLIVEELGGDFTIRNVEGQTPYDSFVENGEDGELIEYMRIKSGVADVHGVDGVQGEGVIDSKLLEEFKDNVRYTLENDPEEGADEATLQRRRQLEQIITGDNAEEELERYIRAMVREQMLGQGSMAGSGDEPDSKRRK>YCR052WMVTQTNPVPVTYPTDAYIPTYLPDDKVSNLADLKKLIEMDSRLDLYLTRRRLDTSINLPTNTKTKDHPPNKEMLRIYVYNTTESSPRSDSGTPADSGKTTWTLRIEGKLLHESANGKHPFSEFLEGVAVDFKRLKPLGMGKKRKRDSSLSLPLNLQQPEYNDQDSTMGDNDNGEDEDSAEAESREEIVDALEWNYDENNVVEFDGIDIKRQGKDNLRCSITIQLRGVDGGKVQYSPNLATLIGMQTGSVNDAVYSIYKYILINNLFVTEQTEAQDGSNDAEDSSNENNNKNGAGDDDGVEGSTPKDKPELGEVKLDSLLQKVLDTNAAHLPLMNVVQTVNKLVSPLPPIILDYTIDLSKDTTYGATTLDVDVSHILHQPQPQPNLQKEEETDAEDTAKLREITKLALQLNSSAQKYQFFHELSLHPRETLTHYLWSSKQNELVLQGDQYFNEDAARTSDIYSNNNNDRSLMGNISLLYSQGRL>YCR053WMPNASQVYRSTRSSSPKTISFEEAIIQGLATDGGLFIPPTIPQVDQATLFNDWSKLSFQDLAFAIMRLYIAQEEIPDADLKDLIKRSYSTFRSDEVTPLVQNVTGDKENLHILELFHGPTYAFKDVALQFVGNLFEYFLQRTNANLPEGEKKQITVVGATSGDTGSAAIYGLRGKKDVSVFILYPTGRISPIQEEQMTTVPDENVQTLSVTGTFDNCQDIVKAIFGDKEFNSKHNVGAVNSINWARILAQMTYYFYSFFQATNGKDSKKVKFVVPSGNFGDILAGYFAKKMGLPIEKLAIATNENDILDRFLKSGLYERSDKVAATLSPAMDILISSNFERLLWYLAREYLANGDDLKAGEIVNNWFQELKTNGKFQVDKSIIEGASKDFTSERVSNEETSETIKKIYESSVNPKHYILDPHTAVGVCATERLIAKDNDKSIQYISLSTAHPAKFADAVNNALSGFSNYSFEKDVLPEELKKLSTLKKKLKFIERADVELVKNAIEEELAKMKL>YCR057CMKSDFKFSNLLGTVYRQGNITFSDDGKQLLSPVGNRVSVFDLINNKSFTFEYEHRKNIAAIDLNKQGTLLISIDEDGRAILVNFKARNVLHHFNFKEKCSAVKFSPDGRLFALASGRFLQIWKTPDVNKDRQFAPFVRHRVHAGHFQDITSLTWSQDSRFILTTSKDLSAKIWSVDSEEKNLAATTFNGHRDYVMGAFFSHDQEKIYTVSKDGAVFVWEFTKRPSDDDDNESEDDDKQEEVDISKYSWRITKKHFFYANQAKVKCVTFHPATRLLAVGFTSGEFRLYDLPDFTLIQQLSMGQNPVNTVSVNQTGEWLAFGSSKLGQLLVYEWQSESYILKQQGHFDSTNSLAYSPDGSRVVTASEDGKIKVWDITSGFCLATFEEHTSSVTAVQFAKRGQVMFSSSLDGTVRAWDLIRYRNFRTFTGTERIQFNCLAVDPSGEVVCAGSLDNFDIHVWSVQTGQLLDALSGHEGPVSCLSFSQENSVLASASWDKTIRIWSIFGRSQQVEPIEVYSDVLALSMRPDGKEVAVSTLKGQISIFNIEDAKQVGNIDCRKDIISGRFNQDRFTAKNSERSKFFTTIHYSFDGMAIVAGGNNNSICLYDVPNEVLLKRFIVSRNMALNGTLEFLNSKKMTEAGSLDLIDDAGENSDLEDRIDNSLPGSQRGGDLSTRKMRPEVRVTSVQFSPTANAFAAASTEGLLIYSTNDTILFDPFDLDVDVTPHSTVEALREKQFLNALVMAFRLNEEYLINKVYEAIPIKEIPLVASNIPAIYLPRILKFIGDFAIESQHIEFNLIWIKALLSASGGYINEHKYLFSTAMRSIQRFIVRVAKEVVNTTTDNKYTYRFLVSTDGSMEDGAADDDEVLLKDDADEDNEENEENDVVMESDDEEGWIGFNGKDNKLPLSNENDSSDEEENEKELP>YCR059CMDDDHEQLVEELEAVEAIYPDLLSKKQEDGSIIVVKVPQHEYMTLQISFPTHYPSEEAPNVIEVGVCTSLAKRDLYDTKYLQHLFQEVMDSVFHRGSVCLFDFLTELDGVLYVEPEEETEPVQQSDIPTDPFEGWTASDPITDRGSTFMAFAAHVTSEEQAFAMLDLLKTDSKMRKANHVMSAWRIKQDGSAATYQDSDDDGETAAGSRMLHLITIMDVWNVIVVVARWFGGAHIGPDRFKHINSTAREAVVRAGFDS>YCR060WMSQFEKQKEQGNSLFKQGLYREAVHCYDQLITAQPQNPVGYSNKAMALIKLGEYTQAIQMCQQGLRYTSTAEHVAIRSKLQYRLELAQGAVGSVQIPVVEVDELPEGYDRS>YCR061WMVRFVSILSLFGCAATLVTAHDDMDMDMDMDMDMDMNIDTTTSQSIDVSSTASIVPVPHEPKHLHGLPILQSPSLTPAERLYWENYNTTTYFTTQAGNRSALRYHIITLLLVAFVLYPVSLALSAARSRWYLPLLFVNLCICISSVMALSVFKNTFPEEDWYAHNIYGTTSVLLLVFMLVHFFAAVLSVPVSLASKKEYRPVDTIPLNDLESTPVMVNSARGSPSPSSNRDTLFSLSSDTTTATATNNNKRRRAEGEDEGDNTSNHDTLRDEDYDNDDDEIASIEAPPLLPQDIPVFRILFTNTKYQMLAAHLSCVANVVFHMLTYPLFMYIFVDLIIGFAVGNLLGKGIRIFNLLAHWIKGGVFFTLGVVSLARYCGFAAKYGWAWNNISFTSQLTQTRSSNLLFRFAPAGTFTMEFVESFLIFFYGSTNIFLEHLAGNGGAWTAKDLQHVSIAFMFIGTGLCGLLTEYKLNHWRFEHARKRPQTDVVAATPGYSPNPFPAFTIFWTGILMSQHAQSSQFSTTIHTQWGYLLSYGSFFRLLTFLILFLVPNTNSAASKPFTELITSFCLLCGGLVFMESTDQSIEAMEYRGFTPMFTFNLSVGFVSLLMAWEMILFIWKDWLIKTRKTSL>YCR063WMPRIKTRRSKPAPDGFEKIKPTLTDFEIQLRDAQKDKSSKLAAKSNEQLWEIMQLHHQRSRYIYTLYYKRKAISKDLYDWLIKEKYADKLLIAKWRKTGYEKLCCLRCIQKNETNNGSTCICRVPRAQLEEEARKKGTQVSFHQCVHCGCRGCASTD>YCR065WMMNEDISIIDGHNSFLTEKSTVLLTQAKRTLEDEKEMITPPSSTVRKTMKEVNKRPSHPLSPDHSSPIAPSKAKRQRSDTCARSNGNLTLEEILQSLERRRINGELAKKPPYSYATLICLAILQSQEGKLTLSQIYHWIHVHFPYYKQKDASWQNSIRHNLSLNDAFIKTEKSCDGKGHFWEVRPGAETKFFKGENRGYEFVKDSLQDIGKYFEIDSTLDELEQVESGEGNDDLPDEEEREEAGKFPSIEIQLNSSPILRVSQLHHIPQLKTDNSVLNPHENLESMRNMIENDVNNIDSLEPPYVMKKYHTSLGLPSLVNAKDHFQAGVKNNNITQANRFNTLPITSAKSPQNFRKYFTSFNSNFEDLSPLRSNVGAGSLLDPLPYSPLKLYDQKNLALMSKPQSQQSYSNSQLPPPPSSHGSDLLKTPKMRHSDGLEKTPSRLISTPKDGNSILRKWQTPSHLFEDLYCSPLFRAIETPIRYITTPGGTLETQISPRKSSAPDVLTSATNSKFASSGLFGVDVYSVWKRATEKISDGNNTTDSNQKHHPYHNHPSNDSGNEKN>YCR066WMDHQITTASDFTTTSIPSLYQLDTLLRCHICKDFLKVPVLTPCGHTFCSLCIRTHLNNQPNCPLCLFEFRESLLRSEFLVSEIIQSYTSLRSSLLDALRIPKPTPVPENEEVPGPENSSWIELISESESDSVNAADDDLQIVATSERKLAKRSMTDILPLSSKPSKRNFAMFRSERIKKKSKPNEQMAQCPICQQFYPLKALEKTHLDECLTLQSLGKKPKISTTFPTESNPHNKSSSRFKVRTPEVDKSSCGETSHVDKYLNSMMSAEHQRLPKINFTSMTQSQIKQKLSSLGLSTNGTRQNMIKRYNHYEMLWNSNFCDSLEPVDEAELKRQLLSWDVSHNKTPQNSSNKGGISKLMIMKSNGKSSSYRKLLENFKNDKFNRKGWMVMFRKDFARLIREAKMKIKTGSSDSSGSVGHSNDGDGVEKVQSDQGTEDQQMEKDQDTVINEDRVAGERNLPNEDSTDADLSRELMDLNEYSKDPPGNN>YCR068WMLHKSPSRKRFASPLHLGCILTLTVLCLIAYYFALPDYLSVGKSSSRGAMDQKSDGTFRLKSIYRHGVGANHRLHQRLEVTPEVISAAGMLYQETTTQGQDFEDQEPLWTTNAEYATTNPFDFEFELRRMPLLMKRMKERDPEFIESYIYGETYMTEEEEHAMWIDDDIVAPNITDRGTVVSLALMSSNAYVRIPQTGDWRNVTEPWNETEPEDFGWDGDGIRGHVFYNEVENIVVLSIKGTSAQGLPGSGEDETTGNDKINDNLLFSCCCARVSYLWTTVCDCYVKSYICDESCLEKELRRKDRFYSAVVDIYKGVLKEYPDAAIWVTGHSLGGALASLLGRTFGLPAVAFESPGELLPSKRLHLPFPPGLPSYMEGIWHFGHNADPIFMGTCNGASSSCSLVGYAMETACHTGRVCVYDVVNDKGWSVNMFNHRIHKVIDEVLLGYEQAAKCVEPEPCVDCYNWKFIPSRDWESSSRLITKTKSHAAPTTTTRTTATTTSSSTCVGRNWLGFCTKYEL>YCR069WMWLKSLLLCLYSLVLCQVHAAPSSGKQITSKDVDLQKKYEPSPPATHRGIITIEYFDPVSKSMKEADLTFELYGTVVPKTVNNFAMLAHGVKAVIEGKDPNDIHTYSYRKTKINKVYPNKYIQGGVVAPDVGPFTVYGPKFDDENFYLKHDRPERLAMAYFGPDSNTSEFIITTKADGNEELDGKSVVFGQITSGLDQLMDAIQYTETDEYGKPQHELRFLYFVLEILKISNILDLHAAYTEKVEKFRNGDVSVGSTLENIFRNDKAYTPLTTSTGTTAYDLNHPISRALMCLTVLGLCFIAYKGMHEKPHTVSLRHK>YCR071CMISSCVTRCFGRGKCLPGPATASIYQTIRCISTNSNKAAEAPIFPKLEDVKMHELIGNNNFGKKTYYVERSRTGNLPVYSAYKNGGNKIITEIRKIEGDVIQLRNDLQEQLPFIPKKSWSVVMQSKKIIIKGNAVEAVKRVLTKKF>YCR073W-AMTTTVPKIFAFHEFSDVAEAVADHVVHAQDGALAPKNERKHSVPNISMNALDMTREASCKSTASAAEGKSGSSGSGSGSSKPKKEKRFKIALSGGSLIEVLHEGLLKRDDVRWGDWDIYFADERLVPFSSNESNYGCAKRKILDLIDTAKYGTPKVYHIDESLIDDPQECADNYEKVLIRGFAGRDSVKLPMFDLFLLGCAPDGHIASLFPNFQDNLREKLAWVVPVENAPSGPSTRISLTIPVICHSHRVTFVVEGATKAPIIKTIMERPEKGLPSSIVNEGAAGRVSWFVDDDALTDVLVTKKKYKFHQGLSI>YCR076CMIENKVELVAELVLESIGKTEVVSRHTEGTKSCQVSFRIKDSPSEKGSTSFLSELVVIQTLDDNDKYTVVIRHGTSITMACVVGYSDFKLPTELKWPLERESLPVEPDLKPIMTQLKRQTAGSADMPKFDDEYQAQARQNQGTAPLNPYPGLTVTEPSFANPAGGYADGDLYPVGTSHPDWSGGLPNPLGNPSSQGGMIFDPNRRPAPRREDMPPGWMPGSKYDEPFGPGSGGFGGSGSGGFGGSGSGFI>YCR077CMSFFGLENSGNARDGPLDFEESYKGYGEHELEENDYLNDETFGDNVQVGTDFDFGNPHSSGSSGNAIGGNGVGATARSYVAATAEGISGPRTDGTAAAGPLDLKPMESLWSTAPPPAMAPSPQSTMAPAPAPQQMAPLQPILSMQDLERQQRQMQQQFMNFHAMGHPQGLPQGPPQQQFPMQPASGQPGPSQFAPPPPPPGVNVNMNQMPMGPVQVPVQASPSPIGMSNTPSPGPVVGATKMPLQSGRRSKRDLSPEEQRRLQIRHAKVEKILKYSGLMTPRDKDFITRYQLSQIVTEDPYNEDFYFQVYKIIQRGGITSESNKGLIARAYLEHSGHRLGGRYKRTDIALQRMQSQVEKAVTVAKERPSKLKDQQAAAGNSSQDNKQANTVLGKISSTLNSKNPRRQLQIPRQQPSDPDALKDVTDSLTNVDLASSGSSSTGSSAAAVASKQRRRSSYAFNNGNGATNLNKSGGKKFILELIETVYEEILDLEANLRNGQQTDSTAMWEALHIDDSSYDVNPFISMLSFDKGIKIMPRIFNFLDKQQKLKILQKIFNELSHLQIIILSSYKTTPKPTLTQLKKVDLFQMIILKIIVSFLSNNSNFIEIMGLLLQLIRNNNVSFLTTSKIGLNLITILISRAALIKQDSSRSNILSSPEISTWNEIYDKLFTSLESKIQLIFPPREYNVHIMRLQNDKFMDEAYIWQFLASLALSGKLNHQRIIIDEVRDEIFATINEAETLQKKEKELSVLPQRSQELDTELKSIIYNKEKLYQDLNLFLNVMGLVYRDGEISELK>YCR082WMITPKGTHDAVAKFQKTDLHQDLDYIVLQQRRTQLETLINERESFVKNLCSLFHKIQNTKNYQEFVDVLAENRDLLREIFTVENGFQKQKWISNDDIPQIDWDKFALDINAYIAENDQLLALYEDGLL>YCR083WMLFYKPVMRMAVRPLKSIRFQSSYTSITKLTNLTEFRNLIKQNDKLVIDFYATWCGPCKMMQPHLTKLIQAYPDVRFVKCDVDESPDIAKECEVTAMPTFVLGKDGQLIGKIIGANPTALEKGIKDL>YCR086WMDPLTVYKNSVKQQIDSADLLVANLVNENFVLSEKLDTKATEIKQLQKQIDSLNAQVKELKTQTSQQAENSEVIKDLYEYLCNVRVHKSYEDDSGLWFDISQGTHSGGSSDDYSIMDYKLGFVKGQAQVTEVIYAPVLKQRSTEELYSLQSKLPEYLFETLSFPLSSLNQFYNKIAKSLNKKREKKDETE>YCR087C-AMVTFNCEVCNDTVPKKNTEKHYYRCPNAYYTCIDCSKTFEDGVSYKNHTSCISEDEKYQKALYKGNKKQKQKQQQKQQQKQHQHQPVATPAKKVEKPVIKKAEKVEKTSNGIELHKGKSLYKILKTMKDKGAKKTFLKSLVVDSEGQIRYAKE>YCR088WMALEPIDYTTHSREIDAEYLKIVRGSDPDTTWLIISPNAKKEYEPESTGSSFHDFLQLFDETKVQYGLARVSPPGSDVEKIIIIGWCPDSAPLKTRASFAANFAAVANNLFKGYHVQVTARDEDDLDENELLMKISNAAGARYSIQTSSKQQGKASTPPVKKSFTPSKSPAPVSKKEPVKTPSPAPAAKISSRVNDNNDDDDWNEPELKERDFDQAPLKPNQSSYKPIGKIDLQKVIAEEKAKEDPRLVQKPTAAGSKIDPSSDIANLKNESKLKRDSEFNSFLGTTKPPSMTESSLKNDDDKVIKGFRNEKSPAQLWAERKAKQNSGNAETKAEAPKPEVPEDEPEGEPDVKDLKSKFEGLAASEKEEEEMENKFAPPPKKSEPTIISPKPFSKPQEPVKAEEAEQPKTDYKKIGNPLPGMHIEADNEEEPEENDDDWDDDEDEAAQPPLPSRNVASGAPVQKEEPEQEEIAPSLPSRNSIPAPKQEEAPEQAPEEEIEEEAEEAAPQLPSRSSAAPPPPPRRATPEKKPKENPWATAEYDYDAAEDNELTFVENDKIINIEFVDDDWWLGELEKDGSKGLFPSNYVSLGN>YCR090CMPLFLVLKATLSENVTKVSIENTNESRAEFAFDLQCTSCRELHDSKVIINTFEEYAMPASKGTASFLMKCKFCSKELSVNLCAFEDEYLTDQSDDKWAKIKDVRKKHGLSKVKEDSFIPLSLDCRGCELIKFYPDTITFEVSLSSGKVMSCQLEDNEWYDYDDNLGEEVTMTDFSSSIIKGK>YCR091WMTQQEYRSPSQRLSKGRSMSLPKIFARNLRSLQNNAPPGKNINVNCLNVNSCSLSASPSSQINMACNGNKQDLPIPFPLHVECNDSWSSSKLNKFKSMFNHNRSKSSGTTDASTSEKGTHKREPRSTIHTELLQSSIIGEPNVHSTTSSTLIPNEAICSTPNEISGSSSPDAELFTFDMPTDPSSFHTPSSPSYIAKDSRNLSNGSLNDINENEELQNFHRKISENGSASPLANLSLSNSPIDSPRKNSETRKDQIPMNITPRLRRAASEPFNTAKDGLMREDYIALKQPPSLGDIVEPRRSRRLRTKSFGNKFQDITVEPQSFEKIRLLGQGDVGKVYLMRERDTNQIFALKVLNKHEMIKRKKIKRVLTEQEILATSDHPFIVTLYHSFQTKDYLYLCMEYCMGGEFFRALQTRKSKCIAEEDAKFYASEVVAALEYLHLLGFIYRDLKPENILLHQSGHVMLSDFDLSIQATGSKKPTMKDSTYLDTKICSDGFRTNSFVGTEEYLAPEVIRGNGHTAAVDWWTLGILIYEMLFGCTPFKGDNSNETFSNILTKDVKFPHDKEVSKNCKDLIKKLLNKNEAKRLGSKSGAADIKRHPFFKKVQWSFLRNQDPPLIPALNDNGCELPFILSCNKHPKRNSVSEQETKMFCEKVANDDEIDEADPFHDFNSMSLTKKDHNILTYSENYTYGKILYKATCTRPRHNSSHRSFFKDIIPEL>YCR095CMLVPPANFGIAEEGIYRCSKVETLNLSFLETLNLKTAIFIGGQEPSKFFKDFFTRSSIKWIVLRMSDFSAAAVPVKSSSVSNANLYSNNNSTLSLQEEKKKSTANGSQNSTTGDPVIQEELAYHLTDNDDLMLIKSTCLKRTFKTLLNVDNYNVLLVDKTALVIGILRKIQKWNIASIINEYRLFSGKNRNYFAETFLEIINIEIEQEKDNKTIVDNKAKKLPLENNRTHSIEYKANSGKLIRVNEDDLCREPEVPQRLLTLINQIETKVKNNKVLQVSGVLGDDLKKTSSDLGIFGHRYRLAFNKKENGDYGYYKARGKDNVKIRIPCDSELPDWFRFQRDLWEKENVPEEHHFYREHIFT>YDL001WMVDSNDDQPERHALLHNDIQTSNNSRPSLVQKKYLPTPQDTRKSRASYTGSAMINPTSKQSRTGSGAQRTSRTAQKLKLLPEEPFQRDSERLTDLKNQEVYSQVNRIKDKPARRDAEKLGKAHRHLLPRSTAYCTASSYNMKELVRWLKDCRKLHHTHPKLFDECLYTPFIYNDWRGDKRFEDEDVIRLDDEGGEIIVSDKHPDLFIFEYGVVVMWGFTEREEKAFLNDIEKFEKEKLAEEDIQVEEFNYYVTKSYQPRIYNDFITLRDGSNYMVKLSISHAIAQSVKISLFEELVDNTIEDTQDIPQEIAYSGKVSMSKEDIMKSIGELFILRININLHGSVLDSPEIMWSEPQLEPIYQATRGYLEINQRVSLLNQRLEVISDLLQMLKEQLGHSHEEYLEFIVILLVGVEVLISVINIVVDMLASQH>YDL002CMSVEEKKRRLEELKDQNVVLGLAIQRSRLSVKRLKLEYGVLLERLESRIELDPELNCEDPLPTLASFKQELLTKPFRKSKTKRHKVKERDPNMPKRPTNAYLLYCEMNKERIRQNGSLDVTRDLAEGWKNLNEQDRKPYYKLYSEDRERYQMEMEIYNKKISNIDADDDKEENEQKIKNNEEGSSTKVADSKGGEDGSLVSSN>YDL003WMVTENPQRLTVLRLATNKGPLAQIWLASNMSNIPRGSVIQTHIAESAKEIAKASGCDDESGDNEYITLRTSGELLQGIVRVYSKQATFLLTDIKDTLTKISMLFKTSQKMTSTVNRLNTVTRVHQLMLEDAVTEREVLVTPGLEFLDDTTIPVGLMAQENSMERKVQGAAPWDTSLEVGRRFSPDEDFEHNNLSSMNLDFDIEEGPITSKSWEEGTRQSSRNFDTHENYIQDDDFPLDDAGTIGWDLGITEKNDQNNDDDDNSVEQGRRLGESIMSEEPTDFGFDLDIEKEAPAGNIDTITDAMTESQPKQTGTRRNSKLLNTKSIQIDEETENSESIASSNTYKEERSNNLLTPQPTNFTTKRLWSEITESMSYLPDPILKNFLSYESLKKRKIHNGREGSIEEPELNVSLNLTDDVISNAGTNDNSFNELTDNMSDFVPIDAGLNEAPFPEENIIDAKTRNEQTTIQTEKVRPTPGEVASKAIVQMAKILRKELSEEKEVIFTDVLKSQANTEPENITKREASRGFFDILSLATEGCIGLSQTEAFGNIKIDAKPALFERFINA>YDL004WMLRSIIGKSASRSLNFVAKRSYAEAAAASSGLKLQFALPHETLYSGSEVTQVNLPAKSGRIGVLANHVPTVEQLLPGVVEVMEGSNSKKFFISGGFATVQPDSQLCVTAIEAFPLESFSQENIKNLLAEAKKNVSSSDAREAAEAAIQVEVLENLQSVLK>YDL005CMVVQNSPVSSVHTANFSERGSNTRTMTYKNKLTVCFDDILKVGAEMMMQQQLKNVQLDSYLVNGFSQSQQKLLKEKVKLFHGILDDLETSLSQSSSYLETLTALGKEKEKEREEAEKKRAEQENMRKVREQEELKKRQELEEASQQQQLQQNSKEKNGLGLNFSTTAPANTTDANGSKENYQELGSLQSSSQTQLENANAANNGAAFSPLTTTRIQSQQAQPSDVMFNDLNSMDISMFSGLDSTGFDSTAFNATVDETKGFDDNDSGNNYNDINISSIENNINNNINSTKNGKDNNNESNKNNNGDEKNKNNNEDNENNNNSSEKNNNNNNNNNNNNDDNGNNNNNNSGNDNNNTTNNDSNNKNNSITTGNDNENIVNNDLPTTVVSNPGDNPPPADNGEEYLTLNDFNDLNIDWSTTGDNGELDLSGFNI>YDL006WMSNHSEILERPETPYDITYRVGVAENKNSKFRRTMEDVHTYVKNFASRLDWGYFAVFDGHAGIQASKWCGKHLHTIIEQNILADETRDVRDVLNDSFLAIDEEINTKLVGNSGCTAAVCVLRWELPDSVSDDSMDLAQHQRKLYTANVGDSRIVLFRNGNSIRLTYDHKASDTLEMQRVEQAGGLIMKSRVNGMLAVTRSLGDKFFDSLVVGSPFTTSVEITSEDKFLILACDGLWDVIDDQDACELIKDITEPNEAAKVLVRYALENGTTDNVTVMVVFL>YDL007WMGQGVSSGQDKKKKKGSNQKPKYEPPVQSKFGRKKRKGGPATAEKLPNIYPSTRCKLKLLRMERIKDHLLLEEEFVSNSEILKPFEKKQEEEKKQLEEIRGNPLSIGTLEEIIDDDHAIVTSPTMPDYYVSILSFVDKELLEPGCSVLLHHKTMSIVGVLQDDADPMVSVMKMDKSPTESYSDIGGLESQIQEIKESVELPLTHPELYEEMGIKPPKGVILYGAPGTGKTLLAKAVANQTSATFLRIVGSELIQKYLGDGPRLCRQIFKVAGENAPSIVFIDEIDAIGTKRYDSNSGGEREIQRTMLELLNQLDGFDDRGDVKVIMATNKIETLDPALIRPGRIDRKILFENPDLSTKKKILGIHTSKMNLSEDVNLETLVTTKDDLSGADIQAMCTEAGLLALRERRMQVTAEDFKQAKERVMKNKVEENLEGLYL>YDL008WMKVKINEVHSVFAWSWHIPSTSDEDAANNDPIGNDEDEDVCGICRASYNGTCPSCKFPGDQCPLVIGLCHHNFHDHCIYRWLDTPTSKGLCPMCRQTFQLQKGLAINDAHVQKFVEIVSRRREEMIEEGVAEEFVDFDEPIRQNTDNPIGRQQVDTILDEDFLLR>YDL010WMIPSNKRNARILSITTLLLLLVFFVAQNANFLTVEIKEETSKAFSTNMDNMAGGSSREYAAMPTSTTNKGSSEVDEEINEIKQKVGLQQPIASVDDSLSAIKNDKGSRITKAFNVQKEYSLILDLSPIIIFSKSTCSYSKGMKELLENEYQFIPNYYIIELDKHGHGEELQEYIKLVTGRGTVPNLLVNGVSRGGNEEIKKLHTQGKLLESLQVWSDGKFSVEQREKPSNN>YDL013WMHSDTNGRTKSNNSPSDNNPNETVILIDSDKEEDASIREANLPVRLYPDRRVGRRRDALNRFVRSDSRSRNSQRTHITASSERPDFQANNDDITIIREVGRFFGDDGPIDPSAHYVDLDQEPGSETLETPRTIQVDNTNGYLNDNGNNNESDDGLTIVEERTTRPRVTLNLPGGERLEVTATTTDIPIRRSFEFQEDLGASRRQLLRRSATRARNLFVDRSDENDEDWTDDTHNLPEAIQRARRESRMRMSRRIAERQRRVQQQRVSSDENISTSIRLQSIRERIQSYTPDIRSAFHRAESLHEFRSILQNVAPITLQECEEELMALFTEFRNQLLQNWAIDRVRNTQEEALRLHREALERQERTAGRVFHRGTLRESITNYLNFNGEDGFLSRLWSGPALSDADEERHTQNIIDMIQEREERERDVVMKNLMNKTRAQQEEFEARAASLPEGYSASFDTTPKMKLDITKNGKEETIIVTDDDLAKTLEDIPVCCLCGAELGVGIPDDFTGISQKDRGVSFEGLVSKYKFHCPYQTLARPSMLDRDLSKRTFIASCGHAFCGRCFARIDNAKKKSKMPKKKLAQLKGSAHPDNYGPKLCPADSCKKLIRSRGRLKEVYF>YDL014WMSFRPGSRGGSRGGSRGGFGGRGGSRGGARGGSRGGFGGRGGSRGGARGGSRGGFGGRGGSRGGARGGSRGGRGGAAGGARGGAKVVIEPHRHAGVYIARGKEDLLVTKNMAPGESVYGEKRISVEEPSKEDGVPPTKVEYRVWNPFRSKLAAGIMGGLDELFIAPGKKVLYLGAASGTSVSHVSDVVGPEGVVYAVEFSHRPGRELISMAKKRPNIIPIIEDARHPQKYRMLIGMVDCVFADVAQPDQARIIALNSHMFLKDQGGVVISIKANCIDSTVDAETVFAREVQKLREERIKPLEQLTLEPYERDHCIVVGRYMRSGLKK>YDL015CMPITIKSRSKGLRDTEIDLSKKPTLDDVLKKISANNHNISKYRIRLTYKKESKQVPVISESFFQEEADDSMEFFIKDLGPQISWRLVFFCEYLGPVLVHSLFYYLSTIPTVVDRWHSASSDYNPFLNRVAYFLILGHYGKRLFETLFVHQFSLATMPIFNLFKNCFHYWVLSGLISFGYFGYGFPFGNAKLFKYYSYLKLDDLSTLIGLFVLSELWNFYCHIKLRLWGDYQKKHGNAKIRVPLNQGIFNLFVAPNYTFEVWSWIWFTFVFKFNLFAVLFLTVSTAQMYAWAQKKNKKYHTRRAFLIPFVF>YDL017WMTSKTKNIDDIPPEIKEEMIQLYHDLPGIENEYKLIDKIGEGTFSSVYKAKDITGKITKKFASHFWNYGSNYVALKKIYVTSSPQRIYNELNLLYIMTGSSRVAPLCDAKRVRDQVIAVLPYYPHEEFRTFYRDLPIKGIKKYIWELLRALKFVHSKGIIHRDIKPTNFLFNLELGRGVLVDFGLAEAQMDYKSMISSQNDYDNYANTNHDGGYSMRNHEQFCPCIMRNQYSPNSHNQTPPMVTIQNGKVVHLNNVNGVDLTKGYPKNETRRIKRANRAGTRGFRAPEVLMKCGAQSTKIDIWSVGVILLSLLGRRFPMFQSLDDADSLLELCTIFGWKELRKCAALHGLGFEASGLIWDKPNGYSNGLKEFVYDLLNKECTIGTFPEYSVAFETFGFLQQELHDRMSIEPQLPDPKTNMDAVDAYELKKYQEEIWSDHYWCFQVLEQCFEMDPQKRSSAEDLLKTPFFNELNENTYLLDGESTDEDDVVSSSEADLLDKDVLLISE>YDL018CMSNLCVLFFQFFFLAQFFAEASPLTFELNKGRKECLYTLTPEIDCTISYYFAVQQGESNDFDVNYEIFAPDDKNKPIIERSGERQGEWSFIGQHKGEYAICFYGGKAHDKIVDLDFKYNCERQDDIRNERRKARKAQRNLRDSKTDPLQDSVENSIDTIERQLHVLERNIQYYKSRNTRNHHTVCSTEHRIVMFSIYGILLIIGMSCAQIAILEFIFRESRKHNV>YDL020CMASTELSLKRTLTDILEDELYHTNPGHSQFTSHYQNYHPNASITPYKLVNKNKENNTFTWNHSLQHQNESSAASIPPQQTYHFPIFNKYADPTLTTTTSFTTSEATANDRQINNVHLIPNEIKGASETPLQKTVNLKNIMKVSDPYVPTRNTFNYDVKISNDFFDNGDNLYGNDEEVLFYEDNYNPKMQWSLQDNSAAINNEDARAIFNNEFDSDDDDISDDEEDEIEENCLQQEQHQEEPLLSLDVTPISMFGSDQKTGRAKSSSHLFNEYSYVDSNMDSISSVVSEDLLDERGHEKIEDEDEDNDLDEDDIYDISLLKNRRKQSFVLNKNTIDFERFPSPSTSANVPSTATTGKRKPAKSSSNRSCVSNSNENGTLERIKKPTSAVVSSNASRRKLINYTKKHLSSHSSTNSNSKPSTASPSAHTSSSDGNNEIFTCQIMNLITNEPCGAQFSRSYDLTRHQNTIHAKRKIVFRCSECIKILGSEGYQKTFSRLDALTRHIKSKHEDLSLEQRQEVTKFAKANIGYVMG>YDL021WMTASTPSNVMTLFLLRHGQSELNHENIFCGWIDAKLTEKGKEQARHSAELIEQYCKANNLRLPQIGYTSRLIRTQQTIETMCEEFKLKPQLQVVYDFNKIKLGDEFGSDDKDNMKIPILQTWRLNERHYGSWQGQRKPNVLKEYGKDKYMFIRRDYEGKPPPVDLDREMIQQENEKGSSTGYEFKEPNRQIKYELECSNHDIVLPDSESLREVVYRLNPFLQNVILKLANQYDESSCLIVGHGSSVRSLLKILEGISDDDIKNVDIPNGIPLVVELDKNNGLKFIRKFYLDPESAKINAEKVRNEGFIKNP>YDL022WMSAAADRLNLTSGHLNAGRKRSSSSVSLKAAEKPFKVTVIGSGNWGTTIAKVVAENCKGYPEVFAPIVQMWVFEEEINGEKLTEIINTRHQNVKYLPGITLPDNLVANPDLIDSVKDVDIIVFNIPHQFLPRICSQLKGHVDSHVRAISCLKGFEVGAKGVQLLSSYITEELGIQCGALSGANIATEVAQEHWSETTVAYHIPKDFRGEGKDVDHKVLKALFHRPYFHVSVIEDVAGISICGALKNVVALGCGFVEGLGWGNNASAAIQRVGLGEIIRFGQMFFPESREETYYQESAGVADLITTCAGGRNVKVARLMATSGKDAWECEKELLNGQSAQGLITCKEVHEWLETCGSVEDFPLFEAVYQIVYNNYPMKNLPDMIEELDLHED>YDL027CMFKVPVGLASRTRELMNSVTLNSLNNGKGFNMYLPGILRAFPKPVPSAITSPAIPKYRGESFQFRKLSCISSNYCSTTHQFLSSLKSSTSRLVGKRAFHSSRRAEIKFIFSSKSPKNGNKPFVKVYKVSPFFIIFATASIFTFILTSTIVVIPLIFHFFFPLLIMFFFFKQFKKWQKNIFYKDVLTSLPKTKLKITLPTMRSLQLQPMVQSWKEISSRMGIPNEFAKGLNVDLVKQEETRKQFLSFLQKRVLESFTKNELGIRSYFLGDSVEKWIKESYDLELDIDNCRSELRKFQTFIFSSVRYKLYLDSMKNLPLNPSKKLEGKKHIADVYVIILDESFPAIMFNGGAYSKADFFKILQESETSNSSKTLNTIIAIKSVNTLLSKHFVITTNGDSGEFFSKYNISKINDKNTEYTLKE>YDL028CMSTNSFHDYVDLKSRTNTRQFSDDEEFTTPPKLSNFGSALLSHTEKTSASEILSSHNNDKIANRLEEMDRSSSRSHPPPSMGNLTSGHTSTSSHSTLFGRYLRNNHQTSMTTMNTSDIEINVGNSLDKSFERIRNLRQNMKEDITAKYAERRSKRFLISNRTTKLGPAKRAMTLTNIFDEDVPNSPNQPINARETVELPLEDSHQTNFKERRENTDYDSIDFGDLNPIQYIKKHNLPTSDLPLISQIYFDKQREENRQAALRKHSSRELLYKSRSSSSSLSSNNLLANKDNSITSNNGSQPRRKVSTGSSSSKSSIEIRRALKENIDTSNNSNFNSPIHKIYKGISRNKDSDSEKREVLRNISINANHADNLLQQENKRLKRSLDDAITNENINSKNLEVFYHRPAPKPPVTKKVEIVEPAKSASLSNNRNIITVNDSQYEKIELLGRGGSSRVYKVKGSGNRVYALKRVSFDAFDDSSIDGFKGEIELLEKLKDQKRVIQLLDYEMGDGLLYLIMECGDHDLSQILNQRSGMPLDFNFVRFYTKEMLLCIKVVHDAGIVHSDLKPANFVLVKGILKIIDFGIANAVPEHTVNIYRETQIGTPNYMAPEALVAMNYTQNSENQHEGNKWKVGRPSDMWSCGCIIYQMIYGKPPYGSFQGQNRLLAIMNPDVKIPFPEHTSNNEKIPKSAIELMKACLYRNPDKRWTVDKVLSSTFLQPFMISGSIMEDLIRNAVRYGSEKPHISQDDLNDVVDTVLRKFADYKI>YDL029WMDPHNPIVLDQGTGFVKIGRAGENFPDYTFPSIVGRPILRAEERASVATPLKDIMIGDEASEVRSYLQISYPMENGIIKNWTDMELLWDYAFFEQMKLPSTSNGKILLTEPPMNPLKNREKMCEVMFEKYDFGGVYVAIQAVLALYAQGLSSGVVVDSGDGVTHIVPVYESVVLSHLTRRLDVAGRDVTRHLIDLLSRRGYAFNRTADFETVRQIKEKLCYVSYDLDLDTKLARETTALVESYELPDGRTIKVGQERFEAPECLFQPGLVDVEQPGVGELLFNTVQSADVDIRSSLYKAIVLSGGSSMYPGLPSRLEKELKQLWFSRVLHNDPSRLDKFKVRIEDPPRRKHMVFIGGAVLASIMADKDHMWLSKQEWQESGPSAMTKFGPR>YDL030WMNLLETRRSLLEEMEIIENAIAERIQRNPELYYHYIQESSKVFPDTKLPRSSLIAENKIYKFKKVKRKRKQIILQQHEINIFLRDYQEKQQTFNKINRPEETQEDDKDLPNFERKLQQLEKELKNEDENFELDINSKKDKYALFSSSSDPSRRTNILSDRARDLDLNEIFTRDEQYGEYMELEQFHSLWLNVIKRGDCSLLQFLDILELFLDDEKYLLTPPMDRKNDRYMAFLLKLSKYVETFFFKSYALLDAAAVENLIKSDFEHSYCRGSLRSEAKGIYCPFCSRWFKTSSVFESHLVGKIHKKNESKRRNFVYSEYKLHRYLKYLNDEFSRTRSFVERKLAFTANERMAEMDILTQKYEAPAYDSTEKEGAEQVDGEQRDGQLQEEHLSGKSFDMPLGPDGLPMPYWLYKLHGLDREYRCEICSNKVYNGRRTFERHFNEERHIYHLRCLGIEPSSVFKGITKIKEAQELWKNMQGQSQLTSIAAVPPKPNPSQLKVPTELELEEEDEEGNVMSKKVYDELKKQGLV>YDL033CMLARYLNLIGRRSASPYRPQRLPAKFDNVIVAMSSGVDSSVAAALFAGEFPNTRGVYMQNWSESQSLDDPGKEPCYERDWRDVNRVAKHLNIRVDKVNFEQDYWIDVFEPMLRGYSEGSTPNPDIGCNKFVKFGKLREWLDEKYGTGNYWLVTGHYARVMQEMNGKGLFHLLRSIYRPKDQSYYLSQINSTVLSSLLLPIGHLTKPEVRDLAKYAGLPTAEKPDSQGICFVNNSQHGKFKNFLKHYLPSSPGDIITVDPQSGAKTTWGRHDGLWSYTIGQKVGISMPQADPNYQGTWFVSEKLRDTNEILIVRGRDNPALYSDTMRIENFSSLGPREDTINAFQNTGALTLQFRSLQVPVQIKSCKLNRSADNLDITIHLASKQRAITPGQSCCLYIDDRVLGSGPISHVNNNDTHA>YDL036CMQRNNRLRNLFTVPVIMARQLKRNALSAGLAFAGNATSNEFDEHLQNEVEREREIQKKKKIKRTQSKKSPDLINKSTFQSRTIGSKKEKHRQLDPEYEIVIDGPLRKIKPYHFTYRTFCKERWRDKKLVDVFISEFRDRESEYYKRTIENGDVHINDETADLSTVIRNGDLITHQVHRHEPPVTSRPIKVIFEDDNIMVIDKPSGIPVHPTGRYRFNTITKMLQNNLGFVVNPCNRLDRLTSGLMFLAKTPKGADNIGDQLKAREVTKEYVAKVVGEFPETEVIVEKPLKLIEPRLALNAVCQMDEKGAKHAKTVFNRISYDGKTSIVKCKPLTGRSHQIRVHLQYLGHPIANDPIYSNDEVWGNNLGKGGQADFDIVITKLDEIGKRKPAKSWFHSNGGYGEVLRQEKCSICESDLYTDPGPNDLDLWLHAYLYESTETEEGTEKKKWCYKTEYPEWALRR>YDL042CMTIPHMKYAVSKTSENKVSNTVSPTQDKDAIRKQPDDIINNDEPSHKKIKVAQPDSLRETNTTDPLGHTKAALGEVASMELKPTNDMDPLAVSAASVVSMSNDVLKPETPKGPIIISKNPSNGIFYGPSFTKRESLNARMFLKYYGAHKFLDTYLPEDLNSLYIYYLIKLLGFEVKDQALIGTINSIVHINSQERVQDLGSAISVTNVEDPLAKKQTVRLIKDLQRAINKVLCTRLRLSNFFTIDHFIQKLHTARKILVLTGAGVSTSLGIPDFRSSEGFYSKIKHLGLDDPQDVFNYNIFMHDPSVFYNIANMVLPPEKIYSPLHSFIKMLQMKGKLLRNYTQNIDNLESYAGISTDKLVQCHGSFATATCVTCHWNLPGERIFNKIRNLELPLCPYCYKKRREYFPEGYNNKVGVAASQGSMSERPPYILNSYGVLKPDITFFGEALPNKFHKSIREDILECDLLICIGTSLKVAPVSEIVNMVPSHVPQVLINRDPVKHAEFDLSLLGYCDDIAAMVAQKCGWTIPHKKWNDLKNKNFKCQEKDKGVYVVTSDEHPKTL>YDL044CMIRTSSILKNCNYRYIHCIHRCLLNEANLKDRKTHNVERVSNEKTFEQALEEERKVFGELFEAGARVENMRHTNASKIIDKYYNGLQDNSEGTSVKKEKIVFNHSQRAQRKLPNKDHEFLKETAGNDYVYERAEPSAISTKTISEQTRTLLEKIFDEDNSINKSNRELLNLNLRKGSGMEALRQPVAHSNVKFSEEVMQEIGNKIRYQTTLDQVLEPHIDYLREAVKSDYDLLRYLKQSLDIYKKRNKDLELKMNAESSNIFEDIRSACINKPAELPKPLAMTLPYIIVKSLRLGDFDFPADRKYTLISYVYNECKNNMDASLYLTICNVDFYNLLVQLLWENFQEIRYLRRVVTEMSVNGVIGNIETVDILDKIVKEMRSLNEDVFLEAGEQLSADEEVSSSANKIVNVGVLWNKDTNNDLLIVENYLKSLKKNLTRDR>YDL045CMQLSKAAEMCYEITNSYLHIDQKSQIIASTQEAIRLTRKYLLSEIFVRWSPLNGEISFSYNGGKDCQVLLLLYLSCLWEYFFIKAQNSQFDFEFQSFPMQRLPTVFIDQEETFPTLENFVLETSERYCLSLYESQRQSGASVNMADAFRDFIKIYPETEAIVIGIRHTDPFGEALKPIQRTDSNWPDFMRLQPLLHWDLTNIWSFLLYSNEPICGLYGKGFTSIGGINNSLPNPHLRKDSNNPALHFEWEIIHAFGKDAEGERSSAINTSPISVVDKERFSKYHDNYYPGWYLVDDTLERAGRIKN>YDL045W-AMSGKPPVYRLPPLPRLKVKKPIIRQEANKCLVLMSNLLQCWSSYGHMSPKCAGLVTELKSCTSESALGKRNNVQKSNINYHAARLYDRINGKPHD>YDL046WMTHSLKALFALLFLYTAAVNAGVIGIFNALPPPNTKPINGESPLYQCDILDKQLVEIKEVNLDPNPPVRGENLTISANGEVFETIEEGAYIDVEVRLGYIRLLSQTFDLCETLEDNDIEGLSCPIEPGEYNIKKIVEIPGEVPPGKYVVVARAYTEKDDLITCLTGEVIFPPR>YDL047WMVSRGPDEWLETIKKCQALTENEMKQLCEMVKELLMEESNIQPVQTPVTVCGDIHGQFHDLLELFRTAGGFPDDINYIFLGDYVDRGYYSLETFTLLMCLKVKYPAKITLVRGNHESRQITQVYGFYEECLNKYGSTTVWKYCCQVFDFLTLAAIIDGKILCVHGGLSPEIRMLDQIRVLSRAQEVPHEGGFSDLLWSDPDNVEAWQVSPRGAGWLFGSKVAREFNHVNGLNLIARAHQLVMEGFKYHFPEKDVVTVWSAPNYCYRCGNVASVMKVDEDLEPTFKIFSAVPDDYIRESTANHNNQRAGYFL>YDL048CMLVSSSFASSIDSVMSHETMSLRRNPPFIDTPEKMPNPTASPNGTIHHLIDPSLPLLSSTTSSSRSTLSSTLNSPPPPPLTTSYSSYNSSACQSITSSPTDNTALAHNSKCYFPHSLSPTPLSSNSSSHVILPPISSFTNLITVAEREFNGRSNSLHANFTSPVPRTVLDHHRHELTFCNPNNTTGFKTITPSPPTQHQSILPTAVDNVPRSKSVSSLPVSGFPPLIVKQQQQQQLNSSSSASALPSIHSPLTNEHTSRYSSSLKDSAKITKQRKKKECPICHNFYANLSTHKSTHLTPEDRPHKCPICQRGFARNNDLIRHKKRHWKDEFMQIYARESDNNSGADDQDDTARTSANNDSDDSNDKLAASSSSEETKLLKKNQLKSLYKIKGAFKCPYNSTLINLDMEVYPHKSRSLYFEPINCHQTGVFSRCDTFKNHLKALHFEYPPKTKKEDRGVVPGKCKHCGLQFPNVDVWLNKHVGKGCGYSYH>YDL049CMLIVLFLTLFCSVVFRTAYCDVAIVAPEPNSVYDLSGTSQAVVKVKWMHTDNTPQEKDFVRYTFTLCSGTNAMIEAMATLQTLSASDLTDNEFNAIIENTVGTDGVYFIQVFAQTAIGYTIHYTNRFKLKGMIGTKAANPSMITIAPEAQTRITTGDVGATIDSKSFTVPYNLQTGVVKYAPMQLQPATKVTAKTWKRKYATSEVTYYYTLRNSVDQHTTVTPGWSYIITADSNYATPAPMPADNGGWYNPRKRLSLTARKVNALRHR>YDL051WMSEKPQQEEQEKPQSRRNSFAVIEFTPEVLDRCLKQVEFYFSEFNFPYDRFLRTTAEKNDGWVPISTIATFNRMKKYRPVDKVIEALRSSEILEVSADGENVKRRVPLDLTAARNARIEQNQRTLAVMNFPHEDVEASQIPELQENLEAFFKKLGEINQVRLRRDHRNKKFNGTVLVEFKTIPECEAFLKSYSNDDESNEILSYEGKKLSVLTKKQFDLQREASKSKNFSGRSRSFNGHKKKNLPKFPKNKKKNGKEESKEDSSAIADDDEEHKE>YDL052CMSVIGRFLYYLRSVLVVLALAGCGFYGVIASILCTLIGKQHLAQWITARCFYHVMKLMLGLDVKVVGEENLAKKPYIMIANHQSTLDIFMLGRIFPPGCTVTAKKSLKYVPFLGWFMALSGTYFLDRSKRQEAIDTLNKGLENVKKNKRALWVFPEGTRSYTSELTMLPFKKGAFHLAQQGKIPIVPVVVSNTSTLVSPKYGVFNRGCMIVRILKPISTENLTKDKIGEFAEKVRDQMVDTLKEIGYSPAINDTTLPPQAIEYAALQHDKKVNKKIKNEPVPSVSISNDVNTHNEGSSVKKMH>YDL053CMTTTSTTSVDGRTSSTLKATLSASGPNSNGPTPAVLPQKPKLTGWAQAAAKALPRQQQQQQQARKDDSVAVQPANTKTKTIASTAPPANIKGSSTANGSSTNKKFKRANKQPYNREEVRSYMHKLFQSYTAGEKSHSMKTYKQVLSETASGRVSTATDWGTVSSSKNKNKKYGCLSDIAKVLRNQ>YDL056WMSNQIYSARYSGVDVYEFIHSTGSIMKRKKDDWVNATHILKAANFAKAKRTRILEKEVLKETHEKVQGGFGKYQGTWVPLNIAKQLAEKFSVYDQLKPLFDFTQTDGSASPPPAPKHHHASKVDRKKAIRSASTSAIMETKRNNKKAEENQFQSSKILGNPTAAPRKRGRPVGSTRGSRRKLGVNLQRSQSDMGFPRPAIPNSSISTTQLPSIRSTMGPQSPTLGILEEERHDSRQQQPQQNNSAQFKEIDLEDGLSSDVEPSQQLQQVFNQNTGFVPQQQSSLIQTQQTESMATSVSSSPSLPTSPGDFADSNPFEERFPGGGTSPIISMIPRYPVTSRPQTSDINDKVNKYLSKLVDYFISNEMKSNKSLPQVLLHPPPHSAPYIDAPIDPELHTAFHWACSMGNLPIAEALYEAGTSIRSTNSQGQTPLMRSSLFHNSYTRRTFPRIFQLLHETVFDIDSQSQTVIHHIVKRKSTTPSAVYYLDVVLSKIKDFSPQYRIELLLNTQDKNGDTALHIASKNGDVVFFNTLVKMGALTTISNKEGLTANEIMNQQYEQMMIQNGTNQHVNSSNTDLNIHVNTNNIETKNDVNSMVIMSPVSPSDYITYPSQIATNISRNIPNVVNSMKQMASIYNDLHEQHDNEIKSLQKTLKSISKTKIQVSLKTLEVLKESSKDENGEAQTNDDFEILSRLQEQNTKKLRKRLIRYKRLIKQKLEYRQTVLLNKLIEDETQATTNNTVEKDNNTLERLELAQELTMLQLQRKNKLSSLVKKFEDNAKIHKYRRIIREGTEMNIEEVDSSLDVILQTLIANNNKNKGAEQIITISNANSHA>YDL057WMEKKHVTVQIQSAPPSYIKLEANEKFVYITSTMNGLSYQIAAIVSYPEKRNSSTANKEDGKLLCKENKLALLLHGSQSHKNAIYQTLLAKRLAEFGYWVLRIDFRGQGDSSDNCDPGLGRTLAQDLEDLSTVYQTVSDRSLRVQLYKTSTISLDVVVAHSRGSLAMFKFCLKLHAAESPLPSHLINCAGRYDGRGLIERCTRLHPHWQAEGGFWANGPRNGEYKDFWIPLSETYSIAGVCVPEFATIPQTCSVMSCYGMCDHIVPISAASNYARLFEGRHSLKLIENADHNYYGIEGDPNALGLPIRRGRVNYSPLVVDLIMEYLQDT>YDL058WMDIIQGLIQQPKIQSVDETIPTLCDRVENSTLISDRRSAVLGLKAFSRQYRESVIASGLKPLLNTLKRDYMDEDSVKAILETILILFIRGDGHDDLTRGWISQQSRLQNGKYPSPLVMKQEKEQVDQFSLWIADALTQSEDLIHLLVEFWEIDNFHIRLYTIQLLEAVMATRPLKARSALISLPTSISTMVSLLDDMHEPIRDEAILLLMAVVNDSPHVQKLVAFENIFERLFSIIEEEGGLRGSLVVNDCLSLINNILKYNTSNQTLFLETGNLPKLAHLLSEPISQDEVFFWNDQRIVNINTALDIVSLTVEPGNTVTTKHQNALLDSSVLMVVLRLAFFHNIPKKVRPVALLTAANMVRSNEHAQLEFSKIDVPYFDPSLPVNSTANGGPIKLIPVVSILINWMLYANSVHTFDTRVACSRLLKAYFMDNFDLQRDFLLKQVQLCNNSTNNVGDNAKENGGSNKSDKESDSDKDTDGKDGTEYEGSFKANLFEVLLNYDAELNLNPFKLFFTTDIFMFFFQQDHKYSEELREITRNVTTGNDLEDEEPLKAIQTISELLTTSLTAADIRIPISYLTFLIYWLFGDFKATNDFLSDKSVIKSLLSFSYQIQDEDVTIKCLVTMLLGVAYEFSSKESPFPRKEYFEFITKTLGKDNYASRIKQFKKDSYFSKVDMNEDSILTPELDETGLPKVYFSTYFIQLFNENIYRIRTALSHDPDEEPINKISFEEVEKLQRQCTKLKGEITSLQTETESTHENLTEKLIALTNEHKELDEKYQILNSSHSSLKENFSILETELKNVRDSLDEMTQLRDVLETKDKENQTALLEYKSTIHKQEDSIKTLEKGLETILSQKKKAEDGINKMGKDLFALSREMQAVEENCKNLQKEKDKSNVNHQKETKSLKEDIAAKITEIKAINENLEEMKIQCNNLSKEKEHISKELVEYKSRFQSHDNLVAKLTEKLKSLANNYKDMQAENESLIKAVEESKNESSIQLSNLQNKIDSMSQEKENFQIERGSIEKNIEQLKKTISDLEQTKEEIISKSDSSKDEYESQISLLKEKLETATTANDENVNKISELTKTREELEAELAAYKNLKNELETKLETSEKALKEVKENEEHLKEEKIQLEKEATETKQQLNSLRANLESLEKEHEDLAAQLKKYEEQIANKERQYNEEISQLNDEITSTQQENESIKKKNDELEGEVKAMKSTSEEQSNLKKSEIDALNLQIKELKKKNETNEASLLESIKSVESETVKIKELQDECNFKEKEVSELEDKLKASEDKNSKYLELQKESEKIKEELDAKTTELKIQLEKITNLSKAKEKSESELSRLKKTSSEERKNAEEQLEKLKNEIQIKNQAFEKERKLLNEGSSTITQEYSEKINTLEDELIRLQNENELKAKEIDNTRSELEKVSLSNDELLEEKQNTIKSLQDEILSYKDKITRNDEKLLSIERDNKRDLESLKEQLRAAQESKAKVEEGLKKLEEESSKEKAELEKSKEMMKKLESTIESNETELKSSMETIRKSDEKLEQSKKSAEEDIKNLQHEKSDLISRINESEKDIEELKSKLRIEAKSGSELETVKQELNNAQEKIRINAEENTVLKSKLEDIERELKDKQAEIKSNQEEKELLTSRLKELEQELDSTQQKAQKSEEERRAEVRKFQVEKSQLDEKAMLLETKYNDLVNKEQAWKRDEDTVKKTTDSQRQEIEKLAKELDNLKAENSKLKEANEDRSEIDDLMLLVTDLDEKNAKYRSKLKDLGVEISSDEEDDEEDDEEDEEEGQVA>YDL059CMTIQAKPSSSISYDSTTYGTAPGLDIKEFQIIEDWNGRPASAWSVQRIGLLQSKIERYTYNIYHNNKYGKHNLSKLIPGHALIQFANETFGYDGWRMDVIDVEARECQPFTAVNNGENTNTSEVKYTVVAEAQVKVTLKDGTNTQCGGLGRITLSSRGECYNRSKKEAVGDALKKALLSFEKIILDYETKITNNYYVDGLYGSKKIKNEANTNYNLLSATNSKPTFIKLEDAKGTHIK>YDL060WMAGHSHRSSLKNGHKSYKSKHASKGALKRLYKGKVEKEPVGTGKPDKQVSKLQRKNKAKQLRAQRILDSIENRKLFEGKNGAAKIITIVPLVNDLDPLDILYKLLKCADDEGIMVQEVDSKRIFNVHIKKFKSNLKIIIPDMTNFLNILDCAKVADFVVFGLSGVQEVDEEFGEQIIRALELQGIASYIGVISNLSAVHEKEKFQLDVKQSLESYFKHFFPSEERVYNLEKNSDALNVLRTLCQRLPRSINWRDNRGYVVADFVDFVETSPDSGDLVIEGTVRGIGFNANRLVHIPDFGDFQLNKIEKISESSQKRKIIKEKATDSLSLELDLQTVFESNMNRDTLDEYAPEGTEDWSDYDEDFEYDGLTTARYDDHGFLPGREQTSKKAAVPKGTSDYQAKWYLDDVIDANEEEEAEQTNGKDETMMEIDDEMMVEQDNEEVAGDEEYDIEDNEGFEELSPEEEERQLREFRDMEKEDREFPDEIELEPSESAIERLKRYRGLKNLYNCDWQVDEKDPSSPAEWKRLLRIGNYKNTKNRIIKETKNEAQAIAGDRIRMFIRFPKFLLEKIQDPKQLLFAVYGLLLHEHKNAVVNFSLQRWEQYDKPVPSQEPIVVQYGVRRYTIQPLFSQGSNSPNNVHKYERFLHPDTVSVATCIAPVDFTQSPAIFFKPSPTDAKNIELIGHGTFLNADHSRILAKRAILTGHPFRFHKTVVTVRYMFFRPEDVEWFKSIPLFTKSGRSGFIKESLGTHGYFKATFDGKLSAQDVVAMSLYKRMWPMPSLPWNGM>YDL063CMGRSKKRSRASSSRLNPLRKAGSNDNNKDTNVVNKKLQPLLQNLSSVVPNDRSIALSSISVLCEDAHMRQLLLKEKLVPIILNKLLNDSNSDIVVESFGLLRNLSLEEGYDVSIYLWRSDIWTSITSNFGRIVESLSALQAAEQQPQLKPAGKSKIESKRLLFDFADNLLSLVVALSNGSDDILNEILTESKINEIFQVISQLLKYGVEKLPINLFNTTLDLIYDLSSESFEFIDHVSNNELLSQFLNGLSPALHPQANELTKVLIEGIHCQFLDMKITYDQCNKMIHSVCHSINNIDPVQLVNDINNPVEIGPATSKDESSKVITKIKDYNAKRNESMIKLQSIEIAIDLITAIIEIVASKYESPESQEVAIPEELINTLTNFLPHVFMILKDTFTSRILIGWNNLIWLFVSLSLTELSGELLTTLWSYVTQLDSQDDLSIKIGRMGCIWALLKLIFPDGAFESENRALINVQMLNNSGFARGIIEEFQNNNDLELQQKCINVLSTYAMIQGQIDANKEIGQFFIQTLTQLNVRPEILIEMTNSLFQIYGDASYDYNEPIFVRGGFLSILKDQVVPNLRQQFKMVDKNKNPELKERCHDCFTTLDSFIHYKMNENSTNQ>YDL064WMSSLCLQRLQEERKKWRKDHPFGFYAKPVKKADGSMDLQKWEAGIPGKEGTNWAGGVYPITVEYPNEYPSKPPKVKFPAGFYHPNVYPSGTICLSILNEDQDWRPAITLKQIVLGVQDLLDSPNPNSPAQEPAWRSFSRNKAEYDKKVLLQAKQYSK>YDL065CMNENEYDNFDDLDDLLDEDPTKLDEAEPDDVQAKGSVYNDSENKEKNAESKDSDGVQVANESEEDPELKEMMVDLQNEFANLMKNNGNENNVKTEDFNKLISALEEAAKVPHQQMEQGCSSLKSNSTDKGTVNGSNPGFKNIVSNTLDRLKENGNKVDTSLAEETKESQRSGQNNNIDDILSQLLDQMVASGGKESAENQFDLKDGEMDDAITKILDQMTSKEVLYEPMKEMRSEFGVWFQENGENEEHKEKIGTYKRQFNIVDEIVNIYELKDYDELKHKDRVTELLDELEQLGDSPIRSANSPLKHGNEEEELMKMLEIDGNDPNLGNLDKELTDGCKQQ>YDL066WMSMLSRRLFSTSRLAAFSKIKVKQPVVELDGDEMTRIIWDKIKKKLILPYLDVDLKYYDLSVESRDATSDKITQDAAEAIKKYGVGIKCATITPDEARVKEFNLHKMWKSPNGTIRNILGGTVFREPIVIPRIPRLVPRWEKPIIIGRHAHGDQYKATDTLIPGPGSLELVYKPSDPTTAQPQTLKVYDYKGSGVAMAMYNTDESIEGFAHSSFKLAIDKKLNLFLSTKNTILKKYDGRFKDIFQEVYEAQYKSKFEQLGIHYEHRLIDDMVAQMIKSKGGFIMALKNYDGDVQSDIVAQGFGSLGLMTSILVTPDGKTFESEAAHGTVTRHYRKYQKGEETSTNSIASIFAWSRGLLKRGELDNTPALCKFANILESATLNTVQQDGIMTKDLALACGNNERSAYVTTEEFLDAVEKRLQKEIKSIE>YDL067CMTIAPITGTIKRRVIMDIVLGFSLGGVMASYWWWGFHMDKINKREKFYAELAERKKQEN>YDL069CMLRTKVFATTVARISGIRRYIPIRTINTVTKKNISKIEKLCEVLEVNPDGYKGKERIPTKELTKLLYTTSRNMLVRVPMTGDLSTGNTFETRNETLQKLGEQLIHLEINKMLTITFTNFNQFNIMNKNFNYIHNLDRARVVNMDSISWLIKNSLKINQLAHLRIPANLPKEMGLTSSSNDFQNLNDWKVILSFIGYLKLLEIKNDNKKFIESIIKTICIPLINYHLRKS>YDL070WMSRTNMDTRHAHSALLAAPQSATANSRSSNSSSESSSNKNNINVGVGDDSGNVSAVSIDDGPHFRDIFHYGHEENYKLASSGITNLNSSSHAHQTLSPISISNASTPESFPEHPLGLERETEPALEAEMEAEELPPHQSKYLLSSIKATKRLKDARPFLKPVDPIALNIPHYFNYVQTPMDLSLIETKLQGNVYHSVEQVTSDFKTMVDNCLNFNGPESSISSMAKRIQKYFEKKLSAMPPRVLPASALKKTSRNRKKNEDMDSPLVIRRSVSTTNDNIGESGNREGVSGGRPKRTIHPPKSKDLFDIYENSKPKSKTLQKKFRTCLKILKVLMSKKNSDINFPFLQPVDPIALNLPNYFDVVKNPMDLGTISNNLMNWKYKTIDQFVDDLNLVFYNCFQFNPEGNEVHSMGKKLKELFNFHWLENQDILNEIETDSDLEEDNYSSSYSSDDEYDDEDINENDITNPAIQYLEQKLKKMEVELQQLKRQELSKLSKERKRKHLGKTLLRRKAMKHSVDDLKKSITDKINELSDLEMNGMIRIIKNSLPADEILTSNEDEIEIDLDILDEATIARIYERYFEKKNNNNSKRKLSGNYSTAPTNKKKKTLKFLEKDEIINNNNYSDSEEDSSDSSDSDSD>YDL072CMSLYYTLVFAILVVEIFMFSILALPIPSRYRRPLTLLLLKPFKSSTVQVAIKCVLGFILLLFIDCINRVYSIDKELQLSSASQNNGAIIAQDRIEVLSRKFFAQRNMYLTGITLFLTFVVVRTFGLVIELLTMKDIYRASPPVASSDVKKNDSVTAEAAAQSGASKDDHGDEKNFELLKKIQDIDDEIARLKEKSESLQEEIN>YDL073WMGPAIHNQLYECGLKTTSQDGFLMDNYETQLFQSLNRFINFINNANQSASNKEQNTKFCKSSSNFLRLKLLTVLRQWCDSSRSNCTLEVRDVLTQWWVTLLNFLNSDTSLQIDTALELSLSIELTSVCLECLSKIMTILIILPFHSSRDMEIYSHHLLLTIHCITNKLILISKNSKKLKRTNSDDKCSINDKKLQYLNKYSSLLRAFIGKLNAYAFFYLPEDFHFDTILLLTVSPQISSSIQTSLFSWKKRQYKFTDDQGQMIRTEAFENKDTKFFKIIVSYIKNDFVLMSFYWHYWYIILQFMKFSDSDVGIKKSTLSCIPGSEILLTHVTTRFLNSDLNKFTRIIKQTPNPRIANENVTESHPNFKSLNSSNALITSERINDYVFSNFKTIKLWECLRSLSGCILKENHPEYLENMLSLHESLLIDYVSTISAYDYIAANVIYNKVLQFIIFQFESLPSLKFIQWRSWYNGLLSMLRTKNVNCQTVSLLCLFNIWKHVTIEDRDEIVKVLLSDFWESLIFENEFPLIRILFMKVLVFKIIPSVQNSSSLRFLPHDRIKQLYEELLVNKEELFEMQKHDSNDIVAHRKNALVFNGNSRLMMIPKKPNTEDHLVYKINHDKNLTTERFPSVSSVANTRPNVILKNGKYAYDILDEMTSKAAFLLAEKKTRLNPKKNHKIMDGYEGGQENEDNDEDSEDSGSHKNKRKEGNSSLSATLNTWLSKFSSTSEDSQKKKEQANELGNDFEYDEDVADFAEILPKQSSSNIEKIFKHGNNSGSMVSYNSSIKLNRRENILIGPPELRFSNEIKEHNSIATIFKLVFIQTNRRVVEKIDLANMKWGTIHGGSKYMKPLPVPKDLVASVAKNESETRNLATLCGNGLDFEIPVPDFNIFGKCMEDEQDVAKIGNQNVEDLKVTGGREVTIWKQIQDMKLRTRIQKICVLIETFNATVREYFEFSNRLEHDGIFIDFEVRKPSSNNSINIKV>YDL076CMSVSEQDPNRAYRETQSQIYKLQETLLNSARTKNKQEEGQESNTHSFPEQYMHYQNGRNSAYDLPNVSSQSVLAFTEKHYPNKLKNLGTLYYNRFKEGSFDEDSTSYSDRHSFPYNLYDNTLPPPFLPAIGIQNINNIATLKITYEDIQASFNNIESPRKRNNEIWGCDIYSDDSDPILVLRHCGFKIGAPSGGSFHKLRRTPVNVTNQDNVTGNLPLLEGTPFDLEVELLFLPTLQKYPSVKRFDITSREWGSEATVIHDGLSYGIYSIVIKQRLDRDKPHEPNGYIKNLKWT>YDL077CMLRAQKLHSLKSSDITAILPTEQSQKLVLAKKNGDVEVYSRDGNTLKLFQVYPDLLQNAKNDPLPPVIENFYFANELSTIFAQCKETLILLSTTNLHEYDRIIDRRGINHCWLFERSHKNKEEKNTYLIYSTINTAKMRVLIWEGRTYKNMMEASLSYRKETIRSIYPGETGITLATDLGIYHWPYNKPSLIRIEKTVKNKFPKDMISALTELKEQAEKVIEKKPKKNSHFDAQSFSSMDRMSRKSSMSSLWYRTIRNERGNKIRYTFELDGNDATPMIIDGATKKIFKVELMHNNEEPFLIATDHATFSESNSEFDHMQYLSSNLLMLYNSSTIKFVDYENGFTFLQQKIPEGIKWVKNLSGTYFLVWTSNDEVQLFSYHVDDGSEDDDQESICGDINDPDFYQLWRKVLFYKFFIDSPHSKELCVSDNPEESLDICAMKLRDLTVMWCLRIFDKFQNYMVQLERSRNSRMIRSKCEEMIIKSIFDLFIKFWAPPQLVILKVFPSAISSLVLEITGQEHHCLLKEAEEVKETYDIPPHLLNRWCLPYLTDTRRHLQNLLSKENDDESRITWCYRDREIKQSFDFFLISNHDDVDLNTMLTLIDTVLFKCYLYYNPPMVGPFIRVENHCDSHVIVTELKIRHMFKDLIDFYYKRGNHEEALKFLTDLVDELENDNTDQKQRQKIDHGVKILVIYYLKKLSNPQLDVIFTYTDWLLNRHNDSIKEILSSIFFYDSQACSSRDHLKVYGYIKKFDKLLAIQYLEFAISTFRLEGNKLHTVLIKLYLENLDIPSTRIKLKSLLETTSVYEPRTILKLLNDAIESGSDQLPTNQLNFVKYLKIFPLSKLENHKEAVHILLDEIDDYKAATSYCNDVYQSDSTKGEELLLYLYSKLVSIYDSNRNSKLILNFLQDHGSKLNSAEIYKNLPQDISLYDIGRVVSQLLKKHTSKMDETRLEKALLQVELVATTYKLNERMSSYGVLSDSHKCPICKKVISNFGTDSISWFTREGRNIITHYNCGKVLQERFNAKNEKSSRIKQKTLGEVINELNNK>YDL078CMVKVAILGASGGVGQPLSLLLKLSPYVSELALYDIRAAEGIGKDLSHINTNSSCVGYDKDSIENTLSNAQVVLIPAGVPRKPGLTRDDLFKMNAGIVKSLVTAVGKFAPNARILVISNPVNSLVPIAVETLKKMGKFKPGNVMGVTNLDLVRAETFLVDYLMLKNPKIGQEQDKTTMHRKVTVIGGHSGETIIPIITDKSLVFQLDKQYEHFIHRVQFGGDEIVKAKQGAGSATLSMAFAGAKFAEEVLRSFHNEKPETESLSAFVYLPGLKNGKKAQQLVGDNSIEYFSLPIVLRNGSVVSIDTSVLEKLSPREEQLVNTAVKELRKNIEKGKSFILDSSKL>YDL080CMNSSYTQRYALPKCIAISDYLFHRLNQLNIHTIFGLSGEFSMPLLDKLYNIPNLRWAGNSNELNAAYAADGYSRLKGLGCLITTFGVGELSAINGVAGSYAEHVGILHIVGMPPTSAQTKQLLLHHTLGNGDFTVFHRIASDVACYTTLIIDSELCADEVDKCIKKAWIEQRPVYMGMPVNQVNLPIESARLNTPLDLQLHKNDPDVEKEVISRILSFIYKSQNPAIIVDACTSRQNLIEETKELCNRLKFPVFVTPMGKGTVNETDPQFGGVFTGSISAPEVREVVDFADFIIVIGCMLSEFSTSTFHFQYKTKNCALLYSTSVKLKNATYPDLSIKLLLQKILANLDESKLSYQPSEQPSMMVPRPYPAGNVLLRQEWVWNEISHWFQPGDIIITETGASAFGVNQTRFPVNTLGISQALWGSVGYTMGACLGAEFAVQEINKDKFPATKHRVILFMGDGAFQLTVQELSTIVKWGLTPYIFVMNNQGYSVDRFLHHRSDASYYDIQPWNYLGLLRVFGCTNYETKKIITVGEFRSMISDPNFATNDKIRMIEIMLPPRDVPQALLDRWVVEKEQSKQVQEENENSSAVNTPTPEFQPLLKKNQVGY>YDL081CMSTESALSYAALILADSEIEISSEKLLTLTNAANVPVENIWADIFAKALDGQNLKDLLVNFSAGAAAPAGVAGGVAGGEAGEAEAEKEEEEAKEESDDDMGFGLFD>YDL084WMSHEGEEDLLEYSDNEQEIQIDASKAAEAGETGAATSATEGDNNNNTAAGDKKGSYVGIHSTGFKDFLLKPELSRAIIDCGFEHPSEVQQHTIPQSIHGTDVLCQAKSGLGKTAVFVLSTLQQLDPVPGEVAVVVICNARELAYQIRNEYLRFSKYMPDVKTAVFYGGTPISKDAELLKNKDTAPHIVVATPGRLKALVREKYIDLSHVKNFVIDECDKVLEELDMRRDVQEIFRATPRDKQVMMFSATLSQEIRPICRRFLQNPLEIFVDDEAKLTLHGLQQYYIKLEEREKNRKLAQLLDDLEFNQVIIFVKSTTRANELTKLLNASNFPAITVHGHMKQEERIARYKAFKDFEKRICVSTDVFGRGIDIERINLAINYDLTNEADQYLHRVGRAGRFGTKGLAISFVSSKEDEEVLAKIQERFDVKIAEFPEEGIDPSTYLNN>YDL085C-AMARGNQRDLARQKNLKKQKDMAKNQKKSGDPKKRMESDAEILRQKQAAADARREAEKLEKLKAEKTRR>YDL085WMLPRLGFARTARSIHRFKMTQISKPFFHSTEVGKPGPQQKLSKSYTAVFKKWFVRGLKLTFYTTLAGTLYVSYELYKESNPPKQVPQSTAFANGLKKKELVILGTGWGAISLLKKLDTSLYNVTVVSPRSFFLFTPLLPSTPVGTIEMKSIVEPVRSIARRTPGEVHYIEAEALDVDPKAKKVMVQSVSEDEYFVSSLSYDYLVVSVGAKTTTFNIPGVYGNANFLKEIEDAQNIRMKLMKTIEQASSFPVNDPERKRLLTFVVVGGGPTGVEFAAELQDYINQDLRKWMPDLSKEMKVILIEALPNILNMFDKTLIKYAEDLFARDEIDLQVNTAVKVVEPTYIRTLQNGQTNTDIEYGMLVWATGNEPIDFSKTLMSRIPEQTNRRGLLINDKLELLGSENSIYAIGDCTAHTGFFPTAQVAHQEGEYLAKILDKKLQIEQLEWDMLNSTDETEVSRLQKEVNLRKSKLDKFNYKHMGALAYIGSETAIADLHMGDSSYQLKGMFAFLFWKSAYLAMCLSIRNRILIAMDWTKVYFLGRDSSV>YDL086WMLITETFHDVQTSYGTTLRIYVYSPKIAGYPQAKFPGVILYSEIYQVTGPVRRFGQRIASEGYVVVAPAIYHNFMGPEALPYDVQGTDIGNEYKIKKPLESYDEDNKLCCDLLFQLPQFDGKRIGSTGMCLGGHLAFRALLDKRVTCATCFFPTDIHSRTLGLGQNDNSLERVSKELGNNQEMVLIFGTADTHVDPQGRDLIRKTLRDHGVKFTFLEILAAQHAFIRDEFSKGRFDSAITQSCLGFLFEQFNRKLRIDLGEFVDDNTPLEHVC>YDL087CMSTMSTPAAEQRKLVEQLMGRDFSFRHNRYSHQKRDLGLHDPKICKSYLVGECPYDLFQGTKQSLGKCPQMHLTKHKIQYEREVKQGKTFPEFEREYLAILSRFVNECNGQISVALQNLKHTAEERMKIQQVTEELDVLDVRIGLMGQEIDSLIRADEVSMGMLQSVKLQELISKRKEVAKRVRNITENVGQSAQQKLQVCEVCGAYLSRLDTDRRLADHFLGKIHLGYVKMREDYDRLMKNNRTTNASKTATTLPGRRFV>YDL088CMFGIRSGNNNGGFTNLTSQAPQTTQMFQSQSQLQPQPQPQPQQQQQHLQFNGSSDASSLRFGNSLSNTVNANNYSSNIGNNSINNNNIKNGTNNISQHGQGNNPSWVNNPKKRFTPHTVIRRKTTKQNSSSDINQNDDSSSMNATMRNFSKQNQDSKHNERNKSAANNDINSLLSNFNDIPPSVTLQDWQREDEFGSIPSLTTQFVTDKYTAKKTNRSAYDSKNTPNVFDKDSYVRIANIEQNHLDNNYNTAETNNKVHETSSKSSSLSAIIVFGYPESISNELIEHFSHFGHIMEDFQVLRLGRGINPNTFRIFHNHDTGCDENDSTVNKSITLKGRNNESNNKKYPIFTGESWVKLTYNSPSSALRALQENGTIFRGSLIGCIPYSKNAVEQLAGCKIDNVDDIGEFNVSMYQNSSTSSTSNTPSPPNVIITDGTLLREDDNTPAGHAGNPTNISSPIVANSPNKRLDVIDGKLPFMQNAGPNSNIPNLLRNLESKMRQQEAKYRNNEPAGFTHKLSNWLFGWNDL>YDL089WMGSNDLINEAYDDSEVVGEERESKSAWMKRWYQLLTSPLDLQLVINEKLEMINWDAYAKSLAKPLGNFLTILFFIIRLLQDNLIKPNYYKLNVKSGAFDLSKSNKLKEFDYLWEISSSFQNNNQFYAFQSWYFVTLRFLNNLFRFTIFILLSLNLYVSCKFMFGYFKTYNLFHLKKEFNSPNLTKHNLKDLSKEYYEDIYKQSLWSMLKHFFRGSRDDGPHVNQNEDEIFFQLRKWIPTNFMINLFVSFSPTAIVFLSFSDVSFTSAIAIVFHQYILDYIITKRFQRSVDDDLILSSAALQEYEDKHIMARINQCSNIDTLSSAMGTRSKTPRIFTTHSLCGEEIREVYNYEKREFEALPKMTESVPGSRETRIKDYGGISQVSDHQSHPIGFHYSPRMSPYYRDKVLDNNLAQSSSNENLEKGGAYLPNQDQNRPSKSLSPLRKTPLSARQKRFEGSEFNVLNKNDINSILRSPKKKKNYHKR>YDL090CMRQRVGRSIARAKFINTALLGRKRPVMERVVDIAHVDSSKAIQPLMKELETDTTEARYKVLQSVLEIYDDEKNIEPALTKEFHKMYLDVAFEISLPPQMTALDASQPWMLYWIANSLKVMDRDWLSDDTKRKIVDKLFTISPSGGPFGGGPGQLSHLASTYAAINALSLCDNIDGCWDRIDRKGIYQWLISLKEPNGGFKTCLEVGEVDTRGIYCALSIATLLNILTEELTEGVLNYLKNCQNYEGGFGSCPHVDEAHGGYTFCATASLAILRSMDQINVEKLLEWSSARQLQEERGFCGRSNKLVDGCYSFWVGGSAAILEAFGYGQCFNKHALRDYILYCCQEKEQPGLRDKPGAHSDFYHTNYCLLGLAVAESSYSCTPNDSPHNIKCTPDRLIGSSKLTDVNPVYGLPIENVRKIIHYFKSNLSSPS>YDL091CMDIFRHTFGNNDDSFIRIPGAFREEPPADLNGRTEDQNSNTNEPTQSRDGRLKSILHFLFQAPLIVLYYLLNFIVRSSRLLKPLLRLHGFYQRKHNRLLDHSSQLHRLLENLENEAQAVTCSEGNGNNDDGSNTDSTSNNESSGVQFSFGSLYNPENGTFSKSIMQNSYTELLDACSEQVKFGVIYLHDPLLDNHMDYVNKILCSEAFVNMIRKYQVLLWYGDVTTSEGLQVSNALKIRQYPLLGIISLKAEKKIELIARVEGSISNYKAQDLEAIFSKNYSRLIQLRQQRQNIEMQRLIRQQQDSRYQDSLRRDQQRESERLEQTQREQMEREHQRIENQWLLWRKSQLKPEPSSDKDASKVAIRLENGQRLVRKFDASLPTEEIYAFVELQLHDMLNSENDTLPVYQPANYQHQYSFKLITPVPRRELDLSTKISDVSGIYPSGNIVMERLDE>YDL097CMSLPGSKLEEARRLVNEKQYNEAEQVYLSLLDKDSSQSSAAAGASVDDKRRNEQETSILELGQLYVTMGAKDKLREFIPHSTEYMMQFAKSKTVKVLKTLIEKFEQVPDSLDDQIFVCEKSIEFAKREKRVFLKHSLSIKLATLHYQKKQYKDSLALINDLLREFKKLDDKPSLVDVHLLESKVYHKLRNLAKSKASLTAARTAANSIYCPTQTVAELDLMSGILHCEDKDYKTAFSYFFESFESYHNLTTHNSYEKACQVLKYMLLSKIMLNLIDDVKNILNAKYTKETYQSRGIDAMKAVAEAYNNRSLLDFNTALKQYEKELMGDELTRSHFNALYDTLLESNLCKIIEPFECVEISHISKIIGLDTQQVEGKLSQMILDKIFYGVLDQGNGWLYVYETPNQDATYDSALELVGQLNKVVDQLFEKASVLY>YDL098CMSNFGRRTWDREEYAEQARSGYDDRSLKATLTPIELQALKSKYTNYDHLIKGSLKDLNKRKLTANTESLSSFKRGKKFGFYCDICNLTFKDTLQYIDHLNHKVHAIKFENLFDEPLIIDIRDNDDVPQEEFELCYHNLIKDFVEVRSMETQSKRKRLLDTDVEKAKKVATKPSIESESKVSQMMGFSNFATSKK>YDL100CMDLTVEPNLHSLITSTTHKWIFVGGKGGVGKTTSSCSIAIQMALSQPNKQFLLISTDPAHNLSDAFGEKFGKDARKVTGMNNLSCMEIDPSAALKDMNDMAVSRANNNGSDGQGDDLGSLLQGGALADLTGSIPGIDEALSFMEVMKHIKRQEQGEGETFDTVIFDTAPTGHTLRFLQLPNTLSKLLEKFGEITNKLGPMLNSFMGAGNVDISGKLNELKANVETIRQQFTDPDLTTFVCVCISEFLSLYETERLIQELISYDMDVNSIIVNQLLFAENDQEHNCKRCQARWKMQKKYLDQIDELYEDFHVVKMPLCAGEIRGLNNLTKFSQFLNKEYNPITDGKVIYELEDKE>YDL101CMSLSTKREHSGDVTDSSFKRQQRSNKPSSEYTCLGHLVNLIPGKEQKVEITNRNVTTIGRSRSCDVILSEPDISTFHAEFHLLQMDVDNFQRNLINVIDKSRNGTFINGNRLVKKDYILKNGDRIVFGKSCSFLFKYASSSSTDIENDDEKVSSESRSYKNDDEVFKKPQISATSSQNATTSAAIRKLNKTRPVSFFDKYLLGKELGAGHYALVKEAKNKKTGQQVAVKIFHAQQNDDQKKNKQFREETNILMRVQHPNIVNLLDSFVEPISKSQIQKYLVLEKIDDGELFERIVRKTCLRQDESKALFKQLLTGLKYLHEQNIIHRDIKPENILLNITRRENPSQVQLGPWDEDEIDIQVKIADFGLAKFTGEMQFTNTLCGTPSYVAPEVLTKKGYTSKVDLWSAGVILYVCLCGFPPFSDQLGPPSLKEQILQAKYAFYSPYWDKIDDSVLHLISNLLVLNPDERYNIDEALNHPWFNDIQQQSSVSLELQRLQITDNKIPKTYSELSCL>YDL102WMSEKRSLPMVDVKIDDEDTPQLEKKIKRQSIDHGVGSEPVSTIEIIPSDSFRKYNSQGFKAKDTDLMGTQLESTFEQDVSQMEHDMADQEEHDLSSFERKKLPTDFDPSLYDISFQQIDAEQSVLNGIKDENTSTVVRFFGVTSEGHSVLCNVTGFKNYLYVPAPNSSDANDQEQINKFVHYLNETFDHAIDSIEVVSKQSIWGYSGDTKLPFWKIYVTYPHMVNKLRTAFERGHLSFNSWFSNGTTTYDNIAYTLRLMVDCGIVGMSWITLPKGKYSMIEPNNRVSSCQLEVSINYRNLIAHPAEGDWSHTAPLRIMSFDIECAGRIGVFPEPEYDPVIQIANVVSIAGAKKPFIRNVFTLNTCSPITGSMIFSHATEEEMLSNWRNFIIKVDPDVIIGYNTTNFDIPYLLNRAKALKVNDFPYFGRLKTVKQEIKESVFSSKAYGTRETKNVNIDGRLQLDLLQFIQREYKLRSYTLNAVSAHFLGEQKEDVHYSIISDLQNGDSETRRRLAVYCLKDAYLPLRLMEKLMALVNYTEMARVTGVPFSYLLARGQQIKVVSQLFRKCLEIDTVIPNMQSQASDDQYEGATVIEPIRGYYDVPIATLDFNSLYPSIMMAHNLCYTTLCNKATVERLNLKIDEDYVITPNGDYFVTTKRRRGILPIILDELISARKRAKKDLRDEKDPFKRDVLNGRQLALKISANSVYGFTGATVGKLPCLAISSSVTAYGRTMILKTKTAVQEKYCIKNGYKHDAVVVYGDTDSVMVKFGTTDLKEAMDLGTEAAKYVSTLFKHPINLEFEKAYFPYLLINKKRYAGLFWTNPDKFDKLDQKGLASVRRDSCSLVSIVMNKVLKKILIERNVDGALAFVRETINDILHNRVDISKLIISKTLAPNYTNPQPHAVLAERMKRREGVGPNVGDRVDYVIIGGNDKLYNRAEDPLFVLENNIQVDSRYYLTNQLQNPIISIVAPIIGDKQANGMFVVKSIKINTGSQKGGLMSFIKKVEACKSCKGPLRKGEGPLCSNCLARSGELYIKALYDVRDLEEKYSRLWTQCQRCAGNLHSEVLCSNKNCDIFYMRVKVKKELQEKVEQLSKW>YDL104CMISIKGTGRFLLDNYRIWQRRAFNRPIQLRKGYKVLAIETSCDDTCVSVLDRFSKSAAPNVLANLKDTLDSIDEGGIIPTKAHIHHQARIGPLTERALIESNAREGIDLICVTRGPGMPGSLSGGLDFAKGLAVAWNKPLIGVHHMLGHLLIPRMGTNGKVPQFPFVSLLVSGGHTTFVLSRAIDDHEILCDTIDIAVGDSLDKCGRELGFKGTMIAREMEKFINQDINDQDFALKLEMPSPLKNSASKRNMLSFSFSAFITALRTNLTKLGKTEIQELPEREIRSIAYQVQESVFDHIINKLKHVLKSQPEKFKNVREFVCSGGVSSNQRLRTKLETELGTLNSTSFFNFYYPPMDLCSDNSIMIGWAGIEIWESLRLVSDLDICPIRQWPLNDLLSVDGWRTDQL>YDL105WMSSTVISRKRRNSTVTEPDSSGETRKQKKSRSDEKSSSSKDGDPQLEFKVLQGYRDLESEMHKGRAQVTRTGDIGVAMDNLNAVDSLFNKVIGIKNNGLFAHDARAMVSISELAQISVRNLKFDDSRSMVNLENIVNSLKRYMLKEHFKLNNIAENRNDLTLAADEQSAADQQEESDGDIDRTPDDNHTDKATSSFKATSMRHSYLQQFSHYNEFSQFNWFRIGALYNTISKNAPITDHLMGPLSIEKKPRVLTQRRRNNDQVGEKITAEKITQHSLNSTQQETTPEQVKKCFKKLSKKLGPEGSINLFKFIIDPNSFSRSIENLFYTSFLIKEGKLLMEHDEEGLPTIKIKQSISHTDSRSKEIERQRRRAAHQNHIIFQMDMPTWRKLIKKYNITSPFLD>YDL106CMMEEFSYDHDFNTHFATDLDYLQHDQQQQQQQQHDQQHNQQQQPQPQPIQTQNLEHDHDQHTNDMSASSNASDSGPQRPKRTRAKGEALDVLKRKFEINPTPSLVERKKISDLIGMPEKNVRIWFQNRRAKLRKKQHGSNKDTIPSSQSRDIANDYDRGSTDNNLVTTTSTSSIFHDEDLTFFDRIPLNSNNNYYFFDICSITVGSWNRMKSGALQRRNFQSIKELRNLSPIKINNIMSNATDLMVLISKKNSEINYFFSAMANNTKILFRIFFPLSSVTNCSLTLETDDDIINSNNTSDKNNSNTNNDDDNDDNSNEDNDNSSEDKRNAKDNFGELKLTVTRSPTFAVYFLNNAPDEDPNLNNQWSICDDFSEGRQVNDAFVGGSNIPHTLKGLQKSLRFMNSLILDYKSSNEILPTINTAIPTAAVPQQNIAPPFLNTNSSATDSNPNTNLEDSLFFDHDLLSSSITNTNNGQGSNNGRQASKDDTLNLLDTTVNSNNNHNANNEENHLAQEHLSNDADIVANPNDHLLSLPTDSELPNTPDFLKNTNELTDEHRWI>YDL107WMQRFVSKFVSTPPVPKKFQEIFPKKRTVNKILFQLDTRLTYHEMYPIFLQVSQNTNEENIPWRKKYPYIRSSDIMQMRNVLITLRTQNKFVHKDLLAMEDKLLNIAAELGNNDAISILSFNVIHEYKKENVKSSYEKDIETANEFIKKLYARNHHLTVKLIGDLFFENKTYDKAEKYYQEFLKLENSTKLAGEVHGKLGEIQIKQVNGFLKAEKSWLSCIELLEIERSSRWYFLLARLYMSSEPMKAKALLENCASIGFKECFKTLGFLELNYFNNYERAKEWFKTGMEIMDLECFFGFFDCCVKEENFKGARDCLESVKKLGNDKDKKTMINVFLESRKDSIKLLDKARL>YDL108WMKVNMEYTKEKKVGEGTYAVVYLGCQHSTGRKIAIKEIKTSEFKDGLDMSAIREVKYLQEMQHPNVIELIDIFMAYDNLNLVLEFLPTDLEVVIKDKSILFTPADIKAWMLMTLRGVYHCHRNFILHRDLKPNNLLFSPDGQIKVADFGLARAIPAPHEILTSNVVTRWYRAPELLFGAKHYTSAIDIWSVGVIFAELMLRIPYLPGQNDVDQMEVTFRALGTPTDRDWPEVSSFMTYNKLQIYPPPSRDELRKRFIAASEYALDFMCGMLTMNPQKRWTAVQCLESDYFKELPPPSDPSSIKIRN>YDL110CMCSAGGIRRPIQIEEFKTAISGMSDMELAQIKTEIENSINHLQRSNARLGKYIAKLEGADDRLEADDSDDLENIDSGDLALYKDSVRENEIVLNNYNERVDALEQETVYRKTGHGKSKHEVEAKDNTNKGPDVDMDNSNVDVVTPNSIFI>YDL111CMSLSVAEKSYLYDSLASTPSIRPDGRLPHQFRPIEIFTDFLPSSNGSSRIIASDGSECIVSIKSKVVDHHVENELLQVDVDIAGQRDDALVVETITSLLNKVLKSGSGVDSSKLQLTKKYSFKIFVDVLVISSHSHPVSLISFAIYSALNSTYLPKLISAFDDLEVEELPTFHDYDMVKLDINPPLVFILAVVGNNMLLDPAANESEVANNGLIISWSNGKITSPIRSVALNDSNVKSFKPHLLKQGLAMVEKYAPDVVRSLENL>YDL114WMVRKNKINRASGTTKHLKDFPSVILSLPSYNPSILSKNATALITGGSSGLGFELAKELSRRINKVIVADIQSFPTFAQVEYNNIFYYQCDITSLDEIKNLKKAIERDHGNINIIINNAGVAHIKKLEHMTNKEVEQLIDINLIGAYRIISTFAEDMIDNREGFIINIASVLGELTPARLTSYGASKGAMIGFHKCMSRHFRSLSTECNKTGIKTLLVCPGKIKTNMFIDVPTPSKLLAPDIIPSQLALAIISAMEHNHLQTLNAPYYVNLVPFFKTLSWPYRHLLKHFSGMDHVTSTSPRAINPKRSA>YDL115CMVNDYLKLNKGVEKTERKKPSRKYFTGDSAKIASLPSLDYVFDIYHLEKIHDDEVARYNNEKNIGFVKIIEHIDLALDEESDPNEARSDDEDSNDENYYQNDYPEDEDDDRSILFGSEGEDIAALGEEIVIGVNKSRFSSWNDDKIQGSNGYHDVEEEYGDLFNRLGGKSDVLKSINSSNFIDLDGQEGEIEISDNEDDSDEGDDIEYPRNEFFPTDVDDPLAHHRDRIFHQLQKKINRS>YDL116WMELSPTYQTERFTKFSDTLKEFKIEQNNEQNPIDPFNIIREFRSAAGQLALDLANSGDESNVISSKDWELEARFWHLVELLLVFRNADLDLDEMELHPYNSRGLFEKKLMQDNKQLYQIWIVMVWLKENTYVMERPKNVPTSKWLNSITSGGLKSCDLDFPLRENTNVLDVKDKEEDHIFFKYIYELILAGAIDEALEEAKLSDNISICMILCGIQEYLNPVIDTQIANEFNTQQGIKKHSLWRRTVYSLSQQAGLDPYERAIYSYLSGAIPNQEVLQYSDWESDLHIHLNQILQTEIENYLLENNQVGTDELILPLPSHALTVQEVLNRVASRHPSESEHPIRVLMASVILDSLPSVIHSSVEMLLDVVKGTEASNDIIDKPYLLRIVTHLAICLDIINPGSVEEVDKSKLITTYISLLKLQGLYENIPIYATFLNESDCLEACSFILSSLEDPQVRKKQIETINFLRLPASNILRRTTQRVFDETEQEYSPSNEISISFDVNNIDMHLIYGVEWLIEGKLYVDAVHSIIALSRRFLLNGRVKALEQFMERNNIGEICKNYELEKIADNISKDENEDQFLEEITQYEHLIKGIREYEEWQKSVSLLSSESNIPTLIEKLQGFSKDTFELIKTFLVDLTSSNFADSADYEILYEIRALYTPFLLMELHKKLVEAAKLLKIPKFISEALAFTSLVANENDKIYLLFQSSGKLKEYLDLVARTATLSN>YDL117WMATNLTSLKPPFKVKARYGWSGQTKGDLGFLEGDIMEVTRIAGSWFYGKLLRNKKCSGYFPHNFVILLEERLNSSTENGRQPSKIVESFEKSNKVVIPPVPSRYSDERPRPKKKLSSSMPNSPKKPVDSLTKARKAKSKEMVNEKNIYNTQSSRHHNNSAPNLPLASHSKPQVRNFEESMNNPLPPLPPLPDLDNMRKTDKRAPKKSYSANDLHMARSSREYNYYKDNQKFYDGFIPEKRYSLEEDSISSGLFSNSQYLNDSACSSENSFALMSDFSATSAGSFARHKYAQSFSDSLQRSQNANGCSTKINDSQEFGDSNASSRNGKMGDILRKIIIPKRNTNIYSSSVSSPKSPKAYPKLPDIQNLNLSATPDEARDWIAVKCHLNRARTLTKYDKHPRYMRALEENRDLILHPQDSIYNGLNTNEVKGNTKPGLVDVELAELNIEYIDKMTWKRCIRDGTMTLDSWAQTTFSARYSTVLEKLRGIYIFCTEMFALTDDNGTSDFSAEPQNLEKILYRKHCTPYELTWLFKKLANSLGITCEIVIGFLKTPSAINWEFKYNHCWLRILVNKEWRFIDVILGNVTNPIHEFVNNRKIKKAENSYFLMAPLEMIYTHIPPREFEQHIVPSIDQLSALYLPLVFPSFFKNELKLYKFSTALSFLEDSEIYECSLEIPNDVEVFASVVIPTDNEEASSAYRNMELALTQIKKQKAESGRRIALIKAVLPPNVNKGSLYIHSGVRGTQTSIANIHPLSMMVPLTHKGSNMKYEFVIKIPSESIQKIELYIVEPQSRYLFVGNEYSFEVIQSPSDGIVYSSDEGPNQNRKQPMAIKSPSGRVHELVKSDPHFPYGTWKGSIKIKEPGVWSALVIADSGIGWSVFAEWLCV>YDL119CMTEQATKPRNSSHLIGGFFGGLTSAVALQPLDLLKTRIQQDKKATLWKNLKEIDSPLQLWRGTLPSALRTSIGSALYLSCLNLMRSSLAKRRNAVPSLTNDSNIVYNKSSSLPRLTMYENLLTGAFARGLVGYITMPITVIKVRYESTLYNYSSLKEAITHIYTKEGLFGFFRGFGATCLRDAPYAGLYVLLYEKSKQLLPMVLPSRFIHYNPEGGFTTYTSTTVNTTSAVLSASLATTVTAPFDTIKTRMQLEPSKFTNSFNTFTSIVKNENVLKLFSGLSMRLARKAFSAGIAWGIYEELVKRFM>YDL120WMIKRSLASLVRVSSVMGRRYMIAAAGGERARFCPAVTNKKNHTVNTFQKRFVESSTDGQVVPQEVLNLPLEKYHEEADDYLDHLLDSLEELSEAHPDCIPDVELSHGVMTLEIPAFGTYVINKQPPNKQIWLASPLSGPNRFDLLNGEWVSLRNGTKLTDILTEEVEKAISKSQ>YDL121CMNLYGYFLLLIIVIAFIALLPLFSGIGTFKLTKPKSSATAQSATGKLGKREYLKKKLDHTNVLKFDLKDTEESLGHDSASASSASRKFEIDSKTGLKRRVIGQYNKDPNDFDFDIDDLINEDELDERREEEKKLKKYNGKKNEAYEGFV>YDL123WMCCYCVCCTVSDFILYIVAFFFPPAAVLLRSGPCSSDFLLNVLLTLLGFLPGMLHAFYYITITSPLRNAEYVYYYQQGWVDSERNVPSNRPQNSQTPQNRPQQGSSARNVYPSVETPLLQGAAPHDNKQSLVESPPPYVP>YDL124WMSFHQQFFTLNNGNKIPAIAIIGTGTRWYKNEETDATFSNSLVEQIVYALKLPGIIHIDAAEIYRTYPEVGKALSLTEKPRNAIFLTDKYSPQIKMSDSPADGLDLALKKMGTDYVDLYLLHSPFVSKEVNGLSLEEAWKDMEQLYKSGKAKNIGVSNFAVEDLQRILKVAEVKPQVNQIEFSPFLQNQTPGIYKFCQEHDILVEAYSPLGPLQKKTAQDDSQPFFEYVKELSEKYIKSEAQIILRWVTKRGVLPVTTSSKPQRISDAQNLFSFDLTAEEVDKITELGLEHEPLRLYWNKLYGKYNYAAQKV>YDL126CMGEEHKPLLDASGVDPREEDKTATAILRRKKKDNMLLVDDAINDDNSVIAINSNTMDKLELFRGDTVLVKGKKRKDTVLIVLIDDELEDGACRINRVVRNNLRIRLGDLVTIHPCPDIKYATRISVLPIADTIEGITGNLFDVFLKPYFVEAYRPVRKGDHFVVRGGMRQVEFKVVDVEPEEYAVVAQDTIIHWEGEPINREDEENNMNEVGYDDIGGCRKQMAQIREMVELPLRHPQLFKAIGIKPPRGVLMYGPPGTGKTLMARAVANETGAFFFLINGPEVMSKMAGESESNLRKAFEEAEKNAPAIIFIDEIDSIAPKRDKTNGEVERRVVSQLLTLMDGMKARSNVVVIAATNRPNSIDPALRRFGRFDREVDIGIPDATGRLEVLRIHTKNMKLADDVDLEALAAETHGYVGADIASLCSEAAMQQIREKMDLIDLDEDEIDAEVLDSLGVTMDNFRFALGNSNPSALRETVVESVNVTWDDVGGLDEIKEELKETVEYPVLHPDQYTKFGLSPSKGVLFYGPPGTGKTLLAKAVATEVSANFISVKGPELLSMWYGESESNIRDIFDKARAAAPTVVFLDELDSIAKARGGSLGDAGGASDRVVNQLLTEMDGMNAKKNVFVIGATNRPDQIDPAILRPGRLDQLIYVPLPDENARLSILNAQLRKTPLEPGLELTAIAKATQGFSGADLLYIVQRAAKYAIKDSIEAHRQHEAEKEVKVEGEDVEMTDEGAKAEQEPEVDPVPYITKEHFAEAMKTAKRSVSDAELRRYEAYSQQMKASRGQFSNFNFNDAPLGTTATDNANSNNSAPSGAGAAFGSNAEEDDDLYS>YDL127WMSNYEALLKFNRKAVSKEMVQYLASTTASIIKIKKTNSMIDIALPAPPLTKFINRLIKHSNVQTPTLMATSVYLAKLRSIIPSNVYGIETTRHRIFLGCLILAAKTLNDSSPLNKHWAEYTDGLLILREVNTIERELLEYFDWDVTISTDDLITCLSPFLKPIKEEQLYKSQRDCRTLKNFSAQEKDIVNKTSISHSRSSSNMSIPSLASTSTLSTLESRRSNLSNYSNRIRTLPELHESNNISDKFSPRTYNIDSKHDNKENRPIPTIKPFNFSKARPVILKTGLNKQIIKEDTKVKKSNWSNYFKS>YDL128WMDATTPLLTVANSHPARNPKHTAWRAAVYDLQYILKASPLNFLLVFVPLGLIWGHFQLSHTLTFLFNFLAIIPLAAILANATEELADKAGNTIGGLLNATFGNAVELIVSIIALKKGQVRIVQASMLGSLLSNLLLVLGLCFIFGGYNRVQQTFNQTAAQTMSSLLAIACASLLIPAAFRATLPHGKEDHFIDGKILELSRGTSIVILIVYVLFLYFQLGSHHALFEQQEEETDEVMSTISRNPHHSLSVKSSLVILLGTTVIISFCADFLVGTIDNVVESTGLSKTFIGLIVIPIVGNAAEHVTSVLVAMKDKMDLALGVAIGSSLQVALFVTPFMVLVGWMIDVPMTLNFSTFETATLFIAVFLSNYLILDGESNWLEGVMSLAMYILIAMAFFYYPDEKTLDSIGNSL>YDL130W-AMLNRCISRNTRLPVNLRIASRFYSDGPLGGAGPGNPQDIFIKRERAKEDYYARQQEREQLAHVKEQLKEHKKKLENLENKINNLSK>YDL131WMSENNEFQSVTESTTAPTTSNPYGPNPADYLSNVKNFQLIDSTLREGEQFANAFFDTEKKIEIARALDDFGVDYIELTSPVASEQSRKDCEAICKLGLKAKILTHIRCHMDDARVAVETGVDGVDVVIGTSKFLRQYSHGKDMNYIAKSAVEVIEFVKSKGIEIRFSSEDSFRSDLVDLLNIYKTVDKIGVNRVGIADTVGCANPRQVYELIRTLKSVVSCDIECHFHNDTGCAIANAYTALEGGARLIDVSVLGIGERNGITPLGGLMARMIVAAPDYVRSKYKLHKIRDIENLVADAVEVNIPFNNPITGFCAFTHKAGIHAKAILANPSTYEILDPHDFGMKRYIHFANRLTGWNAIKSRVDQLNLNLTDDQIKEVTAKIKKLGDVRPLNIDDVDSIIKDFHAELSTPLLKPVNKGTDDDNIDISNGHVSKKAKVTK>YDL132WMSETLPRSDDLEATWNFIEPGINQILGNEKNQASTSKRVYKILSPTMYMEVYTAIYNYCVNKSRSSGHFSTDSRTGQSTILVGSEIYEKLKNYLKNYILNFKQSNSETFLQFYVKRWKRFTIGAIFLNHAFDYMNRYWVQKERSDGKRHIFDVNTLCLMTWKEVMFDPSKDVLINELLDQVTLGREGQIIQRSNISTAIKSLVALGIDPQDLKKLNLNVYIQVFEKPFLKKTQEYYTQYTNDYLEKHSVTEYIFEAHEIIKREEKAMTIYWDDHTKKPLSMALNKVLITDHIEKLENEFVVLLDARDIEKITSLYALIRRDFTLIPRMASVFENYVKKTGENEISSLLAMHKHNIMKNENANPKKLALMTAHSLSPKDYIKKLLEVHDIFSKIFNESFPDDIPLAKALDNACGAFININEFALPAGSPKSATSKTSEMLAKYSDILLKKATKPEVASDMSDEDIITIFKYLTDKDAFETHYRRLFAKRLIHGTSTSAEDEENIIQRLQAANSMEYTGKITKMFQDIRLSKILEDDFAVALKNEPDYSKAKYPDLQPFVLAENMWPFSYQEVEFKLPKELVPSHEKLKESYSQKHNGRILKWLWPLCRGELKADIGKPGRMPFNFTVTLFQMAILLLYNDADVLTLENIQEGTSLTIQHIAAAMVPFIKFKLIQQVPPGLDALVKPETQFKLSRPYKALKTNINFASGVKNDILQSLSGGGHDNHGNKLGNKRLTEDERIEKELNTERQIFLEACIVRIMKAKRNLPHTTLVNECIAQSHQRFNAKVSMVKRAIDSLIQKGYLQRGDDGESYAYLA>YDL133WMGDSNSSQEAYSDTTSTNASRIADQNQLNLNVDLEKNQTVRKSGSLEALQNAKIHVPKHSDGSPLDYPKLNTYTFVPTTVPPYVLEAQFDKLRLQDKGTVDGNVTDDKNLPKEFKWGQFASTIGCHSAYTRDQNYNPSHKSYDGYSLSSSTSSKNAALREILGDMCSEWGGEERLEGVLHSEIGANLEFNTTEERKEWLQYIEKVKDFYYGDNKKNPESPESVHNKVYKSDWVNELNKEREKWRRLKQRKLQQWRPPLTSLLLDNQYLILGLRIFTGILSCISLALAIKIFQNSRSNNTISESKIGQQPSTIMAICVNAVAIAYIIYIAHDEFAGKPVGLRNPLSKLKLILLDLLFIIFSSANLALAFNTRFDKEWVCTSIRRSNGSTYGYPKIPRICRKQEALSAFLFVALFMWVITFSISIVRVVEKVSSITNRN>YDL134CMDTDLDVPMQDAVTEQLTPTVSEDMDLNNNSSDNNAEEFSVDDLKPGSSGIADHKSSKPLELNNTNINQLDQWIEHLSKCEPLSEDDVARLCKMAVDVLQFEENVKPINVPVTICGDVHGQFHDLLELFKIGGPCPDTNYLFMGDYVDRGYYSVETVSYLVAMKVRYPHRITILRGNHESRQITQVYGFYDECLRKYGSANVWKMFTDLFDYFPITALVDNKIFCLHGGLSPMIETIDQVRELNRIQEVPHEGPMCDLLWSDPDDRGGWGISPRGAGFTFGQDVSEQFNHTNDLSLIARAHQLVMEGYAWSHQQNVVTIFSAPNYCYRCGNQAAIMEVDENHNRQFLQYDPSVRPGEPSVSRKTPDYFL>YDL135CMAEESTDFSQFEEERNNDQYKVSAKKTVDEYKNLDAEDESLAKWKESLGLSSDVLPLEFPGDKRKVVVQKIQLLVNTEPNPITFDLTNEKTIKELASKRYKIKENSIYKLKIVFKVQHEIITGLRYVQYIKKAGIAVDKIDDHLGSYAPNTKTKPFYEVELPESEAPSGFLARGNYSAVSKFIDDDKTNHLTLNWGVEIVKK>YDL137WMGLYASKLFSNLFGNKEMRILMVGLDGAGKTTVLYKLKLGEVITTIPTIGFNVETVQYKNISFTVWDVGGQDRIRSLWRHYYRNTEGVIFVIDSNDRSRIGEAREVMQRMLNEDELRNAVWLVFANKQDLPEAMSAAEITEKLGLHSIRNRPWFIQSTCATSGEGLYEGLEWLSNNLKNQS>YDL139CMKTNKKISKRRSLKNLHGALKGLLKESGKKSESKIRKHSDCNPVHRVYPPNIEKRKTKKDDGISRPIAERNGHVYIMSKENHIIPKLTDDEVMERHKLADENMRKVWSNIISKYESIEEQGDLVDLKTGEIVEDNGHIKTLTANNSTKDKRTKYTSVLRDIIDISDEEDGDKNDEYTLWANDSEASDSEVDADNDTEEEKDEKLIDADFKKYEAKLSKRILRD>YDL143WMSAKVPSNATFKNKEKPQEVRKANIIAARSVADAIRTSLGPKGMDKMIKTSRGEIIISNDGHTILKQMAILHPVARMLVEVSAAQDSEAGDGTTSVVILTGALLGAAERLLNKGIHPTIIADSFQSAAKRSVDILLEMCHKVSLSDREQLVRAASTSLSSKIVSQYSSFLAPLAVDSVLKISDENSKNVDLNDIRLVKKVGGTIDDTEMIDGVVLTQTAIKSAGGPTRKEKAKIGLIQFQISPPKPDTENNIIVNDYRQMDKILKEERAYLLNICKKIKKAKCNVLLIQKSILRDAVNDLALHFLSKLNIMVVKDIEREEIEFLSKGLGCKPIADIELFTEDRLGSADLVEEIDSDGSKIVRVTGIRNNNARPTVSVVIRGANNMIIDETERSLHDALCVIRCLVKERGLIAGGGAPEIEISRRLSKEARSMEGVQAFIWQEFASALEVIPTTLAENAGLNSIKVVTELRSKHENGELNDGISVRRSGTTNTYEEHILQPVLVSTSAITLASECVKSILRIDDIAFSR>YDL144CMSRILVIGAGGVGVITALSLWLKKESDVSLVVRSDYDRVLKHGYTIESCDYGRLEGWRPHHIYSSVEDAASAADNQGYNYIVVTTKNIIDGPVNSRVSNIIRPVLEKNKELHGPQLTTHILLVQNGIDIEKEIWAEFPREQYRYTVLSGIQLIGSTKIGSGHISQVGQDHLSCGAFDPQDAAAIQAANDFVRMYSNEGQNFVEFDPRVRYSRWKKLLYNAAINTSTALVGLDVPRCLEFGVNKKSTEIEVFHPAMREIIAIAASEGIIIEEEFITMFTEITRKKVFKPSMCVDCEKGQLMELEVILGNPIRIAKRNGVATPTLSILYNLLVLVQAKLKERKGLLKFDEKTATLVDE>YDL146WMILDPLSPNIENHTQDEIIEFWEKTESIANIPKENLDESHVNSSLVAYLKFATDSYKVFINTDRDLYRMSLILLESSLFEFKKEFCLSKLQSLLNIDLLEMNMKFIIVYILLCEAKKNVYSLEIMLKFQGFTVFYNTLYTQFAYLSKYGKERTVASKHQYNSNNSSTGTSLDSLDRSLTDIDLGIIDEMKQISTVLMDLLFQIMKYCKCVIANLQIVDDFFVYYMMESMRSDTMDDMFNNAEFKLLLALNEQYMMFAKEYDIENKVYKYLINGSVSRCFTELLLLKFNRASDPPLQIMMCKIIYLILTPRGDYSPMNFFYTNDLRVLIDVLIRELQNISEDEEVLRNTLLRVLIPLLKNTQLSKTHYRKDDLNKLLNYLSTLDNICVDSPALHEHQVTVALSRKCLQQIPWLETPSTPSDGGSSVSSNNTSRNSSIVALGTPDNQNILARKGHLYSNRELDVSAESLTKRKAKAPPPPPPPPPSRKCGTPK>YDL148CMAGSQLKNLKAALKARGLTGQTNVKSKNKKNSKRQAKEYDREEKKKAIAEIREEFNPFEIKAARNKRRDGLPSKTADRIAVGKPGISKQIGEEQRKRAFEARKMMKNKRGGVIDKRFGERDKLLTEEEKMLERFTRERQSQSKRNANLFNLEDDEDDGDMFGDGLTHLGQSLSLEDELANDEEDFLASKRFNEDDAELQQPQRKKTKAEVMKEVIAKSKFYKQERQKAQGIMEDQIDNLDDNFEDVMSELMMTQPKKNPMEPKTDLDKEYDIKVKELQLDKRAAPSDRTKTEEEKNAEAEEKKRELEQQRLDRMNGMIELEEGEERGVEDLDDGFWENEEDYEDDNDGIADSDDDIKFEDQGRDEGFSQILKKKNISISCPRTHDALLDQVKKLDLDDHPKIVKNIIKAYQPKLAEGNKEKLGKFTAVLLRHIIFLSNQNYLKNVQSFKRTQNALISILKSLSEKYNRELSEECRDYINEMQARYKKNHFDALSNGDLVFFSIIGILFSTSDQYHLVITPALILMSQFLEQIKFNSLKRIAFGAVLVRIVSQYQRISKRYIPEVVYFFQKILLTFIVEKENQEKPLDFENIRLDSYELGLPLDVDFTKKRSTIIPLHTLSTMDTEAHPVDQCVSVLLNVMESLDATISTVWKSLPAFNEIILPIQQLLSAYTSKYSDFEKPRNILNKVEKLTKFTEHIPLALQNHKPVSIPTHAPKYEENFNPDKKSYDPDRTRSEINKMKAQLKKERKFTMKEIRKDAKFEARQRIEEKNKESSDYHAKMAHIVNTINTEEGAEKNKYERERKLRGGKK>YDL149WMERDEYQLPNSHGKNTFLSRIFGLQSDEVNPSLNSQEMSNFPLPDIERGSSLLHSTNDSREDVDENDLRVPESDQGTSTEEEDEVDEEQVQAYAPQISDGLDGDHQLNSVTSKENVLETEKSNLERLVEGSTDDSVPKVGQLSSEEEEDNEFINNDGFDDDTPLFQKSKIHEFSSKKSNTIEDGKRPLFFRHILQNNRPQRDTQKLFTSSNAIHHDKDKSANNGPRNINGNQKHGTKYFGSATQPRFTGSPLNNTNRFTKLFPLRKPNLLSNISVLNNTPEDRINTLSVKERALWKWANVENLDIFLQDVYNYYLGNGFYCIILEKILNICTLLFVVFVSTYMGHCVDYSKLPTSHRVSDIIIDKCYSNSITGFTKFFLWMFYFFVILKIVQLYFDVQKLSELQNFYKYLLNISDDELQTLPWQNVIQQLMYLKDQNAMTANVVEVKAKNRIDAHDVANRIMRRENYLIALYNSDILNLSLPIPLFRTNVLTKTLEWNINLCVMGFVFNESGFIKQSILKPSQREFTREELQKRFMLAGFLNIILAPFLVTYFVLLYFFRYFNEYKTSPGSIGARQYTPIAEWKFREYNELYHIFKKRISLSTTLANKYVDQFPKEKTNLFLKFVSFICGSFVAILAFLTVFDPENFLNFEITSDRSVIFYITILGAIWSVSRNTITQEYHVFDPEETLKELYEYTHYLPKEWEGRYHKEEIKLEFCKLYNLRIVILLRELTSLMITPFVLWFSLPSSAGRIVDFFRENSEYVDGLGYVCKYAMFNMKNIDGEDTHSMDEDSLTKKIAVNGSHTLNSKRRSKFTAEDHSDKDLANNKMLQSYVYFMDDYSNSENLTGKYQLPAKKGYPNNEGDSFLNNKYSWRKQFQPGQKPELFRIGKHALGPGHNISPAIYSTRNPGKNWDNNNNGDDIKNGTNNATAKNDDNNGNNDHEYVLTESFLDSGAFPNHDVIDHNKMLNSNYNGNGILNKGGVLGLVKEYYKKSDVGR>YDL150WMSSNKGNGRLPSLKDSSSNGGGSAKPSLKFKPKAVARKSKEEREAAASKVKLEEESKRGNDKKHFNNKNKRVTGAGGQQRRMAKYLNNTHVISSGPLAAGNFVSEKGDLRRGFIKSEGSGSSLVQKGLETIDNGAESSENEAEDDDNEGVASKSKKKFNMGKEFEARNLIEDEDDGESEKSSDVDMDDEEWRSKRIEQLFPVRPVRVRHEDVETVKREIQEALSEKPTREPTPSVKTEPVGTGLQSYLEERERQVNEKLADLGLEKEFQSVDGKEAAAELELLNADHQHILRKLKKMNNKPERFMVFQLPTRLPAFERPAVKEEKEDMETQASDPSKKKKNIKKKDTKDALSTRELAGKVGSIRVHKSGKLSVKIGNVVMDIGKGAETTFLQDVIALSIADDASSAELLGRVDGKIVVTPQI>YDL151CMSTSLLFSLSPSSSSSMRLRASNSFPILNFFLDLDATPSLSSSSASFSELSAPLSIVSRPFCTRDDPLPSDLMNPLLKSPFSLTKFPAANGPLEITCVLFKYLAIRLCWPPAPVTLLFLLLKCFLSLPLLDSSSSFTLDAAASLSSLDFLATAFGLNFNEGLADPPPLEEESFNDGKRPFPLLLLILNECYPA>YDL153CMVRKGSNRTKTSEVGDEINPYGLNEVDDFASKREKVLLGQSTFGDSNKDDDHSLLEDEDEEEVLAMDEDDESIDEREDEEEEEEEELDGAAAYKKIFGRNLETDQLPEEDEENGMLDNENAWGSTKGEYYGADDLDDDEAAKEIEKEALRQQKKHLEELNMNDYLDEEEEEEWVKSAKEFDMGEFKNSTKQADTKTSITDILNMDDEARDNYLRTMFPEFAPLSKEFTELAPKFDELKKSEENEFNKLKLIALGSYLGTISCYYSILLHELHNNEDFTSMKGHPVMEKILTTKEIWRQASELPSSFDVNEGDGSESEETANIEAFNEKKLNELQNSEDSDAEDGGKQKQEIDEEERESDEEEEEEDVDIDDFEEYVAQSRLHSKPKTSSMPEADDFIESEIADVDAQDKKARRRTLRFYTSKIDQQENKKTDRFKGDDDIPYKERLFERQQRLLDEARKRGMHDNNGADLDDKDYGSEDEAVSRSINTQGENDYYQQVQRGKQDKKISRKEAHKNAVIAAREGKLAELAENVSGDGKRAINYQILKNKGLTPKRNKDNRNSRVKKRKKYQKAQKKLKSVRAVYSGGQSGVYEGEKTGIKKGLTRSVKFKN>YDL154WMSHEWLISASETMRSIGNGEGLRDKGAVVANNDGEFNEGDTNREEDSSTIFSFDFDEEIVMCIDFSGGKLGCSILDYHTKTLKAFDQDYVVNKTTISSHDLIDDADMSSNDISLLLGLLIMEANPTVCLVPARLEDWIFDYIKTKCDEINCRLELQPIKRFKKWDLLQSLQLRGHDNQTILNDILSNSKFTTTVTLGTVGCILANHEQLGEYNDSTASSNMVTGRLVQNAFEDVIHGIRYIDIRDRMVLDENTISALHIFPTAHKLGHDKMMRNGFFSVFELFNQVSSDYARRILKSWLINPLTNKKRIETRYSIIRTLLDKQNAIIFSDLSQSIKRCPDAFGFINQLRSGKSTLGTWSKVASFLEKGIAIFQLVSSLKLGSDEANILHDIKNKVDISALKECLRKVETVIDFDTSRDTKTLTINTGVDNRLDECRNIYNHLEGILLDVARETQIFLLNTMPQEDCKTTKSLEKLVNAVYIPQLGYLVTISVLMEPLLDGIPNLQWEEIFRSSENIYFKNGRVLELDETYGDIYGAISDFEIEILFSLQEQILRRKTQLTAYNILLSELEILLSFAQVSAERNYAEPQLVEDECILEIINGRHALYETFLDNYIPNSTMIDGGLFSELSWCEQNKGRIIVVTGANASGKSVYLTQNGLIVYLAQIGCFVPAERARIGIADKILTRIRTQETVYKTQSSFLLDSQQMAKSLSLATEKSLILIDEYGKGTDILDGPSLFGSIMLNMSKSEKCPRIIACTHFHELFNENVLTENIKGIKHYCTDILISQKYNLLETAHVGEDHESEGITFLFKVKEGISKQSFGIYCAKVCGLSRDIVERAEELSRMINRGDDVVQQCGNLTEKEMREFQKNQEIVKKFLSWDLDLETTTTSENLRLKLKNFLR>YDL155WMHHNSQSLSSGHIRSPEDENVAPIGNLKHRTGSLSHISSAHPRVALSDVTNIVATNSSNNSISKPKVAPIKERLDSAAIIEEERLDANSVAQRKEADHNDLLTDREQEEPVEDDGESEEDEEEDQEPLLLQHYASDTLVWEHAFRTYYRTTLDPNDDDVYDVVMVAELSNEIFEYMRKLEDLYKPNPYYMDKQPELRWSFRSTLIDWIVQVHEKFQLLPETLYLCINIIDRYLCKEVVPVNKFQLVGAASLFIAAKYEEINCPTIKDFVYMSENCYSRNDLLDAERTILNGLEFELGWPGPMSFLRRISKADDYEHDTRTLAKYLLESTIMDHRLVSAQPSWLAAGAYFLSKIILGQNQWSLAHVYYSNYTQEQILPLATIILENCRYASKRHNAIWRKYSSRRYLHSSQIVAKWIALAEHRVERSN>YDL156WMPELTEFQKKRLENIKRNNDLLKKLHLSGVASQIKHEAGVLEKSRAPAKKKQKTTNTRATKSASPTLPTRRSRRLRGESADDVKGIPNVNDNQLLKMGSPDGQDKNFIDAIKEKPVIGDVKLSDLIKDEDESALLEKFKRFNNGNFSSGDFFEEIKKRQGDVTGMDEFDLDLYDVFQPNEIKITYERISATYFHPAMEKKLIIAGDTSGTVGFWNVRDEPLADSEEDRMEEPDITRVKLFTKNVGRIDCFPADTSKILLTSYDGSIRSVHLNNLQSEEVLTLKNEYDDSLGISDCQFSYENPNVLFLTTLGGEFTTFDTRVKKSEYNLRRLADKKIGSMAINPMRPYEIATGSLDRTLKIWDTRNLVKKPEWSQYEDYPSHEIVSTYDSRLSVSAVSYSPTDGTLVCNGYDDTIRLFDVKSRDHLSAKLEPKLTIQHNCQTGRWTSILKARFKPNKNVFAIANMKRAIDIYNSEGQQLAHLPTATVPAVISWHPLRNWIAGGNSSGKIFLFTDDSGTIKQEE>YDL157CMSNILAVFNPPPQRELEKEETMDCVPCQVMSTMFSVGFGSYLASGKPFKYGKKEAKRGISLTEFEKRNPQWWKVTLRSFGGLLIAFGFVRGTEGWLWHKNKEYKNYKKLSNDGETQAN>YDL159WMFQRKTLQRRNLKGLNLNLHPDVGNNGQLQEKTETHQGQSRIEGHVMSNINAIQNNSNLFLRRGIKKKLTLDAFGDDQAISKPNTVVIQQPQNEPVLVLSSLSQSPCVSSSSSLSTPCIIDAYSNNFGLSPSSTNSTPSTIQGLSNIATPVENEHSISLPPLEESLSPAAADLKDTLSGTSNGNYIQLQDLVQLGKIGAGNSGTVVKALHVPDSKIVAKKTIPVEQNNSTIINQLVRELSIVKNVKPHENIITFYGAYYNQHINNEIIILMEYSDCGSLDKILSVYKRFVQRGTVSSKKTWFNELTISKIAYGVLNGLDHLYRQYKIIHRDIKPSNVLINSKGQIKLCDFGVSKKLINSIADTFVGTSTYMSPERIQGNVYSIKGDVWSLGLMIIELVTGEFPLGGHNDTPDGILDLLQRIVNEPSPRLPKDRIYSKEMTDFVNRCCIKNERERSSIHELLHHDLIMKYVSPSKDDKFRHWCRKIKSKIKEDKRIKREALDRAKLEKKQSERSTH>YDL160CMGSINNNFNTNNNSNTDLDRDWKTALNIPKKDTRPQTDDVLNTKGNTFEDFYLKRELLMGIFEAGFEKPSPIQEEAIPVAITGRDILARAKNGTGKTAAFVIPTLEKVKPKLNKIQALIMVPTRELALQTSQVVRTLGKHCGISCMVTTGGTNLRDDILRLNETVHILVGTPGRVLDLASRKVADLSDCSLFIMDEADKMLSRDFKTIIEQILSFLPPTHQSLLFSATFPLTVKEFMVKHLHKPYEINLMEELTLKGITQYYAFVEERQKLHCLNTLFSKLQINQAIIFCNSTNRVELLAKKITDLGYSCYYSHARMKQQERNKVFHEFRQGKVRTLVCSDLLTRGIDIQAVNVVINFDFPKTAETYLHRIGRSGRFGHLGLAINLINWNDRFNLYKIEQELGTEIAAIPATIDKSLYVAENDETVPVPFPIEQQSYHQQAIPQQQLPSQQQFAIPPQQHHPQFMVPPSHQQQQAYPPPQMPSQQGYPPQQEHFMAMPPGQSQPQY>YDL161WMSKQFVRSAKNLVKGYSSTQVLVRNATSNDNHQVSKDSLIELAEKSYDSADFFEIMDMLDKRLNDKGKYWRHIAKALTVIDYLIRFGSENCVLWCRENLYIIKTLKEFRHEDDEGIDQGQIVRVKAKELTALLSDDERLNEERNMNIKGRNRKGRRRRGTGRSDENDDDLQRAISASRLTAEEDERRRKQDEDYETALQLSKEEEELKRLQDLQRMQQQQGQQQLQQPMYYDIFGNPITPEEYAQFQLQQQQQQQQQQLQQQPMYYDVFGNPITPEELAQFQQQQQLQEQQYLASMQQQQQAMSNNPFAKSEQSSSSPKRNQLVAASSPQQLQQQKQQEPLIQNRTGNQSMTDKYSKLNELLATGTGIDTFGNVGEARIPAQHTKTGTFINSQGTGYRQVSDDPNHNPFLNSQYTGLPSTSVVPTQTGYGFGNQSQQQSQNNGSNNRGYTLIDL>YDL163WMELGAATVCVTAVCFNFLVAYSSSPLTLSILSSSICLDDFLGEGVPSVGLFFMEVKNLAKVACLGFLPAEEGNEDDINNGNLDFKGRADERRQPVSNLRM>YDL165WMEKFGLKALVPLLKLEDKELSSTYDHSMTLGADLSSMLYSLGIPRDSQDHRVLDTFQSPWAETSRSEVEPRFFTPESFTNIPGVLQSTVTPPCFNSIQNDQQRVALFQDETLFFLFYKHPGTVIQELTYLELRKRNWRYHKTLKAWLTKDPMMEPIVSADGLSERGSYVFFDPQRWEKCQRDFLLFYNAIM>YDL166CMEARRYGPNIIVTGTPGCGKSSTCEFLKNKLKDYKYYNISDFAKDNDCFEGYDEGRKSHIVDEDKLLDMLEPLLRQGNSIVDWHVNDVFPERLIDLVVVLRCDNSNLYSRLHARGYHDSKIEENLDAEIMGVVKQDAVESYEPHIVVELQSDTKEDMVSNVSRIVAWEKMWLEQHPDGVTNEYQGPRSDDEDDEDSE>YDL167CMHYVVLELQVAHLPDTPKDQCRIANIAFQIVNAETLVCHYGTNSLPSIEVNGTTKSLESAMVQLDKDIHDVIGNDDFVLVSLYSTWHIRVTLPRQARDDGFILTSYLQHPKVFDLWKEFDRWCVNHPEILGQKKAISNNNCNTKSISINAAKNTKDLDEIVRILEVSIPTEEAGSVPEIYSLLKRTTDILIQLHKKCTSPEDMESVLTKPYDSHTDIRAFLQEKSKILYMNNLPPDTTQSELESWFTQYGVRPVGFWTVKNIVEDTSNVNNNWSLNNSPYVEDQDSISGFVVFQTHEEATEVLALNGRSILSNLANTKQPRVVEHVLELQPSSTGVLDKAQEILSPFPQSKNKPRPGDWNCPSCGFSNFQRRTACFRCSFPAPSNSQIHTANSNNNVNSSRNNLNNRVNSGSSSNISNTAANHPYGAPEFNMIANNTPAALTYNRAHFPAITPLSRQNSLNMAPSNSGSPIIIADHFSGNNNIAPNYRYNNNINNNNNNINNMTNNRYNINNNINGNGNGNGNNSNNNNNHNNNHNNNHHNGSINSNSNTNNNNNNNNGNNSNNCNSNIGMGGCGSNMPFRAGDWKCSTCTYHNFAKNVVCLRCGGPKSISGDASETNHYIDSSTFGPASRTPSNNNISVNTNGGSNAGRTDGNDNKGRDISLMEFMSPPLSMATKSMKEGDGNGSSFNEFKSDKANVNFSNVGDNSAFGNGFNSSIRW>YDL168WMSAATVGKPIKCIAAVAYDAKKPLSVEEITVDAPKAHEVRIKIEYTAVCHTDAYTLSGSDPEGLFPCVLGHEGAGIVESVGDDVITVKPGDHVIALYTAECGKCKFCTSGKTNLCGAVRATQGKGVMPDGTTRFHNAKGEDIYHFMGCSTFSEYTVVADVSVVAIDPKAPLDAACLLGCGVTTGFGAALKTANVQKGDTVAVFGCGTVGLSVIQGAKLRGASKIIAIDINNKKKQYCSQFGATDFVNPKEDLAKDQTIVEKLIEMTDGGLDFTFDCTGNTKIMRDALEACHKGWGQSIIIGVAAAGEEISTRPFQLVTGRVWKGSAFGGIKGRSEMGGLIKDYQKGALKVEEFITHRRPFKEINQAFEDLHNGDCLRTVLKSDEIK>YDL170WMNYGVEKLKLKYSKHGCITCKIRKKRCSEDKPVCRDCRRLSFPCIYISESVDKQSLKKIKADIQHQLISKKRKHAPDSAQKAAVATRTRRVGSDEQDNQVYLSKPLEDCISQKLDSMGLQLYNYYRSHLANIISIAPMNQNYYLNIFLPMAHENDGILFAILAWSANHLSISSSNELRKDEIFVNLANKYTYMSLSHLKTNEGSSACAKLGFLYSLAQILILCGSEICQGDVKFWKILLNIGKNLIENHVGKDVSRILTTTTEEPSLEERIIFPNFNSVVKYWLIVNFIYHDILNFNTTSFPIEQYEKFFQRDQNSLPSSANFIESIDSPIEEIDPLIGINKPILLLLGQVTNLTRFLQTMEQEEMLEHGDKILSLQVEIYKLQPSLMALEHLDDEKKFYYLELFEIMKISTLMFFQLTLLKIDKDSLELQILRNKLDSKLDKVIGTFLEGSLCFPLFIYGVCIQVEDMEKKIDLEAKFDDILKRYKCYNFQNARLLIRKIWQNEADGISEHDLVHMIDELDYNINFA>YDL173WMATFNPQNEMENQARVQEYKVSTGRGGAGNIHKSMSKPSPVLLPLKSNSKTVANNNNNGSNQEKVPRFAIGRGGAGNIFHDPHLTRSAQQLDSNDNINYNDVINDIDDYISPITSDMVDEGGSNPVTNTRSRISATRSHQSLHATTSSPNNNAPIVVGRGGAGNIFFNKKKVASNGGNEEDEIRGGNIEDEDTINANEDNLFVVTSNGNALAAIKSTSKKPKNKLKGKSAPEKFAIGRGGAGNIISPKSSRNTINHNLNDDDEDKFNLKDDNGKEKKKKKKKKSGFFSSLKTMFN>YDL174CMLWKRTCTRLIKPIAQPRGRLVRRSCYRYASTGTGSTDSSSQWLKYSVIASSATLFGYLFAKNLYSRETKEDLIEKLEMVKKIDPVNSTLKLSSLDSPDYLHDPVKIDKVVEDLKQVLGNKPENYSDAKSDLDAHSDTYFNTHHPSPEQRPRIILFPHTTEEVSKILKICHDNNMPVVPFSGGTSLEGHFLPTRIGDTITVDLSKFMNNVVKFDKLDLDITVQAGLPWEDLNDYLSDHGLMFGCDPGPGAQIGGCIANSCSGTNAYRYGTMKENIINMTIVLPDGTIVKTKKRPRKSSAGYNLNGLFVGSEGTLGIVTEATVKCHVKPKAETVAVVSFDTIKDAAACASNLTQSGIHLNAMELLDENMMKLINASESTDRCDWVEKPTMFFKIGGRSPNIVNALVDEVKAVAQLNHCNSFQFAKDDDEKLELWEARKVALWSVLDADKSKDKSAKIWTTDVAVPVSQFDKVIHETKKDMQASKLINAIVGHAGDGNFHAFIVYRTPEEHETCSQLVDRMVKRALNAEGTCTGEHGVGIGKREYLLEELGEAPVDLMRKIKLAIDPKRIMNPDKIFKTDPNEPANDYR>YDL175CMEKNTAPFVVDTAPTTPPDKLVAPSIEEVNSNPNELRALRGQGRYFGVSDDDKDAIKEAAPKCNNCSQRGHLKKDCPHIICSYCGATDDHYSRHCPKAIQCSKCDEVGHYRSQCPHKWKKVQCTLCKSKKHSKERCPSIWRAYILVDDNEKAKPKVLPFHTIYCYNCGGKGHFGDDCKEKRSSRVPNEDGSAFTGSNLSVELKQEYYRHMNRNSDENEDYQFSESIYDEDPLPRPSHKRHSQNDHSHSGRNKRRASNFHPPPYQKSNVIQPTIRGETLSLNNNISKNSRYQNTKVNVSSISENMYGSRYNPSTYVDNNSISNSSNYRNYNSYQPYRSGTLGKRR>YDL176WMATGRIQFAVSTPCNTKGKPSGYRLFEFKNDRLALVPSERGCTKVDVNANIQAFCYLRPNGRDTSISPDATHILDSCDYMVLAKSNGFIEIISNYQYKIKNGLRLAPSYILRCTPEDFESNFFSDYMIAGLEYSQGLLYCCMCSGRIYVFVMNLPTDYIQYKNMYNPMFPDCFFKVHHDNNTTHSSEEEKLFEGSTRYTGRSCSKHICYFLLPIEPSHLRSSPVVSSFCNMYQGLPIYRPSMYLHIERGISTFHINPLDRFCFMTVSPRSPLFIRKIILPLTYVTFLSTFISLKNSIQGDTCGEILSWDNVAQQNGFGSLFSWISNKFTFDTDIINSTIWDDIVKYSGTGMLDSGIVWKQRQGHAKDDIYELFHTQDMLGSSRRNSSFSTASSEPRPLSRRRRESFQALTRDAFRERMDVPCSTKWELDSFIRGLRRNTFMVDFEIVEKISHRNGNDGVNEDDNTTDESDETMTSFLTDNYKKMDIVCIDHFVTLSAFRPRYYDEPIIKIDSLSNKNGSENGTNEEEWAESQMKVDGQVIDDETAQFKQALGNLCSFKKLFMLDDSLCFILDTHGVLLINRFEIKNTKNLLRNSKDTIRIIPHDFGLINDTIVIINDIDVGTDNVCALTFHLVVTSMAGEITVLKGEFFKNCRLGRIKLCDSLKLNRKDRFVDKLALIDYDGLNAQKRRLDYDEKDLYTFIVKKVKRD>YDL177CMSKNVGKLVKIWNESEVLVDRKSKFQARCCPLQNQKDIPSILQELTQNNKSVSKASHMHMYAWRTAEVSNNLHLQQEQKKKGNKANKSNNSHVNKSRNITVQPKNIEQGCADCGEAGAGQRLLTLLERANIFNVLVIVTRWYGGTPLGSSRFRHISTCAVETLKKGGFLP>YDL178WMLRNILVRSTGSNFKFAGRYMKSSALLGYYRRVNYYSTKIQTRLTSENYPDVHRDPRFKKLTSDDLNYFKSILSEQEILRASESEDLSFYNEDWMRKYKGQSKLVLRPKSVEKVSLILNYCNDEKIAVVPQGGNTGLVGGSVPIFDELILSLANLNKIRDFDPVSGILKCDAGVILENANNYVMEQNYMFPLDLGAKGSCHVGGVVATNAGGLRLLRYGSLHGSVLGLEVVMPNGQIVNSMHSMRKDNTGYDLKQLFIGSEGTIGIITGVSILTVPKPKAFNVSYLSVESFEDVQKVFVRARQELSEILSAFEFMDAKSQVLAKSQLKDAAFPLEDEHPFYILIETSGSNKDHDDSKLETFLENVMEEGIVTDGVVAQDETELQNLWKWREMIPEASQANGGVYKYDVSLPLKDLYSLVEATNARLSEAELVGDSPKPVVGAIGYGHVGDGNLHLNVAVREYNKNIEKTLEPFVYEFVSSKHGSVSAEHGLGFQKKNYIGYSKSPEEVKMMKDLKVHYDPNGILNPYKYI>YDL179WMISDYDALLQFNKKPVSQEMIQFLATSTASIIKIRENNNPIQGCRPPDLSIFIKNVVIQSNVQTPTLMATSVYLNKLKSVIPKNVYGINTTRHRIFLGCLILAAKTLNDSSPWNKHWTTYTEGLLRIREVNTIERELLEYLNWDVRITTPDLIDSLSYFLGPIKEQLFLQRRQEMLLFNAPSPGQLKEYINHRRPVSHSRTSSAISVPSLTSMATVSTTDSRSSLLAKYQPSLPLVESDNFNKKNHVPLRNNNDICNNFRAEENIHSVNHIDVTMGSSPVMSHKPTIHQRLNFTRRGWSSFFKQ>YDL180WMVRLNHAASYFMPIFCSTRPHIVILSALFSISLFSLFYASSELLLHQYDDPLMFKPNSQDYFRTFLLGLFSPFLYYFLKTFLFNINQRFLILNLIVDFPINDVFMLLILIGLAYPQVQDHEGGTIKHKECSWHIIPRQAYIFGISWALGEFTICIIGNLFNYQEIADPNINSGFTHQESANTYCNNNDMSHNDDCGCSTEYRPNVVDRSDITLSKCIEVRNDSSSISNNVYSSEYHPIKPLRSSSSTYGSIRQQPHENKKQLHVPDNSQDDTIIMMNPIDNSLKLTTLDTGDLSFPIDEEQPILKKSFGYTWAVPNENTQNTTKSFTPIKRFIAFSTAYQLVTGLLLMILVVGSNIMLTIGESLILSMYFVYVRGHEGLFTPVVNYFGSRTISNFILCVIIPFISLNFLINTSIYLRRELDDWFNNSQGEFEDDDENTISKRVATNQEYQHPLSANYISMDSPDVINSSPGHFGMNSGQLLGNTTLYYGSLNGDDDDMTNDSALLRFCKKLVKNWRALARNDSFVLGVMVSWSLLVFVTGILSTVYI>YDL181WMLPRSALARSLQLQRGVAARFYSEGSTGTPRGSGSEDSFVKRERATEDFFVRQREKEQLRHLKEQLEKQRKKIDSLENKIDSMTK>YDL182WMTAAKPNPYAAKPGDYLSNVNNFQLIDSTLREGEQFANAFFDTEKKIEIARALDDFGVDYIELTSPVASEQSRKDCEAICKLGLKAKILTHIRCHMDDAKVAVETGVDGVDVVIGTSKFLRQYSHGKDMNYIAKSAVEVIEFVKSKGIEIRFSSEDSFRSDLVDLLNIYKTVDKIGVNRVGIADTVGCANPRQVYELIRTLKSVVSCDIECHFHNDTGCAIANAYTALEGGARLIDVSVLGIGERNGITPLGGLMARMIVAAPDYVKSKYKLHKIRDIENLVADAVEVNIPFNNPITGFCAFTHKAGIHAKAILANPSTYEILDPHDFGMKRYIHFANRLTGWNAIKARVDQLNLNLTDDQIKEVTAKIKKLGDVRSLNIDDVDSIIKNFHAEVSTPQVLSAKKNKKNDSDVPELATIPAAKRTKPSA>YDL183CMIRSIFIPPTSITATSRLFYGMRAYSTKLEKASLQKYLHDPVKVTVIPITDKESFIYYKHTDNLFNSQSRILKAEKWIVEKSAKLWRKLKKSPKSYNKKIVSMVQSLLNSTPWSENSLLTIPSESYILKRIKGEKDKTQEIRLTLKDYTVKAEQVDTQPLHVYYPPGISSPDECLRQMKKLYQEGLIYHKKWTLYCLLGLPLTIPLILIPLIPNVPGFYLSYRAYVNIKAYLGAKHLKSLLESSKQNLEFRELLGYTEVYKRGTSSRTQGNQKESKGAPELLLNKKTLPLILDFLEVHELESDLNKVILQESKSQEKNKI>YDL186WMQCNTSIEDGLDGGDITFPSYSPEAEAQNRFLPRSEFPSVRQLVISKQKSRRRREENVFVGKMEDVLVKWRPPASSHRGAIEAINGTHPYQLSTAQFNSRRSDPFGSTKRDESVRSIVESFTDWWKRNSKMFFRDEEEGVRTEHSASQDDQELQLFEEDLFSFHLSPNDMSTRNSEHQVQTPVLFPYEAPTRTKQNPAERKVIEIGDGEELSIYRDVYSNPIPVSYLDTLNLNHSMSVRQTQQQQQQRQQQQYVDSSSAFQCCTDSNWARIFFCHHD>YDL188CMDMEIDDPMHGSDEDQLSPTLDEDMNSDDGKNNTKARSNDEDTDEELEDFNFKPGSSGIADHKSSKPLKLTNTNINQLDQWIEHLSKCEPLSEDDVARLCKMAVDVLQFEENVKPINVPVTICGDVHGQFHDLLELFKIGGPCPDTNYLFMGDYVDRGYYSVETVSYLVAMKVRYPHRITILRGNHESRQITQVYGFYDECLRKYGSANVWKMFTDLFDYFPVTALVDNKIFCLHGGLSPMIETIDQVRDLNRIQEVPHEGPMCDLLWSDPDDRGGWGISPRGAGFTFGQDISEQFNHTNDLSLIARAHQLVMEGYSWSHQQNVVTIFSAPNYCYRCGNQAAIMEVDENHNRQFLQYDPSVRPGEPTVTRKTPDYFL>YDL189WMTALDSRNWGLTPAMETGLFQKPQDRIFIIELENSIVSFINSNTESFQLRPMNSYYRLLSHQIAEYHNLNHVLARTQDSCVILFKGENFQKIEGKPLLQELQLNKKPEECASSSESIEKSNNNRIFRILKRKEVGNECDYKIDGNTRTPNSNLTANSNKDQKIEIDDKSSTDLEQERIEKERLYEQRKQEIFDKLNKSEDDVKSTNSSGSSDSDNEWSDWLNGDDSNTQTSNGSVSSSSPFNSSVTTIQVNKPQQQFYDSRRGRGGRRRGTNNYKDAYRGQSRRNKENGGYQSGYSSPYLVYPPPQMGGNSLPTYPLMYNPAGPAPGPAPSPMVMGNNTVFMNPYMYNMNPQGSCSFGTPIPMYPPYQYQYQYQYNTQYHSGPYSNTPSYNSNNYTRSSANKYHHFQGKNSYSGAIPKRSDDSNSNKNEGIRRASVEGSPSSRDTDSVEMKFDKLNI>YDL192WMGLFASKLFSNLFGNKEMRILMVGLDGAGKTTVLYKLKLGEVITTIPTIGFNVETVQYKNISFTVWDVGGQDRIRSLWRHYYRNTEGVIFVVDSNDRSRIGEAREVMQRMLNEDELRNAAWLVFANKQDLPEAMSAAEITEKLGLHSIRNRPWFIQATCATSGEGLYEGLEWLSNSLKNST>YDL193WMPTMIKKDDKAMEPPNEKPHRKIERDDVPESSNHIPPPESGVLKGGKVNSKTRALKAVTSIIADADENPQKKVNNETNGVQKQKTEDLSKRIGKFEYLFYKFLLVLLYICFGLFRYGQYQYNKMKLRIFSIIYNHAYTPQLIRQDVIPLKKIPKRLAAILEVKPVGDVGGGVTGLLNDASEIVCWTVSAGIKHLMLYDYDGILQRNVPELRMEIHSNLAKYFGPAHVPNYAVKIPHSNKIFYNLDGIETETDVGNEIEANQEKDKIAIEISLLSNRDGRETIVDLTKTMAELCAVNELSVSDITMDLVDSELKQLVGPEPDLLLYFGPSLDLQGFPPWHIRLTEFYWEKDNNEVIYSVFIRGLRQYAGCKVNVGK>YDL198CMPHTDKKQSGLARLLGSASAGIMEIAVFHPVDTISKRLMSNHTKITSGQELNRVIFRDHFSEPLGKRLFTLFPGLGYAASYKVLQRVYKYGGQPFANEFLNKHYKKDFDNLFGEKTGKAMRSAAAGSLIGIGEIVLLPLDVLKIKRQTNPESFKGRGFIKILRDEGLFNLYRGWGWTAARNAPGSFALFGGNAFAKEYILGLKDYSQATWSQNFISSIVGACSSLIVSAPLDVIKTRIQNRNFDNPESGLRIVKNTLKNEGVTAFFKGLTPKLLTTGPKLVFSFALAQSLIPRFDNLLSK>YDL199CMKPPLNMSRSNKPLTQEANSSAHIDRAHQLAQDFNSKQDDTALTSLPHKNPDIFRFENNITAHSSRRGSLYRDSDATVVLPLSEHTPRLSMDDPYRQLLQQAEISQLRSKKKRHSSRVLRTSFISFVVLVSSLSGLDQGLISGNVMTLSFQKYFHYPLTSPLGNIVSIVNLGAFMASLFVYSGILEPCSRKKMLQISTMIYSLGAIVQVLALNQWCLLLGRFLLGVGMGFAFSMVIIYQFEFPLPCIRKRTLISIQCVSSVIAYSFGIWINCAFRYLGFAWRYPLSTHVALGIILNLMSFYLILESPSWLLKQKNDVEALVLISNVFDDGNFEENQTQLKFRVLKRDILLKSHLQKNSYPYAYILKDFSSIIKLLIGFQLLTRTNGVDAFLYYSPLILQQMGRGERKSIYLTGLNALIYSIVILAYVPLVLRKRKEKTNVLLGSIVMCALLFTISFTDWFPKSTTRYISILFAVFLFTHFISWDSIGWVMTIELLPHLSQAPVILLVSNFYWIFKWFVSLITPILIDRLSWKFYLIPSLSSFISIIFVLKIFPIETRDERLDSDDDSTGNGSGNHDDVFDDTGSEFSSSPSFSAYQINTLGSSIKQNNQAYSSIQNEQILPKNGNLSNQTHGSAQNVYFITSDSGPSRTGEFFSFHNRTDPNISDNIAANKPSSGGGQNSPGDMAVA>YDL200CMKELLYYTFIETEVTGAFLVFREKTQNLVFASLGNDKLFLLGKVEGFLKKHEKQDTMYDLQELKEAETYKKSIENYTICLENKMPLPSGAIPFEFLFGTDFQRKVWNELLNVEHGHVVTYGDIAKRIGKPTAARSVGRACGSNNLALLVPCHRIVGSNRKLTGYKWSCKLKEQLLNNEKENSLSLSRL>YDL201WMKAKPLSQDPGSKRYAYRINKEENRKELKHVKINESSLVQEGQKIDLPKKRYYRQRAHSNPFSDHQLEYPVSPQDMDWSKLYPYYKNAENGQMTKKVTIADIGCGFGGLMIDLSPAFPEDLILGMEIRVQVTNYVEDRIIALRNNTASKHGFQNINVLRGNAMKFLPNFFEKGQLSKMFFCFPDPHFKQRKHKARIITNTLLSEYAYVLKEGGVVYTITDVKDLHEWMVKHLEEHPLFERLSKEWEENDECVKIMRNATEEGKKVERKKGDKFVACFTRLPTPAIL>YDL202WMLQLRFMPGWVPRNGFFGLKETIGTVHKRFYALASEQPSRKTVKPLDSRKTFLIDTYKHLMENSSMIFFVHYNNLSKTEDHHFRFKIKQTGGKLTKVRNNLFEVYLRNSHLPDPCGFVKRKEQNWKHPLLPLLKGPTATITYEDTNPQQVAKLLKVLQSAQDKLMVIGAKVENEVLNVEKINTFKTLPTKPEMQSQLVSVLQMLSGLGLVRTLENSSNALYLTLKSHNDNQKPKEDVESTTDAESKGSK>YDL204WMNRNTTTNKNANLNNSRNANAPGEAGHQNKTGLIYWTNPSKSGASFAATLVSLLILRNVNVISVLLKIGYMVLFTSFAVELSTKVLFDKGVVSRFGMQESPDLVGVLKPHIDRELDRLPALEDRIRKLVFAHRTRNNFTIGVSLYFLHGLFAIFSMNTVLIMTTIFLYTVPLIYDRKQARIDRAIDRMKDLVIHRFHKNYNKVVEKTEPYIDKIIPPQTDEGSYSTSISNENKSSTSQRNKSGLSSSEFDNMNDTSASKSGKDSYSTSQYNRAEYPVSQNENIGTLKSGKQEIPTEKDFNNRHENFSKPDVKTYDPRTVDIEEELAAHQRELEQNLKDGDYNLVGSKEIPDPITVPAPTRHTTKPAESQSIPIKNNETLHKTTHGLKQKLQHA>YDL207WMRFVFDEVFNSDTDSPEFEETCSTTSSTSSQCPTPEPSPAIKLPSFTKVGTKKLVNESVVILDPALENALRDLNLQSKLIPINEPIVAASSIIVPHSTNMPLPRASHSSLLDNAKNSNATAPLLEAIEESFQRKMQNLVLANQKEIQSIRENKRRVEEQRKRKEEEERKRKEAEEKAKREQELLRQKKDEEERKRKEAEAKLAQQKQEEERKKIEEQNEKERQLKKEHEAKLLQQKDKLGKAVTNFDKISKMFWHYKDKIAQIKQDIVLPIKKADVNVRNLLSRHKRKINPKFGQLTNSNQQLFKIQNELTQLINDTKGDSLAYHWILNFIAKAVVHQAETEVRVKPESALPLGKLTLYLLVQFPELQELFMARLVKKCPFVIGFTCEIDTEKGRQNMGWKRNNENKWEDNTSYDERMGGILSLFAIITRLQLPQEFITTTSHPFPIALSWHILARICNTPLNLITNTHFVILGSWWDAAAVQFLQAYGNQASKLLILIGEELTSRMAEKKYVGAARLRILLEAWQNNNMESFPEMSP>YDL208WMGKDNKEHKESKESKTVDNYEARMPAVLPFAKPLASKKLNKKVLKTVKKASKAKNVKRGVKEVVKALRKGEKGLVVIAGDISPADVISHIPVLCEDHSVPYIFIPSKQDLGAAGATKRPTSVVFIVPGSNKKKDGKNKEEEYKESFNEVVKEVQAL>YDL209CMTSWRDKSAKVQVKESELPSSIPAQTGLTFNIWYNKWSQGFAGNTRFVSPFALQPQLHSGKTRGDNDGQLFFCLFFAKGMCCLGPKCEYLHHIPDEEDIGKLALRTEVLDCFGREKFADYREDMGGIGSFRKKNKTLYVGGIDGALNSKHLKPAQIESRIRFVFSRLGDIDRIRYVESKNCGFVKFKYQANAEFAKEAMSNQTLLLPSDKEWDDRREGTGLLVKWANEDPDPAAQKRLQEELKLESLNMMVHLINNNTNSAGTEVNNKNNERLDRTFPEASVDNVKKRLLPLDNGMESDDFIEKLKKVKKNISRENISSKPSVGKLGGPLLDYLSSDED>YDL210WMSMSSKNENKISVEQRISTDIGQAYQLQGLGSNLRSIRSKTGAGEVNYIDAAKSVNDNQLLAEIGYKQELKRQFSTLQVFGIAFSIMGLLPSIASVMGGGLGGGPATLVWGWFVAAFFILLVGITMAEHASSIPTAGGLYYWTYYYAPEGYKEIISFIIGCSNSLALAAGVCSIDYGLAEEIAAAVTLTKDGNFEVTSGKLYGIFAGAVVVMCICTCVASGAIARLQTLSIFANLFIIVLLFIALPIGTKHRMGGFNDGDFIFGKYENLSDWNNGWQFCLAGFMPAVWTIGSFDSCVHQSEEAKDAKKSVPIGIISSIAVCWILGWLIIICLMACINPDIDSVLDSKYGFALAQIIYDSLGKKWAIAFMSLIAFCQFLMGASITTAVSRQVWAFSRDNGLPLSKYIKRVDSKYSVPFFAILAACVGSLILGLLCLIDDAATDALFSLAVAGNNLAWSTPTVFRLTSGRDLFRPGPFYLGKIWSPIVAWTGVAFQLFIIILVMFPSQQHGITKSTMNYACVIGPGIWILAGIYYKVYKKKYYHGPATNLSDDDYTEAVGADVIDTIMSKQEP>YDL211CMLLNVTSSQYISTPQSRSLSEIDTSETASLSSATDHIFALSTEVVSSITTNLIEGLESSIQVPISTAYGTTSFRNNTSSPQYLVSNCTSSVQSNITIDRGLLSTLKTFTTSQVPTIEPSTTKLTTPLSTTFTSTSTSEIYSVFTSENSVYIIYDQEYKFTERSTTFNTHFPQTTVLQESNPPLTFTIPSNTITGDAKLYQYLSGALNTQDTSDANNRRTGVIVGSTVGVVIGVVIVIFIGFIIIRNRRNVKNHSKKGFSHDIGKRVSCDEVTETEAPSNPFLNVLNYKVTTNGEGKRDSFENGRDLHRASSSDGIYIAHPYYGMADHESGRFSYVSSYNESAESSIEETSSSASTITRPNIQQTNSFLREII>YDL212WMFSYSDFCSIGTAMILSATTFLMGVFFSNMPYDYHLLFNPNSTQEHFDLALRHYQILHETPLPVIVTLCVVAGIGLVGGTIKVFKPNPELQMFEYCSLGLYVLAICVFLTNVKTGIDCSVSHNWGEVTENQGLAVIASSNIILLVMFAGVIILQIGLWYSNWDLQKRLKEFYAQEEREAANAGKKTEKVDNAKKNDNKSKGAQKRKNAKK>YDL213CMGSEEDKKLTKKQLKAQQFRKSKEEKDQEKDVKKEQAPEGKRPNSAAGNDGEEPVKKKRKTRRGRGGKGKNGKKGNRFIVFVGSLPRDITAVELQNHFKNSSPDQIRLRADKGIAFLEFDADKDRTGIQRRMDIALLQHGTLLKEKKINVELTVGGGGNSQERLEKLKNKNIKLDEERKERLTKMINDGNQKKIAKTTATAAQTSGTDNKPVPAGIHPDRAKLLK>YDL214CMSLSRILRYNQRNNKTTASLTAEHAYSDNWAYSVSLGDPTSVGVNMAAKTGEALNKSYDSVFSSLPVADSVPRTDFTASSRDDENTDVQKLTTSWMEKIDTKMPENISKIDSNIISSPMVSKVEARFIVPKGRLRKNSTDFTSSFSNSLSLPKSYGKLIFFTSKKNSSSTKKNLANDISDNKHNNNSSNTIGHNIPVTTATATCDEIACTSTEHEYNVYEEERMFTTRVYSLEDSVSSLSTNPLDDTYSEAVQVNTRHIEDTESTAHIRKHSYTTSLSSIKRLFKITSFSNNNSNSCDHQESTVADDCAISSSLKETTSSPVSTGSFSLMIENEDSDRDQIIQALYSNIEASTDLVSRKYRDLDVVLGEGSGGKVKLVQRVLDNKVFALKEYRSKKKRESERKYIKNIISEYCIASTLKNPNICETLEILYEKGKIFQILEYCEYDLFSLVMSEKMHYEEICCLFKQLINGVKYLHDIGLSHRDLKLDNCVVTRRGILKLIDFGASSVFHYPLSSQMIEANGIVGSDPYLSPEVFYFNEYDPRALDVWSVGIIFFCMITRRFPWKYPKVKDVQFKAFCSGRGVSSFKDLVTRPATDDSNNYDNDGYEEGVIDMGPNFILHRLPEETHKIMRRILEVSPFRRITINGILQDGWIKEIETCQVVGAASPNEASLRIINKGNHIHTNIDQRYAHIGGLHQRT>YDL216CMSLSNKTVKELRQLLKERYTVEDELTESIALSSMRFKPSQEPEFHALSQSSLLKTKLKQQSSTDIPSYTHVLISKLSCEKITHYAVRGGNIEIMGILMGFTLKDNIVVMDCFNLPVVGTETRVNAQLESYEYMVQYIDEMYNHNDGGDGRDYKGAKLNVVGWFHSHPGYDCWLSNIDIQTQDLNQRFQDPYVAIVVDPLKSLEDKILRMGAFRTIESKSDDNSATSYYELETIIFDSELNRALFETKLNLHCVIEDDESEQISLNRLIDSMKQYSYLMDSKNVRTRIKLATTSERVSNENKKNIDYQNRSTRSQFCLNTQRGDSTETSSFGSMFSGDNTSDVDMEDRNLTEFDSTDTSLCINGEPSIHVNRVERSSRSTDNFHNSKKRMNSNQERCHDEGNDMLQRNVLETDYARAKNRILASKIKQYERLRFYKDTFTL>YDL217CMVYTGFGLEQISPAQKKPYNELTPEEQGERGAEMIMNFMTSCPGKSVVSGVTGFALGGVLGLFMASMAYDTPLHTPTPANTAATATAGNIGVGGISRTVQQISDLPFRQQMKLQFTDMGKKSYSSAKNFGYIGMIYAGVECVIESLRAKNDIYNGVTAGFFTGAGLAYKAGPQAALMGGAGFAAFSAAIDLYMKSEDGRPPQNDFKE>YDL218WMKRVTGVFLTLLRFSQFASSVLVMSLLAYAIHAYGNRGNKKTNFTLATGVISVFYLIALGILCLALPTLIYIGMYFCAELIVCMLWLAAFVVLAKAQGERSCSNTNADGLYYNPYSGQYTADSHRRACNSSQAAIAFSGLCFVLFLISVILLGINVLTPIRKRYQTQGMWRSGASMGTKLHRWSGLALSEPFEETAAYDNTNVRTGDVEAGAGDNAAYTSEPNGDARYATNDPNGQYHTTTTNTRYTTTTADPKTRYTTNDRNPGSANVANSAVDQHAYSTDESGDRSYQEKVTEGAHSGAMSGSTAEPNRNVNQMP>YDL219WMKIVLQKVSQASVVVDSKVISSIKHGYMLLVGISIDDSMAEIDKLSKKVLSLRIFEDESRNLWKKNIKEANGEILSVSQFTLMAKTKKGTKPDFHLAQKGHIAKELYEEFLKLLRSDLGEEKVKDGEFGAMMSCSLTNEGPVTIILDSDQ>YDL222CMIFKRFVNLLVFLFLLGAGLLTFFLILSGGRESGTLKNFYWLQADTNGFNSAPSTTRWYNYNWCGYEDGQLANCSSRAPAKPFSPRDNFGNSVNLPSSFRNNRDTYYYLSRVGWAMLLISLFFIVLALVPGFLATFLPFKAVPVLYCVLSWLAFFFIILAACLYTGCYVKARKTFRNSGRSARLGPKNFAFIWTSVFLMLVNAIWSTIFSATHKAHSTYSDHDMYAQYESPSVDTGAQMEKSTYNSGATDGAGPITAAPVVGQPQPTTTTTPAGNGKFFQKLKTRKQVPSAELEPAGDGGLAGPVTVRD>YDL224CMSLVHNQTNLNESKFLIERAFSSSSETVPLSKEATYPMPTAYSFSAVRSNSETNIKRENPQGFAKEPIMTSMLHNLTMSTGKGNGNDVNSLAPHDVDVGPYCLLLRNLPKDITLRECYCIFSLATGVSSIELKRDDREPFNDNEKVVVVKFGSLSLVTHYANILNSKSEIFGPSFPFRSHIDVVNEQTQLPVSFQEHVSSGTTNSSPKNYQLSSSAQNEIQNQSFNTISYGKTSSSPLGPSAAKPRPSLLSERSLRFSFNDPFGLETISQRKESVPFLRNSISQHDLSNVTTTPVPAGMPPQKDAGKSLLLLEKDEINESIWNGDELVNDVGNSSFGASLQEPPMSSTPVMEWNASSTANIPLFQLSSQENHQSNLLPPSHHSISQDVPHIQSQPNLNNSGVIHSATSLPHYHLLNQINASTKTQSIQQSVSNVPSNLDLNLQTENGHPQSSAPNGSSIFNNQKVNQGFLVSEQDTSTISRQKECSSTASASAFSKNNETNVAGSTTISQADLSLLAKVPPPANPADQNPPCNTLYVGNLPPDATEQELRQLFSNQQGFRRLSFRNKMNSHGHGNGHGHGPICFVEFEDVSFATRALAELYGSQLPHPRPSLNNKGGIRLSFSKNPLGVRGSNSRSKSGYSFNGSYGKS>YDL226CMSDWKVDPDTRRRLLQLQKIGANKKCMDCGAPNPQWATPKFGAFICLECAGIHRGLGVHISFVRSITMDQFKPEELLRMEKGGNEPLTEWFKSHNIDLSLPQKVKYDNPVAEDYKEKLTCLCEDRVFEEREHLDFDASKLSATSQTAASATPGVAQSREGTPLENRRSATPANSSNGANFQKEKNEAYFAELGKKNQSRPDHLPPSQGGKYQGFGSTPAKPPQERSAGSSNTLSLENFQADPLGTLSRGWGLFSSAVTKSFEDVNETVIKPHVQQWQSGELSEETKRAAAQFGQKFQETSSYGFQAFSNFTKNFNGNAEDSSTAGNTTHTEYQKIDNNDKKNEQDEDKWDDF>YDL227CMLSENTTILMANGEIKDIANVTANSYVMCADGSAARVINVTQGYQKIYNIQQKTKHRAFEGEPGRLDPRRRTVYQRLALQCTAGHKLSVRVPTKPLLEKSGRNATKYKVRWRNLQQCQTLDGRIIIIPKNHHKTFPMTVEGEFAAKRFIEEMERSKGEYFNFDIEVRDLDYLDAQLRISSCIRFGPVLAGNGVLSKFLTGRSDLVTPAVKSMAWMLGLWLGDSTTKEPEISVDSLDPKLMESLRENAKIWGLYLTVCDDHVPLRAKHVRLHYGDGPDENRKTRNLRKNNPFWKAVTILKFKRDLDGEKQIPEFMYGEHIEVREAFLAGLIDSDGYVVKKGEGPESYKIAIQTVYSSIMDGIVHISRSLGMSATVTTRSAREEIIEGRKVQCQFTYDCNVAGGTTSQNVLSYCRSGHKTREVPPIIKREPVYFSFTDDFQGESTVYGLTIEGHKNFLLGNKIEVKSCRGCCVGEQLKISQKKNLKHCVACPRKGIKYFYKDWSGKNRVCARCYGRYKFSGHHCINCKYVPEAREVKKAKDKGEKLGITPEGLPVKGPECIKCGGILQFDAVRGPHKSCGNNAGARIC>YDL229WMAEGVFQGAIGIDLGTTYSCVATYESSVEIIANEQGNRVTPSFVAFTPEERLIGDAAKNQAALNPRNTVFDAKRLIGRRFDDESVQKDMKTWPFKVIDVDGNPVIEVQYLEETKTFSPQEISAMVLTKMKEIAEAKIGKKVEKAVITVPAYFNDAQRQATKDAGAISGLNVLRIINEPTAAAIAYGLGAGKSEKERHVLIFDLGGGTFDVSLLHIAGGVYTVKSTSGNTHLGGQDFDTNLLEHFKAEFKKKTGLDISDDARALRRLRTAAERAKRTLSSVTQTTVEVDSLFDGEDFESSLTRARFEDLNAALFKSTLEPVEQVLKDAKISKSQIDEVVLVGGSTRIPKVQKLLSDFFDGKQLEKSINPDEAVAYGAAVQGAILTGQSTSDETKDLLLLDVAPLSLGVGMQGDMFGIVVPRNTTVPTIKRRTFTTCADNQTTVQFPVYQGERVNCKENTLLGEFDLKNIPMMPAGEPVLEAIFEVDANGILKVTAVEKSTGKSSNITISNAVGRLSSEEIEKMVNQAEEFKAADEAFAKKHEARQRLESYVASIEQTVTDPVLSSKLKRGSKSKIEAALSDALAALQIEDPSADELRKAEVGLKRVVTKAMSSR>YDL230WMAAAPWYIRQRDTDLLGKFKFIQNQEDGRLREATNGTVNSRWSLGVSIEPRNDARNRYVNIMPYERNRVHLKTLSGNDYINASYVKVNVPGQSIEPGYYIATQGPTRKTWDQFWQMCYHNCPLDNIVIVMVTPLVEYNREKCYQYWPRGGVDDTVRIASKWESPGGANDMTQFPSDLKIEFVNVHKVKDYYTVTDIKLTPTDPLVGPVKTVHHFYFDLWKDMNKPEEVVPIMELCAHSHSLNSRGNPIIVHCSAGVGRTGTFIALDHLMHDTLDFKNITERSRHSDRATEEYTRDLIEQIVLQLRSQRMKMVQTKDQFLFIYHAAKYLNSLSVNQ>YDL231CMKRYERDRSPTPDPDIVKGSYSQTSLRSLHELNYKNPAGISGLSFAGSPQQSVASLSQMRLENLVKDKHWEEVEDFGLEELRDGFFDAAFTKPDSKARSPNSDIDDDNGAARKKLQSGFTKLSEYVWTAIYRPIIHFPRDIRKNGVSIFKFFIAYFIAIVICVIRPSGRWIGHEFRYFLPIAVLIHHPVRNIGVQLEMTISSIIGASFGLGWSALAWYISTATKPTANYQGGILFQSLTMALLFAIWLRSVYRRFFYFTTSFSISIIFTHTVRLASSKFDLKWQIFWDFGISYLFGLLLSLLVCVCVSPHSGNAELMEHYNKCLQTTKTFLMALVDTELIKSKEQIYLAQVKMVKTLNIDLSQGFRDFVNQLTISRFDLQSLKSLRNSLTAMETSLRVLPIAPKIFNDDELKKMYEELEKYRSDSATLSKEASASPQSSGIPTRENTPSAFKPIGPGLLKNEIYINALKASFSKSIFNLILEMIFVLENLSRVLKKYESPNQKNNLDECVKILSHSHSKLKRKIYKLDVCYRDFVNSSFFSQELLNDEESVDIFLFLRYLRNSARQLVTVIHDCQVLGENIHWRIALPSYPLSRALTRLPKQCVLDEGAGNVLHYFEAKRDVDEIFERVYNTYTSRHKYNKGEEEALRLDSQGGDEKSQNRKNHTISIRAIDHNDFNFHTTQNPWRFKLWKLSRILSGDECKWTLKITFCMIFLCLPTWLPESYHWYQEFHCWWAPLTFYLLAHRRYSGNWALVMRRLICGIVGIFWGWAANQSRHFGSPYVVCTFAGLIVVPFSINFLVYRNTKSSFTALMCFTIIALEPYSKPNRHYNLTTAGIWKSTWVTGLALIIGILVSIPINWIVWPFRARTELRDSMSSLLAHLGQSYQTVADRYLYRDADDAPTDLTFAFSHIREVRLTQSLEAIRELLKKARHEPIIISNFNPEKYASLINSCQFLLSKIIEARISGAFFEIWDQDFDIETTRALLSLRRDSVSSVIFVFYILSNCFRSKNKIPRYLPNPIMSRKKLYHFIKKFSEMKDQSHSNLNSGGNSMEKNLFKKIYQQKASSSGQQQLPLPSVANSSEIDSEKMHWTEVHGIAFARAFTDISEALFQVESCAKDILGEENF>YDL233WMYQGPPQPPPQAVPMPYIVNNNTPPYPNGNINFPPTAQQNIPPTVYPQQVPFPGQPQGGQFPQPSSEQQVFNQLPQVTQTFHNSAQNTNATGGPGSGSMPMFTPVASFPHPMATAATAAAPLPRSASQASLSMLRVPYHVRKYLSNLAMLKLYEIINEVNTAMGKIGLLSFWTELISDIFTPDAVIRYSKKSMTDYREFEFIIPVFPVICSTLGRFGIVRMEVKVLQLKTQVLSNSTIFFNCPRVTFVYYYPDGSYITHFSQMKGAFDLDLKINWLDVSMHSFVPDIEWNAVERLLSDDTKSTEIEQIFRKLKQEDVKEQGNSFAENNATNVPPNFEAITQLRSYFDVFRNVSVFGTQEGLMRVMQISTVMSTLKNLRKFQIEKNIDSPVTALSAYIDADKKDSGSEPLHAKRRRNSGISPRTTTLGPNGNSNTSNEELPTSDVNDINKDMTKKKMKF>YDL234CMSKILFCKSKVFLHPTSDARDNIAGFLLLTLEANKLSHQAILQYIPESGLSTLEISKLLKHEAKVGTCPTSTPFVIENSINFSNLVNTSLGQAFEISLSQIYCIQFRPPSPNGWYVGSLVIYPLTEQFTGFQPPVLFFHDQLCPSTTDKLKRLRKSMNPFDDSDELYWGGVDLRNKINELMELKKSNLEPEFWLVNPSLNDLRNFVSKDLLESYNNSKKDTTELATAGVKLNEKFQEWKWNVMSKIADVTTKSTNFIDSWLTNNSPIQKSQIDNEYLQKLLNNEKVKQIEQDYDSARVYLANWSLGVKQEAERYQKQNKLFDSYRNNIFNDLNLTDELSDTEINNALQRQFPLTEAKWNSLWDENDGRLRVTVNEVKDFIFHGGLENDSLRGKVWGFLLEIYPWDSSQDERVQIDQTLAAEYDQLKLTWSKDFLQFDDEDEEEYWNDQLFRISKDVRRCDRNLEIFQYNTIDGLPPPPQQLPANENNSTSPESANDESDDADDGVRNPHLIHLQNILITYNVYNTNLGYVQGMTDLLSPIYVIMKEEWKTFWCFTHFMDIMERNFLRDQSGIHEQMLTLVELVQLMLPELSEHLNKCDSGNLFFCFRMLLVWFKREFEMEDIMHIWENFWTFYYSSQFQLFFMLAILQKNSQAILQHLNQFDQILKFFNELNGKLDWNDLMVRAELLFKKFEKMMHVMERDLQNVSSSSSSSSTGVLPCQSERLTLLLSKKPIIRHEGQRSKNSVK>YDL235CMSTIPSEIINWTILNEIISMDDDDSDFSKGLIIQFIDQAQTTFAQMQRQLDGEKNLTELDNLGHFLKGSSAALGLQRIAWVCERIQNLGRKMEHFFPNKTELVNTLSDKSIINGINIDEDDEEIKIQVDDKDENSIYLILIAKALNQSRLEFKLARIELSKYYNTNL>YDL237WMLGLKGCLTILIGYVIAVCALFSSRGRNPSLTDWEKLKDQKISDIDNFGLTGQHLLEFFQENLPFLSFSEEKYRHKHVSLYYDVFKEYILRRASSKKCLPVDSAIAKLNKDVNPMPVHSHNDYWRKLPLFEGLAYGASSTEADVWNIDEKILAVGHNEAYLDPVELTLDKLYTGPLLEILDEVNCQDSDADRKNGVFFNSPETSLFFYIDFKSDDNELTYKLLMEQYFKSLIDSGYLTYYDMKKDEIIWRPVTVILTGNYPTSLDILDNGNDNGYFESSQRFAFLDAPLLSLEPKYSKLSVAATVSFSQLMKHCGSDHWKVSLRGRMDSNEISCAKSIIDGAHALKLKTRIWGAPTWPANLVETISRQIIHDLGSDLLNLDNLFMASSLI>YDL246CMSQNSNPAVVLEKVGDIAIEQRPIPTIKDPHYVKLAIKATGICGSDIHYYRSGGIGKYILKAPMVLGHESSGQVVEVGDAVTRVKVGDRVAIEPGVPSRYSDETKEGSYNLCPHMAFAATPPIDGTLVKYYLSPEDFLVKLPEGVSYEEGACVEPLSVGVHSNKLAGVRFGTKVVVFGAGPVGLLTGAVARAFGATDVIFVDVFDNKLQRAKDFGATNTFNSSQFSTDKAQDLADGVQKLLGGNHADVVFECSGADVCIDAAVKTTKVGGTMVQVGMGKNYTNFPIAEVSGKEMKLIGCFRYSFGDYRDAVNLVATGKVNVKPLITHKFKFEDAAKAYDYNIAHGGEVVKTIIFGPE>YDL247WMKNLSFLINRRKENTSDSNVYPGKAKSHEPSWIEMDDQTKKDGLDIVHVEFSPDTRAPSDSNKVITEIFDATEDAKEADESERGMPLATALNTYPKAAAWSLLVSTTLIMEGYDTAILGAFYALPIFQRKFGSQNDKTGEWEISASWQIGLTLCYMAGEIVGLQLTGPSVDLVGNRYTLIIALFFLAAFTFILYFCNSLGMIAVGQALCGMPWGCFQCLTVSYASEICPLALRYYLTTYSNLCWLFGQLFAAGIMKNSQKKYADSELGYKLPFALQWILPVPLALGIFFAPESPWWLVKKGRFDEARRSLRRTLSGKGPEKEILVTLEVDKIKVTIDKEKRLTSKEGSYSDCFEDKINRRRTRITCLCWAGQATCGSILIGYSTYFYEKAGVSTEMSFTFSIIQYCLGICATFLSWWASKYFGRYDLYAFGLAFQTIVFFIIGGLGCSSTHGSKMGSGSLLMAVAFFYNLGIAPVVFCLVSEMPSSRLRTKTIILARNTYNVVSIICSVLILYQLNSKKWNWGAKSGFFWGVLCFCTLIWAVVDLPETAGKTFVEINELFKLGVSARKFKSTKVDPFVVKTPLKTSLITTPREISKLPLQRNSNVSHHL>YDR002WMSSEDKKPVVDKKEEAAPKPPSSAVFSMFGGKKAEKPETKKDEEDTKEETKKEGDDAPESPDIHFEPVVHLEKVDVKTMEEDEEVLYKVRAKLFRFDADAKEWKERGTGDCKFLKNKKTNKVRILMRRDKTLKICANHIIAPEYTLKPNVGSDRSWVYACTADIAEGEAEAFTFAIRFGSKENADKFKEEFEKAQEINKKA>YDR003WMILREQIDFLIHKRQDDNNNNGEAITDDDPFSSSSWRWGRWIFFIFFIVALLILLFSTAKVNRRRRIMGQAPIRGTAWLTPPTYRQSERDYNGTQRCVEDYVPEYTETANENDLGFYDERGEFHPNGKTEYLAPPPLSEEQASSTDKDLQRPVAAVVRIPSESEFDFNLLRPTMNNFVNGQSNRNEQHSPTVESSSFDVNNAPARAKVSK>YDR004WMPRALSIKFDNTYMDLYDELPESKLLYDEEFSYLLDAVRQNGVCVVDFLTLTPKELARLIQRSINEVFRFQQLLVHEYNEKYLEICEKNSISPDNGPECFTTADVAMDELLGGGIFTHGITEIFGESSTGKSQLLMQLALSVQLSEPAGGLGGKCVYITTEGDLPTQRLESMLSSRPAYEKLGITQSNIFTVSCNDLINQEHIINVQLPILLERSKGSIKLVIIDSISHHLRVELQNKSFRESQENKNYLDRMAEKLQILAHDYSLSVVVANQVGDKPLANSPVAHRTYVTDYDYQLGWLVGWKNSTILYRQMNSLLGASSNNDEILSDDEDYMLIERVMSTVNDRNYDFFSKKKPPIIENKTVERNSSSPISRQSKKRKFDYRVPNLGLTWSNHVSTRILLQKSFKASTIIQRGEAHLYKGGDSASFWQVKRTMKVVYSTFAKPGQIAYQITKRGIETA>YDR006CMDQPRTHSGPTTASNPAPSSTNSSSAPSATNSKQERSSSSLSKPSSVVPSKDSPDGDAIAKTQAAALKNDMKSGDTSTLDGSSQNIIPNRASMQKYIDQSSDLLSRSSGVITPSMSLNASTNATNNDSSGNSANSSDLKIPIDRDNTIFKTFDTKTGQFLKNDDNEEEIRRNNKVDSIPPKNIYTNINNPSPSPPPSSKQPPSASAPQLPPATEPHKEQAAQQQPPGNASNFLRIFSNKKMRSHSVPTILHSSLRKLSSHNQYYRNQNILLNHPTPSGISKKKFSRNHHQPYLHSNNPLSSNPLSLKRAIFLNQQISGNASTNANNDNINNSTATSMTNQSFLSSSNFDLTLEDRINYIKATPTPVPFPPINLQGLKEIDLQEILKNPQLRHDIIFDPLLQFRPNLDGERGNKKRQLANIYWNDVQNEIYVYSKRPEIFQYNRSRLVPLFDTLRDVLLTIVPQKESPMINNVLDTELNIQELLKGSLIMSNLSGWLADLFKHHCAPMRDPWVDKMSNKFKEAERDSSLTRLIEGLRLVFQILETMKLDIANHQIRILRPALLSNAVEFEKQYFNTLIASKRVNLNTSLLWFDKKFNENVTAGLVRNPSSITIPDVYNICIRSIINLLSCRKMVREYPTPLSFDHARLILLRADIRQIVCILVCRLLFQQLVANDPSMDKATKEYVIHTYSTKRLKNEIISIITDEHGNCRWTKNTMSIAVHLCKVIDDLHREYDNNGSCEQARRPQLPSLDNSKITFAKSWLSKQTQPLSEVYGVLENRVFKSLEDAIFNRSECTIDGRVKQDFVYLYNTNNGNVGSTNTLSTTTDTASVKISPSLMSPSKTSTTTPTGNAIASRGLFAATELEEFENVYRHLYALINLHWSVFGPHYIEMLGDKVNKKGI>YDR012WMSRPQVTVHSLTGEATANALPLPAVFSAPIRPDIVHTVFTSVNKNKRQAYAVSEKAGHQTSAESWGTGRAVARIPRVGGGGTGRSGQGAFGNMCRGGRMFAPTKTWRKWNVKVNHNEKRYATASAIAATAVASLVLARGHRVEKIPEIPLVVSTDLESIQKTKEAVAALKAVGAHSDLLKVLKSKKLRAGKGKYRNRRWTQRRGPLVVYAEDNGIVKALRNVPGVETANVASLNLLQLAPGAHLGRFVIWTEAAFTKLDQVWGSETVASSKVGYTLPSHIISTSDVTRIINSSEIQSAIRPAGQATQKRTHVLKKNPLKNKQVLLRLNPYAKVFAAEKLGSKKAEKTGTKPAAVFAETLKHD>YDR013WMYGDLGNKLVLEAKRTKQLYARSNQDVNLPMYHEDIIRNILKEVSNLRKNTEYLKEQQQLGMLDDKVAKCQYFVTLLCMERNKRCLLAYQRLRTDILDSMAWNNNGLDLMSSITFSQQDTNNLSHQEQEYLKEYCDLITDLKSGDLVDIDLSGSLVPPSDVFIDVRVLKDAGEIQTEYGVFNLIKDSQFFVRQSDVERLIQQGYLQKI>YDR014WMRAYGKRGPVLRTPFRSNKGLPSSSDVEFSDDDVNSVIPDVSSTISSSIADHPIEGLLDEPRKAQDSSSSFDGANEKPSSQLDSKRNDQNVKIITSSDTSMAFMKDEKLSAFNFLDGSKASKRKRRRTYQKHDANITSSIEPDVQDEDSITMHNEFESIRKIYNDINEFILKLPRADDDILNKMLENEMKMDDSIENNSIRTSKDKKYGKFRTILINKNKENEIMGEEVDQKANTLSLNNADNSNAEKEGLTSTNHYNELKNMGDTIKYQDDIEFLLSNSKSNDNTTVPINEYFKKLLNLSLMIINDEEFFQYAKRYFKKEIIKLSFAQFRSDFPELILLQGYLLHKVSESQSDFPPSFDNFSIELSKDDGKIRTKKNKHIKKLSHLNFEDFLRKTQFKTGLYYSLSLWEMHGNLSLDIIKRISILASNKDLFSRHVKTFIPLLEKLITASEFCHMYIEQPEMFDSLISNLNNQFKDMLDDDSLIKILILLTNMEVHNYTLWKEADMIFQSSMNTILESIHPLTDAKVDNILLHLGLCLNICSRENSRLKLDGKLWYDMKTIFVKMIRDGSDTENRLVQGLFYLNFSFLIKQRKENSNLDPGELNLLLVELEAFKSETSQFNEGISNKIEIALNYLKSIYTSERITI>YDR016CMMASTSNDEEKLISTTDKYFIEQRNIVLQEINETMNSILNGLNGLNISLESSIAVGREFQSVSDLWKTLYDGLESLSDEAPIDEQPTLSQSKTK>YDR018CMKHSQKYRRYGIYEKTGNPFIKGLQRLLIACLFISGSLSIVVFQICLQVLLPWSKIRFQNGINQSKKAFIVLLCMILNMVAPSSLNVTFETSRPLKNSSNAKPCFRFKDRAIIIANHQMYADWIYLWWLSFVSNLGGNVYIILKKALQYIPLLGFGMRNFKFIFLSRNWQKDEKALTNSLVSMDLNARCKGPLTNYKSCYSKTNESIAAYNLIMFPEGTNLSLKTREKSEAFCQRAHLDHVQLRHLLLPHSKGLKFAVEKLAPSLDAIYDVTIGYSPALRTEYVGTKFTLKKIFLMGVYPEKVDFYIREFRVNEIPLQDDEVFFNWLLGVWKEKDQLLEDYYNTGQFKSNAKNDNQSIVVTTQTTGFQHETLTPRILSYYGFFAFLILVFVMKKNH>YDR020CMDRKAVEEKRIVISIGGGHATGVGAIALDLQNTFKSLYNSINIRVINLDNMIEGNIKSYNNNDYDFDNILNLVYEKHAVTSQNDMIQHDYEDPIDLIIVCGCYALYDKRINEISQLKVFLDSDADKRLISLIKKKNVGSNEQLAQLITEYMDHLRPEMQQYIEPTRTFADLIIPSTNENLGRAVLVDGIVKAIEDTKSQIEGNNTNNKIRPRLWDFEAETMDLEKDRYYDLS>YDR022CMNVTVTVYDKNVKYRLEENIKNNKGPSNDDQPAYNNESKSTDGSDYAMFPTNIKYIFEDNNDELVDSSDAALTAGIDKVGDELENVIIVQLDESGSLEDITLISDQYELLSHRTNSLSLEENQMRTLSSHGDDKSNDEEEELSVDSDRFRVDSDIELDVISQFCDLSPFLRDLSLNDLIKLYVTQNEQLQMLSNSV>YDR030CMDPFLEFRVGNISLNEFYRRTIQSEFERILEDPLSNMKNYRFSKQSNYSTKEKTPLSIGVNCLDIDDTGQVLLGGGDDGSLSIWGLDESLHRNDEGEQELINKRLNYIKRQPHQSDDEPAQIMGYKNKRTRINDNNTMRLVHSFQTQRNKYRMYRQSSAAVPVQRSHISNKTDSPIGFSETLSETDSEASISHHKYGITTLKWYKADNGMFFTGSNDKTVKIWDTNRFEAVQDINLGYKINQIDNNVVDDSSLLVVASEDYYPRLIDLRTMNSGVTALGMGNQTRMQSEILCCKFNPVREQIIACGDMEGGVKLWDLRMRNRLYSELKRNKNRFKTINNDDNDDQSDVYFSSNQSKAHLRCCSDIVWNSEGSELCSVGMDGKLNVWRPFTEILQPEGLASYSQLGTQDLSRIKYKKRVSRRLLWFDKFLLCITDNGEVEIYNTEEKKLWNKLEYPMVNQVKKNQASHCQFSSMIVQTNIMNSVGLKLFFGTNNNTVSDGGSIFECS>YDR031WMSDILDEIVIEDVVANCPQEFLQYHKCIRDNEENPGKCKDGRMILSTCIREKVPSVKSIMSECSEPMKKYDQCIRDNMGTRTINENCLGFLQDLRKCAELQVKNKNIKPSINGVNLELIKD>YDR032CMPRVAIIIYTLYGHVAATAEAEKKGIEAAGGSADIYQVEETLSPEVVKALGGAPKPDYPIATQDTLTEYDAFLFGIPTRFGNFPAQWKAFWDRTGGLWAKGALHGKVAGCFVSTGTGGGNEATIMNSLSTLAHHGIIFVPLGYKNVFAELTNMDEVHGGSPWGAGTIAGSDGSRSPSALELQVHEIQGKTFYETVAKF>YDR033WMSTFETLIKRGGNEAIKINPPTGADFHITSRGSDWFWTCFCCYLLFGLILTFLMFRKPVNDRFFYLTGIAPNFFMCIAYFTMASNLGWIPVKAKYNHVQTSTQKEHPGYRQIFYSRFVGWFLALPWPIIQICMLAGTPFWQMAFNVCITEFFTVCWLIAACVHSTYKWGYYTIGLGAAIVVSISVMTTSYNLVKQRDNDIRLTFLVFFSIIMFLWIIAYPTCFGITDGGNVLQPDSAGIFYGIIDLILMCFIPTLLVPIANHFGADKLGYHFGPSDAEAVMAPKAPVASPRPAATPNLSKDKKKKSKKSKKSKKSKKSEE>YDR034CMFESVNLDENSPEDRELAKVLSPPGSYLSPASLDSGSSFTNSGTSTSCFEPKNNLPSLSFLNARAGSLGGIFNHKQMTSPSNSNIGGENVESTTSSNDGSNENAGHPTTSEQDQNADHPTISQADDNGHSSLTPNPAVTSTVTDKKGNTVKRKYSRNGCSECKRRRMKCDETKPTCWQCARLNRQCVYVLNPKNKKRRTSNAQRVKEFRKHSTSLDNDHNNARKRQHSSCKAEKKKKVRQNLSEDTTDPKPITDNGKNVPLDEIESLEIPNLDLTTTMNGYDVNLLMQNLNDMVNMKLHDSYLLNEELKGLDLPDLDIPELLPASNVNSSVPISFLVNNVITFNTKLSSFKLGGIHDKYLKIFYYDCLDSIAPFFQNQGNPLRDILLSFAKNEAYLLSSILATGASIAYRKSNNLEDERNYCAYLSHCLSLLGEQFKNESNVLNRIEPIILTVIMLAWDCIYSMNSQWRSHLKGVTDLFKKINAGNSSKVLNVAKCWFKVMETFASISTVFGGSLIDNNDLDAIFDPYDYQYVDSLKFLNIMTPLNEFNLLRGHKEDFDLVIKEVFKSLNTIRSTEKNYFSKEEGLFTKKLDYLLLSSQTSSEKSKDQISYFNTQKILVEIDKQLDYEFIDKSGIIPSDNQSHPRISNIHDNAIDMVTLKNGEEVAISWYDISHQTQVLSFLLIVLLKLLGMPKESSTIQQVVKKIMSFFKFLDSDSPPQNSRTCYSNFAVLIAGLNAMDEETRAIVKRYYKINGGKFQKLTEHNLNRLEKVWYGKNQNYRLEEQDVLTW>YDR041WMLRNTIALRSFIRTQSTRPYPVNVEAVYYAPLKLPIKYGDLVADIQLRSYDNENLDFYSDFILRTGYYLGIPLTGPKPLPTRRERWTVIKSPFVHAKSKENFERHTHKRLIRAWDTNPEVLQMLIAYITKHSMAGVGMKCNFFQRSEISLDLGSDANGLEKSLSNIDELYSLRNDDKAQTSAVGQKVLELLDSPDFKKHLEKK>YDR043CMFYPYNYSNLNVSTMPALPGISAFDGMQDEENVEISPERKYQTLLPVLTNSHVVENELKHKLNKTAFDFRYQTKSENGSEKWEPKYLITPNLQTRSVSFDNSSVQYNSDSSEKSSLSQLTCNSSIIQQPENGIVSNDAYNKMANSRYSLKTRKQRTDPRNTLSDEEDLEQRRKYICKICARGFTTSGHLARHNRIHTGEKNHCCPYKGCTQRFSRHDNCLQHYRTHLKKGQ>YDR044WMPAPQDPRNLPIRQQMEALIRRKQAEITQGLESIDTVKFHADTWTRGNDGGGGTSMVIQDGTTFEKGGVNVSVVYGQLSPAAVSAMKADHKNLRLPEDPKTGLPVTDGVKFFACGLSMVIHPVNPHAPTTHLNYRYFETWNQDGTPQTWWFGGGADLTPSYLYEEDGQLFHQLHKDALDKHDTALYPRFKKWCDEYFYITHRKETRGIGGIFFDDYDERDPQEILKMVEDCFDAFLPSYLTIVKRRKDMPYTKEEQQWQAIRRGRYVEFNLIYDRGTQFGLRTPGSRVESILMSLPEHASWLYNHHPAPGSREAKLLEVTTKPREWVK>YDR045CMLSFCPSCNNMLLITSGDSGVYTLACRSCPYEFPIEGIEIYDRKKLPRKEVDDVLGGGWDNVDQTKTQCPNYDTCGGESAYFFQLQIRSADEPMTTFYKCVNCGHRWKEN>YDR046CMSDPIVTSSKMEKSAEFEVTDSALYNNFNTSTTASLTPEIKEHSEESRNGLVHRFVDSFRRAESQRLEEDNDLEDGTKSMKSNNHLKKSMKSRHVVMMSLGTGIGTGLLVANAKGLSLAGPGSLVIGYVMVSFVTYFMVQAAGEMGVTYPTLPGNFNAYNSIFISKSFGFATTWLFCIQWLTVLPLELITSSMTVKYWNDTINADVFIVIFYVFLLFIHFFGVKAYGETEFIFNSCKILMVAGFIILSVVINCGGAGVDGYIGGKYWRDPGSFAEGSGATRFKGICYILVSAYFSFGGIELFVLSINEQSNPRKSTPVAAKRSVYRILIIYLLTMILIGFNVPHNNDQLMGSGGSATHASPYVLAASIHKVRVIPHIINAVILISVISVANSALYAAPRLMCSLAQQGYAPKFLNYIDREGRPLRALVVCSLVGVVGFVACSPQEEQAFTWLAAIAGLSELFTWSGIMLSHIRFRKAMKVQGRSLDEVGYKANTGIWGSYYGVFFNMLVFMAQFWVALSPIGNGGKCDAQAFFESYLAAPLWIFMYVGYMVYKRDFTFLNPLDKIDLDFHRRVYDPEIMRQEDEENKERLKNSSIFVRVYKFWC>YDR047WMGNFPAPKNDLILRAAKGEKVERPPCWIMRQAGRYLPEYHEVKNNRDFFQTCRDAEIASEITIQPVRRYRGLIDAAIIFSDILVIPQAMGMRVEMLEGKGPHFPEPLRNPEDLQTVLDYKVDVLKELDWAFKAITMTRIKLDGEVPLFGFCGGPWTLMVYMTEGGGSRLFRFAKQWINMYPELSHKLLQKITDVAVEFLSQQVVAGAQILQVFESWGGELSSVDFDEFSLPYLRQIAERVPKRLQELGIMEQIPMIVFAKGSWYALDKLCCSGFDVVSLDWSWDPREAVKINKNRVTLQGNLDPGVMYGSKEVITKKVKQMIEAFGGGKSRYIVNFGHGTHPFMDPDVIKFFLEECHRIGSK>YDR050CMARTFFVGGNFKLNGSKQSIKEIVERLNTASIPENVEVVICPPATYLDYSVSLVKKPQVTVGAQNAYLKASGAFTGENSVDQIKDVGAKWVILGHSERRSYFHEDDKFIADKTKFALGQGVGVILCIGETLEEKKAGKTLDVVERQLNAVLEEVKDWTNVVVAYEPVWAIGTGLAATPEDAQDIHASIRKFLASKLGDKAASELRILYGGSANGSNAVTFKDKADVDGFLVGGASLKPEFVDIINSRN>YDR051CMCEENVHVSEDVAGSHGSFTNARPRLIVLIRHGESESNKNKEVNGYIPNHLISLTKTGQIQARQAGIDLLRVLNVDDHNLVEDLAKKYIKDESSRRTLPLKDYTRLSREKDTNIVFYTSPYRRARETLKGILDVIDEYNELNSGVRICEDMRYDPHGKQKHAFWPRGLNNTGGVYENNEDNICEGKPGKCYLQYRVKDEPRIREQDFGNFQKINSMQDVMKKRSTYGHFFFRFPHGESAADVYDRVASFQETLFRHFHDRQERRPRDVVVLVTHGIYSRVFLMKWFRWTYEEFESFTNVPNGSVMVMELDESINRYVLRTVLPKWTDCEGDLTT>YDR052CMVSPTKMIIRSPLKETDTNLKHNNGIAASTTAAGHLNVFSNDNNCNNNNTTESFPKKRSLERLELQQQQHLHEKKRARIERARSIEGAVQVSKGTGLKNVEPRVTPKELLEWQTNWKKIMKRDSRIYFDITDDVEMNTYNKSKMDKRRDLLKRGFLTLGAQITQFFDTTVTIVITRRSVENIYLLKDTDILSRAKKNYMKVWSYEKAARFLKNLDVDLDHLSKTKSASLAAPTLSNLLHNEKLYGPTDRDPRTKRDDIHYFKYPHVYLYDLWQTWAPIITLEWKPQELTNLDELPYPILKIGSFGRCPFIGDRNYDESSYKRVVKRYSRDKANKKYALQLRALFQYHADTLLNTSSVNDQTKNLIFIPHTCNDSTKSFKKWMQEKAKNFEKTELKKTDDSAVQDVRNEHADQTDEKNSILLNETETKEPPLKEEKENKQSIAEESNKYPQRKELAATPKLNHPVLATFARQETEEVPDDLCTLKTKSRQAFEIKASGAHQSNDVATSFGNGLGPTRASVMSKNMKSLSRLMVDRKLGVKQTNGNNKNYTATIATTAETSKENRHRLDFNALKKDEAPSKETGKDSAVHLETNRKPQNFPKVATKSVSADSKVHNDIKITTTESPTASKKSTSTNVTLHFNAQTAQTAQPVKKETVKNSGYCENCRVKYESLEQHIVSEKHLSFAENDLNFEAIDSLIENLRFQI>YDR055WMQLHSLIASTALLITSALAATSSSSSIPSSCTISSHATATAQSDLDKYSRCDTLVGNLTIGGGLKTGALANVKEINGSLTIFNATNLTSFAADSLESITDSLNLQSLTILTSASFGSLQSVDSIKLITLPAISSFTSNIKSANNIYISDTSLQSVDGFSALKKVNVFNVNNNKKLTSIKSPVETVSDSLQFSFNGNQTKITFDDLVWANNISLTDVHSVSFANLQKINSSLGFINNSISSLNFTKLNTIGQTFSIVSNDYLKNLSFSNLSTIGGALVVANNTGLQKIGGLDNLTTIGGTLEVVGNFTSLNLDSLKSVKGGADVESKSSNFSCNALKALQKKGGIKGESFVCKNGASSTSVKLSSTSKSQSSQTTAKVSKSSSKAEEKKFTSGDIKAAASASSVSSSGASSSSSKSSKGNAAIMAPIGQTTPLVGLLTAIIMSIM>YDR056CMLVRLLRVILLASMVFCADILQLSYSDDAKDAIPLGTFEIDSTSDGNVTVTTVNIQDVEVSGEYCLNAQIEGKLDMPCFSYMKLRTPLKYDLIVDVDEDNEVKQVSLSYDETNDAITATVRYPEAGPTAPVTKLKKKTKTYADKKASKNKDGSTAQFEEDEEVKEVSWFQKNWKMLLLGLLIYNFVAGSAKKQQQGGAGADQKTE>YDR057WMQAKIIYALSAISALIPLGSSLLAPIEDPIVSNKYLISYIDEDDWSDRILQNQSVMNSGYIVNMGDDLECFIQNASTQLNDVLEDSNEHSNSEKTALLTKTLNQGVKTIFDKLNERCIFYQAGFWIYEYCPGIEFVQFHGRVNTKTGEIVNRDESLVYRLGKPKANVEEREFELLYDDVGYYISEIIGSGDICDVTGAERMVEIQYVCGGSNSGPSTIQWVRETKICVYEAQVTIPELCNLELLAKNEDQKNASPILCRMPAKSKIGSNSIDLITKYEPIFLGSGIYFLRPFNTDERDKLMVTDNAMSNWDEITETYYQKFGNAINKMLSLRLVSLPNGHILQPGDSCVWLAEVVDMKDRFQTTLSLNILNSQRAEIFFNKTFTFNEDNGNFLSYKIGDHGESTELGQITHSNKADINTAEIRSDEYLINTDNELFLRISKEIAEVKELLNEIVSPHEMEVIFENMRNQPNNDFELALMNKLKSSLNDDNKVEQINNARMDDDESTSHTTRDIGEAGSQTTGNTESEVTNVAAGVFIEHDEL>YDR059CMSSSKRIAKELSDLGRDPPASCSAGPVGDDLYHWQASIMGPSDSPYAGGVFFLSIHFPTDYPFKPPKVNFTTKIYHPNINSSGNICLDILKDQWSPALTLSKVLLSICSLLTDANPDDPLVPEIAQIYKTDKAKYEATAKEWTKKYAV>YDR061WMSTNKFVVRITNALFKSSLASNSPPVYPKRIRHFEILPNEKWVIWGPGKGKFLDVLNNKYICEPPLSLRFGFLKESSNILPRIEQVAFKGVMPTAHLSARYEYFKDDYDQTCKQFIFDKASGSNAVSYKVETNNRQINMELYNALVENLNLSSLQDRWVMGLSNGQMRRARLARSILKEPDLLLIDDPFLGLDPAAIATISQFLAKYDSIEVSGGCPIVIGLRYQDTIPAWCTHICCVDEKNGILFEGPIEKLQSKMDETRSRALKELEQLKKASNSKEDISINDLICIHPMYGKKEHEIIKMPHLIELDGLSVSYKGEAVLENLHWKVQPGSKWHIRGDNGSGKSTLLSLLTAEHPQSWNSRVIDNGVPRRTGKTNYFDLNSKIGMSSPELHAIFLKNAGGRLNIRESVATGYHEASSNNYLPIWKRLDKNSQEIVNMYLKYFGLDKDADSVLFEQLSVSDQKLVLFVRSLIKMPQILILDEAFSGMEVEPMMRCHEFLEEWPGTVLVVAHVAEETPKCAHYLRLISPGEYEIGDMEN>YDR063WMSNLYKIGTETRNKIKKFRTSTARTDSIKALSIKIEPKPSYEIIVDEDEQEELDEIEDLSELAEILPDNSPRFVLTAYPTTTKDGFKQTPLVLVYWKPMTVVSQEWKMLYAGALEMIREECGTFKLIEVSSGLEDDSDVEELREQLENC>YDR065WMAQNFGKIPSHKSYVLSLYRTVLRNIPKCCHSYAFQYEIKKTLSIQLFKHKHDKSSWSVYTLLNEFSLLNNCLLEGKLQEIKNLMKPLKKMKKQLKTTKILNSLTSLGDVKTNDPEEVRRFHVLSAYIKRKQDLGLLPAYIPKTYQHKLLLPLALNEHACLKLFHIQQKLKNGPPSAGLSYTKEGRNQIWFVRSPINKGRQQSKKLGILIRKERKDSQKNIDNLNFCEINAAWALHEAIWEEYLESKKIIKVNLPKYLEYAANIPKSTKCNPSSQYQKVKEWVDPVREIMFELHSKSFQRVEYFNKYKEKLLKNGGQLAYFDKKSKEMYAKRLTLFRKMSKETLPYVTLFIEGRDLPSVLAKYGF>YDR068WMEFFYEEQVACIEDDKISNSHTKETGSTENTENNELQSRDDKTNEAFQKLEEEVNKRYEKTTSAFKKLVIEKDDGIEINLPISNETTETAQKYLKKLDENIHSVESLAQSYWSKMKTKNFWSGFSSFDNAAENDSNDKDENSKENEIAVGGNRTEAELRTLSKDKSVYLDNKMDLQLDPFDVDEKTEEICSILQGDKDISKLMNDIVPHKISYKDFWHIYFLQRNKILDKESKRKEILSKKEKETEEKEVEWDDEEEEEDDDKVEAVADNKSKGETKVAVSQEGLKDVSDHVGLANKDESKDDDDDDDWE>YDR070CMLRTTFLRTPRQLMRKSPRASFSIVTRAAFPHLKNNQDEAEKKEQGLFDSNKKRLDTLEHGKNPDYKQPGMEDLKKKGDDARIEQNRPDDGVY>YDR071CMASSSSTLPLHMYIRPLIIEDLKQILNLESQGFPPNERASEEIISFRLINCPELCSGLFIREIEGKEVKKETLIGHIMGTKIPHEYITIESMGKLQVESSNHIGIHSVVIKPEYQKKNLATLLLTDYIQKLSNQEIGNKIVLIAHEPLIPFYERVGFKIIAENTNVAKDKNFAEQKWIDMERELIKEEYDN>YDR073WMSSEIAYSNTNTNTENENRNTGAGVDVNTNANANANATANATANATANATAELNLPTVDEQRQYKVQLLLHINSILLARVIQMNNSLQNNLQNNINNSNNNNIIRIQQLISQFLKRVHANLQCISQINQGVPSAKPLILTPPQLANQQQPPQDILSKLYLLLARVFEIW>YDR074WMTTTAQDNSPKKRQRIINCVTQLPYKIQLGESNDDWKISATTGNSALFSSLEYLQFDSTEYEQHVVGWTGEITRTERNLFTREAKEKPQDLDDDPLYLTKEQINGLTTTLQDHMKSDKEAKTDTTQTAPVTNNVHPVWLLRKNQSRWRNYAEKVIWPTFHYILNPSNEGEQEKNWWYDYVKFNEAYAQKIGEVYRKGDIIWIHDYYLLLLPQLLRMKFNDESIIIGYFHHAPWPSNEYFRCLPRRKQILDGLVGANRICFQNESFSRHFVSSCKRLLDATAKKSKNSSNSDQYQVSVYGGDVLVDSLPIGVNTTQILKDAFTKDIDSKVLSIKQAYQNKKIIIGRDRLDSVRGVVQKLRAFETFLAMYPEWRDQVVLIQVSSPTANRNSPQTIRLEQQVNELVNSINSEYGNLNFSPVQHYYMRIPKDVYLSLLRVADLCLITSVRDGMNTTALEYVTVKSHMSNFLCYGNPLILSEFSGSSNVLKDAIVVNPWDSVAVAKSINMALKLDKEEKSNLESKLWKEVPTIQDWTNKFLSSLKEQASSNDDMERKMTPALNRPVLLENYKQAKRRLFLFDYDGTLTPIVKDPAAAIPSARLYTILQKLCADPHNQIWIISGRDQKFLNKWLGGKLPQLGLSAEHGCFMKDVSCQDWVNLTEKVDMSWQVRVNEVMEEFTTRTPGSFIERKKVALTWHYRRTVPELGEFHAKELKEKLLSFTDDFDLEVMDGKANIEVRPRFVNKGEIVKRLVWHQHGKPQDMLKGISEKLPKDEMPDFVLCLGDDFTDEDMFRQLNTIETCWKEKYPDQKNQWGNYGFYPVTVGSASKKTVAKAHLTDPQQVLETLGLLVGDVSLFQSAGTVDLDSRGHVKNSESSLKSKLASKAYVMKRSASYTGAKV>YDR075WMMDLDKIIASLRDGKHIPEETVFRLCLNSQELLMNEGNVTQVDTPVTICGDIHGQLHDLLTLFEKSGGVEKTRYIFLGDFVDRGFYSLESFLLLLCYKLRYPDRITLIRGNHETRQITKVYGFYDEVVRKYGNSNVWRYCCEVFDYLSLGAIINNSIFCVHGGLSPDMTTVDEIRTIDRKQEVPHEGAMCDLLWSDPEDVDTWSLSPRGAGFLFGKREVDQFLEKNNVELIARAHQLVMEGYKEMFDGGLVTVWSAPNYCYRCGNVAAVLKIDDDLNREYTIFEAVQAQNEVGNAIIPTKKSQMDYFL>YDR078CMSKDVIEYSKLFAKLVNTNDDTKLDDTIASFLYYMFPRELFIRAISLLESSDMFIYILDRVHNKEGNEHTSLIDVLVDEFYKGSSNSLLEYRLIVKDTNDGAPPILVDIAHWFCSCEEFCKYFHEALEKTDEKEELHDVLINEVDDHLQFSDDRFAQLDPHSLSKQWYFKFDKVCCSHLLAFSILLRSSINVLKFFTVNSNKVFVIAIDNIDEWLNLHINIVE>YDR079WMGLFNNFKFKYTRAQLEIFRFSFCLLAPVAVMYYIGTDTDKKLNVPGFWPDPATLNQIPKEPYEIKAELARMKKERLEKRLRLEKKIQEEFGLDLEEEKEKIKRDLALKKG>YDR083WMALFNVEGWSIKTKTVAFDNKTNKSSKDKKKNNRKNGKLTREQKLKEETEAELKEQVEDIPSEGSVAKDIPKKNQEKSDQNETSKKRKHDEEAPLMQVKENIEKPTKKQLTPLQQKMMAKLTGSRFRWINEQLYTISSDEALKLIKEQPQLFDEYHDGFRSQVQAWPENPVDVFVDQIRYRCMKPVNAPGGLPGLKDSKEIVIADMGCGEAQLALEINNFFKNYNKKAKKYLKRRHKVHSFDLKKANERITVADIRNVPLPDESCTIVVFCLALMGTNFLDFIKEAYRILAPRGELWIAEIKSRFSDGKGNEFVDALKLMGFFHKKTFDENKMFTRFEFFKPPAEIIEERRQKLERRQKFIEVETEKEELEKKRRKIAEGKWLLKPCIYKRR>YDR084CMDQARNFYNTILKSSHPLLLSFHLAGKAVPIVFYIIGSMFLNFTPQFITVVLLLSFDFYLTKNITGRKLVQLRWWYDSTDVNKDSNFTFESYKQYAPGPPINAIDSKLFWWSMYVTPVIWGVFAVLCLLRLKIFYLILVIVAMCLTAWNTYGFRCCDRWEPNSGQSDGQDTNNWFALPSVPGFENLSRLANIQSFFQRQ>YDR085CMEGSYLSAQENQPIPERLIPRSNSTSNLFALSSTFSKLNVRNDADYNYSNPNKKRHIYSGEIDCRSVTAARKFPVRSCSMTAAQQRKRTALFTVRERNSYHEGFNNDQDYVSQYQKPQYTFGVYKELTPYQLQRSKMKRSFQFPNGEIYKPKLDGKCTHSLKKPELNSRDSSLFKFSEKKGRNLSKDFVGPHNGTSVIHIPPNDTGYGVNSLELNTSVPSTIKSSVSSTSPISAVNTLTSLPESQTDDDDGYENKTVTISYCFENTVNEKHGSHIEKLDLSTKEKTKPTTNSGLFDRKKKTILGTEKYRCIKSQSKLKLGSVLKKLWRTSGNSNTKHGKKDTKRRRIPIDDMVTHSDGNSEAENDIELMDANLDGIEFDDDETLMDTDSIFDDLLSKENDKYDLRRRQLEIRQKLHETSHNDDGKVSFRDTEKHNVNEGLIDKTIIEEFSKLGEYIIDTRNQPPPRSSKRPSLDDNESARYFYNISTDLRQSLSGPISLPMHVGNDMVNRLRNDWEYIRFEDRRNSLPDSSFDKVETPPKPIKKDVRFAKEVCLASTWSSNAYERANPEFIMNRHRLLWMMKVHPSMNSAMNEIKLELNSYKKNEMVVHENSKCFTHYLI>YDR086CMARASEKGEEKKQSNNQVEKLVEAPVEFVREGTQFLAKCKKPDLKEYTKIVKAVGIGFIAVGIIGYAIKLIHIPIRYVIV>YDR087CMETSNFVKQLSSNNRKTRVNALEALKKYLTAKQFKENKQIEFNKLWKGLYYAMWFSDRPRPQQRLANELGELHGLYFDPKDNSTADELTTNDKAFIKFSRGFWKVMCFEWFNIDRYRLDKYLLLIRRVLFSQLKYLQSRNWDKKLVDEYIKKVLRWLPLSGSPKVYTGIPIHIVDILLDEWERLLKDGDEDDEDEENKEEEMRKIAESAKKTPLADVIAIFQDIVADYNNSKVLREKIKEDLFSDTRLVSWDILEGETQHNDSSNESEEEEEEEWKGF>YDR088CMNNNSRNNENRSTINRNKRQLQQAKEKNENIHIPRYIRNQPWYYKDTPKEQEGKKPGNDDTSTAEGGEKSDYLVHHRQKAKGGALDIDNNSEPKIGMGIKDEFKLIRPQKMSVRDSHSLSFCRNCGEAGHKEKDCMEKPRKMQKLVPDLNSQKNNGTVLVRATDDDWDSRKDRWYGYSGKEYNELISKWERDKRNKIKGKDKSQTDETLWDTDEEIELMKLELYKDSVGSLKKDDADNSQLYRTSTRLREDKAAYLNDINSTESNYDPKSRLYKTETLGAVDEKSKMFRRHLTGEGLKLNELNQFARSHAKEMGIRDEIEDKEKVQHVLVANPTKYEYLKKKREQEETKQPKIVSIGDLEARKVDGTKQSEEQRNHLKDLYG>YDR090CMISEKAATALATIATVCWCVQLIPQIIYNWKKKDCTGLPPLMMFLWVVSGIPFAIYFCVSKGNVILQVQPHLFMFFCSISFVQSCYYPPISMARSKIVMIVAAIIAADVGMEVGFILWLRPLYEKGVKWPDLIFGISASVLLAVGLLPPYFELAKRKGRVIGINFAFLFIDSLGAWLSIISVILGNMDIMGIILYSIVAGMELGIFASHFIWWCRFRFLAKGNTFDEESGQAQKEEPDEKIEQDISKSDRNVTNYNLDNCSIPDDASSFADDFNIYDSTDGGTLSRAQTLHAVHGVVVRTDPDRYSRLSV>YDR098CMCSFQVPSAFSFNYTSYCYKRHQARYYTAAKLFQEMPVIEINDQEQFTYLTTTAAGDKLIVLYFHTSWAEPCKALKQVFEAISNEPSNSNVSFLSIDADENSEISELFEISAVPYFIIIHKGTILKELSGADPKEYVSLLEDCKNSVNSGSSQTHTMENANVNEGSHNDEDDDDEEEEEETEEQINARLTKLVNAAPVMLFMKGSPSEPKCGFSRQLVGILREHQVRFGFFDILRDESVRQNLKKFSEWPTFPQLYINGEFQGGLDIIKESLEEDPDFLQHALQS>YDR099WMSQTREDSVYLAKLAEQAERYEEMVENMKAVASSGQELSVEERNLLSVAYKNVIGARRASWRIVSSIEQKEESKEKSEHQVELIRSYRSKIETELTKISDDILSVLDSHLIPSATTGESKVFYYKMKGDYHRYLAEFSSGDAREKATNSSLEAYKTASEIATTELPPTHPIRLGLALNFSVFYYEIQNSPDKACHLAKQAFDDAIAELDTLSEESYKDSTLIMQLLRDNLTLWTSDISESGQEDQQQQQQQQQQQQQQQQQAPAEQTQGEPTK>YDR100WMSVIPPKFFKIANISIGCIDIIAALSQLTYIFTNLNVFLLAVYGLALSVPIVYLEFKVPSNLYRYASFYFSFLGRGLSYILLSLIISFGGIYNILAGMFTFILGVAFIVFHFSQFVEEPANFRAPGSSLSIGDDDIDDDDDMI>YDR101CMALAISHEDTQILLKDKNILQESVLNKYRTAGQIAQTALKYVTSLINDSYHSKTTQRQLTVPELCLLTDSFILTRLEQYYKNKVNERGIAIPTTIDIDQISGGWCPEIDDTQNLLNWNKGKDSTFASSVTGTLRPGDLVKITLGVHIDGYTSEVSHTMVIYPVDETKPILQPTGPLLGGKADAVAAAHIAMETVVALLACALTPEKLPASLGGTSSGITGQLIRTIVDTIARSYNCGVVPGSRVRRIRRFLAGQNEGIVAEREYKGVVWTESHQEADLLSNTDAKDLTVVDRGQSTPFTNVSAIPSDDFVVQSGEVYLIDLKMASLEHCTKKGLVTLETVDSYTGKSHKAGELIARPGAYVRDFAQTHILKLKTSRQLLTKIDKQGVYPFKLSHLSSNFPFVHENEEELQSLKKDLKSFRLGMSEISNNYLCVESPIQIARWVPWDHILKATNPNGNLSYDATSTLTLPGHELPLPKLGVSAIKLKSLMNSTKESISLPVARECNTIVLCDSSVSTTDRPELLRLTGGSKTCQPSWIHSQHELNPQDSIVQGIFQLATLAKDKRFGLLLKETQPMKQKSVETSNGGVEETMKM>YDR105CMGAVISLPVSMAGSFVASCFGGCCSNLVTKTASSLGSSSLGTRLLYAVWLLLNSLISWVSYSANKSILWPGKTCTGTGECGFFTVHRLNFALGCLHLILALVLTGVKSTNDVRAALQNSWWSLKFILYLCLIVLSFVIPNDFYIFFSKWVSVPSGAIFILVGLILLVDFAHEWAETCISHVESEDEDSSFWQRFLVLGTTSMYTASIIMTVVMYVMFCHQQCNMNQTAVTVNLILTVITLVLSVNPKIQEANPKSGLAQSSMVSVYCTYLTMSAMSSEPDDKMCNPLVRSSGTRKFSIILGSLFTFIAIAYTTTRAAANSAFQGTNTNGAIYLGNDIEYEGLGGQTRNQLRYEAIKQAVEEGSLPESALYDTAWLGTSSPTGAMDNQNDDERTGTKYNYTLFHVIFFLATQWIAILLTINVTQDDVGDFIPVGRTYFYSWVKIVSAWICYALYGWTVVAPAIMPDRFDYENYY>YDR106WMSNTIVIVYLGANRIEIGRSADACPQEIIAWKTGSINEKNREELKKIFEHYFQICNILGNREVQVLILEDIFISVVEKRIICSILFKEFDCAHVSFVPRAIVHCLSCNTRNAIVIDIGTNYTTCVPIFDLRPLQQFIKYSKRGKRQVESRIPLPGSLYMPIFFDEEYNSKNCEVDETPVINLVKNIVESLPIDLRRPLRENIIIVNIEEAYETVIRNLFKLKMDTSKIQFPKNYWQAGSACAKILLHYKGSNIVGIERDEFYNNPHIAPDWFDYYFRTGVKRLQ>YDR107CMKRGVWLLIYCYATLTKGFSLPGLSPTTYHSGDEIPLLVNRLTPSIYFQHQDEEGNDVSGDKEHFLYSYDYYNKRFHFCRPEHVEKQPESLGSVIFGDRIYNSPFQLNMLEEKECVALCKSTIPGKDAKFINTLIKSGFFQNWLVDGLPAARKAYDSRTKTNYYGTGFELGFTDVKQTVDGKAVPSTMEELTSEASNEDVILDARLPKNVKPNLVKTVELPYFVNHFDIEVEFHDRGNDNYRVVGVIVNPVSIERSSPGACSTTGKPLILDEDKDNEVYFTYSVKFVASDTVWATRWDKYLHIYDPQIQWFSLINFSVIVILLSSVVMHSLLRALKSDLARYNELNLDNEFHEDSGWKLGHGDVFRTPSKSMLLSILVGSGMQLFLMVMCSIFFAAVGLVSPVSRGSLPTVMFVLYALFGFVGSYASMGVYKFFRGPYWKANMILTPILLPGAIFLLIVIMNFFLLFAHSSGVIPARSLFFIILLWFLVSVPLSFAGSIVAHKQCNWDEHPTKTNQIARQIPYQPWYLRTAQATLIAGIFSFGSIAVELYFIYSSLWFNKIFYMFGFLLFSFLLLTLTTSLVTILITYYSLCLENWLWQWRSFIIGGLGCSIYTFIHSILFTKFKLGGVITVVLYLGYSLIISALCCVVTGAIGFFSSMFFIRKIYSAIKVE>YDR109CMKSRKRQNNMQNETREPAVLSSQETSISRISPQDPEAKFYVGVDVGTGSARACVIDQSGNMLSLAEKPIKREQLISNFITQSSREIWNAVCYCVRTVVEESGVDPERVRGIGFDATCSLVVVSATNFEEIAVGPDFTNNDQNIILWMDHRAMKETEEINSSGDKCLKYVGGQMSVEMEIPKIKWLKNNLEAGIFQDCKFFDLPDYLTFKATGKENRSFCSAVCKQGFLPVGVEGSDIGWSKEFLNSIGLSELTKNDFERLGGSLREKKNFLTAGECISPLDKKAACQLGLTEHCVVSSGIIDAYAGWVGTVAAKPESAVKGLAETENYKKDFNGAIGRLAAVAGTSTCHILLSKNPIFVHGVWGPYRDVLARGFWAAEGGQSCTGVLLDHLITTHPAFTELSHMANLAGVSKFEYLNKILETLVEKRKVRSVISLAKHLFFYGDYHGNRSPIADPNMRACIIGQSMDNSIEDLAVMYLSACEFISQQTRQIIEVMLKSGHEINAIFMSGGQCRNSLLMRLLADCTGLPIVIPRYVDAAVVFGSALLGAAASEDFDYTREKRTLKGQKSSQTKTERFNDSYSSIQKLSMEDRNSTNGFVSPHNLQLSTPSAPAKINNYSLPICTQQPLDKTSEESSKDASLTVGQESLGEGRYNGTSFLWKVMQELTGNARIVNPNEKTHPDRILLDTKYQIFLDMIETQRKYRRMVDKVEGSFSR>YDR110WMTKPRYNDVLFDDDDSVPSESVTRKSQRRKATSPGESRESSKDRLLILPSMGESYTEYVDSYLNLELLERGERETPIFLESLTRQLTQKIYELIKTKSLTADTLQQISDKYDGVVAENKLLFLQRQYYVDDEGNVRDGRNNDKIYCEPKHVYDMVMATHLMNKHLRGKTLHSFLFSHFANISHAIIDWVQQFCSKCNKKGKIKPLKEYKRPDMYDKLLPMERIHIEVFEPFNGEAIEGKYSYVLLCRDYRSSFMWLLPLKSTKFKHLIPVVSSLFLTFARVPIFVTSSTLDKDDLYDICEEIASKYGLRIGLGLKSSARFHTGGILCIQYALNSYKKECLADWGKCLRYGPYRFNRRRNKRTKRKPVQVLLSEVPGHNAKFETKRERVIENTYSRNMFKMAGGKGLIYLEDVNTFALANEADNSCNNNGILHNNNIGNDNFEEEVQKQFDLTEKNYIDEYDDLAHDSSEGEFEPNTLTPEEKPPHNVDEDRIESTGVAAPMQGTEEPEKGDQKESDGASQVDQSVEITRPETSYYQTLESPSTKRQKLDQQGNGDQTRDFGTSMEL>YDR113CMMPANEDKENNIVYTGNESSGINFPQTPAHLLKRSHSNILKPPVRLDQLKRDANSNNGNTLKYIQGGKEVSPTKRLHTHAQQQGRLPLAAKDNNRSKSFIFPETSNQSKDADLPQLQNTLSIRKNDQLRKLSQISRSRSRANHNDLLSNSRKLQKYGSVLGYNALPKMKSLVLKDLADSGKNEESSDDDEGNEDSESKLGKKLQSALLKQDSSDGENELNGGLGLFNEQGGLQQLIKNSTKNEQKTKNDKSDKTDDYDIEIAPQRQEPLPYVPEGYSPFQQDDIEKLKTFNSPYKLDLEDEDDTPDKVDLLPLEQIDEEGEKDETECITRNQEEGAALPLLSKNFKEVAAVPTMELVYSEEGLDPEELEDLVT>YDR115WMPLFARLCQPQSRRMFSSISSFSALSVLRPQTGMLLNSSPLKTPSFTPLGFGLIGQRRWKSRGNTYQPSTLKRKRTFGFLARAKSKQGSKILKRRKLKGRWFLSH>YDR116CMLSVVAIPKICVTGPARRCFFHTAKKLYADDYKPAAMSSNAPSLTKDQAKKRELKRLVQRKAEAKRPATASPLYMPVTKALRYLRAAEVGRPQSQQTINLTTLVVGERGTAPLSGSVTFPKPLRYIKIAAFTNDESKLEELREKYPNHLIGGADLVAKIKSGEISVDFDKAFATPDIVPALQSQVARILGPRGVLPSVKKGTVSDDISSLLQESLGSMPFRQRGNSISIGVGKCYFTDREILQNIISARAAFKTAVDNQKSKKPNILSKTTLSSTHGPGIVIDFA>YDR117CMFKKEPHIKALSNLKNSERKKLLQTFQKQTNNEEYSFRTSTIKQTNFNGQKSVGTVYTDENNTPILFKEKHKEQLFPTVYSCWEYPALLPIVLTHGFVIEEHLFNGANLMISGSIPPFDPRCKIGTLCGIASKQAPETVLAIGIVELDLPSFDKVIGETGVAVKIIHHFNDGLSKVFKMKLEPPFVLSTQSKDNNISSKQIESSEQIKAVEKEQEDVKEASVDVEEIAEVLDHFTVSDVDYFITRALYYTLTQDKGLELPISASNFISNHIMRNLPPIDHNEVNVKKTSWKKSAKFLKHFEKEGFLKLKGKGDDLTIVGKNTDKDELKNFVPYKLGCSKSATESRESTTSKEKTSGMMYSLTLYKPFNLAKDLLKEVNLASHTYYTSQDIRSAVSQYISVKNLADTKDKGKVIMDDLLFDMVNKKKKVLNASRIIARGEILHPLLTNNFTEFYQIFKSDDTLLFKAPMKGSLPHIKIITEMKIGRKVITRVSNFEVFQVDPESLAADLRKICSGSTTISESQTFKCAEVQVQGPHGQSIIDHLNKLGIPSKWIDFENKLKKKKRK>YDR118WMSSPINDYFIDYNPLFPIFATRIAKGLAIYRVSDHARLAVIPIRNINLVANYDWDTTTGKFLSIFFKDGTIRIHDIFKDGRLVSFLRIPSTKISKGIWDRIPLRYEPNNRDFACNIIDDLPKLIRFVKDSKRINIVPYTQPNSLWRGPDEDDLDSNEKLDVHVVFNEGNDKITVFFNGDYAVFLSVDNIENENSLKSIIKVQDGFYQCFYEDGTVQTLNLGPLLQSKSSVNLLNYIMVIKELIGYMLTHLEFINRELATPYLDFVKRLCDEAYGYGKLKSELEALFLLGEISCDLEDWLCNSVGEKNFKRWKYLGCEAYQKTVQILTLIFVPACERIIIYVEKLRAILQAFSIQNKLSYTSDLTAVEVLLKSSQKLLTMTLNSIIGLGRDETLFEKFFIWFNDRLHEALDEDYKLKFQFEDDLYFGYDLLSYFDRILSKKGTEPSSIIDVKLYRDLINSMSDMEKDIAQSNVNSHIQQHILVDLKTDVFAQKYPSSQINLLDAIKLPKHNYIVYLIQVTKHNSAQEPFSEENKKKLYIGTLKDENLGIISKESSVKIPALFKSYRLSSTRFVPNRVHSLLRDIGLSDSNYHSSHVTDYRGENYENEEDDGTIAIPAYIRENRENDDFIACTAKVSVDGRSASLVFPKEKQNV>YDR120CMEGFFRIPLKRANLHGMLKAAISKIKANFTAYGAPRINIEDFNIVKEGKAEILFPKKETVFYNPIQQFNRDLSVTCIKAWDNLYGEECGQKRNNKKSKKKRCAETNDDSSKRQKMGNGSPKEAVGNSNRNEPYINILEALSATGLRAIRYAHEIPHVREVIANDLLPEAVESIKRNVEYNSVENIVKPNLDDANVLMYRNKATNNKFHVIDLDPYGTVTPFVDAAIQSIEEGGLMLVTCTDLSVLAGNGYPEKCFALYGGANMVSHESTHESALRLVLNLLKQTAAKYKKTVEPLLSLSIDFYVRVFVKVKTSPIEVKNVMSSTMTTYHCSRCGSYHNQPLGRISQREGRNNKTFTKYSVAQGPPVDTKCKFCEGTYHLAGPMYAGPLHNKEFIEEVLRINKEEHRDQDDTYGTRKRIEGMLSLAKNELSDSPFYFSPNHIASVIKLQVPPLKKVVAGLGSLGFECSLTHAQPSSLKTNAPWDAIWYVMQKCDDEKKDLSKMNPNTTGYKILSAMPGWLSGTVKSEYDSKLSFAPNEQSGNIEKLRKLKIVRYQENPTKNWGPKARPNTS>YDR121WMPPKGWRKDAQGNYPTTSYIKEQENITIQDLLFPKSTIVNLAREVPQQSGKKLLINKDASLALQRGATVFVNHLLLFAREIAKSQDKKSCSVDDVLSALDHIGHSALKGPVRDKLDEYQAAVEQRKKEKLDSGEVDADGDIDMGEDKENVPVEKVKEHDEIEEQGDALQDVEESSEKKQKTESQDVETRVQNLEQT>YDR122WMDDYHVNTAFSMGRGNQQDDGNSESNSMHTQPSTMAPATLRMMGKSPQQQQQQNTPLMPPADIKYANNGNSHQAEQKERQVELEGKSRENAPKPNTTSQSRVSSSQGMPKQFHRKSLGDWEFVETVGAGSMGKVKLAKHRYTNEVCAVKIVNRATKAFLHKEQMLPPPKNEQDVLERQKKLEKEISRDKRTIREASLGQILYHPHICRLFEMCTLSNHFYMLFEYVSGGQLLDYIIQHGSIREHQARKFARGIASALIYLHANNIVHRDLKIENIMISDSSEIKIIDFGLSNIYDSRKQLHTFCGSLYFAAPELLKANPYTGPEVDVWSFGVVLFVLVCGKVPFDDENSSVLHEKIKQGKVEYPQHLSIEVISLLSKMLVVDPKRRATLKQVVEHHWMVRGFNGPPPSYLPKRVPLTIEMLDINVLKEMYRLEFIDDVEETRSVLVSIITDPTYVLLSRQYWTLAAKMNAESSDNGNAPNITESFEDPTRAYHPMISIYYLTSEMLDRKHAKIRNQQQRQSHENIEKLSEIPESVKQRDVEVNTTAMKSEPEATLATKDTSVPFTPKNSDGTEPPLHVLIPPRLAMPEQAHTSPTSRKSSDNQRREMEYALSPTPQGNDYQQFRVPSTTGDPSEKAKFGNIFRKLSQRRKKTIEQTSVNSNNSINKPVQKTHSRAVSDFVPGFAKPSYDSNYTMNEPVKTNDSRGGNKGDFPALPADAENMVEKQREKQIEEDIMKLHDINKQNNEVAKGSGREAYAAQKFEGSDDDENHPLPPLNVAKGRKLHPSARAKSVGHARRESLKYMRPPMPSSAYPQQELIDTGFLESSDDNKSDSLGNVTSQTNDSVSVHSVNAHINSPSVEKELTDEEILQEASRAPAGSMPSIDFPRSLFLKGFFSVQTTSSKPLPIVRYKIMFVLRKMNIEFKEVKGGFVCMQRFSSNNVAAKREGTPRSIMPLSHHESIRRQGSNKYSPSSPLTTNSIHQRKTSITETYGDDKHSGTSLENIHQQGDGSEGMTTTEKEPIKFEIHIVKVRIVGLAGVHFKKISGNTWLYKELASSILKELKL>YDR123CMQQATGNELLGILDLDNDIDFETAYQMLSSNFDDQMSAHIHENTFSATSPPLLTHELGIIPNVATVQPSHVETIPADNQTHHAPLHTHAHYLNHNPHQPSMGFDQALGLKLSPSSSGLLSTNESNAIEQFLDNLISQDMMSSNASMNSESHLHIRSPKKQHRYTELNQRYPETHPHSNTGELPTNTADVPTEFTTREGPHQPIGNDHYNPPPFSVPEIRIPDSDIPANIEDDPVKVRKWKHVQMEKIRRINTKEAFERLIKSVRTPPKENGKRIPKHILLTCVMNDIKSIRSANEALQHILDDS>YDR125CMLLIKRYLMDPESLRRQIMNVYKCYMWKRAFHSNRSLLEVKRREKSLQRKILERILRPKEENAVKKSGFKLWSSHLNNPHKTYMRLEELQRRIMEEVHVEGIKKNDKLFNEINQWHFQNENTSTVRTPTLLIHGYAASSMSFFRNYPGLSKHIRNLYSIDMPASGLSSVPSLEINTTTPLPLDIKFIGENKFKVPYTINANHNKFVIQMYEDFYLDRIEQWRIDNKLGKMNVVGHSFGGYLSFKYAVKYPNSVNKLCLVSPLGVERNIWSVNNNFHSNTLYTIDFKNPNSKFYSKRNMIPKYLFEQQFHILRMMGPLGAKLCWNYIMAAYSRVPSLAYKEYIFELFYGKGGIPEVTTDIFKALFSRCILAKDPLMDSLQYLNVKKLLIVYGQYDWMNKKAGMFMVKELNNLKNCLEGASYLEIPSSGHNLFLDNPESFNQSIVSFLSDETKSP>YDR126WMSWNLLFVLLIGFVVLILLSPVFKSTWPFSTFYRNVFQPFLVDDQKYRWKLHLVPLFYTSIYLYLVYTYHMRVESTIKNELFLLERILIVPIIILPPVALGILAMVSRAEDSKDHKSGSTEEYPYDYLLYYPAIKCSTCRIVKPARSKHCSICNRCVLVADHHCIWINNCIGKGNYLQFYLFLISNIFSMCYAFLRLWYISLNSTSTLPRAVLTLTILCGCFTIICAIFTYLQLAIVKEGMTTNEQDKWYTIQEYMREGKLVRSLDDDCPSWFFKCTEQKDDAAEPLQDQHVTFYSTNAYDHKHYNLTHYITIKDASEIPNIYDKGTFLANLTDLI>YDR132CMSNSPTVATLSQEYFDPNIPQILPHEKMYKIQVGKSLFKISGASLSSDGPSFFTEYFSKKRSPSNNDDSNNDTMESNKNEVLFIDRSAEVFEWIYQHLQGYIIEIKDEVQYTMLFADAMYYNLPRLRSLLKETDYYFTNIGGQSFKIAKNLFRREGDSPNYFEIYAATVYIDVEELIISKKLLRPPSHSAPYIPRSSEYFKDLLTLLGGASIDLDDNKRNALIKECRYYRLLNLEQRLIKSHISYNPITRKEEICLLLKDLSKKGITFPASSAFSTSPYFEDDFCSINECDSLSKTREQPANKKIKLDMTEKYNDSWNMLCYKRPFLDKHPRELIFQINSTDCTIILNKESQSIHVDITGESAYKFEALFGSHLPNTPSGAPKLKNYQYRFPSDSTQTKIETHYLLPACIYLCDLDINGIKISQVQTLLTDKNKFNDRVIDVSDPLDLRFCSGLKLYLRKSLWKLAVKDGNIMLIAIKAIAFNGTKEYYKGYEYL>YDR134C>YDR137WMRAHRIDTFLIRENIKLEIIHESNSYFGGEHISIAFRFKHLGSQHELFNYKEKLLTVDKAVEEKLEQQAKVQDDGEGTMENQTWSLKSLLGAFKRTGEPEESVDVDNMKMLNESKMLREKIQKQMYFHQPVTLISGYVQISGVFQYDSEVISESKFKQDEVKMVGLDIVPGHTTNSVLALEDGEHFKGKRNLTNYLNSDYTNVTNGLLFSESGSRGRTGTYNERTLMISNDTSIKTLPLLLIPQTLLFSEISLEPGEVRTFYFKSTKLPKDICPSYSSSKVASINYTLEVGADVLSDDNIEKFSNRVPITIAPYISSNAEQYTSRLDKPAIILKTGNIKELKPRLFTRKVSTASAVSFGRRKSSIIDIDSPLEDNEFVKRVKKNFIELVESNQNVSRDIDELIDLQMGVQFGKDEDSSDPEPNDSHFSNEMVTSAESSLRSDAVTKRRKSYSVRDNISNLEQKMWNDCSLVKSDENSNLLPQLINLQNAYQINRNNETMAKVSLSAPFYKTTDDINLVIELDPITTPLLKVTSLTVSLESFEIINPKYKTEGKGIGSKPKGNSVYEKHFICFDECKSVSVKLLPPRSPTNQITGQFKTDVFQHKWMIGLKFVIIAKTESITLDQFYEDKKGILFHSKENLEGEEFTCYVPIPILCTSEDFMGW>YDR138WMSNTEELIQNSIGFLQKTFKALPVSFDSIRHEPLPSSMLHASVLNFEWEPLEKNISAIHDRDSLIDIILKRFIIDSMTNAIEDEEENNLEKGLLNSCIGLDFVYNSRFNRSNPASWGNTFFELFSTIIDLLNSPSTFLKFWPYAESRIEWFKMNTSVEPVSLGESNLISYKQPLYEKLRHWNDILAKLENNDILNTVKHYNMKYKLENFLSELLPINEESNFNRSASISALQESDNEWNRSARERESNRSSDVIFAADYNFVFYHLIICPIEFAFSDLEYKNDVDRSLSPLLDAILEIEENFYSKIKMNNRTRYSLEEALNTEYYANYDVMTPKLPVYMKHSNAMKMDRNEFWANLQNIKESDDYTLRPTIMDISLSNTTCLYKQLTQEDDDYYRKQFILQLCFTTNLIRNLISSDETRNFYKSCYLRENPLSDIDFENLDEVNKKRGLNLCSYICDNRVLKFYKIKDPDFYRVIRKLMSSDEKFTTAKIDGFKEFQNFRISKEKIPPPAFDETFKKFTFIKMGNKLINNVWKIPTGLDKIEQEVKKPEGVYEAAQAKWESKISSETSGGEAKDEIIRQWQTLRFLRSRYLFDFDKVNEKTGVDGLFEEPRKVEALDDSFKEKLLYKINQEHRKKLQDAREYKIGKERKKRALEEEASFPEREQKIKSQRINSASQTEGDELKSEQTQPKGEISEENTKIKSSEVSSQDPDSGVAGEFAPQNTTAQLENPKTEDNNAATSNISNGSSTQDMK>YDR140WMLPTPYVKCDYDKVYEPAEDSFLILDCLEKEHDFLKQKFGNRLAIVCEIGSGSGIVTTFLMQNKIIPQENSIHLAVDINPWALEATLDTAKLNSCKSSFLEVIQADLNSSIRNNQVDVLIFNPPYVPAECVPDVPGSREEADQWLDLALLGGKDGMAITDKLLRQLEQILSPDGVAYILFCARNKPKEVIKRFVDTYKWNVKLIETRKAGWEVLSVYSFTR>YDR142CMLRYHMQGFSGYGVQYSPFFDNRLAVAAGSNFGLVGNGKLFILEIDRSGRIVEVNSFLTQDCLFDLAWNESHENQVLVAQGDGTLRLFDTTFKEFPIAIFKEHEREVFSCNWNLVNRQNFLSSSWDGSIKIWSPLRKQSLMTLTPRPLEITKMVDPLNAIILKKKSFTGISKNRNCVYQAQFSPHDQNLVLSCSGNSYASLFDIRLPSGKNQNNFLVHSGLEALTCDFNKYRPYVVATGGVDNAIRIWDIRMLNKNESATIKRTVPGQLHNSSCINEIPNAHGLAIRKVTWSPHHSNILMSASYDMTCRIWRDLSNDGAKETYKTNSTDATKGSIFNFTQHSEFVFGADWSLWGKPGYVASTAWDGNLFVWNGLG>YDR143CMSESGQEQNRGTNTSPNNAENNNNSNAASGPLNGGAEQTRNITVSIQYSYFTPERLAHLSNISNNDNNENNSAASGSTIANGTGPSFGIGNGGHQPDGALVLSFRDVPASTPQDRLNSFISVAAQLAMERFNRLLNRPKGISKDEFDKLPVLQVSDLPKAEGPLCSICYDEYEDEVDSTKAKRKRDSENEEESEGTKKRKDNEGAPLRTTADNDSNPSITNATVVEPPSIPLTEQQRTLNDEETNPSYKHSPIKLPCGHIFGRECIYKWSRLENSCPLCRQKISESVGVQRAAQQDTDEVAANEAAFERIRRVLYDPTAVNSTNENSSAPSENTSNTTVPTIGNASSGEQMLSRTGFFLVPQNGQPLHNPVRLPPNDSDRNGVNGPSSTTQNPPSNSGGSNNNQSPRWVPIPLTLFQFHSPNPNPSASDSSASPSAANGPNSNNTSSDATDPHHNRLRAVLDHIFNVAQRGTSDTSATTAPGAQTVHNQGRNDSSSSDTTQGSSFLENISRLTGHFTNGSRDNNNDNNHSNDQQRGGSTGENNRNNLFSSGVASYRNQNGDVTTVELRNNNSAAFPPTDENPSQGQGSSSSDTTIHNDVPNDNNEQRSSQ>YDR147WMYTNYSLTSSDAMPRTYLVGTASPEMSKKKRQSANCDKPTRRVIHIIDTNEHSEVDLKNELPITCTNEDGEMTSSSWTSQTANDFLKLAYVNAKLDPSLPSQYFKQDIINVLQSLEIPGWSVPGSKESSLNKNLLTLTQIKGALTNVIYKIHYPNLPPLLMRIFGDSIDSVIDREYELKVIARLSFYDLGPKLEGFFENGRFEKYIEGSRTSTQADFIDRDTSIKIAKKLKELHCTVPLTHKEITDQPSCWTTFDQWIKLIDSHKEWVSNNVNISENLRCSSWNFFLKSFKNYKRWLYNDSAFTSKLLREDDKDSMINSGLKMVFCHNDLQHGNLLFKSKGKDDISVGDLTIIDFEYAGPNPVVFDLSNHLNEWMQDYNDVQSFKSHIDKYPKEEDILVFAQSYINHMNENHVKIASQEVRILYNLIIEWRPCTQLFWCLWALLQSGRLPQRPLIEGEKLMSEKAGLGDETHLMEHKNKENGKYDCSEDDSFNYLGFCKEKMSVFWGDLITLGVIDKDCPDIGKTDYLDTKLIF>YDR148CMLSRATRTAAAKSLVKSKVARNVMAASFVKRHASTSLFKQANKVESLGSIYLSGKKISVAANPFSITSNRFKSTSIEVPPMAESLTEGSLKEYTKNVGDFIKEDELLATIETDKIDIEVNSPVSGTVTKLNFKPEDTVTVGEELAQVEPGEAPAEGSGESKPEPTEQAEPSQGVAARENSSEETASKKEAAPKKEAAPKKEVTEPKKADQPKKTVSKAQEPPVASNSFTPFPRTETRVKMNRMRLRIAERLKESQNTAASLTTFNEVDMSALMEMRKLYKDEIIKKTGTKFGFMGLFSKACTLAAKDIPAVNGAIEGDQIVYRDYTDISVAVATPKGLVTPVVRNAESLSVLDIENEIVRLSHKARDGKLTLEDMTGGTFTISNGGVFGSLYGTPIINSPQTAVLGLHGVKERPVTVNGQIVSRPMMYLALTYDHRLLDGREAVTFLKTVKELIEDPRKMLLW>YDR151CMMPNVAPNSYYLNIPNANSTSTTTSSIFSDLNKEYESKIKEIEEYYIKTLLNENTDNDDSSSSEGHNINETDILSEYSPRPSPWLPSKPNCYHPLGDFKDLIISDSRPTNTLPINNPFAGNNNISTLATTEKKRKKRSLEVEINPTYTTSAFSLPLTAENLQKLSQVDSQSTGLPYTLPIQKTTKLEPCRRAPLQLPQLVNKTLYKTELCESFTIKGYCKYGNKCQFAHGLNELKFKKKSNNYRTKPCINWSKLGYCPYGKRCCFKHGDDKDVEIYQNANDGRSKDTALTPLPTSLAPSNNDNITNLSKPRNLHTSVKALQRMTW>YDR153CMDSLSKKIQNLGIHDIRNAARFAQNVIVQYEPYQIDIRRATNTDAWGPTPKHLAKVLRNRYQVPLYLMTEYTLKRLVDHIATRPKNLYEKARKDYVNYGSEWRVVLKCLVVIEFLLLNVDTGDELNQIRSCLLTHKHILTREIAQFKVKFSNDGKMEIHERGIRKKGELILQYLEDSQFLKKERAKNKKNALKIRQQGESSIYNANQISTSASYDNIDDDEFDADADGFDSEMDANNVTNFNVPVETEANSNTRRRSHMEEQRRQRREILREQIKNKEQQRKRKQQQDSIPDLIDLDDSTSTTNNITIDNGNNDNKNNNINSNSDDDDDEFGDFQSETSPDTTAPKTSNSKIDDLLDWDGPKSDTDTTAAAQTSLPFAEKKQQKARPQATKDKSKGNDAFSDLFSYSKSLV>YDR156WMMKGSRRTGNNTATTLNTPVVIHATQLPQHVSTDEVLQFLESFIDEKENIIDSTTMNTISGNAADADAAAVANTSLNIDTNLSSSISQLKRIQRDFKGLPPAQDFSAAPIQVSTTEKKETSIGVSATGGKKTTFADE>YDR158WMAGKKIAGVLGATGSVGQRFILLLANHPHFELKVLGASSRSAGKKYVDAVNWKQTDLLPESATDIIVSECKSEFFKECDIVFSGLDADYAGAIEKEFMEAGIAIVSNAKNYRREQDVPLIVPVVNPEHLDIVAQKLDTAKAQGKPRPGFIICISNCSTAGLVAPLKPLIEKFGPIDALTTTTLQAISGAGFSPGVPGIDILDNIIPYIGGEEDKMEWETKKILAPLAEDKTHVKLLTPEEIKVSAQCNRVAVSDGHTECISLRFKNRPAPSVEQVKTCLKEYVCDAYKLGCHSAPKQTIHVLEQPDRPQPRLDRNRDSGYGVSVGRIREDPLLDFKMVVLSHNTIIGAAGSGVLIAEILLARNLI>YDR159WMNTSFGSVVPSTNFNFFKGHGNNDNTSANSTVNNSNFFLNSNETKPSKNVFMVHSTSQKKSQQPLQNLSHSPSYTENKPDKKKKYMINDAKTIQLVGPLISSPDNLGFQKRSHKARELPRFLINQEPQLEKRAFVQDPWDKANQEKMISLEESIDDLNELYETLKKMRNTERSIMEEKGLVDKADSAKDLYDAIVFQGTCLDMCPTFERSRRNVEYTVYSYEKNQPNDKKASRTKALKVFARPAAAAAPPLPSDVRPPHILVKTLDYIVDNLLTTLPESEGFLWDRMRSIRQDFTYQNYSGPEAVDCNERIVRIHLLILHIMVKSNVEFSLQQELEQLHKSLITLSEIYDDVRSSGGTCPNEAEFRAYALLSKIRDPQYDENIQRLPKHIFQDKLVQMALCFRRVISNSAYTERGFVKTENCLNFYARFFQLMQSPSLPLLMGFFLQMHLTDIRFYALRALSHTLNKKHKPIPFIYLENMLLFNNRQEIIEFCNYYSIEIINGDAADLKTLQHYSHKLSETQPLKKTYLTCLERRLQKTTYKGLINGGEDNLASSVYVKDPKKDRIPSIADQSFLMENFQNNYNEKLNQNSSVKPQINTSPKRVATRPNHFPFSQESKQLPQISQSHTLSTNPLLTPQVHGDLSEQKQQQIKTVTDGGSPFVFDQSAQNSTVEASKAHMISTTSNGAYDEKLSSEQEEMRKKEEQRIEEEKTQLKKKQENADKQVITEQIANDLVKEVVNSSVISIVKREFSEANYRKDFIDTMTRELYDAFLHERLYLIYMDSRAELKRNSTLKKKFFEKWQASYSQAKKNRILEEKKREEIKLVSHQLGVPGFKKSTCLFRTPYKGNVNSSFMLSSSDKNLIFSPVNDEFNKFATHLTKISKLWRPLEMQSIYYDNLTKKFPSNSLTPANLFIYAKDWTSLSNRWILSKFNLQTAQDSKKFSNNIISSRIICIDDEYEPSDFSDLQLLIFNTGVTNPDIFDLEMKLKDDGEELIKLITGISLNTNICFSLLIIYWESAENTLSESTIKHLLKLNRISKNYSSVIERIDLMNLTEESPHKCLEDKLSEISHSYVYKLTERGKYDKTLRQKRSLAGIHSRSTQLQTTKDIDQKMKKMLEKEKNKYQQQIGERNTYAHLESHIDASPRSKKRKLPILLSTSHSSQFKTPLASRLNTSGSSTSPPLPSHLAMKFRKNSRVTSLHTVLPVSTPSHSNNIPAASFSGNNTTDIQSQQLIENQKSTSVYLNNVSERILGNQEICQTPINPVTPVLDGADQGKEDIPDSILELKILIDSVKKKVNND>YDR161WMSELEATIRQAKEALAENNAKKALKILKPFKSSLKKENANNVILNEVFADAYLDNGQVEKAYPILARACELDPEGQVGGPDKFFTMGQIMGGQDGVSIITRGIMNISNTGGEMLTNVQVEKIVGGLLSVIEIWMTDLCMEPNAEEQCEELIQKAMELTEGKSPETWSTLGSIKISQQKFGEAYEAFSQAWNFFELKKQEIGSGINENGDTTQKAGLQSEYVDLLQPLLSLTKMCLEVGAYEVALKVIAAVRDIDEDNIEGYYLEGFTYYLMSKLEIFKLNNPEVSLRPENIYEFNQLIQEVPLDLSHEPISQLIYDSRLALSFALQAGVNADSKDEIVQELLGGANALLQEIGGPLDPSELTQIKKGDLVNENEDLEELDIEEEYSD>YDR162CMATMETTTQKDTNILKSGLKKTIGVLNEAVLQNGREVEAVQAGNSDTMEDTETTTIGYISIKDYAYADSNPLHYGYFDGDNEEDEMVSDSSNGEDTYNKRQSITLPDDYIVNQRAVALYDFEPENDNELRLAEGDIVFISYKHGQGWLVAENESGSKTGLVPEEFVSYIQPEDGENEVENKARPFYLTHLITQSVSPKNNIDNTNEDEYDDNDEWEDIDDVAEVEADMKTKLDISD>YDR165WMSVIHPLQNLLTSRDGSLVFAIIKNCILSFKYQSPNHWEFAGKWSDDFDKIQESRNTTAKEQQGQSSENENENKKLKSNKGDSIKRTAAKVPSPGLGAPPIYSYIRNLRLTSDESRLIACADSDKSLLVFDVDKTSKNVLKLRKRFCFSKRPNAISIAEDDTTVIIADKFGDVYSIDINSIPEEKFTQEPILGHVSMLTDVHLIKDSDGHQFIITSDRDEHIKISHYPQCFIVDKWLFGHKHFVSSICCGKDYLLLSAGGDDKIFAWDWKTGKNLSTFDYNSLIKPYLNDQHLAPPRFQNENNDIIEFAVSKIIKSKNLPFVAFFVEATKCIIILEMSEKQKGDLALKQIITFPYNVISLSAHNDEFQVTLDNKESSGVQKNFAKFIEYNLNENSFVVNNEKSNEFDSAIIQSVQGDSNLVTKKEEIYPLYNVSSLRKHGEHYS>YDR167WMDFEEDYDAEFDDNQEGQLETPFPSVAGADDGDNDNDDSVAENMKKKQKREAVVDDGSENAFGIPEFTRKDKTLEEILEMMDSTPPIIPDAVIDYYLTKNGFNVADVRVKRLLALATQKFVSDIAKDAYEYSRIRSSVAVSNANNSQARARQLLQGQQQPGVQQISQQQHQQNEKTTASKVVLTVNDLSSAVAEYGLNIGRPDFYR>YDR169CMSENQKEVSPPQAISVKSEASSSIFSKPISTSSPAGLAAAQRVTPGKLSTLLLEMGPLAIRHITQTLCLDIPCFKDLSSSKQRRLIMSAMESGDKEKSVVFEKIGWGQWSAKRVDPANFDKELEATNFANAKVKDLISQESQRRKSNNSNSNSGGKVEMPMKVEHNITNIDGATTPPTAVASTTIPVNIKRSKSPLAAANVVYIDENALASEDEDEEFDEDDHHLHYQNKSRNSSNNFGKSSNGDPYSFGRRRSQVVFADSTPENIEHEIIAQKIRPLLRNRRRSSIKPHTPFISKLNTHQDSSYLSPNTTSTTTPSNNNSNSNQAKIDLEKLTATSEPTSRRASRLSVSKESSIRSTLFPNKNYLIVTTNPNSKATSVSTSPKLEEQMNVSSNPIVLSDKEKHRVASQHLNGESSPQLVPHSHHQPHSDTDEEDWESIGAASLRNNSLAPNIDSVASSTNGVVSPKPTNPSFTNSQNGDIEPPLQHDQQKHEQQPRNGEDNSAAFLLMSLKS>YDR171WMSFYQPSLSLYDVLNALSNQTGQRGQQGYPRQPQRPQRYHPHYGQVHVGGHHPRHHPLYSRYNGVPNTYYYQFPGQAYYYSPEYGYDDEDGEEEDQDEDMVGDSGTTRQEDGGEDSNSRRYPSYYHCNTARNNRTNQQANSLNDLLTALIGVPPYEGTEPEIEANTEQEGEKGEEKDKKDKSEAPKEEAGETNKEKPLNQLEESSRPPLAKKSSSFAHLQAPSPIPDPLQVSKPETRMDLPFSPEVNVYDTEDTYVVVLALPGANSRAFHIDYHPSSHEMLIKGKIEDRVGIDEKFLKITELKYGAFERTVKFPVLPRIKDEEIKATYNNGLLQIKVPKIVNDTEKPKPKKRIAIEEIPDEELEFEENPNPTVEN>YDR172WMSDSNQGNNQQNYQQYSQNGNQQQGNNRYQGYQAYNAQAQPAGGYYQNYQGYSGYQQGGYQQYNPDAGYQQQYNPQGGYQQYNPQGGYQQQFNPQGGRGNYKNFNYNNNLQGYQAGFQPQSQGMSLNDFQKQQKQAAPKPKKTLKLVSSSGIKLANATKKVGTKPAESDKKEEEKSAETKEPTKEPTKVEEPVKKEEKPVQTEEKTEEKSELPKVEDLKISESTHNTNNANVTSADALIKEQEEEVDDEVVNDMFGGKDHVSLIFMGHVDAGKSTMGGNLLYLTGSVDKRTIEKYEREAKDAGRQGWYLSWVMDTNKEERNDGKTIEVGKAYFETEKRRYTILDAPGHKMYVSEMIGGASQADVGVLVISARKGEYETGFERGGQTREHALLAKTQGVNKMVVVVNKMDDPTVNWSKERYDQCVSNVSNFLRAIGYNIKTDVVFMPVSGYSGANLKDHVDPKECPWYTGPTLLEYLDTMNHVDRHINAPFMLPIAAKMKDLGTIVEGKIESGHIKKGQSTLLMPNKTAVEIQNIYNETENEVDMAMCGEQVKLRIKGVEEEDISPGFVLTSPKNPIKSVTKFVAQIAIVELKSIIAAGFSCVMHVHTAIEEVHIVKLLHKLEKGTNRKSKKPPAFAKKGMKVIAVLETEAPVCVETYQDYPQLGRFTLRDQGTTIAIGKIVKIAE>YDR173CMDTVNNYRVLEHKAAGHDGTLTDGDGLLIFKPAFPQELEFYKAIQVRDVSRRKSSADGDAPLCSWMPTYLGVLNEGAKIEQSGDAALLKIDERLSDSTDNLDSIPVKSEKSKQYLVLENLLYGFSKPNILDIKLGKTLYDSKASLEKRERMKRVSETTTSGSLGFRICGMKIQKNPSVLNQLSLEYYEEEADSDYIFINKLYGRSRTDQNVSDAIELYFNNPHLSDARKHQLKKTFLKRLQLFYNTMLEEEVRMISSSLLFIYEGDPERWELLNDVDKLMRDDFIDDDDDDDDNDDDDDDDAEGSSEGPKDKKTTGSLSSMSLIDFAHSEITPGKGYDENVIEGVETLLDIFMKF>YDR175CMKVPLGLWKVSRGNLWSTQKRVLTMSRCLNSDAGNEAKTVREGPAFSADLYMHPEKWKGLPPQRILELYWERMARLGSEYKPNKDELNALLTTSEYSNVPVNDIKKLYHRGEQGAIDIKGGNVNRDNSLRPFMFDELPSQAQELVAQHREQRFYNRLAAYELPLLAQYRQEYKRPSPESHPVTYRYTSYVGEEHPNSRKVVLSVKTKELGLEEKSLHKFRILARSRYDHTTDIFKMSSDKFEHASQNARYLHDILQRLLAESKDLTEDDFSDVPLDTRHTIAKSLRKKKRDYEFPEHWKRPEDAPKKKFDIVDQLLSTL>YDR176WMPRHGRRGKLPKGEKLPKKEGGDNTPSKLLSSMLKTLDLTFERDIGMLNGKSVRSIPNKKTLLELQSQLDSLNEILGTIARGDQETIEALRKIRDSKNEKQANDEKQETSNADGQHESSTATEETNIIDKGVQSPPKPPPSNEISGTIENDVESIKQAADNMAKEEINEDKDLQVHRDQPREKRPFDSETENRATENENTQRPDNKKQKIDVDKMENDPTVKNPKSEFVVSQTLPRAAAALGLFNEEGLESTGEDFLKKKYNVASYPTNDLKDLLPGELPDMDFSHPKPTNQIQFNTFLAFVENFFKDLSDDNLKFLKMKYIIPDSLQFDKTYDPEVNPFIIPKLGPLYTDVWFKDENDKNSAYKKPSPYSNDASTILPKKSANELDDNALESGSISCGPLLSRLLSAVLKDDNDKSELQSSKIIRDGGLPRTGGEDDIQSFRNNNNDTVDMTLSQENGPSVQTPDNDIDEEASFQAKLAENKGSNGGTTSTLPQQIGWITNGINLDYPTFEERLKRELKYVGIYMNLPKDENNPNSDDPDWVTGREDDEISAELRELQGTLKQVTKKNQKRKAQLIPLVERQLAWQEYSSILEDLDKQIDQAYVKRIRVPKKRKKHHTAASNNVNTGTTSQIAQQKAANSSLKSLLDKRQRWINKIGPLFDKPEIMKRIPNESVFKDMDQEEDEDEADVFAQNTNKDVELN>YDR177WMSRAKRIMKEIQAVKDDPAAHITLEFVSESDIHHLKGTFLGPPGTPYEGGKFVVDIEVPMEYPFKPPKMQFDTKVYHPNISSVTGAICLDILKNAWSPVITLKSALISLQALLQSPEPNDPQDAEVAQHYLRDRESFNKTAALWTRLYASETSNGQKGNVEESDLYGIDHDLIDEFESQGFEKDKIVEVLRRLGVKSLDPNDNNTANRIIEELLK>YDR178WMMLPRSMKFMTGRRIFHTATVRAFQSTAKKSLTIPFLPVLPQKPGGVRGTPNDAYVPPPENKLEGSYHWYMEKIFALSVVPLATTAMLTTGPLSTAADSFFSVMLLGYCYMEFNSCITDYISERVYGVWHKYAMYMLGLGSAVSLFGIYKLETENDGVVGLVKSLWDSSEKDNSQKIEAKK>YDR179CMVMREETIKSLEDPYKYHYKEEWLNTKDPDEQQLFEIFAFGNIKDLPENIILTSLMRSKLEKLTLVTLSEIYNELSYELIKEECQIEDDGIIESHLIQLQNIFKAEMDSVSKSMKFSRRFDCRDVYCHEKELTIIKNPRVTKEYLVQNLRSWETKLKQNILE>YDR182WMVYRNRSKSVLSTHSKKSDDKAHYKSRSKKKSKSRSKKRLRIYWRYISIVWILWLGLISYYESVVVKRAMKKCQWSTWEDWPEGAESHRVGLFADPQIMDEYSYPGRPQIVNYFTRVIVDHYHRRNWKYVQYYLDPDSNFFLGDLFDGGRNWDDKQWIKEYTRFNQIFPKKPLRRTVMSLPGNHDIGFGDTVVESSLQRFSSYFGETSSSLDAGNHTFVLLDTISLSDKTNPNVSRVPRQFLDNFAMGSHPLPRILLTHVPLWRDPEQQTCGQLRESKEPFPIQKGHQYQTVIENDISQEILTKIQPEILFSGDDHDHCQISHSYPFQGKTKNAQEITVKSCAMNMGISRPAIQLLSLYNPSDLTMVNAGGEYASKTYQTELCYMPDPYKAIRMYLWGLLFSAAFIAYMHFFPKSFNNRVATIMNRVFTRPDGNTSDLPLPTSISKSKSKKSLTHSKYAVNDTRSIKQFLVNAIVLFVSVMPIFIYFYTVV>YDR183WMEDKLDRYYTNVLSNAEKDKHTTVDSDDKSSGEENLDELLNELDRELDEDHEFLSAYRSERLQQISDHLKQVKKNVEDDGYGRLQCIDNEADAIQICTKTTMVVIHFELETFGKCQYMNEKLENLAKRYLTTRFIKVNVQTCPFLVNKLNIKVLPFVVGYKNGLEKVRYVGFSKLGNDPNGFDIRRLEQSLAHSGVIEDTFEIRKHSSVNTERFASTNHDRSESDSDLDI>YDR184CMNTNQSNPNTDLTDDANIEHTLHRLLTQANNHFDDTVKIDGQSLDLGKDLEQVMMDNLDCTDIFDSDIASQKHLTLESLFNDEHNTDSSTLLEMQRSANDSLVGIDLDRHKKGYTGKASLDKSTNQNNVHKPDKEQKNYKIDKPTIKKKKSLLKTTNEPMLSPASLSPSSSLASSDANESHLKIESMITDITSKIDSARQDIVSATKPAKFTNEFTISQISEMKARIINTHKLLLNFNFIKEGYARSCIQLKKSMDSLKDSEIHRAHLLVENDDLKQQILELTQKLNEKSSKES>YDR185CMKSFQKSYEFDYPWEKVTTANWMKYPNKISTHVIAVDVLRRELKEHGDVLLTERLITIRQNTPHWMSILVGNTNLAYVREVSTVDRRDRSLTMRSCNMTFPHILKCYETVRYVPHPKNPSNVTLFKQDAKFLSGVPTKTFSEKVENWGVKRFSDNAVKGKVGFDSILAMFNDIWKNANE>YDR188WMSLQLLNPKAESLRRDAALKVNVTSAEGLQSVLETNLGPKGTLKMLVDGAGNIKLTKDGKVLLTEMQIQSPTAVLIARAAAAQDEITGDGTTTVVCLVGELLRQAHRFIQEGVHPRIITDGFEIARKESMKFLDEFKISKTNLSNDREFLLQVARSSLLTKVDADLTEVLTPIVTDAVLSVYDAQADNLDLHMVEIMQMQHLSPKDTTFIKGLVLDHGGRHPDMPTRVKNAYVLILNVSLEYEKTEVNSGFFYSSADQRDKLAASERKFVDAKLKKIIDLKNEVCGMDPDKGFVIINQKGIDPMSLDVFAKHNILALRRAKRRNMERLQLVTGGEAQNSVEDLSPQILGFSGLVYQETIGEEKFTYVTENTDPKSCTILIKGSTHYALAQTKDAVRDGLRAVANVLKDKNIIPGAGAFYIALSRYLRSANMNKLGAKGKTKTGIEAFAEALLVIPKTLVKNSGFDPLDVLAMVEDELDDAQDSDETRYVGVDLNIGDSCDPTIEGIWDSYRVLRNAITGATGIASNLLLCDELLRAGRSTLKETPQ>YDR190CMVAISEVKENPGVNSSNSGAVTRTAAHTHIKGLGLDESGVAKRVEGGFVGQIEAREACGVIVDLIKAKKMSGRAILLAGGPSTGKTALALAISQELGPKVPFCPLVGSELYSVEVKKTETLMENFRRAIGLRIKETKEVYEGEVTELTPEDAENPLGGYGKTISHVIVGLKSAKGTKTLRLDPTIYESIQREKVSIGDVIYIEANTGAVKRVGRSDAYATEFDLETEEYVPLPKGEVHKKKEIVQDVTLHDLDVANARPQGGQDVISMMGQLLKPKKTEITEKLRQEVNKVVAKYIDQGVAELIPGVLFIDEVNMLDIEIFTYLNKALESNIAPVVVLASNRGMTTVRGTEDVISPHGVPPDLIDRLLIVRTLPYDKDEIRTIIERRATVERLQVESSALDLLATMGTETSLRYALQLLAPCGILAQTSNRKEIVVNDVNEAKLLFLDAKRSTKILETSANYL>YDR191WMKQKFVLPITPPSTAEKKPQTENRCNENLKPRRLLPQLKKSVRNRKPRLSYRPELNSVFDLDAYVDSTHLSKSQRHHMDRDAGFISYALNYSKRMVVVSGAGISVAAGIPDFRSSEGIFSTVNGGSGKDLFDYNRVYGDESMSLKFNQLMVSLFRLSKNCQPTKFHEMLNEFARDGRLLRLYTQNIDGLDTQLPHLSTNVPLAKPIPSTVQLHGSIKHMECNKCLNIKPFDPELFKCDDKFDSRTEIIPSCPQCEEYETVRKMAGLRSTGVGKLRPRVILYNEVHPEGDFIGEIANNDLKKRIDCLIIVGTSLKIPGVKNICRQFAAKVHANRGIVLYLNTSMPPKNVLDSLKFVDLVVLGDCQHVTSLL>YDR194CMLTSILIKGRTPVLASRNLLAALSNCNHITWAVSRRLYNDGNRDQRNFGRNQRNNNSNRYRNSRFNSRPRTRSREDDDEVHFDKTTFSKLIHVPKEDNSKEVTLDSLLEEGVLDKEIHKAITRMEFPGLTPVQQKTIKPILSSEDHDVIARAKTGTGKTFAFLIPIFQHLINTKFDSQYMVKAVIVAPTRDLALQIEAEVKKIHDMNYGLKKYACVSLVGGTDFRAAMNKMNKLRPNIVIATPGRLIDVLEKYSNKFFRFVDYKVLDEADRLLEIGFRDDLETISGILNEKNSKSADNIKTLLFSATLDDKVQKLANNIMNKKECLFLDTVDKNEPEAHERIDQSVVISEKFANSIFAAVEHIKKQIKERDSNYKAIIFAPTVKFTSFLCSILKNEFKKDLPILEFHGKITQNKRTSLVKRFKKDESGILVCTDVGARGMDFPNVHEVLQIGVPSELANYIHRIGRTARSGKEGSSVLFICKDELPFVRELEDAKNIVIAKQEKYEPSEEIKSEVLEAVTEEPEDISDIVISLISSYRSCIKEYRFSERRILPEIASTYGVLLNDPQLKIPVSRRFLDKLGLSRSPIGKAMFEIRDYSSRDGNNKSYDYDDDSEISFRGNKNYNNRSQNRDYDDEPFRRSNNNRRSFSRSNDKNNYSSRNSNIY>YDR195WMSAPVPQLVNISHALQASTIQQIRLDMVDFNKDCKLSSIQLARIDKYIDSLQAALNQFTKDNLHIERKEKNVTEADIQLYSGLKSMYLDYLNQLIKLKHEKQHHSTPPIANDVSLDFFVNQLPKFSPEERKNYIDNLILNKNSHNRLSKMDGLVDAVINLCVLDTSVAENVRSYMKLLDTLGFQKGSNSTGTKANLKKKLASSKAKIKDSEKEKEKEKDKSKVKMKTKLKPSPLLNNDDKNSSPSPTASTSSMKKLKSGLFNKNEAKSTESLPTSSKKKLSFSKYLNKDDADMTKLGTKRSIDVDFKVNPEASTVASNIISSSTSGSSTTTVATPASSEEPLKKKTKISVQDSNVQSILRNGKPKKARISSIKFLDDSQLIKVYGDDLPNQGLQVSPTQLKKILKPFKEGEPKEIILFEDMSIKLKPLDLMFLKNTNSDDYMDISETKGGPIHCETRTPLIYRKNFNHFNPDLNKRPPREPIEFDLNGNTNSTPTIAKAFGKNSLLLRKDRGGLPYKHVPIVKRNKYPPRPVH>YDR196CMLVVGLTGGIACGKSTVSRRLRDKYKLPIVDADKIARQVVEPGQNAYDQIVLYFKDKIPNLLLEDGHLNREALGKWVFSHKEDLQALNGITHPAIRYAMFKEIGYYYLKGYRMCVLDVPLLFEGNLDSICGVTVSVICTQELQLERLMTRNPELSEEDAKNRLNSQMSTEERMARSDYILQNNSTLVDLYEQIESVVKKIQPSKLRTVLEYFPPFGAVSASSIVMSRLLMKKLQNKKSSAV>YDR197WMSSSIPRVYSLGNSAMTYLLALRIAQLPSQPKVPSVVLLLNDQKKLNRFLNNDSKIIVKSSNNNKETYHRQFMASCVPPILSNGEIAPIENLIVSDPSSKFITAQLSKYNKSLRPETNILFLNPSLNLLEHLHRYRWRFDEARPNLFMGFTTPVDVGTIHQEFQLSLKVKGRIQFHIAKIDGFPRMSSTGKSASLSLRGDRQKNEKENNAFYKLFREISRLRSGIGSDLVSFDLHVHGFQDLFFTELEKLILESCTEPLLAVYDCVYKKELLKIPGAQDIIKKLISEQLSIIDRSYPSLNTNPNYSVIFDKERIFSLVMRDLEVNGHKRAKLAQSLNQLNQTNINELNGFFVSLGKYKKCNCKWNDILLTLIKGKQFITKQKALDYHYL>YDR201WMTDALEQSVLALEGTVSVLKDSVESLKCANEPSTNLASTMLQTKRVFRLVPEYDVERSKLDLIEEVEPLVRTLGDKLRKSMGRMQRELDTLQQTYELNDLRLKKNISMDDDDALNSPDMGQEYEGRDADDVVMMASSTNEELEELKKLKEKKKQLENKLEILKQK>YDR202CMSVDLFPNDRFGAEDKYDNFKDAVKECSWLIEEIVKPQLPNIIDNFSKCLEMLESDQIFKMPVSNGIPNESNKQNDSPTVKGVITRQGQYIVDFHIVVRFPQFQRGKQVMFRMNTGLNFLLIQFSKIMTHLKNILEILNQLQVATDVSEFVSKFGVAMELLNHSLILLQNPPRDLVFPEDNNFAMKEMFQDCYSVCESTAHILGLELTLCRNELCIELRNLIKVTKKPWCEIDSKTGRSFCDQIRNQVTNERNKTLSKILSENGVQVQDSTLLNHIISSFQSEAITLPEAQELLRRGVTFDNRVVMECEKLIVSTSDPTLISISAKLNSLKASMANHQANLVASKQLSTYK>YDR204WMLRLSLLRSTATLPVKCQRRGLILPAAAMYTLGSLIFGKEARLADAMERGELHNKNVDYAKEAEERTELRIRALANTRPMEPRYNGHVPLHRYEKLLLFAISGWNSFFHPEDGYNIVQLGEATALPVFLENLKQTMLSDSSGRRILKEQPNITTEILHMDKLAKLPHNTFGYVYYQWLKRENVSPDTRAPVKFIDDPMHAYIFKRYRQCHDFYHAITNMPIIIEGEITIKALEGANLGVPMAILGGILAPLRLKKVQRKRLYNIYLPWAVRTGLSCKPLINVYWEEMLEKDVTALRKELKITLPPDLRTMRKERAALRKEIDAKYNSQKRATTPA>YDR205WMNLQELLAKVPLLLSYPTIILSSNLIVPSHNDLISRAASTSAAEYADEKLIFFSTDHAIRLIFLPTFVASSFNLFAHYFNFINYSSRRKYYVLFTAIYFLSILTAIFHPIQSTCITLLIIKLLTTADESSPKIALNFKTILKTFVPFITLTLVILRWDPSFDASSGDVNKISTSLAAYALLILTLRYASPLILSTLSSSIGVVSKDTSVAQHSISRNKRFPLILVLPIFSFVLLYLMTIVNKTYNIQLLMVFVFFGCLSIFFLSLKDLFTEDGNQKKGGQEDEYCRMFDIKYMISYLWLTRFTILLTGIMAIVVHFLSFNEITSSIKTDLLSLLFVVVAEYVSSFSNKQPDSHSHNHAHHHSHLTDSLPLENESMFKQMALNKDTRSIFSFLLLNTAFMFVQLLYSFRSKSLGLLSDSLHMALDCTSLLLGLIAGVLTKKPASDKFPFGLNYLGTLAGFTNGVLLLGIVCGIFVEAIERIFNPIHLHATNELLVVATLGLLVNLVGLFAFDHGAHDHGGTDNENMKGIFLHILADTLGSVGVVISTLLIKLTHWPIFDPIASLLIGSLILLSALPLLKSTSANILLRLDDKKHNLVKSALNQISTTPGITGYTTPRFWPTESGSSGHSHAHTHSHAENHSHEHHHDQKNGSQEHPSLVGYIHVQYVDGENSTIIKKRVEKIFENVSIKAWVQVEPQNSTCWCRATSMNTISANPNSLPLQPIAN>YDR206WMEPSNTQKEDLPTAFNGIKSQLNSILKSNQLFQDYALLNGFLAFVHSKLNAAILTSIESQCGKSFAADLDSFDQSSISSILDFSWESVHYPIFKWFQMWRNYILFEKENKKQQTKFIDFRKMNSKMLKFFKTVQNFYVNVINTVYKKYDISVLLPKRIIQDLKLSDIENTTNVGDILAVKTFNSSSPLAHLIPTLFHRCLLFLGTAYRYKTLLEEISNKYSISNFKKSLDFFRLASLVLPSAGETYSQAGAIFLQTGNLGIAVFNFVKGMMTKMPSPVSIKNFGALMVDNKSSLNRSLHTTIMNTYLQESKGPRTPAKEILEFYFLGLFGSVWSPTSWRDDTKPNQLNNGIKLRHLENALYETMSARYLNNIKTIFHNLIITIGGFHLLLKRRSDVSAKTLKDLRSNELDYLNFAFKYIAHILNDIVKESWSENPEVSEILGMVRIINCWIKANPMVLQYSQSNLEFVNALAYLINDIVKKKPSPSFSITEHIPKRTYWFEEDLMVKGLSFVNFQLSDFDDYEKILEMDHSLDRLIGNPPLCDKLSASSEMLLRLQAVVNISSQLLQNNNCGVEWSDNKSRYIFNKKIGFKETVKNSMKTSKQSNEKAKLQRKNKPSTTNGSISMADLERQMRSSSLDSFSPTMGYSGSSVPMAPDTFNVKPSGTITGNKVNVELLKIELSGQNADGAITNISPGYSNAAISSSNSTDESSFDLNNILSSMQQKHAEKSFAKSMQGVNEQIPANDVCHQAQRPMQGGLYSSQQPSSMSSLNSAYQNASMPPSASMVSYPYPFLNQQGQGVFPPYNAQNLQWQSEAYSLKSMNFANPTWLGDQYQTSAPSSAYAQAQRQMFQQPMQQDVGKYMQFPFDAQSNTDSMRGNSRNNMF>YDR208WMSVLRSQPPSVVPLHLTTSTSRKTEQEPSLLHSAIIERHQDRSVPNSNSNPDSNHRIKKDRNNHTSYHSSSNSESNMESPRLSDGESSTPTSIEELNPTINNSRLVKRNYSISIDPLHDNSNNNTDDDHPNTITSPRPNSTSNKEMQKYSFPEGKESKKITTPSLNSNNCLDLDNSSLVHTDSYIQDLNDDHILLNKRVSRRSSRISAVTATSTTIKQRRNTQDSNLPNIPFHASKHSQILPMDDSDVIKLANGDTSMKPNSATKISHSMTSLPLHPLPQPSQKSKQYHMISKSTTSLPPENDHYYQHSRGTNHNHAANAAAVNNNTTTTTAATGLKRSESATAEIKKMRQSLLHKREMKRKRKTFLVDDDRVLIGNKVSEGHVNFIIAYNMLTGIRVAVSRCSGIMKPLTPADFRFTKKLAFDYHGNELTPSSQYAFKFKDYCPEVFRELRALFGLDPADYLVSLTSKYILSELNSPGKSGSFFYYSRDYKYIIKTIHHSEHIHLRKHIQEYYNHVRDNPNTLICQFYGLHRVKMPISFQNKIKHRKIYFLVMNNLFPPHLDIHITYDLKGSTWGRFTNLDKERLAKDRSYRPVMKDLNWLEEGQKIKFGPLKKKTFLTQLKKDVELLAKLNTMDYSLLIGIHDINKAKEDDLQLADTASIEEQPQTQGPIRTGTGTVVRHFFREFEGGIRASDQFNNDVDLIYYVGIIDFLTNYSVMKKLETFWRSLRHDTKLVSAIPPRDYANRFYEFIEDSVDPLPQKKTQSSYRDDPNQKNYKD>YDR210WMSQQQGYYQQGPPQQGYYQQGPPQQGYYQQGPPQQGYPQQQPVYVQQGQPKEESCLDSCLKCLCCCFLLELVCDN>YDR211WMAGKKGQKKSGLGNHGKNSDMDVEDRLQAVVLTDSYETRFMPLTAVKPRCLLPLANVPLIEYTLEFLAKAGVHEVFLICSSHANQINDYIENSKWNLPWSPFKITTIMSPEARCTGDVMRDLDNRGIITGDFILVSGDVLTNIDFSKMLEFHKKMHLQDKDHISTMCLSKASTYPKTRTIEPAAFVLDKSTSRCIYYQDLPLPSSREKTSIQIDPELLDNVDEFVIRNDLIDCRIDICTSHVPLIFQENFDYQSLRTDFVKGVISSDILGKHIYAYLTDEYAVRVESWQTYDTISQDFLGRWCYPLVLDSNIQDDQTYSYESRHIYKEKDVVLAQSCKIGKCTAIGSGTKIGEGTKIENSVIGRNCQIGENIRIKNSFIWDDCIIGNNSIIDHSLIASNATLGSNVRLNDGCIIGFNVKIDDNMDLDRNTKISASPLKNAGSRMYDNESNEQFDQDLDDQTLAVSIVGDKGVGYIYESEVSDDEDSSTEACKEINTLSNQLDELYLSDDSISSATKKTKKRRTMSVNSIYTDREEIDSEFEDEDFEKEGIATVERAMENNHDLDTALLELNTLRMSMNVTYHEVRIATITALLRRVYHFIATQTLGPKDAVVKVFNQWGLLFKRQAFDEEEYIDLMNIIMEKIVEQSFDKPDLILFSALVSLYDNDIIEEDVIYKWWDNVSTDPRYDEVKKLTVKWVEWLQNADEESSSEEE>YDR212WMSQLFNNSRSDTLFLGGEKISGDDIRNQNVLATMAVANVVKSSLGPVGLDKMLVDDIGDFTVTNDGATILSLLDVQHPAGKILVELAQQQDREIGDGTTSVVIIASELLKRANELVKNKIHPTTIITGFRVALREAIRFINEVLSTSVDTLGKETLINIAKTSMSSKIIGADSDFFSNMVVDALLAVKTQNSKGEIKYPVKAVNVLKAHGKSATESLLVPGYALNCTVASQAMPKRIAGGNVKIACLDLNLQKARMAMGVQINIDDPEQLEQIRKREAGIVLERVKKIIDAGAQVVLTTKGIDDLCLKEFVEAKIMGVRRCKKEDLRRIARATGATLVSSMSNLEGEETFESSYLGLCDEVVQAKFSDDECILIKGTSKHSSSSIILRGANDYSLDEMERSLHDSLSVVKRTLESGNVVPGGGCVEAALNIYLDNFATTVGSREQLAIAEFAAALLIIPKTLAVNAAKDSSELVAKLRSYHAASQMAKPEDVKRRSYRNYGLDLIRGKIVDEIHAGVLEPTISKVKSLKSALEACVAILRIDTMITVDPEPPKEDPHDH>YDR213WMSEVGIQNHKKAVTKPRRREKVIELIEVDGKKVSTTSTGKRKFHNKSKNGCDNCKRRRVKCDEGKPACRKCTNMKLECQYTPIHLRKGRGATVVKYVTRKADGSVESDSSVDLPPTIKKEQTPFNDIQSAVKASGSSNDSFPSSASTTKSESEEKSSAPIEDKNNMTPLSMGLQGTINKKDMMNNFFSQNGTIGFGSPERLNSGIDGLLLPPLPSGNMGAFQLQQQQQVQQQSQPQTQAQQASGTPNERYGSFDLAGSPALQSTGMSLSNSLSGMLLCNRIPSGQNYTQQQLQYQLHQQLQLQQHQQVQLQQYQQLRQEQHQQVQQQQQEQLQQYQQHFLQQQQQVLLQQEQQPNDEEGGVQEENSKKVKEGPLQSQTSETTLNSDAATLQADALSQLSKMGLSLKSLSTFPTAGIGGVSYDFQELLGIKFPINNGNSRATKASNAEEALANMQEHHERAAASVKENDGQLSDTKSPAPSNNAQGGSASIMEPQAADAVSTMAPISMIERNMNRNSNISPSTPSAVLNDRQEMQDSISSLGNLTKAALENNEPTISLQTSQTENEDDASRQDMTSKINNEADRSSVSAGTSNIAKLLDLSTKGNLNLIDMKLFHHYCTKVWPTITAAKVSGPEIWRDYIPELAFDYPFLMHALLAFSATHLSRTETGLEQYVSSHRLDALRLLREAVLEISENNTDALVASALILIMDSLANASGNGTVGNQSLNSMSPSAWIFHVKGAATILTAVWPLSERSKFHNIISVDLSDLGDVINPDVGTITELVCFDESIADLYPVGLDSPYLITLAYLDKLHREKNQGDFILRVFTFPALLDKTFLALLMTGDLGAMRIMRSYYKLLRGFATEVKDKVWFLEGVTQVLPQDVDEYSGGGGMHMMLDFLGGGLPSMTTTNFSDFSL>YDR214WMVVNNPNNWHWVDKNCIGWAKEYFKQKLVGVEAGSVKDKKYAKIKSVSSIEGDCEVNQRKGKVISLFDLKITVLIEGHVDSKDGSALPFEGSINVPEVAFDSEASSYQFDISIFKETSELSEAKPLIRSELLPKLRQIFQQFGKDLLATHGNDIQVPESQVKSNYTRGNQKSSFTEIKDSASKPKKNALPSSTSTSAPVSSTNKVPQNGSGNSTSIYLEPTFNVPSSELYETFLDKQRILAWTRSAQFFNSGPKLETKEKFELFGGNVISELVSCEKDKKLVFHWKLKDWSAPFNSTIEMTFHESQEFHETKLQVKWTGIPVGEEDRVRANFEEYYVRSIKLTFGFGAVL>YDR217CMSGQLVQWKSSPDRVTQSAIKEALHSPLADGDMNEMNVPVDPLENKVNSTNIIEGSPKANPNPVKFMNTSEIFQKSLGLLDESPRHDDELNIEVGDNDRPNANILHNERTPDLDRIANFFKSNRTPGKENLLTKYQSSDLEDTPLMLRKKMTFQTPTDPLEQKTFKKLKSDTGFCYYGEQNDGEENASLEVTEADATFVQMAERSADNYDCALEGIVTPKRYKDELSKSGGMQDERVQKTQIMISAESPNSISSYDKNKITGNGRTTRNVNKVFNNNEDNIGAIEEKNPVKKKSENYSSDDLRERNNQIIQSNESEEINELEKNLNVSGRENDVNNLDIDINSAVSGTPSRNNAEEEMYSSESVNNREPSKKWIFRYSKDKTENNSNRSTQIVNNPRTQEMPLDSISIDTQPLSKSFNTETNNELETQIIVSSLSQGISAQKGPVFHSTGQTEEIKTQIINSPEQNALNATFETPVTLSRINFEPILEVPETSSPSKNTMSKPSNSSPIPKEKDTFNIHEREVETNNVFSNDIQNSSNAATRDDIIIAGSSDFNEQKEITDRIYLQLSGKQISDSGSDETERMSPNELDTKKESTIMSEVELTQELPEVEEQQDLQTSPKKLVVEEETLMEIKKSKGNSLQLHDDNKECNSDKQDGTESLDVALIEHESKGQSSELQKNLMQLFPSESQEIIQNRRTIKRRQKDTIEIGEEEENRSTKTSPTKHLKRNSDLDAASIKREPSCSITIQTGETGSGKDSKEQSYVFPEGIRTADNSFLSKDDIIFGNAVWCQYTWNYKFYPGILLEVDTNQDGCWIYFETGRSLTKDEDIYYLDIRIGDAVTFDGNEYVVVGLECRSHDLNIIRCIRGYDTVHLKKKNASGLLGKRTLIKALSSISLDLSEWAKRAKIILEDNEKNKGDAYRYLRHPIRGRKSMTNVLSPKKHTDDEKDINTHTEVYNNEIESSSEKKEIVKKDSRDALAEHAGAPSLLFSSGEIRTGNVFDKCIFVLTSLFENREELRQTIESQGGTVIESGFSTLFNFTHPLAKSLVNKGNTDNIRELALKLAWKPHSLFADCRFACLITKRHLRSLKYLETLALGWPTLHWKFISACIEKKRIVPHLIYQYLLPSGESFRLSLDSPSKGGIIKSNNIFSFYTQFLRGSNLRDQICGVKKMLNDYIVIVWGRSELDSFVKFAFACLSAGRMLTIDLPNIDVDDTEPLLNALDSLVPRIGSELSNRKLKFLIYANENNGKSQMKLLERLRSQISLKFKKFNYIFHTESKEWLIQTIINEDTGFHDDITDNDIYNTISEVR>YDR219CMTLFSCSVQMPLEERSLTNLPLNLLFRILSHLDMNDLQNIGKTCTLLRMLANENIVYRNAVIGSNGNMWWTKNVLVDVFDVLNFNRKAMKTLNSHNISLVASLRNVQRKYKLGVIDPARKTISYRTNEVESKEKGSVKDLNMDLNEPTEITREQIAHTAILQGMNQFIELNDKAFRTHSADSDDTYIEENNGEIHSLHGLEKNTTFEEDLVKKPPFIPSPTFSNYSRSSTNSVFSSSSPKLLDDDWNNITMDFTKSRDPDYKEMTPTSTESSDSITRLRKSNKVKDKAELFEKLIFRDSRPLKTKKKDNPRLKLSSSLSANDEDFRKIISPPSDILPKVGRRSVSRGYLEEIERHYPDFNGETTNPLAIKRVNSTKIANYEQLIIKENSSNCKGITEKNDENKFQRSHTSPVIELSKPHQRSKLKAVVTDGNKICYRKIELDNPSGSNTNDHVIKRLDANTDFNI>YDR222WMLESSSDKIKFAPIKEVDYKKPVSKSKNYTLINDIQPLEWYCHNDSETGYQHTISNKTDGGRGLFRVMKKSMETRVETQTLYFTDLHTGLCGFVQLLYSTVMGGIYKGFQLNFKVFGSESNNTDYDVWESFKLDDIAEFQPLKFVSRNVIFEFLSNKNEKLGSIGQLSIKCDLPTCNNTIQNLKIDLLVDLFQGFKMNPNGCNYYFDKQISMSDEFVSSDKMIRHVFVPRGKCNGNISYDKKLNSGDFQNKNISLTDVPVVYLDAVQGLLPNKAASKWNFLCFQSENYSVLAIEFTTPRDHDNVTVTVWSITEKNKLISIGSSVQSPKRHVRFRATSTDKESGWVYPTSIKFPGGFSEHDLRLVNRYDVLGELPSMVRSLAQKIVSIKPFIYQYCQPSKYKHEKGISIVESTFIS>YDR224CMSAKAEKKPASKAPAEKKPAAKKTSTSTDGKKRSKARKETYSSYIYKVLKQTHPDTGISQKSMSILNSFVNDIFERIATEASKLAAYNKKSTISAREIQTAVRLILPGELAKHAVSEGTRAVTKYSSSTQA>YDR225WMSGGKGGKAGSAAKASQSRSAKAGLTFPVGRVHRLLRRGNYAQRIGSGAPVYLTAVLEYLAAEILELAGNAARDNKKTRIIPRHLQLAIRNDDELNKLLGNVTIAQGGVLPNIHQNLLPKKSAKATKASQEL>YDR226WMSSSESIRMVLIGPPGAGKGTQAPNLQERFHAAHLATGDMLRSQIAKGTQLGLEAKKIMDQGGLVSDDIMVNMIKDELTNNPACKNGFILDGFPRTIPQAEKLDQMLKEQGTPLEKAIELKVDDELLVARITGRLIHPASGRSYHKIFNPPKEDMKDDVTGEALVQRSDDNADALKKRLAAYHAQTEPIVDFYKKTGIWAGVDASQPPATVWADILNKLGKD>YDR228CMDHDTEVIVKDFNSILEELTFNSRPIITTLTKLAEENISCAQYFVDAIESRIEKCMPKQKLYAFYALDSICKNVGSPYTIYFSRNLFNLYKRTYLLVDNTTRTKLINMFKLWLNPNDTGLPLFEGSALEKIEQFLIKASALHQKNLQAMLPTPTVPLLLRDIDKLTCLTSERLKNQPNDEKLKMKLLVLSQLKQELKREKLTLNALKQVQMQLRQVFSQDQQVLQERMRYHELQQQQQQQQQQQQQQQQQQQQYHETKDMVGSYTQNSNSAIPLFGNNSDTTNQQNSLSSSLFGNISGVESFQEIEKKKSLNKINNLYASLKAEGLIYTPPKESIVTLYKKLNGHSNYSLDSHEKQLMKNLPKIPLLNDILSDCKAYFATVNIDVLNNPSLQLSEQTLLQENPIVQNNLIHLLYRSKPNKCSVCGKRFGNSESEKLLQNEHLDWHFRINTRIKGSQNTANTGISNSNLNTTTTRKNIQSRNWYLSDSQWAAFKDDEITSTKHKNDYTDPHANKNIDKSALNIHADENDEGSVDNTLGSDRSNELEIRGKYVVVPETSQDMAFKCPICKETVTGVYDEESGEWVWKNTIEVNGKYFHSTCYHETSQNSSKSNSGKVGLDDLKKLVTK>YDR229WMPDNNTEQLQGSPSSDQRLRVDWDNGNHFDVSPDRYAPHLSEFYPIVNSKRPVASSAGSENNDHLDDMNHLRSSKVYSKARRASSITSGTSTINDLQTLITKRDVKETQEALSTLLRNSNAYSDSLLKTSQNGAEIAHSLENIAKLKGCNDETAEKLLSASGLFYLLSNHQLIMSKYFNDLLGDNLIDDIDEFELQTKIMENKFKAQSKEQSLKLKLQERHNFDISKRKIRNLISYRESLSSLQARLDQLETLKHDFYMDSYELVENTCNKVLSKVATVSRAQVEISENIARKGWSGGGLDELLCDADDPFSKKADGPYGTIGGDGETAGEAYNSDEETGGNDVVLNELLEGTSQPSTSKTSLPKSKGSSTVSTPNHSQSSSNKDGVRNNGGGKNGEDEDTDNLMGTENSFSLPPTRNSAEETTQTFKQLSIKEDNDNHSSDTDGMQDQSSNI>YDR232WMQRSIFARFGNSSAAVSTLNRLSTTAAPHAKNGYATATGAGAAAATATASSTHAAAAAAAAANHSTQESGFDYEGLIDSELQKKRLDKSYRYFNNINRLAKEFPLAHRQREADKVTVWCSNDYLALSKHPEVLDAMHKTIDKYGCGAGGTRNIAGHNIPTLNLEAELATLHKKEGALVFSSCYVANDAVLSLLGQKMKDLVIFSDELNHASMIVGIKHANVKKHIFKHNDLNELEQLLQSYPKSVPKLIAFESVYSMAGSVADIEKICDLADKYGALTFLDEVHAVGLYGPHGAGVAEHCDFESHRASGIATPKTNDKGGAKTVMDRVDMITGTLGKSFGSVGGYVAASRKLIDWFRSFAPGFIFTTTLPPSVMAGATAAIRYQRCHIDLRTSQQKHTMYVKKAFHELGIPVIPNPSHIVPVLIGNADLAKQASDILINKHQIYVQAINFPTVARGTERLRITPTPGHTNDLSDILINAVDDVFNELQLPRVRDWESQGGLLGVGESGFVEESNLWTSSQLSLTNDDLNPNVRDPIVKQLEVSSGIKQ>YDR233CMSASAQHSQAQQQQQQKSCNCDLLLWRNPVQTGKYFGGSLLALLILKKVNLITFFLKVAYTILFTTGSIEFVSKLFLGQGLITKYGPKECPNIAGFIKPHIDEALKQLPVFQAHIRKTVFAQVPKHTFKTAVALFLLHKFFSWFSIWTIVFVADIFTFTLPVIYHSYKHEIDATVAQGVEISKQKTQEFSQMACEKTKPYLDKVESKLGPISNLVKSKTAPVSSTAGPQTASTSKLAADVPLEPESKAYTSSAQVMPEVPQHEPSTTQEFNVDELSNELKKSTKNLQNELEKNNA>YDR234WMLRSTTFTRSFHSSRAWLKGQNLTEKIVQSYAVNLPEGKVVHSGDYVSIKPAHCMSHDNSWPVALKFMGLGATKIKNPSQIVTTLDHDIQNKSEKNLTKYKNIENFAKKHHIDHYPAGRGIGHQIMIEEGYAFPLNMTVASDSHSNTYGGLGSLGTPIVRTDAAAIWATGQTWWQIPPVAQVELKGQLPQGVSGKDIIVALCGLFNNDQVLNHAIEFTGDSLNALPIDHRLTIANMTTEWGALSGLFPVDKTLIDWYKNRLQKLGTNNHPRINPKTIRALEEKAKIPKADKDAHYAKKLIIDLATLTHYVSGPNSVKVSNTVQDLSQQDIKINKAYLVSCTNSRLSDLQSAADVVCPTGDLNKVNKVAPGVEFYVAAASSEIEADARKSGAWEKLLKAGCIPLPSGCGPCIGLGAGLLEPGEVGISATNRNFKGRMGSKDALAYLASPAVVAASAVLGKISSPAEVLSTSEIPFSGVKTEIIENPVVEEEVNAQTEAPKQSVEILEGFPREFSGELVLCDADNINTDGIYPGKYTYQDDVPKEKMAQVCMENYDAEFRTKVHPGDIVVSGFNFGTGSSREQAATALLAKGINLVVSGSFGNIFSRNSINNALLTLEIPALIKKLREKYQGAPKELTRRTGWFLKWDVADAKVVVTEGSLDGPVILEQKVGELGKNLQEIIVKGGLEGWVKSQL>YDR236CMFTWTIYVSLLLVLAGTFLMNRNTNTDIIDTFKREVDLPIPAQPGPPFPLVTDYCDIVCGFGRGSAELGIPTANVPINQLPKGINDLDLGVYFGFAHIKTVDGQELSVETRRDGRTVVYNYGQYLSEANDDLSVLPMVLSVGKNPFYGNDFKTMELHIIHDFKNDFYGARVKFNILGHIRPELNYTTKEALIEDINIDIRTAQTVLATPPYQVFKQQL>YDR237WMQRFSLVTHRSFSHSCVKPKSACSLVKPVHHLVKIDKSKLSPRFPELKYDKSDIRSPGFKPKDTHADRLNDHYLNTLQSDLLLINYSHNAAVVKGLKQRAWSGDSPYHLNRPPKNPRGSKAQLPDIHPIKWSNIPGLESVVINCFVREARENQLLAITAALQLQQITGCKPHPIFSKNDVPTWKLRKGHQMGAKVELKGKEMSQFLSTLTEIVLPRIREYKGISNQSGNRFGGISFGLTAEDIKFFPEIDANQDSWPKTFGMHININTSAQLDYQARTLLSGFQFPFFGEEK>YDR238CMTSLSSQPAYTLVFDPSPSMETYSSTDFQKALEKGSDEQKIDTMKSILVTMLEGNPMPELLMHIIRFVMPSKNKELKKLLYFYWEIVPKLAEDGKLRHEMILVCNAIQHDLQHPNEYIRGNTLRFLTKLREAELLEQMVPSVLACLEYRHAYVRKYAILAVFSIFKVSEHLLPDAKEIINSFIVAETDPICKRNAFIGLAELDRENALHYLENNIADIENLDPLLQAVFVQFIRQDANRTPALKAQYIELLMELLSTTTSDEVIFETALALTVLSANPNVLVPAVNKLIDLAVKVSDNNIKLIVLDRIQDINANNVGALEELTLDILRVLNAEDLDVRSKALDISMDLATSRNAEDVVQLLKKELQTTVNNPDQDKAMQYRQLLIKTIRTVAVNFVEMAASVVSLLLDFIGDLNSVAASGIIAFIKEVIEKYPQLRANILENMVQTLDKVRSAKAYRGALWIMGEYAEGESEIQHCWKHIRNSVGEVPILQSEIKKLTQNQEHTEENEVDATAKPTGPVILPDGTYATESAFDVKTSQKSVTDEERDSRPPIRRFVLSGDFYTAAILANTIIKLVLKFENVSKNKTVINALKAEALLILVSIVRVGQSSLVEKKIDEDSLERVMTSISILLDEVNPEEKKEEVKLLEVAFLDTTKSSFKRQIEIAKKNKHKRALKDSCKNIEPIDTPISFRQFAGVDSTNVQKDSIEEDLQLAMKGDAIHATSSSSISKLKKIVPLCGFSDPVYAEACITNNQFDVVLDVLLVNQTKETLKNLHVQFATLGDLKIIDTPQKTNVIPHGFHKFTVTVKVSSADTGVIFGNIIYDGAHGEDARYVILNDVHVDIMDYIKPATADDEHFRTMWNAFEWENKISVKSQLPTLHAYLRELVKGTNMGILTPSESLGEDDCRFLSCNLYAKSSFGEDALANLCIEKDSKTNDVIGYVRIRSKGQGLALSLGDRVALIAKKTNKLALTHV>YDR240CMRPRRRGLAYHHTKPKGQLSQGHYPTTSNDGQRRKVGNSEAFQSFDIWKNLDRIRSTKKNAGQFIKGSLLILPMRTEDKQQFDECMDELHKYISKDILRCYPQKEQKDEGMLFYIVLKDFNILDSCFVLSVLLAFQKRLWMAPSEKSYFRVPKNINLTGSFYLPKNIETGRGHIITSYRREQPSSSIVEVGFNVVPDFQQFQVKACHVSKFMNELSNFFSQVEFGKCEANVINYFKREYNRTYSQISLALYELPLIGDGLFDIKSYISKTRPIIETSKAQMIKHISEMKAYNEISGLQGDQFPRQQRPLSNSPSSNSISSSQTIEAGATSYQTQPQRHAVNKPSNVLNSSNRHSGPKTFEDGRYSEGNKPGFMTQDEIKQHCIGTIKASMDAVKKKSSYQILKTYVRCPRQNYIDIVYQNLNDLRSKTNCNIVVLNLNNLHESQMWLESLNTTNYTIFAQAPHPSTIRVISIGGVGEYIVKALELILNILEH>YDR242WMTVHSTWKEKVQLKKDQLNSKIKDEWKLNSTTITRLKNDKKNLIKNIDDLCSSSENQITHSTIMALRQALEAKELSCHEITAAFCHRAALIHQVVNCLSEIMFSEALRLADYYDSNRPAILPPLYGIPISLKDQCNVEGVDTSLGYLCRTFKPKTKNEESLIVSFLRDLGAIIFVKTTVPSSMMATDTQSNTFGYTYNSINLSFSSGGSSGGEGSLIGAHGSLLGLGTDIGGSIRIPSSYQGLFGLKPTFGRVPYLRVDNSFEGRETIPSVIGPLARDLSDLRYFMSCVINICQPWVQDVKCIPYHFDSSTSKLHDNYVVGIWYGDGVIDPPPSDIRALKTCEDLVNKTKGMKAVKWEPSSELSRELFDLANEADVADSGNEIKNEFEISGEPLLDILKPMVLENGRPPYTVNEWWDLTKRVYNAQQLMRDYYLSFPESERPDVIISPTTLMPFRPGDMLKTTLRYILLFNVLNFPSLSIPVGSVDCQIDGLMDTTSALNPEDKMIKTYWNDLIQSGEIDGFPIGLQVVSPTFNDNEVCKFASWLFSKI>YDR243CMARPIDVSQLIAGINKKKGLDENTSGKISKPRFLNKQERSKQERLKENEESLTPTQSDSAKVEIKKVNSRDDSFFNETNDKKRNPSKQNGSKFHFSWNESEDTLSGYDPIVSTRAIDLLWKGKTPKNAAESSYMGKHWTEKSLHEMNERDWRILKEDYAIVTKGGTVENPLRNWEELNIIPRDLLRVIIQELRFPSPTPIQRITIPNVCNMKQYRDFLGVASTGSGKTLAFVIPILIKMSRSPPRPPSLKIIDGPKALILAPTRELVQQIQKETQKVTKIWSKESNYDCKVISIVGGHSLEEISFSLSEGCDILVATPGRLIDSLENHLLVMKQVETLVLDEADKMIDLGFEDQVTNILTKVDINADSAVNRQTLMFTATMTPVIEKIAAGYMQKPVYATIGVETGSEPLIQQVVEYADNDEDKFKKLKPIVAKYDPPIIIFINYKQTADWLAEKFQKETNMKVTILHGSKSQEQREHSLQLFRTNKVQIMIATNVAARGLDIPNVSLVVNFQISKKMDDYIHRIGRTGRAANEGTAVSFVSAAEDESLIRELYKYVRKHDPLNSNIFSEAVKNKYNVGKQLSNEIIY>YDR244WMDVGSCSVGNNPLAQLHKHTQQNKSLQFNQKNNGRLNESPLQGTNKPGISEAFISNVNAISQENMANMQRFINGEPLIDDKRRMEIGPSSGRLPPFSNVHSLQTSANPTQIKGVNDISHWSQEFQGSNSIQNRNADTGNSEKAWQRGSTTASSRFQYPNTMMNNYAYASMNSLSGSRLQSPAFMNQQQSGRSKEGVNEQEQQPWTDQFEKLEKEVSENLDINDEIEKEENVSEVEQNKPETVEKEEGVYGDQYQSDFQEVWDSIHKDAEEVLPSELVNDDLNLGEDYLKYLGGRVNGNIEYAFQSNNEYFNNPNAYKIGCLLMENGAKLSEAALAFEAAVKEKPDHVDAWLRLGLVQTQNEKELNGISALEECLKLDPKNLEAMKTLAISYINEGYDMSAFTMLDKWAETKYPEIWSRIKQQDDKFQKEKGFTHIDMNAHITKQFLQLANNLSTIDPEIQLCLGLLFYTKDDFDKTIDCFESALRVNPNDELMWNRLGASLANSNRSEEAIQAYHRALQLKPSFVRARYNLAVSSMNIGCFKEAAGYLLSVLSMHEVNTNNKKGDVGSLLNTYNDTVIETLKRVFIAMNRDDLLQEVKPGMDLKRFKGEFSF>YDR246WMAIETILVINKSGGLIYQRNFTNDEQKLNSNEYLILASTLHGVFAIASQLTPKALQLTQQTNIENTIPYIPYVGMSSNRSDTRNGGGNNNKHTNNEKLGSFKGDDFFKEPFTNWNKSGLRQLCTDQFTMFIYQTLTGLKFVAISSSVMPQRQPTIATTDKPDRPKSTSNLAIQIADNFLRKVYCLYSDYVMKDPSYSMEMPIRSNLFDEKVKKMVENLQ>YDR247WMMMFHNCRINNYLITSQIGEGAYGLVYRALDIRTDRQYAIKAVVQSYGVSKEADMGNDKIHKNSVKLQKKLAKLFKESKNVVRVPSIDLESIENMSEEDFKKLPHYKEISLHLRVHHHKNIVTIHEVLQSAVCTFIVMDYYPTDLFTSIVDNRHFVTNGLLVKKVFLQICSALNYCHEHGIYHCDIKPENLLLDTEDNVFLCDFGLSTTSTYIKPNVCIGSSYYMPPERISFDGRVSSSKSGGHKLGKVCPSCNGDLWSLGIILINLTCIRNPWLKADKTEDNTYYYFTKDPNILKQILPLSDDFYSLLSKILQVNPKNRMSLQELMKEVSSITSFTNEGPLSKVPPLSKSVYEKFVSPVDNTNENLSPKSYVYMHDSKAAKNLSYTSSSEEEDGIKEGIDDDNGSRSGSFGTLDTDTGLHSSFTSTSCESDNECSKISNKFSLFEKKFNELRMSSSSLTN>YDR248CMTEKHKTMGKFKVIVLAGTAGTGKSTIAGELIHEFKDIYPDLKFIEGDDLHPPANVEKMTRGIPLNDDDRWDWLKKVAVESTKAAASTKEHLSIVACSSLKKKYRDLIRHTCPESEFHFIFLYASKIEVLKRLKTRKGHFMKADMMESQFRDLELPDINDETDCDIVPLDFKTFYQIEKDVIQVVKSKVLNIE>YDR251WMTSALRVLVCGDHPNLILYTSRFQHAKNIEFYLVNNSKNANYEVSSLFYGTERFQIQNHFQSLLDLVDLNNENGGLVFDLIIMSASSLQEIPQVLRDIKPMMNKTTKILFESSGFIYLEPFIKASVDLSLSNIFSIFTDYDIRRLDNGSYKQFTTANAKSFSVSIGQTTSVHENSYSSDIIPILNTFQKLFQKLFPRDVVTLYDHSPSAFLAKEWELALPQICFDPLLIILEEKNPSTLDDHVLAKPLISGLLGESLLIIKKMGIAMNNPNFQNEQTILKHWKNKCEDLPDGPALLYNFIHKASSLNIDLLLLQPILLADDFGVKTPYLECLFTMMTQYQLLNKGDSEWFIRKDENTALTRVDDLQNSIALKDGKIMQLQNSESTLKNEIKELQSQVLSLKQEVSSSKANNGQELEILKKKVQMGDNSLFDRPNSNTNGISPSDNIVDVDLNYERSDQGNNSSGNDSRRQSFFNSTSDTTLSRDETSLKERELEVRMKELELQERELELQRKALQQQQQYQQRPPKQVYSGPSGTPTSGNNNNKSYNPNRKSSYSQPQHVAMMTSRGLHGPSAASSSPVISANNFVDPVSSGTPYSSNSSRFSQQIPSQQYMHTVKPTSRKNRSSVMPNIGYVPGLTNNEYGRKFNGNGMNGTQSRLNSLSNQSTFRSQQGPPITQQKSFQNNGGSMRTNRIPSANYNISNQQSGFVNSISSPNLSNLENRNTVQNSRNADSAPCVNQLNSDSPPQLQSLSQNGTSKVPQINITQPSPIQTNFATSDNPAAVIKLGTPSEDTVSAAATANNISTMGDESRKEDVKEKKKKKFSFFGKRKK>YDR252WMPVDQEKLAKLHKLSAANKVGGTRRKINKKGNLYNNNDKDNTKLQAELHKLHPMTIENVAEANFFKKNGKVLHFNSAVVQIAPQCNLTMIHGQPKENTLNGLYPSVASQLGSQELEYLTGLAHNLENEQTVLDQLGDRCSETKQQVMNS>YDR253CMEDQDAAFIKQATEAIVDVSLNIDNIDPIIKELLERVRNRQNRLQNKKPALIPAENGVDINSQGGNIKVKKENALPKPPKSSKSKPQDRRNSTGEKRFKCAKCSLEFSRSSDLRRHEKTHFAILPNICPQCGKGFARKDALKRHYDTLTCRRNRTKLLTAGGEGINELLKKVKQSNIVHRQDNNHNGSSNG>YDR254WMSNELRLEDNYVPTSDTLVVFKQLMKLPVTVLYDLTLSWFAKFGGSFDGDIYLLTETLDLLIEKGVRRNVIVNRILYVYWPDGLNVFQLAEIDCHLMISKPEKFKWLPSKALRGDGKPYVVKLQPAKFIENLQTDLAKIYHCHVYMFKHPSLPVLITRIQLFDSNNLFLSTPNIGSINKESLYNKLDKFQGKPLISRRPYYVAFPLNSPIIFHSVDKDIYARLVLQSISRTISERETIIFKPVQKIPVKSIHNIMTLLGPSRFAESMGPWECYASANFERSPLHDYKKHQGLTGKKVMVREFDDSFLNDDENFYGKEEPEIRRLRLEKNMIKFKGSANGVMDQKYNDLKEFNEHVHNIRNGKKNEDSGEPVYISRYSSLVPIEKVGFTLKNEINSRIITIKLKFNGNDIFGGLHELCDKNLINIDKVPGWLAGENGSFSGTIMNGDFQREQVAKGGLL>YDR256CMSKLGQEKNEVNYSDVREDRVVTNSTGNPINEPFVTQRIGEHGPLLLQDYNLIDSLAHFNRENIPQRNPHAHGSGAFGYFEVTDDITDICGSAMFSKIGKRTKCLTRFSTVGGDKGSADTVRDPRGFATKFYTEEGNLDWVYNNTPVFFIRDPSKFPHFIHTQKRNPQTNLRDADMFWDFLTTPENQVAIHQVMILFSDRGTPANYRSMHGYSGHTYKWSNKNGDWHYVQVHIKTDQGIKNLTIEEATKIAGSNPDYCQQDLFEAIQNGNYPSWTVYIQTMTERDAKKLPFSVFDLTKVWPQGQFPLRRVGKIVLNENPLNFFAQVEQAAFAPSTTVPYQEASADPVLQARLFSYADAHRYRLGPNFHQIPVNCPYASKFFNPAIRDGPMNVNGNFGSEPTYLANDKSYTYIQQDRPIQQHQEVWNGPAIPYHWATSPGDVDFVQARNLYRVLGKQPGQQKNLAYNIGIHVEGACPQIQQRVYDMFARVDKGLSEAIKKVAEAKHASELSSNSKF>YDR259CMQNPPLIRPDMYNQGSSSMATYNASEKNLNEHPSPQIAQPSTSQKLPYRINPTTTNGDTDISVNSNPIQPPLPNLMHLSGPSDYRSMHQSPIHPSYIIPPHSNERKQSASYNRPQNAHVSIQPSVVFPPKSYSISYAPYQINPPLPNGLPNQSISLNKEYIAEEQLSTLPSRNTSVTTAPPSFQNSADTAKNSADNNDNNDNVTKPVPDKDTQLISSSGKTLRNTRRAAQNRTAQKAFRQRKEKYIKNLEQKSKIFDDLLAENNNFKSLNDSLRNDNNILIAQHEAIRNAITMLRSEYDVLCNENNMLKNENSIIKNEHNMSRNENENLKLENKRFHAEYIRMIEDIENTKRKEQEQRDEIEQLKKKIRSLEEIVGRHSDSAT>YDR260CMSSSSYRDSYFQYRHLPAPHHILYAEWNQDILALPDEVANITMAMKDNTRTDAEEGRAPQDGERNSNVRESAQGKALMTSEQNSNRYWNSFHDEDDWNLFNGMELESNGVVTFAGQAFDHSLNGGTNSRNDGANEPRKETITGSIFDRRITQLAYARNNGWHELALPQSR>YDR262WMIFNLPVSVLLYFSLIWAMEPSFVRGKNVVNLITFKDSNGKLHKRLAPEEIPPRLHNSQVNSYPLGYKGMRDFSRPAVNLDDILGTQQRKQQEFLAELSPLSLESKLSLVNEVQIFASYVRNDVETYNKVSDPNEDLIIIAPTNRAVSQLTLKPWQFPNNIDKLESDGATEKELDTAIQENISKFVRSHIVVYNDDKNSYKKVSPGCTLLQSIDFTESKKSDSETGGDILLKKEGEVYYVASSRDEKFHAVESIENGSNGVILMVDFTLVGP>YDR263CMGIPGLLPQLKRIQKQVSLKKYMYQTLAIDGYAWLHRASCACAFELVMNKPTNKYLQFFIKRLQLLKRLKIKPYIVFDGDSLFVKNHTETRRRKKRLENEMIAKKLWSAGNRYNAMEYFQKSVDITPEMAKCIIDYCKLHSIPYIVAPFEADPQMVYLEKMGLIQGIISEDSDLLVFGCKTLITKLNDQGKALEISKDDFSALPENFPLGELSEQQFRNLVCLAGCDYTSGIWKVGVVTAMKIVKRYSEMKDILIQIERTEKLCFSKAFKQQVEFANYAFQYQRVFCPLSNQITTLNNIPKAVTNSHAEIIKIMKCIGSVVERGSGVRKDVINTKNIDHKVHEMIAKGELHPVDMASKLINRERKLKARKLFKVGLLGGESNSFNKKVEQPLVDTQDVLSERENSLDNKNASSIYMTSPAAISGTVPSIF>YDR265WMKNDNKLQKEALMRLSQLRFPFADAPSIVQAHQKDEQIQGLLIMKVTELCKLIKSQLFVNSYPKELSIFAKLLYLLFTTGRRGRTLGEEYVDLTYTNRKGTRLAGRLKMIVFAFAYPLCPYFITKLYKKIMKNNKESKIEDTESVAAFCKGLLDFILDVHMTLFYFKGAFYSISKRIFGMRYVFKHILSKNEANFREEGSQKYKVLGYILLAQNVMKWYPVLTSTLGSWIYGRKRTNDSITRSSVGLQERSEHESIEGIPKESQLTHINLSDKNQLPFIPEASRKCILCLMNMSDPSCAPCGHLFCWSCLMSWCKERPECPLCRQHCQPQEILVLRQ>YDR268WMSNKQAVLKLISKRWISTVQRADFKLNSEALHSNATVFSMIQPTGCFHLGNYLGATRVWTDLCELKQPGQELIFGVADLHAITVPKPDGEMFRKFRHEAVASILAVGVDPEKASVIYQSAIPQHSELHWLLSTLASMGLLNRMTQWKSKSNIKQSTNGDYLVNDSDVGKVRLGLFSYPVLQAADILLYKSTHVPVGDDQSQHLELTRHLAEKFNKMYKKNFFPKPVTMLAQTKKVLSLSTPEKKMSKSDPNHDSVIFLNDEPKAIQKKIRKALTDSISDRFYYDPVERPGVSNLINIVSGIQRKSIEDVVEDVSRFNNYRDFKDYVSEVIIEELKGPRTEFEKYINEPTYLHSVVESGMRKAREKAAKNLADIHKIMGF>YDR270WMREVILAVHGMTCSACTNTINTQLRALKGVTKCDISLVTNECQVTYDNEVTADSIKEIIEDCGFDCEILRDSEITAISTKEGLLSVQGMTCGSCVSTVTKQVEGIEGVESVVVSLVTEECHVIYEPSKTTLETAREMIEDCGFDSNIIMDGNGNADMTEKTVILKVTKAFEDESPLILSSVSERFQFLLDLGVKSIEISDDMHTLTIKYCCNELGIRDLLRHLERTGYKFTVFSNLDNTTQLRLLSKEDEIRFWKKNSIKSTLLAIICMLLYMIVPMMWPTIVQDRIFPYKETSFVRGLFYRDILGVILASYIQFSVGFYFYKAAWASLKHGSGTMDTLVCVSTTCAYTFSVFSLVHNMFHPSSTGKLPRIVFDTSIMIISYISIGKYLETLAKSQTSTALSKLIQLTPSVCSIISDVERNETKEIPIELLQVNDIVEIKPGMKIPADGIITRGESEIDESLMTGESILVPKKTGFPVIAGSVNGPGHFYFRTTTVGEETKLANIIKVMKEAQLSKAPIQGYADYLASIFVPGILILAVLTFFIWCFILNISANPPVAFTANTKADNFFICLQTATSVVIVACPCALGLATPTAIMVGTGVGAQNGVLIKGGEVLEKFNSITTFVFDKTGTLTTGFMVVKKFLKDSNWVGNVDEDEVLACIKATESISDHPVSKAIIRYCDGLNCNKALNAVVLESEYVLGKGIVSKCQVNGNTYDICIGNEALILEDALKKSGFINSNVDQGNTVSYVSVNGHVFGLFEINDEVKHDSYATVQYLQRNGYETYMITGDNNSAAKRVAREVGISFENVYSDVSPTGKCDLVKKIQDKEGNNKVAVVGDGINDAPALALSDLGIAISTGTEIAIEAADIVILCGNDLNTNSLRGLANAIDISLKTFKRIKLNLFWALCYNIFMIPIAMGVLIPWGITLPPMLAGLAMAFSSVSVVLSSLMLKKWTPPDIESHGISDFKSKFSIGNFWSRLFSTRAIAGEQDIESQAGLMSNEEVL>YDR272WMQVKSIKMRWESGGVNYCYLLSDSKNKKSWLIDPAEPPEVLPELTEDEKISVEAIVNTHHHYDHADGNADILKYLKEKNPTSKVEVIGGSKDCPKVTIIPENLKKLHLGDLEITCIRTPCHTRDSICYYVKDPTTDERCIFTGDTLFTAGCGRFFEGTGEEMDIALNNSILETVGRQNWSKTRVYPGHEYTSDNVKFVRKIYPQVGENKALDELEQFCSKHEVTAGRFTLKDEVEFNPFMRLEDPKVQKAAGDTNNSWDRAQIMDKLRAMKNRM>YDR275WMFFFPKLRKLIGSTVIDHDTKNSSGKEEIMSNSRLALVIINHAFDKVLSLTWHCGILSEIRSGLMLMFGIFQLMCSLGVIVLLLPIIILDAIDLFLYMCRLLDYGCKLFHYNRSSLPVADGKEKTSGPISGKEEIVIDEEIINMLNESSESLINHTTAGLEYDISSGSVNKSRRLNSTSTVTFVKQNKLVNERREDAYYEEEDDDFLSNPNYDKISLIEKSFTSRFEVACEQKAA>YDR276CMDSAKIINIILSLFLPPVAVFLARGWGTDCIVDIILTILAWFPGMLYALYIVLQD>YDR277CMFVSPPPATSKNQVLQRRPLESTNSNHGFASSLQAIPENTMSGSDNASFQSLPLSMSSSQSTTSSRRENFVNAPPEYTDRARDEIKKRLLASSPSRRSHHSSSMHSASRRSSVAESGSLLSDNASSYQSSIFSAPSTVHTQLTNDSSFSEFPNHKLITRVSLDEALPKTFYDMYSPDILLADPSNILCNGRPKFTKRELLDWDLNDIRSLLIVEKLRPEWGNQLPEVITVGDNMPQFRLQLLPLYSSDETIIATLVHSDLYMEANLDYEFKLTSAKYTVATARKRHEHITGRNEAVMNLSKPEWRNIIENYLLNIAVEAQCRFDFKQRCSEYKKWKLQQSNLKRPDMPPPSIIPRKNSTETKSLLKKALLKNIQLKNPNNNLDELMMRSSAATNQQGKNKVSLSKEEKATIWSQCQAQVYQRLGLDWQPDSVS>YDR279WMTVSNIGGEERLIILPDDYETSKTINTFTLPPPSNITSKPRIELFENINGKLYEIRSFQFGKGPSYSHEEDLANDKYHYTKENHPIKSTFIVNTSDPTDGYVFNSSKIHFCSLYDIAFSLIGFYYRNSVSADEQDYSNSSDTGENQKSNSKTNEKFLTVRDYHDFLTDNHDKNWENISLSRLKSGLAKVSETIEEAGDVYYKITSAMITQFLLGKVSKIVENFPPSIPTLKNAPTEIKQCYKVVMATNLLVSLIPRAAYHNLLTFSPTMDSGCLNPDIKASFIELENYETTNELQNAERELLMKSAMNVGLNSNGRVSLPVKKVTKKIVQNKKPKVAIGKGAIDGFFKRK>YDR280WMAKDIEISASESKFILEALRQNYRLDGRSFDQFRDVEITFGKEFGDVSVKMGNTKVHCRISCQIAQPYEDRPFEGLFVISTEISPMAGSQFENGNITGEDEVLCSRIIEKSVRRSGALDVEGLCIVAGSKCWAVRADVHFLDCDGGFIDASCIAVMAGLMHFKKPDITVHGEQIIVHPVNEREPVPLGILHIPICVTFSFFNPQDTEENIKGETNSEISIIDATLKEELLRDGVLTVTLNKNREVVQVSKAGGLPMDALTLMKCCHEAYSIIEKITDQILQLLKEDSEKRNKYAAMLTSENAREI>YDR281CMEDTSRCIDDVLKIGQQEKEIRQAEFSDAQGEREEVKCIDYTVDLEAGLPRHESSGKSNTLKQCYNAVLGFLEELIIVIIIVLLLYSLTMVGLFYVMTMTKFLF>YDR284CMNRVSFIKTPFNIGAKWRLEDVFLLIIMILLNYPVYYQQPFERQFYINDLTISHPYATTERVNNNMLFVYSFVVPSLTILIIGSILADRRHLIFILYTSLLGLSLAWFSTSFFTNFIKNWIGRLRPDFLDRCQPVEGLPLDTLFTAKDVCTTKNHERLLDGFRTTPSGHSSESFAGLGYLYFWLCGQLLTESPLMPLWRKMVAFLPLLGAALIALSRTQDYRHHFVDVILGSMLGYIMAHFFYRRIFPPIDDPLPFKPLMDDSDVTLEEAVTHQRIPDEELHPLSDEGM>YDR285WMSNFFRDSSMGFKPRPNIFAKLRVRDVDSDSSANTVVENSSNCLDVGSSIEGDDTFKKPHKTSTEQELITSMSLSQRNHGYSDDMEIGSPKKTTSTDQYNRILKNDVAAIENDTDEDFEITEVREVSEGVAKETKESHGDPNDSETTLKDSKMHEYTMTNGKAPLHTSINNSSTSSNDVLLEAFTNTQRICSNLKQELQKQQQDNAKLKVRLQSYASNSDKINEKVGKYKSCLETLQERIATLTSHKNNQETKLKDLRQNHQLYQRRISGFKTSIENLNKTINDLGKNKKEADAELMKKGKEIEYLKRELDDCSGQLSEEKIKNSSLIQEMGKNREEMIKSIENFFSEDKAHHLLQFNKFEERVHDLFEKKLQKHFDVAKDTLNVGLRNTTVELSSNTETMLKQQYEDIKENLEQKMSSSKDEMAKTINELSVTQKGLIMGVQEELLTSSGNIQTALVSEMNNTRQELLDDASQTAKNYASLENLVKAYKAEIVQSNEYEERIKHLESERSTLSSQKNQIISSLGTKEAQYEDLVKKLEAKNIEISQISGKEQSLTEKNENLSNELKKVQDQLEKLNNLNITTKSNYENKISSQNEIVKALVSENDTLKQRIQQLVEIKENEQKDHTTKLEAFQKNNEQLQKLNVEVVQLKAHELELEEQNRHLKNCLEKKETGVEESLSDVKTLKQQVIVLKSEKQDITAEKLELQDNLESLEEVTKNLQQKVQSQKRELEQKIKELEEIKNHKRNEPSKKGTQNFTKPSDSPKKNATTSNLFPNNSAAIHSPMKKCPKVDHISKSRINSSKETSKFNDEFDLSSSSNDDLELTNPSPIQIKPVRGKIKKGSNCMKPPISSRKKLLLVEDEDQSLKISKKRRRK>YDR286CMLRAFRCSIHTSRVLLHDAGVKLTFFSKPNCGLCDQAKEVIDDVFERKEFHNKAVSLEIVNITDRRNAKWWKEYCFDIPVLHIEKVGDPKSCTKILHFLEEDDISDKIRRMQSR>YDR287WMVLTRQVLEEVENTFIELLRSKIGPLVKSHAGTNFCSYDDKANGVDLVTALDKQIESIIKENLTAKYPSFKFIGEETYVKGVTKITNGPTFIVDPIDGTTNFIHGYPYSCTSLGLAEMGKPVVGVVFNPHLNQLFHASKGNGAFLNDQEIKVSKRPLILQKSLIALEGGSERTEGSQGNFDKKMNTYKNLLSESGAFVHGFRSAGSAAMNICYVASGMLDAYWEGGCWAWDVCAGWCILEEAGGIMVGGNCGEWNIPLDRRCYLAIRGGCESMEQKRFAESFWPHVAGELEY>YDR288WMSSIDNDSDVDLTEDLAVAKIVKENPVARKMVRYILSRGESQNSIITRNKLQSVIHEAAREENIAKPSFSKMFMDINAILYNVYGFELQGLPSKNNMNAGGNGSNSNTNKSMPEPLGHRAQKFILLNNVPHSKNFDDFKILQSAHTYEELIVTGEYIGDDIASGTSNTLESKLSTDRDLVYKGVLSVILCIVFFSKNNILHQELIKFLETFGIPSDGSKIAILNITIEDLIKSLEKREYIVRLEEKSDTDGEVISYRIGRRTQAELGLESLEKLVQEIMGLEKEQTKSLHDDIIKSIGDSYSI>YDR289CMPFSSEQFTTKLNTLEDSQESISSASKWLLLQYRDAPKVAEMWKEYMLRPSVNTRRKLLGLYLMNHVVQQAKGQKIIQFQDSFGKVAAEVLGRINQEFPRDLKKKLSRVVNILKERNIFSKQVVNDIERSLKTESSPVEALVLPQKLKDFAKDYEKLVKMHHNVCAMKMRFDKSSDELDPSSSVYEENFKTISKIGNMAKDIINESILKRESGIHKLQSTLDDEKRHLDEEQNMLSEIEFVLSAKDPSRLNKNVDEDNIIPTYEVGDGDDDDDDGDNDDDDDDDDDDKNYDDRSNDSNYGVTNISTTDKKNEVVEKTDSEHKNSTHNPSDNQFGMKRTHDMIGHDDANDIPEKKVHLDSKTSEDGTFNSEDGHYELDIEGHVGAQTDEGVENSGGVSSSIQDLLSKLAN>YDR293CMSKNSNVNNNRSQEPNNMFVQTTGGGKNAPKQIHVAHRRSQSELTNLMIEQFTLQKQLEQVQAQQQQLMAQQQQLAQQTGQYLSGNSGSNNHFTPQPPHPHYNSNGNSPGMSAGGSRSRTHSRNNSGYYHNSYDNNNNSNNPGSNSHRKTSSQSSIYGHSRRHSLGLNEAKKAAAEEQAKRISGGEAGVTVKIDSVQADSGSNSTTEQSDFKFPPPPNAHQGHRRATSNLSPPSFKFPPNSHGDNDDEFIATSSTHRRSKTRNNEYSPGINSNWRNQSQQPQQQLSPFRHRGSNSRDYNSFNTLEPPAIFQQGHKHRASNSSVHSFSSQGNNNGGGRKSLFAPYLPQANIPELIQEGRLVAGILRVNKKNRSDAWVSTDGALDADIYICGSKDRNRALEGDLVAVELLVVDDVWESKKEKEEKKRRKDASMQHDLIPLNSSDDYHNDASVTAATSNNFLSSPSSSDSLSKDDLSVRRKRSSTINNDSDSLSSPTKSGVRRRSSLKQRPTQKKNDDVEVEGQSLLLVEEEEINDKYKPLYAGHVVAVLDRIPGQLFSGTLGLLRPSQQANSDNNKPPQSPKIAWFKPTDKKVPLIAIPTELAPKDFVENADKYSEKLFVASIKRWPITSLHPFGILVSELGDIHDPDTEIDSILRDNNFLSNEYLDQKNPQKEKPSFQPLPLTAESLEYRRNFTDTNEYNIFAISELGWVSEFALHVRNNGNGTLELGCHVVDVTSHIEEGSSVDRRARKRSSAVFMPQKLVNLLPQSFNDELSLAPGKESATLSVVYTLDSSTLRIKSTWVGESTISPSNILSLEQLDEKLSTGSPTSYLSTVQEIARSFYARRINDPEATLLPTLSLLESLDDEKVKVDLNILDRTLGFVVINEIKRKVNSTVAEKIYTKLGDLALLRRQMQPIATKMASFRKKIQNFGYNFDTNTADELIKGVLKIKDDDVRVGIEILLFKTMPRARYFIAGKVDPDQYGHYALNLPIYTHFTAPMRRYADHVVHRQLKAVIHDTPYTEDMEALKITSEYCNFKKDCAYQAQEQAIHLLLCKTINDMGNTTGQLLTMATVLQVYESSFDVFIPEFGIEKRVHGDQLPLIKAEFDGTNRVLELHWQPGVDSATFIPADEKNPKSYRNSIKNKFRSTAAEIANIELDKEAESEPLISDPLSKELSDLHLTVPNLRLPSASDNKQNALEKFISTTETRIENDNYIQEIHELQKIPILLRAEVGMALPCLTVRALNPFMKRV>YDR294CMSGVSNKTVSINGWYGMPIHLLREEGDFAQFMILTINELKIAIHGYLRNTPWYNMLKDYLFVIFCYKLISNFFYLLKVYGPVRLAVRTYEHSSRRLFRWLLDSPFLRGTVEKEVTKVKQSIEDELIRSDSQLMNFPQLPSNGIPQDDVIEELNKLNDLIPHTQWKEGKVSGAVYHGGDDLIHLQTIAYEKYCVANQLHPDVFPAVRKMESEVVSMVLRMFNAPSDTGCGTTTSGGTESLLLACLSAKMYALHHRGITEPEIIAPVTAHAGFDKAAYYFGMKLRHVELDPTTYQVDLGKVKKFINKNTILLVGSAPNFPHGIADDIEGLGKIAQKYKLPLHVDSCLGSFIVSFMEKAGYKNLPLLDFRVPGVTSISCDTHKYGFAPKGSSVIMYRNSDLRMHQYYVNPAWTGGLYGSPTLAGSRPGAIVVGCWATMVNMGENGYIESCQEIVGAAMKFKKYIQENIPDLNIMGNPRYSVISFSSKTLNIHELSDRLSKKGWHFNALQKPVALHMAFTRLSAHVVDEICDILRTTVQELKSESNSKPSPDGTSALYGVAGSVKTAGVADKLIVGFLDALYKLGPGEDTATK>YDR296WMKVNHSISRFRPASWFEKTKIIPPQVYIFRNLEYGQVLYSQFPNFSQTQVDKLFVRPNWSNRKPSLRRDIWKCMCVVNLQNYKQSVHLYQNLCRLRYLRDVAQRKESDKLRKKDSNGHVWYSGQYRPTYCQEAVADLRESLLKVFENATPAEKQTVPAKKPSIYWEDPWRMGDKDKHWNYDVFNALGLEHKLIQRVGNIAREESVILKELAKLESHPTEQTEVSSQ>YDR297WMNVTSNATAAGSFPLAFGLKTSFGFMHYAKAPAINLRPKESLLPEMSDGVLALVAPVVAYWALSGIFHVIDTFHLAEKYRIHPSEEVAKRNKASRMHVFLEVILQHIIQTIVGLIFMHFEPIYMTGFEENAMWKLRADLPRIIPDAAIYYGYMYGMSALKIFAGFLFVDTWQYFLHRLMHMNKTLYKWFHSVHHELYVPYAYGALFNNPVEGFLLDTLGTGIAMTLTHLTHREQIILFTFATMKTVDDHCGYALPLDPFQWLFPNNAVYHDIHHQQFGIKTNFAQPFFTFWDNLFQTNFKGFEEYQKKQRRVTIDKYKEFLQERELEKKEKLKNFKAMNAAENEVKKEK>YDR298CMFNRVFTRSFASSLRAAASKAAAPPPVRLFGVEGTYATALYQAAAKNSSIDAAFQSLQKVESTVKKNPKLGHLLLNPALSLKDRNSVIDAIVETHKNLDGYVVNLLKVLSENNRLGCFEKIASDFGVLNDAHNGLLKGTVTSAEPLDPKSFKRIEKALSASKLVGQGKSLKLENVVKPEIKGGLIVELGDKTVDLSISTKIQKLNKVLEDSI>YDR299WMEKSLADQISDIAIKPVNKDFDIEDEENASLFQHNEKNGESDLSDYGNSNTEETKKAHYLEVEKSKLRAEKGLELNDPKYTGVKGSRQALYEEVSENEDEEEEEEEEEEKEEDALSFRTDSEDEEVEIDEEESDADGGETEEAQQKRHALSKLIQQETKQAINKLSQSVQRDASKGYSILQQTKLFDNIIDLRIKLQKAVIAANKLPLTTESWEEAKMDDSEETKRLLKENEKLFNNLFNRLINFRIKFQLGDHITQNEEVAKHKLSKKRSLKELYQETNSLDSELKEYRTAVLNKWSTKVSSASGNAALSSNKFKAINLPADVQVENQLSDMSRLMKRTKLNRRNITPLYFQKDCANGRLPELISPVVKDSVDDNENSDDGLDIPKNYDPRRKDNNAIDITENPYVFDDEDFYRVLLNDLIDKKISNAHNSESAAITITSTNARSNNKLKKNIDTKASKGRKLNYSVQDPIANYEAPITSGYKWSDDQIDEFFAGLLGQRVNFNENEDEEQHARIENDEELEAVKNDDIQIFG>YDR300CMKDANESKSYTIVIKLGSSSLVDEKTKEPKLAIMSLIVETVVKLRRMGHKVIIVSSGGIAVGLRTMRMNKRPKHLAEVQAIAAIGQGRLIGRWDLLFSQFDQRIAQILLTRNDILDWTQYKNAQNTINELLNMGVIPIVNENDTLSVREIKFGDNDTLSAITSALIHADYLFLLTDVDCLYTDNPRTNPDAMPILVVPDLSKGLPGVNTAGGSGSDVGTGGMETKLVAADLATNAGVHTLIMKSDTPANIGRIVEYMQTLELDDENKVKQAYNGDLTDLQKREFEKLKALNVPLHTKFIANDNKHHLKNREFWILHGLVSKGAVVIDQGAYAALTRKNKAGLLPAGVIDVQGTFHELECVDIKVGKKLPDGTLDPDFPLQTVGKARCNYTSSELTKIKGLHSDQIEEELGYNDSEYVAHRENLAFPPR>YDR304CMKLQFFSFITLFACLFTTAIFAKEDTAEDPEITHKVYFDINHGDKQIGRIVMGLYGLTTPQTVENFYQLTISRDPKMGYLNSIFHRVIPNFMIQGGDFTHRSGIGGKSIFGNTFKDENFDVKHDKPGRLSMANRGKNTNGSQFFITTVPCPWLDGKHVVFGEVLDGMDVVHYIENVKTDSRNMPVKEVIIVESGELETVPLDNKDAAKLQEEIKAEASEAAHDEL>YDR306CMANKSRPKKIKAPYRKYVAGEGFSSTRNDNKAKEFTITIPEDAELIETPQGSYYYDETNDTIVKLTRLSNEKKDKKGRKQSPSSSSTSSSKGEKNGKVIESEEARMHSVSVKMVLPWEIQHRIIHYLDIPEKEEKLNKTANGKKTTTGINMNYLLVCRNWYAMCLPKLYYAPALTSKNFNGFVDTIIINKKKNLGHYVFELNLSTILQSGRNSFVSKLLRRCCSNLTKFIAPQTSFGYAPLISLKSCHDLKFLDLGLVSETVKLKELFSAIKNFTKLTHLSFPRSSIDCQGFQDIQWPQNLRYLKLSGGITNEFVIDTKWPTTITTLEFSYCPQITELSIYSLLSQIGDNLKHLFFHYPMPSLAENSLDHVFTYCANLISLQLMVDYCSKWCFSEFMLSKLVEYDRPLKTLYLECSGSLGLASKIHPDDLTIAILESRLPCLKNICVSPKLGWNMKSDEVADLVVSLEDQDGSLYLNY>YDR308CMTDRLTQLQICLDQMTEQFCATLNYIDKNHGFERLTVNEPQMSDKHATVVPPEEFSNTIDELSTDIILKTRQINKLIDSLPGVDVSAEEQLRKIDMLQKKLVEVEDEKIEAIKKKEKLLRHVDSLIEDFVDGIANSKKST>YDR309CMTSASITNTGNETMNLPQMRSIWLDEDEEAEKLYGLQAQQFMGSDDEENLGITFINSDKPVLSNKKNIELPPLSPNSHPSCHHRRSNSNSAKSKESSSSSSSANKTNHKKVFLKLNLLKKKLLGAQPDIRGKGISTPFDFQHISHADTRNGFQDEQLQEPSSLSTEIKDDYTSSSSKRDSKSLNKAFVTERIPANRESKLISRSHENKTSRLSVARSISVTSSNYSKNTQGNNHSINGRVVSTSTMATSIFEYSPNASPKQFKNKSHALGHRYTNSTDSSESSLDFLKNYNFPTLLEDKPILDFLPRSQRSSAYRSLLETPNSNKDSAKAFFPSRQSPLPKRRNSIATPSPQSKFSYSDSPVNHRKSFDDVLYSFNQLEPLQT>YDR311WMSHSGAAIFEKVSGIIAINEDVSPAELTWRSTDGDKVHTVVLSTIDKLQATPASSEKMMLRLIGKVDESKKRKDNEGNEVVPKPQRHMFSFNNRTVMDNIKMTLQQIISRYKDADIYEEKRRREESAQHTETPMSSSSVTAGTPTPHLDTPQLNNGAPLINTAKLDDSLSKEKLLTNLKLQQSLLKGNKVLMKVFQETVINAGLPPSEFWSTRIPLLRAFALSTSQKVGPYNVLSTIKPVASSENKVNVNLSREKILNIFENYPIVKKAYTDNVPKNFKEPEFWARFFSSKLFRKLRGEKIMQNDRGDVIIDRYLTLDQEFDRKDDDMLLHPVKKIIDLDGNIQDDPVVRGNRPDFTMQPGVDINGNSDGTVDILKGMNRLSEKMIMALKNEYSRTNLQNKSNITNDEEDEDNDERNELKIDDLNESYKTNYAIIHLKRNAHEKTTDNDAKSSADSIKNADLKVSNQQMLQQLSLVMDNLINKLDLNQVVPNNEVSNKINKRVITAIKINAKQAKHNNVNSALGSFVDNTSQANELEVKSTLPIDLLESCRMLHTTCCEFLKHFYIHFQSGEQKQASTVKKLYNHLKDCIEKLNELFQDVLNGDGESMSNTCTAYLKPVLNSITLATHKYDEYFNEYNNNSN>YDR312WMAKRRQKKRTHAQITPEQERDIPKSMVIRVGQTSLANHSLNQLVKDFRQIMQPHTAVKLKERKSNKLKDFVVMCGPLGVTHLFMFTQSEKTGNVSLKIARTPQGPTVTFQVLDYSLGRDIKKFLKRPKSLNNDDVLNPPLLVLNGFSTSKRSDEDDQDVNVEKVIVSMFQNIFPPLNPARTSLNSIKRIFMINKDRETGEISMRHYFIDIREVEISRNLKRLYKAKNNLSKTVPNLHRKEDISSLILDHDLGAYTSESEIEDDAIVRVVDNQDVKAKHSQTSLSQKTPVKMTDNEEREKGIEEEDVEMEEPKPSENSQPTPRKKAIKLTELGPRLTLKLVKIEDGICSGKVLHHEFVQKSSEEIKALEKRHAAKMRLKEQRRKEQEENIAKKKAVKDAKKQRKLERRKARAEEQGEGQGKDGAMSDDGSSSSEDEHYSDVPEDLDSDLFSEVE>YDR314CMAKRLLESSQNDQANRKNSKIEKKEVSFYEEEETDDSFDSFYQDEEDDLSDIDWEEVSLDGSLTVTFGNIRRDREKVSKYKRKHNKKAFNYQRLKYGLHLIMIPFMLFLLKSRMKWIDDERLNRRLRRSVPKLIGKKFKDWDVRDPAFKMDSLRTLLLGLVLWFRSNYKMNSNGIRQNFNRLQYLIKYADNQNENSISESTYKKVLENQQEFYGNRPLINHGVEDIRKMAKRKMANRDILTLFFFIILENVLPGPKKLYLCFALPLHDYDIRCNKVKWQIEHGIGKVPNRFDSDLIQPYFWIELEVPTLSDGELYIIDPIAHLGEREMVLKTREDQFVPTYQPSVDMKYNLNQKFHYVVRINHAEKVLQDVSPRYVPNVCYRYFELSESSPILKSKHYTSYQYLSKWLKVLNKKKASVHHYAIMKKIALTNFTLPKSVTEIKRTDNFVIPSLLKSNEVLKACAKQAATFTKGDNSQEPIFWRRDVIQLKSKQHWAILGRSILPNAQPLKRKKYLPMRERMVRNLDKYVIKELFSYEQTMKSPKYPSTYCDHLGQEHVITDLSHYKNKFGNIEIYSKETKPDGFELIPLSKEVDIKCLIKEYNKGKRKMQKIKYLDVVSGFDFKQKKGHAIPKIESILVKETDYKAVQLLKQQTKVLLGLSFWDILLRKLRVNDRLNAEYGNVGNNEETLDDH>YDR317WMSYSVNTFTDYVLLFGSSSLVGKGILENLLDINLYIKNVSDLQGKLDSLSEIKGNVVLNKHVFCVNRRCINEEKSFMKTIDYINMRSVTWQGGRYYLRSRKEKDTEKVPSSPNTFCYDNFEEGFIKNTPEERGKDGYSFVYNQKQFSYTLHYACGKEKGIEIICNFTVTQLIIPRSETWPKLLPRIFSGTQKLEKFDIDNKNYVPGRSLPSLCDISTMVCSLGSTSARVRRTQVPSSFADYYLPFNLAQEFTNTTNKRLVVTTAFNNDFLSKTFEYFRIKAKLENDLDEALPNKLKELVILRPGPMCGQHGNPINVELGKENSTFLEKIFYYPHYLLVYKKKYISEARRIGLRTKLSEIIASSIYRMPGSALLGYAVPVSKVSYVASLMAIERKSKEAGPKLEVISSYQIDMIV>YDR319CMIRQLNYWSRKAYLIYPFQVFVGALLSIVVSSETLNHQKETCALLKSSNIFNVIFAYKANQLWPFLFFSLAFLQIYFHYLARMDILPLPISSTETSSSYLTYTNHWPLLKNRIISIMITQYACKFVLKYLLLFLNFQFIDHVFIWTGGECSSGSKTTSAEKCRLENGKWDGGFDISGHFCFLVSISMILWMELHLFSRFVQAEDMFWVVNKWVRACLAIVCAVLVIWICILWVTAIYYHTILEKVLGCLMGFICPVFIYHILPKIGILHNYLYL>YDR320C-AMENPHEQVQANILSRIIGNVKRLNESVAILNQELVTINNRNKNLEIMGAICDNYHSSVQFNLEATNNKKPPL>YDR321WMKSDSVEITTICPDVENSQFVVQSNCPETIPEILKSQNAAVNGSGIACQQRSLPRIKILGTGGTIASKAIDSSQTAGYHVDLTIQDLLDAIPDISKVCDIEYEQLCNVDSKDINEDILYKIYKGVSESLQAFDGIVITHGTDTLSETAFFIESTIDAGDVPIVFVGSMRPSTSVSADGPMNLYQAICIASNPKSRGRGVLVSLNDQISSGYYITKTNANSLDSFNVRQGYLGNFVNNEIHYYYPPVKPQGCHKFKLRVDGKHFKLPEVCILYAHQAFPPAIVNLVADKYDGIVLATMGAGSLPEEVNETCMKLSLPIVYSKRSMDGMVPIANVPKKGSKEDNLIASGYLSPEKSRILLQLCLAGNYTLEEIKHVFTGVYGG>YDR322C-AMSTVNVLRYSALGLGLFFGFRNDMILKCNAKKKEEQAQYEEKLKLVEEAKKEYAKLHPVVTPKDVPANASFNLEDPNIDFERVILNAVESLKEAST>YDR322WMLRRSIHTTKILQKPNATSHIWSDFTTRPSSLSIQSSKVKNYLFQKKASLDPPSISRRSNRIKYSPPEHIDEIFRMSYDFLEQRSSKFYELANKTKNPLKKDALLIKAEINNPEVQYNFQFNNKLNNVKDIIDYDVPVYRHLGKQHWESYGQMLLMQRLETLAAIPDTLPTLVPRAEVNIKFPFSTGVNKWIEPGEFLSSNVTSMRPIFKIQEYELVNVEKQLYTVLIVNPDVPDLSNDSFKTALCYGLVNINLTYNDNLIDPRKFHSSNIIADYLPPVPEKNAGKQRFVVWVFRQPLIEDKQGPNMLEIDRKELSRDDFDIRQFTKKYNLTAIGAHIWRSEWDAKVAAVREKYGLPPGRVFSRVRR>YDR325WMQDPDGIDINTKIFNSVAEVFQKAQGSYAGHRKHIAVLKKIQSKAVEQGYEDAFNFWFDKLVTKILPLKKNEIIGDRIVKLVAAFIASLERELILAKKQNYKLTNDEEGIFSRFVDQFIRHVLRGVESPDKNVRFRVLQLLAVIMDNIGEIDESLFNLLILSLNKRIYDREPTVRIQAVFCLTKFQDEEQTEHLTELSDNEENFEATRTLVASIQNDPSAEVRRAAMLNLINDNNTRPYILERARDVNIVNRRLVYSRILKSMGRKCFDDIEPHIFDQLIEWGLEDRELSVRNACKRLIAHDWLNALDGDLIELLEKLDVSRSSVCVKAIEALFQSRPDILSKIKFPESIWKDFTVEIAFLFRAIYLYCLDNNITEMLEENFPEASKLSEHLNHYILLRYHHNDISNDSQSHFDYNTLEFIIEQLSIAAERYDYSDEVGRRSMLTVVRNMLALTTLSEPLIKIGIRVMKSLSINEKDFVTMAIEIINDIRDDDIEKQEQEEKIKSKKINRRNETSVDEEDENGTHNDEVNEDEEDDNISSFHSAVENLVQGNGNVSESDIINNLPPEKEASSATIVLCLTRSSYMLELVNTPLTENILIASLMDTLITPAVRNTAPNIRELGVKNLGLCCLLDVKLAIDNMYILGMCVSKGNASLKYIALQVIVDIFSVHGNTVVDGEGKVDSISLHKIFYKVLKNNGLPECQVIAAEGLCKLFLADVFTDDDLFETLVLSYFSPINSSNEALVQAFAFCIPVYCFSHPAHQQRMSRTAADILLRLCVLWDDLQSSVIPEVDREAMLKPNIIFQQLLFWTDPRNLVNQTGSTKKDTVQLTFLIDVLKIYAQIEKKEIKKMIITNINAIFLSSEQDYSTLKELLEYSDDIAENDNLDNVSKNALDKLRNNLNSLIEEINERSETQTKDENNTANDQYSSILGNSFNKSSNDTIEHAADITDGNNTELTKTTVNISAVDNTTEQSNSRKRTRSEAEQIDTSKNLENMSIQDTSTVAKNVSFVLPDEKSDAMSIDEEDKDSESFSEVC>YDR326CMRDEATRKKRSFSDGHFFKKLKLMSRKKQPVMERSKTTRTRKESTNSAAKSSLSLRRANNGRKTIAKRRVLTDIGSTNEGVAGNSGSNSPAQYSHTPHFSDSIPPLPLELPDIVSIRSSRSHISNKSNKNKHGIDLTFIPRRSLQNSKAGLKKPNTSPQGYFNIPVTIDRASEKVKHTDTKNTFNSSSSENERPVLSILQKDDSQSSSHPAIDSMSAPNNINNNNDIENSSNSLFDTILSIAHSAISHVPKISALNTEIQREFSHSGESHTGSTRHPYFHIHHAQQQHPLSQQQGPLPVSENANQNPNDTVLIHSPSANTAHRSSSFLRHLDYLLSPTSGPASDKHTQVEEGDDEEELSPLSKAFLSPSTQLVPTNTSTTPLSGSLTPNNRNVNANSNSETENDNDRDDRSNVGKVKFQPLKVHEPAISTFGKGNLTLEAVAGSSDIDNTTIDLDENNTNNNPNASSTNLSHISKSNVNNNLGPKELNTSYRNSTYIDMARFENSQSNLSSHRARSKTLPANKALENAVGDEGNSKRNSRYSSYSNDMAFDDADERKFRSMSKKFLNRRSFSPSNLGNKVIPGINLRNSFNKNRNSSSDFFSTNQGQQMPRTSTAGSGNIHAIMGLDSGNDDFKLEGIEYASEKKNSEFHTLFKDCDINPNEKLIVDHSCALSRDILLQGRMYISDAHIGFFSNILGWVSTVFIPFKEIVQIEKKTTAGIFPNGIVIDTLHTKYIFASFMSRDATFDLITDVWNQIILGKKYRNGFGNNDDGTISDSSSAFFDDSDDNDDDGDLDDDDPDINSTDMTSSDDIDADVFNESNDLGKNQKSTNYLLGPNKHSPTTADFKPSNNDHLVIEANINAPLGKVVNLLYGEDVSYYERILKAQKNFEISPIPNNFLTKKIRDYAYTKPLSGSIGPSKTKCLITDTLEHYDLEDYVKVLSITKNPDVPSGNIFSVKTVFLFSWDKNNSTKLTVYNSVDWTGKSWIKSMIEKGTFDGVADTTKIMISEIKKILSDEDSNINSKHQASNNESEEEIINLPTIGPPVHDPTEPDFQKGKDDTVIDEKINIPVPLGTVFSLLYGDDTSYIKKIIENQNNFNVCDIPKFVNNAREITYTKKLNNSFGPKQTKCIVTETIEHMDLNSFFMVKQIVRSPDVPYGSSFSVHTRFFYSWGDHNTTNMKVVTNVVWTGKSMLKGTIEKGSIDGQRSSTKQLVDDLKKIISNASSTKKKSRRRGKTVNKRKSSPSTIKNEKNEENFEDTSTKNSFFSAFSMLQQVNITSVQGIMTIISFFICLIFFFRLLFHSKNTSNIQIITPGTILINGNEYNYVPNFKTLYHVYEDNIIKDARRKDSNKNNIVTDTEGLIWDWLIDRGNGTVQNSVLSNHIKESNNKKVKLVNGVSDHKIQQLVESIKITELQLQEMKELLAQTDNTSATNQLL>YDR328CMVTSNVVLVSGEGERFTVDKKIAERSLLLKNYLNDMHDSNLQNNSDSESDSDSETNHKSKDNNNGDDDDEDDDEIVMPVPNVRSSVLQKVIEWAEHHRDSNFPDEDDDDSRKSAPVDSWDREFLKVDQEMLYEIILAANYLNIKPLLDAGCKVVAEMIRGRSPEEIRRTFNIVNDFTPEEEAAIRRENEWAEDR>YDR329CMAPNQRSRSLLQRHRGKVLISLTGIAALFTTGSVVVFFVKRWLYKQQLRITEQHFIKEQIKRRFEQTQEDSLYTIYELLPVWRMVLNENDLNLDSIVTQLKDQKNQLTRAKSSESRESSPLKSKAELWNELELKSLIKLVTVTYTVSSLILLTRLQLNILTRNEYLDSAIKLTMQQENCNKLQNRFYNWVTSWWSDPEDKADDAMVMAAKKSKKEGQEVYINEQAFLSLSWWILNKGWLSYNEIITNQIEIEFDGIHPRDTLTLEEFSSRLTNIFRNTNSQIFQQNNNNLTSILLPKDSSGQEFLLSQTLDADALTSFHSNTLVFNQLVNELTQCIESTATSIVLESLINESFHFIMNKVGIKTIAKKKPGQEDQQQYQMAVFAMSMKDCCQEMLQTTAGSSHSGSVNEYLATLDSVQPLDDLSASVYSNFGVSSSFSFKP>YDR331WMRIAMHLPLLLLYIFLLPLSGANNTDAAHEVIATNTNNWAVLVSTSRFWFNYRHMANVLSMYRTVKRLGIPDSQIILMLSDDVACNSRNLFPGSVFNNKDHAIDLYGDSVEVDYRGYEVTVENFIRLLTDRWTEDHPKSKRLLTDENSNIFIYMTGHGGDDFLKFQDAEEIASEDIADAFQQMYEKKRYNEIFFMIDTCQANTMYSKFYSPNILAVGSSEMDESSYSHHSDVEIGVAVIDRFTYYCLDFLEQIDKNSTLTLQDLFDSFTFEKIHSHVGVRTDLFDRNPSEVLITDFFANVQNVIPDDSKPLSVSHYHHYKDHIDTAQYELNNNVLDLALETYRKNNQSSKIEKKIKDIKSTSVLDVDIDSNECFFTSFKQSATIILALIVTILWFMLRGNTAKATYDLYTN>YDR332WMTSTLATRLSTYSISLILQRIKIIKRCYSAPVLRDYQQDAIDACVNSIRQGTKRIGVSLATGGGKTVIFSNLINQLRQNYFKERQGNFKSLILVHRRELALQATATLKKIFPDLKVHIEMGKYDCDIEDSDVIVASVQTLIRRLHKYDTNSVNLIIIDEAHHSVANSYRSILDHFKASTAETKIPVIGFSATFERADKRALSMVMDKIVYHRGILEMIDDKWLCEAKFTSVKIEADLSDVKSTADDFQLAPLSSLMNTKEINEVILKTYLHKKQEKSLKSTLLFGVDKAHVQSLHKLFKDNGINTDYVTSDTKQIERDNIIQKFKNGETEVLMNCGIFTEGTDMPNIDCILLCRPTKSRSLLIQMIGRGLRLHHSKDHCHIIDFIGASSVGVVSAPTLLGIRSDDIEFDDATVEDLKAIQGEIIAKQQKIDERLRALFQTDEAAMENVTERNSVADWIHSANSVDLTLCSFDSFRNFTQSNNSYPSGKEFDEASEAVKEMELLMNSQYPWVKFASNAWGLPLKGKNHLRIYKEKSEDKLSMVYHLKMYRQLPCFITNKYADYVPKSIIKDANLWNVMSKVEKIINTLNSDLEGQTMQYQAISSKYSKWRQTVPTSKQRDFVFRKLKKVYGESSKDFIRLSLDDVTTYVNTKMTKGDASNLIFASSLAPVYPLKSLLRILEYQKRRSFIK>YDR336WMEQLCKRYVHTPAAFIQNIVANTKRTTLATQLSVEKAKKKVPKTALKKKLNSRPKERLPNWLKLNDVFNIHYEKPSNSDINKVNRFFNKAKVEFEWCAASFDDIPENPFLNKKSHKDILKDHGECGTTLIDTLPEVIFLGGTNVGKSSILNNITTSHVSRDLGSLARVSKTTGFTKTLNCYNVGNRLRMIDSPGYGFNSSKEQGKVTLQYLLERKQLVRCFLLLAGDKEINNTDNMIIQYIHEHGVPFEVVFTKMDKVKDLNKFKKKVMSSGLMDLPTLPRLVLTNSLTSSTSPKRFGIDLLRYVIFQSCGLIL>YDR339CMGKAKKTRKFGLVKRTLNTKKDQRLKKNQENIKTKEDPELTRNIPQVSSALFFQYNQAIKPPYQVLIDTNFINFSIQKKVDIVRGMMDCLLAKCNPLITDCVMAELEKLGPKYRIALKLARDPRIKRLSCSHKGTYADDCLVHRVLQHKCYIVATNDAGLKQRIRKIPGIPLMSVGGHAYVIEKLPDVF>YDR345CMNSTPDLISPQKSSENSNADLPSNSSQVMNMPEEKGVQDDFQAEADQVLTNPNTGKGAYVTVSICCVMVAFGGFVFGWDTGTISGFVAQTDFLRRFGMKHKDGSYYLSKVRTGLIVSIFNIGCAIGGIILAKLGDMYGRKMGLIVVVVIYIIGIIIQIASINKWYQYFIGRIISGLGVGGIAVLSPMLISEVAPKEMRGTLVSCYQLMITLGIFLGYCTNFGTKNYSNSVQWRVPLGLCFAWALFMIGGMTFVPESPRYLVEAGQIDEARASLSKVNKVAPDHPFIQQELEVIEASVEEARAAGSASWGELFTGKPAMFKRTMMGIMIQSLQQLTGDNYFFYYGTTVFNAVGMSDSFETSIVFGVVNFFSTCCSLYTVDRFGRRNCLLYGAIGMVCCYVVYASVGVTRLWPNGEGNGSSKGAGNCMIVFACFYIFCFATTWAPIAYVVISETFPLRVKSKAMSIATAANWLWGFLIGFFTPFITGAINFYYGYVFMGCMVFAYFYVFFFVPETKGLTLEEVNDMYAEGVLPWKSASWVPTSQRGANYDADALMHDDQPFYKKMFGKK>YDR346CMLKWIKGGISAVTGMAEPEYGKDYIHSVADRVKNKQPYRETSREDFFWQAPDHTNVESVIFYFSDLKTGIFGFAQVIHSNIIGLHTASQFTFRIFDSKNPEDLNIWTSTKLENFYIEGPNFYADNLSVELSEDGESYHIQSSVCDLSVVDLHIRRLTPGAKIGDDPATYYGNNINEPWGSMRHVFWPRNACHGTIKVKKEVIPESDEEESSADEDDNEDEDEESGDSEEESGSEEESDSEEVEITYEDRTITFKEEDPAISTFIMAFQGMKPHHAAKAWNFMFFHSEKYSAVLMEFTTPKSYANTKISAGIITDDKEVLAMTTNNLVEHLNSEIDSVGWKVPKDIKITFKGINTKVKDEQLESENGTEQALQGEDEKEDEKEDEEEEEYKNVAEENKICAVVEGPLNNLVERIDVMGEIPSFVKNIVSGVAGTKPFIYQYADPKSSTLQINGGEKIHGVAWTEVTFISESDVISEESYNEA>YDR348CMENRNSSTSSRPFSVNNPFRNATVDSSINQYKNDSQFQEWAKNQSRTNSFDMPQLNTRTSSQLSFPNIPEDEPQRNADQQGAFYSGLESFSSGSLSPPSRPLSSKNPFLDDVSSATDFRRSPPPVSRNKNHPTAKEEKEQLRQRYLEESDVSTVGNTRENTDLPPSYEEITSTNGSRRAYPKEKVSRPSSHREHSNSGTYISRRSSSHHHREASSSSTPSKKGKRKSKVIVPKNVDTIDKLDVTGLFGGSFHHDGPFDAVTPHRNKNNKAAPVLAFPVDGPNSTIGGASTKKSALDEVFGRDDTDDSDIYQYSSQTLRRGGDTQDAIKANVGNVQQMDAKNKTELVHGPVTAGLGSSTFLDGAPASSAAIRNDIKAHSYHNRNGGLQRNKSLSQRLGLGGSGDSNAPMTGVRRNLSLSRDNYDVGHSNEGVRRSKTVNSPNRTHKSNYTTDFDGQDDHNEDEEDVYLGVRYNEPNMKKKSTGSKLLSRVKSLKVGRKSQ>YDR350CMLKCICRVYSQPLAQMVTSPLFKHMGSAGTYTILPITNLRHLSTKNCPLKIKSNRSEPLQFGDFERQVPCSRKSGSSKNVQKRLYELRQLKTVLSETFGVTEYASFFESLRNALHINNCSENEKKKLLYDIILHQHELYPEVARKIGFYLPGEVHRWFWYHIPKSESFNHYLFLLKSDVLLFTSNYCTRFTNRLIKGTEMERQLATFQIFLHDETNIKFIMEKVLKLHTFDSLIALVNGLVKAKNFRFIKVFIQALLQKLEQHCYSGKDGAKQKNLRYVKFNNTLLYYLLKSGNVELFIKTFQEELKFIVSSGLLNHIDGNEHILNFPIHHYLNLLRISNRQEELFNVISCLQSSPLMKYKLFKEFLMGELIASFQAFRDPKLVCKYLLSSYSSKASANILNALGIWGWLYHSKSTTLTAPTLARELKNKNNILPNTMRIGSPVTVPILTELYRSLLSSSSVSLESGQFKNCLLDLYYKYKSFLSEEAHKYRYWRNDTGILNVFLNYIRFQAREPRLAYNVLLDFYSQPFAKKVVLTTTLCPFSIVAYKNHTLTQAELSELLQVMHKNGVPLTFKFCSAMVMHYVKMRDEKEHAPGTIRYSSGALKSGIWL>YDR352WMSCSNGIWPTVSNLCGSLSFFTSVISLFPQIIETYRDKSVDGLSPYFLLAWLCGDITSLIGAKLTGQLLFQILLAIYFLLNDSFVCGQYYYYGVLHENKLATVGHEPKPLLPELVENGELLREEEDMIQGGSSAESPRSSRRRSAITAALAIAHTISTASAYPLNVGSTQSQVGPPGDGKNSQLGTILSWIGASFYVGARIPQLIKNYNRKSTDGLSPFLFATTLLCNITYNLSIFTSCRFLDNQNKREFIVNELPFIFGSAGTIAFDLIYFYQYYILYATDMQLRELERELYSPEEDSAAQLVTERTSLLSGETQT>YDR353WMVHNKVTIIGSGPAAHTAAIYLARAEIKPILYEGMMANGIAAGGQLTTTTEIENFPGFPDGLTGSELMDRMREQSTKFGTEIITETVSKVDLSSKPFKLWTEFNEDAEPVTTDAIILATGASAKRMHLPGEETYWQKGISACAVCDGAVPIFRNKPLAVIGGGDSACEEAQFLTKYGSKVFMLVRKDHLRASTIMQKRAEKNEKIEILYNTVALEAKGDGKLLNALRIKNTKKNEETDLPVSGLFYAIGHTPATKIVAGQVDTDEAGYIKTVPGSSLTSVPGFFAAGDVQDSKYRQAITSAGSGCMAALDAEKYLTSLE>YDR354WMSEATLLSYTKKLLASPPQLSSTDLHDALLVILSLLQKCDTNSDESLSIYTKVSSFLTALRVTKLDHKAEYIAEAAKAVLRHSDLVDLPLPKKDELHPEDGPVILDIVGTGGDGQNTFNVSTSAAIVASGIQGLKICKHGGKASTSNSGAGDLIGTLGCDMFKVNSSTVPKLWPDNTFMFLLAPFFHHGMGHVSKIRKFLGIPTVFNVLGPLLHPVSHVNKRILGVYSKELAPEYAKAAALVYPGSETFIVWGHVGLDEVSPIGKTTVWHIDPTSSELKLKTFQLEPSMFGLEEHELSKCASYGPKENARILKEEVLSGKYHLGDNNPIYDYILMNTAVLYCLSQGHQNWKEGIIKAEESIHSGNALRSLEHFIDSVSSL>YDR357CMQDNSSHSRESASAGDDPLGIDKLTVDYDYLLYKMRDYVQSIQLDTTELCKKQNEVMVNGIIENTIDKNIAKFKELLEKCDTLENHYEMLNQLAIITDTFKERIAEAVNNYNSLKKGASKSK>YDR358WMPQRIELTSEPVRKPRSTESSLLRKIQRACRSTLPEPDLGLNLDVADYINSKQGATPREAVLAIEKLVNNGDTQAAVFALSLLDVLVKNCGYSIHLQISRKEFLNDLVKRFPEQPPLRYSKVQQMILEAIEEWYQTICKHASYKDDLQYINDMHKLLKYKGYTFPKVGSENLAVLRPNDQLRTPSELQEEQERAQAAKLEELLRSGKPDDLKEANKLMKIMAGFKDDTKVAVKQAINNELNKLKRKADLFNEMLTSADEPDLENEAIQELYGDLKSAQPKFKKLIEEERDDDALVSNLSKFNDLVIQLLKRYKSIKGMKEEELNVPDTNEPAKELNLIDFDDDTTANTPSVTSPSKSLQPFDDLLGDFNKVSLSSPKSPQENDTVVDILGDAHSKSSGIDLLDFDSQPGESKTALSAYSNSIVLPNGLLNSSSNSKEITAQSQRHILNQSDHLRIDYELTRESMTKLRLVIFYSNISSDPITNFALLVASPKGTTLSLQPQSGNMLQSNSRDGIKQIASVEGISVNLGKPIKLKWKANYCTKGDSKEESGTTSLPTI>YDR361CMVQAIKLNDLKNRKRKNVEEENGSDESEIDISSTDSENEEEQNGEEEIVNIDFDFFGGNPEVDFHALKNLLRQLFGPQESTRIQLSSLADLILGSPTTTIKTDGKESDPYCFLSFVDFKANHLSDYVKYLQKVDMRLSTFFKTMIDSGNKNCALVLSERLINMPPEVVPPLYKITLEDVATALGDDKHYDFYIIVTRKYEVNFDTDDDTDSGKRNKNKDERSKKRVKADEVDYFHEEDRFFEKYAKIHFESEAKKGVISSYMILDHEGLVKSIDELETEISTW>YDR362CMAVIPAKKRGRPRKSVVAEVPYDSLASPVSENSGSKRPRRNASKKAVANFAQLVHAGRDDVINTTQVNNVDDTDDDDFVLNDEGDGEESDNVEIEFENELESTKNEVADLNSSGSGASVRPSGRRNTVQKLRLKKNSTKNMKSSSPGSSLGQKGRPIRLLKDLSSARDKIERIYGLNKEKLLLLAKVKEGFETSVFDFPFKNIQPDSPYFVCLDPPCKKESAYNKVIGDKNRTVYHEINKTEFENMIKLRTKRLKLLIGEVDAEVSTGDKIEFPVLANGKRRGFIYNVGGLVTDIAWLNIEENTDIGKDIQYLAVAVSQYMDEPLNEHLEMFDKEKHSSCIQIFKMNTSTLHCVKVQTIVHSFGEVWDLKWHEGCHAPHLVGCLSFVSQEGTINFLEIIDNATDVHVFKMCEKPSLTLSLADSLITTFDFLSPTTVVCGFKNGFVAEFDLTDPEVPSFYDQVHDSYILSVSTAYSDFEDTVVSTVAVDGYFYIFNPKDIATTKTTVSRFRGSNLVPVVYCPQIYSYIYSDGASSLRAVPSRAAFAVHPLVSRETTITAIGVSRLHPMVLAGSADGSLIITNAARRLLHGIKNSSATQKSLRLWKWDYSIKDDKYRIDSSYEVYPLTVNDVSKAKIDAHGINITCTKWNETSAGGKCYAFSNSAGLLTLEYLS>YDR363W-AMSTDVAAAQAQSKIDLTKKKNEEINKKSLEEDDEFEDFPIDTWANGETIKSNAVTQTNIWEENWDDVEVDDDFTNELKAELDRYKRENQ>YDR363WMTGDSRSISEPSINLDPDNTSFSDENSDDFFMDNSYDIDEIDHSDESNRQSVIVDSKVTVPPSKHSTLTLSDSEDSDAKEQHQSLSRSSSKNVNIEDITEPKPDKPSGRTRGRSVMKESVVEINSSESDLDEDKNFPRSRSRSRSSIRSISPAGKYKRQKSSLLYTYDENDDFFKELAKEAKKSTTISKESTPDQRKRVYNIKFLSKLEGTINKAVQVKVLGKYEFSKILPAALDGLMKSYKIPKVMKDIYKVENVTLYWNNAKLLTFMTCNSLHIPQDFENEVSDIDVTIVSKEYEKNFEATLESKLKEEEAALLIKERQEMERKLEKKRNEQEESEYREFESELKNVEETQEIKENDTVMNTKLLQEGGSLSGNSSSMEEVMRIALMGQDNKKIYVHVRRSTPFSKIAEYYRIQKQLPQKTRVKLLFDHDELDMNECIADQDMEDEDMVDVIID>YDR364CMGLVDGYDTSSDSDLNFDEGKSVHEKKNGNLHEDTSYEPSSNNIHKRKSHFTKSELKRRRKTRKGDGPWGSWSSSDDETSQASETQKEDQDIFVHALAEDNLDSEQIEVEEVSHFYGKSEKDYQGRGYLYPPNDVDVDLREERISFRCYLPKKVIRNYPGHPEGTTALKFLPKTGHLILSGGNDHTIKIWDFYHDYECLRDFQGHNKPIKALRFTEDCQSFLSSSFDRSVKIWDTETGKVKTRLHLNSTPADVESRPTNPHEFIVGLSNSKILHYDDRVSENQGLVQTYDHHLSSILALKYFPDGSKFISSSEDKTVRIWENQINVPIKQISDTAQHSMPFLNVHPSQNYFCAQSMDNRIYSFSLKPKYKRHPKKIFKGHSSAGYGISLAFSGDGRYICSGDSKSRLFTWDWNTSRLLNNIKIPGNKPITQVDWHPQETSKVICSGAAGKIYVCD>YDR368WMPATLKNSSATLKLNTGASIPVLGFGTWRSVDNNGYHSVIAALKAGYRHIDAAAIYLNEEEVGRAIKDSGVPREEIFITTKLWGTEQRDPEAALNKSLKRLGLDYVDLYLMHWPVPLKTDRVTDGNVLCIPTLEDGTVDIDTKEWNFIKTWELMQELPKTGKTKAVGVSNFSINNIKELLESPNNKVVPATNQIEIHPLLPQDELIAFCKEKGIVVEAYSPFGSANAPLLKEQAIIDMAKKHGVEPAQLIISWSIQRGYVVLAKSVNPERIVSNFKIFTLPEDDFKTISNLSKVHGTKRVVDMKWGSFPIFQ>YDR372CMSTLQRRRVNRADSGDTSSIHSSANNTKGDKIANIAVDGDDDNGTNKKIAYDPEESKLRDNINIPTLTLMEEVLLMGLRDREGYLSFWNDSISYALRGCIIIELALRGKIRILDDSARKRFDLSERLIEVIDSSKTGEVLLDETLQLMKNDEPLSISNWIDLLSGETWNLLKINYQLKQVRERLAKGLVDKGVLRTEMKNFFLFDMATHPIADASCKEAIKRRVLSVLVSRNMELSYNEYFPETTSFKIIRTLALICGSYGANVLENVLTTLEYEKRDKAISRAEEIMAQFSQYPFDLEKETELGVSVNLNKEVKEEIENNPGHDLQLEVIAGVFEVFSRMDMLL>YDR373WMGAKTSKLSKDDLTCLKQSTYFDRREIQQWHKGFLRDCPSGQLAREDFVKIYKQFFPFGSPEDFANHLFTVFDKDNNGFIHFEEFITVLSTTSRGTLEEKLSWAFELYDLNHDGYITFDEMLTIVASVYKMMGSMVTLNEDEATPEMRVKKIFKLMDKNEDGYITLDEFREGSKVDPSIIGALNLYDGLI>YDR374CMNQIWSTGPPNFYFNSEWKENKRNDRTIEDSLRELDGLIHSLERTHIEVQTNPKLKNDVTALNDINKKENKEEITHENYTHQINSIPLTSSNLNRHFSFSRDYNQSDNSNNNYYREYLSKPRYLQQSTKEQTFNEINKRKSAAIIPPWLNIPENSRFFVIKSSSLKHVKRSFYNGIWSSTHFGNKRLSEAYKKLNSGAKVFLFFSINTSGRFCGVAEMVSDLKMDLDTSIWEDEQKYGKAFKVRWVIVRDINNRSLKRFLIPSNEMKPITHSRDTQEIPYSIGISIINLFKTQDSDIFSFLDETYE>YDR375CMSDKPIDIQYDKQATPNLSGVITPPTNETGNDSVREKLSKLVGDAMSNNPYFAAGGGLMILGTGLAVARSGIIKASRVLYRQMIVDLEIQSKDKSYAWFLTWMAKHPQRVSRHLSVRTNYIQHDNGSVSTKFSLVPGPGNHWIRYKGAFILIKRERSAKMIDIANGSPFETVTLTTLYRDKHLFDDILNEAKDIALKTTEGKTVIYTSFGPEWRKFGQPKAKRMLPSVILDSGIKEGILDDVYDFMKNGKWYSDRGIPYRRGYLLYGPPGSGKTSFIQALAGELDYNICILNLSENNLTDDRLNHLMNNMPERSILLLEDIDAAFNKRSQTGEQGFHSSVTFSGLLNALDGVTSSEETITFMTTNHPEKLDAAIMRPGRIDYKVFVGNATPYQVEKMFMKFYPGETDICKKFVNSVKELDITVSTAQLQGLFVMNKDAPHDALKMVSSLRNANHIF>YDR377WMIFKRAVSTLIPPKVVSSKNIGSAPNAKRIANVVHFYKSLPQGPAPAIKANTRLARYKAKYFDGDNASGKPLWHFALGIIAFGYSMEYYFHLRHHKGAEEH>YDR378CMSGKASTEGSVTTEFLSDIIGKTVNVKLASGLLYSGRLESIDGFMNVALSSATEHYESNNNKLLNKFNSDVFLRGTQVMYISEQKI>YDR379C-AMPKRLSGLQKEVLHLYRASIRTAHTKPKENQVNFVNYIHEEFGKYRNLPRKDFTTIEHLLRVGNKKIATFSHPELTNIH>YDR379WMSADPINDQSSLCVRCNKSIASSQVYELESKKWHDQCFTCYKCDKKLNADSDFLVLDIGTLICYDCSDKCTNCGDKIDDTAIILPSSNEAYCSNCFRCCRCSNRIKNLKYAKTKRGLCCMDCHEKLLRKKQLLLENQTKNSSKEDFPIKLPERSVKRPLSPTRINGKSDVSTNNTAISKNLVSSNEDQQLTPQVLVSQERDESSLNDNNDNDNSKDREETSSHARTVSIDDILNSTLEHDSNSIEEQSLVDNEDYINKMGEDVTYRLLKPQRANRDSIVVKDPRIPNSNSNANRFFSIYDKEETDKDDTDNKENEIIVNTPRNSTDKITSPLNSPMAVQMNEEVEPPHGLALTLSEATKENNKSSQGIQTSTSKSMNHVSPITRTDTVEMKTSTSSSTLRLSDNGSFSRPQTADNLLPHKKVAPSPNKKLSRSFSLKSKNFVHNLKSKTSEMLDPKHPHHSTSIQESDTHSGWGVSSTHTNIRKSKAKKNPVSRGQSDSTIYNTLPQHGNFTVPEFNHKKAQSSLGSISKKQNSNDTATNRRINGSFTSSSSGHHIAMFRTPPLESGPLFKRPSLSSESAHHRSSSLQTSRSTNALLEDDSTKVDATDESATSLEKDFYFTELTLRKLKLDVRELEGTKKKLLQDVENLRLAKERLLNDVDNLTREKDKQSASSRESLEQKENIATSITVKSPSSNSDRKGSISNASPKPRFWKIFSSAKDHQVGDLESQQRSPNSSSGGTTNIAQKEISSPKLIRVHDELPSPGKVPLSPSPKRLDYTPDGSHLYGSSLQARCAYEKSTVPIIIRCCIDRIEKDDIGLNMEGLYRKSGSQTLVEEIENEFAQNNSLHSDTLSPKLNALLNQDIHAVASVLKRYLRKLPDPVLSFSIYDALIDLVRNNQLIERLPLNNDKFLDSPQKVTIYEMVLKSLLEIFKILPVEHQEVLKVLAAHIGKVRRCSERNLMNLHNLSLVFAPSLIHDFDGEKDIVDMKERNYIVEFILGNYRDIFKQA>YDR380WMAPVTIEKFVNQEERHLVSNRSATIPFGEYIFKRLLSIDTKSVFGVPGDFNLSLLEYLYSPSVESAGLRWVGTCNELNAAYAADGYSRYSNKIGCLITTYGVGELSALNGIAGSFAENVKVLHIVGVAKSIDSRSSNFSDRNLHHLVPQLHDSNFKGPNHKVYHDMVKDRVACSVAYLEDIETACDQVDNVIRDIYKYSKPGYIFVPADFADMSVTCDNLVNVPRISQQDCIVYPSENQLSDIINKITSWIYSSKTPAILGDVLTDRYGVSNFLNKLICKTGIWNFSTVMGKSVIDESNPTYMGQYNGKEGLKQVYEHFELCDLVLHFGVDINEINNGHYTFTYKPNAKIIQFHPNYIRLVDTRQGNEQMFKGINFAPILKELYKRIDVSKLSLQYDSNVTQYTNETMRLEDPTNGQSSIITQVHLQKTMPKFLNPGDVVVCETGSFQFSVRDFAFPSQLKYISQGFFLSIGMALPAALGVGIAMQDHSNAHINGGNVKEDYKPRLILFEGDGAAQMTIQELSTILKCNIPLEVIIWNNNGYTIERAIMGPTRSYNDVMSWKWTKLFEAFGDFDGKYTNSTLIQCPSKLALKLEELKNSNKRSGIELLEVKLGELDFPEQLKCMVEAAALKRNKK>YDR382WMKYLAAYLLLVQGGNAAPSAADIKAVVESVGAEVDEARINELLSSLEGKGSLEEIIAEGQKKFATVPTGGASSAAAGAAGAAAGGDAAEEEKEEEAKEESDDDMGFGLFD>YDR383CMTDTYNSISNFIENELTALLSSDDYLMDDLAGELPNEVCRLLKAQVIEKRKDAMSRGKQDLLSKEIYDNESELRASQSQQIMELVGDIPKYSLGSELRNRVEGEPQSTSIERLIEDVLKLPQMEVADEEEVEVENDLKVLSEYSNLRKDLILKCQALQIGESKLSDILSQTNSINSLTTSIKEASEDDDISEYFATYNGKLVVALEEMKLLLEEAVKTFGNSPEKREKIKKILSELKK>YDR384CMTSSASSPQDLEKGVNTLENIETLPQQGSIAGVSQGFPNIQEIYSDRDFITLGSSTYRRRDLLNALDRGDGEEGNCAKYTPHQFANPVPLGLASFSLSCLVLSLINANVRGVTDGKWALSLFMFFGGAIELFAGLLCFVIGDTYAMTVFSSFGGFWICYGYGLTDTDNLVSGYTDPTMLNNVIGFFLAGWTVFTFLMLMCTLKSTWGLFLLLTFLDLTFLLLCIGTFIDNNNLKMAGGYFGILSSCCGWYSLYCSVVSPSNSYLAFRAHTMPNAP>YDR385WMVAFTVDQMRSLMDKVTNVRNMSVIAHVDHGKSTLTDSLVQRAGIISAAKAGEARFTDTRKDEQERGITIKSTAISLYSEMSDEDVKEIKQKTDGNSFLINLIDSPGHVDFSSEVTAALRVTDGALVVVDTIEGVCVQTETVLRQALGERIKPVVVINKVDRALLELQVSKEDLYQTFARTVESVNVIVSTYADEVLGDVQVYPARGTVAFGSGLHGWAFTIRQFATRYAKKFGVDKAKMMDRLWGDSFFNPKTKKWTNKDTDAEGKPLERAFNMFILDPIFRLFTAIMNFKKDEIPVLLEKLEIVLKGDEKDLEGKALLKVVMRKFLPAADALLEMIVLHLPSPVTAQAYRAEQLYEGPADDANCIAIKNCDPKADLMLYVSKMVPTSDKGRFYAFGRVFAGTVKSGQKVRIQGPNYVPGKKDDLFIKAIQRVVLMMGRFVEPIDDCPAGNIIGLVGIDQFLLKTGTLTTSETAHNMKVMKFSVSPVVQVAVEVKNANDLPKLVEGLKRLSKSDPCVLTYMSESGEHIVAGTGELHLEICLQDLEHDHAGVPLKISPPVVAYRETVESESSQTALSKSPNKHNRIYLKAEPIDEEVSLAIENGIINPRDDFKARARIMADDYGWDVTDARKIWCFGPDGNGPNLVIDQTKAVQYLHEIKDSVVAAFQWATKEGPIFGEEMRSVRVNILDVTLHADAIHRGGGQIIPTMRRATYAGFLLADPKIQEPVFLVEIQCPEQAVGGIYSVLNKKRGQVVSEEQRPGTPLFTVKAYLPVNESFGFTGELRQATGGQAFPQMVFDHWSTLGSDPLDPTSKAGEIVLAARKRHGMKEEVPGWQEYYDKL>YDR387CMSTDESEDVYSDLYSIISQVTSNTANDIEQLPYALTFKTSLIFVGATIGGLLFGYDTGVISGVLLSLKPEDLSLVVLTDVQKELITSSTSVGSFFGSILAFPLADRYGRRITLAICCSIFILAAIGMAIARTLTFLICGRLLVGIAVGVSAQCVPLFLSEISPSRIRGFMLTLNIIAITGGQLVSYVIASLMKEIDNSWRYLFALSAIPAILFLSILDFIPESPRWSISKGDILYTRDSLRMLYPTASTYHVNSKIKQLIIELDKLRLYEDASEPLLVQSQSVIRYMDSSTSGTLSPPNIKRLSSNTERTSNTMSSSSAYLSALRGPAPNGALASNKKKRHRMEPRTIRALIVGCMLMFFQQITGFNAFMYYAAIIFSKFNIKNPLLPPILIASTNFIFTFFAMYTMDSLGRRAILLRTILIMTVGLLLCSVGFGHDQVNLLLISVVIYVAAYASAMGSVPWTCVEFLPLNRRSFGASCIACTNWLTNAFVSMTYLSTINTIGDENTMLIFAFFTVCAWFFVYFWYPEVKGLSLEEVGRVFDNGIDVHYVFRTYH>YDR388WMSFKGFTKAVSRAPQSFRQKFKMGEQTEDPVYEDAERRFQELEQETKKLSEESKRYSTAVNGMLTHQIGFAKSMEEIFKPISGKMSDPNATIPEDNPQGIEASEQYRAIVAELQETLKPDLALVEEKIVTPCQELLKIITYIRKMATKRNHKKLDLDRHLNTYNKHEKKKEPTAKDEERLYKAQAQVEVAQQEYDYYNDLLKTQLPILFSLEAEFVKPLFVSFYFMQLNIFYTLYNRLQDMKIPYFDLNSDIVESYIAKKGNVEEQTDALTITHFKLGYSKAKLEMTRRKYGVATAEGSPVSGASSGVGYGAGYDPATATSPTPTGYGYGAAAPSYAAQPAAQYGTAAAVGTAAAVGTAAGAAAGAVPGTYPQYAAAQSPPLTGLGFQQSPQQQQGPPPAYSNPLTSPVAGTPAAAVAAAPGVETVTALYDYQAQAAGDLSFPAGAVIEIVQRTPDVNEWWTGRYNGQQGVFPGNYVQLNKN>YDR389WMPNNTLKQGSKIENVSPSKGHVPSFWKQFINNPKSMSSENITVPRSPTSLSRNAQPTTLKRPPLSSRPYSYNTPTKDRKSFSKSAKQNNNNNNANSGTSPHAEFKNYRDMFLSNRNGFTGRVFGVTLAESLSVASAEVIVQSELVSFGRIPIVVAKCGAYLKANGLETSGIFRIAGNGKRVKALQYIFSSPPDYGTKFNDWETYTVHDVASLLRRYLNNLAEPLIPLSLYEQFRNPLRSRPRILRHMLTHEVSHPNANKTNNVTVKSSRQNYNDDGANDGDIEKEDAKDDEEKRRRKIRHKRRLTRDIRAAIKEYEELFVTLSNDTKQLTIYLLDLLSLFARQSQFNLMSGRNLAAIFQPSILSHPQHDMDPKEYELSRLVVEFLIEYSYKLLPHLLKLAKREQQERLSTENKKNNGDKQKTDPIEIPKITSSDSPPIVSSNKNPPAIDNNNKLDHTTLSPISTSIPENSSDLQTSKMLKPPKQRRPHSKSFGSTPVPPDVIASNKRRTSLFPWLHKPGILSDTGDNGDLTATEAEGDDYEEENVDPYGQSPSSVHSGSLPKQHYLPIPRMNRSLSGNSTNSSFNTRPISMILTSGNDNSADQLELLSNTHSNNERSNALPLTEDDGDERNSRSRKRESWFQRLTSRSGSANRA>YDR391CMSFENKLPTPLENNDAKGHMVCTLNKTTDARRAAETLSIAFSNSPAFHFICKKILNIPLAEKVPTRTITTDIISPFLDSPYGEISEVNTFDAVAVWSLPPHVPKARSNDAKFNKDFIDDLNARVKQVIPNGINYYYLFCIGKNLNEKGIRGSVRTIFEEYKRRADEENCAIVLEAIAEHAKSVYEYFGFRNYMTFKYGECEVDSNGNCDPNGEGFTAYLMIYHKDGNKVLKE>YDR392WMMDKHKYRVEIQQMMFVSGEINDPPVETTSLIEDIVRGQVIEILLQSNKTAHLRGSRSILPEDVIFLIRHDKAKVNRLRTYLSWKDLRKNAKDQDASAGVASGTGNPGAGGEDDLKKAGGGEKDEKDGGNMMKVKKSQIKLPWELQFMFNEHPLENNDDNDDMDEDEREANIVTLKRLKMADDRTRNMTKEEYVHWSDCRQASFTFRKNKRFKDWSGISQLTEGKPHDDVIDILGFLTFEIVCSLTETALKIKQREQVLQTQKDKSQQSSQDNTNFEFASSTLHRKKRLFDGPENVINPLKPRHIEEAWRVLQTIDMRHRALTNFKGGRLSSKPIIM>YDR393WMLRYYGATRNLPLVFSINKLMLRASSFTRPFHYSSYSLQNGDTPDKGSTNKNEIRTPNNAVWKENIELQWQHLKKKLNELYSRFNFHRDQLSFQVNKAKKSIQEANRKLSEQENEINDSRLNYNKDELTSAKIEGLPSEREQHRKKWSRKLEFYFDSLQETLFTATRALNDVTGYSGIQKLKSSISLMEKKLEATKKEHKLFKAQYANAIDERAQSQREVNELLQRQSAWSSSDLERFTQLYKNDALNARQEQELKNKVKEIESKEEQLNDDLYRAILTRYHEEQIWSDKIRRTSTWGTFILMGMNIFLFIVLQLLLEPWKRKRLVGSFEDKVKSALNEYAKEQNMKMDKLLPGKSSEVTDQGNTENSIVEEHIEQRGECKINTAEIDRPEVATAETTTTEMKSFRDIWERIKALFVTLKSIQYRKLDAPLVFDTLEFYLYSISLVSMTILVSGLI>YDR394WMEELGIVTPVEKAVEEKPAVKSYASLLAQLNGTVNNNSALSNVNSDIYFKLKKLEKEYELLTLQEDYIKDEQRHLKRELKRAQEEVKRIQSVPLVIGQFLEPIDQNTGIVSSTTGMSYVVRILSTLDRELLKPSMSVALHRHSNALVDILPPDSDSSISVMGENEKPDVTYADVGGLDMQKQEIREAVELPLVQADLYEQIGIDPPRGVLLYGPPGTGKTMLVKAVANSTKAAFIRVNGSEFVHKYLGEGPRMVRDVFRLARENAPSIIFIDEVDSIATKRFDAQTGSDREVQRILIELLTQMDGFDQSTNVKVIMATNRADTLDPALLRPGRLDRKIEFPSLRDRRERRLIFGTIASKMSLAPEADLDSLIIRNDSLSGAVIAAIMQEAGLRAVRKNRYVILQSDLEEAYATQVKTDNTVDKFDFYK>YDR397CMAGDSDNVSLPKATVQKMISEILDQDLMFTKDAREIIINSGIEFIMILSSMASEMADNEAKKTIAPEHVIKALEELEYNEFIPFLEEILLNFKGSQKVKETRDSKFKKSGLSEEELLRQQEELFRQSRSRLHHNSVSDPVKSEDSS>YDR398WMDSPVLQSAYDPSGQYLCYVTVALDKQRVGVQPTQRATSSGVDTVWNENFLYLEDSKLKVTCLKWVNLASSDTVAIILGMNNGEIWLYSVLANEVTYKFTTGNSYEIKDIDLMGNQLWCIDSSDAFYQFDLLQFKLLQHFRINNCVQLNKLTIVPAGDSVAQLLVASHSISLIDIEEKKVVMTFPGHVSPVSTLQVITNEFFISGAEGDRFLNVYDIHSGMTKCVLVAESDIKELSHSGQADSIAVTTEDGSLEIFVDPLVSSSTKKRGNKSKKSSKKIQIVSKDGRKVPIYNAFINKDLLNVSWLQNATMPYFKNLQWREIPNEYTVEISLNWNNKNKSADRDLHGKDLASATNYVEGNARVTSGDNFKHVDDAIKSWERELTSLEQEQAKPPQANELLTETFGDKLESSTVARISGKKTNLKGSNLKTATTTGTVTVILSQALQSNDHSLLETVLNNRDERVIRDTIFRLKPALAVILLERLAERIARQTHRQGPLNVWVKWCLIIHGGYLVSIPNLMSTLSSLHSTLKRRSDLLPRLLALDARLDCTINKFKTLNYEAGDIHSSEPVVEEDEDDVEYNEELDDAGLIEDGEESYGSEEEEEGDSDNEEEQKHTSSKQDGRLETEQSDGEEEAGYSDVEME>YDR399WMSANDKQYISYNNVHQLCQVSAERIKNFKPDLIIAIGGGGFIPARILRTFLKEPGVPTIRIFAIILSLYEDLNSVGSEVEEVGVKVSRTQWIDYEQCKLDLVGKNVLIVDEVDDTRTTLHYALSELEKDAAEQAKAKGIDTEKSPEMKTNFGIFVLHDKQKPKKADLPAEMLNDKNRYFAAKTVPDKWYAYPWESTDIVFHTRMAIEQGNDIFIPEQEHKQ>YDR400WMTVSKIPIWLDCDPGHDDAIAILLGCFHPAFNLLGISTCFGNAPPENTDYNARSLLTAMGKAQAIPVYKGAQRPWKREPHYAPDIHGISGLDGTSLLPKPTFEARTDKTYIEAIEEAILANNGEISFVSTGALTTLATVFRCKPYLKKSVKYISIMGGGLHGLGNCNPNLSAEFNVWIDPDAANYIFRDPDVKDKCIVVPLNLTHKAIATYKVNEMIYNEKNNSKLRELFLELFQFFAHTYKDMQGFESGPPIHDPVALMPLLEFYGWDPSSAVGFRYKRMDISCIDDVFNENSGKIIIEKEYPNDSDVGTIIGLDLNIQYFWDQIFEALNRADKMSTIG>YDR403WMTFTSNLPSSSEQSISPPASSFSSSTDTLKDIDIPHNGADLSTYSKFLALYCRSDKCDDFYSLEEKQNCKFGDQWLDFINTIHNLDFSESEVSGRVSERILPASLANKFTNNLGVAIKISEYTRDDERQIRGCVTTVENENSFNNWFVYHILDQSQLSLSEHPIVTKEVKYHELFADFFEKNLKNTIVNDQWNFGGRDYFIERSRYFTDRYLRIECILPAFPCKSSNEQKVYGSVPDKGEELALKRLIKATQDLVKIYPPGMKIWIVSDGHVFSDCIGVDDDVVSTYTTKLHELYKRVAIPGVDAIGFCGLNDLFFSGAASKVFDPKWVSDVEVAHYTGTQICPKSDLSRQILMKGCDTDAGRLRKQIAIEGHPRLHLYRGFSRFMMEDLSLLEHFQSYSRKKFKKIISMIAFNMIKRNDAYSNLVELIFPHHLRISIHAHTNSGPKFGIKVISNEQCSIVSSLEDLDEPKFEDFLHIPTPWHNCVVKVEDEKEKYFLTKSKVVKEALEKGMYDGVWKDTRFDIGEGGHFVIKKIS>YDR404CMFFIKDLSLNITLHPSFFGPRMKQYLKTKLLEEVEGSCTGKFGYILCVLDYDNIDIQRGRILPTDGSAEFNVKYRAVVFKPFKGEVVDGTVVSCSQHGFEVQVGPMKVFVTKHLMPQDLTFNAGSNPPSYQSSEDVITIKSRIRVKIEGCISQVSSIHAIGSIKEDYLGAI>YDR405WMPRLTVGTKNMLYPLQKTLAVGSCKPEQVPIRSLASVVESSSKILDKSGSDREVDINVSEKIYKWTKAGIEQGKEHFKVGGNKVYFPKARIILLRPNAKHTPYQAKFIVPKSFNKLDLRDYLYHIYGLRAMNITTQLLHGKFNRMNLQTTRFREPQIKKMTIEMEEPFIWPEEPRPDENSFWDSTTPDNMEKYREERLNCLGSDANKPGTAFDGVVGPYERVAQPFIPRFLKREIDNKRERHAAELQRADKLIALNRYIEDLH>YDR408CMARIVVLISGSGSNLQALIDAQKQGQLGEDAHIVSVISSSKKAYGLTRAADNNIPTKVCSLYPYTKGIAKEDKAARAKARSQFENDLAKLVLEEKPDVIICAGWLLILGSTFLSQLQSVPILNLHPALPGCFDGTTHAIEMAWRKCQDENKPLTAGCMVHYVIEEVDKGEPLVVKKLEIIPGEETLEQYEQRVHDAEHIAIVEATYKVLQQLHK>YDR410CMHQDFQEDEHEYPDIRRNPLHEVTMTSYILGILLGIFVGLFPQIRFKNFNLFIIALSLFHFLEYYITAKYNPLKVHSESFLLNNGKSYMAAHSFAILECLVESFLFPDLKIFSYSLATKLCTVLGCLLVILGQYTRTIAMHTAGHSFSHIVKTKKESDHVLVKTGVYSWSRHPSYLGFFWWAIGTQLLLLNPLSLVIFIFVLWKFFSDRIRVEEKYLIEFFSAEYIEYKNKVGVGIPFI>YDR411CMAGPRNVRTLHGNGGRNNDVMGPKEFWLNIPPITRTLFTLAIVMTIVGRLNLINPWYFIYVWNLTFKKVQIWRLLTSCVMLSSRAMPALMELYSIYDRSSQLERGHFGPGLSNRRGPMVTVDYAYYLCFCILAITTATTIIYGSYYPVVLTSGFISCITYTWSIDNANVQIMFYGLIPVWGKYFPLIQLFISFVFNEGDFVISLIGFTTGYLYTCLDTHTLGPIWGMISRKADPTYGISPNGKFSTPWWFTSLYARITGAHNETATFNNNFANVPSSQRETRTFSGRGQRLGTAPATLSQTSGTDSGRASGSQLRSGPSNLNQFQGRGQRVGQTNSPSDSQ>YDR412WMAVHTNRQILTRGKNYATKQSKKFGTDEVTFDKDSRLDYLTGFHKRKLQRQKKAQEFIKEQERLRKIEERQKIRQERKEVMEEQLKTFKESLNLEAEIEDAKNDKTEDLQVESDESWHGFDSDKDDGDNDNNESSVKPILKKGAITEIYDDSTTVELETLEPNDNFEYLAQLNNVKLEKAEKVLKQSINRATKYAKFLGVDEKQKKKPRVKKFRYLTKNERRINQRKANDNKRRR>YDR414CMEKSESNSEGLYLQNILNVPPPQRFIVLIILALWIWTWILKFFLHSNLDVSQVILTRVPHDIRPGYTLQQLHRTARNFALKITRIIIPFHFATVFLFEFMNIIEGPLKNIILIVYFLPLIQCVTIFWFLLKECQIIKYCTRRCLLIESSPRSLRNTYILISDTLTSFGKPLIDFTLFTSLIFREPFTHFDLSVALLPVLVRLLQCLREYRLLHEATLLFNALKYSCNLPILFCTWRSRVYEGSINEERLHHVQRWFMLINSSYTLFWDVRMDWSLDSLTSLRSRSKSAVTLKKKMYHSAILVDFLLRFWWLWVYLSQNLKLVAADSDYIFFQGEMQYFEVIRRGIWVVFKLDAEYYIKFASK>YDR415CMRIQSLFVLFNVAIIAWSYPYEPLRVLQVGENEVMEVPESEKLNLRRRGVKFFDVTKHTSFLPFFNKEEEPTVPTYNYPPEISNKEVVDDSIKNIDKGSMHKNLAKFTSFYTRYYKSDHGFESAEWLAATIANITKDIPQDTLTIEHFDHKEWKQYSIIVRVTGSTTPEDIIIIGSHQDSINLLLPSIMAAPGADDNGSGTVTNMEALRLYTENFLKRGFRPNNTVEFHFYSAEEGGLLGSLDVFTAYAKQKKHVRAMLQQDMTGYVSDPEDEHVGIVTDYTTPALTDFIKLIINSYLSIPYRDTQCGYACSDHGSATRNGFPGSFVIESEFKKTNKYIHSTMDTLDRLSLAHMAEHTKIVLGVIIELGSWSAW>YDR416WMSAYIAMKGVITNVDENIRNDEDVAFEYEIQKTPQNILTWKRYIEYWKEEGRTDKQIRWLYERFCSQFVTDTSIWEDYIRWESTKEVVETSRIFWLFQRCLKSCVRDCDRICLSYLELAIEQYDLAMIRHALASSLMKMEREMHRKVWDPVIKFVEEKVLPLTQLDSTQEDEEESTDEAELINVLLVKGFTKGGFISEEISENGSRGDIWSSHILERYLKVAPQQKRNESLATLALTRDNITIKSVYEKYLPQDENSGKYLPSSELPFELNFNYLASLEKLGLDNQYEEFMRQMNGIYPDKWLFLILSLAKYYISRGRLDSCGDLLKKSLQQTLRYSDFDRIYNFYLLFEQECSQFILGKLKENDSKFFNQKDWTEKLQAHMATFESLINLYDIYLNDVALRQDSNLVETWMKRVSLQKSAAEKCNVYSEAILKIDPRKVGTPGSFGRLWCSYGDLYWRSNAISTARELWTQSLKVPYPYIEDLEEIYLNWADRELDKEGVERAFSILEDALHVPTNPEILLEKYKNGHRKIPAQTVLFNSLRIWSKYIDYLEAYCPKDANSSDKIFNKTKMAYNTVIDLRLITPAMAENFALFLQNHYEVMESFQVYEKTIPLFPPEIQYELWIEYLEVATSHQLSSLSPEHIRFLFEKALKNLCSNGIDCKTIFIAYSVFEERISGLISKSIEILRRGAVIGTVSVSTHLESRLQLWRMCISKAESTLGPSVTRELYQECIQILPNSKAVEFVIKFSDFESSIGETIRAREILAYGAKLLPPSRNTELWDSFEIFELKHGDKETYKDMLKMKKVLESNMLIDSASVSHEEGNINFVAAATSHAPNSHTLTQSTSSYSINPDEIELDI>YDR418WMPPKFDPNEVKYLYLRAVGGEVGASAALAPKIGPLGLSPKKVGEDIAKATKEFKGIKVTVQLKIQNRQAAASVVPSASSLVITALKEPPRDRKKDKNVKHSGNIQLDEIIEIARQMRDKSFGRTLASVTKEILGTAQSVGCRVDFKNPHDIIEGINAGEIEIPEN>YDR419WMSKFTWKELIQLGSPSKAYESSLACIAHIDMNAFFAQVEQMRCGLSKEDPVVCVQWNSIIAVSYAARKYGISRMDTIQEALKKCSNLIPIHTAVFKKGEDFWQYHDGCGSWVQDPAKQISVEDHKVSLEPYRRESRKALKIFKSACDLVERASIDEVFLDLGRICFNMLMFDNEYELTGDLKLKDALSNIREAFIGGNYDINSHLPLIPEKIKSLKFEGDVFNPEGRDLITDWDDVILALGSQVCKGIRDSIKDILGYTTSCGLSSTKNVCKLASNYKKPDAQTIVKNDCLLDFLDCGKFEITSFWTLGGVLGKELIDVLDLPHENSIKHIRETWPDNAGQLKEFLDAKVKQSDYDRSTSNIDPLKTADLAEKLFKLSRGRYGLPLSSRPVVKSMMSNKNLRGKSCNSIVDCISWLEVFCAELTSRIQDLEQEYNKIVIPRTVSISLKTKSYEVYRKSGPVAYKGINFQSHELLKVGIKFVTDLDIKGKNKSYYPLTKLSMTITNFDIIDLQKTVVDMFGNQVHTFKSSAGKEDEEKTTSSKADEKTPKLECCKYQVTFTDQKALQEHADYHLALKLSEGLNGAEESSKNLSFGEKRLLFSRKRPNSQHTATPQKKQVTSSKNILSFFTRKK>YDR421WMSAKKRPSGNAAFELPKRRRTYQACISCRSRKVKCDLGPVDNPHDPPCARCKRELKKCIFSSNKGTSNDLPPNSINAISLPSLGKSKQEIQNDSTSPILSDVPLSRKGISSEKSFKSEGMKWKLELSSMQNALEFLAQAAGTVAKEGAKEIIKEKSTTPKPLKSSLDATNKSATDEGLKRLSKSDSTNTLYENTADMLNHTLNTNRKTSQLMEEIGKVRPPPTRKIDDFDYIGPDSLLTKEEAIELIEAFFLTMHPFFPNIPLQLHDPKELAEYPILFCAILTVSARYHPFDTLGLDNGEDGMRHIEVHDKLWVYCQKLISQTIWAEASTRSIGTVLAFIIFTEWNPRSIHYKWSDYANDPELNNVNARGSKNISTRKDEEGLTGVGAIRRSDRMSWMLTGSAVRLAQDMGFIENSSKVFIVTHISETTSAMNMNQRSLLAESFSVLNLNLGKIENDGNESNEDYLGNEKFYLNEILPDEESKLRWKRVFENSENDHDNEKNFLTDWEREFLNDEYVLYYSNKKDDTNLAQNHIPPFPLRFSFAQRAKIEIIRILSIAYETIYCEKNKRKLATTDQRHNLSVLSVFSPLIEGWLSNYRELLVPLSDVPFSLADRKNKKQIFDNIDRINGESIITDFNYCQLYIFSLALQVDGKTSRLNMNEIVTSARYVELAYRSAKEILSSAKRVSRQGMLKYMPVRWVIRIIRSIAFIVKCYLTLTGSELATNPDARNILKLSAISVDETFDIIRDTAVTLKEATPDELHLCQRYAAILMYLCTEMKLRKKSYLERPPLLRDGTTPLESNRESSLEGQDLTKKPIFSKRIGYNKTETTFEPSERPLTEEINSNSQNSNDTSSKGIVDPFVEQNNDITTALLNNELFQGPSLSDEVTDWFGASEDIGLEFVEPWTELIEQRYMQCGDGDNNNFENLYNLFVNSNNINNDINNSRPITRK>YDR425WMDYNIFEAVHEQQSSTSDMDLSEEDNNPFVGTHHLYASGIGTTIGEARPENENSPPSSSSLPSSPAHSSSAGSSRASTSSSTSSHAVVEADAETEPFVSLSMSTTATISKFTPHDMNGTQQIQIIDAGDFKDPWGKHAIGYVILYENNKIIRRYSEFHSLRQSLTRLLPTIIIPPIPSKHSLLKYIWSPINAANDSKIISTRKKMLNSFLSNCLNIQEISNDIVFQKFLNPEFNWKDVLSSSPIIILPLNNLLAPPLSPTKPSPLHSILPIPSNSSLRNYNSIWQQHITVKSHNEISNLPTEILQNESQFTHIENLFQNYKRIITHLLKNIRSNKSHFHSLSTYFAELGAYYNAFSLENDITMPNSLRESENNSNNPMMEIISHIEKTGHSFDVIYISSEILIEKYTSILEDPINELLQFLNESFKVLNFKKLKFLQFKILERLIIEKETKLSSLTEIENQLQKINESLTRSTILTDENYKDTKAADLTFVKKDVRSLSKSSSNSSSSGHQNEIHIGASKLNYKTSTPTMNLNKLEIKQLTEQERSKQIKQLNQDLSKLKDCLSICISDMLEINNSSYNSLMHTYNHINLTIGKILKLFAASFKAWIKECLKNWKLAKLQIDEAL>YDR427WMFNNHEIDTILSTLRMEADPSLHPLFEQFEKFYEEKLWFQLSESLTKFFDDAKSTPLRLRLYDNFVSKFYDKINQLSVVKYLLASLKDSKDFDESLKYLDDLKAQFQELDSKKQRNNGSKDHGDGILLIDSEIARTYLLKNDLVKARDLLDDLEKTLDKKDSIPLRITNSFYSTNSQYFKFKNDFNSFYYTSLLYLSTLEPSTSITLAERQQLAYDLSISALLGDKIYNFGELLHHPIMETIVNDSNYDWLFQLLNALTVGDFDKFDSLIKVQISKIPILAQHESFLRQKICLMTLIETVFVKNIRMLSFEDISKATHLPKDNVEHLVMRAISLGLLKGSIDQVNELVTISWVQPRIISGDQITKMKDRLVEWNDQVEKLGKKMEARGQSIWV>YDR429CMSEVAPEEIIENADGSRSIITYKIEDGVKYKITQKVKEVKVLEKVHKSVAERKNWHKYGSEKGSPAGPSAVTARLGEEVELRLSRNWKQAEEERIQKEKASLTKTGLQCRLCGNDHMTMNCPFKTILSELSALEDPATNEGGVEAASEEKAGQVGGAGSIPGQYVPPSRRAGARDPSSDAYRDSRERDDMCTLKIMQVNENADENSLREELLFPFAPIPRVSVVRNKETGKSRGLAFVTFSSEEVAEQALRFLDGRGYMNLILRVEWSKPKVKE>YDR434WMSNANLRKWVGFCFVAIYLFLGVPLWYKLTTVYRASLPINYIESLQNNKFQDIHLVIPVYVKSDTYRFPDVHDAIQVQVNHLLNSQEQRVPWSLQVLPYNETIEQMESEGNQFHVVTLKLDEFIGYSSAYDTKETLVYYDDAAVLSNDLPFFVAQTLVEHTFQLEWTHLNKTCEGVSTNNDVAISYDPNIHLSVTLLSGDGNPVAWEIEPTLTDYFSPFRKFLSPLVNFTVDSSIVYHNDLNLHSLNGSCTSVTWFDLSHTIDLSELSSMAYYPEDSALNLAIVFPSASSSPDGLAFINGTRISDEITTLDWNSYLVPQWGVIIINKMPLKPNSVISEDYLEPMMYRFATDIFQLLGLTEGSQDLLSPYITIDSFKRLTILQNLDKATETLWSLVKLTQQFQGMSIPREVSDNVIEALDLRLQIIDLLNDPGKGGDIVWNNALHLSNELVKLCEKAFFNGEMVQQNFFPQEHMIAVYLPLLGPISAVMFFGFYNVMKEKNQKSKKNGTEREVAKEKLELKEAQKLHAIDGEDEL>YDR435CMERIIQQTDYDALSCKLAAISVGYLPSSGLQRLSVDLSKKYTEWHRSYLITLKKFSRRAFGKVDKAMRSSFPVMNYGTYLRTVGIDAAILEFLVANEKVQVVNLGCGSDLRMLPLLQMFPHLAYVDIDYNESVELKNSILRESEILRISLGLSKEDTAKSPFLIDQGRYKLAACDLNDITETTRLLDVCTKREIPTIVISECLLCYMHNNESQLLINTIMSKFSHGLWISYDPIGGSQPNDRFGAIMQSNLKESRNLEMPTLMTYNSKEKYASRWSAAPNVIVNDMWEIFNAQIPESERKRLRSLQFLDELEELKVMQTHYILMKAQW>YDR436WMGNSGSKQHTKHNSKKDDHDGDRKKTLDLPPLTKSDTTHSLKSSRSLRSLRSKRSEASLASNVQAQTQPLSRRSSTLGNGNRNHRRSNNAPITPPNNHYLTSHPSSSRRLSSSSRRSSMGNNNNSELPPSMIQMEPKSPILKNSTSMHSTSSFNSYENALTDDDDDRGDDGGESPSMAKVTRINTSSSADRGSKRTPLRRHNSLQPEKGVTGFSSTSSKLRRRSDNTLPASYPLNAEAGGNGSDYFSNRSNSHASSRKSSFGSTGNTAYSTPLHSPALRKMSSRDNDDSGDNVNGRGTSPIPNLNIDKPSPSASSASKREYLSAYPTLAHRDSSSSLSPRGKGQRSSSSSSSSQRIYVSPPSPTGDFVHGSCADGDNGSRTNTMVEMKRKKPVRPVDIDEIIQRLLDAGYAAKRTKNVCLKNSEIIQICHKARELFLAQPALLELSPSVKIVGDVHGQYADLLRLFTKCGFPPMANYLFLGDYVDRGKQSLETILLLLCYKIKYPENFFLLRGNHECANVTRVYGFYDECKRRCNIKIWKTFVDTFNTLPLAAIVTGKIFCVHGGLSPVLNSMDEIRHVSRPTDVPDFGLINDLLWSDPTDSSNEWEDNERGVSFCYNKVAINKFLNKFGFDLVCRAHMVVEDGYEFFNDRSLVTVFSAPNYCGEFDNWGAVMTVSEGLLCSFELLDPLDSTALKQVMKKGRQERKLANR>YDR437WMYTKEYYWFSQYMIITSTLVLTIIWSILPSSLGEAAPKQFINTLLDIFPQRRWIITLESIMLMGMLCTYIGLLMYNEDTLTPPLDSLSTVTDAGGQLVIEDDPDVFVKKWAFKETSGIYDLSLMDACQLLYLYDNDHTST>YDR438WMNRVGIDVDHMIGVLLLAVVVVFWVGASCLTNELLETNAYNKPFFLTYLNISSFALYLTPDLWRIIQSRRKSLQERTERTLPIHTQESFSEFLPLLSSTPSTSSNLSSIADTKVKDTMRLSLLFCVLWFVANLAANAALSYTTVASSTILSSTSSFFTLFLATSLGIETFSTKKLLGLFVSLFGIILIVMQSSKQQDSVSASSFLVGNTLALLGSLGYSVYTTLLKYEISSKGLRLDIQMFLGYVGIFTFLLFWPILIILDITHMETFELPSNFHISFLVMLNCIIIFVSDYFWCKALILTSPLVVTVALTFTIPLAMFADFVWREAFFTPWYIIGVIFIFVSFFLVNHRGESAVEKDCAAVEKGPILDA>YDR439WMTTLLQLLSNYYKAKLDSERIYNEYVQSQYEFASLDKLNNNKGDPKKVVDETLFLQRQIAQLNKQLQLSFQENEKLLSVQKNQKALYQSKLSSKDAFIDDLKLKLKVEQISVDKHNKERTPSTGRDEQQRNSKAAHTSKPTIHLLSPIVNRDKPNNQTNDRGGNDPDSPTSQRRSRGLRSLLSSGKNTIFDSISKNLDDEINENAHIRNDTTSSKIAGKSPSRLSALQKSPELRKERNNMILKEHILRSKDDQNITSSRKLDNIELSSIGDSTAMTSRSSTVNANDILGNEENDGITKLKRVNKLTSSPVKRDCSTNKKRKLTKQRIATLPNSDEELSNNLNVDEFV>YDR441CMSISESYAKEIKTAFRQFTDFPIEGEQFEDFLPIIGNPTLFQKLVHTFKTHLEEKFGKEKIDFIAGIEARGLLFGPSLALALGVGFVPIRRVGKLPGECASITFTKLDHEEIFEMQVEAIPFDSNVVVVDDVLATGGTAYAAGDLIRQVGAHILEYDFVLVLDSLHGEEKLSAPIFSILHS>YDR446WMTVIKTEPTTEVTLYSPPSKESLSKDDAHRKKQNNKPPSSINSRSGPNKHKLAAKAPEKKINNTDKQDLSAFLLNPSLIVKPSESKKKENIVAYNDTPGIKTEHTAFQPLTPISKKRALKEKAASEKCDSFDLSRDEKPYIQKKSKTLSSVTEINSSEYKLSLNGENTSSPAKEKSQEPIENPGSYQKTRNYLFEKPDPLDTCLQDYSSMLPSNVAEEDQEYFISVADSTLEEWTNKGQEIIDQQFQLYQEIIKKRIELSYKFKGIISVINDRADALEEQGQQLEGKIKKVKTLANEILNII>YDR447CMGRVRTKTVKRASKALIERYYPKLTLDFQTNKRLCDEIATIQSKRLRNKIAGYTTHLMKRIQKGPVRGISFKLQEEERERKDQYVPEVSALDLSRSNGVLNVDNQTSDLVKSLGLKLPLSVINVSAQRDRRYRKRN>YDR448WMSNKFHCDVCSADCTNRVRVSCAICPEYDLCVPCFSQGSYTGKHRPYHDYRIIETNSYPILCPDWGADEELQLIKGAQTLGLGNWQDIADHIGSRGKEEVKEHYLKYYLESKYYPIPDITQNIHVPQDEFLEQRRHRIESFRERPLEPPRKPMASVPSCHEVQGFMPGRLEFETEFENEAEGPVKDMVFEPDDQPLDIELKFAILDIYNSRLTTRAEKKRLLFENHLMDYRKLQAIDKKRSKEAKELYNRIKPFARVMTAQDFEEFSKDILEELHCRARIQQLQEWRSNGLTTLEAGLKYERDKQARISSFEKFGASTAASLSEGNSRYRSNSAHRSNAEYSQNYSENGGRKKNMTISDIQHAPDYALLSNDEQQLCIQLKILPKPYLVLKEVMFRELLKTGGNLSKSACRELLNIDPIKANRIYDFFQSQNWM>YDR449CMSKTRYYLEQCIPEMDDLVEKGLFTKNEVSLIMKKRTDFEHRLNSRGSSINDYIKYINYESNVNKLRAKRCKRILQVKKTNSLSDWSIQQRIGFIYQRGTNKFPQDLKFWAMYLNYMKARGNQTSYKKIHNIYNQLLKLHPTNVDIWISCAKYEYEVHANFKSCRNIFQNGLRFNPDVPKLWYEYVKFELNFITKLINRRKVMGLINEREQELDMQNEQKNNQAPDEEKSHLQVPSTGDSMKDKLNELPEADISVLGNAETNPALRGDIALTIFDVCMKTLGKHYINKHKGYYAISDSKMNIELNKETLNYLFSESLRYIKLFDEFLDLERDYLINHVLQFWKNDMYDLSLRKDLPELYLKTVMIDITLNIRYMPVEKLDIDQLQLSVKKYFAYISKLDSASVKSLKNEYRSYLQDNYLKKMNAEDDPRYKILDLIISKL>YDR451CMESRNTVLPSLPNIITGTSNSPFQLHTLPNTNFPSDDQGDIRLPPLAASAHIVRPVVNIYKSPCDEERPKRKSPQAVDFLSQRVTTSMTPLSKPKKLSSHSPFTPTVRVCSKEQPPQSMHSYKKVNILTPLSAAKAVLTPTTRKEKKRSFAFITHSQETFPKKEPKIDNARLARRKRRRTSSYELGILQTAFDECPTPNKAKRIELSEQCNMSEKSVQIWFQNKRQAAKKHKNSGNTSHCKVHSNDSMSMISYSDAALEITSTPTSTKEAITAELLKTSPANTSSIFEDHHITPCKPGGQLKFHRKSVLVKRTLSNTGHSEIIKSPKGKENRLKFNAYERKPLGEVDLNSFKN>YDR452WMVVVGKSEVRNVSMSRPKKKSLIAILSTCVLFFLVFIIGAKFQYVSVFSKFLDDRGDNESLQLLNDIEFTRLGLTPREPVIIKDVKTGKERKLHGRFLHITDIHPDPYYVEGSSIDAVCHTGKPSKKKDVAPKFGKAMSGCDSPVILMEETLRWIKENLRDKIDFVIWTGDNIRHDNDRKHPRTEAQIFDMNNIVADKMTELFSAGNEEDPRDFDVSVIPSLGNNDVFPHNMFALGPTLQTREYYRIWKNFVPQQQQRTFDRSASFLTEVIPGKLAVLSINTLYLFKANPLVDNCNSKKEPGYQLLLWFGYVLEELRSRGMKVWLSGHVPPIAKNFDQSCYDKFTLWTHEYRDIIIGGLYGHMNIDHFIPTDGKKARKSLLKAMEQSTRVQQGEDSNEEDEETELNRILDHAMAAKEVFLMGAKPSNKEAYMNTVRDTYYRKVWNKLERVDEKNVENEKKKKEKKDKKKKKPITRKELIERYSIVNIGGSVIPTFNPSFRIWEYNITDIVNDSNFAVSEYKPWDEFFESLNKIMEDSLLEDEMDSSNIEVGINREKMGEKKNKKKKKNDKTMPIEMPDKYELGPAYVPQLFTPTRFVQFYADLEKINQELHNSFVESKDIFRYEIEYTSDEKPYSMDSLTVGSYLDLAGRLYENKPAWEKYVEWSFASSGYKDD>YDR453CMVAEVQKQAPPFKKTAVVDGIFEEISLEKYKGKYVVLAFVPLAFSFVCPTEIVAFSDAAKKFEDQGAQVLFASTDSEYSLLAWTNLPRKDGGLGPVKVPLLADKNHSLSRDYGVLIEKEGIALRGLFIIDPKGIIRHITINDLSVGRNVNEALRLVEGFQWTDKNGTVLPCNWTPGAATIKPDVKDSKEYFKNANN>YDR454CMSRPIVISGPSGTGKSTLLKKLFAEYPDSFGFSVSSTTRTPRAGEVNGKDYNFVSVDEFKSMIKNNEFIEWAQFSGNYYGSTVASVKQVSKSGKTCILDIDMQGVKSVKAIPELNARFLFIAPPSVEDLKKRLEGRGTETEESINKRLSAAQAELAYAETGAHDKVIVNDDLDKAYKELKDFIFAEK>YDR456WMLSKVLLNIAFKVLLTTAKRAVDPDDDDELLPSPDLPGSDDPIAGDPDVDLNPVTEEMFSSWALFIMLLLLISALWSSYYLTQKRIRAVHETVLSIFYGMVIGLIIRMSPGHYIQDTVTFNSSYFFNVLLPPIILNSGYELNQVNFFNNMLSILIFAIPGTFISAVVIGIILYIWTFLGLESIDISFADAMSVGATLSATDPVTILSIFNAYKVDPKLYTIIFGESLLNDAISIVMFETCQKFHGQPATFSSVFEGAGLFLMTFSVSLLIGVLIGILVALLLKHTHIRRYPQIESCLILLIAYESYFFSNGCHMSGIVSLLFCGITLKHYAYYNMSRRSQITIKYIFQLLARLSENFIFIYLGLELFTEVELVYKPLLIIVAAISICVARWCAVFPLSQFVNWIYRVKTIRSMSGITGENISVPDEIPYNYQMMTFWAGLRGAVGVALALGIQGEYKFTLLATVLVVVVLTVIIFGGTTAGMLEVLNIKTGCISEEDTSDDEFDIEAPRAINLLNGSSIQTDLGPYSDNNSPDISIDQFAVSSNKNLPNNISTTGGNTFGGLNETENTSPNPARSSMDKRNLRDKLGTIFNSDSQWFQNFDEQVLKPVFLDNVSPSLQDSATQSPADFSSQNH>YDR457WMVLFTRCEKARKEKLAAGYKPLVDYLIDCDTPTFLERIEAIQEWDRSRDDLYVWIPILDRMDGLLLKVAEKYKYKQDPKKECEVKLVEMEAHDVDYCLKMLKFTRRLLLNTENRFVYSSGDVLMYLLNCPNFTIKLAVMRILAILGERFVIAREKIVAHNIFGDHNLRKKTLKLALSLSSSVMDEDGEHFSLVDLYFDKKKVPQKWRKLRFTHYTSNDFKKSSQQKNNINETQTSIKKVTMTTQELCEHSLQQIFDKGMALLPAESWFDFSIKASVAKAFSDDSGENIDLRNIIIETKLNAIAFVNTIFSPPQVSSKLFELDPYAFNSLTDLISLSETKIPKELRTDALFTLECISLKHVWCSDIIRNLGGNISHGLLFQILRYIAKTLREATDEIDEEYNVRFFYLISNLADVKPLHESLFAAGLIPTLLEIVSIRNCPYKRTLASATHLLETFIDNSETTTEFIENDGFTMLITSVANEIDFTLAHPETWQPPKYSVVYYSISFRELAYIRSLLKLVLKLLSTDSGDRIRNLIDSPILVSLKKILENKLVFGLTLITYTLDVVQKVINSEPTIYPVLVEAGLIPYVIDNFPKLIGPSAELLSLLPDVVSAICLNPEGLKQVKEKGLINNLFDFLLDADHARILTGGDRSTEYGTDIDELARHYPDLKANIVEALCNVIRKMPSTFRNEREFLFTSPKDQKYFFHRKNEEILTDKEEHEPAYWELLDKGTMLDTFTSVLFGMSLGNGSFSQVPQHLEARDFLAIIFMENPPYEYFTSVAISNVTEVLQYLDEKYEDYAFMDVMKVLNDQLENLNDFLNSPNDRSFFLERDGENSVRSCHSKLCRLAAILNIVTNVYIDLTTLSCKRIMQIYSYFDKRGFSLIKNLKLLFQKCALEEMYIRQHMPDSVITETMPLPIVDVSGDGPPLQIYIDDPKKGDQKGKITSVKTRNTLQMRTILYTLQSNTAILFRCFLRLSHSRNMDLEHKDLTTEVHIFENVVENVIEMLKATELEGHLPYILVLLNFNTFVFTIPKASPNSTEILQTIPAYIFYQKGGYLLYLHIIRDLFTRMTKIKDLSSLDNINYIDESNGILTLSCLINALTFYNKSMQTETMENVQSIGKYYVSIDDDYNIMKALTVPIKVMALAMILDLDKSDSLFKTQSRNVPYSVFKQLLSMLKNIFTNVNIYTKELYELHWDLIFPPIKKISLFEQVGIPGDVAANYLTDTGDDLPADNSIGLFSPEQWEKYKKLIGEDKSIYYPQPMQAQYYKGCSSKELDELRDTFFNDGLPSRIFTVLPFYPKLVNAFAKTLLQIFTKYDEPTEVFAGRILDRILETDLDDPATLSSLIHLFGIFLNEKYIYQKASHLMQRFIEYLEKSLKPEHVNTPWFSKALYVYEIILAKSELPHLEELSKDVLLRYPLLSMAKVFRIPDPMKQKLFDILIRVSDISNFYSALATSRILIFYSRDELYANNIARSGILSRLLKVIGSFQKLDKINFLESSFLLLTRRCFETTENVDALIRAEINRSFTARPLGGGDDAVRELTTILEEKAHVVMRSPSQFIDVLCETARFHEFDDQGALVDYSLKRFLGEKDKNTQASSTEKSDIYERTGIMHLLLSQLMAASEKDWLSEPANSSDLPENKKAQLDPSRNPVCAYMIFLLKLLVELVSSYNQCKFEFLTFSRRNTYAERPRPRTTAINFFLYRLLDKPVGTDHDKHEAKRREVIGMLARSVIIGFLATVQDDRTTKTDVKLADPHMNFIRKFAIEAIIKAIRNATSSSKLLESNHLKLDMWFRIITSMVYVQAPYLRQLLDSNKVEADQYQLCKLVIDLGLPSVITEAMASIDLNYPFSKKIFNVAVEALNTISSTRNNFSEHFKIEDHDEVEDEVDESDKEEIPDMFKNSALGMYDVEDIEEDDDDDTSLIGDDDAMAFVDSDNGFEVVFSDEDDDMGEEDADDARSDSEENELSSEMQSSTADGTDVDYEVDDADGLIINIDQPSGDDEEMADYDANISHSSHSENEDDASMDVIEVYDDELSSGYDVDLSDYDVDESDWDSGLSSLSISDEDSESSEDEPINSTRMGDSRRRWLIAEGVELTDDSQGESEEDDRGVFRGIEHIFSNENEPLFRVHDEMRHRNHHRSINRTHFHSAMSAPSLSLLNRGRRNQSNLINPLGPTGLEQVENDISDQVTVAGSGSRPRSHHLHFSEVLVSGSFFDEPVLDGIILKSTVSRWKDIFDMFYDSKTYANCIIPTVINRLYKVSLALQKDLENKREQEKLKNKNLLFNEAKVESHNSSDAISVEQDDIQESNVTHDDHEPVYVTIQGSEVDIGGTDIDPEFMNALPDDIRADVFAQHVRERRAEARLNSDHNVHSREIDSDFLEAIPEDIREGILDTEAEEQRMFGRIGSSADVIRADDDVSNNDEEVENGLDHGNSNDRNNADPEKKKPARIYFAPLIDRAGIASLMKSVFISKPYIQREIYHELFYRLCSSKQNRNDLMNTFLFILSEGIIDQHSLEKVYNIISSRAMGHAKTTTVRQLPSDCTPLTVANQTIEILQSLIDADSRLKYFLIAEHDNLIVNKANNKSRKEALPDKKLRWPLWHLFSLLDRKLITDESVLMDLLTRILQVCTKTLAVLSTSSNGKENLSKKFHLPSFDEDDLMKILSIIMLDSCTTRVFQQTLNIIYNLSKLQGCMSIFTKHLVSLAISIMSKLKSALDGLSREVGTITTGMEINSELLQKFTLPSSDQAKLLKILTTVDFLYTHKRKEEERNVKDLQSLYDKMNGGPVWSSLSECLSQFEKSQAINTSATILLPLIESLMVVCRRSDLSQNRNTAVKYEDAKLLDFSKTRVENLFFPFTDAHKKLLNQMIRSNPKLMSGPFALLVKNPKVLDFDNKRYFFNAKLKSDNQERPKLPITVRREQVFLDSYRALFFKTNDEIKNSKLEITFKGESGVDAGGVTREWYQVLSRQMFNPDYALFLPVPSDKTTFHPNRTSGINPEHLSFFKFIGMIIGKAIRDQCFLDCHFSREVYKNILGRPVSLKDMESLDPDYYKSLVWILENDITDIIEETFSVETDDYGEHKVINLIEGGKDIIVTEANKQDYVKKVVEYKLQTSVKEQMDNFLVGFYALISKDLITIFDEQELELLISGLPDIDVDDWKNNTTYVNYTATCKEVSYFWRAVRSFDAEERAKLLQFVTGTSKVPLNGFKELSGVNGVCKFSIHRDFGSSERLPSSHTCFNQLNLPPYESYETLRGSLLLAINEGHEGFGLA>YDR459CMALSWNIRIRRRSWFRFILPIIVLGLLCYGTWAYCHKLCYEQVDKRLRQKSVSVGLICAVCFLDVVVIFIWLQIVILVGPGTQPHVAPFLILPIASEEKTSNTSQNTSVEYDAVVPPKCYQSDPHGYPIWCSECQSLKMERTHHSSELGHCIPRFDHYCMWIGTVIGRDNYRLFVQFAAYFSTLLLIMWVSICVYIRIITQHNHNYSPNLNANIISTLVFAILGWLLTASLLASSIFYMSQNKTSLEAIIDSKRKKFGTRKIFCYYSEANKLRFVVEFDRSEFHSFWDKKSILANIKDFMGSNILMWIIPLGKPYTSRCKSDGKSGSKTTLVEILGPYEETLSDYTIQAIEDKISRGEYLATLRASGDDSDPAY>YDR460WMLMDEYEENKDMCPICKTDRYLSPDVKFLVNPECYHRICESCVDRIFSLGPAQCPYKGCDKILRKNKFKTQIFDDVEVEKEVDIRKRVFNVFNKTIDDFNGDLVEYNKYLEEVEDIIYKLDHGIDVAKTEEKLRTYEELNKQLIMNNLERSRTEIESFEQRQKFEKEMKLKKRLLERQIEEEERMNKEWTKKEIVNRLSTTTQDINETIEGVKNTVKLKKSSARRKLEELNRVLKNNPYFNSNVNVQNSRLKDAVPFTPFNGDREAHPRFTLKGSVYNDPFIKDLEHRKEFIASGFNTNYAYERVLTEAFMGLGCVISEEL>YDR462WMLAQTFKKPHRAVLEQVSGTTVFIRNKRTKSKSSLSPLAQRVVTQLSVMSASRKQPKLLKLAREDLIKHQTIEKCWSIYQQQQRERRNLQLELQYKSIERSMNLLQELSPRLFEAANASEKGKRFPMEMKVPTDFPPNTLWHYNFRK>YDR464WMAYDEDDGEINFNELVGNLLSSHNQEGQEEGEVQGGEQEGDDFEKIYPTSENIEPKHPDDSQHMHNSPDQNIEIPHFVDEEDELVSVVANAVQNIDDEQAKPENHLENGSEHVTSDTADDNHEKEQQQEWAHILQQEILKSDGEPLRENTERRVSTSQHHPSQRTDDALDQDDENLRMAILESLQELNTNEEEEKEPEKHEHAAPNDKLSSKKSSKKKKKDKSKNRESSKDKSSKKSKSSSHSKKHAKDRNKEKQSKPTNNENTLDLSNILENLIHENDNAAIDTAKQTVDIQDNSHTDNTNNEDVEAQALVEATLKAFENELLSSAPTEEPSQEQSIGPVSSRKAVEPPRKPTADDIPLAMLQAFKPKKRPPQEKKKTKSKTSKAASTANKSPASESTSKKKKKKKTVKESNKSQEAYEDDEFSRILADMVNQVVNTSLKETSTHTATQDNKLESESDFTSPVQSQYTTEDASTANDDSLDLNQIMQNAMAMVFQNQNDDEFDENIVEDFNRGLGDLSVSDLLPHDNLSRMEKKSVPKSSSKSEKKTAISRRASKKASRDASSVELTEVPSKPKKPSKTEVSLEKKLRKKYVSIANEAASVARKKRWAKNKELKEKEKLERQTAREERRHKKKLEKQRLAEEQEELKKIVERGPPYPPDLRLTKSGKPKKPYRRWTPEELLKRSQEAEKPRKVKKERKKKEKKMKVPSSALKKIPLFNFVKGNVQPSARHRLNDIEGSLSTIGLHKSPDGVRRILSRPKSEDHEWPLSDSSASQNYDAHLKTVVHKEKIPFHPPWTIPSQPPFALPVARRKKIPNIKKYRKRTNNSFRVSKEGTASTRNRILPAILLPIINTLKAAAKSQTAAGATPEEARKRLATIIQHAKSTVIRAALQARKNSMQAAHSKGTTTELATTASRMKNPLKMIPIFNTSRVKQQLDKQLPARSAGTEISSSESPDKATPDPHSNSTIAGHTLKGVTTPIKIEDSDANVPPVSIAVSTIEPSQDKLELTKRAESVEPVENNVETAKETQSVQEIKENVGTKASEEVTLTEDKTNGDPKNEKRILIESPVEKTDKKKPGEKIATDLNEDASLSDKKDGDEKSTLHSDAAQLTGNEPDSVNTTTGKPKLIDVSLKPLNEAKPKIPIIFPLKRPQIKPEVSVINLVQNLVNTKIPEIKNESVDLGSNITDILSSTITNILPEITATDVKNYQYEDENVKYLKKTPRQVLNLDGLVPPSGRCITKAKRVRRIKKLSADATTAPEADGKANSESITYTFDIPSPEEVQSKRSVVLKFAKARLTEAELSCLKKEINNVRKRRWREMNSTKNWEYDVKSRLKKRANAFFGEGESETKSKWIEERFQEKVSQEKYKDRLETTETQANNTKIVIDDKEILNILAVNMNNLNKARCIEKDIQESFREEKLASLQPKKKRKKSILH>YDR466WMTSRKRSPHDFIFKEELGHGSYSTVFKALDKKSPNKIYAIKVCSKKHIIKEAKVKYVTIEKNTMNLLAQKHHAGIIKLYYTFHDEENLYFVLDFAPGGELLSLLHKMGTFNDIWTRHFTAQLIDALEFIHSHGIIHRDLKPENVLLDRDGRLMITDFGAAATIDPSLSGDSAKFNSDSNGSKDNQNCASFVGTAEYVSPELLLYNQCGYGSDIWALGCMIYQFVQGQPPFRGENELKTFEKIVALDYPWGPNNRINNSTSPINPLVINLVQKILVIEVNERISLEQIKRHPYFSKVDWNDKIKIWRGIWQSQGQSLQQTTLGLPNIPQNILPTRQLHVIDTPARSIQITKQKRKKPTKISNTTSSIVVWRKRLGISTGKDDLGTVPSTTPAVTAPNDTNVLTNTAAHSTANIALPPNSQSNQVKRAQLVAPNRIPPKVPVINDNVRNKSIPRTKPNVPPLQTSSIPQKLSTSSASSALSAPSTEIRNQDLTHTLDGRNSIDIHVLKQDYVFIYGIPYEHEGPAMSLNSYNKIDNDLITSLVAQHKEELKNSESFLQVLTLKKSGMLSYKNTVMEGNDDQENKEHQMANIEDTDLSMYDFEFNELTRKGFLILEKYKNRIWFISLPSYSTLSKIPFNAVKSSTINNNENWVDCFFRARQLLEEKQILDKISNVSFDSKASSEPSSPPPISRKERPLSIGNNVTTLSYTAKNGSQNNAPQNDNVGEEKPFRIPSSTKDRPGANSTPSSRHPRVLSSNNAGETPKKMNGRLPNSAPSTNTYTNGSVPAFNHRPSTNVGNNKHNILTSKKQGSSVFSPSSSTTKPQIKTTGYRQPTPSPPLPQMEFPTTREKYSAPSNMVISSSRYEVLHTLNNSQTNFDREIASRGASAAFRSLQKSKKKK>YDR468CMNNSEDPFQQVVKDTKEQLNRINNYITRHNTAGDDDQEEEIQDILKDVEETIVDLDRSIIVMKRDENEDVSGREAQVKNIKQQLDALKLRFDRRIQESTQTTIPLEETVENSTLNTSMAENNDGGMSNPFQEQMLREQDVHLDGIHKTMQNLHIQAQTMGDELENQGQLLDNMDEGMDGVVNKLARGRRQLEWVYEKNKEKYDDCCIGLLIVVLIVLLVLAFIA>YDR469WMNESENSPQHNEVTVPMVEDTSSNADIPMEQIQREDNKNYDKHDNECFDMNGNHNNNSDNLQFDSVPSSATKDLKNIKSVTNQNVKIEESSSTNSVIEESSEPKISKLENVNLAATVGGSQTRKYLNTNVTPHLLAGMRLIAVQQPEDPLRVLGEYLIEQSNILKSGEKESNASK>YDR472WMSQRIIQPSASDQQFPGKSDGYEYTVGPKQAITSEASTTYIPSRIYSESLLFKRQEASLSAMAFLFQEMISQLHRTCKTAGDFETKLSDYGHNIGIRLLELLNFRASVSPSSLPRASAFLSQNESSSKLSNASNSPGMLANSSTATSASANERLQEKQTESLSNYITKMRRRDLKILDILQFIHGTLWSYLFNHVSDDLVKSSERDNEYMIVDNFPTLTQFIPGENVSCEYFVCGIIKGFLFNAGFPCGVTAHRMPQGGHSQRTVYLIQFDRQVLDREGLRFG>YDR473CMPPRNTYEKGNPKRQNSPYYKPSFLRREETTNDEEKFQGHGLKTELHSALKSSNLNLIRRTYQTGENPYLSDPHDRGSSSRFNRRYERGLKFYQKGEISKRIAQERTLQKQQEEEELKRKLKQEEDEKDKRKLIESGDLPNLELHEDKFLLDLSKFKIYYDNNHGYEWWDTAYLDEKGELMEKYDMNGTSPAEEKLAEDIDEVDDDDDDEHPSIRYVAHPLPEKINEAKVSIKAYLTQHERKRLRRNRRKMAREAREIKIKLGLLPKPEPKVKLSNMMSVFENDQNITDPTAWEKVVKDQVDLRKRKHLEENERRHEDAIKRRKEAVNMNVEKPTVYHCKVFQFKNLQNPKIRFKLKMNSKELSLKGLCLRIRDDGPGIIIVVGNEKSCKFYENLVMKRIKWNEDFELHTNTGDIKMDMHNNSISKTWEGYLQDCKFKGWFMKVCNDQDSLLRTLGQFDSEHFYSPVQT>YDR476CMWDSLIVSINDTHKLGLEDCLAVFGHVPITKAVKHVRLTEIDTQTSTFTLKFLHTETGQNIEKIIYFIDNDTGNDTRTATGIKQIFNKMFRIAAEKRKLSLIQIDTVEYPCTLVDLLILVGVALPPLCYLYRPALHAIFFLVPNPVGSTLEAWLDSDLVLRLIIVAEFLTHALETLIFVVPRLKYYRVPGEFVPEWLLLGLLEGYGPARRLDTKARTLGEGSVN>YDR477WMSSNNNTNTAPANANSSHHHHHHHHHHHHHGHGGSNSTLNNPKSSLADGAHIGNYQIVKTLGEGSFGKVKLAYHTTTGQKVALKIINKKVLAKSDMQGRIEREISYLRLLRHPHIIKLYDVIKSKDEIIMVIEYAGNELFDYIVQRDKMSEQEARRFFQQIISAVEYCHRHKIVHRDLKPENLLLDEHLNVKIADFGLSNIMTDGNFLKTSCGSPNYAAPEVISGKLYAGPEVDVWSCGVILYVMLCRRLPFDDESIPVLFKNISNGVYTLPKFLSPGAAGLIKRMLIVNPLNRISIHEIMQDDWFKVDLPEYLLPPDLKPHPEEENENNDSKKDGSSPDNDEIDDNLVNILSSTMGYEKDEIYESLESSEDTPAFNEIRDAYMLIKENKSLIKDMKANKSVSDELDTFLSQSPPTFQQQSKSHQKSQVDHETAKQHARRMASAITQQRTYHQSPFMDQYKEEDSTVSILPTSLPQIHRANMLAQGSPAASKISPLVTKKSKTRWHFGIRSRSYPLDVMGEIYIALKNLGAEWAKPSEEDLWTIKLRWKYDIGNKTNTNEKIPDLMKMVIQLFQIETNNYLVDFKFDGWESSYGDDTTVSNISEDEMSTFSAYPFLHLTTKLIMELAVNSQSN>YDR479CMDSVTNFFWNDTYNAGTPTRSTLKGKKVQNGIDGKSQAKKESISSGSRTSDPTRGSLPSSSGQPTSGGGFPSTSNIQKMMADTLVEKIIKMALPPSSKTAVDTIHHRMVAGKERPKLSVQITSRNFIQMNSRLGVPFMIMDELIKILNWTNPAYTVSIMFLYTLIILKPFQMLSSLPIFYLLFCVMVPQYLYIHKPNPTSYLDNNQTPAQGPPLRRPEVPKPVPELSQEFVLNLTDLQNHMLLYVKFYDFTLLILQKFAFFTNEAISSFYFIVLLIIATLNFLYMDKFIKLIPMRPVLILLGWGFFIASHPSNREYLLTKLNSEETRLKTLTISTNLESKILQHLKLIEAREHRLVMIFEIQKYLPEYKEWRPVGFSDDDYSLFSSLRIYQRRIEENSVKSLEEIEPPKDWEWEANSHWVLDLDPKEWVEDEFIQYVEIDSETKWVYDLNLDGQRGSYRRRMWTNSCVRKKLDSGISSNLGEEEVVNPLREETYRQGVHGVTKGSMSGGLTHSSDDDRADEESINGTIPNLNNIDADASYPSIEELTDTLNSTI>YDR480WMNKEEQEDPQQEQISTVQENDPRNLQQLGMLLVSPGLDEDRLSEKMISKIKKSRDIEKNQKLLISRLSQKEEDHSGKPPTITTSPAEKTVPFKSLNHSLKRKRVPPALNFSDIQASSHLHGSKSAPPNITRFPQHKNSLRVRYMGRMAPTNQDYHPSVANSYMTATYPYPYTGLPPVPCYPYSSTPTQTHAYEGYYSPMYPGPLYNNGIIPADYHAKRKKLAGRSPHLEDLTSRKRTFVSKHHNGDPIISKTDEDIECSVTKNSLSEGASLNDDADDDNDKERIIIGEISLYDDVFKFEVRDDKNDYMKACETIWTEWHNLKK>YDR482CMSYNGIGLKSAKGSSTSGHVQRSLASNNRRRPQGSQQQRQQRQNAIKKASHDKASRPLAVQKQIETHMEKREIEVQVSELRDRLEEEETLSEEQIDKKCEALRAKLTNEWQEQQRMSSLYTPRKARLTEEQHRHE>YDR483WMALFLSKRLLRFTVIAGAVIVLLLTLNSNSRTQQYIPSSISAAFDFTSGSISPEQQVISEENDAKKLEQSALNSEASEDSEAMDEESKALKAAAEKADAPIDTKTTMDYITPSFANKAGKPKACYVTLVRNKELKGLLSSIKYVENKINKKFPYPWVFLNDEPFTEEFKEAVTKAVSSEVKFGILPKEHWSYPEWINQTKAAEIRADAATKYIYGGSESYRHMCRYQSGFFWRHELLEEYDWYWRVEPDIKLYCDINYDVFKWMQENEKVYGFTVSIHEYEVTIPTLWQTSMDFIKKNPEYLDENNLMSFLSNDNGKTYNLCHFWSNFEIANLNLWRSPAYREYFDTLDHQGGFFYERWGDAPVHSIAAALFLPKDKIHYFSDIGYHHPPYDNCPLDKEVYNSNNCECDQGNDFTFQGYSCGKEYYDAQGLVKPKNWKKFRE>YDR484WMDVLKEVLSLDQDKFDQLKETSRDKTNETDDPFENYLKDCKFKAPSNKDQSPFAKLKSLQETHSNNEAAINIIIPQLIDYLTEFTNRLSNYTQDLDFIKKKSNELQSLLEYNSTKLAHISPMVNDLMIPPELIDDIIKGKINESWQDNITFIADKEEIYNKYRSNNLDQDNKDAENSAMLAPKDFDKLCQLLDILKNVILERSKRLIISKIKTLRSHNPVPSQRIQNKLLKVQKIFPFIRDNNLSLALELRQAYCYTMKWYYREYFSRYIRSLTILQFQQIDSQFALGNGLSTTSVSGFNNSPSLFFSNYLTTSASNAFYNKLPVTDEKIDKYFQIKKRLNILTQEDNTVMVSQIAENNTTKNYIEIGFKNLNLAILDNCTVEYHFLKDFFAMNGDNFEEINGLLEQIFQPTFDEATTYTQQLIQYNYDIFGVLISIRVANQLQFESERRGIPSMFDSFLNGQLIQLWPRFQQLVDFQCESLRKAAITTNVAKYAGNSSTSNSSPLTSPHELTVQFGKFLSSFLTLAITHKQSIDERSEPLYNSIIRLRNDFETVMTKCSKKTKSPERFLATNYMYLYNNLQQLHLHLNINDSDAQNYNFDSAENVGTKVANDDDNDSSVPLIIRETENHFKTLVEAFTRN>YDR486CMNRIFGYGNKKSHDQLLQESNQSMNQAQQSLSNRISQLDTQIAQLNFQLQNIQKNLQRSNNKQPSLRKQALKILNKRKQLENMKDSLDSQSWSMTQAQLTNDNLQNTMITINALKQTNNAMKAQYGKINIDKLQDMQDEMLDLIEQGDELQEVLAMNNNSGELDDISDAELDAELDALAQEDFTLPTSENSLGNDMPSYLLGANAPPAFIDEEPNLDTEDKNKALESAQ>YDR487CMFTPIDQAIEHFKQNKFVIVMDDAGRENEGDLICAAENVSTEQMAFLVRHSSGYVCAPMTNAIADKLDLPLLRTGMKFESNDDDRHGTAYTITVDVAQGTTTGISAHDRSMTCRALADSSSTPKSFLKPGHICPLRAADGGVLQRRGHTEAGVDLCKLSGLSPVAVIGELVNDDEQGTMMRLNDCQAFGKKHGIPLISIEELAQYLKK>YDR488CMERLKQLEEKRRQLKELRERRKQASLFPGSETMGHHPTEVHAKATMVSVSVQTDMEEGSKIQEPQSAYLRRKEVITYDKGIQTDQIEEEQLQENENHTTTDAVAIETTAADENNKDKAENDQPRLELAKPFLVEEAAATLSNASFARLETEVSASGQQAPSNMQQDKDNLMQWNMVSENLQSETDCDCIAQEYDPGKGVLVVVYLRLPPADLQYASSEAAWSVVNVVKCDNASGRNGLLIDMVEFRGTRIMTATILRRYHPESNVISILLATLTGKIILYELRLKQKKPETPVVYVVQRNMVARHYFQHPVVAVIETSSVQDQERVLVAADNGNIMELSCLDLTVLRKPQQLRPVPLSQLLSLENDTCTYTERLQRLAKFDEVGIACMAYTSEDPQYVWIGGEDGGIYKVFWDQPGPLYLSLDNNGFQPAENHSTRVTGLEFHWDDARRLMLLLSCSTDWTVRLWDARAGKAIIGAPLLLGGPVLRARWLEKNNGGENSRTLRCQVWCADGRLVVVNWAFDAKTSLYTATVIS>YDR489WMDINIDDILAELDKETTAVDSTKITQGSSSTTHRDANTIVGSSLDLNDKTQIYVSPQQDFSDLMKSWKNERCSPELLPYPHQLMKRLLNRISMQSQLIENISMGFLDMQNASNANPPMPNESKLPLLCMETELERLKFVIRSYIRCRLSKIDKFSLYLRQLNEDENSLISLTDLLSKDEIKYHDTHSLIWLKLVNDSILKYMPEELQAINDTEGSVNMIDEPDWNKFVFIHVNGPPDGKWNEDPLLQENEFGKPCYTVTIPDLKEEVELTIGSIYVMRYEVIRDLLRDDKVALI>YDR490CMGNRSLTEADHALLSKPLVPTSAEHTQTQEYPRPFVDGSNSQSGSELQASPQGQFGEKALTSTNRFIPLANDDPGMQHEMGLDPSMRRRREEWAERGAAKIVKDVVDPATGELTKHVVKMGIKDFKFGEQLGDGSYSSVVLATARDSGKKYAVKVLSKEYLIRQKKVKYVTVEKLALQKLNGTKGIFKLFFTFQDEASLYFLLEYAPHGDFLGLIKKYGSLNETCARYYASQIIDAVDSLHNIGIIHRDIKPENILLDKNMKVKLTDFGTAKILPEEPSNTADGKPYFDLYAKSKSFVGTAEYVSPELLNDNYTDSRCDIWAFGCILYQMLAGKPPFKAANEYLTFQKVMKIQYAFTAGFPQIVKDLVKKLLVRDPNDRLTIKQIKAHLFFHEVNFEDGSVWDDNPPEIQPYKINAEAMKPLQKVSESDTTVKMANLQLAGNGHADTPLQAPAATSQEHSVISMTAATAAFNKDYTSQPKLGSKSSTSVRSASNNTDREVIQKKVSKNRASVSSPSISTTSRGKDNRSRSSDAFWSRYLQNMDERVLLMKEVALSTRNLEDSPVGLENVALDYKNPLDIEPPTDSAGKFYKKMFLITNLGRALVFVKRRSLSMWEEQEFELQFELELNDVEKIRFISDQVLEIDGSRTIFIGCKERAVLMKLWKLIHNGMTAKPKVVSPKSDHKMFDKFILQKRQNTKKKNQAPPVPQSNRLINGLPDRCILKTPEEGALHTKRPTSLQTRSSSNYSKLLARSTQMRKNMTRTDEK>YDR492WMSITTTRRRNQDSVCCKATRASIKVEAVSGQTVFEKQKLLHNFDELPEWQKDNDKILTGYVRETLSWKKCLYSLFYWNNETVNIYTHLVPAIVYFVFAITLTNYFLIPVFPSTSWSDYTVINIFLMGAFSCLMCSSCFHCMKQHSEKQSNFWSKLDYLGIISLISCSMIPIIYFGYFDHISYFSLFTIVTLVLATFCTVCVLHDKFNTSTFRPFRAMFFILFGFSGLLPLTTGFFKFGIQGVLNRIKVSFVFWEALFYISGAVIYGFRIPETLAPGKFDFFGSSHQIFHIMVVLGSVCHLKAIIDSYKLMHSHIHP>YDR493WMSTRTKALNAYRHGLRATRIAFRNDAEVLLAARAKMRSGMLCPPDPKLTTEDQIQHLEDVAVFLRRNLVQGKKVDGSSTKEPRYHLNIHKDTELGDNETIADPTARVKTNLKARPFKCSDKKQ>YDR494WMRSSMFRCVSRAHYSTSVTEDFINSILARAQEATAKASSNALKLDKMKEGRMQNKRRNGNQNRNSMNNKESRGREGNQGERNMRLKNRSSDSVRANKQQWNKGANTSFVKNPTGNTVVMQPQFKKMQNGKNNLKGDARVEDDLLDVFNSSMEQKPVNFNGTPKSKARFQKKSHILTASKRRKAPQQQLQKVIKRPVSSEYVLEEPTPLSLLEYTPQVFPTKESRLVNFTLDSLKKSNYPIYRSPNLGILKVHDFTLNTPNFGKYTPGSSLIFAKEPQLQNLLIEEDPEDFHRQVTGEYQLLKPYVKKDFEKLTKSKDTVSKLVQNSQVVRLSLQSVVMGSEEKKLVYDVCSGMKPISELQQ>YDR496CMAPLTKKTNGKRSAKEVSHSEKKLAKKPRISIDSSDEESELSKKEDAVSSSSDDDDLDDLSTSDSEAEEEADELDISDDSEEHENENEEKEGKDKSEGGENGNHTEQRKLLKERKMQRKSGTQVQQIKSVWERLRVKTPPLPKQIREKLSNEIWELSKDCISDLVLKHDASRIVQTLVKYSSKDRREQIVDALKGKFYVLATSAYGKYLLVKLLHYGSRSSRQTIINELHGSLRKLMRHREGAYVVEDLFVLYATHEQRQQMIKEFWGSEYAVFRETHKDLTIEKVCESSIEKRNIIARNLIGTITASVEKGSTGFQILHAAMREYVKIANEKEISEMIELLHEQFAELVHTPEGSDVACTLVARANAKERKLILKALKNHAEKLIKNEYGNIVFITILNCVDDTVLVFKTFSPTVKEHLQEFIIDKFGRRPWLYILLGLDGKYFSPIVKNELLRYIELSKATSKKDPLQRRHELLSKFAPMFLSTISKDYSSILTENLGCQFIAEVLINDELYAQLNEKDQEKYQQVLNNILTTFKGDITEEEHPIHRAFSTRLLKALIQGGKWNNKEKKVIPLKNVQGLGVPFAEKLYDEIIDSSNLLEWINNADSSFTIVALYETLKDQKEGKPFLKDLRGVQSKITTDESNKGSQLLAKLLK>YDR499WMRRETVGEFSSDDDDDILLELGTRPPRFTQIPPSSAALQTQIPTTLEVTTTTLNNKQSKNDNQLVNQLNKAQGEASMLRDKINFLNIEREKEKNIQAVKVNELQVKHLQELAKLKQELQKLEDEKKFLQMEARGKSKREVITNVKPPSTTLSTNTNTITPDSSSVAIEAKPQSPQSKKRKISDNLLKKNMVPLNPNRIIPDETSLFLESILLHQIIGADLSTIEILNRLKLDYITEFKFKNFVIAKGAPIGKSIVSLLLRCKKTLTLDRFIDTLLEDIAVLIKEISVHPNESKLAVPFLVALMYQIVQFRPSATHNLALKDCFLFICDLIRIYHHVLKVPIHESNMNLHVEPQIFQYELIDYLIISYSFDLLEGILRVLQSHPKQTYMEFFDENILKSFEFVYKLALTISYKPMVNVIFSAVEVVNIITSIILNMDNSSDLKSLISGSWWRDCITRLYALLEKEIKSGDVYNENVDTTTLHMSKYHDFFGLIRNIGDNELGGLISKLIYTDRLQSVPRVISKEDIGMDSDKFTAPIIGYKMEKWLLKLKDEVLNIFENLLMIYGDDATIVNGEMLIHSSKFLSREQALMIERYVGQDSPNLDLRCHLIEHTLTIIYRLWKDHFKQLREEQIKQVESQLIMSLWRFLVCQTETVTANEREMRDHRHLVDSLHDLTIKDQASYYEDAFEDLPEYIEEELKMQLNKRTGRIMQVKYDEKFQEMARTILESKSFDLTTLEEADSLYISMGL>YDR501WMSHLFPPSSPVAGKPLESPQKEPGKLANTSVLTLGRKRYNYELEEYPTPDPSSSIGRQSSPVKDITSRLNETKSALSSPSKQEKVLAGPIEIELDASDPSRLAIGRKKSVCNIILPCRKNISRQHAFISYAADRNEIKLECNGTNGLSVHLPYSMQLHLVKPFPTRNFYKLVAEEPLTSQNTKQSHGKTLQKNQNFISFVLAKGETVTFPYIQGSFINFTGVTVCLSLKKVAPYPGDGNNNFDEENSTETEDELCLLTTTSDDFSWQKETPSMKFVPVEHSPRTEQISKPLLIASPALVKNSPISYRTTPQTSFVINQPSTPKKLKRKSISLKNNTIQETPLPKDKIIGTLSASTRSGGINEEESFAAVAKKTKELSSTTAIVSPAQKRLKTSLNIIPEISRSLSERGIRFDDLVHVLCNHLAFSNLQQTPLSQLQNINSNTSQLSKDELKKVLETISCIGIIVREGKDASGKPLEDEYYYDVENDDSDERKILYNSLKGRSRLRSCRKKHKQYFWKRPTK>YDR502CMSKSKTFLFTSESVGEGHPDKICDQVSDAILDACLEQDPFSKVACETAAKTGMIMVFGEITTKARLDYQQIVRDTIKKIGYDDSAKGFDYKTCNVLVAIEQQSPDIAQGLHYEKSLEDLGAGDQGIMFGYATDETPEGLPLTILLAHKLNMAMADARRDGSLPWLRPDTKTQVTVEYEDDNGRWVPKRIDTVVISAQHADEISTADLRTQLQKDIVEKVIPKDMLDENTKYFIQPSGRFVIGGPQGDAGLTGRKIIVDAYGGASSVGGGAFSGKDYSKVDRSAAYAARWVAKSLVAAGLCKRVQVQFSYAIGIAEPLSLHVDTYGTATKSDDEIIEIIKKNFDLRPGVLVKELDLARPIYLPTASYGHFTNQEYSWEKPKKLEF>YDR505CMDLPTVNSTTSISDNVDLKNYYEDLLFKNNSGKSLSDLPRKLNDNSNNSGSDTVDPLAGLNNLRNSIKSAGNGMENRRTFDDIDFMGRFPYLPPVPNQQQQPFSHQNGFIQEHPSSNLTSFQMSSSNSEPMSAPPISSNNNNLNSTQMGNYQAQQRSFPQFNGNSFHSNGNDLMGNRMDSDYMRLMNKTNIGFTSNSGSNFAAPSHSAGNPSSMNNQQVPSFNWQQPSHPESTIRRSSYISDTLINHQMPDARQKQTSQVQQQHAQGFNLFNSRFNYDNLNSTHLTAKGVPEFGNGVQPPYPYDNEPNNASISNSNNNNNSHNMVPMQQFRRNTQPVASFNPSLPTFQQQQQQPQQPQQPRNVNVPTSFNGERVDDVQLVQLQRSSSVPSSTNSHNLQNENSNEGNVSLDNGLVLIQGKHLTSSKTLHDLYSDCGSGYFASSAVFEFTDNIKKMLKLHDSNESYDAKNMGLIDEEGNTYQSLLNFLDILRSCNMNYVNDPESNNGIVSNNGGNKNRRKGSFTTELSCRNANNSFLPYTPLVLVALKNGKLELLSTPQATNLLLKRGDLVIIDGDRGRDLVLVVEPSVDLNLALFINFLKKKIHFDSLITSESQHYRNDEFIQMLIDSKNGQKKKLNPKLYDVVELTELIIPSKQVLRFATPWEVTTNLHNKFEDELKALHIAQSKLQALNDNSKSQNTNDSSSNNFTNAATYSKPKLNIKILNAEFQFDRKELTFYYVCEERNDFRDLIKELFKYYKTRIWLCAIPNNLSIDSKYYDKQQKELKLYQNIVKNYNAEDLMNVNEFSQNRGNNRVNFAPPLNEIELDNFQIAVYEELVHELFH>YDR507CMAINGNSIPAIKDNTIGPWKLGETLGLGSTGKVQLARNGSTGQEAAVKVISKAVFNTGNVSGTSIVGSTTPDALPYGIEREIIIMKLLNHPNVLRLYDVWETNTDLYLVLEYAEKGELFNLLVERGPLPEHEAIRFFRQIIIGVSYCHALGIVHRDLKPENLLLDHKYNIKIADFGMAALETEGKLLETSCGSPHYAAPEIVSGIPYQGFASDVWSCGVILFALLTGRLPFDEEDGNIRTLLLKVQKGEFEMPSDDEISREAQDLIRKILTVDPERRIKTRDILKHPLLQKYPSIRDSKSIRGLPREDTYLTPLSESNSSIDATILQNLVILWHGRDPEGIKEKLREPGANAEKTLYALLYRFKCDTQKELIKQQQVKKRQSISSVSVSPSKKVSTTPQRRRNRESLISVTSSRKKPISFNKFTASSASSSNLTTPGSSKRLSKNFSSKKKLSTIVNQSSPTPASRNKRASVINVEKNQKRASIFSTTKKNKRSSRSIKRMSLIPSMKRESVTTKLMSTYAKLAEDDDWEYIEKETKRTSSNFATLIDEIFEYEKYEQIRKEKEELERKVREAKAREELERRRRKQEEKERARKLLEKEDLKRKQEELKKQIEIDISDLEQELSKHKEEKLDGNIRSISAPMENEEKNINHLEVDIDNILRRRNFSLQTRPVSRLDPGIMFSSPTEEVSPVEPKRTENERLTTEKKILETIRRSKFLGSSFNIDKELKLSKMEYPSIIAPQRLSEERVVSDSNDGYESLILPKDGNGVSQLKDSTATTAPVSDGRLRKISEIRVPQFTRKSRHFSESNKRLSVLSMYSTKESFTNLVDILKNGNLDVNNQQSQRIPTPRSADDSEFLFETVNEEAEYTGNSSNDERLYDVGDSTIKDKSALKLNFADRFNGSNEAKQTDNLHLPILPPLNGDNELRKQNSQEGDQAHPKIKSMIPESGSSSHTEKEEENEEKEEKKPEQHKQEEDQEKREKVVDDMEPPLNKSVQKIREKNAGSQAKDHSKDHLKEHKQDKNTAIGNGSFFRKFSKSSDKTMELYAKISAKQLFNGLEKLLRGWTQYGLKNIKSHPNNLTLTGKLSSDNIFSLRSTLFEVNIYPRGKMSVVQFKKVSGSFKAVKKLVNEVENVLNKEGVLQK>YDR508CMTLGNRRHGRNNEGSSNMNMNRNDLDDVSHYEMKEIQPKEKQIGSIEPENEVEYFEKTVEKTIENMEYEGEHHASYLRRFIDSFRRAEGSHANSPDSSNSNGTTPISTKDSSSQLDNELNRKSSYITVDGIKQSPQEQEQKQENLKKSIKPRHTVMMSLGTGIGTGLLVGNSKVLNNAGPGGLIIGYAIMGSCVYCIIQACGELAVIYSDLIGGFNTYPLFLVDPALGFSVAWLFCLQWLCVCPLELVTASMTIKYWTTSVNPDVFVVIFYVLIVVINVFGAKGYAEADFFFNCCKILMIVGFFILAIIIDCGGAGTDGYIGSKYWRDPGAFRGDTPIQRFKGVVATFVTAAFAFGMSEQLAMTASEQSNPRKAIPSAAKKMIYRILFVFLASLTLVGFLVPYTSDQLLGAAGSATKASPYVIAVSSHGVRVVPHFINAVILLSVLSVANGAFYTSSRILMSLAKQGNAPKCFDYIDREGRPAAAMLVSALFGVIAFCASSKKEEDVFTWLLAISGLSQLFTWITICLSHIRFRRAMKVQGRSLGEVGYKSQVGVWGSAYAVLMMVLALIAQFWVAIAPIGGGGKLSAQSFFENYLAMPIWIALYIFYKVWKKDWSLFIPADKVDLVSHRNIFDEELLKQEDEEYKERLRNGPYWKRVLDFWC>YDR510WMSDSEVNQEAKPEVKPEVKPETHINLKVSDGSSEIFFKIKKTTPLRRLMEAFAKRQGKEMDSLRFLYDGIRIQADQTPEDLDMEDNDIIEAHREQIGGATY>YDR511WMNNKLIYRSVRFATHNSQLLLPPLVLYRRILRQHKLLPGPQREMGDQYVRNEFKLHKDIDNPLHIVGFLASWQDYLHMISNGKWKDATLSSETLEKLSPEQTVQLYELMKETQKLHQDNEIESSKDVKRNNKD>YDR513WMETNFSFDSNLIVIIIITLFATRIIAKRFLSTPKMVSQETVAHVKDLIGQKEVFVAAKTYCPYCKATLSTLFQELNVPKSKALVLELDEMSNGSEIQDALEEISGQKTVPNVYINGKHIGGNSDLETLKKNGKLAEILKPVFQ>YDR515WMSSQNLNDNPKNTSSAAEDKKKQTSSLKLAPIPTTSPWKSSSPDSNTVIPVEELRDISKTAKPSKNGSGSIKLTSNTKWTPITPSVIISGSKDTNSKSGKNSKNSKTNKKMKKRGKYNNDINKKDFNGQTNSTSEISNVSNLESKPLDANAKVNIHSSSGATANGNIKRITNNNNSTNGRQSRNYQNRNGKTRYNNNSRHSQAANNAISFPNNYQARPEYIPNASHWLNNNSRNSYKQLSYFRQQQYYNNINYQQQLQTPYYYSMEPIFKSIESIKNQIEFYFSEENLKTDEFLRSKFKKANDGFIPMSLIGKFYRMVNLSLGGDPNLILASMREVLQHKETNHLEIALGSIEGAQKNMADDFNPLENYFIRRENWAEYAMESNFDENDDETEKYNIEKLLGPNDLDNYSYMGYPNFFPSNENGKKSQSYDQGEISRQFEQNLQIND>YDR516CMSFENLHKVNAEALEDAVVEICSSLQVDAAKLDELTAYFIECMEKGLNNTSVGEEKTVDKGLPMIPTYVTSLPNGTERGVLLAADLGGTHFRVCSVTLNGDGTFDMQQLKSKIPEEYLNDKDVTSEELFSYLGRRTRAFVRKHHPELLKSTGENIKPLKMGFTFSYPVDQTSLSSGTLIRWTKSFKIEDTVGKDVVRLYQEQLDIQGLSMINVVALTNDTVGTFLSHCYTSGSRPSSAGEISEPVIGCIFGTGTNGCYMEDIENIKKLPDELRTRLLHEGKTQMCINIEWGSFDNELKHLSATKYDIDIDQKFSPNPGYHLFEKRISGMYLGELLRNILVDLHARGLILGQYRNYDQLPHRLKTPFQLCSEVLSRIEIDDSTNLRETELSFLQSLRLPTTFEERKAIQNLVRSITRRSAYLAAVPIAAILIKTNALNKRYHGEVEIGFDGYVIEYYPGFRSMLRHALALSPIGTEGERKIHLRLAKDGSGVGAALCALVA>YDR517WMFRIAKNLVRTFEQSVQDTLALSQDSSNLDAFFQSIPPNLLSAQLESPVDAVSEGVKHTNVNETLSGLRIVWVDEMQFQLQSFFDYIVGFNDDPVPVVSNQHGFSYPDYRRITSIFNEHCGRTLKVNIWSAKGGTFRDEYISIISKESDDLDDVSLNHDERRPSSGEAHQFQALGFKVQWTPLIASTFTYHILNVNIPDGPAQSAGLIPDEDYIIGCQDGLLATGGETLLQDIVRSRANYDLVLYVYNKVSDCVRPITVHIGPDGRLGCNVGYGFLHRIPTVKHCPQQAQQQGQDDNPVPVPVPVESETAFVPSAFTAPPVPTKKKSKNKKGTQPLAMDDYFNEGRDKSSTAAKSAESDILAPPPQKQSSSD>YDR518WMQVTTRFISAIVSFCLFASFTLAENSARATPGSDLLVLTEKKFKSFIESHPLVLVEFFAPWCLHSQILRPHLEEAASILKEHNVPVVQIDCEANSMVCLQQTINTYPTLKIFKNGRIFDGQVYRGVKITDEITQYMIQLYEASVIYLNSEDEIQPYLENATLPVVINRGLTGLNETYQEVALDLAEDYVFLSLLDSEDKSLSIHLPNTTEPILFDGNVDSLVGNSVALTQWLKVVILPYFTDIEPDLFPKYISSNLPLAYFFYTSEEELEDYTDLFTQLGKENRGQINFIALNSTMFPHHVRFLNMREQFPLFAIHNMINNLKYGLPQLPEEEYAKLEKPQPLDRDMIVQLVKDYREGTAKPIVKSEEIPKEQKSNVYKIVGKTHDDIVHDDDKDVLVKYYATWCIHSKRFAPIYEEIANVLASDESVRDKILIAEVDSGANDILSFPVTGYPTIALYPAGNNSKPIIFNKIRNLEDVFEFIKESGTHHIDGQAIYDKLHQAKDSEVSTEDTVHDEL>YDR519WMMFNIYLFVTFFSTILAGSLSDLEIGIIKRIPVEDCLIKAMPGDKVKVHYTGSLLESGTVFDSSYSRGSPIAFELGVGRVIKGWDQGVAGMCVGEKRKLQIPSSLAYGERGVPGVIPPSADLVFDVELVDVKSAA>YDR520CMDINSNASVSPRPDGLPMTAGYNSASGKVRNSIRSIINHPEDSARAKERSETNSPKNNGNKKPRKKRKTFSCDTCRRVKTRCDFEPFIGKCYRCNVLQLDCSLARNKDNEILNTLREDGLLKKINSINHNLGSFSHLNADSPNESQSSFEKNGTVNFDNYMIDKRLSSLEEHIKSLHQKMDLIITTAKMSYNSDIKGPGDDIQNVDFSSNKTYDSRLTSGSETIRKTGEYRKENLFLNGFKLKESPLKLLHDIDERLFPSKATSKAAKLAGQQRPYAVARVNFLHFYENNQELCHKLAKEFLVRSHFWIIPGGRKEIDVEYAHSHLFITSVFTIIAMSFADNDKYAAEQEILYPLVERLLTNTLTMFEKLTAFDIEAILYCCMFHISRKAKRYRQLKFNSLVLSNFALNSLLHVIDFYQIKDRVLVKEVYNPEDLYHLRILNSLTACYLEYSISYGDIREQDDMLKEFNKLVAKFPQANFGDDIKISEINLGDIVNGIFINLKNYFAQCLDDFNNDRYGGNADTFIFVFPELNYWLKNWEELLAKDGAGVLLFTFDFYHIMICRTFITEFSSTLKSNQRFLKLILNTMKEHSFSLLNGFLRLPPTLIRGAPIFTCHQLVYACLTLCDYLYWFDSSERQRVLSLCTKVYWHLSTIGEKMNEATDNVGKIIKSIIDTSKTRINFGSLSKENSDNDKMSTNANNYTGAGNLHAAKPATSPTNVGTLHENLSSSHFMIPDVDQFNSFEDFFQDFFDSLKPNSQKMFTSDKKTEQTT>YDR522CMPIWKTQTFFTSISVIQIVNKETKVSTKKEKDSMLNQLNTILRFLFLFLQLIKSSAAVEPNGGPNILDHNIMLVNTNATIPKKEQTDFEVISPTKQTQVDEDCKKGLYHIENAGNLIELQAKCWKVVGNIEISSNYSGSLIDLGLIREIEGDLIIKNNKHIFRIQGYNLESLGKLELDSLTSFVSLDFPALKEVETVDWRVLPILSSVVINGNIKKIKNIIISDTALTSIDYFNNVKKVDIFNINNNRFLENLFASLESVTKQLTVHSNAKELELDLSNLHTVENMTIKDVSEIKLAKLSSVNSSLEFIENQFSSLELPLLAKVQGTLGLIDNKNLKKLNFSNATDIQGGLMIANNTELAKIDFFPKLRQIGGAIYFEGSFDKIDLPELKLVKGSAYIKSSSEELNCEEFTSPKAGRSIIRGGKIECTSGMKSKMLNVDEEGNVLGKQETDNDNGKKEKGKNGAKSQGSSKKMENSAPKNIFIDAFKMSVYAVFTVLFSIIF>YDR523CMESKEISIRSRTPPSKLYSIQSCIGRGNFGDVYKAVDRVTQEIVAIKVVNLEHSDEDIELLAQEIFFLAELKSPLITNYIATMLEDVSMWIVMEYCGGGSCSDLLKRSYVNGLPEEKVSFIIHEVTLGLKYLHEQRKIHRDIKAANILLNEEGMVKLGDFGVSGHIRSTLKRDTFVGTPYWMAPEVVCCEVDGYNEKADIWSLGITTYELLKGLPPLSKYDPMKVMTNLPKRKPPKLQGPFSDAAKDFVAGCLVKTPADRPSAYNLLSFEFVKNITITNLKSDVDLIKQKKVQERYTKVPKYPLQNRLYKNSNTVRGKEFWNFESTRLSTTQISKEELSPITQDSPTSSLNMESPYLLHGQTVTPITNPSSSSFRKCTQPVFELDSGMDIDSGCPNAQAETEIVPLSNHNKKHKKNDIQALKIEKFDYLKNIVSHILNRMYDRARDDETRKYVNEMLKQFIKTEANVPGFNEVFIEEISLRIEAIKKGFV>YDR525W-AMHARDWFLVFIAIFIPPLAVWLKRGFFTKDLLINFLLFLLGFFPGLIHALYVISCHPYEENEARYSHLSSSDDNYGSLA>YDR527WMDLLGDIVEKDTSDSVESNDNGTLSTNNCGTGFPELYKPKKISSWKERLREKRAQKKKTSGKDAEKQQTSTDAPLSEAKSIHNENIKVLQGMSDEQIVQEREDLYNSLDPKLIAKLLKNINKRAKDENNTPLFAEIEGASGTWVGGNKQGIYDLPPLDDEDVDVALEIRPMLGKDAKHVQFEEAGKEKDVEGEGKTNDDVDDIAPLDFQMAQCIDHMKNEELFKDVHFIKEESQNEINLEKLDINDPNFNDKLHEKYFPDLPKEVDKLKWMQPVQQKTDKNYIIEDVSECRFDFNGDLVPPTRQIDSTIHSGLHHHSDSPELAGYTIVELEHLARSTFPSQRCIAIQTLGRILYKLGQKSYYQLVPEIDADTYKEDGSISNVMDKIYSMFWDLIKDGKVIESLEISSDEKFTRNLSVRNYAIDALWLWKQGGGDFRTKK>YDR528WMENLCPPPPSQMKDFSTPPRNRHRHKRSFAISGDFEFLKQPASAPVLPSAYDSPTFENTPRRVSGMSVTMPDESQNESALLNSPSPRFFISEASTYSSPIKGVPDAIINLDDVLINKPKMCRSHRKTKSVPVKLDEFYSSHKCSSVPELTINEEIDEDDTNPQLLEPVKPLSSTSLSTDMNEDKKMTLKNARSHNSLKIQAQKQRYYNSARYLPLNSEDRATDPQILTKQSSVTSLFSSRSITPVSCNINNAGRINAISGNYLDDVLYDLDTPATTLIQDIDNLQTSINERVRLSPQSSSIKKYFSKDGKSVSSFNFQSQECDMVSFTEDFAHVTSLSSSILDSEKQTDDEEEESIPEEILRGEPLHVYNETSGSDKSVILPTKQKSAPINKDSKHSSTQYEEKSFKKNRKFKIFAKLFCTRK>YDR530CMIEENLKQKIHDKFVAAKKNGHLKVTHAESKKLKDPQTTTQYWVTFAPSLALKPDANKNSDSKAEDPFANPDEELVVTEDLNGDGEYKLLLNKFPVVPEHSLLVTSEFKDQRSALTPSDLMTAYNVLCSLQGDKDDDVTCERYLVFYNCGPHSGSSQDHKHLQIMQMPEKFIPFQDVLCNGKDHFLPTFNAEPLQDDKVSFAHFVLPLPESSDQVDEDLLAMCYVSLMQRALTFFQDWTNESPELTKSYNVLLTKKWICVVPRSHAKSGPPLMLNINSTGYCGMILVKDREKLENLTEDPHLVDKSLLQCGFPNTAGQKPTEYHY>YDR531WMPRITQEISYNCDYGDNTFNLAIDIGGTLAKVVFSPIHSNRLMFYTIETEKIDKFMELLHSIIKEHNNGCYRMTHIIATGGGAFKFYDLLYENFPQIKGISRFEEMEGLIHGLDFFIHEIPDEVFTYNDQDGERIIPTSSGTMDSKAIYPYLLVNIGSGVSILKVTEPNNFSRVGGSSLGGGTLWGLLSLITGAQTYDQMLDWAQEGDNSSVDMLVGDIYGTDYNKIGLKSSAIASSFGKVFQNRMTSNKSLENNENKLYSSHESIEKNNGQMFKNPDICKSLLFAISNNIGQIAYLQAKINNIQNIYFGGSYTRGHLTTMNTLSYAINFWSQGSKQAFFLKHEGYLGAMGAFLSASRHSSTKKTST>YDR532CMDTGSASIKDYETVLTDIEDSIAVSSEEVLNNQELRLKNTLHEITSSILAINEENKFVNPLRNDESLDVEGKEVFVNPKILSAKIKEFNKLMELLKLTYLEQETLDYFFRFTLSSTKPLQLDSEKDPQFVKLNERVNDLKEEISNVQESKIEQIKAEIQETGHNFAERQDLINELYLEATGDIENCWDSLNELKNLTNKEDKNMMGEKDTILNSSDSDDFVEETYTNWQKLLFLQKQNQRLTKELKEMHEVKNQIIRKGEQSKKEDSGHLMANESELCQSINLLTKFWEKHFLLKGSKTTILNFEIFTQLGKVQFEIKDMQYIIAISLSDLKRPMIKDITILQKAGGNIVTDIEASSKFNNKYRNNTKVQIFEVMDDIISELTNE>YDR533CMAPKKVLLALTSYNDVFYSDGAKTGVFVVEALHPFNTFRKEGFEVDFVSETGKFGWDEHSLAKDFLNGQDETDFKNKDSDFNKTLAKIKTPKEVNADDYQIFFASAGHGTLFDYPKAKDLQDIASEIYANGGVVAAVCHGPAIFDGLTDKKTGRPLIEGKSITGFTDVGETILGVDSILKAKNLATVEDVAKKYGAKYLAPVGPWDDYSITDGRLVTGVNPASAHSTAVRSIDALKN>YDR536WMKDLKLSNFKGKFISRTSHWGLTGKKLRYFITIASMTGFSLFGYDQGLMASLITGKQFNYEFPATKENGDHDRHATVVQGATTSCYELGCFAGSLFVMFCGERIGRKPLILMGSVITIIGAVISTCAFRGYWALGQFIIGRVVTGVGTGLNTSTIPVWQSEMSKAENRGLLVNLEGSTIAFGTMIAYWIDFGLSYTNSSVQWRFPVSMQIVFALFLLAFMIKLPESPRWLISQSRTEEARYLVGTLDDADPNDEEVITEVAMLHDAVNRTKHEKHSLSSLFSRGRSQNLQRALIAASTQFFQQFTGCNAAIYYSTVLFNKTIKLDYRLSMIIGGVFATIYALSTIGSFFLIEKLGRRKLFLLGATGQAVSFTITFACLVKENKENARGAAVGLFLFITFFGLSLLSLPWIYPPEIASMKVRASTNAFSTCTNWLCNFAVVMFTPIFIGQSGWGCYLFFAVMNYLYIPVIFFFYPETAGRSLEEIDIIFAKAYEDGTQPWRVANHLPKLSLQEVEDHANALGSYDDEMEKEDFGEDRVEDTYNQINGDNSSSSSNIKNEDTVNDKANFEG>YDR538WMLLFPRRTNIAFFKTTGIFANFPLLGRTITTSPSFLTHKLSKEVTRASTSPPRPKRIVVAITGATGVALGIRLLQVLKELSVETHLVISKWGAATMKYETDWEPHDVAALATKTYSVRDVSACISSGSFQHDGMIVVPCSMKSLAAIRIGFTEDLITRAADVSIKENRKLLLVTRETPLSSIHLENMLSLCRAGVIIFPPVPAFYTRPKSLHDLLEQSVGRILDCFGIHADTFPRWEGIKSK>YDR539WMRKLNPALEFRDFIQVLKDEDDLIEITEEIDPNLEVGAIMRKAYESHLPAPLFKNLKGASKDLFSILGCPAGLRSKEKGDHGRIAHHLGLDPKTTIKEIIDYLLECKEKEPLPPITVPVSSAPCKTHILSEEKIHLQSLPTPYLHVSDGGKYLQTYGMWILQTPDKKWTNWSIARGMVVDDKHITGLVIKPQHIRQIADSWAAIGKANEIPFALCFGVPPAAILVSSMPIPEGVSESDYVGAILGESVPVVKCETNDLMVPATSEMVFEGTLSLTDTHLEGPFGEMHGYVFKSQGHPCPLYTVKAMSYRDNAILPVSNPGLCTDETHTLIGSLVATEAKELAIESGLPILDAFMPYEAQALWLILKVDLKGLQALKTTPEEFCKKVGDIYFRTKVGFIVHEIILVADDIDIFNFKEVIWAYVTRHTPVADQMAFDDVTSFPLAPFVSQSSRSKTMKGGKCVTNCIFRQQYERSFDYITCNFEKGYPKGLVDKVNENWKRYGYK>YEL001CMRFSMLIGFNLLTALSSFCAAISANNSDNVEHEQEVAEAVAPPSINIEVKYDVVGKESENHDSFLEFYAEDTATLAYNVTNWEDTNITIFGVNGTIVTYPHGYPVADITGASIGPYEMEVNGTSKFGQDVTLNLPEGQYFLIPFLLASRFDEIVRIAAPPTLFEIVSPPISFFNPQFLSVQVIFLAIIGGVSYYYMKSKTNQRPSKKSATVKKVDESWLPETYKK>YEL002CMRTDWNFFFCILLQAIFVVGTQTSRTLVLYDQSTEPLEEYSVYLKDLEQRNYKLEYLDINSTSTTVDLYDKEQRLFDNIIVFPTKGGKNLARQIPVKQLIKFFENEGNILCMSSPGAVPNTIRLFLNELGIYPSPKGHVIRDYFSPSSEELVVSSNHLLNKYVYNARKSEDFVFGESSAALLENREQIVPILNAPRTSFTESKGKCNSWTSGSQGFLVVGFQNLNNARLVWIGSSDFLKNKNQDSNQEFAKELLKWTFNEKSVIKSVHAVHSHADGTSYDEEPYKIKDKVIYSVGFSEWNGEEWLPHIADDIQFELRQVDPYYRLTLSPSGNDSETQYYTTGEFILPDRHGVFTFLTDYRKIGLSFTTDKDVKAIRHLANDEYPRSWEISNSWVYISAICGVIVAWIFFVVSFVTTSSVGKKLETFKKTN>YEL004WMWNSLKAFALVFGGCCSNVITFETLMSNETGSINNLITFCQFLFVTCQGLPEFLDVHQPFPYFKPLKTPLHVYVITVVLFYISSTTNNNVFKYNISIPIHIVFRCFGTVITMFTCWLLNGRKYTKIQILSTLFLTIGAIIASLFKDADFRYQDLKLQAWKIGSDQSVDLTFIFGICILVLSSFTSSLLSAYNERTYQKYGKHWKENIFYSHFLSLPLFLFSRKQLIHEYRVMRKSERILCSNFGGKILVPREETLLLFNVLTQYFCVKGVNILASKTNALTLSITLLVRKFISLLLSVRLFDNNLSYTGYIGVYLVFFGAFIYSLGSIHPRQNDKGAIKKSK>YEL009CMSEYQPSLFALNPMGFSPLDGSKSTNENVSASTSTAKPMVGQLIFDKFIKTEEDPIIKQDTPSNLDFDFALPQTATAPDAKTVLPIPELDDAVVESFFSSSTDSTPMFEYENLEDNSKEWTSLFDNDIPVTTDDVSLADKAIESTEEVSLVPSNLEVSTTSFLPTPVLEDAKLTQTRKVKKPNSVVKKSHHVGKDDESRLDHLGVVAYNRKQRSIPLSPIVPESSDPAALKRARNTEAARRSRARKLQRMKQLEDKVEELLSKNYHLENEVARLKKLVGER>YEL012WMSSSKRRIETDVMKLLMSDHQVDLINDSMQEFHVKFLGPKDTPYENGVWRLHVELPDNYPYKSPSIGFVNKIFHPNIDIASGSICLDVINSTWSPLYDLINIVEWMIPGLLKEPNGSDPLNNEAATLQLRDKKLYEEKIKEYIDKYATKEKYQQMFGGDNDSDDSDSGGDLQEEDSDSDEDMDGTGVSSGDDSVDELSEDLSDIDVSDDDDYDEVANQ>YEL013WMGSCCSCLKDSSDEASVSPIADNEREAVTLLLGYLEDKDQLDFYSGGPLKALTTLVYSDNLNLQRSAALAFAEITEKYVRQVSREVLEPILILLQSQDPQIQVAACAALGNLAVNNENKLLIVEMGGLEPLINQMMGDNVEVQCNAVGCITNLATRDDNKHKIATSGALIPLTKLAKSKHIRVQRNATGALLNMTHSEENRKELVNAGAVPVLVSLLSSTDPDVQYYCTTALSNIAVDEANRKKLAQTEPRLVSKLVSLMDSPSSRVKCQATLALRNLASDTSYQLEIVRAGGLPHLVKLIQSDSIPLVLASVACIRNISIHPLNEGLIVDAGFLKPLVRLLDYKDSEEIQCHAVSTLRNLAASSEKNRKEFFESGAVEKCKELALDSPVSVQSEISACFAILALADVSKLDLLEANILDALIPMTFSQNQEVSGNAAAALANLCSRVNNYTKIIEAWDRPNEGIRGFLIRFLKSDYATFEHIALWTILQLLESHNDKVEDLVKNDDDIINGVRKMADVTFERLQRSGIDVKNPGSNNNPSSNDNNSNNNDTGSEHQPVEDASLELYNITQQILQFLH>YEL015WMSQFVGFGVQVELKDGKLIQGKIAKATSKGLTLNDVQFGDGGKSQAFKVRASRLKDLKVLTVASQSGKRKQQRQQQQQNDYNQNRGEHIDWQDDDVSKIKQQEDFDFQRNLGMFNKKDVFAQLKQNDDILPENRLQGHNRKQTQLQQNNYQNDELVIPDAKKDSWNKISSRNEQSTHQSQPQQDAQDDLVLEDDEHEYDVDDIDDPKYLPITQSLNITHLIHSATNSPSINDKTKGTVINDKDQVLAKLGQMIISQSRSNSTSLPAANKQTTIRSKNTKQNIPMATPVQLLEMESITSEFFSINSAGLLENFAVNASFFLKQKLGGRARLRLQNSNPEPLVVILASDSNRSGAKALALGRHLCQTGHIRVITLFTCSQNELQDSMVKKQTDIYKKCGGKIVNSVSSLESAMETLNSPVEIVIDAMQGYDCTLSDLAGTSEVIESRIKSMISWCNKQRGSTKVWSLDIPNGFDAGSGMPDIFFSDRIEATGIICSGWPLIAINNLIANLPSLEDAVLIDIGIPQGAYSQRTSLRKFQNCDLFVTDGSLLLDL>YEL016CMLLFEQPVDLEKNNEDDTNIKPFAISRHFLLKLLLCGIILIELLLYSKCPKPIDNGPRTIANRSNTYFNGTHDFKTLTILISIDGFHPRLIDAKYTPFLYNLHNLRSPYDMNITTAPYMIPSFPTQTFPNHWSMVTGKYPIEHGIVSNIFWDNFTSSEFRPNNLDARIWSNTADPIWQLLQTESQGEYKVATHMWPGSEVVYEDHGDVPRERMPFYFGKFNQWEKLQDKLAQIFRYIDMPQLKDRPELVISYIPNVDSYGHSFGYDLRDKRLQKLIGEVDGFFLDLIEGLQKRNLLKISNVMIVSDHGMSNVNANDGEHVVVWERVFPADAMSAFISHLYNEGPMMMVCLKNPRDKQWICDLIEAQLEKAYGDEISRKFHVILKEDFDPSWKYFQYDNRKHRYDDRVGDIWILADEYYAIVKEMGDVPIGIMGTHGYNFNNCSDMASIFIGMGPMFNNEVVPPFENIEVYNMLIKASALLGEEKTKKEKSLLQ>YEL017WMPTKSTFSRWKKADLIDLANKLEIDGFPNYAKKSDMIDYLESHLNHLEKPVDFKDDYPELRSFYESMTVDQSKDERNEYGSGSGNGSGSGSCDTATNDSDLEKAYIKEDDDEKPQSGDETSATKPLSSRNANSNAKTNFNLLDFSTDNDSSTSAFTKFKFNFQEYLSDIRYQTQKLNENVQDYLSTISAVDTIFSLLEFSFLVRNILAAGQPTSSSSLASSLEAAVAAHNKYQYTLDFCLPILTWLLFFRGIPTLVSYYINFIRYDLNIELDPMTFNLTKFLISLAIFKTCNNKNIDFHSFRCVNQLWTQLCTVNRSLGMVPLVFSMVSCLLTLYVL>YEL018WMDKEVSELVVLQLIHTLISNKNEELVRNGGGINMIGNNLRISLVKLTNEIQNNLLINELTNLRRQSNVANGNRKLGINDILTIVKNLFPEYRTTLNDGQLSLHGLEMHDIEKLLDEKYDRFKKTQVEQIRMMEDEILKNGIKTGASQLQPHANAGKSGSAGTSATITTTTPHMAHSMDPKREKLLKLYRDTVLNKLESKTGNFQKLFKSPDGSIIKNEINYEDIKNETPGSVHELQLILQKSITDGVMRKVIGTDDWKLARQVQFELDDTVQFMRRALE>YEL019CMALNDNPIPKSVPLHPKSGKYFHNLHARDLSNIYQQCYKQIDETINQLVDSTSPSTIGIEEQVADITSTYKLLSTYESESNSFDEHIKDLKKNFKQSSDACPQIDLSTWDKYRTGELTAPKLSELYLNMPTPEPATMVNNTDTLKILKVLPYIWNDPTCVIPDLQNPADEDDLQIEGGKIELTCPITCKPYEAPLISRKCNHVFDRDGIQNYLQGYTTRDCPQAACSQVVSMRDFVRDPIMELRCKIAKMKESQEQDKRSSQAIDVL>YEL020CMTTTATQHFAQLLQKYGIDTVFGIVGIPIVQLADTMVANGIKFIPCRNEQAASYAASAYGYISDKPGVLLIVGGPGLIHALAGIYNSMSNRWPLLVIAGSSSQSDIHKGGFQELDQVSLLSPFLKFTGKLTPDNIDMITQKALNYCIQGTAGVSYIDVPADFIEYEKPLEGNDRTGNELPMILTPNICGPDPSKIKKVVQLILQHKNKNILIVIGKGAVKNSHEIRRLVNTFNLPFLPTPMAKGIVPDSSPLNVSSARSQALKIADIVLVLGARLNWILHFGTSPKWNSESIFIQFDSNPETLGDNNVSPGADLSIWGDIGLSVTALVEELTRQDSCWKYSGVKQEIREKIQLNQTRLLRKEKTRGAQLNYNQVYGTLRPLIDDYRTILVTEGANTMDIARISFPTDAPRRRLDAGTNATMGIGLGYALACKASHPELDVVLIQGDSAFGFSAMEIETAVRCQLALVIVVMNNSGIYHGEKDIEGDLPPTALSKNCRYDLVGKGLGANDFFVNTISELSRCFQQAVQLSRTKRETSVINVIIEPGEQKQIAFAWQNKPRL>YEL020W-AMDALNSKEQQEFQKVVEQKQMKDFMRLYSNLVERCFTDCVNDFTTSKLTNKEQTCIMKCSEKFLKHSERVGQRFQEQNAALGQGLGR>YEL021WMSKATYKERAATHPSPVAAKLFNIMHEKQTNLCASLDVRTTKELLELVEALGPKICLLKTHVDILTDFSMEGTVKPLKALSAKYNFLLFEDRKFADIGNTVKLQYSAGVYRIAEWADITNAHGVVGPGIVSGLKQAAEEVTKEPRGLLMLAELSCKGSLATGEYTKGTVDIAKSDKDFVIGFIAQRDMGGRDEGYDWLIMTPGVGLDDKGDALGQQYRTVDDVVSTGSDIIIVGRGLFAKGRDAKVEGERYRKAGWEAYLRRCGQQN>YEL023CMDSFNYIHGKYKKNGTGGDRSINPSSHSSSGKNIILCFDGTRENFGPQPFTNILKLYNLLENGDSSEQICYYQPGIGSVGFDAVVDVRRRLTISHLQNLLDSMFAFSLDNHICSAYLFLMKYFEPGDRIYMFGFSRGAFIARVLAGMIERVGLLSKGLEEMVKMAWQIYEKWEYDSQPNELQYTSTLAEEFKKTFSRDYEVKIHFQGLFDSVNSVGILRDRLFPCTQRSNIVEHVRHCVSLDERRGKFKQLCFTPMPYIPKLFSLTYCNHITDQCSPVPTSNALMRDLTPENPLIKYTLKSGAHSISNPSPLIPDNPGRLLSSKSEETTELLLDLNSFLEGNSYARDTECSTRGIEAIFQLQSIQGSGTSSRMTMTPDLIEKWFPGDHSDVGGGWAPDCETEENLSNLTLRWILAEAIKFGVKFKPGAIHDFATKHTSIGSLFADTHDYLSFNSPKKCSLLGVSDNEDGAREDKSGRNERMEDCLKNIKETRLSLKDEKEKVKDAFTLKCGHANKFMRLVWWVLELLPIGIRMENKEGKWQNFHTPNLGRSRYVPEYVSLHWSVYWRIKFDRRYRPDNMPEYVRQLFQDLEGIDLKSNKVSNKYDKQDNSNGSEINGGFFDNEEGQELHMGQKASYFATTYNSRLFDSKYSQLKKKFMDWDSNSWTDIPDDLKIYLQQDESL>YEL024WMLGIRSSVKTCFKPMSLTSKRLISQSLLASKSTYRTPNFDDVLKENNDADKGRSYAYFMVGAMGLLSSAGAKSTVETFISSMTATADVLAMAKVEVNLAAIPLGKNVVVKWQGKPVFIRHRTPHEIQEANSVDMSALKDPQTDADRVKDPQWLIMLGICTHLGCVPIGEAGDFGGWFCPCHGSHYDISGRIRKGPAPLNLEIPAYEFDGDKVIVG>YEL025CMARQDETDCKKLDSNSAFLPLCITSYRSKDEAEKLVSCLHHEGSYFLCFSYGLYTGTSNELKAKVEFALDGPFVVNYATVPMFMSASFCHLLMFNDGSIKGFKCENNKFEIIYDSNVAPKLDTISLLTSSWLNASFEGVLYTCNKNISYDSDLHTLCTTIYSFDFREGIVERFYATDGEEIISFDFISREDLLDDATCTGKSHVYFLCLLKSCYSENLFLKEYNLAGKHGSRKFDLNEYRQYNLLPICDNEFCYMKVVCGHTIVVLTTNYTQIINIKASGLTNGAFFNNKGLPNVENYQFLKDSYDVIYERSVILLTISDVYANKYTAKIHTRSLHRDHVLKELQWTKERVFKPPKHDLCDVILQLPREKYIAVTRINGVNFISKDHRGSKLEKVKSGPAYTNKVYLASQVIGNKGTDIDSLLLGGSFNSRRGFLEKKILVYDKTLFKLVTSSKALVENVTDFWVTDLALNGGDEFAYESGGLIYKNGIFLMDEPYDYGLFVSRTGKVLKAGMDGSTGEFRQVDFLQSNHNSSTMFCYPVQNSKLRISILEAKSGFLRKKEEFFIHGLNSKESIVSCFSDGAEKYLFVIYLDGVFSVWNANQKKVATSHPDYSFIAYDQLIKISWLSKKHKHDSIYVIASSYTGCVRVYKSESNFLRVDLEIHSLYDQKLELLDTIPSLPLVFLYNDKEIILLNLQNMSYGCIQLGLVPRRMRIRPGKALFSLCVLDYDSRISIFEFGSTFYREGFTKQMTSLKPQLENHIFYLPSIPVELYTVPNNLNQAVVCLVDSNSRQYKLMLFNYASMKAVSTFSFSDEKYLHAVVKPLWPEQNSIYLSQRPFYGNKFVVCLGVGEKRTKFWLFEIRNNNITQLYANYLEDCIFSVFIYYECNLVLFSGDSGIAAYKINMLKEGAEILEAYSFPALSSINHIGLPAYMSGDYLVQFEILRDFLRTRFPIRTSIVYPSEYPECPYLGFKRLGSITQVATKIIPKKLREPKNDCLASDKNYLLGRLSDLSCATLKYSNRSYVATIGIDNTLTIYEDSKLSVDKGGLAMPYLKIRLPNKIISLAAIPDGFQNLQICPNFNSQRLEGVIPLFLLCGTEGQIYIISEFIGELWMRTLHDYKKIKLECKRAFRRSEKSMRNVTKQYSVSKETGINESGFDELARNSKRRHIDHSPYKTIDFFDPVKLKR>YEL026WMSAPNPKAFPLADAALTQQILDVVQQAANLRQLKKGANEATKTLNRGISEFIIMAADCEPIEILLHLPLLCEDKNVPYVFVPSRVALGRACGVSRPVIAASITTNDASAIKTQIYAVKDKIETLLI>YEL027WMTELCPVYAPFFGAIGCASAIIFTSLGAAYGTAKSGVGICATCVLRPDLLFKNIVPVIMAGIIAIYGLVVSVLVCYSLGQKQALYTGFIQLGAGLSVGLSGLAAGFAIGIVGDAGVRGSSQQPRLFVGMILILIFAEVLGLYGLIVALLLNSRATQDVVC>YEL029CMPRLLATQSHVVHGYVGNKAATFPLQCLGWDVDCCNSVQFSNHTGYGLDKVFGTITRETDLKELLSGLFDNFSQDYQALLSGYLPNKNSVRCMGTYYAKFKEANPEMIWLMDPVMGDEGQLYVSEDVIPEYRKLALSPKQLVDIITPNQFELEILYGGEIKTKEHLKKALKKLHQTIPVIIVTSCDCKMFDDKDFIYCVASMEGKTPIVYRVPFIDSYFTGVGDLFSALLLDRVYKILSNPTTTLKFEDQVNNVLNVIQKVLKITRSYASGKMKAKMGSALEMKEMELRLIESRDIYETINIHQTDYIYARL>YEL031WMTKKSFVSSPIVRDSTLLVPKSLIAKPYVLPFFPLYATFAQLYFQQYDRYIKGPEWTFVYLGTLVSLNILVMLMPAWNVKIKAKFNYSTTKNVNEATHILIYTTPNNGSDGIVEIQRVTEAGSLQTFFQFQKKRFLWHENEQVFSSPKFLVDESPKIGDFQKCKGHSGDLTHLKRLYGENSFDIPIPTFMELFKEHAVAPLFVFQVFCVALWLLDEFWYYSLFNLFMIISMEAAAVFQRLTALKEFRTMGIKPYTINVFRNKKWVALQTNELLPMDLVSITRTAEESAIPCDLILLDGSAIVNEAMLSGESTPLLKESIKLRPSEDNLQLDGVDKIAVLHGGTKALQVTPPEHKSDIPPPPDGGALAIVTKTGFETSQGSLVRVMIYSAERVSVDNKEALMFILFLLIFAVIASWYVWVEGTKMGRIQSKLILDCILIITSVVPPELPMELTMAVNSSLAALAKFYVYCTEPFRIPFAGRIDVCCFDKTGTLTGEDLVFEGLAGISADSENIRHLYSAAEAPESTILVIGAAHALVKLEDGDIVGDPMEKATLKAVGWAVERKNSNYREGTGKLDIIRRFQFSSALKRSASIASHNDALFAAVKGAPETIRERLSDIPKNYDEIYKSFTRSGSRVLALASKSLPKMSQSKIDDLNRDDVESELTFNGFLIFHCPLKDDAIETIKMLNESSHRSIMITGDNPLTAVHVAKEVGIVFGETLILDRAGKSDDNQLLFRDVEETVSIPFDPSKDTFDHSKLFDRYDIAVTGYALNALEGHSQLRDLLRHTWVYARVSPSQKEFLLNTLKDMGYQTLMCGDGTNDVGALKQAHVGIALLNGTEEGLKKLGEQRRLEGMKMMYIKQTEFMARWNQPQPPVPEPIAHLFPPGPKNPHYLKALESKGTVITPEIRKAVEEANSKPVEVIKPNGLSEKKPADLASLLLNSAGDAQGDEAPALKLGDASCAAPFTSKLANVSAVTNIIRQGRCALVNTIQMYKILALNCLISAYSLSIIYMAGVKFGDGQATVSGLLLSVCFLSISRGKPLEKLSKQRPQSGIFNVYIMGSILSQFAVHIATLVYITTEIYKLEPREPQVDLEKEFAPSLLNTGIFIIQLVQQVSTFAVNYQGEPFRENIRSNKGMYYGLLGVTGLALASATEFLPELNEAMKFVPMTDDFKIKLTLTLLLDFFGSWGVEHFFKFFFMDDKPSDISVQQVKIASK>YEL034WMSDEEHTFETADAGSSATYPMQCSALRKNGFVVIKSRPCKIVDMSTSKTGKHGHAKVHLVAIDIFTGKKLEDLSPSTHNMEVPVVKRNEYQLLDIDDGFLSLMNMDGDTKDDVKAPEGELGDSLQTAFDEGKDLMVTIISAMGEEAAISFKEAARTD>YEL036CMKYNNRKLSFNPTTVSIAGTLLTVFFLTRLVLSFFSISLFQLVTFQGIFKPYVPDFKNTPSVEFYDLRNYQGNKDGWQQGDRILFCVPLRDASEHLPMFFNHLNTMTYPHNLIDLSFLVSDSSDNTMGVLLSNLQMAQSQQDKSKRFGNIEIYEKDFGQIIGQSFSDRHGFGAQGPRRKLMARARNWLGSVALKPYHSWVYWRDVDVETIPTTIMEDLMHHDKDVIVPNVWRPLPDWLGNIQPYDLNSWKESEGGLQLADSLDEDAVIVEGYPEYATWRPHLAYMRDPNGNPEDEMELDGIGGVSILAKAKVFRTGSHFPAFSFEKHAETEAFGRLSRRMNYNVIGLPHYVIWHIYEPSSDDLKHMAWMAEEEKRKLEEERIREFYNKIWEIGFEDVRDQWNEERDSILKNIDSTLNNKVTVDWSEEGDGSELVDSKGDFVSPNNQQQQQQQQQQQQQQQQQQQQQQLDGNPQGKPLDDNDKNKKKHPKEVPLDFDPDRN>YEL037CMVSLTFKNFKKEKVPLDLEPSNTILETKTKLAQSISCEESQIKLIYSGKVLQDSKTVSECGLKDGDQVVFMVSQKKSTKTKVTEPPIAPESATTPGRENSTEASPSTDASAAPAATAPEGSQPQEEQTATTERTESASTPGFVVGTERNETIERIMEMGYQREEVERALRAAFNNPDRAVEYLLMGIPENLRQPEPQQQTAAAAEQPSTAATTAEQPAEDDLFAQAAQGGNASSGALGTTGGATDAAQGGPPGSIGLTVEDLLSLRQVVSGNPEALAPLLENISARYPQLREHIMANPEVFVSMLLEAVGDNMQDVMEGADDMVEGEDIEVTGEAAAAGLGQGEGEGSFQVDYTPEDDQAISRLCELGFERDLVIQVYFACDKNEEAAANILFSDHAD>YEL038WMGDNYSTYLLDIEGTVCPISFVKETLFPYFTNKVPQLVQQDTRDSPVSNILSQFHIDNKEQLQAHILELVAKDVKDPILKQLQGYVWAHGYESGQIKAPVYADAIDFIKRKKRVFIYSSGSVKAQKLLFGYVQDPNAPAHDSLDLNSYIDGYFDINTSGKKTETQSYANILRDIGAKASEVLFLSDNPLELDAAAGVGIATGLASRPGNAPVPDGQKYQVYKNFETL>YEL039CMAKESTGFKPGSAKKGATLFKTRCQQCHTIEEGGPNKVGPNLHGIFGRHSGQVKGYSYTDANINKNVKWDEDSMSEYLTNPKKYIPGTKMAFAGLKKEKDRNDLITYMTKAAK>YEL040WMAIVNSWLICLVSIFSFVVRVEAATFCNATQACPEDKPCCSQYGECGTGQYCLNNCDVRYSFSHDSCMPVPICKSSSTKFKDYSSKLGNANTFLGNVSEADWLYTGDVLDYDDEESLILAMPKNSGGTVLSSTRAVWYGKVSARIKTSHLAGVVTGFILYSGAGDELDYEFVGADLETAQTNFYWESVLNYTNSANISTTDTFENYHTYELDWHEDYVTWSIDGVVGRTLYKNETYNATTQKYQYPQTPSKVDISIWPGGNSTNAPGTIAWSGGEINWDASDISNPGYYYAIVNEVNITCYDPPSDTKKNGTSAYVYTSSSEFLAKDIAITDDEVMMDSDEGSGLDPHKGATTSSTQKSSSSTATSSSKTSSDHSSSTKKSSKTSSTASSSSSSSSSSSSSSSTATKNGDKVVSSVSSSVTSQTQTTSSVSGSASSSTSSMSGNNAGANVAANWRLTVLCVILGYVL>YEL041WMKTDRLLINASPETCTKGDAEMDTMDTIDRMTSVKVLAEGKVLSNFEEPGLMRCGYHDAKNWVRRLSSETIVGEDTSNLYPFYVDTAYDVRRLRKDLINAKVDLQVENLIIICNINDISTVFLMREVVEWILRNFHSITVYVQDIFKKSTQFAVGDLCKDSNCSKNRVKYWSKEFVKKHDSFFDLMITLGGDGTVLFASSIFTKDVPPIVPFALGSLGFLTNFEFQNFKETLKHILTDEVRINLRMRLQCKLYRRNKPEIDAATGRKICYIDFISEHHVLNEVTIDRGPAPCLSLLELYGNDSLMTKVQGDGLIVATPTGSTAYSLSAGGSLISPSVNAIAVTPICPHTLSFRPIILPDSMELKVRVDMNSRGTSWVNFDGKDRVELKQGDYVVITASPYSVPTIESSASEFFESISKNLNWNDREEQKPFAHILSPKNQEKYRLDSSKNGNDTISNPLESSCISSDAQDEERKSVTETETEIVVERTRQAHFAI>YEL042WMAPIFRNYRFAIGAFAVIMLILLIKTSSIGPPSIARTVTPNASIPKTPEDISILPVNDEPGYLQDSKTEQNYPELADAVKSQTSQTCSEEHKYVIMIDAGSTGSRVHIYKFDVCTSPPTLLDEKFDMLEPGLSSFDTDSVGAANSLDPLLKVAMNYVPIKARSCTPVAVKATAGLRLLGDAKSSKILSAVRDHLEKDYPFPVVEGDGVSIMGGDEEGVFAWITTNYLLGNIGANGPKLPTAAVFDLGGGSTQIVFEPTFPINEKMVDGEHKFDLKFGDENYTLYQFSHLGYGLKEGRNKVNSVLVENALKDGKILKGDNTKTHQLSSPCLPPKVNATNEKVTLESKETYTIDFIGPDEPSGAQCRFLTDEILNKDAQCQSPPCSFNGVHQPSLVRTFKESNDIYIFSYFYDRTRPLGMPLSFTLNELNDLARIVCKGEETWNSVFSGIAGSLDELESDSHFCLDLSFQVSLLHTGYDIPLQRELRTGKKIANKEIGWCLGASLPLLKADNWKCKIQSA>YEL044WMSGSRGNSSNSSVSNNSNNNNNNDGGDERLLFLRSVGERNEIGFPSRFKSAHYKKPTRRHKSARQLISDENKRINALLTKANKAAESSTAARRLVPKATYFSVEAPPSIRPAKKYCDVTGLKGFYKSPTNNIRYHNAEIYQLIVKPMAPGVDQEYLKLRGANFVLK>YEL046CMTEFELPPKYITAANDLRSDTFTTPTAEMMEAALEASIGDAVYGEDVDTVRLEQTVARMAGKEAGLFCVSGTLSNQIAIRTHLMQPPYSILCDYRAHVYTHEAAGLAILSQAMVVPVVPSNGDYLTLEDIKSHYVPDDGDIHGAPTRLISLENTLHGIVYPLEELVRIKAWCMENGLKLHCDGARIWNAAAQSGVPLKQYGEIFDSISICLSKSMGAPIGSVLVGNLKFVKKATHFRKQQGGGIRQSGMMARMALVNINNDWKSQLLYSHSLAHELAEYCEAKGIPLESPADTNFVFINLKAARMDPDVLVKKGLKYNVKLMGGRVSFHYQVTRDTLEKVKLAISEAFDYAKEHPFDCNGPTQIYRSESTEVDVDGNAIREIKTYKY>YEL047CMSLSPVVVIGTGLAGLAAANELVNKYNIPVTILEKASSIGGNSIKASSGINGACTETQRHFHIEDSPRLFEDDTIKSAKGKGVQELMAKLANDSPLAIEWLKNEFDLKLDLLAQLGGHSVARTHRSSGKLPPGFEIVSALSNNLKKLAETKPELVKINLDSKVVDIHEKDGSISAVVYEDKNGEKHMVSANDVVFCSGGFGFSKEMLKEYAPELVNLPTTNGQQTTGDGQRLLQKLGADLIDMDQIQVHPTGFIDPNDRSSSWKFLAAESLRGLGGILLNPITGRRFVNELTTRDVVTAAIQKVCPQEDNRALLVMGEKMYTDLKNNLDFYMFKKLVQKLTLSQVVSEYNLPITVAQLCEELQTYSSFTTKADPLGRTVILNEFGSDVTPETVVFIGEVTPVVHFTMGGARINVKAQVIGKNDERLLKGLYAAGEVSGGVHGANRLGGSSLLECVVFGRTAAESIANDRK>YEL048CMSLRPCFVSLIDESDKPILIYVPNEAENEMNDVLKYNVLSNISLDYFESALVEWHSLDSKPLLKSIFQLEGVSVFAMLIKQTGLKIVIGFEQKSLSGADDEFEAINQIFETVRKIYIRVKCNPLLVSGDEKSIIKSLERKFDELFISTEVEL>YEL050CMLVLGSLRSALSCSSTASLISKRNPCYPYGILCRTLSQSVKLWQENTSKDDSSLNITPRLLKIIPNDTDIVTLEKQDELIKRRRKLSKEVTQMKRLKPVSPGLRWYRSPIYPYLYKGRPVRALTVVRKKHGGRNNSGKITVRHQGGGHRNRTRLIDFNRWEGGAQTVQRIEYDPGRSSHIALLKHNTTGELSYIIACDGLRPGDVVESFRRGIPQTLLNEMGGKVDPAILSVKTTQRGNCLPISMIPIGTIIHNVGITPVGPGKFCRSAGTYARVLAKLPEKKKAIVRLQSGEHRYVSLEAVATIGVVSNIDHQNRSLGKAGRSRWLGIRPTVRGVAMNKCDHPHGGGRGKSKSNKLSMSPWGQLAKGYKTRRGKNQNRMKVKDRPRGKDARL>YEL051WMSGNREQVFPTRMTLGLMKTKLKGANQGYSLLKRKSEALTKRFRDITKRIDDAKQKMGRVMQTAAFSLAEVSYATGENIGYQVQESVSTARFKVRARQENVSGVYLSQFESYIDPEINDFRLTGLGRGGQQVQRAKEIYSRAVETLVELASLQTAFIILDEVIKVTNRRVNAIEHVIIPRTENTIAYINSELDELDREEFYRLKKVQEKKQNETAKLDAEMKLKRDRAEQDASEVAADEEPQGETLVADQEDDVIF>YEL052WMIALKPNAVRTFRQVQHCSFRICRYQSTKSNKCLTPLQEYDRLVKLGKLRDDTYQRGIISSLGDLYDSLVKYVPPVVKTPNAVDQVGGWLNGLKSVFSRGKPKNIGAYVDVSKIGNSIPRGVYLYGDVGCGKTMLMDLFYTTIPNHLTKKRIHFHQFMQYVHKRSHEIVREQNLKELGDAKGKEIDTVPFLAAEIANNSHVLCFDEFQVTDVADAMILRRLMTALLSDDYGVVLFATSNRHPDELYINGVQRQSFIPCIELIKHRTKVIFLNSPTDYRKIPRPVSSVYYFPSDTSIKYASKECKTRRETHIKEWYNYFAQASHTDDSTDSHTVHKTFYDYPLTIWGREFKVPKCTPPRVAQFTFKQLCGEPLAAGDYLTLAKNFEAFIVTDIPYLSIYVRDEVRRFITFLDAVYDSGGKLATTGAADFSSLFVEPEQILNDFELRPTTKEPDSVDTGMVDEMVEKHGFSKEIAKKSQMFALDEERFAFARALSRLSQMSSTDWVTKPTY>YEL053CMEVDSILGSLSITDDFDQLVDVTSLFDELCSKLKPEAIVKDPRFDLFEGTHSLEVNNSKLDSSLIELTAEEIEFDVNVAYDPPLASVAAIADRLLRCVISWLNDYQTLPTTVLSCRYTESLLSSLVKGTTAGSSWCTGNILYDKVLGSCILGVCYLTKFVQKLLSAGIVFEEEDLNFNNMGFNTFDNLPGQDVVINSLTESLQILEAYSDDSLHLTMLKHILKIIICLVHLEDHLTDYSTKTSHLDELIENANSVNGIFPQLQLSPPKGAFSTYIQKHRSNQFPPRKITKLPTDYSGFITLANDVKTILLVDKAESALETYQFAKFFNKLEQRHVIARILFPLFFIRDDRTVLGKFSYTQFYLLHVKEFSAQTPSEFESSIGNELIQESSNMLLEWYQNCSQNTCRYRQGFNRQLILWDSLQAQFESVNSQVYCSWTYFMKLSSMIEFSLKGFDLDIYKPFEAYSMFWYVYYLSHHLETFLKDSQNDIESNINAIHSMNKKLKKLKAGEKKDQLRLKYRFAMDNEMEQLQATKQFLNYLLKEINITKSLCLIEVFQFAILKSFGLIDNKNSTPSKFSNERLIHNLRFKPFNSIGVPELPEYEVFQQTLKDFVIEEKGAAFDIKLERATNFIETEVRNVVSSIDEIMQGIKGGDNNGVLVTGTRLVQELSLEYYCKLKHTSKALSVNSKVIVNTLKKNIKNKDSHEYKVELVHTTEGWNYFPIQTLRIKQDRYK>YEL054CMPPKFDPNEVKYLYLRAVGGEVGASAALAPKIGPLGLSPKKVGEDIAKATKEFKGIKVTVQLKIQNRQAAASVVPSASSLVITALKEPPRDRKKDKNVKHSGNIQLDEIIEIARQMRDKSFGRTLASVTKEILGTAQSVGCRVDFKNPHDIIEGINAGEIEIPEN>YEL057CMANDGIQRNDNRKGFKTVQFSAYSKEIDVIMKKISFLERNITQQLDTLPHFPKTLPPNHKDCVSRKHRARRGWSSQLKNLLGIYSKEEIFTLDNLAATLHDQVLKLQATLFPNAILKQVHLDNANIENKRILKEITYKYLSNENCKEENKFGTFIVKRIFFGDLSLGVSVLINRIAFESA
[truncated: 1,200,000 more chars]
