# Supplementary material for: Proteome-wide evidence for enhanced positive Darwinian selection within intrinsically disordered regions in proteins
Source: Genome Biol. 2011 Jul 19;12(7):R65. doi: 10.1186/gb-2011-12-7-r65 (PMC3218827; doi:10.1186/gb-2011-12-7-r65)
Supplement: Additional file 10 — Fraction of amino acid residues for each protein that are predicted by the PSIPRED method to adopt α-helical conformation, using a confidence value threshold of 8. [file gb-2011-12-7-r65-S10.RTF]

YHR055C		0.0327868852459016YPR161C		0.135464231354642YOL138C		0.0417598806860552YGR129W		0.469767441860465YPR165W		0.244019138755981YPL015C		0.218487394957983YCL050C		0.177570093457944YMR193W		0.182170542635659YGR053C		0.0671378091872792YOR280C		0.255639097744361YEL004W		0.54093567251462YJL200C		0.17743979721166YDR348C		0.030060120240481YPL183C		YGR003W		0.568548387096774YBL095W		0.174074074074074YEL009C		0.110320284697509YEL015W		0.141560798548094YDR329C		0.489795918367347YBR252W		YDL202W		0.353413654618474YHR209W		0.285223367697594YPR008W		0.00576368876080692YBR050C		0.0887573964497041YMR197C		0.723502304147465YBL049W		0.0144927536231884YNL201C		0.333333333333333YCR061W		0.335974643423138YPL171C		0.255YHR051W		0.472972972972973YDL116W		0.53030303030303YPR124W		0.187192118226601YJL023C		0.230547550432277YGL167C		0.373684210526316YDL165W		0.172774869109948YIL143C		0.214709371293001YOR094W		0.306010928961749YDR167W		0.233009708737864YER052C		0.301707779886148YPL118W		0.11046511627907YGR178C		0.038781163434903YPR086W		0.379710144927536YJL065C		0.341317365269461YBR122C		0.0790960451977401YBR279W		0.0561797752808989YBR271W		0.243436754176611YMR221C		0.529761904761905YER130C		0.0112866817155756YBR155W		0.371428571428571YDR363W		0.0592105263157895YPL147W		0.439080459770115YLR443W		0.299107142857143YBR067C		0.271428571428571YAL034C		0.11864406779661YLR270W		0.165714285714286YCR095C		0.165745856353591YPR084W		0.164473684210526YMR113W		0.34192037470726YGR035C		0.172413793103448YDL222C		0.239482200647249YNL335W		0.444444444444444YDR232W		0.277372262773723YHR116W		0.245033112582781YFR018C		0.278236914600551YKL007W		0.309701492537313YDL213C		0.204444444444444YNL292W		0.143920595533499YNL134C		0.223404255319149YKL100C		0.291311754684838YPL021W		0.0267379679144385YIL146C		0.0982986767485822YNL218W		0.339011925042589YIL121W		0.588560885608856YDL167C		0.0737134909596662YPL214C		0.357407407407407YOR228C		0.384105960264901YBR270C		0.154128440366972YGR222W		0.232081911262799YGR121C		0.410569105691057YDL003W		0.088339222614841YOR323C		0.317982456140351YMR183C		0.640677966101695YOR274W		0.408878504672897YER146W		0.0645161290322581YPL063W		0.266806722689076YML075C		0.297912713472486YLR273C		0.00462962962962963YDR457W		0.349755201958384YCL056C		0.548611111111111YJL083W		0.097682119205298YPL140C		0.185770750988142YOR026W		YNR016C		0.256605463502015YER123W		0.175572519083969YGR055W		0.477351916376307YMR315W		0.286532951289398YKL187C		0.218666666666667YDR383C		0.584033613445378YKL055C		0.39568345323741YPR019W		0.236870310825295YPR190C		0.36697247706422YPL228W		0.0965391621129326YGR041W		0.0822669104204753YDR353W		0.178683385579937YPL047W		0.292929292929293YPL014W		0.133858267716535YGR007W		0.275541795665635YML116W		0.638376383763838YOL137W		0.557344064386318YGL155W		0.375YER061C		0.257918552036199YKL011C		0.320113314447592YNL257C		0.349877949552482YBR291C		0.662207357859532YDR262W		0.132352941176471YDR179C		0.438271604938272YIL053W		0.332YGR220C		0.033457249070632YMR161W		0.28125YER011W		0.165354330708661YGL162W		0.0133779264214047YNL243W		0.629132231404959YNL047C		0.320121951219512YKL038W		0.153846153846154YFL053W		0.348561759729272YLR411W		0.29045643153527YLR318W		0.316742081447964YHR150W		0.260794473229706YGL219C		0.100217864923747YBR045C		0.1239092495637YMR039C		0.0753424657534247YPL188W		0.0966183574879227YGL147C		0.120418848167539YDR217C		0.0458365164247517YPR026W		0.163501238645747YMR008C		0.23644578313253YDL227C		0.107508532423208YOR197W		0.138888888888889YNL081C		0.230769230769231YKL096W-A		0.228260869565217YDR532C		0.41038961038961YKL170W		0.0144927536231884YGL047W		0.267326732673267YPR171W		YML063W		0.145098039215686YLR178C		0.0867579908675799YHL029C		0.213549337260677YOR184W		0.30379746835443YDR372C		0.310144927536232YBR008C		0.532846715328467YMR203W		0.0361757105943152YBL024W		0.182748538011696YDR317W		0.115942028985507YJL038C		0.401826484018265YDR043C		0.0216450216450216YOR340C		0.0245398773006135YCL044C		0.163069544364508YFR022W		YEL072W		0.0865800865800866YOL008W		0.188405797101449YHR017W		0.228571428571429YMR095C		0.214285714285714YHR198C		0.236760124610592YOR076C		0.0963855421686747YNL219C		0.455855855855856YLR210W		0.321739130434783YIL034C		0.250871080139373YLR148W		0.328976034858388YOR342C		0.225705329153605YIL043C		0.140845070422535YDL160C		0.235177865612648YGR231C		0.441935483870968YNL021W		0.243626062322946YMR266W		0.395592864637985YPL107W		0.189516129032258YGR088W		0.165480427046263YCR063W		0.299363057324841YDL048C		0.0428571428571429YML092C		0.28YPR097W		0.391425908667288YNL056W		0.279187817258883YAL020C		YFL017C		0.251572327044025YHR092C		0.575YDR379C-A		0.569620253164557YNR074C		0.214285714285714YLR189C		0.186978297161937YDR113C		0.0509383378016086YML032C		0.0997876857749469YFL028C		0.249134948096886YDL247W		0.533661740558292YBR256C		0.134453781512605YPL024W		0.0954356846473029YMR257C		0.49375YDL219W		0.173333333333333YKL122C		0.0958083832335329YGL240W		0.06YDL080C		0.288998357963875YEL001C		0.0933333333333333YPR168W		0.560509554140127YHR109W		0.264957264957265YPL061W		0.322YJL060W		0.315315315315315YLR240W		0.331428571428571YGR104C		0.104234527687296YER174C		0.282786885245902YMR146C		YPR057W		0.343108504398827YDR156W		0.175182481751825YJR113C		0.40080971659919YPR131C		0.282051282051282YMR155W		0.491773308957952YNL180C		0.126888217522659YDR272W		0.226277372262774YOR238W		0.231023102310231YLR139C		0.147744945567652YKL142W		0.356164383561644YPR009W		0.041044776119403YHR032W		0.590361445783133YDR429C		0.0766423357664234YNR031C		0.303989867004433YGL079W		0.334862385321101YOR004W		0.271653543307087YMR244W		0.0112676056338028YMR233W		0.31858407079646YNL188W		0.157043879907621YNL104C		0.297253634894992YOR365C		0.324324324324324YNR030W		0.475499092558984YBR265W		0.415625YGL131C		0.0719885958660014YNR040W		0.23046875YOR173W		0.15014164305949YOR337W		0.310935441370224YNL181W		0.351351351351351YGR243W		0.445205479452055YDR464W		0.070383275261324YGR210C		0.250608272506083YOR168W		0.213844252163164YNL002C		0.403726708074534YNL294C		0.212007504690432YHR193C		0.247126436781609YPR147C		0.381578947368421YBR092C		0.269807280513919YOR242C		0.137466307277628YJL176C		0.204848484848485YHR128W		0.25462962962963YOR297C		0.338541666666667YDR225W		0.28030303030303YPR020W		0.365217391304348YPR114W		0.476190476190476YPL189W		0.51559934318555YJL056C		0.0125YPL247C		YDR482C		0.288888888888889YJL122W		0.234285714285714YBL058W		0.137115839243499YKL107W		0.313915857605178YCL017C		0.279678068410463YER085C		0.364161849710983YMR186W		0.253900709219858YCR065W		0.0478723404255319YFL045C		0.275590551181102YNL020C		0.13166144200627YGL125W		0.316666666666667YLR354C		0.42089552238806YFR008W		0.588235294117647YDR284C		0.363321799307958YKL051W		0.220963172804533YPR174C		0.248868778280543YMR091C		0.133333333333333YJL143W		0.588607594936709YGR057C		0.231404958677686YLR308W		0.227564102564103YBR065C		0.162087912087912YMR029C		0.116634799235182YPR121W		0.426573426573427YNL068C		0.0220417633410673YGL178W		0.247962747380675YGL157W		0.311239193083574YMR148W		0.283783783783784YML058W		0.259615384615385YLR249W		0.414750957854406YGR019W		0.290870488322718YBR263W		0.346938775510204YPR044C		YDR286C		0.245614035087719YMR076C		0.499608457321848YJR135W-A		0.632183908045977YDR083W		0.178571428571429YLR109W		0.198863636363636YOR032C		0.0967741935483871YOR091W		0.252173913043478YBR205W		0.26980198019802YLR292C		0.590673575129534YLR216C		0.345013477088949YDR046C		0.460264900662252YML018C		0.49618320610687YOR369C		0.293706293706294YOR138C		0.225037257824143YJR145C		0.0498084291187739YGR246C		0.298657718120805YJR138W		0.107323232323232YHR059W		0.338461538461538YLR138W		0.260913705583756YNL156C		0.311036789297659YNL106C		0.127641589180051YNL286W		0.157894736842105YNL093W		0.222727272727273YIL040W		0.782608695652174YLR186W		0.19047619047619YDR425W		0.384YDR293C		0.136YNR015W		0.3125YLR456W		0.0980392156862745YDR448W		0.382488479262673YER184C		0.335012594458438YJL066C		0.119047619047619YOR194C		0.118881118881119YLR368W		0.29933110367893YKL095W		0.197841726618705YGR060W		0.588996763754045YBR115C		0.250718390804598YBR147W		0.398648648648649YHR086W		0.122370936902486YFL003C		0.350797266514806YOR125C		0.562231759656652YML079W		0.0895522388059701YPL134C		0.606451612903226YNL298W		0.121140142517815YGL225W		0.664688427299703YNL224C		0.11864406779661YNL122C		0.260869565217391YLR129W		0.0551431601272534YGL066W		0.114155251141553YNL217W		0.141104294478528YGL063W		0.243243243243243YML030W		0.616352201257862YAL039C		0.197026022304833YOR261C		0.343195266272189YLR191W		0.225388601036269YCR027C		0.200956937799043YDL046W		0.0809248554913295YMR263W		0.199004975124378YOL113W		0.140458015267176YDR517W		0.0725806451612903YAL001C		0.230172413793103YBR197C		0.184331797235023YJR132W		0.586832061068702YDL106C		0.0661896243291592YNL280C		0.420091324200913YDR505C		0.0642092746730083YDL154W		0.334073251942286YMR211W		0.138947368421053YGL202W		0.314YBL036C		0.350194552529183YPR178W		0.0967741935483871YOL147C		0.661016949152542YFR007W		0.305949008498584YGR072W		0.118863049095607YER022W		0.253275109170306YIL118W		0.216450216450216YJR008W		0.245562130177515YCL010C		0.150579150579151YJR024C		0.295081967213115YFR011C		0.505882352941176YAL047C		0.406752411575563YDR256C		0.170873786407767YNL287W		0.403208556149733YGR103W		0.257851239669421YOR281C		0.265734265734266YBR162C		0.0461538461538462YMR114C		0.0597826086956522YGR251W		0.244897959183673YBR094W		0.185922974767596YKL021C		YOR374W		0.310211946050096YHL026C		0.152380952380952YHR014W		YDL015C		0.383870967741935YCR028C		0.60546875YER058W		0.504672897196262YCR016W		0.148275862068966YER149C		0.323809523809524YJL147C		0.421465968586387YGL222C		0.0685714285714286YDR533C		0.248945147679325YDL090C		0.368909512761021YBR177C		0.199556541019956YJL034W		0.303519061583578YNR059W		0.21551724137931YGR156W		0.185882352941176YBR125C		0.188295165394402YBR173C		0.324324324324324YHR057C		0.131707317073171YOR077W		0.375YLR234W		0.295731707317073YHR107C		0.329238329238329YER112W		0.0641711229946524YLR174W		0.286407766990291YCL026C-A		0.310880829015544YDR346C		0.00831600831600832YLR385C		0.454545454545455YLR117C		0.646288209606987YDR143C		0.0770491803278689YER185W		0.554455445544555YOL013C		0.415607985480944YLR089C		0.277027027027027YKL155C		0.189490445859873YGR154C		0.207865168539326YNL277W		0.211934156378601YOL061W		0.167338709677419YFR013W		0.175349428208386YDL014W		0.0978593272171254YCL064C		0.313888888888889YDR493W		0.398373983739837YLR345W		0.280943025540275YLR192C		0.313207547169811YOR265W		0.679245283018868YDR031W		0.537190082644628YBR141C		0.293768545994065YDR453C		0.255102040816327YAL008W		0.222222222222222YDR490C		0.148825065274151YDR191W		0.2YNL193W		0.385304659498208YLR360W		0.234624145785877YOR142W		0.267477203647416YDR304C		0.142222222222222YPL020C		0.177133655394525YLR165C		0.141732283464567YHL023C		0.140488656195462YOR283W		0.28695652173913YMR255W		0.207446808510638YPL154C		0.0962962962962963YDR033W		0.3625YDR253C		0.0523560209424084YNL014W		0.405172413793103YAL032C		0.203166226912929YNL291C		0.0693430656934307YOR348C		0.437001594896332YMR104C		0.119645494830133YOR097C		0.12YOL126C		0.291777188328912YOL107W		0.39766081871345YHR026W		0.328638497652582YGR028W		0.356353591160221YGL061C		0.263157894736842YJL089W		0.184559710494572YKL116C		0.144787644787645YHR134W		0.245353159851301YBR228W		0.164473684210526YNL320W		0.320422535211268YGR123C		0.296296296296296YMR220W		0.277161862527716YBR269C		0.0942028985507246YMR188C		0.333333333333333YLR290C		0.24187725631769YNL258C		0.427055702917772YNL075W		0.206896551724138YPR159W		0.025YHR089C		0.0146341463414634YNR018W		0.566964285714286YAL044C		0.0823529411764706YKL139W		0.149621212121212YHR129C		0.169270833333333YOR253W		0.244318181818182YIL105C		0.287172011661808YDR403W		0.244402985074627YDL029W		0.161125319693095YDR525W-A		0.329113924050633YPL255W		0.251948051948052YEL012W		0.211009174311927YOR192C		0.482470784641068YGL229C		0.299511002444988YOL005C		0.3YML052W		0.268211920529801YML064C		0.2YHL019C		0.084297520661157YBR167C		0.178571428571429YPR176C		0.446153846153846YFL016C		0.119373776908023YLR383W		0.548473967684022YJL062W		0.437349397590361YDR140W		0.266968325791855YNL252C		0.170818505338078YMR196W		0.194852941176471YOR205C		0.107913669064748YER114C		0.0240384615384615YGL258W		0.087378640776699YIL002C		0.151162790697674YDR518W		0.237911025145068YLR328W		0.179551122194514YKL173W		0.204365079365079YBL003C		0.287878787878788YNR013C		0.480984340044743YJR077C		0.607717041800643YPR078C		0.110215053763441YGL058W		0.27906976744186YFL005W		0.223255813953488YMR284W		0.179401993355482YML061C		0.108265424912689YLR449W		0.0943877551020408YDL007W		0.304347826086957YOR145C		0.255474452554745YOR287C		0.333333333333333YIL122W		0.111111111111111YPL218W		0.305263157894737YJL145W		0.353741496598639YPR198W		0.659300184162063YNR052C		0.23094688221709YOR244W		0.166292134831461YMR314W		0.303418803418803YLR173W		0.0740131578947368YHR190W		0.565315315315315YPL097W		0.357723577235772YNL115C		0.273291925465839YLR103C		0.318461538461538YKL132C		0.327906976744186YPL222W		0.276162790697674YKL012W		0.425385934819897YLR395C		0.179487179487179YAL041W		0.276346604215457YBL052C		0.113116726835138YCL045C		0.0671052631578947YDL135C		0.0643564356435644YMR237W		0.348066298342541YGR046W		0.324675324675325YGR224W		0.592169657422512YDR308C		0.6YJL153C		0.236397748592871YBR035C		0.175438596491228YCR002C		0.263975155279503YDR244W		0.509803921568627YAL016W		0.674015748031496YPR093C		0.180555555555556YMR239C		0.301486199575372YLR163C		0.37012987012987YDL072C		0.660098522167488YPL190C		0.0648379052369077YER059W		0.233333333333333YBR207W		0.264516129032258YIL049W		0.616600790513834YBR069C		0.470113085621971YGL215W		0.165929203539823YPR066W		0.284280936454849YOR135C		0.292035398230089YNL202W		0.356164383561644YLR099C		0.263959390862944YOR080W		0.309651474530831YNL133C		0.46242774566474YJL167W		0.605113636363636YIL097W		0.403100775193798YOR198C		0.559574468085106YNL101W		0.380084151472651YGL241W		0.569721115537849YML055W		0.359550561797753YHR143W-A		YFL052W		0.419354838709677YML035C		0.265432098765432YGR133W		0.278688524590164YDR357C		0.729508196721312YDR006C		0.267480577136515YNL256W		0.239077669902913YML100W		0.219489981785064YHR197W		0.342070773263434YNL325C		0.149032992036405YBR128C		0.447674418604651YPL258C		0.451905626134301YNL216W		0.17049576783555YIL036W		0.117546848381601YLR307W		0.252491694352159YML119W		0.0252100840336134YPL273W		0.372307692307692YCL066W		0.217142857142857YPL201C		0.0216919739696312YHR003C		0.319347319347319YPR041W		0.335802469135802YHR171W		0.187301587301587YER116C		0.0437956204379562YOR136W		0.311653116531165YOL086W-A		0.655555555555556YLR364W		0.403669724770642YGR142W		0.151219512195122YKL186C		0.119565217391304YGR264C		0.343541944074567YDR206W		0.312217194570136YPR042C		0.203720930232558YJR152W		0.572744014732965YBR085W		0.628664495114006YKL106W		0.319290465631929YBR281C		0.126423690205011YDL153C		0.226229508196721YBL078C		0.256410256410256YDL150W		YDR398W		0.132192846034215YIL077C		0.325YGR038W		0.238738738738739YMR153W		0.0589473684210526YPL266W		0.29559748427673YPL264C		0.617563739376771YHR132C		0.286046511627907YLR336C		0.340378197997775YER115C		0.193717277486911YDR441C		0.259668508287293YDR101C		0.18043844856661YMR121C		0.132352941176471YHL014C		0.269135802469136YMR312W		0.164835164835165YOR382W		0.0261437908496732YOR214C		0.220338983050847YLR141W		0.40495867768595YJL030W		0.290816326530612YDR410C		0.602510460251046YOR335C		0.315240083507307YNL087W		0.0730050933786078YHR015W		0.176024279210926YGL037C		0.203703703703704YDR502C		0.21875YAR018C		0.220689655172414YOR112W		0.287779237844941YKL045W		0.373106060606061YBR258C		0.507042253521127YPR074C		0.317647058823529YHR162W		0.449612403100775YLR262C-A		0.34375YML013W		0.287671232876712YPL003W		0.365800865800866YLR297W		0.310077519379845YMR319C		0.248188405797101YDL127W		0.318181818181818YOR152C		0.2421875YDL216C		0.265909090909091YFR028C		0.221415607985481YBR068C		0.461412151067323YHR126C		0.0566037735849057YHR008C		0.416309012875536YOL001W		0.290102389078498YML111W		0.0576086956521739YMR016C		0.0127388535031847YKL023W		0.162454873646209YBR133C		0.133010882708585YGR229C		0.126732673267327YOR092W		0.244698205546493YNR010W		0.536912751677852YPL001W		0.302139037433155YDR047W		0.403314917127072YBR159W		0.487031700288184YGR158C		0.224YOR210W		0.442857142857143YKL174C		0.451456310679612YLR403W		0.00732064421669107YLR286C		0.104982206405694YBR274W		0.212523719165085YDL044C		0.365909090909091YNL305C		0.552188552188552YER081W		0.243070362473348YDR148C		0.16414686825054YJL059W		0.551470588235294YIL130W		0.267634854771784YEL044W		0.234939759036145YGL108C		0.35YOR351C		0.173038229376258YGL023C		0.0204724409448819YBL066C		0.210801393728223YBL051C		0.0434131736526946YJR068W		0.456090651558074YDL168W		0.199481865284974YBR020W		0.329545454545455YDL049C		0.0522388059701493YAL019W		0.226348364279399YDR484W		0.519500780031201YEL037C		0.268844221105528YBR273C		0.153669724770642YDL114W		0.422077922077922YIL062C		0.435064935064935YML091C		0.438435940099834YCL021W-A		0.112YOR372C		0.055956678700361YGL098W		0.595918367346939YNL329C		0.238834951456311YMR199W		0.358974358974359YDL209C		0.117994100294985YMR300C		0.213725490196078YCR039C		0.357142857142857YIL107C		0.172914147521161YIL057C		0.176829268292683YGL136C		0.221875YHR024C		0.360995850622407YLR120C		0.0720562390158172YGR211W		0.137860082304527YLR237W		0.483277591973244YBL007C		0.00482315112540193YNR064C		0.313793103448276YPL075W		0.193630573248408YPL017C		0.232464929859719YBR301W		0.441666666666667YML004C		0.116564417177914YMR202W		0.333333333333333YGR292W		0.207191780821918YNL267W		0.269230769230769YEL029C		0.33974358974359YLR250W		0.358974358974359YGR245C		0.406779661016949YNL040W		0.309210526315789YIL003W		0.245733788395904YGL161C		0.335483870967742YPR144C		0.467391304347826YOR359W		0.112810707456979YFL055W		0.550179211469534YGR181W		0.571428571428571YGR070W		0.164502164502165YPL029W		0.324287652645862YLR116W		0.151260504201681YDR197W		0.311053984575835YPL224C		0.469933184855234YPL172C		0.422077922077922YJR144W		0.107806691449814YMR083W		0.216YGR086C		0.451327433628319YCR048W		0.388524590163934YDL065C		0.304093567251462YMR126C		0.225146198830409YDR175C		0.278996865203762YER032W		0.0319634703196347YGR194C		0.265YGR078C		0.381909547738693YMR265C		0.180043383947939YHR163W		0.261044176706827YOR222W		0.592833876221498YBL103C		0.146090534979424YIL052C		0.256198347107438YBR242W		0.525210084033613YBL021C		0.465277777777778YNL246W		0.238636363636364YOL030W		0.212809917355372YER012W		0.303030303030303YLR301W		0.0122950819672131YPR139C		0.226666666666667YPL202C		0.0504807692307692YOL068C		0.192842942345924YLR114C		0.163612565445026YJL019W		0.162756598240469YLR248W		0.150819672131148YIL020C		0.275862068965517YNL063W		0.296178343949045YOR391C		0.236286919831224YFR044C		0.257796257796258YFL023W		0.158291457286432YDR212W		0.427549194991055YOR103C		0.515384615384615YMR185W		0.420998980632008YJL091C		0.440816326530612YDR065W		0.49041095890411YHR174W		0.299771167048055YLR441C		0.145098039215686YBR204C		0.269333333333333YLR283W		0.410828025477707YGL174W		0.285714285714286YGR145W		0.0678925035360679YGR199W		0.359683794466403YDR061W		0.282003710575139YBL005W		0.302254098360656YIL108W		0.0704022988505747YDR404C		0.105263157894737YOR058C		0.383050847457627YBL104C		0.158959537572254YBR095C		0.223255813953488YBR084W		0.242051282051282YMR173W		YHR085W		0.410179640718563YOR301W		0.252873563218391YDL210W		0.499124343257443YNL308C		0.252115059221658YOR171C		0.123397435897436YIL050W		0.294736842105263YBL099W		0.319266055045872YLR359W		0.556016597510373YPL217C		0.139475908706678YNR009W		0.140562248995984YGL194C		0.247787610619469YOR106W		0.720848056537102YJR056C		0.207627118644068YLR287C-A		0.0317460317460317YDR044W		0.259146341463415YLR118C		0.277533039647577YDR387C		0.598198198198198YOL130W		0.218859138533178YIL071C		0.468468468468468YDR169C		0.0818713450292398YER178W		0.335714285714286YMR210W		0.233853006681514YOR338W		0.15702479338843YER088C		0.0238805970149254YDR115W		0.0666666666666667YOR334W		0.346808510638298YGR256W		0.469512195121951YMR107W		0.156521739130435YDR451C		0.0793201133144476YCL063W		0.198581560283688YJL192C		0.209401709401709YHR177W		0.0286975717439294YBR039W		0.504823151125402YIR027C		0.217391304347826YAL042W		0.137349397590361YPR036W		0.51255230125523YIL007C		0.331818181818182YOR030W		0.258481421647819YPL267W		0.100478468899522YMR243C		0.380090497737557YDR188W		0.41025641025641YNR034W-A		YEL023C		0.165689149560117YNL045W		0.220566318926975YPR141C		0.417009602194787YIL104C		0.238658777120316YNL282W		0.205128205128205YLR424W		0.372881355932203YDR520C		0.216321243523316YBR243C		0.426339285714286YGL137W		0.123734533183352YNL212W		0.0639386189258312YMR234W		0.272988505747126YMR112C		0.549618320610687YHR005C-A		0.537634408602151YGL212W		0.468354430379747YNR023W		0.212014134275618YPL253C		0.332302936630603YBR096W		0.269565217391304YJR042W		0.440860215053763YGR150C		0.435185185185185YLR390W-A		0.15546218487395YGR074W		0.0342465753424658YHR043C		0.341463414634146YGL180W		0.236343366778149YDR085C		0.0564516129032258YBR146W		0.294964028776978YPL043W		0.154744525547445YHR011W		0.309417040358744YDL235C		0.497005988023952YLR278C		0.182699478001491YLR348C		0.62751677852349YGL158W		0.193359375YAL028W		0.248106060606061YDL002C		0.467980295566502YDR099W		0.52014652014652YNL173C		0.0163934426229508YMR216C		0.110512129380054YMR306W		0.304201680672269YLR196W		0.0173611111111111YBR085C-A		0.176470588235294YDL059C		0.235294117647059YOR329C		0.0573394495412844YJR116W		0.50179211469534YHL004W		0.299492385786802YBR073W		0.24025974025974YDR449C		0.55YDL088C		0.0492424242424242YNL161W		0.141534391534392YDR435C		0.295731707317073YDL052C		0.343234323432343YMR144W		0.277777777777778YDR161W		0.607235142118863YER062C		0.336YCR068W		0.128846153846154YPR051W		0.244318181818182YOR285W		0.294964028776978YLR253W		0.521968365553603YJL001W		0.274418604651163YHR067W		0.121428571428571YGL237C		0.124528301886792YDR120C		0.219298245614035YJL012C		0.327323162274619YER126C		0.218390804597701YMR246W		0.285302593659942YPL031C		0.252459016393443YLR201C		0.55YHR091C		0.402799377916019YGR020C		0.245762711864407YML066C		0.29810298102981YDR501W		0.0479846449136276YNL088W		0.19047619047619YNL053W		0.120654396728016YDR400W		0.276470588235294YDR137W		0.0165912518853695YBR231C		0.155115511551155YDL120W		0.201149425287356YLR151C		0.173529411764706YFR021W		YML121W		0.206451612903226YLR310C		0.238514789175582YGR244C		0.285714285714286YGL255W		0.404255319148936YHR046C		0.294915254237288YOR016C		0.386473429951691YML065W		0.185995623632385YPR005C		0.0238095238095238YGR223C		YAR003W		YMR267W		0.129032258064516YNL077W		0.100378787878788YJR034W		0.398148148148148YPL160W		0.303669724770642YGL185C		0.237467018469657YKL175W		0.26441351888668YDR456W		0.44391785150079YGL190C		YOR101W		0.168284789644013YMR262W		0.319488817891374YGL005C		0.516129032258065YNL125C		0.479940564635958YBL019W		0.0807692307692308YBR043C		0.473149492017417YDL231C		0.371555555555556YML101C		0.282051282051282YPR007C		0.0985294117647059YMR108W		0.263464337700146YIL067C		0.150442477876106YPL126W		0.0078125YKL179C		0.543446244477172YJL033W		0.280519480519481YEL041W		0.103030303030303YGL208W		YNL113W		0.246478873239437YGL025C		0.148614609571788YBR171W		0.558252427184466YDL005C		0.0580046403712297YNL031C		0.323529411764706YDL056W		0.277310924369748YML120C		0.208576998050682YGR048W		0.0554016620498615YOL149W		0.121212121212121YML129C		0.1YBR220C		0.494642857142857YBR227C		0.25YNR056C		0.488413547237077YER165W		0.159445407279029YNL327W		0.00288184438040346YOR130C		0.602739726027397YPL099C		0.335164835164835YER016W		0.305232558139535YNL242W		0.131909547738693YMR058W		0.050314465408805YOL078W		0.0399659863945578YAL018C		0.483076923076923YEL002C		0.155813953488372YJL156C		0.152838427947598YMR166C		0.5YCL032W		0.19364161849711YPL050C		0.146835443037975YDR510W		0.158415841584158YLR327C		0.104651162790698YLR142W		0.350840336134454YLR104W		0.229007633587786YNL222W		0.310679611650485YLR291C		0.406824146981627YBR293W		0.742616033755274YLR412W		0.237226277372263YNR067C		0.129811996418979YNL074C		0.0199115044247788YBR021W		0.409162717219589YML021C		0.217270194986072YER038C		0.252155172413793YNL044W		0.136363636363636YER060W-A		0.49622641509434YER173W		0.22154779969651YOR190W		0.285393258426966YML048W		0.300248138957816YBR130C		0.357647058823529YPR072W		0.210714285714286YHR048W		0.587548638132296YHL034C		0.129251700680272YHL006C		0.326666666666667YDR418W		0.272727272727273YLR183C		0.0408997955010225YIR024C		0.375YCR038C		0.422118380062305YJR040W		0.439024390243902YJL037W		0.40625YOR059C		0.215555555555556YNR002C		0.446808510638298YMR172C-A		0.291338582677165YIL158W		0.137254901960784YER048W-A		0.574468085106383YGR278W		0.448873483535529YDR462W		0.401360544217687YMR273C		0.0579234972677596YJL162C		0.171526586620926YPR080W		0.0982532751091703YFL017W-A		0.038961038961039YGL127C		0.519685039370079YGR268C		YHL033C		0.26171875YMR285C		0.0990291262135922YBR104W		0.550151975683891YPL263C		0.0737327188940092YER096W		0.37109375YOR325W		0.0191082802547771YDR311W		0.289719626168224YKL088W		0.101576182136602YJL046W		0.207823960880196YLR228C		0.180589680589681YKL056C		0.179640718562874YPL037C		0.210191082802548YDR459C		0.32620320855615YOR357C		0.228395061728395YNR004W		0.212328767123288YNL192W		0.216622458001768YGL068W		0.319587628865979YDL157C		0.169491525423729YOR007C		0.554913294797688YBR105C		0.00552486187845304YDL013W		0.166397415185784YGL200C		0.38423645320197YDR260C		0.0529411764705882YNR072W		0.558510638297872YBR154C		0.283720930232558YPL156C		0.183098591549296YKL048C		0.128125YJL020C		0.0190146931719965YLR251W		0.685279187817259YGR113W		0.14868804664723YBR253W		0.479338842975207YCR020C		0.711627906976744YJR141W		0.0835734870317003YHR060W		0.370165745856354YDR361C		0.229681978798587YPL009C		0.226396917148362YNL027W		0.0309734513274336YNL065W		0.5580204778157YNL211C		0.325581395348837YHL038C		0.252380952380952YPL070W		0.236928104575163YOL111C		0.202830188679245YDR075W		0.237012987012987YER009W		0.232YFL034C-B		0.337979094076655YIR011C		0.351097178683386YMR297W		0.193609022556391YIL139C		0.257142857142857YDR254W		0.172489082969432YPL005W		0.473597359735974YGL105W		0.143617021276596YDL224C		0.0508474576271186YKL062W		0.0142857142857143YJL158C		0.0616740088105727YDR162C		YCR059C		0.263565891472868YDL183C		0.353125YCL039W		0.103355704697987YER140W		0.431654676258993YPL069C		0.629850746268657YOR386W		0.339823008849558YPL151C		0.00665188470066519YNL042W		0.0883838383838384YHR207C		0.184410646387833YDR171W		0.024YPL157W		0.244444444444444YDR479C		0.236462093862816YAL034W-A		0.525951557093426YOR258W		0.262672811059908YNR057C		0.253164556962025YIL015W		0.0340715502555366YHR127W		0.0905349794238683YNL328C		0.5YCL049C		0.0256410256410256YHR111W		0.231818181818182YGL055W		0.296078431372549YBR288C		0.0745341614906832YOR347C		0.282608695652174YGL065C		0.36779324055666YHR013C		0.23109243697479YOR307C		0.410596026490066YNL144C		0.0202702702702703YIR038C		0.435897435897436YAL046C		0.220338983050847YPL193W		0.0971128608923885YGR208W		0.352750809061489YDR201W		0.581818181818182YML077W		0.283018867924528YGL245W		0.21045197740113YHR058C		0.169491525423729YPR181C		0.220052083333333YBR106W		0.436170212765957YHR018C		0.548596112311015YBR170C		0.179310344827586YDL211C		0.032258064516129YPL078C		0.659836065573771YOL007C		0.0997067448680352YDR399W		0.18552036199095YKL181W		0.22248243559719YGR192C		0.159638554216867YDL078C		0.314868804664723YOL041C		0.0806100217864924YMR226C		0.367041198501873YHR200W		0.294776119402985YPR125W		0.39647577092511YEL046C		0.315245478036176YIR015W		0.215277777777778YMR242C		0.0898876404494382YDR204W		0.405970149253731YPL161C		0.439178515007899YDR300C		0.235981308411215YLR229C		0.267015706806283YLR421C		0.0833333333333333YPL096W		0.319559228650138YML038C		0.461538461538462YJL082W		0.484268125854993YIL006W		0.53887399463807YLR392C		0.0154440154440154YGR075C		0.260330578512397YBR268W		0.247619047619048YHR068W		0.297157622739018YGL160W		0.310526315789474YGR042W		YPR160W		0.35920177383592YER007W		0.0694980694980695YGR095C		0.26457399103139YDL089W		0.229338842975207YDR522C		0.0597609561752988YEL062W		0.123577235772358YBR026C		0.25YLR143W		0.194160583941606YMR125W		0.452961672473868YLR126C		0.247011952191235YGL086W		0.381842456608812YNL129W		0.204166666666667YGL048C		0.330864197530864YER106W		0.0993377483443709YGR169C		0.116336633663366YMR074C		0.468965517241379YMR269W		0.109004739336493YHR138C		0.236842105263158YFL021W		YPL144W		0.168918918918919YEL053C		0.448840381991814YHL020C		0.207920792079208YGR106C		0.215094339622642YLR452C		0.217765042979943YMR005W		0.288659793814433YLR363C		0.215596330275229YIL023C		0.569364161849711YKL138C		0.213740458015267YGL002W		0.328703703703704YOR312C		0.0919540229885057YLR179C		0.0845771144278607YNL230C		0.24802110817942YHL028W		0.0297520661157025YCR015C		0.350157728706625YPR199C		0.27891156462585YMR313C		0.401869158878505YBR077C		0.209876543209877YOR298W		0.308977035490605YDR380W		0.280314960629921YDL177C		0.2YGR149W		0.414351851851852YPL203W		0.221052631578947YNR021W		0.368811881188119YGR080W		0.216867469879518YBR195C		0.023696682464455YPL191C		0.225YMR013C		0.385356454720617YPL148C		0.208092485549133YFL038C		0.233009708737864YPL240C		0.256699576868829YGL145W		0.53922967189729YPR060C		0.421875YNL004W		0.142191142191142YLR232W		0.191304347826087YHR069C		0.122562674094708YEL018W		0.379928315412186YLR323C		0.1003861003861YGL172W		0.288135593220339YDL143W		0.456439393939394YKL162C		0.181592039800995YMR290C		0.310891089108911YDL021W		0.279742765273312YPL211W		0.12707182320442YDR452W		0.163204747774481YAL038W		0.288YMR024W		0.287179487179487YLR147C		0.0792079207920792YDL018C		0.346666666666667YML012W		0.374407582938389YIL061C		0.146666666666667YGR155W		0.244575936883629YLR136C		0.0315789473684211YDL060W		0.120558375634518YIL085C		0.199226305609284YHR075C		0.2225YOL119C		0.57684630738523YNL289W		0.383512544802867YJR105W		0.294117647058824YER161C		0.12012012012012YNR036C		0.0588235294117647YMR111C		0.192640692640693YJL096W		YBR136W		0.503800675675676YMR090W		0.299559471365639YNR061C		0.493150684931507YNL154C		0.166666666666667YGR209C		0.346153846153846YDL181W		0.435294117647059YKL029C		0.242152466367713YPR106W		0.200902934537246YNL039W		0.0993265993265993YDL149W		0.232698094282849YGR187C		0.355329949238579YBR282W		0.0958904109589041YER170W		0.382222222222222YPL269W		0.368012422360248YHR137W		0.300194931773879YGR058W		0.283582089552239YGL009C		0.173299101412067YBL009W		0.116863905325444YDR279W		0.214285714285714YLR243W		0.323529411764706YER029C		0.0204081632653061YJL025W		0.332684824902724YDR246W		0.105022831050228YDR427W		0.582697201017812YER182W		0.155737704918033YNL163C		0.220720720720721YMR228W		0.228739002932551YDR411C		0.316715542521994YNL061W		0.194174757281553YDR309C		0.0469973890339426YER095W		0.255YJL138C		0.288607594936709YDR499W		0.385542168674699YJL063C		0.378151260504202YMR206W		0.0766773162939297YMR154C		0.0866574965612105YLR410W		0.174520069808028YER092W		0.44YBR101C		0.620689655172414YIL042C		0.388324873096447YHR112C		0.288359788359788YGL029W		0.608333333333333YGR160W		0.083743842364532YJR060W		0.270655270655271YDR437W		0.471428571428571YHL022C		0.324120603015075YGL175C		0.188405797101449YLR305C		0.472105263157895YIL124W		0.427609427609428YGR234W		0.293233082706767YCR004C		0.275303643724696YKL028W		0.219917012448133YPR173C		0.382151029748284YPR137W		0.0506108202443281YAL011W		0.0864YNL067W		0.12565445026178YEL016C		0.170385395537525YEL020C		0.3375YPL060W		0.380145278450363YER087W		0.204861111111111YOL087C		0.03584229390681YNL304W		0.129496402877698YHR056C		0.272933182332956YNL092W		0.3425YPL152W		0.371508379888268YKL033W		0.48747591522158YHR214W		0.0443349753694581YGL243W		0.2125YBR025C		0.289340101522843YNL208W		YER055C		0.202020202020202YJR123W		0.333333333333333YHR065C		0.233532934131737YNL128W		0.140552995391705YDR405W		0.250950570342205YMR139W		0.235135135135135YDL217C		0.429951690821256YPL094C		0.14963503649635YJL157C		0.0807228915662651YER120W		0.0532786885245902YLR315W		0.699346405228758YDR516C		0.296YPL106C		0.287157287157287YFR042W		0.57YLR256W		0.156458055925433YPR191W		0.347826086956522YMR241W		0.605095541401274YBR260C		0.441441441441441YLR396C		0.316931982633864YNR037C		0.0879120879120879YPL243W		0.454090150250417YBR018C		0.188524590163934YCR043C		0.251968503937008YPL270W		0.455368693402329YBR054W		0.386627906976744YNL164C		0.056980056980057YNL062C		0.190376569037657YMR298W		0.226666666666667YML106W		0.34070796460177YMR037C		0.0127840909090909YKL094W		0.313099041533546YOR066W		0.0429252782193959YJR147W		0.332402234636872YDR041W		0.211822660098522YMR272C		0.2890625YKL143W		0.103671706263499YDL081C		0.30188679245283YIL019W		0.210982658959538YDL201W		0.195804195804196YNL200C		0.268292682926829YER073W		0.311538461538462YJR007W		0.319078947368421YNL238W		0.0933660933660934YBR053C		YCR073W-A		0.222222222222222YOR269W		0.0323886639676113YPL163C		YML125C		0.176282051282051YOL057W		0.317862165963432YKL145W		0.276231263383298YIL150C		0.131348511383538YGR146C		0.0710900473933649YDL126C		0.27185628742515YDL006W		0.217081850533808YMR025W		0.213559322033898YDL233W		0.14410480349345YCL057C-A		0.371134020618557YOR362C		0.274305555555556YMR276W		0.18230563002681YOR217W		0.267131242740999YML124C		0.229213483146067YPL246C		0.484732824427481YDL178W		0.260377358490566YOR111W		0.237068965517241YNL240C		0.25050916496945YLR152C		0.270833333333333YKL019W		0.528481012658228YGR168C		0.340425531914894YGR027C		0.259259259259259YGL250W		0.161825726141079YEL031W		0.352263374485597YDR320C-A		0.708333333333333YIL099W		0.333333333333333YJR135C		0.426778242677824YIR013C		0.0413223140495868YIL094C		0.31266846361186YMR032W		0.324364723467862YPL112C		0.469543147208122YAL049C		0.272357723577236YMR010W		0.367901234567901YBR193C		0.439461883408072YCR023C		0.518821603927987YHL009C		0.251515151515152YDL086W		0.263736263736264YOR201C		0.162621359223301YPR109W		0.285714285714286YIL038C		0.141148325358852YPL137C		0.186520376175549YML082W		0.238828967642527YPL233W		0.569444444444444YJL004C		0.38423645320197YOR286W		0.268456375838926YOR315W		0.046242774566474YNL010W		0.394190871369295YIL087C		0.331210191082803YHR061C		0.0732484076433121YPL111W		0.255255255255255YGL146C		0.12540192926045YBR182C		0.115044247787611YPR015C		0.0121457489878543YER026C		0.434782608695652YOL036W		0.0354796320630749YPR100W		0.185714285714286YDL139C		0.20627802690583YNR033W		0.172808132147395YKL149C		0.133333333333333YBL093C		0.15YPR122W		0.309602649006623YOL009C		0.107011070110701YER124C		YGR253C		0.3YDR055W		0.0292792792792793YDL110C		0.446666666666667YOR049C		0.494350282485876YMR027W		0.325531914893617YJR032W		0.32824427480916YIL044C		0.161073825503356YBR011C		0.104529616724739YDR419W		0.197784810126582YBR110W		0.363028953229399YLR247C		0.301413881748072YPL208W		0.308747855917667YOR319W		0.169014084507042YLR371W		0.141592920353982YDL051W		0.203636363636364YJL072C		0.366197183098592YHR081W		0.423913043478261YOR061W		0.244837758112094YDR090C		0.393548387096774YPL256C		0.328440366972477YOR070C		0.339089481946625YNL024C		0.215447154471545YJL217W		YGL093W		0.248636859323882YDR523C		0.212244897959184YKL098W		0.240896358543417YCR008W		0.127694859038143YOR298C-A		0.304635761589404YOL059W		0.302272727272727YOR250C		0.170786516853933YGR110W		0.276404494382022YHL036W		0.510989010989011YJR088C		0.633561643835616YLR378C		0.5YHR020W		0.242732558139535YMR235C		0.321867321867322YBL050W		0.688356164383562YNL185C		0.240506329113924YFR036W		0.129032258064516YMR067C		0.137019230769231YML086C		0.174904942965779YMR167W		0.204161248374512YDR016C		0.436170212765957YGR062C		0.572784810126582YIL113W		0.267942583732057YNL234W		0.293427230046948YDR414C		0.533149171270718YPL045W		0.365914786967419YGR189C		0.0591715976331361YCR086W		0.363157894736842YHR039C		0.341614906832298YFL044C		0.229235880398671YIL009W		0.295389048991354YNL046W		0.215116279069767YOR355W		0.0766283524904215YPL265W		0.450657894736842YDL128W		0.564476885644769YHR090C		0.315602836879433YDR214W		0.171428571428571YPR061C		0.3421926910299YGR267C		0.259259259259259YGR185C		0.385786802030457YML050W		0.109324758842444YNL078W		0.12039312039312YMR158W		0.193548387096774YOR262W		0.302593659942363YHR016C		0.0683760683760684YFL004W		0.288647342995169YDL045W-A		0.357894736842105YNL290W		0.517647058823529YMR132C		0.288461538461538YDR358W		0.263913824057451YGL085W		0.18978102189781YBR010W		0.330882352941176YML008C		0.344647519582245YIL119C		0.194103194103194YDR073W		0.313609467455621YOR180C		0.413284132841328YPL170W		0.171052631578947YGL139W		0.269326683291771YLR287C		0.501408450704225YBR248C		0.213768115942029YAL044W-A		0.272727272727273YGL140C		0.370795734208368YGR180C		0.568115942028986YPL229W		0.160194174757282YDR079W		0.405405405405405YDR183W		0.326086956521739YDR205W		0.44475138121547YER118C		0.0708446866485014YKL144C		0.0849056603773585YLR343W		0.218018018018018YMR064W		0.3996138996139YGR193C		0.141463414634146YML041C		0.117857142857143YMR260C		0.0588235294117647YOR060C		0.221789883268482YHR191C		YDL170W		0.401515151515151YDR185C		0.284916201117318YPL153C		0.121802679658953YML072C		0.055663430420712YGR036C		0.539748953974895YKL086W		0.196850393700787YGL226W		0.382113821138211YBR283C		0.491836734693878YGR196C		0.0452876376988984YNL330C		0.267898383371824YLR393W		0.150537634408602YHR189W		0.257894736842105YDR513W		0.440559440559441YPR069C		0.204778156996587YNL064C		0.122249388753056YBR061C		0.235483870967742YPL002C		0.467811158798283YJR053W		0.0731707317073171YHR010W		0.161764705882353YER102W		0.14YKL074C		0.117647058823529YGR126W		0.0782608695652174YAL036C		0.246612466124661YKL082C		0.345622119815668YGL010W		0.614942528735632YHR195W		0.133956386292835YIL131C		0.0578512396694215YDR287W		0.291095890410959YMR002W		0.224358974358974YDR122W		0.104323308270677YIL127C		0.470873786407767YIR042C		0.266949152542373YBL068W		0.247706422018349YPR055W		0.483568075117371YMR281W		0.299342105263158YBR019C		0.171673819742489YOR256C		0.286773794808405YER139C		0.287610619469027YNL174W		0.0526315789473684YBR160W		0.268456375838926YMR006C		0.198300283286119YHR064C		0.213754646840149YMR174C		0.411764705882353YPL120W		0.330341113105925YOR151C		0.165032679738562YPR025C		0.468193384223919YLR121C		0.0649606299212598YNL220W		0.210161662817552YJL186W		0.237201365187713YGR263C		0.219339622641509YIL024C		0.0899470899470899YPR070W		0.144876325088339YMR087W		0.25YDR068W		0.383870967741935YGR059W		0.2890625YKL080W		0.380102040816327YGL246C		0.162790697674419YBL075C		0.278890600924499YHR108W		0.235897435897436YER035W		YMR299C		0.176282051282051YML093W		0.183537263626251YGL143C		0.382566585956416YNR073C		0.378486055776892YMR030W		0.146276595744681YDL117W		0.0485875706214689YOL075C		0.432766615146832YOL056W		0.277227722772277YIL022W		0.364269141531323YMR073C		0.139303482587065YDR224C		0.320610687022901YER171W		0.344473007712082YNL141W		0.440922190201729YDL010W		0.229437229437229YDR106W		0.179577464788732YHR078W		0.509057971014493YDR270W		0.335657370517928YGR009C		0.207373271889401YGL122C		0.0323809523809524YMR128W		0.188634569850039YEL013W		0.612456747404844YLR268W		0.485981308411215YDL069C		0.174672489082969YMR065W		0.640873015873016YIL068C		0.607453416149068YPR183W		0.397003745318352YLR387C		0.256944444444444YGR012W		0.234096692111959YNL095C		0.230529595015576YBR007C		0.150815217391304YGL253W		0.325102880658436YOR196C		0.234299516908213YDR233C		0.461016949152542YEL040W		0.0342612419700214YNL250W		0.634908536585366YMR252C		0.26865671641791YLR312W-A		0.391304347826087YGR206W		0.336633663366337YKL189W		0.431077694235589YER152C		0.329571106094808YCL033C		0.0892857142857143YGR006W		0.438247011952191YPL041C		0.333333333333333YOL131W		0.324074074074074YLR088W		0.338762214983713YCR045C		0.144602851323829YKL163W		0.0307692307692308YPR149W		0.566473988439306YNL306W		0.115207373271889YBR034C		0.140804597701149YPL187W		0.096969696969697YHR030C		0.212809917355372YDR384C		0.454545454545455YMR052W		0.696078431372549YGR124W		0.281468531468531YIL123W		YOR163W		0.132978723404255YJL178C		0.143911439114391YLR352W		0.135068153655514YKL018C-A		0.242424242424242YPR112C		0.129650507328072YNR038W		0.21939586645469YDL192W		0.325966850828729YPR157W		0.0578158458244111YIL109C		0.187904967602592YGL257C		0.206093189964158YER099C		0.254716981132075YNL182C		0.027027027027027YPR163C		0.0389908256880734YML042W		0.338805970149254YGR152C		0.1875YDL124W		0.326923076923077YIL138C		0.782608695652174YDR268W		0.41688654353562YOL064C		0.212885154061625YER091C		0.376792698826597YOL110W		0.215189873417722YHR132W-A		0.137404580152672YMR129W		0.0388930441286462YLR153C		0.24597364568082YOL128C		0.242666666666667YOR085W		0.28YJL190C		0.2YPL204W		0.176113360323887YOR159C		0.0638297872340425YER072W		0.550387596899225YDL004W		0.19375YJL164C		0.219143576826196YNL130C		0.460559796437659YER100W		0.26YMR110C		0.37781954887218YDR228C		0.206070287539936YFL048C		0.197752808988764YBR247C		0.360248447204969YNL091W		0.0701612903225806YDL159W		0.198058252427184YLR347C		0.658536585365854YDR159W		0.282090699461952YOR040W		0.224561403508772YGL187C		0.0709677419354839YJR095W		0.611801242236025YNL029C		0.220306513409962YJL013C		0.231067961165049YNL136W		0.0282352941176471YDL058W		0.46927374301676YBL057C		0.303738317757009YBL087C		0.0510948905109489YGL130W		0.148148148148148YMR035W		0.135593220338983YPL181W		0.0731225296442688YOR075W		0.615606936416185YMR159C		0.48YOR211C		0.417707150964813YKL183W		0.0522875816993464YGL084C		0.557142857142857YPL245W		0.229074889867841YOL049W		0.311608961303462YMR088C		0.672597864768683YJR044C		0.428571428571429YDR436W		0.107042253521127YIL083C		0.246575342465753YHR133C		0.305841924398625YDL064W		0.312101910828025YGR024C		0.358649789029536YNL051W		0.598014888337469YML097C		0.401330376940133YMR097C		0.239782016348774YNL059C		0.290066225165563YMR253C		0.521739130434783YPR050C		0.167883211678832YHL040C		0.609250398724083YBL098W		0.317391304347826YJR009C		0.159638554216867YPR180W		0.262247838616715YJL196C		0.554838709677419YGR148C		0.445161290322581YMR204C		0.0690476190476191YHR105W		0.163551401869159YCR053W		0.348249027237354YLR137W		0.155313351498638YHR114W		0.437598736176935YPR010C		0.149625935162095YGR257C		0.519125683060109YOR279C		0.116129032258065YGR242W		YJL112W		0.0868347338935574YLR398C		0.290598290598291YPL227C		0.338323353293413YGR273C		0.0632183908045977YMR184W		0.257575757575758YBL081W		YJR142W		0.198830409356725YHR194W		0.150259067357513YFR046C		0.199445983379501YGL027C		0.243697478991597YLR423C		0.690647482014389YMR286W		0.267441860465116YDL036C		0.116883116883117YBR213W		0.339416058394161YHR037W		0.311304347826087YLR107W		0.23019801980198YNR045W		0.263803680981595YMR072W		0.497267759562842YDL186W		0.104693140794224YCR082W		0.5546875YER063W		0.215596330275229YNL090W		0.239583333333333YMR280C		0.188415910676902YHR045W		0.157142857142857YPL072W		0.118236472945892YOR330C		0.230462519936204YPL252C		0.133720930232558YNL108C		0.225925925925926YDL073W		0.257113821138211YEL042W		0.223938223938224YKL085W		0.362275449101796YLR219W		0.00686813186813187YNL138W		0.23574144486692YBR082C		0.304054054054054YLR355C		0.329113924050633YHL032C		0.225669957686883YHR002W		0.532212885154062YPL038W		0.0338983050847458YJR085C		0.628571428571429YCL030C		0.314142678347935YLR284C		0.435714285714286YOR017W		0.185YIL095W		0.117283950617284YBR162W-A		0.4YER105C		0.304816678648454YJR080C		0.0558375634517767YIL055C		0.0558213716108453YKL157W		0.33048128342246YKL113C		0.335078534031414YER014W		0.246753246753247YPR164W		0.0177683013503909YER158C		0.075043630017452YNL189W		0.607011070110701YJL079C		0.130434782608696YKL109W		0.0234657039711191YER049W		0.127329192546584YJL171C		0.0707070707070707YBR280C		0.021978021978022YBR259W		0.303779069767442YGL181W		0.108585858585859YHR040W		0.103825136612022YDL226C		0.213068181818182YDR394W		0.299065420560748YLR344W		0.125984251968504YGL198W		0.34468085106383YER128W		0.251231527093596YBR223C		0.101102941176471YMR079W		0.335526315789474YAL022C		0.415860735009671YHR071W		0.344978165938865YMR264W		0.339901477832512YMR200W		0.06640625YBR165W		0.202166064981949YIL027C		0.354609929078014YNR049C		0.080952380952381YPL249C		0.314317673378076YJL035C		0.268YLR131C		0.0558441558441558YAR014C		0.0537482319660538YGL261C		0.4YER156C		0.29585798816568YDR210W		0.24YDL130W-A		0.523255813953488YLR233C		0.369098712446352YPL127C		0.244186046511628YDR147W		0.254681647940075YPL077C		0.2125YPL051W		0.323232323232323YMR157C		0.247058823529412YOR002W		0.507352941176471YMR176W		0.270729978738483YER111C		0.205855443732845YOR175C		0.487883683360259YAL061W		0.211031175059952YGR215W		0.354545454545455YNL118C		0.0845360824742268YNR050C		0.275784753363229YDR489W		0.357142857142857YML096W		0.217142857142857YGL179C		0.125YOR264W		0.0511627906976744YMR089C		0.299393939393939YOL045W		0.11716621253406YPL207W		0.187654320987654YIL070C		0.218045112781955YCR091W		0.122222222222222YGL203C		0.152263374485597YIL117C		0.0817610062893082YMR307W		0.211091234347048YOL152W		0.332258064516129YJR131W		0.366120218579235YJL003W		0.313559322033898YNL082W		0.169530355097365YDR196C		0.518672199170125YBR003W		0.505285412262156YNR032W		0.233695652173913YGL148W		0.188829787234043YOR245C		0.26555023923445YNL299W		0.191588785046729YOL144W		0.12603305785124YGL111W		0.00647948164146868YBR181C		0.216101694915254YCL038C		0.537878787878788YPR158W		0.214285714285714YOR313C		0.171597633136095YGL039W		0.318965517241379YDL207W		0.431226765799257YLR394W		0.170124481327801YNL231C		0.356125356125356YML099C		0.246590909090909YJL159W		0.0217917675544794YOR187W		0.125858123569794YNL160W		0.194915254237288YNL107W		0.146017699115044YJL125C		0.169712793733681YEL036C		0.218YGR013W		0.291935483870968YJL054W		0.177824267782427YMR214W		0.159151193633952YNR008W		0.180030257186082YMR042W		0.0451977401129944YJR019C		0.0888252148997135YDL084W		0.262331838565022YGL077C		0.506216696269982YLR182W		0.330012453300125YAR019C		0.286447638603696YNL121C		0.649918962722853YMR308C		0.6455463728191YMR309C		0.445812807881773YDL182W		0.355140186915888YMR170C		0.316205533596838YLR231C		0.280353200883002YDL208W		0.371794871794872YOR099W		0.256997455470738YAL043C		0.41656050955414YDR531W		0.256130790190736YBR028C		0.165714285714286YNL225C		0.38382099827883YIR023W		0.237113402061856YDR377W		0.346534653465347YDR486C		0.602620087336245YHR115C		0.00240384615384615YPL007C		0.0731292517006803YDR248C		0.341968911917098YNL111C		0.325YPR032W		0.0561471442400774YDR415C		0.259358288770053YJL173C		0.131147540983607YDR298C		0.481132075471698YOR114W		0.408163265306122YNR054C		0.218354430379747YCR071C		0.136986301369863YHR199C		0.183870967741935YOL108C		0.370860927152318YHR154W		0.131775700934579YHR038W		0.439130434782609YNL008C		0.278026905829596YMR191W		0.195710455764075YOR148C		0.0648648648648649YJL194W		0.339181286549708YHR027C		0.411883182275932YLR225C		0.0147420147420147YCR090C		0.010989010989011YDR219C		0.174193548387097YMR168C		0.337171052631579YDR084C		0.462311557788945YGL252C		0.36734693877551YGL221C		0.263888888888889YPL103C		0.106837606837607YLR167W		0.0921052631578947YPR079W		0.005249343832021YJR148W		0.175531914893617YJR102C		0.292079207920792YER117W		0.0510948905109489YPL128C		0.225978647686833YJR075W		0.285353535353535YGR147C		0.215277777777778YPL132W		0.113333333333333YOR176W		0.305343511450382YDR121W		0.392857142857143YKL050C		0.275488069414317YDL204W		0.338422391857506YMR048W		0.195583596214511YDR098C		0.263157894736842YOR177C		0.34051724137931YGR205W		0.331034482758621YLR188W		0.507913669064748YCR050C		0.186274509803922YHR159W		0.0892857142857143YBL090W		0.322033898305085YPL139C		0.00869565217391304YBR203W		0.114718614718615YGR109C		0.392105263157895YBR121C		0.223388305847076YKL165C-A		0.285714285714286YIL120W		0.580817051509769YGR255C		0.240083507306889YER175C		0.280936454849498YMR256C		0.25YDR070C		0.204301075268817YEL064C		0.516666666666667YPR156C		0.463022508038585YER067W		0.173913043478261YOR003W		0.173640167364017YGL067W		0.143229166666667YNL119W		0.235294117647059YNR035C		0.233918128654971YOR232W		0.412280701754386YMR092C		YOR317W		0.285714285714286YAR066W		0.0443349753694581YGR162W		0.167016806722689YMR102C		YDL066W		0.285046728971963YOR387C		0.087378640776699YGL070C		YBR272C		0.464583333333333YDL111C		0.237735849056604YIR030C		0.368852459016393YPR018W		0.247524752475248YGR161C		0.140684410646388YMR068W		0.288732394366197YKL168C		0.104972375690608YIL011W		0.182156133828996YDR476C		0.339285714285714YPL272C		0.174081237911025YJL123C		0.215481171548117YHR080C		0.0862453531598513YMR086W		0.0135416666666667YDR213W		0.15553121577218YNL071W		0.178423236514523YBL086C		0.0343347639484979YGL006W		0.303495311167945YML087C		0.125YLR090W		0.111111111111111YPR196W		0.402127659574468YMR225C		0.23469387755102YOR230W		0.0160183066361556YBR169C		0.291486291486291YDR004W		0.226086956521739YER076C		0.0860927152317881YBR210W		0.76056338028169YPL206C		0.249221183800623YNL322C		YHR155W		0.357491856677524YNR043W		0.244949494949495YGR082W		0.53551912568306YER004W		0.354978354978355YNL232W		0.00342465753424658YMR251W-A		0.0338983050847458YNL209W		0.262642740619902YHR153C		0.297979797979798YPR016C		0.2YNL178W		0.2375YHR206W		0.244372990353698YPL090C		0.211864406779661YLR132C		0.196551724137931YNL025C		0.554179566563467YDR483W		0.212669683257919YPR027C		0.202166064981949YPL067C		0.262626262626263YCR026C		0.126684636118598YDL214C		0.118741058655222YBL082C		0.513100436681223YDL017W		0.16370808678501YGL218W		0.175925925925926YDR527W		0.209567198177677YBR093C		0.278372591006424YGL138C		0.165217391304348YDR391C		0.237068965517241YOR131C		0.380733944954128YDR345C		0.564373897707231YLR303W		0.279279279279279YLR127C		0.39390386869871YOL116W		0.227748691099476YPL155C		0.274787535410765YNL186W		0.0909090909090909YPR110C		0.140298507462687YLR097C		0.223837209302326YMR238W		0.441048034934498YLR414C		0.32319391634981YDR392W		0.409495548961424YBL032W		0.196850393700787YML049C		0.0345334313005143YDR416W		0.607683352735739YDL108W		0.258169934640523YMR240C		0.174311926605505YGL071W		0.0623188405797101YPL115C		0.134751773049645YMR049C		0.0545229244114002YDR243C		0.241496598639456YDL008W		0.115151515151515YNR017W		0.364864864864865YOR383C		YLR095C		0.20935960591133YOR115C		0.287313432835821YHR019C		0.23826714801444YKL033W-A		0.385593220338983YNL123W		0.086258776328987YPL084W		0.492890995260663YNL162W-A		0.236111111111111YGR260W		0.573033707865168YDR012W		0.207182320441989YCL005W		0.14453125YLR417W		0.231448763250883YBR233W-A		0.648936170212766YHR157W		0.0769230769230769YOL072W		0.496703296703297YPL239W		0.375YBR179C		0.381286549707602YFR048W		0.208459214501511YIL074C		0.245202558635394YOR322C		0.00244498777506112YOR033C		0.163817663817664YPR063C		0.292857142857143YKL046C		0.429844097995546YER107C		YKL024C		0.455882352941176YPR054W		0.260309278350515YMR075W		0.0833333333333333YBR262C		0.19811320754717YDR088C		0.167539267015707YLR377C		0.21551724137931YDR118W		0.262269938650307YIR034C		0.241286863270777YMR291W		0.15358361774744YML011C		0.299435028248588YPL048W		0.298795180722892YGL120C		0.301173402868318YAR008W		0.218181818181818YGL116W		0.0147540983606557YJR047C		0.0445859872611465YDR299W		0.301498127340824YOR223W		0.136986301369863YBR298C		0.566775244299674YHR185C		0.143459915611814YPL049C		YKL159C		0.0710900473933649YNL260C		0.535353535353535YJL210W		0.357933579335793YBL015W		0.226235741444867YAL054C		0.253856942496494YMR119W		0.306089743589744YDL020C		0.0546139359698682YBR055C		0.562847608453838YJL105W		0.0392857142857143YGR040W		0.263586956521739YLR293C		0.251141552511416YHR031C		0.116182572614108YJR118C		0.408866995073892YLR177W		0.0859872611464968YGR279C		0.191709844559585YBR290W		0.180685358255452YGL123W		0.181102362204724YOL115W		0.195205479452055YOR231W		0.175196850393701YMR062C		0.233560090702948YPL176C		0.301404853128991YJR134C		0.332390381895332YIL064W		0.229571984435798YDR165W		YNL142W		0.420841683366733YMR222C		0.295964125560538YHR144C		0.259615384615385YNL311C		0.107470511140236YLR242C		0.364485981308411YER057C		0.271317829457364YML029W		0.218377088305489YCR087C-A		0.111111111111111YAL033W		0.335260115606936YNR060W		0.360222531293463YLR333C		0.259259259259259YML037C		0.0941176470588235YDL101C		0.165692007797271YDR195W		0.172607879924953YDR322W		0.190735694822888YDL102W		0.255241567912489YGR276C		0.21880650994575YBR126C		0.339393939393939YJL081C		0.169734151329243YLR363W-A		0.317647058823529YGR076C		0.318471337579618YCR020C-A		0.113636363636364YML010W		0.0385700846660395YPL168W		0.218604651162791YDR328C		0.402061855670103YGR021W		0.341379310344828YPL237W		0.164912280701754YMR224C		0.154624277456647YPR105C		0.51335656213705YPL083C		0.119914346895075YMR172W		0.121001390820584YOL140W		0.297872340425532YGL107C		0.363777089783282YGR128C		0.109396914446003YNR020C		0.407407407407407YPR017C		0.0559440559440559YDL132W		0.542331288343558YNR039C		0.2YCR010C		0.441696113074205YNL309W		0.05YGR179C		0.342364532019704YGR175C		0.300403225806452YNL265C		0.429530201342282YMR192W		0.398611111111111YPR189W		0.620111731843575YNL196C		0.130872483221477YGL135W		0.313364055299539YOL132W		0.199575371549894YGL082W		0.223097112860892YNR048W		0.0763358778625954YDL134C		0.195121951219512YHR083W		0.237082066869301YFR005C		0.149553571428571YEL020W-A		0.747126436781609YEL038W		0.348017621145374YCR035C		0.147208121827411YMR026C		0.320802005012531YJR016C		0.27008547008547YDR176W		0.180911680911681YGL004C		YDR222W		0.0144578313253012YPR162C		0.323251417769376YEL054C		0.272727272727273YDR020C		0.275862068965517YPR129W		0.00859598853868195YDL091C		0.305494505494506YJL154C		0.510593220338983YDR074W		0.246651785714286YBR240C		0.206666666666667YDR251W		0.171084337349398YOL042W		0.132231404958678YHR196W		0.140869565217391YDR494W		0.132963988919668YGL142C		0.443181818181818YPL230W		0.0434782608695652YOR046C		0.259336099585062YBL056W		0.158119658119658YDL175C		0.0174418604651163YOR336W		0.234432234432234YNL139C		0.350657482780213YHR192W		0.388489208633094YNL313C		0.491150442477876YCR051W		0.40990990990991YGR067C		0.192695214105793YHL024W		0.0659186535764376YJL185C		0.180887372013652YER071C		0.198412698412698YPL076W		0.467857142857143YIR002C		0.24269889224572YMR294W		0.490616621983914YML110C		0.280130293159609YPR065W		0.130434782608696YLR288C		0.0654008438818565YCR057C		0.0682556879739978YLR170C		0.423076923076923YJL160C		0.0383275261324042YBR287W		0.444964871194379YDL033C		0.172661870503597YGR239C		0.138888888888889YER031C		0.224215246636771YOR006C		0.201277955271566YNL283C		0.0298210735586481YOL032W		0.186991869918699YLR194C		YER168C		0.406593406593407YFR032C-A		0.254237288135593YJL151C		0.195488721804511YOR332W		0.626609442060086YMR181C		0.12987012987013YDL042C		0.183274021352313YNL148C		0.0748031496062992YMR318C		0.2YGL078C		0.261950286806883YIL065C		0.606451612903226YNL099C		0.222689075630252YPR083W		0.278065630397237YBL033C		0.136231884057971YCL057W		0.474719101123595YDR117C		0.187610619469027YLR245C		0.274647887323944YPL254W		0.23155737704918YNL100W		0.508547008547009YPL184C		0.11437908496732YDR492W		0.563291139240506YER006W		0.248076923076923YOL114C		0.237623762376238YNL288W		0.332439678284182YGR096W		0.614649681528662YER132C		0.0610382201939532YJL008C		0.419014084507042YHR202W		0.186046511627907YHR094C		0.559649122807018YPR140W		0.230971128608924YPR029C		0.421875YNL318C		0.587037037037037YDR276C		0.581818181818182YOL040C		0.147887323943662YGR143W		0.00907911802853437YPL165C		0.25201072386059YNR026C		0.0191082802547771YBL013W		0.177057356608479YGR122W		0.407960199004975YDR275W		0.225531914893617YHR140W		0.338912133891213YKL127W		0.280701754385965YDR382W		0.327272727272727YOL129W		0.195652173913043YNL102W		0.226839237057221YML014W		0.17921146953405YDR237W		0.160958904109589YJR129C		0.28023598820059YPR024W		0.323962516733601YOR289W		0.207171314741036YLR259C		0.372377622377622YDR496C		0.51219512195122YPL042C		0.138738738738739YJL133W		0.601910828025478YOL077W-A		0.352941176470588YPL145C		0.165898617511521YDL146W		0.321792260692464YDR100W		0.496503496503497YJR004C		0.0215384615384615YBR186W		0.210992907801418YKL137W		0.612612612612613YFR041C		0.332203389830508YKL006C-A		0.731958762886598YLR133W		0.22680412371134YPR192W		0.419672131147541YMR189W		0.276595744680851YOR137C		0.114147909967846YNL215W		0.103125YDL105W		0.278606965174129YCL029C		0.179545454545455YDR172W		0.0861313868613139YNL085W		0.330120481927711YDL230W		0.229850746268657YNL264C		0.354285714285714YBL038W		0.125YHR148W		0.371584699453552YPL166W		0.272300469483568YJL005W		0.0913129318854886YPL026C		0.155378486055777YJR006W		0.117043121149897YJL115W		0.003584229390681YJL099W		0.333780160857909YLR262C		0.223255813953488YNL333W		0.338926174496644YFR010W		0.144288577154309YJL161W		0.266666666666667YJR097W		0.267441860465116YIR021W		0.31129476584022YPR103W		0.247386759581882YJR122W		0.0663983903420523YOR390W		0.394666666666667YML105C		0.0805860805860806YKL035W		0.218436873747495YMR056C		0.624595469255663YGL171W		0.24113475177305YOR373W		0.0164512338425382YGR249W		0.100877192982456YAR035W		0.304221251819505YGR198W		0.474908200734394YNR022C		0.223021582733813YDR034C		0.248101265822785YBR033W		0.224156692056583YPL186C		0.0592105263157895YBL084C		0.474934036939314YOR352W		0.154518950437318YDL030W		0.369811320754717YAL013W		0.326190476190476YDL104C		0.302211302211302YHR175W		0.380952380952381YBR002C		0.384615384615385YBR246W		YHR121W		0.251336898395722YEL017W		0.28486646884273YDL067C		0.559322033898305YGR037C		0.620689655172414YML123C		0.611584327086882YDR280W		0.236065573770492YBR191W		0.0875YGL083W		0.294776119402985YMR229C		0.144013880855986YAL060W		0.219895287958115YCL036W		0.204946996466431YPL046C		0.363636363636364YPL136W		0.10655737704918YJL207C		0.485600794438928YBL069W		0.20979020979021YOR361C		0.107470511140236YML031W		0.386259541984733YNR014W		0.0377358490566038YBR166C		0.424778761061947YHR087W		0.198198198198198YDR151C		0.0338461538461538YGL026C		0.339462517680339YER047C		0.208472686733556YEL050C		YGL036W		0.251925192519252YDL087C		0.475095785440613YPL135W		0.393939393939394YJR064W		0.409252669039146YPL074W		0.26657824933687YOR276W		0.142857142857143YGR261C		0.478368355995056YBL102W		0.548837209302326YGR049W		0.213903743315508YHR156C		0.185294117647059YIR012W		YMR283C		0.233918128654971YER083C		0.329824561403509YLR110C		0.0601503759398496YBR137W		0.379888268156425YJR062C		0.12691466083151YNL094W		0.175468483816014YNL281W		0.235294117647059YHR001W-A		0.142857142857143YMR150C		0.115789473684211YDL098C		0.335051546391753YGR010W		0.177215189873418YBR057C		0.284153005464481YPL032C		0.126060606060606YGR157W		0.272727272727273YNL213C		0.261682242990654YMR018W		0.589494163424125YBR284W		0.21831869510665YGR238C		0.0850340136054422YKL130C		0.601626016260163YDR536W		0.560632688927944YIL051C		0.23448275862069YOR305W		0.169421487603306YER079W		0.0571428571428571YPR003C		0.428381962864721YDR107C		0.279761904761905YGL064C		0.213903743315508YML117W		0.0776014109347443YIL082W		0.355172413793103YKL091C		0.348387096774194YKL096W		0.0836820083682008YDR110W		0.174911660777385YDR412W		0.319148936170213YPR082C		0.223776223776224YJL193W		0.527363184079602YDR123C		0.177631578947368YNR003C		0.302839116719243YBR149W		0.311046511627907YNL112W		0.243589743589744YNL261W		0.281837160751566YBR172C		0.0608108108108108YDR226W		0.432432432432432YDR389W		0.159021406727829YIL144W		0.531114327062229YJL080C		0.218494271685761YOR089C		0.233333333333333YJR022W		0.110091743119266YPR034W		0.161425576519916YDR477W		0.197472353870458YKL061W		0.610619469026549YOR219C		0.0966702470461869YBR164C		0.316939890710383YMR060C		0.394495412844037YBR217W		0.0806451612903226YIL076W		0.584459459459459YIR014W		0.367768595041322YPL068C		0.187713310580205YGR117C		0.0504201680672269YDR014W		0.247295208655332YGR275W		0.0573248407643312YNL210W		0.222222222222222YGL233W		0.542857142857143YLR457C		0.322884012539185YBR030W		0.259057971014493YPL225W		0.39041095890411YJR036C		0.320627802690583YDR138W		0.30186170212766YPR134W		0.111940298507463YDL119C		0.635179153094462YGR004W		0.177489177489178YMR301C		0.505797101449275YPR116W		0.140794223826715YNL253W		YLR386W		0.4875YBR037C		0.233898305084746YFR045W		0.589473684210526YPR145W		0.258741258741259YNR024W		0.0591397849462366YMR123W		0.450819672131148YOR303W		0.165450121654501YLR221C		0.240909090909091YNL152W		0.00488997555012225YNL244C		0.203703703703704YJL111W		0.429090909090909YNL135C		0.0350877192982456YHL015W		0.206611570247934YGR236C		0.389473684210526YIL154C		0.124277456647399YDL085W		0.222018348623853YML115C		0.151401869158879YBR278W		0.223880597014925YDR236C		0.0825688073394495YOR349W		0.460552268244576YLR433C		0.264014466546112YCR018C		0.117647058823529YFR037C		0.235188509874327YJL002C		0.0798319327731092YLR264W		YLR353W		0.0746268656716418YHL016C		0.435374149659864YLR135W		0.127005347593583YOL088C		0.353790613718412YPL086C		0.308797127468582YOR209C		0.347319347319347YDR142C		YIR025W		0.160326086956522YKL135C		0.506887052341598YMR223W		0.144373673036093YKL120W		0.626543209876543YLR239C		0.182926829268293YPL019C		0.295808383233533YPL085W		0.069248291571754YLR102C		0.162264150943396YJL071W		0.151567944250871YJR010W		0.272015655577299YCR021C		0.283132530120482YHR063C		0.316622691292876YOL063C		0.0585161964472309YGR172C		0.342741935483871YHR009C		0.112810707456979YGR111W		0.2YOR039W		0.166666666666667YPL087W		0.514195583596215YMR101C		0.405247813411079YNL001W		0.264248704663212YOL146W		0.371134020618557YDL218W		0.214511041009464YNL098C		0.164596273291925YLR332W		0.0212765957446809YGR081C		0.257142857142857YOL156W		0.564373897707231YBR058C-A		0.5125YHR168W		0.0921843687374749YHR062C		0.242320819112628YGR248W		0.258823529411765YOL058W		0.316666666666667YMR138W		0.287958115183246YPL022W		0.273636363636364YJL110C		0.0399274047186933YHR208W		0.173027989821883YIR009W		0.189189189189189YLR384C		0.240919199406968YPR188C		0.47239263803681YGL018C		0.554347826086957YJL198W		0.457434733257662YPL013C		0.140495867768595YMR261C		0.232447817836812YER001W		0.195538057742782YGR002C		0.292016806722689YPR108W		0.575757575757576YDR385W		0.232779097387173YMR271C		0.334801762114537YBR109C		0.530612244897959YDR480W		0.0743034055727554YOR306C		0.571976967370441YBL089W		0.529411764705882YGR112W		0.190231362467866YLR213C		0.0308056872037915YDR439W		0.190201729106628YLR408C		0.491803278688525YJR010C-A		0.297872340425532YNL323W		0.070048309178744YPR067W		0.0810810810810811YGR254W		0.324942791762014YIL145C		0.310679611650485YMR209C		0.352297592997812YPR102C		0.224137931034483YLR168C		0.31304347826087YPL123C		0.105990783410138YJL149W		0.223227752639517YGR159C		0.0821256038647343YML058W-A		0.0294117647058824YDR325W		0.461835748792271YHR006W		0.0609981515711645YGL040C		0.333333333333333YOR259C		0.302059496567506YDL085C-A		0.558823529411765YBL041W		0.257261410788382YNL009W		0.288095238095238YNL070W		0.0833333333333333YJR139C		0.270194986072423YJL044C		0.366812227074236YLR404W		0.175438596491228YIR016W		0.113207547169811YNL206C		0.0857142857142857YML062C		0.272959183673469YDR375C		0.285087719298246YLR375W		0.0612244897959184YPL183W-A		0.0752688172043011YER010C		0.277777777777778YHR149C		0.0272479564032698YJL197W		0.10207336523126YDL212W		0.380952380952381YER025W		0.10246679316888YBR046C		0.275449101796407YGL011C		0.313492063492063YBR052C		0.280952380952381YPR167C		0.275862068965517YER048C		0.48849104859335YER037W		0.32398753894081YGR247W		0.171548117154812YHR169W		0.266821345707657YKL154W		0.270491803278689YLR321C		0.169014084507042YMR063W		0.322175732217573YFL034W		0.236719478098788YIL125W		0.289940828402367YOR270C		0.453571428571429YGL017W		0.115308151093439YFR053C		0.317525773195876YNL110C		0.145454545454545YIL098C		0.580645161290323YML128C		0.524366471734893YOR001W		0.281036834924966YER129W		0.0840630472854641YOR304C-A		0.407894736842105YOR057W		0.281012658227848YDR373W		0.521052631578947YGR091W		0.398785425101215YLR087C		0.0966869506423259YOL002C		0.55205047318612YGL244W		0.272401433691756YCL035C		0.409090909090909YPR152C		0.206451612903226YGR005C		0.13YHL008C		0.338118022328549YKL146W		0.398843930635838YOL092W		0.373376623376623YMR115W		0.42315369261477YLR381W		0.375170532060027YBL028C		0.311320754716981YKL039W		0.313575525812619YGL028C		0.125461254612546YMR127C		0.21301775147929YLR418C		0.190839694656489YDR247W		0.167028199566161YMR205C		0.280500521376434YJL118W		0.164383561643836YFR043C		0.257383966244726YOR255W		0.122302158273381YDR265W		0.376854599406528YNL131W		0.282894736842105YNL312W		0.164835164835165YER101C		0.183720930232558YMR215W		0.162213740458015YJL172W		0.246527777777778YER159C		0.323943661971831YHL031C		0.713004484304933YEL047C		0.227659574468085YHR118C		0.275862068965517YOR160W		0.66358024691358YKL016C		0.545977011494253YPR056W		0.195266272189349YBR201W		0.611374407582938YOR185C		0.213636363636364YER144C		0.171428571428571YBR264C		0.231155778894472YML016C		0.111271676300578YDL180W		0.206581352833638YEL058W		0.287253141831239YLR172C		0.28YBR024W		0.255813953488372YJL168C		0.184174624829468YJL121C		0.365546218487395YPL212C		0.137867647058824YJL036W		0.567375886524823YOL077C		0.22680412371134YJL201W		0.230383973288815YPL141C		0.0855491329479769YDR528W		0.0212765957446809YNL262W		0.279027902790279YBR135W		0.233333333333333YNL317W		YPR104C		0.0299145299145299YPL244C		0.622418879056047YOR069W		0.334814814814815YGL164C		0.284090909090909YGL231C		0.384210526315789YER090W		0.268244575936884YPR049C		0.368421052631579YDL200C		0.292553191489362YIR018W		0.244897959183673YGR026W		0.474820143884892YIL114C		0.00355871886120996YFL027C		0.374245472837022YLR380W		0.372549019607843YNL153C		0.604651162790698YHR036W		0.235668789808917YKL184W		0.197424892703863YGR079W		0.0324324324324324YML009C		YDL133W		0.160183066361556YOL073C		0.307453416149068YMR250W		0.292307692307692YBR098W		0.172214182344428YHR005C		0.328389830508475YDR507C		0.129597197898424YPR094W		0.0841121495327103YMR303C		0.241379310344828YBR237W		0.239104829210836YER018C		0.547511312217195YHR161C		0.207221350078493YDR116C		0.224561403508772YDR242W		0.287795992714026YOL145C		0.610027855153203YOR290C		0.291250733998826YLR281C		0.187096774193548YBR139W		0.206692913385827YPL100W		YER023W		0.461538461538462YPL146C		0.246153846153846YLR439W		0.338557993730408YCL055W		0.143283582089552YBR138C		0.0801526717557252YJL055W		0.281632653061224YDR057W		0.0682656826568266YMR140W		0.120654396728016YLR092W		0.376259798432251YBR016W		0.1171875YFL054C		0.213622291021672YBR031W		0.201657458563536YHR142W		0.360759493670886YNL035C		YPL164C		0.206993006993007YJR101W		0.323308270676692YDR447C		0.367647058823529YIL136W		0.315521628498728YML127W		0.314974182444062YIL016W		0.421383647798742YOR052C		0.1YOR078W		0.322429906542056YMR302C		0.311764705882353YGR166W		0.0160714285714286YNL221C		0.147428571428571YGL128C		0.328621908127208YKL072W		0.265013054830287YHR052W		0.183510638297872YMR182C		0.033175355450237YDR438W		0.548648648648649YPL098C		0.309734513274336YER017C		0.336399474375821YLR113W		0.227586206896552YNL227C		0.283050847457627YIL149C		0.578916021441334YIL157C		0.203045685279188YPR151C		0.058252427184466YFL024C		0.195913461538462YPL125W		0.586240310077519YMR135C		0.263736263736264YMR259C		0.554929577464789YOL071W		0.395061728395062YGL126W		0.347368421052632YCR083W		0.267716535433071YGL113W		0.133233532934132YNR001C		0.432150313152401YMR160W		0.215686274509804YIL116W		0.361038961038961YGR100W		0.301052631578947YNR032C-A		0.0821917808219178YOR252W		0.455056179775281YKL042W		0.264462809917355YGR052W		0.287262872628726YBR060C		0.196774193548387YER141W		0.452674897119342YPR172W		0.09YJL124C		0.203488372093023YER042W		0.201086956521739YGL091C		0.23780487804878YJL100W		0.102141680395387YHR047C		0.376168224299065YDR202C		0.227920227920228YJR150C		YAL035W		0.148702594810379YPR013C		0.028391167192429YNL015W		0.24YKL167C		0.197080291970803YMR070W		0.0244897959183673YFR049W		0.154471544715447YML083C		0.114832535885167YDR296W		0.207964601769911YPL110C		0.25102207686018YLR205C		0.55205047318612YMR009W		0.162011173184358YOR243C		0.22189349112426YNL207W		0.261176470588235YER110C		0.627133872416891YBR239C		0.156899810964083YFR027W		0.142348754448399YPL040C		0.275449101796407YGR283C		0.134897360703812YIL084C		0.379204892966361YDL151C		0.103626943005181YER119C		0.535714285714286YFL030W		0.342857142857143YPR166C		0.460869565217391YML108W		0.20952380952381YDR322C-A		0.447916666666667YIL132C		0.309859154929577YML070W		0.357876712328767YDR013W		0.423076923076923YML095C		0.233333333333333YPL008W		0.332171893147503YDL156W		0.0325670498084291YDR173C		0.180281690140845YDR190C		0.274298056155508YLR399C		0.243440233236152YDR109C		0.293706293706294YLR356W		0.32994923857868YJL058C		0.180478821362799YFR015C		0.247175141242938YDR263C		0.267441860465116YNL233W		0.0179372197309417YNL334C		0.211711711711712YOL021C		0.204795204795205YHR106W		0.166666666666667YMR100W		0.154838709677419YLR325C		0.192307692307692YGL089C		0.141666666666667YMR207C		0.256241168158267YGL103W		0.147651006711409YDL131W		0.347727272727273YBR163W		0.182905982905983YML047C		0.346590909090909YGR167W		0.36480686695279YER145C		0.376237623762376YMR136W		0.0160714285714286YCR076C		0.032YDR379W		0.102081268582755YGL223C		0.388489208633094YIL160C		0.28537170263789YBL020W		0.5801393728223YGL129C		0.200819672131148YKL176C		0.128019323671498YBR276C		0.201982651796778YBR017C		0.631808278867102YLR146C		0.216666666666667YOL044W		0.422976501305483YNL005C		0.23989218328841YPL221W		0.262295081967213YHL039W		0.348717948717949YOR008C		0.0634920634920635YDR393W		0.504385964912281YNL172W		0.203661327231121YGR093W		0.136094674556213YEL025C		0.0353535353535354YFR023W		0.157119476268412YOL065C		0.135416666666667YNL255C		YML109W		0.0371549893842887YJL199C		0.0462962962962963YDR208W		0.114249037227214YNL259C		0.26027397260274YIL021W		0.110062893081761YJL088W		0.322485207100592YDL161W		0.231277533039648YCL028W		0.0839506172839506YLR166C		0.543053960964409YHL013C		0.403908794788274YLR175W		0.115942028985507YPL116W		0.208034433285509YHR117W		0.660406885758998YGL196W		0.219626168224299YGL035C		0.0595238095238095YMR244C-A		0.5YLR222C		0.0232558139534884YPR048W		0.232744783306581YPL109C		0.43531202435312YPL138C		0.161473087818697YKL117W		0.0185185185185185YOL125W		0.178571428571429YEL027W		0.6YJL165C		0.0900584795321637YMR175W		0.10126582278481YLR438W		0.320754716981132YGL209W		0.0287958115183246YBL092W		0.130769230769231YBR145W		0.216524216524217YIL031W		0.0957446808510638YLR319C		0.319796954314721YPR135W		0.0733549083063646YBR233W		0.147699757869249YDR045C		YHR110W		0.358490566037736YKL001C		0.386138613861386YLR181C		0.381818181818182YOL086C		0.238505747126437YBR244W		0.228395061728395YNL016W		0.116997792494481YMR163C		0.286524822695035YHR034C		0.0813953488372093YML006C		0.0529715762273902YCR042C		0.225302061122957YDR466W		0.123608017817372YJR082C		0.371681415929204YPL054W		0.0232558139534884YMR282C		0.339655172413793YGR207C		0.210727969348659YOR134W		0.246943765281174YCR017C		0.33788037775446YJL179W		0.770642201834862YIR004W		0.462962962962963YPL150W		0.109877913429523YCR088W		0.0405405405405405YIR029W		0.0087463556851312YMR094W		0.098326359832636YBR267W		0.269720101781171YGR174C		0.517647058823529YML107C		0.29940119760479YNL117W		0.36101083032491YCL043C		0.229885057471264YFR034C		0.125YPL066W		0.0375782881002088YGL021W		0.101315789473684YJL141C		0.107806691449814YPL180W		0.0312891113892365YBR107C		0.0571428571428571YOL043C		0.336842105263158YDL166C		0.370558375634518YOR288C		0.19496855345912YBR249C		0.313513513513514YHR152W		0.173410404624277YFL041W		0.0610932475884244YOR377W		0.219047619047619YBR041W		0.306427503736921YGR085C		0.229885057471264YPR148C		0.441379310344828YMR278W		0.305466237942122YMR131C		0.00978473581213307YNL251C		0.173913043478261YMR310C		0.167192429022082YNL058C		0.0664556962025316YER104W		0.408653846153846YBR004C		0.540415704387991YLR362W		0.165969316596932YMR105C		0.279437609841828YPL262W		0.495901639344262YDR519W		0.133333333333333YDL193W		0.338666666666667YLR267W		0.126315789473684YCR066W		0.170431211498973YPL199C		0.416666666666667YBR175W		YGR216C		0.440065681444992YLR373C		0.246392896781354YJR043C		0.108571428571429YOR358W		0.34297520661157YBR221C		0.286885245901639YEL052W		0.292730844793713YDL148C		0.434567901234568YNL307C		0.245333333333333YDR378C		0.0697674418604651YMR295C		0.0304568527918782YDR472W		0.190812720848057YIL142W		0.472485768500949YPR004C		0.191860465116279YNL199C		0.149812734082397YMR043W		0.115384615384615YMR034C		0.513824884792627YMR031C		0.272835112692764YGR195W		0.260162601626016YMR055C		0.522875816993464YHR216W		0.196940726577438YLR438C-A		0.0674157303370786YPL260W		0.41923774954628YBR014C		0.280788177339901YGR289C		0.519480519480519YOL039W		0.358490566037736YLR447C		0.568115942028986YOL012C		0.208955223880597YGL153W		0.404692082111437YMR217W		0.257142857142857YGR171C		0.346086956521739YDR078C		0.255605381165919YAL010C		0.0446247464503043YKL166C		0.21608040201005YDR182W		0.207739307535642YML074C		0.0754257907542579YNR011C		0.272831050228311YGR127W		0.131410256410256YDR363W-A		0.134831460674157YBR180W		0.54020979020979YLR372W		0.492753623188406YER002W		0.194805194805195YDR032C		0.292929292929293YCR033W		0.111745513866232YJL014W		0.434456928838951YOR126C		0.30672268907563YLR340W		0.333333333333333YLR193C		0.262857142857143YOR108W		0.304635761589404YBL016W		0.271954674220963YAR002C-A		0.356164383561644YPL179W		0.138433515482696YBR129C		0.0548780487804878YML060W		0.303191489361702YER034W		0.183783783783784YCL001W		0.48936170212766YPR047W		0.208955223880597YGL100W		YDR388W		0.379668049792531YLR203C		0.11697247706422YLR215C		0.213888888888889YOR375C		0.33920704845815YFL046W		0.681159420289855YHR066W		0.178807947019868YLR312C		0.168341708542714YHR122W		0.186147186147186YHR088W		0.196610169491525YPL219W		0.207317073170732YML094W		0.582822085889571YBL061C		0.461206896551724YER154W		0.455223880597015YOR236W		0.113744075829384YNL273W		0.338449111470113YGR084C		0.377581120943953YNL023C		0.0849740932642487YNL275W		0.454861111111111YGR016W		0.463157894736842YNL052W		0.38562091503268YHR076W		0.149732620320856YJL206C		0.325857519788918YER143W		0.231308411214953YPL175W		0.358407079646018YIR035C		0.381889763779528YDL063C		0.535483870967742YBR251W		0.218241042345277YGR252W		0.236902050113895YJL166W		0.276595744680851YNL073W		0.251736111111111YCL068C		0.419230769230769YPR128C		0.600609756097561YDR339C		0.301587301587302YGL075C		0.294573643410853YPL095C		0.219298245614035YCL004W		0.236084452975048YGL170C		0.108958837772397YOR278W		0.349090909090909YJR063W		YPR073C		0.329192546583851YGL224C		0.367857142857143YIL056W		0.2234375YML080W		0.309692671394799YLR108C		0.111340206185567YPL028W		0.28643216080402YLR214W		0.400874635568513YPL034W		0.315151515151515YDL070W		0.258620689655172YPL209C		0.217983651226158YPL174C		0.20852534562212YDR508C		0.431372549019608YDR364C		0.0021978021978022YLR257W		0.0996884735202492YDR281C		0.509615384615385YOL062C		0.0916496945010183YJL126W		0.188925081433225YOL100W		0.100832562442183YER019C-A		0.159090909090909YGR200C		YOL122C		0.43304347826087YLR289W		0.151937984496124YGL038C		0.189583333333333YMR124W		0.0986214209968187YNR063W		0.303130148270181YJL052W		0.159638554216867YGL184C		0.260215053763441YLR145W		0.432835820895522YMR040W		0.68125YER136W		0.197339246119734YLR197W		0.432539682539683YOR344C		0.185567010309278YAL048C		0.308157099697885YLR351C		0.209621993127148YDL198C		0.643333333333333YPR033C		0.311355311355311YML130C		0.374777975133215YJL117W		0.247588424437299YDR003W		0.080952380952381YDR421W		0.189473684210526YOR110W		0.137931034482759YDR312W		0.167770419426049YDR050C		0.326612903225806YOR117W		0.320276497695853YML068W		0.211206896551724YLR195C		0.182417582417582YFR014C		0.255605381165919YFR004W		0.271241830065359YPL133C		0.168161434977578YGR054W		0.0529595015576324YOR044W		0.477707006369427YHR143W		YKL071W		0.375YHR035W		0.198412698412698YGR083C		0.23963133640553YOL022C		0.0416666666666667YGL001C		0.343839541547278YGL090W		0.123515439429929YNL229C		0.251412429378531YJR049C		0.0962264150943396YDL234C		0.313672922252011YML118W		0.104950495049505YLR094C		0.0398406374501992YDR368W		0.307692307692308YOR378W		0.67378640776699YJL011C		0.298136645962733YOR221C		0.377777777777778YAR007C		0.0998389694041868YKL069W		0.272222222222222YLR208W		YBR022W		0.231638418079096YOR320C		0.185336048879837YML078W		0.0824175824175824YDR326C		0.0751043115438109YER080W		0.244019138755981YHR179W		0.2575YMR117C		0.356807511737089YNL272C		0.213438735177866YOR116C		0.218493150684931YBL074C		0.27887323943662YKL093W		0.0442477876106195YML088W		0.142215568862275YIL106W		0.296178343949045YDR059C		0.304054054054054YLR227C		0.40973630831643YOL124C		0.200923787528868YNR041C		0.537634408602151YOR247W		0.0238095238095238YIL110W		0.140583554376658YKL110C		0.348242811501597YML102W		YLR272C		0.57312925170068YEL070W		0.368525896414343YGR213C		0.482649842271293YGR071C		0.259302325581395YPL220W		0.317972350230415YER176W		0.133809099018733YPR068C		0.24468085106383YPL159C		0.102766798418972YER167W		0.00352526439482961YDL115C		0.0333333333333333YGL256W		0.425806451612903YDL189W		0.0919037199124726YPL231W		0.29570747217806YPR045C		0.338297872340426YHR201C		0.282115869017632YNL048W		0.374087591240876YJR058C		0.414965986394558YDL045C		0.261437908496732YOL133W		0.0743801652892562YDR460W		0.411214953271028YDR087C		0.514388489208633YPR006C		0.361739130434783YJR143C		0.353018372703412YPL210C		0.496875YHR124W		0.00159489633173844YBL060W		0.225618631732169YHL025W		0.246987951807229YER103W		0.277258566978193YOL135C		0.427927927927928YGR056W		0.147629310344828YGR131W		0.568965517241379YLR260W		0.104803493449782YOR128C		0.274956217162872YJR065C		0.153674832962138YOR086C		0.0792580101180438YGL020C		0.382978723404255YJL209W		0.388379204892966YOR353C		0.199747155499368YNL237W		0.383442265795207YBR087W		0.457627118644068YLR429W		0.0307219662058372YDR539W		0.192842942345924YGL242C		0.392265193370166YKL160W		0.096551724137931YHR001W		0.1441647597254YMR118C		0.48469387755102YPR120C		0.367816091954023YPR143W		0.324YBR009C		0.41747572815534YOR028C		0.16271186440678YJR112W		0.651741293532338YDL123W		0.15YGL236C		0.263079222720478YLR407W		0.218340611353712YLR154C		0.145454545454545YDR277C		0.228637413394919YDL053C		0.151351351351351YNL169C		0.104YKL063C		0.209580838323353YDR446W		0.298013245033113YIL152W		0.195744680851064YGL054C		0.797101449275362YJL069C		0.0420875420875421YLR405W		0.302452316076294YJL184W		0.260162601626016YPR115W		0.176361957525392YMR171C		0.0618181818181818YDR530C		0.172307692307692YLR295C		0.25YKL141W		0.555555555555556YEL026W		0.452380952380952YNL072W		0.260586319218241YDL107W		0.50997150997151YAL025C		0.30718954248366YAL023C		0.345191040843215YNL214W		0.35678391959799YNL223W		0.157894736842105YPL173W		0.138047138047138YML067C		0.144886363636364YJR153W		0.0415512465373961YKL152C		0.348178137651822YOL112W		0.436991869918699YGL119W		0.44311377245509YNL236W		0.143737166324435YGR132C		0.452961672473868YBR047W		0.194285714285714YHR007C		0.413207547169811YFR009W		0.375YKL009W		0.241525423728814YMR044W		0.168421052631579YJR121W		0.25440313111546YAR015W		0.225490196078431YHR072W-A		0.155172413793103YNL132W		0.279356060606061YOR062C		0.108208955223881YNL149C		0.612403100775194YDR158W		0.178082191780822YBR296C		0.456445993031359YOR339C		0.307692307692308YGR033C		0.0669456066945607YML022W		0.251336898395722YPL234C		0.597560975609756YJR014W		0.166666666666667YCR037C		0.491874322860238YIR007W		0.134816753926702YKL079W		0.367378048780488YBR176W		0.320512820512821YPR076W		0.169354838709677YKL150W		0.19205298013245YDR336W		0.213375796178344YDR086C		0.5625YNL159C		0.328719723183391YER150W		YKL172W		0.229508196721311YFL047W		0.481792717086835YPL071C		0.256410256410256YML098W		0.48502994011976YDR002W		0.0845771144278607YIR037W		0.239263803680982YDR105C		0.41014799154334YPR193C		0.320512820512821YMR023C		0.346007604562738YBR071W		0.109004739336493YML043C		0.341222879684418YML001W		0.221153846153846YKL185W		0.00680272108843537YLR220W		0.518633540372671YPL023C		0.290715372907154YMR149W		0.202797202797203YFL022C		0.332007952286282YAL007C		0.386046511627907YIL001W		0.395711500974659YOR167C		YDL027C		0.166666666666667YOR100C		0.532110091743119YGL192W		0.168333333333333YDR288W		0.3003300330033YIR005W		0.135135135135135YNL204C		0.22YIR036C		0.403041825095057YBL107C		0.362244897959184YHR204W		0.237437185929648YBR088C		0.147286821705426YFR006W		0.220560747663551YFL042C		0.124629080118694YNL300W		0.0980392156862745YPL108W		0.363095238095238YDR022C		0.0816326530612245YPR002W		0.354651162790698YPL158C		0.0725593667546174YMR047C		0.0161725067385445YER131W		0.0504201680672269YPL027W		0.187755102040816YNL293W		0.336492890995261YPR155C		0.530844155844156YPL177C		0.107843137254902YNR046W		0.214814814814815YLR314C		0.263461538461538YKL013C		0.409356725146199YNL003C		0.647887323943662YER153C		0.437007874015748YBL030C		0.610062893081761YLR276C		0.260942760942761YNR055C		0.549488054607508YDL155W		0.358313817330211YPL065W		0.43801652892562YOR295W		0.280701754385965YBR111C		0.155844155844156YGR105W		0.376623376623377YGR066C		0.0102739726027397YGR101W		0.459537572254335YOR067C		0.44367417677643YNL012W		0.213946117274168YFR039C		0.482352941176471YER183C		0.18957345971564YBR151W		0.113924050632911YLR105C		0.135278514588859YDL100C		0.378531073446328YJL006C		0.439628482972136YMR041C		0.367164179104478YPR011C		0.552147239263804YDR511W		0.481203007518797YDR071C		0.214659685863874YIL039W		0.162790697674419YFR047C		0.345762711864407YCR011C		0.311725452812202YCR077C		0.194723618090452YJL203W		0.35YHR139C		0.171779141104294YOR328W		0.368925831202046YDR178W		0.325966850828729YBL045C		0.354485776805252YLR149C		YOR034C		0.383177570093458YMR208W		0.288939051918736YPL235W		0.282377919320594YBR119W		0.130872483221477YFL010C		0.0568720379146919YER068W		0.0613287904599659YOL082W		0.151807228915663YDR469W		0.205714285714286YAL009W		0.305019305019305YJR067C		0.539007092198582YOR065W		0.355987055016181YFR017C		0.107692307692308YHR146W		0.0129032258064516YPL122C		0.366471734892788YIL045W		0.00557620817843866YGL121C		0.373015873015873YOR038C		0.0628571428571429YCR047C		0.258181818181818YIL103W		0.24YJR017C		0.276470588235294YPR113W		0.636363636363636YMR099C		0.00336700336700337YGR120C		0.67175572519084YLR190W		0.112016293279022YPL011C		0.26628895184136YPL082C		0.384574183181575YDR063W		0.23489932885906YMR036C		0.0848375451263538YHL030W		0.5406852248394YDR352W		0.362776025236593YOR090C		0.148601398601399YEL021W		0.344569288389513YBL006C		0.194444444444444YNL183C		0.10253164556962YIL014W		0.203174603174603YLR144C		0.188703465982028YEL071W		0.284274193548387YLR324W		0.179732313575526YLR389C		0.331061343719572YNL263C		0.404458598726115YPR030W		0.0214094558429973YIL073C		0.592820512820513YPR154W		YKL148C		0.284375YBL029W		0.151595744680851YGL186C		0.493955094991364YER046W		YBR005W		0.117370892018779YJR005W		0.531428571428571YPR023C		0.271820448877805YOR367W		0.32YMR071C		0.550898203592814YOR321W		0.345285524568393YHR203C		0.0498084291187739YER147C		0.519230769230769YIL090W		0.358452138492872YJR109C		0.278175313059034YKL068W		0.0187695516162669YIL096C		0.148809523809524YDR454C		0.379679144385027YDR362C		0.0223214285714286YJL187C		0.0964590964590965YGR202C		0.193396226415094YDR056C		0.151219512195122YDR408C		0.327102803738318YDR297W		0.455587392550143YKL112W		0.0738714090287278YOL121C		0.368055555555556YLR420W		0.258241758241758YNL151C		0.111553784860558YCL016C		0.260526315789474YHR100C		0.345945945945946YOR102W		0.146551724137931YBR097W		0.198074277854195YNR028W		0.181818181818182YLR298C		0.181818181818182YJR117W		0.622516556291391YOR271C		0.452599388379205YDR289C		0.334963325183374YDL028C		0.12565445026178YDR319C		0.452554744525547YGL211W		0.353760445682451YKL133C		0.412526997840173YNL137C		0.269547325102881YDL176W		0.0536723163841808YDR487C		0.298076923076923YOR084W		0.255813953488372YIL135C		0.0229357798165138YLR209C		0.237942122186495YLR093C		0.442687747035573YEL061C		0.52YLR254C		0.28042328042328YOR166C		0.2882096069869YBR091C		0.440366972477064YKL049C		0.270742358078603YLR277C		0.201540436456996YNL167C		0.123647604327666YNL006W		YFR052W		0.565693430656934YGR203W		0.236486486486486YGL248W		0.16260162601626YGL228W		0.47313691507799YOR226C		0.346153846153846YPR035W		0.224324324324324YMR015C		0.410780669144981YFL014W		0.247706422018349YFL029C		0.209239130434783YDL076C		0.0680272108843537YGR201C		0.431111111111111YHR049W		0.275720164609054YJL051W		0.0936739659367397YCR024C		0.223577235772358YPL104W		0.193009118541033YDR018C		0.396464646464646YBR072W		0.0467289719626168YML071C		0.377265238879736YKL026C		0.233532934131737YMR218C		0.23502722323049YOR079C		0.511182108626198YGR044C		0.113333333333333YPL223C		0.130952380952381YPL250C		0.132352941176471YGL213C		YGL101W		0.562790697674419YKL017C		0.213762811127379YMR152W		0.227397260273973YNL083W		0.489908256880734YBR157C		0.0941176470588235YMR061W		0.537666174298375YPL117C		0.128472222222222YGL189C		0.0504201680672269YOR311C		0.462068965517241YMR293C		0.265086206896552YDR259C		0.232375979112272YCL031C		0.296296296296296YIR026C		0.162087912087912YHR096C		0.528716216216216YDR488C		0.0300187617260788YPR169W		YPL195W		0.434549356223176YEL051W		0.59765625YMR165C		0.0487238979118329YMR106C		0.179650238473768YBR202W		0.288757396449704YKL052C		0.14041095890411YPL259C		0.0905263157894737YKL140W		0.240875912408759YNL155W		0.102189781021898YBR285W		0.0694444444444444YKL124W		0.0949913644214162YNR007C		0.132258064516129YOL101C		0.483974358974359YDL022W		0.365728900255754YJL183W		0.180094786729858YJR125C		0.264705882352941YMR069W		0.192982456140351YML126C		0.246435845213849YLR258W		0.246808510638298YMR288W		0.512873326467559YHR176W		0.19212962962963YOR327C		0.6YNR027W		0.321766561514196YCR060W		0.711711711711712YLR130C		0.372037914691943YDR234W		0.186147186147186YAL062W		0.332603938730853YFR032C		0.141868512110727YBL014C		0.0626398210290828YHL021C		0.146236559139785YPR133C		0.224390243902439YPL232W		0.682758620689655YPR182W		0.0697674418604651YDL163W		0.08YNL245C		0.346368715083799YPL215W		0.4YIR028W		0.444094488188976YCL059C		0.325949367088608YAL037W		0.277153558052434YER003C		0.186480186480186YML046W		0.542130365659777YCL034W		0.367231638418079YFL039C		0.170666666666667YHR188C		0.0950819672131148YER127W		0.322128851540616YJR090C		0.226759339704605YCL011C		0.126463700234192YPL059W		0.313333333333333YGL050W		0.282051282051282YLR164W		0.386904761904762YAL055W		0.155555555555556YDL174C		0.245315161839864YBR261C		0.232758620689655YEL034W		0.0445859872611465YKL125W		0.395534290271132YML112W		0.391891891891892YHR135C		0.171003717472119YGR077C		0.422750424448217YDR285W		0.314285714285714YGR285C		0.438799076212471YPL236C		0.277472527472527YFL025C		0.232264334305151YJR133W		0.22488038277512YKL171W		0.115301724137931YJR073C		0.543689320388349YPL101W		0.107456140350877YBL025W		0.337931034482759YJR107W		0.207317073170732YDR434W		0.303370786516854YNL326C		0.321428571428571YKL005C		0.121212121212121YOR021C		0.122065727699531YIL079C		YDR177W		0.325581395348837YOL018C		0.508816120906801YBR183W		0.487341772151899YGL124C		0.198757763975155YAR002W		0.0463821892393321YIL063C		0.0397553516819572YPL010W		0.380952380952381YER180C		0.153558052434457YPR186C		YPL079W		0.08125YHR182W		0.331210191082803YMR081C		0.0443786982248521YER137C		0.331081081081081YHR167W		0.513409961685824YBL031W		0.0562130177514793YLR098C		0.373456790123457YEL066W		0.273743016759777YOR056C		0.124183006535948YBL091C		0.206650831353919YFR033C		0.319727891156463YDR194C		0.237951807228916YLR300W		0.272321428571429YDR350C		0.37152209492635YDL173W		0.0169491525423729YMR017W		0.360201511335013YOR141C		0.143019296254257YDR473C		0.232409381663113YDL057W		0.176829268292683YIR031C		0.366425992779783YCL025C		0.451816745655608YKL178C		0.321276595744681YDL077C		0.286939942802669YNL271C		0.325652841781874YGL096W		0.152173913043478YNL270C		0.49738219895288YMR268C		0.173423423423423YOR207C		0.180156657963446YDR030C		YCR009C		0.732075471698113YNL157W		0.107142857142857YKL040C		0.23828125YLR211C		0.398230088495575YLR091W		0.31740614334471YML076C		0.269067796610169YNR051C		0.083495145631068YDR538W		0.285123966942149YGR284C		0.406451612903226YLR134W		0.303730017761989YMR311C		0.139737991266376YDR515W		0.0850111856823266YNL284C		0.124223602484472YNL279W		0.531013615733737YBR236C		0.194954128440367YJR011C		0.432950191570881YGR262C		0.32183908045977YIR032C		0.0102564102564103YJL180C		0.313846153846154YEL059C-A		0.121621621621622YEL024W		0.13953488372093YNL080C		0.325136612021858YNL127W		0.310598111227702YMR011W		0.576709796672828YJR086W		0.327272727272727YCL051W		0.0188679245283019YMR177W		0.409803921568627YBR099C		0.149606299212598YBR212W		0.0848214285714286YOR291W		0.25883152173913YGL069C		0.0909090909090909YJL218W		0.158163265306122YPL130W		0.112107623318386YBL072C		0.14YCR052W		0.233954451345756YPR058W		0.543973941368078YKL060C		0.356545961002786YBR185C		0.201438848920863YGL112C		0.39922480620155YGL019W		0.183453237410072YBR156C		0.0515759312320917YOR368W		0.0997506234413965YGR008C		0.0952380952380952YKL084W		YPL006W		0.383760683760684YKL003C		0.213740458015267YNL191W		0.128851540616246YOL067C		0.310734463276836YJL116C		YLR370C		0.280898876404494YDL121C		0.295302013422819YMR187C		0.387470997679814YLR100W		0.322766570605187YIL111W		0.463576158940397YMR098C		0.26797385620915YCR069W		0.154088050314465YDL047W		0.244372990353698YGL106W		0.51006711409396YKL004W		0.384039900249377YLR241W		0.457800511508951YOL031C		0.358669833729216YKL077W		0.0586734693877551YER053C		0.646666666666667YNL076W		0.0770547945205479YKL053C-A		0.476744186046512YOR257W		0.496894409937888YBL023C		0.214285714285714YER051W		0.117886178861789YJL042W		0.159513590844063YPR075C		0.0111111111111111YEL057C		0.184549356223176YNL175C		0.109181141439206YOR215C		0.616216216216216YNL332W		0.344117647058824YIL030C		0.32524639878696YBR199W		0.247844827586207YIL134W		0.598070739549839YPR177C		0.113821138211382YBL010C		0.260714285714286YDL001W		0.325581395348837YBR056W		0.297405189620758YMR305C		0.22107969151671YOR273C		0.484066767830046YBL054W		0.0723809523809524YGL012W		0.384778012684989YOR133W		0.223277909738717YML057W		0.19205298013245YFL049W		0.0947030497592295YCR005C		0.45YML028W		0.255102040816327YGL032C		0.264367816091954YBR070C		0.320675105485232YOL060C		0.262039660056657YIL010W		0.181395348837209YGL154C		0.308823529411765YMR093W		0.113060428849903YGL191W		0.341085271317829YJL208C		0.197568389057751YDR132C		0.117171717171717YER177W		0.554307116104869YNL158W		0.0909090909090909YOR224C		0.0136986301369863YNL026W		0.0516528925619835YPR179C		0.337404580152672YER019W		0.20335429769392YIL091C		0.181692094313454YDR052C		0.09375YGL110C		0.262820512820513YHL011C		0.2625YDR051C		0.20059880239521YOR370C		0.164179104477612YDR397C		0.575342465753425YBR120C		0.407407407407407YFL011W		0.582417582417582YHR210C		0.00879765395894428YBR089C-A		0.535353535353535YHL002W		0.207964601769911YPR185W		0.0555555555555556YML114C		0.329411764705882YGR280C		0.0922509225092251YDR153C		0.265206812652068YDR229W		0.34878587196468YIR001C		0.064YDR321W		0.199475065616798YHR029C		0.163265306122449YOL091W		0.277504105090312YBR148W		0.197044334975369YLR246W		0.278551532033426YJL140W		0.357466063348416YOR157C		0.226053639846743YML019W		0.355421686746988YEL048C		0.203947368421053YBL080C		0.33456561922366YJR001W		0.410299003322259YER030W		0.169934640522876YER027C		YFR001W		0.21078431372549YIL075C		0.44973544973545YGL254W		0.0234113712374582YJL104W		0.503355704697987YGR165W		0.185507246376812YKL087C		0.227678571428571YNR012W		0.203592814371257YDR468C		0.642857142857143YCR046C		0.100591715976331YHL012W		0.188640973630832YEL039C		0.309734513274336YDL144C		0.376404494382022YLR200W		0.728070175438597YGL247W		0.588832487309645YNR006W		0.2491961414791YGR286C		0.312YIL133C		0.396984924623116YNL124W		0.024390243902439YJR136C		0.427553444180523YAL005C		0.291277258566978YNR047W		0.097424412094065YPL131W		0.259259259259259YHR123W		0.432225063938619YJR025C		0.0903954802259887YLR224W		0.10840108401084YHL010C		0.234188034188034YOR174W		0.359154929577465YER122C		0.176470588235294YHR070W		0.186372745490982YDR184C		0.19047619047619YHR147C		0.130841121495327YJR074W		0.0871559633027523YOR048C		0.211729622266402YNL295W		0.162213740458015YHR025W		0.252100840336134YNL254C		0.246882793017456YAL027W		0.118773946360153YBR132C		0.463087248322148YMR213W		0.383050847457627YML027W		0.0623376623376623YDR211W		0.199438202247191YDR125C		0.324503311258278YPL169C		0.193656093489149YOL038W		0.303149606299213YGL073W		0.116446578631453YPL200W		0.461538461538462YPL271W		0.387096774193548YDR354W		0.4YPL055C		0.0542168674698795YDR331W		0.223844282238443YDR240C		0.191056910569106YDL237W		0.238461538461538YDL137W		0.331491712707182YBR254C		0.28YBR040W		0.251677852348993YPR022C		0.231244483671668YDR238C		0.409044193216855YBR286W		0.206703910614525YIR022W		0.191616766467066YEL019C		0.393258426966292YPL114W		0.338129496402878YAL059W		0.254716981132075YBR168W		0.196125907990315YPR119W		0.338085539714868YBR006W		0.334004024144869YPR118W		0.343065693430657YBL011W		0.291172595520422YOR189W		0.0775862068965517YPL238C		0.0775193798449612YOR292C		0.485436893203884YOR341W		0.230168269230769YMR287C		0.186790505675955YDL097C		0.599078341013825YER040W		0.00958904109589041YPL242C		0.512374581939799YGR277C		0.232786885245902YAL040C		0.306896551724138YMR277W		0.10792349726776YNL310C		0.0344827586206897YLR376C		0.148760330578512YJR104C		YLR180W		0.201570680628272YGL044C		0.277027027027027YDL188C		0.185676392572944YOL139C		0.164319248826291YGL031C		0.425806451612903YBR123C		0.13251155624037YDL246C		0.235294117647059YDR332W		0.268505079825835YDR294C		0.393887945670628YFR003C		YFR050C		0.221804511278195YHR072W		0.369357045143639YNL030W		0.407766990291262YDR374C		0.117647058823529YPL004C		0.422287390029326YOR036W		0.65625YDR126W		0.321428571428571YOL097C		0.363425925925926YDL229W		0.295269168026101YML023C		0.318345323741007YNL197C		0.048411497730711YDR252W		0.23489932885906YDR306C		0.148535564853556YGR191W		0.480928689883914YJL174W		0.0579710144927536YDL179W		0.319078947368421YLR350W		0.268518518518519YOR025W		0.165548098434004YBR066C		YGR177C		0.183177570093458YER134C		0.185393258426966YPR107C		0.0288461538461538YBR257W		0.154121863799283YCL052C		0.0264423076923077YDL199C		0.441048034934498YKL121W		0.00704225352112676YNL321W		0.263215859030837YMR014W		0.184971098265896YKL041W		0.691964285714286YJL010C		0.48948948948949YOR331C		0.502702702702703YER163C		0.189655172413793YKL054C		0.0149051490514905YBR230C		0.559701492537313YBR214W		0.15180265654649YPL268W		0.192174913693901YOR020C		0.0188679245283019YDR314C		0.199421965317919
