# Supplementary material for: Proteome-wide evidence for enhanced positive Darwinian selection within intrinsically disordered regions in proteins
Source: Genome Biol. 2011 Jul 19;12(7):R65. doi: 10.1186/gb-2011-12-7-r65 (PMC3218827; doi:10.1186/gb-2011-12-7-r65)
Supplement: Additional file 11 — Fraction of amino acid residues for each protein that are predicted by the PSIPRED method to adopt β-sheet conformation, using a confidence value threshold of 8. [file gb-2011-12-7-r65-S11.RTF]

YHR055C		YPR161C		0.030441400304414YOL138C		0.0686055182699478YGR129W		YPR165W		0.167464114832536YPL015C		0.0700280112044818YCL050C		0.102803738317757YMR193W		0.0697674418604651YGR053C		0.0848056537102474YOR280C		0.0714285714285714YEL004W		YJL200C		0.0937896070975919YDR348C		YPL183C		0.362290227048371YGR003W		0.0201612903225806YBL095W		0.181481481481481YEL009C		YEL015W		0.058076225045372YDR329C		0.00680272108843537YBR252W		0.306122448979592YDL202W		0.0963855421686747YHR209W		0.103092783505155YPR008W		YBR050C		0.00591715976331361YMR197C		YBL049W		0.115942028985507YNL201C		0.0233100233100233YCR061W		0.0063391442155309YPL171C		0.0625YHR051W		YDL116W		YPR124W		YJL023C		0.0259365994236311YGL167C		0.0936842105263158YDL165W		0.0418848167539267YIL143C		0.0688018979833926YOR094W		0.163934426229508YDR167W		0.00485436893203883YER052C		0.113851992409867YPL118W		0.0319767441860465YGR178C		0.0207756232686981YPR086W		0.0115942028985507YJL065C		YBR122C		0.0395480225988701YBR279W		0.0382022471910112YBR271W		0.0787589498806683YMR221C		YER130C		0.00451467268623025YBR155W		0.038961038961039YDR363W		0.0504385964912281YPL147W		0.0333333333333333YLR443W		0.0334821428571429YBR067C		YAL034C		YLR270W		0.137142857142857YCR095C		0.0276243093922652YPR084W		0.037280701754386YMR113W		0.110070257611241YGR035C		YDL222C		0.029126213592233YNL335W		YDR232W		0.0693430656934307YHR116W		YFR018C		0.0578512396694215YKL007W		0.156716417910448YDL213C		0.0622222222222222YNL292W		0.0918114143920596YNL134C		0.154255319148936YKL100C		0.0119250425894378YPL021W		YIL146C		0.0472589792060491YNL218W		0.0289608177172061YIL121W		YDL167C		0.0305980528511822YPL214C		0.0833333333333333YOR228C		YBR270C		0.0055045871559633YGR222W		0.0580204778156997YGR121C		0.0040650406504065YDL003W		0.00706713780918728YOR323C		0.103070175438596YMR183C		YOR274W		0.0327102803738318YER146W		0.32258064516129YPL063W		0.0525210084033613YML075C		0.047438330170778YLR273C		0.0679012345679012YDR457W		0.00795593635250918YCL056C		YJL083W		YPL140C		0.0494071146245059YOR026W		0.36950146627566YNR016C		0.109270040304523YER123W		0.0706106870229008YGR055W		0.0261324041811847YMR315W		0.171919770773639YKL187C		0.00666666666666667YDR383C		YKL055C		0.0719424460431655YPR019W		0.0964630225080386YPR190C		0.0275229357798165YPL228W		0.0601092896174863YGR041W		0.0109689213893967YDR353W		0.22884012539185YPL047W		YPL014W		YGR007W		0.0959752321981424YML116W		YOL137W		YGL155W		YER061C		0.0791855203619909YKL011C		0.0311614730878187YNL257C		0.065093572009764YBR291C		YDR262W		0.0625YDR179C		YIL053W		0.096YGR220C		0.234200743494424YMR161W		YER011W		YGL162W		0.0334448160535117YNL243W		YNL047C		0.00762195121951219YKL038W		0.00598290598290598YFL053W		0.0541455160744501YLR411W		0.024896265560166YLR318W		0.0667420814479638YHR150W		0.0138169257340242YGL219C		0.0479302832244009YBR045C		0.0104712041884817YMR039C		0.0102739726027397YPL188W		0.173913043478261YGL147C		0.319371727748691YDR217C		0.0328495034377387YPR026W		0.106523534269199YMR008C		0.036144578313253YDL227C		0.116040955631399YOR197W		0.0625YNL081C		0.041958041958042YKL096W-A		YDR532C		0.0233766233766234YKL170W		0.239130434782609YGL047W		0.108910891089109YPR171W		YML063W		0.192156862745098YLR178C		0.150684931506849YHL029C		0.00441826215022091YOR184W		0.0683544303797468YDR372C		0.00869565217391304YBR008C		0.00182481751824818YMR203W		0.20671834625323YBL024W		0.100877192982456YDR317W		0.0917874396135266YJL038C		0.0319634703196347YDR043C		YOR340C		0.184049079754601YCL044C		0.00479616306954436YFR022W		0.132332878581173YEL072W		0.0822510822510823YOL008W		0.202898550724638YHR017W		0.0649350649350649YMR095C		0.174107142857143YHR198C		0.0965732087227414YOR076C		0.0963855421686747YNL219C		0.018018018018018YLR210W		YIL034C		0.101045296167247YLR148W		0.103485838779956YOR342C		0.0313479623824451YIL043C		0.214788732394366YDL160C		0.0909090909090909YGR231C		0.125806451612903YNL021W		0.0509915014164306YMR266W		0.0178384050367261YPL107W		YGR088W		0.0747330960854092YCR063W		0.0127388535031847YDL048C		YML092C		0.176YPR097W		0.0139794967381174YNL056W		0.0609137055837563YAL020C		0.261261261261261YFL017C		0.213836477987421YHR092C		YDR379C-A		YNR074C		0.169312169312169YLR189C		0.0876460767946578YDR113C		YML032C		0.0212314225053079YFL028C		0.131487889273356YDL247W		YBR256C		0.264705882352941YPL024W		0.153526970954357YMR257C		YDL219W		0.233333333333333YKL122C		0.0538922155688623YGL240W		0.188YDL080C		0.0985221674876847YEL001C		0.222222222222222YPR168W		YHR109W		0.052991452991453YPL061W		0.09YJL060W		0.0788288288288288YLR240W		0.0605714285714286YGR104C		0.0879478827361563YER174C		0.122950819672131YMR146C		0.432276657060519YPR057W		0.0117302052785924YDR156W		0.0291970802919708YJR113C		0.0283400809716599YPR131C		0.143589743589744YMR155W		YNL180C		0.0845921450151057YDR272W		0.142335766423358YOR238W		0.033003300330033YLR139C		0.0777604976671851YKL142W		YPR009W		0.0335820895522388YHR032W		YDR429C		0.171532846715328YNR031C		0.0291323622545915YGL079W		YOR004W		0.0590551181102362YMR244W		0.0957746478873239YMR233W		YNL188W		0.0069284064665127YNL104C		0.124394184168013YOR365C		0.103840682788051YNR030W		0.0217785843920145YBR265W		0.084375YGL131C		0.0220955096222381YNR040W		0.07421875YOR173W		0.130311614730878YOR337W		0.00131752305665349YNL181W		0.0638820638820639YGR243W		YDR464W		YGR210C		0.0656934306569343YOR168W		0.0889987639060569YNL002C		0.0341614906832298YNL294C		0.0187617260787993YHR193C		0.0862068965517241YPR147C		0.0690789473684211YBR092C		0.0663811563169165YOR242C		0.0700808625336927YJL176C		YHR128W		0.180555555555556YOR297C		0.015625YDR225W		0.0151515151515152YPR020W		YPR114W		YPL189W		0.00328407224958949YJL056C		0.00227272727272727YPL247C		0.217973231357553YDR482C		YJL122W		YBL058W		0.0520094562647754YKL107W		0.0776699029126214YCL017C		0.0784708249496982YER085C		0.0289017341040462YMR186W		0.0780141843971631YCR065W		0.00354609929078014YFL045C		0.102362204724409YNL020C		0.0517241379310345YGL125W		0.0583333333333333YLR354C		0.0537313432835821YFR008W		YDR284C		0.0173010380622837YKL051W		0.028328611898017YPR174C		YMR091C		0.0413793103448276YJL143W		YGR057C		0.0826446280991736YLR308W		0.0512820512820513YBR065C		0.0412087912087912YMR029C		0.0936902485659656YPR121W		0.048951048951049YNL068C		0.0290023201856148YGL178W		0.00232828870779977YGL157W		0.103746397694524YMR148W		YML058W		YLR249W		0.0478927203065134YGR019W		0.0573248407643312YBR263W		0.0489795918367347YPR044C		0.0598290598290598YDR286C		0.12280701754386YMR076C		YJR135W-A		YDR083W		0.0586734693877551YLR109W		0.159090909090909YOR032C		YOR091W		0.00289855072463768YBR205W		0.0495049504950495YLR292C		YLR216C		0.0673854447439353YDR046C		0.0033112582781457YML018C		0.0178117048346056YOR369C		0.0839160839160839YOR138C		0.0312965722801788YJR145C		0.252873563218391YGR246C		0.00167785234899329YJR138W		0.0763888888888889YHR059W		YLR138W		0.00406091370558376YNL156C		YNL106C		0.0921386306001691YNL286W		0.140350877192982YNL093W		0.172727272727273YIL040W		YLR186W		0.111111111111111YDR425W		0.0192YDR293C		0.0888YNR015W		0.0520833333333333YLR456W		0.142156862745098YDR448W		0.00460829493087558YER184C		0.00377833753148615YJL066C		0.0595238095238095YOR194C		0.041958041958042YLR368W		0.0351170568561873YKL095W		0.0647482014388489YGR060W		YBR115C		0.0833333333333333YBR147W		0.0135135135135135YHR086W		0.0879541108986616YFL003C		0.0865603644646925YOR125C		YML079W		0.18407960199005YPL134C		YNL298W		0.0391923990498812YGL225W		YNL224C		0.015645371577575YNL122C		YLR129W		0.352067868504772YGL066W		0.0030441400304414YNL217W		0.107361963190184YGL063W		0.110810810810811YML030W		YAL039C		0.0371747211895911YOR261C		0.0857988165680473YLR191W		0.0362694300518135YCR027C		0.177033492822967YDL046W		0.294797687861272YMR263W		YOL113W		0.0534351145038168YDR517W		0.112903225806452YAL001C		0.0336206896551724YBR197C		YJR132W		YDL106C		0.0214669051878354YNL280C		0.0091324200913242YDR505C		0.0285374554102259YDL154W		0.0677025527192009YMR211W		0.0442105263157895YGL202W		0.076YBL036C		0.0856031128404669YPR178W		0.281720430107527YOL147C		YFR007W		0.0509915014164306YGR072W		0.0129198966408269YER022W		0.0349344978165939YIL118W		0.151515151515152YJR008W		0.0710059171597633YCL010C		0.127413127413127YJR024C		0.0737704918032787YFR011C		YAL047C		YDR256C		0.0815533980582524YNL287W		0.0470588235294118YGR103W		0.028099173553719YOR281C		0.0769230769230769YBR162C		0.10989010989011YMR114C		0.0842391304347826YGR251W		YBR094W		0.0770252324037185YKL021C		0.344017094017094YOR374W		0.0867052023121387YHL026C		0.0126984126984127YHR014W		YDL015C		0.0548387096774194YCR028C		YER058W		YCR016W		YER149C		YJL147C		0.00523560209424084YGL222C		YDR533C		0.0970464135021097YDL090C		YBR177C		0.0731707317073171YJL034W		0.107038123167155YNR059W		0.0448275862068966YGR156W		0.0117647058823529YBR125C		0.124681933842239YBR173C		YHR057C		0.151219512195122YOR077W		0.0301724137931034YLR234W		0.0929878048780488YHR107C		0.0835380835380835YER112W		0.155080213903743YLR174W		0.0728155339805825YCL026C-A		0.0518134715025907YDR346C		0.155925155925156YLR385C		YLR117C		0.00145560407569141YDR143C		0.00163934426229508YER185W		YOL013C		0.0199637023593466YLR089C		0.0574324324324324YKL155C		0.036624203821656YGR154C		0.0337078651685393YNL277W		0.0596707818930041YOL061W		0.092741935483871YFR013W		0.00254129606099111YDL014W		0.122324159021407YCL064C		0.0916666666666667YDR493W		YLR345W		0.0864440078585462YLR192C		YOR265W		YDR031W		YBR141C		0.0534124629080119YDR453C		0.137755102040816YAL008W		0.0252525252525253YDR490C		0.0417754569190601YDR191W		0.0648648648648649YNL193W		YLR360W		0.0478359908883827YOR142W		0.100303951367781YDR304C		0.133333333333333YPL020C		0.0193236714975845YLR165C		0.15748031496063YHL023C		0.0253054101221641YOR283W		0.11304347826087YMR255W		YPL154C		0.22716049382716YDR033W		0.015625YDR253C		YNL014W		0.053639846743295YAL032C		0.0131926121372032YNL291C		0.0894160583941606YOR348C		0.00478468899521531YMR104C		0.0635155096011817YOR097C		0.0685714285714286YOL126C		0.0875331564986737YOL107W		0.0146198830409357YHR026W		0.0187793427230047YGR028W		0.0303867403314917YGL061C		YJL089W		0.00723763570566948YKL116C		0.0540540540540541YHR134W		0.0408921933085502YBR228W		0.0822368421052632YNL320W		0.133802816901408YGR123C		0.0584795321637427YMR220W		0.1019955654102YBR269C		YMR188C		0.139240506329114YLR290C		0.075812274368231YNL258C		0.00928381962864721YNL075W		0.186206896551724YPR159W		0.075YHR089C		0.0780487804878049YNR018W		YAL044C		0.188235294117647YKL139W		0.0378787878787879YHR129C		0.0859375YOR253W		0.170454545454545YIL105C		0.00583090379008746YDR403W		0.0522388059701493YDL029W		0.0767263427109974YDR525W-A		0.0379746835443038YPL255W		YEL012W		0.0825688073394495YOR192C		YGL229C		YOL005C		0.125YML052W		0.0298013245033113YML064C		0.146938775510204YHL019C		0.214876033057851YBR167C		0.142857142857143YPR176C		YFL016C		0.0978473581213307YLR383W		0.0538599640933573YJL062W		0.0325301204819277YDR140W		0.158371040723982YNL252C		0.117437722419929YMR196W		0.0229779411764706YOR205C		0.079136690647482YER114C		0.0163461538461538YGL258W		0.0533980582524272YIL002C		0.143763213530655YDR518W		0.106382978723404YLR328W		0.0548628428927681YKL173W		0.100198412698413YBL003C		0.0151515151515152YNR013C		YJR077C		YPR078C		0.00806451612903226YGL058W		0.116279069767442YFL005W		0.162790697674419YMR284W		0.109634551495017YML061C		0.080325960419092YLR449W		0.137755102040816YDL007W		0.0709382151029748YOR145C		0.072992700729927YOR287C		YIL122W		YPL218W		0.163157894736842YJL145W		0.0442176870748299YPR198W		YNR052C		0.0392609699769053YOR244W		0.114606741573034YMR314W		0.179487179487179YLR173W		0.129934210526316YHR190W		YPL097W		0.0569105691056911YNL115C		0.0512422360248447YLR103C		0.0584615384615385YKL132C		0.0976744186046512YPL222W		0.0174418604651163YKL012W		0.0154373927958834YLR395C		YAL041W		0.0327868852459016YBL052C		0.036101083032491YCL045C		0.269736842105263YDL135C		0.237623762376238YMR237W		0.0331491712707182YGR046W		0.051948051948052YGR224W		YDR308C		YJL153C		0.112570356472795YBR035C		0.175438596491228YCR002C		0.114906832298137YDR244W		YAL016W		YPR093C		0.00347222222222222YMR239C		0.0382165605095541YLR163C		0.108225108225108YDL072C		YPL190C		0.0211970074812968YER059W		0.00238095238095238YBR207W		0.0172043010752688YIL049W		0.0118577075098814YBR069C		0.00323101777059774YGL215W		YPR066W		0.0903010033444816YOR135C		0.0176991150442478YNL202W		0.0958904109589041YLR099C		0.0812182741116751YOR080W		0.00804289544235925YNL133C		YJL167W		YIL097W		0.00193798449612403YOR198C		YNL101W		YGL241W		YML055W		0.140449438202247YHR143W-A		0.0714285714285714YFL052W		YML035C		0.0160493827160494YGR133W		0.0983606557377049YDR357C		YDR006C		YNL256W		0.101941747572816YML100W		0.0728597449908925YHR197W		0.00393184796854522YNL325C		0.0955631399317406YBR128C		0.00290697674418605YPL258C		0.0562613430127042YNL216W		0.00483675937122128YIL036W		0.00511073253833049YLR307W		0.0631229235880399YML119W		0.00560224089635854YPL273W		0.0769230769230769YCL066W		0.00571428571428571YPL201C		0.13882863340564YHR003C		0.0652680652680653YPR041W		0.037037037037037YHR171W		0.106349206349206YER116C		0.0072992700729927YOR136W		0.0785907859078591YOL086W-A		YLR364W		0.128440366972477YGR142W		0.0390243902439024YKL186C		0.0706521739130435YGR264C		0.033288948069241YDR206W		0.00339366515837104YPR042C		0.00186046511627907YJR152W		YBR085W		YKL106W		0.0620842572062084YBR281C		0.200455580865604YDL153C		YBL078C		0.153846153846154YDL150W		0.0545023696682464YDR398W		0.192846034214619YIL077C		YGR038W		YMR153W		0.0189473684210526YPL266W		0.0660377358490566YPL264C		YHR132C		0.0883720930232558YLR336C		YER115C		YDR441C		0.198895027624309YDR101C		0.0623946037099494YMR121C		0.0637254901960784YHL014C		0.0938271604938272YMR312W		0.102564102564103YOR382W		0.0326797385620915YOR214C		0.0338983050847458YLR141W		YJL030W		0.142857142857143YDR410C		YOR335C		0.092901878914405YNL087W		0.210526315789474YHR015W		0.101669195751138YGL037C		0.087962962962963YDR502C		0.125YAR018C		0.0758620689655172YOR112W		0.0354796320630749YKL045W		0.0170454545454545YBR258C		YPR074C		0.0705882352941176YHR162W		YLR262C-A		YML013W		0.0445205479452055YPL003W		0.0497835497835498YLR297W		YMR319C		0.0271739130434783YDL127W		0.00324675324675325YOR152C		0.03515625YDL216C		0.0545454545454545YFR028C		0.0399274047186933YBR068C		0.00164203612479475YHR126C		0.0314465408805031YHR008C		0.0643776824034335YOL001W		YML111W		0.0847826086956522YMR016C		0.0127388535031847YKL023W		YBR133C		0.094316807738815YGR229C		0.0257425742574257YOR092W		0.0179445350734095YNR010W		YPL001W		0.114973262032086YDR047W		0.0469613259668508YBR159W		0.0749279538904899YGR158C		0.148YOR210W		YKL174C		0.0307443365695793YLR403W		YLR286C		0.0444839857651246YBR274W		0.100569259962049YDL044C		0.00681818181818182YNL305C		YER081W		0.108742004264392YDR148C		0.131749460043197YJL059W		YIL130W		YEL044W		YGL108C		YOR351C		0.110663983903421YGL023C		YBL066C		0.00174216027874564YBL051C		0.0209580838323353YJR068W		0.0311614730878187YDL168W		0.147668393782383YBR020W		0.0890151515151515YDL049C		0.123134328358209YAL019W		0.0335985853227233YDR484W		YEL037C		0.0402010050251256YBR273C		0.0825688073394495YDL114W		0.0909090909090909YIL062C		0.0194805194805195YML091C		0.00249584026622296YCL021W-A		0.04YOR372C		0.0036101083032491YGL098W		YNL329C		0.0961165048543689YMR199W		YDL209C		0.0383480825958702YMR300C		0.113725490196078YCR039C		YIL107C		0.0544135429262394YIL057C		0.0853658536585366YGL136C		0.06875YHR024C		0.0975103734439834YLR120C		0.18804920913884YGR211W		0.1440329218107YLR237W		YBL007C		0.0329581993569132YNR064C		0.117241379310345YPL075W		0.00382165605095541YPL017C		0.170340681362725YBR301W		YML004C		0.223926380368098YMR202W		0.0900900900900901YGR292W		0.0856164383561644YNL267W		0.0215759849906191YEL029C		0.102564102564103YLR250W		0.00427350427350427YGR245C		YNL040W		0.157894736842105YIL003W		0.0921501706484642YGL161C		YPR144C		YOR359W		0.00191204588910134YFL055W		0.00537634408602151YGR181W		YGR070W		0.1004329004329YPL029W		0.0474898236092266YLR116W		0.0252100840336134YDR197W		0.0591259640102828YPL224C		0.0334075723830735YPL172C		YJR144W		0.0260223048327138YMR083W		0.170666666666667YGR086C		YCR048W		YDL065C		YMR126C		0.00292397660818713YDR175C		0.0407523510971787YER032W		0.00570776255707763YGR194C		0.065YGR078C		0.085427135678392YMR265C		0.0542299349240781YHR163W		0.104417670682731YOR222W		YBL103C		YIL052C		0.0661157024793388YBR242W		YBL021C		YNL246W		0.0681818181818182YOL030W		0.0723140495867769YER012W		0.237373737373737YLR301W		0.151639344262295YPR139C		0.09YPL202C		0.0384615384615385YOL068C		0.0576540755467197YLR114C		0.0366492146596859YJL019W		0.0454545454545455YLR248W		0.0491803278688525YIL020C		0.153256704980843YNL063W		0.105095541401274YOR391C		0.0970464135021097YFR044C		0.112266112266112YFL023W		YDR212W		0.0805008944543828YOR103C		YMR185W		0.00305810397553517YJL091C		0.0122448979591837YDR065W		0.00547945205479452YHR174W		0.0869565217391304YLR441C		0.2YBR204C		0.0826666666666667YLR283W		YGL174W		YGR145W		0.173974540311174YGR199W		0.0579710144927536YDR061W		0.11873840445269YBL005W		YIL108W		0.16235632183908YDR404C		0.421052631578947YOR058C		0.00225988700564972YBL104C		0.0491329479768786YBR095C		YBR084W		0.0697435897435897YMR173W		YHR085W		YOR301W		0.0137931034482759YDL210W		0.0175131348511384YNL308C		YOR171C		0.107371794871795YIL050W		0.00350877192982456YBL099W		0.102752293577982YLR359W		0.004149377593361YPL217C		0.0786136939983094YNR009W		YGL194C		0.0530973451327434YOR106W		YJR056C		YLR287C-A		YDR044W		0.0914634146341463YLR118C		0.110132158590308YDR387C		YOL130W		0.030267753201397YIL071C		0.0045045045045045YDR169C		0.00584795321637427YER178W		0.0547619047619048YMR210W		0.089086859688196YOR338W		YER088C		YDR115W		YOR334W		0.0446808510638298YGR256W		0.0345528455284553YMR107W		YDR451C		YCL063W		0.00709219858156028YJL192C		0.0769230769230769YHR177W		0.0242825607064018YBR039W		0.0482315112540193YIR027C		0.156521739130435YAL042W		0.190361445783133YPR036W		YIL007C		0.127272727272727YOR030W		0.0193861066235864YPL267W		YMR243C		0.0294117647058824YDR188W		0.0824175824175824YNR034W-A		0.204081632653061YEL023C		0.0249266862170088YNL045W		0.104321907600596YPR141C		0.0685871056241427YIL104C		0.0453648915187377YNL282W		0.0564102564102564YLR424W		0.0240112994350282YDR520C		0.00518134715025907YBR243C		0.0223214285714286YGL137W		0.289088863892013YNL212W		0.171355498721228YMR234W		0.0718390804597701YMR112C		YHR005C-A		YGL212W		0.0443037974683544YNR023W		0.049469964664311YPL253C		0.0494590417310665YBR096W		0.173913043478261YJR042W		YGR150C		YLR390W-A		YGR074W		0.13013698630137YHR043C		0.105691056910569YGL180W		0.0356744704570792YDR085C		0.0112903225806452YBR146W		0.0611510791366906YPL043W		0.0948905109489051YHR011W		0.0426008968609865YDL235C		YLR278C		YLR348C		YGL158W		0.056640625YAL028W		YDL002C		YDR099W		YNL173C		0.0683060109289618YMR216C		0.0390835579514825YMR306W		0.02296918767507YLR196W		0.223958333333333YBR085C-A		0.0470588235294118YDL059C		0.0546218487394958YOR329C		YJR116W		YHL004W		0.0355329949238579YBR073W		0.0649350649350649YDR449C		YDL088C		0.0170454545454545YNL161W		0.0330687830687831YDR435C		0.0731707317073171YDL052C		0.099009900990099YMR144W		YDR161W		YER062C		0.096YCR068W		0.0480769230769231YPR051W		0.238636363636364YOR285W		0.0719424460431655YLR253W		0.031634446397188YJL001W		0.209302325581395YHR067W		0.182142857142857YGL237C		YDR120C		0.0754385964912281YJL012C		0.0402219140083218YER126C		0.0383141762452107YMR246W		0.11671469740634YPL031C		0.0852459016393443YLR201C		YHR091C		0.057542768273717YGR020C		0.110169491525424YML066C		0.024390243902439YDR501W		0.036468330134357YNL088W		0.0728291316526611YNL053W		0.0224948875255624YDR400W		0.0970588235294118YDR137W		0.143288084464555YBR231C		0.0363036303630363YDL120W		0.0862068965517241YLR151C		0.0676470588235294YFR021W		0.29YML121W		0.164516129032258YLR310C		0.0151038388923851YGR244C		0.112412177985948YGL255W		0.00265957446808511YHR046C		0.142372881355932YOR016C		0.21256038647343YML065W		0.0568927789934354YPR005C		0.0408163265306122YGR223C		0.287946428571429YAR003W		0.342723004694836YMR267W		0.116129032258065YNL077W		0.115530303030303YJR034W		YPL160W		0.0623853211009174YGL185C		0.100263852242744YKL175W		YDR456W		YGL190C		0.218631178707224YOR101W		0.110032362459547YMR262W		0.0830670926517572YGL005C		YNL125C		YBL019W		0.0673076923076923YBR043C		YDL231C		0.00622222222222222YML101C		YPR007C		0.00294117647058824YMR108W		0.0815138282387191YIL067C		0.0368731563421829YPL126W		0.277901785714286YKL179C		YJL033W		0.0532467532467532YEL041W		0.153535353535354YGL208W		0.103614457831325YNL113W		0.105633802816901YGL025C		YBR171W		YDL005C		YNL031C		YDL056W		0.0132052821128451YML120C		0.128654970760234YGR048W		0.149584487534626YOL149W		0.0649350649350649YML129C		0.0714285714285714YBR220C		0.025YBR227C		0.0307692307692308YNR056C		0.0142602495543672YER165W		0.103986135181976YNL327W		0.00288184438040346YOR130C		YPL099C		YER016W		YNL242W		0.137562814070352YMR058W		0.132075471698113YOL078W		0.0280612244897959YAL018C		0.0215384615384615YEL002C		0.176744186046512YJL156C		0.0334788937409025YMR166C		YCL032W		0.0115606936416185YPL050C		0.0683544303797468YDR510W		0.227722772277228YLR327C		YLR142W		0.0399159663865546YLR104W		0.0381679389312977YNL222W		0.0533980582524272YLR291C		0.057742782152231YBR293W		YLR412W		0.062043795620438YNR067C		0.0635631154879141YNL074C		0.00221238938053097YBR021W		0.00789889415481832YML021C		0.0389972144846797YER038C		YNL044W		0.0113636363636364YER060W-A		0.00377358490566038YER173W		0.0151745068285281YOR190W		0.0651685393258427YML048W		0.0198511166253102YBR130C		YPR072W		0.00714285714285714YHR048W		0.00389105058365759YHL034C		0.105442176870748YHL006C		0.0733333333333333YDR418W		0.096969696969697YLR183C		0.0265848670756646YIR024C		0.00462962962962963YCR038C		0.00311526479750779YJR040W		0.0192554557124519YJL037W		YOR059C		0.0711111111111111YNR002C		0.0141843971631206YMR172C-A		0.102362204724409YIL158W		0.00980392156862745YER048W-A		YGR278W		YDR462W		YMR273C		YJL162C		0.0102915951972556YPR080W		0.231441048034935YFL017W-A		0.363636363636364YGL127C		YGR268C		0.0202020202020202YHL033C		0.0546875YMR285C		0.0679611650485437YBR104W		YPL263C		0.153609831029186YER096W		YOR325W		0.0700636942675159YDR311W		0.0155763239875389YKL088W		0.0332749562171629YJL046W		0.149144254278729YLR228C		YKL056C		0.209580838323353YPL037C		0.0636942675159236YDR459C		0.00802139037433155YOR357C		0.0987654320987654YNR004W		YNL192W		0.0318302387267905YGL068W		0.0360824742268041YDL157C		YOR007C		YBR105C		0.0939226519337017YDL013W		0.012924071082391YGL200C		0.226600985221675YDR260C		0.0235294117647059YNR072W		YBR154C		0.106976744186047YPL156C		0.0563380281690141YKL048C		0.0484375YJL020C		0.0285220397579948YLR251W		YGR113W		YBR253W		YCR020C		YJR141W		0.207492795389049YHR060W		YDR361C		0.0954063604240283YPL009C		0.0712909441233141YNL027W		YNL065W		YNL211C		YHL038C		0.00952380952380952YPL070W		YOL111C		0.0660377358490566YDR075W		0.0876623376623377YER009W		0.312YFL034C-B		0.0139372822299652YIR011C		YMR297W		0.0695488721804511YIL139C		0.102040816326531YDR254W		0.0502183406113537YPL005W		YGL105W		0.0904255319148936YDL224C		0.0292758089368259YKL062W		YJL158C		0.079295154185022YDR162C		0.0677966101694915YCR059C		0.116279069767442YDL183C		0.046875YCL039W		0.209395973154362YER140W		0.00359712230215827YPL069C		YOR386W		0.0353982300884956YPL151C		0.34589800443459YNL042W		YHR207C		0.0608365019011407YDR171W		0.0986666666666667YPL157W		0.073015873015873YDR479C		0.0270758122743682YAL034W-A		YOR258W		0.0506912442396313YNR057C		0.0759493670886076YIL015W		0.187393526405451YHR127W		0.102880658436214YNL328C		YCL049C		0.0288461538461538YHR111W		0.0840909090909091YGL055W		0.0156862745098039YBR288C		0.229813664596273YOR347C		0.16403162055336YGL065C		0.0934393638170974YHR013C		0.159663865546218YOR307C		0.022075055187638YNL144C		0.0202702702702703YIR038C		0.0341880341880342YAL046C		0.101694915254237YPL193W		0.0026246719160105YGR208W		0.100323624595469YDR201W		YML077W		0.132075471698113YGL245W		0.100282485875706YHR058C		0.0474576271186441YPR181C		0.110677083333333YBR106W		0.0212765957446809YHR018C		0.00215982721382289YBR170C		0.0844827586206897YDL211C		0.0241935483870968YPL078C		0.00819672131147541YOL007C		0.0117302052785924YDR399W		0.113122171945701YKL181W		0.128805620608899YGR192C		0.150602409638554YDL078C		0.119533527696793YOL041C		0.065359477124183YMR226C		0.108614232209738YHR200W		0.0858208955223881YPR125W		YEL046C		0.0775193798449612YIR015W		0.0625YMR242C		0.174157303370787YDR204W		YPL161C		YDR300C		0.100467289719626YLR229C		0.193717277486911YLR421C		0.211538461538462YPL096W		0.046831955922865YML038C		YJL082W		YIL006W		YLR392C		0.0945945945945946YGR075C		0.0165289256198347YBR268W		YHR068W		0.0568475452196382YGL160W		0.107017543859649YGR042W		0.0664206642066421YPR160W		0.0343680709534368YER007W		0.108108108108108YGR095C		0.2152466367713YDL089W		0.0268595041322314YDR522C		0.109561752988048YEL062W		0.0422764227642276YBR026C		0.147368421052632YLR143W		0.172262773722628YMR125W		0.00348432055749129YLR126C		0.155378486055777YGL086W		0.0293724966622163YNL129W		0.0791666666666667YGL048C		0.0493827160493827YER106W		YGR169C		0.128712871287129YMR074C		0.0137931034482759YMR269W		0.004739336492891YHR138C		0.0614035087719298YFL021W		0.0117647058823529YPL144W		0.128378378378378YEL053C		0.00272851296043656YHL020C		YGR106C		0.0452830188679245YLR452C		0.0128939828080229YMR005W		YLR363C		0.0458715596330275YIL023C		YKL138C		YGL002W		0.231481481481481YOR312C		0.17816091954023YLR179C		0.164179104477612YNL230C		YHL028W		YCR015C		0.0473186119873817YPR199C		YMR313C		0.0218068535825545YBR077C		0.0555555555555556YOR298W		0.0438413361169102YDR380W		0.0866141732283465YDL177C		0.0882352941176471YGR149W		0.0138888888888889YPL203W		0.068421052631579YNR021W		0.0643564356435644YGR080W		0.129518072289157YBR195C		0.272511848341232YPL191C		0.0555555555555556YMR013C		0.0346820809248555YPL148C		0.138728323699422YFL038C		0.169902912621359YPL240C		0.07475317348378YGL145W		0.00427960057061341YPR060C		YNL004W		0.128205128205128YLR232W		YHR069C		0.167130919220056YEL018W		YLR323C		YGL172W		YDL143W		0.0928030303030303YKL162C		0.0870646766169154YMR290C		0.0792079207920792YDL021W		0.0482315112540193YPL211W		0.132596685082873YDR452W		0.0697329376854599YAL038W		0.164YMR024W		0.0282051282051282YLR147C		0.316831683168317YDL018C		0.195555555555556YML012W		0.208530805687204YIL061C		0.0433333333333333YGR155W		0.0966469428007889YLR136C		YDL060W		0.119289340101523YIL085C		0.0367504835589942YHR075C		0.06YOL119C		YNL289W		0.00716845878136201YJR105W		0.152941176470588YER161C		YNR036C		0.130718954248366YMR111C		0.00432900432900433YJL096W		0.273291925465839YBR136W		0.0105574324324324YMR090W		0.13215859030837YNR061C		YNL154C		0.0677655677655678YGR209C		0.240384615384615YDL181W		YKL029C		0.0418535127055306YPR106W		0.0699774266365688YNL039W		0.00336700336700337YDL149W		0.00200601805416249YGR187C		YBR282W		0.0205479452054795YER170W		0.0755555555555556YPL269W		YHR137W		0.0721247563352826YGR058W		0.00895522388059702YGL009C		0.0808729139922978YBL009W		0.0355029585798817YDR279W		0.0685714285714286YLR243W		0.0845588235294118YER029C		0.13265306122449YJL025W		0.00194552529182879YDR246W		0.127853881278539YDR427W		0.0229007633587786YER182W		0.204918032786885YNL163C		0.0927927927927928YMR228W		0.0645161290322581YDR411C		0.0234604105571848YNL061W		0.0566343042071197YDR309C		0.00522193211488251YER095W		0.0975YJL138C		0.124050632911392YDR499W		YJL063C		0.0168067226890756YMR206W		YMR154C		0.173314993122421YLR410W		0.0462478184991274YER092W		YBR101C		YIL042C		0.0558375634517767YHR112C		0.100529100529101YGL029W		YGR160W		YJR060W		YDR437W		YHL022C		0.0552763819095477YGL175C		0.00289855072463768YLR305C		0.01YIL124W		0.0909090909090909YGR234W		0.12280701754386YCR004C		0.0809716599190283YKL028W		0.0435684647302905YPR173C		0.022883295194508YPR137W		0.300174520069808YAL011W		0.032YNL067W		0.31413612565445YEL016C		0.0649087221095335YEL020C		0.103571428571429YPL060W		0.0484261501210654YER087W		0.0920138888888889YOL087C		0.130824372759857YNL304W		0.0695443645083933YHR056C		YNL092W		0.06YPL152W		0.00279329608938547YKL033W		YHR214W		0.113300492610837YGL243W		0.0575YBR025C		0.104060913705584YNL208W		YER055C		0.202020202020202YJR123W		0.0444444444444444YHR065C		0.0858283433133733YNL128W		0.1036866359447YDR405W		0.0532319391634981YMR139W		0.0648648648648649YDL217C		YPL094C		0.0291970802919708YJL157C		0.0614457831325301YER120W		0.147540983606557YLR315W		YDR516C		0.084YPL106C		0.0995670995670996YFR042W		YLR256W		0.00066577896138482YPR191W		0.119565217391304YMR241W		YBR260C		YLR396C		0.0463096960926194YNR037C		0.120879120879121YPL243W		0.00667779632721202YBR018C		0.10655737704918YCR043C		0.015748031496063YPL270W		0.0413971539456662YBR054W		0.00581395348837209YNL164C		0.0284900284900285YNL062C		0.096234309623431YMR298W		0.08YML106W		0.123893805309735YMR037C		YKL094W		0.115015974440895YOR066W		0.0127186009538951YJR147W		0.0223463687150838YDR041W		0.12807881773399YMR272C		0.0260416666666667YKL143W		0.0107991360691145YDL081C		YIL019W		0.00867052023121387YDL201W		0.0909090909090909YNL200C		0.0934959349593496YER073W		0.0942307692307692YJR007W		0.177631578947368YNL238W		0.0761670761670762YBR053C		0.184357541899441YCR073W-A		0.0888888888888889YOR269W		0.281376518218623YPL163C		0.103846153846154YML125C		0.173076923076923YOL057W		0.0436005625879044YKL145W		0.0513918629550321YIL150C		0.0490367775831874YGR146C		0.004739336492891YDL126C		0.0946107784431138YDL006W		0.195729537366548YMR025W		0.0101694915254237YDL233W		0.0545851528384279YCL057C-A		YOR362C		0.15625YMR276W		0.0375335120643432YOR217W		0.024390243902439YML124C		0.0359550561797753YPL246C		0.0229007633587786YDL178W		0.130188679245283YOR111W		0.155172413793103YNL240C		0.0590631364562118YLR152C		0.0138888888888889YKL019W		YGR168C		YGR027C		0.0555555555555556YGL250W		0.0746887966804979YEL031W		0.0897119341563786YDR320C-A		YIL099W		0.00910746812386157YJR135C		0.00836820083682008YIR013C		YIL094C		0.0862533692722372YMR032W		0.0179372197309417YPL112C		YAL049C		0.126016260162602YMR010W		0.00246913580246914YBR193C		YCR023C		YHL009C		YDL086W		0.142857142857143YOR201C		0.0825242718446602YPR109W		0.0306122448979592YIL038C		0.00358851674641148YPL137C		0.00626959247648903YML082W		0.0677966101694915YPL233W		YJL004C		YOR286W		0.0604026845637584YOR315W		0.0289017341040462YNL010W		0.0912863070539419YIL087C		YHR061C		0.00636942675159236YPL111W		0.102102102102102YGL146C		0.112540192926045YBR182C		0.0243362831858407YPR015C		0.00404858299595142YER026C		YOL036W		0.00919842312746386YPR100W		0.135714285714286YDL139C		0.0224215246636771YNR033W		0.143583227445997YKL149C		0.0765432098765432YBL093C		0.00454545454545455YPR122W		0.0736754966887417YOL009C		0.173431734317343YER124C		0.12739965095986YGR253C		0.176923076923077YDR055W		0.0990990990990991YDL110C		YOR049C		YMR027W		0.0446808510638298YJR032W		0.0661577608142494YIL044C		0.0134228187919463YBR011C		0.132404181184669YDR419W		0.0490506329113924YBR110W		0.10913140311804YLR247C		0.0334190231362468YPL208W		0.0428816466552316YOR319W		0.169014084507042YLR371W		0.0833333333333333YDL051W		0.0436363636363636YJL072C		0.0234741784037559YHR081W		YOR061W		0.0914454277286136YDR090C		0.0193548387096774YPL256C		YOR070C		YNL024C		0.138211382113821YJL217W		0.409090909090909YGL093W		0.0229007633587786YDR523C		0.0612244897959184YKL098W		0.0504201680672269YCR008W		0.0398009950248756YOR298C-A		0.00662251655629139YOL059W		0.0590909090909091YOR250C		0.193258426966292YGR110W		0.0808988764044944YHL036W		0.0384615384615385YJR088C		YLR378C		0.0125YHR020W		0.0857558139534884YMR235C		0.058968058968059YBL050W		YNL185C		0.145569620253165YFR036W		YMR067C		0.0841346153846154YML086C		0.108365019011407YMR167W		0.0793237971391417YDR016C		YGR062C		YIL113W		0.0717703349282297YNL234W		0.0117370892018779YDR414C		0.0276243093922652YPL045W		0.105263157894737YGR189C		0.120315581854043YCR086W		0.0526315789473684YHR039C		0.0729813664596273YFL044C		0.0863787375415282YIL009W		0.0979827089337176YNL046W		YOR355W		0.00574712643678161YPL265W		0.00657894736842105YDL128W		0.0024330900243309YHR090C		0.0141843971631206YDR214W		0.211428571428571YPR061C		YGR267C		0.0905349794238683YGR185C		0.0380710659898477YML050W		0.067524115755627YNL078W		0.00982800982800983YMR158W		0.141935483870968YOR262W		0.0634005763688761YHR016C		0.126068376068376YFL004W		0.0410628019323672YDL045W-A		YNL290W		0.0352941176470588YMR132C		0.100961538461538YDR358W		0.0610412926391382YGL085W		0.142335766423358YBR010W		YML008C		0.0913838120104439YIL119C		0.00245700245700246YDR073W		YOR180C		0.0811808118081181YPL170W		0.0723684210526316YGL139W		0.0760598503740648YLR287C		YBR248C		0.115942028985507YAL044W-A		0.127272727272727YGL140C		0.007383100902379YGR180C		YPL229W		YDR079W		YDR183W		0.108695652173913YDR205W		0.0331491712707182YER118C		0.0708446866485014YKL144C		0.330188679245283YLR343W		0.0666666666666667YMR064W		YGR193C		0.0853658536585366YML041C		0.0107142857142857YMR260C		0.156862745098039YOR060C		0.0894941634241245YHR191C		0.218045112781955YDL170W		YDR185C		0.301675977653631YPL153C		0.0657734470158343YML072C		0.168284789644013YGR036C		YKL086W		0.0866141732283465YGL226W		YBR283C		0.0142857142857143YGR196C		YNL330C		0.0531177829099307YLR393W		0.0681003584229391YHR189W		0.126315789473684YDR513W		0.104895104895105YPR069C		0.184300341296928YNL064C		0.161369193154034YBR061C		0.0645161290322581YPL002C		0.0643776824034335YJR053W		0.00348432055749129YHR010W		0.147058823529412YER102W		0.12YKL074C		0.047438330170778YGR126W		YAL036C		0.0975609756097561YKL082C		YGL010W		YHR195W		0.00934579439252336YIL131C		0.0599173553719008YDR287W		0.143835616438356YMR002W		YDR122W		0.0366541353383459YIL127C		YIR042C		0.216101694915254YBL068W		0.128440366972477YPR055W		YMR281W		0.0756578947368421YBR019C		0.161659513590844YOR256C		0.0593325092707046YER139C		0.00442477876106195YNL174W		0.0473684210526316YBR160W		0.063758389261745YMR006C		0.0325779036827195YHR064C		0.137546468401487YMR174C		YPL120W		0.0251346499102334YOR151C		0.116013071895425YPR025C		YLR121C		0.198818897637795YNL220W		0.12933025404157YJL186W		0.0392491467576792YGR263C		0.0660377358490566YIL024C		0.116402116402116YPR070W		0.049469964664311YMR087W		0.0845070422535211YDR068W		YGR059W		0.080078125YKL080W		0.076530612244898YGL246C		0.147286821705426YBL075C		0.110939907550077YHR108W		0.0564102564102564YER035W		0.0413793103448276YMR299C		0.0801282051282051YML093W		0.00222469410456062YGL143C		0.0823244552058111YNR073C		0.049800796812749YMR030W		YDL117W		0.0926553672316384YOL075C		0.0525502318392581YOL056W		0.0495049504950495YIL022W		0.0765661252900232YMR073C		0.0298507462686567YDR224C		YER171W		0.0655526992287918YNL141W		0.0576368876080692YDL010W		0.0865800865800866YDR106W		0.126760563380282YHR078W		0.00905797101449275YDR270W		0.110557768924303YGR009C		YGL122C		YMR128W		0.0441988950276243YEL013W		YLR268W		0.126168224299065YDL069C		0.0305676855895196YMR065W		YIL068C		0.00372670807453416YPR183W		0.116104868913858YLR387C		0.00694444444444444YGR012W		0.099236641221374YNL095C		0.0155763239875389YBR007C		YGL253W		0.088477366255144YOR196C		0.0314009661835749YDR233C		0.0135593220338983YEL040W		0.132762312633833YNL250W		0.0442073170731707YMR252C		YLR312W-A		YGR206W		YKL189W		0.0050125313283208YER152C		0.0835214446952596YCL033C		0.0773809523809524YGR006W		YPL041C		0.00966183574879227YOL131W		YLR088W		0.0716612377850163YCR045C		0.0814663951120163YKL163W		0.0523076923076923YPR149W		YNL306W		0.133640552995392YBR034C		0.189655172413793YPL187W		0.0424242424242424YHR030C		0.0433884297520661YDR384C		0.0181818181818182YMR052W		YGR124W		0.0961538461538462YIL123W		0.0505263157894737YOR163W		0.202127659574468YJL178C		0.107011070110701YLR352W		0.00867410161090459YKL018C-A		0.0404040404040404YPR112C		0.0992108229988726YNR038W		0.0667726550079491YDL192W		0.165745856353591YPR157W		0.0792291220556745YIL109C		0.0907127429805616YGL257C		0.0537634408602151YER099C		0.138364779874214YNL182C		0.230630630630631YPR163C		0.0344036697247706YML042W		0.0611940298507463YGR152C		0.125YDL124W		0.0544871794871795YIL138C		YDR268W		0.0343007915567282YOL064C		0.112044817927171YER091C		0.0651890482398957YOL110W		0.029535864978903YHR132W-A		YMR129W		0.220643231114435YLR153C		0.108345534407028YOL128C		0.0826666666666667YOR085W		0.0685714285714286YJL190C		0.192307692307692YPL204W		0.0668016194331984YOR159C		0.308510638297872YER072W		YDL004W		0.20625YJL164C		0.0680100755667506YNL130C		0.0330788804071247YER100W		0.08YMR110C		0.0695488721804511YDR228C		YFL048C		0.11685393258427YBR247C		YNL091W		0.000806451612903226YDL159W		0.0446601941747573YLR347C		YDR159W		0.01076095311299YOR040W		0.133333333333333YGL187C		0.0774193548387097YJR095W		YNL029C		0.0421455938697318YJL013C		0.00194174757281553YNL136W		YDL058W		YBL057C		0.0841121495327103YBL087C		0.226277372262774YGL130W		0.128540305010893YMR035W		0.231638418079096YPL181W		0.00592885375494071YOR075W		YMR159C		YOR211C		0.0283768444948922YKL183W		0.065359477124183YGL084C		YPL245W		0.092511013215859YOL049W		0.0631364562118126YMR088C		YJR044C		YDR436W		0.0352112676056338YIL083C		0.0849315068493151YHR133C		YDL064W		0.121019108280255YGR024C		0.0759493670886076YNL051W		YML097C		YMR097C		0.0572207084468665YNL059C		0.0370860927152318YMR253C		YPR050C		0.0218978102189781YHL040C		YBL098W		0.11304347826087YJR009C		0.153614457831325YPR180W		0.0951008645533141YJL196C		YGR148C		0.0451612903225806YMR204C		0.0404761904761905YHR105W		0.0700934579439252YCR053W		0.0525291828793774YLR137W		0.103542234332425YHR114W		0.0347551342812006YPR010C		0.116375727348296YGR257C		YOR279C		YGR242W		0.156862745098039YJL112W		0.184873949579832YLR398C		0.0458430458430458YPL227C		0.0898203592814371YGR273C		YMR184W		YBL081W		YJR142W		0.128654970760234YHR194W		0.122625215889465YFR046C		YGL027C		0.0588235294117647YLR423C		YMR286W		0.186046511627907YDL036C		0.116883116883117YBR213W		0.0875912408759124YHR037W		0.0939130434782609YLR107W		0.0420792079207921YNR045W		0.0122699386503067YMR072W		YDL186W		0.0036101083032491YCR082W		YER063W		YNL090W		0.171875YMR280C		YHR045W		0.0642857142857143YPL072W		0.0460921843687375YOR330C		0.0215311004784689YPL252C		0.13953488372093YNL108C		0.0851851851851852YDL073W		0.00609756097560976YEL042W		0.0849420849420849YKL085W		0.11377245508982YLR219W		YNL138W		0.096958174904943YBR082C		0.148648648648649YLR355C		0.0708860759493671YHL032C		0.0648801128349788YHR002W		YPL038W		YJR085C		YCL030C		0.0863579474342929YLR284C		0.0821428571428571YOR017W		0.025YIL095W		0.045679012345679YBR162W-A		YER105C		0.0718907260963336YJR080C		0.129441624365482YIL055C		0.0207336523125997YKL157W		0.108021390374332YKL113C		0.0445026178010471YER014W		0.11317254174397YPR164W		0.150675195451315YER158C		0.00698080279232112YNL189W		YJL079C		0.040133779264214YKL109W		0.0036101083032491YER049W		0.0574534161490683YJL171C		0.106060606060606YBR280C		0.120879120879121YBR259W		0.0159883720930233YGL181W		0.0101010101010101YHR040W		0.0601092896174863YDL226C		0.00568181818181818YDR394W		0.0514018691588785YLR344W		0.149606299212598YGL198W		0.0170212765957447YER128W		0.0344827586206897YBR223C		0.0661764705882353YMR079W		0.0526315789473684YAL022C		0.00773694390715667YHR071W		0.00436681222707424YMR264W		YMR200W		0.109375YBR165W		0.0722021660649819YIL027C		YNR049C		YPL249C		YJL035C		0.088YLR131C		0.0038961038961039YAR014C		0.0155586987270156YGL261C		YER156C		0.0621301775147929YDR210W		YDL130W-A		YLR233C		0.00143061516452074YPL127C		YDR147W		0.0411985018726592YPL077C		YPL051W		0.156565656565657YMR157C		0.0431372549019608YOR002W		0.00551470588235294YMR176W		0.00992204110559887YER111C		0.00823421774931381YOR175C		YAL061W		0.134292565947242YGR215W		YNL118C		0.0195876288659794YNR050C		0.13677130044843YDR489W		0.0442176870748299YML096W		0.0780952380952381YGL179C		0.0607142857142857YOR264W		0.00930232558139535YMR089C		0.0303030303030303YOL045W		0.0663033605812897YPL207W		0.054320987654321YIL070C		0.135338345864662YCR091W		0.0361111111111111YGL203C		0.0507544581618656YIL117C		0.0251572327044025YMR307W		0.0590339892665474YOL152W		0.0854838709677419YJR131W		0.00910746812386157YJL003W		YNL082W		0.0836197021764032YDR196C		0.0746887966804979YBR003W		YNR032W		0.0815217391304348YGL148W		0.109042553191489YOR245C		0.0574162679425837YNL299W		0.0311526479750779YOL144W		0.0309917355371901YGL111W		0.181425485961123YBR181C		0.101694915254237YCL038C		0.00946969696969697YPR158W		0.0833333333333333YOR313C		0.00591715976331361YGL039W		0.10632183908046YDL207W		YLR394W		0.012448132780083YNL231C		0.0341880341880342YML099C		YJL159W		0.062953995157385YOR187W		0.169336384439359YNL160W		0.0847457627118644YNL107W		0.20353982300885YJL125C		0.133159268929504YEL036C		0.034YGR013W		0.0145161290322581YJL054W		0.0146443514644351YMR214W		0.19893899204244YNR008W		0.0287443267776097YMR042W		0.0508474576271186YJR019C		0.169054441260745YDL084W		0.103139013452915YGL077C		0.0142095914742451YLR182W		0.0012453300124533YAR019C		0.0338809034907598YNL121C		YMR308C		YMR309C		0.0110837438423645YDL182W		0.0771028037383178YMR170C		0.0869565217391304YLR231C		0.0860927152317881YDL208W		0.0833333333333333YOR099W		0.0610687022900763YAL043C		YDR531W		0.106267029972752YBR028C		0.0514285714285714YNL225C		YIR023W		YDR377W		YDR486C		YHR115C		0.0480769230769231YPL007C		0.164965986394558YDR248C		0.0932642487046632YNL111C		0.0833333333333333YPR032W		0.204259438528558YDR415C		0.0882352941176471YJL173C		0.122950819672131YDR298C		0.0943396226415094YOR114W		YNR054C		0.0253164556962025YCR071C		0.123287671232877YHR199C		0.103225806451613YOL108C		YHR154W		0.0429906542056075YHR038W		0.0260869565217391YNL008C		0.0179372197309417YMR191W		0.0160857908847185YOR148C		YJL194W		0.0448343079922027YHR027C		0.00704934541792548YLR225C		0.211302211302211YCR090C		0.247252747252747YDR219C		0.0021505376344086YMR168C		0.00493421052631579YDR084C		0.0301507537688442YGL252C		0.0850340136054422YGL221C		0.0972222222222222YPL103C		0.115384615384615YLR167W		0.171052631578947YPR079W		0.102362204724409YJR148W		0.178191489361702YJR102C		0.0544554455445545YER117W		0.226277372262774YPL128C		YJR075W		0.0454545454545455YGR147C		YPL132W		0.116666666666667YOR176W		0.0712468193384224YDR121W		YKL050C		0.00108459869848156YDL204W		YMR048W		YDR098C		0.115789473684211YOR177C		YGR205W		0.0620689655172414YLR188W		0.0460431654676259YCR050C		0.0490196078431373YHR159W		YBL090W		YPL139C		0.241304347826087YBR203W		0.012987012987013YGR109C		YBR121C		0.0854572713643178YKL165C-A		YIL120W		YGR255C		0.123173277661795YER175C		0.0802675585284281YMR256C		YDR070C		YEL064C		YPR156C		0.00160771704180064YER067W		0.093167701863354YOR003W		0.0794979079497908YGL067W		0.0963541666666667YNL119W		0.0304259634888438YNR035C		0.114035087719298YOR232W		0.043859649122807YMR092C		0.413008130081301YOR317W		0.105714285714286YAR066W		0.113300492610837YGR162W		YMR102C		0.203836930455636YDL066W		0.0607476635514019YOR387C		0.0533980582524272YGL070C		0.19672131147541YBR272C		YDL111C		0.211320754716981YIR030C		0.0942622950819672YPR018W		0.0132013201320132YGR161C		YMR068W		YKL168C		0.0359116022099447YIL011W		YDR476C		0.03125YPL272C		0.0657640232108317YJL123C		0.0418410041841004YHR080C		0.0855018587360595YMR086W		YDR213W		YNL071W		0.134854771784232YBL086C		0.0600858369098712YGL006W		0.0699062233589088YML087C		0.195512820512821YLR090W		0.137254901960784YPR196W		YMR225C		0.142857142857143YOR230W		0.221967963386728YBR169C		0.113997113997114YDR004W		0.0521739130434783YER076C		0.0860927152317881YBR210W		YPL206C		0.0841121495327103YNL322C		0.012779552715655YHR155W		0.0570032573289902YNR043W		0.101010101010101YGR082W		YER004W		0.112554112554113YNL232W		0.198630136986301YMR251W-A		YNL209W		0.099510603588907YHR153C		0.0454545454545455YPR016C		0.191836734693878YNL178W		0.154166666666667YHR206W		0.0257234726688103YPL090C		0.0932203389830508YLR132C		0.0758620689655172YNL025C		YDR483W		0.0452488687782805YPR027C		0.0288808664259928YPL067C		0.0606060606060606YCR026C		0.0390835579514825YDL214C		0.0400572246065808YBL082C		0.0131004366812227YDL017W		0.0631163708086785YGL218W		0.0416666666666667YDR527W		YBR093C		0.0620985010706638YGL138C		0.0811594202898551YDR391C		0.137931034482759YOR131C		0.0825688073394495YDR345C		YLR303W		0.0833333333333333YLR127C		0.0211019929660023YOL116W		YPL155C		0.0679886685552408YNL186W		0.0429292929292929YPR110C		0.164179104477612YLR097C		0.0959302325581395YMR238W		0.00655021834061135YLR414C		0.0380228136882129YDR392W		YBL032W		0.062992125984252YML049C		0.243203526818516YDR416W		YDL108W		0.0718954248366013YMR240C		YGL071W		0.0202898550724638YPL115C		0.0186170212765957YMR049C		0.14002478314746YDR243C		0.0782312925170068YDL008W		0.0848484848484849YNR017W		YOR383C		0.0245098039215686YLR095C		0.0160098522167488YOR115C		0.0447761194029851YHR019C		0.115523465703971YKL033W-A		0.0677966101694915YNL123W		0.228686058174524YPL084W		YNL162W-A		0.0277777777777778YGR260W		YDR012W		0.0331491712707182YCL005W		0.0234375YLR417W		0.03886925795053YBR233W-A		YHR157W		0.021978021978022YOL072W		0.0153846153846154YPL239W		YBR179C		0.0385964912280702YFR048W		0.0181268882175227YIL074C		0.110874200426439YOR322C		0.0660146699266504YOR033C		0.0242165242165242YPR063C		YKL046C		0.0044543429844098YER107C		0.380821917808219YKL024C		0.0833333333333333YPR054W		0.0515463917525773YMR075W		0.0043859649122807YBR262C		0.0660377358490566YDR088C		0.00785340314136126YLR377C		0.114942528735632YDR118W		0.0812883435582822YIR034C		0.0991957104557641YMR291W		0.0460750853242321YML011C		0.0282485875706215YPL048W		0.0530120481927711YGL120C		0.0717079530638853YAR008W		0.101818181818182YGL116W		0.213114754098361YJR047C		0.382165605095541YDR299W		YOR223W		0.0273972602739726YBR298C		YHR185C		0.0126582278481013YPL049C		YKL159C		0.0900473933649289YNL260C		YJL210W		0.003690036900369YBL015W		0.0893536121673004YAL054C		0.103786816269285YMR119W		0.0144230769230769YDL020C		YBR055C		YJL105W		0.0517857142857143YGR040W		0.0597826086956522YLR293C		0.164383561643836YHR031C		0.0774550484094053YJR118C		YLR177W		0.0605095541401274YGR279C		0.0621761658031088YBR290W		YGL123W		0.0905511811023622YOL115W		0.023972602739726YOR231W		0.0511811023622047YMR062C		0.133786848072562YPL176C		0.0574712643678161YJR134C		YIL064W		0.140077821011673YDR165W		0.29954954954955YNL142W		YMR222C		0.0941704035874439YHR144C		0.0865384615384615YNL311C		0.0432503276539974YLR242C		0.0311526479750779YER057C		0.209302325581395YML029W		0.0656324582338902YCR087C-A		0.0196078431372549YAL033W		0.104046242774566YNR060W		0.0792767732962448YLR333C		0.0740740740740741YML037C		0.00882352941176471YDL101C		0.0916179337231969YDR195W		0.00187617260787992YDR322W		0.0899182561307902YDL102W		0.0929808568824066YGR276C		0.0705244122965642YBR126C		0.109090909090909YJL081C		0.0715746421267894YLR363W-A		YGR076C		YCR020C-A		0.204545454545455YML010W		0.0959548447789276YPL168W		0.0418604651162791YDR328C		0.0257731958762887YGR021W		0.1YPL237W		0.0701754385964912YMR224C		0.0780346820809249YPR105C		YPL083C		0.0685224839400428YMR172W		0.0125173852573018YOL140W		0.08274231678487YGL107C		0.00619195046439629YGR128C		0.103786816269285YNR020C		0.0148148148148148YPR017C		0.0839160839160839YDL132W		0.0208588957055215YNR039C		0.0247933884297521YCR010C		0.0141342756183746YNL309W		YGR179C		YGR175C		0.118951612903226YNL265C		YMR192W		YPR189W		YNL196C		YGL135W		0.152073732718894YOL132W		0.0912951167728238YGL082W		0.057742782152231YNR048W		0.0788804071246819YDL134C		0.0758807588075881YHR083W		0.0151975683890578YFR005C		0.120535714285714YEL020W-A		YEL038W		0.066079295154185YCR035C		0.109137055837563YMR026C		YJR016C		0.064957264957265YDR176W		YGL004C		0.390887290167866YDR222W		0.209638554216867YPR162C		0.0396975425330813YEL054C		0.096969696969697YDR020C		0.0862068965517241YPR129W		0.0286532951289398YDL091C		0.0549450549450549YJL154C		YDR074W		0.100446428571429YBR240C		YDR251W		0.027710843373494YOL042W		0.118457300275482YHR196W		0.135652173913043YDR494W		0.00277008310249307YGL142C		0.0292207792207792YPL230W		YOR046C		0.0933609958506224YBL056W		0.0961538461538462YDL175C		YOR336W		0.0725274725274725YNL139C		YHR192W		0.0107913669064748YNL313C		0.00774336283185841YCR051W		0.00900900900900901YGR067C		0.0100755667506297YHL024W		0.0561009817671809YJL185C		0.0136518771331058YER071C		0.134920634920635YPL076W		YIR002C		0.0513595166163142YMR294W		YML110C		0.100977198697068YPR065W		YLR288C		0.109704641350211YCR057C		0.310942578548212YLR170C		0.166666666666667YJL160C		0.0731707317073171YBR287W		0.00936768149882904YDL033C		0.170263788968825YGR239C		YER031C		0.170403587443946YOR006C		0.0159744408945687YNL283C		0.00596421471172962YOL032W		0.101626016260163YLR194C		YER168C		0.0201465201465201YFR032C-A		YJL151C		0.0526315789473684YOR332W		0.0429184549356223YMR181C		YDL042C		0.0409252669039146YNL148C		0.145669291338583YMR318C		0.172222222222222YGL078C		0.0879541108986616YIL065C		YNL099C		0.046218487394958YPR083W		YBL033C		0.0898550724637681YCL057W		0.0224719101123595YDR117C		0.107964601769912YLR245C		0.190140845070423YPL254W		YNL100W		YPL184C		0.0898692810457516YDR492W		0.00632911392405063YER006W		0.0480769230769231YOL114C		0.0544554455445545YNL288W		YGR096W		YER132C		0.0256702795208214YJL008C		0.0792253521126761YHR202W		0.104651162790698YHR094C		YPR140W		0.0288713910761155YPR029C		0.03125YNL318C		YDR276C		YOL040C		0.0774647887323944YGR143W		0.0635538261997406YPL165C		0.0723860589812333YNR026C		0.271762208067941YBL013W		0.0997506234413965YGR122W		0.00995024875621891YDR275W		0.0170212765957447YHR140W		0.0460251046025105YKL127W		0.107017543859649YDR382W		YOL129W		0.0271739130434783YNL102W		0.0769754768392371YML014W		0.0896057347670251YDR237W		0.0582191780821918YJR129C		0.103244837758112YPR024W		0.0227576974564926YOR289W		0.103585657370518YLR259C		0.0664335664335664YDR496C		0.00152439024390244YPL042C		0.0468468468468468YJL133W		YOL077W-A		YPL145C		0.17741935483871YDL146W		YDR100W		YJR004C		0.109230769230769YBR186W		0.0390070921985816YKL137W		YFR041C		0.0305084745762712YKL006C-A		YLR133W		0.0343642611683849YPR192W		YMR189W		0.0522243713733075YOR137C		0.0996784565916399YNL215W		0.003125YDL105W		0.0223880597014925YCL029C		0.0363636363636364YDR172W		0.144525547445255YNL085W		0.0313253012048193YDL230W		0.119402985074627YNL264C		0.0514285714285714YBL038W		0.0732758620689655YHR148W		0.0382513661202186YPL166W		0.0234741784037559YJL005W		0.0508390918065153YPL026C		0.0756972111553785YJR006W		0.158110882956879YJL115W		0.150537634408602YJL099W		0.032171581769437YLR262C		0.172093023255814YNL333W		0.0302013422818792YFR010W		0.094188376753507YJL161W		0.0388888888888889YJR097W		0.0232558139534884YIR021W		0.0385674931129477YPR103W		0.13588850174216YJR122W		0.0744466800804829YOR390W		YML105C		0.032967032967033YKL035W		0.11623246492986YMR056C		YGL171W		0.0797872340425532YOR373W		0.0470035252643948YGR249W		0.0109649122807018YAR035W		0.0567685589519651YGR198W		0.00489596083231334YNR022C		0.0503597122302158YDR034C		YBR033W		0.00108813928182807YPL186C		0.0263157894736842YBL084C		YOR352W		0.0116618075801749YDL030W		YAL013W		YDL104C		0.0786240786240786YHR175W		0.037037037037037YBR002C		0.0839160839160839YBR246W		0.250645994832041YHR121W		0.171122994652406YEL017W		YDL067C		YGR037C		YML123C		YDR280W		0.170491803278689YBR191W		0.14375YGL083W		0.0385572139303483YMR229C		0.218045112781955YAL060W		0.159685863874346YCL036W		0.0424028268551237YPL046C		0.0606060606060606YPL136W		0.040983606557377YJL207C		YBL069W		0.111888111888112YOR361C		0.224115334207077YML031W		YNR014W		0.00943396226415094YBR166C		0.0597345132743363YHR087W		0.0990990990990991YDR151C		YGL026C		0.0792079207920792YER047C		0.0100334448160535YEL050C		0.132315521628499YGL036W		YDL087C		YPL135W		0.103030303030303YJR064W		0.0889679715302491YPL074W		0.0119363395225464YOR276W		YGR261C		YBL102W		YGR049W		0.0160427807486631YHR156C		0.0294117647058824YIR012W		0.368909512761021YMR283C		0.0331384015594542YER083C		YLR110C		0.0601503759398496YBR137W		0.100558659217877YJR062C		0.12035010940919YNL094W		0.0732538330494037YNL281W		0.248366013071895YHR001W-A		YMR150C		0.194736842105263YDL098C		0.0206185567010309YGR010W		0.0556962025316456YBR057C		0.00273224043715847YPL032C		0.0315151515151515YGR157W		0.0276179516685846YNL213C		YMR018W		YBR284W		0.0238393977415307YGR238C		0.109977324263039YKL130C		YDR536W		YIL051C		0.158620689655172YOR305W		0.0495867768595041YER079W		YPR003C		0.0238726790450928YDR107C		0.0833333333333333YGL064C		0.0802139037433155YML117W		0.0414462081128748YIL082W		YKL091C		0.0483870967741935YKL096W		0.133891213389121YDR110W		0.0371024734982332YDR412W		0.0127659574468085YPR082C		0.160839160839161YJL193W		0.00995024875621891YDR123C		YNR003C		0.056782334384858YBR149W		0.0377906976744186YNL112W		0.0824175824175824YNL261W		0.0480167014613779YBR172C		0.00405405405405405YDR226W		0.0675675675675676YDR389W		YIL144W		YJL080C		0.0785597381342062YOR089C		0.180952380952381YJR022W		0.293577981651376YPR034W		0.0670859538784067YDR477W		0.0679304897314376YKL061W		YOR219C		0.15359828141783YBR164C		0.169398907103825YMR060C		0.0305810397553517YBR217W		0.0806451612903226YIL076W		YIR014W		0.0165289256198347YPL068C		YGR117C		0.222689075630252YDR014W		0.00154559505409583YGR275W		YNL210W		0.0555555555555556YGL233W		0.0010989010989011YLR457C		YBR030W		0.0289855072463768YPL225W		YJR036C		0.0123318385650224YDR138W		YPR134W		0.0261194029850746YDL119C		YGR004W		0.0346320346320346YMR301C		0.0594202898550725YPR116W		0.0577617328519856YNL253W		0.348341232227488YLR386W		YBR037C		0.0779661016949153YFR045W		YPR145W		0.0751748251748252YNR024W		YMR123W		YOR303W		0.128953771289538YLR221C		YNL152W		0.0855745721271394YNL244C		0.148148148148148YJL111W		0.0836363636363636YNL135C		0.315789473684211YHL015W		0.206611570247934YGR236C		YIL154C		0.0809248554913295YDL085W		0.11743119266055YML115C		0.0429906542056075YBR278W		YDR236C		0.169724770642202YOR349W		0.00788954635108481YLR433C		0.0488245931283906YCR018C		YFR037C		0.00179533213644524YJL002C		0.310924369747899YLR264W		0.283582089552239YLR353W		0.00497512437810945YHL016C		0.0108843537414966YLR135W		0.00935828877005348YOL088C		0.0505415162454874YPL086C		0.0969479353680431YOR209C		0.0559440559440559YDR142C		0.296YIR025W		YKL135C		YMR223W		0.133757961783439YKL120W		YLR239C		0.0579268292682927YPL019C		0.029940119760479YPL085W		YLR102C		YJL071W		0.0574912891986063YJR010W		0.0841487279843444YCR021C		0.0301204819277108YHR063C		0.0765171503957784YOL063C		0.0867293625914316YGR172C		0.00403225806451613YHR009C		0.0650095602294455YGR111W		0.1225YOR039W		0.0193798449612403YPL087W		0.0189274447949527YMR101C		0.075801749271137YNL001W		0.246113989637306YOL146W		0.0154639175257732YDL218W		0.00315457413249211YNL098C		0.102484472049689YLR332W		0.00797872340425532YGR081C		YOL156W		YBR058C-A		YHR168W		0.12625250501002YHR062C		0.116040955631399YGR248W		0.101960784313725YOL058W		0.1YMR138W		0.172774869109948YPL022W		0.0518181818181818YJL110C		0.00181488203266788YHR208W		0.173027989821883YIR009W		0.162162162162162YLR384C		0.140845070422535YPR188C		0.00613496932515337YGL018C		YJL198W		YPL013C		0.0743801652892562YMR261C		0.0740037950664137YER001W		0.0354330708661417YGR002C		0.00840336134453781YPR108W		0.013986013986014YDR385W		0.122327790973872YMR271C		0.127753303964758YBR109C		0.0204081632653061YDR480W		0.00309597523219814YOR306C		YBL089W		YGR112W		0.0848329048843188YLR213C		0.132701421800948YDR439W		0.00288184438040346YLR408C		YJR010C-A		0.0212765957446809YNL323W		0.0917874396135266YPR067W		0.108108108108108YGR254W		0.0823798627002288YIL145C		0.0906148867313916YMR209C		0.0547045951859956YPR102C		0.126436781609195YLR168C		0.21304347826087YPL123C		0.0921658986175115YJL149W		0.024132730015083YGR159C		0.0845410628019324YML058W-A		0.0441176470588235YDR325W		YHR006W		YGL040C		0.0555555555555556YOR259C		0.068649885583524YDL085C-A		YBL041W		0.182572614107884YNL009W		0.080952380952381YNL070W		0.0666666666666667YJR139C		0.142061281337047YJL044C		YLR404W		0.087719298245614YIR016W		YNL206C		0.118681318681319YML062C		YDR375C		0.111842105263158YLR375W		YPL183W-A		0.0860215053763441YER010C		0.128205128205128YHR149C		YJL197W		0.0773524720893142YDL212W		YER025W		0.144212523719165YBR046C		0.182634730538922YGL011C		0.162698412698413YBR052C		0.104761904761905YPR167C		0.0574712643678161YER048C		YER037W		0.043613707165109YGR247W		0.0753138075313808YHR169W		0.0974477958236659YKL154W		0.159836065573771YLR321C		0.0610328638497653YMR063W		0.0292887029288703YFL034W		0.0167753960857409YIL125W		0.0532544378698225YOR270C		0.0345238095238095YGL017W		0.0715705765407555YFR053C		0.088659793814433YNL110C		0.0818181818181818YIL098C		YML128C		YOR001W		0.0218281036834925YER129W		0.0288966725043783YOR304C-A		YOR057W		0.0658227848101266YDR373W		YGR091W		0.00404858299595142YLR087C		0.189655172413793YOL002C		YGL244W		0.0268817204301075YCL035C		0.145454545454545YPR152C		0.032258064516129YGR005C		0.04YHL008C		YKL146W		YOL092W		0.00974025974025974YMR115W		0.00798403193612774YLR381W		0.00818553888130969YBL028C		YKL039W		0.0669216061185468YGL028C		0.0424354243542435YMR127C		0.0857988165680473YLR418C		0.0508905852417303YDR247W		0.0715835140997831YMR205C		0.104275286757039YJL118W		0.0502283105022831YFR043C		0.0717299578059072YOR255W		0.0287769784172662YDR265W		0.00593471810089021YNL131W		0.00657894736842105YNL312W		0.175824175824176YER101C		0.118604651162791YMR215W		0.0572519083969466YJL172W		0.0763888888888889YER159C		YHL031C		YEL047C		0.0872340425531915YHR118C		0.00459770114942529YOR160W		YKL016C		YPR056W		0.0562130177514793YBR201W		0.023696682464455YOR185C		0.159090909090909YER144C		0.0608695652173913YBR264C		0.175879396984925YML016C		0.0346820809248555YDL180W		0.0237659963436929YEL058W		0.0646319569120287YLR172C		0.116666666666667YBR024W		0.0830564784053156YJL168C		0.0381991814461119YJL121C		0.113445378151261YPL212C		0.0790441176470588YJL036W		0.033096926713948YOL077C		0.123711340206186YJL201W		0.0267111853088481YPL141C		0.0335260115606936YDR528W		YNL262W		0.0751575157515752YBR135W		0.06YNL317W		0.354838709677419YPR104C		0.0192307692307692YPL244C		YOR069W		0.0207407407407407YGL164C		0.0590909090909091YGL231C		YER090W		0.157790927021696YPR049C		0.0178268251273345YDL200C		0.0851063829787234YIR018W		0.00816326530612245YGR026W		0.00359712230215827YIL114C		0.437722419928826YFL027C		YLR380W		0.0367647058823529YNL153C		0.00775193798449612YHR036W		YKL184W		0.120171673819742YGR079W		0.0243243243243243YML009C		0.0428571428571429YDL133W		0.0091533180778032YOL073C		0.0341614906832298YMR250W		0.0615384615384615YBR098W		0.0376266280752533YHR005C		0.0572033898305085YDR507C		0.042907180385289YPR094W		0.0373831775700935YMR303C		0.189655172413793YBR237W		0.0647820965842167YER018C		YHR161C		YDR116C		0.115789473684211YDR242W		0.0437158469945355YOL145C		0.000928505106778087YOR290C		0.0217263652378156YLR281C		0.129032258064516YBR139W		0.0728346456692913YPL100W		0.25YER023W		0.0769230769230769YPL146C		0.0021978021978022YLR439W		YCL055W		0.0597014925373134YBR138C		0.00572519083969466YJL055W		0.0897959183673469YDR057W		0.118081180811808YMR140W		0.00613496932515337YLR092W		0.0201567749160134YBR016W		YFL054C		0.00154798761609907YBR031W		0.0331491712707182YHR142W		YNL035C		0.316195372750643YPL164C		0.0881118881118881YJR101W		0.0451127819548872YDR447C		0.0147058823529412YIL136W		YML127W		0.0103270223752151YIL016W		YOR052C		0.00666666666666667YOR078W		0.0186915887850467YMR302C		0.0541176470588235YGR166W		0.155357142857143YNL221C		0.0697142857142857YGL128C		0.0247349823321555YKL072W		0.0195822454308094YHR052W		0.0638297872340425YMR182C		YDR438W		YPL098C		YER017C		0.0486202365308804YLR113W		0.064367816091954YNL227C		0.00338983050847458YIL149C		YIL157C		0.111675126903553YPR151C		YFL024C		0.00360576923076923YPL125W		YMR135C		YMR259C		YOL071W		YGL126W		0.0157894736842105YCR083W		0.196850393700787YGL113W		0.0149700598802395YNR001C		0.0104384133611691YMR160W		0.0343137254901961YIL116W		0.0805194805194805YGR100W		0.0126315789473684YNR032C-A		0.150684931506849YOR252W		0.0112359550561798YKL042W		YGR052W		0.0785907859078591YBR060C		0.0403225806451613YER141W		YPR172W		0.145YJL124C		0.151162790697674YER042W		0.146739130434783YGL091C		0.0762195121951219YJL100W		0.0164744645799012YHR047C		0.110981308411215YDR202C		0.102564102564103YJR150C		0.0134228187919463YAL035W		0.0898203592814371YPR013C		0.00315457413249211YNL015W		0.173333333333333YKL167C		0.131386861313869YMR070W		YFR049W		YML083C		0.0598086124401914YDR296W		0.0398230088495575YPL110C		0.0400654129190515YLR205C		YMR009W		0.206703910614525YOR243C		0.0710059171597633YNL207W		0.0658823529411765YER110C		YBR239C		0.0359168241965974YFR027W		0.124555160142349YPL040C		0.0648702594810379YGR283C		0.0791788856304985YIL084C		YDL151C		0.00518134715025907YER119C		0.00892857142857143YFL030W		0.0961038961038961YPR166C		YML108W		0.0666666666666667YDR322C-A		YIL132C		0.0375586854460094YML070W		0.0530821917808219YDR013W		0.0625YML095C		0.080952380952381YPL008W		0.0569105691056911YDL156W		0.210727969348659YDR173C		0.0732394366197183YDR190C		0.0885529157667387YLR399C		0.00145772594752187YDR109C		0.0615384615384615YLR356W		0.0203045685279188YJL058C		0.00920810313075507YFR015C		0.0508474576271186YDR263C		0.0465116279069767YNL233W		0.00224215246636771YNL334C		0.175675675675676YOL021C		0.154845154845155YHR106W		0.207602339181287YMR100W		0.0032258064516129YLR325C		0.153846153846154YGL089C		0.1YMR207C		0.120584079133302YGL103W		0.0805369127516778YDL131W		0.0795454545454545YBR163W		0.0358974358974359YML047C		0.00852272727272727YGR167W		YER145C		0.0297029702970297YMR136W		0.00357142857142857YCR076C		0.044YDR379W		YGL223C		YIL160C		0.0767386091127098YBL020W		YGL129C		0.0655737704918033YKL176C		0.0458937198067633YBR276C		0.0309789343246592YBR017C		YLR146C		0.193333333333333YOL044W		YNL005C		0.0646900269541779YPL221W		0.0769230769230769YHL039W		0.052991452991453YOR008C		0.0185185185185185YDR393W		YNL172W		0.0451945080091533YGR093W		0.104536489151874YEL025C		0.113636363636364YFR023W		0.117839607201309YOL065C		0.109375YNL255C		YML109W		YJL199C		0.138888888888889YDR208W		0.0397946084724005YNL259C		0.232876712328767YIL021W		0.179245283018868YJL088W		0.100591715976331YDL161W		0.00440528634361234YCL028W		YLR166C		YHL013C		0.0390879478827362YLR175W		0.107660455486542YPL116W		0.03012912482066YHR117W		YGL196W		0.135514018691589YGL035C		YMR244C-A		YLR222C		0.369645042839657YPR048W		0.0898876404494382YPL109C		0.0289193302891933YPL138C		0.0169971671388102YKL117W		0.12962962962963YOL125W		0.0609243697478992YEL027W		YJL165C		0.0280701754385965YMR175W		YLR438W		0.0919811320754717YGL209W		YBL092W		0.0461538461538462YBR145W		0.179487179487179YIL031W		0.0251450676982592YLR319C		0.00380710659898477YPR135W		0.227615965480043YBR233W		0.0726392251815981YDR045C		0.209090909090909YHR110W		0.231132075471698YKL001C		0.0891089108910891YLR181C		YOL086C		0.181034482758621YBR244W		0.12962962962963YNL016W		0.108167770419426YMR163C		YHR034C		0.101744186046512YML006C		0.0116279069767442YCR042C		0.0788912579957356YDR466W		0.0345211581291759YJR082C		YPL054W		YMR282C		YGR207C		0.218390804597701YOR134W		YCR017C		0.0598111227701994YJL179W		YIR004W		YPL150W		0.0332963374028857YCR088W		0.0320945945945946YIR029W		0.19533527696793YMR094W		0.0460251046025105YBR267W		0.0101781170483461YGR174C		0.0176470588235294YML107C		0.00898203592814371YNL117W		0.0288808664259928YCL043C		0.0919540229885057YFR034C		YPL066W		0.194154488517745YGL021W		0.0368421052631579YJL141C		0.0371747211895911YPL180W		YBR107C		0.122448979591837YOL043C		YDL166C		0.101522842639594YOR288C		0.0786163522012579YBR249C		0.0567567567567568YHR152W		YFL041W		0.163987138263666YOR377W		0.0895238095238095YBR041W		0.133034379671151YGR085C		0.126436781609195YPR148C		YMR278W		0.0980707395498392YMR131C		0.209393346379648YNL251C		0.0173913043478261YMR310C		0.100946372239748YNL058C		0.00949367088607595YER104W		YBR004C		YLR362W		0.0543933054393305YMR105C		0.105448154657293YPL262W		0.00409836065573771YDR519W		0.259259259259259YDL193W		0.0533333333333333YLR267W		0.0701754385964912YCR066W		YPL199C		0.0416666666666667YBR175W		0.419047619047619YGR216C		0.0410509031198686YLR373C		0.00776914539400666YJR043C		0.0342857142857143YOR358W		YBR221C		0.0956284153005464YEL052W		0.0589390962671906YDL148C		YNL307C		0.0773333333333333YDR378C		0.302325581395349YMR295C		YDR472W		0.0318021201413428YIL142W		0.0872865275142315YPR004C		0.162790697674419YNL199C		0.00187265917602996YMR043W		0.027972027972028YMR034C		0.0115207373271889YMR031C		YGR195W		0.207317073170732YMR055C		YHR216W		0.0936902485659656YLR438C-A		0.348314606741573YPL260W		YBR014C		0.0689655172413793YGR289C		YOL039W		YLR447C		YOL012C		YGL153W		YMR217W		0.121904761904762YGR171C		0.0330434782608696YDR078C		0.0582959641255605YAL010C		0.0567951318458418YKL166C		0.0703517587939698YDR182W		0.0794297352342159YML074C		0.128953771289538YNR011C		0.0662100456621005YGR127W		0.125YDR363W-A		YBR180W		YLR372W		YER002W		0.0216450216450216YDR032C		0.101010101010101YCR033W		0.000815660685154976YJL014W		0.0936329588014981YOR126C		0.0714285714285714YLR340W		0.0865384615384615YLR193C		0.308571428571429YOR108W		0.134105960264901YBL016W		0.0679886685552408YAR002C-A		0.242009132420091YPL179W		0.0491803278688525YBR129C		0.0914634146341463YML060W		0.0664893617021277YER034W		YCL001W		YPR047W		0.110874200426439YGL100W		0.36676217765043YDR388W		0.033195020746888YLR203C		0.0527522935779817YLR215C		0.0805555555555556YOR375C		0.0969162995594714YFL046W		YHR066W		0.105960264900662YLR312C		0.0326633165829146YHR122W		0.0476190476190476YHR088W		0.179661016949153YPL219W		YML094W		0.0552147239263804YBL061C		YER154W		YOR236W		0.170616113744076YNL273W		0.00807754442649435YGR084C		0.00884955752212389YNL023C		0.0124352331606218YNL275W		YGR016W		YNL052W		YHR076W		0.120320855614973YJL206C		YER143W		0.130841121495327YPL175W		0.110619469026549YIR035C		0.102362204724409YDL063C		YBR251W		0.0912052117263844YGR252W		0.0455580865603645YJL166W		0.0319148936170213YNL073W		0.0659722222222222YCL068C		YPR128C		YDR339C		0.111111111111111YGL075C		YPL095C		0.0723684210526316YCL004W		0.0844529750479846YGL170C		YOR278W		0.130909090909091YJR063W		0.136YPR073C		0.0745341614906832YGL224C		0.0642857142857143YIL056W		0.015625YML080W		0.0472813238770686YLR108C		0.0845360824742268YPL028W		0.0703517587939698YLR214W		0.0830903790087464YPL034W		0.0545454545454545YDL070W		0.00313479623824451YPL209C		0.0735694822888283YPL174C		0.032258064516129YDR508C		0.00301659125188537YDR364C		0.316483516483516YLR257W		YDR281C		YOL062C		0.238289205702648YJL126W		0.169381107491857YOL100W		0.0471785383903793YER019C-A		0.0454545454545455YGR200C		0.323604060913706YOL122C		0.00695652173913044YLR289W		0.15968992248062YGL038C		0.0354166666666667YMR124W		YNR063W		YJL052W		0.150602409638554YGL184C		0.0860215053763441YLR145W		YMR040W		YER136W		0.121951219512195YLR197W		0.0138888888888889YOR344C		YAL048C		0.0845921450151057YLR351C		0.164948453608247YDL198C		YPR033C		0.0860805860805861YML130C		0.00177619893428064YJL117W		0.112540192926045YDR003W		YDR421W		0.00421052631578947YOR110W		0.071264367816092YDR312W		0.0971302428256071YDR050C		0.108870967741935YOR117W		0.0529953917050691YML068W		0.0387931034482759YLR195C		0.103296703296703YFR014C		0.0695067264573991YFR004W		0.104575163398693YPL133C		0.0426008968609865YGR054W		0.21183800623053YOR044W		YHR143W		0.00615384615384615YKL071W		0.11328125YHR035W		0.117460317460317YGR083C		0.0307219662058372YOL022C		0.0882352941176471YGL001C		0.0802292263610315YGL090W		0.0380047505938242YNL229C		0.0282485875706215YJR049C		0.147169811320755YDL234C		0.0254691689008043YML118W		0.0673267326732673YLR094C		0.0119521912350598YDR368W		0.0544871794871795YOR378W		YJL011C		YOR221C		0.0666666666666667YAR007C		0.249597423510467YKL069W		0.144444444444444YLR208W		0.393939393939394YBR022W		0.11864406779661YOR320C		0.0264765784114053YML078W		0.175824175824176YDR326C		0.0785813630041725YER080W		0.0350877192982456YHR179W		0.0575YMR117C		0.00469483568075117YNL272C		0.00263504611330698YOR116C		0.0513698630136986YBL074C		0.0450704225352113YKL093W		YML088W		0.119760479041916YIL106W		0.00636942675159236YDR059C		0.135135135135135YLR227C		0.0202839756592292YOL124C		0.136258660508083YNR041C		YOR247W		0.1YIL110W		0.0822281167108753YKL110C		0.0511182108626198YML102W		0.286324786324786YLR272C		YEL070W		0.0557768924302789YGR213C		0.00630914826498423YGR071C		0.013953488372093YPL220W		0.147465437788018YER176W		0.0428189116859946YPR068C		0.048936170212766YPL159C		0.00395256916996047YER167W		0.00235017626321974YDL115C		0.0291666666666667YGL256W		0.0623655913978495YDL189W		0.00656455142231947YPL231W		0.045045045045045YPR045C		0.00212765957446809YHR201C		0.0856423173803526YNL048W		0.0821167883211679YJR058C		0.19047619047619YDL045C		0.0392156862745098YOL133W		0.0578512396694215YDR460W		0.00311526479750779YDR087C		YPR006C		0.0260869565217391YJR143C		0.0616797900262467YPL210C		YHR124W		0.087719298245614YBL060W		0.0262008733624454YHL025W		YER103W		0.113707165109034YOL135C		YGR056W		0.0290948275862069YGR131W		YLR260W		0.107714701601164YOR128C		0.15061295971979YJR065C		0.0579064587973274YOR086C		0.204890387858347YGL020C		YJL209W		0.0122324159021407YOR353C		0.0303413400758533YNL237W		0.00871459694989107YBR087W		0.0282485875706215YLR429W		0.181259600614439YDR539W		0.135188866799205YGL242C		YKL160W		0.103448275862069YHR001W		0.194508009153318YMR118C		YPR120C		YPR143W		YBR009C		YOR028C		0.00677966101694915YJR112W		YDL123W		0.0285714285714286YGL236C		0.0343796711509716YLR407W		YLR154C		0.0636363636363636YDR277C		0.0069284064665127YDL053C		YNL169C		0.1YKL063C		YDR446W		YIL152W		0.0212765957446809YGL054C		YJL069C		0.173400673400673YLR405W		0.0626702997275204YJL184W		YPR115W		0.00738688827331487YMR171C		0.0927272727272727YDR530C		0.101538461538462YLR295C		YKL141W		YEL026W		0.111111111111111YNL072W		0.0651465798045603YDL107W		YAL025C		0.042483660130719YAL023C		0.069828722002635YNL214W		YNL223W		0.048582995951417YPL173W		0.0875420875420875YML067C		0.193181818181818YJR153W		0.326869806094183YKL152C		0.0728744939271255YOL112W		YGL119W		0.0379241516966068YNL236W		0.0698151950718686YGR132C		0.132404181184669YBR047W		0.04YHR007C		0.0433962264150943YFR009W		0.0877659574468085YKL009W		0.139830508474576YMR044W		0.0147368421052632YJR121W		0.111545988258317YAR015W		0.101307189542484YHR072W-A		0.120689655172414YNL132W		0.0681818181818182YOR062C		0.00746268656716418YNL149C		YDR158W		0.142465753424658YBR296C		YOR339C		0.128205128205128YGR033C		0.142259414225941YML022W		0.240641711229947YPL234C		YJR014W		0.0909090909090909YCR037C		YIR007W		0.0719895287958115YKL079W		0.086890243902439YBR176W		0.0673076923076923YPR076W		0.0161290322580645YKL150W		0.185430463576159YDR336W		0.0700636942675159YDR086C		YNL159C		0.0484429065743945YER150W		0.0608108108108108YKL172W		YFL047W		YPL071C		YML098W		YDR002W		0.17910447761194YIR037W		0.141104294478528YDR105C		0.00634249471458774YPR193C		0.211538461538462YMR023C		0.0855513307984791YBR071W		0.014218009478673YML043C		YML001W		0.177884615384615YKL185W		YLR220W		YPL023C		0.0487062404870624YMR149W		0.0839160839160839YFL022C		0.073558648111332YAL007C		0.2YIL001W		0.00779727095516569YOR167C		0.283582089552239YDL027C		0.0690476190476191YOR100C		YGL192W		0.0516666666666667YDR288W		0.0363036303630363YIR005W		0.101351351351351YNL204C		0.00666666666666667YIR036C		0.102661596958175YBL107C		YHR204W		0.0351758793969849YBR088C		0.368217054263566YFR006W		0.0766355140186916YFL042C		0.0934718100890208YNL300W		0.0294117647058824YPL108W		0.0178571428571429YDR022C		0.0663265306122449YPR002W		0.0368217054263566YPL158C		0.00923482849604222YMR047C		0.0170709793351303YER131W		0.0336134453781513YPL027W		0.0122448979591837YNL293W		YPR155C		YPL177C		YNR046W		0.0444444444444444YLR314C		0.0557692307692308YKL013C		0.0818713450292398YNL003C		YER153C		YBL030C		YLR276C		0.0740740740740741YNR055C		0.00511945392491468YDL155W		YPL065W		YOR295W		YBR111C		0.268398268398268YGR105W		YGR066C		0.123287671232877YGR101W		YOR067C		YNL012W		0.0301109350237718YFR039C		0.0117647058823529YER183C		0.118483412322275YBR151W		0.0632911392405063YLR105C		0.0795755968169761YDL100C		0.0649717514124294YJL006C		0.00309597523219814YMR041C		0.0417910447761194YPR011C		YDR511W		YDR071C		0.136125654450262YIL039W		0.06553911205074YFR047C		0.125423728813559YCR011C		0.0457578646329838YCR077C		0.00376884422110553YJL203W		0.0178571428571429YHR139C		0.0858895705521472YOR328W		0.0460358056265985YDR178W		0.0165745856353591YBL045C		0.113785557986871YLR149C		0.0904109589041096YOR034C		0.00267022696929239YMR208W		0.126410835214447YPL235W		0.0849256900212314YBR119W		0.0973154362416107YFL010C		0.004739336492891YER068W		0.0221465076660988YOL082W		0.0530120481927711YDR469W		YAL009W		0.0308880308880309YJR067C		YOR065W		YFR017C		YHR146W		0.0666666666666667YPL122C		0.0487329434697856YIL045W		0.0817843866171004YGL121C		YOR038C		0.182857142857143YCR047C		0.0981818181818182YIL103W		0.105882352941176YJR017C		0.0294117647058824YPR113W		YMR099C		0.272727272727273YGR120C		YLR190W		0.00407331975560081YPL011C		YPL082C		0.0235672201392608YDR063W		0.174496644295302YMR036C		0.01985559566787YHL030W		YDR352W		YOR090C		0.0891608391608392YEL021W		0.0973782771535581YBL006C		0.0222222222222222YNL183C		0.0367088607594937YIL014W		0.0492063492063492YLR144C		0.0975609756097561YEL071W		0.120967741935484YLR324W		0.0325047801147228YLR389C		0.0954235637779942YNL263C		0.00318471337579618YPR030W		0.0597680642283675YIL073C		YPR154W		0.0697674418604651YKL148C		0.0875YBL029W		YGL186C		YER046W		0.118881118881119YBR005W		YJR005W		YPR023C		0.0224438902743142YOR367W		YMR071C		YOR321W		0.0664010624169987YHR203C		0.252873563218391YER147C		0.00801282051282051YIL090W		YJR109C		0.113595706618962YKL068W		0.0187695516162669YIL096C		0.0714285714285714YDR454C		0.133689839572193YDR362C		0.144345238095238YJL187C		0.0415140415140415YGR202C		0.0377358490566038YDR056C		0.0682926829268293YDR408C		0.130841121495327YDR297W		YKL112W		0.00136798905608755YOL121C		0.0138888888888889YLR420W		0.0741758241758242YNL151C		YCL016C		0.1YHR100C		0.102702702702703YOR102W		0.112068965517241YBR097W		0.0880330123796424YNR028W		0.0714285714285714YLR298C		0.00865800865800866YJR117W		0.0132450331125828YOR271C		0.00917431192660551YDR289C		YDL028C		0.0418848167539267YDR319C		0.0109489051094891YGL211W		0.0557103064066852YKL133C		0.0237580993520518YNL137C		0.0164609053497942YDL176W		0.104519774011299YDR487C		0.110576923076923YOR084W		0.0852713178294574YIL135C		0.00458715596330275YLR209C		0.112540192926045YLR093C		0.0711462450592885YEL061C		0.047YLR254C		YOR166C		0.0262008733624454YBR091C		YKL049C		YLR277C		0.143774069319641YNL167C		YNL006W		0.442244224422442YFR052W		0.0218978102189781YGR203W		0.0810810810810811YGL248W		0.130081300813008YGL228W		0.0103986135181976YOR226C		0.0961538461538462YPR035W		0.12972972972973YMR015C		0.0446096654275093YFL014W		YFL029C		0.0760869565217391YDL076C		0.0612244897959184YGR201C		0.0222222222222222YHR049W		0.0864197530864197YJL051W		0.00608272506082725YCR024C		0.121951219512195YPL104W		0.0820668693009119YDR018C		0.0681818181818182YBR072W		0.186915887850467YML071C		YKL026C		0.107784431137725YMR218C		0.137931034482759YOR079C		YGR044C		YPL223C		YPL250C		YGL213C		0.299748110831234YGL101W		YKL017C		0.105417276720351YMR152W		0.164383561643836YNL083W		YBR157C		YMR061W		YPL117C		0.121527777777778YGL189C		0.0336134453781513YOR311C		YMR293C		0.0538793103448276YDR259C		YCL031C		0.0841750841750842YIR026C		0.0521978021978022YHR096C		YDR488C		0.118198874296435YPR169W		0.221544715447154YPL195W		YEL051W		0.046875YMR165C		0.0464037122969838YMR106C		0.106518282988871YBR202W		0.101775147928994YKL052C		YPL259C		0.263157894736842YKL140W		0.0547445255474453YNL155W		0.0583941605839416YBR285W		0.0208333333333333YKL124W		0.0898100172711572YNR007C		0.0741935483870968YOL101C		YDL022W		0.0639386189258312YJL183W		0.0592417061611374YJR125C		0.00490196078431373YMR069W		0.147368421052632YML126C		0.0753564154786151YLR258W		0.0482269503546099YMR288W		YHR176W		0.113425925925926YOR327C		YNR027W		0.0977917981072555YCR060W		YLR130C		YDR234W		0.101010101010101YAL062W		0.0940919037199125YFR032C		0.100346020761246YBL014C		0.0771812080536913YHL021C		0.0989247311827957YPR133C		YPL232W		YPR182W		0.290697674418605YDL163W		YNL245C		YPL215W		YIR028W		0.0031496062992126YCL059C		0.0759493670886076YAL037W		0.0374531835205993YER003C		0.137529137529138YML046W		YCL034W		YFL039C		0.072YHR188C		0.157377049180328YER127W		YJR090C		0.0243266724587315YCL011C		0.131147540983607YPL059W		0.08YGL050W		0.0769230769230769YLR164W		0.0178571428571429YAL055W		0.0722222222222222YDL174C		0.117546848381601YBR261C		0.112068965517241YEL034W		0.337579617834395YKL125W		YML112W		YHR135C		0.0706319702602231YGR077C		YDR285W		YGR285C		YPL236C		0.123626373626374YFL025C		0.0748299319727891YJR133W		0.148325358851675YKL171W		0.0301724137931034YJR073C		YPL101W		0.0614035087719298YBL025W		YJR107W		0.0914634146341463YDR434W		0.0674157303370786YNL326C		YKL005C		0.031986531986532YOR021C		0.0845070422535211YIL079C		YDR177W		0.0930232558139535YOL018C		0.00503778337531486YBR183W		0.00949367088607595YGL124C		0.0807453416149068YAR002W		YIL063C		0.0886850152905199YPL010W		0.164021164021164YER180C		YPR186C		0.00466200466200466YPL079W		0.13125YHR182W		0.00382165605095541YMR081C		YER137C		0.00675675675675676YHR167W		YBL031W		YLR098C		YEL066W		0.173184357541899YOR056C		0.0479302832244009YBL091C		0.114014251781473YFR033C		YDR194C		0.0632530120481928YLR300W		0.0424107142857143YDR350C		0.0114566284779051YDL173W		0.00677966101694915YMR017W		0.00503778337531486YOR141C		0.0329171396140749YDR473C		0.0447761194029851YDL057W		0.0701219512195122YIR031C		0.0270758122743682YCL025C		0.00315955766192733YKL178C		0.0191489361702128YDL077C		0.0753098188751192YNL271C		YGL096W		YNL270C		YMR268C		0.141891891891892YOR207C		0.118363794604003YDR030C		0.278656126482213YCR009C		YNL157W		YKL040C		0.09375YLR211C		YLR091W		0.0068259385665529YML076C		0.00105932203389831YNR051C		0.0524271844660194YDR538W		0.0702479338842975YGR284C		0.00645161290322581YLR134W		0.104795737122558YMR311C		YDR515W		0.00447427293064877YNL284C		0.0496894409937888YNL279W		YBR236C		0.0802752293577982YJR011C		0.00766283524904215YGR262C		0.0919540229885057YIR032C		0.138461538461538YJL180C		0.0461538461538462YEL059C-A		0.0675675675675676YEL024W		0.0790697674418605YNL080C		0.0109289617486339YNL127W		0.00209863588667366YMR011W		YJR086W		YCL051W		YMR177W		0.0392156862745098YBR099C		0.102362204724409YBR212W		0.0639880952380952YOR291W		0.0801630434782609YGL069C		0.142857142857143YJL218W		0.173469387755102YPL130W		0.0493273542600897YBL072C		0.12YCR052W		0.05175983436853YPR058W		YKL060C		0.0696378830083565YBR185C		0.0683453237410072YGL112C		0.00581395348837209YGL019W		0.0143884892086331YBR156C		YOR368W		0.197007481296758YGR008C		YKL084W		0.0344827586206897YPL006W		0.0427350427350427YKL003C		0.16030534351145YNL191W		0.120448179271709YOL067C		YJL116C		0.0741839762611276YLR370C		0.00561797752808989YDL121C		YMR187C		0.0208816705336427YLR100W		0.0720461095100865YIL111W		YMR098C		0.0196078431372549YCR069W		0.106918238993711YDL047W		0.109324758842444YGL106W		0.0134228187919463YKL004W		0.00498753117206983YLR241W		0.0166240409207161YOL031C		0.00712589073634204YKL077W		0.0739795918367347YER053C		YNL076W		YKL053C-A		YOR257W		0.0062111801242236YBL023C		0.0737327188940092YER051W		0.0711382113821138YJL042W		0.00643776824034335YPR075C		0.0138888888888889YEL057C		0.0429184549356223YNL175C		0.086848635235732YOR215C		YNL332W		0.0970588235294118YIL030C		0.0136467020470053YBR199W		0.0387931034482759YIL134W		YPR177C		0.0894308943089431YBL010C		YDL001W		0.0418604651162791YBR056W		0.0459081836327345YMR305C		0.06426735218509YOR273C		YBL054W		YGL012W		0.0126849894291755YOR133W		0.128266033254157YML057W		0.043046357615894YFL049W		0.0256821829855538YCR005C		0.00434782608695652YML028W		0.147959183673469YGL032C		0.0229885057471264YBR070C		0.135021097046413YOL060C		0.0509915014164306YIL010W		0.116279069767442YGL154C		0.102941176470588YMR093W		0.292397660818713YGL191W		YJL208C		0.0729483282674772YDR132C		0.0868686868686869YER177W		YNL158W		0.156565656565657YOR224C		0.287671232876712YNL026W		0.258264462809917YPR179C		0.0259541984732824YER019W		0.109014675052411YIL091C		0.0527045769764216YDR052C		0.0198863636363636YGL110C		0.0016025641025641YHL011C		0.15YDR051C		0.0479041916167665YOR370C		0.0829187396351575YDR397C		YBR120C		YFL011W		YHR210C		0.284457478005865YBR089C-A		YHL002W		0.0287610619469027YPR185W		0.0311653116531165YML114C		0.00392156862745098YGR280C		0.014760147601476YDR153C		0.0024330900243309YDR229W		YIR001C		0.048YDR321W		0.110236220472441YHR029C		0.244897959183673YOL091W		0.00328407224958949YBR148W		YLR246W		0.0362116991643454YJL140W		YOR157C		0.229885057471264YML019W		0.0753012048192771YEL048C		0.138157894736842YBL080C		0.0609981515711645YJR001W		YER030W		YER027C		0.103117505995204YFR001W		YIL075C		0.0137566137566138YGL254W		YJL104W		YGR165W		0.00869565217391304YKL087C		0.03125YNR012W		0.101796407185629YDR468C		YCR046C		0.171597633136095YHL012W		0.125760649087221YEL039C		YDL144C		0.0814606741573034YLR200W		YGL247W		YNR006W		YGR286C		0.064YIL133C		0.0452261306532663YNL124W		0.0345528455284553YJR136C		YAL005C		0.113707165109034YNR047W		0.0257558790593505YPL131W		0.063973063973064YHR123W		0.0332480818414322YJR025C		0.186440677966102YLR224W		0.0650406504065041YHL010C		0.0632478632478632YOR174W		YER122C		0.00608519269776876YHR070W		0.140280561122244YDR184C		YHR147C		0.266355140186916YJR074W		0.128440366972477YOR048C		0.0288270377733598YNL295W		0.0190839694656489YHR025W		0.123249299719888YNL254C		YAL027W		0.10727969348659YBR132C		0.00671140939597315YMR213W		YML027W		YDR211W		0.0997191011235955YDR125C		0.0618101545253863YPL169C		0.0801335559265442YOL038W		0.169291338582677YGL073W		0.00720288115246098YPL200W		YPL271W		YDR354W		0.0526315789473684YPL055C		YDR331W		0.0510948905109489YDR240C		0.0426829268292683YDL237W		0.0743589743589744YDL137W		0.165745856353591YBR254C		0.114285714285714YBR040W		0.0469798657718121YPR022C		YDR238C		0.0575539568345324YBR286W		0.0595903165735568YIR022W		0.269461077844311YEL019C		YPL114W		YAL059W		YBR168W		0.0314769975786925YPR119W		YBR006W		0.0804828973843058YPR118W		0.0851581508515815YBL011W		0.0513833992094862YOR189W		0.0258620689655172YPL238C		0.0155038759689922YOR292C		YOR341W		0.0492788461538462YMR287C		0.111455108359133YDL097C		0.0115207373271889YER040W		YPL242C		0.011371237458194YGR277C		0.101639344262295YAL040C		YMR277W		0.0655737704918033YNL310C		0.0574712643678161YLR376C		0.0867768595041322YJR104C		0.272727272727273YLR180W		0.128272251308901YGL044C		0.0405405405405405YDL188C		0.0822281167108753YOL139C		0.136150234741784YGL031C		0.0451612903225806YBR123C		0.049306625577812YDL246C		0.165266106442577YDR332W		0.0725689404934688YDR294C		0.0560271646859083YFR003C		0.0193548387096774YFR050C		0.169172932330827YHR072W		0.00410396716826265YNL030W		YDR374C		0.0849673202614379YPL004C		YOR036W		YDR126W		0.0238095238095238YOL097C		0.0277777777777778YDL229W		0.115823817292007YML023C		0.0143884892086331YNL197C		0.0332829046898638YDR252W		0.0604026845637584YDR306C		0.0313807531380753YGR191W		YJL174W		0.134057971014493YDL179W		YLR350W		YOR025W		0.0559284116331096YBR066C		0.00454545454545455YGR177C		0.0822429906542056YER134C		0.101123595505618YPR107C		YBR257W		0.0824372759856631YCL052C		0.141826923076923YDL199C		YKL121W		0.193661971830986YNL321W		0.00770925110132159YMR014W		YKL041W		YJL010C		YOR331C		0.0108108108108108YER163C		0.125YKL054C		YBR230C		YBR214W		0.079696394686907YPL268W		0.0782508630609896YOR020C		0.320754716981132YDR314C		0.0491329479768786
