# Supplementary material for: Proteome-wide evidence for enhanced positive Darwinian selection within intrinsically disordered regions in proteins
Source: Genome Biol. 2011 Jul 19;12(7):R65. doi: 10.1186/gb-2011-12-7-r65 (PMC3218827; doi:10.1186/gb-2011-12-7-r65)
Supplement: Additional file 12 — Fraction of amino acid residues for each protein that are predicted by the VSL2 method to adopt intrinsically disordered conformation, using a confidence value threshold of 0.8. [file gb-2011-12-7-r65-S12.RTF]

YHR055C		1YPR161C		0.515981735159817YOL138C		0.451155853840418YGR129W		0.469767441860465YPR165W		0.138755980861244YPL015C		0.170868347338936YCL050C		0.0560747663551402YMR193W		0YGR053C		0.187279151943463YOR280C		0.093984962406015YEL004W		0.0321637426900585YJL200C		0.00506970849176172YDR348C		0.923847695390782YPL183C		0.00197433366238894YGR003W		0.0470430107526882YBL095W		0.037037037037037YEL009C		0.950177935943061YEL015W		0.393829401088929YDR329C		0.142857142857143YBR252W		0.0272108843537415YDL202W		0.120481927710843YHR209W		0.0481099656357388YPR008W		0.886167146974063YBR050C		0.396449704142012YMR197C		0YBL049W		0YNL201C		0.148018648018648YCR061W		0.19175911251981YPL171C		0.0275YHR051W		0.0337837837837838YDL116W		0.0495867768595041YPR124W		0.566502463054187YJL023C		0.0259365994236311YGL167C		0.0610526315789474YDL165W		0.130890052356021YIL143C		0.258600237247924YOR094W		0.0163934426229508YDR167W		0.495145631067961YER052C		0.0113851992409867YPL118W		0.159883720930233YGR178C		0.775623268698061YPR086W		0.205797101449275YJL065C		0.413173652694611YBR122C		0.621468926553672YBR279W		0.337078651685393YBR271W		0.145584725536993YMR221C		0.101190476190476YER130C		0.688487584650113YBR155W		0.254545454545455YDR363W		0.519736842105263YPL147W		0.106896551724138YLR443W		0.254464285714286YBR067C		0.4YAL034C		0.573849878934625YLR270W		0.0457142857142857YCR095C		0.218232044198895YPR084W		0.228070175438596YMR113W		0.00702576112412178YGR035C		0.75YDL222C		0.252427184466019YNL335W		0.0533333333333333YDR232W		0.0821167883211679YHR116W		0.529801324503311YFR018C		0.0220385674931129YKL007W		0.0111940298507463YDL213C		0.631111111111111YNL292W		0.114143920595533YNL134C		0.0132978723404255YKL100C		0.207836456558773YPL021W		0.614973262032086YIL146C		0.410207939508507YNL218W		0.258943781942078YIL121W		0.0940959409594096YDL167C		0.44923504867872YPL214C		0.0351851851851852YOR228C		0.122516556291391YBR270C		0.548623853211009YGR222W		0.184300341296928YGR121C		0.119918699186992YDL003W		0.45583038869258YOR323C		0.0087719298245614YMR183C		0.155932203389831YOR274W		0.0490654205607477YER146W		0.0860215053763441YPL063W		0.470588235294118YML075C		0.105313092979127YLR273C		0.481481481481481YDR457W		0.185128518971848YCL056C		0.0416666666666667YJL083W		0.662251655629139YPL140C		0.397233201581028YOR026W		0.00879765395894428YNR016C		0.0250783699059561YER123W		0.398854961832061YGR055W		0.0888501742160279YMR315W		0.0200573065902579YKL187C		0.485333333333333YDR383C		0.180672268907563YKL055C		0YPR019W		0.187566988210075YPR190C		0.209480122324159YPL228W		0.482695810564663YGR041W		0.749542961608775YDR353W		0YPL047W		0.131313131313131YPL014W		0.758530183727034YGR007W		0.0154798761609907YML116W		0.0996309963099631YOL137W		0.146881287726358YGL155W		0YER061C		0YKL011C		0.121813031161473YNL257C		0.13506916192026YBR291C		0.0167224080267559YDR262W		0.169117647058824YDR179C		0YIL053W		0.02YGR220C		0.0780669144981413YMR161W		0.477678571428571YER011W		0.641732283464567YGL162W		0.615384615384615YNL243W		0.301652892561983YNL047C		0.407012195121951YKL038W		0.513675213675214YFL053W		0.0423011844331641YLR411W		0.0829875518672199YLR318W		0.0723981900452489YHR150W		0.359240069084629YGL219C		0.477124183006536YBR045C		0.68586387434555YMR039C		0.811643835616438YPL188W		0.0772946859903382YGL147C		0YDR217C		0.616501145912911YPR026W		0.0478943022295623YMR008C		0.0647590361445783YDL227C		0.0307167235494881YOR197W		0.277777777777778YNL081C		0.20979020979021YKL096W-A		0.597826086956522YDR532C		0.161038961038961YKL170W		0YGL047W		0.0099009900990099YPR171W		1YML063W		0.149019607843137YLR178C		0.0684931506849315YHL029C		0.244477172312224YOR184W		0.0177215189873418YDR372C		0.176811594202899YBR008C		0.102189781021898YMR203W		0.147286821705426YBL024W		0.138888888888889YDR317W		0.0265700483091787YJL038C		0.118721461187215YDR043C		0.696969696969697YOR340C		0.349693251533742YCL044C		0.215827338129496YFR022W		0.474761255115962YEL072W		0YOL008W		0.00966183574879227YHR017W		0.0285714285714286YMR095C		0.0401785714285714YHR198C		0.0249221183800623YOR076C		0.334672021419009YNL219C		0.0144144144144144YLR210W		0.391304347826087YIL034C		0.0452961672473868YLR148W		0.0163398692810458YOR342C		0.141065830721003YIL043C		0.0105633802816901YDL160C		0.24901185770751YGR231C		0.158064516129032YNL021W		0.0609065155807365YMR266W		0.159496327387198YPL107W		0.596774193548387YGR088W		0.0640569395017794YCR063W		0.254777070063694YDL048C		0.714285714285714YML092C		0.004YPR097W		0.169617893755825YNL056W		0.106598984771574YAL020C		0.036036036036036YFL017C		0.00628930817610063YHR092C		0.0982142857142857YDR379C-A		0YNR074C		0.00529100529100529YLR189C		0.247913188647746YDR113C		0.815013404825737YML032C		0.668789808917197YFL028C		0.00346020761245675YDL247W		0.136288998357964YBR256C		0.046218487394958YPL024W		0.128630705394191YMR257C		0.0775YDL219W		0YKL122C		0.502994011976048YGL240W		0.008YDL080C		0.0738916256157636YEL001C		0.0488888888888889YPR168W		0.121019108280255YHR109W		0.0102564102564103YPL061W		0.006YJL060W		0.0675675675675676YLR240W		0.0228571428571429YGR104C		0.195439739413681YER174C		0.163934426229508YMR146C		0.0201729106628242YPR057W		0.0909090909090909YDR156W		0.627737226277372YJR113C		0.311740890688259YPR131C		0.123076923076923YMR155W		0.135283363802559YNL180C		0.341389728096677YDR272W		0YOR238W		0.0627062706270627YLR139C		0.222395023328149YKL142W		0.438356164383562YPR009W		0.23134328358209YHR032W		0.209982788296041YDR429C		0.317518248175182YNR031C		0.258391386953768YGL079W		0.486238532110092YOR004W		0.377952755905512YMR244W		0.138028169014085YMR233W		0.442477876106195YNL188W		0.771362586605081YNL104C		0.0936995153473344YOR365C		0.00568990042674253YNR030W		0.0127041742286751YBR265W		0.040625YGL131C		0.339272986457591YNR040W		0.16015625YOR173W		0.0623229461756374YOR337W		0.226613965744401YNL181W		0.0786240786240786YGR243W		0.116438356164384YDR464W		0.902439024390244YGR210C		0.0097323600973236YOR168W		0.0716934487021014YNL002C		0.273291925465839YNL294C		0.189493433395872YHR193C		0.362068965517241YPR147C		0.0230263157894737YBR092C		0.0107066381156317YOR242C		0.393530997304582YJL176C		0.638787878787879YHR128W		0YOR297C		0.114583333333333YDR225W		0.363636363636364YPR020W		0.0260869565217391YPR114W		0.0253968253968254YPL189W		0.0410509031198686YJL056C		0.517045454545455YPL247C		0.1434034416826YDR482C		1YJL122W		0.811428571428571YBL058W		0.515366430260047YKL107W		0.0194174757281553YCL017C		0.0523138832997988YER085C		0.046242774566474YMR186W		0.154609929078014YCR065W		0.764184397163121YFL045C		0.00393700787401575YNL020C		0.515673981191223YGL125W		0.00833333333333333YLR354C		0.0417910447761194YFR008W		0.312217194570136YDR284C		0.103806228373702YKL051W		0.107648725212465YPR174C		0.588235294117647YMR091C		0.331034482758621YJL143W		0.132911392405063YGR057C		0.0785123966942149YLR308W		0.00961538461538462YBR065C		0.203296703296703YMR029C		0.250478011472275YPR121W		0.0174825174825175YNL068C		0.675174013921114YGL178W		0.545983701979045YGL157W		0.0144092219020173YMR148W		0.317567567567568YML058W		0.846153846153846YLR249W		0.053639846743295YGR019W		0.0424628450106157YBR263W		0.0122448979591837YPR044C		0.427350427350427YDR286C		0.0350877192982456YMR076C		0.132341425215348YJR135W-A		0.402298850574713YDR083W		0.443877551020408YLR109W		0YOR032C		0.774193548387097YOR091W		0.47536231884058YBR205W		0.00495049504950495YLR292C		0YLR216C		0.0754716981132075YDR046C		0.165562913907285YML018C		0.0763358778625954YOR369C		0.20979020979021YOR138C		0.184798807749627YJR145C		0.0574712643678161YGR246C		0.416107382550336YJR138W		0.320075757575758YHR059W		0.261538461538462YLR138W		0.551269035532995YNL156C		0.354515050167224YNL106C		0.252747252747253YNL286W		0.319298245614035YNL093W		0.177272727272727YIL040W		0.072463768115942YLR186W		0.138888888888889YDR425W		0.4144YDR293C		0.3912YNR015W		0.0859375YLR456W		0.127450980392157YDR448W		0.195852534562212YER184C		0.141057934508816YJL066C		0.0555555555555556YOR194C		0.622377622377622YLR368W		0.0217391304347826YKL095W		0.460431654676259YGR060W		0.0453074433656958YBR115C		0.0301724137931034YBR147W		0.0540540540540541YHR086W		0.41491395793499YFL003C		0.10250569476082YOR125C		0.206008583690987YML079W		0.174129353233831YPL134C		0.0419354838709677YNL298W		0.475059382422803YGL225W		0YNL224C		0.59973924380704YNL122C		0.408695652173913YLR129W		0.00424178154825027YGL066W		0.785388127853881YNL217W		0.0552147239263804YGL063W		0YML030W		0.415094339622642YAL039C		0.360594795539033YOR261C		0.112426035502959YLR191W		0.484455958549223YCR027C		0.076555023923445YDL046W		0.0404624277456647YMR263W		0.611940298507463YOL113W		0.299236641221374YDR517W		0.32258064516129YAL001C		0.212068965517241YBR197C		0.654377880184332YJR132W		0.00190839694656489YDL106C		0.652951699463327YNL280C		0.0319634703196347YDR505C		0.623067776456599YDL154W		0.0610432852386237YMR211W		0.117894736842105YGL202W		0.07YBL036C		0.0194552529182879YPR178W		0.139784946236559YOL147C		0.0720338983050847YFR007W		0.0056657223796034YGR072W		0.731266149870801YER022W		0.336244541484716YIL118W		0.0779220779220779YJR008W		0.0355029585798817YCL010C		0.193050193050193YJR024C		0.069672131147541YFR011C		0.741176470588235YAL047C		0.416398713826367YDR256C		0.0563106796116505YNL287W		0.0983957219251337YGR103W		0.383471074380165YOR281C		0.43006993006993YBR162C		0.175824175824176YMR114C		0.320652173913043YGR251W		0.928571428571429YBR094W		0.0637450199203187YKL021C		0.117521367521368YOR374W		0.0269749518304432YHL026C		0.146031746031746YHR014W		0.529209621993127YDL015C		0.0935483870967742YCR028C		0.111328125YER058W		0.542056074766355YCR016W		0.66551724137931YER149C		0.69047619047619YJL147C		0.18848167539267YGL222C		1YDR533C		0YDL090C		0.0951276102088167YBR177C		0.0155210643015521YJL034W		0.0601173020527859YNR059W		0.00172413793103448YGR156W		0.604705882352941YBR125C		0.0407124681933842YBR173C		0.337837837837838YHR057C		0.00975609756097561YOR077W		0.288793103448276YLR234W		0.00457317073170732YHR107C		0.157248157248157YER112W		0.524064171122995YLR174W		0.00485436893203883YCL026C-A		0YDR346C		0.158004158004158YLR385C		0.0378787878787879YLR117C		0.0116448326055313YDR143C		0.783606557377049YER185W		0.0165016501650165YOL013C		0.214156079854809YLR089C		0.128378378378378YKL155C		0.179936305732484YGR154C		0.0393258426966292YNL277W		0.0823045267489712YOL061W		0.00403225806451613YFR013W		0.433290978398984YDL014W		0.262996941896024YCL064C		0.00555555555555556YDR493W		0.24390243902439YLR345W		0.0923379174852652YLR192C		0.664150943396226YOR265W		0.254716981132075YDR031W		0.0165289256198347YBR141C		0.109792284866469YDR453C		0.0102040816326531YAL008W		0.0101010101010101YDR490C		0.486945169712794YDR191W		0.151351351351351YNL193W		0.161290322580645YLR360W		0.0842824601366743YOR142W		0.0182370820668693YDR304C		0.0844444444444444YPL020C		0.557165861513688YLR165C		0.0078740157480315YHL023C		0.471204188481675YOR283W		0.0347826086956522YMR255W		0.946808510638298YPL154C		0.00493827160493827YDR033W		0.1375YDR253C		0.743455497382199YNL014W		0.0660919540229885YAL032C		0.477572559366755YNL291C		0.0182481751824818YOR348C		0.18341307814992YMR104C		0.41506646971935YOR097C		0.491428571428571YOL126C		0.0291777188328912YOL107W		0.0760233918128655YHR026W		0.0563380281690141YGR028W		0.143646408839779YGL061C		0.538461538461538YJL089W		0.283474065138721YKL116C		0.341698841698842YHR134W		0.219330855018587YBR228W		0.0986842105263158YNL320W		0.0352112676056338YGR123C		0.103313840155945YMR220W		0.0266075388026608YBR269C		0.840579710144927YMR188C		0.185654008438819YLR290C		0.0180505415162455YNL258C		0.176392572944297YNL075W		0.2YPR159W		0.334722222222222YHR089C		0.560975609756098YNR018W		0.160714285714286YAL044C		0.00588235294117647YKL139W		0.388257575757576YHR129C		0.0130208333333333YOR253W		0.0397727272727273YIL105C		0.43731778425656YDR403W		0.0634328358208955YDL029W		0YDR525W-A		0.240506329113924YPL255W		0.503896103896104YEL012W		0.31651376146789YOR192C		0.125208681135225YGL229C		0.278728606356968YOL005C		0.0416666666666667YML052W		0.23841059602649YML064C		0.204081632653061YHL019C		0.203305785123967YBR167C		0.257142857142857YPR176C		0YFL016C		0.320939334637965YLR383W		0.0601436265709156YJL062W		0.00120481927710843YDR140W		0YNL252C		0.0818505338078292YMR196W		0.153492647058824YOR205C		0.0611510791366906YER114C		0.772115384615385YGL258W		0.00970873786407767YIL002C		0.100422832980973YDR518W		0.0483558994197292YLR328W		0.356608478802993YKL173W		0.0863095238095238YBL003C		0.356060606060606YNR013C		0.138702460850112YJR077C		0.0257234726688103YPR078C		0.56989247311828YGL058W		0.151162790697674YFL005W		0.134883720930233YMR284W		0.0465116279069767YML061C		0.217694994179278YLR449W		0.372448979591837YDL007W		0.187643020594966YOR145C		0.339416058394161YOR287C		0.946666666666667YIL122W		0.834757834757835YPL218W		0YJL145W		0.0204081632653061YPR198W		0.0441988950276243YNR052C		0.337182448036952YOR244W		0.195505617977528YMR314W		0.00854700854700855YLR173W		0.0411184210526316YHR190W		0.0202702702702703YPL097W		0.0182926829268293YNL115C		0.125776397515528YLR103C		0.133846153846154YKL132C		0.0162790697674419YPL222W		0.061046511627907YKL012W		0.205831903945111YLR395C		0.0769230769230769YAL041W		0.413348946135831YBL052C		0.404332129963899YCL045C		0.0171052631578947YDL135C		0.198019801980198YMR237W		0.107734806629834YGR046W		0.0883116883116883YGR224W		0.0962479608482871YDR308C		0.0785714285714286YJL153C		0.0187617260787993YBR035C		0.0394736842105263YCR002C		0.0838509316770186YDR244W		0.320261437908497YAL016W		0.0551181102362205YPR093C		0.100694444444444YMR239C		0.337579617834395YLR163C		0.0670995670995671YDL072C		0.261083743842365YPL190C		0.756857855361596YER059W		0.535714285714286YBR207W		0.247311827956989YIL049W		0.0118577075098814YBR069C		0.159935379644588YGL215W		0.559734513274336YPR066W		0.00334448160535117YOR135C		0.079646017699115YNL202W		0.0273972602739726YLR099C		0.0203045685279188YOR080W		0.049597855227882YNL133C		1YJL167W		0.0170454545454545YIL097W		0.0872093023255814YOR198C		0.514893617021277YNL101W		0.359046283309958YGL241W		0.0448207171314741YML055W		0.0393258426966292YHR143W-A		0.357142857142857YFL052W		0.133333333333333YML035C		0.230864197530864YGR133W		0.00546448087431694YDR357C		0.163934426229508YDR006C		0.450610432852386YNL256W		0.0121359223300971YML100W		0.298724954462659YHR197W		0.243774574049803YNL325C		0.178612059158134YBR128C		0.188953488372093YPL258C		0.0308529945553539YNL216W		0.551390568319226YIL036W		0.908006814310051YLR307W		0.00664451827242525YML119W		0.509803921568627YPL273W		0.0184615384615385YCL066W		0.434285714285714YPL201C		0.0954446854663774YHR003C		0.0559440559440559YPR041W		0.380246913580247YHR171W		0.0253968253968254YER116C		0.766423357664234YOR136W		0.024390243902439YOL086W-A		0.0444444444444444YLR364W		0.018348623853211YGR142W		0.541463414634146YKL186C		0.168478260869565YGR264C		0.00133155792276964YDR206W		0.343891402714932YPR042C		0.610232558139535YJR152W		0.106813996316759YBR085W		0.0162866449511401YKL106W		0.0155210643015521YBR281C		0.0159453302961276YDL153C		0.649180327868852YBL078C		0.205128205128205YDL150W		0.838862559241706YDR398W		0.219284603421462YIL077C		0.45625YGR038W		0.274774774774775YMR153W		0.707368421052632YPL266W		0.0660377358490566YPL264C		0.0991501416430595YHR132C		0.0162790697674419YLR336C		0.406006674082314YER115C		0.141361256544503YDR441C		0YDR101C		0.0708263069139966YMR121C		0.254901960784314YHL014C		0YMR312W		0.0366300366300366YOR382W		0.522875816993464YOR214C		0.0127118644067797YLR141W		0.327823691460055YJL030W		0.0408163265306122YDR410C		0.0460251046025105YOR335C		0YNL087W		0.134125636672326YHR015W		0.244309559939302YGL037C		0.0648148148148148YDR502C		0.0130208333333333YAR018C		0.268965517241379YOR112W		0.304862023653088YKL045W		0.138257575757576YBR258C		0.253521126760563YPR074C		0.0147058823529412YHR162W		0.0387596899224806YLR262C-A		1YML013W		0.138698630136986YPL003W		0.00865800865800866YLR297W		0.193798449612403YMR319C		0.134057971014493YDL127W		0.418831168831169YOR152C		0.2421875YDL216C		0.420454545454545YFR028C		0.361161524500907YBR068C		0.169129720853859YHR126C		0.113207547169811YHR008C		0.0858369098712446YOL001W		0.290102389078498YML111W		0.333695652173913YMR016C		0.850955414012739YKL023W		0.891696750902527YBR133C		0.124546553808948YGR229C		0.475247524752475YOR092W		0.362153344208809YNR010W		0.449664429530201YPL001W		0.0267379679144385YDR047W		0.00276243093922652YBR159W		0.0230547550432277YGR158C		0.148YOR210W		0YKL174C		0.153721682847896YLR403W		0.784773060029283YLR286C		0.382562277580071YBR274W		0.16888045540797YDL044C		0.288636363636364YNL305C		0.0942760942760943YER081W		0.106609808102345YDR148C		0.321814254859611YJL059W		0.0906862745098039YIL130W		0.411825726141079YEL044W		0.632530120481928YGL108C		1YOR351C		0.134808853118712YGL023C		0.84251968503937YBL066C		0.551393728222996YBL051C		0.781437125748503YJR068W		0.0679886685552408YDL168W		0.0103626943005181YBR020W		0.0170454545454545YDL049C		0YAL019W		0.475685234305924YDR484W		0.109204368174727YEL037C		0.49748743718593YBR273C		0.467889908256881YDL114W		0.00324675324675325YIL062C		0.123376623376623YML091C		0.0740432612312812YCL021W-A		0YOR372C		0.862815884476534YGL098W		0.289795918367347YNL329C		0.0398058252427184YMR199W		0.32967032967033YDL209C		0.35693215339233YMR300C		0.0215686274509804YCR039C		0.685714285714286YIL107C		0.423216444981862YIL057C		0.128048780487805YGL136C		0YHR024C		0.0601659751037344YLR120C		0.0878734622144113YGR211W		0.127572016460905YLR237W		0.118729096989967YBL007C		0.654340836012862YNR064C		0.0137931034482759YPL075W		0.463694267515924YPL017C		0.0240480961923848YBR301W		0YML004C		0.0766871165644172YMR202W		0YGR292W		0.0102739726027397YNL267W		0.401500938086304YEL029C		0.0224358974358974YLR250W		0.00854700854700855YGR245C		0.358539765319426YNL040W		0.00657894736842105YIL003W		0.0614334470989761YGL161C		0.287096774193548YPR144C		0.0561594202898551YOR359W		0.812619502868069YFL055W		0.0806451612903226YGR181W		0.504761904761905YGR070W		0.289177489177489YPL029W		0.0230664857530529YLR116W		0.584033613445378YDR197W		0.0077120822622108YPL224C		0.142538975501114YPL172C		0.164502164502165YJR144W		0.241635687732342YMR083W		0.0506666666666667YGR086C		0.339233038348083YCR048W		0.188524590163934YDL065C		0.804093567251462YMR126C		0.280701754385965YDR175C		0.0470219435736677YER032W		0.848173515981735YGR194C		0.0333333333333333YGR078C		0.0301507537688442YMR265C		0.0433839479392625YHR163W		0.0160642570281124YOR222W		0.0456026058631922YBL103C		0.753086419753086YIL052C		0.15702479338843YBR242W		0.0168067226890756YBL021C		0.354166666666667YNL246W		0.170454545454545YOL030W		0.243801652892562YER012W		0YLR301W		0YPR139C		0.133333333333333YPL202C		0.442307692307692YOL068C		0.101391650099404YLR114C		0.391361256544503YJL019W		0.231671554252199YLR248W		0.459016393442623YIL020C		0.00383141762452107YNL063W		0YOR391C		0YFR044C		0.0103950103950104YFL023W		0.743718592964824YDR212W		0.0447227191413238YOR103C		0.176923076923077YMR185W		0.110091743119266YJL091C		0.0489795918367347YDR065W		0.0684931506849315YHR174W		0.0045766590389016YLR441C		0.149019607843137YBR204C		0.0853333333333333YLR283W		0.378980891719745YGL174W		0.646616541353383YGR145W		0.371994342291372YGR199W		0.069828722002635YDR061W		0.0166975881261596YBL005W		0.289959016393443YIL108W		0.0373563218390805YDR404C		0YOR058C		0.361581920903955YBL104C		0.171483622350674YBR095C		0.334883720930233YBR084W		0.0041025641025641YMR173W		1YHR085W		0.296407185628743YOR301W		0.245977011494253YDL210W		0.0788091068301226YNL308C		0.697123519458545YOR171C		0.150641025641026YIL050W		0.396491228070175YBL099W		0.0422018348623853YLR359W		0.012448132780083YPL217C		0.34742180896027YNR009W		0.91566265060241YGL194C		0.0995575221238938YOR106W		0.438162544169611YJR056C		0.724576271186441YLR287C-A		1YDR044W		0.0457317073170732YLR118C		0.0220264317180617YDR387C		0.0432432432432432YOL130W		0.509895227008149YIL071C		0.137387387387387YDR169C		0.713450292397661YER178W		0.14047619047619YMR210W		0.0601336302895323YOR338W		0.443526170798898YER088C		0.943283582089552YDR115W		0.895238095238095YOR334W		0.146808510638298YGR256W		0.00609756097560976YMR107W		0.730434782608696YDR451C		0.875354107648725YCL063W		0.607565011820331YJL192C		0.0341880341880342YHR177W		0.631346578366446YBR039W		0YIR027C		0.0152173913043478YAL042W		0.0578313253012048YPR036W		0.00209205020920502YIL007C		0.0818181818181818YOR030W		0.234248788368336YPL267W		0.344497607655502YMR243C		0.289592760180996YDR188W		0.00915750915750916YNR034W-A		0.173469387755102YEL023C		0.164222873900293YNL045W		0.022354694485842YPR141C		0.414266117969822YIL104C		0.155818540433925YNL282W		0.0461538461538462YLR424W		0.305084745762712YDR520C		0.446891191709845YBR243C		0.00223214285714286YGL137W		0.093363329583802YNL212W		0.120204603580563YMR234W		0.146551724137931YMR112C		0.251908396946565YHR005C-A		0.258064516129032YGL212W		0.183544303797468YNR023W		0.30565371024735YPL253C		0.228748068006182YBR096W		0.0260869565217391YJR042W		0.0577956989247312YGR150C		0.103009259259259YLR390W-A		0.663865546218487YGR074W		0.23972602739726YHR043C		0.00813008130081301YGL180W		0.40133779264214YDR085C		0.664516129032258YBR146W		0.165467625899281YPL043W		0.41021897810219YHR011W		0YDL235C		0.137724550898204YLR278C		0.65324384787472YLR348C		0.0268456375838926YGL158W		0.294921875YAL028W		0.685606060606061YDL002C		0.541871921182266YDR099W		0.201465201465201YNL173C		0.729508196721312YMR216C		0.477088948787062YMR306W		0.0661064425770308YLR196W		0.135416666666667YBR085C-A		0.105882352941176YDL059C		0.0714285714285714YOR329C		0.868119266055046YJR116W		0YHL004W		0.370558375634518YBR073W		0.230519480519481YDR449C		0.0795454545454545YDL088C		0.676136363636364YNL161W		0.514550264550265YDR435C		0.0121951219512195YDL052C		0.115511551155116YMR144W		0.769005847953216YDR161W		0.0439276485788114YER062C		0.024YCR068W		0.153846153846154YPR051W		0.0511363636363636YOR285W		0.0575539568345324YLR253W		0.0562390158172232YJL001W		0.0372093023255814YHR067W		0.0464285714285714YGL237C		0.883018867924528YDR120C		0.103508771929825YJL012C		0.0970873786407767YER126C		0.486590038314176YMR246W		0.00864553314121038YPL031C		0.0295081967213115YLR201C		0.0269230769230769YHR091C		0.00933125972006221YGR020C		0YML066C		0.0894308943089431YDR501W		0.54510556621881YNL088W		0.207282913165266YNL053W		0.662576687116564YDR400W		0.0117647058823529YDR137W		0.351432880844646YBR231C		0.66996699669967YDL120W		0.0977011494252874YLR151C		0.0794117647058823YFR021W		0.036YML121W		0.0645161290322581YLR310C		0.375707992448081YGR244C		0.0655737704918033YGL255W		0.0984042553191489YHR046C		0.0542372881355932YOR016C		0.0193236714975845YML065W		0.281181619256017YPR005C		0.340136054421769YGR223C		0.0200892857142857YAR003W		0.00234741784037559YMR267W		0.0483870967741935YNL077W		0.422348484848485YJR034W		0.240740740740741YPL160W		0.0412844036697248YGL185C		0.0158311345646438YKL175W		0.407554671968191YDR456W		0.18957345971564YGL190C		0.0798479087452472YOR101W		0.436893203883495YMR262W		0.00638977635782748YGL005C		0.150537634408602YNL125C		0.285289747399703YBL019W		0.238461538461538YBR043C		0.284470246734398YDL231C		0.155555555555556YML101C		0.384615384615385YPR007C		0.405882352941176YMR108W		0.0858806404657933YIL067C		0.0619469026548673YPL126W		0.046875YKL179C		0.56259204712813YJL033W		0.406493506493507YEL041W		0.216161616161616YGL208W		0.563855421686747YNL113W		0.352112676056338YGL025C		0.73551637279597YBR171W		0.0776699029126214YDL005C		0.844547563805104YNL031C		0.404411764705882YDL056W		0.301320528211285YML120C		0.0467836257309941YGR048W		0.426592797783933YOL149W		0.151515151515152YML129C		0.242857142857143YBR220C		0.0160714285714286YBR227C		0.271153846153846YNR056C		0.017825311942959YER165W		0.327556325823224YNL327W		0.857829010566763YOR130C		0.0102739726027397YPL099C		0.307692307692308YER016W		0.354651162790698YNL242W		0.175879396984925YMR058W		0.0613207547169811YOL078W		0.585884353741497YAL018C		0.0369230769230769YEL002C		0.027906976744186YJL156C		0.285298398835517YMR166C		0.152173913043478YCL032W		0.468208092485549YPL050C		0.0278481012658228YDR510W		0.267326732673267YLR327C		0.186046511627907YLR142W		0.111344537815126YLR104W		0.152671755725191YNL222W		0.029126213592233YLR291C		0.047244094488189YBR293W		0.0168776371308017YLR412W		0.0766423357664234YNR067C		0.354521038495971YNL074C		0.820796460176991YBR021W		0.105845181674566YML021C		0.286908077994429YER038C		0.265086206896552YNL044W		0.232954545454545YER060W-A		0.0716981132075472YER173W		0.241274658573596YOR190W		0.00224719101123596YML048W		0.0942928039702233YBR130C		0.783529411764706YPR072W		0.617857142857143YHR048W		0.0875486381322957YHL034C		0.32312925170068YHL006C		0.0866666666666667YDR418W		0.0545454545454545YLR183C		0.539877300613497YIR024C		0.203703703703704YCR038C		0.0638629283489097YJR040W		0.0436456996148909YJL037W		0.0892857142857143YOR059C		0YNR002C		0.134751773049645YMR172C-A		0YIL158W		0.406862745098039YER048W-A		0YGR278W		0.211438474870017YDR462W		0.26530612244898YMR273C		0.86120218579235YJL162C		0.500857632933105YPR080W		0.0262008733624454YFL017W-A		0.025974025974026YGL127C		0.133858267716535YGR268C		0.737373737373737YHL033C		0.29296875YMR285C		0.240776699029126YBR104W		0.103343465045593YPL263C		0.30568356374808YER096W		0.296875YOR325W		0.0445859872611465YDR311W		0.341121495327103YKL088W		0.632224168126095YJL046W		0.0171149144254279YLR228C		0.597051597051597YKL056C		0.0179640718562874YPL037C		0.528662420382166YDR459C		0.0909090909090909YOR357C		0.179012345679012YNR004W		0.00684931506849315YNL192W		0.351016799292661YGL068W		0.190721649484536YDL157C		0.101694915254237YOR007C		0.208092485549133YBR105C		0.232044198895028YDL013W		0.489499192245557YGL200C		0.00492610837438424YDR260C		0.223529411764706YNR072W		0.104609929078014YBR154C		0.00930232558139535YPL156C		0.387323943661972YKL048C		0.475YJL020C		0.727744165946413YLR251W		0.0710659898477157YGR113W		0.740524781341108YBR253W		0.15702479338843YCR020C		0YJR141W		0.0576368876080692YHR060W		0.353591160220994YDR361C		0.243816254416961YPL009C		0.290944123314065YNL027W		0.824483775811209YNL065W		0.16382252559727YNL211C		0.174418604651163YHL038C		0.0111111111111111YPL070W		0.483660130718954YOL111C		0.405660377358491YDR075W		0YER009W		0YFL034C-B		0.344947735191638YIR011C		0.410658307210031YMR297W		0.0432330827067669YIL139C		0YDR254W		0.15938864628821YPL005W		0.242574257425743YGL105W		0.202127659574468YDL224C		0.731895223420647YKL062W		0.938095238095238YJL158C		0.242290748898678YDR162C		0.572033898305085YCR059C		0.0155038759689922YDL183C		0.10625YCL039W		0.0120805369127517YER140W		0.289568345323741YPL069C		0.00597014925373134YOR386W		0.0761061946902655YPL151C		0.0155210643015521YNL042W		0.813131313131313YHR207C		0.129277566539924YDR171W		0.466666666666667YPL157W		0.196825396825397YDR479C		0.296028880866426YAL034W-A		0.422145328719723YOR258W		0.0230414746543779YNR057C		0.0506329113924051YIL015W		0.155025553662692YHR127W		0.329218106995885YNL328C		0YCL049C		0.182692307692308YHR111W		0.0840909090909091YGL055W		0.119607843137255YBR288C		0.10351966873706YOR347C		0YGL065C		0.0159045725646123YHR013C		0.109243697478992YOR307C		0.134657836644592YNL144C		0.504054054054054YIR038C		0.0128205128205128YAL046C		0.152542372881356YPL193W		0.640419947506562YGR208W		0.0161812297734628YDR201W		0.533333333333333YML077W		0.10062893081761YGL245W		0.0268361581920904YHR058C		0.301694915254237YPR181C		0.05859375YBR106W		0.207446808510638YHR018C		0.00647948164146868YBR170C		0.194827586206897YDL211C		0.336021505376344YPL078C		0.180327868852459YOL007C		0.621700879765396YDR399W		0.0678733031674208YKL181W		0.0163934426229508YGR192C		0YDL078C		0.00291545189504373YOL041C		0.625272331154684YMR226C		0.0299625468164794YHR200W		0.287313432835821YPR125W		0.337004405286344YEL046C		0.0103359173126615YIR015W		0.145833333333333YMR242C		0.00561797752808989YDR204W		0.0835820895522388YPL161C		0.0126382306477093YDR300C		0.0186915887850467YLR229C		0YLR421C		0.185897435897436YPL096W		0.154269972451791YML038C		0.14027149321267YJL082W		0.177838577291382YIL006W		0.0697050938337802YLR392C		0.133204633204633YGR075C		0.103305785123967YBR268W		0.0380952380952381YHR068W		0.0387596899224806YGL160W		0.107017543859649YGR042W		0.616236162361624YPR160W		0.0521064301552106YER007W		0YGR095C		0YDL089W		0.326446280991736YDR522C		0.0896414342629482YEL062W		0.305691056910569YBR026C		0.00526315789473684YLR143W		0.0408759124087591YMR125W		0.0452961672473868YLR126C		0YGL086W		0.45260347129506YNL129W		0YGL048C		0.0296296296296296YER106W		0.533112582781457YGR169C		0.0272277227722772YMR074C		0.655172413793103YMR269W		0.630331753554502YHR138C		0.0175438596491228YFL021W		0.866666666666667YPL144W		0.00675675675675676YEL053C		0.0518417462482947YHL020C		0.655940594059406YGR106C		0.116981132075472YLR452C		0.163323782234957YMR005W		0.621134020618557YLR363C		0.0688073394495413YIL023C		0.0491329479768786YKL138C		0.0687022900763359YGL002W		0YOR312C		0.00574712643678161YLR179C		0.0248756218905473YNL230C		0.651715039577836YHL028W		0.803305785123967YCR015C		0.0126182965299685YPR199C		0.26530612244898YMR313C		0.00934579439252336YBR077C		0YOR298W		0.0396659707724426YDR380W		0.0425196850393701YDL177C		0.111764705882353YGR149W		0.173611111111111YPL203W		0.181578947368421YNR021W		0.121287128712871YGR080W		0.144578313253012YBR195C		0.0165876777251185YPL191C		0.241666666666667YMR013C		0YPL148C		0.0520231213872832YFL038C		0.184466019417476YPL240C		0.155148095909732YGL145W		0.0485021398002853YPR060C		0.01171875YNL004W		0.370629370629371YLR232W		0.234782608695652YHR069C		0.103064066852368YEL018W		0.150537634408602YLR323C		0.447876447876448YGL172W		0.625YDL143W		0.0397727272727273YKL162C		0.00497512437810945YMR290C		0.132673267326733YDL021W		0.0160771704180064YPL211W		0YDR452W		0.140949554896142YAL038W		0YMR024W		0.156410256410256YLR147C		0.158415841584158YDL018C		0YML012W		0.004739336492891YIL061C		0.46YGR155W		0.0236686390532544YLR136C		0.750877192982456YDL060W		0.263959390862944YIL085C		0.00967117988394584YHR075C		0.2125YOL119C		0.155688622754491YNL289W		0.333333333333333YJR105W		0.0176470588235294YER161C		0.975975975975976YNR036C		0.444444444444444YMR111C		0.586580086580087YJL096W		0.0869565217391304YBR136W		0.00717905405405405YMR090W		0YNR061C		0.264840182648402YNL154C		0.461538461538462YGR209C		0.0192307692307692YDL181W		1YKL029C		0.122571001494768YPR106W		0.275395033860045YNL039W		0.765993265993266YDL149W		0.413239719157472YGR187C		0.124365482233503YBR282W		0.0410958904109589YER170W		0.00444444444444444YPL269W		0.417701863354037YHR137W		0.0448343079922027YGR058W		0.459701492537313YGL009C		0.0962772785622593YBL009W		0.52810650887574YDR279W		0.145714285714286YLR243W		0.00735294117647059YER029C		0.540816326530612YJL025W		0.0466926070038911YDR246W		0.150684931506849YDR427W		0.00508905852417303YER182W		0.0368852459016393YNL163C		0.100900900900901YMR228W		0.0263929618768328YDR411C		0.255131964809384YNL061W		0.398058252427184YDR309C		0.757180156657963YER095W		0.2YJL138C		0.0430379746835443YDR499W		0.248995983935743YJL063C		0.180672268907563YMR206W		0.623003194888179YMR154C		0.00412654745529574YLR410W		0.242582897033159YER092W		0.2YBR101C		0.0448275862068966YIL042C		0.0532994923857868YHR112C		0YGL029W		1YGR160W		0.83743842364532YJR060W		0.735042735042735YDR437W		0.0357142857142857YHL022C		0.0527638190954774YGL175C		0.518840579710145YLR305C		0.0336842105263158YIL124W		0.0336700336700337YGR234W		0.0150375939849624YCR004C		0.186234817813765YKL028W		0.547717842323652YPR173C		0.258581235697941YPR137W		0.174520069808028YAL011W		0.6224YNL067W		0.00523560209424084YEL016C		0.0202839756592292YEL020C		0.0142857142857143YPL060W		0.0968523002421307YER087W		0.00347222222222222YOL087C		0.282258064516129YNL304W		0.434052757793765YHR056C		0.229898074745187YNL092W		0.06YPL152W		0.128491620111732YKL033W		0.10019267822736YHR214W		0.133004926108374YGL243W		0.1625YBR025C		0.0228426395939086YNL208W		1YER055C		0.0101010101010101YJR123W		0.0888888888888889YHR065C		0.249500998003992YNL128W		0.0345622119815668YDR405W		0.418250950570342YMR139W		0.143243243243243YDL217C		0.0628019323671498YPL094C		0.164233576642336YJL157C		0.380722891566265YER120W		0.35655737704918YLR315W		0.0130718954248366YDR516C		0.024YPL106C		0.132756132756133YFR042W		0.03YLR256W		0.486684420772304YPR191W		0.0217391304347826YMR241W		0.0254777070063694YBR260C		0.355855855855856YLR396C		0.101302460202605YNR037C		0YPL243W		0.125208681135225YBR018C		0.00819672131147541YCR043C		0.188976377952756YPL270W		0.232858990944373YBR054W		0.194767441860465YNL164C		0.458689458689459YNL062C		0.228033472803347YMR298W		0.04YML106W		0.0619469026548673YMR037C		0.917613636363636YKL094W		0.0511182108626198YOR066W		0.75516693163752YJR147W		0.363128491620112YDR041W		0.187192118226601YMR272C		0.143229166666667YKL143W		0.693304535637149YDL081C		0.386792452830189YIL019W		0.667630057803468YDL201W		0.15034965034965YNL200C		0.016260162601626YER073W		0.0288461538461538YJR007W		0.0789473684210526YNL238W		0.192874692874693YBR053C		0.0195530726256983YCR073W-A		0.0603174603174603YOR269W		0.0121457489878543YPL163C		0.288461538461538YML125C		0.032051282051282YOL057W		0.0126582278481013YKL145W		0.137044967880086YIL150C		0.569176882661997YGR146C		0.710900473933649YDL126C		0.152095808383234YDL006W		0.0498220640569395YMR025W		0.0169491525423729YDL233W		0.541484716157205YCL057C-A		0.309278350515464YOR362C		0.149305555555556YMR276W		0.699731903485255YOR217W		0.337979094076655YML124C		0.0224719101123595YPL246C		0.118320610687023YDL178W		0YOR111W		0.021551724137931YNL240C		0.0122199592668024YLR152C		0.317708333333333YKL019W		0.0189873417721519YGR168C		0.0398936170212766YGR027C		0.314814814814815YGL250W		0.319502074688797YEL031W		0.0650205761316872YDR320C-A		0.0416666666666667YIL099W		0.0218579234972678YJR135C		0.158995815899582YIR013C		0.760330578512397YIL094C		0.0107816711590297YMR032W		0.478325859491779YPL112C		0.233502538071066YAL049C		0.0040650406504065YMR010W		0.148148148148148YBR193C		0.31390134529148YCR023C		0.180032733224223YHL009C		0.772727272727273YDL086W		0.021978021978022YOR201C		0.276699029126214YPR109W		0.129251700680272YIL038C		0.688995215311005YPL137C		0.513322884012539YML082W		0.103235747303544YPL233W		0.087962962962963YJL004C		0.182266009852217YOR286W		0YOR315W		0.398843930635838YNL010W		0.004149377593361YIL087C		0.43312101910828YHR061C		0.719745222929936YPL111W		0.021021021021021YGL146C		0.0418006430868167YBR182C		0.619469026548673YPR015C		0.692307692307692YER026C		0.257246376811594YOL036W		0.813403416557162YPR100W		0.0214285714285714YDL139C		0.645739910313901YNR033W		0.0343074968233799YKL149C		0.0839506172839506YBL093C		0.468181818181818YPR122W		0.0504966887417219YOL009C		0.151291512915129YER124C		0.116928446771379YGR253C		0.0615384615384615YDR055W		0.184684684684685YDL110C		0.426666666666667YOR049C		0.19774011299435YMR027W		0.00851063829787234YJR032W		0.0483460559796438YIL044C		0.533557046979866YBR011C		0.0696864111498258YDR419W		0.197784810126582YBR110W		0YLR247C		0.132390745501285YPL208W		0.00514579759862779YOR319W		0.169014084507042YLR371W		0.429941002949852YDL051W		0.312727272727273YJL072C		0.0751173708920188YHR081W		0.494565217391304YOR061W		0.056047197640118YDR090C		0.145161290322581YPL256C		0.354128440366972YOR070C		0.34850863422292YNL024C		0.024390243902439YJL217W		0.0353535353535354YGL093W		0.526717557251908YDR523C		0.328571428571429YKL098W		0.0504201680672269YCR008W		0.507462686567164YOR298C-A		0.695364238410596YOL059W		0.05YOR250C		0.0314606741573034YGR110W		0.125842696629213YHL036W		0.102564102564103YJR088C		0.0650684931506849YLR378C		0YHR020W		0.0828488372093023YMR235C		0.199017199017199YBL050W		0.0376712328767123YNL185C		0.0506329113924051YFR036W		0.766129032258065YMR067C		0.286057692307692YML086C		0.026615969581749YMR167W		0.202860858257477YDR016C		0.425531914893617YGR062C		0.0854430379746835YIL113W		0.244019138755981YNL234W		0.476525821596244YDR414C		0.0414364640883978YPL045W		0.0012531328320802YGR189C		0.467455621301775YCR086W		0.126315789473684YHR039C		0.0232919254658385YFL044C		0.149501661129568YIL009W		0.0273775216138329YNL046W		0.430232558139535YOR355W		0.689655172413793YPL265W		0.152960526315789YDL128W		0.0997566909975669YHR090C		0.397163120567376YDR214W		0.131428571428571YPR061C		0.0664451827242525YGR267C		0.189300411522634YGR185C		0.101522842639594YML050W		0.067524115755627YNL078W		0.616707616707617YMR158W		0YOR262W		0.146974063400576YHR016C		0.391025641025641YFL004W		0.158212560386473YDL045W-A		0.294736842105263YNL290W		0.0441176470588235YMR132C		0.120192307692308YDR358W		0.387791741472172YGL085W		0.0109489051094891YBR010W		0.419117647058824YML008C		0.0391644908616188YIL119C		0.614250614250614YDR073W		0.437869822485207YOR180C		0.022140221402214YPL170W		0.118421052631579YGL139W		0.261845386533666YLR287C		0.166197183098592YBR248C		0.016304347826087YAL044W-A		0.145454545454545YGL140C		0.162428219852338YGR180C		0.150724637681159YPL229W		0.703883495145631YDR079W		0.36036036036036YDR183W		0.195652173913043YDR205W		0.107734806629834YER118C		0.425068119891008YKL144C		0.113207547169811YLR343W		0.16036036036036YMR064W		0.00193050193050193YGR193C		0.14390243902439YML041C		0.528571428571429YMR260C		0.34640522875817YOR060C		0.143968871595331YHR191C		0.0150375939849624YDL170W		0.0890151515151515YDR185C		0.00558659217877095YPL153C		0.345919610231425YML072C		0.248543689320388YGR036C		0.0376569037656904YKL086W		0.015748031496063YGL226W		0.024390243902439YBR283C		0.00816326530612245YGR196C		0.82374541003672YNL330C		0.0993071593533487YLR393W		0.21505376344086YHR189W		0YDR513W		0.034965034965035YPR069C		0.0307167235494881YNL064C		0.254278728606357YBR061C		0.0612903225806452YPL002C		0YJR053W		0.745644599303136YHR010W		0YER102W		0.105YKL074C		0.497153700189753YGR126W		0.969565217391304YAL036C		0.013550135501355YKL082C		0.930875576036866YGL010W		0.0114942528735632YHR195W		0.423676012461059YIL131C		0.429752066115702YDR287W		0.0308219178082192YMR002W		0.801282051282051YDR122W		0.554511278195489YIL127C		0.805825242718447YIR042C		0YBL068W		0.0152905198776758YPR055W		0.155868544600939YMR281W		0.0559210526315789YBR019C		0.0243204577968526YOR256C		0.112484548825711YER139C		0.314159292035398YNL174W		0.0157894736842105YBR160W		0.0100671140939597YMR006C		0.128895184135977YHR064C		0.033457249070632YMR174C		1YPL120W		0.308797127468582YOR151C		0.0130718954248366YPR025C		0.272264631043257YLR121C		0.108267716535433YNL220W		0.0069284064665127YJL186W		0.00853242320819113YGR263C		0.0141509433962264YIL024C		0.158730158730159YPR070W		0.319787985865724YMR087W		0.0352112676056338YDR068W		0.567741935483871YGR059W		0.31640625YKL080W		0.0102040816326531YGL246C		0.0439276485788114YBL075C		0.0600924499229584YHR108W		0.393162393162393YER035W		0.737931034482759YMR299C		0.0224358974358974YML093W		0.85650723025584YGL143C		0.036319612590799YNR073C		0.0199203187250996YMR030W		0.452127659574468YDL117W		0.351412429378531YOL075C		0.0301391035548686YOL056W		0.0033003300330033YIL022W		0.382830626450116YMR073C		0.567164179104478YDR224C		0.366412213740458YER171W		0.0758354755784062YNL141W		0.00288184438040346YDL010W		0.281385281385281YDR106W		0.0140845070422535YHR078W		0.0851449275362319YDR270W		0.0149402390438247YGR009C		0.792626728110599YGL122C		0.739047619047619YMR128W		0.265193370165746YEL013W		0.0536332179930796YLR268W		0YDL069C		0.00436681222707424YMR065W		0.0138888888888889YIL068C		0.0819875776397515YPR183W		0YLR387C		0.375YGR012W		0.0229007633587786YNL095C		0.383177570093458YBR007C		0.729619565217391YGL253W		0.0308641975308642YOR196C		0.140096618357488YDR233C		0.342372881355932YEL040W		0.244111349036403YNL250W		0.32469512195122YMR252C		0.0298507462686567YLR312W-A		0.0316205533596838YGR206W		0.0792079207920792YKL189W		0.175438596491228YER152C		0.0496613995485327YCL033C		0YGR006W		0.302788844621514YPL041C		0.318840579710145YOL131W		0.398148148148148YLR088W		0.0537459283387622YCR045C		0.00814663951120163YKL163W		0.544615384615385YPR149W		0.121387283236994YNL306W		0.161290322580645YBR034C		0.0517241379310345YPL187W		0YHR030C		0.276859504132231YDR384C		0.105454545454545YMR052W		0.397058823529412YGR124W		0.0034965034965035YIL123W		0.418947368421053YOR163W		0.122340425531915YJL178C		0.0959409594095941YLR352W		0.154894671623296YKL018C-A		0.282828282828283YPR112C		0.393461104847802YNR038W		0.297297297297297YDL192W		0.0110497237569061YPR157W		0.0278372591006424YIL109C		0.208423326133909YGL257C		0.014336917562724YER099C		0.00943396226415094YNL182C		0.0630630630630631YPR163C		0.704128440366973YML042W		0.0223880597014925YGR152C		0.375YDL124W		0.032051282051282YIL138C		1YDR268W		0.0765171503957784YOL064C		0.0280112044817927YER091C		0YOL110W		0.0717299578059072YHR132W-A		1YMR129W		0.081525804038893YLR153C		0.0351390922401171YOL128C		0.0586666666666667YOR085W		0.00285714285714286YJL190C		0YPL204W		0.419028340080972YOR159C		0.106382978723404YER072W		0.116279069767442YDL004W		0YJL164C		0.168765743073048YNL130C		0.00508905852417303YER100W		0.216YMR110C		0.0206766917293233YDR228C		0.472843450479233YFL048C		0.101123595505618YBR247C		0.416149068322981YNL091W		0.826612903225806YDL159W		0.431067961165049YLR347C		0.00696864111498258YDR159W		0.479631053036126YOR040W		0.0140350877192982YGL187C		0.032258064516129YJR095W		0.0124223602484472YNL029C		0.00383141762452107YJL013C		0.516504854368932YNL136W		0.882352941176471YDL058W		0.635754189944134YBL057C		0.214953271028037YBL087C		0.0072992700729927YGL130W		0.108932461873638YMR035W		0.0508474576271186YPL181W		0.798418972332016YOR075W		0.26878612716763YMR159C		0.206666666666667YOR211C		0.104426787741203YKL183W		0.15359477124183YGL084C		0.0714285714285714YPL245W		0.0903083700440529YOL049W		0.00814663951120163YMR088C		0.0676156583629893YJR044C		0.05YDR436W		0.571830985915493YIL083C		0.0465753424657534YHR133C		0.219931271477663YDL064W		0YGR024C		0.0126582278481013YNL051W		0.0570719602977667YML097C		0.339246119733925YMR097C		0.00817438692098093YNL059C		0.27682119205298YMR253C		0.202898550724638YPR050C		0.0218978102189781YHL040C		0.146730462519936YBL098W		0.00652173913043478YJR009C		0YPR180W		0.0893371757925072YJL196C		0.0129032258064516YGR148C		0.354838709677419YMR204C		0.402380952380952YHR105W		0.289719626168224YCR053W		0.00972762645914397YLR137W		0.160762942779292YHR114W		0.325434439178515YPR010C		0.0182876142975894YGR257C		0.0273224043715847YOR279C		0.648387096774194YGR242W		0.0392156862745098YJL112W		0.239495798319328YLR398C		0.125874125874126YPL227C		0.0119760479041916YGR273C		0.396551724137931YMR184W		0.424242424242424YBL081W		0.929347826086957YJR142W		0.0263157894736842YHR194W		0.0846286701208981YFR046C		0.506925207756233YGL027C		0.00240096038415366YLR423C		0.0215827338129496YMR286W		0YDL036C		0.158008658008658YBR213W		0.0145985401459854YHR037W		0.0156521739130435YLR107W		0.150990099009901YNR045W		0.102249488752556YMR072W		0.316939890710383YDL186W		0.523465703971119YCR082W		0.0390625YER063W		0.669724770642202YNL090W		0.0104166666666667YMR280C		0.605722260990928YHR045W		0YPL072W		0.064128256513026YOR330C		0.161084529505582YPL252C		0.25YNL108C		0.0666666666666667YDL073W		0.225609756097561YEL042W		0.0694980694980695YKL085W		0.0269461077844311YLR219W		0.898351648351648YNL138W		0.306083650190114YBR082C		0.00675675675675676YLR355C		0.0784810126582278YHL032C		0.0465444287729196YHR002W		0.0224089635854342YPL038W		0.638418079096045YJR085C		0YCL030C		0.0350438047559449YLR284C		0.0535714285714286YOR017W		0.24625YIL095W		0.628395061728395YBR162W-A		0.523076923076923YER105C		0.0927390366642703YJR080C		0.124365482233503YIL055C		0.507177033492823YKL157W		0.0545454545454545YKL113C		0.120418848167539YER014W		0.0204081632653061YPR164W		0.0291400142146411YER158C		0.654450261780105YNL189W		0.202952029520295YJL079C		0.521739130434783YKL109W		0.931407942238267YER049W		0.105590062111801YJL171C		0.0151515151515152YBR280C		0.189952904238619YBR259W		0.00726744186046512YGL181W		0.686868686868687YHR040W		0.319672131147541YDL226C		0.576704545454545YDR394W		0.0747663551401869YLR344W		0.173228346456693YGL198W		0.182978723404255YER128W		0.433497536945813YBR223C		0.213235294117647YMR079W		0.0361842105263158YAL022C		0.170212765957447YHR071W		0.327510917030568YMR264W		0.58128078817734YMR200W		0.140625YBR165W		0.231046931407942YIL027C		0YNR049C		0.838095238095238YPL249C		0.552572706935123YJL035C		0.024YLR131C		0.758441558441558YAR014C		0.727015558698727YGL261C		0YER156C		0.0207100591715976YDR210W		0.826666666666667YDL130W-A		0.372093023255814YLR233C		0.200286123032904YPL127C		0.60077519379845YDR147W		0.168539325842697YPL077C		0.558333333333333YPL051W		0.0353535353535354YMR157C		0.0313725490196078YOR002W		0.0367647058823529YMR176W		0.143160878809355YER111C		0.498627630375114YOR175C		0.163166397415186YAL061W		0.0983213429256595YGR215W		0.263636363636364YNL118C		0.754639175257732YNR050C		0YDR489W		0.105442176870748YML096W		0.0342857142857143YGL179C		0.4125YOR264W		0.409302325581395YMR089C		0.271515151515152YOL045W		0.316984559491371YPL207W		0.246913580246914YIL070C		0.131578947368421YCR091W		0.551388888888889YGL203C		0.246913580246914YIL117C		0.584905660377358YMR307W		0.207513416815742YOL152W		0.0629032258064516YJR131W		0.0418943533697632YJL003W		0.466101694915254YNL082W		0.274914089347079YDR196C		0.029045643153527YBR003W		0.0380549682875264YNR032W		0.10054347826087YGL148W		0.023936170212766YOR245C		0.0980861244019139YNL299W		0.465732087227414YOL144W		0.448347107438017YGL111W		0.166306695464363YBR181C		0.300847457627119YCL038C		0.109848484848485YPR158W		0.178571428571429YOR313C		0.210059171597633YGL039W		0.0114942528735632YDL207W		0.45910780669145YLR394W		0.639004149377593YNL231C		0.142450142450142YML099C		0.428409090909091YJL159W		0.61501210653753YOR187W		0.05720823798627YNL160W		0.00564971751412429YNL107W		0.0619469026548673YJL125C		0.143603133159269YEL036C		0.162YGR013W		0.383870967741935YJL054W		0.347280334728033YMR214W		0.151193633952255YNR008W		0.122541603630862YMR042W		0.610169491525424YJR019C		0.0573065902578797YDL084W		0.112107623318386YGL077C		0.122557726465364YLR182W		0.210460772104608YAR019C		0.196098562628337YNL121C		0.0551053484602917YMR308C		0YMR309C		0.198275862068966YDL182W		0.0467289719626168YMR170C		0.00592885375494071YLR231C		0.00662251655629139YDL208W		0.16025641025641YOR099W		0.0636132315521628YAL043C		0.266242038216561YDR531W		0.0354223433242507YBR028C		0.276190476190476YNL225C		0.519793459552496YIR023W		0.457731958762887YDR377W		0.0594059405940594YDR486C		0.720524017467249YHR115C		0.454326923076923YPL007C		0.0187074829931973YDR248C		0.0414507772020725YNL111C		0.125YPR032W		0.116166505324298YDR415C		0.00802139037433155YJL173C		0.114754098360656YDR298C		0.0188679245283019YOR114W		0.159863945578231YNR054C		0.534810126582278YCR071C		0.00684931506849315YHR199C		0.0193548387096774YOL108C		0.642384105960265YHR154W		0.351401869158878YHR038W		0.0782608695652174YNL008C		0.207772795216741YMR191W		0.136729222520107YOR148C		0.827027027027027YJL194W		0.128654970760234YHR027C		0.172205438066465YLR225C		0.0171990171990172YCR090C		0YDR219C		0.41505376344086YMR168C		0.115131578947368YDR084C		0.0603015075376884YGL252C		0.122448979591837YGL221C		0.0243055555555556YPL103C		0.0747863247863248YLR167W		0.164473684210526YPR079W		0.207349081364829YJR148W		0.0212765957446809YJR102C		0.0148514851485149YER117W		0.0072992700729927YPL128C		0.329181494661922YJR075W		0.128787878787879YGR147C		0.34375YPL132W		0.18YOR176W		0.083969465648855YDR121W		0.489795918367347YKL050C		0.566160520607375YDL204W		0.521628498727735YMR048W		0.429022082018927YDR098C		0.143859649122807YOR177C		0.424568965517241YGR205W		0.0172413793103448YLR188W		0.110791366906475YCR050C		0.00980392156862745YHR159W		0.875YBL090W		0.553672316384181YPL139C		0.0326086956521739YBR203W		0.427489177489177YGR109C		0.252631578947368YBR121C		0.0299850074962519YKL165C-A		0.012987012987013YIL120W		0.0959147424511545YGR255C		0.0146137787056367YER175C		0.020066889632107YMR256C		0.1YDR070C		1YEL064C		0.13125YPR156C		0.245980707395498YER067W		0.130434782608696YOR003W		0.0251046025104603YGL067W		0.0130208333333333YNL119W		0.0223123732251521YNR035C		0.0672514619883041YOR232W		0.31140350877193YMR092C		0YOR317W		0.00428571428571429YAR066W		0.133004926108374YGR162W		0.755252100840336YMR102C		0.146282973621103YDL066W		0.00934579439252336YOR387C		0.00970873786407767YGL070C		0YBR272C		0.0229166666666667YDL111C		0YIR030C		0YPR018W		0.618811881188119YGR161C		0.923954372623574YMR068W		0.0751173708920188YKL168C		0.564917127071823YIL011W		0.58364312267658YDR476C		0.0133928571428571YPL272C		0.102514506769826YJL123C		0.368200836820084YHR080C		0.565055762081784YMR086W		1YDR213W		0.660460021905805YNL071W		0.182572614107884YBL086C		0.439914163090129YGL006W		0.0639386189258312YML087C		0.00961538461538462YLR090W		0.24400871459695YPR196W		0.138297872340426YMR225C		0.0816326530612245YOR230W		0.0205949656750572YBR169C		0.122655122655123YDR004W		0.176086956521739YER076C		0.0264900662251656YBR210W		0.0352112676056338YPL206C		0.0249221183800623YNL322C		0.664536741214057YHR155W		0.11400651465798YNR043W		0.128787878787879YGR082W		0.316939890710383YER004W		0.00865800865800866YNL232W		0.123287671232877YMR251W-A		0.288135593220339YNL209W		0.0130505709624796YHR153C		0.00505050505050505YPR016C		0YNL178W		0.154166666666667YHR206W		0.435691318327974YPL090C		0.377118644067797YLR132C		0.148275862068966YNL025C		0.0464396284829721YDR483W		0.124434389140271YPR027C		0.0505415162454874YPL067C		0.0404040404040404YCR026C		0.133423180592992YDL214C		0.473533619456366YBL082C		0.091703056768559YDL017W		0.0453648915187377YGL218W		0.00462962962962963YDR527W		0.52619589977221YBR093C		0.0107066381156317YGL138C		0.11304347826087YDR391C		0.0732758620689655YOR131C		0.0458715596330275YDR345C		0.0776014109347443YLR303W		0.00675675675675676YLR127C		0.0363423212192263YOL116W		0.526178010471204YPL155C		0.338526912181303YNL186W		0.563131313131313YPR110C		0YLR097C		0.0755813953488372YMR238W		0.0349344978165939YLR414C		0.0190114068441065YDR392W		0.299703264094956YBL032W		0.249343832020997YML049C		0.0617193240264511YDR416W		0.0488940628637951YDL108W		0.0163398692810458YMR240C		0.51605504587156YGL071W		0.471014492753623YPL115C		0.555851063829787YMR049C		0.24907063197026YDR243C		0.168367346938776YDL008W		0.272727272727273YNR017W		0.387387387387387YOR383C		0.691176470588235YLR095C		0.323891625615764YOR115C		0.164179104477612YHR019C		0.104693140794224YKL033W-A		0.0127118644067797YNL123W		0.0712136409227683YPL084W		0.182464454976303YNL162W-A		0.208333333333333YGR260W		0.117977528089888YDR012W		0.157458563535912YCL005W		0.33203125YLR417W		0.356890459363958YBR233W-A		0.319148936170213YHR157W		0.258241758241758YOL072W		0.00879120879120879YPL239W		0.065YBR179C		0.12280701754386YFR048W		0.410876132930514YIL074C		0.110874200426439YOR322C		0.431540342298289YOR033C		0.494301994301994YPR063C		0.25YKL046C		0.0489977728285078YER107C		0.0164383561643836YKL024C		0.0294117647058824YPR054W		0.0850515463917526YMR075W		0.494152046783626YBR262C		0.0471698113207547YDR088C		0.523560209424084YLR377C		0.0316091954022989YDR118W		0.0429447852760736YIR034C		0YMR291W		0.450511945392491YML011C		0.0677966101694915YPL048W		0.113253012048193YGL120C		0.117340286831812YAR008W		0.207272727272727YGL116W		0.283606557377049YJR047C		0.0318471337579618YDR299W		0.47940074906367YOR223W		0.297945205479452YBR298C		0.0928338762214984YHR185C		0.248945147679325YPL049C		0.911504424778761YKL159C		0.241706161137441YNL260C		0.0454545454545455YJL210W		0.0627306273062731YBL015W		0.0171102661596958YAL054C		0.0687237026647966YMR119W		0.174679487179487YDL020C		0.564971751412429YBR055C		0.137931034482759YJL105W		0.482142857142857YGR040W		0.144021739130435YLR293C		0.17351598173516YHR031C		0.275242047026279YJR118C		0.241379310344828YLR177W		0.45859872611465YGR279C		0.235751295336788YBR290W		0.545171339563863YGL123W		0.173228346456693YOL115W		0.410958904109589YOR231W		0.405511811023622YMR062C		0.018140589569161YPL176C		0.0842911877394636YJR134C		0.701555869872702YIL064W		0.11284046692607YDR165W		0.0698198198198198YNL142W		0.12625250501002YMR222C		0.00896860986547085YHR144C		0YNL311C		0.121887287024902YLR242C		0.0404984423676012YER057C		0.0310077519379845YML029W		0.109785202863962YCR087C-A		0.522875816993464YAL033W		0.190751445086705YNR060W		0.0278164116828929YLR333C		0.324074074074074YML037C		0.702941176470588YDL101C		0.175438596491228YDR195W		0.49718574108818YDR322W		0.177111716621253YDL102W		0.103919781221513YGR276C		0.188065099457505YBR126C		0.0323232323232323YJL081C		0.0674846625766871YLR363W-A		1YGR076C		0.522292993630573YCR020C-A		0YML010W		0.505174035747883YPL168W		0.223255813953488YDR328C		0.283505154639175YGR021W		0.0137931034482759YPL237W		0.484210526315789YMR224C		0.263005780346821YPR105C		0.0789779326364692YPL083C		0.254817987152034YMR172W		0.72461752433936YOL140W		0.0189125295508274YGL107C		0.106811145510836YGR128C		0.0785413744740533YNR020C		0.0703703703703704YPR017C		0.0769230769230769YDL132W		0.0662576687116564YNR039C		0.371900826446281YCR010C		0.123674911660777YNL309W		1YGR179C		0.433497536945813YGR175C		0.0241935483870968YNL265C		0.426174496644295YMR192W		0.361111111111111YPR189W		0.0370111731843575YNL196C		0.439597315436242YGL135W		0YOL132W		0.0976645435244161YGL082W		0.291338582677165YNR048W		0.106870229007634YDL134C		0.173441734417344YHR083W		0.0516717325227964YFR005C		0.0669642857142857YEL020W-A		0.448275862068966YEL038W		0.0616740088105727YCR035C		0.0152284263959391YMR026C		0.135338345864662YJR016C		0.0290598290598291YDR176W		0.572649572649573YGL004C		0YDR222W		0.0144578313253012YPR162C		0.126654064272212YEL054C		0.0545454545454545YDR020C		0.0172413793103448YPR129W		0.750716332378224YDL091C		0.23956043956044YJL154C		0.0370762711864407YDR074W		0.0881696428571429YBR240C		0.355555555555556YDR251W		0.565060240963855YOL042W		0.0220385674931129YHR196W		0.175652173913043YDR494W		0.609418282548476YGL142C		0.0308441558441558YPL230W		0.91304347826087YOR046C		0.184647302904564YBL056W		0.346153846153846YDL175C		0.86046511627907YOR336W		0.00512820512820513YNL139C		0.3055729492799YHR192W		0.140287769784173YNL313C		0.0719026548672566YCR051W		0.0855855855855856YGR067C		0.188916876574307YHL024W		0.607293127629734YJL185C		0.37542662116041YER071C		0.365079365079365YPL076W		0YIR002C		0.259818731117825YMR294W		0.501340482573727YML110C		0.0749185667752443YPR065W		0.75YLR288C		0.19620253164557YCR057C		0.0877573131094258YLR170C		0.0705128205128205YJL160C		0.344947735191638YBR287W		0.107728337236534YDL033C		0.0311750599520384YGR239C		0.569444444444444YER031C		0.188340807174888YOR006C		0.453674121405751YNL283C		0.842942345924453YOL032W		0.126016260162602YLR194C		0.834645669291339YER168C		0.0512820512820513YFR032C-A		1YJL151C		0.368421052631579YOR332W		0.0128755364806867YMR181C		0.538961038961039YDL042C		0.270462633451957YNL148C		0.12992125984252YMR318C		0.00277777777777778YGL078C		0.198852772466539YIL065C		0.0258064516129032YNL099C		0.239495798319328YPR083W		0.516407599309154YBL033C		0.168115942028985YCL057W		0YDR117C		0.182300884955752YLR245C		0.028169014084507YPL254W		0.284836065573771YNL100W		0.047008547008547YPL184C		0.330065359477124YDR492W		0.0348101265822785YER006W		0.292307692307692YOL114C		0.198019801980198YNL288W		0.260053619302949YGR096W		0.035031847133758YER132C		0.584141471762692YJL008C		0.0492957746478873YHR202W		0.0215946843853821YHR094C		0.087719298245614YPR140W		0.0656167979002625YPR029C		0.106971153846154YNL318C		0.0166666666666667YDR276C		0YOL040C		0.176056338028169YGR143W		0.390402075226978YPL165C		0.0348525469168901YNR026C		0.0127388535031847YBL013W		0.00748129675810474YGR122W		0.146766169154229YDR275W		0.0595744680851064YHR140W		0.0585774058577406YKL127W		0.0105263157894737YDR382W		0.481818181818182YOL129W		0.0760869565217391YNL102W		0.217302452316076YML014W		0.247311827956989YDR237W		0.219178082191781YJR129C		0.0176991150442478YPR024W		0.220883534136546YOR289W		0.0199203187250996YLR259C		0.0472027972027972YDR496C		0.231707317073171YPL042C		0.318918918918919YJL133W		0.035031847133758YOL077W-A		0.5YPL145C		0.0806451612903226YDL146W		0.195519348268839YDR100W		0.0769230769230769YJR004C		0.409230769230769YBR186W		0.152482269503546YKL137W		0.18018018018018YFR041C		0.145762711864407YKL006C-A		0.0103092783505155YLR133W		0.225085910652921YPR192W		0.160655737704918YMR189W		0.04642166344294YOR137C		0.0241157556270096YNL215W		0.934375YDL105W		0.296019900497512YCL029C		0.581818181818182YDR172W		0.362043795620438YNL085W		0.0939759036144578YDL230W		0YNL264C		0.102857142857143YBL038W		0.28448275862069YHR148W		0.0710382513661202YPL166W		0.582159624413146YJL005W		0.465942744323791YPL026C		0.334661354581673YJR006W		0.0698151950718686YJL115W		0.433691756272401YJL099W		0.0790884718498659YLR262C		0.176744186046512YNL333W		0.00335570469798658YFR010W		0.064128256513026YJL161W		0.222222222222222YJR097W		0.191860465116279YIR021W		0.0220385674931129YPR103W		0.10801393728223YJR122W		0.0422535211267606YOR390W		0.0106666666666667YML105C		0.593406593406593YKL035W		0.106212424849699YMR056C		0.0194174757281553YGL171W		0.25177304964539YOR373W		0.438307873090482YGR249W		0.74780701754386YAR035W		0.131004366812227YGR198W		0.0514075887392901YNR022C		0.460431654676259YDR034C		0.417721518987342YBR033W		0.368879216539717YPL186C		0.592105263157895YBL084C		0.258575197889182YOR352W		0.434402332361516YDL030W		0.245283018867925YAL013W		0.416666666666667YDL104C		0.00737100737100737YHR175W		0.201058201058201YBR002C		0.0769230769230769YBR246W		0.020671834625323YHR121W		0.0267379679144385YEL017W		0.27893175074184YDL067C		0.11864406779661YGR037C		0YML123C		0.0715502555366269YDR280W		0.0163934426229508YBR191W		0.10625YGL083W		0.272388059701493YMR229C		0.131868131868132YAL060W		0.0183246073298429YCL036W		0.243816254416961YPL046C		0YPL136W		0.0163934426229508YJL207C		0.0322740814299901YBL069W		0.0909090909090909YOR361C		0.0458715596330275YML031W		0.23206106870229YNR014W		0.75YBR166C		0.0641592920353982YHR087W		0.279279279279279YDR151C		0.710769230769231YGL026C		0.0325318246110325YER047C		0.573021181716834YEL050C		0.244274809160305YGL036W		0.291529152915292YDL087C		0.130268199233716YPL135W		0.0242424242424242YJR064W		0.0587188612099644YPL074W		0.43368700265252YOR276W		0.590062111801242YGR261C		0.207663782447466YBL102W		0.27906976744186YGR049W		0.181818181818182YHR156C		0.355882352941176YIR012W		0.00696055684454756YMR283C		0.0487329434697856YER083C		0.449122807017544YLR110C		0.428571428571429YBR137W		0.0670391061452514YJR062C		0.0590809628008753YNL094W		0.252129471890971YNL281W		0.0196078431372549YHR001W-A		0.103896103896104YMR150C		0.0421052631578947YDL098C		0.170103092783505YGR010W		0.329113924050633YBR057C		0.73224043715847YPL032C		0.581818181818182YGR157W		0.0575373993095512YNL213C		0.672897196261682YMR018W		0YBR284W		0.119196988707654YGR238C		0.445578231292517YKL130C		0.0569105691056911YDR536W		0.086115992970123YIL051C		0.117241379310345YOR305W		0.0785123966942149YER079W		0.695238095238095YPR003C		0.147214854111406YDR107C		0.00148809523809524YGL064C		0.117647058823529YML117W		0.556437389770723YIL082W		0.375862068965517YKL091C		0.0193548387096774YKL096W		0.326359832635983YDR110W		0.34452296819788YDR412W		0.774468085106383YPR082C		0.034965034965035YJL193W		0.0597014925373134YDR123C		0.5625YNR003C		0.0788643533123028YBR149W		0.0406976744186047YNL112W		0.206959706959707YNL261W		0.058455114822547YBR172C		0.801351351351351YDR226W		0.00900900900900901YDR389W		0.617737003058104YIL144W		0.287988422575977YJL080C		0.170212765957447YOR089C		0.166666666666667YJR022W		0.036697247706422YPR034W		0.0482180293501048YDR477W		0.339652448657188YKL061W		0.283185840707965YOR219C		0.113856068743287YBR164C		0.00546448087431694YMR060C		0.0214067278287462YBR217W		0.537634408602151YIL076W		0YIR014W		0.0661157024793388YPL068C		0.406143344709898YGR117C		0.023109243697479YDR014W		0.401854714064915YGR275W		0.605095541401274YNL210W		0.037037037037037YGL233W		0.134065934065934YLR457C		0.80564263322884YBR030W		0.144927536231884YPL225W		0.0205479452054795YJR036C		0.146860986547085YDR138W		0.214095744680851YPR134W		0.0932835820895522YDL119C		0.0260586319218241YGR004W		0.207792207792208YMR301C		0.0681159420289855YPR116W		0.0938628158844765YNL253W		0.0734597156398104YLR386W		0.1875YBR037C		0.155932203389831YFR045W		0.0105263157894737YPR145W		0YNR024W		0.720430107526882YMR123W		0.336065573770492YOR303W		0.0583941605839416YLR221C		0.754545454545455YNL152W		0.657701711491443YNL244C		0.0277777777777778YJL111W		0.0581818181818182YNL135C		0YHL015W		0.12396694214876YGR236C		0.294736842105263YIL154C		0.393063583815029YDL085W		0.0880733944954128YML115C		0.104672897196262YBR278W		0.587064676616915YDR236C		0.0642201834862385YOR349W		0.00591715976331361YLR433C		0.363471971066908YCR018C		0.642533936651584YFR037C		0.556552962298025YJL002C		0YLR264W		0.0298507462686567YLR353W		0.749585406301824YHL016C		0.0857142857142857YLR135W		0.676470588235294YOL088C		0.137184115523466YPL086C		0.0359066427289048YOR209C		0.020979020979021YDR142C		0.0346666666666667YIR025W		0.638586956521739YKL135C		0.166666666666667YMR223W		0.00636942675159236YKL120W		0.0740740740740741YLR239C		0.00914634146341463YPL019C		0.134131736526946YPL085W		0.851025056947608YLR102C		0.471698113207547YJL071W		0.0435540069686411YJR010W		0.0136986301369863YCR021C		0.126506024096386YHR063C		0.0369393139841689YOL063C		0.101358411703239YGR172C		0.25YHR009C		0.0401529636711281YGR111W		0.0325YOR039W		0.213178294573643YPL087W		0.0410094637223975YMR101C		0YNL001W		0.0155440414507772YOL146W		0.0824742268041237YDL218W		0.372239747634069YNL098C		0.462732919254658YLR332W		0.694148936170213YGR081C		0.966666666666667YOL156W		0.0793650793650794YBR058C-A		0.175YHR168W		0.0280561122244489YHR062C		0.146757679180887YGR248W		0.00392156862745098YOL058W		0.0142857142857143YMR138W		0YPL022W		0.231818181818182YJL110C		0.923774954627949YHR208W		0.00508905852417303YIR009W		0.243243243243243YLR384C		0.0719051148999259YPR188C		0YGL018C		0.0434782608695652YJL198W		0.170261066969353YPL013C		0.148760330578512YMR261C		0.259013282732448YER001W		0.0498687664041995YGR002C		0.420168067226891YPR108W		0.0396270396270396YDR385W		0.00118764845605701YMR271C		0.052863436123348YBR109C		0YDR480W		0.727554179566563YOR306C		0.172744721689059YBL089W		0.0217864923747277YGR112W		0.138817480719794YLR213C		0.0165876777251185YDR439W		0.864553314121037YLR408C		0YJR010C-A		0.0425531914893617YNL323W		0.101449275362319YPR067W		0.210810810810811YGR254W		0.0022883295194508YIL145C		0.00647249190938511YMR209C		0.00437636761487965YPR102C		0.0632183908045977YLR168C		0.0130434782608696YPL123C		0.0368663594470046YJL149W		0.147812971342383YGR159C		0.606280193236715YML058W-A		0.102941176470588YDR325W		0.189371980676329YHR006W		0.707948243992606YGL040C		0.0204678362573099YOR259C		0.183066361556064YDL085C-A		1YBL041W		0.037344398340249YNL009W		0.0404761904761905YNL070W		0.15YJR139C		0YJL044C		0.203056768558952YLR404W		0.0350877192982456YIR016W		0.509433962264151YNL206C		0.391208791208791YML062C		0.469387755102041YDR375C		0.109649122807018YLR375W		0.498542274052478YPL183W-A		0.172043010752688YER010C		0.0213675213675214YHR149C		0.837874659400545YJL197W		0.354066985645933YDL212W		0.147619047619048YER025W		0.157495256166983YBR046C		0.00598802395209581YGL011C		0.0396825396825397YBR052C		0YPR167C		0.0268199233716475YER048C		0.378516624040921YER037W		0.059190031152648YGR247W		0.0334728033472803YHR169W		0.0997679814385151YKL154W		0.0450819672131148YLR321C		0.288732394366197YMR063W		0.00836820083682008YFL034W		0.425908667287978YIL125W		0.0207100591715976YOR270C		0.0738095238095238YGL017W		0.0636182902584493YFR053C		0.0268041237113402YNL110C		0.45YIL098C		0.0451612903225806YML128C		0.0116959064327485YOR001W		0.345156889495225YER129W		0.655866900175131YOR304C-A		0.671052631578947YOR057W		0.29873417721519YDR373W		0.068421052631579YGR091W		0.388663967611336YLR087C		0.115618661257606YOL002C		0.0883280757097792YGL244W		0.600358422939068YCL035C		0.0272727272727273YPR152C		0.356989247311828YGR005C		0.535YHL008C		0.456140350877193YKL146W		0.338150289017341YOL092W		0.113636363636364YMR115W		0.287425149700599YLR381W		0.00818553888130969YBL028C		0.962264150943396YKL039W		0.110898661567878YGL028C		0.527675276752767YMR127C		0.115384615384615YLR418C		0.185750636132316YDR247W		0.292841648590022YMR205C		0.0510948905109489YJL118W		0.200913242009132YFR043C		0.00421940928270042YOR255W		0.165467625899281YDR265W		0.195845697329377YNL131W		0.335526315789474YNL312W		0.32967032967033YER101C		0.0883720930232558YMR215W		0.179389312977099YJL172W		0.0399305555555556YER159C		0.415492957746479YHL031C		0.165919282511211YEL047C		0YHR118C		0.416091954022988YOR160W		0.0195473251028807YKL016C		0.00574712643678161YPR056W		0.13905325443787YBR201W		0YOR185C		0.163636363636364YER144C		0.234782608695652YBR264C		0.120603015075377YML016C		0.569364161849711YDL180W		0.221206581352834YEL058W		0.00538599640933573YLR172C		0.03YBR024W		0.142857142857143YJL168C		0.391541609822647YJL121C		0YPL212C		0.216911764705882YJL036W		0.0520094562647754YOL077C		0.213058419243986YJL201W		0.357262103505843YPL141C		0.641618497109827YDR528W		0.832151300236407YNL262W		0.0648064806480648YBR135W		0.326666666666667YNL317W		0.0688172043010753YPR104C		0.75534188034188YPL244C		0.0353982300884956YOR069W		0.45037037037037YGL164C		0.270454545454545YGL231C		0.284210526315789YER090W		0.0374753451676529YPR049C		0.291171477079796YDL200C		0.0265957446808511YIR018W		0.86530612244898YGR026W		0.122302158273381YIL114C		0YFL027C		0.0764587525150905YLR380W		0.0808823529411765YNL153C		0.0542635658914729YHR036W		0.560509554140127YKL184W		0.0300429184549356YGR079W		0.6YML009C		0.157142857142857YDL133W		0.304347826086957YOL073C		0.273291925465839YMR250W		0.0547008547008547YBR098W		0.477568740955137YHR005C		0.0911016949152542YDR507C		0.563922942206655YPR094W		0.0560747663551402YMR303C		0YBR237W		0.347467608951708YER018C		0.420814479638009YHR161C		0.590266875981162YDR116C		0.136842105263158YDR242W		0.029143897996357YOL145C		0.15041782729805YOR290C		0.46095126247798YLR281C		0.361290322580645YBR139W		0.0728346456692913YPL100W		0.0483870967741935YER023W		0.013986013986014YPL146C		0.762637362637363YLR439W		0.244514106583072YCL055W		0.17910447761194YBR138C		0.641221374045801YJL055W		0.0979591836734694YDR057W		0.143911439114391YMR140W		0.627811860940695YLR092W		0.131019036954087YBR016W		0.78125YFL054C		0.540247678018576YBR031W		0.174033149171271YHR142W		0.0158227848101266YNL035C		0.0668380462724936YPL164C		0.0195804195804196YJR101W		0.0112781954887218YDR447C		0.470588235294118YIL136W		0.300254452926209YML127W		0.175559380378657YIL016W		0.377358490566038YOR052C		0.84YOR078W		0.939252336448598YMR302C		0.0482352941176471YGR166W		0.192857142857143YNL221C		0.277714285714286YGL128C		0.141342756183746YKL072W		0.168407310704961YHR052W		0.321808510638298YMR182C		0.720379146919431YDR438W		0.135135135135135YPL098C		0.079646017699115YER017C		0.232588699080158YLR113W		0.2YNL227C		0.457627118644068YIL149C		0.603335318642049YIL157C		0.223350253807107YPR151C		0.330097087378641YFL024C		0.438701923076923YPL125W		0.00872093023255814YMR135C		0.184615384615385YMR259C		0.0133802816901408YOL071W		0.0617283950617284YGL126W		0.0315789473684211YCR083W		0.0551181102362205YGL113W		0.523952095808383YNR001C		0.0417536534446764YMR160W		0.167892156862745YIL116W		0.012987012987013YGR100W		0.210526315789474YNR032C-A		0YOR252W		0.370786516853933YKL042W		0.776859504132231YGR052W		0.02710027100271YBR060C		0.451612903225806YER141W		0.0576131687242798YPR172W		0.085YJL124C		0.319767441860465YER042W		0.103260869565217YGL091C		0.179878048780488YJL100W		0.224052718286656YHR047C		0.00350467289719626YDR202C		0.0997150997150997YJR150C		0.560402684563758YAL035W		0.388223552894212YPR013C		0.712933753943218YNL015W		0.106666666666667YKL167C		0.116788321167883YMR070W		0.926530612244898YFR049W		0.333333333333333YML083C		0.129186602870813YDR296W		0.0929203539823009YPL110C		0.0188062142273099YLR205C		0.0220820189274448YMR009W		0YOR243C		0.14792899408284YNL207W		0.195294117647059YER110C		0.0188679245283019YBR239C		0.570888468809074YFR027W		0.120996441281139YPL040C		0.0379241516966068YGR283C		0.0967741935483871YIL084C		0.495412844036697YDL151C		0.523316062176166YER119C		0.0401785714285714YFL030W		0.0233766233766234YPR166C		0YML108W		0.219047619047619YDR322C-A		0.125YIL132C		0.0563380281690141YML070W		0.0308219178082192YDR013W		0.0144230769230769YML095C		0.385714285714286YPL008W		0.174216027874564YDL156W		0.222222222222222YDR173C		0.11830985915493YDR190C		0YLR399C		0.549562682215743YDR109C		0.0685314685314685YLR356W		0.218274111675127YJL058C		0.41804788213628YFR015C		0.0875706214689266YDR263C		0.172093023255814YNL233W		0.897982062780269YNL334C		0.0135135135135135YOL021C		0.037962037962038YHR106W		0.0292397660818713YMR100W		0.248387096774194YLR325C		0YGL089C		0.00833333333333333YMR207C		0.0273198304286387YGL103W		0.288590604026846YDL131W		0.0613636363636364YBR163W		0.11965811965812YML047C		0.110795454545455YGR167W		0.759656652360515YER145C		0.133663366336634YMR136W		0.65YCR076C		0.588YDR379W		0.654112983151635YGL223C		0.0863309352517986YIL160C		0.0575539568345324YBL020W		0.0696864111498258YGL129C		0.114754098360656YKL176C		0.306763285024155YBR276C		0.0198265179677819YBR017C		0.0348583877995643YLR146C		0.02YOL044W		0.276762402088773YNL005C		0.30188679245283YPL221W		0.221941992433796YHL039W		0.0205128205128205YOR008C		0.592592592592593YDR393W		0.256578947368421YNL172W		0.12070938215103YGR093W		0.0729783037475345YEL025C		0.0378787878787879YFR023W		0.2569558101473YOL065C		0.0078125YNL255C		0.843137254901961YML109W		0.835456475583864YJL199C		0.0740740740740741YDR208W		0.534017971758665YNL259C		0.0547945205479452YIL021W		0.154088050314465YJL088W		0.0502958579881657YDL161W		0.702643171806167YCL028W		0.871604938271605YLR166C		0.12743972445465YHL013C		0.485342019543974YLR175W		0.271221532091097YPL116W		0.307030129124821YHR117W		0.0500782472613459YGL196W		0.00700934579439252YGL035C		1YMR244C-A		0.288461538461538YLR222C		0.00367197062423501YPR048W		0.00160513643659711YPL109C		0.0182648401826484YPL138C		0.246458923512748YKL117W		0.435185185185185YOL125W		0.149159663865546YEL027W		0.025YJL165C		0.588304093567251YMR175W		1YLR438W		0.0141509433962264YGL209W		1YBL092W		0.315384615384615YBR145W		0.0056980056980057YIL031W		0.585106382978723YLR319C		0.463197969543147YPR135W		0.0366774541531823YBR233W		0.23728813559322YDR045C		0.00909090909090909YHR110W		0.0141509433962264YKL001C		0.0346534653465347YLR181C		0.403030303030303YOL086C		0YBR244W		0YNL016W		0.364238410596026YMR163C		0.24113475177305YHR034C		0.284883720930233YML006C		0.60594315245478YCR042C		0.132196162046908YDR466W		0.426503340757238YJR082C		0.548672566371681YPL054W		0.621262458471761YMR282C		0.0637931034482759YGR207C		0.00383141762452107YOR134W		0.491442542787286YCR017C		0.0335781741867786YJL179W		0.36697247706422YIR004W		0.37037037037037YPL150W		0.635960044395117YCR088W		0.684121621621622YIR029W		0.0174927113702624YMR094W		0.100418410041841YBR267W		0.325699745547074YGR174C		0.117647058823529YML107C		0.0359281437125748YNL117W		0.00902527075812274YCL043C		0.0862068965517241YFR034C		0.775641025641026YPL066W		0.0480167014613779YGL021W		0.590789473684211YJL141C		0.526641883519207YPL180W		0.877346683354193YBR107C		0YOL043C		0.218421052631579YDL166C		0.0558375634517767YOR288C		0.110062893081761YBR249C		0.0432432432432432YHR152W		1YFL041W		0.0112540192926045YOR377W		0.0285714285714286YBR041W		0.00597907324364723YGR085C		0.0804597701149425YPR148C		0.597701149425287YMR278W		0.0112540192926045YMR131C		0.2426614481409YNL251C		0.500869565217391YMR310C		0.0473186119873817YNL058C		0.667721518987342YER104W		0.754807692307692YBR004C		0.0184757505773672YLR362W		0.354253835425384YMR105C		0.00878734622144113YPL262W		0.0348360655737705YDR519W		0YDL193W		0.189333333333333YLR267W		0.0210526315789474YCR066W		0.655030800821355YPL199C		0.233333333333333YBR175W		0YGR216C		0.0574712643678161YLR373C		0.169811320754717YJR043C		0.66YOR358W		0.512396694214876YBR221C		0.112021857923497YEL052W		0.0432220039292731YDL148C		0.504938271604938YNL307C		0.04YDR378C		0.104651162790698YMR295C		0.472081218274112YDR472W		0.346289752650177YIL142W		0.0094876660341556YPR004C		0.0261627906976744YNL199C		0.631086142322097YMR043W		0.758741258741259YMR034C		0.0990783410138249YMR031C		0.677342823250297YGR195W		0.024390243902439YMR055C		0.0457516339869281YHR216W		0YLR438C-A		0.0224719101123595YPL260W		0.310344827586207YBR014C		0.236453201970443YGR289C		0.154220779220779YOL039W		0.556603773584906YLR447C		0YOL012C		0.41044776119403YGL153W		0.63049853372434YMR217W		0.00952380952380952YGR171C		0.0226086956521739YDR078C		0.00896860986547085YAL010C		0.196754563894523YKL166C		0.248743718592965YDR182W		0.112016293279022YML074C		0.452554744525547YNR011C		0.202054794520548YGR127W		0.0224358974358974YDR363W-A		0.539325842696629YBR180W		0.157342657342657YLR372W		0.101449275362319YER002W		0.541125541125541YDR032C		0.0202020202020202YCR033W		0.791190864600326YJL014W		0.0112359550561798YOR126C		0.0126050420168067YLR340W		0.128205128205128YLR193C		0.00571428571428571YOR108W		0.0811258278145695YBL016W		0.0141643059490085YAR002C-A		0YPL179W		0.446265938069217YBR129C		0.253048780487805YML060W		0.127659574468085YER034W		0.881081081081081YCL001W		0.186170212765957YPR047W		0YGL100W		0.0114613180515759YDR388W		0.4149377593361YLR203C		0.0986238532110092YLR215C		0.113888888888889YOR375C		0YFL046W		0YHR066W		0.355408388520971YLR312C		0.35427135678392YHR122W		0.415584415584416YHR088W		0.206779661016949YPL219W		0.567073170731707YML094W		0.239263803680982YBL061C		0.265804597701149YER154W		0.278606965174129YOR236W		0.018957345971564YNL273W		0.229402261712439YGR084C		0.153392330383481YNL023C		0.658031088082902YNL275W		0.0972222222222222YGR016W		0.184210526315789YNL052W		0.0784313725490196YHR076W		0.101604278074866YJL206C		0.271767810026385YER143W		0.273364485981308YPL175W		0.0420353982300885YIR035C		0.0078740157480315YDL063C		0.116129032258065YBR251W		0.133550488599349YGR252W		0.280182232346241YJL166W		0.0638297872340425YNL073W		0.0173611111111111YCL068C		0.0269230769230769YPR128C		0.0487804878048781YDR339C		0.201058201058201YGL075C		0.418604651162791YPL095C		0.0197368421052632YCL004W		0.00767754318618042YGL170C		0.423728813559322YOR278W		0.0181818181818182YJR063W		0YPR073C		0.0496894409937888YGL224C		0YIL056W		0.4140625YML080W		0.104018912529551YLR108C		0.0845360824742268YPL028W		0YLR214W		0.00583090379008746YPL034W		0.290909090909091YDL070W		0.586206896551724YPL209C		0.267029972752044YPL174C		0.399769585253456YDR508C		0.232277526395173YDR364C		0.169230769230769YLR257W		0.956386292834891YDR281C		0.144230769230769YOL062C		0.105906313645621YJL126W		0.0488599348534202YOL100W		0.548566142460685YER019C-A		0.443181818181818YGR200C		0.00253807106598985YOL122C		0.142608695652174YLR289W		0.0124031007751938YGL038C		0.1125YMR124W		0.951219512195122YNR063W		0.306425041186161YJL052W		0YGL184C		0.0021505376344086YLR145W		0.263681592039801YMR040W		0.10625YER136W		0.0177383592017738YLR197W		0.168650793650794YOR344C		0.714776632302406YAL048C		0.0287009063444109YLR351C		0.0171821305841924YDL198C		0.0366666666666667YPR033C		0.119047619047619YML130C		0.150976909413854YJL117W		0.118971061093248YDR003W		0.59047619047619YDR421W		0.401052631578947YOR110W		0.137931034482759YDR312W		0.344370860927152YDR050C		0YOR117W		0.124423963133641YML068W		0.0129310344827586YLR195C		0.149450549450549YFR014C		0.302690582959641YFR004W		0.264705882352941YPL133C		0.533632286995516YGR054W		0.292834890965732YOR044W		0.0955414012738854YHR143W		0.895384615384615YKL071W		0.0078125YHR035W		0.0380952380952381YGR083C		0.453149001536098YOL022C		0.313725490196078YGL001C		0YGL090W		0.529691211401425YNL229C		0.259887005649718YJR049C		0.345283018867925YDL234C		0.134048257372654YML118W		0.279207920792079YLR094C		0.390438247011952YDR368W		0.00320512820512821YOR378W		0.0912621359223301YJL011C		0.173913043478261YOR221C		0.00833333333333333YAR007C		0.0917874396135266YKL069W		0.0777777777777778YLR208W		0.0168350168350168YBR022W		0.0112994350282486YOR320C		0.0386965376782077YML078W		0.0274725274725275YDR326C		0.592489568845619YER080W		0.0287081339712919YHR179W		0.0425YMR117C		0.394366197183099YNL272C		0.561264822134387YOR116C		0.023972602739726YBL074C		0.0647887323943662YKL093W		0.952802359882006YML088W		0.320359281437126YIL106W		0.398089171974522YDR059C		0.00675675675675676YLR227C		0.0608519269776876YOL124C		0.0115473441108545YNR041C		0.0887096774193548YOR247W		0.328571428571429YIL110W		0.135278514588859YKL110C		0.0575079872204473YML102W		0.0961538461538462YLR272C		0.102891156462585YEL070W		0.0199203187250996YGR213C		0.0788643533123028YGR071C		0.0918604651162791YPL220W		0YER176W		0.331846565566459YPR068C		0.0340425531914894YPL159C		0.691699604743083YER167W		0.851938895417156YDL115C		0.379166666666667YGL256W		0.010752688172043YDL189W		0.796498905908096YPL231W		0.100158982511924YPR045C		0.368085106382979YHR201C		0.0125944584382872YNL048W		0.0383211678832117YJR058C		0.0136054421768707YDL045C		0.0686274509803922YOL133W		0.223140495867769YDR460W		0.370716510903427YDR087C		0.197841726618705YPR006C		0.0991304347826087YJR143C		0.0603674540682415YPL210C		0.165625YHR124W		0.32695374800638YBL060W		0.326055312954876YHL025W		0.319277108433735YER103W		0.0514018691588785YOL135C		0.207207207207207YGR056W		0.37823275862069YGR131W		0.14367816091954YLR260W		0.326055312954876YOR128C		0.021015761821366YJR065C		0.0022271714922049YOR086C		0.126475548060708YGL020C		0.127659574468085YJL209W		0.0198776758409786YOR353C		0.420986093552465YNL237W		0.0915032679738562YBR087W		0YLR429W		0.393241167434716YDR539W		0.00795228628230616YGL242C		0.138121546961326YKL160W		0.551724137931034YHR001W		0.173913043478261YMR118C		0.127551020408163YPR120C		0.328735632183908YPR143W		0.6YBR009C		0.281553398058252YOR028C		0.535593220338983YJR112W		0.0845771144278607YDL123W		0.364285714285714YGL236C		0.0179372197309417YLR407W		0.353711790393013YLR154C		0.209090909090909YDR277C		0.501154734411085YDL053C		0.675675675675676YNL169C		0.146YKL063C		0.688622754491018YDR446W		0.645695364238411YIL152W		0.565957446808511YGL054C		0.0289855072463768YJL069C		0.269360269360269YLR405W		0.0844686648501362YJL184W		0.609756097560976YPR115W		0.594644506001847YMR171C		0.349090909090909YDR530C		0.04YLR295C		0.548387096774194YKL141W		0.338383838383838YEL026W		0.00793650793650794YNL072W		0.130293159609121YDL107W		0.0911680911680912YAL025C		0.480392156862745YAL023C		0.0711462450592885YNL214W		0.0301507537688442YNL223W		0.0910931174089069YPL173W		0.272727272727273YML067C		0.0426136363636364YJR153W		0.00277008310249307YKL152C		0YOL112W		0.209349593495935YGL119W		0.127744510978044YNL236W		0.0749486652977413YGR132C		0.0731707317073171YBR047W		0.0342857142857143YHR007C		0.0207547169811321YFR009W		0.0558510638297872YKL009W		0.0635593220338983YMR044W		0.448421052631579YJR121W		0.0273972602739726YAR015W		0.042483660130719YHR072W-A		0.189655172413793YNL132W		0.180871212121212YOR062C		0.23134328358209YNL149C		0.581395348837209YDR158W		0.00273972602739726YBR296C		0.0905923344947735YOR339C		0.0384615384615385YGR033C		0.129707112970711YML022W		0.0213903743315508YPL234C		0.0548780487804878YJR014W		0.444444444444444YCR037C		0.210184182015168YIR007W		0.0942408376963351YKL079W		0.455792682926829YBR176W		0.0641025641025641YPR076W		0.0967741935483871YKL150W		0.043046357615894YDR336W		0.14968152866242YDR086C		0.275YNL159C		0.328719723183391YER150W		0.027027027027027YKL172W		0.782201405152225YFL047W		0.124649859943978YPL071C		0.25YML098W		0.437125748502994YDR002W		0.388059701492537YIR037W		0.00613496932515337YDR105C		0.0591966173361522YPR193C		0.032051282051282YMR023C		0.0171102661596958YBR071W		0.777251184834123YML043C		0.331360946745562YML001W		0.134615384615385YKL185W		0.748299319727891YLR220W		0.254658385093168YPL023C		0.0958904109589041YMR149W		0.0034965034965035YFL022C		0.00397614314115308YAL007C		0.0232558139534884YIL001W		0.0350877192982456YOR167C		0.0298507462686567YDL027C		0.0214285714285714YOR100C		0.100917431192661YGL192W		0.25YDR288W		0.132013201320132YIR005W		0.222972972972973YNL204C		0.496666666666667YIR036C		0.0304182509505703YBL107C		0.25YHR204W		0.0942211055276382YBR088C		0YFR006W		0.0598130841121495YFL042C		0.449554896142433YNL300W		0.205882352941176YPL108W		0.357142857142857YDR022C		0.306122448979592YPR002W		0.0271317829457364YPL158C		0.715039577836412YMR047C		0.869721473495058YER131W		0.378151260504202YPL027W		0.244897959183673YNL293W		0.31911532385466YPR155C		0.086038961038961YPL177C		0.545751633986928YNR046W		0.0148148148148148YLR314C		0.403846153846154YKL013C		0YNL003C		0.0246478873239437YER153C		0YBL030C		0.0660377358490566YLR276C		0.205387205387205YNR055C		0.10580204778157YDL155W		0.327868852459016YPL065W		0.128099173553719YOR295W		0.460526315789474YBR111C		0.0909090909090909YGR105W		0.194805194805195YGR066C		0.0273972602739726YGR101W		0.0346820809248555YOR067C		0.0641247833622184YNL012W		0.00475435816164818YFR039C		0.105882352941176YER183C		0.037914691943128YBR151W		0.183544303797468YLR105C		0.251989389920424YDL100C		0.00282485875706215YJL006C		0.188854489164087YMR041C		0.0149253731343284YPR011C		0.0613496932515337YDR511W		0.300751879699248YDR071C		0.0418848167539267YIL039W		0YFR047C		0.0271186440677966YCR011C		0.117254528122021YCR077C		0.474874371859296YJL203W		0.278571428571429YHR139C		0.00613496932515337YOR328W		0.123401534526854YDR178W		0.165745856353591YBL045C		0.0196936542669584YLR149C		0.183561643835616YOR034C		0.054739652870494YMR208W		0.0112866817155756YPL235W		0.0764331210191083YBR119W		0.335570469798658YFL010C		0.767772511848341YER068W		0.643952299829642YOL082W		0.325301204819277YDR469W		0.685714285714286YAL009W		0.185328185328185YJR067C		0.0141843971631206YOR065W		0.00647249190938511YFR017C		0.882051282051282YHR146W		0.772043010752688YPL122C		0.130604288499025YIL045W		0.420074349442379YGL121C		0.111111111111111YOR038C		0.178285714285714YCR047C		0.236363636363636YIL103W		0.115294117647059YJR017C		0YPR113W		0.0954545454545455YMR099C		0.0168350168350168YGR120C		0.00763358778625954YLR190W		0.794297352342159YPL011C		0.337110481586402YPL082C		0.133369041242635YDR063W		0YMR036C		0.633574007220217YHL030W		0.0481798715203426YDR352W		0.151419558359621YOR090C		0.236013986013986YEL021W		0.0112359550561798YBL006C		0.183333333333333YNL183C		0.617721518987342YIL014W		0.0603174603174603YLR144C		0.133504492939666YEL071W		0.0161290322580645YLR324W		0.323135755258126YLR389C		0.0282375851996105YNL263C		0.305732484076433YPR030W		0.498661909009813YIL073C		0.0153846153846154YPR154W		0.613953488372093YKL148C		0.0453125YBL029W		0.5YGL186C		0.0725388601036269YER046W		0.041958041958042YBR005W		0.624413145539906YJR005W		0.132857142857143YPR023C		0.261845386533666YOR367W		0.305YMR071C		0.0419161676646707YOR321W		0.0637450199203187YHR203C		0.0574712643678161YER147C		0.0144230769230769YIL090W		0.0468431771894094YJR109C		0YKL068W		0.844629822732012YIL096C		0.273809523809524YDR454C		0.0053475935828877YDR362C		0.168154761904762YJL187C		0.63003663003663YGR202C		0.476415094339623YDR056C		0.180487804878049YDR408C		0.0327102803738318YDR297W		0.100286532951289YKL112W		0.647058823529412YOL121C		0.0625YLR420W		0.0192307692307692YNL151C		0.49800796812749YCL016C		0.0157894736842105YHR100C		0YOR102W		0.0603448275862069YBR097W		0.151994497936726YNR028W		0.100649350649351YLR298C		0.454545454545455YJR117W		0.0022075055187638YOR271C		0.00917431192660551YDR289C		0.54278728606357YDL028C		0.579842931937173YDR319C		0.072992700729927YGL211W		0.125348189415042YKL133C		0.0323974082073434YNL137C		0.251028806584362YDL176W		0.0946327683615819YDR487C		0.00480769230769231YOR084W		0.0310077519379845YIL135C		0.878440366972477YLR209C		0.0385852090032154YLR093C		0.0434782608695652YEL061C		0.404YLR254C		0.671957671957672YOR166C		0.231441048034935YBR091C		0.119266055045872YKL049C		0.624454148471616YLR277C		0.0629011553273428YNL167C		0.805255023183926YNL006W		0YFR052W		0.0474452554744526YGR203W		0YGL248W		0.013550135501355YGL228W		0.206239168110919YOR226C		0.0512820512820513YPR035W		0.0540540540540541YMR015C		0.00557620817843866YFL014W		0.917431192660551YFL029C		0.00815217391304348YDL076C		0.408163265306122YGR201C		0.00444444444444444YHR049W		0.0164609053497942YJL051W		0.608272506082725YCR024C		0.00813008130081301YPL104W		0.013677811550152YDR018C		0.047979797979798YBR072W		0.177570093457944YML071C		0.309719934102142YKL026C		0.0239520958083832YMR218C		0.0190562613430127YOR079C		0.0830670926517572YGR044C		0.47YPL223C		1YPL250C		0.448529411764706YGL213C		0.0226700251889169YGL101W		0.0418604651162791YKL017C		0.00292825768667643YMR152W		0.00547945205479452YNL083W		0.0458715596330275YBR157C		0.756862745098039YMR061W		0.115214180206795YPL117C		0.0798611111111111YGL189C		0.378151260504202YOR311C		0.186206896551724YMR293C		0.0021551724137931YDR259C		0.825065274151436YCL031C		0.313131313131313YIR026C		0.269230769230769YHR096C		0.131756756756757YDR488C		0.279549718574109YPR169W		0.195121951219512YPL195W		0.32725321888412YEL051W		0.203125YMR165C		0.532482598607889YMR106C		0.141494435612083YBR202W		0.072189349112426YKL052C		0.60958904109589YPL259C		0.0989473684210526YKL140W		0.202554744525547YNL155W		0.386861313868613YBR285W		0.326388888888889YKL124W		0.212435233160622YNR007C		0.141935483870968YOL101C		0.150641025641026YDL022W		0.0767263427109974YJL183W		0.033175355450237YJR125C		0.617647058823529YMR069W		0.129824561403509YML126C		0.0224032586558045YLR258W		0.0936170212765957YMR288W		0.160659114315139YHR176W		0.0324074074074074YOR327C		0.226086956521739YNR027W		0.0157728706624606YCR060W		0.00900900900900901YLR130C		0.281990521327014YDR234W		0.0216450216450216YAL062W		0YFR032C		0.342560553633218YBL014C		0.223713646532438YHL021C		0.0473118279569892YPR133C		0.641463414634146YPL232W		0.131034482758621YPR182W		0.0813953488372093YDL163W		0.26YNL245C		0.972067039106145YPL215W		0.247761194029851YIR028W		0.119685039370079YCL059C		0.424050632911392YAL037W		0.138576779026217YER003C		0.00466200466200466YML046W		0.0254372019077901YCL034W		0.330508474576271YFL039C		0.00266666666666667YHR188C		0.040983606557377YER127W		0.434173669467787YJR090C		0.278887923544744YCL011C		0.36768149882904YPL059W		0.226666666666667YGL050W		0.183150183150183YLR164W		0.0357142857142857YAL055W		0.105555555555556YDL174C		0.0187393526405451YBR261C		0.0172413793103448YEL034W		0.0382165605095541YKL125W		0.205741626794258YML112W		0.128378378378378YHR135C		0.460966542750929YGR077C		0.0067911714770798YDR285W		0.820571428571429YGR285C		0.260969976905312YPL236C		0.0549450549450549YFL025C		0.032069970845481YJR133W		0.0717703349282297YKL171W		0.573275862068966YJR073C		0.0436893203883495YPL101W		0.421052631578947YBL025W		0.0413793103448276YJR107W		0.0274390243902439YDR434W		0.0599250936329588YNL326C		0.0833333333333333YKL005C		0.407407407407407YOR021C		0.0234741784037559YIL079C		0.830555555555556YDR177W		0.00465116279069767YOL018C		0.347607052896725YBR183W		0.0189873417721519YGL124C		0.243788819875776YAR002W		0.961038961038961YIL063C		0.617737003058104YPL010W		0.0582010582010582YER180C		0.528089887640449YPR186C		0.412587412587413YPL079W		0.125YHR182W		0.194904458598726YMR081C		0.736686390532544YER137C		0.587837837837838YHR167W		0.203065134099617YBL031W		0.792899408284024YLR098C		0.242283950617284YEL066W		0.134078212290503YOR056C		0.38562091503268YBL091C		0.173396674584323YFR033C		0.435374149659864YDR194C		0.287650602409639YLR300W		0.0133928571428571YDR350C		0.0474631751227496YDL173W		0.766101694915254YMR017W		0.465994962216625YOR141C		0.36322360953462YDR473C		0.477611940298507YDL057W		0.0152439024390244YIR031C		0.0108303249097473YCL025C		0.186413902053712YKL178C		0.238297872340426YDL077C		0.0571973307912297YNL271C		0.528929851510497YGL096W		0.702898550724638YNL270C		0.12565445026178YMR268C		0.204954954954955YOR207C		0.0191470844212359YDR030C		0.00988142292490119YCR009C		0.0792452830188679YNL157W		1YKL040C		0.0703125YLR211C		0.442477876106195YLR091W		0YML076C		0.50635593220339YNR051C		0.584466019417476YDR538W		0.107438016528926YGR284C		0.12258064516129YLR134W		0.0159857904085258YMR311C		0.847161572052402YDR515W		0.677852348993289YNL284C		0.170807453416149YNL279W		0.0741301059001513YBR236C		0.284403669724771YJR011C		0.0574712643678161YGR262C		0.0383141762452107YIR032C		0YJL180C		0.0738461538461539YEL059C-A		0.0675675675675676YEL024W		0.0418604651162791YNL080C		0.289617486338798YNL127W		0.312696747114376YMR011W		0.066543438077634YJR086W		0.409090909090909YCL051W		0.794168096054888YMR177W		0.190196078431373YBR099C		0.0236220472440945YBR212W		0.50297619047619YOR291W		0.181385869565217YGL069C		0.12987012987013YJL218W		0.0153061224489796YPL130W		0.125560538116592YBL072C		0.105YCR052W		0.304347826086957YPR058W		0.0325732899022801YKL060C		0.0334261838440111YBR185C		0.20863309352518YGL112C		0.0988372093023256YGL019W		0.169064748201439YBR156C		0.86676217765043YOR368W		0.206982543640898YGR008C		0.761904761904762YKL084W		0.0517241379310345YPL006W		0.0282051282051282YKL003C		0.152671755725191YNL191W		0.103641456582633YOL067C		0.553672316384181YJL116C		0.264094955489614YLR370C		0YDL121C		0.657718120805369YMR187C		0.0348027842227378YLR100W		0.0144092219020173YIL111W		0.132450331125828YMR098C		0.199346405228758YCR069W		0.0471698113207547YDL047W		0.00321543408360129YGL106W		0.00671140939597315YKL004W		0.107231920199501YLR241W		0.0639386189258312YOL031C		0.0950118764845606YKL077W		0.155612244897959YER053C		0.0233333333333333YNL076W		0.791095890410959YKL053C-A		0.22093023255814YOR257W		0.111801242236025YBL023C		0.241935483870968YER051W		0.0711382113821138YJL042W		0.562947067238913YPR075C		0.755555555555556YEL057C		0.0300429184549356YNL175C		0.553349875930521YOR215C		0.0378378378378378YNL332W		0.00588235294117647YIL030C		0.214556482183472YBR199W		0.140086206896552YIL134W		0.0578778135048231YPR177C		0.227642276422764YBL010C		0.403571428571429YDL001W		0.234883720930233YBR056W		0.0219560878243513YMR305C		0.210796915167095YOR273C		0.241274658573596YBL054W		0.859047619047619YGL012W		0.0634249471458774YOR133W		0.00712589073634204YML057W		0.399006622516556YFL049W		0.298555377207063YCR005C		0.0130434782608696YML028W		0.0102040816326531YGL032C		0.103448275862069YBR070C		0.0168776371308017YOL060C		0.307365439093484YIL010W		0.293023255813953YGL154C		0.0294117647058824YMR093W		0.0233918128654971YGL191W		0.0155038759689922YJL208C		0.0668693009118541YDR132C		0.139393939393939YER177W		0.187265917602996YNL158W		0.0202020202020202YOR224C		0YNL026W		0.0537190082644628YPR179C		0.337404580152672YER019W		0.0587002096436059YIL091C		0.324549237170596YDR052C		0.586647727272727YGL110C		0.241987179487179YHL011C		0.009375YDR051C		0.0269461077844311YOR370C		0.0945273631840796YDR397C		0.335616438356164YBR120C		0.345679012345679YFL011W		0.0860805860805861YHR210C		0.0146627565982405YBR089C-A		0.333333333333333YHL002W		0.429203539823009YPR185W		0.630081300813008YML114C		0.574509803921569YGR280C		0.649446494464945YDR153C		0.552311435523114YDR229W		0.573951434878587YIR001C		0.66YDR321W		0.036745406824147YHR029C		0YOL091W		0.559934318555008YBR148W		0.61576354679803YLR246W		0.0947075208913649YJL140W		0.524886877828054YOR157C		0YML019W		0YEL048C		0.0197368421052632YBL080C		0.0314232902033272YJR001W		0.267441860465116YER030W		1YER027C		0.515587529976019YFR001W		1YIL075C		0.124867724867725YGL254W		0.317725752508361YJL104W		0.422818791946309YGR165W		0.194202898550725YKL087C		0.334821428571429YNR012W		0.0359281437125748YDR468C		0.1875YCR046C		0.14792899408284YHL012W		0.026369168356998YEL039C		0YDL144C		0.047752808988764YLR200W		0.578947368421053YGL247W		0.16751269035533YNR006W		0.548231511254019YGR286C		0.0826666666666667YIL133C		0YNL124W		0.754065040650406YJR136C		0.0546318289786223YAL005C		0.0576323987538941YNR047W		0.647256438969765YPL131W		0.269360269360269YHR123W		0.0051150895140665YJR025C		0YLR224W		0.024390243902439YHL010C		0.297435897435897YOR174W		0.496478873239437YER122C		0.659229208924949YHR070W		0.0160320641282565YDR184C		0.58843537414966YHR147C		0.0186915887850467YJR074W		0.0321100917431193YOR048C		0.300198807157058YNL295W		0.185114503816794YHR025W		0.0196078431372549YNL254C		0.44139650872818YAL027W		0.130268199233716YBR132C		0.135906040268456YMR213W		0.503389830508475YML027W		0.896103896103896YDR211W		0.10814606741573YDR125C		0.033112582781457YPL169C		0.143572621035058YOL038W		0.062992125984252YGL073W		0.843937575030012YPL200W		0.0128205128205128YPL271W		0.17741935483871YDR354W		0.0236842105263158YPL055C		0.873493975903614YDR331W		0.0437956204379562YDR240C		0.227642276422764YDL237W		0.00256410256410256YDL137W		0.0110497237569061YBR254C		0.142857142857143YBR040W		0.0671140939597315YPR022C		0.409532215357458YDR238C		0.0483042137718397YBR286W		0.0577281191806331YIR022W		0.0179640718562874YEL019C		0.108614232209738YPL114W		0.273381294964029YAL059W		0.64622641509434YBR168W		0.106537530266344YPR119W		0.435845213849287YBR006W		0.0160965794768612YPR118W		0.0097323600973236YBL011W		0.247694334650856YOR189W		0.629310344827586YPL238C		0.0387596899224806YOR292C		0.0517799352750809YOR341W		0.0811298076923077YMR287C		0.0515995872033024YDL097C		0.0276497695852535YER040W		0.963013698630137YPL242C		0.181270903010033YGR277C		0.0524590163934426YAL040C		0.406896551724138YMR277W		0.308743169398907YNL310C		0.28735632183908YLR376C		0.012396694214876YJR104C		0.038961038961039YLR180W		0.0104712041884817YGL044C		0.432432432432432YDL188C		0.185676392572944YOL139C		0.173708920187793YGL031C		0.335483870967742YBR123C		0.218798151001541YDL246C		0.00840336134453781YDR332W		0.02322206095791YDR294C		0.0271646859083192YFR003C		1YFR050C		0YHR072W		0.0191518467852257YNL030W		0.300970873786408YDR374C		0.274509803921569YPL004C		0.348973607038123YOR036W		0.204861111111111YDR126W		0.0297619047619048YOL097C		0.0949074074074074YDL229W		0.00978792822185971YML023C		0.0143884892086331YNL197C		0.745839636913767YDR252W		0.335570469798658YDR306C		0.192468619246862YGR191W		0.15257048092869YJL174W		0.141304347826087YDL179W		0.391447368421053YLR350W		0.25462962962963YOR025W		0.259507829977629YBR066C		0.904545454545455YGR177C		0.0186915887850467YER134C		0.0561797752808989YPR107C		0.278846153846154YBR257W		0.401433691756272YCL052C		0.00480769230769231YDL199C		0.209606986899563YKL121W		0.194835680751174YNL321W		0.251101321585903YMR014W		0.691714836223507YKL041W		0.571428571428571YJL010C		0.141141141141141YOR331C		0.0756756756756757YER163C		0.0603448275862069YKL054C		0.982384823848238YBR230C		0.164179104477612YBR214W		0.324478178368121YPL268W		0.15765247410817YOR020C		0YDR314C		0.140173410404624
