# Supplementary material for: Proteome-wide evidence for enhanced positive Darwinian selection within intrinsically disordered regions in proteins
Source: Genome Biol. 2011 Jul 19;12(7):R65. doi: 10.1186/gb-2011-12-7-r65 (PMC3218827; doi:10.1186/gb-2011-12-7-r65)
Supplement: Additional file 13 — Fraction of amino acid residues for each protein that are predicted by the VSL2 method to adopt intrinsically disordered conformation, using a confidence value threshold of 0.5. [file gb-2011-12-7-r65-S13.RTF]

YHR055C		1YPR161C		0.595129375951294YOL138C		0.636838180462342YGR129W		0.967441860465116YPR165W		0.287081339712919YPL015C		0.316526610644258YCL050C		0.311526479750779YMR193W		0.403100775193798YGR053C		0.491166077738516YOR280C		0.300751879699248YEL004W		0.137426900584795YJL200C		0.224334600760456YDR348C		1YPL183C		0.0473840078973346YGR003W		0.283602150537634YBL095W		0.3YEL009C		1YEL015W		0.55535390199637YDR329C		0.507936507936508YBR252W		0.217687074829932YDL202W		0.29718875502008YHR209W		0.130584192439863YPR008W		0.972622478386167YBR050C		0.849112426035503YMR197C		0.456221198156682YBL049W		0.289855072463768YNL201C		0.421911421911422YCR061W		0.381933438985737YPL171C		0.215YHR051W		0.351351351351351YDL116W		0.380165289256198YPR124W		0.704433497536946YJL023C		0.510086455331412YGL167C		0.211578947368421YDL165W		0.549738219895288YIL143C		0.430604982206406YOR094W		0.103825136612022YDR167W		0.645631067961165YER052C		0.159392789373814YPL118W		0.671511627906977YGR178C		0.93213296398892YPR086W		0.397101449275362YJL065C		0.598802395209581YBR122C		0.790960451977401YBR279W		0.62247191011236YBR271W		0.343675417661098YMR221C		0.218253968253968YER130C		0.986455981941309YBR155W		0.444155844155844YDR363W		0.725877192982456YPL147W		0.26551724137931YLR443W		0.441964285714286YBR067C		0.614285714285714YAL034C		0.811138014527845YLR270W		0.222857142857143YCR095C		0.472375690607735YPR084W		0.557017543859649YMR113W		0.0562060889929742YGR035C		1YDL222C		0.398058252427184YNL335W		0.217777777777778YDR232W		0.319343065693431YHR116W		0.71523178807947YFR018C		0.286501377410468YKL007W		0.130597014925373YDL213C		0.768888888888889YNL292W		0.342431761786601YNL134C		0.143617021276596YKL100C		0.40885860306644YPL021W		0.887700534759358YIL146C		0.638941398865784YNL218W		0.434412265758092YIL121W		0.199261992619926YDL167C		0.688456189151599YPL214C		0.12962962962963YOR228C		0.33112582781457YBR270C		0.671559633027523YGR222W		0.453924914675768YGR121C		0.199186991869919YDL003W		0.802120141342756YOR323C		0.149122807017544YMR183C		0.661016949152542YOR274W		0.257009345794392YER146W		0.258064516129032YPL063W		0.623949579831933YML075C		0.367172675521822YLR273C		0.796296296296296YDR457W		0.447674418604651YCL056C		0.125YJL083W		0.882450331125828YPL140C		0.492094861660079YOR026W		0.0909090909090909YNR016C		0.238244514106583YER123W		0.524809160305344YGR055W		0.193379790940767YMR315W		0.0974212034383954YKL187C		0.669333333333333YDR383C		0.920168067226891YKL055C		0.0683453237410072YPR019W		0.453376205787781YPR190C		0.510703363914373YPL228W		0.644808743169399YGR041W		0.802559414990859YDR353W		0.0658307210031348YPL047W		0.858585858585859YPL014W		0.837270341207349YGR007W		0.229102167182663YML116W		0.169741697416974YOL137W		0.223340040241449YGL155W		0.265957446808511YER061C		0.147058823529412YKL011C		0.461756373937677YNL257C		0.350691619202604YBR291C		0.20066889632107YDR262W		0.522058823529412YDR179C		0.41358024691358YIL053W		0.176YGR220C		0.368029739776952YMR161W		0.763392857142857YER011W		0.811023622047244YGL162W		0.989966555183946YNL243W		0.584710743801653YNL047C		0.670731707317073YKL038W		0.752991452991453YFL053W		0.241962774957699YLR411W		0.390041493775934YLR318W		0.311085972850679YHR150W		0.557858376511226YGL219C		0.671023965141612YBR045C		0.972076788830716YMR039C		0.917808219178082YPL188W		0.306763285024155YGL147C		0.0785340314136126YDR217C		0.792207792207792YPR026W		0.262592898431049YMR008C		0.289156626506024YDL227C		0.298634812286689YOR197W		0.564814814814815YNL081C		0.391608391608392YKL096W-A		0.83695652173913YDR532C		0.755844155844156YKL170W		0.123188405797101YGL047W		0.193069306930693YPR171W		1YML063W		0.309803921568627YLR178C		0.315068493150685YHL029C		0.592047128129602YOR184W		0.19746835443038YDR372C		0.443478260869565YBR008C		0.200729927007299YMR203W		0.284237726098191YBL024W		0.35233918128655YDR317W		0.306763285024155YJL038C		0.429223744292237YDR043C		1YOR340C		0.503067484662577YCL044C		0.570743405275779YFR022W		0.627557980900409YEL072W		0.212121212121212YOL008W		0.314009661835749YHR017W		0.220779220779221YMR095C		0.138392857142857YHR198C		0.504672897196262YOR076C		0.4190093708166YNL219C		0.106306306306306YLR210W		0.460869565217391YIL034C		0.317073170731707YLR148W		0.240740740740741YOR342C		0.448275862068966YIL043C		0.0563380281690141YDL160C		0.320158102766798YGR231C		0.348387096774194YNL021W		0.281869688385269YMR266W		0.318992654774397YPL107W		0.866935483870968YGR088W		0.277580071174377YCR063W		0.509554140127389YDL048C		0.920408163265306YML092C		0.148YPR097W		0.530288909599254YNL056W		0.213197969543147YAL020C		0.258258258258258YFL017C		0.0817610062893082YHR092C		0.210714285714286YDR379C-A		0.291139240506329YNR074C		0.0793650793650794YLR189C		0.427378964941569YDR113C		1YML032C		0.736730360934183YFL028C		0.231833910034602YDL247W		0.22824302134647YBR256C		0.176470588235294YPL024W		0.506224066390041YMR257C		0.3475YDL219W		0.12YKL122C		0.676646706586826YGL240W		0.328YDL080C		0.177339901477833YEL001C		0.248888888888889YPR168W		0.388535031847134YHR109W		0.403418803418803YPL061W		0.042YJL060W		0.166666666666667YLR240W		0.284571428571429YGR104C		0.52442996742671YER174C		0.286885245901639YMR146C		0.0893371757925072YPR057W		0.633431085043988YDR156W		1YJR113C		0.45748987854251YPR131C		0.194871794871795YMR155W		0.281535648994516YNL180C		0.534743202416918YDR272W		0.113138686131387YOR238W		0.412541254125413YLR139C		0.547433903576983YKL142W		0.904109589041096YPR009W		0.75YHR032W		0.282271944922547YDR429C		0.565693430656934YNR031C		0.506649778340722YGL079W		0.86697247706422YOR004W		0.47244094488189YMR244W		0.549295774647887YMR233W		0.65929203539823YNL188W		0.92378752886836YNL104C		0.216478190630048YOR365C		0.174964438122333YNR030W		0.0744101633393829YBR265W		0.190625YGL131C		0.682822523164647YNR040W		0.37890625YOR173W		0.232294617563739YOR337W		0.58498023715415YNL181W		0.223587223587224YGR243W		0.315068493150685YDR464W		1YGR210C		0.233576642335766YOR168W		0.250927070457355YNL002C		0.596273291925466YNL294C		0.386491557223265YHR193C		0.586206896551724YPR147C		0.161184210526316YBR092C		0.220556745182013YOR242C		0.644204851752022YJL176C		0.827878787878788YHR128W		0.0277777777777778YOR297C		0.432291666666667YDR225W		0.659090909090909YPR020W		0.165217391304348YPR114W		0.196825396825397YPL189W		0.201970443349754YJL056C		0.881818181818182YPL247C		0.307839388145315YDR482C		1YJL122W		0.994285714285714YBL058W		0.697399527186761YKL107W		0.207119741100324YCL017C		0.241448692152918YER085C		0.433526011560694YMR186W		0.346099290780142YCR065W		0.870567375886525YFL045C		0.149606299212598YNL020C		0.606583072100313YGL125W		0.195YLR354C		0.17910447761194YFR008W		0.597285067873303YDR284C		0.207612456747405YKL051W		0.30028328611898YPR174C		0.914027149321267YMR091C		0.572413793103448YJL143W		0.291139240506329YGR057C		0.409090909090909YLR308W		0.224358974358974YBR065C		0.590659340659341YMR029C		0.520076481835564YPR121W		0.120629370629371YNL068C		0.812064965197216YGL178W		0.623981373690338YGL157W		0.146974063400576YMR148W		0.547297297297297YML058W		1YLR249W		0.316091954022989YGR019W		0.176220806794055YBR263W		0.148979591836735YPR044C		0.991452991452991YDR286C		0.254385964912281YMR076C		0.35708692247455YJR135W-A		0.988505747126437YDR083W		0.553571428571429YLR109W		0.142045454545455YOR032C		1YOR091W		0.831884057971015YBR205W		0.180693069306931YLR292C		0.233160621761658YLR216C		0.31266846361186YDR046C		0.218543046357616YML018C		0.256997455470738YOR369C		0.321678321678322YOR138C		0.661698956780924YJR145C		0.24904214559387YGR246C		0.647651006711409YJR138W		0.505050505050505YHR059W		0.6YLR138W		0.601015228426396YNL156C		0.454849498327759YNL106C		0.450549450549451YNL286W		0.463157894736842YNL093W		0.318181818181818YIL040W		0.115942028985507YLR186W		0.261904761904762YDR425W		0.6016YDR293C		0.5016YNR015W		0.247395833333333YLR456W		0.269607843137255YDR448W		0.483870967741935YER184C		0.482367758186398YJL066C		0.654761904761905YOR194C		0.79020979020979YLR368W		0.222408026755853YKL095W		0.726618705035971YGR060W		0.148867313915858YBR115C		0.235632183908046YBR147W		0.320945945945946YHR086W		0.586998087954111YFL003C		0.249430523917995YOR125C		0.309012875536481YML079W		0.308457711442786YPL134C		0.167741935483871YNL298W		0.630641330166271YGL225W		0.14540059347181YNL224C		0.863102998696219YNL122C		1YLR129W		0.11558854718982YGL066W		0.960426179604262YNL217W		0.162576687116564YGL063W		0.108108108108108YML030W		0.578616352201258YAL039C		0.576208178438662YOR261C		0.384615384615385YLR191W		0.699481865284974YCR027C		0.258373205741627YDL046W		0.115606936416185YMR263W		0.935323383084577YOL113W		0.502290076335878YDR517W		0.532258064516129YAL001C		0.474137931034483YBR197C		0.976958525345622YJR132W		0.180343511450382YDL106C		0.799642218246869YNL280C		0.0936073059360731YDR505C		0.78359096313912YDL154W		0.264150943396226YMR211W		0.471578947368421YGL202W		0.218YBL036C		0.1284046692607YPR178W		0.279569892473118YOL147C		0.156779661016949YFR007W		0.175637393767705YGR072W		0.824289405684755YER022W		0.672489082969432YIL118W		0.32034632034632YJR008W		0.227810650887574YCL010C		0.50965250965251YJR024C		0.19672131147541YFR011C		0.882352941176471YAL047C		0.837620578778135YDR256C		0.209708737864078YNL287W		0.253475935828877YGR103W		0.583471074380165YOR281C		0.692307692307692YBR162C		0.498901098901099YMR114C		0.445652173913043YGR251W		1YBR094W		0.361221779548473YKL021C		0.217948717948718YOR374W		0.0847784200385356YHL026C		0.336507936507937YHR014W		0.9553264604811YDL015C		0.3YCR028C		0.171875YER058W		0.719626168224299YCR016W		0.831034482758621YER149C		1YJL147C		0.426701570680628YGL222C		1YDR533C		0.10126582278481YDL090C		0.287703016241299YBR177C		0.197339246119734YJL034W		0.173020527859238YNR059W		0.255172413793103YGR156W		0.809411764705882YBR125C		0.307888040712468YBR173C		0.77027027027027YHR057C		0.253658536585366YOR077W		0.625YLR234W		0.205792682926829YHR107C		0.471744471744472YER112W		0.625668449197861YLR174W		0.152912621359223YCL026C-A		0.181347150259067YDR346C		0.326403326403326YLR385C		0.431818181818182YLR117C		0.0655021834061135YDR143C		0.942622950819672YER185W		0.155115511551155YOL013C		0.422867513611615YLR089C		0.273648648648649YKL155C		0.547770700636943YGR154C		0.27247191011236YNL277W		0.316872427983539YOL061W		0.282258064516129YFR013W		0.687420584498094YDL014W		0.415902140672783YCL064C		0.113888888888889YDR493W		0.536585365853659YLR345W		0.265225933202358YLR192C		0.932075471698113YOR265W		0.933962264150943YDR031W		0.619834710743802YBR141C		0.44213649851632YDR453C		0.0663265306122449YAL008W		0.383838383838384YDR490C		0.612271540469974YDR191W		0.364864864864865YNL193W		0.376344086021505YLR360W		0.485193621867882YOR142W		0.167173252279635YDR304C		0.306666666666667YPL020C		0.711755233494364YLR165C		0.165354330708661YHL023C		0.68586387434555YOR283W		0.147826086956522YMR255W		1YPL154C		0.204938271604938YDR033W		0.253125YDR253C		1YNL014W		0.32183908045977YAL032C		0.936675461741425YNL291C		0.295620437956204YOR348C		0.251993620414673YMR104C		0.555391432791728YOR097C		0.588571428571429YOL126C		0.161803713527851YOL107W		0.216374269005848YHR026W		0.183098591549296YGR028W		0.345303867403315YGL061C		0.777327935222672YJL089W		0.635705669481303YKL116C		0.511583011583012YHR134W		0.50185873605948YBR228W		0.404605263157895YNL320W		0.105633802816901YGR123C		0.300194931773879YMR220W		0.319290465631929YBR269C		1YMR188C		0.662447257383966YLR290C		0.24187725631769YNL258C		0.453580901856764YNL075W		0.337931034482759YPR159W		0.484722222222222YHR089C		0.682926829268293YNR018W		0.348214285714286YAL044C		0.235294117647059YKL139W		0.503787878787879YHR129C		0.0911458333333333YOR253W		0.136363636363636YIL105C		0.641399416909621YDR403W		0.313432835820896YDL029W		0.0920716112531969YDR525W-A		0.291139240506329YPL255W		0.935064935064935YEL012W		0.376146788990826YOR192C		0.232053422370618YGL229C		0.552567237163814YOL005C		0.166666666666667YML052W		0.317880794701987YML064C		0.33469387755102YHL019C		0.434710743801653YBR167C		0.478571428571429YPR176C		0.138461538461538YFL016C		0.649706457925636YLR383W		0.468581687612208YJL062W		0.153012048192771YDR140W		0.0769230769230769YNL252C		0.565836298932384YMR196W		0.316176470588235YOR205C		0.404676258992806YER114C		0.915384615384615YGL258W		0.0533980582524272YIL002C		0.330866807610994YDR518W		0.243713733075435YLR328W		0.488778054862843YKL173W		0.378968253968254YBL003C		0.659090909090909YNR013C		0.304250559284116YJR077C		0.183279742765273YPR078C		0.948924731182796YGL058W		0.226744186046512YFL005W		0.246511627906977YMR284W		0.410299003322259YML061C		0.474970896391153YLR449W		0.528061224489796YDL007W		0.453089244851259YOR145C		0.445255474452555YOR287C		1YIL122W		0.945868945868946YPL218W		0.126315789473684YJL145W		0.248299319727891YPR198W		0.178637200736648YNR052C		0.526558891454965YOR244W		0.319101123595506YMR314W		0.11965811965812YLR173W		0.192434210526316YHR190W		0.173423423423423YPL097W		0.258130081300813YNL115C		0.349378881987578YLR103C		0.312307692307692YKL132C		0.0837209302325581YPL222W		0.284883720930233YKL012W		0.564322469982847YLR395C		0.474358974358974YAL041W		0.615925058548009YBL052C		0.645006016847172YCL045C		0.201315789473684YDL135C		0.450495049504951YMR237W		0.339779005524862YGR046W		0.322077922077922YGR224W		0.228384991843393YDR308C		0.685714285714286YJL153C		0.138836772983114YBR035C		0.12280701754386YCR002C		0.279503105590062YDR244W		0.382352941176471YAL016W		0.108661417322835YPR093C		0.496527777777778YMR239C		0.556263269639066YLR163C		0.16017316017316YDL072C		0.408866995073892YPL190C		0.817955112219451YER059W		0.702380952380952YBR207W		0.365591397849462YIL049W		0.0671936758893281YBR069C		0.252019386106624YGL215W		0.732300884955752YPR066W		0.123745819397993YOR135C		0.309734513274336YNL202W		0.13013698630137YLR099C		0.241116751269036YOR080W		0.357908847184987YNL133C		1YJL167W		0.127840909090909YIL097W		0.368217054263566YOR198C		0.865957446808511YNL101W		0.481065918653576YGL241W		0.166334661354582YML055W		0.168539325842697YHR143W-A		0.885714285714286YFL052W		0.382795698924731YML035C		0.402469135802469YGR133W		0.224043715846995YDR357C		0.254098360655738YDR006C		0.622641509433962YNL256W		0.229368932038835YML100W		0.390710382513661YHR197W		0.440366972477064YNL325C		0.379977246871445YBR128C		0.502906976744186YPL258C		0.107078039927405YNL216W		0.782345828295042YIL036W		1YLR307W		0.259136212624585YML119W		0.932773109243697YPL273W		0.110769230769231YCL066W		0.605714285714286YPL201C		0.318872017353579YHR003C		0.254079254079254YPR041W		0.535802469135802YHR171W		0.241269841269841YER116C		0.839416058394161YOR136W		0.105691056910569YOL086W-A		0.211111111111111YLR364W		0.220183486238532YGR142W		0.660975609756098YKL186C		0.41304347826087YGR264C		0.276964047936085YDR206W		0.565610859728507YPR042C		0.713488372093023YJR152W		0.191528545119705YBR085W		0.182410423452769YKL106W		0.106430155210643YBR281C		0.152619589977221YDL153C		0.898360655737705YBL078C		0.393162393162393YDL150W		0.867298578199052YDR398W		0.43701399688958YIL077C		0.721875YGR038W		0.346846846846847YMR153W		0.818947368421053YPL266W		0.166666666666667YPL264C		0.201133144475921YHR132C		0.155813953488372YLR336C		0.648498331479422YER115C		0.638743455497382YDR441C		0.132596685082873YDR101C		0.470489038785835YMR121C		0.490196078431373YHL014C		0.195061728395062YMR312W		0.311355311355311YOR382W		0.888888888888889YOR214C		0.63135593220339YLR141W		0.68870523415978YJL030W		0.188775510204082YDR410C		0.125523012552301YOR335C		0.192066805845511YNL087W		0.330220713073005YHR015W		0.544764795144158YGL037C		0.24537037037037YDR502C		0.130208333333333YAR018C		0.468965517241379YOR112W		0.488830486202365YKL045W		0.511363636363636YBR258C		1YPR074C		0.119117647058824YHR162W		0.193798449612403YLR262C-A		1YML013W		0.578767123287671YPL003W		0.199134199134199YLR297W		0.751937984496124YMR319C		0.344202898550725YDL127W		0.561688311688312YOR152C		0.32421875YDL216C		0.606818181818182YFR028C		0.464609800362976YBR068C		0.221674876847291YHR126C		0.685534591194969YHR008C		0.201716738197425YOL001W		0.525597269624573YML111W		0.705434782608696YMR016C		0.89171974522293YKL023W		0.989169675090253YBR133C		0.370012091898428YGR229C		0.647524752475248YOR092W		0.427406199021207YNR010W		0.89261744966443YPL001W		0.197860962566845YDR047W		0.0635359116022099YBR159W		0.15850144092219YGR158C		0.376YOR210W		0.228571428571429YKL174C		0.229773462783172YLR403W		0.953147877013177YLR286C		0.519572953736655YBR274W		0.40607210626186YDL044C		0.659090909090909YNL305C		0.208754208754209YER081W		0.162046908315565YDR148C		0.403887688984881YJL059W		0.193627450980392YIL130W		0.649377593360996YEL044W		0.813253012048193YGL108C		1YOR351C		0.356136820925553YGL023C		0.933858267716535YBL066C		0.731707317073171YBL051C		0.862275449101796YJR068W		0.192634560906516YDL168W		0.0854922279792746YBR020W		0.210227272727273YDL049C		0.257462686567164YAL019W		0.634836427939876YDR484W		0.447737909516381YEL037C		0.733668341708543YBR273C		0.644495412844037YDL114W		0.136363636363636YIL062C		0.435064935064935YML091C		0.337770382695507YCL021W-A		0.48YOR372C		0.996389891696751YGL098W		0.620408163265306YNL329C		0.295145631067961YMR199W		0.542124542124542YDL209C		0.68141592920354YMR300C		0.0862745098039216YCR039C		0.814285714285714YIL107C		0.52962515114873YIL057C		0.51219512195122YGL136C		0.390625YHR024C		0.176348547717842YLR120C		0.307557117750439YGR211W		0.329218106995885YLR237W		0.230769230769231YBL007C		0.778938906752412YNR064C		0.0862068965517241YPL075W		0.703184713375796YPL017C		0.062124248496994YBR301W		0.3YML004C		0.288343558282209YMR202W		0.157657657657658YGR292W		0.155821917808219YNL267W		0.615384615384615YEL029C		0.102564102564103YLR250W		0.709401709401709YGR245C		0.560625814863103YNL040W		0.114035087719298YIL003W		0.358361774744027YGL161C		0.438709677419355YPR144C		0.454710144927536YOR359W		0.908221797323136YFL055W		0.168458781362007YGR181W		0.60952380952381YGR070W		0.490909090909091YPL029W		0.373134328358209YLR116W		0.785714285714286YDR197W		0.264781491002571YPL224C		0.342984409799555YPL172C		0.363636363636364YJR144W		0.479553903345725YMR083W		0.0853333333333333YGR086C		0.539823008849557YCR048W		0.4YDL065C		0.964912280701754YMR126C		0.526315789473684YDR175C		0.495297805642633YER032W		0.987442922374429YGR194C		0.21YGR078C		0.412060301507538YMR265C		0.308026030368764YHR163W		0.0522088353413655YOR222W		0.166123778501629YBL103C		0.952674897119342YIL052C		0.867768595041322YBR242W		0.260504201680672YBL021C		0.576388888888889YNL246W		0.435606060606061YOL030W		0.353305785123967YER012W		0.0707070707070707YLR301W		0.315573770491803YPR139C		0.406666666666667YPL202C		0.793269230769231YOL068C		0.37375745526839YLR114C		0.545811518324607YJL019W		0.571847507331378YLR248W		0.60327868852459YIL020C		0.0421455938697318YNL063W		0.140127388535032YOR391C		0.109704641350211YFR044C		0.108108108108108YFL023W		0.914572864321608YDR212W		0.171735241502683YOR103C		0.253846153846154YMR185W		0.495412844036697YJL091C		0.193877551020408YDR065W		0.413698630136986YHR174W		0.157894736842105YLR441C		0.294117647058824YBR204C		0.317333333333333YLR283W		0.592356687898089YGL174W		0.890977443609023YGR145W		0.438472418670438YGR199W		0.235836627140975YDR061W		0.246753246753247YBL005W		0.558401639344262YIL108W		0.28735632183908YDR404C		0.0409356725146199YOR058C		0.67683615819209YBL104C		0.466281310211946YBR095C		0.704651162790698YBR084W		0.145641025641026YMR173W		1YHR085W		0.568862275449102YOR301W		0.510344827586207YDL210W		0.157618213660245YNL308C		0.966159052453469YOR171C		0.386217948717949YIL050W		0.6YBL099W		0.258715596330275YLR359W		0.118257261410788YPL217C		0.615384615384615YNR009W		1YGL194C		0.252212389380531YOR106W		0.685512367491166YJR056C		0.779661016949153YLR287C-A		1YDR044W		0.167682926829268YLR118C		0.162995594713656YDR387C		0.133333333333333YOL130W		0.664726426076834YIL071C		0.40990990990991YDR169C		0.9317738791423YER178W		0.319047619047619YMR210W		0.271714922048998YOR338W		0.768595041322314YER088C		1YDR115W		1YOR334W		0.41063829787234YGR256W		0.109756097560976YMR107W		1YDR451C		0.934844192634561YCL063W		0.988179669030733YJL192C		0.247863247863248YHR177W		0.799116997792494YBR039W		0.334405144694534YIR027C		0.130434782608696YAL042W		0.448192771084337YPR036W		0.253138075313808YIL007C		0.331818181818182YOR030W		0.423263327948304YPL267W		0.933014354066986YMR243C		0.407239819004525YDR188W		0.146520146520147YNR034W-A		0.326530612244898YEL023C		0.495601173020528YNL045W		0.135618479880775YPR141C		0.669410150891632YIL104C		0.47534516765286YNL282W		0.246153846153846YLR424W		0.556497175141243YDR520C		0.509067357512953YBR243C		0.142857142857143YGL137W		0.132733408323959YNL212W		0.352941176470588YMR234W		0.370689655172414YMR112C		0.816793893129771YHR005C-A		0.591397849462366YGL212W		0.715189873417722YNR023W		0.600706713780919YPL253C		0.69242658423493YBR096W		0.204347826086957YJR042W		0.325268817204301YGR150C		0.381944444444444YLR390W-A		0.840336134453782YGR074W		0.479452054794521YHR043C		0.113821138211382YGL180W		0.59866220735786YDR085C		0.938709677419355YBR146W		0.467625899280576YPL043W		0.648175182481752YHR011W		0.15695067264574YDL235C		0.413173652694611YLR278C		0.76510067114094YLR348C		0.134228187919463YGL158W		0.490234375YAL028W		0.821969696969697YDL002C		0.876847290640394YDR099W		0.556776556776557YNL173C		0.775956284153005YMR216C		0.622641509433962YMR306W		0.254901960784314YLR196W		0.319444444444444YBR085C-A		0.529411764705882YDL059C		0.42436974789916YOR329C		0.913990825688073YJR116W		0.157706093189964YHL004W		0.576142131979695YBR073W		0.478354978354978YDR449C		0.363636363636364YDL088C		0.821969696969697YNL161W		0.686507936507937YDR435C		0.24390243902439YDL052C		0.237623762376238YMR144W		0.961988304093567YDR161W		0.129198966408269YER062C		0.112YCR068W		0.396153846153846YPR051W		0.0965909090909091YOR285W		0.237410071942446YLR253W		0.173989455184534YJL001W		0.134883720930233YHR067W		0.160714285714286YGL237C		1YDR120C		0.371929824561403YJL012C		0.371705963938974YER126C		0.670498084291188YMR246W		0.145533141210375YPL031C		0.226229508196721YLR201C		0.288461538461538YHR091C		0.1850699844479YGR020C		0.245762711864407YML066C		0.2710027100271YDR501W		0.744721689059501YNL088W		0.337535014005602YNL053W		0.719836400817996YDR400W		0.102941176470588YDR137W		0.586726998491704YBR231C		0.841584158415842YDL120W		0.408045977011494YLR151C		0.397058823529412YFR021W		0.24YML121W		0.261290322580645YLR310C		0.591567023285085YGR244C		0.161592505854801YGL255W		0.268617021276596YHR046C		0.203389830508475YOR016C		0.173913043478261YML065W		0.526258205689278YPR005C		0.789115646258503YGR223C		0.285714285714286YAR003W		0.107981220657277YMR267W		0.290322580645161YNL077W		0.647727272727273YJR034W		0.833333333333333YPL160W		0.253211009174312YGL185C		0.174142480211082YKL175W		0.560636182902585YDR456W		0.345971563981043YGL190C		0.243346007604563YOR101W		0.472491909385113YMR262W		0.182108626198083YGL005C		0.874551971326165YNL125C		0.387815750371471YBL019W		0.488461538461538YBR043C		0.37300435413643YDL231C		0.343111111111111YML101C		0.760683760683761YPR007C		0.792647058823529YMR108W		0.243085880640466YIL067C		0.306784660766962YPL126W		0.204241071428571YKL179C		0.904270986745214YJL033W		0.520779220779221YEL041W		0.397979797979798YGL208W		0.708433734939759YNL113W		0.471830985915493YGL025C		0.863979848866499YBR171W		0.349514563106796YDL005C		0.930394431554524YNL031C		0.514705882352941YDL056W		0.440576230492197YML120C		0.210526315789474YGR048W		0.512465373961219YOL149W		0.432900432900433YML129C		0.442857142857143YBR220C		0.167857142857143YBR227C		0.530769230769231YNR056C		0.133689839572193YER165W		0.47313691507799YNL327W		0.958693563880884YOR130C		0.13013698630137YPL099C		0.791208791208791YER016W		0.604651162790698YNL242W		0.429020100502513YMR058W		0.19496855345912YOL078W		0.797619047619048YAL018C		0.2YEL002C		0.16046511627907YJL156C		0.489082969432314YMR166C		0.320652173913043YCL032W		0.690751445086705YPL050C		0.30379746835443YDR510W		0.673267326732673YLR327C		1YLR142W		0.25YLR104W		0.50381679389313YNL222W		0.203883495145631YLR291C		0.26246719160105YBR293W		0.0886075949367089YLR412W		0.346715328467153YNR067C		0.528200537153089YNL074C		0.980088495575221YBR021W		0.255924170616114YML021C		0.37883008356546YER038C		0.521551724137931YNL044W		0.357954545454545YER060W-A		0.132075471698113YER173W		0.576631259484067YOR190W		0.103370786516854YML048W		0.349875930521092YBR130C		0.941176470588235YPR072W		0.844642857142857YHR048W		0.155642023346304YHL034C		0.520408163265306YHL006C		0.4YDR418W		0.272727272727273YLR183C		0.78118609406953YIR024C		0.652777777777778YCR038C		0.299065420560748YJR040W		0.191270860077022YJL037W		0.495535714285714YOR059C		0.346666666666667YNR002C		0.226950354609929YMR172C-A		0.062992125984252YIL158W		0.808823529411765YER048W-A		0.212765957446809YGR278W		0.419410745233969YDR462W		0.741496598639456YMR273C		0.955191256830601YJL162C		0.801029159519726YPR080W		0.11353711790393YFL017W-A		0.155844155844156YGL127C		0.236220472440945YGR268C		0.893939393939394YHL033C		0.58203125YMR285C		0.497087378640777YBR104W		0.252279635258359YPL263C		0.480798771121352YER096W		0.470703125YOR325W		0.197452229299363YDR311W		0.630841121495327YKL088W		0.691768826619965YJL046W		0.224938875305623YLR228C		0.708845208845209YKL056C		0.119760479041916YPL037C		0.67515923566879YDR459C		0.331550802139037YOR357C		0.450617283950617YNR004W		0.198630136986301YNL192W		0.474801061007958YGL068W		0.855670103092783YDL157C		0.542372881355932YOR007C		0.543352601156069YBR105C		0.610497237569061YDL013W		0.859450726978998YGL200C		0.197044334975369YDR260C		0.523529411764706YNR072W		0.210992907801418YBR154C		0.311627906976744YPL156C		0.559859154929577YKL048C		0.621875YJL020C		0.821953327571305YLR251W		0.16751269035533YGR113W		0.810495626822157YBR253W		0.669421487603306YCR020C		0.0651162790697674YJR141W		0.37463976945245YHR060W		0.635359116022099YDR361C		0.512367491166078YPL009C		0.582851637764933YNL027W		1YNL065W		0.286689419795222YNL211C		0.662790697674419YHL038C		0.322222222222222YPL070W		0.718954248366013YOL111C		0.867924528301887YDR075W		0.188311688311688YER009W		0.024YFL034C-B		0.439024390243902YIR011C		0.639498432601881YMR297W		0.280075187969925YIL139C		0.318367346938775YDR254W		0.456331877729258YPL005W		0.488448844884488YGL105W		0.515957446808511YDL224C		0.85824345146379YKL062W		1YJL158C		0.383259911894273YDR162C		0.741525423728814YCR059C		0.232558139534884YDL183C		0.546875YCL039W		0.197315436241611YER140W		0.393884892086331YPL069C		0.226865671641791YOR386W		0.235398230088496YPL151C		0.108647450110865YNL042W		0.987373737373737YHR207C		0.475285171102662YDR171W		0.730666666666667YPL157W		0.342857142857143YDR479C		0.51985559566787YAL034W-A		0.761245674740484YOR258W		0.419354838709677YNR057C		0.147679324894515YIL015W		0.427597955706985YHR127W		0.617283950617284YNL328C		0.73972602739726YCL049C		0.413461538461538YHR111W		0.254545454545455YGL055W		0.294117647058824YBR288C		0.287784679089027YOR347C		0.124505928853755YGL065C		0.137176938369781YHR013C		0.281512605042017YOR307C		0.346578366445916YNL144C		0.77972972972973YIR038C		0.0897435897435897YAL046C		0.440677966101695YPL193W		0.968503937007874YGR208W		0.0841423948220065YDR201W		0.866666666666667YML077W		0.320754716981132YGL245W		0.186440677966102YHR058C		0.583050847457627YPR181C		0.283854166666667YBR106W		0.367021276595745YHR018C		0.129589632829374YBR170C		0.494827586206897YDL211C		0.731182795698925YPL078C		0.377049180327869YOL007C		0.759530791788856YDR399W		0.235294117647059YKL181W		0.238875878220141YGR192C		0.00903614457831325YDL078C		0.0932944606413994YOL041C		0.751633986928105YMR226C		0.134831460674157YHR200W		0.444029850746269YPR125W		0.559471365638767YEL046C		0.118863049095607YIR015W		0.625YMR242C		0.140449438202247YDR204W		0.340298507462687YPL161C		0.315955766192733YDR300C		0.252336448598131YLR229C		0.157068062827225YLR421C		0.333333333333333YPL096W		0.385674931129477YML038C		0.341628959276018YJL082W		0.421340629274966YIL006W		0.319034852546917YLR392C		0.480694980694981YGR075C		0.367768595041322YBR268W		0.704761904761905YHR068W		0.266149870801034YGL160W		0.254385964912281YGR042W		0.749077490774908YPR160W		0.17960088691796YER007W		0.698841698841699YGR095C		0.188340807174888YDL089W		0.605371900826446YDR522C		0.252988047808765YEL062W		0.48130081300813YBR026C		0.152631578947368YLR143W		0.213138686131387YMR125W		0.328687572590012YLR126C		0.0438247011952191YGL086W		0.811748998664886YNL129W		0.245833333333333YGL048C		0.345679012345679YER106W		0.76158940397351YGR169C		0.23019801980198YMR074C		0.903448275862069YMR269W		0.962085308056872YHR138C		0.43859649122807YFL021W		0.896078431372549YPL144W		0.324324324324324YEL053C		0.418826739427012YHL020C		0.846534653465347YGR106C		0.49811320754717YLR452C		0.531518624641834YMR005W		0.847938144329897YLR363C		0.348623853211009YIL023C		0.257225433526012YKL138C		0.740458015267176YGL002W		0.194444444444444YOR312C		0.132183908045977YLR179C		0.298507462686567YNL230C		0.79155672823219YHL028W		0.927272727272727YCR015C		0.441640378548896YPR199C		0.622448979591837YMR313C		0.21183800623053YBR077C		0.487654320987654YOR298W		0.298538622129436YDR380W		0.174803149606299YDL177C		0.382352941176471YGR149W		0.344907407407407YPL203W		0.273684210526316YNR021W		0.316831683168317YGR080W		0.424698795180723YBR195C		0.203791469194313YPL191C		0.413888888888889YMR013C		0.092485549132948YPL148C		0.265895953757225YFL038C		0.281553398058252YPL240C		0.337094499294781YGL145W		0.303851640513552YPR060C		0.296875YNL004W		0.470862470862471YLR232W		0.773913043478261YHR069C		0.389972144846797YEL018W		0.645161290322581YLR323C		0.733590733590734YGL172W		0.741525423728814YDL143W		0.113636363636364YKL162C		0.271144278606965YMR290C		0.24950495049505YDL021W		0.257234726688103YPL211W		0.110497237569061YDR452W		0.369436201780415YAL038W		0.098YMR024W		0.528205128205128YLR147C		0.306930693069307YDL018C		0.231111111111111YML012W		0.270142180094787YIL061C		0.736666666666667YGR155W		0.224852071005917YLR136C		0.926315789473684YDL060W		0.436548223350254YIL085C		0.381044487427466YHR075C		0.32YOL119C		0.227544910179641YNL289W		0.53405017921147YJR105W		0.0588235294117647YER161C		1YNR036C		0.660130718954248YMR111C		0.909090909090909YJL096W		0.428571428571429YBR136W		0.276182432432432YMR090W		0.118942731277533YNR061C		0.429223744292237YNL154C		0.549450549450549YGR209C		0.0961538461538462YDL181W		1YKL029C		0.246636771300448YPR106W		0.489841986455982YNL039W		0.883838383838384YDL149W		0.53259779338014YGR187C		0.355329949238579YBR282W		0.561643835616438YER170W		0.16YPL269W		0.624223602484472YHR137W		0.255360623781676YGR058W		0.602985074626866YGL009C		0.20795892169448YBL009W		0.702662721893491YDR279W		0.491428571428571YLR243W		0.0919117647058824YER029C		0.678571428571429YJL025W		0.464980544747082YDR246W		0.465753424657534YDR427W		0.173027989821883YER182W		0.30327868852459YNL163C		0.338738738738739YMR228W		0.202346041055718YDR411C		0.404692082111437YNL061W		0.470873786407767YDR309C		0.976501305483029YER095W		0.3675YJL138C		0.108860759493671YDR499W		0.431057563587684YJL063C		0.390756302521008YMR206W		0.987220447284345YMR154C		0.237964236588721YLR410W		0.5YER092W		1YBR101C		0.344827586206897YIL042C		0.33502538071066YHR112C		0.0476190476190476YGL029W		1YGR160W		0.896551724137931YJR060W		0.971509971509972YDR437W		0.1YHL022C		0.339195979899498YGL175C		0.852173913043478YLR305C		0.280526315789474YIL124W		0.144781144781145YGR234W		0.0902255639097744YCR004C		0.384615384615385YKL028W		0.695020746887967YPR173C		0.443935926773455YPR137W		0.232111692844677YAL011W		0.8YNL067W		0.0785340314136126YEL016C		0.217038539553753YEL020C		0.114285714285714YPL060W		0.368038740920097YER087W		0.125YOL087C		0.459677419354839YNL304W		0.633093525179856YHR056C		0.616081540203851YNL092W		0.3YPL152W		0.290502793296089YKL033W		0.438342967244701YHR214W		0.714285714285714YGL243W		0.4625YBR025C		0.197969543147208YNL208W		1YER055C		0.0707070707070707YJR123W		0.413333333333333YHR065C		0.303393213572854YNL128W		0.334101382488479YDR405W		0.752851711026616YMR139W		0.318918918918919YDL217C		0.386473429951691YPL094C		0.448905109489051YJL157C		0.633734939759036YER120W		0.545081967213115YLR315W		0.647058823529412YDR516C		0.25YPL106C		0.186147186147186YFR042W		0.11YLR256W		0.720372836218375YPR191W		0.125YMR241W		0.226114649681529YBR260C		0.552552552552553YLR396C		0.390738060781476YNR037C		0.406593406593407YPL243W		0.47245409015025YBR018C		0.229508196721311YCR043C		0.31496062992126YPL270W		0.283311772315653YBR054W		0.284883720930233YNL164C		0.871794871794872YNL062C		0.47489539748954YMR298W		0.286666666666667YML106W		0.190265486725664YMR037C		1YKL094W		0.12779552715655YOR066W		0.97933227344992YJR147W		0.653631284916201YDR041W		0.423645320197044YMR272C		0.28125YKL143W		0.928725701943845YDL081C		0.509433962264151YIL019W		1YDL201W		0.335664335664336YNL200C		0.126016260162602YER073W		0.0826923076923077YJR007W		0.217105263157895YNL238W		0.404176904176904YBR053C		0.195530726256983YCR073W-A		0.263492063492063YOR269W		0.176113360323887YPL163C		0.780769230769231YML125C		0.102564102564103YOL057W		0.185654008438819YKL145W		0.50321199143469YIL150C		0.756567425569177YGR146C		0.919431279620853YDL126C		0.4YDL006W		0.163701067615658YMR025W		0.386440677966102YDL233W		0.655021834061135YCL057C-A		0.422680412371134YOR362C		0.333333333333333YMR276W		0.798927613941019YOR217W		0.571428571428571YML124C		0.191011235955056YPL246C		0.206106870229008YDL178W		0.171698113207547YOR111W		0.129310344827586YNL240C		0.331975560081466YLR152C		0.388888888888889YKL019W		0.164556962025316YGR168C		0.268617021276596YGR027C		0.472222222222222YGL250W		0.522821576763486YEL031W		0.253497942386831YDR320C-A		0.180555555555556YIL099W		0.207650273224044YJR135C		0.610878661087866YIR013C		1YIL094C		0.0943396226415094YMR032W		0.63677130044843YPL112C		0.33502538071066YAL049C		0.0447154471544715YMR010W		0.348148148148148YBR193C		0.600896860986547YCR023C		0.317512274959083YHL009C		0.83030303030303YDL086W		0.131868131868132YOR201C		0.444174757281553YPR109W		0.482993197278912YIL038C		0.850478468899522YPL137C		0.682601880877743YML082W		0.281972265023112YPL233W		0.439814814814815YJL004C		0.334975369458128YOR286W		0.288590604026846YOR315W		0.979768786127168YNL010W		0.0954356846473029YIL087C		0.547770700636943YHR061C		0.968152866242038YPL111W		0.0810810810810811YGL146C		0.282958199356913YBR182C		0.869469026548673YPR015C		0.91497975708502YER026C		0.340579710144928YOL036W		0.964520367936925YPR100W		0.335714285714286YDL139C		0.905829596412556YNR033W		0.198221092757306YKL149C		0.407407407407407YBL093C		0.913636363636364YPR122W		0.347682119205298YOL009C		0.431734317343173YER124C		0.37347294938918YGR253C		0.173076923076923YDR055W		0.448198198198198YDL110C		0.933333333333333YOR049C		0.336158192090395YMR027W		0.18936170212766YJR032W		0.290076335877863YIL044C		0.664429530201342YBR011C		0.254355400696864YDR419W		0.47626582278481YBR110W		0.102449888641425YLR247C		0.464652956298201YPL208W		0.276157804459691YOR319W		0.267605633802817YLR371W		0.610619469026549YDL051W		0.570909090909091YJL072C		0.352112676056338YHR081W		0.744565217391304YOR061W		0.218289085545723YDR090C		0.3YPL256C		0.561467889908257YOR070C		0.529042386185243YNL024C		0.24390243902439YJL217W		0.0808080808080808YGL093W		0.731733914940022YDR523C		0.487755102040816YKL098W		0.358543417366947YCR008W		0.611940298507463YOR298C-A		1YOL059W		0.281818181818182YOR250C		0.195505617977528YGR110W		0.312359550561798YHL036W		0.133699633699634YJR088C		0.284246575342466YLR378C		0.170833333333333YHR020W		0.289244186046512YMR235C		0.380835380835381YBL050W		0.167808219178082YNL185C		0.360759493670886YFR036W		1YMR067C		0.677884615384615YML086C		0.165399239543726YMR167W		0.374512353706112YDR016C		0.829787234042553YGR062C		0.392405063291139YIL113W		0.344497607655502YNL234W		0.666666666666667YDR414C		0.146408839779006YPL045W		0.131578947368421YGR189C		0.603550295857988YCR086W		0.484210526315789YHR039C		0.130434782608696YFL044C		0.3421926910299YIL009W		0.151296829971182YNL046W		0.552325581395349YOR355W		0.835249042145594YPL265W		0.225328947368421YDL128W		0.172749391727494YHR090C		0.617021276595745YDR214W		0.271428571428571YPR061C		0.541528239202658YGR267C		0.34156378600823YGR185C		0.276649746192893YML050W		0.446945337620579YNL078W		0.857493857493858YMR158W		0.109677419354839YOR262W		0.291066282420749YHR016C		0.527777777777778YFL004W		0.515700483091787YDL045W-A		0.694736842105263YNL290W		0.129411764705882YMR132C		0.341346153846154YDR358W		0.615798922800718YGL085W		0.27007299270073YBR010W		0.514705882352941YML008C		0.245430809399478YIL119C		0.793611793611794YDR073W		0.828402366863905YOR180C		0.107011070110701YPL170W		0.335526315789474YGL139W		0.290523690773067YLR287C		0.492957746478873YBR248C		0.144927536231884YAL044W-A		0.309090909090909YGL140C		0.425758818703856YGR180C		0.214492753623188YPL229W		0.893203883495146YDR079W		0.558558558558559YDR183W		0.530434782608696YDR205W		0.283149171270718YER118C		0.520435967302452YKL144C		0.193396226415094YLR343W		0.340540540540541YMR064W		0.403474903474904YGR193C		0.392682926829268YML041C		0.764285714285714YMR260C		0.496732026143791YOR060C		0.447470817120623YHR191C		0.458646616541353YDL170W		0.439393939393939YDR185C		0.0391061452513966YPL153C		0.594397076735688YML072C		0.445954692556634YGR036C		0.163179916317992YKL086W		0.566929133858268YGL226W		0.308943089430894YBR283C		0.118367346938776YGR196C		0.957160342717258YNL330C		0.265588914549654YLR393W		0.369175627240143YHR189W		0.189473684210526YDR513W		0.181818181818182YPR069C		0.0784982935153584YNL064C		0.662591687041565YBR061C		0.370967741935484YPL002C		0.0686695278969957YJR053W		0.952961672473868YHR010W		0.455882352941176YER102W		0.455YKL074C		0.74573055028463YGR126W		1YAL036C		0.249322493224932YKL082C		0.967741935483871YGL010W		0.0804597701149425YHR195W		0.791277258566978YIL131C		0.650826446280992YDR287W		0.198630136986301YMR002W		0.935897435897436YDR122W		0.710526315789474YIL127C		1YIR042C		0.152542372881356YBL068W		0.0978593272171254YPR055W		0.490140845070423YMR281W		0.177631578947368YBR019C		0.0743919885550787YOR256C		0.320148331273177YER139C		0.685840707964602YNL174W		0.221052631578947YBR160W		0.11744966442953YMR006C		0.392351274787535YHR064C		0.122676579925651YMR174C		1YPL120W		0.599640933572711YOR151C		0.277777777777778YPR025C		0.506361323155216YLR121C		0.281496062992126YNL220W		0.0877598152424942YJL186W		0.445392491467577YGR263C		0.200471698113208YIL024C		0.455026455026455YPR070W		0.487632508833922YMR087W		0.183098591549296YDR068W		0.861290322580645YGR059W		0.5078125YKL080W		0.191326530612245YGL246C		0.322997416020672YBL075C		0.172573189522342YHR108W		0.673504273504274YER035W		1YMR299C		0.538461538461538YML093W		1YGL143C		0.312348668280872YNR073C		0.111553784860558YMR030W		0.800531914893617YDL117W		0.510734463276836YOL075C		0.205564142194745YOL056W		0.257425742574257YIL022W		0.587006960556845YMR073C		0.611940298507463YDR224C		0.541984732824427YER171W		0.224935732647815YNL141W		0.0259365994236311YDL010W		0.497835497835498YDR106W		0.130281690140845YHR078W		0.311594202898551YDR270W		0.134462151394422YGR009C		0.898617511520737YGL122C		0.931428571428571YMR128W		0.501183898973954YEL013W		0.157439446366782YLR268W		0.294392523364486YDL069C		0.344978165938865YMR065W		0.380952380952381YIL068C		0.350310559006211YPR183W		0.0262172284644195YLR387C		0.678240740740741YGR012W		0.170483460559796YNL095C		0.456386292834891YBR007C		0.77445652173913YGL253W		0.218106995884774YOR196C		0.415458937198068YDR233C		0.444067796610169YEL040W		0.451820128479657YNL250W		0.676067073170732YMR252C		0.447761194029851YLR312W-A		0.379446640316206YGR206W		0.594059405940594YKL189W		0.363408521303258YER152C		0.225733634311512YCL033C		0.238095238095238YGR006W		0.49800796812749YPL041C		0.405797101449275YOL131W		0.731481481481482YLR088W		0.206840390879479YCR045C		0.236252545824847YKL163W		0.661538461538462YPR149W		0.260115606936416YNL306W		0.433179723502304YBR034C		0.146551724137931YPL187W		0.442424242424242YHR030C		0.400826446280992YDR384C		0.214545454545455YMR052W		0.799019607843137YGR124W		0.124125874125874YIL123W		0.614736842105263YOR163W		0.25YJL178C		0.383763837638376YLR352W		0.426270136307311YKL018C-A		0.404040404040404YPR112C		0.54227733934611YNR038W		0.445151033386327YDL192W		0.0883977900552486YPR157W		0.39186295503212YIL109C		0.42548596112311YGL257C		0.229390681003584YER099C		0.0817610062893082YNL182C		0.275675675675676YPR163C		0.827981651376147YML042W		0.237313432835821YGR152C		0.400735294117647YDL124W		0.092948717948718YIL138C		1YDR268W		0.269129287598945YOL064C		0.350140056022409YER091C		0.0925684485006519YOL110W		0.590717299578059YHR132W-A		1YMR129W		0.294689603590127YLR153C		0.0966325036603221YOL128C		0.301333333333333YOR085W		0.165714285714286YJL190C		0.0615384615384615YPL204W		0.508097165991903YOR159C		0.212765957446809YER072W		0.248062015503876YDL004W		0.29375YJL164C		0.370277078085642YNL130C		0.0788804071246819YER100W		0.328YMR110C		0.116541353383459YDR228C		0.736421725239617YFL048C		0.447191011235955YBR247C		0.544513457556936YNL091W		0.921774193548387YDL159W		0.566990291262136YLR347C		0.150987224157956YDR159W		0.661029976940815YOR040W		0.129824561403509YGL187C		0.516129032258065YJR095W		0.239130434782609YNL029C		0.438697318007663YJL013C		0.718446601941748YNL136W		0.915294117647059YDL058W		0.728491620111732YBL057C		0.336448598130841YBL087C		0.204379562043796YGL130W		0.361655773420479YMR035W		0.163841807909605YPL181W		0.893280632411067YOR075W		0.592485549132948YMR159C		0.786666666666667YOR211C		0.48694665153235YKL183W		0.555555555555556YGL084C		0.173214285714286YPL245W		0.403083700440529YOL049W		0.107942973523422YMR088C		0.144128113879004YJR044C		0.214285714285714YDR436W		0.652112676056338YIL083C		0.364383561643836YHR133C		0.402061855670103YDL064W		0.114649681528662YGR024C		0.0970464135021097YNL051W		0.54590570719603YML097C		0.534368070953437YMR097C		0.277929155313351YNL059C		0.463576158940397YMR253C		0.314009661835749YPR050C		0.291970802919708YHL040C		0.228070175438596YBL098W		0.143478260869565YJR009C		0.00903614457831325YPR180W		0.293948126801153YJL196C		0.0935483870967742YGR148C		0.690322580645161YMR204C		0.8YHR105W		0.537383177570093YCR053W		0.229571984435798YLR137W		0.514986376021798YHR114W		0.622432859399684YPR010C		0.276807980049875YGR257C		0.243169398907104YOR279C		0.909677419354839YGR242W		0.0686274509803922YJL112W		0.5YLR398C		0.372960372960373YPL227C		0.173652694610778YGR273C		1YMR184W		0.787878787878788YBL081W		0.97554347826087YJR142W		0.14327485380117YHR194W		0.302245250431779YFR046C		0.908587257617729YGL027C		0.322929171668667YLR423C		0.268585131894484YMR286W		0.267441860465116YDL036C		0.335497835497836YBR213W		0.193430656934307YHR037W		0.132173913043478YLR107W		0.517326732673267YNR045W		0.59918200408998YMR072W		0.721311475409836YDL186W		0.949458483754513YCR082W		0.3046875YER063W		0.954128440366973YNL090W		0.130208333333333YMR280C		0.77529658060014YHR045W		0.253571428571429YPL072W		0.527054108216433YOR330C		0.472089314194577YPL252C		0.447674418604651YNL108C		0.266666666666667YDL073W		0.547764227642276YEL042W		0.335907335907336YKL085W		0.104790419161677YLR219W		0.995879120879121YNL138W		0.435361216730038YBR082C		0.0540540540540541YLR355C		0.159493670886076YHL032C		0.231311706629055YHR002W		0.271708683473389YPL038W		1YJR085C		0.0666666666666667YCL030C		0.196495619524406YLR284C		0.132142857142857YOR017W		0.67625YIL095W		0.717283950617284YBR162W-A		0.646153846153846YER105C		0.354421279654925YJR080C		0.50761421319797YIL055C		0.732057416267943YKL157W		0.160427807486631YKL113C		0.384816753926702YER014W		0.217068645640074YPR164W		0.321961620469083YER158C		0.930191972076789YNL189W		0.29520295202952YJL079C		0.642140468227425YKL109W		1YER049W		0.501552795031056YJL171C		0.396464646464646YBR280C		0.510204081632653YBR259W		0.311046511627907YGL181W		0.782828282828283YHR040W		0.770491803278688YDL226C		0.744318181818182YDR394W		0.488317757009346YLR344W		0.425196850393701YGL198W		0.268085106382979YER128W		0.610837438423645YBR223C		0.466911764705882YMR079W		0.296052631578947YAL022C		0.295938104448743YHR071W		0.550218340611354YMR264W		0.891625615763547YMR200W		0.2421875YBR165W		0.483754512635379YIL027C		0.312056737588652YNR049C		1YPL249C		0.728187919463087YJL035C		0.268YLR131C		0.936363636363636YAR014C		0.87977369165488YGL261C		0.216666666666667YER156C		0.266272189349112YDR210W		1YDL130W-A		0.976744186046512YLR233C		0.450643776824034YPL127C		0.89922480620155YDR147W		0.404494382022472YPL077C		1YPL051W		0.131313131313131YMR157C		0.423529411764706YOR002W		0.148897058823529YMR176W		0.486888731396173YER111C		0.699908508691674YOR175C		0.324717285945073YAL061W		0.191846522781775YGR215W		0.418181818181818YNL118C		0.788659793814433YNR050C		0.143497757847534YDR489W		0.534013605442177YML096W		0.224761904761905YGL179C		0.566071428571429YOR264W		0.86046511627907YMR089C		0.475151515151515YOL045W		0.518619436875568YPL207W		0.416049382716049YIL070C		0.578947368421053YCR091W		0.668055555555556YGL203C		0.449931412894376YIL117C		0.89622641509434YMR307W		0.341681574239714YOL152W		0.143548387096774YJR131W		0.234972677595628YJL003W		0.61864406779661YNL082W		0.455899198167239YDR196C		0.103734439834025YBR003W		0.291754756871036YNR032W		0.271739130434783YGL148W		0.196808510638298YOR245C		0.203349282296651YNL299W		0.596573208722741YOL144W		0.692148760330578YGL111W		0.380129589632829YBR181C		0.584745762711864YCL038C		0.210227272727273YPR158W		0.388888888888889YOR313C		0.378698224852071YGL039W		0.175287356321839YDL207W		0.656133828996282YLR394W		0.87551867219917YNL231C		0.398860398860399YML099C		0.6875YJL159W		0.73365617433414YOR187W		0.128146453089245YNL160W		0.271186440677966YNL107W		0.566371681415929YJL125C		0.360313315926893YEL036C		0.456YGR013W		0.743548387096774YJL054W		0.575313807531381YMR214W		0.435013262599469YNR008W		0.367624810892587YMR042W		0.711864406779661YJR019C		0.415472779369628YDL084W		0.208520179372197YGL077C		0.161634103019538YLR182W		0.524283935242839YAR019C		0.412731006160164YNL121C		0.106969205834684YMR308C		0.133149678604224YMR309C		0.443349753694581YDL182W		0.126168224299065YMR170C		0.0632411067193676YLR231C		0.141280353200883YDL208W		0.435897435897436YOR099W		0.216284987277354YAL043C		0.545222929936306YDR531W		0.228882833787466YBR028C		0.539047619047619YNL225C		0.805507745266781YIR023W		0.684536082474227YDR377W		0.603960396039604YDR486C		1YHR115C		0.673076923076923YPL007C		0.188775510204082YDR248C		0.233160621761658YNL111C		0.216666666666667YPR032W		0.350435624394966YDR415C		0.179144385026738YJL173C		0.467213114754098YDR298C		0.155660377358491YOR114W		0.598639455782313YNR054C		0.715189873417722YCR071C		0.479452054794521YHR199C		0.496774193548387YOL108C		0.860927152317881YHR154W		0.577570093457944YHR038W		0.504347826086956YNL008C		0.460388639760837YMR191W		0.512064343163539YOR148C		1YJL194W		0.421052631578947YHR027C		0.309164149043303YLR225C		0.250614250614251YCR090C		0.181318681318681YDR219C		0.763440860215054YMR168C		0.389802631578947YDR084C		0.291457286432161YGL252C		0.302721088435374YGL221C		0.184027777777778YPL103C		0.373931623931624YLR167W		0.375YPR079W		0.509186351706037YJR148W		0.0930851063829787YJR102C		0.316831683168317YER117W		0.204379562043796YPL128C		0.617437722419929YJR075W		0.383838383838384YGR147C		0.645833333333333YPL132W		0.426666666666667YOR176W		0.203562340966921YDR121W		0.668367346938776YKL050C		0.850325379609544YDL204W		0.618320610687023YMR048W		0.766561514195584YDR098C		0.294736842105263YOR177C		0.920258620689655YGR205W		0.227586206896552YLR188W		0.20863309352518YCR050C		0.176470588235294YHR159W		1YBL090W		1YPL139C		0.26304347826087YBR203W		0.695887445887446YGR109C		0.360526315789474YBR121C		0.184407796101949YKL165C-A		0.532467532467532YIL120W		0.24866785079929YGR255C		0.1419624217119YER175C		0.234113712374582YMR256C		0.2YDR070C		1YEL064C		0.2625YPR156C		0.308681672025723YER067W		0.53416149068323YOR003W		0.274058577405858YGL067W		0.234375YNL119W		0.525354969574036YNR035C		0.315789473684211YOR232W		0.434210526315789YMR092C		0.0439024390243902YOR317W		0.161428571428571YAR066W		0.714285714285714YGR162W		0.877100840336134YMR102C		0.429256594724221YDL066W		0.200934579439252YOR387C		0.0485436893203883YGL070C		0.516393442622951YBR272C		0.23125YDL111C		0.230188679245283YIR030C		0.118852459016393YPR018W		0.907590759075908YGR161C		1YMR068W		0.380281690140845YKL168C		0.693370165745856YIL011W		0.698884758364312YDR476C		0.138392857142857YPL272C		0.55705996131528YJL123C		0.669456066945607YHR080C		0.706319702602231YMR086W		1YDR213W		0.740416210295728YNL071W		0.29253112033195YBL086C		0.65450643776824YGL006W		0.289002557544757YML087C		0.102564102564103YLR090W		0.498910675381264YPR196W		0.374468085106383YMR225C		0.622448979591837YOR230W		0.327231121281465YBR169C		0.236652236652237YDR004W		0.473913043478261YER076C		0.248344370860927YBR210W		0.0774647887323944YPL206C		0.0809968847352025YNL322C		0.891373801916933YHR155W		0.350162866449511YNR043W		0.255050505050505YGR082W		0.546448087431694YER004W		0.12987012987013YNL232W		0.465753424657534YMR251W-A		0.983050847457627YNL209W		0.150081566068515YHR153C		0.272727272727273YPR016C		0.142857142857143YNL178W		0.225YHR206W		0.692926045016077YPL090C		0.572033898305085YLR132C		0.372413793103448YNL025C		0.229102167182663YDR483W		0.341628959276018YPR027C		0.451263537906137YPL067C		0.318181818181818YCR026C		0.438005390835579YDL214C		0.678111587982833YBL082C		0.152838427947598YDL017W		0.455621301775148YGL218W		0.166666666666667YDR527W		0.769931662870159YBR093C		0.224839400428266YGL138C		0.405797101449275YDR391C		0.245689655172414YOR131C		0.151376146788991YDR345C		0.215167548500882YLR303W		0.11036036036036YLR127C		0.375146541617819YOL116W		0.790575916230366YPL155C		0.664305949008499YNL186W		0.710858585858586YPR110C		0.235820895522388YLR097C		0.26453488372093YMR238W		0.131004366812227YLR414C		0.121673003802281YDR392W		0.608308605341246YBL032W		0.587926509186352YML049C		0.276267450404115YDR416W		0.178114086146682YDL108W		0.163398692810458YMR240C		0.772935779816514YGL071W		0.88695652173913YPL115C		0.719858156028369YMR049C		0.421313506815366YDR243C		0.340136054421769YDL008W		0.533333333333333YNR017W		0.563063063063063YOR383C		0.803921568627451YLR095C		0.688423645320197YOR115C		0.477611940298507YHR019C		0.328519855595668YKL033W-A		0.127118644067797YNL123W		0.256770310932798YPL084W		0.472748815165877YNL162W-A		0.361111111111111YGR260W		0.194756554307116YDR012W		0.5YCL005W		0.4765625YLR417W		0.636042402826855YBR233W-A		0.787234042553192YHR157W		0.5YOL072W		0.276923076923077YPL239W		0.35YBR179C		0.375438596491228YFR048W		0.617824773413897YIL074C		0.1727078891258YOR322C		0.65158924205379YOR033C		0.58974358974359YPR063C		0.6YKL046C		0.142538975501114YER107C		0.126027397260274YKL024C		0.161764705882353YPR054W		0.280927835051546YMR075W		0.865497076023392YBR262C		0.339622641509434YDR088C		0.900523560209424YLR377C		0.135057471264368YDR118W		0.334355828220859YIR034C		0.160857908847185YMR291W		0.568259385665529YML011C		0.305084745762712YPL048W		0.197590361445783YGL120C		0.241199478487614YAR008W		0.429090909090909YGL116W		0.440983606557377YJR047C		0.210191082802548YDR299W		0.911985018726592YOR223W		0.616438356164384YBR298C		0.245928338762215YHR185C		0.784810126582278YPL049C		0.988938053097345YKL159C		0.601895734597156YNL260C		0.671717171717172YJL210W		0.328413284132841YBL015W		0.169201520912548YAL054C		0.138849929873773YMR119W		0.469551282051282YDL020C		0.934086629001883YBR055C		0.261401557285873YJL105W		0.682142857142857YGR040W		0.255434782608696YLR293C		0.296803652968037YHR031C		0.413554633471646YJR118C		0.334975369458128YLR177W		0.651273885350318YGR279C		0.414507772020725YBR290W		0.682242990654206YGL123W		0.330708661417323YOL115W		0.583904109589041YOR231W		0.501968503937008YMR062C		0.0793650793650794YPL176C		0.232439335887612YJR134C		1YIL064W		0.155642023346304YDR165W		0.286036036036036YNL142W		0.206412825651303YMR222C		0.188340807174888YHR144C		0.330128205128205YNL311C		0.479685452162516YLR242C		0.186915887850467YER057C		0.0775193798449612YML029W		0.334128878281623YCR087C-A		0.810457516339869YAL033W		0.346820809248555YNR060W		0.151599443671766YLR333C		0.5YML037C		0.835294117647059YDL101C		0.362573099415205YDR195W		0.821763602251407YDR322W		0.376021798365123YDL102W		0.257976298997265YGR276C		0.562386980108499YBR126C		0.109090909090909YJL081C		0.224948875255624YLR363W-A		1YGR076C		1YCR020C-A		0.25YML010W		0.632173095014111YPL168W		0.706976744186047YDR328C		0.675257731958763YGR021W		0.182758620689655YPL237W		0.645614035087719YMR224C		0.562138728323699YPR105C		0.394889663182346YPL083C		0.558886509635974YMR172W		0.915159944367177YOL140W		0.0685579196217494YGL107C		0.360681114551084YGR128C		0.413744740532959YNR020C		0.355555555555556YPR017C		0.440559440559441YDL132W		0.341104294478528YNR039C		0.51900826446281YCR010C		0.240282685512367YNL309W		1YGR179C		0.879310344827586YGR175C		0.0967741935483871YNL265C		0.590604026845638YMR192W		0.602777777777778YPR189W		0.0858938547486034YNL196C		0.98993288590604YGL135W		0.179723502304147YOL132W		0.295116772823779YGL082W		0.480314960629921YNR048W		0.231552162849873YDL134C		0.365853658536585YHR083W		0.571428571428571YFR005C		0.285714285714286YEL020W-A		1YEL038W		0.162995594713656YCR035C		0.375634517766497YMR026C		0.458646616541353YJR016C		0.124786324786325YDR176W		0.861823361823362YGL004C		0.0743405275779376YDR222W		0.228915662650602YPR162C		0.429111531190926YEL054C		0.272727272727273YDR020C		0.280172413793103YPR129W		0.819484240687679YDL091C		0.531868131868132YJL154C		0.310381355932203YDR074W		0.285714285714286YBR240C		0.573333333333333YDR251W		0.643373493975904YOL042W		0.253443526170799YHR196W		0.4YDR494W		0.941828254847645YGL142C		0.149350649350649YPL230W		1YOR046C		0.261410788381743YBL056W		0.493589743589744YDL175C		1YOR336W		0.290842490842491YNL139C		0.509079524107702YHR192W		0.618705035971223YNL313C		0.248893805309735YCR051W		0.292792792792793YGR067C		0.530226700251889YHL024W		0.713884992987377YJL185C		0.815699658703072YER071C		0.603174603174603YPL076W		0.117857142857143YIR002C		0.542799597180262YMR294W		0.774798927613941YML110C		0.257328990228013YPR065W		0.864130434782609YLR288C		0.483122362869198YCR057C		0.151679306608884YLR170C		0.166666666666667YJL160C		0.519163763066202YBR287W		0.234192037470726YDL033C		0.20863309352518YGR239C		0.979166666666667YER031C		0.282511210762332YOR006C		0.562300319488818YNL283C		0.884691848906561YOL032W		0.48780487804878YLR194C		0.956692913385827YER168C		0.197802197802198YFR032C-A		1YJL151C		0.526315789473684YOR332W		0.150214592274678YMR181C		0.896103896103896YDL042C		0.508896797153025YNL148C		0.47244094488189YMR318C		0.0805555555555556YGL078C		0.304015296367113YIL065C		0.225806451612903YNL099C		0.348739495798319YPR083W		0.77720207253886YBL033C		0.32463768115942YCL057W		0.13623595505618YDR117C		0.527433628318584YLR245C		0.0845070422535211YPL254W		0.719262295081967YNL100W		0.341880341880342YPL184C		0.540849673202614YDR492W		0.113924050632911YER006W		0.536538461538462YOL114C		0.47029702970297YNL288W		0.375335120643432YGR096W		0.203821656050955YER132C		0.724472333143183YJL008C		0.167253521126761YHR202W		0.204318936877076YHR094C		0.22280701754386YPR140W		0.461942257217848YPR029C		0.318509615384615YNL318C		0.192592592592593YDR276C		0.0545454545454545YOL040C		0.661971830985915YGR143W		0.512321660181582YPL165C		0.203753351206434YNR026C		0.159235668789809YBL013W		0.194513715710723YGR122W		0.475124378109453YDR275W		0.595744680851064YHR140W		0.184100418410042YKL127W		0.121052631578947YDR382W		0.654545454545455YOL129W		0.146739130434783YNL102W		0.412125340599455YML014W		0.333333333333333YDR237W		0.448630136986301YJR129C		0.24188790560472YPR024W		0.414993306559572YOR289W		0.270916334661355YLR259C		0.0996503496503496YDR496C		0.36280487804878YPL042C		0.540540540540541YJL133W		0.200636942675159YOL077W-A		0.661764705882353YPL145C		0.329493087557604YDL146W		0.427698574338086YDR100W		0.230769230769231YJR004C		0.507692307692308YBR186W		0.563829787234043YKL137W		0.459459459459459YFR041C		0.379661016949153YKL006C-A		0.350515463917526YLR133W		0.434707903780069YPR192W		0.272131147540984YMR189W		0.161508704061896YOR137C		0.326366559485531YNL215W		1YDL105W		0.587064676616915YCL029C		0.843181818181818YDR172W		0.405839416058394YNL085W		0.368674698795181YDL230W		0.232835820895522YNL264C		0.377142857142857YBL038W		0.551724137931034YHR148W		0.262295081967213YPL166W		0.666666666666667YJL005W		0.768509378084896YPL026C		0.563745019920319YJR006W		0.308008213552361YJL115W		0.562724014336918YJL099W		0.365951742627346YLR262C		0.297674418604651YNL333W		0.258389261744966YFR010W		0.503006012024048YJL161W		0.611111111111111YJR097W		0.517441860465116YIR021W		0.336088154269972YPR103W		0.310104529616725YJR122W		0.420523138832998YOR390W		0.234666666666667YML105C		0.73992673992674YKL035W		0.232464929859719YMR056C		0.187702265372168YGL171W		0.313829787234043YOR373W		0.920094007050529YGR249W		0.861842105263158YAR035W		0.321688500727802YGR198W		0.263157894736842YNR022C		0.60431654676259YDR034C		0.636708860759494YBR033W		0.603917301414581YPL186C		0.769736842105263YBL084C		0.357519788918206YOR352W		0.685131195335277YDL030W		0.571698113207547YAL013W		0.723809523809524YDL104C		0.199017199017199YHR175W		0.444444444444444YBR002C		0.181818181818182YBR246W		0.113695090439276YHR121W		0.283422459893048YEL017W		0.44213649851632YDL067C		0.220338983050847YGR037C		0.344827586206897YML123C		0.247018739352641YDR280W		0.334426229508197YBR191W		0.41875YGL083W		0.533582089552239YMR229C		0.333718912666281YAL060W		0.0942408376963351YCL036W		0.565371024734982YPL046C		0.262626262626263YPL136W		0.524590163934426YJL207C		0.251737835153923YBL069W		0.237762237762238YOR361C		0.260812581913499YML031W		0.419847328244275YNR014W		0.962264150943396YBR166C		0.230088495575221YHR087W		0.576576576576577YDR151C		0.926153846153846YGL026C		0.118811881188119YER047C		0.713489409141583YEL050C		0.60559796437659YGL036W		0.54015401540154YDL087C		0.57088122605364YPL135W		0.309090909090909YJR064W		0.144128113879004YPL074W		0.616710875331565YOR276W		0.894409937888199YGR261C		0.404202719406675YBL102W		0.404651162790698YGR049W		0.342245989304813YHR156C		0.714705882352941YIR012W		0.109048723897912YMR283C		0.423001949317739YER083C		0.614035087719298YLR110C		0.917293233082707YBR137W		0.240223463687151YJR062C		0.317286652078775YNL094W		0.536626916524702YNL281W		0.169934640522876YHR001W-A		0.376623376623377YMR150C		0.0736842105263158YDL098C		0.670103092783505YGR010W		0.481012658227848YBR057C		0.994535519125683YPL032C		0.653333333333333YGR157W		0.287686996547756YNL213C		0.94392523364486YMR018W		0.17704280155642YBR284W		0.392722710163112YGR238C		0.614512471655329YKL130C		0.191056910569106YDR536W		0.209138840070299YIL051C		0.206896551724138YOR305W		0.504132231404959YER079W		0.90952380952381YPR003C		0.282493368700265YDR107C		0.188988095238095YGL064C		0.402852049910873YML117W		0.717813051146384YIL082W		0.520689655172414YKL091C		0.183870967741935YKL096W		0.573221757322176YDR110W		0.641342756183746YDR412W		1YPR082C		0.104895104895105YJL193W		0.248756218905473YDR123C		0.868421052631579YNR003C		0.466876971608833YBR149W		0.156976744186047YNL112W		0.307692307692308YNL261W		0.298538622129436YBR172C		0.928378378378378YDR226W		0.144144144144144YDR389W		0.773700305810398YIL144W		0.714905933429812YJL080C		0.646481178396072YOR089C		0.342857142857143YJR022W		0.431192660550459YPR034W		0.316561844863732YDR477W		0.484992101105845YKL061W		0.876106194690266YOR219C		0.211600429645542YBR164C		0.103825136612022YMR060C		0.327217125382263YBR217W		0.586021505376344YIL076W		0.135135135135135YIR014W		0.297520661157025YPL068C		0.791808873720137YGR117C		0.313025210084034YDR014W		0.573415765069552YGR275W		0.878980891719745YNL210W		0.311111111111111YGL233W		0.385714285714286YLR457C		1YBR030W		0.445652173913043YPL225W		0.315068493150685YJR036C		0.36322869955157YDR138W		0.586436170212766YPR134W		0.593283582089552YDL119C		0.221498371335505YGR004W		0.508658008658009YMR301C		0.185507246376812YPR116W		0.523465703971119YNL253W		0.163507109004739YLR386W		0.373863636363636YBR037C		0.4YFR045W		0.196491228070175YPR145W		0.148601398601399YNR024W		1YMR123W		0.418032786885246YOR303W		0.172749391727494YLR221C		0.954545454545455YNL152W		0.718826405867971YNL244C		0.25YJL111W		0.16YNL135C		0.0701754385964912YHL015W		0.214876033057851YGR236C		0.652631578947368YIL154C		0.569364161849711YDL085W		0.198165137614679YML115C		0.463551401869159YBR278W		0.751243781094527YDR236C		0.188073394495413YOR349W		0.212031558185404YLR433C		0.513562386980108YCR018C		0.97737556561086YFR037C		0.820466786355476YJL002C		0.147058823529412YLR264W		0.298507462686567YLR353W		0.840796019900497YHL016C		0.180952380952381YLR135W		0.915775401069519YOL088C		0.472924187725632YPL086C		0.233393177737882YOR209C		0.149184149184149YDR142C		0.149333333333333YIR025W		0.89945652173913YKL135C		0.329201101928375YMR223W		0.286624203821656YKL120W		0.219135802469136YLR239C		0.246951219512195YPL019C		0.444311377245509YPL085W		0.90751708428246YLR102C		0.811320754716981YJL071W		0.411149825783972YJR010W		0.13307240704501YCR021C		0.243975903614458YHR063C		0.179419525065963YOL063C		0.401253918495298YGR172C		0.358870967741935YHR009C		0.261950286806883YGR111W		0.295YOR039W		0.406976744186047YPL087W		0.17981072555205YMR101C		0.19533527696793YNL001W		0.194300518134715YOL146W		0.443298969072165YDL218W		0.621451104100946YNL098C		0.506211180124224YLR332W		0.856382978723404YGR081C		1YOL156W		0.216931216931217YBR058C-A		0.35YHR168W		0.390781563126253YHR062C		0.327645051194539YGR248W		0.0627450980392157YOL058W		0.138095238095238YMR138W		0.25130890052356YPL022W		0.465454545454545YJL110C		1YHR208W		0.137404580152672YIR009W		0.342342342342342YLR384C		0.288361749444033YPR188C		0.300613496932515YGL018C		0.309782608695652YJL198W		0.353007945516459YPL013C		0.28099173553719YMR261C		0.362428842504744YER001W		0.471128608923885YGR002C		0.848739495798319YPR108W		0.207459207459207YDR385W		0.21021377672209YMR271C		0.193832599118943YBR109C		0.122448979591837YDR480W		0.888544891640867YOR306C		0.234165067178503YBL089W		0.213507625272331YGR112W		0.473007712082262YLR213C		0.267772511848341YDR439W		0.962536023054755YLR408C		0.245901639344262YJR010C-A		0.138297872340426YNL323W		0.376811594202899YPR067W		0.508108108108108YGR254W		0.135011441647597YIL145C		0.110032362459547YMR209C		0.319474835886214YPR102C		0.264367816091954YLR168C		0.256521739130435YPL123C		0.384792626728111YJL149W		0.440422322775264YGR159C		0.681159420289855YML058W-A		0.544117647058823YDR325W		0.370048309178744YHR006W		0.937153419593346YGL040C		0.10233918128655YOR259C		0.437070938215103YDL085C-A		1YBL041W		0.203319502074689YNL009W		0.19047619047619YNL070W		0.483333333333333YJR139C		0.0974930362116992YJL044C		0.449781659388646YLR404W		0.308771929824561YIR016W		0.849056603773585YNL206C		0.540659340659341YML062C		0.826530612244898YDR375C		0.285087719298246YLR375W		0.985422740524781YPL183W-A		0.645161290322581YER010C		0.179487179487179YHR149C		0.965940054495913YJL197W		0.58133971291866YDL212W		0.242857142857143YER025W		0.273244781783681YBR046C		0.0149700598802395YGL011C		0.162698412698413YBR052C		0.157142857142857YPR167C		0.256704980842912YER048C		0.782608695652174YER037W		0.252336448598131YGR247W		0.359832635983264YHR169W		0.185614849187935YKL154W		0.340163934426229YLR321C		0.565727699530516YMR063W		0.317991631799163YFL034W		0.600186393289842YIL125W		0.258382642998028YOR270C		0.270238095238095YGL017W		0.407554671968191YFR053C		0.220618556701031YNL110C		0.645454545454546YIL098C		0.683870967741935YML128C		0.243664717348928YOR001W		0.586630286493861YER129W		0.799474605954466YOR304C-A		1YOR057W		0.483544303797468YDR373W		0.373684210526316YGR091W		0.637651821862348YLR087C		0.314063556457066YOL002C		0.132492113564669YGL244W		0.790322580645161YCL035C		0.1YPR152C		0.759139784946237YGR005C		0.74YHL008C		0.58213716108453YKL146W		0.48121387283237YOL092W		0.298701298701299YMR115W		0.562874251497006YLR381W		0.336971350613915YBL028C		1YKL039W		0.267686424474187YGL028C		0.590405904059041YMR127C		0.27810650887574YLR418C		0.534351145038168YDR247W		0.568329718004338YMR205C		0.228362877997914YJL118W		0.5662100456621YFR043C		0.345991561181435YOR255W		0.758992805755396YDR265W		0.397626112759644YNL131W		0.598684210526316YNL312W		0.498168498168498YER101C		0.209302325581395YMR215W		0.433206106870229YJL172W		0.256944444444444YER159C		0.612676056338028YHL031C		0.439461883408072YEL047C		0.051063829787234YHR118C		0.577011494252874YOR160W		0.16358024691358YKL016C		0.660919540229885YPR056W		0.443786982248521YBR201W		0.042654028436019YOR185C		0.290909090909091YER144C		0.46583850931677YBR264C		0.180904522613065YML016C		0.651734104046243YDL180W		0.455210237659963YEL058W		0.179533213644524YLR172C		0.16YBR024W		0.40531561461794YJL168C		0.630286493860846YJL121C		0.092436974789916YPL212C		0.424632352941176YJL036W		0.368794326241135YOL077C		0.436426116838488YJL201W		0.510851419031719YPL141C		0.715606936416185YDR528W		1YNL262W		0.323582358235824YBR135W		0.58YNL317W		0.174193548387097YPR104C		0.862179487179487YPL244C		0.182890855457227YOR069W		0.651851851851852YGL164C		0.547727272727273YGL231C		0.521052631578947YER090W		0.145956607495069YPR049C		0.574702886247878YDL200C		0.234042553191489YIR018W		1YGR026W		0.219424460431655YIL114C		0YFL027C		0.424547283702213YLR380W		0.384803921568627YNL153C		0.372093023255814YHR036W		0.709129511677282YKL184W		0.225321888412017YGR079W		0.87027027027027YML009C		0.4YDL133W		0.649885583524027YOL073C		0.350931677018634YMR250W		0.305982905982906YBR098W		0.749638205499276YHR005C		0.38135593220339YDR507C		0.731173380035026YPR094W		0.476635514018692YMR303C		0.0201149425287356YBR237W		0.517078916372203YER018C		0.742081447963801YHR161C		0.676609105180534YDR116C		0.343859649122807YDR242W		0.191256830601093YOL145C		0.220984215413185YOR290C		0.66001174398121YLR281C		0.6YBR139W		0.226377952755905YPL100W		0.326612903225806YER023W		0.108391608391608YPL146C		1YLR439W		0.557993730407524YCL055W		0.388059701492537YBR138C		0.83206106870229YJL055W		0.269387755102041YDR057W		0.476014760147601YMR140W		0.793456032719836YLR092W		0.387458006718925YBR016W		0.8359375YFL054C		0.617647058823529YBR031W		0.497237569060773YHR142W		0.186708860759494YNL035C		0.179948586118252YPL164C		0.332867132867133YJR101W		0.25187969924812YDR447C		0.632352941176471YIL136W		0.931297709923664YML127W		0.390705679862306YIL016W		0.949685534591195YOR052C		1YOR078W		1YMR302C		0.317647058823529YGR166W		0.398214285714286YNL221C		0.604571428571429YGL128C		0.445229681978799YKL072W		0.592689295039164YHR052W		0.571808510638298YMR182C		1YDR438W		0.251351351351351YPL098C		0.36283185840708YER017C		0.409986859395532YLR113W		0.363218390804598YNL227C		0.784745762711864YIL149C		0.944014294222752YIL157C		0.436548223350254YPR151C		0.922330097087379YFL024C		0.895432692307692YPL125W		0.167635658914729YMR135C		0.703296703296703YMR259C		0.211267605633803YOL071W		0.475308641975309YGL126W		0.334210526315789YCR083W		0.259842519685039YGL113W		0.712574850299401YNR001C		0.340292275574113YMR160W		0.448529411764706YIL116W		0.0571428571428571YGR100W		0.514736842105263YNR032C-A		0YOR252W		0.764044943820225YKL042W		1YGR052W		0.37940379403794YBR060C		0.633870967741936YER141W		0.290123456790123YPR172W		0.28YJL124C		0.598837209302326YER042W		0.217391304347826YGL091C		0.326219512195122YJL100W		0.57331136738056YHR047C		0.0899532710280374YDR202C		0.39031339031339YJR150C		0.865771812080537YAL035W		0.521956087824351YPR013C		1YNL015W		0.4YKL167C		0.394160583941606YMR070W		1YFR049W		0.739837398373984YML083C		0.538277511961722YDR296W		0.407079646017699YPL110C		0.309076042518397YLR205C		0.214511041009464YMR009W		0.134078212290503YOR243C		0.406804733727811YNL207W		0.418823529411765YER110C		0.188679245283019YBR239C		0.655954631379962YFR027W		0.441281138790036YPL040C		0.149700598802395YGR283C		0.457478005865103YIL084C		0.82262996941896YDL151C		0.792746113989637YER119C		0.205357142857143YFL030W		0.0753246753246753YPR166C		0.121739130434783YML108W		0.485714285714286YDR322C-A		0.541666666666667YIL132C		0.413145539906103YML070W		0.196917808219178YDR013W		0.379807692307692YML095C		0.519047619047619YPL008W		0.354239256678281YDL156W		0.409961685823755YDR173C		0.442253521126761YDR190C		0.222462203023758YLR399C		0.715743440233236YDR109C		0.247552447552448YLR356W		0.355329949238579YJL058C		0.662983425414365YFR015C		0.279661016949153YDR263C		0.341860465116279YNL233W		0.949551569506726YNL334C		0.117117117117117YOL021C		0.214785214785215YHR106W		0.108187134502924YMR100W		0.653225806451613YLR325C		0.474358974358974YGL089C		0.266666666666667YMR207C		0.190767781441357YGL103W		0.395973154362416YDL131W		0.15YBR163W		0.514529914529914YML047C		0.286931818181818YGR167W		0.974248927038627YER145C		0.304455445544554YMR136W		1YCR076C		0.828YDR379W		0.800792864222002YGL223C		0.546762589928058YIL160C		0.146282973621103YBL020W		0.219512195121951YGL129C		0.42827868852459YKL176C		0.621980676328502YBR276C		0.441140024783147YBR017C		0.200435729847495YLR146C		0.0766666666666667YOL044W		0.488250652741514YNL005C		0.768194070080862YPL221W		0.301387137452711YHL039W		0.152136752136752YOR008C		0.891534391534392YDR393W		0.50219298245614YNL172W		0.481693363844394YGR093W		0.317554240631164YEL025C		0.218855218855219YFR023W		0.438625204582651YOL065C		0.270833333333333YNL255C		1YML109W		0.949044585987261YJL199C		0.138888888888889YDR208W		0.697047496790757YNL259C		0.178082191780822YIL021W		0.314465408805031YJL088W		0.159763313609467YDL161W		0.766519823788546YCL028W		1YLR166C		0.413318025258324YHL013C		0.625407166123778YLR175W		0.399585921325052YPL116W		0.523672883787661YHR117W		0.111111111111111YGL196W		0.088785046728972YGL035C		1YMR244C-A		0.961538461538462YLR222C		0.0771113831089351YPR048W		0.186195826645265YPL109C		0.258751902587519YPL138C		0.71671388101983YKL117W		0.541666666666667YOL125W		0.569327731092437YEL027W		0.06875YJL165C		0.727485380116959YMR175W		1YLR438W		0.0542452830188679YGL209W		1YBL092W		0.553846153846154YBR145W		0.0313390313390313YIL031W		0.745647969052224YLR319C		0.854060913705584YPR135W		0.327939590075512YBR233W		0.554479418886199YDR045C		0.263636363636364YHR110W		0.169811320754717YKL001C		0.168316831683168YLR181C		0.581818181818182YOL086C		0.00862068965517241YBR244W		0.185185185185185YNL016W		0.529801324503311YMR163C		0.608510638297872YHR034C		0.555232558139535YML006C		0.763565891472868YCR042C		0.388059701492537YDR466W		0.642538975501114YJR082C		1YPL054W		1YMR282C		0.41551724137931YGR207C		0.229885057471264YOR134W		0.640586797066015YCR017C		0.195173137460651YJL179W		0.917431192660551YIR004W		0.666666666666667YPL150W		0.72031076581576YCR088W		0.751689189189189YIR029W		0.166180758017493YMR094W		0.278242677824268YBR267W		0.653944020356234YGR174C		0.694117647058824YML107C		0.350299401197605YNL117W		0.126353790613718YCL043C		0.354406130268199YFR034C		0.96474358974359YPL066W		0.457202505219207YGL021W		0.740789473684211YJL141C		0.657992565055762YPL180W		0.97747183979975YBR107C		0.122448979591837YOL043C		0.410526315789474YDL166C		0.294416243654822YOR288C		0.317610062893082YBR249C		0.132432432432432YHR152W		1YFL041W		0.186495176848875YOR377W		0.329523809523809YBR041W		0.0896860986547085YGR085C		0.304597701149425YPR148C		0.822988505747126YMR278W		0.110932475884244YMR131C		0.401174168297456YNL251C		0.666086956521739YMR310C		0.34384858044164YNL058C		0.854430379746835YER104W		1YBR004C		0.1270207852194YLR362W		0.516039051603905YMR105C		0.131810193321617YPL262W		0.180327868852459YDR519W		0.0518518518518519YDL193W		0.405333333333333YLR267W		0.208771929824561YCR066W		0.84394250513347YPL199C		0.395833333333333YBR175W		0.0412698412698413YGR216C		0.182266009852217YLR373C		0.469478357380688YJR043C		0.697142857142857YOR358W		0.768595041322314YBR221C		0.155737704918033YEL052W		0.37524557956778YDL148C		0.739506172839506YNL307C		0.293333333333333YDR378C		0.337209302325581YMR295C		0.903553299492386YDR472W		0.607773851590106YIL142W		0.115749525616698YPR004C		0.127906976744186YNL199C		0.816479400749064YMR043W		0.818181818181818YMR034C		0.184331797235023YMR031C		0.894424673784104YGR195W		0.24390243902439YMR055C		0.238562091503268YHR216W		0.265774378585086YLR438C-A		0.224719101123595YPL260W		0.62431941923775YBR014C		0.443349753694581YGR289C		0.253246753246753YOL039W		0.688679245283019YLR447C		0.153623188405797YOL012C		0.738805970149254YGL153W		0.847507331378299YMR217W		0.08YGR171C		0.210434782608696YDR078C		0.286995515695067YAL010C		0.413793103448276YKL166C		0.339195979899498YDR182W		0.287169042769857YML074C		0.603406326034063YNR011C		0.323059360730594YGR127W		0.217948717948718YDR363W-A		1YBR180W		0.26048951048951YLR372W		0.231884057971014YER002W		0.991341991341991YDR032C		0.131313131313131YCR033W		0.870309951060359YJL014W		0.125468164794007YOR126C		0.126050420168067YLR340W		0.25YLR193C		0.08YOR108W		0.19205298013245YBL016W		0.235127478753541YAR002C-A		0.164383561643836YPL179W		0.550091074681239YBR129C		0.484756097560976YML060W		0.263297872340426YER034W		1YCL001W		0.313829787234043YPR047W		0.232409381663113YGL100W		0.0372492836676218YDR388W		0.58298755186722YLR203C		0.327981651376147YLR215C		0.369444444444444YOR375C		0.129955947136564YFL046W		0.516908212560386YHR066W		0.660044150110375YLR312C		0.698492462311558YHR122W		0.558441558441558YHR088W		0.366101694915254YPL219W		0.747967479674797YML094W		0.49079754601227YBL061C		0.399425287356322YER154W		0.532338308457711YOR236W		0.109004739336493YNL273W		0.512924071082391YGR084C		0.525073746312684YNL023C		0.813471502590674YNL275W		0.192708333333333YGR016W		0.252631578947368YNL052W		0.535947712418301YHR076W		0.350267379679144YJL206C		0.554089709762533YER143W		0.55607476635514YPL175W		0.103982300884956YIR035C		0.122047244094488YDL063C		0.35YBR251W		0.482084690553746YGR252W		0.403189066059225YJL166W		0.234042553191489YNL073W		0.255208333333333YCL068C		0.407692307692308YPR128C		0.246951219512195YDR339C		0.26984126984127YGL075C		0.656330749354005YPL095C		0.265350877192982YCL004W		0.218809980806142YGL170C		0.811138014527845YOR278W		0.149090909090909YJR063W		0.328YPR073C		0.267080745341615YGL224C		0.185714285714286YIL056W		0.709375YML080W		0.295508274231678YLR108C		0.389690721649485YPL028W		0.0552763819095477YLR214W		0.0889212827988338YPL034W		0.557575757575758YDL070W		0.689655172413793YPL209C		0.4141689373297YPL174C		0.692396313364055YDR508C		0.298642533936652YDR364C		0.259340659340659YLR257W		1YDR281C		0.528846153846154YOL062C		0.262729124236253YJL126W		0.133550488599349YOL100W		0.66049953746531YER019C-A		0.556818181818182YGR200C		0.0621827411167513YOL122C		0.278260869565217YLR289W		0.189147286821705YGL038C		0.483333333333333YMR124W		1YNR063W		0.481054365733114YJL052W		0.00301204819277108YGL184C		0.0881720430107527YLR145W		0.427860696517413YMR040W		0.2375YER136W		0.184035476718404YLR197W		0.369047619047619YOR344C		0.893470790378007YAL048C		0.202416918429003YLR351C		0.106529209621993YDL198C		0.173333333333333YPR033C		0.293040293040293YML130C		0.440497335701599YJL117W		0.254019292604502YDR003W		0.804761904761905YDR421W		0.694736842105263YOR110W		0.47816091954023YDR312W		0.653421633554084YDR050C		0.0362903225806452YOR117W		0.587557603686636YML068W		0.209051724137931YLR195C		0.331868131868132YFR014C		0.446188340807175YFR004W		0.483660130718954YPL133C		0.60762331838565YGR054W		0.41588785046729YOR044W		0.560509554140127YHR143W		0.950769230769231YKL071W		0.109375YHR035W		0.265079365079365YGR083C		0.572964669738863YOL022C		0.53921568627451YGL001C		0.106017191977077YGL090W		0.712589073634204YNL229C		0.409604519774011YJR049C		0.458490566037736YDL234C		0.453083109919571YML118W		0.499009900990099YLR094C		0.762948207171315YDR368W		0.137820512820513YOR378W		0.128155339805825YJL011C		0.478260869565217YOR221C		0.133333333333333YAR007C		0.27536231884058YKL069W		0.172222222222222YLR208W		0.0673400673400673YBR022W		0.209039548022599YOR320C		0.25050916496945YML078W		0.175824175824176YDR326C		0.702364394993046YER080W		0.381180223285486YHR179W		0.225YMR117C		0.723004694835681YNL272C		0.805006587615283YOR116C		0.265068493150685YBL074C		0.383098591549296YKL093W		1YML088W		0.458083832335329YIL106W		0.496815286624204YDR059C		0.0540540540540541YLR227C		0.259634888438134YOL124C		0.277136258660508YNR041C		0.228494623655914YOR247W		0.528571428571429YIL110W		0.395225464190981YKL110C		0.338658146964856YML102W		0.196581196581197YLR272C		0.311224489795918YEL070W		0.0737051792828685YGR213C		0.211356466876972YGR071C		0.508139534883721YPL220W		0.244239631336406YER176W		0.489741302408564YPR068C		0.28936170212766YPL159C		1YER167W		0.978848413631022YDL115C		0.875YGL256W		0.116129032258065YDL189W		0.853391684901532YPL231W		0.342342342342342YPR045C		0.502127659574468YHR201C		0.221662468513854YNL048W		0.167883211678832YJR058C		0.17687074829932YDL045C		0.30718954248366YOL133W		0.421487603305785YDR460W		0.64797507788162YDR087C		0.47841726618705YPR006C		0.260869565217391YJR143C		0.224409448818898YPL210C		0.334375YHR124W		0.760765550239234YBL060W		0.63901018922853YHL025W		0.858433734939759YER103W		0.14797507788162YOL135C		0.545045045045045YGR056W		0.594827586206897YGR131W		0.281609195402299YLR260W		0.48471615720524YOR128C		0.0858143607705779YJR065C		0.21826280623608YOR086C		0.306913996627319YGL020C		0.480851063829787YJL209W		0.307339449541284YOR353C		0.763590391908976YNL237W		0.185185185185185YBR087W		0.129943502824859YLR429W		0.482334869431644YDR539W		0.133200795228628YGL242C		0.215469613259669YKL160W		0.675862068965517YHR001W		0.290617848970252YMR118C		0.362244897959184YPR120C		0.450574712643678YPR143W		1YBR009C		0.427184466019417YOR028C		0.96271186440678YJR112W		0.517412935323383YDL123W		0.492857142857143YGL236C		0.144992526158445YLR407W		0.838427947598253YLR154C		0.663636363636364YDR277C		0.709006928406467YDL053C		1YNL169C		0.348YKL063C		0.81437125748503YDR446W		0.73841059602649YIL152W		0.774468085106383YGL054C		0.0434782608695652YJL069C		0.474747474747475YLR405W		0.212534059945504YJL184W		1YPR115W		0.78393351800554YMR171C		0.638181818181818YDR530C		0.316923076923077YLR295C		0.82258064516129YKL141W		0.393939393939394YEL026W		0.166666666666667YNL072W		0.224755700325733YDL107W		0.316239316239316YAL025C		0.627450980392157YAL023C		0.214756258234519YNL214W		0.462311557788945YNL223W		0.491902834008097YPL173W		0.548821548821549YML067C		0.340909090909091YJR153W		0.074792243767313YKL152C		0.137651821862348YOL112W		0.560975609756098YGL119W		0.231536926147705YNL236W		0.365503080082136YGR132C		0.250871080139373YBR047W		0.44YHR007C		0.181132075471698YFR009W		0.333776595744681YKL009W		0.343220338983051YMR044W		0.673684210526316YJR121W		0.193737769080235YAR015W		0.251633986928105YHR072W-A		0.655172413793103YNL132W		0.395833333333333YOR062C		0.619402985074627YNL149C		0.643410852713178YDR158W		0.178082191780822YBR296C		0.200348432055749YOR339C		0.0833333333333333YGR033C		0.418410041841004YML022W		0.0802139037433155YPL234C		0.140243902439024YJR014W		0.782828282828283YCR037C		0.368364030335861YIR007W		0.215968586387435YKL079W		0.655487804878049YBR176W		0.16025641025641YPR076W		0.693548387096774YKL150W		0.165562913907285YDR336W		0.5YDR086C		0.4125YNL159C		0.480968858131488YER150W		0.621621621621622YKL172W		0.920374707259953YFL047W		0.387955182072829YPL071C		0.621794871794872YML098W		0.640718562874252YDR002W		0.477611940298507YIR037W		0.159509202453988YDR105C		0.247357293868922YPR193C		0.0769230769230769YMR023C		0.212927756653992YBR071W		0.933649289099526YML043C		0.652859960552268YML001W		0.25YKL185W		0.94047619047619YLR220W		0.37888198757764YPL023C		0.286149162861492YMR149W		0.300699300699301YFL022C		0.276341948310139YAL007C		0.213953488372093YIL001W		0.230019493177388YOR167C		0.238805970149254YDL027C		0.516666666666667YOR100C		0.250764525993884YGL192W		0.496666666666667YDR288W		0.726072607260726YIR005W		0.452702702702703YNL204C		0.666666666666667YIR036C		0.091254752851711YBL107C		0.714285714285714YHR204W		0.334170854271357YBR088C		0.116279069767442YFR006W		0.214953271028037YFL042C		0.572700296735905YNL300W		0.843137254901961YPL108W		0.595238095238095YDR022C		0.719387755102041YPR002W		0.174418604651163YPL158C		0.949868073878628YMR047C		0.919137466307278YER131W		0.546218487394958YPL027W		0.820408163265306YNL293W		0.639810426540284YPR155C		0.397727272727273YPL177C		0.892156862745098YNR046W		0.207407407407407YLR314C		0.567307692307692YKL013C		0.175438596491228YNL003C		0.144366197183099YER153C		0.440944881889764YBL030C		0.210691823899371YLR276C		0.387205387205387YNR055C		0.244027303754266YDL155W		0.440281030444965YPL065W		0.351239669421488YOR295W		0.728070175438597YBR111C		0.199134199134199YGR105W		0.25974025974026YGR066C		0.441780821917808YGR101W		0.398843930635838YOR067C		0.168110918544194YNL012W		0.328050713153724YFR039C		0.429411764705882YER183C		0.241706161137441YBR151W		0.484177215189873YLR105C		0.493368700265252YDL100C		0.27683615819209YJL006C		0.340557275541796YMR041C		0.128358208955224YPR011C		0.226993865030675YDR511W		0.556390977443609YDR071C		0.172774869109948YIL039W		0.221987315010571YFR047C		0.0542372881355932YCR011C		0.372735938989514YCR077C		0.697236180904523YJL203W		0.539285714285714YHR139C		0.279141104294479YOR328W		0.271739130434783YDR178W		0.419889502762431YBL045C		0.164113785557987YLR149C		0.605479452054794YOR034C		0.166889185580774YMR208W		0.203160270880361YPL235W		0.346072186836518YBR119W		0.651006711409396YFL010C		0.909952606635071YER068W		0.793867120954003YOL082W		0.6YDR469W		0.777142857142857YAL009W		0.332046332046332YJR067C		0.347517730496454YOR065W		0.352750809061489YFR017C		1YHR146W		0.808602150537634YPL122C		0.35672514619883YIL045W		0.775092936802974YGL121C		0.579365079365079YOR038C		0.370285714285714YCR047C		0.290909090909091YIL103W		0.235294117647059YJR017C		0.352941176470588YPR113W		0.168181818181818YMR099C		0.131313131313131YGR120C		0.446564885496183YLR190W		0.971486761710794YPL011C		0.691218130311615YPL082C		0.365827530798072YDR063W		0.516778523489933YMR036C		0.759927797833935YHL030W		0.264989293361884YDR352W		0.350157728706625YOR090C		0.520979020979021YEL021W		0.134831460674157YBL006C		0.616666666666667YNL183C		0.710126582278481YIL014W		0.336507936507937YLR144C		0.218228498074454YEL071W		0.0725806451612903YLR324W		0.546845124282983YLR389C		0.237585199610516YNL263C		0.420382165605096YPR030W		0.763603925066905YIL073C		0.273846153846154YPR154W		0.767441860465116YKL148C		0.1859375YBL029W		0.845744680851064YGL186C		0.200345423143351YER046W		0.377622377622378YBR005W		0.877934272300469YJR005W		0.285714285714286YPR023C		0.483790523690773YOR367W		0.53YMR071C		0.191616766467066YOR321W		0.204515272244356YHR203C		0.24904214559387YER147C		0.282051282051282YIL090W		0.238289205702648YJR109C		0.117173524150268YKL068W		0.893639207507821YIL096C		0.505952380952381YDR454C		0.155080213903743YDR362C		0.447916666666667YJL187C		0.733821733821734YGR202C		0.629716981132076YDR056C		0.385365853658537YDR408C		0.0934579439252336YDR297W		0.255014326647564YKL112W		0.820793433652531YOL121C		0.520833333333333YLR420W		0.0879120879120879YNL151C		0.97609561752988YCL016C		0.310526315789474YHR100C		0.151351351351351YOR102W		0.5YBR097W		0.376203576341128YNR028W		0.493506493506494YLR298C		0.783549783549784YJR117W		0.0883002207505519YOR271C		0.226299694189602YDR289C		0.770171149144254YDL028C		0.708115183246073YDR319C		0.211678832116788YGL211W		0.348189415041783YKL133C		0.453563714902808YNL137C		0.631687242798354YDL176W		0.34180790960452YDR487C		0.100961538461538YOR084W		0.281653746770026YIL135C		0.963302752293578YLR209C		0.202572347266881YLR093C		0.217391304347826YEL061C		0.75YLR254C		1YOR166C		0.510917030567686YBR091C		0.477064220183486YKL049C		0.676855895196507YLR277C		0.31193838254172YNL167C		0.9629057187017YNL006W		0.0297029702970297YFR052W		0.259124087591241YGR203W		0.256756756756757YGL248W		0.327913279132791YGL228W		0.431542461005199YOR226C		0.275641025641026YPR035W		0.137837837837838YMR015C		0.217472118959108YFL014W		1YFL029C		0.35054347826087YDL076C		0.646258503401361YGR201C		0.0666666666666667YHR049W		0.267489711934156YJL051W		0.854014598540146YCR024C		0.154471544715447YPL104W		0.234042553191489YDR018C		0.207070707070707YBR072W		0.523364485981308YML071C		0.683690280065898YKL026C		0.167664670658683YMR218C		0.241379310344828YOR079C		0.284345047923323YGR044C		0.81YPL223C		1YPL250C		0.897058823529412YGL213C		0.143576826196474YGL101W		0.130232558139535YKL017C		0.210834553440703YMR152W		0.0767123287671233YNL083W		0.289908256880734YBR157C		0.952941176470588YMR061W		0.317577548005908YPL117C		0.329861111111111YGL189C		0.563025210084034YOR311C		0.279310344827586YMR293C		0.084051724137931YDR259C		0.906005221932115YCL031C		0.653198653198653YIR026C		0.445054945054945YHR096C		0.246621621621622YDR488C		0.461538461538462YPR169W		0.367886178861789YPL195W		0.431330472103004YEL051W		0.41796875YMR165C		0.65661252900232YMR106C		0.483306836248013YBR202W		0.4YKL052C		0.787671232876712YPL259C		0.273684210526316YKL140W		0.312043795620438YNL155W		0.572992700729927YBR285W		0.819444444444444YKL124W		0.583765112262522YNR007C		0.438709677419355YOL101C		0.189102564102564YDL022W		0.225063938618926YJL183W		0.33175355450237YJR125C		0.659313725490196YMR069W		0.428070175438596YML126C		0.260692464358452YLR258W		0.24822695035461YMR288W		0.270854788877446YHR176W		0.152777777777778YOR327C		0.521739130434783YNR027W		0.132492113564669YCR060W		0.0720720720720721YLR130C		0.443127962085308YDR234W		0.187590187590188YAL062W		0.140043763676149YFR032C		0.536332179930796YBL014C		0.638702460850112YHL021C		0.313978494623656YPR133C		0.75609756097561YPL232W		0.575862068965517YPR182W		0.22093023255814YDL163W		0.38YNL245C		1YPL215W		0.453731343283582YIR028W		0.275590551181102YCL059C		0.493670886075949YAL037W		0.460674157303371YER003C		0.165501165501166YML046W		0.213036565977742YCL034W		0.601694915254237YFL039C		0.0906666666666667YHR188C		0.311475409836066YER127W		0.913165266106443YJR090C		0.441355343179844YCL011C		0.473067915690867YPL059W		0.38YGL050W		0.406593406593407YLR164W		0.386904761904762YAL055W		0.394444444444444YDL174C		0.214650766609881YBR261C		0.237068965517241YEL034W		0.229299363057325YKL125W		0.38755980861244YML112W		0.648648648648649YHR135C		0.563197026022305YGR077C		0.174872665534805YDR285W		1YGR285C		0.646651270207852YPL236C		0.258241758241758YFL025C		0.282798833819242YJR133W		0.258373205741627YKL171W		0.756465517241379YJR073C		0.106796116504854YPL101W		0.614035087719298YBL025W		0.524137931034483YJR107W		0.192073170731707YDR434W		0.353932584269663YNL326C		0.342261904761905YKL005C		0.749158249158249YOR021C		0.483568075117371YIL079C		1YDR177W		0.288372093023256YOL018C		0.695214105793451YBR183W		0.145569620253165YGL124C		0.445652173913043YAR002W		1YIL063C		0.675840978593272YPL010W		0.386243386243386YER180C		0.659176029962547YPR186C		0.822843822843823YPL079W		0.41875YHR182W		0.560509554140127YMR081C		0.985207100591716YER137C		0.932432432432432YHR167W		0.601532567049808YBL031W		1YLR098C		0.524691358024691YEL066W		0.184357541899441YOR056C		0.596949891067538YBL091C		0.268408551068884YFR033C		0.707482993197279YDR194C		0.451807228915663YLR300W		0.151785714285714YDR350C		0.353518821603928YDL173W		1YMR017W		0.730478589420655YOR141C		0.600454029511918YDR473C		0.727078891257996YDL057W		0.271341463414634YIR031C		0.160649819494585YCL025C		0.259083728278041YKL178C		0.385106382978723YDL077C		0.299332697807436YNL271C		0.731182795698925YGL096W		0.844202898550725YNL270C		0.167539267015707YMR268C		0.333333333333333YOR207C		0.269799825935596YDR030C		0.24901185770751YCR009C		0.339622641509434YNL157W		1YKL040C		0.42578125YLR211C		0.911504424778761YLR091W		0.416382252559727YML076C		0.63771186440678YNR051C		0.724271844660194YDR538W		0.268595041322314YGR284C		0.241935483870968YLR134W		0.124333925399645YMR311C		1YDR515W		0.850111856823266YNL284C		0.552795031055901YNL279W		0.275340393343419YBR236C		0.408256880733945YJR011C		0.314176245210728YGR262C		0.298850574712644YIR032C		0.179487179487179YJL180C		0.347692307692308YEL059C-A		0.337837837837838YEL024W		0.316279069767442YNL080C		0.505464480874317YNL127W		0.538300104931794YMR011W		0.179297597042514YJR086W		0.772727272727273YCL051W		1YMR177W		0.376470588235294YBR099C		0.196850393700787YBR212W		0.677083333333333YOR291W		0.402173913043478YGL069C		0.279220779220779YJL218W		0.142857142857143YPL130W		0.565022421524664YBL072C		0.455YCR052W		0.56935817805383YPR058W		0.211726384364821YKL060C		0.194986072423398YBR185C		0.442446043165468YGL112C		0.436046511627907YGL019W		0.535971223021583YBR156C		0.98567335243553YOR368W		0.428927680798005YGR008C		1YKL084W		0.275862068965517YPL006W		0.224786324786325YKL003C		0.305343511450382YNL191W		0.338935574229692YOL067C		0.966101694915254YJL116C		0.543026706231454YLR370C		0.207865168539326YDL121C		0.832214765100671YMR187C		0.164733178654292YLR100W		0.178674351585014YIL111W		0.66887417218543YMR098C		0.527777777777778YCR069W		0.308176100628931YDL047W		0.170418006430868YGL106W		0.134228187919463YKL004W		0.241895261845387YLR241W		0.228900255754476YOL031C		0.560570071258907YKL077W		0.469387755102041YER053C		0.14YNL076W		0.912671232876712YKL053C-A		0.406976744186047YOR257W		0.198757763975155YBL023C		0.5YER051W		0.447154471544715YJL042W		0.73032904148784YPR075C		0.941666666666667YEL057C		0.309012875536481YNL175C		0.689826302729528YOR215C		0.52972972972973YNL332W		0.102941176470588YIL030C		0.363912054586808YBR199W		0.316810344827586YIL134W		0.221864951768489YPR177C		0.666666666666667YBL010C		0.95YDL001W		0.476744186046512YBR056W		0.193612774451098YMR305C		0.323907455012853YOR273C		0.349013657056146YBL054W		0.994285714285714YGL012W		0.120507399577167YOR133W		0.191211401425178YML057W		0.544701986754967YFL049W		0.629213483146067YCR005C		0.241304347826087YML028W		0.0663265306122449YGL032C		0.402298850574713YBR070C		0.185654008438819YOL060C		0.558073654390935YIL010W		0.367441860465116YGL154C		0.150735294117647YMR093W		0.152046783625731YGL191W		0.527131782945736YJL208C		0.331306990881459YDR132C		0.424242424242424YER177W		0.51685393258427YNL158W		0.282828282828283YOR224C		0.171232876712329YNL026W		0.198347107438017YPR179C		0.618320610687023YER019W		0.257861635220126YIL091C		0.533980582524272YDR052C		0.762784090909091YGL110C		0.599358974358974YHL011C		0.078125YDR051C		0.491017964071856YOR370C		0.368159203980099YDR397C		0.458904109589041YBR120C		0.648148148148148YFL011W		0.186813186813187YHR210C		0.0703812316715543YBR089C-A		0.555555555555556YHL002W		0.672566371681416YPR185W		0.789972899728997YML114C		0.831372549019608YGR280C		0.948339483394834YDR153C		0.661800486618005YDR229W		0.673289183222958YIR001C		0.712YDR321W		0.244094488188976YHR029C		0.0612244897959184YOL091W		0.80952380952381YBR148W		0.860426929392447YLR246W		0.356545961002786YJL140W		0.764705882352941YOR157C		0.157088122605364YML019W		0.147590361445783YEL048C		0.269736842105263YBL080C		0.269870609981516YJR001W		0.393687707641196YER030W		1YER027C		0.693045563549161YFR001W		1YIL075C		0.299470899470899YGL254W		0.852842809364548YJL104W		0.74496644295302YGR165W		0.788405797101449YKL087C		0.441964285714286YNR012W		0.209580838323353YDR468C		0.625YCR046C		0.455621301775148YHL012W		0.170385395537525YEL039C		0.433628318584071YDL144C		0.154494382022472YLR200W		1YGL247W		0.304568527918782YNR006W		0.717041800643087YGR286C		0.210666666666667YIL133C		0.321608040201005YNL124W		0.83130081300813YJR136C		0.32541567695962YAL005C		0.163551401869159YNR047W		0.724524076147816YPL131W		0.508417508417508YHR123W		0.0792838874680307YJR025C		0.101694915254237YLR224W		0.292682926829268YHL010C		0.647863247863248YOR174W		0.774647887323944YER122C		0.797160243407708YHR070W		0.324649298597194YDR184C		0.904761904761905YHR147C		0.205607476635514YJR074W		0.279816513761468YOR048C		0.550695825049702YNL295W		0.687022900763359YHR025W		0.182072829131653YNL254C		0.683291770573566YAL027W		0.363984674329502YBR132C		0.219798657718121YMR213W		0.794915254237288YML027W		1YDR211W		0.346910112359551YDR125C		0.251655629139073YPL169C		0.425709515859766YOL038W		0.196850393700787YGL073W		0.929171668667467YPL200W		0.166666666666667YPL271W		0.548387096774194YDR354W		0.202631578947368YPL055C		1YDR331W		0.326034063260341YDR240C		0.471544715447154YDL237W		0.284615384615385YDL137W		0.0994475138121547YBR254C		0.4YBR040W		0.328859060402685YPR022C		0.653133274492498YDR238C		0.286742034943474YBR286W		0.353817504655493YIR022W		0.0958083832335329YEL019C		0.730337078651685YPL114W		0.62589928057554YAL059W		0.924528301886792YBR168W		0.404358353510896YPR119W		0.517311608961303YBR006W		0.0402414486921529YPR118W		0.206812652068127YBL011W		0.36231884057971YOR189W		0.801724137931034YPL238C		0.813953488372093YOR292C		0.330097087378641YOR341W		0.406850961538462YMR287C		0.288957688338493YDL097C		0.214285714285714YER040W		1YPL242C		0.393311036789298YGR277C		0.255737704918033YAL040C		0.603448275862069YMR277W		0.546448087431694YNL310C		0.706896551724138YLR376C		0.223140495867769YJR104C		0.181818181818182YLR180W		0.162303664921466YGL044C		0.608108108108108YDL188C		0.342175066312997YOL139C		0.305164319248826YGL031C		0.645161290322581YBR123C		0.61633281972265YDL246C		0.0392156862745098YDR332W		0.317851959361393YDR294C		0.173174872665535YFR003C		1YFR050C		0.266917293233083YHR072W		0.143638850889193YNL030W		0.485436893203884YDR374C		0.529411764705882YPL004C		0.571847507331378YOR036W		0.53125YDR126W		0.160714285714286YOL097C		0.310185185185185YDL229W		0.110929853181077YML023C		0.221223021582734YNL197C		0.89409984871407YDR252W		0.590604026845638YDR306C		0.370292887029289YGR191W		0.227197346600332YJL174W		0.344202898550725YDL179W		0.536184210526316YLR350W		0.342592592592593YOR025W		0.420581655480984YBR066C		1YGR177C		0.366355140186916YER134C		0.174157303370787YPR107C		0.793269230769231YBR257W		0.551971326164875YCL052C		0.206730769230769YDL199C		0.366812227074236YKL121W		0.4518779342723YNL321W		0.431718061674009YMR014W		0.89402697495183YKL041W		0.90625YJL010C		0.372372372372372YOR331C		0.356756756756757YER163C		0.254310344827586YKL054C		1YBR230C		0.305970149253731YBR214W		0.582542694497154YPL268W		0.432681242807825YOR020C		0.0660377358490566YDR314C		0.48121387283237
