# Supplementary material for: Proteome-wide evidence for enhanced positive Darwinian selection within intrinsically disordered regions in proteins
Source: Genome Biol. 2011 Jul 19;12(7):R65. doi: 10.1186/gb-2011-12-7-r65 (PMC3218827; doi:10.1186/gb-2011-12-7-r65)
Supplement: Additional file 14 — Fixation index of each investigated protein coding gene along with the associated P-value. The FI was calculated using the MKtest program and is defined as the ratio (dN/dS)/(pN/pS). [file gb-2011-12-7-r65-S14.RTF]

YAL001C	FI:0.605028017567772	p:0.0180752YAL005C	FI:0.387096774193548	p:0.0800057YAL007C	FI:0.338461538461538	p:0.0850948YAL008W	FI:1.55102040816327	p:0.144047YAL009W	FI:0.552631578947368	p:0.200901YAL010C	FI:0.805357142857143	p:0.11105YAL011W	FI:1.13570127504554	p:0.118476YAL013W	FI:0.6	p:0.131462YAL016W	FI:0.52801724137931	p:0.163601YAL018C	FI:0.947916666666667	p:0.185734YAL019W	FI:0.592546583850932	p:0.104165YAL020C	FI:0.512091038406828	p:0.0348976YAL022C	FI:1.6875	p:0.142745YAL023C	FI:1.05344827586207	p:0.215947YAL025C	FI:1.94444444444444	p:0.331732YAL027W	FI:1.20545073375262	p:0.167891YAL028W	FI:0.763157894736842	p:0.0903352YAL032C	FI:1.13636363636364	p:0.157189YAL033W	FI:4.30434782608696	p:0.135651YAL034C	FI:0.837209302325581	p:0.195817YAL034W-A	FI:1.77272727272727	p:0.179619YAL035W	FI:1.75690607734807	p:0.0692527YAL036C	FI:	p:0.374632YAL037W	FI:0.463636363636364	p:0.0569064YAL038W	FI:0.526315789473684	p:0.229586YAL039C	FI:2.32258064516129	p:0.0980955YAL040C	FI:0.997732426303855	p:0.198341YAL041W	FI:0.962566844919786	p:0.204338YAL042W	FI:1.755	p:0.116595YAL043C	FI:0.84	p:0.117934YAL044C	FI:0.0833333333333333	p:0.0138022YAL044W-A	FI:0.0952380952380952	p:0.22YAL046C	FI:0.761904761904762	p:0.237055YAL047C	FI:0.762973352033661	p:0.0855617YAL048C	FI:1.21078431372549	p:0.247482YAL049C	FI:0.833333333333333	p:0.182836YAL054C	FI:0.704225352112676	p:0.2132YAL055W	FI:0.86996336996337	p:0.163675YAL059W	FI:0.611111111111111	p:0.148771YAL060W	FI:0.550819672131148	p:0.0889976YAL061W	FI:0.37987012987013	p:0.0502436YAL062W	FI:0.794117647058824	p:0.230584YAR002C-A	FI:2.1	p:0.217434YAR002W	FI:0.56241426611797	p:0.0280432YAR003W	FI:0.236486486486487	p:0.00286522YAR007C	FI:2.97142857142857	p:0.0903678YAR008W	FI:0.793604651162791	p:0.18667YAR014C	FI:1.1140456182473	p:0.11898YAR015W	FI:3.33333333333333	p:0.206199YAR018C	FI:0.867857142857143	p:0.244232YAR019C	FI:0.746769562096195	p:0.0596572YAR035W	FI:3.744	p:0.0134514YAR062W	FI:	p:YAR066W	FI:	p:0.34507YBL003C	FI:	p:0.423077YBL005W	FI:1.09407507914971	p:0.106466YBL006C	FI:0.535714285714286	p:0.124799YBL007C	FI:1.26330532212885	p:0.102797YBL009W	FI:2.16	p:0.00481126YBL010C	FI:0.622540250447227	p:0.110549YBL011W	FI:1.58707643814027	p:0.0799694YBL013W	FI:0.907460545193687	p:0.139321YBL014C	FI:1.52631578947368	p:0.0634489YBL015W	FI:	p:0.467487YBL016W	FI:1.2972972972973	p:0.252152YBL019W	FI:0.897382633984213	p:0.112362YBL020W	FI:1.8046875	p:0.124454YBL021C	FI:0.423076923076923	p:0.437247YBL023C	FI:1.02601156069364	p:0.236838YBL024W	FI:0.755494505494505	p:0.120889YBL025W	FI:0.525	p:0.183046YBL028C	FI:1.07692307692308	p:0.400445YBL029W	FI:1.17727272727273	p:0.156774YBL030C	FI:1.08620689655172	p:0.272342YBL031W	FI:0.655737704918033	p:0.131383YBL032W	FI:0.457142857142857	p:0.106287YBL033C	FI:0.957207207207207	p:0.20671YBL036C	FI:0.782608695652174	p:0.181253YBL038W	FI:0.809523809523809	p:0.2284YBL041W	FI:0.174242424242424	p:0.1003YBL045C	FI:0.45	p:0.0426305YBL049W	FI:0	p:0.0541872YBL050W	FI:2.46296296296296	p:0.146621YBL051C	FI:2.09090909090909	p:0.0178187YBL052C	FI:0.933524629363941	p:0.0848023YBL054W	FI:0.717777777777778	p:0.095096YBL056W	FI:2.38461538461539	p:0.067613YBL057C	FI:0.668989547038327	p:0.171008YBL058W	FI:0.909090909090909	p:0.127139YBL060W	FI:0.970921255790015	p:0.108455YBL061C	FI:1.01880877742947	p:0.121048YBL066C	FI:0.730239818362424	p:0.0519968YBL068W	FI:0.23728813559322	p:0.112919YBL069W	FI:1.27058823529412	p:0.21616YBL072C	FI:1	p:0.535714YBL074C	FI:1.30994152046784	p:0.108466YBL075C	FI:0.909090909090909	p:0.236244YBL078C	FI:	p:0.703704YBL080C	FI:0.706140350877193	p:0.0839787YBL081W	FI:0.663983903420523	p:0.0981197YBL082C	FI:1.23697916666667	p:0.161034YBL084C	FI:0.689519306540583	p:0.119697YBL086C	FI:0.761904761904762	p:0.146823YBL087C	FI:	p:1YBL089W	FI:0.864086408640864	p:0.150909YBL090W	FI:1.05882352941176	p:0.259079YBL091C	FI:0.692541856925419	p:0.142062YBL092W	FI:	p:0.461539YBL093C	FI:0.735294117647059	p:0.328222YBL095W	FI:1.903125	p:0.101191YBL098W	FI:1.57843137254902	p:0.132084YBL099W	FI:0.217105263157895	p:0.201014YBL102W	FI:0.547008547008547	p:0.243158YBL103C	FI:1.05142857142857	p:0.170559YBL104C	FI:0.587606837606838	p:0.0343711YBL107C	FI:0.6	p:0.104044YBR002C	FI:0.261904761904762	p:0.114635YBR003W	FI:0.673684210526316	p:0.119772YBR004C	FI:0.757954545454545	p:0.136844YBR005W	FI:1.06666666666667	p:0.19151YBR006W	FI:0.87032967032967	p:0.218334YBR007C	FI:0.487836438923395	p:0.00777791YBR008C	FI:1.04700854700855	p:0.168839YBR009C	FI:	p:1YBR010W	FI:	p:1YBR011C	FI:3.33333333333333	p:0.219443YBR014C	FI:1.04395604395604	p:0.267229YBR016W	FI:	p:0.163749YBR017C	FI:0.79959100204499	p:0.124806YBR018C	FI:1.67763157894737	p:0.0879065YBR019C	FI:0.510835913312694	p:0.0189741YBR020W	FI:1.02317290552585	p:0.15831YBR021W	FI:0.963983050847458	p:0.234883YBR022W	FI:0.673992673992674	p:0.181458YBR024W	FI:0.425454545454545	p:0.0448046YBR025C	FI:0.851851851851852	p:0.337216YBR026C	FI:1.0625	p:0.187723YBR028C	FI:1.05913043478261	p:0.159896YBR030W	FI:1.17647058823529	p:0.118854YBR031W	FI:1.58333333333333	p:0.428195YBR033W	FI:0.64704474754726	p:0.0117364YBR034C	FI:0.248756218905473	p:0.119255YBR035C	FI:0.526315789473684	p:0.462712YBR037C	FI:0.453703703703704	p:0.0853127YBR039W	FI:3.81818181818182	p:0.1535YBR040W	FI:1.54929577464789	p:0.214623YBR041W	FI:0.733333333333333	p:0.129622YBR043C	FI:2.4	p:0.0341373YBR045C	FI:1.41843971631206	p:0.0657484YBR046C	FI:0.682203389830509	p:0.118942YBR047W	FI:0.642857142857143	p:0.224884YBR050C	FI:1.16923076923077	p:0.178052YBR052C	FI:2.07692307692308	p:0.115586YBR053C	FI:0.836538461538462	p:0.148024YBR054W	FI:0.633333333333333	p:0.427055YBR055C	FI:0.80068060281964	p:0.0584466YBR056W	FI:1.89111111111111	p:0.0947764YBR057C	FI:0.482758620689655	p:0.0824002YBR058C-A	FI:0.583333333333333	p:0.495238YBR060C	FI:1.24386252045826	p:0.116339YBR061C	FI:1.66101694915254	p:0.26129YBR065C	FI:0.941840277777778	p:0.144862YBR066C	FI:0.464285714285714	p:0.0536785YBR067C	FI:2.77083333333333	p:0.08218YBR068C	FI:0.495867768595041	p:0.0585522YBR069C	FI:0.975	p:0.13614YBR070C	FI:0.7	p:0.132109YBR071W	FI:1.46718146718147	p:0.156215YBR072W	FI:0.771428571428571	p:0.257353YBR073W	FI:0.746148092744951	p:0.0507296YBR077C	FI:1	p:0.266267YBR082C	FI:	p:1YBR084W	FI:1.0419621749409	p:0.15917YBR085C-A	FI:0.266666666666667	p:0.380953YBR085W	FI:1.25714285714286	p:0.303807YBR087W	FI:2.85990338164251	p:0.0575762YBR088C	FI:	p:1YBR089C-A	FI:0.264705882352941	p:0.270936YBR091C	FI:0.818181818181818	p:0.353689YBR092C	FI:7.48837209302326	p:0.0175823YBR093C	FI:0.477272727272727	p:0.112454YBR094W	FI:0.581671415004748	p:0.0442482YBR095C	FI:1.30909090909091	p:0.127041YBR096W	FI:0.871794871794872	p:0.23658YBR097W	FI:0.861332647285824	p:0.0875012YBR098W	FI:0.968858131487889	p:0.106673YBR099C	FI:0.0594594594594595	p:0.00642672YBR101C	FI:0.346153846153846	p:0.0483132YBR104W	FI:0.510416666666667	p:0.200353YBR105C	FI:1.2200956937799	p:0.143276YBR106W	FI:0.289473684210526	p:0.285924YBR107C	FI:0.822510822510822	p:0.168758YBR109C	FI:	p:1YBR110W	FI:0.548888888888889	p:0.081157YBR111C	FI:1.78378378378378	p:0.197922YBR115C	FI:1.12815198618307	p:0.121519YBR119W	FI:0.887812752219532	p:0.133854YBR120C	FI:1.16071428571429	p:0.258434YBR121C	FI:1.18811881188119	p:0.227135YBR122C	FI:1.95555555555556	p:0.125487YBR123C	FI:0.921259842519685	p:0.138519YBR125C	FI:3.55384615384615	p:0.155107YBR126C	FI:0.927835051546392	p:0.427828YBR128C	FI:0.576612903225806	p:0.0638026YBR129C	FI:0.149882903981265	p:0.000212615YBR130C	FI:1.40972222222222	p:0.129038YBR132C	FI:0.940594059405941	p:0.172979YBR133C	FI:1.009375	p:0.124762YBR135W	FI:	p:1YBR136W	FI:0.763206162876009	p:0.0534797YBR137W	FI:2.14285714285714	p:0.218708YBR138C	FI:0.762471395881007	p:0.0901275YBR139W	FI:1.0423197492163	p:0.154552YBR141C	FI:1.30128205128205	p:0.128147YBR145W	FI:1.91549295774648	p:0.122185YBR146W	FI:0.445269016697588	p:0.0524615YBR147W	FI:0.519230769230769	p:0.0607744YBR148W	FI:1.19444444444444	p:0.0834281YBR149W	FI:0.751515151515151	p:0.203763YBR151W	FI:1.1864406779661	p:0.168137YBR154C	FI:	p:0.469697YBR155W	FI:0.949771689497717	p:0.160217YBR156C	FI:1.38888888888889	p:0.0510755YBR157C	FI:0.899470899470899	p:0.190169YBR159W	FI:0.49486301369863	p:0.0796246YBR160W	FI:	p:0.75YBR162C	FI:0.285714285714286	p:0.0248895YBR162W-A	FI:5.625	p:0.0893963YBR163W	FI:0.69187145557656	p:0.0606344YBR164C	FI:	p:1YBR165W	FI:0.643491124260355	p:0.108016YBR166C	FI:0.507692307692308	p:0.0493271YBR167C	FI:0.616	p:0.157419YBR168W	FI:0.642424242424242	p:0.0860897YBR169C	FI:0.904494382022472	p:0.202914YBR170C	FI:1.50630630630631	p:0.0904131YBR171W	FI:0.325581395348837	p:0.108135YBR172C	FI:0.980824544582934	p:0.0944567YBR173C	FI:1.11111111111111	p:0.354445YBR175W	FI:1.7816091954023	p:0.186022YBR176W	FI:0.685714285714286	p:0.158503YBR177C	FI:1.11036036036036	p:0.153273YBR179C	FI:0.944571428571429	p:0.117313YBR180W	FI:0.862155388471178	p:0.115704YBR181C	FI:2	p:0.474308YBR182C	FI:2.16338259441708	p:0.0120611YBR183W	FI:1.02450980392157	p:0.271299YBR185C	FI:0.978723404255319	p:0.219874YBR186W	FI:2.15485278080698	p:0.0404696YBR191W	FI:	p:1YBR193C	FI:0.141304347826087	p:0.000647475YBR195C	FI:0.582995951417004	p:0.0744917YBR197C	FI:0.826475849731664	p:0.16816YBR199W	FI:1.59	p:0.0585348YBR201W	FI:0.84375	p:0.194871YBR202W	FI:0.996875	p:0.171231YBR203W	FI:0.786084381939304	p:0.0715628YBR204C	FI:0.84228473998295	p:0.132493YBR205W	FI:1.45833333333333	p:0.151569YBR207W	FI:0.660412757973734	p:0.0971035YBR210W	FI:0.478947368421053	p:0.145709YBR212W	FI:0.793103448275862	p:0.124305YBR213W	FI:1.02127659574468	p:0.196863YBR214W	FI:0.694736842105263	p:0.184494YBR217W	FI:0.292207792207792	p:0.0159632YBR220C	FI:0.840384615384616	p:0.22271YBR221C	FI:0.283333333333333	p:0.119939YBR223C	FI:1.25322997416021	p:0.153049YBR227C	FI:0.725057121096725	p:0.110742YBR228W	FI:1.61458333333333	p:0.11235YBR230C	FI:0.448	p:0.164675YBR231C	FI:1.34272300469484	p:0.119412YBR233W	FI:1.22352941176471	p:0.18442YBR233W-A	FI:0.833333333333333	p:0.319214YBR236C	FI:0.590163934426229	p:0.282345YBR237W	FI:0.781316348195329	p:0.0719508YBR239C	FI:0.666666666666667	p:0.110352YBR240C	FI:2.75757575757576	p:0.0569773YBR242W	FI:0.388888888888889	p:0.0527332YBR243C	FI:0.120300751879699	p:0.00164539YBR244W	FI:0.254545454545455	p:0.054256YBR246W	FI:1.34796238244514	p:0.118485YBR247C	FI:0.260215053763441	p:0.00587693YBR248C	FI:1.52727272727273	p:0.170364YBR249C	FI:0.516304347826087	p:0.188203YBR251W	FI:	p:0.131991YBR252W	FI:0.444444444444444	p:0.207647YBR253W	FI:0.324675324675325	p:0.0744422YBR254C	FI:1.01190476190476	p:0.336809YBR256C	FI:0.867924528301887	p:0.229067YBR257W	FI:1.3037037037037	p:0.200507YBR258C	FI:0.673469387755102	p:0.200517YBR259W	FI:0.725793650793651	p:0.0536019YBR260C	FI:3.2	p:0.0273672YBR261C	FI:1.0188679245283	p:0.278584YBR262C	FI:1.95555555555556	p:0.208744YBR263W	FI:1.41025641025641	p:0.204195YBR264C	FI:0.583333333333333	p:0.137616YBR265W	FI:0.293367346938775	p:0.0383873YBR267W	FI:0.780189959294437	p:0.146558YBR268W	FI:0.49	p:0.179841YBR269C	FI:0.363636363636364	p:0.135619YBR270C	FI:1.16504854368932	p:0.145212YBR271W	FI:1.34615384615385	p:0.0888979YBR272C	FI:0.79689366786141	p:0.115569YBR273C	FI:0.790909090909091	p:0.122341YBR274W	FI:1.05494505494506	p:0.153498YBR276C	FI:0.635593220338983	p:0.0210558YBR278W	FI:1.125	p:0.168573YBR279W	FI:1.39354838709677	p:0.116114YBR280C	FI:1.06971428571429	p:0.138059YBR281C	FI:0.96	p:0.145175YBR282W	FI:1.09803921568627	p:0.288949YBR283C	FI:1.8	p:0.131808YBR284W	FI:1.58763586956522	p:0.0428802YBR285W	FI:0.86231884057971	p:0.244247YBR286W	FI:0.520433694745621	p:0.0543752YBR287W	FI:0.767045454545455	p:0.228219YBR288C	FI:0.8015625	p:0.169073YBR290W	FI:1.22727272727273	p:0.230721YBR291C	FI:1.20149253731343	p:0.31188YBR293W	FI:0.726315789473684	p:0.131165YBR296C	FI:0.901960784313726	p:0.272285YBR298C	FI:2.57472527472527	p:0.00158383YBR301W	FI:3.85714285714286	p:0.159993YCL001W	FI:0.3375	p:0.0914991YCL004W	FI:0.989010989010989	p:0.152503YCL005W	FI:1.37142857142857	p:0.164575YCL010C	FI:0.446511627906977	p:0.062663YCL011C	FI:0.259259259259259	p:0.00406034YCL016C	FI:0.36986301369863	p:0.0257363YCL017C	FI:0.4296875	p:0.142993YCL021W-A	FI:0.291666666666667	p:0.0591677YCL025C	FI:0.244897959183673	p:0.0156748YCL026C-A	FI:0.755555555555556	p:0.17733YCL028W	FI:0.835390946502058	p:0.178755YCL029C	FI:0.512890094979647	p:0.0643048YCL030C	FI:1.34052757793765	p:0.157847YCL031C	FI:2.35658914728682	p:0.111572YCL032W	FI:2.205	p:0.092103YCL033C	FI:0.85	p:0.341648YCL034W	FI:0.571428571428571	p:0.126168YCL035C	FI:0.518518518518518	p:0.260796YCL036W	FI:1.24519230769231	p:0.120395YCL038C	FI:0.833333333333333	p:0.194128YCL039W	FI:0.555555555555556	p:0.0392587YCL043C	FI:2.125	p:0.0888709YCL044C	FI:0.582352941176471	p:0.178329YCL045C	FI:0.677837837837838	p:0.052263YCL049C	FI:0.548076923076923	p:0.151281YCL050C	FI:0.901515151515151	p:0.285841YCL051W	FI:0.983118172790467	p:0.13081YCL052C	FI:1.41176470588235	p:0.112535YCL055W	FI:0.191780821917808	p:0.0785285YCL056C	FI:0.4	p:0.100043YCL057C-A	FI:	p:0.191453YCL057W	FI:0.988082340195016	p:0.143317YCL059C	FI:0.642857142857143	p:0.429351YCL063W	FI:0.651017214397496	p:0.129625YCL064C	FI:0.406444906444906	p:0.022489YCL066W	FI:2.06060606060606	p:0.178626YCL068C	FI:1.39130434782609	p:0.221771YCR002C	FI:0.310457516339869	p:0.0557968YCR004C	FI:0.121276595744681	p:0.00148749YCR005C	FI:0.5	p:0.0924631YCR008W	FI:0.386363636363636	p:0.0205569YCR009C	FI:0	p:0.00452863YCR010C	FI:0	p:0.00495071YCR011C	FI:1.03034789045152	p:0.177244YCR015C	FI:1.05241935483871	p:0.215211YCR016W	FI:0.814583333333333	p:0.140487YCR017C	FI:0.356382018414876	p:0.00026541YCR018C	FI:0.523255813953488	p:0.0707937YCR020C	FI:0.18801652892562	p:0.00302244YCR020C-A	FI:0.285714285714286	p:0.0813948YCR021C	FI:0.566666666666667	p:0.0921432YCR023C	FI:0.814895947426068	p:0.147992YCR024C	FI:0.945794392523365	p:0.164536YCR026C	FI:0.553924994176566	p:0.0175701YCR027C	FI:1.27272727272727	p:0.261092YCR028C	FI:1.03529411764706	p:0.21045YCR033W	FI:1.20832520959251	p:0.0583532YCR035C	FI:0.880281690140845	p:0.155897YCR037C	FI:0.431442080378251	p:0.00890094YCR038C	FI:0.901030927835052	p:0.141492YCR039C	FI:	p:0.170242YCR042C	FI:1.12800565770863	p:0.124106YCR043C	FI:0	p:0.243243YCR045C	FI:0.314814814814815	p:0.00594037YCR046C	FI:0.102564102564103	p:0.0045505YCR047C	FI:0.181159420289855	p:0.0318346YCR048W	FI:0.779541446208113	p:0.145291YCR050C	FI:0.803571428571429	p:0.296413YCR051W	FI:0.362790697674419	p:0.035422YCR052W	FI:0.65979381443299	p:0.0605303YCR053W	FI:0.17032967032967	p:0.00176525YCR057C	FI:0.367560041768187	p:0.0035643YCR059C	FI:0.245283018867925	p:0.0198513YCR060W	FI:0.942857142857143	p:0.260523YCR061W	FI:0.547826086956522	p:0.0677917YCR063W	FI:0.394736842105263	p:0.170174YCR065W	FI:1.18867924528302	p:0.201586YCR066W	FI:0.9375	p:0.152015YCR068W	FI:1.10539215686275	p:0.129539YCR069W	FI:0.636363636363636	p:0.106949YCR071C	FI:0.6875	p:0.212058YCR073W-A	FI:0.26984126984127	p:0.0173615YCR076C	FI:0.686456400742115	p:0.143266YCR077C	FI:0.40251572327044	p:0.012311YCR082W	FI:1.46666666666667	p:0.2643YCR083W	FI:0.916666666666667	p:0.280983YCR086W	FI:3.75	p:0.0726351YCR087C-A	FI:0.415384615384615	p:0.135989YCR088W	FI:1.13666666666667	p:0.144151YCR090C	FI:0.972972972972973	p:0.241499YCR091W	FI:0.695187165775401	p:0.0664999YCR095C	FI:0.525606469002695	p:0.113445YDL001W	FI:2.47058823529412	p:0.140388YDL002C	FI:0.878048780487805	p:0.328989YDL003W	FI:0.910910910910911	p:0.110752YDL004W	FI:0.185185185185185	p:0.0695084YDL005C	FI:0.721544715447154	p:0.0867299YDL006W	FI:1.59574468085106	p:0.277984YDL007W	FI:	p:1YDL008W	FI:1.03448275862069	p:0.293671YDL010W	FI:0.911877394636015	p:0.203584YDL013W	FI:1.3875	p:0.152101YDL014W	FI:0.227642276422764	p:0.11243YDL015C	FI:2.77477477477478	p:0.0816295YDL017W	FI:0.329896907216495	p:0.0277307YDL018C	FI:0.507317073170732	p:0.152295YDL020C	FI:1.74140752864157	p:0.0424868YDL021W	FI:1.40344827586207	p:0.190203YDL022W	FI:	p:0.503371YDL027C	FI:0.575757575757576	p:0.043107YDL028C	FI:1.11748251748252	p:0.150888YDL029W	FI:	p:0.611226YDL030W	FI:0.768695652173913	p:0.0946486YDL033C	FI:0.743434343434343	p:0.130391YDL036C	FI:0.532535885167464	p:0.0277278YDL042C	FI:0.621153846153846	p:0.0993591YDL044C	FI:1.4126582278481	p:0.0914167YDL045C	FI:0.282786885245902	p:0.0241292YDL045W-A	FI:0.5	p:0.46798YDL046W	FI:1.66666666666667	p:0.221515YDL047W	FI:	p:0.734177YDL048C	FI:0.772173913043478	p:0.138519YDL049C	FI:1.95555555555556	p:0.152811YDL051W	FI:3.76923076923077	p:0.159299YDL052C	FI:1.67424242424242	p:0.18066YDL053C	FI:0.829268292682927	p:0.231378YDL056W	FI:1.6729797979798	p:0.0623564YDL057W	FI:0.707070707070707	p:0.0990787YDL058W	FI:1.17327829238824	p:0.0543081YDL059C	FI:2.2	p:0.100286YDL060W	FI:0.558747697974217	p:0.0378753YDL063C	FI:0.620648259303721	p:0.0462734YDL064W	FI:	p:0.0293683YDL065C	FI:0.561643835616438	p:0.0582139YDL066W	FI:0.825396825396825	p:0.333364YDL067C	FI:0	p:0.666667YDL069C	FI:1.04112554112554	p:0.183789YDL070W	FI:0.824050632911392	p:0.116915YDL072C	FI:0.444444444444444	p:0.202579YDL073W	FI:0.628571428571429	p:0.0162862YDL076C	FI:1.11272727272727	p:0.148324YDL077C	FI:0.65158371040724	p:0.0350034YDL078C	FI:1.70238095238095	p:0.12508YDL080C	FI:1.404	p:0.190155YDL081C	FI:0.5	p:0.494506YDL084W	FI:0	p:0.0395812YDL085C-A	FI:1.5	p:0.453297YDL085W	FI:1.96100917431193	p:0.106076YDL086W	FI:0.333333333333333	p:0.151511YDL087C	FI:0.817777777777778	p:0.232051YDL088C	FI:1.30454545454545	p:0.087744YDL089W	FI:1.21487603305785	p:0.10697YDL090C	FI:1.1029810298103	p:0.170378YDL091C	FI:1.56701030927835	p:0.0907331YDL097C	FI:0	p:0.096292YDL098C	FI:1.20192307692308	p:0.239064YDL100C	FI:0.648148148148148	p:0.256899YDL101C	FI:0.736842105263158	p:0.142067YDL102W	FI:0.699342498505678	p:0.139008YDL104C	FI:1.09090909090909	p:0.154434YDL105W	FI:1.26682692307692	p:0.129489YDL106C	FI:0.697010869565218	p:0.0918608YDL107W	FI:0.927027027027027	p:0.149507YDL108W	FI:0.62962962962963	p:0.305998YDL110C	FI:4.52941176470588	p:0.137251YDL111C	FI:0.491228070175439	p:0.159472YDL114W	FI:1.15207373271889	p:0.14741YDL115C	FI:0.68	p:0.14555YDL116W	FI:0.777777777777778	p:0.0876671YDL117W	FI:1.16949152542373	p:0.103995YDL119C	FI:0.37037037037037	p:0.0209016YDL120W	FI:1.56521739130435	p:0.298099YDL121C	FI:0.358974358974359	p:0.0406519YDL123W	FI:1.97435897435897	p:0.179146YDL124W	FI:0.353535353535354	p:0.0168507YDL126C	FI:0.975	p:0.425774YDL127W	FI:0.931451612903226	p:0.188635YDL128W	FI:0.513157894736842	p:0.132645YDL130W-A	FI:0.4375	p:0.453333YDL131W	FI:1.47560975609756	p:0.223132YDL132W	FI:2.03313253012048	p:0.179999YDL133W	FI:1.9375	p:0.0860664YDL134C	FI:3.34328358208955	p:0.178077YDL135C	FI:0.727272727272727	p:0.447213YDL137W	FI:0	p:0.129445YDL139C	FI:0.789473684210526	p:0.180354YDL143W	FI:0.336842105263158	p:0.222932YDL144C	FI:0.360387192851824	p:0.00683079YDL146W	FI:1.61240310077519	p:0.086206YDL148C	FI:0.688311688311688	p:0.141209YDL149W	FI:0.789925925925926	p:0.106133YDL150W	FI:1.05882352941176	p:0.189722YDL151C	FI:1.58024691358025	p:0.238253YDL153C	FI:0.488297013720742	p:0.0165459YDL154W	FI:0.67750988646511	p:0.028529YDL155W	FI:1.30352303523035	p:0.141919YDL156W	FI:1.02752293577982	p:0.169797YDL157C	FI:1.06666666666667	p:0.319214YDL159W	FI:0.55045871559633	p:0.134665YDL160C	FI:	p:0.249759YDL161W	FI:0.719858156028369	p:0.137828YDL163W	FI:0.371428571428571	p:0.146925YDL165W	FI:0.319444444444444	p:0.293496YDL166C	FI:1.25714285714286	p:0.279399YDL167C	FI:0.727272727272727	p:0.0807285YDL168W	FI:0.818181818181818	p:0.19278YDL170W	FI:0.772946859903382	p:0.151727YDL173W	FI:0.571428571428571	p:0.159821YDL174C	FI:1.31048387096774	p:0.202234YDL175C	FI:1.97916666666667	p:0.1217YDL176W	FI:0.492424242424242	p:0.0347453YDL177C	FI:0.335664335664336	p:0.0385388YDL178W	FI:0.984633569739953	p:0.176742YDL179W	FI:0.772727272727273	p:0.142462YDL180W	FI:1.54132539091586	p:0.0589075YDL181W	FI:	p:0.684211YDL182W	FI:0.313253012048193	p:0.161206YDL183C	FI:1.53962264150943	p:0.104415YDL186W	FI:1.25409836065574	p:0.188958YDL188C	FI:0.576	p:0.162572YDL189W	FI:1.04934210526316	p:0.126853YDL192W	FI:	p:1YDL193W	FI:1.18055555555556	p:0.180352YDL198C	FI:1.39655172413793	p:0.298892YDL199C	FI:0.661538461538462	p:0.0802825YDL200C	FI:0.256410256410256	p:0.0153746YDL201W	FI:0.518518518518518	p:0.153414YDL202W	FI:2.35714285714286	p:0.160904YDL204W	FI:1.05452674897119	p:0.156867YDL207W	FI:0.820388349514563	p:0.114707YDL208W	FI:	p:0.755556YDL209C	FI:1.13317191283293	p:0.148535YDL210W	FI:2.19230769230769	p:0.0811014YDL211C	FI:1.29166666666667	p:0.0900229YDL212W	FI:1.23188405797101	p:0.265787YDL213C	FI:3.0625	p:0.212166YDL214C	FI:0.800865800865801	p:0.076884YDL216C	FI:1.8096256684492	p:0.0455718YDL217C	FI:	p:0.324991YDL218W	FI:1.13432835820896	p:0.265063YDL219W	FI:2.33333333333333	p:0.170785YDL222C	FI:0.369230769230769	p:0.064695YDL224C	FI:1.59153952843273	p:0.0559355YDL226C	FI:1.14754098360656	p:0.180098YDL227C	FI:0.617096018735363	p:0.12278YDL229W	FI:	p:0.656717YDL230W	FI:0.467320261437909	p:0.0663641YDL231C	FI:0.609085591733281	p:0.0285363YDL233W	FI:0.602678571428571	p:0.0730925YDL234C	FI:0.796743402582819	p:0.122386YDL235C	FI:	p:0.433144YDL237W	FI:0.826666666666667	p:0.137654YDL246C	FI:0.968944099378882	p:0.196861YDL247W	FI:3.02521008403361	p:0.00388436YDR002W	FI:0.789473684210526	p:0.365275YDR003W	FI:0.565714285714286	p:0.178521YDR004W	FI:0.588075880758808	p:0.0527549YDR006C	FI:0.985209923664122	p:0.134692YDR012W	FI:0.72	p:0.373896YDR013W	FI:0.32520325203252	p:0.141724YDR014W	FI:1.24574209245742	p:0.107107YDR016C	FI:0.0769230769230769	p:0.0372143YDR018C	FI:1.875	p:0.0409668YDR020C	FI:0.0797101449275362	p:0.000351046YDR022C	FI:0.686070686070686	p:0.132722YDR030C	FI:0.636792452830189	p:0.0479936YDR031W	FI:0.19047619047619	p:0.0489671YDR032C	FI:0	p:0.0199161YDR033W	FI:0.15	p:0.0223226YDR034C	FI:0.485943775100402	p:0.0330347YDR041W	FI:3.03333333333333	p:0.0581931YDR043C	FI:0.685975609756098	p:0.163734YDR044W	FI:0.576923076923077	p:0.418268YDR045C	FI:0	p:0.0902256YDR046C	FI:0.236928104575163	p:0.00212153YDR047W	FI:0.426439232409382	p:0.0753057YDR050C	FI:0	p:0.00748908YDR051C	FI:0.251231527093596	p:0.0216127YDR052C	FI:0.652418447694038	p:0.0556118YDR055W	FI:0.902777777777778	p:0.178264YDR056C	FI:0.7	p:0.183349YDR057W	FI:1.14089347079038	p:0.118812YDR059C	FI:	p:1YDR061W	FI:0.73805060918463	p:0.084535YDR063W	FI:0.321428571428571	p:0.0917614YDR065W	FI:0.352702702702703	p:0.00847815YDR068W	FI:0.775862068965517	p:0.159394YDR070C	FI:1.375	p:0.256704YDR071C	FI:0.266666666666667	p:0.230378YDR073W	FI:0.916666666666667	p:0.23973YDR074W	FI:0.21875	p:0.000165811YDR075W	FI:0	p:0.000277568YDR078C	FI:0.293877551020408	p:0.0162891YDR079W	FI:0	p:0.00131752YDR083W	FI:0.483870967741935	p:0.0390933YDR084C	FI:0.0172413793103448	p:0.00119366YDR085C	FI:0.709831254585473	p:0.0459405YDR086C	FI:3	p:0.302198YDR087C	FI:0.297619047619048	p:0.0652318YDR088C	FI:0.672768878718536	p:0.0868112YDR090C	FI:1.15	p:0.201531YDR098C	FI:0.407407407407407	p:0.0815392YDR099W	FI:0.0319148936170213	p:0.000122698YDR100W	FI:0.291666666666667	p:0.066428YDR101C	FI:0.53822629969419	p:0.0548678YDR105C	FI:0.659574468085106	p:0.179951YDR106W	FI:0.878468899521531	p:0.142902YDR107C	FI:0.671917018824433	p:0.0646134YDR109C	FI:0.437557816836263	p:0.00876733YDR110W	FI:1.42241379310345	p:0.121824YDR113C	FI:0.58980044345898	p:0.0478935YDR115W	FI:1.16666666666667	p:0.336479YDR116C	FI:0.426229508196721	p:0.0423994YDR117C	FI:0.627272727272727	p:0.0551192YDR118W	FI:0.653609831029186	p:0.0868257YDR120C	FI:0.258461538461538	p:0.000779444YDR121W	FI:0.99	p:0.24133YDR122W	FI:0.693520642201835	p:0.0369609YDR123C	FI:0.593220338983051	p:0.0963422YDR125C	FI:0.777777777777778	p:0.099357YDR126W	FI:0.651988636363636	p:0.114292YDR132C	FI:1.43114311431143	p:0.100474YDR134C	FI:	p:YDR137W	FI:0.565891472868217	p:0.025929YDR138W	FI:0.634146341463415	p:0.0264126YDR140W	FI:1.02790697674419	p:0.234134YDR142C	FI:0.822784810126582	p:0.172585YDR143C	FI:1.44642857142857	p:0.0764034YDR147W	FI:0.673469387755102	p:0.0608011YDR148C	FI:0.638078902229846	p:0.0944521YDR151C	FI:0.602429149797571	p:0.0611155YDR153C	FI:0.601136363636364	p:0.0893087YDR156W	FI:0.333333333333333	p:0.0705415YDR158W	FI:0.2	p:0.0244674YDR159W	FI:0.787095088819227	p:0.0960392YDR161W	FI:0.34140625	p:0.00597971YDR162C	FI:0.339089481946625	p:0.0199126YDR165W	FI:0.594951923076923	p:0.0854737YDR167W	FI:5.5	p:0.0684714YDR169C	FI:0.767045454545455	p:0.130748YDR171W	FI:0.742913000977517	p:0.13153YDR172W	FI:0.555555555555555	p:0.062611YDR173C	FI:0.895522388059702	p:0.160284YDR175C	FI:0.72	p:0.14044YDR176W	FI:0.8	p:0.102179YDR177W	FI:0.179487179487179	p:0.0556889YDR178W	FI:0.928571428571429	p:0.222546YDR179C	FI:1.925	p:0.126874YDR182W	FI:0.856496444731739	p:0.12963YDR183W	FI:0.59765625	p:0.150609YDR184C	FI:0.670833333333333	p:0.107672YDR185C	FI:0.078125	p:0.000821675YDR188W	FI:0.216	p:0.00119096YDR190C	FI:0.153061224489796	p:0.00668761YDR191W	FI:0.641891891891892	p:0.130701YDR194C	FI:0.687619047619048	p:0.177898YDR195W	FI:0.574418604651163	p:0.0426303YDR196C	FI:0.278985507246377	p:0.00971531YDR197W	FI:0.14453125	p:2.68176e-07YDR201W	FI:0.3375	p:0.0525693YDR202C	FI:0.317460317460317	p:0.0169132YDR204W	FI:0.584375	p:0.0979753YDR205W	FI:0.584795321637427	p:0.0322858YDR206W	FI:0.861844197138315	p:0.0862179YDR208W	FI:0.486973180076628	p:0.0169535YDR210W	FI:0.100840336134454	p:0.00848211YDR211W	FI:0.530075187969925	p:0.0276719YDR212W	FI:0.040133779264214	p:1.72852e-07YDR213W	FI:0.607106598984772	p:0.0504981YDR214W	FI:1.08024691358025	p:0.200584YDR217C	FI:0.902149321266968	p:0.0747549YDR219C	FI:0.598148148148148	p:0.0598499YDR222W	FI:0.409090909090909	p:0.0379673YDR224C	FI:0.363636363636364	p:0.350649YDR225W	FI:0	p:0.2YDR226W	FI:0.375	p:0.237236YDR228C	FI:1.83760683760684	p:0.048053YDR229W	FI:0.529411764705882	p:0.0843141YDR232W	FI:0.436893203883495	p:0.0260114YDR233C	FI:0.706521739130435	p:0.165984YDR234W	FI:0.661764705882353	p:0.21005YDR236C	FI:0.68733850129199	p:0.153483YDR237W	FI:0.28448275862069	p:0.0125213YDR238C	FI:0.267515923566879	p:0.00535036YDR240C	FI:0.768421052631579	p:0.131469YDR242W	FI:0.693753790175864	p:0.0864978YDR243C	FI:1.04827586206897	p:0.149539YDR244W	FI:0.69327731092437	p:0.0844888YDR246W	FI:0.558139534883721	p:0.150496YDR247W	FI:1.77777777777778	p:0.0606701YDR248C	FI:1.12820512820513	p:0.325327YDR251W	FI:0.668534482758621	p:0.0403525YDR252W	FI:0.169230769230769	p:0.0261606YDR253C	FI:0.518518518518518	p:0.160915YDR254W	FI:0.921052631578947	p:0.202967YDR256C	FI:0.674074074074074	p:0.163648YDR259C	FI:0.612409347300564	p:0.0689062YDR260C	FI:2.86363636363636	p:0.138898YDR262W	FI:0.776515151515151	p:0.179549YDR263C	FI:0.779746835443038	p:0.110264YDR265W	FI:0.919191919191919	p:0.184765YDR268W	FI:0.828828828828829	p:0.166403YDR270W	FI:0.797596457938014	p:0.0703389YDR272W	FI:0.6	p:0.101792YDR275W	FI:0.554733727810651	p:0.0766421YDR276C	FI:0	p:0.109091YDR277C	FI:0.421768707482993	p:0.0694824YDR279W	FI:1.36842105263158	p:0.121465YDR280W	FI:0.19047619047619	p:0.0286071YDR281C	FI:0.272727272727273	p:0.098446YDR284C	FI:0.311890838206628	p:0.022149YDR285W	FI:0.585555555555555	p:0.0160385YDR286C	FI:1.44642857142857	p:0.252853YDR287W	FI:0.678571428571429	p:0.114828YDR288W	FI:2.03396226415094	p:0.0411793YDR289C	FI:0.5807962529274	p:0.0762184YDR293C	FI:0.496193226568653	p:0.0235847YDR294C	FI:0.417572857764245	p:0.00742137YDR296W	FI:0.777777777777778	p:0.173006YDR297W	FI:0.601990049751244	p:0.150113YDR298C	FI:0.227272727272727	p:0.0200374YDR299W	FI:0.644	p:0.0604725YDR300C	FI:0.852272727272727	p:0.197938YDR304C	FI:0.331018518518518	p:0.0296551YDR306C	FI:0.675105485232067	p:0.0729378YDR308C	FI:0.491228070175439	p:0.284495YDR309C	FI:0.726643598615917	p:0.105181YDR311W	FI:0.354671280276817	p:0.00370453YDR312W	FI:0.801315789473684	p:0.119476YDR314C	FI:0.427350427350427	p:0.00132843YDR317W	FI:0.526241134751773	p:0.0478397YDR319C	FI:0.703703703703704	p:0.153462YDR320C-A	FI:	p:0.714286YDR321W	FI:0.786885245901639	p:0.198829YDR322C-A	FI:4.05263157894737	p:0.161412YDR322W	FI:0.258706467661692	p:0.000709383YDR325W	FI:0.668218085106383	p:0.0686056YDR326C	FI:0.495483870967742	p:0.00296602YDR328C	FI:0.0514285714285714	p:0.00453548YDR329C	FI:0.338461538461538	p:0.0105322YDR331W	FI:1.23943661971831	p:0.202047YDR332W	FI:0.405253283302064	p:0.0199083YDR336W	FI:1.45785123966942	p:0.115387YDR339C	FI:0.311111111111111	p:0.0724547YDR345C	FI:0.0158730158730159	p:3.23573e-05YDR346C	FI:0.799276672694394	p:0.179573YDR348C	FI:0.48314606741573	p:0.0307476YDR350C	FI:1.44378698224852	p:0.0957725YDR352W	FI:0.42827868852459	p:0.0224354YDR353W	FI:0.574468085106383	p:0.202591YDR354W	FI:0.592105263157895	p:0.0546078YDR357C	FI:0.131578947368421	p:0.00507698YDR358W	FI:1.31216931216931	p:0.0936425YDR361C	FI:0.917378917378917	p:0.19473YDR362C	FI:0.79730557256583	p:0.0903132YDR363W	FI:0.956521739130435	p:0.129216YDR363W-A	FI:0.642857142857143	p:0.358366YDR364C	FI:0.539473684210526	p:0.0345669YDR368W	FI:0.318181818181818	p:0.011793YDR372C	FI:0.298850574712644	p:0.0410141YDR373W	FI:0.355263157894737	p:0.22559YDR374C	FI:0.793190416141236	p:0.144332YDR375C	FI:0.321531100478469	p:0.0111027YDR377W	FI:0.394736842105263	p:0.272325YDR378C	FI:0.157894736842105	p:0.0368957YDR379C-A	FI:0.3125	p:0.115619YDR379W	FI:0.47478354978355	p:0.00107903YDR380W	FI:0.686088527551942	p:0.0745269YDR382W	FI:0.75	p:0.454546YDR383C	FI:0.333333333333333	p:0.0421966YDR384C	FI:0.406451612903226	p:0.100058YDR385W	FI:0.0495867768595041	p:0.000248198YDR387C	FI:0.725274725274725	p:0.108117YDR388W	FI:0.229933110367893	p:0.00140151YDR389W	FI:0.379259259259259	p:0.0328877YDR391C	FI:5.77777777777778	p:0.123994YDR392W	FI:0.12	p:0.0121533YDR393W	FI:1.28218243819267	p:0.10988YDR394W	FI:0.529661016949152	p:0.188275YDR397C	FI:0	p:0.110837YDR398W	FI:0.578512396694215	p:0.038386YDR399W	FI:0.0487012987012987	p:0.000204403YDR400W	FI:0.405365126676602	p:0.0334747YDR403W	FI:0.518518518518518	p:0.0302821YDR404C	FI:0	p:0.00582868YDR405W	FI:0.875	p:0.212499YDR408C	FI:0.544871794871795	p:0.203329YDR410C	FI:0.436065573770492	p:0.0484192YDR411C	FI:1.08474576271186	p:0.180028YDR412W	FI:1.33333333333333	p:0.254986YDR414C	FI:0.73828125	p:0.110165YDR415C	FI:0.473457675753228	p:0.0251338YDR416W	FI:0.71207729468599	p:0.0721089YDR418W	FI:0	p:0.421053YDR419W	FI:0.588778146917682	p:0.0366153YDR421W	FI:0.977546549835706	p:0.115682YDR425W	FI:0.770833333333333	p:0.105023YDR427W	FI:0.445628997867804	p:0.0770631YDR429C	FI:1.84615384615385	p:0.201814YDR434W	FI:0.2109375	p:0.000260795YDR435C	FI:0.615384615384615	p:0.0924783YDR436W	FI:0.495160468670402	p:0.0315616YDR437W	FI:0.740384615384615	p:0.258079YDR438W	FI:1.08108108108108	p:0.21298YDR439W	FI:0.596273291925466	p:0.0562618YDR441C	FI:0.510204081632653	p:0.117572YDR446W	FI:1.16934046345811	p:0.164924YDR447C	FI:3	p:0.367133YDR448W	FI:2.8695652173913	p:0.0947097YDR449C	FI:0.617283950617284	p:0.0945174YDR451C	FI:0.789473684210526	p:0.1364YDR452W	FI:0.802768166089965	p:0.11277YDR453C	FI:0.511627906976744	p:0.131051YDR454C	FI:0.219512195121951	p:0.0248786YDR456W	FI:0.769722814498934	p:0.163154YDR457W	FI:0.514200298953662	p:0.00479952YDR459C	FI:0.973656480505795	p:0.158364YDR460W	FI:0.0482954545454545	p:1.52919e-05YDR462W	FI:0.515625	p:0.193257YDR464W	FI:0.79036420992257	p:0.0508905YDR466W	FI:0.713286713286713	p:0.0412801YDR468C	FI:0.191721132897604	p:0.00466006YDR469W	FI:1.39090909090909	p:0.174388YDR472W	FI:1.06666666666667	p:0.237794YDR473C	FI:1.12475759534583	p:0.130212YDR476C	FI:0.230769230769231	p:0.015648YDR477W	FI:0.283636363636364	p:0.00707375YDR479C	FI:0.64957264957265	p:0.0968567YDR480W	FI:1.96960486322188	p:0.0837146YDR482C	FI:1.2	p:0.258132YDR483W	FI:0.52112676056338	p:0.0191977YDR484W	FI:0.353448275862069	p:0.000493461YDR486C	FI:0.07	p:0.00644016YDR487C	FI:0.449324324324324	p:0.0930047YDR488C	FI:1.31370091896408	p:0.08558YDR489W	FI:0.57	p:0.150254YDR490C	FI:0.668055555555556	p:0.0390954YDR492W	FI:0.410869565217391	p:0.0516423YDR493W	FI:6.31578947368421	p:0.0608309YDR494W	FI:0.658340767172168	p:0.0856752YDR496C	FI:0.709978070175439	p:0.0899031YDR499W	FI:0.777777777777778	p:0.0918975YDR501W	FI:0.98	p:0.124804YDR502C	FI:0.172413793103448	p:0.0462703YDR505C	FI:0.542044767090139	p:0.0112951YDR507C	FI:0.593220338983051	p:0.0278055YDR508C	FI:0.482758620689655	p:0.0614652YDR510W	FI:0	p:0.230769YDR511W	FI:0.205882352941176	p:0.0246892YDR513W	FI:0.114583333333333	p:0.0518611YDR515W	FI:0.576659038901602	p:0.0552597YDR516C	FI:0.25	p:0.0670352YDR517W	FI:0.537634408602151	p:0.0791356YDR518W	FI:0.469072164948454	p:0.022953YDR519W	FI:1.55172413793103	p:0.288024YDR520C	FI:0.730434782608696	p:0.0708478YDR522C	FI:1.4375	p:0.0957333YDR523C	FI:0.6	p:0.0730433YDR525W-A	FI:0.222222222222222	p:0.134394YDR527W	FI:0.762310606060606	p:0.0918302YDR528W	FI:0.795205479452055	p:0.113227YDR530C	FI:0.304347826086957	p:0.0133027YDR531W	FI:0.766666666666667	p:0.202906YDR532C	FI:0.637898686679174	p:0.0927668YDR533C	FI:0.308	p:0.0188698YDR536W	FI:0.34375	p:0.0294341YDR538W	FI:0.602439024390244	p:0.121019YDR539W	FI:0.816326530612245	p:0.205475YEL001C	FI:1.14024390243902	p:0.191702YEL002C	FI:0.693150684931507	p:0.128773YEL004W	FI:1.33636363636364	p:0.131943YEL009C	FI:0.502901353965184	p:0.0732228YEL012W	FI:0.804597701149425	p:0.29715YEL013W	FI:0.191919191919192	p:0.0529304YEL015W	FI:0.774757281553398	p:0.143618YEL016C	FI:0.820540540540541	p:0.11166YEL017W	FI:0.529411764705882	p:0.0859693YEL018W	FI:2.29565217391304	p:0.0645683YEL019C	FI:1.28165374677003	p:0.164934YEL020C	FI:1.00840336134454	p:0.145969YEL020W-A	FI:	p:1YEL021W	FI:0.148148148148148	p:0.0341293YEL023C	FI:0.895357406042741	p:0.113242YEL024W	FI:0.633333333333333	p:0.486275YEL025C	FI:1.49215965787598	p:0.008785YEL026W	FI:	p:1YEL027W	FI:	p:1YEL029C	FI:1.5609756097561	p:0.223733YEL031W	FI:2.02040816326531	p:0.120579YEL034W	FI:	p:0.266667YEL036C	FI:2.58426966292135	p:0.242621YEL037C	FI:0.63556338028169	p:0.129921YEL038W	FI:0.477810650887574	p:0.049589YEL039C	FI:	p:0.852941YEL040W	FI:1.27010309278351	p:0.136647YEL041W	FI:1.0596546310832	p:0.125344YEL042W	FI:1.35585585585586	p:0.105126YEL044W	FI:0.296511627906977	p:0.0331677YEL046C	FI:0.950819672131147	p:0.285949YEL047C	FI:0.770833333333333	p:0.159431YEL048C	FI:2.47272727272727	p:0.0733509YEL050C	FI:1.47413793103448	p:0.154722YEL051W	FI:0.275862068965517	p:0.206626YEL052W	FI:0.522	p:0.0644485YEL053C	FI:0.626086956521739	p:0.0398814YEL054C	FI:0	p:0.00105402YEL057C	FI:0.63905325443787	p:0.104457YEL058W	FI:0.81060116354234	p:0.128109YEL059C-A	FI:3.5	p:0.141742YEL061C	FI:0.742424242424243	p:0.0811792YEL062W	FI:1.52413793103448	p:0.0899651YEL064C	FI:0.694017094017094	p:0.116576YEL066W	FI:2.18390804597701	p:0.0757945YEL070W	FI:0.617142857142857	p:0.161166YEL071W	FI:1.03409090909091	p:0.238143YEL072W	FI:1.32391304347826	p:0.132749YER001W	FI:0.953778429933969	p:0.106787YER002W	FI:0.282051282051282	p:0.0087744YER003C	FI:1.02413793103448	p:0.22966YER004W	FI:0.930091185410334	p:0.205541YER006W	FI:0.947916666666667	p:0.208374YER007W	FI:1.39817629179331	p:0.0731806YER009W	FI:0	p:0.347826YER010C	FI:0.72	p:0.204765YER011W	FI:2.58412698412698	p:0.0247815YER012W	FI:	p:0.320517YER014W	FI:0.504201680672269	p:0.0174424YER016W	FI:1.2375	p:0.204915YER017C	FI:2.65	p:0.0327849YER018C	FI:0.585365853658537	p:0.103513YER019C-A	FI:0	p:0.239131YER019W	FI:1.445	p:0.26991YER022W	FI:1.0780487804878	p:0.134121YER023W	FI:0.25	p:0.0758688YER025W	FI:	p:0.219764YER026C	FI:0.673170731707317	p:0.215443YER027C	FI:1.41230769230769	p:0.17936YER029C	FI:1.85714285714286	p:0.124625YER030W	FI:0.447204968944099	p:0.0705323YER031C	FI:	p:1YER032W	FI:1.19491525423729	p:0.0739021YER034W	FI:1.23295454545455	p:0.180902YER035W	FI:1.40350877192982	p:0.249804YER037W	FI:1.25357142857143	p:0.208587YER038C	FI:1.6374269005848	p:0.0393918YER040W	FI:0.783333333333333	p:0.118294YER042W	FI:0.628205128205128	p:0.220262YER046W	FI:1.265625	p:0.319002YER047C	FI:0.861675126903553	p:0.098637YER048C	FI:1.20481927710843	p:0.412307YER048W-A	FI:1	p:0.455957YER049W	FI:0.394957983193277	p:0.0142291YER051W	FI:0.935837245696401	p:0.127417YER052C	FI:0.433734939759036	p:0.15177YER053C	FI:1.76470588235294	p:0.360433YER055C	FI:0.7421875	p:0.227473YER057C	FI:0.611111111111111	p:0.276255YER058W	FI:1.42105263157895	p:0.32189YER059W	FI:0.45867460908414	p:0.0220164YER060W-A	FI:0.651724137931035	p:0.103608YER061C	FI:0.703883495145631	p:0.109354YER062C	FI:0.577777777777778	p:0.336187YER063W	FI:0.821109123434705	p:0.158637YER067W	FI:1.2037037037037	p:0.336206YER068W	FI:1.29166666666667	p:0.169626YER071C	FI:7.36666666666667	p:0.00142933YER072W	FI:0	p:0.275862YER073W	FI:1.73399014778325	p:0.089499YER076C	FI:0.909090909090909	p:0.156181YER079W	FI:0.931034482758621	p:0.212889YER080W	FI:0.892857142857143	p:0.131093YER081W	FI:0.488888888888889	p:0.255425YER083C	FI:1.88888888888889	p:0.15861YER085C	FI:0.764267990074442	p:0.164488YER087W	FI:0.588235294117647	p:0.0550378YER088C	FI:0.657004830917874	p:0.0456023YER090W	FI:0.271551724137931	p:0.0226254YER091C	FI:0.588034188034188	p:0.160117YER092W	FI:0.773809523809524	p:0.215574YER095W	FI:0.38671875	p:0.148935YER096W	FI:0.889952153110048	p:0.120866YER099C	FI:0.597014925373134	p:0.294456YER100W	FI:0.504807692307692	p:0.16999YER101C	FI:0.886363636363636	p:0.143476YER102W	FI:10.6666666666667	p:0.11039YER103W	FI:0.353053435114504	p:0.0280717YER104W	FI:0.825	p:0.216784YER105C	FI:0.994939038417299	p:0.0747673YER106W	FI:1.84090909090909	p:0.0585645YER107C	FI:0.958333333333333	p:0.273646YER110C	FI:0.556306306306306	p:0.150757YER111C	FI:0.824638233054075	p:0.0843224YER112W	FI:1	p:0.338168YER114C	FI:0.939673913043478	p:0.105717YER115C	FI:1.20634920634921	p:0.204767YER116C	FI:0.704918032786885	p:0.0958681YER117W	FI:	p:1YER118C	FI:0.882352941176471	p:0.222415YER119C	FI:0.869300911854103	p:0.184171YER120W	FI:1.16666666666667	p:0.259957YER122C	FI:1.26506024096386	p:0.15846YER123W	FI:0.321348314606742	p:0.0147036YER124C	FI:1.41958041958042	p:0.102375YER126C	FI:	p:1YER127W	FI:0.99047619047619	p:0.226327YER128W	FI:0.802325581395349	p:0.257108YER129W	FI:0.802329144958627	p:0.0577168YER130C	FI:1.25614591593973	p:0.121373YER131W	FI:	p:1YER132C	FI:1.25595738716008	p:0.059323YER134C	FI:1.05882352941176	p:0.260994YER136W	FI:0.214285714285714	p:0.301531YER137C	FI:0.818181818181818	p:0.189224YER139C	FI:2.1	p:0.213519YER140W	FI:1.8989898989899	p:0.0446327YER141W	FI:0.573208722741433	p:0.0874767YER143W	FI:0.838709677419355	p:0.170753YER144C	FI:1.17873651771957	p:0.0961482YER145C	FI:2.58064516129032	p:0.249608YER146W	FI:	p:0.416809YER147C	FI:0.777777777777778	p:0.189872YER149C	FI:0.88	p:0.147814YER150W	FI:0.9	p:0.265773YER152C	FI:1.30645161290323	p:0.170297YER153C	FI:0.914772727272727	p:0.198698YER154W	FI:1.22222222222222	p:0.213661YER156C	FI:0.984615384615385	p:0.224109YER158C	FI:1.1252427184466	p:0.118636YER159C	FI:0.694444444444445	p:0.21327YER161C	FI:0.335483870967742	p:0.0208858YER163C	FI:1.00975609756098	p:0.186412YER165W	FI:0.272727272727273	p:0.253117YER167W	FI:0.600415923945336	p:0.0333001YER168C	FI:1.24038461538462	p:0.139809YER170W	FI:1.94021739130435	p:0.12873YER171W	FI:0.838235294117647	p:0.259343YER173W	FI:0.759868421052632	p:0.103323YER174C	FI:0.894736842105263	p:0.192944YER175C	FI:1.00308641975309	p:0.17797YER176W	FI:1.17948717948718	p:0.0963451YER177W	FI:0.422222222222222	p:0.428905YER178W	FI:2.31884057971015	p:0.294143YER180C	FI:0.73469387755102	p:0.125398YER182W	FI:0.653846153846154	p:0.191172YER183C	FI:0.641975308641975	p:0.167098YER184C	FI:1.55761078998073	p:0.049322YER185W	FI:2.31481481481481	p:0.0453401YFL003C	FI:1.17004219409283	p:0.11818YFL004W	FI:0.869565217391304	p:0.136464YFL005W	FI:0.225806451612903	p:0.222017YFL010C	FI:0.266666666666667	p:0.0155095YFL011W	FI:1.00813008130081	p:0.184237YFL014W	FI:0.22	p:0.162319YFL016C	FI:0.849056603773585	p:0.194615YFL017C	FI:0.366666666666667	p:0.0521298YFL017W-A	FI:0.916666666666667	p:0.478948YFL021W	FI:0.940568475452196	p:0.173478YFL022C	FI:1.66233766233766	p:0.194764YFL023W	FI:1.30783699059561	p:0.0636098YFL024C	FI:0.724528301886793	p:0.118796YFL025C	FI:1.10220994475138	p:0.105705YFL027C	FI:0.857058823529412	p:0.125969YFL028C	FI:3	p:0.206253YFL029C	FI:1.33670886075949	p:0.130527YFL030W	FI:1.78508771929825	p:0.178645YFL034C-B	FI:	p:0.216925YFL034W	FI:1.54017857142857	p:0.0698494YFL038C	FI:0	p:0.316667YFL039C	FI:	p:1YFL041W	FI:1.12777777777778	p:0.159773YFL042C	FI:0.874	p:0.107069YFL044C	FI:2.23770491803279	p:0.0831794YFL045C	FI:0.458333333333333	p:0.388683YFL046W	FI:0.549019607843137	p:0.293224YFL047W	FI:0.988425925925926	p:0.149254YFL048C	FI:0.524002704530088	p:0.0357848YFL049W	FI:2.03149606299213	p:0.0128344YFL052W	FI:0.538172715894869	p:0.0306911YFL053W	FI:0.617760617760618	p:0.149879YFL054C	FI:1.95789473684211	p:0.0133071YFL055W	FI:0.987460815047022	p:0.163952YFR001W	FI:4.02631578947368	p:0.137016YFR003C	FI:0.807692307692308	p:0.248269YFR004W	FI:0.5	p:0.399753YFR005C	FI:0.84472049689441	p:0.11484YFR006W	FI:1.43680709534368	p:0.103063YFR007W	FI:0.869936034115139	p:0.147748YFR008W	FI:0.704061895551257	p:0.141575YFR009W	FI:1.03888888888889	p:0.209933YFR010W	FI:0.682648401826484	p:0.170506YFR011C	FI:0.740740740740741	p:0.205032YFR013W	FI:1.61517857142857	p:0.0397617YFR014C	FI:1.13131313131313	p:0.154218YFR015C	FI:0.576470588235294	p:0.159444YFR017C	FI:0.541666666666667	p:0.131446YFR018C	FI:0.941176470588235	p:0.148599YFR021W	FI:1.28030303030303	p:0.245402YFR022W	FI:1.34541984732824	p:0.103696YFR023W	FI:1.08791208791209	p:0.186177YFR027W	FI:2.1875	p:0.0742714YFR028C	FI:1.1	p:0.2142YFR032C	FI:1.00588235294118	p:0.2225YFR032C-A	FI:0	p:0.75YFR033C	FI:5.5	p:0.0199161YFR034C	FI:0.775793650793651	p:0.170662YFR036W	FI:1.5	p:0.255155YFR037C	FI:0.80952380952381	p:0.177514YFR039C	FI:0.787155963302752	p:0.103618YFR041C	FI:0.816	p:0.174187YFR042W	FI:0.466666666666667	p:0.0852988YFR043C	FI:0.713846153846154	p:0.130352YFR044C	FI:0.590909090909091	p:0.28915YFR045W	FI:1.71428571428571	p:0.153434YFR046C	FI:0.9375	p:0.143793YFR047C	FI:0.426282051282051	p:0.0965435YFR048W	FI:1.01744186046512	p:0.148919YFR049W	FI:0.953333333333333	p:0.238849YFR050C	FI:0.572916666666667	p:0.208452YFR052W	FI:0.266666666666667	p:0.0683625YFR053C	FI:0.485294117647059	p:0.103955YGL001C	FI:0.634920634920635	p:0.18578YGL002W	FI:0.593650793650794	p:0.133581YGL004C	FI:0.696774193548387	p:0.070505YGL005C	FI:1.23809523809524	p:0.170541YGL006W	FI:0.892857142857143	p:0.10813YGL009C	FI:0.76027397260274	p:0.214279YGL010W	FI:1.22222222222222	p:0.23332YGL011C	FI:0.666666666666667	p:0.35764YGL012W	FI:0.868571428571429	p:0.209165YGL017W	FI:0.946831364124597	p:0.130095YGL018C	FI:0.597560975609756	p:0.209765YGL019W	FI:0	p:0.151515YGL020C	FI:2.24390243902439	p:0.0730778YGL021W	FI:0.848378615249781	p:0.124764YGL023C	FI:0.861244019138756	p:0.114607YGL025C	FI:1.05263157894737	p:0.153746YGL026C	FI:0.130952380952381	p:0.00330173YGL027C	FI:0.643527204502814	p:0.0755372YGL028C	FI:1.09133858267717	p:0.133393YGL029W	FI:0.307692307692308	p:0.0720015YGL031C	FI:0	p:0.082353YGL032C	FI:0.784313725490196	p:0.266108YGL035C	FI:0.603328710124827	p:0.066658YGL036W	FI:0.614095238095238	p:0.0263364YGL037C	FI:	p:0.0419732YGL038C	FI:2.84057971014493	p:0.00366566YGL039W	FI:0.802083333333333	p:0.174953YGL040C	FI:0.385714285714286	p:0.22738YGL044C	FI:2.02380952380952	p:0.211301YGL047W	FI:0.960365853658537	p:0.201466YGL048C	FI:	p:1YGL050W	FI:0.857142857142857	p:0.170843YGL054C	FI:0	p:0.452381YGL055W	FI:3.30909090909091	p:0.0736885YGL058W	FI:	p:0.35942YGL061C	FI:0.867208672086721	p:0.18845YGL063W	FI:0.363067292644757	p:0.0355599YGL064C	FI:1.38046875	p:0.111668YGL065C	FI:0.371967654986523	p:0.00334481YGL066W	FI:0.965467625899281	p:0.161848YGL067W	FI:0.931216931216931	p:0.148348YGL068W	FI:0.5	p:0.19427YGL069C	FI:0.676691729323308	p:0.207733YGL070C	FI:	p:1YGL071W	FI:0.791666666666667	p:0.0935439YGL073W	FI:0.750366389838788	p:0.0724595YGL075C	FI:0.846768336964415	p:0.131802YGL077C	FI:0.846153846153846	p:0.184801YGL078C	FI:0.287037037037037	p:0.00626737YGL079W	FI:1.04395604395604	p:0.210154YGL082W	FI:0.833333333333333	p:0.198442YGL083W	FI:0.908653846153846	p:0.138471YGL084C	FI:0.916666666666667	p:0.209725YGL085W	FI:1.71	p:0.160445YGL086W	FI:0.888888888888889	p:0.135504YGL089C	FI:2.68253968253968	p:0.105545YGL090W	FI:1.24285714285714	p:0.11357YGL091C	FI:2.23214285714286	p:0.306304YGL093W	FI:0.97848955880044	p:0.103214YGL096W	FI:0.63894523326572	p:0.08351YGL098W	FI:1.46938775510204	p:0.147503YGL100W	FI:0.796875	p:0.205882YGL101W	FI:1.16666666666667	p:0.21276YGL103W	FI:	p:1YGL105W	FI:0.373333333333333	p:0.0747428YGL106W	FI:1.6551724137931	p:0.388079YGL107C	FI:1.10852713178295	p:0.131387YGL108C	FI:0.634920634920635	p:0.179103YGL110C	FI:1.35538592027142	p:0.0776574YGL111W	FI:1.11572535991141	p:0.122652YGL112C	FI:0.494117647058824	p:0.129507YGL113W	FI:1.31637519872814	p:0.0862183YGL116W	FI:0.339130434782609	p:0.0170521YGL119W	FI:1.66666666666667	p:0.0780323YGL120C	FI:1.46031746031746	p:0.275097YGL121C	FI:0.260869565217391	p:0.059695YGL122C	FI:1.51692307692308	p:0.145322YGL123W	FI:	p:1YGL124C	FI:1.14685314685315	p:0.134102YGL125W	FI:1.625	p:0.193546YGL126W	FI:1.56756756756757	p:0.131676YGL127C	FI:0.666666666666667	p:0.352243YGL128C	FI:1.9296875	p:0.0829346YGL129C	FI:2.66304347826087	p:0.0390315YGL130W	FI:1.30120481927711	p:0.291746YGL131C	FI:1.1256038647343	p:0.0723687YGL135W	FI:	p:0.421053YGL136C	FI:0.525252525252525	p:0.0910718YGL137W	FI:0.984340044742729	p:0.194851YGL138C	FI:0.693196405648267	p:0.0900865YGL139W	FI:1.56484962406015	p:0.102482YGL140C	FI:1.19248366013072	p:0.122026YGL142C	FI:0.734901462174189	p:0.0761013YGL143C	FI:1.62921348314607	p:0.158634YGL145W	FI:0.709905660377358	p:0.0903334YGL146C	FI:1.14406779661017	p:0.158529YGL147C	FI:	p:0.35YGL148W	FI:	p:0.736842YGL153W	FI:1.47887323943662	p:0.123771YGL154C	FI:0.633962264150943	p:0.101199YGL155W	FI:2.12019230769231	p:0.0366856YGL157W	FI:1.88311688311688	p:0.0728954YGL158W	FI:1.22129226648812	p:0.0882816YGL160W	FI:0.63768115942029	p:0.0472957YGL161C	FI:1.26388888888889	p:0.154092YGL162W	FI:0.756521739130435	p:0.14819YGL164C	FI:1.36475869809203	p:0.126428YGL167C	FI:0.375	p:0.0315261YGL170C	FI:1.32761437908497	p:0.103561YGL171W	FI:0.671328671328671	p:0.0949186YGL172W	FI:0.366315789473684	p:0.00389823YGL174W	FI:0.549019607843137	p:0.0623555YGL175C	FI:1.03703703703704	p:0.177512YGL178W	FI:0.588183421516755	p:0.0889801YGL179C	FI:1.62857142857143	p:0.0751174YGL180W	FI:0.892857142857143	p:0.163372YGL181W	FI:2.9620253164557	p:0.0458412YGL184C	FI:0.457142857142857	p:0.163736YGL185C	FI:0.935483870967742	p:0.136051YGL186C	FI:1.55347871235722	p:0.096113YGL187C	FI:	p:0.432479YGL189C	FI:	p:1YGL190C	FI:0.836158192090395	p:0.297028YGL191W	FI:	p:0.499289YGL192W	FI:1.11111111111111	p:0.124375YGL194C	FI:1.70394736842105	p:0.252248YGL196W	FI:0.847975553857907	p:0.130148YGL198W	FI:0.62862669245648	p:0.11893YGL200C	FI:0.296875	p:0.279391YGL202W	FI:1.01111111111111	p:0.184134YGL203C	FI:1.74188311688312	p:0.0397341YGL208W	FI:1.50545454545455	p:0.0943436YGL209W	FI:0.493506493506494	p:0.0950594YGL211W	FI:0.864197530864197	p:0.315822YGL212W	FI:0.421978021978022	p:0.0255159YGL213C	FI:1.33333333333333	p:0.179277YGL215W	FI:0.9375	p:0.157971YGL218W	FI:1.01149425287356	p:0.295077YGL219C	FI:0.329896907216495	p:0.0294795YGL221C	FI:0.610169491525424	p:0.0960001YGL222C	FI:0.918367346938775	p:0.22251YGL223C	FI:1.14027149321267	p:0.128646YGL224C	FI:0.474576271186441	p:0.105044YGL225W	FI:3.52	p:0.168986YGL226W	FI:0.496894409937888	p:0.140333YGL228W	FI:0.652464494569758	p:0.061945YGL229C	FI:1.40909090909091	p:0.0679885YGL231C	FI:0.712962962962963	p:0.200242YGL233W	FI:0.795031055900621	p:0.109978YGL236C	FI:1.30555555555556	p:0.109749YGL237C	FI:0.82051282051282	p:0.177455YGL240W	FI:0.431372549019608	p:0.0684547YGL241W	FI:0.576668557383248	p:0.0058067YGL242C	FI:1.09090909090909	p:0.260023YGL243W	FI:1.3167701863354	p:0.116803YGL244W	FI:0.683495145631068	p:0.177807YGL245W	FI:0.404371584699454	p:0.0688816YGL246C	FI:2.11280487804878	p:0.0907627YGL247W	FI:0.65	p:0.127021YGL248W	FI:0.666023166023166	p:0.150687YGL250W	FI:0.465116279069767	p:0.0716216YGL252C	FI:1.03469387755102	p:0.218573YGL253W	FI:0.915254237288136	p:0.451199YGL254W	FI:1.52414398595259	p:0.068761YGL255W	FI:1.14795008912656	p:0.12848YGL256W	FI:1.83838383838384	p:0.0822081YGL257C	FI:1.51865671641791	p:0.0376074YGL258W	FI:0.65	p:0.143557YGL261C	FI:5	p:0.142143YGR002C	FI:0.513333333333333	p:0.0683611YGR003W	FI:1.11979334840168	p:0.116318YGR004W	FI:0.512820512820513	p:0.0494654YGR005C	FI:0.860759493670886	p:0.417595YGR006W	FI:0.723333333333333	p:0.13992YGR007W	FI:0.466880341880342	p:0.0378415YGR008C	FI:0.291666666666667	p:0.130398YGR009C	FI:0.542036910457963	p:0.0667096YGR010W	FI:0.0666666666666667	p:0.00664309YGR012W	FI:0.545454545454546	p:0.0661952YGR013W	FI:0.696649029982363	p:0.0795583YGR016W	FI:1.54545454545455	p:0.177842YGR019W	FI:0.5390625	p:0.102317YGR020C	FI:0.333333333333333	p:0.375494YGR021W	FI:1.56	p:0.162988YGR024C	FI:0.972972972972973	p:0.287752YGR026W	FI:0.448979591836735	p:0.0831324YGR027C	FI:	p:1YGR028W	FI:	p:0.315108YGR033C	FI:0.916666666666667	p:0.217979YGR035C	FI:1.2	p:0.256837YGR036C	FI:0.286764705882353	p:0.0234198YGR037C	FI:0	p:0.526316YGR038W	FI:0.0957446808510638	p:0.00306875YGR040W	FI:	p:0.718182YGR041W	FI:1.4294670846395	p:0.0514451YGR042W	FI:1.23176361957525	p:0.121528YGR044C	FI:1.35357624831309	p:0.124638YGR046W	FI:0.565656565656566	p:0.135359YGR048W	FI:0.347736625514403	p:0.0484505YGR049W	FI:0.12962962962963	p:0.0142155YGR052W	FI:1.7037037037037	p:0.18272YGR053C	FI:1.04910714285714	p:0.156312YGR054W	FI:0.435897435897436	p:0.0226408YGR055W	FI:1.10514018691589	p:0.235085YGR056W	FI:1.28174603174603	p:0.0869011YGR057C	FI:1.02702702702703	p:0.293697YGR058W	FI:0.91991341991342	p:0.196698YGR059W	FI:1.05324074074074	p:0.175737YGR060W	FI:	p:0.21068YGR062C	FI:0.230263157894737	p:0.000732449YGR066C	FI:0.906716417910448	p:0.186142YGR067C	FI:0.763157894736842	p:0.091442YGR070W	FI:1.03383458646617	p:0.0900885YGR071C	FI:0.603015075376884	p:0.03705YGR072W	FI:1.071875	p:0.135659YGR074W	FI:	p:0.45234YGR075C	FI:1.80878552971576	p:0.0780628YGR076C	FI:1.40625	p:0.405955YGR077C	FI:1.05870206489676	p:0.109778YGR078C	FI:1.04347826086957	p:0.282514YGR079W	FI:0.355113636363636	p:0.0229205YGR080W	FI:1.11538461538462	p:0.154995YGR081C	FI:0.764705882352941	p:0.148474YGR082W	FI:1	p:0.39308YGR083C	FI:1.56862745098039	p:0.11789YGR084C	FI:0.547588005215124	p:0.0716426YGR085C	FI:	p:1YGR086C	FI:0.68	p:0.292348YGR088W	FI:0.389830508474576	p:0.0998845YGR091W	FI:0.859802431610942	p:0.106695YGR093W	FI:1.05982905982906	p:0.128338YGR095C	FI:1.8	p:0.246589YGR096W	FI:1.14285714285714	p:0.194987YGR100W	FI:0.575757575757576	p:0.057055YGR101W	FI:0.815068493150685	p:0.17428YGR103W	FI:0.417271407837446	p:0.0190512YGR104C	FI:0.833172613307618	p:0.133795YGR105W	FI:0.75	p:0.465652YGR106C	FI:0.866666666666667	p:0.17686YGR109C	FI:0.595238095238095	p:0.0701563YGR110W	FI:0.96969696969697	p:0.159575YGR111W	FI:0.382239382239382	p:0.00610649YGR112W	FI:0.910843373493976	p:0.168888YGR113W	FI:0.761029411764706	p:0.136122YGR117C	FI:1.09985935302391	p:0.115764YGR120C	FI:0.443076923076923	p:0.0402193YGR121C	FI:0.432432432432432	p:0.0517503YGR122W	FI:0.947368421052632	p:0.132517YGR123C	FI:2.07272727272727	p:0.0723306YGR124W	FI:1.01149425287356	p:0.282247YGR126W	FI:0.804597701149425	p:0.256111YGR127W	FI:0.62890625	p:0.089914YGR128C	FI:1.28893662728249	p:0.0849027YGR129W	FI:0.957446808510638	p:0.190724YGR131W	FI:3.66666666666667	p:0.168399YGR132C	FI:	p:0.592304YGR133W	FI:0.514285714285714	p:0.122358YGR142W	FI:0.69874686716792	p:0.089335YGR143W	FI:0.676571428571429	p:0.102677YGR145W	FI:1.00534759358289	p:0.156329YGR146C	FI:0.465686274509804	p:0.0678174YGR147C	FI:0.704402515723271	p:0.120615YGR148C	FI:0.7	p:0.51462YGR149W	FI:1.04634146341463	p:0.220565YGR150C	FI:1.20901205838798	p:0.0818174YGR152C	FI:1.36363636363636	p:0.249802YGR154C	FI:0.575854700854701	p:0.0836884YGR155W	FI:0.598290598290598	p:0.179052YGR156W	FI:2.1	p:0.0798275YGR157W	FI:0.84610472541507	p:0.109493YGR158C	FI:1.25581395348837	p:0.160832YGR159C	FI:0.758928571428571	p:0.193944YGR160W	FI:0.634920634920635	p:0.18197YGR161C	FI:0.627717391304348	p:0.145022YGR162W	FI:1.09712230215827	p:0.111628YGR165W	FI:0.613333333333333	p:0.110473YGR166W	FI:1.35416666666667	p:0.0902652YGR167W	FI:1.44761904761905	p:0.174852YGR168C	FI:1.66754850088183	p:0.0455541YGR169C	FI:0.386363636363636	p:0.00881393YGR171C	FI:0.7	p:0.086859YGR172C	FI:0.0833333333333333	p:0.0215858YGR174C	FI:0.2	p:0.100478YGR175C	FI:0.847826086956522	p:0.348413YGR177C	FI:0.803212851405623	p:0.109651YGR178C	FI:0.833499501495513	p:0.117534YGR179C	FI:0.851461988304093	p:0.122647YGR180C	FI:0.71969696969697	p:0.276019YGR181W	FI:1.83333333333333	p:0.390029YGR185C	FI:0.619047619047619	p:0.131934YGR187C	FI:0.972789115646259	p:0.191978YGR189C	FI:0.853033145716072	p:0.139198YGR191W	FI:1.45714285714286	p:0.135112YGR192C	FI:0.952380952380952	p:0.236418YGR193C	FI:1.05820105820106	p:0.166961YGR194C	FI:1.05442176870748	p:0.140337YGR195W	FI:0.407407407407407	p:0.230038YGR196C	FI:1.41197604790419	p:0.0339081YGR198W	FI:0.871817909553759	p:0.109107YGR199W	FI:1.13286713286713	p:0.133314YGR200C	FI:0.51037037037037	p:0.0220229YGR201C	FI:1.35658914728682	p:0.184294YGR202C	FI:0.37037037037037	p:0.0318897YGR203W	FI:0.785714285714286	p:0.222808YGR205W	FI:1.9047619047619	p:0.158309YGR206W	FI:9.38888888888889	p:0.00557753YGR207C	FI:0.338345864661654	p:0.0635429YGR208W	FI:1.49808429118774	p:0.116906YGR209C	FI:1.90909090909091	p:0.398086YGR210C	FI:0.328767123287671	p:0.0993775YGR211W	FI:6.08510638297872	p:0.037488YGR213C	FI:0.58974358974359	p:0.0798698YGR215W	FI:1.85294117647059	p:0.269599YGR216C	FI:1.5501976284585	p:0.0757954YGR220C	FI:1.33333333333333	p:0.24922YGR222W	FI:1.42424242424242	p:0.11937YGR223C	FI:0.726126126126126	p:0.12711YGR224W	FI:1.12408759124088	p:0.133972YGR229C	FI:0.925531914893617	p:0.120121YGR231C	FI:0.119047619047619	p:0.0079152YGR234W	FI:0.353801169590643	p:0.00522585YGR236C	FI:1.5	p:0.241703YGR238C	FI:0.946455026455026	p:0.086633YGR239C	FI:0.814299900695134	p:0.131067YGR242W	FI:1.42857142857143	p:0.299839YGR243W	FI:1.02439024390244	p:0.332699YGR244C	FI:0.227450980392157	p:0.0242873YGR245C	FI:1.6	p:0.129922YGR246C	FI:1.14251207729469	p:0.155408YGR247W	FI:0.609756097560976	p:0.204647YGR248W	FI:0.377840909090909	p:0.0258373YGR249W	FI:1.40074906367041	p:0.122233YGR251W	FI:2.16363636363636	p:0.0891403YGR252W	FI:0.712328767123288	p:0.178223YGR253C	FI:0.765957446808511	p:0.331527YGR254W	FI:0	p:0.4YGR255C	FI:1.14336917562724	p:0.260288YGR256W	FI:0.405263157894737	p:0.0762208YGR257C	FI:0.875	p:0.174223YGR260W	FI:0.305263157894737	p:0.0292596YGR261C	FI:0.998719590268886	p:0.118055YGR262C	FI:0.414545454545455	p:0.110618YGR263C	FI:0.489028213166144	p:0.0179284YGR264C	FI:1.05576923076923	p:0.148871YGR267C	FI:0	p:0.145955YGR268C	FI:2.07373271889401	p:0.0739843YGR273C	FI:0.814285714285714	p:0.196951YGR275W	FI:0.748768472906404	p:0.207942YGR276C	FI:0.989206691851052	p:0.13435YGR277C	FI:0.672316384180791	p:0.137703YGR278W	FI:0.898983050847458	p:0.112391YGR279C	FI:0.547619047619048	p:0.0817281YGR280C	FI:0.295081967213115	p:0.0513998YGR283C	FI:1	p:0.155187YGR284C	FI:0.534883720930233	p:0.252613YGR285C	FI:1.625	p:0.170529YGR286C	FI:0.676616915422885	p:0.246313YGR289C	FI:0.616194865042791	p:0.0603474YGR292W	FI:0.92974358974359	p:0.135354YHL002W	FI:0.833333333333333	p:0.138168YHL004W	FI:0.536585365853659	p:0.0735152YHL006C	FI:1.35	p:0.241624YHL008C	FI:1.38753687315634	p:0.0598046YHL009C	FI:0.715665976535542	p:0.0976948YHL010C	FI:1.23623853211009	p:0.110204YHL011C	FI:0.26984126984127	p:0.346884YHL012W	FI:0.587155963302752	p:0.0426054YHL013C	FI:0.777272727272727	p:0.153488YHL014C	FI:1.03125	p:0.182911YHL015W	FI:	p:1YHL016C	FI:0.267676767676768	p:0.00660465YHL019C	FI:5.02830188679245	p:0.00217132YHL020C	FI:1.19318181818182	p:0.165489YHL021C	FI:0.645161290322581	p:0.151345YHL022C	FI:1.0752688172043	p:0.153081YHL023C	FI:0.925087983911513	p:0.100027YHL024W	FI:1.24004424778761	p:0.109433YHL025W	FI:2.48584905660377	p:0.0201693YHL026C	FI:0.941176470588235	p:0.186727YHL028W	FI:1.32	p:0.0826523YHL029C	FI:1.45547073791349	p:0.104706YHL030W	FI:1.06336901685739	p:0.0771959YHL031C	FI:0.25	p:0.239334YHL032C	FI:0.929032258064516	p:0.155781YHL033C	FI:1.96825396825397	p:0.22189YHL034C	FI:2.63157894736842	p:0.142017YHL036W	FI:0.912280701754386	p:0.160062YHL038C	FI:0.699838000462856	p:0.0518797YHL039W	FI:1.20287769784173	p:0.115892YHL040C	FI:1.61417322834646	p:0.13007YHR001W	FI:0.857142857142857	p:0.175273YHR001W-A	FI:0.181818181818182	p:0.168421YHR002W	FI:1.90243902439024	p:0.172382YHR003C	FI:0.731944444444445	p:0.141685YHR005C	FI:0.898765432098765	p:0.218435YHR005C-A	FI:0	p:0.0328407YHR006W	FI:1.15508021390374	p:0.122946YHR007C	FI:1.86290322580645	p:0.233649YHR008C	FI:0.311111111111111	p:0.168046YHR009C	FI:0.479381443298969	p:0.0998791YHR010W	FI:1.625	p:0.254811YHR011W	FI:1.70833333333333	p:0.0581163YHR013C	FI:0.991666666666667	p:0.289104YHR014W	FI:0.601851851851852	p:0.110081YHR015W	FI:1.37231968810916	p:0.133507YHR016C	FI:0.785046728971963	p:0.138018YHR017W	FI:0.771929824561404	p:0.123209YHR018C	FI:2.11111111111111	p:0.129755YHR019C	FI:1.2962962962963	p:0.305494YHR020W	FI:0.689855072463768	p:0.162534YHR024C	FI:0.9375	p:0.203076YHR025W	FI:0.762295081967213	p:0.341349YHR026W	FI:1.73333333333333	p:0.423582YHR027C	FI:0.453709575142676	p:0.00975447YHR029C	FI:1.24861265260821	p:0.124278YHR030C	FI:0.909465020576132	p:0.206429YHR031C	FI:0.607305936073059	p:0.0559623YHR032W	FI:23.1439393939394	p:9.1817e-07YHR034C	FI:0.97737556561086	p:0.153384YHR035W	FI:0.916307161345988	p:0.1002YHR036W	FI:1.4027149321267	p:0.0893076YHR037W	FI:0.477714285714286	p:0.0779781YHR038W	FI:0.879120879120879	p:0.208549YHR039C	FI:0.962962962962963	p:0.193253YHR040W	FI:0.667735042735043	p:0.102549YHR043C	FI:2.04716981132075	p:0.113088YHR045W	FI:1.20022123893805	p:0.11686YHR046C	FI:0.286549707602339	p:0.0384757YHR047C	FI:0.721417069243156	p:0.0531148YHR048W	FI:0.54	p:0.0435192YHR049W	FI:0.857142857142857	p:0.29199YHR051W	FI:0.636363636363636	p:0.277335YHR052W	FI:1.40625	p:0.163315YHR055C	FI:5.71428571428571	p:0.131358YHR056C	FI:0.838709677419355	p:0.0912965YHR057C	FI:0.95625	p:0.201605YHR058C	FI:0.255208333333333	p:0.0268852YHR059W	FI:1.04761904761905	p:0.257752YHR060W	FI:0.674193548387097	p:0.156653YHR061C	FI:0.885245901639344	p:0.17174YHR062C	FI:2.26086956521739	p:0.175527YHR063C	FI:1.02857142857143	p:0.207656YHR064C	FI:1.29230769230769	p:0.184918YHR065C	FI:0.790123456790123	p:0.184605YHR066W	FI:0.786516853932584	p:0.141329YHR067W	FI:1.26666666666667	p:0.143385YHR068W	FI:1.625	p:0.394185YHR069C	FI:0.666666666666667	p:0.207387YHR070W	FI:3.20392156862745	p:0.00553604YHR071W	FI:0.866562009419152	p:0.159763YHR072W	FI:1.41843971631206	p:0.183676YHR072W-A	FI:2	p:0.454546YHR075C	FI:0.717592592592593	p:0.0974568YHR076W	FI:1.00574712643678	p:0.165977YHR078W	FI:0.841121495327103	p:0.146353YHR080C	FI:0.878513356562137	p:0.0746507YHR081W	FI:1.14883720930233	p:0.226142YHR083W	FI:1.80714285714286	p:0.0485369YHR085W	FI:1.07407407407407	p:0.173763YHR086W	FI:0.788530465949821	p:0.110013YHR087W	FI:	p:0.185204YHR088W	FI:	p:0.583642YHR089C	FI:1.61764705882353	p:0.305457YHR090C	FI:2.22727272727273	p:0.16992YHR091C	FI:0.712209302325581	p:0.0526981YHR092C	FI:10.8705882352941	p:0.00211798YHR094C	FI:0.993788819875776	p:0.202591YHR096C	FI:0.902654867256637	p:0.234442YHR100C	FI:0.84	p:0.242942YHR105W	FI:1.53061224489796	p:0.190231YHR106W	FI:2.88461538461538	p:0.100354YHR107C	FI:0.444444444444444	p:0.2816YHR108W	FI:1.5	p:0.109017YHR109W	FI:1.13821138211382	p:0.103448YHR110W	FI:1.25	p:0.23022YHR111W	FI:0.882075471698113	p:0.146836YHR112C	FI:0.852941176470588	p:0.223058YHR114W	FI:1.17056856187291	p:0.132143YHR115C	FI:0.860215053763441	p:0.175357YHR116W	FI:6.28571428571429	p:0.0510276YHR117W	FI:0.725146198830409	p:0.0826404YHR118C	FI:0.977301387137453	p:0.160709YHR121W	FI:0.62962962962963	p:0.250244YHR122W	FI:0.489795918367347	p:0.136863YHR123W	FI:0.723684210526316	p:0.170155YHR124W	FI:1.79310344827586	p:0.053664YHR126C	FI:1.21527777777778	p:0.188286YHR127W	FI:0.998459167950693	p:0.172703YHR128W	FI:0.428571428571429	p:0.262784YHR129C	FI:0.941558441558441	p:0.184381YHR132C	FI:2.14024390243902	p:0.0865932YHR132W-A	FI:0	p:0.3YHR133C	FI:1.85185185185185	p:0.113091YHR134W	FI:1.07090909090909	p:0.172691YHR135C	FI:4.60526315789474	p:0.0766448YHR137W	FI:0.383838383838384	p:0.0141553YHR138C	FI:1.28571428571429	p:0.284639YHR139C	FI:0.996428571428571	p:0.248675YHR140W	FI:2.25563909774436	p:0.0489383YHR142W	FI:0.78125	p:0.238477YHR143W	FI:1.32882882882883	p:0.124811YHR143W-A	FI:	p:1YHR144C	FI:2	p:0.0548554YHR146W	FI:0.924	p:0.128426YHR147C	FI:0.798245614035088	p:0.281807YHR148W	FI:0.192307692307692	p:0.194989YHR149C	FI:0.729953917050691	p:0.0811945YHR150W	FI:0.890909090909091	p:0.120413YHR152W	FI:	p:0.0275677YHR153C	FI:1.07027027027027	p:0.191485YHR154W	FI:0.994901960784314	p:0.0995835YHR155W	FI:1.23231154810102	p:0.0825589YHR156C	FI:0.474898236092266	p:0.0240394YHR157W	FI:1.18857142857143	p:0.180155YHR159W	FI:0.807017543859649	p:0.115524YHR161C	FI:0.766006097560976	p:0.0767263YHR162W	FI:0.263157894736842	p:0.369231YHR163W	FI:2.6448087431694	p:0.0734875YHR167W	FI:1.55675675675676	p:0.171846YHR168W	FI:1.71130104463438	p:0.0495387YHR169W	FI:0.864197530864197	p:0.315822YHR171W	FI:0.768115942028985	p:0.0995301YHR174W	FI:	p:0.529412YHR175W	FI:1.27272727272727	p:0.341989YHR176W	FI:0.701123595505618	p:0.0986233YHR177W	FI:0.905882352941176	p:0.140367YHR179W	FI:1.06206896551724	p:0.228212YHR182W	FI:0.622953558302706	p:0.0213524YHR185C	FI:0.681818181818182	p:0.225372YHR188C	FI:1.06794871794872	p:0.138323YHR189W	FI:1.00757575757576	p:0.268093YHR190W	FI:0.334302325581395	p:0.0912839YHR191C	FI:0.942857142857143	p:0.260523YHR192W	FI:0.496732026143791	p:0.0834276YHR193C	FI:0.285714285714286	p:0.37931YHR194W	FI:1.13414634146341	p:0.187908YHR195W	FI:1.04817708333333	p:0.162461YHR196W	FI:1.0822966507177	p:0.120925YHR197W	FI:0.966126230457441	p:0.116456YHR198C	FI:1.14230769230769	p:0.178788YHR199C	FI:0.6875	p:0.165154YHR200W	FI:0.169230769230769	p:0.0188493YHR201C	FI:0.94017094017094	p:0.147373YHR202W	FI:1.15652173913043	p:0.126544YHR203C	FI:0	p:0.478261YHR204W	FI:1.14245951417004	p:0.0988569YHR206W	FI:2.23728813559322	p:0.102623YHR207C	FI:0.66955266955267	p:0.0892928YHR208W	FI:0.873417721518987	p:0.427853YHR209W	FI:1.30820105820106	p:0.124359YHR210C	FI:1.98932926829268	p:0.0468949YHR214W	FI:0.357142857142857	p:0.231458YHR216W	FI:0.589285714285714	p:0.145257YIL001W	FI:1.20442708333333	p:0.162694YIL002C	FI:0.338655462184874	p:0.00566042YIL003W	FI:0.945454545454545	p:0.247867YIL006W	FI:1.08888888888889	p:0.208924YIL007C	FI:0.317254174397032	p:0.0189231YIL009W	FI:0.763492063492063	p:0.134208YIL010W	FI:1.8972972972973	p:0.122123YIL011W	FI:0.694989106753813	p:0.154583YIL014W	FI:1.08035714285714	p:0.144359YIL015W	FI:1.6672268907563	p:0.0428014YIL016W	FI:0.416666666666667	p:0.147472YIL019W	FI:1.89	p:0.0506466YIL020C	FI:1.38297872340426	p:0.194295YIL021W	FI:0.272727272727273	p:0.114394YIL022W	FI:0.560578661844485	p:0.120336YIL023C	FI:1.6150234741784	p:0.0920964YIL024C	FI:1.02857142857143	p:0.431337YIL027C	FI:0.964285714285714	p:0.279675YIL030C	FI:0.481203007518797	p:0.0145864YIL031W	FI:1.80782230806374	p:0.0123788YIL034C	FI:1.3125	p:0.223679YIL036W	FI:0.9112	p:0.10344YIL038C	FI:0.67177033492823	p:0.0602843YIL039W	FI:0.713513513513514	p:0.0912585YIL040W	FI:1.44642857142857	p:0.302817YIL042C	FI:1.08641975308642	p:0.235177YIL043C	FI:0.666666666666667	p:0.207979YIL044C	FI:0.91304347826087	p:0.188198YIL045W	FI:0.73970473970474	p:0.115331YIL049W	FI:0.650406504065041	p:0.122563YIL050W	FI:0.819327731092437	p:0.189212YIL051C	FI:	p:0.481271YIL052C	FI:	p:0.0589051YIL053W	FI:	p:1YIL055C	FI:0.624274099883856	p:0.0334537YIL056W	FI:0.752802359882006	p:0.107217YIL057C	FI:0.297297297297297	p:0.213493YIL061C	FI:1.27272727272727	p:0.165838YIL062C	FI:0.43859649122807	p:0.147232YIL063C	FI:0.861538461538462	p:0.150262YIL064W	FI:0.525	p:0.0888553YIL065C	FI:0.0857142857142857	p:0.0407031YIL067C	FI:0.496551724137931	p:0.0167746YIL068C	FI:1.05095541401274	p:0.25541YIL070C	FI:0.772881355932204	p:0.212184YIL071C	FI:0.617224880382775	p:0.105111YIL073C	FI:1.01734104046243	p:0.113778YIL074C	FI:0.421875	p:0.203049YIL075C	FI:0.633716993906567	p:0.119514YIL076W	FI:1.33333333333333	p:0.217289YIL077C	FI:1.49152542372881	p:0.120827YIL079C	FI:1.39393939393939	p:0.119627YIL082W	FI:0.742857142857143	p:0.168535YIL083C	FI:1.125	p:0.327624YIL084C	FI:0.3	p:0.0149053YIL085C	FI:1.48148148148148	p:0.107756YIL087C	FI:0.159090909090909	p:0.028957YIL090W	FI:1.23456790123457	p:0.303895YIL091C	FI:2.46128680479826	p:0.0157637YIL094C	FI:1.08695652173913	p:0.413964YIL095W	FI:1.34720616570328	p:0.086636YIL096C	FI:0.966101694915254	p:0.200131YIL097W	FI:1.42745098039216	p:0.11898YIL098C	FI:1.57635467980296	p:0.147464YIL099W	FI:0.767543859649123	p:0.109081YIL103W	FI:0.501204819277108	p:0.12362YIL104C	FI:1.40056022408964	p:0.140236YIL105C	FI:0.644628099173554	p:0.136763YIL106W	FI:0.375	p:0.408692YIL107C	FI:0.989711934156378	p:0.162759YIL108W	FI:0.925636007827789	p:0.181557YIL109C	FI:0.504658385093168	p:0.0800336YIL110W	FI:0.780063291139241	p:0.118747YIL111W	FI:0.196428571428571	p:0.197038YIL113W	FI:0.490566037735849	p:0.153107YIL114C	FI:1.21333333333333	p:0.166267YIL116W	FI:0.921052631578947	p:0.239741YIL117C	FI:0.82480173035328	p:0.122577YIL118W	FI:0.454545454545455	p:0.275208YIL119C	FI:1.03286384976526	p:0.178387YIL120W	FI:0.807322654462242	p:0.109077YIL121W	FI:1.74836173001311	p:0.0804817YIL122W	FI:2.16140350877193	p:0.0724133YIL123W	FI:5.14018691588785	p:0.00195865YIL124W	FI:0.320422535211268	p:0.0251266YIL125W	FI:0.515094339622642	p:0.110975YIL127C	FI:0.343137254901961	p:0.0385923YIL130W	FI:0.914022842639594	p:0.124183YIL131C	FI:0.380787037037037	p:0.00825364YIL132C	FI:0.451871657754011	p:0.0662525YIL133C	FI:2.8	p:0.16485YIL134W	FI:0.496732026143791	p:0.0730462YIL135C	FI:0.37359900373599	p:0.0224844YIL136W	FI:1.2	p:0.232274YIL138C	FI:1.57407407407407	p:0.297433YIL139C	FI:0.391836734693877	p:0.103002YIL142W	FI:0.277173913043478	p:0.252232YIL143C	FI:1.53020134228188	p:0.170016YIL144W	FI:0.941860465116279	p:0.133289YIL145C	FI:0.516746411483254	p:0.070457YIL146C	FI:1.62857142857143	p:0.0578022YIL149C	FI:1.00161377084454	p:0.0664372YIL150C	FI:0.982346832814122	p:0.131887YIL152W	FI:1.05442176870748	p:0.188066YIL154C	FI:0.88135593220339	p:0.228056YIL157C	FI:1.8	p:0.136222YIL158W	FI:0.619834710743802	p:0.123698YIL160C	FI:0.918367346938776	p:0.266429YIL167W	FI:	p:YIL170W	FI:	p:YIR001C	FI:0.394463667820069	p:0.0169569YIR002C	FI:1.93798449612403	p:0.0127968YIR004W	FI:0.8775	p:0.163754YIR005W	FI:2.24691358024691	p:0.141318YIR007W	FI:1.17458823529412	p:0.115568YIR009W	FI:0.4375	p:0.202675YIR011C	FI:1.26708074534161	p:0.182062YIR012W	FI:0.404761904761905	p:0.0519205YIR013C	FI:1.8	p:0.216059YIR014W	FI:0.576329331046312	p:0.0943287YIR015W	FI:1.32222222222222	p:0.200202YIR016W	FI:1.38300653594771	p:0.108813YIR018W	FI:0.958333333333333	p:0.160126YIR021W	FI:0.929292929292929	p:0.142745YIR022W	FI:0.7	p:0.343346YIR023W	FI:1.00242718446602	p:0.133595YIR024C	FI:1.0875	p:0.193883YIR025W	FI:1.59119496855346	p:0.103525YIR026C	FI:0.685185185185185	p:0.168087YIR027C	FI:1.11650485436893	p:0.193003YIR028W	FI:3.64705882352941	p:0.0511754YIR029W	FI:1.58688524590164	p:0.151021YIR030C	FI:1.88837209302326	p:0.0591913YIR031C	FI:1.46788990825688	p:0.21532YIR032C	FI:0.970588235294118	p:0.193368YIR034C	FI:1.72307692307692	p:0.251659YIR035C	FI:1.02439024390244	p:0.238247YIR036C	FI:1.8	p:0.136855YIR037W	FI:	p:0.154723YIR038C	FI:1.9349593495935	p:0.167254YIR042C	FI:1.03333333333333	p:0.173001YJL001W	FI:	p:0.637288YJL002C	FI:0.534090909090909	p:0.0581637YJL003W	FI:0.35	p:0.19188YJL004C	FI:0.702127659574468	p:0.205257YJL005W	FI:0.353187042842215	p:0.000382279YJL006C	FI:0.496894409937888	p:0.0937382YJL008C	FI:0.494565217391304	p:0.115247YJL010C	FI:0.695187165775401	p:0.109843YJL011C	FI:0.370588235294118	p:0.102589YJL012C	FI:0.571428571428571	p:0.214579YJL013C	FI:0.55331541218638	p:0.0319724YJL014W	FI:0.238636363636364	p:0.137131YJL019W	FI:0.619354838709677	p:0.077033YJL020C	FI:0.752944188428059	p:0.03637YJL023C	FI:0.703448275862069	p:0.0904153YJL025W	FI:1.04239766081871	p:0.150368YJL030W	FI:0.599078341013825	p:0.16189YJL033W	FI:1.01488095238095	p:0.241828YJL034W	FI:0.229749631811487	p:0.0184797YJL035C	FI:0.780780780780781	p:0.18096YJL036W	FI:0.211930926216641	p:0.00073009YJL037W	FI:0.559139784946237	p:0.103466YJL038C	FI:0.875379939209726	p:0.204987YJL042W	FI:0.794211677603904	p:0.0817768YJL044C	FI:0.617239300783605	p:0.0545979YJL046W	FI:2.08490566037736	p:0.109877YJL051W	FI:0.646008403361344	p:0.0343734YJL052W	FI:1.84782608695652	p:0.26722YJL054W	FI:0.42386403052376	p:0.00346881YJL055W	FI:1.15151515151515	p:0.25762YJL056C	FI:1.51313131313131	p:0.0272777YJL058C	FI:0.37962962962963	p:0.00391731YJL059W	FI:0.295138888888889	p:0.00190816YJL060W	FI:0.176470588235294	p:0.000148198YJL062W	FI:0.615544041450777	p:0.0560145YJL063C	FI:0.863636363636364	p:0.175762YJL065C	FI:0.714285714285714	p:0.198082YJL066C	FI:0.777777777777778	p:0.221553YJL069C	FI:0.666666666666667	p:0.0888852YJL071W	FI:0.857142857142857	p:0.118626YJL072C	FI:3.5625	p:0.183633YJL079C	FI:0.346938775510204	p:0.011551YJL080C	FI:0.485175202156334	p:0.00903064YJL081C	FI:0.156716417910448	p:0.00243893YJL082W	FI:0.185542168674699	p:6.62284e-05YJL083W	FI:1.31926406926407	p:0.150812YJL088W	FI:0.296296296296296	p:0.0185368YJL089W	FI:0.503625209146682	p:0.0161353YJL091C	FI:0.748235294117647	p:0.103471YJL096W	FI:0.30791788856305	p:0.0270723YJL099W	FI:0.846153846153846	p:0.127195YJL100W	FI:0.792235801581596	p:0.0894864YJL104W	FI:0.426086956521739	p:0.145807YJL105W	FI:0.416083916083916	p:0.00576754YJL110C	FI:0.555984555984556	p:0.0117125YJL111W	FI:0.2875	p:0.0455368YJL112W	FI:2.08252427184466	p:0.0241083YJL115W	FI:0.566666666666667	p:0.159454YJL116C	FI:0.564102564102564	p:0.104276YJL117W	FI:0.421052631578947	p:0.0528069YJL118W	FI:0.5	p:0.112448YJL121C	FI:0.266666666666667	p:0.143203YJL122W	FI:0.482142857142857	p:0.131778YJL123C	FI:1.48809523809524	p:0.175425YJL124C	FI:1.4	p:0.412959YJL125C	FI:1.52073732718894	p:0.131849YJL126W	FI:1.56887755102041	p:0.130629YJL133W	FI:0.444444444444444	p:0.198806YJL138C	FI:0.15	p:0.0790209YJL140W	FI:0.021875	p:1.71813e-05YJL141C	FI:0.344387755102041	p:0.00630648YJL143W	FI:0.5	p:0.470769YJL145W	FI:0.392324093816631	p:0.0186025YJL147C	FI:0.878787878787879	p:0.124521YJL149W	FI:0.346320346320346	p:0.019YJL151C	FI:0.541666666666667	p:0.144185YJL153C	FI:0.254312251216276	p:0.000267972YJL154C	FI:0.983850931677019	p:0.186039YJL156C	FI:0.363098440021517	p:0.00951687YJL157C	FI:1.06212765957447	p:0.11206YJL158C	FI:1.03888888888889	p:0.239734YJL159W	FI:0.0555555555555556	p:1.8176e-07YJL160C	FI:0.90738423028786	p:0.151037YJL161W	FI:2.43243243243243	p:0.0791603YJL162C	FI:1.16458704693999	p:0.0935087YJL164C	FI:0.293333333333333	p:0.0119002YJL165C	FI:0.590357598978289	p:0.0377864YJL166W	FI:0	p:0.416667YJL167W	FI:0.333333333333333	p:0.0999656YJL168C	FI:0.680597014925373	p:0.0810753YJL171C	FI:0.738396624472574	p:0.181677YJL172W	FI:0.535244922341697	p:0.0811273YJL173C	FI:0.266666666666667	p:0.160595YJL174W	FI:2.16149068322981	p:0.0521241YJL176C	FI:1.87848932676519	p:0.00926866YJL178C	FI:0.402930402930403	p:0.0352105YJL179W	FI:0.823529411764706	p:0.467692YJL180C	FI:1.03567318757192	p:0.162518YJL183W	FI:1.37162162162162	p:0.165432YJL184W	FI:0.489130434782609	p:0.200575YJL185C	FI:1.45107794361526	p:0.12092YJL186W	FI:1.44342672413793	p:0.0701181YJL187C	FI:1.08108108108108	p:0.144567YJL190C	FI:	p:YJL192C	FI:0.734567901234568	p:0.264774YJL193W	FI:1.09090909090909	p:0.189455YJL194W	FI:1.07032590051458	p:0.154524YJL196C	FI:1.24897959183673	p:0.21023YJL197W	FI:1.35774613899614	p:0.034807YJL198W	FI:0.525096525096525	p:0.0812446YJL199C	FI:6.88235294117647	p:0.0516052YJL200C	FI:0.46494708994709	p:0.0439809YJL201W	FI:0.992156862745098	p:0.165705YJL203W	FI:1.8956043956044	p:0.0447262YJL206C	FI:0.4375	p:0.0121261YJL207C	FI:0.926929095943181	p:0.0784549YJL208C	FI:0.612745098039216	p:0.115365YJL209W	FI:1.48235294117647	p:0.0584763YJL210W	FI:0.830769230769231	p:0.184528YJL217W	FI:2.43243243243243	p:0.0592958YJL218W	FI:0.95	p:0.261623YJR001W	FI:0.330383480825959	p:0.0085432YJR004C	FI:0.901589912280702	p:0.112548YJR005W	FI:0.367274569402229	p:0.00704124YJR006W	FI:0.90702947845805	p:0.19649YJR007W	FI:0	p:0.0115528YJR008W	FI:1.07441860465116	p:0.181364YJR009C	FI:0.555555555555556	p:0.323386YJR010C-A	FI:0.3125	p:0.165644YJR010W	FI:0.248275862068966	p:0.0326295YJR011C	FI:0.481132075471698	p:0.0854515YJR014W	FI:0.324074074074074	p:0.069474YJR016C	FI:0.225	p:0.0542422YJR017C	FI:0.888888888888889	p:0.293671YJR019C	FI:0.160714285714286	p:0.00642153YJR022W	FI:0.253968253968254	p:0.0597534YJR024C	FI:0.501519756838906	p:0.109319YJR025C	FI:0.344827586206897	p:0.174162YJR032W	FI:0.473255813953488	p:0.0575871YJR034W	FI:0	p:0.25YJR036C	FI:0.613636363636364	p:0.040014YJR040W	FI:0.423076923076923	p:0.01988YJR042W	FI:1.06132075471698	p:0.162017YJR043C	FI:1.39240506329114	p:0.165141YJR044C	FI:0.296296296296296	p:0.141768YJR047C	FI:0.0615384615384615	p:0.0174745YJR049C	FI:0.232664233576642	p:0.00493376YJR053W	FI:1.70138888888889	p:0.106628YJR056C	FI:0.505952380952381	p:0.104644YJR058C	FI:0	p:0.0990991YJR060W	FI:0.373626373626374	p:0.0204702YJR062C	FI:1.05769230769231	p:0.143797YJR063W	FI:	p:0.453333YJR064W	FI:0.178571428571429	p:0.0371062YJR065C	FI:0.205714285714286	p:0.0336811YJR067C	FI:0.242424242424242	p:0.0367252YJR068W	FI:0.21505376344086	p:0.00708377YJR073C	FI:0.668571428571428	p:0.208883YJR074W	FI:0.864864864864865	p:0.221538YJR075W	FI:0.415742793791574	p:0.030921YJR077C	FI:0.4	p:0.276594YJR080C	FI:0.361702127659574	p:0.0213127YJR082C	FI:0.352941176470588	p:0.167654YJR085C	FI:0	p:0.00144928YJR086W	FI:4	p:0.251748YJR088C	FI:2.06349206349206	p:0.0767388YJR090C	FI:0.647430830039526	p:0.0395504YJR095W	FI:0.392156862745098	p:0.138912YJR097W	FI:0.370656370656371	p:0.073089YJR101W	FI:0.762345679012346	p:0.196434YJR102C	FI:0.958974358974359	p:0.23897YJR104C	FI:0.491228070175439	p:0.248733YJR105W	FI:0.260720411663808	p:0.00886305YJR107W	FI:0.545454545454545	p:0.111645YJR109C	FI:0.172297297297297	p:0.00225023YJR112W	FI:0.162162162162162	p:0.00307416YJR113C	FI:2.77551020408163	p:0.116529YJR116W	FI:1.69811320754717	p:0.249142YJR117W	FI:0.634680134680135	p:0.12583YJR118C	FI:0.166666666666667	p:0.149282YJR121W	FI:0	p:0.000649256YJR122W	FI:0.785714285714286	p:0.0845618YJR123W	FI:0	p:0.222222YJR125C	FI:0.288006756756757	p:0.00337717YJR129C	FI:0.608465608465608	p:0.0576021YJR131W	FI:0.928571428571429	p:0.13886YJR132W	FI:0.552631578947368	p:0.0217219YJR133W	FI:0.181818181818182	p:0.0171209YJR134C	FI:0.787878787878788	p:0.107288YJR135C	FI:0.609230769230769	p:0.131543YJR135W-A	FI:1	p:0.469556YJR136C	FI:0.838330393885949	p:0.120501YJR138W	FI:0.482881136950904	p:0.0162942YJR139C	FI:0.384615384615385	p:0.0887762YJR141W	FI:0.61734693877551	p:0.0797534YJR142W	FI:1.485	p:0.109813YJR143C	FI:0.692307692307692	p:0.191951YJR144W	FI:1.06666666666667	p:0.215927YJR145C	FI:2.6	p:0.296377YJR147W	FI:0.506493506493507	p:0.0897472YJR148W	FI:0.76056338028169	p:0.120621YJR150C	FI:1.25	p:0.174149YJR152W	FI:0.1455525606469	p:0.00709384YJR153W	FI:0.319602272727273	p:0.0243505YKL001C	FI:0	p:0.290909YKL003C	FI:0.642857142857143	p:0.342911YKL004W	FI:0.430769230769231	p:0.153826YKL005C	FI:0.840909090909091	p:0.10767YKL006C-A	FI:	p:0.0380896YKL007W	FI:0.451388888888889	p:0.0732049YKL009W	FI:1.66666666666667	p:0.285993YKL011C	FI:0.96604600219058	p:0.164476YKL012W	FI:0.816964285714286	p:0.114735YKL013C	FI:	p:1YKL016C	FI:0.285714285714286	p:0.0928931YKL017C	FI:0.566929133858268	p:0.0379927YKL018C-A	FI:0.357142857142857	p:0.17367YKL019W	FI:1.47368421052632	p:0.154754YKL021C	FI:	p:YKL023W	FI:0.872340425531915	p:0.151107YKL024C	FI:0.6	p:0.178826YKL026C	FI:0.491891891891892	p:0.151994YKL028W	FI:3.00925925925926	p:0.0448366YKL029C	FI:0.458015267175573	p:0.128545YKL033W	FI:0.961271367521368	p:0.0942872YKL033W-A	FI:0.751552795031056	p:0.188737YKL035W	FI:	p:0.425467YKL038W	FI:1.0625	p:0.125478YKL039W	FI:0.965853658536585	p:0.16895YKL040C	FI:0.405	p:0.0530987YKL041W	FI:0.347222222222222	p:0.0800072YKL042W	FI:1.42857142857143	p:0.174017YKL045W	FI:0.35964035964036	p:0.0182146YKL046C	FI:0.170542635658915	p:0.00205011YKL048C	FI:0.957264957264957	p:0.105764YKL049C	FI:2.01923076923077	p:0.20362YKL050C	FI:1.17175454825779	p:0.0677853YKL051W	FI:0.607142857142857	p:0.135772YKL052C	FI:0.962962962962963	p:0.179175YKL053C-A	FI:0.0952380952380952	p:0.0717704YKL054C	FI:0.529100529100529	p:0.0231661YKL055C	FI:0.643434343434343	p:0.0924675YKL056C	FI:0	p:0.110526YKL060C	FI:1	p:0.296433YKL061W	FI:1.96	p:0.350133YKL062W	FI:0.921428571428571	p:0.139676YKL063C	FI:0.6	p:0.173638YKL068W	FI:0.84627410109432	p:0.119221YKL069W	FI:0.8203125	p:0.199798YKL071W	FI:1.19298245614035	p:0.11769YKL072W	FI:0.613333333333333	p:0.0471366YKL074C	FI:1.03927068723703	p:0.112703YKL077W	FI:0.915740740740741	p:0.148044YKL079W	FI:0.478405315614618	p:0.0068465YKL080W	FI:0.15	p:0.113519YKL082C	FI:1.76555023923445	p:0.053111YKL084W	FI:0.25	p:0.0351277YKL085W	FI:0.143589743589744	p:0.00199939YKL086W	FI:	p:0.167095YKL087C	FI:1.22051282051282	p:0.220699YKL088W	FI:0.703125	p:0.0865085YKL091C	FI:0.0583333333333333	p:0.0120258YKL093W	FI:1.22222222222222	p:0.143969YKL094W	FI:0.397058823529412	p:0.0232485YKL095W	FI:0.466666666666667	p:0.037058YKL096W	FI:0.712053571428572	p:0.165084YKL096W-A	FI:0.416666666666667	p:0.25241YKL098W	FI:1.99038461538462	p:0.0481928YKL100C	FI:0.595161290322581	p:0.0400983YKL106W	FI:0.982905982905983	p:0.205087YKL107W	FI:0.139689578713969	p:0.000248409YKL109W	FI:0.563428571428572	p:0.0266209YKL110C	FI:0.304761904761905	p:0.0548334YKL112W	FI:0.654545454545455	p:0.0508878YKL113C	FI:0.253521126760563	p:0.039903YKL116C	FI:0.692307692307692	p:0.125415YKL117W	FI:0.121621621621622	p:0.020772YKL120W	FI:0.45	p:0.310484YKL121W	FI:0.497237569060773	p:0.040546YKL122C	FI:2.59259259259259	p:0.0751772YKL124W	FI:0.222772277227723	p:0.00033684YKL125W	FI:0.374025974025974	p:0.0029476YKL127W	FI:0.501474926253687	p:0.0176323YKL130C	FI:0.2109375	p:0.0153129YKL132C	FI:0.545031055900621	p:0.0596126YKL133C	FI:0.735393258426966	p:0.101358YKL135C	FI:0.551470588235294	p:0.0375496YKL137W	FI:0.181818181818182	p:0.0221941YKL138C	FI:	p:0.22673YKL139W	FI:1.77142857142857	p:0.0673855YKL140W	FI:0.576230492196879	p:0.109555YKL141W	FI:0.428571428571429	p:0.101126YKL142W	FI:1.21276595744681	p:0.319881YKL143W	FI:1.27323162274619	p:0.160789YKL144C	FI:0.205882352941176	p:0.155649YKL145W	FI:1.93103448275862	p:0.330008YKL146W	FI:2.90445859872611	p:0.0390365YKL148C	FI:0.428571428571429	p:0.114034YKL149C	FI:0.837209302325581	p:0.211676YKL150W	FI:1.56284153005464	p:0.215282YKL152C	FI:0	p:0.285714YKL154W	FI:1.02325581395349	p:0.22121YKL155C	FI:1.23035714285714	p:0.0883428YKL157W	FI:1.16192411924119	p:0.150298YKL159C	FI:1.51914893617021	p:0.170241YKL160W	FI:1.11111111111111	p:0.464961YKL162C	FI:0.831081081081081	p:0.120071YKL163W	FI:6.17647058823529	p:0.0536969YKL165C-A	FI:1.66666666666667	p:0.368937YKL166C	FI:0.438356164383562	p:0.103744YKL167C	FI:0.386206896551724	p:0.116443YKL168C	FI:0.905895691609977	p:0.100618YKL170W	FI:0	p:0.282051YKL171W	FI:1.09393346379648	p:0.128675YKL172W	FI:0.604133545310016	p:0.065794YKL173W	FI:1.96446700507614	p:0.00223818YKL174C	FI:1.1443661971831	p:0.185029YKL175W	FI:1.18867924528302	p:0.112965YKL176C	FI:1.13214108296075	p:0.139186YKL178C	FI:2.14756671899529	p:0.0382727YKL179C	FI:1.03384207033842	p:0.152076YKL181W	FI:0.986301369863014	p:0.430451YKL183W	FI:0.842105263157895	p:0.206165YKL184W	FI:1.4	p:0.0984625YKL185W	FI:0.788032454361055	p:0.107747YKL186C	FI:	p:0.0433115YKL187C	FI:0.885989010989011	p:0.134138YKL189W	FI:0.3625	p:0.0505496YLR087C	FI:0.849278979478647	p:0.079785YLR088W	FI:0.46890756302521	p:0.0223994YLR089C	FI:0.68	p:0.211113YLR090W	FI:1.91574279379157	p:0.0360509YLR091W	FI:0.786780383795309	p:0.193026YLR092W	FI:0.645	p:0.092779YLR093C	FI:0.477272727272727	p:0.16724YLR094C	FI:0.63975628332064	p:0.086624YLR095C	FI:0.664646464646465	p:0.0326906YLR097C	FI:0.75	p:0.130837YLR098C	FI:1.41520467836257	p:0.149867YLR099C	FI:0.752941176470588	p:0.229323YLR100W	FI:0.485074626865672	p:0.16741YLR102C	FI:0.522727272727273	p:0.103246YLR103C	FI:1.22644628099174	p:0.135637YLR104W	FI:2.58064516129032	p:0.279162YLR105C	FI:1.17482517482518	p:0.153667YLR107W	FI:1.98795180722892	p:0.046195YLR108C	FI:0.882178217821782	p:0.121775YLR109W	FI:1.15384615384615	p:0.439808YLR110C	FI:0.714285714285714	p:0.527473YLR113W	FI:3.28205128205128	p:0.181031YLR114C	FI:1.06456838694747	p:0.103835YLR116W	FI:0.766826923076923	p:0.126333YLR117C	FI:1.1953125	p:0.107894YLR118C	FI:1.17361111111111	p:0.268975YLR120C	FI:2.68571428571429	p:0.0114593YLR121C	FI:0.836538461538462	p:0.146258YLR126C	FI:0.530920060331825	p:0.0682029YLR127C	FI:1.23481781376518	p:0.0797301YLR129W	FI:0.632432432432433	p:0.0593653YLR130C	FI:0.59328968903437	p:0.0705883YLR131C	FI:0.521874392377989	p:0.00620185YLR132C	FI:1.34736842105263	p:0.113088YLR133W	FI:0.799145299145299	p:0.160068YLR134W	FI:4.37735849056604	p:0.112718YLR135W	FI:1.08579749103943	p:0.100606YLR136C	FI:1.83703703703704	p:0.117397YLR137W	FI:0.798190045248869	p:0.12972YLR138W	FI:2.06632653061224	p:0.0383777YLR139C	FI:0.810077519379845	p:0.0931719YLR141W	FI:0.863636363636364	p:0.170235YLR142W	FI:0.545655375552283	p:0.0518814YLR143W	FI:1.06333333333333	p:0.123277YLR144C	FI:0.752836484983315	p:0.0634203YLR145W	FI:0.581196581196581	p:0.194749YLR146C	FI:0.666666666666667	p:0.116528YLR147C	FI:0	p:0.0541872YLR148W	FI:1.42601267566823	p:0.0543515YLR149C	FI:1.6141235813367	p:0.0542343YLR151C	FI:0.764587525150905	p:0.175153YLR152C	FI:0.657894736842105	p:0.0690175YLR153C	FI:1.09322033898305	p:0.425594YLR154C	FI:0.852173913043478	p:0.256572YLR163C	FI:0.666666666666667	p:0.161233YLR164W	FI:1.75	p:0.17212YLR165C	FI:1.15819209039548	p:0.175693YLR166C	FI:0.986686390532544	p:0.170557YLR167W	FI:0.666666666666667	p:0.506494YLR168C	FI:0.666666666666667	p:0.412799YLR170C	FI:0.634920634920635	p:0.286674YLR172C	FI:0.305882352941176	p:0.0696506YLR173W	FI:0.784516129032258	p:0.0922495YLR174W	FI:1.82	p:0.236897YLR175W	FI:0.736842105263158	p:0.264038YLR177W	FI:1.20789779326365	p:0.167543YLR178C	FI:1.25	p:0.262947YLR179C	FI:0.483950617283951	p:0.081963YLR180W	FI:0.111111111111111	p:0.0564752YLR181C	FI:1.3739224137931	p:0.102809YLR182W	FI:1.28333333333333	p:0.100083YLR183C	FI:0.931730769230769	p:0.126538YLR186W	FI:0.171428571428571	p:0.167143YLR188W	FI:1.20512820512821	p:0.128968YLR189C	FI:1.21953488372093	p:0.089292YLR190W	FI:1.4936170212766	p:0.0981538YLR191W	FI:0.916666666666667	p:0.177934YLR192C	FI:1.07142857142857	p:0.330652YLR193C	FI:1.6969696969697	p:0.381181YLR194C	FI:	p:0.0778683YLR195C	FI:0.910416666666667	p:0.202834YLR196W	FI:0.762107051826678	p:0.131549YLR197W	FI:2.34375	p:0.286011YLR200W	FI:0.8	p:0.355542YLR201C	FI:1.43255813953488	p:0.188046YLR203C	FI:0.414814814814815	p:0.0457745YLR205C	FI:0.928571428571429	p:0.250502YLR208W	FI:0.517241379310345	p:0.286339YLR209C	FI:0.977941176470588	p:0.242955YLR210W	FI:1.25	p:0.143042YLR211C	FI:1.6	p:0.145582YLR213C	FI:0.778688524590164	p:0.195158YLR214W	FI:1.12820512820513	p:0.135345YLR215C	FI:0.334637964774951	p:0.00840565YLR216C	FI:0.33	p:0.0292017YLR219W	FI:1.7880701754386	p:0.0163904YLR220W	FI:1.09289617486339	p:0.271476YLR221C	FI:1.23529411764706	p:0.189643YLR222C	FI:0.726315789473684	p:0.161306YLR224W	FI:1.27678571428571	p:0.164297YLR225C	FI:2.82558139534884	p:0.0983901YLR227C	FI:0.901149425287356	p:0.128395YLR228C	FI:0.882716049382716	p:0.117852YLR229C	FI:	p:1YLR231C	FI:1.6988416988417	p:0.0991472YLR232W	FI:1.08333333333333	p:0.224758YLR233C	FI:1.15710382513661	p:0.0887065YLR234W	FI:1.125	p:0.224486YLR237W	FI:1.08428571428571	p:0.184731YLR239C	FI:0.410353535353535	p:0.0302178YLR240W	FI:1.1475	p:0.200908YLR241W	FI:1.2497430626927	p:0.163748YLR242C	FI:1.06280193236715	p:0.186864YLR243W	FI:0.906976744186046	p:0.435818YLR245C	FI:0.648148148148148	p:0.258828YLR246W	FI:0.855855855855856	p:0.200251YLR247C	FI:0.880294117647059	p:0.0681133YLR248W	FI:0.643382352941177	p:0.0716525YLR249W	FI:2.52702702702703	p:0.0903277YLR250W	FI:1.10322580645161	p:0.24286YLR251W	FI:0.756756756756757	p:0.224359YLR253W	FI:0.973790322580645	p:0.23038YLR254C	FI:0.421052631578947	p:0.10122YLR256W	FI:1.24159021406728	p:0.114772YLR257W	FI:0.594086021505376	p:0.147534YLR258W	FI:2.56488549618321	p:0.245109YLR259C	FI:0.556818181818182	p:0.25141YLR260W	FI:0.898774983881367	p:0.148511YLR262C	FI:2.11764705882353	p:0.334953YLR262C-A	FI:3.75	p:0.293706YLR264W	FI:	p:YLR267W	FI:1.45272435897436	p:0.0889065YLR268W	FI:0.303030303030303	p:0.169189YLR270W	FI:	p:0.19283YLR272C	FI:0.723387096774194	p:0.0659379YLR273C	FI:0.905106707317073	p:0.0951768YLR276C	FI:1.59657320872274	p:0.118867YLR277C	FI:1.0426731078905	p:0.163876YLR278C	FI:1.18426501035197	p:0.0904391YLR281C	FI:0.899470899470899	p:0.224575YLR283W	FI:1.48118279569892	p:0.0970204YLR284C	FI:1.07276119402985	p:0.184228YLR286C	FI:1.56955017301038	p:0.0530574YLR287C	FI:0.695704779189353	p:0.0839889YLR287C-A	FI:0.25	p:0.409091YLR288C	FI:1.11932773109244	p:0.186587YLR289W	FI:0.787515006002401	p:0.170932YLR290C	FI:3.234375	p:0.0234292YLR291C	FI:0.159090909090909	p:0.0119619YLR292C	FI:1.02857142857143	p:0.438848YLR293C	FI:	p:0.67742YLR295C	FI:0.9	p:0.261623YLR297W	FI:1.64197530864198	p:0.232668YLR298C	FI:0.748299319727891	p:0.19223YLR300W	FI:1.39130434782609	p:0.206958YLR301W	FI:1.14285714285714	p:0.199055YLR303W	FI:0.904109589041096	p:0.429527YLR305C	FI:0.986182519280206	p:0.119744YLR307W	FI:0.858064516129032	p:0.220726YLR308W	FI:0.756363636363636	p:0.210548YLR310C	FI:1.5982428115016	p:0.0309422YLR312C	FI:1.52816901408451	p:0.0901011YLR312W-A	FI:1.10192837465565	p:0.181726YLR314C	FI:1.04012841091493	p:0.189302YLR315W	FI:0.786324786324786	p:0.297402YLR318W	FI:1.18761904761905	p:0.0926759YLR319C	FI:1.02378048780488	p:0.119737YLR321C	FI:1.03571428571429	p:0.218694YLR323C	FI:1.14230769230769	p:0.171207YLR324W	FI:0.757246376811594	p:0.130186YLR325C	FI:	p:YLR327C	FI:	p:1YLR328W	FI:0.714285714285714	p:0.224662YLR332W	FI:0.967032967032967	p:0.173916YLR333C	FI:	p:0.705883YLR336C	FI:0.771166269976199	p:0.0612726YLR340W	FI:1.28787878787879	p:0.29267YLR343W	FI:1.84896584896585	p:0.0135032YLR344W	FI:0.571428571428571	p:0.441177YLR345W	FI:1.54814814814815	p:0.131347YLR347C	FI:	p:0.581359YLR348C	FI:1.51515151515152	p:0.235292YLR350W	FI:4.95238095238095	p:0.0898556YLR351C	FI:0.293785310734463	p:0.152149YLR352W	FI:0.524765729585007	p:0.0229327YLR353W	FI:1.0818018018018	p:0.113736YLR354C	FI:1.4468085106383	p:0.289813YLR355C	FI:2.21052631578947	p:0.349762YLR356W	FI:0.694980694980695	p:0.18153YLR359W	FI:1.20833333333333	p:0.410967YLR360W	FI:0.596143824908807	p:0.0483231YLR362W	FI:0.585714285714286	p:0.04669YLR363C	FI:0.228758169934641	p:0.0798071YLR363W-A	FI:0.333333333333333	p:0.415585YLR364W	FI:0.476190476190476	p:0.220955YLR368W	FI:1.02857142857143	p:0.138231YLR370C	FI:	p:0.457959YLR371W	FI:1.14070162202942	p:0.134929YLR372W	FI:1.83006535947712	p:0.176167YLR373C	FI:0.700638880438089	p:0.0679296YLR375W	FI:0.9	p:0.27519YLR376C	FI:0.474509803921569	p:0.0688691YLR377C	FI:	p:0.506139YLR378C	FI:0.78	p:0.420374YLR380W	FI:1.29411764705882	p:0.222096YLR381W	FI:1.07544836116265	p:0.115601YLR383W	FI:0.486631016042781	p:0.0127917YLR384C	FI:0.7673343605547	p:0.075373YLR385C	FI:0.617647058823529	p:0.17218YLR386W	FI:1.08571428571429	p:0.204264YLR387C	FI:0.704545454545454	p:0.123395YLR389C	FI:0.818112633181126	p:0.123463YLR390W-A	FI:0.9	p:0.257533YLR392C	FI:0.851327433628319	p:0.153372YLR393W	FI:2.85714285714286	p:0.014852YLR394W	FI:1.20599613152805	p:0.141206YLR395C	FI:0.238095238095238	p:0.132031YLR396C	FI:1.20691358024691	p:0.114761YLR398C	FI:1.07386363636364	p:0.13994YLR399C	FI:1.01720430107527	p:0.137534YLR403W	FI:0.730188679245283	p:0.0731802YLR404W	FI:1.02978723404255	p:0.233618YLR405W	FI:0.878787878787879	p:0.163019YLR407W	FI:0.416666666666667	p:0.0901033YLR408C	FI:0.7	p:0.239602YLR410W	FI:0.365853658536585	p:0.00756007YLR411W	FI:0.240384615384615	p:0.141544YLR412W	FI:0.972222222222222	p:0.171772YLR414C	FI:0.981818181818182	p:0.22824YLR417W	FI:0.73610361575823	p:0.0956378YLR418C	FI:0.854033290653009	p:0.158067YLR420W	FI:0.708211143695015	p:0.13109YLR421C	FI:0.363636363636364	p:0.126392YLR423C	FI:0.393650793650794	p:0.0153139YLR424W	FI:0.941176470588235	p:0.109052YLR429W	FI:0.408522464103752	p:0.0115676YLR433C	FI:0.559440559440559	p:0.0583455YLR438C-A	FI:0.126984126984127	p:0.0621378YLR438W	FI:0.118421052631579	p:0.00507618YLR439W	FI:0.148194271481943	p:0.000489862YLR441C	FI:0	p:0.17931YLR443W	FI:0.923383084577114	p:0.148775YLR447C	FI:0	p:0.000552752YLR449W	FI:0.585074626865672	p:0.172304YLR452C	FI:0.828571428571429	p:0.104994YLR456W	FI:0.141843971631206	p:0.00383315YLR457C	FI:1.75694444444444	p:0.102142YML001W	FI:0	p:0.131092YML004C	FI:0.873563218390805	p:0.178697YML006C	FI:1.44932432432432	p:0.0652117YML008C	FI:0.654761904761905	p:0.171527YML009C	FI:0	p:0.333333YML010W	FI:0.73905652358691	p:0.102119YML011C	FI:0.775862068965517	p:0.221721YML012W	FI:0.619047619047619	p:0.255079YML013W	FI:1.01449275362319	p:0.13939YML014W	FI:0.866666666666667	p:0.179868YML016C	FI:0.767307692307692	p:0.141709YML018C	FI:0.867768595041322	p:0.162031YML019W	FI:0.543859649122807	p:0.078807YML021C	FI:1.15409836065574	p:0.164521YML022W	FI:1.13793103448276	p:0.25887YML023C	FI:1.38461538461538	p:0.0920342YML027W	FI:0.7	p:0.131234YML028W	FI:3.82352941176471	p:0.189279YML029W	FI:1.17733627667402	p:0.108135YML030W	FI:0.155555555555556	p:0.108336YML031W	FI:0.847058823529412	p:0.12496YML032C	FI:1.70454545454545	p:0.0804833YML035C	FI:1.72394366197183	p:0.111185YML037C	FI:1.11519607843137	p:0.16649YML038C	FI:0.766071428571429	p:0.124409YML041C	FI:0.236065573770492	p:0.00818286YML042W	FI:0.926153846153846	p:0.158536YML043C	FI:0.834365325077399	p:0.115628YML046W	FI:0.888157894736842	p:0.131433YML047C	FI:2.95652173913044	p:0.0175107YML048W	FI:0.708571428571429	p:0.165787YML049C	FI:1.37562437562438	p:0.0404168YML050W	FI:0.656779661016949	p:0.0928625YML052W	FI:2.07547169811321	p:0.197354YML055W	FI:1.52173913043478	p:0.278121YML057W	FI:1.2375	p:0.158404YML058W	FI:0.941176470588235	p:0.365791YML058W-A	FI:	p:0.647059YML060W	FI:0.734463276836158	p:0.119316YML061C	FI:0.7053125	p:0.0689773YML062C	FI:0.728506787330317	p:0.103438YML063W	FI:0	p:0.082353YML064C	FI:2.86363636363636	p:0.233013YML065W	FI:1.46450304259635	p:0.0554619YML066C	FI:1.09565217391304	p:0.217851YML067C	FI:0.472049689440994	p:0.103139YML068W	FI:0.93140589569161	p:0.129008YML070W	FI:0.859504132231405	p:0.193057YML071C	FI:1.1980491942324	p:0.114672YML072C	FI:0.828360673786194	p:0.0718117YML074C	FI:0.831967213114754	p:0.153783YML075C	FI:1.15041208791209	p:0.168757YML076C	FI:1.55471574104502	p:0.0412209YML077W	FI:	p:0.336479YML078W	FI:0.609375	p:0.324353YML079W	FI:0.486486486486487	p:0.128212YML080W	FI:0.633077765607886	p:0.101523YML082W	FI:1.10964912280702	p:0.172034YML083C	FI:0.459770114942529	p:0.0181475YML086C	FI:0.726495726495727	p:0.142465YML087C	FI:1	p:0.205727YML088W	FI:1.39644607843137	p:0.0935134YML091C	FI:0.440025252525252	p:0.000353623YML092C	FI:0	p:0.413793YML093W	FI:1.33950617283951	p:0.0743579YML094W	FI:	p:0.322365YML095C	FI:2.12121212121212	p:0.0802116YML096W	FI:1.21710526315789	p:0.106612YML097C	FI:1.13888888888889	p:0.136946YML098W	FI:0.35	p:0.107723YML099C	FI:0.515151515151515	p:0.0129295YML100W	FI:1.44152046783626	p:0.205306YML101C	FI:0.75	p:0.200629YML102W	FI:0.78375	p:0.113615YML105C	FI:1.13333333333333	p:0.272737YML106W	FI:	p:0.497341YML107C	FI:1.20879120879121	p:0.137234YML108W	FI:0.125	p:0.0190919YML109W	FI:1.14214789652469	p:0.0766288YML110C	FI:0.751851851851852	p:0.222097YML111W	FI:1.27619047619048	p:0.129272YML112W	FI:0.622222222222222	p:0.121549YML114C	FI:0.584347826086956	p:0.0565423YML115C	FI:0.828571428571429	p:0.181105YML116W	FI:1.41132075471698	p:0.167995YML117W	FI:0.769113924050633	p:0.0697116YML118W	FI:1.19496855345912	p:0.105363YML119W	FI:0.506024096385542	p:0.0515505YML120C	FI:0.407986111111111	p:0.0905484YML121W	FI:0.362068965517241	p:0.24369YML123C	FI:3	p:0.0146042YML124C	FI:1.58333333333333	p:0.203395YML125C	FI:0.315270935960591	p:0.0356561YML126C	FI:0.64	p:0.266027YML127W	FI:3.98076923076923	p:0.0323555YML128C	FI:0.404312668463612	p:0.0517963YML129C	FI:0	p:0.476191YML130C	FI:1.60910518053375	p:0.106637YMR002W	FI:0.603448275862069	p:0.310861YMR005W	FI:0.898395721925134	p:0.163881YMR006C	FI:0.774125874125874	p:0.134265YMR008C	FI:0.745011086474501	p:0.128378YMR009W	FI:0.763265306122449	p:0.199055YMR010W	FI:0.241758241758242	p:0.0165245YMR011W	FI:1.53846153846154	p:0.216871YMR013C	FI:0.604395604395604	p:0.05234YMR014W	FI:0.747243772968558	p:0.078162YMR015C	FI:0.671052631578947	p:0.22269YMR016C	FI:0.938271604938272	p:0.106927YMR017W	FI:1.13953488372093	p:0.139515YMR018W	FI:1.34722222222222	p:0.0888221YMR023C	FI:1.3610747051114	p:0.0969664YMR024W	FI:0.549295774647887	p:0.127692YMR025W	FI:0.35546875	p:0.00437608YMR026C	FI:1.14147286821705	p:0.125841YMR027W	FI:1.13888888888889	p:0.19371YMR029C	FI:0.642857142857143	p:0.0565698YMR030W	FI:0.731455399061033	p:0.115799YMR031C	FI:1.12122448979592	p:0.094551YMR032W	FI:0.579449747332959	p:0.0235295YMR034C	FI:0.812199036918138	p:0.177903YMR035W	FI:2.77777777777778	p:0.138111YMR036C	FI:1.3545	p:0.0780745YMR037C	FI:1.41714285714286	p:0.0778678YMR039C	FI:0.444444444444444	p:0.0756845YMR040W	FI:0.954545454545455	p:0.223198YMR041C	FI:0.832282471626734	p:0.148972YMR042W	FI:0.62015503875969	p:0.13256YMR043W	FI:0.982222222222222	p:0.233941YMR044W	FI:1.05617283950617	p:0.132049YMR047C	FI:0.952380952380952	p:0.110442YMR048W	FI:1.49308755760369	p:0.146871YMR049C	FI:0.676691729323308	p:0.0844482YMR052W	FI:1.13513513513514	p:0.205468YMR055C	FI:0.259154929577465	p:0.0475969YMR056C	FI:0.732203389830508	p:0.209264YMR058W	FI:0.309139784946237	p:0.0312296YMR060C	FI:1.52255639097744	p:0.0833416YMR061W	FI:0.513761467889908	p:0.0673224YMR062C	FI:0.431673052362708	p:0.0467981YMR063W	FI:0.525	p:0.17893YMR064W	FI:0.9272030651341	p:0.112282YMR065W	FI:1.4765625	p:0.0891342YMR067C	FI:0.991304347826087	p:0.151325YMR068W	FI:2.10674157303371	p:0.0888243YMR069W	FI:1.26666666666667	p:0.125922YMR070W	FI:1.16166666666667	p:0.124323YMR071C	FI:0.387096774193548	p:0.26938YMR072W	FI:0.683760683760684	p:0.26243YMR073C	FI:1.07142857142857	p:0.223157YMR074C	FI:0.952380952380952	p:0.309608YMR075W	FI:1.38372093023256	p:0.0796184YMR076C	FI:0.919981060606061	p:0.101541YMR077C	FI:0.348837209302326	p:0.0273017YMR079W	FI:0.357142857142857	p:0.2565YMR081C	FI:1.23214285714286	p:0.16791YMR083W	FI:1	p:0.43051YMR086W	FI:1.1573275862069	p:0.072582YMR087W	FI:0.62962962962963	p:0.163917YMR088C	FI:0.357894736842105	p:0.0285686YMR089C	FI:1.28301886792453	p:0.134509YMR090W	FI:0.650510204081633	p:0.147077YMR091C	FI:0.534246575342466	p:0.0534654YMR092C	FI:0.719738276990185	p:0.0981943YMR093W	FI:0.415841584158416	p:0.0731905YMR094W	FI:0.537265660722451	p:0.023183YMR095C	FI:0.486486486486487	p:0.0555642YMR097C	FI:1.921875	p:0.064624YMR098C	FI:1.715625	p:0.0165198YMR099C	FI:2	p:0.214273YMR100W	FI:1.27946127946128	p:0.1255YMR101C	FI:3.0952380952381	p:0.0149383YMR102C	FI:1.16705882352941	p:0.142994YMR104C	FI:0.766059443911793	p:0.108698YMR105C	FI:0.924528301886792	p:0.315217YMR106C	FI:0.882352941176471	p:0.132011YMR107W	FI:0.444444444444444	p:0.237881YMR108W	FI:0.84973544973545	p:0.176347YMR110C	FI:0.892452830188679	p:0.121229YMR111C	FI:1.53846153846154	p:0.0804853YMR112C	FI:	p:YMR113W	FI:1.1	p:0.163271YMR114C	FI:1.43111111111111	p:0.104062YMR115W	FI:0.700471698113208	p:0.0887765YMR117C	FI:0.973913043478261	p:0.233087YMR118C	FI:0.609756097560976	p:0.150018YMR119W	FI:2.61224489795918	p:0.0113894YMR121C	FI:0.625	p:0.325383YMR123W	FI:0.872727272727273	p:0.257455YMR124W	FI:0.823348445595855	p:0.0782787YMR125W	FI:0.903846153846154	p:0.124946YMR126C	FI:1.04761904761905	p:0.223487YMR127C	FI:0.75	p:0.186238YMR128W	FI:1.01626016260163	p:0.094565YMR129W	FI:0.733542319749216	p:0.0486669YMR131C	FI:0.360119047619048	p:0.0396396YMR132C	FI:1.41891891891892	p:0.219201YMR135C	FI:1.0989898989899	p:0.155167YMR136W	FI:0.646923076923077	p:0.0491858YMR138W	FI:1.27439024390244	p:0.173904YMR139W	FI:0.415584415584416	p:0.11947YMR140W	FI:1.22093023255814	p:0.12568YMR144W	FI:0.867768595041322	p:0.162031YMR146C	FI:	p:0.710527YMR148W	FI:0.666666666666667	p:0.267983YMR149W	FI:0.450980392156863	p:0.0269825YMR150C	FI:0.507936507936508	p:0.125657YMR152W	FI:0.324305060584462	p:0.00303793YMR153W	FI:1.30356164383562	p:0.0850378YMR154C	FI:0.967741935483871	p:0.106934YMR155W	FI:0.786255096097845	p:0.111152YMR157C	FI:1.05660377358491	p:0.257965YMR158W	FI:2	p:0.366296YMR159C	FI:1.11111111111111	p:0.260067YMR160W	FI:0.732652342374075	p:0.0866696YMR161W	FI:	p:0.210077YMR163C	FI:0.998759305210918	p:0.110412YMR165C	FI:1.22713414634146	p:0.0833252YMR166C	FI:1.33101851851852	p:0.172261YMR167W	FI:0.642101784534038	p:0.058042YMR168C	FI:1.08211143695015	p:0.150625YMR170C	FI:0.948623853211009	p:0.164386YMR171C	FI:1.32063492063492	p:0.133068YMR172C-A	FI:3.11111111111111	p:0.0782519YMR172W	FI:0.728003784295175	p:0.0611876YMR173W	FI:2.38167938931298	p:0.0364891YMR174C	FI:0.588235294117647	p:0.206706YMR175W	FI:0.181818181818182	p:0.0281875YMR176W	FI:1.15350877192982	p:0.0670748YMR177W	FI:1.13489736070381	p:0.151414YMR181C	FI:1.16666666666667	p:0.342638YMR182C	FI:0.32258064516129	p:0.0286342YMR183C	FI:0.295081967213115	p:0.36358YMR184W	FI:1.03618421052632	p:0.202335YMR185W	FI:0.95	p:0.0891181YMR186W	FI:1.05769230769231	p:0.279758YMR187C	FI:0.928270042194093	p:0.1464YMR188C	FI:0.485714285714286	p:0.102136YMR189W	FI:0.834937083641747	p:0.119402YMR191W	FI:0.905651340996169	p:0.117604YMR192W	FI:1.13936491935484	p:0.0992646YMR193W	FI:2.36065573770492	p:0.102104YMR196W	FI:0.555555555555555	p:0.0609993YMR197C	FI:0.634146341463415	p:0.31249YMR199W	FI:0.86890243902439	p:0.181167YMR200W	FI:0.483516483516484	p:0.104642YMR202W	FI:	p:0.175021YMR203W	FI:0.586666666666667	p:0.250928YMR204C	FI:0.715873015873016	p:0.105389YMR205C	FI:1.07462686567164	p:0.309123YMR206W	FI:1.0534188034188	p:0.185339YMR207C	FI:0.518649014164709	p:0.000327946YMR208W	FI:0.619469026548673	p:0.138259YMR209C	FI:1.94662921348315	p:0.0178865YMR210W	FI:0.778378378378378	p:0.1499YMR211W	FI:1.23232323232323	p:0.104603YMR213W	FI:1.48214285714286	p:0.05693YMR214W	FI:2.07142857142857	p:0.0672726YMR215W	FI:0.459195402298851	p:0.0233341YMR216C	FI:1.59398496240601	p:0.0596996YMR217W	FI:0.444444444444444	p:0.432884YMR218C	FI:0.817972350230415	p:0.0675192YMR220W	FI:1.18222222222222	p:0.126932YMR221C	FI:1.45408163265306	p:0.128078YMR222C	FI:0.404312668463612	p:0.0682511YMR223W	FI:1.32602739726027	p:0.189057YMR224C	FI:0.664961636828644	p:0.0717656YMR225C	FI:1.5	p:0.230559YMR226C	FI:0.75	p:0.268352YMR228W	FI:0.191176470588235	p:0.00215404YMR229C	FI:0.57469387755102	p:0.00410842YMR233W	FI:0.297619047619048	p:0.0104221YMR234W	FI:1.30434782608696	p:0.116789YMR235C	FI:0.785714285714286	p:0.177688YMR237W	FI:1.13366750208855	p:0.159808YMR238W	FI:1.53424657534247	p:0.179823YMR239C	FI:1.50537634408602	p:0.154207YMR240C	FI:1.38373655913978	p:0.0892416YMR241W	FI:0.431818181818182	p:0.432692YMR242C	FI:7.33333333333333	p:0.0538932YMR243C	FI:0.625210084033613	p:0.138018YMR244C-A	FI:0.171428571428571	p:0.0397304YMR244W	FI:0.388505747126437	p:0.0284918YMR246W	FI:0.372047244094488	p:0.0287343YMR250W	FI:2.26865671641791	p:0.0684044YMR251W-A	FI:0.714285714285714	p:0.415385YMR252C	FI:0.482758620689655	p:0.182875YMR253C	FI:0.764966740576497	p:0.137932YMR255W	FI:1.41176470588235	p:0.254925YMR256C	FI:0	p:0.357143YMR257C	FI:0.894409937888199	p:0.104552YMR259C	FI:1.04964717741935	p:0.0732625YMR260C	FI:	p:1YMR261C	FI:2.17580213903743	p:0.021921YMR262W	FI:0.622641509433962	p:0.0970935YMR263W	FI:0.674074074074074	p:0.223339YMR264W	FI:1.20645161290323	p:0.230743YMR265C	FI:1.1716388996513	p:0.0987294YMR266W	FI:0.601092896174863	p:0.0640812YMR267W	FI:1.66037735849057	p:0.0797961YMR268C	FI:0.824552429667519	p:0.111126YMR269W	FI:0.49390243902439	p:0.0539425YMR271C	FI:0.708333333333333	p:0.186034YMR272C	FI:	p:0.175991YMR273C	FI:0.518065268065268	p:0.013933YMR276W	FI:1.1910447761194	p:0.209364YMR277W	FI:0.293706293706294	p:0.0121796YMR278W	FI:1.01709401709402	p:0.175647YMR280C	FI:1.175	p:0.0639726YMR281W	FI:0.819711538461538	p:0.145564YMR282C	FI:1.26047068209015	p:0.0885272YMR283C	FI:0.6578073089701	p:0.0650871YMR284W	FI:0.997144022847817	p:0.126033YMR285C	FI:0.58494623655914	p:0.0432797YMR286W	FI:0.666666666666667	p:0.279446YMR287C	FI:0.610079575596817	p:0.0213543YMR288W	FI:0.629441624365482	p:0.0332163YMR290C	FI:0.952941176470588	p:0.245852YMR291W	FI:1.36554621848739	p:0.201746YMR293C	FI:1.24208375893769	p:0.136163YMR294W	FI:1.08904109589041	p:0.136232YMR295C	FI:	p:0.184808YMR297W	FI:0.96	p:0.320529YMR298W	FI:0.3125	p:0.109827YMR299C	FI:0.601651186790506	p:0.08454YMR300C	FI:0.168674698795181	p:0.151956YMR301C	FI:0.748201438848921	p:0.116554YMR302C	FI:1.09377738825592	p:0.183069YMR303C	FI:3.35593220338983	p:0.0332523YMR305C	FI:1.66153846153846	p:0.121758YMR306W	FI:0.675091383812011	p:0.0349624YMR307W	FI:0.931034482758621	p:0.197849YMR308C	FI:0.940755208333333	p:0.175005YMR309C	FI:1.23940677966102	p:0.216023YMR310C	FI:0.380952380952381	p:0.0155231YMR311C	FI:0.705882352941177	p:0.203019YMR312W	FI:0.662077596996245	p:0.0978268YMR313C	FI:0.876456876456877	p:0.192816YMR314W	FI:0.303571428571429	p:0.286714YMR315W	FI:0.389873417721519	p:0.0297234YMR318C	FI:1.11934156378601	p:0.199653YMR319C	FI:0.492063492063492	p:0.0604081YNL001W	FI:0.825	p:0.246829YNL002C	FI:0.830903790087464	p:0.196877YNL003C	FI:0.839506172839506	p:0.275268YNL004W	FI:0.675	p:0.0922103YNL005C	FI:1.51260504201681	p:0.129685YNL006W	FI:	p:1YNL008C	FI:0.896551724137931	p:0.123951YNL009W	FI:0.736531986531987	p:0.106161YNL010W	FI:0.15625	p:0.0837471YNL012W	FI:0.714	p:0.141974YNL014W	FI:2.34864864864865	p:0.0341464YNL015W	FI:0.4	p:0.372181YNL016W	FI:1.2	p:0.30876YNL020C	FI:0.931372549019608	p:0.128201YNL021W	FI:1.54589371980676	p:0.197357YNL023C	FI:0.890527950310559	p:0.111189YNL024C	FI:1.86046511627907	p:0.233025YNL025C	FI:0.857142857142857	p:0.32613YNL026W	FI:0.714619883040936	p:0.143785YNL027W	FI:1.09009812667261	p:0.121933YNL029C	FI:1.03189300411523	p:0.174283YNL030W	FI:	p:1YNL031C	FI:	p:1YNL032W	FI:0.673469387755102	p:0.172373YNL035C	FI:1.26086956521739	p:0.13907YNL039W	FI:2.59847328244275	p:0.0241266YNL040W	FI:1.03623188405797	p:0.144416YNL042W	FI:1.24657534246575	p:0.224001YNL044W	FI:	p:0.265457YNL045W	FI:0.816129032258065	p:0.104209YNL046W	FI:1.69921875	p:0.114892YNL047C	FI:1.36544250451535	p:0.103367YNL048W	FI:1.05446623093682	p:0.131975YNL051W	FI:0.746296296296296	p:0.096783YNL052W	FI:1.81818181818182	p:0.373023YNL053W	FI:1.19772727272727	p:0.145452YNL056W	FI:0.727272727272727	p:0.279149YNL058C	FI:0.50989010989011	p:0.0504235YNL059C	FI:1.25891472868217	p:0.1873YNL061W	FI:0.829268292682927	p:0.259441YNL062C	FI:1.03504043126685	p:0.186353YNL063W	FI:2.10905349794239	p:0.038377YNL064C	FI:0.631578947368421	p:0.196861YNL065W	FI:0.835714285714286	p:0.17015YNL067W	FI:	p:0.247357YNL068C	FI:0.541189931350114	p:0.0226138YNL070W	FI:0	p:0.5YNL071W	FI:0.461538461538462	p:0.0357232YNL072W	FI:1.2	p:0.210368YNL073W	FI:0.915234375	p:0.122109YNL074C	FI:0.537037037037037	p:0.0621543YNL075W	FI:0.51219512195122	p:0.458333YNL076W	FI:1.26458157227388	p:0.121415YNL077W	FI:3.06666666666667	p:0.0194728YNL078W	FI:0.931372549019608	p:0.124631YNL080C	FI:0.822448979591837	p:0.189129YNL081C	FI:0.294117647058823	p:0.0981862YNL082W	FI:1.36585968379447	p:0.0578418YNL083W	FI:1.11661166116612	p:0.16384YNL085W	FI:1.42991202346041	p:0.0906957YNL087W	FI:0.904306220095694	p:0.110976YNL088W	FI:0.809183064997019	p:0.0769328YNL090W	FI:0.186046511627907	p:0.178001YNL091W	FI:0.774276252537122	p:0.0552296YNL092W	FI:0.704819277108434	p:0.151348YNL093W	FI:0.735459662288931	p:0.144135YNL094W	FI:1.20689655172414	p:0.130752YNL095C	FI:1.57407407407407	p:0.0888718YNL098C	FI:1.11111111111111	p:0.209261YNL099C	FI:1.06944444444444	p:0.264145YNL100W	FI:0.638888888888889	p:0.105483YNL101W	FI:0.612903225806452	p:0.104219YNL102W	FI:0.732631578947368	p:0.0831898YNL104C	FI:0.862745098039216	p:0.218449YNL106C	FI:0.92998510321345	p:0.0870077YNL107W	FI:0.802083333333333	p:0.220092YNL108C	FI:1.38666666666667	p:0.194787YNL110C	FI:0.791666666666667	p:0.189247YNL111C	FI:	p:0.261539YNL112W	FI:16.28	p:0.000115923YNL113W	FI:0.954545454545454	p:0.449658YNL115C	FI:0.6975	p:0.129894YNL117W	FI:0.377777777777778	p:0.058517YNL118C	FI:0.809311224489796	p:0.0660544YNL119W	FI:0.818713450292398	p:0.11265YNL121C	FI:0.641509433962264	p:0.0953113YNL122C	FI:0.373333333333333	p:0.152271YNL123W	FI:1.19159159159159	p:0.145579YNL124W	FI:1.30929264909847	p:0.107135YNL125C	FI:1.53409090909091	p:0.0761423YNL127W	FI:1.26805213103205	p:0.0927157YNL128W	FI:0.6875	p:0.0909566YNL129W	FI:0.857142857142857	p:0.224432YNL130C	FI:1.42857142857143	p:0.177021YNL131W	FI:0.827586206896552	p:0.338859YNL132W	FI:0.627450980392157	p:0.120187YNL133C	FI:0.931263858093126	p:0.182976YNL134C	FI:0.45	p:0.105211YNL135C	FI:	p:0.5625YNL136W	FI:0.487272727272727	p:0.0219559YNL137C	FI:0.818181818181818	p:0.121049YNL138W	FI:1.46320754716981	p:0.0995397YNL139C	FI:0.7429614181439	p:0.0451169YNL141W	FI:1.23582089552239	p:0.150954YNL142W	FI:0.225	p:0.0311242YNL144C	FI:1.25034482758621	p:0.0731437YNL148C	FI:0.871459694989107	p:0.142168YNL149C	FI:3.375	p:0.102096YNL151C	FI:1.68	p:0.25321YNL152W	FI:2	p:0.0444944YNL153C	FI:0.631578947368421	p:0.289701YNL154C	FI:1.40625	p:0.194768YNL155W	FI:2.29090909090909	p:0.0388959YNL156C	FI:1.84210526315789	p:0.226132YNL157W	FI:3.70588235294118	p:0.166948YNL158W	FI:1.51111111111111	p:0.12406YNL159C	FI:0.694214876033058	p:0.133264YNL160W	FI:1.625	p:0.143716YNL161W	FI:0.419408812046849	p:0.0234329YNL162W-A	FI:0.7	p:0.305416YNL163C	FI:0.751179245283019	p:0.06779YNL164C	FI:2.01412429378531	p:0.0726362YNL167C	FI:0.754901960784314	p:0.0932573YNL169C	FI:0.704861111111111	p:0.169714YNL172W	FI:0.86492771841609	p:0.0650835YNL173C	FI:1.11392405063291	p:0.127831YNL174W	FI:1.55555555555556	p:0.139292YNL175C	FI:1.68421052631579	p:0.071652YNL178W	FI:0	p:0.5YNL180C	FI:0.750617283950617	p:0.167004YNL181W	FI:0.777142857142857	p:0.206947YNL182C	FI:1.4792899408284	p:0.076075YNL183C	FI:0.715264187866927	p:0.150189YNL185C	FI:1.89655172413793	p:0.356479YNL186W	FI:0.750579150579151	p:0.0830713YNL188W	FI:1.64252179745138	p:0.0403988YNL189W	FI:1.64705882352941	p:0.374784YNL191W	FI:1.9672131147541	p:0.215863YNL192W	FI:1.11604774535809	p:0.128019YNL193W	FI:0.855263157894737	p:0.109876YNL196C	FI:0.626865671641791	p:0.0755295YNL197C	FI:1.21212121212121	p:0.165483YNL199C	FI:0.829482948294829	p:0.159162YNL200C	FI:0.663157894736842	p:0.209692YNL201C	FI:0.577777777777778	p:0.0364859YNL202W	FI:0.608695652173913	p:0.17026YNL204C	FI:0.902777777777778	p:0.17863YNL206C	FI:1.97542735042735	p:0.0360823YNL207W	FI:0.696117804551539	p:0.134425YNL208W	FI:0.95625	p:0.259048YNL209W	FI:0.714285714285714	p:0.493521YNL210W	FI:1.65413533834586	p:0.0659187YNL211C	FI:1.6	p:0.446617YNL212W	FI:0.617073170731707	p:0.08673YNL213C	FI:0.814968814968815	p:0.164458YNL214W	FI:0.909560723514212	p:0.195951YNL215W	FI:1.49152542372881	p:0.110739YNL216W	FI:1.18944281524927	p:0.13399YNL217W	FI:1.004329004329	p:0.198622YNL218W	FI:1.734375	p:0.0918434YNL219C	FI:1.07089947089947	p:0.168487YNL220W	FI:0.310344827586207	p:0.106947YNL221C	FI:1.34450261780105	p:0.0686624YNL222W	FI:0.0357142857142857	p:0.00158423YNL223W	FI:0.863553113553113	p:0.143956YNL224C	FI:1.04212678936605	p:0.103509YNL225C	FI:1.02941176470588	p:0.132205YNL227C	FI:0.963937621832359	p:0.130346YNL229C	FI:0.529100529100529	p:0.227237YNL230C	FI:0.659722222222222	p:0.0926188YNL231C	FI:0.578389830508475	p:0.118063YNL232W	FI:1.18598382749326	p:0.188487YNL233W	FI:1.17687661777394	p:0.0737043YNL234W	FI:1.24249084249084	p:0.117595YNL236W	FI:0.752941176470588	p:0.103988YNL237W	FI:0.753640776699029	p:0.152775YNL238W	FI:0.781609195402299	p:0.099471YNL240C	FI:1.17647058823529	p:0.16467YNL242W	FI:1.16521739130435	p:0.0781376YNL243W	FI:2.14432989690722	p:0.103089YNL244C	FI:0	p:0.538462YNL245C	FI:1.1875	p:0.233047YNL246W	FI:0.727272727272727	p:0.195998YNL250W	FI:0.84	p:0.0856672YNL251C	FI:0.359504132231405	p:0.014983YNL252C	FI:0.814814814814815	p:0.16992YNL253W	FI:1.01767676767677	p:0.17334YNL254C	FI:1.24470588235294	p:0.118894YNL255C	FI:0.466666666666667	p:0.463768YNL256W	FI:0.47953216374269	p:0.00356627YNL257C	FI:0.568934863423307	p:0.0173728YNL258C	FI:0.792571428571429	p:0.0863241YNL259C	FI:0.4375	p:0.202675YNL260C	FI:0.553359683794466	p:0.0976382YNL261W	FI:1.37142857142857	p:0.196287YNL262W	FI:0.805077327108258	p:0.073783YNL263C	FI:2.03389830508475	p:0.199394YNL264C	FI:1.41538461538462	p:0.200849YNL265C	FI:0.875576036866359	p:0.194624YNL267W	FI:1.45454545454545	p:0.0760112YNL270C	FI:0.605769230769231	p:0.0540256YNL271C	FI:1.3644801980198	p:0.0437788YNL272C	FI:0.887464387464387	p:0.107034YNL273W	FI:1.2604257438068	p:0.0606539YNL275W	FI:0.520522388059702	p:0.0296069YNL277W	FI:0.292452830188679	p:0.00950865YNL279W	FI:0.663982102908277	p:0.0751167YNL280C	FI:4.20779220779221	p:0.107372YNL281W	FI:0.421052631578947	p:0.229422YNL282W	FI:1.25	p:0.261424YNL283C	FI:1.17073170731707	p:0.140189YNL284C	FI:1.37878787878788	p:0.142734YNL286W	FI:1.00683994528044	p:0.156753YNL287W	FI:5.18831168831169	p:0.0529912YNL288W	FI:0.634375	p:0.18961YNL289W	FI:3.69230769230769	p:0.148733YNL290W	FI:1.06666666666667	p:0.269389YNL291C	FI:0.964218455743879	p:0.167861YNL292W	FI:3.45205479452055	p:0.0254362YNL293W	FI:0.79646017699115	p:0.159724YNL294C	FI:1.0702479338843	p:0.153706YNL295W	FI:0.628600823045268	p:0.0599926YNL298W	FI:2.96381578947368	p:0.00605671YNL299W	FI:0.489738805970149	p:0.0289165YNL300W	FI:1.57575757575758	p:0.245686YNL304W	FI:3.21621621621622	p:0.0182196YNL305C	FI:0.8	p:0.214739YNL306W	FI:1.57407407407407	p:0.15283YNL307C	FI:0.319298245614035	p:0.0642448YNL308C	FI:0.675551470588235	p:0.0729264YNL309W	FI:0.808362369337979	p:0.180937YNL310C	FI:3.07758620689655	p:0.0404397YNL311C	FI:0.763473053892216	p:0.0904304YNL312W	FI:3.65853658536585	p:0.171343YNL313C	FI:0.56	p:0.0250649YNL315C	FI:0.971111111111111	p:0.20604YNL317W	FI:1.83529411764706	p:0.22902YNL318C	FI:0.82995951417004	p:0.133113YNL320W	FI:0.9	p:0.24921YNL321W	FI:0	p:0.63373YNL322C	FI:2.65909090909091	p:0.169908YNL323W	FI:0	p:0.944445YNL325C	FI:	p:YNL326C	FI:	p:YNL327W	FI:2.3671875	p:0.225684YNL328C	FI:	p:0.142857YNL329C	FI:0.895833333333333	p:0.41622YNL330C	FI:0	p:0.969697YNL332W	FI:0	p:0.133965YNL333W	FI:0.75765306122449	p:0.185266YNL334C	FI:2.16049382716049	p:0.0434881YNL335W	FI:0.4	p:0.0251353YNR001C	FI:1.1	p:0.326468YNR002C	FI:0.287179487179487	p:0.109406YNR003C	FI:0.818181818181818	p:0.326654YNR004W	FI:0.563049853372434	p:0.111623YNR006W	FI:0.940781440781441	p:0.145329YNR007C	FI:2.19230769230769	p:0.181518YNR008W	FI:0.939221272554606	p:0.168645YNR009W	FI:1.98412698412698	p:0.0787932YNR010W	FI:0.46875	p:0.12719YNR011C	FI:0.729064039408867	p:0.0563553YNR012W	FI:0.30377358490566	p:0.00953176YNR013C	FI:0.8862897985705	p:0.160243YNR014W	FI:1.2375	p:0.242607YNR015W	FI:0.461538461538462	p:0.037278YNR016C	FI:1.06029850746269	p:0.204015YNR017W	FI:0.643939393939394	p:0.259985YNR018W	FI:0	p:0.044757YNR020C	FI:3.94545454545455	p:0.016462YNR021W	FI:0.98972602739726	p:0.187639YNR022C	FI:0.683908045977011	p:0.208202YNR023W	FI:1.2974358974359	p:0.137497YNR024W	FI:0.790123456790123	p:0.187211YNR026C	FI:0.621664050235479	p:0.0763167YNR027W	FI:3.5	p:0.0013354YNR028W	FI:2.09852216748768	p:0.0214296YNR030W	FI:0.71484593837535	p:0.0951086YNR031C	FI:0.640776699029126	p:0.0596764YNR032C-A	FI:0	p:0.272727YNR032W	FI:0.963855421686747	p:0.316193YNR033W	FI:0.766488413547237	p:0.0825065YNR034W-A	FI:	p:0.761905YNR035C	FI:	p:0.0473066YNR036C	FI:2.36842105263158	p:0.333808YNR037C	FI:0	p:0.32353YNR038W	FI:1.21311475409836	p:0.11303YNR039C	FI:0.775401069518717	p:0.13123YNR040W	FI:0.682098765432099	p:0.122909YNR041C	FI:0.781954887218045	p:0.177828YNR043W	FI:0.206572769953052	p:0.0194101YNR045W	FI:0.397707231040564	p:0.00518413YNR046W	FI:1.52	p:0.432933YNR047W	FI:1.21749755620723	p:0.126406YNR048W	FI:1.30784708249497	p:0.163404YNR049C	FI:0.384146341463415	p:0.0590846YNR050C	FI:0.741573033707865	p:0.251636YNR051C	FI:0.8	p:0.111765YNR052C	FI:1.43283582089552	p:0.285391YNR054C	FI:0.721393034825871	p:0.149444YNR055C	FI:1.19166666666667	p:0.253366YNR056C	FI:0.638655462184874	p:0.139287YNR057C	FI:1.1	p:0.241851YNR059W	FI:1.51975683890577	p:0.0362533YNR060W	FI:1.87187187187187	p:0.045968YNR061C	FI:0.302469135802469	p:0.00887452YNR063W	FI:0.76	p:0.122909YNR064C	FI:0.466666666666667	p:0.160463YNR067C	FI:0.932295352677518	p:0.105932YNR072W	FI:1.96363636363636	p:0.19341YNR073C	FI:2.27368421052632	p:0.109897YNR074C	FI:0.852713178294574	p:0.150268YOL001W	FI:0.491489361702128	p:0.0775125YOL002C	FI:0.555555555555556	p:0.158563YOL005C	FI:0	p:0.0241936YOL007C	FI:0.391640866873065	p:0.0108613YOL008W	FI:0.947368421052632	p:0.214137YOL009C	FI:1.43181818181818	p:0.136828YOL012C	FI:0.4375	p:0.453333YOL013C	FI:1.01258894362343	p:0.12918YOL018C	FI:0.767543859649123	p:0.158042YOL021C	FI:0.703812316715542	p:0.108961YOL022C	FI:0.987179487179487	p:0.30031YOL030W	FI:0.670731707317073	p:0.236628YOL031C	FI:1.2	p:0.223982YOL032W	FI:0.332225913621262	p:0.0481368YOL036W	FI:0.847252481239409	p:0.0964395YOL038W	FI:0.0833333333333333	p:0.03599YOL039W	FI:0.285714285714286	p:0.20362YOL040C	FI:	p:0.082353YOL041C	FI:1.00941176470588	p:0.168856YOL042W	FI:0.287179487179487	p:0.018146YOL043C	FI:0.352226720647773	p:0.00480165YOL044W	FI:1.38125	p:0.0941142YOL045W	FI:1.0625	p:0.127382YOL049W	FI:0.654444444444444	p:0.0655926YOL056W	FI:0.774410774410774	p:0.151866YOL057W	FI:0.578925095217943	p:0.0414078YOL058W	FI:0.320987654320988	p:0.0270916YOL059W	FI:0.827586206896552	p:0.279802YOL060C	FI:0.859416445623342	p:0.133152YOL061W	FI:0.463768115942029	p:0.206842YOL062C	FI:0.0915384615384616	p:6.01689e-06YOL063C	FI:0.398768033716972	p:0.00058803YOL064C	FI:0.616071428571429	p:0.133973YOL065C	FI:0.408163265306122	p:0.0282268YOL067C	FI:0.2625	p:0.0865597YOL068C	FI:0.414245548266167	p:0.027284YOL071W	FI:0.369230769230769	p:0.0991629YOL072W	FI:0.834697217675941	p:0.137905YOL073C	FI:0.381172839506173	p:0.034755YOL075C	FI:0.763358778625954	p:0.0861005YOL077C	FI:0.235294117647059	p:0.0667797YOL077W-A	FI:0.15	p:0.196429YOL078W	FI:0.87136086662153	p:0.0906711YOL082W	FI:1.24789915966387	p:0.108379YOL086C	FI:0.686274509803922	p:0.230329YOL086W-A	FI:1.01680672268908	p:0.242428YOL087C	FI:0.54140127388535	p:0.0109777YOL088C	FI:0.779487179487179	p:0.138635YOL091W	FI:0.603603603603604	p:0.0447086YOL092W	FI:0.326086956521739	p:0.0147176YOL097C	FI:0.658536585365854	p:0.356359YOL100W	FI:0.769692186266772	p:0.0591171YOL101C	FI:0.609523809523809	p:0.148273YOL107W	FI:0.735607675906183	p:0.171616YOL108C	FI:0.684210526315789	p:0.177471YOL110W	FI:0.538461538461539	p:0.104321YOL111C	FI:1.29699248120301	p:0.183995YOL112W	FI:0.902610441767068	p:0.154247YOL113W	FI:0.895927601809955	p:0.137261YOL114C	FI:0.866666666666667	p:0.169739YOL115W	FI:0.675544794188862	p:0.0869212YOL116W	FI:0.253521126760563	p:0.00293774YOL119C	FI:0.216346153846154	p:0.000336972YOL121C	FI:3.33333333333333	p:0.165039YOL122C	FI:0.606060606060606	p:0.0955254YOL124C	FI:0.269662921348315	p:0.00261742YOL125W	FI:0.393858477970628	p:0.00650496YOL126C	FI:0.269230769230769	p:0.0153127YOL128C	FI:0.71830985915493	p:0.105674YOL129W	FI:0.333333333333333	p:0.208525YOL130W	FI:1.16741182314953	p:0.134369YOL131W	FI:1.29813664596273	p:0.203448YOL132W	FI:1.44329896907216	p:0.121817YOL133W	FI:0.5625	p:0.336083YOL135C	FI:0.33806146572104	p:0.0320364YOL137W	FI:0.401913875598086	p:0.00653913YOL138C	FI:1.4535864978903	p:0.0419856YOL139C	FI:	p:1YOL140W	FI:0.613333333333333	p:0.121699YOL144W	FI:0.615168539325843	p:0.0369399YOL145C	FI:0.215311004784689	p:0.00105189YOL146W	FI:0.587096774193548	p:0.193649YOL147C	FI:0.642857142857143	p:0.211752YOL149W	FI:0.105263157894737	p:0.237154YOL152W	FI:0.911764705882353	p:0.11515YOL153C	FI:	p:YOL156W	FI:1.18518518518519	p:0.249766YOR001W	FI:1.04983639567078	p:0.107384YOR002W	FI:0.710470085470085	p:0.134012YOR003W	FI:0.454293628808864	p:0.0280878YOR004W	FI:1.4	p:0.252708YOR006C	FI:0.818181818181818	p:0.15826YOR007C	FI:0.343915343915344	p:0.0756407YOR008C	FI:3.64788732394366	p:0.0207865YOR016C	FI:0.215686274509804	p:0.0312334YOR017W	FI:1.203125	p:0.0889282YOR020C	FI:0	p:0.217391YOR021C	FI:0.34468085106383	p:0.0784828YOR023C	FI:0.912399492170969	p:0.126132YOR025W	FI:0.576576576576577	p:0.0996673YOR026W	FI:0.678125	p:0.10087YOR028C	FI:0.430434782608696	p:0.0465787YOR030W	FI:0.464285714285714	p:0.0251321YOR032C	FI:0.8625	p:0.139867YOR033C	FI:0.545977011494253	p:0.0931181YOR034C	FI:1.32993197278912	p:0.0754504YOR036W	FI:0.403846153846154	p:0.0423524YOR038C	FI:0.665509259259259	p:0.0486452YOR039W	FI:0.163265306122449	p:0.263004YOR040W	FI:0.517857142857143	p:0.0689797YOR044W	FI:1.02857142857143	p:0.241966YOR046C	FI:0.391608391608392	p:0.0239569YOR048C	FI:0.708433734939759	p:0.0856093YOR049C	FI:0.253164556962025	p:0.00255844YOR052C	FI:0	p:0.00728292YOR056C	FI:0.547008547008547	p:0.060681YOR057W	FI:0.514285714285714	p:0.162737YOR058C	FI:0.617647058823529	p:0.0251691YOR059C	FI:0.952380952380952	p:0.329561YOR060C	FI:0.664893617021277	p:0.123157YOR061W	FI:0.104166666666667	p:0.0346646YOR062C	FI:0.881481481481482	p:0.158781YOR065W	FI:0.308108108108108	p:0.0948766YOR066W	FI:0.410509031198686	p:0.00630356YOR067C	FI:0.456730769230769	p:0.0644686YOR069W	FI:1.0413744740533	p:0.119974YOR070C	FI:0.630208333333333	p:0.103342YOR075W	FI:1.34865900383142	p:0.143239YOR076C	FI:0.994736842105263	p:0.0992584YOR077W	FI:1.84166666666667	p:0.107876YOR078W	FI:0.476190476190476	p:0.0609352YOR079C	FI:0.492307692307692	p:0.0727434YOR080W	FI:1.67905405405405	p:0.0474153YOR084W	FI:0.522727272727273	p:0.046315YOR085W	FI:0.324324324324324	p:0.103176YOR086C	FI:0.532786885245902	p:0.037239YOR089C	FI:0.376344086021505	p:0.0911976YOR090C	FI:0.254959349593496	p:5.85674e-05YOR091W	FI:0.34375	p:0.0276149YOR092W	FI:0.670168067226891	p:0.074935YOR094W	FI:0	p:0.254791YOR097C	FI:0.266862170087977	p:0.0170366YOR099W	FI:0.59973924380704	p:0.0864615YOR100C	FI:1.17446808510638	p:0.222401YOR101W	FI:0.390804597701149	p:0.0107309YOR102W	FI:1.66666666666667	p:0.318841YOR103C	FI:0.333333333333333	p:0.118555YOR106W	FI:0.557377049180328	p:0.0898064YOR108W	FI:0.233590733590734	p:0.000966818YOR110W	FI:0.559289790741915	p:0.0429722YOR111W	FI:0.477040816326531	p:0.0871238YOR112W	FI:0.930704898446834	p:0.108167YOR114W	FI:1.03309692671395	p:0.18802YOR115C	FI:0.704166666666667	p:0.148379YOR116C	FI:0.157800358637179	p:1.15071e-07YOR117W	FI:0.151898734177215	p:0.134771YOR125C	FI:0.841836734693878	p:0.246364YOR126C	FI:0.574468085106383	p:0.121438YOR128C	FI:2.46551724137931	p:0.249036YOR130C	FI:0.247619047619048	p:0.0862426YOR131C	FI:0.222857142857143	p:0.01053YOR133W	FI:0.120527306967985	p:0.00101357YOR134W	FI:0.555882352941176	p:0.0563078YOR135C	FI:0.8125	p:0.239802YOR136W	FI:0.166071428571429	p:0.00729168YOR137C	FI:0.623501199040767	p:0.0440363YOR138C	FI:0.579075425790754	p:0.0270005YOR141C	FI:0.813666784078901	p:0.106644YOR142W	FI:2.33333333333333	p:0.283335YOR145C	FI:1.44444444444444	p:0.23602YOR148C	FI:0.96969696969697	p:0.181722YOR151C	FI:0.156716417910448	p:0.013555YOR152C	FI:1.105	p:0.231733YOR157C	FI:0.0909090909090909	p:0.00460463YOR159C	FI:0.875	p:0.385376YOR160W	FI:1.14043392504931	p:0.127224YOR163W	FI:0.107142857142857	p:0.00247909YOR166C	FI:0.892456676860347	p:0.137742YOR167C	FI:0	p:0.714286YOR168W	FI:0.91	p:0.172547YOR171C	FI:0.456140350877193	p:0.0115135YOR173W	FI:0.43064182194617	p:0.0764551YOR174W	FI:1.065625	p:0.226386YOR175C	FI:1.03030303030303	p:0.173235YOR176W	FI:0.203636363636364	p:0.00238735YOR177C	FI:1.31313131313131	p:0.126253YOR180C	FI:0.39375	p:0.0170131YOR184W	FI:0.932835820895522	p:0.209023YOR185C	FI:0	p:0.30303YOR187W	FI:0.627450980392157	p:0.166507YOR189W	FI:0.270833333333333	p:0.216157YOR190W	FI:1.17732558139535	p:0.168094YOR192C	FI:1.43362831858407	p:0.145267YOR194C	FI:0.261538461538461	p:0.00492811YOR196C	FI:0.791208791208791	p:0.134716YOR197W	FI:0.765217391304348	p:0.216176YOR198C	FI:0.394736842105263	p:0.0950877YOR201C	FI:0.65382557517389	p:0.0644372YOR205C	FI:0.955656108597285	p:0.132041YOR207C	FI:0.458333333333333	p:0.166418YOR209C	FI:0.51	p:0.0795677YOR210W	FI:0	p:0.307692YOR211C	FI:1.88258706467662	p:0.0797968YOR214C	FI:1.11616161616162	p:0.168586YOR215C	FI:0.503225806451613	p:0.16647YOR217W	FI:0.743589743589744	p:0.10945YOR219C	FI:1.19493908153702	p:0.0922586YOR221C	FI:0.328638497652582	p:0.0215499YOR222W	FI:0.345454545454545	p:0.0502961YOR223W	FI:0.455710955710956	p:0.0369882YOR224C	FI:0.346153846153846	p:0.252763YOR226C	FI:0.277777777777778	p:0.0619995YOR228C	FI:0.431034482758621	p:0.0211527YOR230W	FI:0.405448717948718	p:0.049381YOR231W	FI:0.579639889196676	p:0.0493346YOR232W	FI:2.76923076923077	p:0.131969YOR236W	FI:1.77777777777778	p:0.200628YOR238W	FI:1.03846153846154	p:0.23484YOR242C	FI:0.315897435897436	p:0.0131282YOR243C	FI:1.09454545454545	p:0.149446YOR244W	FI:0.818604651162791	p:0.213236YOR245C	FI:0.779220779220779	p:0.149022YOR247W	FI:0.626373626373626	p:0.164019YOR250C	FI:0.305555555555556	p:0.0198054YOR252W	FI:0.387096774193548	p:0.119638YOR253W	FI:0.246305418719212	p:0.0340876YOR255W	FI:0.760344827586207	p:0.115479YOR256C	FI:0.891304347826087	p:0.105675YOR257W	FI:0.121212121212121	p:0.0730144YOR258W	FI:1.496	p:0.17886YOR259C	FI:0.641025641025641	p:0.283603YOR261C	FI:0.612021857923497	p:0.153607YOR262W	FI:0.284810126582278	p:0.0394093YOR264W	FI:1.26482213438735	p:0.0952195YOR265W	FI:0.340909090909091	p:0.149685YOR269W	FI:0.627906976744186	p:0.0644034YOR270C	FI:0.388888888888889	p:0.0286062YOR271C	FI:0.428571428571429	p:0.372676YOR273C	FI:1.54521963824289	p:0.0680325YOR274W	FI:0.72742474916388	p:0.113624YOR276W	FI:2.35294117647059	p:0.321896YOR278W	FI:0.4	p:0.0893699YOR279C	FI:0.974025974025974	p:0.197829YOR280C	FI:0.570532915360502	p:0.0909668YOR281C	FI:0.141509433962264	p:9.56547e-05YOR283W	FI:0.4375	p:0.119987YOR285W	FI:1.56481481481481	p:0.205083YOR286W	FI:0.729166666666667	p:0.278062YOR287C	FI:0.728571428571429	p:0.129374YOR288C	FI:1.11885245901639	p:0.182795YOR289W	FI:1.41489361702128	p:0.147114YOR290C	FI:0.81766430912541	p:0.0659366YOR291W	FI:0.482030548068284	p:0.00279465YOR292C	FI:0.436781609195402	p:0.0578191YOR295W	FI:1.37009803921569	p:0.144563YOR297C	FI:0.692307692307692	p:0.195362YOR298C-A	FI:0	p:0.00995733YOR298W	FI:0.483516483516484	p:0.0174583YOR301W	FI:0.339230769230769	p:0.0122197YOR303W	FI:0.826086956521739	p:0.179895YOR304C-A	FI:1.11111111111111	p:0.328503YOR305W	FI:0.76048951048951	p:0.147204YOR306C	FI:0.273684210526316	p:0.000422106YOR307C	FI:0.597802197802198	p:0.0938274YOR311C	FI:0.914814814814815	p:0.218628YOR312C	FI:	p:1YOR313C	FI:1.04072398190045	p:0.145235YOR315W	FI:2.80701754385965	p:0.0398164YOR317W	FI:0.41779497098646	p:0.0222539YOR319W	FI:1.03779069767442	p:0.198154YOR320C	FI:0.76140350877193	p:0.0977368YOR321W	FI:0.547945205479452	p:0.0421732YOR322C	FI:0.870229007633588	p:0.132276YOR323C	FI:0.183333333333333	p:0.0192075YOR325W	FI:0.578512396694215	p:0.142866YOR327C	FI:0.529411764705882	p:0.343186YOR328W	FI:0.711229946524064	p:0.0342872YOR329C	FI:1.01382488479263	p:0.116871YOR330C	FI:0.45468509984639	p:0.00152793YOR331C	FI:1.38888888888889	p:0.289224YOR332W	FI:0.275862068965517	p:0.127409YOR334W	FI:0.567056705670567	p:0.0720501YOR335C	FI:0.48502994011976	p:0.0656271YOR336W	FI:1.20924985899605	p:0.0679785YOR337W	FI:0.323076923076923	p:0.00541339YOR338W	FI:0.30976430976431	p:0.0434097YOR339C	FI:0.777777777777778	p:0.224509YOR340C	FI:0.893617021276596	p:0.215831YOR341W	FI:0.539498141263941	p:0.0277592YOR342C	FI:0.832535885167464	p:0.161874YOR344C	FI:0.719148936170213	p:0.208263YOR347C	FI:1.39449541284404	p:0.193058YOR348C	FI:0.174657534246575	p:0.000175974YOR349W	FI:1.00401606425703	p:0.114199YOR351C	FI:0.440613026819923	p:0.0418441YOR352W	FI:0.391705069124424	p:0.0194902YOR353C	FI:0.86144578313253	p:0.138604YOR355W	FI:0.365532879818594	p:0.00291411YOR357C	FI:2.10526315789474	p:0.350055YOR358W	FI:0.4	p:0.0413631YOR359W	FI:1.25263157894737	p:0.139217YOR361C	FI:0.474489795918367	p:0.057222YOR362C	FI:0.212053571428571	p:0.0110005YOR365C	FI:0.616161616161616	p:0.0291859YOR367W	FI:0.476190476190476	p:0.106802YOR368W	FI:0.715447154471545	p:0.13978YOR369C	FI:0	p:0.0649351YOR370C	FI:0.220338983050847	p:0.0489066YOR372C	FI:0.472280998730427	p:0.0165664YOR373W	FI:0.613207547169811	p:0.0332579YOR374W	FI:0.692307692307692	p:0.225794YOR375C	FI:0.832835820895522	p:0.223544YOR377W	FI:1.21847389558233	p:0.122901YOR378W	FI:0.226415094339623	p:0.00294554YOR382W	FI:0.266666666666667	p:0.0613294YOR383C	FI:0.672514619883041	p:0.158275YOR386W	FI:0.768382352941177	p:0.109263YOR387C	FI:0.666666666666667	p:0.289701YOR390W	FI:0.62962962962963	p:0.137023YOR391C	FI:0.21301775147929	p:0.00220811YPL001W	FI:0.698412698412698	p:0.118264YPL002C	FI:0.581395348837209	p:0.24327YPL003W	FI:0.510144927536232	p:0.024876YPL004C	FI:0.857142857142857	p:0.247789YPL005W	FI:1.12570356472795	p:0.140266YPL006W	FI:0.853615520282187	p:0.0978942YPL007C	FI:1.01641414141414	p:0.123948YPL008W	FI:0.895956607495069	p:0.105732YPL009C	FI:1.24285714285714	p:0.0710366YPL010W	FI:2.4	p:0.316905YPL011C	FI:1.33246753246753	p:0.187374YPL013C	FI:0.757894736842105	p:0.256017YPL014W	FI:0.657142857142857	p:0.113749YPL015C	FI:1.1875	p:0.153318YPL017C	FI:0.949433962264151	p:0.118932YPL019C	FI:0.855704697986577	p:0.163629YPL020C	FI:1.59123055162659	p:0.059571YPL021W	FI:0.58125	p:0.0861972YPL022W	FI:0.931818181818182	p:0.105332YPL023C	FI:0.692640692640693	p:0.110747YPL024W	FI:0.944444444444444	p:0.223475YPL026C	FI:0.372932330827068	p:0.0114638YPL027W	FI:1.13174603174603	p:0.154101YPL028W	FI:0.347826086956522	p:0.110273YPL029W	FI:1.4910394265233	p:0.0945523YPL031C	FI:1.24293785310734	p:0.259636YPL032C	FI:1.57211870255349	p:0.0812212YPL034W	FI:0.8	p:0.224151YPL037C	FI:	p:1YPL038W	FI:0.263157894736842	p:0.0183793YPL040C	FI:0.881133800670527	p:0.0868913YPL041C	FI:1.38947368421053	p:0.199793YPL042C	FI:0.58958958958959	p:0.0933673YPL043W	FI:1.03349875930521	p:0.144801YPL045W	FI:0.41948051948052	p:0.00177895YPL046C	FI:0.857142857142857	p:0.307303YPL047W	FI:0.145833333333333	p:0.116239YPL048W	FI:1.05	p:0.184866YPL049C	FI:0.809847198641766	p:0.1139YPL050C	FI:0.373333333333333	p:0.0635536YPL051W	FI:1.33333333333333	p:0.417542YPL054W	FI:1.02813852813853	p:0.196672YPL055C	FI:1.2014652014652	p:0.141805YPL059W	FI:2.91666666666667	p:0.245067YPL060W	FI:0.417391304347826	p:0.0366465YPL061W	FI:0.454212454212454	p:0.0839455YPL063W	FI:2.81081081081081	p:0.0342429YPL065W	FI:1.36888888888889	p:0.196599YPL066W	FI:0.607966457023061	p:0.0529794YPL067C	FI:0.838961038961039	p:0.177496YPL068C	FI:1.04166666666667	p:0.141915YPL069C	FI:0.488888888888889	p:0.0853173YPL070W	FI:1.724399494311	p:0.0829004YPL071C	FI:0.547368421052632	p:0.140688YPL072W	FI:1.04081632653061	p:0.114359YPL074W	FI:0.978723404255319	p:0.17416YPL075W	FI:1.02534965034965	p:0.131638YPL076W	FI:1.17021276595745	p:0.224127YPL077C	FI:0.816326530612245	p:0.200644YPL078C	FI:0.476190476190476	p:0.214352YPL079W	FI:0	p:0.121212YPL082C	FI:0.878598247809762	p:0.107399YPL083C	FI:2.69230769230769	p:0.00237434YPL084W	FI:0.808201058201058	p:0.082532YPL085W	FI:0.700176411290323	p:0.0101616YPL086C	FI:0.0833333333333333	p:0.0176796YPL087W	FI:2.66666666666667	p:0.244369YPL090C	FI:1.57142857142857	p:0.505263YPL094C	FI:0.875	p:0.327896YPL095C	FI:0.530769230769231	p:0.0687616YPL096W	FI:1.26470588235294	p:0.191157YPL097W	FI:1.31313131313131	p:0.148048YPL098C	FI:0.857142857142857	p:0.474308YPL099C	FI:0.336538461538462	p:0.0242571YPL100W	FI:0.489690721649485	p:0.0428622YPL101W	FI:0.798728813559322	p:0.159066YPL103C	FI:1.23762376237624	p:0.166834YPL104W	FI:0.555236728837877	p:0.0378324YPL106C	FI:1.30454545454545	p:0.297372YPL107W	FI:0.608510638297872	p:0.118551YPL108W	FI:0.6875	p:0.174789YPL109C	FI:3.58333333333333	p:0.00644308YPL110C	FI:0.968499315202504	p:0.115484YPL111W	FI:0.847457627118644	p:0.327787YPL112C	FI:1.30528375733855	p:0.163369YPL114W	FI:2.95454545454545	p:0.129638YPL115C	FI:1.66875	p:0.0188916YPL116W	FI:1.408	p:0.123162YPL117C	FI:0.485106382978723	p:0.146039YPL118W	FI:0.298305084745763	p:0.0167339YPL120W	FI:1.10566893424036	p:0.117852YPL122C	FI:0.331395348837209	p:0.113899YPL123C	FI:0.478260869565217	p:0.0215806YPL125W	FI:1.42945544554455	p:0.112171YPL126W	FI:0.520389371218101	p:0.0154377YPL127C	FI:0.511363636363636	p:0.10525YPL128C	FI:1.7	p:0.094666YPL130W	FI:1.04132231404959	p:0.177168YPL131W	FI:	p:1YPL132W	FI:3.40476190476191	p:0.0311273YPL133C	FI:1.15012886597938	p:0.127131YPL134C	FI:1.17279411764706	p:0.235583YPL135W	FI:0.897435897435898	p:0.306187YPL136W	FI:0.838095238095238	p:0.245991YPL137C	FI:1.13093004556419	p:0.0890164YPL138C	FI:4.68852459016393	p:0.00581923YPL139C	FI:0.890721649484536	p:0.166792YPL140C	FI:0.363636363636364	p:0.00986263YPL141C	FI:1.58066860465116	p:0.0417781YPL144W	FI:0.944444444444444	p:0.196974YPL145C	FI:1.62162162162162	p:0.378497YPL146C	FI:0.852941176470588	p:0.13338YPL147W	FI:0.714539007092199	p:0.100499YPL148C	FI:0.584795321637427	p:0.129011YPL150W	FI:1.25139664804469	p:0.101857YPL151C	FI:1.22687439143135	p:0.130896YPL152W	FI:1.74242424242424	p:0.123613YPL153C	FI:0.938461538461539	p:0.141054YPL154C	FI:0.430379746835443	p:0.125411YPL155C	FI:1.10875912408759	p:0.152866YPL156C	FI:0.859649122807018	p:0.163029YPL157W	FI:1.178125	p:0.214789YPL158C	FI:0.996904024767802	p:0.127252YPL159C	FI:0.692307692307692	p:0.132515YPL160W	FI:1.01098901098901	p:0.227434YPL161C	FI:0.819542947202522	p:0.0789227YPL163C	FI:0.824414715719064	p:0.157306YPL164C	FI:0.668673489865543	p:0.038417YPL165C	FI:0.809716599190283	p:0.146524YPL166W	FI:0.558139534883721	p:0.0856077YPL168W	FI:0.885542168674699	p:0.122474YPL169C	FI:0.588675213675214	p:0.101352YPL170W	FI:0.5	p:0.166341YPL171C	FI:0.650826446280992	p:0.103857YPL172C	FI:1.09465020576132	p:0.171721YPL173W	FI:0.8	p:0.225322YPL174C	FI:1.22568322068763	p:0.0678839YPL175W	FI:0.8	p:0.205245YPL176C	FI:1.29826695044086	p:0.0738963YPL177C	FI:1.22448979591837	p:0.139746YPL179W	FI:1.4480198019802	p:0.188796YPL180W	FI:1.28571428571429	p:0.0841321YPL181W	FI:0.931304347826087	p:0.124888YPL183C	FI:0.817460317460317	p:0.0938718YPL183W-A	FI:0.320855614973262	p:0.0550637YPL184C	FI:0.851254480286738	p:0.158768YPL186C	FI:0.823529411764706	p:0.141768YPL187W	FI:0.905882352941176	p:0.241606YPL188W	FI:0.593567251461988	p:0.106817YPL189W	FI:0.571580063626723	p:0.0284188YPL190C	FI:1.22144927536232	p:0.0890465YPL191C	FI:0.544600938967136	p:0.0543364YPL193W	FI:1.35	p:0.114288YPL195W	FI:1.00932320441989	p:0.130083YPL196W	FI:0.714285714285714	p:0.310603YPL199C	FI:1.97142857142857	p:0.0710395YPL200W	FI:4.28571428571429	p:0.00326647YPL201C	FI:2.71428571428571	p:0.0029026YPL202C	FI:0.703779366700715	p:0.0784124YPL203W	FI:0.252927400468384	p:0.0288949YPL204W	FI:1.0224358974359	p:0.242716YPL206C	FI:0.462962962962963	p:0.057368YPL207W	FI:1.67307692307692	p:0.0457146YPL208W	FI:1.07339791356185	p:0.117073YPL209C	FI:0.541176470588235	p:0.121062YPL210C	FI:0.751346871114795	p:0.0899164YPL211W	FI:0.369565217391304	p:0.33255YPL212C	FI:1.35294117647059	p:0.199666YPL214C	FI:0.517241379310345	p:0.0418858YPL215W	FI:1.94285714285714	p:0.102044YPL217C	FI:0.91169671752196	p:0.109739YPL218W	FI:	p:1YPL219W	FI:0.58628841607565	p:0.0975688YPL220W	FI:	p:0.578948YPL221W	FI:1.74375	p:0.10423YPL222W	FI:0.47992259313014	p:0.00690244YPL223C	FI:0.540540540540541	p:0.159226YPL224C	FI:1.5	p:0.0814729YPL225W	FI:0	p:0.0766551YPL227C	FI:1.20879120879121	p:0.186111YPL228W	FI:0.619585687382297	p:0.0547734YPL229W	FI:0.541666666666667	p:0.0853702YPL230W	FI:1.69230769230769	p:0.0729543YPL231W	FI:1.16538461538462	p:0.152276YPL232W	FI:1.04	p:0.452737YPL233W	FI:2.19512195121951	p:0.0994925YPL234C	FI:	p:1YPL235W	FI:0.540540540540541	p:0.46102YPL236C	FI:1.63235294117647	p:0.110678YPL237W	FI:0.512820512820513	p:0.243229YPL238C	FI:0.363636363636364	p:0.149504YPL239W	FI:0.438405797101449	p:0.0995085YPL240C	FI:0.45985401459854	p:0.102163YPL242C	FI:1.53503427265803	p:0.0199328YPL243W	FI:1.24508519003932	p:0.11651YPL244C	FI:0.523076923076923	p:0.0603985YPL245W	FI:1.30285714285714	p:0.165338YPL246C	FI:1.16	p:0.216323YPL247C	FI:0.557142857142857	p:0.0448235YPL249C	FI:0.858505564387917	p:0.0943585YPL250C	FI:0.557142857142857	p:0.175377YPL252C	FI:0.166666666666667	p:0.0120756YPL253C	FI:0.564568462037638	p:0.026122YPL254W	FI:1.5	p:0.135169YPL255W	FI:0.626865671641791	p:0.117218YPL256C	FI:1.30693069306931	p:0.15723YPL258C	FI:0.673050615595075	p:0.11383YPL259C	FI:1.40639269406393	p:0.246316YPL260W	FI:0.557798165137615	p:0.0768913YPL262W	FI:	p:0.131774YPL263C	FI:0.584824624194703	p:0.0749137YPL264C	FI:1.88888888888889	p:0.175071YPL265W	FI:0.238095238095238	p:0.0107634YPL266W	FI:	p:0.738095YPL267W	FI:1.08527131782946	p:0.190017YPL268W	FI:0.63768115942029	p:0.0366949YPL269W	FI:1.40576923076923	p:0.0638867YPL270W	FI:0.79662447257384	p:0.111969YPL271W	FI:0	p:0.277778YPL272C	FI:0.704081632653061	p:0.129967YPL273W	FI:1.46268656716418	p:0.284923YPR002W	FI:0.74468085106383	p:0.0984627YPR003C	FI:1.22	p:0.137424YPR004C	FI:0.911487758945386	p:0.181005YPR005C	FI:2.15686274509804	p:0.0229756YPR006C	FI:0.470940170940171	p:0.0135844YPR007C	FI:1.36842105263158	p:0.0732933YPR008W	FI:0.418269230769231	p:0.00494586YPR009W	FI:2.36296296296296	p:0.0338493YPR010C	FI:0.1875	p:0.00206161YPR011C	FI:0.806451612903226	p:0.188054YPR013C	FI:1.63522012578616	p:0.0979389YPR015C	FI:0.615384615384615	p:0.131738YPR016C	FI:	p:0.708334YPR017C	FI:0.666666666666667	p:0.166637YPR018W	FI:0.796296296296296	p:0.090815YPR019W	FI:0.839939024390244	p:0.164128YPR020W	FI:0.25	p:0.122656YPR022C	FI:1.05871212121212	p:0.139023YPR023C	FI:0.521262002743484	p:0.0765709YPR024W	FI:0.5375	p:0.0693298YPR025C	FI:0.980592441266599	p:0.164184YPR026W	FI:0.428571428571429	p:0.00232794YPR027C	FI:1.52941176470588	p:0.135119YPR029C	FI:1.18285714285714	p:0.123514YPR030W	FI:0.896829810901001	p:0.0936412YPR032W	FI:0.669789227166276	p:0.0529322YPR033C	FI:3.27604166666667	p:0.0140208YPR034W	FI:1.13333333333333	p:0.200319YPR035W	FI:0.6	p:0.313534YPR036W	FI:0.370967741935484	p:0.0939298YPR041W	FI:0.842105263157895	p:0.33586YPR042C	FI:1.52352941176471	p:0.0422491YPR044C	FI:	p:0.645162YPR045C	FI:0.758082497212932	p:0.131089YPR047W	FI:0.653846153846154	p:0.0918306YPR048W	FI:0.734892787524367	p:0.0913681YPR049C	FI:0.98404452690167	p:0.0940379YPR050C	FI:1.03703703703704	p:0.312036YPR051W	FI:0.928571428571428	p:0.351644YPR054W	FI:1.28472222222222	p:0.183054YPR055W	FI:1.13483749664249	p:0.111726YPR056W	FI:0.941520467836257	p:0.282339YPR057W	FI:0.925786163522012	p:0.155554YPR058W	FI:1.5	p:0.148794YPR060C	FI:0.851063829787234	p:0.33911YPR061C	FI:1.26	p:0.161784YPR063C	FI:	p:0.0107188YPR065W	FI:0.588697017268446	p:0.120311YPR066W	FI:0.96875	p:0.175174YPR067W	FI:0.777439024390244	p:0.184268YPR068C	FI:1.08070175438596	p:0.14336YPR069C	FI:2.60869565217391	p:0.268625YPR070W	FI:0.589970501474926	p:0.0473451YPR072W	FI:1.06019417475728	p:0.211881YPR073C	FI:0.46551724137931	p:0.177436YPR074C	FI:0.552380952380952	p:0.210722YPR075C	FI:0.74719800747198	p:0.0950914YPR076W	FI:1.27450980392157	p:0.20062YPR078C	FI:1.62950819672131	p:0.0481629YPR079W	FI:1.14285714285714	p:0.262997YPR080W	FI:	p:0.26087YPR082C	FI:0.4	p:0.301028YPR083W	FI:0.956637168141593	p:0.163685YPR084W	FI:0.93	p:0.187788YPR086W	FI:0	p:0.0820794YPR093C	FI:0.819607843137255	p:0.144968YPR094W	FI:	p:0.268145YPR097W	FI:1.08484304932735	p:0.100473YPR100W	FI:1.77777777777778	p:0.37665YPR102C	FI:0	p:0.444445YPR103W	FI:2.8	p:0.236795YPR104C	FI:1.18158567774936	p:0.0912765YPR105C	FI:0.89011663597299	p:0.116237YPR106W	FI:0.697368421052632	p:0.0829887YPR107C	FI:0.485714285714286	p:0.237602YPR108W	FI:1.33333333333333	p:0.208546YPR109W	FI:0.585516178736518	p:0.0934129YPR110C	FI:1.26229508196721	p:0.307989YPR112C	FI:0.910700338453528	p:0.108952YPR113W	FI:0.77027027027027	p:0.348756YPR114W	FI:0.540983606557377	p:0.173349YPR115W	FI:1.26303780964798	p:0.0508887YPR116W	FI:0.628571428571428	p:0.0703573YPR118W	FI:1.00690448791715	p:0.163975YPR119W	FI:0.678492239467849	p:0.116033YPR120C	FI:0.989010989010989	p:0.151361YPR121W	FI:1.06060606060606	p:0.129424YPR122W	FI:1.62446400361092	p:0.0223917YPR124W	FI:1.49477351916376	p:0.127995YPR125W	FI:2.19047619047619	p:0.0371083YPR128C	FI:0.56078431372549	p:0.0597269YPR129W	FI:2.12865497076023	p:0.0606212YPR131C	FI:0.694444444444444	p:0.275769YPR133C	FI:1.78313253012048	p:0.0948642YPR134W	FI:0.607894736842105	p:0.0810451YPR135W	FI:0.936170212765957	p:0.115684YPR137W	FI:0.382072829131653	p:0.0069828YPR139C	FI:1.17735849056604	p:0.164704YPR140W	FI:1.18421052631579	p:0.177355YPR141C	FI:1.04627766599598	p:0.137636YPR143W	FI:1.43617021276596	p:0.146751YPR144C	FI:1.17971014492754	p:0.153698YPR145W	FI:0.833333333333333	p:0.268858YPR147C	FI:0.404761904761905	p:0.037828YPR148C	FI:1.21596244131455	p:0.117388YPR149W	FI:	p:0.774194YPR151C	FI:4.77272727272727	p:0.0919262YPR152C	FI:0.412921348314607	p:0.00835379YPR154W	FI:0.676923076923077	p:0.205684YPR155C	FI:0.858778625954199	p:0.140146YPR156C	FI:0.535537190082645	p:0.15149YPR157W	FI:0.965463108320251	p:0.15002YPR158W	FI:1.5224358974359	p:0.0995318YPR159W	FI:0.566037735849057	p:0.071074YPR160W	FI:0.523255813953488	p:0.110748YPR161C	FI:1.59349593495935	p:0.0815517YPR162C	FI:0.936139332365748	p:0.147429YPR163C	FI:3.37662337662338	p:0.0139978YPR164W	FI:1.21846153846154	p:0.0516642YPR165W	FI:0	p:0.357143YPR166C	FI:	p:0.30914YPR167C	FI:0.314814814814815	p:0.0521811YPR168W	FI:1.79310344827586	p:0.37347YPR169W	FI:0.538461538461538	p:0.0254563YPR171W	FI:1.84027777777778	p:0.0130332YPR172W	FI:0.526315789473684	p:0.175898YPR173C	FI:0.642361111111111	p:0.223791YPR174C	FI:0.856589147286822	p:0.215857YPR176C	FI:0.445161290322581	p:0.0524373YPR177C	FI:5.0625	p:0.0308069YPR178W	FI:1.0171875	p:0.143297YPR179C	FI:0.808823529411765	p:0.100945YPR180W	FI:1.01694915254237	p:0.15747YPR181C	FI:1.08064516129032	p:0.33291YPR182W	FI:0.692307692307692	p:0.507247YPR183W	FI:0.373983739837398	p:0.208907YPR185W	FI:1.38028169014085	p:0.108449YPR186C	FI:1.4566265060241	p:0.106183YPR188C	FI:1.1	p:0.267193YPR189W	FI:0.856918032786885	p:0.0876738YPR190C	FI:1.10925196850394	p:0.170172YPR191W	FI:0.444444444444444	p:0.0527313YPR192W	FI:0.378306878306878	p:0.0575119YPR193C	FI:0.791666666666667	p:0.212841YPR196W	FI:0.711570247933884	p:0.122909YPR198W	FI:0.895238095238095	p:0.158236YPR199C	FI:0.857142857142857	p:0.271718YPR200C	FI:	p:0.307692
