# Supplementary material for: Proteome-wide evidence for enhanced positive Darwinian selection within intrinsically disordered regions in proteins
Source: Genome Biol. 2011 Jul 19;12(7):R65. doi: 10.1186/gb-2011-12-7-r65 (PMC3218827; doi:10.1186/gb-2011-12-7-r65)
Supplement: Additional file 15 — Fixation index calculated for the merged aligned regions in α-helical, β-strand, or intrinsically disordered conformation from all analyzed proteins, with or without removal of genes with a fixation index deviating more than three standard deviations from the mean of the entire data set. [file gb-2011-12-7-r65-S15.RTF]

WHOLE DATA SET* SUMMARY TABLE FOR ALPHA-HELICAL SUBSEQUENCESdN:17731dS:79866pN:7495pS:25412RATIO (dN/dS)/(pN/pS):0.752728752615267* SUMMARY TABLE FOR BETA-STRAND SUBSEQUENCESdN:3036dS:17898pN:1448pS:6028RATIO (dN/dS)/(pN/pS):0.706158100321713* SUMMARY TABLE FOR INTRINSICALLY DISORDERED SUBSEQUENCESdN:47578dS:73626pN:14043pS:21162RATIO (dN/dS)/(pN/pS):0.973804528209812DATA SET AFTER REMOVAL OF OUTLIERS* SUMMARY TABLE FOR ALPHA-HELICAL SUBSEQUENCESdN:27888dS:116190pN:11226pS:35595RATIO (dN/dS)/(pN/pS):0.761048926064217* SUMMARY TABLE FOR BETA-STRAND SUBSEQUENCESdN:4655dS:24083pN:2098pS:7972RATIO (dN/dS)/(pN/pS):0.734464663375987* SUMMARY TABLE FOR INTRINSICALLY DISORDERED SUBSEQUENCESdN:45323dS:70916pN:13652pS:20102RATIO (dN/dS)/(pN/pS):0.941060200356386
