# Supplementary material for: Proteome-wide evidence for enhanced positive Darwinian selection within intrinsically disordered regions in proteins
Source: Genome Biol. 2011 Jul 19;12(7):R65. doi: 10.1186/gb-2011-12-7-r65 (PMC3218827; doi:10.1186/gb-2011-12-7-r65)
Supplement: Additional file 16 — Amino acid positions of functional sites in each investigated protein as predicted by the Limacs method for known pfam domains mapped to the proteins. [file gb-2011-12-7-r65-S16.RTF]

>YAL001C1626323762647888103119137163171175194200210219223225231239258279285292300305339365366379402404>YAL005C13314256707379109129131148157183184194201202213221227229236241251257281288293297336385400417423441470478487509518538582>YAL008W105118125150>YAL009W48517475109138235239240>YAL019W575582597598599638679709710731736741748>YAL022C195225248259272358379382404418422442448458461490>YAL023C919699111190198203215250269347368423425456472474479602648653665668719741>YAL025C41635404261637592100107110111114123144145149165175187189233>YAL032C108126130134159162178183194199212222238>YAL033W1127313235120122124>YAL034C340396>YAL034W-A1425264467117129152>YAL035W476265676974778083102103106108120122124129132136137141143149152158163165169177178184191193208214232236265301344363366371404409411412419420421426428433439440442444445448456465466467473474477480482483484485486490495505506508510511516517518520528530532535537544545561571576579581582592598606610617623645647648652655672675676692693705706719728731753754757760763764788798811837840841842872902919921925960>YAL036C6323441587375808385868996114118138141149151158164165172174183212217219227229230233242281285288297319322328335336344350>YAL038W192021314658858992107112116129130136146149157169172175177180192201208209220231249251252257273275288292296299300302303306316324326335340346348355356384392401402404407409412413414415424431436437455459465477488491492493>YAL039C26698184101102104149167169215220249>YAL040C139198>YAL041W190191219222233285289293303335356369412427>YAL042W222258305326360361371377>YAL044C475267117124134141162>YAL044W-A5759637071101105>YAL046C6874109>YAL048C1319202528618590159180286289300310324387399415429449451462463468488504509510511518519522524525529530532533536537568602>YAL049C5079129153154207>YAL054C105110126153156158164165220225233242275277293310311315319322332333337340349354369378383398404408410412430440453477484492501508510527538539540543545548553557558562565575577581582608613625628642656664685690694696699>YAL062W63646674808398114115125131133161167185200295304305335355397425449>YAR007C111466502534538581583>YAR015W22242736374042436175124150160199216234240243244261>YAR018C2530323335363738405153575864738388909192939596100103104105110113117124133136143168172173178186189198230232233234236242245248249251253254255256262264265267271272273274279280286288297308312>YAR019C94140144145175181182184192194197201>YAR035W157181164168188198242281325344349407438457467483485490491497528599>YBL005W488495>YBL006C194274759699104>YBL007C493504510512>YBL013W68138155159164174184291>YBL015W1625465993129148217221>YBL016W132024252839647075828588909495117120131135136141156158166179182185188190196199206207214215305306308309>YBL020W193437646570727580104115119129151179225248260281307315337338341342347348413416429438450485572>YBL021C529495>YBL023C328341382404422449506536555563566567570571583586588616617625627628630641645656657658703770786790802811>YBL024W366396399427428432436455503505509573576>YBL025W257493106>YBL030C81>YBL033C152158214221235251>YBL036C72181243>YBL041W303839565961124134142144149160205209217>YBL045C597174147>YBL049W262843638789119122124>YBL052C332340352370371385406425430437442506>YBL056W156189>YBL057C103106123180184192>YBL058W244247248252254259262293294296354357377403>YBL060W125128129134140146156162166170176188193197198201209211212213220224228231258344353>YBL066C489496>YBL068W229>YBL072C112425364784>YBL074C13224347100106118141169178193228231238261269289320>YBL075C314256109129131148157183184195202203214222228230237242252258282289294298337386401418424442471479488510519539584>YBL078C172228354954606679100>YBL080C79108112114117120152154157178189202243245265280281292293301303305325349358375432476491497>YBL082C546972108110153161168206214287290323353377378384387410412413>YBL087C1621282956616871758283858689110112120121123126>YBL089W69214216>YBL091C141162175176189198200202209225249253265279311326342353387391402415416>YBL092W2734445764698183105>YBL093C33385962114115203207>YBL098W3132384968788788131139162171193229236246258269274309348352356357>YBL099W46889294104105126128138146153176178198202207210213219238245246248249263266271278281285290294305309311313317331337340341346348349355357359368372373374388391403411413421428431433434436438440445446450451467470473481483522523526536>YBL102W76117145149152>YBR002C538082848689125165167173203209216231239251253260267269>YBR003W149186189192207212223231234249250255258261264316321333350368372388390395399432435457>YBR004C81348798391149155163169176178204212262267268271274305310312364376382418>YBR006W3866194258308332385408>YBR011C4554727482103139149171218>YBR018C17333568717475808187106115120126146167170176177178186198205216232235236240241243245247270279284287292294313318326328334336349>YBR019C1720223844656869829495109110113120122123130132133135137139158159165166169184185190193204214218258263272278290328334336415422426451465467469486510513551562598605612630646648651659667672696>YBR021W120130182201246247249286324325330334335336388416455487500516517557>YBR024W108125151152154184186191193198200204208212219225226229240246255262>YBR025C2122283035373840414750545760708890104112118125128135136140144147151153157166169212229235249266267283284302310311321322331338339340352354375381386389>YBR028C127128132133135136138139140141143145146147148149150152153154156160161162163167175177181189192194195197199200201203204210211213215216218219220221222223224225226228231232233238239241244245246247248249250251252253255257260262266267270271273275276281287288290292294296297298304305306311312314317318319320321322325327328329330332334335336337338339341345346347350353354356357359360362363364365366367368369370371372373375376377378379380383384385386387388389390391392393395397400401402404405406410411420421425427429431434436437440441443444445446448449450453455456458459465466468469471472473475476477479480481482485486489490494496498503505506510512513514515516>YBR031W3761100114117128160163187220234249251>YBR034C103125>YBR037C102119145146148178180185187192194198202206213219220223234240249256>YBR039W3742495158636668110112120198201230250255272>YBR041W113193250256270281361364382398404460479503504506510528530531537539558568642>YBR053C141175194207251>YBR054W82123149162171174204>YBR055C2756586061667098110113>YBR060C259301304317364373426455458473475481483525582584598>YBR061C1227575899119136182189194197201>YBR067C747679879798>YBR068C172185313336393404>YBR069C171184306329387398>YBR070C187190198200>YBR072W118148158181>YBR073W265272312313314370415444445466471476483>YBR082C6104546517078>YBR084W87108122201218226346349357374376379384397401403414415417418422439440451456463464465466467470478489493553559567582586591593610612614618621622630632633641645646649655658691704705718720723725729743748768770782787789802803804812829834841853855858862865866878881882883903904905909912913914915924925931935940950951955960961964966>YBR085W70>YBR088C17212865758689115118122130141174184192207216232>YBR089C-A232529343742657376>YBR092C327>YBR093C329>YBR094W659115144185567577579591612613677684>YBR095C6293132164174185221>YBR097W738091949799103104105106112117118119124127130136137141145146151157160164166177181184189195196198214216223224226228231232249255256260265267271280290>YBR098W578628639>YBR101C2372846606281>YBR104W292>YBR111C134>YBR115C31627486108115119142151157163167169176177179180186204211213235244245248249250252253254259265266267269270271272274276277280281282285286287292294295300302304306307308309310311312315321322325326327328329331332335336337339342343344348349351352353355358359360361369371372376378379382383385388389390391407411413414415416417418420422424427432434436437439440441442445446447448449451452453454455456457460461462463466468469470473474476477480481482486487488489492494495497499500501502503506507508510513516517518519520521525526527528529532533534537538539541543544545546548549550551552554555556557558559560561563565568570571572573574575577579582583585587588589591592595597599601604606607608609610612613615618619621622623626628630631634635638639640642644645646647657659660661670677678680685686687688689691692693694696697698700701702704705707708710712713716718719720721722723726732736737738739742744747748750759761764765768769778779781783784786788789793795796797800801802804806807810811813817819823825833834839847849858866870889897908912953961976979981101710311041104210721085109611041107110911301134114111511153115411551174118011821183118811921197120912211224124412451252>YBR125C214247>YBR126C23102105128150153160167182188190191192195293298303383388392400401433>YBR132C164177312335395406>YBR133C202245263286301327338341344368411418452462479497498499500506521524542566583586598631638670673>YBR135W37394980101>YBR136W887924164418242004200620332054205620582060206120632064206620672069207020712075207720792080208220832085208620872089209020922093209420972098210421072110211121142117211921222124212721282130213121332134213521362137213921402143214821512157215821602173217421752184218821892193219521972198219922002203220422062209221322142215221722182220222422282229223122332235223622382240224122432244224522462248224922552256225822622264226522672270227522782279228122822284228522862288228922972299230223032304230523062308230923102311231323162317232223252327233523522367>YBR137W148>YBR139W94105164173197310339341401>YBR146W159160162210233264276>YBR147W3641>YBR151W261278>YBR154C613213465159160168194211>YBR156C630632633663668678>YBR159W717274767882858894105106108119124125128135139151153160161164165166167171174175177181182183188209211212215217219220221223224226227228229230232233234235236239243244249252255258259265267272274>YBR160W83130134135165168174177185187195>YBR164C2037535559646783869195125129152169172>YBR166C2743107126139187188218220222253255>YBR168W2953111180213314318379385402406408>YBR169C314256110130132149184185201220236248258264288300343389402419426445477485494515553573618>YBR170C437578100107115116147152154174182187189201217218231246255262264267271285294311321326327328343348370372374376377379380381387399412417436438448457461484487525526527528554>YBR172C221225226234>YBR173C597383868793115130139143>YBR176W3545476178839096107111112122180181197240264278287>YBR179C201202>YBR180W153175229234235271291>YBR181C617276174798496105>YBR182C20212357>YBR183W222531404586104111114243252>YBR185C63889096103106109112115130153165169193196198223241243244245271273274>YBR191W4343847579497>YBR199W145152157160168173196234241247257258259262270294342349358359367372378389409>YBR201W19548894142143182198>YBR202W249262307329347374423453472480483484487488500503505533534542544545547558562573574575620645660664681690>YBR205W98105110113121126147185192198208209210213221245285292301302310315321332352>YBR207W184237282369>YBR210W9323742548398127>YBR212W212238>YBR217W89118139153159166169182185>YBR223C104107122125128180183201208211212217219244262263277301303342345369374376379387410414420436468475479481487521>YBR227C193841586573155167168169174178180203204206212232236238240265270310312326328329330340346353359380396398402405420421436440456>YBR231C236239241244248256266269275279285288>YBR233W-A76>YBR236C138141152161175182244247255256310314336348408410419424>YBR237W256265280289299302304310322323325336351356358364372376379381394399402403405410421422431433435436449450452458459463473476477483484489490498523526527536543546554556558595598605609622627633642672>YBR243C156222>YBR244W835427678108>YBR247C143224247255260261279289312317331346364369377396424431435438455457466>YBR248C8111622577991929394111112117121122125139142145149150173176178179184190196208238253261262274278281286287296300302307314335348351359362365379386394406407431433435436437438439446459463469472473480481486489490499506510516518524528530531532539547>YBR249C116157158180261281>YBR251W146174189196212215218238254257265275>YBR252W326987114>YBR254C163495101102140157>YBR256C149179>YBR258C67158586>YBR260C697376468470473475476477479480481482483488489490491492494495497498499502503504507508510512513514515517518520521522523524525526527528529530531534535536540541542543545546548552553554555557560563564565566568569570571573575578579581582583587588589596597598599600601603604605606607608611613614615617618619620622623624625629630633635636639640641642643644645646647648651653654657658661>YBR261C2426326978799398119124126153171218220225>YBR263W637094111128135143157175186219221266268297330331354362381412>YBR264C4679121318192021222324252627282930323334353639404142434445474849505153555657585960616264656970717273747576777879808182838485868890919293949596979899100102103104105106107109110111112113114117122123124125126127128129130131132133134136137138140141142144145146147148149150152153154156158159161162165167169178185191193200>YBR265W1641144148172180184186187210>YBR268W2846525460>YBR270C455458>YBR273C222257272>YBR274W15202325262728424447495660667782848586878990929697102105113121124131136140141146155159161162163171173174181183185186187188196198199205206213214220222247255274278280281>YBR276C594730739741770>YBR279W6121322375359617578859495107108117120121126132137148153157164199200201232236259260271275278313348349366383389421422>YBR284W482522645678689695698730737>YBR286W192234261326380>YBR287W29318>YBR291C264>YBR293W31>YBR296C2728>YBR298C177295380465>YBR301W7678818999100>YCL001W53647383106108135153163164>YCL011C239263>YCL017C107148166196248296298310404449451>YCL025C197210338361418429>YCL029C245667>YCL030C168185186189190204236238255265269304383400406409410411429441450453464475487494514517524532540546551553555556562563569571577578588596597604605631635640647654657658663673674675682698701703704706710712713714721728735738740746747752753755758759773778791>YCL032W3638395254628091102257260261265273314>YCL033C526887939597116142156160165>YCL043C426286467479>YCL044C417333638515876115182183235242248253261266270273312352368377>YCL045C514546561563598610618619649655689692706708715718>YCL052C258261262263360>YCL055W154157248270288290>YCL057C-A2735596179>YCL057W78106134178195238264287294297306331357358361362364371382399403415418422439440444454479480495503508538540556574590594631640643648649660672682689692693704>YCL064C82833356593139144153154232272319>YCL066W414244485261718096>YCR002C33343645518694101103106111122125165208232237259261262272282295>YCR004C10>YCR005C395765676974757686909394111113114123126131133145149153168186187192193210214223228232243247256261266278279280297302303316323329330332335337343344349355374382386388390397404410411412425430431445>YCR008W321323324326327328329331334336337346348350352357358359362364365367369371373374384389390391392393394396397398399400403404405410411413416417419421422423424425427429432435438439443445447448453460462464466468469478482483485486488491493494495496500502504505508512513514520521524526527533535536537538539545549560564587589592>YCR009C252730126>YCR010C144155174176178255264267>YCR011C386393425430431434451461470472483486534536539542543552558586588592595598599600611614>YCR020C244148208>YCR024C44144146167172181206210212213215222227232238251252255256261268324341353359361382385389394424437438441442443445446>YCR026C176224404405>YCR027C253031363851647577919596102103104129148167171>YCR035C7785>YCR037C3141519222627278296304305315318321322490497536551554560586592596598600603605623638640642644651653664668692727757765772776785803818821823848861863865867872875877892>YCR038C419421424428455460475487488489490501530535548551555557558578580>YCR045C183228268346>YCR048W349500507513>YCR053W85897114122158161190199218253254269276278279295305317322328332340390418423449>YCR057C741762781806825826833840844848>YCR059C184185229230>YCR065W112131138143145172191392402404410>YCR069W55115123143146157166169171183204>YCR071C667188108>YCR083W365678>YCR088W325266899697101125>YCR091W306323324328329331332334335336337339341342343344345346348349350352356357358359363364367369370371373374376379380381383385386388389390391392393394395396398401402403408409410411412414415416417418419420421422423424425427429432434438439442443445447448453459460462464466468469470472474481484486487489494495496497498499500501502505507508509510512513514515516517518519521525526527530533534536537539540542543544545546548549550551552553554555557558559561563564565566567568569570571572573575577578580581582583584585586589590592594595597598599601602603604605606607608609611613614617618620624627632>YCR095C67778586113151159160182185197201>YDL001W133134150206218223248250263276280288290303341345348360368377>YDL003W935798182848791521529548>YDL006W165184198>YDL014W122148165180206248289302309311320>YDL017W334044454648525354555668707375768285919798102107109112113115117121122128130133134141143146152157159161162167174179183185200206222262265281290302304310327328331347351353356>YDL022W45150174186234239259262269308339343>YDL028C422439440445447448449450451452453455457458459460461462465470472474477478484485487496498503505506507508510511513516517518522523524525528529533534541543546552553557559561562567575577579581583589591592593594599602604605607612613615616623631633637638639645646649651654655657658661663669671675678679682686689690698702704708709713715>YDL029W1013202381109110115120127146153157164166170176189212217262275277307319320323354376377>YDL033C8298103115153182184209222224294350>YDL036C195214218227231239241242273336353>YDL042C99118125131134141152154158198219226255274281300308309323342366367439444465472492498>YDL045C61107108171202220>YDL049C3336396586107108112124144151163167200206207208210219231260>YDL051W39404271>YDL052C8187>YDL056W31354547505594>YDL058W371401403408421425473474517547549576587625635644647648650653663696697731739744758762770771799812832836837838840845846859868870871878883886893894900910918922930931949966969977988995100510071011101210151022102510281031103610381040104410481049105010511054105610581060106110671069107210751076107810791081108210841086108810891090109510971099110011011102110411101111111411181124112611281130113311341139114011421144114911521153115611581160116111621165116911701175117611781181118212071208121312161217121912201222122312241227122812291233123612371241124312441249125112531254125512561259126212641265127012711272127612781280128112851287128912901292129512971299130213031306130713081309131013131314131513181323132413261327132813301331133213411346134813511356135713601366137513761378138213921396139814021407141014141420142214231425142814301431143214351437143914401441144514461455145814601464146614681471147414771478148114821483148514911495149714981499150115021504150515061507150815101511151315181523152515261527152915331535153615371538154415451548155115521554155515621564156615681569157115761577157915821592159416071623162716301632163416371641164316451646164816511653166016611664167116831685168716911698169917011711171617201727173217351740174217501752177317751776>YDL059C6567879092168178>YDL060W53557687949698124153215234240258268275305321355397399406424431447452484485496497500505525526535562578582590603604605607610620621626630638642644650652655670671676709715718723726731736738742744753757771772773>YDL064W8125253587785>YDL065C216222223230231236238239241251256261270271292293>YDL066W33505875769596102115135151159172176200201205216225227230236237240242251256265266280288304314328333351355356362>YDL067C344547>YDL072C252932646772102143202203>YDL073W494530533539>YDL076C163>YDL078C3333444475770858897117121137141146153154156173187190191195197205216227232235247260288289301315319324>YDL080C4046616770858991102127129132171423428505555>YDL084W66728695105108110116123127129136151156157158164172176180182197198200202203205210221225232234236237250251253259260264273274279284286289290299304307308317324327335337339376379386390403408414417420421>YDL085W200240299404>YDL086W4371129151152218>YDL087C1639414855616480819496107109151170197202214219221229234250259>YDL088C263274276282354355>YDL091C366>YDL100C3361128142160167172173250285293304>YDL101C200205207208210211212213215220221222224226228233240246249256259262264266267270273274276279282283284289290292296300306308311317318322326327332337341360362364366367368375377378381384386387388389394395397398400404405406412413416419421423424426429431432441449450452454466473477479480481483485>YDL102W229279281315318321323324338376379386389394399443455470471473483489497501502503511534536537538547549550553587594599606615620624625626644647650653664666667681682685694703707708710717722724728729730731734737738746755765767770774779783784788790793797799800802805810813815822825828829830831837839840841858876880886887890891898903909910922926936947953956957>YDL105W48657999117131142143176238246313320326333373385392396>YDL106C8388>YDL108W18336578112123127128148158161167170178180188197295>YDL111C4756>YDL114W394042444748495051525354565758606163646667686970717273758081828385868788899092939495969798100102103105106108110112113115116117120121122123124125126127128129130131132133134135136138139140141142143144145146147149150152154155156157158159160162163166168169170171173174176178179180182183184185186187188189190191192193194195197198199200201202205206207210213214215216217218221222223224225226227229231232233234235239240241242244245250>YDL115C539404252667174787980889098120159180208213217219230>YDL116W748285113136142162175183187192193199206216235242255257259262278280293297299307308314322323336345384440442450456512534575584594596607623624626645665674693710>YDL119C166>YDL120W7378868999110124129131134138141142164173>YDL123W4958>YDL126C101108169170172187213214224231279288290297299301323329332342368383386417419420422434437446485493504513560567578635645690711>YDL127W77138>YDL130W-A404449515264>YDL131W4955131156169172199229230237259293300320333355>YDL135C3037107109118133136142168187>YDL137W17181920222631323334353637383940414244454647515254555860616365666768717273747576777980818283848586909293949596979899100102103104106108109110113114115116117123124126127128129134138140141143144147151155156158159161162167168170171172176>YDL143W14193337384450586068778086889395105118124125157167176192198205207233242245249255260263267276295298299303304309314317320322328337338339362376378384389407436439443448455457464468470499512520522523>YDL144C91527366971118151157191222226246250257290306320>YDL146W279289292301337346>YDL148C5964677888939697108136137143148152163176177195203217222229234237271276280287288293297299324327329333335338343351353354360361362364367379402417421422436482483497498505516527533542551556567603620632642690703708713714716718731733735738742744746750751754763776789>YDL149W417456478508515518558565574583585588595624626632642643650661683686716720733739756758760764768772795798>YDL150W367381390392419421>YDL153C559560>YDL154W268282312341342513514539543589602603607612613616617642646647650651652654655659661662664668680683684685688689690691697699700702705714716717722724728729731732733742745751752754755756757759760761763771778782796807809810816818819820830837>YDL155W191197207210226227232244245289292303>YDL159W266324329330354360361367370378380387390>YDL160C50567079899294100107108113120135140141147155159162164177178180182183185190201202211213215216229230232238239243252256262263267268276281284285294301304312314316353356363367380385391393396397>YDL161W25304671108117123135>YDL165W16171921335678103109114116129131135137139144149168178182>YDL174C489>YDL177C6364141142>YDL178W445>YDL179W77138>YDL182W3541117142155158185215216223245279286306319341>YDL192W17181920222631323334353637383940414244454647515254555860616365666768717273747576777980818283848586909293949596979899100102103104106108109110113114115116117123124126127128129130134138140141143144147151155156158159161162167168170171172176>YDL198C260>YDL199C190306390476>YDL200C101143>YDL201W82101219259277>YDL204W37425459638287100148169176>YDL207W141142149198210221225297326369379381404415423457486492>YDL210W302325379389>YDL212W12202732343847538495101108110113124126159174>YDL214C366368369371372373374376387389395396403412422427429430431432434435437441442446448449457464465467474478482483488497501503504505517520523526528529530531537539540547548555556562564574583586>YDL216C161163165212>YDL219W536669788094128138>YDL222C4761677591104106110114140195214230283284>YDL224C553584>YDL226C2328363853596169719091114>YDL227C25424579133135152180190214219230289317325352366367395462>YDL229W1824252935465060697075808287909798100104108112113114115120124127131132133135136139142152153161170174185187188190194195200201207208217218219227229230232233234235239242243247255257260261263270271272276285287291293294295297298299302304309310315318321332333335336342343344360361364365391392393394400407415419421423424425426428429430432433438448452456464475477483484485487492494495496501502504506508513514516518519520521525527529540541543545558559563571575579583587590>YDL230W265965676878798399112116136145212214221251256259264305307311316318>YDL234C540548591609611616638651652>YDL247W175293379464>YDR002W56808287949699103105119133152166168178182187190191192>YDR004W117124127129132137157163172182193199200201203213231234235262264265>YDR006C421423439452476495504508545546548568569574638639643761789826887>YDR012W3761100114117128160163187220234249251>YDR013W1226323435394249578283103129130145159162169171175188193201206>YDR016C24303650585969>YDR018C116>YDR032C10>YDR041W505156100102>YDR044W25556087136164170189200222225251258273283302318325>YDR045C10317681829195107>YDR046C167180308331388399>YDR047W3948566274150155198202213214215225258303335338>YDR050C737598104128129167185239>YDR052C107125127146166176200205224239241242248255257261267283296660668677690>YDR059C6104546517078>YDR061W161167177181190217240273316333346347375405408412414451462464467493501506511513515516522>YDR074W68147150176198201208215227233235236237240307338347352357392400401403404410438443447455456494578579581617619694780781786>YDR083W120122131159181210212218241264282291305313319339368>YDR084C22264058596580828587105123135149>YDR086C38>YDR098C497094>YDR100W717279132>YDR156W43818288132>YDR167W102122>YDR172W263270274275281284288289293297301302307308309313314318320321322325327328330332333334335337340342345348349351352353355357360363372373386387391394395404408409415417419420421426427428429430432435436437441444453454456468471477479487490491494497499500501503505506508510511517518523528529530532533537539541542548549550552553558559560562565566567574580582585588589592593599604606607614617622629630640642654655661665666671673674679>YDR177W6104647527179161162166171176>YDR178W506673108110141155166>YDR191W112113119132136141143145146154159163182183211215216257275277280281283304305311338344>YDR204W113115116121123148151153169177184193195206208221258284296>YDR205W535>YDR246W127134136176188196>YDR252W4143506188>YDR254W266893128201279423>YDR260C111326365495100149>YDR265W85157>YDR284C114118164171179183212216226227>YDR286C72>YDR304C37119130139154174>YDR322C-A5918>YDR339C139151165180183>YDR345C139256340433>YDR352W3439>YDR353W48113114134141155190198219238294>YDR374C252259266284>YDR387C109223>YDR388W272932138>YDR408C13104116123127132158163>YDR411C3772107123173174213244>YDR418W34464753697189103114119130133134135137138144160>YDR437W2227485286131>YDR469W145148152156>YDR492W124278>YDR502C475162738188105122127129130147164171173174194200231236238258259277281282289291293308318323324341359362363366369373374380>YDR517W177179180181183187229235236238256258273275>YDR519W647391108>YDR525W-A4251>YEL012W7114344496876>YEL013W8889125156158171174282322348350>YEL040W102130132142155184199206245248268277301>YEL052W354052130133136137139140203204207214277347376377384395406415423425430488497498>YEL054C34464753697189103114119130133134135137138144160>YEL064C130268270>YER011W7678818999100>YER012W323359638398145148179>YER018C141161209>YER019C-A525879>YER027C360403407413414>YER031C91012141516182122262728293031323334353637383940414243444548495051525354555658596061626364656768707273747576777879808182838485868788899193949596979899100101102103105106107108109110112113114116117118121124126127128129130131132133134135137138139140142143144145146147148150151152153154155156158159160161162164165166167168169170171172173175176177182184196222>YER042W829697118156166>YER057C7982>YER081W109113132135137153193255260267276283319322334337376418440>YER083C51016222731323988101104141144157165167203212233262263>YER090W40414651586061626566676872747583848586879299105107110112121125128129131132135136137138140146147148153154157158160162163165166171176177183184188189195207224226227231233234237238242243245248250255257258259262265266267268270275276278279281283284285286287289291295296297300301311316317322323325328332333335337338339340341344345348349353356357360361362363365367368369370371373374375377382386390391394395397403406409412417421423425426427430431437439440441443444446447450452454455458462463464472474476477479480482483484488499503>YER131W7192455>YER159C104105>YER161C234244270276283284290295325329>YER163C353946158167190210>YER165W146170503511514536537541>YER178W118138216221225226229247251275303307354>YFL028C1648505355100112127153154156171188190201205206211233284>YFL039C1114212479106107112117124143150154161163167173186209214259272274304316317320340361362>YFL041W80127164274416487>YFL045C43617173100109147151154163179180184187190212218228236237>YFL054C354357360362388412487491537541570585>YFL055W129142265288343354>YFR003C53748191>YFR047C2430313233343944474849505860636676788384104106107108109111116121123124126128134136138142144145150151153154165166167168171172173176177178185187189191194199200202204208209212214222225228230232233237240241248252254256260262267269270272273274276277278281283284286290291292>YGL001C101315616285102109111119122124126147154157173183189247248251257>YGL010W2165118139>YGL012W92102105170194205213281292308343398402413416422428429430433457458466473>YGL017W3570788791107111114115165167170187188191259260293307319>YGL019W38454663134137148152158164181191202206208213>YGL020C10174865676870727387104109112113125168174>YGL031C8213436475561>YGL040C172022344349526098103137138139155157182190206211215221232244261293306308328>YGL044C3863>YGL054C6293439518095124>YGL055W169199206218313321323325332341352435436438440444458460482>YGL058W8124748537280>YGL070C123494>YGL089C224366371>YGL135W3133465083108113131143157166202>YGL175C191194225259261264268272273298313>YGL179C495055575860616263656870727678838893959798103105112115120122123124125126127128132135136137142145148151154161164167169172178179183187188193202204206208209210212218219224227229231233236238239240241246250251252255259260261262267268271274276278279280282284285291292295299300306314316321330337339341343344>YGL181W26273240425764707880100101122343>YGL187C585961627981123133141>YGL189C7192455>YGL191W52566971106114122>YGL225W58111121202286292>YGL231C36728992101124125144158172>YGL240W909196110112115116127139146149163211212215>YGL261C7678818999100>YGR019W7981134144189193200265277285298303304308351355406>YGR020C131516105106>YGR021W435299125126131134135136141145150154189204269278>YGR027C34426167>YGR033C98100103109139149154172188200204>YGR036C3874118148>YGR048W909195124136151155169175177189195196>YGR080W200220249256257261>YGR082W5808496112137144160161173>YGR091W211222228240258260266281286>YGR104C4162171181213220250>YGR109C144150162165181182187199200244247258>YGR148C8213436475561>YGR159C188213>YGR175C5866155162203212267286317330344366379380381399402448449454>YGR181W40798081>YGR199W9297100112191199204216251270348368427429460483485490612658662674677723745>YGR206W101518294371727377839798>YGR209C113254>YGR213C72838699102118121142168190197199240258270280>YGR215W334757848593>YGR234W220>YGR243W212224326466677699>YGR267C747577787992119139142152166177198211214218219230234>YGR283C6772116154284327>YHL002W1423243033678389103117126>YHL004W209221225252270275283324327328347348349350353356362363368>YHL014C2631323334363740465053548385110113120126135139142146159164172210227233264282284295309316317327328337344345346358360384390398>YHL015W218082115>YHR002W89>YHR008C293541555986100106110137139141143153188204206208211215220228>YHR013C167>YHR019C142180241243251264269278294298300301303308310312315318320326333339340343344349355390399401407418424426446449451453458480483488501502504505506508509522545>YHR051W4853567189146>YHR059W59718694>YHR063C198>YHR070W178242260261280292307324394407436>YHR072W-A21273435>YHR087W1640>YHR100C4873119123124>YHR106W2771136137157164178213221242260317>YHR107C353638475253879599102104107108112123126130148149161165180185193208237242243248267268271273274284294299301307>YHR129C1821283186114115120125132151158162169171175181194218223269282284314326327330350371372>YHR132W-A26333949575960129>YHR137W152156159163169172199220253261264268288294319333346347350418>YHR148W14676894108>YHR175W63100145154155>YHR189W327287100109111124125136154158>YHR210C106238287>YIL036W427445>YIL043C105112248>YIL044C2025343651575967698990112>YIL051C9598>YIL061C127152>YIL062C56911899294109114133>YIL094C3780120146173213217237248267279289301322323>YIL104C276289290309311312314316347385403421427434437>YIL106W152155164165183195233243256257283286>YIL111W30626667707374142>YIL113W69109145154156183>YIL114C61193152168189190260>YIL131C306325332337339340350352358366387>YIR001C84109>YIR005W5176>YIR025W81106116127156218220222>YIR026C20122131133160>YIR028W122132184203248249251288326327332336337338390418457489502518519559>YIR036C34568111214161718202122242526272829313233343536373839404243444647484950515354555758596162636465666768697071727577798081828485868889909192939495979899100101102103104105106107108109110112113114115116117118119120121122123124125126127129130131132133134135136137138141143144145146147148149150151153154155157158159160161162163164165166167168169170171172173174175177178179183184185186189190191192193194195196197198199200201205206207208212213214216220224225226228229232233236237239240244245247249250252>YIR037W73441425262656671757786107130142>YJL001W21273739405071130152156169173>YJL030W10323794>YJL035C4354105>YJL063C35363944485562919798106110>YJL104W81980120>YJL151C5867>YJL164C676971748687919295979899100102104105106107108109111112113115119120121122126127130132133134136137139143144146148149151152153154155156157158159161164165166171172173174176177178180181182183184185186188190193195199200203204206208209214220221223225227229230231234235236237239240241242245247248249250252254255256257258259261265266267270273274276277279280282283284285286288289290291292293296297298301302303304305306307308309310311313315316318319320322323324329331332333334336337338340341342343344345346347348350352355358359361364366367369370371373374375378>YJL178C314655133220222231234252265>YJL180C557680103106127>YJR007W1833374345495760646772848894102103138142145146189192202229234236>YJR010C-A184447637174>YJR019C2556828993114246258282286288305320>YJR024C3441556087888997163191220>YJR044C82124>YJR063W1535105>YJR077C67>YJR082C22336373747692>YJR085C82289>YJR095W66>YJR097W10659499109122163>YJR102C8101314153742767883104125131138153185201>YJR116W6382123140141163173174182184222226>YJR117W306384432>YJR123W4346737685118145151173182185213214215216218219220224225>YJR135W-A27707172>YJR144W94105128151176190205225231233234235244253254260261268>YJR153W75106144186218250252258271273287316331350>YKL001C3839456162707477818394103105129157158165171>YKL007W2430366675848895111121124126135145150153156157158187193196199206227232245250258>YKL016C18253449565973757989114123135137>YKL041W105143162166>YKL046C254765757779104122127144152153158160182183184186187191201206262272286297310328369>YKL053C-A212527283234606770>YKL056C41824282940568390124130135138146>YKL087C10566871878890130148150179184216>YKL139W194209241254289300304305325335337343346354356364373>YKL141W9699163>YKL157W193223236278281286342362403416419475>YKL159C1853555762110122128197>YLR090W111866105134>YLR098C373380>YLR118C195072143>YLR120C192198199238294295310372462465>YLR167W214552105137>YLR170C172021226264788090929598103129130>YLR175W356371727374788081838487939799108110111115118126127128129132133136146155157158161167170172176178186190191202219221224226232240268274283311341>YLR180W454960717986103120125127128145162169171172192198229234236256257275279280287289291306316321322339357360361364367371372378>YLR186W93101113115132133134136162167213243246>YLR192C2330353877101146149150173194206212>YLR193C110123147153>YLR197W1871185216255285293296302314328329332334336337340354356359369385389408485493>YLR215C273137558485104122125126133139142147155161187199211216232234242247250252264277291321326>YLR225C6399122159199222256273277283287288289291294295336359367379381404>YLR241W388390424450518523531533576579652657661683736>YLR245C324246495661106140>YLR251W137148163174177>YLR262C10111213151819232425262728293031323334353637383940414245464748495051525355565758596061626465676970717273747576777879808182838485868890919293949596979899100101102103104105106107108109110111112113114115116117119120124125126127128129130131132133134135136137138140141142143144145146148149150151152153154155157158159160161163164165166167168169170171173174175180188195197215>YLR264W1418364959>YLR308W124146>YLR315W9102125>YLR325C7102952>YLR332W202204206286287289316320328>YLR333C34426167>YLR343W4453110147189214238239245250264276293301309332333>YLR348C62>YLR377C79141194203219222236256259263265274286291298301324326>YLR380W175200227252268272278286309>YLR395C154445565774>YLR417W453>YLR441C131719243072104108116119122133142157168205>YLR447C1218304080102147156235236312317322336>YML050W255279>YML063W131719243072104108116119122133142157168205>YML064C283435373940414243454648495051525657585960616365686970717479808182838486888990929394959698100101102103104105107108109110113116117118119122124132134135136137141144145146153154155157162163165166167169170171172173175176178180184186196>YML078W25496689106117126139141161179>YML080W415859158162191204245248>YML098W11141920243237386670>YML105C102107110111117125146149>YML120C169229268377>YML121W134167170172173175190209212274290>YMR011W133250334427>YMR040W25293264677299140>YMR042W9798100134>YMR043W182732353637384048495154646572>YMR063W254245148157>YMR072W394145505358818992>YMR074C91012155560678891100112>YMR077C106142157161>YMR101C88115117121124162204256270292299305307>YMR114C491161>YMR118C9497161>YMR123W182562>YMR132C4096>YMR138W8161848545657656875798485879296133161172178186>YMR166C104>YMR188C384172>YMR222C1138465355566678103121207219224>YMR234W7814192122243237384049106128191230234263>YMR272C3961>YMR284W37427685191195287335386456494501505526>YMR293C7074102109129150168170198285286296331414439452>YNL003C238>YNL004W156179>YNL016W95119>YNL025C106168>YNL056W82129305895108109132135147151>YNL062C3849124140141147154165169172180183185198208218239242251310322>YNL070W153032>YNL081C2149517073848889106107109>YNL085W45547999406452476529532544547583>YNL090W7891012151620212223242526272829303132333435363738394042434446474849515254555657586061636566676869707172737475767778798081828486878889909192939495969899100101102103104105106108109110111114117119120121122123124125126127128129131132135136137139140141142144145146147148149150152153154155156157158159161162163164165167168169170171172173174175179187>YNL095C28>YNL111C3153>YNL135C46557390>YNL136W101228297382125>YNL141W63100132145177185241252256273284286290293322329331>YNL142W80145248263337422432>YNL157W27344050586061>YNL180C3568111216171819202122232425262728293031323334353839404142434445464856616277808182838486879192939495969899100101102103104105106107108110112113114115116117118119120121122124125126127128129130132134136138140143151154156158161162163164165166168169174176177179181182184185186187188189190192193194195196197198199201202203204205207208209210211212214215221223227>YNL204C25303840556171>YNL222W343941666870108119143150160180205206>YNL244C4052646783>YNL281W13536768>YNL282W172025272829407374140162168>YNL290W1819202425263032353638394042454849525560616264686970717273747578798283868889919294969899100102103104105108110111112113114115116117122123124125126127128133136137140142149151152154161164167168171172176177183184186189190191199202204205207210213218233238247251255258261264265267274276277280293295298301302305307308309316>YNL307C464765819198104106110144147158162163184186194197199202205207213216223231232324326327>YNL315C414774121133136152164170199223224239241251265314>YNL326C109119120133>YNL334C27457898123128136146202206>YNR002C143154173175177254263266>YNR010W7893118122136>YNR015W405859154158192205247250>YNR036C30333959636972868794103104122123>YNR037C11143234353954555966717276787982>YNR048W148152187203204229233249308313325352>YNR052C191261302345383393401>YOL002C121275>YOL032W133068708791130204206216235>YOL071W687197104106114116>YOL121C55455608086109125>YOL129W2234506094175176178>YOL139C35363740546365869099107115135140155176189>YOL146W374979103104130162>YOL147C252627303485103106224>YOL152W251357372376394410497561563>YOR004W101117131146149>YOR039W50575875117120131135141147164174185189191196>YOR056C230269276308320331334338359363>YOR057W188192196202245259281324332334337354355356361362372375393>YOR065W769799150156175186195219220223267>YOR078W115158171192204>YOR089C7910121516202122232425262728293031323334353637383942434445464748495052535455565758596162646667686970717273747576777879808182838587888990919293949596979899100101102103104106107108110111112115118120121122123124125126127128129131132134135136137139140141142143144145147148149150151152153155156157158159161162163164165166168169171172173174177179183194201203>YOR136W40505388117120125145153171175176182186201211214215216231242248263276286298301315319320335343352>YOR167C1418364959>YOR173W375185139216224242275295>YOR180C34129144>YOR210W6921435255>YOR215C43858893100115>YOR224C81019204753619192100116133>YOR228C178>YOR236W384263128141155>YOR258W124>YOR265W1923264461688488>YOR319W2954>YOR339C12165253587785>YOR347C212223334860879194109118122131132138148151159171174177179182194203210211222233251253254259275277290294298301302304305308318326328337342348350357358386394403404406409411414415416417426433438439453457467479490493494495>YOR370C44578996129141151155160203207216221226236238257271276337409425>YOR390W256318325329>YPL013C272933354368828384>YPL023C1920234560132152171176184195197200219224285>YPL050C7599115131141157188199213248249254286288301322326331344349365370371373374377388>YPL079W4343847579497>YPL087W212430394485103110113243252>YPL127C6061112>YPL152W1216194980828588114123125131133180183191201203207232250267268280289290291293>YPL159C95100105109120123124163173185190>YPL187W229416876>YPL189W331451458464>YPL191C617183101104111113131146>YPL196W102110142158167205>YPL199C26>YPL203W6970747578808182838587888990919294959698102103104105108109110113115116117119120122126127129131132134135136137138139140141142144147148149154155156157159160161162163164165166167168169171173176178182183186187189191192197203204206208210212213214217218219220221222223224225228230231232233235237238239240241242244248249250253256257259260262263265266267268269270271272273274275276278279280281284285286288289290291292293294296298299301302303305306307312314315316317319320321323324325326327328329330331333334335338341342344347348349350352353354355356357358361364370>YPL214C93106114135141143145266314345365383414415423461463495510511517518526>YPL215W155157184191195217226230235248260265267269>YPL218W252858606972889196100134164181183>YPL220W3133465083108113131143157166202>YPL236C30374142456686101103106109116120121149209213214219236244247262264265267276278285286288293361>YPL244C51106116193288294>YPL258C284647536566100232241242269355372379498533543>YPL272C32334889112146158169173219251301306341361395442464481487>YPR004C30152173258272275276285>YPR017C78878892101106108114>YPR056W31336087118159174193204205231235272275277278279287296297>YPR065W12172021616484>YPR066W41926313536373940414546505259606465727883848587899093100102104107108110112128129130131133136137141148149150151152154162164165177178180183185186189193194201202204214222225227229239240251268271278281286>YPR067W155164166>YPR073C12192946596789132>YPR082C7186105108112118121122126>YPR100W4156>YPR133C165205212214230232239249257263266270274275278294297300338349368371405>YPR151C50556064757879162172184189>YPR166C91112>YPR181C66728795102110122124126130132139159160161162170178187195226233250255283287293301303304315321333335342346349350363365371373375378381383384385398424443458459487491492494495501503522532548561563564565566571573581584586589606607622630645656672676704708709721729746747753>YPR190C57106199283287292476509518>YPR191W394952130>YPR192W56596267229251>YPR193C141
